# Equine herpesvirus myeloencephalopathy-associated mutations in equine herpesvirus 1 DNA polymerase confer the ability to replicate at elevated temperatures

**DOI:** 10.1128/spectrum.03055-25

**Published:** 2026-08-21

**Authors:** Noriko Fukushi, Rikio Kirisawa, Fuka Nishimura, Koji Tsujimura, Yuko Toishi, Hideto Fukushi

**Affiliations:** 1 The United Graduate School of Veterinary Sciences, Gifu University, Gifu, Japan; 2 Rakuno Gakuen University 13022, Hokkaido, Japan; 3 Faculty of Applied Biological Sciences, Gifu University 12785, Gifu, Japan; 4 Japan Racing Association 68373, Tochigi, Japan; 5 Shadai Stallion Station, Hokkaido, Japan; Howard University College of Medicine, Washington, DC, USA

**Keywords:** equine herpesvirus myeloencephalopathy, equine herpesvirus, DNA polymerase, fever

## Abstract

Equine herpesvirus myeloencephalopathy (EHM), caused by equine herpesvirus type 1 (EHV-1), poses a major threat to the equine industry because of its devastating impact on animal welfare and athletic performance. A single-nucleotide polymorphism (SNP), G2254, in ORF30 (UL30), which encodes the viral DNA polymerase, has been used as a marker of neuropathogenic EHV-1 strains, although its contribution to EHM pathogenesis remains controversial. In addition, other ORF30 SNPs have been reported in association with EHM, but their functional significance is unclear. Clinical observations indicate that fever and high levels of viremia are closely associated with EHM onset. Here, we investigated EHV-1 replication under elevated-temperature conditions that mimic febrile states using a fetal horse kidney cell line, equine peripheral blood mononuclear cells (PBMCs), and equine vascular endothelial cells (EVECs). EHV-1 isolates derived from horses with EHM consistently retained the ability to replicate at elevated temperature in permissive cells and remained competent for infection despite restricted replication in PBMCs, whereas replication of many non-EHM-derived isolates was suppressed. Specific ORF30 SNPs were associated with replication at elevated temperatures, and a molecular epidemiological association analysis of these SNPs suggested an association with EHM. Together, these findings suggest that replication at febrile temperature is a viral property relevant to EHV-1 pathogenesis and EHM risk.

**IMPORTANCE:**

Equine herpesvirus myeloencephalopathy (EHM), caused by equine herpesvirus type 1 (EHV-1), represents a serious threat to the equine industry. Here, we show that EHV-1 field isolates and recombinant viruses harboring EHM-associated UL30 variants retain replication capacity at elevated temperatures in fetal horse kidney cells and equine vascular endothelial cells, and that peripheral blood mononuclear cells (PBMCs) infected with EHM-associated viruses can mediate cell-to-cell transfer under these conditions. In contrast, replication of many non-EHM-derived isolates and recombinant viruses in these cells, as well as PBMC-mediated cell-to-cell transfer of EHV-1, is suppressed at elevated temperatures. Notably, we show that single nucleotide polymorphism (SNPs) in ORF30, which encodes the viral DNA polymerase UL30, and which have been implicated in EHM, are linked to the ability of EHV-1 to replicate at elevated temperatures. These findings suggest that replication at febrile temperature is a viral property relevant to EHV-1 pathogenesis and EHM risk.

## INTRODUCTION

Equine herpesvirus type 1 (EHV-1; family *Orthoherpesviridae*, subfamily *Alphaherpesvirinae*, genus *Varicellovirus*, species *equidalpha1*) is a ubiquitous equine pathogen worldwide (1). Primary infection with EHV-1 typically causes respiratory disease, followed by the establishment of latent infection. Reactivation of latent virus and subsequent horizontal transmission may result in abortion and neurological disease (1, 2). The neurological disease caused by EHV-1, termed equine herpesvirus myeloencephalopathy (EHM), results from infection of vascular endothelial cells, leading to thrombosis and ischemic damage to the spinal cord and brain (2–4). Saxegaard first reported EHV-1 (at that time, equine rhinopneumonitis virus) in association with a disease of the nervous system in horses (5). Since then, there have been additional reports of a neurologic syndrome of horses associated with respiratory diseases and abortion due to EHV-1 (2, 6). In Japan, the first neurological disease caused by EHV-1 occurred at Ritto Training Center in 1989 (7). The incidence of EHM has increased in Europe and the United States in recent years (8). Neurological deficits may persist after recovery from EHM (9). Following an outbreak at an international event in Valencia, Spain in 2021, many exposed horses were transported back to their respective countries, where they subsequently developed EHM (10, 11). Fever is a common clinical sign of EHM (12–16). The onset of neurological signs typically occurs 1-3 days after resolution of fever (2). Horses with EHM exhibit prolonged viremia and higher levels of virus in peripheral blood leukocytes compared with infected horses that do not develop neurological disease (17–19).

A single nucleotide polymorphism (SNP) involving an adenine-to-guanine substitution at nucleotide position 2254 (A2254G), resulting in an asparagine-to-aspartic acid substitution at amino acid position 752 (N752D) in the viral DNA polymerase UL30, has been implicated in EHM (20). This SNP has been widely used as a molecular marker to differentiate neuropathogenic and non-neuropathogenic EHV-1 strains (1, 2, 19, 21–23). Goodman et al. (24) and Van de Walle et al. (25) reported that the D752 SNP is a key determinant of EHV-1 neuropathogenicity. The neuropathogenic genotype G2254/D752 has been associated with several viral and clinical characteristics, including fever and higher levels of viremia (18, 19, 24, 25). However, this genotype has not been consistently associated with differences in clinical signs or disease outcome (21, 26, 27). Moreover, EHM has been reported in horses infected with EHV-1 strains encoding asparagine (N) at position 752 of UL30 (2, 20, 22). Ma et al. demonstrated that residue 752 in UL30 is not essential for virus growth *in vitro* (28). In addition, an alternative SNP at the same nucleotide position 2254 (C2254), resulting in an N752H substitution in UL30, has been identified in EHM-associated isolates from France (29) and the United States (30). Therefore, whether residue 752 contributes to the neuropathogenicity of EHV-1 remains speculative and continues to be a subject of debate (1, 2, 27, 28). Taken together, these findings suggest that additional viral and/or host factors contribute to the development of EHM, and that further studies are required to clarify the roles of the G2254/D752 and C2254/H752 genotypes in EHM outbreaks.

Here, we investigated the ability of EHM-derived EHV-1 to replicate at elevated temperatures. We hypothesized that EHV-1 replicates efficiently in the respiratory tract at physiological temperatures, but that viral replication is inhibited at higher temperatures (≥39°C) corresponding to febrile conditions in infected horses. To date, however, no studies have systematically examined EHV-1 replication under elevated-temperature conditions. We further hypothesized that certain EHV-1 strains harboring specific SNPs in UL30 retain the ability to replicate efficiently under febrile conditions, resulting in higher viral loads and enhanced dissemination, including infection of vascular endothelial cells, thereby contributing to the development of EHM. Accordingly, the aim of this study was to elucidate the relationship between EHV-1 replication at elevated temperatures and EHM-associated SNPs in ORF30, which encodes the viral DNA polymerase UL30.

## RESULTS

### Effect of elevated temperature on viral replication in fetal horse kidney cells

**Fig 1:**
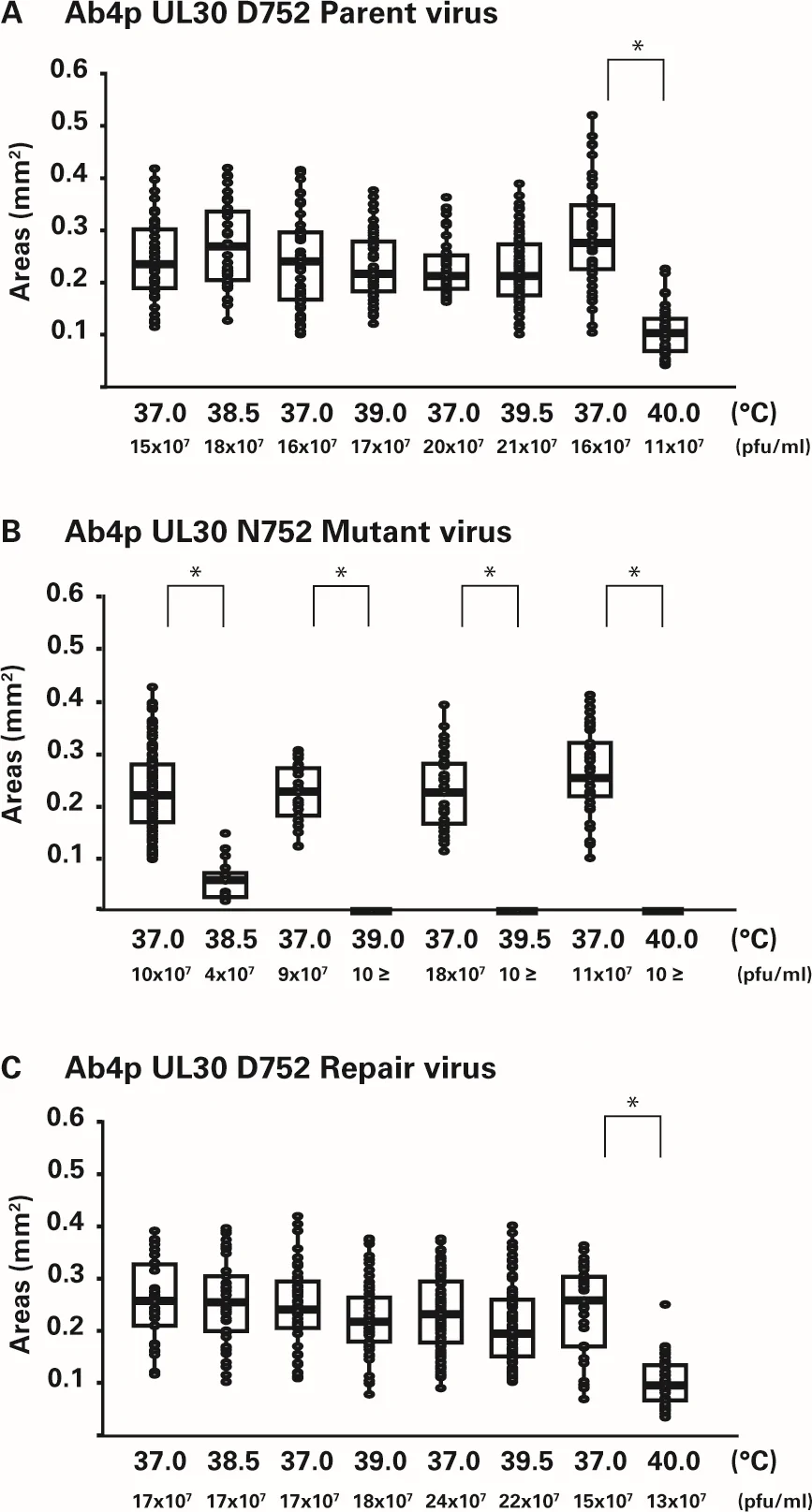
Plaque formation of Ab4p UL30 D752, UL30 N752, and repaired UL30 D752 viruses at elevated temperatures. Plaque assays were performed on FHKCs infected with Ab4p UL30 D752 parent (**A**), Ab4p UL30 N752 mutant (**B**), or Ab4p UL30 D752 repair viruses (**C**) and incubated at 37.0°C or at elevated temperatures (38.5°C, 39.0°C, 39.5°C, and 40.0°C), as indicated. The graphs show the areas of plaques formed by each virus at each temperature. Infectious titers (PFU/mL) at each temperature are shown beneath the corresponding temperature on the x-axis. Titers represent the mean of four wells per dilution. Boxplots were generated using the ggplot2 package in RStudio version 2024.12.1+563, with dots representing individual data points. Data normality was assessed using the Shapiro-Wilk test; because the data did not follow a normal distribution, nonparametric statistical analysis was performed. Statistical comparisons were conducted using the Wilcoxon rank-sum test for pairwise comparisons and the Kruskal–Wallis rank-sum test followed by Dunn’s post hoc test with Bonferroni correction for multiple group comparisons. Consistent results were obtained with both statistical approaches. *P*-values < 0.05 are indicated.

We compared the effects of temperatures ranging from 37.0°C to 40.5°C on the replication of three recombinant viruses: a parental virus (EHM-derived Ab4p UL30 D752), a mutant virus (Ab4p UL30 N752), and a repaired virus (Ab4p UL30 D752), in which the N752D substitution was previously restored (31). The recombinant viruses were generated in the genetic background of the EHV-1 Ab4p strain and were grown in fetal horse kidney cells (FHKCs). All three viruses formed plaques at 38.5°C but failed to form plaques at 40.5°C. The mutant virus carrying UL30 N752 did not form plaques at temperatures of 39.0°C or higher. In contrast, the parental and repaired viruses, both carrying UL30 D752, formed plaques at temperatures up to 40.0°C. Plaque sizes of the parental and repaired viruses at 38.5°C to 39.5°C were comparable to those observed at 37.0°C but were reduced at 40.0°C (Fig. 1). In contrast, plaques formed by the UL30 N752 mutant virus at 38.5°C were significantly smaller than those observed at 37.0°C. The numbers of plaques formed by the parental and repaired viruses at 38.5°C and higher temperatures were similar to those observed at 37.0°C. However, plaque formation by the mutant virus at 39.0°C and above was approximately six orders of magnitude less than it was at 37.0°C (*P* < 0.05) (Table 1).

**TABLE 1:** Plating efficiency of the parent, mutant, and repair viruses at elevated temperatures^*b*^

Viruses	Amino acid at position 752 of UL30	38.5°C	39.0°C	39.5°C	40.0°C
Ab4p UL30 D752 parent	D	1.03	1.00	1.08	1.02
Ab4p UL30 N752 mutant	N	0.63	≤10^−6*a*^	≤10^−6*a*^	≤10^−6*a*^
Ab4p UL30 D752 repair	D	1.00	1.21	1.00	1.07

^*a*^ *: Plaque formation at elevated temperatures was below the limit of detection (≤10^−6^ relative to 37.0°C).

^*b*^ Values represent the ratio of plaque numbers obtained at elevated temperatures to those obtained at 37.0°C.

**Fig 2:**
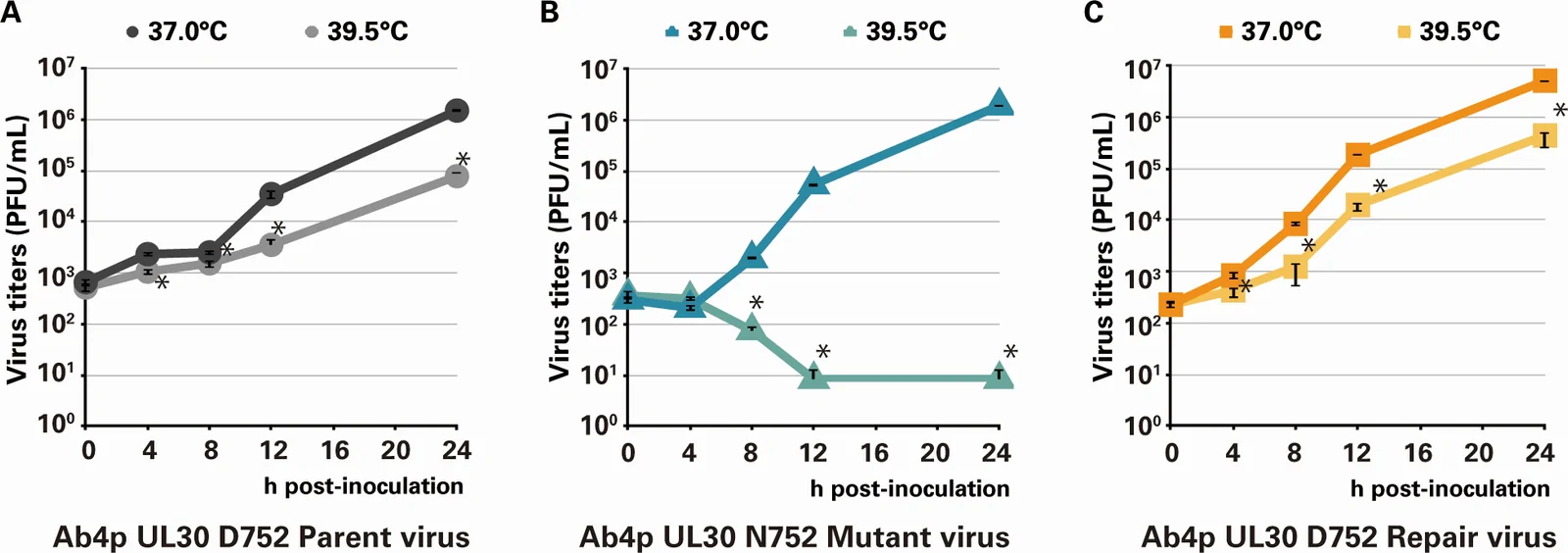
Growth kinetics of Ab4p viruses at 37.0°C and 39.5°C. FHKCs were inoculated with the indicated viruses and incubated at the temperatures shown. Culture supernatants were collected at 0, 4, 8, 12, and 24 hpi, and virus titers were determined by plaque assay. (**A**) Ab4p UL30 D752 parent virus, (**B**) Ab4p UL30 N752 mutant virus, and (**C**) Ab4p UL30 D752 repair virus. Both the parental and repaired UL30 D752 viruses replicated at 39.5°C, whereas the UL30 N752 mutant virus failed to replicate at this elevated temperature. Viral titers represent the mean values obtained from four wells per dilution. Vertical bars indicate the standard error (SE) (*n* = 4). Statistical comparisons between 37.0°C and 39.5°C at each time point were performed. *P < 0.05.

**Fig 3:**
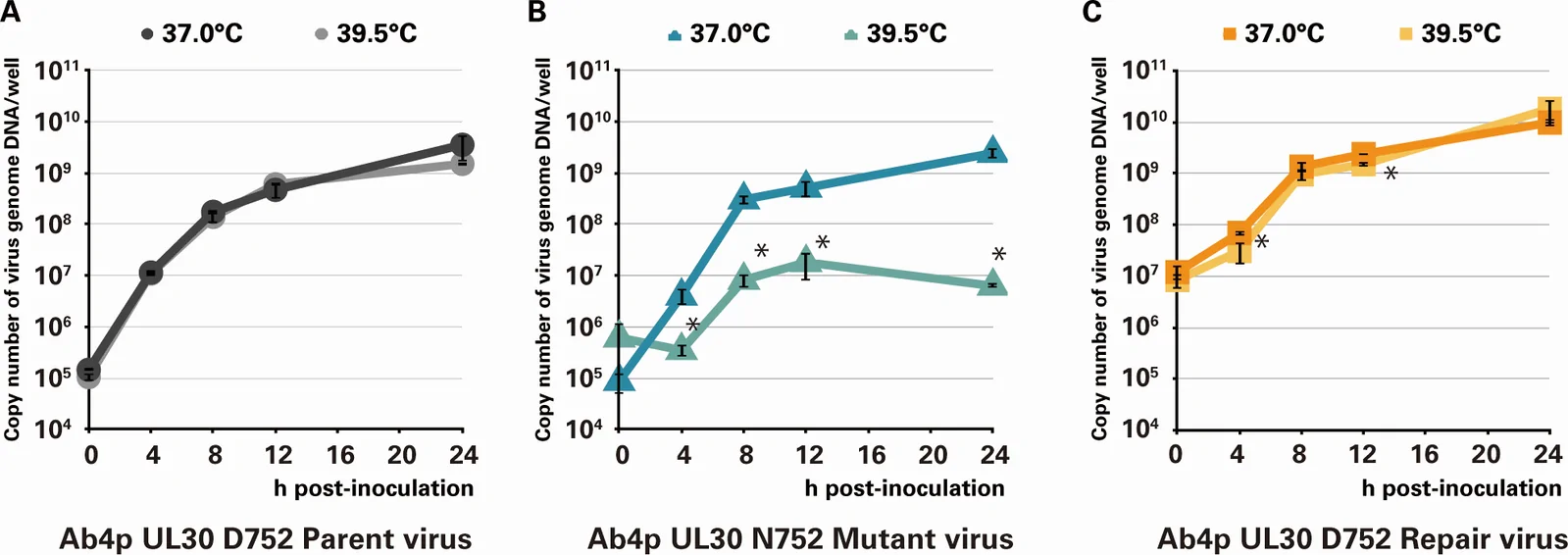
Viral genomic DNA replication kinetics of Ab4p viruses at 37.0℃ and 39.5℃. FHKCs were inoculated with the indicated viruses and incubated at the indicated temperatures. Infected cells were collected at 0, 4, 8, 12, and 24 hpi, and total DNA was extracted. Viral genomic DNA copy numbers were quantified by real-time PCR targeting ORF30. (**A**) Ab4p UL30 D752 parent virus, (**B**) Ab4p UL30 N752 mutant virus, and (**C**) Ab4p UL30 D752 repair virus. Viral DNA synthesis by the parental and repaired viruses was comparable at both 37.0°C and 39.5°C. In contrast, viral DNA synthesis by the UL30 N752 mutant virus was markedly reduced at 39.5°C compared with 37.0°C. Viral genomic DNA copy numbers represent the mean values obtained from four wells. Vertical bars indicate the standard error (SE) (*n* = 4). Statistical comparisons between 37.0°C and 39.5°C at each time point were performed. *P < 0.05.

Viral replication kinetics were further evaluated by measuring both infectious virus production using plaque formation assays and viral genomic DNA copy numbers using quantitative real-time PCR. At 39.5°C, the parental and repaired viruses retained the ability to replicate, although their viral titers at 24 h post-inoculation (hpi) were approximately one-tenth of those observed at 37.0°C (*P* < 0.05) (Fig. 2A and C). In contrast, the mutant virus carrying the UL30 N752 substitution failed to replicate at 39.5°C (*P* < 0.05) (Fig. 2B). Quantitative real-time PCR targeting the ORF30 gene revealed that genome copy numbers of the parental UL30 D752 virus at 39.5°C were comparable to those at 37.0°C, with no significant differences observed (Fig. 3A). Genome copy numbers of the repaired UL30 D752 virus were also largely comparable to those at 37°C, except at 4 and 12 hpi (Fig. 3C). By contrast, the mutant virus exhibited at least a 100-fold reduction in genome copy number at 39.5°C compared with 37.0°C (*P* < 0.05), and no significant increase was observed between 8 and 24 hpi (Fig. 3B). Taken together, these results demonstrate that the UL30 N752 substitution markedly impairs viral replication at elevated temperatures *in vitro*.

### Replication capacity of EHV-1 field isolates at 39.5°C in FHKCs

The replication capacity of 49 EHV-1 field isolates, including 4 EHM-derived isolates and 45 non-EHM-derived isolates, was evaluated at 39.5°C in FHKCs (Table 2). Virus replication was assessed qualitatively based on the presence or absence of cytopathic effects (CPEs) observed after 72 h of incubation at 39.5°C. Four EHM-derived isolates (89c105, 01c1, 7759, and 7855) and three non-EHM-derived isolates (8343, 9204, and 13757) replicated at 39.5°C, whereas the remaining 42 non-EHM-derived isolates did not replicate under these conditions. Among the isolates that replicated at 39.5°C, one isolate (01c1) encoded an aspartic acid at position 752 (D752) in UL30, whereas the remaining six isolates (89c105, 7855, 7759, 8343, 9204, and 13757) encoded an asparagine at this position (N752). All six N752 isolates that replicated at elevated temperatures harbored additional UL30 SNPs that resulted in amino acid substitutions at other positions: A742 in isolates 89c105 and 7855; R250 in 7759; G760 in 8343; I291 in 9204; and S151, H625, K683, and T1107 in 13757 (Table 3). Therefore, specific amino-acid residues at multiple UL30 positions, rather than residue 752 alone, affect the ability of the virus to replicate at elevated temperature. Among the 42 isolates that failed to replicate at 39.5°C, two N752 isolates carried additional UL30 SNPs (K683 and N769 in 06-I-70; V946 in 08-I-812). The remaining 40 isolates consisted of 39 N752 isolates and 1 D752 isolate (06-I-80), none of which exhibited detectable replication at 39.5°C or harbored additional SNPs in UL30.

**TABLE 2:** EHV-1 field isolates in Japan*^a^*^,^*^b^*

Strains	Replication at 37.0°C	Replication at 39.5°C	Clinical signs	Year	Prefecture	Tissues or organs	Accession number	Remarks
89C105	+	+	EHM	1989	Shiga	Nasal swab	KF644577	EHM outbreak isolate (1989)
90C16	+	–	Respiratory	1990	Shiga	Nasal swab	KF644566	
3292	+	–	Abortion	1993	Hokkaido	Aborted fetus	LC834900 ^*c*^	
4370	+	–	Abortion	1995	Hokkaido	Aborted fetus	LC834901 ^*c*^	
5030	+	–	Abortion	2000	Hokkaido	Aborted fetus	LC834902 ^*c*^	
01-I-241	+	–	Abortion	2001	Hokkaido	Lungs	LC109626	
01C1	+	+	EHM	2001	Hokkaido	PBMC	KF644578	EHM outbreak isolate (2001)
02-I-136	+	–	Abortion	2002	Hokkaido	Lungs	LC109627	
02-I-152	+	–	Abortion	2002	Hokkaido	Lungs	LC109628	
03-I-104	+	–	Abortion	2003	Hokkaido	Thymus	LC109629	
03-I-107	+	–	Abortion	2003	Hokkaido	Lungs	LC109630	
03-I-124	+	–	Abortion	2003	Hokkaido	Thymus	LC109631	
04-I-68	+	–	Abortion	2004	Hokkaido	Lungs	LC109632	
04-I-70	+	–	Abortion	2004	Hokkaido	Lungs	LC109633	
04-I-77	+	–	Abortion	2004	Hokkaido	Thymus	LC109634	
04-I-89	+	–	Abortion	2004	Hokkaido	Thymus	LC109635	
05-I-239	+	–	Abortion	2005	Hokkaido	Thymus	LC109636	
05-I-255	+	–	Abortion	2005	Hokkaido	Thymus	LC109637	
6383	+	–	Abortion	2005	Hokkaido	Aborted fetus	LC834903 ^*c*^	
06-I-24	+	–	Abortion	2006	Hokkaido	Thymus	LC109640	
06-I-70	+	–	Abortion	2006	Hokkaido	Lungs	LC109641	
06-I-76	+	–	Abortion	2006	Hokkaido	Lungs	LC109642	
06-I-80	+	–	Abortion	2006	Hokkaido	Lungs	LC109643	
07-I-100	+	–	Abortion	2007	Hokkaido	Lungs	LC109644	
07-I-114	+	–	Abortion	2007	Hokkaido	Lungs	LC109645	
6786	+	–	Abortion	2007	Hokkaido	Aborted fetus	LC834904 ^*c*^	
08-I-793	+	–	Abortion	2008	Hokkaido	Thymus	LC109646	
08-I-797	+	–	Abortion	2008	Hokkaido	Thymus	LC109647	
08-I-812	+	–	Abortion	2008	Hokkaido	Lungs	LC109648	
09-I-474	+	–	Abortion	2009	Hokkaido	Lungs	LC109649	
09-I-478	+	–	Abortion	2009	Hokkaido	Lungs	LC109650	
09-I-743	+	–	Respiratory	2009	Ibaraki	Nasal swab	LC109651	
10-I-224	+	–	Respiratory	2010	Tokyo	Nasal swab	LC109652	
10-I-713	+	–	Abortion	2010	Hokkaido	Thymus	LC109653	
7759	+	+	EHM	2010	Hokkaido	Spinal cord	LC834905 ^*c*^	EHM outbreak isolate (2010)
7855	+	+	EHM	2010	Hokkaido	PBMC	LC834906 ^*c*^	EHM outbreak isolate (2010)
11-I-475	+	–	Abortion	2011	Hokkaido	Lungs	LC109654	
11-I-487	+	–	Abortion	2011	Hokkaido	Lungs	LC109655	
12-I-306	+	–	Abortion	2012	Hokkaido	Lungs	LC109656	
12-I-324	+	–	Abortion	2012	Hokkaido	Lungs	LC109657	
12-I-330	+	–	Abortion	2012	Hokkaido	Lungs	LC109658	
12-I-336	+	–	Abortion	2012	Hokkaido	Lungs	LC109659	
12-I-344	+	–	Abortion	2012	Hokkaido	Lungs	LC109660	
8343	+	+	Abortion	2012	Hokkaido	Aborted fetus	LC834908 ^*c*^	Same ORF30 SNP as strain VA02
8394	+	–	Abortion	2012	Hokkaido	Aborted fetus	LC834909 ^*c*^	
9204	+	+	Abortion	2014	Hokkaido	Aborted fetus	LC834910 ^*c*^	Same ORF30 SNP as Valencia isolates
12222	+	–	Abortion	2018	Hokkaido	Aborted fetus	LC834911 ^*c*^	
12693	+	–	Abortion	2018	Hokkaido	Aborted fetus	LC834912 ^*c*^	
13757	+	+	Abortion	2021	Hokkaido	Aborted fetus	LC834913 ^*c*^	

^*a*^ +, detectable replication.

^*b*^ –, no detectable replication at the indicated temperature.

^*c*^ *ORF30 sequence available; all other isolates have complete genome sequences.

**TABLE 3:** Replication at 39.5°C and UL30 amino acid substitutions in Ab4p, Japanese field isolates, and chimeric BAC viruses in FHKCs^*e*^^,^*^f^*

Virus	Clinical signs	Replication at 39.5°C	Amino acids of UL30										
			151	250	291	625	683	742	752	760	769	946	1107
Ab4p^*a*^	EHM	+	R	H	S	R	E	V	D	D	H	I	A
01c1	EHM	+	•	•	•	•	•	•	•	•	•	•	•
H752^*b*^	EHM	+	•	•	•	•	•	•	H	•	•	•	•
13757	Abortion	+	S	•	•	H	K	•	N	•	•	•	T
7759^*a*^	EHM	+	•	R	•	•	•	•	N	•	•	•	•
9204^*a*^	Abortion	+	•	•	I	•	•	•	N	•	•	•	•
Valencia^*b*^	EHM	+	•	•	I	•	•	•	N	•	•	•	•
06-I-70^*c*^	Respiratory	+	•	•	•	•	K	•	N	•	N	•	•
89c105^*a*^	EHM	+	•	•	•	•	•	A	N	•	•	•	•
7855^*a*^	EHM	+	•	•	•	•	•	A	N	•	•	•	•
8343	Abortion	+	•	•	•	•	•	•	N	G	•	•	•
06-I-80	Abortion	−	•	•	•	•	•	•	•	•	•	•	•
06-I-70^*d*^	Respiratory	−	•	•	•	•	K	•	N	•	N	•	•
08-I-812^*a*^	Respiratory	−	•	•	•	•	•	•	N	•	•	V	•

^*a*^ *, Replication at 39.5°C was evaluated using both original virus stocks and corresponding chimeric BAC viruses.

^*b*^ **, Replication at 39.5°C was evaluated using the chimeric viruses only.

^*c*^ #1, Replication at 39.5°C was evaluated using the chimeric virus.

^*d*^ #2, Replication at 39.5°C was evaluated using the original virus stock.

^*e*^ • indicates the same amino acid as that of Ab4p.

^*f*^ Additional isolates not listed in this table (e.g., 90c16 and others) carried UL30 N752 without additional substitutions and did not replicate at 39.5°C.

### UL30 SNPs other than D752 and replication capacity at elevated temperatures using chimeric BAC viruses in FHKCs

N752 field isolates that replicated at elevated temperatures have additional amino acid substitutions in UL30. To examine their contribution to growth at elevated temperatures, we constructed chimeric BAC-derived viruses. These BAC viruses encode a modified enhanced green fluorescent protein (mEGFP) (Fig. S1), which served as a marker for virus-infected cells.

**Fig 4:**
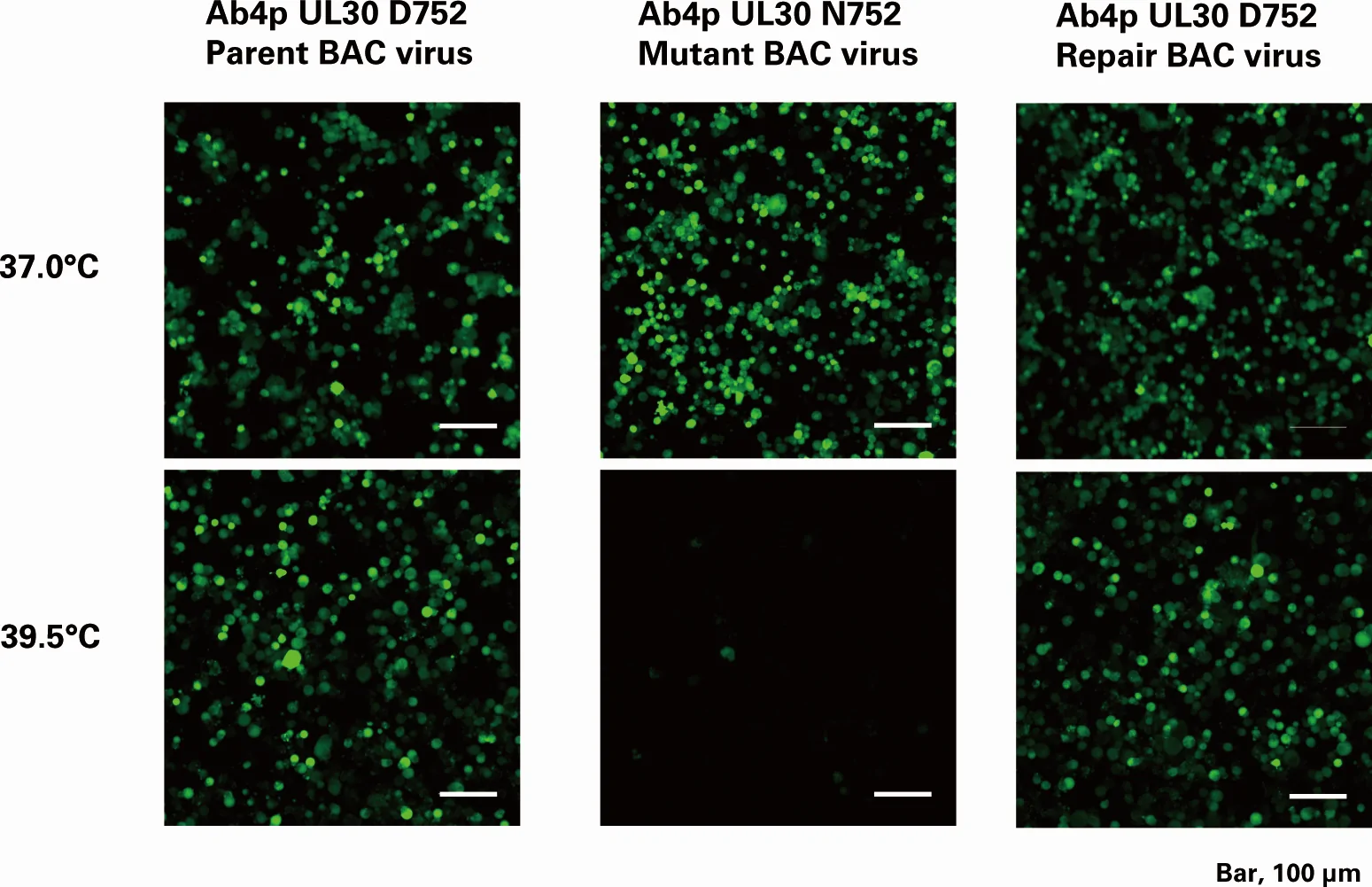
Growth of Ab4p BAC parent, mutant, and repaired viruses at 37.0°C and 39.5°C. FHKCs were inoculated with BAC-derived viruses (MOI = 0.1) and incubated at the temperatures indicated. Live-cell fluorescence images were acquired at 48 hpi. The BAC vector inserted into the EHV-1 genome contains the mEGFP gene, which is expressed in infected cells (Fig. S1). At 37.0°C, cells inoculated with each of the three viruses exhibited widespread green fluorescence throughout the monolayer, accompanied by CPE, including cell rounding, syncytium formation, and detachment of cells from the substrate. At 39.5°C, widespread green fluorescence was observed in cells inoculated with the parental and repaired Ab4p UL30 D752 BAC viruses, whereas only scattered single cells showing green fluorescence were detected in monolayers inoculated with the mutant Ab4p UL30 N752 BAC virus. This experiment was designed as a qualitative assessment of spread (focus formation versus single-cell confinement); therefore, no statistical analysis was applied.

Before analyzing these substitutions, we first assessed whether insertion of the BAC vector sequence affects replication at elevated temperatures using Ab4p-based BAC-derived viruses (parental Ab4p UL30 D752 BAC, mutant Ab4p UL30 N752 BAC, and repaired Ab4p UL30 D752 BAC) in FHKCs. At 37.0°C, cells inoculated with all three viruses exhibited widespread green fluorescence throughout the monolayer, accompanied by CPE, including cell rounding, syncytium formation, and detachment of cells from the substrate, indicating efficient viral replication (Fig. 4). In contrast, at 39.5°C, the UL30 D752 BAC viruses retained the ability to replicate and spread across the monolayer, as evidenced by mEGFP-positive focus formation, whereas the UL30 N752 BAC virus was largely confined to scattered single mEGFP-positive cells, indicating that infection was initiated but subsequent amplification and cell-to-cell spread were strongly restricted at elevated temperatures. These findings were consistent with those obtained using the corresponding viruses lacking the BAC vector sequence.

**Fig 5:**
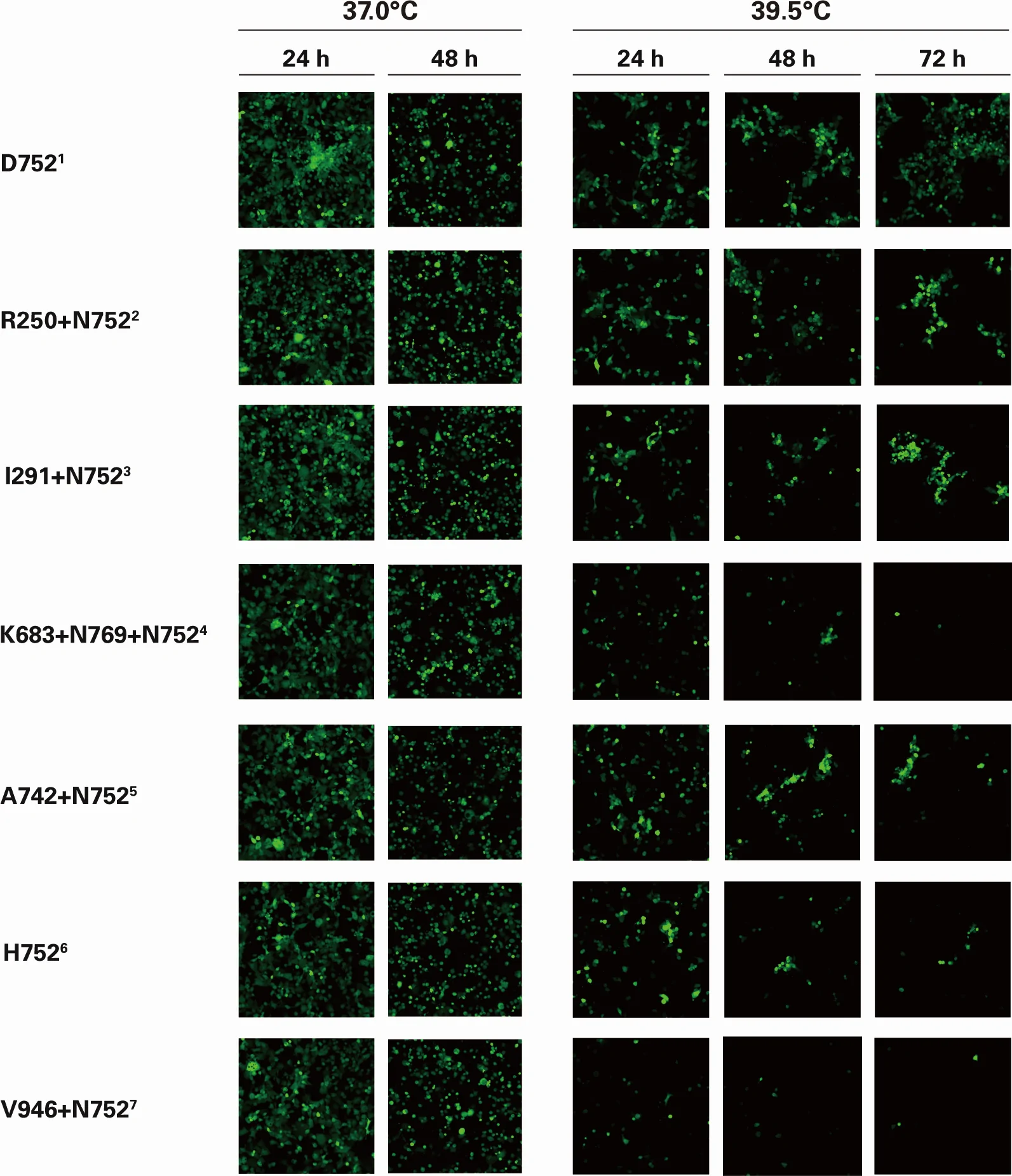
Effects of UL30 SNPs on proliferation of chimeric BAC viruses at elevated temperatures. The D752 parent BAC and the six chimeric BAC viruses are as follows: 1, Ab4p D752 parent; 2, 7759; 3, Valencia; 4, 06-I-70; 5, 89c105; 6, H752; 7, 08-I-812. FHKCs were transfected with chimeric BAC DNAs and incubated for 72 h at 37.0°C. Culture supernatants were collected from each virus culture and stored at −80°C. Fresh FHKCs were subsequently inoculated with the recovered viruses and incubated for 72 h at either 37.0°C or 39.5°C. Live-cell fluorescence images were acquired by laser confocal microscopy at 24, 48, and 72 hpi. Widespread green fluorescence with mEGFP-positive focus formation, indicative of productive viral replication and cell-to-cell spread, was observed in cells inoculated with chimeric BAC viruses capable of replicating under the indicated conditions. In contrast, only scattered single green fluorescent cells were detected in monolayers inoculated with chimeric BAC viruses that failed to replicate at 39.5°C. The N752-only Ab4p BAC virus is already included in Fig. 4 and serves as the negative control for febrile-temperature spread in the same genetic background. The same qualitative pattern (focus formation versus single-cell confinement) was reproduced in at least three independent experiments. This experiment was designed as a qualitative assessment of spread (focus formation versus single-cell confinement); therefore, no statistical analysis was applied.

We next compared the effects of D752 with seven additional UL30 SNPs (R250, I291, K683, and N769, A742, H752, and V946) on viral replication at 37.0°C and 39.5°C (Fig. 5; Table 3). For this purpose, each SNP was individually introduced into chimeric BAC viruses constructed on the Ab4p genetic background by *in situ* mutagenesis, incorporating the ORF30 sequence derived from the corresponding field isolate. At 37.0°C, all chimeric BAC viruses exhibited widespread mEGFP fluorescence accompanied by CPE, indicating efficient replication (Fig. 5). In contrast, at 39.5°C, mEGFP-positive focus formation was observed in cells infected with four chimeric BAC viruses harboring the UL30 substitutions R250 (Ab4p UL30 7759 BAC), I291 (Ab4p UL30 Valencia BAC), A742 (Ab4p UL30 89c105 BAC), and H752 (Ab4p UL30 H752 BAC). Among these viruses, the Ab4p UL30 H752 BAC virus replicated more weakly than the other three (Fig. 5). Cells inoculated with chimeric Ab4p UL30 06-I-70 BAC (K683, N752, and N769) showed only small clusters of mEGFP-positive cells at 48 hpi, which diminished to scattered single cells by 72 hpi. By comparison, only scattered single mEGFP-positive cells were detected in monolayers infected with the remaining chimeric BAC virus, Ab4p UL30 08-I-812 BAC, which harbors the V946 substitution, at 48 hpi onward (Fig. 5; Table 3). Therefore, individual amino-acid substitutions at multiple UL30 positions, rather than residue 752 alone, can independently affect the ability of the virus to replicate at elevated temperatures.

### UL30 SNPs and EHV-1 replication capacity at elevated temperatures in equine PBMCs

**Fig 6:**
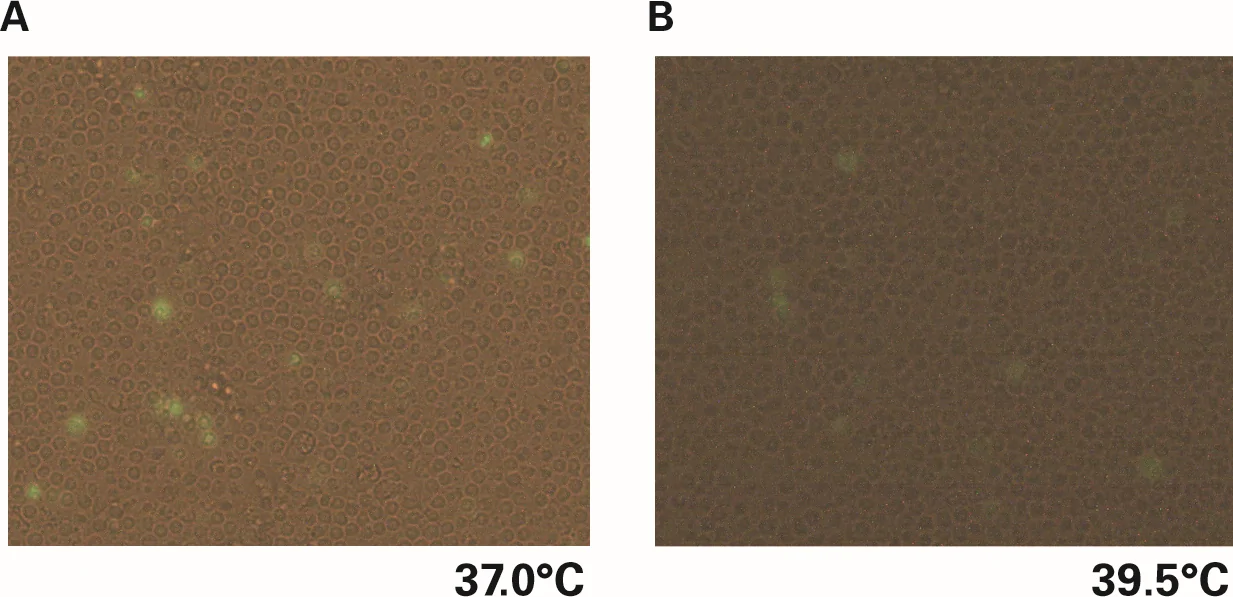
Replication of Ab4p UL30 7759 BAC (R250) virus in equine PBMCs at 37℃ and 39.5℃. Equine PBMCs were inoculated with Ab4p UL30 7759 BAC virus harboring the R250 substitution and incubated at 37.0°C (**A**) or 39.5°C (**B**). Expression of mEGFP indicates that the Ab4p UL30 7759 BAC (R250) virus infected PBMCs at both temperatures. Fluorescence images were acquired at 48 hpi.

We next examined whether EHV-1 replication capacity at elevated temperatures is also observed in PBMCs. Both field isolates and BAC-derived viruses (Table S1) were used in these experiments. PBMCs were inoculated with EHV-1 at a multiplicity of infection (MOI) of 0.1–0.3 and incubated at either 37.0°C or 39.5°C (Table 4). Expression of the EHV-1 glycoprotein B (gB) was detected in PBMCs inoculated with all of the viruses shown in Table 4 by indirect immunofluorescence assay at 48 hpi at both temperatures. For BAC-derived viruses, expression of mEGFP was also detected in infected PBMCs, confirming successful viral infection (Fig. 6).

**TABLE 4:** Expression of mEGFP in PBMCs infected with EHV-1 field isolates and chimeric BAC viruses at 37.0°C and 39.5°C*^a^*^,^*^b^*^,^*^c^*

Viruses	SNP	mEGFP expression	
		37.0°C	39.5°C
7759	R250, N752	N.A	N.A
9204	I291, N752	N.A	N.A
7855	A742, N752	N.A	N.A
6383	N752	N.A	N.A
3292	N752	N.A	N.A
8343	N752, G760	N.A	N.A
Ab4p UL30 D752 parent^*d*^	D752	2.4^*e*^	1.1
Ab4p UL30 N752 mutant^*d*^	N752	1.5	1.1
Ab4p UL30 D752 repair^*d*^	D752	2.2	1.6
Ab4p UL30 R250 + N752 (7759)^*d*^	R250, N752	1.5	0.9
Ab4p UL30 I291 + N752 (Valencia)^*d*^	I291, N752	2.4	0.8
Ab4p UL30 A752 + N752 (89c105)^*d*^	A742, N752	3.8	0.5
Ab4p UL30 H752^*d*^	H752	2.6	0.8
Ab4p UL30 K683 + N752+N769 (06-I-70)^*d*^	K683, N752, N769	3.9	1.9
Ab4p UL30 N752 + V946 (08-I-812)^*d*^	N752, V946	1.4	1.0

^*a*^ mEGFP expression indicates successful infection with chimeric BAC-derived viruses encoding the mEGFP reporter gene.

^*b*^ +, positive detection of antigen expression or mEGFP fluorescence.

^*c*^ N.A., not applicable.

^*d*^ #, Chimeric BAC-derived viruses.

^*e*^ *, Values indicate the percentage of mEGFP-positive cells at 48 hpi.

At both 37.0°C and 39.5°C, only low titers of infectious virus (0.56–13.49 50% tissue culture infectious dose [TCID_50_] per 25 µL) were detected in PBMC supernatants within 48 hpi (Table 5). Infectious titers did not increase over time and instead declined, consistent with residual input virus rather than *de novo* production of infectious progeny. Under febrile conditions (39.5°C), residual infectivity in the supernatant was rapidly lost, with only isolate 7759 retaining detectable infectivity at 24 hpi. Collectively, these results indicate that equine PBMCs support viral antigen expression (Table 4) but do not support productive replication accompanied by release of infectious virus under the conditions tested.

**TABLE 5:** Viral titers in the supernatants of PBMCs infected with EHV-1 field isolates and chimeric BAC viruses at 37.0°C and 39.5°C^*c*^

Viruses	SNP	Inoculated viral titer^*a*^ (×10^4^ TCID_50_/25 µL) and MOI	Incubation temperature (°C)	Viral titer in supernatant (TCID_50_ per 25 µL)^*b*^		
				0 h	24 h	48 h
Ab4p UL30 D752 parent^*d*^	D752	22.39 (MOI 0.1)	37.0	5.62	4.27	1.35
			39.5	5.62	–	–
Ab4p UL30 N752 mutant^*d*^	N752	70.79 (MOI 0.3)	37.0	5.62	0.74	–
			39.5	4.27	–	–
Ab4p UL30 D752 repair^*d*^	D752	39.81 (MOI 0.2)	37.0	1.35	1.78	0.56
			39.5	7.59	–	–
7759	R250, N752	9.55 (MOI 0.1)	37.0	0.74	3.16	3.16
			39.5	4.27	0.56	–
Ab4p UL30 R250 + N752 (7759)^*d*^	R250, N752	53.70 (MOI 0.3)	37.0	4.27	3.16	1
			39.5	10	–	–
9204	I291, N752	7.08 (MOI 0.1)	37.0	3.16	0.56	–
			39.5	1.35	–	–
Ab4p UL30 I291 + N752 (Valencia)^#^	I291, N752	70.79 (MOI 0.3)	37.0	3.16	1.78	0.74
			39.5	5.62	–	–
Ab4p UL30 A742 + N752 (89c105)^*d*^	A742, N752	16.98 (MOI 0.1)	37.0	7.59	1.78	1.78
			39.5	13.49	–	–
7855	A742, N752	22.39 (MOI 0.1)	37.0	5.62	4.27	1.35
			39.5	7.59	–	–
Ab4p UL30 H752^*d*^	H752	70.79 (MOI 0.3)	37.0	4.27	1.78	1
			39.5	4.27	–	–
6383	N752	22.39 (MOI 0.1)	37.0	1.35	1.78	2.4
			39.5	3.16	–	–
3292	N752	9.55 (MOI 0.1)	37.0	0.74	0.56	–
			39.5	3.16	–	–
8343	N752, G760	22.39 (MOI 0.1)	37.0	0.74	2.4	3.16
			39.5	1	–	–
Ab4p UL30 K683 + N752+N769 (06-I-70)^*d*^	K683, N752, N769	39.81 (MOI 0.2)	37.0	5.62	5.62	1
			39.5	5.62	–	–
Ab4p UL30 N752 + V946 (08-I-812)^*d*^	N752, V946	39.81 (MOI 0.2)	37.0	3.16	2.4	0.74
			39.5	5.62	–	–

^*a*^ *, inoculated viral titers represent the mean of duplicate assays and expressed as ×10^4^ TCID_50 _per 25 µL.

^*b*^ **, viral titers in supernatants represent the mean of duplicate assays. Values at 0 h reflect residual virus remaining immediately after inoculation.

^*c*^ “–” indicates values below the experimentally defined limit of detection (<0.56 TCID₅₀ per 25 µL).

^*d*^ #, chimeric BAC-derived viruses.

### PBMC-mediated cell-to-cell transfer of EHV-1 to FHKCs at elevated temperatures

The ability of infected PBMCs to initiate infection in susceptible FHKCs upon co-culture (cell-to-cell transfer) was evaluated at 39.5°C using the same set of viruses described in the previous section. At 37.0°C, cell-to-cell transfer was observed in PBMCs infected with all viruses examined between 24 and 48 hpi (Table 6). The number of PBMCs capable of transferring virus to FHKCs at 37.0°C ranged from 31.62 to 316.23 cells per 8 × 10^4^ PBMCs. At 39.5°C, cell-to-cell transfer was detected in PBMCs infected with EHM-associated field isolates (7759 and 7855) and one non-EHM-associated isolate (8343), as well as in PBMCs infected with several chimeric BAC-derived viruses (Table S1) at 24 hpi. However, the efficiency of cell-to-cell transfer at 39.5°C was markedly reduced compared with that observed at 37.0°C. Under febrile conditions, the number of PBMCs capable of transferring virus to FHKCs ranged from 0.56 to 13.49 cells per 8 × 10^4^ PBMCs at 24 hpi. Two notable exceptions were observed. PBMCs infected with isolate 9204, which replicated efficiently in FHKCs at 39.5°C, did not mediate detectable cell-to-cell transfer. In contrast, PBMCs infected with the chimeric Ab4p UL30 06-I-70 BAC virus, of which the original isolate failed to replicate in FHKCs at 39.5°C, retained the ability to transfer virus to FHKCs. These findings indicate that replication capacity in FHKCs at elevated temperatures does not strictly correlate with the ability of infected PBMCs to mediate cell-to-cell transfer.

**TABLE 6:** Number of PBMCs capable of transferring EHV-1 to FHKCs at 37.0°C and 39.5°C^*b*^

Viruses	SNP	Inoculated viral titer^*a*^ and MOI	Incubation temperature (°C)	Cells capable of transferring virus (cells per 8 × 10^4^ PBMCs)	
				24 h	48 h
Ab4p UL30 D752 parent ^*c*^	D752	22.39 (MOI 0.1)	37.0	100	23.99
			39.5	10	–
Ab4p UL30 N752 mutant ^*c*^	N752	70.79 (MOI 0.3)	37.0	100	31.62
			39.5	–	–
Ab4p UL30 D752 repair^*c*^	D752	39.81 (MOI 0.2)	37.0	316.23	31.62
			39.5	13.49	–
7759	R250, N752	9.55 (MOI 0.1)	37.0	177.83	17.78
			39.5	4.27	–
Ab4p UL30 R250 + N752 (7759)^*c*^	R250, N752	53.70 (MOI 0.3)	37.0	75.86	31.62
			39.5	2.4	–
9204	I219, N752	7.08 (MOI 0.1)	37.0	31.62	1.78
			39.5	–	–
Ab4p UL30 I291 + N752 (Valencia)^*c*^	I291, N752	70.79 (MOI 0.3)	37.0	239.88	17.78
			39.5	1	–
Ab4p UL30 A742 + N752 (89c105)^*c*^	A742, N752	16.98 (MOI 0.1)	37.0	134.9	17.78
			39.5	1	–
7855	A742, N752	22.39 (MOI 0.1)	37.0	177.83	31.62
			39.5	7.59	–
Ab4p UL30 H752^*c*^	H752	70.79 (MOI 0.3)	37.0	100	23.99
			39.5	1.33	–
6383	N752	22.39 (MOI 0.1)	37.0	56.23	13.49
			39.5	–	–
3292	N752	9.55 (MOI 0.1)	37.0	31.62	3.16
			39.5	–	–
8343	N752, G760	22.39 (MOI 0.1)	37.0	31.62	10
			39.5	0.56	–
Ab4p UL30 K683 + N752+N769 (06-I-70)^*c*^	K683, N752, N769	39.81 (MOI 0.2)	37.0	134.9	17.78
			39.5	1	–
Ab4p UL30 N752 + V946 (08-I-812)^*c*^	N752, V946	39.81 (MOI 0.2)	37.0	56.23	42.66
			39.5	–	–

^*a*^ *, Inoculated viral titers represent the mean of duplicate assays and are expressed as ×10^4^ TCID_50 _per 25 µL.

^*b*^ “–” indicates values below the experimentally defined limit of detection (<0.56 TCID₅₀ per 8 × 10^4^ PBMCs).

^*c*^ # Chimeric BAC-derived viruses.

### UL30 SNPs and EHV-1 replication capacity at elevated temperatures in equine vascular endothelial cells

**Fig 7:**
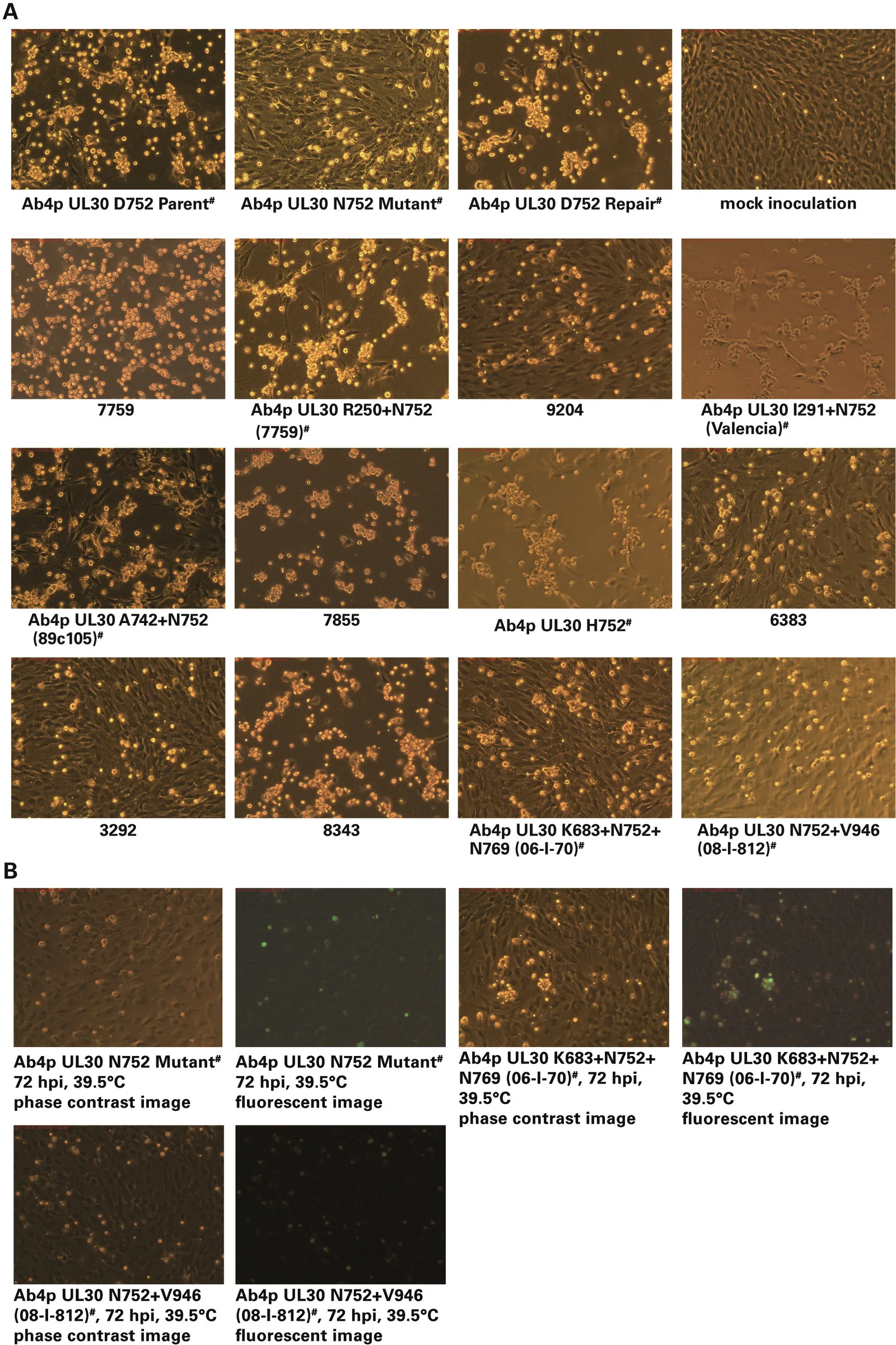
Cytopathic effects and infection patterns of EHV-1 in equine vascular endothelial cells (EVECs) at 39.5°C. EVEC monolayers were inoculated with the indicated EHV-1 field isolates and/or BAC-derived viruses and incubated at 39.5°C. (**A**) Phase-contrast images acquired at 48 hpi. (**B**) Representative paired phase-contrast and fluorescence images acquired at 72 hpi for the indicated BAC-derived viruses that did not show detectable CPE clusters at 48 hpi; green fluorescence indicates mEGFP expression from the BAC cassette. Because field isolates do not encode mEGFP, they were not included in panel B. At 37.0°C, infection with all viruses resulted in rapid progression of CPE and near-complete monolayer detachment by 48 hpi; therefore, 37.0°C images are not shown. Mock-inoculated EVECs maintained comparable morphology and confluence at 39.5°C and 37.0°C on microscopic examination.

**Fig 8:**
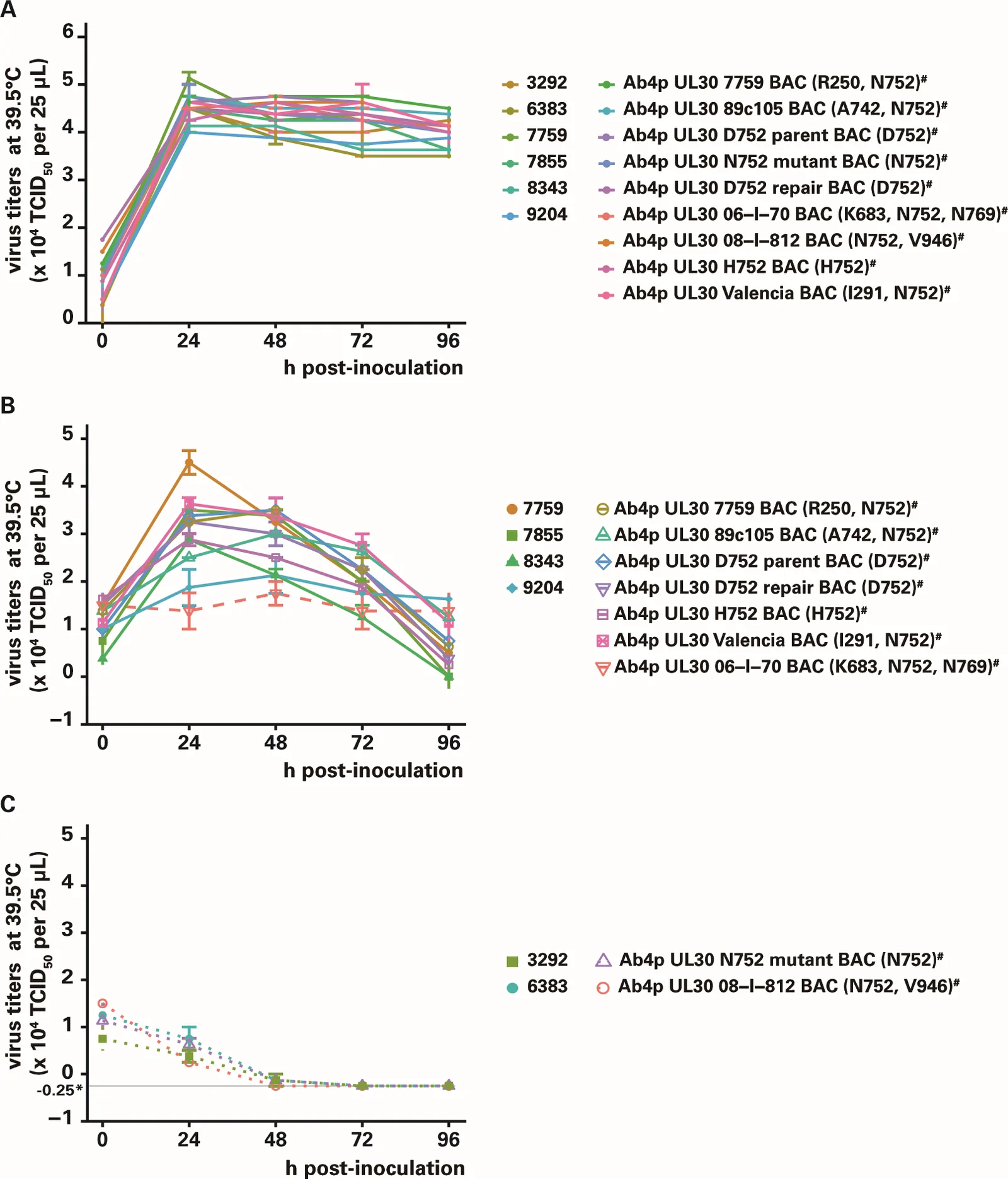
Replication of EHV-1 field isolates and chimeric BAC viruses in EVECs at 39.5°C. EVECs were inoculated with EHV-1 field isolates (3292, 6383, 7759, 7855, 8343, and 9204) and chimeric BAC viruses (Ab4p UL30 7759 BAC, Ab4p Ab4p UL30 D752 BAC parent, Ab4p UL30 N752 BAC mutant, Ab4p UL30 D752 BAC repair, Ab4p UL30 89c105 BAC, Ab4p UL30 H752 BAC, Ab4p UL30 Valencia BAC, Ab4p UL30 06-I-70 BAC, and Ab4p UL30 08-I-812 BAC; indicated by #) and incubated at either 37.0°C or 39.5°C. The incubation temperature for each panel is indicated on the *y*-axis. At 37.0°C, all viruses examined replicated efficiently in EVECs, reaching peak viral titers at 24 hpi and maintaining comparable titers between 48 and 96 hpi (**A**). At 39.5°C, EHV-1 field isolates (7759, 7855, 8343, and 9204) and chimeric BAC viruses (Ab4p UL30 7759 BAC, Ab4p UL30 D752 BAC parent, Ab4p UL30 D752 BAC repair, Ab4p UL30 89c105 BAC, Ab4p UL30 H752 BAC, and Ab4p UL30 Valencia BAC), all of which were capable of replicating in FHKCs at elevated temperatures, replicated in EVECs with peak viral titers observed between 24 and 48 hpi (**B**, solid line). The chimeric 06-I-70 BAC virus, which failed to replicate in FHKCs at elevated temperatures, maintained low but detectable viral titers throughout the experiment at 39.5°C (**B**, dashed line). In contrast, field isolates (3292 and 6383) and chimeric BAC-derived viruses (Ab4p UL30 N752 BAC mutant and Ab4p UL30 08-I-812 BAC), which did not replicate in FHKCs at elevated temperatures, showed no detectable replication in EVECs at 39.5°C (**C**, dotted lines). Vertical bars represent standard error of the mean (SE) (*n* = 2).

We next examined whether replication capacity at elevated temperatures was also observed in equine vascular endothelial cells (EVECs). Replication of EHV-1 in vascular endothelial cells is considered to contribute to vasculitis and thrombosis, which are characteristic pathological features of EHM. Both field isolates and BAC-derived viruses (Table S1) were used in these experiments. EVECs were inoculated with EHV-1 at an MOI of 0.1–0.3 and incubated at either 37.0°C or 39.5°C. At 37.0°C, virus replication accompanied by CPE was detected in EVEC cultures inoculated with all viruses by 24 hpi. At 39.5°C, CPE clusters became evident by 48 hpi in EVECs inoculated with viruses that replicated at elevated temperatures in FHKCs (Fig. 7A), whereas no comparable clustering was observed up to 72 h in EVECs inoculated with viruses that failed to replicate at elevated temperatures in FHKCs (Fig. 7B). Viral titers at 24 hpi ranged from 10^4.13^ to 10^5.13^ TCID_50_ per 25 µL, and comparable levels of infectious virus were maintained in EVEC cultures until 96 hpi (Fig. 8A). At 39.5°C, an increase in infectious virus titers peaking at 24–48 hpi was observed in EVEC cultures inoculated with viruses that exhibited replication capacity at elevated temperatures in FHKCs (Fig. 8B). Viral titers at 24 hpi ranged from 10^1.8^ to 10^4.5^ TCID_50_ per 25 µL. In contrast, EVEC cultures inoculated with viruses that did not show replication capacity at elevated temperatures in FHKCs (6383, 3292, mutant Ab4p UL30 N752 BAC, Ab4p UL30 08-I-812 BAC) exhibited only low titers at 24 hpi (10^0.25^ to 10^0.75^ TCID_50_ per 25 µL) with no subsequent increase; infectious virus levels declined to below the limit of detection by 72 hpi (Fig. 8C). Notably, for chimeric Ab4p UL30 06-I-70 BAC virus, infectious virus was detected in EVEC cultures until 96 hpi at 39.5°C; however, viral titers remained largely unchanged over time, indicating minimal viral replication under these conditions (Fig. 7B; Fig. 8B). Collectively, single amino acid substitutions at multiple UL30 positions, rather than residue 752 alone, can each independently confer the ability of EHV-1 to replicate and achieve cell-to-cell transfer in physiologically relevant cell types at febrile temperatures (Table 7).

**TABLE 7:** Summary of UL30 substitutions, domain location, replication, and cell-to-cell transfer at 39.5°C

Position	Substitutions^*a*^	Domain^*b*^	Productive replication^*c*^	Cell-to-cell transfer	EHM link (reported)^*d*^	Remarks
250	R	NH_2_-terminal	+	+	Present in EHM-derived isolate(s) in this study	BAC (single SNP); ORF30 from EHM-derived isolate 7759
291	I	NH_2_-terminal	+	+	Reported in EHM-associated outbreaks/lineages	BAC (single SNP); ORF30 variant shared with Valencia outbreak lineage
625	H	NH_2_-terminal	+	+	Not specifically linked to EHM	BAC (single SNP); ORF30 variant from field isolate 13757 (non-EHM-derived)
683 + 769	K + N	Linker (NH_2_-terminal–Palm) and Finger	field isolate: -; BAC: +	field isolate: N.D.^*e*^; BAC: +	Not specifically linked to EHM	Field isolate 06-I-70: no detectable replication at 39.5°C; Ab4p-based chimeric BAC carrying 06-I-70 ORF30: positive phenotype at 39.5°C
742	A	Palm	+	+	Present in EHM-derived isolate(s) in this study	BAC (single SNP); ORF30 variant from EHM-derived isolate 89c105
752	N	Palm	–	–	Not specific to EHM; common genotype	BAC (single SNP) in Ab4p background
752	H	Palm	+	+	Reported in EHM outbreaks	BAC (single SNP); H752 genotype reported in EHM outbreaks
760	G	Palm	+	+	Reported in EHM-associated isolate (VA02)	Field isolate 8343 replicated at 39.5°C; same UL30 G760 reported in VA02; not tested as single SNP in Ab4p
946	V	Thumb	–	–	Not specifically linked to EHM	BAC (single SNP); ORF30 variant from field isolate 08-I-812 (non-EHM-derived)

^*a*^ Different from Ab4p.

^*b*^ Domain assignment based on HSV-1 UL30 domain annotation (32) and mapping in Fig. 9.

^*c*^ Productive replication in FHKCs and EVECs.

^*d*^ EHM link (reported) summarizes published associations and/or isolate origin and does not imply causality.

^*e*^ N.D., not determined (not assessed under the indicated condition/assay).

### A molecular epidemiologic association analysis of UL30 amino acid variants associated with EHM in EHV-1

Based on these results, we conducted a molecular epidemiologic association analysis to assess relationships between variation at UL30 positions 291, 250, 742, 752, and 760 and clinical outcomes (EHM versus non-EHM phenotypes such as abortion) using an observational data set of publicly available EHV-1 UL30 sequences with clinical phenotype annotations (*n* = 266). Comparison of these sequences revealed an additional substitution at UL30 residue 990 in addition to those summarized in Table 3. Notably, all Japanese isolates examined for elevated-temperature replication, as well as the Ab4p strain, encoded E at position 990. Accordingly, strains possessing UL30 sequences with N752 (i.e., sequences lacking the 250/291/742/760 substitutions) could be subdivided into two groups depending on whether they encoded E or K at position 990 of UL30. The percentage of these strains that were associated with EHM was 2.4% for the E strains and 38.1% for the K strains (Table 8). Using the number of strains harboring N752 and E990 in UL30 as the reference background (164 strains), EHM was significantly enriched in the D752 category (25/55, 45.5%), and stronger associations with EHM were observed in the I291 (6/7, 85.7%), A742 (4/5, 80.0%), R250 (4/6, 66.7%), and G760 (2/3, 66.7%) categories (Table 8). The substantially higher background proportion of EHM cases in the N752-K990 suggests that UL30–EHM associations are sensitive to underlying genetic background. Consistent with this, when analyses were restricted to the N752-K990 UL30 background group, none of the variant-versus-background comparisons remained significant (*P*-values > 0.05 and *q* values > 0.10) after multiple-testing corrections (Table S2), likely due to more frequent EHM in this group and the small sample size. These results indicate that EHM cannot be attributed to D752 alone and are consistent with multiple UL30 genetic backgrounds contributing to EHM risk.

**TABLE 8:** Enrichment of EHM in UL30 categories relative to the N752–E990 background*^a^*^,^*^b^*^,^*^c^*

UL30 category	EHM/Total (EHM%)	Reference background (N752–E990) EHM/Total	OR (Haldane)	P	Q (BH-FDR)
N752–E990	4/164 (2.4%)	–	–	–	–
N752–K990	8/21 (38.1%)	4/164	–	–	–
D752	25/55 (45.5%)	4/164	29.82	7.61 × 10^−14^	3.80 × 10^−13^
I291	6/7 (85.7%)	4/164	154.56	4.53 × 10⁻⁸	1.13 × 10⁻⁷
A742	4/5 (80.0%)	4/164	107.00	1.05 × 10⁻⁵	1.74 × 10⁻⁵
R250	4/6 (66.7%)	4/164	64.20	3.01 × 10⁻⁵	3.76 × 10⁻⁵
G760	2/3 (66.7%)	4/164	59.44	3.20 × 10⁻³	3.20 × 10⁻³

^*a*^ A total of 266 ORF30 amino-acid sequences were retrieved from public databases (GenBank/DDBJ/EMBL). Five sequences carried a double substitution (R250 plus D752) and were excluded because this combination could not be evaluated within the present analysis framework. *N752–E990* denotes N752 sequences lacking the additional substitutions at UL30 positions 250/291/742/760 and carrying E at position 990; *N752–K990* denotes the analogous N752 group carrying K at position 990. Odds ratios (OR) are reported with Haldane–Anscombe correction (+0.5 to all cells). *P*-values are from two-sided Fisher’s exact tests; *q* values are BH-FDR adjusted across the five comparisons using the same reference background group. OR/*P*/q are not reported for N752–K990 because it is shown descriptively (baseline EHM frequency) rather than as a marker-vs-background test in this table.

^*b*^ S151, H625, K683, N769, V946, and T1107 were not observed among EHM-associated sequences, and their frequencies were too low to yield statistically significant associations.

^*c*^ H752 was not included because only one H752 ORF30 nucleotide sequence with clinical phenotype annotation was retrieved from public databases in this dataset, precluding meaningful statistical comparison within this framework.

### Phylogenetic evidence for repeated independent emergence of UL30 variants

To account for synonymous nucleotide variation, we evaluated independent emergence on a nucleotide-based ORF30 phylogeny inferred from 266 ORF30 nucleotide sequences from isolates with annotated clinical phenotypes. Using this nucleotide-based phylogeny, we estimated the minimum number of independent gains of the UL30 amino-acid variants examined in this study by parsimony. R250, H625, K683, G760, and V946 each required at least one independent gain, whereas I291, A742, and N769 each required at least two independent gains across the phylogeny (Table 9). D752 showed the greatest homoplasy, requiring at least seven independent gains. Residue 990 varied between E and K (E: 244/266; K: 22/266), and when the ancestral state was constrained to E990, K990 required at least three independent gains (Table 9). Together, these phylogenetic patterns provide an evolutionary context for the enrichment of EHM among UL30 variant groups (Table 8; Table S2), indicating that several UL30 variants have arisen repeatedly on distinct nucleotide backgrounds.

**TABLE 9:** Nucleotide-tree–based parsimony estimates of the minimum number of independent gains of UL30 variants^*a*^

UL30 position (Ab4p)	Target amino-acid state	Tip counts	Minimum changes (Fitch)	Minimum independent gains to target state	Root constraint
250	R	H:255; R:11	1	1	None
291	I	S:259; I:7	2	2	None
625	H	R:265; H:1	1	1	None
683	K	E:264; K:2	1	1	None
742	A	V:261; A:5	2	2	None
752	D	N:205; D:60; H:1	8	7	None
760	G	D:263; G:3	1	1	None
769	N	H:264; N:2	2	2	None
946	V	I:264; V:1	1	1	None
990	K	E:244; K:22	1	3	Root fixed to E

^*a*^ Independent-origin analysis was performed on a nucleotide-based ORF30 phylogeny inferred from 266 codon-aligned nucleotide sequences corresponding to isolates with annotated clinical phenotypes. Minimum changes and minimum independent gains were estimated by Fitch parsimony on the fixed nucleotide-based tree. “Minimum independent gains” denotes the minimum number of parent≠variant → child = variant transitions for the focal UL30 variant. For residue 990, the root state was constrained to E to estimate the minimum number of independent gains of K990.

### SNP mapping in a three-dimensional model of EHV-1 DNA polymerase UL30

**Fig 9:**
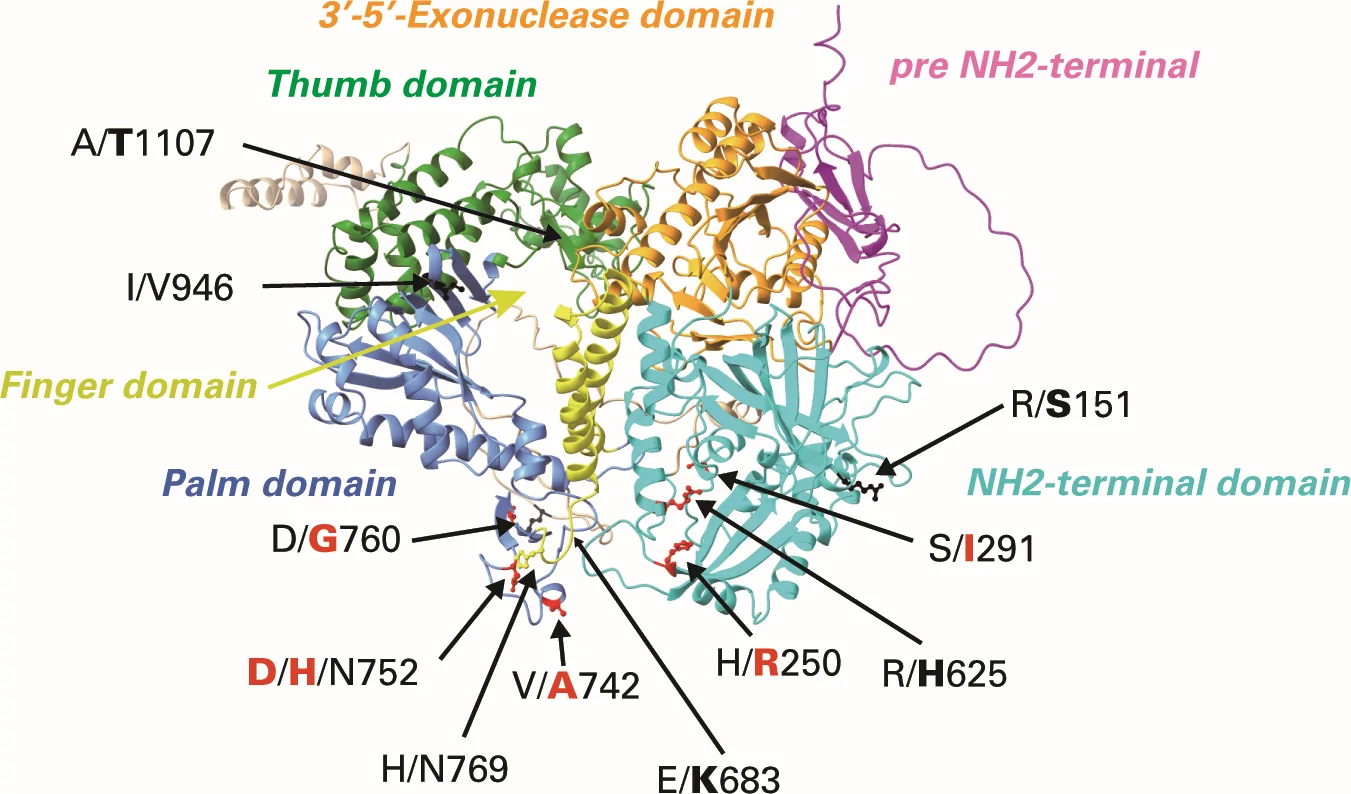
SNPs mapping on 3D modeling of EHV-1 UL30. A three-dimensional model of EHV-1 Ab4p UL30 D752 was predicted using AlphaFold3. Functional domains of UL30 are annotated based on the three-dimensional structure of herpes simplex virus type 1 (HSV-1) UL30 (32). Amino acid substitutions present in viruses capable of replicating at elevated temperatures are shown in red bold type. The positions of individual SNPs were mapped onto the UL30 structure. EHM-associated SNPs (red) are clustered within the NH2-terminal domain (turquoise) (S/I291, H/R250, R/H625) and Palm domain (blue) (V/A742, D/H/N752, D/G760) domains. In contrast, SNPs (black) not associated with replication capacity at elevated temperatures are distributed outside these regions, including the N-terminal domain (R/S151), the joining loop between the NH2-terminal and Palm domains (E/K683), Finger domain (H/N769), Palm domain (I/V946), and Thumb domain (A/T1107)

Three-dimensional (3D) structure models of UL30 from eight variants (Ab4p, 06-I-70, 89c105, 7759, 8343, 13757, H752, and Valencia) were predicted using AlphaFold3. The predicted structures were similar to one another and to the X-ray crystallography-determined structure of the herpes simplex virus 1 (HSV-1) DNA polymerase (POL) (32), which has 67% similarity with EHV-1 UL30. Despite sequence divergence, the overall architecture and functional domains of the UL30 proteins were highly conserved relative to those of HSV-1 POL. Structural domains were designated according to Liu et al. (32) (Fig. 9; Fig. S2). All UL30 SNPs analyzed in the present study were mapped onto the 3D model of EHV-1 Ab4p UL30 (Fig. 9). SNPs associated with replication capacity at elevated temperatures were predominantly clustered in a region adjacent to the NH2-terminal and Palm domains of UL30 (turquoise and blue domains, respectively, in Fig. 9). In contrast, SNPs not associated with replication capacity at elevated temperatures were distributed across other regions of UL30. Although UL30 SNPs associated with replication at elevated temperatures likely affect functions of the viral DNA polymerase, our data do not distinguish whether this reflects altered enzymatic activity *per se* or effects on other stages of the viral replication cycle.

## DISCUSSION

The present study identifies the ability of EHV-1 to replicate at elevated temperature as a UL30-associated viral phenotype (hereafter referred to as elevated-temperature replication). Using recombinant viruses, field isolates, and chimeric BAC constructs, we demonstrate that specific UL30 polymorphisms are associated with replication at 39.5°C in permissive cells. The contribution of UL30 amino-acid substitutions outside position 752, identified in N752 field isolates, to elevated-temperature replication was evaluated using chimeric BAC viruses in the Ab4p genetic background, and these chimeric BAC-derived viruses also replicated efficiently under elevated-temperature conditions. Viruses exhibiting this phenotype also retained the capacity for PBMC-mediated cell-to-cell transfer and endothelial infection under the same conditions, albeit with restricted productive replication in PBMCs. The molecular epidemiologic association analysis further indicated that the UL30 amino-acid variants identified in this study were significantly associated with EHM relative to the N752–E990 reference background. Collectively, these findings demonstrate that the ability to replicate at elevated temperatures and the risk of EHM cannot be attributed to D752 alone and that specific amino-acid residues at multiple UL30 positions are probably involved.

Pyrexia is consistently reported during EHM outbreaks and often precedes the onset of neurological deficits (11, 13, 14, 33–35). Although fever may resolve before neurologic signs become apparent (33–35), the febrile phase likely represents a critical stage of systemic infection. Horses that develop EHM have been reported to exhibit prolonged viremia and higher leukocyte-associated viral loads compared with infected horses that do not develop neurologic disease (17, 18). Our data suggest that elevated temperatures impose a restrictive environment on many EHV-1 strains, suppressing replication in permissive cells and limiting PBMC-mediated dissemination. In contrast, strains retaining the ability to replicate at elevated temperatures were able to maintain viral genome synthesis in permissive cells and preserve PBMC-mediated transfer to susceptible target cells despite limited release of infectious progeny from PBMCs. Such properties could facilitate continued vascular dissemination during febrile episodes. EHM can occur without clearly documented fever (1). Therefore, replication at elevated temperatures should not be interpreted as a prerequisite for neurologic disease, but rather as a viral property that may confer resilience under host-imposed thermal stress.

The D752 substitution in UL30 has been widely used as a molecular marker for neuropathogenic strains (1, 2, 22, 24, 25). However, multiple studies have shown that D752 is neither necessary nor sufficient for EHM development (1, 26). Our findings are consistent with this view. Replication at elevated temperatures was not strictly determined by residue 752 alone. Several N752 isolates retained replication capacity when additional UL30 substitutions were present, and corresponding chimeric BAC viruses reproduced this phenotype in a controlled genetic background. Thus, specific amino-acid residues at multiple UL30 positions, rather than residue 752 alone, can each independently confer the ability of EHV-1 to replicate at febrile temperatures.

Our molecular epidemiologic association analysis of public EHV-1 UL30 sequences suggests that EHM is not attributable to D752 alone and is consistent with enrichment of multiple UL30 amino-acid variants (R250, I291, A742, and G760) among EHM-associated sequences relative to the N752–E990 background (Table 8; Table S2). In addition, the N752 background is genetically structured and can be stratified into the N752–E990 and N752–K990 groups, underscoring that UL30–phenotype associations should be interpreted in the context of genetic background and lineage structure (Table 8; Table S2). Consistent with this view, our nucleotide-based independent-origin analysis showed that several UL30 variants have arisen repeatedly on distinct nucleotide backgrounds. D752 exhibited the greatest homoplasy, requiring at least seven independent gains, whereas K990 required at least three independent gains when the ancestral state was constrained to E990 (Table 9). I291, A742, and N769 each required at least two independent gains, while R250, H625, K683, G760, and V946 were each compatible with a single emergence within this data set (Table 9). Given that UL30 encodes the viral DNA polymerase, variants at positions 250/291/742/752/760 could plausibly affect replication kinetics and/or cell-to-cell spread under elevated temperature, aligning with our experimental observations; however, because these inferences are drawn from observational public data sets and may be affected by confounding (e.g., sampling bias and linkage to broader genomic background), confirmation using standardized cohorts, whole-genome context, and targeted functional assays will be important.

Among abortion-derived isolates, strain 8343 replicated efficiently at 39.5°C and possessed the G760 substitution, identical to that reported in the EHM-associated strain VA02 (20). Although the G760 substitution was not independently engineered into the Ab4p background in the present study, its occurrence in viruses capable of replication at elevated temperatures, together with prior reports linking this substitution to neuropathogenic lineages previously associated with EHM outbreaks, suggests that G760 may contribute to replication at elevated temperatures. Definitive functional validation will require targeted mutagenesis and biochemical analysis.

Endothelial infection is central to the pathogenesis of both EHM and abortion (1, 3, 17, 36). In the central nervous system, vascular injury can result in ischemic damage and neurologic deficits; in pregnant mares, placental vascular compromise contributes to fetal loss. In the present study, several abortion-derived isolates demonstrated replication at elevated temperatures and shared UL30 genotypes with strains implicated in EHM outbreaks outside Japan. In addition, because only four EHM-derived field isolates were available for analysis, larger collections of neurologic isolates will be required to determine the generalizability of this phenotype across diverse outbreaks. These findings indicate that the ability to replicate at febrile temperature is not exclusive to EHM-associated viruses. Rather than dictating clinical outcome, this phenotype may support sustained vascular dissemination. The ultimate clinical manifestation—EHM, abortion, both, or neither—likely depends on the interaction between viral determinants and host context, including pregnancy status, vascular susceptibility, and immune response dynamics (1, 17, 18, 37–39).

Discordance between permissive-cell replication and PBMC-mediated transfer further supports a multifactorial model. For example, isolate 9204 replicated efficiently at 39.5°C in permissive cells but failed to mediate detectable PBMC-to-cell transfer. Conversely, the chimeric UL30 06-I-70 BAC virus retained partial transfer-associated phenotypes despite limited replication in FHKCs at elevated temperature. These observations suggest that UL30 strongly influences temperature-dependent replication but does not alone determine leukocyte-associated dissemination. Additional viral genes and host-cell interactions likely modulate these processes.

Replication-associated substitutions, including residues 250, 291, 742, 752, and 760, were localized predominantly within or adjacent to the NH2-terminal and Palm domains of UL30. These domains are functionally important in herpesvirus DNA polymerases (32). Although structural modeling did not predict major global rearrangements, localized effects on thermal stability or catalytic efficiency may underlie the observed phenotype. However, we cannot exclude the possibility that these substitutions influence upstream events in the viral replication cycle that secondarily affect viral DNA synthesis at elevated temperature. Experimental validation at the biochemical level remains necessary.

Cell-to-cell transmission from PBMCs infected with EHV-1 to vascular endothelial cells likely represents key events occurring *in vivo* during EHV-1 infection in horses (2). Under febrile conditions, EHV-1 strains that retain replication capacity at elevated temperatures can continue to maintain viral gene expression and cell-associated infection in PBMCs, resulting in sustained viremia with high viral loads. This suggests that early administration of antiviral agents to reduce viral load during the initial phase of infection may be critical for preventing progression to EHM. Previous clinical studies have shown that early administration of valacyclovir significantly decreased viral replication and signs of disease in EHV-1-infected horses (40), but that antiviral treatments are largely ineffective once horses exhibit clinical signs of EHM, suggesting that intervention at this stage is unlikely to alter disease outcome (1). The development of effective and affordable antiviral strategies is therefore urgently needed.

Our findings raise the possibility that certain EHM-associated amino acid residues at multiple UL30 positions (D752, I291, etc.) can independently modulate UL30 function at elevated temperatures. While our chimeric BAC viruses support this link at the viral level, further mechanistic studies—such as biochemical characterization of polymerase activity at elevated temperatures—may help to clarify how these residues influence the ability to replicate at elevated temperatures and to define their relationship to EHM.

## MATERIALS AND METHODS

### Cell line

Fetal horse kidney-derived FHK Tcl3.1 cells (FHKCs) were cultured in Dulbecco’s modified Eagle’s medium (DMEM) (FUJIFILM Wako Pure Chemical Corporation, Osaka, Japan) containing 10% fetal bovine serum (FBS) without antibiotics (41). The cells were maintained in a humidified 5% CO_2_ atmosphere at 37.0°C.

### Viruses

EHV-1 Ab4p strain was kindly provided by Dr. A. J. Davison (Glasgow University, Scotland) (42, 43). A bacterial artificial chromosome (BAC) vector, pZC320mEGFP, was inserted into the Ab4p virus genome to generate pAb4p BAC (parental BAC plasmid) in our laboratory, as described previously (Fig. S1) (44). The pZC320mEGFP vector was constructed from the mini-F plasmid pZC320, obtained from the National BioResource Project (NBRP) *Escherichia coli* Strain Collection (National Institute of Genetics, Mishima, Japan), and contained a modified enhanced green fluorescent protein (mEGFP) gene as a selection marker (31, 44). Using pAb4p BAC, a single nucleotide substitution was introduced at nucleotide position 2254 of ORF30 to generate a mutant BAC, designated pAb4p UL30 N752 BAC, in which the codon for the 752nd amino acid was changed from aspartic acid (GAC, D752) to asparagine (AAC, N752). A repaired pAb4p UL30 D752 Repair BAC virus was also constructed by reverting this codon to the original aspartic acid sequence (GAC, D752). Recombinant viruses lacking the BAC sequence—Ab4p UL30 D752 (parental virus), Ab4p UL30 N752 (mutant virus), and Ab4p UL30 D752 Repair (repair virus)—as well as recombinant viruses retaining the BAC sequence—Ab4p UL30 D752 BAC (parental BAC virus), Ab4p UL30 N752 BAC (mutant BAC virus), and Ab4p UL30 D752 Repair BAC (repair BAC virus)—were reconstituted as described previously (31). Although we did not sequence the whole genomes of Ab4p UL30 N752 BAC and Ab4p UL30 D752 Repair BAC, we used Sanger sequencing to confirm that the UL30 locus in the repaired virus was identical to that of the parental strain. The possibility of additional mutations elsewhere in the genome cannot be excluded, although a restriction fragment length polymorphism (RFLP) analysis using EcoRI, HindIII, and SalI did not show any major insertions or deletions in the genomic sequence of the constructed virus (31).

Forty-nine EHV-1 field isolates collected in Japan, including four EHM isolates (89c105, 01c1, 7759, and 7855) are listed in Table 2. Viruses used in the present study were propagated in FHKCs in a humidified cell culture incubator with 5% CO_2_ at 37°C.

The full genome sequences (KF644566, KF644577, KF644578, LC109626 to LC109660) and ORF30 sequences (LC834900 to LC84906, LC834908 to LC834913) of the field isolates in Japan have been registered in the DNA Data Bank of Japan (DDBJ) (Table 2). Additional sequence data will be reported elsewhere.

### Evaluation of viral replication capacity at elevated temperatures

Ab4p UL30 D752 parent, Ab4p UL30 N752 mutant, and Ab4p UL30 D752 repair viruses lacking BAC vector sequence were subjected to plaque formation assay at elevated temperatures. Two cell culture incubators with humidified atmospheres containing 5% CO_2_ were used: one maintained at 37.0°C (MCO-170A/CUVD-PJ, Panasonic, Tokyo, Japan), and the other set at 38.5°C, 39.0°C, 39.5°C, 40.0°C, or 40.5°C (CPO2-2301, KK-Hirasawa, Tokyo, Japan). The temperature fluctuation was maintained within ±0.1°C.

Confluent FHKCs were prepared in duplicate 24-well plates at 37.0°C. Viral stock solution (10^7^ PFU/mL) was serially diluted in DMEM. Cells were inoculated with 10^−4^, 10^−5^, 10^−6^ dilutions of virus and incubated at 37.0°C or 38.5°C. For the Ab4p UL30 N752 mutant virus at 39.0°C or higher temperatures, 10^−1^, 10^−2^, and 10^−3^ dilutions were used. One hundred microliters of each diluted virus suspension was added per well, and each dilution was inoculated into four wells. After adsorption for 1 h at 37.0°C, the inoculum was removed, and cells were overlaid with 0.5 mL per well of DMEM containing 1% methylcellulose and 1% FBS. One plate was incubated at 37.0°C, and the corresponding plate was incubated at the designated elevated temperature. After 3 days of incubation, cells were stained by directly adding 0.5 mL per well of 0.15% crystal violet in 20% ethanol, followed by incubation for 3 days at room temperature. Plates were then washed with tap water and air-dried. Images of the plates were acquired using a digital camera. Plaque areas were measured using ImageJ software (version 1.54M), and plaque numbers were counted visually.

### Viral growth kinetics

Monolayers of FHKCs in four-well plates were inoculated with viruses at a multiplicity of infection (MOI) of 0.1. Virus stock solutions were treated with DNase I (1 µL per 1.5 mL of virus stock) and incubated for 15 min at 37°C prior to inoculation. After adsorption for 1 h at 37°C, the inoculum was removed and 0.5 mL per well of DMEM containing 1% FBS was added. Infected cells were incubated at 37.0°C or 39.5°C. Culture supernatants and cells were harvested at 0, 4, 8, 12 and 24 hpi. Culture media were collected, centrifuged to remove cell debris, and the clarified supernatants were stored at −80°C. Cells remaining in the wells were washed three times with Dulbecco’s phosphate-buffered saline without Ca^2+^ and Mg^2+^ (D-PBS(-)), treated once with 0.25% trypsin-0.02% EDTA (Sigma-Aldrich Japan, Tokyo, Japan), and incubated for 5 min. Cells were then suspended in 0.5 mL D-PBS(-) and centrifuged at 12,000 rpm for 1 min at 4°C. After removal of the supernatant, cell pellets were resuspended in 0.5 mL D-PBS(-) and stored at −80°C until analysis.

### Isolation of equine PBMCs and infection with EHV-1

Peripheral blood mononuclear cells (PBMCs) were isolated from heparinized blood collected from a horse by density gradient centrifugation using Lymphocyte Separation Solution (*d* = 1.077, Nakarai Tesque, Inc, Kyoto, Japan). Immediately after isolation, PBMCs were inoculated with various EHV-1 strains at MOI of 0.1–0.3. After adsorption for 1 h at either 37.0°C or 39.5°C to mimic febrile conditions throughout the infection process, unadsorbed virus was removed by three rounds of centrifugation and washing at 3,000 rpm for 2 min each. Cells were then resuspended in RPMI-1640 (FUJIFILM Wako Pure Chemical Corporation, Osaka, Japan), supplemented with 10% FBS and incubated at 37.0°C or 39.5°C in a humidified atmosphere containing 5% CO_2_. Mock-infected cells were used as negative controls. At designated time points, culture supernatants and cells were collected for quantification of extracellular virus, detection of glycoprotein B (gB) antigen (45) and mEGFP, and evaluation of cell-to-cell infection.

### Cell-to-cell infection assay

For preparation of PBMC samples, a total of 3 × 10^6^ PBMCs were used for virus adsorption. After adsorption, cells were washed three times by centrifugation and resuspended in 1.5 mL of maintenance medium. Of this suspension, 500 µL was centrifuged and separated into cell pellets and supernatants, which were used as samples for 0 hpi. The supernatants were directly subjected to virus titration using two serial dilution columns. The remaining 1.0 mL of the PBMC suspension was divided equally into two wells of a 24-well plate (500 µL per well), incubated, and harvested at 24 h and 48 hpi to assess viral infectivity in both supernatant and cells. Cell pellets collected at 24 and 48 hpi were suspended in 300 µL of maintenance medium, serially diluted in two steps, and inoculated onto FHKC monolayers as described below. The calculated maximum number of PBMCs inoculated was 8 × 10^4^ per well.

Monolayers of FHKCs grown in 96-well plates were washed and overlaid 125 µL per well of maintenance medium consisting of DMEM supplemented with 4% FBS. PBMCs infected with EHV-1 were diluted 10-fold, and 25 µL of the diluted PBMC suspension was inoculated into four replicate wells per condition. FHKCs inoculated with PBMCs at 37.0°C and 39.5°C were cultured at the inoculation temperatures. After 5 days, cell-to-cell infection was evaluated based on the presence or absence of CPE.

### Confirmation of EHV-1 infection in PBMCs

EHV-1 infection was confirmed by detecting both mEGFP and gB expression. mEGFP expression in cells infected with BAC-derived viruses was monitored daily after infection using a fluorescence microscope (Olympus, Tokyo, Japan). For BAC-derived viruses, the percentage of mEGFP-positive PBMCs at 48 hpi was recorded as a semiquantitative indicator of infection (Table 4). For detection of gB, PBMCs were harvested at 48 hpi and fixed in cold acetone on ice for 30 min. After fixation, cells were incubated with a monoclonal anti-EHV-1 gB antibody (clone 121-3) (45), for 30 min at 37°C in a humidified chamber, followed by incubation with a fluorescein isothiocyanate (FITC)-conjugated donkey anti-mouse IgG antibody (Jackson Immuno Research Laboratories, Inc., West Grove, PA, USA) under the same conditions. gB expression was visualized using a fluorescence microscope.

### Preparation of equine vascular endothelial cells and infection with EHV-1

Aortic tissue was collected from a euthanized foal. One end of the aorta was clamped with forceps, and the lumen was washed three times with phosphate-buffered saline (PBS). The aorta was then filled with 0.25% trypsin and incubated at room temperature for 30 min; this procedure was repeated twice. Trypsinized cells were collected and centrifuged at 1,500 rpm for 5 min. The resulting cell pellets were resuspended in DMEM supplemented with 10% FBS and designated as EVECs. EVECs were cultured in DMEM with 10% FBS at 37 °C in a humidified atmosphere with 5% CO_2_.

Monolayers of EVECs grown in six-well plates were infected with EHV-1 at MOI of 0.1–0.3 and allowed to adsorb at either 37.0°C or 39.5°C. After adsorption, the cell surfaces were washed three times with DMEM to remove unadsorbed virus, and DMEM supplemented with 4% FBS was added to each well. Cells were then incubated at either 37.0°C or 39.5°C in a humidified atmosphere with 5% CO_2_. Culture supernatants were collected at 0, 24, 48, 72, and 96 hpi and used for virus titration.

### Virus titration

Virus-containing culture supernatants were subjected to either a plaque formation assay or a conventional TCID_50_ assay. For plaque formation assay, confluent FHKC monolayers were prepared in 24-well plates and inoculated with 0.1 mL per well of serially diluted virus samples. Four wells were used for each dilution. After adsorption for 1 h at 37°C, the inoculum was removed and cells were overlaid with 0.5 mL per well of DMEM containing 1% methylcellulose and 1% FBS. After incubation for 3 days, an equal volume of 0.15% crystal violet in 20% ethanol was added to each well, and the plates were incubated for an additional 3 days. The cells were then washed with tap water and air-dried, and plaques were counted visually.

A conventional TCID_50_ assay was also performed to evaluate the infectivity of culture supernatants and cell-associated virus obtained from PBMCs and EVECs experiments, utilizing FHKC monolayers cultured in 96-well plates. Viral titers were calculated using the Behrens–Kärber’s method (46), based on the presence of CPEs observed after 5 days of incubation.

### DNA extraction

Genomic DNA was extracted from infected cells using SepaGene (Sekisui Medical Co., Ltd. Tokyo, Japan) according to the manufacturer’s protocol. The extracted DNA was dissolved in TE (10 mM Tris-HCl, pH 8.0, 1 mM EDTA) and stored at −20°C until use. DNA concentrations were determined using a Qubit 2.0 Fluorometer (Thermo Fisher Scientific K.K., Tokyo, Japan) with a Qubit dsDNA BR Assay kit.

### Real-time PCR

Viral DNA copy numbers were quantified using a Thermal Cycler Dice Real Time System (TaKaRa Bio Inc) and TB Green Premix Ex Taq II (Tli RNaseH Plus) (TaKaRa Bio, Bio Inc). Real-time PCR was performed using primers targeting the EHV-1 ORF30 gene: UL30F (5′-GTCAGGCCCACAAACTTGAT-3′) and UL30R (5′-ACTCGGTTTACGGATTCACG-3′). The PCR conditions consisted of an initial denaturation at 95°C for 30 s, followed by 40 cycles of denaturation at 95°C for 5 s and annealing/extension at 60°C for 30 s. A dissociation (melting curve) analysis was subsequently performed at 95°C for 15 s, 60°C for 30 s, and 95°C for 15 s. Purified pAb4p BAC DNA was used as a reference standard for quantitative analysis.

### Construction of chimeric BAC viruses

Chimeric BAC viruses containing specific nucleotide mutations in the ORF30 gene were constructed based on the Ab4p BAC and pAb4p ∆ORF30 rpsLneo BAC using BAC technology established in our previous studies (31, 44). The introduced mutations corresponded SNPs identified in Japanese field isolates (i) T2225C (V742A) and G2254A (D752N); (ii) G2047A (E683K), G2254A (D752N), and C2305A (H769N); (iii) C748G (H250R) and G2254A (D752N); and (iv) G2254A (D752N) and A2836G (I946V). SNPs shared between a Japanese field isolate and the Valencia outbreak lineage, G872T (S291I) and G2254A (D752N), were also introduced. In addition, a novel SNP, G2254C (D752H), reported in isolates from the United States and Europe, was introduced. For the construction of additional recombinant BACs, a pAb4p ORF30 Δaa55–494 rpsLneo BAC plasmid was generated in the present study. A rpsLneo cassette flanked by homologous arms was amplified using a rpsLneo DNA as a template and PrimeSTAR MAX DNA polymerase (TaKaRa Bio Inc.), with the primers ∆30(55-494)rpsL-neo F (5′-tcaccttgctcttcttctgaaaatggttcgtggcgatgtcccacaccttGGCCTGGTGATGATGGCG-3′) and ∆30(55-494)rpsL-neo R (5′-gcccaaggcccccccaacactcgtactgcacagaggtgggtagctttaagTCAGAAGAACTCGTCAAGAAGGCG-3′). In these primer sequences, lowercase letters indicate ORF30 homologous arm sequences, and uppercase letters indicate sequences homologous to the rpsLneo cassette. The amplified rpsLneo cassette was electroporated into *Escherichia coli* DH10beta cells harboring the pAb4p BAC and pRED/ET plasmids. Replacement of the corresponding region of the ORF30 gene with the rpsLneo cassette was achieved using RED/ET gene recombination system (Counter Selection BAC Modification Kit; Gene Cambridge GmbH, Heidelberg, Germany). All BAC constructs contained pZC320mEGFP as the BAC vector backbone.

### Site-directed mutagenesis of ORF30

Site-directed mutagenesis was performed to introduce either G872T (S291I) or G2254C (D752H) substitution into the ORF30 gene using inverse PCR with primers containing the desired nucleotide changes. Briefly, an ORF30 fragment containing the A2254 mutation, as previously described (31), was amplified from Ab4p UL30 N752 DNA using the primer pair infusion ORF30 pUC19F (5′-acgaggccctttcgtcggccacatcctcgtcgtaca-3′) and infusion ORF30 pUC19R (5′-caatacgcaaaccgcacgggtacggcaagttcaac-3′) and PrimeSTAR Max DNA polymerase (TaKaRa Bio Inc.). PCR amplification was performed for 30 cycles under the following conditions: 98°C for 10 s, 60°C for 5 s and 72°C for 8 s. The PCR product was purified using a NucleoSpin kit (TaKaRa Bio Inc.), and DNA concentration was measured using a Qubit 2.0 Fluorometer. The amplified UL30 fragment was cloned into pUC19 vector using the In-Fusion cloning system according to the manufacturer’s instruction (TaKaRa Bio Inc.). The In-Fusion reaction mixture was transformed into *E. coli* DH5alpha competent cells (TaKaRa Bio Inc.) and plated on Luria-Bertani (LB) agar containing 50 µg/mL ampicillin. The resulting plasmid was designated pUC-UL30. Site-directed mutagenesis of pUC-UL30 was carried out using a PrimeSTAR Mutagenesis Kit (TaKaRa Bio Inc.). For the G872T (S291I) mutation, the primers ORF30_G872T_ F (5’-gaggggaTcgtggacgtgaccacgcg-3’) and ORF30_G872T_R (5′-gtccacgAtcccctcgaattttgtaatc-3′) were used. For the G2254C (D752H) mutation, the primers ORF30_G2254C_F (5′-gagtagtggacggatggttgaagccc-3′) and ORF30_G2254C_R (5′-tccgtccactactcgacgttcgagg-3′) were used. The resulting plasmid DNA was purified and subjected to Sanger sequencing to confirm the presence of the intended nucleotide substitutions and the absence of unintended mutations.

### Amplification of ORF30 fragments for BAC recombination

PCR fragments of the ORF30 gene were amplified using DNA extracted from field isolates (89c105, 06-I-70, 08-I-812) or mutagenized plasmid DNA carrying C2254 (H752) as template DNA. Amplification was performed using the primer pair ΔrpsL ORF30 F (5′-ggccacatcctcgtcgta-3′) and ΔrpsL ORF30 R (5′- tttaaggtgtgggacatcg-3′) with PrimeSTAR Max DNA polymerase (TaKaRa Bio Inc.). A second PCR fragment corresponding to another region of ORF30 (nucleotide positions 62 to 1575, encoding amino acids 55 to 494 of UL30) was amplified using genomic DNA from the field isolate 7759 DNA or mutagenized plasmid DNA carrying the T872 (I291) substitution as template DNA. The primers ORF30 62-82-F (5′-gcaagaggccatttttcaggc-3′) and OR30 1575-1555-R (5′-cgtcgccacagaatacatgtc-3′) were used together with PrimeSTAR max DNA polymerase (TaKaRa Bio Inc., Kusatsu, Japan). All PCR products were purified using a NucleoSpin Kit (TaKaRa Bio Inc., Kusatsu, Japan), and DNA concentrations were measured using a Qubit 2.0 Fluorometer using a Qubit dsDNA BR Assay kit.

### Construction and confirmation of chimeric BACs

Each PCR fragment was introduced into *E. coli* DH10beta cells harboring pAb4p ∆ORF30 rpsLneo BAC or pAb4p ORF30∆aa55-494_rpsLneo BAC together with the pRED/ET plasmid. The rpsLneo cassette in the BAC plasmid was replaced with an ORF30 fragment carrying the desired mutation using the Red/ET gene replacement system (Counter Selection BAC Modification Kit; Gene Cambridge GmbH, Heidelberg, Germany). Introduction of the intended mutations into the ORF30 gene of each BAC plasmid DNA was confirmed by Sanger sequencing. The resulting chimeric BAC DNAs were digested with EcoRI, HindIII or SalI, and the digested DNAs were analyzed by 0.5% agarose gel electrophoresis in 40 mM Tris-acetate, 1 mM EDTA (pH8.3). Gels were stained with ethidium bromide (0.5 µg/mL), and the fluorescence images were acquired for analysis. No large deletions or insertions were detected in the chimeric BAC DNA (Fig. S3).

### Recovery and replication assessments of recombinant chimeric BAC viruses

Each recombinant chimeric BAC virus was recovered as described previously (31, 44). Briefly, 200 ng BAC DNA purified using NucleoBond BAC100 Kit (TaKaRa Bio Inc., Kusatsu, Japan) was transfected into FHKCs seeded in 24-well plates using Lipofectamine 3000 (ThermoFisher Scientific K.K., Tokyo, Japan). Transfected cells were cultured at 37˚C for 3–5 days. After confirming green fluorescent protein expression and the presence of CPE by confocal laser scanning microscopy, the culture supernatants were harvested and used to inoculate fresh FHKCs. The recovered viruses were subjected to three rounds of plaque purification. Replication of recombinant chimeric BAC viruses was assessed qualitatively at 37.0°C and 39.5°C by monitoring mEGFP expression in live cells. The phenotype (focus formation, defined as clusters of ≥3 adjacent mEGFP-positive cells, versus single-cell confinement) was observed in at least three independent experiments.

### Statistical analyses

Data normality was assessed using the Shapiro–Wilk test. For data that followed a normal distribution, parametric statistical analyses were performed using Student’s *t*-test. For data that did not follow a normal distribution, nonparametric statistical analyses were performed using the Wilcoxon rank-sum test. For comparisons involving multiple groups, the Kruskal–Wallis rank-sum test followed by Dunn’s post hoc test with Bonferroni correction was used. *P*-values of <0.05 were considered statistically significant. For experiments designed to yield qualitative all-or-none outcomes, no statistical analysis was performed. All statistical analyses were conducted using RStudio (version 2024.12.1_563; Posit Software, PBC). Graphs were generated using the ggplot2 package in R, and figure labels were edited using Adobe Illustrator 2025 (Adobe, CA, USA).

### Confocal laser scanning microscopy

Cells were observed with a confocal laser scanning microscope (Zeiss LSM700; Carl Zeiss, Tokyo, Japan). Image acquisition was performed using Zeiss ZEN 2009 software (Carl Zeiss).

### UL30 sequence curation, reference-coordinate mapping, association testing, and phylogenetic analysis

Public EHV-1 UL30/ORF30 sequences retrieved from GenBank, DDBJ, and EMBL were compiled from complete-genome records and ORF30-only (partial) records. Clinical information was curated and standardized into a single variable (clin_sign) with the following categories: EHM, abortion, respiratory disease, ocular disease, and neonatal mortality. Phenotype annotations were harmonized by integrating accession-linked metadata and literature tables, with normalization of inconsistent terminology. Details of data sources, inclusion and exclusion criteria, and curation rules are provided in the Supplemental Methods.

#### UL30 coordinate mapping, alignment construction, and haplotype reduction

All analyses were conducted in an Ab4p (GenBank AY665713) reference-coordinate framework. UL30 amino-acid sequences were mapped to the reference by reference-to-query pairwise global alignment and processed using Biopython utilities (47), and the window corresponding to UL30 reference positions 151-1107 was extracted for each sequence. A “common-sites” alignment was generated by retaining only positions with complete coverage across all labeled sequences. To improve interpretability of phylogenetic visualization, identical amino-acid sequences in the common-sites alignment were collapsed into representative haplotypes (dedup), while preserving the mapping between haplotypes and member accessions.

#### Marker definitions and statistical analysis

UL30 marker categories were defined by amino-acid states at positions 250 (R), 291 (I), 742 (A), 752 (D), and 760 (G) in Ab4p coordinates, and sequences were assigned to mutually exclusive marker categories (with a MULTI category for co-occurring markers). The N752 background (i.e., sequences lacking the 250/291/742/760 substitutions) was further stratified by residue 990 into N752–E990 and N752–K990 subgroups, based on whether residue 990 was E or K, respectively. Associations between UL30 categories and EHM were tested using two-sided Fisher’s exact tests (48); odds ratios were reported with the Haldane–Anscombe correction (+0.5 to all cells) to accommodate zero counts (49, 50). Multiple testing was controlled using the Benjamini–Hochberg false discovery rate (BH-FDR) (51). Reproducible command lines, parameter settings, and scripts are provided in the Supplemental Methods and Supplementary Data.

#### Nucleotide-based phylogenetic reconstruction and independent-origin analysis

To account for synonymous nucleotide variation, independent-origin analysis was performed on nucleotide-based phylogenies rather than on amino-acid phylogenies. For each accession, candidate coding sequences were retrieved from the corresponding GenBank record, translated, and compared with the reference-mapped common-site amino-acid sequence to identify the matching ORF30 nucleotide sequence. The matched ORF30 nucleotide sequences were aligned with MAFFT (52), and a maximum-likelihood phylogeny was inferred using IQ-TREE (53) with ModelFinder (54) and ultrafast bootstrap approximation (55). Using this nucleotide-based phylogeny, we estimated the minimum number of independent gains of the UL30 amino-acid variants examined in this study by parsimony (56). For each position, gains were counted as parent-to-child transitions from a non-target amino acid to the target amino acid. The positions examined were 250, 291, 625, 683, 742, 752, 760, 769, 946, and 990. For residue 990, the ancestral state was constrained to E to estimate the minimum number of independent gains of K990.

### Protein structure predictions

The three-dimensional (3D) structure of the EHV-1 UL30 protein was predicted using AlphaFold 3 (https://alphafoldserver.com) with default parameters (57). The predicted models were visualized with the open-source software UCSF ChimeraX 1.9 (https://www.cgl.ucsf.edu/chimerax/) (58). Structural alignment and superimposition of the predicted models were performed using the MatchMaker tool in Chimera-X. Structural domains and secondary structure elements were mapped onto Ab4p UL30 amino acid sequence based on Liu et al. (32), using pair-wise amino acid alignment between HSV-1 KOS UL30 (GenBank accession no. JQ673480) and EHV-1 Ab4p UL30 (GenBank accession no. AY665713.1) generated with AliView 1.30 (https://github.com/AliView/AliView) (59) (Fig. S2).

## Supplementary Material

Reviewer comments

## Data Availability

All data are available in the main text or the supplemental materials.

## SUPPLEMENTAL MATERIAL

Data S1 spectrum.03055-25-s0001.docx Scripts for analysis.

Data S2 spectrum.03055-25-s0002.docx Data for analyses.

Data S3 spectrum.03055-25-s0003.docx Sequences for analyses.

Data S4 spectrum.03055-25-s0004.txt A phylogenetic tree file.

Supplemental Text spectrum.03055-25-s0005.docx Tables S1 and S2; Fig. S1 and S2.

Supplemental Methods spectrum.03055-25-s0006.docx Methods S1 to S9.

OPEN PEER REVIEW reviewer-comments.pdf An accounting of the reviewer comments and feedback.

ASM does not own the copyrights to Supplemental Material that may be linked to, or accessed through, an article. The authors have granted ASM a non-exclusive, world-wide license to publish the Supplemental Material files. Please contact the corresponding author directly for reuse.
