# Supplementary material for: Equine herpesvirus myeloencephalopathy-associated mutations in equine herpesvirus 1 DNA polymerase confer the ability to replicate at elevated temperatures
Source: Microbiol Spectr. 2026 Aug 21;14(10):e03055-25. doi: 10.1128/spectrum.03055-25 (PMC13689140; doi:10.1128/spectrum.03055-25)
Supplement: Supplementary file 1 [file reviewer-comments.pdf]

AMERICAN SOCIETY FOR MICROBIOLOGY | Microbiology Spectrum

# Equine herpesvirus myeloencephalopathy-associated mutations in equine herpesvirus 1 DNA polymerase confer the ability to replicate at elevated temperatures

Noriko Fukushi, Rikio Kirisawa, Fuka Nishimura, Koji Tsujimura, Yuko Toishi, and Hideto Fukushi

*Corresponding Author(s): Hideto Fukushi, Hoso Daigaku Gifu Gakushu Center (Gifu, Gifu Prefecture, JP)*

**Review Timeline:**

| | |
|---|---|
| Submission Date: | March 16, 2026 |
| Editorial Decision: | April 29, 2026 |
| Revision Received: | May 25, 2026 |
| Accepted: | May 27, 2026 |

*Editor: Qiyi Tang*

*Reviewer(s): The reviewers have opted to remain anonymous.*

## Transaction Report:

(Note: With the exception of the correction of typographical or spelling errors that could be a source of ambiguity, letters and reports are not edited. The original formatting of letters and referee reports may not be reflected in this compilation.)

**DOI: https://doi.org/10.1128/spectrum.03055-25**

Re: Spectrum03055-25 (Equine herpesvirus myeloencephalopathy-associated mutations in equine herpesvirus 1 DNA polymerase confer the ability to replicate at elevated temperatures)

Dear Prof. Hideto Fukushi:

Thank you for the privilege of reviewing your work. Below you will find my comments, instructions from the Spectrum editorial office, and the reviewer comments.

Please return the manuscript within 60 days; if you cannot complete the modification within this time period, please contact me. If you do not wish to modify the manuscript and prefer to submit it to another journal, notify me immediately so that the manuscript may be formally withdrawn from consideration by Spectrum.

**Revision Guidelines**
To submit your modified manuscript, log into the submission site at https://spectrum.msubmit.net/cgi-bin/main.plex. Go to Author Tasks and click the appropriate manuscript title to begin. The information you entered when you first submitted the paper will be displayed; update this as necessary. Note the following requirements:

• Upload point-by-point responses to the issues raised by the reviewers in a file named "Response to Reviewers," NOT in your cover letter.
• Upload a compare copy of the manuscript (without figures) as a "Marked-Up Manuscript" file.
• Upload a clean .DOC/.DOCX version of the revised manuscript and remove the previous version.
• Each figure must be uploaded as a separate, editable, high-resolution file (TIFF or EPS preferred), and any multipanel figures must be assembled into one file.
• Any supplemental material intended for posting by ASM should be uploaded with their legends separate from the main manuscript. You can combine all supplemental material into one file (preferred) or split it into a maximum of 10 files with all associated legends included.

For complete guidelines on revision requirements, see our Submission and Review Process webpage. Submission of a paper that does not conform to guidelines may delay acceptance of your manuscript.

**Data availability:** ASM policy requires that data be available to the public upon online posting of the article, so please verify all links to sequence records, if present, and make sure that each number retrieves the full record of the data. If a new accession number is not linked or a link is broken, provide Spectrum production staff with the correct URL for the record. If the accession numbers for new data are not publicly accessible before the expected online posting of the article, publication may be delayed; please contact production staff (Spectrum@asmusa.org) immediately with the expected release date.

**Publication Fees:** For information on publication fees and which article types are subject to charges, visit our website. If your manuscript is accepted for publication and any fees apply, you will be contacted separately about payment during the production process; please follow the instructions in that e-mail. Arrangements for payment must be made before your article is published.

**ASM Membership:** Corresponding authors may join or renew ASM membership to obtain discounts on publication fees. Need to upgrade your membership level? Please contact Customer Service at Service@asmusa.org.

The ASM Journals program strives for constant improvement in our submission and publication process. Please tell us how we can improve your experience by taking this quick Author Survey.

Thank you for submitting your paper to Spectrum.

Sincerely,
Qiyi Tang
Editor
Microbiology Spectrum

Reviewer #1 (Comments for the Author):

I have reviewed this manuscript before for JVI.
I appreciate the way how the authors have reworked their manuscript based on the comments, adding careful nuances as well as additional data (that led to further nuancing, seen the lack of apparent phenotype e.g. in PBMC).
Overall, I think this manuscript will be a very valuable contribution to the field.

Reviewer #2 (Comments for the Author):

The study focuses on assessing UL30 SNPs that may favor EHV-1 replication under febrile conditions and attempts to correlate replication at elevated temperatures and cell-to-cell transfer with the EHM-associated phenotype. The use of both clinical isolates and BAC-derived recombinant viruses to evaluate viral growth under febrile conditions is a strength and adds robustness to the study. However, the methods employed are largely qualitative rather than quantitative. For example, viral replication of the clinical isolates at febrile temperatures is primarily assessed through observation of CPE, which limits precise interpretation. Additionally, the study reads more as a collection of observations rather than leading to a clear conclusion supporting the hypothesis that the ability to replicate under febrile conditions contributes to the development of EHM. The presentation is somewhat confusing and difficult to follow. Including a summary table outlining the identified mutations, their locations within DNA polymerase (UL30), and their association with EHM would greatly improve clarity for readers.
Here are my additional comments:

1) Figure 1: Did the authors assess cell viability at febrile temperatures? Please also provide viral titers (PFU/mL) at different temperatures for the three recombinant viruses used in this figure, in addition to the fold change relative to 37 {degree sign}C shown in Table 1.
2) Table 2: A qualitative assessment may overlook isolates with low levels of replication.
3) Repaired UL30 D752 virus: Does this virus have an identical genome sequence to the parental strain, or are additional mutations present?
4) Quantification of the immunofluorescence images is needed across biological replicates.
5) Line 164: The D752 isolate that did not replicate at 39.5 {degree sign}C: did it harbor additional mutations in UL30?
6) Please clarify what is meant by "cell-to-cell transfer." The authors state (Line 218) that PBMCs are not capable of de novo virus production. How, then, is the virus transferred from PBMCs to FHCK cells? Are the authors suggesting cell-cell fusion?
7) Figure 7: Please include phase-contrast images showing CPE for all viruses used to infect EVECs.
8) Lines 262-265: Which chimeric BAC virus is being referred to here?

Minor point:
1) Figure 8: Please clearly label which graphs correspond to 37 {degree sign}C and 39.5 {degree sign}C.

**We thank the reviewers for their helpful comments. Our responses to comments are shown in bold.**

Reviewer #1 (Comments for the Author):

I have reviewed this manuscript before for JVI.
I appreciate the way how the authors have reworked their manuscript based on the comments, adding careful nuances as well as additional data (that led to further nuancing, seen the lack of apparent phenotype e.g. in PBMC).
Overall, I think this manuscript will be a very valuable contribution to the field.

**Thank you for the positive comments.**

Reviewer #2 (Comments for the Author):

The study focuses on assessing UL30 SNPs that may favor EHV-1 replication under febrile conditions and attempts to correlate replication at elevated temperatures and cell-to-cell transfer with the EHM-associated phenotype. The use of both clinical isolates and BAC-derived recombinant viruses to evaluate viral growth under febrile conditions is a strength and adds robustness to the study. However, the methods employed are largely qualitative rather than quantitative. For example, viral replication of the clinical isolates at febrile temperatures is primarily assessed through observation of CPE, which limits precise interpretation.

**We agree that it would be helpful to have more quantitative data. This could be achieved by doing titrations of viral growth to obtain PFU/mL or $TCID_{50}$/mL for each clinical isolate, but the cost and time needed to do this is beyond our budget. Experimental infection of healthy horses might also provide stronger results but this would be expensive and difficult to get approved.**

Additionally, the study reads more as a collection of observations rather than leading to a clear conclusion supporting the hypothesis that the ability to replicate under febrile conditions contributes to the development of EHM.

**In this study we found that some neuropathogenic markers and other amino acid substitutions were associated with viral replication under febrile conditions in cell culture. These observations led to our hypothesis that viral replication at elevated temperatures is the cause of EHM. Although our results are not definitive, we feel they are significant and worth reporting.**

The presentation is somewhat confusing and difficult to follow. Including a summary table outlining

the identified mutations, their locations within DNA polymerase (UL30), and their association with EHM would greatly improve clarity for readers.

**We greatly expanded Table 7, adding rows for the K+N and D SNPs, and columns for the domain locations of each SNP, whether they are linked to EHM and remarks. The revised table should help readers to quickly integrate the genotype–phenotype relationships across the study.**

**We also made changes to three other parts that some readers might find confusing and difficult to follow:**

**Table 4 may have been unclear. Virus names were changed to reflect the SNPs and derived strain names in the case of BAC viruses. "N752" was added to the SNPs in the table to reflect differences between Ab4p and the other viruses. Columns for gB have repeated data and were replaced with a sentence in the text. The percentages of mEGFP-positive cells at 48 hpi are added to make the data more quantitative.**

**Table S2 was not in standard table form and had much repeated data. The table was thoroughly revised to remove the repeated data and standardize the numerical data.**

**A molecular epidemiologic association analysis of UL30 amino-acid variants associated with EHM in EHV-1 (lines 277-300): This section was thoroughly revised to make it easier to read.**

Here are my additional comments:

1) Figure 1: Did the authors assess cell viability at febrile temperatures?

**No, but no overt temperature-dependent cytotoxicity was observed in uninfected FHK Tcl3.1 monolayers under the incubation conditions used for the plaque assays.**

Please also provide viral titers (PFU/mL) at different temperatures for the three recombinant viruses used in this figure, in addition to the fold change relative to 37 {degree sign}C shown in Table 1.

**Done.**

2) Table 2: A qualitative assessment may overlook isolates with low levels of replication.

**We agree that a CPE-based screen could miss very low-level replication. Accordingly, Table 2 is intended to serve as a screening classification ("replication detectable" vs "no detectable replication" at 39.5°C under our assay conditions), rather than a quantitative comparison. Importantly, our main conclusions are supported by controlled recombinant and chimeric BAC experiments in a common genetic background, which reproduced the temperature-dependent phenotypes under standardized conditions.**

3) Repaired UL30 D752 virus: Does this virus have an identical genome sequence to the parental strain, or are additional mutations present?

**We do not know. We added the following clarification to line 488 (Materials and Methods section) of the manuscript (lines 483-489):**

**"Although we did not sequence the whole genomes of Ab4p UL30 N752 BAC and Ab4p UL30 D752 Repair BAC virus, we used Sanger sequencing to confirm that the UL30 locus in the repaired virus was identical to that of the parental strain. The possibility of additional mutations elsewhere in the genome cannot be excluded, although a restriction fragment length polymorphism (RFLP) analysis using EcoRI, HindIII, and SalI did not show any major insertions or deletions in the genomic sequence of the constructed virus (31)."**

4) Quantification of the immunofluorescence images is needed across biological replicates.

**Quantitative immunofluorescence data have now been added to Table 4 by reporting the percentage of mEGFP-positive PBMCs at 48 h post-inoculation for each BAC-derived virus at 37.0°C and 39.5°C, together with qualitative gB positivity as confirmation of infection.**
**A sentence was added to the Methods to describe the quantification (line 569):**

**"For BAC-derived viruses, the percentage of mEGFP-positive PBMCs at 48 h post-inoculation was recorded as a semiquantitative indicator of infection (Table 4)."**

5) Line 164: The D752 isolate that did not replicate at 39.5 {degree sign}C: did it harbor additional mutations in UL30?

**No. To clarify, we added the underlined words to the sentence (line 168):**

**"The remaining 40 isolates consisted of 39 N752 isolates and one D752 isolate (06-I-80), none of which exhibited detectable replication at 39.5°C or harbored additional SNPs in UL30."**

6) Please clarify what is meant by "cell-to-cell transfer." The authors state (Line 218) that PBMCs are not capable of de novo virus production. How, then, is the virus transferred from PBMCs to FHCK cells? Are the authors suggesting cell-cell fusion?

**No, we are not suggesting cell-cell fusion. Although infectious virus was not detectably released into PBMC supernatants, PBMCs when in direct contact with FHCK cells can transfer the virus to the FHCK cells. This is what we mean by cell-to-cell transfer. (line 230)**

7) Figure 7: Please include phase-contrast images showing CPE for all viruses used to infect EVECs.

**Done. This should facilitate direct comparison across viruses.**
**Also, we found some errors in the description of Fig. 7 in lines 251-252 of the original manuscript:**

**"At 37.0°C, virus replication accompanied by CPE was detected in EVEC cultures inoculated with all viruses by 24 h post-inoculation (Fig. 7)."**

**To correct the errors, the sentence was changed to**

**"At 39.5°C, CPE clusters became evident by 48 h post-inoculation in EVECs inoculated with viruses that replicated at elevated temperatures in FHKCs (Fig. 7A), whereas no comparable clustering was observed up to 72 h in EVECs inoculated with viruses that failed to replicate at elevated temperatures in FHKCs (Fig. 7B)." (lines 256-260)**

8) Lines 262-265: Which chimeric BAC virus is being referred to here?

**As stated in the sentence, it is Ab4p UL30 06-I-70 BAC.**

Minor point:

1) Figure 8: Please clearly label which graphs correspond to 37 {degree sign}C and 39.5 {degree

sign}C.

**Done.**

Re: Spectrum03055-25R1 (Equine herpesvirus myeloencephalopathy-associated mutations in equine herpesvirus 1 DNA polymerase confer the ability to replicate at elevated temperatures)

Dear Prof. Hideto Fukushi:

I have read the revised manuscript and authors' responses to the both reviewers. I found that authors have addressed all the concerns properly. I suggest the manuscript be accepted.

Your manuscript has been accepted, and I am forwarding it to the ASM production staff for publication. Your paper will first be checked to make sure all elements meet the technical requirements. ASM staff will contact you if anything needs to be revised before copyediting and production can begin. Otherwise, you will be notified when your proofs are ready to be viewed.

**Data Availability:** ASM policy requires that data be available to the public upon online posting of the article, so please verify all links to sequence records, if present, and make sure that each number retrieves the full record of the data. If a new accession number is not linked or a link is broken, provide production staff with the correct URL for the record. If the accession numbers for new data are not publicly accessible before the expected online posting of the article, publication may be delayed; please contact ASM production staff immediately with the expected release date.

**Publication Fees:** For information on publication fees and which article types have charges, please visit our website. We have partnered with Copyright Clearance Center (CCC) to collect author charges. If fees apply to your paper, you will receive a message from no-reply@copyright.com with further instructions. For questions related to paying charges through RightsLink, please contact CCC at ASM_Support@copyright.com or toll free at +1-877-622-5543. CCC makes every attempt to respond to all emails within 24 hours.

**ASM Membership:** Corresponding authors may join or renew ASM membership to obtain discounts on publication fees. Need to upgrade your membership level? Please contact Customer Service at Service@asmusa.org.

**Embargo Policy:** A press release may be issued as soon as the manuscript is posted on the Spectrum Latest Articles webpage. The corresponding author will receive an email with the subject line "ASM Journals Author Services Notification" when the article is available online.

The ASM Journals program strives for constant improvement in our submission and publication process. Please tell us how we can improve your experience by taking this quick Author Survey.

Thank you for submitting your paper to Spectrum.

Sincerely,
Qiyi Tang
Editor
Microbiology Spectrum
