# Supplementary material for: Equine herpesvirus myeloencephalopathy-associated mutations in equine herpesvirus 1 DNA polymerase confer the ability to replicate at elevated temperatures
Source: Microbiol Spectr. 2026 Aug 21;14(10):e03055-25. doi: 10.1128/spectrum.03055-25 (PMC13689140; doi:10.1128/spectrum.03055-25)
Supplement: Supplementary file 2 [file spectrum.03055-25-s0001.docx]

```
ul30_epi_pipeline.py

#!/usr/bin/env python3

ul30_epi_pipeline.py

Reproducible pipeline for:
1) Mapping UL30 amino-acid sequences to Ab4p reference coordinates (AY665713)
2) Extracting UL30 window (positions 151–1107) and building a complete-coverage "common-sites" alignment
3) Deduplicating identical sequences (haplotypes)
4) Assigning UL30 categories including N752-E990 / N752-K990 (ASCII labels)
5) Summarizing EHM rates by category
6) Testing EHM enrichment for variant groups vs N752-E990 background (Fisher + BH-FDR)
7) (Optional) Running IQ-TREE on deduplicated alignment

Requires: biopython, pandas, scipy. IQ-TREE optional.

Example:
  python3 ul30_epi_pipeline.py \
    --ref Ab4p_UL30_ref.aa.fasta \
    --full UL30_full145.aa.fasta \
    --part UL30_part194.aa.fasta \
    --meta EHV1_ORF30_accession_clin_sign01.CLEAN.tsv \
    --outprefix UL30_243

Outputs (prefix = UL30_243 by default):
  UL30_243_mapped151_1107.fasta
  UL30_243_commonSites.fasta
  UL30_243_commonSites.mask.tsv
  UL30_243_commonSites.dedup.fasta
  UL30_243_commonSites.dedup.map.tsv
  UL30_243_category_with_N752E990K990.tsv
  UL30_243_category_EHM_rate.tsv
  UL30_243_variant_vs_N752E990_Fisher_BH.tsv
  UL30_243_Table7.tsv
"""

from __future__ import annotations

import argparse
import csv
import subprocess
from collections import Counter, OrderedDict
from typing import Dict, List, Tuple

import pandas as pd
from Bio import SeqIO
from Bio import pairwise2
from Bio.Seq import Seq
from Bio.SeqRecord import SeqRecord
from scipy.stats import fisher_exact


AAS = set("ACDEFGHIKLMNPQRSTVWY")
START = 151
END = 1107
WIN_LEN = END - START + 1  # 957


def strip_version(acc: str) -> str:
    return acc.split(".", 1)[0]


def load_clin_sign(meta_tsv: str) -> Dict[str, str]:
    m: Dict[str, str] = {}
    with open(meta_tsv, newline="") as f:
        r = csv.DictReader(f, delimiter="	")
        for row in r:
            acc = (row.get("accession") or "").strip()
            cs = (row.get("clin_sign") or "").strip()
            if acc and cs:
                m[acc] = cs
                m[strip_version(acc)] = cs
    return m


def md5(path: str, chunk: int = 1024 * 1024) -> str:
    import hashlib
    h = hashlib.md5()
    with open(path, "rb") as fh:
        while True:
            b = fh.read(chunk)
            if not b:
                break
            h.update(b)
    return h.hexdigest()


def map_to_ref_window(aln_ref: str, aln_q: str) -> str:
    out: List[str] = []
    refpos = 0
    for rch, qch in zip(aln_ref, aln_q):
        if rch != "-":
            refpos += 1
            if START <= refpos <= END:
                out.append(qch if qch in AAS else "-")
    if len(out) < WIN_LEN:
        out.extend(["-"] * (WIN_LEN - len(out)))
    return "".join(out[:WIN_LEN])


def build_mapped_alignment(ref_fa: str, full_fa: str, part_fa: str, meta_tsv: str, out_mapped: str) -> List[SeqRecord]:
    clin = load_clin_sign(meta_tsv)
    ref = str(next(SeqIO.parse(ref_fa, "fasta")).seq).upper()

    seqs: List[Tuple[str, str]] = []
    for fn in (full_fa, part_fa):
        for rec in SeqIO.parse(fn, "fasta"):
            acc = rec.id
            cs = clin.get(acc) or clin.get(strip_version(acc))
            if not cs:
                continue
            s = str(rec.seq).upper().replace("*", "")
            if len(s) < 200:
                continue
            seqs.append((acc, s))

    mapped: List[SeqRecord] = []
    for acc, s in seqs:
        aln = pairwise2.align.globalms(ref, s, 2, -1, -5, -0.5, one_alignment_only=True)[0]
        win = map_to_ref_window(aln.seqA, aln.seqB)
        mapped.append(SeqRecord(Seq(win), id=acc, description=""))

    SeqIO.write(mapped, out_mapped, "fasta")
    return mapped


def build_common_sites(mapped: List[SeqRecord], out_common: str, out_mask: str) -> List[SeqRecord]:
    cols_keep: List[int] = []
    for j in range(WIN_LEN):
        if all(str(rec.seq)[j] != "-" for rec in mapped):
            cols_keep.append(j)

    common: List[SeqRecord] = []
    for rec in mapped:
        s = str(rec.seq)
        seq = "".join(s[j] for j in cols_keep)
        common.append(SeqRecord(Seq(seq), id=rec.id, description=""))

    SeqIO.write(common, out_common, "fasta")

    with open(out_mask, "w") as f:
        f.write("ref_pos\\twindow_index0\\n")
        for j in cols_keep:
            f.write(f"{START + j}\\t{j}\\n")

    return common


def dedup_alignment(in_fa: str, out_fa: str, out_map: str) -> Tuple[int, int]:
    seq_to_rep: "OrderedDict[str, str]" = OrderedDict()
    seq_to_members: "OrderedDict[str, List[str]]" = OrderedDict()
    n_in = 0

    for rec in SeqIO.parse(in_fa, "fasta"):
        n_in += 1
        s = str(rec.seq)
        if s not in seq_to_rep:
            seq_to_rep[s] = rec.id
            seq_to_members[s] = [rec.id]
        else:
            seq_to_members[s].append(rec.id)

    recs = [SeqRecord(Seq(s), id=rep, description=f"dedup_n={len(seq_to_members[s])}")
            for s, rep in seq_to_rep.items()]
    SeqIO.write(recs, out_fa, "fasta")

    with open(out_map, "w", newline="") as f:
        w = csv.writer(f, delimiter="	")
        w.writerow(["rep", "count", "members"])
        for s, rep in seq_to_rep.items():
            mem = seq_to_members[s]
            w.writerow([rep, len(mem), ",".join(mem)])

    return n_in, len(recs)


def assign_categories(full_fa: str, part_fa: str, meta_tsv: str, out_tsv: str) -> pd.DataFrame:
    clin = load_clin_sign(meta_tsv)

    markers = [("D752", 752, "D"),
               ("R250", 250, "R"),
               ("I291", 291, "I"),
               ("A742", 742, "A"),
               ("G760", 760, "G")]

    rows = []
    for fn in (full_fa, part_fa):
        for rec in SeqIO.parse(fn, "fasta"):
            acc = rec.id
            cs = clin.get(acc) or clin.get(strip_version(acc))
            if not cs:
                continue
            s = str(rec.seq).replace("*", "").upper()
            if len(s) < 990:
                continue

            hits = []
            for name, pos, alt in markers:
                if len(s) >= pos and s[pos - 1] == alt:
                    hits.append(name)

            if len(hits) == 0:
                aa990 = s[990 - 1]
                if aa990 == "E":
                    cat = "N752-E990"
                elif aa990 == "K":
                    cat = "N752-K990"
                else:
                    cat = f"N752-{aa990}"
            elif len(hits) == 1:
                cat = hits[0]
            else:
                cat = "MULTI:" + "+".join(hits)

            rows.append((acc, cs, cat))

    df = pd.DataFrame(rows, columns=["accession", "clin_sign", "UL30_category"])
    df.to_csv(out_tsv, sep="\t", index=False)
    return df


def ehm_rate_table(df: pd.DataFrame, out_tsv: str) -> pd.DataFrame:
    df = df.copy()
    df["cat_base"] = df["UL30_category"].str.split(":", n=1).str[0]
    g = df.groupby("cat_base").apply(lambda x: pd.Series({
        "total": len(x),
        "EHM": int((x["clin_sign"] == "EHM").sum()),
    }))
    g["nonEHM"] = g["total"] - g["EHM"]
    g["EHM_rate"] = g["EHM"] / g["total"]
    g = g.reset_index().rename(columns={"cat_base": "category"})

    order = ["N752-E990", "N752-K990", "D752", "I291", "A742", "R250", "G760", "MULTI"]
    g["order"] = g["category"].apply(lambda c: order.index(c) if c in order else 999)
    g = g.sort_values(["order", "category"]).drop(columns=["order"])

    g.to_csv(out_tsv, sep="\t", index=False)
    return g


def bh_fdr(pvals: List[float]) -> List[float]:
    m = len(pvals)
    order = sorted(range(m), key=lambda i: pvals[i])
    q = [0.0] * m
    prev = 1.0
    for rank, _ in enumerate(reversed(order), start=1):
        i = m - rank
        pv = pvals[order[i]]
        qv = pv * m / (i + 1)
        if qv > prev:
            qv = prev
        prev = qv
        q[order[i]] = qv
    return q


def fisher_vs_background(df: pd.DataFrame, out_tsv: str) -> pd.DataFrame:
    df = df.copy()
    df["cat_base"] = df["UL30_category"].str.split(":", n=1).str[0]
    df["is_ehm"] = (df["clin_sign"] == "EHM")

    variants = ["D752", "R250", "I291", "A742", "G760"]
    bg_groups = {
        "N752_all": {"N752-E990", "N752-K990"},
        "N752-E990": {"N752-E990"},
        "N752-K990": {"N752-K990"},
    }

    rows = []
    for bg_name, bg_set in bg_groups.items():
        for vg in variants:
            sub = df[df["cat_base"].isin({vg, *bg_set})]
            a = int(((sub["cat_base"] == vg) & (sub["is_ehm"])).sum())
            b = int(((sub["cat_base"] == vg) & (~sub["is_ehm"])).sum())
            c = int(((sub["cat_base"] != vg) & (sub["is_ehm"])).sum())
            d = int(((sub["cat_base"] != vg) & (~sub["is_ehm"])).sum())
            OR = ((a + 0.5) * (d + 0.5)) / ((b + 0.5) * (c + 0.5))
            p = float(fisher_exact([[a, b], [c, d]], alternative="two-sided")[1])
            rows.append([vg, bg_name, a, b, c, d, OR, p])

    res = pd.DataFrame(rows, columns=[
        "variant_group", "background_group",
        "EHM_in_group", "nonEHM_in_group",
        "EHM_in_background", "nonEHM_in_background",
        "OR_Haldane", "p"
    ])

    res["q_BH"] = 0.0
    for bg_name in res["background_group"].unique():
        sub = res[res["background_group"] == bg_name].copy()
        q = bh_fdr(sub["p"].tolist())
        res.loc[sub.index, "q_BH"] = q

    res = res.sort_values(["background_group", "p"])
    res.to_csv(out_tsv, sep="\t", index=False)
    return res


def make_table7(df: pd.DataFrame, fisher_df: pd.DataFrame, out_tsv: str) -> pd.DataFrame:
    df2 = df.copy()
    df2["cat_base"] = df2["UL30_category"].str.split(":", n=1).str[0]
    totals = df2.groupby("cat_base").apply(lambda x: pd.Series({
        "EHM": int((x["clin_sign"] == "EHM").sum()),
        "total": len(x)
    })).reset_index().rename(columns={"cat_base": "UL30_category"})

    def fmt_rate(e, t):
        return f"{e}/{t} ({(100*e/t):.1f}%)"

    baseline = totals[totals["UL30_category"] == "N752-E990"].iloc[0]
    baseline_str = f"{baseline['EHM']}/{baseline['total']}"

    rows = []
    rows.append({
        "UL30 category": "N752-E990",
        "EHM/Total (EHM%)": fmt_rate(int(baseline["EHM"]), int(baseline["total"])),
        "Reference background (N752-E990) EHM/Total": "—",
        "OR (Haldane)": "—",
        "p": "—",
        "q (BH-FDR)": "—",
    })

    nk = totals[totals["UL30_category"] == "N752-K990"]
    if len(nk):
        nk = nk.iloc[0]
        rows.append({
            "UL30 category": "N752-K990",
            "EHM/Total (EHM%)": fmt_rate(int(nk["EHM"]), int(nk["total"])),
            "Reference background (N752-E990) EHM/Total": baseline_str,
            "OR (Haldane)": "—",
            "p": "—",
            "q (BH-FDR)": "—",
        })

    sub = fisher_df[fisher_df["background_group"] == "N752-E990"].copy()
    order = ["D752", "I291", "A742", "R250", "G760"]
    sub["variant_group"] = pd.Categorical(sub["variant_group"], categories=order, ordered=True)
    sub = sub.sort_values("variant_group")

    for _, r in sub.iterrows():
        vg = str(r["variant_group"])
        tot = totals[totals["UL30_category"] == vg]
        if len(tot) == 0:
            continue
        tot = tot.iloc[0]
        rows.append({
            "UL30 category": vg,
            "EHM/Total (EHM%)": fmt_rate(int(tot["EHM"]), int(tot["total"])),
            "Reference background (N752-E990) EHM/Total": baseline_str,
            "OR (Haldane)": f"{float(r['OR_Haldane']):.2f}",
            "p": f"{float(r['p']):.3g}",
            "q (BH-FDR)": f"{float(r['q_BH']):.3g}",
        })

    out = pd.DataFrame(rows)
    out.to_csv(out_tsv, sep="\t", index=False)
    return out


def run_iqtree(aln_fa: str, threads: str = "AUTO", boots: int = 1000) -> None:
    cmd = ["iqtree", "-s", aln_fa, "-m", "MFP", "-B", str(boots), "-T", threads]
    subprocess.check_call(cmd)


def main():
    ap = argparse.ArgumentParser()
    ap.add_argument("--ref", required=True, help="Reference UL30 AA fasta (Ab4p; AY665713).")
    ap.add_argument("--full", required=True, help="UL30 AA fasta from genome-derived set.")
    ap.add_argument("--part", required=True, help="UL30 AA fasta from ORF30-only set.")
    ap.add_argument("--meta", required=True, help="TSV with columns: accession, clin_sign.")
    ap.add_argument("--outprefix", default="UL30_243", help="Prefix for outputs.")
    ap.add_argument("--run-iqtree", action="store_true", help="Run IQ-TREE on deduplicated alignment.")
    ap.add_argument("--iqtree-threads", default="AUTO")
    ap.add_argument("--iqtree-boots", type=int, default=1000)
    args = ap.parse_args()

    out_mapped = f"{args.outprefix}_mapped151_1107.fasta"
    out_common = f"{args.outprefix}_commonSites.fasta"
    out_mask = f"{args.outprefix}_commonSites.mask.tsv"
    out_dedup = f"{args.outprefix}_commonSites.dedup.fasta"
    out_dedup_map = f"{args.outprefix}_commonSites.dedup.map.tsv"
    out_cat = f"{args.outprefix}_category_with_N752E990K990.tsv"
    out_rate = f"{args.outprefix}_category_EHM_rate.tsv"
    out_fisher = f"{args.outprefix}_variant_vs_N752E990_Fisher_BH.tsv"
    out_table7 = f"{args.outprefix}_Table7.tsv"

    print("[1] Mapping sequences to reference window...")
    mapped = build_mapped_alignment(args.ref, args.full, args.part, args.meta, out_mapped)
    print("  wrote:", out_mapped, "n=", len(mapped), "md5=", md5(out_mapped))

    print("[2] Building common-sites alignment...")
    common = build_common_sites(mapped, out_common, out_mask)
    print("  wrote:", out_common, "len=", len(common[0].seq) if common else 0, "md5=", md5(out_common))
    print("  wrote:", out_mask, "md5=", md5(out_mask))

    print("[3] Deduplicating alignment...")
    n_in, n_dedup = dedup_alignment(out_common, out_dedup, out_dedup_map)
    print(f"  original n={n_in} dedup n={n_dedup}")
    print("  wrote:", out_dedup, "md5=", md5(out_dedup))
    print("  wrote:", out_dedup_map, "md5=", md5(out_dedup_map))

    if args.run_iqtree:
        print("[4] Running IQ-TREE on deduplicated alignment...")
        run_iqtree(out_dedup, threads=args.iqtree_threads, boots=args.iqtree_boots)
        print("  IQ-TREE done. treefile:", out_dedup + ".treefile")

    print("[5] Assigning categories (ASCII labels: N752-E990/N752-K990)...")
    df_cat = assign_categories(args.full, args.part, args.meta, out_cat)
    print("  wrote:", out_cat, "rows=", len(df_cat), "md5=", md5(out_cat))
    print("  category counts:", Counter(df_cat["UL30_category"].str.split(":", n=1).str[0]).most_common(10))

    print("[6] Summarizing EHM rates by category...")
    df_rate = ehm_rate_table(df_cat, out_rate)
    print("  wrote:", out_rate, "md5=", md5(out_rate))

    print("[7] Fisher tests (variant groups vs background groups)...")
    df_fisher = fisher_vs_background(df_cat, out_fisher)
    print("  wrote:", out_fisher, "md5=", md5(out_fisher))

    print("[8] Creating Table 7 TSV...")
    df_t7 = make_table7(df_cat, df_fisher, out_table7)
    print("  wrote:", out_table7, "md5=", md5(out_table7))

    print("Done.")


if __name__ == "__main__":
    main()
```

```
extract_orf30_by_commonsites_match.py

#!/usr/bin/env python3

from Bio import SeqIO, pairwise2
from Bio.Seq import Seq
from Bio.SeqRecord import SeqRecord
import csv
from pathlib import Path

AA_FASTA = "UL30_clin02_labeled_rerun_commonSites.fasta"
GB_FILE = "commonSites_records.gb"
REF_AA_FASTA = "Ab4p_UL30_ref.aa.fasta"
MASK_FILE = "UL30_clin02_labeled_rerun_commonSites.mask.tsv"

OUT_NT = "ORF30_nt_matched_by_commonsites.fasta"
OUT_REPORT = "ORF30_nt_matched_by_commonsites.report.tsv"
OUT_FAILED = "ORF30_nt_matched_by_commonsites.failed.txt"


def normalize_aa(seq: str) -> str:
    return seq.upper().replace("-", "").replace("*", "").strip()


def load_mask(mask_path):
    """
    The uploaded mask file is escaped as one long line containing literal \\t and \\n.
    Decode it and return ordered ref_pos list.
    """
    raw = Path(mask_path).read_text().strip()

    # if file contains literal escapes, decode them
    if "\\t" in raw or "\\n" in raw:
        decoded = raw.encode("utf-8").decode("unicode_escape")
    else:
        decoded = raw

    lines = [x for x in decoded.strip().splitlines() if x.strip()]
    header = lines[0].split("\t")
    assert header[0] == "ref_pos", f"Unexpected mask header: {header}"

    ref_positions = []
    for line in lines[1:]:
        cols = line.split("\t")
        ref_pos = int(cols[0])
        ref_positions.append(ref_pos)
    return ref_positions


def project_to_ref_positions(ref_seq, query_seq, ref_positions):
    """
    Align query AA to ref AA and project query residues onto requested ref positions.
    Returns projected AA string of len(ref_positions), with '-' for uncovered positions.
    """
    aln = pairwise2.align.globalms(ref_seq, query_seq, 2, -1, -10, -0.5, one_alignment_only=True)[0]
    ref_aln, qry_aln, score, start, end = aln

    ref_index = 0  # 1-based reference coordinate after increment
    refpos_to_qaa = {}

    for r, q in zip(ref_aln, qry_aln):
        if r != "-":
            ref_index += 1
            if q != "-":
                refpos_to_qaa[ref_index] = q
            else:
                refpos_to_qaa[ref_index] = "-"

    proj = []
    for rp in ref_positions:
        proj.append(refpos_to_qaa.get(rp, "-"))
    return "".join(proj)


# 1) load truth AA common-sites sequences
aa_truth = {rec.id: normalize_aa(str(rec.seq)) for rec in SeqIO.parse(AA_FASTA, "fasta")}
truth_len_set = sorted(set(len(v) for v in aa_truth.values()))
print("Truth common-sites lengths:", truth_len_set)

# 2) load reference AA
ref_records = list(SeqIO.parse(REF_AA_FASTA, "fasta"))
assert len(ref_records) == 1, "Reference AA FASTA should contain exactly one sequence."
ref_aa = normalize_aa(str(ref_records[0].seq))

# 3) load mask ref positions
mask_ref_positions = load_mask(MASK_FILE)
print("Mask positions:", len(mask_ref_positions), "first=", mask_ref_positions[:5], "last=", mask_ref_positions[-5:])

# 4) load GenBank records
gb_records = {rec.id: rec for rec in SeqIO.parse(GB_FILE, "genbank")}

matched = []
report_rows = []
failed = []

for acc, truth_common in aa_truth.items():
    if acc not in gb_records:
        failed.append((acc, "genbank_record_not_found"))
        continue

    rec = gb_records[acc]
    candidates = []

    for feat in rec.features:
        if feat.type != "CDS":
            continue
        try:
            nt_seq = feat.extract(rec.seq)
        except Exception:
            continue

        nt_str = str(nt_seq).upper().replace("-", "")
        if len(nt_str) < 3 or len(nt_str) % 3 != 0:
            continue

        try:
            aa_full = normalize_aa(str(Seq(nt_str).translate(to_stop=False)))
        except Exception:
            continue

        projected_common = project_to_ref_positions(ref_aa, aa_full, mask_ref_positions)
        projected_common_norm = normalize_aa(projected_common)

        if projected_common_norm == truth_common:
            qualifiers = feat.qualifiers
            gene = ";".join(qualifiers.get("gene", []))
            product = ";".join(qualifiers.get("product", []))
            note = ";".join(qualifiers.get("note", []))

            candidates.append({
                "nt": nt_str,
                "aa_full_len": len(aa_full),
                "gene": gene,
                "product": product,
                "note": note,
            })

    if len(candidates) == 0:
        failed.append((acc, "no_commonsites_translation_match"))
        continue

    # Prefer longest nucleotide CDS if multiple matches
    candidates.sort(key=lambda x: -len(x["nt"]))
    best = candidates[0]

    matched.append(SeqRecord(Seq(best["nt"]), id=acc, description=""))
    report_rows.append({
        "Accession": acc,
        "NT_len": len(best["nt"]),
        "AA_full_len": best["aa_full_len"],
        "gene": best["gene"],
        "product": best["product"],
        "note": best["note"],
    })

# 5) write outputs
SeqIO.write(matched, OUT_NT, "fasta")

with open(OUT_REPORT, "w", newline="", encoding="utf-8") as f:
    w = csv.DictWriter(f,
                       fieldnames=["Accession", "NT_len", "AA_full_len", "gene", "product", "note"],
                       delimiter="\t")
    w.writeheader()
    w.writerows(report_rows)

with open(OUT_FAILED, "w", encoding="utf-8") as f:
    for acc, reason in failed:
        f.write(f"{acc}\t{reason}\n")

print(f"Matched: {len(matched)}")
print(f"Failed: {len(failed)}")
print(f"Wrote: {OUT_NT}")
print(f"Wrote: {OUT_REPORT}")
print(f"Wrote: {OUT_FAILED}")
```

```
ul30_clin02_E990K990_marker_assoc.py

#!/usr/bin/env python3
# -*- coding: utf-8 -*-

import pandas as pd
from Bio import SeqIO
from collections import defaultdict
from math import isnan
from scipy.stats import fisher_exact

META = "EHV1_ORF30_accession_clin_sign02.CLEAN.LABELED.tsv"
FA   = "UL30_clin02_labeled_rerun_mapped151_1107.fasta"

# mapped window is Ab4p coords 151–1107 (length=957)
WIN_START = 151

# markers you requested: target residue at Ab4p position
TARGETS = {
    "R250": (250, "R"),
    "I291": (291, "I"),
    "H625": (625, "H"),
    "K683": (683, "K"),
    "A742": (742, "A"),
    "N752": (752, "N"),
    "H752": (752, "H"),
    "D752": (752, "D"),
    "G760": (760, "G"),
    "V946": (946, "V"),
}

STRAT_POS = 990  # E990 / K990

def haldane_or(a,b,c,d):
    # a=EHM carrier, b=nonEHM carrier, c=EHM noncarrier, d=nonEHM noncarrier
    a+=0.5; b+=0.5; c+=0.5; d+=0.5
    return (a*d)/(b*c)

def bh_fdr(pvals):
    # returns q-values (BH) in original order
    m=len(pvals)
    order=sorted(range(m), key=lambda i: pvals[i])
    q=[None]*m
    prev=1.0
    for rank, i in enumerate(order, start=1):
        val=pvals[i]*m/rank
        if val>prev: val=prev
        prev=val
        q[i]=min(val,1.0)
    return q

# load meta
meta = pd.read_csv(META, sep="\t")
meta["accession"]=meta["accession"].astype(str).str.strip()
meta["clin_sign"]=meta["clin_sign"].astype(str).str.strip()

def is_ehm(x):
    return x.upper()=="EHM"

# load sequences
seqs={}
for r in SeqIO.parse(FA,"fasta"):
    acc=r.id.strip()
    seqs[acc]=str(r.seq)

# merge
rows=[]
for _,r in meta.iterrows():
    acc=r["accession"]
    if acc not in seqs:
        continue
    seq=seqs[acc]
    # residue at 990 for stratification
    idx = STRAT_POS - WIN_START
    if idx<0 or idx>=len(seq):
        continue
    aa990=seq[idx]
    if aa990 not in ("E","K"):
        # keep but mark other
        group="other_990"
    else:
        group=f"{aa990}990"
    rows.append((acc, r["clin_sign"], group, seq))

df = pd.DataFrame(rows, columns=["accession","clin_sign","grp990","seq"])
print("used n=", len(df))
print(df["grp990"].value_counts())

# function to compute per-group association
def assoc_for_group(subdf, label):
    out=[]
    for name,(pos,target) in TARGETS.items():
        i = pos - WIN_START
        if i<0 or i>=len(subdf.iloc[0]["seq"]):
            continue
        carriers = subdf[subdf["seq"].str[i]==target]
        noncar  = subdf[subdf["seq"].str[i]!=target]
        a = carriers["clin_sign"].apply(is_ehm).sum()
        b = len(carriers) - a
        c = noncar["clin_sign"].apply(is_ehm).sum()
        d = len(noncar) - c
        # Fisher
        p = fisher_exact([[a,b],[c,d]], alternative="two-sided")[1]
        OR = haldane_or(a,b,c,d)
        out.append({
            "grp990": label,
            "marker": name,
            "pos": pos,
            "target_AA": target,
            "carriers": len(carriers),
            "total": len(subdf),
            "carrier_rate": len(carriers)/len(subdf) if len(subdf)>0 else float("nan"),
            "EHM_carrier": int(a),
            "nonEHM_carrier": int(b),
            "EHM_noncarrier": int(c),
            "nonEHM_noncarrier": int(d),
            "OR_Haldane": OR,
            "p_Fisher": p,
        })
    # BH within this stratum
    ps=[x["p_Fisher"] for x in out]
    qs=bh_fdr(ps) if ps else []
    for x,q in zip(out,qs):
        x["q_BH_within_grp"]=q
    return out

res=[]
for grp in ["E990","K990","other_990"]:
    sub=df[df["grp990"]==grp]
    if len(sub)==0:
        continue
    res += assoc_for_group(sub, grp)

outdf=pd.DataFrame(res)
outdf["carrier_rate"]=outdf["carrier_rate"].map(lambda x: f"{x:.4f}")
outdf["OR_Haldane"]=outdf["OR_Haldane"].map(lambda x: f"{x:.6g}")
outdf["p_Fisher"]=outdf["p_Fisher"].map(lambda x: f"{x:.6g}")
outdf["q_BH_within_grp"]=outdf["q_BH_within_grp"].map(lambda x: f"{x:.6g}")

OUT="UL30_clin02_E990K990_marker_counts_and_assoc.tsv"
outdf.to_csv(OUT, sep="\t", index=False)
print("wrote", OUT)
```

```
nt_tree_aa_gain_parsimony.py

#!/usr/bin/env python3

import argparse
import csv
from collections import Counter
from typing import Dict, List, Set, Optional
from Bio import Phylo, SeqIO

AA_ALPHABET = set("ACDEFGHIKLMNPQRSTVWY")

CODON_TABLE = {
    "TTT":"F","TTC":"F","TTA":"L","TTG":"L",
    "TCT":"S","TCC":"S","TCA":"S","TCG":"S",
    "TAT":"Y","TAC":"Y","TAA":"*","TAG":"*",
    "TGT":"C","TGC":"C","TGA":"*","TGG":"W",
    "CTT":"L","CTC":"L","CTA":"L","CTG":"L",
    "CCT":"P","CCC":"P","CCA":"P","CCG":"P",
    "CAT":"H","CAC":"H","CAA":"Q","CAG":"Q",
    "CGT":"R","CGC":"R","CGA":"R","CGG":"R",
    "ATT":"I","ATC":"I","ATA":"I","ATG":"M",
    "ACT":"T","ACC":"T","ACA":"T","ACG":"T",
    "AAT":"N","AAC":"N","AAA":"K","AAG":"K",
    "AGT":"S","AGC":"S","AGA":"R","AGG":"R",
    "GTT":"V","GTC":"V","GTA":"V","GTG":"V",
    "GCT":"A","GCC":"A","GCA":"A","GCG":"A",
    "GAT":"D","GAC":"D","GAA":"E","GAG":"E",
    "GGT":"G","GGC":"G","GGA":"G","GGG":"G"
}

def parse_positions(items: List[str]) -> Dict[int, str]:
    out = {}
    for it in items:
        pos_s, aa = it.split(":")
        out[int(pos_s)] = aa.upper()
    return out

def parse_root_fixed(items: List[str]) -> Dict[int, str]:
    out = {}
    for it in items:
        pos_s, aa = it.split(":")
        out[int(pos_s)] = aa.upper()
    return out

def load_alignment(path: str) -> Dict[str, str]:
    seqs = {}
    for r in SeqIO.parse(path, "fasta"):
        seqs[r.id] = str(r.seq).upper()
    if not seqs:
        raise ValueError("No sequences found.")
    lengths = {len(v) for v in seqs.values()}
    if len(lengths) != 1:
        raise ValueError(f"Alignment lengths differ: {sorted(lengths)}")
    aln_len = next(iter(lengths))
    if aln_len % 3 != 0:
        raise ValueError(f"Alignment length {aln_len} is not divisible by 3.")
    return seqs

def translate_codon(codon: str) -> Optional[str]:
    codon = codon.upper().replace("U", "T")
    if len(codon) != 3:
        return None
    if any(b not in "ACGT" for b in codon):
        return None
    aa = CODON_TABLE.get(codon)
    if aa is None or aa == "*":
        return None
    return aa

def fitch_bottom_up(root, seed_sets):
    state_sets = dict(seed_sets)
    min_changes = 0

    def postorder(node):
        nonlocal min_changes
        for ch in node.clades:
            postorder(ch)
        if node.is_terminal():
            return
        child_sets = [state_sets[ch] for ch in node.clades]
        inter = set.intersection(*child_sets)
        if inter:
            state_sets[node] = inter
        else:
            state_sets[node] = set.union(*child_sets)
            min_changes += 1

    postorder(root)
    return state_sets, min_changes

def assign_top_down_states(root, state_sets, root_fixed=None):
    assigned = {}
    if root_fixed is not None:
        assigned[root] = root_fixed
    else:
        assigned[root] = next(iter(state_sets[root]))

    def preorder(node):
        pstate = assigned[node]
        for ch in node.clades:
            if pstate in state_sets[ch]:
                assigned[ch] = pstate
            else:
                assigned[ch] = next(iter(state_sets[ch]))
            preorder(ch)

    preorder(root)
    return assigned

def count_target_gains(root, assigned, target):
    gains = 0
    def walk(node):
        nonlocal gains
        for ch in node.clades:
            if assigned[node] != target and assigned[ch] == target:
                gains += 1
            walk(ch)
    walk(root)
    return gains

def main():
    ap = argparse.ArgumentParser()
    ap.add_argument("--aln", required=True, help="Codon alignment FASTA")
    ap.add_argument("--tree", required=True, help="Treefile from nucleotide/codon phylogeny")
    ap.add_argument("--positions", nargs="+", required=True, help="e.g. 291:I 752:D 990:K")
    ap.add_argument("--aa_start", type=int, default=1,
                    help="Amino-acid coordinate of the first codon in the alignment. "
                         "Use 1 for full ORF30 CDS, or 151 if alignment starts at aa151.")
    ap.add_argument("--root_fixed", nargs="*", default=[],
                    help="Optional root constraints, e.g. 990:E")
    ap.add_argument("--out_tsv", default=None)
    args = ap.parse_args()

    seqs = load_alignment(args.aln)
    tree = Phylo.read(args.tree, "newick")

    tips = [t.name for t in tree.get_terminals()]
    missing = [t for t in tips if t not in seqs]
    if missing:
        raise SystemExit(f"Tips missing from alignment: {missing[:10]}")

    pos2target = parse_positions(args.positions)
    root_fixed_map = parse_root_fixed(args.root_fixed)

    print(f"tips_in_tree:\t{len(tips)}")
    print(f"seqs_in_aln:\t{len(seqs)}")
    print(f"nt_aln_len:\t{len(next(iter(seqs.values())))}\n")
    print("aa_pos\ttarget\tmin_changes\tmin_target_gains\troot_fixed\troot_assigned\ttip_counts")

    rows = []
    root = tree.root

    for aa_pos, target in pos2target.items():
        codon_index0 = aa_pos - args.aa_start
        nt_start = codon_index0 * 3
        nt_end = nt_start + 3

        seed_sets = {}
        counts = Counter()

        for tip in tree.get_terminals():
            codon = seqs[tip.name][nt_start:nt_end]
            aa = translate_codon(codon)
            if aa is None:
                seed_sets[tip] = set(AA_ALPHABET)
            else:
                seed_sets[tip] = {aa}
                counts[aa] += 1

        state_sets, min_changes = fitch_bottom_up(root, seed_sets)
        rf = root_fixed_map.get(aa_pos)
        assigned = assign_top_down_states(root, state_sets, root_fixed=rf)
        min_gains = count_target_gains(root, assigned, target)

        root_fixed_flag = "yes" if rf else "no"
        root_assigned = assigned[root]
        tip_counts = "; ".join(f"{k}:{v}" for k, v in sorted(counts.items()))

        print(f"{aa_pos}\t{target}\t{min_changes}\t{min_gains}\t{root_fixed_flag}\t{root_assigned}\t{{{tip_counts}}}")

        rows.append({
            "UL30_position_Ab4p": aa_pos,
            "Target_amino_acid": target,
            "Tip_counts": tip_counts,
            "Min_changes_Fitch_on_nt_tree": min_changes,
            "Min_independent_gains_to_target_on_nt_tree": min_gains,
            "Root_constraint": f"root fixed to {rf}" if rf else "none"
        })

    if args.out_tsv:
        with open(args.out_tsv, "w", newline="", encoding="utf-8") as f:
            w = csv.DictWriter(
                f,
                fieldnames=[
                    "UL30_position_Ab4p",
                    "Target_amino_acid",
                    "Tip_counts",
                    "Min_changes_Fitch_on_nt_tree",
                    "Min_independent_gains_to_target_on_nt_tree",
                    "Root_constraint",
                ],
                delimiter="\t"
            )
            w.writeheader()
            w.writerows(rows)
        print(f"\nWrote TSV:\t{args.out_tsv}")

if __name__ == "__main__":
    main()
```

```
fetch_genbank_from_commonsites.py

#!/usr/bin/env python3
from Bio import Entrez, SeqIO
import time
import sys

Entrez.email = "your_email@example.com"
# Entrez.api_key = "YOUR_NCBI_API_KEY"

aa_fasta = "UL30_clin02_labeled_rerun_commonSites.fasta"
out_gb = "commonSites_records.gb"

accs = [rec.id for rec in SeqIO.parse(aa_fasta, "fasta")]
accs = list(dict.fromkeys(accs))

print(f"n_accessions = {len(accs)}", file=sys.stderr)

batch_size = 50
with open(out_gb, "w") as out:
    for i in range(0, len(accs), batch_size):
        batch = accs[i:i+batch_size]
        print(f"Fetching {i+1}-{i+len(batch)} / {len(accs)}", file=sys.stderr)
        handle = Entrez.efetch(
            db="nuccore",
            id=",".join(batch),
            rettype="gb",
            retmode="text"
        )
        out.write(handle.read())
        handle.close()
        time.sleep(0.4)

print(f"Wrote {out_gb}", file=sys.stderr)
```
