# Supplementary material for: Equine herpesvirus myeloencephalopathy-associated mutations in equine herpesvirus 1 DNA polymerase confer the ability to replicate at elevated temperatures
Source: Microbiol Spectr. 2026 Aug 21;14(10):e03055-25. doi: 10.1128/spectrum.03055-25 (PMC13689140; doi:10.1128/spectrum.03055-25)
Supplement: Supplementary file 3 [file spectrum.03055-25-s0002.docx]

```
EHV1_ORF30_accession_clin_sign02.FIXED.tsv

accession	clin_sign	name	year	Note
AB992258.1	abortion	HH1	1967	Japan
AY464052.1	abortion	V592	1985	UK
AY665713.1	EHM	Ab4p	1980	UK
KC924759.1		Fr_101		France
KC924760.1		Fr_104		France
KC924761.1		Fr_14242	2006	France
KC924762.1		Fr_14659	2006	France
KC924763.1		Fr_14664	2006	France
KC924764.1		Fr_15768	2006	France
KC924765.1		Fr_16086	2006	France
KC924766.1		Fr_17186	2006	France
KC924767.1		Fr_20111	2006	France
KC924768.1		Fr_22936	2005	France
KC924769.1		Fr_29500	2005	France
KC924770.1		Fr_3134	2006	France
KC924771.1		Fr_3730	2006	France
KC924772.1		Fr_3989	2006	France
KC924773.1		Fr_41		France
KC924774.1		Fr_41663	2005	France
KC924775.1		Fr_4263	2005	France
KC924776.1		Fr_44724	2006	France
KC924777.1		Fr_51		France
KC924778.1		Fr_5666	2006	France
KC924779.1		Fr_98		France
KC924780.1		Bella_CSU	2011	USA
KC924781.1		Rosie_CSU	2011	USA
KC924783.1	EHM	Ab4	1980	UK
KC924784.1	EHM	Ca_Brain	2007	USA
KC924785.1		Ca_liver	2007	USA
KC924786.1		Ca_lung	2008	USA, zebra
KC924787.1	abortion	Ca_Placent	2007	USA
KC924788.1		DE_81	2008	USA
KC924789.1		IL_16	2008	USA
KC924790.1		MD_19	2008	USA
KC924791.1		NJ_40	2008	USA
KC924792.1		NJ_42	2008	USA
KC924793.1		RHVac		vaccine
KC924794.1	abortion	T186	1974	USA
KC924795.1	abortion	T188	1974	USA
KC924796.1	abortion	T220	1976	USA
KC924797.1	abortion	T262	1975	USA
KC924798.1	EHM	T313	1979	USA
KC924799.1	abortion	T480	1983	USA
KC924800.1	abortion	T493		USA
KC924801.1	EHM	T510	1983	USA
KC924802.1	abortion	T547		USA
KC924803.1	abortion	T572		USA
KC924804.1	abortion	T61	1981	USA
KC924805.1	abortion	T637	1997	USA
KC924806.1	EHM	T672	1986	USA
KC924807.1	abortion	T677	1986	USA
KC924808.1	abortion	T75	1979	USA
KC924809.1	EHM	T935	1999	USA
KC924810.1	EHM	T946	2001	USA
KC924812.1	abortion	T954		USA
KC924813.1	abortion	T955		USA
KC924814.1	abortion	T956		USA
KC924815.1	abortion	T964		USA
KC924816.1	abortion	T967		USA
KC924817.1	abortion	T970		USA
KC924818.1		TX_06	2006	USA
KC924820.1		VR_2003		USA
KC924821.1		VR_2229		USA
KC924822.1		VR_700		USA
KF434369.1	abortion	438/77	1977	Australia
KF434370.1	abortion	838/89	1989	Australia
KF434371.1	abortion	970A/90	1990	Australia
KF434372.1	abortion	1026A/93	1993	Australia
KF434373.1	abortion	1029/93	1993	Australia
KF434374.1	abortion	1053A/94	1994	Australia
KF434375.1	abortion	1623/99	1999	Australia
KF434376.1	EHM	1756/01	2001	Australia
KF434377.1	abortion	1805/01	2001	Australia
KF434378.1	abortion	1985/02	2002	Australia
KF434379.1	abortion	2222/03	2003	Australia
KF434380.1	abortion	2371/04	2004	Australia
KF434381.1	abortion	3040/07	2007	Australia
KF434382.1	EHM	3144/10	2010	Australia
KF515985.1	abortion	G433	1999	Australia
KF515986.1	abortion	G058	2012	Australia
KF644566.1	respiratory	90C16	1990
KF644567.1	EHM	FL06	2006	USA
KF644568.1	EHM	NMKT04	2004	UK
KF644569.1	EHM	NY03	2003	USA
KF644570.1	EHM	NY05	2005	USA
KF644572.1	EHM	VA02	2002	USA
KF644576.1	respiratory	00c19	2000
KF644577.1	EHM	89C105	1989	Japan
KF644578.1	EHM	01C1	2001	Japan
KF644579.1	EHM	89c25	1989	Japan
KM593996.1	EHM	T953	2003	USA
KT324724.1	abortion	NZA77	1977
KT324725.1	abortion	3045-07	2007
KT324726.1	EHM	3038-07	2007
KT324728.1	abortion	2019-02	2002
KT324729.1	abortion	1966-02	2002
KT324730.1	abortion	1074-94	1994
KT324733.1	EHM	717A-82	1982
KU206404.1	abortion	Nottinghamshire/10/2004	2004	UK
KU206405.1	abortion	Shropshire/167/2006	2006	UK
KU206406.1	abortion	Cheshire/275/2010	2010	UK
KU206407.1	abortion	Shropshire/38/1996	1996	UK
KU206408.1	abortion	Shropshire/68/1989	1989	UK
KU206409.1	respiratory	Suffolk/82/1994	1994	UK
KU206410.1	abortion	Essex/199/2005	2005	UK
KU206411.1	abortion	Essex/200/2005	2005	UK
KU206412.1	abortion	Lincolnshire/10/1996	1996	UK
KU206413.1	abortion	Leicestershire/13/2000	2000	UK
KU206414.1	abortion	United Kingdom/58/1995	1995	UK
KU206415.1	ocular	United Kingdom/106/1985	1985	UK
KU206416.1	abortion	Lincolnshire/13/1996	1996	UK
KU206417.1	abortion	Suffolk/16/1996	1996	UK
KU206418.1	abortion	Yorkshire/1/1993	1993	UK
KU206419.1	abortion	Hampshire/77/2009	2009	UK
KU206420.1	abortion	Nottinghamshire/70/2004	2004	UK
KU206421.1	abortion	Wiltshire/40/1996	1996	UK
KU206422.1	abortion	Suffolk/60/1996	1996	UK
KU206423.1	abortion	Leicestershire/59/1996	1996	UK
KU206424.1	abortion	Bristol/55/2004	2004	UK
KU206425.1	abortion	Suffolk/48/2013	2013	UK
KU206426.1	abortion	Suffolk/125/2013	2013	UK
KU206427.1	abortion	Norfolk/124/2010	2010	UK
KU206428.1	abortion	Buckinghamshire/9/1993	1993	UK
KU206429.1	abortion	Derbyshire/39/2002	2002	UK
KU206430.1	respiratory	Suffolk/48/2005	2005	UK
KU206431.1	abortion	Buckinghamshire/24/2012	2012	UK
KU206432.1	abortion	Cambridgeshire/3/1995	1995	UK
KU206433.1	abortion	Buckinghamshire/114/2010	2010	UK
KU206434.1	abortion	United Kingdom/109/1994	1994	UK
KU206435.1	abortion	Kent/177/1991	1991	UK
KU206436.1	abortion	Staffordshire/80/1989	1989	UK
KU206437.1	abortion	Yorkshire/12/1990	1990	UK
KU206438.1	abortion	Berkshire/64/2009	2009	UK
KU206439.1	abortion	Cambridgeshire/96/2013	2013	UK
KU206440.1	respiratory	Devon/28/2003	2003	UK
KU206441.1	abortion	Suffolk/82/2013	2013	UK
KU206442.1	abortion	Suffolk/89/2013	2013	UK
KU206443.1	abortion	Suffolk/87/2009	2009	UK
KU206444.1	EHM	United Kingdom/58/2003	2003	UK
KU206445.1	EHM	Gloucestershire/127/1998	1998	UK
KU206446.1	EHM	Gloucestershire/77/2013	2013	UK
KU206447.1	EHM	Gloucestershire/54/2013	2013	UK
KU206448.1	EHM	Gloucestershire/70/2013	2013	UK
KU206449.1	EHM	Gloucestershire/108/2013	2013	UK
KU206450.1	EHM	Gloucestershire/114/2013	2013	UK
KU206451.1	abortion	Bristol/2/1993	1993	UK
KU206452.1	abortion	Suffolk/45/2013	2013	UK
KU206453.1	abortion	Kent/43/1994	1994	UK
KU206454.1	EHM	Oxfordshire/27/2011	2011	UK
KU206455.1	abortion	Buckinghamshire/93/2011	2011	UK
KU206456.1	abortion	Oxfordshire/31/2011	2011	UK
KU206457.1	abortion	Oxfordshire/34/2011	2011	UK
KU206458.1	abortion	Suffolk/41/2013	2013	UK
KU206459.1	abortion	Suffolk/73/1985	1985	UK
KU206460.1	abortion	Suffolk/110/1994	1994	UK
KU206461.1	abortion	Aberdeenshire/84/2013	2013	UK
KU206462.1	abortion	Hampshire/1/2008	2008	UK
KU206463.1	abortion	Berkshire/7/1996	1996	UK
KU206464.1	EHM	Leicestershire/22/1996	1996	UK
KU206465.1	abortion	United Kingdom/32/1982	1982	UK
KU206466.1	EHM	Hertfordshire/188/2010	2010	UK
KU206467.1	respiratory	Hong Kong/57/1984	1984	UK
KU206468.1	abortion	Ab1		UK
KU206469.1	EHM	Devonshire/97/2012	2012	UK
KU206470.1	EHM	Oxfordshire/206/2013	2013	UK
KU206471.1	EHM	Oxfordshire/207/2013	2013	UK
KU206472.1	abortion	Essex/81/1994	1994	UK
KU206473.1	abortion	Yorkshire/114/1999	1999	UK
KU206474.1	abortion	Suffolk/10/2012	2012	UK
KU206475.1	abortion	Lincolnshire/2/2012	2012	UK
KU206476.1	abortion	Hertfordshire/109/2009	2009	UK
KU206477.1	respiratory	Army 183	1941	USA
KU206479.1	abortion	Suffolk/91/94	1994	UK
KU206480.1	abortion	Suffolk/123/2005	2005	UK
KY852346.1	abortion	Hertfordshire/150/2016	2016	UK
LC109623.1	abortion	F1	1978	Japan
LC109624.1	abortion	F4	1983	Japan
LC109625.1	abortion	F7	1987	Japan
LC109626.1	abortion	01-I-241	2001	Japan
LC109627.1	abortion	02-I-136	2002	Japan
LC109628.1	abortion	02-I-152	2002	Japan
LC109629.1	abortion	03-I-104	2003	Japan
LC109630.1	abortion	03-I-107	2003	Japan
LC109631.1	abortion	03-I-124	2003	Japan
LC109632.1	abortion	04-I-68	2004	Japan
LC109633.1	abortion	04-I-70	2004	Japan
LC109634.1	abortion	04-I-77	2004	Japan
LC109635.1	abortion	04-I-89	2004	Japan
LC109636.1	abortion	05-I-239	2005	Japan
LC109637.1	abortion	05-I-255	2005	Japan
LC109638.1	abortion	05-I-257	2005	Japan
LC109639.1	abortion	05-I-261	2005	Japan
LC109640.1	abortion	06-I-24	2006	Japan
LC109641.1	abortion	06-I-70	2006	Japan
LC109642.1	abortion	06-I-76	2006	Japan
LC109643.1	abortion	06-I-80	2006	Japan
LC109644.1	abortion	07-I-100	2007	Japan
LC109645.1	abortion	07-I-114	2007	Japan
LC109646.1	abortion	08-I-793	2008	Japan
LC109647.1	abortion	08-I-797	2008	Japan
LC109648.1	abortion	08-I-812	2008	Japan
LC109649.1	abortion	09-I-474	2009	Japan
LC109650.1	abortion	09-I-478	2009	Japan
LC109651.1	respiratory	09-I-743	2009	Japan
LC109652.1	respiratory	10-I-224	2010	Japan
LC109653.1	abortion	10-I-713	2010	Japan
LC109654.1	abortion	11-I-475	2011	Japan
LC109655.1	abortion	11-I-487	2011	Japan
LC109656.1	abortion	12-I-306	2012	Japan
LC109657.1	abortion	12-I-324	2012	Japan
LC109658.1	abortion	12-I-330	2012	Japan
LC109659.1	abortion	12-I-336	2012	Japan
LC109660.1	abortion	12-I-344	2012	Japan
LC109662.1	abortion	KyA	1954	USA
LC109663.1	abortion	KyD	1962	USA
LC834900.1	abortion	3292	1993	JAPAN
LC834901.1	abortion	4370	1995	JAPAN
LC834902.1	abortion	5030	2000	JAPAN
LC834903.1	abortion	6383	2005	JAPAN
LC834904.1	abortion	6786	2006	JAPAN
LC834905.1	EHM	7759	2010	JAPAN
LC834906.1	EHM	7855	2010	JAPAN
LC834907.1	abortion	7872	2010	JAPAN
LC834908.1	abortion	8343	2012	JAPAN
LC834909.1	abortion	8394	2012	JAPAN
LC834910.1	abortion	9204	2014	JAPAN
LC834911.1	abortion	12222	2018	JAPAN
LC834912.1	abortion	12693	2018	JAPAN
LC834913.1	abortion	13757	2021	JAPAN
MF975656.1	abortion	RacL11	1965	Poland
MN688617.1	abortion	NORM/2/2010	2010	France
MN688618.1	abortion	ILEDEFR/3/2012	2012	France
MN688619.1	abortion	NORM/4/2012	2012	France
MN688620.1	abortion	NORM/5/2012	2012	France
MN688621.1	abortion	NORM/6/2013	2013	France
MN688622.1	abortion	NORM/8/2015	2015	France
MN688623.1		NELLEAQU/9/2015	2015	France
MN688624.1	abortion	BELG/10/2016	2016	Belgium
MN688625.1	abortion	BELG/12/2017	2017	Belgium
MN688626.1	respiratory	ILEDEFR/14/2018	2018	France
MN688627.1	abortion	RHONEALPES/16/2018	2018	France
MN688628.1	abortion	NORM/17/2018	2018	France
MN688629.1	abortion	NORM/18/2018	2018	France
MN688630.1		BRE/20/2018	2018	France
MN912433.1	abortion	Hisar-14	2014	India
MT063054.1	abortion	YM2019	2019	China
MT077857.1	abortion	Meerut/2014	2014	India
MT968035.1	EHM	FR-56628	2018	France
MW396756.1	abortion	Delhi-3/2007	2007	India
MW396757.1	abortion	Tohana-2/2013	2013	India
MW396758.1	neonatal_mortality	Hisar-9/2014	2014	India
MW396759.1	abortion	Hisar-1/2017	2017	India
MW396760.1	abortion	Hisar-1/2018	2018	India
MW855958.1	EHM	BE/21P40	2021	Valencia
MW855959.1	EHM	BE/21P41	2021	Valencia
MW855960.1	EHM	BE/21P43	2021	Valencia
MW855961.1	EHM	FR/Valencia1	2021	Valencia
MW855962.1	EHM	FR/Valencia2	2021	Valencia
MZ318700.1	EHM	IRL/626	2009	Ireland
MZ318701.1	EHM	IRL/348	2002	Ireland
MZ318702.1	EHM	IRL/867	2006	Ireland
MZ318703.1	EHM	IRL/394	2009	Ireland
MZ318704.1	EHM	IRL/774	2019	Ireland
MZ318705.1	EHM	IRL/372	2013	Ireland
MZ318706.1	EHM	IRL/009	2017	Ireland
MZ357402.1	EHM	H3_Allg_92_21/CH	2021	Valencia
OR085496	abortion	LS050627
OR085497	abortion	LS080124
OR085498	abortion	LS100106
OR085499	abortion	LS100106	2011
OR085500	abortion	LS100106	2011
OR085501	abortion	LS100106	2011
OR085502	abortion	LS111457	2011
OR085503	abortion	LS111457	2011
OR085504	abortion	LS119764	2011
OR085505	abortion	LS130922	2013
OR085506	abortion	LS140310
OR085507	abortion	LS140325
OR085508	abortion	LS143101
OR085509	abortion	LS161221
OR085510	abortion	LS161227
OR085511	abortion	LS170109
OR085512	abortion	LS170307
OR085513	abortion	LS180416
OR085514	abortion	LS213627	2021
OR085515	EHM	LS0433182
OR085516	abortion	LS9719959
OR085517	abortion	LS9816616
OR085518	abortion	LS9922376
OR801338.1	EHM	ZS-29	2021	China
PP084325.1	abortion	E/745/99	1999	Argentina, close to NY03
PP084326.1	abortion	E/1297/07	2007	Argentina, NY03
PP481413.1	abortion	126764	2023	Italy
PP856396.1	abortion	97P70	1997	Belgium
PP856397.1	EHM	03P37	2003	Belgium
PQ630625.1	respiratory	KY17UT30	2017	USA
PQ630626.1	abortion	KY21UT70	2021	USA
PQ630627.1	abortion	KY23UT75	2023	USA
PQ630628.1	abortion	KY24UT16	2024	USA
PQ630629.1	abortion	KY24UT26	2024	USA
PQ630630.1	abortion	KY24UT38	2024	USA
PQ630631.1	respiratory	KY25UT61	2024	USA
```

```
Supplementary_Table_S2_UL30_Fisher_LABELED_clin02.tsv

variant_group	background_group	EHM_in_group	nonEHM_in_group	EHM_in_background	nonEHM_in_background	OR_Haldane	p	q_BH
A742	N752-E990	4	1	4	160	107.0	1.0465339307192209e-05	1.744223217865368e-05
D752	N752-E990	25	30	4	160	29.81967213114754	7.607400594378709e-14	3.8037002971893553e-13
G760	N752-E990	2	1	4	160	59.44444444444444	0.0031940500160686	0.0031940500160686
I291	N752-E990	6	1	4	160	154.55555555555554	4.529652384526396e-08	1.1324130961315993e-07
R250	N752-E990	4	2	4	160	64.2	3.006739998873596e-05	3.758424998591995e-05
A742	N752-K990	4	1	8	13	4.764705882352941	0.1478260869565217	0.3695652173913044
D752	N752-K990	25	30	8	13	1.3278688524590163	0.6133145515235107	0.6133145515235107
G760	N752-K990	2	1	8	13	2.6470588235294117	0.5503952569169961	0.6133145515235107
I291	N752-K990	6	1	8	13	6.882352941176471	0.0768115942028985	0.3695652173913044
R250	N752-K990	4	2	8	13	2.8588235294117648	0.357487922705314	0.5958132045088568
A742	N752_all	4	1	12	173	41.64	0.0001640678207949	0.0002734463679915
D752	N752_all	25	30	12	173	11.604590163934429	1.7166618297267775e-10	8.583309148633889e-10
G760	N752_all	2	1	12	173	23.133333333333333	0.0148627866945118	0.0148627866945118
I291	N752_all	6	1	12	173	60.14666666666667	1.9091795179450236e-06	4.772948794862559e-06
R250	N752_all	4	2	12	173	24.984	0.0004572359858154	0.0005715449822693
```

```
Table8_UL30_enrichment_vs_N752E990_LABELED_clin02.tsv

UL30 category	EHM/Total (EHM%)	Reference background (N752–E990) EHM/Total	OR (Haldane)	p	q (BH-FDR)
N752-E990	4/164 (2.4%)	—		—	—	—
N752-K990	8/21 (38.1%)	4/164	—	—	—
D752	25/55 (45.5%)	4/164	29.819672	7.607e-14	3.804e-13
I291	6/7 (85.7%)	4/164	154.555556	4.530e-08	1.132e-07
A742	4/5 (80.0%)	4/164	107	1.047e-05	1.744e-05
R250	4/6 (66.7%)	4/164	64.2	3.007e-05	3.758e-05
G760	2/3 (66.7%)	4/164	59.444444	0.003194	0.003194
MULTI	0/5 (0.0%)	4/164	—	—	—
```

```
UL30_clin02_E990K990_marker_counts_and_assoc.tsv

grp990	marker	pos	target_AA	carriers	total	carrier_rate	EHM_carrier	nonEHM_carrier	EHM_noncarrier	nonEHM_noncarrier	OR_Haldane	p_Fisher	q_BH_within_grp
E990	R250	250	R	11	222	0.0495	4	7	40	171	2.54074	0.234596	6.53582e-06
E990	I291	291	I	7	222	0.0315	6	1	38	177	19.9784	0.000270185	6.53582e-06
E990	H625	625	H	1	222	0.0045	0	1	44	177	1.32959	1	6.53582e-06
E990	K683	683	K	2	222	0.0090	0	2	44	176	0.793258	1	6.53582e-06
E990	A742	742	A	5	222	0.0225	4	1	40	177	13.1481	0.00587988	6.53582e-06
E990	N752	752	N	164	222	0.7387	19	145	25	33	0.176066	1.30716e-06	1.30716e-05
E990	H752	752	H	0	222	0.0000	0	0	44	178	4.01124	1	6.53582e-06
E990	D752	752	D	58	222	0.2613	25	33	19	145	5.67968	1.30716e-06	6.53582e-06
E990	G760	760	G	2	222	0.0090	1	1	43	177	4.08046	0.357833	6.53582e-06
E990	V946	946	V	1	222	0.0045	0	1	44	177	1.32959	1	6.53582e-06
K990	R250	250	R	0	21	0.0000	0	0	8	13	1.58824	1	1
K990	I291	291	I	0	21	0.0000	0	0	8	13	1.58824	1	1
K990	H625	625	H	0	21	0.0000	0	0	8	13	1.58824	1	1
K990	K683	683	K	0	21	0.0000	0	0	8	13	1.58824	1	1
K990	A742	742	A	0	21	0.0000	0	0	8	13	1.58824	1	1
K990	N752	752	N	19	21	0.9048	7	12	1	1	0.6	1	1
K990	H752	752	H	1	21	0.0476	1	0	7	13	5.4	0.380952	1
K990	D752	752	D	1	21	0.0476	0	1	8	12	0.490196	1	1
K990	G760	760	G	0	21	0.0000	0	0	8	13	1.58824	1	1
K990	V946	946	V	0	21	0.0000	0	0	8	13	1.58824	1	1
```

```
UL30_clin02_labeled_rerun_category_with_N752E990K990.tsv

accession	clin_sign	UL30_category
AY464052.1	abortion	N752-K990
AY665713.1	EHM	D752
KF644566.1	respiratory	N752-E990
KF644567.1	EHM	D752
KF644568.1	EHM	D752
KF644569.1	EHM	N752-E990
KF644570.1	EHM	D752
KF644572.1	EHM	G760
KF644576.1	respiratory	N752-E990
KF644577.1	EHM	A742
KF644578.1	EHM	D752
KF644579.1	EHM	A742
AB992258.1	abortion	N752-E990
KM593996.1	EHM	D752
KT324724.1	abortion	N752-E990
KT324725.1	abortion	N752-E990
KT324726.1	EHM	N752-E990
KT324728.1	abortion	N752-E990
KT324729.1	abortion	N752-E990
KT324730.1	abortion	N752-E990
KT324733.1	EHM	N752-E990
MF975656.1	abortion	MULTI:D752+R250
MN912433.1	abortion	N752-E990
MT077857.1	abortion	N752-E990
MT063054.1	abortion	N752-E990
MW855958.1	EHM	I291
MW855959.1	EHM	I291
MW855960.1	EHM	I291
MW855961.1	EHM	I291
MW855962.1	EHM	I291
MZ357402.1	EHM	I291
MZ318700.1	EHM	N752-K990
MZ318701.1	EHM	N752-K990
MZ318702.1	EHM	N752-K990
MZ318703.1	EHM	N752-K990
MZ318704.1	EHM	N752-K990
MZ318705.1	EHM	N752-K990
MZ318706.1	EHM	N752-K990
OR085496.1	abortion	D752
OR085497.1	abortion	N752-E990
OR085498.1	abortion	N752-E990
OR085499.1	abortion	N752-E990
OR085500.1	abortion	N752-E990
OR085501.1	abortion	N752-E990
OR085502.1	abortion	N752-E990
OR085503.1	abortion	N752-E990
OR085504.1	abortion	N752-E990
OR085505.1	abortion	N752-E990
OR085506.1	abortion	N752-E990
OR085507.1	abortion	N752-E990
OR085508.1	abortion	N752-E990
OR085509.1	abortion	N752-E990
OR085510.1	abortion	N752-E990
OR085511.1	abortion	N752-E990
OR085512.1	abortion	N752-E990
OR085513.1	abortion	N752-E990
OR085514.1	abortion	N752-E990
OR085515.1	EHM	G760
OR085516.1	abortion	N752-K990
OR085517.1	abortion	N752-E990
OR085518.1	abortion	N752-E990
PP481413.1	abortion	D752
OR801338.1	EHM	D752
PP084325.1	abortion	N752-E990
PP084326.1	abortion	N752-E990
LC109623.1	abortion	N752-E990
LC109624.1	abortion	N752-E990
LC109625.1	abortion	N752-E990
LC109626.1	abortion	N752-E990
LC109627.1	abortion	N752-E990
LC109628.1	abortion	N752-E990
LC109629.1	abortion	N752-E990
LC109630.1	abortion	N752-E990
LC109631.1	abortion	N752-E990
LC109632.1	abortion	N752-E990
LC109633.1	abortion	N752-E990
LC109634.1	abortion	N752-E990
LC109635.1	abortion	N752-E990
LC109636.1	abortion	N752-E990
LC109637.1	abortion	N752-E990
LC109638.1	abortion	N752-E990
LC109639.1	abortion	N752-E990
LC109640.1	abortion	N752-E990
LC109641.1	abortion	N752-E990
LC109642.1	abortion	N752-E990
LC109643.1	abortion	D752
LC109644.1	abortion	N752-E990
LC109645.1	abortion	N752-E990
LC109646.1	abortion	N752-E990
LC109647.1	abortion	N752-E990
LC109648.1	abortion	N752-E990
LC109649.1	abortion	N752-E990
LC109650.1	abortion	N752-E990
LC109651.1	respiratory	N752-E990
LC109652.1	respiratory	N752-E990
LC109653.1	abortion	N752-E990
LC109654.1	abortion	N752-E990
LC109655.1	abortion	N752-E990
LC109656.1	abortion	N752-E990
LC109657.1	abortion	N752-E990
LC109658.1	abortion	N752-E990
LC109659.1	abortion	N752-E990
LC109660.1	abortion	N752-E990
LC109662.1	abortion	MULTI:D752+R250
LC109663.1	abortion	MULTI:D752+R250
PP856396.1	abortion	N752-E990
PQ630625.1	respiratory	N752-E990
PQ630626.1	abortion	N752-E990
PQ630627.1	abortion	D752
PQ630628.1	abortion	N752-E990
PQ630629.1	abortion	N752-E990
PQ630630.1	abortion	N752-E990
PQ630631.1	respiratory	N752-E990
KF434369.1	abortion	N752-E990
KF434370.1	abortion	N752-K990
KF434371.1	abortion	N752-E990
KF434372.1	abortion	N752-E990
KF434373.1	abortion	N752-E990
KF434374.1	abortion	N752-E990
KF434375.1	abortion	N752-E990
KF434376.1	EHM	D752
KF434377.1	abortion	D752
KF434378.1	abortion	N752-E990
KF434379.1	abortion	R250
KF434380.1	abortion	N752-E990
KF434381.1	abortion	N752-E990
KF434382.1	EHM	A742
KF515985.1	abortion	N752-E990
KF515986.1	abortion	D752
KC924782.1	abortion	D752
KC924783.1	EHM	D752
KC924784.1	EHM	N752-E990
KC924787.1	abortion	D752
KC924794.1	abortion	MULTI:D752+R250
KC924795.1	abortion	MULTI:D752+R250
KC924796.1	abortion	N752-E990
KC924797.1	abortion	N752-E990
KC924798.1	EHM	D752
KC924799.1	abortion	N752-E990
KC924800.1	abortion	N752-E990
KC924801.1	EHM	D752
KC924802.1	abortion	N752-E990
KC924803.1	abortion	N752-E990
KC924804.1	abortion	N752-E990
KC924805.1	abortion	N752-E990
KC924806.1	EHM	D752
KC924807.1	abortion	N752-E990
KC924808.1	abortion	N752-E990
KC924809.1	EHM	D752
KC924810.1	EHM	D752
KC924812.1	abortion	D752
KC924813.1	abortion	D752
KC924814.1	abortion	D752
KC924815.1	abortion	D752
KC924816.1	abortion	D752
KC924817.1	abortion	D752
KU206404.1	abortion	N752-E990
KU206405.1	abortion	N752-E990
KU206406.1	abortion	N752-E990
KU206407.1	abortion	N752-E990
KU206408.1	abortion	N752-E990
KU206409.1	respiratory	N752-E990
KU206410.1	abortion	N752-E990
KU206411.1	abortion	N752-E990
KU206412.1	abortion	N752-E990
KU206413.1	abortion	N752-E990
KU206414.1	abortion	N752-E990
KU206415.1	ocular	N752-E990
KU206416.1	abortion	N752-E990
KU206417.1	abortion	N752-E990
KU206418.1	abortion	N752-E990
KU206419.1	abortion	N752-E990
KU206420.1	abortion	N752-E990
KU206421.1	abortion	N752-E990
KU206422.1	abortion	N752-E990
KU206423.1	abortion	N752-E990
KU206424.1	abortion	N752-E990
KU206425.1	abortion	N752-E990
KU206426.1	abortion	N752-E990
KU206427.1	abortion	N752-E990
KU206428.1	abortion	N752-E990
KU206429.1	abortion	N752-E990
KU206430.1	respiratory	N752-E990
KU206431.1	abortion	N752-E990
KU206432.1	abortion	N752-E990
KU206433.1	abortion	N752-E990
KU206434.1	abortion	N752-E990
KU206435.1	abortion	N752-E990
KU206436.1	abortion	N752-E990
KU206437.1	abortion	N752-E990
KU206438.1	abortion	N752-E990
KU206439.1	abortion	N752-K990
KU206440.1	respiratory	D752
KU206441.1	abortion	N752-K990
KU206442.1	abortion	N752-K990
KU206443.1	abortion	N752-K990
KU206444.1	EHM	D752
KU206445.1	EHM	D752
KU206446.1	EHM	D752
KU206447.1	EHM	D752
KU206448.1	EHM	D752
KU206449.1	EHM	D752
KU206450.1	EHM	D752
KU206451.1	abortion	N752-K990
KU206452.1	abortion	N752-K990
KU206453.1	abortion	D752
KU206454.1	EHM	D752
KU206455.1	abortion	D752
KU206456.1	abortion	D752
KU206457.1	abortion	D752
KU206458.1	abortion	D752
KU206459.1	abortion	D752
KU206460.1	abortion	D752
KU206461.1	abortion	N752-E990
KU206462.1	abortion	N752-E990
KU206463.1	abortion	N752-E990
KU206464.1	EHM	D752
KU206465.1	abortion	D752
KU206466.1	EHM	D752
KU206467.1	respiratory	N752-E990
KU206468.1	abortion	D752
KU206469.1	EHM	R250
KU206470.1	EHM	R250
KU206471.1	EHM	R250
KU206472.1	abortion	N752-E990
KU206473.1	abortion	D752
KU206474.1	abortion	N752-E990
KU206475.1	abortion	N752-E990
KU206476.1	abortion	N752-E990
KU206477.1	abortion	D752
KU206479.1	abortion	D752
KU206480.1	abortion	N752-E990
KY852346.1	abortion	D752
MN688617.1	abortion	N752-K990
MN688618.1	abortion	N752-E990
MN688619.1	abortion	D752
MN688620.1	abortion	N752-E990
MN688621.1	abortion	N752-E990
MN688622.1	abortion	N752-E990
MN688624.1	abortion	N752-E990
MN688625.1	abortion	N752-K990
MN688626.1	respiratory	D752
MN688627.1	abortion	N752-E990
MN688628.1	abortion	N752-K990
MN688629.1	abortion	N752-K990
MT968035.1	EHM	N752-K990
MW396756.1	abortion	N752-E990
MW396757.1	abortion	N752-E990
MW396758.1	neonatal_mortality	N752-E990
MW396759.1	abortion	N752-E990
MW396760.1	abortion	R250
PP856397.1	EHM	D752
LC834900.1	abortion	N752-E990
LC834901.1	abortion	N752-E990
LC834902.1	abortion	N752-E990
LC834903.1	abortion	N752-E990
LC834904.1	abortion	N752-E990
LC834905.1	EHM	R250
LC834906.1	EHM	A742
LC834907.1	abortion	A742
LC834908.1	abortion	G760
LC834909.1	abortion	N752-E990
LC834910.1	abortion	I291
LC834911.1	abortion	N752-E990
LC834912.1	abortion	N752-E990
LC834913.1	abortion	N752-E990
```

```
UL30_clin02_labeled_rerun_commonSites.dedup.map.tsv

rep	count	members
AY464052.1	18	AY464052.1,MZ318700.1,MZ318701.1,MZ318702.1,MZ318703.1,MZ318704.1,MZ318705.1,MZ318706.1,OR085516.1,KU206439.1,KU206441.1,KU206442.1,KU206443.1,KU206451.1,MN688617.1,MN688625.1,MN688628.1,MN688629.1
AY665713.1	41	AY665713.1,KF644567.1,KF644570.1,KF644578.1,KM593996.1,OR085496.1,OR801338.1,LC109643.1,KF434377.1,KF515986.1,KC924782.1,KC924783.1,KC924798.1,KC924801.1,KC924806.1,KC924809.1,KC924810.1,KC924812.1,KC924813.1,KC924814.1,KC924815.1,KC924816.1,KC924817.1,KU206444.1,KU206445.1,KU206446.1,KU206447.1,KU206448.1,KU206449.1,KU206450.1,KU206458.1,KU206459.1,KU206464.1,KU206465.1,KU206466.1,KU206468.1,KU206473.1,KU206477.1,KU206479.1,MN688619.1,PP856397.1
KF644566.1	138	KF644566.1,KF644576.1,AB992258.1,KT324724.1,KT324726.1,KT324728.1,KT324729.1,KT324730.1,MT077857.1,OR085498.1,OR085499.1,OR085500.1,OR085501.1,OR085502.1,OR085503.1,OR085504.1,OR085505.1,OR085507.1,OR085509.1,OR085510.1,OR085511.1,OR085513.1,OR085514.1,OR085518.1,PP084325.1,PP084326.1,LC109623.1,LC109624.1,LC109625.1,LC109626.1,LC109627.1,LC109628.1,LC109629.1,LC109630.1,LC109631.1,LC109632.1,LC109633.1,LC109634.1,LC109635.1,LC109636.1,LC109637.1,LC109638.1,LC109639.1,LC109640.1,LC109642.1,LC109644.1,LC109645.1,LC109646.1,LC109647.1,LC109648.1,LC109649.1,LC109650.1,LC109651.1,LC109652.1,LC109653.1,LC109654.1,LC109655.1,LC109656.1,LC109657.1,LC109658.1,LC109659.1,LC109660.1,PP856396.1,PQ630626.1,PQ630628.1,PQ630630.1,PQ630631.1,KF434371.1,KF434373.1,KF434374.1,KF434380.1,KF515985.1,KC924784.1,KC924796.1,KC924797.1,KC924799.1,KC924800.1,KC924802.1,KC924804.1,KC924808.1,KU206404.1,KU206407.1,KU206408.1,KU206409.1,KU206410.1,KU206411.1,KU206412.1,KU206413.1,KU206414.1,KU206415.1,KU206416.1,KU206417.1,KU206418.1,KU206419.1,KU206420.1,KU206421.1,KU206422.1,KU206423.1,KU206424.1,KU206425.1,KU206426.1,KU206427.1,KU206428.1,KU206429.1,KU206430.1,KU206431.1,KU206432.1,KU206433.1,KU206434.1,KU206435.1,KU206436.1,KU206437.1,KU206438.1,KU206461.1,KU206462.1,KU206463.1,KU206467.1,KU206472.1,KU206474.1,KU206475.1,KU206476.1,KU206480.1,MN688618.1,MN688620.1,MN688622.1,MN688624.1,MN688627.1,MW396756.1,MW396757.1,MW396758.1,LC834900.1,LC834901.1,LC834902.1,LC834903.1,LC834904.1,LC834909.1,LC834911.1,LC834912.1
KF644568.1	7	KF644568.1,PP481413.1,KU206454.1,KU206455.1,KU206456.1,KU206457.1,MN688626.1
KF644569.1	2	KF644569.1,KC924805.1
KF644572.1	3	KF644572.1,OR085515.1,LC834908.1
KF644577.1	5	KF644577.1,KF644579.1,KF434382.1,LC834906.1,LC834907.1
KT324725.1	3	KT324725.1,KT324733.1,KF434369.1
MF975656.1	3	MF975656.1,LC109662.1,LC109663.1
MN912433.1	2	MN912433.1,MW396759.1
MT063054.1	1	MT063054.1
MW855958.1	6	MW855958.1,MW855959.1,MW855960.1,MW855961.1,MW855962.1,LC834910.1
MZ357402.1	1	MZ357402.1
OR085497.1	2	OR085497.1,OR085512.1
OR085506.1	3	OR085506.1,OR085508.1,MN688621.1
OR085517.1	2	OR085517.1,PQ630625.1
LC109641.1	1	LC109641.1
PQ630627.1	1	PQ630627.1
PQ630629.1	1	PQ630629.1
KF434370.1	1	KF434370.1
KF434372.1	1	KF434372.1
KF434375.1	1	KF434375.1
KF434376.1	1	KF434376.1
KF434378.1	1	KF434378.1
KF434379.1	5	KF434379.1,KU206469.1,KU206470.1,KU206471.1,LC834905.1
KF434381.1	1	KF434381.1
KC924787.1	1	KC924787.1
KC924794.1	2	KC924794.1,KC924795.1
KC924803.1	1	KC924803.1
KC924807.1	1	KC924807.1
KU206405.1	2	KU206405.1,KU206406.1
KU206440.1	1	KU206440.1
KU206452.1	1	KU206452.1
KU206453.1	1	KU206453.1
KU206460.1	1	KU206460.1
KY852346.1	1	KY852346.1
MT968035.1	1	MT968035.1
MW396760.1	1	MW396760.1
LC834913.1	1	LC834913.1
```

UL30_clin02_labeled_rerun_commonSites.mask.tsv

ref_pos\twindow_index0\n151\t0\n152\t1\n153\t2\n154\t3\n155\t4\n156\t5\n157\t6\n158\t7\n159\t8\n160\t9\n161\t10\n162\t11\n163\t12\n164\t13\n165\t14\n166\t15\n167\t16\n168\t17\n169\t18\n170\t19\n171\t20\n172\t21\n173\t22\n174\t23\n175\t24\n176\t25\n177\t26\n178\t27\n179\t28\n180\t29\n181\t30\n182\t31\n183\t32\n184\t33\n185\t34\n186\t35\n187\t36\n188\t37\n189\t38\n190\t39\n191\t40\n192\t41\n193\t42\n194\t43\n195\t44\n196\t45\n197\t46\n198\t47\n199\t48\n200\t49\n201\t50\n202\t51\n203\t52\n204\t53\n205\t54\n206\t55\n207\t56\n208\t57\n209\t58\n210\t59\n211\t60\n212\t61\n213\t62\n214\t63\n215\t64\n216\t65\n217\t66\n218\t67\n219\t68\n220\t69\n221\t70\n222\t71\n223\t72\n224\t73\n225\t74\n226\t75\n227\t76\n228\t77\n229\t78\n232\t81\n233\t82\n234\t83\n235\t84\n236\t85\n237\t86\n238\t87\n239\t88\n240\t89\n241\t90\n242\t91\n243\t92\n244\t93\n245\t94\n246\t95\n247\t96\n248\t97\n249\t98\n250\t99\n251\t100\n252\t101\n253\t102\n254\t103\n255\t104\n256\t105\n257\t106\n258\t107\n259\t108\n260\t109\n261\t110\n262\t111\n263\t112\n264\t113\n265\t114\n266\t115\n267\t116\n268\t117\n269\t118\n270\t119\n271\t120\n272\t121\n273\t122\n274\t123\n275\t124\n276\t125\n277\t126\n278\t127\n279\t128\n280\t129\n281\t130\n282\t131\n283\t132\n284\t133\n285\t134\n286\t135\n287\t136\n288\t137\n289\t138\n290\t139\n291\t140\n292\t141\n293\t142\n294\t143\n295\t144\n296\t145\n297\t146\n298\t147\n299\t148\n300\t149\n301\t150\n302\t151\n303\t152\n304\t153\n305\t154\n306\t155\n361\t210\n362\t211\n363\t212\n364\t213\n365\t214\n366\t215\n367\t216\n368\t217\n369\t218\n370\t219\n371\t220\n372\t221\n373\t222\n374\t223\n375\t224\n376\t225\n377\t226\n378\t227\n379\t228\n380\t229\n381\t230\n382\t231\n383\t232\n384\t233\n385\t234\n386\t235\n387\t236\n388\t237\n389\t238\n390\t239\n391\t240\n392\t241\n393\t242\n394\t243\n395\t244\n396\t245\n397\t246\n398\t247\n399\t248\n400\t249\n401\t250\n402\t251\n403\t252\n404\t253\n405\t254\n406\t255\n407\t256\n408\t257\n409\t258\n410\t259\n424\t273\n425\t274\n426\t275\n427\t276\n428\t277\n429\t278\n430\t279\n431\t280\n432\t281\n433\t282\n434\t283\n435\t284\n436\t285\n437\t286\n438\t287\n439\t288\n440\t289\n441\t290\n442\t291\n443\t292\n444\t293\n445\t294\n446\t295\n447\t296\n448\t297\n449\t298\n450\t299\n451\t300\n452\t301\n453\t302\n454\t303\n455\t304\n456\t305\n457\t306\n458\t307\n459\t308\n460\t309\n461\t310\n462\t311\n463\t312\n464\t313\n465\t314\n466\t315\n467\t316\n468\t317\n469\t318\n470\t319\n471\t320\n472\t321\n473\t322\n483\t332\n484\t333\n485\t334\n486\t335\n487\t336\n488\t337\n489\t338\n490\t339\n492\t341\n493\t342\n494\t343\n495\t344\n496\t345\n497\t346\n498\t347\n499\t348\n500\t349\n501\t350\n502\t351\n503\t352\n504\t353\n505\t354\n506\t355\n507\t356\n508\t357\n509\t358\n510\t359\n511\t360\n512\t361\n513\t362\n514\t363\n515\t364\n516\t365\n517\t366\n518\t367\n519\t368\n520\t369\n521\t370\n522\t371\n523\t372\n524\t373\n525\t374\n526\t375\n527\t376\n528\t377\n529\t378\n530\t379\n531\t380\n532\t381\n533\t382\n534\t383\n535\t384\n536\t385\n537\t386\n538\t387\n539\t388\n540\t389\n541\t390\n542\t391\n543\t392\n544\t393\n545\t394\n546\t395\n547\t396\n548\t397\n549\t398\n550\t399\n551\t400\n552\t401\n553\t402\n554\t403\n555\t404\n556\t405\n557\t406\n558\t407\n559\t408\n560\t409\n561\t410\n562\t411\n563\t412\n564\t413\n565\t414\n566\t415\n567\t416\n568\t417\n569\t418\n570\t419\n571\t420\n572\t421\n573\t422\n574\t423\n575\t424\n576\t425\n577\t426\n578\t427\n579\t428\n580\t429\n581\t430\n582\t431\n583\t432\n585\t434\n586\t435\n587\t436\n588\t437\n589\t438\n590\t439\n591\t440\n592\t441\n593\t442\n594\t443\n595\t444\n596\t445\n597\t446\n598\t447\n599\t448\n600\t449\n601\t450\n602\t451\n603\t452\n604\t453\n605\t454\n606\t455\n607\t456\n608\t457\n609\t458\n610\t459\n611\t460\n612\t461\n613\t462\n614\t463\n615\t464\n616\t465\n617\t466\n618\t467\n619\t468\n620\t469\n621\t470\n622\t471\n623\t472\n624\t473\n625\t474\n626\t475\n627\t476\n628\t477\n629\t478\n630\t479\n631\t480\n632\t481\n633\t482\n634\t483\n635\t484\n636\t485\n637\t486\n638\t487\n639\t488\n640\t489\n641\t490\n642\t491\n643\t492\n644\t493\n645\t494\n646\t495\n647\t496\n648\t497\n649\t498\n650\t499\n651\t500\n652\t501\n653\t502\n654\t503\n655\t504\n656\t505\n657\t506\n658\t507\n659\t508\n660\t509\n661\t510\n662\t511\n663\t512\n664\t513\n665\t514\n666\t515\n667\t516\n668\t517\n669\t518\n670\t519\n671\t520\n672\t521\n673\t522\n674\t523\n675\t524\n676\t525\n677\t526\n678\t527\n679\t528\n680\t529\n681\t530\n682\t531\n683\t532\n684\t533\n685\t534\n686\t535\n687\t536\n688\t537\n689\t538\n690\t539\n691\t540\n692\t541\n693\t542\n694\t543\n695\t544\n696\t545\n697\t546\n698\t547\n699\t548\n700\t549\n701\t550\n702\t551\n703\t552\n704\t553\n705\t554\n706\t555\n707\t556\n708\t557\n709\t558\n710\t559\n711\t560\n712\t561\n713\t562\n714\t563\n715\t564\n716\t565\n717\t566\n718\t567\n719\t568\n720\t569\n721\t570\n722\t571\n723\t572\n724\t573\n725\t574\n726\t575\n727\t576\n728\t577\n729\t578\n730\t579\n731\t580\n732\t581\n733\t582\n734\t583\n735\t584\n736\t585\n737\t586\n738\t587\n739\t588\n740\t589\n741\t590\n742\t591\n743\t592\n744\t593\n745\t594\n746\t595\n747\t596\n748\t597\n749\t598\n750\t599\n751\t600\n752\t601\n753\t602\n754\t603\n755\t604\n756\t605\n757\t606\n758\t607\n759\t608\n760\t609\n761\t610\n762\t611\n763\t612\n765\t614\n766\t615\n767\t616\n768\t617\n769\t618\n770\t619\n771\t620\n772\t621\n773\t622\n774\t623\n775\t624\n776\t625\n777\t626\n778\t627\n779\t628\n780\t629\n781\t630\n782\t631\n783\t632\n784\t633\n785\t634\n786\t635\n787\t636\n788\t637\n789\t638\n790\t639\n791\t640\n792\t641\n793\t642\n794\t643\n795\t644\n796\t645\n797\t646\n798\t647\n799\t648\n800\t649\n801\t650\n802\t651\n803\t652\n804\t653\n805\t654\n806\t655\n807\t656\n808\t657\n809\t658\n810\t659\n811\t660\n812\t661\n813\t662\n814\t663\n815\t664\n816\t665\n817\t666\n818\t667\n819\t668\n820\t669\n821\t670\n822\t671\n823\t672\n824\t673\n825\t674\n826\t675\n827\t676\n828\t677\n829\t678\n830\t679\n831\t680\n832\t681\n833\t682\n834\t683\n835\t684\n836\t685\n837\t686\n838\t687\n839\t688\n840\t689\n841\t690\n842\t691\n843\t692\n844\t693\n845\t694\n846\t695\n847\t696\n848\t697\n849\t698\n850\t699\n851\t700\n852\t701\n853\t702\n854\t703\n855\t704\n856\t705\n857\t706\n858\t707\n859\t708\n860\t709\n861\t710\n862\t711\n863\t712\n864\t713\n865\t714\n866\t715\n867\t716\n868\t717\n869\t718\n870\t719\n871\t720\n872\t721\n873\t722\n874\t723\n875\t724\n876\t725\n877\t726\n878\t727\n879\t728\n880\t729\n881\t730\n882\t731\n883\t732\n884\t733\n885\t734\n886\t735\n887\t736\n888\t737\n889\t738\n890\t739\n891\t740\n892\t741\n893\t742\n894\t743\n895\t744\n896\t745\n897\t746\n898\t747\n899\t748\n900\t749\n901\t750\n902\t751\n903\t752\n904\t753\n905\t754\n906\t755\n907\t756\n908\t757\n909\t758\n910\t759\n911\t760\n912\t761\n913\t762\n914\t763\n915\t764\n916\t765\n917\t766\n918\t767\n919\t768\n920\t769\n921\t770\n922\t771\n923\t772\n924\t773\n925\t774\n926\t775\n927\t776\n928\t777\n929\t778\n930\t779\n931\t780\n932\t781\n933\t782\n934\t783\n935\t784\n936\t785\n937\t786\n938\t787\n939\t788\n940\t789\n941\t790\n956\t805\n957\t806\n958\t807\n960\t809\n961\t810\n962\t811\n963\t812\n964\t813\n965\t814\n966\t815\n967\t816\n968\t817\n969\t818\n970\t819\n971\t820\n972\t821\n973\t822\n974\t823\n975\t824\n976\t825\n977\t826\n978\t827\n979\t828\n980\t829\n981\t830\n982\t831\n983\t832\n984\t833\n985\t834\n986\t835\n987\t836\n988\t837\n989\t838\n990\t839\n991\t840\n992\t841\n993\t842\n994\t843\n995\t844\n996\t845\n997\t846\n998\t847\n999\t848\n1000\t849\n1001\t850\n1002\t851\n1003\t852\n1004\t853\n1005\t854\n1006\t855\n1007\t856\n1008\t857\n1009\t858\n1010\t859\n1011\t860\n1012\t861\n1013\t862\n1014\t863\n1015\t864\n1016\t865\n1017\t866\n1018\t867\n1019\t868\n1020\t869\n1021\t870\n1022\t871\n1023\t872\n1024\t873\n1025\t874\n1026\t875\n1027\t876\n1028\t877\n1029\t878\n1030\t879\n1031\t880\n1032\t881\n1033\t882\n1034\t883\n1035\t884\n1036\t885\n1037\t886\n1038\t887\n1039\t888\n1040\t889\n1041\t890\n1042\t891\n1043\t892\n1044\t893\n1045\t894\n1046\t895\n1047\t896\n1048\t897\n1049\t898\n1050\t899\n1051\t900\n1052\t901\n1053\t902\n1054\t903\n1055\t904\n1056\t905\n1057\t906\n1058\t907\n1059\t908\n1060\t909\n1061\t910\n1062\t911\n1063\t912\n1064\t913\n1065\t914\n1066\t915\n1067\t916\n1068\t917\n1069\t918\n1070\t919\n1071\t920\n1072\t921\n1073\t922\n1074\t923\n1075\t924\n1076\t925\n1077\t926\n1078\t927\n1079\t928\n1080\t929\n1081\t930\n1082\t931\n1083\t932\n1084\t933\n1085\t934\n1086\t935\n1087\t936\n1088\t937\n1089\t938\n1090\t939\n1091\t940\n1092\t941\n1093\t942\n1094\t943\n1095\t944\n1096\t945\n1097\t946\n1098\t947\n1099\t948\n1100\t949\n1101\t950\n1102\t951\n1103\t952\n1104\t953\n1105\t954\n1106\t955\n1107\t956\n

```
UL30_clin02_labeled_rerun_Table8.tsv

UL30 category	EHM/Total (EHM%)	Reference background (N752-E990) EHM/Total	OR (Haldane)	p	q (BH-FDR)
N752-E990	4/164 (2.4%)	—	—	—	—
N752-K990	8/21 (38.1%)	4/164	—	—	—
D752	25/55 (45.5%)	4/164	29.82	7.61e-14	3.8e-13
I291	6/7 (85.7%)	4/164	154.56	4.53e-08	1.13e-07
A742	4/5 (80.0%)	4/164	107.00	1.05e-05	1.74e-05
R250	4/6 (66.7%)	4/164	64.20	3.01e-05	3.76e-05
G760	2/3 (66.7%)	4/164	59.44	0.00319	0.00319
```

```
UL30_clin02_labeled_rerun_variant_vs_N752E990_Fisher_BH.tsv

variant_group	background_group	EHM_in_group	nonEHM_in_group	EHM_in_background	nonEHM_in_background	OR_Haldane	p	q_BH
D752	N752-E990	25	30	4	160	29.81967213114754	7.607400594378709e-14	3.803700297189355e-13
I291	N752-E990	6	1	4	160	154.55555555555554	4.529652384526396e-08	1.1324130961315991e-07
A742	N752-E990	4	1	4	160	107.0	1.0465339307192209e-05	1.744223217865368e-05
R250	N752-E990	4	2	4	160	64.2	3.0067399988735962e-05	3.7584249985919956e-05
G760	N752-E990	2	1	4	160	59.44444444444444	0.003194050016068629	0.0031940500160686286
I291	N752-K990	6	1	8	13	6.882352941176471	0.07681159420289854	0.3695652173913044
A742	N752-K990	4	1	8	13	4.764705882352941	0.14782608695652175	0.3695652173913044
R250	N752-K990	4	2	8	13	2.8588235294117648	0.35748792270531404	0.5958132045088568
G760	N752-K990	2	1	8	13	2.6470588235294117	0.5503952569169961	0.6133145515235107
D752	N752-K990	25	30	8	13	1.3278688524590163	0.6133145515235107	0.6133145515235107
D752	N752_all	25	30	12	173	11.604590163934427	1.7166618297267777e-10	8.583309148633889e-10
I291	N752_all	6	1	12	173	60.14666666666667	1.9091795179450236e-06	4.772948794862559e-06
A742	N752_all	4	1	12	173	41.64	0.00016406782079492663	0.0002734463679915444
R250	N752_all	4	2	12	173	24.984	0.00045723598581545825	0.0005715449822693228
G760	N752_all	2	1	12	173	23.133333333333333	0.014862786694511835	0.014862786694511835
```
