# Supplementary material for: Equine herpesvirus myeloencephalopathy-associated mutations in equine herpesvirus 1 DNA polymerase confer the ability to replicate at elevated temperatures
Source: Microbiol Spectr. 2026 Aug 21;14(10):e03055-25. doi: 10.1128/spectrum.03055-25 (PMC13689140; doi:10.1128/spectrum.03055-25)
Supplement: Supplementary file 5 [file spectrum.03055-25-s0005.docx]

**Table S1. Viruses used in experiments using PBMCs and EVECs**

| Field viruses | 3292, 6383,7759, 7855, 8343, 9204 |
| --- | --- |
| Parental BAC virus | Ab4p UL30 D752 BAC (D752) |
| Mutant BAC virus | Ab4p UL30 N752 BAC (N752) |
| Repaired BAC virus | Ab4p UL30 D752 BAC (D752) |
| Chimeric BAC viruses | Ab4p UL30 Valencia BAC (I291, N752)  Ab4p UL30 H752 BAC (H752)  Ab4p UL30 08-I-812 BAC (N752, V946)  Ab4p UL30 89c105 BAC (A742, N752)  Ab4p UL30 7759 BAC (R250, N752)  Ab4p UL30 06-I-70 BAC (K683, N752, N769) |

**Table S2. Enrichment of EHM in UL30 amino-acid variant groups (Fisher tests)**

* Numbers in parentheses indicate the number of strains with EHM and non-EHM backgrounds, respectively, in each genetic reference background.

Two-sided Fisher’s exact tests comparing each UL30 variant group against the specified N752 background group. Odds ratios (OR) are reported with the Haldane–Anscombe correction (+0.5 to all cells). BH-FDR q-values are computed within each background block (five tests per block).

Significance was defined as q < 0.05 after BH-FDR correction (two-sided Fisher’s exact tests).

|  |  |  |  |  |  | Background | |  |  |  |  |
| --- | --- | --- | --- | --- | --- | --- | --- | --- | --- | --- | --- |
| UL30 variant group | Number of strains | | N752-E990 and N752-K990  (12, 173)* | | | N752-E990  (4,160)* | | | N752-K990  (8,13)* | | |
|  | EHM | nonEHM | OR | p | q (BH-FDR) | OR | p | q (BH-FDR) | OR | p | q (BH-FDR) |
| D752 | 25 | 30 | 11.6 | 1.71E-10 | 8.58E-10 | 29.8 | 7.61E-14 | 3.80E-13 | 1.3 | 6.13E-01 | 6.13E-01 |
| I291 | 6 | 1 | 60.1 | 1.90E-06 | 4.77E-06 | 154.6 | 4.53E-08 | 1.13E-07 | 6.9 | 7.68E-02 | 3.70E-01 |
| A742 | 4 | 1 | 41.6 | 1.64E-04 | 2.73E-04 | 107.0 | 1.05E-05 | 1.74E-05 | 4.8 | 1.48E-01 | 3.70E-01 |
| R250 | 4 | 2 | 25.0 | 4.57E-04 | 5.71E-04 | 64.2 | 3.01E-05 | 3.76E-05 | 2.9 | 3.57E-01 | 5.96E-01 |
| G760 | 2 | 1 | 23.1 | 1.48E-02 | 1.48E-02 | 59.4 | 3.19E-03 | 3.19E-03 | 2.6 | 5.50E-01 | 6.13E-01 |

Nominal p values are shown; FDR-adjusted q values were used to determine significance.

Notes: N752-E990 denotes N752 sequences lacking additional substitutions at UL30 positions 250/291/742/760 and carrying E at position 990; N752-K990 denotes the analogous N752 group carrying K at position 990. ORs are Haldane–Anscombe corrected; q-values are BH-FDR adjusted within each background block.

(A)

(B)

**Appendix Fig. A1. pAb4p BAC.** (A) Circular map of pAb4p BAC. The BAC vector cassette (pZC320-mEGFP) is shown in green, ORF30 in brown, and other ORFs in white. The BAC cassette was inserted between positions 27,825 and 27,826 located between ORF19 and ORF20 of the Ab4p genome. A region spanning nucleotide 6,770 to 7,201 of pZC320 (accession number U26464) was replaced with nucleotide 61 to 1,642 of pEGFP-N1 (accession number U55762) in which an alanine codon at amino acid position 206 of the EGFP gene was substituted with a lysine codon to prevent dimerization. (B) Schematic representation of pZC320mEGFP. oriS: replication origin derived from F-plasmid; repE: replication initiator protein; sopA, sopB, sopC: plasmid partition; bla: ampicillin resistance gene; CMV enhancer, CMV promoter, mEGFP: mEGFP expression gene derived from pEGFP-N1. Seq origin: the sequence numbering origin of pZC320mEGFP.

**KOS MFSGGGGPLS PGGKSAARAA ......SGFF APAGPRGAGR GPPPCLRQNF**

**Ab4p M......... .....AAREQ ANSVRRSGFF NPF....IGK RP.......F**

Pre-NH2-terminal domain

```
KOS  YNPYLAPVGT QQKPTGPTQR HTYYSECDEF RFIAPRVLDE DAPPEKRAGV 100
Ab4p FRPGSGQTAE TERPRPP..Q HSYCTEVGSF KFIAPRCLDE EAPADQRRGV
```

pN1

pN2

**KOS HDGHLKRAPK VYCGGDERDV LRVGSGGFWP RRSRLWGGVD HAPAGFNPTV**

**Ab4p HVGTLERPPK VYCDGSEYDV LNFASGGCWP RRIRVWNGQD FRGDGFNPRF**

pN2

**KOS TVFHVYDILE NVEHAYGMRA AQFHARFMDA ITPTGTVITL LGLTPEGHRV 200**

**Ab4p ERFHVYDIVE TSESA....S HDDPSRFAEL SRPSGSVVTL LGMSECGKRV**

N4

N3

**R151S**

NA

N2

N1

**KOS AVHVYGTRQY FYMNKEEVDR HLQCRAPRDL CERM...... ...AAALRES**

N5

NB

**Ab4p AVHVYGVRHY FYMAKAEVDS ACGITTEAEL VRAMVDCAHS SALSAALGNG**

NH2-terminal domain

NC

N4

**KOS PGASFRG... ........IS ADHFEAEVVE RTDVYYYETR PALFYRVYVR 300**

**Ab4p NGGKQSGGSG GGWWGGKHVS ADCFKVETVC HTTLYYFGSK PALYYRVSAS**

N6

**H250R**

NE

**KOS SGRVLSYLCD NFCPAIKKYE GGVDATTRFI LDNPGFVTFG WYRLKPGRNN**

**Ab4p SSRLGGFICD NFHPEITKFE GSVDVTTRLL LDNENFTSFG WYRLRPGTHG**

**S291I**

N9

```
KOS  TLAQPRAPMA FGTSSDVEFN CTADNLAIEG GMSDLPAYKL MCFDIECKAG 400
Ab4p ERVQLRPVER HVTSSDVEIN CTPDNLEPIP DEAAWPDYKL MCFDIECKAG
```

E1

N10

N11

**KOS GEDELAFPVA GHPEDLVIQI SCLLYDLSTT ALEHVLLFSL GSCDLPESHL**

**Ab4p TGNEMAFPVA TNQEDLVIQI SCLLYSLATQ NHEHTLLFSL GSCDISEEYS**

EA

E3

E2

```
KOS  NELAARGLPT PVVLEFDSEF EMLLAFMTLV KQYGPEFVTG YNIINFDWPF 500
Ab4p FACVQRGEPR PTVLEFDSEY ELLVAFLTFL KQYSPEFATG YNIVNFDWAY
```

E5

EC

EB

E4

EA

3’-5’-exonuclease domain

```
KOS  LLAKLTDIYK VPLDGYGRMN GRGVFRVWDI GQSHFQKRSK IKVNGMVNID
Ab4p IVNKVTSVYN IKLDGYGKFN KGGLFKVWDI ATNHFQKKSK VKINGLISLD
```

E6

EC

```
KOS  MYGIITDKIK LSSYKLNAVA EAVLKDKKKD LSYRDIPAYY ATGPAQRGVI 600
Ab4p MYSVATEKLK LPSYKLDAVV GDVLGEHKID LPYKEIPSYY AGGPDRRGVI
```

EG

EF

EE

ED

**KOS GEYCIQDSLL VGQLFFKFLP HLELSAVARL AGINITRTIY DGQQIRVFTC**

NH2 domain

**Ab4p GEYCIQDSRL VGKLFFKYLP HLELSAVAKL ARITLTRVIF DGQQIRVYTC**

NG

NH

NF

EG

**KOS LLRLADQKGF ILPDTQGRFR GAGGEAPKRP AAAREDEERP EEEGEDED.. 700**

**Ab4p LLKLARERNF ILPDNRRRFD SQADAASETS ELAMDSQSHA FDSTDEPDGV**

**R625H**

NH

**KOS ....EREEGG GEREPEGARE TAGRHVGYQG ARVLDPTSGF HVNPVVVFDF**

Palm domain

**Ab4p DGTPDAAGSG ATSENGGGKP GVGRAVGYQG AKVLDPVSGF HVDPVVVFDF**

P2

P1

**E683K**

**KOS ASLYPSIIQA HNLCFSTLSL RADAVAHLEA GKDYLEIEVG GRRLFFVKAH 800**

**Ab4p ASLYPSIIQA HNLCFTTLAL DEVDLAGLQP SVDYSTFEVG DQKLFFVHAH**

**H769N**

P4

P3

**D760G**

**D752N**

PB

**V742A**

PA

**KOS VRESLLSILL RDWLAMRKQI RSRIPQSSPE EAVLLDKQQA AIKVVCNSVY**

Finger domain

**Ab4p IRESLLGILL RDWLAMRKAV RARIPTSTPE EAVLLDKQQS AIKVICNSVY**

FB

FB

FA

**KOS GFTGVQHGLL PCLHVAATVT TIGREMLLAT REYVHARWAA FEQLLADFPE 900**

**Ab4p GFTGVANGLL PCLRIAATVT TIGRDMLLKT RDYVHSRWAT RELLEDNFPG**

PD

PC

FB

PE

P6

P5

**KOS AADMRAPGPY SMRIIYGDTD SIFVLCRGLT AAGLTAMGDK MASHISRALF**

Palm domain

**Ab4p AIGFRNHKPY SVRVIYGDTD SVFIKFVGLT YEGVSELGDA MSRQISADLF**

P7

**KOS LPPIKLECEK TFTKLLLIAK KKYIGVIYGG KMLIKGVDLV RKNNCAFINR 1000**

**Ab4p RAPIKLECEK TFQRLLLITK KKYIGVINGG KMLMKGVDLV RKNNCSFINL**

TA

P11

P9

P8

I946V

P10

**KOS TSRALVDLLF YDDTVSGAAA ALAERPAEEW LARPLPEGLQ AFGAVLVDAH**

**Ab4p YARHLVDLLL YDEDVATAAA EVTDVPPAEW VGRPLPSGFD KFGRVLVEAY**

TC

TA

Thumb domain

TB

**KOS RRITDPERDI QDFVLTAELS RHPRAYTNKR LAHLTVYYKL MARRAQVPSI 1100**

TD

T1

**Ab4p NRITAPNLDV REFVMTAELS RSPESYTNKR LPHLTVYFKL AMRNEELPSV**

TC

**KOS KDRIPYVIVA QTREVEETVA RLAALRELDA AAPGDEPAPP AALPSPAKRP**

**Ab4p KERIPYVIVA QTEAAEREAG VVNSMRG... .........T AQNPVVTKTA**

TE

T2

A1107T

**KOS RETPSHADPP GGASKPRKLL VSELAEDPAY AIAHGVALNT DYYFSHLLGA 1200**

TF

TG

**Ab4p RPQPK..... ......RKLL VSDLAEDPTY VSENDVPLNT DYYFSHLLGT**

**KOS ACVTFKALFG NNAKITESLL KRFIPEVWHP .PDDVAARLR AAGFGAVGAG**

TG

**Ab4p ISVTFKALFG NDVRTTENLL KRFIPETPHK TPTKTQALLE RAGFEKLTPF**

TH

```
KOS  ATAEETRRML HRAFDTLA.. ....
Ab4p TPEEESRRIL HTVFCTLEAA PHQS
```

**Appendix Fig. A2. Pairwise alignment of UL30 amino acid sequences for domain mapping.** Pairwise alignment of the EHV-1 Ab4p UL30 amino acid sequence (Ab4p) with HSV-1 KOS UL30 amino acid sequence (KOS). Structural domains are indicated by color coding corresponding to Fig. 6, and secondary structure elements are shown as dotted rectangles, based on the structural analysis by Liu *et al.* (24). SNPs examined in the present study are enclosed in rectangles. SNPs associated with replication capacity at elevated temperatures are highlighted in brown. Dots indicate gaps introduced for optimal alignment.

Domain boundaries of Ab4p UL30 are as follows (amino acid positions): pre NH_2_-terminal domain, 1–119; NH_2_-terminal domain, 120–357, 589–634; 3’-5’-Exonuclease domain, 358–588; Palm domain, 702–767, 827–956; Finger domain, 768–826; Thumb domain, 958–1175.

**Appendix Fig. A3. Restriction fragment length polymorphism analysis of the chimera BAC DNAs.** Restriction fragment length polymorphism (RFLP) analysis of chimera BAC DNAs. Chimeric BAC DNAs were digested with EcoRI, HindIII or SalI, and the resulting fragments were separated electrophoresis on a 0.5% agarose gel in 40mM Tris-acetate, 1mM EDTA (pH8.3). Gels were stained with ethidium bromide (0.5µg/mL), and the fluorescence images were acquired for analysis. No large deletion or insertions were detected in any of the chimeric BAC DNAs.

Lane M, 1 kbp ladder; lane 2, Ab4p UL30 89c105 BAC; lane 3, Ab4p UL30 06-I-70 BAC; lane 4, Ab4p UL30 08-I-812 BAC; lane 5, Ab4p UL30 7759 BAC; lane 6, Ab4p UL30 H752 BAC; lane 7, Ab4p UL30 Valencia BAC.
