# Supplementary material for: Equine herpesvirus myeloencephalopathy-associated mutations in equine herpesvirus 1 DNA polymerase confer the ability to replicate at elevated temperatures
Source: Microbiol Spectr. 2026 Aug 21;14(10):e03055-25. doi: 10.1128/spectrum.03055-25 (PMC13689140; doi:10.1128/spectrum.03055-25)
Supplement: Supplementary file 6 [file spectrum.03055-25-s0006.docx]

**Supplementary Methods**

**S1. Sequence collection and clinical metadata curation**

Public EHV-1 UL30/ORF30 sequence records were collected from (i) complete-genome accessions and (ii) ORF30-only or partial records. Clinical phenotype labels were curated into a unified variable (clin_sign) with categories EHM, abortion, respiratory, ocular, and neonatal_mortality. Label harmonization included normalization of spelling variants and mapping of synonymous descriptions into the above categories. When multiple sources provided phenotype information for the same accession, genome-derived metadata were prioritized. Accession matching supported both versioned and versionless accessions.

**S2. UL30 amino-acid sequence preparation and coordinate framework**

UL30 amino-acid sequences were analyzed in a reference-coordinate framework anchored to the Ab4p reference (GenBank AY665713). A reference UL30 amino-acid sequence (Ab4p_UL30_ref.aa.fasta) defined residue numbering (1–1220 aa). Analyses focused on the UL30 window corresponding to reference positions 151–1107.

**S3. Construction of the “common-sites” alignment**

From the mapped window alignment (positions 151–1107; length 957 aa), we retained only sites with complete coverage across all labeled sequences (i.e., no gaps or missing residues in any of the 266 sequences). This produced a “common-sites” alignment of 861 amino-acid positions for downstream phylogenetic inference and comparative analyses. A mapping table of retained reference positions was saved to enable back-conversion of alignment columns to reference coordinates.

**S4. Deduplication into representative UL30 haplotypes**

To reduce redundancy from identical UL30 amino-acid sequences, we deduplicated the common-sites alignment by collapsing identical sequences into representative haplotypes (dedup). For each unique amino-acid sequence, one representative accession was retained and the list of member accessions mapping to that haplotype was recorded. In this dataset, 266 labeled sequences collapsed into 39 unique UL30 haplotypes, reflecting strong conservation of UL30 across many records.

**S5. Maximum-likelihood phylogenetic inference from amino-acid data**

Maximum-likelihood phylogenies were inferred from the common-sites alignment using IQ-TREE with automated model selection (ModelFinder) and bootstrap support assessment. For visualization and interpretability, phylogenetic inference was also performed on the deduplicated alignment (39 unique UL30 haplotypes).

**S6. Definition of UL30 marker categories and N752 background stratification**

Marker categories were defined in the Ab4p coordinate system using amino-acid states at positions 250 (R), 291 (I), 742 (A), 752 (D), and 760 (G). Each accession was assigned to a mutually exclusive category: if exactly one marker state was present, the accession was assigned to that marker category; if none were present, the accession was assigned to the “N752” background category; and if multiple marker states co-occurred, the accession was assigned to a MULTI category (recorded as the set of co-occurring markers).

Within the N752 background (i.e., sequences lacking the additional substitutions at UL30 positions 250/291/742/760), sequences were further stratified by residue 990 into two groups: N752–E990 and N752–K990. This stratification was motivated by the observation that, in our UL30 common-sites alignment, these two N752 groups differed at residue 990 while sharing the same states at the other UL30 marker sites, and that their baseline EHM frequencies differed.

**S7. Association testing: marker-versus-background framework**

To quantify enrichment of EHM within each marker category, we performed two-sided Fisher’s exact tests using a “marker versus background” framework. For each marker category, a 2×2 contingency table was constructed with rows defined by (marker category) versus (specified N752 background group) and columns defined by EHM versus non-EHM. Odds ratios were reported with the Haldane–Anscombe correction (+0.5 to all cells). Multiple testing correction across marker categories was performed using the Benjamini–Hochberg false discovery rate (BH-FDR). Tests were performed against three background definitions: (i) combined N752 background (N752–E990 + N752–K990), (ii) N752–E990 only, and (iii) N752–K990 only, to assess robustness to background-risk stratification.

**S8. Summary measures**

For each UL30 category (N752–E990, N752–K990, D752, I291, A742, R250, G760, MULTI), we computed total number of sequences, number of EHM sequences, and EHM rate (EHM/total), as descriptive measures complementing the inferential tests.

**S9. Nucleotide-based phylogenetic reconstruction and independent-origin analysis of UL30 variants**

To account for synonymous nucleotide variation, independent-origin analysis was performed on a nucleotide-based ORF30 phylogeny. For each accession in the 266-sequence common-sites amino-acid dataset, candidate coding sequences were retrieved from the corresponding GenBank record, translated, and compared with the reference-mapped common-sites amino-acid sequence to identify the matching ORF30 nucleotide sequence. This procedure recovered matched ORF30 nucleotide sequences for all 266 accessions.

The matched ORF30 nucleotide sequences comprised 265 full-length coding sequences of 3,663 nt and one coding sequence of 3,651 nt. After alignment with MAFFT, the final codon alignment had a uniform length of 3,663 nt. A maximum-likelihood phylogeny was inferred using IQ-TREE with ModelFinder and UFBoot2. Using this nucleotide-based phylogeny, the minimum number of independent gains of UL30 variants at positions 250, 291, 625, 683, 742, 752, 760, 769, 946, and 990 was estimated by Fitch parsimony. Minimum independent gains were defined as the minimum number of parent≠variant → child=variant transitions compatible with the parsimony state sets. For residue 990, the ancestral/root state was constrained to E to estimate a conservative lower bound on independent gains of K990. Output summaries are provided in Table 9 (main text).

**Commands used (reproducible workflow)**

Software

Python 3 with Biopython; MAFFT v7; IQ-TREE.

**Primary inputs**

Ab4p_UL30_ref.aa.fasta; UL30_clin02_labeled_rerun_commonSites.fasta; UL30_clin02_labeled_rerun_commonSites.mask.tsv; commonSites_records.gb.

**Workflow**

```
# 1) Fetch GenBank records for the 266 accessions in the common-sites dataset
python3 fetch_genbank_from_commonsites.py

# 2) Identify the matching ORF30 nucleotide sequence for each accession by
#    translation-based comparison against the reference-mapped common-sites
#    amino-acid sequence
python3 extract_orf30_by_commonsites_match.py

# 3) Align the matched ORF30 nucleotide sequences
mafft --auto ORF30_nt_matched_by_commonsites.fasta > ORF30_nt_matched_by_commonsites.aln.fasta

# 4) Infer a codon-based maximum-likelihood phylogeny
iqtree3 \
  -s ORF30_nt_matched_by_commonsites.aln.fasta \
  -st CODON \
  -m MFP \
  -B 1000 \
  -nt AUTO \
  -pre ORF30_nt266_codon

# 5) Estimate the minimum number of independent gains of UL30 variants on the
#    nucleotide-based tree
python3 nt_tree_aa_gain_parsimony.py \
  --aln ORF30_nt_matched_by_commonsites.aln.fasta \
  --tree ORF30_nt266_codon.treefile \
  --positions 250:R 291:I 625:H 683:K 742:A 752:D 760:G 769:N 946:V 990:K \
  --aa_start 1 \
  --root_fixed 990:E \
  --out_tsv Table_S3_nt_tree_aa_gains_266.tsv \
| tee nt_tree_aa_gains_266.log
```
