# Supplementary material for: Equine herpesvirus myeloencephalopathy-associated mutations in equine herpesvirus 1 DNA polymerase confer the ability to replicate at elevated temperatures
Source: Microbiol Spectr. 2026 Aug 21;14(10):e03055-25. doi: 10.1128/spectrum.03055-25 (PMC13689140; doi:10.1128/spectrum.03055-25)
Supplement: Supplementary file 7 [file spectrum.03055-25-s0003.docx]

Ab4p_UL30_ref.aa.fasta

>AY665713.1 Ab4p_UL30_ref

MAAREQANSVRRSGFFNPFIGKRPFFRPGSGQTAETERPRPPQHSYCTEVGSFKFIAPRC

LDEEAPADQRRGVHVGTLERPPKVYCDGSEYDVLNFASGGCWPRRIRVWNGQDFRGDGFN

PRFERFHVYDIVETSESASHDDPSRFAELSRPSGSVVTLLGMSECGKRVAVHVYGVRHYF

YMAKAEVDSACGITTEAELVRAMVDCAHSSALSAALGNGNGGKQSGGSGGGWWGGKHVSA

DCFKVETVCHTTLYYFGSKPALYYRVSASSSRLGGFICDNFHPEITKFEGSVDVTTRLLL

DNENFTSFGWYRLRPGTHGERVQLRPVERHVTSSDVEINCTPDNLEPIPDEAAWPDYKLM

CFDIECKAGTGNEMAFPVATNQEDLVIQISCLLYSLATQNHEHTLLFSLGSCDISEEYSF

ACVQRGEPRPTVLEFDSEYELLVAFLTFLKQYSPEFATGYNIVNFDWAYIVNKVTSVYNI

KLDGYGKFNKGGLFKVWDIATNHFQKKSKVKINGLISLDMYSVATEKLKLPSYKLDAVVG

DVLGEHKIDLPYKEIPSYYAGGPDRRGVIGEYCIQDSRLVGKLFFKYLPHLELSAVAKLA

RITLTRVIFDGQQIRVYTCLLKLARERNFILPDNRRRFDSQADAASETSELAMDSQSHAF

DSTDEPDGVDGTPDAAGSGATSENGGGKPGVGRAVGYQGAKVLDPVSGFHVDPVVVFDFA

SLYPSIIQAHNLCFTTLALDEVDLAGLQPSVDYSTFEVGDQKLFFVHAHIRESLLGILLR

DWLAMRKAVRARIPTSTPEEAVLLDKQQSAIKVICNSVYGFTGVANGLLPCLRIAATVTT

IGRDMLLKTRDYVHSRWATRELLEDNFPGAIGFRNHKPYSVRVIYGDTDSVFIKFVGLTY

EGVSELGDAMSRQISADLFRAPIKLECEKTFQRLLLITKKKYIGVINGGKMLMKGVDLVR

KNNCSFINLYARHLVDLLLYDEDVATAAAEVTDVPPAEWVGRPLPSGFDKFGRVLVEAYN

RITAPNLDVREFVMTAELSRSPESYTNKRLPHLTVYFKLAMRNEELPSVKERIPYVIVAQ

TEAAEREAGVVNSMRGTAQNPVVTKTARPQPKRKLLVSDLAEDPTYVSENDVPLNTDYYF

SHLLGTISVTFKALFGNDVRTTENLLKRFIPETPHKTPTKTQALLERAGFEKLTPFTPEE

ESRRILHTVFCTLEAAPHQS

ORF30_nt_matched_by_commonsites.fasta

>AY464052.1

ATGGCGGCGCGCGAACAGGCCAACTCCGTTCGCAGGAGCGGGTTTTTTAATCCGTTTATT

GGCAAGAGGCCATTTTTCAGGCCGGGAAGCGGGCAGACCGCGGAGACAGAGCGCCCAAGG

CCCCCCCAACACTCGTACTGCACAGAGGTGGGTAGCTTTAAGTTTATAGCTCCAAGATGC

CTCGATGAAGAAGCCCCCGCCGACCAGCGACGCGGTGTACACGTGGGCACCTTGGAGCGC

CCACCCAAAGTGTACTGCGATGGCTCAGAGTACGACGTGCTGAACTTTGCCTCCGGAGGT

TGCTGGCCTCGCAGGATTCGGGTTTGGAACGGCCAGGATTTTCGGGGCGATGGATTCAAC

CCCAGATTTGAGAGATTTCACGTGTACGACATAGTAGAGACTTCGGAGAGCGCCTCACAC

GATGACCCATCCAGGTTTGCTGAGCTATCCCGCCCAAGCGGGTCTGTGGTAACACTGCTG

GGAATGAGCGAGTGTGGAAAGCGAGTCGCGGTTCACGTCTATGGTGTGCGCCATTATTTT

TACATGGCAAAGGCGGAGGTGGATAGCGCTTGTGGAATAACCACCGAGGCAGAACTCGTG

CGCGCAATGGTGGACTGCGCGCACAGCTCGGCTTTGAGCGCGGCCCTGGGAAATGGCAAC

GGCGGCAAACAGAGCGGCGGCAGCGGCGGGGGATGGTGGGGCGGAAAGCACGTGTCTGCG

GACTGCTTCAAAGTGGAGACCGTGTGCCACACGACGCTGTACTACTTTGGATCTAAGCCA

GCTCTCTACTATAGAGTATCTGCCTCCAGCAGCCGCCTGGGAGGGTTCATCTGCGACAAC

TTTCACCCGGAGATTACAAAATTCGAGGGGAGCGTGGACGTGACCACGCGGCTGTTGTTG

GACAATGAAAATTTTACCAGTTTTGGGTGGTACCGCCTGCGACCCGGCACCCACGGAGAG

CGTGTTCAACTTCGCCCCGTTGAGCGACACGTCACCTCAAGCGACGTGGAGATTAACTGT

ACTCCCGATAACCTGGAGCCGATACCAGACGAGGCTGCCTGGCCCGACTATAAGCTCATG

TGCTTTGATATAGAGTGTAAAGCTGGAACGGGTAACGAAATGGCGTTCCCAGTGGCAACT

AACCAAGAGGACCTGGTCATCCAGATCTCCTGTCTGCTGTACTCGCTTGCTACTCAGAAC

CACGAACACACCCTGCTGTTTTCCCTCGGGTCATGCGATATCTCTGAGGAATACTCGTTT

GCATGCGTCCAGCGCGGCGAGCCCAGACCGACGGTTTTGGAGTTTGACAGCGAGTACGAG

CTGCTGGTTGCCTTCCTGACCTTTCTCAAGCAGTACTCTCCCGAGTTCGCCACCGGCTAC

AACATCGTTAATTTTGACTGGGCGTACATAGTTAACAAGGTAACGTCGGTGTATAACATC

AAGCTGGACGGGTACGGCAAGTTCAACAAAGGGGGGCTGTTTAAGGTGTGGGACATCGCC

ACGAACCATTTTCAGAAGAAGAGCAAGGTGAAAATCAATGGCCTGATATCTCTAGACATG

TATTCTGTGGCGACGGAAAAGCTAAAGCTACCCAGCTACAAACTCGACGCGGTCGTGGGA

GACGTCCTCGGCGAGCATAAGATAGACCTTCCCTATAAAGAAATACCCTCCTATTACGCG

GGAGGGCCTGACCGGAGGGGCGTAATAGGAGAGTATTGTATCCAGGACTCTAGGCTGGTG

GGCAAGCTGTTTTTTAAGTACCTCCCCCATCTGGAACTATCGGCGGTGGCCAAACTCGCC

CGTATCACCCTAACGCGGGTAATTTTTGACGGTCAGCAAATTCGCGTGTACACGTGCTTG

CTGAAACTCGCCCGCGAGAGAAATTTCATTTTGCCAGACAACAGACGCCGGTTTGACAGT

CAGGCAGACGCCGCGTCAGAGACTTCGGAGTTGGCTATGGATAGCCAAAGCCACGCCTTC

GACAGTACAGACGAACCCGACGGTGTGGACGGTACCCCGGACGCCGCAGGATCTGGCGCT

ACTTCTGAAAACGGAGGCGGGAAGCCCGGCGTCGGGAGGGCCGTGGGCTACCAGGGAGCA

AAGGTTCTAGACCCCGTATCCGGCTTTCATGTGGACCCCGTGGTTGTGTTTGACTTCGCT

AGCTTATACCCAAGCATTATCCAGGCCCATAACCTCTGTTTCACCACCCTGGCGCTCGAT

GAAGTGGATCTGGCCGGGCTTCAACCATCCGTCAACTACTCGACGTTCGAGGTGGGTGAC

CAAAAGTTATTTTTTGTCCACGCCCATATTCGCGAAAGCCTGCTTGGCATCTTGCTGCGC

GACTGGCTGGCCATGCGAAAGGCGGTGAGGGCGCGAATCCCCACCAGCACCCCCGAGGAG

GCAGTTTTACTAGATAAGCAGCAGTCTGCGATTAAGGTGATATGCAACTCGGTTTACGGA

TTCACGGGGGTGGCAAACGGCCTGTTGCCGTGTCTGAGGATAGCGGCTACCGTTACCACG

ATAGGACGCGACATGCTCCTCAAGACCCGAGATTACGTTCACTCTCGTTGGGCGACGCGC

GAGCTGCTGGAGGACAATTTTCCAGGGGCTATAGGTTTCCGAAACCACAAGCCTTACTCC

GTCAGGGTTATCTACGGAGACACCGACTCCGTGTTTATCAAGTTTGTGGGCCTGACGTAC

GAGGGGGTATCGGAGCTGGGGGATGCTATGTCGCGTCAGATTTCAGCGGACCTCTTTAGA

GCGCCCATCAAACTGGAGTGTGAGAAGACCTTTCAGCGACTGCTGCTGATCACCAAGAAG

AAGTACATAGGTGTCATAAACGGGGGGAAGATGCTCATGAAGGGGGTCGACCTGGTCCGC

AAAAATAACTGCTCTTTCATAAACTTGTACGCGCGACATCTGGTAGATCTTTTGTTGTAC

GACGAGGATGTGGCCACGGCGGCAGCAAAGGTGACAGACGTGCCTCCCGCAGAATGGGTG

GGGCGCCCGCTACCGAGCGGCTTTGACAAGTTTGGGCGAGTGCTGGTAGAGGCGTACAAC

CGTATCACTGCCCCCAACTTGGACGTGCGCGAGTTCGTTATGACTGCTGAGCTGAGCCGC

TCACCCGAATCGTATACCAACAAGCGCCTGCCGCACCTCACCGTCTACTTTAAGCTCGCC

ATGAGGAATGAAGAACTGCCCAGCGTAAAAGAGAGAATTCCGTATGTGATAGTTGCGCAG

ACCGAGGCCGCGGAACGCGAAGCGGGTGTAGTAAACTCAATGCGCGGTACCGCCCAAAAC

CCCGTGGTAACCAAGACCGCACGCCCCCAACCTAAACGCAAACTGCTGGTTTCCGACCTC

GCCGAAGACCCGACCTATGTTTCCGAGAATGACGTACCGCTAAACACAGACTACTATTTC

TCCCACCTGTTGGGTACCATAAGCGTGACCTTTAAGGCTCTATTCGGAAATGATGTGAGA

ACAACAGAAAATCTTTTAAAGCGGTTTATTCCGGAAACCCCCCACAAGACCCCCACGAAA

ACCCAGGCACTGCTTGAGCGCGCCGGCTTTGAAAAGCTGACGCCCTTTACACCGGAGGAA

GAAAGTCGTCGAATACTGCATACAGTTTTTTGTACTCTAGAAGCAGCTCCCCATCAAAGC

TGA

>AY665713.1

ATGGCGGCGCGCGAACAGGCCAACTCCGTTCGCAGGAGCGGGTTTTTTAATCCGTTTATT

GGCAAGAGGCCATTTTTCAGGCCGGGAAGCGGGCAGACCGCGGAGACAGAGCGCCCAAGG

CCCCCCCAACACTCGTACTGCACAGAGGTGGGTAGCTTTAAGTTTATAGCTCCAAGATGC

CTCGATGAAGAAGCCCCCGCCGACCAGCGACGCGGTGTACACGTGGGCACCTTGGAGCGC

CCACCCAAAGTGTACTGCGATGGCTCAGAGTACGACGTGCTGAACTTTGCCTCCGGAGGT

TGCTGGCCTCGCAGGATTCGGGTTTGGAACGGCCAGGATTTTCGGGGCGATGGATTCAAC

CCCAGATTTGAGAGATTTCACGTGTACGACATAGTAGAGACTTCGGAGAGCGCCTCACAC

GATGACCCATCCAGGTTTGCTGAGCTATCCCGCCCAAGCGGGTCTGTGGTAACACTGCTG

GGAATGAGCGAGTGTGGAAAGCGAGTCGCGGTTCACGTCTATGGTGTGCGCCATTATTTT

TACATGGCAAAGGCGGAGGTGGATAGCGCTTGTGGAATAACCACCGAGGCAGAACTCGTG

CGCGCAATGGTGGACTGCGCGCACAGCTCGGCTTTGAGCGCGGCCCTGGGAAATGGCAAC

GGCGGCAAACAGAGCGGCGGCAGCGGCGGGGGATGGTGGGGCGGAAAGCACGTGTCTGCG

GACTGCTTCAAAGTGGAGACCGTGTGCCACACGACGCTGTACTACTTTGGATCTAAGCCA

GCTCTCTACTATAGAGTATCTGCCTCCAGCAGCCGCCTGGGAGGGTTCATCTGCGACAAC

TTTCACCCGGAGATTACAAAATTCGAGGGGAGCGTGGACGTGACCACGCGGCTGTTGTTG

GACAATGAAAATTTTACCAGTTTCGGGTGGTACCGCCTGCGACCCGGCACCCACGGAGAG

CGTGTTCAACTTCGCCCCGTTGAGCGACACGTCACCTCAAGCGACGTGGAGATTAACTGT

ACTCCCGATAACCTGGAGCCGATACCAGACGAGGCTGCCTGGCCCGACTATAAGCTCATG

TGCTTTGATATAGAGTGTAAAGCTGGAACGGGTAACGAAATGGCGTTCCCAGTGGCAACT

AACCAAGAGGACCTGGTCATCCAGATCTCCTGTCTGCTGTACTCGCTTGCTACTCAGAAC

CACGAACACACCCTGCTGTTTTCCCTCGGGTCATGCGATATCTCTGAGGAATACTCGTTT

GCATGCGTCCAGCGCGGCGAGCCCAGACCGACGGTTTTGGAGTTTGACAGCGAGTACGAG

CTGCTGGTTGCCTTCCTGACCTTTCTCAAGCAGTACTCTCCCGAGTTCGCCACCGGCTAC

AACATCGTTAATTTTGACTGGGCGTACATAGTTAACAAGGTAACGTCGGTGTATAACATC

AAGCTGGACGGGTACGGCAAGTTCAACAAAGGGGGGCTGTTTAAGGTGTGGGACATCGCC

ACGAACCATTTTCAGAAGAAGAGCAAGGTGAAAATCAATGGCCTGATATCTCTAGACATG

TATTCTGTGGCGACGGAAAAGCTAAAGCTACCCAGCTACAAACTCGACGCGGTCGTGGGA

GACGTCCTCGGCGAGCATAAGATAGACCTTCCCTATAAAGAAATACCCTCCTATTACGCG

GGAGGGCCTGACCGGAGGGGCGTAATAGGAGAGTATTGTATCCAGGACTCTAGGCTGGTG

GGCAAGCTGTTTTTTAAGTACCTCCCCCATCTGGAACTATCGGCGGTGGCCAAACTCGCC

CGTATCACCCTAACGCGGGTAATTTTTGACGGTCAGCAAATTCGCGTGTACACGTGCTTG

CTGAAACTCGCCCGCGAGAGAAATTTCATTTTGCCAGACAACAGACGCCGGTTTGACAGT

CAGGCAGACGCCGCGTCAGAGACTTCGGAGTTGGCTATGGATAGCCAAAGCCACGCCTTC

GACAGTACAGACGAACCCGACGGTGTGGACGGTACCCCGGACGCCGCAGGATCTGGCGCT

ACTTCTGAAAACGGAGGCGGGAAGCCCGGCGTCGGGAGGGCCGTGGGCTACCAGGGAGCA

AAGGTTCTAGACCCCGTATCCGGCTTTCATGTGGACCCCGTGGTTGTGTTTGACTTCGCT

AGCTTATACCCAAGCATTATCCAGGCCCATAACCTCTGTTTCACCACCCTGGCGCTCGAT

GAAGTGGATCTGGCCGGGCTTCAACCATCCGTCGACTACTCGACGTTCGAGGTGGGTGAC

CAAAAGTTATTTTTTGTCCACGCCCATATTCGCGAAAGCCTGCTTGGCATCTTGCTGCGC

GACTGGCTGGCCATGCGAAAGGCGGTGAGGGCGCGAATCCCCACCAGCACCCCCGAGGAG

GCAGTTTTACTAGATAAGCAGCAGTCTGCGATTAAGGTGATATGCAACTCGGTTTACGGA

TTCACGGGGGTGGCAAACGGCCTGTTGCCGTGTCTGAGGATAGCGGCTACCGTTACCACG

ATAGGACGCGACATGCTCCTCAAGACCCGAGATTACGTTCACTCTCGTTGGGCGACGCGC

GAGCTGCTGGAGGACAATTTTCCAGGGGCTATAGGTTTCCGAAACCACAAGCCTTACTCC

GTCAGGGTTATCTACGGAGACACCGACTCCGTGTTTATCAAGTTTGTGGGCCTGACGTAC

GAGGGGGTATCGGAGCTGGGGGATGCTATGTCGCGTCAGATTTCAGCGGACCTCTTTAGA

GCGCCCATCAAACTGGAGTGTGAGAAGACCTTTCAGCGACTGCTGCTGATCACCAAGAAG

AAGTACATAGGTGTCATAAACGGGGGGAAGATGCTCATGAAGGGGGTCGACCTGGTCCGC

AAAAATAACTGCTCTTTCATAAACTTGTACGCGCGACATCTGGTAGATCTTTTGTTGTAC

GACGAGGATGTGGCCACGGCGGCAGCAGAGGTGACAGACGTGCCTCCCGCAGAATGGGTG

GGGCGCCCGCTACCGAGCGGCTTTGACAAGTTTGGGCGAGTGCTGGTAGAGGCGTACAAC

CGTATCACTGCCCCCAACTTGGACGTGCGCGAGTTCGTTATGACTGCTGAGCTGAGCCGC

TCACCCGAATCGTATACCAACAAGCGCCTGCCGCACCTCACCGTCTACTTTAAGCTCGCC

ATGAGGAATGAAGAACTGCCCAGCGTAAAAGAGAGAATTCCGTATGTGATAGTTGCGCAG

ACCGAGGCCGCGGAACGCGAAGCGGGTGTAGTAAACTCAATGCGCGGTACCGCCCAAAAC

CCCGTGGTAACCAAGACCGCACGCCCCCAACCTAAACGCAAACTGCTGGTTTCCGACCTC

GCCGAAGACCCGACCTATGTTTCCGAGAATGACGTACCGCTAAACACAGACTACTATTTC

TCCCACCTGTTGGGTACCATAAGCGTGACCTTTAAGGCTCTATTCGGAAATGATGTGAGA

ACAACAGAAAATCTTTTAAAGCGGTTTATTCCGGAAACCCCCCACAAGACCCCCACGAAA

ACCCAGGCACTGCTTGAGCGCGCCGGCTTTGAAAAGCTGACGCCCTTTACACCGGAGGAA

GAAAGTCGTCGAATACTGCATACAGTTTTTTGTACTCTAGAAGCAGCTCCCCATCAAAGC

TGA

>KF644566.1

ATGGCGGCGCGCGAACAGGCCAACTCCGTTCGCAGGAGCGGGTTTTTTAATCCGTTTATT

GGCAAGAGGCCATTTTTCAGGCCGGGAAGCGGGCAGACCGCGGAGACAGAGCGCCCAAGG

CCCCCCCAACACTCGTACTGCACAGAGGTGGGTAGCTTTAAGTTTATAGCTCCAAGATGC

CTCGATGAAGAAGCCCCCGCCGACCAGCGACGCGGTGTACACGTGGGCACCTTGGAGCGC

CCACCCAAAGTGTACTGCGATGGCTCAGAGTACGACGTGCTGAACTTTGCCTCCGGAGGT

TGCTGGCCTCGCAGGATTCGGGTTTGGAACGGCCAGGATTTTCGGGGCGATGGATTCAAC

CCCAGATTTGAGAGATTTCACGTGTACGACATAGTAGAGACTTCGGAGAGCGCCTCACAC

GATGACCCATCCAGGTTTGCTGAGCTATCCCGCCCAAGCGGGTCTGTGGTAACACTGCTG

GGAATGAGCGAGTGTGGAAAGCGAGTCGCGGTTCACGTCTATGGTGTGCGCCATTATTTT

TACATGGCAAAGGCGGAGGTGGATAGCGCTTGTGGAATAACCACCGAGGCAGAACTCGTG

CGCGCAATGGTGGACTGCGCGCACAGCTCGGCTTTGAGCGCGGCCCTGGGAAATGGCAAC

GGCGGCAAACAGAGCGGCGGCAGCGGCGGGGGATGGTGGGGCGGAAAGCACGTGTCCGCG

GACTGCTTCAAAGTGGAGACCGTGTGCCACACGACGCTGTACTACTTTGGATCTAAGCCA

GCTCTCTACTATAGAGTATCTGCCTCCAGCAGCCGCCTGGGAGGGTTCATCTGCGACAAC

TTTCACCCGGAGATTACAAAATTCGAGGGGAGCGTGGACGTGACCACGCGGCTGTTGTTG

GACAATGAAAATTTTACCAGTTTCGGGTGGTACCGCCTGCGACCCGGCACCCACGGAGAG

CGTGTTCAACTTCGCCCCGTTGAGCGACACGTCACCTCAAGCGACGTGGAGATTAACTGT

ACTCCCGATAACCTGGAGCCGATACCAGACGAGGCTGCCTGGCCCGACTATAAGCTCATG

TGCTTTGATATAGAGTGTAAAGCTGGAACGGGTAACGAAATGGCGTTCCCAGTGGCAACT

AACCAAGAGGACCTGGTCATCCAGATCTCCTGTCTGCTGTACTCGCTTGCTACTCAGAAC

CACGAACACACCCTGCTGTTTTCCCTCGGGTCATGCGATATCTCTGAGGAATACTCGTTT

GCATGCGTCCAGCGCGGCGAGCCCAGACCGACGGTTTTGGAGTTTGACAGCGAGTACGAG

CTGCTGGTTGCCTTCCTGACCTTTCTCAAGCAGTACTCTCCCGAGTTCGCCACCGGCTAC

AACATCGTTAATTTTGACTGGGCGTACATAGTTAACAAGGTAACGTCGGTGTATAACATC

AAGCTGGACGGGTACGGCAAGTTCAACAAAGGGGGGCTGTTTAAGGTGTGGGACATCGCC

ACGAACCATTTTCAGAAGAAGAGCAAGGTGAAAATCAATGGCCTGATATCTCTAGACATG

TATTCTGTGGCGACGGAAAAGCTAAAGCTACCCAGCTACAAACTCGACGCGGTCGTGGGA

GACGTCCTCGGCGAGCATAAGATAGACCTTCCCTATAAAGAAATACCCTCCTATTACGCG

GGAGGGCCTGACCGGAGGGGCGTAATAGGAGAGTATTGTATCCAGGACTCTAGGCTGGTG

GGCAAGCTGTTTTTTAAGTACCTCCCCCATCTGGAACTATCGGCGGTGGCCAAACTCGCC

CGTATCACCCTAACGCGGGTAATTTTTGACGGTCAGCAAATTCGCGTGTACACGTGCTTG

CTGAAACTCGCCCGCGAGAGAAATTTCATTTTGCCAGACAACAGACGCCGGTTTGACAGT

CAGGCAGACGCCGCGTCAGAGACTTCGGAGTTGGCTATGGATAGCCAAAGCCACGCCTTC

GACAGTACAGACGAACCCGACGGTGTGGACGGTACCCCGGACGCCGCAGGATCTGGCGCT

ACTTCTGAAAACGGAGGCGGGAAGCCCGGCGTCGGGAGGGCCGTGGGCTACCAGGGAGCA

AAGGTTCTAGACCCCGTATCCGGCTTTCATGTGGACCCCGTGGTTGTGTTTGACTTCGCT

AGCTTATACCCAAGCATTATCCAGGCCCATAACCTCTGTTTCACCACCCTGGCGCTCGAT

GAAGTGGATCTGGCCGGGCTTCAACCATCCGTCAACTACTCGACGTTCGAGGTGGGTGAC

CAAAAGTTATTTTTTGTCCACGCCCATATTCGCGAAAGCCTGCTTGGCATCTTGCTGCGC

GACTGGCTGGCCATGCGAAAGGCGGTGAGGGCGCGAATCCCCACCAGCACCCCCGAGGAG

GCAGTTTTACTAGATAAGCAGCAGTCTGCGATTAAGGTGATATGCAACTCGGTTTACGGA

TTCACGGGGGTGGCAAACGGCCTGTTGCCGTGTCTGAGGATAGCGGCTACCGTTACCACG

ATAGGACGCGACATGCTCCTCAAGACCCGAGATTACGTTCACTCTCGTTGGGCGACGCGC

GAGCTGCTGGAGGACAATTTTCCAGGGGCTATAGGTTTCCGAAACCACAAGCCTTACTCC

GTCAGGGTTATCTACGGAGACACCGACTCCGTGTTTATCAAGTTTGTGGGCCTGACGTAC

GAGGGGGTATCGGAGCTGGGGGATGCTATGTCGCGTCAGATTTCAGCGGACCTCTTTAGA

GCCCCCATCAAACTGGAGTGTGAGAAGACCTTTCAGCGACTGCTGCTGATCACCAAGAAG

AAGTACATAGGTGTCATAAACGGGGGGAAGATGCTCATGAAGGGGGTCGACCTGGTCCGC

AAAAATAACTGCTCTTTCATAAACTTGTACGCGCGACATCTGGTAGATCTTTTGTTGTAC

GACGAGGATGTGGCCACGGCGGCAGCAGAGGTGACAGACGTGCCTCCCGCAGAATGGGTG

GGGCGCCCGCTACCGAGCGGCTTTGACAAGTTTGGGCGAGTGCTGGTAGAGGCGTACAAC

CGTATCACTGCCCCCAACTTGGACGTGCGCGAGTTCGTTATGACTGCTGAGCTGAGCCGC

TCACCCGAATCGTATACCAACAAGCGCCTGCCGCACCTCACCGTCTACTTTAAGCTCGCC

ATGAGGAATGAAGAACTGCCCAGCGTAAAAGAGAGAATTCCGTATGTGATAGTTGCGCAG

ACCGAGGCCGCGGAACGCGAAGCGGGTGTAGTAAACTCAATGCGCGGTACCGCCCAAAAC

CCCGTGGTAACCAAGACCGCACGCCCCCAACCTAAACGCAAACTGCTGGTTTCCGACCTC

GCCGAAGACCCGACCTATGTTTCCGAGAATGACGTACCGCTAAACACAGACTACTATTTC

TCCCACCTGTTGGGTACCATAAGCGTGACCTTTAAGGCTCTATTCGGAAATGATGTGAGA

ACAACAGAAAATCTTTTAAAGCGGTTTATTCCGGAAACCCCCCACAAGACCCCCACGAAA

ACCCAGGCACTGCTTGAGCGCGCCGGCTTTGAAAAGCTGACGCCCTTTACACCGGAGGAA

GAAAGTCGTCGAATACTGCATACAGTTTTTTGTACTCTAGAAGCAGCTCCCCATCAAAGC

TGA

>KF644567.1

ATGGCGGCGCGCGAACAGGCCAACTCCGTTCGCAGGAGCGGGTTTTTTAATCCGTTTATT

GGCAAGAGGCCATTTTTCAGGCCGGGAAGCGGGCAAACCGCGGAGACAGAGCGCCCAAGG

CCCCCCCAACACTCGTACTGCACAGAGGTGGGTAGCTTTAAGTTTATAGCTCCAAGATGC

CTCGATGAAGAAGCCCCCGCCGACCAGCGACGCGGTGTACACGTGGGCACCTTGGAGCGC

CCACCCAAAGTGTACTGCGATGGCTCAGAGTACGACGTGCTGAACTTTGCCTCCGGAGGT

TGCTGGCCTCGCAGGATTCGGGTTTGGAACGGCCAGGATTTTCGGGGCGATGGATTCAAC

CCCAGATTTGAGAGATTTCACGTGTACGACATAGTAGAGACTTCGGAGAGCGCCTCACAC

GATGACCCATCCAGGTTTGCTGAGCTATCCCGCCCAAGCGGGTCTGTGGTAACACTGCTG

GGAATGAGCGAGTGTGGAAAGCGAGTCGCGGTTCACGTCTATGGTGTGCGCCATTATTTT

TACATGGCAAAGGCGGAGGTGGATAGCGCTTGTGGAATAACCACCGAGGCAGAACTCGTG

CGCGCAATGGTGGACTGCGCGCACAGCTCGGCTTTGAGCGCGGCCCTGGGAAATGGCAAC

GGCGGCAAACAGAGCGGCGGCAGCGGCGGGGGATGGTGGGGCGGAAAGCACGTGTCTGCG

GACTGCTTCAAAGTGGAGACCGTGTGCCACACGACGCTGTACTACTTTGGATCTAAGCCA

GCTCTCTACTATAGAGTATCTGCCTCCAGCAGCCGCCTGGGAGGGTTCATCTGCGACAAC

TTTCACCCGGAGATTACAAAATTCGAGGGGAGCGTGGACGTGACCACGCGGCTGTTGTTG

GACAATGAAAATTTTACCAGTTTCGGGTGGTACCGCCTGCGACCCGGCACCCACGGAGAG

CGTGTTCAACTTCGCCCCGTTGAGCGACACGTCACCTCAAGCGACGTGGAGATTAACTGT

ACTCCCGATAACCTGGAGCCGATACCAGACGAGGCTGCCTGGCCCGACTATAAGCTCATG

TGCTTTGATATAGAGTGTAAAGCTGGAACGGGTAACGAAATGGCGTTCCCAGTGGCAACT

AACCAAGAGGACCTGGTCATCCAGATCTCCTGTCTGCTGTACTCGCTTGCTACTCAGAAC

CACGAACACACCCTGCTGTTTTCCCTCGGGTCATGCGATATCTCTGAGGAATACTCGTTT

GCATGCGTCCAGCGCGGCGAGCCCAGACCGACGGTTTTGGAGTTTGACAGCGAGTACGAG

CTGCTGGTTGCCTTCCTGACCTTTCTCAAGCAGTACTCTCCCGAGTTCGCCACCGGCTAC

AACATCGTTAATTTTGACTGGGCGTACATAGTTAACAAGGTAACGTCGGTGTATAACATC

AAGCTGGACGGGTACGGCAAGTTCAACAAAGGGGGGCTGTTTAAGGTGTGGGACATCGCC

ACGAACCATTTTCAGAAGAAGAGCAAGGTGAAAATCAATGGCCTGATATCTCTAGACATG

TATTCTGTGGCGACGGAAAAGCTAAAGCTACCCAGCTACAAACTCGACGCGGTCGTGGGA

GACGTCCTCGGCGAGCATAAGATAGACCTTCCCTATAAAGAAATACCCTCCTATTACGCG

GGAGGGCCTGACCGGAGGGGCGTAATAGGAGAGTATTGTATCCAGGACTCTAGGCTGGTG

GGCAAGCTGTTTTTTAAGTACCTCCCCCATCTGGAACTATCGGCGGTGGCCAAACTCGCC

CGTATCACCCTAACGCGGGTAATTTTTGACGGTCAGCAAATTCGCGTGTACACGTGCTTG

CTGAAACTCGCCCGCGAGAGAAATTTCATTTTGCCAGACAACAGACGCCGGTTTGACAGT

CAGGCAGACGCCGCGTCAGAGACTTCGGAGTTGGCTATGGATAGCCAAAGCCACGCCTTC

GACAGTACAGACGAACCCGACGGTGTGGACGGTACCCCGGACGCCGCAGGATCTGGCGCT

ACTTCTGAAAACGGAGGCGGGAAGCCCGGCGTCGGGAGGGCCGTGGGCTACCAGGGAGCA

AAGGTTCTAGACCCCGTATCCGGCTTTCATGTGGACCCCGTGGTTGTGTTTGACTTCGCT

AGCTTATACCCAAGCATTATCCAGGCCCATAACCTCTGTTTCACCACCCTGGCGCTCGAT

GAAGTGGATCTGGCCGGGCTTCAACCATCCGTCGACTACTCGACGTTCGAGGTGGGTGAC

CAAAAGTTATTTTTTGTCCACGCCCATATTCGCGAAAGCCTGCTTGGCATCTTGCTGCGC

GACTGGCTGGCCATGCGAAAGGCGGTGAGGGCGCGAATCCCCACCAGCACCCCCGAGGAG

GCAGTTTTACTAGATAAGCAGCAGTCTGCGATTAAGGTGATATGCAACTCGGTTTACGGA

TTCACGGGGGTGGCAAACGGCCTGTTGCCGTGTCTGAGGATAGCGGCTACCGTTACCACG

ATAGGACGCGACATGCTCCTCAAGACCCGAGATTACGTTCACTCTCGTTGGGCGACGCGC

GAGCTGCTGGAGGACAATTTTCCAGGGGCTATAGGTTTCCGAAACCACAAGCCTTACTCC

GTCAGGGTTATCTACGGAGACACCGACTCCGTGTTTATCAAGTTTGTGGGCCTGACGTAC

GAGGGGGTATCGGAGCTGGGGGATGCTATGTCGCGTCAGATTTCAGCGGACCTCTTTAGA

GCGCCCATCAAACTGGAGTGTGAGAAGACCTTTCAGCGACTGCTGCTGATCACCAAGAAG

AAGTACATAGGTGTCATAAACGGGGGGAAGATGCTCATGAAGGGGGTCGACCTGGTCCGC

AAAAATAACTGCTCTTTCATAAACTTGTACGCGCGACATCTGGTAGATCTTTTGTTGTAC

GACGAGGATGTGGCCACGGCGGCAGCAGAGGTGACAGACGTGCCTCCCGCAGAATGGGTG

GGGCGCCCGCTACCGAGCGGCTTTGACAAGTTTGGGCGAGTGCTGGTAGAGGCGTACAAC

CGTATCACTGCCCCCAACTTGGACGTGCGCGAGTTCGTTATGACTGCTGAGCTGAGCCGC

TCACCCGAATCGTATACCAACAAGCGCCTGCCGCACCTCACCGTCTACTTTAAGCTCGCC

ATGAGGAATGAAGAACTGCCCAGCGTAAAAGAGAGAATTCCGTATGTGATAGTTGCGCAG

ACCGAGGCCGCGGAACGCGAAGCGGGTGTAGTAAACTCAATGCGCGGTACCGCCCAAAAC

CCCGTGGTAACCAAGACCGCACGCCCCCAACCTAAACGCAAACTGCTGGTTTCCGACCTC

GCCGAAGACCCGACCTATGTTTCCGAGAATGACGTACCGCTAAACACAGACTACTATTTC

TCCCACCTGTTGGGTACCATAAGCGTGACCTTTAAGGCTCTATTCGGAAATGATGTGAGA

ACAACAGAAAATCTTTTAAAGCGGTTTATTCCGGAAACCCCCCACAAGACCCCCACGAAA

ACCCAGGCACTGCTTGAGCGCGCCGGCTTTGAAAAGCTGACGCCCTTTACACCGGAGGAA

GAAAGTCGTCGAATACTGCATACAGTTTTTTGTACTCTAGAAGCAGCTCCCCATCAAAGC

TGA

>KF644568.1

ATGGCGGCGCGCGAACAGGCCAACTCCGTTCGCAGGAGCGGGTTTTTTAATCCGTTTATT

GGCAAGAGGCCATTTTTCAGGCCGGGAAGCGGGCAGACCGCGGAGACAGAGCGCCCAAGG

CCCCCCCAACACTCGTACTGCACAGAGGTGGGTAGCTTTAAGTTTATAGCTCCAAGATGC

CTCGATGAAGAAGCCCCCGCCGACCAGCGACGCGGTGTACACGTGGGCACCTTGGAGCGC

CCACCCAAAGTGTACTGCGATGGCTCAGAGTACGACGTGCTGAACTTTGCCTCCGGAGGT

TGCTGGCCTCGCAGGATTCGGGTTTGGAACGGCCAGGATTTTCGGGGCGATGGATTCAAC

CCCAGATTTGAGAGATTTCACGTGTACGACATAGTAGAGACTTCGGAGAGCGCCTCACAC

GATGACCCATCCAGGTTTGCTGAGCTATCCCGCCCAAGCGGGTCTGTGGTAACACTGCTG

GGAATGAGCGAGTGTGGAAAGCGAGTCGCGGTTCACGTCTATGGTGTGCGCCATTATTTT

TACATGGCAAAGGCGGAGGTGGATAGCGCTTGTGGAATAACCACCGAGGCAGAACTCGTG

CGCGCAATGGTGGACTGCGCGCACAGCTCGGCTTTGAGCGCGGCCCTGGGAAATGGCAAC

GGCGGCAAACAGAGCGGCGGCAGCGGCGGGGGATGGTGGGGCGGAAAGCACGTGTCTGCG

GACTGCTTCAAAGTGGAGACCGTGTGCCACACGACGCTGTACTACTTTGGATCTAAGCCA

GCTCTCTACTATAGAGTATCTGCCTCCAGCAGCCGCCTGGGAGGGTTCATCTGCGACAAC

TTTCACCCGGAGATTACAAAATTCGAGGGGAGCGTGGACGTGACCACGCGGCTGTTGTTG

GACAATGAAAATTTTACCAGTTTCGGGTGGTACCGCCTGCGACCCGGCACCCACGGAGAG

CGTGTTCAACTTCGCCCCGTTGAGCGACACGTCACCTCAAGCGACGTGGAGATTAACTGT

ACTCCCGATAACCTGGAGCCGATACCAGACGAGGCTGCCTGGCCCGACTATAAGCTCATG

TGCTTTGATATAGAGTGTAAAGCTGGAACGGGTAACGAAATGGCGTTCCCAGTGGCAACT

AACCAAGAGGACCTGGTCATCCAGATCTCCTGTCTGCTGTACTCGCTTGCTACTCAGAAC

CACGAACACACCCTGCTGTTTTCCCTCGGGTCATGCGATATCTCTGAGGAATACTCGTTT

GCATGCGTCCAGCGCGGCGAGCCCAGACCGACGGTTTTGGAGTTTGACAGCGAGTACGAG

CTGCTGGTTGCCTTCCTGACCTTTCTCAAGCAGTACTCTCCCGAGTTCGCCACCGGCTAC

AACATCGTTAATTTTGACTGGGCGTACATAGTTAACAAGGTAACGTCGGTGTATAACATC

AAGCTGGACGGGTACGGCAAGTTCAACAAAGGGGGGCTGTTTAAGGTGTGGGACATCGCC

ACGAACCATTTTCAGAAGAAGAGCAAGGTGAAAATCAATGGCCTGATATCTCTAGACATG

TATTCTGTGGCGACGGAAAAGCTAAAGCTACCCAGCTACAAACTCGACGCGGTCGTGGGA

GACGTCCTCGGCGAGCATAAGATAGACCTTCCCTATAAAGAAATACCCTCCTATTACGCG

GGAGGGCCTGACCGGAGGGGCGTAATAGGAGAGTATTGTATCCAGGACTCTAGGCTGGTG

GGCAAGCTGTTTTTTAAGTACCTCCCCCATCTGGAACTATCGGCGGTGGCCAAACTCGCC

CGTATCACCCTAACGCGGGTAATTTTTGACGGTCAGCAAATTCGCGTGTACACGTGCTTG

CTGAAACTCGCCCGCGAGAGAAATTTCATTTTGCCAGACAACAGACGCCGGTTTGACAGT

CAGGCAGACGCCGCGTCAGAGACTTCGGAGTTGGCTATGGATAGCCAAAGCCACGCCTTC

GACAGTACAGACGAACCCGACGGTGTGGACGGTACCCCGGACGCCGCAGGATCTGGCGCT

ACTTCTGAAAACGGAGGCGGGAAGCCCGGCGTCGGGAGGGTCGTGGGCTACCAGGGAGCA

AAGGTTCTAGACCCCGTATCCGGCTTTCATGTGGACCCCGTGGTTGTGTTTGACTTCGCT

AGCTTATACCCAAGCATTATCCAGGCCCATAACCTCTGTTTCACCACCCTGGCGCTCGAT

GAAGTGGATCTGGCCGGGCTTCAACCATCCGTCGACTACTCGACGTTCGAGGTGGGTGAC

CAAAAGTTATTTTTTGTCCACGCCCATATTCGCGAAAGCCTGCTTGGCATCTTGCTGCGC

GACTGGCTGGCCATGCGAAAGGCGGTGAGGGCGCGAATCCCCACCAGCACCCCCGAGGAG

GCAGTTTTACTAGATAAGCAGCAGTCTGCGATTAAGGTGATATGCAACTCGGTTTACGGA

TTCACGGGGGTGGCAAACGGCCTGTTGCCGTGTCTGAGGATAGCGGCTACCGTTACCACG

ATAGGACGCGACATGCTCCTCAAGACCCGAGATTACGTTCACTCTCGTTGGGCGACGCGC

GAGCTGCTGGAGGACAATTTTCCAGGGGCTATAGGTTTCCGAAACCACAAGCCTTACTCC

GTCAGGGTTATCTACGGAGACACCGACTCCGTGTTTATCAAGTTTGTGGGCCTGACGTAC

GAGGGGGTATCGGAGCTGGGGGATGCTATGTCGCGTCAGATTTCAGCGGACCTCTTTAGA

GCGCCCATCAAACTGGAGTGTGAGAAGACCTTTCAGCGACTGCTGCTGATCACCAAGAAG

AAGTACATAGGTGTCATAAACGGGGGGAAGATGCTCATGAAGGGGGTCGACCTGGTCCGC

AAAAATAACTGCTCTTTCATAAACTTGTACGCGCGACATCTGGTAGATCTTTTGTTGTAC

GACGAGGATGTGGCCACGGCGGCAGCAGAGGTGACAGACGTGCCTCCCGCAGAATGGGTG

GGGCGCCCGCTACCGAGCGGCTTTGACAAGTTTGGGCGAGTGCTGGTAGAGGCGTACAAC

CGTATCACTGCCCCCAACTTGGACGTGCGCGAGTTCGTTATGACTGCTGAGCTGAGCCGC

TCACCCGAATCGTATACCAACAAGCGCCTGCCGCACCTCACCGTCTACTTTAAGCTCGCC

ATGAGGAATGAAGAACTGCCCAGCGTAAAAGAGAGAATTCCGTATGTGATAGTTGCGCAG

ACCGAGGCCGCGGAACGCGAAGCGGGTGTAGTAAACTCAATGCGCGGTACCGCCCAAAAC

CCCGTGGTAACCAAGACCGCACGCCCCCAACCTAAACGCAAACTGCTGGTTTCCGACCTC

GCCGAAGACCCGACCTATGTTTCCGAGAATGACGTACCGCTAAACACAGACTACTATTTC

TCCCACCTGTTGGGTACCATAAGCGTGACCTTTAAGGCTCTATTCGGAAATGATGTGAGA

ACAACAGAAAATCTTTTAAAGCGGTTTATTCCGGAAACCCCCCACAAGACCCCCACGAAA

ACCCAGGCACTGCTTGAGCGCGCCGGCTTTGAAAAGCTGACGCCCTTTACACCGGAGGAA

GAAAGTCGTCGAATACTGCATACAGTTTTTTGTACTCTAGAAGCAGCTCCCCATCAAAGC

TGA

>KF644569.1

ATGGCGGCGCGCGAACAGGCCAACTCCGTTCGCAGGAGCGGGTTTTTTAATCCGTTTATT

GGCAAGAGGCCATTTTTCAGGCCGGGAAGCGGGCAGACCGCGGAGACAGAGCGCCCAAGG

CCCCCCCAACACTCGTACTGCACAGAGGTGGGTAGCTTTAAGTTTATAGCTCCAAGATGC

CTCGATGAAGAAGCCCCCGCCGACCAGCGACGCGGTGTACACGTGGGCACCTTGGAGCGC

CCACCCAAAGTGTACTGCGATGGCTCAGAGTACGACGTGCTGAACTTTGCCTCCGGAGGT

TGCTGGCCTCGCAGGATTCGGGTTTGGAACGGCCAGGATTTTCGGGGCGATGGATTCAAC

CCCAGATTTGAGAGATTTCACGTGTACGACATAGTAGAGACTTCGGAGAGCGCCTCACAC

GATGACCCATCCAGGTTTGCTGAGCTATCCCGCCCAAGCGGGTCTGTGGTAACACTGCTG

GGAATGAGCGAGTGTGGAAAGCGAGTCGCGGTTCACGTCTATGGTGTGCGCCATTATTTT

TACATGGCAAAGGCGGAGGTGGATAGCGCTTGTGGAATAACCACCGAGGCAGAACTCGTG

CGCGCAATGGTGGACTGCGCGCACAGCTCGGCTTTGAGCGCGGCCCTGGGAAATGGCAAC

GGCGGCAAACAGAGCGGCGGCAGCGGCGGGGGATGGTGGGGCGGAAAGCACGTGTCTGCG

GACTGCTTCAAAGTGGAGACCGTGTGCCACACGACGCTGTACTACTTTGGATCTAAGCCA

GCTCTCTACTATAGAGTATCTGCCTCCAGCAGCCGCCTGGGAGGGTTCATCTGCGACAAC

TTTCACCCGGAGATTACAAAATTCGAGGGGAGCGTGGACGTGACCACGCGGCTGTTGTTG

GACAATGAAAATTTTACCAGTTTCGGGTGGTACCGCCTGCGACCCGGCACCCACGGAGAG

CGTGTTCAACTTCGCCCCGTTGAGCGACACGTCACCTCAAGCGACGTGGAGATTAACTGT

ACTCCCGATAACCTGGAGCCGATACCAGACGAGGCTGCCTGGCCCGACTATAAGCTCATG

TGCTTTGATATAGAGTGTAAAGCTGGAACGGGTAACGAAATGGCGTTCCCAGTGGCAACT

AACCAAGAGGACCTGGTCATCCAGATCTCCTGTCTGCTGTACTCGCTTGCTACTCAGAAC

CACGAACACACCCTGCTGTTTTCCCTCGGGTCATGCGATATCTCTGAGGAATACTCGTTT

GCATGCGTCCAGCGCGGCGAGCCCAGACCGACGGTTTTGGAGTTTGACAGCGAGTACGAG

CTGCTGGTTGCCTTCCTGACCTTTCTCAAGCAGTACTCTCCCGAGTTCGCCACCGGCTAC

AACATCGTTAATTTTGACTGGGCGTACATAGTTAACAAGGTAACGTCGGTGTATAACATC

AAGCTGGACGGGTACGGCAAGTTCAACAAAGGGGGGCTGTTTAAGGTGTGGGACATCGCC

ACGAACCATTTTCAGAAGAAGAGCAAGGTGAAAATCAATGGCCTGATATCTCTAGACATG

TATTCTGTGGCGACGGAAAAGCTAAAGCTACCCAGCTACAAACTCGACGCGGTCGTGGGA

GACGTCCTCGGCGAGCATAAGATAGACCTTCCCTATAAAGAAATACCCTCCTATTACGCG

GGAGGGCCTGACCGGAGGGGCGTAATAGGAGAGTATTGTATCCAGGACTCTAGGCTGGTG

GGCAAGCTGTTTTTTAAGTACCTCCCCCATCTGGAACTATCGGCGGTGGCCAAACTCGCC

CGTATCACCCTAACGCGGGTAATTTTTGACGGTCAGCAAATTCGCGTGTACACGTGCTTG

CTGAAACTCGCCCGCGAGAGAAATTTCATTTTGCCAGACAACAGACGCCGGTTTGACAGT

CAGGCAGACGCCGCGTCAGAGACTTCGGAGTTGGCTATGGATAGCCAAAGCCACGCCTTC

GACAGTACAGACGAACCCGACGGTGTGGACGGTACCCCGGACGCCGCAGGATCTGGCGCT

ACTTCTGAAAACGGAGGCGGGAAGCCCGGCGTCGGGAGGGCCGTGGGCTACCAGGGAGCA

AAGGTTCTAGACCCCGTATCCGGCTTTCATGTGGACCCCGTGGTTGTGTTTGACTTCGCT

AGCTTATACCCAAGCATTATCCAGGCCCATAACCTCTGTTTCACCACCCTGGCGCTCGAT

GAAGTGGATCTGGCCGGGCTTCAACCATCCGTCAACTACTCGACGTTCGAGGTGGGTGAC

CAAAAGTTATTTTTTGTCCACGCCCATATTCGCGAAAGCCTGCTTGGCATCTTGCTGCGC

GACTGGCTGGCCATGCGAAAGGCGGTGAGGGCGCGAATCCCCACCAGCACCCCCGAGGAG

GCAGTTTTACTAGATAAGCAGCAGTCTGCGATTAAGGTGATATGCAACTCGGTTTACGGA

TTCACGGGGGTGGCAAACGGCCTGTTGCCGTGTCTGAGGATAGCGGCTACCGTTACCACG

ATAGGACGCGACATGCTCCTCAAGACCCGAGATTACGTTCACTCTCGTTGGGCGACGCGC

GAGCTGCTGGAGGACAATTTTCCAGGGGCTATAGGTTTCCGAAACCACAAGCCTTACTCC

GTCAGGGTTATCTACGGAGACACCGACTCCGTGTTTATCAAGTTTGTGGGCCTGACGTAC

GAGGGGGTATCGGAGCTGGGGGATGCTATGTCGCGTCAGATTTCAGCGGACCTCTTTAGA

GCGCCCATCAAACTGGAGTGTGAGAAGACCTTTCAGCGACTGCTGCTGATCACCAAGAAG

AAGTACATAGGTGTCATAAACGGGGGGAAGATGCTCATGAAGGGGGTCGACCTGGTCCGC

AAAAATAACTGCTCTTTCATAAACTTGTACGCGCGACATCTGGTAGATCTTTTGTTGTAC

GACGAGGATGTGGCCACGGCGGCAGCAGAGGTGACAGACGTGCCTCCCGCAGAATGGGTG

GGGCGCCCGCTACCGAGCGGCTTTGACAAGTTTGGGCGAGTGCTGGTAGAGGCGTACAAC

CGTATCACTGCCCCCAACTTGGACGTGCGCGAGTTCGTTATGACTGCTGAGCTGAGCCGC

TCACCCGAATTGTATACCAACAAGCGCCTGCCGCACCTCACCGTCTACTTTAAGCTCGCC

ATGAGGAATGAAGAACTGCCCAGCGTAAAAGAGAGAATTCCGTATGTGATAGTTGCGCAG

ACCGAGGCCGCGGAACGCGAAGCGGGTGTAGTAAACTCAATGCGCGGTACCGCCCAAAAC

CCCGTGGTAACCAAGACCGCACGCCCCCAACCTAAACGCAAACTGCTGGTTTCCGACCTC

GCCGAAGACCCGACCTATGTTTCCGAGAATGACGTACCGCTAAACACAGACTACTATTTC

TCCCACCTGTTGGGTACCATAAGCGTGACCTTTAAGGCTCTATTCGGAAATGATGTGAGA

ACAACAGAAAATCTTTTAAAGCGGTTTATTCCGGAAACCCCCCACAAGACCCCCACGAAA

ACCCAGGCACTGCTTGAGCGCGCCGGCTTTGAAAAGCTGACGCCCTTTACACCGGAGGAA

GAAAGTCGTCGAATACTGCATACAGTTTTTTGTACTCTAGAAGCAGCTCCCCATCAAAGC

TGA

>KF644570.1

ATGGCGGCGCGCGAACAGGCCAACTCCGTTCGCAGGAGCGGGTTTTTTAATCCGTTTATT

GGCAAGAGGCCATTTTTCAGGCCGGGAAGCGGGCAGACCGCGGAGACAGAGCGCCCAAGG

CCCCCCCAACACTCGTACTGCACAGAGGTGGGTAGCTTTAAGTTTATAGCTCCAAGATGC

CTCGATGAAGAAGCCCCCGCCGACCAGCGACGCGGTGTACACGTGGGCACCTTGGAGCGC

CCACCCAAAGTGTACTGCGATGGCTCAGAGTACGACGTGCTGAACTTTGCCTCCGGAGGT

TGCTGGCCTCGCAGGATTCGGGTTTGGAACGGCCAGGATTTTCGGGGCGATGGATTCAAC

CCCAGATTTGAGAGATTTCACGTGTACGACATAGTAGAGACTTCGGAGAGCGCCTCACAC

GATGACCCATCCAGGTTTGCTGAGCTATCCCGCCCAAGCGGGTCTGTGGTAACACTGCTG

GGAATGAGCGAGTGTGGAAAGCGAGTCGCGGTTCACGTCTATGGTGTGCGCCATTATTTT

TACATGGCAAAGGCGGAGGTGGATAGCGCTTGTGGAATAACCACCGAGGCAGAACTCGTG

CGCGCAATGGTGGACTGCGCGCACAGCTCGGCTTTGAGCGCGGCCCTGGGAAATGGCAAC

GGCGGCAAACAGAGCGGCGGCAGCGGCGGGGGATGGTGGGGCGGAAAGCACGTGTCTGCG

GACTGCTTCAAAGTGGAGACCGTGTGCCACACGACGCTGTACTACTTTGGATCTAAGCCA

GCTCTCTACTATAGAGTATCTGCCTCCAGCAGCCGCCTGGGAGGGTTCATCTGCGACAAC

TTTCACCCGGAGATTACAAAATTCGAGGGGAGCGTGGACGTGACCACGCGGCTGTTGTTG

GACAATGAAAATTTTACCAGTTTCGGGTGGTACCGCCTGCGACCCGGCACCCACGGAGAG

CGTGTTCAACTTCGCCCCGTTGAGCGACACGTCACCTCAAGCGACGTGGAGATTAACTGT

ACTCCCGATAACCTGGAGCCGATACCAGACGAGGCTGCCTGGCCCGACTATAAGCTCATG

TGCTTTGATATAGAGTGTAAAGCTGGAACGGGTAACGAAATGGCGTTCCCAGTGGCAACT

AACCAAGAGGACCTGGTCATCCAGATCTCCTGTCTGCTGTACTCGCTTGCTACTCAGAAC

CACGAACACACCCTGCTGTTTTCCCTCGGGTCATGCGATATCTCTGAGGAATACTCGTTT

GCATGCGTCCAGCGCGGCGAGCCCAGACCGACGGTTTTGGAGTTTGACAGCGAGTACGAG

CTGCTGGTTGCCTTCCTGACCTTTCTCAAGCAGTACTCTCCCGAGTTCGCCACCGGCTAC

AACATCGTTAATTTTGACTGGGCGTACATAGTTAACAAGGTAACGTCGGTGTATAACATC

AAGCTGGACGGGTACGGCAAGTTCAACAAAGGGGGGCTGTTTAAGGTGTGGGACATCGCC

ACGAACCATTTTCAGAAGAAGAGCAAGGTGAAAATCAATGGCCTGATATCTCTAGACATG

TATTCTGTGGCGACGGAAAAGCTAAAGCTACCCAGCTACAAACTCGACGCGGTCGTGGGA

GACGTCCTCGGCGAGCATAAGATAGACCTTCCCTATAAAGAAATACCCTCCTATTACGCG

GGAGGGCCTGACCGGAGGGGCGTAATAGGAGAGTATTGTATCCAGGACTCTAGGCTGGTG

GGCAAGCTGTTTTTTAAGTACCTCCCCCATCTGGAACTATCGGCGGTGGCCAAACTCGCC

CGTATCACCCTAACGCGGGTAATTTTTGACGGTCAGCAAATTCGCGTGTACACGTGCTTG

CTGAAACTCGCCCGCGAGAGAAATTTCATTTTGCCAGACAACAGACGCCGGTTTGACAGT

CAGGCAGACGCCGCGTCAGAGACTTCGGAGTTGGCTATGGATAGCCAAAGCCACGCCTTC

GACAGTACAGACGAACCCGACGGTGTGGACGGTACCCCGGACGCCGCAGGATCTGGCGCT

ACTTCTGAAAACGGAGGCGGGAAGCCCGGCGTCGGGAGGGCCGTGGGCTACCAGGGAGCA

AAGGTTCTAGACCCCGTATCCGGCTTTCATGTGGACCCCGTGGTTGTGTTTGACTTCGCT

AGCTTATACCCAAGCATTATCCAGGCCCATAACCTCTGTTTCACCACCCTGGCGCTCGAT

GAAGTGGATCTGGCCGGGCTTCAACCATCCGTCGACTACTCGACGTTCGAGGTGGGTGAC

CAAAAGTTATTTTTTGTCCACGCCCATATTCGCGAAAGCCTGCTTGGCATCTTGCTGCGC

GACTGGCTGGCCATGCGAAAGGCGGTGAGGGCGCGAATCCCCACCAGCACCCCCGAGGAG

GCAGTTTTACTAGATAAGCAGCAGTCTGCGATTAAGGTGATATGCAACTCGGTTTACGGA

TTCACGGGGGTGGCAAACGGCCTGTTGCCGTGTCTGAGGATAGCGGCTACCGTTACCACG

ATAGGACGCGACATGCTCCTCAAGACCCGAGATTACGTTCACTCTCGTTGGGCGACGCGC

GAGCTGCTGGAGGACAATTTTCCAGGGGCTATAGGTTTCCGAAACCACAAGCCTTACTCC

GTCAGGGTTATCTACGGAGACACCGACTCCGTGTTTATCAAGTTTGTGGGCCTGACGTAC

GAGGGGGTATCGGAGCTGGGGGATGCTATGTCGCGTCAGATTTCAGCGGACCTCTTTAGA

GCGCCCATCAAACTGGAGTGTGAGAAGACCTTTCAGCGACTGCTGCTGATCACCAAGAAG

AAGTACATAGGTGTCATAAACGGGGGGAAGATGCTCATGAAGGGGGTCGACCTGGTCCGC

AAAAATAACTGCTCTTTCATAAACTTGTACGCGCGACATCTGGTAGATCTTTTGTTGTAC

GACGAGGATGTGGCCACGGCGGCAGCAGAGGTGACAGACGTGCCTCCCGCAGAATGGGTG

GGGCGCCCGCTACCGAGCGGCTTTGACAAGTTTGGGCGAGTGCTGGTAGAGGCGTACAAC

CGTATCACTGCCCCCAACTTGGACGTGCGCGAGTTCGTTATGACTGCTGAGCTGAGCCGC

TCACCCGAATCGTATACCAACAAGCGCCTGCCGCACCTCACCGTCTACTTTAAGCTCGCC

ATGAGGAATGAAGAACTGCCCAGCGTAAAAGAGAGAATTCCGTATGTGATAGTTGCGCAG

ACCGAGGCCGCGGAACGCGAAGCGGGTGTAGTAAACTCAATGCGCGGTACCGCCCAAAAC

CCCGTGGTAACCAAGACCGCACGCCCCCAACCTAAACGCAAACTGCTGGTTTCCGACCTC

GCCGAAGACCCGACCTATGTTTCCGAGAATGACGTACCGCTAAACACAGACTACTATTTC

TCCCACCTGTTGGGTACCATAAGCGTGACCTTTAAGGCTCTATTCGGAAATGATGTGAGA

ACAACAGAAAATCTTTTAAAGCGGTTTATTCCGGAAACCCCCCACAAGACCCCCACGAAA

ACCCAGGCACTGCTTGAGCGCGCCGGCTTTGAAAAGCTGACGCCCTTTACACCGGAGGAA

GAAAGTCGTCGAATACTGCATACAGTTTTTTGTACTCTAGAAGCAGCTCCCCATCAAAGC

TGA

>KF644572.1

ATGGCGGCGCGCGAACAGGCCAACTCCGTTCGCAGGAGCGGGTTTTTTAATCCGTTTATT

GGCAAGAGGCCATTTTTCAGGCCGGGAAGCGGGCAGACCGCGGAGACAGAGCGCCCAAGG

CCCCCCCAACACTCGTACTGCACAGAGGTGGGTAGCTTTAAGTTTATAGCTCCAAGATGC

CTCGATGAAGAAGCCCCCGCCGACCAGCGACGCGGTGTACACGTGGGCACCTTGGAGCGC

CCACCCAAAGTGTACTGCGATGGCTCAGAGTACGACGTGCTGAACTTTGCCTCCGGAGGT

TGCTGGCCTCGCAGGATTCGGGTTTGGAACGGCCAGGATTTTCGGGGCGATGGATTCAAC

CCCAGATTTGAGAGATTTCACGTGTACGACATAGTAGAGACTTCGGAGAGCGCCTCACAC

GATGACCCATCCAGGTTTGCTGAGCTATCCCGCCCAAGCGGGTCTGTGGTAACACTGCTG

GGAATGAGCGAGTGTGGAAAGCGAGTCGCGGTTCACGTCTATGGTGTGCGCCATTATTTT

TACATGGCAAAGGCGGAGGTGGATAGCGCTTGTGGAATAACCACCGAGGCAGAACTCGTG

CGCGCAATGGTGGACTGCGCGCACAGCTCGGCTTTGAGCGCGGCCCTGGGAAATGGCAAC

GGCGGCAAACAGAGCGGCGGCAGCGGCGGGGGATGGTGGGGCGGAAAGCACGTGTCTGCG

GACTGCTTCAAAGTGGAGACCGTGTGCCACACGACGCTGTACTACTTTGGATCTAAGCCA

GCTCTCTACTATAGAGTATCTGCCTCCAGCAGCCGCCTGGGAGGGTTCATCTGCGACAAC

TTTCACCCGGAGATTACAAAATTCGAGGGGAGCGTGGACGTGACCACGCGGCTGTTGTTG

GACAATGAAAATTTTACCAGTTTCGGGTGGTACCGCCTGCGACCCGGCACCCACGGAGAG

CGTGTTCAACTTCGCCCCGTTGAGCGACACGTCACCTCAAGCGACGTGGAGATTAACTGT

ACTCCCGATAACCTGGAGCCGATACCAGACGAGGCTGCCTGGCCCGACTATAAGCTCATG

TGCTTTGATATAGAGTGTAAAGCTGGAACGGGTAACGAAATGGCGTTCCCAGTGGCAACT

AACCAAGAGGACCTGGTCATCCAGATCTCCTGTCTGCTGTACTCGCTTGCTACTCAGAAC

CACGAACACACCCTGCTGTTTTCCCTCGGGTCATGCGATATCTCTGAGGAATACTCGTTT

GCATGCGTCCAGCGCGGCGAGCCCAGACCGACGGTTTTGGAGTTTGACAGCGAGTACGAG

CTGCTGGTTGCCTTCCTGACCTTTCTCAAGCAGTACTCTCCCGAGTTCGCCACCGGCTAC

AACATCGTTAATTTTGACTGGGCGTACATAGTTAACAAGGTAACGTCGGTGTATAACATC

AAGCTGGACGGGTACGGCAAGTTCAACAAAGGGGGGCTGTTTAAGGTGTGGGACATCGCC

ACGAACCATTTTCAGAAGAAGAGCAAGGTGAAAATCAATGGCCTGATATCTCTAGACATG

TATTCTGTGGCGACGGAAAAGCTAAAGCTACCCAGCTACAAACTCGACGCGGTCGTGGGA

GACGTCCTCGGCGAGCATAAGATAGACCTTCCCTATAAAGAAATACCCTCCTATTACGCG

GGAGGGCCTGACCGGAGGGGCGTAATAGGAGAGTATTGTATCCAGGACTCTAGGCTGGTG

GGCAAGCTGTTTTTTAAGTACCTCCCCCATCTGGAACTATCGGCGGTGGCCAAACTCGCC

CGTATCACCCTAACGCGGGTAATTTTTGACGGTCAGCAAATTCGCGTGTACACGTGCTTG

CTGAAACTCGCCCGCGAGAGAAATTTCATTTTGCCAGACAACAGACGCCGGTTTGACAGT

CAGGCAGACGCCGCGTCAGAGACTTCGGAGTTGGCTATGGATAGCCAAAGCCACGCCTTC

GACAGTACAGACGAACCCGACGGTGTGGACGGTACCCCGGACGCCGCAGGATCTGGCGCT

ACTTCTGAAAACGGAGGCGGGAAGCCCGGCGTCGGGAGGGCCGTGGGCTACCAGGGAGCA

AAGGTTCTAGACCCCGTATCCGGCTTTCATGTGGACCCCGTGGTTGTGTTTGACTTCGCT

AGCTTATACCCAAGCATTATCCAGGCCCATAACCTCTGTTTCACCACCCTGGCGCTCGAT

GAAGTGGATCTGGCCGGGCTTCAACCATCCGTCAACTACTCGACGTTCGAGGTGGGTGGC

CAAAAGTTATTTTTTGTCCACGCCCATATTCGCGAAAGCCTGCTTGGCATCTTGCTGCGC

GACTGGCTGGCCATGCGAAAGGCGGTGAGGGCGCGAATCCCCACCAGCACCCCCGAGGAG

GCAGTTTTACTAGATAAGCAGCAGTCTGCGATTAAGGTGATATGCAACTCGGTTTACGGA

TTCACGGGGGTGGCAAACGGCCTGTTGCCGTGTCTGAGGATAGCGGCTACCGTTACCACG

ATAGGACGCGACATGCTCCTCAAGACCCGAGATTACGTTCACTCTCGTTGGGCGACGCGC

GAGCTGCTGGAGGACAATTTTCCAGGGGCTATAGGTTTCCGAAACCACAAGCCTTACTCC

GTCAGGGTTATCTACGGAGACACCGACTCCGTGTTTATCAAGTTTGTGGGCCTGACGTAC

GAGGGGGTATCGGAGCTGGGGGATGCTATGTCGCGTCAGATTTCAGCGGACCTCTTTAGA

GCGCCCATCAAACTGGAGTGTGAGAAGACCTTTCAGCGACTGCTGCTGATCACCAAGAAG

AAGTACATAGGTGTCATAAACGGGGGGAAGATGCTCATGAAGGGGGTCGACCTGGTCCGC

AAAAATAACTGCTCTTTCATAAACTTGTACGCGCGACATCTGGTAGATCTTTTGTTGTAC

GACGAGGATGTGGCCACGGCGGCAGCAGAGGTGACAGACGTGCCTCCCGCAGAATGGGTG

GGGCGCCCGCTACCGAGCGGCTTTGACAAGTTTGGGCGAGTGCTGGTAGAGGCGTACAAC

CGTATCACTGCCCCCAACTTGGACGTGCGCGAGTTCGTTATGACTGCTGAGCTGAGCCGC

TCACCCGAATCGTATACCAACAAGCGCCTGCCGCACCTCACCGTCTACTTTAAGCTCGCC

ATGAGGAATGAAGAACTGCCCAGCGTAAAAGAGAGAATTCCGTATGTGATAGTTGCGCAG

ACCGAGGCCGCGGAACGCGAAGCGGGTGTAGTAAACTCAATGCGCGGTACCGCCCAAAAC

CCCGTGGTAACCAAGACCGCACGCCCCCAACCTAAACGCAAACTGCTGGTTTCCGACCTC

GCCGAAGACCCGACCTATGTTTCCGAGAATGACGTACCGCTAAACACAGACTACTATTTC

TCCCACCTGTTGGGTACCATAAGCGTGACCTTTAAGGCTCTATTCGGAAATGATGTGAGA

ACAACAGAAAATCTTTTAAAGCGGTTTATTCCGGAAACCCCCCACAAGACCCCCACGAAA

ACCCAGGCACTGCTTGAGCGCGCCGGCTTTGAAAAGCTGACGCCCTTTACACCGGAGGAA

GAAAGTCGTCGAATACTGCATACAGTTTTTTGTACTCTAGAAGCAGCTCCCCATCAAAGC

TGA

>KF644576.1

ATGGCGGCGCGCGAACAGGCCAACTCCGTTCGCAGGAGCGGGTTTTTTAATCCGTTTATT

GGCAAGAGGCCATTTTTCAGGCCGGGAAGCGGGCAGACCGCGGAGACAGAGCGCCCAAGG

CCCCCCCAACACTCGTACTGCACAGAGGTGGGTAGCTTTAAGTTTATAGCTCCAAGATGC

CTCGATGAAGAAGCCCCCGCCGACCAGCGACGCGGTGTACACGTGGGCACCTTGGAGCGC

CCACCCAAAGTGTACTGCGATGGCTCAGAGTACGACGTGCTGAACTTTGCCTCCGGAGGT

TGCTGGCCTCGCAGGATTCGGGTTTGGAACGGCCAGGATTTTCGGGGCGATGGATTCAAC

CCCAGATTTGAGAGATTTCACGTGTACGACATAGTAGAGACTTCGGAGAGCGCCTCACAC

GATGACCCATCCAGGTTTGCTGAGCTATCCCGCCCAAGCGGGTCTGTGGTAACACTGCTG

GGAATGAGCGAGTGTGGAAAGCGAGTCGCGGTTCACGTCTATGGTGTGCGCCATTATTTT

TACATGGCAAAGGCGGAGGTGGATAGCGCTTGTGGAATAACCACCGAGGCAGAACTCGTG

CGCGCAATGGTGGACTGCGCGCACAGCTCGGCTTTGAGCGCGGCCCTGGGAAATGGCAAC

GGCGGCAAACAGAGCGGCGGCAGCGGCGGGGGATGGTGGGGCGGAAAGCACGTGTCTGCG

GACTGCTTCAAAGTGGAGACCGTGTGCCACACGACGCTGTACTACTTTGGATCTAAGCCA

GCTCTCTACTATAGAGTATCTGCCTCCAGCAGCCGCCTGGGAGGGTTCATCTGCGACAAC

TTTCACCCGGAGATTACAAAATTCGAGGGGAGCGTGGACGTGACCACGCGGCTGTTGTTG

GACAATGAAAATTTTACCAGTTTCGGGTGGTACCGCCTGCGACCCGGCACCCACGGAGAG

CGTGTTCAACTTCGCCCCGTTGAGCGACACGTCACCTCAAGCGACGTGGAGATTAACTGT

ACTCCCGATAACCTGGAGCCGATACCAGACGAGGCTGCCTGGCCCGACTATAAGCTCATG

TGCTTTGATATAGAGTGTAAAGCTGGAACGGGTAACGAAATGGCGTTCCCAGTGGCAACT

AACCAAGAGGACCTGGTCATCCAGATCTCCTGTCTGCTGTACTCGCTTGCTACTCAGAAC

CACGAACACACCCTGCTGTTTTCCCTCGGGTCATGCGATATCTCTGAGGAATACTCGTTT

GCATGCGTCCAGCGCGGCGAGCCCAGACCGACGGTTTTGGAGTTTGACAGCGAGTACGAG

CTGCTGGTTGCCTTCCTGACCTTTCTCAAGCAGTACTCTCCCGAGTTCGCCACCGGCTAC

AACATCGTTAATTTTGACTGGGCGTACATAGTTAACAAGGTAACGTCGGTGTATAACATC

AAGCTGGACGGGTACGGCAAGTTCAACAAAGGGGGGCTGTTTAAGGTGTGGGACATCGCC

ACGAACCATTTTCAGAAGAAGAGCAAGGTGAAAATCAATGGCCTGATATCTCTAGACATG

TATTCTGTGGCGACGGAAAAGCTAAAGCTACCCAGCTACAAACTCGACGCGGTCGTGGGA

GACGTCCTCGGCGAGCATAAGATAGACCTTCCCTATAAAGAAATACCCTCCTATTACGCG

GGAGGGCCTGACCGGAGGGGCGTAATAGGAGAGTATTGTATCCAGGACTCTAGGCTGGTG

GGCAAGCTGTTTTTTAAGTACCTCCCCCATCTGGAACTATCGGCGGTGGCCAAACTCGCC

CGTATCACCCTAACGCGGGTAATTTTTGACGGTCAGCAAATTCGCGTGTACACGTGCTTG

CTGAAACTCGCCCGCGAGAGAAATTTCATTTTGCCAGACAACAGACGCCGGTTTGACAGT

CAGGCAGACGCCGCGTCAGAGACTTCGGAGTTGGCTATGGATAGCCAAAGCCACGCCTTC

GACAGTACAGACGAACCCGACGGTGTGGACGGTACCCCGGACGCCGCAGGATCTGGCGCT

ACTTCTGAAAACGGAGGCGGGAAGCCCGGCGTCGGGAGGGCCGTGGGCTACCAGGGAGCA

AAGGTTCTAGACCCCGTATCCGGCTTTCATGTGGACCCCGTGGTTGTGTTTGACTTCGCT

AGCTTATACCCAAGCATTATCCAGGCCCATAACCTCTGTTTCACCACCCTGGCGCTCGAT

GAAGTGGATCTGGCCGGGCTTCAACCATCCGTCAACTACTCGACGTTCGAGGTGGGTGAC

CAAAAGTTATTTTTTGTCCACGCCCATATTCGCGAAAGCCTGCTTGGCATCTTGCTGCGC

GACTGGCTGGCCATGCGAAAGGCGGTGAGGGCGCGAATCCCCACCAGCACCCCCGAGGAG

GCAGTTTTACTAGATAAGCAGCAGTCTGCGATTAAGGTGATATGCAACTCGGTTTACGGA

TTCACGGGGGTGGCAAACGGCCTGTTGCCGTGTCTGAGGATAGCGGCTACCGTTACCACG

ATAGGACGCGACATGCTCCTCAAAACCCGAGATTACGTTCACTCTCGTTGGGCGACGCGC

GAGCTGCTGGAGGACAATTTTCCAGGGGCTATAGGTTTCCGAAACCACAAGCCTTACTCC

GTCAGGGTTATCTACGGAGACACCGACTCCGTGTTTATCAAGTTTGTGGGCCTGACGTAC

GAGGGGGTATCGGAGCTGGGGGATGCTATGTCGCGTCAGATTTCAGCGGACCTCTTTAGA

GCCCCCATCAAACTGGAGTGTGAGAAGACCTTTCAGCGACTGCTGCTGATCACCAAGAAG

AAGTACATAGGTGTCATAAACGGGGGGAAGATGCTCATGAAGGGGGTCGACCTGGTCCGC

AAAAATAACTGCTCTTTCATAAACTTGTACGCGCGACATCTGGTAGATCTTTTGTTGTAC

GACGAGGATGTGGCCACGGCGGCAGCAGAGGTGACAGACGTGCCTCCCGCAGAATGGGTG

GGGCGCCCGCTACCGAGCGGCTTTGACAAGTTTGGGCGAGTGCTGGTAGAGGCGTACAAC

CGTATCACTGCCCCCAACTTGGACGTGCGCGAGTTCGTTATGACTGCTGAGCTGAGCCGC

TCACCCGAATCGTATACCAACAAGCGCCTGCCGCACCTCACCGTCTACTTTAAGCTCGCC

ATGAGGAATGAAGAACTGCCCAGCGTAAAAGAGAGAATTCCGTATGTGATAGTTGCGCAG

ACCGAGGCCGCGGAACGCGAAGCGGGTGTAGTAAACTCAATGCGCGGTACCGCCCAAAAC

CCCGTGGTAACCAAGACCGCACGCCCCCAACCTAAACGCAAACTGCTGGTTTCCGACCTC

GCCGAAGACCCGACCTATGTTTCCGAGAATGACGTACCGCTAAACACAGACTACTATTTC

TCCCACCTGTTGGGTACCATAAGCGTGACCTTTAAGGCTCTATTCGGAAATGATGTGAGA

ACAACAGAAAATCTTTTAAAGCGGTTTATTCCGGAAACCCCCCACAAGACCCCCACGAAA

ACCCAGGCACTGCTTGAGCGCGCCGGCTTTGAAAAGCTGACGCCCTTTACACCGGAGGAA

GAAAGTCGTCGAATACTGCATACAGTTTTTTGTACTCTAGAAGCAGCTCCCCATCAAAGC

TGA

>KF644577.1

ATGGCGGCGCGCGAACAGGCCAACTCCGTTCGCAGGAGCGGGTTTTTTAATCCGTTTATT

GGCAAGAGGCCATTTTTCAGGCCGGGAAGCGGGCAGACCGCGGAGACAGAGCGCCCAAGG

CCCCCCCAACACTCGTACTGCACAGAGGTGGGTAGCTTTAAGTTTATAGCTCCAAGATGC

CTCGATGAAGAAGCCCCCGCCGACCAGCGACGCGGTGTACACGTGGGCACCTTGGAGCGC

CCACCCAAAGTGTACTGCGATGGCTCAGAGTACGACGTGCTGAACTTTGCCTCCGGAGGT

TGCTGGCCTCGCAGGATTCGGGTTTGGAACGGCCAGGATTTTCGGGGCGATGGATTCAAC

CCCAGATTTGAGAGATTTCACGTGTACGACATAGTAGAGACTTCGGAGAGCGCCTCACAC

GATGACCCATCCAGGTTTGCTGAGCTATCCCGCCCAAGCGGGTCTGTGGTAACACTGCTG

GGAATGAGCGAGTGTGGAAAGCGAGTCGCGGTTCACGTCTATGGTGTGCGCCATTATTTT

TACATGGCAAAGGCGGAGGTGGATAGCGCTTGTGGAATAACCACCGAGGCAGAACTCGTG

CGCGCAATGGTGGACTGCGCGCACAGCTCGGCTTTGAGCGCGGCCCTGGGAAATGGCAAC

GGCGGCAAACAGAGCGGCGGCAGCGGCGGGGGATGGTGGGGCGGAAAGCACGTGTCTGCG

GACTGCTTCAAAGTGGAGACCGTGTGCCACACGACGCTGTACTACTTTGGATCTAAGCCA

GCTCTCTACTATAGAGTATCTGCCTCCAGCAGCCGCCTGGGAGGGTTCATCTGCGACAAC

TTTCACCCGGAGATTACAAAATTCGAGGGGAGCGTGGACGTGACCACGCGGCTGTTGTTG

GACAATGAAAATTTTACCAGTTTCGGGTGGTACCGCCTGCGACCCGGCACCCACGGAGAG

CGTGTTCAACTTCGCCCCGTTGAGCGACACGTCACCTCAAGCGACGTGGAGATTAACTGT

ACTCCCGATAACCTGGAGCCGATACCAGACGAGGCTGCCTGGCCCGACTATAAGCTCATG

TGCTTTGATATAGAGTGTAAAGCTGGAACGGGTAACGAAATGGCGTTCCCAGTGGCAACT

AACCAAGAGGACCTGGTCATCCAGATCTCCTGTCTGCTGTACTCGCTTGCTACTCAGAAC

CACGAACACACCCTGCTGTTTTCCCTCGGGTCATGCGATATCTCTGAGGAATACTCGTTT

GCATGCGTCCAGCGCGGCGAGCCCAGACCGACGGTTTTGGAGTTTGACAGCGAGTACGAG

CTGCTGGTTGCCTTCCTGACCTTTCTCAAGCAGTACTCTCCCGAGTTCGCCACCGGCTAC

AACATCGTTAATTTTGACTGGGCGTACATAGTTAACAAGGTAACGTCGGTGTATAACATC

AAGCTGGACGGGTACGGCAAGTTCAACAAAGGGGGGCTGTTTAAGGTGTGGGACATCGCC

ACGAACCATTTTCAGAAGAAGAGCAAGGTGAAAATCAATGGCCTGATATCTCTAGACATG

TATTCTGTGGCGACGGAAAAGCTAAAGCTACCCAGCTACAAACTCGACGCGGTCGTGGGA

GACGTCCTCGGCGAGCATAAGATAGACCTTCCCTATAAAGAAATACCCTCCTATTACGCG

GGAGGGCCTGACCGGAGGGGCGTAATAGGAGAGTATTGTATCCAGGACTCTAGGCTGGTG

GGCAAGCTGTTTTTTAAGTACCTCCCCCATCTGGAACTATCGGCGGTGGCCAAACTCGCC

CGTATCACCCTAACGCGGGTAATTTTTGACGGTCAGCAAATTCGCGTGTACACGTGCTTG

CTGAAACTCGCCCGCGAGAGAAATTTCATTTTGCCAGACAACAGACGCCGGTTTGACAGT

CAGGCAGACGCCGCGTCAGAGACTTCGGAGTTGGCTATGGATAGCCAAAGCCACGCCTTC

GACAGTACAGACGAACCCGACGGTGTGGACGGTACCCCGGACGCCGCAGGATCTGGCGCT

ACTTCTGAAAACGGAGGCGGGAAGCCCGGCGTCGGGAGGGCCGTGGGCTACCAGGGAGCA

AAGGTTCTAGACCCCGTATCCGGCTTTCATGTGGACCCCGTGGTTGTGTTTGACTTCGCT

AGCTTATACCCAAGCATTATCCAGGCCCATAACCTCTGTTTCACCACCCTGGCGCTCGAT

GAAGCGGATCTGGCCGGGCTTCAACCATCCGTCAACTACTCGACGTTCGAGGTGGGTGAC

CAAAAGTTATTTTTTGTCCACGCCCATATTCGCGAAAGCCTGCTTGGCATCTTGCTGCGC

GACTGGCTGGCCATGCGAAAGGCGGTGAGGGCGCGAATCCCCACCAGCACCCCCGAGGAG

GCAGTTTTACTAGATAAGCAGCAGTCTGCGATTAAGGTGATATGCAACTCGGTTTACGGA

TTCACGGGGGTGGCAAACGGCCTGTTGCCGTGTCTGAGGATAGCGGCTACCGTTACCACG

ATAGGACGCGACATGCTCCTCAAGACCCGAGATTACGTTCACTCTCGTTGGGCGACGCGC

GAGCTGCTGGAGGACAATTTTCCAGGGGCTATAGGTTTCCGAAACCACAAGCCTTACTCC

GTCAGGGTTATCTACGGAGACACCGACTCCGTGTTTATCAAGTTTGTGGGCCTGACGTAC

GAGGGGGTATCGGAGCTGGGGGATGCTATGTCGCGTCAGATTTCAGCGGACCTCTTTAGA

GCCCCCATCAAACTGGAGTGTGAGAAGACCTTTCAGCGACTGCTGCTGATCACCAAGAAG

AAGTACATAGGTGTCATAAACGGGGGGAAGATGCTCATGAAGGGGGTCGACCTGGTCCGC

AAAAATAACTGCTCTTTCATAAACTTGTACGCGCGACATCTGGTAGATCTTTTGTTGTAC

GACGAGGATGTGGCCACGGCGGCAGCAGAGGTGACAGACGTGCCTCCCGCAGAATGGGTG

GGGCGCCCGCTACCGAGCGGCTTTGACAAGTTTGGGCGAGTGCTGGTAGAGGCGTACAAC

CGTATCACTGCCCCCAACTTGGACGTGCGCGAGTTCGTTATGACTGCTGAGCTGAGCCGC

TCACCCGAATCGTATACCAACAAGCGCCTGCCGCACCTCACCGTCTACTTTAAGCTCGCC

ATGAGGAATGAAGAACTGCCCAGCGTAAAAGAGAGAATTCCGTATGTGATAGTTGCGCAG

ACCGAGGCCGCGGAACGCGAAGCGGGTGTAGTAAACTCAATGCGCGGTACCGCCCAAAAC

CCCGTGGTAACCAAGACCGCACGCCCCCAACCTAAACGCAAACTGCTGGTTTCCGACCTC

GCCGAAGACCCGACCTATGTTTCCGAGAATGACGTACCGCTAAACACAGACTACTATTTC

TCCCACCTGTTGGGTACCATAAGCGTGACCTTTAAGGCTCTATTCGGAAATGATGTGAGA

ACAACAGAAAATCTTTTAAAGCGGTTTATTCCGGAAACCCCCCACAAGACCCCCACGAAA

ACCCAGGCACTGCTTGAGCGCGCCGGCTTTGAAAAGCTGACGCCCTTTACACCGGAGGAA

GAAAGTCGTCGAATACTGCATACAGTTTTTTGTACTCTAGAAGCAGCTCCCCATCAAAGC

TGA

>KF644578.1

ATGGCGGCGCGCGAACAGGCCAACTCCGTTCGCAGGAGCGGGTTTTTTAATCCGTTTATT

GGCAAGAGGCCATTTTTCAGGCCGGGAAGCGGGCAGACCGCGGAGACAGAGCGCCCAAGG

CCCCCCCAACACTCGTACTGCACAGAGGTGGGTAGCTTTAAGTTTATAGCTCCAAGATGC

CTCGATGAAGAAGCCCCCGCCGACCAGCGACGCGGTGTACACGTGGGCACCTTGGAGCGC

CCACCCAAAGTGTACTGCGATGGCTCAGAGTACGACGTGCTGAACTTTGCCTCCGGAGGT

TGCTGGCCTCGCAGGATTCGGGTTTGGAACGGCCAGGATTTTCGGGGCGATGGATTCAAC

CCCAGATTTGAGAGATTTCACGTGTACGACATAGTAGAGACTTCGGAGAGCGCCTCACAC

GATGACCCATCCAGGTTTGCTGAGCTATCCCGCCCAAGCGGGTCTGTGGTAACACTGCTG

GGAATGAGCGAGTGTGGAAAGCGAGTCGCGGTTCACGTCTATGGTGTGCGCCATTATTTT

TACATGGCAAAGGCGGAGGTGGATAGCGCTTGTGGAATAACCACCGAGGCAGAACTCGTG

CGCGCAATGGTGGACTGCGCGCACAGCTCGGCTTTGAGCGCGGCCCTGGGAAATGGCAAC

GGCGGCAAACAGAGCGGCGGCAGCGGCGGGGGATGGTGGGGCGGAAAGCACGTGTCCGCG

GACTGCTTCAAAGTGGAGACCGTGTGCCACACGACGCTGTACTACTTTGGATCTAAGCCA

GCTCTCTACTATAGAGTATCTGCCTCCAGCAGCCGCCTGGGAGGGTTCATCTGCGACAAC

TTTCACCCGGAGATTACAAAATTCGAGGGGAGCGTGGACGTGACCACGCGGCTGTTGTTG

GACAATGAAAATTTTACCAGTTTCGGGTGGTACCGCCTGCGACCCGGCACCCACGGAGAG

CGTGTTCAACTTCGCCCCGTTGAGCGACACGTCACCTCAAGCGACGTGGAGATTAACTGT

ACTCCCGATAACCTGGAGCCGATACCAGACGAGGCTGCCTGGCCCGACTATAAGCTCATG

TGCTTTGATATAGAGTGTAAAGCTGGAACGGGTAACGAAATGGCGTTCCCAGTGGCAACT

AACCAAGAGGACCTGGTCATCCAGATCTCCTGTCTGCTGTACTCGCTTGCTACTCAGAAC

CACGAACACACCCTGCTGTTTTCCCTCGGGTCATGCGATATCTCTGAGGAATACTCGTTT

GCATGCGTCCAGCGCGGCGAGCCCAGACCGACGGTTTTGGAGTTTGACAGCGAGTACGAG

CTGCTGGTTGCCTTCCTGACCTTTCTCAAGCAGTACTCTCCCGAGTTCGCCACCGGCTAC

AACATCGTTAATTTTGACTGGGCGTACATAGTTAACAAGGTAACGTCGGTGTATAACATC

AAGCTGGACGGGTACGGCAAGTTCAACAAAGGGGGGCTGTTTAAGGTGTGGGACATCGCC

ACGAACCATTTTCAGAAGAAGAGCAAGGTGAAAATCAATGGCCTGATATCTCTAGACATG

TATTCTGTGGCGACGGAAAAGCTAAAGCTACCCAGCTACAAACTCGACGCGGTCGTGGGA

GACGTCCTCGGCGAGCATAAGATAGACCTTCCCTATAAAGAAATACCCTCCTATTACGCG

GGAGGGCCTGACCGGAGGGGCGTAATAGGAGAGTATTGTATCCAGGACTCTAGGCTGGTG

GGCAAGCTGTTTTTTAAGTACCTCCCCCATCTGGAACTATCGGCGGTGGCCAAACTCGCC

CGTATCACCCTAACGCGGGTAATTTTTGACGGTCAGCAAATTCGCGTGTACACGTGCTTG

CTGAAACTCGCCCGCGAGAGAAATTTCATTTTGCCAGACAACAGACGCCGGTTTGACAGT

CAGGCAGACGCCGCGTCAGAGACTTCGGAGTTGGCTATGGATAGCCAAAGCCACGCCTTC

GACAGTACAGACGAACCCGACGGTGTGGACGGTACCCCGGACGCCGCAGGATCTGGCGCT

ACTTCTGAAAACGGAGGCGGGAAGCCCGGCGTCGGGAGGGCCGTGGGCTACCAGGGAGCA

AAGGTTCTAGACCCCGTATCCGGCTTTCATGTGGACCCCGTGGTTGTGTTTGACTTCGCT

AGCTTATACCCAAGCATTATCCAGGCCCATAACCTCTGTTTCACCACCCTGGCGCTCGAT

GAAGTGGATCTGGCCGGGCTTCAACCATCCGTCGACTACTCGACGTTCGAGGTGGGTGAC

CAAAAGTTATTTTTTGTCCACGCCCATATTCGCGAAAGCCTGCTTGGCATCTTGCTGCGC

GACTGGCTGGCCATGCGAAAGGCGGTGAGGGCGCGAATCCCCACCAGCACCCCCGAGGAG

GCAGTTTTACTAGATAAGCAGCAGTCTGCGATTAAGGTGATATGCAACTCGGTTTACGGA

TTCACGGGGGTGGCAAACGGCCTGTTGCCGTGTCTGAGGATAGCGGCTACCGTTACCACG

ATAGGACGCGACATGCTCCTCAAGACCCGAGATTACGTTCACTCTCGTTGGGCGACGCGC

GAGCTGCTGGAGGACAATTTTCCAGGGGCTATAGGTTTCCGAAACCACAAGCCTTACTCC

GTCAGGGTTATCTACGGAGACACCGACTCCGTGTTTATCAAGTTTGTGGGCCTGACGTAC

GAGGGGGTATCGGAGCTGGGGGATGCTATGTCGCGTCAGATTTCAGCGGACCTCTTTAGA

GCCCCCATCAAACTGGAGTGTGAGAAGACCTTTCAGCGACTGCTGCTGATCACCAAGAAG

AAGTACATAGGTGTCATAAACGGGGGGAAGATGCTCATGAAGGGGGTCGACCTGGTCCGC

AAAAATAACTGCTCTTTCATAAACTTGTACGCGCGACATCTGGTAGATCTTTTGTTGTAC

GACGAGGATGTGGCCACGGCGGCAGCAGAGGTGACAGACGTGCCTCCCGCAGAATGGGTG

GGGCGCCCGCTACCGAGCGGCTTTGACAAGTTTGGGCGAGTGCTGGTAGAGGCGTACAAC

CGTATCACTGCCCCCAACTTGGACGTGCGCGAGTTCGTTATGACTGCTGAGCTGAGCCGC

TCACCCGAATCGTATACCAACAAGCGCCTGCCGCACCTCACCGTCTACTTTAAGCTCGCC

ATGAGGAATGAAGAACTGCCCAGCGTAAAAGAGAGAATTCCGTATGTGATAGTTGCGCAG

ACCGAGGCCGCGGAACGCGAAGCGGGTGTAGTAAACTCAATGCGCGGTACCGCCCAAAAC

CCCGTGGTAACCAAGACCGCACGCCCCCAACCTAAACGCAAACTGCTGGTTTCCGACCTC

GCCGAAGACCCGACCTATGTTTCCGAGAATGACGTACCGCTAAACACAGACTACTATTTC

TCCCACCTGTTGGGTACCATAAGCGTGACCTTTAAGGCTCTATTCGGAAATGATGTGAGA

ACAACAGAAAATCTTTTAAAGCGGTTTATTCCGGAAACCCCCCACAAGACCCCCACGAAA

ACCCAGGCACTGCTTGAGCGCGCCGGCTTTGAAAAGCTGACGCCCTTTACACCGGAGGAA

GAAAGTCGTCGAATACTGCATACAGTTTTTTGTACTCTAGAAGCAGCTCCCCATCAAAGC

TGA

>KF644579.1

ATGGCGGCGCGCGAACAGGCCAACTCCGTTCGCAGGAGCGGGTTTTTTAATCCGTTTATT

GGCAAGAGGCCATTTTTCAGGCCGGGAAGCGGGCAGACCGCGGAGACAGAGCGCCCAAGG

CCCCCCCAACACTCGTACTGCACAGAGGTGGGTAGCTTTAAGTTTATAGCTCCAAGATGC

CTCGATGAAGAAGCCCCCGCCGACCAGCGACGCGGTGTACACGTGGGCACCTTGGAGCGC

CCACCCAAAGTGTACTGCGATGGCTCAGAGTACGACGTGCTGAACTTTGCCTCCGGAGGT

TGCTGGCCTCGCAGGATTCGGGTTTGGAACGGCCAGGATTTTCGGGGCGATGGATTCAAC

CCCAGATTTGAGAGATTTCACGTGTACGACATAGTAGAGACTTCGGAGAGCGCCTCACAC

GATGACCCATCCAGGTTTGCTGAGCTATCCCGCCCAAGCGGGTCTGTGGTAACACTGCTG

GGAATGAGCGAGTGTGGAAAGCGAGTCGCGGTTCACGTCTATGGTGTGCGCCATTATTTT

TACATGGCAAAGGCGGAGGTGGATAGCGCTTGTGGAATAACCACCGAGGCAGAACTCGTG

CGCGCAATGGTGGACTGCGCGCACAGCTCGGCTTTGAGCGCGGCCCTGGGAAATGGCAAC

GGCGGCAAACAGAGCGGCGGCAGCGGCGGGGGATGGTGGGGCGGAAAGCACGTGTCTGCG

GACTGCTTCAAAGTGGAGACCGTGTGCCACACGACGCTGTACTACTTTGGATCTAAGCCA

GCTCTCTACTATAGAGTATCTGCCTCCAGCAGCCGCCTGGGAGGGTTCATCTGCGACAAC

TTTCACCCGGAGATTACAAAATTCGAGGGGAGCGTGGACGTGACCACGCGGCTGTTGTTG

GACAATGAAAATTTTACCAGTTTCGGGTGGTACCGCCTGCGACCCGGCACCCACGGAGAG

CGTGTTCAACTTCGCCCCGTTGAGCGACACGTCACCTCAAGCGACGTGGAGATTAACTGT

ACTCCCGATAACCTGGAGCCGATACCAGACGAGGCTGCCTGGCCCGACTATAAGCTCATG

TGCTTTGATATAGAGTGTAAAGCTGGAACGGGTAACGAAATGGCGTTCCCAGTGGCAACT

AACCAAGAGGACCTGGTCATCCAGATCTCCTGTCTGCTGTACTCGCTTGCTACTCAGAAC

CACGAACACACCCTGCTGTTTTCCCTCGGGTCATGCGATATCTCTGAGGAATACTCGTTT

GCATGCGTCCAGCGCGGCGAGCCCAGACCGACGGTTTTGGAGTTTGACAGCGAGTACGAG

CTGCTGGTTGCCTTCCTGACCTTTCTCAAGCAGTACTCTCCCGAGTTCGCCACCGGCTAC

AACATCGTTAATTTTGACTGGGCGTACATAGTTAACAAGGTAACGTCGGTGTATAACATC

AAGCTGGACGGGTACGGCAAGTTCAACAAAGGGGGGCTGTTTAAGGTGTGGGACATCGCC

ACGAACCATTTTCAGAAGAAGAGCAAGGTGAAAATCAATGGCCTGATATCTCTAGACATG

TATTCTGTGGCGACGGAAAAGCTAAAGCTACCCAGCTACAAACTCGACGCGGTCGTGGGA

GACGTCCTCGGCGAGCATAAGATAGACCTTCCCTATAAAGAAATACCCTCCTATTACGCG

GGAGGGCCTGACCGGAGGGGCGTAATAGGAGAGTATTGTATCCAGGACTCTAGGCTGGTG

GGCAAGCTGTTTTTTAAGTACCTCCCCCATCTGGAACTATCGGCGGTGGCCAAACTCGCC

CGTATCACCCTAACGCGGGTAATTTTTGACGGTCAGCAAATTCGCGTGTACACGTGCTTG

CTGAAACTCGCCCGCGAGAGAAATTTCATTTTGCCAGACAACAGACGCCGGTTTGACAGT

CAGGCAGACGCCGCGTCAGAGACTTCGGAGTTGGCTATGGATAGCCAAAGCCACGCCTTC

GACAGTACAGACGAACCCGACGGTGTGGACGGTACCCCGGACGCCGCAGGATCTGGCGCT

ACTTCTGAAAACGGAGGCGGGAAGCCCGGCGTCGGGAGGGCCGTGGGCTACCAGGGAGCA

AAGGTTCTAGACCCCGTATCCGGCTTTCATGTGGACCCCGTGGTTGTGTTTGACTTCGCT

AGCTTATACCCAAGCATTATCCAGGCCCATAACCTCTGTTTCACCACCCTGGCGCTCGAT

GAAGCGGATCTGGCCGGGCTTCAACCATCCGTCAACTACTCGACGTTCGAGGTGGGTGAC

CAAAAGTTATTTTTTGTCCACGCCCATATTCGCGAAAGCCTGCTTGGCATCTTGCTGCGC

GACTGGCTGGCCATGCGAAAGGCGGTGAGGGCGCGAATCCCCACCAGCACCCCCGAGGAG

GCAGTTTTACTAGATAAGCAGCAGTCTGCGATTAAGGTGATATGCAACTCGGTTTACGGA

TTCACGGGGGTGGCAAACGGCCTGTTGCCGTGTCTGAGGATAGCGGCTACCGTTACCACG

ATAGGACGCGACATGCTCCTCAAGACCCGAGATTACGTTCACTCTCGTTGGGCGACGCGC

GAGCTGCTGGAGGACAATTTTCCAGGGGCTATAGGTTTCCGAAACCACAAGCCTTACTCC

GTCAGGGTTATCTACGGAGACACCGACTCCGTGTTTATCAAGTTTGTGGGCCTGACGTAC

GAGGGGGTATCGGAGCTGGGGGATGCTATGTCGCGTCAGATTTCAGCGGACCTCTTTAGA

GCCCCCATCAAACTGGAGTGTGAGAAGACCTTTCAGCGACTGCTGCTGATCACCAAGAAG

AAGTACATAGGTGTCATAAACGGGGGGAAGATGCTCATGAAGGGGGTCGACCTGGTCCGC

AAAAATAACTGCTCTTTCATAAACTTGTACGCGCGACATCTGGTAGATCTTTTGTTGTAC

GACGAGGATGTGGCCACGGCGGCAGCAGAGGTGACAGACGTGCCTCCCGCAGAATGGGTG

GGGCGCCCGCTACCGAGCGGCTTTGACAAGTTTGGGCGAGTGCTGGTAGAGGCGTACAAC

CGTATCACTGCCCCCAACTTGGACGTGCGCGAGTTCGTTATGACTGCTGAGCTGAGCCGC

TCACCCGAATCGTATACCAACAAGCGCCTGCCGCACCTCACCGTCTACTTTAAGCTCGCC

ATGAGGAATGAAGAACTGCCCAGCGTAAAAGAGAGAATTCCGTATGTGATAGTTGCGCAG

ACCGAGGCCGCGGAACGCGAAGCGGGTGTAGTAAACTCAATGCGCGGTACCGCCCAAAAC

CCCGTGGTAACCAAGACCGCACGCCCCCAACCTAAACGCAAACTGCTGGTTTCCGACCTC

GCCGAAGACCCGACCTATGTTTCCGAGAATGACGTACCGCTAAACACAGACTACTATTTC

TCCCACCTGTTGGGTACCATAAGCGTGACCTTTAAGGCTCTATTCGGAAATGATGTGAGA

ACAACAGAAAATCTTTTAAAGCGGTTTATTCCGGAAACCCCCCACAAGACCCCCACGAAA

ACCCAGGCACTGCTTGAGCGCGCCGGCTTTGAAAAGCTGACGCCCTTTACACCGGAGGAA

GAAAGTCGTCGAATACTGCATACAGTTTTTTGTACTCTAGAAGCAGCTCCCCATCAAAGC

TGA

>AB992258.1

ATGGCGGCGCGCGAACAGGCCAACTCCGTTCGCAGGAGCGGGTTTTTTAATCCGTTTATT

GGCAAGAGGCCATTTTTCAGGCCGGGAAGCGGGCAGACCGCGGAGACAGAGCGCCCAAGG

CCCCCCCAACACTCGTACTGCACAGAGGTGGGTAGCTTTAAGTTTATAGCTCCAAGATGC

CTCGATGAAGAAGCCCCCGCCGACCAGCGACGCGGTGTACACGTGGGCACCTTGGAGCGC

CCACCCAAAGTGTACTGCGATGGCTCAGAGTACGACGTGCTGAACTTTGCCTCCGGAGGT

TGCTGGCCTCGCAGGATTCGGGTTTGGAACGGCCAGGATTTTCGGGGCGATGGATTCAAC

CCCAGATTTGAGAGATTTCACGTGTACGACATAGTAGAGACTTCGGAGAGCGCCTCACAC

GATGACCCATCCAGGTTTGCTGAGCTATCCCGCCCAAGCGGGTCTGTGGTAACACTGCTG

GGAATGAGCGAGTGTGGAAAGCGAGTCGCGGTTCACGTCTATGGTGTGCGCCATTATTTT

TACATGGCAAAGGCGGAGGTGGATAGCGCTTGTGGAATAACCACCGAGGCAGAACTCGTG

CGCGCAATGGTGGACTGCGCGCACAGCTCGGCTTTGAGCGCGGCCCTGGGAAATGGCAAC

GGCGGCAAACAGAGCGGCGGCAGCGGCGGGGGATGGTGGGGCGGAAAGCACGTGTCTGCG

GACTGCTTCAAAGTGGAGACCGTGTGCCACACGACGCTGTACTACTTTGGATCTAAGCCA

GCTCTCTACTATAGAGTATCTGCCTCCAGCAGCCGCCTGGGAGGGTTCATCTGCGACAAC

TTTCACCCGGAGATTACAAAATTCGAGGGGAGCGTGGACGTGACCACGCGGCTGTTGTTG

GACAATGAAAATTTTACCAGTTTCGGGTGGTACCGCCTGCGACCCGGCACCCACGGAGAG

CGTGTTCAACTTCGCCCCGTTGAGCGACACGTCACCTCAAGCGACGTGGAGATTAACTGT

ACTCCCGATAACCTGGAGCCGATACCAGACGAGGCTGCCTGGCCCGACTATAAGCTCATG

TGCTTTGATATAGAGTGTAAAGCTGGAACGGGTAACGAAATGGCGTTCCCAGTGGCAACT

AACCAAGAGGACCTGGTCATCCAGATCTCCTGTCTGCTGTACTCGCTTGCTACTCAGAAC

CACGAACACACCCTGCTGTTTTCCCTCGGGTCATGCGATATCTCTGAGGAATACTCGTTT

GCATGCGTCCAGCGCGGCGAGCCCAGACCGACGGTTTTGGAGTTTGACAGCGAGTACGAG

CTGCTGGTTGCCTTCCTGACCTTTCTCAAGCAGTACTCTCCCGAGTTCGCCACCGGCTAC

AACATCGTTAATTTTGACTGGGCGTACATAGTTAACAAGGTAACGTCGGTGTATAACATC

AAGCTGGACGGGTACGGCAAGTTCAACAAAGGGGGGCTGTTTAAGGTGTGGGACATCGCC

ACGAACCATTTTCAGAAGAAGAGCAAGGTGAAAATCAATGGCCTGATATCTCTAGACATG

TATTCTGTGGCGACGGAAAAGCTAAAGCTACCCAGCTACAAACTCGACGCGGTCGTGGGA

GACGTCCTCGGCGAGCATAAGATAGACCTTCCCTATAAAGAAATACCCTCCTATTACGCG

GGAGGGCCTGACCGGAGGGGCGTAATAGGAGAGTATTGTATCCAGGACTCTAGGCTGGTG

GGCAAGCTGTTTTTTAAGTACCTCCCCCATCTGGAACTATCGGCGGTGGCCAAACTCGCC

CGTATCACCCTAACGCGGGTAATTTTTGACGGTCAGCAAATTCGCGTGTACACGTGCTTG

CTGAAACTCGCCCGCGAGAGAAATTTCATTTTGCCAGACAACAGACGCCGGTTTGACAGT

CAGGCAGACGCCGCGTCAGAGACTTCGGAGTTGGCTATGGATAGCCAAAGCCACGCCTTC

GACAGTACAGACGAACCCGACGGTGTGGACGGTACCCCGGACGCCGCAGGATCTGGCGCT

ACTTCTGAAAACGGAGGCGGGAAGCCCGGCGTCGGGAGGGCCGTGGGCTACCAGGGAGCA

AAGGTTCTAGACCCCGTATCCGGCTTTCATGTGGACCCCGTGGTTGTGTTTGACTTCGCT

AGCTTATACCCAAGCATTATCCAGGCCCATAACCTCTGTTTCACCACCCTGGCGCTCGAT

GAAGTGGATCTGGCCGGGCTTCAACCATCCGTCAACTACTCGACGTTCGAGGTGGGTGAC

CAAAAGTTATTTTTTGTCCACGCCCATATTCGCGAAAGCCTGCTTGGCATCTTGCTGCGC

GACTGGCTGGCCATGCGAAAGGCGGTGAGGGCGCGAATCCCCACCAGCACCCCCGAGGAG

GCAGTTTTACTAGATAAGCAGCAGTCTGCGATTAAGGTGATATGCAACTCGGTTTACGGA

TTCACGGGGGTGGCAAACGGCCTGTTGCCGTGTCTGAGGATAGCGGCTACCGTTACCACG

ATAGGACGCGACATGCTCCTCAAGACCCGAGATTACGTTCACTCTCGTTGGGCGACGCGC

GAGCTGCTGGAGGACAATTTTCCAGGGGCTATAGGTTTCCGAAACCACAAGCCTTACTCC

GTCAGGGTTATCTACGGAGACACCGACTCCGTGTTTATCAAGTTTGTGGGCCTGACGTAC

GAGGGGGTATCGGAGCTGGGGGATGCTATGTCGCGTCAGATTTCAGCGGACCTCTTTAGA

GCGCCCATCAAACTGGAGTGTGAGAAGACCTTTCAGCGACTGCTGCTGATCACCAAGAAG

AAGTACATAGGTGTCATAAACGGGGGGAAGATGCTCATGAAGGGGGTCGACCTGGTCCGC

AAAAATAACTGCTCTTTCATAAACTTGTACGCGCGACATCTGGTAGATCTTTTGTTGTAC

GACGAGGATGTGGCCACGGCGGCAGCAGAGGTGACAGACGTGCCTCCCGCAGAATGGGTG

GGGCGCCCGCTACCGAGCGGCTTTGACAAGTTTGGGCGAGTGCTGGTAGAGGCGTACAAC

CGTATCACTGCCCCCAACTTGGACGTGCGCGAGTTCGTTATGACTGCTGAGCTGAGCCGC

TCACCCGAATCGTATACCAACAAGCGCCTGCCGCACCTCACCGTCTACTTTAAGCTCGCC

ATGAGGAATGAAGAACTGCCCAGCGTAAAAGAGAGAATTCCGTATGTGATAGTTGCGCAG

ACCGAGGCCGCGGAACGCGAAGCGGGTGTAGTAAACTCAATGCGCGGTACCGCCCAAAAC

CCCGTGGTAACCAAGACCGCACGCCCCCAACCTAAACGCAAACTGCTGGTTTCCGACCTC

GCCGAAGACCCGACCTATGTTTCCGAGAATGACGTACCGCTAAACACAGACTACTATTTC

TCCCACCTGTTGGGTACCATAAGCGTGACCTTTAAGGCTCTATTCGGAAATGATGTGAGA

ACAACAGAAAATCTTTTAAAGCGGTTTATTCCGGAAACCCCCCACAAGACCCCCACGAAA

ACCCAGGCACTGCTTGAGCGCGCCGGCTTTGAAAAGCTGACGCCCTTTACACCGGAGGAA

GAAAGTCGTCGAATACTGCATACAGTTTTTTGTACTCTAGAAGCAGCTCCCCATCAAAGC

TGA

>KM593996.1

ATGGCGGCGCGCGAACAGGCCAACTCCGTTCGCAGGAGCGGGTTTTTTAATCCGTTTATT

GGCAAGAGGCCATTTTTCAGGCCGGGAAGCGGGCAGACCGCGGAGACAGAGCGCCCAAGG

CCCCCCCAACACTCGTACTGCACAGAGGTGGGTAGCTTTAAGTTTATAGCTCCAAGATGC

CTCGATGAAGAAGCCCCCGCCGACCAGCGACGCGGTGTACACGTGGGCACCTTGGAGCGC

CCACCCAAAGTGTACTGCGATGGCTCAGAGTACGACGTGCTGAACTTTGCCTCCGGAGGT

TGCTGGCCTCGCAGGATTCGGGTTTGGAACGGCCAGGATTTTCGGGGCGATGGATTCAAC

CCCAGATTTGAGAGATTTCACGTGTACGACATAGTAGAGACTTCGGAGAGCGCCTCACAC

GATGACCCATCCAGGTTTGCTGAGCTATCCCGCCCAAGCGGGTCTGTGGTAACACTGCTG

GGAATGAGCGAGTGTGGAAAGCGAGTCGCGGTTCACGTCTATGGTGTGCGCCATTATTTT

TACATGGCAAAGGCGGAGGTGGATAGCGCTTGTGGAATAACCACCGAGGCAGAACTCGTG

CGCGCAATGGTGGACTGCGCGCACAGCTCGGCTTTGAGCGCGGCCCTGGGAAATGGCAAC

GGCGGCAAACAGAGCGGCGGCAGCGGCGGGGGATGGTGGGGCGGAAAGCACGTGTCTGCG

GACTGCTTCAAAGTGGAGACCGTGTGCCACACGACGCTGTACTACTTTGGATCTAAGCCA

GCTCTCTACTATAGAGTATCTGCCTCCAGCAGCCGCCTGGGAGGGTTCATCTGCGACAAC

TTTCACCCGGAGATTACAAAATTCGAGGGGAGCGTGGACGTGACCACGCGGCTGTTGTTG

GACAATGAAAATTTTACCAGTTTCGGGTGGTACCGCCTGCGACCCGGCACCCACGGAGAG

CGTGTTCAACTTCGCCCCGTTGAGCGACACGTCACCTCAAGCGACGTGGAGATTAACTGT

ACTCCCGATAACCTGGAGCCGATACCAGACGAGGCTGCCTGGCCCGACTATAAGCTCATG

TGCTTTGATATAGAGTGTAAAGCTGGAACGGGTAACGAAATGGCGTTCCCAGTGGCAACT

AACCAAGAGGACCTGGTCATCCAGATCTCCTGTCTGCTGTACTCGCTTGCTACTCAGAAC

CACGAACACACCCTGCTGTTTTCCCTCGGGTCATGCGATATCTCTGAGGAATACTCGTTT

GCATGCGTCCAGCGCGGCGAGCCCAGACCGACGGTTTTGGAGTTTGACAGCGAGTACGAG

CTGCTGGTTGCCTTCCTGACCTTTCTCAAGCAGTACTCTCCCGAGTTCGCCACCGGCTAC

AACATCGTTAATTTTGACTGGGCGTACATAGTTAACAAGGTAACGTCGGTGTATAACATC

AAGCTGGACGGGTACGGCAAGTTCAACAAAGGGGGGCTGTTTAAGGTGTGGGACATCGCC

ACGAACCATTTTCAGAAGAAGAGCAAGGTGAAAATCAATGGCCTGATATCTCTAGACATG

TATTCTGTGGCGACGGAAAAGCTAAAGCTACCCAGCTACAAACTCGACGCGGTCGTGGGA

GACGTCCTCGGCGAGCATAAGATAGACCTTCCCTATAAAGAAATACCCTCCTATTACGCG

GGAGGGCCTGACCGGAGGGGCGTAATAGGAGAGTATTGTATCCAGGACTCTAGGCTGGTG

GGCAAGCTGTTTTTTAAGTACCTCCCCCATCTGGAACTATCGGCGGTGGCCAAACTCGCC

CGTATCACCCTAACGCGGGTAATTTTTGACGGTCAGCAAATTCGCGTGTACACGTGCTTG

CTGAAACTCGCCCGCGAGAGAAATTTCATTTTGCCAGACAACAGACGCCGGTTTGACAGT

CAGGCAGACGCCGCGTCAGAGACTTCGGAGTTGGCTATGGATAGCCAAAGCCACGCCTTC

GACAGTACAGACGAACCCGACGGTGTGGACGGTACCCCGGACGCCGCAGGATCTGGCGCT

ACTTCTGAAAACGGAGGCGGGAAGCCCGGCGTCGGGAGGGCCGTGGGCTACCAGGGAGCA

AAGGTTCTAGACCCCGTATCCGGCTTTCATGTGGACCCCGTGGTTGTGTTTGACTTCGCT

AGCTTATACCCAAGCATTATCCAGGCCCATAACCTCTGTTTCACCACCCTGGCGCTCGAT

GAAGTGGATCTGGCCGGGCTTCAACCATCCGTCGACTACTCGACGTTCGAGGTGGGTGAC

CAAAAGTTATTTTTTGTCCACGCCCATATTCGCGAAAGCCTGCTTGGCATCTTGCTGCGC

GACTGGCTGGCCATGCGAAAGGCGGTGAGGGCGCGAATCCCCACCAGCACCCCCGAGGAG

GCAGTTTTACTAGATAAGCAGCAGTCTGCGATTAAGGTGATATGCAACTCGGTTTACGGA

TTCACGGGGGTGGCAAACGGCCTGTTGCCGTGTCTGAGGATAGCGGCTACCGTTACCACG

ATAGGACGCGACATGCTCCTCAAGACCCGAGATTACGTTCACTCTCGTTGGGCGACGCGC

GAGCTGCTGGAGGACAATTTTCCAGGGGCTATAGGTTTCCGAAACCACAAGCCTTACTCC

GTCAGGGTTATCTACGGAGACACCGACTCCGTGTTTATCAAGTTTGTGGGCCTGACGTAC

GAGGGGGTATCGGAGCTGGGGGATGCTATGTCGCGTCAGATTTCAGCGGACCTCTTTAGA

GCGCCCATCAAACTGGAGTGTGAGAAGACCTTTCAGCGACTGCTGCTGATCACCAAGAAG

AAGTACATAGGTGTCATAAACGGGGGGAAGATGCTCATGAAGGGGGTCGACCTAGTCCGC

AAAAATAACTGCTCTTTCATAAACTTGTACGCGCGACATCTGGTAGATCTTTTGTTGTAC

GACGAGGATGTGGCCACGGCGGCAGCAGAGGTGACAGACGTGCCTCCCGCAGAATGGGTG

GGGCGCCCGCTACCGAGCGGCTTTGACAAGTTTGGGCGAGTGCTGGTAGAGGCGTACAAC

CGTATCACTGCCCCCAACTTGGACGTGCGCGAGTTCGTTATGACTGCTGAGCTGAGCCGC

TCACCCGAATCGTATACCAACAAGCGCCTGCCGCACCTCACCGTCTACTTTAAGCTCGCC

ATGAGGAATGAAGAACTGCCCAGCGTAAAAGAGAGAATTCCGTATGTGATAGTTGCGCAG

ACCGAGGCCGCGGAACGCGAAGCGGGTGTAGTAAACTCAATGCGCGGTACCGCCCAAAAC

CCCGTGGTAACCAAGACCGCACGCCCCCAACCTAAACGCAAACTGCTGGTTTCCGACCTC

GCCGAAGACCCGACCTATGTTTCCGAGAATGACGTACCGCTAAACACAGACTACTATTTC

TCCCACCTGTTGGGTACCATAAGCGTGACCTTTAAGGCTCTATTCGGAAATGATGTGAGA

ACAACAGAAAATCTTTTAAAGCGGTTTATTCCGGAAACCCCCCACAAGACCCCCACGAAA

ACCCAGGCACTGCTTGAGCGCGCCGGCTTTGAAAAGCTGACGCCCTTTACACCGGAGGAA

GAAAGTCGTCGAATACTGCATACAGTTTTTTGTACTCTAGAAGCAGCTCCCCATCAAAGC

TGA

>KT324724.1

ATGGCGGCGCGCGAACAGGCCAACTCCGTTCGCAGGAGCGGGTTTTTTAATCCGTTTATT

GGCAAGAGGCCATTTTTCAGGCCGGGAAGCGGGCAGACCGCGGAGACAGAGCGCCCAAGG

CCCCCCCAACACTCGTACTGCACAGAGGTGGGTAGCTTTAAGTTTATAGCTCCAAGATGC

CTCGATGAAGAAGCCCCCGCCGACCAGCGACGCGGTGTACACGTGGGCACCTTGGAGCGC

CCACCCAAAGTGTACTGCGATGGCTCAGAGTACGACGTGCTGAACTTTGCCTCCGGAGGT

TGCTGGCCTCGCAGGATTCGGGTTTGGAACGGCCAGGATTTTCGGGGCGATGGATTCAAC

CCCAGATTTGAGAGATTTCACGTGTACGACATAGTAGAGACTTCGGAGAGCGCCTCACAC

GATGACCCATCCAGGTTTGCTGAGCTATCCCGCCCAAGCGGGTCTGTGGTAACACTGCTG

GGAATGAGCGAGTGTGGAAAGCGAGTCGCGGTTCACGTCTATGGTGTGCGCCATTATTTT

TACATGGCAAAGGCGGAGGTGGATAGCGCTTGTGGAATAACCACCGAGGCAGAACTCGTG

CGCGCAATGGTGGACTGCGCGCACAGCTCGGCTTTGAGCGCGGCCCTGGGAAATGGCAAC

GGCGGCAAACAGAGCGGCGGCAGCGGCGGGGGATGGTGGGGCGGAAAGCACGTGTCTGCG

GACTGCTTCAAAGTGGAGACCGTGTGCCACACGACGCTGTACTACTTTGGATCTAAGCCA

GCTCTCTACTATAGAGTATCTGCCTCCAGCAGCCGCCTGGGAGGGTTCATCTGCGACAAC

TTTCACCCGGAGATTACAAAATTCGAGGGGAGCGTGGACGTGACCACGCGGCTGTTGTTG

GACAATGAAAATTTTACCAGTTTCGGGTGGTACCGCCTGCGACCCGGCACCCACGGAGAG

CGTGTTCAACTTCGCCCCGTTGAGCGACACGTCACCTCAAGCGACGTGGAGATTAACTGT

ACTCCCGATAACCTGGAGCCGATACCAGACGAGGCTGCCTGGCCCGACTATAAGCTCATG

TGCTTTGATATAGAGTGTAAAGCTGGAACGGGTAACGAAATGGCGTTCCCAGTGGCAACT

AACCAAGAGGACCTGGTCATCCAGATCTCCTGTCTGCTGTACTCGCTTGCTACTCAGAAC

CACGAACACACCCTGCTGTTTTCCCTCGGGTCATGCGATATCTCTGAGGAATACTCGTTT

GCATGCGTCCAGCGCGGCGAGCCCAGACCGACGGTTTTGGAGTTTGACAGCGAGTACGAG

CTGCTGGTTGCCTTCCTGACCTTTCTCAAGCAGTACTCTCCCGAGTTCGCCACCGGCTAC

AACATCGTTAATTTTGACTGGGCGTACATAGTTAACAAGGTAACGTCGGTGTATAACATC

AAGCTGGACGGGTACGGCAAGTTCAACAAAGGGGGGCTGTTTAAGGTGTGGGACATCGCC

ACGAACCATTTTCAGAAGAAGAGCAAGGTGAAAATCAATGGCCTGATATCTCTAGACATG

TATTCTGTGGCGACGGAAAAGCTAAAGCTACCCAGCTACAAACTCGACGCGGTCGTGGGA

GACGTCCTCGGCGAGCATAAGATAGACCTTCCCTATAAAGAAATACCCTCCTATTACGCG

GGAGGGCCTGACCGGAGGGGCGTAATAGGAGAGTATTGTATCCAGGACTCTAGGCTGGTG

GGCAAGCTGTTTTTTAAGTACCTCCCCCATCTGGAACTATCGGCGGTGGCCAAACTCGCC

CGTATCACCCTAACGCGGGTAATTTTTGACGGTCAGCAAATTCGCGTGTACACGTGCTTG

CTGAAACTCGCCCGCGAGAGAAATTTCATTTTGCCAGACAACAGACGCCGGTTTGACAGT

CAGGCAGACGCCGCGTCAGAGACTTCGGAGTTGGCTATGGATAGCCAAAGCCACGCCTTC

GACAGTACAGACGAACCCGACGGTGTGGACGGTACCCCGGACGCCGCAGGATCTGGCGCT

ACTTCTGAAAACGGAGGCGGGAAGCCCGGCGTCGGGAGGGCCGTGGGCTACCAGGGAGCA

AAGGTTCTAGACCCCGTATCCGGCTTTCATGTGGACCCCGTGGTTGTGTTTGACTTCGCT

AGCTTATACCCAAGCATTATCCAGGCCCATAACCTCTGTTTCACCACCCTGGCGCTCGAT

GAAGTGGATCTGGCCGGGCTTCAACCATCCGTCAACTACTCGACGTTCGAGGTGGGTGAC

CAAAAGTTATTTTTTGTCCACGCCCATATTCGCGAAAGCCTGCTTGGCATCTTGCTGCGC

GACTGGCTGGCCATGCGAAAGGCGGTGAGGGCGCGAATCCCCACCAGCACCCCCGAGGAG

GCAGTTTTACTAGATAAGCAGCAGTCTGCGATTAAGGTGATATGCAACTCGGTTTACGGA

TTCACGGGGGTGGCAAACGGCCTGTTGCCGTGTCTGAGGATAGCGGCTACCGTTACCACG

ATAGGACGCGACATGCTCCTCAAGACCCGAGATTACGTTCACTCTCGTTGGGCGACGCGC

GAGCTGCTGGAGGACAATTTTCCAGGGGCTATAGGTTTCCGAAACCACAAGCCTTACTCC

GTCAGGGTTATCTACGGAGACACCGACTCCGTGTTTATCAAGTTTGTGGGCCTGACGTAC

GAGGGGGTATCGGAGCTGGGGGATGCTATGTCGCGTCAGATTTCAGCGGACCTCTTTAGA

GCGCCCATCAAACTGGAGTGTGAGAAGACCTTTCAGCGACTGCTGCTGATCACCAAGAAG

AAGTACATAGGTGTCATAAACGGGGGGAAGATGCTCATGAAGGGGGTCGACCTGGTCCGC

AAAAATAACTGCTCTTTCATAAACTTGTACGCGCGACATCTGGTAGATCTTTTGTTGTAC

GACGAGGATGTGGCCACGGCGGCAGCAGAGGTGACAGACGTGCCTCCCGCAGAATGGGTG

GGGCGCCCGCTACCGAGCGGCTTTGACAAGTTTGGGCGAGTGCTGGTAGAGGCGTACAAC

CGTATCACTGCCCCCAACTTGGACGTGCGCGAGTTCGTTATGACTGCTGAGCTGAGCCGC

TCACCCGAATCGTATACCAACAAGCGCCTGCCGCACCTCACCGTCTACTTTAAGCTCGCC

ATGAGGAATGAAGAACTGCCCAGCGTAAAAGAGAGAATTCCGTATGTGATAGTTGCGCAG

ACCGAGGCCGCGGAACGCGAAGCGGGTGTAGTAAACTCAATGCGCGGTACCGCCCAAAAC

CCCGTGGTAACCAAGACCGCACGCCCCCAACCTAAACGCAAACTGCTGGTTTCCGACCTC

GCCGAAGACCCGACCTATGTTTCCGAGAATGACGTACCGCTAAACACAGACTACTATTTC

TCCCACCTGTTGGGTACCATAAGCGTGACCTTTAAGGCTCTATTCGGAAATGATGTGAGA

ACAACAGAAAATCTTTTAAAGCGGTTTATTCCGGAAACCCCCCACAAGACCCCCACGAAA

ACCCAGGCACTGCTTGAGCGCGCCGGCTTTGAAAAGCTGACGCCCTTTACACCGGAGGAA

GAAAGTCGTCGAATACTGCATACAGTTTTTTGTACTCTAGAAGCAGCTCCCCATCAAAGC

TGA

>KT324725.1

ATGGCGGCGCGCGAACAGGCCAACTCCGTTCGCAGGAGCGGGTTTTTTAATCCGTTTATT

GGCAAGAGGCCATTTTTCAGGCCGGGAAGCGGGCAGACCGCGGAGACAGAGCGCCCAAGG

CCCCCCCAACACTCGTACTGCACAGAGGTGGGTAGCTTTAAGTTTATAGCTCCAAGATGC

CTCGATGAAGAAGCCCCCGCCGACCAGCGACGCGGTGTACACGTGGGCACCTTGGAGCGC

CCACCCAAAGTGTACTGCGATGGCTCAGAGTACGACGTGCTGAACTTTGCCTCCGGAGGT

TGCTGGCCTCGCAGGATTCGGGTTTGGAACGGCCAGGATTTTCGGGGCGATGGATTCAAC

CCCAGATTTGAGAGATTTCACGTGTACGACATAGTAGAGACTTCGGAGAGCGCCTCACAC

GATGACCCATCCAGGTTTGCTGAGCTATCCCGCCCAAGCGGGTCTGTGGTAACACTGCTG

GGAATGAGCGAGTGTGGAAAGCGAGTCGCGGTTCACGTCTATGGTGTGCGCCATTATTTT

TACATGGCAAAGGCGGAGGTGGATAGCGCTTGTGGAATAACCACCGAGGCAGAACTCGTG

CGCGCAATGGTGGACTGCGCGCACAGCTCGGCTTTGAGCGCGGCCCTGGGAAATGGCAAC

GGCGGCAAACAGAGCGGCGGCAGCGGCGGGGGATGGTGGGGCGGAAAGCACGTGTCTGCG

GACTGCTTCAAAGTGGAGACCGTGTGCCACACGACGCTGTACTACTTTGGATCTAAGCCA

GCTCTCTACTATAGAGTATCTGCCTCCAGCAGCCGCCTGGGAGGGTTCATCTGCGACAAC

TTTCACCCGGAGATTACAAAATTCGAGGGGAGCGTGGACGTGACCACGCGGCTGTTGTTG

GACAATGAAAATTTTACCAGTTTCGGGTGGTACCGCCTGCGACCCGGCACCCACGGAGAG

CGTGTTCAACTTCGCCCCGTTGAGCGACACGTCACCTCAAGCGACGTGGAGATTAACTGT

ACTCCCGATAACCTGGAGCCGATACCAGACGAGGCTGCCTGGCCCGACTATAAGCTCATG

TGCTTTGATATAGAGTGTAAAGCTGGAACGGGTAACGAAATGGCGTTCCCAGTGGCAACT

AACCAAGAGGACCTGGTCATCCAGATCTCCTGTCTGCTGTACTCGCTTGCTACTCAGAAC

CACGAACACACCCTGCTGTTTTCCCTCGGGTCATGCGATATCTCTGAGGAATACTCGTTT

GCATGCGTCCAGCGCGGCGAGCCCAGACCGACGGTTTTGGAGTTTGACAGCGAGTACGAG

CTGCTGGTTGCCTTCCTGACCTTTCTCAAGCAGTACTCTCCCGAGTTCGCCACCGGCTAC

AACATCGTTAATTTTGACTGGGCGTACATAGTTAACAAGGTAACGTCGGTGTATAACATC

AAGCTGGACGGGTACGGCAAGTTCAACAAAGGGGGGCTGTTTAAGGTGTGGGACATCGCC

ACGAACCATTTTCAGAAGAAGAGCAAGGTGAAAATCAATGGCCTGATATCTCTAGACATG

TATTCTGTGGCGACGGAAAAGCTAAAGCTACCCAGCTACAAACTCGACGCGGTCGTGGGA

GACGTCCTCGGCGAGCATAAGATAGACCTTCCCTATAAAGAAATACCCTCCTATTACGCG

GGAGGGCCTGACCGGAGGGGCGTAATAGGAGAGTATTGTATCCAGGACTCTAGGCTGGTG

GGCAAGCTGTTTTTTAAGTACCTCCCCCATCTGGAACTATCGGCGGTGGCCAAACTCGCC

CGTATCACCCTAACGCGGGTAATTTTTGACGGTCAGCAAATTCGCGTGTACACGTGCTTG

CTGAAACTCGCCCGCGAGAGAAATTTCATTTTGCCAGACAACAGACGCCGGTTTGACAGT

CAGGCAGACGCCGCGTCAGAGACTTCGGAGTTGGCTATGGATAGCCAAAGCCACGCCTTC

GACAGTACAGACGAACCCGACGGTGTGGACGGTACCCCGGACGCCGCAGGATCTGGCGCT

ACTTCTGAAAACGGAGGCGGGAAGCCCGGCGTCGGGAGGGCCGTGGGCTACCAGGGAGCA

AAGGTTCTAGACCCCGTATCCGGCTTTCATGTGGACCCCGTGGTTGTGTTTGACTTCGCT

AGCTTATACCCAAGCATTATCCAGGCCCATAACCTCTGTTTCACCACCCTGGCGCTCGAT

GAAGTGGATCTGGCCGGGCTTCAACCATCCGTCAACTACTCGACGTTCGAGGTGGGTGAC

CAAAAGTTATTTTTTGTCCACGCCCATATTCGCGAAAGCCTGCTTGGCATCTTGCTGCGC

GACTGGCTGGCCATGCGAAAGGCGGTGAGGGCGCGAATCCCCACCAGCACCCCCGAGGAG

GCAGTTTTACTAGATAAGCAGCAGTCTGCGATTAAGGTGATATGCAACTCGGTTTACGGA

TTCACGGGGGTGGCAAACGGCCTGTTGCCGTGTCTGAGGATAGCGGCTACCGTTACCACG

ATAGGACGCGACATGCTCCTCAAGACCCGAGATTACGTTCACTCTCGTTGGGCGACGCGC

GAGCTGCTGGAGGACAATTTTCCAGGGGCTATAGGTTTCCGAAACCACAAGCCTTACTCC

GTCAGGGTTATCTACGGAGACACCGACTCCGTGTTTATCAAGTTTGTGGGCCTGACGTAC

GAGGGGGTATCGGAGCTGGGGGATGCTATGTCGCGTCAGATTTCAGCGGACCTCTTTAGA

GCGCCCATCAAACTGGAGTGTGAGAAGACCTTTCAGCGACTGCTGCTGATCACCAAGAAG

AAGTACATAGGTGTCATAAACGGGGGGAAGATGCTCATGAAGGGGGTCGACCTGGTCCGC

AAAAATAACTGCTCTTTCATAAACTTGTACGCGCGACATCTGGTAGATCTTTTGTTGTAC

GACGAGGATGTGGCCACGGCGGCAGCAGAGGTGACAGACGTGCCTCCCGCAGAATGGGTG

GGGCGCCCGCTACCGAGCGGCTTTGACAAGTTTGGGCGAGTGCTGGTAGAGGCGTACAAC

CGTATCACTGCCCCCAACTTGGACGTGCGCAAGTTCGTTATGACTGCTGAGCTGAGCCGC

TCACCCGAATCGTATACCAACAAGCGCCTGCCGCACCTCACCGTCTACTTTAAGCTCGCC

ATGAGGAATGAAGAACTGCCCAGCGTAAAAGAGAGAATTCCGTATGTGATAGTTGCGCAG

ACCGAGGCCGCGGAACGCGAAGCGGGTGTAGTAAACTCAATGCGCGGTACCGCCCAAAAC

CCCGTGGTAACCAAGACCGCACGCCCCCAACCTAAACGCAAACTGCTGGTTTCCGACCTC

GCCGAAGACCCGACCTATGTTTCCGAGAATGACGTACCGCTAAACACAGACTACTATTTC

TCCCACCTGTTGGGTACCATAAGCGTGACCTTTAAGGCTCTATTCGGAAATGATGTGAGA

ACAACAGAAAATCTTTTAAAGCGGTTTATTCCGGAAACCCCCCACAAGACCCCCACGAAA

ACCCAGGCACTGCTTGAGCGCGCCGGCTTTGAAAAGCTGACGCCCTTTACACCGGAGGAA

GAAAGTCGTCGAATACTGCATACAGTTTTTTGTACTCTAGAAGCAGCTCCCCATCAAAGC

TGA

>KT324726.1

ATGGCGGCGCGCGAACAGGCCAACTCCGTTCGCAGGAGCGGGTTTTTTAATCCGTTTATT

GGCAAGAGGCCATTTTTCAGGCCGGGAAGCGGGCAAACCGCGGAGACAGAGCGCCCAAGG

CCCCCCCAACACTCGTACTGCACAGAGGTGGGTAGCTTTAAGTTTATAGCTCCAAGATGC

CTCGATGAAGAAGCCCCCGCCGACCAGCGACGCGGTGTACACGTGGGCACCTTGGAGCGC

CCACCCAAAGTGTACTGCGATGGCTCAGAGTACGACGTGCTGAACTTTGCCTCCGGAGGT

TGCTGGCCTCGCAGGATTCGGGTTTGGAACGGCCAGGATTTTCGGGGCGATGGATTCAAC

CCCAGATTTGAGAGATTTCACGTGTACGACATAGTAGAGACTTCGGAGAGCGCCTCACAC

GATGACCCATCCAGGTTTGCTGAGCTATCCCGCCCAAGCGGGTCTGTGGTAACACTGCTG

GGAATGAGCGAGTGTGGAAAGCGAGTCGCGGTTCACGTCTATGGTGTGCGCCATTATTTT

TACATGGCAAAGGCGGAGGTGGATAGCGCTTGTGGAATAACCACCGAGGCAGAACTCGTG

CGCGCAATGGTGGACTGCGCGCACAGCTCGGCTTTGAGCGCGGCCCTGGGAAATGGCAAC

GGCGGCAAACAGAGCGGCGGCAGCGGCGGGGGATGGTGGGGCGGAAAGCACGTGTCTGCG

GACTGCTTCAAAGTGGAGACCGTGTGCCACACGACGCTGTACTACTTTGGATCTAAGCCA

GCTCTCTACTATAGAGTATCTGCCTCCAGCAGCCGCCTGGGAGGGTTCATCTGCGACAAC

TTTCACCCGGAGATTACAAAATTCGAGGGGAGCGTGGACGTGACCACGCGGCTGTTGTTG

GACAATGAAAATTTTACCAGTTTCGGGTGGTACCGCCTGCGACCCGGCACCCACGGAGAG

CGTGTTCAACTTCGCCCCGTTGAGCGACACGTCACCTCAAGCGACGTGGAGATTAACTGT

ACTCCCGATAACCTGGAGCCGATACCAGACGAGGCTGCCTGGCCCGACTATAAGCTCATG

TGCTTTGATATAGAGTGTAAAGCTGGAACGGGTAACGAAATGGCGTTCCCAGTGGCAACT

AACCAAGAGGACCTGGTCATCCAGATCTCCTGTCTGCTGTACTCGCTTGCTACTCAGAAC

CACGAACACACCCTGCTGTTTTCCCTCGGGTCATGCGATATCTCTGAGGAATACTCGTTT

GCATGCGTCCAGCGCGGCGAGCCCAGACCGACGGTTTTGGAGTTTGACAGCGAGTACGAG

CTGCTGGTTGCCTTCCTGACCTTTCTCAAGCAGTACTCTCCCGAGTTCGCCACCGGCTAC

AACATCGTTAATTTTGACTGGGCGTACATAGTTAACAAGGTAACGTCGGTGTATAACATC

AAGCTGGACGGGTACGGCAAGTTCAACAAAGGGGGGCTGTTTAAGGTGTGGGACATCGCC

ACGAACCATTTTCAGAAGAAGAGCAAGGTGAAAATCAATGGCCTGATATCTCTAGACATG

TATTCTGTGGCGACGGAAAAGCTAAAGCTACCCAGCTACAAACTCGACGCGGTCGTGGGA

GACGTCCTCGGCGAGCATAAGATAGACCTTCCCTATAAAGAAATACCCTCCTATTACGCG

GGAGGGCCTGACCGGAGGGGCGTAATAGGAGAGTATTGTATCCAGGACTCTAGGCTGGTG

GGCAAGCTGTTTTTTAAGTACCTCCCCCATCTGGAACTATCGGCGGTGGCCAAACTCGCC

CGTATCACCCTAACGCGGGTAATTTTTGACGGTCAGCAAATTCGCGTGTACACGTGCTTG

CTGAAACTCGCCCGCGAGAGAAATTTCATTTTGCCAGACAACAGACGCCGGTTTGACAGT

CAGGCAGACGCCGCGTCAGAGACTTCGGAGTTGGCTATGGATAGCCAAAGCCACGCCTTC

GACAGTACAGACGAACCCGACGGTGTGGACGGTACCCCGGACGCCGCAGGATCTGGCGCT

ACTTCTGAAAACGGAGGCGGGAAGCCCGGCGTCGGGAGGGCCGTGGGCTACCAGGGAGCA

AAGGTTCTAGACCCCGTATCCGGCTTTCATGTGGACCCCGTGGTTGTGTTTGACTTCGCT

AGCTTATACCCAAGCATTATCCAGGCCCATAACCTCTGTTTCACCACCCTGGCGCTCGAT

GAAGTGGATCTGGCCGGGCTTCAACCATCCGTCAACTACTCGACGTTCGAGGTGGGTGAC

CAAAAGTTATTTTTTGTCCACGCCCATATTCGCGAAAGCCTGCTTGGCATCTTGCTGCGC

GACTGGCTGGCCATGCGAAAGGCGGTGAGGGCGCGAATCCCCACCAGCACCCCCGAGGAG

GCAGTTTTACTAGATAAGCAGCAGTCTGCGATTAAGGTGATATGCAACTCGGTTTACGGA

TTCACGGGGGTGGCAAACGGCCTGTTGCCGTGTCTGAGGATAGCGGCTACCGTTACCACG

ATAGGACGCGACATGCTCCTCAAGACCCGAGATTACGTTCACTCTCGTTGGGCGACGCGC

GAGCTGCTGGAGGACAATTTTCCAGGGGCTATAGGTTTCCGAAACCACAAGCCTTACTCC

GTCAGGGTTATCTACGGAGACACCGACTCCGTGTTTATCAAGTTTGTGGGCCTGACGTAC

GAGGGGGTATCGGAGCTGGGGGATGCTATGTCGCGTCAGATTTCAGCGGACCTCTTTAGA

GCGCCCATCAAACTGGAGTGTGAGAAGACCTTTCAGCGACTGCTGCTGATCACCAAGAAG

AAGTACATAGGTGTCATAAACGGGGGGAAGATGCTCATGAAGGGGGTCGACCTGGTCCGC

AAAAATAACTGCTCTTTCATAAACTTGTACGCGCGACATCTGGTAGATCTTTTGTTGTAC

GACGAGGATGTGGCCACGGCGGCAGCAGAGGTGACAGACGTGCCTCCCGCAGAATGGGTG

GGGCGCCCGCTACCGAGCGGCTTTGACAAGTTTGGGCGAGTGCTGGTAGAGGCGTACAAC

CGTATCACTGCCCCCAACTTGGACGTGCGCGAGTTCGTTATGACTGCTGAGCTGAGCCGC

TCACCCGAATCGTATACCAACAAGCGCCTGCCGCACCTCACCGTCTACTTTAAGCTCGCC

ATGAGGAATGAAGAACTGCCCAGCGTAAAAGAGAGAATTCCGTATGTGATAGTTGCGCAG

ACCGAGGCCGCGGAACGCGAAGCGGGTGTAGTAAACTCAATGCGCGGTACCGCCCAAAAC

CCCGTGGTAACCAAGACCGCACGCCCCCAACCTAAACGCAAACTGCTGGTTTCCGACCTC

GCCGAAGACCCGACCTATGTTTCCGAGAATGACGTACCGCTAAACACAGACTACTATTTC

TCCCACCTGTTGGGTACCATAAGCGTGACCTTTAAGGCTCTATTCGGAAATGATGTGAGA

ACAACAGAAAATCTTTTAAAGCGGTTTATTCCGGAAACCCCCCACAAGACCCCCACGAAA

ACCCAGGCACTGCTTGAGCGCGCCGGCTTTGAAAAGCTGACGCCCTTTACACCGGAGGAA

GAAAGTCGTCGAATACTGCATACAGTTTTTTGTACTCTAGAAGCAGCTCCCCATCAAAGC

TGA

>KT324728.1

ATGGCGGCGCGCGAACAGGCCAACTCCGTTCGCAGGAGCGGGTTTTTTAATCCGTTTATT

GGCAAGAGGCCATTTTTCAGGCCGGGAAGCGGGCAAACCGCGGAGACAGAGCGCCCAAGG

CCCCCCCAACACTCGTACTGCACAGAGGTGGGTAGCTTTAAGTTTATAGCTCCAAGATGC

CTCGATGAAGAAGCCCCCGCCGACCAGCGACGCGGTGTACACGTGGGCACCTTGGAGCGC

CCACCCAAAGTGTACTGCGATGGCTCAGAGTACGACGTGCTGAACTTTGCCTCCGGAGGT

TGCTGGCCTCGCAGGATTCGGGTTTGGAACGGCCAGGATTTTCGGGGCGATGGATTCAAC

CCCAGATTTGAGAGATTTCACGTGTACGACATAGTAGAGACTTCGGAGAGCGCCTCACAC

GATGACCCATCCAGGTTTGCTGAGCTATCCCGCCCAAGCGGGTCTGTGGTAACACTGCTG

GGAATGAGCGAGTGTGGAAAGCGAGTCGCGGTTCACGTCTATGGTGTGCGCCATTATTTT

TACATGGCAAAGGCGGAGGTGGATAGCGCTTGTGGAATAACCACCGAGGCAGAACTCGTG

CGCGCAATGGTGGACTGCGCGCACAGCTCGGCTTTGAGCGCGGCCCTGGGAAATGGCAAC

GGCGGCAAACAGAGCGGCGGCAGCGGCGGGGGATGGTGGGGCGGAAAGCACGTGTCTGCG

GACTGCTTCAAAGTGGAGACCGTGTGCCACACGACGCTGTACTACTTTGGATCTAAGCCA

GCTCTCTACTATAGAGTATCTGCCTCCAGCAGCCGCCTGGGAGGGTTCATCTGCGACAAC

TTTCACCCGGAGATTACAAAATTCGAGGGGAGCGTGGACGTGACCACGCGGCTGTTGTTG

GACAATGAAAATTTTACCAGTTTCGGGTGGTACCGCCTGCGACCCGGCACCCACGGAGAG

CGTGTTCAACTTCGCCCCGTTGAGCGACACGTCACCTCAAGCGACGTGGAGATTAACTGT

ACTCCCGATAACCTGGAGCCGATACCAGACGAGGCTGCCTGGCCCGACTATAAGCTCATG

TGCTTTGATATAGAGTGTAAAGCTGGAACGGGTAACGAAATGGCGTTCCCAGTGGCAACT

AACCAAGAGGACCTGGTCATCCAGATCTCCTGTCTGCTGTACTCGCTTGCTACTCAGAAC

CACGAACACACCCTGCTGTTTTCCCTCGGGTCATGCGATATCTCTGAGGAATACTCGTTT

GCATGCGTCCAGCGCGGCGAGCCCAGACCGACGGTTTTGGAGTTTGACAGCGAGTACGAG

CTGCTGGTTGCCTTCCTGACCTTTCTCAAGCAGTACTCTCCCGAGTTCGCCACCGGCTAC

AACATCGTTAATTTTGACTGGGCGTACATAGTTAACAAGGTAACGTCGGTGTATAACATC

AAGCTGGACGGGTACGGCAAGTTCAACAAAGGGGGGCTGTTTAAGGTGTGGGACATCGCC

ACGAACCATTTTCAGAAGAAGAGCAAGGTGAAAATCAATGGCCTGATATCTCTAGACATG

TATTCTGTGGCGACGGAAAAGCTAAAGCTACCCAGCTACAAACTCGACGCGGTCGTGGGA

GACGTCCTCGGCGAGCATAAGATAGACCTTCCCTATAAAGAAATACCCTCCTATTACGCG

GGAGGGCCTGACCGGAGGGGCGTAATAGGAGAGTATTGTATCCAGGACTCTAGGCTGGTG

GGCAAGCTGTTTTTTAAGTACCTCCCCCATCTGGAACTATCGGCGGTGGCCAAACTCGCC

CGTATCACCCTAACGCGGGTAATTTTTGACGGTCAGCAAATTCGCGTGTACACGTGCTTG

CTGAAACTCGCCCGCGAGAGAAATTTCATTTTGCCAGACAACAGACGCCGGTTTGACAGT

CAGGCAGACGCCGCGTCAGAGACTTCGGAGTTGGCTATGGATAGCCAAAGCCACGCCTTC

GACAGTACAGACGAACCCGACGGTGTGGACGGTACCCCGGACGCCGCAGGATCTGGCGCT

ACTTCTGAAAACGGAGGCGGGAAGCCCGGCGTCGGGAGGGCCGTGGGCTACCAGGGAGCA

AAGGTTCTAGACCCCGTATCCGGCTTTCATGTGGACCCCGTGGTTGTGTTTGACTTCGCT

AGCTTATACCCAAGCATTATCCAGGCCCATAACCTCTGTTTCACCACCCTGGCGCTCGAT

GAAGTGGATCTGGCCGGGCTTCAACCATCCGTCAACTACTCGACGTTCGAGGTGGGTGAC

CAAAAGTTATTTTTTGTCCACGCCCATATTCGCGAAAGCCTGCTTGGCATCTTGCTGCGC

GACTGGCTGGCCATGCGAAAGGCGGTGAGGGCGCGAATCCCCACCAGCACCCCCGAGGAG

GCAGTTTTACTAGATAAGCAGCAGTCTGCGATTAAGGTGATATGCAACTCGGTTTACGGA

TTCACGGGGGTGGCAAACGGCCTGTTGCCGTGTCTGAGGATAGCGGCTACCGTTACCACG

ATAGGACGCGACATGCTCCTCAAGACCCGAGATTACGTTCACTCTCGTTGGGCGACGCGC

GAGCTGCTGGAGGACAATTTTCCAGGGGCTATAGGTTTCCGAAACCACAAGCCTTACTCC

GTCAGGGTTATCTACGGAGACACCGACTCCGTGTTTATCAAGTTTGTGGGCCTGACGTAC

GAGGGGGTATCGGAGCTGGGGGATGCTATGTCGCGTCAGATTTCAGCGGACCTCTTTAGA

GCGCCCATCAAACTGGAGTGTGAGAAGACCTTTCAGCGACTGCTGCTGATCACCAAGAAG

AAGTACATAGGTGTCATAAACGGGGGGAAGATGCTCATGAAGGGGGTCGACCTGGTCCGC

AAAAATAACTGCTCTTTCATAAACTTGTACGCGCGACATCTGGTAGATCTTTTGTTGTAC

GACGAGGATGTGGCCACGGCGGCAGCAGAGGTGACAGACGTGCCTCCCGCAGAATGGGTG

GGGCGCCCGCTACCGAGCGGCTTTGACAAGTTTGGGCGAGTGCTGGTAGAGGCGTACAAC

CGTATCACTGCCCCCAACTTGGACGTGCGCGAGTTCGTTATGACTGCTGAGCTGAGCCGC

TCACCCGAATCGTATACCAACAAGCGCCTGCCGCACCTCACCGTCTACTTTAAGCTCGCC

ATGAGGAATGAAGAACTGCCCAGCGTAAAAGAGAGAATTCCGTATGTGATAGTTGCGCAG

ACCGAGGCCGCGGAACGCGAAGCGGGTGTAGTAAACTCAATGCGCGGTACCGCCCAAAAC

CCCGTGGTAACCAAGACCGCACGCCCCCAACCTAAACGCAAACTGCTGGTTTCCGACCTC

GCCGAAGACCCGACCTATGTTTCCGAGAATGACGTACCGCTAAACACAGACTACTATTTC

TCCCACCTGTTGGGTACCATAAGCGTGACCTTTAAGGCTCTATTCGGAAATGATGTGAGA

ACAACAGAAAATCTTTTAAAGCGGTTTATTCCGGAAACCCCCCACAAGACCCCCACGAAA

ACCCAGGCACTGCTTGAGCGCGCCGGCTTTGAAAAGCTGACGCCCTTTACACCGGAGGAA

GAAAGTCGTCGAATACTGCATACAGTTTTTTGTACTCTAGAAGCAGCTCCCCATCAAAGC

TGA

>KT324729.1

ATGGCGGCGCGCGAACAGGCCAACTCCGTTCGCAGGAGCGGGTTTTTTAATCCGTTTATT

GGCAAGAGGCCATTTTTCAGGCCGGGAAGCGGGCAAACCGCGGAGACAGAGCGCCCAAGG

CCCCCCCAACACTCGTACTGCACAGAGGTGGGTAGCTTTAAGTTTATAGCTCCAAGATGC

CTCGATGAAGAAGCCCCCGCCGACCAGCGACGCGGTGTACACGTGGGCACCTTGGAGCGC

CCACCCAAAGTGTACTGCGATGGCTCAGAGTACGACGTGCTGAACTTTGCCTCCGGAGGT

TGCTGGCCTCGCAGGATTCGGGTTTGGAACGGCCAGGATTTTCGGGGCGATGGATTCAAC

CCCAGATTTGAGAGATTTCACGTGTACGACATAGTAGAGACTTCGGAGAGCGCCTCACAC

GATGACCCATCCAGGTTTGCTGAGCTATCCCGCCCAAGCGGGTCTGTGGTAACACTGCTG

GGAATGAGCGAGTGTGGAAAGCGAGTCGCGGTTCACGTCTATGGTGTGCGCCATTATTTT

TACATGGCAAAGGCGGAGGTGGATAGCGCTTGTGGAATAACCACCGAGGCAGAACTCGTG

CGCGCAATGGTGGACTGCGCGCACAGCTCGGCTTTGAGCGCGGCCCTGGGAAATGGCAAC

GGCGGCAAACAGAGCGGCGGCAGCGGCGGGGGATGGTGGGGCGGAAAGCACGTGTCTGCG

GACTGCTTCAAAGTGGAGACCGTGTGCCACACGACGCTGTACTACTTTGGATCTAAGCCA

GCTCTCTACTATAGAGTATCTGCCTCCAGCAGCCGCCTGGGAGGGTTCATCTGCGACAAC

TTTCACCCGGAGATTACAAAATTCGAGGGGAGCGTGGACGTGACCACGCGGCTGTTGTTG

GACAATGAAAATTTTACCAGTTTCGGGTGGTACCGCCTGCGACCCGGCACCCACGGAGAG

CGTGTTCAACTTCGCCCCGTTGAGCGACACGTCACCTCAAGCGACGTGGAGATTAACTGT

ACTCCCGATAACCTGGAGCCGATACCAGACGAGGCTGCCTGGCCCGACTATAAGCTCATG

TGCTTTGATATAGAGTGTAAAGCTGGAACGGGTAACGAAATGGCGTTCCCAGTGGCAACT

AACCAAGAGGACCTGGTCATCCAGATCTCCTGTCTGCTGTACTCGCTTGCTACTCAGAAC

CACGAACACACCCTGCTGTTTTCCCTCGGGTCATGCGATATCTCTGAGGAATACTCGTTT

GCATGCGTCCAGCGCGGCGAGCCCAGACCGACGGTTTTGGAGTTTGACAGCGAGTACGAG

CTGCTGGTTGCCTTCCTGACCTTTCTCAAGCAGTACTCTCCCGAGTTCGCCACCGGCTAC

AACATCGTTAATTTTGACTGGGCGTACATAGTTAACAAGGTAACGTCGGTGTATAACATC

AAGCTGGACGGGTACGGCAAGTTCAACAAAGGGGGGCTGTTTAAGGTGTGGGACATCGCC

ACGAACCATTTTCAGAAGAAGAGCAAGGTGAAAATCAATGGCCTGATATCTCTAGACATG

TATTCTGTGGCGACGGAAAAGCTAAAGCTACCCAGCTACAAACTCGACGCGGTCGTGGGA

GACGTCCTCGGCGAGCATAAGATAGACCTTCCCTATAAAGAAATACCCTCCTATTACGCG

GGAGGGCCTGACCGGAGGGGCGTAATAGGAGAGTATTGTATCCAGGACTCTAGGCTGGTG

GGCAAGCTGTTTTTTAAGTACCTCCCCCATCTGGAACTATCGGCGGTGGCCAAACTCGCC

CGTATCACCCTAACGCGGGTAATTTTTGACGGTCAGCAAATTCGCGTGTACACGTGCTTG

CTGAAACTCGCCCGCGAGAGAAATTTCATTTTGCCAGACAACAGACGCCGGTTTGACAGT

CAGGCAGACGCCGCGTCAGAGACTTCGGAGTTGGCTATGGATAGCCAAAGCCACGCCTTC

GACAGTACAGACGAACCCGACGGTGTGGACGGTACCCCGGACGCCGCAGGATCTGGCGCT

ACTTCTGAAAACGGAGGCGGGAAGCCCGGCGTCGGGAGGGCCGTGGGCTACCAGGGAGCA

AAGGTTCTAGACCCCGTATCCGGCTTTCATGTGGACCCCGTGGTTGTGTTTGACTTCGCT

AGCTTATACCCAAGCATTATCCAGGCCCATAACCTCTGTTTCACCACCCTGGCGCTCGAT

GAAGTGGATCTGGCCGGGCTTCAACCATCCGTCAACTACTCGACGTTCGAGGTGGGTGAC

CAAAAGTTATTTTTTGTCCACGCCCATATTCGCGAAAGCCTGCTTGGCATCTTGCTGCGC

GACTGGCTGGCCATGCGAAAGGCGGTGAGGGCGCGAATCCCCACCAGCACCCCCGAGGAG

GCAGTTTTACTAGATAAGCAGCAGTCTGCGATTAAGGTGATATGCAACTCGGTTTACGGA

TTCACGGGGGTGGCAAACGGCCTGTTGCCGTGTCTGAGGATAGCGGCTACCGTTACCACG

ATAGGACGCGACATGCTCCTCAAGACCCGAGATTACGTTCACTCTCGTTGGGCGACGCGC

GAGCTGCTGGAGGACAATTTTCCAGGGGCTATAGGTTTCCGAAACCACAAGCCTTACTCC

GTCAGGGTTATCTACGGAGACACCGACTCCGTGTTTATCAAGTTTGTGGGCCTGACGTAC

GAGGGGGTATCGGAGCTGGGGGATGCTATGTCGCGTCAGATTTCAGCGGACCTCTTTAGA

GCGCCCATCAAACTGGAGTGTGAGAAGACCTTTCAGCGACTGCTGCTGATCACCAAGAAG

AAGTACATAGGTGTCATAAACGGGGGGAAGATGCTCATGAAGGGGGTCGACCTGGTCCGC

AAAAATAACTGCTCTTTCATAAACTTGTACGCGCGACATCTGGTAGATCTTTTGTTGTAC

GACGAGGATGTGGCCACGGCGGCAGCAGAGGTGACAGACGTGCCTCCCGCAGAATGGGTG

GGGCGCCCGCTACCGAGCGGCTTTGACAAGTTTGGGCGAGTGCTGGTAGAGGCGTACAAC

CGTATCACTGCCCCCAACTTGGACGTGCGCGAGTTCGTTATGACTGCTGAGCTGAGCCGC

TCACCCGAATCGTATACCAACAAGCGCCTGCCGCACCTCACCGTCTACTTTAAGCTCGCC

ATGAGGAATGAAGAACTGCCCAGCGTAAAAGAGAGAATTCCGTATGTGATAGTTGCGCAG

ACCGAGGCCGCGGAACGCGAAGCGGGTGTAGTAAACTCAATGCGCGGTACCGCCCAAAAC

CCCGTGGTAACCAAGACCGCACGCCCCCAACCTAAACGCAAACTGCTGGTTTCCGACCTC

GCCGAAGACCCGACCTATGTTTCCGAGAATGACGTACCGCTAAACACAGACTACTATTTC

TCCCACCTGTTGGGTACCATAAGCGTGACCTTTAAGGCTCTATTCGGAAATGATGTGAGA

ACAACAGAAAATCTTTTAAAGCGGTTTATTCCGGAAACCCCCCACAAGACCCCCACGAAA

ACCCAGGCACTGCTTGAGCGCGCCGGCTTTGAAAAGCTGACGCCCTTTACACCGGAGGAA

GAAAGTCGTCGAATACTGCATACAGTTTTTTGTACTCTAGAAGCAGCTCCCCATCAAAGC

TGA

>KT324730.1

ATGGCGGCGCGCGAACAGGCCAACTCCGTTCGCAGGAGCGGGTTTTTTAATCCGTTTATT

GGCAAGAGGCCATTTTTCAGGCCGGGAAGCGGGCAGACCGCGGAGACAGAGCGCCCAAGG

CCCCCCCAACACTCGTACTGCACAGAGGTGGGTAGCTTTAAGTTTATAGCTCCAAGATGC

CTCGATGAAGAAGCCCCCGCCGACCAGCGACGCGGTGTACACGTGGGCACCTTGGAGCGC

CCACCCAAAGTGTACTGCGATGGCTCAGAGTACGACGTGCTGAACTTTGCCTCCGGAGGT

TGCTGGCCTCGCAGGATTCGGGTTTGGAACGGCCAGGATTTTCGGGGCGATGGATTCAAC

CCCAGATTTGAGAGATTTCACGTGTACGACATAGTAGAGACTTCGGAGAGCGCCTCACAC

GATGACCCATCCAGGTTTGCTGAGCTATCCCGCCCAAGTGGGTCTGTGGTAACACTGCTG

GGAATGAGCGAGTGTGGAAAGCGAGTCGCGGTTCACGTCTATGGTGTGCGCCATTATTTT

TACATGGCAAAGGCGGAGGTGGATAGCGCTTGTGGAATAACCACCGAGGCAGAACTCGTG

CGCGCAATGGTGGACTGCGCGCACAGCTCGGCTTTGAGCGCGGCCCTGGGAAATGGCAAC

GGCGGCAAACAGAGCGGCGGCAGCGGCGGGGGATGGTGGGGCGGAAAGCACGTGTCTGCG

GACTGCTTCAAAGTGGAGACCGTGTGCCACACGACGCTGTACTACTTTGGATCTAAGCCA

GCTCTCTACTATAGAGTATCTGCCTCCAGCAGCCGCCTGGGAGGGTTCATCTGCGACAAC

TTTCACCCGGAGATTACAAAATTCGAGGGGAGCGTGGACGTGACCACGCGGCTGTTGTTG

GACAATGAAAATTTTACCAGTTTCGGGTGGTACCGCCTGCGACCCGGCACCCACGGAGAG

CGTGTTCAACTTCGCCCCGTTGAGCGACACGTCACCTCAAGCGACGTGGAGATTAACTGT

ACTCCCGATAACCTGGAGCCGATACCAGACGAGGCTGCCTGGCCCGACTATAAGCTCATG

TGCTTTGATATAGAGTGTAAAGCTGGAACGGGTAACGAAATGGCGTTCCCAGTGGCAACT

AACCAAGAGGACCTGGTCATCCAGATCTCCTGTCTGCTGTACTCGCTTGCTACTCAGAAC

CACGAACACACCCTGCTGTTTTCCCTCGGGTCATGCGATATCTCTGAGGAATACTCGTTT

GCATGCGTCCAGCGCGGCGAGCCCAGACCGACGGTTTTGGAGTTTGACAGCGAGTACGAG

CTGCTGGTTGCCTTCCTGACCTTTCTCAAGCAGTACTCTCCCGAGTTCGCCACCGGCTAC

AACATCGTTAATTTTGACTGGGCGTACATAGTTAACAAGGTAACGTCGGTGTATAACATC

AAGCTGGACGGGTACGGCAAGTTCAACAAAGGGGGGCTGTTTAAGGTGTGGGACATCGCC

ACGAACCATTTTCAGAAGAAGAGCAAGGTGAAAATCAATGGCCTGATATCTCTAGACATG

TATTCTGTGGCGACGGAAAAGCTAAAGCTACCCAGCTACAAACTCGACGCGGTCGTGGGA

GACGTCCTCGGCGAGCATAAGATAGACCTTCCCTATAAAGAAATACCCTCCTATTACGCG

GGAGGGCCTGACCGGAGGGGCGTAATAGGAGAGTATTGTATCCAGGACTCTAGGCTGGTG

GGCAAGCTGTTTTTTAAGTACCTCCCCCATCTGGAACTATCGGCGGTGGCCAAACTCGCC

CGTATCACCCTAACGCGGGTAATTTTTGACGGTCAGCAAATTCGCGTGTACACGTGCTTG

CTGAAACTCGCCCGCGAGAGAAATTTCATTTTGCCAGACAACAGACGCCGGTTTGACAGT

CAGGCAGACGCCGCGTCAGAGACTTCGGAGTTGGCTATGGATAGCCAAAGCCACGCCTTC

GACAGTACAGACGAACCCGACGGTGTGGACGGTACCCCGGACGCCGCAGGATCTGGCGCT

ACTTCTGAAAACGGAGGCGGGAAGCCCGGCGTCGGGAGGGCCGTGGGCTACCAGGGAGCA

AAGGTTCTAGACCCCGTATCCGGCTTTCATGTGGACCCCGTGGTTGTGTTTGACTTCGCT

AGCTTATACCCAAGCATTATCCAGGCCCATAACCTCTGTTTCACCACCCTGGCGCTCGAT

GAAGTGGATCTGGCCGGGCTTCAACCATCCGTCAACTACTCGACGTTCGAGGTGGGTGAC

CAAAAGTTATTTTTTGTCCACGCCCATATTCGCGAAAGCCTGCTTGGCATCTTGCTGCGC

GACTGGCTGGCCATGCGAAAGGCGGTGAGGGCGCGAATCCCCACCAGCACCCCCGAGGAG

GCAGTTTTACTAGATAAGCAGCAGTCTGCGATTAAGGTGATATGCAACTCGGTTTACGGA

TTCACGGGGGTGGCAAACGGCCTGTTGCCGTGTCTGAGGATAGCGGCTACCGTTACCACG

ATAGGACGCGACATGCTCCTCAAGACCCGAGATTACGTTCACTCTCGTTGGGCGACGCGC

GAGCTGCTGGAGGACAATTTTCCAGGGGCTATAGGTTTCCGAAACCACAAGCCTTACTCC

GTCAGGGTTATCTACGGAGACACCGACTCCGTGTTTATCAAGTTTGTGGGCCTGACGTAC

GAGGGGGTATCGGAGCTGGGGGATGCTATGTCGCGTCAGATTTCAGCGGACCTCTTTAGA

GCGCCCATCAAACTGGAGTGTGAGAAGACCTTTCAGCGACTGCTGCTGATCACCAAGAAG

AAGTACATAGGTGTCATAAACGGGGGGAAGATGCTCATGAAGGGGGTCGACCTGGTCCGC

AAAAATAACTGCTCTTTCATAAACTTGTACGCGCGACATCTGGTAGATCTTTTGTTGTAC

GACGAGGATGTGGCCACGGCGGCAGCAGAGGTGACAGACGTGCCTCCCGCAGAATGGGTG

GGGCGCCCGCTACCGAGCGGCTTTGACAAGTTTGGGCGAGTGCTGGTAGAGGCGTACAAC

CGTATCACTGCCCCCAACTTGGACGTGCGCGAGTTCGTTATGACTGCTGAGCTGAGCCGC

TCACCCGAATCGTATACCAACAAGCGCCTGCCGCACCTCACCGTCTACTTTAAGCTCGCC

ATGAGGAATGAAGAACTGCCCAGCGTAAAAGAGAGAATTCCGTATGTGATAGTTGCGCAG

ACCGAGGCCGCGGAACGCGAAGCGGGTGTAGTAAACTCAATGCGCGGTACCGCCCAAAAC

CCCGTGGTAACCAAGACCGCACGCCCCCAACCTAAACGCAAACTGCTGGTTTCCGACCTC

GCCGAAGACCCGACCTATGTTTCCGAGAATGACGTACCGCTAAACACAGACTACTATTTC

TCCCACCTGTTGGGTACCATAAGCGTGACCTTTAAGGCTCTATTCGGAAATGATGTGAGA

ACAACAGAAAATCTTTTAAAGCGGTTTATTCCGGAAACCCCCCACAAGACCCCCACGAAA

ACCCAGGCACTGCTTGAGCGCGCCGGCTTTGAAAAGCTGACGCCCTTTACACCGGAGGAA

GAAAGTCGTCGAATACTGCATACAGTTTTTTGTACTCTAGAAGCAGCTCCCCATCAAAGC

TGA

>KT324733.1

ATGGCGGCGCGCGAACAGGCCAACTCCGTTCGCAGGAGCGGGTTTTTTAATCCGTTTATT

GGCAAGAGGCCATTTTTCAGGCCGGGAAGCGGGCAGACCGCGGAGACAGAGCGCCCAAGG

CCCCCCCAACACTCGTACTGCACAGAGGTGGGTAGCTTTAAGTTTATAGCTCCAAGATGC

CTCGATGAAGAAGCCCCCGCCGACCAGCGACGCGGTGTACACGTGGGCACCTTGGAGCGC

CCACCCAAAGTGTACTGCGATGGCTCAGAGTACGACGTGCTGAACTTTGCCTCCGGAGGT

TGCTGGCCTCGCAGGATTCGGGTTTGGAACGGCCAGGATTTTCGGGGCGATGGATTCAAC

CCCAGATTTGAGAGATTTCACGTGTACGACATAGTAGAGACTTCGGAGAGCGCCTCACAC

GATGACCCATCCAGGTTTGCTGAGCTATCCCGCCCAAGCGGGTCTGTGGTAACACTGCTG

GGAATGAGCGAGTGTGGAAAGCGAGTCGCGGTTCACGTCTATGGTGTGCGCCATTATTTT

TACATGGCAAAGGCGGAGGTGGATAGCGCTTGTGGAATAACCACCGAGGCAGAACTCGTG

CGCGCAATGGTGGACTGCGCGCACAGCTCGGCTTTGAGCGCGGCCCTGGGAAATGGCAAC

GGCGGCAAACAGAGCGGCGGCAGCGGCGGGGGATGGTGGGGCGGAAAGCACGTGTCTGCG

GACTGCTTCAAAGTGGAGACCGTGTGCCACACGACGCTGTACTACTTTGGATCTAAGCCA

GCTCTCTACTATAGAGTATCTGCCTCCAGCAGCCGCCTGGGAGGGTTCATCTGCGACAAC

TTTCACCCGGAGATTACAAAATTCGAGGGGAGCGTGGACGTGACCACGCGGCTGTTGTTG

GACAATGAAAATTTTACCAGTTTCGGGTGGTACCGCCTGCGACCCGGCACCCACGGAGAG

CGTGTTCAACTTCGCCCCGTTGAGCGACACGTCACCTCAAGCGACGTGGAGATTAACTGT

ACTCCCGATAACCTGGAGCCGATACCAGACGAGGCTGCCTGGCCCGACTATAAGCTCATG

TGCTTTGATATAGAGTGTAAAGCTGGAACGGGTAACGAAATGGCGTTCCCAGTGGCAACT

AACCAAGAGGACCTGGTCATCCAGATCTCCTGTCTGCTGTACTCGCTTGCTACTCAGAAC

CACGAACACACCCTGCTGTTTTCCCTCGGGTCATGCGATATCTCTGAGGAATACTCGTTT

GCATGCGTCCAGCGCGGCGAGCCCAGACCGACGGTTTTGGAGTTTGACAGCGAGTACGAG

CTGCTGGTTGCCTTCCTGACCTTTCTCAAGCAGTACTCTCCCGAGTTCGCCACCGGCTAC

AACATCGTTAATTTTGACTGGGCGTACATAGTTAACAAGGTAACGTCGGTGTATAACATC

AAGCTGGACGGGTACGGCAAGTTCAACAAAGGGGGGCTGTTTAAGGTGTGGGACATCGCC

ACGAACCATTTTCAGAAGAAGAGCAAGGTGAAAATCAATGGCCTGATATCTCTAGACATG

TATTCTGTGGCGACGGAAAAGCTAAAGCTACCCAGCTACAAACTCGACGCGGTCGTGGGA

GACGTCCTCGGCGAGCATAAGATAGACCTTCCCTATAAAGAAATACCCTCCTATTACGCG

GGAGGGCCTGACCGGAGGGGCGTAATAGGAGAGTATTGTATCCAGGACTCTAGGCTGGTG

GGCAAGCTGTTTTTTAAGTACCTCCCCCATCTGGAACTATCGGCGGTGGCCAAACTCGCC

CGTATCACCCTAACGCGGGTAATTTTTGACGGTCAGCAAATTCGCGTGTACACGTGCTTG

CTGAAACTCGCCCGCGAGAGAAATTTCATTTTGCCAGACAACAGACGCCGGTTTGACAGT

CAGGCAGACGCCGCGTCAGAGACTTCGGAGTTGGCTATGGATAGCCAAAGCCACGCCTTC

GACAGTACAGACGAACCCGACGGTGTGGACGGTACCCCGGACGCCGCAGGATCTGGCGCT

ACTTCTGAAAACGGAGGCGGGAAGCCCGGCGTCGGGAGGGCCGTGGGCTACCAGGGAGCA

AAGGTTCTAGACCCCGTATCCGGCTTTCATGTGGACCCCGTGGTTGTGTTTGACTTCGCT

AGCTTATACCCAAGCATTATCCAGGCCCATAACCTCTGTTTCACCACCCTGGCGCTCGAT

GAAGTGGATCTGGCCGGGCTTCAACCATCCGTCAACTACTCGACGTTCGAGGTGGGTGAC

CAAAAGTTATTTTTTGTCCACGCCCATATTCGCGAAAGCCTGCTTGGCATCTTGCTGCGC

GACTGGCTGGCCATGCGAAAGGCGGTGAGGGCGCGAATCCCCACCAGCACCCCCGAGGAG

GCAGTTTTACTAGATAAGCAGCAGTCTGCGATTAAGGTGATATGCAACTCGGTTTACGGA

TTCACGGGGGTGGCAAACGGCCTGTTGCCGTGTCTGAGGATAGCGGCTACCGTTACCACG

ATAGGACGCGACATGCTCCTCAAGACCCGAGATTACGTTCACTCTCGTTGGGCGACGCGC

GAGCTGCTGGAGGACAATTTTCCAGGGGCTATAGGTTTCCGAAACCACAAGCCTTACTCC

GTCAGGGTTATCTACGGAGACACCGACTCCGTGTTTATCAAGTTTGTGGGCCTGACGTAC

GAGGGGGTATCGGAGCTGGGGGATGCTATGTCGCGTCAGATTTCAGCGGACCTCTTTAGA

GCGCCCATCAAACTGGAGTGTGAGAAGACCTTTCAGCGACTGCTGCTGATCACCAAGAAG

AAGTACATAGGTGTCATAAACGGGGGGAAGATGCTCATGAAGGGGGTCGACCTGGTCCGC

AAAAATAACTGCTCTTTCATAAACTTGTACGCGCGACATCTGGTAGATCTTTTGTTGTAC

GACGAGGATGTGGCCACGGCGGCAGCAGAGGTGACAGACGTGCCTCCCGCAGAATGGGTG

GGGCGCCCGCTACCGAGCGGCTTTGACAAGTTTGGGCGAGTGCTGGTAGAGGCGTACAAC

CGTATCACTGCCCCCAACTTGGACGTGCGCAAGTTCGTTATGACTGCTGAGCTGAGCCGC

TCACCCGAATCGTATACCAACAAGCGCCTGCCGCACCTCACCGTCTACTTTAAGCTCGCC

ATGAGGAATGAAGAACTGCCCAGCGTAAAAGAGAGAATTCCGTATGTGATAGTTGCGCAG

ACCGAGGCCGCGGAACGCGAAGCGGGTGTAGTAAACTCAATGCGCGGTACCGCCCAAAAC

CCCGTGGTAACCAAGACCGCACGCCCCCAACCTAAACGCAAACTGCTGGTTTCCGACCTC

GCCGAAGACCCGACCTATGTTTCCGAGAATGACGTACCGCTAAACACAGACTACTATTTC

TCCCACCTGTTGGGTACCATAAGCGTGACCTTTAAGGCTCTATTCGGAAATGATGTGAGA

ACAACAGAAAATCTTTTAAAGCGGTTTATTCCGGAAACCCCCCACAAGACCCCCACGAAA

ACCCAGGCACTGCTTGAGCGCGCCGGCTTTGAAAAGCTGACGCCCTTTACACCGGAGGAA

GAAAGTCGTCGAATACTGCATACAGTTTTTTGTACTCTAGAAGCAGCTCCCCATCAAAGC

TGA

>MF975656.1

ATGGCGGCGCGCGAACAGGCCAACTCCGTTCGCAGGAGCGGGTTTTTTAATCCGTTTATT

GGCAAGAGGCCATTTTTCAGGCCGGGAAGCGGGCAGACCGCGGAGACAGAGCGCCCAAGG

CCCCCCCAACACTCGTACTGCACAGAGGTGGGTAGCTTTAAGTTTATAGCTCCAAGATGC

CTCGATGAAGAAGCCCCCGCCGACCAGCGACGCGGTGTACACGTGGGCACCTTGGAGCGC

CCACCCAAAGTGTACTGCGATGGCTCAGAGTACGACGTGCTGAACTTTGCCTCCGGAGGT

TGCTGGCCTCGCAGGATTCGGGTTTGGAACGGCCAGGATTTTCGGGGCGATGGATTCAAC

CCCAGATTTGAGAGATTTCACGTGTACGACATAGTAGAGACTTCGGAGAGCGCCTCACAC

GATGACCCATCCAGGTTTGCTGAGCTATCCCGCCCAAGCGGGTCTGTGGTAACACTGCTG

GGAATGAGCGAGTGTGGAAAGCGAGTCGCGGTTCACGTCTATGGTGTGCGCCATTATTTT

TACATGGCAAAGGCGGAGGTGGATAGCGCTTGTGGAATAACCACCGAGGCAGAACTCGTG

CGCGCAATGGTGGACTGCGCGCACAGCTCGGCTTTGAGCGCGGCCCTGGGAAATGGCAAC

GGCGGCAAACAGAGCGGCGGCAGCGGCGGGGGATGGTGGGGCGGAAAGCACGTGTCTGCG

GACTGCTTCAAAGTGGAGACCGTGTGCCGCACGACGCTGTACTACTTTGGATCTAAGCCA

GCTCTCTACTATAGAGTATCTGCCTCCAGCAGCCGCCTGGGAGGGTTCATCTGCGACAAC

TTTCACCCGGAGATTACAAAATTCGAGGGGAGCGTGGACGTGACCACGCGGCTGTTGTTG

GACAATGAAAATTTTACCAGTTTCGGGTGGTACCGCCTGCGACCCGGCACCCACGGAGAG

CGTGTTCAACTTCGCCCCGTTGAGCGACACGTCACCTCAAGCGACGTGGAGATTAACTGT

ACTCCCGATAACCTGGAGCCGATACCAGACGAGGCTGCCTGGCCCGACTATAAGCTCATG

TGCTTTGATATAGAGTGTAAAGCTGGAACGGGTAACGAAATGGCGTTCCCAGTGGCAACT

AACCAAGAGGACCTGGTCATCCAGATCTCCTGTCTGCTGTACTCGCTTGCTACTCAGAAC

CACGAACACACCCTGCTGTTTTCCCTCGGGTCATGCGATATCTCTGAGGAATACTCGTTT

GCATGCGTCCAGCGCGGCGAGCCCAGACCGACGGTTTTGGAGTTTGACAGCGAGTACGAG

CTGCTGGTTGCCTTCCTGACCTTTCTCAAGCAGTACTCTCCCGAGTTCGCCACCGGCTAC

AACATCGTTAATTTTGACTGGGCGTACATAGTTAACAAGGTAACGTCGGTGTATAACATC

AAGCTGGACGGGTACGGCAAGTTCAACAAAGGGGGGCTGTTTAAGGTGTGGGACATCGCC

ACGAACCATTTTCAGAAGAAGAGCAAGGTGAAAATCAATGGCCTGATATCTCTAGACATG

TATTCTGTGGCGACGGAAAAGCTAAAGCTACCCAGCTACAAACTCGACGCGGTCGTGGGA

GACGTCCTCGGCGAGCATAAGATAGACCTTCCCTATAAAGAAATACCCTCCTATTACGCG

GGAGGGCCTGACCGGAGGGGCGTAATAGGAGAGTATTGTATCCAGGACTCTAGGCTGGTG

GGCAAGCTGTTTTTTAAGTACCTCCCCCATCTGGAACTATCGGCGGTGGCCAAACTCGCC

CGTATCACCCTAACGCGGGTAATTTTTGACGGTCAGCAAATTCGCGTGTACACGTGCTTG

CTGAAACTCGCCCGCGAGAGAAATTTCATTTTGCCAGACAACAGACGCCGGTTTGACAGT

CAGGCAGACGCCGCGTCAGAGACTTCGGAGTTGGCTATGGATAGCCAAAGCCACGCCTTC

GACAGTACAGACGAACCCGACGGTGTGGACGGTACCCCGGACGCCGCAGGATCTGGCGCT

ACTTCTGAAAACGGAGGCGGGAAGCCCGGCGTCGGGAGGGCCGTGGGCTACCAGGGAGCA

AAGGTTCTAGACCCCGTATCCGGCTTTCATGTGGACCCCGTGGTTGTGTTTGACTTCGCT

AGCTTATACCCAAGCATTATCCAGGCCCATAACCTCTGTTTCACCACCCTGGCGCTCGAT

GAAGTGGATCTGGCCGGGCTTCAACCATCCGTCGACTACTCGACGTTCGAGGTGGGTGAC

CAAAAGTTATTTTTTGTCCACGCCCATATTCGCGAAAGCCTGCTTGGCATCTTGCTGCGC

GACTGGCTGGCCATGCGAAAGGCGGTGAGGGCGCGAATCCCCACCAGCACCCCCGAGGAG

GCAGTTTTACTAGATAAGCAGCAGTCTGCGATTAAGGTGATATGCAACTCGGTTTACGGA

TTCACGGGGGTGGCAAACGGCCTGTTGCCGTGTCTGAGGATAGCGGCTACCGTTACCACG

ATAGGACGCGACATGCTCCTCAAGACCCGAGATTACGTTCACTCTCGTTGGGCGACGCGC

GAGCTGCTGGAGGACAATTTTCCAGGGGCTATAGGTTTCCGAAACCACAAGCCTTACTCC

GTCAGGGTTATCTACGGAGACACCGACTCCGTGTTTATCAAGTTTGTGGGCCTGACGTAC

GAGGGGGTATCGGAGCTGGGGGATGCTATGTCGCGTCAGATTTCAGCGGACCTCTTTAGA

GCGCCCATCAAACTGGAGTGTGAGAAGACCTTTCAGCGACTGCTGCTGATCACCAAGAAG

AAGTACATAGGTGTCATAAACGGGGGGAAGATGCTCATGAAGGGGGTCGACCTGGTCCGC

AAAAATAACTGCTCTTTCATAAACTTGTACGCGCGACATCTGGTAGATCTTTTGTTGTAC

GACGAGGATGTGGCCACGGCGGCAGCAGAGGTGACAGACGTGCCTCCCGCAGAATGGGTG

GGGCGCCCGCTACCGAGCGGCTTTGACAAGTTTGGGCGAGTGCTGGTAGAGGCGTACAAC

CGTATCACTGCCCCCAACTTGGACGTGCGCGAGTTCGTTATGACTGCTGAGCTGAGCCGC

TCACCCGAATCGTATACCAACAAGCGCCTGCCGCACCTCACCGTCTACTTTAAGCTCGCC

ATGAGGAATGAAGAACTGCCCAGCGTAAAAGAGAGAATTCCGTATGTGATAGTTGCGCAG

ACCGAGGCCGCGGAACGCGAAGCGGGTGTAGTAAACTCAATGCGCGGTACCGCCCAAAAC

CCCGTGGTAACCAAGACCGCACGCCCCCAACCTAAACGCAAACTGCTGGTTTCCGACCTC

GCCGAAGACCCGACCTATGTTTCCGAGAATGACGTACCGCTAAACACAGACTACTATTTC

TCCCACCTGTTGGGTACCATAAGCGTGACCTTTAAGGCTCTATTCGGAAATGATGTGAGA

ACAACAGAAAATCTTTTAAAGCGGTTTATTCCGGAAACCCCCCACAAGACCCCCACGAAA

ACCCAGGCACTGCTTGAGCGCGCCGGCTTTGAAAAGCTGACGCCCTTTACACCGGAGGAA

GAAAGTCGTCGAATACTGCATACAGTTTTTTGTACTCTAGAAGCAGCTCCCCATCAAAGC

TGA

>MN912433.1

ATGGCGGCGCGCGAACAGGCCAACTCCGTTCGCAGGAGCGGGTTTTTTAATCCGTTTATT

GGCAAGAGGCCATTTTTCAGGCCGGGAAGCGGGCAGACCGCGGAGACAGAGCGCCCAAGG

CCCCCCCAACACTCGTACTGCACAGAGGTGGGTAGCTTTAAGTTTATAGCTCCAAGATGC

CTCGATGAAGAAGCCCCCGCCGACCAGCGACGCGGTGTACACGTGGGCACCTTGGAGCGC

CCACCCAAAGTGTACTGCGATGGCTCAGAGTACGACGTGCTGAACTTTGCCTCCGGAGGT

TGCTGGCCTCGCAGGATTCGGGTTTGGAACGGCCAGGATTTTCGGGGCGATGGATTCAAC

CCCAGATTTGAGAGATTTCACGTGTACGACATAGTAGAGACTTCGGAGAGCGCCTCACAC

GATGACCCATCCAGGTTTGCTGAGCTATCCCGCCCAAGCGGGTCTGTGGTAACACTGCTG

GGAATGAGCGAGTGTGGAAAGCGAGTCGCGGTTCACGTCTATGGTGTGCGCCATTATTTT

TACATGGCAAAGGCGGAGGTGGATAGCGCTTGTGGAATAACCACCGAGGCAGAACTCGTG

CGCGCAATGGTGGACTGCGCGCACAGCTCGGCTTTGAGCGCGGCCCTGGGAAATGGCAAC

GGCGGCAAACAGAGCGGCGGCAGCGGCGGGGGATGGTGGGGCGGAAAGCACGTGTCTGCG

GACTGCTTCAAAGTGGAGACCGTGTGCCACACGACGCTGTACTACTTTGGATCTAAGCCA

GCTCTCTACTATAGAGTATCTGCCTCCAGCAGCCGCCTGGGAGGGTTCATCTGCGACAAC

TTTCACCCGGAGATTACAAAATTCGAGGGGAGCGTGGACGTGACCACGCGGCTGTTGTTG

GACAATGAAAATTTTACCAGTTTCGGGTGGTACCGCCTGCGACCCGGCACCCACGGAGAG

CGTGTTCAACTTCGCCCCGTTGAGCGACACGTCACCTCAAGCGACGTGGAGATTAACTGT

ACTCCCGATAACCTGGAGCCGATACCAGACGAGGCTGCCTGGCCCGACTATAAGCTCATG

TGCTTTGATATAGAGTGTAAAGCTGGAACGGGTAACGAAATGGCGTTCCCAGTGGCAACT

AACCAAGAGGACCTGGTCATCCAGATCTCCTGTCTGCTGTACTCGCTTGCTACTCAGAAC

CACGAACACACCCTGCTGTTTTCCCTCGGGTCATGCGATATCTCTGAGGAATACTCGTTT

GCATGCGTCCAGCGCGGCGAGCCCAGACCGACGGTTTTGGAGTTTGACAGCGAGTACGAG

CTGCTGGTTGCCTTCCTGACCTTTCTCAAGCAGTACTCTCCCGAGTTCGCCACCGGCTAC

AACATCGTTAATTTTGACTGGGCGTACATAGTTAACAAGGTAACGTCGGTGTATAACATC

AAGCTGGACGGGTACGGCAAGTTCAACAAAGGGGGGCTGTTTAAGGTGTGGGACATCGCC

ACGAACCATTTTCAGAAGAAGAGCAAGGTGAAAATCAATGGCCTGATATCTCTAGACATG

TATTCTGTGGCGACGGAAAAGCTAAAGCTACCCAGCTACAAACTCGACGCGGTCGTGGGA

GACGTCCTCGGCGAGCATAAGATAGACCTTCCCTATAAAGAAATACCCTCCTATTACGCG

GGAGGGCCTGACCGGAGGGGCGTAATAGGAGAGTATTGTATCCAGGACTCTAGGCTGGTG

GGCAAGCTGTTTTTTAAGTACCTCCCCCATCTGGAACTATCGGCGGTGGCCAAACTCGCC

CGTATCACCCTAACGCGGGTAATTTTTGACGGTCAGCAAATTCGCGTGTACACGTGCTTG

CTGAAACTCGCCCGCGAGAGAAATTTCATTTTGCCAGACAACAAACGCCGGTTTGACAGT

CAGGCAGACGCCGCGTCAGAGACTTCGGAGTTGGCTATGGATAGCCAAAGCCACGCCTTC

GACAGTACAGACGAACCCGACGGTGTGGACGGTACCCCGGACGCCGCAGGATCTGGCGCT

ACTTCTGAAAACGGAGGCGGGAAGCCCGGCGTCGGGAGGGCCGTGGGCTACCAGGGAGCA

AAGGTTCTAGACCCCGTATCCGGCTTTCATGTGGACCCCGTGGTTGTGTTTGACTTCGCT

AGCTTATACCCAAGCATTATCCAGGCCCATAACCTCTGTTTCACCACCCTGGCGCTCGAT

GAAGTGGATCTGGCCGGGCTTCAACCATCCGTCAACTACTCGACGTTCGAGGTGGGTGAC

CAAAAGTTATTTTTTGTCCACGCCCATATTCGCGAAAGCCTGCTTGGCATCTTGCTGCGC

GACTGGCTGGCCATGCGAAAGGCGGTGAGGGCGCGAATCCCCACCAGCACCCCCGAGGAG

GCAGTTTTACTAGATAAGCAGCAGTCTGCGATTAAGGTGATATGCAACTCGGTTTACGGA

TTCACGGGGGTGGCAAACGGCCTGTTGCCGTGTCTGAGGATAGCGGCTACCGTTACCACG

ATAGGACGCGACATGCTCCTCAAGACCCGAGATTACGTTCACTCTCGTTGGGCGACGCGC

GAGCTGCTGGAGGACAATTTTCCAGGGGCTATAGGTTTCCGAAACCACAAGCCTTACTCC

GTCAGGGTTATCTACGGAGACACCGACTCCGTGTTTATCAAGTTTGTGGGCCTGACGTAC

GAGGGGGTATCGGAGCTGGGGGATGCTATGTCGCGTCAGATTTCAGCGGACCTCTTTAGA

GCGCCCATCAAACTGGAGTGTGAGAAGACCTTTCAGCGACTGCTGCTGATCACCAAGAAG

AAGTACATAGGTGTCATAAACGGGGGGAAGATGCTCATGAAGGGGGTCGACCTAGTCCGC

AAAAATAACTGCTCTTTCATAAACTTGTACGCGCGACATCTGGTAGATCTTTTGTTGTAC

GACGAGGATGTGGCCACGGCGGCAGCAGAGGTGACAGACGTGCCTCCCGCAGAATGGGTG

GGGCGCCCGCTACCGAGCGGCTTTGACAAGTTTGGGCGAGTGCTGGTAGAGGCGTACAAC

CGTATCACTGCCCCCAACTTGGACGTGCGCGAGTTCGTTATGACTGCTGAGCTGAGCCGC

TCACCCGAATCGTATACCAACAAGCGCCTGCCGCACCTCACCGTCTACTTTAAGCTCGCC

ATGAGGAATGAAGAACTGCCCAGCGTAAAAGAGAGAATTCCGTATGTGATAGTTGCGCAG

ACCGAGGCCGCGGAACGCGAAGCGGGTGTAGTAAACTCAATGCGCGGTACCGCCCAAAAC

CCCGTGGTAACCAAGACCGCACGCCCCCAACCTAAACGCAAACTGCTGGTTTCCGACCTC

GCCGAAGACCCGACCTATGTTTCCGAGAATGACGTACCGCTAAACACAGACTACTATTTC

TCCCACCTGTTGGGTACCATAAGCGTGACCTTTAAGGCTCTATTCGGAAATGATGTGAGA

ACAACAGAAAATCTTTTAAAGCGGTTTATTCCGGAAACCCCCCACAAGACCCCCACGAAA

ACCCAGGCACTGCTTGAGCGCGCCGGCTTTGAAAAGCTGACGCCCTTTACACCGGAGGAA

GAAAGTCGTCGAATACTGCATACAGTTTTTTGTACTCTAGAAGCAGCTCCCCATCAAAGC

TGA

>MT077857.1

ATGGCGGCGCGCGAACAGGCCAACTCCGTTCGCAGGAGCGGGTTTTTTAATCCGTTTATT

GGCAAGAGGCCATTTTTCAGGCCGGGAAGCGGGCAGACCGCGGAGACAGAGCGCCCAAGG

CCCCCCCAACACTCGTACTGCACAGAGGTGGGTAGCTTTAAGTTTATAGCTCCAAGATGC

CTCGATGAAGAAGCCCCCGCCGACCAGCGACGCGGTGTACACGTGGGCACCTTGGAGCGC

CCACCCAAAGTGTACTGCGATGGCTCAGAGTACGACGTGCTGAACTTTGCCTCCGGAGGT

TGCTGGCCTCGCAGGATTCGGGTTTGGAACGGCCAGGATTTTCGGGGCGATGGATTCAAC

CCCAGATTTGAGAGATTTCACGTGTACGACATAGTAGAGACTTCGGAGAGCGCCTCACAC

GATGACCCATCCAGGTTTGCTGAGCTATCCCGCCCAAGCGGGTCTGTGGTAACACTGCTG

GGAATGAGCGAGTGTGGAAAGCGAGTCGCGGTTCACGTCTATGGTGTGCGCCATTATTTT

TACATGGCAAAGGCGGAGGTGGATAGCGCTTGTGGAATAACCACCGAGGCAGAACTCGTG

CGCGCAATGGTGGACTGCGCGCACAGCTCGGCTTTGAGCGCGGCCCTGGGAAATGGCAAC

GGCGGCAAACAGAGCGGCGGCAGCGGCGGGGGATGGTGGGGCGGAAAGCACGTGTCTGCG

GACTGCTTCAAAGTGGAGACCGTGTGCCACACGACGCTGTACTACTTTGGATCTAAGCCA

GCTCTCTACTATAGAGTATCTGCCTCCAGCAGCCGCCTGGGAGGGTTCATCTGCGACAAC

TTTCACCCGGAGATTACAAAATTCGAGGGGAGCGTGGACGTGACCACGCGGCTGTTGTTG

GACAATGAAAATTTTACCAGTTTCGGGTGGTACCGCCTGCGACCCGGCACCCACGGAGAG

CGTGTTCAACTTCGCCCCGTTGAGCGACACGTCACCTCAAGCGACGTGGAGATTAACTGT

ACTCCCGATAACCTGGAGCCGATACCAGACGAGGCTGCCTGGCCCGACTATAAGCTCATG

TGCTTTGATATAGAGTGTAAAGCTGGAACGGGTAACGAAATGGCGTTCCCAGTGGCAACT

AACCAAGAGGACCTGGTCATCCAGATCTCCTGTCTGCTGTACTCGCTTGCTACTCAGAAC

CACGAACACACCCTGCTGTTTTCCCTCGGGTCATGCGATATCTCTGAGGAATACTCGTTT

GCATGCGTCCAGCGCGGCGAGCCCAGACCGACGGTTTTGGAGTTTGACAGCGAGTACGAG

CTGCTGGTTGCCTTCCTGACCTTTCTCAAGCAGTACTCTCCCGAGTTCGCCACCGGCTAC

AACATCGTTAATTTTGACTGGGCGTACATAGTTAACAAGGTAACGTCGGTGTATAACATC

AAGCTGGACGGGTACGGCAAGTTCAACAAAGGGGGGCTGTTTAAGGTGTGGGACATCGCC

ACGAACCATTTTCAGAAGAAGAGCAAGGTGAAAATCAATGGCCTGATATCTCTAGACATG

TATTCTGTGGCGACGGAAAAGCTAAAGCTACCCAGCTACAAACTCGACGCGGTCGTGGGA

GACGTCCTCGGCGAGCATAAGATAGACCTTCCCTATAAAGAAATACCCTCCTATTACGCG

GGAGGGCCTGACCGGAGGGGCGTAATAGGAGAGTATTGTATCCAGGACTCTAGGCTGGTG

GGCAAGCTGTTTTTTAAGTACCTCCCCCATCTGGAACTATCGGCGGTGGCCAAACTCGCC

CGTATCACCCTAACGCGGGTAATTTTTGACGGTCAGCAAATTCGCGTGTACACGTGCTTG

CTGAAACTCGCCCGCGAGAGAAATTTCATTTTGCCAGACAACAGACGCCGGTTTGACAGT

CAGGCAGACGCCGCGTCAGAGACTTCGGAGTTGGCTATGGATAGCCAAAGCCACGCCTTC

GACAGTACAGACGAACCCGACGGTGTGGACGGTACCCCGGACGCCGCAGGATCTGGCGCT

ACTTCTGAAAACGGAGGCGGGAAGCCCGGCGTCGGGAGGGCCGTGGGCTACCAGGGAGCA

AAGGTTCTAGACCCCGTATCCGGCTTTCATGTGGACCCCGTGGTTGTGTTTGACTTCGCT

AGCTTATACCCAAGCATTATCCAGGCCCATAACCTCTGTTTCACCACCCTGGCGCTCGAT

GAAGTGGATCTGGCCGGGCTTCAACCATCCGTCAACTACTCGACGTTCGAGGTGGGTGAC

CAAAAGTTATTTTTTGTCCACGCCCATATTCGCGAAAGCCTGCTTGGCATCTTGCTGCGC

GACTGGCTGGCCATGCGAAAGGCGGTGAGGGCGCGAATCCCCACCAGCACCCCCGAGGAG

GCAGTTTTACTAGATAAGCAGCAGTCTGCGATTAAGGTGATATGCAACTCGGTTTACGGA

TTCACGGGGGTGGCAAACGGCCTGTTGCCGTGTCTGAGGATAGCGGCTACCGTTACCACG

ATAGGACGCGACATGCTCCTCAAGACCCGAGATTACGTTCACTCTCGTTGGGCGACGCGC

GAGCTGCTGGAGGACAATTTTCCAGGGGCTATAGGTTTCCGAAACCACAAGCCTTACTCC

GTCAGGGTTATCTACGGAGACACCGACTCCGTGTTTATCAAGTTTGTGGGCCTGACGTAC

GAGGGGGTATCGGAGCTGGGGGATGCTATGTCGCGTCAGATTTCAGCGGACCTCTTTAGA

GCGCCCATCAAACTGGAGTGTGAGAAGACCTTTCAGCGACTGCTGCTGATCACCAAGAAG

AAGTACATAGGTGTCATAAACGGGGGGAAGATGCTCATGAAGGGGGTCGACCTAGTCCGC

AAAAATAACTGCTCTTTCATAAACTTGTACGCGCGACATCTGGTAGATCTTTTGTTGTAC

GACGAGGATGTGGCCACGGCGGCAGCAGAGGTGACAGACGTGCCTCCCGCAGAATGGGTG

GGGCGCCCGCTACCGAGCGGCTTTGACAAGTTTGGGCGAGTGCTGGTAGAGGCGTACAAC

CGTATCACTGCCCCCAACTTGGACGTGCGCGAGTTCGTTATGACTGCTGAGCTGAGCCGC

TCACCCGAATCGTATACCAACAAGCGCCTGCCGCACCTCACCGTCTACTTTAAGCTCGCC

ATGAGGAATGAAGAACTGCCCAGCGTAAAAGAGAGAATTCCGTATGTGATAGTTGCGCAG

ACCGAGGCCGCGGAACGCGAAGCGGGTGTAGTAAACTCAATGCGCGGTACCGCCCAAAAC

CCCGTGGTAACCAAGACCGCACGCCCCCAACCTAAACGCAAACTGCTGGTTTCCGACCTC

GCCGAAGACCCGACCTATGTTTCCGAGAATGACGTACCGCTAAACACAGACTACTATTTC

TCCCACCTGTTGGGTACCATAAGCGTGACCTTTAAGGCTCTATTCGGAAATGATGTGAGA

ACAACAGAAAATCTTTTAAAGCGGTTTATTCCGGAAACCCCCCACAAGACCCCCACGAAA

ACCCAGGCACTGCTTGAGCGCGCCGGCTTTGAAAAGCTGACGCCCTTTACACCGGAGGAA

GAAAGTCGTCGAATACTGCATACAGTTTTTTGTACTCTAGAAGCAGCTCCCCATCAAAGC

TGA

>MT063054.1

ATGGCGGCGCGCGAACAGGCCAACTCCGTTCGCAGGAACGGGTTTTTTAATCCGTTTATT

GGCAAGAGGCCATTTTTCAGGCCGGGAAGCGGGCAGACCGCGGAGACAGAGCGCCCAAGG

CCCCCCCAACACTCGTACTGCACAGAGGTGGGTAGCTTTAAGTTTATAGCTCCAAGATGC

CTCGATGAAGAAGCCCCCGCCGACCAGCGACGCGGTGTACACGTGGGCACCTTGGAGCGC

CCACCCAAAGTGTACTGCGATGGCTCAGAGTACGACGTGCTGAACTTTGCCTCCGGAGGT

TGCTGGCCTCGCAGGATTCGGGTTTGGAACGGCCAGGATTTTCGGGGCGATGGATTCAAC

CCCAGATTTGAGAGATTTCACGTGTACGACATAGTAGAGACTTCGGAGAGCGCCTCACAC

GATGACCCATCCAGGTTTGCTGAGCTATCCCGCCCAAGCGGGTCTGTGGTAACACTGCTG

GGAATGAGCGAGTGTGGAAAGCGAGTCGCGGTTCACGTCTATGGTGTGCGCCATTATTTT

TACATGGCAAAGGCGGAGGTGGATAGCGCTTGTGGAATAACCACCGAGGCAGAACTCGTG

CGCGCAATGGTGGACTGCGCGCACAGCTCGGCTTTGAGCGCGGCCCTGGGAAATGGCAAC

GGCGGCAAACAGAGCGGCGGCAGCGGCGGGGGATGGTGGGGCGGAAAGCACGTGTCTGCG

GACTGCTTCAAGGTGGAGACCGTGTGCCACACGACGCTGTACTACTTTGGATCTAAGCCA

GCTCTCTACTATAGAGTATCTGCCTCCAGCAGCCGCCTGGGAGGATTCATCTGCGACAAC

TTTCACCCGGAGATTACAAAATTCGAGGGGAGCGTGGACGTGACCACGCGGCTGTTGTTG

GACAATGAAAATTTTACCAGTTTCGGGTGGTACCGCCTGCGACCCGGCACCCACGGAGAG

CGTGTTCAACTTCGCCCCGTTGAGCGACACGTCACCTCAAGCGACGTGGAGATTAACTGT

ACTCCCGATAACCTGGAGCCGATACCAGACGAGGCTGCCTGGCCCGACTATAAGCTCATG

TGCTTTGATATAGAGTGTAAAGCTGGAACGGGTAACGAAATGGCGTTCCCAGTGGCAACT

AACCAAGAGGACCTGGTCATCCAGATCTCCTGTCTGCTGTACTCGCTTGCTACTCAGAAC

CACGAACACACCCTGCTGTTTTCCCTCGGGTCATGCGATATCTCTGAGGAATACTCGTTT

GCATGCGTCCAGCGCGGCGAGCCCAGACAGACGGTTTTGGAGTTTGACAGCGAGTACGAG

CTGCTGGTTGCCTTCCTGACCTTTCTCAAGCAGTACTCTCCCGAGTTCGCCACCGGCTAC

AACATCGTTAATTTTGACTGGGCGTACATAGTTAACAAGGTAACGTCGGTGTATAACATC

AAGCTGGACGGGTACGGCAAGTTCAACAAAGGGGGGCTGTTTAAGGTGTGGGACATCGCC

ACGAACCATTTTCAGAAGAAGAGCAAGGTGAAAATCAATGGCCTGATATCTCTAGACATG

TATTCTGTGGCGACGGAAAAGCTAAAGCTACCCAGCTACAAACTCGACGCGGTCGTGGGA

GACGTCCTCGGCGAGCATAAGATAGACCTTCCCTATAAAGAAATACCCTCCTATTACGCG

GGAGGGCCTGACCGGAGGGGCGTAATAGGAGAGTATTGTATCCAGGACTCTAGGCTGGTG

GGCAAGCTGTTTTTTAAGTACCTCCCCCATCTGGAACTATCGGCGGTGGCCAAACTCGCC

CGTATCACCCTAACGCGGGTAATTTTTGACGGTCAGCAAATTCGCGTGTCCACGTGCTTG

CTGAAACTCGCCCGCGAGAGAAATTTCATTTTGCCAGACAACAGACGCCGGTTTGACAGT

CAGGCAGACGCCGCGTCAGAGACTTCGGAGTTGGCTATGGATAGCCAAAGCCACGCCTTC

GACAGTACAGACGAACCCGACGGTGTGGACGGTACCCCGGACGCCGCAGGATCTGGCGCT

ACTTCTGAAAACGGAGGCGGGAAGCCCGGCGTCGGGAGGGCCGTGGGCTACCAGGGAGCA

AAGGTTCTAGACCCCGTATCCGGCTTTCATGTGGACCCCGTGGTTGTGTTTGACTTCGCT

AGCTTATACCCAAGCATTATCCAGGCCCATAACCTCTGTTTCACCACCCTGGCGCTCGAT

GAAGTGGATCTGGCCGGGCTTCAACCATCCGTCAACTACTCGACGTTCGAGGTGGGTGAC

CAAAAGTTATTTTTTGTCCACGCCCATATTCGCGAAAGCCTGCTTGGCATCTTGCTGCGC

GACTGGCTGGCCATGCGAAAGGCGGTGAGGGCGCGAATCCCCACCAGCACCCCCGAGGAG

GCAGTTTTACTAGATAAGCAGCAGTCTGCGATTAAGGTGATATGCAACTCGGTTTACGGA

TTCACGGGGGTGGCAAACGGCCTGTTGCCGTGTCTGAGGATAGCGGCTACCGTTACCACG

ATAGGACGCGACATGCTCCTCAAGACCCGAGATTACGTTCACTCTCGTTGGGCGACGCGC

GAGCTGCTGGAGGACAATTTTCCAGGGGCTATAGGTTTCCGAAACCACAAGCCTTACTCC

GTCAGGGTTATCTACGGAGACACCGACTCCGTGTTTATCAAGTTTGTGGGCCTGACGTAC

GAGGGGGTATCGGAGCTGGGGGATGCTATGTCGCGTCAGATTTCAGCGGACCTCTTTAGA

GCGCCCATCAAACTGGAGTGTGAGAAGACCTTTCAGCGACTGCTGCTGATCACCAAGAAG

AAGTACATAGGTGTCATAAACGGGGGGAAGATGCTCATGAAGGGGGTCGACCTGGTCCGC

AAAAATAACTGCTCTTTCATAAACTTGTACGCGCGACATCTGGTAGATCTTTTGTTGTAC

GACGAGGATGTGGCCACGGCGGCAGCAGAGGTGACAGACGTGCCTCCCGCAGAATGGGTG

GGGCGCCCGCTACCGAGCGGCTTTGACAAGTTTGGGCGAGTGCTGGTAGAGGCGTACAAC

CGTATCACTGCCCCCAACTTGGACGTGCGCGAGTTCGTTATGACTGCTGAGCTGAGCCGC

TCACCCGAATCGTATACCAACAAGCGCCTGCCGCACCTCACCGTCTACTTTAAGCTCGCC

ATGAGGAATGAAGAACTGCCCAGCGTAAAAGAGAGAATTCCGTATGTGATAGTTGCGCAG

ACCGAGGCCGCGGAACGCGAAGCGGGTGTAGTAAACTCAATGCGCGGTACCGCCCAAAAC

CCCGTGGTAACCAAGACCGCACGCCCCCAACCTAAACGCAAACTGCTGGTTTCCGACCTC

GCCGAAGACCCGACCTATGTTTCCGAGAATGACGTACCGCTAAACACAGACTACTATTTC

TCCCACCTGTTGGGTACCATAAGCGTGACCTTTAAGGCTCTATTCGGAAATGATGTGAGA

ACAACAGAAAATCTTTTAAAGCGGTTTATTCCGGAAACCCCCCACAAGACCCCCACGAAA

ACCCAGGCACTGCTTGAGCGCGCCGGCTTTGAAAAGCTGACGCCCTTTACACCGGAGGAA

GAAAGTCGTCGAATACTGCATACAGTTTTTTGTACTCTAGAAGCAGCTCCCCATCAAAGC

TGA

>MW855958.1

ATGGCGGCGCGCGAACAGGCCAACTCCGTTCGCAGGAGCGGGTTTTTTAATCCGTTTATT

GGCAAGAGGCCATTTTTCAGGCCGGGAAGCGGGCAAACCGCGGAGACAGAGCGCCCAAGG

CCCCCCCAACACTCGTACTGCACAGAGGTGGGTAGCTTTAAGTTTATAGCTCCAAGATGC

CTCGATGAAGAAGCCCCCGCCGACCAGCGACGCGGTGTACACGTGGGCACCTTGGAGCGC

CCACCCAAAGTGTACTGCGATGGCTCAGAGTACGACGTGCTGAACTTTGCCTCCGGAGGT

TGCTGGCCTCGCAGGATTCGGGTTTGGAACGGCCAGGATTTTCGGGGCGATGGATTCAAC

CCCAGATTTGAGAGATTTCACGTGTACGACATAGTAGAGACTTCGGAGAGCGCCTCACAC

GATGACCCATCCAGGTTTGCTGAGCTATCCCGCCCAAGCGGGTCTGTGGTAACACTGCTG

GGAATGAGCGAGTGTGGAAAGCGAGTCGCGGTTCACGTCTATGGTGTGCGCCATTATTTT

TACATGGCAAAGGCGGAGGTGGATAGCGCTTGTGGAATAACCACCGAGGCAGAACTCGTG

CGCGCAATGGTGGACTGCGCGCACAGCTCGGCTTTGAGCGCGGCCCTGGGAAATGGCAAC

GGCGGCAAACAGAGCGGCGGCAGCGGCGGGGGATGGTGGGGCGGAAAGCACGTGTCTGCG

GACTGCTTCAAAGTGGAGACCGTGTGCCACACGACGCTGTACTACTTTGGATCTAAGCCA

GCTCTCTACTATAGAGTATCTGCCTCCAGCAGCCGCCTGGGAGGGTTCATCTGCGACAAC

TTTCACCCGGAGATTACAAAATTCGAGGGGATCGTGGACGTGACCACGCGGCTGTTGTTG

GACAATGAAAATTTTACCAGTTTCGGGTGGTACCGCCTGCGACCCGGCACCCACGGAGAG

CGTGTTCAACTTCGCCCCGTTGAGCGACACGTCACCTCAAGCGACGTGGAGATTAACTGT

ACTCCCGATAACCTGGAGCCGATACCAGACGAGGCTGCCTGGCCCGACTATAAGCTCATG

TGCTTTGATATAGAGTGTAAAGCTGGAACGGGTAACGAAATGGCGTTCCCAGTGGCAACT

AACCAAGAGGACCTGGTCATCCAGATCTCCTGTCTGCTGTACTCGCTTGCTACTCAGAAC

CACGAACACACCCTGCTGTTTTCCCTCGGGTCATGCGATATCTCTGAGGAATACTCGTTT

GCATGCGTCCAGCGCGGCGAGCCCAGACCGACGGTTTTGGAGTTTGACAGCGAGTACGAG

CTGCTGGTTGCCTTCCTGACCTTTCTCAAGCAGTACTCTCCCGAGTTCGCCACCGGCTAC

AACATCGTTAATTTTGACTGGGCGTACATAGTTAACAAGGTAACGTCGGTGTATAACATC

AAGCTGGACGGGTACGGCAAGTTCAACAAAGGGGGNCTGTTTAAGGTGTGGGACATCGCC

ACGAACCATTTTCAGAAGAAGAGCAAGGTGAAAATCAATGGCCTGATATCTCTAGACATG

TATTCTGTGGCGACGGAAAAGCTAAAGCTACCCAGCTACAAACTCGACGCGGTCGTGGGA

GACGTCCTCGGCGAGCATAAGATAGACCTTCCCTATAAAGAAATACCCTCCTATTACGCG

GGAGGGCCTGACCGGAGGGGCGTAATAGGAGAGTATTGTATCCAGGACTCTAGGCTGGTG

GGCAAGCTGTTTTTTAAGTACCTCCCCCATCTGGAACTATCGGCGGTGGCCAAACTCGCC

CGTATCACCCTAACGCGGGTAATTTTTGACGGTCAGCAAATTCGCGTGTACACGTGCTTG

CTGAAACTCGCCCGCGAGAGAAATTTCATTTTGCCAGACAACAGACGCCGGTTTGACAGT

CAGGCAGACGCCGCGTCAGAGACTTCGGAGTTGGCTATGGATAGCCAAAGCCACGCCTTC

GACAGTACAGACGAACCCGACGGTGTGGACGGTACCCCGGACGCCGCAGGATCTGGCGCT

ACTTCTGAAAACGGAGGCGGGAAGCCCGGCGTCGGGAGGGCCGTGGGCTACCAGGGAGCA

AAGGTTCTAGACCCCGTATCCGGCTTTCATGTGGACCCCGTGGTTGTGTTTGACTTCGCT

AGCTTATACCCAAGCATTATCCAGGCCCATAACCTCTGTTTCACCACCCTGGCGCTCGAT

GAAGTGGATCTGGCCGGGCTTCAACCATCCGTCAACTACTCGACGTTCGAGGTGGGTGAC

CAAAAGTTATTTTTTGTCCACGCCCATATTCGCGAAAGCCTGCTTGGCATCTTGCTGCGC

GACTGGCTGGCCATGCGAAAGGCGGTGAGGGCGCGAATCCCCACCAGCACCCCCGAGGAG

GCAGTTTTACTAGATAAGCAGCAGTCTGCGATTAAGGTGATATGCAACTCGGTTTACGGA

TTCACGGGGGTGGCAAACGGCCTGTTGCCGTGTCTGAGGATAGCGGCTACCGTTACCACG

ATAGGACGCGACATGCTCCTCAAGACCCGAGATTACGTTCACTCTCGTTGGGCGACGCGC

GAGCTGCTGGAGGACAATTTTCCAGGGGCTATAGGTTTCCGAAACCACAAGCCTTACTCC

GTCAGGGTTATCTACGGAGACACCGACTCCGTGTTTATCAAGTTTGTGGGCCTGACGTAC

GAGGGGGTATCGGAGCTGGGGGATGCTATGTCGCGTCAGATTTCAGCGGACCTCTTTAGA

GCGCCCATCAAACTGGAGTGTGAGAAGACCTTTCAGCGACTGCTTCTGATCACCAAGAAG

AAGTACATAGGTGTCATAAACGGGGGGAAGATGCTCATGAAGGGGGTCGACCTGGTCCGC

AAAAATAACTGCTCTTTCATAAACTTGTACGCGCGACATCTGGTAGATCTTTTGTTGTAC

GACGAGGATGTGGCCACGGCGGCAGCAGAGGTGACAGACGTGCCTCCCGCAGAATGGGTG

GGGCGCCCGCTACCGAGCGGCTTTGACAAGTTTGGGCGAGTGCTGGTAGAGGCGTACAAC

CGTATCACTGCCCCCAACTTGGACGTGCGCGAGTTCGTTATGACTGCTGAGCTGAGCCGC

TCACCCGAATCGTATACCAACAAGCGCCTGCCGCACCTCACCGTCTACTTTAAGCTCGCC

ATGAGGAATGAAGAACTGCCCAGCGTAAAAGAGAGAATTCCGTATGTGATAGTTGCGCAG

ACCGAGGCCGCGGAACGCGAAGCGGGTGTAGTAAACTCAATGCGCGGTACCGCCCAAAAC

CCCGTGGTAACCAAGACCGCACGCCCCCAACCTAAACGCAAACTGCTGGTTTCCGACCTC

GCCGAAGACCCGACCTATGTTTCCGAGAATGACGTACCGCTAAACACAGACTACTATTTC

TCCCACCTGTTGGGTACCATAAGCGTGACCTTTAAGGCTCTATTCGGAAATGATGTGAGA

ACAACAGAAAATCTTTTAAAGCGGTTTATTCCGGAAACCCCCCACAAGACCCCCACGAAA

ACCCAGGCACTGCTTGAGCGCGCCGGCTTTGAAAAGCTGACGCCCTTTACACCGGAGGAA

GAAAGTCGTCGAATACTGCATACAGTTTTTTGTACTCTAGAAGCAGCTCCCCATCAAAGC

TGA

>MW855959.1

ATGGCGGCGCGCGAACAGGCCAACTCCGTTCGCAGGAGCGGGTTTTTTAATCCGTTTATT

GGCAAGAGGCCATTTTTCAGGCCGGGAAGCGGGCAAACCGCGGAGACAGAGCGCCCAAGG

CCCCCCCAACACTCGTACTGCACAGAGGTGGGTAGCTTTAAGTTTATAGCTCCAAGATGC

CTCGATGAAGAAGCCCCCGCCGACCAGCGACGCGGTGTACACGTGGGCACCTTGGAGCGC

CCACCCAAAGTGTACTGCGATGGCTCAGAGTACGACGTGCTGAACTTTGCCTCCGGAGGT

TGCTGGCCTCGCAGGATTCGGGTTTGGAACGGCCAGGATTTTCGGGGCGATGGATTCAAC

CCCAGATTTGAGAGATTTCACGTGTACGACATAGTAGAGACTTCGGAGAGCGCCTCACAC

GATGACCCATCCAGGTTTGCTGAGCTATCCCGCCCAAGCGGGTCTGTGGTAACACTGCTG

GGAATGAGCGAGTGTGGAAAGCGAGTCGCGGTTCACGTCTATGGTGTGCGCCATTATTTT

TACATGGCAAAGGCGGAGGTGGATAGCGCTTGTGGAATAACCACCGAGGCAGAACTCGTG

CGCGCAATGGTGGACTGCGCGCACAGCTCGGCTTTGAGCGCGGCCCTGGGAAATGGCAAC

GGCGGCAAACAGAGCGGCGGCAGCGGCGGGGGATGGTGGGGCGGAAAGCACGTGTCTGCG

GACTGCTTCAAAGTGGAGACCGTGTGCCACACGACGCTGTACTACTTTGGATCTAAGCCA

GCTCTCTACTATAGAGTATCTGCCTCCAGCAGCCGCCTGGGAGGGTTCATCTGCGACAAC

TTTCACCCGGAGATTACAAAATTCGAGGGGATCGTGGACGTGACCACGCGGCTGTTGTTG

GACAATGAAAATTTTACCAGTTTCGGGTGGTACCGCCTGCGACCCGGCACCCACGGAGAG

CGTGTTCAACTTCGCCCCGTTGAGCGACACGTCACCTCAAGCGACGTGGAGATTAACTGT

ACTCCCGATAACCTGGAGCCGATACCAGACGAGGCTGCCTGGCCCGACTATAAGCTCATG

TGCTTTGATATAGAGTGTAAAGCTGGAACGGGTAACGAAATGGCGTTCCCAGTGGCAACT

AACCAAGAGGACCTGGTCATCCAGATCTCCTGTCTGCTGTACTCGCTTGCTACTCAGAAC

CACGAACACACCCTGCTGTTTTCCCTCGGGTCATGCGATATCTCTGAGGAATACTCGTTT

GCATGCGTCCAGCGCGGCGAGCCCAGACCGACGGTTTTGGAGTTTGACAGCGAGTACGAG

CTGCTGGTTGCCTTCCTGACCTTTCTCAAGCAGTACTCTCCCGAGTTCGCCACCGGCTAC

AACATCGTTAATTTTGACTGGGCGTACATAGTTAACAAGGTAACGTCGGTGTATAACATC

AAGCTGGACGGGTACGGCAAGTTCAACAAAGGGGGGCTGTTTAAGGTGTGGGACATCGCC

ACGAACCATTTTCAGAAGAAGAGCAAGGTGAAAATCAATGGCCTGATATCTCTAGACATG

TATTCTGTGGCGACGGAAAAGCTAAAGCTACCCAGCTACAAACTCGACGCGGTCGTGGGA

GACGTCCTCGGCGAGCATAAGATAGACCTTCCCTATAAAGAAATACCCTCCTATTACGCG

GGAGGGCCTGACCGGAGGGGCGTAATAGGAGAGTATTGTATCCAGGACTCTAGGCTGGTG

GGCAAGCTGTTTTTTAAGTACCTCCCCCATCTGGAACTATCGGCGGTGGCCAAACTCGCC

CGTATCACCCTAACGCGGGTAATTTTTGACGGTCAGCAAATTCGCGTGTACACGTGCTTG

CTGAAACTCGCCCGCGAGAGAAATTTCATTTTGCCAGACAACAGACGCCGGTTTGACAGT

CAGGCAGACGCCGCGTCAGAGACTTCGGAGTTGGCTATGGATAGCCAAAGCCACGCCTTC

GACAGTACAGACGAACCCGACGGTGTGGACGGTACCCCGGACGCCGCAGGATCTGGCGCT

ACTTCTGAAAACGGAGGCGGGAAGCCCGGCGTCGGGAGGGCCGTGGGCTACCAGGGAGCA

AAGGTTCTAGACCCCGTATCCGGCTTTCATGTGGACCCCGTGGTTGTGTTTGACTTCGCT

AGCTTATACCCAAGCATTATCCAGGCCCATAACCTCTGTTTCACCACCCTGGCGCTCGAT

GAAGTGGATCTGGCCGGGCTTCAACCATCCGTCAACTACTCGACGTTCGAGGTGGGTGAC

CAAAAGTTATTTTTTGTCCACGCCCATATTCGCGAAAGCCTGCTTGGCATCTTGCTGCGC

GACTGGCTGGCCATGCGAAAGGCGGTGAGGGCGCGAATCCCCACCAGCACCCCCGAGGAG

GCAGTTTTACTAGATAAGCAGCAGTCTGCGATTAAGGTGATATGCAACTCGGTTTACGGA

TTCACGGGGGTGGCAAACGGCCTGTTGCCGTGTCTGAGGATAGCGGCTACCGTTACCACG

ATAGGACGCGACATGCTCCTCAAGACCCGAGATTACGTTCACTCTCGTTGGGCGACGCGC

GAGCTGCTGGAGGACAATTTTCCAGGGGCTATAGGTTTCCGAAACCACAAGCCTTACTCC

GTCAGGGTTATCTACGGAGACACCGACTCCGTGTTTATCAAGTTTGTGGGCCTGACGTAC

GAGGGGGTATCGGAGCTGGGGGATGCTATGTCGCGTCAGATTTCAGCGGACCTCTTTAGA

GCGCCCATCAAACTGGAGTGTGAGAAGACCTTTCAGCGACTGCTTCTGATCACCAAGAAG

AAGTACATAGGTGTCATAAACGGGGGGAAGATGCTCATGAAGGGGGTCGACCTGGTCCGC

AAAAATAACTGCTCTTTCATAAACTTGTACGCGCGACATCTGGTAGATCTTTTGTTGTAC

GACGAGGATGTGGCCACGGCGGCAGCAGAGGTGACAGACGTGCCTCCCGCAGAATGGGTG

GGGCGCCCGCTACCGAGCGGCTTTGACAAGTTTGGGCGAGTGCTGGTAGAGGCGTACAAC

CGTATCACTGCCCCCAACTTGGACGTGCGCGAGTTCGTTATGACTGCTGAGCTGAGCCGC

TCACCCGAATCGTATACCAACAAGCGCCTGCCGCACCTCACCGTCTACTTTAAGCTCGCC

ATGAGGAATGAAGAACTGCCCAGCGTAAAAGAGAGAATTCCGTATGTGATAGTTGCGCAG

ACCGAGGCCGCGGAACGCGAAGCGGGTGTAGTAAACTCAATGCGCGGTACCGCCCAAAAC

CCCGTGGTAACCAAGACCGCACGCCCCCAACCTAAACGCAAACTGCTGGTTTCCGACCTC

GCCGAAGACCCGACCTATGTTTCCGAGAATGACGTACCGCTAAACACAGACTACTATTTC

TCCCACCTGTTGGGTACCATAAGCGTGACCTTTAAGGCTCTATTCGGAAATGATGTGAGA

ACAACAGAAAATCTTTTAAAGCGGTTTATTCCGGAAACCCCCCACAAGACCCCCACGAAA

ACCCAGGCACTGCTTGAGCGCGCCGGCTTTGAAAAGCTGACGCCCTTTACACCGGAGGAA

GAAAGTCGTCGAATACTGCATACAGTTTTTTGTACTCTAGAAGCAGCTCCCCATCAAAGC

TGA

>MW855960.1

ATGGCGGCGCGCGAACAGGCCAACTCCGTTCGCAGGAGCGGGTTTTTTAATCCGTTTATT

GGCAAGAGGCCATTTTTCAGGCCGGGAAGCGGGCAAACCGCGGAGACAGAGCGCCCAAGG

CCCCCCNAACACTCGTACTGCACAGAGGTGGGTAGCTTTAAGTTTATAGCTCCAAGATGC

CTCGATGAAGAAGCCCCCGCCGACCAGCGACGCGGTGTACACGTGGGCACCTTGGAGCGC

CCACCCAAAGTGTACTGCGATGGCTCAGAGTACGACGTGCTGAACTTTGCCTCCGGAGGT

TGCTGGCCTCGCAGGATTCGGGTTTGGAACGGCCAGGATTTTCGGGGCGATGGATTCAAC

CCCAGATTTGAGAGATTTCACGTGTACGACATAGTAGAGACTTCGGAGAGCGCCTCACAC

GATGACCCATCCAGGTTTGCTGAGCTATCCCGCCCAAGCGGGTCTGTGGTAACACTGCTG

GGAATGAGCGAGTGTGGAAAGCGAGTCGCGGTTCACGTCTATGGTGTGCGCCATTATTTT

TACATGGCAAAGGCGGAGGTGGATAGCGCTTGTGGAATAACCACCGAGGCAGAACTCGTG

CGCGCAATGGTGGACTGCGCGCACAGCTCGGCTTTGAGCGCGGCCCTGGGAAATGGCAAC

GGCGGCAAACAGAGCGGCGGCAGCGGCGGGGNATGGTGGGGCGGAAAGCACGTGTCTGCG

GACTGCTTCAAAGTGGAGACCGTGTGCCACACGACGCTGTACTACTTTGGATCTAAGCCA

GCTCTCTACTATAGAGTATCTGCCTCCAGCAGCCGCCTGGGAGGGTTCATCTGCGACAAC

TTTCACCCGGAGATTACAAAATTCGAGGGGATCGTGGACGTGACCACGCGGCTGTTGTTG

GACAATGAAAATTTTACCAGTTTCGGGTGGTACCGCCTGCGACCCGGCACCCACGGAGAG

CGTGTTCAACTTCGCCCCGTTGAGCGACACGTCACCTCAAGCGACGTGGAGATTAACTGT

ACTCCCGATAACCTGGAGCCGATACCAGACGAGGCTGCCTGGCCCGACTATAAGCTCATG

TGCTTTGATATAGAGTGTAAAGCTGGAACGGGTAACGAAATGGCGTTCCCAGTGGCAACT

AACCAAGAGGACCTGGTCATCCAGATCTCCTGTCTGCTGTACTCGCTTGCTACTCAGAAC

CACGAACACACCCTGCTGTTTTCCCTCGGGTCATGCGATATCTCTGAGGAATACTCGTTT

GCATGCGTCCAGCGCGGCGAGCCCAGACCGACGGTTTTGGAGTTTGACAGCGAGTACGAG

CTGCTGGTTGCCTTCCTGACCTTTCTCAAGCAGTACTCTCCCGAGTTCGCCACCGGCTAC

AACATCGTTAATTTTGACTGGGCGTACATAGTTAACAAGGTAACGTCGGTGTATAACATC

AAGCTGGACGGGTACGGCAAGTTCAACAAAGGGGGGCTGTTTAAGGTGTGGGACATCGCC

ACGAACCATTTTCAGAAGAAGAGCAAGGTGAAAATCAATGGCCTGATATCTCTAGACATG

TATTCTGTGGCGACGGAAAAGCTAAAGCTACCCAGCTACAAACTCGACGCGGTCGTGGGA

GACGTCCTCGGCGAGCATAAGATAGACCTTCCCTATAAAGAAATACCCTCCTATTACGCG

GGAGGGCCTGACCGGAGGGGCGTAATAGGAGAGTATTGTATCCAGGACTCTAGGCTGGTG

GGCAAGCTGTTTTTTAAGTACCTCCCCCATCTGGAACTATCGGCGGTGGCCAAACTCGCC

CGTATCACCCTAACGCGGGTAATTTTTGACGGTCAGCAAATTCGCGTGTACACGTGCTTG

CTGAAACTCGCCCGCGAGAGAAATTTCATTTTGCCAGACAACAGACGCCGGTTTGACAGT

CAGGCAGACGCCGCGTCAGAGACTTCGGAGTTGGCTATGGATAGCCAAAGCCACGCCTTC

GACAGTACAGACGAACCCGACGGTGTGGACGGTACCCCGGACGCCGCAGGATCTGGCGCT

ACTTCTGAAAACGGAGGCGGGAAGCCCGGCGTCGGGAGGGCCGTGGGCTACCAGGGAGCA

AAGGTTCTAGACCCCGTATCCGGCTTTCATGTGGACCCCGTGGTTGTGTTTGACTTCGCT

AGCTTATACCCAAGCATTATCCAGGCCCATAACCTCTGTTTCACCACCCTGGCGCTCGAT

GAAGTGGATCTGGCCGGGCTTCAACCATCCGTCAACTACTCGACGTTCGAGGTGGGTGAC

CAAAAGTTATTTTTTGTCCACGCCCATATTCGCGAAAGCCTGCTTGGCATCTTGCTGCGC

GACTGGCTGGCCATGCGAAAGGCGGTGAGGGCGCGAATCCCCACCAGCACCCCCGAGGAG

GCAGTTTTACTAGATAAGCAGCAGTCTGCGATTAAGGTGATATGCAACTCGGTTTACGGA

TTCACGGGGGTGGCAAACGGCCTGTTGCCGTGTCTGAGGATAGCGGCTACCGTTACCACG

ATAGGACGCGACATGCTCCTCAAGACCCGAGATTACGTTCACTCTCGTTGGGCGACGCGC

GAGCTGCTGGAGGACAATTTTCCAGGGGCTATAGGTTTCCGAAACCACAAGCCTTACTCC

GTCAGGGTTATCTACGGAGACACCGACTCCGTGTTTATCAAGTTTGTGGGCCTGACGTAC

GAGGGGGTATCGGAGCTGGGGGATGCTATGTCGCGTCAGATTTCAGCGGACCTCTTTAGA

GCGCCCATCAAACTGGAGTGTGAGAAGACCTTTCAGCGACTGCTTCTGATCACCAAGAAG

AAGTACATAGGTGTCATAAACGGGGGGAAGATGCTCATGAAGGGGGTCGACCTGGTCCGC

AAAAATAACTGCTCTTTCATAAACTTGTACGCGCGACATCTGGTAGATCTTTTGTTGTAC

GACGAGGATGTGGCCACGGCGGCAGCAGAGGTGACAGACGTGCCTCCCGCAGAATGGGTG

GGGCGCCCGCTACCGAGCGGCTTTGACAAGTTTGGGCGAGTGCTGGTAGAGGCGTACAAC

CGTATCACTGCCCCCAACTTGGACGTGCGCGAGTTCGTTATGACTGCTGAGCTGAGCCGC

TCACCCGAATCGTATACCAACAAGCGCCTGCCGCACCTCACCGTCTACTTTAAGCTCGCC

ATGAGGAATGAAGAACTGCCCAGCGTAAAAGAGAGAATTCCGTATGTGATAGTTGCGCAG

ACCGAGGCCGCGGAACGCGAAGCGGGTGTAGTAAACTCAATGCGCGGTACCGCCCAAAAC

CCCGTGGTAACCAAGACCGCACGCCCCCAACCTAAACGCAAACTGCTGGTTTCCGACCTC

GCCGAAGACCCGACCTATGTTTCCGAGAATGACGTACCGCTAAACACAGACTACTATTTC

TCCCACCTGTTGGGTACCATAAGCGTGACCTTTAAGGCTCTATTCGGAAATGATGTGAGA

ACAACAGAAAATCTTTTAAAGCGGTTTATTCCGGAAACCCCCCACAAGACCCCCACGAAA

ACCCAGGCACTGCTTGAGCGCGCCGGCTTTGAAAAGCTGACGCCCTTTACACCGGAGGAA

GAAAGTCGTCGAATACTGCATACAGTTTTTTGTACTCTAGAAGCAGCTCCCCATCAAAGC

TGA

>MW855961.1

ATGGCGGCGCGCGAACAGGCCAACTCCGTTCGCAGGAGCGGGTTTTTTAATCCGTTTATT

GGCAAGAGGCCATTTTTCAGGCCGGGAAGCGGGCAAACCGCGGAGACAGAGCGCCCAAGG

CCCCCCCAACACTCGTACTGCACAGAGGTGGGTAGCTTTAAGTTTATAGCTCCAAGATGC

CTCGATGAAGAAGCCCCCGCCGACCAGCGACGCGGTGTACACGTGGGCACCTTGGAGCGC

CCACCCAAAGTGTACTGCGATGGCTCAGAGTACGACGTGCTGAACTTTGCCTCCGGAGGT

TGCTGGCCTCGCAGGATTCGGGTTTGGAACGGCCAGGATTTTCGGGGCGATGGATTCAAC

CCCAGATTTGAGAGATTTCACGTGTACGACATAGTAGAGACTTCGGAGAGCGCCTCACAC

GATGACCCATCCAGGTTTGCTGAGCTATCCCGCCCAAGCGGGTCTGTGGTAACACTGCTG

GGAATGAGCGAGTGTGGAAAGCGAGTCGCGGTTCACGTCTATGGTGTGCGCCATTATTTT

TACATGGCAAAGGCGGAGGTGGATAGCGCTTGTGGAATAACCACCGAGGCAGAACTCGTG

CGCGCAATGGTGGACTGCGCGCACAGCTCGGCTTTGAGCGCGGCCCTGGGAAATGGCAAC

GGCGGCAAACAGAGCGGCGGCAGCGGCGGGGGATGGTGGGGCGGAAAGCACGTGTCTGCG

GACTGCTTCAAAGTGGAGACCGTGTGCCACACGACGCTGTACTACTTTGGATCTAAGCCA

GCTCTCTACTATAGAGTATCTGCCTCCAGCAGCCGCCTGGGAGGGTTCATCTGCGACAAC

TTTCACCCGGAGATTACAAAATTCGAGGGGATCGTGGACGTGACCACGCGGCTGTTGTTG

GACAATGAAAATTTTACCAGTTTCGGGTGGTACCGCCTGCGACCCGGCACCCACGGAGAG

CGTGTTCAACTTCGCCCCGTTGAGCGACACGTCACCTCAAGCGACGTGGAGATTAACTGT

ACTCCCGATAACCTGGAGCCGATACCAGACGAGGCTGCCTGGCCCGACTATAAGCTCATG

TGCTTTGATATAGAGTGTAAAGCTGGAACGGGTAACGAAATGGCGTTCCCAGTGGCAACT

AACCAAGAGGACCTGGTCATCCAGATCTCCTGTCTGCTGTACTCGCTTGCTACTCAGAAC

CACGAACACACCCTGCTGTTTTCCCTCGGGTCATGCGATATCTCTGAGGAATACTCGTTT

GCATGCGTCCAGCGCGGCGAGCCCAGACCGACGGTTTTGGAGTTTGACAGCGAGTACGAG

CTGCTGGTTGCCTTCCTGACCTTTCTCAAGCAGTACTCTCCCGAGTTCGCCACCGGCTAC

AACATCGTTAATTTTGACTGGGCGTACATAGTTAACAAGGTAACGTCGGTGTATAACATC

AAGCTGGACGGGTACGGCAAGTTCAACAAAGGGGGGCTGTTTAAGGTGTGGGACATCGCC

ACGAACCATTTTCAGAAGAAGAGCAAGGTGAAAATCAATGGCCTGATATCTCTAGACATG

TATTCTGTGGCGACGGAAAAGCTAAAGCTACCCAGCTACAAACTCGACGCGGTCGTGGGA

GACGTCCTCGGCGAGCATAAGATAGACCTTCCCTATAAAGAAATACCCTCCTATTACGCG

GGAGGGCCTGACCGGAGGGGCGTAATAGGAGAGTATTGTATCCAGGACTCTAGGCTGGTG

GGCAAGCTGTTTTTTAAGTACCTCCCCCATCTGGAACTATCGGCGGTGGCCAAACTCGCC

CGTATCACCCTAACGCGGGTAATTTTTGACGGTCAGCAAATTCGCGTGTACACGTGCTTG

CTGAAACTCGCCCGCGAGAGAAATTTCATTTTGCCAGACAACAGACGCCGGTTTGACAGT

CAGGCAGACGCCGCGTCAGAGACTTCGGAGTTGGCTATGGATAGCCAAAGCCACGCCTTC

GACAGTACAGACGAACCCGACGGTGTGGACGGTACCCCGGACGCCGCAGGATCTGGCGCT

ACTTCTGAAAACGGAGGCGGGAAGCCCGGCGTCGGGAGGGCCGTGGGCTACCAGGGAGCA

AAGGTTCTAGACCCCGTATCCGGCTTTCATGTGGACCCCGTGGTTGTGTTTGACTTCGCT

AGCTTATACCCAAGCATTATCCAGGCCCATAACCTCTGTTTCACCACCCTGGCGCTCGAT

GAAGTGGATCTGGCCGGGCTTCAACCATCCGTCAACTACTCGACGTTCGAGGTGGGTGAC

CAAAAGTTATTTTTTGTCCACGCCCATATTCGCGAAAGCCTGCTTGGCATCTTGCTGCGC

GACTGGCTGGCCATGCGAAAGGCGGTGAGGGCGCGAATCCCCACCAGCACCCCCGAGGAG

GCAGTTTTACTAGATAAGCAGCAGTCTGCGATTAAGGTGATATGCAACTCGGTTTACGGA

TTCACGGGGGTGGCAAACGGCCTGTTGCCGTGTCTGAGGATAGCGGCTACCGTTACCACG

ATAGGACGCGACATGCTCCTCAAGACCCGAGATTACGTTCACTCTCGTTGGGCGACGCGC

GAGCTGCTGGAGGACAATTTTCCAGGGGCTATAGGTTTCCGAAACCACAAGCCTTACTCC

GTCAGGGTTATCTACGGAGACACCGACTCCGTGTTTATCAAGTTTGTGGGCCTGACGTAC

GAGGGGGTATCGGAGCTGGGGGATGCTATGTCGCGTCAGATTTCAGCGGACCTCTTTAGA

GCGCCCATCAAACTGGAGTGTGAGAAGACCTTTCAGCGACTGCTTCTGATCACCAAGAAG

AAGTACATAGGTGTCATAAACGGGGGGAAGATGCTCATGAAGGGGGTCGACCTGGTCCGC

AAAAATAACTGCTCTTTCATAAACTTGTACGCGCGACATCTGGTAGATCTTTTGTTGTAC

GACGAGGATGTGGCCACGGCGGCAGCAGAGGTGACAGACGTGCCTCCCGCAGAATGGGTG

GGGCGCCCGCTACCGAGCGGCTTTGACAAGTTTGGGCGAGTGCTGGTAGAGGCGTACAAC

CGTATCACTGCCCCCAACTTGGACGTGCGCGAGTTCGTTATGACTGCTGAGCTGAGCCGC

TCACCCGAATCGTATACCAACAAGCGCCTGCCGCACCTCACCGTCTACTTTAAGCTCGCC

ATGAGGAATGAAGAACTGCCCAGCGTAAAAGAGAGAATTCCGTATGTGATAGTTGCGCAG

ACCGAGGCCGCGGAACGCGAAGCGGGTGTAGTAAACTCAATGCGCGGTACCGCCCAAAAC

CCCGTGGTAACCAAGACCGCACGCCCCCAACCTAAACGCAAACTGCTGGTTTCCGACCTC

GCCGAAGACCCGACCTATGTTTCCGAGAATGACGTACCGCTAAACACAGACTACTATTTC

TCCCACCTGTTGGGTACCATAAGCGTGACCTTTAAGGCTCTATTCGGAAATGATGTGAGA

ACAACAGAAAATCTTTTAAAGCGGTTTATTCCGGAAACCCCCCACAAGACCCCCACGAAA

ACCCAGGCACTGCTTGAGCGCGCCGGCTTTGAAAAGCTGACGCCCTTTACACCGGAGGAA

GAAAGTCGTCGAATACTGCATACAGTTTTTTGTACTCTAGAAGCAGCTCCCCATCAAAGC

TGA

>MW855962.1

ATGGCGGCGCGCGAACAGGCCAACTCCGTTCGCAGGAGCGGGTTTTTTAATCCGTTTATT

GGCAAGAGGCCATTTTTCAGGCCGGGAAGCGGGCAAACCGCGGAGACAGAGCGCCCAAGG

CCCCCCCAACACTCGTACTGCACAGAGGTGGGTAGCTTTAAGTTTATAGCTCCAAGATGC

CTCGATGAAGAAGCCCCCGCCGACCAGCGACGCGGTGTACACGTGGGCACCTTGGAGCGC

CCACCCAAAGTGTACTGCGATGGCTCAGAGTACGACGTGCTGAACTTTGCCTCCGGAGGT

TGCTGGCCTCGCAGGATTCGGGTTTGGAACGGCCAGGATTTTCGGGGCGATGGATTCAAC

CCCAGATTTGAGAGATTTCACGTGTACGACATAGTAGAGACTTCGGAGAGCGCCTCACAC

GATGACCCATCCAGGTTTGCTGAGCTATCCCGCCCAAGCGGGTCTGTGGTAACACTGCTG

GGAATGAGCGAGTGTGGAAAGCGAGTCGCGGTTCACGTCTATGGTGTGCGCCATTATTTT

TACATGGCAAAGGCGGAGGTGGATAGCGCTTGTGGAATAACCACCGAGGCAGAACTCGTG

CGCGCAATGGTGGACTGCGCGCACAGCTCGGCTTTGAGCGCGGCCCTGGGAAATGGCAAC

GGCGGCAAACAGAGCGGCGGCAGCGGCGGGGGATGGTGGGGCGGAAAGCACGTGTCTGCG

GACTGCTTCAAAGTGGAGACCGTGTGCCACACGACGCTGTACTACTTTGGATCTAAGCCA

GCTCTCTACTATAGAGTATCTGCCTCCAGCAGCCGCCTGGGAGGGTTCATCTGCGACAAC

TTTCACCCGGAGATTACAAAATTCGAGGGGATCGTGGACGTGACCACGCGGCTGTTGTTG

GACAATGAAAATTTTACCAGTTTCGGGTGGTACCGCCTGCGACCCGGCACCCACGGAGAG

CGTGTTCAACTTCGCCCCGTTGAGCGACACGTCACCTCAAGCGACGTGGAGATTAACTGT

ACTCCCGATAACCTGGAGCCGATACCAGACGAGGCTGCCTGGCCCGACTATAAGCTCATG

TGCTTTGATATAGAGTGTAAAGCTGGAACGGGTAACGAAATGGCGTTCCCAGTGGCAACT

AACCAAGAGGACCTGGTCATCCAGATCTCCTGTCTGCTGTACTCGCTTGCTACTCAGAAC

CACGAACACACCCTGCTGTTTTCCCTCGGGTCATGCGATATCTCTGAGGAATACTCGTTT

GCATGCGTCCAGCGCGGCGAGCCCAGACCGACGGTTTTGGAGTTTGACAGCGAGTACGAG

CTGCTGGTTGCCTTCCTGACCTTTCTCAAGCAGTACTCTCCCGAGTTCGCCACCGGCTAC

AACATCGTTAATTTTGACTGGGCGTACATAGTTAACAAGGTAACGTCGGTGTATAACATC

AAGCTGGACGGGTACGGCAAGTTCAACAAAGGGGGGCTGTTTAAGGTGTGGGACATCGCC

ACGAACCATTTTCAGAAGAAGAGCAAGGTGAAAATCAATGGCCTGATATCTCTAGACATG

TATTCTGTGGCGACGGAAAAGCTAAAGCTACCCAGCTACAAACTCGACGCGGTCGTGGGA

GACGTCCTCGGCGAGCATAAGATAGACCTTCCCTATAAAGAAATACCCTCCTATTACGCG

GGAGGGCCTGACCGGAGGGGCGTAATAGGAGAGTATTGTATCCAGGACTCTAGGCTGGTG

GGCAAGCTGTTTTTTAAGTACCTCCCCCATCTGGAACTATCGGCGGTGGCCAAACTCGCC

CGTATCACCCTAACGCGGGTAATTTTTGACGGTCAGCAAATTCGCGTGTACACGTGCTTG

CTGAAACTCGCCCGCGAGAGAAATTTCATTTTGCCAGACAACAGACGCCGGTTTGACAGT

CAGGCAGACGCCGCGTCAGAGACTTCGGAGTTGGCTATGGATAGCCAAAGCCACGCCTTC

GACAGTACAGACGAACCCGACGGTGTGGACGGTACCCCGGACGCCGCAGGATCTGGCGCT

ACTTCTGAAAACGGAGGCGGGAAGCCCGGCGTCGGGAGGGCCGTGGGCTACCAGGGAGCA

AAGGTTCTAGACCCCGTATCCGGCTTTCATGTGGACCCCGTGGTTGTGTTTGACTTCGCT

AGCTTATACCCAAGCATTATCCAGGCCCATAACCTCTGTTTCACCACCCTGGCGCTCGAT

GAAGTGGATCTGGCCGGGCTTCAACCATCCGTCAACTACTCGACGTTCGAGGTGGGTGAC

CAAAAGTTATTTTTTGTCCACGCCCATATTCGCGAAAGCCTGCTTGGCATCTTGCTGCGC

GACTGGCTGGCCATGCGAAAGGCGGTGAGGGCGCGAATCCCCACCAGCACCCCCGAGGAG

GCAGTTTTACTAGATAAGCAGCAGTCTGCGATTAAGGTGATATGCAACTCGGTTTACGGA

TTCACGGGGGTGGCAAACGGCCTGTTGCCGTGTCTGAGGATAGCGGCTACCGTTACCACG

ATAGGACGCGACATGCTCCTCAAGACCCGAGATTACGTTCACTCTCGTTGGGCGACGCGC

GAGCTGCTGGAGGACAATTTTCCAGGGGCTATAGGTTTCCGAAACCACAAGCCTTACTCC

GTCAGGGTTATCTACGGAGACACCGACTCCGTGTTTATCAAGTTTGTGGGCCTGACGTAC

GAGGGGGTATCGGAGCTGGGGGATGCTATGTCGCGTCAGATTTCAGCGGACCTCTTTAGA

GCGCCCATCAAACTGGAGTGTGAGAAGACCTTTCAGCGACTGCTTCTGATCACCAAGAAG

AAGTACATAGGTGTCATAAACGGGGGGAAGATGCTCATGAAGGGGGTCGACCTGGTCCGC

AAAAATAACTGCTCTTTCATAAACTTGTACGCGCGACATCTGGTAGATCTTTTGTTGTAC

GACGAGGATGTGGCCACGGCGGCAGCAGAGGTGACAGACGTGCCTCCCGCAGAATGGGTG

GGGCGCCCGCTACCGAGCGGCTTTGACAAGTTTGGGCGAGTGCTGGTAGAGGCGTACAAC

CGTATCACTGCCCCCAACTTGGACGTGCGCGAGTTCGTTATGACTGCTGAGCTGAGCCGC

TCACCCGAATCGTATACCAACAAGCGCCTGCCGCACCTCACCGTCTACTTTAAGCTCGCC

ATGAGGAATGAAGAACTGCCCAGCGTAAAAGAGAGAATTCCGTATGTGATAGTTGCGCAG

ACCGAGGCCGCGGAACGCGAAGCGGGTGTAGTAAACTCAATGCGCGGTACCGCCCAAAAC

CCCGTGGTAACCAAGACCGCACGCCCCCAACCTAAACGCAAACTGCTGGTTTCCGACCTC

GCCGAAGACCCGACCTATGTTTCCGAGAATGACGTACCGCTAAACACAGACTACTATTTC

TCCCACCTGTTGGGTACCATAAGCGTGACCTTTAAGGCTCTATTCGGAAATGATGTGAGA

ACAACAGAAAATCTTTTAAAGCGGTTTATTCCGGAAACCCCCCACAAGACCCCCACGAAA

ACCCAGGCACTGCTTGAGCGCGCCGGCTTTGAAAAGCTGACGCCCTTTACACCGGAGGAA

GAAAGTCGTCGAATACTGCATACAGTTTTTTGTACTCTAGAAGCAGCTCCCCATCAAAGC

TGA

>MZ357402.1

ATGGCGGCGCGCGAACAGGCCAACTCCGTTCGCAGGAGCGGGTTTTTTAATCCGTTTATT

GGCAAGAGGCCATTTTTCAGGCCGGGAAGCGGGCAAACCGCGGAGACAGAGCGCCCAAGG

CCCCCCCAACACTCGTACTGCACAGAGGTGGGTAGCTTTAAGTTTATAGCTCCAAGATGC

CTCGATGAAGAAGCCCCCGCCGACCAGCGACGCGGTGTACACGTGGGCACCTTGGAGCGC

CCACCCAAAGTGTACTGCGATGGCTCAGAGTACGACGTGCTGAACTTTGCCTCCGGAGGT

TGCTGGCCTCGCAGGATTCGGGTTTGGAACGGCCAGGATTTTCGGGGCGATGGATTCAAC

CCCAGATTTGAGAGATTTCACGTGTACGACATAGTAGAGACTTCGGAGAGCGCCTCACAC

GATGACCCATCCAGGTTTGCTGAGCTATCCCGCCCAAGCGGGTCTGTGGTAACACTGCTG

GGAATGAGCGAGTGTGGAAAGCGAGTCGCGGTTCACGTCTATGGTGTGCGCCATTATTTT

TACATGGCAAAGGCGGAGGTGGATAGCGCTTGTGGAATAACCACCGAGGCAGAACTCGTG

CGCGCAATGGTGGACTGCGCGCACAGCTCGGCTTTGAGCGCGGCCCTGGGAAATGGCAAC

GGCGGCAAACAGAGCGGCGGCAGCGGCGGGGGATGGTGGGGCGGAAAGCACGTGTCTGCG

GACTGCTTCAAAGTGGAGACCGTGTGCCACACGACGCTGTACTACTTTGGATCTAAGCCA

GCTCTCTACTATAGAGTATCTGCCTCCAGCAGCCGCCTGGGAGGGTTCATCTGCGACAAC

TTTCACCCGGAGATTACAAAATTCGAGGGGATCGTGGACGTGACCACGCGGCTGTTGTTG

GACAATGAAAATTTTACCAGNNNNNNNNNNNNNNNNNNNNNNNNNNNNNNNNNNNNNNNN

NNNNNNNNNNNNNNNNNNNNNNNNNNNNNNNNNNNNNNNNNNNNNNNNNNNNNNNNNNNN

NNNNNNNNNNNNNNNNNNNNNNNNNNNNNNNNNNNNNNNNNNNNNNNNNNNNNNNNNNTG

TGCTTTGATATAGAGTGTAAAGCTGGAACGGGTAACGAAATGGCGTTCCCAGTGGCAACT

AACCAAGAGGACCTGGTCATCCAGATCTCCTGTCTGCTGTACTCGCTTGCTACTCAGAAC

CACGAACACACCCTGCTGTTTTCCCTCGGNNNNNNNNNNNNNNNNNNNNNNNNNNNNNNN

NNNNNNNNNCAGCGCGGCGAGCCCAGACCGACGGTTTTGGAGTTTGACAGCGAGTACGAG

CTGCTGGTTGCCTTCCTGACCTTTCTCAAGCAGTACTCTCCCGAGTTCGCCACCGGCTAC

AACATCGTTAATTTTGACTGGGCGTACATAGTTAACAAGGNNNNNNNNNNNNNNNNNNNN

NNNNNNGACGGGTACGGCAAGTTCAACAAAGGGGGGCTGTTTAAGGTGTGGGACATCGCC

ACGAACCATTTTCAGAAGAAGAGCAAGGTGAAAATCAATGGCCTGAGATCTCTAGACATG

TATTCTGTGGCGACGGAAAAGCTAAAGCTACCCAGCTACAAACTCGACGCGGTCGTGGGA

GACGTCCTCGGCGAGCATAAGATAGACCTTCCCTATAAAGAAATACCCTCCTATTACGCG

GGAGGGCCTGACCGGAGGGGCGTAATAGGAGAGTATTGTATCCAGGACTCTAGGCTGGTG

GGCAAGCTGTTTTTTAAGTACCTCCCCCATCTGGAACTATCGGCGGTGGCCAAACTCGCC

CGTATCACCCTAACGCGGGTAATTTTTGACGGTCAGCAAATTCGCGTGTACACGTGCTTG

CTGAAACTCGCCCGCGAGAGAAATTTCATTTTGCCAGACAACAGACGCCGGTTTGACAGT

CAGGCAGACGCCGCGTCAGAGACTTCGGAGTTGGCTATGGATAGCCAAAGCCACGCCTTC

GACAGTACAGACGAACCCGACGGTGTGGACGGTACCCCGGACGCCGCAGGATCTGGCGCT

ACTTCTGAAAACGGAGGCGGGAAGCCCGGCGTCGGGAGGGCCGTGGGCTACCAGGGAGCA

AAGGTTCTAGACCCCGTATCCGGCTTTCATGTGGACCCCGTGGTTGTGTTTGACTTCGCT

AGCTTATACCCAAGCATTATCCAGGCCCATAACCTCTGTTTCACCACCCTGGCGCTCGAT

GAAGTGGATCTGGCCGGGCTTCAACCATCCGTCAACTACTCGACGTTCGAGGTGGGTGAC

CAAAAGTTATTTTTTGTCCACGCCCATATTCGCGAAAGCCTGCTTGGCATCTTGCTGCGC

GACTGGCTGGCCATGCGAAAGGCGGTGAGGGCGCGAATCCCCACCAGCACCCCCGAGGAG

GCAGTTTTACTAGATAAGCAGCAGTCTGCGATTAAGGTGATATGCAACTCGGTTTACGGA

TTCACGGGGGTGGCAAACGGCCTGTTGCCGTGTCTGAGGATAGCGGCTACCGTTACCACG

ATAGGACGCGACATGCTCCTCAAGACCCGAGATTACGTTCACTCTCGTTGGGCGACGCGC

GAGCTGCTGGAGGACAATTTTCCAGGGGCTATAGGTTTCCGAAACCACAAGCCTTACTCC

GTCAGGGTTATCTACGGAGACACCGACTCCGTGTTTATCAAGTTTGTGGGCCTGACGTAC

GAGGGGGTATCGGAGCTGGGGGATGCTATGTCGCGTCAGATTTCAGCGGACCTCTTTAGA

GCGCCCATCAAACTGGAGTGTGAGAAGACCTTTCAGCGACTGCTTCTGATCACCAAGAAG

AAGNNNNNNNNNNNNNNNNNNNNNNNNNNNNNNNNNNNNNNNNNNGTCGACCTGGTCCGC

AAAAATAACTGCTCTTTCATAAACTTGTACGCGCGACATCTGGTAGATCTTTTGTTGTAC

GACGAGGATGTGGCCACGGCGGCAGCAGAGGTGACAGACGTGCCTCCCGCAGAATGGGTG

GGGCGCCCGCTACCGAGCGGCTTTGACAAGTTTGGGCGAGTGCTGGTAGAGGCGTACAAC

CGTATCACTGCCCCCAACTTGGACGTGCGCGAGTTCGTTATGACTGCTGAGCTGAGCCGC

TCACCCGAATCGTATACCAACAAGCGCCTGCCGCACCTCACCGTCTACTTTAAGCTCGCC

ATGAGGAATGAAGAACTGCCCAGCGTAAAAGAGAGAATTCCGTATGTGATAGTTGCGCAG

ACCGAGGCCGCGGAACGCGAAGCGGGTGTAGTAAACTCAATGCGCGGTACCGCCCAAAAC

CCCGTGGTAACCAAGACCGCACGCCCCCNNNNNNNNNNNNNNNNNNNNNNNNNNNNNNNN

NNNNNNNNNNNNNNCTATGTTTCCGAGAATGACGTACCGCTAAACACAGACTACTATTTC

TCCCACCTGTTGGGTACCATAAGCGTGACCTTTAAGGCTCTATTCGGAAATGATGTGAGA

ACAACAGAAAATCTTTTAAAGCGGTTTATTCCGGAAACCCCCCACAAGACCCCCACGAAA

ACCCAGGCACTGCTTGAGCGCGCCGGCTTTGAAAAGCTGACGCCCTTTACACCGGAGGAA

GAAAGTCGTCGAATACTGCATACAGTTTTTTGTACTCTAGAAGCAGCTCCCCATCAAAGC

TGA

>MZ318700.1

ATGGCGGCGCGCGAACAGGCCAACTCCGTTCGCAGGAGCGGGTTTTTTAATCCGTTTATT

GGCAAGAGGCCATTTTTCAGGCCGGGAAGCGGGCAAACCGCGGAGACAGAGCGCCCAAGG

CCCCCCCAACACTCGTACTGCACAGAGGTGGGTAGCTTTAAGTTTATAGCTCCAAGATGC

CTCGATGAAGAAGCCCCCGCCGACCAGCGACGCGGTGTACACGTGGGCACCTTGGAGCGC

CCACCCAAAGTGTACTGCGATGGCTCAGAGTACGACGTGCTGAACTTTGCCTCCGGAGGT

TGCTGGCCTCGCAGGATTCGGGTTTGGAACGGCCAGGATTTTCGGGGCGATGGATTCAAC

CCCAGATTTGAGAGATTTCACGTGTACGACATAGTAGAGACTTCGGAGAGCGCCTCACAC

GATGACCCATCCAGGTTTGCTGAGCTATCCCGCCCAAGCGGGTCTGTGGTAACACTGCTG

GGAATGAGCGAGTGTGGAAAGCGAGTCGCGGTTCACGTCTATGGTGTGCGCCATTATTTT

TACATGGCAAAGGCGGAGGTGGATAGCGCTTGTGGAATAACCACCGAGGCAGAACTCGTG

CGCGCAATGGTGGACTGCGCGCACAGCTCGGCTTTGAGCGCGGCCCTGGGAAATGGCAAC

GGCGGCAAACAGAGCGGCGGCAGCGGCGGGGGATGGTGGGGCGGAAAGCACGTGTCTGCG

GACTGCTTCAAAGTGGAGACCGTGTGCCACACGACGCTGTACTACTTTGGATCTAAGCCA

GCTCTCTACTATAGAGTATCTGCCTCCAGCAGCCGCCTGGGAGGGTTCATCTGCGACAAC

TTTCACCCGGAGATTACAAAATTCGAGGGGAGCGTGGACGTGACCACGCGGCTGTTGTTG

GACAATGAAAATTTTACCAGTTTCGGGTGGTACCGCCTGCGACCCGGCACCCACGGAGAG

CGTGTTCAACTTCGCCCCGTTGAGCGACACGTCACCTCAAGCGACGTGGAGATTAACTGT

ACTCCCGATAACCTGGAGCCGATACCAGACGAGGCTGCCTGGCCCGACTATAAGCTCATG

TGCTTTGATATAGAGTGTAAAGCTGGAACGGGTAACGAAATGGCGTTCCCAGTGGCAACT

AACCAAGAGGACCTGGTCATCCAGATCTCCTGTCTGCTGTACTCGCTTGCTACTCAGAAC

CACGAACACACCCTGCTGTTTTCCCTCGGGTCATGCGATATCTCTGAGGAATACTCGTTT

GCATGCGTCCAGCGCGGCGAGCCCAGACCGACGGTTTTGGAGTTTGACAGCGAGTACGAG

CTGCTGGTTGCCTTCCTGACCTTTCTCAAGCAGTACTCTCCCGAGTTCGCCACCGGCTAC

AACATCGTTAATTTTGACTGGGCGTACATAGTTAACAAGGTAACGTCGGTGTATAACATC

AAGCTGGACGGGTACGGCAAGTTCAACAAAGGGGGGCTGTTTAAGGTGTGGGACATCGCC

ACGAACCATTTTCAGAAGAAGAGCAAGGTGAAAATCAATGGCCTGATATCTCTAGACATG

TATTCTGTGGCGACGGAAAAGCTAAAGCTACCCAGCTACAAACTCGACGCGGTCGTGGGA

GACGTCCTCGGCGAGCATAAGATAGACCTTCCCTATAAAGAAATACCCTCCTATTACGCG

GGAGGGCCTGACCGGAGGGGCGTAATAGGAGAGTATTGTATCCAGGACTCTAGGCTGGTG

GGCAAGCTGTTTTTTAAGTACCTCCCCCATCTGGAACTATCGGCGGTGGCCAAACTCGCC

CGTATCACCCTAACGCGGGTAATTTTTGACGGTCAGCAAATTCGCGTGTACACGTGCTTG

CTGAAACTCGCCCGCGAGAGAAATTTCATTTTGCCAGACAACAGACGCCGGTTTGACAGT

CAGGCAGACGCCGCGTCAGAGACTTCGGAGTTGGCTATGGATAGCCAAAGCCACGCCTTC

GACAGTACAGACGAACCCGACGGTGTGGACGGTACCCCGGACGCCGCAGGATCTGGCGCT

ACTTCTGAAAACGGAGGCGGGAAGCCCGGCGTCGGGAGGGCCGTGGGCTACCAGGGAGCA

AAGGTTCTAGACCCCGTATCCGGCTTTCATGTGGACCCCGTGGTTGTGTTTGACTTCGCT

AGCTTATACCCAAGCATTATCCAGGCCCATAACCTCTGTTTCACCACCCTGGCGCTCGAT

GAAGTGGATCTGGCCGGGCTTCAACCATCCGTCAACTACTCGACGTTCGAGGTGGGTGAC

CAAAAGTTATTTTTTGTCCACGCCCATATTCGCGAAAGCCTGCTTGGCATCTTGCTGCGC

GACTGGCTGGCCATGCGAAAGGCGGTGAGGGCGCGAATCCCCACCAGCACCCCCGAGGAG

GCAGTTTTACTAGATAAGCAGCAGTCTGCGATTAAGGTGATATGCAACTCGGTTTACGGA

TTCACGGGGGTGGCAAACGGCCTGTTGCCGTGTCTGAGGATAGCGGCTACCGTTACCACG

ATAGGACGCGACATGCTCCTCAAGACCCGAGATTACGTTCACTCTCGTTGGGCGACGCGC

GAGCTGCTGGAGGACAATTTTCCAGGGGCTATAGGTTTCCGAAACCACAAGCCTTACTCC

GTCAGGGTTATCTACGGAGACACCGACTCCGTGTTTATCAAGTTTGTGGGCCTGACGTAC

GAGGGGGTATCGGAGCTGGGGGATGCTATGTCGCGTCAGATTTCAGCGGACCTCTTTAGA

GCGCCCATCAAACTGGAGTGTGAGAAGACCTTTCAGCGACTGCTGCTGATCACCAAGAAG

AAGTACATAGGTGTCATAAACGGGGGGAAGATGCTCATGAAGGGGGTCGACCTGGTCCGC

AAAAATAACTGCTCTTTCATAAACTTGTACGCGCGACATCTGGTAGATCTTTTGTTGTAC

GACGAGGATGTGGCCACGGCGGCAGCAAAGGTGACAGACGTGCCTCCCGCAGAATGGGTG

GGGCGCCCGCTACCGAGCGGCTTTGACAAGTTTGGGCGAGTGCTGGTAGAGGCGTACAAC

CGTATCACTGCCCCCAACTTGGACGTGCGCGAGTTCGTTATGACTGCTGAGCTGAGCCGC

TCACCCGAATCGTATACCAACAAGCGCCTGCCGCACCTCACCGTCTACTTTAAGCTCGCC

ATGAGGAATGAAGAACTGCCCAGCGTAAAAGAGAGAATTCCGTATGTGATAGTTGCGCAG

ACCGAGGCCGCGGAACGCGAAGCGGGTGTAGTAAACTCAATGCGCGGTACCGCCCAAAAC

CCCGTGGTAACCAAGACCGCACGCCCCCAACCTAAACGCAAACTGCTGGTTTCCGACCTC

GCCGAAGACCCGACCTATGTTTCCGAGAATGACGTACCGCTAAACACAGACTACTATTTC

TCCCACCTGTTGGGTACCATAAGCGTGACCTTTAAGGCTCTATTCGGAAATGATGTGAGA

ACAACAGAAAATCTTTTAAAGCGGTTTATTCCGGAAACCCCCCACAAGACCCCCACGAAA

ACCCAGGCACTGCTTGAGCGCGCCGGCTTTGAAAAGCTGACGCCCTTTACACCGGAGGAA

GAAAGTCGTCGAATACTGCATACAGTTTTTTGTACTCTAGAAGCAGCTCCCCATCAAAGC

TGA

>MZ318701.1

ATGGCGGCGCGCGAACAGGCCAACTCCGTTCGCAGGAGCGGGTTTTTTAATCCGTTTATT

GGCAAGAGGCCATTTTTCAGGCCGGGAAGCGGGCAAACCGCGGAGACAGAGCGCCCAAGG

CCCCCCCAACACTCGTACTGCACAGAGGTGGGTAGCTTTAAGTTTATAGCTCCAAGATGC

CTCGATGAAGAAGCCCCCGCCGACCAGCGACGCGGTGTACACGTGGGCACCTTGGAGCGC

CCACCCAAAGTGTACTGCGATGGCTCAGAGTACGACGTGCTGAACTTTGCCTCCGGAGGT

TGCTGGCCTCGCAGGATTCGGGTTTGGAACGGCCAGGATTTTCGGGGCGATGGATTCAAC

CCCAGATTTGAGAGATTTCACGTGTACGACATAGTAGAGACTTCGGAGAGCGCCTCACAC

GATGACCCATCCAGGTTTGCTGAGCTATCCCGCCCAAGCGGGTCTGTGGTAACACTGCTG

GGAATGAGCGAGTGTGGAAAGCGAGTCGCGGTTCACGTCTATGGTGTGCGCCATTATTTT

TACATGGCAAAGGCGGAGGTGGATAGCGCTTGTGGAATAACCACCGAGGCAGAACTCGTG

CGCGCAATGGTGGACTGCGCGCACAGCTCGGCTTTGAGCGCGGCCCTGGGAAATGGCAAC

GGCGGCAAACAGAGCGGCGGCAGCGGCGGGGGATGGTGGGGCGGAAAGCACGTGTCTGCG

GACTGCTTCAAAGTGGAGACCGTGTGCCACACGACGCTGTACTACTTTGGATCTAAGCCA

GCTCTCTACTATAGAGTATCTGCCTCCAGCAGCCGCCTGGGAGGGTTCATCTGCGACAAC

TTTCACCCGGAGATTACAAAATTCGAGGGGAGCGTGGACGTGACCACGCGGCTGTTGTTG

GACAATGAAAATTTTACCAGTTTCGGGTGGTACCGCCTGCGACCCGGCACCCACGGAGAG

CGTGTTCAACTTCGCCCCGTTGAGCGACACGTCACCTCAAGCGACGTGGAGATTAACTGT

ACTCCCGATAACCTGGAGCCGATACCAGACGAGGCTGCCTGGCCCGACTATAAGCTCATG

TGCTTTGATATAGAGTGTAAAGCTGGAACGGGTAACGAAATGGCGTTCCCAGTGGCAACT

AACCAAGAGGACCTGGTCATCCAGATCTCCTGTCTGCTGTACTCGCTTGCTACTCAGAAC

CACGAACACACCCTGCTGTTTTCCCTCGGGTCATGCGATATCTCTGAGGAATACTCGTTT

GCATGCGTCCAGCGCGGCGAGCCCAGACCGACGGTTTTGGAGTTTGACAGCGAGTACGAG

CTGCTGGTTGCCTTCCTGACCTTTCTCAAGCAGTACTCTCCCGAGTTCGCCACCGGCTAC

AACATCGTTAATTTTGACTGGGCGTACATAGTTAACAAGGTAACGTCGGTGTATAACATC

AAGCTGGACGGGTACGGCAAGTTCAACAAANGGGGGCTGTTTAAGGTGTGGGACATCGCC

ACGAACCATTTTCAGAAGAAGAGCAAGGTGAAAATCAATGGCCTGATATCTCTAGACATG

TATTCTGTGGCGACGGAAAAGCTAAAGCTACCCAGCTACAAACTCGACGCGGTCGTGGGA

GACGTCCTCGGCGAGCATAAGATAGACCTTCCCTATAAAGAAATACCCTCCTATTACGCG

GGAGGGCCTGACCGGAGGGGCGTAATAGGAGAGTATTGTATCCAGGACTCTAGGCTGGTG

GGCAAGCTGNTTTTTAAGTACCTCCCCCATCTGGAACTATCGGCGGTGGCCAAACTCGCC

CGTATCACCCTAACGCGGGTAATTTTTGACGGTCAGCAAATTCGCGTGTACACGTGCTTG

CTGAAACTCGCCCGCGAGAGAAATTTCATTTTGCCAGACAACAGACGCCGGTTTGACAGT

CAGGCAGACGCCGCGTCAGAGACTTCGGAGTTGGCTATGGATAGCCAAAGCCACGCCTTC

GACAGTACAGACGAACCCGACGGTGTGGACGGTACCCCGGACGCCGCAGGATCTGGCGCT

ACTTCTGAAAACGGAGGCGGGAAGCCCGGCGTCGGGAGGGCCGTGGGCTACCAGGGAGCA

AAGGTTCTAGACCCCGTATCCGGCTTTCATGTGGACCCCGTGGTTGTGTTTGACTTCGCT

AGCTTATACCCAAGCATTATCCAGGCCCATAACCTCTGTTTCACCACCCTGGCGCTCGAT

GAAGTGGATCTGGCCGGGCTTCAACCATCCGTCAACTACTCGACGTTCGAGGTGGGTGAC

CAAAAGTTATTTTTTGTCCACGCCCATATTCGCGAAAGCCTGCTTGGCATCTTGCTGCGC

GACTGGCTGGCCATGCGAAAGGCGGTGAGGGCGCGAATCCCCACCAGCACCCCCGAGGAG

GCAGTTTTACTAGATAAGCAGCAGTCTGCGATTAAGGTGATATGCAACTCGGTTTACGGA

TTCACGGGGGTGGCAAACGGCCTGTTGCCGTGTCTGAGGATAGCGGCTACCGTTACCACG

ATAGGACGCGACATGCTCCTCAAGACCCGAGATTACGTTCACTCTCGTTGGGCGACGCGC

GAGCTGCTGGAGGACAATTTTCCAGGGGCTATAGGTTTCCGAAACCACAAGCCTTACTCC

GTCAGGGTTATCTACGGAGACACCGACTCCGTGTTTATCAAGTTTGTGGGCCTGACGTAC

GAGGGGGTATCGGAGCTGGGGGATGCTATGTCGCGTCAGATTTCAGCGGACCTCTTTAGA

GCGCCCATCAAACTGGAGTGTGAGAAGACCTTTCAGCGACTGCTGCTGATCACCAAGAAG

AAGTACATAGGTGTCATAAACGGGGGGAAGATGCTCATGAAGGGGGTCGACCTGGTCCGC

AAAAATAACTGCTCTTTCATAAACTTGTACGCGCGACATCTGGTAGATCTTTTGTTGTAC

GACGAGGATGTGGCCACGGCGGCAGCAAAGGTGACAGACGTGCCTCCCGCAGAATGGGTG

GGGCGCCCGCTACCGAGCGGCTTTGACAAGTTTGGGCGAGTGCTGGTAGAGGCGTACAAC

CGTATCACTGCCCCCAACTTGGACGTGCGCGAGTTCGTTATGACTGCTGAGCTGAGCCGC

TCACCCGAATCGTATACCAACAAGCGCCTGCCGCACCTCACCGTCTACTTTAAGCTCGCC

ATGAGGAATGAAGAACTGCCCAGCGTAAAAGAGAGAATTCCGTATGTGATAGTTGCGCAG

ACCGAGGCCGCGGAACGCGAAGCGGGTGTAGTAAACTCAATGCGCGGTACCGCCCAAAAC

CCCGTGGTAACCAAGACCGCACGCCCCCAACCTAAACGCAAACTGCTGGTTTCCGACCTC

GCCGAAGACCCGACCTATGTTTCCGAGAATGACGTACCGCTAAACACAGACTACTATTTC

TCCCACCTGTTGGGTACCATAAGCGTGACCTTTAAGGCTCTATTCGGAAATGATGTGAGA

ACAACAGAAAATCTTTTAAAGCGGTTTATTCCGGAAACCCCCCACAAGACCCCCACGAAA

ACCCAGGCACTGCTTGAGCGCGCCGGCTTTGAAAAGCTGACGCCCTTTACACCGGAGGAA

GAAAGTCGTCGAATACTGCATACAGTTTTTTGTACTCTAGAAGCAGCTCCCCATCAAAGC

TGA

>MZ318702.1

ATGGCGGCGCGCGAACAGGCCAACTCCGTTCGCAGGAGCGGGTTTTTTAATCCGTTTATT

GGCAAGAGGCCATTTTTCAGGCCGGGAAGCGGGCAAACCGCGGAGACAGAGCGCCCAAGG

NCCCCCCAACACTCGTACTGCACAGAGGTGGGTAGCTTTAAGTTTATAGCTCCAAGATGC

CTCGATGAAGAAGCCCCCGCCGACCAGCGACGCGGTGTACACGTGGGCACCTTGGAGCGC

CCACCCAAAGTGTACTGCGATGGCTCAGAGTACGACGTGCTGAACTTTGCCTCCGGAGGT

TGCTGGCCTCGCAGGATTCGGGTTTGGAACGGCCAGGATTTTCGGGGCGATGGATTCAAC

CCCAGATTTGAGAGATTTCACGTGTACGACATAGTAGAGACTTCGGAGAGCGCCTCACAC

GATGACCCATCCAGGTTTGCTGAGCTATCCCGCCCAAGCGGGTCTGTGGTAACACTGCTG

GGAATGAGCGAGTGTGGAAAGCGAGTCGCGGTTCACGTCTATGGTGTGCGCCATTATTTT

TACATGGCAAAGGCGGAGGTGGATAGCGCTTGTGGAATAACCACCGAGGCAGAACTCGTG

CGCGCAATGGTGGACTGCGCGCACAGCTCGGCTTTGAGCGCGGCCCTGGGAAATGGCAAC

GGCGGCAAACAGAGCGGCGGCAGCGGCNGGGGATGGTGGGGCGGAAAGCACGTGTCTGCG

GACTGCTTCAAAGTGGAGACCGTGTGCCACACGACGCTGTACTACTTTGGATCTAAGCCA

GCTCTCTACTATAGAGTATCTGCCTCCAGCAGCCGCCTGGGAGGGTTCATCTGCGACAAC

TTTCACCCGGAGATTACAAAATTCGAGGGGAGCGTGGACGTGACCACGCGGCTGTTGTTG

GACAATGAAAATTTTACCAGTTTCGGGTGGTACCGCCTGCGACCCGGCACCCACGGAGAG

CGTGTTCAACTTCGCCCCGTTGAGCGACACGTCACCTCAAGCGACGTGGAGATTAACTGT

ACTCCCGATAACCTGGAGCCGATACCAGACGAGGCTGCCTGGCCCGACTATAAGCTCATG

TGCTTTGATATAGAGTGTAAAGCTGGAACGGGTAACGAAATGGCGTTCCCAGTGGCAACT

AACCAAGAGGACCTGGTCATCCAGATCTCCTGTCTGCTGTACTCGCTTGCTACTCAGAAC

CACGAACACACCCTGCTGTTTTCCCTCGGGTCATGCGATATCTCTGAGGAATACTCGTTT

GCATGCGTCCAGCGCGGCGAGCCCAGACCGACGGTTTTGGAGTTTGACAGCGAGTACGAG

CTGCTGGTTGCCTTCCTGACCTTTCTCAAGCAGTACTCTCCCGAGTTCGCCACCGGCTAC

AACATCGTTAATTTTGACTGGGCGTACATAGTTAACAAGGTAACGTCGGTGTATAACATC

AAGCTGGACGGGTACGGCAAGTTCAACAAANGGGGGCTGTTTAAGGTGTGGGACATCGCC

ACGAACCATTTTCAGAAGAAGAGCAAGGTGAAAATCAATGGCCTGATATCTCTAGACATG

TATTCTGTGGCGACGGAAAAGCTAAAGCTACCCAGCTACAAACTCGACGCGGTCGTGGGA

GACGTCCTCGGCGAGCATAAGATAGACCTTCCCTATAAAGAAATACCCTCCTATTACGCG

GGAGGGCCTGACCGGAGGGGCGTAATAGGAGAGTATTGTATCCAGGACTCTAGGCTGGTG

GGCAAGCTGTTTTTTAAGTACCTCCCCCATCTGGAACTATCGGCGGTGGCCAAACTCGCC

CGTATCACCCTAACGCGGGTAATTTTTGACGGTCAGCAAATTCGCGTGTACACGTGCTTG

CTGAAACTCGCCCGCGAGAGAAATTTCATTTTGCCAGACAACAGACGCCGGTTTGACAGT

CAGGCAGACGCCGCGTCAGAGACTTCGGAGTTGGCTATGGATAGCCAAAGCCACGCCTTC

GACAGTACAGACGAACCCGACGGTGTGGACGGTACCCCGGACGCCGCAGGATCTGGCGCT

ACTTCTGAAAACGGAGGCGGGAAGCCCGGCGTCGGGAGGGCCGTGGGCTACCAGGGAGCA

AAGGTTCTAGACCCCGTATCCGGCTTTCATGTGGACCCCGTGGTTGTGTTTGACTTCGCT

AGCTTATACCCAAGCATTATCCAGGCCCATAACCTCTGTTTCACCACCCTGGCGCTCGAT

GAAGTGGATCTGGCCGGGCTTCAACCATCCGTCAACTACTCGACGTTCGAGGTGGGTGAC

CAAAAGTTATTTTTTGTCCACGCCCATATTCGCGAAAGCCTGCTTGGCATCTTGCTGCGC

GACTGGCTGGCCATGCGAAAGGCGGTGAGGGCGCGAATCCCCACCAGCACCCCCGAGGAG

GCAGTTTTACTAGATAAGCAGCAGTCTGCGATTAAGGTGATATGCAACTCGGTTTACGGA

TTCACGGGGGTGGCAAACGGCCTGTTGCCGTGTCTGAGGATAGCGGCTACCGTTACCACG

ATAGGACGCGACATGCTCCTCAAGACCCGAGATTACGTTCACTCTCGTTGGGCGACGCGC

GAGCTGCTGGAGGACAATTTTCCAGGGGCTATAGGTTTCCGAAACCACAAGCCTTACTCC

GTCAGGGTTATCTACGGAGACACCGACTCCGTGTTTATCAAGTTTGTGGGCCTGACGTAC

GAGGGGGTATCGGAGCTGGGGGATGCTATGTCGCGTCAGATTTCAGCGGACCTCTTTAGA

GCGCCCATCAAACTGGAGTGTGAGAAGACCTTTCAGCGACTGCTGCTGATCACCAAGAAG

AAGTACATAGGTGTCATAAACGGGGGGAAGATGCTCATGAAGGGGGTCGACCTGGTCCGC

AAAAATAACTGCTCTTTCATAAACTTGTACGCGCGACATCTGGTAGATCTTTTGTTGTAC

GACGAGGATGTGGCCACGGCGGCAGCAAAGGTGACAGACGTGCCTCCCGCAGAATGGGTG

GGGCGCCCGCTACCGAGCGGCTTTGACAAGTTTGGGCGAGTGCTGGTAGAGGCGTACAAC

CGTATCACTGCCCCCAACTTGGACGTGCGCGAGTTCGTTATGACTGCTGAGCTGAGCCGC

TCACCCGAATCGTATACCAACAAGCGCCTGCCGCACCTCACCGTCTACTTTAAGCTCGCC

ATGAGGAATGAAGAACTGCCCAGCGTAAAAGAGAGAATTCCGTATGTGATAGTTGCGCAG

ACCGAGGCCGCGGAACGCGAAGCGGGTGTAGTAAACTCAATGCGCGGTACCGCCCAAAAC

CCCGTGGTAACCAAGACCGCACGCCCCCAACCTAAACGCAAACTGCTGGTTTCCGACCTC

GCCGAAGACCCGACCTATGTTTCCGAGAATGACGTACCGCTAAACACAGACTACTATTTC

TCCCACCTGTTGGGTACCATAAGCGTGACCTTTAAGGCTCTATTCGGAAATGATGTGAGA

ACAACAGAAAATCTTTTAAAGCGGTTTATTCCGGAAACCCCCCACAAGACCCCCACGAAA

ACCCAGGCACTGCTTGAGCGCGCCGGCTTTGAAAAGCTGACGCCCTTTACACCGGAGGAA

GAAAGTCGTCGAATACTGCATACAGTTTTTTGTACTCTAGAAGCAGCTCCCCATCAAAGC

TGA

>MZ318703.1

ATGGCGGCGCGCGAACAGGCCAACTCCGTTCGCAGGAGCGGGTTTTTTAATCCGTTTATT

GGCAAGAGGCCATTTTTCAGGCCGGGAAGCGGGCAAACCGCGGAGACAGAGCGCCCAAGG

CCCCCCCAACACTCGTACTGCACAGAGGTGGGTAGCTTTAAGTTTATAGCTCCAAGATGC

CTCGATGAAGAAGCCCCCGCCGACCAGCGACGCGGTGTACACGTGGGCACCTTGGAGCGC

CCACCCAAAGTGTACTGCGATGGCTCAGAGTACGACGTGCTGAACTTTGCCTCCGGAGGT

TGCTGGCCTCGCAGGATTCGGGTTTGGAACGGCCAGGATTTTCGGGGCGATGGATTCAAC

CCCAGATTTGAGAGATTTCACGTGTACGACATAGTAGAGACTTCGGAGAGCGCCTCACAC

GATGACCCATCCAGGTTTGCTGAGCTATCCCGCCCAAGCGGGTCTGTGGTAACACTGCTG

GGAATGAGCGAGTGTGGAAAGCGAGTCGCGGTTCACGTCTATGGTGTGCGCCATTATTTT

TACATGGCAAAGGCGGAGGTGGATAGCGCTTGTGGAATAACCACCGAGGCAGAACTCGTG

CGCGCAATGGTGGACTGCGCGCACAGCTCGGCTTTGAGCGCGGCCCTGGGAAATGGCAAC

GGCGGCAAACAGAGCGGCGGCAGCGGCGGGGGATGGTGGGGCGGAAAGCACGTGTCTGCG

GACTGCTTCAAAGTGGAGACCGTGTGCCACACGACGCTGTACTACTTTGGATCTAAGCCA

GCTCTCTACTATAGAGTATCTGCCTCCAGCAGCCGCCTGGGAGGGTTCATCTGCGACAAC

TTTCACCCGGAGATTACAAAATTCGAGGGGAGCGTGGACGTGACCACGCGGCTGTTGTTG

GACAATGAAAATTTTACCAGTTTCGGGTGGTACCGCCTGCGACCCGGCACCCACGGAGAG

CGTGTTCAACTTCGCCCCGTTGAGCGACACGTCACCTCAAGCGACGTGGAGATTAACTGT

ACTCCCGATAACCTGGAGCCGATACCAGACGAGGCTGCCTGGCCCGACTATAAGCTCATG

TGCTTTGATATAGAGTGTAAAGCTGGAACGGGTAACGAAATGGCGTTCCCAGTGGCAACT

AACCAAGAGGACCTGGTCATCCAGATCTCCTGTCTGCTGTACTCGCTTGCTACTCAGAAC

CACGAACACACCCTGCTGTTTTCCCTCGGGTCATGCGATATCTCTGAGGAATACTCGTTT

GCATGCGTCCAGCGCGGCGAGCCCAGACCGACGGTTTTGGAGTTTGACAGCGAGTACGAG

CTGCTGGTTGCCTTCCTGACCTTTCTCAAGCAGTACTCTCCCGAGTTCGCCACCGGCTAC

AACATCGTTAATTTTGACTGGGCGTACATAGTTAACAAGGTAACGTCGGTGTATAACATC

AAGCTGGACGGGTACGGCAAGTTCAACAAANGGGGGCTGTTTAAGGTGTGGGACATCGCC

ACGAACCATTTTCAGAAGAAGAGCAAGGTGAAAATCAATGGCCTGATATCTCTAGACATG

TATTCTGTGGCGACGGAAAAGCTAAAGCTACCCAGCTACAAACTCGACGCGGTCGTGGGA

GACGTCCTCGGCGAGCATAAGATAGACCTTCCCTATAAAGAAATACCCTCCTATTACGCG

GGAGGGCCTGACCGGAGGGGCGTAATAGGAGAGTATTGTATCCAGGACTCTAGGCTGGTG

GGCAAGCTGTTTTTTAAGTACCTCCCCCATCTGGAACTATCGGCGGTGGCCAAACTCGCC

CGTATCACCCTAACGCGGGTAATTTTTGACGGTCAGCAAATTCGCGTGTACACGTGCTTG

CTGAAACTCGCCCGCGAGAGAAATTTCATTTTGCCAGACAACAGACGCCGGTTTGACAGT

CAGGCAGACGCCGCGTCAGAGACTTCGGAGTTGGCTATGGATAGCCAAAGCCACGCCTTC

GACAGTACAGACGAACCCGACGGTGTGGACGGTACCCCGGACGCCGCAGGATCTGGCGCT

ACTTCTGAAAACGGAGGCGGGAAGCCCGGCGTCGGGAGGGCCGTGGGCTACCAGGGAGCA

AAGGTTCTAGACCCCGTATCCGGCTTTCATGTGGACCCCGTGGTTGTGTTTGACTTCGCT

AGCTTATACCCAAGCATTATCCAGGCCCATAACCTCTGTTTCACCACCCTGGCGCTCGAT

GAAGTGGATCTGGCCGGGCTTCAACCATCCGTCAACTACTCGACGTTCGAGGTGGGTGAC

CAAAAGTTATTTTTTGTCCACGCCCATATTCGCGAAAGCCTGCTTGGCATCTTGCTGCGC

GACTGGCTGGCCATGCGAAAGGCGGTGAGGGCGCGAATCCCCACCAGCACCCCCGAGGAG

GCAGTTTTACTAGATAAGCAGCAGTCTGCGATTAAGGTGATATGCAACTCGGTTTACGGA

TTCACGGGGGTGGCAAACGGCCTGTTGCCGTGTCTGAGGATAGCGGCTACCGTTACCACG

ATAGGACGCGACATGCTCCTCAAGACCCGAGATTACGTTCACTCTCGTTGGGCGACGCGC

GAGCTGCTGGAGGACAATTTTCCAGGGGCTATAGGTTTCCGAAACCACAAGCCTTACTCC

GTCAGGGTTATCTACGGAGACACCGACTCCGTGTTTATCAAGTTTGTGGGCCTGACGTAC

GAGGGGGTATCGGAGCTGGGGGATGCTATGTCGCGTCAGATTTCAGCGGACCTCTTTAGA

GCGCCCATCAAACTGGAGTGTGAGAAGACCTTTCAGCGACTGCTGCTGATCACCAAGAAG

AAGTACATAGGTGTCATAAACNGGGGGAAGATGCTCATGAAGGGGGTCGACCTGGTCCGC

AAAAATAACTGCTCTTTCATAAACTTGTACGCGCGACATCTGGTAGATCTTTTGTTGTAC

GACGAGGATGTGGCCACGGCGGCAGCAAAGGTGACAGACGTGCCTCCCGCAGAATGGGTG

GGGCGCCCGCTACCGAGCGGCTTTGACAAGTTTGGGCGAGTGCTGGTAGAGGCGTACAAC

CGTATCACTGCCCCCAACTTGGACGTGCGCGAGTTCGTTATGACTGCTGAGCTGAGCCGC

TCACCCGAATCGTATACCAACAAGCGCCTGCCGCACCTCACCGTCTACTTTAAGCTCGCC

ATGAGGAATGAAGAACTGCCCAGCGTNAAAGAGAGAATTCCGTATGTGATAGTTGCGCAG

ACCGAGGCCGCGGAACGCGAAGCGGGTGTAGTAAACTCAATGCGCGGTACCGCCCAAAAC

CCCGTGGTAACCAAGACCGCACGCCCCCAACCTAAACGCAAACTGCTGGTTTCCGACCTC

GCCGAAGACCCGACCTATGTTTCCGAGAATGACGTACCGCTAAACACAGACTACTATTTC

TCCCACCTGTTGGGTACCATAAGCGTGACCTTTAAGGCTCTATTCGGAAATGATGTGAGA

ACAACAGAAAATCTTTTAAAGCGGTTTATTCCGGAAANCCCCCACAAGACCCCCACGAAA

ACCCAGGCACTGCTTGAGCGCGCCGGCTTTGAAAAGCTGACGCCCTTTACACCGGAGGAA

GAAAGTCGTCGAATACTGCATACAGTTTTTTGTACTCTAGAAGCAGCTCCCCATCAAAGC

TGA

>MZ318704.1

ATGGCGGCGCGCGAACAGGCCAACTCCGTTCGCAGGAGCGGGTTTTTTAATCCGTTTATT

GGCAAGAGGCCATTTTTCAGGCCGGGAAGCGGGCAAACCGCGGAGACAGAGCGCCCAAGG

CCCCCCCAACACTCGTACTGCACAGAGGTGGGTAGCTTTAAGTTTATAGCTCCAAGATGC

CTCGATGAAGAAGCCCCCGCCGACCAGCGACGCGGTGTACACGTGGGCACCTTGGAGCGC

CCACCCAAAGTGTACTGCGATGGCTCAGAGTACGACGTGCTGAACTTTGCCTCCGGAGGT

TGCTGGCCTCGCAGGATTCGGGTTTGGAACGGCCAGGATTTTCGGGGCGATGGATTCAAC

CCCAGATTTGAGAGATTTCACGTGTACGACATAGTAGAGACTTCGGAGAGCGCCTCACAC

GATGACCCATCCAGGTTTGCTGAGCTATCCCGCCCAAGCGGGTCTGTGGTAACACTGCTG

GGAATGAGCGAGTGTGGAAAGCGAGTCGCGGTTCACGTCTATGGTGTGCGCCATTATTTT

TACATGGCAAAGGCGGAGGTGGATAGCGCTTGTGGAATAACCACCGAGGCAGAACTCGTG

CGCGCAATGGTGGACTGCGCGCACAGCTCGGCTTTGAGCGCGGCCCTGGGAAATGGCAAC

GGCGGCAAACAGAGCGGCGGCAGCGGCGGGGGATGGTGGGGCGGAAAGCACGTGTCTGCG

GACTGCTTCAAAGTGGAGACCGTGTGCCACACGACGCTGTACTACTTTGGATCTAAGCCA

GCTCTCTACTATAGAGTATCTGCCTCCAGCAGCCGCCTGGGAGGGTTCATCTGCGACAAC

TTTCACCCGGAGATTACAAAATTCGAGGGGAGCGTGGACGTGACCACGCGGCTGTTGTTG

GACAATGAAAATTTTACCAGTTTCGGGTGGTACCGCCTGCGACCCGGCACCCACGGAGAG

CGTGTTCAACTTCGCCCCGTTGAGCGACACGTCACCTCAAGCGACGTGGAGATTAACTGT

ACTCCCGATAACCTGGAGCCGATACCAGACGAGGCTGCCTGGCCCGACTATAAGCTCATG

TGCTTTGATATAGAGTGTAAAGCTGGAACGGGTAACGAAATGGCGTTCCCAGTGGCAACT

AACCAAGAGGACCTGGTCATCCAGATCTCCTGTCTGCTGTACTCGCTTGCTACTCAGAAC

CACGAACACACCCTGCTGTTTTCCCTCGGGTCATGCGATATCTCTGAGGAATACTCGTTT

GCATGCGTCCAGCGCGGCGAGCCCAGACCGACGGTTTTGGAGTTTGACAGCGAGTACGAG

CTGCTGGTTGCCTTCCTGACCTTTCTCAAGCAGTACTCTCCCGAGTTCGCCACCGGCTAC

AACATCGTTAATTTTGACTGGGCGTACATAGTTAACAAGGTAACGTCGGTGTATAACATC

AAGCTGGACGGGTACGGCAAGTTCAACAAAGGGGGGCTGTTTAAGGTGTGGGACATCGCC

ACGAACCATTTTCAGAAGAAGAGCAAGGTGAAAATCAATGGCCTGATATCTCTAGACATG

TATTCTGTGGCGACGGAAAAGCTAAAGCTACCCAGCTACAAACTCGACGCGGTCGTGGGA

GACGTCCTCGGCGAGCATAAGATAGACCTTCCCTATAAAGAAATACCCTCCTATTACGCG

GGAGGGCCTGACCGGAGGGGCGTAATAGGAGAGTATTGTATCCAGGACTCTAGGCTGGTG

GGCAAGCTGTTTTTTAAGTACCTCCCCCATCTGGAACTATCGGCGGTGGCCAAACTCGCC

CGTATCACCCTAACGCGGGTAATTTTTGACGGTCAGCAAATTCGCGTGTACACGTGCTTG

CTGAAACTCGCCCGCGAGAGAAATTTCATTTTGCCAGACAACAGACGCCGGTTTGACAGT

CAGGCAGACGCCGCGTCAGAGACTTCGGAGTTGGCTATGGATAGCCAAAGCCACGCCTTC

GACAGTACAGACGAACCCGACGGTGTGGACGGTACCCCGGACGCCGCAGGATCTGGCGCT

ACTTCTGAAAACGGAGGCGGGAAGCCCGGCGTCGGGAGGGCCGTGGGCTACCAGGGAGCA

AAGGTTCTAGACCCCGTATCCGGCTTTCATGTGGACCCCGTGGTTGTGTTTGACTTCGCT

AGCTTATACCCAAGCATTATCCAGGCCCATAACCTCTGTTTCACCACCCTGGCGCTCGAT

GAAGTGGATCTGGCCGGGCTTCAACCATCCGTCAACTACTCGACGTTCGAGGTGGGTGAC

CAAAAGTTATTTTTTGTCCACGCCCATATTCGCGAAAGCCTGCTTGGCATCTTGCTGCGC

GACTGGCTGGCCATGCGAAAGGCGGTGAGGGCGCGAATCCCCACCAGCACCCCCGAGGAG

GCAGTTTTACTAGATAAGCAGCAGTCTGCGATTAAGGTGATATGCAACTCGGTTTACGGA

TTCACGGGGGTGGCAAACGGCCTGTTGCCGTGTCTGAGGATAGCGGCTACCGTTACCACG

ATAGGACGCGACATGCTCCTCAAGACCCGAGATTACGTTCACTCTCGTTGGGCGACGCGC

GAGCTGCTGGAGGACAATTTTCCAGGGGCTATAGGTTTCCGAAACCACAAGCCTTACTCC

GTCAGGGTTATCTACGGAGACACCGACTCCGTGTTTATCAAGTTTGTGGGCCTGACGTAC

GAGGGGGTATCGGAGCTGGGGGATGCTATGTCGCGTCAGATTTCAGCGGACCTCTTTAGA

GCGCCCATCAAACTGGAGTGTGAGAAGACCTTTCAGCGACTGCTGCTGATCACCAAGAAG

AAGTACATAGGTGTCATAAACGGGGGGAAGATGCTCATGAAGGGGGTCGACCTGGTCCGC

AAAAATAACTGCTCTTTCATAAACTTGTACGCGCGACATCTGGTAGATCTTTTGTTGTAC

GACGAGGATGTGGCCACGGCGGCAGCAAAGGTGACAGACGTGCCTCCCGCAGAATGGGTG

GGGCGCCCGCTACCGAGCGGCTTTGACAAGTTTGGGCGAGTGCTGGTAGAGGCGTACAAC

CGTATCACTGCCCCCAACTTGGACGTGCGCGAGTTCGTTATGACTGCTGAGCTGAGCCGC

TCACCCGAATCGTATACCAACAAGCGCCTGCCGCACCTCACCGTCTACTTTAAGCTCGCC

ATGAGGAATGAAGAACTGCCCAGCGTAAAAGAGAGAATTCCGTATGTGATAGTTGCGCAG

ACCGAGGCCGCGGAACGCGAAGCGGGTGTAGTAAACTCAATGCGCGGTACCGCCCAAAAC

CCCGTGGTAACCAAGACCGCACGCCCCCAACCTAAACGCAAACTGCTGGTTTCCGACCTC

GCCGAAGACCCGACCTATGTTTCCGAGAATGACGTACCGCTAAACACAGACTACTATTTC

TCCCACCTGTTGGGTACCATAAGCGTGACCTTTAAGGCTCTATTCGGAAATGATGTGAGA

ACAACAGAAAATCTTTTAAAGCGGTTTATTCCGGAAANCCCCCACAAGACCCCCACGAAA

ACCCAGGCACTGCTTGAGCGCGCCGGCTTTGAAAAGCTGACGCCCTTTACACCGGAGGAA

GAAAGTCGTCGAATACTGCATACAGTTTTTTGTACTCTAGAAGCAGCTCCCCATCAAAGC

TGA

>MZ318705.1

ATGGCGGCGCGCGAACAGGCCAACTCCGTTCGCAGGAGCGGGTTTTTTAATCCGTTTATT

GGCAAGAGGCCATTTTTCAGGCCGGGAAGCGGGCAAACCGCGGAGACAGAGCGCCCAAGG

NCCCCCCAACACTCGTACTGCACAGAGGTGGGTAGCTTTAAGTTTATAGCTCCAAGATGC

CTCGATGAAGAAGCCCCCGCCGACCAGCGACGCGGTGTACACGTGGGCACCTTGGAGCGC

CCACCCAAAGTGTACTGCGATGGCTCAGAGTACGACGTGCTGAACTTTGCCTCCGGAGGT

TGCTGGCCTCGCAGGATTCGGGTTTGGAACGGCCAGGATTTTCGGGGCGATGGATTCAAC

CCCAGATTTGAGAGATTTCACGTGTACGACATAGTAGAGACTTCGGAGAGCGCCTCACAC

GATGACCCATCCAGGTTTGCTGAGCTATCCCGCCCAAGCGGGTCTGTGGTAACACTGCTG

GGAATGAGCGAGTGTGGAAAGCGAGTCGCGGTTCACGTCTATGGTGTGCGCCATTATTTT

TACATGGCAAAGGCGGAGGTGGATAGCGCTTGTGGAATAACCACCGAGGCAGAACTCGTG

CGCGCAATGGTGGACTGCGCGCACAGCTCGGCTTTGAGCGCGGCCCTGGGAAATGGCAAC

GGCGGCAAACAGAGCGGCGGCAGCGGCGGGGGATGGTGGGGCGGAAAGCACGTGTCTGCG

GACTGCTTCAAAGTGGAGACCGTGTGCCACACGACGCTGTACTACTTTGGATCTAAGCCA

GCTCTCTACTATAGAGTATCTGCCTCCAGCAGCCGCCTGGGAGGGTTCATCTGCGACAAC

TTTCACCCGGAGATTACAAAATTCGAGGGGAGCGTGGACGTGACCACGCGGCTGTTGTTG

GACAATGAAAATTTTACCAGTTTCGGGTGGTACCGCCTGCGACCCGGCACCCACGGAGAG

CGTGTTCAACTTCGCCCCGTTGAGCGACACGTCACCTCAAGCGACGTGGAGATTAACTGT

ACTCCCGATAACCTGGAGCCGATACCAGACGAGGCTGCCTGGCCCGACTATAAGCTCATG

TGCTTTGATATAGAGTGTAAAGCTGGAACGGGTAACGAAATGGCGTTCCCAGTGGCAACT

AACCAAGAGGACCTGGTCATCCAGATCTCCTGTCTGCTGTACTCGCTTGCTACTCAGAAC

CACGAACACACCCTGCTGTTTTCCCTCGGGTCATGCGATATCTCTGAGGAATACTCGTTT

GCATGCGTCCAGCGCGGCGAGCCCAGACCGACGGTTTTGGAGTTTGACAGCGAGTACGAG

CTGCTGGTTGCCTTCCTGACCTTTCTCAAGCAGTACTCTCCCGAGTTCGCCACCGGCTAC

AACATCGTTAATTTTGACTGGGCGTACATAGTTAACAAGGTAACGTCGGTGTATAACATC

AAGCTGGACGGGTACGGCAAGTTCAACAAAGGGGGGCTGTTTAAGGTGTGGGACATCGCC

ACGAACCATTTTCAGAAGAAGAGCAAGGTGAAAATCAATGGCCTGATATCTCTAGACATG

TATTCTGTGGCGACGGAAAAGCTAAAGCTACCCAGCTACAAACTCGACGCGGTCGTGGGA

GACGTCCTCGGCGAGCATAAGATAGACCTTCCCTATAAAGAAATACCCTCCTATTACGCG

GGAGGGCCTGACCGGAGGGGCGTAATAGGAGAGTATTGTATCCAGGACTCTAGGCTGGTG

GGCAAGCTGTTTTTTAAGTACCTCCCCCATCTGGAACTATCGGCGGTGGCCAAACTCGCC

CGTATCACCCTAACGCGGGTAATTTTTGACGGTCAGCAAATTCGCGTGTACACGTGCTTG

CTGAAACTCGCCCGCGAGAGAAATTTCATTTTGCCAGACAACAGACGCCGGTTTGACAGT

CAGGCAGACGCCGCGTCAGAGACTTCGGAGTTGGCTATGGATAGCCAAAGCCACGCCTTC

GACAGTACAGACGAACCCGACGGTGTGGACGGTACCCCGGACGCCGCAGGATCTGGCGCT

ACTTCTGAAAACGGAGGCGGGAAGCCCGGCGTCGGGAGGGCCGTGGGCTACCAGGGAGCA

AAGGTTCTAGACCCCGTATCCGGCTTTCATGTGGACCCCGTGGTTGTGTTTGACTTCGCT

AGCTTATACCCAAGCATTATCCAGGCCCATAACCTCTGTTTCACCACCCTGGCGCTCGAT

GAAGTGGATCTGGCCGGGCTTCAACCATCCGTCAACTACTCGACGTTCGAGGTGGGTGAC

CAAAAGTTATTTTTTGTCCACGCCCATATTCGCGAAAGCCTGCTTGGCATCTTGCTGCGC

GACTGGCTGGCCATGCGAAAGGCGGTGAGGGCGCGAATCCCCACCAGCACCCCCGAGGAG

GCAGTTTTACTAGATAAGCAGCAGTCTGCGATTAAGGTGATATGCAACTCGGTTTACGGA

TTCACGGGGGTGGCAAACGGCCTGTTGCCGTGTCTGAGGATAGCGGCTACCGTTACCACG

ATAGGACGCGACATGCTCCTCAAGACCCGAGATTACGTTCACTCTCGTTGGGCGACGCGC

GAGCTGCTGGAGGACAATTTTCCAGGGGCTATAGGTTTCCGAAACCACAAGCCTTACTCC

GTCAGGGTTATCTACGGAGACACCGACTCCGTGTTTATCAAGTTTGTGGGCCTGACGTAC

GAGGGGGTATCGGAGCTGGGGGATGCTATGTCGCGTCAGATTTCAGCGGACCTCTTTAGA

GCGCCCATCAAACTGGAGTGTGAGAAGACCTTTCAGCGACTGCTGCTGATCACCAAGAAG

AAGTACATAGGTGTCATAAACGGGGGGAAGATGCTCATGAAGGGGGTCGACCTGGTCCGC

AAAAATAACTGCTCTTTCATAAACTTGTACGCGCGACATCTGGTAGATCTTTTGTTGTAC

GACGAGGATGTGGCCACGGCGGCAGCAAAGGTGACAGACGTGCCTCCCGCAGAATGGGTG

GGGCGCCCGCTACCGAGCGGCTTTGACAAGTTTGGGCGAGTGCTGGTAGAGGCGTACAAC

CGTATCACTGCCCCCAACTTGGACGTGCGCGAGTTCGTTATGACTGCTGAGCTGAGCCGC

TCACCCGAATCGTATACCAACAAGCGCCTGCCGCACCTCACCGTCTACTTTAAGCTCGCC

ATGAGGAATGAAGAACTGCCCAGCGTAAAAGAGAGAATTCCGTATGTGATAGTTGCGCAG

ACCGAGGCCGCGGAACGCGAAGCGGGTGTAGTAAACTCAATGCGCGGTACCGCCCAAAAC

CCCGTGGTAACCAAGACCGCACGCCCCCAACCTAAACGCAAACTGCTGGTTTCCGACCTC

GCCGAAGACCCGACCTATGTTTCCGAGAATGACGTACCGCTAAACACAGACTACTATTTC

TCCCACCTGTTGGGTACCATAAGCGTGACCTTTAAGGCTCTATTCGGAAATGATGTGAGA

ACAACAGAAAATCTTTTAAAGCGGTTTATTCCGGAAACCCCCCACAAGACCCCCACGAAA

ACCCAGGCACTGCTTGAGCGCGCCGGCTTTGAAAAGCTGACGCCCTTTACACCGGAGGAA

GAAAGTCGTCGAATACTGCATACAGTTTTTTGTACTCTAGAAGCAGCTCCCCATCAAAGC

TGA

>MZ318706.1

ATGGCGGCGCGCGAACAGGCCAACTCCGTTCGCAGGAGCGGGTTTTTTAATCCGTTTATT

GGCAAGAGGCCATTTTTCAGGCCGGGAAGCGGGCAAACCGCGGAGACAGAGCGCCCAAGG

NCCCCCCAACACTCGTACTGCACAGAGGTGGGTAGCTTTAAGTTTATAGCTCCAAGGTGC

CTCGATGAAGAAGCCCCCGCCGACCAGCGACGCGGTGTACACGTGGGCACCTTGGAGCGC

CCACCCAAAGTGTACTGCGATGGCTCAGAGTACGACGTGCTGAACTTTGCCTCCGGAGGT

TGCTGGCCTCGCAGGATTCGGGTTTGGAACGGCCAGGATTTTCGGGGCGATGGATTCAAC

CCCAGATTTGAGAGATTTCACGTGTACGACATAGTAGAGACTTCGGAGAGCGCCTCACAC

GATGACCCATCCAGGTTTGCTGAGCTATCCCGCCCAAGCGGGTCTGTGGTAACACTGCTG

GGAATGAGCGAGTGTGGAAAGCGAGTCGCGGTTCACGTCTATGGTGTGCGCCATTATTTT

TACATGGCAAAGGCGGAGGTGGATAGCGCTTGTGGAATAACCACCGAGGCAGAACTCGTG

CGCGCAATGGTGGACTGCGCGCACAGCTCGGCTTTGAGCGCGGCCCTGGGAAATGGCAAC

GGCGGCAAACAGAGCGGCGGCAGCGGCNGGGGATGGTGGGGCGGAAAGCACGTGTCTGCG

GACTGCTTCAAAGTGGAGACCGTGTGCCACACGACGCTGTACTACTTTGGATCTAAGCCA

GCTCTCTACTATAGAGTATCTGCCTCCAGCAGCCGCCTGGGAGGGTTCATCTGCGACAAC

TTTCACCCGGAGATTACAAAATTCGAGGGGAGCGTGGACGTGACCACGCGGCTGTTGTTG

GACAATGAAAATTTTACCAGTTTCGGGTGGTACCGCCTGCGACCCGGCACCCACGGAGAG

CGTGTTCAACTTCGCCCCGTTGAGCGACACGTCACCTCAAGCGACGTGGAGATTAACTGT

ACTCCCGATAACCTGGAGCCGATACCAGACGAGGCTGCCTGGCCCGACTATAAGCTCATG

TGCTTTGATATAGAGTGTAAAGCTGGAACGGGTAACGAAATGGCGTTCCCAGTGGCAACT

AACCAAGAGGACCTGGTCATCCAGATCTCCTGTCTGCTGTACTCGCTTGCTACTCAGAAC

CACGAACACACCCTGCTGTTTTCCCTCGGGTCATGCGATATCTCTGAGGAATACTCGTTT

GCATGCGTCCAGCGCGGCGAGCCCAGACCGACGGTTTTGGAGTTTGACAGCGAGTACGAG

CTGCTGGTTGCCTTCCTGACCTTTCTCAAGCAGTACTCTCCCGAGTTCGCCACCGGCTAC

AACATCGTTAATTTTGACTGGGCGTACATAGTTAACAAGGTAACGTCGGTGTATAACATC

AAGCTGGACGGGTACGGCAAGTTCAACAAAGGGGGGCTGTTTAAGGTGTGGGACATCGCC

ACGAACCATTTTCAGAAGAAGAGCAAGGTGAAAATCAATGGCCTGATATCTCTAGACATG

TATTCTGTGGCGACGGAAAAGCTAAAGCTACCCAGCTACAAACTCGACGCGGTCGTGGGA

GACGTCCTCGGCGAGCATAAGATAGACCTTCCCTATAAAGAAATACCCTCCTATTACGCG

GGAGGGCCTGACCGGAGGGGCGTAATAGGAGAGTATTGTATCCAGGACTCTAGGCTGGTG

GGCAAGCTGTTTTTTAAGTACCTCCCCCATCTGGAACTATCGGCGGTGGCCAAACTCGCC

CGTATCACCCTAACGCGGGTAATTTTTGACGGTCAGCAAATTCGCGTGTACACGTGCTTG

CTGAAACTCGCCCGCGAGAGAAATTTCATTTTGCCAGACAACAGACGCCGGTTTGACAGT

CAGGCAGACGCCGCGTCAGAGACTTCGGAGTTGGCTATGGATAGCCAAAGCCACGCCTTC

GACAGTACAGACGAACCCGACGGTGTGGACGGTACCCCGGACGCCGCAGGATCTGGCGCT

ACTTCTGAAAACGGAGGCGGGAAGCCCGGCGTCGGGAGGGCCGTGGGCTACCAGGGAGCA

AAGGTTCTAGACCCCGTATCCGGCTTTCATGTGGACCCCGTGGTTGTGTTTGACTTCGCT

AGCTTATACCCAAGCATTATCCAGGCCCATAACCTCTGTTTCACCACCCTGGCGCTCGAT

GAAGTGGATCTGGCCGGGCTTCAACCATCCGTCAACTACTCGACGTTCGAGGTGGGTGAC

CAAAAGTTATNNTTTGTCCACGCCCATATTCGCGAAAGCCTGCTTGGCATCTTGCTGCGC

GACTGGCTGGCCATGCGAAAGGCGGTGAGGGCGCGAATCCCCACCAGCACCCCCGAGGAG

GCAGTTTTACTAGATAAGCAGCAGTCTGCGATTAAGGTGATATGCAACTCGGTTTACGGA

TTCACGGGGGTGGCAAACGGCCTGTTGCCGTGTCTGAGGATAGCGGCTACCGTTACCACG

ATAGGACGCGACATGCTCCTCAAGACCCGAGATTACGTTCACTCTCGTTGGGCGACGCGC

GAGCTGCTGGAGGACAATTTTCCAGGGGCTATAGGTTTCCGAAACCACAAGCCTTACTCC

GTCAGGGTTATCTACGGAGACACCGACTCCGTGTTTATCAAGTTTGTGGGCCTGACGTAC

GAGGGGGTATCGGAGCTGGGGGATGCTATGTCGCGTCAGATTTCAGCGGACCTCTTTAGA

GCGCCCATCAAACTGGAGTGTGAGAAGACCTTTCAGCGACTGCTGCTGATCACCAAGAAG

AAGTACATAGGTGTCATAAACGGGGGGAAGATGCTCATGAAGGGGGTCGACCTGGTCCGC

AAAAATAACTGCTCTTTCATAAACTTGTACGCGCGACATCTGGTAGATCTTTTGTTGTAC

GACGAGGATGTGGCCACGGCGGCAGCAAAGGTGACAGACGTGCCTCCCGCAGAATGGGTG

GGGCGCCCGCTACCGAGCGGCTTTGACAAGTTTGGGCGAGTGCTGGTAGAGGCGTACAAC

CGTATCACTGCCCCCAACTTGGACGTGCGCGAGTTCGTTATGACTGCTGAGCTGAGCCGC

TCACCCGAATCGTATACCAACAAGCGCCTGCCGCACCTCACCGTCTACTTTAAGCTCGCC

ATGAGGAATGAAGAACTGCCCAGCGTAAAAGAGAGAATTCCGTATGTGATAGTTGCGCAG

ACCGAGGCCGCGGAACGCGAAGCGGGTGTAGTAAACTCAATGCGCGGTACCGCCCAAAAC

CCCGTGGTAACCAAGACCGCACGCCCCCAACCTAAACGCAAACTGCTGGTTTCCGACCTC

GCCGAAGACCCGACCTATGTTTCCGAGAATGACGTACCGCTAAACACAGACTACTATTTC

TCCCACCTGTTGGGTACCATAAGCGTGACCTTTAAGGCTCTATTCGGAAATGATGTGAGA

ACAACAGAAAATCTTTTAAAGCGGTTTATTCCGGAAACCCCCCACAAGACCCCCACGAAA

ACCCAGGCACTGCTTGAGCGCGCCGGCTTTGAAAAGCTGACGCCCTTTACACCGGAGGAA

GAAAGTCGTCGAATACTGCATACAGTTTTTTGTACTCTAGAAGCAGCTCCCCATCAAAGC

TGA

>OR085496.1

ATGGCGGCGCGCGAACAGGCCAACTCCGTTCGCAGGAGCGGGTTTTTTAATCCGTTTATT

GGCAAGAGGCCATTTTTCAGGCCGGGAAGCGGGCAGACCGCGGAGACAGAGCGCCCAAGG

CCCCCCCAACACTCGTACTGCACAGAGGTGGGTAGCTTTAAGTTTATAGCTCCAAGATGC

CTCGATGAAGAAGCCCCCGCCGACCAGCGACGCGGTGTACACGTGGGCACCTTGGAGCGC

CCACCCAAAGTGTACTGCGATGGCTCAGAGTACGACGTGCTGAACTTTGCCTCCGGAGGT

TGCTGGCCTCGCAGGATTCGGGTTTGGAACGGCCAGGATTTTCGGGGCGATGGATTCAAC

CCCAGATTTGAGAGATTTCACGTGTACGACATAGTAGAGACTTCGGAGAGCGCCTCACAC

GATGACCCATCCAGGTTTGCTGAGCTATCCCGCCCAAGCGGGTCTGTGGTAACACTGCTG

GGAATGAGCGAGTGTGGAAAGCGAGTCGCGGTTCACGTCTATGGTGTGCGCCATTATTTT

TACATGGCAAAGGCGGAGGTGGATAGCGCTTGTGGAATAACCACCGAGGCAGAACTCGTG

CGCGCAATGGTGGACTGCGCGCACAGCTCGGCTTTGAGCGCGGCCCTGGGAAATGGCAAC

GGCGGCAAACAGAGCGGCGGCAGCGGCGGGGGATGGTGGGGCGGAAAGCACGTGTCTGCG

GACTGCTTCAAAGTGGAGACCGTGTGCCACACGACGCTGTACTACTTTGGATCTAAGCCA

GCTCTCTACTATAGAGTATCTGCCTCCAGCAGCCGCCTGGGAGGGTTCATCTGCGACAAC

TTTCACCCGGAGATTACAAAATTCGAGGGGAGCGTGGACGTGACCACGCGGCTGTTGTTG

GACAATGAAAATTTTACCAGTTTCGGGTGGTACCGCCTGCGACCCGGCACCCACGGAGAG

CGTGTTCAACTTCGCCCCGTTGAGCGACACGTCACCTCAAGCGACGTGGAGATTAACTGT

ACTCCCGATAACCTGGAGCCGATACCAGACGAGGCTGCCTGGCCCGACTATAAGCTCATG

TGCTTTGATATAGAGTGTAAAGCTGGAACGGGTAACGAAATGGCGTTCCCAGTGGCAACT

AACCAAGAGGACCTGGTCATCCAGATCTCCTGTCTGCTGTACTCGCTTGCTACTCAGAAC

CACGAACACACCCTGCTGTTTTCCCTCGGGTCATGCGATATCTCTGAGGAATACTCGTTT

GCATGCGTCCAGCGCGGCGAGCCCAGACCGACGGTTTTGGAGTTTGACAGCGAGTACGAG

CTGCTGGTTGCCTTCCTGACCTTTCTCAAGCAGTACTCTCCCGAGTTCGCCACCGGCTAC

AACATCGTTAATTTTGACTGGGCGTACATAGTTAACAAGGTAACGTCGGTGTATAACATC

AAGCTGGACGGGTACGGCAAGTTCAACAAAGGGGGGCTGTTTAAGGTGTGGGACATCGCC

ACGAACCATTTTCAGAAGAAGAGCAAGGTGAAAATCAATGGCCTGATATCTCTAGACATG

TATTCTGTGGCGACGGAAAAGCTAAAGCTACCCAGCTACAAACTCGACGCGGTCGTGGGA

GACGTCCTCGGCGAGCATAAGATAGACCTTCCCTATAAAGAAATACCCTCCTATTACGCG

GGAGGGCCTGACCGGAGGGGCGTAATAGGAGAGTATTGTATCCAGGACTCTAGGCTGGTG

GGCAAGCTGTTTTTTAAGTACCTCCCCCATCTGGAACTATCGGCGGTGGCCAAACTCGCC

CGTATCACCCTAACGCGGGTAATTTTTGACGGTCAGCAAATTCGCGTGTACACGTGCTTG

CTGAAACTCGCCCGCGAGAGAAATTTCATTTTGCCAGACAACAGACGCCGGTTTGACAGT

CAGGCAGACGCCGCGTCAGAGACTTCGGAGTTGGCTATGGATAGCCAAAGCCACGCCTTC

GACAGTACAGACGAACCCGACGGTGTGGACGGTACCCCGGACGCCGCAGGATCTGGCGCT

ACTTCTGAAAACGGAGGCGGGAAGCCCGGCGTCGGGAGGGCCGTGGGCTACCAGGGAGCA

AAGGTTCTAGACCCCGTATCCGGCTTTCATGTGGACCCCGTGGTTGTGTTTGACTTCGCT

AGCTTATACCCAAGCATTATCCAGGCCCATAACCTCTGTTTCACCACCCTGGCGCTCGAT

GAAGTGGATCTGGCCGGGCTTCAACCATCCGTCGACTACTCGACGTTCGAGGTGGGTGAC

CAAAAGTTATTTTTTGTCCACGCCCATATTCGCGAAAGCCTGCTTGGCATCTTGCTGCGC

GACTGGCTGGCCATGCGAAAGGCGGTGAGGGCGCGAATCCCCACCAGCACCCCCGAGGAG

GCAGTTTTACTAGATAAGCAGCAGTCTGCGATTAAGGTGATATGCAACTCGGTTTACGGA

TTCACGGGGGTGGCAAACGGCCTGTTGCCGTGTCTGAGGATAGCGGCTACCGTTACCACG

ATAGGACGCGACATGCTCCTCAAGACCCGAGATTACGTTCACTCTCGTTGGGCGACGCGC

GAGCTGCTGGAGGACAATTTTCCAGGGGCTATAGGTTTCCGAAACCACAAGCCTTACTCC

GTCAGGGTTATCTACGGAGACACCGACTCCGTGTTTATCAAGTTTGTGGGCCTGACGTAC

GAGGGGGTATCGGAGCTGGGGGATGCTATGTCGCGTCAGATTTCAGCGGACCTCTTTAGA

GCGCCCATCAAACTGGAGTGTGAGAAGACCTTTCAGCGACTGCTGCTGATCACCAAGAAG

AAGTACATAGGTGTCATAAACGGGGGGAAGATGCTCATGAAGGGGGTCGACCTGGTCCGC

AAAAATAACTGCTCTTTCATAAACTTGTACGCGCGACATCTGGTAGATCTTTTGTTGTAC

GACGAGGATGTGGCCACGGCGGCAGCAGAGGTGACAGACGTGCCTCCCGCAGAATGGGTG

GGGCGCCCGCTACCGAGCGGCTTTGACAAGTTTGGGCGAGTGCTGGTAGAGGCGTACAAC

CGTATCACTGCCCCCAACTTGGACGTGCGCGAGTTCGTTATGACTGCTGAGCTGAGCCGC

TCACCCGAATCGTATACCAACAAGCGCCTGCCGCACCTCACCGTCTACTTTAAGCTCGCC

ATGAGGAATGAAGAACTGCCCAGCGTAAAAGAGAGAATTCCGTATGTGATAGTTGCGCAG

ACCGAGGCCGCGGAACGCGAAGCGGGTGTAGTAAACTCAATGCGCGGTACCGCCCAAAAC

CCCGTGGTAACCAAGACCGCACGCCCCCAACCTAAACGCAAACTGCTGGTTTCCGACCTC

GCCGAAGACCCGACCTATGTTTCCGAGAATGACGTACCGCTAAACACAGACTACTATTTC

TCCCACCTGTTGGGTACCATAAGCGTGACCTTTAAGGCTCTATTCGGAAATGATGTGAGA

ACAACAGAAAATCTTTTAAAGCGGTTTATTCCGGAAACCCCCCACAAGACCCCCACGAAA

ACCCAGGCACTGCTTGAGCGCGCCGGCTTTGAAAAGCTGACGCCCTTTACACCGGAGGAA

GAAAGTCGTCGAATACTGCATACAGTTTTTTGTACTCTAGAAGCAGCTCCCCATCAAAGC

TGA

>OR085497.1

ATGGCGGCGCGCGAACAGGCCAACTCCGTTCGCAGGAGCGGGTTTTTTAATCCGTTTATT

GGCAAGAGGCCATTTTTCAGGCCGGGAAGCGGGCAGACCGCGGAGACAGAGCGCCCAAGG

CCCCCCCAACACTCGTACTGCACAGAGGTGGGTAGCTTTAAGTTTATAGCTCCAAGATGC

CTCGATGAAGAAGCCCCCGCCGACCAGCGACGCGGTGTACACGTGGGCACCTTGGAGCGC

CCACCCAAAGTGTACTGCGATGGCTCAGAGTACGACGTGCTGAACTTTGCCTCCGGAGGT

TGCTGGCCTCGCAGGATTCGGGTTTGGAACGGCCAGGATTTTCGGGGCGATGGATTCAAC

CCCAGATTTGAGAGATTTCACGTGTACGACATAGTAGAGACTTCGGAGAGCGCCTCACAC

GATGACCCATCCAGGTTTGCTGAGCTATCCCGCCCAAGCGGGTCTGTGGTAACACTGCTG

GGAATGAGCGAGTGTGGAAAGCGAGTCGCGGTTCACGTCTATGGTGTGCGCCATTATTTT

TACATGGCAAAGGCGGAGGTGGATAGCGCTTGTGGAATAACCACCGAGGCAGAACTCGTG

CGCGCAATGGTGGACTGCGCGCACAGCTCGGCTTTGAGCGCGGCCCTGGGAAATGGCAAC

GGCGGCAAACAGAGCGGCGGCAGCGGCGGGGGATGGTGGGGCGGAAAGCACGTGTCTGCG

GACTGCTTCAAAGTGGAGACCGTGTGCCACACGACGCTGTACTACTTTGGATCTAAGCCA

GCTCTCTACTATAGAGTATCTGCCTCCAGCAGCCGCCTGGGAGGGTTCATCTGCGACAAC

TTTCACCCGGAGATTACAAAATTCGAGGGGAGCGTGGACGTGACCACGCGGCTGTTGTTG

GACAATGAAAATTTTACCAGTTTCGGGTGGTACCGCCTGCGACCCGGCACCCACGGAGAG

CGTGTTCAACTTCGCCCCGTTGAGCGACACGTCACCTCAAGCGACGTGGAGATTAACTGT

ACTCCCGATAACCTGGAGCCGATACCAGACGAGGCTGCCTGGCCCGACTATAAGCTCATG

TGCTTTGATATAGAGTGTAAAGCTGGAACGGGTAACGAAATGGCGTTCCCAGTGGCAACT

AACCAAGAGGACCTGGTCATCCAGATCTCCTGTCTGCTGTACTCGCTTGCTACTCAGAAC

CACGAACACACCCTGCTGTTTTCCCTCGGGTCATGCGATATCTCTGAGGAATACTCGTTT

GCATGCGTCCAGCGCGGCGAGCCCAGACCGACGGTTTTGGAGTTTGACAGCGAGTACGAG

CTGCTGGTTGCCTTCCTGACCTTTCTCAAGCAGTACTCTCCCGAGTTCGCCACCGGCTAC

AACATCGTTAATTTTGACTGGGCGTACATAGTTAACAAGGTAACGTCGGTGTATAACATC

AAGCTGGACGGGTACGGCAAGTTCAACAAAGGGGGGCTGTTTAAGGTGTGGGACATCGCC

ACGAACCATTTTCAGAAGAAGAGCAAGGTGAAAATCAATGGCCTGATATCTCTAGACATG

TATTCTGTGGCGACGGAAAAGCTAAAGCTACCCAGCTACAAACTCGACGCGGTCGTGGGA

GACGTCCTCGGCGAGCATAAGATAGACCTTCCCTATAAAGAAATACCCTCCTATTACGCG

GGAGGGCCTGACCGGAGGGGCGTAATAGGAGAGTATTGTATCCAGGACTCTAGGCTGGTG

GGCAAGCTGTTTTTTAAGTACCTCCCCCATCTGGAACTATCGGCGGTGGCCAAACTCGCC

CGTATCACCCTAACGCGGGTAATTTTTGACGGTCAGCAAATTCGCGTGTACACGTGCTTG

CTGAAACTCGCCCGCGAGAGAAATTTCATTTTGCCAGACAACAGACGCCGGTTTGACAGT

CAGGCAGACGCCGCGTCAGAGACTTCGGAGTTGGCTATGGATAGCCAAAGCCACGCCTTC

GACCGTACAGACGAACCCGACGGTGTGGACGGTACCCCGGACGCCGCAGGATCTGGCGCT

ACTTCTGAAAACGGAGGCGGGAAGCCCGGCGTCGGGAGGGCCGTGGGCTACCAGGGAGCA

AAGGTTCTAGACCCCGTATCCGGCTTTCATGTGGACCCCGTGGTTGTGTTTGACTTCGCT

AGCTTATACCCAAGCATTATCCAGGCCCATAACCTCTGTTTCACCACCCTGGCGCTCGAT

GAAGTGGATCTGGCCGGGCTTCAACCATCCGTCAACTACTCGACGTTCGAGGTGGGTGAC

CAAAAGTTATTTTTTGTCCACGCCCATATTCGCGAAAGCCTGCTTGGCATCTTGCTGCGC

GACTGGCTGGCCATGCGAAAGGCGGTGAGGGCGCGAATCCCCACCAGCACCCCCGAGGAG

GCAGTTTTACTAGATAAGCAGCAGTCTGCGATTAAGGTGATATGCAACTCGGTTTACGGA

TTCACGGGGGTGGCAAACGGCCTGTTGCCGTGTCTGAGGATAGCGGCTACCGTTACCACG

ATAGGACGCGACATGCTCCTCAAGACCCGAGATTACGTTCACTCTCGTTGGGCGACGCGC

GAGCTGCTGGAGGACAATTTTCCAGGGGCTATAGGTTTCCGAAACCACAAGCCTTACTCC

GTCAGGGTTATCTACGGAGACACCGACTCCGTGTTTATCAAGTTTGTGGGCCTGACGTAC

GAGGGGGTATCGGAGCTGGGGGATGCTATGTCGCGTCAGATTTCAGCGGACCTCTTTAGA

GCGCCCATCAAACTGGAGTGTGAGAAGACCTTTCAGCGACTGCTGCTGATCACCAAGAAG

AAGTACATAGGTGTCATAAACGGGGGGAAGATGCTCATGAAGGGGGTCGACCTGGTCCGC

AAAAATAACTGCTCTTTCATAAACTTGTACGCGCGACATCTGGTAGATCTTTTGTTGTAC

GACGAGGATGTGGCCACGGCGGCAGCAGAGGTGACAGACGTGCCTCCCGCAGAATGGGTG

GGGCGCCCGCTACCGAGCGGCTTTGACAAGTTTGGGCGAGTGCTGGTAGAGGCGTACAAC

CGTATCACTGCCCCCAACTTGGACGTGCGCGAGTTCGTTATGACTGCTGAGCTGAGCCGC

TCACCCGAATCGTATACCAACAAGCGCCTGCCGCACCTCACCGTCTACTTTAAGCTCGCC

ATGAGGAATGAAGAACTGCCCAGCGTAAAAGAGAGAATTCCGTATGTGATAGTTGCGCAG

ACCGAGGCCGCGGAACGCGAAGCGGGTGTAGTAAACTCAATGCGCGGTACCGCCCAAAAC

CCCGTGGTAACCAAGACCGCACGCCCCCAACCTAAACGCAAACTGCTGGTTTCCGACCTC

GCCGAAGACCCGACCTATGTTTCCGAGAATGACGTACCGCTAAACACAGACTACTATTTC

TCCCACCTGTTGGGTACCATAAGCGTGACCTTTAAGGCTCTATTCGGAAATGATGTGAGA

ACAACAGAAAATCTTTTAAAGCGGTTTATTCCGGAAACCCCCCACAAGACCCCCACGAAA

ACCCAGGCACTGCTTGAGCGCGCCGGCTTTGAAAAGCTGACGCCCTTTACACCGGAGGAA

GAAAGTCGTCGAATACTGCATACAGTTTTTTGTACTCTAGAAGCAGCTCCCCATCAAAGC

TGA

>OR085498.1

ATGGCGGCGCGCGAACAGGCCAACTCCGTTCGCAGGAGCGGGTTTTTTAATCCGTTTATT

GGCAAGAGGCCATTTTTCAGGCCGGGAAGCGGGCAGACCGCGGAGACAGAGCGCCCAAGG

CCCCCCCAACACTCGTACTGCACAGAGGTGGGTAGCTTTAAGTTTATAGCTCCAAGATGC

CTCGATGAAGAAGCCCCCGCCGACCAGCGACGCGGTGTACACGTGGGCACCTTGGAGCGC

CCACCCAAAGTGTACTGCGATGGCTCAGAGTACGACGTGCTGAACTTTGCCTCCGGAGGT

TGCTGGCCTCGCAGGATTCGGGTTTGGAACGGCCAGGATTTTCGGGGCGATGGATTCAAC

CCCAGATTTGAGAGATTTCACGTGTACGACATAGTAGAGACTTCGGAGAGCGCCTCACAC

GATGACCCATCCAGGTTTGCTGAGCTATCCCGCCCAAGCGGGTCTGTGGTAACACTGCTG

GGAATGAGCGAGTGTGGAAAGCGAGTCGCGGTTCACGTCTATGGTGTGCGCCATTATTTT

TACATGGCAAAGGCGGAGGTGGATAGCGCTTGTGGAATAACCACCGAGGCAGAACTCGTG

CGCGCAATGGTGGACTGCGCGCACAGCTCGGCTTTGAGCGCGGCCCTGGGAAATGGCAAC

GGCGGCAAACAGAGCGGCGGCAGCGGCGGGGGATGGTGGGGCGGAAAGCACGTGTCTGCG

GACTGCTTCAAAGTGGAGACCGTGTGCCACACGACGCTGTACTACTTTGGATCTAAGCCA

GCTCTCTACTATAGAGTATCTGCCTCCAGCAGCCGCCTGGGAGGGTTCATCTGCGACAAC

TTTCACCCGGAGATTACAAAATTCGAGGGGAGCGTGGACGTGACCACGCGGCTGTTGTTG

GACAATGAAAATTTTACCAGTTTCGGGTGGTACCGCCTGCGACCCGGCACCCACGGAGAG

CGTGTTCAACTTCGCCCCGTTGAGCGACACGTCACCTCAAGCGACGTGGAGATTAACTGT

ACTCCCGATAACCTGGAGCCGATACCAGACGAGGCTGCCTGGCCCGACTATAAGCTCATG

TGCTTTGATATAGAGTGTAAAGCTGGAACGGGTAACGAAATGGCGTTCCCAGTGGCAACT

AACCAAGAGGACCTGGTCATCCAGATCTCCTGTCTGCTGTACTCGCTTGCTACTCAGAAC

CACGAACACACCCTGCTGTTTTCCCTCGGGTCATGCGATATCTCTGAGGAATACTCGTTT

GCATGCGTCCAGCGCGGCGAGCCCAGACCGACGGTTTTGGAGTTTGACAGCGAGTACGAG

CTGCTGGTTGCCTTCCTGACCTTTCTCAAGCAGTACTCTCCCGAGTTCGCCACCGGCTAC

AACATCGTTAATTTTGACTGGGCGTACATAGTTAACAAGGTAACGTCGGTGTATAACATC

AAGCTGGACGGGTACGGCAAGTTCAACAAAGGGGGGCTGTTTAAGGTGTGGGACATCGCC

ACGAACCATTTTCAGAAGAAGAGCAAGGTGAAAATCAATGGCCTGATATCTCTAGACATG

TATTCTGTGGCGACGGAAAAGCTAAAGCTACCCAGCTACAAACTCGACGCGGTCGTGGGA

GACGTCCTCGGCGAGCATAAGATAGACCTTCCCTATAAAGAAATACCCTCCTATTACGCG

GGAGGGCCTGACCGGAGGGGCGTAATAGGAGAGTATTGTATCCAGGACTCTAGGCTGGTG

GGCAAGCTGTTTTTTAAGTACCTCCCCCATCTGGAACTATCGGCGGTGGCCAAACTCGCC

CGTATCACCCTAACGCGGGTAATTTTTGACGGTCAGCAAATTCGCGTGTACACGTGCTTG

CTGAAACTCGCCCGCGAGAGAAATTTCATTTTGCCAGACAACAGACGCCGGTTTGACAGT

CAGGCAGACGCCGCGTCAGAGACTTCGGAGTTGGCTATGGATAGCCAAAGCCACGCCTTC

GACAGTACAGACGAACCCGACGGTGTGGACGGTACCCCGGACGCCGCAGGATCTGGCGCT

ACTTCTGAAAACGGAGGCGGGAAGCCCGGCGTCGGGAGGGCCGTGGGCTACCAGGGAGCA

AAGGTTCTAGACCCCGTATCCGGCTTTCATGTGGACCCCGTGGTTGTGTTTGACTTCGCT

AGCTTATACCCAAGCATTATCCAGGCCCATAACCTCTGTTTCACCACCCTGGCGCTCGAT

GAAGTGGATCTGGCCGGGCTTCAACCATCCGTCAACTACTCGACGTTCGAGGTGGGTGAC

CAAAAGTTATTTTTTGTCCACGCCCATATTCGCGAAAGCCTGCTTGGCATCTTGCTGCGC

GACTGGCTGGCCATGCGAAAGGCGGTGAGGGCGCGAATCCCCACCAGCACCCCCGAGGAG

GCAGTTTTACTAGATAAGCAGCAGTCTGCGATTAAGGTGATATGCAACTCGGTTTACGGA

TTCACGGGGGTGGCAAACGGCCTGTTGCCGTGTCTGAGGATAGCGGCTACCGTTACCACG

ATAGGACGCGACATGCTCCTCAAGACCCGAGATTACGTTCACTCTCGTTGGGCGACGCGC

GAGCTGCTGGAGGACAATTTTCCAGGGGCTATAGGTTTCCGAAACCACAAGCCTTACTCC

GTCAGGGTTATCTACGGAGACACCGACTCCGTGTTTATCAAGTTTGTGGGCCTGACGTAC

GAGGGGGTATCGGAGCTGGGGGATGCTATGTCGCGTCAGATTTCAGCGGACCTCTTTAGA

GCGCCCATCAAACTGGAGTGTGAGAAGACCTTTCAGCGACTGCTGCTGATCACCAAGAAG

AAGTACATAGGTGTCATAAACGGGGGGAAGATGCTCATGAAGGGGGTCGACCTGGTCCGC

AAAAATAACTGCTCTTTCATAAACTTGTACGCGCGACATCTGGTAGATCTTTTGTTGTAC

GACGAGGATGTGGCCACGGCGGCAGCAGAGGTGACAGACGTGCCTCCCGCAGAATGGGTG

GGGCGCCCGCTACCGAGCGGCTTTGACAAGTTTGGGCGAGTGCTGGTAGAGGCGTACAAC

CGTATCACTGCCCCCAACTTGGACGTGCGCGAGTTCGTTATGACTGCTGAGCTGAGCCGC

TCACCCGAATCGTATACCAACAAGCGCCTGCCGCACCTCACCGTCTACTTTAAGCTCGCC

ATGAGGAATGAAGAACTGCCCAGCGTAAAAGAGAGAATTCCGTATGTGATAGTTGCGCAG

ACCGAGGCCGCGGAACGCGAAGCGGGTGTAGTAAACTCAATGCGCGGTACCGCCCAAAAC

CCCGTGGTAACCAAGACCGCACGCCCCCAACCTAAACGCAAACTGCTGGTTTCCGACCTC

GCCGAAGACCCGACCTATGTTTCCGAGAATGACGTACCGCTAAACACAGACTACTATTTC

TCCCACCTGTTGGGTACCATAAGCGTGACCTTTAAGGCTCTATTCGGAAATGATGTGAGA

ACAACAGAAAATCTTTTAAAGCGGTTTATTCCGGAAACCCCCCACAAGACCCCCACGAAA

ACCCAGGCACTGCTTGAGCGCGCCGGCTTTGAAAAGCTGACGCCCTTTACACCGGAGGAA

GAAAGTCGTCGAATACTGCATACAGTTTTTTGTACTCTAGAAGCAGCTCCCCATCAAAGC

TGA

>OR085499.1

ATGGCGGCGCGCGAACAGGCCAACTCCGTTCGCAGGAGCGGGTTTTTTAATCCGTTTATT

GGCAAGAGGCCATTTTTCAGGCCGGGAAGCGGGCAAACCGCGGAGACAGAGCGCCCAAGG

CCCCCCCAACACTCGTACTGCACAGAGGTGGGTAGCTTTAAGTTTATAGCTCCAAGATGC

CTCGATGAAGAAGCCCCCGCCGACCAGCGACGCGGTGTACACGTGGGCACCTTGGAGCGC

CCACCCAAAGTGTACTGCGATGGCTCAGAGTACGACGTGCTGAACTTTGCCTCCGGAGGT

TGCTGGCCTCGCAGGATTCGGGTTTGGAACGGCCAGGATTTTCGGGGCGATGGATTCAAC

CCCAGATTTGAGAGATTTCACGTGTACGACATAGTAGAGACTTCGGAGAGCGCCTCACAC

GATGACCCATCCAGGTTTGCTGAGCTATCCCGCCCAAGCGGGTCTGTGGTAACACTGCTG

GGAATGAGCGAGTGTGGAAAGCGAGTCGCGGTTCACGTCTATGGTGTGCGCCATTATTTT

TACATGGCAAAGGCGGAGGTGGATAGCGCTTGTGGAATAACCACCGAGGCAGAACTCGTG

CGCGCAATGGTGGACTGCGCGCACAGCTCGGCTTTGAGCGCGGCCCTGGGAAATGGCAAC

GGCGGCAAACAGAGCGGCGGCAGCGGCGGGGGATGGTGGGGCGGAAAGCACGTGTCTGCG

GACTGCTTCAAAGTGGAGACCGTGTGCCACACGACGCTGTACTACTTTGGATCTAAGCCA

GCTCTCTACTATAGAGTATCTGCCTCCAGCAGCCGCCTGGGAGGGTTCATCTGCGACAAC

TTTCACCCGGAGATTACAAAATTCGAGGGGAGCGTGGACGTGACCACGCGGCTGTTGTTG

GACAATGAAAATTTTACCAGTTTCGGGTGGTACCGCCTGCGACCCGGCACCCACGGAGAG

CGTGTTCAACTTCGCCCCGTTGAGCGACACGTCACCTCAAGCGACGTGGAGATTAACTGT

ACTCCCGATAACCTGGAGCCGATACCAGACGAGGCTGCCTGGCCCGACTATAAGCTCATG

TGCTTTGATATAGAGTGTAAAGCTGGAACGGGTAACGAAATGGCGTTCCCAGTGGCAACT

AACCAAGAGGACCTGGTCATCCAGATCTCCTGTCTGCTGTACTCGCTTGCTACTCAGAAC

CACGAACACACCCTGCTGTTTTCCCTCGGGTCATGCGATATCTCTGAGGAATACTCGTTT

GCATGCGTCCAGCGCGGCGAGCCCAGACCGACGGTTTTGGAGTTTGACAGCGAGTACGAG

CTGCTGGTTGCCTTCCTGACCTTTCTCAAGCAGTACTCTCCCGAGTTCGCCACCGGCTAC

AACATCGTTAATTTTGACTGGGCGTACATAGTTAACAAGGTAACGTCGGTGTATAACATC

AAGCTGGACGGGTACGGCAAGTTCAACAAAGGGGGGCTGTTTAAGGTGTGGGACATCGCC

ACGAACCATTTTCAGAAGAAGAGCAAGGTGAAAATCAATGGCCTGATATCTCTAGACATG

TATTCTGTGGCGACGGAAAAGCTAAAGCTACCCAGCTACAAACTCGACGCGGTCGTGGGA

GACGTCCTCGGCGAGCATAAGATAGACCTTCCCTATAAAGAAATACCCTCCTATTACGCG

GGAGGGCCTGACCGGAGGGGCGTAATAGGAGAGTATTGTATCCAGGACTCTAGGCTGGTG

GGCAAGCTGTTTTTTAAGTACCTCCCCCATCTGGAACTATCGGCGGTGGCCAAACTCGCC

CGTATCACCCTAACGCGGGTAATTTTTGACGGTCAGCAAATTCGCGTGTACACGTGCTTG

CTGAAACTCGCCCGCGAGAGAAATTTCATTTTGCCAGACAACAGACGCCGGTTTGACAGT

CAGGCAGACGCCGCGTCAGAGACTTCGGAGTTGGCTATGGATAGCCAAAGCCACGCCTTC

GACAGTACAGACGAACCCGACGGTGTGGACGGTACCCCGGACGCCGCAGGATCTGGCGCT

ACTTCTGAAAACGGAGGCGGGAAGCCCGGCGTCGGGAGGGCCGTGGGCTACCAGGGAGCA

AAGGTTCTAGACCCCGTATCCGGCTTTCATGTGGACCCCGTGGTTGTGTTTGACTTCGCT

AGCTTATACCCAAGCATTATCCAGGCCCATAACCTCTGTTTCACCACCCTGGCGCTCGAT

GAAGTGGATCTGGCCGGGCTTCAACCATCCGTCAACTACTCGACGTTCGAGGTGGGTGAC

CAAAAGTTATTTTTTGTCCACGCCCATATTCGCGAAAGCCTGCTTGGCATCTTGCTGCGC

GACTGGCTGGCCATGCGAAAGGCGGTGAGGGCGCGAATCCCCACCAGCACCCCCGAGGAG

GCAGTTTTACTAGATAAGCAGCAGTCTGCGATTAAGGTGATATGCAACTCGGTTTACGGA

TTCACGGGGGTGGCAAACGGCCTGTTGCCGTGTCTGAGGATAGCGGCTACCGTTACCACG

ATAGGACGCGACATGCTCCTCAAGACCCGAGATTACGTTCACTCTCGTTGGGCGACGCGC

GAGCTGCTGGAGGACAATTTTCCAGGGGCTATAGGTTTCCGAAACCACAAGCCTTACTCC

GTCAGGGTTATCTACGGAGACACCGACTCCGTGTTTATCAAGTTTGTGGGCCTGACGTAC

GAGGGGGTATCGGAGCTGGGGGATGCTATGTCGCGTCAGATTTCAGCGGACCTCTTTAGA

GCGCCCATCAAACTGGAGTGTGAGAAGACCTTTCAGCGACTGCTTCTGATCACCAAGAAG

AAGTACATAGGTGTCATAAACGGGGGGAAGATGCTCATGAAGGGGGTCGACCTGGTCCGC

AAAAATAACTGCTCTTTCATAAACTTGTACGCGCGACATCTGGTAGATCTTTTGTTGTAC

GACGAGGATGTGGCCACGGCGGCAGCAGAGGTGACAGACGTGCCTCCCGCAGAATGGGTG

GGGCGCCCGCTACCGAGCGGCTTTGACAAGTTTGGGCGAGTGCTGGTAGAGGCGTACAAC

CGTATCACTGCCCCCAACTTGGACGTGCGCGAGTTCGTTATGACTGCTGAGCTGAGCCGC

TCACCCGAATCGTATACCAACAAGCGCCTGCCGCACCTCACCGTCTACTTTAAGCTCGCC

ATGAGGAATGAAGAACTGCCCAGCGTAAAAGAGAGAATTCCGTATGTGATAGTTGCGCAG

ACCGAGGCCGCGGAACGCGAAGCGGGTGTAGTAAACTCAATGCGCGGTACCGCCCAAAAC

CCCGTGGTAACCAAGACCGCACGCCCCCAACCTAAACGCAAACTGCTGGTTTCCGACCTC

GCCGAAGACCCGACCTATGTTTCCGAGAATGACGTACCGCTAAACACAGACTACTATTTC

TCCCACCTGTTGGGTACCATAAGCGTGACCTTTAAGGCTCTATTCGGAAATGATGTGAGA

ACAACAGAAAATCTTTTAAAGCGGTTTATTCCGGAAACCCCCCACAAGACCCCCACGAAA

ACCCAGGCACTGCTTGAGCGCGCCGGCTTTGAAAAGCTGACGCCCTTTACACCGGAGGAA

GAAAGTCGTCGAATACTGCATACAGTTTTTTGTACTCTAGAAGCAGCTCCCCATCAAAGC

TGA

>OR085500.1

ATGGCGGCGCGCGAACAGGCCAACTCCGTTCGCAGGAGCGGGTTTTTTAATCCGTTTATT

GGCAAGAGGCCATTTTTCAGGCCGGGAAGCGGGCAAACCGCGGAGACAGAGCGCCCAAGG

CCCCCCCAACACTCGTACTGCACAGAGGTGGGTAGCTTTAAGTTTATAGCTCCAAGATGC

CTCGATGAAGAAGCCCCCGCCGACCAGCGACGCGGTGTACACGTGGGCACCTTGGAGCGC

CCACCCAAAGTGTACTGCGATGGCTCAGAGTACGACGTGCTGAACTTTGCCTCCGGAGGT

TGCTGGCCTCGCAGGATTCGGGTTTGGAACGGCCAGGATTTTCGGGGCGATGGATTCAAC

CCCAGATTTGAGAGATTTCACGTGTACGACATAGTAGAGACTTCGGAGAGCGCCTCACAC

GATGACCCATCCAGGTTTGCTGAGCTATCCCGCCCAAGCGGGTCTGTGGTAACACTGCTG

GGAATGAGCGAGTGTGGAAAGCGAGTCGCGGTTCACGTCTATGGTGTGCGCCATTATTTT

TACATGGCAAAGGCGGAGGTGGATAGCGCTTGTGGAATAACCACCGAGGCAGAACTCGTG

CGCGCAATGGTGGACTGCGCGCACAGCTCGGCTTTGAGCGCGGCCCTGGGAAATGGCAAC

GGCGGCAAACAGAGCGGCGGCAGCGGCGGGGGATGGTGGGGCGGAAAGCACGTGTCTGCG

GACTGCTTCAAAGTGGAGACCGTGTGCCACACGACGCTGTACTACTTTGGATCTAAGCCA

GCTCTCTACTATAGAGTATCTGCCTCCAGCAGCCGCCTGGGAGGGTTCATCTGCGACAAC

TTTCACCCGGAGATTACAAAATTCGAGGGGAGCGTGGACGTGACCACGCGGCTGTTGTTG

GACAATGAAAATTTTACCAGTTTCGGGTGGTACCGCCTGCGACCCGGCACCCACGGAGAG

CGTGTTCAACTTCGCCCCGTTGAGCGACACGTCACCTCAAGCGACGTGGAGATTAACTGT

ACTCCCGATAACCTGGAGCCGATACCAGACGAGGCTGCCTGGCCCGACTATAAGCTCATG

TGCTTTGATATAGAGTGTAAAGCTGGAACGGGTAACGAAATGGCGTTCCCAGTGGCAACT

AACCAAGAGGACCTGGTCATCCAGATCTCCTGTCTGCTGTACTCGCTTGCTACTCAGAAC

CACGAACACACCCTGCTGTTTTCCCTCGGGTCATGCGATATCTCTGAGGAATACTCGTTT

GCATGCGTCCAGCGCGGCGAGCCCAGACCGACGGTTTTGGAGTTTGACAGCGAGTACGAG

CTGCTGGTTGCCTTCCTGACCTTTCTCAAGCAGTACTCTCCCGAGTTCGCCACCGGCTAC

AACATCGTTAATTTTGACTGGGCGTACATAGTTAACAAGGTAACGTCGGTGTATAACATC

AAGCTGGACGGGTACGGCAAGTTCAACAAAGGGGGGCTGTTTAAGGTGTGGGACATCGCC

ACGAACCATTTTCAGAAGAAGAGCAAGGTGAAAATCAATGGCCTGATATCTCTAGACATG

TATTCTGTGGCGACGGAAAAGCTAAAGCTACCCAGCTACAAACTCGACGCGGTCGTGGGA

GACGTCCTCGGCGAGCATAAGATAGACCTTCCCTATAAAGAAATACCCTCCTATTACGCG

GGAGGGCCTGACCGGAGGGGCGTAATAGGAGAGTATTGTATCCAGGACTCTAGGCTGGTG

GGCAAGCTGTTTTTTAAGTACCTCCCCCATCTGGAACTATCGGCGGTGGCCAAACTCGCC

CGTATCACCCTAACGCGGGTAATTTTTGACGGTCAGCAAATTCGCGTGTACACGTGCTTG

CTGAAACTCGCCCGCGAGAGAAATTTCATTTTGCCAGACAACAGACGCCGGTTTGACAGT

CAGGCAGACGCCGCGTCAGAGACTTCGGAGTTGGCTATGGATAGCCAAAGCCACGCCTTC

GACAGTACAGACGAACCCGACGGTGTGGACGGTACCCCGGACGCCGCAGGATCTGGCGCT

ACTTCTGAAAACGGAGGCGGGAAGCCCGGCGTCGGGAGGGCCGTGGGCTACCAGGGAGCA

AAGGTTCTAGACCCCGTATCCGGCTTTCATGTGGACCCCGTGGTTGTGTTTGACTTCGCT

AGCTTATACCCAAGCATTATCCAGGCCCATAACCTCTGTTTCACCACCCTGGCGCTCGAT

GAAGTGGATCTGGCCGGGCTTCAACCATCCGTCAACTACTCGACGTTCGAGGTGGGTGAC

CAAAAGTTATTTTTTGTCCACGCCCATATTCGCGAAAGCCTGCTTGGCATCTTGCTGCGC

GACTGGCTGGCCATGCGAAAGGCGGTGAGGGCGCGAATCCCCACCAGCACCCCCGAGGAG

GCAGTTTTACTAGATAAGCAGCAGTCTGCGATTAAGGTGATATGCAACTCGGTTTACGGA

TTCACGGGGGTGGCAAACGGCCTGTTGCCGTGTCTGAGGATAGCGGCTACCGTTACCACG

ATAGGACGCGACATGCTCCTCAAGACCCGAGATTACGTTCACTCTCGTTGGGCGACGCGC

GAGCTGCTGGAGGACAATTTTCCAGGGGCTATAGGTTTCCGAAACCACAAGCCTTACTCC

GTCAGGGTTATCTACGGAGACACCGACTCCGTGTTTATCAAGTTTGTGGGCCTGACGTAC

GAGGGGGTATCGGAGCTGGGGGATGCTATGTCGCGTCAGATTTCAGCGGACCTCTTTAGA

GCGCCCATCAAACTGGAGTGTGAGAAGACCTTTCAGCGACTGCTTCTGATCACCAAGAAG

AAGTACATAGGTGTCATAAACGGGGGGAAGATGCTCATGAAGGGGGTCGACCTGGTCCGC

AAAAATAACTGCTCTTTCATAAACTTGTACGCGCGACATCTGGTAGATCTTTTGTTGTAC

GACGAGGATGTGGCCACGGCGGCAGCAGAGGTGACAGACGTGCCTCCCGCAGAATGGGTG

GGGCGCCCGCTACCGAGCGGCTTTGACAAGTTTGGGCGAGTGCTGGTAGAGGCGTACAAC

CGTATCACTGCCCCCAACTTGGACGTGCGCGAGTTCGTTATGACTGCTGAGCTGAGCCGC

TCACCCGAATCGTATACCAACAAGCGCCTGCCGCACCTCACCGTCTACTTTAAGCTCGCC

ATGAGGAATGAAGAACTGCCCAGCGTAAAAGAGAGAATTCCGTATGTGATAGTTGCGCAG

ACCGAGGCCGCGGAACGCGAAGCGGGTGTAGTAAACTCAATGCGCGGTACCGCCCAAAAC

CCCGTGGTAACCAAGACCGCACGCCCCCAACCTAAACGCAAACTGCTGGTTTCCGACCTC

GCCGAAGACCCGACCTATGTTTCCGAGAATGACGTACCGCTAAACACAGACTACTATTTC

TCCCACCTGTTGGGTACCATAAGCGTGACCTTTAAGGCTCTATTCGGAAATGATGTGAGA

ACAACAGAAAATCTTTTAAAGCGGTTTATTCCGGAAACCCCCCACAAGACCCCCACGAAA

ACCCAGGCACTGCTTGAGCGCGCCGGCTTTGAAAAGCTGACGCCCTTTACACCGGAGGAA

GAAAGTCGTCGAATACTGCATACAGTTTTTTGTACTCTAGAAGCAGCTCCCCATCAAAGC

TGA

>OR085501.1

ATGGCGGCGCGCGAACAGGCCAACTCCGTTCGCAGGAGCGGGTTTTTTAATCCGTTTATT

GGCAAGAGGCCATTTTTCAGGCCGGGAAGCGGGCAAACCGCGGAGACAGAGCGCCCAAGG

CCCCCCCAACACTCGTACTGCACAGAGGTGGGTAGCTTTAAGTTTATAGCTCCAAGATGC

CTCGATGAAGAAGCCCCCGCCGACCAGCGACGCGGTGTACACGTGGGCACCTTGGAGCGC

CCACCCAAAGTGTACTGCGATGGCTCAGAGTACGACGTGCTGAACTTTGCCTCCGGAGGT

TGCTGGCCTCGCAGGATTCGGGTTTGGAACGGCCAGGATTTTCGGGGCGATGGATTCAAC

CCCAGATTTGAGAGATTTCACGTGTACGACATAGTAGAGACTTCGGAGAGCGCCTCACAC

GATGACCCATCCAGGTTTGCTGAGCTATCCCGCCCAAGCGGGTCTGTGGTAACACTGCTG

GGAATGAGCGAGTGTGGAAAGCGAGTCGCGGTTCACGTCTATGGTGTGCGCCATTATTTT

TACATGGCAAAGGCGGAGGTGGATAGCGCTTGTGGAATAACCACCGAGGCAGAACTCGTG

CGCGCAATGGTGGACTGCGCGCACAGCTCGGCTTTGAGCGCGGCCCTGGGAAATGGCAAC

GGCGGCAAACAGAGCGGCGGCAGCGGCGGGGGATGGTGGGGCGGAAAGCACGTGTCTGCG

GACTGCTTCAAAGTGGAGACCGTGTGCCACACGACGCTGTACTACTTTGGATCTAAGCCA

GCTCTCTACTATAGAGTATCTGCCTCCAGCAGCCGCCTGGGAGGGTTCATCTGCGACAAC

TTTCACCCGGAGATTACAAAATTCGAGGGGAGCGTGGACGTGACCACGCGGCTGTTGTTG

GACAATGAAAATTTTACCAGTTTCGGGTGGTACCGCCTGCGACCCGGCACCCACGGAGAG

CGTGTTCAACTTCGCCCCGTTGAGCGACACGTCACCTCAAGCGACGTGGAGATTAACTGT

ACTCCCGATAACCTGGAGCCGATACCAGACGAGGCTGCCTGGCCCGACTATAAGCTCATG

TGCTTTGATATAGAGTGTAAAGCTGGAACGGGTAACGAAATGGCGTTCCCAGTGGCAACT

AACCAAGAGGACCTGGTCATCCAGATCTCCTGTCTGCTGTACTCGCTTGCTACTCAGAAC

CACGAACACACCCTGCTGTTTTCCCTCGGGTCATGCGATATCTCTGAGGAATACTCGTTT

GCATGCGTCCAGCGCGGCGAGCCCAGACCGACGGTTTTGGAGTTTGACAGCGAGTACGAG

CTGCTGGTTGCCTTCCTGACCTTTCTCAAGCAGTACTCTCCCGAGTTCGCCACCGGCTAC

AACATCGTTAATTTTGACTGGGCGTACATAGTTAACAAGGTAACGTCGGTGTATAACATC

AAGCTGGACGGGTACGGCAAGTTCAACAAAGGGGGGCTGTTTAAGGTGTGGGACATCGCC

ACGAACCATTTTCAGAAGAAGAGCAAGGTGAAAATCAATGGCCTGATATCTCTAGACATG

TATTCTGTGGCGACGGAAAAGCTAAAGCTACCCAGCTACAAACTCGACGCGGTCGTGGGA

GACGTCCTCGGCGAGCATAAGATAGACCTTCCCTATAAAGAAATACCCTCCTATTACGCG

GGAGGGCCTGACCGGAGGGGCGTAATAGGAGAGTATTGTATCCAGGACTCTAGGCTGGTG

GGCAAGCTGTTTTTTAAGTACCTCCCCCATCTGGAACTATCGGCGGTGGCCAAACTCGCC

CGTATCACCCTAACGCGGGTAATTTTTGACGGTCAGCAAATTCGCGTGTACACGTGCTTG

CTGAAACTCGCCCGCGAGAGAAATTTCATTTTGCCAGACAACAGACGCCGGTTTGACAGT

CAGGCAGACGCCGCGTCAGAGACTTCGGAGTTGGCTATGGATAGCCAAAGCCACGCCTTC

GACAGTACAGACGAACCCGACGGTGTGGACGGTACCCCGGACGCCGCAGGATCTGGCGCT

ACTTCTGAAAACGGAGGCGGGAAGCCCGGCGTCGGGAGGGCCGTGGGCTACCAGGGAGCA

AAGGTTCTAGACCCCGTATCCGGCTTTCATGTGGACCCCGTGGTTGTGTTTGACTTCGCT

AGCTTATACCCAAGCATTATCCAGGCCCATAACCTCTGTTTCACCACCCTGGCGCTCGAT

GAAGTGGATCTGGCCGGGCTTCAACCATCCGTCAACTACTCGACGTTCGAGGTGGGTGAC

CAAAAGTTATTTTTTGTCCACGCCCATATTCGCGAAAGCCTGCTTGGCATCTTGCTGCGC

GACTGGCTGGCCATGCGAAAGGCGGTGAGGGCGCGAATCCCCACCAGCACCCCCGAGGAG

GCAGTTTTACTAGATAAGCAGCAGTCTGCGATTAAGGTGATATGCAACTCGGTTTACGGA

TTCACGGGGGTGGCAAACGGCCTGTTGCCGTGTCTGAGGATAGCGGCTACCGTTACCACG

ATAGGACGCGACATGCTCCTCAAGACCCGAGATTACGTTCACTCTCGTTGGGCGACGCGC

GAGCTGCTGGAGGACAATTTTCCAGGGGCTATAGGTTTCCGAAACCACAAGCCTTACTCC

GTCAGGGTTATCTACGGAGACACCGACTCCGTGTTTATCAAGTTTGTGGGCCTGACGTAC

GAGGGGGTATCGGAGCTGGGGGATGCTATGTCGCGTCAGATTTCAGCGGACCTCTTTAGA

GCGCCCATCAAACTGGAGTGTGAGAAGACCTTTCAGCGACTGCTTCTGATCACCAAGAAG

AAGTACATAGGTGTCATAAACGGGGGGAAGATGCTCATGAAGGGGGTCGACCTGGTCCGC

AAAAATAACTGCTCTTTCATAAACTTGTACGCGCGACATCTGGTAGATCTTTTGTTGTAC

GACGAGGATGTGGCCACGGCGGCAGCAGAGGTGACAGACGTGCCTCCCGCAGAATGGGTG

GGGCGCCCGCTACCGAGCGGCTTTGACAAGTTTGGGCGAGTGCTGGTAGAGGCGTACAAC

CGTATCACTGCCCCCAACTTGGACGTGCGCGAGTTCGTTATGACTGCTGAGCTGAGCCGC

TCACCCGAATCGTATACCAACAAGCGCCTGCCGCACCTCACCGTCTACTTTAAGCTCGCC

ATGAGGAATGAAGAACTGCCCAGCGTAAAAGAGAGAATTCCGTATGTGATAGTTGCGCAG

ACCGAGGCCGCGGAACGCGAAGCGGGTGTAGTAAACTCAATGCGCGGTACCGCCCAAAAC

CCCGTGGTAACCAAGACCGCACGCCCCCAACCTAAACGCAAACTGCTGGTTTCCGACCTC

GCCGAAGACCCGACCTATGTTTCCGAGAATGACGTACCGCTAAACACAGACTACTATTTC

TCCCACCTGTTGGGTACCATAAGCGTGACCTTTAAGGCTCTATTCGGAAATGATGTGAGA

ACAACAGAAAATCTTTTAAAGCGGTTTATTCCGGAAACCCCCCACAAGACCCCCACGAAA

ACCCAGGCACTGCTTGAGCGCGCCGGCTTTGAAAAGCTGACGCCCTTTACACCGGAGGAA

GAAAGTCGTCGAATACTGCATACAGTTTTTTGTACTCTAGAAGCAGCTCCCCATCAAAGC

TGA

>OR085502.1

ATGGCGGCGCGCGAACAGGCCAACTCCGTTCGCAGGAGCGGGTTTTTTAATCCGTTTATT

GGCAAGAGGCCATTTTTCAGGCCGGGAAGCGGGCAAACCGCGGAGACAGAGCGCCCAAGG

CCCCCCCAACACTCGTACTGCACAGAGGTGGGTAGCTTTAAGTTTATAGCTCCAAGATGC

CTCGATGAAGAAGCCCCCGCCGACCAGCGACGCGGTGTACACGTGGGCACCTTGGAGCGC

CCACCCAAAGTGTACTGCGATGGCTCAGAGTACGACGTGCTGAACTTTGCCTCCGGAGGT

TGCTGGCCTCGCAGGATTCGGGTTTGGAACGGCCAGGATTTTCGGGGCGATGGATTCAAC

CCCAGATTTGAGAGATTTCACGTGTACGACATAGTAGAGACTTCGGAGAGCGCCTCACAC

GATGACCCATCCAGGTTTGCTGAGCTATCCCGCCCAAGCGGGTCTGTGGTAACACTGCTG

GGAATGAGCGAGTGTGGAAAGCGAGTCGCGGTTCACGTCTATGGTGTGCGCCATTATTTT

TACATGGCAAAGGCGGAGGTGGATAGCGCTTGTGGAATAACCACCGAGGCAGAACTCGTG

CGCGCAATGGTGGACTGCGCGCACAGCTCGGCTTTGAGCGCGGCCCTGGGAAATGGCAAC

GGCGGCAAACAGAGCGGCGGCAGCGGCGGGGGATGGTGGGGCGGAAAGCACGTGTCTGCG

GACTGCTTCAAAGTGGAGACCGTGTGCCACACGACGCTGTACTACTTTGGATCTAAGCCA

GCTCTCTACTATAGAGTATCTGCCTCCAGCAGCCGCCTGGGAGGGTTCATCTGCGACAAC

TTTCACCCGGAGATTACAAAATTCGAGGGGAGCGTGGACGTGACCACGCGGCTGTTGTTG

GACAATGAAAATTTTACCAGTTTCGGGTGGTACCGCCTGCGACCCGGCACCCACGGAGAG

CGTGTTCAACTTCGCCCCGTTGAGCGACACGTCACCTCAAGCGACGTGGAGATTAACTGT

ACTCCCGATAACCTGGAGCCGATACCAGACGAGGCTGCCTGGCCCGACTATAAGCTCATG

TGCTTTGATATAGAGTGTAAAGCTGGAACGGGTAACGAAATGGCGTTCCCAGTGGCAACT

AACCAAGAGGACCTGGTCATCCAGATCTCCTGTCTGCTGTACTCGCTTGCTACTCAGAAC

CACGAACACACCCTGCTGTTTTCCCTCGGGTCATGCGATATCTCTGAGGAATACTCGTTT

GCATGCGTCCAGCGCGGCGAGCCCAGACCGACGGTTTTGGAGTTTGACAGCGAGTACGAG

CTGCTGGTTGCCTTCCTGACCTTTCTCAAGCAGTACTCTCCCGAGTTCGCCACCGGCTAC

AACATCGTTAATTTTGACTGGGCGTACATAGTTAACAAGGTAACGTCGGTGTATAACATC

AAGCTGGACGGGTACGGCAAGTTCAACAAAGGGGGGCTGTTTAAGGTGTGGGACATCGCC

ACGAACCATTTTCAGAAGAAGAGCAAGGTGAAAATCAATGGCCTGATATCTCTAGACATG

TATTCTGTGGCGACGGAAAAGCTAAAGCTACCCAGCTACAAACTCGACGCGGTCGTGGGA

GACGTCCTCGGCGAGCATAAGATAGACCTTCCCTATAAAGAAATACCCTCCTATTACGCG

GGAGGGCCTGACCGGAGGGGCGTAATAGGAGAGTATTGTATCCAGGACTCTAGGCTGGTG

GGCAAGCTGTTTTTTAAGTACCTCCCCCATCTGGAACTATCGGCGGTGGCCAAACTCGCC

CGTATCACCCTAACGCGGGTAATTTTTGACGGTCAGCAAATTCGCGTGTACACGTGCTTG

CTGAAACTCGCCCGCGAGAGAAATTTCATTTTGCCAGACAACAGACGCCGGTTTGACAGT

CAGGCAGACGCCGCGTCAGAGACTTCGGAGTTGGCTATGGATAGCCAAAGCCACGCCTTC

GACAGTACAGACGAACCCGACGGTGTGGACGGTACCCCGGACGCCGCAGGATCTGGCGCT

ACTTCTGAAAACGGAGGCGGGAAGCCCGGCGTCGGGAGGGCCGTGGGCTACCAGGGAGCA

AAGGTTCTAGACCCCGTATCCGGCTTTCATGTGGACCCCGTGGTTGTGTTTGACTTCGCT

AGCTTATACCCAAGCATTATCCAGGCCCATAACCTCTGTTTCACCACCCTGGCGCTCGAT

GAAGTGGATCTGGCCGGGCTTCAACCATCCGTCAACTACTCGACGTTCGAGGTGGGTGAC

CAAAAGTTATTTTTTGTCCACGCCCATATTCGCGAAAGCCTGCTTGGCATCTTGCTGCGC

GACTGGCTGGCCATGCGAAAGGCGGTGAGGGCGCGAATCCCCACCAGCACCCCCGAGGAG

GCAGTTTTACTAGATAAGCAGCAGTCTGCGATTAAGGTGATATGCAACTCGGTTTACGGA

TTCACGGGGGTGGCAAACGGCCTGTTGCCGTGTCTGAGGATAGCGGCTACCGTTACCACG

ATAGGACGCGACATGCTCCTCAAGACCCGAGATTACGTTCACTCTCGTTGGGCGACGCGC

GAGCTGCTGGAGGACAATTTTCCAGGGGCTATAGGTTTCCGAAACCACAAGCCTTACTCC

GTCAGGGTTATCTACGGAGACACCGACTCCGTGTTTATCAAGTTTGTGGGCCTGACGTAC

GAGGGGGTATCGGAGCTGGGGGATGCTATGTCGCGTCAGATTTCAGCGGACCTCTTTAGA

GCGCCCATCAAACTGGAGTGTGAGAAGACCTTTCAGCGACTGCTTCTGATCACCAAGAAG

AAGTACATAGGTGTCATAAACGGGGGGAAGATGCTCATGAAGGGGGTCGACCTGGTCCGC

AAAAATAACTGCTCTTTCATAAACTTGTACGCGCGACATCTGGTAGATCTTTTGTTGTAC

GACGAGGATGTGGCCACGGCGGCAGCAGAGGTGACAGACGTGCCTCCCGCAGAATGGGTG

GGGCGCCCGCTACCGAGCGGCTTTGACAAGTTTGGGCGAGTGCTGGTAGAGGCGTACAAC

CGTATCACTGCCCCCAACTTGGACGTGCGCGAGTTCGTTATGACTGCTGAGCTGAGCCGC

TCACCCGAATCGTATACCAACAAGCGCCTGCCGCACCTCACCGTCTACTTTAAGCTCGCC

ATGAGGAATGAAGAACTGCCCAGCGTAAAAGAGAGAATTCCGTATGTGATAGTTGCGCAG

ACCGAGGCCGCGGAACGCGAAGCGGGTGTAGTAAACTCAATGCGCGGTACCGCCCAAAAC

CCCGTGGTAACCAAGACCGCACGCCCCCAACCTAAACGCAAACTGCTGGTTTCCGACCTC

GCCGAAGACCCGACCTATGTTTCCGAGAATGACGTACCGCTAAACACAGACTACTATTTC

TCCCACCTGTTGGGTACCATAAGCGTGACCTTTAAGGCTCTATTCGGAAATGATGTGAGA

ACAACAGAAAATCTTTTAAAGCGGTTTATTCCGGAAACCCCCCACAAGACCCCCACGAAA

ACCCAGGCACTGCTTGAGCGCGCCGGCTTTGAAAAGCTGACGCCCTTTACACCGGAGGAA

GAAAGTCGTCGAATACTGCATACAGTTTTTTGTACTCTAGAAGCAGCTCCCCATCAAAGC

TGA

>OR085503.1

ATGGCGGCGCGCGAACAGGCCAACTCCGTTCGCAGGAGCGGGTTTTTTAATCCGTTTATT

GGCAAGAGGCCATTTTTCAGGCCGGGAAGCGGGCAGACCGCGGAGACAGAGCGCCCAAGG

CCCCCCCAACACTCGTACTGCACAGAGGTGGGTAGCTTTAAGTTTATAGCTCCAAGATGC

CTCGATGAAGAAGCCCCCGCCGACCAGCGACGCGGTGTACACGTGGGCACCTTGGAGCGC

CCACCCAAAGTGTACTGCGATGGCTCAGAGTACGACGTGCTGAACTTTGCCTCCGGAGGT

TGCTGGCCTCGCAGGATTCGGGTTTGGAACGGCCAGGATTTTCGGGGCGATGGATTCAAC

CCCAGATTTGAGAGATTTCACGTGTACGACATAGTAGAGACTTCGGAGAGCGCCTCACAC

GATGACCCATCCAGGTTTGCTGAGCTATCCCGCCCAAGCGGGTCTGTGGTAACACTGCTG

GGAATGAGCGAGTGTGGAAAGCGAGTCGCGGTTCACGTCTATGGTGTGCGCCATTATTTT

TACATGGCAAAGGCGGAGGTGGATAGCGCTTGTGGAATAACCACCGAGGCAGAACTCGTG

CGCGCAATGGTGGACTGCGCGCACAGCTCGGCTTTGAGCGCGGCCCTGGGAAATGGCAAC

GGCGGCAAACAGAGCGGCGGCAGCGGCGGGGGATGGTGGGGCGGAAAGCACGTGTCTGCG

GACTGCTTCAAAGTGGAGACCGTGTGCCACACGACGCTGTACTACTTTGGATCTAAGCCA

GCTCTCTACTATAGAGTATCTGCCTCCAGCAGCCGCCTGGGAGGGTTCATCTGCGACAAC

TTTCACCCGGAGATTACAAAATTCGAGGGGAGCGTGGACGTGACCACGCGGCTGTTGTTG

GACAATGAAAATTTTACCAGTTTCGGGTGGTACCGCCTGCGACCCGGCACCCACGGAGAG

CGTGTTCAACTTCGCCCCGTTGAGCGACACGTCACCTCAAGCGACGTGGAGATTAACTGT

ACTCCCGATAACCTGGAGCCGATACCAGACGAGGCTGCCTGGCCCGACTATAAGCTCATG

TGCTTTGATATAGAGTGTAAAGCTGGAACGGGTAACGAAATGGCGTTCCCAGTGGCAACT

AACCAAGAGGACCTGGTCATCCAGATCTCCTGTCTGCTGTACTCGCTTGCTACTCAGAAC

CACGAACACACCCTGCTGTTTTCCCTCGGGTCATGCGATATCTCTGAGGAATACTCGTTT

GCATGCGTCCAGCGCGGCGAGCCCAGACCGACGGTTTTGGAGTTTGACAGCGAGTACGAG

CTGCTGGTTGCCTTCCTGACCTTTCTCAAGCAGTACTCTCCCGAGTTCGCCACCGGCTAC

AACATCGTTAATTTTGACTGGGCGTACATAGTTAACAAGGTAACGTCGGTGTATAACATC

AAGCTGGACGGGTACGGCAAGTTCAACAAAGGGGGGCTGTTTAAGGTGTGGGACATCGCC

ACGAACCATTTTCAGAAGAAGAGCAAGGTGAAAATCAATGGCCTGATATCTCTAGACATG

TATTCTGTGGCGACGGAAAAGCTAAAGCTACCCAGCTACAAACTCGACGCGGTCGTGGGA

GACGTCCTCGGCGAGCATAAGATAGACCTTCCCTATAAAGAAATACCCTCCTATTACGCG

GGAGGGCCTGACCGGAGGGGCGTAATAGGAGAGTATTGTATCCAGGACTCTAGGCTGGTG

GGCAAGCTGTTTTTTAAGTACCTCCCCCATCTGGAACTATCGGCGGTGGCCAAACTCGCC

CGTATCACCCTAACGCGGGTAATTTTTGACGGTCAGCAAATTCGCGTGTACACGTGCTTG

CTGAAACTCGCCCGCGAGAGAAATTTCATTTTGCCAGACAACAGACGCCGGTTTGACAGT

CAGGCAGACGCCGCGTCAGAGACTTCGGAGTTGGCTATGGATAGCCAAAGCCACGCCTTC

GACAGTACAGACGAACCCGACGGTGTGGACGGTACCCCGGACGCCGCAGGATCTGGCGCT

ACTTCTGAAAACGGAGGCGGGAAGCCCGGCGTCGGGAGGGCCGTGGGCTACCAGGGAGCA

AAGGTTCTAGACCCCGTATCCGGCTTTCATGTGGACCCCGTGGTTGTGTTTGACTTCGCT

AGCTTATACCCAAGCATTATCCAGGCCCATAACCTCTGTTTCACCACCCTGGCGCTCGAT

GAAGTGGATCTGGCCGGGCTTCAACCATCCGTCAACTACTCGACGTTCGAGGTGGGTGAC

CAAAAGTTATTTTTTGTCCACGCCCATATTCGCGAAAGCCTGCTTGGCATCTTGCTGCGC

GACTGGCTGGCCATGCGAAAGGCGGTGAGGGCGCGAATCCCCACCAGCACCCCCGAGGAG

GCAGTTTTACTAGATAAGCAGCAGTCTGCGATTAAGGTGATATGCAACTCGGTTTACGGA

TTCACGGGGGTGGCAAACGGCCTGTTGCCGTGTCTGAGGATAGCGGCTACCGTTACCACG

ATAGGACGCGACATGCTCCTCAAGACCCGAGATTACGTTCACTCTCGTTGGGCGACGCGC

GAGCTGCTGGAGGACAATTTTCCAGGGGCTATAGGTTTCCGAAACCACAAGCCTTACTCC

GTCAGGGTTATCTACGGAGACACCGACTCCGTGTTTATCAAGTTTGTGGGCCTGACGTAC

GAGGGGGTATCGGAGCTGGGGGATGCTATGTCGCGTCAGATTTCAGCGGACCTCTTTAGA

GCGCCCATCAAACTGGAGTGTGAGAAGACCTTTCAGCGACTGCTGCTGATCACCAAGAAG

AAGTACATAGGTGTCATAAACGGGGGGAAGATGCTCATGAAGGGGGTCGACCTGGTCCGC

AAAAATAACTGCTCTTTCATAAACTTGTACGCGCGACATCTGGTAGATCTTTTGTTGTAC

GACGAGGATGTGGCCACGGCGGCAGCAGAGGTGACAGACGTGCCTCCCGCAGAATGGGTG

GGGCGCCCGCTACCGAGCGGCTTTGACAAGTTTGGGCGAGTGCTGGTAGAGGCGTACAAC

CGTATCACTGCCCCCAACTTGGACGTGCGCGAGTTCGTTATGACTGCTGAGCTGAGCCGC

TCACCCGAATCGTATACCAACAAGCGCCTGCCGCACCTCACCGTCTACTTTAAGCTCGCC

ATGAGGAATGAAGAACTGCCCAGCGTAAAAGAGAGAATTCCGTATGTGATAGTTGCGCAG

ACCGAGGCCGCGGAACGCGAAGCGGGTGTAGTAAACTCAATGCGCGGTACCGCCCAAAAC

CCCGTGGTAACCAAGACCGCACGCCCCCAACCTAAACGCAAACTGCTGGTTTCCGACCTC

GCCGAAGACCCGACCTATGTTTCCGAGAATGACGTACCGCTAAACACAGACTACTATTTC

TCCCACCTGTTGGGTACCATAAGCGTGACCTTTAAGGCTCTATTCGGAAATGATGTGAGA

ACAACAGAAAATCTTTTAAAGCGGTTTATTCCGGAAACCCCCCACAAGACCCCCACGAAA

ACCCAGGCACTGCTTGAGCGCGCCGGCTTTGAAAAGCTGACGCCCTTTACACCGGAGGAA

GAAAGTCGTCGAATACTGCATACAGTTTTTTGTACTCTAGAAGCAGCTCCCCATCAAAGC

TGA

>OR085504.1

ATGGCGGCGCGCGAACAGGCCAACTCCGTTCGCAGGAGCGGGTTTTTTAATCCGTTTATT

GGCAAGAGGCCATTTTTCAGGCCGGGAAGCGGGCAAACCGCGGAGACAGAGCGCCCAAGG

CCCCCCCAACACTCGTACTGCACAGAGGTGGGTAGCTTTAAGTTTATAGCTCCAAGATGC

CTCGATGAAGAAGCCCCCGCCGACCAGCGACGCGGTGTACACGTGGGCACCTTGGAGCGC

CCACCCAAAGTGTACTGCGATGGCTCAGAGTACGACGTGCTGAACTTTGCCTCCGGAGGT

TGCTGGCCTCGCAGGATTCGGGTTTGGAACGGCCAGGATTTTCGGGGCGATGGATTCAAC

CCCAGATTTGAGAGATTTCACGTGTACGACATAGTAGAGACTTCGGAGAGCGCCTCACAC

GATGACCCATCCAGGTTTGCTGAGCTATCCCGCCCAAGCGGGTCTGTGGTAACACTGCTG

GGAATGAGCGAGTGTGGAAAGCGAGTCGCGGTTCACGTCTATGGTGTGCGCCATTATTTT

TACATGGCAAAGGCGGAGGTGGATAGCGCTTGTGGAATAACCACCGAGGCAGAACTCGTG

CGCGCAATGGTGGACTGCGCGCACAGCTCGGCTTTGAGCGCGGCCCTGGGAAATGGCAAC

GGCGGCAAACAGAGCGGCGGCAGCGGCGGGGGATGGTGGGGCGGAAAGCACGTGTCTGCG

GACTGCTTCAAAGTGGAGACCGTGTGCCACACGACGCTGTACTACTTTGGATCTAAGCCA

GCTCTCTACTATAGAGTATCTGCCTCCAGCAGCCGCCTGGGAGGGTTCATCTGCGACAAC

TTTCACCCGGAGATTACAAAATTCGAGGGGAGCGTGGACGTGACCACGCGGCTGTTGTTG

GACAATGAAAATTTTACCAGTTTCGGGTGGTACCGCCTGCGACCCGGCACCCACGGAGAG

CGTGTTCAACTTCGCCCCGTTGAGCGACACGTCACCTCAAGCGACGTGGAGATTAACTGT

ACTCCCGATAACCTGGAGCCGATACCAGACGAGGCTGCCTGGCCCGACTATAAGCTCATG

TGCTTTGATATAGAGTGTAAAGCTGGAACGGGTAACGAAATGGCGTTCCCAGTGGCAACT

AACCAAGAGGACCTGGTCATCCAGATCTCCTGTCTGCTGTACTCGCTTGCTACTCAGAAC

CACGAACACACCCTGCTGTTTTCCCTCGGGTCATGCGATATCTCTGAGGAATACTCGTTT

GCATGCGTCCAGCGCGGCGAGCCCAGACCGACGGTTTTGGAGTTTGACAGCGAGTACGAG

CTGCTGGTTGCCTTCCTGACCTTTCTCAAGCAGTACTCTCCCGAGTTCGCCACCGGCTAC

AACATCGTTAATTTTGACTGGGCGTACATAGTTAACAAGGTAACGTCGGTGTATAACATC

AAGCTGGACGGGTACGGCAAGTTCAACAAAGGGGGGCTGTTTAAGGTGTGGGACATCGCC

ACGAACCATTTTCAGAAGAAGAGCAAGGTGAAAATCAATGGCCTGATATCTCTAGACATG

TATTCTGTGGCGACGGAAAAGCTAAAGCTACCCAGCTACAAACTCGACGCGGTCGTGGGA

GACGTCCTCGGCGAGCATAAGATAGACCTTCCCTATAAAGAAATACCCTCCTATTACGCG

GGAGGGCCTGACCGGAGGGGCGTAATAGGAGAGTATTGTATCCAGGACTCTAGGCTGGTG

GGCAAGCTGTTTTTTAAGTACCTCCCCCATCTGGAACTATCGGCGGTGGCCAAACTCGCC

CGTATCACCCTAACGCGGGTAATTTTTGACGGTCAGCAAATTCGCGTGTACACGTGCTTG

CTGAAACTCGCCCGCGAGAGAAATTTCATTTTGCCAGACAACAGACGCCGGTTTGACAGT

CAGGCAGACGCCGCGTCAGAGACTTCGGAGTTGGCTATGGATAGCCAAAGCCACGCCTTC

GACAGTACAGACGAACCCGACGGTGTGGACGGTACCCCGGACGCCGCAGGATCTGGCGCT

ACTTCTGAAAACGGAGGCGGGAAGCCCGGCGTCGGGAGGGCCGTGGGCTACCAGGGAGCA

AAGGTTCTAGACCCCGTATCCGGCTTTCATGTGGACCCCGTGGTTGTGTTTGACTTCGCT

AGCTTATACCCAAGCATTATCCAGGCCCATAACCTCTGTTTCACCACCCTGGCGCTCGAT

GAAGTGGATCTGGCCGGGCTTCAACCATCCGTCAACTACTCGACGTTCGAGGTGGGTGAC

CAAAAGTTATTTTTTGTCCACGCCCATATTCGCGAAAGCCTGCTTGGCATCTTGCTGCGC

GACTGGCTGGCCATGCGAAAGGCGGTGAGGGCGCGAATCCCCACCAGCACCCCCGAGGAG

GCAGTTTTACTAGATAAGCAGCAGTCTGCGATTAAGGTGATATGCAACTCGGTTTACGGA

TTCACGGGGGTGGCAAACGGCCTGTTGCCGTGTCTGAGGATAGCGGCTACCGTTACCACG

ATAGGACGCGACATGCTCCTCAAGACCCGAGATTACGTTCACTCTCGTTGGGCGACGCGC

GAGCTGCTGGAGGACAATTTTCCAGGGGCTATAGGTTTCCGAAACCACAAGCCTTACTCC

GTCAGGGTTATCTACGGAGACACCGACTCCGTGTTTATCAAGTTTGTGGGCCTGACGTAC

GAGGGGGTATCGGAGCTGGGGGATGCTATGTCGCGTCAGATTTCAGCGGACCTCTTTAGA

GCGCCCATCAAACTGGAGTGTGAGAAGACCTTTCAGCGACTGCTTCTGATCACCAAGAAG

AAGTACATAGGTGTCATAAACGGGGGGAAGATGCTCATGAAGGGGGTCGACCTGGTCCGC

AAAAATAACTGCTCTTTCATAAACTTGTACGCGCGACATCTGGTAGATCTTTTGTTGTAC

GACGAGGATGTGGCCACGGCGGCAGCAGAGGTGACAGACGTGCCTCCCGCAGAATGGGTG

GGGCGCCCGCTACCGAGCGGCTTTGACAAGTTTGGGCGAGTGCTGGTAGAGGCGTACAAC

CGTATCACTGCCCCCAACTTGGACGTGCGCGAGTTCGTTATGACTGCTGAGCTGAGCCGC

TCACCCGAATCGTATACCAACAAGCGCCTGCCGCACCTCACCGTCTACTTTAAGCTCGCC

ATGAGGAATGAAGAACTGCCCAGCGTAAAAGAGAGAATTCCGTATGTGATAGTTGCGCAG

ACCGAGGCCGCGGAACGCGAAGCGGGTGTAGTAAACTCAATGCGCGGTACCGCCCAAAAC

CCCGTGGTAACCAAGACCGCACGCCCCCAACCTAAACGCAAACTGCTGGTTTCCGACCTC

GCCGAAGACCCGACCTATGTTTCCGAGAATGACGTACCGCTAAACACAGACTACTATTTC

TCCCACCTGTTGGGTACCATAAGCGTGACCTTTAAGGCTCTATTCGGAAATGATGTGAGA

ACAACAGAAAATCTTTTAAAGCGGTTTATTCCGGAAACCCCCCACAAGACCCCCACGAAA

ACCCAGGCACTGCTTGAGCGCGCCGGCTTTGAAAAGCTGACGCCCTTTACACCGGAGGAA

GAAAGTCGTCGAATACTGCATACAGTTTTTTGTACTCTAGAAGCAGCTCCCCATCAAAGC

TGA

>OR085505.1

ATGGCGGCGCGCGAACAGGCCAACTCCGTTCGCAGGAGCGGGTTTTTTAATCCGTTTATT

GGCAAGAGGCCATTTTTCAGGCCGGGAAGCGGGCAAACCGCGGAGACAGAGCGCCCAAGG

CCCCCCCAACACTCGTACTGCACAGAGGTGGGTAGCTTTAAGTTTATAGCTCCAAGATGC

CTCGATGAAGAAGCCCCCGCCGACCAGCGACGCGGTGTACACGTGGGCACCTTGGAGCGC

CCACCCAAAGTGTACTGCGATGGCTCAGAGTACGACGTGCTGAACTTTGCCTCCGGAGGT

TGCTGGCCTCGCAGGATTCGGGTTTGGAACGGCCAGGATTTTCGGGGCGATGGATTCAAC

CCCAGATTTGAGAGATTTCACGTGTACGACATAGTAGAGACTTCGGAGAGCGCCTCACAC

GATGACCCATCCAGGTTTGCTGAGCTATCCCGCCCAAGCGGGTCTGTGGTAACACTGCTG

GGAATGAGCGAGTGTGGAAAGCGAGTCGCGGTTCACGTCTATGGTGTGCGCCATTATTTT

TACATGGCAAAGGCGGAGGTGGATAGCGCTTGTGGAATAACCACCGAGGCAGAACTCGTG

CGCGCAATGGTGGACTGCGCGCACAGCTCGGCTTTGAGCGCGGCCCTGGGAAATGGCAAC

GGCGGCAAACAGAGCGGCGGCAGCGGCGGGGGATGGTGGGGCGGAAAGCACGTGTCTGCG

GACTGCTTCAAAGTGGAGACCGTGTGCCACACGACGCTGTACTACTTTGGATCTAAGCCA

GCTCTCTACTATAGAGTATCTGCCTCCAGCAGCCGCCTGGGAGGGTTCATCTGCGACAAC

TTTCACCCGGAGATTACAAAATTCGAGGGGAGCGTGGACGTGACCACGCGGCTGTTGTTG

GACAATGAAAATTTTACCAGTTTCGGGTGGTACCGCCTGCGACCCGGCACCCACGGAGAG

CGTGTTCAACTTCGCCCCGTTGAGCGACACGTCACCTCAAGCGACGTGGAGATTAACTGT

ACTCCCGATAACCTGGAGCCGATACCAGACGAGGCTGCCTGGCCCGACTATAAGCTCATG

TGCTTTGATATAGAGTGTAAAGCTGGAACGGGTAACGAAATGGCGTTCCCAGTGGCAACT

AACCAAGAGGACCTGGTCATCCAGATCTCCTGTCTGCTGTACTCGCTTGCTACTCAGAAC

CACGAACACACCCTGCTGTTTTCCCTCGGGTCATGCGATATCTCTGAGGAATACTCGTTT

GCATGCGTCCAGCGCGGCGAGCCCAGACCGACGGTTTTGGAGTTTGACAGCGAGTACGAG

CTGCTGGTTGCCTTCCTGACCTTTCTCAAGCAGTACTCTCCCGAGTTCGCCACCGGCTAC

AACATCGTTAATTTTGACTGGGCGTACATAGTTAACAAGGTAACGTCGGTGTATAACATC

AAGCTGGACGGGTACGGCAAGTTCAACAAAGGGGGGCTGTTTAAGGTGTGGGACATCGCC

ACGAACCATTTTCAGAAGAAGAGCAAGGTGAAAATCAATGGCCTGATATCTCTAGACATG

TATTCTGTGGCGACGGAAAAGCTAAAGCTACCCAGCTACAAACTCGACGCGGTCGTGGGA

GACGTCCTCGGCGAGCATAAGATAGACCTTCCCTATAAAGAAATACCCTCCTATTACGCG

GGAGGGCCTGACCGGAGGGGCGTAATAGGAGAGTATTGTATCCAGGACTCTAGGCTGGTG

GGCAAGCTGTTTTTTAAGTACCTCCCCCATCTGGAACTATCGGCGGTGGCCAAACTCGCC

CGTATCACCCTAACGCGGGTAATTTTTGACGGTCAGCAAATTCGCGTGTACACGTGCTTG

CTGAAACTCGCCCGCGAGAGAAATTTCATTTTGCCAGACAACAGACGCCGGTTTGACAGT

CAGGCAGACGCCGCGTCAGAGACTTCGGAGTTGGCTATGGATAGCCAAAGCCACGCCTTC

GACAGTACAGACGAACCCGACGGTGTGGACGGTACCCCGGACGCCGCAGGATCTGGCGCT

ACTTCTGAAAACGGAGGCGGGAAGCCCGGCGTCGGGAGGGCCGTGGGCTACCAGGGAGCA

AAGGTTCTAGACCCCGTATCCGGCTTTCATGTGGACCCCGTGGTTGTGTTTGACTTCGCT

AGCTTATACCCAAGCATTATCCAGGCCCATAACCTCTGTTTCACCACCCTGGCGCTCGAT

GAAGTGGATCTGGCCGGGCTTCAACCATCCGTCAACTACTCGACGTTCGAGGTGGGTGAC

CAAAAGTTATTTTTTGTCCACGCCCATATTCGCGAAAGCCTGCTTGGCATCTTGCTGCGC

GACTGGCTGGCCATGCGAAAGGCGGTGAGGGCGCGAATCCCCACCAGCACCCCCGAGGAG

GCAGTTTTACTAGATAAGCAGCAGTCTGCGATTAAGGTGATATGCAACTCGGTTTACGGA

TTCACGGGGGTGGCAAACGGCCTGTTGCCGTGTCTGAGGATAGCGGCTACCGTTACCACG

ATAGGACGCGACATGCTCCTCAAGACCCGAGATTACGTTCACTCTCGTTGGGCGACGCGC

GAGCTGCTGGAGGACAATTTTCCAGGGGCTATAGGTTTCCGAAACCACAAGCCTTACTCC

GTCAGGGTTATCTACGGAGACACCGACTCCGTGTTTATCAAGTTTGTGGGCCTGACGTAC

GAGGGGGTATCGGAGCTGGGGGATGCTATGTCGCGTCAGATTTCAGCGGACCTCTTTAGA

GCGCCCATCAAACTGGAGTGTGAGAAGACCTTTCAGCGACTGCTGCTGATCACCAAGAAG

AAGTACATAGGTGTCATAAACGGGGGGAAGATGCTCATGAAGGGGGTCGACCTAGTCCGC

AAAAATAACTGCTCTTTCATAAACTTGTACGCGCGACATCTGGTAGATCTTTTGTTGTAC

GACGAGGATGTGGCCACGGCGGCAGCAGAGGTGACAGACGTGCCTCCCGCAGAATGGGTG

GGGCGCCCGCTACCGAGCGGCTTTGACAAGTTTGGGCGAGTGCTGGTAGAGGCGTACAAC

CGTATCACTGCCCCCAACTTGGACGTGCGCGAGTTCGTTATGACTGCTGAGCTGAGCCGC

TCACCCGAATCGTATACCAACAAGCGCCTGCCGCACCTCACCGTCTACTTTAAGCTCGCC

ATGAGGAATGAAGAACTGCCCAGCGTAAAAGAGAGAATTCCGTATGTGATAGTTGCGCAG

ACCGAGGCCGCGGAACGCGAAGCGGGTGTAGTAAACTCAATGCGCGGTACCGCCCAAAAC

CCCGTGGTAACCAAGACCGCACGCCCCCAACCTAAACGCAAACTGCTGGTTTCCGACCTC

GCCGAAGACCCGACCTATGTTTCCGAGAATGACGTACCGCTAAACACAGACTACTATTTC

TCCCACCTGTTGGGTACCATAAGCGTGACCTTTAAGGCTCTATTCGGAAATGATGTGAGA

ACAACAGAAAATCTTTTAAAGCGGTTTATTCCGGAAACCCCCCACAAGACCCCCACGAAA

ACCCAGGCACTGCTTGAGCGCGCCGGCTTTGAAAAGCTGACGCCCTTTACACCGGAGGAA

GAAAGTCGTCGAATACTGCATACAGTTTTTTGTACTCTAGAAGCAGCTCCCCATCAAAGC

TGA

>OR085506.1

ATGGCGGCGCGCGAACAGGCCAACTCCGTTCGCAGGAGCGGGTTTTTTAATCCGTTTATT

GGCAAGAGGCCATTTTTCAGGCCGGGAAGCGGGCAGACCGCGGAGACAGAGCGCCCAAGG

CCCCCCCAACACTCGTACTGCACAGAGGTGGGTAGCTTTAAGTTTATAGCTCCAAGATGC

CTCGATGAAGAAGCCCCCGCCGACCAGCGACGCGGTGTACACGTGGGCACCTTGGAGCGC

CCACCCAAAGTGTACTGCGATGGCTCAGAGTACGACGTGCTGAACTTTGCCTCCGGAGGT

TGCTGGCCTCGCAGGATTCGGGTTTGGAACGGCCAGGATTTTCGGGGCGATGGATTCAAC

CCCAGATTTGAGAGATTTCACGTGTACGACATAGTAGAGACTTCGGAGAGCGCCTCACAC

GATGACCCATCCAGGTTTGCTGAGCTATCCCGCCCAAGCGGGTCTGTGGTAACACTGCTG

GGAATGAGCGAGTGTGGAAAGCGAGTCGCGGTTCACGTCTATGGTGTGCGCCATTATTTT

TACATGGCAAAGGCGGAGGTGGATAGCGCTTGTGGAATAACCACCGAGGCAGAACTCGTG

CGCGCAATGGTGGACTGCGCGCACAGCTCGGCTTTGAGCGCGGCCCTGGGAAATGGCAAC

GGCGGCAAACAGAGCGGCGGCAGCGGCGGGGGATGGTGGGGCGGAAAGCACGTGTCTGCG

GACTGCTTCAAAGTGGAGACCGTGTGCCACACGACGCTGTACTACTTTGGATCTAAGCCA

GCTCTCTACTATAGAGTATCTGCCTCCAGCAGCCGCCTGGGAGGGTTCATCTGCGACAAC

TTTCACCCGGAGATTACAAAATTCGAGGGGAGCGTGGACGTGACCACGCGGCTGTTGTTG

GACAATGAAAATTTTACCAGTTTCGGGTGGTACCGCCTGCGACCCGGCACCCACGGAGAG

CGTGTTCAACTTCGCCCCGTTGAGCGACACGTCACCTCAAGCGACGTGGAGATTAACTGT

ACTCCCGATAACCTGGAGCCGATACCAGACGAGGCTGCCTGGCCCGACTATAAGCTCATG

TGCTTTGATATAGAGTGTAAAGCTGGAACGGGTAACGAAATGGCGTTCCCAGTGGCAACT

AACCAAGAGGACCTGGTCATCCAGATCTCCTGTCTGCTGTACTCGCTTGCTACTCAGAAC

CACGAACACACCCTGCTGTTTTCCCTCGGGTCATGCGATATCTCTGAGGAATACTCGTTT

GCATGCGTCCAGCGCGGCGAGCCCAAACCGACGGTTTTGGAGTTTGACAGCGAGTACGAG

CTGCTGGTTGCCTTCCTGACCTTTCTCAAGCAGTACTCTCCCGAGTTCGCCACCGGCTAC

AACATCGTTAATTTTGACTGGGCGTACATAGTTAACAAGGTAACGTCGGTGTATAACATC

AAGCTGGACGGGTACGGCAAGTTCAACAAAGGGGGGCTGTTTAAGGTGTGGGACATCGCC

ACGAACCATTTTCAGAAGAAGAGCAAGGTGAAAATCAATGGCCTGATATCTCTAGACATG

TATTCTGTGGCGACGGAAAAGCTAAAGCTACCCAGCTACAAACTCGACGCGGTCGTGGGA

GACGTCCTCGGCGAGCATAAGATAGACCTTCCCTATAAAGAAATACCCTCCTATTACGCG

GGAGGGCCTGACCGGAGGGGCGTAATAGGAGAGTATTGTATCCAGGACTCTAGGCTGGTG

GGCAAGCTGTTTTTTAAGTACCTCCCCCATCTGGAACTATCGGCGGTGGCCAAACTCGCC

CGTATCACCCTAACGCGGGTAATTTTTGACGGTCAGCAAATTCGCGTGTACACGTGCTTG

CTGAAACTCGCCCGCGAGAGAAATTTCATTTTGCCAGACAACAGACGCCGGTTTGACAGT

CAGGCAGACGCCGCGTCAGAGACTTCGGAGTTGGCTATGGATAGCCAAAGCCACGCCTTC

GACAGTACAGACGAACCCGACGGTGTGGACGGTACCCCGGACGCCGCAGGATCTGGCGCT

ACTTCTGAAAACGGAGGCGGGAAGCCCGGCGTCGGGAGGGCCGTGGGCTACCAGGGAGCA

AAGGTTCTAGACCCCGTATCCGGCTTTCATGTGGACCCCGTGGTTGTGTTTGACTTCGCT

AGCTTATACCCAAGCATTATCCAGGCCCATAACCTCTGTTTCACCACCCTGGCGCTCGAT

GAAGTGGATCTGGCCGGGCTTCAACCATCCGTCAACTACTCGACGTTCGAGGTGGGTGAC

CAAAAGTTATTTTTTGTCCACGCCCATATTCGCGAAAGCCTGCTTGGCATCTTGCTGCGC

GACTGGCTGGCCATGCGAAAGGCGGTGAGGGCGCGAATCCCCACCAGCACCCCCGAGGAG

GCAGTTTTACTAGATAAGCAGCAGTCTGCGATTAAGGTGATATGCAACTCGGTTTACGGA

TTCACGGGGGTGGCAAACGGCCTGTTGCCGTGTCTGAGGATAGCGGCTACCGTTACCACG

ATAGGACGCGACATGCTCCTCAAGACCCGAGATTACGTTCACTCTCGTTGGGCGACGCGC

GAGCTGCTGGAGGACAATTTTCCAGGGGCTATAGGTTTCCGAAACCACAAGCCTTACTCC

GTCAGGGTTATCTACGGAGACACCGACTCCGTGTTTATCAAGTTTGTGGGCCTGACGTAC

GAGGGGGTATCGGAGCTGGGGGATGCTATGTCGCGTCAGATTTCAGCGGACCTCTTTAGA

GCGCCCATCAAACTGGAGTGTGAGAAGACCTTTCAGCGACTGCTGCTGATCACCAAGAAG

AAGTACATAGGTGTCATAAACGGGGGGAAGATGCTCATGAAGGGGGTCGACCTGGTCCGC

AAAAATAACTGCTCTTTCATAAACTTGTACGCGCGACATCTGGTAGATCTTTTGTTGTAC

GACGAGGATGTGGCCACGGCGGCAGCAGAGGTGACAGACGTGCCTCCCGCAGAATGGGTG

GGGCGCCCGCTACCGAGCGGCTTTGACAAGTTTGGGCGAGTGCTGGTAGAGGCGTACAAC

CGTATCACTGCCCCCAACTTGGACGTGCGCGAGTTCGTTATGACTGCTGAGCTGAGCCGC

TCACCCGAATCGTATACCAACAAGCGCCTGCCGCACCTCACCGTCTACTTTAAGCTCGCC

ATGAGGAATGAAGAACTGCCCAGCGTAAAAGAGAGAATTCCGTATGTGATAGTTGCGCAG

ACCGAGGCCGCGGAACGCGAAGCGGGTGTAGTAAACTCAATGCGCGGTACCGCCCAAAAC

CCCGTGGTAACCAAGACCGCACGCCCCCAACCTAAACGCAAACTGCTGGTTTCCGACCTC

GCCGAAGACCCGACCTATGTTTCCGAGAATGACGTACCGCTAAACACAGACTACTATTTC

TCCCACCTGTTGGGTACCATAAGCGTGACCTTTAAGGCTCTATTCGGAAATGATGTGAGA

ACAACAGAAAATCTTTTAAAGCGGTTTATTCCGGAAACCCCCCACAAGACCCCCACGAAA

ACCCAGGCACTGCTTGAGCGCGCCGGCTTTGAAAAGCTGACGCCCTTTACACCGGAGGAA

GAAAGTCGTCGAATACTGCATACAGTTTTTTGTACTCTAGAAGCAGCTCCCCATCAAAGC

TGA

>OR085507.1

ATGGCGGCGCGCGAACAGGCCAACTCCGTTCGCAGGAGCGGGTTTTTTAATCCGTTTATT

GGCAAGAGGCCATTTTTCAGGCCGGGAAGCGGGCAGACCGCGGAGACAGAGCGCCCAAGG

CCCCCCCAACACTCGTACTGCACAGAGGTGGGTAGCTTTAAGTTTATAGCTCCAAGATGC

CTCGATGAAGAAGCCCCCGCCGACCAGCGACGCGGTGTACACGTGGGCACCTTGGAGCGC

CCACCCAAAGTGTACTGCGATGGCTCAGAGTACGACGTGCTGAACTTTGCCTCCGGAGGT

TGCTGGCCTCGCAGGATTCGGGTTTGGAACGGCCAGGATTTTCGGGGCGATGGATTCAAC

CCCAGATTTGAGAGATTTCACGTGTACGACATAGTAGAGACTTCGGAGAGCGCCTCACAC

GATGACCCATCCAGGTTTGCTGAGCTATCCCGCCCAAGCGGGTCTGTGGTAACACTGCTG

GGAATGAGCGAGTGTGGAAAGCGAGTCGCGGTTCACGTCTATGGTGTGCGCCATTATTTT

TACATGGCAAAGGCGGAGGTGGATAGCGCTTGTGGAATAACCACCGAGGCAGAACTCGTG

CGCGCAATGGTGGACTGCGCGCACAGCTCGGCTTTGAGCGCGGCCCTGGGAAATGGCAAC

GGCGGCAAACAGAGCGGCGGCAGCGGCGGGGGATGGTGGGGCGGAAAGCACGTGTCTGCG

GACTGCTTCAAAGTGGAGACCGTGTGCCACACGACGCTGTACTACTTTGGATCTAAGCCA

GCTCTCTACTATAGAGTATCTGCCTCCAGCAGCCGCCTGGGAGGGTTCATCTGCGACAAC

TTTCACCCGGAGATTACAAAATTCGAGGGGAGCGTGGACGTGACCACGCGGCTGTTGTTG

GACAATGAAAATTTTACCAGTTTCGGGTGGTACCGCCTGCGACCCGGCACCCACGGAGAG

CGTGTTCAACTTCGCCCCGTTGAGCGACACGTCACCTCAAGCGACGTGGAGATTAACTGT

ACTCCCGATAACCTGGAGCCGATACCAGACGAGGCTGCCTGGCCCGACTATAAGCTCATG

TGCTTTGATATAGAGTGTAAAGCTGGAACGGGTAACGAAATGGCGTTCCCAGTGGCAACT

AACCAAGAGGACCTGGTCATCCAGATCTCCTGTCTGCTGTACTCGCTTGCTACTCAGAAC

CACGAACACACCCTGCTGTTTTCCCTCGGGTCATGCGATATCTCTGAGGAATACTCGTTT

GCATGCGTCCAGCGCGGCGAGCCCAGACCGACGGTTTTGGAGTTTGACAGCGAGTACGAG

CTGCTGGTTGCCTTCCTGACCTTTCTCAAGCAGTACTCTCCCGAGTTCGCCACCGGCTAC

AACATCGTTAATTTTGACTGGGCGTACATAGTTAACAAGGTAACGTCGGTGTATAACATC

AAGCTGGACGGGTACGGCAAGTTCAACAAAGGGGGGCTGTTTAAGGTGTGGGACATCGCC

ACGAACCATTTTCAGAAGAAGAGCAAGGTGAAAATCAATGGCCTGATATCTCTAGACATG

TATTCTGTGGCGACGGAAAAGCTAAAGCTACCCAGCTACAAACTCGACGCGGTCGTGGGA

GACGTCCTCGGCGAGCATAAGATAGACCTTCCCTATAAAGAAATACCCTCCTATTACGCG

GGAGGGCCTGACCGGAGGGGCGTAATAGGAGAGTATTGTATCCAGGACTCTAGGCTGGTG

GGCAAGCTGTTTTTTAAGTACCTCCCCCATCTGGAACTATCGGCGGTGGCCAAACTCGCC

CGTATCACCCTAACGCGGGTAATTTTTGACGGTCAGCAAATTCGCGTGTACACGTGCTTG

CTGAAACTCGCCCGCGAGAGAAATTTCATTTTGCCAGACAACAGACGCCGGTTTGACAGT

CAGGCAGACGCCGCGTCAGAGACTTCGGAGTTGGCTATGGATAGCCAAAGCCACGCCTTC

GACAGTACAGACGAACCCGACGGTGTGGACGGTACCCCGGACGCCGCAGGATCTGGCGCT

ACTTCTGAAAACGGAGGCGGGAAGCCCGGCGTCGGGAGGGCCGTGGGCTACCAGGGAGCA

AAGGTTCTAGACCCCGTATCCGGCTTTCATGTGGACCCCGTGGTTGTGTTTGACTTCGCT

AGCTTATACCCAAGCATTATCCAGGCCCATAACCTCTGTTTCACCACCCTGGCGCTCGAT

GAAGTGGATCTGGCCGGGCTTCAACCATCCGTCAACTACTCGACGTTCGAGGTGGGTGAC

CAAAAGTTATTTTTTGTCCACGCCCATATTCGCGAAAGCCTGCTTGGCATCTTGCTGCGC

GACTGGCTGGCCATGCGAAAGGCGGTGAGGGCGCGAATCCCCACCAGCACCCCCGAGGAG

GCAGTTTTACTAGATAAGCAGCAGTCTGCGATTAAGGTGATATGCAACTCGGTTTACGGA

TTCACGGGGGTGGCAAACGGCCTGTTGCCGTGTCTGAGGATAGCGGCTACCGTTACCACG

ATAGGACGCGACATGCTCCTCAAGACCCGAGATTACGTTCACTCTCGTTGGGCGACGCGC

GAGCTGCTGGAGGACAATTTTCCAGGGGCTATAGGTTTCCGAAACCACAAGCCTTACTCC

GTCAGGGTTATCTACGGAGACACCGACTCCGTGTTTATCAAGTTTGTGGGCCTGACGTAC

GAGGGGGTATCGGAGCTGGGGGATGCTATGTCGCGTCAGATTTCAGCGGACCTCTTTAGA

GCGCCCATCAAACTGGAGTGTGAGAAGACCTTTCAGCGACTGCTGCTGATCACCAAGAAG

AAGTACATAGGTGTCATAAACGGGGGGAAGATGCTCATGAAGGGGGTCGACCTGGTCCGC

AAAAATAACTGCTCTTTCATAAACTTGTACGCGCGACATCTGGTAGATCTTTTGTTGTAC

GACGAGGATGTGGCCACGGCGGCAGCAGAGGTGACAGACGTGCCTCCCGCAGAATGGGTG

GGGCGCCCGCTACCGAGCGGCTTTGACAAGTTTGGGCGAGTGCTGGTAGAGGCGTACAAC

CGTATCACTGCCCCCAACTTGGACGTGCGCGAGTTCGTTATGACTGCTGAGCTGAGCCGC

TCACCCGAATCGTATACCAACAAGCGCCTGCCGCACCTCACCGTCTACTTTAAGCTCGCC

ATGAGGAATGAAGAACTGCCCAGCGTAAAAGAGAGAATTCCGTATGTGATAGTTGCGCAG

ACCGAGGCCGCGGAACGCGAAGCGGGTGTAGTAAACTCAATGCGCGGTACCGCCCAAAAC

CCCGTGGTAACCAAGACCGCACGCCCCCAACCTAAACGCAAACTGCTGGTTTCCGACCTC

GCCGAAGACCCGACCTATGTTTCCGAGAATGACGTACCGCTAAACACAGACTACTATTTC

TCCCACCTGTTGGGTACCATAAGCGTGACCTTTAAGGCTCTATTCGGAAATGATGTGAGA

ACAACAGAAAATCTTTTAAAGCGGTTTATTCCGGAAACCCCCCACAAGACCCCCACGAAA

ACCCAGGCACTGCTTGAGCGCGCCGGCTTTGAAAAGCTGACGCCCTTTACACCGGAGGAA

GAAAGTCGTCGAATACTGCATACAGTTTTTTGTACTCTAGAAGCAGCTCCCCATCAAAGC

TGA

>OR085508.1

ATGGCGGCGCGCGAACAGGCCAACTCCGTTCGCAGGAGCGGGTTTTTTAATCCGTTTATT

GGCAAGAGGCCATTTTTCAGGCCGGGAAGCGGGCAGACCGCGGAGACAGAGCGCCCAAGG

CCCCCCCAACACTCGTACTGCACAGAGGTGGGTAGCTTTAAGTTTATAGCTCCAAGATGC

CTCGATGAAGAAGCCCCCGCCGACCAGCGACGCGGTGTACACGTGGGCACCTTGGAGCGC

CCACCCAAAGTGTACTGCGATGGCTCAGAGTACGACGTGCTGAACTTTGCCTCCGGAGGT

TGCTGGCCTCGCAGGATTCGGGTTTGGAACGGCCAGGATTTTCGGGGCGATGGATTCAAC

CCCAGATTTGAGAGATTTCACGTGTACGACATAGTAGAGACTTCGGAGAGCGCCTCACAC

GATGACCCATCCAGGTTTGCTGAGCTATCCCGCCCAAGCGGGTCTGTGGTAACACTGCTG

GGAATGAGCGAGTGTGGAAAGCGAGTCGCGGTTCACGTCTATGGTGTGCGCCATTATTTT

TACATGGCAAAGGCGGAGGTGGATAGCGCTTGTGGAATAACCACCGAGGCAGAACTCGTG

CGCGCAATGGTGGACTGCGCGCACAGCTCGGCTTTGAGCGCGGCCCTGGGAAATGGCAAC

GGCGGCAAACAGAGCGGCGGCAGCGGCGGGGGATGGTGGGGCGGAAAGCACGTGTCTGCG

GACTGCTTCAAAGTGGAGACCGTGTGCCACACGACGCTGTACTACTTTGGATCTAAGCCA

GCTCTCTACTATAGAGTATCTGCCTCCAGCAGCCGCCTGGGAGGGTTCATCTGCGACAAC

TTTCACCCGGAGATTACAAAATTCGAGGGGAGCGTGGACGTGACCACGCGGCTGTTGTTG

GACAATGAAAATTTTACCAGTTTCGGGTGGTACCGCCTGCGACCCGGCACCCACGGAGAG

CGTGTTCAACTTCGCCCCGTTGAGCGACACGTCACCTCAAGCGACGTGGAGATTAACTGT

ACTCCCGATAACCTGGAGCCGATACCAGACGAGGCTGCCTGGCCCGACTATAAGCTCATG

TGCTTTGATATAGAGTGTAAAGCTGGAACGGGTAACGAAATGGCGTTCCCAGTGGCAACT

AACCAAGAGGACCTGGTCATCCAGATCTCCTGTCTGCTGTACTCGCTTGCTACTCAGAAC

CACGAACACACCCTGCTGTTTTCCCTCGGGTCATGCGATATCTCTGAGGAATACTCGTTT

GCATGCGTCCAGCGCGGCGAGCCCAAACCGACGGTTTTGGAGTTTGACAGCGAGTACGAG

CTGCTGGTTGCCTTCCTGACCTTTCTCAAGCAGTACTCTCCCGAGTTCGCCACCGGCTAC

AACATCGTTAATTTTGACTGGGCGTACATAGTTAACAAGGTAACGTCGGTGTATAACATC

AAGCTGGACGGGTACGGCAAGTTCAACAAAGGGGGGCTGTTTAAGGTGTGGGACATCGCC

ACGAACCATTTTCAGAAGAAGAGCAAGGTGAAAATCAATGGCCTGATATCTCTAGACATG

TATTCTGTGGCGACGGAAAAGCTAAAGCTACCCAGCTACAAACTCGACGCGGTCGTGGGA

GACGTCCTCGGCGAGCATAAGATAGACCTTCCCTATAAAGAAATACCCTCCTATTACGCG

GGAGGGCCTGACCGGAGGGGCGTAATAGGAGAGTATTGTATCCAGGACTCTAGGCTGGTG

GGCAAGCTGTTTTTTAAGTACCTCCCCCATCTGGAACTATCGGCGGTGGCCAAACTCGCC

CGTATCACCCTAACGCGGGTAATTTTTGACGGTCAGCAAATTCGCGTGTACACGTGCTTG

CTGAAACTCGCCCGCGAGAGAAATTTCATTTTGCCAGACAACAGACGCCGGTTTGACAGT

CAGGCAGACGCCGCGTCAGAGACTTCGGAGTTGGCTATGGATAGCCAAAGCCACGCCTTC

GACAGTACAGACGAACCCGACGGTGTGGACGGTACCCCGGACGCCGCAGGATCTGGCGCT

ACTTCTGAAAACGGAGGCGGGAAGCCCGGCGTCGGGAGGGCCGTGGGCTACCAGGGAGCA

AAGGTTCTAGACCCCGTATCCGGCTTTCATGTGGACCCCGTGGTTGTGTTTGACTTCGCT

AGCTTATACCCAAGCATTATCCAGGCCCATAACCTCTGTTTCACCACCCTGGCGCTCGAT

GAAGTGGATCTGGCCGGGCTTCAACCATCCGTCAACTACTCGACGTTCGAGGTGGGTGAC

CAAAAGTTATTTTTTGTCCACGCCCATATTCGCGAAAGCCTGCTTGGCATCTTGCTGCGC

GACTGGCTGGCCATGCGAAAGGCGGTGAGGGCGCGAATCCCCACCAGCACCCCCGAGGAG

GCAGTTTTACTAGATAAGCAGCAGTCTGCGATTAAGGTGATATGCAACTCGGTTTACGGA

TTCACGGGGGTGGCAAACGGCCTGTTGCCGTGTCTGAGGATAGCGGCTACCGTTACCACG

ATAGGACGCGACATGCTCCTCAAGACCCGAGATTACGTTCACTCTCGTTGGGCGACGCGC

GAGCTGCTGGAGGACAATTTTCCAGGGGCTATAGGTTTCCGAAACCACAAGCCTTACTCC

GTCAGGGTTATCTACGGAGACACCGACTCCGTGTTTATCAAGTTTGTGGGCCTGACGTAC

GAGGGGGTATCGGAGCTGGGGGATGCTATGTCGCGTCAGATTTCAGCGGACCTCTTTAGA

GCGCCCATCAAACTGGAGTGTGAGAAGACCTTTCAGCGACTGCTGCTGATCACCAAGAAG

AAGTACATAGGTGTCATAAACGGGGGGAAGATGCTCATGAAGGGGGTCGACCTGGTCCGC

AAAAATAACTGCTCTTTCATAAACTTGTACGCGCGACATCTGGTAGATCTTTTGTTGTAC

GACGAGGATGTGGCCACGGCGGCAGCAGAGGTGACAGACGTGCCTCCCGCAGAATGGGTG

GGGCGCCCGCTACCGAGCGGCTTTGACAAGTTTGGGCGAGTGCTGGTAGAGGCGTACAAC

CGTATCACTGCCCCCAACTTGGACGTGCGCGAGTTCGTTATGACTGCTGAGCTGAGCCGC

TCACCCGAATCGTATACCAACAAGCGCCTGCCGCACCTCACCGTCTACTTTAAGCTCGCC

ATGAGGAATGAAGAACTGCCCAGCGTAAAAGAGAGAATTCCGTATGTGATAGTTGCGCAG

ACCGAGGCCGCGGAACGCGAAGCGGGTGTAGTAAACTCAATGCGCGGTACCGCCCAAAAC

CCCGTGGTAACCAAGACCGCACGCCCCCAACCTAAACGCAAACTGCTGGTTTCCGACCTC

GCCGAAGACCCGACCTATGTTTCCGAGAATGACGTACCGCTAAACACAGACTACTATTTC

TCCCACCTGTTGGGTACCATAAGCGTGACCTTTAAGGCTCTATTCGGAAATGATGTGAGA

ACAACAGAAAATCTTTTAAAGCGGTTTATTCCGGAAACCCCCCACAAGACCCCCACGAAA

ACCCAGGCACTGCTTGAGCGCGCCGGCTTTGAAAAGCTGACGCCCTTTACACCGGAGGAA

GAAAGTCGTCGAATACTGCATACAGTTTTTTGTACTCTAGAAGCAGCTCCCCATCAAAGC

TGA

>OR085509.1

ATGGCGGCGCGCGAACAGGCCAACTCCGTTCGCAGGAGCGGGTTTTTTAATCCGTTTATT

GGCAAGAGGCCATTTTTCAGGCCGGGAAGCGGGCAGACCGCGGAGACAGAGCGCCCAAGG

CCCCCCCAACACTCGTACTGCACAGAGGTGGGTAGCTTTAAGTTTATAGCTCCAAGATGC

CTCGATGAAGAAGCCCCCGCCGACCAGCGACGCGGTGTACACGTGGGCACCTTGGAGCGC

CCACCCAAAGTGTACTGCGATGGCTCAGAGTACGACGTGCTGAACTTTGCCTCCGGAGGT

TGCTGGCCTCGCAGGATTCGGGTTTGGAACGGCCAGGATTTTCGGGGCGATGGATTCAAC

CCCAGATTTGAGAGATTTCACGTGTACGACATAGTAGAGACTTCGGAGAGCGCCTCACAC

GATGACCCATCCAGGTTTGCTGAGCTATCCCGCCCAAGCGGGTCTGTGGTAACACTGCTG

GGAATGAGCGAGTGTGGAAAGCGAGTCGCGGTTCACGTCTATGGTGTGCGCCATTATTTT

TACATGGCAAAGGCGGAGGTGGATAGCGCTTGTGGAATAACCACCGAGGCAGAACTCGTG

CGCGCAATGGTGGACTGCGCGCACAGCTCGGCTTTGAGCGCGGCCCTGGGAAATGGCAAC

GGCGGCAAACAGAGCGGCGGCAGCGGCGGGGGATGGTGGGGCGGAAAGCACGTGTCTGCG

GACTGCTTCAAAGTGGAGACCGTGTGCCACACGACGCTGTACTACTTTGGATCTAAGCCA

GCTCTCTACTATAGAGTATCTGCCTCCAGCAGCCGCCTGGGAGGGTTCATCTGCGACAAC

TTTCACCCGGAGATTACAAAATTCGAGGGGAGCGTGGACGTGACCACGCGGCTGTTGTTG

GACAATGAAAATTTTACCAGTTTCGGGTGGTACCGCCTGCGACCCGGCACCCACGGAGAG

CGTGTTCAACTTCGCCCCGTTGAGCGACACGTCACCTCAAGCGACGTGGAGATTAACTGT

ACTCCCGATAACCTGGAGCCGATACCAGACGAGGCTGCCTGGCCCGACTATAAGCTCATG

TGCTTTGATATAGAGTGTAAAGCTGGAACGGGTAACGAAATGGCGTTCCCAGTGGCAACT

AACCAAGAGGACCTGGTCATCCAGATCTCCTGTCTGCTGTACTCGCTTGCTACTCAGAAC

CACGAACACACCCTGCTGTTTTCCCTCGGGTCATGCGATATCTCTGAGGAATACTCGTTT

GCATGCGTCCAGCGCGGCGAGCCCAGACCGACGGTTTTGGAGTTTGACAGCGAGTACGAG

CTGCTGGTTGCCTTCCTGACCTTTCTCAAGCAGTACTCTCCCGAGTTCGCCACCGGCTAC

AACATCGTTAATTTTGACTGGGCGTACATAGTTAACAAGGTAACGTCGGTGTATAACATC

AAGCTGGACGGGTACGGCAAGTTCAACAAAGGGGGGCTGTTTAAGGTGTGGGACATCGCC

ACGAACCATTTTCAGAAGAAGAGCAAGGTGAAAATCAATGGCCTGATATCTCTAGACATG

TATTCTGTGGCGACGGAAAAGCTAAAGCTACCCAGCTACAAACTCGACGCGGTCGTGGGA

GACGTCCTCGGCGAGCATAAGATAGACCTTCCCTATAAAGAAATACCCTCCTATTACGCG

GGAGGGCCTGACCGGAGGGGCGTAATAGGAGAGTATTGTATCCAGGACTCTAGGCTGGTG

GGCAAGCTGTTTTTTAAGTACCTCCCCCATCTGGAACTATCGGCGGTGGCCAAACTCGCC

CGTATCACCCTAACGCGGGTAATTTTTGACGGTCAGCAAATTCGCGTGTACACGTGCTTG

CTGAAACTCGCCCGCGAGAGAAATTTCATTTTGCCAGACAACAGACGCCGGTTTGACAGT

CAGGCAGACGCCGCGTCAGAGACTTCGGAGTTGGCTATGGATAGCCAAAGCCACGCCTTC

GACAGTACAGACGAACCCGACGGTGTGGACGGTACCCCGGACGCCGCAGGATCTGGCGCT

ACTTCTGAAAACGGAGGCGGGAAGCCCGGCGTCGGGAGGGCCGTGGGCTACCAGGGAGCA

AAGGTTCTAGACCCCGTATCCGGCTTTCATGTGGACCCCGTGGTTGTGTTTGACTTCGCT

AGCTTATACCCAAGCATTATCCAGGCCCATAACCTCTGTTTCACCACCCTGGCGCTCGAT

GAAGTGGATCTGGCCGGGCTTCAACCATCCGTCAACTACTCGACGTTCGAGGTGGGTGAC

CAAAAGTTATTTTTTGTCCACGCCCATATTCGCGAAAGCCTGCTTGGCATCTTGCTGCGC

GACTGGCTGGCCATGCGAAAGGCGGTGAGGGCGCGAATCCCCACCAGCACCCCCGAGGAG

GCAGTTTTACTAGATAAGCAGCAGTCTGCGATTAAGGTGATATGCAACTCGGTTTACGGA

TTCACGGGGGTGGCAAACGGCCTGTTGCCGTGTCTGAGGATAGCGGCTACCGTTACCACG

ATAGGACGCGACATGCTCCTCAAGACCCGAGATTACGTTCACTCTCGTTGGGCGACGCGC

GAGCTGCTGGAGGACAATTTTCCAGGGGCTATAGGTTTCCGAAACCACAAGCCTTACTCC

GTCAGGGTTATCTACGGAGACACCGACTCCGTGTTTATCAAGTTTGTGGGCCTGACGTAC

GAGGGGGTATCGGAGCTGGGGGATGCTATGTCGCGTCAGATTTCAGCGGACCTCTTTAGA

GCGCCCATCAAACTGGAGTGTGAGAAGACCTTTCAGCGACTGCTGCTGATCACCAAGAAG

AAGTACATAGGTGTCATAAACGGGGGGAAGATGCTCATGAAGGGGGTCGACCTGGTCCGC

AAAAATAACTGCTCTTTCATAAACTTGTACGCGCGACATCTGGTAGATCTTTTGTTGTAC

GACGAGGATGTGGCCACGGCGGCAGCAGAGGTGACAGACGTGCCTCCCGCAGAATGGGTG

GGGCGCCCGCTACCGAGCGGCTTTGACAAGTTTGGGCGAGTGCTGGTAGAGGCGTACAAC

CGTATCACTGCCCCCAACTTGGACGTGCGCGAGTTCGTTATGACTGCTGAGCTGAGCCGC

TCACCCGAATCGTATACCAACAAGCGCCTGCCGCACCTCACCGTCTACTTTAAGCTCGCC

ATGAGGAATGAAGAACTGCCCAGCGTAAAAGAGAGAATTCCGTATGTGATAGTTGCGCAG

ACCGAGGCCGCGGAACGCGAAGCGGGTGTAGTAAACTCAATGCGCGGTACCGCCCAAAAC

CCCGTGGTAACCAAGACCGCACGCCCCCAACCTAAACGCAAACTGCTGGTTTCCGACCTC

GCCGAAGACCCGACCTATGTTTCCGAGAATGACGTACCGCTAAACACAGACTACTATTTC

TCCCACCTGTTGGGTACCATAAGCGTGACCTTTAAGGCTCTATTCGGAAATGATGTGAGA

ACAACAGAAAATCTTTTAAAGCGGTTTATTCCGGAAACCCCCCACAAGACCCCCACGAAA

ACCCAGGCACTGCTTGAGCGCGCCGGCTTTGAAAAGCTGACGCCCTTTACACCGGAGGAA

GAAAGTCGTCGAATACTGCATACAGTTTTTTGTACTCTAGAAGCAGCTCCCCATCAAAGC

TGA

>OR085510.1

ATGGCGGCGCGCGAACAGGCCAACTCCGTTCGCAGGAGCGGGTTTTTTAATCCGTTTATT

GGCAAGAGGCCATTTTTCAGGCCGGGAAGCGGGCAGACCGCGGAGACAGAGCGCCCAAGG

CCCCCCCAACACTCGTACTGCACAGAGGTGGGTAGCTTTAAGTTTATAGCTCCAAGATGC

CTCGATGAAGAAGCCCCCGCCGACCAGCGACGCGGTGTACACGTGGGCACCTTGGAGCGC

CCACCCAAAGTGTACTGCGATGGCTCAGAGTACGACGTGCTGAACTTTGCCTCCGGAGGT

TGCTGGCCTCGCAGGATTCGGGTTTGGAACGGCCAGGATTTTCGGGGCGATGGATTCAAC

CCCAGATTTGAGAGATTTCACGTGTACGACATAGTAGAGACTTCGGAGAGCGCCTCACAC

GATGACCCATCCAGGTTTGCTGAGCTATCCCGCCCAAGCGGGTCTGTGGTAACACTGCTG

GGAATGAGCGAGTGTGGAAAGCGAGTCGCGGTTCACGTCTATGGTGTGCGCCATTATTTT

TACATGGCAAAGGCGGAGGTGGATAGCGCTTGTGGAATAACCACCGAGGCAGAACTCGTG

CGCGCAATGGTGGACTGCGCGCACAGCTCGGCTTTGAGCGCGGCCCTGGGAAATGGCAAC

GGCGGCAAACAGAGCGGCGGCAGCGGCGGGGGATGGTGGGGCGGAAAGCACGTGTCTGCG

GACTGCTTCAAAGTGGAGACCGTGTGCCACACGACGCTGTACTACTTTGGATCTAAGCCA

GCTCTCTACTATAGAGTATCTGCCTCCAGCAGCCGCCTGGGAGGGTTCATCTGCGACAAC

TTTCACCCGGAGATTACAAAATTCGAGGGGAGCGTGGACGTGACCACGCGGCTGTTGTTG

GACAATGAAAATTTTACCAGTTTCGGGTGGTACCGCCTGCGACCCGGCACCCACGGAGAG

CGTGTTCAACTTCGCCCCGTTGAGCGACACGTCACCTCAAGCGACGTGGAGATTAACTGT

ACTCCCGATAACCTGGAGCCGATACCAGACGAGGCTGCCTGGCCCGACTATAAGCTCATG

TGCTTTGATATAGAGTGTAAAGCTGGAACGGGTAACGAAATGGCGTTCCCAGTGGCAACT

AACCAAGAGGACCTGGTCATCCAGATCTCCTGTCTGCTGTACTCGCTTGCTACTCAGAAC

CACGAACACACCCTGCTGTTTTCCCTCGGGTCATGCGATATCTCTGAGGAATACTCGTTT

GCATGCGTCCAGCGCGGCGAGCCCAGACCGACGGTTTTGGAGTTTGACAGCGAGTACGAG

CTGCTGGTTGCCTTCCTGACCTTTCTCAAGCAGTACTCTCCCGAGTTCGCCACCGGCTAC

AACATCGTTAATTTTGACTGGGCGTACATAGTTAACAAGGTAACGTCGGTGTATAACATC

AAGCTGGACGGGTACGGCAAGTTCAACAAAGGGGGGCTGTTTAAGGTGTGGGACATCGCC

ACGAACCATTTTCAGAAGAAGAGCAAGGTGAAAATCAATGGCCTGATATCTCTAGACATG

TATTCTGTGGCGACGGAAAAGCTAAAGCTACCCAGCTACAAACTCGACGCGGTCGTGGGA

GACGTCCTCGGCGAGCATAAGATAGACCTTCCCTATAAAGAAATACCCTCCTATTACGCG

GGAGGGCCTGACCGGAGGGGCGTAATAGGAGAGTATTGTATCCAGGACTCTAGGCTGGTG

GGCAAGCTGTTTTTTAAGTACCTCCCCCATCTGGAACTATCGGCGGTGGCCAAACTCGCC

CGTATCACCCTAACGCGGGTAATTTTTGACGGTCAGCAAATTCGCGTGTACACGTGCTTG

CTGAAACTCGCCCGCGAGAGAAATTTCATTTTGCCAGACAACAGACGCCGGTTTGACAGT

CAGGCAGACGCCGCGTCAGAGACTTCGGAGTTGGCTATGGATAGCCAAAGCCACGCCTTC

GACAGTACAGACGAACCCGACGGTGTGGACGGTACCCCGGACGCCGCAGGATCTGGCGCT

ACTTCTGAAAACGGAGGCGGGAAGCCCGGCGTCGGGAGGGCCGTGGGCTACCAGGGAGCA

AAGGTTCTAGACCCCGTATCCGGCTTTCATGTGGACCCCGTGGTTGTGTTTGACTTCGCT

AGCTTATACCCAAGCATTATCCAGGCCCATAACCTCTGTTTCACCACCCTGGCGCTCGAT

GAAGTGGATCTGGCCGGGCTTCAACCATCCGTCAACTACTCGACGTTCGAGGTGGGTGAC

CAAAAGTTATTTTTTGTCCACGCCCATATTCGCGAAAGCCTGCTTGGCATCTTGCTGCGC

GACTGGCTGGCCATGCGAAAGGCGGTGAGGGCGCGAATCCCCACCAGCACCCCCGAGGAG

GCAGTTTTACTAGATAAGCAGCAGTCTGCGATTAAGGTGATATGCAACTCGGTTTACGGA

TTCACGGGGGTGGCAAACGGCCTGTTGCCGTGTCTGAGGATAGCGGCTACCGTTACCACG

ATAGGACGCGACATGCTCCTCAAGACCCGAGATTACGTTCACTCTCGTTGGGCGACGCGC

GAGCTGCTGGAGGACAATTTTCCAGGGGCTATAGGTTTCCGAAACCACAAGCCTTACTCC

GTCAGGGTTATCTACGGAGACACCGACTCCGTGTTTATCAAGTTTGTGGGCCTGACGTAC

GAGGGGGTATCGGAGCTGGGGGATGCTATGTCGCGTCAGATTTCAGCGGACCTCTTTAGA

GCGCCCATCAAACTGGAGTGTGAGAAGACCTTTCAGCGACTGCTGCTGATCACCAAGAAG

AAGTACATAGGTGTCATAAACGGGGGGAAGATGCTCATGAAGGGGGTCGACCTGGTCCGC

AAAAATAACTGCTCTTTCATAAACTTGTACGCGCGACATCTGGTAGATCTTTTGTTGTAC

GACGAGGATGTGGCCACGGCGGCAGCAGAGGTGACAGACGTGCCTCCCGCAGAATGGGTG

GGGCGCCCGCTACCGAGCGGCTTTGACAAGTTTGGGCGAGTGCTGGTAGAGGCGTACAAC

CGTATCACTGCCCCCAACTTGGACGTGCGCGAGTTCGTTATGACTGCTGAGCTGAGCCGC

TCACCCGAATCGTATACCAACAAGCGCCTGCCGCACCTCACCGTCTACTTTAAGCTCGCC

ATGAGGAATGAAGAACTGCCCAGCGTAAAAGAGAGAATTCCGTATGTGATAGTTGCGCAG

ACCGAGGCCGCGGAACGCGAAGCGGGTGTAGTAAACTCAATGCGCGGTACCGCCCAAAAC

CCCGTGGTAACCAAGACCGCACGCCCCCAACCTAAACGCAAACTGCTGGTTTCCGACCTC

GCCGAAGACCCGACCTATGTTTCCGAGAATGACGTACCGCTAAACACAGACTACTATTTC

TCCCACCTGTTGGGTACCATAAGCGTGACCTTTAAGGCTCTATTCGGAAATGATGTGAGA

ACAACAGAAAATCTTTTAAAGCGGTTTATTCCGGAAACCCCCCACAAGACCCCCACGAAA

ACCCAGGCACTGCTTGAGCGCGCCGGCTTTGAAAAGCTGACGCCCTTTACACCGGAGGAA

GAAAGTCGTCGAATACTGCATACAGTTTTTTGTACTCTAGAAGCAGCTCCCCATCAAAGC

TGA

>OR085511.1

ATGGCGGCGCGCGAACAGGCCAACTCCGTTCGCAGGAGCGGGTTTTTTAATCCGTTTATT

GGCAAGAGGCCATTTTTCAGGCCGGGAAGCGGGCAGACCGCGGAGACAGAGCGCCCAAGG

CCCCCCCAACACTCGTACTGCACAGAGGTGGGTAGCTTTAAGTTTATAGCTCCAAGATGC

CTCGATGAAGAAGCCCCCGCCGACCAGCGACGCGGTGTACACGTGGGCACCTTGGAGCGC

CCACCCAAAGTGTACTGCGATGGCTCAGAGTACGACGTGCTGAACTTTGCCTCCGGAGGT

TGCTGGCCTCGCAGGATTCGGGTTTGGAACGGCCAGGATTTTCGGGGCGATGGATTCAAC

CCCAGATTTGAGAGATTTCACGTGTACGACATAGTAGAGACTTCGGAGAGCGCCTCACAC

GATGACCCATCCAGGTTTGCTGAGCTATCCCGCCCAAGCGGGTCTGTGGTAACACTGCTG

GGAATGAGCGAGTGTGGAAAGCGAGTCGCGGTTCACGTCTATGGTGTGCGCCATTATTTT

TACATGGCAAAGGCGGAGGTGGATAGCGCTTGTGGAATAACCACCGAGGCAGAACTCGTG

CGCGCAATGGTGGACTGCGCGCACAGCTCGGCTTTGAGCGCGGCCCTGGGAAATGGCAAC

GGCGGCAAACAGAGCGGCGGCAGCGGCGGGGGATGGTGGGGCGGAAAGCACGTGTCTGCG

GACTGCTTCAAAGTGGAGACCGTGTGCCACACGACGCTGTACTACTTTGGATCTAAGCCA

GCTCTCTACTATAGAGTATCTGCCTCCAGCAGCCGCCTGGGAGGGTTCATCTGCGACAAC

TTTCACCCGGAGATTACAAAATTCGAGGGGAGCGTGGACGTGACCACGCGGCTGTTGTTG

GACAATGAAAATTTTACCAGTTTCGGGTGGTACCGCCTGCGACCCGGCACCCACGGAGAG

CGTGTTCAACTTCGCCCCGTTGAGCGACACGTCACCTCAAGCGACGTGGAGATTAACTGT

ACTCCCGATAACCTGGAGCCGATACCAGACGAGGCTGCCTGGCCCGACTATAAGCTCATG

TGCTTTGATATAGAGTGTAAAGCTGGAACGGGTAACGAAATGGCGTTCCCAGTGGCAACT

AACCAAGAGGACCTGGTCATCCAGATCTCCTGTCTGCTGTACTCGCTTGCTACTCAGAAC

CACGAACACACCCTGCTGTTTTCCCTCGGGTCATGCGATATCTCTGAGGAATACTCGTTT

GCATGCGTCCAGCGCGGCGAGCCCAGACCGACGGTTTTGGAGTTTGACAGCGAGTACGAG

CTGCTGGTTGCCTTCCTGACCTTTCTCAAGCAGTACTCTCCCGAGTTCGCCACCGGCTAC

AACATCGTTAATTTTGACTGGGCGTACATAGTTAACAAGGTAACGTCGGTGTATAACATC

AAGCTGGACGGGTACGGCAAGTTCAACAAAGGGGGGCTGTTTAAGGTGTGGGACATCGCC

ACGAACCATTTTCAGAAGAAGAGCAAGGTGAAAATCAATGGCCTGATATCTCTAGACATG

TATTCTGTGGCGACGGAAAAGCTAAAGCTACCCAGCTACAAACTCGACGCGGTCGTGGGA

GACGTCCTCGGCGAGCATAAGATAGACCTTCCCTATAAAGAAATACCCTCCTATTACGCG

GGAGGGCCTGACCGGAGGGGCGTAATAGGAGAGTATTGTATCCAGGACTCTAGGCTGGTG

GGCAAGCTGTTTTTTAAGTACCTCCCCCATCTGGAACTATCGGCGGTGGCCAAACTCGCC

CGTATCACCCTAACGCGGGTAATTTTTGACGGTCAGCAAATTCGCGTGTACACGTGCTTG

CTGAAACTCGCCCGCGAGAGAAATTTCATTTTGCCAGACAACAGACGCCGGTTTGACAGT

CAGGCAGACGCCGCGTCAGAGACTTCGGAGTTGGCTATGGATAGCCAAAGCCACGCCTTC

GACAGTACAGACGAACCCGACGGTGTGGACGGTACCCCGGACGCCGCAGGATCTGGCGCT

ACTTCTGAAAACGGAGGCGGGAAGCCCGGCGTCGGGAGGGCCGTGGGCTACCAGGGAGCA

AAGGTTCTAGACCCCGTATCCGGCTTTCATGTGGACCCCGTGGTTGTGTTTGACTTCGCT

AGCTTATACCCAAGCATTATCCAGGCCCATAACCTCTGTTTCACCACCCTGGCGCTCGAT

GAAGTGGATCTGGCCGGGCTTCAACCATCCGTCAACTACTCGACGTTCGAGGTGGGTGAC

CAAAAGTTATTTTTTGTCCACGCCCATATTCGCGAAAGCCTGCTTGGCATCTTGCTGCGC

GACTGGCTGGCCATGCGAAAGGCGGTGAGGGCGCGAATCCCCACCAGCACCCCCGAGGAG

GCAGTTTTACTAGATAAGCAGCAGTCTGCGATTAAGGTGATATGCAACTCGGTTTACGGA

TTCACGGGGGTGGCAAACGGCCTGTTGCCGTGTCTGAGGATAGCGGCTACCGTTACCACG

ATAGGACGCGACATGCTCCTCAAGACCCGAGATTACGTTCACTCTCGTTGGGCGACGCGC

GAGCTGCTGGAGGACAATTTTCCAGGGGCTATAGGTTTCCGAAACCACAAGCCTTACTCC

GTCAGGGTTATCTACGGAGACACCGACTCCGTGTTTATCAAGTTTGTGGGCCTGACGTAC

GAGGGGGTATCGGAGCTGGGGGATGCTATGTCGCGTCAGATTTCAGCGGACCTCTTTAGA

GCGCCCATCAAACTGGAGTGTGAGAAGACCTTTCAGCGACTGCTGCTGATCACCAAGAAG

AAGTACATAGGTGTCATAAACGGGGGGAAGATGCTCATGAAGGGGGTCGACCTGGTCCGC

AAAAATAACTGCTCTTTCATAAACTTGTACGCGCGACATCTGGTAGATCTTTTGTTGTAC

GACGAGGATGTGGCCACGGCGGCAGCAGAGGTGACAGACGTGCCTCCCGCAGAATGGGTG

GGGCGCCCGCTACCGAGCGGCTTTGACAAGTTTGGGCGAGTGCTGGTAGAGGCGTACAAC

CGTATCACTGCCCCCAACTTGGACGTGCGCGAGTTCGTTATGACTGCTGAGCTGAGCCGC

TCACCCGAATCGTATACCAACAAGCGCCTGCCGCACCTCACCGTCTACTTTAAGCTCGCC

ATGAGGAATGAAGAACTGCCCAGCGTAAAAGAGAGAATTCCGTATGTGATAGTTGCGCAG

ACCGAGGCCGCGGAACGCGAAGCGGGTGTAGTAAACTCAATGCGCGGTACCGCCCAAAAC

CCCGTGGTAACCAAGACCGCACGCCCCCAACCTAAACGCAAACTGCTGGTTTCCGACCTC

GCCGAAGACCCGACCTATGTTTCCGAGAATGACGTACCGCTAAACACAGACTACTATTTC

TCCCACCTGTTGGGTACCATAAGCGTGACCTTTAAGGCTCTATTCGGAAATGATGTGAGA

ACAACAGAAAATCTTTTAAAGCGGTTTATTCCGGAAACCCCCCACAAGACCCCCACGAAA

ACCCAGGCACTGCTTGAGCGCGCCGGCTTTGAAAAGCTGACGCCCTTTACACCGGAGGAA

GAAAGTCGTCGAATACTGCATACAGTTTTTTGTACTCTAGAAGCAGCTCCCCATCAAAGC

TGA

>OR085512.1

ATGGCGGCGCGCGAACAGGCCAACTCCGTTCGCAGGAGCGGGTTTTTTAATCCGTTTATT

GGCAAGAGGCCATTTTTCAGGCCGGGAAGCGGGCAGACCGCGGAGACAGAGCGCCCAAGG

CCCCCCCAACACTCGTACTGCACAGAGGTGGGTAGCTTTAAGTTTATAGCTCCAAGATGC

CTCGATGAAGAAGCCCCCGCCGACCAGCGACGCGGTGTACACGTGGGCACCTTGGAGCGC

CCACCCAAAGTGTACTGCGATGGCTCAGAGTACGACGTGCTGAACTTTGCCTCCGGAGGT

TGCTGGCCTCGCAGGATTCGGGTTTGGAACGGCCAGGATTTTCGGGGCGATGGATTCAAC

CCCAGATTTGAGAGATTTCACGTGTACGACATAGTAGAGACTTCGGAGAGCGCCTCACAC

GATGACCCATCCAGGTTTGCTGAGCTATCCCGCCCAAGCGGGTCTGTGGTAACACTGCTG

GGAATGAGCGAGTGTGGAAAGCGAGTCGCGGTTCACGTCTATGGTGTGCGCCATTATTTT

TACATGGCAAAGGCGGAGGTGGATAGCGCTTGTGGAATAACCACCGAGGCAGAACTCGTG

CGCGCAATGGTGGACTGCGCGCACAGCTCGGCTTTGAGCGCGGCCCTGGGAAATGGCAAC

GGCGGCAAACAGAGCGGCGGCAGCGGCGGGGGATGGTGGGGCGGAAAGCACGTGTCTGCG

GACTGCTTCAAAGTGGAGACCGTGTGCCACACGACGCTGTACTACTTTGGATCTAAGCCA

GCTCTCTACTATAGAGTATCTGCCTCCAGCAGCCGCCTGGGAGGGTTCATCTGCGACAAC

TTTCACCCGGAGATTACAAAATTCGAGGGGAGCGTGGACGTGACCACGCGGCTGTTGTTG

GACAATGAAAATTTTACCAGTTTCGGGTGGTACCGCCTGCGACCCGGCACCCACGGAGAG

CGTGTTCAACTTCGCCCCGTTGAGCGACACGTCACCTCAAGCGACGTGGAGATTAACTGT

ACTCCCGATAACCTGGAGCCGATACCAGACGAGGCTGCCTGGCCCGACTATAAGCTCATG

TGCTTTGATATAGAGTGTAAAGCTGGAACGGGTAACGAAATGGCGTTCCCAGTGGCAACT

AACCAAGAGGACCTGGTCATCCAGATCTCCTGTCTGCTGTACTCGCTTGCTACTCAGAAC

CACGAACACACCCTGCTGTTTTCCCTCGGGTCATGCGATATCTCTGAGGAATACTCGTTT

GCATGCGTCCAGCGCGGCGAGCCCAGACCGACGGTTTTGGAGTTTGACAGCGAGTACGAG

CTGCTGGTTGCCTTCCTGACCTTTCTCAAGCAGTACTCTCCCGAGTTCGCCACCGGCTAC

AACATCGTTAATTTTGACTGGGCGTACATAGTTAACAAGGTAACGTCGGTGTATAACATC

AAGCTGGACGGGTACGGCAAGTTCAACAAAGGGGGGCTGTTTAAGGTGTGGGACATCGCC

ACGAACCATTTTCAGAAGAAGAGCAAGGTGAAAATCAATGGCCTGATATCTCTAGACATG

TATTCTGTGGCGACGGAAAAGCTAAAGCTACCCAGCTACAAACTCGACGCGGTCGTGGGA

GACGTCCTCGGCGAGCATAAGATAGACCTTCCCTATAAAGAAATACCCTCCTATTACGCG

GGAGGGCCTGACCGGAGGGGCGTAATAGGAGAGTATTGTATCCAGGACTCTAGGCTGGTG

GGCAAGCTGTTTTTTAAGTACCTCCCCCATCTGGAACTATCGGCGGTGGCCAAACTCGCC

CGTATCACCCTAACGCGGGTAATTTTTGACGGTCAGCAAATTCGCGTGTACACGTGCTTG

CTGAAACTCGCCCGCGAGAGAAATTTCATTTTGCCAGACAACAGACGCCGGTTTGACAGT

CAGGCAGACGCCGCGTCAGAGACTTCGGAGTTGGCTATGGATAGCCAAAGCCACGCCTTC

GACCGTACAGACGAACCCGACGGTGTGGACGGTACCCCGGACGCCGCAGGATCTGGCGCT

ACTTCTGAAAACGGAGGCGGGAAGCCCGGCGTCGGGAGGGCCGTGGGCTACCAGGGAGCA

AAGGTTCTAGACCCCGTATCCGGCTTTCATGTGGACCCCGTGGTTGTGTTTGACTTCGCT

AGCTTATACCCAAGCATTATCCAGGCCCATAACCTCTGTTTCACCACCCTGGCGCTCGAT

GAAGTGGATCTGGCCGGGCTTCAACCATCCGTCAACTACTCGACGTTCGAGGTGGGTGAC

CAAAAGTTATTTTTTGTCCACGCCCATATTCGCGAAAGCCTGCTTGGCATCTTGCTGCGC

GACTGGCTGGCCATGCGAAAGGCGGTGAGGGCGCGAATCCCCACCAGCACCCCCGAGGAG

GCAGTTTTACTAGATAAGCAGCAGTCTGCGATTAAGGTGATATGCAACTCGGTTTACGGA

TTCACGGGGGTGGCAAACGGCCTGTTGCCGTGTCTGAGGATAGCGGCTACCGTTACCACG

ATAGGACGCGACATGCTCCTCAAGACCCGAGATTACGTTCACTCTCGTTGGGCGACGCGC

GAGCTGCTGGAGGACAATTTTCCAGGGGCTATAGGTTTCCGAAACCACAAGCCTTACTCC

GTCAGGGTTATCTACGGAGACACCGACTCCGTGTTTATCAAGTTTGTGGGCCTGACGTAC

GAGGGGGTATCGGAGCTGGGGGATGCTATGTCGCGTCAGATTTCAGCGGACCTCTTTAGA

GCGCCCATCAAACTGGAGTGTGAGAAGACCTTTCAGCGACTGCTGCTGATCACCAAGAAG

AAGTACATAGGTGTCATAAACGGGGGGAAGATGCTCATGAAGGGGGTCGACCTGGTCCGC

AAAAATAACTGCTCTTTCATAAACTTGTACGCGCGACATCTGGTAGATCTTTTGTTGTAC

GACGAGGATGTGGCCACGGCGGCAGCAGAGGTGACAGACGTGCCTCCCGCAGAATGGGTG

GGGCGCCCGCTACCGAGCGGCTTTGACAAGTTTGGGCGAGTGCTGGTAGAGGCGTACAAC

CGTATCACTGCCCCCAACTTGGACGTGCGCGAGTTCGTTATGACTGCTGAGCTGAGCCGC

TCACCCGAATCGTATACCAACAAGCGCCTGCCGCACCTCACCGTCTACTTTAAGCTCGCC

ATGAGGAATGAAGAACTGCCCAGCGTAAAAGAGAGAATTCCGTATGTGATAGTTGCGCAG

ACCGAGGCCGCGGAACGCGAAGCGGGTGTAGTAAACTCAATGCGCGGTACCGCCCAAAAC

CCCGTGGTAACCAAGACCGCACGCCCCCAACCTAAACGCAAACTGCTGGTTTCCGACCTC

GCCGAAGACCCGACCTATGTTTCCGAGAATGACGTACCGCTAAACACAGACTACTATTTC

TCCCACCTGTTGGGTACCATAAGCGTGACCTTTAAGGCTCTATTCGGAAATGATGTGAGA

ACAACAGAAAATCTTTTAAAGCGGTTTATTCCGGAAACCCCCCACAAGACCCCCACGAAA

ACCCAGGCACTGCTTGAGCGCGCCGGCTTTGAAAAGCTGACGCCCTTTACACCGGAGGAA

GAAAGTCGTCGAATACTGCATACAGTTTTTTGTACTCTAGAAGCAGCTCCCCATCAAAGC

TGA

>OR085513.1

ATGGCGGCGCGCGAACAGGCCAACTCCGTTCGCAGGAGCGGGTTTTTTAATCCGTTTATT

GGCAAGAGGCCATTTTTCAGGCCGGGAAGCGGGCAGACCGCGGAGACAGAGCGCCCAAGG

CCCCCCCAACACTCGTACTGCACAGAGGTGGGTAGCTTTAAGTTTATAGCTCCAAGATGC

CTCGATGAAGAAGCCCCCGCCGACCAGCGACGCGGTGTACACGTGGGCACCTTGGAGCGC

CCACCCAAAGTGTACTGCGATGGCTCAGAGTACGACGTGCTGAACTTTGCCTCCGGAGGT

TGCTGGCCTCGCAGGATTCGGGTTTGGAACGGCCAGGATTTTCGGGGCGATGGATTCAAC

CCCAGATTTGAGAGATTTCACGTGTACGACATAGTAGAGACTTCGGAGAGCGCCTCACAC

GATGACCCATCCAGGTTTGCTGAGCTATCCCGCCCAAGCGGGTCTGTGGTAACACTGCTG

GGAATGAGCGAGTGTGGAAAGCGAGTCGCGGTTCACGTCTATGGTGTGCGCCATTATTTT

TACATGGCAAAGGCGGAGGTGGATAGCGCTTGTGGAATAACCACCGAGGCAGAACTCGTG

CGCGCAATGGTGGACTGCGCGCACAGCTCGGCTTTGAGCGCGGCCCTGGGAAATGGCAAC

GGCGGCAAACAGAGCGGCGGCAGCGGCGGGGGATGGTGGGGCGGAAAGCACGTGTCTGCG

GACTGCTTCAAAGTGGAGACCGTGTGCCACACGACGCTGTACTACTTTGGATCTAAGCCA

GCTCTCTACTATAGAGTATCTGCCTCCAGCAGCCGCCTGGGAGGGTTCATCTGCGACAAC

TTTCACCCGGAGATTACAAAATTCGAGGGGAGCGTGGACGTGACCACGCGGCTGTTGTTG

GACAATGAAAATTTTACCAGTTTCGGGTGGTACCGCCTGCGACCCGGCACCCACGGAGAG

CGTGTTCAACTTCGCCCCGTTGAGCGACACGTCACCTCAAGCGACGTGGAGATTAACTGT

ACTCCCGATAACCTGGAGCCGATACCAGACGAGGCTGCCTGGCCCGACTATAAGCTCATG

TGCTTTGATATAGAGTGTAAAGCTGGAACGGGTAACGAAATGGCGTTCCCAGTGGCAACT

AACCAAGAGGACCTGGTCATCCAGATCTCCTGTCTGCTGTACTCGCTTGCTACTCAGAAC

CACGAACACACCCTGCTGTTTTCCCTCGGGTCATGCGATATCTCTGAGGAATACTCGTTT

GCATGCGTCCAGCGCGGCGAGCCCAGACCGACGGTTTTGGAGTTTGACAGCGAGTACGAG

CTGCTGGTTGCCTTCCTGACCTTTCTCAAGCAGTACTCTCCCGAGTTCGCCACCGGCTAC

AACATCGTTAATTTTGACTGGGCGTACATAGTTAACAAGGTAACGTCGGTGTATAACATC

AAGCTGGACGGGTACGGCAAGTTCAACAAAGGGGGGCTGTTTAAGGTGTGGGACATCGCC

ACGAACCATTTTCAGAAGAAGAGCAAGGTGAAAATCAATGGCCTGATATCTCTAGACATG

TATTCTGTGGCGACGGAAAAGCTAAAGCTACCCAGCTACAAACTCGACGCGGTCGTGGGA

GACGTCCTCGGCGAGCATAAGATAGACCTTCCCTATAAAGAAATACCCTCCTATTACGCG

GGAGGGCCTGACCGGAGGGGCGTAATAGGAGAGTATTGTATCCAGGACTCTAGGCTGGTG

GGCAAGCTGTTTTTTAAGTACCTCCCCCATCTGGAACTATCGGCGGTGGCCAAACTCGCC

CGTATCACCCTAACGCGGGTAATTTTTGACGGTCAGCAAATTCGCGTGTACACGTGCTTG

CTGAAACTCGCCCGCGAGAGAAATTTCATTTTGCCAGACAACAGACGCCGGTTTGACAGT

CAGGCAGACGCCGCGTCAGAGACTTCGGAGTTGGCTATGGATAGCCAAAGCCACGCCTTC

GACAGTACAGACGAACCCGACGGTGTGGACGGTACCCCGGACGCCGCAGGATCTGGCGCT

ACTTCTGAAAACGGAGGCGGGAAGCCCGGCGTCGGGAGGGCCGTGGGCTACCAGGGAGCA

AAGGTTCTAGACCCCGTATCCGGCTTTCATGTGGACCCCGTGGTTGTGTTTGACTTCGCT

AGCTTATACCCAAGCATTATCCAGGCCCATAACCTCTGTTTCACCACCCTGGCGCTCGAT

GAAGTGGATCTGGCCGGGCTTCAACCATCCGTCAACTACTCGACGTTCGAGGTGGGTGAC

CAAAAGTTATTTTTTGTCCACGCCCATATTCGCGAAAGCCTGCTTGGCATCTTGCTGCGC

GACTGGCTGGCTATGCGAAAGGCGGTGAGGGCGCGAATCCCCACCAGCACCCCCGAGGAG

GCAGTTTTACTAGATAAGCAGCAGTCTGCGATTAAGGTGATATGCAACTCGGTTTACGGA

TTCACGGGGGTGGCAAACGGCCTGTTGCCGTGTCTGAGGATAGCGGCTACCGTTACCACG

ATAGGACGCGACATGCTCCTCAAGACCCGAGATTACGTTCACTCTCGTTGGGCGACGCGC

GAGCTGCTGGAGGACAATTTTCCAGGGGCTATAGGTTTCCGAAACCACAAGCCTTACTCC

GTCAGGGTTATCTACGGAGACACCGACTCCGTGTTTATCAAGTTTGTGGGCCTGACGTAC

GAGGGGGTATCGGAGCTGGGGGATGCTATGTCGCGTCAGATTTCAGCGGACCTCTTTAGA

GCGCCCATCAAACTGGAGTGTGAGAAGACCTTTCAGCGACTGCTGCTGATCACCAAGAAG

AAGTACATAGGTGTCATAAACGGGGGGAAGATGCTCATGAAGGGGGTCGACCTGGTCCGC

AAAAATAACTGCTCTTTCATAAACTTGTACGCGCGACATCTGGTAGATCTTTTGTTGTAC

GACGAGGATGTGGCCACGGCGGCAGCAGAGGTGACAGACGTGCCTCCCGCAGAATGGGTG

GGGCGCCCGCTACCGAGCGGCTTTGACAAGTTTGGGCGAGTGCTGGTAGAGGCGTACAAC

CGTATCACTGCCCCCAACTTGGACGTGCGCGAGTTCGTTATGACTGCTGAGCTGAGCCGC

TCACCCGAATCGTATACCAACAAGCGCCTGCCGCACCTCACCGTCTACTTTAAGCTCGCC

ATGAGGAATGAAGAACTGCCCAGCGTAAAAGAGAGAATTCCGTATGTGATAGTTGCGCAG

ACCGAGGCCGCGGAACGCGAAGCGGGTGTAGTAAACTCAATGCGCGGTACCGCCCAAAAC

CCCGTGGTAACCAAGACCGCACGCCCCCAACCTAAACGCAAACTGCTGGTTTCCGACCTC

GCCGAAGACCCGACCTATGTTTCCGAGAATGACGTACCGCTAAACACAGACTACTATTTC

TCCCACCTGTTGGGTACCATAAGCGTGACCTTTAAGGCTCTATTCGGAAATGATGTGAGA

ACAACAGAAAATCTTTTAAAGCGGTTTATTCCGGAAACCCCCCACAAGACCCCCACGAAA

ACCCAGGCACTGCTTGAGCGCGCCGGCTTTGAAAAGCTGACGCCCTTTACACCGGAGGAA

GAAAGTCGTCGAATACTGCATACAGTTTTTTGTACTCTAGAAGCAGCTCCCCATCAAAGC

TGA

>OR085514.1

ATGGCGGCGCGCGAACAGGCCAACTCCGTTCGCAGGAGCGGGTTTTTTAATCCGTTTATT

GGCAAGAGGCCATTTTTCAGGCCGGGAAGCGGGCAGACCGCGGAGACAGAGCGCCCAAGG

CCCCCCCAACACTCGTACTGCACAGAGGTGGGTAGCTTTAAGTTTATAGCTCCAAGATGC

CTCGATGAAGAAGCCCCCGCCGACCAGCGACGCGGTGTACACGTGGGCACCTTGGAGCGC

CCACCCAAAGTGTACTGCGATGGCTCAGAGTACGACGTGCTGAACTTTGCCTCCGGAGGT

TGCTGGCCTCGCAGGATTCGGGTTTGGAACGGCCAGGATTTTCGGGGCGATGGATTCAAC

CCCAGATTTGAGAGATTTCACGTGTACGACATAGTAGAGACTTCGGAGAGCGCCTCACAC

GATGACCCATCCAGGTTTGCTGAGCTATCCCGCCCAAGCGGGTCTGTGGTAACACTGCTG

GGAATGAGCGAGTGTGGAAAGCGAGTCGCGGTTCACGTCTATGGTGTGCGCCATTATTTT

TACATGGCAAAGGCGGAGGTGGATAGCGCTTGTGGAATAACCACCGAGGCAGAACTCGTG

CGCGCAATGGTGGACTGCGCGCACAGCTCGGCTTTGAGCGCGGCCCTGGGAAATGGCAAC

GGCGGCAAACAGAGCGGCGGCAGCGGCGGGGGATGGTGGGGCGGAAAGCACGTGTCTGCG

GACTGCTTCAAAGTGGAGACCGTGTGCCACACGACGCTGTACTACTTTGGATCTAAGCCA

GCTCTCTACTATAGAGTATCTGCCTCCAGCAGCCGCCTGGGAGGGTTCATCTGCGACAAC

TTTCACCCGGAGATTACAAAATTCGAGGGGAGCGTGGACGTGACCACGCGGCTGTTGTTG

GACAATGAAAATTTTACCAGTTTCGGGTGGTACCGCCTGCGACCCGGCACCCACGGAGAG

CGTGTTCAACTTCGCCCCGTTGAGCGACACGTCACCTCAAGCGACGTGGAGATTAACTGT

ACTCCCGATAACCTGGAGCCGATACCAGACGAGGCTGCCTGGCCCGACTATAAGCTCATG

TGCTTTGATATAGAGTGTAAAGCTGGAACGGGTAACGAAATGGCGTTCCCAGTGGCAACT

AACCAAGAGGACCTGGTCATCCAGATCTCCTGTCTGCTGTACTCGCTTGCTACTCAGAAC

CACGAACACACCCTGCTGTTTTCCCTCGGGTCATGCGATATCTCTGAGGAATACTCGTTT

GCATGCGTCCAGCGCGGCGAGCCCAGACCGACGGTTTTGGAGTTTGACAGCGAGTACGAG

CTGCTGGTTGCCTTCCTGACCTTTCTCAAGCAGTACTCTCCCGAGTTCGCCACCGGCTAC

AACATCGTTAATTTTGACTGGGCGTACATAGTTAACAAGGTAACGTCGGTGTATAACATC

AAGCTGGACGGGTACGGCAAGTTCAACAAAGGGGGGCTGTTTAAGGTGTGGGACATCGCC

ACGAACCATTTTCAGAAGAAGAGCAAGGTGAAAATCAATGGCCTGATATCTCTAGACATG

TATTCTGTGGCGACGGAAAAGCTAAAGCTACCCAGCTACAAACTCGACGCGGTCGTGGGA

GACGTCCTCGGCGAGCATAAGATAGACCTTCCCTATAAAGAAATACCCTCCTATTACGCG

GGAGGGCCTGACCGGAGGGGCGTAATAGGAGAGTATTGTATCCAGGACTCTAGGCTGGTG

GGCAAGCTGTTTTTTAAGTACCTCCCCCATCTGGAACTATCGGCGGTGGCCAAACTCGCC

CGTATCACCCTAACGCGGGTAATTTTTGACGGTCAGCAAATTCGCGTGTACACGTGCTTG

CTGAAACTCGCCCGCGAGAGAAATTTCATTTTGCCAGACAACAGACGCCGGTTTGACAGT

CAGGCAGACGCCGCGTCAGAGACTTCGGAGTTGGCTATGGATAGCCAAAGCCACGCCTTC

GACAGTACAGACGAACCCGACGGTGTGGACGGTACCCCGGACGCCGCAGGATCTGGCGCT

ACTTCTGAAAACGGAGGCGGGAAGCCCGGCGTCGGGAGGGCCGTGGGCTACCAGGGAGCA

AAGGTTCTAGACCCCGTATCCGGCTTTCATGTGGACCCCGTGGTTGTGTTTGACTTCGCT

AGCTTATACCCAAGCATTATCCAGGCCCATAACCTCTGTTTCACCACCCTGGCGCTCGAT

GAAGTGGATCTGGCCGGGCTTCAACCATCCGTCAACTACTCGACGTTCGAGGTGGGTGAC

CAAAAGTTATTTTTTGTCCACGCCCATATTCGCGAAAGCCTGCTTGGCATCTTGCTGCGC

GACTGGCTGGCCATGCGAAAGGCGGTGAGGGCGCGAATCCCCACCAGCACCCCCGAGGAG

GCAGTTTTACTAGATAAGCAGCAGTCTGCGATTAAGGTGATATGCAACTCGGTTTACGGA

TTCACGGGGGTGGCAAACGGCCTGTTGCCGTGTCTGAGGATAGCGGCTACCGTTACCACG

ATAGGACGCGACATGCTCCTCAAGACCCGAGATTACGTTCACTCTCGTTGGGCGACGCGC

GAGCTGCTGGAGGACAATTTTCCAGGGGCTATAGGTTTCCGAAACCACAAGCCTTACTCC

GTCAGGGTTATCTACGGAGACACCGACTCCGTGTTTATCAAGTTTGTGGGCCTGACGTAC

GAGGGGGTATCGGAGCTGGGGGATGCTATGTCGCGTCAGATTTCAGCGGACCTCTTTAGA

GCGCCCATCAAACTGGAGTGTGAGAAGACCTTTCAGCGACTGCTGCTGATCACCAAGAAG

AAGTACATAGGTGTCATAAACGGGGGGAAGATGCTCATGAAGGGGGTCGACCTGGTCCGC

AAAAATAACTGCTCTTTCATAAACTTGTACGCGCGACATCTGGTAGATCTTTTGTTGTAC

GACGAGGATGTGGCCACGGCGGCAGCAGAGGTGACAGACGTGCCTCCCGCAGAATGGGTG

GGGCGCCCGCTACCGAGCGGCTTTGACAAGTTTGGGCGAGTGCTGGTAGAGGCGTACAAC

CGTATCACTGCCCCCAACTTGGACGTGCGCGAGTTCGTTATGACTGCTGAGCTGAGCCGC

TCACCCGAATCGTATACCAACAAGCGCCTGCCGCACCTCACCGTCTACTTTAAGCTCGCC

ATGAGGAATGAAGAACTGCCCAGCGTAAAAGAGAGAATTCCGTATGTGATAGTTGCGCAG

ACCGAGGCCGCGGAACGCGAAGCGGGTGTAGTAAACTCAATGCGCGGTACCGCCCAAAAC

CCCGTGGTAACCAAGACCGCACGCCCCCAACCTAAACGCAAACTGCTGGTTTCCGACCTC

GCCGAAGACCCGACCTATGTTTCCGAGAATGACGTACCGCTAAACACAGACTACTATTTC

TCCCACCTGTTGGGTACCATAAGCGTGACCTTTAAGGCTCTATTCGGAAATGATGTGAGA

ACAACAGAAAATCTTTTAAAGCGGTTTATTCCGGAAACCCCCCACAAGACCCCCACGAAA

ACCCAGGCACTGCTTGAGCGCGCCGGCTTTGAAAAGCTGACGCCCTTTACACCGGAGGAA

GAAAGTCGTCGAATACTGCATACAGTTTTTTGTACTCTAGAAGCAGCTCCCCATCAAAGC

TGA

>OR085515.1

ATGGCGGCGCGCGAACAGGCCAACTCCGTTCGCAGGAGCGGGTTTTTTAATCCGTTTATT

GGCAAGAGGCCATTTTTCAGGCCGGGAAGCGGGCAGACCGCGGAGACAGAGCGCCCAAGG

CCCCCCCAACACTCGTACTGCACAGAGGTGGGTAGCTTTAAGTTTATAGCTCCAAGATGC

CTCGATGAAGAAGCCCCCGCCGACCAGCGACGCGGTGTACACGTGGGCACCTTGGAGCGC

CCACCCAAAGTGTACTGCGATGGCTCAGAGTACGACGTGCTGAACTTTGCCTCCGGAGGT

TGCTGGCCTCGCAGGATTCGGGTTTGGAACGGCCAGGATTTTCGGGGCGATGGATTCAAC

CCCAGATTTGAGAGATTTCACGTGTACGACATAGTAGAGACTTCGGAGAGCGCCTCACAC

GATGACCCATCCAGGTTTGCTGAGCTATCCCGCCCAAGCGGGTCTGTGGTAACACTGCTG

GGAATGAGCGAGTGTGGAAAGCGAGTCGCGGTTCACGTCTATGGTGTGCGCCATTATTTT

TACATGGCAAAGGCGGAGGTGGATAGCGCTTGTGGAATAACCACCGAGGCAGAACTCGTG

CGCGCAATGGTGGACTGCGCGCACAGCTCGGCTTTGAGCGCGGCCCTGGGAAATGGCAAC

GGCGGCAAACAGAGCGGCGGCAGCGGCGGGGGATGGTGGGGCGGAAAGCACGTGTCTGCG

GACTGCTTCAAAGTGGAGACCGTGTGCCACACGACGCTGTACTACTTTGGATCTAAGCCA

GCTCTCTACTATAGAGTATCTGCCTCCAGCAGCCGCCTGGGAGGGTTCATCTGCGACAAC

TTTCACCCGGAGATTACAAAATTCGAGGGGAGCGTGGACGTGACCACGCGGCTGTTGTTG

GACAATGAAAATTTTACCAGTTTCGGGTGGTACCGCCTGCGACCCGGCACCCACGGAGAG

CGTGTTCAACTTCGCCCCGTTGAGCGACACGTCACCTCAAGCGACGTGGAGATTAACTGT

ACTCCCGATAACCTGGAGCCGATACCAGACGAGGCTGCCTGGCCCGACTATAAGCTCATG

TGCTTTGATATAGAGTGTAAAGCTGGAACGGGTAACGAAATGGCGTTCCCAGTGGCAACT

AACCAAGAGGACCTGGTCATCCAGATCTCCTGTCTGCTGTACTCGCTTGCTACTCAGAAC

CACGAACACACCCTGCTGTTTTCCCTCGGGTCATGCGATATCTCTGAGGAATACTCGTTT

GCATGCGTCCAGCGCGGCGAGCCCAGACCGACGGTTTTGGAGTTTGACAGCGAGTACGAG

CTGCTGGTTGCCTTCCTGACCTTTCTCAAGCAGTACTCTCCCGAGTTCGCCACCGGCTAC

AACATCGTTAATTTTGACTGGGCGTACATAGTTAACAAGGTAACGTCGGTGTATAACATC

AAGCTGGACGGGTACGGCAAGTTCAACAAAGGGGGGCTGTTTAAGGTGTGGGACATCGCC

ACGAACCATTTTCAGAAGAAGAGCAAGGTGAAAATCAATGGCCTGATATCTCTAGACATG

TATTCTGTGGCGACGGAAAAGCTAAAGCTACCCAGCTACAAACTCGACGCGGTCGTGGGA

GACGTCCTCGGCGAGCATAAGATAGACCTTCCCTATAAAGAAATACCCTCCTATTACGCG

GGAGGGCCTGACCGGAGGGGCGTAATAGGAGAGTATTGTATCCAGGACTCTAGGCTGGTG

GGCAAGCTGTTTTTTAAGTACCTCCCCCATCTGGAACTATCGGCGGTGGCCAAACTCGCC

CGTATCACCCTAACGCGGGTAATTTTTGACGGTCAGCAAATTCGCGTGTACACGTGCTTG

CTGAAACTCGCCCGCGAGAGAAATTTCATTTTGCCAGACAACAGACGCCGGTTTGACAGT

CAGGCAGACGCCGCGTCAGAGACTTCGGAGTTGGCTATGGATAGCCAAAGCCACGCCTTC

GACAGTACAGACGAACCCGACGGTGTGGACGGTACCCCGGACGCCGCAGGATCTGGCGCT

ACTTCTGAAAACGGAGGCGGGAAGCCCGGCGTCGGGAGGGCCGTGGGCTACCAGGGAGCA

AAGGTTCTAGACCCCGTATCCGGCTTTCATGTGGACCCCGTGGTTGTGTTTGACTTCGCT

AGCTTATACCCAAGCATTATCCAGGCCCATAACCTCTGTTTCACCACCCTGGCGCTCGAT

GAAGTGGATCTGGCCGGGCTTCAACCATCCGTCAACTACTCGACGTTCGAGGTGGGTGGC

CAAAAGTTATTTTTTGTCCACGCCCATATTCGCGAAAGCCTGCTTGGCATCTTGCTGCGC

GACTGGCTGGCCATGCGAAAGGCGGTGAGGGCGCGAATCCCCACCAGCACCCCCGAGGAG

GCAGTTTTACTAGATAAGCAGCAGTCTGCGATTAAGGTGATATGCAACTCGGTTTACGGA

TTCACGGGGGTGGCAAACGGCCTGTTGCCGTGTCTGAGGATAGCGGCTACCGTTACCACG

ATAGGACGCGACATGCTCCTCAAGACCCGAGATTACGTTCACTCTCGTTGGGCGACGCGC

GAGCTGCTGGAGGACAATTTTCCAGGGGCTATAGGTTTCCGAAACCACAAGCCTTACTCC

GTCAGGGTTATCTACGGAGACACCGACTCCGTGTTTATCAAGTTTGTGGGCCTGACGTAC

GAGGGGGTATCGGAGCTGGGGGATGCTATGTCGCGTCAGATTTCAGCGGACCTCTTTAGA

GCGCCCATCAAACTGGAGTGTGAGAAGACCTTTCAGCGACTGCTGCTGATCACCAAGAAG

AAGTACATAGGTGTCATAAACGGGGGGAAGATGCTCATGAAGGGGGTCGACCTGGTCCGC

AAAAATAACTGCTCTTTCATAAACTTGTACGCGCGACATCTGGTAGATCTTTTGTTGTAC

GACGAGGATGTGGCCACGGCGGCAGCAGAGGTGACAGACGTGCCTCCCGCAGAATGGGTG

GGGCGCCCGCTACCGAGCGGCTTTGACAAGTTTGGGCGAGTGCTGGTAGAGGCGTACAAC

CGTATCACTGCCCCCAACTTGGACGTGCGCGAGTTCGTTATGACTGCTGAGCTGAGCCGC

TCACCCGAATCGTATACCAACAAGCGCCTGCCGCACCTCACCGTCTACTTTAAGCTCGCC

ATGAGGAATGAAGAACTGCCCAGCGTAAAAGAGAGAATTCCGTATGTGATAGTTGCGCAG

ACCGAGGCCGCGGAACGCGAAGCGGGTGTAGTAAACTCAATGCGCGGTACCGCCCAAAAC

CCCGTGGTAACCAAGACCGCACGCCCCCAACCTAAACGCAAACTGCTGGTTTCCGACCTC

GCCGAAGACCCGACCTATGTTTCCGAGAATGACGTACCGCTAAACACAGACTACTATTTC

TCCCACCTGTTGGGTACCATAAGCGTGACCTTTAAGGCTCTATTCGGAAATGATGTGAGA

ACAACAGAAAATCTTTTAAAGCGGTTTATTCCGGAAACCCCCCACAAGACCCCCACGAAA

ACCCAGGCACTGCTTGAGCGCGCCGGCTTTGAAAAGCTGACGCCCTTTACACCGGAGGAA

GAAAGTCGTCGAATACTGCATACAGTTTTTTGTACTCTAGAAGCAGCTCCCCATCAAAGC

TGA

>OR085516.1

ATGGCGGCGCGCGAACAGGCCAACTCCGTTCGCAGGAGCGGGTTTTTTAATCCGTTTATT

GGCAAGAGGCCATTTTTCAGGCCGGGAAGCGGGCAAACCGCGGAGACAGAGCGCCCAAGG

CCCCCCCAACACTCGTACTGCACAGAGGTGGGTAGCTTTAAGTTTATAGCTCCAAGATGC

CTCGATGAAGAAGCCCCCGCCGACCAGCGACGCGGTGTACACGTGGGCACCTTGGAGCGC

CCACCCAAAGTGTACTGCGATGGCTCAGAGTACGACGTGCTGAACTTTGCCTCCGGAGGT

TGCTGGCCTCGCAGGATTCGGGTTTGGAACGGCCAGGATTTTCGGGGCGATGGATTCAAC

CCCAGATTTGAGAGATTTCACGTGTACGACATAGTAGAGACTTCGGAGAGCGCCTCACAC

GATGACCCATCCAGGTTTGCTGAGCTATCCCGCCCAAGCGGGTCTGTGGTAACACTGCTG

GGAATGAGCGAGTGTGGAAAGCGAGTCGCGGTTCACGTCTATGGTGTGCGCCATTATTTT

TACATGGCAAAGGCGGAGGTGGATAGCGCTTGTGGAATAACCACCGAGGCAGAACTCGTG

CGCGCAATGGTGGACTGCGCGCACAGCTCGGCTTTGAGCGCGGCCCTGGGAAATGGCAAC

GGCGGCAAACAGAGCGGCGGCAGCGGCGGGGGATGGTGGGGCGGAAAGCACGTGTCTGCG

GACTGCTTCAAAGTGGAGACCGTGTGCCACACGACGCTGTACTACTTTGGATCTAAGCCA

GCTCTCTACTATAGAGTATCTGCCTCCAGCAGCCGCCTGGGAGGGTTCATCTGCGACAAC

TTTCACCCGGAGATTACAAAATTCGAGGGGAGCGTGGACGTGACCACGCGGCTGTTGTTG

GACAATGAAAATTTTACCAGTTTCGGGTGGTACCGCCTGCGACCCGGCACCCACGGAGAG

CGTGTTCAACTTCGCCCCGTTGAGCGACACGTCACCTCAAGCGACGTGGAGATTAACTGT

ACTCCCGATAACCTGGAGCCGATACCAGACGAGGCTGCCTGGCCCGACTATAAGCTCATG

TGCTTTGATATAGAGTGTAAAGCTGGAACGGGTAACGAAATGGCGTTCCCAGTGGCAACT

AACCAAGAGGACCTGGTCATCCAGATCTCCTGTCTGCTGTACTCGCTTGCTACTCAGAAC

CACGAACACACCCTGCTGTTTTCCCTCGGGTCATGCGATATCTCTGAGGAATACTCGTTT

GCATGCGTCCAGCGCGGCGAGCCCAGACCGACGGTTTTGGAGTTTGACAGCGAGTACGAG

CTGCTGGTTGCCTTCCTGACCTTTCTCAAGCAGTACTCTCCCGAGTTCGCCACCGGCTAC

AACATCGTTAATTTTGACTGGGCGTACATAGTTAACAAGGTAACGTCGGTGTATAACATC

AAGCTGGACGGGTACGGCAAGTTCAACAAAGGGGGGCTGTTTAAGGTGTGGGACATCGCC

ACGAACCATTTTCAGAAGAAGAGCAAGGTGAAAATCAATGGCCTGATATCTCTAGACATG

TATTCTGTGGCGACGGAAAAGCTAAAGCTACCCAGCTACAAACTCGACGCGGTCGTGGGA

GACGTCCTCGGCGAGCATAAGATAGACCTTCCCTATAAAGAAATACCCTCCTATTACGCG

GGAGGGCCTGACCGGAGGGGCGTAATAGGAGAGTATTGTATCCAGGACTCTAGGCTGGTG

GGCAAGCTGTTTTTTAAGTACCTCCCCCATCTGGAACTATCGGCGGTGGCCAAACTCGCC

CGTATCACCCTAACGCGGGTAATTTTTGACGGTCAGCAAATTCGCGTGTACACGTGCTTG

CTGAAACTCGCCCGCGAGAGAAATTTCATTTTGCCAGACAACAGACGCCGGTTTGACAGT

CAGGCAGACGCCGCGTCAGAGACTTCGGAGTTGGCTATGGATAGCCAAAGCCACGCCTTC

GACAGTACAGACGAACCCGACGGTGTGGACGGTACCCCGGACGCCGCAGGATCTGGCGCT

ACTTCTGAAAACGGAGGCGGGAAGCCCGGCGTCGGGAGGGCCGTGGGCTACCAGGGAGCA

AAGGTTCTAGACCCCGTATCCGGCTTTCATGTGGACCCCGTGGTTGTGTTTGACTTCGCT

AGCTTATACCCAAGCATTATCCAGGCCCATAACCTCTGTTTCACCACCCTGGCGCTCGAT

GAAGTGGATCTGGCCGGGCTTCAACCATCCGTCAACTACTCGACGTTCGAGGTGGGTGAC

CAAAAGTTATTTTTTGTCCACGCCCATATTCGCGAAAGCCTGCTTGGCATCTTGCTGCGC

GACTGGCTGGCCATGCGAAAGGCGGTGAGGGCGCGAATCCCCACCAGCACCCCCGAGGAG

GCAGTTTTACTAGATAAGCAGCAGTCTGCGATTAAGGTGATATGCAACTCGGTTTACGGA

TTCACGGGGGTGGCAAACGGCCTGTTGCCGTGTCTGAGGATAGCGGCTACCGTTACCACG

ATAGGACGCGACATGCTCCTCAAGACCCGAGATTACGTTCACTCTCGTTGGGCGACGCGC

GAGCTGCTGGAGGACAATTTTCCAGGGGCTATAGGTTTCCGAAACCACAAGCCTTACTCC

GTCAGGGTTATCTACGGAGACACCGACTCCGTGTTTATCAAGTTTGTGGGCCTGACGTAC

GAGGGGGTATCGGAGCTGGGGGATGCTATGTCGCGTCAGATTTCAGCGGACCTCTTTAGA

GCGCCCATCAAACTGGAGTGTGAGAAGACCTTTCAGCGACTGCTGCTGATCACCAAGAAG

AAGTACATAGGTGTCATAAACGGGGGGAAGATGCTCATGAAGGGGGTCGACCTGGTCCGC

AAAAATAACTGCTCTTTCATAAACTTGTACGCGCGACATCTGGTAGATCTTTTGTTGTAC

GACGAGGATGTGGCCACGGCGGCAGCAAAGGTGACAGACGTGCCTCCCGCAGAATGGGTG

GGGCGCCCGCTACCGAGCGGCTTTGACAAGTTTGGGCGAGTGCTGGTAGAGGCGTACAAC

CGTATCACTGCCCCCAACTTGGACGTGCGCGAGTTCGTTATGACTGCTGAGCTGAGCCGC

TCACCCGAATCGTATACCAACAAGCGCCTGCCGCACCTCACCGTCTACTTTAAGCTCGCC

ATGAGGAATGAAGAACTGCCCAGCGTAAAAGAGAGAATTCCGTATGTGATAGTTGCGCAG

ACCGAGGCCGCGGAACGCGAAGCGGGTGTAGTAAACTCAATGCGCGGTACCGCCCAAAAC

CCCGTGGTAACCAAGACCGCACGCCCCCAACCTAAACGCAAACTGCTGGTTTCCGACCTC

GCCGAAGACCCGACCTATGTTTCCGAGAATGACGTACCGCTAAACACAGACTACTATTTC

TCCCACCTGTTGGGTACCATAAGCGTGACCTTTAAGGCTCTATTCGGAAATGATGTGAGA

ACAACAGAAAATCTTTTAAAGCGGTTTATTCCGGAAACCCCCCACAAGACCCCCACGAAA

ACCCAGGCACTGCTTGAGCGCGCCGGCTTTGAAAAGCTGACGCCCTTTACACCGGAGGAA

GAAAGTCGTCGAATACTGCATACAGTTTTTTGTACTCTAGAAGCAGCTCCCCATCAAAGC

TGA

>OR085517.1

ATGGCGGCGCGCGAACAGGCCAACTCCGTTCGCAGGAGCGGGTTTTTTAATCCGTTTATT

GGCAAGAGGCCATTTTTCAGGCCGGGAAGCGGGCAGACCGCGGAGACAGAGCGCCCAAGG

CCCCCCCAACACTCGTACTGCACAGAGGTGGGTAGCTTTAAGTTTATAGCTCCAAGATGC

CTCGATGAAGAAGCCCCCGCCGACCAGCGACGCGGTGTACACGTGGGCACCTTGGAGCGC

CCACCCAAAGTGTACTGCGATGGCTCAGAGTACGACGTGCTGAACTTTGCCTCCGGAGGT

TGCTGGCCTCGCAGGATTCGGGTTTGGAACGGCCAGGATTTTCGGGGCGATGGATTCAAC

CCCAGATTTGAGAGATTTCACGTGTACGACATAGTAGAGACTTCGGAGAGCGCCTCACAC

GATGACCCATCCAGGTTTGCTGAGCTATCCCGCCCAAGCGGGTCTGTGGTAACACTGCTG

GGAATGAGCGAGTGTGGAAAGCGAGTCGCGGTTCACGTCTATGGTGTGCGCCATTATTTT

TACATGGCAAAGGCGGAGGTGGATAGCGCTTGTGGAATAACCACCGAGGCAGAACTCGTG

CGCGCAATGGTGGACTGCGCGCACAGCTCGGCTTTGAGCGCGGCCCTGGGAAATGGCAAC

GGCGGCAAACAGAGCGGCGGCAGCGGCGGGGGATGGTGGGGCGGAAAGCACGTGTCTGCG

GACTGCTTCAAAGTGGAGACCGTGTGCCACACGACGCTGTACTACTTTGGATCTAAGCCA

GCTCTCTACTATAGAGTATCTGCCTCCAGCAGCCGCCTGGGAGGGTTCATCTGCGACAAC

TTTCACCCGGAGATTACAAAATTCGAGGGGAGCGTGGACGTGACCACGCGGCTGTTGTTG

GACAATGAAAATTTTACCAGTTTCGGGTGGTACCGCCTGCGACCCGGCACCCACGGAGAG

CGTGTTCAACTTCGCCCCGTTGAGCGACACGTCACCTCAAGCGACGTGGAGATTAACTGT

ACTCCCGATAACCTGGAGCCGATACCAGACGAGGCTGCCTGGCCCGACTATAAGCTCATG

TGCTTTGATATAGAGTGTAAAGCTGGAACGGGTAACGAAATGGCGTTCCCAGTGGCAACT

AACCAAGAGGACCTGGTCATCCAGATCTCCTGTCTGCTGTACTCGCTTGCTACTCAGAAC

CACGAACACACCCTGCTGTTTTCCCTCGGGTCATGCGATATCTCTGAGGAATACTCGTTT

GCATGCGTCCAGCGCGGCGAGCCCAGACCGACGGTTTTGGAGTTTGACAGCGAGTACGAG

CTGCTGGTTGCCTTCCTGACCTTTCTCAAGCAGTACTCTCCCGAGTTCGCCACCGGCTAC

AACATCGTTAATTTTGACTGGGCGTACATAGTTAACAAGGTAACGTCGGTGTATAACATC

AAGCTGGACGGGTACGGCAAGTTCAACAAAGGGGGGCTGTTTAAGGTGTGGGACATCGCC

ACGAACCATTTTCAGAAGAAGAGCAAGGTGAAAATCAATGGCCTGATATCTCTAGACATG

TATTCTGTGGCGACGGAAAAGCTAAAGCTACCCAGCTACAAACTCGACGCGGTCGTGGGA

GACGTCCTCGGCGAGCATAAGATAGACCTTCCCTATAAAGAAATACCCTCCTATTACGCG

GGAGGGCCTGACCGGAGGGGCGTAATAGGAGAGTATTGTATCCAGGACTCTAGGCTGGTG

GGCAAGCTGTTTTTTAAGTACCTCCCCCATCTGGAACTATCGGCGGTGGCCAAACTCGCC

CGTATCACCCTAACGCGGGTAATTTTTGACGGTCAGCAAATTCGCGTGTACACGTGCTTG

CTGAAACTCGCCCGCGAGAGAAATTTCATTTTGCCAGACAACAGACGCCGGTTTGACAGT

CAGGCAGACGCCGCGTCAGAGACTTCGGAGTTGGCTATGGATAGCCAAAGCCACGCCTTC

GACAGTACAGACGAACCCGACGGTGTGGACGGTACCCCGGACGCCGCAGGATCTGGCGCT

ACTTCTGAAAACGGAGGCGGGAAGCCCGGCGTCGGGAGGGCCGTGGGCTACCAGGGAGCA

AAGGTTCTAGACCCCGTATCCGGCTTTCATGTGGACCCCGTGGTTGTGCTTGACTTCGCT

AGCTTATACCCAAGCATTATCCAGGCCCATAACCTCTGTTTCACCACCCTGGCGCTCGAT

GAAGTGGATCTGGCCGGGCTTCAACCATCCGTCAACTACTCGACGTTCGAGGTGGGTGAC

CAAAAGTTATTTTTTGTCCACGCCCATATTCGCGAAAGCCTGCTTGGCATCTTGCTGCGC

GACTGGCTGGCCATGCGAAAGGCGGTGAGGGCGCGAATCCCCACCAGCACCCCCGAGGAG

GCAGTTTTACTAGATAAGCAGCAGTCTGCGATTAAGGTGATATGCAACTCGGTTTACGGA

TTCACGGGGGTGGCAAACGGCCTGTTGCCGTGTCTGAGGATAGCGGCTACCGTTACCACG

ATAGGACGCGACATGCTCCTCAAGACCCGAGATTACGTTCACTCTCGTTGGGCGACGCGC

GAGCTGCTGGAGGACAATTTTCCAGGGGCTATAGGTTTCCGAAACCACAAGCCTTACTCC

GTCAGGGTTATCTACGGAGACACCGACTCCGTGTTTATCAAGTTTGTGGGCCTGACGTAC

GAGGGGGTATCGGAGCTGGGGGATGCTATGTCGCGTCAGATTTCAGCGGACCTCTTTAGA

GCGCCCATCAAACTGGAGTGTGAGAAGACCTTTCAGCGACTGCTGCTGATCACCAAGAAG

AAGTACATAGGTGTCATAAACGGGGGGAAGATGCTCATGAAGGGGGTCGACCTGGTCCGC

AAAAATAACTGCTCTTTCATAAACTTGTACGCGCGACATCTGGTAGATCTTTTGTTGTAC

GACGAGGATGTGGCCACGGCGGCAGCAGAGGTGACAGACGTGCCTCCCGCAGAATGGGTG

GGGCGCCCGCTACCGAGCGGCTTTGACAAGTTTGGGCGAGTGCTGGTAGAGGCGTACAAC

CGTATCACTGCCCCCAACTTGGACGTGCGCGAGTTCGTTATGACTGCTGAGCTGAGCCGC

TCACCCGAATTGTATACCAACAAGCGCCTGCCGCACCTCACCGTCTACTTTAAGCTCGCC

ATGAGGAATGAAGAACTGCCCAGCGTAAAAGAGAGAATTCCGTATGTGATAGTTGCGCAG

ACCGAGGCCGCGGAACGCGAAGCGGGTGTAGTAAACTCAATGCGCGGTACCGCCCAAAAC

CCCGTGGTAACCAAGACCGCACGCCCCCAACCTAAACGCAAACTGCTGGTTTCCGACCTC

GCCGAAGACCCGACCTATGTTTCCGAGAATGACGTACCGCTAAACACAGACTACTATTTC

TCCCACCTGTTGGGTACCATAAGCGTGACCTTTAAGGCTCTATTCGGAAATGATGTGAGA

ACAACAGAAAATCTTTTAAAGCGGTTTATTCCGGAAACCCCCCACAAGACCCCCACGAAA

ACCCAGGCACTGCTTGAGCGCGCCGGCTTTGAAAAGCTGACGCCCTTTACACCGGAGGAA

GAAAGTCGTCGAATACTGCATACAGTTTTTTGTACTCTAGAAGCAGCTCCCCATCAAAGC

TGA

>OR085518.1

ATGGCGGCGCGCGAACAGGCCAACTCCGTTCGCAGGAGCGGGTTTTTTAATCCGTTTATT

GGCAAGAGGCCATTTTTCAGGCCGGGAAGCGGGCAGACCGCGGAGACAGAGCGCCCAAGG

CCCCCCCAACACTCGTACTGCACAGAGGTGGGTAGCTTTAAGTTTATAGCTCCAAGATGC

CTCGATGAAGAAGCCCCCGCCGACCAGCGACGCGGTGTACACGTGGGCACCTTGGAGCGC

CCACCCAAAGTGTACTGCGATGGCTCAGAGTACGACGTGCTGAACTTTGCCTCCGGAGGT

TGCTGGCCTCGCAGGATTCGGGTTTGGAACGGCCAGGATTTTCGGGGCGATGGATTCAAC

CCCAGATTTGAGAGATTTCACGTGTACGACATAGTAGAGACTTCGGAGAGCGCCTCACAC

GATGACCCATCCAGGTTTGCTGAGCTATCCCGCCCAAGCGGGTCTGTGGTAACACTGCTG

GGAATGAGCGAGTGTGGAAAGCGAGTCGCGGTTCACGTCTATGGTGTGCGCCATTATTTT

TACATGGCAAAGGCGGAGGTGGATAGCGCTTGTGGAATAACCACCGAGGCAGAACTCGTG

CGCGCAATGGTGGACTGCGCGCACAGCTCGGCTTTGAGCGCGGCCCTGGGAAATGGCAAC

GGCGGCAAACAGAGCGGCGGCAGCGGCGGGGGATGGTGGGGCGGAAAGCACGTGTCTGCG

GACTGCTTCAAAGTGGAGACCGTGTGCCACACGACGCTGTACTACTTTGGATCTAAGCCA

GCTCTCTACTATAGAGTATCTGCCTCCAGCAGCCGCCTGGGAGGGTTCATCTGCGACAAC

TTTCACCCGGAGATTACAAAATTCGAGGGGAGCGTGGACGTGACCACGCGGCTGTTGTTG

GACAATGAAAATTTTACCAGTTTCGGGTGGTACCGCCTGCGACCCGGCACCCACGGAGAG

CGTGTTCAACTTCGCCCCGTTGAGCGACACGTCACCTCAAGCGACGTGGAGATTAACTGT

ACTCCCGATAACCTGGAGCCGATACCAGACGAGGCTGCCTGGCCCGACTATAAGCTCATG

TGCTTTGATATAGAGTGTAAAGCTGGAACGGGTAACGAAATGGCGTTCCCAGTGGCAACT

AACCAAGAGGACCTGGTCATCCAGATCTCCTGTCTGCTGTACTCGCTTGCTACTCAGAAC

CACGAACACACCCTGCTGTTTTCCCTCGGGTCATGCGATATCTCTGAGGAATACTCGTTT

GCATGCGTCCAGCGCGGCGAGCCCAGACCGACGGTTTTGGAGTTTGACAGCGAGTACGAG

CTGCTGGTTGCCTTCCTGACCTTTCTCAAGCAGTACTCTCCCGAGTTCGCCACCGGCTAC

AACATCGTTAATTTTGACTGGGCGTACATAGTTAACAAGGTAACGTCGGTGTATAACATC

AAGCTGGACGGGTACGGCAAGTTCAACAAAGGGGGGCTGTTTAAGGTGTGGGACATCGCC

ACGAACCATTTTCAGAAGAAGAGCAAGGTGAAAATCAATGGCCTGATATCTCTAGACATG

TATTCTGTGGCGACGGAAAAGCTAAAGCTACCCAGCTACAAACTCGACGCGGTCGTGGGA

GACGTCCTCGGCGAGCATAAGATAGACCTTCCCTATAAAGAAATACCCTCCTATTACGCG

GGAGGGCCTGACCGGAGGGGCGTAATAGGAGAGTATTGTATCCAGGACTCTAGGCTGGTG

GGCAAGCTGTTTTTTAAGTACCTCCCCCATCTGGAACTATCGGCGGTGGCCAAACTCGCC

CGTATCACCCTAACGCGGGTAATTTTTGACGGTCAGCAAATTCGCGTGTACACGTGCTTG

CTGAAACTCGCCCGCGAGAGAAATTTCATTTTGCCAGACAACAGACGCCGGTTTGACAGT

CAGGCAGACGCCGCGTCAGAGACTTCGGAGTTGGCTATGGATAGCCAAAGCCACGCCTTC

GACAGTACAGACGAACCCGACGGTGTGGACGGTACCCCGGACGCCGCAGGATCTGGCGCT

ACTTCTGAAAACGGAGGCGGGAAGCCCGGCGTCGGGAGGGCCGTGGGCTACCAGGGAGCA

AAGGTTCTAGACCCCGTATCCGGCTTTCATGTGGACCCCGTGGTTGTGTTTGACTTCGCT

AGCTTATACCCAAGCATTATCCAGGCCCATAACCTCTGTTTCACCACCCTGGCGCTCGAT

GAAGTGGATCTGGCCGGGCTTCAACCATCCGTCAACTACTCGACGTTCGAGGTGGGTGAC

CAAAAGTTATTTTTTGTCCACGCCCATATTCGCGAAAGCCTGCTTGGCATCTTGCTGCGC

GACTGGCTGGCCATGCGAAAGGCGGTGAGGGCGCGAATCCCCACCAGCACCCCCGAGGAG

GCAGTTTTACTAGATAAGCAGCAGTCTGCGATTAAGGTGATATGCAACTCGGTTTACGGA

TTCACGGGGGTGGCAAACGGCCTGTTGCCGTGTCTGAGGATAGCGGCTACCGTTACCACG

ATAGGACGCGACATGCTCCTCAAGACCCGAGATTACGTTCACTCTCGTTGGGCGACGCGC

GAGCTGCTGGAGGACAATTTTCCAGGGGCTATAGGTTTCCGAAACCACAAGCCTTACTCC

GTCAGGGTTATCTACGGAGACACCGACTCCGTGTTTATCAAGTTTGTGGGCCTGACGTAC

GAGGGGGTATCGGAGCTGGGGGATGCTATGTCGCGTCAGATTTCAGCGGACCTCTTTAGA

GCGCCCATCAAACTGGAGTGTGAGAAGACCTTTCAGCGACTGCTGCTGATCACCAAGAAG

AAGTACATAGGTGTCATAAACGGGGGGAAGATGCTCATGAAGGGGGTCGACCTGGTCCGC

AAAAATAACTGCTCTTTCATAAACTTGTACGCGCGACATCTGGTAGATCTTTTGTTGTAC

GACGAGGATGTGGCCACGGCGGCAGCAGAGGTGACAGACGTGCCTCCCGCAGAATGGGTG

GGGCGCCCGCTACCGAGCGGCTTTGACAAGTTTGGGCGAGTGCTGGTAGAGGCGTACAAC

CGTATCACTGCCCCCAACTTGGACGTGCGCGAGTTCGTTATGACTGCTGAGCTGAGCCGC

TCACCCGAATCGTATACCAACAAGCGCCTGCCGCACCTCACCGTCTACTTTAAGCTCGCC

ATGAGGAATGAAGAACTGCCCAGCGTAAAAGAGAGAATTCCGTATGTGATAGTTGCGCAG

ACCGAGGCCGCGGAACGCGAAGCGGGTGTAGTAAACTCAATGCGCGGTACCGCCCAAAAC

CCCGTGGTAACCAAGACCGCACGCCCCCAACCTAAACGCAAACTGCTGGTTTCCGACCTC

GCCGAAGACCCGACCTATGTTTCCGAGAATGACGTACCGCTAAACACAGACTACTATTTC

TCCCACCTGTTGGGTACCATAAGCGTGACCTTTAAGGCTCTATTCGGAAATGATGTGAGA

ACAACAGAAAATCTTTTAAAGCGGTTTATTCCGGAAACCCCCCACAAGACCCCCACGAAA

ACCCAGGCACTGCTTGAGCGCGCCGGCTTTGAAAAGCTGACGCCCTTTACACCGGAGGAA

GAAAGTCGTCGAATACTGCATACAGTTTTTTGTACTCTAGAAGCAGCTCCCCATCAAAGC

TGA

>PP481413.1

ATGGCGGCGCGCGAACAGGCCAACTCCGTTCGCAGGAGCGGGTTTTTTAATCCGTTTATT

GGCAAGAGGCCATTTTTCAGGCCGGGAAGCGGGCAGACCGCGGAGACAGAGCGCCCAAGG

CCCCCCCAACACTCGTACTGCACAGAGGTGGGTAGCTTTAAGTTTATAGCTCCAAGATGC

CTCGATGAAGAAGCCCCCGCCGACCAGCGACGCGGTGTACACGTGGGCACCTTGGAGCGC

CCACCCAAAGTGTACTGCGATGGCTCAGAGTACGACGTGCTGAACTTTGCCTCCGGAGGT

TGCTGGCCTCGCAGGATTCGGGTTTGGAACGGCCAGGATTTTCGGGGCGATGGATTCAAC

CCCAGATTTGAGAGATTTCACGTGTACGACATAGTAGAGACTTCGGAGAGCGCCTCACAC

GATGACCCATCCAGGTTTGCTGAGCTATCCCGCCCAAGCGGGTCTGTGGTAACACTGCTG

GGAATGAGCGAGTGTGGAAAGCGAGTCGCGGTTCACGTCTATGGTGTGCGCCATTATTTT

TACATGGCAAAGGCGGAGGTGGATAGCGCTTGTGGAATAACCACCGAGGCAGAACTCGTG

CGCGCAATGGTGGACTGCGCGCACAGCTCGGCTTTGAGCGCGGCCCTGGGAAATGGCAAC

GGCGGCAAACAGAGCGGCGGCAGCGGCGGGGGATGGTGGGGCGGAAAGCACGTGTCTGCG

GACTGCTTCAAAGTGGAGACCGTGTGCCACACGACGCTGTACTACTTTGGATCTAAGCCA

GCTCTCTACTATAGAGTATCTGCCTCCAGCAGCCGCCTGGGAGGGTTCATCTGCGACAAC

TTTCACCCGGAGATTACAAAATTCGAGGGGAGCGTGGACGTGACCACGCGGCTGTTGTTG

GACAATGAAAATTTTACCAGTTTCGGGTGGTACCGCCTGCGACCCGGCACCCACGGAGAG

CGTGTTCAACTTCGCCCCGTTGAGCGACACGTCACCTCAAGCGACGTGGAGATTAACTGT

ACTCCCGATAACCTGGAGCCGATACCAGACGAGGCTGCCTGGCCCGACTATAAGCTCATG

TGCTTTGATATAGAGTGTAAAGCTGGAACGGGTAACGAAATGGCGTTCCCAGTGGCAACT

AACCAAGAGGACCTGGTCATCCAGATCTCCTGTCTGCTGTACTCGCTTGCTACTCAGAAC

CACGAACACACCCTGCTGTTTTCCCTCGGGTCATGCGATATCTCTGAGGAATACTCGTTT

GCATGCGTCCAGCGCGGCGAGCCCAGACCGACGGTTTTGGAGTTTGACAGCGAGTACGAG

CTGCTGGTTGCCTTCCTGACCTTTCTCAAGCAGTACTCTCCCGAGTTCGCCACCGGCTAC

AACATCGTTAATTTTGACTGGGCGTACATAGTTAACAAGGTAACGTCGGTGTATAACATC

AAGCTGGACGGGTACGGCAAGTTCAACAAAGGGGGGCTGTTTAAGGTGTGGGACATCGCC

ACGAACCATTTTCAGAAGAAGAGCAAGGTGAAAATCAATGGCCTGATATCTCTAGACATG

TATTCTGTGGCGACGGAAAAGCTAAAGCTACCCAGCTACAAACTCGACGCGGTCGTGGGA

GACGTCCTCGGCGAGCATAAGATAGACCTTCCCTATAAAGAAATACCCTCCTATTACGCG

GGAGGGCCTGACCGGAGGGGCGTAATAGGAGAGTATTGTATCCAGGACTCTAGGCTGGTG

GGCAAGCTGTTTTTTAAGTACCTCCCCCATCTGGAACTATCGGCGGTGGCCAAACTCGCC

CGTATCACCCTAACGCGGGTAATTTTTGACGGTCAGCAAATTCGCGTGTACACGTGCTTG

CTGAAACTCGCCCGCGAGAGAAATTTCATTTTGCCAGACAACAGACGCCGGTTTGACAGT

CAGGCAGACGCCGCGTCAGAGACTTCGGAGTTGGCTATGGATAGCCAAAGCCACGCCTTC

GACAGTACAGACGAACCCGACGGTGTGGACGGTACCCCGGACGCCGCAGGATCTGGCGCT

ACTTCTGAAAACGGAGGCGGGAAGCCCGGCGTCGGGAGGGTCGTGGGCTACCAGGGAGCA

AAGGTTCTAGACCCCGTATCCGGCTTTCATGTGGACCCCGTGGTTGTGTTTGACTTCGCT

AGCTTATACCCAAGCATTATCCAGGCCCATAACCTCTGTTTCACCACCCTGGCGCTCGAT

GAAGTGGATCTGGCCGGGCTTCAACCATCCGTCGACTACTCGACGTTCGAGGTGGGTGAC

CAAAAGTTATTTTTTGTCCACGCCCATATTCGCGAAAGCCTGCTTGGCATCTTGCTGCGC

GACTGGCTGGCCATGCGAAAGGCGGTGAGGGCGCGAATCCCCACCAGCACCCCCGAGGAG

GCAGTTTTACTAGATAAGCAGCAGTCTGCGATTAAGGTGATATGCAACTCGGTTTACGGA

TTCACGGGGGTGGCAAACGGCCTGTTGCCGTGTCTGAGGATAGCGGCTACCGTTACCACG

ATAGGACGCGACATGCTCCTCAAGACCCGAGATTACGTTCACTCTCGTTGGGCGACGCGC

GAGCTGCTGGAGGACAATTTTCCAGGGGCTATAGGTTTCCGAAACCACAAGCCTTACTCC

GTCAGGGTTATCTACGGAGACACCGACTCCGTGTTTATCAAGTTTGTGGGCCTGACGTAC

GAGGGGGTATCGGAGCTGGGGGATGCTATGTCGCGTCAGATTTCAGCGGACCTCTTTAGA

GCGCCCATCAAACTGGAGTGTGAGAAGACCTTTCAGCGACTGCTGCTGATCACCAAGAAG

AAGTACATAGGTGTCATAAACGGGGGGAAGATGCTCATGAAGGGGGTCGACCTGGTCCGC

AAAAATAACTGCTCTTTCATAAACTTGTACGCGCGACATCTGGTAGATCTTTTGTTGTAC

GACGAGGATGTGGCCACGGCGGCAGCAGAGGTGACAGACGTGCCTCCCGCAGAATGGGTG

GGGCGCCCGCTACCGAGCGGCTTTGACAAGTTTGGGCGAGTGCTGGTAGAGGCGTACAAC

CGTATCACTGCCCCCAACTTGGACGTGCGCGAGTTCGTTATGACTGCTGAGCTGAGCCGC

TCACCCGAATCGTATACCAACAAGCGCCTGCCGCACCTCACCGTCTACTTTAAGCTCGCC

ATGAGGAATGAAGAACTGCCCAGCGTAAAAGAGAGAATTCCGTATGTGATAGTTGCGCAG

ACCGAGGCCGCGGAACGCGAAGCGGGTGTAGTAAACTCAATGCGCGGTACCGCCCAAAAC

CCCGTGGTAACCAAGACCGCACGCCCCCAACCTAAACGCAAACTGCTGGTTTCCGACCTC

GCCGAAGACCCGACCTATGTTTCCGAGAATGACGTACCGCTAAACACAGACTACTATTTC

TCCCACCTGTTGGGTACCATAAGCGTGACCTTTAAGGCTCTATTCGGAAATGATGTGAGA

ACAACAGAAAATCTTTTAAAGCGGTTTATTCCGGAAACCCCCCACAAGACCCCCACGAAA

ACCCAGGCACTGCTTGAGCGCGCCGGCTTTGAAAAGCTGACGCCCTTTACACCGGAGGAA

GAAAGTCGTCGAATACTGCATACAGTTTTTTGTACTCTAGAAGCAGCTCCCCATCAAAGC

TGA

>OR801338.1

ATGGCGGCGCGCGAACAGGCCAACTCCGTTCGCAGGAGCGGGTTTTTTAATCCGTTTATT

GGCAAGAGGCCATTTTTCAGGCCGGGAAGCGGGCAGACCGCGGAGACAGAGCGCCCAAGG

CCCCCCCAACACTCGTACTGCACAGAGGTGGGTAGCTTTAAGTTTATAGCTCCAAGATGC

CTCGATGAAGAAGCCCCCGCCGACCAGCGACGCGGTGTACACGTGGGCACCTTGGAGCGC

CCACCCAAAGTGTACTGCGATGGCTCAGAGTACGACGTGCTGAACTTTGCCTCCGGAGGT

TGCTGGCCTCGCAGGATTCGGGTTTGGAACGGCCAGGATTTTCGGGGCGATGGATTCAAC

CCCAGATTTGAGAGATTTCACGTGTACGACATAGTAGAGACTTCGGAGAGCGCCTCACAC

GATGACCCATCCAGGTTTGCTGAGCTATCCCGCCCAAGCGGGTCTGTGGTAACACTGCTG

GGAATGAGCGAGTGTGGAAAGCGAGTCGCGGTTCACGTCTATGGTGTGCGCCATTATTTT

TACATGGCAAAGGCGGAGGTGGATAGCGCTTGTGGAATAACCACCGAGGCAGAACTCGTG

CGCGCAATGGTGGACTGCGCGCACAGCTCGGCTTTGAGCGCGGCCCTGGGAAATGGCAAC

GGCGGCAAACAGAGCGGCGGCAGCGGCGGGGGATGGTGGGGCGGAAAGCACGTGTCTGCG

GACTGCTTCAAAGTGGAGACCGTGTGCCACACGACGCTGTACTACTTTGGATCTAAGCCA

GCTCTCTACTATAGAGTATCTGCCTCCAGCAGCCGCCTGGGAGGGTTCATCTGCGACAAC

TTTCACCCGGAGATTACAAAATTCGAGGGGAGCGTGGACGTGACCACGCGGCTGTTGTTG

GACAATGAAAATTTTACCAGTTTCGGGTGGTACCGCCTGCGACCCGGCACCCACGGAGAG

CGTGTTCAACTTCGCCCCGTTGAGCGACACGTCACCTCAAGCGACGTGGAGATTAACTGT

ACTCCCGATAACCTGGAGCCGATACCAGACGAGGCTGCCTGGCCCGACTATAAGCTCATG

TGCTTTGATATAGAGTGTAAAGCTGGAACGGGTAACGAAATGGCGTTCCCAGTGGCAACT

AACCAAGAGGACCTGGTCATCCAGATCTCCTGTCTGCTGTACTCGCTTGCTACTCAGAAC

CACGAACACACCCTGCTGTTTTCCCTCGGGTCATGCGATATCTCTGAGGAATACTCGTTT

GCATGCGTCCAGCGCGGCGAGCCCAGACCGACGGTTTTGGAGTTTGACAGCGAGTACGAG

CTGCTGGTTGCCTTCCTGACCTTTCTCAAGCAGTACTCTCCCGAGTTCGCCACCGGCTAC

AACATCGTTAATTTTGACTGGGCGTACATAGTTAACAAGGTAACGTCGGTGTATAACATC

AAGCTGGACGGGTACGGCAAGTTCAACAAAGGGGGGCTGTTTAAGGTGTGGGACATCGCC

ACGAACCATTTTCAGAAGAAGAGCAAGGTGAAAATCAATGGCCTGATATCTCTAGACATG

TATTCTGTGGCGACGGAAAAGCTAAAGCTACCCAGCTACAAACTCGACGCGGTCGTGGGA

GACGTCCTCGGCGAGCATAAGATAGACCTTCCCTATAAAGAAATACCCTCCTATTACGCG

GGAGGGCCTGACCGGAGGGGCGTAATAGGAGAGTATTGTATCCAGGACTCTAGGCTGGTG

GGCAAGCTGTTTTTTAAGTACCTCCCCCATCTGGAACTATCGGCGGTGGCCAAACTCGCC

CGTATCACCCTAACGCGGGTAATTTTTGACGGTCAGCAAATTCGCGTGTACACGTGCTTG

CTGAAACTCGCCCGCGAGAGAAATTTCATTTTGCCAGACAACAGACGCCGGTTTGACAGT

CAGGCAGACGCCGCGTCAGAGACTTCGGAGTTGGCTATGGATAGCCAAAGCCACGCCTTC

GACAGTACAGACGAACCCGACGGTGTGGACGGTACCCCGGACGCCGCAGGATCTGGCGCT

ACTTCTGAAAACGGAGGCGGGAAGCCCGGCGTCGGGAGGGCCGTGGGCTACCAGGGAGCA

AAGGTTCTAGACCCCGTATCCGGCTTTCATGTGGACCCCGTGGTTGTGTTTGACTTCGCT

AGCTTATACCCAAGCATTATCCAGGCCCATAACCTCTGTTTCACCACCCTGGCGCTCGAT

GAAGTGGATCTGGCCGGGCTTCAACCATCCGTCGACTACTCGACGTTCGAGGTGGGTGAC

CAAAAGTTATTTTTTGTCCACGCCCATATTCGCGAAAGCCTGCTTGGCATCTTGCTGCGC

GACTGGCTGGCCATGCGAAAGGCGGTGAGGGCGCGAATCCCCACCAGCACCCCCGAGGAG

GCAGTTTTACTAGATAAGCAGCAGTCTGCGATTAAGGTGATATGCAACTCGGTTTACGGA

TTCACGGGGGTGGCAAACGGCCTGTTGCCGTGTCTGAGGATAGCGGCTACCGTTACCACG

ATAGGACGCGACATGCTCCTCAAGACCCGAGATTACGTTCACTCTCGTTGGGCGACGCGC

GAGCTGCTGGAGGACAATTTTCCAGGGGCTATAGGTTTCCGAAACCACAAGCCTTACTCC

GTCAGGGTTATCTACGGAGACACCGACTCCGTGTTTATCAAGTTTGTGGGCCTGACGTAC

GAGGGGGTATCGGAGCTGGGGGATGCTATGTCGCGTCAGATTTCAGCGGACCTCTTTAGA

GCGCCCATCAAACTGGAGTGTGAGAAGACCTTTCAGCGACTGCTGCTGATCACCAAGAAG

AAGTACATAGGTGTCATAAACGGGGGGAAGATGCTCATGAAGGGGGTCGACCTGGTCCGC

AAAAATAACTGCTCTTTCATAAACTTGTACGCGCGACATCTGGTAGATCTTTTGTTGTAC

GACGAGGATGTGGCCACGGCGGCAGCAGAGGTGACAGACGTGCCTCCCGCAGAATGGGTG

GGGCGCCCGCTACCGAGCGGCTTTGACAAGTTTGGGCGAGTGCTGGTAGAGGCGTACAAC

CGTATCACTGCCCCCAACTTGGACGTGCGCGAGTTCGTTATGACTGCTGAGCTGAGCCGC

TCACCCGAATCGTATACCAACAAGCGCCTGCCGCACCTCACCGTCTACTTTAAGCTCGCC

ATGAGGAATGAAGAACTGCCCAGCGTAAAAGAGAGAATTCCGTATGTGATAGTTGCGCAG

ACCGAGGCCGCGGAACGCGAAGCGGGTGTAGTAAACTCAATGCGCGGTACCGCCCAAAAC

CCCGTGGTAACCAAGACCGCACGCCCCCAACCTAAACGCAAACTGCTGGTTTCCGACCTC

GCCGAAGACCCGACCTATGTTTCCGAGAATGACGTACCGCTAAACACAGACTACTATTTC

TCCCACCTGTTGGGTACCATAAGCGTGACCTTTAAGGCTCTATTCGGAAATGATGTGAGA

ACAACAGAAAATCTTTTAAAGCGGTTTATTCCGGAAACCCCCCACAAGACCCCCACGAAA

ACCCAGGCACTGCTTGAGCGCGCCGGCTTTGAAAAGCTGACGCCCTTTACACCGGAGGAA

GAAAGTCGTCGAATACTGCATACAGTTTTTTGTACTCTAGAAGCAGCTCCCCATCAAAGC

TGA

>PP084325.1

ATGGCGGCGCGCGAACAGGCCAACTCCGTTCGCAGGAGCGGGTTTTTTAATCCGTTTATT

GGCAAGAGGCCATTTTTCAGGCCGGGAAGCGGGCAGACCGCGGAGACAGAGCGCCCAAGG

CCCCCCCAACACTCGTACTGCACAGAGGTGGGTAGCTTTAAGTTTATAGCTCCAAGATGC

CTCGATGAAGAAGCCCCCGCCGACCAGCGACGCGGTGTACACGTGGGCACCTTGGAGCGC

CCACCCAAAGTGTACTGCGATGGCTCAGAGTACGACGTGCTGAACTTTGCCTCCGGAGGT

TGCTGGCCTCGCAGGATTCGGGTTTGGAACGGCCAGGATTTTCGGGGCGATGGATTCAAC

CCCAGATTTGAGAGATTTCACGTGTACGACATAGTAGAGACTTCGGAGAGCGCCTCACAC

GATGACCCATCCAGGTTTGCTGAGCTATCCCGCCCAAGCGGGTCTGTGGTAACACTGCTG

GGAATGAGCGAGTGTGGAAAGCGAGTCGCGGTTCACGTCTATGGTGTGCGCCATTATTTT

TACATGGCAAAGGCGGAGGTGGATAGCGCTTGTGGAATAACCACCGAGGCAGAACTCGTG

CGCGCAATGGTGGACTGCGCGCACAGCTCGGCTTTGAGCGCGGCCCTGGGAAATGGCAAC

GGCGGCAAACAGAGCGGCGGCAGCGGCGGGGGATGGTGGGGCGGAAAGCACGTGTCTGCG

GACTGCTTCAAAGTGGAGACCGTGTGCCACACGACGCTGTACTACTTTGGATCTAAGCCA

GCTCTCTACTATAGAGTATCTGCCTCCAGCAGCCGCCTGGGAGGGTTCATCTGCGACAAC

TTTCACCCGGAGATTACAAAATTCGAGGGGAGCGTGGACGTGACCACGCGGCTGTTGTTG

GACAATGAAAATTTTACCAGTTTCGGGTGGTACCGCCTGCGACCCGGCACCCACGGAGAG

CGTGTTCAACTTCGCCCCGTTGAGCGACACGTCACCTCAAGCGACGTGGAGATTAACTGT

ACTCCCGATAACCTGGAGCCGATACCAGACGAGGCTGCCTGGCCCGACTATAAGCTCATG

TGCTTTGATATAGAGTGTAAAGCTGGAACGGGTAACGAAATGGCGTTCCCAGTGGCAACT

AACCAAGAGGACCTGGTCATCCAGATCTCCTGTCTGCTGTACTCGCTTGCTACTCAGAAC

CACGAACACACCCTGCTGTTTTCCCTCGGGTCATGCGATATCTCTGAGGAATACTCGTTT

GCATGCGTCCAGCGCGGCGAGCCCAGACCGACGGTTTTGGAGTTTGACAGCGAGTACGAG

CTGCTGGTTGCCTTCCTGACCTTTCTCAAGCAGTACTCTCCCGAGTTCGCCACCGGCTAC

AACATCGTTAATTTTGACTGGGCGTACATAGTTAACAAGGTAACGTCGGTGTATAACATC

AAGCTGGACGGGTACGGCAAGTTCAACAAAGGGGGGCTGTTTAAGGTGTGGGACATCGCC

ACGAACCATTTTCAGAAGAAGAGCAAGGTGAAAATCAATGGCCTGATATCTCTAGACATG

TATTCTGTGGCGACGGAAAAGCTAAAGCTACCCAGCTACAAACTCGACGCGGTCGTGGGA

GACGTCCTCGGCGAGCATAAGATAGACCTTCCCTATAAAGAAATACCCTCCTATTACGCG

GGAGGGCCTGACCGGAGGGGCGTAATAGGAGAGTATTGTATCCAGGACTCTAGGCTGGTG

GGCAAGCTGTTTTTTAAGTACCTCCCCCATCTGGAACTATCGGCGGTGGCCAAACTCGCC

CGTATCACCCTAACGCGGGTAATTTTTGACGGTCAGCAAATTCGCGTGTACACGTGCTTG

CTGAAACTCGCCCGCGAGAGAAATTTCATTTTGCCAGACAACAGACGCCGGTTTGACAGT

CAGGCAGACGCCGCGTCAGAGACTTCGGAGTTGGCTATGGATAGCCAAAGCCACGCCTTC

GACAGTACAGACGAACCCGACGGTGTGGACGGTACCCCGGACGCCGCAGGATCTGGCGCT

ACTTCTGAAAACGGAGGCGGGAAGCCCGGCGTCGGGAGGGCCGTGGGCTACCAGGGAGCA

AAGGTTCTAGACCCCGTATCCGGCTTTCATGTGGACCCCGTGGTTGTGTTTGACTTCGCT

AGCTTATACCCAAGCATTATCCAGGCCCATAACCTCTGTTTCACCACCCTGGCGCTCGAT

GAAGTGGATCTGGCCGGGCTTCAACCATCCGTCAACTACTCGACGTTCGAGGTGGGTGAC

CAAAAGTTATTTTTTGTCCACGCCCATATTCGCGAAAGCCTGCTTGGCATCTTGCTGCGC

GACTGGCTGGCCATGCGAAAGGCGGTGAGGGCGCGAATCCCCACCAGCACCCCCGAGGAG

GCAGTTTTACTAGATAAGCAGCAGTCTGCGATTAAGGTGATATGCAACTCGGTTTACGGA

TTCACGGGGGTGGCAAACGGCCTGTTGCCGTGTCTGAGGATAGCGGCTACCGTTACCACG

ATAGGACGCGACATGCTCCTCAAGACCCGAGATTACGTTCACTCTCGTTGGGCGACGCGC

GAGCTGCTGGAGGACAATTTTCCAGGGGCTATAGGTTTCCGAAACCACAAGCCTTACTCC

GTCAGGGTTATCTACGGAGACACCGACTCCGTGTTTATCAAGTTTGTGGGCCTGACGTAC

GAGGGGGTATCGGAGCTGGGGGATGCTATGTCGCGTCAGATTTCAGCGGACCTCTTTAGA

GCGCCCATCAAACTGGAGTGTGAGAAGACCTTTCAGCGACTGCTGCTGATCACCAAGAAG

AAGTACATAGGTGTCATAAACGGGGGGAAGATGCTCATGAAGGGGGTCGACCTGGTCCGC

AAAAATAACTGCTCTTTCATAAACTTGTACGCGCGACATCTGGTAGATCTTTTGTTGTAC

GACGAGGATGTGGCCACGGCGGCAGCAGAGGTGACAGACGTGCCTCCCGCAGAATGGGTG

GGGCGCCCGCTACCGAGCGGCTTTGACAAGTTTGGGCGAGTGCTGGTAGAGGCGTACAAC

CGTATCACTGCCCCCAACTTGGACGTGCGCGAGTTCGTTATGACTGCTGAGCTGAGCCGC

TCACCCGAATCGTATACCAACAAGCGCCTGCCGCACCTCACCGTCTACTTTAAGCTCGCC

ATGAGGAATGAAGAACTGCCCAGCGTAAAAGAGAGAATTCCGTATGTGATAGTTGCGCAG

ACCGAGGCCGCGGAACGCGAAGCGGGTGTAGTAAACTCAATGCGCGGTACCGCCCAAAAC

CCCGTGGTAACCAAGACCGCACGCCCCCAACCTAAACGCAAACTGCTGGTTTCCGACCTC

GCCGAAGACCCGACCTATGTTTCCGAGAATGACGTACCGCTAAACACAGACTACTATTTC

TCCCACCTGTTGGGTACCATAAGCGTGACCTTTAAGGCTCTATTCGGAAATGATGTGAGA

ACAACAGAAAATCTTTTAAAGCGGTTTATTCCGGAAACCCCCCACAAGACCCCCACGAAA

ACCCAGGCACTGCTTGAGCGCGCCGGCTTTGAAAAGCTGACGCCCTTTACACCGGAGGAA

GAAAGTCGTCGAATACTGCATACAGTTTTTTGTACTCTAGAAGCAGCTCCCCATCAAAGC

TGA

>PP084326.1

ATGGCGGCGCGCGAACAGGCCAACTCCGTTCGCAGGAGCGGGTTTTTTAATCCGTTTATT

GGCAAGAGGCCATTTTTCAGGCCGGGAAGCGGGCAGACCGCGGAGACAGAGCGCCCAAGG

CCCCCCCAACACTCGTACTGCACAGAGGTGGGTAGCTTTAAGTTTATAGCTCCAAGATGC

CTCGATGAAGAAGCCCCCGCCGACCAGCGACGCGGTGTACACGTGGGCACCTTGGAGCGC

CCACCCAAAGTGTACTGCGATGGCTCAGAGTACGACGTGCTGAACTTTGCCTCCGGAGGT

TGCTGGCCTCGCAGGATTCGGGTTTGGAACGGCCAGGATTTTCGGGGCGATGGATTCAAC

CCCAGATTTGAGAGATTTCACGTGTACGACATAGTAGAGACTTCGGAGAGCGCCTCACAC

GATGACCCATCCAGGTTTGCTGAGCTATCCCGCCCAAGCGGGTCTGTGGTAACACTGCTG

GGAATGAGCGAGTGTGGAAAGCGAGTCGCGGTTCACGTCTATGGTGTGCGCCATTATTTT

TACATGGCAAAGGCGGAGGTGGATAGCGCTTGTGGAATAACCACCGAGGCAGAACTCGTG

CGCGCAATGGTGGACTGCGCGCACAGCTCGGCTTTGAGCGCGGCCCTGGGAAATGGCAAC

GGCGGCAAACAGAGCGGCGGCAGCGGCGGGGGATGGTGGGGCGGAAAGCACGTGTCTGCG

GACTGCTTCAAAGTGGAGACCGTGTGCCACACGACGCTGTACTACTTTGGATCTAAGCCA

GCTCTCTACTATAGAGTATCTGCCTCCAGCAGCCGCCTGGGAGGGTTCATCTGCGACAAC

TTTCACCCGGAGATTACAAAATTCGAGGGGAGCGTGGACGTGACCACGCGGCTGTTGTTG

GACAATGAAAATTTTACCAGTTTCGGGTGGTACCGCCTGCGACCCGGCACCCACGGAGAG

CGTGTTCAACTTCGCCCCGTTGAGCGACACGTCACCTCAAGCGACGTGGAGATTAACTGT

ACTCCCGATAACCTGGAGCCGATACCAGACGAGGCTGCCTGGCCCGACTATAAGCTCATG

TGCTTTGATATAGAGTGTAAAGCTGGAACGGGTAACGAAATGGCGTTCCCAGTGGCAACT

AACCAAGAGGACCTGGTCATCCAGATCTCCTGTCTGCTGTACTCGCTTGCTACTCAGAAC

CACGAACACACCCTGCTGTTTTCCCTCGGGTCATGCGATATCTCTGAGGAATACTCGTTT

GCATGCGTCCAGCGCGGCGAGCCCAGACCGACGGTTTTGGAGTTTGACAGCGAGTACGAG

CTGCTGGTTGCCTTCCTGACCTTTCTCAAGCAGTACTCTCCCGAGTTCGCCACCGGCTAC

AACATCGTTAATTTTGACTGGGCGTACATAGTTAACAAGGTAACGTCGGTGTATAACATC

AAGCTGGACGGGTACGGCAAGTTCAACAAAGGGGGGCTGTTTAAGGTGTGGGACATCGCC

ACGAACCATTTTCAGAAGAAGAGCAAGGTGAAAATCAATGGCCTGATATCTCTAGACATG

TATTCTGTGGCGACGGAAAAGCTAAAGCTACCCAGCTACAAACTCGACGCGGTCGTGGGA

GACGTCCTCGGCGAGCATAAGATAGACCTTCCCTATAAAGAAATACCCTCCTATTACGCG

GGAGGGCCTGACCGGAGGGGCGTAATAGGAGAGTATTGTATCCAGGACTCTAGGCTGGTG

GGCAAGCTGTTTTTTAAGTACCTCCCCCATCTGGAACTATCGGCGGTGGCCAAACTCGCC

CGTATCACCCTAACGCGGGTAATTTTTGACGGTCAGCAAATTCGCGTGTACACGTGCTTG

CTGAAACTCGCCCGCGAGAGAAATTTCATTTTGCCAGACAACAGACGCCGGTTTGACAGT

CAGGCAGACGCCGCGTCAGAGACTTCGGAGTTGGCTATGGATAGCCAAAGCCACGCCTTC

GACAGTACAGACGAACCCGACGGTGTGGACGGTACCCCGGACGCCGCAGGATCTGGCGCT

ACTTCTGAAAACGGAGGCGGGAAGCCCGGCGTCGGGAGGGCCGTGGGCTACCAGGGAGCA

AAGGTTCTAGACCCCGTATCCGGCTTTCATGTGGACCCCGTGGTTGTGTTTGACTTCGCT

AGCTTATACCCAAGCATTATCCAGGCCCATAACCTCTGTTTCACCACCCTGGCGCTCGAT

GAAGTGGATCTGGCCGGGCTTCAACCATCCGTCAACTACTCGACGTTCGAGGTGGGTGAC

CAAAAGTTATTTTTTGTCCACGCCCATATTCGCGAAAGCCTGCTTGGCATCTTGCTGCGC

GACTGGCTGGCCATGCGAAAGGCGGTGAGGGCGCGAATCCCCACCAGCACCCCCGAGGAG

GCAGTTTTACTAGATAAGCAGCAGTCTGCGATTAAGGTGATATGCAACTCGGTTTACGGA

TTCACGGGGGTGGCAAACGGCCTGTTGCCGTGTCTGAGGATAGCGGCTACCGTTACCACG

ATAGGACGCGACATGCTCCTCAAGACCCGAGATTACGTTCACTCTCGTTGGGCGACGCGC

GAGCTGCTGGAGGACAATTTTCCAGGGGCTATAGGTTTCCGAAACCACAAGCCTTACTCC

GTCAGGGTTATCTACGGAGACACCGACTCCGTGTTTATCAAGTTTGTGGGCCTGACGTAC

GAGGGGGTATCGGAGCTGGGGGATGCTATGTCGCGTCAGATTTCAGCGGACCTCTTTAGA

GCGCCCATCAAACTGGAGTGTGAGAAGACCTTTCAGCGACTGCTGCTGATCACCAAGAAG

AAGTACATAGGTGTCATAAACGGGGGGAAGATGCTCATGAAGGGGGTCGACCTGGTCCGC

AAAAATAACTGCTCTTTCATAAACTTGTACGCGCGACATCTGGTAGATCTTTTGTTGTAC

GACGAGGATGTGGCCACGGCGGCAGCAGAGGTGACAGACGTGCCTCCCGCAGAATGGGTG

GGGCGCCCGCTACCGAGCGGCTTTGACAAGTTTGGGCGAGTGCTGGTAGAGGCGTACAAC

CGTATCACTGCCCCCAACTTGGACGTGCGCGAGTTCGTTATGACTGCTGAGCTGAGCCGC

TCACCCGAATCGTATACCAACAAGCGCCTGCCGCACCTCACCGTCTACTTTAAGCTCGCC

ATGAGGAATGAAGAACTGCCCAGCGTAAAAGAGAGAATTCCGTATGTGATAGTTGCGCAG

ACCGAGGCCGCGGAACGCGAAGCGGGTGTAGTAAACTCAATGCGCGGTACCGCCCAAAAC

CCCGTGGTAACCAAGACCGCACGCCCCCAACCTAAACGCAAACTGCTGGTTTCCGACCTC

GCCGAAGACCCGACCTATGTTTCCGAGAATGACGTACCGCTAAACACAGACTACTATTTC

TCCCACCTGTTGGGTACCATAAGCGTGACCTTTAAGGCTCTATTCGGAAATGATGTGAGA

ACAACAGAAAATCTTTTAAAGCGGTTTATTCCGGAAACCCCCCACAAGACCCCCACGAAA

ACCCAGGCACTGCTTGAGCGCGCCGGCTTTGAAAAGCTGACGCCCTTTACACCGGAGGAA

GAAAGTCGTCGAATACTGCATACAGTTTTTTGTACTCTAGAAGCAGCTCCCCATCAAAGC

TGA

>LC109623.1

ATGGCGGCGCGCGAACAGGCCAACTCCGTTCGCAGGAGCGGGTTTTTTAATCCGTTTATT

GGCAAGAGGCCATTTTTCAGGCCGGGAAGCGGGCAGACCGCGGAGACAGAGCGCCCAAGG

CCCCCCCAACACTCGTACTGCACAGAGGTGGGTAGCTTTAAGTTTATAGCTCCAAGATGC

CTCGATGAAGAAGCCCCCGCCGACCAGCGACGCGGTGTACACGTGGGCACCTTGGAGCGC

CCACCCAAAGTGTACTGCGATGGCTCAGAGTACGACGTGCTGAACTTTGCCTCCGGAGGT

TGCTGGCCTCGCAGGATTCGGGTTTGGAACGGCCAGGATTTTCGGGGCGATGGATTCAAC

CCCAGATTTGAGAGATTTCACGTGTACGACATAGTAGAGACTTCGGAGAGCGCCTCACAC

GATGACCCATCCAGGTTTGCTGAGCTATCCCGCCCAAGCGGGTCTGTGGTAACACTGCTG

GGAATGAGCGAGTGTGGAAAGCGAGTCGCGGTTCACGTCTATGGTGTGCGCCATTATTTT

TACATGGCAAAGGCGGAGGTGGATAGCGCTTGTGGAATAACCACCGAGGCAGAACTCGTG

CGCGCAATGGTGGACTGCGCGCACAGCTCGGCTTTGAGCGCGGCCCTGGGAAATGGCAAC

GGCGGCAAACAGAGCGGCGGCAGCGGCGGGGGATGGTGGGGCGGAAAGCACGTGTCTGCG

GACTGCTTCAAAGTGGAGACCGTGTGCCACACGACGCTGTACTACTTTGGATCTAAGCCA

GCTCTCTACTATAGAGTATCTGCCTCCAGCAGCCGCCTGGGAGGGTTCATCTGCGACAAC

TTTCACCCGGAGATTACAAAATTCGAGGGGAGCGTGGACGTGACCACGCGGCTGTTGTTG

GACAATGAAAATTTTACCAGTTTCGGGTGGTACCGCCTGCGACCCGGCACCCACGGAGAG

CGTGTTCAACTTCGCCCCGTTGAGCGACACGTCACCTCAAGCGACGTGGAGATTAACTGT

ACTCCCGATAACCTGGAGCCGATACCAGACGAGGCTGCCTGGCCCGACTATAAGCTCATG

TGCTTTGATATAGAGTGTAAAGCTGGAACGGGTAACGAAATGGCGTTCCCAGTGGCAACT

AACCAAGAGGACCTGGTCATCCAGATCTCCTGTCTGCTGTACTCGCTTGCTACTCAGAAC

CACGAACACACCCTGCTGTTTTCCCTCGGGTCATGCGATATCTCTGAGGAATACTCGTTT

GCATGCGTCCAGCGCGGCGAGCCCAGACCGACGGTTTTGGAGTTTGACAGCGAGTACGAG

CTGCTGGTTGCCTTCCTGACCTTTCTCAAGCAGTACTCTCCCGAGTTCGCCACCGGCTAC

AACATCGTTAATTTTGACTGGGCGTACATAGTTAACAAGGTAACGTCGGTGTATAACATC

AAGCTGGACGGGTACGGCAAGTTCAACAAAGGGGGGCTGTTTAAGGTGTGGGACATCGCC

ACGAACCATTTTCAGAAGAAGAGCAAGGTGAAAATCAATGGCCTGATATCTCTAGACATG

TATTCTGTGGCGACGGAAAAGCTAAAGCTACCCAGCTACAAACTCGACGCGGTCGTGGGA

GACGTCCTCGGCGAGCATAAGATAGACCTTCCCTATAAAGAAATACCCTCCTATTACGCG

GGAGGGCCTGACCGGAGGGGCGTAATAGGAGAGTATTGTATCCAGGACTCTAGGCTGGTG

GGCAAGCTGTTTTTTAAGTACCTCCCCCATCTGGAACTATCGGCGGTGGCCAAACTCGCC

CGTATCACCCTAACGCGGGTAATTTTTGACGGTCAGCAAATTCGCGTGTACACGTGCTTG

CTGAAACTCGCCCGCGAGAGAAATTTCATTTTGCCAGACAACAGACGCCGGTTTGACAGT

CAGGCAGACGCCGCGTCAGAGACTTCGGAGTTGGCTATGGATAGCCAAAGCCACGCCTTC

GACAGTACAGACGAACCCGACGGTGTGGACGGTACCCCGGACGCCGCAGGATCTGGCGCT

ACTTCTGAAAACGGAGGCGGGAAGCCCGGCGTCGGGAGGGCCGTGGGCTACCAGGGAGCA

AAGGTTCTAGACCCCGTATCCGGCTTTCATGTGGACCCCGTGGTTGTGTTTGACTTCGCT

AGCTTATACCCAAGCATTATCCAGGCCCATAACCTCTGTTTCACCACCCTGGCGCTCGAT

GAAGTGGATCTGGCCGGGCTTCAACCATCCGTCAACTACTCGACGTTCGAGGTGGGTGAC

CAAAAGTTATTTTTTGTCCACGCCCATATTCGCGAAAGCCTGCTTGGCATCTTGCTGCGC

GACTGGCTGGCCATGCGAAAGGCGGTGAGGGCGCGAATCCCCACCAGCACCCCCGAGGAG

GCAGTTTTACTAGATAAGCAGCAGTCTGCGATTAAGGTGATATGCAACTCGGTTTACGGA

TTCACGGGGGTGGCAAACGGCCTGTTGCCGTGTCTGAGGATAGCGGCTACCGTTACCACG

ATAGGACGCGACATGCTCCTCAAGACCCGAGATTACGTTCACTCTCGTTGGGCGACGCGC

GAGCTGCTGGAGGACAATTTTCCAGGGGCTATAGGTTTCCGAAACCACAAGCCTTACTCC

GTCAGGGTTATCTACGGAGACACCGACTCCGTGTTTATCAAGTTTGTGGGCCTGACGTAC

GAGGGGGTATCGGAGCTGGGGGATGCTATGTCGCGTCAGATTTCAGCGGACCTCTTTAGA

GCCCCCATCAAACTGGAGTGTGAGAAGACCTTTCAGCGACTGCTGCTGATCACCAAGAAG

AAGTACATAGGTGTCATAAACGGGGGGAAGATGCTCATGAAGGGGGTCGACCTGGTCCGC

AAAAATAACTGCTCTTTCATAAACTTGTACGCGCGACATCTGGTAGATCTTTTGTTGTAC

GACGAGGATGTGGCCACGGCGGCAGCAGAGGTGACAGACGTGCCTCCCGCAGAATGGGTG

GGGCGCCCGCTACCGAGCGGCTTTGACAAGTTTGGGCGAGTGCTGGTAGAGGCGTACAAC

CGTATCACTGCCCCCAACTTGGACGTGCGCGAGTTCGTTATGACTGCTGAGCTGAGCCGC

TCACCCGAATCGTATACCAACAAGCGCCTGCCGCACCTCACCGTCTACTTTAAGCTCGCC

ATGAGGAATGAAGAACTGCCCAGCGTAAAAGAGAGAATTCCGTATGTGATAGTTGCGCAG

ACCGAGGCCGCGGAACGCGAAGCGGGTGTAGTAAACTCAATGCGCGGTACCGCCCAAAAC

CCCGTGGTAACCAAGACCGCACGCCCCCAACCTAAACGCAAACTGCTGGTTTCCGACCTC

GCCGAAGACCCGACCTATGTTTCCGAGAATGACGTACCGCTAAACACAGACTACTATTTC

TCCCACCTGTTGGGTACCATAAGCGTGACCTTTAAGGCTCTATTCGGAAATGATGTGAGA

ACAACAGAAAATCTTTTAAAGCGGTTTATTCCGGAAACCCCCCACAAGACCCCCACGAAA

ACCCAGGCACTGCTTGAGCGCGCCGGCTTTGAAAAGCTGACGCCCTTTACACCGGAGGAA

GAAAGTCGTCGAATACTGCATACAGTTTTTTGTACTCTAGAAGCAGCTCCCCATCAAAGC

TGA

>LC109624.1

ATGGCGGCGCGCGAACAGGCCAACTCCGTTCGCAGGAGCGGGTTTTTTAATCCGTTTATT

GGCAAGAGGCCATTTTTCAGGCCGGGAAGCGGGCAGACCGCGGAGACAGAGCGCCCAAGG

CCCCCCCAACACTCGTACTGCACAGAGGTGGGTAGCTTTAAGTTTATAGCTCCAAGATGC

CTCGATGAAGAAGCCCCCGCCGACCAGCGACGCGGTGTACACGTGGGCACCTTGGAGCGC

CCACCCAAAGTGTACTGCGATGGCTCAGAGTACGACGTGCTGAACTTTGCCTCCGGAGGT

TGCTGGCCTCGCAGGATTCGGGTTTGGAACGGCCAGGATTTTCGGGGCGATGGATTCAAC

CCCAGATTTGAGAGATTCCACGTGTACGACATAGTAGAGACTTCGGAGAGCGCCTCACAC

GATGACCCATCCAGGTTTGCTGAGCTATCCCGCCCAAGCGGGTCTGTGGTAACACTGCTG

GGAATGAGCGAGTGTGGAAAGCGAGTCGCGGTTCACGTCTATGGTGTGCGCCATTATTTT

TACATGGCAAAGGCGGAGGTGGATAGCGCTTGTGGAATAACCACCGAGGCAGAACTCGTG

CGCGCAATGGTGGACTGCGCGCACAGCTCGGCTTTGAGCGCGGCCCTGGGAAATGGCAAC

GGCGGCAAACAGAGCGGCGGCAGCGGCGGGGGATGGTGGGGCGGAAAGCACGTGTCTGCG

GACTGCTTCAAAGTGGAGACCGTGTGCCACACGACGCTGTACTACTTTGGATCTAAGCCA

GCTCTCTACTATAGAGTATCTGCCTCCAGCAGCCGCCTGGGAGGGTTCATCTGCGACAAC

TTTCACCCGGAGATTACAAAATTCGAGGGGAGCGTGGACGTGACCACGCGGCTGTTGTTG

GACAATGAAAATTTTACCAGTTTCGGGTGGTACCGCCTGCGACCCGGCACCCACGGAGAG

CGTGTTCAACTTCGCCCCGTTGAGCGACACGTCACCTCAAGCGACGTGGAGATTAACTGT

ACTCCCGATAACCTGGAGCCGATACCAGACGAGGCTGCCTGGCCCGACTATAAGCTCATG

TGCTTTGATATAGAGTGTAAAGCTGGAACGGGTAACGAAATGGCGTTCCCAGTGGCAACT

AACCAAGAGGACCTGGTCATCCAGATCTCCTGTCTGCTGTACTCGCTTGCTACTCAGAAC

CACGAACACACCCTGCTGTTTTCCCTCGGGTCATGCGATATCTCTGAGGAATACTCGTTT

GCATGCGTCCAGCGCGGCGAGCCCAGACCGACGGTTTTGGAGTTTGACAGCGAGTACGAG

CTGCTGGTTGCCTTCCTGACCTTTCTCAAGCAGTACTCTCCCGAGTTCGCCACCGGCTAC

AACATCGTTAATTTTGACTGGGCGTACATAGTTAACAAGGTAACGTCGGTGTATAACATC

AAGCTGGACGGGTACGGCAAGTTCAACAAAGGGGGGCTGTTTAAGGTGTGGGACATCGCC

ACGAACCATTTTCAGAAGAAGAGCAAGGTGAAAATCAATGGCCTGATATCTCTAGACATG

TATTCTGTGGCGACGGAAAAGCTAAAGCTACCCAGCTACAAACTCGACGCGGTCGTGGGA

GACGTCCTCGGCGAGCATAAGATAGACCTTCCCTATAAAGAAATACCCTCCTATTACGCG

GGAGGGCCTGACCGGAGGGGCGTAATAGGAGAGTATTGTATCCAGGACTCTAGGCTGGTG

GGCAAGCTGTTTTTTAAGTACCTCCCCCATCTGGAACTATCGGCGGTGGCCAAACTCGCC

CGTATCACCCTAACGCGGGTAATTTTTGACGGTCAGCAAATTCGCGTGTACACGTGCTTG

CTGAAACTCGCCCGCGAGAGAAATTTCATTTTGCCAGACAACAGACGCCGGTTTGACAGT

CAGGCAGACGCCGCGTCAGAGACTTCGGAGTTGGCTATGGATAGCCAAAGCCACGCCTTC

GACAGTACAGACGAACCCGACGGTGTGGACGGTACCCCGGACGCCGCAGGATCTGGCGCT

ACTTCTGAAAACGGAGGCGGGAAGCCCGGCGTCGGGAGGGCCGTGGGCTACCAGGGAGCA

AAGGTTCTAGACCCCGTATCCGGCTTTCATGTGGACCCCGTGGTTGTGTTTGACTTCGCT

AGCTTATACCCAAGCATTATCCAGGCCCATAACCTCTGTTTCACCACCCTGGCGCTCGAT

GAAGTGGATCTGGCCGGGCTTCAACCATCCGTCAACTACTCGACGTTCGAGGTGGGTGAC

CAAAAGTTATTTTTTGTCCACGCCCATATTCGCGAAAGCCTGCTTGGCATCTTGCTGCGC

GACTGGCTGGCCATGCGAAAGGCGGTGAGGGCGCGAATCCCCACCAGCACCCCCGAGGAG

GCAGTTTTACTAGATAAGCAGCAGTCTGCGATTAAGGTGATATGCAACTCGGTTTACGGA

TTCACGGGGGTGGCAAACGGCCTGTTGCCGTGTCTGAGGATAGCGGCTACCGTTACCACG

ATAGGACGCGACATGCTCCTCAAGACCCGAGATTACGTTCACTCTCGTTGGGCGACGCGC

GAGCTGCTGGAGGACAATTTTCCAGGGGCTATAGGTTTCCGAAACCACAAGCCTTACTCC

GTCAGGGTTATCTACGGAGACACCGACTCCGTGTTTATCAAGTTTGTGGGCCTGACGTAC

GAGGGGGTATCGGAGCTGGGGGATGCTATGTCGCGTCAGATTTCAGCGGACCTCTTTAGA

GCCCCCATCAAACTGGAGTGTGAGAAGACCTTTCAGCGACTGCTGCTGATCACCAAGAAG

AAGTACATAGGTGTCATAAACGGGGGGAAGATGCTCATGAAGGGGGTCGACCTGGTCCGC

AAAAATAACTGCTCTTTCATAAACTTGTACGCGCGACATCTGGTAGATCTTTTGTTGTAC

GACGAGGATGTGGCCACGGCGGCAGCAGAGGTGACAGACGTGCCTCCCGCAGAATGGGTG

GGGCGCCCGCTACCGAGCGGCTTTGACAAGTTTGGGCGAGTGCTGGTAGAGGCGTACAAC

CGTATCACTGCCCCCAACTTGGACGTGCGCGAGTTCGTTATGACTGCTGAGCTGAGCCGC

TCACCCGAATCGTATACCAACAAGCGCCTGCCGCACCTCACCGTCTACTTTAAGCTCGCC

ATGAGGAATGAAGAACTGCCCAGCGTAAAAGAGAGAATTCCGTATGTGATAGTTGCGCAG

ACCGAGGCCGCGGAACGCGAAGCGGGTGTAGTAAACTCAATGCGCGGTACCGCCCAAAAC

CCCGTGGTAACCAAGACCGCACGCCCCCAACCTAAACGCAAACTGCTGGTTTCCGACCTC

GCCGAAGACCCGACCTATGTTTCCGAGAATGACGTACCGCTAAACACAGACTACTATTTC

TCCCACCTGTTGGGTACCATAAGCGTGACCTTTAAGGCTCTATTCGGAAATGATGTGAGA

ACAACAGAAAATCTTTTAAAGCGGTTTATTCCGGAAACCCCCCACAAGACCCCCACGAAA

ACCCAGGCACTGCTTGAGCGCGCCGGCTTTGAAAAGCTGACGCCCTTTACACCGGAGGAA

GAAAGTCGTCGAATACTGCATACAGTTTTTTGTACTCTAGAAGCAGCTCCCCATCAAAGC

TGA

>LC109625.1

ATGGCGGCGCGCGAACAGGCCAACTCCGTTCGCAGGAGCGGGTTTTTTAATCCGTTTATT

GGCAAGAGGCCATTTTTCAGGCCGGGAAGCGGGCAGACCGCGGAGACAGAGCGCCCAAGG

CCCCCCCAACACTCGTACTGCACAGAGGTGGGTAGCTTTAAGTTTATAGCTCCAAGATGC

CTCGATGAAGAAGCCCCCGCCGACCAGCGACGCGGTGTACACGTGGGCACCTTGGAGCGC

CCACCCAAAGTGTACTGCGATGGCTCAGAGTACGACGTGCTGAACTTTGCCTCCGGAGGT

TGCTGGCCTCGCAGGATTCGGGTTTGGAACGGCCAGGATTTTCGGGGCGATGGATTCAAC

CCCAGATTTGAGAGATTTCACGTGTACGACATAGTAGAGACTTCGGAGAGCGCCTCACAC

GATGACCCATCCAGGTTTGCTGAGCTATCCCGCCCAAGCGGGTCTGTGGTAACACTGCTG

GGAATGAGCGAGTGTGGAAAGCGAGTCGCGGTTCACGTCTATGGTGTGCGCCATTATTTT

TACATGGCAAAGGCGGAGGTGGATAGCGCTTGTGGAATAACCACCGAGGCAGAACTCGTG

CGCGCAATGGTGGACTGCGCGCACAGCTCGGCTTTGAGCGCGGCCCTGGGAAATGGCAAC

GGCGGCAAACAGAGCGGCGGCAGCGGCGGGGGATGGTGGGGCGGAAAGCACGTGTCTGCG

GACTGCTTCAAAGTGGAGACCGTGTGCCACACGACGCTGTACTACTTTGGATCTAAGCCA

GCTCTCTACTATAGAGTATCTGCCTCCAGCAGCCGCCTGGGAGGGTTCATCTGCGACAAC

TTTCACCCGGAGATTACAAAATTCGAGGGGAGCGTGGACGTGACCACGCGGCTGTTGTTG

GACAATGAAAATTTTACCAGTTTCGGGTGGTACCGCCTGCGACCCGGCACCCACGGAGAG

CGTGTTCAACTTCGCCCCGTTGAGCGACACGTCACCTCAAGCGACGTGGAGATTAACTGT

ACTCCCGATAACCTGGAGCCGATACCAGACGAGGCTGCCTGGCCCGACTATAAGCTCATG

TGCTTTGATATAGAGTGTAAAGCTGGAACGGGTAACGAAATGGCGTTCCCAGTGGCAACT

AACCAAGAGGACCTGGTCATCCAGATCTCCTGTCTGCTGTACTCGCTTGCTACTCAGAAC

CACGAACACACCCTGCTGTTTTCCCTCGGGTCATGCGATATCTCTGAGGAATACTCGTTT

GCATGCGTCCAGCGCGGCGAGCCCAGACCGACGGTTTTGGAGTTTGACAGCGAGTACGAG

CTGCTGGTTGCCTTCCTGACCTTTCTCAAGCAGTACTCTCCCGAGTTCGCCACCGGCTAC

AACATCGTTAATTTTGACTGGGCGTACATAGTTAACAAGGTAACGTCGGTGTATAACATC

AAGCTGGACGGGTACGGCAAGTTCAACAAAGGGGGGCTGTTTAAGGTGTGGGACATCGCC

ACGAACCATTTTCAGAAGAAGAGCAAGGTGAAAATCAATGGCCTGATATCTCTAGACATG

TATTCTGTGGCGACGGAAAAGCTAAAGCTACCCAGCTACAAACTCGACGCGGTCGTGGGA

GACGTCCTCGGCGAGCATAAGATAGACCTTCCCTATAAAGAAATACCCTCCTATTACGCG

GGAGGGCCTGACCGGAGGGGCGTAATAGGAGAGTATTGTATCCAGGACTCTAGGCTGGTG

GGCAAGCTGTTTTTTAAGTACCTCCCCCATCTGGAACTATCGGCGGTGGCCAAACTCGCC

CGTATCACCCTAACGCGGGTAATTTTTGACGGTCAGCAAATTCGCGTGTACACGTGCTTG

CTGAAACTCGCCCGCGAGAGAAATTTCATTTTGCCAGACAACAGACGCCGGTTTGACAGT

CAGGCAGACGCCGCGTCAGAGACTTCGGAGTTGGCTATGGATAGCCAAAGCCACGCCTTC

GACAGTACAGACGAACCCGACGGTGTGGACGGTACCCCGGACGCCGCAGGATCTGGCGCT

ACTTCTGAAAACGGAGGCGGGAAGCCCGGCGTCGGGAGGGCCGTGGGCTACCAGGGAGCA

AAGGTTCTAGACCCCGTATCCGGCTTTCATGTGGACCCCGTGGTTGTGTTTGACTTCGCT

AGCTTATACCCAAGCATTATCCAGGCCCATAACCTCTGTTTCACCACCCTGGCGCTCGAT

GAAGTGGATCTGGCCGGGCTTCAACCATCCGTCAACTACTCGACGTTCGAGGTGGGTGAC

CAAAAGTTATTTTTTGTCCACGCCCATATTCGCGAAAGCCTGCTTGGCATCTTGCTGCGC

GACTGGCTGGCCATGCGAAAGGCGGTGAGGGCGCGAATCCCCACCAGCACCCCCGAGGAG

GCAGTTTTACTAGATAAGCAGCAGTCTGCGATTAAGGTGATATGCAACTCGGTTTACGGA

TTCACGGGGGTGGCAAACGGCCTGTTGCCGTGTCTGAGGATAGCGGCTACCGTTACCACG

ATAGGACGCGACATGCTCCTCAAGACCCGAGATTACGTTCACTCTCGTTGGGCGACGCGC

GAGCTGCTGGAGGACAATTTTCCAGGGGCTATAGGTTTCCGAAACCACAAGCCTTACTCC

GTCAGGGTTATCTACGGAGACACCGACTCCGTGTTTATCAAGTTTGTGGGCCTGACGTAC

GAGGGGGTATCGGAGCTGGGGGATGCTATGTCGCGTCAGATTTCAGCGGACCTCTTTAGA

GCCCCCATCAAACTGGAGTGTGAGAAGACCTTTCAGCGACTGCTGCTGATCACCAAGAAG

AAGTACATAGGTGTCATAAACGGGGGGAAGATGCTCATGAAGGGGGTCGACCTGGTCCGC

AAAAATAACTGCTCTTTCATAAACTTGTACGCGCGACATCTGGTAGATCTTTTGTTGTAC

GACGAGGATGTGGCCACGGCGGCAGCAGAGGTGACAGACGTGCCTCCCGCAGAATGGGTG

GGGCGCCCGCTACCGAGCGGCTTTGACAAGTTTGGGCGAGTGCTGGTAGAGGCGTACAAC

CGTATCACTGCCCCCAACTTGGACGTGCGCGAGTTCGTTATGACTGCTGAGCTGAGCCGC

TCACCCGAATCGTATACCAACAAGCGCCTGCCGCACCTCACCGTCTACTTTAAGCTCGCC

ATGAGGAATGAAGAACTGCCCAGCGTAAAAGAGAGAATTCCGTATGTGATAGTTGCGCAG

ACCGAGGCCGCGGAACGCGAAGCGGGTGTAGTAAACTCAATGCGCGGTACCGCCCAAAAC

CCCGTGGTAACCAAGACCGCACGCCCCCAACCTAAACGCAAACTGCTGGTTTCCGACCTC

GCCGAAGACCCGACCTATGTTTCCGAGAATGACGTACCGCTAAACACAGACTACTATTTC

TCCCACCTGTTGGGTACCATAAGCGTGACCTTTAAGGCTCTATTCGGAAATGATGTGAGA

ACAACAGAAAATCTTTTAAAGCGGTTTATTCCGGAAACCCCCCACAAGACCCCCACGAAA

ACCCAGGCACTGCTTGAGCGCGCCGGCTTTGAAAAGCTGACGCCCTTTACACCGGAGGAA

GAAAGTCGTCGAATACTGCATACAGTTTTTTGTACTCTAGAAGCAGCTCCCCATCAAAGC

TGA

>LC109626.1

ATGGCGGCGCGCGAACAGGCCAACTCCGTTCGCAGGAGCGGGTTTTTTAATCCGTTTATT

GGCAAGAGGCCATTTTTCAGGCCGGGAAGCGGGCAGACCGCGGAGACAGAGCGCCCAAGG

CCCCCCCAACACTCGTACTGCACAGAGGTGGGTAGCTTTAAGTTTATAGCTCCAAGATGC

CTCGATGAAGAAGCCCCCGCCGACCAGCGACGCGGTGTACACGTGGGCACCTTGGAGCGC

CCACCCAAAGTGTACTGCGATGGCTCAGAGTACGACGTGCTGAACTTTGCCTCCGGAGGT

TGCTGGCCTCGCAGGATTCGGGTTTGGAACGGCCAGGATTTTCGGGGCGATGGATTCAAC

CCCAGATTTGAGAGATTTCACGTGTACGACATAGTAGAGACTTCGGAGAGCGCCTCACAC

GATGACCCATCCAGGTTTGCTGAGCTATCCCGCCCAAGCGGGTCTGTGGTAACACTGCTG

GGAATGAGCGAGTGTGGAAAGCGAGTCGCGGTTCACGTCTATGGTGTGCGCCATTATTTT

TACATGGCAAAGGCGGAGGTGGATAGCGCTTGTGGAATAACCACCGAGGCAGAACTCGTG

CGCGCAATGGTGGACTGCGCGCACAGCTCGGCTTTGAGCGCGGCCCTGGGAAATGGCAAC

GGCGGCAAACAGAGCGGCGGCAGCGGCGGGGGATGGTGGGGCGGAAAGCACGTGTCTGCG

GACTGCTTCAAAGTGGAGACCGTGTGCCACACGACGCTGTACTACTTTGGATCTAAGCCA

GCTCTCTACTATAGAGTATCTGCCTCCAGCAGCCGCCTGGGAGGGTTCATCTGCGACAAC

TTTCACCCGGAGATTACAAAATTCGAGGGGAGCGTGGACGTGACCACGCGGCTGTTGTTG

GACAATGAAAATTTTACCAGTTTCGGGTGGTACCGCCTGCGACCCGGCACCCACGGAGAG

CGTGTTCAACTTCGCCCCGTTGAGCGACACGTCACCTCAAGCGACGTGGAGATTAACTGT

ACTCCCGATAACCTGGAGCCGATACCAGACGAGGCTGCCTGGCCCGACTATAAGCTCATG

TGCTTTGATATAGAGTGTAAAGCTGGAACGGGTAACGAAATGGCGTTCCCAGTGGCAACT

AACCAAGAGGACCTGGTCATCCAGATCTCCTGTCTGCTGTACTCGCTTGCTACTCAGAAC

CACGAACACACCCTGCTGTTTTCCCTCGGGTCATGCGATATCTCTGAGGAATACTCGTTT

GCATGCGTCCAGCGCGGCGAGCCCAGACCGACGGTTTTGGAGTTTGACAGCGAGTACGAG

CTGCTGGTTGCCTTCCTGACCTTTCTCAAGCAGTACTCTCCCGAGTTCGCCACCGGCTAC

AACATCGTTAATTTTGACTGGGCGTACATAGTTAACAAGGTAACGTCGGTGTATAACATC

AAGCTGGACGGGTACGGCAAGTTCAACAAAGGGGGGCTGTTTAAGGTGTGGGACATCGCC

ACGAACCATTTTCAGAAGAAGAGCAAGGTGAAAATCAATGGCCTGATATCTCTAGACATG

TATTCTGTGGCGACGGAAAAGCTAAAGCTACCCAGCTACAAACTCGACGCGGTCGTGGGA

GACGTCCTCGGCGAGCATAAGATAGACCTTCCCTATAAAGAAATACCCTCCTATTACGCG

GGAGGGCCTGACCGGAGGGGCGTAATAGGAGAGTATTGTATCCAGGACTCTAGGCTGGTG

GGCAAGCTGTTTTTTAAGTACCTCCCCCATCTGGAACTATCGGCGGTGGCCAAACTCGCC

CGTATCACCCTAACGCGGGTAATTTTTGACGGTCAGCAAATTCGCGTGTACACGTGCTTG

CTGAAACTCGCCCGCGAGAGAAATTTCATTTTGCCAGACAACAGACGCCGGTTTGACAGT

CAGGCAGACGCCGCGTCAGAGACTTCGGAGTTGGCTATGGATAGCCAAAGCCACGCCTTC

GACAGTACAGACGAACCCGACGGTGTGGACGGTACCCCGGACGCCGCAGGATCTGGCGCT

ACTTCTGAAAACGGAGGCGGGAAGCCCGGCGTCGGGAGGGCCGTGGGCTACCAGGGAGCA

AAGGTTCTAGACCCCGTATCCGGCTTTCATGTGGACCCCGTGGTTGTGTTTGACTTCGCT

AGCTTATACCCAAGCATTATCCAGGCCCATAACCTCTGTTTCACCACCCTGGCGCTCGAT

GAAGTGGATCTGGCCGGGCTTCAACCATCCGTCAACTACTCGACGTTCGAGGTGGGTGAC

CAAAAGTTATTTTTTGTCCACGCCCATATTCGCGAAAGCCTGCTTGGCATCTTGCTGCGC

GACTGGCTGGCCATGCGAAAGGCGGTGAGGGCGCGAATCCCCACCAGCACCCCCGAGGAG

GCAGTTTTACTAGATAAGCAGCAGTCTGCGATTAAGGTGATATGCAACTCGGTTTACGGA

TTCACGGGGGTGGCAAACGGCCTGTTGCCGTGTCTGAGGATAGCGGCTACCGTTACCACG

ATAGGACGCGACATGCTCCTCAAGACCCGAGATTACGTTCACTCTCGTTGGGCGACGCGC

GAGCTGCTGGAGGACAATTTTCCAGGGGCTATAGGTTTCCGAAACCACAAGCCTTACTCC

GTCAGGGTTATCTACGGAGACACCGACTCCGTGTTTATCAAGTTTGTGGGCCTGACGTAC

GAGGGGGTATCGGAGCTGGGGGATGCTATGTCGCGTCAGATTTCAGCGGACCTCTTTAGA

GCCCCCATCAAACTGGAGTGTGAGAAGACCTTTCAGCGACTGCTGCTGATCACCAAGAAG

AAGTACATAGGTGTCATAAACGGGGGGAAGATGCTCATGAAGGGGGTCGACCTGGTCCGC

AAAAATAACTGCTCTTTCATAAACTTGTACGCGCGACATCTGGTAGATCTTTTGTTGTAC

GACGAGGATGTGGCCACGGCGGCAGCAGAGGTGACAGACGTGCCTCCCGCAGAATGGGTG

GGGCGCCCGCTACCGAGCGGCTTTGACAAGTTTGGGCGAGTGCTGGTAGAGGCGTACAAC

CGTATCACTGCCCCCAACTTGGACGTGCGCGAGTTCGTTATGACTGCTGAGCTGAGCCGC

TCACCCGAATCGTATACCAACAAGCGCCTGCCGCACCTCACCGTCTACTTTAAGCTCGCC

ATGAGGAATGAAGAACTGCCCAGCGTAAAAGAGAGAATTCCGTATGTGATAGTTGCGCAG

ACCGAGGCCGCGGAACGCGAAGCGGGTGTAGTAAACTCAATGCGCGGTACCGCCCAAAAC

CCCGTGGTAACCAAGACCGCACGCCCCCAACCTAAACGCAAACTGCTGGTTTCCGACCTC

GCCGAAGACCCGACCTATGTTTCCGAGAATGACGTACCGCTAAACACAGACTACTATTTC

TCCCACCTGTTGGGTACCATAAGCGTGACCTTTAAGGCTCTATTCGGAAATGATGTGAGA

ACAACAGAAAATCTTTTAAAGCGGTTTATTCCGGAAACCCCCCACAAGACCCCCACGAAA

ACCCAGGCACTGCTTGAGCGCGCCGGCTTTGAAAAGCTGACGCCCTTTACACCGGAGGAA

GAAAGTCGTCGAATACTGCATACAGTTTTTTGTACTCTAGAAGCAGCTCCCCATCAAAGC

TGA

>LC109627.1

ATGGCGGCGCGCGAACAGGCCAACTCCGTTCGCAGGAGCGGGTTTTTTAATCCGTTTATT

GGCAAGAGGCCATTTTTCAGGCCGGGAAGCGGGCAGACCGCGGAGACAGAGCGCCCAAGG

CCCCCCCAACACTCGTACTGCACAGAGGTGGGTAGCTTTAAGTTTATAGCTCCAAGATGC

CTCGATGAAGAAGCCCCCGCCGACCAGCGACGCGGTGTACACGTGGGCACCTTGGAGCGC

CCACCCAAAGTGTACTGCGATGGCTCAGAGTACGACGTGCTGAACTTTGCCTCCGGAGGT

TGCTGGCCTCGCAGGATTCGGGTTTGGAACGGCCAGGATTTTCGGGGCGATGGATTCAAC

CCCAGATTTGAGAGATTTCACGTGTACGACATAGTAGAGACTTCGGAGAGCGCCTCACAC

GATGACCCATCCAGGTTTGCTGAGCTATCCCGCCCAAGCGGGTCTGTGGTAACACTGCTG

GGAATGAGCGAGTGTGGAAAGCGAGTCGCGGTTCACGTCTATGGTGTGCGCCATTATTTT

TACATGGCAAAGGCGGAGGTGGATAGCGCTTGTGGAATAACCACCGAGGCAGAACTCGTG

CGCGCAATGGTGGACTGCGCGCACAGCTCGGCTTTGAGCGCGGCCCTGGGAAATGGCAAC

GGCGGCAAACAGAGCGGCGGCAGCGGCGGGGGATGGTGGGGCGGAAAGCACGTGTCTGCG

GACTGCTTCAAAGTGGAGACCGTGTGCCACACGACGCTGTACTACTTTGGATCTAAGCCA

GCTCTCTACTATAGAGTATCTGCCTCCAGCAGCCGCCTGGGAGGGTTCATCTGCGACAAC

TTTCACCCGGAGATTACAAAATTCGAGGGGAGCGTGGACGTGACCACGCGGCTGTTGTTG

GACAATGAAAATTTTACCAGTTTCGGGTGGTACCGCCTGCGACCCGGCACCCACGGAGAG

CGTGTTCAACTTCGCCCCGTTGAGCGACACGTCACCTCAAGCGACGTGGAGATTAACTGT

ACTCCCGATAACCTGGAGCCGATACCAGACGAGGCTGCCTGGCCCGACTATAAGCTCATG

TGCTTTGATATAGAGTGTAAAGCTGGAACGGGTAACGAAATGGCGTTCCCAGTGGCAACT

AACCAAGAGGACCTGGTCATCCAGATCTCCTGTCTGCTGTACTCGCTTGCTACTCAGAAC

CACGAACACACCCTGCTGTTTTCCCTCGGGTCATGCGATATCTCTGAGGAATACTCGTTT

GCATGCGTCCAGCGCGGCGAGCCCAGACCGACGGTTTTGGAGTTTGACAGCGAGTACGAG

CTGCTGGTTGCCTTCCTGACCTTTCTCAAGCAGTACTCTCCCGAGTTCGCCACCGGCTAC

AACATCGTTAATTTTGACTGGGCGTACATAGTTAACAAGGTAACGTCGGTGTATAACATC

AAGCTGGACGGGTACGGCAAGTTCAACAAAGGGGGGCTGTTTAAGGTGTGGGACATCGCC

ACGAACCATTTTCAGAAGAAGAGCAAGGTGAAAATCAATGGCCTGATATCTCTAGACATG

TATTCTGTGGCGACGGAAAAGCTAAAGCTACCCAGCTACAAACTCGACGCGGTCGTGGGA

GACGTCCTCGGCGAGCATAAGATAGACCTTCCCTATAAAGAAATACCCTCCTATTACGCG

GGAGGGCCTGACCGGAGGGGCGTAATAGGAGAGTATTGTATCCAGGACTCTAGGCTGGTG

GGCAAGCTGTTTTTTAAGTACCTCCCCCATCTGGAACTATCGGCGGTGGCCAAACTCGCC

CGTATCACCCTAACGCGGGTAATTTTTGACGGTCAGCAAATTCGCGTGTACACGTGCTTG

CTGAAACTCGCCCGCGAGAGAAATTTCATTTTGCCAGACAACAGACGCCGGTTTGACAGT

CAGGCAGACGCCGCGTCAGAGACTTCGGAGTTGGCTATGGATAGCCAAAGCCACGCCTTC

GACAGTACAGACGAACCCGACGGTGTGGACGGTACCCCGGACGCCGCAGGATCTGGCGCT

ACTTCTGAAAACGGAGGCGGGAAGCCCGGCGTCGGGAGGGCCGTGGGCTACCAGGGAGCA

AAGGTTCTAGACCCCGTATCCGGCTTTCATGTGGACCCCGTGGTTGTGTTTGACTTCGCT

AGCTTATACCCAAGCATTATCCAGGCCCATAACCTCTGTTTCACCACCCTGGCGCTCGAT

GAAGTGGATCTGGCCGGGCTTCAACCATCCGTCAACTACTCGACGTTCGAGGTGGGTGAC

CAAAAGTTATTTTTTGTCCACGCCCATATTCGCGAAAGCCTGCTTGGCATCTTGCTGCGC

GACTGGCTGGCCATGCGAAAGGCGGTGAGGGCGCGAATCCCCACCAGCACCCCCGAGGAG

GCAGTTTTACTAGATAAGCAGCAGTCTGCGATTAAGGTGATATGCAACTCGGTTTACGGA

TTCACGGGGGTGGCAAACGGCCTGTTGCCGTGTCTGAGGATAGCGGCTACCGTTACCACG

ATAGGACGCGACATGCTCCTCAAGACCCGAGATTACGTTCACTCTCGTTGGGCGACGCGC

GAGCTGCTGGAGGACAATTTTCCAGGGGCTATAGGTTTCCGAAACCACAAGCCTTACTCC

GTCAGGGTTATCTACGGAGACACCGACTCCGTGTTTATCAAGTTTGTGGGCCTGACGTAC

GAGGGGGTATCGGAGCTGGGGGATGCTATGTCGCGTCAGATTTCAGCGGACCTCTTTAGA

GCCCCCATCAAACTGGAGTGTGAGAAGACCTTTCAGCGACTGCTGCTGATCACCAAGAAG

AAGTACATAGGTGTCATAAACGGGGGGAAGATGCTCATGAAGGGGGTCGACCTGGTCCGC

AAAAATAACTGCTCTTTCATAAACTTGTACGCGCGACATCTGGTAGATCTTTTGTTGTAC

GACGAGGATGTGGCCACGGCGGCAGCAGAGGTGACAGACGTGCCTCCCGCAGAATGGGTG

GGGCGCCCGCTACCGAGCGGCTTTGACAAGTTTGGGCGAGTGCTGGTAGAGGCGTACAAC

CGTATCACTGCCCCCAACTTGGACGTGCGCGAGTTCGTTATGACTGCTGAGCTGAGCCGC

TCACCCGAATCGTATACCAACAAGCGCCTGCCGCACCTCACCGTCTACTTTAAGCTCGCC

ATGAGGAATGAAGAACTGCCCAGCGTAAAAGAGAGAATTCCGTATGTGATAGTTGCGCAG

ACCGAGGCCGCGGAACGCGAAGCGGGTGTAGTAAACTCAATGCGCGGTACCGCCCAAAAC

CCCGTGGTAACCAAGACCGCACGCCCCCAACCTAAACGCAAACTGCTGGTTTCCGACCTC

GCCGAAGACCCGACCTATGTTTCCGAGAATGACGTACCGCTAAACACAGACTACTATTTC

TCCCACCTGTTGGGTACCATAAGCGTGACCTTTAAGGCTCTATTCGGAAATGATGTGAGA

ACAACAGAAAATCTTTTAAAGCGGTTTATTCCGGAAACCCCCCACAAGACCCCCACGAAA

ACCCAGGCACTGCTTGAGCGCGCCGGCTTTGAAAAGCTGACGCCCTTTACACCGGAGGAA

GAAAGTCGTCGAATACTGCATACAGTTTTTTGTACTCTAGAAGCAGCTCCCCATCAAAGC

TGA

>LC109628.1

ATGGCGGCGCGCGAACAGGCCAACTCCGTTCGCAGGAGCGGGTTTTTTAATCCGTTTATT

GGCAAGAGGCCATTTTTCAGGCCGGGAAGCGGGCAGACCGCGGAGACAGAGCGCCCAAGG

CCCCCCCAACACTCGTACTGCACAGAGGTGGGTAGCTTTAAGTTTATAGCTCCAAGATGC

CTCGATGAAGAAGCCCCCGCCGACCAGCGACGCGGTGTACACGTGGGCACCTTGGAGCGC

CCACCCAAAGTGTACTGCGATGGCTCAGAGTACGACGTGCTGAACTTTGCCTCCGGAGGT

TGCTGGCCTCGCAGGATTCGGGTTTGGAACGGCCAGGATTTTCGGGGCGATGGATTCAAC

CCCAGATTTGAGAGATTTCACGTGTACGACATAGTAGAGACTTCGGAGAGCGCCTCACAC

GATGACCCATCCAGGTTTGCTGAGCTATCCCGCCCAAGCGGGTCTGTGGTAACACTGCTG

GGAATGAGCGAGTGTGGAAAGCGAGTCGCGGTTCACGTCTATGGTGTGCGCCATTATTTT

TACATGGCAAAGGCGGAGGTGGATAGCGCTTGTGGAATAACCACCGAGGCAGAACTCGTG

CGCGCAATGGTGGACTGCGCGCACAGCTCGGCTTTGAGCGCGGCCCTGGGAAATGGCAAC

GGCGGCAAACAGAGCGGCGGCAGCGGCGGGGGATGGTGGGGCGGAAAGCACGTGTCTGCG

GACTGCTTCAAAGTGGAGACCGTGTGCCACACGACGCTGTACTACTTTGGATCTAAGCCA

GCTCTCTACTATAGAGTATCTGCCTCCAGCAGCCGCCTGGGAGGGTTCATCTGCGACAAC

TTTCACCCGGAGATTACAAAATTCGAGGGGAGCGTGGACGTGACCACGCGGCTGTTGTTG

GACAATGAAAATTTTACCAGTTTCGGGTGGTACCGCCTGCGACCCGGCACCCACGGAGAG

CGTGTTCAACTTCGCCCCGTTGAGCGACACGTCACCTCAAGCGACGTGGAGATTAACTGT

ACTCCCGATAACCTGGAGCCGATACCAGACGAGGCTGCCTGGCCCGACTATAAGCTCATG

TGCTTTGATATAGAGTGTAAAGCTGGAACGGGTAACGAAATGGCGTTCCCAGTGGCAACT

AACCAAGAGGACCTGGTCATCCAGATCTCCTGTCTGCTGTACTCGCTTGCTACTCAGAAC

CACGAACACACCCTGCTGTTTTCCCTCGGGTCATGCGATATCTCTGAGGAATACTCGTTT

GCATGCGTCCAGCGCGGCGAGCCCAGACCGACGGTTTTGGAGTTTGACAGCGAGTACGAG

CTGCTGGTTGCCTTCCTGACCTTTCTCAAGCAGTACTCTCCCGAGTTCGCCACCGGCTAC

AACATCGTTAATTTTGACTGGGCGTACATAGTTAACAAGGTAACGTCGGTGTATAACATC

AAGCTGGACGGGTACGGCAAGTTCAACAAAGGGGGGCTGTTTAAGGTGTGGGACATCGCC

ACGAACCATTTTCAGAAGAAGAGCAAGGTGAAAATCAATGGCCTGATATCTCTAGACATG

TATTCTGTGGCGACGGAAAAGCTAAAGCTACCCAGCTACAAACTCGACGCGGTCGTGGGA

GACGTCCTCGGCGAGCATAAGATAGACCTTCCCTATAAAGAAATACCCTCCTATTACGCG

GGAGGGCCTGACCGGAGGGGCGTAATAGGAGAGTATTGTATCCAGGACTCTAGGCTGGTG

GGCAAGCTGTTTTTTAAGTACCTCCCCCATCTGGAACTATCGGCGGTGGCCAAACTCGCC

CGTATCACCCTAACGCGGGTAATTTTTGACGGTCAGCAAATTCGCGTGTACACGTGCTTG

CTGAAACTCGCCCGCGAGAGAAATTTCATTTTGCCAGACAACAGACGCCGGTTTGACAGT

CAGGCAGACGCCGCGTCAGAGACTTCGGAGTTGGCTATGGATAGCCAAAGCCACGCCTTC

GACAGTACAGACGAACCCGACGGTGTGGACGGTACCCCGGACGCCGCAGGATCTGGCGCT

ACTTCTGAAAACGGAGGCGGGAAGCCCGGCGTCGGGAGGGCCGTGGGCTACCAGGGAGCA

AAGGTTCTAGACCCCGTATCCGGCTTTCATGTGGACCCCGTGGTTGTGTTTGACTTCGCT

AGCTTATACCCAAGCATTATCCAGGCCCATAACCTCTGTTTCACCACCCTGGCGCTCGAT

GAAGTGGATCTGGCCGGGCTTCAACCATCCGTCAACTACTCGACGTTCGAGGTGGGTGAC

CAAAAGTTATTTTTTGTCCACGCCCATATTCGCGAAAGCCTGCTTGGCATCTTGCTGCGC

GACTGGCTGGCCATGCGAAAGGCGGTGAGGGCGCGAATCCCCACCAGCACCCCCGAGGAG

GCAGTTTTACTAGATAAGCAGCAGTCTGCGATTAAGGTGATATGCAACTCGGTTTACGGA

TTCACGGGGGTGGCAAACGGCCTGTTGCCGTGTCTGAGGATAGCGGCTACCGTTACCACG

ATAGGACGCGACATGCTCCTCAAGACCCGAGATTACGTTCACTCTCGTTGGGCGACGCGC

GAGCTGCTGGAGGACAATTTTCCAGGGGCTATAGGTTTCCGAAACCACAAGCCTTACTCC

GTCAGGGTTATCTACGGAGACACCGACTCCGTGTTTATCAAGTTTGTGGGCCTGACGTAC

GAGGGGGTATCGGAGCTGGGGGATGCTATGTCGCGTCAGATTTCAGCGGACCTCTTTAGA

GCCCCCATCAAACTGGAGTGTGAGAAGACCTTTCAGCGACTGCTGCTGATCACCAAGAAG

AAGTACATAGGTGTCATAAACGGGGGGAAGATGCTCATGAAGGGGGTCGACCTGGTCCGC

AAAAATAACTGCTCTTTCATAAACTTGTACGCGCGACATCTGGTAGATCTTTTGTTGTAC

GACGAGGATGTGGCCACGGCGGCAGCAGAGGTGACAGACGTGCCTCCCGCAGAATGGGTG

GGGCGCCCGCTACCGAGCGGCTTTGACAAGTTTGGGCGAGTGCTGGTAGAGGCGTACAAC

CGTATCACTGCCCCCAACTTGGACGTGCGCGAGTTCGTTATGACTGCTGAGCTGAGCCGC

TCACCCGAATCGTATACCAACAAGCGCCTGCCGCACCTCACCGTCTACTTTAAGCTCGCC

ATGAGGAATGAAGAACTGCCCAGCGTAAAAGAGAGAATTCCGTATGTGATAGTTGCGCAG

ACCGAGGCCGCGGAACGCGAAGCGGGTGTAGTAAACTCAATGCGCGGTACCGCCCAAAAC

CCCGTGGTAACCAAGACCGCACGCCCCCAACCTAAACGCAAACTGCTGGTTTCCGACCTC

GCCGAAGACCCGACCTATGTTTCCGAGAATGACGTACCGCTAAACACAGACTACTATTTC

TCCCACCTGTTGGGTACCATAAGCGTGACCTTTAAGGCTCTATTCGGAAATGATGTGAGA

ACAACAGAAAATCTTTTAAAGCGGTTTATTCCGGAAACCCCCCACAAGACCCCCACGAAA

ACCCAGGCACTGCTTGAGCGCGCCGGCTTTGAAAAGCTGACGCCCTTTACACCGGAGGAA

GAAAGTCGTCGAATACTGCATACAGTTTTTTGTACTCTAGAAGCAGCTCCCCATCAAAGC

TGA

>LC109629.1

ATGGCGGCGCGCGAACAGGCCAACTCCGTTCGCAGGAGCGGGTTTTTTAATCCGTTTATT

GGCAAGAGGCCATTTTTCAGGCCGGGAAGCGGGCAGACCGCGGAGACAGAGCGCCCAAGG

CCCCCCCAACACTCGTACTGCACAGAGGTGGGTAGCTTTAAGTTTATAGCTCCAAGATGC

CTCGATGAAGAAGCCCCCGCCGACCAGCGACGCGGTGTACACGTGGGCACCTTGGAGCGC

CCACCCAAAGTGTACTGCGATGGCTCAGAGTACGACGTGCTGAACTTTGCCTCCGGAGGT

TGCTGGCCTCGCAGGATTCGGGTTTGGAACGGCCAGGATTTTCGGGGCGATGGATTCAAC

CCCAGATTTGAGAGATTTCACGTGTACGACATAGTAGAGACTTCGGAGAGCGCCTCACAC

GATGACCCATCCAGGTTTGCTGAGCTATCCCGCCCAAGCGGGTCTGTGGTAACACTGCTG

GGAATGAGCGAGTGTGGAAAGCGAGTCGCGGTTCACGTCTATGGTGTGCGCCATTATTTT

TACATGGCAAAGGCGGAGGTGGATAGCGCTTGTGGAATAACCACCGAGGCAGAACTCGTG

CGCGCAATGGTGGACTGCGCGCACAGCTCGGCTTTGAGCGCGGCCCTGGGAAATGGCAAC

GGCGGCAAACAGAGCGGCGGCAGCGGCGGGGGATGGTGGGGCGGAAAGCACGTGTCCGCG

GACTGCTTCAAAGTGGAGACCGTGTGCCACACGACGCTGTACTACTTTGGATCTAAGCCA

GCTCTCTACTATAGAGTATCTGCCTCCAGCAGCCGCCTGGGAGGGTTCATCTGCGACAAC

TTTCACCCGGAGATTACAAAATTCGAGGGGAGCGTGGACGTGACCACGCGGCTGTTGTTG

GACAATGAAAATTTTACCAGTTTCGGGTGGTACCGCCTGCGACCCGGCACCCACGGAGAG

CGTGTTCAACTTCGCCCCGTTGAGCGACACGTCACCTCAAGCGACGTGGAGATTAACTGT

ACTCCCGATAACCTGGAGCCGATACCAGACGAGGCTGCCTGGCCCGACTATAAGCTCATG

TGCTTTGATATAGAGTGTAAAGCTGGAACGGGTAACGAAATGGCGTTCCCAGTGGCAACT

AACCAAGAGGACCTGGTCATCCAGATCTCCTGTCTGCTGTACTCGCTTGCTACTCAGAAC

CACGAACACACCCTGCTGTTTTCCCTCGGGTCATGCGATATCTCTGAGGAATACTCGTTT

GCATGCGTCCAGCGCGGCGAGCCCAGACCGACGGTTTTGGAGTTTGACAGCGAGTACGAG

CTGCTGGTTGCCTTCCTGACCTTTCTCAAGCAGTACTCTCCCGAGTTCGCCACCGGCTAC

AACATCGTTAATTTTGACTGGGCGTACATAGTTAACAAGGTAACGTCGGTGTATAACATC

AAGCTGGACGGGTACGGCAAGTTCAACAAAGGGGGGCTGTTTAAGGTGTGGGACATCGCC

ACGAACCATTTTCAGAAGAAGAGCAAGGTGAAAATCAATGGCCTGATATCTCTAGACATG

TATTCTGTGGCGACGGAAAAGCTAAAGCTACCCAGCTACAAACTCGACGCGGTCGTGGGA

GACGTCCTCGGCGAGCATAAGATAGACCTTCCCTATAAAGAAATACCCTCCTATTACGCG

GGAGGGCCTGACCGGAGGGGCGTAATAGGAGAGTATTGTATCCAGGACTCTAGGCTGGTG

GGCAAGCTGTTTTTTAAGTACCTCCCCCATCTGGAACTATCGGCGGTGGCCAAACTCGCC

CGTATCACCCTAACGCGGGTAATTTTTGACGGTCAGCAAATTCGCGTGTACACGTGCTTG

CTGAAACTCGCCCGCGAGAGAAATTTCATTTTGCCAGACAACAGACGCCGGTTTGACAGT

CAGGCAGACGCCGCGTCAGAGACTTCGGAGTTGGCTATGGATAGCCAAAGCCACGCCTTC

GACAGTACAGACGAACCCGACGGTGTGGACGGTACCCCGGACGCCGCAGGATCTGGCGCT

ACTTCTGAAAACGGAGGCGGGAAGCCCGGCGTCGGGAGGGCCGTGGGCTACCAGGGAGCA

AAGGTTCTAGACCCCGTATCCGGCTTTCATGTGGACCCCGTGGTTGTGTTTGACTTCGCT

AGCTTATACCCAAGCATTATCCAGGCCCATAACCTCTGTTTCACCACCCTGGCGCTCGAT

GAAGTGGATCTGGCCGGGCTTCAACCATCCGTCAACTACTCGACGTTCGAGGTGGGTGAC

CAAAAGTTATTTTTTGTCCACGCCCATATTCGCGAAAGCCTGCTTGGCATCTTGCTGCGC

GACTGGCTGGCCATGCGAAAGGCGGTGAGGGCGCGAATCCCCACCAGCACCCCCGAGGAG

GCAGTTTTACTAGATAAGCAGCAGTCTGCGATTAAGGTGATATGCAACTCGGTTTACGGA

TTCACGGGGGTGGCAAACGGCCTGTTGCCGTGTCTGAGGATAGCGGCTACCGTTACCACG

ATAGGACGCGACATGCTCCTCAAGACCCGAGATTACGTTCACTCTCGTTGGGCGACGCGC

GAGCTGCTGGAGGACAATTTTCCAGGGGCTATAGGTTTCCGAAACCACAAGCCTTACTCC

GTCAGGGTTATCTACGGAGACACCGACTCCGTGTTTATCAAGTTTGTGGGCCTGACGTAC

GAGGGGGTATCGGAGCTGGGGGATGCTATGTCGCGTCAGATTTCAGCGGACCTCTTTAGA

GCCCCCATCAAACTGGAGTGTGAGAAGACCTTTCAGCGACTGCTGCTGATCACCAAGAAG

AAGTACATAGGTGTCATAAACGGGGGGAAGATGCTCATGAAGGGGGTCGACCTGGTCCGC

AAAAATAACTGCTCTTTCATAAACTTGTACGCGCGACATCTGGTAGATCTTTTGTTGTAC

GACGAGGATGTGGCCACGGCGGCAGCAGAGGTGACAGACGTGCCTCCCGCAGAATGGGTG

GGGCGCCCGCTACCGAGCGGCTTTGACAAGTTTGGGCGAGTGCTGGTAGAGGCGTACAAC

CGTATCACTGCCCCCAACTTGGACGTGCGCGAGTTCGTTATGACTGCTGAGCTGAGCCGC

TCACCCGAATCGTATACCAACAAGCGCCTGCCGCACCTCACCGTCTACTTTAAGCTCGCC

ATGAGGAATGAAGAACTGCCCAGCGTAAAAGAGAGAATTCCGTATGTGATAGTTGCGCAG

ACCGAGGCCGCGGAACGCGAAGCGGGTGTAGTAAACTCAATGCGCGGTACCGCCCAAAAC

CCCGTGGTAACCAAGACCGCACGCCCCCAACCTAAACGCAAACTGCTGGTTTCCGACCTC

GCCGAAGACCCGACCTATGTTTCCGAGAATGACGTACCGCTAAACACAGACTACTATTTC

TCCCACCTGTTGGGTACCATAAGCGTGACCTTTAAGGCTCTATTCGGAAATGATGTGAGA

ACAACAGAAAATCTTTTAAAGCGGTTTATTCCGGAAACCCCCCACAAGACCCCCACGAAA

ACCCAGGCACTGCTTGAGCGCGCCGGCTTTGAAAAGCTGACGCCCTTTACACCGGAGGAA

GAAAGTCGTCGAATACTGCATACAGTTTTTTGTACTCTAGAAGCAGCTCCCCATCAAAGC

TGA

>LC109630.1

ATGGCGGCGCGCGAACAGGCCAACTCCGTTCGCAGGAGCGGGTTTTTTAATCCGTTTATT

GGCAAGAGGCCATTTTTCAGGCCGGGAAGCGGGCAGACCGCGGAGACAGAGCGCCCAAGG

CCCCCCCAACACTCGTACTGCACAGAGGTGGGTAGCTTTAAGTTTATAGCTCCAAGATGC

CTCGATGAAGAAGCCCCCGCCGACCAGCGACGCGGTGTACACGTGGGCACCTTGGAGCGC

CCACCCAAAGTGTACTGCGATGGCTCAGAGTACGACGTGCTGAACTTTGCCTCCGGAGGT

TGCTGGCCTCGCAGGATTCGGGTTTGGAACGGCCAGGATTTTCGGGGCGATGGATTCAAC

CCCAGATTTGAGAGATTTCACGTGTACGACATAGTAGAGACTTCGGAGAGCGCCTCACAC

GATGACCCATCCAGGTTTGCTGAGCTATCCCGCCCAAGCGGGTCTGTGGTAACACTGCTG

GGAATGAGCGAGTGTGGAAAGCGAGTCGCGGTTCACGTCTATGGTGTGCGCCATTATTTT

TACATGGCAAAGGCGGAGGTGGATAGCGCTTGTGGAATAACCACCGAGGCAGAACTCGTG

CGCGCAATGGTGGACTGCGCGCACAGCTCGGCTTTGAGCGCGGCCCTGGGAAATGGCAAC

GGCGGCAAACAGAGCGGCGGCAGCGGCGGGGGATGGTGGGGCGGAAAGCACGTGTCTGCG

GACTGCTTCAAAGTGGAGACCGTGTGCCACACGACGCTGTACTACTTTGGATCTAAGCCA

GCTCTCTACTATAGAGTATCTGCCTCCAGCAGCCGCCTGGGAGGGTTCATCTGCGACAAC

TTTCACCCGGAGATTACAAAATTCGAGGGGAGCGTGGACGTGACCACGCGGCTGTTGTTG

GACAATGAAAATTTTACCAGTTTCGGGTGGTACCGCCTGCGACCCGGCACCCACGGAGAG

CGTGTTCAACTTCGCCCCGTTGAGCGACACGTCACCTCAAGCGACGTGGAGATTAACTGT

ACTCCCGATAACCTGGAGCCGATACCAGACGAGGCTGCCTGGCCCGACTATAAGCTCATG

TGCTTTGATATAGAGTGTAAAGCTGGAACGGGTAACGAAATGGCGTTCCCAGTGGCAACT

AACCAAGAGGACCTGGTCATCCAGATCTCCTGTCTGCTGTACTCGCTTGCTACTCAGAAC

CACGAACACACCCTGCTGTTTTCCCTCGGGTCATGCGATATCTCTGAGGAATACTCGTTT

GCATGCGTCCAGCGCGGCGAGCCCAGACCGACGGTTTTGGAGTTTGACAGCGAGTACGAG

CTGCTGGTTGCCTTCCTGACCTTTCTCAAGCAGTACTCTCCCGAGTTCGCCACCGGCTAC

AACATCGTTAATTTTGACTGGGCGTACATAGTTAACAAGGTAACGTCGGTGTATAACATC

AAGCTGGACGGGTACGGCAAGTTCAACAAAGGGGGGCTGTTTAAGGTGTGGGACATCGCC

ACGAACCATTTTCAGAAGAAGAGCAAGGTGAAAATCAATGGCCTGATATCTCTAGACATG

TATTCTGTGGCGACGGAAAAGCTAAAGCTACCCAGCTACAAACTCGACGCGGTCGTGGGA

GACGTCCTCGGCGAGCATAAGATAGACCTTCCCTATAAAGAAATACCCTCCTATTACGCG

GGAGGGCCTGACCGGAGGGGCGTAATAGGAGAGTATTGTATCCAGGACTCTAGGCTGGTG

GGCAAGCTGTTTTTTAAGTACCTCCCCCATCTGGAACTATCGGCGGTGGCCAAACTCGCC

CGTATCACCCTAACGCGGGTAATTTTTGACGGTCAGCAAATTCGCGTGTACACGTGCTTG

CTGAAACTCGCCCGCGAGAGAAATTTCATTTTGCCAGACAACAGACGCCGGTTTGACAGT

CAGGCAGACGCCGCGTCAGAGACTTCGGAGTTGGCTATGGATAGCCAAAGCCACGCCTTC

GACAGTACAGACGAACCCGACGGTGTGGACGGTACCCCGGACGCCGCAGGATCTGGCGCT

ACTTCTGAAAACGGAGGCGGGAAGCCCGGCGTCGGGAGGGCCGTGGGCTACCAGGGAGCA

AAGGTTCTAGACCCCGTATCCGGCTTTCATGTGGACCCCGTGGTTGTGTTTGACTTCGCT

AGCTTATACCCAAGCATTATCCAGGCCCATAACCTCTGTTTCACCACCCTGGCGCTCGAT

GAAGTGGATCTGGCCGGGCTTCAACCATCCGTCAACTACTCGACGTTCGAGGTGGGTGAC

CAAAAGTTATTTTTTGTCCACGCCCATATTCGCGAAAGCCTGCTTGGCATCTTGCTGCGC

GACTGGCTGGCCATGCGAAAGGCGGTGAGGGCGCGAATCCCCACCAGCACCCCCGAGGAG

GCAGTTTTACTAGATAAGCAGCAGTCTGCGATTAAGGTGATATGCAACTCGGTTTACGGA

TTCACGGGGGTGGCAAACGGCCTGTTGCCGTGTCTGAGGATAGCGGCTACCGTTACCACG

ATAGGACGCGACATGCTCCTCAAGACCCGAGATTACGTTCACTCTCGTTGGGCGACGCGC

GAGCTGCTGGAGGACAATTTTCCAGGGGCTATAGGTTTCCGAAACCACAAGCCTTACTCC

GTCAGGGTTATCTACGGAGACACCGACTCCGTGTTTATCAAGTTTGTGGGCCTGACGTAC

GAGGGGGTATCGGAGCTGGGGGATGCTATGTCGCGTCAGATTTCAGCGGACCTCTTTAGA

GCGCCCATCAAACTGGAGTGTGAGAAGACCTTTCAGCGACTGCTGCTGATCACCAAGAAG

AAGTACATAGGTGTCATAAACGGGGGGAAGATGCTCATGAAGGGGGTCGACCTGGTCCGC

AAAAATAACTGCTCTTTCATAAACTTGTACGCGCGACATCTGGTAGATCTTTTGTTGTAC

GACGAGGATGTGGCCACGGCGGCAGCAGAGGTGACAGACGTGCCTCCCGCAGAATGGGTG

GGGCGCCCGCTACCGAGCGGCTTTGACAAGTTTGGGCGAGTGCTGGTAGAGGCGTACAAC

CGTATCACTGCCCCCAACTTGGACGTGCGCGAGTTCGTTATGACTGCTGAGCTGAGCCGC

TCACCCGAATCGTATACCAACAAGCGCCTGCCGCACCTCACCGTCTACTTTAAGCTCGCC

ATGAGGAATGAAGAACTGCCCAGCGTAAAAGAGAGAATTCCGTATGTGATAGTTGCGCAG

ACCGAGGCCGCGGAACGCGAAGCGGGTGTAGTAAACTCAATGCGCGGTACCGCCCAAAAC

CCCGTGGTAACCAAGACCGCACGCCCCCAACCTAAACGCAAACTGCTGGTTTCCGACCTC

GCCGAAGACCCGACCTATGTTTCCGAGAATGACGTACCGCTAAACACAGACTACTATTTC

TCCCACCTGTTGGGTACCATAAGCGTGACCTTTAAGGCTCTATTCGGAAATGATGTGAGA

ACAACAGAAAATCTTTTAAAGCGGTTTATTCCGGAAACCCCCCACAAGACCCCCACGAAA

ACCCAGGCACTGCTTGAGCGCGCCGGCTTTGAAAAGCTGACGCCCTTTACACCGGAGGAA

GAAAGTCGTCGAATACTGCATACAGTTTTTTGTACTCTAGAAGCAGCTCCCCATCAAAGC

TGA

>LC109631.1

ATGGCGGCGCGCGAACAGGCCAACTCCGTTCGCAGGAGCGGGTTTTTTAATCCGTTTATT

GGCAAGAGGCCATTTTTCAGGCCGGGAAGCGGGCAAACCGCGGAGACAGAGCGCCCAAGG

CCCCCCCAACACTCGTACTGCACAGAGGTGGGTAGCTTTAAGTTTATAGCTCCAAGATGC

CTCGATGAAGAAGCCCCCGCCGACCAGCGACGCGGTGTACACGTGGGCACCTTGGAGCGC

CCACCCAAAGTGTACTGCGATGGCTCAGAGTACGACGTGCTGAACTTTGCCTCCGGAGGT

TGCTGGCCTCGCAGGATTCGGGTTTGGAACGGCCAGGATTTTCGGGGCGATGGATTCAAC

CCCAGATTTGAGAGATTTCACGTGTACGACATAGTAGAGACTTCGGAGAGCGCCTCACAC

GATGACCCATCCAGGTTTGCTGAGCTATCCCGCCCAAGCGGGTCTGTGGTAACACTGCTG

GGAATGAGCGAGTGTGGAAAGCGAGTCGCGGTTCACGTCTATGGTGTGCGCCATTATTTT

TACATGGCAAAGGCGGAGGTGGATAGCGCTTGTGGAATAACCACCGAGGCAGAACTCGTG

CGCGCAATGGTGGACTGCGCGCACAGCTCGGCTTTGAGCGCGGCCCTGGGAAATGGCAAC

GGCGGCAAACAGAGCGGCGGCAGCGGCGGGGGATGGTGGGGCGGAAAGCACGTGTCTGCG

GACTGCTTCAAAGTGGAGACCGTGTGCCACACGACGCTGTACTACTTTGGATCTAAGCCA

GCTCTCTACTATAGAGTATCTGCCTCCAGCAGCCGCCTGGGAGGGTTCATCTGCGACAAC

TTTCACCCGGAGATTACAAAATTCGAGGGGAGCGTGGACGTGACCACGCGGCTGTTGTTG

GACAATGAAAATTTTACCAGTTTCGGGTGGTACCGCCTGCGACCCGGCACCCACGGAGAG

CGTGTTCAACTTCGCCCCGTTGAGCGACACGTCACCTCAAGCGACGTGGAGATTAACTGT

ACTCCCGATAACCTGGAGCCGATACCAGACGAGGCTGCCTGGCCCGACTATAAGCTCATG

TGCTTTGATATAGAGTGTAAAGCTGGAACGGGTAACGAAATGGCGTTCCCAGTGGCAACT

AACCAAGAGGACCTGGTCATCCAGATCTCCTGTCTGCTGTACTCGCTTGCTACTCAGAAC

CACGAACACACCCTGCTGTTTTCCCTCGGGTCATGCGATATCTCTGAGGAATACTCGTTT

GCATGCGTCCAGCGCGGCGAGCCCAGACCGACGGTTTTGGAGTTTGACAGCGAGTACGAG

CTGCTGGTTGCCTTCCTGACCTTTCTCAAGCAGTACTCTCCCGAGTTCGCCACCGGCTAC

AACATCGTTAATTTTGACTGGGCGTACATAGTTAACAAGGTAACGTCGGTGTATAACATC

AAGCTGGACGGGTACGGCAAGTTCAACAAAGGGGGGCTGTTTAAGGTGTGGGACATCGCC

ACGAACCATTTTCAGAAGAAGAGCAAGGTGAAAATCAATGGCCTGATATCTCTAGACATG

TATTCTGTGGCGACGGAAAAGCTAAAGCTACCCAGCTACAAACTCGACGCGGTCGTGGGA

GACGTCCTCGGCGAGCATAAGATAGACCTTCCCTATAAAGAAATACCCTCCTATTACGCG

GGAGGGCCTGACCGGAGGGGCGTAATAGGAGAGTATTGTATCCAGGACTCTAGGCTGGTG

GGCAAGCTGTTTTTTAAGTACCTCCCCCATCTGGAACTATCGGCGGTGGCCAAACTCGCC

CGTATCACCCTAACGCGGGTAATTTTTGACGGTCAGCAAATTCGCGTGTACACGTGCTTG

CTGAAACTCGCCCGCGAGAGAAATTTCATTTTGCCAGACAACAGACGCCGGTTTGACAGT

CAGGCAGACGCCGCGTCAGAGACTTCGGAGTTGGCTATGGATAGCCAAAGCCACGCCTTC

GACAGTACAGACGAACCCGACGGTGTGGACGGTACCCCGGACGCCGCAGGATCTGGCGCT

ACTTCTGAAAACGGAGGCGGGAAGCCCGGCGTCGGGAGGGCCGTGGGCTACCAGGGAGCA

AAGGTTCTAGACCCCGTATCCGGCTTTCATGTGGACCCCGTGGTTGTGTTTGACTTCGCT

AGCTTATACCCAAGCATTATCCAGGCCCATAACCTCTGTTTCACCACCCTGGCGCTCGAT

GAAGTGGATCTGGCCGGGCTTCAACCATCCGTCAACTACTCGACGTTCGAGGTGGGTGAC

CAAAAGTTATTTTTTGTCCACGCCCATATTCGCGAAAGCCTGCTTGGCATCTTGCTGCGC

GACTGGCTGGCCATGCGAAAGGCGGTGAGGGCGCGAATCCCCACCAGCACCCCCGAGGAG

GCAGTTTTACTAGATAAGCAGCAGTCTGCGATTAAGGTGATATGCAACTCGGTTTACGGA

TTCACGGGGGTGGCAAACGGCCTGTTGCCGTGTCTGAGGATAGCGGCTACCGTTACCACG

ATAGGACGCGACATGCTCCTCAAGACCCGAGATTACGTTCACTCTCGTTGGGCGACGCGC

GAGCTGCTGGAGGACAATTTTCCAGGGGCTATAGGTTTCCGAAACCACAAGCCTTACTCC

GTCAGGGTTATCTACGGAGACACCGACTCCGTGTTTATCAAGTTTGTGGGCCTGACGTAC

GAGGGGGTATCGGAGCTGGGGGATGCTATGTCGCGTCAGATTTCAGCGGACCTCTTTAGA

GCGCCCATCAAACTGGAGTGTGAGAAGACCTTTCAGCGACTGCTGCTGATCACCAAGAAG

AAGTACATAGGTGTCATAAACGGGGGGAAGATGCTCATGAAGGGGGTCGACCTGGTCCGC

AAAAATAACTGCTCTTTCATAAACTTGTACGCGCGACATCTGGTAGATCTTTTGTTGTAC

GACGAGGATGTGGCCACGGCGGCAGCAGAGGTGACAGACGTGCCTCCCGCAGAATGGGTG

GGGCGCCCGCTACCGAGCGGCTTTGACAAGTTTGGGCGAGTGCTGGTAGAGGCGTACAAC

CGTATCACTGCCCCCAACTTGGACGTGCGCGAGTTCGTTATGACTGCTGAGCTGAGCCGC

TCACCCGAATCGTATACCAACAAGCGCCTGCCGCACCTCACCGTCTACTTTAAGCTCGCC

ATGAGGAATGAAGAACTGCCCAGCGTAAAAGAGAGAATTCCGTATGTGATAGTTGCGCAG

ACCGAGGCCGCGGAACGCGAAGCGGGTGTAGTAAACTCAATGCGCGGTACCGCCCAAAAC

CCCGTGGTAACCAAGACCGCACGCCCCCAACCTAAACGCAAACTGCTGGTTTCCGACCTC

GCCGAAGACCCGACCTATGTTTCCGAGAATGACGTACCGCTAAACACAGACTACTATTTC

TCCCACCTGTTGGGTACCATAAGCGTGACCTTTAAGGCTCTATTCGGAAATGATGTGAGA

ACAACAGAAAATCTTTTAAAGCGGTTTATTCCGGAAACCCCCCACAAGACCCCCACGAAA

ACCCAGGCACTGCTTGAGCGCGCCGGCTTTGAAAAGCTGACGCCCTTTACACCGGAGGAA

GAAAGTCGTCGAATACTGCATACAGTTTTTTGTACTCTAGAAGCAGCTCCCCATCAAAGC

TGA

>LC109632.1

ATGGCGGCGCGCGAACAGGCCAACTCCGTTCGCAGGAGCGGGTTTTTTAATCCGTTTATT

GGCAAGAGGCCATTTTTCAGGCCGGGAAGCGGGCAAACCGCGGAGACAGAGCGCCCAAGG

CCCCCCCAACACTCGTACTGCACAGAGGTGGGTAGCTTTAAGTTTATAGCTCCAAGATGC

CTCGATGAAGAAGCCCCCGCCGACCAGCGACGCGGTGTACACGTGGGCACCTTGGAGCGC

CCACCCAAAGTGTACTGCGATGGCTCAGAGTACGACGTGCTGAACTTTGCCTCCGGAGGT

TGCTGGCCTCGCAGGATTCGGGTTTGGAACGGCCAGGATTTTCGGGGCGATGGATTCAAC

CCCAGATTTGAGAGATTTCACGTGTACGACATAGTAGAGACTTCGGAGAGCGCCTCACAC

GATGACCCATCCAGGTTTGCTGAGCTATCCCGCCCAAGCGGGTCTGTGGTAACACTGCTG

GGAATGAGCGAGTGTGGAAAGCGAGTCGCGGTTCACGTCTATGGTGTGCGCCATTATTTT

TACATGGCAAAGGCGGAGGTGGATAGCGCTTGTGGAATAACCACCGAGGCAGAACTCGTG

CGCGCAATGGTGGACTGCGCGCACAGCTCGGCTTTGAGCGCGGCCCTGGGAAATGGCAAC

GGCGGCAAACAGAGCGGCGGCAGCGGCGGGGGATGGTGGGGCGGAAAGCACGTGTCTGCG

GACTGCTTCAAAGTGGAGACCGTGTGCCACACGACGCTGTACTACTTTGGATCTAAGCCA

GCTCTCTACTATAGAGTATCTGCCTCCAGCAGCCGCCTGGGAGGGTTCATCTGCGACAAC

TTTCACCCGGAGATTACAAAATTCGAGGGGAGCGTGGACGTGACCACGCGGCTGTTGTTG

GACAATGAAAATTTTACCAGTTTCGGGTGGTACCGCCTGCGACCCGGCACCCACGGAGAG

CGTGTTCAACTTCGCCCCGTTGAGCGACACGTCACCTCAAGCGACGTGGAGATTAACTGT

ACTCCCGATAACCTGGAGCCGATACCAGACGAGGCTGCCTGGCCCGACTATAAGCTCATG

TGCTTTGATATAGAGTGTAAAGCTGGAACGGGTAACGAAATGGCGTTCCCAGTGGCAACT

AACCAAGAGGACCTGGTCATCCAGATCTCCTGTCTGCTGTACTCGCTTGCTACTCAGAAC

CACGAACACACCCTGCTGTTTTCCCTCGGGTCATGCGATATCTCTGAGGAATACTCGTTT

GCATGCGTCCAGCGCGGCGAGCCCAGACCGACGGTTTTGGAGTTTGACAGCGAGTACGAG

CTGCTGGTTGCCTTCCTGACCTTTCTCAAGCAGTACTCTCCCGAGTTCGCCACCGGCTAC

AACATCGTTAATTTTGACTGGGCGTACATAGTTAACAAGGTAACGTCGGTGTATAACATC

AAGCTGGACGGGTACGGCAAGTTCAACAAAGGGGGGCTGTTTAAGGTGTGGGACATCGCC

ACGAACCATTTTCAGAAGAAGAGCAAGGTGAAAATCAATGGCCTGATATCTCTAGACATG

TATTCTGTGGCGACGGAAAAGCTAAAGCTACCCAGCTACAAACTCGACGCGGTCGTGGGA

GACGTCCTCGGCGAGCATAAGATAGACCTTCCCTATAAAGAAATACCCTCCTATTACGCG

GGAGGGCCTGACCGGAGGGGCGTAATAGGAGAGTATTGTATCCAGGACTCTAGGCTGGTG

GGCAAGCTGTTTTTTAAGTACCTCCCCCATCTGGAACTATCGGCGGTGGCCAAACTCGCC

CGTATCACCCTAACGCGGGTAATTTTTGACGGTCAGCAAATTCGCGTGTACACGTGCTTG

CTGAAACTCGCCCGCGAGAGAAATTTCATTTTGCCAGACAACAGACGCCGGTTTGACAGC

CAGGCAGACGCCGCGTCAGAGACTTCGGAGTTGGCTATGGATAGCCAAAGCCACGCCTTC

GACAGTACAGACGAACCCGACGGTGTGGACGGTACCCCGGACGCCGCAGGATCTGGCGCT

ACTTCTGAAAACGGAGGCGGGAAGCCCGGCGTCGGGAGGGCCGTGGGCTACCAGGGAGCA

AAGGTTCTAGACCCCGTATCCGGCTTTCATGTGGACCCCGTGGTTGTGTTTGACTTCGCT

AGCTTATACCCAAGCATTATCCAGGCCCATAACCTCTGTTTCACCACCCTGGCGCTCGAT

GAAGTGGATCTGGCCGGGCTTCAACCATCCGTCAACTACTCGACGTTCGAGGTGGGTGAC

CAAAAGTTATTTTTTGTCCACGCCCATATTCGCGAAAGCCTGCTTGGCATCTTGCTGCGC

GACTGGCTGGCCATGCGAAAGGCGGTGAGGGCGCGAATCCCCACCAGCACCCCCGAGGAG

GCAGTTTTACTAGATAAGCAGCAGTCTGCGATTAAGGTGATATGCAACTCGGTTTACGGA

TTCACGGGGGTGGCAAACGGCCTGTTGCCGTGTCTGAGGATAGCGGCTACCGTTACCACG

ATAGGACGCGACATGCTCCTCAAGACCCGAGATTACGTTCACTCTCGTTGGGCGACGCGC

GAGCTGCTGGAGGACAATTTTCCAGGGGCTATAGGTTTCCGAAACCACAAGCCTTACTCC

GTCAGGGTTATCTACGGAGACACCGACTCCGTGTTTATCAAGTTTGTGGGCCTGACGTAC

GAGGGGGTATCGGAGCTGGGGGATGCTATGTCGCGTCAGATTTCAGCGGACCTCTTTAGA

GCGCCCATCAAACTGGAGTGTGAGAAGACCTTTCAGCGACTGCTGCTGATCACCAAGAAG

AAGTACATAGGTGTCATAAACGGGGGGAAGATGCTCATGAAGGGGGTCGACCTGGTCCGC

AAAAATAACTGCTCTTTCATAAACTTGTACGCGCGACATCTGGTAGATCTTTTGTTGTAC

GACGAGGATGTGGCCACGGCGGCAGCAGAGGTGACAGACGTGCCTCCCGCAGAATGGGTG

GGGCGCCCGCTACCGAGCGGCTTTGACAAGTTTGGGCGAGTGCTGGTAGAGGCGTACAAC

CGTATCACTGCCCCCAACTTGGACGTGCGCGAGTTCGTTATGACTGCTGAGCTGAGCCGC

TCACCCGAATCGTATACCAACAAGCGCCTGCCGCACCTCACCGTCTACTTTAAGCTCGCC

ATGAGGAATGAAGAACTGCCCAGCGTAAAAGAGAGAATTCCGTATGTGATAGTTGCGCAG

ACCGAGGCCGCGGAACGCGAAGCGGGTGTAGTAAACTCAATGCGCGGTACCGCCCAAAAC

CCCGTGGTAACCAAGACCGCACGCCCCCAACCTAAACGCAAACTGCTGGTTTCCGACCTC

GCCGAAGACCCGACCTATGTTTCCGAGAATGACGTACCGCTAAACACAGACTACTATTTC

TCCCACCTGTTGGGTACCATAAGCGTGACCTTTAAGGCTCTATTCGGAAATGATGTGAGA

ACAACAGAAAATCTTTTAAAGCGGTTTATTCCGGAAACCCCCCACAAGACCCCCACGAAA

ACCCAGGCACTGCTTGAGCGCGCCGGCTTTGAAAAGCTGACGCCCTTTACACCGGAGGAA

GAAAGTCGTCGAATACTGCATACAGTTTTTTGTACTCTAGAAGCAGCTCCCCATCAAAGC

TGA

>LC109633.1

ATGGCGGCGCGCGAACAGGCCAACTCCGTTCGCAGGAGCGGGTTTTTTAATCCGTTTATT

GGCAAGAGGCCATTTTTCAGGCCGGGAAGCGGGCAGACCGCGGAGACAGAGCGCCCAAGG

CCCCCCCAACACTCGTACTGCACAGAGGTGGGTAGCTTTAAATTTATAGCTCCAAGATGC

CTCGATGAAGAAGCCCCCGCCGACCAGCGACGCGGTGTACACGTGGGCACCTTGGAGCGC

CCACCCAAAGTGTACTGCGATGGCTCAGAGTACGACGTGCTGAACTTTGCCTCCGGAGGT

TGCTGGCCTCGCAGGATTCGGGTTTGGAACGGCCAGGATTTTCGGGGCGATGGATTCAAC

CCCAGATTTGAGAGATTTCACGTGTACGACATAGTAGAGACTTCGGAGAGCGCCTCACAC

GATGACCCATCCAGGTTTGCTGAGCTATCCCGCCCAAGCGGGTCTGTGGTAACACTGCTG

GGAATGAGCGAGTGTGGAAAGCGAGTCGCGGTTCACGTCTATGGTGTGCGCCATTATTTT

TACATGGCAAAGGCGGAGGTGGATAGCGCTTGTGGAATAACCACCGAGGCAGAACTCGTG

CGCGCAATGGTGGACTGCGCGCACAGCTCGGCTTTGAGCGCGGCCCTGGGAAATGGCAAC

GGCGGCAAACAGAGCGGCGGCAGCGGCGGGGGATGGTGGGGCGGAAAGCACGTGTCCGCG

GACTGCTTCAAAGTGGAGACCGTGTGCCACACGACGCTGTACTACTTTGGATCTAAGCCA

GCTCTCTACTATAGAGTATCTGCCTCCAGCAGCCGCCTGGGAGGGTTCATCTGCGACAAC

TTTCACCCGGAGATTACAAAATTCGAGGGGAGCGTGGACGTGACCACGCGGCTGTTGTTG

GACAATGAAAATTTTACCAGTTTCGGGTGGTACCGCCTGCGACCCGGCACCCACGGAGAG

CGTGTTCAACTTCGCCCCGTTGAGCGACACGTCACCTCAAGCGACGTGGAGATTAACTGT

ACTCCCGATAACCTGGAGCCGATACCAGACGAGGCTGCCTGGCCCGACTATAAGCTCATG

TGCTTTGATATAGAGTGTAAAGCTGGAACGGGTAACGAAATGGCGTTCCCAGTGGCAACT

AACCAAGAGGACCTGGTCATCCAGATCTCCTGTCTGCTGTACTCGCTTGCTACTCAGAAC

CACGAACACACCCTGCTGTTTTCCCTCGGGTCATGCGATATCTCTGAGGAATACTCGTTT

GCATGCGTCCAGCGCGGCGAGCCCAGACCGACGGTTTTGGAGTTTGACAGCGAGTACGAG

CTGCTGGTTGCCTTCCTGACCTTTCTCAAGCAGTACTCTCCCGAGTTCGCCACCGGCTAC

AACATCGTTAATTTTGACTGGGCGTACATAGTTAACAAGGTAACGTCGGTGTATAACATC

AAGCTGGACGGGTACGGCAAGTTCAACAAAGGGGGGCTGTTTAAGGTGTGGGACATCGCC

ACGAACCATTTTCAGAAGAAGAGCAAGGTGAAAATCAATGGCCTGATATCTCTAGACATG

TATTCTGTGGCGACGGAAAAGCTAAAGCTACCCAGCTACAAACTCGACGCGGTCGTGGGA

GACGTCCTCGGCGAGCATAAGATAGACCTTCCCTATAAAGAAATACCCTCCTATTACGCG

GGAGGGCCTGACCGGAGGGGCGTAATAGGAGAGTATTGTATCCAGGACTCTAGGCTGGTG

GGCAAGCTGTTTTTTAAGTACCTCCCCCATCTGGAACTATCGGCGGTGGCCAAACTCGCC

CGTATCACCCTAACGCGGGTAATTTTTGACGGTCAGCAAATTCGCGTGTACACGTGCTTG

CTGAAACTCGCCCGCGAGAGAAATTTCATTTTGCCAGACAACAGACGCCGGTTTGACAGT

CAGGCAGACGCCGCGTCAGAGACTTCGGAGTTGGCTATGGATAGCCAAAGCCACGCCTTC

GACAGTACAGACGAACCCGACGGTGTGGACGGTACCCCGGACGCCGCAGGATCTGGCGCT

ACTTCTGAAAACGGAGGCGGGAAGCCCGGCGTCGGGAGGGCCGTGGGCTACCAGGGAGCA

AAGGTTCTAGACCCCGTATCCGGCTTTCATGTGGACCCCGTGGTTGTGTTTGACTTCGCT

AGCTTATACCCAAGCATTATCCAGGCCCATAACCTCTGTTTCACCACCCTGGCGCTCGAT

GAAGTGGATCTGGCCGGGCTTCAACCATCCGTCAACTACTCGACGTTCGAGGTGGGTGAC

CAAAAGTTATTTTTTGTCCACGCCCATATTCGCGAAAGCCTGCTTGGCATCTTGCTGCGC

GACTGGCTGGCCATGCGAAAGGCGGTGAGGGCGCGAATCCCCACCAGCACCCCCGAGGAG

GCAGTTTTACTAGATAAGCAGCAGTCTGCGATTAAGGTGATATGCAACTCGGTTTACGGA

TTCACGGGGGTGGCAAACGGCCTGTTGCCGTGTCTGAGGATAGCGGCTACCGTTACCACG

ATAGGACGCGACATGCTCCTCAAGACCCGAGATTACGTTCACTCTCGTTGGGCGACGCGC

GAGCTGCTGGAGGACAATTTTCCAGGGGCTATAGGTTTCCGAAACCACAAGCCTTACTCC

GTCAGGGTTATCTACGGAGACACCGACTCCGTGTTTATCAAGTTTGTGGGCCTGACGTAC

GAGGGGGTATCGGAGCTGGGGGATGCTATGTCGCGTCAGATTTCAGCGGACCTCTTTAGA

GCCCCCATCAAACTGGAGTGTGAGAAGACCTTTCAGCGACTGCTGCTGATCACCAAGAAG

AAGTACATAGGTGTCATAAACGGGGGGAAGATGCTCATGAAGGGGGTCGACCTGGTCCGC

AAAAATAACTGCTCTTTCATAAACTTGTACGCGCGACATCTGGTAGATCTTTTGTTGTAC

GACGAGGATGTGGCCACGGCGGCAGCAGAGGTGACAGACGTGCCTCCCGCAGAATGGGTG

GGGCGCCCGCTACCGAGCGGCTTTGACAAGTTTGGGCGAGTGCTGGTAGAGGCGTACAAC

CGTATCACTGCCCCCAACTTGGACGTGCGCGAGTTCGTTATGACTGCTGAGCTGAGCCGC

TCACCCGAATCGTATACCAACAAGCGCCTGCCGCACCTCACCGTCTACTTTAAGCTCGCC

ATGAGGAATGAAGAACTGCCCAGCGTAAAAGAGAGAATTCCGTATGTGATAGTTGCGCAG

ACCGAGGCCGCGGAACGCGAAGCGGGTGTAGTAAACTCAATGCGCGGTACCGCCCAAAAC

CCCGTGGTAACCAAGACCGCACGCCCCCAACCTAAACGCAAACTGCTGGTTTCCGACCTC

GCCGAAGACCCGACCTATGTTTCCGAGAATGACGTACCGCTAAACACAGACTACTATTTC

TCCCACCTGTTGGGTACCATAAGCGTGACCTTTAAGGCTCTATTCGGAAATGATGTGAGA

ACAACAGAAAATCTTTTAAAGCGGTTTATTCCGGAAACCCCCCACAAGACCCCCACGAAA

ACCCAGGCACTGCTTGAGCGCGCCGGCTTTGAAAAGCTGACGCCCTTTACACCGGAGGAA

GAAAGTCGTCGAATACTGCATACAGTTTTTTGTACTCTAGAAGCAGCTCCCCATCAAAGC

TGA

>LC109634.1

ATGGCGGCGCGCGAACAGGCCAACTCCGTTCGCAGGAGCGGGTTTTTTAATCCGTTTATT

GGCAAGAGGCCATTTTTCAGGCCGGGAAGCGGGCAGACCGCGGAGACAGAGCGCCCAAGG

CCCCCCCAACACTCGTACTGCACAGAGGTGGGTAGCTTTAAGTTTATAGCTCCAAGATGC

CTCGATGAAGAAGCCCCCGCCGACCAGCGACGCGGTGTACACGTGGGCACCTTGGAGCGC

CCACCCAAAGTGTACTGCGATGGCTCAGAGTACGACGTGCTGAACTTTGCCTCCGGAGGT

TGCTGGCCTCGCAGGATTCGGGTTTGGAACGGCCAGGATTTTCGGGGCGATGGATTCAAC

CCCAGATTTGAGAGATTTCACGTGTACGACATAGTAGAGACTTCGGAGAGCGCCTCACAC

GATGACCCATCCAGGTTTGCTGAGCTATCCCGCCCAAGCGGGTCTGTGGTAACACTGCTG

GGAATGAGCGAGTGTGGAAAGCGAGTCGCGGTTCACGTCTATGGTGTGCGCCATTATTTT

TACATGGCAAAGGCGGAGGTGGATAGCGCTTGTGGAATAACCACCGAGGCAGAACTCGTG

CGCGCAATGGTGGACTGCGCGCACAGCTCGGCTTTGAGCGCGGCCCTGGGAAATGGCAAC

GGCGGCAAACAGAGCGGCGGCAGCGGCGGGGGATGGTGGGGCGGAAAGCACGTGTCTGCG

GACTGCTTCAAAGTGGAGACCGTGTGCCACACGACGCTGTACTACTTTGGATCTAAGCCA

GCTCTCTACTATAGAGTATCTGCCTCCAGCAGCCGCCTGGGAGGGTTCATCTGCGACAAC

TTTCACCCGGAGATTACAAAATTCGAGGGGAGCGTGGACGTGACCACGCGGCTGTTGTTG

GACAATGAAAATTTTACCAGTTTCGGGTGGTACCGCCTGCGACCCGGCACCCACGGAGAG

CGTGTTCAACTTCGCCCCGTTGAGCGACACGTCACCTCAAGCGACGTGGAGATTAACTGT

ACTCCCGATAACCTGGAGCCGATACCAGACGAGGCTGCCTGGCCCGACTATAAGCTCATG

TGCTTTGATATAGAGTGTAAAGCTGGAACGGGTAACGAAATGGCGTTCCCAGTGGCAACT

AACCAAGAGGACCTGGTCATCCAGATCTCCTGTCTGCTGTACTCGCTTGCTACTCAGAAC

CACGAACACACCCTGCTGTTTTCCCTCGGGTCATGCGATATCTCTGAGGAATACTCGTTT

GCATGCGTCCAGCGCGGCGAGCCCAGACCGACGGTTTTGGAGTTTGACAGCGAGTACGAG

CTGCTGGTTGCCTTCCTGACCTTTCTCAAGCAGTACTCTCCCGAGTTCGCCACCGGCTAC

AACATCGTTAATTTTGACTGGGCGTACATAGTTAACAAGGTAACGTCGGTGTATAACATC

AAGCTGGACGGGTACGGCAAGTTCAACAAAGGGGGGCTGTTTAAGGTGTGGGACATCGCC

ACGAACCATTTTCAGAAGAAGAGCAAGGTGAAAATCAATGGCCTGATATCTCTAGACATG

TATTCTGTGGCGACGGAAAAGCTAAAGCTACCCAGCTACAAACTCGACGCGGTCGTGGGA

GACGTCCTCGGCGAGCATAAGATAGACCTTCCCTATAAAGAAATACCCTCCTATTACGCG

GGAGGGCCTGACCGGAGGGGCGTAATAGGAGAGTATTGTATCCAGGACTCTAGGCTGGTG

GGCAAGCTGTTTTTTAAGTACCTCCCCCATCTGGAACTATCGGCGGTGGCCAAACTCGCC

CGTATCACCCTAACGCGGGTAATTTTTGACGGTCAGCAAATTCGCGTGTACACGTGCTTG

CTGAAACTCGCCCGCGAGAGAAATTTCATTTTGCCAGACAACAGACGCCGGTTTGACAGT

CAGGCAGACGCCGCGTCAGAGACTTCGGAGTTGGCTATGGATAGCCAAAGCCACGCCTTC

GACAGTACAGACGAACCCGACGGTGTGGACGGTACCCCGGACGCCGCAGGATCTGGCGCT

ACTTCTGAAAACGGAGGCGGGAAGCCCGGCGTCGGGAGGGCCGTGGGCTACCAGGGAGCA

AAGGTTCTAGACCCCGTATCCGGCTTTCATGTGGACCCCGTGGTTGTGTTTGACTTCGCT

AGCTTATACCCAAGCATTATCCAGGCCCATAACCTCTGTTTCACCACCCTGGCGCTCGAT

GAAGTGGATCTGGCCGGGCTTCAACCATCCGTCAACTACTCGACGTTCGAGGTGGGTGAC

CAAAAGTTATTTTTTGTCCACGCCCATATTCGCGAAAGCCTGCTTGGCATCTTGCTGCGC

GACTGGCTGGCCATGCGAAAGGCGGTGAGGGCGCGAATCCCCACCAGCACCCCCGAGGAG

GCAGTTTTACTAGATAAGCAGCAGTCTGCGATTAAGGTGATATGCAACTCGGTTTACGGA

TTCACGGGGGTGGCAAACGGCCTGTTGCCGTGTCTGAGGATAGCGGCTACCGTTACCACG

ATAGGACGCGACATGCTCCTCAAGACCCGAGATTACGTTCACTCTCGTTGGGCGACGCGC

GAGCTGCTGGAGGACAATTTTCCAGGGGCTATAGGTTTCCGAAACCACAAGCCTTACTCC

GTCAGGGTTATCTACGGAGACACCGACTCCGTGTTTATCAAGTTTGTGGGCCTGACGTAC

GAGGGGGTATCGGAGCTGGGGGATGCTATGTCGCGTCAGATTTCAGCGGACCTCTTTAGA

GCCCCCATCAAACTGGAGTGTGAGAAGACCTTTCAGCGACTGCTGCTGATCACCAAGAAG

AAGTACATAGGTGTCATAAACGGGGGGAAGATGCTCATGAAGGGGGTCGACCTGGTCCGC

AAAAATAACTGCTCTTTCATAAACTTGTACGCGCGACATCTGGTAGATCTTTTGTTGTAC

GACGAGGATGTGGCCACGGCGGCAGCAGAGGTGACAGACGTGCCTCCCGCAGAATGGGTG

GGGCGCCCGCTACCGAGCGGCTTTGACAAGTTTGGGCGAGTGCTGGTAGAGGCGTACAAC

CGTATCACTGCCCCCAACTTGGACGTGCGCGAGTTCGTTATGACTGCTGAGCTGAGCCGC

TCACCCGAATCGTATACCAACAAGCGCCTGCCGCACCTCACCGTCTACTTTAAGCTCGCC

ATGAGGAATGAAGAACTGCCCAGCGTAAAAGAGAGAATTCCGTATGTGATAGTTGCGCAG

ACCGAGGCCGCGGAACGCGAAGCGGGTGTAGTAAACTCAATGCGCGGTACCGCCCAAAAC

CCCGTGGTAACCAAGACCGCACGCCCCCAACCTAAACGCAAACTGCTGGTTTCCGACCTC

GCCGAAGACCCGACCTATGTTTCCGAGAATGACGTACCGCTAAACACAGACTACTATTTC

TCCCACCTGTTGGGTACCATAAGCGTGACCTTTAAGGCTCTATTCGGAAATGATGTGAGA

ACAACAGAAAATCTTTTAAAGCGGTTTATTCCGGAAACCCCCCACAAGACCCCCACGAAA

ACCCAGGCACTGCTTGAGCGCGCCGGCTTTGAAAAGCTGACGCCCTTTACACCGGAGGAA

GAAAGTCGTCGAATACTGCATACAGTTTTTTGTACTCTAGAAGCAGCTCCCCATCAAAGC

TGA

>LC109635.1

ATGGCGGCGCGCGAACAGGCCAACTCCGTTCGCAGGAGCGGGTTTTTTAATCCGTTTATT

GGCAAGAGGCCATTTTTCAGGCCGGGAAGCGGGCAGACCGCGGAGACAGAGCGCCCAAGG

CCCCCCCAACACTCGTACTGCACAGAGGTGGGTAGCTTTAAGTTTATAGCTCCAAGATGC

CTCGATGAAGAAGCCCCCGCCGACCAGCGACGCGGTGTACACGTGGGCACCTTGGAGCGC

CCACCCAAAGTGTACTGCGATGGCTCAGAGTACGACGTGCTGAACTTTGCCTCCGGAGGT

TGCTGGCCTCGCAGGATTCGGGTTTGGAACGGCCAGGATTTTCGGGGCGATGGATTCAAC

CCCAGATTTGAGAGATTTCACGTGTACGACATAGTAGAGACTTCGGAGAGCGCCTCACAC

GATGACCCATCCAGGTTTGCTGAGCTATCCCGCCCAAGCGGGTCTGTGGTAACACTGCTG

GGAATGAGCGAGTGTGGAAAGCGAGTCGCGGTTCACGTCTATGGTGTGCGCCATTATTTT

TACATGGCAAAGGCGGAGGTGGATAGCGCTTGTGGAATAACCACCGAGGCAGAACTCGTG

CGCGCAATGGTGGACTGCGCGCACAGCTCGGCTTTGAGCGCGGCCCTGGGAAATGGCAAC

GGCGGCAAACAGAGCGGCGGCAGCGGCGGGGGATGGTGGGGCGGAAAGCACGTGTCTGCG

GACTGCTTCAAAGTGGAGACCGTGTGCCACACGACGCTGTACTACTTTGGATCTAAGCCA

GCTCTCTACTATAGAGTATCTGCCTCCAGCAGCCGCCTGGGAGGGTTCATCTGCGACAAC

TTTCACCCGGAGATTACAAAATTCGAGGGGAGCGTGGACGTGACCACGCGGCTGTTGTTG

GACAATGAAAATTTTACCAGTTTCGGGTGGTACCGCCTGCGACCCGGCACCCACGGAGAG

CGTGTTCAACTTCGCCCCGTTGAGCGACACGTCACCTCAAGCGACGTGGAGATTAACTGT

ACTCCCGATAACCTGGAGCCGATACCAGACGAGGCTGCCTGGCCCGACTATAAGCTCATG

TGCTTTGATATAGAGTGTAAAGCTGGAACGGGTAACGAAATGGCGTTCCCAGTGGCAACT

AACCAAGAGGACCTGGTCATCCAGATCTCCTGTCTGCTGTACTCGCTTGCTACTCAGAAC

CACGAACACACCCTGCTGTTTTCCCTCGGGTCATGCGATATCTCTGAGGAATACTCGTTT

GCATGCGTCCAGCGCGGCGAGCCCAGACCGACGGTTTTGGAGTTTGACAGCGAGTACGAG

CTGCTGGTTGCCTTCCTGACCTTTCTCAAGCAGTACTCTCCCGAGTTCGCCACCGGCTAC

AACATCGTTAATTTTGACTGGGCGTACATAGTTAACAAGGTAACGTCGGTGTATAACATC

AAGCTGGACGGGTACGGCAAGTTCAACAAAGGGGGGCTGTTTAAGGTGTGGGACATCGCC

ACGAACCATTTTCAGAAGAAGAGCAAGGTGAAAATCAATGGCCTGATATCTCTAGACATG

TATTCTGTGGCGACGGAAAAGCTAAAGCTACCCAGCTACAAACTCGACGCGGTCGTGGGA

GACGTCCTCGGCGAGCATAAGATAGACCTTCCCTATAAAGAAATACCCTCCTATTACGCG

GGAGGGCCTGACCGGAGGGGCGTAATAGGAGAGTATTGTATCCAGGACTCTAGGCTGGTG

GGCAAGCTGTTTTTTAAGTACCTCCCCCATCTGGAACTATCGGCGGTGGCCAAACTCGCC

CGTATCACCCTAACGCGGGTAATTTTTGACGGTCAGCAAATTCGCGTGTACACGTGCTTG

CTGAAACTCGCCCGCGAGAGAAATTTCATTTTGCCAGACAACAGACGCCGGTTTGACAGT

CAGGCAGACGCCGCGTCAGAGACTTCGGAGTTGGCTATGGATAGCCAAAGCCACGCCTTC

GACAGTACAGACGAACCCGACGGTGTGGACGGTACCCCGGACGCCGCAGGATCTGGCGCT

ACTTCTGAAAACGGAGGCGGGAAGCCCGGCGTCGGGAGGGCCGTGGGCTACCAGGGAGCA

AAGGTTCTAGACCCCGTATCCGGCTTTCATGTGGACCCCGTGGTTGTGTTTGACTTCGCT

AGCTTATACCCAAGCATTATCCAGGCCCATAACCTCTGTTTCACCACCCTGGCGCTCGAT

GAAGTGGATCTGGCCGGGCTTCAACCATCCGTCAACTACTCGACGTTCGAGGTGGGTGAC

CAAAAGTTATTTTTTGTCCACGCCCATATTCGCGAAAGCCTGCTTGGCATCTTGCTGCGC

GACTGGCTGGCCATGCGAAAGGCGGTGAGGGCGCGAATCCCCACCAGCACCCCCGAGGAG

GCAGTTTTACTAGATAAGCAGCAGTCTGCGATTAAGGTGATATGCAACTCGGTTTACGGA

TTCACGGGGGTGGCAAACGGCCTGTTGCCGTGTCTGAGGATAGCGGCTACCGTTACCACG

ATAGGACGCGACATGCTCCTCAAGACCCGAGATTACGTTCACTCTCGTTGGGCGACGCGC

GAGCTGCTGGAGGACAATTTTCCAGGGGCTATAGGTTTCCGAAACCACAAGCCTTACTCC

GTCAGGGTTATCTACGGAGACACCGACTCCGTGTTTATCAAGTTTGTGGGCCTGACGTAC

GAGGGGGTATCGGAGCTGGGGGATGCTATGTCGCGTCAGATTTCAGCGGACCTCTTTAGA

GCGCCCATCAAACTGGAGTGTGAGAAGACCTTTCAGCGACTGCTGCTGATCACCAAGAAG

AAGTACATAGGTGTCATAAACGGGGGGAAGATGCTCATGAAGGGGGTCGACCTGGTCCGC

AAAAATAACTGCTCTTTCATAAACTTGTACGCGCGACATCTGGTAGATCTTTTGTTGTAC

GACGAGGATGTGGCCACGGCGGCAGCAGAGGTGACAGACGTGCCTCCCGCAGAATGGGTG

GGGCGCCCGCTACCGAGCGGCTTTGACAAGTTTGGGCGAGTGCTGGTAGAGGCGTACAAC

CGTATCACTGCCCCCAACTTGGACGTGCGCGAGTTCGTTATGACTGCTGAGCTGAGCCGC

TCACCCGAATCGTATACCAACAAGCGCCTGCCGCACCTCACCGTCTACTTTAAGCTCGCC

ATGAGGAATGAAGAACTGCCCAGCGTAAAAGAGAGAATTCCGTATGTGATAGTTGCGCAG

ACCGAGGCCGCGGAACGCGAAGCGGGTGTAGTAAACTCAATGCGCGGTACCGCCCAAAAC

CCCGTGGTAACCAAGACCGCACGCCCCCAACCTAAACGCAAACTGCTGGTTTCCGACCTC

GCCGAAGACCCGACCTATGTTTCCGAGAATGACGTACCGCTAAACACAGACTACTATTTC

TCCCACCTGTTGGGTACCATAAGCGTGACCTTTAAGGCTCTATTCGGAAATGATGTGAGA

ACAACAGAAAATCTTTTAAAGCGGTTTATTCCGGAAACCCCCCACAAGACCCCCACGAAA

ACCCAGGCACTGCTTGAGCGCGCCGGCTTTGAAAAGCTGACGCCCTTTACACCGGAGGAA

GAAAGTCGTCGAATACTGCATACAGTTTTTTGTACTCTAGAAGCAGCTCCCCATCAAAGC

TGA

>LC109636.1

ATGGCGGCGCGCGAACAGGCCAACTCCGTTCGCAGGAGCGGGTTTTTTAATCCGTTTATT

GGCAAGAGGCCATTTTTCAGGCCGGGAAGCGGGCAGACCGCGGAGACAGAGCGCCCAAGG

CCCCCCCAACACTCGTACTGCACAGAGGTGGGTAGCTTTAAGTTTATAGCTCCAAGATGC

CTCGATGAAGAAGCCCCCGCCGACCAGCGACGCGGTGTACACGTGGGCACCTTGGAGCGC

CCACCCAAAGTGTACTGCGATGGCTCAGAGTACGACGTGCTGAACTTTGCCTCCGGAGGT

TGCTGGCCTCGCAGGATTCGGGTTTGGAACGGCCAGGATTTTCGGGGCGATGGATTCAAC

CCCAGATTTGAGAGATTTCACGTGTACGACATAGTAGAGACTTCGGAGAGCGCCTCACAC

GATGACCCATCCAGGTTTGCTGAGCTATCCCGCCCAAGCGGGTCTGTGGTAACACTGCTG

GGAATGAGCGAGTGTGGAAAGCGAGTCGCGGTTCACGTCTATGGTGTGCGCCATTATTTT

TACATGGCAAAGGCGGAGGTGGATAGCGCTTGTGGAATAACCACCGAGGCAGAACTCGTG

CGCGCAATGGTGGACTGCGCGCACAGCTCGGCTTTGAGCGCGGCCCTGGGAAATGGCAAC

GGCGGCAAACAGAGCGGCGGCAGCGGCGGGGGATGGTGGGGCGGAAAGCACGTGTCTGCG

GACTGCTTCAAAGTGGAGACCGTGTGCCACACGACGCTGTACTACTTTGGATCTAAGCCA

GCTCTCTACTATAGAGTATCTGCCTCCAGCAGCCGCCTGGGAGGGTTCATCTGCGACAAC

TTTCACCCGGAGATTACAAAATTCGAGGGGAGCGTGGACGTGACCACGCGGCTGTTGTTG

GACAATGAAAATTTTACCAGTTTCGGGTGGTACCGCCTGCGACCCGGCACCCACGGAGAG

CGTGTTCAACTTCGCCCCGTTGAGCGACACGTCACCTCAAGCGACGTGGAGATTAACTGT

ACTCCCGATAACCTGGAGCCGATACCAGACGAGGCTGCCTGGCCCGACTATAAGCTCATG

TGCTTTGATATAGAGTGTAAAGCTGGAACGGGTAACGAAATGGCGTTCCCAGTGGCAACT

AACCAAGAGGACCTGGTCATCCAGATCTCCTGTCTGCTGTACTCGCTTGCTACTCAGAAC

CACGAACACACCCTGCTGTTTTCCCTCGGGTCATGCGATATCTCTGAGGAATACTCGTTT

GCATGCGTCCAGCGCGGCGAGCCCAGACCGACGGTTTTGGAGTTTGACAGCGAGTACGAG

CTGCTGGTTGCCTTCCTGACCTTTCTCAAGCAGTACTCTCCCGAGTTCGCCACCGGCTAC

AACATCGTTAATTTTGACTGGGCGTACATAGTTAACAAGGTAACGTCGGTGTATAACATC

AAGCTGGACGGGTACGGCAAGTTCAACAAAGGGGGGCTGTTTAAGGTGTGGGACATCGCC

ACGAACCATTTTCAGAAGAAGAGCAAGGTGAAAATCAATGGCCTGATATCTCTAGACATG

TATTCTGTGGCGACGGAAAAGCTAAAGCTACCCAGCTACAAACTCGACGCGGTCGTGGGA

GACGTCCTCGGCGAGCATAAGATAGACCTTCCCTATAAAGAAATACCCTCCTATTACGCG

GGAGGGCCTGACCGGAGGGGCGTAATAGGAGAGTATTGTATCCAGGACTCTAGGCTGGTG

GGCAAGCTGTTTTTTAAGTACCTCCCCCATCTGGAACTATCGGCGGTGGCCAAACTCGCC

CGTATCACCCTAACGCGGGTAATTTTTGACGGTCAGCAAATTCGCGTGTACACGTGCTTG

CTGAAACTCGCCCGCGAGAGAAATTTCATTTTGCCAGACAACAGACGCCGGTTTGACAGT

CAGGCAGACGCCGCGTCAGAGACTTCGGAGTTGGCTATGGATAGCCAAAGCCACGCCTTC

GACAGTACAGACGAACCCGACGGTGTGGACGGTACCCCGGACGCCGCAGGATCTGGCGCT

ACTTCTGAAAACGGAGGCGGGAAGCCCGGCGTCGGGAGGGCCGTGGGCTACCAGGGAGCA

AAGGTTCTAGACCCCGTATCCGGCTTTCATGTGGACCCCGTGGTTGTGTTTGACTTCGCT

AGCTTATACCCAAGCATTATCCAGGCCCATAACCTCTGTTTCACCACCCTGGCGCTCGAT

GAAGTGGATCTGGCCGGGCTTCAACCATCCGTCAACTACTCGACGTTCGAGGTGGGTGAC

CAAAAGTTATTTTTTGTCCACGCCCATATTCGCGAAAGCCTGCTTGGCATCTTGCTGCGC

GACTGGCTGGCCATGCGAAAGGCGGTGAGGGCGCGAATCCCCACCAGCACCCCCGAGGAG

GCAGTTTTACTAGATAAGCAGCAGTCTGCGATTAAGGTGATATGCAACTCGGTTTACGGA

TTCACGGGGGTGGCAAACGGCCTGTTGCCGTGTCTGAGGATAGCGGCTACCGTTACCACG

ATAGGACGCGACATGCTCCTCAAGACCCGAGATTACGTTCACTCTCGTTGGGCGACGCGC

GAGCTGCTGGAGGACAATTTTCCAGGGGCTATAGGTTTCCGAAACCACAAGCCTTACTCC

GTCAGGGTTATCTACGGAGACACCGACTCCGTGTTTATCAAGTTTGTGGGCCTGACGTAC

GAGGGGGTATCGGAGCTGGGGGATGCTATGTCGCGTCAGATTTCAGCGGACCTCTTTAGA

GCCCCCATCAAACTGGAGTGTGAGAAGACCTTTCAGCGACTGCTGCTGATCACCAAGAAG

AAGTACATAGGTGTCATAAACGGGGGGAAGATGCTCATGAAGGGGGTCGACCTGGTCCGC

AAAAATAACTGCTCTTTCATAAACTTGTACGCGCGACATCTGGTAGATCTTTTGTTGTAC

GACGAGGATGTGGCCACGGCGGCAGCAGAGGTGACAGACGTGCCTCCCGCAGAATGGGTG

GGGCGCCCGCTACCGAGCGGCTTTGACAAGTTTGGGCGAGTGCTGGTAGAGGCGTACAAC

CGTATCACTGCCCCCAACTTGGACGTGCGCGAGTTCGTTATGACTGCTGAGCTGAGCCGC

TCACCCGAATCGTATACCAACAAGCGCCTGCCGCACCTCACCGTCTACTTTAAGCTCGCC

ATGAGGAATGAAGAACTGCCCAGCGTAAAAGAGAGAATTCCGTATGTGATAGTTGCGCAG

ACCGAGGCCGCGGAACGCGAAGCGGGTGTAGTAAACTCAATGCGCGGTACCGCCCAAAAC

CCCGTGGTAACCAAGACCGCACGCCCCCAACCTAAACGCAAACTGCTGGTTTCCGACCTC

GCCGAAGACCCGACCTATGTTTCCGAGAATGACGTACCGCTAAACACAGACTACTATTTC

TCCCACCTGTTGGGTACCATAAGCGTGACCTTTAAGGCTCTATTCGGAAATGATGTGAGA

ACAACAGAAAATCTTTTAAAGCGGTTTATTCCGGAAACCCCCCACAAGACCCCCACGAAA

ACCCAGGCACTGCTTGAGCGCGCCGGCTTTGAAAAGCTGACGCCCTTTACACCGGAGGAA

GAAAGTCGTCGAATACTGCATACAGTTTTTTGTACTCTAGAAGCAGCTCCCCATCAAAGC

TGA

>LC109637.1

ATGGCGGCGCGCGAACAGGCCAACTCCGTTCGCAGGAGCGGGTTTTTTAATCCGTTTATT

GGCAAGAGGCCATTTTTCAGGCCGGGAAGCGGGCAGACCGCGGAGACAGAGCGCCCAAGG

CCCCCCCAACACTCGTACTGCACAGAGGTGGGTAGCTTTAAGTTTATAGCTCCAAGATGC

CTCGATGAAGAAGCCCCCGCCGACCAGCGACGCGGTGTACACGTGGGCACCTTGGAGCGC

CCACCCAAAGTGTACTGCGATGGCTCAGAGTACGACGTGCTGAACTTTGCCTCCGGAGGT

TGCTGGCCTCGCAGGATTCGGGTTTGGAACGGCCAGGATTTTCGGGGCGATGGATTCAAC

CCCAGATTTGAGAGATTTCACGTGTACGACATAGTAGAGACTTCGGAGAGCGCCTCACAC

GATGACCCATCCAGGTTTGCTGAGCTATCCCGCCCAAGCGGGTCTGTGGTAACACTGCTG

GGAATGAGCGAGTGTGGAAAGCGAGTCGCGGTTCACGTCTATGGTGTGCGCCATTATTTT

TACATGGCAAAGGCGGAGGTGGATAGCGCTTGTGGAATAACCACCGAGGCAGAACTCGTG

CGCGCAATGGTGGACTGCGCGCACAGCTCGGCTTTGAGCGCGGCCCTGGGAAATGGCAAC

GGCGGCAAACAGAGCGGCGGCAGCGGCGGGGGATGGTGGGGCGGAAAGCACGTGTCTGCG

GACTGCTTCAAAGTGGAGACCGTGTGCCACACGACGCTGTACTACTTTGGATCTAAGCCA

GCTCTCTACTATAGAGTATCTGCCTCCAGCAGCCGCCTGGGAGGGTTCATCTGCGACAAC

TTTCACCCGGAGATTACAAAATTCGAGGGGAGCGTGGACGTGACCACGCGGCTGTTGTTG

GACAATGAAAATTTTACCAGTTTCGGGTGGTACCGCCTGCGACCCGGCACCCACGGAGAG

CGTGTTCAACTTCGCCCCGTTGAGCGACACGTCACCTCAAGCGACGTGGAGATTAACTGT

ACTCCCGATAACCTGGAGCCGATACCAGACGAGGCTGCCTGGCCCGACTATAAGCTCATG

TGCTTTGATATAGAGTGTAAAGCTGGAACGGGTAACGAAATGGCGTTCCCAGTGGCAACT

AACCAAGAGGACCTGGTCATCCAGATCTCCTGTCTGCTGTACTCGCTTGCTACTCAGAAC

CACGAACACACCCTGCTGTTTTCCCTCGGGTCATGCGATATCTCTGAGGAATACTCGTTT

GCATGCGTCCAGCGCGGCGAGCCCAGACCGACGGTTTTGGAGTTTGACAGCGAGTACGAG

CTGCTGGTTGCCTTCCTGACCTTTCTCAAGCAGTACTCTCCCGAGTTCGCCACCGGCTAC

AACATCGTTAATTTTGACTGGGCGTACATAGTTAACAAGGTAACGTCGGTGTATAACATC

AAGCTGGACGGGTACGGCAAGTTCAACAAAGGGGGGCTGTTTAAGGTGTGGGACATCGCC

ACGAACCATTTTCAGAAGAAGAGCAAGGTGAAAATCAATGGCCTGATATCTCTAGACATG

TATTCTGTGGCGACGGAAAAGCTAAAGCTACCCAGCTACAAACTCGACGCGGTCGTGGGA

GACGTCCTCGGCGAGCATAAGATAGACCTTCCCTATAAAGAAATACCCTCCTATTACGCG

GGAGGGCCTGACCGGAGGGGCGTAATAGGAGAGTATTGTATCCAGGACTCTAGGCTGGTG

GGCAAGCTGTTTTTTAAGTACCTCCCCCATCTGGAACTATCGGCGGTGGCCAAACTCGCC

CGTATCACCCTAACGCGGGTAATTTTTGACGGTCAGCAAATTCGCGTGTACACGTGCTTG

CTGAAACTCGCCCGCGAGAGAAATTTCATTTTGCCAGACAACAGACGCCGGTTTGACAGT

CAGGCAGACGCCGCGTCAGAGACTTCGGAGTTGGCTATGGATAGCCAAAGCCACGCCTTC

GACAGTACAGACGAACCCGACGGTGTGGACGGTACCCCGGACGCCGCAGGATCTGGCGCT

ACTTCTGAAAACGGAGGCGGGAAGCCCGGCGTCGGGAGGGCCGTGGGCTACCAGGGAGCA

AAGGTTCTAGACCCCGTATCCGGCTTTCATGTGGACCCCGTGGTTGTGTTTGACTTCGCT

AGCTTATACCCAAGCATTATCCAGGCCCATAACCTCTGTTTCACCACCCTGGCGCTCGAT

GAAGTGGATCTGGCCGGGCTTCAACCATCCGTCAACTACTCGACGTTCGAGGTGGGTGAC

CAAAAGTTATTTTTTGTCCACGCCCATATTCGCGAAAGCCTGCTTGGCATCTTGCTGCGC

GACTGGCTGGCCATGCGAAAGGCGGTGAGGGCGCGAATCCCCACCAGCACCCCCGAGGAG

GCAGTTTTACTAGATAAGCAGCAGTCTGCGATTAAGGTGATATGCAACTCGGTTTACGGA

TTCACGGGGGTGGCAAACGGCCTGTTGCCGTGTCTGAGGATAGCGGCTACCGTTACCACG

ATAGGACGCGACATGCTCCTCAAGACCCGAGATTACGTTCACTCTCGTTGGGCGACGCGC

GAGCTGCTGGAGGACAATTTTCCAGGGGCTATAGGTTTCCGAAACCACAAGCCTTACTCC

GTCAGGGTTATCTACGGAGACACCGACTCCGTGTTTATCAAGTTTGTGGGCCTGACGTAC

GAGGGGGTATCGGAGCTGGGGGATGCTATGTCGCGTCAGATTTCAGCGGACCTCTTTAGA

GCGCCCATCAAACTGGAGTGTGAGAAGACCTTTCAGCGACTGCTGCTGATCACCAAGAAG

AAGTACATAGGTGTCATAAACGGGGGGAAGATGCTCATGAAGGGGGTCGACCTGGTCCGC

AAAAATAACTGCTCTTTCATAAACTTGTACGCGCGACATCTGGTAGATCTTTTGTTGTAC

GACGAGGATGTGGCCACGGCGGCAGCAGAGGTGACAGACGTGCCTCCCGCAGAATGGGTG

GGGCGCCCGCTACCGAGCGGCTTTGACAAGTTTGGGCGAGTGCTGGTAGAGGCGTACAAC

CGTATCACTGCCCCCAACTTGGACGTGCGCGAGTTCGTTATGACTGCTGAGCTGAGCCGC

TCACCCGAATCGTATACCAACAAGCGCCTGCCGCACCTCACCGTCTACTTTAAGCTCGCC

ATGAGGAATGAAGAACTGCCCAGCGTAAAAGAGAGAATTCCGTATGTGATAGTTGCGCAG

ACCGAGGCCGCGGAACGCGAAGCGGGTGTAGTAAACTCAATGCGCGGTACCGCCCAAAAC

CCCGTGGTAACCAAGACCGCACGCCCCCAACCTAAACGCAAACTGCTGGTTTCCGACCTC

GCCGAAGACCCGACCTATGTTTCCGAGAATGACGTACCGCTAAACACAGACTACTATTTC

TCCCACCTGTTGGGTACCATAAGCGTGACCTTTAAGGCTCTATTCGGAAATGATGTGAGA

ACAACAGAAAATCTTTTAAAGCGGTTTATTCCGGAAACCCCCCACAAGACCCCCACGAAA

ACCCAGGCACTGCTTGAGCGCGCCGGCTTTGAAAAGCTGACGCCCTTTACACCGGAGGAA

GAAAGTCGTCGAATACTGCATACAGTTTTTTGTACTCTAGAAGCAGCTCCCCATCAAAGC

TGA

>LC109638.1

ATGGCGGCGCGCGAACAGGCCAACTCCGTTCGCAGGAGCGGGTTTTTTAATCCGTTTATT

GGCAAGAGGCCATTTTTCAGGCCGGGAAGCGGGCAAACCGCGGAGACAGAGCGCCCAAGG

CCCCCCCAACACTCGTACTGCACAGAGGTGGGTAGCTTTAAGTTTATAGCTCCAAGATGC

CTCGATGAAGAAGCCCCCGCCGACCAGCGACGCGGTGTACACGTGGGCACCTTGGAGCGC

CCACCCAAAGTGTACTGCGATGGCTCAGAGTACGACGTGCTGAACTTTGCCTCCGGAGGT

TGCTGGCCTCGCAGGATTCGGGTTTGGAACGGCCAGGATTTTCGGGGCGATGGATTCAAC

CCCAGATTTGAGAGATTTCACGTGTACGACATAGTAGAGACTTCGGAGAGCGCCTCACAC

GATGACCCATCCAGGTTTGCTGAGCTATCCCGCCCAAGCGGGTCTGTGGTAACACTGCTG

GGAATGAGCGAGTGTGGAAAGCGAGTCGCGGTTCACGTCTATGGTGTGCGCCATTATTTT

TACATGGCAAAGGCGGAGGTGGATAGCGCTTGTGGAATAACCACCGAGGCAGAACTCGTG

CGCGCAATGGTGGACTGCGCGCACAGCTCGGCTTTGAGCGCGGCCCTGGGAAATGGCAAC

GGCGGCAAACAGAGCGGCGGCAGCGGCGGGGGATGGTGGGGCGGAAAGCACGTGTCTGCG

GACTGCTTCAAAGTGGAGACCGTGTGCCACACGACGCTGTACTACTTTGGATCTAAGCCA

GCTCTCTACTATAGAGTATCTGCCTCCAGCAGCCGCCTGGGAGGGTTCATCTGCGACAAC

TTTCACCCGGAGATTACAAAATTCGAGGGGAGCGTGGACGTGACCACGCGGCTGTTGTTG

GACAATGAAAATTTTACCAGTTTCGGGTGGTACCGCCTGCGACCCGGCACCCACGGAGAG

CGTGTTCAACTTCGCCCCGTTGAGCGACACGTCACCTCAAGCGACGTGGAGATTAACTGT

ACTCCCGATAACCTGGAGCCGATACCAGACGAGGCTGCCTGGCCCGACTATAAGCTCATG

TGCTTTGATATAGAGTGTAAAGCTGGAACGGGTAACGAAATGGCGTTCCCAGTGGCAACT

AACCAAGAGGACCTGGTCATCCAGATCTCCTGTCTGCTGTACTCGCTTGCTACTCAGAAC

CACGAACACACCCTGCTGTTTTCCCTCGGGTCATGCGATATCTCTGAGGAATACTCGTTT

GCATGCGTCCAGCGCGGCGAGCCCAGACCGACGGTTTTGGAGTTTGACAGCGAGTACGAG

CTGCTGGTTGCCTTCCTGACCTTTCTCAAGCAGTACTCTCCCGAGTTCGCCACCGGCTAC

AACATCGTTAATTTTGACTGGGCGTACATAGTTAACAAGGTAACGTCGGTGTATAACATC

AAGCTGGACGGGTACGGCAAGTTCAACAAAGGGGGGCTGTTTAAGGTGTGGGACATCGCC

ACGAACCATTTTCAGAAGAAGAGCAAGGTGAAAATCAATGGCCTGATATCTCTAGACATG

TATTCTGTGGCGACGGAAAAGCTAAAGCTACCCAGCTACAAACTCGACGCGGTCGTGGGA

GACGTCCTCGGCGAGCATAAGATAGACCTTCCCTATAAAGAAATACCCTCCTATTACGCG

GGAGGGCCTGACCGGAGGGGCGTAATAGGAGAGTATTGTATCCAGGACTCTAGGCTGGTG

GGCAAGCTGTTTTTTAAGTACCTCCCCCATCTGGAACTATCGGCGGTGGCCAAACTCGCC

CGTATCACCCTAACGCGGGTAATTTTTGACGGTCAGCAAATTCGCGTGTACACGTGCTTG

CTGAAACTCGCCCGCGAGAGAAATTTCATTTTGCCAGACAACAGACGCCGGTTTGACAGT

CAGGCAGACGCCGCGTCAGAGACTTCGGAGTTGGCTATGGATAGCCAAAGCCACGCCTTC

GACAGTACAGACGAACCCGACGGTGTGGACGGTACCCCGGACGCCGCAGGATCTGGCGCT

ACTTCTGAAAACGGAGGCGGGAAGCCCGGCGTCGGGAGGGCCGTGGGCTACCAGGGAGCA

AAGGTTCTAGACCCCGTATCCGGCTTTCATGTGGACCCCGTGGTTGTGTTTGACTTCGCT

AGCTTATACCCAAGCATTATCCAGGCCCATAACCTCTGTTTCACCACCCTGGCGCTCGAT

GAAGTGGATCTGGCCGGGCTTCAACCATCCGTCAACTACTCGACGTTCGAGGTGGGTGAC

CAAAAGTTATTTTTTGTCCACGCCCATATTCGCGAAAGCCTGCTTGGCATCTTGCTGCGC

GACTGGCTGGCCATGCGAAAGGCGGTGAGGGCGCGAATCCCCACCAGCACCCCCGAGGAG

GCAGTTTTACTAGATAAGCAGCAGTCTGCGATTAAGGTGATATGCAACTCGGTTTACGGA

TTCACGGGGGTGGCAAACGGCCTGTTGCCGTGTCTGAGGATAGCGGCTACCGTTACCACG

ATAGGACGCGACATGCTCCTCAAGACCCGAGATTACGTTCACTCTCGTTGGGCGACGCGC

GAGCTGCTGGAGGACAATTTTCCAGGGGCTATAGGTTTCCGAAACCACAAGCCTTACTCC

GTCAGGGTTATCTACGGAGACACCGACTCCGTGTTTATCAAGTTTGTGGGCCTGACGTAC

GAGGGGGTATCGGAGCTGGGGGATGCTATGTCGCGTCAGATTTCAGCGGACCTCTTTAGA

GCGCCCATCAAACTGGAGTGTGAGAAGACCTTTCAGCGACTGCTGCTGATCACCAAGAAG

AAGTACATAGGTGTCATAAACGGGGGGAAGATGCTCATGAAGGGGGTCGACCTGGTCCGC

AAAAATAACTGCTCTTTCATAAACTTGTACGCGCGACATCTGGTAGATCTTTTGTTGTAC

GACGAGGATGTGGCCACGGCGGCAGCAGAGGTGACAGACGTGCCTCCCGCAGAATGGGTG

GGGCGCCCGCTACCGAGCGGCTTTGACAAGTTTGGGCGAGTGCTGGTAGAGGCGTACAAC

CGTATCACTGCCCCCAACTTGGACGTGCGCGAGTTCGTTATGACTGCTGAGCTGAGCCGC

TCACCCGAATCGTATACCAACAAGCGCCTGCCGCACCTCACCGTCTACTTTAAGCTCGCC

ATGAGGAATGAAGAACTGCCCAGCGTAAAAGAGAGAATTCCGTATGTGATAGTTGCGCAG

ACCGAGGCCGCGGAACGCGAAGCGGGTGTAGTAAACTCAATGCGCGGTACCGCCCAAAAC

CCCGTGGTAACCAAGACCGCACGCCCCCAACCTAAACGCAAACTGCTGGTTTCCGACCTC

GCCGAAGACCCGACCTATGTTTCCGAGAATGACGTACCGCTAAACACAGACTACTATTTC

TCCCACCTGTTGGGTACCATAAGCGTGACCTTTAAGGCTCTATTCGGAAATGATGTGAGA

ACAACAGAAAATCTTTTAAAGCGGTTTATTCCGGAAACCCCCCACAAGACCCCCACGAAA

ACCCAGGCACTGCTTGAGCGCGCCGGCTTTGAAAAGCTGACGCCCTTTACACCGGAGGAA

GAAAGTCGTCGAATACTGCATACAGTTTTTTGTACTCTAGAAGCAGCTCCCCATCAAAGC

TGA

>LC109639.1

ATGGCGGCGCGCGAACAGGCCAACTCCGTTCGCAGGAGCGGGTTTTTTAATCCGTTTATT

GGCAAGAGGCCATTTTTCAGGCCGGGAAGCGGGCAGACCGCGGAGACAGAGCGCCCAAGG

CCCCCCCAACACTCGTACTGCACAGAGGTGGGTAGCTTTAAGTTTATAGCTCCAAGATGC

CTCGATGAAGAAGCCCCCGCCGACCAGCGACGCGGTGTACACGTGGGCACCTTGGAGCGC

CCACCCAAAGTGTACTGCGATGGCTCAGAGTACGACGTGCTGAACTTTGCCTCCGGAGGT

TGCTGGCCTCGCAGGATTCGGGTTTGGAACGGCCAGGATTTTCGGGGCGATGGATTCAAC

CCCAGATTTGAGAGATTTCACGTGTACGACATAGTAGAGACTTCGGAGAGCGCCTCACAC

GATGACCCATCCAGGTTTGCTGAGCTATCCCGCCCAAGCGGGTCTGTGGTAACACTGCTG

GGAATGAGCGAGTGTGGAAAGCGAGTCGCGGTTCACGTCTATGGTGTGCGCCATTATTTT

TACATGGCAAAGGCGGAGGTGGATAGCGCTTGTGGAATAACCACCGAGGCAGAACTCGTG

CGCGCAATGGTGGACTGCGCGCACAGCTCGGCTTTGAGCGCGGCCCTGGGAAATGGCAAC

GGCGGCAAACAGAGCGGCGGCAGCGGCGGGGGATGGTGGGGCGGAAAGCACGTGTCCGCG

GACTGCTTCAAAGTGGAGACCGTGTGCCACACGACGCTGTACTACTTTGGATCTAAGCCA

GCTCTCTACTATAGAGTATCTGCCTCCAGCAGCCGCCTGGGAGGGTTCATCTGCGACAAC

TTTCACCCGGAGATTACAAAATTCGAGGGGAGCGTGGACGTGACCACGCGGCTGTTGTTG

GACAATGAAAATTTTACCAGTTTCGGGTGGTACCGCCTGCGACCCGGCACCCACGGAGAG

CGTGTTCAACTTCGCCCCGTTGAGCGACACGTCACCTCAAGCGACGTGGAGATTAACTGT

ACTCCCGATAACCTGGAGCCGATACCAGACGAGGCTGCCTGGCCCGACTATAAGCTCATG

TGCTTTGATATAGAGTGTAAAGCTGGAACGGGTAACGAAATGGCGTTCCCAGTGGCAACT

AACCAAGAGGACCTGGTCATCCAGATCTCCTGTCTGCTGTACTCGCTTGCTACTCAGAAC

CACGAACACACCCTGCTGTTTTCCCTCGGGTCATGCGATATCTCTGAGGAATACTCGTTT

GCATGCGTCCAGCGCGGCGAGCCCAGACCGACGGTTTTGGAGTTTGACAGCGAGTACGAG

CTGCTGGTTGCCTTCCTGACCTTTCTCAAGCAGTACTCTCCCGAGTTCGCCACCGGCTAC

AACATCGTTAATTTTGACTGGGCGTACATAGTTAACAAGGTAACGTCGGTGTATAACATC

AAGCTGGACGGGTACGGCAAGTTCAACAAAGGGGGGCTGTTTAAGGTGTGGGACATCGCC

ACGAACCATTTTCAGAAGAAGAGCAAGGTGAAAATCAATGGCCTGATATCTCTAGACATG

TATTCTGTGGCGACGGAAAAGCTAAAGCTACCCAGCTACAAACTCGACGCGGTCGTGGGA

GACGTCCTCGGCGAGCATAAGATAGACCTTCCCTATAAAGAAATACCCTCCTATTACGCG

GGAGGGCCTGACCGGAGGGGCGTAATAGGAGAGTATTGTATCCAGGACTCTAGGCTGGTG

GGCAAGCTGTTTTTTAAGTACCTCCCCCATCTGGAACTATCGGCGGTGGCCAAACTCGCC

CGTATCACCCTAACGCGGGTAATTTTTGACGGTCAGCAAATTCGCGTGTACACGTGCTTG

CTGAAACTCGCCCGCGAGAGAAATTTCATTTTGCCAGACAACAGACGCCGGTTTGACAGT

CAGGCAGACGCCGCGTCAGAGACTTCGGAGTTGGCTATGGATAGCCAAAGCCACGCCTTC

GACAGTACAGACGAACCCGACGGTGTGGACGGTACCCCGGACGCCGCAGGATCTGGCGCT

ACTTCTGAAAACGGAGGCGGGAAGCCCGGCGTCGGGAGGGCCGTGGGCTACCAGGGAGCA

AAGGTTCTAGACCCCGTATCCGGCTTTCATGTGGACCCCGTGGTTGTGTTTGACTTCGCT

AGCTTATACCCAAGCATTATCCAGGCCCATAACCTCTGTTTCACCACCCTGGCGCTCGAT

GAAGTGGATCTGGCCGGGCTTCAACCATCCGTCAACTACTCGACGTTCGAGGTGGGTGAC

CAAAAGTTATTTTTTGTCCACGCCCATATTCGCGAAAGCCTGCTTGGCATCTTGCTGCGC

GACTGGCTGGCCATGCGAAAGGCGGTGAGGGCGCGAATCCCCACCAGCACCCCCGAGGAG

GCAGTTTTACTAGATAAGCAGCAGTCTGCGATTAAGGTGATATGCAACTCGGTTTACGGA

TTCACGGGGGTGGCAAACGGCCTGTTGCCGTGTCTGAGGATAGCGGCTACCGTTACCACG

ATAGGACGCGACATGCTCCTCAAGACCCGAGATTACGTTCACTCTCGTTGGGCGACGCGC

GAGCTGCTGGAGGACAATTTTCCAGGGGCTATAGGTTTCCGAAACCACAAGCCTTACTCC

GTCAGGGTTATCTACGGAGACACCGACTCCGTGTTTATCAAGTTTGTGGGCCTGACGTAC

GAGGGGGTATCGGAGCTGGGGGATGCTATGTCGCGTCAGATTTCAGCGGACCTCTTTAGA

GCCCCCATCAAACTGGAGTGTGAGAAGACCTTTCAGCGACTGCTGCTGATCACCAAGAAG

AAGTACATAGGTGTCATAAACGGGGGGAAGATGCTCATGAAGGGGGTCGACCTGGTCCGC

AAAAATAACTGCTCTTTCATAAACTTGTACGCGCGACATCTGGTAGATCTTTTGTTGTAC

GACGAGGATGTGGCCACGGCGGCAGCAGAGGTGACAGACGTGCCTCCCGCAGAATGGGTG

GGGCGCCCGCTACCGAGCGGCTTTGACAAGTTTGGGCGAGTGCTGGTAGAGGCGTACAAC

CGTATCACTGCCCCCAACTTGGACGTGCGCGAGTTCGTTATGACTGCTGAGCTGAGCCGC

TCACCCGAATCGTATACCAACAAGCGCCTGCCGCACCTCACCGTCTACTTTAAGCTCGCC

ATGAGGAATGAAGAACTGCCCAGCGTAAAAGAGAGAATTCCGTATGTGATAGTTGCGCAG

ACCGAGGCCGCGGAACGCGAAGCGGGTGTAGTAAACTCAATGCGCGGTACCGCCCAAAAC

CCCGTGGTAACCAAGACCGCACGCCCCCAACCTAAACGCAAACTGCTGGTTTCCGACCTC

GCCGAAGACCCGACCTATGTTTCCGAGAATGACGTACCGCTAAACACAGACTACTATTTC

TCCCACCTGTTGGGTACCATAAGCGTGACCTTTAAGGCTCTATTCGGAAATGATGTGAGA

ACAACAGAAAATCTTTTAAAGCGGTTTATTCCGGAAACCCCCCACAAGACCCCCACGAAA

ACCCAGGCACTGCTTGAGCGCGCCGGCTTTGAAAAGCTGACGCCCTTTACACCGGAGGAA

GAAAGTCGTCGAATACTGCATACAGTTTTTTGTACTCTAGAAGCAGCTCCCCATCAAAGC

TGA

>LC109640.1

ATGGCGGCGCGCGAACAGGCCAACTCCGTTCGCAGGAGCGGGTTTTTTAATCCGTTTATT

GGCAAGAGGCCATTTTTCAGGCCGGGAAGCGGGCAGACCGCGGAGACAGAGCGCCCAAGG

CCCCCCCAACACTCGTACTGCACAGAGGTGGGTAGCTTTAAGTTTATAGCTCCAAGATGC

CTCGATGAAGAAGCCCCCGCCGACCAGCGACGCGGTGTACACGTGGGCACCTTGGAGCGC

CCACCCAAAGTGTACTGCGATGGCTCAGAGTACGACGTGCTGAACTTTGCCTCCGGAGGT

TGCTGGCCTCGCAGGATTCGGGTTTGGAACGGCCAGGATTTTCGGGGCGATGGATTCAAC

CCCAGATTTGAGAGATTTCACGTGTACGACATAGTAGAGACTTCGGAGAGCGCCTCACAC

GATGACCCATCCAGGTTTGCTGAGCTATCCCGCCCAAGCGGGTCTGTGGTAACACTGCTG

GGAATGAGCGAGTGTGGAAAGCGAGTCGCGGTTCACGTCTATGGTGTGCGCCATTATTTT

TACATGGCAAAGGCGGAGGTGGATAGCGCTTGTGGAATAACCACCGAGGCAGAACTCGTG

CGCGCAATGGTGGACTGCGCGCACAGCTCGGCTTTGAGCGCGGCCCTGGGAAATGGCAAC

GGCGGCAAACAGAGCGGCGGCAGCGGCGGGGGATGGTGGGGCGGAAAGCACGTGTCCGCG

GACTGCTTCAAAGTGGAGACCGTGTGCCACACGACGCTGTACTACTTTGGATCTAAGCCA

GCTCTCTACTATAGAGTATCTGCCTCCAGCAGCCGCCTGGGAGGGTTCATCTGCGACAAC

TTTCACCCGGAGATTACAAAATTCGAGGGGAGCGTGGACGTGACCACGCGGCTGTTGTTG

GACAATGAAAATTTTACCAGTTTCGGGTGGTACCGCCTGCGACCCGGCACCCACGGAGAG

CGTGTTCAACTTCGCCCCGTTGAGCGACACGTCACCTCAAGCGACGTGGAGATTAACTGT

ACTCCCGATAACCTGGAGCCGATACCAGACGAGGCTGCCTGGCCCGACTATAAGCTCATG

TGCTTTGATATAGAGTGTAAAGCTGGAACGGGTAACGAAATGGCGTTCCCAGTGGCAACT

AACCAAGAGGACCTGGTCATCCAGATCTCCTGTCTGCTGTACTCGCTTGCTACTCAGAAC

CACGAACACACCCTGCTGTTTTCCCTCGGGTCATGCGATATCTCTGAGGAATACTCGTTT

GCATGCGTCCAGCGCGGCGAGCCCAGACCGACGGTTTTGGAGTTTGACAGCGAGTACGAG

CTGCTGGTTGCCTTCCTGACCTTTCTCAAGCAGTACTCTCCCGAGTTCGCCACCGGCTAC

AACATCGTTAATTTTGACTGGGCGTACATAGTTAACAAGGTAACGTCGGTGTATAACATC

AAGCTGGACGGGTACGGCAAGTTCAACAAAGGGGGGCTGTTTAAGGTGTGGGACATCGCC

ACGAACCATTTTCAGAAGAAGAGCAAGGTGAAAATCAATGGCCTGATATCTCTAGACATG

TATTCTGTGGCGACGGAAAAGCTAAAGCTACCCAGCTACAAACTCGACGCGGTCGTGGGA

GACGTCCTCGGCGAGCATAAGATAGACCTTCCCTATAAAGAAATACCCTCCTATTACGCG

GGAGGGCCTGACCGGAGGGGCGTAATAGGAGAGTATTGTATCCAGGACTCTAGGCTGGTG

GGCAAGCTGTTTTTTAAGTACCTCCCCCATCTGGAACTATCGGCGGTGGCCAAACTCGCC

CGTATCACCCTAACGCGGGTAATTTTTGACGGTCAGCAAATTCGCGTGTACACGTGCTTG

CTGAAACTCGCCCGCGAGAGAAATTTCATTTTGCCAGACAACAGACGCCGGTTTGACAGT

CAGGCAGACGCCGCGTCAGAGACTTCGGAGTTGGCTATGGATAGCCAAAGCCACGCCTTC

GACAGTACAGACGAACCCGACGGTGTGGACGGTACCCCGGACGCCGCAGGATCTGGCGCT

ACTTCTGAAAACGGAGGCGGGAAGCCCGGCGTCGGGAGGGCCGTGGGCTACCAGGGAGCA

AAGGTTCTAGACCCCGTATCCGGCTTTCATGTGGACCCCGTGGTTGTGTTTGACTTCGCT

AGCTTATACCCAAGCATTATCCAGGCCCATAACCTCTGTTTCACCACCCTGGCGCTCGAT

GAAGTGGATCTGGCCGGGCTTCAACCATCCGTCAACTACTCGACGTTCGAGGTGGGTGAC

CAAAAGTTATTTTTTGTCCACGCCCATATTCGCGAAAGCCTGCTTGGCATCTTGCTGCGC

GACTGGCTGGCCATGCGAAAGGCGGTGAGGGCGCGAATCCCCACCAGCACCCCCGAGGAG

GCAGTTTTACTAGATAAGCAGCAGTCTGCGATTAAGGTGATATGCAACTCGGTTTACGGA

TTCACGGGGGTGGCAAACGGCCTGTTGCCGTGTCTGAGGATAGCGGCTACCGTTACCACG

ATAGGACGCGACATGCTCCTCAAGACCCGAGATTACGTTCACTCTCGTTGGGCGACGCGC

GAGCTGCTGGAGGACAATTTTCCAGGGGCTATAGGTTTCCGAAACCACAAGCCTTACTCC

GTCAGGGTTATCTACGGAGACACCGACTCCGTGTTTATCAAGTTTGTGGGCCTGACGTAC

GAGGGGGTATCGGAGCTGGGGGATGCTATGTCGCGTCAGATTTCAGCGGACCTCTTTAGA

GCCCCCATCAAACTGGAGTGTGAGAAGACCTTTCAGCGACTGCTGCTGATCACCAAGAAG

AAGTACATAGGTGTCATAAACGGGGGGAAGATGCTCATGAAGGGGGTCGACCTGGTCCGC

AAAAATAACTGCTCTTTCATAAACTTGTACGCGCGACATCTGGTAGATCTTTTGTTGTAC

GACGAGGATGTGGCCACGGCGGCAGCAGAGGTGACAGACGTGCCTCCCGCAGAATGGGTG

GGGCGCCCGCTACCGAGCGGCTTTGACAAGTTTGGGCGAGTGCTGGTAGAGGCGTACAAC

CGTATCACTGCCCCCAACTTGGACGTGCGCGAGTTCGTTATGACTGCTGAGCTGAGCCGC

TCACCCGAATCGTATACCAACAAGCGCCTGCCGCACCTCACCGTCTACTTTAAGCTCGCC

ATGAGGAATGAAGAACTGCCCAGCGTAAAAGAGAGAATTCCGTATGTGATAGTTGCGCAG

ACCGAGGCCGCGGAACGCGAAGCGGGTGTAGTAAACTCAATGCGCGGTACCGCCCAAAAC

CCCGTGGTAACCAAGACCGCACGCCCCCAACCTAAACGCAAACTGCTGGTTTCCGACCTC

GCCGAAGACCCGACCTATGTTTCCGAGAATGACGTACCGCTAAACACAGACTACTATTTC

TCCCACCTGTTGGGTACCATAAGCGTGACCTTTAAGGCTCTATTCGGAAATGATGTGAGA

ACAACAGAAAATCTTTTAAAGCGGTTTATTCCGGAAACCCCCCACAAGACCCCCACGAAA

ACCCAGGCACTGCTTGAGCGCGCCGGCTTTGAAAAGCTGACGCCCTTTACACCGGAGGAA

GAAAGTCGTCGAATACTGCATACAGTTTTTTGTACTCTAGAAGCAGCTCCCCATCAAAGC

TGA

>LC109641.1

ATGGCGGCGCGCGAACAGGCCAACTCCGTTCGCAGGAGCGGGTTTTTTAATCCGTTTATT

GGCAAGAGGCCATTTTTCAGGCCGGGAAGCGGGCAGACCGCGGAGACAGAGCGCCCAAGG

CCCCCCCAACACTCGTACTGCACAGAGGTGGGTAGCTTTAAGTTTATAGCTCCAAGATGC

CTCGATGAAGAAGCCCCCGCCGACCAGCGACGCGGTGTACACGTGGGCACCTTGGAGCGC

CCACCCAAAGTGTACTGCGATGGCTCAGAGTACGACGTGCTGAACTTTGCCTCCGGAGGT

TGCTGGCCTCGCAGGATTCGGGTTTGGAACGGCCAGGATTTTCGGGGCGATGGATTCAAC

CCCAGATTTGAGAGATTTCACGTGTACGACATAGTAGAGACTTCGGAGAGCGCCTCACAC

GATGACCCATCCAGGTTTGCTGAGCTATCCCGCCCAAGCGGGTCTGTGGTAACACTGCTG

GGAATGAGCGAGTGTGGAAAGCGAGTCGCGGTTCACGTCTATGGTGTGCGCCATTATTTT

TACATGGCAAAGGCGGAGGTGGATAGCGCTTGTGGAATAACCACCGAGGCAGAACTCGTG

CGCGCAATGGTGGACTGCGCGCACAGCTCGGCTTTGAGCGCGGCCCTGGGAAATGGCAAC

GGCGGCAAACAGAGCGGCGGCAGCGGCGGGGGATGGTGGGGCGGAAAGCACGTGTCTGCG

GACTGCTTCAAAGTGGAGACCGTGTGCCACACGACGCTGTACTACTTTGGATCTAAGCCA

GCTCTCTACTATAGAGTATCTGCCTCCAGCAGCCGCCTGGGAGGGTTCATCTGCGACAAC

TTTCACCCGGAGATTACAAAATTCGAGGGGAGCGTGGACGTGACCACGCGGCTGTTGTTG

GACAATGAAAATTTTACCAGTTTCGGGTGGTACCGCCTGCGACCCGGCACCCACGGAGAG

CGTGTTCAACTTCGCCCCGTTGAGCGACACGTCACCTCAAGCGACGTGGAGATTAACTGT

ACTCCCGATAACCTGGAGCCGATACCAGACGAGGCTGCCTGGCCCGACTATAAGCTCATG

TGCTTTGATATAGAGTGTAAAGCTGGAACGGGTAACGAAATGGCGTTCCCAGTGGCAACT

AACCAAGAGGACCTGGTCATCCAGATCTCCTGTCTGCTGTACTCGCTTGCTACTCAGAAC

CACGAACACACCCTGCTGTTTTCCCTCGGGTCATGCGATATCTCTGAGGAATACTCGTTT

GCATGCGTCCAGCGCGGCGAGCCCAGACCGACGGTTTTGGAGTTTGACAGCGAGTACGAG

CTGCTGGTTGCCTTCCTGACCTTTCTCAAGCAGTACTCTCCCGAGTTCGCCACCGGCTAC

AACATCGTTAATTTTGACTGGGCGTACATAGTTAACAAGGTAACGTCGGTGTATAACATC

AAGCTGGACGGGTACGGCAAGTTCAACAAAGGGGGGCTGTTTAAGGTGTGGGACATCGCC

ACGAACCATTTTCAGAAGAAGAGCAAGGTGAAAATCAATGGCCTGATATCTCTAGACATG

TATTCTGTGGCGACGGAAAAGCTAAAGCTACCCAGCTACAAACTCGACGCGGTCGTGGGA

GACGTCCTCGGCGAGCATAAGATAGACCTTCCCTATAAAGAAATACCCTCCTATTACGCG

GGAGGGCCTGACCGGAGGGGCGTAATAGGAGAGTATTGTATCCAGGACTCTAGGCTGGTG

GGCAAGCTGTTTTTTAAGTACCTCCCCCATCTGGAACTATCGGCGGTGGCCAAACTCGCC

CGTATCACCCTAACGCGGGTAATTTTTGACGGTCAGCAAATTCGCGTGTACACGTGCTTG

CTGAAACTCGCCCGCGAGAGAAATTTCATTTTGCCAGACAACAGACGCCGGTTTGACAGT

CAGGCAGACGCCGCGTCAGAGACTTCGGAGTTGGCTATGGATAGCCAAAGCCACGCCTTC

GACAGTACAGACGAACCCGACGGTGTGGACGGTACCCCGGACGCCGCAGGATCTGGCGCT

ACTTCTAAAAACGGAGGCGGGAAGCCCGGCGTCGGGAGGGCCGTGGGCTACCAGGGAGCA

AAGGTTCTAGACCCCGTATCCGGCTTTCATGTGGACCCCGTGGTTGTGTTTGACTTCGCT

AGCTTATACCCAAGCATTATCCAGGCCCATAACCTCTGTTTCACCACCCTGGCGCTCGAT

GAAGTGGATCTGGCCGGGCTTCAACCATCCGTCAACTACTCGACGTTCGAGGTGGGTGAC

CAAAAGTTATTTTTTGTCCACGCCAATATTCGCGAAAGCCTGCTTGGCATCTTGCTGCGC

GACTGGCTGGCCATGCGAAAGGCGGTGAGGGCGCGAATCCCCACCAGCACCCCCGAGGAG

GCAGTTTTACTAGATAAGCAGCAGTCTGCGATTAAGGTGATATGCAACTCGGTTTACGGA

TTCACGGGGGTGGCAAACGGCCTGTTGCCGTGTCTGAGGATAGCGGCTACCGTTACCACG

ATAGGACGCGACATGCTCCTCAAGACCCGAGATTACGTTCACTCTCGTTGGGCGACGCGC

GAGCTGCTGGAGGACAATTTTCCAGGGGCTATAGGTTTCCGAAACCACAAGCCTTACTCC

GTCAGGGTTATCTACGGAGACACCGACTCCGTGTTTATCAAGTTTGTGGGCCTGACGTAC

GAGGGGGTATCGGAGCTGGGGGATGCTATGTCGCGTCAGATTTCAGCGGACCTCTTTAGA

GCCCCCATCAAACTGGAGTGTGAGAAGACCTTTCAGCGACTGCTGCTGATCACCAAGAAG

AAGTACATAGGTGTCATAAACGGGGGGAAGATGCTCATGAAGGGGGTCGACCTGGTCCGC

AAAAATAACTGCTCTTTCATAAACTTGTACGCGCGACATCTGGTAGATCTTTTGTTGTAC

GACGAGGATGTGGCCACGGCGGCAGCAGAGGTGACAGACGTGCCTCCCGCAGAATGGGTG

GGGCGCCCGCTACCGAGCGGCTTTGACAAGTTTGGGCGAGTGCTGGTAGAGGCGTACAAC

CGTATCACTGCCCCCAACTTGGACGTGCGCGAGTTCGTTATGACTGCTGAGCTGAGCCGC

TCACCCGAATCGTATACCAACAAGCGCCTGCCGCACCTCACCGTCTACTTTAAGCTCGCC

ATGAGGAATGAAGAACTGCCCAGCGTAAAAGAGAGAATTCCGTATGTGATAGTTGCGCAG

ACCGAGGCCGCGGAACGCGAAGCGGGTGTAGTAAACTCAATGCGCGGTACCGCCCAAAAC

CCCGTGGTAACCAAGACCACACGCCCCCAACCTAAACGCAAACTGCTGGTTTCCGACCTC

GCCGAAGACCCGACCTATGTTTCCGAGAATGACGTACCGCTAAACACAGACTACTATTTC

TCCCACCTGTTGGGTACCATAAGCGTGACCTTTAAGGCTCTATTCGGAAATGATGTGAGA

ACAACAGAAAATCTTTTAAAGCGGTTTATTCCGGAAACCCCCCACAAGACCCCCACGAAA

ACCCAGGCACTGCTTGAGCGCGCCGGCTTTGAAAAGCTGACGCCCTTTACACCGGAGGAA

GAAAGTCGTCGAATACTGCATACAGTTTTTTGTACTCTAGAAGCAGCTCCCCATCAAAGC

TGA

>LC109642.1

ATGGCGGCGCGCGAACAGGCCAACTCCGTTCGCAGGAGCGGGTTTTTTAATCCGTTTATT

GGCAAGAGGCCATTTTTCAGGCCGGGAAGCGGGCAGACCGCGGAGACAGAGCGCCCAAGG

CCCCCCCAACACTCGTACTGCACAGAGGTGGGTAGCTTTAAGTTTATAGCTCCAAGATGC

CTCGATGAAGAAGCCCCCGCCGACCAGCGACGCGGTGTACACGTGGGCACCTTGGAGCGC

CCACCCAAAGTGTACTGCGATGGCTCAGAGTACGACGTGCTGAACTTTGCCTCCGGAGGT

TGCTGGCCTCGCAGGATTCGGGTTTGGAACGGCCAGGATTTTCGGGGCGATGGATTCAAC

CCCAGATTTGAGAGATTTCACGTGTACGACATAGTAGAGACTTCGGAGAGCGCCTCACAC

GATGACCCATCCAGGTTTGCTGAGCTATCCCGCCCAAGCGGGTCTGTGGTAACACTGCTG

GGAATGAGCGAGTGTGGAAAGCGAGTCGCGGTTCACGTCTATGGTGTGCGCCATTATTTT

TACATGGCAAAGGCGGAGGTGGATAGCGCTTGTGGAATAACCACCGAGGCAGAACTCGTG

CGCGCAATGGTGGACTGCGCGCACAGCTCGGCTTTGAGCGCGGCCCTGGGAAATGGCAAC

GGCGGCAAACAGAGCGGCGGCAGCGGCGGGGGATGGTGGGGCGGAAAGCACGTGTCTGCG

GACTGCTTCAAAGTGGAGACCGTGTGCCACACGACGCTGTACTACTTTGGATCTAAGCCA

GCTCTCTACTATAGAGTATCTGCCTCCAGCAGCCGCCTGGGAGGGTTCATCTGCGACAAC

TTTCACCCGGAGATTACAAAATTCGAGGGGAGCGTGGACGTGACCACGCGGCTGTTGTTG

GACAATGAAAATTTTACCAGTTTCGGGTGGTACCGCCTGCGACCCGGCACCCACGGAGAG

CGTGTTCAACTTCGCCCCGTTGAGCGACACGTCACCTCAAGCGACGTGGAGATTAACTGT

ACTCCCGATAACCTGGAGCCGATACCAGACGAGGCTGCCTGGCCCGACTATAAGCTCATG

TGCTTTGATATAGAGTGTAAAGCTGGAACGGGTAACGAAATGGCGTTCCCAGTGGCAACT

AACCAAGAGGACCTGGTCATCCAGATCTCCTGTCTGCTGTACTCGCTTGCTACTCAGAAC

CACGAACACACCCTGCTGTTTTCCCTCGGGTCATGCGATATCTCTGAGGAATACTCGTTT

GCATGCGTCCAGCGCGGCGAGCCCAGACCGACGGTTTTGGAGTTTGACAGCGAGTACGAG

CTGCTGGTTGCCTTCCTGACCTTTCTCAAGCAGTACTCTCCCGAGTTCGCCACCGGCTAC

AACATCGTTAATTTTGACTGGGCGTACATAGTTAACAAGGTAACGTCGGTGTATAACATC

AAGCTGGACGGGTACGGCAAGTTCAACAAAGGGGGGCTGTTTAAGGTGTGGGACATCGCC

ACGAACCATTTTCAGAAGAAGAGCAAGGTGAAAATCAATGGCCTGATATCTCTAGACATG

TATTCTGTGGCGACGGAAAAGCTAAAGCTACCCAGCTACAAACTCGACGCGGTCGTGGGA

GACGTCCTCGGCGAGCATAAGATAGACCTTCCCTATAAAGAAATACCCTCCTATTACGCG

GGAGGGCCTGACCGGAGGGGCGTAATAGGAGAGTATTGTATCCAGGACTCTAGGCTGGTG

GGCAAGCTGTTTTTTAAGTACCTCCCCCATCTGGAACTATCGGCGGTGGCCAAACTCGCC

CGTATCACCCTAACGCGGGTAATTTTTGACGGTCAGCAAATTCGCGTGTACACGTGCTTG

CTGAAACTCGCCCGCGAGAGAAATTTCATTTTGCCAGACAACAGACGCCGGTTTGACAGT

CAGGCAGACGCCGCGTCAGAGACTTCGGAGTTGGCTATGGATAGCCAAAGCCACGCCTTC

GACAGTACAGACGAACCCGACGGTGTGGACGGTACCCCGGACGCCGCAGGATCTGGCGCT

ACTTCTGAAAACGGAGGCGGGAAGCCCGGCGTCGGGAGGGCCGTGGGCTACCAGGGAGCA

AAGGTTCTAGACCCCGTATCCGGCTTTCATGTGGACCCCGTGGTTGTGTTTGACTTCGCT

AGCTTATACCCAAGCATTATCCAGGCCCATAACCTCTGTTTCACCACCCTGGCGCTCGAT

GAAGTGGATCTGGCCGGGCTTCAACCATCCGTCAACTACTCGACGTTCGAGGTGGGTGAC

CAAAAGTTATTTTTTGTCCACGCCCATATTCGCGAAAGCCTGCTTGGCATCTTGCTGCGC

GACTGGCTGGCCATGCGAAAGGCGGTGAGGGCGCGAATCCCCACCAGCACCCCCGAGGAG

GCAGTTTTACTAGATAAGCAGCAGTCTGCGATTAAGGTGATATGCAACTCGGTTTACGGA

TTCACGGGGGTGGCAAACGGCCTGTTGCCGTGTCTGAGGATAGCGGCTACCGTTACCACG

ATAGGACGCGACATGCTCCTCAAGACCCGAGATTACGTTCACTCTCGTTGGGCGACGCGC

GAGCTGCTGGAGGACAATTTTCCAGGGGCTATAGGTTTCCGAAACCACAAGCCTTACTCC

GTCAGGGTTATCTACGGAGACACCGACTCCGTGTTTATCAAGTTTGTGGGCCTGACGTAC

GAGGGGGTATCGGAGCTGGGGGATGCTATGTCGCGTCAGATTTCAGCGGACCTCTTTAGA

GCGCCCATCAAACTGGAGTGTGAGAAGACCTTTCAGCGACTGCTGCTGATCACCAAGAAG

AAGTACATAGGTGTCATAAACGGGGGGAAGATGCTCATGAAGGGGGTCGACCTGGTCCGC

AAAAATAACTGCTCTTTCATAAACTTGTACGCGCGACATCTGGTAGATCTTTTGTTGTAC

GACGAGGATGTGGCCACGGCGGCAGCAGAGGTGACAGACGTGCCTCCCGCAGAATGGGTG

GGGCGCCCGCTACCGAGCGGCTTTGACAAGTTTGGGCGAGTGCTGGTAGAGGCGTACAAC

CGTATCACTGCCCCCAACTTGGACGTGCGCGAGTTCGTTATGACTGCTGAGCTGAGCCGC

TCACCCGAATCGTATACCAACAAGCGCCTGCCGCACCTCACCGTCTACTTTAAGCTCGCC

ATGAGGAATGAAGAACTGCCCAGCGTAAAAGAGAGAATTCCGTATGTGATAGTTGCGCAG

ACCGAGGCCGCGGAACGCGAAGCGGGTGTAGTAAACTCAATGCGCGGTACCGCCCAAAAC

CCCGTGGTAACCAAGACCGCACGCCCCCAACCTAAACGCAAACTGCTGGTTTCCGACCTC

GCCGAAGACCCGACCTATGTTTCCGAGAATGACGTACCGCTAAACACAGACTACTATTTC

TCCCACCTGTTGGGTACCATAAGCGTGACCTTTAAGGCTCTATTCGGAAATGATGTGAGA

ACAACAGAAAATCTTTTAAAGCGGTTTATTCCGGAAACCCCCCACAAGACCCCCACGAAA

ACCCAGGCACTGCTTGAGCGCGCCGGCTTTGAAAAGCTGACGCCCTTTACACCGGAGGAA

GAAAGTCGTCGAATACTGCATACAGTTTTTTGTACTCTAGAAGCAGCTCCCCATCAAAGC

TGA

>LC109643.1

ATGGCGGCGCGCGAACAGGCCAACTCCGTTCGCAGGAGCGGGTTTTTTAATCCGTTTATT

GGCAAGAGGCCATTTTTCAGGCCGGGAAGCGGGCAGACCGCGGAGACAGAGCGCCCAAGG

CCCCCCCAACACTCGTACTGCACAGAGGTGGGTAGCTTTAAGTTTATAGCTCCAAGATGC

CTCGATGAAGAAGCCCCCGCCGACCAGCGACGCGGTGTACACGTGGGCACCTTGGAGCGC

CCACCCAAAGTGTACTGCGATGGCTCAGAGTACGACGTGCTGAACTTTGCCTCCGGAGGT

TGCTGGCCTCGCAGGATTCGGGTTTGGAACGGCCAGGATTTTCGGGGCGATGGATTCAAC

CCCAGATTTGAGAGATTTCACGTGTACGACATAGTAGAGACTTCGGAGAGCGCCTCACAC

GATGACCCATCCAGGTTTGCTGAGCTATCCCGCCCAAGCGGGTCTGTGGTAACACTGCTG

GGAATGAGCGAGTGTGGAAAGCGAGTCGCGGTTCACGTCTATGGTGTGCGCCATTATTTT

TACATGGCAAAGGCGGAGGTGGATAGCGCTTGTGGAATAACCACCGAGGCAGAACTCGTG

CGCGCAATGGTGGACTGCGCGCACAGCTCGGCTTTGAGCGCGGCCCTGGGAAATGGCAAC

GGCGGCAAACAGAGCGGCGGCAGCGGCGGGGGATGGTGGGGCGGAAAGCACGTGTCTGCG

GACTGCTTCAAAGTGGAGACCGTGTGCCACACGACGCTGTACTACTTTGGATCTAAGCCA

GCTCTCTACTATAGAGTATCTGCCTCCAGCAGCCGCCTGGGAGGGTTCATCTGCGACAAC

TTTCACCCGGAGATTACAAAATTCGAGGGGAGCGTGGACGTGACCACGCGGCTGTTGTTG

GACAATGAAAATTTTACCAGTTTCGGGTGGTACCGCCTGCGACCCGGCACCCACGGAGAG

CGTGTTCAACTTCGCCCCGTTGAGCGACACGTCACCTCAAGCGACGTGGAGATTAACTGT

ACTCCCGATAACCTGGAGCCGATACCAGACGAGGCTGCCTGGCCCGACTATAAGCTCATG

TGCTTTGATATAGAGTGTAAAGCTGGAACGGGTAACGAAATGGCGTTCCCAGTGGCAACT

AACCAAGAGGACCTGGTCATCCAGATCTCCTGTCTGCTGTACTCGCTTGCTACTCAGAAC

CACGAACACACCCTGCTGTTTTCCCTCGGGTCATGCGATATCTCTGAGGAATACTCGTTT

GCATGCGTCCAGCGCGGCGAGCCCAGACCGACGGTTTTGGAGTTTGACAGCGAGTACGAG

CTGCTGGTTGCCTTCCTGACCTTTCTCAAGCAGTACTCTCCCGAGTTCGCCACCGGCTAC

AACATCGTTAATTTTGACTGGGCGTACATAGTTAACAAGGTAACGTCGGTGTATAACATC

AAGCTGGACGGGTACGGCAAGTTCAACAAAGGGGGGCTGTTTAAGGTGTGGGACATCGCC

ACGAACCATTTTCAGAAGAAGAGCAAGGTGAAAATCAATGGCCTGATATCTCTAGACATG

TATTCTGTGGCGACGGAAAAGCTAAAGCTACCCAGCTACAAACTCGACGCGGTCGTGGGA

GACGTCCTCGGCGAGCATAAGATAGACCTTCCCTATAAAGAAATACCCTCCTATTACGCG

GGAGGGCCTGACCGGAGGGGCGTAATAGGAGAGTATTGTATCCAGGACTCTAGGCTGGTG

GGCAAGCTGTTTTTTAAGTACCTCCCCCATCTGGAACTATCGGCGGTGGCCAAACTCGCC

CGTATCACCCTAACGCGGGTAATTTTTGACGGTCAGCAAATTCGCGTGTACACGTGCTTG

CTGAAACTCGCCCGCGAGAGAAATTTCATTTTGCCAGACAACAGACGCCGGTTTGACAGT

CAGGCAGACGCCGCGTCAGAGACTTCGGAGTTGGCTATGGATAGCCAAAGCCACGCCTTC

GACAGTACAGACGAACCCGACGGTGTGGACGGTACCCCGGACGCCGCAGGATCTGGCGCT

ACTTCTGAAAACGGAGGCGGGAAGCCCGGCGTCGGGAGGGCCGTGGGCTACCAGGGAGCA

AAGGTTCTAGACCCCGTATCCGGCTTTCATGTGGACCCCGTGGTTGTGTTTGACTTCGCT

AGCTTATACCCAAGCATTATCCAGGCCCATAACCTCTGTTTCACCACCCTGGCGCTCGAT

GAAGTGGATCTGGCCGGGCTTCAACCATCCGTCGACTACTCGACGTTCGAGGTGGGTGAC

CAAAAGTTATTTTTTGTCCACGCCCATATTCGCGAAAGCCTGCTTGGCATCTTGCTGCGC

GACTGGCTGGCCATGCGAAAGGCGGTGAGGGCGCGAATCCCCACCAGCACCCCCGAGGAG

GCAGTTTTACTAGATAAGCAGCAGTCTGCGATTAAGGTGATATGCAACTCGGTTTACGGA

TTCACGGGGGTGGCAAACGGCCTGTTGCCGTGTCTGAGGATAGCGGCTACCGTTACCACG

ATAGGACGCGACATGCTCCTCAAGACCCGAGATTACGTTCACTCTCGTTGGGCGACGCGC

GAGCTGCTGGAGGACAATTTTCCAGGGGCTATAGGTTTCCGAAACCACAAGCCTTACTCC

GTCAGGGTTATCTACGGAGACACCGACTCCGTGTTTATCAAGTTTGTGGGCCTGACGTAC

GAGGGGGTATCGGAGCTGGGGGATGCTATGTCGCGTCAGATTTCAGCGGACCTCTTTAGA

GCCCCCATCAAACTGGAGTGTGAGAAGACCTTTCAGCGACTGCTGCTGATCACCAAGAAG

AAGTACATAGGTGTCATAAACGGGGGGAAGATGCTCATGAAGGGGGTCGACCTGGTCCGC

AAAAATAACTGCTCTTTCATAAACTTGTACGCGCGACATCTGGTAGATCTTTTGTTGTAC

GACGAGGATGTGGCCACGGCGGCAGCAGAGGTGACAGACGTGCCTCCCGCAGAATGGGTG

GGGCGCCCGCTACCGAGCGGCTTTGACAAGTTTGGGCGAGTGCTGGTAGAGGCGTACAAC

CGTATCACTGCCCCCAACTTGGACGTGCGCGAGTTCGTTATGACTGCTGAGCTGAGCCGC

TCACCCGAATCGTATACCAACAAGCGCCTGCCGCACCTCACCGTCTACTTTAAGCTCGCC

ATGAGGAATGAAGAACTGCCCAGCGTAAAAGAGAGAATTCCGTATGTGATAGTTGCGCAG

ACCGAGGCCGCGGAACGCGAAGCGGGTGTAGTAAACTCAATGCGCGGTACCGCCCAAAAC

CCCGTGGTAACCAAGACCGCACGCCCCCAACCTAAACGCAAACTGCTGGTTTCCGACCTC

GCCGAAGACCCGACCTATGTTTCCGAGAATGACGTACCGCTAAACACAGACTACTATTTC

TCCCACCTGTTGGGTACCATAAGCGTGACCTTTAAGGCTCTATTCGGAAATGATGTGAGA

ACAACAGAAAATCTTTTAAAGCGGTTTATTCCGGAAACCCCCCACAAGACCCCCACGAAA

ACCCAGGCACTGCTTGAGCGCGCCGGCTTTGAAAAGCTGACGCCCTTTACACCGGAGGAA

GAAAGTCGTCGAATACTGCATACAGTTTTTTGTACTCTAGAAGCAGCTCCCCATCAAAGC

TGA

>LC109644.1

ATGGCGGCGCGCGAACAGGCCAACTCCGTTCGCAGGAGCGGGTTTTTTAATCCGTTTATT

GGCAAGAGGCCATTTTTCAGGCCGGGAAGCGGGCAGACCGCGGAGACAGAGCGCCCAAGG

CCCCCCCAACACTCGTACTGCACAGAGGTGGGTAGCTTTAAGTTTATAGCTCCAAGATGC

CTCGATGAAGAAGCCCCCGCCGACCAGCGACGCGGTGTACACGTGGGCACCTTGGAGCGC

CCACCCAAAGTGTACTGCGATGGCTCAGAGTACGACGTGCTGAACTTTGCCTCCGGAGGT

TGCTGGCCTCGCAGGATTCGGGTTTGGAACGGCCAGGATTTTCGGGGCGATGGATTCAAC

CCCAGATTTGAGAGATTTCACGTGTACGACATAGTAGAGACTTCGGAGAGCGCCTCACAC

GATGACCCATCCAGGTTTGCTGAGCTATCCCGCCCAAGCGGGTCTGTGGTAACACTGCTG

GGAATGAGCGAGTGTGGAAAGCGAGTCGCGGTTCACGTCTATGGTGTGCGCCATTATTTT

TACATGGCAAAGGCGGAGGTGGATAGCGCTTGTGGAATAACCACCGAGGCAGAACTCGTG

CGCGCAATGGTGGACTGCGCGCACAGCTCGGCTTTGAGCGCGGCCCTGGGAAATGGCAAC

GGCGGCAAACAGAGCGGCGGCAGCGGCGGGGGATGGTGGGGCGGAAAGCACGTGTCTGCG

GACTGCTTCAAAGTGGAGACCGTGTGCCACACGACGCTGTACTACTTTGGATCTAAGCCA

GCTCTCTACTATAGAGTATCTGCCTCCAGCAGCCGCCTGGGAGGGTTCATCTGCGACAAC

TTTCACCCGGAGATTACAAAATTCGAGGGGAGCGTGGACGTGACCACGCGGCTGTTGTTG

GACAATGAAAATTTTACCAGTTTCGGGTGGTACCGCCTGCGACCCGGCACCCACGGAGAG

CGTGTTCAACTTCGCCCCGTTGAGCGACACGTCACCTCAAGCGACGTGGAGATTAACTGT

ACTCCCGATAACCTGGAGCCGATACCAGACGAGGCTGCCTGGCCCGACTATAAGCTCATG

TGCTTTGATATAGAGTGTAAAGCTGGAACGGGTAACGAAATGGCGTTCCCAGTGGCAACT

AACCAAGAGGACCTGGTCATCCAGATCTCCTGTCTGCTGTACTCGCTTGCTACTCAGAAC

CACGAACACACCCTGCTGTTTTCCCTCGGGTCATGCGATATCTCTGAGGAATACTCGTTT

GCATGCGTCCAGCGCGGCGAGCCCAGACCGACGGTTTTGGAGTTTGACAGCGAGTACGAG

CTGCTGGTTGCCTTCCTGACCTTTCTCAAGCAGTACTCTCCCGAGTTCGCCACCGGCTAC

AACATCGTTAATTTTGACTGGGCGTACATAGTTAACAAGGTAACGTCGGTGTATAACATC

AAGCTGGACGGGTACGGCAAGTTCAACAAAGGGGGGCTGTTTAAGGTGTGGGACATCGCC

ACGAACCATTTTCAGAAGAAGAGCAAGGTGAAAATCAATGGCCTGATATCTCTAGACATG

TATTCTGTGGCGACGGAAAAGCTAAAGCTACCCAGCTACAAACTCGACGCGGTCGTGGGA

GACGTCCTCGGCGAGCATAAGATAGACCTTCCCTATAAAGAAATACCCTCCTATTACGCG

GGAGGGCCTGACCGGAGGGGCGTAATAGGAGAGTATTGTATCCAGGACTCTAGGCTGGTG

GGCAAGCTGTTTTTTAAGTACCTCCCCCATCTGGAACTATCGGCGGTGGCCAAACTCGCC

CGTATCACCCTAACGCGGGTAATTTTTGACGGTCAGCAAATTCGCGTGTACACGTGCTTG

CTGAAACTCGCCCGCGAGAGAAATTTCATTTTGCCAGACAACAGACGCCGGTTTGACAGT

CAGGCAGACGCCGCGTCAGAGACTTCGGAGTTGGCTATGGATAGCCAAAGCCACGCCTTC

GACAGTACAGACGAACCCGACGGTGTGGACGGTACCCCGGACGCCGCAGGATCTGGCGCT

ACTTCTGAAAACGGAGGCGGGAAGCCCGGCGTCGGGAGGGCCGTGGGCTACCAGGGAGCA

AAGGTTCTAGACCCCGTATCCGGCTTTCATGTGGACCCCGTGGTTGTGTTTGACTTCGCT

AGCTTATACCCAAGCATTATCCAGGCCCATAACCTCTGTTTCACCACCCTGGCGCTCGAT

GAAGTGGATCTGGCCGGGCTTCAACCATCCGTCAACTACTCGACGTTCGAGGTGGGTGAC

CAAAAGTTATTTTTTGTCCACGCCCATATTCGCGAAAGCCTGCTTGGCATCTTGCTGCGC

GACTGGCTGGCCATGCGAAAGGCGGTGAGGGCGCGAATCCCCACCAGCACCCCCGAGGAG

GCAGTTTTACTAGATAAGCAGCAGTCTGCGATTAAGGTGATATGCAACTCGGTTTACGGA

TTCACGGGGGTGGCAAACGGCCTGTTGCCGTGTCTGAGGATAGCGGCTACCGTTACCACG

ATAGGACGCGACATGCTCCTCAAGACCCGAGATTACGTTCACTCTCGTTGGGCGACGCGC

GAGCTGCTGGAGGACAATTTTCCAGGGGCTATAGGTTTCCGAAACCACAAGCCTTACTCC

GTCAGGGTTATCTACGGAGACACCGACTCCGTGTTTATCAAGTTTGTGGGCCTGACGTAC

GAGGGGGTATCGGAGCTGGGGGATGCTATGTCGCGTCAGATTTCAGCGGACCTCTTTAGA

GCCCCCATCAAACTGGAGTGTGAGAAGACCTTTCAGCGACTGCTGCTGATCACCAAGAAG

AAGTACATAGGTGTCATAAACGGGGGGAAGATGCTCATGAAGGGGGTCGACCTGGTCCGC

AAAAATAACTGCTCTTTCATAAACTTGTACGCGCGACATCTGGTAGATCTTTTGTTGTAC

GACGAGGATGTGGCCACGGCGGCAGCAGAGGTGACAGACGTGCCTCCCGCAGAATGGGTG

GGGCGCCCGCTACCGAGCGGCTTTGACAAGTTTGGGCGAGTGCTGGTAGAGGCGTACAAC

CGTATCACTGCCCCCAACTTGGACGTGCGCGAGTTCGTTATGACTGCTGAGCTGAGCCGC

TCACCCGAATCGTATACCAACAAGCGCCTGCCGCACCTCACCGTCTACTTTAAGCTCGCC

ATGAGGAATGAAGAACTGCCCAGCGTAAAAGAGAGAATTCCGTATGTGATAGTTGCGCAG

ACCGAGGCCGCGGAACGCGAAGCGGGTGTAGTAAACTCAATGCGCGGTACCGCCCAAAAC

CCCGTGGTAACCAAGACCGCACGCCCCCAACCTAAACGCAAACTGCTGGTTTCCGACCTC

GCCGAAGACCCGACCTATGTTTCCGAGAATGACGTACCGCTAAACACAGACTACTATTTC

TCCCACCTGTTGGGTACCATAAGCGTGACCTTTAAGGCTCTATTCGGAAATGATGTGAGA

ACAACAGAAAATCTTTTAAAGCGGTTTATTCCGGAAACCCCCCACAAGACCCCCACGAAA

ACCCAGGCACTGCTTGAGCGCGCCGGCTTTGAAAAGCTGACGCCCTTTACACCGGAGGAA

GAAAGTCGTCGAATACTGCATACAGTTTTTTGTACTCTAGAAGCAGCTCCCCATCAAAGC

TGA

>LC109645.1

ATGGCGGCGCGCGAACAGGCCAACTCCGTTCGCAGGAGCGGGTTTTTTAATCCGTTTATT

GGCAAGAGGCCATTTTTCAGGCCGGGAAGCGGGCAGACCGCGGAGACAGAGCGCCCAAGG

CCCCCCCAACACTCGTACTGCACAGAGGTGGGTAGCTTTAAGTTTATAGCTCCAAGATGC

CTCGATGAAGAAGCCCCCGCCGACCAGCGACGCGGTGTACACGTGGGCACCTTGGAGCGC

CCACCCAAAGTGTACTGCGATGGCTCAGAGTACGACGTGCTGAACTTTGCCTCCGGAGGT

TGCTGGCCTCGCAGGATTCGGGTTTGGAACGGCCAGGATTTTCGGGGCGATGGATTCAAC

CCCAGATTTGAGAGATTTCACGTGTACGACATAGTAGAGACTTCGGAGAGCGCCTCACAC

GATGACCCATCCAGGTTTGCTGAGCTATCCCGCCCAAGCGGGTCTGTGGTAACACTGCTG

GGAATGAGCGAGTGTGGAAAGCGAGTCGCGGTTCACGTCTATGGTGTGCGCCATTATTTT

TACATGGCAAAGGCGGAGGTGGATAGCGCTTGTGGAATAACCACCGAGGCAGAACTCGTG

CGCGCAATGGTGGACTGCGCGCACAGCTCGGCTTTGAGCGCGGCCCTGGGAAATGGCAAC

GGCGGCAAACAGAGCGGCGGCAGCGGCGGGGGATGGTGGGGCGGAAAGCACGTGTCTGCG

GACTGCTTCAAAGTGGAGACCGTGTGCCACACGACGCTGTACTACTTTGGATCTAAGCCA

GCTCTCTACTATAGAGTATCTGCCTCCAGCAGCCGCCTGGGAGGGTTCATCTGCGACAAC

TTTCACCCGGAGATTACAAAATTCGAGGGGAGCGTGGACGTGACCACGCGGCTGTTGTTG

GACAATGAAAATTTTACCAGTTTCGGGTGGTACCGCCTGCGACCCGGCACCCACGGAGAG

CGTGTTCAACTTCGCCCCGTTGAGCGACACGTCACCTCAAGCGACGTGGAGATTAACTGT

ACTCCCGATAACCTGGAGCCGATACCAGACGAGGCTGCCTGGCCCGACTATAAGCTCATG

TGCTTTGATATAGAGTGTAAAGCTGGAACGGGTAACGAAATGGCGTTCCCAGTGGCAACT

AACCAAGAGGACCTGGTCATCCAGATCTCCTGTCTGCTGTACTCGCTTGCTACTCAGAAC

CACGAACACACCCTGCTGTTTTCCCTCGGGTCATGCGATATCTCTGAGGAATACTCGTTT

GCATGCGTCCAGCGCGGCGAGCCCAGACCGACGGTTTTGGAGTTTGACAGCGAGTACGAG

CTGCTGGTTGCCTTCCTGACCTTTCTCAAGCAGTACTCTCCCGAGTTCGCCACCGGCTAC

AACATCGTTAATTTTGACTGGGCGTACATAGTTAACAAGGTAACGTCGGTGTATAACATC

AAGCTGGACGGGTACGGCAAGTTCAACAAAGGGGGGCTGTTTAAGGTGTGGGACATCGCC

ACGAACCATTTTCAGAAGAAGAGCAAGGTGAAAATCAATGGCCTGATATCTCTAGACATG

TATTCTGTGGCGACGGAAAAGCTAAAGCTACCCAGCTACAAACTCGACGCGGTCGTGGGA

GACGTCCTCGGCGAGCATAAGATAGACCTTCCCTATAAAGAAATACCCTCCTATTACGCG

GGAGGGCCTGACCGGAGGGGCGTAATAGGAGAGTATTGTATCCAGGACTCTAGGCTGGTG

GGCAAGCTGTTTTTTAAGTACCTCCCCCATCTGGAACTATCGGCGGTGGCCAAACTCGCC

CGTATCACCCTAACGCGGGTAATTTTTGACGGTCAGCAAATTCGCGTGTACACGTGCTTG

CTGAAACTCGCCCGCGAGAGAAATTTCATTTTGCCAGACAACAGACGCCGGTTTGACAGT

CAGGCAGACGCCGCGTCAGAGACTTCGGAGTTGGCTATGGATAGCCAAAGCCACGCCTTC

GACAGTACAGACGAACCCGACGGTGTGGACGGTACCCCGGACGCCGCAGGATCTGGCGCT

ACTTCTGAAAACGGAGGCGGGAAGCCCGGCGTCGGGAGGGCCGTGGGCTACCAGGGAGCA

AAGGTTCTAGACCCCGTATCCGGCTTTCATGTGGACCCCGTGGTTGTGTTTGACTTCGCT

AGCTTATACCCAAGCATTATCCAGGCCCATAACCTCTGTTTCACCACCCTGGCGCTCGAT

GAAGTGGATCTGGCCGGGCTTCAACCATCCGTCAACTACTCGACGTTCGAGGTGGGTGAC

CAAAAGTTATTTTTTGTCCACGCCCATATTCGCGAAAGCCTGCTTGGCATCTTGCTGCGC

GACTGGCTGGCCATGCGAAAGGCGGTGAGGGCGCGAATCCCCACCAGCACCCCCGAGGAG

GCAGTTTTACTAGATAAGCAGCAGTCTGCGATTAAGGTGATATGCAACTCGGTTTACGGA

TTCACGGGGGTGGCAAACGGCCTGTTGCCGTGTCTGAGGATAGCGGCTACCGTTACCACG

ATAGGACGCGACATGCTCCTCAAGACCCGAGATTACGTTCACTCTCGTTGGGCGACGCGC

GAGCTGCTGGAGGACAATTTTCCAGGGGCTATAGGTTTCCGAAACCACAAGCCTTACTCC

GTCAGGGTTATCTACGGAGACACCGACTCCGTGTTTATCAAGTTTGTGGGCCTGACGTAC

GAGGGGGTATCGGAGCTGGGGGATGCTATGTCGCGTCAGATTTCAGCGGACCTCTTTAGA

GCGCCCATCAAACTGGAGTGTGAGAAGACCTTTCAGCGACTGCTGCTGATCACCAAGAAG

AAGTACATAGGTGTCATAAACGGGGGGAAGATGCTCATGAAGGGGGTCGACCTGGTCCGC

AAAAATAACTGCTCTTTCATAAACTTGTACGCGCGACATCTGGTAGATCTTTTGTTGTAC

GACGAGGATGTGGCCACGGCGGCAGCAGAGGTGACAGACGTGCCTCCCGCAGAATGGGTG

GGGCGCCCGCTACCGAGCGGCTTTGACAAGTTTGGGCGAGTGCTGGTAGAGGCGTACAAC

CGTATCACTGCCCCCAACTTGGACGTGCGCGAGTTCGTTATGACTGCTGAGCTGAGCCGC

TCACCCGAATCGTATACCAACAAGCGCCTGCCGCACCTCACCGTCTACTTTAAGCTCGCC

ATGAGGAATGAAGAACTGCCCAGCGTAAAAGAGAGAATTCCGTATGTGATAGTTGCGCAG

ACCGAGGCCGCGGAACGCGAAGCGGGTGTAGTAAACTCAATGCGCGGTACCGCCCAAAAC

CCCGTGGTAACCAAGACCGCACGCCCCCAACCTAAACGCAAACTGCTGGTTTCCGACCTC

GCCGAAGACCCGACCTATGTTTCCGAGAATGACGTACCGCTAAACACAGACTACTATTTC

TCCCACCTGTTGGGTACCATAAGCGTGACCTTTAAGGCTCTATTCGGAAATGATGTGAGA

ACAACAGAAAATCTTTTAAAGCGGTTTATTCCGGAAACCCCCCACAAGACCCCCACGAAA

ACCCAGGCACTGCTTGAGCGCGCCGGCTTTGAAAAGCTGACGCCCTTTACACCGGAGGAA

GAAAGTCGTCGAATACTGCATACAGTTTTTTGTACTCTAGAAGCAGCTCCCCATCAAAGC

TGA

>LC109646.1

ATGGCGGCGCGCGAACAGGCCAACTCCGTTCGCAGGAGCGGGTTTTTTAATCCGTTTATT

GGCAAGAGGCCATTTTTCAGGCCGGGAAGCGGGCAAACCGCGGAGACAGAGCGCCCAAGG

CCCCCCCAACACTCGTACTGCACAGAGGTGGGTAGCTTTAAGTTTATAGCTCCAAGATGC

CTCGATGAAGAAGCCCCCGCCGACCAGCGACGCGGTGTACACGTGGGCACCTTGGAGCGC

CCACCCAAAGTGTACTGCGATGGCTCAGAGTACGACGTGCTGAACTTTGCCTCCGGAGGT

TGCTGGCCTCGCAGGATTCGGGTTTGGAACGGCCAGGATTTTCGGGGCGATGGATTCAAC

CCCAGATTTGAGAGATTTCACGTGTACGACATAGTAGAGACTTCGGAGAGCGCCTCACAC

GATGACCCATCCAGGTTTGCTGAGCTATCCCGCCCAAGCGGGTCTGTGGTAACACTGCTG

GGAATGAGCGAGTGTGGAAAGCGAGTCGCGGTTCACGTCTATGGTGTGCGCCATTATTTT

TACATGGCAAAGGCGGAGGTGGATAGCGCTTGTGGAATAACCACCGAGGCAGAACTCGTG

CGCGCAATGGTGGACTGCGCGCACAGCTCGGCTTTGAGCGCGGCCCTGGGAAATGGCAAC

GGCGGCAAACAGAGCGGCGGCAGCGGCGGGGGATGGTGGGGCGGAAAGCACGTGTCTGCG

GACTGCTTCAAAGTGGAGACCGTGTGCCACACGACGCTGTACTACTTTGGATCTAAGCCA

GCTCTCTACTATAGAGTATCTGCCTCCAGCAGCCGCCTGGGAGGGTTCATCTGCGACAAC

TTTCACCCGGAGATTACAAAATTCGAGGGGAGCGTGGACGTGACCACGCGGCTGTTGTTG

GACAATGAAAATTTTACCAGTTTCGGGTGGTACCGCCTGCGACCCGGCACCCACGGAGAG

CGTGTTCAACTTCGCCCCGTTGAGCGACACGTCACCTCAAGCGACGTGGAGATTAACTGT

ACTCCCGATAACCTGGAGCCGATACCAGACGAGGCTGCCTGGCCCGACTATAAGCTCATG

TGCTTTGATATAGAGTGTAAAGCTGGAACGGGTAACGAAATGGCGTTCCCAGTGGCAACT

AACCAAGAGGACCTGGTCATCCAGATCTCCTGTCTGCTGTACTCGCTTGCTACTCAGAAC

CACGAACACACCCTGCTGTTTTCCCTCGGGTCATGCGATATCTCTGAGGAATACTCGTTT

GCATGCGTCCAGCGCGGCGAGCCCAGACCGACGGTTTTGGAGTTTGACAGCGAGTACGAG

CTGCTGGTTGCCTTCCTGACCTTTCTCAAGCAGTACTCTCCCGAGTTCGCCACCGGCTAC

AACATCGTTAATTTTGACTGGGCGTACATAGTTAACAAGGTAACGTCGGTGTATAACATC

AAGCTGGACGGGTACGGCAAGTTCAACAAAGGGGGGCTGTTTAAGGTGTGGGACATCGCC

ACGAACCATTTTCAGAAGAAGAGCAAGGTGAAAATCAATGGCCTGATATCTCTAGACATG

TATTCTGTGGCGACGGAAAAGCTAAAGCTACCCAGCTACAAACTCGACGCGGTCGTGGGA

GACGTCCTCGGCGAGCATAAGATAGACCTTCCCTATAAAGAAATACCCTCCTATTACGCG

GGAGGGCCTGACCGGAGGGGCGTAATAGGAGAGTATTGTATCCAGGACTCTAGGCTGGTG

GGCAAGCTGTTTTTTAAGTACCTCCCCCATCTGGAACTATCGGCGGTGGCCAAACTCGCC

CGTATCACCCTAACGCGGGTAATTTTTGACGGTCAGCAAATTCGCGTGTACACGTGCTTG

CTGAAACTCGCCCGCGAGAGAAATTTCATTTTGCCAGACAACAGACGCCGGTTTGACAGT

CAGGCAGACGCCGCGTCAGAGACTTCGGAGTTGGCTATGGATAGCCAAAGCCACGCCTTC

GACAGTACAGACGAACCCGACGGTGTGGACGGTACCCCGGACGCCGCAGGATCTGGCGCT

ACTTCTGAAAACGGAGGCGGGAAGCCCGGCGTCGGGAGGGCCGTGGGCTACCAGGGAGCA

AAGGTTCTAGACCCCGTATCCGGCTTTCATGTGGACCCCGTGGTTGTGTTTGACTTCGCT

AGCTTATACCCAAGCATTATCCAGGCCCATAACCTCTGTTTCACCACCCTGGCGCTCGAT

GAAGTGGATCTGGCCGGGCTTCAACCATCCGTCAACTACTCGACGTTCGAGGTGGGTGAC

CAAAAGTTATTTTTTGTCCACGCCCATATTCGCGAAAGCCTGCTTGGCATCTTGCTGCGC

GACTGGCTGGCCATGCGAAAGGCGGTGAGGGCGCGAATCCCCACCAGCACCCCCGAGGAG

GCAGTTTTACTAGATAAGCAGCAGTCTGCGATTAAGGTGATATGCAACTCGGTTTACGGA

TTCACGGGGGTGGCAAACGGCCTGTTGCCGTGTCTGAGGATAGCGGCTACCGTTACCACG

ATAGGACGCGACATGCTCCTCAAGACCCGAGATTACGTTCACTCTCGTTGGGCGACGCGC

GAGCTGCTGGAGGACAATTTTCCAGGGGCTATAGGTTTCCGAAACCACAAGCCTTACTCC

GTCAGGGTTATCTACGGAGACACCGACTCCGTGTTTATCAAGTTTGTGGGCCTGACGTAC

GAGGGGGTATCGGAGCTGGGGGATGCTATGTCGCGTCAGATTTCAGCGGACCTCTTTAGA

GCGCCCATCAAACTGGAGTGTGAGAAGACCTTTCAGCGACTGCTGCTGATCACCAAGAAG

AAGTACATAGGTGTCATAAACGGGGGGAAGATGCTCATGAAGGGGGTCGACCTGGTCCGC

AAAAATAACTGCTCTTTCATAAACTTGTACGCGCGACATCTGGTAGATCTTTTGTTGTAC

GACGAGGATGTGGCCACGGCGGCAGCAGAGGTGACAGACGTGCCTCCCGCAGAATGGGTG

GGGCGCCCGCTACCGAGCGGCTTTGACAAGTTTGGGCGAGTGCTGGTAGAGGCGTACAAC

CGTATCACTGCCCCCAACTTGGACGTGCGCGAGTTCGTTATGACTGCTGAGCTGAGCCGC

TCACCCGAATCGTATACCAACAAGCGCCTGCCGCACCTCACCGTCTACTTTAAGCTCGCC

ATGAGGAATGAAGAACTGCCCAGCGTAAAAGAGAGAATTCCGTATGTGATAGTTGCGCAG

ACCGAGGCCGCGGAACGCGAAGCGGGTGTAGTAAACTCAATGCGCGGTACCGCCCAAAAC

CCCGTGGTAACCAAGACCGCACGCCCCCAACCTAAACGCAAACTGCTGGTTTCCGACCTC

GCCGAAGACCCGACCTATGTTTCCGAGAATGACGTACCGCTAAACACAGACTACTATTTC

TCCCACCTGTTGGGTACCATAAGCGTGACCTTTAAGGCTCTATTCGGAAATGATGTGAGA

ACAACAGAAAATCTTTTAAAGCGGTTTATTCCGGAAACCCCCCACAAGACCCCCACGAAA

ACCCAGGCACTGCTTGAGCGCGCCGGCTTTGAAAAGCTGACGCCCTTTACACCGGAGGAA

GAAAGTCGTCGAATACTGCATACAGTTTTTTGTACTCTAGAAGCAGCTCCCCATCAAAGC

TGA

>LC109647.1

ATGGCGGCGCGCGAACAGGCCAACTCCGTTCGCAGGAGCGGGTTTTTTAATCCGTTTATT

GGCAAGAGGCCATTTTTCAGGCCGGGAAGCGGGCAGACCGCGGAGACAGAGCGCCCAAGG

CCCCCCCAACACTCGTACTGCACAGAGGTGGGTAGCTTTAAGTTTATAGCTCCAAGATGC

CTCGATGAAGAAGCCCCCGCCGACCAGCGACGCGGTGTACACGTGGGCACCTTGGAGCGC

CCACCCAAAGTGTACTGCGATGGCTCAGAGTACGACGTGCTGAACTTTGCCTCCGGAGGT

TGCTGGCCTCGCAGGATTCGGGTTTGGAACGGCCAGGATTTTCGGGGCGATGGATTCAAC

CCCAGATTTGAGAGATTTCACGTGTACGACATAGTAGAGACTTCGGAGAGCGCCTCACAC

GATGACCCATCCAGGTTTGCTGAGCTATCCCGCCCAAGCGGGTCTGTGGTAACACTGCTG

GGAATGAGCGAGTGTGGAAAGCGAGTCGCGGTTCACGTCTATGGTGTGCGCCATTATTTT

TACATGGCAAAGGCGGAGGTGGATAGCGCTTGTGGAATAACCACCGAGGCAGAACTCGTG

CGCGCAATGGTGGACTGCGCGCACAGCTCGGCTTTGAGCGCGGCCCTGGGAAATGGCAAC

GGCGGCAAACAGAGCGGCGGCAGCGGCGGGGGATGGTGGGGCGGAAAGCACGTGTCTGCG

GACTGCTTCAAAGTGGAGACCGTGTGCCACACGACGCTGTACTACTTTGGATCTAAGCCA

GCTCTCTACTATAGAGTATCTGCCTCCAGCAGCCGCCTGGGAGGGTTCATCTGCGACAAC

TTTCACCCGGAGATTACAAAATTCGAGGGGAGCGTGGACGTGACCACGCGGCTGTTGTTG

GACAATGAAAATTTTACCAGTTTCGGGTGGTACCGCCTGCGACCCGGCACCCACGGAGAG

CGTGTTCAACTTCGCCCCGTTGAGCGACACGTCACCTCAAGCGACGTGGAGATTAACTGT

ACTCCCGATAACCTGGAGCCGATACCAGACGAGGCTGCCTGGCCCGACTATAAGCTCATG

TGCTTTGATATAGAGTGTAAAGCTGGAACGGGTAACGAAATGGCGTTCCCAGTGGCAACT

AACCAAGAGGACCTGGTCATCCAGATCTCCTGTCTGCTGTACTCGCTTGCTACTCAGAAC

CACGAACACACCCTGCTGTTTTCCCTCGGGTCATGCGATATCTCTGAGGAATACTCGTTT

GCATGCGTCCAGCGCGGCGAGCCCAGACCGACGGTTTTGGAGTTTGACAGCGAGTACGAG

CTGCTGGTTGCCTTCCTGACCTTTCTCAAGCAGTACTCTCCCGAGTTCGCCACCGGCTAC

AACATCGTTAATTTTGACTGGGCGTACATAGTTAACAAGGTAACGTCGGTGTATAACATC

AAGCTGGACGGGTACGGCAAGTTCAACAAAGGGGGGCTGTTTAAGGTGTGGGACATCGCC

ACGAACCATTTTCAGAAGAAGAGCAAGGTGAAAATCAATGGCCTGATATCTCTAGACATG

TATTCTGTGGCGACGGAAAAGCTAAAGCTACCCAGCTACAAACTCGACGCGGTCGTGGGA

GACGTCCTCGGCGAGCATAAGATAGACCTTCCCTATAAAGAAATACCCTCCTATTACGCG

GGAGGGCCTGACCGGAGGGGCGTAATAGGAGAGTATTGTATCCAGGACTCTAGGCTGGTG

GGCAAGCTGTTTTTTAAGTACCTCCCCCATCTGGAACTATCGGCGGTGGCCAAACTCGCC

CGTATCACCCTAACGCGGGTAATTTTTGACGGTCAGCAAATTCGCGTGTACACGTGCTTG

CTGAAACTCGCCCGCGAGAGAAATTTCATTTTGCCAGACAACAGACGCCGGTTTGACAGT

CAGGCAGACGCCGCGTCAGAGACTTCGGAGTTGGCTATGGATAGCCAAAGCCACGCCTTC

GACAGTACAGACGAACCCGACGGTGTGGACGGTACCCCGGACGCCGCAGGATCTGGCGCT

ACTTCTGAAAACGGAGGCGGGAAGCCCGGCGTCGGGAGGGCCGTGGGCTACCAGGGAGCA

AAGGTTCTAGACCCCGTATCCGGCTTTCATGTGGACCCCGTGGTTGTGTTTGACTTCGCT

AGCTTATACCCAAGCATTATCCAGGCCCATAACCTCTGTTTCACCACCCTGGCGCTCGAT

GAAGTGGATCTGGCCGGGCTTCAACCATCCGTCAACTACTCGACGTTCGAGGTGGGTGAC

CAAAAGTTATTTTTTGTCCACGCCCATATTCGCGAAAGCCTGCTTGGCATCTTGCTGCGC

GACTGGCTGGCCATGCGAAAGGCGGTGAGGGCGCGAATCCCCACCAGCACCCCCGAGGAG

GCAGTTTTACTAGATAAGCAGCAGTCTGCGATTAAGGTGATATGCAACTCGGTTTACGGA

TTCACGGGGGTGGCAAACGGCCTGTTGCCGTGTCTGAGGATAGCGGCTACCGTTACCACG

ATAGGACGCGACATGCTCCTCAAGACCCGAGATTACGTTCACTCTCGTTGGGCGACGCGC

GAGCTGCTGGAGGACAATTTTCCAGGGGCTATAGGTTTCCGAAACCACAAGCCTTACTCC

GTCAGGGTTATCTACGGAGACACCGACTCCGTGTTTATCAAGTTTGTGGGCCTGACGTAC

GAGGGGGTATCGGAGCTGGGGGATGCTATGTCGCGTCAGATTTCAGCGGACCTCTTTAGA

GCCCCCATCAAACTGGAGTGTGAGAAGACCTTTCAGCGACTGCTGCTGATCACCAAGAAG

AAGTACATAGGTGTCATAAACGGGGGGAAGATGCTCATGAAGGGGGTCGACCTGGTCCGC

AAAAATAACTGCTCTTTCATAAACTTGTACGCGCGACATCTGGTAGATCTTTTGTTGTAC

GACGAGGATGTGGCCACGGCGGCAGCAGAGGTGACAGACGTGCCTCCCGCAGAATGGGTG

GGGCGCCCGCTACCGAGCGGCTTTGACAAGTTTGGGCGAGTGCTGGTAGAGGCGTACAAC

CGTATCACTGCCCCCAACTTGGACGTGCGCGAGTTCGTTATGACTGCTGAGCTGAGCCGC

TCACCCGAATCGTATACCAACAAGCGCCTGCCGCACCTCACCGTCTACTTTAAGCTCGCC

ATGAGGAATGAAGAACTGCCCAGCGTAAAAGAGAGAATTCCGTATGTGATAGTTGCGCAG

ACCGAGGCCGCGGAACGCGAAGCGGGTGTAGTAAACTCAATGCGCGGTACCGCCCAAAAC

CCCGTGGTAACCAAGACCGCACGCCCCCAACCTAAACGCAAACTGCTGGTTTCCGACCTC

GCCGAAGACCCGACCTATGTTTCCGAGAATGACGTACCGCTAAACACAGACTACTATTTC

TCCCACCTGTTGGGTACCATAAGCGTGACCTTTAAGGCTCTATTCGGAAATGATGTGAGA

ACAACAGAAAATCTTTTAAAGCGGTTTATTCCGGAAACCCCCCACAAGACCCCCACGAAA

ACCCAGGCACTGCTTGAGCGCGCCGGCTTTGAAAAGCTGACGCCCTTTACACCGGAGGAA

GAAAGTCGTCGAATACTGCATACAGTTTTTTGTACTCTAGAAGCAGCTCCCCATCAAAGC

TGA

>LC109648.1

ATGGCGGCGCGCGAACAGGCCAACTCCGTTCGCAGGAGCGGGTTTTTTAATCCGTTTATT

GGCAAGAGGCCATTTTTCAGGCCGGGAAGCGGGCAGACCGCGGAGACAGAGCGCCCAAGG

CCCCCCCAACACTCGTACTGCACAGAGGTGGGTAGCTTTAAGTTTATAGCTCCAAGATGC

CTCGATGAAGAAGCCCCCGCCGACCAGCGACGCGGTGTACACGTGGGCACCTTGGAGCGC

CCACCCAAAGTGTACTGCGATGGCTCAGAGTACGACGTGCTGAACTTTGCCTCCGGAGGT

TGCTGGCCTCGCAGGATTCGGGTTTGGAACGGCCAGGATTTTCGGGGCGATGGATTCAAC

CCCAGATTTGAGAGATTTCACGTGTACGACATAGTAGAGACTTCGGAGAGCGCCTCACAC

GATGACCCATCCAGGTTTGCTGAGCTATCCCGCCCAAGCGGGTCTGTGGTAACACTGCTG

GGAATGAGCGAGTGTGGAAAGCGAGTCGCGGTTCACGTCTATGGTGTGCGCCATTATTTT

TACATGGCAAAGGCGGAGGTGGATAGCGCTTGTGGAATAACCACCGAGGCAGAACTCGTG

CGCGCAATGGTGGACTGCGCGCACAGCTCGGCTTTGAGCGCGGCCCTGGGAAATGGCAAC

GGCGGCAAACAGAGCGGCGGCAGCGGCGGGGGATGGTGGGGCGGAAAGCACGTGTCTGCG

GACTGCTTCAAAGTGGAGACCGTGTGCCACACGACGCTGTACTACTTTGGATCTAAGCCA

GCTCTCTACTATAGAGTATCTGCCTCCAGCAGCCGCCTGGGAGGGTTCATCTGCGACAAC

TTTCACCCGGAGATTACAAAATTCGAGGGGAGCGTGGACGTGACCACGCGGCTGTTGTTG

GACAATGAAAATTTTACCAGTTTCGGGTGGTACCGCCTGCGACCCGGCACCCACGGAGAG

CGTGTTCAACTTCGCCCCGTTGAGCGACACGTCACCTCAAGCGACGTGGAGATTAACTGT

ACTCCCGATAACCTGGAGCCGATACCAGACGAGGCTGCCTGGCCCGACTATAAGCTCATG

TGCTTTGATATAGAGTGTAAAGCTGGAACGGGTAACGAAATGGCGTTCCCAGTGGCAACT

AACCAAGAGGACCTGGTCATCCAGATCTCCTGTCTGCTGTACTCGCTTGCTACTCAGAAC

CACGAACACACCCTGCTGTTTTCCCTCGGGTCATGCGATATCTCTGAGGAATACTCGTTT

GCATGCGTCCAGCGCGGCGAGCCCAGACCGACGGTTTTGGAGTTTGACAGCGAGTACGAG

CTGCTGGTTGCCTTCCTGACCTTTCTCAAGCAGTACTCTCCCGAGTTCGCCACCGGCTAC

AACATCGTTAATTTTGACTGGGCGTACATAGTTAACAAGGTAACGTCGGTGTATAACATC

AAGCTGGACGGGTACGGCAAGTTCAACAAAGGGGGGCTGTTTAAGGTGTGGGACATCGCC

ACGAACCATTTTCAGAAGAAGAGCAAGGTGAAAATCAATGGCCTGATATCTCTAGACATG

TATTCTGTGGCGACGGAAAAGCTAAAGCTACCCAGCTACAAACTCGACGCGGTCGTGGGA

GACGTCCTCGGCGAGCATAAGATAGACCTTCCCTATAAAGAAATACCCTCCTATTACGCG

GGAGGGCCTGACCGGAGGGGCGTAATAGGAGAGTATTGTATCCAGGACTCTAGGCTGGTG

GGCAAGCTGTTTTTTAAGTACCTCCCCCATCTGGAACTATCGGCGGTGGCCAAACTCGCC

CGTATCACCCTAACGCGGGTAATTTTTGACGGTCAGCAAATTCGCGTGTACACGTGCTTG

CTGAAACTCGCCCGCGAGAGAAATTTCATTTTGCCAGACAACAGACGCCGGTTTGACAGT

CAGGCAGACGCCGCGTCAGAGACTTCGGAGTTGGCTATGGATAGCCAAAGCCACGCCTTC

GACAGTACAGACGAACCCGACGGTGTGGACGGTACCCCGGACGCCGCAGGATCTGGCGCT

ACTTCTGAAAACGGAGGCGGGAAGCCCGGCGTCGGGAGGGCCGTGGGCTACCAGGGAGCA

AAGGTTCTAGACCCCGTATCCGGCTTTCATGTGGACCCCGTGGTTGTGTTTGACTTCGCT

AGCTTATACCCAAGCATTATCCAGGCCCATAACCTCTGTTTCACCACCCTGGCGCTCGAT

GAAGTGGATCTGGCCGGGCTTCAACCATCCGTCAACTACTCGACGTTCGAGGTGGGTGAC

CAAAAGTTATTTTTTGTCCACGCCCATATTCGCGAAAGCCTGCTTGGCATCTTGCTGCGC

GACTGGCTGGCCATGCGAAAGGCGGTGAGGGCGCGAATCCCCACCAGCACCCCCGAGGAG

GCAGTTTTACTAGATAAGCAGCAGTCTGCGATTAAGGTGATATGCAACTCGGTTTACGGA

TTCACGGGGGTGGCAAACGGCCTGTTGCCGTGTCTGAGGATAGCGGCTACCGTTACCACG

ATAGGACGCGACATGCTCCTCAAGACCCGAGATTACGTTCACTCTCGTTGGGCGACGCGC

GAGCTGCTGGAGGACAATTTTCCAGGGGCTATAGGTTTCCGAAACCACAAGCCTTACTCC

GTCAGGGTTATCTACGGAGACACCGACTCCGTGTTTATCAAGTTTGTGGGCCTGACGTAC

GAGGGGGTATCGGAGCTGGGGGATGCTATGTCGCGTCAGATTTCAGCGGACCTCTTTAGA

GCGCCCATCAAACTGGAGTGTGAGAAGACCTTTCAGCGACTGCTGCTGATCACCAAGAAG

AAGTACATAGGTGTCGTAAACGGGGGGAAGATGCTCATGAAGGGGGTCGACCTGGTCCGC

AAAAATAACTGCTCTTTCATAAACTTGTACGCGCGACATCTGGTAGATCTTTTGTTGTAC

GACGAGGATGTGGCCACGGCGGCAGCAGAGGTGACAGACGTGCCTCCCGCAGAATGGGTG

GGGCGCCCGCTACCGAGCGGCTTTGACAAGTTTGGGCGAGTGCTGGTAGAGGCGTACAAC

CGTATCACTGCCCCCAACTTGGACGTGCGCGAGTTCGTTATGACTGCTGAGCTGAGCCGC

TCACCCGAATCGTATACCAACAAGCGCCTGCCGCACCTCACCGTCTACTTTAAGCTCGCC

ATGAGGAATGAAGAACTGCCCAGCGTAAAAGAGAGAATTCCGTATGTGATAGTTGCGCAG

ACCGAGGCCGCGGAACGCGAAGCGGGTGTAGTAAACTCAATGCGCGGTACCGCCCAAAAC

CCCGTGGTAACCAAGACCGCACGCCCCCAACCTAAACGCAAACTGCTGGTTTCCGACCTC

GCCGAAGACCCGACCTATGTTTCCGAGAATGACGTACCGCTAAACACAGACTACTATTTC

TCCCACCTGTTGGGTACCATAAGCGTGACCTTTAAGGCTCTATTCGGAAATGATGTGAGA

ACAACAGAAAATCTTTTAAAGCGGTTTATTCCGGAAACCCCCCACAAGACCCCCACGAAA

ACCCAGGCACTGCTTGAGCGCGCCGGCTTTGAAAAGCTGACGCCCTTTACACCGGAGGAA

GAAAGTCGTCGAATACTGCATACAGTTTTTTGTACTCTAGAAGCAGCTCCCCATCAAAGC

TGA

>LC109649.1

ATGGCGGCGCGCGAACAGGCCAACTCCGTTCGCAGGAGCGGGTTTTTTAATCCGTTTATT

GGCAAGAGGCCATTTTTCAGGCCGGGAAGCGGGCAGACCGCGGAGACAGAGCGCCCAAGG

CCCCCCCAACACTCGTACTGCACAGAGGTGGGTAGCTTTAAGTTTATAGCTCCAAGATGC

CTCGATGAAGAAGCCCCCGCCGACCAGCGACGCGGTGTACACGTGGGCACCTTGGAGCGC

CCACCCAAAGTGTACTGCGATGGCTCAGAGTACGACGTGCTGAACTTTGCCTCCGGAGGT

TGCTGGCCTCGCAGGATTCGGGTTTGGAACGGCCAGGATTTTCGGGGCGATGGATTCAAC

CCCAGATTTGAGAGATTTCACGTGTACGACATAGTAGAGACTTCGGAGAGCGCCTCACAC

GATGACCCATCCAGGTTTGCTGAGCTATCCCGCCCAAGCGGGTCTGTGGTAACACTGCTG

GGAATGAGCGAGTGTGGAAAGCGAGTCGCGGTTCACGTCTATGGTGTGCGCCATTATTTT

TACATGGCAAAGGCGGAGGTGGATAGCGCTTGTGGAATAACCACCGAGGCAGAACTCGTG

CGCGCAATGGTGGACTGCGCGCACAGCTCGGCTTTGAGCGCGGCCCTGGGAAATGGCAAC

GGCGGCAAACAGAGCGGCGGCAGCGGCGGGGGATGGTGGGGCGGAAAGCACGTGTCTGCG

GACTGCTTCAAAGTGGAGACCGTGTGCCACACGACGCTGTACTACTTTGGATCTAAGCCA

GCTCTCTACTATAGAGTATCTGCCTCCAGCAGCCGCCTGGGAGGGTTCATCTGCGACAAC

TTTCACCCGGAGATTACAAAATTCGAGGGGAGCGTGGACGTGACCACGCGGCTGTTGTTG

GACAATGAAAATTTTACCAGTTTCGGGTGGTACCGCCTGCGACCCGGCACCCACGGAGAG

CGTGTTCAACTTCGCCCCGTTGAGCGACACGTCACCTCAAGCGACGTGGAGATTAACTGT

ACTCCCGATAACCTGGAGCCGATACCAGACGAGGCTGCCTGGCCCGACTATAAGCTCATG

TGCTTTGATATAGAGTGTAAAGCTGGAACGGGTAACGAAATGGCGTTCCCAGTGGCAACT

AACCAAGAGGACCTGGTCATCCAGATCTCCTGTCTGCTGTACTCGCTTGCTACTCAGAAC

CACGAACACACCCTGCTGTTTTCCCTCGGGTCATGCGATATCTCTGAGGAATACTCGTTT

GCATGCGTCCAGCGCGGCGAGCCCAGACCGACGGTTTTGGAGTTTGACAGCGAGTACGAG

CTGCTGGTTGCCTTCCTGACCTTTCTCAAGCAGTACTCTCCCGAGTTCGCCACCGGCTAC

AACATCGTTAATTTTGACTGGGCGTACATAGTTAACAAGGTAACGTCGGTGTATAACATC

AAGCTGGACGGGTACGGCAAGTTCAACAAAGGGGGGCTGTTTAAGGTGTGGGACATCGCC

ACGAACCATTTTCAGAAGAAGAGCAAGGTGAAAATCAATGGCCTGATATCTCTAGACATG

TATTCTGTGGCGACGGAAAAGCTAAAGCTACCCAGCTACAAACTCGACGCGGTCGTGGGA

GACGTCCTCGGCGAGCATAAGATAGACCTTCCCTATAAAGAAATACCCTCCTATTACGCG

GGAGGGCCTGACCGGAGGGGCGTAATAGGAGAGTATTGTATCCAGGACTCTAGGCTGGTG

GGCAAGCTGTTTTTTAAGTACCTCCCCCATCTGGAACTATCGGCGGTGGCCAAACTCGCC

CGTATCACCCTAACGCGGGTAATTTTTGACGGTCAGCAAATTCGCGTGTACACGTGCTTG

CTGAAACTCGCCCGCGAGAGAAATTTCATTTTGCCAGACAACAGACGCCGGTTTGACAGT

CAGGCAGACGCCGCGTCAGAGACTTCGGAGTTGGCTATGGATAGCCAAAGCCACGCCTTC

GACAGTACAGACGAACCCGACGGTGTGGACGGTACCCCGGACGCCGCAGGATCTGGCGCT

ACTTCTGAAAACGGAGGCGGGAAGCCCGGCGTCGGGAGGGCCGTGGGCTACCAGGGAGCA

AAGGTTCTAGACCCCGTATCCGGCTTTCATGTGGACCCCGTGGTTGTGTTTGACTTCGCT

AGCTTATACCCAAGCATTATCCAGGCCCATAACCTCTGTTTCACCACCCTGGCGCTCGAT

GAAGTGGATCTGGCCGGGCTTCAACCATCCGTCAACTACTCGACGTTCGAGGTGGGTGAC

CAAAAGTTATTTTTTGTCCACGCCCATATTCGCGAAAGCCTGCTTGGCATCTTGCTGCGC

GACTGGCTGGCCATGCGAAAGGCGGTGAGGGCGCGAATCCCCACCAGCACCCCCGAGGAG

GCAGTTTTACTAGATAAGCAGCAGTCTGCGATTAAGGTGATATGCAACTCGGTTTACGGA

TTCACGGGGGTGGCAAACGGCCTGTTGCCGTGTCTGAGGATAGCGGCTACCGTTACCACG

ATAGGACGCGACATGCTCCTCAAGACCCGAGATTACGTTCACTCTCGTTGGGCGACGCGC

GAGCTGCTGGAGGACAATTTTCCAGGGGCTATAGGTTTCCGAAACCACAAGCCTTACTCC

GTCAGGGTTATCTACGGAGACACCGACTCCGTGTTTATCAAGTTTGTGGGCCTGACGTAC

GAGGGGGTATCGGAGCTGGGGGATGCTATGTCGCGTCAGATTTCAGCGGACCTCTTTAGA

GCGCCCATCAAACTGGAGTGTGAGAAGACCTTTCAGCGACTGCTGCTGATCACCAAGAAG

AAGTACATAGGTGTCATAAACGGGGGGAAGATGCTCATGAAGGGGGTCGACCTGGTCCGC

AAAAATAACTGCTCTTTCATAAACTTGTACGCGCGACATCTGGTAGATCTTTTGTTGTAC

GACGAGGATGTGGCCACGGCGGCAGCAGAGGTGACAGACGTGCCTCCCGCAGAATGGGTG

GGGCGCCCGCTACCGAGCGGCTTTGACAAGTTTGGGCGAGTGCTGGTAGAGGCGTACAAC

CGTATCACTGCCCCCAACTTGGACGTGCGCGAGTTCGTTATGACTGCTGAGCTGAGCCGC

TCACCCGAATCGTATACCAACAAGCGCCTGCCGCACCTCACCGTCTACTTTAAGCTCGCC

ATGAGGAATGAAGAACTGCCCAGCGTAAAAGAGAGAATTCCGTATGTGATAGTTGCGCAG

ACCGAGGCCGCGGAACGCGAAGCGGGTGTAGTAAACTCAATGCGCGGTACCGCCCAAAAC

CCCGTGGTAACCAAGACCGCACGCCCCCAACCTAAACGCAAACTGCTGGTTTCCGACCTC

GCCGAAGACCCGACCTATGTTTCCGAGAATGACGTACCGCTAAACACAGACTACTATTTC

TCCCACCTGTTGGGTACCATAAGCGTGACCTTTAAGGCTCTATTCGGAAATGATGTGAGA

ACAACAGAAAATCTTTTAAAGCGGTTTATTCCGGAAACCCCCCACAAGACCCCCACGAAA

ACCCAGGCACTGCTTGAGCGCGCCGGCTTTGAAAAGCTGACGCCCTTTACACCGGAGGAA

GAAAGTCGTCGAATACTGCATACAGTTTTTTGTACTCTAGAAGCAGCTCCCCATCAAAGC

TGA

>LC109650.1

ATGGCGGCGCGCGAACAGGCCAACTCCGTTCGCAGGAGCGGGTTTTTTAATCCGTTTATT

GGCAAGAGGCCATTTTTCAGGCCGGGAAGCGGGCAGACCGCGGAGACAGAGCGCCCAAGG

CCCCCCCAACACTCGTACTGCACAGAGGTGGGTAGCTTTAAGTTTATAGCTCCAAGATGC

CTCGATGAAGAAGCCCCCGCCGACCAGCGACGCGGTGTACACGTGGGCACCTTGGAGCGC

CCACCCAAAGTGTACTGCGATGGCTCAGAGTACGACGTGCTGAACTTTGCCTCCGGAGGT

TGCTGGCCTCGCAGGATTCGGGTTTGGAACGGCCAGGATTTTCGGGGCGATGGATTCAAC

CCCAGATTTGAGAGATTTCACGTGTACGACATAGTAGAGACTTCGGAGAGCGCCTCACAC

GATGACCCATCCAGGTTTGCTGAGCTATCCCGCCCAAGCGGGTCTGTGGTAACACTGCTG

GGAATGAGCGAGTGTGGAAAGCGAGTCGCGGTTCACGTCTATGGTGTGCGCCATTATTTT

TACATGGCAAAGGCGGAGGTGGATAGCGCTTGTGGAATAACCACCGAGGCAGAACTCGTG

CGCGCAATGGTGGACTGCGCGCACAGCTCGGCTTTGAGCGCGGCCCTGGGAAATGGCAAC

GGCGGCAAACAGAGCGGCGGCAGCGGCGGGGGATGGTGGGGCGGAAAGCACGTGTCTGCG

GACTGCTTCAAAGTGGAGACCGTGTGCCACACGACGCTGTACTACTTTGGATCTAAGCCA

GCTCTCTACTATAGAGTATCTGCCTCCAGCAGCCGCCTGGGAGGGTTCATCTGCGACAAC

TTTCACCCGGAGATTACAAAATTCGAGGGGAGCGTGGACGTGACCACGCGGCTGTTGTTG

GACAATGAAAATTTTACCAGTTTCGGGTGGTACCGCCTGCGACCCGGCACCCACGGAGAG

CGTGTTCAACTTCGCCCCGTTGAGCGACACGTCACCTCAAGCGACGTGGAGATTAACTGT

ACTCCCGATAACCTGGAGCCGATACCAGACGAGGCTGCCTGGCCCGACTATAAGCTCATG

TGCTTTGATATAGAGTGTAAAGCTGGAACGGGTAACGAAATGGCGTTCCCAGTGGCAACT

AACCAAGAGGACCTGGTCATCCAGATCTCCTGTCTGCTGTACTCGCTTGCTACTCAGAAC

CACGAACACACCCTGCTGTTTTCCCTCGGGTCATGCGATATCTCTGAGGAATACTCGTTT

GCATGCGTCCAGCGCGGCGAGCCCAGACCGACGGTTTTGGAGTTTGACAGCGAGTACGAG

CTGCTGGTTGCCTTCCTGACCTTTCTCAAGCAGTACTCTCCCGAGTTCGCCACCGGCTAC

AACATCGTTAATTTTGACTGGGCGTACATAGTTAACAAGGTAACGTCGGTGTATAACATC

AAGCTGGACGGGTACGGCAAGTTCAACAAAGGGGGGCTGTTTAAGGTGTGGGACATCGCC

ACGAACCATTTTCAGAAGAAGAGCAAGGTGAAAATCAATGGCCTGATATCTCTAGACATG

TATTCTGTGGCGACGGAAAAGCTAAAGCTACCCAGCTACAAACTCGACGCGGTCGTGGGA

GACGTCCTCGGCGAGCATAAGATAGACCTTCCCTATAAAGAAATACCCTCCTATTACGCG

GGAGGGCCTGACCGGAGGGGCGTAATAGGAGAGTATTGTATCCAGGACTCTAGGCTGGTG

GGCAAGCTGTTTTTTAAGTACCTCCCCCATCTGGAACTATCGGCGGTGGCCAAACTCGCC

CGTATCACCCTAACGCGGGTAATTTTTGACGGTCAGCAAATTCGCGTGTACACGTGCTTG

CTGAAACTCGCCCGCGAGAGAAATTTCATTTTGCCAGACAACAGACGCCGGTTTGACAGT

CAGGCAGACGCCGCGTCAGAGACTTCGGAGTTGGCTATGGATAGCCAAAGCCACGCCTTC

GACAGTACAGACGAACCCGACGGTGTGGACGGTACCCCGGACGCCGCAGGATCTGGCGCT

ACTTCTGAAAACGGAGGCGGGAAGCCCGGCGTCGGGAGGGCCGTGGGCTACCAGGGAGCA

AAGGTTCTAGACCCCGTATCCGGCTTTCATGTGGACCCCGTGGTTGTGTTTGACTTCGCT

AGCTTATACCCAAGCATTATCCAGGCCCATAACCTCTGTTTCACCACCCTGGCGCTCGAT

GAAGTGGATCTGGCCGGGCTTCAACCATCCGTCAACTACTCGACGTTCGAGGTGGGTGAC

CAAAAGTTATTTTTTGTCCACGCCCATATTCGCGAAAGCCTGCTTGGCATCTTGCTGCGC

GACTGGCTGGCCATGCGAAAGGCGGTGAGGGCGCGAATCCCCACCAGCACCCCCGAGGAG

GCAGTTTTACTAGATAAGCAGCAGTCTGCGATTAAGGTGATATGCAACTCGGTTTACGGA

TTCACGGGGGTGGCAAACGGCCTGTTGCCGTGTCTGAGGATAGCGGCTACCGTTACCACG

ATAGGACGCGACATGCTCCTCAAGACCCGAGATTACGTTCACTCTCGTTGGGCGACGCGC

GAGCTGCTGGAGGACAATTTTCCAGGGGCTATAGGTTTCCGAAACCACAAGCCTTACTCC

GTCAGGGTTATCTACGGAGACACCGACTCCGTGTTTATCAAGTTTGTGGGCCTGACGTAC

GAGGGGGTATCGGAGCTGGGGGATGCTATGTCGCGTCAGATTTCAGCGGACCTCTTTAGA

GCCCCCATCAAACTGGAGTGTGAGAAGACCTTTCAGCGACTGCTGCTGATCACCAAGAAG

AAGTACATAGGTGTCATAAACGGGGGGAAGATGCTCATGAAGGGGGTCGACCTGGTCCGC

AAAAATAACTGCTCTTTCATAAACTTGTACGCGCGACATCTGGTAGATCTTTTGTTGTAC

GACGAGGATGTGGCCACGGCGGCAGCAGAGGTGACAGACGTGCCTCCCGCAGAATGGGTG

GGGCGCCCGCTACCGAGCGGCTTTGACAAGTTTGGGCGAGTGCTGGTAGAGGCGTACAAC

CGTATCACTGCCCCCAACTTGGACGTGCGCGAGTTCGTTATGACTGCTGAGCTGAGCCGC

TCACCCGAATCGTATACCAACAAGCGCCTGCCGCACCTCACCGTCTACTTTAAGCTCGCC

ATGAGGAATGAAGAACTGCCCAGCGTAAAAGAGAGAATTCCGTATGTGATAGTTGCGCAG

ACCGAGGCCGCGGAACGCGAAGCGGGTGTAGTAAACTCAATGCGCGGTACCGCCCAAAAC

CCCGTGGTAACCAAGACCGCACGCCCCCAACCTAAACGCAAACTGCTGGTTTCCGACCTC

GCCGAAGACCCGACCTATGTTTCCGAGAATGACGTACCGCTAAACACAGACTACTATTTC

TCCCACCTGTTGGGTACCATAAGCGTGACCTTTAAGGCTCTATTCGGAAATGATGTGAGA

ACAACAGAAAATCTTTTAAAGCGGTTTATTCCGGAAACCCCCCACAAGACCCCCACGAAA

ACCCAGGCACTGCTTGAGCGCGCCGGCTTTGAAAAGCTGACGCCCTTTACACCGGAGGAA

GAAAGTCGTCGAATACTGCATACAGTTTTTTGTACTCTAGAAGCAGCTCCCCATCAAAGC

TGA

>LC109651.1

ATGGCGGCGCGCGAACAGGCCAACTCCGTTCGCAGGAGCGGGTTTTTTAATCCGTTTATT

GGCAAGAGGCCATTTTTCAGGCCGGGAAGCGGGCAGACCGCGGAGACAGAGCGCCCAAGG

CCCCCCCAACACTCGTACTGCACAGAGGTGGGTAGCTTTAAGTTTATAGCTCCAAGATGC

CTCGATGAAGAAGCCCCCGCCGACCAGCGACGCGGTGTACACGTGGGCACCTTGGAGCGC

CCACCCAAAGTGTACTGCGATGGCTCAGAGTACGACGTGCTGAACTTTGCCTCCGGAGGT

TGCTGGCCTCGCAGGATTCGGGTTTGGAACGGCCAGGATTTTCGGGGCGATGGATTCAAC

CCCAGATTTGAGAGATTTCACGTGTACGACATAGTAGAGACTTCGGAGAGCGCCTCACAC

GATGACCCATCCAGGTTTGCTGAGCTATCCCGCCCAAGCGGGTCTGTGGTAACACTGCTG

GGAATGAGCGAGTGTGGAAAGCGAGTCGCGGTTCACGTCTATGGTGTGCGCCATTATTTT

TACATGGCAAAGGCGGAGGTGGATAGCGCTTGTGGAATAACCACCGAGGCAGAACTCGTG

CGCGCAATGGTGGACTGCGCGCACAGCTCGGCTTTGAGCGCGGCCCTGGGAAATGGCAAC

GGCGGCAAACAGAGCGGCGGCAGCGGCGGGGGATGGTGGGGCGGAAAGCACGTGTCTGCG

GACTGCTTCAAAGTGGAGACCGTGTGCCACACGACGCTGTACTACTTTGGATCTAAGCCA

GCTCTCTACTATAGAGTATCTGCCTCCAGCAGCCGCCTGGGAGGGTTCATCTGCGACAAC

TTTCACCCGGAGATTACAAAATTCGAGGGGAGCGTGGACGTGACCACGCGGCTGTTGTTG

GACAATGAAAATTTTACCAGTTTCGGGTGGTACCGCCTGCGACCCGGCACCCACGGAGAG

CGTGTTCAACTTCGCCCCGTTGAGCGACACGTCACCTCAAGCGACGTGGAGATTAACTGT

ACTCCCGATAACCTGGAGCCGATACCAGACGAGGCTGCCTGGCCCGACTATAAGCTCATG

TGCTTTGATATAGAGTGTAAAGCTGGAACGGGTAACGAAATGGCGTTCCCAGTGGCAACT

AACCAAGAGGACCTGGTCATCCAGATCTCCTGTCTGCTGTACTCGCTTGCTACTCAGAAC

CACGAACACACCCTGCTGTTTTCCCTCGGGTCATGCGATATCTCTGAGGAATACTCGTTT

GCATGCGTCCAGCGCGGCGAGCCCAGACCGACGGTTTTGGAGTTTGACAGCGAGTACGAG

CTGCTGGTTGCCTTCCTGACCTTTCTCAAGCAGTACTCTCCCGAGTTCGCCACCGGCTAC

AACATCGTTAATTTTGACTGGGCGTACATAGTTAACAAGGTAACGTCGGTGTATAACATC

AAGCTGGACGGGTACGGCAAGTTCAACAAAGGGGGGCTGTTTAAGGTGTGGGACATCGCC

ACGAACCATTTTCAGAAGAAGAGCAAGGTGAAAATCAATGGCCTGATATCTCTAGACATG

TATTCTGTGGCGACGGAAAAGCTAAAGCTACCCAGCTACAAACTCGACGCGGTCGTGGGA

GACGTCCTCGGCGAGCATAAGATAGACCTTCCCTATAAAGAAATACCCTCCTATTACGCG

GGAGGGCCTGACCGGAGGGGCGTAATAGGAGAGTATTGTATCCAGGACTCTAGGCTGGTG

GGCAAGCTGTTTTTTAAGTACCTCCCCCATCTGGAACTATCGGCGGTGGCCAAACTCGCC

CGTATCACCCTAACGCGGGTAATTTTTGACGGTCAGCAAATTCGCGTGTACACGTGCTTG

CTGAAACTCGCCCGCGAGAGAAATTTCATTTTGCCAGACAACAGACGCCGGTTTGACAGT

CAGGCAGACGCCGCGTCAGAGACTTCGGAGTTGGCTATGGATAGCCAAAGCCACGCCTTC

GACAGTACAGACGAACCCGACGGTGTGGACGGTACCCCGGACGCCGCAGGATCTGGCGCT

ACTTCTGAAAACGGAGGCGGGAAGCCCGGCGTCGGGAGGGCCGTGGGCTACCAGGGAGCA

AAGGTTCTAGACCCCGTATCCGGCTTTCATGTGGACCCCGTGGTTGTGTTTGACTTCGCT

AGCTTATACCCAAGCATTATCCAGGCCCATAACCTCTGTTTCACCACCCTGGCGCTCGAT

GAAGTGGATCTGGCCGGGCTTCAACCATCCGTCAACTACTCGACGTTCGAGGTGGGTGAC

CAAAAGTTATTTTTTGTCCACGCCCATATTCGCGAAAGCCTGCTTGGCATCTTGCTGCGC

GACTGGCTGGCCATGCGAAAGGCGGTGAGGGCGCGAATCCCCACCAGCACCCCCGAGGAG

GCAGTTTTACTAGATAAGCAGCAGTCTGCGATTAAGGTGATATGCAACTCGGTTTACGGA

TTCACGGGGGTGGCAAACGGCCTGTTGCCGTGTCTGAGGATAGCGGCTACCGTTACCACG

ATAGGACGCGACATGCTCCTCAAGACCCGAGATTACGTTCACTCTCGTTGGGCGACGCGC

GAGCTGCTGGAGGACAATTTTCCAGGGGCTATAGGTTTCCGAAACCACAAGCCTTACTCC

GTCAGGGTTATCTACGGAGACACCGACTCCGTGTTTATCAAGTTTGTGGGCCTGACGTAC

GAGGGGGTATCGGAGCTGGGGGATGCTATGTCGCGTCAGATTTCAGCGGACCTCTTTAGA

GCGCCCATCAAACTGGAGTGTGAGAAGACCTTTCAGCGACTGCTGCTGATCACCAAGAAG

AAGTACATAGGTGTCATAAACGGGGGGAAGATGCTCATGAAGGGGGTCGACCTGGTCCGC

AAAAATAACTGCTCTTTCATAAACTTGTACGCGCGACATCTGGTAGATCTTTTGTTGTAC

GACGAGGATGTGGCCACGGCGGCAGCAGAGGTGACAGACGTGCCTCCCGCAGAATGGGTG

GGGCGCCCGCTACCGAGCGGCTTTGACAAGTTTGGGCGAGTGCTGGTAGAGGCGTACAAC

CGTATCACTGCCCCCAACTTGGACGTGCGCGAGTTCGTTATGACTGCTGAGCTGAGCCGC

TCACCCGAATCGTATACCAACAAGCGCCTGCCGCACCTCACCGTCTACTTTAAGCTCGCC

ATGAGGAATGAAGAACTGCCCAGCGTAAAAGAGAGAATTCCGTATGTGATAGTTGCGCAG

ACCGAGGCCGCGGAACGCGAAGCGGGTGTAGTAAACTCAATGCGCGGTACCGCCCAAAAC

CCCGTGGTAACCAAGACCGCACGCCCCCAACCTAAACGCAAACTGCTGGTTTCCGACCTC

GCCGAAGACCCGACCTATGTTTCCGAGAATGACGTACCGCTAAACACAGACTACTATTTC

TCCCACCTGTTGGGTACCATAAGCGTGACCTTTAAGGCTCTATTCGGAAATGATGTGAGA

ACAACAGAAAATCTTTTAAAGCGGTTTATTCCGGAAACCCCCCACAAGACCCCCACGAAA

ACCCAGGCACTGCTTGAGCGCGCCGGCTTTGAAAAGCTGACGCCCTTTACACCGGAGGAA

GAAAGTCGTCGAATACTGCATACAGTTTTTTGTACTCTAGAAGCAGCTCCCCATCAAAGC

TGA

>LC109652.1

ATGGCGGCGCGCGAACAGGCCAACTCCGTTCGCAGGAGCGGGTTTTTTAATCCGTTTATT

GGCAAGAGGCCATTTTTCAGGCCGGGAAGCGGGCAGACCGCGGAGACAGAGCGCCCAAGG

CCCCCCCAACACTCGTACTGCACAGAGGTGGGTAGCTTTAAGTTTATAGCTCCAAGATGC

CTCGATGAAGAAGCCCCCGCCGACCAGCGACGCGGTGTACACGTGGGCACCTTGGAGCGC

CCACCCAAAGTGTACTGCGATGGCTCAGAGTACGACGTGCTGAACTTTGCCTCCGGAGGT

TGCTGGCCTCGCAGGATTCGGGTTTGGAACGGCCAGGATTTTCGGGGCGATGGATTCAAC

CCCAGATTTGAGAGATTTCACGTGTACGACATAGTAGAGACTTCGGAGAGCGCCTCACAC

GATGACCCATCCAGGTTTGCTGAGCTATCCCGCCCAAGCGGGTCTGTGGTAACACTGCTG

GGAATGAGCGAGTGTGGAAAGCGAGTCGCGGTTCACGTCTATGGTGTGCGCCATTATTTT

TACATGGCAAAGGCGGAGGTGGATAGCGCTTGTGGAATAACCACCGAGGCAGAACTCGTG

CGCGCAATGGTGGACTGCGCGCACAGCTCGGCTTTGAGCGCGGCCCTGGGAAATGGCAAC

GGCGGCAAACAGAGCGGCGGCAGCGGCGGGGGATGGTGGGGCGGAAAGCACGTGTCTGCG

GACTGCTTCAAAGTGGAGACCGTGTGCCACACGACGCTGTACTACTTTGGATCTAAGCCA

GCTCTCTACTATAGAGTATCTGCCTCCAGCAGCCGCCTGGGAGGGTTCATCTGCGACAAC

TTTCACCCGGAGATTACAAAATTCGAGGGGAGCGTGGACGTGACCACGCGGCTGTTGTTG

GACAATGAAAATTTTACCAGTTTCGGGTGGTACCGCCTGCGACCCGGCACCCACGGAGAG

CGTGTTCAACTTCGCCCCGTTGAGCGACACGTCACCTCAAGCGACGTGGAGATTAACTGT

ACTCCCGATAACCTGGAGCCGATACCAGACGAGGCTGCCTGGCCCGACTATAAGCTCATG

TGCTTTGATATAGAGTGTAAAGCTGGAACGGGTAACGAAATGGCGTTCCCAGTGGCAACT

AACCAAGAGGACCTGGTCATCCAGATCTCCTGTCTGCTGTACTCGCTTGCTACTCAGAAC

CACGAACACACCCTGCTGTTTTCCCTCGGGTCATGCGATATCTCTGAGGAATACTCGTTT

GCATGCGTCCAGCGCGGCGAGCCCAGACCGACGGTTTTGGAGTTTGACAGCGAGTACGAG

CTGCTGGTTGCCTTCCTGACCTTTCTCAAGCAGTACTCTCCCGAGTTCGCCACCGGCTAC

AACATCGTTAATTTTGACTGGGCGTACATAGTTAACAAGGTAACGTCGGTGTATAACATC

AAGCTGGACGGGTACGGCAAGTTCAACAAAGGGGGGCTGTTTAAGGTGTGGGACATCGCC

ACGAACCATTTTCAGAAGAAGAGCAAGGTGAAAATCAATGGCCTGATATCTCTAGACATG

TATTCTGTGGCGACGGAAAAGCTAAAGCTACCCAGCTACAAACTCGACGCGGTCGTGGGA

GACGTCCTCGGCGAGCATAAGATAGACCTTCCCTATAAAGAAATACCCTCCTATTACGCG

GGAGGGCCTGACCGGAGGGGCGTAATAGGAGAGTATTGTATCCAGGACTCTAGGCTGGTG

GGCAAGCTGTTTTTTAAGTACCTCCCCCATCTGGAACTATCGGCGGTGGCCAAACTCGCC

CGTATCACCCTAACGCGGGTAATTTTTGACGGTCAGCAAATTCGCGTGTACACGTGCTTG

CTGAAACTCGCCCGCGAGAGAAATTTCATTTTGCCAGACAACAGACGCCGGTTTGACAGT

CAGGCAGACGCCGCGTCAGAGACTTCGGAGTTGGCTATGGATAGCCAAAGCCACGCCTTC

GACAGTACAGACGAACCCGACGGTGTGGACGGTACCCCGGACGCCGCAGGATCTGGCGCT

ACTTCTGAAAACGGAGGCGGGAAGCCCGGCGTCGGGAGGGCCGTGGGCTACCAGGGAGCA

AAGGTTCTAGACCCCGTATCCGGCTTTCATGTGGACCCCGTGGTTGTGTTTGACTTCGCT

AGCTTATACCCAAGCATTATCCAGGCCCATAACCTCTGTTTCACCACCCTGGCGCTCGAT

GAAGTGGATCTGGCCGGGCTTCAACCATCCGTCAACTACTCGACGTTCGAGGTGGGTGAC

CAAAAGTTATTTTTTGTCCACGCCCATATTCGCGAAAGCCTGCTTGGCATCTTGCTGCGC

GACTGGCTGGCCATGCGAAAGGCGGTGAGGGCGCGAATCCCCACCAGCACCCCCGAGGAG

GCAGTTTTACTAGATAAGCAGCAGTCTGCGATTAAGGTGATATGCAACTCGGTTTACGGA

TTCACGGGGGTGGCAAACGGCCTGTTGCCGTGTCTGAGGATAGCGGCTACCGTTACCACG

ATAGGACGCGACATGCTCCTCAAGACCCGAGATTACGTTCACTCTCGTTGGGCGACGCGC

GAGCTGCTGGAGGACAATTTTCCAGGGGCTATAGGTTTCCGAAACCACAAGCCTTACTCC

GTCAGGGTTATCTACGGAGACACCGACTCCGTGTTTATCAAGTTTGTGGGCCTGACGTAC

GAGGGGGTATCGGAGCTGGGGGATGCTATGTCGCGTCAGATTTCAGCGGACCTCTTTAGA

GCGCCCATCAAACTGGAGTGTGAGAAGACCTTTCAGCGACTGCTGCTGATCACCAAGAAG

AAGTACATAGGTGTCATAAACGGGGGGAAGATGCTCATGAAGGGGGTCGACCTGGTCCGC

AAAAATAACTGCTCTTTCATAAACTTGTACGCGCGACATCTGGTAGATCTTTTGTTGTAC

GACGAGGATGTGGCCACGGCGGCAGCAGAGGTGACAGACGTGCCTCCCGCAGAATGGGTG

GGGCGCCCGCTACCGAGCGGCTTTGACAAGTTTGGGCGAGTGCTGGTAGAGGCGTACAAC

CGTATCACTGCCCCCAACTTGGACGTGCGCGAGTTCGTTATGACTGCTGAGCTGAGCCGC

TCACCCGAATCGTATACCAACAAGCGCCTGCCGCACCTCACCGTCTACTTTAAGCTCGCC

ATGAGGAATGAAGAACTGCCCAGCGTAAAAGAGAGAATTCCGTATGTGATAGTTGCGCAG

ACCGAGGCCGCGGAACGCGAAGCGGGTGTAGTAAACTCAATGCGCGGTACCGCCCAAAAC

CCCGTGGTAACCAAGACCGCACGCCCCCAACCTAAACGCAAACTGCTGGTTTCCGACCTC

GCCGAAGACCCGACCTATGTTTCCGAGAATGACGTACCGCTAAACACAGACTACTATTTC

TCCCACCTGTTGGGTACCATAAGCGTGACCTTTAAGGCTCTATTCGGAAATGATGTGAGA

ACAACAGAAAATCTTTTAAAGCGGTTTATTCCGGAAACCCCCCACAAGACCCCCACGAAA

ACCCAGGCACTGCTTGAGCGCGCCGGCTTTGAAAAGCTGACGCCCTTTACACCGGAGGAA

GAAAGTCGTCGAATACTGCATACAGTTTTTTGTACTCTAGAAGCAGCTCCCCATCAAAGC

TGA

>LC109653.1

ATGGCGGCGCGCGAACAGGCCAACTCCGTTCGCAGGAGCGGGTTTTTTAATCCGTTTATT

GGCAAGAGGCCATTTTTCAGGCCGGGAAGCGGGCAGACCGCGGAGACAGAGCGCCCAAGG

CCCCCCCAACACTCGTACTGCACAGAGGTGGGTAGCTTTAAGTTTATAGCTCCAAGATGC

CTCGATGAAGAAGCCCCCGCCGACCAGCGACGCGGTGTACACGTGGGCACCTTGGAGCGC

CCACCCAAAGTGTACTGCGATGGCTCAGAGTACGACGTGCTGAACTTTGCCTCCGGAGGT

TGCTGGCCTCGCAGGATTCGGGTTTGGAACGGCCAGGATTTTCGGGGCGATGGATTCAAC

CCCAGATTTGAGAGATTTCACGTGTACGACATAGTAGAGACTTCGGAGAGCGCCTCACAC

GATGACCCATCCAGGTTTGCTGAGCTATCCCGCCCAAGCGGGTCTGTGGTAACACTGCTG

GGAATGAGCGAGTGTGGAAAGCGAGTCGCGGTTCACGTCTATGGTGTGCGCCATTATTTT

TACATGGCAAAGGCGGAGGTGGATAGCGCTTGTGGAATAACCACCGAGGCAGAACTCGTG

CGCGCAATGGTGGACTGCGCGCACAGCTCGGCTTTGAGCGCGGCCCTGGGAAATGGCAAC

GGCGGCAAACAGAGCGGCGGCAGCGGCGGGGGATGGTGGGGCGGAAAGCACGTGTCTGCG

GACTGCTTCAAAGTGGAGACCGTGTGCCACACGACGCTGTACTACTTTGGATCTAAGCCA

GCTCTCTACTATAGAGTATCTGCCTCCAGCAGCCGCCTGGGAGGGTTCATCTGCGACAAC

TTTCACCCGGAGATTACAAAATTCGAGGGGAGCGTGGACGTGACCACGCGGCTGTTGTTG

GACAATGAAAATTTTACCAGTTTCGGGTGGTACCGCCTGCGACCCGGCACCCACGGAGAG

CGTGTTCAACTTCGCCCCGTTGAGCGACACGTCACCTCAAGCGACGTGGAGATTAACTGT

ACTCCCGATAACCTGGAGCCGATACCAGACGAGGCTGCCTGGCCCGACTATAAGCTCATG

TGCTTTGATATAGAGTGTAAAGCTGGAACGGGTAACGAAATGGCGTTCCCAGTGGCAACT

AACCAAGAGGACCTGGTCATCCAGATCTCCTGTCTGCTGTACTCGCTTGCTACTCAGAAC

CACGAACACACCCTGCTGTTTTCCCTCGGGTCATGCGATATCTCTGAGGAATACTCGTTT

GCATGCGTCCAGCGCGGCGAGCCCAGACCGACGGTTTTGGAGTTTGACAGCGAGTACGAG

CTGCTGGTTGCCTTCCTGACCTTTCTCAAGCAGTACTCTCCCGAGTTCGCCACCGGCTAC

AACATCGTTAATTTTGACTGGGCGTACATAGTTAACAAGGTAACGTCGGTGTATAACATC

AAGCTGGACGGGTACGGCAAGTTCAACAAAGGGGGGCTGTTTAAGGTGTGGGACATCGCC

ACGAACCATTTTCAGAAGAAGAGCAAGGTGAAAATCAATGGCCTGATATCTCTAGACATG

TATTCTGTGGCGACGGAAAAGCTAAAGCTACCCAGCTACAAACTCGACGCGGTCGTGGGA

GACGTCCTCGGCGAGCATAAGATAGACCTTCCCTATAAAGAAATACCCTCCTATTACGCG

GGAGGGCCTGACCGGAGGGGCGTAATAGGAGAGTATTGTATCCAGGACTCTAGGCTGGTG

GGCAAGCTGTTTTTTAAGTACCTCCCCCATCTGGAACTATCGGCGGTGGCCAAACTCGCC

CGTATCACCCTAACGCGGGTAATTTTTGACGGTCAGCAAATTCGCGTGTACACGTGCTTG

CTGAAACTCGCCCGCGAGAGAAATTTCATTTTGCCAGACAACAGACGCCGGTTTGACAGT

CAGGCAGACGCCGCGTCAGAGACTTCGGAGTTGGCTATGGATAGCCAAAGCCACGCCTTC

GACAGTACAGACGAACCCGACGGTGTGGACGGTACCCCGGACGCCGCAGGATCTGGCGCT

ACTTCTGAAAACGGAGGCGGGAAGCCCGGCGTCGGGAGGGCCGTGGGCTACCAGGGAGCA

AAGGTTCTAGACCCCGTATCCGGCTTTCATGTGGACCCCGTGGTTGTGTTTGACTTCGCT

AGCTTATACCCAAGCATTATCCAGGCCCATAACCTCTGTTTCACCACCCTGGCGCTCGAT

GAAGTGGATCTGGCCGGGCTTCAACCATCCGTCAACTACTCGACGTTCGAGGTGGGTGAC

CAAAAGTTATTTTTTGTCCACGCCCATATTCGCGAAAGCCTGCTTGGCATCTTGCTGCGC

GACTGGCTGGCCATGCGAAAGGCGGTGAGGGCGCGAATCCCCACCAGCACCCCCGAGGAG

GCAGTTTTACTAGATAAGCAGCAGTCTGCGATTAAGGTGATATGCAACTCGGTTTACGGA

TTCACGGGGGTGGCAAACGGCCTGTTGCCGTGTCTGAGGATAGCGGCTACCGTTACCACG

ATAGGACGCGACATGCTCCTCAAGACCCGAGATTACGTTCACTCTCGTTGGGCGACGCGC

GAGCTGCTGGAGGACAATTTTCCAGGGGCTATAGGTTTCCGAAACCACAAGCCTTACTCC

GTCAGGGTTATCTACGGAGACACCGACTCCGTGTTTATCAAGTTTGTGGGCCTGACGTAC

GAGGGGGTATCGGAGCTGGGGGATGCTATGTCGCGTCAGATTTCAGCGGACCTCTTTAGA

GCCCCCATCAAACTGGAGTGTGAGAAGACCTTTCAGCGACTGCTGCTGATCACCAAGAAG

AAGTACATAGGTGTCATAAACGGGGGGAAGATGCTCATGAAGGGGGTCGACCTGGTCCGC

AAAAATAACTGCTCTTTCATAAACTTGTACGCGCGACATCTGGTAGATCTTTTGTTGTAC

GACGAGGATGTGGCCACGGCGGCAGCAGAGGTGACAGACGTGCCTCCCGCAGAATGGGTG

GGGCGCCCGCTACCGAGCGGCTTTGACAAGTTTGGGCGAGTGCTGGTAGAGGCGTACAAC

CGTATCACTGCCCCCAACTTGGACGTGCGCGAGTTCGTTATGACTGCTGAGCTGAGCCGC

TCACCCGAATCGTATACCAACAAGCGCCTGCCGCACCTCACCGTCTACTTTAAGCTCGCC

ATGAGGAATGAAGAACTGCCCAGCGTAAAAGAGAGAATTCCGTATGTGATAGTTGCGCAG

ACCGAGGCCGCGGAACGCGAAGCGGGTGTAGTAAACTCAATGCGCGGTACCGCCCAAAAC

CCCGTGGTAACCAAGACCGCACGCCCCCAACCTAAACGCAAACTGCTGGTTTCCGACCTC

GCCGAAGACCCGACCTATGTTTCCGAGAATGACGTACCGCTAAACACAGACTACTATTTC

TCCCACCTGTTGGGTACCATAAGCGTGACCTTTAAGGCTCTATTCGGAAATGATGTGAGA

ACAACAGAAAATCTTTTAAAGCGGTTTATTCCGGAAACCCCCCACAAGACCCCCACGAAA

ACCCAGGCACTGCTTGAGCGCGCCGGCTTTGAAAAGCTGACGCCCTTTACACCGGAGGAA

GAAAGTCGTCGAATACTGCATACAGTTTTTTGTACTCTAGAAGCAGCTCCCCATCAAAGC

TGA

>LC109654.1

ATGGCGGCGCGCGAACAGGCCAACTCCGTTCGCAGGAGCGGGTTTTTTAATCCGTTTATT

GGCAAGAGGCCATTTTTCAGGCCGGGAAGCGGGCAGACCGCGGAGACAGAGCGCCCAAGG

CCCCCCCAACACTCGTACTGCACAGAGGTGGGTAGCTTTAAGTTTATAGCTCCAAGATGC

CTCGATGAAGAAGCCCCCGCCGACCAGCGACGCGGTGTACACGTGGGCACCTTGGAGCGC

CCACCCAAAGTGTACTGCGATGGCTCAGAGTACGACGTGCTGAACTTTGCCTCCGGAGGT

TGCTGGCCTCGCAGGATTCGGGTTTGGAACGGCCAGGATTTTCGGGGCGATGGATTCAAC

CCCAGATTTGAGAGATTTCACGTGTACGACATAGTAGAGACTTCGGAGAGCGCCTCACAC

GATGACCCATCCAGGTTTGCTGAGCTATCCCGCCCAAGCGGGTCTGTGGTAACACTGCTG

GGAATGAGCGAGTGTGGAAAGCGAGTCGCGGTTCACGTCTATGGTGTGCGCCATTATTTT

TACATGGCAAAGGCGGAGGTGGATAGCGCTTGTGGAATAACCACCGAGGCAGAACTCGTG

CGCGCAATGGTGGACTGCGCGCACAGCTCGGCTTTGAGCGCGGCCCTGGGAAATGGCAAC

GGCGGCAAACAGAGCGGCGGCAGCGGCGGGGGATGGTGGGGCGGAAAGCACGTGTCTGCG

GACTGCTTCAAAGTGGAGACCGTGTGCCACACGACGCTGTACTACTTTGGATCTAAGCCA

GCTCTCTACTATAGAGTATCTGCCTCCAGCAGCCGCCTGGGAGGGTTCATCTGCGACAAC

TTTCACCCGGAGATTACAAAATTCGAGGGGAGCGTGGACGTGACCACGCGGCTGTTGTTG

GACAATGAAAATTTTACCAGTTTCGGGTGGTACCGCCTGCGACCCGGCACCCACGGAGAG

CGTGTTCAACTTCGCCCCGTTGAGCGACACGTCACCTCAAGCGACGTGGAGATTAACTGT

ACTCCCGATAACCTGGAGCCGATACCAGACGAGGCTGCCTGGCCCGACTATAAGCTCATG

TGCTTTGATATAGAGTGTAAAGCTGGAACGGGTAACGAAATGGCGTTCCCAGTGGCAACT

AACCAAGAGGACCTGGTCATCCAGATCTCCTGTCTGCTGTACTCGCTTGCTACTCAGAAC

CACGAACACACCCTGCTGTTTTCCCTCGGGTCATGCGATATCTCTGAGGAATACTCGTTT

GCATGCGTCCAGCGCGGCGAGCCCAGACCGACGGTTTTGGAGTTTGACAGCGAGTACGAG

CTGCTGGTTGCCTTCCTGACCTTTCTCAAGCAGTACTCTCCCGAGTTCGCCACCGGCTAC

AACATCGTTAATTTTGACTGGGCGTACATAGTTAACAAGGTAACGTCGGTGTATAACATC

AAGCTGGACGGGTACGGCAAGTTCAACAAAGGGGGGCTGTTTAAGGTGTGGGACATCGCC

ACGAACCATTTTCAGAAGAAGAGCAAGGTGAAAATCAATGGCCTGATATCTCTAGACATG

TATTCTGTGGCGACGGAAAAGCTAAAGCTACCCAGCTACAAACTCGACGCGGTCGTGGGA

GACGTCCTCGGCGAGCATAAGATAGACCTTCCCTATAAAGAAATACCCTCCTATTACGCG

GGAGGGCCTGACCGGAGGGGCGTAATAGGAGAGTATTGTATCCAGGACTCTAGGCTGGTG

GGCAAGCTGTTTTTTAAGTACCTCCCCCATCTGGAACTATCGGCGGTGGCCAAACTCGCC

CGTATCACCCTAACGCGGGTAATTTTTGACGGTCAGCAAATTCGCGTGTACACGTGCTTG

CTGAAACTCGCCCGCGAGAGAAATTTCATTTTGCCAGACAACAGACGCCGGTTTGACAGT

CAGGCAGACGCCGCGTCAGAGACTTCGGAGTTGGCTATGGATAGCCAAAGCCACGCCTTC

GACAGTACAGACGAACCCGACGGTGTGGACGGTACCCCGGACGCCGCAGGATCTGGCGCT

ACTTCTGAAAACGGAGGCGGGAAGCCCGGCGTCGGGAGGGCCGTGGGCTACCAGGGAGCA

AAGGTTCTAGACCCCGTATCCGGCTTTCATGTGGACCCCGTGGTTGTGTTTGACTTCGCT

AGCTTATACCCAAGCATTATCCAGGCCCATAACCTCTGTTTCACCACCCTGGCGCTCGAT

GAAGTGGATCTGGCCGGGCTTCAACCATCCGTCAACTACTCGACGTTCGAGGTGGGTGAC

CAAAAGTTATTTTTTGTCCACGCCCATATTCGCGAAAGCCTGCTTGGCATCTTGCTGCGC

GACTGGCTGGCCATGCGAAAGGCGGTGAGGGCGCGAATCCCCACCAGCACCCCCGAGGAG

GCAGTTTTACTAGATAAGCAGCAGTCTGCGATTAAGGTGATATGCAACTCGGTTTACGGA

TTCACGGGGGTGGCAAACGGCCTGTTGCCGTGTCTGAGGATAGCGGCTACCGTTACCACG

ATAGGACGCGACATGCTCCTCAAGACCCGAGATTACGTTCACTCTCGTTGGGCGACGCGC

GAGCTGCTGGAGGACAATTTTCCAGGGGCTATAGGTTTCCGAAACCACAAGCCTTACTCC

GTCAGGGTTATCTACGGAGACACCGACTCCGTGTTTATCAAGTTTGTGGGCCTGACGTAC

GAGGGGGTATCGGAGCTGGGGGATGCTATGTCGCGTCAGATTTCAGCGGACCTCTTTAGA

GCGCCCATCAAACTGGAGTGTGAGAAGACCTTTCAGCGACTGCTGCTGATCACCAAGAAG

AAGTACATAGGTGTCATAAACGGGGGGAAGATGCTCATGAAGGGGGTCGACCTGGTCCGC

AAAAATAACTGCTCTTTCATAAACTTGTACGCGCGACATCTGGTAGATCTTTTGTTGTAC

GACGAGGATGTGGCCACGGCGGCAGCAGAGGTGACAGACGTGCCTCCCGCAGAATGGGTG

GGGCGCCCGCTACCGAGCGGCTTTGACAAGTTTGGGCGAGTGCTGGTAGAGGCGTACAAC

CGTATCACTGCCCCCAACTTGGACGTGCGCGAGTTCGTTATGACTGCTGAGCTGAGCCGC

TCACCCGAATCGTATACCAACAAGCGCCTGCCGCACCTCACCGTCTACTTTAAGCTCGCC

ATGAGGAATGAAGAACTGCCCAGCGTAAAAGAGAGAATTCCGTATGTGATAGTTGCGCAG

ACCGAGGCCGCGGAACGCGAAGCGGGTGTAGTAAACTCAATGCGCGGTACCGCCCAAAAC

CCCGTGGTAACCAAGACCGCACGCCCCCAACCTAAACGCAAACTGCTGGTTTCCGACCTC

GCCGAAGACCCGACCTATGTTTCCGAGAATGACGTACCGCTAAACACAGACTACTATTTC

TCCCACCTGTTGGGTACCATAAGCGTGACCTTTAAGGCTCTATTCGGAAATGATGTGAGA

ACAACAGAAAATCTTTTAAAGCGGTTTATTCCGGAAACCCCCCACAAGACCCCCACGAAA

ACCCAGGCACTGCTTGAGCGCGCCGGCTTTGAAAAGCTGACGCCCTTTACACCGGAGGAA

GAAAGTCGTCGAATACTGCATACAGTTTTTTGTACTCTAGAAGCAGCTCCCCATCAAAGC

TGA

>LC109655.1

ATGGCGGCGCGCGAACAGGCCAACTCCGTTCGCAGGAGCGGGTTTTTTAATCCGTTTATT

GGCAAGAGGCCATTTTTCAGGCCGGGAAGCGGGCAGACCGCGGAGACAGAGCGCCCAAGG

CCCCCCCAACACTCGTACTGCACAGAGGTGGGTAGCTTTAAGTTTATAGCTCCAAGATGC

CTCGATGAAGAAGCCCCCGCCGACCAGCGACGCGGTGTACACGTGGGCACCTTGGAGCGC

CCACCCAAAGTGTACTGCGATGGCTCAGAGTACGACGTGCTGAACTTTGCCTCCGGAGGT

TGCTGGCCTCGCAGGATTCGGGTTTGGAACGGCCAGGATTTTCGGGGCGATGGATTCAAC

CCCAGATTTGAGAGATTTCACGTGTACGACATAGTAGAGACTTCGGAGAGCGCCTCACAC

GATGACCCATCCAGGTTTGCTGAGCTATCCCGCCCAAGCGGGTCTGTGGTAACACTGCTG

GGAATGAGCGAGTGTGGAAAGCGAGTCGCGGTTCACGTCTATGGTGTGCGCCATTATTTT

TACATGGCAAAGGCGGAGGTGGATAGCGCTTGTGGAATAACCACCGAGGCAGAACTCGTG

CGCGCAATGGTGGACTGCGCGCACAGCTCGGCTTTGAGCGCGGCCCTGGGAAATGGCAAC

GGCGGCAAACAGAGCGGCGGCAGCGGCGGGGGATGGTGGGGCGGAAAGCACGTGTCTGCG

GACTGCTTCAAAGTGGAGACCGTGTGCCACACGACGCTGTACTACTTTGGATCTAAGCCA

GCTCTCTACTATAGAGTATCTGCCTCCAGCAGCCGCCTGGGAGGGTTCATCTGCGACAAC

TTTCACCCGGAGATTACAAAATTCGAGGGGAGCGTGGACGTGACCACGCGGCTGTTGTTG

GACAATGAAAATTTTACCAGTTTCGGGTGGTACCGCCTGCGACCCGGCACCCACGGAGAG

CGTGTTCAACTTCGCCCCGTTGAGCGACACGTCACCTCAAGCGACGTGGAGATTAACTGT

ACTCCCGATAACCTGGAGCCGATACCAGACGAGGCTGCCTGGCCCGACTATAAGCTCATG

TGCTTTGATATAGAGTGTAAAGCTGGAACGGGTAACGAAATGGCGTTCCCAGTGGCAACT

AACCAAGAGGACCTGGTCATCCAGATCTCCTGTCTGCTGTACTCGCTTGCTACTCAGAAC

CACGAACACACCCTGCTGTTTTCCCTCGGGTCATGCGATATCTCTGAGGAATACTCGTTT

GCATGCGTCCAGCGCGGCGAGCCCAGACCGACGGTTTTGGAGTTTGACAGCGAGTACGAG

CTGCTGGTTGCCTTCCTGACCTTTCTCAAGCAGTACTCTCCCGAGTTCGCCACCGGCTAC

AACATCGTTAATTTTGACTGGGCGTACATAGTTAACAAGGTAACGTCGGTGTATAACATC

AAGCTGGACGGGTACGGCAAGTTCAACAAAGGGGGGCTGTTTAAGGTGTGGGACATCGCC

ACGAACCATTTTCAGAAGAAGAGCAAGGTGAAAATCAATGGCCTGATATCTCTAGACATG

TATTCTGTGGCGACGGAAAAGCTAAAGCTACCCAGCTACAAACTCGACGCGGTCGTGGGA

GACGTCCTCGGCGAGCATAAGATAGACCTTCCCTATAAAGAAATACCCTCCTATTACGCG

GGAGGGCCTGACCGGAGGGGCGTAATAGGAGAGTATTGTATCCAGGACTCTAGGCTGGTG

GGCAAGCTGTTTTTTAAGTACCTCCCCCATCTGGAACTATCGGCGGTGGCCAAACTCGCC

CGTATCACCCTAACGCGGGTAATTTTTGACGGTCAGCAAATTCGCGTGTACACGTGCTTG

CTGAAACTCGCCCGCGAGAGAAATTTCATTTTGCCAGACAACAGACGCCGGTTTGACAGT

CAGGCAGACGCCGCGTCAGAGACTTCGGAGTTGGCTATGGATAGCCAAAGCCACGCCTTC

GACAGTACAGACGAACCCGACGGTGTGGACGGTACCCCGGACGCCGCAGGATCTGGCGCT

ACTTCTGAAAACGGAGGCGGGAAGCCCGGCGTCGGGAGGGCCGTGGGCTACCAGGGAGCA

AAGGTTCTAGACCCCGTATCCGGCTTTCATGTGGACCCCGTGGTTGTGTTTGACTTCGCT

AGCTTATACCCAAGCATTATCCAGGCCCATAACCTCTGTTTCACCACCCTGGCGCTCGAT

GAAGTGGATCTGGCCGGGCTTCAACCATCCGTCAACTACTCGACGTTCGAGGTGGGTGAC

CAAAAGTTATTTTTTGTCCACGCCCATATTCGCGAAAGCCTGCTTGGCATCTTGCTGCGC

GACTGGCTGGCCATGCGAAAGGCGGTGAGGGCGCGAATCCCCACCAGCACCCCCGAGGAG

GCAGTTTTACTAGATAAGCAGCAGTCTGCGATTAAGGTGATATGCAACTCGGTTTACGGA

TTCACGGGGGTGGCAAACGGCCTGTTGCCGTGTCTGAGGATAGCGGCTACCGTTACCACG

ATAGGACGCGACATGCTCCTCAAGACCCGAGATTACGTTCACTCTCGTTGGGCGACGCGC

GAGCTGCTGGAGGACAATTTTCCAGGGGCTATAGGTTTCCGAAACCACAAGCCTTACTCC

GTCAGGGTTATCTACGGAGACACCGACTCCGTGTTTATCAAGTTTGTGGGCCTGACGTAC

GAGGGGGTATCGGAGCTGGGGGATGCTATGTCGCGTCAGATTTCAGCGGACCTCTTTAGA

GCCCCCATCAAACTGGAGTGTGAGAAGACCTTTCAGCGACTGCTGCTGATCACCAAGAAG

AAGTACATAGGTGTCATAAACGGGGGGAAGATGCTCATGAAGGGGGTCGACCTGGTCCGC

AAAAATAACTGCTCTTTCATAAACTTGTACGCGCGACATCTGGTAGATCTTTTGTTGTAC

GACGAGGATGTGGCCACGGCGGCAGCAGAGGTGACAGACGTGCCTCCCGCAGAATGGGTG

GGGCGCCCGCTACCGAGCGGCTTTGACAAGTTTGGGCGAGTGCTGGTAGAGGCGTACAAC

CGTATCACTGCCCCCAACTTGGACGTGCGCGAGTTCGTTATGACTGCTGAGCTGAGCCGC

TCACCCGAATCGTATACCAACAAGCGCCTGCCGCACCTCACCGTCTACTTTAAGCTCGCC

ATGAGGAATGAAGAACTGCCCAGCGTAAAAGAGAGAATTCCGTATGTGATAGTTGCGCAG

ACCGAGGCCGCGGAACGCGAAGCGGGTGTAGTAAACTCAATGCGCGGTACCGCCCAAAAC

CCCGTGGTAACCAAGACCGCACGCCCCCAACCTAAACGCAAACTGCTGGTTTCCGACCTC

GCCGAAGACCCGACCTATGTTTCCGAGAATGACGTACCGCTAAACACAGACTACTATTTC

TCCCACCTGTTGGGTACCATAAGCGTGACCTTTAAGGCTCTATTCGGAAATGATGTGAGA

ACAACAGAAAATCTTTTAAAGCGGTTTATTCCGGAAACCCCCCACAAGACCCCCACGAAA

ACCCAGGCACTGCTTGAGCGCGCCGGCTTTGAAAAGCTGACGCCCTTTACACCGGAGGAA

GAAAGTCGTCGAATACTGCATACAGTTTTTTGTACTCTAGAAGCAGCTCCCCATCAAAGC

TGA

>LC109656.1

ATGGCGGCGCGCGAACAGGCCAACTCCGTTCGCAGGAGCGGGTTTTTTAATCCGTTTATT

GGCAAGAGGCCATTTTTCAGGCCGGGAAGCGGGCAGACCGCGGAGACAGAGCGCCCAAGG

CCCCCCCAACACTCGTACTGCACAGAGGTGGGTAGCTTTAAGTTTATAGCTCCAAGATGC

CTCGATGAAGAAGCCCCCGCCGACCAGCGACGCGGTGTACACGTGGGCACCTTGGAGCGC

CCACCCAAAGTGTACTGCGATGGCTCAGAGTACGACGTGCTGAACTTTGCCTCCGGAGGT

TGCTGGCCTCGCAGGATTCGGGTTTGGAACGGCCAGGATTTTCGGGGCGATGGATTCAAC

CCCAGATTTGAGAGATTTCACGTGTACGACATAGTAGAGACTTCGGAGAGCGCCTCACAC

GATGACCCATCCAGGTTTGCTGAGCTATCCCGCCCAAGCGGGTCTGTGGTAACACTGCTG

GGAATGAGCGAGTGTGGAAAGCGAGTCGCGGTTCACGTCTATGGTGTGCGCCATTATTTT

TACATGGCAAAGGCGGAGGTGGATAGCGCTTGTGGAATAACCACCGAGGCAGAACTCGTG

CGCGCAATGGTGGACTGCGCGCACAGCTCGGCTTTGAGCGCGGCCCTGGGAAATGGCAAC

GGCGGCAAACAGAGCGGCGGCAGCGGCGGGGGATGGTGGGGCGGAAAGCACGTGTCTGCG

GACTGCTTCAAAGTGGAGACCGTGTGCCACACGACGCTGTACTACTTTGGATCTAAGCCA

GCTCTCTACTATAGAGTATCTGCCTCCAGCAGCCGCCTGGGAGGGTTCATCTGCGACAAC

TTTCACCCGGAGATTACAAAATTCGAGGGGAGCGTGGACGTGACCACGCGGCTGTTGTTG

GACAATGAAAATTTTACCAGTTTCGGGTGGTACCGCCTGCGACCCGGCACCCACGGAGAG

CGTGTTCAACTTCGCCCCGTTGAGCGACACGTCACCTCAAGCGACGTGGAGATTAACTGT

ACTCCCGATAACCTGGAGCCGATACCAGACGAGGCTGCCTGGCCCGACTATAAGCTCATG

TGCTTTGATATAGAGTGTAAAGCTGGAACGGGTAACGAAATGGCGTTCCCAGTGGCAACT

AACCAAGAGGACCTGGTCATCCAGATCTCCTGTCTGCTGTACTCGCTTGCTACTCAGAAC

CACGAACACACCCTGCTGTTTTCCCTCGGGTCATGCGATATCTCTGAGGAATACTCGTTT

GCATGCGTCCAGCGCGGCGAGCCCAGACCGACGGTTTTGGAGTTTGACAGCGAGTACGAG

CTGCTGGTTGCCTTCCTGACCTTTCTCAAGCAGTACTCTCCCGAGTTCGCCACCGGCTAC

AACATCGTTAATTTTGACTGGGCGTACATAGTTAACAAGGTAACGTCGGTGTATAACATC

AAGCTGGACGGGTACGGCAAGTTCAACAAAGGGGGGCTGTTTAAGGTGTGGGACATCGCC

ACGAACCATTTTCAGAAGAAGAGCAAGGTGAAAATCAATGGCCTGATATCTCTAGACATG

TATTCTGTGGCGACGGAAAAGCTAAAGCTACCCAGCTACAAACTCGACGCGGTCGTGGGA

GACGTCCTCGGCGAGCATAAGATAGACCTTCCCTATAAAGAAATACCCTCCTATTACGCG

GGAGGGCCTGACCGGAGGGGCGTAATAGGAGAGTATTGTATCCAGGACTCTAGGCTGGTG

GGCAAGCTGTTTTTTAAGTACCTCCCCCATCTGGAACTATCGGCGGTGGCCAAACTCGCC

CGTATCACCCTAACGCGGGTAATTTTTGACGGTCAGCAAATTCGCGTGTACACGTGCTTG

CTGAAACTCGCCCGCGAGAGAAATTTCATTTTGCCAGACAACAGACGCCGGTTTGACAGT

CAGGCAGACGCCGCGTCAGAGACTTCGGAGTTGGCTATGGATAGCCAAAGCCACGCCTTC

GACAGTACAGACGAACCCGACGGTGTGGACGGTACCCCGGACGCCGCAGGATCTGGCGCT

ACTTCTGAAAACGGAGGCGGGAAGCCCGGCGTCGGGAGGGCCGTGGGCTACCAGGGAGCA

AAGGTTCTAGACCCCGTATCCGGCTTTCATGTGGACCCCGTGGTTGTGTTTGACTTCGCT

AGCTTATACCCAAGCATTATCCAGGCCCATAACCTCTGTTTCACCACCCTGGCGCTCGAT

GAAGTGGATCTGGCCGGGCTTCAACCATCCGTCAACTACTCGACGTTCGAGGTGGGTGAC

CAAAAGTTATTTTTTGTCCACGCCCATATTCGCGAAAGCCTGCTTGGCATCTTGCTGCGC

GACTGGCTGGCCATGCGAAAGGCGGTGAGGGCGCGAATCCCCACCAGCACCCCCGAGGAG

GCAGTTTTACTAGATAAGCAGCAGTCTGCGATTAAGGTGATATGCAACTCGGTTTACGGA

TTCACGGGGGTGGCAAACGGCCTGTTGCCGTGTCTGAGGATAGCGGCTACCGTTACCACG

ATAGGACGCGACATGCTCCTCAAGACCCGAGATTACGTTCACTCTCGTTGGGCGACGCGC

GAGCTGCTGGAGGACAATTTTCCAGGGGCTATAGGTTTCCGAAACCACAAGCCTTACTCC

GTCAGGGTTATCTACGGAGACACCGACTCCGTGTTTATCAAGTTTGTGGGCCTGACGTAC

GAGGGGGTATCGGAGCTGGGGGATGCTATGTCGCGTCAGATTTCAGCGGACCTCTTTAGA

GCGCCCATCAAACTGGAGTGTGAGAAGACCTTTCAGCGACTGCTGCTGATCACCAAGAAG

AAGTACATAGGTGTCATAAACGGGGGGAAGATGCTCATGAAGGGGGTCGACCTGGTCCGC

AAAAATAACTGCTCTTTCATAAACTTGTACGCGCGACATCTGGTAGATCTTTTGTTGTAC

GACGAGGATGTGGCCACGGCGGCAGCAGAGGTGACAGACGTGCCTCCCGCAGAATGGGTG

GGGCGCCCGCTACCGAGCGGCTTTGACAAGTTTGGGCGAGTGCTGGTAGAGGCGTACAAC

CGTATCACTGCCCCCAACTTGGACGTGCGCGAGTTCGTTATGACTGCTGAGCTGAGCCGC

TCACCCGAATCGTATACCAACAAGCGCCTGCCGCACCTCACCGTCTACTTTAAGCTCGCC

ATGAGGAATGAAGAACTGCCCAGCGTAAAAGAGAGAATTCCGTATGTGATAGTTGCGCAG

ACCGAGGCCGCGGAACGCGAAGCGGGTGTAGTAAACTCAATGCGCGGTACCGCCCAAAAC

CCCGTGGTAACCAAGACCGCACGCCCCCAACCTAAACGCAAACTGCTGGTTTCCGACCTC

GCCGAAGACCCGACCTATGTTTCCGAGAATGACGTACCGCTAAACACAGACTACTATTTC

TCCCACCTGTTGGGTACCATAAGCGTGACCTTTAAGGCTCTATTCGGAAATGATGTGAGA

ACAACAGAAAATCTTTTAAAGCGGTTTATTCCGGAAACCCCCCACAAGACCCCCACGAAA

ACCCAGGCACTGCTTGAGCGCGCCGGCTTTGAAAAGCTGACGCCCTTTACACCGGAGGAA

GAAAGTCGTCGAATACTGCATACAGTTTTTTGTACTCTAGAAGCAGCTCCCCATCAAAGC

TGA

>LC109657.1

ATGGCGGCGCGCGAACAGGCCAACTCCGTTCGCAGGAGCGGGTTTTTTAATCCGTTTATT

GGCAAGAGGCCATTTTTCAGGCCGGGAAGCGGGCAGACCGCGGAGACAGAGCGCCCAAGG

CCCCCCCAACACTCGTACTGCACAGAGGTGGGTAGCTTTAAGTTTATAGCTCCAAGATGC

CTCGATGAAGAAGCCCCCGCCGACCAGCGACGCGGTGTACACGTGGGCACCTTGGAGCGC

CCACCCAAAGTGTACTGCGATGGCTCAGAGTACGACGTGCTGAACTTTGCCTCCGGAGGT

TGCTGGCCTCGCAGGATTCGGGTTTGGAACGGCCAGGATTTTCGGGGCGATGGATTCAAC

CCCAGATTTGAGAGATTTCACGTGTACGACATAGTAGAGACTTCGGAGAGCGCCTCACAC

GATGACCCATCCAGGTTTGCTGAGCTATCCCGCCCAAGCGGGTCTGTGGTAACACTGCTG

GGAATGAGCGAGTGTGGAAAGCGAGTCGCGGTTCACGTCTATGGTGTGCGCCATTATTTT

TACATGGCAAAGGCGGAGGTGGATAGCGCTTGTGGAATAACCACCGAGGCAGAACTCGTG

CGCGCAATGGTGGACTGCGCGCACAGCTCGGCTTTGAGCGCGGCCCTGGGAAATGGCAAC

GGCGGCAAACAGAGCGGCGGCAGCGGCGGGGGATGGTGGGGCGGAAAGCACGTGTCTGCG

GACTGCTTCAAAGTGGAGACCGTGTGCCACACGACGCTGTACTACTTTGGATCTAAGCCA

GCTCTCTACTATAGAGTATCTGCCTCCAGCAGCCGCCTGGGAGGGTTCATCTGCGACAAC

TTTCACCCGGAGATTACAAAATTCGAGGGGAGCGTGGACGTGACCACGCGGCTGTTGTTG

GACAATGAAAATTTTACCAGTTTCGGGTGGTACCGCCTGCGACCCGGCACCCACGGAGAG

CGTGTTCAACTTCGCCCCGTTGAGCGACACGTCACCTCAAGCGACGTGGAGATTAACTGT

ACTCCCGATAACCTGGAGCCGATACCAGACGAGGCTGCCTGGCCCGACTATAAGCTCATG

TGCTTTGATATAGAGTGTAAAGCTGGAACGGGTAACGAAATGGCGTTCCCAGTGGCAACT

AACCAAGAGGACCTGGTCATCCAGATCTCCTGTCTGCTGTACTCGCTTGCTACTCAGAAC

CACGAACACACCCTGCTGTTTTCCCTCGGGTCATGCGATATCTCTGAGGAATACTCGTTT

GCATGCGTCCAGCGCGGCGAGCCCAGACCGACGGTTTTGGAGTTTGACAGCGAGTACGAG

CTGCTGGTTGCCTTCCTGACCTTTCTCAAGCAGTACTCTCCCGAGTTCGCCACCGGCTAC

AACATCGTTAATTTTGACTGGGCGTACATAGTTAACAAGGTAACGTCGGTGTATAACATC

AAGCTGGACGGGTACGGCAAGTTCAACAAAGGGGGGCTGTTTAAGGTGTGGGACATCGCC

ACGAACCATTTTCAGAAGAAGAGCAAGGTGAAAATCAATGGCCTGATATCTCTAGACATG

TATTCTGTGGCGACGGAAAAGCTAAAGCTACCCAGCTACAAACTCGACGCGGTCGTGGGA

GACGTCCTCGGCGAGCATAAGATAGACCTTCCCTATAAAGAAATACCCTCCTATTACGCG

GGAGGGCCTGACCGGAGGGGCGTAATAGGAGAGTATTGTATCCAGGACTCTAGGCTGGTG

GGCAAGCTGTTTTTTAAGTACCTCCCCCATCTGGAACTATCGGCGGTGGCCAAACTCGCC

CGTATCACCCTAACGCGGGTAATTTTTGACGGTCAGCAAATTCGCGTGTACACGTGCTTG

CTGAAACTCGCCCGCGAGAGAAATTTCATTTTGCCAGACAACAGACGCCGGTTTGACAGT

CAGGCAGACGCCGCGTCAGAGACTTCGGAGTTGGCTATGGATAGCCAAAGCCACGCCTTC

GACAGTACAGACGAACCCGACGGTGTGGACGGTACCCCGGACGCCGCAGGATCTGGCGCT

ACTTCTGAAAACGGAGGCGGGAAGCCCGGCGTCGGGAGGGCCGTGGGCTACCAGGGAGCA

AAGGTTCTAGACCCCGTATCCGGCTTTCATGTGGACCCCGTGGTTGTGTTTGACTTCGCT

AGCTTATACCCAAGCATTATCCAGGCCCATAACCTCTGTTTCACCACCCTGGCGCTCGAT

GAAGTGGATCTGGCCGGGCTTCAACCATCCGTCAACTACTCGACGTTCGAGGTGGGTGAC

CAAAAGTTATTTTTTGTCCACGCCCATATTCGCGAAAGCCTGCTTGGCATCTTGCTGCGC

GACTGGCTGGCCATGCGAAAGGCGGTGAGGGCGCGAATCCCCACCAGCACCCCCGAGGAG

GCAGTTTTACTAGATAAGCAGCAGTCTGCGATTAAGGTGATATGCAACTCGGTTTACGGA

TTCACGGGGGTGGCAAACGGCCTGTTGCCGTGTCTGAGGATAGCGGCTACCGTTACCACG

ATAGGACGCGACATGCTCCTCAAGACCCGAGATTACGTTCACTCTCGTTGGGCGACGCGC

GAGCTGCTGGAGGACAATTTTCCAGGGGCTATAGGTTTCCGAAACCACAAGCCTTACTCC

GTCAGGGTTATCTACGGAGACACCGACTCCGTGTTTATCAAGTTTGTGGGCCTGACGTAC

GAGGGGGTATCGGAGCTGGGGGATGCTATGTCGCGTCAGATTTCAGCGGACCTCTTTAGA

GCCCCCATCAAACTGGAGTGTGAGAAGACCTTTCAGCGACTGCTGCTGATCACCAAGAAG

AAGTACATAGGTGTCATAAACGGGGGGAAGATGCTCATGAAGGGGGTCGACCTGGTCCGC

AAAAATAACTGCTCTTTCATAAACTTGTACGCGCGACATCTGGTAGATCTTTTGTTGTAC

GACGAGGATGTGGCCACGGCGGCAGCAGAGGTGACAGACGTGCCTCCCGCAGAATGGGTG

GGGCGCCCGCTACCGAGCGGCTTTGACAAGTTTGGGCGAGTGCTGGTAGAGGCGTACAAC

CGTATCACTGCCCCCAACTTGGACGTGCGCGAGTTCGTTATGACTGCTGAGCTGAGCCGC

TCACCCGAATCGTATACCAACAAGCGCCTGCCGCACCTCACCGTCTACTTTAAGCTCGCC

ATGAGGAATGAAGAACTGCCCAGCGTAAAAGAGAGAATTCCGTATGTGATAGTTGCGCAG

ACCGAGGCCGCGGAACGCGAAGCGGGTGTAGTAAACTCAATGCGCGGTACCGCCCAAAAC

CCCGTGGTAACCAAGACCGCACGCCCCCAACCTAAACGCAAACTGCTGGTTTCCGACCTC

GCCGAAGACCCGACCTATGTTTCCGAGAATGACGTACCGCTAAACACAGACTACTATTTC

TCCCACCTGTTGGGTACCATAAGCGTGACCTTTAAGGCTCTATTCGGAAATGATGTGAGA

ACAACAGAAAATCTTTTAAAGCGGTTTATTCCGGAAACCCCCCACAAGACCCCCACGAAA

ACCCAGGCACTGCTTGAGCGCGCCGGCTTTGAAAAGCTGACGCCCTTTACACCGGAGGAA

GAAAGTCGTCGAATACTGCATACAGTTTTTTGTACTCTAGAAGCAGCTCCCCATCAAAGC

TGA

>LC109658.1

ATGGCGGCGCGCGAACAGGCCAACTCCGTTCGCAGGAGCGGGTTTTTTAATCCGTTTATT

GGCAAGAGGCCATTTTTCAGGCCGGGAAGCGGGCAGACCGCGGAGACAGAGCGCCCAAGG

CCCCCCCAACACTCGTACTGCACAGAGGTGGGTAGCTTTAAGTTTATAGCTCCAAGATGC

CTCGATGAAGAAGCCCCCGCCGACCAGCGACGCGGTGTACACGTGGGCACCTTGGAGCGC

CCACCCAAAGTGTACTGCGATGGCTCAGAGTACGACGTGCTGAACTTTGCCTCCGGAGGT

TGCTGGCCTCGCAGGATTCGGGTTTGGAACGGCCAGGATTTTCGGGGCGATGGATTCAAC

CCCAGATTTGAGAGATTTCACGTGTACGACATAGTAGAGACTTCGGAGAGCGCCTCACAC

GATGACCCATCCAGGTTTGCTGAGCTATCCCGCCCAAGCGGGTCTGTGGTAACACTGCTG

GGAATGAGCGAGTGTGGAAAGCGAGTCGCGGTTCACGTCTATGGTGTGCGCCATTATTTT

TACATGGCAAAGGCGGAGGTGGATAGCGCTTGTGGAATAACCACCGAGGCAGAACTCGTG

CGCGCAATGGTGGACTGCGCGCACAGCTCGGCTTTGAGCGCGGCCCTGGGAAATGGCAAC

GGCGGCAAACAGAGCGGCGGCAGCGGCGGGGGATGGTGGGGCGGAAAGCACGTGTCTGCG

GACTGCTTCAAAGTGGAGACCGTGTGCCACACGACGCTGTACTACTTTGGATCTAAGCCA

GCTCTCTACTATAGAGTATCTGCCTCCAGCAGCCGCCTGGGAGGGTTCATCTGCGACAAC

TTTCACCCGGAGATTACAAAATTCGAGGGGAGCGTGGACGTGACCACGCGGCTGTTGTTG

GACAATGAAAATTTTACCAGTTTCGGGTGGTACCGCCTGCGACCCGGCACCCACGGAGAG

CGTGTTCAACTTCGCCCCGTTGAGCGACACGTCACCTCAAGCGACGTGGAGATTAACTGT

ACTCCCGATAACCTGGAGCCGATACCAGACGAGGCTGCCTGGCCCGACTATAAGCTCATG

TGCTTTGATATAGAGTGTAAAGCTGGAACGGGTAACGAAATGGCGTTCCCAGTGGCAACT

AACCAAGAGGACCTGGTCATCCAGATCTCCTGTCTGCTGTACTCGCTTGCTACTCAGAAC

CACGAACACACCCTGCTGTTTTCCCTCGGGTCATGCGATATCTCTGAGGAATACTCGTTT

GCATGCGTCCAGCGCGGCGAGCCCAGACCGACGGTTTTGGAGTTTGACAGCGAGTACGAG

CTGCTGGTTGCCTTCCTGACCTTTCTCAAGCAGTACTCTCCCGAGTTCGCCACCGGCTAC

AACATCGTTAATTTTGACTGGGCGTACATAGTTAACAAGGTAACGTCGGTGTATAACATC

AAGCTGGACGGGTACGGCAAGTTCAACAAAGGGGGGCTGTTTAAGGTGTGGGACATCGCC

ACGAACCATTTTCAGAAGAAGAGCAAGGTGAAAATCAATGGCCTGATATCTCTAGACATG

TATTCTGTGGCGACGGAAAAGCTAAAGCTACCCAGCTACAAACTCGACGCGGTCGTGGGA

GACGTCCTCGGCGAGCATAAGATAGACCTTCCCTATAAAGAAATACCCTCCTATTACGCG

GGAGGGCCTGACCGGAGGGGCGTAATAGGAGAGTATTGTATCCAGGACTCTAGGCTGGTG

GGCAAGCTGTTTTTTAAGTACCTCCCCCATCTGGAACTATCGGCGGTGGCCAAACTCGCC

CGTATCACCCTAACGCGGGTAATTTTTGACGGTCAGCAAATTCGCGTGTACACGTGCTTG

CTGAAACTCGCCCGCGAGAGAAATTTCATTTTGCCAGACAACAGACGCCGGTTTGACAGT

CAGGCAGACGCCGCGTCAGAGACTTCGGAGTTGGCTATGGATAGCCAAAGCCACGCCTTC

GACAGTACAGACGAACCCGACGGTGTGGACGGTACCCCGGACGCCGCAGGATCTGGCGCT

ACTTCTGAAAACGGAGGCGGGAAGCCCGGCGTCGGGAGGGCCGTGGGCTACCAGGGAGCA

AAGGTTCTAGACCCCGTATCCGGCTTTCATGTGGACCCCGTGGTTGTGTTTGACTTCGCT

AGCTTATACCCAAGCATTATCCAGGCCCATAACCTCTGTTTCACCACCCTGGCGCTCGAT

GAAGTGGATCTGGCCGGGCTTCAACCATCCGTCAACTACTCGACGTTCGAGGTGGGTGAC

CAAAAGTTATTTTTTGTCCACGCCCATATTCGCGAAAGCCTGCTTGGCATCTTGCTGCGC

GACTGGCTGGCCATGCGAAAGGCGGTGAGGGCGCGAATCCCCACCAGCACCCCCGAGGAG

GCAGTTTTACTAGATAAGCAGCAGTCTGCGATTAAGGTGATATGCAACTCGGTTTACGGA

TTCACGGGGGTGGCAAACGGCCTGTTGCCGTGTCTGAGGATAGCGGCTACCGTTACCACG

ATAGGACGCGACATGCTCCTCAAGACCCGAGATTACGTTCACTCTCGTTGGGCGACGCGC

GAGCTGCTGGAGGACAATTTTCCAGGGGCTATAGGTTTCCGAAACCACAAGCCTTACTCC

GTCAGGGTTATCTACGGAGACACCGACTCCGTGTTTATCAAGTTTGTGGGCCTGACGTAC

GAGGGGGTATCGGAGCTGGGGGATGCTATGTCGCGTCAGATTTCAGCGGACCTCTTTAGA

GCGCCCATCAAACTGGAGTGTGAGAAGACCTTTCAGCGACTGCTGCTGATCACCAAGAAG

AAGTACATAGGTGTCATAAACGGGGGGAAGATGCTCATGAAGGGGGTCGACCTGGTCCGC

AAAAATAACTGCTCTTTCATAAACTTGTACGCGCGACATCTGGTAGATCTTTTGTTGTAC

GACGAGGATGTGGCCACGGCGGCAGCAGAGGTGACAGACGTGCCTCCCGCAGAATGGGTG

GGGCGCCCGCTACCGAGCGGCTTTGACAAGTTTGGGCGAGTGCTGGTAGAGGCGTACAAC

CGTATCACTGCCCCCAACTTGGACGTGCGCGAGTTCGTTATGACTGCTGAGCTGAGCCGC

TCACCCGAATCGTATACCAACAAGCGCCTGCCGCACCTCACCGTCTACTTTAAGCTCGCC

ATGAGGAATGAAGAACTGCCCAGCGTAAAAGAGAGAATTCCGTATGTGATAGTTGCGCAG

ACCGAGGCCGCGGAACGCGAAGCGGGTGTAGTAAACTCAATGCGCGGTACCGCCCAAAAC

CCCGTGGTAACCAAGACCGCACGCCCCCAACCTAAACGCAAACTGCTGGTTTCCGACCTC

GCCGAAGACCCGACCTATGTTTCCGAGAATGACGTACCGCTAAACACAGACTACTATTTC

TCCCACCTGTTGGGTACCATAAGCGTGACCTTTAAGGCTCTATTCGGAAATGATGTGAGA

ACAACAGAAAATCTTTTAAAGCGGTTTATTCCGGAAACCCCCCACAAGACCCCCACGAAA

ACCCAGGCACTGCTTGAGCGCGCCGGCTTTGAAAAGCTGACGCCCTTTACACCGGAGGAA

GAAAGTCGTCGAATACTGCATACAGTTTTTTGTACTCTAGAAGCAGCTCCCCATCAAAGC

TGA

>LC109659.1

ATGGCGGCGCGCGAACAGGCCAACTCCGTTCGCAGGAGCGGGTTTTTTAATCCGTTTATT

GGCAAGAGGCCATTTTTCAGGCCGGGAAGCGGGCAAACCGCGGAGACAGAGCGCCCAAGG

CCCCCCCAACACTCGTACTGCACAGAGGTGGGTAGCTTTAAGTTTATAGCTCCAAGATGC

CTCGATGAAGAAGCCCCCGCCGACCAGCGACGCGGTGTACACGTGGGCACCTTGGAGCGC

CCACCCAAAGTGTACTGCGATGGCTCAGAGTACGACGTGCTGAACTTTGCCTCCGGAGGT

TGCTGGCCTCGCAGGATTCGGGTTTGGAACGGCCAGGATTTTCGGGGCGATGGATTCAAC

CCCAGATTTGAGAGATTTCACGTGTACGACATAGTAGAGACTTCGGAGAGCGCCTCACAC

GATGACCCATCCAGGTTTGCTGAGCTATCCCGCCCAAGCGGGTCTGTGGTAACACTGCTG

GGAATGAGCGAGTGTGGAAAGCGAGTCGCGGTTCACGTCTATGGTGTGCGCCATTATTTT

TACATGGCAAAGGCGGAGGTGGATAGCGCTTGTGGAATAACCACCGAGGCAGAACTCGTG

CGCGCAATGGTGGACTGCGCGCACAGCTCGGCTTTGAGCGCGGCCCTGGGAAATGGCAAC

GGCGGCAAACAGAGCGGCGGCAGCGGCGGGGGATGGTGGGGCGGAAAGCACGTGTCTGCG

GACTGCTTCAAAGTGGAGACCGTGTGCCACACGACGCTGTACTACTTTGGATCTAAGCCA

GCTCTCTACTATAGAGTATCTGCCTCCAGCAGCCGCCTGGGAGGGTTCATCTGCGACAAC

TTTCACCCGGAGATTACAAAATTCGAGGGGAGCGTGGACGTGACCACGCGGCTGTTGTTG

GACAATGAAAATTTTACCAGTTTCGGGTGGTACCGCCTGCGACCCGGCACCCACGGAGAG

CGTGTTCAACTTCGCCCCGTTGAGCGACACGTCACCTCAAGCGACGTGGAGATTAACTGT

ACTCCCGATAACCTGGAGCCGATACCAGACGAGGCTGCCTGGCCCGACTATAAGCTCATG

TGCTTTGATATAGAGTGTAAAGCTGGAACGGGTAACGAAATGGCGTTCCCAGTGGCAACT

AACCAAGAGGACCTGGTCATCCAGATCTCCTGTCTGCTGTACTCGCTTGCTACTCAGAAC

CACGAACACACCCTGCTGTTTTCCCTCGGGTCATGCGATATCTCTGAGGAATACTCGTTT

GCATGCGTCCAGCGCGGCGAGCCCAGACCGACGGTTTTGGAGTTTGACAGCGAGTACGAG

CTGCTGGTTGCCTTCCTGACCTTTCTCAAGCAGTACTCTCCCGAGTTCGCCACCGGCTAC

AACATCGTTAATTTTGACTGGGCGTACATAGTTAACAAGGTAACGTCGGTGTATAACATC

AAGCTGGACGGGTACGGCAAGTTCAACAAAGGGGGGCTGTTTAAGGTGTGGGACATCGCC

ACGAACCATTTTCAGAAGAAGAGCAAGGTGAAAATCAATGGCCTGATATCTCTAGACATG

TATTCTGTGGCGACGGAAAAGCTAAAGCTACCCAGCTACAAACTCGACGCGGTCGTGGGA

GACGTCCTCGGCGAGCATAAGATAGACCTTCCCTATAAAGAAATACCCTCCTATTACGCG

GGAGGGCCTGACCGGAGGGGCGTAATAGGAGAGTATTGTATCCAGGACTCTAGGCTGGTG

GGCAAGCTGTTTTTTAAGTACCTCCCCCATCTGGAACTATCGGCGGTGGCCAAACTCGCC

CGTATCACCCTAACGCGGGTAATTTTTGACGGTCAGCAAATTCGCGTGTACACGTGCTTG

CTGAAACTCGCCCGCGAGAGAAATTTCATTTTGCCAGACAACAGACGCCGGTTTGACAGT

CAGGCAGACGCCGCGTCAGAGACTTCGGAGTTGGCTATGGATAGCCAAAGCCACGCCTTC

GACAGTACAGACGAACCCGACGGTGTGGACGGTACCCCGGACGCCGCAGGATCTGGCGCT

ACTTCTGAAAACGGAGGCGGGAAGCCCGGCGTCGGGAGGGCCGTGGGCTACCAGGGAGCA

AAGGTTCTAGACCCCGTATCCGGCTTTCATGTGGACCCCGTGGTTGTGTTTGACTTCGCT

AGCTTATACCCAAGCATTATCCAGGCCCATAACCTCTGTTTCACCACCCTGGCGCTCGAT

GAAGTGGATCTGGCCGGGCTTCAACCATCCGTCAACTACTCGACGTTCGAGGTGGGTGAC

CAAAAGTTATTTTTTGTCCACGCCCATATTCGCGAAAGCCTGCTTGGCATCTTGCTGCGC

GACTGGCTGGCCATGCGAAAGGCGGTGAGGGCGCGAATCCCCACCAGCACCCCCGAGGAG

GCAGTTTTACTAGATAAGCAGCAGTCTGCGATTAAGGTGATATGCAACTCGGTTTACGGA

TTCACGGGGGTGGCAAACGGCCTGTTGCCGTGTCTGAGGATAGCGGCTACCGTTACCACG

ATAGGACGCGACATGCTCCTCAAGACCCGAGATTACGTTCACTCTCGTTGGGCGACGCGC

GAGCTGCTGGAGGACAATTTTCCAGGGGCTATAGGTTTCCGAAACCACAAGCCTTACTCC

GTCAGGGTTATCTACGGAGACACCGACTCCGTGTTTATCAAGTTTGTGGGCCTGACGTAC

GAGGGGGTATCGGAGCTGGGGGATGCTATGTCGCGTCAGATTTCAGCGGACCTCTTTAGA

GCGCCCATCAAACTGGAGTGTGAGAAGACCTTTCAGCGACTGCTGCTGATCACCAAGAAG

AAGTACATAGGTGTCATAAACGGGGGGAAGATGCTCATGAAGGGGGTCGACCTGGTCCGC

AAAAATAACTGCTCTTTCATAAACTTGTACGCGCGACATCTGGTAGATCTTTTGTTGTAC

GACGAGGATGTGGCCACGGCGGCAGCAGAGGTGACAGACGTGCCTCCCGCAGAATGGGTG

GGGCGCCCGCTACCGAGCGGCTTTGACAAGTTTGGGCGAGTGCTGGTAGAGGCGTACAAC

CGTATCACTGCCCCCAACTTGGACGTGCGCGAGTTCGTTATGACTGCTGAGCTGAGCCGC

TCACCCGAATCGTATACCAACAAGCGCCTGCCGCACCTCACCGTCTACTTTAAGCTCGCC

ATGAGGAATGAAGAACTGCCCAGCGTAAAAGAGAGAATTCCGTATGTGATAGTTGCGCAG

ACCGAGGCCGCGGAACGCGAAGCGGGTGTAGTAAACTCAATGCGCGGTACCGCCCAAAAC

CCCGTGGTAACCAAGACCGCACGCCCCCAACCTAAACGCAAACTGCTGGTTTCCGACCTC

GCCGAAGACCCGACCTATGTTTCCGAGAATGACGTACCGCTAAACACAGACTACTATTTC

TCCCACCTGTTGGGTACCATAAGCGTGACCTTTAAGGCTCTATTCGGAAATGATGTGAGA

ACAACAGAAAATCTTTTAAAGCGGTTTATTCCGGAAACCCCCCACAAGACCCCCACGAAA

ACCCAGGCACTGCTTGAGCGCGCCGGCTTTGAAAAGCTGACGCCCTTTACACCGGAGGAA

GAAAGTCGTCGAATACTGCATACAGTTTTTTGTACTCTAGAAGCAGCTCCCCATCAAAGC

TGA

>LC109660.1

ATGGCGGCGCGCGAACAGGCCAACTCCGTTCGCAGGAGCGGGTTTTTTAATCCGTTTATT

GGCAAGAGGCCATTTTTCAGGCCGGGAAGCGGGCAGACCGCGGAGACAGAGCGCCCAAGG

CCCCCCCAACACTCGTACTGCACAGAGGTGGGTAGCTTTAAGTTTATAGCTCCAAGATGC

CTCGATGAAGAAGCCCCCGCCGACCAGCGACGCGGTGTACACGTGGGCACCTTGGAGCGC

CCACCCAAAGTGTACTGCGATGGCTCAGAGTACGACGTGCTGAACTTTGCCTCCGGAGGT

TGCTGGCCTCGCAGGATTCGGGTTTGGAACGGCCAGGATTTTCGGGGCGATGGATTCAAC

CCCAGATTTGAGAGATTTCACGTGTACGACATAGTAGAGACTTCGGAGAGCGCCTCACAC

GATGACCCATCCAGGTTTGCTGAGCTATCCCGCCCAAGCGGGTCTGTGGTAACACTGCTG

GGAATGAGCGAGTGTGGAAAGCGAGTCGCGGTTCACGTCTATGGTGTGCGCCATTATTTT

TACATGGCAAAGGCGGAGGTGGATAGCGCTTGTGGAATAACCACCGAGGCAGAACTCGTG

CGCGCAATGGTGGACTGCGCGCACAGCTCGGCTTTGAGCGCGGCCCTGGGAAATGGCAAC

GGCGGCAAACAGAGCGGCGGCAGCGGCGGGGGATGGTGGGGCGGAAAGCACGTGTCCGCG

GACTGCTTCAAAGTGGAGACCGTGTGCCACACGACGCTGTACTACTTTGGATCTAAGCCA

GCTCTCTACTATAGAGTATCTGCCTCCAGCAGCCGCCTGGGAGGGTTCATCTGCGACAAC

TTTCACCCGGAGATTACAAAATTCGAGGGGAGCGTGGACGTGACCACGCGGCTGTTGTTG

GACAATGAAAATTTTACCAGTTTCGGGTGGTACCGCCTGCGACCCGGCACCCACGGAGAG

CGTGTTCAACTTCGCCCCGTTGAGCGACACGTCACCTCAAGCGACGTGGAGATTAACTGT

ACTCCCGATAACCTGGAGCCGATACCAGACGAGGCTGCCTGGCCCGACTATAAGCTCATG

TGCTTTGATATAGAGTGTAAAGCTGGAACGGGTAACGAAATGGCGTTCCCAGTGGCAACT

AACCAAGAGGACCTGGTCATCCAGATCTCCTGTCTGCTGTACTCGCTTGCTACTCAGAAC

CACGAACACACCCTGCTGTTTTCCCTCGGGTCATGCGATATCTCTGAGGAATACTCGTTT

GCATGCGTCCAGCGCGGCGAGCCCAGACCGACGGTTTTGGAGTTTGACAGCGAGTACGAG

CTGCTGGTTGCCTTCCTGACCTTTCTCAAGCAGTACTCTCCCGAGTTCGCCACCGGCTAC

AACATCGTTAATTTTGACTGGGCGTACATAGTTAACAAGGTAACGTCGGTGTATAACATC

AAGCTGGACGGGTACGGCAAGTTCAACAAAGGGGGGCTGTTTAAGGTGTGGGACATCGCC

ACGAACCATTTTCAGAAGAAGAGCAAGGTGAAAATCAATGGCCTGATATCTCTAGACATG

TATTCTGTGGCGACGGAAAAGCTAAAGCTACCCAGCTACAAACTCGACGCGGTCGTGGGA

GACGTCCTCGGCGAGCATAAGATAGACCTTCCCTATAAAGAAATACCCTCCTATTACGCG

GGAGGGCCTGACCGGAGGGGCGTAATAGGAGAGTATTGTATCCAGGACTCTAGGCTGGTG

GGCAAGCTGTTTTTTAAGTACCTCCCCCATCTGGAACTATCGGCGGTGGCCAAACTCGCC

CGTATCACCCTAACGCGGGTAATTTTTGACGGTCAGCAAATTCGCGTGTACACGTGCTTG

CTGAAACTCGCCCGCGAGAGAAATTTCATTTTGCCAGACAACAGACGCCGGTTTGACAGT

CAGGCAGACGCCGCGTCAGAGACTTCGGAGTTGGCTATGGATAGCCAAAGCCACGCCTTC

GACAGTACAGACGAACCCGACGGTGTGGACGGTACCCCGGACGCCGCAGGATCTGGCGCT

ACTTCTGAAAACGGAGGCGGGAAGCCCGGCGTCGGGAGGGCCGTGGGCTACCAGGGAGCA

AAGGTTCTAGACCCCGTATCCGGCTTTCATGTGGACCCCGTGGTTGTGTTTGACTTCGCT

AGCTTATACCCAAGCATTATCCAGGCCCATAACCTCTGTTTCACCACCCTGGCGCTCGAT

GAAGTGGATCTGGCCGGGCTTCAACCATCCGTCAACTACTCGACGTTCGAGGTGGGTGAC

CAAAAGTTATTTTTTGTCCACGCCCATATTCGCGAAAGCCTGCTTGGCATCTTGCTGCGC

GACTGGCTGGCCATGCGAAAGGCGGTGAGGGCGCGAATCCCCACCAGCACCCCCGAGGAG

GCAGTTTTACTAGATAAGCAGCAGTCTGCGATTAAGGTGATATGCAACTCGGTTTACGGA

TTCACGGGGGTGGCAAACGGCCTGTTGCCGTGTCTGAGGATAGCGGCTACCGTTACCACG

ATAGGACGCGACATGCTCCTCAAGACCCGAGATTACGTTCACTCTCGTTGGGCGACGCGC

GAGCTGCTGGAGGACAATTTTCCAGGGGCTATAGGTTTCCGAAACCACAAGCCTTACTCC

GTCAGGGTTATCTACGGAGACACCGACTCCGTGTTTATCAAGTTTGTGGGCCTGACGTAC

GAGGGGGTATCGGAGCTGGGGGATGCTATGTCGCGTCAGATTTCAGCGGACCTCTTTAGA

GCCCCCATCAAACTGGAGTGTGAGAAGACCTTTCAGCGACTGCTGCTGATCACCAAGAAG

AAGTACATAGGTGTCATAAACGGGGGGAAGATGCTCATGAAGGGGGTCGACCTGGTCCGC

AAAAATAACTGCTCTTTCATAAACTTGTACGCGCGACATCTGGTAGATCTTTTGTTGTAC

GACGAGGATGTGGCCACGGCGGCAGCAGAGGTGACAGACGTGCCTCCCGCAGAATGGGTG

GGGCGCCCGCTACCGAGCGGCTTTGACAAGTTTGGGCGAGTGCTGGTAGAGGCGTACAAC

CGTATCACTGCCCCCAACTTGGACGTGCGCGAGTTCGTTATGACTGCTGAGCTGAGCCGC

TCACCCGAATCGTATACCAACAAGCGCCTGCCGCACCTCACCGTCTACTTTAAGCTCGCC

ATGAGGAATGAAGAACTGCCCAGCGTAAAAGAGAGAATTCCGTATGTGATAGTTGCGCAG

ACCGAGGCCGCGGAACGCGAAGCGGGTGTAGTAAACTCAATGCGCGGTACCGCCCAAAAC

CCCGTGGTAACCAAGACCGCACGCCCCCAACCTAAACGCAAACTGCTGGTTTCCGACCTC

GCCGAAGACCCGACCTATGTTTCCGAGAATGACGTACCGCTAAACACAGACTACTATTTC

TCCCACCTGTTGGGTACCATAAGCGTGACCTTTAAGGCTCTATTCGGAAATGATGTGAGA

ACAACAGAAAATCTTTTAAAGCGGTTTATTCCGGAAACCCCCCACAAGACCCCCACGAAA

ACCCAGGCACTGCTTGAGCGCGCCGGCTTTGAAAAGCTGACGCCCTTTACACCGGAGGAA

GAAAGTCGTCGAATACTGCATACAGTTTTTTGTACTCTAGAAGCAGCTCCCCATCAAAGC

TGA

>LC109662.1

ATGGCGGCGCGCGAACAGGCCAACTCCGTTCGCAGGAGCGGGTTTTTTAATCCGTTTATT

GGCAAGAGGCCATTTTTCAGGCCGGGAAGCGGGCAGACCGCGGAGACAGAGCGCCCAAGG

CCCCCCCAACACTCGTACTGCACAGAGGTGGGTAGCTTTAAGTTTATAGCTCCAAGATGC

CTCGATGAAGAAGCCCCCGCCGACCAGCGACGCGGTGTACACGTGGGCACCTTGGAGCGC

CCACCCAAAGTGTACTGCGATGGCTCAGAGTACGACGTGCTGAACTTTGCCTCCGGAGGT

TGCTGGCCTCGCAGGATTCGGGTTTGGAACGGCCAGGATTTTCGGGGCGATGGATTCAAC

CCCAGATTTGAGAGATTTCACGTGTACGACATAGTAGAGACTTCGGAGAGCGCCTCACAC

GATGACCCATCCAGGTTTGCTGAGCTATCCCGCCCAAGCGGGTCTGTGGTAACACTGCTG

GGAATGAGCGAGTGTGGAAAGCGAGTCGCGGTTCACGTCTATGGTGTGCGCCATTATTTT

TACATGGCAAAGGCGGAGGTGGATAGCGCTTGTGGAATAACCACCGAGGCAGAACTCGTG

CGCGCAATGGTGGACTGCGCGCACAGCTCGGCTTTGAGCGCGGCCCTGGGAAATGGCAAC

GGCGGCAAACAGAGCGGCGGCAGCGGCGGGGGATGGTGGGGCGGAAAGCACGTGTCTGCG

GACTGCTTCAAAGTGGAGACCGTGTGCCGCACGACGCTGTACTACTTTGGATCTAAGCCA

GCTCTCTACTATAGAGTATCTGCCTCCAGCAGCCGCCTGGGAGGGTTCATCTGCGACAAC

TTTCACCCGGAGATTACAAAATTCGAGGGGAGCGTGGACGTGACCACGCGGCTGTTGTTG

GACAATGAAAATTTTACCAGTTTCGGGTGGTACCGCCTGCGACCCGGCACCCACGGAGAG

CGTGTTCAACTTCGCCCCGTTGAGCGACACGTCACCTCAAGCGACGTGGAGATTAACTGT

ACTCCCGATAACCTGGAGCCGATACCAGACGAGGCTGCCTGGCCCGACTATAAGCTCATG

TGCTTTGATATAGAGTGTAAAGCTGGAACGGGTAACGAAATGGCGTTCCCAGTGGCAACT

AACCAAGAGGACCTGGTCATCCAGATCTCCTGTCTGCTGTACTCGCTTGCTACTCAGAAC

CACGAACACACCCTGCTGTTTTCCCTCGGGTCATGCGATATCTCTGAGGAATACTCGTTT

GCATGCGTCCAGCGCGGCGAGCCCAGACCGACGGTTTTGGAGTTTGACAGCGAGTACGAG

CTGCTGGTTGCCTTCCTGACCTTTCTCAAGCAGTACTCTCCCGAGTTCGCCACCGGCTAC

AACATCGTTAATTTTGACTGGGCGTACATAGTTAACAAGGTAACGTCGGTGTATAACATC

AAGCTGGACGGGTACGGCAAGTTCAACAAAGGGGGGCTGTTTAAGGTGTGGGACATCGCC

ACGAACCATTTTCAGAAGAAGAGCAAGGTGAAAATCAATGGCCTGATATCTCTAGACATG

TATTCTGTGGCGACGGAAAAGCTAAAGCTACCCAGCTACAAACTCGACGCGGTCGTGGGA

GACGTCCTCGGCGAGCATAAGATAGACCTTCCCTATAAAGAAATACCCTCCTATTACGCG

GGAGGGCCTGACCGGAGGGGCGTAATAGGAGAGTATTGTATCCAGGACTCTAGGCTGGTG

GGCAAGCTGTTTTTTAAGTACCTCCCCCATCTGGAACTATCGGCGGTGGCCAAACTCGCC

CGTATCACCCTAACGCGGGTAATTTTTGACGGTCAGCAAATTCGCGTGTACACGTGCTTG

CTGAAACTCGCCCGCGAGAGAAATTTCATTTTGCCAGACAACAGACGCCGGTTTGACAGT

CAGGCAGACGCCGCGTCAGAGACTTCGGAGTTGGCTATGGATAGCCAAAGCCACGCCTTC

GACAGTACAGACGAACCCGACGGTGTGGACGGTACCCCGGACGCCGCAGGATCTGGCGCT

ACTTCTGAAAACGGAGGCGGGAAGCCCGGCGTCGGGAGGGCCGTGGGCTACCAGGGAGCA

AAGGTTCTAGACCCCGTATCCGGCTTTCATGTGGACCCCGTGGTTGTGTTTGACTTCGCT

AGCTTATACCCAAGCATTATCCAGGCCCATAACCTCTGTTTCACCACCCTGGCGCTCGAT

GAAGTGGATCTGGCCGGGCTTCAACCATCCGTCGACTACTCGACGTTCGAGGTGGGTGAC

CAAAAGTTATTTTTTGTCCACGCCCATATTCGCGAAAGCCTGCTTGGCATCTTGCTGCGC

GACTGGCTGGCCATGCGAAAGGCGGTGAGGGCGCGAATCCCCACCAGCACCCCCGAGGAG

GCAGTTTTACTAGATAAGCAGCAGTCTGCGATTAAGGTGATATGCAACTCGGTTTACGGA

TTCACGGGGGTGGCAAACGGCCTGTTGCCGTGTCTGAGGATAGCGGCTACCGTTACCACG

ATAGGACGCGACATGCTCCTCAAGACCCGAGATTACGTTCACTCTCGTTGGGCGACGCGC

GAGCTGCTGGAGGACAATTTTCCAGGGGCTATAGGTTTCCGAAACCACAAGCCTTACTCC

GTCAGGGTTATCTACGGAGACACCGACTCCGTGTTTATCAAGTTTGTGGGCCTGACGTAC

GAGGGGGTATCGGAGCTGGGGGATGCTATGTCGCGTCAGATTTCAGCGGACCTCTTTAGA

GCGCCCATCAAACTGGAGTGTGAGAAGACCTTTCAGCGACTGCTGCTGATCACCAAGAAG

AAGTACATAGGTGTCATAAACGGGGGGAAGATGCTCATGAAGGGGGTCGACCTGGTCCGC

AAAAATAACTGCTCTTTCATAAACTTGTACGCGCGACATCTGGTAGATCTTTTGTTGTAC

GACGAGGATGTGGCCACGGCGGCAGCAGAGGTGACAGACGTGCCTCCCGCAGAATGGGTG

GGGCGCCCGCTACCGAGCGGCTTTGACAAGTTTGGGCGAGTGCTGGTAGAGGCGTACAAC

CGTATCACTGCCCCCAACTTGGACGTGCGCGAGTTCGTTATGACTGCTGAGCTGAGCCGC

TCACCCGAATCGTATACCAACAAGCGCCTGCCGCACCTCACCGTCTACTTTAAGCTCGCC

ATGAGGAATGAAGAACTGCCCAGCGTAAAAGAGAGAATTCCGTATGTGATAGTTGCGCAG

ACCGAGGCCGCGGAACGCGAAGCGGGTGTAGTAAACTCAATGCGCGGTACCGCCCAAAAC

CCCGTGGTAACCAAGACCGCACGCCCCCAACCTAAACGCAAACTGCTGGTTTCCGACCTC

GCCGAAGACCCGACCTATGTTTCCGAGAATGACGTACCGCTAAACACAGACTACTATTTC

TCCCACCTGTTGGGTACCATAAGCGTGACCTTTAAGGCTCTATTCGGAAATGATGTGAGA

ACAACAGAAAATCTTTTAAAGCGGTTTATTCCGGAAACCCCCCACAAGACCCCCACGAAA

ACCCAGGCACTGCTTGAGCGCGCCGGCTTTGAAAAGCTGACGCCCTTTACACCGGAGGAA

GAAAGTCGTCGAATACTGCATACAGTTTTTTGTACTCTAGAAGCAGCTCCCCATCAAAGC

TGA

>LC109663.1

ATGGCGGCGCGCGAACAGGCCAACTCCGTTCGCAGGAGCGGGTTTTTTAATCCGTTTATT

GGCAAGAGGCCATTTTTCAGGCCGGGAAGCGGGCAGACCGCGGAGACAGAGCGCCCAAGG

CCCCCCCAACACTCGTACTGCACAGAGGTGGGTAGCTTTAAGTTTATAGCTCCAAGATGC

CTCGATGAAGAAGCCCCCGCCGACCAGCGACGCGGTGTACACGTGGGCACCTTGGAGCGC

CCACCCAAAGTGTACTGCGATGGCTCAGAGTACGACGTGCTGAACTTTGCCTCCGGAGGT

TGCTGGCCTCGCAGGATTCGGGTTTGGAACGGCCAGGATTTTCGGGGCGATGGATTCAAC

CCCAGATTTGAGAGATTTCACGTGTACGACATAGTAGAGACTTCGGAGAGCGCCTCACAC

GATGACCCATCCAGGTTTGCTGAGCTATCCCGCCCAAGCGGGTCTGTGGTAACACTGCTG

GGAATGAGCGAGTGTGGAAAGCGAGTCGCGGTTCACGTCTATGGTGTGCGCCATTATTTT

TACATGGCAAAGGCGGAGGTGGATAGCGCTTGTGGAATAACCACCGAGGCAGAACTCGTG

CGCGCAATGGTGGACTGCGCGCACAGCTCGGCTTTGAGCGCGGCCCTGGGAAATGGCAAC

GGCGGCAAACAGAGCGGCGGCAGCGGCGGGGGATGGTGGGGCGGAAAGCACGTGTCTGCG

GACTGCTTCAAAGTGGAGACCGTGTGCCGCACGACGCTGTACTACTTTGGATCTAAGCCA

GCTCTCTACTATAGAGTATCTGCCTCCAGCAGCCGCCTGGGAGGGTTCATCTGCGACAAC

TTTCACCCGGAGATTACAAAATTCGAGGGGAGCGTGGACGTGACCACGCGGCTGTTGTTG

GACAATGAAAATTTTACCAGTTTCGGGTGGTACCGCCTGCGACCCGGCACCCACGGAGAG

CGTGTTCAACTTCGCCCCGTTGAGCGACACGTCACCTCAAGCGACGTGGAGATTAACTGT

ACTCCCGATAACCTGGAGCCGATACCAGACGAGGCTGCCTGGCCCGACTATAAGCTCATG

TGCTTTGATATAGAGTGTAAAGCTGGAACGGGTAACGAAATGGCGTTCCCAGTGGCAACT

AACCAAGAGGACCTGGTCATCCAGATCTCCTGTCTGCTGTACTCGCTTGCTACTCAGAAC

CACGAACACACCCTGCTGTTTTCCCTCGGGTCATGCGATATCTCTGAGGAATACTCGTTT

GCATGCGTCCAGCGCGGCGAGCCCAGACCGACGGTTTTGGAGTTTGACAGCGAGTACGAG

CTGCTGGTTGCCTTCCTGACCTTTCTCAAGCAGTACTCTCCCGAGTTCGCCACCGGCTAC

AACATCGTTAATTTTGACTGGGCGTACATAGTTAACAAGGTAACGTCGGTGTATAACATC

AAGCTGGACGGGTACGGCAAGTTCAACAAAGGGGGGCTGTTTAAGGTGTGGGACATCGCC

ACGAACCATTTTCAGAAGAAGAGCAAGGTGAAAATCAATGGCCTGATATCTCTAGACATG

TATTCTGTGGCGACGGAAAAGCTAAAGCTACCCAGCTACAAACTCGACGCGGTCGTGGGA

GACGTCCTCGGCGAGCATAAGATAGACCTTCCCTATAAAGAAATACCCTCCTATTACGCG

GGAGGGCCTGACCGGAGGGGCGTAATAGGAGAGTATTGTATCCAGGACTCTAGGCTGGTG

GGCAAGCTGTTTTTTAAGTACCTCCCCCATCTGGAACTATCGGCGGTGGCCAAACTCGCC

CGTATCACCCTAACGCGGGTAATTTTTGACGGTCAGCAAATTCGCGTGTACACGTGCTTG

CTGAAACTCGCCCGCGAGAGAAATTTCATTTTGCCAGACAACAGACGCCGGTTTGACAGT

CAGGCAGACGCCGCGTCAGAGACTTCGGAGTTGGCTATGGATAGCCAAAGCCACGCCTTC

GACAGTACAGACGAACCCGACGGTGTGGACGGTACCCCGGACGCCGCAGGATCTGGCGCT

ACTTCTGAAAACGGAGGCGGGAAGCCCGGCGTCGGGAGGGCCGTGGGCTACCAGGGAGCA

AAGGTTCTAGACCCCGTATCCGGCTTTCATGTGGACCCCGTGGTTGTGTTTGACTTCGCT

AGCTTATACCCAAGCATTATCCAGGCCCATAACCTCTGTTTCACCACCCTGGCGCTCGAT

GAAGTGGATCTGGCCGGGCTTCAACCATCCGTCGACTACTCGACGTTCGAGGTGGGTGAC

CAAAAGTTATTTTTTGTCCACGCCCATATTCGCGAAAGCCTGCTTGGCATCTTGCTGCGC

GACTGGCTGGCCATGCGAAAGGCGGTGAGGGCGCGAATCCCCACCAGCACCCCCGAGGAG

GCAGTTTTACTAGATAAGCAGCAGTCTGCGATTAAGGTGATATGCAACTCGGTTTACGGA

TTCACGGGGGTGGCAAACGGCCTGTTGCCGTGTCTGAGGATAGCGGCTACCGTTACCACG

ATAGGACGCGACATGCTCCTCAAGACCCGAGATTACGTTCACTCTCGTTGGGCGACGCGC

GAGCTGCTGGAGGACAATTTTCCAGGGGCTATAGGTTTCCGAAACCACAAGCCTTACTCC

GTCAGGGTTATCTACGGAGACACCGACTCCGTGTTTATCAAGTTTGTGGGCCTGACGTAC

GAGGGGGTATCGGAGCTGGGGGATGCTATGTCGCGTCAGATTTCAGCGGACCTCTTTAGA

GCGCCCATCAAACTGGAGTGTGAGAAGACCTTTCAGCGACTGCTGCTGATCACCAAGAAG

AAGTACATAGGTGTCATAAACGGGGGGAAGATGCTCATGAAGGGGGTCGACCTGGTCCGC

AAAAATAACTGCTCTTTCATAAACTTGTACGCGCGACATCTGGTAGATCTTTTGTTGTAC

GACGAGGATGTGGCCACGGCGGCAGCAGAGGTGACAGACGTGCCTCCCGCAGAATGGGTG

GGGCGCCCGCTACCGAGCGGCTTTGACAAGTTTGGGCGAGTGCTGGTAGAGGCGTACAAC

CGTATCACTGCCCCCAACTTGGACGTGCGCGAGTTCGTTATGACTGCTGAGCTGAGCCGC

TCACCCGAATCGTATACCAACAAGCGCCTGCCGCACCTCACCGTCTACTTTAAGCTCGCC

ATGAGGAATGAAGAACTGCCCAGCGTAAAAGAGAGAATTCCGTATGTGATAGTTGCGCAG

ACCGAGGCCGCGGAACGCGAAGCGGGTGTAGTAAACTCAATGCGCGGTACCGCCCAAAAC

CCCGTGGTAACCAAGACCGCACGCCCCCAACCTAAACGCAAACTGCTGGTTTCCGACCTC

GCCGAAGACCCGACCTATGTTTCCGAGAATGACGTACCGCTAAACACAGACTACTATTTC

TCCCACCTGTTGGGTACCATAAGCGTGACCTTTAAGGCTCTATTCGGAAATGATGTGAGA

ACAACAGAAAATCTTTTAAAGCGGTTTATTCCGGAAACCCCCCACAAGACCCCCACGAAA

ACCCAGGCACTGCTTGAGCGCGCCGGCTTTGAAAAGCTGACGCCCTTTACACCGGAGGAA

GAAAGTCGTCGAATACTGCATACAGTTTTTTGTACTCTAGAAGCAGCTCCCCATCAAAGC

TGA

>PP856396.1

ATGGCGGCGCGCGAACAGGCCAACTCCGTTCGCAGGAGCGGGTTTTTTAATCCGTTTATT

GGCAAGAGGCCATTTTTCAGGCCGGGAAGCGGGCAAACCGCGGAGACAGAGCGCCCAAGG

CCCCCCCAACACTCGTACTGCACAGAGGTGGGTAGCTTTAAGTTTATAGCTCCAAGATGC

CTCGATGAAGAAGCCCCCGCCGACCAGCGACGCGGTGTACACGTGGGCACCTTGGAGCGC

CCACCCAAAGTGTACTGCGATGGCTCAGAGTACGACGTGCTGAACTTTGCCTCCGGAGGT

TGCTGGCCTCGCAGGATTCGGGTTTGGAACGGCCAGGATTTTCGGGGCGATGGATTCAAC

CCCAGATTTGAGAGATTTCACGTGTACGACATAGTAGAGACTTCGGAGAGCGCCTCACAC

GATGACCCATCCAGGTTTGCTGAGCTATCCCGCCCAAGCGGGTCTGTGGTAACACTGCTG

GGAATGAGCGAGTGTGGAAAGCGAGTCGCGGTTCACGTCTATGGTGTGCGCCATTATTTT

TACATGGCAAAGGCGGAGGTGGATAGCGCTTGTGGAATAACCACCGAGGCAGAACTCGTG

CGCGCAATGGTGGACTGCGCGCACAGCTCGGCTTTGAGCGCGGCCCTGGGAAATGGCAAC

GGCGGCAAACAGAGCGGCGGCAGCGGCGGGGGATGGTGGGGCGGAAAGCACGTGTCTGCG

GACTGCTTCAAAGTGGAGACCGTGTGCCACACGACGCTGTACTACTTTGGATCTAAGCCA

GCTCTCTACTATAGAGTATCTGCCTCCAGCAGCCGCCTGGGAGGGTTCATCTGCGACAAC

TTTCACCCGGAGATTACAAAATTCGAGGGGAGCGTGGACGTGACCACGCGGCTGTTGTTG

GACAATGAAAATTTTACCAGTTTCGGGTGGTACCGCCTGCGACCCGGCACCCACGGAGAG

CGTGTTCAACTTCGCCCCGTTGAGCGACACGTCACCTCAAGCGACGTGGAGATTAACTGT

ACTCCCGATAACCTGGAGCCGATACCAGACGAGGCTGCCTGGCCCGACTATAAGCTCATG

TGCTTTGATATAGAGTGTAAAGCTGGAACGGGTAACGAAATGGCGTTCCCAGTGGCAACT

AACCAAGAGGACCTGGTCATCCAGATCTCCTGTCTGCTGTACTCGCTTGCTACTCAGAAC

CACGAACACACCCTGCTGTTTTCCCTCGGGTCATGCGATATCTCTGAGGAATACTCGTTT

GCATGCGTCCAGCGCGGCGAGCCCAGACCGACGGTTTTGGAGTTTGACAGCGAGTACGAG

CTGCTGGTTGCCTTCCTGACCTTTCTCAAGCAGTACTCTCCCGAGTTCGCCACCGGCTAC

AACATCGTTAATTTTGACTGGGCGTACATAGTTAACAAGGTAACGTCGGTGTATAACATC

AAGCTGGACGGGTACGGCAAGTTCAACAAAGGGGGGCTGTTTAAGGTGTGGGACATCGCC

ACGAACCATTTTCAGAAGAAGAGCAAGGTGAAAATCAATGGCCTGATATCTCTAGACATG

TATTCTGTGGCGACGGAAAAGCTAAAGCTACCCAGCTACAAACTCGACGCGGTCGTGGGA

GACGTCCTCGGCGAGCATAAGATAGACCTTCCCTATAAAGAAATACCCTCCTATTACGCG

GGAGGGCCTGACCGGAGGGGCGTAATAGGAGAGTATTGTATCCAGGACTCTAGGCTGGTG

GGCAAGCTGTTTTTTAAGTACCTCCCCCATCTGGAACTATCGGCGGTGGCCAAACTCGCC

CGTATCACCCTAACGCGGGTAATTTTTGACGGTCAGCAAATTCGCGTGTACACGTGCTTG

CTGAAACTCGCCCGCGAGAGAAATTTCATTTTGCCAGACAACAGACGCCGGTTTGACAGT

CAGGCAGACGCCGCGTCAGAGACTTCGGAGTTGGCTATGGATAGCCAAAGCCACGCCTTC

GACAGTACAGACGAACCCGACGGTGTGGACGGTACCCCGGACGCCGCAGGATCTGGCGCT

ACTTCTGAAAACGGAGGCGGGAAGCCCGGCGTCGGGAGGGCCGTGGGCTACCAGGGAGCA

AAGGTTCTAGACCCCGTATCCGGCTTTCATGTGGACCCCGTGGTTGTGTTTGACTTCGCT

AGCTTATACCCAAGCATTATCCAGGCCCATAACCTCTGTTTCACCACCCTGGCGCTCGAT

GAAGTGGATCTGGCCGGGCTTCAACCATCCGTCAACTACTCGACGTTCGAGGTGGGTGAC

CAAAAGTTATTTTTTGTCCACGCCCATATTCGCGAAAGCCTGCTTGGCATCTTGCTGCGC

GACTGGCTGGCCATGCGAAAGGCGGTGAGGGCGCGAATCCCCACCAGCACCCCCGAGGAG

GCAGTTTTACTAGATAAGCAGCAGTCTGCGATTAAGGTGATATGCAACTCGGTTTACGGA

TTCACGGGGGTGGCAAACGGCCTGTTGCCGTGTCTGAGGATAGCGGCTACCGTTACCACG

ATAGGACGCGACATGCTCCTCAAGACCCGAGATTACGTTCACTCTCGTTGGGCGACGCGC

GAGCTGCTGGAGGACAATTTTCCAGGGGCTATAGGTTTCCGAAACCACAAGCCTTACTCC

GTCAGGGTTATCTACGGAGACACCGACTCCGTGTTTATCAAGTTTGTGGGCCTGACGTAC

GAGGGGGTATCGGAGCTGGGGGATGCTATGTCGCGTCAGATTTCAGCGGACCTCTTTAGA

GCGCCCATCAAACTGGAGTGTGAGAAGACCTTTCAGCGACTGCTTCTGATCACCAAGAAG

AAGTACATAGGTGTCATAAACGGGGGGAAGATGCTCATGAAGGGGGTCGACCTGGTCCGC

AAAAATAACTGCTCTTTCATAAACTTGTACGCGCGACATCTGGTAGATCTTTTGTTGTAC

GACGAGGATGTGGCCACGGCGGCAGCAGAGGTGACAGACGTGCCTCCCGCAGAATGGGTG

GGGCGCCCGCTACCGAGCGGCTTTGACAAGTTTGGGCGAGTGCTGGTAGAGGCGTACAAC

CGTATCACTGCCCCCAACTTGGACGTGCGCGAGTTCGTTATGACTGCTGAGCTGAGCCGC

TCACCCGAATCGTATACCAACAAGCGCCTGCCGCACCTCACCGTCTACTTTAAGCTCGCC

ATGAGGAATGAAGAACTGCCCAGCGTAAAAGAGAGAATTCCGTATGTGATAGTTGCGCAG

ACCGAGGCCGCGGAACGCGAAGCGGGTGTAGTAAACTCAATGCGCGGTACCGCCCAAAAC

CCCGTGGTAACCAAGACCGCACGCCCCCAACCTAAACGCAAACTGCTGGTTTCCGACCTC

GCCGAAGACCCGACCTATGTTTCCGAGAATGACGTACCGCTAAACACAGACTACTATTTC

TCCCACCTGTTGGGTACCATAAGCGTGACCTTTAAGGCTCTATTCGGAAATGATGTGAGA

ACAACAGAAAATCTTTTAAAGCGGTTTATTCCGGAAACCCCCCACAAGACCCCCACGAAA

ACCCAGGCACTGCTTGAGCGCGCCGGCTTTGAAAAGCTGACGCCCTTTACACCGGAGGAA

GAAAGTCGTCGAATACTGCATACAGTTTTTTGTACTCTAGAAGCAGCTCCCCATCAAAGC

TGA

>PQ630625.1

ATGGCGGCGCGCGAACAGGCCAACTCCGTTCGCAGGAGCGGGTTTTTTAATCCGTTTATT

GGCAAGAGGCCATTTTTCAGGCCGGGAAGCGGGCAGACCGCGGAGACAGAGCGCCCAAGG

CCCCCCCAACACTCGTACTGCACAGAGGTGGGTAGCTTTAAGTTTATAGCTCCAAGATGC

CTCGATGAAGAAGCCCCCGCCGACCAGCGACGCGGTGTACACGTGGGCACCTTGGAGCGC

CCACCCAAAGTGTACTGCGATGGCTCAGAGTACGACGTGCTGAACTTTGCCTCCGGAGGT

TGCTGGCCTCGCAGGATTCGGGTTTGGAACGGCCAGGATTTTCGGGGCGATGGATTCAAC

CCCAGATTTGAGAGATTTCACGTGTACGACATAGTAGAGACTTCGGAGAGCGCCTCACAC

GATGACCCATCCAGGTTTGCTGAGCTATCCCGCCCAAGCGGGTCTGTGGTAACACTGCTG

GGAATGAGCGAGTGTGGAAAGCGAGTCGCGGTTCACGTCTATGGTGTGCGCCATTATTTT

TACATGGCAAAGGCGGAGGTGGATAGCGCTTGTGGAATAACCACCGAGGCAGAACTCGTG

CGCGCAATGGTGGACTGCGCGCACAGCTCGGCTTTGAGCGCGGCCCTGGGAAATGGCAAC

GGCGGCAAACAGAGCGGCGGCAGCGGCGGGGGATGGTGGGGCGGAAAGCACGTGTCTGCG

GACTGCTTCAAAGTGGAGACCGTGTGCCACACGACGCTGTACTACTTTGGATCTAAGCCA

GCTCTCTACTATAGAGTATCTGCCTCCAGCAGCCGCCTGGGAGGGTTCATCTGCGACAAC

TTTCACCCGGAGATTACAAAATTCGAGGGGAGCGTGGACGTGACCACGCGGCTGTTGTTG

GACAATGAAAATTTTACCAGTTTCGGGTGGTACCGCCTGCGACCCGGCACCCACGGAGAG

CGTGTTCAACTTCGCCCCGTTGAGCGACACGTCACCTCAAGCGACGTGGAGATTAACTGT

ACTCCCGATAACCTGGAGCCGATACCAGACGAGGCTGCCTGGCCCGACTATAAGCTCATG

TGCTTTGATATAGAGTGTAAAGCTGGAACGGGTAACGAAATGGCGTTCCCAGTGGCAACT

AACCAAGAGGACCTGGTCATCCAGATCTCCTGTCTGCTGTACTCGCTTGCTACTCAGAAC

CACGAACACACCCTGCTGTTTTCCCTCGGGTCATGCGATATCTCTGAGGAATACTCGTTT

GCATGCGTCCAGCGCGGCGAGCCCAGACCGACGGTTTTGGAGTTTGACAGCGAGTACGAG

CTGCTGGTTGCCTTCCTGACCTTTCTCAAGCAGTACTCTCCCGAGTTCGCCACCGGCTAC

AACATCGTTAATTTTGACTGGGCGTACATAGTTAACAAGGTAACGTCGGTGTATAACATC

AAGCTGGACGGGTACGGCAAGTTCAACAAAGGGGGGCTGTTTAAGGTGTGGGACATCGCC

ACGAACCATTTTCAGAAGAAGAGCAAGGTGAAAATCAATGGCCTGATATCTCTAGACATG

TATTCTGTGGCGACGGAAAAGCTAAAGCTACCCAGCTACAAACTCGACGCGGTCGTGGGA

GACGTCCTCGGCGAGCATAAGATAGACCTTCCCTATAAAGAAATACCCTCCTATTACGCG

GGAGGGCCTGACCGGAGGGGCGTAATAGGAGAGTATTGTATCCAGGACTCTAGGCTGGTG

GGCAAGCTGTTTTTTAAGTACCTCCCCCATCTGGAACTATCGGCGGTGGCCAAACTCGCC

CGTATCACCCTAACGCGGGTAATTTTTGACGGTCAGCAAATTCGCGTGTACACGTGCTTG

CTGAAACTCGCCCGCGAGAGAAATTTCATTTTGCCAGACAACAGACGCCGGTTTGACAGT

CAGGCAGACGCCGCGTCAGAGACTTCGGAGTTGGCTATGGATAGCCAAAGCCACGCCTTC

GACAGTACAGACGAACCCGACGGTGTGGACGGTACCCCGGACGCCGCAGGATCTGGCGCT

ACTTCTGAAAACGGAGGCGGGAAGCCCGGCGTCGGGAGGGCCGTGGGCTACCAGGGAGCA

AAGGTTCTAGACCCCGTATCCGGCTTTCATGTGGACCCCGTGGTTGTGCTTGACTTCGCT

AGCTTATACCCAAGCATTATCCAGGCCCATAACCTCTGTTTCACCACCCTGGCGCTCGAT

GAAGTGGATCTGGCCGGGCTTCAACCATCCGTCAACTACTCGACGTTCGAGGTGGGTGAC

CAAAAGTTATTTTTTGTCCACGCCCATATTCGCGAAAGCCTGCTTGGCATCTTGCTGCGC

GACTGGCTGGCCATGCGAAAGGCGGTGAGGGCGCGAATCCCCACCAGCACCCCCGAGGAG

GCAGTTTTACTAGATAAGCAGCAGTCTGCGATTAAGGTGATATGCAACTCGGTTTACGGA

TTCACGGGGGTGGCAAACGGCCTGTTGCCGTGTCTGAGGATAGCGGCTACCGTTACCACG

ATAGGACGCGACATGCTCCTCAAGACCCGAGATTACGTTCACTCTCGTTGGGCGACGCGC

GAGCTGCTGGAGGACAATTTTCCAGGGGCTATAGGTTTCCGAAACCACAAGCCTTACTCC

GTCAGGGTTATCTACGGAGACACCGACTCCGTGTTTATCAAGTTTGTGGGCCTGACGTAC

GAGGGGGTATCGGAGCTGGGGGATGCTATGTCGCGTCAGATTTCAGCGGACCTCTTTAGA

GCGCCCATCAAACTGGAGTGTGAGAAGACCTTTCAGCGACTGCTGCTGATCACCAAGAAG

AAGTACATAGGTGTCATAAACGGGGGGAAGATGCTCATGAAGGGGGTCGACCTGGTCCGC

AAAAATAACTGCTCTTTCATAAACTTGTACGCGCGACATCTGGTAGATCTTTTGTTGTAC

GACGAGGATGTGGCCACGGCGGCAGCAGAGGTGACAGACGTGCCTCCCGCAGAATGGGTG

GGGCGCCCGCTACCGAGCGGCTTTGACAAGTTTGGGCGAGTGCTGGTAGAGGCGTACAAC

CGTATCACTGCCCCCAACTTGGACGTGCGCGAGTTCGTTATGACTGCTGAGCTGAGCCGC

TCACCCGAATTGTATACCAACAAGCGCCTGCCGCACCTCACCGTCTACTTTAAGCTCGCC

ATGAGGAATGAAGAACTGCCCAGCGTAAAAGAGAGAATTCCGTATGTGATAGTTGCGCAG

ACCGAGGCCGCGGAACGCGAAGCGGGTGTAGTAAACTCAATGCGCGGTACCGCCCAAAAC

CCCGTGGTAACCAAGACCGCACGCCCCCAACCTAAACGCAAACTGCTGGTTTCCGACCTC

GCCGAAGACCCGACCTATGTTTCCGAGAATGACGTACCGCTAAACACAGACTACTATTTC

TCCCACCTGTTGGGTACCATAAGCGTGACCTTTAAGGCTCTATTCGGAAATGATGTGAGA

ACAACAGAAAATCTTTTAAAGCGGTTTATTCCGGAAACCCCCCACAAGACCCCCACGAAA

ACCCAGGCACTGCTTGAGCGCGCCGGCTTTGAAAAGCTGACGCCCTTTACACCGGAGGAA

GAAAGTCGTCGAATACTGCATACAGTTTTTTGTACTCTAGAAGCAGCTCCCCATCAAAGC

TGA

>PQ630626.1

ATGGCGGCGCGCGAACAGGCCAACTCCGTTCGCAGGAGCGGGTTTTTTAATCCGTTTATT

GGCAAGAGGCCATTTTTCAGGCCGGGAAGCGGGCAGACCGCGGAGACAGAGCGCCCAAGG

CCCCCCCAACACTCGTACTGCACAGAGGTGGGTAGCTTTAAGTTTATAGCTCCAAGATGC

CTCGATGAAGAAGCCCCCGCCGACCAGCGACGCGGTGTACACGTGGGCACCTTGGAGCGC

CCACCCAAAGTGTACTGCGATGGCTCAGAGTACGACGTGCTGAACTTTGCCTCCGGAGGT

TGCTGGCCTCGCAGGATTCGGGTTTGGAACGGCCAGGATTTTCGGGGCGATGGATTCAAC

CCCAGATTTGAGAGATTTCACGTGTACGACATAGTAGAGACTTCGGAGAGCGCCTCACAC

GATGACCCATCCAGGTTTGCTGAGCTATCCCGCCCAAGCGGGTCTGTGGTAACACTGCTG

GGAATGAGCGAGTGTGGAAAGCGAGTCGCGGTTCACGTCTATGGTGTGCGCCATTATTTT

TACATGGCAAAGGCGGAGGTGGATAGCGCTTGTGGAATAACCACCGAGGCAGAACTCGTG

CGCGCAATGGTGGACTGCGCGCACAGCTCGGCTTTGAGCGCGGCCCTGGGAAATGGCAAC

GGCGGCAAACAGAGCGGCGGCAGCGGCGGGGGATGGTGGGGCGGAAAGCACGTGTCTGCG

GACTGCTTCAAAGTGGAGACCGTGTGCCACACGACGCTGTACTACTTTGGATCTAAGCCA

GCTCTCTACTATAGAGTATCTGCCTCCAGCAGCCGCCTGGGAGGGTTCATCTGCGACAAC

TTTCACCCGGAGATTACAAAATTCGAGGGGAGCGTGGACGTGACCACGCGGCTGTTGTTG

GACAATGAAAATTTTACCAGTTTCGGGTGGTACCGCCTGCGACCCGGCACCCACGGAGAG

CGTGTTCAACTTCGCCCCGTTGAGCGACACGTCACCTCAAGCGACGTGGAGATTAACTGT

ACTCCCGATAACCTGGAGCCGATACCAGACGAGGCTGCCTGGCCCGACTATAAGCTCATG

TGCTTTGATATAGAGTGTAAAGCTGGAACGGGTAACGAAATGGCGTTCCCAGTGGCAACT

AACCAAGAGGACCTGGTCATCCAGATCTCCTGTCTGCTGTACTCGCTTGCTACTCAGAAC

CACGAACACACCCTGCTGTTTTCCCTCGGGTCATGCGATATCTCTGAGGAATACTCGTTT

GCATGCGTCCAGCGCGGCGAGCCCAGACCGACGGTTTTGGAGTTTGACAGCGAGTACGAG

CTGCTGGTTGCCTTCCTGACCTTTCTCAAGCAGTACTCTCCCGAGTTCGCCACCGGCTAC

AACATCGTTAATTTTGACTGGGCGTACATAGTTAACAAGGTAACGTCGGTGTATAACATC

AAGCTGGACGGGTACGGCAAGTTCAACAAAGGGGGGCTGTTTAAGGTGTGGGACATCGCC

ACGAACCATTTTCAGAAGAAGAGCAAGGTGAAAATCAATGGCCTGATATCTCTAGACATG

TATTCTGTGGCGACGGAAAAGCTAAAGCTACCCAGCTACAAACTCGACGCGGTCGTGGGA

GACGTCCTCGGCGAGCATAAGATAGACCTTCCCTATAAAGAAATACCCTCCTATTACGCG

GGAGGGCCTGACCGGAGGGGCGTAATAGGAGAGTATTGTATCCAGGACTCTAGGCTGGTG

GGCAAGCTGTTTTTTAAGTACCTCCCCCATCTGGAACTATCGGCGGTGGCCAAACTCGCC

CGTATCACCCTAACGCGGGTAATTTTTGACGGTCAGCAAATTCGCGTGTACACGTGCTTG

CTGAAACTCGCCCGCGAGAGAAATTTCATTTTGCCAGACAACAGACGCCGGTTTGACAGT

CAGGCAGACGCCGCGTCAGAGACTTCGGAGTTGGCTATGGATAGCCAAAGCCACGCCTTC

GACAGTACAGACGAACCCGACGGTGTGGACGGTACCCCGGACGCCGCAGGATCTGGCGCT

ACTTCTGAAAACGGAGGCGGGAAGCCCGGCGTCGGGAGGGCCGTGGGCTACCAGGGAGCA

AAGGTTCTAGACCCCGTATCCGGCTTTCATGTGGACCCCGTGGTTGTGTTTGACTTCGCT

AGCTTATACCCAAGCATTATCCAGGCCCATAACCTCTGTTTCACCACCCTGGCGCTCGAT

GAAGTGGATCTGGCCGGGCTTCAACCATCCGTCAACTACTCGACGTTCGAGGTGGGTGAC

CAAAAGTTATTTTTTGTCCACGCCCATATTCGCGAAAGCCTGCTTGGCATCTTGCTGCGC

GACTGGCTGGCCATGCGAAAGGCGGTGAGGGCGCGAATCCCCACCAGCACCCCCGAGGAG

GCAGTTTTACTAGATAAGCAGCAGTCTGCGATTAAGGTGATATGCAACTCGGTTTACGGA

TTCACGGGGGTGGCAAACGGCCTGTTGCCGTGTCTGAGGATAGCGGCTACCGTTACCACG

ATAGGACGCGACATGCTCCTCAAGACCCGAGATTACGTTCACTCTCGTTGGGCGACGCGC

GAGCTGCTGGAGGACAATTTTCCAGGGGCTATAGGTTTCCGAAACCACAAGCCTTACTCC

GTCAGGGTTATCTACGGAGACACCGACTCCGTGTTTATCAAGTTTGTGGGCCTGACGTAC

GAGGGGGTATCGGAGCTGGGGGATGCTATGTCGCGTCAGATTTCAGCGGACCTCTTTAGA

GCGCCCATCAAACTGGAGTGTGAGAAGACCTTTCAGCGACTGCTGCTGATCACCAAGAAG

AAGTACATAGGTGTCATAAACGGGGGGAAGATGCTCATGAAGGGGGTCGACCTGGTCCGC

AAAAATAACTGCTCTTTCATAAACTTGTACGCGCGACATCTGGTAGATCTTTTGTTGTAC

GACGAGGATGTGGCCACGGCGGCAGCAGAGGTGACAGACGTGCCTCCCGCAGAATGGGTG

GGGCGCCCGCTACCGAGCGGCTTTGACAAGTTTGGGCGAGTGCTGGTAGAGGCGTACAAC

CGTATCACTGCCCCCAACTTGGACGTGCGCGAGTTCGTTATGACTGCTGAGCTGAGCCGC

TCACCCGAATCGTATACCAACAAGCGCCTGCCGCACCTCACCGTCTACTTTAAGCTCGCC

ATGAGGAATGAAGAACTGCCCAGCGTAAAAGAGAGAATTCCGTATGTGATAGTTGCGCAG

ACCGAGGCCGCGGAACGCGAAGCGGGTGTAGTAAACTCAATGCGCGGTACCGCCCAAAAC

CCCGTGGTAACCAAGACCGCACGCCCCCAACCTAAACGCAAACTGCTGGTTTCCGACCTC

GCCGAAGACCCGACCTATGTTTCCGAGAATGACGTACCGCTAAACACAGACTACTATTTC

TCCCACCTGTTGGGTACCATAAGCGTGACCTTTAAGGCTCTATTCGGAAATGATGTGAGA

ACAACAGAAAATCTTTTAAAGCGGTTTATTCCGGAAACCCCCCACAAGACCCCCACGAAA

ACCCAGGCACTGCTTGAGCGCGCCGGCTTTGAAAAGCTGACGCCCTTTACACCGGAGGAA

GAAAGTCGTCGAATACTGCATACAGTTTTTTGTACTCTAGAAGCAGCTCCCCATCAAAGC

TGA

>PQ630627.1

ATGGCGGCGCGCGAACAGGCCAACTCCGTTCGCAGGAGCGGGTTTTTTAATCCGTTTATT

GGCAAGAGGCCATTTTTCAGGCCGGGAAGCGGGCAGACCGCGGAGACAGAGCGCCCAAGG

CCCCCCCAACACTCGTACTGCACAGAGGTGGGTAGCTTTAAGTTTATAGCTCCAAGATGC

CTCGATGAAGAAGCCCCCGCCGACCAGCGACGCGGTGTACACGTGGGCACCTTGGAGCGC

CCACCCAAAGTGTACTGCGATGGCTCAGAGTACGACGTGCTGAACTTTGCCTCCGGAGGT

TGCTGGCCTCGCAGGATTCGGGTTTGGAACGGCCAGGATTTTCGGGGCGATGGATTCAAC

CCCAGATTTGAGAGATTTCACGTGTACGACATAGTAGAGACTTCGGAGAGCGCCTCACAC

GATGACCCATCCAGGTTTGCTGAGCTATCCCGCCCAAGCGGGTCTGTGGTAACACTGCTG

GGAATGAGCGAGTGTGGAAAGCGAGTCGCGGTTCACGTCTATGGTGTGCGCCATTATTTT

TACATGGCAAAGGCGGAGGTGGATAGCGCTTGTGGAATAACCACCGAGGCAGAACTCGTG

CGCGCAATGGTGGACTGCGCGCACAGCTCGGCTTTGAGCGCGGCCCTGGGAAATGGCAAC

GGCGGCAAACAGAGCGGCGGCAGCGGCGGGGGATGGTGGGGCGGAAAGCACGTGTCTGCG

GACTGCTTCAAAGTGGAGACCGTGTGCCACACGACGCTGTACTACTTTGGATCTAAGCCA

GCTCTCTACTATAGAGTATCTGCCTCCAGCAGCCGCCTGGGAGGGTTCATCTGCGACAAC

TTTCACCCGGAGATTACAAAATTCGAGGGGAGCGTGGACGTGACCACGCGGCTGTTGTTG

GACAATGAAAATTTTACCAGTTTCGGGTGGTACCGCCTGCGACCCGGCACCCACGGAGAG

CGTGTTCAACTTCGCCCCGTTGAGCGACACGTCACCTCAAGCGACGTGGAGATTAACTGT

ACTCCCGATAACCTGGAGCCGATACCAGACGAGGCTGCCTGGCCCGACTATAAGCTCATG

TGCTTTGATATAGAGTGTAAAGCTGGAACGGGTAACGAAATGGCGTTCCCAGTGGCAACT

AACCAAGAGGACCTGGTCATCCAGATCTCCTGTCTGCTGTACTCGCTTGCTACTCAGAAC

CACGAACACACCCTGCTGTTTTCCCTCGGGTCATGCGATATCTCTGAGGAATACTCGTTT

GCATGCGTCCAGCGCGGCGAGCCCAGACCGACGGTTTTGGAGTTTGACAGCGAGTACGAG

CTGCTGGTTGCCTTCCTGACCTTTCTCAAGCAGTACTCTCCCGAGTTCGCCACCGGCTAC

AACATCGTTAATTTTGACTGGGCGTACATAGTTAACAAGGTAACGTCGGTGTATAACATC

AAGCTGGACGGGTACGGCAAGTTCAACAAAGGGGGGCTGTTTAAGGTGTGGGACATCGCC

ACGAACCATTTTCAGAAGAAGAGCAAGGTGAAAATCAATGGCCTGATATCTCTAGACATG

TATTCTGTGGCGACGGAAAAGCTAAAGCTACCCAGCTACAAACTCGACGCGGTCGTGGGA

GACGTCCTCGGCGAGCATAAGATAGACCTTCCCTATAAAGAAATACCCTCCTATTACGCG

GGAGGGCCTGACCGGAGGGGCGTAATAGGAGAGTATTGTATCCAGGACTCTAGGCTGGTG

GGCAAGCTGTTTTTTAAGTACCTCCCCCATCTGGAACTATCGGCGGTGGCCAAACTCGCC

CGTATCACCCTAACGCGGGTAATTTTTGACGGTCAGCAAATTCGCGTGTACACGTGCTTG

CTGAAACTCGCCCGCGAGAGAAATTTCATTTTGCCAGACAACAGACGCCGGTTTGACAGT

CAGGCAGACGCCGCGTCAGAGACTTCGGAGTTGGCTATGGATAGCCAAAGCCACGCCTTC

GACCGTACAGACGAACCCGACGGTGTGGACGGTACCCCGGACGCCGCAGGATCTGGCGCT

ACTTCTGAAAACGGAGGCGGGAAGCCCGGCGTCGGGAGGGCCGTGGGCTACCAGGGAGCA

AAGGTTCTAGACCCCGTATCCGGCTTTCATGTGGACCCCGTGGTTGTGTTTGACTTCGCT

AGCTTATACCCAAGCATTATCCAGGCCCATAACCTCTGTTTCACCACCCTGGCGCTCGAT

GAAGTGGATCTGGCCGGGCTTCAACCATCCGTCGACTACTCGACGTTCGAGGTGGGTGAC

CAAAAGTTATTTTTTGTCCACGCCCATATTCGCGAAAGCCTGCTTGGCATCTTGCTGCGC

GACTGGCTGGCCATGCGAAAGGCGGTGAGGGCGCGAATCCCCACCAGCACCCCCGAGGAG

GCAGTTTTACTAGATAAGCAGCAGTCTGCGATTAAGGTGATATGCAACTCGGTTTACGGA

TTCACGGGGGTGGCAAACGGCCTGTTGCCGTGTCTGAGGATAGCGGCTACCGTTACCACG

ATAGGACGCGACATGCTCCTCAAGACCCGAGATTACGTTCACTCTCGTTGGGCGACGCGC

GAGCTGCTGGAGGACAATTTTCCAGGGGCTATAGGTTTCCGAAACCACAAGCCTTACTCC

GTCAGGGTTATCTACGGAGACACCGACTCCGTGTTTATCAAGTTTGTGGGCCTGACGTAC

GAGGGGGTATCGGAGCTGGGGGATGCTATGTCGCGTCAGATTTCAGCGGACCTCTTTAGA

GCGCCCATCAAACTGGAGTGTGAGAAGACCTTTCAGCGACTGCTGCTGATCACCAAGAAG

AAGTACATAGGTGTCATAAACGGGGGGAAGATGCTCATGAAGGGGGTCGACCTGGTCCGC

AAAAATAACTGCTCTTTCATAAACTTGTACGCGCGACATCTGGTAGATCTTTTGTTGTAC

GACGAGGATGTGGCCACGGCGGCAGCAGAGGTGACAGACGTGCCTCCCGCAGAATGGGTG

GGGCGCCCGCTACCGAGCGGCTTTGACAAGTTTGGGCGAGTGCTGGTAGAGGCGTACAAC

CGTATCACTGCCCCCAACTTGGACGTGCGCGAGTTCGTTATGACTGCTGAGCTGAGCCGC

TCACCCGAATCGTATACCAACAAGCGCCTGCCGCACCTCACCGTCTACTTTAAGCTCGCC

ATGAGGAATGAAGAACTGCCCAGCGTAAAAGAGAGAATTCCGTATGTGATAGTTGCGCAG

ACCGAGGCCGCGGAACGCGAAGCGGGTGTAGTAAACTCAATGCGCGGTACCGCCCAAAAC

CCCGTGGTAACCAAGACCGCACGCCCCCAACCTAAACGCAAACTGCTGGTTTCCGACCTC

GCCGAAGACCCGACCTATGTTTCCGAGAATGACGTACCGCTAAACACAGACTACTATTTC

TCCCACCTGTTGGGTACCATAAGCGTGACCTTTAAGGCTCTATTCGGAAATGATGTGAGA

ACAACAGAAAATCTTTTAAAGCGGTTTATTCCGGAAACCCCCCACAAGACCCCCACGAAA

ACCCAGGCACTGCTTGAGCGCGCCGGCTTTGAAAAGCTGACGCCCTTTACACCGGAGGAA

GAAAGTCGTCGAATACTGCATACAGTTTTTTGTACTCTAGAAGCAGCTCCCCATCAAAGC

TGA

>PQ630628.1

ATGGCGGCGCGCGAACAGGCCAACTCCGTTCGCAGGAGCGGGTTTTTTAATCCGTTTATT

GGCAAGAGGCCATTTTTCAGGCCGGGAAGCGGGCAGACCGCGGAGACAGAGCGCCCAAGG

CCCCCCCAACACTCGTACTGCACAGAGGTGGGTAGCTTTAAGTTTATAGCTCCAAGATGC

CTCGATGAAGAAGCCCCCGCCGACCAGCGACGCGGTGTACACGTGGGCACCTTGGAGCGC

CCACCCAAAGTGTACTGCGATGGCTCAGAGTACGACGTGCTGAACTTTGCCTCCGGAGGT

TGCTGGCCTCGCAGGATTCGGGTTTGGAACGGCCAGGATTTTCGGGGCGATGGATTCAAC

CCCAGATTTGAGAGATTTCACGTGTACGACATAGTAGAGACTTCGGAGAGCGCCTCACAC

GATGACCCATCCAGGTTTGCTGAGCTATCCCGCCCAAGCGGGTCTGTGGTAACACTGCTG

GGAATGAGCGAGTGTGGAAAGCGAGTCGCGGTTCACGTCTATGGTGTGCGCCATTATTTT

TACATGGCAAAGGCGGAGGTGGATAGCGCTTGTGGAATAACCACCGAGGCAGAACTCGTG

CGCGCAATGGTGGACTGCGCGCACAGCTCGGCTTTGAGCGCGGCCCTGGGAAATGGCAAC

GGCGGCAAACAGAGCGGCGGCAGCGGCGGGGGATGGTGGGGCGGAAAGCACGTGTCTGCG

GACTGCTTCAAAGTGGAGACCGTGTGCCACACGACGCTGTACTACTTTGGATCTAAGCCA

GCTCTCTACTATAGAGTATCTGCCTCCAGCAGCCGCCTGGGAGGGTTCATCTGCGACAAC

TTTCACCCGGAGATTACAAAATTCGAGGGGAGCGTGGACGTGACCACGCGGCTGTTGTTG

GACAATGAAAATTTTACCAGTTTCGGGTGGTACCGCCTGCGACCCGGCACCCACGGAGAG

CGTGTTCAACTTCGCCCCGTTGAGCGACACGTCACCTCAAGCGACGTGGAGATTAACTGT

ACTCCCGATAACCTGGAGCCGATACCAGACGAGGCTGCCTGGCCCGACTATAAGCTCATG

TGCTTTGATATAGAGTGTAAAGCTGGAACGGGTAACGAAATGGCGTTCCCAGTGGCAACT

AACCAAGAGGACCTGGTCATCCAGATCTCCTGTCTGCTGTACTCGCTTGCTACTCAGAAC

CACGAACACACCCTGCTGTTTTCCCTCGGGTCATGCGATATCTCTGAGGAATACTCGTTT

GCATGCGTCCAGCGCGGCGAGCCCAGACCGACGGTTTTGGAGTTTGACAGCGAGTACGAG

CTGCTGGTTGCCTTCCTGACCTTTCTCAAGCAGTACTCTCCCGAGTTCGCCACCGGCTAC

AACATCGTTAATTTTGACTGGGCGTACATAGTTAACAAGGTAACGTCGGTGTATAACATC

AAGCTGGACGGGTACGGCAAGTTCAACAAAGGGGGGCTGTTTAAGGTGTGGGACATCGCC

ACGAACCATTTTCAGAAGAAGAGCAAGGTGAAAATCAATGGCCTGATATCTCTAGACATG

TATTCTGTGGCGACGGAAAAGCTAAAGCTACCCAGCTACAAACTCGACGCGGTCGTGGGA

GACGTCCTCGGCGAGCATAAGATAGACCTTCCCTATAAAGAAATACCCTCCTATTACGCG

GGAGGGCCTGACCGGAGGGGCGTAATAGGAGAGTATTGTATCCAGGACTCTAGGCTGGTG

GGCAAGCTGTTTTTTAAGTACCTCCCCCATCTGGAACTATCGGCGGTGGCCAAACTCGCC

CGTATCACCCTAACGCGGGTAATTTTTGACGGTCAGCAAATTCGCGTGTACACGTGCTTG

CTGAAACTCGCCCGCGAGAGAAATTTCATTTTGCCAGACAACAGACGCCGGTTTGACAGT

CAGGCAGACGCCGCGTCAGAGACTTCGGAGTTGGCTATGGATAGCCAAAGCCACGCCTTC

GACAGTACAGACGAACCCGACGGTGTGGACGGTACCCCGGACGCCGCAGGATCTGGCGCT

ACTTCTGAAAACGGAGGCGGGAAGCCCGGCGTCGGGAGGGCCGTGGGCTACCAGGGAGCA

AAGGTTCTAGACCCCGTATCCGGCTTTCATGTGGACCCCGTGGTTGTGTTTGACTTCGCT

AGCTTATACCCAAGCATTATCCAGGCCCATAACCTCTGTTTCACCACCCTGGCGCTCGAT

GAAGTGGATCTGGCCGGGCTTCAACCATCCGTCAACTACTCGACGTTCGAGGTGGGTGAC

CAAAAGTTATTTTTTGTCCACGCCCATATTCGCGAAAGCCTGCTTGGCATCTTGCTGCGC

GACTGGCTGGCCATGCGAAAGGCGGTGAGGGCGCGAATCCCCACCAGCACCCCCGAGGAG

GCAGTTTTACTAGATAAGCAGCAGTCTGCGATTAAGGTGATATGCAACTCGGTTTACGGA

TTCACGGGGGTGGCAAACGGCCTGTTGCCGTGTCTGAGGATAGCGGCTACCGTTACCACG

ATAGGACGCGACATGCTCCTCAAGACCCGAGATTACGTTCACTCTCGTTGGGCGACGCGC

GAGCTGCTGGAGGACAATTTTCCAGGGGCTATAGGTTTCCGAAACCACAAGCCTTACTCC

GTCAGGGTTATCTACGGAGACACCGACTCCGTGTTTATCAAGTTTGTGGGCCTGACGTAC

GAGGGGGTATCGGAGCTGGGGGATGCTATGTCGCGTCAGATTTCAGCGGACCTCTTTAGA

GCGCCCATCAAACTGGAGTGTGAGAAGACCTTTCAGCGACTGCTGCTGATCACCAAGAAG

AAGTACATAGGTGTCATAAACGGGGGGAAGATGCTCATGAAGGGGGTCGACCTGGTCCGC

AAAAATAACTGCTCTTTCATAAACTTGTACGCGCGACATCTGGTAGATCTTTTGTTGTAC

GACGAGGATGTGGCCACGGCGGCAGCAGAGGTGACAGACGTGCCTCCCGCAGAATGGGTG

GGGCGCCCGCTACCGAGCGGCTTTGACAAGTTTGGGCGAGTGCTGGTAGAGGCGTACAAC

CGTATCACTGCCCCCAACTTGGACGTGCGCGAGTTCGTTATGACTGCTGAGCTGAGCCGC

TCACCCGAATCGTATACCAACAAGCGCCTGCCGCACCTCACCGTCTACTTTAAGCTCGCC

ATGAGGAATGAAGAACTGCCCAGCGTAAAAGAGAGAATTCCGTATGTGATAGTTGCGCAG

ACCGAGGCCGCGGAACGCGAAGCGGGTGTAGTAAACTCAATGCGCGGTACCGCCCAAAAC

CCCGTGGTAACCAAGACCGCACGCCCCCAACCTAAACGCAAACTGCTGGTTTCCGACCTC

GCCGAAGACCCGACCTATGTTTCCGAGAATGACGTACCGCTAAACACAGACTACTATTTC

TCCCACCTGTTGGGTACCATAAGCGTGACCTTTAAGGCTCTATTCGGAAATGATGTGAGA

ACAACAGAAAATCTTTTAAAGCGGTTTATTCCGGAAACCCCCCACAAGACCCCCACGAAA

ACCCAGGCACTGCTTGAGCGCGCCGGCTTTGAAAAGCTGACGCCCTTTACACCGGAGGAA

GAAAGTCGTCGAATACTGCATACAGTTTTTTGTACTCTAGAAGCAGCTCCCCATCAAAGC

TGA

>PQ630629.1

ATGGCGGCGCGCGAACAGGCCAACTCCGTTCGCAGGAGCGGGTTTTTTAATCCGTTTATT

GGCAAGAGGCCATTTTTCAGGCCGGGAAGCGGGCAGACCGCGGAGACAGAGCGCCCAAGG

CCCCCCCAACACTCGTACTGCACAGAGGTGGGTAGCTTTAAGTTTATAGCTCCAAGATGC

CTCGATGAAGAAGCCCCCGCCGACCAGCGACGCGGTGTACACGTGGGCACCTTGGAGCGC

CCACCCAAAGTGTACTGCGATGGCTCAGAGTACGACGTGCTGAACTTTGCCTCCGGAGGT

TGCTGGCCTCGCAGGATTCGGGTTTGGAACGGCCAGGATTTTCGGGGCGATGGATTCAAC

CCCAGATTTGAGAGATTTCACGTGTACGACATAGTAGAGACTTCGGAGAGCGCCTCACAC

GATGACCCATCCAGGTTTGCTGAGCTATCCCGCCCAAGCGGGTCTGTGGTAACACTGCTG

GGAATGAGCGAGTGTGGAAAGCGAGTCGCGGTTCACGTCTATGGTGTGCGCCATTATTTT

TACATGGCAAAGGCGGAGGTGGATAGCGCTTGTGGAATAACCACCGAGGCAGAACTCGTG

CGCGCAATGGTGGACTGCGCGCACAGCTCGGCTTTGAGCGCGGCCCTGGGAAATGGCAAC

GGCGGCAAACAGAGCGGCGGCAGCGGCGGGGGATGGTGGGGCGGAAAGCACGTGTCTGCG

GACTGCTTCAAAGTGGAGACCGTGTGCCACACGACGCTGTACTACTTTGGATCTAAGCCA

GCTCTCTACTATAGAGTATCTGCCTCCAGCAGCCGCCTGGGAGGGTTCATCTGCGACAAC

TTTCACCCGGAGATTACAAAATTCGAGGGGAGCGTGGACGTGACCACGCGGCTGTTGTTG

GACAATGAAAATTTTACCAGTTTCGGGTGGTACCGCCTGCGACCCGGCACCCACGGAGAG

CGTGTTCAACTTCGCCCCGTTGAGCGACACGTCACCTCAAGCGACGTGGAGATTAACTGT

ACTCCCGATAACCTGGAGCCGATACCAGACGAGGCTGCCTGGCCCGACTATAAGCTCATG

TGCTTTGATATAGAGTGTAAAGCTGGAACGGGTAACGAAATGGCGTTCCCAGTGGCAACT

AACCAAGAGGACCTGGTCATCCAGATCTCCTGTCTGCTGTACTCGCTTGCTACTCAGAAC

CACGAACACACCCTGCTGTTTTCCCTCGGGTCATGCGATATCTCTGAGGAATACTCGTTT

GCATGCGTCCAGCGCGGCGAGCCCAGACCGACGGTTTTGGAGTTTGACAGCGAGTACGAG

CTGCTGGTTGCCTTCCTGACCTTTCTCAAGCAGTACTCTCCCGAGTTCGCCACCGGCTAC

AACATCGTTAATTTTGACTGGGCGTACATAGTTAACAAGGTAACGTCGGTGTATAACATC

AAGCTGGACGGGTACGGCAAGTTCAACAAAGGGGGGCTGTTTAAGGTGTGGGACATCGCC

ACGAACCATTTTCAGAAGAAGAGCAAGGTGAAAATCAATGGCCTGATATCTCTAGACATG

TATTCTGTGGCGACGGAAAAGCTAAAGCTACCCAGCTACAAACTCGACGCGGTCGTGGGA

GACGTCCTCGGCGAGCATAAGATAGACCTTCCCTATAAAGAAATACCCTCCTATTACGCG

GGAGGGCCTGACCGGAGGGGCGTAATAGGAGAGTATTGTATCCAGGACTCTAGGCTGGTG

GGCAAGCTGTTTTTTAAGTACCTCCCCCATCTGGAACTATCGGCGGTGGCCAAACTCGCC

CGTATCACCCTAACGCGGGTAATTTTTGACGGTCAGCAAATTCGCGTGTACACGTGCTTG

CTGAAACTCGCCCGCGAGAGAAATTTCATTTTGCCAGACAACAGACGCCGGTTTGACAGT

CAGGCAGACGCCGCGTCAGAGACTTCGGAGTTGGCTATGGATAGCCAAAGCCACGCCTTC

GACAGTACAGACGAACCCGACGGTGTGGACGGTACCCCGGACGCCGCAGGATCTGGCGCT

ACTTCTGAAAACGGAGGCGGGAAGCCCGGCGTCGGGAGGGCCGTGGGCTACCAGGGAGCA

AAGGTTCTAGACCCCGTATCCGGCTTTCATGTGGACCCCGTGGTTGTGTTTGACTTCGCT

AGCTTATACCCAAGCATTATCCAGGCCCATAACCTCTGTTTCACCACCCTGGCGCTCGAT

GAAGTGGATCTGGCCGGGCTTCAACCATCCGTCAACTACTCGACGTTCGAGGTGGGTGAC

CAAAAGTTATTTTTTGTCCACGCCCATATTCGCGAAAGCCTGCTTGGCATCTTGCTGCGC

GACTGGCTGGCCATGCGAAAGGCGGTGAGGGCGCGAATCCCCACCAGCACCCCCGAGGAG

GCAGTTTTACTAGATAAGCAGCAGTCTGCGATTAAGGTGATATGCAACTCGGTTTACGGA

TTCACGGGGGTGGCAAACGGCCTGTTGCCGTGTCTGAGGATAGCGGCTACCGTTACCACG

ATAGGACGCGACATGCTCCTCAAGACCCGAGATTACGTTCACTCTCGTTGGGCGACGCGC

GAGCTGCTGGAGGACAATTTTCCAGGGGCTATAGGTTTCCGAAACCACAAGCCTTACTCC

GTCAGGGTTATCTACGGAGACACCGACTCCGTGTTTATCAAGTTTGTGGGCCTGACGTAC

GAGGGGGTATCGGAGCTGGGGGATGCTATGTCGCGTCAGATTTCAGCGGACCTCTTTAGA

GCGCCCATCAAACTGGAGTGTGAGAAGACCTTTCAGCGACTGCTGCTGATCACCAAGAAG

AAGTACATAGGTGTCATAAACGGGGGGAAGATGCTCATGAAGGGGGTCGACCTGGTCCGC

AAAAATAACTGCTCTTTCATAAACTTGTACGCGCGACATCTGGTAGATCTTTTGTTGTAC

GACGAGGATGTGGCCACGGCGGCAGCAGAGGTGACAGACGTGCCTCCCGCAGAATGGGTG

GGGCGCCCGCTACCGAGCGGCTTTGACAAGTTTGGGCGAGTGCTGGTAGAGGCGTACAAC

CGTATCACTGCCCCCAACTTGGACGTGCGCGAGTTCGTTATGACTGCTGAGCTGAGCCGC

TCACCCGAATCGTATACCAACAAGCGCCTGCCGCACCTCACCGTCTACTTTAAGCTCGCC

ATGAGGAATGAAGAACTGCCCAGCGTAAAAGAGAGAATTCCGTATGTGATAGTTGCGCAG

ACCGAGGCCGCGGAACGCGAAGCGGGTGTAGTAAACTCAATGCGCGGTACCGCCCAAAAC

CCCGTGGTAACCAGGACCGCACGCCCCCAACCTAAACGCAAACTGCTGGTTTCCGACCTC

GCCGAAGACCCGACCTATGTTTCCGAGAATGACGTACCGCTAAACACAGACTACTATTTC

TCCCACCTGTTGGGTACCATAAGCGTGACCTTTAAGGCTCTATTCGGAAATGATGTGAGA

ACAACAGAAAATCTTTTAAAGCGGTTTATTCCGGAAACCCCCCACAAGACCCCCACGAAA

ACCCAGGCACTGCTTGAGCGCGCCGGCTTTGAAAAGCTGACGCCCTTTACACCGGAGGAA

GAAAGTCGTCGAATACTGCATACAGTTTTTTGTACTCTAGAAGCAGCTCCCCATCAAAGC

TGA

>PQ630630.1

ATGGCGGCGCGCGAACAGGCCAACTCCGTTCGCAGGAGCGGGTTTTTTAATCCGTTTATT

GGCAAGAGGCCATTTTTCAGGCCGGGAAGCGGGCAGACCGCGGAGACAGAGCGCCCAAGG

CCCCCCCAACACTCGTACTGCACAGAGGTGGGTAGCTTTAAGTTTATAGCTCCAAGATGC

CTCGATGAAGAAGCCCCCGCCGACCAGCGACGCGGTGTACACGTGGGCACCTTGGAGCGC

CCACCCAAAGTGTACTGCGATGGCTCAGAGTACGACGTGCTGAACTTTGCCTCCGGAGGT

TGCTGGCCTCGCAGGATTCGGGTTTGGAACGGCCAGGATTTTCGGGGCGATGGATTCAAC

CCCAGATTTGAGAGATTTCACGTGTACGACATAGTAGAGACTTCGGAGAGCGCCTCACAC

GATGACCCATCCAGGTTTGCTGAGCTATCCCGCCCAAGCGGGTCTGTGGTAACACTGCTG

GGAATGAGCGAGTGTGGAAAGCGAGTCGCGGTTCACGTCTATGGTGTGCGCCATTATTTT

TACATGGCAAAGGCGGAGGTGGATAGCGCTTGTGGAATAACCACCGAGGCAGAACTCGTG

CGCGCAATGGTGGACTGCGCGCACAGCTCGGCTTTGAGCGCGGCCCTGGGAAATGGCAAC

GGCGGCAAACAGAGCGGCGGCAGCGGCGGGGGATGGTGGGGCGGAAAGCACGTGTCTGCG

GACTGCTTCAAAGTGGAGACCGTGTGCCACACGACGCTGTACTACTTTGGATCTAAGCCA

GCTCTCTACTATAGAGTATCTGCCTCCAGCAGCCGCCTGGGAGGGTTCATCTGCGACAAC

TTTCACCCGGAGATTACAAAATTCGAGGGGAGCGTGGACGTGACCACGCGGCTGTTGTTG

GACAATGAAAATTTTACCAGTTTCGGGTGGTACCGCCTGCGACCCGGCACCCACGGAGAG

CGTGTTCAACTTCGCCCCGTTGAGCGACACGTCACCTCAAGCGACGTGGAGATTAACTGT

ACTCCCGATAACCTGGAGCCGATACCAGACGAGGCTGCCTGGCCCGACTATAAGCTCATG

TGCTTTGATATAGAGTGTAAAGCTGGAACGGGTAACGAAATGGCGTTCCCAGTGGCAACT

AACCAAGAGGACCTGGTCATCCAGATCTCCTGTCTGCTGTACTCGCTTGCTACTCAGAAC

CACGAACACACCCTGCTGTTTTCCCTCGGGTCATGCGATATCTCTGAGGAATACTCGTTT

GCATGCGTCCAGCGCGGCGAGCCCAGACCGACGGTTTTGGAGTTTGACAGCGAGTACGAG

CTGCTGGTTGCCTTCCTGACCTTTCTCAAGCAGTACTCTCCCGAGTTCGCCACCGGCTAC

AACATCGTTAATTTTGACTGGGCGTACATAGTTAACAAGGTAACGTCGGTGTATAACATC

AAGCTGGACGGGTACGGCAAGTTCAACAAAGGGGGGCTGTTTAAGGTGTGGGACATCGCC

ACGAACCATTTTCAGAAGAAGAGCAAGGTGAAAATCAATGGCCTGATATCTCTAGACATG

TATTCTGTGGCGACGGAAAAGCTAAAGCTACCCAGCTACAAACTCGACGCGGTCGTGGGA

GACGTCCTCGGCGAGCATAAGATAGACCTTCCCTATAAAGAAATACCCTCCTATTACGCG

GGAGGGCCTGACCGGAGGGGCGTAATAGGAGAGTATTGTATCCAGGACTCTAGGCTGGTG

GGCAAGCTGTTTTTTAAGTACCTCCCCCATCTGGAACTATCGGCGGTGGCCAAACTCGCC

CGTATCACCCTAACGCGGGTAATTTTTGACGGTCAGCAAATTCGCGTGTACACGTGCTTG

CTGAAACTCGCCCGCGAGAGAAATTTCATTTTGCCAGACAACAGACGCCGGTTTGACAGT

CAGGCAGACGCCGCGTCAGAGACTTCGGAGTTGGCTATGGATAGCCAAAGCCACGCCTTC

GACAGTACAGACGAACCCGACGGTGTGGACGGTACCCCGGACGCCGCAGGATCTGGCGCT

ACTTCTGAAAACGGAGGCGGGAAGCCCGGCGTCGGGAGGGCCGTGGGCTACCAGGGAGCA

AAGGTTCTAGACCCCGTATCCGGCTTTCATGTGGACCCCGTGGTTGTGTTTGACTTCGCT

AGCTTATACCCAAGCATTATCCAGGCCCATAACCTCTGTTTCACCACCCTGGCGCTCGAT

GAAGTGGATCTGGCCGGGCTTCAACCATCCGTCAACTACTCGACGTTCGAGGTGGGTGAC

CAAAAGTTATTTTTTGTCCACGCCCATATTCGCGAAAGCCTGCTTGGCATCTTGCTGCGC

GACTGGCTGGCCATGCGAAAGGCGGTGAGGGCGCGAATCCCCACCAGCACCCCCGAGGAG

GCAGTTTTACTAGATAAGCAGCAGTCTGCGATTAAGGTGATATGCAACTCGGTTTACGGA

TTCACGGGGGTGGCAAACGGCCTGTTGCCGTGTCTGAGGATAGCGGCTACCGTTACCACG

ATAGGACGCGACATGCTCCTCAAGACCCGAGATTACGTTCACTCTCGTTGGGCGACGCGC

GAGCTGCTGGAGGACAATTTTCCAGGGGCTATAGGTTTCCGAAACCACAAGCCTTACTCC

GTCAGGGTTATCTACGGAGACACCGACTCCGTGTTTATCAAGTTTGTGGGCCTGACGTAC

GAGGGGGTATCGGAGCTGGGGGATGCTATGTCGCGTCAGATTTCAGCGGACCTCTTTAGA

GCGCCCATCAAACTGGAGTGTGAGAAGACCTTTCAGCGACTGCTGCTGATCACCAAGAAG

AAGTACATAGGTGTCATAAACGGGGGGAAGATGCTCATGAAGGGGGTCGACCTGGTCCGC

AAAAATAACTGCTCTTTCATAAACTTGTACGCGCGACATCTGGTAGATCTTTTGTTGTAC

GACGAGGATGTGGCCACGGCGGCAGCAGAGGTGACAGACGTGCCTCCCGCAGAATGGGTG

GGGCGCCCGCTACCGAGCGGCTTTGACAAGTTTGGGCGAGTGCTGGTAGAGGCGTACAAC

CGTATCACTGCCCCCAACTTGGACGTGCGCGAGTTCGTTATGACTGCTGAGCTGAGCCGC

TCACCCGAATCGTATACCAACAAGCGCCTGCCGCACCTCACCGTCTACTTTAAGCTCGCC

ATGAGGAATGAAGAACTGCCCAGCGTAAAAGAGAGAATTCCGTATGTGATAGTTGCGCAG

ACCGAGGCCGCGGAACGCGAAGCGGGTGTAGTAAACTCAATGCGCGGTACCGCCCAAAAC

CCCGTGGTAACCAAGACCGCACGCCCCCAACCTAAACGCAAACTGCTGGTTTCCGACCTC

GCCGAAGACCCGACCTATGTTTCCGAGAATGACGTACCGCTAAACACAGACTACTATTTC

TCCCACCTGTTGGGTACCATAAGCGTGACCTTTAAGGCTCTATTCGGAAATGATGTGAGA

ACAACAGAAAATCTTTTAAAGCGGTTTATTCCGGAAACCCCCCACAAGACCCCCACGAAA

ACCCAGGCACTGCTTGAGCGCGCCGGCTTTGAAAAGCTGACGCCCTTTACACCGGAGGAA

GAAAGTCGTCGAATACTGCATACAGTTTTTTGTACTCTAGAAGCAGCTCCCCATCAAAGC

TGA

>PQ630631.1

ATGGCGGCGCGCGAACAGGCCAACTCCGTTCGCAGGAGCGGGTTTTTTAATCCGTTTATT

GGCAAGAGGCCATTTTTCAGGCCGGGAAGCGGGCAGACCGCGGAGACAGAGCGCCCAAGG

CCCCCCCAACACTCGTACTGCACAGAGGTGGGTAGCTTTAAGTTTATAGCTCCAAGATGC

CTCGATGAAGAAGCCCCCGCCGACCAGCGACGCGGTGTACACGTGGGCACCTTGGAGCGC

CCACCCAAAGTGTACTGCGATGGCTCAGAGTACGACGTGCTGAACTTTGCCTCCGGAGGT

TGCTGGCCTCGCAGGATTCGGGTTTGGAACGGCCAGGATTTTCGGGGCGATGGATTCAAC

CCCAGATTTGAGAGATTTCACGTGTACGACATAGTAGAGACTTCGGAGAGCGCCTCACAC

GATGACCCATCCAGGTTTGCTGAGCTATCCCGCCCAAGCGGGTCTGTGGTAACACTGCTG

GGAATGAGCGAGTGTGGAAAGCGAGTCGCGGTTCACGTCTATGGTGTGCGCCATTATTTT

TACATGGCAAAGGCGGAGGTGGATAGCGCTTGTGGAATAACCACCGAGGCAGAACTCGTG

CGCGCAATGGTGGACTGCGCGCACAGCTCGGCTTTGAGCGCGGCCCTGGGAAATGGCAAC

GGCGGCAAACAGAGCGGCGGCAGCGGCGGGGGATGGTGGGGCGGAAAGCACGTGTCTGCG

GACTGCTTCAAAGTGGAGACCGTGTGCCACACGACGCTGTACTACTTTGGATCTAAGCCA

GCTCTCTACTATAGAGTATCTGCCTCCAGCAGCCGCCTGGGAGGGTTCATCTGCGACAAC

TTTCACCCGGAGATTACAAAATTCGAGGGGAGCGTGGACGTGACCACGCGGCTGTTGTTG

GACAATGAAAATTTTACCAGTTTCGGGTGGTACCGCCTGCGACCCGGCACCCACGGAGAG

CGTGTTCAACTTCGCCCCGTTGAGCGACACGTCACCTCAAGCGACGTGGAGATTAACTGT

ACTCCCGATAACCTGGAGCCGATACCAGACGAGGCTGCCTGGCCCGACTATAAGCTCATG

TGCTTTGATATAGAGTGTAAAGCTGGAACGGGTAACGAAATGGCGTTCCCAGTGGCAACT

AACCAAGAGGACCTGGTCATCCAGATCTCCTGTCTGCTGTACTCGCTTGCTACTCAGAAC

CACGAACACACCCTGCTGTTTTCCCTCGGGTCATGCGATATCTCTGAGGAATACTCGTTT

GCATGCGTCCAGCGCGGCGAGCCCAGACCGACGGTTTTGGAGTTTGACAGCGAGTACGAG

CTGCTGGTTGCCTTCCTGACCTTTCTCAAGCAGTACTCTCCCGAGTTCGCCACCGGCTAC

AACATCGTTAATTTTGACTGGGCGTACATAGTTAACAAGGTAACGTCGGTGTATAACATC

AAGCTGGACGGGTACGGCAAGTTCAACAAAGGGGGGCTGTTTAAGGTGTGGGACATCGCC

ACGAACCATTTTCAGAAGAAGAGCAAGGTGAAAATCAATGGCCTGATATCTCTAGACATG

TATTCTGTGGCGACGGAAAAGCTAAAGCTACCCAGCTACAAACTCGACGCGGTCGTGGGA

GACGTCCTCGGCGAGCATAAGATAGACCTTCCCTATAAAGAAATACCCTCCTATTACGCG

GGAGGGCCTGACCGGAGGGGCGTAATAGGAGAGTATTGTATCCAGGACTCTAGGCTGGTG

GGCAAGCTGTTTTTTAAGTACCTCCCCCATCTGGAACTATCGGCGGTGGCCAAACTCGCC

CGTATCACCCTAACGCGGGTAATTTTTGACGGTCAGCAAATTCGCGTGTACACGTGCTTG

CTGAAACTCGCCCGCGAGAGAAATTTCATTTTGCCAGACAACAGACGCCGGTTTGACAGT

CAGGCAGACGCCGCGTCAGAGACTTCGGAGTTGGCTATGGATAGCCAAAGCCACGCCTTC

GACAGTACAGACGAACCCGACGGTGTGGACGGTACCCCGGACGCCGCAGGATCTGGCGCT

ACTTCTGAAAACGGAGGCGGGAAGCCCGGCGTCGGGAGGGCCGTGGGCTACCAGGGAGCA

AAGGTTCTAGACCCCGTATCCGGCTTTCATGTGGACCCCGTGGTTGTGTTTGACTTCGCT

AGCTTATACCCAAGCATTATCCAGGCCCATAACCTCTGTTTCACCACCCTGGCGCTCGAT

GAAGTGGATCTGGCCGGGCTTCAACCATCCGTCAACTACTCGACGTTCGAGGTGGGTGAC

CAAAAGTTATTTTTTGTCCACGCCCATATTCGCGAAAGCCTGCTTGGCATCTTGCTGCGC

GACTGGCTGGCCATGCGAAAGGCGGTGAGGGCGCGAATCCCCACCAGCACCCCCGAGGAG

GCAGTTTTACTAGATAAGCAGCAGTCTGCGATTAAGGTGATATGCAACTCGGTTTACGGA

TTCACGGGGGTGGCAAACGGCCTGTTGCCGTGTCTGAGGATAGCGGCTACCGTTACCACG

ATAGGACGCGACATGCTCCTCAAGACCCGAGATTACGTTCACTCTCGTTGGGCGACGCGC

GAGCTGCTGGAGGACAATTTTCCAGGGGCTATAGGTTTCCGAAACCACAAGCCTTACTCC

GTCAGGGTTATCTACGGAGACACCGACTCCGTGTTTATCAAGTTTGTGGGCCTGACGTAC

GAGGGGGTATCGGAGCTGGGGGATGCTATGTCGCGTCAGATTTCAGCGGACCTCTTTAGA

GCGCCCATCAAACTGGAGTGTGAGAAGACCTTTCAGCGACTGCTGCTGATCACCAAGAAG

AAGTACATAGGTGTCATAAACGGGGGGAAGATGCTCATGAAGGGGGTCGACCTGGTCCGC

AAAAATAACTGCTCTTTCATAAACTTGTACGCGCGACATCTGGTAGATCTTTTGTTGTAC

GACGAGGATGTGGCCACGGCGGCAGCAGAGGTGACAGACGTGCCTCCCGCAGAATGGGTG

GGGCGCCCGCTACCGAGCGGCTTTGACAAGTTTGGGCGAGTGCTGGTAGAGGCGTACAAC

CGTATCACTGCCCCCAACTTGGACGTGCGCGAGTTCGTTATGACTGCTGAGCTGAGCCGC

TCACCCGAATCGTATACCAACAAGCGCCTGCCGCACCTCACCGTCTACTTTAAGCTCGCC

ATGAGGAATGAAGAACTGCCCAGCGTAAAAGAGAGAATTCCGTATGTGATAGTTGCGCAG

ACCGAGGCCGCGGAACGCGAAGCGGGTGTAGTAAACTCAATGCGCGGTACCGCCCAAAAC

CCCGTGGTAACCAAGACCGCACGCCCCCAACCTAAACGCAAACTGCTGGTTTCCGACCTC

GCCGAAGACCCGACCTATGTTTCCGAGAATGACGTACCGCTAAACACAGACTACTATTTC

TCCCACCTGTTGGGTACCATAAGCGTGACCTTTAAGGCTCTATTCGGAAATGATGTGAGA

ACAACAGAAAATCTTTTAAAGCGGTTTATTCCGGAAACCCCCCACAAGACCCCCACGAAA

ACCCAGGCACTGCTTGAGCGCGCCGGCTTTGAAAAGCTGACGCCCTTTACACCGGAGGAA

GAAAGTCGTCGAATACTGCATACAGTTTTTTGTACTCTAGAAGCAGCTCCCCATCAAAGC

TGA

>KF434369.1

ATGGCGGCGCGCGAACAGGCCAACTCCGTTCGCAGGAGCGGGTTTTTTAATCCGTTTATT

GGCAAGAGGCCATTTTTCAGGCCGGGAAGCGGGCAGACCGCGGAGACAGAGCGCCCAAGG

CCCCCCCAACACTCGTACTGCACAGAGGTGGGTAGCTTTAAGTTTATAGCTCCAAGATGC

CTCGATGAAGAAGCCCCCGCCGACCAGCGACGCGGTGTACACGTGGGCACCTTGGAGCGC

CCACCCAAAGTGTACTGCGATGGCTCAGAGTACGACGTGCTGAACTTTGCCTCCGGAGGT

TGCTGGCCTCGCAGGATTCGGGTTTGGAACGGCCAGGATTTTCGGGGCGATGGATTCAAC

CCCAGATTTGAGAGATTTCACGTGTACGACATAGTAGAGACTTCGGAGAGCGCCTCACAC

GATGACCCATCCAGGTTTGCTGAGCTATCCCGCCCAAGCGGGTCTGTGGTAACACTGCTG

GGAATGAGCGAGTGTGGAAAGCGAGTCGCGGTTCACGTCTATGGTGTGCGCCATTATTTT

TACATGGCAAAGGCGGAGGTGGATAGCGCTTGTGGAATAACCACCGAGGCAGAACTCGTG

CGCGCAATGGTGGACTGCGCGCACAGCTCGGCTTTGAGCGCGGCCCTGGGAAATGGCAAC

GGCGGCAAACAGAGCGGCGGCAGCGGCGGGGGATGGTGGGGCGGAAAGCACGTGTCTGCG

GACTGCTTCAAAGTGGAGACCGTGTGCCACACGACGCTGTACTACTTTGGATCTAAGCCA

GCTCTCTACTATAGAGTATCTGCCTCCAGCAGCCGCCTGGGAGGGTTCATCTGCGACAAC

TTTCACCCGGAGATTACAAAATTCGAGGGGAGCGTGGACGTGACCACGCGGCTGTTGTTG

GACAATGAAAATTTTACCAGTTTCGGGTGGTACCGCCTGCGACCCGGCACCCACGGAGAG

CGTGTTCAACTTCGCCCCGTTGAGCGACACGTCACCTCAAGCGACGTGGAGATTAACTGT

ACTCCCGATAACCTGGAGCCGATACCAGACGAGGCTGCCTGGCCCGACTATAAGCTCATG

TGCTTTGATATAGAGTGTAAAGCTGGAACGGGTAACGAAATGGCGTTCCCAGTGGCAACT

AACCAAGAGGACCTGGTCATCCAGATCTCCTGTCTGCTGTACTCGCTTGCTACTCAGAAC

CACGAACACACCCTGCTGTTTTCCCTCGGGTCATGCGATATCTCTGAGGAATACTCGTTT

GCATGCGTCCAGCGCGGCGAGCCCAGACCGACGGTTTTGGAGTTTGACAGCGAGTACGAG

CTGCTGGTTGCCTTCCTGACCTTTCTCAAGCAGTACTCTCCCGAGTTCGCCACCGGCTAC

AACATCGTTAATTTTGACTGGGCGTACATAGTTAACAAGGTAACGTCGGTGTATAACATC

AAGCTGGACGGGTACGGCAAGTTCAACAAAGGGGGGCTGTTTAAGGTGTGGGACATCGCC

ACGAACCATTTTCAGAAGAAGAGCAAGGTGAAAATCAATGGCCTGATATCTCTAGACATG

TATTCTGTGGCGACGGAAAAGCTAAAGCTACCCAGCTACAAACTCGACGCGGTCGTGGGA

GACGTCCTCGGCGAGCATAAGATAGACCTTCCCTATAAAGAAATACCCTCCTATTACGCG

GGAGGGCCTGACCGGAGGGGCGTAATAGGAGAGTATTGTATCCAGGACTCTAGGCTGGTG

GGCAAGCTGTTTTTTAAGTACCTCCCCCATCTGGAACTATCGGCGGTGGCCAAACTCGCC

CGTATCACCCTAACGCGGGTAATTTTTGACGGTCAGCAAATTCGCGTGTACACGTGCTTG

CTGAAACTCGCCCGCGAGAGAAATTTCATTTTGCCAGACAACAGACGCCGGTTTGACAGT

CAGGCAGACGCCGCGTCAGAGACTTCGGAGTTGGCTATGGATAGCCAAAGCCACGCCTTC

GACAGTACAGACGAACCCGACGGTGTGGACGGTACCCCGGACGCCGCAGGATCTGGCGCT

ACTTCTGAAAACGGAGGCGGGAAGCCCGGCGTCGGGAGGGCCGTGGGCTACCAGGGAGCA

AAGGTTCTAGACCCCGTATCCGGCTTTCATGTGGACCCCGTGGTTGTGTTTGACTTCGCT

AGCTTATACCCAAGCATTATCCAGGCCCATAACCTCTGTTTCACCACCCTGGCGCTCGAT

GAAGTGGATCTGGCCGGGCTTCAACCATCCGTCAACTACTCGACGTTCGAGGTGGGTGAC

CAAAAGTTATTTTTTGTCCACGCCCATATTCGCGAAAGCCTGCTTGGCATCTTGCTGCGC

GACTGGCTGGCCATGCGAAAGGCGGTGAGGGCGCGAATCCCCACCAGCACCCCCGAGGAG

GCAGTTTTACTAGATAAGCAGCAGTCTGCGATTAAGGTGATATGCAACTCGGTTTACGGA

TTCACGGGGGTGGCAAACGGCCTGTTGCCGTGTCTGAGGATAGCGGCTACCGTTACCACG

ATAGGACGCGACATGCTCCTCAAGACCCGAGATTACGTTCACTCTCGTTGGGCGACGCGC

GAGCTGCTGGAGGACAATTTTCCAGGGGCTATAGGTTTCCGAAACCACAAGCCTTACTCC

GTCAGGGTTATCTACGGAGACACCGACTCCGTGTTTATCAAGTTTGTGGGCCTGACGTAC

GAGGGGGTATCGGAGCTGGGGGATGCTATGTCGCGTCAGATTTCAGCGGACCTCTTTAGA

GCGCCCATCAAACTGGAGTGTGAGAAGACCTTTCAGCGACTGCTGCTGATCACCAAGAAG

AAGTACATAGGTGTCATAAACGGGGGGAAGATGCTCATGAAGGGGGTCGACCTGGTCCGC

AAAAATAACTGCTCTTTCATAAACTTGTACGCGCGACATCTGGTAGATCTTTTGTTGTAC

GACGAGGATGTGGCCACGGCGGCAGCAGAGGTGACAGACGTGCCTCCCGCAGAATGGGTG

GGGCGCCCGCTACCGAGCGGCTTTGACAAGTTTGGGCGAGTGCTGGTAGAGGCGTACAAC

CGTATCACTGCCCCCAACTTGGACGTGCGCAAGTTCGTTATGACTGCTGAGCTGAGCCGC

TCACCCGAATCGTATACCAACAAGCGCCTGCCGCACCTCACCGTCTACTTTAAGCTCGCC

ATGAGGAATGAAGAACTGCCCAGCGTAAAAGAGAGAATTCCGTATGTGATAGTTGCGCAG

ACCGAGGCCGCGGAACGCGAAGCGGGTGTAGTAAACTCAATGCGCGGTACCGCCCAAAAC

CCCGTGGTAACCAAGACCGCACGCCCCCAACCTAAACGCAAACTGCTGGTTTCCGACCTC

GCCGAAGACCCGACCTATGTTTCCGAGAATGACGTACCGCTAAACACAGACTACTATTTC

TCCCACCTGTTGGGTACCATAAGCGTGACCTTTAAGGCTCTATTCGGAAATGATGTGAGA

ACAACAGAAAATCTTTTAAAGCGGTTTATTCCGGAAACCCCCCACAAGACCCCCACGAAA

ACCCAGGCACTGCTTGAGCGCGCCGGCTTTGAAAAGCTGACGCCCTTTACACCGGAGGAA

GAAAGTCGTCGAATACTGCATACAGTTTTTTGTACTCTAGAAGCAGCTCCCCATCAAAGC

TGA

>KF434370.1

ATGGCGGCGCGCGAACAGGCCAACTCCGTTCGCAGGAGCGGGTTTTTTAATCCGTTTATT

GGCAAGAGGCCATTTTTCAGGCCGGGAAGCGGGCAGACCGCGGAGACAGAGCGCCCAAGG

CCCCCCCAACACTCGTACTGCACAGAGGTGGGTAGCTTTAAGTTTATAGCTCCAAGATGC

CTCGATGAAGAAGCCCCCGCCGACCAGCGACGCGGTGTACACGTGGGCACCTTGGAGCGC

CCACCCAAAGTGTACTGCGATGGCTCAGAGTACGACGTGCTGAACTTTGCCTCCGGAGGT

TGCTGGCCTCGCAGGATTCGGGTTTGGAACGGCCAGGATTTTCGGGGCGATGGATTCAAC

CCCAGATTTGAGAGATTTCACGTGTACGACATAGTAGAGACTTCGGAGAGCGCCTCACAC

GATGACCCATCCAGGTTTGCTGAGCTATCCCGCCCAAGCGGGTCTGTGGTAACACTGCTG

GGAATGAGCGAGTGTGGAAAGCGAGTCGCGGTTCACGTCTATGGTGTGCGCCATTATTTT

TACATGGCAAAGGCGGAGGTGGATAGCGCTTGTGGAATAACCACCGAGGCAGAACTCGTG

CGCGCAATGGTGGACTGCGCGCACAGCTCGGCTTTGAGCGCGGCCCTGGGAAATGGCAAC

GGCGGCAAACAGAGCGGCGGCAGCGGCGGGGGATGGTGGGGCGGAAAGCACGTGTCTGCG

GACTGCTTCAAAGTGGAGACCGTGTGCCACACGACGCTGTACTACTTTGGATCTAAGCCA

GCTCTCTACTATAGAGTATCTGCCTCCAGCAGCCGCCTGGGAGGGTTCATCTGCGACAAC

TTTCACCCGGAGATTACAAAATTCGAGGGGAGCGTGGACGTGACCACGCGGCTGTTGTTG

GACAATGAAAATTTTACCAGTTTTGGGTGGTACCGCCTGCGACCCGGCACCCACGGAGAG

CGTGTTCAACTTCGCCCCGTTGAGCGACACGTCACCTCAAGCGACGTGGAGATTAACTGT

ACTCCCGATAACCTGGAGCCGATACCAGACGAGGCTGCCTGGCCCGACTATAAGCTCATG

TGCTTTGATATAGAGTGTAAAGCTGGAACGGGTAACGAAATGGCGTTCCCAGTGGCAACT

AACCAAGAGGACCTGGTCATCCAGATCTCCTGTCTGCTGTACTCGCTTGCTACTCAGAAC

CACGAACACACCCTGCTGTTTTCCCTCGGGTCATGCGATATCTCTGAGGAATACTCGTTT

GCATGCGTCCAGCGCGGCGAGCCCAGACCGACGGTTTTGGAGTTTGACAGCGAGTACGAG

CTGCTGGTTGCCTTCCTGACCTTTCTCAAGCAGTACTCTCCCGAGTTCGCCACCGGCTAC

AACATCGTTAATTTTGACTGGGCGTACATAGTTAACAAGGTAACGTCGGTGTATAACATC

AAGCTGGACGGGTACGGCAAGTTCAACAAAGGGGGGCTGTTTAAGGTGTGGGACATCGCC

ACGAACCATTTTCAGAAGAAGAGCAAGGTGAAAATCAATGGCCTGATATCTCTAGACATG

TATTCTGTGGCGACGGAAAAGCTAAAGCTACCCAGCTACAAACTCGACGCGGTCGTGGGA

GACGTCCTCGGCGAGCATAAGATAGACCTTCCCTATAAAGAAATACCCTCCTATTACGCG

GGAGGGCCTGACCGGAGGGGCGTAATAGGAGAGTATTGTATCCAGGACTCTAGGCTGGTG

GGCAAGCTGTTTTTTAAGTACCTCCCCCATCTGGAACTATCGGAGGTGGCCAAACTCGCC

CGTATCACCCTAACGCGGGTAATTTTTGACGGTCAGCAAATTCGCGTGTACACGTGCTTG

CTGAAACTCGCCCGCGAGAGAAATTTCATTTTGCCAGACAACAGACGCCGGTTTGACAGT

CAGGCAGACGCCGCGTCAGAGACTTCGGAGTTGGCTATGGATAGCCAAAGCCACGCCTTC

GACAGTACAGACGAACCCGACGGTGTGGACGGTACCCCGGACGCCGCAGGATCTGGCGCT

ACTTCTGAAAACGGAGGCGGGAAGCCCGGCGTCGGGAGGGCCGTGGGCTACCAGGGAGCA

AAGGTTCTAGACCCCGTATCCGGCTTTCATGTGGACCCCGTGGTTGTGTTTGACTTCGCT

AGCTTATACCCAAGCATTATCCAGGCCCATAACCTCTGTTTCACCACCCTGGCGCTCGAT

GAAGTGGATCTGGCCGGGCTTCAACCATCCGTCAACTACTCGACGTTCGAGGTGGGTGAC

CAAAAGTTATTTTTTGTCCACGCCCATATTCGCGAAAGCCTGCTTGGCATCTTGCTGCGC

GACTGGCTGGCCATGCGAAAGGCGGTGAGGGCGCGAATCCCCACCAGCACCCCCGAGGAG

GCAGTTTTACTAGATAAGCAGCAGTCTGCGATTAAGGTGATATGCAACTCGGTTTACGGA

TTCACGGGGGTGGCAAACGGCCTGTTGCCGTGTCTGAGGATAGCGGCTACCGTTACCACG

ATAGGACGCGACATGCTCCTCAAGACCCGAGATTACGTTCACTCTCGTTGGGCGACGCGC

GAGCTGCTGGAGGACAATTTTCCAGGGGCTATAGGTTTCCGAAACCACAAGCCTTACTCC

GTCAGGGTTATCTACGGAGACACCGACTCCGTGTTTATCAAGTTTGTGGGCCTGACGTAC

GAGGGGGTATCGGAGCTGGGGGATGCTATGTCGCGTCAGATTTCAGCGGACCTCTTTAGA

GCGCCCATCAAACTGGAGTGTGAGAAGACCTTTCAGCGACTGCTGCTGATCACCAAGAAG

AAGTACATAGGTGTCATAAACGGGGGGAAGATGCTCATGAAGGGGGTCGACCTGGTCCGC

AAAAATAACTGCTCTTTCATAAACTTGTACGCGCGACATCTGGTAGATCTTTTGTTGTAC

GACGAGGATGTGGCCACGGCGGCAGCAAAGGTGACAGACGTGCCTCCCGCAGAATGGGTG

GGGCGCCCGCTACCGAGCGGCTTTGACAAGTTTGGGCGAGTGCTGGTAGAGGCGTACAAC

CGTATCACTGCCCCCAACTTGGACGTGCGCGAGTTCGTTATGACTGCTGAGCTGAGCCGC

TCACCCGAATCGTATACCAACAAGCGCCTGCCGCACCTCACCGTCTACTTTAAGCTCGCC

ATGAGGAATGAAGAACTGCCCAGCGTAAAAGAGAGAATTCCGTATGTGATAGTTGCGCAG

ACCGAGGCCGCGGAACGCGAAGCGGGTGTAGTAAACTCAATGCGCGGTACCGCCCAAAAC

CCCGTGGTAACCAAGACCGCACGCCCCCAACCTAAACGCAAACTGCTGGTTTCCGACCTC

GCCGAAGACCCGACCTATGTTTCCGAGAATGACGTACCGCTAAACACAGACTACTATTTC

TCCCACCTGTTGGGTACCATAAGCGTGACCTTTAAGGCTCTATTCGGAAATGATGTGAGA

ACAACAGAAAATCTTTTAAAGCGGTTTATTCCGGAAACCCCCCACAAGACCCCCACGAAA

ACCCAGGCACTGCTTGAGCGCGCCGGCTTTGAAAAGCTGACGCCCTTTACACCGGAGGAA

GAAAGTCGTCGAATACTGCATACAGTTTTTTGTACTCTAGAAGCAGCTCCCCATCAAAGC

TGA

>KF434371.1

ATGGCGGCGCGCGAACAGGCCAACTCCGTTCGCAGGAGCGGGTTTTTTAATCCGTTTATT

GGCAAGAGGCCATTTTTCAGGCCGGGAAGCGGGCAAACCGCGGAGACAGAGCGCCCAAGG

CCCCCCCAACACTCGTACTGCACAGAGGTGGGTAGCTTTAAGTTTATAGCTCCAAGATGC

CTCGATGAAGAAGCCCCCGCCGACCAGCGACGCGGTGTACACGTGGGCACCTTGGAGCGC

CCACCCAAAGTGTACTGCGATGGCTCAGAGTACGACGTGCTGAACTTTGCCTCCGGAGGT

TGCTGGCCTCGCAGGATTCGGGTTTGGAACGGCCAGGATTTTCGGGGCGATGGATTCAAC

CCCAGATTTGAGAGATTTCACGTGTACGACATAGTAGAGACTTCGGAGAGCGCCTCACAC

GATGACCCATCCAGGTTTGCTGAGCTATCCCGCCCAAGCGGGTCTGTGGTAACACTGCTG

GGAATGAGCGAGTGTGGAAAGCGAGTCGCGGTTCACGTCTATGGTGTGCGCCATTATTTT

TACATGGCAAAGGCGGAGGTGGATAGCGCTTGTGGAATAACCACCGAGGCAGAACTCGTG

CGCGCAATGGTGGACTGCGCGCACAGCTCGGCTTTGAGCGCGGCCCTGGGAAATGGCAAC

GGCGGCAAACAGAGCGGCGGCAGCGGCGGGGGATGGTGGGGCGGAAAGCACGTGTCTGCG

GACTGCTTCAAAGTGGAGACCGTGTGCCACACGACGCTGTACTACTTTGGATCTAAGCCA

GCTCTCTACTATAGAGTATCTGCCTCCAGCAGCCGCCTGGGAGGGTTCATCTGCGACAAC

TTTCACCCGGAGATTACAAAATTCGAGGGGAGCGTGGACGTGACCACGCGGCTGTTGTTG

GACAATGAAAATTTTACCAGTTTCGGGTGGTACCGCCTGCGACCCGGCACCCACGGAGAG

CGTGTTCAACTTCGCCCCGTTGAGCGACACGTCACCTCAAGCGACGTGGAGATTAACTGT

ACTCCCGATAACCTGGAGCCGATACCAGACGAGGCTGCCTGGCCCGACTATAAGCTCATG

TGCTTTGATATAGAGTGTAAAGCTGGAACGGGTAACGAAATGGCGTTCCCAGTGGCAACT

AACCAAGAGGACCTGGTCATCCAGATCTCCTGTCTGCTGTACTCGCTTGCTACTCAGAAC

CACGAACACACCCTGCTGTTTTCCCTCGGGTCATGCGATATCTCTGAGGAATACTCGTTT

GCATGCGTCCAGCGCGGCGAGCCCAGACCGACGGTTTTGGAGTTTGACAGCGAGTACGAG

CTGCTGGTTGCCTTCCTGACCTTTCTCAAGCAGTACTCTCCCGAGTTCGCCACCGGCTAC

AACATCGTTAATTTTGACTGGGCGTACATAGTTAACAAGGTAACGTCGGTGTATAACATC

AAGCTGGACGGGTACGGCAAGTTCAACAAAGGGGGGCTGTTTAAGGTGTGGGACATCGCC

ACGAACCATTTTCAGAAGAAGAGCAAGGTGAAAATCAATGGCCTGATATCTCTAGACATG

TATTCTGTGGCGACGGAAAAGCTAAAGCTACCCAGCTACAAACTCGACGCGGTCGTGGGA

GACGTCCTCGGCGAGCATAAGATAGACCTTCCCTATAAAGAAATACCCTCCTATTACGCG

GGAGGGCCTGACCGGAGGGGCGTAATAGGAGAGTATTGTATCCAGGACTCTAGGCTGGTG

GGCAAGCTGTTTTTTAAGTACCTCCCCCATCTGGAACTATCGGCGGTGGCCAAACTCGCC

CGTATCACCCTAACGCGGGTAATTTTTGACGGTCAGCAAATTCGCGTGTACACGTGCTTG

CTGAAACTCGCCCGCGAGAGAAATTTCATTTTGCCAGACAACAGACGCCGGTTTGACAGT

CAGGCAGACGCCGCGTCAGAGACTTCGGAGTTGGCTATGGATAGCCAAAGCCACGCCTTC

GACAGTACAGACGAACCCGACGGTGTGGACGGTACCCCGGACGCCGCAGGATCTGGCGCT

ACTTCTGAAAACGGAGGCGGGAAGCCCGGCGTCGGGAGGGCCGTGGGCTACCAGGGAGCA

AAGGTTCTAGACCCCGTATCCGGCTTTCATGTGGACCCCGTGGTTGTGTTTGACTTCGCT

AGCTTATACCCAAGCATTATCCAGGCCCATAACCTCTGTTTCACCACCCTGGCGCTCGAT

GAAGTGGATCTGGCCGGGCTTCAACCATCCGTCAACTACTCGACGTTCGAGGTGGGTGAC

CAAAAGTTATTTTTTGTCCACGCCCATATTCGCGAAAGCCTGCTTGGCATCTTGCTGCGC

GACTGGCTGGCCATGCGAAAGGCGGTGAGGGCGCGAATCCCCACCAGCACCCCCGAGGAG

GCAGTTTTACTAGATAAGCAGCAGTCTGCGATTAAGGTGATATGCAACTCGGTTTACGGA

TTCACGGGGGTGGCAAACGGCCTGTTGCCGTGTCTGAGGATAGCGGCTACCGTTACCACG

ATAGGACGCGACATGCTCCTCAAGACCCGAGATTACGTTCACTCTCGTTGGGCGACGCGC

GAGCTGCTGGAGGACAATTTTCCAGGGGCTATAGGTTTCCGAAACCACAAGCCTTACTCC

GTCAGGGTTATCTACGGAGACACCGACTCCGTGTTTATCAAGTTTGTGGGCCTGACGTAC

GAGGGGGTATCGGAGCTGGGGGATGCTATGTCGCGTCAGATTTCAGCGGACCTCTTTAGA

GCGCCCATCAAACTGGAGTGTGAGAAGACCTTTCAGCGACTGCTGCTGATCACCAAGAAG

AAGTACATAGGTGTCATAAACGGGGGGAAGATGCTCATGAAGGGGGTCGACCTGGTCCGC

AAAAATAACTGCTCTTTCATAAACTTGTACGCGCGACATCTGGTAGATCTTTTGTTGTAC

GACGAGGATGTGGCCACGGCGGCAGCAGAGGTGACAGACGTGCCTCCCGCAGAATGGGTG

GGGCGCCCGCTACCGAGCGGCTTTGACAAGTTTGGGCGAGTGCTGGTAGAGGCGTACAAC

CGTATCACTGCCCCCAACTTGGACGTGCGCGAGTTCGTTATGACTGCTGAGCTGAGCCGC

TCACCCGAATCGTATACCAACAAGCGCCTGCCGCACCTCACCGTCTACTTTAAGCTCGCC

ATGAGGAATGAAGAACTGCCCAGCGTAAAAGAGAGAATTCCGTATGTGATAGTTGCGCAG

ACCGAGGCCGCGGAACGCGAAGCGGGTGTAGTAAACTCAATGCGCGGTACCGCCCAAAAC

CCCGTGGTAACCAAGACCGCACGCCCCCAACCTAAACGCAAACTGCTGGTTTCCGACCTC

GCCGAAGACCCGACCTATGTTTCCGAGAATGACGTACCGCTAAACACAGACTACTATTTC

TCCCACCTGTTGGGTACCATAAGCGTGACCTTTAAGGCTCTATTCGGAAATGATGTGAGA

ACAACAGAAAATCTTTTAAAGCGGTTTATTCCGGAAACCCCCCACAAGACCCCCACGAAA

ACCCAGGCACTGCTTGAGCGCGCCGGCTTTGAAAAGCTGACGCCCTTTACACCGGAGGAA

GAAAGTCGTCGAATACTGCATACAGTTTTTTGTACTCTAGAAGCAGCTCCCCATCAAAGC

TGA

>KF434372.1

ATGGCGGCGCGCGAACAGGCCAACTCCGTTCGCAGGAGCGGGTTTTTTAATCCGTTTATT

GGCAAGAGGCCATTTTTCAGGCCGGGAAGCGGGCAGACCGCGGAGACAGAGCGCCCAAGG

CCCCCCCAACACTCGTACTGCACAGAGGTGGGTAGCTTTAAGTTTATAGCTCCAAGATGC

CTCGATGAAGAAGCCCCCGCCGACCAGCGACGCGGTGTACACGTGGGCACCTTGGAGCGC

CCACCCAAAGTGTACTGCGATGGCTCAGAGTACGACGTGCTGAACTTTGCCTCCGGAGGT

TGCTGGCCTCGCAGGATTCGGGTTTGGAACGGCCAGGATTTTCGGGGCGATGGATTCAAC

CCCAGATTTGAGAGATTTCACGTGTACGACATAGTAGAGACTTCGGAGAGCGCCTCACAC

GATGACCCATCCAGGTTTGCTGAGCTATCCCGCCCAAGCGGGTCTGTGGTAACACTGCTG

GGAATGAGCGAGTGTGGAAAGCGAGTCGCGGTTCACGTCTATGGTGTGCGCCATTATTTT

TACATGGCAAAGGCGGAGGTGGATAGCGCTTGTGGAATAACCACCGAGGCAGAACTCGTG

CGCGCAATGGTGGACTGCGCGCACAGCTCGGCTTTGAGCGCGGCCCTGGGAAATGGCAAC

GGCGGCAAACAGAGCGGCGGCAGCGGCGGGGGATGGTGGGGCGGAAAGCACGTGTCTGCG

GACTGCTTCAAAGTGGAGACCGTGTGCCACACGACGCTGTACTACTTTGGATCTAAGCCA

GCTCTCTACTATAGAGTATCTGCCTCCAGCAGCCGCCTGGGAGGGTTCATCTGCGACAAC

TTTCACCCGGAGATTACAAAATTCGAGGGGAGCGTGGACGTGACCACGCGGCTGTTGTTG

GACAATGAAAATTTTACCAGTTTCGGGTGGTACCGCCTGCGACCCGGCACCCACGGAGAG

CGTGTTCAACTTCGCCCCGTTGAGCGACACGTCACCTCAAGCGACGTGGAGATTAACTGT

ACTCCCGATAACCTGGAGCCGATACCAGACGAGGCTGCCTGGCCCGACTATAAGCTCATG

TGCTTTGATATAGAGTGTAAAGCTGGAACGGGTAACGAAATGGCGTTCCCAGTGGCAACT

AACCAAGAGGACCTGGTCATCCAGATCTCCTGTCTGCTGTACTCGCTTGCTACTCAGAAC

CACGAACACACCCTGCTGTTTTCCCTCGGGTCATGCGATATCTCTGAGGAATACTCGTTT

GCATGCGTCCAGCGCGGCGAGCCCAGACCGACGGTTTTGGAGTTTGACAGCGAGTACGAG

CTGCTGGTTGCCTTCCTGACCTTTCTCAAGCAGTACTCTCCCGAGTTCGCCACCGGCTAC

AACATCGTTAATTTTGACTGGGCGTACATAGTTAACAAGGTAACGTCGGTGTATAACATC

AAGCTGGACGGGTACGGCAAGTTCAACAAAGGGGGGCTGTTTAAGGTGTGGGACATCGCC

ACGAACCATTTTCAGAAGAAGAGCAAGGTGAAAATCAATGGCCTGATATCTCTAGACATG

TATTCTGTGGCGACGGAAAAGCTAAAGCTACCCAGCTACAAACTCGACGCGGTCGTGGGA

GACGTCCTCGGCGAGCATAAGATAGACCTTCCCTATAAAGAAATACCCTCCTATTACGCG

GGAGGGCCTGACCGGAGGGGCGTAATAGGAGAGTATTGTATCCAGGACTCTAGGCTGGTG

GGCAAGCTGTTTTTTAAGTACCTCCCCCATCTGGAACTATCGGCGGTGGCCAAACTCGCC

CATATCACCCTAACGCGGGTAATTTTTGACGGTCAGCAAATTCGCGTGTACACGTGCTTG

CTGAAACTCGCCCGCGAGAGAAATTTCATTTTGCCAGACAACAGACGCCGGTTTGACAGT

CAGGCAGACGCCGCGTCAGAGACTTCGGAGTTGGCTATGGATAGCCAAAGCCACGCCTTC

GACAGTACAGACGAACCCGACGGTGTGGACGGTACCCCGGACGCCGCAGGATCTGGCGCT

ACTTCTGAAAACGGAGGCGGGAAGCCCGGCGTCGGGAGGGCCGTGGGCTACCAGGGAGCA

AAGGTTCTAGACCCCGTATCCGGCTTTCATGTGGACCCCGTGGTTGTGTTTGACTTCGCT

AGCTTATACCCAAGCATTATCCAGGCCCATAACCTCTGTTTCACCACCCTGGCGCTCGAT

GAAGTGGATCTGGCCGGGCTTCAACCATCCGTCAACTACTCGACGTTCGAGGTGGGTGAC

CAAAAGTTATTTTTTGTCCACGCCCATATTCGCGAAAGCCTGCTTGGCATCTTGCTGCGC

GACTGGCTGGCCATGCGAAAGGCGGTGAGGGCGCGAATCCCCACCAGCACCCCCGAGGAG

GCAGTTTTACTAGATAAGCAGCAGTCTGCGATTAAGGTGATATGCAACTCGGTTTACGGA

TTCACGGGGGTGGCAAACGGCCTGTTGCCGTGTCTGAGGATAGCGGCTACCGTTACCACG

ATAGGACGCGACATGCTCCTCAAGACCCGAGATTACGTTCACTCTCGTTGGGCGACGCGC

GAGCTGCTGGAGGACAATTTTCCAGGGGCTATAGGTTTCCGAAACCACAAGCCTTACTCC

GTCAGGGTTATCTACGGAGACACCGACTCCGTGTTTATCAAGTTTGTGGGCCTGACGTAC

GAGGGGGTATCGGAGCTGGGGGATGCTATGTCGCGTCAGATTTCAGCGGACCTCTTTAGA

GCGCCCATCAAACTGGAGTGTGAGAAGACCTTTCAGCGACTGCTGCTGATCACCAAGAAG

AAGTACATAGGTGTCATAAACGGGGGGAAGATGCTCATGAAGGGGGTCGACCTGGTCCGC

AAAAATAACTGCTCTTTCATAAACTTGTACGCGCGACATCTGGTAGATCTTTTGTTGTAC

GACGAGGATGTGGCCACGGCGGCAGCAGAGGTGACAGACGTGCCTCCCGCAGAATGGGTG

GGGCGCCCGCTACCGAGCGGCTTTGACAAGTTTGGGCGAGTGCTGGTAGAGGCGTACAAC

CGTATCACTGCCCCCAACTTGGACGTGCGCGAGTTCGTTATGACTGCTGAGCTGAGCCGC

TCACCCGAATCGTATACCAACAAGCGCCTGCCGCACCTCACCGTCTACTTTAAGCTCGCC

ATGAGGAATGAAGAACTGCCCAGCGTAAAAGAGAGAATTCCGTATGTGATAGTTGCGCAG

ACCGAGGCCGCGGAACGCGAAGCGGGTGTAGTAAACTCAATGCGCGGTACCGCCCAAAAC

CCCGTGGTAACCAAGACCGCACGCCCCCAACCTAAACGCAAACTGCTGGTTTCCGACCTC

GCCGAAGACCCGACCTATGTTTCCGAGAATGACGTACCGCTAAACACAGACTACTATTTC

TCCCACCTGTTGGGTACCATAAGCGTGACCTTTAAGGCTCTATTCGGAAATGATGTGAGA

ACAACAGAAAATCTTTTAAAGCGGTTTATTCCGGAAACCCCCCACAAGACCCCCACGAAA

ACCCAGGCACTGCTTGAGCGCGCCGGCTTTGAAAAGCTGACGCCCTTTACACCGGAGGAA

GAAAGTCGTCGAATACTGCATACAGTTTTTTGTACTCTAGAAGCAGCTCCCCATCAAAGC

TGA

>KF434373.1

ATGGCGGCGCGCGAACAGGCCAACTCCGTTCGCAGGAGCGGGTTTTTTAATCCGTTTATT

GGCAAGAGGCCATTTTTCAGGCCGGGAAGCGGGCAGACCGCGGAGACAGAGCGCCCAAGG

CCCCCCCAACACTCGTACTGCACAGAGGTGGGTAGCTTTAAGTTTATAGCTCCAAGATGC

CTCGATGAAGAAGCCCCCGCCGACCAGCGACGCGGTGTACACGTGGGCACCTTGGAGCGC

CCACCCAAAGTGTACTGCGATGGCTCAGAGTACGACGTGCTGAACTTTGCCTCCGGAGGT

TGCTGGCCTCGCAGGATTCGGGTTTGGAACGGCCAGGATTTTCGGGGCGATGGATTCAAC

CCCAGATTTGAGAGATTTCACGTGTACGACATAGTAGAGACTTCGGAGAGCGCCTCACAC

GATGACCCATCCAGGTTTGCTGAGCTATCCCGCCCAAGCGGGTCTGTGGTAACACTGCTG

GGAATGAGCGAGTGTGGAAAGCGAGTCGCGGTTCACGTCTATGGTGTGCGCCATTATTTT

TACATGGCAAAGGCGGAGGTGGATAGCGCTTGTGGAATAACCACCGAGGCAGAACTCGTG

CGCGCAATGGTGGACTGCGCGCACAGCTCGGCTTTGAGCGCGGCCCTGGGAAATGGCAAC

GGCGGCAAACAGAGCGGCGGCAGCGGCGGGGGATGGTGGGGCGGAAAGCACGTGTCTGCG

GACTGCTTCAAAGTGGAGACCGTGTGCCACACGACGCTGTACTACTTTGGATCTAAGCCA

GCTCTCTACTATAGAGTATCTGCCTCCAGCAGCCGCCTGGGAGGGTTCATCTGCGACAAC

TTTCACCCGGAGATTACAAAATTCGAGGGGAGCGTGGACGTGACCACGCGGCTGTTGTTG

GACAATGAAAATTTTACCAGTTTCGGGTGGTACCGCCTGCGACCCGGCACCCACGGAGAG

CGTGTTCAACTTCGCCCCGTTGAGCGACACGTCACCTCAAGCGACGTGGAGATTAACTGT

ACTCCCGATAACCTGGAGCCGATACCAGACGAGGCTGCCTGGCCCGACTATAAGCTCATG

TGCTTTGATATAGAGTGTAAAGCTGGAACGGGTAACGAAATGGCGTTCCCAGTGGCAACT

AACCAAGAGGACCTGGTCATCCAGATCTCCTGTCTGCTGTACTCGCTTGCTACTCAGAAC

CACGAACACACCCTGCTGTTTTCCCTCGGGTCATGCGATATCTCTGAGGAATACTCGTTT

GCATGCGTCCAGCGCGGCGAGCCCAGACCGACGGTTTTGGAGTTTGACAGCGAGTACGAG

CTGCTGGTTGCCTTCCTGACCTTTCTCAAGCAGTACTCTCCCGAGTTCGCCACCGGCTAC

AACATCGTTAATTTTGACTGGGCGTACATAGTTAACAAGGTAACGTCGGTGTATAACATC

AAGCTGGACGGGTACGGCAAGTTCAACAAAGGGGGGCTGTTTAAGGTGTGGGACATCGCC

ACGAACCATTTTCAGAAGAAGAGCAAGGTGAAAATCAATGGCCTGATATCTCTAGACATG

TATTCTGTGGCGACGGAAAAGCTAAAGCTACCCAGCTACAAACTCGACGCGGTCGTGGGA

GACGTCCTCGGCGAGCATAAGATAGACCTTCCCTATAAAGAAATACCCTCCTATTACGCG

GGAGGGCCTGACCGGAGGGGCGTAATAGGAGAGTATTGTATCCAGGACTCTAGGCTGGTG

GGCAAGCTGTTTTTTAAGTACCTCCCCCATCTGGAACTATCGGCGGTGGCCAAACTCGCC

CGTATCACCCTAACGCGGGTAATTTTTGACGGTCAGCAAATTCGCGTGTACACGTGCTTG

CTGAAACTCGCCCGCGAGAGAAATTTCATTTTGCCAGACAACAGACGCCGGTTTGACAGT

CAGGCAGACGCCGCGTCAGAGACTTCGGAGTTGGCTATGGATAGCCAAAGCCACGCCTTC

GACAGTACAGACGAACCCGACGGTGTGGACGGTACCCCGGACGCCGCAGGATCTGGCGCT

ACTTCTGAAAACGGAGGCGGGAAGCCCGGCGTCGGGAGGGCCGTGGGCTACCAGGGAGCA

AAGGTTCTAGACCCCGTATCCGGCTTTCATGTGGACCCCGTGGTTGTGTTTGACTTCGCT

AGCTTATACCCAAGCATTATCCAGGCCCATAACCTCTGTTTCACCACCCTGGCGCTCGAT

GAAGTGGATCTGGCCGGGCTTCAACCATCCGTCAACTACTCGACGTTCGAGGTGGGTGAC

CAAAAGTTATTTTTTGTCCACGCCCATATTCGCGAAAGCCTGCTTGGCATCTTGCTGCGC

GACTGGCTGGCCATGCGAAAGGCGGTGAGGGCGCGAATCCCCACCAGCACCCCCGAGGAG

GCAGTTTTACTAGATAAGCAGCAGTCTGCGATTAAGGTGATATGCAACTCGGTTTACGGA

TTCACGGGGGTGGCAAACGGCCTGTTGCCGTGTCTGAGGATAGCGGCTACCGTTACCACG

ATAGGACGCGACATGCTCCTCAAGACCCGAGATTACGTTCACTCTCGTTGGGCGACGCGC

GAGCTGCTGGAGGACAATTTTCCAGGGGCTATAGGTTTCCGAAACCACAAGCCTTACTCC

GTCAGGGTTATCTACGGAGACACCGACTCCGTGTTTATCAAGTTTGTGGGCCTGACGTAC

GAGGGGGTATCGGAGCTGGGGGATGCTATGTCGCGTCAGATTTCAGCGGACCTCTTTAGA

GCGCCCATCAAACTGGAGTGTGAGAAGACCTTTCAGCGACTGCTGCTGATCACCAAGAAG

AAGTACATAGGTGTCATAAACGGGGGGAAGATGCTCATGAAGGGGGTCGACCTGGTCCGC

AAAAATAACTGCTCTTTCATAAACTTGTACGCGCGACATCTGGTAGATCTTTTGTTGTAC

GACGAGGATGTGGCCACGGCGGCAGCAGAGGTGACAGACGTGCCTCCCGCAGAATGGGTG

GGGCGCCCGCTACCGAGCGGCTTTGACAAGTTTGGGCGAGTGCTGGTAGAGGCGTACAAC

CGTATCACTGCCCCCAACTTGGACGTGCGCGAGTTCGTTATGACTGCTGAGCTGAGCCGC

TCACCCGAATCGTATACCAACAAGCGCCTGCCGCACCTCACCGTCTACTTTAAGCTCGCC

ATGAGGAATGAAGAACTGCCCAGCGTAAAAGAGAGAATTCCGTATGTGATAGTTGCGCAG

ACCGAGGCCGCGGAACGCGAAGCGGGTGTAGTAAACTCAATGCGCGGTACCGCCCAAAAC

CCCGTGGTAACCAAGACCGCACGCCCCCAACCTAAACGCAAACTGCTGGTTTCCGACCTC

GCCGAAGACCCGACCTATGTTTCCGAGAATGACGTACCGCTAAACACAGACTACTATTTC

TCCCACCTGTTGGGTACCATAAGCGTGACCTTTAAGGCTCTATTCGGAAATGATGTGAGA

ACAACAGAAAATCTTTTAAAGCGGTTTATTCCGGAAACCCCCCACAAGACCCCCACGAAA

ACCCAGGCACTGCTTGAGCGCGCCGGCTTTGAAAAGCTGACGCCCTTTACACCGGAGGAA

GAAAGTCGTCGAATACTGCATACAGTTTTTTGTACTCTAGAAGCAGCTCCCCATCAAAGC

TGA

>KF434374.1

ATGGCGGCGCGCGAACAGGCCAACTCCGTTCGCAGGAGCGGGTTTTTTAATCCGTTTATT

GGCAAGAGGCCATTTTTCAGGCCGGGAAGCGGGCAGACCGCGGAGACAGAGCGCCCAAGG

CCCCCCCAACACTCGTACTGCACAGAGGTGGGTAGCTTTAAGTTTATAGCTCCAAGATGC

CTCGATGAAGAAGCCCCCGCCGACCAGCGACGCGGTGTACACGTGGGCACCTTGGAGCGC

CCACCCAAAGTGTACTGCGATGGCTCAGAGTACGACGTGCTGAACTTTGCCTCCGGAGGT

TGCTGGCCTCGCAGGATTCGGGTTTGGAACGGCCAGGATTTTCGGGGCGATGGATTCAAC

CCCAGATTTGAGAGATTTCACGTGTACGACATAGTAGAGACTTCGGAGAGCGCCTCACAC

GATGACCCATCCAGGTTTGCTGAGCTATCCCGCCCAAGTGGGTCTGTGGTAACACTGCTG

GGAATGAGCGAGTGTGGAAAGCGAGTCGCGGTTCACGTCTATGGTGTGCGCCATTATTTT

TACATGGCAAAGGCGGAGGTGGATAGCGCTTGTGGAATAACCACCGAGGCAGAACTCGTG

CGCGCAATGGTGGACTGCGCGCACAGCTCGGCTTTGAGCGCGGCCCTGGGAAATGGCAAC

GGCGGCAAACAGAGCGGCGGCAGCGGCGGGGGATGGTGGGGCGGAAAGCACGTGTCTGCG

GACTGCTTCAAAGTGGAGACCGTGTGCCACACGACGCTGTACTACTTTGGATCTAAGCCA

GCTCTCTACTATAGAGTATCTGCCTCCAGCAGCCGCCTGGGAGGGTTCATCTGCGACAAC

TTTCACCCGGAGATTACAAAATTCGAGGGGAGCGTGGACGTGACCACGCGGCTGTTGTTG

GACAATGAAAATTTTACCAGTTTCGGGTGGTACCGCCTGCGACCCGGCACCCACGGAGAG

CGTGTTCAACTTCGCCCCGTTGAGCGACACGTCACCTCAAGCGACGTGGAGATTAACTGT

ACTCCCGATAACCTGGAGCCGATACCAGACGAGGCTGCCTGGCCCGACTATAAGCTCATG

TGCTTTGATATAGAGTGTAAAGCTGGAACGGGTAACGAAATGGCGTTCCCAGTGGCAACT

AACCAAGAGGACCTGGTCATCCAGATCTCCTGTCTGCTGTACTCGCTTGCTACTCAGAAC

CACGAACACACCCTGCTGTTTTCCCTCGGGTCATGCGATATCTCTGAGGAATACTCGTTT

GCATGCGTCCAGCGCGGCGAGCCCAGACCGACGGTTTTGGAGTTTGACAGCGAGTACGAG

CTGCTGGTTGCCTTCCTGACCTTTCTCAAGCAGTACTCTCCCGAGTTCGCCACCGGCTAC

AACATCGTTAATTTTGACTGGGCGTACATAGTTAACAAGGTAACGTCGGTGTATAACATC

AAGCTGGACGGGTACGGCAAGTTCAACAAAGGGGGGCTGTTTAAGGTGTGGGACATCGCC

ACGAACCATTTTCAGAAGAAGAGCAAGGTGAAAATCAATGGCCTGATATCTCTAGACATG

TATTCTGTGGCGACGGAAAAGCTAAAGCTACCCAGCTACAAACTCGACGCGGTCGTGGGA

GACGTCCTCGGCGAGCATAAGATAGACCTTCCCTATAAAGAAATACCCTCCTATTACGCG

GGAGGGCCTGACCGGAGGGGCGTAATAGGAGAGTATTGTATCCAGGACTCTAGGCTGGTG

GGCAAGCTGTTTTTTAAGTACCTCCCCCATCTGGAACTATCGGCGGTGGCCAAACTCGCC

CGTATCACCCTAACGCGGGTAATTTTTGACGGTCAGCAAATTCGCGTGTACACGTGCTTG

CTGAAACTCGCCCGCGAGAGAAATTTCATTTTGCCAGACAACAGACGCCGGTTTGACAGT

CAGGCAGACGCCGCGTCAGAGACTTCGGAGTTGGCTATGGATAGCCAAAGCCACGCCTTC

GACAGTACAGACGAACCCGACGGTGTGGACGGTACCCCGGACGCCGCAGGATCTGGCGCT

ACTTCTGAAAACGGAGGCGGGAAGCCCGGCGTCGGGAGGGCCGTGGGCTACCAGGGAGCA

AAGGTTCTAGACCCCGTATCCGGCTTTCATGTGGACCCCGTGGTTGTGTTTGACTTCGCT

AGCTTATACCCAAGCATTATCCAGGCCCATAACCTCTGTTTCACCACCCTGGCGCTCGAT

GAAGTGGATCTGGCCGGGCTTCAACCATCCGTCAACTACTCGACGTTCGAGGTGGGTGAC

CAAAAGTTATTTTTTGTCCACGCCCATATTCGCGAAAGCCTGCTTGGCATCTTGCTGCGC

GACTGGCTGGCCATGCGAAAGGCGGTGAGGGCGCGAATCCCCACCAGCACCCCCGAGGAG

GCAGTTTTACTAGATAAGCAGCAGTCTGCGATTAAGGTGATATGCAACTCGGTTTACGGA

TTCACGGGGGTGGCAAACGGCCTGTTGCCGTGTCTGAGGATAGCGGCTACCGTTACCACG

ATAGGACGCGACATGCTCCTCAAGACCCGAGATTACGTTCACTCTCGTTGGGCGACGCGC

GAGCTGCTGGAGGACAATTTTCCAGGGGCTATAGGTTTCCGAAACCACAAGCCTTACTCC

GTCAGGGTTATCTACGGAGACACCGACTCCGTGTTTATCAAGTTTGTGGGCCTGACGTAC

GAGGGGGTATCGGAGCTGGGGGATGCTATGTCGCGTCAGATTTCAGCGGACCTCTTTAGA

GCGCCCATCAAACTGGAGTGTGAGAAGACCTTTCAGCGACTGCTGCTGATCACCAAGAAG

AAGTACATAGGTGTCATAAACGGGGGGAAGATGCTCATGAAGGGGGTCGACCTGGTCCGC

AAAAATAACTGCTCTTTCATAAACTTGTACGCGCGACATCTGGTAGATCTTTTGTTGTAC

GACGAGGATGTGGCCACGGCGGCAGCAGAGGTGACAGACGTGCCTCCCGCAGAATGGGTG

GGGCGCCCGCTACCGAGCGGCTTTGACAAGTTTGGGCGAGTGCTGGTAGAGGCGTACAAC

CGTATCACTGCCCCCAACTTGGACGTGCGCGAGTTCGTTATGACTGCTGAGCTGAGCCGC

TCACCCGAATCGTATACCAACAAGCGCCTGCCGCACCTCACCGTCTACTTTAAGCTCGCC

ATGAGGAATGAAGAACTGCCCAGCGTAAAAGAGAGAATTCCGTATGTGATAGTTGCGCAG

ACCGAGGCCGCGGAACGCGAAGCGGGTGTAGTAAACTCAATGCGCGGTACCGCCCAAAAC

CCCGTGGTAACCAAGACCGCACGCCCCCAACCTAAACGCAAACTGCTGGTTTCCGACCTC

GCCGAAGACCCGACCTATGTTTCCGAGAATGACGTACCGCTAAACACAGACTACTATTTC

TCCCACCTGTTGGGTACCATAAGCGTGACCTTTAAGGCTCTATTCGGAAATGATGTGAGA

ACAACAGAAAATCTTTTAAAGCGGTTTATTCCGGAAACCCCCCACAAGACCCCCACGAAA

ACCCAGGCACTGCTTGAGCGCGCCGGCTTTGAAAAGCTGACGCCCTTTACACCGGAGGAA

GAAAGTCGTCGAATACTGCATACAGTTTTTTGTACTCTAGAAGCAGCTCCCCATCAAAGC

TGA

>KF434375.1

ATGGCGGCGCGCGAACAGGCCAACTCCGTTCGCAGGAGCGGGTTTTTTAATCCGTTTATT

GGCAAGAGGCCATTTTTCAGGCCGGGAAGCGGGCAGACCGCGGAGACAGAGCGCCCAAGG

CCCCCCCAACACTCGTACTGCACAGAGGTGGGTAGCTTTAAGTTTATAGCTCCAAGATGC

CTCGATGAAGAAGCCCCCGCCGACCAGCGACGCGGTGTACACGTGGGCACCTTGGAGCGC

CCACCCAAAGTGTACTGCGATGGCTCAGAGTACGACGTGCTGAACTTTGCCTCCGGAGGT

TGCTGGCCTCGCAGGATTCGGGTTTGGAACGGCCAGGATTTTCGGGGCGATGGATTCAAC

CCCAGATTTGAGAGATTTCACGTGTACGACATAGTAGAGACTTCGGAGAGCGCCTCACAC

GATGACCCATCCAGGTTTGCTGAGCTATCCCGCCCAAGCGGGTCTGTGGTAACACTGCTG

GGAATGAGCGAGTGTGGAAAGCGAGTCGCGGTTCACGTCTATGGTGTGCGCCATTATTTT

TACATGGCAAAGGCGGAGGTGGATAGCGCTTGTGGAATAACCACCGAGGCAGAACTCGTG

CGCGCAATGGTGGACTGCGCGCACAGCTCGGCTTTGAGCGCGGCCCTGGGAAATGGCAAC

GGCGGCAAACAGAGCGGCGGCAGCGGCGGGGGATGGTGGGGCGGAAAGCACGTGTCTGCG

GACTGCTTCAAAGTGGAGACCGTGTGCCACACGACGCTGTACTACTTTGGATCTAAGCCA

GCTCTCTACTATAGAGTATCTGCCTCCAGCAGCCGCCTGGGAGGGTTCATCTGCGACAAC

TTTCACCCGGAGATTACAAAATTCGAGGGGAGCGTGGACGTGACCACGCGGCTGTTGTTG

GACAATGAAAATTTTACCAGTTTCGGGTGGTACCGCCTGCGACCCGGCACCCACGGAGAG

CGTGTTCAACTTCGCCCCGTTGAGCGACACGTCACCTCAAGCGACGTGGAGATTAACTGT

ACTCCCGATAACCTGGAGCCGATACCAGACGAGGCTGCCTGGCCCGACTATAAGCTCATG

TGCTTTGATATAGAGTGTAAAGCTGGAACGGGTAACGAAATGGCGTTCCCAGTGGCAACT

AACCAAGAGGACCTGGTCATCCAGATCTCCTGTCTGCTGTACTCGCTTGCTACTCAGAAC

CACGAACACACCCTGCTGTTTTCCCTCGGGTCATGCGATATCTCTGAGGAATACTCGTTT

GCATGCGTCCAGCGCGGCGAGCCCAGACCGACGGTTTTGGAGTTTGACAGCGAGTACGAG

CTGCTGGTTGCCTTCCTGACCTTTCTCAAGCAGTACTCTCCCGAGTTCGCCACCGGCTAC

AACATCGTTAATTTTGACTGGGCGTACATAGTTAACAAGGTAACGTCGGTGTATAACATC

AAGCTGGACGGGTACGGCAAGTTCAACAAAGGGGGGCTGTTTAAGGTGTGGGACATCGCC

ACGAACCATTTTCAGAAGAAGAGCAAGGTGAAAATCAATGGCCTGATATCTATAGACATG

TATTCTGTGGCGACGGAAAAGCTAAAGCTACCCAGCTACAAACTCGACGCGGTCGTGGGA

GACGTCCTCGGCGAGCATAAGATAGACCTTCCCTATAAAGAAATACCCTCCTATTACGCG

GGAGGGCCTGACCGGAGGGGCGTAATAGGAGAGTATTGTATCCAGGACTCTAGGCTGGTG

GGCAAGCTGTTTTTTAAGTACCTCCCCCATCTGGAACTATCGGCGGTGGCCAAACTCGCC

CGTATCACCCTAACGCGGGTAATTTTTGACGGTCAGCAAATTCGCGTGTACACGTGCTTG

CTGAAACTCGCCCGCGAGAGAAATTTCATTTTGCCAGACAACAGACGCCGGTTTGACAGT

CAGGCAGACGCCGCGTCAGAGACTTCGGAGTTGGCTATGGATAGCCAAAGCCACGCCTTC

GACAGTACAGACGAACCCGACGGTGTGGACGGTACCCCGGACGCCGCAGGATCTGGCGCT

ACTTCTGAAAACGGAGGCGGGAAGCCCGGCGTCGGGAGGGCCGTGGGCTACCAGGGAGCA

AAGGTTCTAGACCCCGTATCCGGCTTTCATGTGGACCCCGTGGTTGTGTTTGACTTCGCT

AGCTTATACCCAAGCATTATCCAGGCCCATAACCTCTGTTTCACCACCCTGGCGCTCGAT

GAAGTGGATCTGGCCGGGCTTCAACCATCCGTCAACTACTCGACGTTCGAGGTGGGTGAC

CAAAAGTTATTTTTTGTCCACGCCCATATTCGCGAAAGCCTGCTTGGCATCTTGCTGCGC

GACTGGCTGGCCATGCGAAAGGCGGTGAGGGCGCGAATCCCCACCAGCACCCCCGAGGAG

GCAGTTTTACTAGATAAGCAGCAGTCTGCGATTAAGGTGATATGCAACTCGGTTTACGGA

TTCACGGGGGTGGCAAACGGCCTGTTGCCGTGTCTGAGGATAGCGGCTACCGTTACCACG

ATAGGACGCGACATGCTCCTCAAGACCCGAGATTACGTTCACTCTCGTTGGGCGACGCGC

GAGCTGCTGGAGGACAATTTTCCAGGGGCTATAGGTTTCCGAAACCACAAGCCTTACTCC

GTCAGGGTTATCTACGGAGACACCGACTCCGTGTTTATCAAGTTTGTGGGCCTGACGTAC

GAGGGGGTATCGGAGCTGGGGGATGCTATGTCGCGTCAGATTTCAGCGGACCTCTTTAGA

GCGCCCATCAAACTGGAGTGTGAGAAGACCTTTCAGCGACTGCTGCTGATCACCAAGAAG

AAGTACATAGGTGTCATAAACGGGGGGAAGATGCTCATGAAGGGGGTCGACCTGGTCCGC

AAAAATAACTGCTCTTTCATAAACTTGTACGCGCGACATCTGGTAGATCTTTTGTTGTAC

GACGAGGATGTGGCCACGGCGGCAGCAGAGGTGACAGACGTGCCTCCCGCAGAATGGGTG

GGGCGCCCGCTACCGAGCGGCTTTGACAAGTTTGGGCGAGTGCTGGTAGAGGCGTACAAC

CGTATCACTGCCCCCAACTTGGACGTGCGCGAGTTCGTTATGACTGCTGAGCTGAGCCGC

TCACCCGAATCGTATACCAACAAGCGCCTGCCGCACCTCACCGTCTACTTTAAGCTCGCC

ATGAGGAATGAAGAACTGCCCAGCGTAAAAGAGAGAATTCCGTATGTGATAGTTGCGCAG

ACCGAGGCCGCGGAACGCGAAGCGGGTGTAGTAAACTCAATGCGCGGTACCGCCCAAAAC

CCCGTGGTAACCAAGACCGCACGCCCCCAACCTAAACGCAAACTGCTGGTTTCCGACCTC

GCCGAAGACCCGACCTATGTTTCCGAGAATGACGTACCGCTAAACACAGACTACTATTTC

TCCCACCTGTTGGGTACCATAAGCGTGACCTTTAAGGCTCTATTCGGAAATGATGTGAGA

ACAACAGAAAATCTTTTAAAGCGGTTTATTCCGGAAACCCCCCACAAGACCCCCACGAAA

ACCCAGGCACTGCTTGAGCGCGCCGGCTTTGAAAAGCTGACGCCCTTTACACCGGAGGAA

GAAAGTCGTCGAATACTGCATACAGTTTTTTGTACTCTAGAAGCAGCTCCCCATCAAAGC

TGA

>KF434376.1

ATGGCGGCGCGCGAACAGGCCAACTCCGTTCGCAGGAGCGGGTTTTTTAATCCGTTTATT

GGCAAGAGGCCATTTTTCAGGCCGGGAAGCGGGCAGACCGCGGAGACAGAGCGCCCAAGG

CCCCCCCAACACTCGTACTGCACAGAGGTGGGTAGCTTTAAGTTTATAGCTCCAAGATGC

CTCGATGAAGAAGCCCCCGCCGACCAGCGACGCGGTGTACACGTGGGCACCTTGGAGCGC

CCACCCAAAGTGTACTGCGATGGCTCAGAGTACGACGTGCTGAACTTTGCCTCCGGAGGT

TGCTGGCCTCGCAGGATTCGGGTTTGGAACGGCCAGGATTTTCGGGGCGATGGATTCAAC

CCCAGATTTGAGAGATTTCACGTGTACGACATAGTAGAGACTTCGGAGAGCGCCTCACAC

GATGACCCATCCAGGTTTGCTGAGCTATCCCGCCCAAGCGGGTCTGTGGTAACACTGCTG

GGAATGAGCGAGTGTGGAAAGCGAGTCGCGGTTCACGTCTATGGTGTGCGCCATTATTTT

TACATGGCAAAGGCGGAGGTGGATAGCGCTTGTGGAATAACCACCGAGGCAGAACTCGTG

CGCGCAATGGTGGACTGCGCGCACAGCTCGGCTTTGAGCGCGGCCCTGGGAAATGGCAAC

GGCGGCAAACAGAGCGGCGGCAGCGGCGGGGGATGGTGGGGCGGAAAGCACGTGTCTGCG

GACTGCTTCAAAGTGGAGACCGTGTGCCACACGACGCTGTACTACTTTGGATCTAAGCCA

GCTCTCTACTATAGAGTATCTGCCTCCAGCAGCCGCCTGGGAGGGTTCATCTGCGACAAC

TTTCACCCGGAGATTACAAAATTCGAGGGGAGCGTGGACGTGACCACGCGGCTGTTGTTG

GACAATGAAAATTTTACCAGTTTCGGGTGGTACCGCCTGCGACCCGGCACCCACGGAGAG

CGTGTTCAACTTCGCCCCGTTGAGCGACACGTCACCTCAAGCGACGTGGAGATTAACTGT

ACTCCCGATAACCTGGAGCCGATACCAGACGAGGCTGCCTGGCCCGACTATAAGCTCATG

TGCTTTGATATAGAGTGTAAAGCTGGAACGGGTAACGAAATGGCGTTCCCAGTGGCAACT

AACCAAGAGGACCTGGTCATCCAGATCTCCTGTCTGCTGTACTCGCTTGCTACTCAGAAC

CACGAACACACCCTGCTGTTTTCCCTCGGGTCATGCGATATCTCTGAGGAATACTCGTTT

GCATGCGTCCAGCGCGGCGAGCCCAGACCGACGGTTTTGGAGTTTGACAGCGAGTACGAG

CTGCTGGTTGCCTTCCTGACCTTTCTCAAGCAGTACTCTCCCGAGTTCGCCACCGGCTAC

AACATCGTTAATTTTGACTGGGCGTACATAGTTAACAAGGTAACGTCGGTGTATAACATC

AAGCTGGACGGGTACGGCAAGTTCAACAAAGGGGGGCTGTTTAAGGTGTGGGACATCGCC

ACGAACCATTTTCAGAAGAAGAGCAAGGTGAAAATCAATGGCCTGATATCTCTAGACATG

TATTCTGTGGCGACGGAAAAGCTAAAGCTACCCAGCTACAAACTCGACGCGGTCGTGGGA

GACGTCCTCGGCGAGCATAAGATAGACCTTCCCTATAAAGAAATACCCTCCTATTACGCG

GGAGGGCCTGACCGGAGGGGCGTAATAGGAGAGTATTGTATCCAGGACTCTAGGCTGGTG

GGCAAGCTGTTTTTTAAGTACCTCCCCCATCTGGAACTATCGGCGGTGGCCAAACTCGCC

CGTATCACCCTAACGCGGGTAATTTTTGACGGTCAGCAAATTCGCGTGTACACGTGCTTG

CTGAAACTCGCCCGCGAGAGAAATTTCATTTTGCCAGACAACAGACGCCGGTTTGACAGT

CAGGCAGACGCCGCGTCAGAGACTTCGGAGTTGGCTATGGATAGCCAAAGCCACGCCTTC

GACAGTACAGACGAACCCGACGGTGTGGACGGTACCCCGGACGCCGCAGGATCTGGCGCT

ACTTCTGAAAACGGAGGCGGGAAGCCCGGCGTCGGGAGGGCCGTGGGCTACCAGGGAGCA

AAGGTTCTAGACCCCGTATCCGGCTTTCATGTGGACCCCGTGGTTGTGTTTGACTTCGCT

AGCTTATACCCAAGCATTATCCAGGCCCATAACCTCTGTTTCACCACCCTGGCGCTCGAT

GAAGTGGATCTGGCCGGGCTTCAACCATCCGTCGACTACTCGACGTTCGAGGTGGGTGAC

CAAAAGTTATTTTTTGTCCACGCCCATATTCGCGAAAGCCTGCTTGGCATCTTGCTGCGC

GACTGGCTGGCCATGCGAAAGGCGGTGAGGGCGCGAATCCCCACCAGCACCCCCGAGGAG

GCAGTTTTACTAGATAAGCAGCAGTCTGCGATTAAGGTGATATGCAACTCGGTTTACGGA

TTCACGGGGGTGGCAAACGGCCTGTTGCCGTGTCTGAGGATAGCGGCTACCGTTACCACG

ATAGGACGCGACATGCTCCTCAAGACCCGAGATTACGTTCACTCTCGTTGGGCGACGCGC

GAGCTGCTGGAGGACAATTTTCCAGGGGCTATAGGTTTCCGAAACCACAAGCCTTACTCC

GTCAGGGTTATCTACGGAGACACCGACTCCGTGTTTATCAAGTTTGTGGGCCTGACGTAC

GAGGGGGTATCGGAGCTGGGGGATGCTATGTCGCGTCAGATTTCAGCGGACCTCTTTAGA

GCGCCCATCAAACTGGAGTGTGAGAAGACCTTTCAGCGACTGCTGCTGATCACCAAGAAG

AAGTACATAGGTGTCATAAACGGGGGGAAGATGCTCATGAAGGGGGTCGACCTGGTCCGC

AAAAATAACTGCTCTTTCATAAACTTGTACGCGCGACATCTGGTAGATCTTTTGTTGTAC

GACGAGGATGTGGCCACGGCGGCAGCAGAGGTGACAGACGTGCCTCCCGCAGAATGGGTG

GGGCGCCCGCTACCGAGCGGCTTTGACAAGTTTGGGCGAGTGCTGGTAGAGGCGTACAAC

CGTATCACTGCCCCCAACTTGGACGTGCGCGAGTTCGTTATGACTGCTGAGCTGAGCCGC

TCACCCGAATTGTATACCAACAAGCGCCTGCCGCACCTCACCGTCTACTTTAAGCTCGCC

ATGAGGAATGAAGAACTGCCCAGCGTAAAAGAGAGAATTCCGTATGTGATAGTTGCGCAG

ACCGAGGCCGCGGAACGCGAAGCGGGTGTAGTAAACTCAATGCGCGGTACCGCCCAAAAC

CCCGTGGTAACCAAGACCGCACGCCCCCAACCTAAACGCAAACTGCTGGTTTCCGACCTC

GCCGAAGACCCGACCTATGTTTCCGAGAATGACGTACCGCTAAACACAGACTACTATTTC

TCCCACCTGTTGGGTACCATAAGCGTGACCTTTAAGGCTCTATTCGGAAATGATGTGAGA

ACAACAGAAAATCTTTTAAAGCGGTTTATTCCGGAAACCCCCCACAAGACCCCCACGAAA

ACCCAGGCACTGCTTGAGCGCGCCGGCTTTGAAAAGCTGACGCCCTTTACACCGGAGGAA

GAAAGTCGTCGAATACTGCATACAGTTTTTTGTACTCTAGAAGCAGCTCCCCATCAAAGC

TGA

>KF434377.1

ATGGCGGCGCGCGAACAGGCCAACTCCGTTCGCAGGAGCGGGTTTTTTAATCCGTTTATT

GGCAAGAGGCCATTTTTCAGGCCGGGAAGCGGGCAAACCGCGGAGACAGAGCGCCCAAGG

CCCCCCCAACACTCGTACTGCACAGAGGTGGGTAGCTTTAAGTTTATAGCTCCAAGATGC

CTCGATGAAGAAGCCCCCGCCGACCAGCGACGCGGTGTACACGTGGGCACCTTGGAGCGC

CCACCCAAAGTGTACTGCGATGGCTCAGAGTACGACGTGCTGAACTTTGCCTCCGGAGGT

TGCTGGCCTCGCAGGATTCGGGTTTGGAACGGCCAGGATTTTCGGGGCGATGGATTCAAC

CCCAGATTTGAGAGATTTCACGTGTACGACATAGTAGAGACTTCGGAGAGCGCCTCACAC

GATGACCCATCCAGGTTTGCTGAGCTATCCCGCCCAAGCGGGTCTGTGGTAACACTGCTG

GGAATGAGCGAGTGTGGAAAGCGAGTCGCGGTTCACGTCTATGGTGTGCGCCATTATTTT

TACATGGCAAAGGCGGAGGTGGATAGCGCTTGTGGAATAACCACCGAGGCAGAACTCGTG

CGCGCAATGGTGGACTGCGCGCACAGCTCGGCTTTGAGCGCGGCCCTGGGAAATGGCAAC

GGCGGCAAACAGAGCGGCGGCAGCGGCGGGGGATGGTGGGGCGGAAAGCACGTGTCTGCG

GACTGCTTCAAAGTGGAGACCGTGTGCCACACGACGCTGTACTACTTTGGATCTAAGCCA

GCTCTCTACTATAGAGTATCTGCCTCCAGCAGCCGCCTGGGAGGGTTCATCTGCGACAAC

TTTCACCCGGAGATTACAAAATTCGAGGGGAGCGTGGACGTGACCACGCGGCTGTTGTTG

GACAATGAAAATTTTACCAGTTTCGGGTGGTACCGCCTGCGACCCGGCACCCACGGAGAG

CGTGTTCAACTTCGCCCCGTTGAGCGACACGTCACCTCAAGCGACGTGGAGATTAACTGT

ACTCCCGATAACCTGGAGCCGATACCAGACGAGGCTGCCTGGCCCGACTATAAGCTCATG

TGCTTTGATATAGAGTGTAAAGCTGGAACGGGTAACGAAATGGCGTTCCCAGTGGCAACT

AACCAAGAGGACCTGGTCATCCAGATCTCCTGTCTGCTGTACTCGCTTGCTACTCAGAAC

CACGAACACACCCTGCTGTTTTCCCTCGGGTCATGCGATATCTCTGAGGAATACTCGTTT

GCATGCGTCCAGCGCGGCGAGCCCAGACCGACGGTTTTGGAGTTTGACAGCGAGTACGAG

CTGCTGGTTGCCTTCCTGACCTTTCTCAAGCAGTACTCTCCCGAGTTCGCCACCGGCTAC

AACATCGTTAATTTTGACTGGGCGTACATAGTTAACAAGGTAACGTCGGTGTATAACATC

AAGCTGGACGGGTACGGCAAGTTCAACAAAGGGGGGCTGTTTAAGGTGTGGGACATCGCC

ACGAACCATTTTCAGAAGAAGAGCAAGGTGAAAATCAATGGCCTGATATCTCTAGACATG

TATTCTGTGGCGACGGAAAAGCTAAAGCTACCCAGCTACAAACTCGACGCGGTCGTGGGA

GACGTCCTCGGCGAGCATAAGATAGACCTTCCCTATAAAGAAATACCCTCCTATTACGCG

GGAGGGCCTGACCGGAGGGGCGTAATAGGAGAGTATTGTATCCAGGACTCTAGGCTGGTG

GGCAAGCTGTTTTTTAAGTACCTCCCCCATCTGGAACTATCGGCGGTGGCCAAACTCGCC

CGTATCACCCTAACGCGGGTAATTTTTGACGGTCAGCAAATTCGCGTGTACACGTGCTTG

CTGAAACTCGCCCGCGAGAGAAATTTCATTTTGCCAGACAACAGACGCCGGTTTGACAGT

CAGGCAGACGCCGCGTCAGAGACTTCGGAGTTGGCTATGGATAGCCAAAGCCACGCCTTC

GACAGTACAGACGAACCCGACGGTGTGGACGGTACCCCGGACGCCGCAGGATCTGGCGCT

ACTTCTGAAAACGGAGGCGGGAAGCCCGGCGTCGGGAGGGCCGTGGGCTACCAGGGAGCA

AAGGTTCTAGACCCCGTATCCGGCTTTCATGTGGACCCCGTGGTTGTGTTTGACTTCGCT

AGCTTATACCCAAGCATTATCCAGGCCCATAACCTCTGTTTCACCACCCTGGCGCTCGAT

GAAGTGGATCTGGCCGGGCTTCAACCATCCGTCGACTACTCGACGTTCGAGGTGGGTGAC

CAAAAGTTATTTTTTGTCCACGCCCATATTCGCGAAAGCCTGCTTGGCATCTTGCTGCGC

GACTGGCTGGCCATGCGAAAGGCGGTGAGGGCGCGAATCCCCACCAGCACCCCCGAGGAG

GCAGTTTTACTAGATAAGCAGCAGTCTGCGATTAAGGTGATATGCAACTCGGTTTACGGA

TTCACGGGGGTGGCAAACGGCCTGTTGCCGTGTCTGAGGATAGCGGCTACCGTTACCACG

ATAGGACGCGACATGCTCCTCAAGACCCGAGATTACGTTCACTCTCGTTGGGCGACGCGC

GAGCTGCTGGAGGACAATTTTCCAGGGGCTATAGGTTTCCGAAACCACAAGCCTTACTCC

GTCAGGGTTATCTACGGAGACACCGACTCCGTGTTTATCAAGTTTGTGGGCCTGACGTAC

GAGGGGGTATCGGAGCTGGGGGATGCTATGTCGCGTCAGATTTCAGCGGACCTCTTTAGA

GCGCCCATCAAACTGGAGTGTGAGAAGACCTTTCAGCGACTGCTGCTGATCACCAAGAAG

AAGTACATAGGTGTCATAAACGGGGGGAAGATGCTCATGAAGGGGGTCGACCTGGTCCGC

AAAAATAACTGCTCTTTCATAAACTTGTACGCGCGACATCTGGTAGATCTTTTGTTGTAC

GACGAGGATGTGGCCACGGCGGCAGCAGAGGTGACAGACGTGCCTCCCGCAGAATGGGTG

GGGCGCCCGCTACCGAGCGGCTTTGACAAGTTTGGGCGAGTGCTGGTAGAGGCGTACAAC

CGTATCACTGCCCCCAACTTGGACGTGCGCGAGTTCGTTATGACTGCTGAGCTGAGCCGC

TCACCCGAATCGTATACCAACAAGCGCCTGCCGCACCTCACCGTCTACTTTAAGCTCGCC

ATGAGGAATGAAGAACTGCCCAGCGTAAAAGAGAGAATTCCGTATGTGATAGTTGCGCAG

ACCGAGGCCGCGGAACGCGAAGCGGGTGTAGTAAACTCAATGCGCGGTACCGCCCAAAAC

CCCGTGGTAACCAAGACCGCACGCCCCCAACCTAAACGCAAACTGCTGGTTTCCGACCTC

GCCGAAGACCCGACCTATGTTTCCGAGAATGACGTACCGCTAAACACAGACTACTATTTC

TCCCACCTGTTGGGTACCATAAGCGTGACCTTTAAGGCTCTATTCGGAAATGATGTGAGA

ACAACAGAAAATCTTTTAAAGCGGTTTATTCCGGAAACCCCCCACAAGACCCCCACGAAA

ACCCAGGCACTGCTTGAGCGCGCCGGCTTTGAAAAGCTGACGCCCTTTACACCGGAGGAA

GAAAGTCGTCGAATACTGCATACAGTTTTTTGTACTCTAGAAGCAGCTCCCCATCAAAGC

TGA

>KF434378.1

ATGGCGGCGCGCGAACAGGCCAACTCCGTTCGCAGGAGCGGGTTTTTTAATCCGTTTATT

GGCAAGAGGCCATTTTTCAGGCCGGGAAGCGGGCAAACCGCGGAGACAGAGCGCCCAAGG

CCCCCCCAACACTCGTACTGCACAGAGGTGGGTAGCTTTAAGTTTATAGCTCCAAGATGC

CTCGATGAAGAAGCCCCCGCCGACCAGCGACGCGGTGTACACGTGGGCACCTTGGAGCGC

CCACCCAAAGTGTACTGCGATGGCTCAGAGTACGACGTGCTGAACTTTGCCTCCGGAGGT

TGCTGGCCTCGCAGGATTCGGGTTTGGAACGGCCAGGATTTTCGGGGCGATGGATTCAAC

CCCAGATTTGAGAGATTTCACGTGTACGACATAGTAGAGACTTCGGAGAGCGCCTCACAC

GATGACCCATCCAGGTTTGCTGAGCTATCCCGCCCAAGCGGGTCTGTGGTAACACTGCTG

GGAATGAGCGAGTGTGGAAAGCGAGTCGCGGTTCACGTCTATGGTGTGCGCCATTATTTT

TACATGGCAAAGGCGGAGGTGGATAGCGCTTGTGGAATAACCACCGAGGCAGAACTCGTG

CGCGCAATGGTGGACTGCGCGCACAGCTCGGCTTTGAGCGCGGCCCTGGGAAATGGCAAC

GGCGGCAAACAGAGCGGCGGCAGCGGCGGGGGATGGTGGGGCGGAAAGCACGTGTCTGCG

GACTGCTTCAAAGTGGAGACCGTGTGCCACACGACGCTGTACTACTTTGGATCTAAGCCA

GCTCTCTACTATAGAGTATCTGCCTCCAGCAGCCGCCTGGGAGGGTTCATCTGCGACAAC

TTTCACCCGGAGATTACAAAATTCGAGGGGAGCGTGGACGTGACCACGCGGCTGTTGTTG

GACAATGAAAATTTTACCAGTTTCGGGTGGTACCGCCTGCGACCCGGCACCCACGGAGAG

CGTGTTCAACTTCGCCCCGTTGAGCGACACGTCACCTCAAGCGACGTGGAGATTAACTGT

ACTCCCGATAACCTGGAGCCGATACCAGACGAGGCTGCCTGGCCCGACTATAAGCTCATG

TGCTTTGATATAGAGTGTAAAGCTGGAACGGGTAACGAAATGGCGTTCCCAGTGGCAACT

AACCAAGAGGACCTGGTCATCCAGATCTCCTGTCTGCTGTACTCGCTTGCTACTCAGAAC

CACGAACACACCCTGCTGTTTTCCCTCGGGTCATGCGATATCTCTGAGGAATACTCGTTT

GCATGCGTCCAGCGCGGCGAGCCCAGACCGACGGTTTTGGAGTTTGACAGCGAGTACGAG

CTGCTGGTTGCCTTCCTGACCTTTCTCAAGCAGTACTCTCCCGAGTTCGCCACCGGCTAC

AACATCGTTAATTTTGACTGGGCGTACATAGTTAACAAGGTAACGTCGGTGTATAACATC

AAGCTGGACGGGTACGGCAAGTTCAACAAAGGGGGGCTGTTTAAGGTGTGGGACATCGCC

ACGAACCATTTTCAGAAGAAGAGCAAGGTGAAAATCAATGGCCTGATATCTCTAGACATG

TATTCTGTGGCGACGGAAAAGCTAAAGCTACCCAGCTACAAACTCGACGCGGTCGTGGGA

GACGTCCTCGGCGAGCATAAGATAGACCTTCCCTATAAAGAAATACCCTCCTATTACGCG

GGAGGGCCTGACCGGAGGGGCGTAATAGGAGAGTATTGTATCCAGGACTCTAGGCTGGTG

GGCAAGCTGTTTTTTAAGTACCTCCCCCATCTGGAACTATCGGCGGTGGCCAAACTCGCC

CGTATCACCCTAACGCGGGTAATTTTTGACGGTCAGCAAATTCGCGTGTACACGTGCTTG

CTGAAACTCGCCCGCGAGAGAAATTTCATTTTGCCAGACAACAGACGCCGGTTTGACAGT

CAGGCAGACGCCGCGTCAGAGACTTCGGAGTTGGCTATGGATAGCCAAAGCCACGCCTTC

GACAGTACAGACGAACCCGACGGTGTGGACGGTACCCCGGACGCCGCAGGATCTGGCGCT

ACTTCTGAAAACGGAGGCGGGAAGCCCGGCGTCGGGAGGGCCGTGGGCTACCAGGGAGCA

AAGGTTCTAGACCCCGTATCCGGCTTTCATGTGGACCCCGTGGTTGTGTTTGACTTCGCT

AGCTTATACCCAAGCATTATCCAGGCCCATAACCTCTGTTTCACCACCCTGGCGCTCGAT

GAAGTGGATCTGGCCGGGCTTCAACCATCCGTCAACTACTCGACGTTCGAGGTGGGTGAC

CAAAAGTTATTTTTTGTCCACGCCCATATTCGCGAAAGCCTGCTTGGCATCTTGCTGCGC

GACTGGCTGGCCATGCGAAAGGCGGTGAGGGCGCGAATCCCCACCAGCACCCCCGAGGAG

GCAGTTTTACTAGATAAGCAGCAGTCTGCGATTAAGGTGATATGCAACTCGGTTTACGGA

TTCACGGGGGTGGCAAACGGCCTGTTGCCGTGTCTGGGGATAGCGGCTACCGTTACCACG

ATAGGACGCGACATGCTCCTCAAGACCCGAGATTACGTTCACTCTCGTTGGGCGACGCGC

GAGCTGCTGGAGGACAATTTTCCAGGGGCTATAGGTTTCCGAAACCACAAGCCTTACTCC

GTCAGGGTTATCTACGGAGACACCGACTCCGTGTTTATCAAGTTTGTGGGCCTGACGTAC

GAGGGGGTATCGGAGCTGGGGGATGCTATGTCGCGTCAGATTTCAGCGGACCTCTTTAGA

GCGCCCATCAAACTGGAGTGTGAGAAGACCTTTCAGCGACTGCTGCTGATCACCAAGAAG

AAGTACATAGGTGTCATAAACGGGGGGAAGATGCTCATGAAGGGGGTCGACCTGGTCCGC

AAAAATAACTGCTCTTTCATAAACTTGTACGCGCGACATCTGGTAGATCTTTTGTTGTAC

GACGAGGATGTGGCCACGGCGGCAGCAGAGGTGACAGACGTGCCTCCCGCAGAATGGGTG

GGGCGCCCGCTACCGAGCGGCTTTGACAAGTTTGGGCGAGTGCTGGTAGAGGCGTACAAC

CGTATCACTGCCCCCAACTTGGACGTGCGCGAGTTCGTTATGACTGCTGAGCTGAGCCGC

TCACCCGAATCGTATACCAACAAGCGCCTGCCGCACCTCACCGTCTACTTTAAGCTCGCC

ATGAGGAATGAAGAACTGCCCAGCGTAAAAGAGAGAATTCCGTATGTGATAGTTGCGCAG

ACCGAGGCCGCGGAACGCGAAGCGGGTGTAGTAAACTCAATGCGCGGTACCGCCCAAAAC

CCCGTGGTAACCAAGACCGCACGCCCCCAACCTAAACGCAAACTGCTGGTTTCCGACCTC

GCCGAAGACCCGACCTATGTTTCCGAGAATGACGTACCGCTAAACACAGACTACTATTTC

TCCCACCTGTTGGGTACCATAAGCGTGACCTTTAAGGCTCTATTCGGAAATGATGTGAGA

ACAACAGAAAATCTTTTAAAGCGGTTTATTCCGGAAACCCCCCACAAGACCCCCACGAAA

ACCCAGGCACTGCTTGAGCGCGCCGGCTTTGAAAAGCTGACGCCCTTTACACCGGAGGAA

GAAAGTCGTCGAATACTGCATACAGTTTTTTGTACTCTAGAAGCAGCTCCCCATCAAAGC

TGA

>KF434379.1

ATGGCGGCGCGCGAACAGGCCAACTCCGTTCGCAGGAGCGGGTTTTTTAATCCGTTTATT

GGCAAGAGGCCATTTTTCAGGCCGGGAAGCGGGCAGACCGCGGAGACAGAGCGCCCAAGG

CCCCCCCAACACTCGTACTGCACAGAGGTGGGTAGCTTTAAGTTTATAGCTCCAAGATGC

CTCGATGAAGAAGCCCCCGCCGACCAGCGACGCGGTGTACACGTGGGCACCTTGGAGCGC

CCACCCAAAGTGTACTGCGATGGCTCAGAGTACGACGTGCTGAACTTTGCCTCCGGAGGT

TGCTGGCCTCGCAGGATTCGGGTTTGGAACGGCCAGGATTTTCGGGGCGATGGATTCAAC

CCCAGATTTGAGAGATTTCACGTGTACGACATAGTAGAGACTTCGGAGAGCGCCTCACAC

GATGACCCATCCAGGTTTGCTGAGCTATCCCGCCCAAGCGGGTCTGTGGTAACACTGCTG

GGAATGAGCGAGTGTGGAAAGCGAGTCGCGGTTCACGTCTATGGTGTGCGCCATTATTTT

TACATGGCAAAGGCGGAGGTGGATAGCGCTTGTGGAATAACCACCGAGGCAGAACTCGTG

CGCGCAATGGTGGACTGCGCGCACAGCTCGGCTTTGAGCGCGGCCCTGGGAAATGGCAAC

GGCGGCAAACAGAGCGGCGGCAGCGGCGGGGGATGGTGGGGCGGAAAGCACGTGTCTGCG

GACTGCTTCAAAGTGGAGACCGTGTGCCGCACGACGCTGTACTACTTTGGATCTAAGCCA

GCTCTCTACTATAGAGTATCTGCCTCCAGCAGCCGCCTGGGAGGGTTCATCTGCGACAAC

TTTCACCCGGAGATTACAAAATTCGAGGGGAGCGTGGACGTGACCACGCGGCTGTTGTTG

GACAATGAAAATTTTACCAGTTTCGGGTGGTACCGCCTGCGACCCGGCACCCACGGAGAG

CGTGTTCAACTTCGCCCCGTTGAGCGACACGTCACCTCAAGCGACGTGGAGATTAACTGT

ACTCCCGATAACCTGGAGCCGATACCAGACGAGGCTGCCTGGCCCGACTATAAGCTCATG

TGCTTTGATATAGAGTGTAAAGCTGGAACGGGTAACGAAATGGCGTTCCCAGTGGCAACT

AACCAAGAGGACCTGGTCATCCAGATCTCCTGTCTGCTGTACTCGCTTGCTACTCAGAAC

CACGAACACACCCTGCTGTTTTCCCTCGGGTCATGCGATATCTCTGAGGAATACTCGTTT

GCATGCGTCCAGCGCGGCGAGCCCAGACCGACGGTTTTGGAGTTTGACAGCGAGTACGAG

CTGCTGGTTGCCTTCCTGACCTTTCTCAAGCAGTACTCTCCCGAGTTCGCCACCGGCTAC

AACATCGTTAATTTTGACTGGGCGTACATAGTTAACAAGGTAACGTCGGTGTATAACATC

AAGCTGGACGGGTACGGCAAGTTCAACAAAGGGGGGCTGTTTAAGGTGTGGGACATCGCC

ACGAACCATTTTCAGAAGAAGAGCAAGGTGAAAATCAATGGCCTGATATCTCTAGACATG

TATTCTGTGGCGACGGAAAAGCTAAAGCTACCCAGCTACAAACTCGACGCGGTCGTGGGA

GACGTCCTCGGCGAGCATAAGATAGACCTTCCCTATAAAGAAATACCCTCCTATTACGCG

GGAGGGCCTGACCGGAGGGGCGTAATAGGAGAGTATTGTATCCAGGACTCTAGGCTGGTG

GGCAAGCTGTTTTTTAAGTACCTCCCCCATCTGGAACTATCGGCGGTGGCCAAACTCGCC

CGTATCACCCTAACGCGGGTAATTTTTGACGGTCAGCAAATTCGCGTGTACACGTGCTTG

CTGAAACTCGCCCGCGAGAGAAATTTCATTTTGCCAGACAACAGACGCCGGTTTGACAGT

CAGGCAGACGCCGCGTCAGAGACTTCGGAGTTGGCTATGGATAGCCAAAGCCACGCCTTC

GACAGTACAGACGAACCCGACGGTGTGGACGGTACCCCGGACGCCGCAGGATCTGGCGCT

ACTTCTGAAAACGGAGGCGGGAAGCCCGGCGTCGGGAGGGCCGTGGGCTACCAGGGAGCA

AAGGTTCTAGACCCCGTATCCGGCTTTCATGTGGACCCCGTGGTTGTGTTTGACTTCGCT

AGCTTATACCCAAGCATTATCCAGGCCCATAACCTCTGTTTCACCACCCTGGCGCTCGAT

GAAGTGGATCTGGCCGGGCTTCAACCATCCGTCAACTACTCGACGTTCGAGGTGGGTGAC

CAAAAGTTATTTTTTGTCCACGCCCATATTCGCGAAAGCCTGCTTGGCATCTTGCTGCGC

GACTGGCTGGCCATGCGAAAGGCGGTGAGGGCGCGAATCCCCACCAGCACCCCCGAGGAG

GCAGTTTTACTAGATAAGCAGCAGTCTGCGATTAAGGTGATATGCAACTCGGTTTACGGA

TTCACGGGGGTGGCAAACGGCCTGTTGCCGTGTCTGAGGATAGCGGCTACCGTTACCACG

ATAGGACGCGACATGCTCCTCAAGACCCGAGATTACGTTCACTCTCGTTGGGCGACGCGC

GAGCTGCTGGAGGACAATTTTCCAGGGGCTATAGGTTTCCGAAACCACAAGCCTTACTCC

GTCAGGGTTATCTACGGAGACACCGACTCCGTGTTTATCAAGTTTGTGGGCCTGACGTAC

GAGGGGGTATCGGAGCTGGGGGATGCTATGTCGCGTCAGATTTCAGCGGACCTCTTTAGA

GCGCCCATCAAACTGGAGTGTGAGAAGACCTTTCAGCGACTGCTGCTGATCACCAAGAAG

AAGTACATAGGTGTCATAAACGGGGGGAAGATGCTCATGAAGGGGGTCGACCTGGTCCGC

AAAAATAACTGCTCTTTCATAAACTTGTACGCGCGACATCTGGTAGATCTTTTGTTGTAC

GACGAGGATGTGGCCACGGCGGCAGCAGAGGTGACAGACGTGCCTCCCGCAGAATGGGTG

GGGCGCCCGCTACCGAGCGGCTTTGACAAGTTTGGGCGAGTGCTGGTAGAGGCGTACAAC

CGTATCACTGCCCCCAACTTGGACGTGCGCGAGTTCGTTATGACTGCTGAGCTGAGCCGC

TCACCCGAATCGTATACCAACAAGCGCCTGCCGCACCTCACCGTCTACTTTAAGCTCGCC

ATGAGGAATGAAGAACTGCCCAGCGTAAAAGAGAGAATTCCGTATGTGATAGTTGCGCAG

ACCGAGGCCGCGGAACGCGAAGCGGGTGTAGTAAACTCAATGCGCGGTACCGCCCAAAAC

CCCGTGGTAACCAAGACCGCACGCCCCCAACCTAAACGCAAACTGCTGGTTTCCGACCTC

GCCGAAGACCCGACCTATGTTTCCGAGAATGACGTACCGCTAAACACAGACTACTATTTC

TCCCACCTGTTGGGTACCATAAGCGTGACCTTTAAGGCTCTATTCGGAAATGATGTGAGA

ACAACAGAAAATCTTTTAAAGCGGTTTATTCCGGAAACCCCCCACAAGACCCCCACGAAA

ACCCAGGCACTGCTTGAGCGCGCCGGCTTTGAAAAGCTGACGCCCTTTACACCGGAGGAA

GAAAGTCGTCGAATACTGCATACAGTTTTTTGTACTCTAGAAGCAGCTCCCCATCAAAGC

TGA

>KF434380.1

ATGGCGGCGCGCGAACAGGCCAACTCCGTTCGCAGGAGCGGGTTTTTTAATCCGTTTATT

GGCAAGAGGCCATTTTTCAGGCCGGGAAGCGGGCAGACCGCGGAGACAGAGCGCCCAAGG

CCCCCCCAACACTCGTACTGCACAGAGGTGGGCAGCTTTAAGTTTATAGCTCCAAGATGC

CTCGATGAAGAAGCCCCCGCCGACCAGCGACGCGGTGTACACGTGGGCACCTTGGAGCGC

CCACCCAAAGTGTACTGCGATGGCTCAGAGTACGACGTGCTGAACTTTGCCTCCGGAGGT

TGCTGGCCTCGCAGGATTCGGGTTTGGAACGGCCAGGATTTTCGGGGCGATGGATTCAAC

CCCAGATTTGAGAGATTTCACGTGTACGACATAGTAGAGACTTCGGAGAGCGCCTCACAC

GATGACCCATCCAGGTTTGCTGAGCTATCCCGCCCAAGCGGGTCTGTGGTAACACTGCTG

GGAATGAGCGAGTGTGGAAAGCGAGTCGCGGTTCACGTCTATGGTGTGCGCCATTATTTT

TACATGGCAAAGGCGGAGGTGGATAGCGCTTGTGGAATAACCACCGAGGCAGAACTCGTG

CGCGCAATGGTGGACTGCGCGCACAGCTCGGCTTTGAGCGCGGCCCTGGGAAATGGCAAC

GGCGGCAAACAGAGCGGCGGCAGCGGCGGGGGATGGTGGGGCGGAAAGCACGTGTCTGCG

GACTGCTTCAAAGTGGAGACCGTGTGCCACACGACGCTGTACTACTTTGGATCTAAGCCA

GCTCTCTACTATAGAGTATCTGCCTCCAGCAGCCGCCTGGGAGGGTTCATCTGCGACAAC

TTTCACCCGGAGATTACAAAATTCGAGGGGAGCGTGGACGTGACCACGCGGCTGTTGTTG

GACAATGAAAATTTTACCAGTTTCGGGTGGTACCGCCTGCGACCCGGCACCCACGGAGAG

CGTGTTCAACTTCGCCCCGTTGAGCGACACGTCACCTCAAGCGACGTGGAGATTAACTGT

ACTCCCGATAACCTGGAGCCGATACCAGACGAGGCTGCCTGGCCCGACTATAAGCTCATG

TGCTTTGATATAGAGTGTAAAGCTGGAACGGGTAACGAAATGGCGTTCCCAGTGGCAACT

AACCAAGAGGACCTGGTCATCCAGATCTCCTGTCTGCTGTACTCGCTTGCTACTCAGAAC

CACGAACACACCCTGCTGTTTTCCCTCGGGTCATGCGATATCTCTGAGGAATACTCGTTT

GCATGCGTCCAGCGCGGCGAGCCCAGACCGACGGTTTTGGAGTTTGACAGCGAGTACGAG

CTGCTGGTTGCCTTCCTGACCTTTCTCAAGCAGTACTCTCCCGAGTTCGCCACCGGCTAC

AACATCGTTAATTTTGACTGGGCGTACATAGTTAACAAGGTAACGTCGGTGTATAACATC

AAGCTGGACGGGTACGGCAAGTTCAACAAAGGGGGGCTGTTTAAGGTGTGGGACATCGCC

ACGAACCATTTTCAGAAGAAGAGCAAGGTGAAAATCAATGGCCTGATATCTCTAGACATG

TATTCTGTGGCGACGGAAAAGCTAAAGCTACCCAGCTACAAACTCGACGCGGTCGTGGGA

GACGTCCTCGGCGAGCATAAGATAGACCTTCCCTATAAAGAAATACCCTCCTATTACGCG

GGAGGGCCTGACCGGAGGGGCGTAATAGGAGAGTATTGTATCCAGGACTCTAGGCTGGTG

GGCAAGCTGTTTTTTAAGTACCTCCCCCATCTGGAACTATCGGCGGTGGCCAAACTCGCC

CGTATCACCCTAACGCGGGTAATTTTTGACGGTCAGCAAATTCGCGTGTACACGTGCTTG

CTGAAACTCGCCCGCGAGAGAAATTTCATTTTGCCAGACAACAGACGCCGGTTTGACAGT

CAGGCAGACGCCGCGTCAGAGACTTCGGAGTTGGCTATGGATAGCCAAAGCCACGCCTTC

GACAGTACAGACGAACCCGACGGTGTGGACGGTACCCCGGACGCCGCAGGATCTGGCGCT

ACTTCTGAAAACGGAGGCGGGAAGCCCGGCGTCGGGAGGGCCGTGGGCTACCAGGGAGCA

AAGGTTCTAGACCCCGTATCCGGCTTTCATGTGGACCCCGTGGTTGTGTTTGACTTCGCT

AGCTTATACCCAAGCATTATCCAGGCCCATAACCTCTGTTTCACCACCCTGGCGCTCGAT

GAAGTGGATCTGGCCGGGCTTCAACCATCCGTCAACTACTCGACGTTCGAGGTGGGTGAC

CAAAAGTTATTTTTTGTCCACGCCCATATTCGCGAAAGCCTGCTTGGCATCTTGCTGCGC

GACTGGCTGGCCATGCGAAAGGCGGTGAGGGCGCGAATCCCCACCAGCACCCCCGAGGAG

GCAGTTTTACTAGATAAGCAGCAGTCTGCGATTAAGGTGATATGCAACTCGGTTTACGGA

TTCACGGGGGTGGCAAACGGCCTGTTGCCGTGTCTGAGGATAGCGGCTACCGTTACCACG

ATAGGACGCGACATGCTCCTCAAGACCCGAGATTACGTTCACTCTCGTTGGGCGACGCGC

GAGCTGCTGGAGGACAATTTTCCAGGGGCTATAGGTTTCCGAAACCACAAGCCTTACTCC

GTCAGGGTTATCTACGGAGACACCGACTCCGTGTTTATCAAGTTTGTGGGCCTGACGTAC

GAGGGGGTATCGGAGCTGGGGGATGCTATGTCGCGTCAGATTTCAGCGGACCTCTTTAGA

GCGCCCATCAAACTGGAGTGTGAGAAGACCTTTCAGCGACTGCTGCTGATCACCAAGAAG

AAGTACATAGGTGTCATAAACGGGGGGAAGATGCTCATGAAGGGGGTCGACCTGGTCCGC

AAAAATAACTGCTCTTTCATAAACTTGTACGCGCGACATCTGGTAGATCTTTTGTTGTAC

GACGAGGATGTGGCCACGGCGGCAGCAGAGGTGACAGACGTGCCTCCCGCAGAATGGGTG

GGGCGCCCGCTACCGAGCGGCTTTGACAAGTTTGGGCGAGTGCTGGTAGAGGCGTACAAC

CGTATCACTGCCCCCAACTTGGACGTGCGCGAGTTCGTTATGACTGCTGAGCTGAGCCGC

TCACCCGAATCGTATACCAACAAGCGCCTGCCGCACCTCACCGTCTACTTTAAGCTCGCC

ATGAGGAATGAAGAACTGCCCAGCGTAAAAGAGAGAATTCCGTATGTGATAGTTGCGCAG

ACCGAGGCCGCGGAACGCGAAGCGGGTGTAGTAAACTCAATGCGCGGTACCGCCCAAAAC

CCCGTGGTAACCAAGACCGCACGCCCCCAACCTAAACGCAAACTGCTGGTTTCCGACCTC

GCCGAAGACCCGACCTATGTTTCCGAGAATGACGTACCGCTAAACACAGACTACTATTTC

TCCCACCTGTTGGGTACCATAAGCGTGACCTTTAAGGCTCTATTCGGAAATGATGTGAGA

ACAACAGAAAATCTTTTAAAGCGGTTTATTCCGGAAACCCCCCACAAGACCCCCACGAAA

ACCCAGGCACTGCTTGAGCGCGCCGGCTTTGAAAAGCTGACGCCCTTTACACCGGAGGAA

GAAAGTCGTCGAATACTGCATACAGTTTTTTGTACTCTAGAAGCAGCTCCCCATCAAAGC

TGA

>KF434381.1

ATGGCGGCGCGCGAACAGGCCAACTCCGTTCGCAGGAGCGGGTTTTTTAATCCGTTTATT

GGCAAGAGGCCATTTTTCAGGCCGGGAAGCGGGCAGACCGCGGAGACAGAGCGCCCAAGG

CCCCCCCAACACTCGTACTGCACAGAGGTGGGTAGCTTTAAGTTTATAGCTCCAAGATGC

CTCGATGAAGAAGCCCCCGCCGACCAGCGACGCGGTGTACACGTGGGCACCTTGGAGCGC

CCACCCAAAGTGTACTGCGATGGCTCAGAGTACGACGTGCTGAACTTTGCCTCCGGAGGT

TGCTGGCCTCGCAGGATTCGGGTTTGGAACGGCCAGGATTTTCGGGGCGATGGATTCAAC

CCCAGATTTGAGAGATTTCACGTGTACGACATAGTAGAGACTTCGGAGAGCGCCTCACAC

GATGACCCATCCAGGTTTGCTGAGCTATCCCGCCCAAGCGGGTCTGTGGTAACACTGCTG

GGAATGAGCGAGTGTGGAAAGCGAGTCGCGGTTCACGTCTATGGTGTGCGCCATTATTTT

TACATGGCAAAGGCGGAGGTGGATAGCGCTTGTGGAATAACCACCGAGGCAGAACTCGTG

CGCGCAATGGTGGACTGCGCGCACAGCTCGGCTTTGAGCGCGGCCCTGGGAAATGGCAAC

GGCGGCAAACAGAGCGGCGGCAGCGGCGGGGGATGGTGGGGCGGAAAGCACGTGTCTGCG

GACTGCTTCAAAGTGGAGACCGTGTGCCACACGACGCTGTACTACTTTGGATCTAAGCCA

GCTCTCTACTATAGAGTATCTGCCTCCAGCAGCCGCCTGGGAGGGTTCATCTGCGACAAC

TTTCACCCGGAGATTACAAAATTCGAGGGGAGCGTGGACGTGACCACGCGGCTGTTGTTG

GACAATGAAAATTTTACCAGTTTCGGGTGGTACCGCCTGCGACCCGGCACCCACGGAGAG

CGTGTTCAACTTCGCCCCGTTGAGCGACACGTCACCTCAAGCGACGTGGAGATTAACTGT

ACTCCCGATAACCTGGAGCCGATACCAGACGAGGCTGCCTGGCCCGACTATAAGCTCATG

TGCTTTGATATAGAGTGTAAAGCTGGAACGGGTAACGAAATGGCGTTCCCAGTGGCAACT

AACCAAGAGGACCTGGTCATCCAGATCTCCTGTCTGCTGTACTCGCTTGCTACTCAGAAC

CACGAACACACCCTGCTGTTTTCCCTCGGGTCATGCGATATCTCTGAGGAATACTCGTTT

GCATGCGTCCAGCGCGGCGAGCCCAGACCGACGGTTTTGGAGTTTGACAGCGAGTACGAG

ATGCTGGTTGCCTTCCTGACCTTTCTCAAGCAGTACTCTCCCGAGTTCGCCACCGGCTAC

AACATCGTTAATTTTGACTGGGCGTACATAGTTAACAAGGTAACGTCGGTGTATAACATC

AAGCTGGACGGGTACGGCAAGTTCAACAAAGGGGGGCTGTTTAAGGTGTGGGACATCGCC

ACGAACCATTTTCAGAAGAAGAGCAAGGTGAAAATCAATGGCCTGATATCTCTAGACATG

TATTCTGTGGCGACGGAAAAGCTAAAGCTACCCAGCTACAAACTCGACGCGGTCGTGGGA

GACGTCCTCGGCGAGCATAAGATAGACCTTCCCTATAAAGAAATACCCTCCTATTACGCG

GGAGGGCCTGACCGGAGGGGCGTAATAGGAGAGTATTGTATCCAGGACTCTAGGCTGGTG

GGCAAGCTGTTTTTTAAGTACCTCCCCCATCTGGAACTATCGGCGGTGGCCAAACTCGCC

CGTATCACCCTAACGCGGGTAATTTTTGACGGTCAGCAAATTCGCGTGTACACGTGCTTG

CTGAAACTCGCCCGCGAGAGAAATTTCATTTTGCCAGACAACAGACGCCGGTTTGACAGT

CAGGCAGACGCCGCGTCAGAGACTTCGGAGTTGGCTATGGATAGCCAAAGCCACGCCTTC

GACAGTACAGACGAACCCGACGGTGTGGACGGTACCCCGGACGCCGCAGGATCTGGCGCT

ACTTCTGAAAACGGAGGCGGGAAGCCCGGCGTCGGGAGGGCCGTGGGCTACCAGGGAGCA

AAGGTTCTAGACCCCGTATCCGGCTTTCATGTGGACCCCGTGGTTGTGTTTGACTTCGCT

AGCTTATACCCAAGCATTATCCAGGCCCATAACCTCTGTTTCACCACCCTGGCGCTCGAT

GAAGTGGATCTGGCCGGGCTTCAACCATCCGTCAACTACTCGACGTTCGAGGTGGGTGAC

CAAAAGTTATTTTTTGTCCACGCCCATATTCGCGAAAGCCTGCTTGGCATCTTGCTGCGC

GACTGGCTGGCCATGCGAAAGGCGGTGAGGGCGCGAATCCCCACCAGCACCCCCGAGGAG

GCAGTTTTACTAGATAAGCAGCAGTCTGCGATTAAGGTGATATGCAACTCGGTTTACGGA

TTCACGGGGGTGGCAAACGGCCTGTTGCCGTGTCTGAGGATAGCGGCTACCGTTACCACG

ATAGGACGCGACATGCTCCTCAAGACCCGAGATTACGTTCACTCTCGTTGGGCGACGCGC

GAGCTGCTGGAGGACAATTTTCCAGGGGCTATAGGTTTCCGAAACCACAAGCCTTACTCC

GTCAGGGTTATCTACGGAGACACCGACTCCGTGTTTATCAAGTTTGTGGGCCTGACGTAC

GAGGGGGTATCGGAGCTGGGGGATGCTATGTCGCGTCAGATTTCAGCGGACCTCTTTAGA

GCGCCCATCAAACTGGAGTGTGAGAAGACCTTTCAGCGACTGCTGCTGATCACCAAGAAG

AAGTACATAGGTGTCATAAACGGGGGGAAGATGCTCATGAAGGGGGTCGACCTGGTCCGC

AAAAATAACTGCTCTTTCATAAACTTGTACGCGCGACATCTGGTAGATCTTTTGTTGTAC

GACGAGGATGTGGCCACGGCGGCAGCAGAGGTGACAGACGTGCCTCCCGCAGAATGGGTG

GGGCGCCCGCTACCGAGCGGCTTTGACAAGTTTGGGCGAGTGCTGGTAGAGGCGTACAAC

CGTATCACTGCCCCCAACTTGGACGTGCGCGAGTTCGTTATGACTGCTGAGCTGAGCCGC

TCACCCGAATCGTATACCAACAAGCGCCTGCCGCACCTCACCGTCTACTTTAAGCTCGCC

ATGAGGAATGAAGAACTGCCCAGCGTAAAAGAGAGAATTCCGTATGTGATAGTTGCGCAG

ACCGAGGCCGCGGAACGCGAAGCGGGTGTAGTAAACTCAATGCGCGGTACCGCCCAAAAC

CCCGTGGTAACCAAGACCGCACGCCCCCAACCTAAACGCAAACTGCTGGTTTCCGACCTC

GCCGAAGACCCGACCTATGTTTCCGAGAATGACGTACCGCTAAACACAGACTACTATTTC

TCCCACCTGTTGGGTACCATAAGCGTGACCTTTAAGGCTCTATTCGGAAATGATGTGAGA

ACAACAGAAAATCTTTTAAAGCGGTTTATTCCGGAAACCCCCCACAAGACCCCCACGAAA

ACCCAGGCACTGCTTGAGCGCGCCGGCTTTGAAAAGCTGACGCCCTTTACACCGGAGGAA

GAAAGTCGTCGAATACTGCATACAGTTTTTTGTACTCTAGAAGCAGCTCCCCATCAAAGC

TGA

>KF434382.1

ATGGCGGCGCGCGAACAGGCCAACTCCGTTCGCAGGAGCGGGTTTTTTAATCCGTTTATT

GGCAAGAGGCCATTTTTCAGGCCGGGAAGCGGGCAAACCGCGGAGACAGAGCGCCCAAGG

CCCCCCCAACACTCGTACTGCACAGAGGTGGGTAGCTTTAAGTTTATAGCTCCAAGATGC

CTCGATGAAGAAGCCCCCGCCGACCAGCGACGCGGTGTACACGTGGGCACCTTGGAGCGC

CCACCCAAAGTGTACTGCGATGGCTCAGAGTACGACGTGCTGAACTTTGCCTCCGGAGGT

TGCTGGCCTCGCAGGATTCGGGTTTGGAACGGCCAGGATTTTCGGGGCGATGGATTCAAC

CCCAGATTTGAGAGATTTCACGTGTACGACATAGTAGAGACTTCGGAGAGCGCCTCACAC

GATGACCCATCCAGGTTTGCTGAGCTATCCCGCCCAAGCGGGTCTGTGGTAACACTGCTG

GGAATGAGCGAGTGTGGAAAGCGAGTCGCGGTTCACGTCTATGGTGTGCGCCATTATTTT

TACATGGCAAAGGCGGAGGTGGATAGCGCTTGTGGAATAACCACCGAGGCAGAACTCGTG

CGCGCAATGGTGGACTGCGCGCACAGCTCGGCTTTGAGCGCGGCCCTGGGAAATGGCAAC

GGCGGCAAACAGAGCGGCGGCAGCGGCGGGGGATGGTGGGGCGGAAAGCACGTGTCTGCG

GACTGCTTCAAAGTGGAGACCGTGTGCCACACGACGCTGTACTACTTTGGATCTAAGCCA

GCTCTCTACTATAGAGTATCTGCCTCCAGCAGCCGCCTGGGAGGGTTCATCTGCGACAAC

TTTCACCCGGAGATTACAAAATTCGAGGGGAGCGTGGACGTGACCACGCGGCTGTTGTTG

GACAATGAAAATTTTACCAGTTTCGGGTGGTACCGCCTGCGACCCGGCACCCACGGAGAG

CGTGTTCAACTTCGCCCCGTTGAGCGACACGTCACCTCAAGCGACGTGGAGATTAACTGT

ACTCCCGATAACCTGGAGCCGATACCAGACGAGGCTGCCTGGCCCGACTATAAGCTCATG

TGCTTTGATATAGAGTGTAAAGCTGGAACGGGTAACGAAATGGCGTTCCCAGTGGCAACT

AACCAAGAGGACCTGGTCATCCAGATCTCCTGTCTGCTGTACTCGCTTGCTACTCAGAAC

CACGAACACACCCTGCTGTTTTCCCTCGGGTCATGCGATATCTCTGAGGAATACTCGTTT

GCATGCGTCCAGCGCGGCGAGCCCAGACCGACGGTTTTGGAGTTTGACAGCGAGTACGAG

CTGCTGGTTGCCTTCCTGACCTTTCTCAAGCAGTACTCTCCCGAGTTCGCCACCGGCTAC

AACATCGTTAATTTTGACTGGGCGTACATAGTTAACAAGGTAACGTCGGTGTATAACATC

AAGCTGGACGGGTACGGCAAGTTCAACAAAGGGGGGCTGTTTAAGGTGTGGGACATCGCC

ACGAACCATTTTCAGAAGAAGAGCAAGGTGAAAATCAATGGCCTGATATCTCTAGACATG

TATTCTGTGGCGACGGAAAAGCTAAAGCTACCCAGCTACAAACTCGACGCGGTCGTGGGA

GACGTCCTCGGCGAGCATAAGATAGACCTTCCCTATAAAGAAATACCCTCCTATTACGCG

GGAGGGCCTGACCGGAGGGGCGTAATAGGAGAGTATTGTATCCAGGACTCTAGGCTGGTG

GGCAAGCTGTTTTTTAAGTACCTCCCCCATCTGGAACTATCGGCGGTGGCCAAACTCGCC

CGTATCACCCTAACGCGGGTAATTTTTGACGGTCAGCAAATTCGCGTGTACACGTGCTTG

CTGAAACTCGCCCGCGAGAGAAATTTCATTTTGCCAGACAACAGACGCCGGTTTGACAGT

CAGGCAGACGCCGCGTCAGAGACTTCGGAGTTGGCTATGGATAGCCAAAGCCACGCCTTC

GACAGTACAGACGAACCCGACGGTGTGGACGGTACCCCGGACGCCGCAGGATCTGGCGCT

ACTTCTGAAAACGGAGGCGGGAAGCCCGGCGTCGGGAGGGCCGTGGGCTACCAGGGAGCA

AAGGTTCTAGACCCCGTATCCGGCTTTCATGTGGACCCCGTGGTTGTGTTTGACTTCGCT

AGCTTATACCCAAGCATTATCCAGGCCCATAACCTCTGTTTCACCACCCTGGCGCTCGAT

GAAGCGGATCTGGCCGGGCTTCAACCATCCGTCAACTACTCGACGTTCGAGGTGGGTGAC

CAAAAGTTATTTTTTGTCCACGCCCATATTCGCGAAAGCCTGCTTGGCATCTTGCTGCGC

GACTGGCTGGCCATGCGAAAGGCGGTGAGGGCGCGAATCCCCACCAGCACCCCCGAGGAG

GCAGTTTTACTAGATAAGCAGCAGTCTGCGATTAAGGTGATATGCAACTCGGTTTACGGA

TTCACGGGGGTGGCAAACGGCCTGTTGCCGTGTCTGAGGATAGCGGCTACCGTTACCACG

ATAGGACGCGACATGCTCCTCAAGACCCGAGATTACGTTCACTCTCGTTGGGCGACGCGC

GAGCTGCTGGAGGACAATTTTCCAGGGGCTATAGGTTTCCGAAACCACAAGCCTTACTCC

GTCAGGGTTATCTACGGAGACACCGACTCCGTGTTTATCAAGTTTGTGGGCCTGACGTAC

GAGGGGGTATCGGAGCTGGGGGATGCTATGTCGCGTCAGATTTCAGCGGACCTCTTTAGA

GCGCCCATCAAACTGGAGTGTGAGAAGACCTTTCAGCGACTGCTGCTGATCACCAAGAAG

AAGTACATAGGTGTCATAAACGGGGGGAAGATGCTCATGAAGGGGGTCGACCTGGTCCGC

AAAAATAACTGCTCTTTCATAAACTTGTACGCGCGACATCTGGTAGATCTTTTGTTGTAC

GACGAGGATGTGGCCACGGCGGCAGCAGAGGTGACAGACGTGCCTCCCGCAGAATGGGTG

GGGCGCCCGCTACCGAGCGGCTTTGACAAGTTTGGGCGAGTGCTGGTAGAGGCGTACAAC

CGTATCACTGCCCCCAACTTGGACGTGCGCGAGTTCGTTATGACTGCTGAGCTGAGCCGC

TCACCCGAATCGTATACCAACAAGCGCCTGCCGCACCTCACCGTCTACTTTAAGCTCGCC

ATGAGGAATGAAGAACTGCCCAGCGTAAAAGAGAGAATTCCGTATGTGATAGTTGCGCAG

ACCGAGGCCGCGGAACGCGAAGCGGGTGTAGTAAACTCAATGCGCGGTACCGCCCAAAAC

CCCGTGGTAACCAAGACCGCACGCCCCCAACCTAAACGCAAACTGCTGGTTTCCGACCTC

GCCGAAGACCCGACCTATGTTTCCGAGAATGACGTACCGCTAAACACAGACTACTATTTC

TCCCACCTGTTGGGTACCATAAGCGTGACCTTTAAGGCTCTATTCGGAAATGATGTGAGA

ACAACAGAAAATCTTTTAAAGCGGTTTATTCCGGAAACCCCCCACAAGACCCCCACGAAA

ACCCAGGCACTGCTTGAGCGCGCCGGCTTTGAAAAGCTGACGCCCTTTACACCGGAGGAA

GAAAGTCGTCGAATACTGCATACAGTTTTTTGTACTCTAGAAGCAGCTCCC

>KF515985.1

ATGGCGGCGCGCGAACAGGCCAACTCCGTTCGCAGGAGCGGGTTTTTTAATCCGTTTATT

GGCAAGAGGCCATTTTTCAGGCCGGGAAGCGGGCAGACCGCGGAGACAGAGCGCCCAAGG

CCCCCCCAACACTCGTACTGCACAGAGGTGGGTAGCTTTAAGTTTATAGCTCCAAGATGC

CTCGATGAAGAAGCCCCCGCCGACCAGCGACGCGGTGTACACGTGGGCACCTTGGAGCGC

CCACCCAAAGTGTACTGCGATGGCTCAGAGTACGACGTGCTGAACTTTGCCTCCGGAGGT

TGCTGGCCTCGCAGGATTCGGGTTTGGAACGGCCAGGATTTTCGGGGCGATGGATTCAAC

CCCAGATTTGAGAGATTTCACGTGTACGACATAGTAGAGACTTCGGAGAGCGCCTCACAC

GATGACCCATCCAGGTTTGCTGAGCTATCCCGCCCAAGTGGGTCTGTGGTAACACTGCTG

GGAATGAGCGAGTGTGGAAAGCGAGTCGCGGTTCACGTCTATGGTGTGCGCCATTATTTT

TACATGGCAAAGGCGGAGGTGGATAGCGCTTGTGGAATAACCACCGAGGCAGAACTCGTG

CGCGCAATGGTGGACTGCGCGCACAGCTCGGCTTTGAGCGCGGCCCTGGGAAATGGCAAC

GGCGGCAAACAGAGCGGCGGCAGCGGCGGGGGATGGTGGGGCGGAAAGCACGTGTCTGCG

GACTGCTTCAAAGTGGAGACCGTGTGCCACACGACGCTGTACTACTTTGGATCTAAGCCA

GCTCTCTACTATAGAGTATCTGCCTCCAGCAGCCGCCTGGGAGGGTTCATCTGCGACAAC

TTTCACCCGGAGATTACAAAATTCGAGGGGAGCGTGGACGTGACCACGCGGCTGTTGTTG

GACAATGAAAATTTTACCAGTTTCGGGTGGTACCGCCTGCGACCCGGCACCCACGGAGAG

CGTGTTCAACTTCGCCCCGTTGAGCGACACGTCACCTCAAGCGACGTGGAGATTAACTGT

ACTCCCGATAACCTGGAGCCGATACCAGACGAGGCTGCCTGGCCCGACTATAAGCTCATG

TGCTTTGATATAGAGTGTAAAGCTGGAACGGGTAACGAAATGGCGTTCCCAGTGGCAACT

AACCAAGAGGACCTGGTCATCCAGATCTCCTGTCTGCTGTACTCGCTTGCTACTCAGAAC

CACGAACACACCCTGCTGTTTTCCCTCGGGTCATGCGATATCTCTGAGGAATACTCGTTT

GCATGCGTCCAGCGCGGCGAGCCCAGACCGACGGTTTTGGAGTTTGACAGCGAGTACGAG

CTGCTGGTTGCCTTCCTGACCTTTCTCAAGCAGTACTCTCCCGAGTTCGCCACCGGCTAC

AACATCGTTAATTTTGACTGGGCGTACATAGTTAACAAGGTAACGTCGGTGTATAACATC

AAGCTGGACGGGTACGGCAAGTTCAACAAAGGGGGGCTGTTTAAGGTGTGGGACATCGCC

ACGAACCATTTTCAGAAGAAGAGCAAGGTGAAAATCAATGGCCTGATATCTCTAGACATG

TATTCTGTGGCGACGGAAAAGCTAAAGCTACCCAGCTACAAACTCGACGCGGTCGTGGGA

GACGTCCTCGGCGAGCATAAGATAGACCTTCCCTATAAAGAAATACCCTCCTATTACGCG

GGAGGGCCTGACCGGAGGGGCGTAATAGGAGAGTATTGTATCCAGGACTCTAGGCTGGTG

GGCAAGCTGTTTTTTAAGTACCTCCCCCATCTGGAACTATCGGCGGTGGCCAAACTCGCC

CGTATCACCCTAACGCGGGTAATTTTTGACGGTCAGCAAATTCGCGTGTACACGTGCTTG

CTGAAACTCGCCCGCGAGAGAAATTTCATTTTGCCAGACAACAGACGCCGGTTTGACAGT

CAGGCAGACGCCGCGTCAGAGACTTCGGAGTTGGCTATGGATAGCCAAAGCCACGCCTTC

GACAGTACAGACGAACCCGACGGTGTGGACGGTACCCCGGACGCCGCAGGATCTGGCGCT

ACTTCTGAAAACGGAGGCGGGAAGCCCGGCGTCGGGAGGGCCGTGGGCTACCAGGGAGCA

AAGGTTCTAGACCCCGTATCCGGCTTTCATGTGGACCCCGTGGTTGTGTTTGACTTCGCT

AGCTTATACCCAAGCATTATCCAGGCCCATAACCTCTGTTTCACCACCCTGGCGCTCGAT

GAAGTGGATCTGGCCGGGCTTCAACCATCCGTCAACTACTCGACGTTCGAGGTGGGTGAC

CAAAAGTTATTTTTTGTCCACGCCCATATTCGCGAAAGCCTGCTTGGCATCTTGCTGCGC

GACTGGCTGGCCATGCGAAAGGCGGTGAGGGCGCGAATCCCCACCAGCACCCCCGAGGAG

GCAGTTTTACTAGATAAGCAGCAGTCTGCGATTAAGGTGATATGCAACTCGGTTTACGGA

TTCACGGGGGTGGCAAACGGCCTGTTGCCGTGTCTGAGGATAGCGGCTACCGTTACCACG

ATAGGACGCGACATGCTCCTCAAGACCCGAGATTACGTTCACTCTCGTTGGGCGACGCGC

GAGCTGCTGGAGGACAATTTTCCAGGGGCTATAGGTTTCCGAAACCACAAGCCTTACTCC

GTCAGGGTTATCTACGGAGACACCGACTCCGTGTTTATCAAGTTTGTGGGCCTGACGTAC

GAGGGGGTATCGGAGCTGGGGGATGCTATGTCGCGTCAGATTTCAGCGGACCTCTTTAGA

GCGCCCATCAAACTGGAGTGTGAGAAGACCTTTCAGCGACTGCTGCTGATCACCAAGAAG

AAGTACATAGGTGTCATAAACGGGGGGAAGATGCTCATGAAGGGGGTCGACCTGGTCCGC

AAAAATAACTGCTCTTTCATAAACTTGTACGCGCGACATCTGGTAGATCTTTTGTTGTAC

GACGAGGATGTGGCCACGGCGGCAGCAGAGGTGACAGACGTGCCTCCCGCAGAATGGGTG

GGGCGCCCGCTACCGAGCGGCTTTGACAAGTTTGGGCGAGTGCTGGTAGAGGCGTACAAC

CGTATCACTGCCCCCAACTTGGACGTGCGCGAGTTCGTTATGACTGCTGAGCTGAGCCGC

TCACCCGAATCGTATACCAACAAGCGCCTGCCGCACCTCACCGTCTACTTTAAGCTCGCC

ATGAGGAATGAAGAACTGCCCAGCGTAAAAGAGAGAATTCCGTATGTGATAGTTGCGCAG

ACCGAGGCCGCGGAACGCGAAGCGGGTGTAGTAAACTCAATGCGCGGTACCGCCCAAAAC

CCCGTGGTAACCAAGACCGCACGCCCCCAACCTAAACGCAAACTGCTGGTTTCCGACCTC

GCCGAAGACCCGACCTATGTTTCCGAGAATGACGTACCGCTAAACACAGACTACTATTTC

TCCCACCTGTTGGGTACCATAAGCGTGACCTTTAAGGCTCTATTCGGAAATGATGTGAGA

ACAACAGAAAATCTTTTAAAGCGGTTTATTCCGGAAACCCCCCACAAGACCCCCACGAAA

ACCCAGGCACTGCTTGAGCGCGCCGGCTTTGAAAAGCTGACGCCTTTTACACCGGAGGAA

GAAAGTCGTCGAATACTGCATACAGTTTTTTGTACTCTAGAAGCAGCTCCCCATCAAAGC

TGA

>KF515986.1

ATGGCGGCGCGCGAACAGGCCAACTCCGTTCGCAGGAGCGGGTTTTTTAATCCGTTTATT

GGCAAGAGGCCATTTTTCAGGCCGGGAAGCGGGCAAACCGCGGAGACAGAGCGCCCAAGG

CCCCCCCAACACTCGTACTGCACAGAGGTGGGTAGCTTTAAGTTTATAGCTCCAAGATGC

CTCGATGAAGAAGCCCCCGCCGACCAGCGACGCGGTGTACACGTGGGCACCTTGGAGCGC

CCACCCAAAGTGTACTGCGATGGCTCAGAGTACGACGTGCTGAACTTTGCCTCCGGAGGT

TGCTGGCCTCGCAGGATTCGGGTTTGGAACGGCCAGGATTTTCGGGGCGATGGATTCAAC

CCCAGATTTGAGAGATTTCACGTGTACGACATAGTAGAGACTTCGGAGAGCGCCTCACAC

GATGACCCATCCAGGTTTGCTGAGCTATCCCGCCCAAGCGGGTCTGTGGTAACACTGCTG

GGAATGAGCGAGTGTGGAAAGCGAGTCGCGGTTCACGTCTATGGTGTGCGCCATTATTTT

TACATGGCAAAGGCGGAGGTGGATAGCGCTTGTGGAATAACCACCGAGGCAGAACTCGTG

CGCGCAATGGTGGACTGCGCGCACAGCTCGGCTTTGAGCGCGGCCCTGGGAAATGGCAAC

GGCGGCAAACAGAGCGGCGGCAGCGGCGGGGGATGGTGGGGCGGAAAGCACGTGTCTGCG

GACTGCTTCAAAGTGGAGACCGTGTGCCACACGACGCTGTACTACTTTGGATCTAAGCCA

GCTCTCTACTATAGAGTATCTGCCTCCAGCAGCCGCCTGGGAGGGTTCATCTGCGACAAC

TTTCACCCGGAGATTACAAAATTCGAGGGGAGCGTGGACGTGACCACGCGGCTGTTGTTG

GACAATGAAAATTTTACCAGTTTCGGGTGGTACCGCCTGCGACCCGGCACCCACGGAGAG

CGTGTTCAACTTCGCCCCGTTGAGCGACACGTCACCTCAAGCGACGTGGAGATTAACTGT

ACTCCCGATAACCTGGAGCCGATACCAGACGAGGCTGCCTGGCCCGACTATAAGCTCATG

TGCTTTGATATAGAGTGTAAAGCTGGAACGGGTAACGAAATGGCGTTCCCAGTGGCAACT

AACCAAGAGGACCTGGTCATCCAGATCTCCTGTCTGCTGTACTCGCTTGCTACTCAGAAC

CACGAACACACCCTGCTGTTTTCCCTCGGGTCATGCGATATCTCTGAGGAATACTCGTTT

GCATGCGTCCAGCGCGGCGAGCCCAGACCGACGGTTTTGGAGTTTGACAGCGAGTACGAG

CTGCTGGTTGCCTTCCTGACCTTTCTCAAGCAGTACTCTCCCGAGTTCGCCACCGGCTAC

AACATCGTTAATTTTGACTGGGCGTACATAGTTAACAAGGTAACGTCGGTGTATAACATC

AAGCTGGACGGGTACGGCAAGTTCAACAAAGGGGGGCTGTTTAAGGTGTGGGACATCGCC

ACGAACCATTTTCAGAAGAAGAGCAAGGTGAAAATCAATGGCCTGATATCTCTAGACATG

TATTCTGTGGCGACGGAAAAGCTAAAGCTACCCAGCTACAAACTCGACGCGGTCGTGGGA

GACGTCCTCGGCGAGCATAAGATAGACCTTCCCTATAAAGAAATACCCTCCTATTACGCG

GGAGGGCCTGACCGGAGGGGCGTAATAGGAGAGTATTGTATCCAGGACTCTAGGCTGGTG

GGCAAGCTGTTTTTTAAGTACCTCCCCCATCTGGAACTATCGGCGGTGGCCAAACTCGCC

CGTATCACCCTAACGCGGGTAATTTTTGACGGTCAGCAAATTCGCGTGTACACGTGCTTG

CTGAAACTCGCCCGCGAGAGAAATTTCATTTTGCCAGACAACAGACGCCGGTTTGACAGT

CAGGCAGACGCCGCGTCAGAGACTTCGGAGTTGGCTATGGATAGCCAAAGCCACGCCTTC

GACAGTACAGACGAACCCGACGGTGTGGACGGTACCCCGGACGCCGCAGGATCTGGCGCT

ACTTCTGAAAACGGAGGCGGGAAGCCCGGCGTCGGGAGGGCCGTGGGCTACCAGGGAGCA

AAGGTTCTAGACCCCGTATCCGGCTTTCATGTGGACCCCGTGGTTGTGTTTGACTTCGCT

AGCTTATACCCAAGCATTATCCAGGCCCATAACCTCTGTTTCACCACCCTGGCGCTCGAT

GAAGTGGATCTGGCCGGGCTTCAACCATCCGTCGACTACTCGACGTTCGAGGTGGGTGAC

CAAAAGTTATTTTTTGTCCACGCCCATATTCGCGAAAGCCTGCTTGGCATCTTGCTGCGC

GACTGGCTGGCCATGCGAAAGGCGGTGAGGGCGCGAATCCCCACCAGCACCCCCGAGGAG

GCAGTTTTACTAGATAAGCAGCAGTCTGCGATTAAGGTGATATGCAACTCGGTTTACGGA

TTCACGGGGGTGGCAAACGGCCTGTTGCCGTGTCTGAGGATAGCGGCTACCGTTACCACG

ATAGGACGCGACATGCTCCTCAAGACCCGAGATTACGTTCACTCTCGTTGGGCGACGCGC

GAGCTGCTGGAGGACAATTTTCCAGGGGCTATAGGTTTCCGAAACCACAAGCCTTACTCC

GTCAGGGTTATCTACGGAGACACCGACTCCGTGTTTATCAAGTTTGTGGGCCTGACGTAC

GAGGGGGTATCGGAGCTGGGGGATGCTATGTCGCGTCAGATTTCAGCGGACCTCTTTAGA

GCGCCCATCAAACTGGAGTGTGAGAAGACCTTTCAGCGACTGCTGCTGATCACCAAGAAG

AAGTACATAGGTGTCATAAACGGGGGGAAGATGCTCATGAAGGGGGTCGACCTGGTCCGC

AAAAATAACTGCTCTTTCATAAACTTGTACGCGCGACATCTGGTAGATCTTTTGCTGTAC

GACGAGGATGTGGCCACGGCGGCAGCAGAGGTGACAGACGTGCCTCCCGCAGAATGGGTG

GGGCGCCCGCTACCGAGCGGCTTTGACAAGTTTGGGCGAGTGCTGGTAGAGGCGTACAAC

CGTATCACTGCCCCCAACTTGGACGTGCGCGAGTTCGTTATGACTGCTGAGCTGAGCCGC

TCACCCGAATCGTATACCAACAAGCGCCTGCCGCACCTCACCGTCTACTTTAAGCTCGCC

ATGAGGAATGAAGAACTGCCCAGCGTAAAAGAGAGAATTCCGTATGTGATAGTTGCGCAG

ACCGAGGCCGCGGAACGCGAAGCGGGTGTAGTAAACTCAATGCGCGGTACCGCCCAAAAC

CCCGTGGTAACCAAGACCGCACGCCCCCAACCTAAACGCAAACTGCTGGTTTCCGACCTC

GCCGAAGACCCGACCTATGTTTCCGAGAATGACGTACCGCTAAACACAGACTACTATTTC

TCCCACCTGTTGGGTACCATAAGCGTGACCTTTAAGGCTCTATTCGGAAATGATGTGAGA

ACAACAGAAAATCTTTTAAAGCGGTTTATTCCGGAAACCCCCCACAAGACCCCCACGAAA

ACCCAGGCACTGCTTGAGCGCGCCGGCTTTGAAAAGCTGACGCCCTTTACACCGGAGGAA

GAAAGTCGTCGAATACTGCATACAGTTTTTTGTACTCTAGAAGCAGCTCCCCATCAAAGC

TGA

>KC924782.1

ATGGCGGCGCGCGAACAGGCCAACTCCGTTCGCAGGAGCGGGTTTTTTAATCCGTTTATT

GGCAAGAGGCCATTTTTCAGGCCGGGAAGCGGGCAGACCGCGGAGACAGAGCGCCCAAGG

CCCCCCCAACACTCGTACTGCACAGAGGTGGGTAGCTTTAAGTTTATAGCTCCAAGATGC

CTCGATGAAGAAGCCCCCGCCGACCAGCGACGCGGTGTACACGTGGGCACCTTGGAGCGC

CCACCCAAAGTGTACTGCGATGGCTCAGAGTACGACGTGCTGAACTTTGCCTCCGGAGGT

TGCTGGCCTCGCAGGATTCGGGTTTGGAACGGCCAGGATTTTCGGGGCGATGGATTCAAC

CCCAGATTTGAGAGATTTCACGTGTACGACATAGTAGAGACTTCGGAGAGCGCCTCACAC

GATGACCCATCCAGGTTTGCTGAGCTATCCCGCCCAAGCGGGTCTGTGGTAACACTGCTG

GGAATGAGCGAGTGTGGAAAGCGAGTCGCGGTTCACGTCTATGGTGTGCGCCATTATTTT

TACATGGCAAAGGCGGAGGTGGATAGCGCTTGTGGAATAACCACCGAGGCAGAACTCGTG

CGCGCAATGGTGGACTGCGCGCACAGCTCGGCTTTGAGCGCGGCCCTGGGAAATGGCAAC

GGCGGCAAACAGAGCGGCGGCAGCGGCGGGGGATGGTGGGGCGGAAAGCACGTGTCTGCG

GACTGCTTCAAAGTGGAGACCGTGTGCCACACGACGCTGTACTACTTTGGATCTAAGCCA

GCTCTCTACTATAGAGTATCTGCCTCCAGCAGCCGCCTGGGAGGGTTCATCTGCGACAAC

TTTCACCCGGAGATTACAAAATTCGAGGGGAGCGTGGACGTGACCACGCGGCTGTTGTTG

GACAATGAAAATTTTACCAGTTTCGGGTGGTACCGCCTGCGACCCGGCACCCACGGAGAG

CGTGTTCAACTTCGCCCCGTTGAGCGACACGTCACCTCAAGCGACGTGGAGATTAACTGT

ACTCCCGATAACCTGGAGCCGATACCAGACGAGGCTGCCTGGCCCGACTATAAGCTCATG

TGCTTTGATATAGAGTGTAAAGCTGGAACGGGTAACGAAATGGCGTTCCCAGTGGCAACT

AACCAAGAGGACCTGGTCATCCAGATCTCCTGTCTGCTGTACTCGCTTGCTACTCAGAAC

CACGAACACACCCTGCTGTTTTCCCTCGGGTCATGCGATATCTCTGAGGAATACTCGTTT

GCATGCGTCCAGCGCGGCGAGCCCAGACCGACGGTTTTGGAGTTTGACAGCGAGTACGAG

CTGCTGGTTGCCTTCCTGACCTTTCTCAAGCAGTACTCTCCCGAGTTCGCCACCGGCTAC

AACATCGTTAATTTTGACTGGGCGTACATAGTTAACAAGGTAACGTCGGTGTATAACATC

AAGCTGGACGGGTACGGCAAGTTCAACAAAGGGGGGCTGTTTAAGGTGTGGGACATCGCC

ACGAACCATTTTCAGAAGAAGAGCAAGGTGAAAATCAATGGCCTGATATCTCTAGACATG

TATTCTGTGGCGACGGAAAAGCTAAAGCTACCCAGCTACAAACTCGACGCGGTCGTGGGA

GACGTCCTCGGCGAGCATAAGATAGACCTTCCCTATAAAGAAATACCCTCCTATTACGCG

GGAGGGCCTGACCGGAGGGGCGTAATAGGAGAGTATTGTATCCAGGACTCTAGGCTGGTG

GGCAAGCTGTTTTTTAAGTACCTCCCCCATCTGGAACTATCGGCGGTGGCCAAACTCGCC

CGTATCACCCTAACGCGGGTAATTTTTGACGGTCAGCAAATTCGCGTGTACACGTGCTTG

CTGAAACTCGCCCGCGAGAGAAATTTCATTTTGCCAGACAACAGACGCCGGTTTGACAGT

CAGGCAGACGCCGCGTCAGAGACTTCGGAGTTGGCTATGGATAGCCAAAGCCACGCCTTC

GACAGTACAGACGAACCCGACGGTGTGGACGGTACCCCGGACGCCGCAGGATCTGGCGCT

ACTTCTGAAAACGGAGGCGGGAAGCCCGGCGTCGGGAGGGCCGTGGGCTACCAGGGAGCA

AAGGTTCTAGACCCCGTATCCGGCTTTCATGTGGACCCCGTGGTTGTGTTTGACTTCGCT

AGCTTATACCCAAGCATTATCCAGGCCCATAACCTCTGTTTCACCACCCTGGCGCTCGAT

GAAGTGGATCTGGCCGGGCTTCAACCATCCGTCGACTACTCGACGTTCGAGGTGGGTGAC

CAAAAGTTATTTTTTGTCCACGCCCATATTCGCGAAAGCCTGCTTGGCATCTTGCTGCGC

GACTGGCTGGCCATGCGAAAGGCGGTGAGGGCGCGAATCCCCACCAGCACCCCCGAGGAG

GCAGTTTTACTAGATAAGCAGCAGTCTGCGATTAAGGTGATATGCAACTCGGTTTACGGA

TTCACGGGGGTGGCAAACGGCCTGTTGCCGTGTCTGAGGATAGCGGCTACCGTTACCACG

ATAGGACGCGACATGCTCCTCAAGACCCGAGATTACGTTCACTCTCGTTGGGCGACGCGC

GAGCTGCTGGAGGACAATTTTCCAGGGGCTATAGGTTTCCGAAACCACAAGCCTTACTCC

GTCAGGGTTATCTACGGAGACACCGACTCCGTGTTTATCAAGTTTGTGGGCCTGACGTAC

GAGGGGGTATCGGAGCTGGGGGATGCTATGTCGCGTCAGATTTCAGCGGACCTCTTTAGA

GCGCCCATCAAACTGGAGTGTGAGAAGACCTTTCAGCGACTGCTGCTGATCACCAAGAAG

AAGTACATAGGTGTCATAAACGGGGGGAAGATGCTCATGAAGGGGGTCGACCTGGTCCGC

AAAAATAACTGCTCTTTCATAAACTTGTACGCGCGACATCTGGTAGATCTTTTGTTGTAC

GACGAGGATGTGGCCACGGCGGCAGCAGAGGTGACAGACGTGCCTCCCGCAGAATGGGTG

GGGCGCCCGCTACCGAGCGGCTTTGACAAGTTTGGGCGAGTGCTGGTAGAGGCGTACAAC

CGTATCACTGCCCCCAACTTGGACGTGCGCGAGTTCGTTATGACTGCTGAGCTGAGCCGC

TCACCCGAATCGTATACCAACAAGCGCCTGCCGCACCTCACCGTCTACTTTAAGCTCGCC

ATGAGGAATGAAGAACTGCCCAGCGTAAAAGAGAGAATTCCGTATGTGATAGTTGCGCAG

ACCGAGGCCGCGGAACGCGAAGCGGGTGTAGTAAACTCAATGCGCGGTACCGCCCAAAAC

CCCGTGGTAACCAAGACCGCACGCCCCCAACCTAAACGCAAACTGCTGGTTTCCGACCTC

GCCGAAGACCCGACCTATGTTTCCGAGAATGACGTACCGCTAAACACAGACTACTATTTC

TCCCACCTGTTGGGTACCATAAGCGTGACCTTTAAGGCTCTATTCGGAAATGATGTGAGA

ACAACAGAAAATCTTTTAAAGCGGTTTATTCCGGAAACCCCCCACAAGACCCCCACGAAA

ACCCAGGCACTGCTTGAGCGCGCCGGCTTTGAAAAGCTGACGCCCTTTACACCGGAGGAA

GAAAGTCGTCGAATACTGCATACAGTTTTTTGTACTCTAGAAGCAGCTCCCCATCAAAGC

TGA

>KC924783.1

ATGGCGGCGCGCGAACAGGCCAACTCCGTTCGCAGGAGCGGGTTTTTTAATCCGTTTATT

GGCAAGAGGCCATTTTTCAGGCCGGGAAGCGGGCAGACCGCGGAGACAGAGCGCCCAAGG

CCCCCCCAACACTCGTACTGCACAGAGGTGGGTAGCTTTAAGTTTATAGCTCCAAGATGC

CTCGATGAAGAAGCCCCCGCCGACCAGCGACGCGGTGTACACGTGGGCACCTTGGAGCGC

CCACCCAAAGTGTACTGCGATGGCTCAGAGTACGACGTGCTGAACTTTGCCTCCGGAGGT

TGCTGGCCTCGCAGGATTCGGGTTTGGAACGGCCAGGATTTTCGGGGCGATGGATTCAAC

CCCAGATTTGAGAGATTTCACGTGTACGACATAGTAGAGACTTCGGAGAGCGCCTCACAC

GATGACCCATCCAGGTTTGCTGAGCTATCCCGCCCAAGCGGGTCTGTGGTAACACTGCTG

GGAATGAGCGAGTGTGGAAAGCGAGTCGCGGTTCACGTCTATGGTGTGCGCCATTATTTT

TACATGGCAAAGGCGGAGGTGGATAGCGCTTGTGGAATAACCACCGAGGCAGAACTCGTG

CGCGCAATGGTGGACTGCGCGCACAGCTCGGCTTTGAGCGCGGCCCTGGGAAATGGCAAC

GGCGGCAAACAGAGCGGCGGCAGCGGCGGGGGATGGTGGGGCGGAAAGCACGTGTCTGCG

GACTGCTTCAAAGTGGAGACCGTGTGCCACACGACGCTGTACTACTTTGGATCTAAGCCA

GCTCTCTACTATAGAGTATCTGCCTCCAGCAGCCGCCTGGGAGGGTTCATCTGCGACAAC

TTTCACCCGGAGATTACAAAATTCGAGGGGAGCGTGGACGTGACCACGCGGCTGTTGTTG

GACAATGAAAATTTTACCAGTTTCGGGTGGTACCGCCTGCGACCCGGCACCCACGGAGAG

CGTGTTCAACTTCGCCCCGTTGAGCGACACGTCACCTCAAGCGACGTGGAGATTAACTGT

ACTCCCGATAACCTGGAGCCGATACCAGACGAGGCTGCCTGGCCCGACTATAAGCTCATG

TGCTTTGATATAGAGTGTAAAGCTGGAACGGGTAACGAAATGGCGTTCCCAGTGGCAACT

AACCAAGAGGACCTGGTCATCCAGATCTCCTGTCTGCTGTACTCGCTTGCTACTCAGAAC

CACGAACACACCCTGCTGTTTTCCCTCGGGTCATGCGATATCTCTGAGGAATACTCGTTT

GCATGCGTCCAGCGCGGCGAGCCCAGACCGACGGTTTTGGAGTTTGACAGCGAGTACGAG

CTGCTGGTTGCCTTCCTGACCTTTCTCAAGCAGTACTCTCCCGAGTTCGCCACCGGCTAC

AACATCGTTAATTTTGACTGGGCGTACATAGTTAACAAGGTAACGTCGGTGTATAACATC

AAGCTGGACGGGTACGGCAAGTTCAACAAAGGGGGGCTGTTTAAGGTGTGGGACATCGCC

ACGAACCATTTTCAGAAGAAGAGCAAGGTGAAAATCAATGGCCTGATATCTCTAGACATG

TATTCTGTGGCGACGGAAAAGCTAAAGCTACCCAGCTACAAACTCGACGCGGTCGTGGGA

GACGTCCTCGGCGAGCATAAGATAGACCTTCCCTATAAAGAAATACCCTCCTATTACGCG

GGAGGGCCTGACCGGAGGGGCGTAATAGGAGAGTATTGTATCCAGGACTCTAGGCTGGTG

GGCAAGCTGTTTTTTAAGTACCTCCCCCATCTGGAACTATCGGCGGTGGCCAAACTCGCC

CGTATCACCCTAACGCGGGTAATTTTTGACGGTCAGCAAATTCGCGTGTACACGTGCTTG

CTGAAACTCGCCCGCGAGAGAAATTTCATTTTGCCAGACAACAGACGCCGGTTTGACAGT

CAGGCAGACGCCGCGTCAGAGACTTCGGAGTTGGCTATGGATAGCCAAAGCCACGCCTTC

GACAGTACAGACGAACCCGACGGTGTGGACGGTACCCCGGACGCCGCAGGATCTGGCGCT

ACTTCTGAAAACGGAGGCGGGAAGCCCGGCGTCGGGAGGGCCGTGGGCTACCAGGGAGCA

AAGGTTCTAGACCCCGTATCCGGCTTTCATGTGGACCCCGTGGTTGTGTTTGACTTCGCT

AGCTTATACCCAAGCATTATCCAGGCCCATAACCTCTGTTTCACCACCCTGGCGCTCGAT

GAAGTGGATCTGGCCGGGCTTCAACCATCCGTCGACTACTCGACGTTCGAGGTGGGTGAC

CAAAAGTTATTTTTTGTCCACGCCCATATTCGCGAAAGCCTGCTTGGCATCTTGCTGCGC

GACTGGCTGGCCATGCGAAAGGCGGTGAGGGCGCGAATCCCCACCAGCACCCCCGAGGAG

GCAGTTTTACTAGATAAGCAGCAGTCTGCGATTAAGGTGATATGCAACTCGGTTTACGGA

TTCACGGGGGTGGCAAACGGCCTGTTGCCGTGTCTGAGGATAGCGGCTACCGTTACCACG

ATAGGACGCGACATGCTCCTCAAGACCCGAGATTACGTTCACTCTCGTTGGGCGACGCGC

GAGCTGCTGGAGGACAATTTTCCAGGGGCTATAGGTTTCCGAAACCACAAGCCTTACTCC

GTCAGGGTTATCTACGGAGACACCGACTCCGTGTTTATCAAGTTTGTGGGCCTGACGTAC

GAGGGGGTATCGGAGCTGGGGGATGCTATGTCGCGTCAGATTTCAGCGGACCTCTTTAGA

GCGCCCATCAAACTGGAGTGTGAGAAGACCTTTCAGCGACTGCTGCTGATCACCAAGAAG

AAGTACATAGGTGTCATAAACGGGGGGAAGATGCTCATGAAGGGGGTCGACCTGGTCCGC

AAAAATAACTGCTCTTTCATAAACTTGTACGCGCGACATCTGGTAGATCTTTTGTTGTAC

GACGAGGATGTGGCCACGGCGGCAGCAGAGGTGACAGACGTGCCTCCCGCAGAATGGGTG

GGGCGCCCGCTACCGAGCGGCTTTGACAAGTTTGGGCGAGTGCTGGTAGAGGCGTACAAC

CGTATCACTGCCCCCAACTTGGACGTGCGCGAGTTCGTTATGACTGCTGAGCTGAGCCGC

TCACCCGAATCGTATACCAACAAGCGCCTGCCGCACCTCACCGTCTACTTTAAGCTCGCC

ATGAGGAATGAAGAACTGCCCAGCGTAAAAGAGAGAATTCCGTATGTGATAGTTGCGCAG

ACCGAGGCCGCGGAACGCGAAGCGGGTGTAGTAAACTCAATGCGCGGTACCGCCCAAAAC

CCCGTGGTAACCAAGACCGCACGCCCCCAACCTAAACGCAAACTGCTGGTTTCCGACCTC

GCCGAAGACCCGACCTATGTTTCCGAGAATGACGTACCGCTAAACACAGACTACTATTTC

TCCCACCTGTTGGGTACCATAAGCGTGACCTTTAAGGCTCTATTCGGAAATGATGTGAGA

ACAACAGAAAATCTTTTAAAGCGGTTTATTCCGGAAACCCCCCACAAGACCCCCACGAAA

ACCCAGGCACTGCTTGAGCGCGCCGGCTTTGAAAAGCTGACGCCCTTTACACCGGAGGAA

GAAAGTCGTCGAATACTGCATACAGTTTTTTGTACTCTAGAAGCAGCTCCCCATCAAAGC

TGA

>KC924784.1

ATGGCGGCGCGCGAACAGGCCAACTCCGTTCGCAGGAGCGGGTTTTTTAATCCGTTTATT

GGCAAGAGGCCATTTTTCAGGCCGGGAAGCGGGCAGACCGCGGAGACAGAGCGCCCAAGG

CCCCCCCAACACTCGTACTGCACAGAGGTGGGTAGCTTTAAGTTTATAGCTCCAAGATGC

CTCGATGAAGAAGCCCCCGCCGACCAGCGACGCGGTGTACACGTGGGCACCTTGGAGCGC

CCACCCAAAGTGTACTGCGATGGCTCAGAGTACGACGTGCTGAACTTTGCCTCCGGAGGT

TGCTGGCCTCGCAGGATTCGGGTTTGGAACGGCCAGGATTTTCGGGGCGATGGATTCAAC

CCCAGATTTGAGAGATTTCACGTGTACGACATAGTAGAGACTTCGGAGAGCGCCTCACAC

GATGACCCATCCAGGTTTGCTGAGCTATCCCGCCCAAGCGGGTCTGTGGTAACACTGCTG

GGAATGAGCGAGTGTGGAAAGCGAGTCGCGGTTCACGTCTATGGTGTGCGCCATTATTTT

TACATGGCAAAGGCGGAGGTGGATAGCGCTTGTGGAATAACCACCGAGGCAGAACTCGTG

CGCGCAATGGTGGACTGCGCGCACAGCTCGGCTTTGAGCGCGGCCCTGGGAAATGGCAAC

GGCGGCAAACAGAGCGGCGGCAGCGGCGGGGGATGGTGGGGCGGAAAGCACGTGTCTGCG

GACTGCTTCAAAGTGGAGACCGTGTGCCACACGACGCTGTACTACTTTGGATCTAAGCCA

GCTCTCTACTATAGAGTATCTGCCTCCAGCAGCCGCCTGGGAGGGTTCATCTGCGACAAC

TTTCACCCGGAGATTACAAAATTCGAGGGGAGCGTGGACGTGACCACGCGGCTGTTGTTG

GACAATGAAAATTTTACCAGTTTCGGGTGGTACCGCCTGCGACCCGGCACCCACGGAGAG

CGTGTTCAACTTCGCCCCGTTGAGCGACACGTCACCTCAAGCGACGTGGAGATTAACTGT

ACTCCCGATAACCTGGAGCCGATACCAGACGAGGCTGCCTGGCCCGACTATAAGCTCATG

TGCTTTGATATAGAGTGTAAAGCTGGAACGGGTAACGAAATGGCGTTCCCAGTGGCAACT

AACCAAGAGGACCTGGTCATCCAGATCTCCTGTCTGCTGTACTCGCTTGCTACTCAGAAC

CACGAACACACCCTGCTGTTTTCCCTCGGGTCATGCGATATCTCTGAGGAATACTCGTTT

GCATGCGTCCAGCGCGGCGAGCCCAGACCGACGGTTTTGGAGTTTGACAGCGAGTACGAG

CTGCTGGTTGCCTTCCTGACCTTTCTCAAGCAGTACTCTCCCGAGTTCGCCACCGGCTAC

AACATCGTTAATTTTGACTGGGCGTACATAGTTAACAAGGTAACGTCGGTGTATAACATC

AAGCTGGACGGGTACGGCAAGTTCAACAAAGGGGGGCTGTTTAAGGTGTGGGACATCGCC

ACGAACCATTTTCAGAAGAAGAGCAAGGTGAAAATCAATGGCCTGATATCTCTAGACATG

TATTCTGTGGCGACGGAAAAGCTAAAGCTACCCAGCTACAAACTCGACGCGGTCGTGGGA

GACGTCCTCGGCGAGCATAAGATAGACCTTCCCTATAAAGAAATACCCTCCTATTACGCG

GGAGGGCCTGACCGGAGGGGCGTAATAGGAGAGTATTGTATCCAGGACTCTAGGCTGGTG

GGCAAGCTGTTTTTTAAGTACCTCCCCCATCTGGAACTATCGGCGGTGGCCAAACTCGCC

CGTATCACCCTAACGCGGGTAATTTTTGACGGTCAGCAAATTCGCGTGTACACGTGCTTG

CTGAAACTCGCCCGCGAGAGAAATTTCATTTTGCCAGACAACAGACGCCGGTTTGACAGT

CAGGCAGACGCCGCGTCAGAGACTTCGGAGTTGGCTATGGATAGCCAAAGCCACGCCTTC

GACAGTACAGACGAACCCGACGGTGTGGACGGTACCCCGGACGCCGCAGGATCTGGCGCT

ACTTCTGAAAACGGAGGCGGGAAGCCCGGCGTCGGGAGGGCCGTGGGCTACCAGGGAGCA

AAGGTTCTAGACCCCGTATCCGGCTTTCATGTGGACCCCGTGGTTGTGTTTGACTTCGCT

AGCTTATACCCAAGCATTATCCAGGCCCATAACCTCTGTTTCACCACCCTGGCGCTCGAT

GAAGTGGATCTGGCCGGGCTTCAACCATCCGTCAACTACTCGACGTTCGAGGTGGGTGAC

CAAAAGTTATTTTTTGTCCACGCCCATATTCGCGAAAGCCTGCTTGGCATCTTGCTGCGC

GACTGGCTGGCCATGCGAAAGGCGGTGAGGGCGCGAATCCCCACCAGCACCCCCGAGGAG

GCAGTTTTACTAGATAAGCAGCAGTCTGCGATTAAGGTGATATGCAACTCGGTTTACGGA

TTCACGGGGGTGGCAAACGGCCTGTTGCCGTGTCTGAGGATAGCGGCTACCGTTACCACG

ATAGGACGCGACATGCTCCTCAAGACCCGAGATTACGTTCACTCTCGTTGGGCGACGCGC

GAGCTGCTGGAGGACAATTTTCCAGGGGCTATAGGTTTCCGAAACCACAAGCCTTACTCC

GTCAGGGTTATCTACGGAGACACCGACTCCGTGTTTATCAAGTTTGTGGGCCTGACGTAC

GAGGGGGTATCGGAGCTGGGGGATGCTATGTCGCGTCAGATTTCAGCGGACCTCTTTAGA

GCGCCCATCAAACTGGAGTGTGAGAAGACCTTTCAGCGACTGCTGCTGATCACCAAGAAG

AAGTACATAGGTGTCATAAACGGGGGGAAGATGCTCATGAAGGGGGTCGACCTGGTCCGC

AAAAATAACTGCTCTTTCATAAACTTGTACGCGCGACATCTGGTAGATCTTTTGTTGTAC

GACGAGGATGTGGCCACGGCGGCAGCAGAGGTGACAGACGTGCCTCCCGCAGAATGGGTG

GGGCGCCCGCTACCGAGCGGCTTTGACAAGTTTGGGCGAGTGCTGGTAGAGGCGTACAAC

CGTATCACTGCCCCCAACTTGGACGTGCGCGAGTTCGTTATGACTGCTGAGCTGAGCCGC

TCACCCGAATCGTATACCAACAAGCGCCTGCCGCACCTCACCGTCTACTTTAAGCTCGCC

ATGAGGAATGAAGAACTGCCCAGCGTAAAAGAGAGAATTCCGTATGTGATAGTTGCGCAG

ACCGAGGCCGCGGAACGCGAAGCGGGTGTAGTAAACTCAATGCGCGGTACCGCCCAAAAC

CCCGTGGTAACCAAGACCGCACGCCCCCAACCTAAACGCAAACTGCTGGTTTCCGACCTC

GCCGAAGACCCGACCTATGTTTCCGAGAATGACGTACCGCTAAACACAGACTACTATTTC

TCCCACCTGTTGGGTACCATAAGCGTGACCTTTAAGGCTCTATTCGGAAATGATGTGAGA

ACAACAGAAAATCTTTTAAAGCGGTTTATTCCGGAAACCCCCCACAAGACCCCCACGAAA

ACCCAGGCACTGCTTGAGCGCGCCGGCTTTGAAAAGCTGACGCCCTTTACACCGGAGGAA

GAAAGTCGTCGAATACTGCATACAGTTTTTTGTACTCTAGAAGCAGCTCCCCATCAAAGC

TGA

>KC924787.1

ATGGCGGCGCGCGAACAGGCCAACTCCGTTCGCAGGAGCGGGTTTTTTAATCCGTTTATT

GGCAAGAGGCCATGTTTCAGGCCAGGAGGCGGGCAGACCGCGGAGACCGATCGCCCCAGG

CCCCCCCAACACTCGTACTGCACCGAGGTGGGTAGCTTTAAGTTTATAGCCCCCAGATGC

CTCGATGAAGAAGCCCCCGCAGACCAGCGATGTGGTGTACACGTGGGCACCTTGGAGCGC

CCACCCAAAGTGTACTGCGATGGCACAGAGTACGACGTGCTTGACTTTGCCGCCGGATGT

TGCTGGCCTCGCAGGATTCGGGTTTGGAACGGCCAGGATTTTTGGGGCGATGGATTCAAC

CCCAGATTTGAGAGATTTCACGTGTACGACATAGTAGAGACTTCGGAGAGCGCCGCCCAC

GATGACCCATCCAGGTTTGCTGAGCTATCCCGCCCAAGCGGGTCTGTGGTAACGCTGCTG

GGAATGAGCGAGTGCGGAAAGCGAGTCGCGGTTCACGTCTATGGCGTGCGCCATTATTTT

TACATGGCAAAGGCGGAGGTTGACAGCGCTTGCGGAATAACCACCGAGGCCGAACTCGTG

CGCGCCATGGTGGACTGCGCGCACAGCTCGGCTTTGAGCGCGGCCCTGGGAAATGGCAAC

GGCGGCAAACAGAGCGGCGGCAGCGGCGGGGGATGGTGGGGCGGAAAGCACGTGTCTGCG

GACTGCTTCAGAGTTGATACTGTGTGCCACACGACGCTGTACTACTTTGGATCTAAGCCA

GCTCTCTACTATAGAGTTTCTGCATCCAGCAGCCGCCTGGGCGGGTTCATTTGCGACAAC

TTTCACCCGGAGATTACAAAATTCGAGGGGAGCGTGGACGTGACCACGCGGCTGTTGTTG

GACAATGAAAATTTTACCAGTTTCGGGTGGTACCGCCTGCGGCCCGGCGCCCACGGAGAG

CGTGTTCAACTTCGCCCCGTTGAGCGACACGTCACCTCAAGCGACGTGGAGATTAACTGT

ACTCCCGATAACCTGGAGCCGATACCCGACGAGGCTGCCTGGCCCGACTATAAGCTCATG

TGCTTTGATATAGAGTGTAAAGCTGGAACGGGGAACGAAATGGCGTTCCCCGTGGCAACT

AACCAAGAGGACCTGGTCATCCAGATATCCTGTCTGCTGTACTCGCTTGCTACTCAGAAA

CACGAACACACCCTGCTGTTTTCCCTAGGGTCGTGCGACATCTCTGAGGAATGCTCGTCC

GCATGCGTCCAACGCGGCGAGCCCAGACCGACGGTTTTGGAGTTTGACAGCGAGTACGAG

CTGCTGGTTGCCTTCCTGACCTTTCTCAAGCAGTACTCTCCCGAGTTCGCCACCGGCTAC

AACATCGTGAATTTTGACTGGGCGTACATAGTTAACAAGGTAACGTCGGTGTATAACATC

AAGCTGGACGGGTACGGCAAGTTCAACAAAGGGGGGCTGTTCAAGGTGTGGGACATCGCC

ACGAACCACTTTCAGAAGAAGAGCAAGGTGAAGATCAACGGCCTGATATCTCTAGACATG

TATTCTGTGGCGACGGAAAAGCTAAAGCTACCCAGCTACAAACTCGACGCGGTCGTGGGA

GACGTCCTCGGCGAGCATAAGATAGACCTTCCCTATAAAGAAATACCCTCCTATTACGCG

GGAGGGCCTGACCGGAGGGGCGTAATAGGAGAGTATTGTATCCAGGACTCTAGGCTGGTG

GGCAAGCTGTTTTTTAAGTACCTCCCCCATCTGGAACTATCCGCGGTGGCCAAACTCGCC

CGTATAACCCTAACGCGGGTGATTTTTGACGGTCAGCAAATTCGCGTGTACACGTGCTTG

CTGAAACTCGCCCGCGAGAGAAATTTCATTTTGCCCGACAACCGACGCCGGTTTGACGGT

CAGGCAGACGCCGCGTCCGAGACTTCGGAGTTGGCTCTGGATAGCCAGAGCCACGCCTTC

GACAGTACCGACGAACCAGACGGTGTGGACGGTACCCCGGACGCCGCGGGATCTGGCGCG

ACTTCTGAAAATGGAGGCGGAAAGCCCGGCGTCGGGAGGGCCGTGGGCTACCAGGGAGCA

AAGGTGCTAGACCCCGTATCCGGCTTTCATGTGGACCCCGTGGTTGTGTTTGACTTCGCT

AGCTTATACCCCAGCATTATCCAGGCCCATAACCTCTGTTTCACCACCCTGGCGCTTGAT

GAAGTGGATCTGGCCGGGCTTCAACCATCCGTCGACTACTCGACGTTCGAGGTGGGTGAC

CAAAAGTTATTTTTTGTCCACGCCCATATTCGCGAAAGCCTGCTTGGCATCTTGCTGCGC

GACTGGCTGGCCATGCGAAAGTCCGTAAGGGCGCGGATCCCCACCAGCACCCCCGAGGAG

GCAGTTTTACTAGATAAACAGCAGTCTGCGATTAAGGTGATATGCAACTCCGTTTACGGA

TTCACGGGGGTGGCAAACGGCCTGTTGCCGTGTCTGAGGATAGCGGCTACCGTTACCACG

ATAGGACGCGACATGCTCCTCAAGACCCGAGATTACGTTCACTCTCGTTGGGCGACGCGC

GAGCTGCTGGAGGACAATTTCCCAGGGGCTACAGCTTTCCGAAACCACAAGCCTTACTCC

GTCAGGGTTATCTACGGAGACACCGACTCCGTGTTTATCAAGTTTGTGGGCCTGACGTAC

GAGGGGGTATCGGAGCTGGGGGATGCTATGTCGCGTCAGATTTCAGCGGACCTCTTTAGA

GCGCCCATCAAACTGGAGTGTGAGAAGACCTTTCAGCGCCTGCTGCTGATCACCAAAAAG

AAGTACATAGGTGTCATAAACGGGGGGAAGATGCTCATGAAGGGGGTCGACCTGGTCCGC

AAAAATAACTGCTCTTTCATAAACTCGTACGCGCGGCATCTGGTAGATCTTTTGTTGTAC

GACGAGGATGTGGCCACGGCGGCCGCAGAGGTGACAGACGTGCCTCCCTCAGAGTGGGTG

GGGCGTCCGCTACCGAGCGGGTTTGACAAGTTTGGGCGAGTGCTGGTAGAGGCGTACAAC

CGTATCACTGCGCCCAACTTGGACGTGCGCGAGTTCGTTATGACGGCTGAGCTGAGCCGC

TCGCCCGAATCGTATACTAACAAGCGCCTGCCGCACCTCACCGTCTACTTTAAGCTAGCC

ATGAGGAATGAAGAACTGCCCAGCGTAAAAGAGAGAATTCCGTACGTGATAGTTGCTCAG

ACCGAGGCCGCAGAACGCGAAGCGGGTGTGGTAAACTCAATGCGCGGTACCGCCCAAAAC

CCCTTGGTCACCAATACCGCACGCCCCCAACCTAAACGCAAACTGCTTGTTTCCGACCTC

GCCGAAGACCCGACCTATGTTTCCGAGAATGACATACCGCTAAACACAGACTACTATTTC

TCCCACCTGTTGGGTACCATAAGCGTAACCTTTAAGGCTCTATTCGGAAATGATGTGAGA

ACAACAGAAAATCTTTTGAAGCGGTTTATTCCGGAAACCCCCCACAAGACCTCCACAAAA

ACCCAGGCACTGCTGGAGCGCGCAGGCTTTGAAAAGCTGACGCCCTCTACACCGGAGGAA

GAAAGTCGTCGAATACTGCATACAGTTTTTTGTACTCTAGAAGCAGCTCCCCATCAAAGC

TGA

>KC924794.1

ATGGCGGCGCGCGAACAGGCCAACTCCGTTCGCAGGAGCGGGTTTTTTAATCCGTTTATT

GGCAAGAGGCCATTTTTCAGGCCGGGAAGCGGGCAGACCGCGGAGACAGAGCGCCCAAGG

CCCCCCCAACACTCGTACTGCACAGAGGTGGGTAGCTTTAAGTTTATAGCTCCAAGATGC

CTCGATGAAGAAGCCCCCGCCGACCAGCGACGCGGTGTACACGTGGGCACCTTGGAGCGC

CCACCCAAAGTGTACTGCGATGGCTCAGAGTACGACGTGCTGAACTTTGCCTCCGGAGGT

TGCTGGCCTCGCAGGATTCGGGTTTGGAACGGCCAGGATTTTCGGGGCGATGGATTCAAC

CCCAGATTTGAGAGATTTCACGTGTACGACATAGTAGAGACTTCGGAGAGCGCCTCACAC

GATGACCCATCCAGGTTTGCTGAGCTATCCCGCCCAAGCGGGTCTGTGGTAACACTGCTG

GGAATGAGCGAGTGTGGAAAGCGAGTCGCGGTTCACGTCTATGGTGTGCGCCATTATTTT

TACATGGCAAAGGCGGAGGTGGATAGCGCTTGTGGAATAACCACCGAGGCAGAACTCGTG

CGCGCAATGGTGGACTGCGCGCACAGCTCGGCTTTGAGCGCGGCCCTGGGAAATGGCAAC

GGCGGCAAACAGAGCGGCGGCAGCGGCGGGGGATGGTGGGGCGGAAAGCACGTGTCTGCG

GACTGCTTCAAAGTGGAGACCGTGTGCCGCACGACGCTGTACTACTTTGGATCTAAGCCA

GCTCTCTACTATAGAGTATCTGCCTCCAGCGGCCGCCTGGGAGGGTTCATCTGCGACAAC

TTTCACCCGGAGATTACAAAATTCGAGGGGAGCGTGGACGTGACCACGCGGCTGTTGTTG

GACAATGAAAATTTTACCAGTTTCGGGTGGTACCGCCTGCGACCCGGCACCCACGGAGAG

CGTGTTCAACTTCGCCCCGTTGAGCGACACGTCACCTCAAGCGACGTGGAGATTAACTGT

ACTCCCGATAACCTGGAGCCGATACCAGACGAGGCTGCCTGGCCCGACTATAAGCTCATG

TGCTTTGATATAGAGTGTAAAGCTGGAACGGGTAACGAAATGGCGTTCCCAGTGGCAACT

AACCAAGAGGACCTGGTCATCCAGATCTCCTGTCTGCTGTACTCGCTTGCTACTCAGAAC

CACGAACACACCCTGCTGTTTTCCCTCGGGTCATGCGATATCTCTGAGGAATACTCGTTT

GCATGCGTCCAGCGCGGCGAGCCCAGACCGACGGTTTTGGAGTTTGACAGCGAGTACGAG

CTGCTGGTTGCCTTCCTGACCTTTCTCAAGCAGTACTCTCCCGAGTTCGCCACCGGCTAC

AACATCGTTAATTTTGACTGGGCGTACATAGTTAACAAGGTAACGTCGGTGTATAACATC

AAGCTGGACGGGTACGGCAAGTTCAACAAAGGGGGGCTGTTTAAGGTGTGGGACATCGCC

ACGAACCATTTTCAGAAGAAGAGCAAGGTGAAAATCAATGGCCTGATATCTCTAGACATG

TATTCTGTGGCGACGGAAAAGCTAAAGCTACCCAGCTACAAACTCGACGCGGTCGTGGGA

GACGTCCTCGGCGAGCATAAGATAGACCTTCCCTATAAAGAAATACCCTCCTATTACGCG

GGAGGGCCTGACCGGAGGGGCGTAATAGGAGAGTATTGTATCCAGGACTCTAGGCTGGTG

GGCAAGCTGTTTTTTAAGTACCTCCCCCATCTGGAACTATCGGCGGTGGCCAAACTCGCC

CGTATCACCCTAACGCGGGTAATTTTTGACGGTCAGCAAATTCGCGTGTACACGTGCTTG

CTGAAACTCGCCCGCGAGAGAAATTTCATTTTGCCAGACAACAGACGCCGGTTTGACAGT

CAGGCAGACGCCGCGTCAGAGACTTCGGAGTTGGCTATGGATAGCCAAAGCCACGCCTTC

GACAGTACAGACGAACCCGACGGTGTGGACGGTACCCCGGACGCCGCAGGATCTGGCGCT

ACTTCTGAAAACGGAGGCGGGAAGCCCGGCGTCGGGAGGGCCGTGGGCTACCAGGGAGCA

AAGGTTCTAGACCCCGTATCCGGCTTTCATGTGGACCCCGTGGTTGTGTTTGACTTCGCT

AGCTTATACCCAAGCATTATCCAGGCCCATAACCTCTGTTTCACCACCCTGGCGCTCGAT

GAAGTGGATCTGGCCGGGCTTCAACCATCCGTCGACTCCTCGACGTTCGAGGTGGGTGAC

CAAAAGTTATTTTTTGTCCACGCCCATATTCGCGAAAGCCTGCTTGGCATCTTGCTGCGC

GACTGGCTGGCCATGCGAAAGGCGGTGAGGGCGCGAATCCCCACCAGCACCCCCGAGGAG

GCAGTTTTACTAGATAAGCAGCAGTCTGCGATTAAGGTGATATGCAACTCGGTTTACGGA

TTCACGGGGGTGGCAAACGGCCTGTTGCCGTGTCTGAGGATAGCGGCTACCGTTACCACG

ATAGGACGCGACATGCTCCTCAAGACCCGAGATTACGTTCACTCTCGTTGGGCGACGCGC

GAGCTGCTGGAGGACAATTTTCCAGGGGCTATAGGTTTCCGAAACCACAAGCCTTACTCC

GTCAGGGTTATCTACGGAGACACCGACTCCGTGTTTATCAAGTTTGTGGGCCTGACGTAC

GAGGGGGTATCGGAGCTGGGGGATGCTATGTCGCGTCAGATTTCAGCGGACCTCTTTAGA

GCGCCCATCAAACTGGAGTGTGAGAAGACCTTTCAGCGACTGCTGCTGATCACCAAGAAG

AAGTACATAGGTGTCATAAACGGGGGGAAGATGCTCATGAAGGGGGTCGACCTGGTCCGC

AAAAATAACTGCTCTTTCATAAACTTGTACGCGCGACATCTGGTAGATCTTTTGTTGTAC

GACGAGGATGTGGCCACGGCGGCAGCAGAGGTGACAGACGTGCCTCCCGCAGAATGGGTG

GGGCGCCCGCTACCGAGCGGCTTTGACAAGTTTGGGCGAGTGCTGGTAGAGGCGTACAAC

CGTATCACTGCCCCCAACTTGGACGTGCGCGAGTTCGTTATGACTGCTGAGCTGAGCCGC

TCACCCGAATCGTATACCAACAAGCGCCTGCCGCACCTCACCGTCTACTTTAAGCTCGCC

ATGAGGAATGAAGAACTGCCCAGCGTAAAAGAGAGAATTCCGTATGTGATAGTTGCGCAG

ACCGAGGCCGCGGAACGCGAAGCGGGTGTAGTAAACTCAATGCGCGGTACCGCCCAAAAC

CCCGTGGTAACCAAGACCGCACGCCCCCAACCTAAACGCAAACTGCTGGTTTCCGACCTC

GCCGAAGACCCGACCTATGTTTCCGAGAATGACGTACCGCTAAACACAGACTACTATTTC

TCCCACCTGTTGGGTACCATAAGCGTGACCTTTAAGGCTCTATTCGGAAATGATGTGAGA

ACAACAGAAAATCTTTTAAAGCGGTTTATTCCGGAAACCCCCCACAAGACCCCCACGAAA

ACCCAGGCACTGCTTGAGCGCGCCGGCTTTGAAAAGCTGACGCCCTTTACACCGGAGGAA

GAAAGTCGTCGAATACTGCATACAGTTTTTTGTACTCTAGAAGCAGCTCCCCATCAAAGC

TGA

>KC924795.1

ATGGCGGCGCGCGAACAGGCCAACTCCGTTCGCAGGAGCGGGTTTTTTAATCCGTTTATT

GGCAAGAGGCCATTTTTCAGGCCGGGAAGCGGGCAGACCGCGGAGACAGAGCGCCCAAGG

CCCCCCCAACACTCGTACTGCACAGAGGTGGGTAGCTTTAAGTTTATAGCTCCAAGATGC

CTCGATGAAGAAGCCCCCGCCGACCAGCGACGCGGTGTACACGTGGGCACCTTGGAGCGC

CCACCCAAAGTGTACTGCGATGGCTCAGAGTACGACGTGCTGAACTTTGCCTCCGGAGGT

TGCTGGCCTCGCAGGATTCGGGTTTGGAACGGCCAGGATTTTCGGGGCGATGGATTCAAC

CCCAGATTTGAGAGATTTCACGTGTACGACATAGTAGAGACTTCGGAGAGCGCCTCACAC

GATGACCCATCCAGGTTTGCTGAGCTATCCCGCCCAAGCGGGTCTGTGGTAACACTGCTG

GGAATGAGCGAGTGTGGAAAGCGAGTCGCGGTTCACGTCTATGGTGTGCGCCATTATTTT

TACATGGCAAAGGCGGAGGTGGATAGCGCTTGTGGAATAACCACCGAGGCAGAACTCGTG

CGCGCAATGGTGGACTGCGCGCACAGCTCGGCTTTGAGCGCGGCCCTGGGAAATGGCAAC

GGCGGCAAACAGAGCGGCGGCAGCGGCGGGGGATGGTGGGGCGGAAAGCACGTGTCTGCG

GACTGCTTCAAAGTGGAGACCGTGTGCCGCACGACGCTGTACTACTTTGGATCTAAGCCA

GCTCTCTACTATAGAGTATCTGCCTCCAGCGGCCGCCTGGGAGGGTTCATCTGCGACAAC

TTTCACCCGGAGATTACAAAATTCGAGGGGAGCGTGGACGTGACCACGCGGCTGTTGTTG

GACAATGAAAATTTTACCAGTTTCGGGTGGTACCGCCTGCGACCCGGCACCCACGGAGAG

CGTGTTCAACTTCGCCCCGTTGAGCGACACGTCACCTCAAGCGACGTGGAGATTAACTGT

ACTCCCGATAACCTGGAGCCGATACCAGACGAGGCTGCCTGGCCCGACTATAAGCTCATG

TGCTTTGATATAGAGTGTAAAGCTGGAACGGGTAACGAAATGGCGTTCCCAGTGGCAACT

AACCAAGAGGACCTGGTCATCCAGATCTCCTGTCTGCTGTACTCGCTTGCTACTCAGAAC

CACGAACACACCCTGCTGTTTTCCCTCGGGTCATGCGATATCTCTGAGGAATACTCGTTT

GCATGCGTCCAGCGCGGCGAGCCCAGACCGACGGTTTTGGAGTTTGACAGCGAGTACGAG

CTGCTGGTTGCCTTCCTGACCTTTCTCAAGCAGTACTCTCCCGAGTTCGCCACCGGCTAC

AACATCGTTAATTTTGACTGGGCGTACATAGTTAACAAGGTAACGTCGGTGTATAACATC

AAGCTGGACGGGTACGGCAAGTTCAACAAAGGGGGGCTGTTTAAGGTGTGGGACATCGCC

ACGAACCATTTTCAGAAGAAGAGCAAGGTGAAAATCAATGGCCTGATATCTCTAGACATG

TATTCTGTGGCGACGGAAAAGCTAAAGCTACCCAGCTACAAACTCGACGCGGTCGTGGGA

GACGTCCTCGGCGAGCATAAGATAGACCTTCCCTATAAAGAAATACCCTCCTATTACGCG

GGAGGGCCTGACCGGAGGGGCGTAATAGGAGAGTATTGTATCCAGGACTCTAGGCTGGTG

GGCAAGCTGTTTTTTAAGTACCTCCCCCATCTGGAACTATCGGCGGTGGCCAAACTCGCC

CGTATCACCCTAACGCGGGTAATTTTTGACGGTCAGCAAATTCGCGTGTACACGTGCTTG

CTGAAACTCGCCCGCGAGAGAAATTTCATTTTGCCAGACAACAGACGCCGGTTTGACAGT

CAGGCAGACGCCGCGTCAGAGACTTCGGAGTTGGCTATGGATAGCCAAAGCCACGCCTTC

GACAGTACAGACGAACCCGACGGTGTGGACGGTACCCCGGACGCCGCAGGATCTGGCGCT

ACTTCTGAAAACGGAGGCGGGAAGCCCGGCGTCGGGAGGGCCGTGGGCTACCAGGGAGCA

AAGGTTCTAGACCCCGTATCCGGCTTTCATGTGGACCCCGTGGTTGTGTTTGACTTCGCT

AGCTTATACCCAAGCATTATCCAGGCCCATAACCTCTGTTTCACCACCCTGGCGCTCGAT

GAAGTGGATCTGGCCGGGCTTCAACCATCCGTCGACTCCTCGACGTTCGAGGTGGGTGAC

CAAAAGTTATTTTTTGTCCACGCCCATATTCGCGAAAGCCTGCTTGGCATCTTGCTGCGC

GACTGGCTGGCCATGCGAAAGGCGGTGAGGGCGCGAATCCCCACCAGCACCCCCGAGGAG

GCAGTTTTACTAGATAAGCAGCAGTCTGCGATTAAGGTGATATGCAACTCGGTTTACGGA

TTCACGGGGGTGGCAAACGGCCTGTTGCCGTGTCTGAGGATAGCGGCTACCGTTACCACG

ATAGGACGCGACATGCTCCTCAAGACCCGAGATTACGTTCACTCTCGTTGGGCGACGCGC

GAGCTGCTGGAGGACAATTTTCCAGGGGCTATAGGTTTCCGAAACCACAAGCCTTACTCC

GTCAGGGTTATCTACGGAGACACCGACTCCGTGTTTATCAAGTTTGTGGGCCTGACGTAC

GAGGGGGTATCGGAGCTGGGGGATGCTATGTCGCGTCAGATTTCAGCGGACCTCTTTAGA

GCGCCCATCAAACTGGAGTGTGAGAAGACCTTTCAGCGACTGCTGCTGATCACCAAGAAG

AAGTACATAGGTGTCATAAACGGGGGGAAGATGCTCATGAAGGGGGTCGACCTGGTCCGC

AAAAATAACTGCTCTTTCATAAACTTGTACGCGCGACATCTGGTAGATCTTTTGTTGTAC

GACGAGGATGTGGCCACGGCGGCAGCAGAGGTGACAGACGTGCCTCCCGCAGAATGGGTG

GGGCGCCCGCTACCGAGCGGCTTTGACAAGTTTGGGCGAGTGCTGGTAGAGGCGTACAAC

CGTATCACTGCCCCCAACTTGGACGTGCGCGAGTTCGTTATGACTGCTGAGCTGAGCCGC

TCACCCGAATCGTATACCAACAAGCGCCTGCCGCACCTCACCGTCTACTTTAAGCTCGCC

ATGAGGAATGAAGAACTGCCCAGCGTAAAAGAGAGAATTCCGTATGTGATAGTTGCGCAG

ACCGAGGCCGCGGAACGCGAAGCGGGTGTAGTAAACTCAATGCGCGGTACCGCCCAAAAC

CCCGTGGTAACCAAGACCGCACGCCCCCAACCTAAACGCAAACTGCTGGTTTCCGACCTC

GCCGAAGACCCGACCTATGTTTCCGAGAATGACGTACCGCTAAACACAGACTACTATTTC

TCCCACCTGTTGGGTACCATAAGCGTGACCTTTAAGGCTCTATTCGGAAATGATGTGAGA

ACAACAGAAAATCTTTTAAAGCGGTTTATTCCGGAAACCCCCCACAAGACCCCCACGAAA

ACCCAGGCACTGCTTGAGCGCGCCGGCTTTGAAAAGCTGACGCCCTTTACACCGGAGGAA

GAAAGTCGTCGAATACTGCATACAGTTTTTTGTACTCTAGAAGCAGCTCCCCATCAAAGC

TGA

>KC924796.1

ATGGCGGCGCGCGAACAGGCCAACTCCGTTCGCAGGAGCGGGTTTTTTAATCCGTTTATT

GGCAAGAGGCCATTTTTCAGGCCGGGAAGCGGGCAGACCGCGGAGACAGAGCGCCCAAGG

CCCCCCCAACACTCGTACTGCACAGAGGTGGGTAGCTTTAAGTTTATAGCTCCAAGATGC

CTCGATGAAGAAGCCCCCGCCGACCAGCGACGCGGTGTACACGTGGGCACCTTGGAGCGC

CCACCCAAAGTGTACTGCGATGGCTCAGAGTACGACGTGCTGAACTTTGCCTCCGGAGGT

TGCTGGCCTCGCAGGATTCGGGTTTGGAACGGCCAGGATTTTCGGGGCGATGGATTCAAC

CCCAGATTTGAGAGATTTCACGTGTACGACATAGTAGAGACTTCGGAGAGCGCCTCACAC

GATGACCCATCCAGGTTTGCTGAGCTATCCCGCCCAAGCGGGTCTGTGGTAACACTGCTG

GGAATGAGCGAGTGTGGAAAGCGAGTCGCGGTTCACGTCTATGGTGTGCGCCATTATTTT

TACATGGCAAAGGCGGAGGTGGATAGCGCTTGTGGAATAACCACCGAGGCAGAACTCGTG

CGCGCAATGGTGGACTGCGCGCACAGCTCGGCTTTGAGCGCGGCCCTGGGAAATGGCAAC

GGCGGCAAACAGAGCGGCGGCAGCGGCGGGGGATGGTGGGGCGGAAAGCACGTGTCTGCG

GACTGCTTCAAAGTGGAGACCGTGTGCCACACGACGCTGTACTACTTTGGATCTAAGCCA

GCTCTCTACTATAGAGTATCTGCCTCCAGCAGCCGCCTGGGAGGGTTCATCTGCGACAAC

TTTCACCCGGAGATTACAAAATTCGAGGGGAGCGTGGACGTGACCACGCGGCTGTTGTTG

GACAATGAAAATTTTACCAGTTTCGGGTGGTACCGCCTGCGACCCGGCACCCACGGAGAG

CGTGTTCAACTTCGCCCCGTTGAGCGACACGTCACCTCAAGCGACGTGGAGATTAACTGT

ACTCCCGATAACCTGGAGCCGATACCAGACGAGGCTGCCTGGCCCGACTATAAGCTCATG

TGCTTTGATATAGAGTGTAAAGCTGGAACGGGTAACGAAATGGCGTTCCCAGTGGCAACT

AACCAAGAGGACCTGGTCATCCAGATCTCCTGTCTGCTGTACTCGCTTGCTACTCAGAAC

CACGAACACACCCTGCTGTTTTCCCTCGGGTCATGCGATATCTCTGAGGAATACTCGTTT

GCATGCGTCCAGCGCGGCGAGCCCAGACCGACGGTTTTGGAGTTTGACAGCGAGTACGAG

CTGCTGGTTGCCTTCCTGACCTTTCTCAAGCAGTACTCTCCCGAGTTCGCCACCGGCTAC

AACATCGTTAATTTTGACTGGGCGTACATAGTTAACAAGGTAACGTCGGTGTATAACATC

AAGCTGGACGGGTACGGCAAGTTCAACAAAGGGGGGCTGTTTAAGGTGTGGGACATCGCC

ACGAACCATTTTCAGAAGAAGAGCAAGGTGAAAATCAATGGCCTGATATCTCTAGACATG

TATTCTGTGGCGACGGAAAAGCTAAAGCTACCCAGCTACAAACTCGACGCGGTCGTGGGA

GACGTCCTCGGCGAGCATAAGATAGACCTTCCCTATAAAGAAATACCCTCCTATTACGCG

GGAGGGCCTGACCGGAGGGGCGTAATAGGAGAGTATTGTATCCAGGACTCTAGGCTGGTG

GGCAAGCTGTTTTTTAAGTACCTCCCCCATCTGGAACTATCGGCGGTGGCCAAACTCGCC

CGTATCACCCTAACGCGGGTAATTTTTGACGGTCAGCAAATTCGCGTGTACACGTGCTTG

CTGAAACTCGCCCGCGAGAGAAATTTCATTTTGCCAGACAACAGACGCCGGTTTGACAGT

CAGGCAGACGCCGCGTCAGAGACTTCGGAGTTGGCTATGGATAGCCAAAGCCACGCCTTC

GACAGTACAGACGAACCCGACGGTGTGGACGGTACCCCGGACGCCGCAGGATCTGGCGCT

ACTTCTGAAAACGGAGGCGGGAAGCCCGGCGTCGGGAGGGCCGTGGGCTACCAGGGAGCA

AAGGTTCTAGACCCCGTATCCGGCTTTCATGTGGACCCCGTGGTTGTGTTTGACTTCGCT

AGCTTATACCCAAGCATTATCCAGGCCCATAACCTCTGTTTCACCACCCTGGCGCTCGAT

GAAGTGGATCTGGCCGGGCTTCAACCATCCGTCAACTACTCGACGTTCGAGGTGGGTGAC

CAAAAGTTATTTTTTGTCCACGCCCATATTCGCGAAAGCCTGCTTGGCATCTTGCTGCGC

GACTGGCTGGCCATGCGAAAGGCGGTGAGGGCGCGAATCCCCACCAGCACCCCCGAGGAG

GCAGTTTTACTAGATAAGCAGCAGTCTGCGATTAAGGTGATATGCAACTCGGTTTACGGA

TTCACGGGGGTGGCAAACGGCCTGTTGCCGTGTCTGAGGATAGCGGCTACCGTTACCACG

ATAGGACGCGACATGCTCCTCAAGACCCGAGATTACGTTCACTCTCGTTGGGCGACGCGC

GAGCTGCTGGAGGACAATTTTCCAGGGGCTATAGGTTTCCGAAACCACAAGCCTTACTCC

GTCAGGGTTATCTACGGAGACACCGACTCCGTGTTTATCAAGTTTGTGGGCCTGACGTAC

GAGGGGGTATCGGAGCTGGGGGATGCTATGTCGCGTCAGATTTCAGCGGACCTCTTTAGA

GCGCCCATCAAACTGGAGTGTGAGAAGACCTTTCAGCGACTGCTGCTGATCACCAAGAAG

AAGTACATAGGTGTCATAAACGGGGGGAAGATGCTCATGAAGGGGGTCGACCTGGTCCGC

AAAAATAACTGCTCTTTCATAAACTTGTACGCGCGACATCTGGTAGATCTTTTGTTGTAC

GACGAGGATGTGGCCACGGCGGCAGCAGAGGTGACAGACGTGCCTCCCGCAGAATGGGTG

GGGCGCCCGCTACCGAGCGGCTTTGACAAGTTTGGGCGAGTGCTGGTAGAGGCGTACAAC

CGTATCACTGCCCCCAACTTGGACGTGCGCGAGTTCGTTATGACTGCTGAGCTGAGCCGC

TCACCCGAATCGTATACCAACAAGCGCCTGCCGCACCTCACCGTCTACTTTAAGCTCGCC

ATGAGGAATGAAGAACTGCCCAGCGTAAAAGAGAGAATTCCGTATGTGATAGTTGCGCAG

ACCGAGGCCGCGGAACGCGAAGCGGGTGTAGTAAACTCAATGCGCGGTACCGCCCAAAAC

CCCGTGGTAACCAAGACCGCACGCCCCCAACCTAAACGCAAACTGCTGGTTTCCGACCTC

GCCGAAGACCCGACCTATGTTTCCGAGAATGACGTACCGCTAAACACAGACTACTATTTC

TCCCACCTGTTGGGTACCATAAGCGTGACCTTTAAGGCTCTATTCGGAAATGATGTGAGA

ACAACAGAAAATCTTTTAAAGCGGTTTATTCCGGAAACCCCCCACAAGACCCCCACGAAA

ACCCAGGCACTGCTTGAGCGCGCCGGCTTTGAAAAGCTGACGCCCTTTACACCGGAGGAA

GAAAGTCGTCGAATACTGCATACAGTTTTTTGTACTCTAGAAGCAGCTCCCCATCAAAGC

TGA

>KC924797.1

ATGGCGGCGCGCGAACAGGCCAACTCCGTTCGCAGGAGCGGGTTTTTTAATCCGTTTATT

GGCAAGAGGCCATTTTTCAGGCCGGGAAGCGGGCAGACCGCGGAGACAGAGCGCCCAAGG

CCCCCCCAACACTCGTACTGCACAGAGGTGGGTAGCTTTAAGTTTATAGCTCCAAGATGC

CTCGATGAAGAAGCCCCCGCCGACCAGCGACGCGGTGTACACGTGGGCACCTTGGAGCGC

CCACCCAAAGTGTACTGCGATGGCTCAGAGTACGACGTGCTGAACTTTGCCTCCGGAGGT

TGCTGGCCTCGCAGGATTCGGGTTTGGAACGGCCAGGATTTTCGGGGCGATGGATTCAAC

CCCAGATTTGAGAGATTTCACGTGTACGACATAGTAGAGACTTCGGAGAGCGCCTCACAC

GATGACCCATCCAGGTTTGCTGAGCTATCCCGCCCAAGCGGGTCTGTGGTAACACTGCTG

GGAATGAGCGAGTGTGGAAAGCGAGTCGCGGTTCACGTCTATGGTGTGCGCCATTATTTT

TACATGGCAAAGGCGGAGGTGGATAGCGCTTGTGGAATAACCACCGAGGCAGAACTCGTG

CGCGCAATGGTGGACTGCGCGCACAGCTCGGCTTTGAGCGCGGCCCTGGGAAATGGCAAC

GGCGGCAAACAGAGCGGCGGCAGCGGCGGGGGATGGTGGGGCGGAAAGCACGTGTCTGCG

GACTGCTTCAAAGTGGAGACCGTGTGCCACACGACGCTGTACTACTTTGGATCTAAGCCA

GCTCTCTACTATAGAGTATCTGCCTCCAGCAGCCGCCTGGGAGGGTTCATCTGCGACAAC

TTTCACCCGGAGATTACAAAATTCGAGGGGAGCGTGGACGTGACCACGCGGCTGTTGTTG

GACAATGAAAATTTTACCAGTTTCGGGTGGTACCGCCTGCGACCCGGCACCCACGGAGAG

CGTGTTCAACTTCGCCCCGTTGAGCGACACGTCACCTCAAGCGACGTGGAGATTAACTGT

ACTCCCGATAACCTGGAGCCGATACCAGACGAGGCTGCCTGGCCCGACTATAAGCTCATG

TGCTTTGATATAGAGTGTAAAGCTGGAACGGGTAACGAAATGGCGTTCCCAGTGGCAACT

AACCAAGAGGACCTGGTCATCCAGATCTCCTGTCTGCTGTACTCGCTTGCTACTCAGAAC

CACGAACACACCCTGCTGTTTTCCCTCGGGTCATGCGATATCTCTGAGGAATACTCGTTT

GCATGCGTCCAGCGCGGCGAGCCCAGACCGACGGTTTTGGAGTTTGACAGCGAGTACGAG

CTGCTGGTTGCCTTCCTGACCTTTCTCAAGCAGTACTCTCCCGAGTTCGCCACCGGCTAC

AACATCGTTAATTTTGACTGGGCGTACATAGTTAACAAGGTAACGTCGGTGTATAACATC

AAGCTGGACGGGTACGGCAAGTTCAACAAAGGGGGGCTGTTTAAGGTGTGGGACATCGCC

ACGAACCATTTTCAGAAGAAGAGCAAGGTGAAAATCAATGGCCTGATATCTCTAGACATG

TATTCTGTGGCGACGGAAAAGCTAAAGCTACCCAGCTACAAACTCGACGCGGTCGTGGGA

GACGTCCTCGGCGAGCATAAGATAGACCTTCCCTATAAAGAAATACCCTCCTATTACGCG

GGAGGGCCTGACCGGAGGGGCGTAATAGGAGAGTATTGTATCCAGGACTCTAGGCTGGTG

GGCAAGCTGTTTTTTAAGTACCTCCCCCATCTGGAACTATCGGCGGTGGCCAAACTCGCC

CGTATCACCCTAACGCGGGTAATTTTTGACGGTCAGCAAATTCGCGTGTACACGTGCTTG

CTGAAACTCGCCCGCGAGAGAAATTTCATTTTGCCAGACAACAGACGCCGGTTTGACAGT

CAGGCAGACGCCGCGTCAGAGACTTCGGAGTTGGCTATGGATAGCCAAAGCCACGCCTTC

GACAGTACAGACGAACCCGACGGTGTGGACGGTACCCCGGACGCCGCAGGATCTGGCGCT

ACTTCTGAAAACGGAGGCGGGAAGCCCGGCGTCGGGAGGGCCGTGGGCTACCAGGGAGCA

AAGGTTCTAGACCCCGTATCCGGCTTTCATGTGGACCCCGTGGTTGTGTTTGACTTCGCT

AGCTTATACCCAAGCATTATCCAGGCCCATAACCTCTGTTTCACCACCCTGGCGCTCGAT

GAAGTGGATCTGGCCGGGCTTCAACCATCCGTCAACTACTCGACGTTCGAGGTGGGTGAC

CAAAAGTTATTTTTTGTCCACGCCCATATTCGCGAAAGCCTGCTTGGCATCTTGCTGCGC

GACTGGCTGGCCATGCGAAAGGCGGTGAGGGCGCGAATCCCCACCAGCACCCCCGAGGAG

GCAGTTTTACTAGATAAGCAGCAGTCTGCGATTAAGGTGATATGCAACTCGGTTTACGGA

TTCACGGGGGTGGCAAACGGCCTGTTGCCGTGTCTGAGGATAGCGGCTACCGTTACCACG

ATAGGACGCGACATGCTCCTCAAGACCCGAGATTACGTTCACTCTCGTTGGGCGACGCGC

GAGCTGCTGGAGGACAATTTTCCAGGGGCTATAGGTTTCCGAAACCACAAGCCTTACTCC

GTCAGGGTTATCTACGGAGACACCGACTCCGTGTTTATCAAGTTTGTGGGCCTGACGTAC

GAGGGGGTATCGGAGCTGGGGGATGCTATGTCGCGTCAGATTTCAGCGGACCTCTTTAGA

GCGCCCATCAAACTGGAGTGTGAGAAGACCTTTCAGCGACTGCTGCTGATCACCAAGAAG

AAGTACATAGGTGTCATAAACGGGGGGAAGATGCTCATGAAGGGGGTCGACCTGGTCCGC

AAAAATAACTGCTCTTTCATAAACTTGTACGCGCGACATCTGGTAGATCTTTTGTTGTAC

GACGAGGATGTGGCCACGGCGGCAGCAGAGGTGACAGACGTGCCTCCCGCAGAATGGGTG

GGGCGCCCGCTACCGAGCGGCTTTGACAAGTTTGGGCGAGTGCTGGTAGAGGCGTACAAC

CGTATCACTGCCCCCAACTTGGACGTGCGCGAGTTCGTTATGACTGCTGAGCTGAGCCGC

TCACCCGAATCGTATACCAACAAGCGCCTGCCGCACCTCACCGTCTACTTTAAGCTCGCC

ATGAGGAATGAAGAACTGCCCAGCGTAAAAGAGAGAATTCCGTATGTGATAGTTGCGCAG

ACCGAGGCCGCGGAACGCGAAGCGGGTGTAGTAAACTCAATGCGCGGTACCGCCCAAAAC

CCCGTGGTAACCAAGACCGCACGCCCCCAACCTAAACGCAAACTGCTGGTTTCCGACCTC

GCCGAAGACCCGACCTATGTTTCCGAGAATGACGTACCGCTAAACACAGACTACTATTTC

TCCCACCTGTTGGGTACCATAAGCGTGACCTTTAAGGCTCTATTCGGAAATGATGTGAGA

ACAACAGAAAATCTTTTAAAGCGGTTTATTCCGGAAACCCCCCACAAGACCCCCACGAAA

ACCCAGGCACTGCTTGAGCGCGCCGGCTTTGAAAAGCTGACGCCCTTTACACCGGAGGAA

GAAAGTCGTCGAATACTGCATACAGTTTTTTGTACTCTAGAAGCAGCTCCCCATCAAAGC

TGA

>KC924798.1

ATGGCGGCGCGCGAACAGGCCAACTCCGTTCGCAGGAGCGGGTTTTTTAATCCGTTTATT

GGCAAGAGGCCATTTTTCAGGCCGGGAAGCGGGCAGACCGCGGAGACAGAGCGCCCAAGG

CCCCCCCAACACTCGTACTGCACAGAGGTGGGTAGCTTTAAGTTTATAGCTCCAAGATGC

CTCGATGAAGAAGCCCCCGCCGACCAGCGACGCGGTGTACACGTGGGCACCTTGGAGCGC

CCACCCAAAGTGTACTGCGATGGCTCAGAGTACGACGTGCTGAACTTTGCCTCCGGAGGT

TGCTGGCCTCGCAGGATTCGGGTTTGGAACGGCCAGGATTTTCGGGGCGATGGATTCAAC

CCCAGATTTGAGAGATTTCACGTGTACGACATAGTAGAGACTTCGGAGAGCGCCTCACAC

GATGACCCATCCAGGTTTGCTGAGCTATCCCGCCCAAGCGGGTCTGTGGTAACACTGCTG

GGAATGAGCGAGTGTGGAAAGCGAGTCGCGGTTCACGTCTATGGTGTGCGCCATTATTTT

TACATGGCAAAGGCGGAGGTGGATAGCGCTTGTGGAATAACCACCGAGGCAGAACTCGTG

CGCGCAATGGTGGACTGCGCGCACAGCTCGGCTTTGAGCGCGGCCCTGGGAAATGGCAAC

GGCGGCAAACAGAGCGGCGGCAGCGGCGGGGGATGGTGGGGCGGAAAGCACGTGTCTGCG

GACTGCTTCAAAGTGGAGACCGTGTGCCACACGACGCTGTACTACTTTGGATCTAAGCCA

GCTCTCTACTATAGAGTATCTGCCTCCAGCAGCCGCCTGGGAGGGTTCATCTGCGACAAC

TTTCACCCGGAGATTACAAAATTCGAGGGGAGCGTGGACGTGACCACGCGGCTGTTGTTG

GACAATGAAAATTTTACCAGTTTCGGGTGGTACCGCCTGCGACCCGGCACCCACGGAGAG

CGTGTTCAACTTCGCCCCGTTGAGCGACACGTCACCTCAAGCGACGTGGAGATTAACTGT

ACTCCCGATAACCTGGAGCCGATACCAGACGAGGCTGCCTGGCCCGACTATAAGCTCATG

TGCTTTGATATAGAGTGTAAAGCTGGAACGGGTAACGAAATGGCGTTCCCAGTGGCAACT

AACCAAGAGGACCTGGTCATCCAGATCTCCTGTCTGCTGTACTCGCTTGCTACTCAGAAC

CACGAACACACCCTGCTGTTTTCCCTCGGGTCATGCGATATCTCTGAGGAATACTCGTTT

GCATGCGTCCAGCGCGGCGAGCCCAGACCGACGGTTTTGGAGTTTGACAGCGAGTACGAG

CTGCTGGTTGCCTTCCTGACCTTTCTCAAGCAGTACTCTCCCGAGTTCGCCACCGGCTAC

AACATCGTTAATTTTGACTGGGCGTACATAGTTAACAAGGTAACGTCGGTGTATAACATC

AAGCTGGACGGGTACGGCAAGTTCAACAAAGGGGGGCTGTTTAAGGTGTGGGACATCGCC

ACGAACCATTTTCAGAAGAAGAGCAAGGTGAAAATCAATGGCCTGATATCTCTAGACATG

TATTCTGTGGCGACGGAAAAGCTAAAGCTACCCAGCTACAAACTCGACGCGGTCGTGGGA

GACGTCCTCGGCGAGCATAAGATAGACCTTCCCTATAAAGAAATACCCTCCTATTACGCG

GGAGGGCCTGACCGGAGGGGCGTAATAGGAGAGTATTGTATCCAGGACTCTAGGCTGGTG

GGCAAGCTGTTTTTTAAGTACCTCCCCCATCTGGAACTATCGGCGGTGGCCAAACTCGCC

CGTATCACCCTAACGCGGGTAATTTTTGACGGTCAGCAAATTCGCGTGTACACGTGCTTG

CTGAAACTCGCCCGCGAGAGAAATTTCATTTTGCCAGACAACAGACGCCGGTTTGACAGT

CAGGCAGACGCCGCGTCAGAGACTTCGGAGTTGGCTATGGATAGCCAAAGCCACGCCTTC

GACAGTACAGACGAACCCGACGGTGTGGACGGTACCCCGGACGCCGCAGGATCTGGCGCT

ACTTCTGAAAACGGAGGCGGGAAGCCCGGCGTCGGGAGGGCCGTGGGCTACCAGGGAGCA

AAGGTTCTAGACCCCGTATCCGGCTTTCATGTGGACCCCGTGGTTGTGTTTGACTTCGCT

AGCTTATACCCAAGCATTATCCAGGCCCATAACCTCTGTTTCACCACCCTGGCGCTCGAT

GAAGTGGATCTGGCCGGGCTTCAACCATCCGTCGACTACTCGACGTTCGAGGTGGGTGAC

CAAAAGTTATTTTTTGTCCACGCCCATATTCGCGAAAGCCTGCTTGGCATCTTGCTGCGC

GACTGGCTGGCCATGCGAAAGGCGGTGAGGGCGCGAATCCCCACCAGCACCCCCGAGGAG

GCAGTTTTACTAGATAAGCAGCAGTCTGCGATTAAGGTGATATGCAACTCGGTTTACGGA

TTCACGGGGGTGGCAAACGGCCTGTTGCCGTGTCTGAGGATAGCGGCTACCGTTACCACG

ATAGGACGCGACATGCTCCTCAAGACCCGAGATTACGTTCACTCTCGTTGGGCGACGCGC

GAGCTGCTGGAGGACAATTTTCCAGGGGCTATAGGTTTCCGAAACCACAAGCCTTACTCC

GTCAGGGTTATCTACGGAGACACCGACTCCGTGTTTATCAAGTTTGTGGGCCTGACGTAC

GAGGGGGTATCGGAGCTGGGGGATGCTATGTCGCGTCAGATTTCAGCGGACCTCTTTAGA

GCGCCCATCAAACTGGAGTGTGAGAAGACCTTTCAGCGACTGCTGCTGATCACCAAGAAG

AAGTACATAGGTGTCATAAACGGGGGGAAGATGCTCATGAAGGGGGTCGACCTGGTCCGC

AAAAATAACTGCTCTTTCATAAACTTGTACGCGCGACATCTGGTAGATCTTTTGTTGTAC

GACGAGGATGTGGCCACGGCGGCAGCAGAGGTGACAGACGTGCCTCCCGCAGAATGGGTG

GGGCGCCCGCTACCGAGCGGCTTTGACAAGTTTGGGCGAGTGCTGGTAGAGGCGTACAAC

CGTATCACTGCCCCCAACTTGGACGTGCGCGAGTTCGTTATGACTGCTGAGCTGAGCCGC

TCACCCGAATCGTATACCAACAAGCGCCTGCCGCACCTCACCGTCTACTTTAAGCTCGCC

ATGAGGAATGAAGAACTGCCCAGCGTAAAAGAGAGAATTCCGTATGTGATAGTTGCGCAG

ACCGAGGCCGCGGAACGCGAAGCGGGTGTAGTAAACTCAATGCGCGGTACCGCCCAAAAC

CCCGTGGTAACCAAGACCGCACGCCCCCAACCTAAACGCAAACTGCTGGTTTCCGACCTC

GCCGAAGACCCGACCTATGTTTCCGAGAATGACGTACCGCTAAACACAGACTACTATTTC

TCCCACCTGTTGGGTACCATAAGCGTGACCTTTAAGGCTCTATTCGGAAATGATGTGAGA

ACAACAGAAAATCTTTTAAAGCGGTTTATTCCGGAAACCCCCCACAAGACCCCCACGAAA

ACCCAGGCACTGCTTGAGCGCGCCGGCTTTGAAAAGCTGACGCCCTTTACACCGGAGGAA

GAAAGTCGTCGAATACTGCATACAGTTTTTTGTACTCTAGAAGCAGCTCCCCATCAAAGC

TGA

>KC924799.1

ATGGCGGCGCGCGAACAGGCCAACTCCGTTCGCAGGAGCGGGTTTTTTAATCCGTTTATT

GGCAAGAGGCCATTTTTCAGGCCGGGAAGCGGGCAGACCGCGGAGACAGAGCGCCCAAGG

CCCCCCCAACACTCGTACTGCACAGAGGTGGGTAGCTTTAAGTTTATAGCTCCAAGATGC

CTCGATGAAGAAGCCCCCGCCGACCAGCGACGCGGTGTACACGTGGGCACCTTGGAGCGC

CCACCCAAAGTGTACTGCGATGGCTCAGAGTACGACGTGCTGAACTTTGCCTCCGGAGGT

TGCTGGCCTCGCAGGATTCGGGTTTGGAACGGCCAGGATTTTCGGGGCGATGGATTCAAC

CCCAGATTTGAGAGATTTCACGTGTACGACATAGTAGAGACTTCGGAGAGCGCCTCACAC

GATGACCCATCCAGGTTTGCTGAGCTATCCCGCCCAAGCGGGTCTGTGGTAACACTGCTG

GGAATGAGCGAGTGTGGAAAGCGAGTCGCGGTTCACGTCTATGGTGTGCGCCATTATTTT

TACATGGCAAAGGCGGAGGTGGATAGCGCTTGTGGAATAACCACCGAGGCAGAACTCGTG

CGCGCAATGGTGGACTGCGCGCACAGCTCGGCTTTGAGCGCGGCCCTGGGAAATGGCAAC

GGCGGCAAACAGAGCGGCGGCAGCGGCGGGGGATGGTGGGGCGGAAAGCACGTGTCTGCG

GACTGCTTCAAAGTGGAGACCGTGTGCCACACGACGCTGTACTACTTTGGATCTAAGCCA

GCTCTCTACTATAGAGTATCTGCCTCCAGCAGCCGCCTGGGAGGGTTCATCTGCGACAAC

TTTCACCCGGAGATTACAAAATTCGAGGGGAGCGTGGACGTGACCACGCGGCTGTTGTTG

GACAATGAAAATTTTACCAGTTTCGGGTGGTACCGCCTGCGACCCGGCACCCACGGAGAG

CGTGTTCAACTTCGCCCCGTTGAGCGACACGTCACCTCAAGCGACGTGGAGATTAACTGT

ACTCCCGATAACCTGGAGCCGATACCAGACGAGGCTGCCTGGCCCGACTATAAGCTCATG

TGCTTTGATATAGAGTGTAAAGCTGGAACGGGTAACGAAATGGCGTTCCCAGTGGCAACT

AACCAAGAGGACCTGGTCATCCAGATCTCCTGTCTGCTGTACTCGCTTGCTACTCAGAAC

CACGAACACACCCTGCTGTTTTCCCTCGGGTCATGCGATATCTCTGAGGAATACTCGTTT

GCATGCGTCCAGCGCGGCGAGCCCAGACCGACGGTTTTGGAGTTTGACAGCGAGTACGAG

CTGCTGGTTGCCTTCCTGACCTTTCTCAAGCAGTACTCTCCCGAGTTCGCCACCGGCTAC

AACATCGTTAATTTTGACTGGGCGTACATAGTTAACAAGGTAACGTCGGTGTATAACATC

AAGCTGGACGGGTACGGCAAGTTCAACAAAGGGGGGCTGTTTAAGGTGTGGGACATCGCC

ACGAACCATTTTCAGAAGAAGAGCAAGGTGAAAATCAATGGCCTGATATCTCTAGACATG

TATTCTGTGGCGACGGAAAAGCTAAAGCTACCCAGCTACAAACTCGACGCGGTCGTGGGA

GACGTCCTCGGCGAGCATAAGATAGACCTTCCCTATAAAGAAATACCCTCCTATTACGCG

GGAGGGCCTGACCGGAGGGGCGTAATAGGAGAGTATTGTATCCAGGACTCTAGGCTGGTG

GGCAAGCTGTTTTTTAAGTACCTCCCCCATCTGGAACTATCGGCGGTGGCCAAACTCGCC

CGTATCACCCTAACGCGGGTAATTTTTGACGGTCAGCAAATTCGCGTGTACACGTGCTTG

CTGAAACTCGCCCGCGAGAGAAATTTCATTTTGCCAGACAACAGACGCCGGTTTGACAGT

CAGGCAGACGCCGCGTCAGAGACTTCGGAGTTGGCTATGGATAGCCAAAGCCACGCCTTC

GACAGTACAGACGAACCCGACGGTGTGGACGGTACCCCGGACGCCGCAGGATCTGGCGCT

ACTTCTGAAAACGGAGGCGGGAAGCCCGGCGTCGGGAGGGCCGTGGGCTACCAGGGAGCA

AAGGTTCTAGACCCCGTATCCGGCTTTCATGTGGACCCCGTGGTTGTGTTTGACTTCGCT

AGCTTATACCCAAGCATTATCCAGGCCCATAACCTCTGTTTCACCACCCTGGCGCTCGAT

GAAGTGGATCTGGCCGGGCTTCAACCATCCGTCAACTACTCGACGTTCGAGGTGGGTGAC

CAAAAGTTATTTTTTGTCCACGCCCATATTCGCGAAAGCCTGCTTGGCATCTTGCTGCGC

GACTGGCTGGCCATGCGAAAGGCGGTGAGGGCGCGAATCCCCACCAGCACCCCCGAGGAG

GCAGTTTTACTAGATAAGCAGCAGTCTGCGATTAAGGTGATATGCAACTCGGTTTACGGA

TTCACGGGGGTGGCAAACGGCCTGTTGCCGTGTCTGAGGATAGCGGCTACCGTTACCACG

ATAGGACGCGACATGCTCCTCAAGACCCGAGATTACGTTCACTCTCGTTGGGCGACGCGC

GAGCTGCTGGAGGACAATTTTCCAGGGGCTATAGGTTTCCGAAACCACAAGCCTTACTCC

GTCAGGGTTATCTACGGAGACACCGACTCCGTGTTTATCAAGTTTGTGGGCCTGACGTAC

GAGGGGGTATCGGAGCTGGGGGATGCTATGTCGCGTCAGATTTCAGCGGACCTCTTTAGA

GCGCCCATCAAACTGGAGTGTGAGAAGACCTTTCAGCGACTGCTGCTGATCACCAAGAAG

AAGTACATAGGTGTCATAAACGGGGGGAAGATGCTCATGAAGGGGGTCGACCTGGTCCGC

AAAAATAACTGCTCTTTCATAAACTTGTACGCGCGACATCTGGTAGATCTTTTGTTGTAC

GACGAGGATGTGGCCACGGCGGCAGCAGAGGTGACAGACGTGCCTCCCGCAGAATGGGTG

GGGCGCCCGCTACCGAGCGGCTTTGACAAGTTTGGGCGAGTGCTGGTAGAGGCGTACAAC

CGTATCACTGCCCCCAACTTGGACGTGCGCGAGTTCGTTATGACTGCTGAGCTGAGCCGC

TCACCCGAATCGTATACCAACAAGCGCCTGCCGCACCTCACCGTCTACTTTAAGCTCGCC

ATGAGGAATGAAGAACTGCCCAGCGTAAAAGAGAGAATTCCGTATGTGATAGTTGCGCAG

ACCGAGGCCGCGGAACGCGAAGCGGGTGTAGTAAACTCAATGCGCGGTACCGCCCAAAAC

CCCGTGGTAACCAAGACCGCACGCCCCCAACCTAAACGCAAACTGCTGGTTTCCGACCTC

GCCGAAGACCCGACCTATGTTTCCGAGAATGACGTACCGCTAAACACAGACTACTATTTC

TCCCACCTGTTGGGTACCATAAGCGTGACCTTTAAGGCTCTATTCGGAAATGATGTGAGA

ACAACAGAAAATCTTTTAAAGCGGTTTATTCCGGAAACCCCCCACAAGACCCCCACGAAA

ACCCAGGCACTGCTTGAGCGCGCCGGCTTTGAAAAGCTGACGCCCTTTACACCGGAGGAA

GAAAGTCGTCGAATACTGCATACAGTTTTTTGTACTCTAGAAGCAGCTCCCCATCAAAGC

TGA

>KC924800.1

ATGGCGGCGCGCGAACAGGCCAACTCCGTTCGCAGGAGCGGGTTTTTTAATCCGTTTATT

GGCAAGAGGCCATTTTTCAGGCCGGGAAGCGGGCAGACCGCGGAGACAGAGCGCCCAAGG

CCCCCCCAACACTCGTACTGCACAGAGGTGGGTAGCTTTAAGTTTATAGCTCCAAGATGC

CTCGATGAAGAAGCCCCCGCCGACCAGCGACGCGGTGTACACGTGGGCACCTTGGAGCGC

CCACCCAAAGTGTACTGCGATGGCTCAGAGTACGACGTGCTGAACTTTGCCTCCGGAGGT

TGCTGGCCTCGCAGGATTCGGGTTTGGAACGGCCAGGATTTTCGGGGCGATGGATTCAAC

CCCAGATTTGAGAGATTTCACGTGTACGACATAGTAGAGACTTCGGAGAGCGCCTCACAC

GATGACCCATCCAGGTTTGCTGAGCTATCCCGCCCAAGCGGGTCTGTGGTAACACTGCTG

GGAATGAGCGAGTGTGGAAAGCGAGTCGCGGTTCACGTCTATGGTGTGCGCCATTATTTT

TACATGGCAAAGGCGGAGGTGGATAGCGCTTGTGGAATAACCACCGAGGCAGAACTCGTG

CGCGCAATGGTGGACTGCGCGCACAGCTCGGCTTTGAGCGCGGCCCTGGGAAATGGCAAC

GGCGGCAAACAGAGCGGCGGCAGCGGCGGGGGATGGTGGGGCGGAAAGCACGTGTCTGCG

GACTGCTTCAAAGTGGAGACCGTGTGCCACACGACGCTGTACTACTTTGGATCTAAGCCA

GCTCTCTACTATAGAGTATCTGCCTCCAGCAGCCGCCTGGGAGGGTTCATCTGCGACAAC

TTTCACCCGGAGATTACAAAATTCGAGGGGAGCGTGGACGTGACCACGCGGCTGTTGTTG

GACAATGAAAATTTTACCAGTTTCGGGTGGTACCGCCTGCGACCCGGCACCCACGGAGAG

CGTGTTCAACTTCGCCCCGTTGAGCGACACGTCACCTCAAGCGACGTGGAGATTAACTGT

ACTCCCGATAACCTGGAGCCGATACCAGACGAGGCTGCCTGGCCCGACTATAAGCTCATG

TGCTTTGATATAGAGTGTAAAGCTGGAACGGGTAACGAAATGGCGTTCCCAGTGGCAACT

AACCAAGAGGACCTGGTCATCCAGATCTCCTGTCTGCTGTACTCGCTTGCTACTCAGAAC

CACGAACACACCCTGCTGTTTTCCCTCGGGTCATGCGATATCTCTGAGGAATACTCGTTT

GCATGCGTCCAGCGCGGCGAGCCCAGACCGACGGTTTTGGAGTTTGACAGCGAGTACGAG

CTGCTGGTTGCCTTCCTGACCTTTCTCAAGCAGTACTCTCCCGAGTTCGCCACCGGCTAC

AACATCGTTAATTTTGACTGGGCGTACATAGTTAACAAGGTAACGTCGGTGTATAACATC

AAGCTGGACGGGTACGGCAAGTTCAACAAAGGGGGGCTGTTTAAGGTGTGGGACATCGCC

ACGAACCATTTTCAGAAGAAGAGCAAGGTGAAAATCAATGGCCTGATATCTCTAGACATG

TATTCTGTGGCGACGGAAAAGCTAAAGCTACCCAGCTACAAACTCGACGCGGTCGTGGGA

GACGTCCTCGGCGAGCATAAGATAGACCTTCCCTATAAAGAAATACCCTCCTATTACGCG

GGAGGGCCTGACCGGAGGGGCGTAATAGGAGAGTATTGTATCCAGGACTCTAGGCTGGTG

GGCAAGCTGTTTTTTAAGTACCTCCCCCATCTGGAACTATCGGCGGTGGCCAAACTCGCC

CGTATCACCCTAACGCGGGTAATTTTTGACGGTCAGCAAATTCGCGTGTACACGTGCTTG

CTGAAACTCGCCCGCGAGAGAAATTTCATTTTGCCAGACAACAGACGCCGGTTTGACAGT

CAGGCAGACGCCGCGTCAGAGACTTCGGAGTTGGCTATGGATAGCCAAAGCCACGCCTTC

GACAGTACAGACGAACCCGACGGTGTGGACGGTACCCCGGACGCCGCAGGATCTGGCGCT

ACTTCTGAAAACGGAGGCGGGAAGCCCGGCGTCGGGAGGGCCGTGGGCTACCAGGGAGCA

AAGGTTCTAGACCCCGTATCCGGCTTTCATGTGGACCCCGTGGTTGTGTTTGACTTCGCT

AGCTTATACCCAAGCATTATCCAGGCCCATAACCTCTGTTTCACCACCCTGGCGCTCGAT

GAAGTGGATCTGGCCGGGCTTCAACCATCCGTCAACTACTCGACGTTCGAGGTGGGTGAC

CAAAAGTTATTTTTTGTCCACGCCCATATTCGCGAAAGCCTGCTTGGCATCTTGCTGCGC

GACTGGCTGGCCATGCGAAAGGCGGTGAGGGCGCGAATCCCCACCAGCACCCCCGAGGAG

GCAGTTTTACTAGATAAGCAGCAGTCTGCGATTAAGGTGATATGCAACTCGGTTTACGGA

TTCACGGGGGTGGCAAACGGCCTGTTGCCGTGTCTGAGGATAGCGGCTACCGTTACCACG

ATAGGACGCGACATGCTCCTCAAGACCCGAGATTACGTTCACTCTCGTTGGGCGACGCGC

GAGCTGCTGGAGGACAATTTTCCAGGGGCTATAGGTTTCCGAAACCACAAGCCTTACTCC

GTCAGGGTTATCTACGGAGACACCGACTCCGTGTTTATCAAGTTTGTGGGCCTGACGTAC

GAGGGGGTATCGGAGCTGGGGGATGCTATGTCGCGTCAGATTTCAGCGGACCTCTTTAGA

GCGCCCATCAAACTGGAGTGTGAGAAGACCTTTCAGCGACTGCTGCTGATCACCAAGAAG

AAGTACATAGGTGTCATAAACGGGGGGAAGATGCTCATGAAGGGGGTCGACCTGGTCCGC

AAAAATAACTGCTCTTTCATAAACTTGTACGCGCGACATCTGGTAGATCTTTTGTTGTAC

GACGAGGATGTGGCCACGGCGGCAGCAGAGGTGACAGACGTGCCTCCCGCAGAATGGGTG

GGGCGCCCGCTACCGAGCGGCTTTGACAAGTTTGGGCGAGTGCTGGTAGAGGCGTACAAC

CGTATCACTGCCCCCAACTTGGACGTGCGCGAGTTCGTTATGACTGCTGAGCTGAGCCGC

TCACCCGAATCGTATACCAACAAGCGCCTGCCGCACCTCACCGTCTACTTTAAGCTCGCC

ATGAGGAATGAAGAACTGCCCAGCGTAAAAGAGAGAATTCCGTATGTGATAGTTGCGCAG

ACCGAGGCCGCGGAACGCGAAGCGGGTGTAGTAAACTCAATGCGCGGTACCGCCCAAAAC

CCCGTGGTAACCAAGACCGCACGCCCCCAACCTAAACGCAAACTGCTGGTTTCCGACCTC

GCCGAAGACCCGACCTATGTTTCCGAGAATGACGTACCGCTAAACACAGACTACTATTTC

TCCCACCTGTTGGGTACCATAAGCGTGACCTTTAAGGCTCTATTCGGAAATGATGTGAGA

ACAACAGAAAATCTTTTAAAGCGGTTTATTCCGGAAACCCCCCACAAGACCCCCACGAAA

ACCCAGGCACTGCTTGAGCGCGCCGGCTTTGAAAAGCTGACGCCCTTTACACCGGAGGAA

GAAAGTCGTCGAATACTGCATACAGTTTTTTGTACTCTAGAAGCAGCTCCCCATCAAAGC

TGA

>KC924801.1

ATGGCGGCGCGCGAACAGGCCAACTCCGTTCGCAGGAGCGGGTTTTTTAATCCGTTTATT

GGCAAGAGGCCATTTTTCAGGCCGGGAAGCGGGCAGACCGCGGAGACAGAGCGCCCAAGG

CCCCCCCAACACTCGTACTGCACAGAGGTGGGTAGCTTTAAGTTTATAGCTCCAAGATGC

CTCGATGAAGAAGCCCCCGCCGACCAGCGACGCGGTGTACACGTGGGCACCTTGGAGCGC

CCACCCAAAGTGTACTGCGATGGCTCAGAGTACGACGTGCTGAACTTTGCCTCCGGAGGT

TGCTGGCCTCGCAGGATTCGGGTTTGGAACGGCCAGGATTTTCGGGGCGATGGATTCAAC

CCCAGATTTGAGAGATTTCACGTGTACGACATAGTAGAGACTTCGGAGAGCGCCTCACAC

GATGACCCATCCAGGTTTGCTGAGCTATCCCGCCCAAGCGGGTCTGTGGTAACACTGCTG

GGAATGAGCGAGTGTGGAAAGCGAGTCGCGGTTCACGTCTATGGTGTGCGCCATTATTTT

TACATGGCAAAGGCGGAGGTGGATAGCGCTTGTGGAATAACCACCGAGGCAGAACTCGTG

CGCGCAATGGTGGACTGCGCGCACAGCTCGGCTTTGAGCGCGGCCCTGGGAAATGGCAAC

GGCGGCAAACAGAGCGGCGGCAGCGGCGGGGGATGGTGGGGCGGAAAGCACGTGTCTGCG

GACTGCTTCAAAGTGGAGACCGTGTGCCACACGACGCTGTACTACTTTGGATCTAAGCCA

GCTCTCTACTATAGAGTATCTGCCTCCAGCAGCCGCCTGGGAGGGTTCATCTGCGACAAC

TTTCACCCGGAGATTACAAAATTCGAGGGGAGCGTGGACGTGACCACGCGGCTGTTGTTG

GACAATGAAAATTTTACCAGTTTCGGGTGGTACCGCCTGCGACCCGGCACCCACGGAGAG

CGTGTTCAACTTCGCCCCGTTGAGCGACACGTCACCTCAAGCGACGTGGAGATTAACTGT

ACTCCCGATAACCTGGAGCCGATACCAGACGAGGCTGCCTGGCCCGACTATAAGCTCATG

TGCTTTGATATAGAGTGTAAAGCTGGAACGGGTAACGAAATGGCGTTCCCAGTGGCAACT

AACCAAGAGGACCTGGTCATCCAGATCTCCTGTCTGCTGTACTCGCTTGCTACTCAGAAC

CACGAACACACCCTGCTGTTTTCCCTCGGGTCATGCGATATCTCTGAGGAATACTCGTTT

GCATGCGTCCAGCGCGGCGAGCCCAGACCGACGGTTTTGGAGTTTGACAGCGAGTACGAG

CTGCTGGTTGCCTTCCTGACCTTTCTCAAGCAGTACTCTCCCGAGTTCGCCACCGGCTAC

AACATCGTTAATTTTGACTGGGCGTACATAGTTAACAAGGTAACGTCGGTGTATAACATC

AAGCTGGACGGGTACGGCAAGTTCAACAAAGGGGGGCTGTTTAAGGTGTGGGACATCGCC

ACGAACCATTTTCAGAAGAAGAGCAAGGTGAAAATCAATGGCCTGATATCTCTAGACATG

TATTCTGTGGCGACGGAAAAGCTAAAGCTACCCAGCTACAAACTCGACGCGGTCGTGGGA

GACGTCCTCGGCGAGCATAAGATAGACCTTCCCTATAAAGAAATACCCTCCTATTACGCG

GGAGGGCCTGACCGGAGGGGCGTAATAGGAGAGTATTGTATCCAGGACTCTAGGCTGGTG

GGCAAGCTGTTTTTTAAGTACCTCCCCCATCTGGAACTATCGGCGGTGGCCAAACTCGCC

CGTATCACCCTAACGCGGGTAATTTTTGACGGTCAGCAAATTCGCGTGTACACGTGCTTG

CTGAAACTCGCCCGCGAGAGAAATTTCATTTTGCCAGACAACAGACGCCGGTTTGACAGT

CAGGCAGACGCCGCGTCAGAGACTTCGGAGTTGGCTATGGATAGCCAAAGCCACGCCTTC

GACAGTACAGACGAACCCGACGGTGTGGACGGTACCCCGGACGCCGCAGGATCTGGCGCT

ACTTCTGAAAACGGAGGCGGGAAGCCCGGCGTCGGGAGGGCCGTGGGCTACCAGGGAGCA

AAGGTTCTAGACCCCGTATCCGGCTTTCATGTGGACCCCGTGGTTGTGTTTGACTTCGCT

AGCTTATACCCAAGCATTATCCAGGCCCATAACCTCTGTTTCACCACCCTGGCGCTCGAT

GAAGTGGATCTGGCCGGGCTTCAACCATCCGTCGACTACTCGACGTTCGAGGTGGGTGAC

CAAAAGTTATTTTTTGTCCACGCCCATATTCGCGAAAGCCTGCTTGGCATCTTGCTGCGC

GACTGGCTGGCCATGCGAAAGGCGGTGAGGGCGCGAATCCCCACCAGCACCCCCGAGGAG

GCAGTTTTACTAGATAAGCAGCAGTCTGCGATTAAGGTGATATGCAACTCGGTTTACGGA

TTCACGGGGGTGGCAAACGGCCTGTTGCCGTGTCTGAGGATAGCGGCTACCGTTACCACG

ATAGGACGCGACATGCTCCTCAAGACCCGAGATTACGTTCACTCTCGTTGGGCGACGCGC

GAGCTGCTGGAGGACAATTTTCCAGGGGCTATAGGTTTCCGAAACCACAAGCCTTACTCC

GTCAGGGTTATCTACGGAGACACCGACTCCGTGTTTATCAAGTTTGTGGGCCTGACGTAC

GAGGGGGTATCGGAGCTGGGGGATGCTATGTCGCGTCAGATTTCAGCGGACCTCTTTAGA

GCGCCCATCAAACTGGAGTGTGAGAAGACCTTTCAGCGACTGCTGCTGATCACCAAGAAG

AAGTACATAGGTGTCATAAACGGGGGGAAGATGCTCATGAAGGGGGTCGACCTGGTCCGC

AAAAATAACTGCTCTTTCATAAACTTGTACGCGCGACATCTGGTAGATCTTTTGTTGTAC

GACGAGGATGTGGCCACGGCGGCAGCAGAGGTGACAGACGTGCCTCCCGCAGAATGGGTG

GGGCGCCCGCTACCGAGCGGCTTTGACAAGTTTGGGCGAGTGCTGGTAGAGGCGTACAAC

CGTATCACTGCCCCCAACTTGGACGTGCGCGAGTTCGTTATGACTGCTGAGCTGAGCCGC

TCACCCGAATCGTATACCAACAAGCGCCTGCCGCACCTCACCGTCTACTTTAAGCTCGCC

ATGAGGAATGAAGAACTGCCCAGCGTAAAAGAGAGAATTCCGTATGTGATAGTTGCGCAG

ACCGAGGCCGCGGAACGCGAAGCGGGTGTAGTAAACTCAATGCGCGGTACCGCCCAAAAC

CCCGTGGTAACCAAGACCGCACGCCCCCAACCTAAACGCAAACTGCTGGTTTCCGACCTC

GCCGAAGACCCGACCTATGTTTCCGAGAATGACGTACCGCTAAACACAGACTACTATTTC

TCCCACCTGTTGGGTACCATAAGCGTGACCTTTAAGGCTCTATTCGGAAATGATGTGAGA

ACAACAGAAAATCTTTTAAAGCGGTTTATTCCGGAAACCCCCCACAAGACCCCCACGAAA

ACCCAGGCACTGCTTGAGCGCGCCGGCTTTGAAAAGCTGACGCCCTTTACACCGGAGGAA

GAAAGTCGTCGAATACTGCATACAGTTTTTTGTACTCTAGAAGCAGCTCCCCATCAAAGC

TGA

>KC924802.1

ATGGCGGCGCGCGAACAGGCCAACTCCGTTCGCAGGAGCGGGTTTTTTAATCCGTTTATT

GGCAAGAGGCCATTTTTCAGGCCGGGAAGCGGGCAGACCGCGGAGACAGAGCGCCCAAGG

CCCCCCCAACACTCGTACTGCACAGAGGTGGGTAGCTTTAAGTTTATAGCTCCAAGATGC

CTCGATGAAGAAGCCCCCGCCGACCAGCGACGCGGTGTACACGTGGGCACCTTGGAGCGC

CCACCCAAAGTGTACTGCGATGGCTCAGAGTACGACGTGCTGAACTTTGCCTCCGGAGGT

TGCTGGCCTCGCAGGATTCGGGTTTGGAACGGCCAGGATTTTCGGGGCGATGGATTCAAC

CCCAGATTTGAGAGATTTCACGTGTACGACATAGTAGAGACTTCGGAGAGCGCCTCACAC

GATGACCCATCCAGGTTTGCTGAGCTATCCCGCCCAAGCGGGTCTGTGGTAACACTGCTG

GGAATGAGCGAGTGTGGAAAGCGAGTCGCGGTTCACGTCTATGGTGTGCGCCATTATTTT

TACATGGCAAAGGCGGAGGTGGATAGCGCTTGTGGAATAACCACCGAGGCAGAACTCGTG

CGCGCAATGGTGGACTGCGCGCACAGCTCGGCTTTGAGCGCGGCCCTGGGAAATGGCAAC

GGCGGCAAACAGAGCGGCGGCAGCGGCGGGGGATGGTGGGGCGGAAAGCACGTGTCTGCG

GACTGCTTCAAAGTGGAGACCGTGTGCCACACGACGCTGTACTACTTTGGATCTAAGCCA

GCTCTCTACTATAGAGTATCTGCCTCCAGCAGCCGCCTGGGAGGGTTCATCTGCGACAAC

TTTCACCCGGAGATTACAAAATTCGAGGGGAGCGTGGACGTGACCACGCGGCTGTTGTTG

GACAATGAAAATTTTACCAGTTTCGGGTGGTACCGCCTGCGACCCGGCACCCACGGAGAG

CGTGTTCAACTTCGCCCCGTTGAGCGACACGTCACCTCAAGCGACGTGGAGATTAACTGT

ACTCCCGATAACCTGGAGCCGATACCAGACGAGGCTGCCTGGCCCGACTATAAGCTCATG

TGCTTTGATATAGAGTGTAAAGCTGGAACGGGTAACGAAATGGCGTTCCCAGTGGCAACT

AACCAAGAGGACCTGGTCATCCAGATCTCCTGTCTGCTGTACTCGCTTGCTACTCAGAAC

CACGAACACACCCTGCTGTTTTCCCTCGGGTCATGCGATATCTCTGAGGAATACTCGTTT

GCATGCGTCCAGCGCGGCGAGCCCAGACCGACGGTTTTGGAGTTTGACAGCGAGTACGAG

CTGCTGGTTGCCTTCCTGACCTTTCTCAAGCAGTACTCTCCCGAGTTCGCCACCGGCTAC

AACATCGTTAATTTTGACTGGGCGTACATAGTTAACAAGGTAACGTCGGTGTATAACATC

AAGCTGGACGGGTACGGCAAGTTCAACAAAGGGGGGCTGTTTAAGGTGTGGGACATCGCC

ACGAACCATTTTCAGAAGAAGAGCAAGGTGAAAATCAATGGCCTGATATCTCTAGACATG

TATTCTGTGGCGACGGAAAAGCTAAAGCTACCCAGCTACAAACTCGACGCGGTCGTGGGA

GACGTCCTCGGCGAGCATAAGATAGACCTTCCCTATAAAGAAATACCCTCCTATTACGCG

GGAGGGCCTGACCGGAGGGGCGTAATAGGAGAGTATTGTATCCAGGACTCTAGGCTGGTG

GGCAAGCTGTTTTTTAAGTACCTCCCCCATCTGGAACTATCGGCGGTGGCCAAACTCGCC

CGTATCACCCTAACGCGGGTAATTTTTGACGGTCAGCAAATTCGCGTGTACACGTGCTTG

CTGAAACTCGCCCGCGAGAGAAATTTCATTTTGCCAGACAACAGACGCCGGTTTGACAGT

CAGGCAGACGCCGCGTCAGAGACTTCGGAGTTGGCTATGGATAGCCAAAGCCACGCCTTC

GACAGTACAGACGAACCCGACGGTGTGGACGGTACCCCGGACGCCGCAGGATCTGGCGCT

ACTTCTGAAAACGGAGGCGGGAAGCCCGGCGTCGGGAGGGCCGTGGGCTACCAGGGAGCA

AAGGTTCTAGACCCCGTATCCGGCTTTCATGTGGACCCCGTGGTTGTGTTTGACTTCGCT

AGCTTATACCCAAGCATTATCCAGGCCCATAACCTCTGTTTCACCACCCTGGCGCTCGAT

GAAGTGGATCTGGCCGGGCTTCAACCATCCGTCAACTACTCGACGTTCGAGGTGGGTGAC

CAAAAGTTATTTTTTGTCCACGCCCATATTCGCGAAAGCCTGCTTGGCATCTTGCTGCGC

GACTGGCTGGCCATGCGAAAGGCGGTGAGGGCGCGAATCCCCACCAGCACCCCCGAGGAG

GCAGTTTTACTAGATAAGCAGCAGTCTGCGATTAAGGTGATATGCAACTCGGTTTACGGA

TTCACGGGGGTGGCAAACGGCCTGTTGCCGTGTCTGAGGATAGCGGCTACCGTTACCACG

ATAGGACGCGACATGCTCCTCAAGACCCGAGATTACGTTCACTCTCGTTGGGCGACGCGC

GAGCTGCTGGAGGACAATTTTCCAGGGGCTATAGGTTTCCGAAACCACAAGCCTTACTCC

GTCAGGGTTATCTACGGAGACACCGACTCCGTGTTTATCAAGTTTGTGGGCCTGACGTAC

GAGGGGGTATCGGAGCTGGGGGATGCTATGTCGCGTCAGATTTCAGCGGACCTCTTTAGA

GCGCCCATCAAACTGGAGTGTGAGAAGACCTTTCAGCGACTGCTGCTGATCACCAAGAAG

AAGTACATAGGTGTCATAAACGGGGGGAAGATGCTCATGAAGGGGGTCGACCTGGTCCGC

AAAAATAACTGCTCTTTCATAAACTTGTACGCGCGACATCTGGTAGATCTTTTGTTGTAC

GACGAGGATGTGGCCACGGCGGCAGCAGAGGTGACAGACGTGCCTCCCGCAGAATGGGTG

GGGCGCCCGCTACCGAGCGGCTTTGACAAGTTTGGGCGAGTGCTGGTAGAGGCGTACAAC

CGTATCACTGCCCCCAACTTGGACGTGCGCGAGTTCGTTATGACTGCTGAGCTGAGCCGC

TCACCCGAATCGTATACCAACAAGCGCCTGCCGCACCTCACCGTCTACTTTAAGCTCGCC

ATGAGGAATGAAGAACTGCCCAGCGTAAAAGAGAGAATTCCGTATGTGATAGTTGCGCAG

ACCGAGGCCGCGGAACGCGAAGCGGGTGTAGTAAACTCAATGCGCGGTACCGCCCAAAAC

CCCGTGGTAACCAAGACCGCACGCCCCCAACCTAAACGCAAACTGCTGGTTTCCGACCTC

GCCGAAGACCCGACCTATGTTTCCGAGAATGACGTACCGCTAAACACAGACTACTATTTC

TCCCACCTGTTGGGTACCATAAGCGTGACCTTTAAGGCTCTATTCGGAAATGATGTGAGA

ACAACAGAAAATCTTTTAAAGCGGTTTATTCCGGAAACCCCCCACAAGACCCCCACGAAA

ACCCAGGCACTGCTTGAGCGCGCCGGCTTTGAAAAGCTGACGCCCTTTACACCGGAGGAA

GAAAGTCGTCGAATACTGCATACAGTTTTTTGTACTCTAGAAGCAGCTCCCCATCAAAGC

TGA

>KC924803.1

ATGGCGGCGCGCGAACAGGCCAACTCCGTTCGCAGGAGCGGGTTTTTTAATCCGTTTATT

GGCAAGAGGCCATTTTTCAGGCCGGGAAGCGGGCAGACCGCGGAGACAGAGCGCCCAAGG

CCCCCCCAACACTCGTACTGCACAGAGGTGGGTAGCTTTAAGTTTATAGCTCCAAGATGC

CTCGATGAAGAAGCCCCCGCCGACCAGCGACGCGGTGTACACGTGGGCACCTTGGAGCGC

CCACCCAAAGTGTACTGCGATGGCTCAGAGTACGACGTGCTGAACTTTGCCTCCGGAGGT

TGCTGGCCTCGCAGGATTCGGGTTTGGAACGGCCAGGATTTTCGGGGCGATGGATTCAAC

CCCAGATTTGAGAGATTTCACGTGTACGACATAGTAGAGACTTCGGAGAGCGCCTCACAC

GATGACCCATCCAGGTTTGCTGAGCTATCCCGCCCAAGCGGGTCTGTGGTAACACTGCTG

GGAATGAGCGAGTGTGGAAAGCGAGTCGCGGTTCACGTCTATGGTGTGCGCCATTATTTT

TACATGGCAAAGGCGGAGGTGGATAGCGCTTGTGGAATAACCACCGAGGCAGAACTCGTG

CGCGCAATGGTGGACTGCGCGCACAGCTCGGCTTTGAGCGCGGCCCTGGGAAATGGCAAC

GGCGGCAAACAGAGCGGCGGCAGCGGCGGGGGATGGTGGGGCGGAAAGCACGTGTCTGCG

GACTGCTTCAAAGTGGAGACCGTGTGCCACACGACGCTGTACTACTTTGGATCTAAGCCA

GCTCTCTACTATAGAGTATCTGCCTCCAGCAGCCGCCTGGGAGGGTTCATCTGCGACAAC

TTTCACCCGGAGATTACAAAATTCGAGGGGAGCGTGGACGTGACCACGCGGCTGTTGTTG

GACAATGAAAATTTTACCAGTTTCGGGTGGTACCGCCTGCGACCCGGCACCCACGGAGAG

CGTGTTCAACTTCGCCCCGTTGAGCGACACGTCACCTCAAGCGACGTGGAGATTAACTGT

ACTCCCGATAACCTGGAGCCGATACCAGACGAGGCTGCCTGGCCCGACTATAAGCTCATG

TGCTTTGATATAGAGTGTAAAGCTGGAACGGGTAACGAAATGGCGTTCCCAGTGGCAACT

AACCAAGAGGACCTGGTCATCCAGATCTCCTGTCTGCTGTACTCGCTTGCTACTCAGAAC

CACGAACACACCCTGCTGTTTTCCCTCGGGTCATGCGATATCTCTGAGGAATACTCGTTT

GCATGCGTCCAGCGCGGCGAGCCCAGACCGACGGTTTTGGAGTTTGACAGCGAGTACGAG

CTGCTGGTTGCCTTCCTGACCTTTCTCAAGCAGTACTCTCCCGAGTTCGCCACCGGCTAC

AACATCGTTAATTTTGACTGGGCGTACATAGTTAACAAGGTAACGTCGGTGTATAACATC

AAGCTGGACGGGTACGGCAAGTTCAACAAAGGGGGGCTGTTTAAGGTGTGGGACATCGCC

ACGAACCATTTTCAGAAGAAGAGCAAGGTGAAAATCAATGGCCTGATATCTCTAGACATG

TATTCTGTGGCGACGGAAAAGCTAAAGCTACCCAGCTACAAACTCGACGCGGTCGTGGGA

GACGTCCTCGGCGAGCATAAGATAGACCTTCCCTATAAAGAAATACCCTCCTATTACGCG

GGAGGGCCTGACCGGAGGGGCGTAATAGGAGAGTATTGTATCCAGGACTCTAGGCTGGTG

GGCAAGCTGTTTTTTAAGTACCTCCCCCATCTGGAACTATCGGCGGTGGCCAAACTCGCC

CGTATCACCCTAACGCGGGTAATTTTTGACGGTCAGCAAATTCGCGTGTACACGTGCTTG

CTGAAACTCGCCCGCGAGAGAAATTTCATTTTGCCAGACAACAGACGCCGGTTTGACAGT

CAGGCAGACGCCGCGTCAGAGACTTCGGAGTTGGCTATGGATAGCCAAAGCCACGCCTTC

GACAGTACAGACGAACCCGACGGTGTGGACGGTACCCCGGACGCCGCAGGATCTGGCGCT

ACTTCTGAAAACGGAGGCGGGAAGCCCGGCGTCGGGAGGGCCGTGGGCTACCAGGGAGCA

AAGGTTCTAGACCCCGTATCCGGCTTTCATGTGGACCCCGTGGTTGTGTTTGACTTCGCT

AGCTTATACCCAAGCATTATCCAGGCCCATAACCTCTGTTTCACCACCCTGGCGCTCGAT

GAAGTGGATCTGGCCGGGCTTCAACCATCCGTCAACTACTCGACGTTCGAGGTGGGTGAC

CAAAAGTTATTTTTTGTCCACGCCCATATTCGCGAAAGCCTGCTTGGCATCTTGCTGCGC

GACTGGCTGGCCATGCGAAAGGCGGTGAGGGCGCGAATCCCCACCAGCACCCCCGAGGAG

GCAGTTTTACTAGATAAGCAGCAGTCTGCGATTAAGGTGATATGCAACTCGGTTTACGGA

TTCACGGGGGTGGCAAACGGCCTGTTGCCGTGTCTGAGGATAGCGGCTACCGTTACCACG

ATAGGACGCGACATGCTCCTCAAGACCCGAGATTACGTTCACTCTCGTTGGGCGACGCGC

GAGCTGCTGGAGAACAATTTTCCAGGGGCTATAGGTTTCCGAAACCACAAGCCTTACTCC

GTCAGGGTTATCTACGGAGACACCGACTCCGTGTTTATCAAGTTTGTGGGCCTGACGTAC

GAGGGGGTATCGGAGCTGGGGGATGCTATGTCGCGTCAGATTTCAGCGGACCTCTTTAGA

GCGCCCATCAAACTGGAGTGTGAGAAGACCTTTCAGCGACTGCTGCTGATCACCAAGAAG

AAGTACATAGGTGTCATAAACGGGGGGAAGATGCTCATGAAGGGGGTCGACCTGGTCCGC

AAAAATAACTGCTCTTTCATAAACTTGTACGCGCGACATCTGGTAGATCTTTTGTTGTAC

GACGAGGATGTGGCCACGGCGGCAGCAGAGGTGACAGACGTGCCTCCCGCAGAATGGGTG

GGGCGCCCGCTACCGAGCGGCTTTGACAAGTTTGGGCGAGTGCTGGTAGAGGCGTACAAC

CGTATCACTGCCCCCAACTTGGACGTGCGCGAGTTCGTTATGACTGCTGAGCTGAGCCGC

TCACCCGAATCGTATACCAACAAGCGCCTGCCGCACCTCACCGTCTACTTTAAGCTCGCC

ATGAGGAATGAAGAACTGCCCAGCGTAAAAGAGAGAATTCCGTATGTGATAGTTGCGCAG

ACCGAGGCCGCGGAACGCGAAGCGGGTGTAGTAAACTCAATGCGCGGTACCGCCCAAAAC

CCCGTGGTAACCAAGACCGCACGCCCCCAACCTAAACGCAAACTGCTGGTTTCCGACCTC

GCCGAAGACCCGACCTATGTTTCCGAGAATGACGTACCGCTAAACACAGACTACTATTTC

TCCCACCTGTTGGGTACCATAAGCGTGACCTTTAAGGCTCTATTCGGAAATGATGTGAGA

ACAACAGAAAATCTTTTAAAGCGGTTTATTCCGGAAACCCCCCACAAGACCCCCACGAAA

ACCCAGGCACTGCTTGAGCGCGCCGGCTTTGAAAAGCTGACGCCCTTTACACCGGAGGAA

GAAAGTCGTCGAATACTGCATACAGTTTTTTGTACTCTAGAAGCAGCTCCCCATCAAAGC

TGA

>KC924804.1

ATGGCGGCGCGCGAACAGGCCAACTCCGTTCGCAGGAGCGGGTTTTTTAATCCGTTTATT

GGCAAGAGGCCATTTTTCAGGCCGGGAAGCGGGCAGACCGCGGAGACAGAGCGCCCAAGG

CCCCCCCAACACTCGTACTGCACAGAGGTGGGTAGCTTTAAGTTTATAGCTCCAAGATGC

CTCGATGAAGAAGCCCCCGCCGACCAGCGACGCGGTGTACACGTGGGCACCTTGGAGCGC

CCACCCAAAGTGTACTGCGATGGCTCAGAGTACGACGTGCTGAACTTTGCCTCCGGAGGT

TGCTGGCCTCGCAGGATTCGGGTTTGGAACGGCCAGGATTTTCGGGGCGATGGATTCAAC

CCCAGATTTGAGAGATTTCACGTGTACGACATAGTAGAGACTTCGGAGAGCGCCTCACAC

GATGACCCATCCAGGTTTGCTGAGCTATCCCGCCCAAGCGGGTCTGTGGTAACACTGCTG

GGAATGAGCGAGTGTGGAAAGCGAGTCGCGGTTCACGTCTATGGTGTGCGCCATTATTTT

TACATGGCAAAGGCGGAGGTGGATAGCGCTTGTGGAATAACCACCGAGGCAGAACTCGTG

CGCGCAATGGTGGACTGCGCGCACAGCTCGGCTTTGAGCGCGGCCCTGGGAAATGGCAAC

GGCGGCAAACAGAGCGGCGGCAGCGGCGGGGGATGGTGGGGCGGAAAGCACGTGTCTGCG

GACTGCTTCAAAGTGGAGACCGTGTGCCACACGACGCTGTACTACTTTGGATCTAAGCCA

GCTCTCTACTATAGAGTATCTGCCTCCAGCAGCCGCCTGGGAGGGTTCATCTGCGACAAC

TTTCACCCGGAGATTACAAAATTCGAGGGGAGCGTGGACGTGACCACGCGGCTGTTGTTG

GACAATGAAAATTTTACCAGTTTCGGGTGGTACCGCCTGCGACCCGGCACCCACGGAGAG

CGTGTTCAACTTCGCCCCGTTGAGCGACACGTCACCTCAAGCGACGTGGAGATTAACTGT

ACTCCCGATAACCTGGAGCCGATACCAGACGAGGCTGCCTGGCCCGACTATAAGCTCATG

TGCTTTGATATAGAGTGTAAAGCTGGAACGGGTAACGAAATGGCGTTCCCAGTGGCAACT

AACCAAGAGGACCTGGTCATCCAGATCTCCTGTCTGCTGTACTCGCTTGCTACTCAGAAC

CACGAACACACCCTGCTGTTTTCCCTCGGGTCATGCGATATCTCTGAGGAATACTCGTTT

GCATGCGTCCAGCGCGGCGAGCCCAGACCGACGGTTTTGGAGTTTGACAGCGAGTACGAG

CTGCTGGTTGCCTTCCTGACCTTTCTCAAGCAGTACTCTCCCGAGTTCGCCACCGGCTAC

AACATCGTTAATTTTGACTGGGCGTACATAGTTAACAAGGTAACGTCGGTGTATAACATC

AAGCTGGACGGGTACGGCAAGTTCAACAAAGGGGGGCTGTTTAAGGTGTGGGACATCGCC

ACGAACCATTTTCAGAAGAAGAGCAAGGTGAAAATCAATGGCCTGATATCTCTAGACATG

TATTCTGTGGCGACGGAAAAGCTAAAGCTACCCAGCTACAAACTCGACGCGGTCGTGGGA

GACGTCCTCGGCGAGCATAAGATAGACCTTCCCTATAAAGAAATACCCTCCTATTACGCG

GGAGGGCCTGACCGGAGGGGCGTAATAGGAGAGTATTGTATCCAGGACTCTAGGCTGGTG

GGCAAGCTGTTTTTTAAGTACCTCCCCCATCTGGAACTATCGGCGGTGGCCAAACTCGCC

CGTATCACCCTAACGCGGGTAATTTTTGACGGTCAGCAAATTCGCGTGTACACGTGCTTG

CTGAAACTCGCCCGCGAGAGAAATTTCATTTTGCCAGACAACAGACGCCGGTTTGACAGT

CAGGCAGACGCCGCGTCAGAGACTTCGGAGTTGGCTATGGATAGCCAAAGCCACGCCTTC

GACAGTACAGACGAACCCGACGGTGTGGACGGTACCCCGGACGCCGCAGGATCTGGCGCT

ACTTCTGAAAACGGAGGCGGGAAGCCCGGCGTCGGGAGGGCCGTGGGCTACCAGGGAGCA

AAGGTTCTAGACCCCGTATCCGGCTTTCATGTGGACCCCGTGGTTGTGTTTGACTTCGCT

AGCTTATACCCAAGCATTATCCAGGCCCATAACCTCTGTTTCACCACCCTGGCGCTCGAT

GAAGTGGATCTGGCCGGGCTTCAACCATCCGTCAACTACTCGACGTTCGAGGTGGGTGAC

CAAAAGTTATTTTTTGTCCACGCCCATATTCGCGAAAGCCTGCTTGGCATCTTGCTGCGC

GACTGGCTGGCCATGCGAAAGGCGGTGAGGGCGCGAATCCCCACCAGCACCCCCGAGGAG

GCAGTTTTACTAGATAAGCAGCAGTCTGCGATTAAGGTGATATGCAACTCGGTTTACGGA

TTCACGGGGGTGGCAAACGGCCTGTTGCCGTGTCTGAGGATAGCGGCTACCGTTACCACG

ATAGGACGCGACATGCTCCTCAAGACCCGAGATTACGTTCACTCTCGTTGGGCGACGCGC

GAGCTGCTGGAGGACAATTTTCCAGGGGCTATAGGTTTCCGAAACCACAAGCCTTACTCC

GTCAGGGTTATCTACGGAGACACCGACTCCGTGTTTATCAAGTTTGTGGGCCTGACGTAC

GAGGGGGTATCGGAGCTGGGGGATGCTATGTCGCGTCAGATTTCAGCGGACCTCTTTAGA

GCGCCCATCAAACTGGAGTGTGAGAAGACCTTTCAGCGACTGCTGCTGATCACCAAGAAG

AAGTACATAGGTGTCATAAACGGGGGGAAGATGCTCATGAAGGGGGTCGACCTGGTCCGC

AAAAATAACTGCTCTTTCATAAACTTGTACGCGCGACATCTGGTAGATCTTTTGTTGTAC

GACGAGGATGTGGCCACGGCGGCAGCAGAGGTGACAGACGTGCCTCCCGCAGAATGGGTG

GGGCGCCCGCTACCGAGCGGCTTTGACAAGTTTGGGCGAGTGCTGGTAGAGGCGTACAAC

CGTATCACTGCCCCCAACTTGGACGTGCGCGAGTTCGTTATGACTGCTGAGCTGAGCCGC

TCACCCGAATCGTATACCAACAAGCGCCTGCCGCACCTCACCGTCTACTTTAAGCTCGCC

ATGAGGAATGAAGAACTGCCCAGCGTAAAAGAGAGAATTCCGTATGTGATAGTTGCGCAG

ACCGAGGCCGCGGAACGCGAAGCGGGTGTAGTAAACTCAATGCGCGGTACCGCCCAAAAC

CCCGTGGTAACCAAGACCGCACGCCCCCAACCTAAACGCAAACTGCTGGTTTCCGACCTC

GCCGAAGACCCGACCTATGTTTCCGAGAATGACGTACCGCTAAACACAGACTACTATTTC

TCCCACCTGTTGGGTACCATAAGCGTGACCTTTAAGGCTCTATTCGGAAATGATGTGAGA

ACAACAGAAAATCTTTTAAAGCGGTTTATTCCGGAAACCCCCCACAAGACCCCCACGAAA

ACCCAGGCACTGCTTGAGCGCGCCGGCTTTGAAAAGCTGACGCCCTTTACACCGGAGGAA

GAAAGTCGTCGAATACTGCATACAGTTTTTTGTACTCTAGAAGCAGCTCCCCATCAAAGC

TGA

>KC924805.1

ATGGCGGCGCGCGAACAGGCCAACTCCGTTCGCAGGAGCGGGTTTTTTAATCCGTTTATT

GGCAAGAGGCCATTTTTCAGGCCGGGAAGCGGGCAGACCGCGGAGACAGAGCGCCCAAGG

CCCCCCCAACACTCGTACTGCACAGAGGTGGGTAGCTTTAAGTTTATAGCTCCAAGATGC

CTCGATGAAGAAGCCCCCGCCGACCAGCGACGCGGTGTACACGTGGGCACCTTGGAGCGC

CCACCCAAAGTGTACTGCGATGGCTCAGAGTACGACGTGCTGAACTTTGCCTCCGGAGGT

TGCTGGCCTCGCAGGATTCGGGTTTGGAACGGCCAGGATTTTCGGGGCGATGGATTCAAC

CCCAGATTTGAGAGATTTCACGTGTACGACATAGTAGAGACTTCGGAGAGCGCCTCACAC

GATGACCCATCCAGGTTTGCTGAGCTATCCCGCCCAAGCGGGTCTGTGGTAACACTGCTG

GGAATGAGCGAGTGTGGAAAGCGAGTCGCGGTTCACGTCTATGGTGTGCGCCATTATTTT

TACATGGCAAAGGCGGAGGTGGATAGCGCTTGTGGAATAACCACCGAGGCAGAACTCGTG

CGCGCAATGGTGGACTGCGCGCACAGCTCGGCTTTGAGCGCGGCCCTGGGAAATGGCAAC

GGCGGCAAACAGAGCGGCGGCAGCGGCGGGGGATGGTGGGGCGGAAAGCACGTGTCTGCG

GACTGCTTCAAAGTGGAGACCGTGTGCCACACGACGCTGTACTACTTTGGATCTAAGCCA

GCTCTCTACTATAGAGTATCTGCCTCCAGCAGCCGCCTGGGAGGGTTCATCTGCGACAAC

TTTCACCCGGAGATTACAAAATTCGAGGGGAGCGTGGACGTGACCACGCGGCTGTTGTTG

GACAATGAAAATTTTACCAGTTTCGGGTGGTACCGCCTGCGACCCGGCACCCACGGAGAG

CGTGTTCAACTTCGCCCCGTTGAGCGACACGTCACCTCAAGCGACGTGGAGATTAACTGT

ACTCCCGATAACCTGGAGCCGATACCAGACGAGGCTGCCTGGCCCGACTATAAGCTCATG

TGCTTTGATATAGAGTGTAAAGCTGGAACGGGTAACGAAATGGCGTTCCCAGTGGCAACT

AACCAAGAGGACCTGGTCATCCAGATCTCCTGTCTGCTGTACTCGCTTGCTACTCAGAAC

CACGAACACACCCTGCTGTTTTCCCTCGGGTCATGCGATATCTCTGAGGAATACTCGTTT

GCATGCGTCCAGCGCGGCGAGCCCAGACCGACGGTTTTGGAGTTTGACAGCGAGTACGAG

CTGCTGGTTGCCTTCCTGACCTTTCTCAAGCAGTACTCTCCCGAGTTCGCCACCGGCTAC

AACATCGTTAATTTTGACTGGGCGTACATAGTTAACAAGGTAACGTCGGTGTATAACATC

AAGCTGGACGGGTACGGCAAGTTCAACAAAGGGGGGCTGTTTAAGGTGTGGGACATCGCC

ACGAACCATTTTCAGAAGAAGAGCAAGGTGAAAATCAATGGCCTGATATCTCTAGACATG

TATTCTGTGGCGACGGAAAAGCTAAAGCTACCCAGCTACAAACTCGACGCGGTCGTGGGA

GACGTCCTCGGCGAGCATAAGATAGACCTTCCCTATAAAGAAATACCCTCCTATTACGCG

GGAGGGCCTGACCGGAGGGGCGTAATAGGAGAGTATTGTATCCAGGACTCTAGGCTGGTG

GGCAAGCTGTTTTTTAAGTACCTCCCCCATCTGGAACTATCGGCGGTGGCCAAACTCGCC

CGTATCACCCTAACGCGGGTAATTTTTGACGGTCAGCAAATTCGCGTGTACACGTGCTTG

CTGAAACTCGCCCGCGAGAGAAATTTCATTTTGCCAGACAACAGACGCCGGTTTGACAGT

CAGGCAGACGCCGCGTCAGAGACTTCGGAGTTGGCTATGGATAGCCAAAGCCACGCCTTC

GACAGTACAGACGAACCCGACGGTGTGGACGGTACCCCGGACGCCGCAGGATCTGGCGCT

ACTTCTGAAAACGGAGGCGGGAAGCCCGGCGTCGGGAGGGCCGTGGGCTACCAGGGAGCA

AAGGTTCTAGACCCCGTATCCGGCTTTCATGTGGACCCCGTGGTTGTGTTTGACTTCGCT

AGCTTATACCCAAGCATTATCCAGGCCCATAACCTCTGTTTCACCACCCTGGCGCTCGAT

GAAGTGGATCTGGCCGGGCTTCAACCATCCGTCAACTACTCGACGTTCGAGGTGGGTGAC

CAAAAGTTATTTTTTGTCCACGCCCATATTCGCGAAAGCCTGCTTGGCATCTTGCTGCGC

GACTGGCTGGCCATGCGAAAGGCGGTGAGGGCGCGAATCCCCACCAGCACCCCCGAGGAG

GCAGTTTTACTAGATAAGCAGCAGTCTGCGATTAAGGTGATATGCAACTCGGTTTACGGA

TTCACGGGGGTGGCAAACGGCCTGTTGCCGTGTCTGAGGATAGCGGCTACCGTTACCACG

ATAGGACGCGACATGCTCCTCAAGACCCGAGATTACGTTCACTCTCGTTGGGCGACGCGC

GAGCTGCTGGAGGACAATTTTCCAGGGGCTATAGGTTTCCGAAACCACAAGCCTTACTCC

GTCAGGGTTATCTACGGAGACACCGACTCCGTGTTTATCAAGTTTGTGGGCCTGACGTAC

GAGGGGGTATCGGAGCTGGGGGATGCTATGTCGCGTCAGATTTCAGCGGACCTCTTTAGA

GCGCCCATCAAACTGGAGTGTGAGAAGACCTTTCAGCGACTGCTGCTGATCACCAAGAAG

AAGTACATAGGTGTCATAAACGGGGGGAAGATGCTCATGAAGGGGGTCGACCTGGTCCGC

AAAAATAACTGCTCTTTCATAAACTTGTACGCGCGACATCTGGTAGATCTTTTGTTGTAC

GACGAGGATGTGGCCACGGCGGCAGCAGAGGTGACAGACGTGCCTCCCGCAGAATGGGTG

GGGCGCCCGCTACCGAGCGGCTTTGACAAGTTTGGGCGAGTGCTGGTAGAGGCGTACAAC

CGTATCACTGCCCCCAACTTGGACGTGCGCGAGTTCGTTATGACTGCTGAGCTGAGCCGC

TCACCCGAATTGTATACCAACAAGCGCCTGCCGCACCTCACCGTCTACTTTAAGCTCGCC

ATGAGGAATGAAGAACTGCCCAGCGTAAAAGAGAGAATTCCGTATGTGATAGTTGCGCAG

ACCGAGGCCGCGGAACGCGAAGCGGGTGTAGTAAACTCAATGCGCGGTACCGCCCAAAAC

CCCGTGGTAACCAAGACCGCACGCCCCCAACCTAAACGCAAACTGCTGGTTTCCGACCTC

GCCGAAGACCCGACCTATGTTTCCGAGAATGACGTACCGCTAAACACAGACTACTATTTC

TCCCACCTGTTGGGTACCATAAGCGTGACCTTTAAGGCTCTATTCGGAAATGATGTGAGA

ACAACAGAAAATCTTTTAAAGCGGTTTATTCCGGAAACCCCCCACAAGACCCCCACGAAA

ACCCAGGCACTGCTTGAGCGCGCCGGCTTTGAAAAGCTGACGCCCTTTACACCGGAGGAA

GAAAGTCGTCGAATACTGCATACAGTTTTTTGTACTCTAGAAGCAGCTCCCCATCAAAGC

TGA

>KC924806.1

ATGGCGGCGCGCGAACAGGCCAACTCCGTTCGCAGGAGCGGGTTTTTTAATCCGTTTATT

GGCAAGAGGCCATTTTTCAGGCCGGGAAGCGGGCAGACCGCGGAGACAGAGCGCCCAAGG

CCCCCCCAACACTCGTACTGCACAGAGGTGGGTAGCTTTAAGTTTATAGCTCCAAGATGC

CTCGATGAAGAAGCCCCCGCCGACCAGCGACGCGGTGTACACGTGGGCACCTTGGAGCGC

CCACCCAAAGTGTACTGCGATGGCTCAGAGTACGACGTGCTGAACTTTGCCTCCGGAGGT

TGCTGGCCTCGCAGGATTCGGGTTTGGAACGGCCAGGATTTTCGGGGCGATGGATTCAAC

CCCAGATTTGAGAGATTTCACGTGTACGACATAGTAGAGACTTCGGAGAGCGCCTCACAC

GATGACCCATCCAGGTTTGCTGAGCTATCCCGCCCAAGCGGGTCTGTGGTAACACTGCTG

GGAATGAGCGAGTGTGGAAAGCGAGTCGCGGTTCACGTCTATGGTGTGCGCCATTATTTT

TACATGGCAAAGGCGGAGGTGGATAGCGCTTGTGGAATAACCACCGAGGCAGAACTCGTG

CGCGCAATGGTGGACTGCGCGCACAGCTCGGCTTTGAGCGCGGCCCTGGGAAATGGCAAC

GGCGGCAAACAGAGCGGCGGCAGCGGCGGGGGATGGTGGGGCGGAAAGCACGTGTCTGCG

GACTGCTTCAAAGTGGAGACCGTGTGCCACACGACGCTGTACTACTTTGGATCTAAGCCA

GCTCTCTACTATAGAGTATCTGCCTCCAGCAGCCGCCTGGGAGGGTTCATCTGCGACAAC

TTTCACCCGGAGATTACAAAATTCGAGGGGAGCGTGGACGTGACCACGCGGCTGTTGTTG

GACAATGAAAATTTTACCAGTTTCGGGTGGTACCGCCTGCGACCCGGCACCCACGGAGAG

CGTGTTCAACTTCGCCCCGTTGAGCGACACGTCACCTCAAGCGACGTGGAGATTAACTGT

ACTCCCGATAACCTGGAGCCGATACCAGACGAGGCTGCCTGGCCCGACTATAAGCTCATG

TGCTTTGATATAGAGTGTAAAGCTGGAACGGGTAACGAAATGGCGTTCCCAGTGGCAACT

AACCAAGAGGACCTGGTCATCCAGATCTCCTGTCTGCTGTACTCGCTTGCTACTCAGAAC

CACGAACACACCCTGCTGTTTTCCCTCGGGTCATGCGATATCTCTGAGGAATACTCGTTT

GCATGCGTCCAGCGCGGCGAGCCCAGACCGACGGTTTTGGAGTTTGACAGCGAGTACGAG

CTGCTGGTTGCCTTCCTGACCTTTCTCAAGCAGTACTCTCCCGAGTTCGCCACCGGCTAC

AACATCGTTAATTTTGACTGGGCGTACATAGTTAACAAGGTAACGTCGGTGTATAACATC

AAGCTGGACGGGTACGGCAAGTTCAACAAAGGGGGGCTGTTTAAGGTGTGGGACATCGCC

ACGAACCATTTTCAGAAGAAGAGCAAGGTGAAAATCAATGGCCTGATATCTCTAGACATG

TATTCTGTGGCGACGGAAAAGCTAAAGCTACCCAGCTACAAACTCGACGCGGTCGTGGGA

GACGTCCTCGGCGAGCATAAGATAGACCTTCCCTATAAAGAAATACCCTCCTATTACGCG

GGAGGGCCTGACCGGAGGGGCGTAATAGGAGAGTATTGTATCCAGGACTCTAGGCTGGTG

GGCAAGCTGTTTTTTAAGTACCTCCCCCATCTGGAACTATCGGCGGTGGCCAAACTCGCC

CGTATCACCCTAACGCGGGTAATTTTTGACGGTCAGCAAATTCGCGTGTACACGTGCTTG

CTGAAACTCGCCCGCGAGAGAAATTTCATTTTGCCAGACAACAGACGCCGGTTTGACAGT

CAGGCAGACGCCGCGTCAGAGACTTCGGAGTTGGCTATGGATAGCCAAAGCCACGCCTTC

GACAGTACAGACGAACCCGACGGTGTGGACGGTACCCCGGACGCCGCAGGATCTGGCGCT

ACTTCTGAAAACGGAGGCGGGAAGCCCGGCGTCGGGAGGGCCGTGGGCTACCAGGGAGCA

AAGGTTCTAGACCCCGTATCCGGCTTTCATGTGGACCCCGTGGTTGTGTTTGACTTCGCT

AGCTTATACCCAAGCATTATCCAGGCCCATAACCTCTGTTTCACCACCCTGGCGCTCGAT

GAAGTGGATCTGGCCGGGCTTCAACCATCCGTCGACTACTCGACGTTCGAGGTGGGTGAC

CAAAAGTTATTTTTTGTCCACGCCCATATTCGCGAAAGCCTGCTTGGCATCTTGCTGCGC

GACTGGCTGGCCATGCGAAAGGCGGTGAGGGCGCGAATCCCCACCAGCACCCCCGAGGAG

GCAGTTTTACTAGATAAGCAGCAGTCTGCGATTAAGGTGATATGCAACTCGGTTTACGGA

TTCACGGGGGTGGCAAACGGCCTGTTGCCGTGTCTGAGGATAGCGGCTACCGTTACCACG

ATAGGACGCGACATGCTCCTCAAGACCCGAGATTACGTTCACTCTCGTTGGGCGACGCGC

GAGCTGCTGGAGGACAATTTTCCAGGGGCTATAGGTTTCCGAAACCACAAGCCTTACTCC

GTCAGGGTTATCTACGGAGACACCGACTCCGTGTTTATCAAGTTTGTGGGCCTGACGTAC

GAGGGGGTATCGGAGCTGGGGGATGCTATGTCGCGTCAGATTTCAGCGGACCTCTTTAGA

GCGCCCATCAAACTGGAGTGTGAGAAGACCTTTCAGCGACTGCTGCTGATCACCAAGAAG

AAGTACATAGGTGTCATAAACGGGGGGAAGATGCTCATGAAGGGGGTCGACCTGGTCCGC

AAAAATAACTGCTCTTTCATAAACTTGTACGCGCGACATCTGGTAGATCTTTTGTTGTAC

GACGAGGATGTGGCCACGGCGGCAGCAGAGGTGACAGACGTGCCTCCCGCAGAATGGGTG

GGGCGCCCGCTACCGAGCGGCTTTGACAAGTTTGGGCGAGTGCTGGTAGAGGCGTACAAC

CGTATCACTGCCCCCAACTTGGACGTGCGCGAGTTCGTTATGACTGCTGAGCTGAGCCGC

TCACCCGAATCGTATACCAACAAGCGCCTGCCGCACCTCACCGTCTACTTTAAGCTCGCC

ATGAGGAATGAAGAACTGCCCAGCGTAAAAGAGAGAATTCCGTATGTGATAGTTGCGCAG

ACCGAGGCCGCGGAACGCGAAGCGGGTGTAGTAAACTCAATGCGCGGTACCGCCCAAAAC

CCCGTGGTAACCAAGACCGCACGCCCCCAACCTAAACGCAAACTGCTGGTTTCCGACCTC

GCCGAAGACCCGACCTATGTTTCCGAGAATGACGTACCGCTAAACACAGACTACTATTTC

TCCCACCTGTTGGGTACCATAAGCGTGACCTTTAAGGCTCTATTCGGAAATGATGTGAGA

ACAACAGAAAATCTTTTAAAGCGGTTTATTCCGGAAACCCCCCACAAGACCCCCACGAAA

ACCCAGGCACTGCTTGAGCGCGCCGGCTTTGAAAAGCTGACGCCCTTTACACCGGAGGAA

GAAAGTCGTCGAATACTGCATACAGTTTTTTGTACTCTAGAAGCAGCTCCCCATCAAAGC

TGA

>KC924807.1

ATGGCGGCGCGCGAACAGGCCAACTCCGTTCGCAGGAGCGGGTTTTTTAATCCGTTTATT

GGCAAGAGGCCATTTTTCAGGCCGGGAAGCGGGCAGACCGCGGAGACAGAGCGCCCAAGG

CCCCCCCAACACTCGTACTGCACAGAGGTGGGTAGCTTTAAGTTTATAGCTCCAAGATGC

CTCGATGAAGAAGCCCCCGCCGACCAGCGACGCGGTGTACACGTGGGCACCTTGGAGCGC

CCACCCAAAGTGTACTGCGATGGCTCAGAGTACGACGTGTTGAACTTTGCCTCCGGAGGT

TGCTGGCCTCGCAGGATTCGGGTTTGGAACGGCCAGGATTTTCGGGGCGATGGATTCAAC

CCCAGATTTGAGAGATTTCACGTGTACGACATAGTAGAGACTTCGGAGAGCGCCTCACAC

GATGACCCATCCAGGTTTGCTGAGCTATCCCGCCCAAGCGGGTCTGTGGTAACACTGCTG

GGAATGAGCGAGTGTGGAAAGCGAGTCGCGGTTCACGTCTATGGTGTGCGCCATTATTTT

TACATGGCAAAGGCGGAGGTGGATAGCGCTTGTGGAATAACCACCGAGGCAGAACTCGTG

CGCGCAATGGTGGACTGCGCGCACAGCTCGGCTTTGAGCGCGGCCCTGGGAAATGGCAAC

GGCGGCAAACAGAGCGGCGGCAGCGGCGGGGGATGGTGGGGCGGAAAGCACGTGTCTGCG

GACTGCTTCAAAGTGGAGACCGTGTGCCACACGACGCTGTACTACTTTGGATCTAAGCCA

GCTCTCTACTATAGAGTATCTGCCTCCAGCAGCCGCCTGGGAGGGTTCATCTGCGACAAC

TTTCACCCGGAGATTACAAAATTCGAGGGGAGCGTGGACGTGACCACGCGGCTGTTGTTG

GACAATGAAAATTTTACCAGTTTCGGGTGGTACCGCCTGCGACCCGGCACCCACGGAGAG

CGTGTTCAACTTCGCCCCGTTGAGCGACACGTCACCTCAAGCGACGTGGAGATTAACTGT

ACTCCCGATAACCTGGAGCCGATACCAGACGAGGCTGCCTGGCCCGACTATAAGCTCATG

TGCTTTGATATAGAGTGTAAAGCTGGAACGGGTAACGAAATGGCGTTCCCAGTGGCAACT

AACCAAGAGGACCTGGTCATCCAGATCTCCTGTCTGCTGTACTCGCTTGCTACTCAGAAC

CACGAACACACCCTGCTGTTTTCCCTCGGGTCATGCGATATCTCTGAGGAATACTCGTTT

GCATGCGTCCAGCGCGGCGAGCCCAGACCGACGGTTTTGGAGTTTGACAGCGAGTACGAG

CTGCTGGTTGCCTTCCTGACCTTTCTCAAGCAGTACTCTCCCGAGTTCGCCACCGGCTAC

AACATCGTTAATTTTGACTGGGCGTACATAGTTAACAAGGTAACGTCGGTGTATAACATC

AAGCTGGACGGGTACGGCAAGTTCAACAAAGGGGGGCTGTTTAAGGTGTGGGACATCGCC

ACGAACCATTTTCAGAAGAAGAGCAAGGTGAAAATCAATGGCCTGATATCTCTAGACATG

TATTCTGTGGCGACGGAAAAGCTAAAGCTACCCAGCTACAAACTCGACGCGGTCGTGGGA

GACGTCCTCGGCGAGCATAAGATAGACCTTCCCTATAAAGAAATACCCTCCTATTACGCG

GGAGGGCCTGACCGGAGGGGCGTAATAGGAGAGTATTGTATCCAGGACTCTAGGCTGGTG

GGCAAGCTGTTTTTTAAGTACCTCCCCCATCTGGAACTATCGGCGGTGGCCAAACTCGCC

CGTATCACCCTAACGCGGGTAATTTTTGACGGTCAGCAAATTCGCGTGTACACGTGCTTG

CTGAAACTCGCCCGCGAGAGAAATTTCATTTTGCCAGACAACAGACGCCGGTTTGACAGT

CAGGCAGACGCCGCGTCAGAGACTTCGGAGTTGGCTATGGATAGCCAAAGCCACGCCTTC

GACAGTACAGACGAACCCGACGGTGTGGACGGTACCCCGGACGCCGCAGGATCTGGCGCT

ACTTCTGAAAACGGAGGCGGGAAGCCCGGCGTCGGGAGGGTCGTGGGCTACCAGGGAGCA

AAGGTTCTAGACCCCGTATCCGGCTTTCATGTGGACCCCGTGGTTGTGTTTGACTTCGCT

AGCTTATACCCAAGCATTATCCAGGCCCATAACCTCTGTTTCACCACCCTGGCGCTCGAT

GAAGTGGATCTGGCCGGGCTTCAACCATCCGTCAACTACTCGACGTTCGAGGTGGGTGAC

CAAAAGTTATTTTTTGTCCACGCCCATATTCGCGAAAGCCTGCTTGGCATCTTGCTGCGC

GACTGGCTGGCCATGCGAAAGGCGGTGAGGGCGCGAATCCCCACCAGCACCCCCGAGGAG

GCAGTTTTACTAGATAAGCAGCAGTCTGCGATTAAGGTGATATGCAACTCGGTTTACGGA

TTCACGGGGGTGGCAAACGGCCTGTTGCCGTGTCTGAGGATAGCGGCTACCGTTACCACG

ATAGGACGCGACATGCTCCTCAAGACCCGAGATTACGTTCACTCTCGTTGGGCGACGCGC

GAGCTGCTGGAGGACAATTTTCCAGGGGCTATAGGTTTCCGAAACCACAAGCCTTACTCC

GTCAGGGTTATCTACGGAGACACCGACTCCGTGTTTATCAAGTTTGTGGGCCTGACGTAC

GAGGGGGTATCGGAGCTGGGGGATGCTATGTCGCGTCAGATTTCAGCGGACCTCTTTAGA

GCGCCCATCAAACTGGAGTGTGAGAAGACCTTTCAGCGACTGCTGCTGATCACCAAGAAG

AAGTACATAGGTGTCATAAACGGGGGGAAGATGCTCATGAAGGGGGTCGACCTGGTCCGC

AAAAATAACTGCTCTTTCATAAACTTGTACGCGCGACATCTGGTAGATCTTTTGTTGTAC

GACGAGGATGTGGCCACGGCGGCAGCAGAGGTGACAGACGTGCCTCCCGCAGAATGGGTG

GGGCGCCCGCTACCGAGCGGCTTTGACAAGTTTGGGCGAGTGCTGGTAGAGGCGTACAAC

CGTATCACTGCCCCCAACTTGGACGTGCGCGAGTTCGTTATGACTGCTGAGCTGAGCCGC

TCACCCGAATCGTATACCAACAAGCGCCTGCCGCACCTCACCGTCTACTTTAAGCTCGCC

ATGAGGAATGAAGAACTGCCCAGCGTAAAAGAGAGAATTCCGTATGTGATAGTTGCGCAG

ACCGAGGCCGCGGAACGCGAAGCGGGTGTAGTAAACTCAATGCGCGGTACCGCCCAAAAC

CCCGTGGTAACCAAGACCGCACGCCCCCAACCTAAACGCAAACTGCTGGTTTCCGACCTC

GCCGAAGACCCGACCTATGTTTCCGAGAATGACGTACCGCTAAACACAGACTACTATTTC

TCCCACCTGTTGGGTACCATAAGCGTGACCTTTAAGGCTCTATTCGGAAATGATGTGAGA

ACAACAGAAAATCTTTTAAAGCGGTTTATTCCGGAAACCCCCCACAAGACCCCCACGAAA

ACCCAGGCACTGCTTGAGCGCGCCGGCTTTGAAAAGCTGACGCCCTTTACACCGGAGGAA

GAAAGTCGTCGAATACTGCATACAGTTTTTTGTACTCTAGAAGCAGCTCCCCATCAAAGC

TGA

>KC924808.1

ATGGCGGCGTGCGAACAGGCCAACTCCGTTCGCAGGAGCGGGTTTTTTAATCCGTTTATT

GGCAAGAGGCCATTTTTCAGGCCGGGAAGCGGGCAGACCGCGGAGACAGAGCGCCCAAGG

CCCCCCCAACACTCGTACTGCACAGAGGTGGGTAGCTTTAAGTTTATAGCTCCAAGATGC

CTCGATGAAGAAGCCCCCGCCGACCAGCGACGCGGTGTACACGTGGGCACCTTGGAGCGC

CCACCCAAAGTGTACTGCGATGGCTCAGAGTACGACGTGCTGAACTTTGCCTCCGGAGGT

TGCTGGCCTCGCAGGATTCGGGTTTGGAACGGCCAGGATTTTCGGGGCGATGGATTCAAC

CCCAGATTTGAGAGATTTCACGTGTACGACATAGTAGAGACTTCGGAGAGCGCCTCACAC

GATGACCCATCCAGGTTTGCTGAGCTATCCCGCCCAAGCGGGTCTGTGGTAACACTGCTG

GGAATGAGCGAGTGTGGAAAGCGAGTCGCGGTTCACGTCTATGGTGTGCGCCATTATTTT

TACATGGCAAAGGCGGAGGTGGATAGCGCTTGTGGAATAACCACCGAGGCAGAACTCGTG

CGCGCAATGGTGGACTGCGCGCACAGCTCGGCTTTGAGCGCGGCCCTGGGAAATGGCAAC

GGCGGCAAACAGAGCGGCGGCAGCGGCGGGGGATGGTGGGGCGGAAAGCACGTGTCTGCG

GACTGCTTCAAAGTGGAGACCGTGTGCCACACGACGCTGTACTACTTTGGATCTAAGCCA

GCTCTCTACTATAGAGTATCTGCCTCCAGCAGCCGCCTGGGAGGGTTCATCTGCGACAAC

TTTCACCCGGAGATTACAAAATTCGAGGGGAGCGTGGACGTGACCACGCGGCTGTTGTTG

GACAATGAAAATTTTACCAGTTTCGGGTGGTACCGCCTGCGACCCGGCACCCACGGAGAG

CGTGTTCAACTTCGCCCCGTTGAGCGACACGTCACCTCAAGCGACGTGGAGATTAACTGT

ACTCCCGATAACCTGGAGCCGATACCAGACGAGGCTGCCTGGCCCGACTATAAGCTCATG

TGCTTTGATATAGAGTGTAAAGCTGGAACGGGTAACGAAATGGCGTTCCCAGTGGCAACT

AACCAAGAGGACCTGGTCATCCAGATCTCCTGTCTGCTGTACTCGCTTGCTACTCAGAAC

CACGAACACACCCTGCTGTTTTCCCTCGGGTCATGCGATATCTCTGAGGAATACTCGTTT

GCATGCGTCCAGCGCGGCGAGCCCAGACCGACGGTTTTGGAGTTTGACAGCGAGTACGAG

CTGCTGGTTGCCTTCCTGACCTTTCTCAAGCAGTACTCTCCCGAGTTCGCCACCGGCTAC

AACATCGTTAATTTTGACTGGGCGTACATAGTTAACAAGGTAACGTCGGTGTATAACATC

AAGCTGGACGGGTACGGCAAGTTCAACAAAGGGGGGCTGTTTAAGGTGTGGGACATCGCC

ACGAACCATTTTCAGAAGAAGAGCAAGGTGAAAATCAATGGCCTGATATCTCTAGACATG

TATTCTGTGGCGACGGAAAAGCTAAAGCTACCCAGCTACAAACTCGACGCGGTCGTGGGA

GACGTCCTCGGCGAGCATAAGATAGACCTTCCCTATAAAGAAATACCCTCCTATTACGCG

GGAGGGCCTGACCGGAGGGGCGTAATAGGAGAGTATTGTATCCAGGACTCTAGGCTGGTG

GGCAAGCTGTTTTTTAAGTACCTCCCCCATCTGGAACTATCGGCGGTGGCCAAACTCGCC

CGTATCACCCTAACGCGGGTAATTTTTGACGGTCAGCAAATTCGCGTGTACACGTGCTTG

CTGAAACTCGCCCGCGAGAGAAATTTCATTTTGCCAGACAACAGACGCCGGTTTGACAGT

CAGGCAGACGCCGCGTCAGAGACTTCGGAGTTGGCTATGGATAGCCAAAGCCACGCCTTC

GACAGTACAGACGAACCCGACGGTGTGGACGGTACCCCGGACGCCGCAGGATCTGGCGCT

ACTTCTGAAAACGGAGGCGGGAAGCCCGGCGTCGGGAGGGCCGTGGGCTACCAGGGAGCA

AAGGTTCTAGACCCCGTATCCGGCTTTCATGTGGACCCCGTGGTTGTGTTTGACTTCGCT

AGCTTATACCCAAGCATTATCCAGGCCCATAACCTCTGTTTCACCACCCTGGCGCTCGAT

GAAGTGGATCTGGCCGGGCTTCAACCATCCGTCAACTACTCGACGTTCGAGGTGGGTGAC

CAAAAGTTATTTTTTGTCCACGCCCATATTCGCGAAAGCCTGCTTGGCATCTTGCTGCGC

GACTGGCTGGCCATGCGAAAGGCGGTGAGGGCGCGAATCCCCACCAGCACCCCCGAGGAG

GCAGTTTTACTAGATAAGCAGCAGTCTGCGATTAAGGTGATATGCAACTCGGTTTACGGA

TTCACGGGGGTGGCAAACGGCCTGTTGCCGTGTCTGAGGATAGCGGCTACCGTTACCACG

ATAGGACGCGACATGCTCCTCAAGACCCGAGATTACGTTCACTCTCGTTGGGCGACGCGC

GAGCTGCTGGAGGACAATTTTCCAGGGGCTATAGGTTTCCGAAACCACAAGCCTTACTCC

GTCAGGGTTATCTACGGAGACACCGACTCCGTGTTTATCAAGTTTGTGGGCCTGACGTAC

GAGGGGGTATCGGAGCTGGGGGATGCTATGTCGCGTCAGATTTCAGCGGACCTCTTTAGA

GCGCCCATCAAACTGGAGTGTGAGAAGACCTTTCAGCGACTGCTGCTGATCACCAAGAAG

AAGTACATAGGTGTCATAAACGGGGGGAAGATGCTCATGAAGGGGGTCGACCTGGTCCGC

AAAAATAACTGCTCTTTCATAAACTTGTACGCGCGACATCTGGTAGATCTTTTGTTGTAC

GACGAGGATGTGGCCACGGCGGCAGCAGAGGTGACAGACGTGCCTCCCGCAGAATGGGTG

GGGCGCCCGCTACCGAGCGGCTTTGACAAGTTTGGGCGAGTGCTGGTAGAGGCGTACAAC

CGTATCACTGCCCCCAACTTGGACGTGCGCGAGTTCGTTATGACTGCTGAGCTGAGCCGC

TCACCCGAATCGTATACCAACAAGCGCCTGCCGCACCTCACCGTCTACTTTAAGCTCGCC

ATGAGGAATGAAGAACTGCCCAGCGTAAAAGAGAGAATTCCGTATGTGATAGTTGCGCAG

ACCGAGGCCGCGGAACGCGAAGCGGGTGTAGTAAACTCAATGCGCGGTACCGCCCAAAAC

CCCGTGGTAACCAAGACCGCACGCCCCCAACCTAAACGCAAACTGCTGGTTTCCGACCTC

GCCGAAGACCCGACCTATGTTTCCGAGAATGACGTACCGCTAAACACAGACTACTATTTC

TCCCACCTGTTGGGTACCATAAGCGTGACCTTTAAGGCTCTATTCGGAAATGATGTGAGA

ACAACAGAAAATCTTTTAAAGCGGTTTATTCCGGAAACCCCCCACAAGACCCCCACGAAA

ACCCAGGCACTGCTTGAGCGCGCCGGCTTTGAAAAGCTGACGCCCTTTACACCGGAGGAA

GAAAGTCGTCGAATACTGCATACAGTTTTTTGTACTCTAGAAGCAGCTCCCCATCAAAGC

TGA

>KC924809.1

ATGGCGGCGCGCGAACAGGCCAACTCCGTTCGCAGGAGCGGGTTTTTTAATCCGTTTATT

GGCAAGAGGCCATTTTTCAGGCCGGGAAGCGGGCAGACCGCGGAGACAGAGCGCCCAAGG

CCCCCCCAACACTCGTACTGCACAGAGGTGGGTAGCTTTAAGTTTATAGCTCCAAGATGC

CTCGATGAAGAAGCCCCCGCCGACCAGCGACGCGGTGTACACGTGGGCACCTTGGAGCGC

CCACCCAAAGTGTACTGCGATGGCTCAGAGTACGACGTGCTGAACTTTGCCTCCGGAGGT

TGCTGGCCTCGCAGGATTCGGGTTTGGAACGGCCAGGATTTTCGGGGCGATGGATTCAAC

CCCAGATTTGAGAGATTTCACGTGTACGACATAGTAGAGACTTCGGAGAGCGCCTCACAC

GATGACCCATCCAGGTTTGCTGAGCTATCCCGCCCAAGCGGGTCTGTGGTAACACTGCTG

GGAATGAGCGAGTGTGGAAAGCGAGTCGCGGTTCACGTCTATGGTGTGCGCCATTATTTT

TACATGGCAAAGGCGGAGGTGGATAGCGCTTGTGGAATAACCACCGAGGCAGAACTCGTG

CGCGCAATGGTGGACTGCGCGCACAGCTCGGCTTTGAGCGCGGCCCTGGGAAATGGCAAC

GGCGGCAAACAGAGCGGCGGCAGCGGCGGGGGATGGTGGGGCGGAAAGCACGTGTCTGCG

GACTGCTTCAAAGTGGAGACCGTGTGCCACACGACGCTGTACTACTTTGGATCTAAGCCA

GCTCTCTACTATAGAGTATCTGCCTCCAGCAGCCGCCTGGGAGGGTTCATCTGCGACAAC

TTTCACCCGGAGATTACAAAATTCGAGGGGAGCGTGGACGTGACCACGCGGCTGTTGTTG

GACAATGAAAATTTTACCAGTTTCGGGTGGTACCGCCTGCGACCCGGCACCCACGGAGAG

CGTGTTCAACTTCGCCCCGTTGAGCGACACGTCACCTCAAGCGACGTGGAGATTAACTGT

ACTCCCGATAACCTGGAGCCGATACCAGACGAGGCTGCCTGGCCCGACTATAAGCTCATG

TGCTTTGATATAGAGTGTAAAGCTGGAACGGGTAACGAAATGGCGTTCCCAGTGGCAACT

AACCAAGAGGACCTGGTCATCCAGATCTCCTGTCTGCTGTACTCGCTTGCTACTCAGAAC

CACGAACACACCCTGCTGTTTTCCCTCGGGTCATGCGATATCTCTGAGGAATACTCGTTT

GCATGCGTCCAGCGCGGCGAGCCCAGACCGACGGTTTTGGAGTTTGACAGCGAGTACGAG

CTGCTGGTTGCCTTCCTGACCTTTCTCAAGCAGTACTCTCCCGAGTTCGCCACCGGCTAC

AACATCGTTAATTTTGACTGGGCGTACATAGTTAACAAGGTAACGTCGGTGTATAACATC

AAGCTGGACGGGTACGGCAAGTTCAACAAAGGGGGGCTGTTTAAGGTGTGGGACATCGCC

ACGAACCATTTTCAGAAGAAGAGCAAGGTGAAAATCAATGGCCTGATATCTCTAGACATG

TATTCTGTGGCGACGGAAAAGCTAAAGCTACCCAGCTACAAACTCGACGCGGTCGTGGGA

GACGTCCTCGGCGAGCATAAGATAGACCTTCCCTATAAAGAAATACCCTCCTATTACGCG

GGAGGGCCTGACCGGAGGGGCGTAATAGGAGAGTATTGTATCCAGGACTCTAGGCTGGTG

GGCAAGCTGTTTTTTAAGTACCTCCCCCATCTGGAACTATCGGCGGTGGCCAAACTCGCC

CGTATCACCCTAACGCGGGTAATTTTTGACGGTCAGCAAATTCGCGTGTACACGTGCTTG

CTGAAACTCGCCCGCGAGAGAAATTTCATTTTGCCAGACAACAGACGCCGGTTTGACAGT

CAGGCAGACGCCGCGTCAGAGACTTCGGAGTTGGCTATGGATAGCCAAAGCCACGCCTTC

GACAGTACAGACGAACCCGACGGTGTGGACGGTACCCCGGACGCCGCAGGATCTGGCGCT

ACTTCTGAAAACGGAGGCGGGAAGCCCGGCGTCGGGAGGGCCGTGGGCTACCAGGGAGCA

AAGGTTCTAGACCCCGTATCCGGCTTTCATGTGGACCCCGTGGTTGTGTTTGACTTCGCT

AGCTTATACCCAAGCATTATCCAGGCCCATAACCTCTGTTTCACCACCCTGGCGCTCGAT

GAAGTGGATCTGGCCGGGCTTCAACCATCCGTCGACTACTCGACGTTCGAGGTGGGTGAC

CAAAAGTTATTTTTTGTCCACGCCCATATTCGCGAAAGCCTGCTTGGCATCTTGCTGCGC

GACTGGCTGGCCATGCGAAAGGCGGTGAGGGCGCGAATCCCCACCAGCACCCCCGAGGAG

GCAGTTTTACTAGATAAGCAGCAGTCTGCGATTAAGGTGATATGCAACTCGGTTTACGGA

TTCACGGGGGTGGCAAACGGCCTGTTGCCGTGTCTGAGGATAGCGGCTACCGTTACCACG

ATAGGACGCGACATGCTCCTCAAGACCCGAGATTACGTTCACTCTCGTTGGGCGACGCGC

GAGCTGCTGGAGGACAATTTTCCAGGGGCTATAGGTTTCCGAAACCACAAGCCTTACTCC

GTCAGGGTTATCTACGGAGACACCGACTCCGTGTTTATCAAGTTTGTGGGCCTGACGTAC

GAGGGGGTATCGGAGCTGGGGGATGCTATGTCGCGTCAGATTTCAGCGGACCTCTTTAGA

GCGCCCATCAAACTGGAGTGTGAGAAGACCTTTCAGCGACTGCTGCTGATCACCAAGAAG

AAGTACATAGGTGTCATAAACGGGGGGAAGATGCTCATGAAGGGGGTCGACCTGGTCCGC

AAAAATAACTGCTCTTTCATAAACTTGTACGCGCGACATCTGGTAGATCTTTTGTTGTAC

GACGAGGATGTGGCCACGGCGGCAGCAGAGGTGACAGACGTGCCTCCCGCAGAATGGGTG

GGGCGCCCGCTACCGAGCGGCTTTGACAAGTTTGGGCGAGTGCTGGTAGAGGCGTACAAC

CGTATCACTGCCCCCAACTTGGACGTGCGCGAGTTCGTTATGACTGCTGAGCTGAGCCGC

TCACCCGAATCGTATACCAACAAGCGCCTGCCGCACCTCACCGTCTACTTTAAGCTCGCC

ATGAGGAATGAAGAACTGCCCAGCGTAAAAGAGAGAATTCCGTATGTGATAGTTGCGCAG

ACCGAGGCCGCGGAACGCGAAGCGGGTGTAGTAAACTCAATGCGCGGTACCGCCCAAAAC

CCCGTGGTAACCAAGACCGCACGCCCCCAACCTAAACGCAAACTGCTGGTTTCCGACCTC

GCCGAAGACCCGACCTATGTTTCCGAGAATGACGTACCGCTAAACACAGACTACTATTTC

TCCCACCTGTTGGGTACCATAAGCGTGACCTTTAAGGCTCTATTCGGAAATGATGTGAGA

ACAACAGAAAATCTTTTAAAGCGGTTTATTCCGGAAACCCCCCACAAGACCCCCACGAAA

ACCCAGGCACTGCTTGAGCGCGCCGGCTTTGAAAAGCTGACGCCCTTTACACCGGAGGAA

GAAAGTCGTCGAATACTGCATACAGTTTTTTGTACTCTAGAAGCAGCTCCCCATCAAAGC

TGA

>KC924810.1

ATGGCGGCGCGCGAACAGGCCAACTCCGTTCGCAGGAGCGGGTTTTTTAATCCGTTTATT

GGCAAGAGGCCATTTTTCAGGCCGGGAAGCGGGCAGACCGCGGAGACAGAGCGCCCAAGG

CCCCCCCAACACTCGTACTGCACAGAGGTGGGTAGCTTTAAGTTTATAGCTCCAAGATGC

CTCGATGAAGAAGCCCCCGCCGACCAGCGACGCGGTGTACACGTGGGCACCTTGGAGCGC

CCACCCAAAGTGTACTGCGATGGCTCAGAGTACGACGTGCTGAACTTTGCCTCCGGAGGT

TGCTGGCCTCGCAGGATTCGGGTTTGGAACGGCCAGGATTTTCGGGGCGATGGATTCAAC

CCCAGATTTGAGAGATTTCACGTGTACGACATAGTAGAGACTTCGGAGAGCGCCTCACAC

GATGACCCATCCAGGTTTGCTGAGCTATCCCGCCCAAGCGGGTCTGTGGTAACACTGCTG

GGAATGAGCGAGTGTGGAAAGCGAGTCGCGGTTCACGTCTATGGTGTGCGCCATTATTTT

TACATGGCAAAGGCGGAGGTGGATAGCGCTTGTGGAATAACCACCGAGGCAGAACTCGTG

CGCGCAATGGTGGACTGCGCGCACAGCTCGGCTTTGAGCGCGGCCCTGGGAAATGGCAAC

GGCGGCAAACAGAGCGGCGGCAGCGGCGGGGGATGGTGGGGCGGAAAGCACGTGTCTGCG

GACTGCTTCAAAGTGGAGACCGTGTGCCACACGACGCTGTACTACTTTGGATCTAAGCCA

GCTCTCTACTATAGAGTATCTGCCTCCAGCAGCCGCCTGGGAGGGTTCATCTGCGACAAC

TTTCACCCGGAGATTACAAAATTCGAGGGGAGCGTGGACGTGACCACGCGGCTGTTGTTG

GACAATGAAAATTTTACCAGTTTCGGGTGGTACCGCCTGCGACCCGGCACCCACGGAGAG

CGTGTTCAACTTCGCCCCGTTGAGCGACACGTCACCTCAAGCGACGTGGAGATTAACTGT

ACTCCCGATAACCTGGAGCCGATACCAGACGAGGCTGCCTGGCCCGACTATAAGCTCATG

TGCTTTGATATAGAGTGTAAAGCTGGAACGGGTAACGAAATGGCGTTCCCAGTGGCAACT

AACCAAGAGGACCTGGTCATCCAGATCTCCTGTCTGCTGTACTCGCTTGCTACTCAGAAC

CACGAACACACCCTGCTGTTTTCCCTCGGGTCATGCGATATCTCTGAGGAATACTCGTTT

GCATGCGTCCAGCGCGGCGAGCCCAGACCGACGGTTTTGGAGTTTGACAGCGAGTACGAG

CTGCTGGTTGCCTTCCTGACCTTTCTCAAGCAGTACTCTCCCGAGTTCGCCACCGGCTAC

AACATCGTTAATTTTGACTGGGCGTACATAGTTAACAAGGTAACGTCGGTGTATAACATC

AAGCTGGACGGGTACGGCAAGTTCAACAAAGGGGGGCTGTTTAAGGTGTGGGACATCGCC

ACGAACCATTTTCAGAAGAAGAGCAAGGTGAAAATCAATGGCCTGATATCTCTAGACATG

TATTCTGTGGCGACGGAAAAGCTAAAGCTACCCAGCTACAAACTCGACGCGGTCGTGGGA

GACGTCCTCGGCGAGCATAAGATAGACCTTCCCTATAAAGAAATACCCTCCTATTACGCG

GGAGGGCCTGACCGGAGGGGCGTAATAGGAGAGTATTGTATCCAGGACTCTAGGCTGGTG

GGCAAGCTGTTTTTTAAGTACCTCCCCCATCTGGAACTATCGGCGGTGGCCAAACTCGCC

CGTATCACCCTAACGCGGGTAATTTTTGACGGTCAGCAAATTCGCGTGTACACGTGCTTG

CTGAAACTCGCCCGCGAGAGAAATTTCATTTTGCCAGACAACAGACGCCGGTTTGACAGT

CAGGCAGACGCCGCGTCAGAGACTTCGGAGTTGGCTATGGATAGCCAAAGCCACGCCTTC

GACAGTACAGACGAACCCGACGGTGTGGACGGTACCCCGGACGCCGCAGGATCTGGCGCT

ACTTCTGAAAACGGAGGCGGGAAGCCCGGCGTCGGGAGGGCCGTGGGCTACCAGGGAGCA

AAGGTTCTAGACCCCGTATCCGGCTTTCATGTGGACCCCGTGGTTGTGTTTGACTTCGCT

AGCTTATACCCAAGCATTATCCAGGCCCATAACCTCTGTTTCACCACCCTGGCGCTCGAT

GAAGTGGATCTGGCCGGGCTTCAACCATCCGTCGACTACTCGACGTTCGAGGTGGGTGAC

CAAAAGTTATTTTTTGTCCACGCCCATATTCGCGAAAGCCTGCTTGGCATCTTGCTGCGC

GACTGGCTGGCCATGCGAAAGGCGGTGAGGGCGCGAATCCCCACCAGCACCCCCGAGGAG

GCAGTTTTACTAGATAAGCAGCAGTCTGCGATTAAGGTGATATGCAACTCGGTTTACGGA

TTCACGGGGGTGGCAAACGGCCTGTTGCCGTGTCTGAGGATAGCGGCTACCGTTACCACG

ATAGGACGCGACATGCTCCTCAAGACCCGAGATTACGTTCACTCTCGTTGGGCGACGCGC

GAGCTGCTGGAGGACAATTTTCCAGGGGCTATAGGTTTCCGAAACCACAAGCCTTACTCC

GTCAGGGTTATCTACGGAGACACCGACTCCGTGTTTATCAAGTTTGTGGGCCTGACGTAC

GAGGGGGTATCGGAGCTGGGGGATGCTATGTCGCGTCAGATTTCAGCGGACCTCTTTAGA

GCGCCCATCAAACTGGAGTGTGAGAAGACCTTTCAGCGACTGCTGCTGATCACCAAGAAG

AAGTACATAGGTGTCATAAACGGGGGGAAGATGCTCATGAAGGGGGTCGACCTGGTCCGC

AAAAATAACTGCTCTTTCATAAACTTGTACGCGCGACATCTGGTAGATCTTTTGTTGTAC

GACGAGGATGTGGCCACGGCGGCAGCAGAGGTGACAGACGTGCCTCCCGCAGAATGGGTG

GGGCGCCCGCTACCGAGCGGCTTTGACAAGTTTGGGCGAGTGCTGGTAGAGGCGTACAAC

CGTATCACTGCCCCCAACTTGGACGTGCGCGAGTTCGTTATGACTGCTGAGCTGAGCCGC

TCACCCGAATCGTATACCAACAAGCGCCTGCCGCACCTCACCGTCTACTTTAAGCTCGCC

ATGAGGAATGAAGAACTGCCCAGCGTAAAAGAGAGAATTCCGTATGTGATAGTTGCGCAG

ACCGAGGCCGCGGAACGCGAAGCGGGTGTAGTAAACTCAATGCGCGGTACCGCCCAAAAC

CCCGTGGTAACCAAGACCGCACGCCCCCAACCTAAACGCAAACTGCTGGTTTCCGACCTC

GCCGAAGACCCGACCTATGTTTCCGAGAATGACGTACCGCTAAACACAGACTACTATTTC

TCCCACCTGTTGGGTACCATAAGCGTGACCTTTAAGGCTCTATTCGGAAATGATGTGAGA

ACAACAGAAAATCTTTTAAAGCGGTTTATTCCGGAAACCCCCCACAAGACCCCCACGAAA

ACCCAGGCACTGCTTGAGCGCGCCGGCTTTGAAAAGCTGACGCCCTTTACACCGGAGGAA

GAAAGTCGTCGAATACTGCATACAGTTTTTTGTACTCTAGAAGCAGCTCCCCATCAAAGC

TGA

>KC924812.1

ATGGCGGCGCGCGAACAGGCCAACTCCGTTCGCAGGAGCGGGTTTTTTAATCCGTTTATT

GGCAAGAGGCCATTTTTCAGGCCGGGAAGCGGGCAGACCGCGGAGACAGAGCGCCCAAGG

CCCCCCCAACACTCGTACTGCACAGAGGTGGGTAGCTTTAAGTTTATAGCTCCAAGATGC

CTCGATGAAGAAGCCCCCGCCGACCAGCGACGCGGTGTACACGTGGGCACCTTGGAGCGC

CCACCCAAAGTGTACTGCGATGGCTCAGAGTACGACGTGCTGAACTTTGCCTCCGGAGGT

TGCTGGCCTCGCAGGATTCGGGTTTGGAACGGCCAGGATTTTCGGGGCGATGGATTCAAC

CCCAGATTTGAGAGATTTCACGTGTACGACATAGTAGAGACTTCGGAGAGCGCCTCACAC

GATGACCCATCCAGGTTTGCTGAGCTATCCCGCCCAAGCGGGTCTGTGGTAACACTGCTG

GGAATGAGCGAGTGTGGAAAGCGAGTCGCGGTTCACGTCTATGGTGTGCGCCATTATTTT

TACATGGCAAAGGCGGAGGTGGATAGCGCTTGTGGAATAACCACCGAGGCAGAACTCGTG

CGCGCAATGGTGGACTGCGCGCACAGCTCGGCTTTGAGCGCGGCCCTGGGAAATGGCAAC

GGCGGCAAACAGAGCGGCGGCAGCGGCGGGGGATGGTGGGGCGGAAAGCACGTGTCTGCG

GACTGCTTCAAAGTGGAGACCGTGTGCCACACGACGCTGTACTACTTTGGATCTAAGCCA

GCTCTCTACTATAGAGTATCTGCCTCCAGCAGCCGCCTGGGAGGGTTCATCTGCGACAAC

TTTCACCCGGAGATTACAAAATTCGAGGGGAGCGTGGACGTGACCACGCGGCTGTTGTTG

GACAATGAAAATTTTACCAGTTTCGGGTGGTACCGCCTGCGACCCGGCACCCACGGAGAG

CGTGTTCAACTTCGCCCCGTTGAGCGACACGTCACCTCAAGCGACGTGGAGATTAACTGT

ACTCCCGATAACCTGGAGCCGATACCAGACGAGGCTGCCTGGCCCGACTATAAGCTCATG

TGCTTTGATATAGAGTGTAAAGCTGGAACGGGTAACGAAATGGCGTTCCCAGTGGCAACT

AACCAAGAGGACCTGGTCATCCAGATCTCCTGTCTGCTGTACTCGCTTGCTACTCAGAAC

CACGAACACACCCTGCTGTTTTCCCTCGGGTCATGCGATATCTCTGAGGAATACTCGTTT

GCATGCGTCCAGCGCGGCGAGCCCAGACCGACGGTTTTGGAGTTTGACAGCGAGTACGAG

CTGCTGGTTGCCTTCCTGACCTTTCTCAAGCAGTACTCTCCCGAGTTCGCCACCGGCTAC

AACATCGTTAATTTTGACTGGGCGTACATAGTTAACAAGGTAACGTCGGTGTATAACATC

AAGCTGGACGGGTACGGCAAGTTCAACAAAGGGGGGCTGTTTAAGGTGTGGGACATCGCC

ACGAACCATTTTCAGAAGAAGAGCAAGGTGAAAATCAATGGCCTGATATCTCTAGACATG

TATTCTGTGGCGACGGAAAAGCTAAAGCTACCCAGCTACAAACTCGACGCGGTCGTGGGA

GACGTCCTCGGCGAGCATAAGATAGACCTTCCCTATAAAGAAATACCCTCCTATTACGCG

GGAGGGCCTGACCGGAGGGGCGTAATAGGAGAGTATTGTATCCAGGACTCTAGGCTGGTG

GGCAAGCTGTTTTTTAAGTACCTCCCCCATCTGGAACTATCGGCGGTGGCCAAACTCGCC

CGTATCACCCTAACGCGGGTAATTTTTGACGGTCAGCAAATTCGCGTGTACACGTGCTTG

CTGAAACTCGCCCGCGAGAGAAATTTCATTTTGCCAGACAACAGACGCCGGTTTGACAGT

CAGGCAGACGCCGCGTCAGAGACTTCGGAGTTGGCTATGGATAGCCAAAGCCACGCCTTC

GACAGTACAGACGAACCCGACGGTGTGGACGGTACCCCGGACGCCGCAGGATCTGGCGCT

ACTTCTGAAAACGGAGGCGGGAAGCCCGGCGTCGGGAGGGCCGTGGGCTACCAGGGAGCA

AAGGTTCTAGACCCCGTATCCGGCTTTCATGTGGACCCCGTGGTTGTGTTTGACTTCGCT

AGCTTATACCCAAGCATTATCCAGGCCCATAACCTCTGTTTCACCACCCTGGCGCTCGAT

GAAGTGGATCTGGCCGGGCTTCAACCATCCGTCGACTACTCGACGTTCGAGGTGGGTGAC

CAAAAGTTATTTTTTGTCCACGCCCATATTCGCGAAAGCCTGCTTGGCATCTTGCTGCGC

GACTGGCTGGCCATGCGAAAGGCGGTGAGGGCGCGAATCCCCACCAGCACCCCCGAGGAG

GCAGTTTTACTAGATAAGCAGCAGTCTGCGATTAAGGTGATATGCAACTCGGTTTACGGA

TTCACGGGGGTGGCAAACGGCCTGTTGCCGTGTCTGAGGATAGCGGCTACCGTTACCACG

ATAGGACGCGACATGCTCCTCAAGACCCGAGATTACGTTCACTCTCGTTGGGCGACGCGC

GAGCTGCTGGAGGACAATTTTCCAGGGGCTATAGGTTTCCGAAACCACAAGCCTTACTCC

GTCAGGGTTATCTACGGAGACACCGACTCCGTGTTTATCAAGTTTGTGGGCCTGACGTAC

GAGGGGGTATCGGAGCTGGGGGATGCTATGTCGCGTCAGATTTCAGCGGACCTCTTTAGA

GCGCCCATCAAACTGGAGTGTGAGAAGACCTTTCAGCGACTGCTGCTGATCACCAAGAAG

AAGTACATAGGTGTCATAAACGGGGGGAAGATGCTCATGAAGGGGGTCGACCTAGTCCGC

AAAAATAACTGCTCTTTCATAAACTTGTACGCGCGACATCTGGTAGATCTTTTGTTGTAC

GACGAGGATGTGGCCACGGCGGCAGCAGAGGTGACAGACGTGCCTCCCGCAGAATGGGTG

GGGCGCCCGCTACCGAGCGGCTTTGACAAGTTTGGGCGAGTGCTGGTAGAGGCGTACAAC

CGTATCACTGCCCCCAACTTGGACGTGCGCGAGTTCGTTATGACTGCTGAGCTGAGCCGC

TCACCCGAATCGTATACCAACAAGCGCCTGCCGCACCTCACCGTCTACTTTAAGCTCGCC

ATGAGGAATGAAGAACTGCCCAGCGTAAAAGAGAGAATTCCGTATGTGATAGTTGCGCAG

ACCGAGGCCGCGGAACGCGAAGCGGGTGTAGTAAACTCAATGCGCGGTACCGCCCAAAAC

CCCGTGGTAACCAAGACCGCACGCCCCCAACCTAAACGCAAACTGCTGGTTTCCGACCTC

GCCGAAGACCCGACCTATGTTTCCGAGAATGACGTACCGCTAAACACAGACTACTATTTC

TCCCACCTGTTGGGTACCATAAGCGTGACCTTTAAGGCTCTATTCGGAAATGATGTGAGA

ACAACAGAAAATCTTTTAAAGCGGTTTATTCCGGAAACCCCCCACAAGACCCCCACGAAA

ACCCAGGCACTGCTTGAGCGCGCCGGCTTTGAAAAGCTGACGCCCTTTACACCGGAGGAA

GAAAGTCGTCGAATACTGCATACAGTTTTTTGTACTCTAGAAGCAGCTCCCCATCAAAGC

TGA

>KC924813.1

ATGGCGGCGCGCGAACAGGCCAACTCCGTTCGCAGGAGCGGGTTTTTTAATCCGTTTATT

GGCAAGAGGCCATTTTTCAGGCCGGGAAGCGGGCAGACCGCGGAGACAGAGCGCCCAAGG

CCCCCCCAACACTCGTACTGCACAGAGGTGGGTAGCTTTAAGTTTATAGCTCCAAGATGC

CTCGATGAAGAAGCCCCCGCCGACCAGCGACGCGGTGTACACGTGGGCACCTTGGAGCGC

CCACCCAAAGTGTACTGCGATGGCTCAGAGTACGACGTGCTGAACTTTGCCTCCGGAGGT

TGCTGGCCTCGCAGGATTCGGGTTTGGAACGGCCAGGATTTTCGGGGCGATGGATTCAAC

CCCAGATTTGAGAGATTTCACGTGTACGACATAGTAGAGACTTCGGAGAGCGCCTCACAC

GATGACCCATCCAGGTTTGCTGAGCTATCCCGCCCAAGCGGGTCTGTGGTAACACTGCTG

GGAATGAGCGAGTGTGGAAAGCGAGTCGCGGTTCACGTCTATGGTGTGCGCCATTATTTT

TACATGGCAAAGGCGGAGGTGGATAGCGCTTGTGGAATAACCACCGAGGCAGAACTCGTG

CGCGCAATGGTGGACTGCGCGCACAGCTCGGCTTTGAGCGCGGCCCTGGGAAATGGCAAC

GGCGGCAAACAGAGCGGCGGCAGCGGCGGGGGATGGTGGGGCGGAAAGCACGTGTCTGCG

GACTGCTTCAAAGTGGAGACCGTGTGCCACACGACGCTGTACTACTTTGGATCTAAGCCA

GCTCTCTACTATAGAGTATCTGCCTCCAGCAGCCGCCTGGGAGGGTTCATCTGCGACAAC

TTTCACCCGGAGATTACAAAATTCGAGGGGAGCGTGGACGTGACCACGCGGCTGTTGTTG

GACAATGAAAATTTTACCAGTTTCGGGTGGTACCGCCTGCGACCCGGCACCCACGGAGAG

CGTGTTCAACTTCGCCCCGTTGAGCGACACGTCACCTCAAGCGACGTGGAGATTAACTGT

ACTCCCGATAACCTGGAGCCGATACCAGACGAGGCTGCCTGGCCCGACTATAAGCTCATG

TGCTTTGATATAGAGTGTAAAGCTGGAACGGGTAACGAAATGGCGTTCCCAGTGGCAACT

AACCAAGAGGACCTGGTCATCCAGATCTCCTGTCTGCTGTACTCGCTTGCTACTCAGAAC

CACGAACACACCCTGCTGTTTTCCCTCGGGTCATGCGATATCTCTGAGGAATACTCGTTT

GCATGCGTCCAGCGCGGCGAGCCCAGACCGACGGTTTTGGAGTTTGACAGCGAGTACGAG

CTGCTGGTTGCCTTCCTGACCTTTCTCAAGCAGTACTCTCCCGAGTTCGCCACCGGCTAC

AACATCGTTAATTTTGACTGGGCGTACATAGTTAACAAGGTAACGTCGGTGTATAACATC

AAGCTGGACGGGTACGGCAAGTTCAACAAAGGGGGGCTGTTTAAGGTGTGGGACATCGCC

ACGAACCATTTTCAGAAGAAGAGCAAGGTGAAAATCAATGGCCTGATATCTCTAGACATG

TATTCTGTGGCGACGGAAAAGCTAAAGCTACCCAGCTACAAACTCGACGCGGTCGTGGGA

GACGTCCTCGGCGAGCATAAGATAGACCTTCCCTATAAAGAAATACCCTCCTATTACGCG

GGAGGGCCTGACCGGAGGGGCGTAATAGGAGAGTATTGTATCCAGGACTCTAGGCTGGTG

GGCAAGCTGTTTTTTAAGTACCTCCCCCATCTGGAACTATCGGCGGTGGCCAAACTCGCC

CGTATCACCCTAACGCGGGTAATTTTTGACGGTCAGCAAATTCGCGTGTACACGTGCTTG

CTGAAACTCGCCCGCGAGAGAAATTTCATTTTGCCAGACAACAGACGCCGGTTTGACAGT

CAGGCAGACGCCGCGTCAGAGACTTCGGAGTTGGCTATGGATAGCCAAAGCCACGCCTTC

GACAGTACAGACGAACCCGACGGTGTGGACGGTACCCCGGACGCCGCAGGATCTGGCGCT

ACTTCTGAAAACGGAGGCGGGAAGCCCGGCGTCGGGAGGGCCGTGGGCTACCAGGGAGCA

AAGGTTCTAGACCCCGTATCCGGCTTTCATGTGGACCCCGTGGTTGTGTTTGACTTCGCT

AGCTTATACCCAAGCATTATCCAGGCCCATAACCTCTGTTTCACCACCCTGGCGCTCGAT

GAAGTGGATCTGGCCGGGCTTCAACCATCCGTCGACTACTCGACGTTCGAGGTGGGTGAC

CAAAAGTTATTTTTTGTCCACGCCCATATTCGCGAAAGCCTGCTTGGCATCTTGCTGCGC

GACTGGCTGGCCATGCGAAAGGCGGTGAGGGCGCGAATCCCCACCAGCACCCCCGAGGAG

GCAGTTTTACTAGATAAGCAGCAGTCTGCGATTAAGGTGATATGCAACTCGGTTTACGGA

TTCACGGGGGTGGCAAACGGCCTGTTGCCGTGTCTGAGGATAGCGGCTACCGTTACCACG

ATAGGACGCGACATGCTCCTCAAGACCCGAGATTACGTTCACTCTCGTTGGGCGACGCGC

GAGCTGCTGGAGGACAATTTTCCAGGGGCTATAGGTTTCCGAAACCACAAGCCTTACTCC

GTCAGGGTTATCTACGGAGACACCGACTCCGTGTTTATCAAGTTTGTGGGCCTGACGTAC

GAGGGGGTATCGGAGCTGGGGGATGCTATGTCGCGTCAGATTTCAGCGGACCTCTTTAGA

GCGCCCATCAAACTGGAGTGTGAGAAGACCTTTCAGCGACTGCTGCTGATCACCAAGAAG

AAGTACATAGGTGTCATAAACGGGGGGAAGATGCTCATGAAGGGGGTCGACCTAGTCCGC

AAAAATAACTGCTCTTTCATAAACTTGTACGCGCGACATCTGGTAGATCTTTTGTTGTAC

GACGAGGATGTGGCCACGGCGGCAGCAGAGGTGACAGACGTGCCTCCCGCAGAATGGGTG

GGGCGCCCGCTACCGAGCGGCTTTGACAAGTTTGGGCGAGTGCTGGTAGAGGCGTACAAC

CGTATCACTGCCCCCAACTTGGACGTGCGCGAGTTCGTTATGACTGCTGAGCTGAGCCGC

TCACCCGAATCGTATACCAACAAGCGCCTGCCGCACCTCACCGTCTACTTTAAGCTCGCC

ATGAGGAATGAAGAACTGCCCAGCGTAAAAGAGAGAATTCCGTATGTGATAGTTGCGCAG

ACCGAGGCCGCGGAACGCGAAGCGGGTGTAGTAAACTCAATGCGCGGTACCGCCCAAAAC

CCCGTGGTAACCAAGACCGCACGCCCCCAACCTAAACGCAAACTGCTGGTTTCCGACCTC

GCCGAAGACCCGACCTATGTTTCCGAGAATGACGTACCGCTAAACACAGACTACTATTTC

TCCCACCTGTTGGGTACCATAAGCGTGACCTTTAAGGCTCTATTCGGAAATGATGTGAGA

ACAACAGAAAATCTTTTAAAGCGGTTTATTCCGGAAACCCCCCACAAGACCCCCACGAAA

ACCCAGGCACTGCTTGAGCGCGCCGGCTTTGAAAAGCTGACGCCCTTTACACCGGAGGAA

GAAAGTCGTCGAATACTGCATACAGTTTTTTGTACTCTAGAAGCAGCTCCCCATCAAAGC

TGA

>KC924814.1

ATGGCGGCGCGCGAACAGGCCAACTCCGTTCGCAGGAGCGGGTTTTTTAATCCGTTTATT

GGCAAGAGGCCATTTTTCAGGCCGGGAAGCGGGCAGACCGCGGAGACAGAGCGCCCAAGG

CCCCCCCAACACTCGTACTGCACAGAGGTGGGTAGCTTTAAGTTTATAGCTCCAAGATGC

CTCGATGAAGAAGCCCCCGCCGACCAGCGACGCGGTGTACACGTGGGCACCTTGGAGCGC

CCACCCAAAGTGTACTGCGATGGCTCAGAGTACGACGTGCTGAACTTTGCCTCCGGAGGT

TGCTGGCCTCGCAGGATTCGGGTTTGGAACGGCCAGGATTTTCGGGGCGATGGATTCAAC

CCCAGATTTGAGAGATTTCACGTGTACGACATAGTAGAGACTTCGGAGAGCGCCTCACAC

GATGACCCATCCAGGTTTGCTGAGCTATCCCGCCCAAGCGGGTCTGTGGTAACACTGCTG

GGAATGAGCGAGTGTGGAAAGCGAGTCGCGGTTCACGTCTATGGTGTGCGCCATTATTTT

TACATGGCAAAGGCGGAGGTGGATAGCGCTTGTGGAATAACCACCGAGGCAGAACTCGTG

CGCGCAATGGTGGACTGCGCGCACAGCTCGGCTTTGAGCGCGGCCCTGGGAAATGGCAAC

GGCGGCAAACAGAGCGGCGGCAGCGGCGGGGGATGGTGGGGCGGAAAGCACGTGTCTGCG

GACTGCTTCAAAGTGGAGACCGTGTGCCACACGACGCTGTACTACTTTGGATCTAAGCCA

GCTCTCTACTATAGAGTATCTGCCTCCAGCAGCCGCCTGGGAGGGTTCATCTGCGACAAC

TTTCACCCGGAGATTACAAAATTCGAGGGGAGCGTGGACGTGACCACGCGGCTGTTGTTG

GACAATGAAAATTTTACCAGTTTCGGGTGGTACCGCCTGCGACCCGGCACCCACGGAGAG

CGTGTTCAACTTCGCCCCGTTGAGCGACACGTCACCTCAAGCGACGTGGAGATTAACTGT

ACTCCCGATAACCTGGAGCCGATACCAGACGAGGCTGCCTGGCCCGACTATAAGCTCATG

TGCTTTGATATAGAGTGTAAAGCTGGAACGGGTAACGAAATGGCGTTCCCAGTGGCAACT

AACCAAGAGGACCTGGTCATCCAGATCTCCTGTCTGCTGTACTCGCTTGCTACTCAGAAC

CACGAACACACCCTGCTGTTTTCCCTCGGGTCATGCGATATCTCTGAGGAATACTCGTTT

GCATGCGTCCAGCGCGGCGAGCCCAGACCGACGGTTTTGGAGTTTGACAGCGAGTACGAG

CTGCTGGTTGCCTTCCTGACCTTTCTCAAGCAGTACTCTCCCGAGTTCGCCACCGGCTAC

AACATCGTTAATTTTGACTGGGCGTACATAGTTAACAAGGTAACGTCGGTGTATAACATC

AAGCTGGACGGGTACGGCAAGTTCAACAAAGGGGGGCTGTTTAAGGTGTGGGACATCGCC

ACGAACCATTTTCAGAAGAAGAGCAAGGTGAAAATCAATGGCCTGATATCTCTAGACATG

TATTCTGTGGCGACGGAAAAGCTAAAGCTACCCAGCTACAAACTCGACGCGGTCGTGGGA

GACGTCCTCGGCGAGCATAAGATAGACCTTCCCTATAAAGAAATACCCTCCTATTACGCG

GGAGGGCCTGACCGGAGGGGCGTAATAGGAGAGTATTGTATCCAGGACTCTAGGCTGGTG

GGCAAGCTGTTTTTTAAGTACCTCCCCCATCTGGAACTATCGGCGGTGGCCAAACTCGCC

CGTATCACCCTAACGCGGGTAATTTTTGACGGTCAGCAAATTCGCGTGTACACGTGCTTG

CTGAAACTCGCCCGCGAGAGAAATTTCATTTTGCCAGACAACAGACGCCGGTTTGACAGT

CAGGCAGACGCCGCGTCAGAGACTTCGGAGTTGGCTATGGATAGCCAAAGCCACGCCTTC

GACAGTACAGACGAACCCGACGGTGTGGACGGTACCCCGGACGCCGCAGGATCTGGCGCT

ACTTCTGAAAACGGAGGCGGGAAGCCCGGCGTCGGGAGGGCCGTGGGCTACCAGGGAGCA

AAGGTTCTAGACCCCGTATCCGGCTTTCATGTGGACCCCGTGGTTGTGTTTGACTTCGCT

AGCTTATACCCAAGCATTATCCAGGCCCATAACCTCTGTTTCACCACCCTGGCGCTCGAT

GAAGTGGATCTGGCCGGGCTTCAACCATCCGTCGACTACTCGACGTTCGAGGTGGGTGAC

CAAAAGTTATTTTTTGTCCACGCCCATATTCGCGAAAGCCTGCTTGGCATCTTGCTGCGC

GACTGGCTGGCCATGCGAAAGGCGGTGAGGGCGCGAATCCCCACCAGCACCCCCGAGGAG

GCAGTTTTACTAGATAAGCAGCAGTCTGCGATTAAGGTGATATGCAACTCGGTTTACGGA

TTCACGGGGGTGGCAAACGGCCTGTTGCCGTGTCTGAGGATAGCGGCTACCGTTACCACG

ATAGGACGCGACATGCTCCTCAAGACCCGAGATTACGTTCACTCTCGTTGGGCGACGCGC

GAGCTGCTGGAGGACAATTTTCCAGGGGCTATAGGTTTCCGAAACCACAAGCCTTACTCC

GTCAGGGTTATCTACGGAGACACCGACTCCGTGTTTATCAAGTTTGTGGGCCTGACGTAC

GAGGGGGTATCGGAGCTGGGGGATGCTATGTCGCGTCAGATTTCAGCGGACCTCTTTAGA

GCGCCCATCAAACTGGAGTGTGAGAAGACCTTTCAGCGACTGCTGCTGATCACCAAGAAG

AAGTACATAGGTGTCATAAACGGGGGGAAGATGCTCATGAAGGGGGTCGACCTAGTCCGC

AAAAATAACTGCTCTTTCATAAACTTGTACGCGCGACATCTGGTAGATCTTTTGTTGTAC

GACGAGGATGTGGCCACGGCGGCAGCAGAGGTGACAGACGTGCCTCCCGCAGAATGGGTG

GGGCGCCCGCTACCGAGCGGCTTTGACAAGTTTGGGCGAGTGCTGGTAGAGGCGTACAAC

CGTATCACTGCCCCCAACTTGGACGTGCGCGAGTTCGTTATGACTGCTGAGCTGAGCCGC

TCACCCGAATCGTATACCAACAAGCGCCTGCCGCACCTCACCGTCTACTTTAAGCTCGCC

ATGAGGAATGAAGAACTGCCCAGCGTAAAAGAGAGAATTCCGTATGTGATAGTTGCGCAG

ACCGAGGCCGCGGAACGCGAAGCGGGTGTAGTAAACTCAATGCGCGGTACCGCCCAAAAC

CCCGTGGTAACCAAGACCGCACGCCCCCAACCTAAACGCAAACTGCTGGTTTCCGACCTC

GCCGAAGACCCGACCTATGTTTCCGAGAATGACGTACCGCTAAACACAGACTACTATTTC

TCCCACCTGTTGGGTACCATAAGCGTGACCTTTAAGGCTCTATTCGGAAATGATGTGAGA

ACAACAGAAAATCTTTTAAAGCGGTTTATTCCGGAAACCCCCCACAAGACCCCCACGAAA

ACCCAGGCACTGCTTGAGCGCGCCGGCTTTGAAAAGCTGACGCCCTTTACACCGGAGGAA

GAAAGTCGTCGAATACTGCATACAGTTTTTTGTACTCTAGAAGCAGCTCCCCATCAAAGC

TGA

>KC924815.1

ATGGCGGCGCGCGAACAGGCCAACTCCGTTCGCAGGAGCGGGTTTTTTAATCCGTTTATT

GGCAAGAGGCCATTTTTCAGGCCGGGAAGCGGGCAGACCGCGGAGACAGAGCGCCCAAGG

CCCCCCCAACACTCGTACTGCACAGAGGTGGGTAGCTTTAAGTTTATAGCTCCAAGATGC

CTCGATGAAGAAGCCCCCGCCGACCAGCGACGCGGTGTACACGTGGGCACCTTGGAGCGC

CCACCCAAAGTGTACTGCGATGGCTCAGAGTACGACGTGCTGAACTTTGCCTCCGGAGGT

TGCTGGCCTCGCAGGATTCGGGTTTGGAACGGCCAGGATTTTCGGGGCGATGGATTCAAC

CCCAGATTTGAGAGATTTCACGTGTACGACATAGTAGAGACTTCGGAGAGCGCCTCACAC

GATGACCCATCCAGGTTTGCTGAGCTATCCCGCCCAAGCGGGTCTGTGGTAACACTGCTG

GGAATGAGCGAGTGTGGAAAGCGAGTCGCGGTTCACGTCTATGGTGTGCGCCATTATTTT

TACATGGCAAAGGCGGAGGTGGATAGCGCTTGTGGAATAACCACCGAGGCAGAACTCGTG

CGCGCAATGGTGGACTGCGCGCACAGCTCGGCTTTGAGCGCGGCCCTGGGAAATGGCAAC

GGCGGCAAACAGAGCGGCGGCAGCGGCGGGGGATGGTGGGGCGGAAAGCACGTGTCTGCG

GACTGCTTCAAAGTGGAGACCGTGTGCCACACGACGCTGTACTACTTTGGATCTAAGCCA

GCTCTCTACTATAGAGTATCTGCCTCCAGCAGCCGCCTGGGAGGGTTCATCTGCGACAAC

TTTCACCCGGAGATTACAAAATTCGAGGGGAGCGTGGACGTGACCACGCGGCTGTTGTTG

GACAATGAAAATTTTACCAGTTTCGGGTGGTACCGCCTGCGACCCGGCACCCACGGAGAG

CGTGTTCAACTTCGCCCCGTTGAGCGACACGTCACCTCAAGCGACGTGGAGATTAACTGT

ACTCCCGATAACCTGGAGCCGATACCAGACGAGGCTGCCTGGCCCGACTATAAGCTCATG

TGCTTTGATATAGAGTGTAAAGCTGGAACGGGTAACGAAATGGCGTTCCCAGTGGCAACT

AACCAAGAGGACCTGGTCATCCAGATCTCCTGTCTGCTGTACTCGCTTGCTACTCAGAAC

CACGAACACACCCTGCTGTTTTCCCTCGGGTCATGCGATATCTCTGAGGAATACTCGTTT

GCATGCGTCCAGCGCGGCGAGCCCAGACCGACGGTTTTGGAGTTTGACAGCGAGTACGAG

CTGCTGGTTGCCTTCCTGACCTTTCTCAAGCAGTACTCTCCCGAGTTCGCCACCGGCTAC

AACATCGTTAATTTTGACTGGGCGTACATAGTTAACAAGGTAACGTCGGTGTATAACATC

AAGCTGGACGGGTACGGCAAGTTCAACAAAGGGGGGCTGTTTAAGGTGTGGGACATCGCC

ACGAACCATTTTCAGAAGAAGAGCAAGGTGAAAATCAATGGCCTGATATCTCTAGACATG

TATTCTGTGGCGACGGAAAAGCTAAAGCTACCCAGCTACAAACTCGACGCGGTCGTGGGA

GACGTCCTCGGCGAGCATAAGATAGACCTTCCCTATAAAGAAATACCCTCCTATTACGCG

GGAGGGCCTGACCGGAGGGGCGTAATAGGAGAGTATTGTATCCAGGACTCTAGGCTGGTG

GGCAAGCTGTTTTTTAAGTACCTCCCCCATCTGGAACTATCGGCGGTGGCCAAACTCGCC

CGTATCACCCTAACGCGGGTAATTTTTGACGGTCAGCAAATTCGCGTGTACACGTGCTTG

CTGAAACTCGCCCGCGAGAGAAATTTCATTTTGCCAGACAACAGACGCCGGTTTGACAGT

CAGGCAGACGCCGCGTCAGAGACTTCGGAGTTGGCTATGGATAGCCAAAGCCACGCCTTC

GACAGTACAGACGAACCCGACGGTGTGGACGGTACCCCGGACGCCGCAGGATCTGGCGCT

ACTTCTGAAAACGGAGGCGGGAAGCCCGGCGTCGGGAGGGCCGTGGGCTACCAGGGAGCA

AAGGTTCTAGACCCCGTATCCGGCTTTCATGTGGACCCCGTGGTTGTGTTTGACTTCGCT

AGCTTATACCCAAGCATTATCCAGGCCCATAACCTCTGTTTCACCACCCTGGCGCTCGAT

GAAGTGGATCTGGCCGGGCTTCAACCATCCGTCGACTACTCGACGTTCGAGGTGGGTGAC

CAAAAGTTATTTTTTGTCCACGCCCATATTCGCGAAAGCCTGCTTGGCATCTTGCTGCGC

GACTGGCTGGCCATGCGAAAGGCGGTGAGGGCGCGAATCCCCACCAGCACCCCCGAGGAG

GCAGTTTTACTAGATAAGCAGCAGTCTGCGATTAAGGTGATATGCAACTCGGTTTACGGA

TTCACGGGGGTGGCAAACGGCCTGTTGCCGTGTCTGAGGATAGCGGCTACCGTTACCACG

ATAGGACGCGACATGCTCCTCAAGACCCGAGATTACGTTCACTCTCGTTGGGCGACGCGC

GAGCTGCTGGAGGACAATTTTCCAGGGGCTATAGGTTTCCGAAACCACAAGCCTTACTCC

GTCAGGGTTATCTACGGAGACACCGACTCCGTGTTTATCAAGTTTGTGGGCCTGACGTAC

GAGGGGGTATCGGAGCTGGGGGATGCTATGTCGCGTCAGATTTCAGCGGACCTCTTTAGA

GCGCCCATCAAACTGGAGTGTGAGAAGACCTTTCAGCGACTGCTGCTGATCACCAAGAAG

AAGTACATAGGTGTCATAAACGGGGGGAAGATGCTCATGAAGGGGGTCGACCTGGTCCGC

AAAAATAACTGCTCTTTCATAAACTTGTACGCGCGACATCTGGTAGATCTTTTGTTGTAC

GACGAGGATGTGGCCACGGCGGCAGCAGAGGTGACAGACGTGCCTCCCGCAGAATGGGTG

GGGCGCCCGCTACCGAGCGGCTTTGACAAGTTTGGGCGAGTGCTGGTAGAGGCGTACAAC

CGTATCACTGCCCCCAACTTGGACGTGCGCGAGTTCGTTATGACTGCTGAGCTGAGCCGC

TCACCCGAATCGTATACCAACAAGCGCCTGCCGCACCTCACCGTCTACTTTAAGCTCGCC

ATGAGGAATGAAGAACTGCCCAGCGTAAAAGAGAGAATTCCGTATGTGATAGTTGCGCAG

ACCGAGGCCGCGGAACGCGAAGCGGGTGTAGTAAACTCAATGCGCGGTACCGCCCAAAAC

CCCGTGGTAACCAAGACCGCACGCCCCCAACCTAAACGCAAACTGCTGGTTTCCGACCTC

GCCGAAGACCCGACCTATGTTTCCGAGAATGACGTACCGCTAAACACAGACTACTATTTC

TCCCACCTGTTGGGTACCATAAGCGTGACCTTTAAGGCTCTATTCGGAAATGATGTGAGA

ACAACAGAAAATCTTTTAAAGCGGTTTATTCCGGAAACCCCCCACAAGACCCCCACGAAA

ACCCAGGCACTGCTTGAGCGCGCCGGCTTTGAAAAGCTGACGCCCTTTACACCGGAGGAA

GAAAGTCGTCGAATACTGCATACAGTTTTTTGTACTCTAGAAGCAGCTCCCCATCAAAGC

TGA

>KC924816.1

ATGGCGGCGCGCGAACAGGCCAACTCCGTTCGCAGGAGCGGGTTTTTTAATCCGTTTATT

GGCAAGAGGCCATTTTTCAGGCCGGGAAGCGGGCAGACCGCGGAGACAGAGCGCCCAAGG

CCCCCCCAACACTCGTACTGCACAGAGGTGGGTAGCTTTAAGTTTATAGCTCCAAGATGC

CTCGATGAAGAAGCCCCCGCCGACCAGCGACGCGGTGTACACGTGGGCACCTTGGAGCGC

CCACCCAAAGTGTACTGCGATGGCTCAGAGTACGACGTGCTGAACTTTGCCTCCGGAGGT

TGCTGGCCTCGCAGGATTCGGGTTTGGAACGGCCAGGATTTTCGGGGCGATGGATTCAAC

CCCAGATTTGAGAGATTTCACGTGTACGACATAGTAGAGACTTCGGAGAGCGCCTCACAC

GATGACCCATCCAGGTTTGCTGAGCTATCCCGCCCAAGCGGGTCTGTGGTAACACTGCTG

GGAATGAGCGAGTGTGGAAAGCGAGTCGCGGTTCACGTCTATGGTGTGCGCCATTATTTT

TACATGGCAAAGGCGGAGGTGGATAGCGCTTGTGGAATAACCACCGAGGCAGAACTCGTG

CGCGCAATGGTGGACTGCGCGCACAGCTCGGCTTTGAGCGCGGCCCTGGGAAATGGCAAC

GGCGGCAAACAGAGCGGCGGCAGCGGCGGGGGATGGTGGGGCGGAAAGCACGTGTCTGCG

GACTGCTTCAAAGTGGAGACCGTGTGCCACACGACGCTGTACTACTTTGGATCTAAGCCA

GCTCTCTACTATAGAGTATCTGCCTCCAGCAGCCGCCTGGGAGGGTTCATCTGCGACAAC

TTTCACCCGGAGATTACAAAATTCGAGGGGAGCGTGGACGTGACCACGCGGCTGTTGTTG

GACAATGAAAATTTTACCAGTTTCGGGTGGTACCGCCTGCGACCCGGCACCCACGGAGAG

CGTGTTCAACTTCGCCCCGTTGAGCGACACGTCACCTCAAGCGACGTGGAGATTAACTGT

ACTCCCGATAACCTGGAGCCGATACCAGACGAGGCTGCCTGGCCCGACTATAAGCTCATG

TGCTTTGATATAGAGTGTAAAGCTGGAACGGGTAACGAAATGGCGTTCCCAGTGGCAACT

AACCAAGAGGACCTGGTCATCCAGATCTCCTGTCTGCTGTACTCGCTTGCTACTCAGAAC

CACGAACACACCCTGCTGTTTTCCCTCGGGTCATGCGATATCTCTGAGGAATACTCGTTT

GCATGCGTCCAGCGCGGCGAGCCCAGACCGACGGTTTTGGAGTTTGACAGCGAGTACGAG

CTGCTGGTTGCCTTCCTGACCTTTCTCAAGCAGTACTCTCCCGAGTTCGCCACCGGCTAC

AACATCGTTAATTTTGACTGGGCGTACATAGTTAACAAGGTAACGTCGGTGTATAACATC

AAGCTGGACGGGTACGGCAAGTTCAACAAAGGGGGGCTGTTTAAGGTGTGGGACATCGCC

ACGAACCATTTTCAGAAGAAGAGCAAGGTGAAAATCAATGGCCTGATATCTCTAGACATG

TATTCTGTGGCGACGGAAAAGCTAAAGCTACCCAGCTACAAACTCGACGCGGTCGTGGGA

GACGTCCTCGGCGAGCATAAGATAGACCTTCCCTATAAAGAAATACCCTCCTATTACGCG

GGAGGGCCTGACCGGAGGGGCGTAATAGGAGAGTATTGTATCCAGGACTCTAGGCTGGTG

GGCAAGCTGTTTTTTAAGTACCTCCCCCATCTGGAACTATCGGCGGTGGCCAAACTCGCC

CGTATCACCCTAACGCGGGTAATTTTTGACGGTCAGCAAATTCGCGTGTACACGTGCTTG

CTGAAACTCGCCCGCGAGAGAAATTTCATTTTGCCAGACAACAGACGCCGGTTTGACAGT

CAGGCAGACGCCGCGTCAGAGACTTCGGAGTTGGCTATGGATAGCCAAAGCCACGCCTTC

GACAGTACAGACGAACCCGACGGTGTGGACGGTACCCCGGACGCCGCAGGATCTGGCGCT

ACTTCTGAAAACGGAGGCGGGAAGCCCGGCGTCGGGAGGGCCGTGGGCTACCAGGGAGCA

AAGGTTCTAGACCCCGTATCCGGCTTTCATGTGGACCCCGTGGTTGTGTTTGACTTCGCT

AGCTTATACCCAAGCATTATCCAGGCCCATAACCTCTGTTTCACCACCCTGGCGCTCGAT

GAAGTGGATCTGGCCGGGCTTCAACCATCCGTCGACTACTCGACGTTCGAGGTGGGTGAC

CAAAAGTTATTTTTTGTCCACGCCCATATTCGCGAAAGCCTGCTTGGCATCTTGCTGCGC

GACTGGCTGGCCATGCGAAAGGCGGTGAGGGCGCGAATCCCCACCAGCACCCCCGAGGAG

GCAGTTTTACTAGATAAGCAGCAGTCTGCGATTAAGGTGATATGCAACTCGGTTTACGGA

TTCACGGGGGTGGCAAACGGCCTGTTGCCGTGTCTGAGGATAGCGGCTACCGTTACCACG

ATAGGACGCGACATGCTCCTCAAGACCCGAGATTACGTTCACTCTCGTTGGGCGACGCGC

GAGCTGCTGGAGGACAATTTTCCAGGGGCTATAGGTTTCCGAAACCACAAGCCTTACTCC

GTCAGGGTTATCTACGGAGACACCGACTCCGTGTTTATCAAGTTTGTGGGCCTGACGTAC

GAGGGGGTATCGGAGCTGGGGGATGCTATGTCGCGTCAGATTTCAGCGGACCTCTTTAGA

GCGCCCATCAAACTGGAGTGTGAGAAGACCTTTCAGCGACTGCTGCTGATCACCAAGAAG

AAGTACATAGGTGTCATAAACGGGGGGAAGATGCTCATGAAGGGGGTCGACCTGGTCCGC

AAAAATAACTGCTCTTTCATAAACTTGTACGCGCGACATCTGGTAGATCTTTTGTTGTAC

GACGAGGATGTGGCCACGGCGGCAGCAGAGGTGACAGACGTGCCTCCCGCAGAATGGGTG

GGGCGCCCGCTACCGAGCGGCTTTGACAAGTTTGGGCGAGTGCTGGTAGAGGCGTACAAC

CGTATCACTGCCCCCAACTTGGACGTGCGCGAGTTCGTTATGACTGCTGAGCTGAGCCGC

TCACCCGAATCGTATACCAACAAGCGCCTGCCGCACCTCACCGTCTACTTTAAGCTCGCC

ATGAGGAATGAAGAACTGCCCAGCGTAAAAGAGAGAATTCCGTATGTGATAGTTGCGCAG

ACCGAGGCCGCGGAACGCGAAGCGGGTGTAGTAAACTCAATGCGCGGTACCGCCCAAAAC

CCCGTGGTAACCAAGACCGCACGCCCCCAACCTAAACGCAAACTGCTGGTTTCCGACCTC

GCCGAAGACCCGACCTATGTTTCCGAGAATGACGTACCGCTAAACACAGACTACTATTTC

TCCCACCTGTTGGGTACCATAAGCGTGACCTTTAAGGCTCTATTCGGAAATGATGTGAGA

ACAACAGAAAATCTTTTAAAGCGGTTTATTCCGGAAACCCCCCACAAGACCCCCACGAAA

ACCCAGGCACTGCTTGAGCGCGCCGGCTTTGAAAAGCTGACGCCCTTTACACCGGAGGAA

GAAAGTCGTCGAATACTGCATACAGTTTTTTGTACTCTAGAAGCAGCTCCCCATCAAAGC

TGA

>KC924817.1

ATGGCGGCGCGCGAACAGGCCAACTCCGTTCGCAGGAGCGGGTTTTTTAATCCGTTTATT

GGCAAGAGGCCATTTTTCAGGCCGGGAAGCGGGCAGACCGCGGAGACAGAGCGCCCAAGG

CCCCCCCAACACTCGTACTGCACAGAGGTGGGTAGCTTTAAGTTTATAGCTCCAAGATGC

CTCGATGAAGAAGCCCCCGCCGACCAGCGACGCGGTGTACACGTGGGCACCTTGGAGCGC

CCACCCAAAGTGTACTGCGATGGCTCAGAGTACGACGTGCTGAACTTTGCCTCCGGAGGT

TGCTGGCCTCGCAGGATTCGGGTTTGGAACGGCCAGGATTTTCGGGGCGATGGATTCAAC

CCCAGATTTGAGAGATTTCACGTGTACGACATAGTAGAGACTTCGGAGAGCGCCTCACAC

GATGACCCATCCAGGTTTGCTGAGCTATCCCGCCCAAGCGGGTCTGTGGTAACACTGCTG

GGAATGAGCGAGTGTGGAAAGCGAGTCGCGGTTCACGTCTATGGTGTGCGCCATTATTTT

TACATGGCAAAGGCGGAGGTGGATAGCGCTTGTGGAATAACCACCGAGGCAGAACTCGTG

CGCGCAATGGTGGACTGCGCGCACAGCTCGGCTTTGAGCGCGGCCCTGGGAAATGGCAAC

GGCGGCAAACAGAGCGGCGGCAGCGGCGGGGGATGGTGGGGCGGAAAGCACGTGTCTGCG

GACTGCTTCAAAGTGGAGACCGTGTGCCACACGACGCTGTACTACTTTGGATCTAAGCCA

GCTCTCTACTATAGAGTATCTGCCTCCAGCAGCCGCCTGGGAGGGTTCATCTGCGACAAC

TTTCACCCGGAGATTACAAAATTCGAGGGGAGCGTGGACGTGACCACGCGGCTGTTGTTG

GACAATGAAAATTTTACCAGTTTCGGGTGGTACCGCCTGCGACCCGGCACCCACGGAGAG

CGTGTTCAACTTCGCCCCGTTGAGCGACACGTCACCTCAAGCGACGTGGAGATTAACTGT

ACTCCCGATAACCTGGAGCCGATACCAGACGAGGCTGCCTGGCCCGACTATAAGCTCATG

TGCTTTGATATAGAGTGTAAAGCTGGAACGGGTAACGAAATGGCGTTCCCAGTGGCAACT

AACCAAGAGGACCTGGTCATCCAGATCTCCTGTCTGCTGTACTCGCTTGCTACTCAGAAC

CACGAACACACCCTGCTGTTTTCCCTCGGGTCATGCGATATCTCTGAGGAATACTCGTTT

GCATGCGTCCAGCGCGGCGAGCCCAGACCGACGGTTTTGGAGTTTGACAGCGAGTACGAG

CTGCTGGTTGCCTTCCTGACCTTTCTCAAGCAGTACTCTCCCGAGTTCGCCACCGGCTAC

AACATCGTTAATTTTGACTGGGCGTACATAGTTAACAAGGTAACGTCGGTGTATAACATC

AAGCTGGACGGGTACGGCAAGTTCAACAAAGGGGGGCTGTTTAAGGTGTGGGACATCGCC

ACGAACCATTTTCAGAAGAAGAGCAAGGTGAAAATCAATGGCCTGATATCTCTAGACATG

TATTCTGTGGCGACGGAAAAGCTAAAGCTACCCAGCTACAAACTCGACGCGGTCGTGGGA

GACGTCCTCGGCGAGCATAAGATAGACCTTCCCTATAAAGAAATACCCTCCTATTACGCG

GGAGGGCCTGACCGGAGGGGCGTAATAGGAGAGTATTGTATCCAGGACTCTAGGCTGGTG

GGCAAGCTGTTTTTTAAGTACCTCCCCCATCTGGAACTATCGGCGGTGGCCAAACTCGCC

CGTATCACCCTAACGCGGGTAATTTTTGACGGTCAGCAAATTCGCGTGTACACGTGCTTG

CTGAAACTCGCCCGCGAGAGAAATTTCATTTTGCCAGACAACAGACGCCGGTTTGACAGT

CAGGCAGACGCCGCGTCAGAGACTTCGGAGTTGGCTATGGATAGCCAAAGCCACGCCTTC

GACAGTACAGACGAACCCGACGGTGTGGACGGTACCCCGGACGCCGCAGGATCTGGCGCT

ACTTCTGAAAACGGAGGCGGGAAGCCCGGCGTCGGGAGGGCCGTGGGCTACCAGGGAGCA

AAGGTTCTAGACCCCGTATCCGGCTTTCATGTGGACCCCGTGGTTGTGTTTGACTTCGCT

AGCTTATACCCAAGCATTATCCAGGCCCATAACCTCTGTTTCACCACCCTGGCGCTCGAT

GAAGTGGATCTGGCCGGGCTTCAACCATCCGTCGACTACTCGACGTTCGAGGTGGGTGAC

CAAAAGTTATTTTTTGTCCACGCCCATATTCGCGAAAGCCTGCTTGGCATCTTGCTGCGC

GACTGGCTGGCCATGCGAAAGGCGGTGAGGGCGCGAATCCCCACCAGCACCCCCGAGGAG

GCAGTTTTACTAGATAAGCAGCAGTCTGCGATTAAGGTGATATGCAACTCGGTTTACGGA

TTCACGGGGGTGGCAAACGGCCTGTTGCCGTGTCTGAGGATAGCGGCTACCGTTACCACG

ATAGGACGCGACATGCTCCTCAAGACCCGAGATTACGTTCACTCTCGTTGGGCGACGCGC

GAGCTGCTGGAGGACAATTTTCCAGGGGCTATAGGTTTCCGAAACCACAAGCCTTACTCC

GTCAGGGTTATCTACGGAGACACCGACTCCGTGTTTATCAAGTTTGTGGGCCTGACGTAC

GAGGGGGTATCGGAGCTGGGGGATGCTATGTCGCGTCAGATTTCAGCGGACCTCTTTAGA

GCGCCCATCAAACTGGAGTGTGAGAAGACCTTTCAGCGACTGCTGCTGATCACCAAGAAG

AAGTACATAGGTGTCATAAACGGGGGGAAGATGCTCATGAAGGGGGTCGACCTGGTCCGC

AAAAATAACTGCTCTTTCATAAACTTGTACGCGCGACATCTGGTAGATCTTTTGTTGTAC

GACGAGGATGTGGCCACGGCGGCAGCAGAGGTGACAGACGTGCCTCCCGCAGAATGGGTG

GGGCGCCCGCTACCGAGCGGCTTTGACAAGTTTGGGCGAGTGCTGGTAGAGGCGTACAAC

CGTATCACTGCCCCCAACTTGGACGTGCGCGAGTTCGTTATGACTGCTGAGCTGAGCCGC

TCACCCGAATCGTATACCAACAAGCGCCTGCCGCACCTCACCGTCTACTTTAAGCTCGCC

ATGAGGAATGAAGAACTGCCCAGCGTAAAAGAGAGAATTCCGTATGTGATAGTTGCGCAG

ACCGAGGCCGCGGAACGCGAAGCGGGTGTAGTAAACTCAATGCGCGGTACCGCCCAAAAC

CCCGTGGTAACCAAGACCGCACGCCCCCAACCTAAACGCAAACTGCTGGTTTCCGACCTC

GCCGAAGACCCGACCTATGTTTTCGAGAATGACGTACCGCTAAACACAGACTACTATTTC

TCCCACCTGTTGGGTACCATAAGCGTGACCTTTAAGGCTCTATTCGGAAATGATGTGAGA

ACAACAGAAAATCTTTTAAAGCGGTTTATTCCGGAAACCCCCCACAAGACCCCCACGAAA

ACCCAGGCACTGCTTGAGCGCGCCGGCTTTGAAAAGCTGACGCCCTTTACACCGGAGGAA

GAAAGTCGTCGAATACTGCATACAGTTTTTTGTACTCTAGAAGCAGCTCCCCATCAAAGC

TGA

>KU206404.1

ATGGCGGCGCGCGAACAGGCCAACTCCGTTCGCAGGAGCGGGTTTTTTAATCCGTTTATT

GGCAAGAGGCCATTTTTCAGGCCGGGAAGCGGGCAAACCGCGGAGACAGAGCGCCCAAGG

CCCCCCCAACACTCGTACTGCACAGAGGTGGGTAGCTTTAAGTTTATAGCTCCAAGATGC

CTCGATGAAGAAGCCCCCGCCGACCAGCGACGCGGTGTACACGTGGGCACCTTGGAGCGC

CCACCCAAAGTGTACTGCGATGGCTCAGAGTACGACGTGCTGAACTTTGCCTCCGGAGGT

TGCTGGCCTCGCAGGATTCGGGTTTGGAACGGCCAGGATTTTCGGGGCGATGGATTCAAC

CCCAGATTTGAGAGATTTCACGTGTACGACATAGTAGAGACTTCGGAGAGCGCCTCACAC

GATGACCCATCCAGGTTTGCTGAGCTATCCCGCCCAAGCGGGTCTGTGGTAACACTGCTG

GGAATGAGCGAGTGTGGAAAGCGAGTCGCGGTTCACGTCTATGGTGTGCGCCATTATTTT

TACATGGCAAAGGCGGAGGTGGATAGCGCTTGTGGAATAACCACCGAGGCAGAACTCGTG

CGCGCAATGGTGGACTGCGCGCACAGCTCGGCTTTGAGCGCGGCCCTGGGAAATGGCAAC

GGCGGCAAACAGAGCGGCGGCAGCGGCGGGGGATGGTGGGGCGGAAAGCACGTGTCTGCG

GACTGCTTCAAAGTGGAGACCGTGTGCCACACGACGCTGTACTACTTTGGATCTAAGCCA

GCTCTCTACTATAGAGTATCTGCCTCCAGCAGCCGCCTGGGAGGGTTCATCTGCGACAAC

TTTCACCCGGAGATTACAAAATTCGAGGGGAGCGTGGACGTGACCACGCGGCTGTTGTTG

GACAATGAAAATTTTACCAGTTTCGGGTGGTACCGCCTGCGACCCGGCACCCACGGAGAG

CGTGTTCAACTTCGCCCCGTTGAGCGACACGTCACCTCAAGCGACGTGGAGATTAACTGT

ACTCCCGATAACCTGGAGCCGATACCAGACGAGGCTGCCTGGCCCGACTATAAGCTCATG

TGCTTTGATATAGAGTGTAAAGCTGGAACGGGTAACGAAATGGCGTTCCCAGTGGCAACT

AACCAAGAGGACCTGGTCATCCAGATCTCCTGTCTGCTGTACTCGCTTGCTACTCAGAAC

CACGAACACACCCTGCTGTTTTCCCTCGGGTCATGCGATATCTCTGAGGAATACTCGTTT

GCATGCGTCCAGCGCGGCGAGCCCAGACCGACGGTTTTGGAGTTTGACAGCGAGTACGAG

CTGCTGGTTGCCTTCCTGACCTTTCTCAAGCAGTACTCTCCCGAGTTCGCCACCGGCTAC

AACATCGTTAATTTTGACTGGGCGTACATAGTTAACAAGGTAACGTCGGTGTATAACATC

AAGCTGGACGGGTACGGCAAGTTCAACAAAGGGGGGCTGTTTAAGGTGTGGGACATCGCC

ACGAACCATTTTCAGAAGAAGAGCAAGGTGAAAATCAATGGCCTGATATCTCTAGACATG

TATTCTGTGGCGACGGAAAAGCTAAAGCTACCCAGCTACAAACTCGACGCGGTCGTGGGA

GACGTCCTCGGCGAGCATAAGATAGACCTTCCCTATAAAGAAATACCCTCCTATTACGCG

GGAGGGCCTGACCGGAGGGGCGTAATAGGAGAGTATTGTATCCAGGACTCTAGGCTGGTG

GGCAAGCTGTTTTTTAAGTACCTCCCCCATCTGGAACTATCGGCGGTGGCCAAACTCGCC

CGTATCACCCTAACGCGGGTAATTTTTGACGGTCAGCAAATTCGCGTGTACACGTGCTTG

CTGAAACTCGCCCGCGAGAGAAATTTCATTTTGCCAGACAACAGACGCCGGTTTGACAGT

CAGGCAGACGCCGCGTCAGAGACTTCGGAGTTGGCTATGGATAGCCAAAGCCACGCCTTC

GACAGTACAGACGAACCCGACGGTGTGGACGGTACCCCGGACGCCGCAGGATCTGGCGCT

ACTTCTGAAAACGGAGGCGGGAAGCCCGGCGTCGGGAGGGCCGTGGGCTACCAGGGAGCA

AAGGTTCTAGACCCCGTATCCGGCTTTCATGTGGACCCCGTGGTTGTGTTTGACTTCGCT

AGCTTATACCCAAGCATTATCCAGGCCCATAACCTCTGTTTCACCACCCTGGCGCTCGAT

GAAGTGGATCTGGCCGGGCTTCAACCATCCGTCAACTACTCGACGTTCGAGGTGGGTGAC

CAAAAGTTATTTTTTGTCCACGCCCATATTCGCGAAAGCCTGCTTGGCATCTTGCTGCGC

GACTGGCTGGCCATGCGAAAGGCGGTGAGGGCGCGAATCCCCACCAGCACCCCCGAGGAG

GCAGTTTTACTAGATAAGCAGCAGTCTGCGATTAAGGTGATATGCAACTCGGTTTACGGA

TTCACGGGGGTGGCAAACGGCCTGTTGCCGTGTCTGAGGATAGCGGCTACCGTTACCACG

ATAGGACGCGACATGCTCCTCAAGACCCGAGATTACGTTCACTCTCGTTGGGCGACGCGC

GAGCTGCTGGAGGACAATTTTCCAGGGGCTATAGGTTTCCGAAACCACAAGCCTTACTCC

GTCAGGGTTATCTACGGAGACACCGACTCCGTGTTTATCAAGTTTGTGGGCCTGACGTAC

GAGGGGGTATCGGAGCTGGGGGATGCTATGTCGCGTCAGATTTCAGCGGACCTCTTTAGA

GCGCCCATCAAACTGGAGTGTGAGAAGACCTTTCAGCGACTGCTGCTGATCACCAAGAAG

AAGTACATAGGTGTCATAAACGGGGGGAAGATGCTCATGAAGGGGGTCGACCTGGTCCGC

AAAAATAACTGCTCTTTCATAAACTTGTACGCGCGACATCTGGTAGATCTTTTGTTGTAC

GACGAGGATGTGGCCACGGCGGCAGCAGAGGTGACAGACGTGCCTCCCGCAGAATGGGTG

GGGCGCCCGCTACCGAGCGGCTTTGACAAGTTTGGGCGAGTGCTGGTAGAGGCGTACAAC

CGTATCACTGCCCCCAACTTGGACGTGCGCGAGTTCGTTATGACTGCTGAGCTGAGCCGC

TCACCCGAATCGTATACCAACAAGCGCCTGCCGCACCTCACCGTCTACTTTAAGCTCGCC

ATGAGGAATGAAGAACTGCCCAGCGTAAAAGAGAGAATTCCGTATGTGATAGTTGCGCAG

ACCGAGGCCGCGGAACGCGAAGCGGGTGTAGTAAACTCAATGCGCGGTACCGCCCAAAAC

CCCGTGGTAACCAAGACCGCACGCCCCCAACCTAAACGCAAACTGCTGGTTTCCGACCTC

GCCGAAGACCCGACCTATGTTTCCGAGAATGACGTACCGCTAAACACAGACTACTATTTC

TCCCACCTGTTGGGTACCATAAGCGTGACCTTTAAGGCTCTATTCGGAAATGATGTGAGA

ACAACAGAAAATCTTTTAAAGCGGTTTATTCCGGAAACCCCCCACAAGACCCCCACGAAA

ACCCAGGCACTGCTTGAGCGCGCCGGCTTTGAAAAGCTGACGCCCTTTACACCGGAGGAA

GAAAGTCGTCGAATACTGCATACAGTTTTTTGTACTCTAGAAGCAGCTCCCCATCAAAGC

TGA

>KU206405.1

ATGGCGGCGCGCGAACAGGCCAACTCCGTTCGCAGGAGCGGGTTTTTTAATCCGTTTATT

GGCAAGAGGCCATTTTTCAGGCCGGGAAGCGGGCAAACCGCGGAGACAGAGCGCCCAAGG

CCCCCCCAACACTCGTACTGCACAGAGGTGGGTAGCTTTAAGTTTATAGCTCCAAGATGC

CTCGATGAAGAAGCCCCCGCCGACCAGCGACGCGGTGTACACGTGGGCACCTTGGAGCGC

CCACCCAAAGTGTACTGCGATGGCTCAGAGTACGACGTGCTGAACTTTGCCTCCGGAGGT

TGCTGGCCTCGCAGGATTCGGGTTTGGAACGGCCAGGATTTTCGGGGCGATGGATTCAAC

CCCAGATTTGAGAGATTTCACGTGTACGACATAGTAGAGACTTCGGAGAGCGCCTCACAC

GATGACCCATCCAGGTTTGCTGAGCTATCCCGCCCAAGCGGGTCTGTGGTAACACTGCTG

GGAATGAGCGAGTGTGGAAAGCGAGTCGCGGTTCACGTCTATGGTGTGCGCCATTATTTT

TACATGGCAAAGGCGGAGGTGGATAGCGCTTGTGGAATAACCACCGAGGCAGAACTCGTG

CGCGCAATGGTGGACTGCGCGCACAGCTCGGCTTTGAGCGCGGCCCTGGGAAATGGCAAC

GGCGGCAAACAGAGCGGCGGCAGCGGCGGGGGATGGTGGGGCGGAAAGCACGTGTCTGCG

GACTGCTTCAAAGTGGAGACCGTGTGCCACACGACGCTGTACTACTTTGGATCTAAGCCA

GCTCTCTACTATAGAGTATCTGCCTCCAGCAGCCGCCTGGGAGGGTTCATCTGCGACAAC

TTTCACCCGGAGATTACAAAATTCGAGGGGAGCGTGGACGTGACCACGCGGCTGTTGTTG

GACAATGAAAATTTTACCAGTTTCGGGTGGTACCGCCTGCGACCCGGCACCCACGGAGAG

CGTGTTCAACTTCGCCCCGTTGAGCGACACGTCACCTCAAGCGACGTGGAGATTAACTGT

ACTCCCGATAACCTGGAGCCGATACCAGACGAGGCTGCCTGGCCCGACTATAAGCTCATG

TGCTTTGATATAGAGTGTAAAGCTGGAACGGGTAACGAAATGGCGTTCCCAGTGGCAACT

AACCAAGAGGACCTGGTCATCCAGATCTCCTGTCTGCTGTACTCGCTTGCTACTCAGAAC

CACGAACACACCCTGCTGTTTTCCCTCGGGTCATGCGATATCTCTGAGGAATACTCGTTT

GCATGCGTCCAGCGCGGCGAGCCCAGACCGACGGTTTTGGAGTTTGACAGCGAGTACGAG

CTGCTGGTTGCCTTCCTGACCTTTCTCAAGCAGTACTCTCCCGAGTTCGCCACCGGCTAC

AACATCGTTAATTTTGACTGGGCGTACATAGTTAACAAGGTAACGTCGGTGTATAACATC

AAGCTGGACGGGTACGGCAAGTTCAACAAAGGGGGGCTGTTTAAGGTGTGGGACATCGCC

ACGAACCATTTTCAGAAGAAGAGCAAGGTGAAAATCAATGGCCTGATATCTCTAGACATG

TATTCTGTGGCGACGGAAAAGCTAAAGCTACCCAGCTACAAACTCGACGCGGTCGTGGGA

GACGTCCTCGGCGAGCATAAGATAGACCTTCCCTATAAAGAAATACCCTCCTATTACGCG

GGAGGGCCTGACCGGAGGGGCGTAATAGGAGAGTATTGTATCCAGGACTCTAGGCTGGTG

GGCAAGCTGTTTTTTAAGTACCTCCCCCATCTGGAACTATCGGCGGTGGCCAAACTCGCC

CGTATCACCCTAACGCGGGTAATTTTTGACGGTCAGCAAATTCGCGTGTACACGTGCTTG

CTGAAACTCGCCCGCGAGAGAAATTTCATTTTGCCAGACAACAGACGCCGGTTTGACAGT

CAGGCAAACGCCGCGTCAGAGACTTCGGAGTTGGCTATGGATAGCCAAAGCCACGCCTTC

GACAGTACAGACGAACCCGACGGTGTGGACGGTACCCCGGACGCCGCAGGATCTGGCGCT

ACTTCTGAAAACGGAGGCGGGAAGCCCGGCGTCGGGAGGGCCGTGGGCTACCAGGGAGCA

AAGGTTCTAGACCCCGTATCCGGCTTTCATGTGGACCCCGTGGTTGTGTTTGACTTCGCT

AGCTTATACCCAAGCATTATCCAGGCCCATAACCTCTGTTTCACCACCCTGGCGCTCGAT

GAAGTGGATCTGGCCGGGCTTCAACCATCCGTCAACTACTCGACGTTCGAGGTGGGTGAC

CAAAAGTTATTTTTTGTCCACGCCCATATTCGCGAAAGCCTGCTTGGCATCTTGCTGCGC

GACTGGCTGGCCATGCGAAAGGCGGTGAGGGCGCGAATCCCCACCAGCACCCCCGAGGAG

GCAGTTTTACTAGATAAGCAGCAGTCTGCGATTAAGGTGATATGCAACTCGGTTTACGGA

TTCACGGGGGTGGCAAACGGCCTGTTGCCGTGTCTGAGGATAGCGGCTACCGTTACCACG

ATAGGACGCGACATGCTCCTCAAGACCCGAGATTACGTTCACTCTCGTTGGGCGACGCGC

GAGCTGCTGGAGGACAATTTTCCAGGGGCTATAGGTTTCCGAAACCACAAGCCTTACTCC

GTCAGGGTTATCTACGGAGACACCGACTCCGTGTTTATCAAGTTTGTGGGCCTGACGTAC

GAGGGGGTATCGGAGCTGGGGGATGCTATGTCGCGTCAGATTTCAGCGGACCTCTTTAGA

GCGCCCATCAAACTGGAGTGTGAGAAGACCTTTCAGCGACTGCTGCTGATCACCAAGAAG

AAGTACATAGGTGTCATAAACGGGGGGAAGATGCTCATGAAGGGGGTCGACCTGGTCCGC

AAAAATAACTGCTCTTTCATAAACTTGTACGCGCGACATCTGGTAGATCTTTTGTTGTAC

GACGAGGATGTGGCCACGGCGGCAGCAGAGGTGACAGACGTGCCTCCCGCAGAATGGGTG

GGGCGCCCGCTACCGAGCGGCTTTGACAAGTTTGGGCGAGTGCTGGTAGAGGCGTACAAC

CGTATCACTGCCCCCAACTTGGACGTGCGCGAGTTCGTTATGACTGCTGAGCTGAGCCGC

TCACCCGAATCGTATACCAACAAGCGCCTGCCGCACCTCACCGTCTACTTTAAGCTCGCC

ATGAGGAATGAAGAACTGCCCAGCGTAAAAGAGAGAATTCCGTATGTGATAGTTGCGCAG

ACCGAGGCCGCGGAACGCGAAGCGGGTGTAGTAAACTCAATGCGCGGTACCGCCCAAAAC

CCCGTGGTAACCAAGACCGCACGCCCCCAACCTAAACGCAAACTGCTGGTTTCCGACCTC

GCCGAAGACCCGACCTATGTTTCCGAGAATGACGTACCGCTAAACACAGACTACTATTTC

TCCCACCTGTTGGGTACCATAAGCGTGACCTTTAAGGCTCTATTCGGAAATGATGTGAGA

ACAACAGAAAATCTTTTAAAGCGGTTTATTCCGGAAACCCCCCACAAGACCCCCACGAAA

ACCCAGGCACTGCTTGAGCGCGCCGGCTTTGAAAAGCTGACGCCCTTTACACCGGAGGAA

GAAAGTCGTCGAATACTGCATACAGTTTTTTGTACTCTAGAAGCAGCTCCCCATCAAAGC

TGA

>KU206406.1

ATGGCGGCGCGCGAACAGGCCAACTCCGTTCGCAGGAGCGGGTTTTTTAATCCGTTTATT

GGCAAGAGGCCATTTTTCAGGCCGGGAAGCGGGCAAACCGCGGAGACAGAGCGCCCAAGG

CCCCCCCAACACTCGTACTGCACAGAGGTGGGTAGCTTTAAGTTTATAGCTCCAAGATGC

CTCGATGAAGAAGCCCCCGCCGACCAGCGACGCGGTGTACACGTGGGCACCTTGGAGCGC

CCACCCAAAGTGTACTGCGATGGCTCAGAGTACGACGTGCTGAACTTTGCCTCCGGAGGT

TGCTGGCCTCGCAGGATTCGGGTTTGGAACGGCCAGGATTTTCGGGGCGATGGATTCAAC

CCCAGATTTGAGAGATTTCACGTGTACGACATAGTAGAGACTTCGGAGAGCGCCTCACAC

GATGACCCATCCAGGTTTGCTGAGCTATCCCGCCCAAGCGGGTCTGTGGTAACACTGCTG

GGAATGAGCGAGTGTGGAAAGCGAGTCGCGGTTCACGTCTATGGTGTGCGCCATTATTTT

TACATGGCAAAGGCGGAGGTGGATAGCGCTTGTGGAATAACCACCGAGGCAGAACTCGTG

CGCGCAATGGTGGACTGCGCGCACAGCTCGGCTTTGAGCGCGGCCCTGGGAAATGGCAAC

GGCGGCAAACAGAGCGGCGGCAGCGGCGGGGGATGGTGGGGCGGAAAGCACGTGTCTGCG

GACTGCTTCAAAGTGGAGACCGTGTGCCACACGACGCTGTACTACTTTGGATCTAAGCCA

GCTCTCTACTATAGAGTATCTGCCTCCAGCAGCCGCCTGGGAGGGTTCATCTGCGACAAC

TTTCACCCGGAGATTACAAAATTCGAGGGGAGCGTGGACGTGACCACGCGGCTGTTGTTG

GACAATGAAAATTTTACCAGTTTCGGGTGGTACCGCCTGCGACCCGGCACCCACGGAGAG

CGTGTTCAACTTCGCCCCGTTGAGCGACACGTCACCTCAAGCGACGTGGAGATTAACTGT

ACTCCCGATAACCTGGAGCCGATACCAGACGAGGCTGCCTGGCCCGACTATAAGCTCATG

TGCTTTGATATAGAGTGTAAAGCTGGAACGGGTAACGAAATGGCGTTCCCAGTGGCAACT

AACCAAGAGGACCTGGTCATCCAGATCTCCTGTCTGCTGTACTCGCTTGCTACTCAGAAC

CACGAACACACCCTGCTGTTTTCCCTCGGGTCATGCGATATCTCTGAGGAATACTCGTTT

GCATGCGTCCAGCGCGGCGAGCCCAGACCGACGGTTTTGGAGTTTGACAGCGAGTACGAG

CTGCTGGTTGCCTTCCTGACCTTTCTCAAGCAGTACTCTCCCGAGTTCGCCACCGGCTAC

AACATCGTTAATTTTGACTGGGCGTACATAGTTAACAAGGTAACGTCGGTGTATAACATC

AAGCTGGACGGGTACGGCAAGTTCAACAAAGGGGGGCTGTTTAAGGTGTGGGACATCGCC

ACGAACCATTTTCAGAAGAAGAGCAAGGTGAAAATCAATGGCCTGATATCTCTAGACATG

TATTCTGTGGCGACGGAAAAGCTAAAGCTACCCAGCTACAAACTCGACGCGGTCGTGGGA

GACGTCCTCGGCGAGCATAAGATAGACCTTCCCTATAAAGAAATACCCTCCTATTACGCG

GGAGGGCCTGACCGGAGGGGCGTAATAGGAGAGTATTGTATCCAGGACTCTAGGCTGGTG

GGCAAGCTGTTTTTTAAGTACCTCCCCCATCTGGAACTATCGGCGGTGGCCAAACTCGCC

CGTATCACCCTAACGCGGGTAATTTTTGACGGTCAGCAAATTCGCGTGTACACGTGCTTG

CTGAAACTCGCCCGCGAGAGAAATTTCATTTTGCCAGACAACAGACGCCGGTTTGACAGT

CAGGCAAACGCCGCGTCAGAGACTTCGGAGTTGGCTATGGATAGCCAAAGCCACGCCTTC

GACAGTACAGACGAACCCGACGGTGTGGACGGTACCCCGGACGCCGCAGGATCTGGCGCT

ACTTCTGAAAACGGAGGCGGGAAGCCCGGCGTCGGGAGGGCCGTGGGCTACCAGGGAGCA

AAGGTTCTAGACCCCGTATCCGGCTTTCATGTGGACCCCGTGGTTGTGTTTGACTTCGCT

AGCTTATACCCAAGCATTATCCAGGCCCATAACCTCTGTTTCACCACCCTGGCGCTCGAT

GAAGTGGATCTGGCCGGGCTTCAACCATCCGTCAACTACTCGACGTTCGAGGTGGGTGAC

CAAAAGTTATTTTTTGTCCACGCCCATATTCGCGAAAGCCTGCTTGGCATCTTGCTGCGC

GACTGGCTGGCCATGCGAAAGGCGGTGAGGGCGCGAATCCCCACCAGCACCCCCGAGGAG

GCAGTTTTACTAGATAAGCAGCAGTCTGCGATTAAGGTGATATGCAACTCGGTTTACGGA

TTCACGGGGGTGGCAAACGGCCTGTTGCCGTGTCTGAGGATAGCGGCTACCGTTACCACG

ATAGGACGCGACATGCTCCTCAAGACCCGAGATTACGTTCACTCTCGTTGGGCGACGCGC

GAGCTGCTGGAGGACAATTTTCCAGGGGCTATAGGTTTCCGAAACCACAAGCCTTACTCC

GTCAGGGTTATCTACGGAGACACCGACTCCGTGTTTATCAAGTTTGTGGGCCTGACGTAC

GAGGGGGTATCGGAGCTGGGGGATGCTATGTCGCGTCAGATTTCAGCGGACCTCTTTAGA

GCGCCCATCAAACTGGAGTGTGAGAAGACCTTTCAGCGACTGCTGCTGATCACCAAGAAG

AAGTACATAGGTGTCATAAACGGGGGGAAGATGCTCATGAAGGGGGTCGACCTGGTCCGC

AAAAATAACTGCTCTTTCATAAACTTGTACGCGCGACATCTGGTAGATCTTTTGTTGTAC

GACGAGGATGTGGCCACGGCGGCAGCAGAGGTGACAGACGTGCCTCCCGCAGAATGGGTG

GGGCGCCCGCTACCGAGCGGCTTTGACAAGTTTGGGCGAGTGCTGGTAGAGGCGTACAAC

CGTATCACTGCCCCCAACTTGGACGTGCGCGAGTTCGTTATGACTGCTGAGCTGAGCCGC

TCACCCGAATCGTATACCAACAAGCGCCTGCCGCACCTCACCGTCTACTTTAAGCTCGCC

ATGAGGAATGAAGAACTGCCCAGCGTAAAAGAGAGAATTCCGTATGTGATAGTTGCGCAG

ACCGAGGCCGCGGAACGCGAAGCGGGTGTAGTAAACTCAATGCGCGGTACCGCCCAAAAC

CCCGTGGTAACCAAGACCGCACGCCCCCAACCTAAACGCAAACTGCTGGTTTCCGACCTC

GCCGAAGACCCGACCTATGTTTCCGAGAATGACGTACCGCTAAACACAGACTACTATTTC

TCCCACCTGTTGGGTACCATAAGCGTGACCTTTAAGGCTCTATTCGGAAATGATGTGAGA

ACAACAGAAAATCTTTTAAAGCGGTTTATTCCGGAAACCCCCCACAAGACCCCCACGAAA

ACCCAGGCACTGCTTGAGCGCGCCGGCTTTGAAAAGCTGACGCCCTTTACACCGGAGGAA

GAAAGTCGTCGAATACTGCATACAGTTTTTTGTACTCTAGAAGCAGCTCCCCATCAAAGC

TGA

>KU206407.1

ATGGCGGCGCGCGAACAGGCCAACTCCGTTCGCAGGAGCGGGTTTTTTAATCCGTTTATT

GGCAAGAGGCCATTTTTCAGGCCGGGAAGCGGGCAAACCGCGGAGACAGAGCGCCCAAGG

CCCCCCCAACACTCGTACTGCACAGAGGTGGGTAGCTTTAAGTTTATAGCTCCAAGATGC

CTCGATGAAGAAGCCCCCGCCGACCAGCGACGCGGTGTACACGTGGGCACCTTGGAGCGC

CCACCCAAAGTGTACTGCGATGGCTCAGAGTACGACGTGCTGAACTTTGCCTCCGGAGGT

TGCTGGCCTCGCAGGATTCGGGTTTGGAACGGCCAGGATTTTCGGGGCGATGGATTCAAC

CCCAGATTTGAGAGATTTCACGTGTACGACATAGTAGAGACTTCGGAGAGCGCCTCACAC

GATGACCCATCCAGGTTTGCTGAGCTATCCCGCCCAAGCGGGTCTGTGGTAACACTGCTG

GGAATGAGCGAGTGTGGAAAGCGAGTCGCGGTTCACGTCTATGGTGTGCGCCATTATTTT

TACATGGCAAAGGCGGAGGTGGATAGCGCTTGTGGAATAACCACCGAGGCAGAACTCGTG

CGCGCAATGGTGGACTGCGCGCACAGCTCGGCTTTGAGCGCGGCCCTGGGAAATGGCAAC

GGCGGCAAACAGAGCGGCGGCAGCGGCGGGGGATGGTGGGGCGGAAAGCACGTGTCTGCG

GACTGCTTCAAAGTGGAGACCGTGTGCCACACGACGCTGTACTACTTTGGATCTAAGCCA

GCTCTCTACTATAGAGTATCTGCCTCCAGCAGCCGCCTGGGAGGGTTCATCTGCGACAAC

TTTCACCCGGAGATTACAAAATTCGAGGGGAGCGTGGACGTGACCACGCGGCTGTTGTTG

GACAATGAAAATTTTACCAGTTTCGGGTGGTACCGCCTGCGACCCGGCACCCACGGAGAG

CGTGTTCAACTTCGCCCCGTTGAGCGACACGTCACCTCAAGCGACGTGGAGATTAACTGT

ACTCCCGATAACCTGGAGCCGATACCAGACGAGGCTGCCTGGCCCGACTATAAGCTCATG

TGCTTTGATATAGAGTGTAAAGCTGGAACGGGTAACGAAATGGCGTTCCCAGTGGCAACT

AACCAAGAGGACCTGGTCATCCAGATCTCCTGTCTGCTGTACTCGCTTGCTACTCAGAAC

CACGAACACACCCTGCTGTTTTCCCTCGGGTCATGCGATATCTCTGAGGAATACTCGTTT

GCATGCGTCCAGCGCGGCGAGCCCAGACCGACGGTTTTGGAGTTTGACAGCGAGTACGAG

CTGCTGGTTGCCTTCCTGACCTTTCTCAAGCAGTACTCTCCCGAGTTCGCCACCGGCTAC

AACATCGTTAATTTTGACTGGGCGTACATAGTTAACAAGGTAACGTCGGTGTATAACATC

AAGCTGGACGGGTACGGCAAGTTCAACAAAGGGGGGCTGTTTAAGGTGTGGGACATCGCC

ACGAACCATTTTCAGAAGAAGAGCAAGGTGAAAATCAATGGCCTGATATCTCTAGACATG

TATTCTGTGGCGACGGAAAAGCTAAAGCTACCCAGCTACAAACTCGACGCGGTCGTGGGA

GACGTCCTCGGCGAGCATAAGATAGACCTTCCCTATAAAGAAATACCCTCCTATTACGCG

GGAGGGCCTGACCGGAGGGGCGTAATAGGAGAGTATTGTATCCAGGACTCTAGGCTGGTG

GGCAAGCTGTTTTTTAAGTACCTCCCCCATCTGGAACTATCGGCGGTGGCCAAACTCGCC

CGTATCACCCTAACGCGGGTAATTTTTGACGGTCAGCAAATTCGCGTGTACACGTGCTTG

CTGAAACTCGCCCGCGAGAGAAATTTCATTTTGCCAGACAACAGACGCCGGTTTGACAGT

CAGGCAGACGCCGCGTCAGAGACTTCGGAGTTGGCTATGGATAGCCAAAGCCACGCCTTC

GACAGTACAGACGAACCCGACGGTGTGGACGGTACCCCGGACGCCGCAGGATCTGGCGCT

ACTTCTGAAAACGGAGGCGGGAAGCCCGGCGTCGGGAGGGCCGTGGGCTACCAGGGAGCA

AAGGTTCTAGACCCCGTATCCGGCTTTCATGTGGACCCCGTGGTTGTGTTTGACTTCGCT

AGCTTATACCCAAGCATTATCCAGGCCCATAACCTCTGTTTCACCACCCTGGCGCTCGAT

GAAGTGGATCTGGCCGGGCTTCAACCATCCGTCAACTACTCGACGTTCGAGGTGGGTGAC

CAAAAGTTATTTTTTGTCCACGCCCATATTCGCGAAAGCCTGCTTGGCATCTTGCTGCGC

GACTGGCTGGCCATGCGAAAGGCGGTGAGGGCGCGAATCCCCACCAGCACCCCCGAGGAG

GCAGTTTTACTAGATAAGCAGCAGTCTGCGATTAAGGTGATATGCAACTCGGTTTACGGA

TTCACGGGGGTGGCAAACGGCCTGTTGCCGTGTCTGAGGATAGCGGCTACCGTTACCACG

ATAGGACGCGACATGCTCCTCAAGACCCGAGATTACGTTCACTCTCGTTGGGCGACGCGC

GAGCTGCTGGAGGACAATTTTCCAGGGGCTATAGGTTTCCGAAACCACAAGCCTTACTCC

GTCAGGGTTATCTACGGAGACACCGACTCCGTGTTTATCAAGTTTGTGGGCCTGACGTAC

GAGGGGGTATCGGAGCTGGGGGATGCTATGTCGCGTCAGATTTCAGCGGACCTCTTTAGA

GCGCCCATCAAACTGGAGTGTGAGAAGACCTTTCAGCGACTGCTGCTGATCACCAAGAAG

AAGTACATAGGTGTCATAAACGGGGGGAAGATGCTCATGAAGGGGGTCGACCTGGTCCGC

AAAAATAACTGCTCTTTCATAAACTTGTACGCGCGACATCTGGTAGATCTTTTGTTGTAC

GACGAGGATGTGGCCACGGCGGCAGCAGAGGTGACAGACGTGCCTCCCGCAGAATGGGTG

GGGCGCCCGCTACCGAGCGGCTTTGACAAGTTTGGGCGAGTGCTGGTAGAGGCGTACAAC

CGTATCACTGCCCCCAACTTGGACGTGCGCGAGTTCGTTATGACTGCTGAGCTGAGCCGC

TCACCCGAATCGTATACCAACAAGCGCCTGCCGCACCTCACCGTCTACTTTAAGCTCGCC

ATGAGGAATGAAGAACTGCCCAGCGTAAAAGAGAGAATTCCGTATGTGATAGTTGCGCAG

ACCGAGGCCGCGGAACGCGAAGCGGGTGTAGTAAACTCAATGCGCGGTACCGCCCAAAAC

CCCGTGGTAACCAAGACCGCACGCCCCCAACCTAAACGCAAACTGCTGGTTTCCGACCTC

GCCGAAGACCCGACCTATGTTTCCGAGAATGACGTACCGCTAAACACAGACTACTATTTC

TCCCACCTGTTGGGTACCATAAGCGTGACCTTTAAGGCTCTATTCGGAAATGATGTGAGA

ACAACAGAAAATCTTTTAAAGCGGTTTATTCCGGAAACCCCCCACAAGACCCCCACGAAA

ACCCAGGCACTGCTTGAGCGCGCCGGCTTTGAAAAGCTGACGCCCTTTACACCGGAGGAA

GAAAGTCGTCGAATACTGCATACAGTTTTTTGTACTCTAGAAGCAGCTCCCCATCAAAGC

TGA

>KU206408.1

ATGGCGGCGCGCGAACAGGCCAACTCCGTTCGCAGGAGCGGGTTTTTTAATCCGTTTATT

GGCAAGAGGCCATTTTTCAGGCCGGGAAGCGGGCAAACCGCGGAGACAGAGCGCCCAAGG

CCCCCCCAACACTCGTACTGCACAGAGGTGGGTAGCTTTAAGTTTATAGCTCCAAGATGC

CTCGATGAAGAAGCCCCCGCCGACCAGCGACGCGGTGTACACGTGGGCACCTTGGAGCGC

CCACCCAAAGTGTACTGCGATGGCTCAGAGTACGACGTGCTGAACTTTGCCTCCGGAGGT

TGCTGGCCTCGCAGGATTCGGGTTTGGAACGGCCAGGATTTTCGGGGCGATGGATTCAAC

CCCAGATTTGAGAGATTTCACGTGTACGACATAGTAGAGACTTCGGAGAGCGCCTCACAC

GATGACCCATCCAGGTTTGCTGAGCTATCCCGCCCAAGCGGGTCTGTGGTAACACTGCTG

GGAATGAGCGAGTGTGGAAAGCGAGTCGCGGTTCACGTCTATGGTGTGCGCCATTATTTT

TACATGGCAAAGGCGGAGGTGGATAGCGCTTGTGGAATAACCACCGAGGCAGAACTCGTG

CGCGCAATGGTGGACTGCGCGCACAGCTCGGCTTTGAGCGCGGCCCTGGGAAATGGCAAC

GGCGGCAAACAGAGCGGCGGCAGCGGCGGGGGATGGTGGGGCGGAAAGCACGTGTCTGCG

GACTGCTTCAAAGTGGAGACCGTGTGCCACACGACGCTGTACTACTTTGGATCTAAGCCA

GCTCTCTACTATAGAGTATCTGCCTCCAGCAGCCGCCTGGGAGGGTTCATCTGCGACAAC

TTTCACCCGGAGATTACAAAATTCGAGGGGAGCGTGGACGTGACCACGCGGCTGTTGTTG

GACAATGAAAATTTTACCAGTTTCGGGTGGTACCGCCTGCGACCCGGCACCCACGGAGAG

CGTGTTCAACTTCGCCCCGTTGAGCGACACGTCACCTCAAGCGACGTGGAGATTAACTGT

ACTCCCGATAACCTGGAGCCGATACCAGACGAGGCTGCCTGGCCCGACTATAAGCTCATG

TGCTTTGATATAGAGTGTAAAGCTGGAACGGGTAACGAAATGGCGTTCCCAGTGGCAACT

AACCAAGAGGACCTGGTCATCCAGATCTCCTGTCTGCTGTACTCGCTTGCTACTCAGAAC

CACGAACACACCCTGCTGTTTTCCCTCGGGTCATGCGATATCTCTGAGGAATACTCGTTT

GCATGCGTCCAGCGCGGCGAGCCCAGACCGACGGTTTTGGAGTTTGACAGCGAGTACGAG

CTGCTGGTTGCCTTCCTGACCTTTCTCAAGCAGTACTCTCCCGAGTTCGCCACCGGCTAC

AACATCGTTAATTTTGACTGGGCGTACATAGTTAACAAGGTAACGTCGGTGTATAACATC

AAGCTGGACGGGTACGGCAAGTTCAACAAAGGGGGGCTGTTTAAGGTGTGGGACATCGCC

ACGAACCATTTTCAGAAGAAGAGCAAGGTGAAAATCAATGGCCTGATATCTCTAGACATG

TATTCTGTGGCGACGGAAAAGCTAAAGCTACCCAGCTACAAACTCGACGCGGTCGTGGGA

GACGTCCTCGGCGAGCATAAGATAGACCTTCCCTATAAAGAAATACCCTCCTATTACGCG

GGAGGGCCTGACCGGAGGGGCGTAATAGGAGAGTATTGTATCCAGGACTCTAGGCTGGTG

GGCAAGCTGTTTTTTAAGTACCTCCCCCATCTGGAACTATCGGCGGTGGCCAAACTCGCC

CGTATCACCCTAACGCGGGTAATTTTTGACGGTCAGCAAATTCGCGTGTACACGTGCTTG

CTGAAACTCGCCCGCGAGAGAAATTTCATTTTGCCAGACAACAGACGCCGGTTTGACAGT

CAGGCAGACGCCGCGTCAGAGACTTCGGAGTTGGCTATGGATAGCCAAAGCCACGCCTTC

GACAGTACAGACGAACCCGACGGTGTGGACGGTACCCCGGACGCCGCAGGATCTGGCGCT

ACTTCTGAAAACGGAGGCGGGAAGCCCGGCGTCGGGAGGGCCGTGGGCTACCAGGGAGCA

AAGGTTCTAGACCCCGTATCCGGCTTTCATGTGGACCCCGTGGTTGTGTTTGACTTCGCT

AGCTTATACCCAAGCATTATCCAGGCCCATAACCTCTGTTTCACCACCCTGGCGCTCGAT

GAAGTGGATCTGGCCGGGCTTCAACCATCCGTCAACTACTCGACGTTCGAGGTGGGTGAC

CAAAAGTTATTTTTTGTCCACGCCCATATTCGCGAAAGCCTGCTTGGCATCTTGCTGCGC

GACTGGCTGGCCATGCGAAAGGCGGTGAGGGCGCGAATCCCCACCAGCACCCCCGAGGAG

GCAGTTTTACTAGATAAGCAGCAGTCTGCGATTAAGGTGATATGCAACTCGGTTTACGGA

TTCACGGGGGTGGCAAACGGCCTGTTGCCGTGTCTGAGGATAGCGGCTACCGTTACCACG

ATAGGACGCGACATGCTCCTCAAGACCCGAGATTACGTTCACTCTCGTTGGGCGACGCGC

GAGCTGCTGGAGGACAATTTTCCAGGGGCTATAGGTTTCCGAAACCACAAGCCTTACTCC

GTCAGGGTTATCTACGGAGACACCGACTCCGTGTTTATCAAGTTTGTGGGCCTGACGTAC

GAGGGGGTATCGGAGCTGGGGGATGCTATGTCGCGTCAGATTTCAGCGGACCTCTTTAGA

GCGCCCATCAAACTGGAGTGTGAGAAGACCTTTCAGCGACTGCTGCTGATCACCAAGAAG

AAGTACATAGGTGTCATAAACGGGGGGAAGATGCTCATGAAGGGGGTCGACCTGGTCCGC

AAAAATAACTGCTCTTTCATAAACTTGTACGCGCGACATCTGGTAGATCTTTTGTTGTAC

GACGAGGATGTGGCCACGGCGGCAGCAGAGGTGACAGACGTGCCTCCCGCAGAATGGGTG

GGGCGCCCGCTACCGAGCGGCTTTGACAAGTTTGGGCGAGTGCTGGTAGAGGCGTACAAC

CGTATCACTGCCCCCAACTTGGACGTGCGCGAGTTCGTTATGACTGCTGAGCTGAGCCGC

TCACCCGAATCGTATACCAACAAGCGCCTGCCGCACCTCACCGTCTACTTTAAGCTCGCC

ATGAGGAATGAAGAACTGCCCAGCGTAAAAGAGAGAATTCCGTATGTGATAGTTGCGCAG

ACCGAGGCCGCGGAACGCGAAGCGGGTGTAGTAAACTCAATGCGCGGTACCGCCCAAAAC

CCCGTGGTAACCAAGACCGCACGCCCCCAACCTAAACGCAAACTGCTGGTTTCCGACCTC

GCCGAAGACCCGACCTATGTTTCCGAGAATGACGTACCGCTAAACACAGACTACTATTTC

TCCCACCTGTTGGGTACCATAAGCGTGACCTTTAAGGCTCTATTCGGAAATGATGTGAGA

ACAACAGAAAATCTTTTAAAGCGGTTTATTCCGGAAACCCCCCACAAGACCCCCACGAAA

ACCCAGGCACTGCTTGAGCGCGCCGGCTTTGAAAAGCTGACGCCCTTTACACCGGAGGAA

GAAAGTCGTCGAATACTGCATACAGTTTTTTGTACTCTAGAAGCAGCTCCCCATCAAAGC

TGA

>KU206409.1

ATGGCGGCGCGCGAACAGGCCAACTCCGTTCGCAGGAGCGGGTTTTTTAATCCGTTTATT

GGCAAGAGGCCATTTTTCAGGCCGGGAAGCGGGCAAACCGCGGAGACAGAGCGCCCAAGG

CCCCCCCAACACTCGTACTGCACAGAGGTGGGTAGCTTTAAGTTTATAGCTCCAAGATGC

CTCGATGAAGAAGCCCCCGCCGACCAGCGACGCGGTGTACACGTGGGCACCTTGGAGCGC

CCACCCAAAGTGTACTGCGATGGCTCAGAGTACGACGTGCTGAACTTTGCCTCCGGAGGT

TGCTGGCCTCGCAGGATTCGGGTTTGGAACGGCCAGGATTTTCGGGGCGATGGATTCAAC

CCCAGATTTGAGAGATTTCACGTGTACGACATAGTAGAGACTTCGGAGAGCGCCTCACAC

GATGACCCATCCAGGTTTGCTGAGCTATCCCGCCCAAGCGGGTCTGTGGTAACACTGCTG

GGAATGAGCGAGTGTGGAAAGCGAGTCGCGGTTCACGTCTATGGTGTGCGCCATTATTTT

TACATGGCAAAGGCGGAGGTGGATAGCGCTTGTGGAATAACCACCGAGGCAGAACTCGTG

CGCGCAATGGTGGACTGCGCGCACAGCTCGGCTTTGAGCGCGGCCCTGGGAAATGGCAAC

GGCGGCAAACAGAGCGGCGGCAGCGGCGGGGGATGGTGGGGCGGAAAGCACGTGTCTGCG

GACTGCTTCAAAGTGGAGACCGTGTGCCACACGACGCTGTACTACTTTGGATCTAAGCCA

GCTCTCTACTATAGAGTATCTGCCTCCAGCAGCCGCCTGGGAGGGTTCATCTGCGACAAC

TTTCACCCGGAGATTACAAAATTCGAGGGGAGCGTGGACGTGACCACGCGGCTGTTGTTG

GACAATGAAAATTTTACCAGTTTCGGGTGGTACCGCCTGCGACCCGGCACCCACGGAGAG

CGTGTTCAACTTCGCCCCGTTGAGCGACACGTCACCTCAAGCGACGTGGAGATTAACTGT

ACTCCCGATAACCTGGAGCCGATACCAGACGAGGCTGCCTGGCCCGACTATAAGCTCATG

TGCTTTGATATAGAGTGTAAAGCTGGAACGGGTAACGAAATGGCGTTCCCAGTGGCAACT

AACCAAGAGGACCTGGTCATCCAGATCTCCTGTCTGCTGTACTCGCTTGCTACTCAGAAC

CACGAACACACCCTGCTGTTTTCCCTCGGGTCATGCGATATCTCTGAGGAATACTCGTTT

GCATGCGTCCAGCGCGGCGAGCCCAGACCGACGGTTTTGGAGTTTGACAGCGAGTACGAG

CTGCTGGTTGCCTTCCTGACCTTTCTCAAGCAGTACTCTCCCGAGTTCGCCACCGGCTAC

AACATCGTTAATTTTGACTGGGCGTACATAGTTAACAAGGTAACGTCGGTGTATAACATC

AAGCTGGACGGGTACGGCAAGTTCAACAAAGGGGGGCTGTTTAAGGTGTGGGACATCGCC

ACGAACCATTTTCAGAAGAAGAGCAAGGTGAAAATCAATGGCCTGATATCTCTAGACATG

TATTCTGTGGCGACGGAAAAGCTAAAGCTACCCAGCTACAAACTCGACGCGGTCGTGGGA

GACGTCCTCGGCGAGCATAAGATAGACCTTCCCTATAAAGAAATACCCTCCTATTACGCG

GGAGGGCCTGACCGGAGGGGCGTAATAGGAGAGTATTGTATCCAGGACTCTAGGCTGGTG

GGCAAGCTGTTTTTTAAGTACCTCCCCCATCTGGAACTATCGGCGGTGGCCAAACTCGCC

CGTATCACCCTAACGCGGGTAATTTTTGACGGTCAGCAAATTCGCGTGTACACGTGCTTG

CTGAAACTCGCCCGCGAGAGAAATTTCATTTTGCCAGACAACAGACGCCGGTTTGACAGT

CAGGCAGACGCCGCGTCAGAGACTTCGGAGTTGGCTATGGATAGCCAAAGCCACGCCTTC

GACAGTACAGACGAACCCGACGGTGTGGACGGTACCCCGGACGCCGCAGGATCTGGCGCT

ACTTCTGAAAACGGAGGCGGGAAGCCCGGCGTCGGGAGGGCCGTGGGCTACCAGGGAGCA

AAGGTTCTAGACCCCGTATCCGGCTTTCATGTGGACCCCGTGGTTGTGTTTGACTTCGCT

AGCTTATACCCAAGCATTATCCAGGCCCATAACCTCTGTTTCACCACCCTGGCGCTCGAT

GAAGTGGATCTGGCCGGGCTTCAACCATCCGTCAACTACTCGACGTTCGAGGTGGGTGAC

CAAAAGTTATTTTTTGTCCACGCCCATATTCGCGAAAGCCTGCTTGGCATCTTGCTGCGC

GACTGGCTGGCCATGCGAAAGGCGGTGAGGGCGCGAATCCCCACCAGCACCCCCGAGGAG

GCAGTTTTACTAGATAAGCAGCAGTCTGCGATTAAGGTGATATGCAACTCGGTTTACGGA

TTCACGGGGGTGGCAAACGGCCTGTTGCCGTGTCTGAGGATAGCGGCTACCGTTACCACG

ATAGGACGCGACATGCTCCTCAAGACCCGAGATTACGTTCACTCTCGTTGGGCGACGCGC

GAGCTGCTGGAGGACAATTTTCCAGGGGCTATAGGTTTCCGAAACCACAAGCCTTACTCC

GTCAGGGTTATCTACGGAGACACCGACTCCGTGTTTATCAAGTTTGTGGGCCTGACGTAC

GAGGGGGTATCGGAGCTGGGGGATGCTATGTCGCGTCAGATTTCAGCGGACCTCTTTAGA

GCGCCCATCAAACTGGAGTGTGAGAAGACCTTTCAGCGACTGCTGCTGATCACCAAGAAG

AAGTACATAGGTGTCATAAACGGGGGGAAGATGCTCATGAAGGGGGTCGACCTGGTCCGC

AAAAATAACTGCTCTTTCATAAACTTGTACGCGCGACATCTGGTAGATCTTTTGTTGTAC

GACGAGGATGTGGCCACGGCGGCAGCAGAGGTGACAGACGTGCCTCCCGCAGAATGGGTG

GGGCGCCCGCTACCGAGCGGCTTTGACAAGTTTGGGCGAGTGCTGGTAGAGGCGTACAAC

CGTATCACTGCCCCCAACTTGGACGTGCGCGAGTTCGTTATGACTGCTGAGCTGAGCCGC

TCACCCGAATCGTATACCAACAAGCGCCTGCCGCACCTCACCGTCTACTTTAAGCTCGCC

ATGAGGAATGAAGAACTGCCCAGCGTAAAAGAGAGAATTCCGTATGTGATAGTTGCGCAG

ACCGAGGCCGCGGAACGCGAAGCGGGTGTAGTAAACTCAATGCGCGGTACCGCCCAAAAC

CCCGTGGTAACCAAGACCGCACGCCCCCAACCTAAACGCAAACTGCTGGTTTCCGACCTC

GCCGAAGACCCGACCTATGTTTCCGAGAATGACGTACCGCTAAACACAGACTACTATTTC

TCCCACCTGTTGGGTACCATAAGCGTGACCTTTAAGGCTCTATTCGGAAATGATGTGAGA

ACAACAGAAAATCTTTTAAAGCGGTTTATTCCGGAAACCCCCCACAAGACCCCCACGAAA

ACCCAGGCACTGCTTGAGCGCGCCGGCTTTGAAAAGCTGACGCCCTTTACACCGGAGGAA

GAAAGTCGTCGAATACTGCATACAGTTTTTTGTACTCTAGAAGCAGCTCCCCATCAAAGC

TGA

>KU206410.1

ATGGCGGCGCGCGAACAGGCCAACTCCGTTCGCAGGAGCGGGTTTTTTAATCCGTTTATT

GGCAAGAGGCCATTTTTCAGGCCGGGAAGCGGGCAAACCGCGGAGACAGAGCGCCCAAGG

CCCCCCCAACACTCGTACTGCACAGAGGTGGGTAGCTTTAAGTTTATAGCTCCAAGATGC

CTCGATGAAGAAGCCCCCGCCGACCAGCGACGCGGTGTACACGTGGGCACCTTGGAGCGC

CCACCCAAAGTGTACTGCGATGGCTCAGAGTACGACGTGCTGAACTTTGCCTCCGGAGGT

TGCTGGCCTCGCAGGATTCGGGTTTGGAACGGCCAGGATTTTCGGGGCGATGGATTCAAC

CCCAGATTTGAGAGATTTCACGTGTACGACATAGTAGAGACTTCGGAGAGCGCCTCACAC

GATGACCCATCCAGGTTTGCTGAGCTATCCCGCCCAAGCGGGTCTGTGGTAACACTGCTG

GGAATGAGCGAGTGTGGAAAGCGAGTCGCGGTTCACGTCTATGGTGTGCGCCATTATTTT

TACATGGCAAAGGCGGAGGTGGATAGCGCTTGTGGAATAACCACCGAGGCAGAACTCGTG

CGCGCAATGGTGGACTGCGCGCACAGCTCGGCTTTGAGCGCGGCCCTGGGAAATGGCAAC

GGCGGCAAACAGAGCGGCGGCAGCGGCGGGGGATGGTGGGGCGGAAAGCACGTGTCTGCG

GACTGCTTCAAAGTGGAGACCGTGTGCCACACGACGCTGTACTACTTTGGATCTAAGCCA

GCTCTCTACTATAGAGTATCTGCCTCCAGCAGCCGCCTGGGAGGGTTCATCTGCGACAAC

TTTCACCCGGAGATTACAAAATTCGAGGGGAGCGTGGACGTGACCACGCGGCTGTTGTTG

GACAATGAAAATTTTACCAGTTTCGGGTGGTACCGCCTGCGACCCGGCACCCACGGAGAG

CGTGTTCAACTTCGCCCCGTTGAGCGACACGTCACCTCAAGCGACGTGGAGATTAACTGT

ACTCCCGATAACCTGGAGCCGATACCAGACGAGGCTGCCTGGCCCGACTATAAGCTCATG

TGCTTTGATATAGAGTGTAAAGCTGGAACGGGTAACGAAATGGCGTTCCCAGTGGCAACT

AACCAAGAGGACCTGGTCATCCAGATCTCCTGTCTGCTGTACTCGCTTGCTACTCAGAAC

CACGAACACACCCTGCTGTTTTCCCTCGGGTCATGCGATATCTCTGAGGAATACTCGTTT

GCATGCGTCCAGCGCGGCGAGCCCAGACCGACGGTTTTGGAGTTTGACAGCGAGTACGAG

CTGCTGGTTGCCTTCCTGACCTTTCTCAAGCAGTACTCTCCCGAGTTCGCCACCGGCTAC

AACATCGTTAATTTTGACTGGGCGTACATAGTTAACAAGGTAACGTCGGTGTATAACATC

AAGCTGGACGGGTACGGCAAGTTCAACAAAGGGGGGCTGTTTAAGGTGTGGGACATCGCC

ACGAACCATTTTCAGAAGAAGAGCAAGGTGAAAATCAATGGCCTGATATCTCTAGACATG

TATTCTGTGGCGACGGAAAAGCTAAAGCTACCCAGCTACAAACTCGACGCGGTCGTGGGA

GACGTCCTCGGCGAGCATAAGATAGACCTTCCCTATAAAGAAATACCCTCCTATTACGCG

GGAGGGCCTGACCGGAGGGGCGTAATAGGAGAGTATTGTATCCAGGACTCTAGGCTGGTG

GGCAAGCTGTTTTTTAAGTACCTCCCCCATCTGGAACTATCGGCGGTGGCCAAACTCGCC

CGTATCACCCTAACGCGGGTAATTTTTGACGGTCAGCAAATTCGCGTGTACACGTGCTTG

CTGAAACTCGCCCGCGAGAGAAATTTCATTTTGCCAGACAACAGACGCCGGTTTGACAGT

CAGGCAGACGCCGCGTCAGAGACTTCGGAGTTGGCTATGGATAGCCAAAGCCACGCCTTC

GACAGTACAGACGAACCCGACGGTGTGGACGGTACCCCGGACGCCGCAGGATCTGGCGCT

ACTTCTGAAAACGGAGGCGGGAAGCCCGGCGTCGGGAGGGCCGTGGGCTACCAGGGAGCA

AAGGTTCTAGACCCCGTATCCGGCTTTCATGTGGACCCCGTGGTTGTGTTTGACTTCGCT

AGCTTATACCCAAGCATTATCCAGGCCCATAACCTCTGTTTCACCACCCTGGCGCTCGAT

GAAGTGGATCTGGCCGGGCTTCAACCATCCGTCAACTACTCGACGTTCGAGGTGGGTGAC

CAAAAGTTATTTTTTGTCCACGCCCATATTCGCGAAAGCCTGCTTGGCATCTTGCTGCGC

GACTGGCTGGCCATGCGAAAGGCGGTGAGGGCGCGAATCCCCACCAGCACCCCCGAGGAG

GCAGTTTTACTAGATAAGCAGCAGTCTGCGATTAAGGTGATATGCAACTCGGTTTACGGA

TTCACGGGGGTGGCAAACGGCCTGTTGCCGTGTCTGAGGATAGCGGCTACCGTTACCACG

ATAGGACGCGACATGCTCCTCAAGACCCGAGATTACGTTCACTCTCGTTGGGCGACGCGC

GAGCTGCTGGAGGACAATTTTCCAGGGGCTATAGGTTTCCGAAACCACAAGCCTTACTCC

GTCAGGGTTATCTACGGAGACACCGACTCCGTGTTTATCAAGTTTGTGGGCCTGACGTAC

GAGGGGGTATCGGAGCTGGGGGATGCTATGTCGCGTCAGATTTCAGCGGACCTCTTTAGA

GCGCCCATCAAACTGGAGTGTGAGAAGACCTTTCAGCGACTGCTGCTGATCACCAAGAAG

AAGTACATAGGTGTCATAAACGGGGGGAAGATGCTCATGAAGGGGGTCGACCTGGTCCGC

AAAAATAACTGCTCTTTCATAAACTTGTACGCGCGACATCTGGTAGATCTTTTGTTGTAC

GACGAGGATGTGGCCACGGCGGCAGCAGAGGTGACAGACGTGCCTCCCGCAGAATGGGTG

GGGCGCCCGCTACCGAGCGGCTTTGACAAGTTTGGGCGAGTGCTGGTAGAGGCGTACAAC

CGTATCACTGCCCCCAACTTGGACGTGCGCGAGTTCGTTATGACTGCTGAGCTGAGCCGC

TCACCCGAATCGTATACCAACAAGCGCCTGCCGCACCTCACCGTCTACTTTAAGCTCGCC

ATGAGGAATGAAGAACTGCCCAGCGTAAAAGAGAGAATTCCGTATGTGATAGTTGCGCAG

ACCGAGGCCGCGGAACGCGAAGCGGGTGTAGTAAACTCAATGCGCGGTACCGCCCAAAAC

CCCGTGGTAACCAAGACCGCACGCCCCCAACCTAAACGCAAACTGCTGGTTTCCGACCTC

GCCGAAGACCCGACCTATGTTTCCGAGAATGACGTACCGCTAAACACAGACTACTATTTC

TCCCACCTGTTGGGTACCATAAGCGTGACCTTTAAGGCTCTATTCGGAAATGATGTGAGA

ACAACAGAAAATCTTTTAAAGCGGTTTATTCCGGAAACCCCCCACAAGACCCCCACGAAA

ACCCAGGCACTGCTTGAGCGCGCCGGCTTTGAAAAGCTGACGCCCTTTACACCGGAGGAA

GAAAGTCGTCGAATACTGCATACAGTTTTTTGTACTCTAGAAGCAGCTCCCCATCAAAGC

TGA

>KU206411.1

ATGGCGGCGCGCGAACAGGCCAACTCCGTTCGCAGGAGCGGGTTTTTTAATCCGTTTATT

GGCAAGAGGCCATTTTTCAGGCCGGGAAGCGGGCAAACCGCGGAGACAGAGCGCCCAAGG

CCCCCCCAACACTCGTACTGCACAGAGGTGGGTAGCTTTAAGTTTATAGCTCCAAGATGC

CTCGATGAAGAAGCCCCCGCCGACCAGCGACGCGGTGTACACGTGGGCACCTTGGAGCGC

CCACCCAAAGTGTACTGCGATGGCTCAGAGTACGACGTGCTGAACTTTGCCTCCGGAGGT

TGCTGGCCTCGCAGGATTCGGGTTTGGAACGGCCAGGATTTTCGGGGCGATGGATTCAAC

CCCAGATTTGAGAGATTTCACGTGTACGACATAGTAGAGACTTCGGAGAGCGCCTCACAC

GATGACCCATCCAGGTTTGCTGAGCTATCCCGCCCAAGCGGGTCTGTGGTAACACTGCTG

GGAATGAGCGAGTGTGGAAAGCGAGTCGCGGTTCACGTCTATGGTGTGCGCCATTATTTT

TACATGGCAAAGGCGGAGGTGGATAGCGCTTGTGGAATAACCACCGAGGCAGAACTCGTG

CGCGCAATGGTGGACTGCGCGCACAGCTCGGCTTTGAGCGCGGCCCTGGGAAATGGCAAC

GGCGGCAAACAGAGCGGCGGCAGCGGCGGGGGATGGTGGGGCGGAAAGCACGTGTCTGCG

GACTGCTTCAAAGTGGAGACCGTGTGCCACACGACGCTGTACTACTTTGGATCTAAGCCA

GCTCTCTACTATAGAGTATCTGCCTCCAGCAGCCGCCTGGGAGGGTTCATCTGCGACAAC

TTTCACCCGGAGATTACAAAATTCGAGGGGAGCGTGGACGTGACCACGCGGCTGTTGTTG

GACAATGAAAATTTTACCAGTTTCGGGTGGTACCGCCTGCGACCCGGCACCCACGGAGAG

CGTGTTCAACTTCGCCCCGTTGAGCGACACGTCACCTCAAGCGACGTGGAGATTAACTGT

ACTCCCGATAACCTGGAGCCGATACCAGACGAGGCTGCCTGGCCCGACTATAAGCTCATG

TGCTTTGATATAGAGTGTAAAGCTGGAACGGGTAACGAAATGGCGTTCCCAGTGGCAACT

AACCAAGAGGACCTGGTCATCCAGATCTCCTGTCTGCTGTACTCGCTTGCTACTCAGAAC

CACGAACACACCCTGCTGTTTTCCCTCGGGTCATGCGATATCTCTGAGGAATACTCGTTT

GCATGCGTCCAGCGCGGCGAGCCCAGACCGACGGTTTTGGAGTTTGACAGCGAGTACGAG

CTGCTGGTTGCCTTCCTGACCTTTCTCAAGCAGTACTCTCCCGAGTTCGCCACCGGCTAC

AACATCGTTAATTTTGACTGGGCGTACATAGTTAACAAGGTAACGTCGGTGTATAACATC

AAGCTGGACGGGTACGGCAAGTTCAACAAAGGGGGGCTGTTTAAGGTGTGGGACATCGCC

ACGAACCATTTTCAGAAGAAGAGCAAGGTGAAAATCAATGGCCTGATATCTCTAGACATG

TATTCTGTGGCGACGGAAAAGCTAAAGCTACCCAGCTACAAACTCGACGCGGTCGTGGGA

GACGTCCTCGGCGAGCATAAGATAGACCTTCCCTATAAAGAAATACCCTCCTATTACGCG

GGAGGGCCTGACCGGAGGGGCGTAATAGGAGAGTATTGTATCCAGGACTCTAGGCTGGTG

GGCAAGCTGTTTTTTAAGTACCTCCCCCATCTGGAACTATCGGCGGTGGCCAAACTCGCC

CGTATCACCCTAACGCGGGTAATTTTTGACGGTCAGCAAATTCGCGTGTACACGTGCTTG

CTGAAACTCGCCCGCGAGAGAAATTTCATTTTGCCAGACAACAGACGCCGGTTTGACAGT

CAGGCAGACGCCGCGTCAGAGACTTCGGAGTTGGCTATGGATAGCCAAAGCCACGCCTTC

GACAGTACAGACGAACCCGACGGTGTGGACGGTACCCCGGACGCCGCAGGATCTGGCGCT

ACTTCTGAAAACGGAGGCGGGAAGCCCGGCGTCGGGAGGGCCGTGGGCTACCAGGGAGCA

AAGGTTCTAGACCCCGTATCCGGCTTTCATGTGGACCCCGTGGTTGTGTTTGACTTCGCT

AGCTTATACCCAAGCATTATCCAGGCCCATAACCTCTGTTTCACCACCCTGGCGCTCGAT

GAAGTGGATCTGGCCGGGCTTCAACCATCCGTCAACTACTCGACGTTCGAGGTGGGTGAC

CAAAAGTTATTTTTTGTCCACGCCCATATTCGCGAAAGCCTGCTTGGCATCTTGCTGCGC

GACTGGCTGGCCATGCGAAAGGCGGTGAGGGCGCGAATCCCCACCAGCACCCCCGAGGAG

GCAGTTTTACTAGATAAGCAGCAGTCTGCGATTAAGGTGATATGCAACTCGGTTTACGGA

TTCACGGGGGTGGCAAACGGCCTGTTGCCGTGTCTGAGGATAGCGGCTACCGTTACCACG

ATAGGACGCGACATGCTCCTCAAGACCCGAGATTACGTTCACTCTCGTTGGGCGACGCGC

GAGCTGCTGGAGGACAATTTTCCAGGGGCTATAGGTTTCCGAAACCACAAGCCTTACTCC

GTCAGGGTTATCTACGGAGACACCGACTCCGTGTTTATCAAGTTTGTGGGCCTGACGTAC

GAGGGGGTATCGGAGCTGGGGGATGCTATGTCGCGTCAGATTTCAGCGGACCTCTTTAGA

GCGCCCATCAAACTGGAGTGTGAGAAGACCTTTCAGCGACTGCTGCTGATCACCAAGAAG

AAGTACATAGGTGTCATAAACGGGGGGAAGATGCTCATGAAGGGGGTCGACCTGGTCCGC

AAAAATAACTGCTCTTTCATAAACTTGTACGCGCGACATCTGGTAGATCTTTTGTTGTAC

GACGAGGATGTGGCCACGGCGGCAGCAGAGGTGACAGACGTGCCTCCCGCAGAATGGGTG

GGGCGCCCGCTACCGAGCGGCTTTGACAAGTTTGGGCGAGTGCTGGTAGAGGCGTACAAC

CGTATCACTGCCCCCAACTTGGACGTGCGCGAGTTCGTTATGACTGCTGAGCTGAGCCGC

TCACCCGAATCGTATACCAACAAGCGCCTGCCGCACCTCACCGTCTACTTTAAGCTCGCC

ATGAGGAATGAAGAACTGCCCAGCGTAAAAGAGAGAATTCCGTATGTGATAGTTGCGCAG

ACCGAGGCCGCGGAACGCGAAGCGGGTGTAGTAAACTCAATGCGCGGTACCGCCCAAAAC

CCCGTGGTAACCAAGACCGCACGCCCCCAACCTAAACGCAAACTGCTGGTTTCCGACCTC

GCCGAAGACCCGACCTATGTTTCCGAGAATGACGTACCGCTAAACACAGACTACTATTTC

TCCCACCTGTTGGGTACCATAAGCGTGACCTTTAAGGCTCTATTCGGAAATGATGTGAGA

ACAACAGAAAATCTTTTAAAGCGGTTTATTCCGGAAACCCCCCACAAGACCCCCACGAAA

ACCCAGGCACTGCTTGAGCGCGCCGGCTTTGAAAAGCTGACGCCCTTTACACCGGAGGAA

GAAAGTCGTCGAATACTGCATACAGTTTTTTGTACTCTAGAAGCAGCTCCCCATCAAAGC

TGA

>KU206412.1

ATGGCGGCGCGCGAACAGGCCAACTCCGTTCGCAGGAGCGGGTTTTTTAATCCGTTTATT

GGCAAGAGGCCATTTTTCAGGCCGGGAAGCGGGCAAACCGCGGAGACAGAGCGCCCAAGG

CCCCCCCAACACTCGTACTGCACAGAGGTGGGTAGCTTTAAGTTTATAGCTCCAAGATGC

CTCGATGAAGAAGCCCCCGCCGACCAGCGACGCGGTGTACACGTGGGCACCTTGGAGCGC

CCACCCAAAGTGTACTGCGATGGCTCAGAGTACGACGTGCTGAACTTTGCCTCCGGAGGT

TGCTGGCCTCGCAGGATTCGGGTTTGGAACGGCCAGGATTTTCGGGGCGATGGATTCAAC

CCCAGATTTGAGAGATTTCACGTGTACGACATAGTAGAGACTTCGGAGAGCGCCTCACAC

GATGACCCATCCAGGTTTGCTGAGCTATCCCGCCCAAGCGGGTCTGTGGTAACACTGCTG

GGAATGAGCGAGTGTGGAAAGCGAGTCGCGGTTCACGTCTATGGTGTGCGCCATTATTTT

TACATGGCAAAGGCGGAGGTGGATAGCGCTTGTGGAATAACCACCGAGGCAGAACTCGTG

CGCGCAATGGTGGACTGCGCGCACAGCTCGGCTTTGAGCGCGGCCCTGGGAAATGGCAAC

GGCGGCAAACAGAGCGGCGGCAGCGGCGGGGGATGGTGGGGCGGAAAGCACGTGTCTGCG

GACTGCTTCAAAGTGGAGACCGTGTGCCACACGACGCTGTACTACTTTGGATCTAAGCCA

GCTCTCTACTATAGAGTATCTGCCTCCAGCAGCCGCCTGGGAGGGTTCATCTGCGACAAC

TTTCACCCGGAGATTACAAAATTCGAGGGGAGCGTGGACGTGACCACGCGGCTGTTGTTG

GACAATGAAAATTTTACCAGTTTCGGGTGGTACCGCCTGCGACCCGGCACCCACGGAGAG

CGTGTTCAACTTCGCCCCGTTGAGCGACACGTCACCTCAAGCGACGTGGAGATTAACTGT

ACTCCCGATAACCTGGAGCCGATACCAGACGAGGCTGCCTGGCCCGACTATAAGCTCATG

TGCTTTGATATAGAGTGTAAAGCTGGAACGGGTAACGAAATGGCGTTCCCAGTGGCAACT

AACCAAGAGGACCTGGTCATCCAGATCTCCTGTCTGCTGTACTCGCTTGCTACTCAGAAC

CACGAACACACCCTGCTGTTTTCCCTCGGGTCATGCGATATCTCTGAGGAATACTCGTTT

GCATGCGTCCAGCGCGGCGAGCCCAGACCGACGGTTTTGGAGTTTGACAGCGAGTACGAG

CTGCTGGTTGCCTTCCTGACCTTTCTCAAGCAGTACTCTCCCGAGTTCGCCACCGGCTAC

AACATCGTTAATTTTGACTGGGCGTACATAGTTAACAAGGTAACGTCGGTGTATAACATC

AAGCTGGACGGGTACGGCAAGTTCAACAAAGGGGGGCTGTTTAAGGTGTGGGACATCGCC

ACGAACCATTTTCAGAAGAAGAGCAAGGTGAAAATCAATGGCCTGATATCTCTAGACATG

TATTCTGTGGCGACGGAAAAGCTAAAGCTACCCAGCTACAAACTCGACGCGGTCGTGGGA

GACGTCCTCGGCGAGCATAAGATAGACCTTCCCTATAAAGAAATACCCTCCTATTACGCG

GGAGGGCCTGACCGGAGGGGCGTAATAGGAGAGTATTGTATCCAGGACTCTAGGCTGGTG

GGCAAGCTGTTTTTTAAGTACCTCCCCCATCTGGAACTATCGGCGGTGGCCAAACTCGCC

CGTATCACCCTAACGCGGGTAATTTTTGACGGTCAGCAAATTCGCGTGTACACGTGCTTG

CTGAAACTCGCCCGCGAGAGAAATTTCATTTTGCCAGACAACAGACGCCGGTTTGACAGT

CAGGCAGACGCCGCGTCAGAGACTTCGGAGTTGGCTATGGATAGCCAAAGCCACGCCTTC

GACAGTACAGACGAACCCGACGGTGTGGACGGTACCCCGGACGCCGCAGGATCTGGCGCT

ACTTCTGAAAACGGAGGCGGGAAGCCCGGCGTCGGGAGGGCCGTGGGCTACCAGGGAGCA

AAGGTTCTAGACCCCGTATCCGGCTTTCATGTGGACCCCGTGGTTGTGTTTGACTTCGCT

AGCTTATACCCAAGCATTATCCAGGCCCATAACCTCTGTTTCACCACCCTGGCGCTCGAT

GAAGTGGATCTGGCCGGGCTTCAACCATCCGTCAACTACTCGACGTTCGAGGTGGGTGAC

CAAAAGTTATTTTTTGTCCACGCCCATATTCGCGAAAGCCTGCTTGGCATCTTGCTGCGC

GACTGGCTGGCCATGCGAAAGGCGGTGAGGGCGCGAATCCCCACCAGCACCCCCGAGGAG

GCAGTTTTACTAGATAAGCAGCAGTCTGCGATTAAGGTGATATGCAACTCGGTTTACGGA

TTCACGGGGGTGGCAAACGGCCTGTTGCCGTGTCTGAGGATAGCGGCTACCGTTACCACG

ATAGGACGCGACATGCTCCTCAAGACCCGAGATTACGTTCACTCTCGTTGGGCGACGCGC

GAGCTGCTGGAGGACAATTTTCCAGGGGCTATAGGTTTCCGAAACCACAAGCCTTACTCC

GTCAGGGTTATCTACGGAGACACCGACTCCGTGTTTATCAAGTTTGTGGGCCTGACGTAC

GAGGGGGTATCGGAGCTGGGGGATGCTATGTCGCGTCAGATTTCAGCGGACCTCTTTAGA

GCGCCCATCAAACTGGAGTGTGAGAAGACCTTTCAGCGACTGCTGCTGATCACCAAGAAG

AAGTACATAGGTGTCATAAACGGGGGGAAGATGCTCATGAAGGGGGTCGACCTGGTCCGC

AAAAATAACTGCTCTTTCATAAACTTGTACGCGCGACATCTGGTAGATCTTTTGTTGTAC

GACGAGGATGTGGCCACGGCGGCAGCAGAGGTGACAGACGTGCCTCCCGCAGAATGGGTG

GGGCGCCCGCTACCGAGCGGCTTTGACAAGTTTGGGCGAGTGCTGGTAGAGGCGTACAAC

CGTATCACTGCCCCCAACTTGGACGTGCGCGAGTTCGTTATGACTGCTGAGCTGAGCCGC

TCACCCGAATCGTATACCAACAAGCGCCTGCCGCACCTCACCGTCTACTTTAAGCTCGCC

ATGAGGAATGAAGAACTGCCCAGCGTAAAAGAGAGAATTCCGTATGTGATAGTTGCGCAG

ACCGAGGCCGCGGAACGCGAAGCGGGTGTAGTAAACTCAATGCGCGGTACCGCCCAAAAC

CCCGTGGTAACCAAGACCGCACGCCCCCAACCTAAACGCAAACTGCTGGTTTCCGACCTC

GCCGAAGACCCGACCTATGTTTCCGAGAATGACGTACCGCTAAACACAGACTACTATTTC

TCCCACCTGTTGGGTACCATAAGCGTGACCTTTAAGGCTCTATTCGGAAATGATGTGAGA

ACAACAGAAAATCTTTTAAAGCGGTTTATTCCGGAAACCCCCCACAAGACCCCCACGAAA

ACCCAGGCACTGCTTGAGCGCGCCGGCTTTGAAAAGCTGACGCCCTTTACACCGGAGGAA

GAAAGTCGTCGAATACTGCATACAGTTTTTTGTACTCTAGAAGCAGCTCCCCATCAAAGC

TGA

>KU206413.1

ATGGCGGCGCGCGAACAGGCCAACTCCGTTCGCAGGAGCGGGTTTTTTAATCCGTTTATT

GGCAAGAGGCCATTTTTCAGGCCGGGAAGCGGGCAAACCGCGGAGACAGAGCGCCCAAGG

CCCCCCCAACACTCGTACTGCACAGAGGTGGGTAGCTTTAAGTTTATAGCTCCAAGATGC

CTCGATGAAGAAGCCCCCGCCGACCAGCGACGCGGTGTACACGTGGGCACCTTGGAGCGC

CCACCCAAAGTGTACTGCGATGGCTCAGAGTACGACGTGCTGAACTTTGCCTCCGGAGGT

TGCTGGCCTCGCAGGATTCGGGTTTGGAACGGCCAGGATTTTCGGGGCGATGGATTCAAC

CCCAGATTTGAGAGATTTCACGTGTACGACATAGTAGAGACTTCGGAGAGCGCCTCACAC

GATGACCCATCCAGGTTTGCTGAGCTATCCCGCCCAAGCGGGTCTGTGGTAACACTGCTG

GGAATGAGCGAGTGTGGAAAGCGAGTCGCGGTTCACGTCTATGGTGTGCGCCATTATTTT

TACATGGCAAAGGCGGAGGTGGATAGCGCTTGTGGAATAACCACCGAGGCAGAACTCGTG

CGCGCAATGGTGGACTGCGCGCACAGCTCGGCTTTGAGCGCGGCCCTGGGAAATGGCAAC

GGCGGCAAACAGAGCGGCGGCAGCGGCGGGGGATGGTGGGGCGGAAAGCACGTGTCTGCG

GACTGCTTCAAAGTGGAGACCGTGTGCCACACGACGCTGTACTACTTTGGATCTAAGCCA

GCTCTCTACTATAGAGTATCTGCCTCCAGCAGCCGCCTGGGAGGGTTCATCTGCGACAAC

TTTCACCCGGAGATTACAAAATTCGAGGGGAGCGTGGACGTGACCACGCGGCTGTTGTTG

GACAATGAAAATTTTACCAGTTTCGGGTGGTACCGCCTGCGACCCGGCACCCACGGAGAG

CGTGTTCAACTTCGCCCCGTTGAGCGACACGTCACCTCAAGCGACGTGGAGATTAACTGT

ACTCCCGATAACCTGGAGCCGATACCAGACGAGGCTGCCTGGCCCGACTATAAGCTCATG

TGCTTTGATATAGAGTGTAAAGCTGGAACGGGTAACGAAATGGCGTTCCCAGTGGCAACT

AACCAAGAGGACCTGGTCATCCAGATCTCCTGTCTGCTGTACTCGCTTGCTACTCAGAAC

CACGAACACACCCTGCTGTTTTCCCTCGGGTCATGCGATATCTCTGAGGAATACTCGTTT

GCATGCGTCCAGCGCGGCGAGCCCAGACCGACGGTTTTGGAGTTTGACAGCGAGTACGAG

CTGCTGGTTGCCTTCCTGACCTTTCTCAAGCAGTACTCTCCCGAGTTCGCCACCGGCTAC

AACATCGTTAATTTTGACTGGGCGTACATAGTTAACAAGGTAACGTCGGTGTATAACATC

AAGCTGGACGGGTACGGCAAGTTCAACAAAGGGGGGCTGTTTAAGGTGTGGGACATCGCC

ACGAACCATTTTCAGAAGAAGAGCAAGGTGAAAATCAATGGCCTGATATCTCTAGACATG

TATTCTGTGGCGACGGAAAAGCTAAAGCTACCCAGCTACAAACTCGACGCGGTCGTGGGA

GACGTCCTCGGCGAGCATAAGATAGACCTTCCCTATAAAGAAATACCCTCCTATTACGCG

GGAGGGCCTGACCGGAGGGGCGTAATAGGAGAGTATTGTATCCAGGACTCTAGGCTGGTG

GGCAAGCTGTTTTTTAAGTACCTCCCCCATCTGGAACTATCGGCGGTGGCCAAACTCGCC

CGTATCACCCTAACGCGGGTAATTTTTGACGGTCAGCAAATTCGCGTGTACACGTGCTTG

CTGAAACTCGCCCGCGAGAGAAATTTCATTTTGCCAGACAACAGACGCCGGTTTGACAGT

CAGGCAGACGCCGCGTCAGAGACTTCGGAGTTGGCTATGGATAGCCAAAGCCACGCCTTC

GACAGTACAGACGAACCCGACGGTGTGGACGGTACCCCGGACGCCGCAGGATCTGGCGCT

ACTTCTGAAAACGGAGGCGGGAAGCCCGGCGTCGGGAGGGCCGTGGGCTACCAGGGAGCA

AAGGTTCTAGACCCCGTATCCGGCTTTCATGTGGACCCCGTGGTTGTGTTTGACTTCGCT

AGCTTATACCCAAGCATTATCCAGGCCCATAACCTCTGTTTCACCACCCTGGCGCTCGAT

GAAGTGGATCTGGCCGGGCTTCAACCATCCGTCAACTACTCGACGTTCGAGGTGGGTGAC

CAAAAGTTATTTTTTGTCCACGCCCATATTCGCGAAAGCCTGCTTGGCATCTTGCTGCGC

GACTGGCTGGCCATGCGAAAGGCGGTGAGGGCGCGAATCCCCACCAGCACCCCCGAGGAG

GCAGTTTTACTAGATAAGCAGCAGTCTGCGATTAAGGTGATATGCAACTCGGTTTACGGA

TTCACGGGGGTGGCAAACGGCCTGTTGCCGTGTCTGAGGATAGCGGCTACCGTTACCACG

ATAGGACGCGACATGCTCCTCAAGACCCGAGATTACGTTCACTCTCGTTGGGCGACGCGC

GAGCTGCTGGAGGACAATTTTCCAGGGGCTATAGGTTTCCGAAACCACAAGCCTTACTCC

GTCAGGGTTATCTACGGAGACACCGACTCCGTGTTTATCAAGTTTGTGGGCCTGACGTAC

GAGGGGGTATCGGAGCTGGGGGATGCTATGTCGCGTCAGATTTCAGCGGACCTCTTTAGA

GCGCCCATCAAACTGGAGTGTGAGAAGACCTTTCAGCGACTGCTGCTGATCACCAAGAAG

AAGTACATAGGTGTCATAAACGGGGGGAAGATGCTCATGAAGGGGGTCGACCTGGTCCGC

AAAAATAACTGCTCTTTCATAAACTTGTACGCGCGACATCTGGTAGATCTTTTGTTGTAC

GACGAGGATGTGGCCACGGCGGCAGCAGAGGTGACAGACGTGCCTCCCGCAGAATGGGTG

GGGCGCCCGCTACCGAGCGGCTTTGACAAGTTTGGGCGAGTGCTGGTAGAGGCGTACAAC

CGTATCACTGCCCCCAACTTGGACGTGCGCGAGTTCGTTATGACTGCTGAGCTGAGCCGC

TCACCCGAATCGTATACCAACAAGCGCCTGCCGCACCTCACCGTCTACTTTAAGCTCGCC

ATGAGGAATGAAGAACTGCCCAGCGTAAAAGAGAGAATTCCGTATGTGATAGTTGCGCAG

ACCGAGGCCGCGGAACGCGAAGCGGGTGTAGTAAACTCAATGCGCGGTACCGCCCAAAAC

CCCGTGGTAACCAAGACCGCACGCCCCCAACCTAAACGCAAACTGCTGGTTTCCGACCTC

GCCGAAGACCCGACCTATGTTTCCGAGAATGACGTACCGCTAAACACAGACTACTATTTC

TCCCACCTGTTGGGTACCATAAGCGTGACCTTTAAGGCTCTATTCGGAAATGATGTGAGA

ACAACAGAAAATCTTTTAAAGCGGTTTATTCCGGAAACCCCCCACAAGACCCCCACGAAA

ACCCAGGCACTGCTTGAGCGCGCCGGCTTTGAAAAGCTGACGCCCTTTACACCGGAGGAA

GAAAGTCGTCGAATACTGCATACAGTTTTTTGTACTCTAGAAGCAGCTCCCCATCAAAGC

TGA

>KU206414.1

ATGGCGGCGCGCGAACAGGCCAACTCCGTTCGCAGGAGCGGGTTTTTTAATCCGTTTATT

GGCAAGAGGCCATTTTTCAGGCCGGGAAGCGGGCAAACCGCGGAGACAGAGCGCCCAAGG

CCCCCCCAACACTCGTACTGCACAGAGGTGGGTAGCTTTAAGTTTATAGCTCCAAGATGC

CTCGATGAAGAAGCCCCCGCCGACCAGCGACGCGGTGTACACGTGGGCACCTTGGAGCGC

CCACCCAAAGTGTACTGCGATGGCTCAGAGTACGACGTGCTGAACTTTGCCTCCGGAGGT

TGCTGGCCTCGCAGGATTCGGGTTTGGAACGGCCAGGATTTTCGGGGCGATGGATTCAAC

CCCAGATTTGAGAGATTTCACGTGTACGACATAGTAGAGACTTCGGAGAGCGCCTCACAC

GATGACCCATCCAGGTTTGCTGAGCTATCCCGCCCAAGCGGGTCTGTGGTAACACTGCTG

GGAATGAGCGAGTGTGGAAAGCGAGTCGCGGTTCACGTCTATGGTGTGCGCCATTATTTT

TACATGGCAAAGGCGGAGGTGGATAGCGCTTGTGGAATAACCACCGAGGCAGAACTCGTG

CGCGCAATGGTGGACTGCGCGCACAGCTCGGCTTTGAGCGCGGCCCTGGGAAATGGCAAC

GGCGGCAAACAGAGCGGCGGCAGCGGCGGGGGATGGTGGGGCGGAAAGCACGTGTCTGCG

GACTGCTTCAAAGTGGAGACCGTGTGCCACACGACGCTGTACTACTTTGGATCTAAGCCA

GCTCTCTACTATAGAGTATCTGCCTCCAGCAGCCGCCTGGGAGGGTTCATCTGCGACAAC

TTTCACCCGGAGATTACAAAATTCGAGGGGAGCGTGGACGTGACCACGCGGCTGTTGTTG

GACAATGAAAATTTTACCAGTTTCGGGTGGTACCGCCTGCGACCCGGCACCCACGGAGAG

CGTGTTCAACTTCGCCCCGTTGAGCGACACGTCACCTCAAGCGACGTGGAGATTAACTGT

ACTCCCGATAACCTGGAGCCGATACCAGACGAGGCTGCCTGGCCCGACTATAAGCTCATG

TGCTTTGATATAGAGTGTAAAGCTGGAACGGGTAACGAAATGGCGTTCCCAGTGGCAACT

AACCAAGAGGACCTGGTCATCCAGATCTCCTGTCTGCTGTACTCGCTTGCTACTCAGAAC

CACGAACACACCCTGCTGTTTTCCCTCGGGTCATGCGATATCTCTGAGGAATACTCGTTT

GCATGCGTCCAGCGCGGCGAGCCCAGACCGACGGTTTTGGAGTTTGACAGCGAGTACGAG

CTGCTGGTTGCCTTCCTGACCTTTCTCAAGCAGTACTCTCCCGAGTTCGCCACCGGCTAC

AACATCGTTAATTTTGACTGGGCGTACATAGTTAACAAGGTAACGTCGGTGTATAACATC

AAGCTGGACGGGTACGGCAAGTTCAACAAAGGGGGGCTGTTTAAGGTGTGGGACATCGCC

ACGAACCATTTTCAGAAGAAGAGCAAGGTGAAAATCAATGGCCTGATATCTCTAGACATG

TATTCTGTGGCGACGGAAAAGCTAAAGCTACCCAGCTACAAACTCGACGCGGTCGTGGGA

GACGTCCTCGGCGAGCATAAGATAGACCTTCCCTATAAAGAAATACCCTCCTATTACGCG

GGAGGGCCTGACCGGAGGGGCGTAATAGGAGAGTATTGTATCCAGGACTCTAGGCTGGTG

GGCAAGCTGTTTTTTAAGTACCTCCCCCATCTGGAACTATCGGCGGTGGCCAAACTCGCC

CGTATCACCCTAACGCGGGTAATTTTTGACGGTCAGCAAATTCGCGTGTACACGTGCTTG

CTGAAACTCGCCCGCGAGAGAAATTTCATTTTGCCAGACAACAGACGCCGGTTTGACAGT

CAGGCAGACGCCGCGTCAGAGACTTCGGAGTTGGCTATGGATAGCCAAAGCCACGCCTTC

GACAGTACAGACGAACCCGACGGTGTGGACGGTACCCCGGACGCCGCAGGATCTGGCGCT

ACTTCTGAAAACGGAGGCGGGAAGCCCGGCGTCGGGAGGGCCGTGGGCTACCAGGGAGCA

AAGGTTCTAGACCCCGTATCCGGCTTTCATGTGGACCCCGTGGTTGTGTTTGACTTCGCT

AGCTTATACCCAAGCATTATCCAGGCCCATAACCTCTGTTTCACCACCCTGGCGCTCGAT

GAAGTGGATCTGGCCGGGCTTCAACCATCCGTCAACTACTCGACGTTCGAGGTGGGTGAC

CAAAAGTTATTTTTTGTCCACGCCCATATTCGCGAAAGCCTGCTTGGCATCTTGCTGCGC

GACTGGCTGGCCATGCGAAAGGCGGTGAGGGCGCGAATCCCCACCAGCACCCCCGAGGAG

GCAGTTTTACTAGATAAGCAGCAGTCTGCGATTAAGGTGATATGCAACTCGGTTTACGGA

TTCACGGGGGTGGCAAACGGCCTGTTGCCGTGTCTGAGGATAGCGGCTACCGTTACCACG

ATAGGACGCGACATGCTCCTCAAGACCCGAGATTACGTTCACTCTCGTTGGGCGACGCGC

GAGCTGCTGGAGGACAATTTTCCAGGGGCTATAGGTTTCCGAAACCACAAGCCTTACTCC

GTCAGGGTTATCTACGGAGACACCGACTCCGTGTTTATCAAGTTTGTGGGCCTGACGTAC

GAGGGGGTATCGGAGCTGGGGGATGCTATGTCGCGTCAGATTTCAGCGGACCTCTTTAGA

GCGCCCATCAAACTGGAGTGTGAGAAGACCTTTCAGCGACTGCTGCTGATCACCAAGAAG

AAGTACATAGGTGTCATAAACGGGGGGAAGATGCTCATGAAGGGGGTCGACCTGGTCCGC

AAAAATAACTGCTCTTTCATAAACTTGTACGCGCGACATCTGGTAGATCTTTTGTTGTAC

GACGAGGATGTGGCCACGGCGGCAGCAGAGGTGACAGACGTGCCTCCCGCAGAATGGGTG

GGGCGCCCGCTACCGAGCGGCTTTGACAAGTTTGGGCGAGTGCTGGTAGAGGCGTACAAC

CGTATCACTGCCCCCAACTTGGACGTGCGCGAGTTCGTTATGACTGCTGAGCTGAGCCGC

TCACCCGAATCGTATACCAACAAGCGCCTGCCGCACCTCACCGTCTACTTTAAGCTCGCC

ATGAGGAATGAAGAACTGCCCAGCGTAAAAGAGAGAATTCCGTATGTGATAGTTGCGCAG

ACCGAGGCCGCGGAACGCGAAGCGGGTGTAGTAAACTCAATGCGCGGTACCGCCCAAAAC

CCCGTGGTAACCAAGACCGCACGCCCCCAACCTAAACGCAAACTGCTGGTTTCCGACCTC

GCCGAAGACCCGACCTATGTTTCCGAGAATGACGTACCGCTAAACACAGACTACTATTTC

TCCCACCTGTTGGGTACCATAAGCGTGACCTTTAAGGCTCTATTCGGAAATGATGTGAGA

ACAACAGAAAATCTTTTAAAGCGGTTTATTCCGGAAACCCCCCACAAGACCCCCACGAAA

ACCCAGGCACTGCTTGAGCGCGCCGGCTTTGAAAAGCTGACGCCCTTTACACCGGAGGAA

GAAAGTCGTCGAATACTGCATACAGTTTTTTGTACTCTAGAAGCAGCTCCCCATCAAAGC

TGA

>KU206415.1

ATGGCGGCGCGCGAACAGGCCAACTCCGTTCGCAGGAGCGGGTTTTTTAATCCGTTTATT

GGCAAGAGGCCATTTTTCAGGCCGGGAAGCGGGCAAACCGCGGAGACAGAGCGCCCAAGG

CCCCCCCAACACTCGTACTGCACAGAGGTGGGTAGCTTTAAGTTTATAGCTCCAAGATGC

CTCGATGAAGAAGCCCCCGCCGACCAGCGACGCGGTGTACACGTGGGCACCTTGGAGCGC

CCACCCAAAGTGTACTGCGATGGCTCAGAGTACGACGTGCTGAACTTTGCCTCCGGAGGT

TGCTGGCCTCGCAGGATTCGGGTTTGGAACGGCCAGGATTTTCGGGGCGATGGATTCAAC

CCCAGATTTGAGAGATTTCACGTGTACGACATAGTAGAGACTTCGGAGAGCGCCTCACAC

GATGACCCATCCAGGTTTGCTGAGCTATCCCGCCCAAGCGGGTCTGTGGTAACACTGCTG

GGAATGAGCGAGTGTGGAAAGCGAGTCGCGGTTCACGTCTATGGTGTGCGCCATTATTTT

TACATGGCAAAGGCGGAGGTGGATAGCGCTTGTGGAATAACCACCGAGGCAGAACTCGTG

CGCGCAATGGTGGACTGCGCGCACAGCTCGGCTTTGAGCGCGGCCCTGGGAAATGGCAAC

GGCGGCAAACAGAGCGGCGGCAGCGGCGGGGGATGGTGGGGCGGAAAGCACGTGTCTGCG

GACTGCTTCAAAGTGGAGACCGTGTGCCACACGACGCTGTACTACTTTGGATCTAAGCCA

GCTCTCTACTATAGAGTATCTGCCTCCAGCAGCCGCCTGGGAGGGTTCATCTGCGACAAC

TTTCACCCGGAGATTACAAAATTCGAGGGGAGCGTGGACGTGACCACGCGGCTGTTGTTG

GACAATGAAAATTTTACCAGTTTCGGGTGGTACCGCCTGCGACCCGGCACCCACGGAGAG

CGTGTTCAACTTCGCCCCGTTGAGCGACACGTCACCTCAAGCGACGTGGAGATTAACTGT

ACTCCCGATAACCTGGAGCCGATACCAGACGAGGCTGCCTGGCCCGACTATAAGCTCATG

TGCTTTGATATAGAGTGTAAAGCTGGAACGGGTAACGAAATGGCGTTCCCAGTGGCAACT

AACCAAGAGGACCTGGTCATCCAGATCTCCTGTCTGCTGTACTCGCTTGCTACTCAGAAC

CACGAACACACCCTGCTGTTTTCCCTCGGGTCATGCGATATCTCTGAGGAATACTCGTTT

GCATGCGTCCAGCGCGGCGAGCCCAGACCGACGGTTTTGGAGTTTGACAGCGAGTACGAG

CTGCTGGTTGCCTTCCTGACCTTTCTCAAGCAGTACTCTCCCGAGTTCGCCACCGGCTAC

AACATCGTTAATTTTGACTGGGCGTACATAGTTAACAAGGTAACGTCGGTGTATAACATC

AAGCTGGACGGGTACGGCAAGTTCAACAAAGGGGGGCTGTTTAAGGTGTGGGACATCGCC

ACGAACCATTTTCAGAAGAAGAGCAAGGTGAAAATCAATGGCCTGATATCTCTAGACATG

TATTCTGTGGCGACGGAAAAGCTAAAGCTACCCAGCTACAAACTCGACGCGGTCGTGGGA

GACGTCCTCGGCGAGCATAAGATAGACCTTCCCTATAAAGAAATACCCTCCTATTACGCG

GGAGGGCCTGACCGGAGGGGCGTAATAGGAGAGTATTGTATCCAGGACTCTAGGCTGGTG

GGCAAGCTGTTTTTTAAGTACCTCCCCCATCTGGAACTATCGGCGGTGGCCAAACTCGCC

CGTATCACCCTAACGCGGGTAATTTTTGACGGTCAGCAAATTCGCGTGTACACGTGCTTG

CTGAAACTCGCCCGCGAGAGAAATTTCATTTTGCCAGACAACAGACGCCGGTTTGACAGT

CAGGCAGACGCCGCGTCAGAGACTTCGGAGTTGGCTATGGATAGCCAAAGCCACGCCTTC

GACAGTACAGACGAACCCGACGGTGTGGACGGTACCCCGGACGCCGCAGGATCTGGCGCT

ACTTCTGAAAACGGAGGCGGGAAGCCCGGCGTCGGGAGGGCCGTGGGCTACCAGGGAGCA

AAGGTTCTAGACCCCGTATCCGGCTTTCATGTGGACCCCGTGGTTGTGTTTGACTTCGCT

AGCTTATACCCAAGCATTATCCAGGCCCATAACCTCTGTTTCACCACCCTGGCGCTCGAT

GAAGTGGATCTGGCCGGGCTTCAACCATCCGTCAACTACTCGACGTTCGAGGTGGGTGAC

CAAAAGTTATTTTTTGTCCACGCCCATATTCGCGAAAGCCTGCTTGGCATCTTGCTGCGC

GACTGGCTGGCCATGCGAAAGGCGGTGAGGGCGCGAATCCCCACCAGCACCCCCGAGGAG

GCAGTTTTACTAGATAAGCAGCAGTCTGCGATTAAGGTGATATGCAACTCGGTTTACGGA

TTCACGGGGGTGGCAAACGGCCTGTTGCCGTGTCTGAGGATAGCGGCTACCGTTACCACG

ATAGGACGCGACATGCTCCTCAAGACCCGAGATTACGTTCACTCTCGTTGGGCGACGCGC

GAGCTGCTGGAGGACAATTTTCCAGGGGCTATAGGTTTCCGAAACCACAAGCCTTACTCC

GTCAGGGTTATCTACGGAGACACCGACTCCGTGTTTATCAAGTTTGTGGGCCTGACGTAC

GAGGGGGTATCGGAGCTGGGGGATGCTATGTCGCGTCAGATTTCAGCGGACCTCTTTAGA

GCGCCCATCAAACTGGAGTGTGAGAAGACCTTTCAGCGACTGCTGCTGATCACCAAGAAG

AAGTACATAGGTGTCATAAACGGGGGGAAGATGCTCATGAAGGGGGTCGACCTGGTCCGC

AAAAATAACTGCTCTTTCATAAACTTGTACGCGCGACATCTGGTAGATCTTTTGTTGTAC

GACGAGGATGTGGCCACGGCGGCAGCAGAGGTGACAGACGTGCCTCCCGCAGAATGGGTG

GGGCGCCCGCTACCGAGCGGCTTTGACAAGTTTGGGCGAGTGCTGGTAGAGGCGTACAAC

CGTATCACTGCCCCCAACTTGGACGTGCGCGAGTTCGTTATGACTGCTGAGCTGAGCCGC

TCACCCGAATCGTATACCAACAAGCGCCTGCCGCACCTCACCGTCTACTTTAAGCTCGCC

ATGAGGAATGAAGAACTGCCCAGCGTAAAAGAGAGAATTCCGTATGTGATAGTTGCGCAG

ACCGAGGCCGCGGAACGCGAAGCGGGTGTAGTAAACTCAATGCGCGGTACCGCCCAAAAC

CCCGTGGTAACCAAGACCGCACGCCCCCAACCTAAACGCAAACTGCTGGTTTCCGACCTC

GCCGAAGACCCGACCTATGTTTCCGAGAATGACGTACCGCTAAACACAGACTACTATTTC

TCCCACCTGTTGGGTACCATAAGCGTGACCTTTAAGGCTCTATTCGGAAATGATGTGAGA

ACAACAGAAAATCTTTTAAAGCGGTTTATTCCGGAAACCCCCCACAAGACCCCCACGAAA

ACCCAGGCACTGCTTGAGCGCGCCGGCTTTGAAAAGCTGACGCCCTTTACACCGGAGGAA

GAAAGTCGTCGAATACTGCATACAGTTTTTTGTACTCTAGAAGCAGCTCCCCATCAAAGC

TGA

>KU206416.1

ATGGCGGCGCGCGAACAGGCCAACTCCGTTCGCAGGAGCGGGTTTTTTAATCCGTTTATT

GGCAAGAGGCCATTTTTCAGGCCGGGAAGCGGGCAAACCGCGGAGACAGAGCGCCCAAGG

CCCCCCCAACACTCGTACTGCACAGAGGTGGGTAGCTTTAAGTTTATAGCTCCAAGATGC

CTCGATGAAGAAGCCCCCGCCGACCAGCGACGCGGTGTACACGTGGGCACCTTGGAGCGC

CCACCCAAAGTGTACTGCGATGGCTCAGAGTACGACGTGCTGAACTTTGCCTCCGGAGGT

TGCTGGCCTCGCAGGATTCGGGTTTGGAACGGCCAGGATTTTCGGGGCGATGGATTCAAC

CCCAGATTTGAGAGATTTCACGTGTACGACATAGTAGAGACTTCGGAGAGCGCCTCACAC

GATGACCCATCCAGGTTTGCTGAGCTATCCCGCCCAAGCGGGTCTGTGGTAACACTGCTG

GGAATGAGCGAGTGTGGAAAGCGAGTCGCGGTTCACGTCTATGGTGTGCGCCATTATTTT

TACATGGCAAAGGCGGAGGTGGATAGCGCTTGTGGAATAACCACCGAGGCAGAACTCGTG

CGCGCAATGGTGGACTGCGCGCACAGCTCGGCTTTGAGCGCGGCCCTGGGAAATGGCAAC

GGCGGCAAACAGAGCGGCGGCAGCGGCGGGGGATGGTGGGGCGGAAAGCACGTGTCTGCG

GACTGCTTCAAAGTGGAGACCGTGTGCCACACGACGCTGTACTACTTTGGATCTAAGCCA

GCTCTCTACTATAGAGTATCTGCCTCCAGCAGCCGCCTGGGAGGGTTCATCTGCGACAAC

TTTCACCCGGAGATTACAAAATTCGAGGGGAGCGTGGACGTGACCACGCGGCTGTTGTTG

GACAATGAAAATTTTACCAGTTTCGGGTGGTACCGCCTGCGACCCGGCACCCACGGAGAG

CGTGTTCAACTTCGCCCCGTTGAGCGACACGTCACCTCAAGCGACGTGGAGATTAACTGT

ACTCCCGATAACCTGGAGCCGATACCAGACGAGGCTGCCTGGCCCGACTATAAGCTCATG

TGCTTTGATATAGAGTGTAAAGCTGGAACGGGTAACGAAATGGCGTTCCCAGTGGCAACT

AACCAAGAGGACCTGGTCATCCAGATCTCCTGTCTGCTGTACTCGCTTGCTACTCAGAAC

CACGAACACACCCTGCTGTTTTCCCTCGGGTCATGCGATATCTCTGAGGAATACTCGTTT

GCATGCGTCCAGCGCGGCGAGCCCAGACCGACGGTTTTGGAGTTTGACAGCGAGTACGAG

CTGCTGGTTGCCTTCCTGACCTTTCTCAAGCAGTACTCTCCCGAGTTCGCCACCGGCTAC

AACATCGTTAATTTTGACTGGGCGTACATAGTTAACAAGGTAACGTCGGTGTATAACATC

AAGCTGGACGGGTACGGCAAGTTCAACAAAGGGGGGCTGTTTAAGGTGTGGGACATCGCC

ACGAACCATTTTCAGAAGAAGAGCAAGGTGAAAATCAATGGCCTGATATCTCTAGACATG

TATTCTGTGGCGACGGAAAAGCTAAAGCTACCCAGCTACAAACTCGACGCGGTCGTGGGA

GACGTCCTCGGCGAGCATAAGATAGACCTTCCCTATAAAGAAATACCCTCCTATTACGCG

GGAGGGCCTGACCGGAGGGGCGTAATAGGAGAGTATTGTATCCAGGACTCTAGGCTGGTG

GGCAAGCTGTTTTTTAAGTACCTCCCCCATCTGGAACTATCGGCGGTGGCCAAACTCGCC

CGTATCACCCTAACGCGGGTAATTTTTGACGGTCAGCAAATTCGCGTGTACACGTGCTTG

CTGAAACTCGCCCGCGAGAGAAATTTCATTTTGCCAGACAACAGACGCCGGTTTGACAGC

CAGGCAGACGCCGCGTCAGAGACTTCGGAGTTGGCTATGGATAGCCAAAGCCACGCCTTC

GACAGTACAGACGAACCCGACGGTGTGGACGGTACCCCGGACGCCGCAGGATCTGGCGCT

ACTTCTGAAAACGGAGGCGGGAAGCCCGGCGTCGGGAGGGCCGTGGGCTACCAGGGAGCA

AAGGTTCTAGACCCCGTATCCGGCTTTCATGTGGACCCCGTGGTTGTGTTTGACTTCGCT

AGCTTATACCCAAGCATTATCCAGGCCCATAACCTCTGTTTCACCACCCTGGCGCTCGAT

GAAGTGGATCTGGCCGGGCTTCAACCATCCGTCAACTACTCGACGTTCGAGGTGGGTGAC

CAAAAGTTATTTTTTGTCCACGCCCATATTCGCGAAAGCCTGCTTGGCATCTTGCTGCGC

GACTGGCTGGCCATGCGAAAGGCGGTGAGGGCGCGAATCCCCACCAGCACCCCCGAGGAG

GCAGTTTTACTAGATAAGCAGCAGTCTGCGATTAAGGTGATATGCAACTCGGTTTACGGA

TTCACGGGGGTGGCAAACGGCCTGTTGCCGTGTCTGAGGATAGCGGCTACCGTTACCACG

ATAGGACGCGACATGCTCCTCAAGACCCGAGATTACGTTCACTCTCGTTGGGCGACGCGC

GAGCTGCTGGAGGACAATTTTCCAGGGGCTATAGGTTTCCGAAACCACAAGCCTTACTCC

GTCAGGGTTATCTACGGAGACACCGACTCCGTGTTTATCAAGTTTGTGGGCCTGACGTAC

GAGGGGGTATCGGAGCTGGGGGATGCTATGTCGCGTCAGATTTCAGCGGACCTCTTTAGA

GCGCCCATCAAACTGGAGTGTGAGAAGACCTTTCAGCGACTGCTGCTGATCACCAAGAAG

AAGTACATAGGTGTCATAAACGGGGGGAAGATGCTCATGAAGGGGGTCGACCTGGTCCGC

AAAAATAACTGCTCTTTCATAAACTTGTACGCGCGACATCTGGTAGATCTTTTGTTGTAC

GACGAGGATGTGGCCACGGCGGCAGCAGAGGTGACAGACGTGCCTCCCGCAGAATGGGTG

GGGCGCCCGCTACCGAGCGGCTTTGACAAGTTTGGGCGAGTGCTGGTAGAGGCGTACAAC

CGTATCACTGCCCCCAACTTGGACGTGCGCGAGTTCGTTATGACTGCTGAGCTGAGCCGC

TCACCCGAATCGTATACCAACAAGCGCCTGCCGCACCTCACCGTCTACTTTAAGCTCGCC

ATGAGGAATGAAGAACTGCCCAGCGTAAAAGAGAGAATTCCGTATGTGATAGTTGCGCAG

ACCGAGGCCGCGGAACGCGAAGCGGGTGTAGTAAACTCAATGCGCGGTACCGCCCAAAAC

CCCGTGGTAACCAAGACCGCACGCCCCCAACCTAAACGCAAACTGCTGGTTTCCGACCTC

GCCGAAGACCCGACCTATGTTTCCGAGAATGACGTACCGCTAAACACAGACTACTATTTC

TCCCACCTGTTGGGTACCATAAGCGTGACCTTTAAGGCTCTATTCGGAAATGATGTGAGA

ACAACAGAAAATCTTTTAAAGCGGTTTATTCCGGAAACCCCCCACAAGACCCCCACGAAA

ACCCAGGCACTGCTTGAGCGCGCCGGCTTTGAAAAGCTGACGCCCTTTACACCGGAGGAA

GAAAGTCGTCGAATACTGCATACAGTTTTTTGTACTCTAGAAGCAGCTCCCCATCAAAGC

TGA

>KU206417.1

ATGGCGGCGCGCGAACAGGCCAACTCCGTTCGCAGGAGCGGGTTTTTTAATCCGTTTATT

GGCAAGAGGCCATTTTTCAGGCCGGGAAGCGGGCAAACCGCGGAGACAGAGCGCCCAAGG

CCCCCCCAACACTCGTACTGCACAGAGGTGGGTAGCTTTAAGTTTATAGCTCCAAGATGC

CTCGATGAAGAAGCCCCCGCCGACCAGCGACGCGGTGTACACGTGGGCACCTTGGAGCGC

CCACCCAAAGTGTACTGCGATGGCTCAGAGTACGACGTGCTGAACTTTGCCTCCGGAGGT

TGCTGGCCTCGCAGGATTCGGGTTTGGAACGGCCAGGATTTTCGGGGCGATGGATTCAAC

CCCAGATTTGAGAGATTTCACGTGTACGACATAGTAGAGACTTCGGAGAGCGCCTCACAC

GATGACCCATCCAGGTTTGCTGAGCTATCCCGCCCAAGCGGGTCTGTGGTAACACTGCTG

GGAATGAGCGAGTGTGGAAAGCGAGTCGCGGTTCACGTCTATGGTGTGCGCCATTATTTT

TACATGGCAAAGGCGGAGGTGGATAGCGCTTGTGGAATAACCACCGAGGCAGAACTCGTG

CGCGCAATGGTGGACTGCGCGCACAGCTCGGCTTTGAGCGCGGCCCTGGGAAATGGCAAC

GGCGGCAAACAGAGCGGCGGCAGCGGCGGGGGATGGTGGGGCGGAAAGCACGTGTCTGCG

GACTGCTTCAAAGTGGAGACCGTGTGCCACACGACGCTGTACTACTTTGGATCTAAGCCA

GCTCTCTACTATAGAGTATCTGCCTCCAGCAGCCGCCTGGGAGGGTTCATCTGCGACAAC

TTTCACCCGGAGATTACAAAATTCGAGGGGAGCGTGGACGTGACCACGCGGCTGTTGTTG

GACAATGAAAATTTTACCAGTTTCGGGTGGTACCGCCTGCGACCCGGCACCCACGGAGAG

CGTGTTCAACTTCGCCCCGTTGAGCGACACGTCACCTCAAGCGACGTGGAGATTAACTGT

ACTCCCGATAACCTGGAGCCGATACCAGACGAGGCTGCCTGGCCCGACTATAAGCTCATG

TGCTTTGATATAGAGTGTAAAGCTGGAACGGGTAACGAAATGGCGTTCCCAGTGGCAACT

AACCAAGAGGACCTGGTCATCCAGATCTCCTGTCTGCTGTACTCGCTTGCTACTCAGAAC

CACGAACACACCCTGCTGTTTTCCCTCGGGTCATGCGATATCTCTGAGGAATACTCGTTT

GCATGCGTCCAGCGCGGCGAGCCCAGACCGACGGTTTTGGAGTTTGACAGCGAGTACGAG

CTGCTGGTTGCCTTCCTGACCTTTCTCAAGCAGTACTCTCCCGAGTTCGCCACCGGCTAC

AACATCGTTAATTTTGACTGGGCGTACATAGTTAACAAGGTAACGTCGGTGTATAACATC

AAGCTGGACGGGTACGGCAAGTTCAACAAAGGGGGGCTGTTTAAGGTGTGGGACATCGCC

ACGAACCATTTTCAGAAGAAGAGCAAGGTGAAAATCAATGGCCTGATATCTCTAGACATG

TATTCTGTGGCGACGGAAAAGCTAAAGCTACCCAGCTACAAACTCGACGCGGTCGTGGGA

GACGTCCTCGGCGAGCATAAGATAGACCTTCCCTATAAAGAAATACCCTCCTATTACGCG

GGAGGGCCTGACCGGAGGGGCGTAATAGGAGAGTATTGTATCCAGGACTCTAGGCTGGTG

GGCAAGCTGTTTTTTAAGTACCTCCCCCATCTGGAACTATCGGCGGTGGCCAAACTCGCC

CGTATCACCCTAACGCGGGTAATTTTTGACGGTCAGCAAATTCGCGTGTACACGTGCTTG

CTGAAACTCGCCCGCGAGAGAAATTTCATTTTGCCAGACAACAGACGCCGGTTTGACAGT

CAGGCAGACGCCGCGTCAGAGACTTCGGAGTTGGCTATGGATAGCCAAAGCCACGCCTTC

GACAGTACAGACGAACCCGACGGTGTGGACGGTACCCCGGACGCCGCAGGATCTGGCGCT

ACTTCTGAAAACGGAGGCGGGAAGCCCGGCGTCGGGAGGGCCGTGGGCTACCAGGGAGCA

AAGGTTCTAGACCCCGTATCCGGCTTTCATGTGGACCCCGTGGTTGTGTTTGACTTCGCT

AGCTTATACCCAAGCATTATCCAGGCCCATAACCTCTGTTTCACCACCCTGGCGCTCGAT

GAAGTGGATCTGGCCGGGCTTCAACCATCCGTCAACTACTCGACGTTCGAGGTGGGTGAC

CAAAAGTTATTTTTTGTCCACGCCCATATTCGCGAAAGCCTGCTTGGCATCTTGCTGCGC

GACTGGCTGGCCATGCGAAAGGCGGTGAGGGCGCGAATCCCCACCAGCACCCCCGAGGAG

GCAGTTTTACTAGATAAGCAGCAGTCTGCGATTAAGGTGATATGCAACTCGGTTTACGGA

TTCACGGGGGTGGCAAACGGCCTGTTGCCGTGTCTGAGGATAGCGGCTACCGTTACCACG

ATAGGACGCGACATGCTCCTCAAGACCCGAGATTACGTTCACTCTCGTTGGGCGACGCGC

GAGCTGCTGGAGGACAATTTTCCAGGGGCTATAGGTTTCCGAAACCACAAGCCTTACTCC

GTCAGGGTTATCTACGGAGACACCGACTCCGTGTTTATCAAGTTTGTGGGCCTGACGTAC

GAGGGGGTATCGGAGCTGGGGGATGCTATGTCGCGTCAGATTTCAGCGGACCTCTTTAGA

GCGCCCATCAAACTGGAGTGTGAGAAGACCTTTCAGCGACTGCTGCTGATCACCAAGAAG

AAGTACATAGGTGTCATAAACGGGGGGAAGATGCTCATGAAGGGGGTCGACCTGGTCCGC

AAAAATAACTGCTCTTTCATAAACTTGTACGCGCGACATCTGGTAGATCTTTTGTTGTAC

GACGAGGATGTGGCCACGGCGGCAGCAGAGGTGACAGACGTGCCTCCCGCAGAATGGGTG

GGGCGCCCGCTACCGAGCGGCTTTGACAAGTTTGGGCGAGTGCTGGTAGAGGCGTACAAC

CGTATCACTGCCCCCAACTTGGACGTGCGCGAGTTCGTTATGACTGCTGAGCTGAGCCGC

TCACCCGAATCGTATACCAACAAGCGCCTGCCGCACCTCACCGTCTACTTTAAGCTCGCC

ATGAGGAATGAAGAACTGCCCAGCGTAAAAGAGAGAATTCCGTATGTGATAGTTGCGCAG

ACCGAGGCCGCGGAACGCGAAGCGGGTGTAGTAAACTCAATGCGCGGTACCGCCCAAAAC

CCCGTGGTAACCAAGACCGCACGCCCCCAACCTAAACGCAAACTGCTGGTTTCCGACCTC

GCCGAAGACCCGACCTATGTTTCCGAGAATGACGTACCGCTAAACACAGACTACTATTTC

TCCCACCTGTTGGGTACCATAAGCGTGACCTTTAAGGCTCTATTCGGAAATGATGTGAGA

ACAACAGAAAATCTTTTAAAGCGGTTTATTCCGGAAACCCCCCACAAGACCCCCACGAAA

ACCCAGGCACTGCTTGAGCGCGCCGGCTTTGAAAAGCTGACGCCCTTTACACCGGAGGAA

GAAAGTCGTCGAATACTGCATACAGTTTTTTGTACTCTAGAAGCAGCTCCCCATCAAAGC

TGA

>KU206418.1

ATGGCGGCGCGCGAACAGGCCAACTCCGTTCGCAGGAGCGGGTTTTTTAATCCGTTTATT

GGCAAGAGGCCATTTTTCAGGCCGGGAAGCGGGCAAACCGCGGAGACAGAGCGCCCAAGG

CCCCCCCAACACTCGTACTGCACAGAGGTGGGTAGCTTTAAGTTTATAGCTCCAAGATGC

CTCGATGAAGAAGCCCCCGCCGACCAGCGACGCGGTGTACACGTGGGCACCTTGGAGCGC

CCACCCAAAGTGTACTGCGATGGCTCAGAGTACGACGTGCTGAACTTTGCCTCCGGAGGT

TGCTGGCCTCGCAGGATTCGGGTTTGGAACGGCCAGGATTTTCGGGGCGATGGATTCAAC

CCCAGATTTGAGAGATTTCACGTGTACGACATAGTAGAGACTTCGGAGAGCGCCTCACAC

GATGACCCATCCAGGTTTGCTGAGCTATCCCGCCCAAGCGGGTCTGTGGTAACACTGCTG

GGAATGAGCGAGTGTGGAAAGCGAGTCGCGGTTCACGTCTATGGTGTGCGCCATTATTTT

TACATGGCAAAGGCGGAGGTGGATAGCGCTTGTGGAATAACCACCGAGGCAGAACTCGTG

CGCGCAATGGTGGACTGCGCGCACAGCTCGGCTTTGAGCGCGGCCCTGGGAAATGGCAAC

GGCGGCAAACAGAGCGGCGGCAGCGGCGGGGGATGGTGGGGCGGAAAGCACGTGTCTGCG

GACTGCTTCAAAGTGGAGACCGTGTGCCACACGACGCTGTACTACTTTGGATCTAAGCCA

GCTCTCTACTATAGAGTATCTGCCTCCAGCAGCCGCCTGGGAGGGTTCATCTGCGACAAC

TTTCACCCGGAGATTACAAAATTCGAGGGGAGCGTGGACGTGACCACGCGGCTGTTGTTG

GACAATGAAAATTTTACCAGTTTCGGGTGGTACCGCCTGCGACCCGGCACCCACGGAGAG

CGTGTTCAACTTCGCCCCGTTGAGCGACACGTCACCTCAAGCGACGTGGAGATTAACTGT

ACTCCCGATAACCTGGAGCCGATACCAGACGAGGCTGCCTGGCCCGACTATAAGCTCATG

TGCTTTGATATAGAGTGTAAAGCTGGAACGGGTAACGAAATGGCGTTCCCAGTGGCAACT

AACCAAGAGGACCTGGTCATCCAGATCTCCTGTCTGCTGTACTCGCTTGCTACTCAGAAC

CACGAACACACCCTGCTGTTTTCCCTCGGGTCATGCGATATCTCTGAGGAATACTCGTTT

GCATGCGTCCAGCGCGGCGAGCCCAGACCGACGGTTTTGGAGTTTGACAGCGAGTACGAG

CTGCTGGTTGCCTTCCTGACCTTTCTCAAGCAGTACTCTCCCGAGTTCGCCACCGGCTAC

AACATCGTTAATTTTGACTGGGCGTACATAGTTAACAAGGTAACGTCGGTGTATAACATC

AAGCTGGACGGGTACGGCAAGTTCAACAAAGGGGGGCTGTTTAAGGTGTGGGACATCGCC

ACGAACCATTTTCAGAAGAAGAGCAAGGTGAAAATCAATGGCCTGATATCTCTAGACATG

TATTCTGTGGCGACGGAAAAGCTAAAGCTACCCAGCTACAAACTCGACGCGGTCGTGGGA

GACGTCCTCGGCGAGCATAAGATAGACCTTCCCTATAAAGAAATACCCTCCTATTACGCG

GGAGGGCCTGACCGGAGGGGCGTAATAGGAGAGTATTGTATCCAGGACTCTAGGCTGGTG

GGCAAGCTGTTTTTTAAGTACCTCCCCCATCTGGAACTATCGGCGGTGGCCAAACTCGCC

CGTATCACCCTAACGCGGGTAATTTTTGACGGTCAGCAAATTCGCGTGTACACGTGCTTG

CTGAAACTCGCCCGCGAGAGAAATTTCATTTTGCCAGACAACAGACGCCGGTTTGACAGT

CAGGCAGACGCCGCGTCAGAGACTTCGGAGTTGGCTATGGATAGCCAAAGCCACGCCTTC

GACAGTACAGACGAACCCGACGGTGTGGACGGTACCCCGGACGCCGCAGGATCTGGCGCT

ACTTCTGAAAACGGAGGCGGGAAGCCCGGCGTCGGGAGGGCCGTGGGCTACCAGGGAGCA

AAGGTTCTAGACCCCGTATCCGGCTTTCATGTGGACCCCGTGGTTGTGTTTGACTTCGCT

AGCTTATACCCAAGCATTATCCAGGCCCATAACCTCTGTTTCACCACCCTGGCGCTCGAT

GAAGTGGATCTGGCCGGGCTTCAACCATCCGTCAACTACTCGACGTTCGAGGTGGGTGAC

CAAAAGTTATTTTTTGTCCACGCCCATATTCGCGAAAGCCTGCTTGGCATCTTGCTGCGC

GACTGGCTGGCCATGCGAAAGGCGGTGAGGGCGCGAATCCCCACCAGCACCCCCGAGGAG

GCAGTTTTACTAGATAAGCAGCAGTCTGCGATTAAGGTGATATGCAACTCGGTTTACGGA

TTCACGGGGGTGGCAAACGGCCTGTTGCCGTGTCTGAGGATAGCGGCTACCGTTACCACG

ATAGGACGCGACATGCTCCTCAAGACCCGAGATTACGTTCACTCTCGTTGGGCGACGCGC

GAGCTGCTGGAGGACAATTTTCCAGGGGCTATAGGTTTCCGAAACCACAAGCCTTACTCC

GTCAGGGTTATCTACGGAGACACCGACTCCGTGTTTATCAAGTTTGTGGGCCTGACGTAC

GAGGGGGTATCGGAGCTGGGGGATGCTATGTCGCGTCAGATTTCAGCGGACCTCTTTAGA

GCGCCCATCAAACTGGAGTGTGAGAAGACCTTTCAGCGACTGCTGCTGATCACCAAGAAG

AAGTACATAGGTGTCATAAACGGGGGGAAGATGCTCATGAAGGGGGTCGACCTGGTCCGC

AAAAATAACTGCTCTTTCATAAACTTGTACGCGCGACATCTGGTAGATCTTTTGTTGTAC

GACGAGGATGTGGCCACGGCGGCAGCAGAGGTGACAGACGTGCCTCCCGCAGAATGGGTG

GGGCGCCCGCTACCGAGCGGCTTTGACAAGTTTGGGCGAGTGCTGGTAGAGGCGTACAAC

CGTATCACTGCCCCCAACTTGGACGTGCGCGAGTTCGTTATGACTGCTGAGCTGAGCCGC

TCACCCGAATCGTATACCAACAAGCGCCTGCCGCACCTCACCGTCTACTTTAAGCTCGCC

ATGAGGAATGAAGAACTGCCCAGCGTAAAAGAGAGAATTCCGTATGTGATAGTTGCGCAG

ACCGAGGCCGCGGAACGCGAAGCGGGTGTAGTAAACTCAATGCGCGGTACCGCCCAAAAC

CCCGTGGTAACCAAGACCGCACGCCCCCAACCTAAACGCAAACTGCTGGTTTCCGACCTC

GCCGAAGACCCGACCTATGTTTCCGAGAATGACGTACCGCTAAACACAGACTACTATTTC

TCCCACCTGTTGGGTACCATAAGCGTGACCTTTAAGGCTCTATTCGGAAATGATGTGAGA

ACAACAGAAAATCTTTTAAAGCGGTTTATTCCGGAAACCCCCCACAAGACCCCCACGAAA

ACCCAGGCACTGCTTGAGCGCGCCGGCTTTGAAAAGCTGACGCCCTTTACACCGGAGGAA

GAAAGTCGTCGAATACTGCATACAGTTTTTTGTACTCTAGAAGCAGCTCCCCATCAAAGC

TGA

>KU206419.1

ATGGCGGCGCGCGAACAGGCCAACTCCGTTCGCAGGAGCGGGTTTTTTAATCCGTTTATT

GGCAAGAGGCCATTTTTCAGGCCGGGAAGCGGGCAAACCGCGGAGACAGAGCGCCCAAGG

CCCCCCCAACACTCGTACTGCACAGAGGTGGGTAGCTTTAAGTTTATAGCTCCAAGATGC

CTCGATGAAGAAGCCCCCGCCGACCAGCGACGCGGTGTACACGTGGGCACCTTGGAGCGC

CCACCCAAAGTGTACTGCGATGGCTCAGAGTACGACGTGCTGAACTTTGCCTCCGGAGGT

TGCTGGCCTCGCAGGATTCGGGTTTGGAACGGCCAGGATTTTCGGGGCGATGGATTCAAC

CCCAGATTTGAGAGATTTCACGTGTACGACATAGTAGAGACTTCGGAGAGCGCCTCACAC

GATGACCCATCCAGGTTTGCTGAGCTATCCCGCCCAAGCGGGTCTGTGGTAACACTGCTG

GGAATGAGCGAGTGTGGAAAGCGAGTCGCGGTTCACGTCTATGGTGTGCGCCATTATTTT

TACATGGCAAAGGCGGAGGTGGATAGCGCTTGTGGAATAACCACCGAGGCAGAACTCGTG

CGCGCAATGGTGGACTGCGCGCACAGCTCGGCTTTGAGCGCGGCCCTGGGAAATGGCAAC

GGCGGCAAACAGAGCGGCGGCAGCGGCGGGGGATGGTGGGGCGGAAAGCACGTGTCTGCG

GACTGCTTCAAAGTGGAGACCGTGTGCCACACGACGCTGTACTACTTTGGATCTAAGCCA

GCTCTCTACTATAGAGTATCTGCCTCCAGCAGCCGCCTGGGAGGGTTCATCTGCGACAAC

TTTCACCCGGAGATTACAAAATTCGAGGGGAGCGTGGACGTGACCACGCGGCTGTTGTTG

GACAATGAAAATTTTACCAGTTTCGGGTGGTACCGCCTGCGACCCGGCACCCACGGAGAG

CGTGTTCAACTTCGCCCCGTTGAGCGACACGTCACCTCAAGCGACGTGGAGATTAACTGT

ACTCCCGATAACCTGGAGCCGATACCAGACGAGGCTGCCTGGCCCGACTATAAGCTCATG

TGCTTTGATATAGAGTGTAAAGCTGGAACGGGTAACGAAATGGCGTTCCCAGTGGCAACT

AACCAAGAGGACCTGGTCATCCAGATCTCCTGTCTGCTGTACTCGCTTGCTACTCAGAAC

CACGAACACACCCTGCTGTTTTCCCTCGGGTCATGCGATATCTCTGAGGAATACTCGTTT

GCATGCGTCCAGCGCGGCGAGCCCAGACCGACGGTTTTGGAGTTTGACAGCGAGTACGAG

CTGCTGGTTGCCTTCCTGACCTTTCTCAAGCAGTACTCTCCCGAGTTCGCCACCGGCTAC

AACATCGTTAATTTTGACTGGGCGTACATAGTTAACAAGGTAACGTCGGTGTATAACATC

AAGCTGGACGGGTACGGCAAGTTCAACAAAGGGGGGCTGTTTAAGGTGTGGGACATCGCC

ACGAACCATTTTCAGAAGAAGAGCAAGGTGAAAATCAATGGCCTGATATCTCTAGACATG

TATTCTGTGGCGACGGAAAAGCTAAAGCTACCCAGCTACAAACTCGACGCGGTCGTGGGA

GACGTCCTCGGCGAGCATAAGATAGACCTTCCCTATAAAGAAATACCCTCCTATTACGCG

GGAGGGCCTGACCGGAGGGGCGTAATAGGAGAGTATTGTATCCAGGACTCTAGGCTGGTG

GGCAAGCTGTTTTTTAAGTACCTCCCCCATCTGGAACTATCGGCGGTGGCCAAACTCGCC

CGTATCACCCTAACGCGGGTAATTTTTGACGGTCAGCAAATTCGCGTGTACACGTGCTTG

CTGAAACTCGCCCGCGAGAGAAATTTCATTTTGCCAGACAACAGACGCCGGTTTGACAGT

CAGGCAGACGCCGCGTCAGAGACTTCGGAGTTGGCTATGGATAGCCAAAGCCACGCCTTC

GACAGTACAGACGAACCCGACGGTGTGGACGGTACCCCGGACGCCGCAGGATCTGGCGCT

ACTTCTGAAAACGGAGGCGGGAAGCCCGGCGTCGGGAGGGCCGTGGGCTACCAGGGAGCA

AAGGTTCTAGACCCCGTATCCGGCTTTCATGTGGACCCCGTGGTTGTGTTTGACTTCGCT

AGCTTATACCCAAGCATTATCCAGGCCCATAACCTCTGTTTCACCACCCTGGCGCTCGAT

GAAGTGGATCTGGCCGGGCTTCAACCATCCGTCAACTACTCGACGTTCGAGGTGGGTGAC

CAAAAGTTATTTTTTGTCCACGCCCATATTCGCGAAAGCCTGCTTGGCATCTTGCTGCGC

GACTGGCTGGCCATGCGAAAGGCGGTGAGGGCGCGAATCCCCACCAGCACCCCCGAGGAG

GCAGTTTTACTAGATAAGCAGCAGTCTGCGATTAAGGTGATATGCAACTCGGTTTACGGA

TTCACGGGGGTGGCAAACGGCCTGTTGCCGTGTCTGAGGATAGCGGCTACCGTTACCACG

ATAGGACGCGACATGCTCCTCAAGACCCGAGATTACGTTCACTCTCGTTGGGCGACGCGC

GAGCTGCTGGAGGACAATTTTCCAGGGGCTATAGGTTTCCGAAACCACAAGCCTTACTCC

GTCAGGGTTATCTACGGAGACACCGACTCCGTGTTTATCAAGTTTGTGGGCCTGACGTAC

GAGGGGGTATCGGAGCTGGGGGATGCTATGTCGCGTCAGATTTCAGCGGACCTCTTTAGA

GCGCCCATCAAACTGGAGTGTGAGAAGACCTTTCAGCGACTGCTGCTGATCACCAAGAAG

AAGTACATAGGTGTCATAAACGGGGGGAAGATGCTCATGAAGGGGGTCGACCTGGTCCGC

AAAAATAACTGCTCTTTCATAAACTTGTACGCGCGACATCTGGTAGATCTTTTGTTGTAC

GACGAGGATGTGGCCACGGCGGCAGCAGAGGTGACAGACGTGCCTCCCGCAGAATGGGTG

GGGCGCCCGCTACCGAGCGGCTTTGACAAGTTTGGGCGAGTGCTGGTAGAGGCGTACAAC

CGTATCACTGCCCCCAACTTGGACGTGCGCGAGTTCGTTATGACTGCTGAGCTGAGCCGC

TCACCCGAATCGTATACCAACAAGCGCCTGCCGCACCTCACCGTCTACTTTAAGCTCGCC

ATGAGGAATGAAGAACTGCCCAGCGTAAAAGAGAGAATTCCGTATGTGATAGTTGCGCAG

ACCGAGGCCGCGGAACGCGAAGCGGGTGTAGTAAACTCAATGCGCGGTACCGCCCAAAAC

CCCGTGGTAACCAAGACCGCACGCCCCCAACCTAAACGCAAACTGCTGGTTTCCGACCTC

GCCGAAGACCCGACCTATGTTTCCGAGAATGACGTACCGCTAAACACAGACTACTATTTC

TCCCACCTGTTGGGTACCATAAGCGTGACCTTTAAGGCTCTATTCGGAAATGATGTGAGA

ACAACAGAAAATCTTTTAAAGCGGTTTATTCCGGAAACCCCCCACAAGACCCCCACGAAA

ACCCAGGCACTGCTTGAGCGCGCCGGCTTTGAAAAGCTGACGCCCTTTACACCGGAGGAA

GAAAGTCGTCGAATACTGCATACAGTTTTTTGTACTCTAGAAGCAGCTCCCCATCAAAGC

TGA

>KU206420.1

ATGGCGGCGCGCGAACAGGCCAACTCCGTTCGCAGGAGCGGGTTTTTTAATCCGTTTATT

GGCAAGAGGCCATTTTTCAGGCCGGGAAGCGGGCAAACCGCGGAGACAGAGCGCCCAAGG

CCCCCCCAACACTCGTACTGCACAGAGGTGGGTAGCTTTAAGTTTATAGCTCCAAGATGC

CTCGATGAAGAAGCCCCCGCCGACCAGCGACGCGGTGTACACGTGGGCACCTTGGAGCGC

CCACCCAAAGTGTACTGCGATGGCTCAGAGTACGACGTGCTGAACTTTGCCTCCGGAGGT

TGCTGGCCTCGCAGGATTCGGGTTTGGAACGGCCAGGATTTTCGGGGCGATGGATTCAAC

CCCAGATTTGAGAGATTTCACGTGTACGACATAGTAGAGACTTCGGAGAGCGCCTCACAC

GATGACCCATCCAGGTTTGCTGAGCTATCCCGCCCAAGCGGGTCTGTGGTAACACTGCTG

GGAATGAGCGAGTGTGGAAAGCGAGTCGCGGTTCACGTCTATGGTGTGCGCCATTATTTT

TACATGGCAAAGGCGGAGGTGGATAGCGCTTGTGGAATAACCACCGAGGCAGAACTCGTG

CGCGCAATGGTGGACTGCGCGCACAGCTCGGCTTTGAGCGCGGCCCTGGGAAATGGCAAC

GGCGGCAAACAGAGCGGCGGCAGCGGCGGGGGATGGTGGGGCGGAAAGCACGTGTCTGCG

GACTGCTTCAAAGTGGAGACCGTGTGCCACACGACGCTGTACTACTTTGGATCTAAGCCA

GCTCTCTACTATAGAGTATCTGCCTCCAGCAGCCGCCTGGGAGGGTTCATCTGCGACAAC

TTTCACCCGGAGATTACAAAATTCGAGGGGAGCGTGGACGTGACCACGCGGCTGTTGTTG

GACAATGAAAATTTTACCAGTTTCGGGTGGTACCGCCTGCGACCCGGCACCCACGGAGAG

CGTGTTCAACTTCGCCCCGTTGAGCGACACGTCACCTCAAGCGACGTGGAGATTAACTGT

ACTCCCGATAACCTGGAGCCGATACCAGACGAGGCTGCCTGGCCCGACTATAAGCTCATG

TGCTTTGATATAGAGTGTAAAGCTGGAACGGGTAACGAAATGGCGTTCCCAGTGGCAACT

AACCAAGAGGACCTGGTCATCCAGATCTCCTGTCTGCTGTACTCGCTTGCTACTCAGAAC

CACGAACACACCCTGCTGTTTTCCCTCGGGTCATGCGATATCTCTGAGGAATACTCGTTT

GCATGCGTCCAGCGCGGCGAGCCCAGACCGACGGTTTTGGAGTTTGACAGCGAGTACGAG

CTGCTGGTTGCCTTCCTGACCTTTCTCAAGCAGTACTCTCCCGAGTTCGCCACCGGCTAC

AACATCGTTAATTTTGACTGGGCGTACATAGTTAACAAGGTAACGTCGGTGTATAACATC

AAGCTGGACGGGTACGGCAAGTTCAACAAAGGGGGGCTGTTTAAGGTGTGGGACATCGCC

ACGAACCATTTTCAGAAGAAGAGCAAGGTGAAAATCAATGGCCTGATATCTCTAGACATG

TATTCTGTGGCGACGGAAAAGCTAAAGCTACCCAGCTACAAACTCGACGCGGTCGTGGGA

GACGTCCTCGGCGAGCATAAGATAGACCTTCCCTATAAAGAAATACCCTCCTATTACGCG

GGAGGGCCTGACCGGAGGGGCGTAATAGGAGAGTATTGTATCCAGGACTCTAGGCTGGTG

GGCAAGCTGTTTTTTAAGTACCTCCCCCATCTGGAACTATCGGCGGTGGCCAAACTCGCC

CGTATCACCCTAACGCGGGTAATTTTTGACGGTCAGCAAATTCGCGTGTACACGTGCTTG

CTGAAACTCGCCCGCGAGAGAAATTTCATTTTGCCAGACAACAGACGCCGGTTTGACAGT

CAGGCAGACGCCGCGTCAGAGACTTCGGAGTTGGCTATGGATAGCCAAAGCCACGCCTTC

GACAGTACAGACGAACCCGACGGTGTGGACGGTACCCCGGACGCCGCAGGATCTGGCGCT

ACTTCTGAAAACGGAGGCGGGAAGCCCGGCGTCGGGAGGGCCGTGGGCTACCAGGGAGCA

AAGGTTCTAGACCCCGTATCCGGCTTTCATGTGGACCCCGTGGTTGTGTTTGACTTCGCT

AGCTTATACCCAAGCATTATCCAGGCCCATAACCTCTGTTTCACCACCCTGGCGCTCGAT

GAAGTGGATCTGGCCGGGCTTCAACCATCCGTCAACTACTCGACGTTCGAGGTGGGTGAC

CAAAAGTTATTTTTTGTCCACGCCCATATTCGCGAAAGCCTGCTTGGCATCTTGCTGCGC

GACTGGCTGGCCATGCGAAAGGCGGTGAGGGCGCGAATCCCCACCAGCACCCCCGAGGAG

GCAGTTTTACTAGATAAGCAGCAGTCTGCGATTAAGGTGATATGCAACTCGGTTTACGGA

TTCACGGGGGTGGCAAACGGCCTGTTGCCGTGTCTGAGGATAGCGGCTACCGTTACCACG

ATAGGACGCGACATGCTCCTCAAGACCCGAGATTACGTTCACTCTCGTTGGGCGACGCGC

GAGCTGCTGGAGGACAATTTTCCAGGGGCTATAGGTTTCCGAAACCACAAGCCTTACTCC

GTCAGGGTTATCTACGGAGACACCGACTCCGTGTTTATCAAGTTTGTGGGCCTGACGTAC

GAGGGGGTATCGGAGCTGGGGGATGCTATGTCGCGTCAGATTTCAGCGGACCTCTTTAGA

GCGCCCATCAAACTGGAGTGTGAGAAGACCTTTCAGCGACTGCTGCTGATCACCAAGAAG

AAGTACATAGGTGTCATAAACGGGGGGAAGATGCTCATGAAGGGGGTCGACCTGGTCCGC

AAAAATAACTGCTCTTTCATAAACTTGTACGCGCGACATCTGGTAGATCTTTTGTTGTAC

GACGAGGATGTGGCCACGGCGGCAGCAGAGGTGACAGACGTGCCTCCCGCAGAATGGGTG

GGGCGCCCGCTACCGAGCGGCTTTGACAAGTTTGGGCGAGTGCTGGTAGAGGCGTACAAC

CGTATCACTGCCCCCAACTTGGACGTGCGCGAGTTCGTTATGACTGCTGAGCTGAGCCGC

TCACCCGAATCGTATACCAACAAGCGCCTGCCGCACCTCACCGTCTACTTTAAGCTCGCC

ATGAGGAATGAAGAACTGCCCAGCGTAAAAGAGAGAATTCCGTATGTGATAGTTGCGCAG

ACCGAGGCCGCGGAACGCGAAGCGGGTGTAGTAAACTCAATGCGCGGTACCGCCCAAAAC

CCCGTGGTAACCAAGACCGCACGCCCCCAACCTAAACGCAAACTGCTGGTTTCCGACCTC

GCCGAAGACCCGACCTATGTTTCCGAGAATGACGTACCGCTAAACACAGACTACTATTTC

TCCCACCTGTTGGGTACCATAAGCGTGACCTTTAAGGCTCTATTCGGAAATGATGTGAGA

ACAACAGAAAATCTTTTAAAGCGGTTTATTCCGGAAACCCCCCACAAGACCCCCACGAAA

ACCCAGGCACTGCTTGAGCGCGCCGGCTTTGAAAAGCTGACGCCCTTTACACCGGAGGAA

GAAAGTCGTCGAATACTGCATACAGTTTTTTGTACTCTAGAAGCAGCTCCCCATCAAAGC

TGA

>KU206421.1

ATGGCGGCGCGCGAACAGGCCAACTCCGTTCGCAGGAGCGGGTTTTTTAATCCGTTTATT

GGCAAGAGGCCATTTTTCAGGCCGGGAAGCGGGCAAACCGCGGAGACAGAGCGCCCAAGG

CCCCCCCAACACTCGTACTGCACAGAGGTGGGTAGCTTTAAGTTTATAGCTCCAAGATGC

CTCGATGAAGAAGCCCCCGCCGACCAGCGACGCGGTGTACACGTGGGCACCTTGGAGCGC

CCACCCAAAGTGTACTGCGATGGCTCAGAGTACGACGTGCTGAACTTTGCCTCCGGAGGT

TGCTGGCCTCGCAGGATTCGGGTTTGGAACGGCCAGGATTTTCGGGGCGATGGATTCAAC

CCCAGATTTGAGAGATTTCACGTGTACGACATAGTAGAGACTTCGGAGAGCGCCTCACAC

GATGACCCATCCAGGTTTGCTGAGCTATCCCGCCCAAGCGGGTCTGTGGTAACACTGCTG

GGAATGAGCGAGTGTGGAAAGCGAGTCGCGGTTCACGTCTATGGTGTGCGCCATTATTTT

TACATGGCAAAGGCGGAGGTGGATAGCGCTTGTGGAATAACCACCGAGGCAGAACTCGTG

CGCGCAATGGTGGACTGCGCGCACAGCTCGGCTTTGAGCGCGGCCCTGGGAAATGGCAAC

GGCGGCAAACAGAGCGGCGGCAGCGGCGGGGGATGGTGGGGCGGAAAGCACGTGTCTGCG

GACTGCTTCAAAGTGGAGACCGTGTGCCACACGACGCTGTACTACTTTGGATCTAAGCCA

GCTCTCTACTATAGAGTATCTGCCTCCAGCAGCCGCCTGGGAGGGTTCATCTGCGACAAC

TTTCACCCGGAGATTACAAAATTCGAGGGGAGCGTGGACGTGACCACGCGGCTGTTGTTG

GACAATGAAAATTTTACCAGTTTCGGGTGGTACCGCCTGCGACCCGGCACCCACGGAGAG

CGTGTTCAACTTCGCCCCGTTGAGCGACACGTCACCTCAAGCGACGTGGAGATTAACTGT

ACTCCCGATAACCTGGAGCCGATACCAGACGAGGCTGCCTGGCCCGACTATAAGCTCATG

TGCTTTGATATAGAGTGTAAAGCTGGAACGGGTAACGAAATGGCGTTCCCAGTGGCAACT

AACCAAGAGGACCTGGTCATCCAGATCTCCTGTCTGCTGTACTCGCTTGCTACTCAGAAC

CACGAACACACCCTGCTGTTTTCCCTCGGGTCATGCGATATCTCTGAGGAATACTCGTTT

GCATGCGTCCAGCGCGGCGAGCCCAGACCGACGGTTTTGGAGTTTGACAGCGAGTACGAG

CTGCTGGTTGCCTTCCTGACCTTTCTCAAGCAGTACTCTCCCGAGTTCGCCACCGGCTAC

AACATCGTTAATTTTGACTGGGCGTACATAGTTAACAAGGTAACGTCGGTGTATAACATC

AAGCTGGACGGGTACGGCAAGTTCAACAAAGGGGGGCTGTTTAAGGTGTGGGACATCGCC

ACGAACCATTTTCAGAAGAAGAGCAAGGTGAAAATCAATGGCCTGATATCTCTAGACATG

TATTCTGTGGCGACGGAAAAGCTAAAGCTACCCAGCTACAAACTCGACGCGGTCGTGGGA

GACGTCCTCGGCGAGCATAAGATAGACCTTCCCTATAAAGAAATACCCTCCTATTACGCG

GGAGGGCCTGACCGGAGGGGCGTAATAGGAGAGTATTGTATCCAGGACTCTAGGCTGGTG

GGCAAGCTGTTTTTTAAGTACCTCCCCCATCTGGAACTATCGGCGGTGGCCAAACTCGCC

CGTATCACCCTAACGCGGGTAATTTTTGACGGTCAGCAAATTCGCGTGTACACGTGCTTG

CTGAAACTCGCCCGCGAGAGAAATTTCATTTTGCCAGACAACAGACGCCGGTTTGACAGT

CAGGCAGACGCCGCGTCAGAGACTTCGGAGTTGGCTATGGATAGCCAAAGCCACGCCTTC

GACAGTACAGACGAACCCGACGGTGTGGACGGTACCCCGGACGCCGCAGGATCTGGCGCT

ACTTCTGAAAACGGAGGCGGGAAGCCCGGCGTCGGGAGGGCCGTGGGCTACCAGGGAGCA

AAGGTTCTAGACCCCGTATCCGGCTTTCATGTGGACCCCGTGGTTGTGTTTGACTTCGCT

AGCTTATACCCAAGCATTATCCAGGCCCATAACCTCTGTTTCACCACCCTGGCGCTCGAT

GAAGTGGATCTGGCCGGGCTTCAACCATCCGTCAACTACTCGACGTTCGAGGTGGGTGAC

CAAAAGTTATTTTTTGTCCACGCCCATATTCGCGAAAGCCTGCTTGGCATCTTGCTGCGC

GACTGGCTGGCCATGCGAAAGGCGGTGAGGGCGCGAATCCCCACCAGCACCCCCGAGGAG

GCAGTTTTACTAGATAAGCAGCAGTCTGCGATTAAGGTGATATGCAACTCGGTTTACGGA

TTCACGGGGGTGGCAAACGGCCTGTTGCCGTGTCTGAGGATAGCGGCTACCGTTACCACG

ATAGGACGCGACATGCTCCTCAAGACCCGAGATTACGTTCACTCTCGTTGGGCGACGCGC

GAGCTGCTGGAGGACAATTTTCCAGGGGCTATAGGTTTCCGAAACCACAAGCCTTACTCC

GTCAGGGTTATCTACGGAGACACCGACTCCGTGTTTATCAAGTTTGTGGGCCTGACGTAC

GAGGGGGTATCGGAGCTGGGGGATGCTATGTCGCGTCAGATTTCAGCGGACCTCTTTAGA

GCGCCCATCAAACTGGAGTGTGAGAAGACCTTTCAGCGACTGCTGCTGATCACCAAGAAG

AAGTACATAGGTGTCATAAACGGGGGGAAGATGCTCATGAAGGGGGTCGACCTGGTCCGC

AAAAATAACTGCTCTTTCATAAACTTGTACGCGCGACATCTGGTAGATCTTTTGTTGTAC

GACGAGGATGTGGCCACGGCGGCAGCAGAGGTGACAGACGTGCCTCCCGCAGAATGGGTG

GGGCGCCCGCTACCGAGCGGCTTTGACAAGTTTGGGCGAGTGCTGGTAGAGGCGTACAAC

CGTATCACTGCCCCCAACTTGGACGTGCGCGAGTTCGTTATGACTGCTGAGCTGAGCCGC

TCACCCGAATCGTATACCAACAAGCGCCTGCCGCACCTCACCGTCTACTTTAAGCTCGCC

ATGAGGAATGAAGAACTGCCCAGCGTAAAAGAGAGAATTCCGTATGTGATAGTTGCGCAG

ACCGAGGCCGCGGAACGCGAAGCGGGTGTAGTAAACTCAATGCGCGGTACCGCCCAAAAC

CCCGTGGTAACCAAGACCGCACGCCCCCAACCTAAACGCAAACTGCTGGTTTCCGACCTC

GCCGAAGACCCGACCTATGTTTCCGAGAATGACGTACCGCTAAACACAGACTACTATTTC

TCCCACCTGTTGGGTACCATAAGCGTGACCTTTAAGGCTCTATTCGGAAATGATGTGAGA

ACAACAGAAAATCTTTTAAAGCGGTTTATTCCGGAAACCCCCCACAAGACCCCCACGAAA

ACCCAGGCACTGCTTGAGCGCGCCGGCTTTGAAAAGCTGACGCCCTTTACACCGGAGGAA

GAAAGTCGTCGAATACTGCATACAGTTTTTTGTACTCTAGAAGCAGCTCCCCATCAAAGC

TGA

>KU206422.1

ATGGCGGCGCGCGAACAGGCCAACTCCGTTCGCAGGAGCGGGTTTTTTAATCCGTTTATT

GGCAAGAGGCCATTTTTCAGGCCGGGAAGCGGGCAAACCGCGGAGACAGAGCGCCCAAGG

CCCCCCCAACACTCGTACTGCACAGAGGTGGGTAGCTTTAAGTTTATAGCTCCAAGATGC

CTCGATGAAGAAGCCCCCGCCGACCAGCGACGCGGTGTACACGTGGGCACCTTGGAGCGC

CCACCCAAAGTGTACTGCGATGGCTCAGAGTACGACGTGCTGAACTTTGCCTCCGGAGGT

TGCTGGCCTCGCAGGATTCGGGTTTGGAACGGCCAGGATTTTCGGGGCGATGGATTCAAC

CCCAGATTTGAGAGATTTCACGTGTACGACATAGTAGAGACTTCGGAGAGCGCCTCACAC

GATGACCCATCCAGGTTTGCTGAGCTATCCCGCCCAAGCGGGTCTGTGGTAACACTGCTG

GGAATGAGCGAGTGTGGAAAGCGAGTCGCGGTTCACGTCTATGGTGTGCGCCATTATTTT

TACATGGCAAAGGCGGAGGTGGATAGCGCTTGTGGAATAACCACCGAGGCAGAACTCGTG

CGCGCAATGGTGGACTGCGCGCACAGCTCGGCTTTGAGCGCGGCCCTGGGAAATGGCAAC

GGCGGCAAACAGAGCGGCGGCAGCGGCGGGGGATGGTGGGGCGGAAAGCACGTGTCTGCG

GACTGCTTCAAAGTGGAGACCGTGTGCCACACGACGCTGTACTACTTTGGATCTAAGCCA

GCTCTCTACTATAGAGTATCTGCCTCCAGCAGCCGCCTGGGAGGGTTCATCTGCGACAAC

TTTCACCCGGAGATTACAAAATTCGAGGGGAGCGTGGACGTGACCACGCGGCTGTTGTTG

GACAATGAAAATTTTACCAGTTTCGGGTGGTACCGCCTGCGACCCGGCACCCACGGAGAG

CGTGTTCAACTTCGCCCCGTTGAGCGACACGTCACCTCAAGCGACGTGGAGATTAACTGT

ACTCCCGATAACCTGGAGCCGATACCAGACGAGGCTGCCTGGCCCGACTATAAGCTCATG

TGCTTTGATATAGAGTGTAAAGCTGGAACGGGTAACGAAATGGCGTTCCCAGTGGCAACT

AACCAAGAGGACCTGGTCATCCAGATCTCCTGTCTGCTGTACTCGCTTGCTACTCAGAAC

CACGAACACACCCTGCTGTTTTCCCTCGGGTCATGCGATATCTCTGAGGAATACTCGTTT

GCATGCGTCCAGCGCGGCGAGCCCAGACCGACGGTTTTGGAGTTTGACAGCGAGTACGAG

CTGCTGGTTGCCTTCCTGACCTTTCTCAAGCAGTACTCTCCCGAGTTCGCCACCGGCTAC

AACATCGTTAATTTTGACTGGGCGTACATAGTTAACAAGGTAACGTCGGTGTATAACATC

AAGCTGGACGGGTACGGCAAGTTCAACAAAGGGGGGCTGTTTAAGGTGTGGGACATCGCC

ACGAACCATTTTCAGAAGAAGAGCAAGGTGAAAATCAATGGCCTGATATCTCTAGACATG

TATTCTGTGGCGACGGAAAAGCTAAAGCTACCCAGCTACAAACTCGACGCGGTCGTGGGA

GACGTCCTCGGCGAGCATAAGATAGACCTTCCCTATAAAGAAATACCCTCCTATTACGCG

GGAGGGCCTGACCGGAGGGGCGTAATAGGAGAGTATTGTATCCAGGACTCTAGGCTGGTG

GGCAAGCTGTTTTTTAAGTACCTCCCCCATCTGGAACTATCGGCGGTGGCCAAACTCGCC

CGTATCACCCTAACGCGGGTAATTTTTGACGGTCAGCAAATTCGCGTGTACACGTGCTTG

CTGAAACTCGCCCGCGAGAGAAATTTCATTTTGCCAGACAACAGACGCCGGTTTGACAGT

CAGGCAGACGCCGCGTCAGAGACTTCGGAGTTGGCTATGGATAGCCAAAGCCACGCCTTC

GACAGTACAGACGAACCCGACGGTGTGGACGGTACCCCGGACGCCGCAGGATCTGGCGCT

ACTTCTGAAAACGGAGGCGGGAAGCCCGGCGTCGGGAGGGCCGTGGGCTACCAGGGAGCA

AAGGTTCTAGACCCCGTATCCGGCTTTCATGTGGACCCCGTGGTTGTGTTTGACTTCGCT

AGCTTATACCCAAGCATTATCCAGGCCCATAACCTCTGTTTCACCACCCTGGCGCTCGAT

GAAGTGGATCTGGCCGGGCTTCAACCATCCGTCAACTACTCGACGTTCGAGGTGGGTGAC

CAAAAGTTATTTTTTGTCCACGCCCATATTCGCGAAAGCCTGCTTGGCATCTTGCTGCGC

GACTGGCTGGCCATGCGAAAGGCGGTGAGGGCGCGAATCCCCACCAGCACCCCCGAGGAG

GCAGTTTTACTAGATAAGCAGCAGTCTGCGATTAAGGTGATATGCAACTCGGTTTACGGA

TTCACGGGGGTGGCAAACGGCCTGTTGCCGTGTCTGAGGATAGCGGCTACCGTTACCACG

ATAGGACGCGACATGCTCCTCAAGACCCGAGATTACGTTCACTCTCGTTGGGCGACGCGC

GAGCTGCTGGAGGACAATTTTCCAGGGGCTATAGGTTTCCGAAACCACAAGCCTTACTCC

GTCAGGGTTATCTACGGAGACACCGACTCCGTGTTTATCAAGTTTGTGGGCCTGACGTAC

GAGGGGGTATCGGAGCTGGGGGATGCTATGTCGCGTCAGATTTCAGCGGACCTCTTTAGA

GCGCCCATCAAACTGGAGTGTGAGAAGACCTTTCAGCGACTGCTGCTGATCACCAAGAAG

AAGTACATAGGTGTCATAAACGGGGGGAAGATGCTCATGAAGGGGGTCGACCTGGTCCGC

AAAAATAACTGCTCTTTCATAAACTTGTACGCGCGACATCTGGTAGATCTTTTGTTGTAC

GACGAGGATGTGGCCACGGCGGCAGCAGAGGTGACAGACGTGCCTCCCGCAGAATGGGTG

GGGCGCCCGCTACCGAGCGGCTTTGACAAGTTTGGGCGAGTGCTGGTAGAGGCGTACAAC

CGTATCACTGCCCCCAACTTGGACGTGCGCGAGTTCGTTATGACTGCTGAGCTGAGCCGC

TCACCCGAATCGTATACCAACAAGCGCCTGCCGCACCTCACCGTCTACTTTAAGCTCGCC

ATGAGGAATGAAGAACTGCCCAGCGTAAAAGAGAGAATTCCGTATGTGATAGTTGCGCAG

ACCGAGGCCGCGGAACGCGAAGCGGGTGTAGTAAACTCAATGCGCGGTACCGCCCAAAAC

CCCGTGGTAACCAAGACCGCACGCCCCCAACCTAAACGCAAACTGCTGGTTTCCGACCTC

GCCGAAGACCCGACCTATGTTTCCGAGAATGACGTACCGCTAAACACAGACTACTATTTC

TCCCACCTGTTGGGTACCATAAGCGTGACCTTTAAGGCTCTATTCGGAAATGATGTGAGA

ACAACAGAAAATCTTTTAAAGCGGTTTATTCCGGAAACCCCCCACAAGACCCCCACGAAA

ACCCAGGCACTGCTTGAGCGCGCCGGCTTTGAAAAGCTGACGCCCTTTACACCGGAGGAA

GAAAGTCGTCGAATACTGCATACAGTTTTTTGTACTCTAGAAGCAGCTCCCCATCAAAGC

TGA

>KU206423.1

ATGGCGGCGCGCGAACAGGCCAACTCCGTTCGCAGGAGCGGGTTTTTTAATCCGTTTATT

GGCAAGAGGCCATTTTTCAGGCCGGGAAGCGGGCAAACCGCGGAGACAGAGCGCCCAAGG

CCCCCCCAACACTCGTACTGCACAGAGGTGGGTAGCTTTAAGTTTATAGCTCCAAGATGC

CTCGATGAAGAAGCCCCCGCCGACCAGCGACGCGGTGTACACGTGGGCACCTTGGAGCGC

CCACCCAAAGTGTACTGCGATGGCTCAGAGTACGACGTGCTGAACTTTGCCTCCGGAGGT

TGCTGGCCTCGCAGGATTCGGGTTTGGAACGGCCAGGATTTTCGGGGCGATGGATTCAAC

CCCAGATTTGAGAGATTTCACGTGTACGACATAGTAGAGACTTCGGAGAGCGCCTCACAC

GATGACCCATCCAGGTTTGCTGAGCTATCCCGCCCAAGCGGGTCTGTGGTAACACTGCTG

GGAATGAGCGAGTGTGGAAAGCGAGTCGCGGTTCACGTCTATGGTGTGCGCCATTATTTT

TACATGGCAAAGGCGGAGGTGGATAGCGCTTGTGGAATAACCACCGAGGCAGAACTCGTG

CGCGCAATGGTGGACTGCGCGCACAGCTCGGCTTTGAGCGCGGCCCTGGGAAATGGCAAC

GGCGGCAAACAGAGCGGCGGCAGCGGCGGGGGATGGTGGGGCGGAAAGCACGTGTCTGCG

GACTGCTTCAAAGTGGAGACCGTGTGCCACACGACGCTGTACTACTTTGGATCTAAGCCA

GCTCTCTACTATAGAGTATCTGCCTCCAGCAGCCGCCTGGGAGGGTTCATCTGCGACAAC

TTTCACCCGGAGATTACAAAATTCGAGGGGAGCGTGGACGTGACCACGCGGCTGTTGTTG

GACAATGAAAATTTTACCAGTTTCGGGTGGTACCGCCTGCGACCCGGCACCCACGGAGAG

CGTGTTCAACTTCGCCCCGTTGAGCGACACGTCACCTCAAGCGACGTGGAGATTAACTGT

ACTCCCGATAACCTGGAGCCGATACCAGACGAGGCTGCCTGGCCCGACTATAAGCTCATG

TGCTTTGATATAGAGTGTAAAGCTGGAACGGGTAACGAAATGGCGTTCCCAGTGGCAACT

AACCAAGAGGACCTGGTCATCCAGATCTCCTGTCTGCTGTACTCGCTTGCTACTCAGAAC

CACGAACACACCCTGCTGTTTTCCCTCGGGTCATGCGATATCTCTGAGGAATACTCGTTT

GCATGCGTCCAGCGCGGCGAGCCCAGACCGACGGTTTTGGAGTTTGACAGCGAGTACGAG

CTGCTGGTTGCCTTCCTGACCTTTCTCAAGCAGTACTCTCCCGAGTTCGCCACCGGCTAC

AACATCGTTAATTTTGACTGGGCGTACATAGTTAACAAGGTAACGTCGGTGTATAACATC

AAGCTGGACGGGTACGGCAAGTTCAACAAAGGGGGGCTGTTTAAGGTGTGGGACATCGCC

ACGAACCATTTTCAGAAGAAGAGCAAGGTGAAAATCAATGGCCTGATATCTCTAGACATG

TATTCTGTGGCGACGGAAAAGCTAAAGCTACCCAGCTACAAACTCGACGCGGTCGTGGGA

GACGTCCTCGGCGAGCATAAGATAGACCTTCCCTATAAAGAAATACCCTCCTATTACGCG

GGAGGGCCTGACCGGAGGGGCGTAATAGGAGAGTATTGTATCCAGGACTCTAGGCTGGTG

GGCAAGCTGTTTTTTAAGTACCTCCCCCATCTGGAACTATCGGCGGTGGCCAAACTCGCC

CGTATCACCCTAACGCGGGTAATTTTTGACGGTCAGCAAATTCGCGTGTACACGTGCTTG

CTGAAACTCGCCCGCGAGAGAAATTTCATTTTGCCAGACAACAGACGCCGGTTTGACAGT

CAGGCAGACGCCGCGTCAGAGACTTCGGAGTTGGCTATGGATAGCCAAAGCCACGCCTTC

GACAGTACAGACGAACCCGACGGTGTGGACGGTACCCCGGACGCCGCAGGATCTGGCGCT

ACTTCTGAAAACGGAGGCGGGAAGCCCGGCGTCGGGAGGGCCGTGGGCTACCAGGGAGCA

AAGGTTCTAGACCCCGTATCCGGCTTTCATGTGGACCCCGTGGTTGTGTTTGACTTCGCT

AGCTTATACCCAAGCATTATCCAGGCCCATAACCTCTGTTTCACCACCCTGGCGCTCGAT

GAAGTGGATCTGGCCGGGCTTCAACCATCCGTCAACTACTCGACGTTCGAGGTGGGTGAC

CAAAAGTTATTTTTTGTCCACGCCCATATTCGCGAAAGCCTGCTTGGCATCTTGCTGCGC

GACTGGCTGGCCATGCGAAAGGCGGTGAGGGCGCGAATCCCCACCAGCACCCCCGAGGAG

GCAGTTTTACTAGATAAGCAGCAGTCTGCGATTAAGGTGATATGCAACTCGGTTTACGGA

TTCACGGGGGTGGCAAACGGCCTGTTGCCGTGTCTGAGGATAGCGGCTACCGTTACCACG

ATAGGACGCGACATGCTCCTCAAGACCCGAGATTACGTTCACTCTCGTTGGGCGACGCGC

GAGCTGCTGGAGGACAATTTTCCAGGGGCTATAGGTTTCCGAAACCACAAGCCTTACTCC

GTCAGGGTTATCTACGGAGACACCGACTCCGTGTTTATCAAGTTTGTGGGCCTGACGTAC

GAGGGGGTATCGGAGCTGGGGGATGCTATGTCGCGTCAGATTTCAGCGGACCTCTTTAGA

GCGCCCATCAAACTGGAGTGTGAGAAGACCTTTCAGCGACTGCTGCTGATCACCAAGAAG

AAGTACATAGGTGTCATAAACGGGGGGAAGATGCTCATGAAGGGGGTCGACCTGGTCCGC

AAAAATAACTGCTCTTTCATAAACTTGTACGCGCGACATCTGGTAGATCTTTTGTTGTAC

GACGAGGATGTGGCCACGGCGGCAGCAGAGGTGACAGACGTGCCTCCCGCAGAATGGGTG

GGGCGCCCGCTACCGAGCGGCTTTGACAAGTTTGGGCGAGTGCTGGTAGAGGCGTACAAC

CGTATCACTGCCCCCAACTTGGACGTGCGCGAGTTCGTTATGACTGCTGAGCTGAGCCGC

TCACCCGAATCGTATACCAACAAGCGCCTGCCGCACCTCACCGTCTACTTTAAGCTCGCC

ATGAGGAATGAAGAACTGCCCAGCGTAAAAGAGAGAATTCCGTATGTGATAGTTGCGCAG

ACCGAGGCCGCGGAACGCGAAGCGGGTGTAGTAAACTCAATGCGCGGTACCGCCCAAAAC

CCCGTGGTAACCAAGACCGCACGCCCCCAACCTAAACGCAAACTGCTGGTTTCCGACCTC

GCCGAAGACCCGACCTATGTTTCCGAGAATGACGTACCGCTAAACACAGACTACTATTTC

TCCCACCTGTTGGGTACCATAAGCGTGACCTTTAAGGCTCTATTCGGAAATGATGTGAGA

ACAACAGAAAATCTTTTAAAGCGGTTTATTCCGGAAACCCCCCACAAGACCCCCACGAAA

ACCCAGGCACTGCTTGAGCGCGCCGGCTTTGAAAAGCTGACGCCCTTTACACCGGAGGAA

GAAAGTCGTCGAATACTGCATACAGTTTTTTGTACTCTAGAAGCAGCTCCCCATCAAAGC

TGA

>KU206424.1

ATGGCGGCGCGCGAACAGGCCAACTCCGTTCGCAGGAGCGGGTTTTTTAATCCGTTTATT

GGCAAGAGGCCATTTTTCAGGCCGGGAAGCGGGCAAACCGCGGAGACAGAGCGCCCAAGG

CCCCCCCAACACTCGTACTGCACAGAGGTGGGTAGCTTTAAGTTTATAGCTCCAAGATGC

CTCGATGAAGAAGCCCCCGCCGACCAGCGACGCGGTGTACACGTGGGCACCTTGGAGCGC

CCACCCAAAGTGTACTGCGATGGCTCAGAGTACGACGTGCTGAACTTTGCCTCCGGAGGT

TGCTGGCCTCGCAGGATTCGGGTTTGGAACGGCCAGGATTTTCGGGGCGATGGATTCAAC

CCCAGATTTGAGAGATTTCACGTGTACGACATAGTAGAGACTTCGGAGAGCGCCTCACAC

GATGACCCATCCAGGTTTGCTGAGCTATCCCGCCCAAGCGGGTCTGTGGTAACACTGCTG

GGAATGAGCGAGTGTGGAAAGCGAGTCGCGGTTCACGTCTATGGTGTGCGCCATTATTTT

TACATGGCAAAGGCGGAGGTGGATAGCGCTTGTGGAATAACCACCGAGGCAGAACTCGTG

CGCGCAATGGTGGACTGCGCGCACAGCTCGGCTTTGAGCGCGGCCCTGGGAAATGGCAAC

GGCGGCAAACAGAGCGGCGGCAGCGGCGGGGGATGGTGGGGCGGAAAGCACGTGTCTGCG

GACTGCTTCAAAGTGGAGACCGTGTGCCACACGACGCTGTACTACTTTGGATCTAAGCCA

GCTCTCTACTATAGAGTATCTGCCTCCAGCAGCCGCCTGGGAGGGTTCATCTGCGACAAC

TTTCACCCGGAGATTACAAAATTCGAGGGGAGCGTGGACGTGACCACGCGGCTGTTGTTG

GACAATGAAAATTTTACCAGTTTCGGGTGGTACCGCCTGCGACCCGGCACCCACGGAGAG

CGTGTTCAACTTCGCCCCGTTGAGCGACACGTCACCTCAAGCGACGTGGAGATTAACTGT

ACTCCCGATAACCTGGAGCCGATACCAGACGAGGCTGCCTGGCCCGACTATAAGCTCATG

TGCTTTGATATAGAGTGTAAAGCTGGAACGGGTAACGAAATGGCGTTCCCAGTGGCAACT

AACCAAGAGGACCTGGTCATCCAGATCTCCTGTCTGCTGTACTCGCTTGCTACTCAGAAC

CACGAACACACCCTGCTGTTTTCCCTCGGGTCATGCGATATCTCTGAGGAATACTCGTTT

GCATGCGTCCAGCGCGGCGAGCCCAGACCGACGGTTTTGGAGTTTGACAGCGAGTACGAG

CTGCTGGTTGCCTTCCTGACCTTTCTCAAGCAGTACTCTCCCGAGTTCGCCACCGGCTAC

AACATCGTTAATTTTGACTGGGCGTACATAGTTAACAAGGTAACGTCGGTGTATAACATC

AAGCTGGACGGGTACGGCAAGTTCAACAAAGGGGGGCTGTTTAAGGTGTGGGACATCGCC

ACGAACCATTTTCAGAAGAAGAGCAAGGTGAAAATCAATGGCCTGATATCTCTAGACATG

TATTCTGTGGCGACGGAAAAGCTAAAGCTACCCAGCTACAAACTCGACGCGGTCGTGGGA

GACGTCCTCGGCGAGCATAAGATAGACCTTCCCTATAAAGAAATACCCTCCTATTACGCG

GGAGGGCCTGACCGGAGGGGCGTAATAGGAGAGTATTGTATCCAGGACTCTAGGCTGGTG

GGCAAGCTGTTTTTTAAGTACCTCCCCCATCTGGAACTATCGGCGGTGGCCAAACTCGCC

CGTATCACCCTAACGCGGGTAATTTTTGACGGTCAGCAAATTCGCGTGTACACGTGCTTG

CTGAAACTCGCCCGCGAGAGAAATTTCATTTTGCCAGACAACAGACGCCGGTTTGACAGT

CAGGCAGACGCCGCGTCAGAGACTTCGGAGTTGGCTATGGATAGCCAAAGCCACGCCTTC

GACAGTACAGACGAACCCGACGGTGTGGACGGTACCCCGGACGCCGCAGGATCTGGCGCT

ACTTCTGAAAACGGAGGCGGGAAGCCCGGCGTCGGGAGGGCCGTGGGCTACCAGGGAGCA

AAGGTTCTAGACCCCGTATCCGGCTTTCATGTGGACCCCGTGGTTGTGTTTGACTTCGCT

AGCTTATACCCAAGCATTATCCAGGCCCATAACCTCTGTTTCACCACCCTGGCGCTCGAT

GAAGTGGATCTGGCCGGGCTTCAACCATCCGTCAACTACTCGACGTTCGAGGTGGGTGAC

CAAAAGTTATTTTTTGTCCACGCCCATATTCGCGAAAGCCTGCTTGGCATCTTGCTGCGC

GACTGGCTGGCCATGCGAAAGGCGGTGAGGGCGCGAATCCCCACCAGCACCCCCGAGGAG

GCAGTTTTACTAGATAAGCAGCAGTCTGCGATTAAGGTGATATGCAACTCGGTTTACGGA

TTCACGGGGGTGGCAAACGGCCTGTTGCCGTGTCTGAGGATAGCGGCTACCGTTACCACG

ATAGGACGCGACATGCTCCTCAAGACCCGAGATTACGTTCACTCTCGTTGGGCGACGCGC

GAGCTGCTGGAGGACAATTTTCCAGGGGCTATAGGTTTCCGAAACCACAAGCCTTACTCC

GTCAGGGTTATCTACGGAGACACCGACTCCGTGTTTATCAAGTTTGTGGGCCTGACGTAC

GAGGGGGTATCGGAGCTGGGGGATGCTATGTCGCGTCAGATTTCAGCGGACCTCTTTAGA

GCGCCCATCAAACTGGAGTGTGAGAAGACCTTTCAGCGACTGCTGCTGATCACCAAGAAG

AAGTACATAGGTGTCATAAACGGGGGGAAGATGCTCATGAAGGGGGTCGACCTGGTCCGC

AAAAATAACTGCTCTTTCATAAACTTGTACGCGCGACATCTGGTAGATCTTTTGTTGTAC

GACGAGGATGTGGCCACGGCGGCAGCAGAGGTGACAGACGTGCCTCCCGCAGAATGGGTG

GGGCGCCCGCTACCGAGCGGCTTTGACAAGTTTGGGCGAGTGCTGGTAGAGGCGTACAAC

CGTATCACTGCCCCCAACTTGGACGTGCGCGAGTTCGTTATGACTGCTGAGCTGAGCCGC

TCACCCGAATCGTATACCAACAAGCGCCTGCCGCACCTCACCGTCTACTTTAAGCTCGCC

ATGAGGAATGAAGAACTGCCCAGCGTAAAAGAGAGAATTCCGTATGTGATAGTTGCGCAG

ACCGAGGCCGCGGAACGCGAAGCGGGTGTAGTAAACTCAATGCGCGGTACCGCCCAAAAC

CCCGTGGTAACCAAGACCGCACGCCCCCAACCTAAACGCAAACTGCTGGTTTCCGACCTC

GCCGAAGACCCGACCTATGTTTCCGAGAATGACGTACCGCTAAACACAGACTACTATTTC

TCCCACCTGTTGGGTACCATAAGCGTGACCTTTAAGGCTCTATTCGGAAATGATGTGAGA

ACAACAGAAAATCTTTTAAAGCGGTTTATTCCGGAAACCCCCCACAAGACCCCCACGAAA

ACCCAGGCACTGCTTGAGCGCGCCGGCTTTGAAAAGCTGACGCCCTTTACACCGGAGGAA

GAAAGTCGTCGAATACTGCATACAGTTTTTTGTACTCTAGAAGCAGCTCCCCATCAAAGC

TGA

>KU206425.1

ATGGCGGCGCGCGAACAGGCCAACTCCGTTCGCAGGAGCGGGTTTTTTAATCCGTTTATT

GGCAAGAGGCCATTTTTCAGGCCGGGAAGCGGGCAAACCGCGGAGACAGAGCGCCCAAGG

CCCCCCCAACACTCGTACTGCACAGAGGTGGGTAGCTTTAAGTTTATAGCTCCAAGATGC

CTCGATGAAGAAGCCCCCGCCGACCAGCGACGCGGTGTACACGTGGGCACCTTGGAGCGC

CCACCCAAAGTGTACTGCGATGGCTCAGAGTACGACGTGCTGAACTTTGCCTCCGGAGGT

TGCTGGCCTCGCAGGATTCGGGTTTGGAACGGCCAGGATTTTCGGGGCGATGGATTCAAC

CCCAGATTTGAGAGATTTCACGTGTACGACATAGTAGAGACTTCGGAGAGCGCCTCACAC

GATGACCCATCCAGGTTTGCTGAGCTATCCCGCCCAAGCGGGTCTGTGGTAACACTGCTG

GGAATGAGCGAGTGTGGAAAGCGAGTCGCGGTTCACGTCTATGGTGTGCGCCATTATTTT

TACATGGCAAAGGCGGAGGTGGATAGCGCTTGTGGAATAACCACCGAGGCAGAACTCGTG

CGCGCAATGGTGGACTGCGCGCACAGCTCGGCTTTGAGCGCGGCCCTGGGAAATGGCAAC

GGCGGCAAACAGAGCGGCGGCAGCGGCGGGGGATGGTGGGGCGGAAAGCACGTGTCTGCG

GACTGCTTCAAAGTGGAGACCGTGTGCCACACGACGCTGTACTACTTTGGATCTAAGCCA

GCTCTCTACTATAGAGTATCTGCCTCCAGCAGCCGCCTGGGAGGGTTCATCTGCGACAAC

TTTCACCCGGAGATTACAAAATTCGAGGGGAGCGTGGACGTGACCACGCGGCTGTTGTTG

GACAATGAAAATTTTACCAGTTTCGGGTGGTACCGCCTGCGACCCGGCACCCACGGAGAG

CGTGTTCAACTTCGCCCCGTTGAGCGACACGTCACCTCAAGCGACGTGGAGATTAACTGT

ACTCCCGATAACCTGGAGCCGATACCAGACGAGGCTGCCTGGCCCGACTATAAGCTCATG

TGCTTTGATATAGAGTGTAAAGCTGGAACGGGTAACGAAATGGCGTTCCCAGTGGCAACT

AACCAAGAGGACCTGGTCATCCAGATCTCCTGTCTGCTGTACTCGCTTGCTACTCAGAAC

CACGAACACACCCTGCTGTTTTCCCTCGGGTCATGCGATATCTCTGAGGAATACTCGTTT

GCATGCGTCCAGCGCGGCGAGCCCAGACCGACGGTTTTGGAGTTTGACAGCGAGTACGAG

CTGCTGGTTGCCTTCCTGACCTTTCTCAAGCAGTACTCTCCCGAGTTCGCCACCGGCTAC

AACATCGTTAATTTTGACTGGGCGTACATAGTTAACAAGGTAACGTCGGTGTATAACATC

AAGCTGGACGGGTACGGCAAGTTCAACAAAGGGGGGCTGTTTAAGGTGTGGGACATCGCC

ACGAACCATTTTCAGAAGAAGAGCAAGGTGAAAATCAATGGCCTGATATCTCTAGACATG

TATTCTGTGGCGACGGAAAAGCTAAAGCTACCCAGCTACAAACTCGACGCGGTCGTGGGA

GACGTCCTCGGCGAGCATAAGATAGACCTTCCCTATAAAGAAATACCCTCCTATTACGCG

GGAGGGCCTGACCGGAGGGGCGTAATAGGAGAGTATTGTATCCAGGACTCTAGGCTGGTG

GGCAAGCTGTTTTTTAAGTACCTCCCCCATCTGGAACTATCGGCGGTGGCCAAACTCGCC

CGTATCACCCTAACGCGGGTAATTTTTGACGGTCAGCAAATTCGCGTGTACACGTGCTTG

CTGAAACTCGCCCGCGAGAGAAATTTCATTTTGCCAGACAACAGACGCCGGTTTGACAGT

CAGGCAGACGCCGCGTCAGAGACTTCGGAGTTGGCTATGGATAGCCAAAGCCACGCCTTC

GACAGTACAGACGAACCCGACGGTGTGGACGGTACCCCGGACGCCGCAGGATCTGGCGCT

ACTTCTGAAAACGGAGGCGGGAAGCCCGGCGTCGGGAGGGCCGTGGGCTACCAGGGAGCA

AAGGTTCTAGACCCCGTATCCGGCTTTCATGTGGACCCCGTGGTTGTGTTTGACTTCGCT

AGCTTATACCCAAGCATTATCCAGGCCCATAACCTCTGTTTCACCACCCTGGCGCTCGAT

GAAGTGGATCTGGCCGGGCTTCAACCATCCGTCAACTACTCGACGTTCGAGGTGGGTGAC

CAAAAGTTATTTTTTGTCCACGCCCATATTCGCGAAAGCCTGCTTGGCATCTTGCTGCGC

GACTGGCTGGCCATGCGAAAGGCGGTGAGGGCGCGAATCCCCACCAGCACCCCCGAGGAG

GCAGTTTTACTAGATAAGCAGCAGTCTGCGATTAAGGTGATATGCAACTCGGTTTACGGA

TTCACGGGGGTGGCAAACGGCCTGTTGCCGTGTCTGAGGATAGCGGCTACCGTTACCACG

ATAGGACGCGACATGCTCCTCAAGACCCGAGATTACGTTCACTCTCGTTGGGCGACGCGC

GAGCTGCTGGAGGACAATTTTCCAGGGGCTATAGGTTTCCGAAACCACAAGCCTTACTCC

GTCAGGGTTATCTACGGAGACACCGACTCCGTGTTTATCAAGTTTGTGGGCCTGACGTAC

GAGGGGGTATCGGAGCTGGGGGATGCTATGTCGCGTCAGATTTCAGCGGACCTCTTTAGA

GCGCCCATCAAACTGGAGTGTGAGAAGACCTTTCAGCGACTGCTGCTGATCACCAAGAAG

AAGTACATAGGTGTCATAAACGGGGGGAAGATGCTCATGAAGGGGGTCGACCTGGTCCGC

AAAAATAACTGCTCTTTCATAAACTTGTACGCGCGACATCTGGTAGATCTTTTGTTGTAC

GACGAGGATGTGGCCACGGCGGCAGCAGAGGTGACAGACGTGCCTCCCGCAGAATGGGTG

GGGCGCCCGCTACCGAGCGGCTTTGACAAGTTTGGGCGAGTGCTGGTAGAGGCGTACAAC

CGTATCACTGCCCCCAACTTGGACGTGCGCGAGTTCGTTATGACTGCTGAGCTGAGCCGC

TCACCCGAATCGTATACCAACAAGCGCCTGCCGCACCTCACCGTCTACTTTAAGCTCGCC

ATGAGGAATGAAGAACTGCCCAGCGTAAAAGAGAGAATTCCGTATGTGATAGTTGCGCAG

ACCGAGGCCGCGGAACGCGAAGCGGGTGTAGTAAACTCAATGCGCGGTACCGCCCAAAAC

CCCGTGGTAACCAAGACCGCACGCCCCCAACCTAAACGCAAACTGCTGGTTTCCGACCTC

GCCGAAGACCCGACCTATGTTTCCGAGAATGACGTACCGCTAAACACAGACTACTATTTC

TCCCACCTGTTGGGTACCATAAGCGTGACCTTTAAGGCTCTATTCGGAAATGATGTGAGA

ACAACAGAAAATCTTTTAAAGCGGTTTATTCCGGAAACCCCCCACAAGACCCCCACGAAA

ACCCAGGCACTGCTTGAGCGCGCCGGCTTTGAAAAGCTGACGCCCTTTACACCGGAGGAA

GAAAGTCGTCGAATACTGCATACAGTTTTTTGTACTCTAGAAGCAGCTCCCCATCAAAGC

TGA

>KU206426.1

ATGGCGGCGCGCGAACAGGCCAACTCCGTTCGCAGGAGCGGGTTTTTTAATCCGTTTATT

GGCAAGAGGCCATTTTTCAGGCCGGGAAGCGGGCAAACCGCGGAGACAGAGCGCCCAAGG

CCCCCCCAACACTCGTACTGCACAGAGGTGGGTAGCTTTAAGTTTATAGCTCCAAGATGC

CTCGATGAAGAAGCCCCCGCCGACCAGCGACGCGGTGTACACGTGGGCACCTTGGAGCGC

CCACCCAAAGTGTACTGCGATGGCTCAGAGTACGACGTGCTGAACTTTGCCTCCGGAGGT

TGCTGGCCTCGCAGGATTCGGGTTTGGAACGGCCAGGATTTTCGGGGCGATGGATTCAAC

CCCAGATTTGAGAGATTTCACGTGTACGACATAGTAGAGACTTCGGAGAGCGCCTCACAC

GATGACCCATCCAGGTTTGCTGAGCTATCCCGCCCAAGCGGGTCTGTGGTAACACTGCTG

GGAATGAGCGAGTGTGGAAAGCGAGTCGCGGTTCACGTCTATGGTGTGCGCCATTATTTT

TACATGGCAAAGGCGGAGGTGGATAGCGCTTGTGGAATAACCACCGAGGCAGAACTCGTG

CGCGCAATGGTGGACTGCGCGCACAGCTCGGCTTTGAGCGCGGCCCTGGGAAATGGCAAC

GGCGGCAAACAGAGCGGCGGCAGCGGCGGGGGATGGTGGGGCGGAAAGCACGTGTCTGCG

GACTGCTTCAAAGTGGAGACCGTGTGCCACACGACGCTGTACTACTTTGGATCTAAGCCA

GCTCTCTACTATAGAGTATCTGCCTCCAGCAGCCGCCTGGGAGGGTTCATCTGCGACAAC

TTTCACCCGGAGATTACAAAATTCGAGGGGAGCGTGGACGTGACCACGCGGCTGTTGTTG

GACAATGAAAATTTTACCAGTTTCGGGTGGTACCGCCTGCGACCCGGCACCCACGGAGAG

CGTGTTCAACTTCGCCCCGTTGAGCGACACGTCACCTCAAGCGACGTGGAGATTAACTGT

ACTCCCGATAACCTGGAGCCGATACCAGACGAGGCTGCCTGGCCCGACTATAAGCTCATG

TGCTTTGATATAGAGTGTAAAGCTGGAACGGGTAACGAAATGGCGTTCCCAGTGGCAACT

AACCAAGAGGACCTGGTCATCCAGATCTCCTGTCTGCTGTACTCGCTTGCTACTCAGAAC

CACGAACACACCCTGCTGTTTTCCCTCGGGTCATGCGATATCTCTGAGGAATACTCGTTT

GCATGCGTCCAGCGCGGCGAGCCCAGACCGACGGTTTTGGAGTTTGACAGCGAGTACGAG

CTGCTGGTTGCCTTCCTGACCTTTCTCAAGCAGTACTCTCCCGAGTTCGCCACCGGCTAC

AACATCGTTAATTTTGACTGGGCGTACATAGTTAACAAGGTAACGTCGGTGTATAACATC

AAGCTGGACGGGTACGGCAAGTTCAACAAAGGGGGGCTGTTTAAGGTGTGGGACATCGCC

ACGAACCATTTTCAGAAGAAGAGCAAGGTGAAAATCAATGGCCTGATATCTCTAGACATG

TATTCTGTGGCGACGGAAAAGCTAAAGCTACCCAGCTACAAACTCGACGCGGTCGTGGGA

GACGTCCTCGGCGAGCATAAGATAGACCTTCCCTATAAAGAAATACCCTCCTATTACGCG

GGAGGGCCTGACCGGAGGGGCGTAATAGGAGAGTATTGTATCCAGGACTCTAGGCTGGTG

GGCAAGCTGTTTTTTAAGTACCTCCCCCATCTGGAACTATCGGCGGTGGCCAAACTCGCC

CGTATCACCCTAACGCGGGTAATTTTTGACGGTCAGCAAATTCGCGTGTACACGTGCTTG

CTGAAACTCGCCCGCGAGAGAAATTTCATTTTGCCAGACAACAGACGCCGGTTTGACAGT

CAGGCAGACGCCGCGTCAGAGACTTCGGAGTTGGCTATGGATAGCCAAAGCCACGCCTTC

GACAGTACAGACGAACCCGACGGTGTGGACGGTACCCCGGACGCCGCAGGATCTGGCGCT

ACTTCTGAAAACGGAGGCGGGAAGCCCGGCGTCGGGAGGGCCGTGGGCTACCAGGGAGCA

AAGGTTCTAGACCCCGTATCCGGCTTTCATGTGGACCCCGTGGTTGTGTTTGACTTCGCT

AGCTTATACCCAAGCATTATCCAGGCCCATAACCTCTGTTTCACCACCCTGGCGCTCGAT

GAAGTGGATCTGGCCGGGCTTCAACCATCCGTCAACTACTCGACGTTCGAGGTGGGTGAC

CAAAAGTTATTTTTTGTCCACGCCCATATTCGCGAAAGCCTGCTTGGCATCTTGCTGCGC

GACTGGCTGGCCATGCGAAAGGCGGTGAGGGCGCGAATCCCCACCAGCACCCCCGAGGAG

GCAGTTTTACTAGATAAGCAGCAGTCTGCGATTAAGGTGATATGCAACTCGGTTTACGGA

TTCACGGGGGTGGCAAACGGCCTGTTGCCGTGTCTGAGGATAGCGGCTACCGTTACCACG

ATAGGACGCGACATGCTCCTCAAGACCCGAGATTACGTTCACTCTCGTTGGGCGACGCGC

GAGCTGCTGGAGGACAATTTTCCAGGGGCTATAGGTTTCCGAAACCACAAGCCTTACTCC

GTCAGGGTTATCTACGGAGACACCGACTCCGTGTTTATCAAGTTTGTGGGCCTGACGTAC

GAGGGGGTATCGGAGCTGGGGGATGCTATGTCGCGTCAGATTTCAGCGGACCTCTTTAGA

GCGCCCATCAAACTGGAGTGTGAGAAGACCTTTCAGCGACTGCTGCTGATCACCAAGAAG

AAGTACATAGGTGTCATAAACGGGGGGAAGATGCTCATGAAGGGGGTCGACCTGGTCCGC

AAAAATAACTGCTCTTTCATAAACTTGTACGCGCGACATCTGGTAGATCTTTTGTTGTAC

GACGAGGATGTGGCCACGGCGGCAGCAGAGGTGACAGACGTGCCTCCCGCAGAATGGGTG

GGGCGCCCGCTACCGAGCGGCTTTGACAAGTTTGGGCGAGTGCTGGTAGAGGCGTACAAC

CGTATCACTGCCCCCAACTTGGACGTGCGCGAGTTCGTTATGACTGCTGAGCTGAGCCGC

TCACCCGAATCGTATACCAACAAGCGCCTGCCGCACCTCACCGTCTACTTTAAGCTCGCC

ATGAGGAATGAAGAACTGCCCAGCGTAAAAGAGAGAATTCCGTATGTGATAGTTGCGCAG

ACCGAGGCCGCGGAACGCGAAGCGGGTGTAGTAAACTCAATGCGCGGTACCGCCCAAAAC

CCCGTGGTAACCAAGACCGCACGCCCCCAACCTAAACGCAAACTGCTGGTTTCCGACCTC

GCCGAAGACCCGACCTATGTTTCCGAGAATGACGTACCGCTAAACACAGACTACTATTTC

TCCCACCTGTTGGGTACCATAAGCGTGACCTTTAAGGCTCTATTCGGAAATGATGTGAGA

ACAACAGAAAATCTTTTAAAGCGGTTTATTCCGGAAACCCCCCACAAGACCCCCACGAAA

ACCCAGGCACTGCTTGAGCGCGCCGGCTTTGAAAAGCTGACGCCCTTTACACCGGAGGAA

GAAAGTCGTCGAATACTGCATACAGTTTTTTGTACTCTAGAAGCAGCTCCCCATCAAAGC

TGA

>KU206427.1

ATGGCGGCGCGCGAACAGGCCAACTCCGTTCGCAGGAGCGGGTTTTTTAATCCGTTTATT

GGCAAGAGGCCATTTTTCAGGCCGGGAAGCGGGCAAACCGCGGAGACAGAGCGCCCAAGG

CCCCCCCAACACTCGTACTGCACAGAGGTGGGTAGCTTTAAGTTTATAGCTCCAAGATGC

CTCGATGAAGAAGCCCCCGCCGACCAGCGACGCGGTGTACACGTGGGCACCTTGGAGCGC

CCACCCAAAGTGTACTGCGATGGCTCAGAGTACGACGTGCTGAACTTTGCCTCCGGAGGT

TGCTGGCCTCGCAGGATTCGGGTTTGGAACGGCCAGGATTTTCGGGGCGATGGATTCAAC

CCCAGATTTGAGAGATTTCACGTGTACGACATAGTAGAGACTTCGGAGAGCGCCTCACAC

GATGACCCATCCAGGTTTGCTGAGCTATCCCGCCCAAGCGGGTCTGTGGTAACACTGCTG

GGAATGAGCGAGTGTGGAAAGCGAGTCGCGGTTCACGTCTATGGTGTGCGCCATTATTTT

TACATGGCAAAGGCGGAGGTGGATAGCGCTTGTGGAATAACCACCGAGGCAGAACTCGTG

CGCGCAATGGTGGACTGCGCGCACAGCTCGGCTTTGAGCGCGGCCCTGGGAAATGGCAAC

GGCGGCAAACAGAGCGGCGGCAGCGGCGGGGGATGGTGGGGCGGAAAGCACGTGTCTGCG

GACTGCTTCAAAGTGGAGACCGTGTGCCACACGACGCTGTACTACTTTGGATCTAAGCCA

GCTCTCTACTATAGAGTATCTGCCTCCAGCAGCCGCCTGGGAGGGTTCATCTGCGACAAC

TTTCACCCGGAGATTACAAAATTCGAGGGGAGCGTGGACGTGACCACGCGGCTGTTGTTG

GACAATGAAAATTTTACCAGTTTCGGGTGGTACCGCCTGCGACCCGGCACCCACGGAGAG

CGTGTTCAACTTCGCCCCGTTGAGCGACACGTCACCTCAAGCGACGTGGAGATTAACTGT

ACTCCCGATAACCTGGAGCCGATACCAGACGAGGCTGCCTGGCCCGACTATAAGCTCATG

TGCTTTGATATAGAGTGTAAAGCTGGAACGGGTAACGAAATGGCGTTCCCAGTGGCAACT

AACCAAGAGGACCTGGTCATCCAGATCTCCTGTCTGCTGTACTCGCTTGCTACTCAGAAC

CACGAACACACCCTGCTGTTTTCCCTCGGGTCATGCGATATCTCTGAGGAATACTCGTTT

GCATGCGTCCAGCGCGGCGAGCCCAGACCGACGGTTTTGGAGTTTGACAGCGAGTACGAG

CTGCTGGTTGCCTTCCTGACCTTTCTCAAGCAGTACTCTCCCGAGTTCGCCACCGGCTAC

AACATCGTTAATTTTGACTGGGCGTACATAGTTAACAAGGTAACGTCGGTGTATAACATC

AAGCTGGACGGGTACGGCAAGTTCAACAAAGGGGGGCTGTTTAAGGTGTGGGACATCGCC

ACGAACCATTTTCAGAAGAAGAGCAAGGTGAAAATCAATGGCCTGATATCTCTAGACATG

TATTCTGTGGCGACGGAAAAGCTAAAGCTACCCAGCTACAAACTCGACGCGGTCGTGGGA

GACGTCCTCGGCGAGCATAAGATAGACCTTCCCTATAAAGAAATACCCTCCTATTACGCG

GGAGGGCCTGACCGGAGGGGCGTAATAGGAGAGTATTGTATCCAGGACTCTAGGCTGGTG

GGCAAGCTGTTTTTTAAGTACCTCCCCCATCTGGAACTATCGGCGGTGGCCAAACTCGCC

CGTATCACCCTAACGCGGGTAATTTTTGACGGTCAGCAAATTCGCGTGTACACGTGCTTG

CTGAAACTCGCCCGCGAGAGAAATTTCATTTTGCCAGACAACAGACGCCGGTTTGACAGT

CAGGCAGACGCCGCGTCAGAGACTTCGGAGTTGGCTATGGATAGCCAAAGCCACGCCTTC

GACAGTACAGACGAACCCGACGGTGTGGACGGTACCCCGGACGCCGCAGGATCTGGCGCT

ACTTCTGAAAACGGAGGCGGGAAGCCCGGCGTCGGGAGGGCCGTGGGCTACCAGGGAGCA

AAGGTTCTAGACCCCGTATCCGGCTTTCATGTGGACCCCGTGGTTGTGTTTGACTTCGCT

AGCTTATACCCAAGCATTATCCAGGCCCATAACCTCTGTTTCACCACCCTGGCGCTCGAT

GAAGTGGATCTGGCCGGGCTTCAACCATCCGTCAACTACTCGACGTTCGAGGTGGGTGAC

CAAAAGTTATTTTTTGTCCACGCCCATATTCGCGAAAGCCTGCTTGGCATCTTGCTGCGC

GACTGGCTGGCCATGCGAAAGGCGGTGAGGGCGCGAATCCCCACCAGCACCCCCGAGGAG

GCAGTTTTACTAGATAAGCAGCAGTCTGCGATTAAGGTGATATGCAACTCGGTTTACGGA

TTCACGGGGGTGGCAAACGGCCTGTTGCCGTGTCTGAGGATAGCGGCTACCGTTACCACG

ATAGGACGCGACATGCTCCTCAAGACCCGAGATTACGTTCACTCTCGTTGGGCGACGCGC

GAGCTGCTGGAGGACAATTTTCCAGGGGCTATAGGTTTCCGAAACCACAAGCCTTACTCC

GTCAGGGTTATCTACGGAGACACCGACTCCGTGTTTATCAAGTTTGTGGGCCTGACGTAC

GAGGGGGTATCGGAGCTGGGGGATGCTATGTCGCGTCAGATTTCAGCGGACCTCTTTAGA

GCGCCCATCAAACTGGAGTGTGAGAAGACCTTTCAGCGACTGCTGCTGATCACCAAGAAG

AAGTACATAGGTGTCATAAACGGGGGGAAGATGCTCATGAAGGGGGTCGACCTGGTCCGC

AAAAATAACTGCTCTTTCATAAACTTGTACGCGCGACATCTGGTAGATCTTTTGTTGTAC

GACGAGGATGTGGCCACGGCGGCAGCAGAGGTGACAGACGTGCCTCCCGCAGAATGGGTG

GGGCGCCCGCTACCGAGCGGCTTTGACAAGTTTGGGCGAGTGCTGGTAGAGGCGTACAAC

CGTATCACTGCCCCCAACTTGGACGTGCGCGAGTTCGTTATGACTGCTGAGCTGAGCCGC

TCACCCGAATCGTATACCAACAAGCGCCTGCCGCACCTCACCGTCTACTTTAAGCTCGCC

ATGAGGAATGAAGAACTGCCCAGCGTAAAAGAGAGAATTCCGTATGTGATAGTTGCGCAG

ACCGAGGCCGCGGAACGCGAAGCGGGTGTAGTAAACTCAATGCGCGGTACCGCCCAAAAC

CCCGTGGTAACCAAGACCGCACGCCCCCAACCTAAACGCAAACTGCTGGTTTCCGACCTC

GCCGAAGACCCGACCTATGTTTCCGAGAATGACGTACCGCTAAACACAGACTACTATTTC

TCCCACCTGTTGGGTACCATAAGCGTGACCTTTAAGGCTCTATTCGGAAATGATGTGAGA

ACAACAGAAAATCTTTTAAAGCGGTTTATTCCGGAAACCCCCCACAAGACCCCCACGAAA

ACCCAGGCACTGCTTGAGCGCGCCGGCTTTGAAAAGCTGACGCCCTTTACACCGGAGGAA

GAAAGTCGTCGAATACTGCATACAGTTTTTTGTACTCTAGAAGCAGCTCCCCATCAAAGC

TGA

>KU206428.1

ATGGCGGCGCGCGAACAGGCCAACTCCGTTCGCAGGAGCGGGTTTTTTAATCCGTTTATT

GGCAAGAGGCCATTTTTCAGGCCGGGAAGCGGGCAAACCGCGGAGACAGAGCGCCCAAGG

CCCCCCCAACACTCGTACTGCACAGAGGTGGGTAGCTTTAAGTTTATAGCTCCAAGATGC

CTCGATGAAGAAGCCCCCGCCGACCAGCGACGCGGTGTACACGTGGGCACCTTGGAGCGC

CCACCCAAAGTGTACTGCGATGGCTCAGAGTACGACGTGCTGAACTTTGCCTCCGGAGGT

TGCTGGCCTCGCAGGATTCGGGTTTGGAACGGCCAGGATTTTCGGGGCGATGGATTCAAC

CCCAGATTTGAGAGATTTCACGTGTACGACATAGTAGAGACTTCGGAGAGCGCCTCACAC

GATGACCCATCCAGGTTTGCTGAGCTATCCCGCCCAAGCGGGTCTGTGGTAACACTGCTG

GGAATGAGCGAGTGTGGAAAGCGAGTCGCGGTTCACGTCTATGGTGTGCGCCATTATTTT

TACATGGCAAAGGCGGAGGTGGATAGCGCTTGTGGAATAACCACCGAGGCAGAACTCGTG

CGCGCAATGGTGGACTGCGCGCACAGCTCGGCTTTGAGCGCGGCCCTGGGAAATGGCAAC

GGCGGCAAACAGAGCGGCGGCAGCGGCGGGGGATGGTGGGGCGGAAAGCACGTGTCTGCG

GACTGCTTCAAAGTGGAGACCGTGTGCCACACGACGCTGTACTACTTTGGATCTAAGCCA

GCTCTCTACTATAGAGTATCTGCCTCCAGCAGCCGCCTGGGAGGGTTCATCTGCGACAAC

TTTCACCCGGAGATTACAAAATTCGAGGGGAGCGTGGACGTGACCACGCGGCTGTTGTTG

GACAATGAAAATTTTACCAGTTTCGGGTGGTACCGCCTGCGACCCGGCACCCACGGAGAG

CGTGTTCAACTTCGCCCCGTTGAGCGACACGTCACCTCAAGCGACGTGGAGATTAACTGT

ACTCCCGATAACCTGGAGCCGATACCAGACGAGGCTGCCTGGCCCGACTATAAGCTCATG

TGCTTTGATATAGAGTGTAAAGCTGGAACGGGTAACGAAATGGCGTTCCCAGTGGCAACT

AACCAAGAGGACCTGGTCATCCAGATCTCCTGTCTGCTGTACTCGCTTGCTACTCAGAAC

CACGAACACACCCTGCTGTTTTCCCTCGGGTCATGCGATATCTCTGAGGAATACTCGTTT

GCATGCGTCCAGCGCGGCGAGCCCAGACCGACGGTTTTGGAGTTTGACAGCGAGTACGAG

CTGCTGGTTGCCTTCCTGACCTTTCTCAAGCAGTACTCTCCCGAGTTCGCCACCGGCTAC

AACATCGTTAATTTTGACTGGGCGTACATAGTTAACAAGGTAACGTCGGTGTATAACATC

AAGCTGGACGGGTACGGCAAGTTCAACAAAGGGGGGCTGTTTAAGGTGTGGGACATCGCC

ACGAACCATTTTCAGAAGAAGAGCAAGGTGAAAATCAATGGCCTGATATCTCTAGACATG

TATTCTGTGGCGACGGAAAAGCTAAAGCTACCCAGCTACAAACTCGACGCGGTCGTGGGA

GACGTCCTCGGCGAGCATAAGATAGACCTTCCCTATAAAGAAATACCCTCCTATTACGCG

GGAGGGCCTGACCGGAGGGGCGTAATAGGAGAGTATTGTATCCAGGACTCTAGGCTGGTG

GGCAAGCTGTTTTTTAAGTACCTCCCCCATCTGGAACTATCGGCGGTGGCCAAACTCGCC

CGTATCACCCTAACGCGGGTAATTTTTGACGGTCAGCAAATTCGCGTGTACACGTGCTTG

CTGAAACTCGCCCGCGAGAGAAATTTCATTTTGCCAGACAACAGACGCCGGTTTGACAGT

CAGGCAGACGCCGCGTCAGAGACTTCGGAGTTGGCTATGGATAGCCAAAGCCACGCCTTC

GACAGTACAGACGAACCCGACGGTGTGGACGGTACCCCGGACGCCGCAGGATCTGGCGCT

ACTTCTGAAAACGGAGGCGGGAAGCCCGGCGTCGGGAGGGCCGTGGGCTACCAGGGAGCA

AAGGTTCTAGACCCCGTATCCGGCTTTCATGTGGACCCCGTGGTTGTGTTTGACTTCGCT

AGCTTATACCCAAGCATTATCCAGGCCCATAACCTCTGTTTCACCACCCTGGCGCTCGAT

GAAGTGGATCTGGCCGGGCTTCAACCATCCGTCAACTACTCGACGTTCGAGGTGGGTGAC

CAAAAGTTATTTTTTGTCCACGCCCATATTCGCGAAAGCCTGCTTGGCATCTTGCTGCGC

GACTGGCTGGCCATGCGAAAGGCGGTGAGGGCGCGAATCCCCACCAGCACCCCCGAGGAG

GCAGTTTTACTAGATAAGCAGCAGTCTGCGATTAAGGTGATATGCAACTCGGTTTACGGA

TTCACGGGGGTGGCAAACGGCCTGTTGCCGTGTCTGAGGATAGCGGCTACCGTTACCACG

ATAGGACGCGACATGCTCCTCAAGACCCGAGATTACGTTCACTCTCGTTGGGCGACGCGC

GAGCTGCTGGAGGACAATTTTCCAGGGGCTATAGGTTTCCGAAACCACAAGCCTTACTCC

GTCAGGGTTATCTACGGAGACACCGACTCCGTGTTTATCAAGTTTGTGGGCCTGACGTAC

GAGGGGGTATCGGAGCTGGGGGATGCTATGTCGCGTCAGATTTCAGCGGACCTCTTTAGA

GCGCCCATCAAACTGGAGTGTGAGAAGACCTTTCAGCGACTGCTGCTGATCACCAAGAAG

AAGTACATAGGTGTCATAAACGGGGGGAAGATGCTCATGAAGGGGGTCGACCTGGTCCGC

AAAAATAACTGCTCTTTCATAAACTTGTACGCGCGACATCTGGTAGATCTTTTGTTGTAC

GACGAGGATGTGGCCACGGCGGCAGCAGAGGTGACAGACGTGCCTCCCGCAGAATGGGTG

GGGCGCCCGCTACCGAGCGGCTTTGACAAGTTTGGGCGAGTGCTGGTAGAGGCGTACAAC

CGTATCACTGCCCCCAACTTGGACGTGCGCGAGTTCGTTATGACTGCTGAGCTGAGCCGC

TCACCCGAATCGTATACCAACAAGCGCCTGCCGCACCTCACCGTCTACTTTAAGCTCGCC

ATGAGGAATGAAGAACTGCCCAGCGTAAAAGAGAGAATTCCGTATGTGATAGTTGCGCAG

ACCGAGGCCGCGGAACGCGAAGCGGGTGTAGTAAACTCAATGCGCGGTACCGCCCAAAAC

CCCGTGGTAACCAAGACCGCACGCCCCCAACCTAAACGCAAACTGCTGGTTTCCGACCTC

GCCGAAGACCCGACCTATGTTTCCGAGAATGACGTACCGCTAAACACAGACTACTATTTC

TCCCACCTGTTGGGTACCATAAGCGTGACCTTTAAGGCTCTATTCGGAAATGATGTGAGA

ACAACAGAAAATCTTTTAAAGCGGTTTATTCCGGAAACCCCCCACAAGACCCCCACGAAA

ACCCAGGCACTGCTTGAGCGCGCCGGCTTTGAAAAGCTGACGCCCTTTACACCGGAGGAA

GAAAGTCGTCGAATACTGCATACAGTTTTTTGTACTCTAGAAGCAGCTCCCCATCAAAGC

TGA

>KU206429.1

ATGGCGGCGCGCGAACAGGCCAACTCCGTTCGCAGGAGCGGGTTTTTTAATCCGTTTATT

GGCAAGAGGCCATTTTTCAGGCCGGGAAGCGGGCAAACCGCGGAGACAGAGCGCCCAAGG

CCCCCCCAACACTCGTACTGCACAGAGGTGGGTAGCTTTAAGTTTATAGCTCCAAGATGC

CTCGATGAAGAAGCCCCCGCCGACCAGCGACGCGGTGTACACGTGGGCACCTTGGAGCGC

CCACCCAAAGTGTACTGCGATGGCTCAGAGTACGACGTGCTGAACTTTGCCTCCGGAGGT

TGCTGGCCTCGCAGGATTCGGGTTTGGAACGGCCAGGATTTTCGGGGCGATGGATTCAAC

CCCAGATTTGAGAGATTTCACGTGTACGACATAGTAGAGACTTCGGAGAGCGCCTCACAC

GATGACCCATCCAGGTTTGCTGAGCTATCCCGCCCAAGCGGGTCTGTGGTAACACTGCTG

GGAATGAGCGAGTGTGGAAAGCGAGTCGCGGTTCACGTCTATGGTGTGCGCCATTATTTT

TACATGGCAAAGGCGGAGGTGGATAGCGCTTGTGGAATAACCACCGAGGCAGAACTCGTG

CGCGCAATGGTGGACTGCGCGCACAGCTCGGCTTTGAGCGCGGCCCTGGGAAATGGCAAC

GGCGGCAAACAGAGCGGCGGCAGCGGCGGGGGATGGTGGGGCGGAAAGCACGTGTCTGCG

GACTGCTTCAAAGTGGAGACCGTGTGCCACACGACGCTGTACTACTTTGGATCTAAGCCA

GCTCTCTACTATAGAGTATCTGCCTCCAGCAGCCGCCTGGGAGGGTTCATCTGCGACAAC

TTTCACCCGGAGATTACAAAATTCGAGGGGAGCGTGGACGTGACCACGCGGCTGTTGTTG

GACAATGAAAATTTTACCAGTTTCGGGTGGTACCGCCTGCGACCCGGCACCCACGGAGAG

CGTGTTCAACTTCGCCCCGTTGAGCGACACGTCACCTCAAGCGACGTGGAGATTAACTGT

ACTCCCGATAACCTGGAGCCGATACCAGACGAGGCTGCCTGGCCCGACTATAAGCTCATG

TGCTTTGATATAGAGTGTAAAGCTGGAACGGGTAACGAAATGGCGTTCCCAGTGGCAACT

AACCAAGAGGACCTGGTCATCCAGATCTCCTGTCTGCTGTACTCGCTTGCTACTCAGAAC

CACGAACACACCCTGCTGTTTTCCCTCGGGTCATGCGATATCTCTGAGGAATACTCGTTT

GCATGCGTCCAGCGCGGCGAGCCCAGACCGACGGTTTTGGAGTTTGACAGCGAGTACGAG

CTGCTGGTTGCCTTCCTGACCTTTCTCAAGCAGTACTCTCCCGAGTTCGCCACCGGCTAC

AACATCGTTAATTTTGACTGGGCGTACATAGTTAACAAGGTAACGTCGGTGTATAACATC

AAGCTGGACGGGTACGGCAAGTTCAACAAAGGGGGGCTGTTTAAGGTGTGGGACATCGCC

ACGAACCATTTTCAGAAGAAGAGCAAGGTGAAAATCAATGGCCTGATATCTCTAGACATG

TATTCTGTGGCGACGGAAAAGCTAAAGCTACCCAGCTACAAACTCGACGCGGTCGTGGGA

GACGTCCTCGGCGAGCATAAGATAGACCTTCCCTATAAAGAAATACCCTCCTATTACGCG

GGAGGGCCTGACCGGAGGGGCGTAATAGGAGAGTATTGTATCCAGGACTCTAGGCTGGTG

GGCAAGCTGTTTTTTAAGTACCTCCCCCATCTGGAACTATCGGCGGTGGCCAAACTCGCC

CGTATCACCCTAACGCGGGTAATTTTTGACGGTCAGCAAATTCGCGTGTACACGTGCTTG

CTGAAACTCGCCCGCGAGAGAAATTTCATTTTGCCAGACAACAGACGCCGGTTTGACAGT

CAGGCAGACGCCGCGTCAGAGACTTCGGAGTTGGCTATGGATAGCCAAAGCCACGCCTTC

GACAGTACAGACGAACCCGACGGTGTGGACGGTACCCCGGACGCCGCAGGATCTGGCGCT

ACTTCTGAAAACGGAGGCGGGAAGCCCGGCGTCGGGAGGGCCGTGGGCTACCAGGGAGCA

AAGGTTCTAGACCCCGTATCCGGCTTTCATGTGGACCCCGTGGTTGTGTTTGACTTCGCT

AGCTTATACCCAAGCATTATCCAGGCCCATAACCTCTGTTTCACCACCCTGGCGCTCGAT

GAAGTGGATCTGGCCGGGCTTCAACCATCCGTCAACTACTCGACGTTCGAGGTGGGTGAC

CAAAAGTTATTTTTTGTCCACGCCCATATTCGCGAAAGCCTGCTTGGCATCTTGCTGCGC

GACTGGCTGGCCATGCGAAAGGCGGTGAGGGCGCGAATCCCCACCAGCACCCCCGAGGAG

GCAGTTTTACTAGATAAGCAGCAGTCTGCGATTAAGGTGATATGCAACTCGGTTTACGGA

TTCACGGGGGTGGCAAACGGCCTGTTGCCGTGTCTGAGGATAGCGGCTACCGTTACCACG

ATAGGACGCGACATGCTCCTCAAGACCCGAGATTACGTTCACTCTCGTTGGGCGACGCGC

GAGCTGCTGGAGGACAATTTTCCAGGGGCTATAGGTTTCCGAAACCACAAGCCTTACTCC

GTCAGGGTTATCTACGGAGACACCGACTCCGTGTTTATCAAGTTTGTGGGCCTGACGTAC

GAGGGGGTATCGGAGCTGGGGGATGCTATGTCGCGTCAGATTTCAGCGGACCTCTTTAGA

GCGCCCATCAAACTGGAGTGTGAGAAGACCTTTCAGCGACTGCTGCTGATCACCAAGAAG

AAGTACATAGGTGTCATAAACGGGGGGAAGATGCTCATGAAGGGGGTCGACCTGGTCCGC

AAAAATAACTGCTCTTTCATAAACTTGTACGCGCGACATCTGGTAGATCTTTTGTTGTAC

GACGAGGATGTGGCCACGGCGGCAGCAGAGGTGACAGACGTGCCTCCCGCAGAATGGGTG

GGGCGCCCGCTACCGAGCGGCTTTGACAAGTTTGGGCGAGTGCTGGTAGAGGCGTACAAC

CGTATCACTGCCCCCAACTTGGACGTGCGCGAGTTCGTTATGACTGCTGAGCTGAGCCGC

TCACCCGAATCGTATACCAACAAGCGCCTGCCGCACCTCACCGTCTACTTTAAGCTCGCC

ATGAGGAATGAAGAACTGCCCAGCGTAAAAGAGAGAATTCCGTATGTGATAGTTGCGCAG

ACCGAGGCCGCGGAACGCGAAGCGGGTGTAGTAAACTCAATGCGCGGTACCGCCCAAAAC

CCCGTGGTAACCAAGACCGCACGCCCCCAACCTAAACGCAAACTGCTGGTTTCCGACCTC

GCCGAAGACCCGACCTATGTTTCCGAGAATGACGTACCGCTAAACACAGACTACTATTTC

TCCCACCTGTTGGGTACCATAAGCGTGACCTTTAAGGCTCTATTCGGAAATGATGTGAGA

ACAACAGAAAATCTTTTAAAGCGGTTTATTCCGGAAACCCCCCACAAGACCCCCACGAAA

ACCCAGGCACTGCTTGAGCGCGCCGGCTTTGAAAAGCTGACGCCCTTTACACCGGAGGAA

GAAAGTCGTCGAATACTGCATACAGTTTTTTGTACTCTAGAAGCAGCTCCCCATCAAAGC

TGA

>KU206430.1

ATGGCGGCGCGCGAACAGGCCAACTCCGTTCGCAGGAGCGGGTTTTTTAATCCGTTTATT

GGCAAGAGGCCATTTTTCAGGCCGGGAAGCGGGCAAACCGCGGAGACAGAGCGCCCAAGG

CCCCCCCAACACTCGTACTGCACAGAGGTGGGTAGCTTTAAGTTTATAGCTCCAAGATGC

CTCGATGAAGAAGCCCCCGCCGACCAGCGACGCGGTGTACACGTGGGCACCTTGGAGCGC

ACACCCAAAGTGTACTGCGATGGCTCAGAGTACGACGTGCTGAACTTTGCCTCCGGAGGT

TGCTGGCCTCGCAGGATTCGGGTTTGGAACGGCCAGGATTTTCGGGGCGATGGATTCAAC

CCCAGATTTGAGAGATTTCACGTGTACGACATAGTAGAGACTTCGGAGAGCGCCTCACAC

GATGACCCATCCAGGTTTGCTGAGCTATCCCGCCCAAGCGGGTCTGTGGTAACACTGCTG

GGAATGAGCGAGTGTGGAAAGCGAGTCGCGGTTCACGTCTATGGTGTGCGCCATTATTTT

TACATGGCAAAGGCGGAGGTGGATAGCGCTTGTGGAATAACCACCGAGGCAGAACTCGTG

CGCGCAATGGTGGACTGCGCGCACAGCTCGGCTTTGAGCGCGGCCCTGGGAAATGGCAAC

GGCGGCAAACAGAGCGGCGGCAGCGGCGGGGGATGGTGGGGCGGAAAGCACGTGTCTGCG

GACTGCTTCAAAGTGGAGACCGTGTGCCACACGACGCTGTACTACTTTGGATCTAAGCCA

GCTCTCTACTATAGAGTATCTGCCTCCAGCAGCCGCCTGGGAGGGTTCATCTGCGACAAC

TTTCACCCAGAGATTACAAAATTCGAGGGGAGCGTGGACGTGACCACGCGGCTGTTGTTG

GACAATGAAAATTTTACCAGTTTCGGGTGGTACCGCCTGCGACCCGGCACCCACGGAGAG

CGTGTTCAACTTCGCCCCGTTGAGCGACACGTCACCTCAAGCGACGTGGAGATTAACTGT

ACTCCCGATAACCTGGAGCCGATACCAGACGAGGCTGCCTGGCCCGACTATAAGCTCATG

TGCTTTGATATAGAGTGTAAAGCTGGAACGGGTAACGAAATGGCGTTCCCAGTGGCAACT

AACCAAGAGGACCTGGTCATCCAGATCTCCTGTCTGCTGTACTCGCTTGCTACTCAGAAC

CACGAACACACCCTGCTGTTTTCCCTCGGGTCATGCGATATCTCTGAGGAATACTCGTTT

GCATGCGTCCAGCGCGGCGAGCCCAGACCGACGGTTTTGGAGTTTGACAGCGAGTACGAG

CTGCTGGTTGCCTTCCTGACCTTTCTCAAGCAGTACTCTCCCGAGTTCGCCACCGGCTAC

AACATCGTTAATTTTGACTGGGCGTACATAGTTAACAAGGTAACGTCGGTGTATAACATC

AAGCTGGACGGGTACGGCAAGTTCAACAAAGGGGGGCTGTTTAAGGTGTGGGACATCGCC

ACGAACCATTTTCAGAAGAAGAGCAAGGTGAAAATCAATGGCCTGATATCTCTAGACATG

TATTCTGTGGCGACGGAAAAGCTAAAGCTACCCAGCTACAAACTCGACGCGGTCGTGGGA

GACGTCCTCGGCGAGCATAAGATAGACCTTCCCTATAAAGAAATACCCTCCTATTACGCG

GGAGGGCCTGACCGGAGGGGCGTAATAGGAGAGTATTGTATCCAGGACTCTAGGCTGGTG

GGCAAGCTGTTTTTTAAGTACCTCCCCCATCTGGAACTATCGGCGGTGGCCAAACTCGCC

CGTATCACCCTAACGCGGGTAATTTTTGACGGTCAGCAAATTCGCGTGTACACGTGCTTG

CTGAAACTCGCCCGCGAGAGAAATTTCATTTTGCCAGACAACAGACGCCGGTTTGACAGT

CAGGCAGACGCCGCGTCAGAGACTTCGGAGTTGGCTATGGATAGCCAAAGCCACGCCTTC

GACAGTACAGACGAACCCGACGGTGTGGACGGTACCCCGGACGCCGCAGGATCTGGCGCT

ACTTCTGAAAACGGAGGCGGGAAGCCCGGCGTCGGGAGGGCCGTGGGCTACCAGGGAGCA

AAGGTTCTAGACCCCGTATCCGGCTTTCATGTGGACCCCGTGGTTGTGTTTGACTTCGCT

AGCTTATACCCAAGCATTATCCAGGCCCATAACCTCTGTTTCACCACCCTGGCGCTCGAT

GAAGTGGATCTGGCCGGGCTTCAACCATCCGTCAACTACTCGACGTTCGAGGTGGGTGAC

CAAAAGTTATTTTTTGTCCACGCCCATATTCGCGAAAGCCTGCTTGGCATCTTGCTGCGC

GACTGGCTGGCCATGCGAAAGGCGGTGAGGGCGCGAATCCCCACCAGCACCCCCGAGGAG

GCAGTTTTACTAGATAAGCAGCAGTCTGCGATTAAGGTGATATGCAACTCGGTTTACGGA

TTCACGGGGGTGGCAAACGGCCTGTTGCCGTGTCTGAGGATAGCGGCTACCGTTACCACG

ATAGGACGCGACATGCTCCTCAAGACCCGAGATTACGTTCACTCTCGTTGGGCGACGCGC

GAGCTGCTGGAGGACAATTTTCCAGGGGCTATAGGTTTCCGAAACCACAAGCCTTACTCC

GTCAGGGTTATCTACGGAGACACCGACTCCGTGTTTATCAAGTTTGTGGGCCTGACGTAC

GAGGGGGTATCGGAGCTGGGGGATGCTATGTCGCGTCAGATTTCAGCGGACCTCTTTAGA

GCGCCCATCAAACTGGAGTGTGAGAAGACCTTTCAGCGACTGCTGCTGATCACCAAGAAG

AAGTACATAGGTGTCATAAACGGGGGGAAGATGCTCATGAAGGGGGTCGACCTGGTCCGC

AAAAATAACTGCTCTTTCATAAACTTGTACGCGCGACATCTGGTAGATCTTTTGTTGTAC

GACGAGGATGTGGCCACGGCGGCAGCAGAGGTGACAGACGTGCCTCCCGCAGAATGGGTG

GGGCGCCCGCTACCGAGCGGCTTTGACAAGTTTGGGCGAGTGCTGGTAGAGGCGTACAAC

CGTATCACTGCCCCCAACTTGGACGTGCGCGAGTTCGTTATGACTGCTGAGCTGAGCCGC

TCACCCGAATCGTATACCAACAAGCGCCTGCCGCACCTCACCGTCTACTTTAAGCTCGCC

ATGAGGAATGAAGAACTGCCCAGCGTAAAAGAGAGAATTCCGTATGTGATAGTTGCGCAG

ACCGAGGCCGCGGAACGCGAAGCGGGTGTAGTAAACTCAATGCGCGGTACCGCCCAAAAC

CCCGTGGTAACCAAGACCGCACGCCCCCAACCTAAACGCAAACTGCTGGTTTCCGACCTC

GCCGAAGACCCGACCTATGTTTCCGAGAATGACGTACCGCTAAACACAGACTACTATTTC

TCCCACCTGTTGGGTACCATAAGCGTGACCTTTAAGGCTCTATTCGGAAATGATGTGAGA

ACAACAGAAAATCTTTTAAAGCGGTTTATTCCGGAAACCCCCCACAAGACCCCCACGAAA

ACCCAGGCACTGCTTGAGCGCGCCGGCTTTGAAAAGCTGACGCCCTTTACACCGGAGGAA

GAAAGTCGTCGAATACTGCATACAGTTTTTTGTACTCTAGAAGCAGCTCCCCATCAAAGC

TGA

>KU206431.1

ATGGCGGCGCGCGAACAGGCCAACTCCGTTCGCAGGAGCGGGTTTTTTAATCCGTTTATT

GGCAAGAGGCCATTTTTCAGGCCGGGAAGCGGGCAAACCGCGGAGACAGAGCGCCCAAGG

CCCCCCCAACACTCGTACTGCACAGAGGTGGGTAGCTTTAAGTTTATAGCTCCAAGATGC

CTCGATGAAGAAGCCCCCGCCGACCAGCGACGCGGTGTACACGTGGGCACCTTGGAGCGC

CCACCCAAAGTGTACTGCGATGGCTCAGAGTACGACGTGCTGAACTTTGCCTCCGGAGGT

TGCTGGCCTCGCAGGATTCGGGTTTGGAACGGCCAGGATTTTCGGGGCGATGGATTCAAC

CCCAGATTTGAGAGATTTCACGTGTACGACATAGTAGAGACTTCGGAGAGCGCCTCACAC

GATGACCCATCCAGGTTTGCTGAGCTATCCCGCCCAAGCGGGTCTGTGGTAACACTGCTG

GGAATGAGCGAGTGTGGAAAGCGAGTCGCGGTTCACGTCTATGGTGTGCGCCATTATTTT

TACATGGCAAAGGCGGAGGTGGATAGCGCTTGTGGAATAACCACCGAGGCAGAACTCGTG

CGCGCAATGGTGGACTGCGCGCACAGCTCGGCTTTGAGCGCGGCCCTGGGAAATGGCAAC

GGCGGCAAACAGAGCGGCGGCAGCGGCGGGGGATGGTGGGGCGGAAAGCACGTGTCTGCG

GACTGCTTCAAAGTGGAGACCGTGTGCCACACGACGCTGTACTACTTTGGATCTAAGCCA

GCTCTCTACTATAGAGTATCTGCCTCCAGCAGCCGCCTGGGAGGGTTCATCTGCGACAAC

TTTCACCCGGAGATTACAAAATTCGAGGGGAGCGTGGACGTGACCACGCGGCTGTTGTTG

GACAATGAAAATTTTACCAGTTTCGGGTGGTACCGCCTGCGACCCGGCACCCACGGAGAG

CGTGTTCAACTTCGCCCCGTTGAGCGACACGTCACCTCAAGCGACGTGGAGATTAACTGT

ACTCCCGATAACCTGGAGCCGATACCAGACGAGGCTGCCTGGCCCGACTATAAGCTCATG

TGCTTTGATATAGAGTGTAAAGCTGGAACGGGTAACGAAATGGCGTTCCCAGTGGCAACT

AACCAAGAGGACCTGGTCATCCAGATCTCCTGTCTGCTGTACTCGCTTGCTACTCAGAAC

CACGAACACACCCTGCTGTTTTCCCTCGGGTCATGCGATATCTCTGAGGAATACTCGTTT

GCATGCGTCCAGCGCGGCGAGCCCAGACCGACGGTTTTGGAGTTTGACAGCGAGTACGAG

CTGCTGGTTGCCTTCCTGACCTTTCTCAAGCAGTACTCTCCCGAGTTCGCCACCGGCTAC

AACATCGTTAATTTTGACTGGGCGTACATAGTTAACAAGGTAACGTCGGTGTATAACATC

AAGCTGGACGGGTACGGCAAGTTCAACAAAGGGGGGCTGTTTAAGGTGTGGGACATCGCC

ACGAACCATTTTCAGAAGAAGAGCAAGGTGAAAATCAATGGCCTGATATCTCTAGACATG

TATTCTGTGGCGACGGAAAAGCTAAAGCTACCCAGCTACAAACTCGACGCGGTCGTGGGA

GACGTCCTCGGCGAGCATAAGATAGACCTTCCCTATAAAGAAATACCCTCCTATTACGCG

GGAGGGCCTGACCGGAGGGGCGTAATAGGAGAGTATTGTATCCAGGACTCTAGGCTGGTG

GGCAAGCTGTTTTTTAAGTACCTCCCCCATCTGGAACTATCGGCGGTGGCCAAACTCGCC

CGTATCACCCTAACGCGGGTAATTTTTGACGGTCAGCAAATTCGCGTGTACACGTGCTTG

CTGAAACTCGCCCGCGAGAGAAATTTCATTTTGCCAGACAACAGACGCCGGTTTGACAGT

CAGGCAGACGCCGCGTCAGAGACTTCGGAGTTGGCTATGGATAGCCAAAGCCACGCCTTC

GACAGTACAGACGAACCCGACGGTGTGGACGGTACCCCGGACGCCGCAGGATCTGGCGCT

ACTTCTGAAAACGGAGGCGGGAAGCCCGGCGTCGGGAGGGCCGTGGGCTACCAGGGAGCA

AAGGTTCTAGACCCCGTATCCGGCTTTCATGTGGACCCCGTGGTTGTGTTTGACTTCGCT

AGCTTATACCCAAGCATTATCCAGGCCCATAACCTCTGTTTCACCACCCTGGCGCTCGAT

GAAGTGGATCTGGCCGGGCTTCAACCATCCGTCAACTACTCGACGTTCGAGGTGGGTGAC

CAAAAGTTATTTTTTGTCCACGCCCATATTCGCGAAAGCCTGCTTGGCATCTTGCTGCGC

GACTGGCTGGCCATGCGAAAGGCGGTGAGGGCGCGAATCCCCACCAGCACCCCCGAGGAG

GCAGTTTTACTAGATAAGCAGCAGTCTGCGATTAAGGTGATATGCAACTCGGTTTACGGA

TTCACGGGGGTGGCAAACGGCCTGTTGCCGTGTCTGAGGATAGCGGCTACCGTTACCACG

ATAGGACGCGACATGCTCCTCAAGACCCGAGATTACGTTCACTCTCGTTGGGCGACGCGC

GAGCTGCTGGAGGACAATTTTCCAGGGGCTATAGGTTTCCGAAACCACAAGCCTTACTCC

GTCAGGGTTATCTACGGAGACACCGACTCCGTGTTTATCAAGTTTGTGGGCCTGACGTAC

GAGGGGGTATCGGAGCTGGGGGATGCTATGTCGCGTCAGATTTCAGCGGACCTCTTTAGA

GCGCCCATCAAACTGGAGTGTGAGAAGACCTTTCAGCGACTGCTGCTGATCACCAAGAAG

AAGTACATAGGTGTCATAAACGGGGGGAAGATGCTCATGAAGGGGGTCGACCTGGTCCGC

AAAAATAACTGCTCTTTCATAAACTTGTACGCGCGACATCTGGTAGATCTTTTGTTGTAC

GACGAGGATGTGGCCACGGCGGCAGCAGAGGTGACAGACGTGCCTCCCGCAGAATGGGTG

GGGCGCCCGCTACCGAGCGGCTTTGACAAGTTTGGGCGAGTGCTGGTAGAGGCGTACAAC

CGTATCACTGCCCCCAACTTGGACGTGCGCGAGTTCGTTATGACTGCTGAGCTGAGCCGC

TCACCCGAATCGTATACCAACAAGCGCCTGCCGCACCTCACCGTCTACTTTAAGCTCGCC

ATGAGGAATGAAGAACTGCCCAGCGTAAAAGAGAGAATTCCGTATGTGATAGTTGCGCAG

ACCGAGGCCGCGGAACGCGAAGCGGGTGTAGTAAACTCAATGCGCGGTACCGCCCAAAAC

CCCGTGGTAACCAAGACCGCACGCCCCCAACCTAAACGCAAACTGCTGGTTTCCGACCTC

GCCGAAGACCCGACCTATGTTTCCGAGAATGACGTACCGCTAAACACAGACTACTATTTC

TCCCACCTGTTGGGTACCATAAGCGTGACCTTTAAGGCTCTATTCGGAAATGATGTGAGA

ACAACAGAAAATCTTTTAAAGCGGTTTATTCCGGAAACCCCCCACAAGACCCCCACGAAA

ACCCAGGCACTGCTTGAGCGCGCCGGCTTTGAAAAGCTGACGCCCTTTACACCGGAGGAA

GAAAGTCGTCGAATACTGCATACAGTTTTTTGTACTCTAGAAGCAGCTCCCCATCAAAGC

TGA

>KU206432.1

ATGGCGGCGCGCGAACAGGCCAACTCCGTTCGCAGGAGCGGGTTTTTTAATCCGTTTATT

GGCAAGAGGCCATTTTTCAGGCCGGGAAGCGGGCAAACCGCGGAGACAGAGCGCCCAAGG

CCCCCCCAACACTCGTACTGCACAGAGGTGGGTAGCTTTAAGTTTATAGCTCCAAGATGC

CTCGATGAAGAAGCCCCCGCCGACCAGCGACGCGGTGTACACGTGGGCACCTTGGAGCGC

CCACCCAAAGTGTACTGCGATGGCTCAGAGTACGACGTGCTGAACTTTGCCTCCGGAGGT

TGCTGGCCTCGCAGGATTCGGGTTTGGAACGGCCAGGATTTTCGGGGCGATGGATTCAAC

CCCAGATTTGAGAGATTTCACGTGTACGACATAGTAGAGACTTCGGAGAGCGCCTCACAC

GATGACCCATCCAGGTTTGCTGAGCTATCCCGCCCAAGCGGGTCTGTGGTAACACTGCTG

GGAATGAGCGAGTGTGGAAAGCGAGTCGCGGTTCACGTCTATGGTGTGCGCCATTATTTT

TACATGGCAAAGGCGGAGGTGGATAGCGCTTGTGGAATAACCACCGAGGCAGAACTCGTG

CGCGCAATGGTGGACTGCGCGCACAGCTCGGCTTTGAGCGCGGCCCTGGGAAATGGCAAC

GGCGGCAAACAGAGCGGCGGCAGCGGCGGGGGATGGTGGGGCGGAAAGCACGTGTCTGCG

GACTGCTTCAAAGTGGAGACCGTGTGCCACACGACGCTGTACTACTTTGGATCTAAGCCA

GCTCTCTACTATAGAGTATCTGCCTCCAGCAGCCGCCTGGGAGGGTTCATCTGCGACAAC

TTTCACCCGGAGATTACAAAATTCGAGGGGAGCGTGGACGTGACCACGCGGCTGTTGTTG

GACAATGAAAATTTTACCAGTTTCGGGTGGTACCGCCTGCGACCCGGCACCCACGGAGAG

CGTGTTCAACTTCGCCCCGTTGAGCGACACGTCACCTCAAGCGACGTGGAGATTAACTGT

ACTCCCGATAACCTGGAGCCGATACCAGACGAGGCTGCCTGGCCCGACTATAAGCTCATG

TGCTTTGATATAGAGTGTAAAGCTGGAACGGGTAACGAAATGGCGTTCCCAGTGGCAACT

AACCAAGAGGACCTGGTCATCCAGATCTCCTGTCTGCTGTACTCGCTTGCTACTCAGAAC

CACGAACACACCCTGCTGTTTTCCCTCGGGTCATGCGATATCTCTGAGGAATACTCGTTT

GCATGCGTCCAGCGCGGCGAGCCCAGACCGACGGTTTTGGAGTTTGACAGCGAGTACGAG

CTGCTGGTTGCCTTCCTGACCTTTCTCAAGCAGTACTCTCCCGAGTTCGCCACCGGCTAC

AACATCGTTAATTTTGACTGGGCGTACATAGTTAACAAGGTAACGTCGGTGTATAACATC

AAGCTGGACGGGTACGGCAAGTTCAACAAAGGGGGGCTGTTTAAGGTGTGGGACATCGCC

ACGAACCATTTTCAGAAGAAGAGCAAGGTGAAAATCAATGGCCTGATATCTCTAGACATG

TATTCTGTGGCGACGGAAAAGCTAAAGCTACCCAGCTACAAACTCGACGCGGTCGTGGGA

GACGTCCTCGGCGAGCATAAGATAGACCTTCCCTATAAAGAAATACCCTCCTATTACGCG

GGAGGGCCTGACCGGAGGGGCGTAATAGGAGAGTATTGTATCCAGGACTCTAGGCTGGTG

GGCAAGCTGTTTTTTAAGTACCTCCCCCATCTGGAACTATCGGCGGTGGCCAAACTCGCC

CGTATCACCCTAACGCGGGTAATTTTTGACGGTCAGCAAATTCGCGTGTACACGTGCTTG

CTGAAACTCGCCCGCGAGAGAAATTTCATTTTGCCAGACAACAGACGCCGGTTTGACAGT

CAGGCAGACGCCGCGTCAGAGACTTCGGAGTTGGCTATGGATAGCCAAAGCCACGCCTTC

GACAGTACAGACGAACCCGACGGTGTGGACGGTACCCCGGACGCCGCAGGATCTGGCGCT

ACTTCTGAAAACGGAGGCGGGAAGCCCGGCGTCGGGAGGGCCGTGGGCTACCAGGGAGCA

AAGGTTCTAGACCCCGTATCCGGCTTTCATGTGGACCCCGTGGTTGTGTTTGACTTCGCT

AGCTTATACCCAAGCATTATCCAGGCCCATAACCTCTGTTTCACCACCCTGGCGCTCGAT

GAAGTGGATCTGGCCGGGCTTCAACCATCCGTCAACTACTCGACGTTCGAGGTGGGTGAC

CAAAAGTTATTTTTTGTCCACGCCCATATTCGCGAAAGCCTGCTTGGCATCTTGCTGCGC

GACTGGCTGGCCATGCGAAAGGCGGTGAGGGCGCGAATCCCCACCAGCACCCCCGAGGAG

GCAGTTTTACTAGATAAGCAGCAGTCTGCGATTAAGGTGATATGCAACTCGGTTTACGGA

TTCACGGGGGTGGCAAACGGCCTGTTGCCGTGTCTGAGGATAGCGGCTACCGTTACCACG

ATAGGACGCGACATGCTCCTCAAGACCCGAGATTACGTTCACTCTCGTTGGGCGACGCGC

GAGCTGCTGGAGGACAATTTTCCAGGGGCTATAGGTTTCCGAAACCACAAGCCTTACTCC

GTCAGGGTTATCTACGGAGACACCGACTCCGTGTTTATCAAGTTTGTGGGCCTGACGTAC

GAGGGGGTATCGGAGCTGGGGGATGCTATGTCGCGTCAGATTTCAGCGGACCTCTTTAGA

GCGCCCATCAAACTGGAGTGTGAGAAGACCTTTCAGCGACTGCTGCTGATCACCAAGAAG

AAGTACATAGGTGTCATAAACGGGGGGAAGATGCTCATGAAGGGGGTCGACCTGGTCCGC

AAAAATAACTGCTCTTTCATAAACTTGTACGCGCGACATCTGGTAGATCTTTTGTTGTAC

GACGAGGATGTGGCCACGGCGGCAGCAGAGGTGACAGACGTGCCTCCCGCAGAATGGGTG

GGGCGCCCGCTACCGAGCGGCTTTGACAAGTTTGGGCGAGTGCTGGTAGAGGCGTACAAC

CGTATCACTGCCCCCAACTTGGACGTGCGCGAGTTCGTTATGACTGCTGAGCTGAGCCGC

TCACCCGAATCGTATACCAACAAGCGCCTGCCGCACCTCACCGTCTACTTTAAGCTCGCC

ATGAGGAATGAAGAACTGCCCAGCGTAAAAGAGAGAATTCCGTATGTGATAGTTGCGCAG

ACCGAGGCCGCGGAACGCGAAGCGGGTGTAGTAAACTCAATGCGCGGTACCGCCCAAAAC

CCCGTGGTAACCAAGACCGCACGCCCCCAACCTAAACGCAAACTGCTGGTTTCCGACCTC

GCCGAAGACCCGACCTATGTTTCCGAGAATGACGTACCGCTAAACACAGACTACTATTTC

TCCCACCTGTTGGGTACCATAAGCGTGACCTTTAAGGCTCTATTCGGAAATGATGTGAGA

ACAACAGAAAATCTTTTAAAGCGGTTTATTCCGGAAACCCCCCACAAGACCCCCACGAAA

ACCCAGGCACTGCTTGAGCGCGCCGGCTTTGAAAAGCTGACGCCCTTTACACCGGAGGAA

GAAAGTCGTCGAATACTGCATACAGTTTTTTGTACTCTAGAAGCAGCTCCCCATCAAAGC

TGA

>KU206433.1

ATGGCGGCGCGCGAACAGGCCAACTCCGTTCGCAGGAGCGGGTTTTTTAATCCGTTTATT

GGCAAGAGGCCATTTTTCAGGCCGGGAAGCGGGCAAACCGCGGAGACAGAGCGCCCAAGG

CCCCCCCAACACTCGTACTGCACAGAGGTGGGTAGCTTTAAGTTTATAGCTCCAAGATGC

CTCGATGAAGAAGCCCCCGCCGACCAGCGACGCGGTGTACACGTGGGCACCTTGGAGCGC

CCACCCAAAGTGTACTGCGATGGCTCAGAGTACGACGTGCTGAACTTTGCCTCCGGAGGT

TGCTGGCCTCGCAGGATTCGGGTTTGGAACGGCCAGGATTTTCGGGGCGATGGATTCAAC

CCCAGATTTGAGAGATTTCACGTGTACGACATAGTAGAGACTTCGGAGAGCGCCTCACAC

GATGACCCATCCAGGTTTGCTGAGCTATCCCGCCCAAGCGGGTCTGTGGTAACACTGCTG

GGAATGAGCGAGTGTGGAAAGCGAGTCGCGGTTCACGTCTATGGTGTGCGCCATTATTTT

TACATGGCAAAGGCGGAGGTGGATAGCGCTTGTGGAATAACCACCGAGGCAGAACTCGTG

CGCGCAATGGTGGACTGCGCGCACAGCTCGGCTTTGAGCGCGGCCCTGGGAAATGGCAAC

GGCGGCAAACAGAGCGGCGGCAGCGGCGGGGGATGGTGGGGCGGAAAGCACGTGTCTGCG

GACTGCTTCAAAGTGGAGACCGTGTGCCACACGACGCTGTACTACTTTGGATCTAAGCCA

GCTCTCTACTATAGAGTATCTGCCTCCAGCAGCCGCCTGGGAGGGTTCATCTGCGACAAC

TTTCACCCGGAGATTACAAAATTCGAGGGGAGCGTGGACGTGACCACGCGGCTGTTGTTG

GACAATGAAAATTTTACCAGTTTCGGGTGGTACCGCCTGCGACCCGGCACCCACGGAGAG

CGTGTTCAACTTCGCCCCGTTGAGCGACACGTCACCTCAAGCGACGTGGAGATTAACTGT

ACTCCCGATAACCTGGAGCCGATACCAGACGAGGCTGCCTGGCCCGACTATAAGCTCATG

TGCTTTGATATAGAGTGTAAAGCTGGAACGGGTAACGAAATGGCGTTCCCAGTGGCAACT

AACCAAGAGGACCTGGTCATCCAGATCTCCTGTCTGCTGTACTCGCTTGCTACTCAGAAC

CACGAACACACCCTGCTGTTTTCCCTCGGGTCATGCGATATCTCTGAGGAATACTCGTTT

GCATGCGTCCAGCGCGGCGAGCCCAGACCGACGGTTTTGGAGTTTGACAGCGAGTACGAG

CTGCTGGTTGCCTTCCTGACCTTTCTCAAGCAGTACTCTCCCGAGTTCGCCACCGGCTAC

AACATCGTTAATTTTGACTGGGCGTACATAGTTAACAAGGTAACGTCGGTGTATAACATC

AAGCTGGACGGGTACGGCAAGTTCAACAAAGGGGGGCTGTTTAAGGTGTGGGACATCGCC

ACGAACCATTTTCAGAAGAAGAGCAAGGTGAAAATCAATGGCCTGATATCTCTAGACATG

TATTCTGTGGCGACGGAAAAGCTAAAGCTACCCAGCTACAAACTCGACGCGGTCGTGGGA

GACGTCCTCGGCGAGCATAAGATAGACCTTCCCTATAAAGAAATACCCTCCTATTACGCG

GGAGGGCCTGACCGGAGGGGCGTAATAGGAGAGTATTGTATCCAGGACTCTAGGCTGGTG

GGCAAGCTGTTTTTTAAGTACCTCCCCCATCTGGAACTATCGGCGGTGGCCAAACTCGCC

CGTATCACCCTAACGCGGGTAATTTTTGACGGTCAGCAAATTCGCGTGTACACGTGCTTG

CTGAAACTCGCCCGCGAGAGAAATTTCATTTTGCCAGACAACAGACGCCGGTTTGACAGT

CAGGCAGACGCCGCGTCAGAGACTTCGGAGTTGGCTATGGATAGCCAAAGCCACGCCTTC

GACAGTACAGACGAACCCGACGGTGTGGACGGTACCCCGGACGCCGCAGGATCTGGCGCT

ACTTCTGAAAACGGAGGCGGGAAGCCCGGCGTCGGGAGGGCCGTGGGCTACCAGGGAGCA

AAGGTTCTAGACCCCGTATCCGGCTTTCATGTGGACCCCGTGGTTGTGTTTGACTTCGCT

AGCTTATACCCAAGCATTATCCAGGCCCATAACCTCTGTTTCACCACCCTGGCGCTCGAT

GAAGTGGATCTGGCCGGGCTTCAACCATCCGTCAACTACTCGACGTTCGAGGTGGGTGAC

CAAAAGTTATTTTTTGTCCACGCCCATATTCGCGAAAGCCTGCTTGGCATCTTGCTGCGC

GACTGGCTGGCCATGCGAAAGGCGGTGAGGGCGCGAATCCCCACCAGCACCCCCGAGGAG

GCAGTTTTACTAGATAAGCAGCAGTCTGCGATTAAGGTGATATGCAACTCGGTTTACGGA

TTCACGGGGGTGGCAAACGGCCTGTTGCCGTGTCTGAGGATAGCGGCTACCGTTACCACG

ATAGGACGCGACATGCTCCTCAAGACCCGAGATTACGTTCACTCTCGTTGGGCGACGCGC

GAGCTGCTGGAGGACAATTTTCCAGGGGCTATAGGTTTCCGAAACCACAAGCCTTACTCC

GTCAGGGTTATCTACGGAGACACCGACTCCGTGTTTATCAAGTTTGTGGGCCTGACGTAC

GAGGGGGTATCGGAGCTGGGGGATGCTATGTCGCGTCAGATTTCAGCGGACCTCTTTAGA

GCGCCCATCAAACTGGAGTGTGAGAAGACCTTTCAGCGACTGCTGCTGATCACCAAGAAG

AAGTACATAGGTGTCATAAACGGGGGGAAGATGCTCATGAAGGGGGTCGACCTGGTCCGC

AAAAATAACTGCTCTTTCATAAACTTGTACGCGCGACATCTGGTAGATCTTTTGTTGTAC

GACGAGGATGTGGCCACGGCGGCAGCAGAGGTGACAGACGTGCCTCCCGCAGAATGGGTG

GGGCGCCCGCTACCGAGCGGCTTTGACAAGTTTGGGCGAGTGCTGGTAGAGGCGTACAAC

CGTATCACTGCCCCCAACTTGGACGTGCGCGAGTTCGTTATGACTGCTGAGCTGAGCCGC

TCACCCGAATCGTATACCAACAAGCGCCTGCCGCACCTCACCGTCTACTTTAAGCTCGCC

ATGAGGAATGAAGAACTGCCCAGCGTAAAAGAGAGAATTCCGTATGTGATAGTTGCGCAG

ACCGAGGCCGCGGAACGCGAAGCGGGTGTAGTAAACTCAATGCGCGGTACCGCCCAAAAC

CCCGTGGTAACCAAGACCGCACGCCCCCAACCTAAACGCAAACTGCTGGTTTCCGACCTC

GCCGAAGACCCGACCTATGTTTCCGAGAATGACGTACCGCTAAACACAGACTACTATTTC

TCCCACCTGTTGGGTACCATAAGCGTGACCTTTAAGGCTCTATTCGGAAATGATGTGAGA

ACAACAGAAAATCTTTTAAAGCGGTTTATTCCGGAAACCCCCCACAAGACCCCCACGAAA

ACCCAGGCACTGCTTGAGCGCGCCGGCTTTGAAAAGCTGACGCCCTTTACACCGGAGGAA

GAAAGTCGTCGAATACTGCATACAGTTTTTTGTACTCTAGAAGCAGCTCCCCATCAAAGC

TGA

>KU206434.1

ATGGCGGCGCGCGAACAGGCCAACTCCGTTCGCAGGAGCGGGTTTTTTAATCCGTTTATT

GGCAAGAGGCCATTTTTCAGGCCGGGAAGCGGGCAAACCGCGGAGACAGAGCGCCCAAGG

CCCCCCCAACACTCGTACTGCACAGAGGTGGGTAGCTTTAAGTTTATAGCTCCAAGATGC

CTCGATGAAGAAGCCCCCGCCGACCAGCGACGCGGTGTACACGTGGGCACCTTGGAGCGC

CCACCCAAAGTGTACTGCGATGGCTCAGAGTACGACGTGCTGAACTTTGCCTCCGGAGGT

TGCTGGCCTCGCAGGATTCGGGTTTGGAACGGCCAGGATTTTCGGGGCGATGGATTCAAC

CCCAGATTTGAGAGATTTCACGTGTACGACATAGTAGAGACTTCGGAGAGCGCCTCACAC

GATGACCCATCCAGGTTTGCTGAGCTATCCCGCCCAAGCGGGTCTGTGGTAACACTGCTG

GGAATGAGCGAGTGTGGAAAGCGAGTCGCGGTTCACGTCTATGGTGTGCGCCATTATTTT

TACATGGCAAAGGCGGAGGTGGATAGCGCTTGTGGAATAACCACCGAGGCAGAACTCGTG

CGCGCAATGGTGGACTGCGCGCACAGCTCGGCTTTGAGCGCGGCCCTGGGAAATGGCAAC

GGCGGCAAACAGAGCGGCGGCAGCGGCGGGGGATGGTGGGGCGGAAAGCACGTGTCTGCG

GACTGCTTCAAAGTGGAGACCGTGTGCCACACGACGCTGTACTACTTTGGATCTAAGCCA

GCTCTCTACTATAGAGTATCTGCCTCCAGCAGCCGCCTGGGAGGGTTCATCTGCGACAAC

TTTCACCCGGAGATTACAAAATTCGAGGGGAGCGTGGACGTGACCACGCGGCTGTTGTTG

GACAATGAAAATTTTACCAGTTTCGGGTGGTACCGCCTGCGACCCGGCACCCACGGAGAG

CGTGTTCAACTTCGCCCCGTTGAGCGACACGTCACCTCAAGCGACGTGGAGATTAACTGT

ACTCCCGATAACCTGGAGCCGATACCAGACGAGGCTGCCTGGCCCGACTATAAGCTCATG

TGCTTTGATATAGAGTGTAAAGCTGGAACGGGTAACGAAATGGCGTTCCCAGTGGCAACT

AACCAAGAGGACCTGGTCATCCAGATCTCCTGTCTGCTGTACTCGCTTGCTACTCAGAAC

CACGAACACACCCTGCTGTTTTCCCTCGGGTCATGCGATATCTCTGAGGAATACTCGTTT

GCATGCGTCCAGCGCGGCGAGCCCAGACCGACGGTTTTGGAGTTTGACAGCGAGTACGAG

CTGCTGGTTGCCTTCCTGACCTTTCTCAAGCAGTACTCTCCCGAGTTCGCCACCGGCTAC

AACATCGTTAATTTTGACTGGGCGTACATAGTTAACAAGGTAACGTCGGTGTATAACATC

AAGCTGGACGGGTACGGCAAGTTCAACAAAGGGGGGCTGTTTAAGGTGTGGGACATCGCC

ACGAACCATTTTCAGAAGAAGAGCAAGGTGAAAATCAATGGCCTGATATCTCTAGACATG

TATTCTGTGGCGACGGAAAAGCTAAAGCTACCCAGCTACAAACTCGACGCGGTCGTGGGA

GACGTCCTCGGCGAGCATAAGATAGACCTTCCCTATAAAGAAATACCCTCCTATTACGCG

GGAGGGCCTGACCGGAGGGGCGTAATAGGAGAGTATTGTATCCAGGACTCTAGGCTGGTG

GGCAAGCTGTTTTTTAAGTACCTCCCCCATCTGGAACTATCGGCGGTGGCCAAACTCGCC

CGTATCACCCTAACGCGGGTAATTTTTGACGGTCAGCAAATTCGCGTGTACACGTGCTTG

CTGAAACTCGCCCGCGAGAGAAATTTCATTTTGCCAGACAACAGACGCCGGTTTGACAGT

CAGGCAGACGCCGCGTCAGAGACTTCGGAGTTGGCTATGGATAGCCAAAGCCACGCCTTC

GACAGTACAGACGAACCCGACGGTGTGGACGGTACCCCGGACGCCGCAGGATCTGGCGCT

ACTTCTGAAAACGGAGGCGGGAAGCCCGGCGTCGGGAGGGCCGTGGGCTACCAGGGAGCA

AAGGTTCTAGACCCCGTATCCGGCTTTCATGTGGACCCCGTGGTTGTGTTTGACTTCGCT

AGCTTATACCCAAGCATTATCCAGGCCCATAACCTCTGTTTCACCACCCTGGCGCTCGAT

GAAGTGGATCTGGCCGGGCTTCAACCATCCGTCAACTACTCGACGTTCGAGGTGGGTGAC

CAAAAGTTATTTTTTGTCCACGCCCATATTCGCGAAAGCCTGCTTGGCATCTTGCTGCGC

GACTGGCTGGCCATGCGAAAGGCGGTGAGGGCGCGAATCCCCACCAGCACCCCCGAGGAG

GCAGTTTTACTAGATAAGCAGCAGTCTGCGATTAAGGTGATATGCAACTCGGTTTACGGA

TTCACGGGGGTGGCAAACGGCCTGTTGCCGTGTCTGAGGATAGCGGCTACCGTTACCACG

ATAGGACGCGACATGCTCCTCAAGACCCGAGATTACGTTCACTCTCGTTGGGCGACGCGC

GAGCTGCTGGAGGACAATTTTCCAGGGGCTATAGGTTTCCGAAACCACAAGCCTTACTCC

GTCAGGGTTATCTACGGAGACACCGACTCCGTGTTTATCAAGTTTGTGGGCCTGACGTAC

GAGGGGGTATCGGAGCTGGGGGATGCTATGTCGCGTCAGATTTCAGCGGACCTCTTTAGA

GCGCCCATCAAACTGGAGTGTGAGAAGACCTTTCAGCGACTGCTGCTGATCACCAAGAAG

AAGTACATAGGTGTCATAAACGGGGGGAAGATGCTCATGAAGGGGGTCGACCTGGTCCGC

AAAAATAACTGCTCTTTCATAAACTTGTACGCGCGACATCTGGTAGATCTTTTGTTGTAC

GACGAGGATGTGGCCACGGCGGCAGCAGAGGTGACAGACGTGCCTCCCGCAGAATGGGTG

GGGCGCCCGCTACCGAGCGGCTTTGACAAGTTTGGGCGAGTGCTGGTAGAGGCGTACAAC

CGTATCACTGCCCCCAACTTGGACGTGCGCGAGTTCGTTATGACTGCTGAGCTGAGCCGC

TCACCCGAATCGTATACCAACAAGCGCCTGCCGCACCTCACCGTCTACTTTAAGCTCGCC

ATGAGGAATGAAGAACTGCCCAGCGTAAAAGAGAGAATTCCGTATGTGATAGTTGCGCAG

ACCGAGGCCGCGGAACGCGAAGCGGGTGTAGTAAACTCAATGCGCGGTACCGCCCAAAAC

CCCGTGGTAACCAAGACCGCACGCCCCCAACCTAAACGCAAACTGCTGGTTTCCGACCTC

GCCGAAGACCCGACCTATGTTTCCGAGAATGACGTACCGCTAAACACAGACTACTATTTC

TCCCACCTGTTGGGTACCATAAGCGTGACCTTTAAGGCTCTATTCGGAAATGATGTGAGA

ACAACAGAAAATCTTTTAAAGCGGTTTATTCCGGAAACCCCCCACAAGACCCCCACGAAA

ACCCAGGCACTGCTTGAGCGCGCCGGCTTTGAAAAGCTGACGCCCTTTACACCGGAGGAA

GAAAGTCGTCGAATACTGCATACAGTTTTTTGTACTCTAGAAGCAGCTCCCCATCAAAGC

TGA

>KU206435.1

ATGGCGGCGCGCGAACAGGCCAACTCCGTTCGCAGGAGCGGGTTTTTTAATCCGTTTATT

GGCAAGAGGCCATTTTTCAGGCCGGGAAGCGGGCAAACCGCGGAGACAGAGCGCCCAAGG

CCCCCCCAACACTCGTACTGCACAGAGGTGGGTAGCTTTAAGTTTATAGCTCCAAGATGC

CTCGATGAAGAAGCCCCCGCCGACCAGCGACGCGGTGTACACGTGGGCACCTTGGAGCGC

CCACCCAAAGTGTACTGCGATGGCTCAGAGTACGACGTGCTGAACTTTGCCTCCGGAGGT

TGCTGGCCTCGCAGGATTCGGGTTTGGAACGGCCAGGATTTTCGGGGCGATGGATTCAAC

CCCAGATTTGAGAGATTTCACGTGTACGACATAGTAGAGACTTCGGAGAGCGCCTCACAC

GATGACCCATCCAGGTTTGCTGAGCTATCCCGCCCAAGCGGGTCTGTGGTAACACTGCTG

GGAATGAGCGAGTGTGGAAAGCGAGTCGCGGTTCACGTCTATGGTGTGCGCCATTATTTT

TACATGGCAAAGGCGGAGGTGGATAGCGCTTGTGGAATAACCACCGAGGCAGAACTCGTG

CGCGCAATGGTGGACTGCGCGCACAGCTCGGCTTTGAGCGCGGCCCTGGGAAATGGCAAC

GGCGGCAAACAGAGCGGCGGCAGCGGCGGGGGATGGTGGGGCGGAAAGCACGTGTCTGCG

GACTGCTTCAAAGTGGAGACCGTGTGCCACACGACGCTGTACTACTTTGGATCTAAGCCA

GCTCTCTACTATAGAGTATCTGCCTCCAGCAGCCGCCTGGGAGGGTTCATCTGCGACAAC

TTTCACCCGGAGATTACAAAATTCGAGGGGAGCGTGGACGTGACCACGCGGCTGTTGTTG

GACAATGAAAATTTTACCAGTTTCGGGTGGTACCGCCTGCGACCCGGCACCCACGGAGAG

CGTGTTCAACTTCGCCCCGTTGAGCGACACGTCACCTCAAGCGACGTGGAGATTAACTGT

ACTCCCGATAACCTGGAGCCGATACCAGACGAGGCTGCCTGGCCCGACTATAAGCTCATG

TGCTTTGATATAGAGTGTAAAGCTGGAACGGGTAACGAAATGGCGTTCCCAGTGGCAACT

AACCAAGAGGACCTGGTCATCCAGATCTCCTGTCTGCTGTACTCGCTTGCTACTCAGAAC

CACGAACACACCCTGCTGTTTTCCCTCGGGTCATGCGATATCTCTGAGGAATACTCGTTT

GCATGCGTCCAGCGCGGCGAGCCCAGACCGACGGTTTTGGAGTTTGACAGCGAGTACGAG

CTGCTGGTTGCCTTCCTGACCTTTCTCAAGCAGTACTCTCCCGAGTTCGCCACCGGCTAC

AACATCGTTAATTTTGACTGGGCGTACATAGTTAACAAGGTAACGTCGGTGTATAACATC

AAGCTGGACGGGTACGGCAAGTTCAACAAAGGGGGGCTGTTTAAGGTGTGGGACATCGCC

ACGAACCATTTTCAGAAGAAGAGCAAGGTGAAAATCAATGGCCTGATATCTCTAGACATG

TATTCTGTGGCGACGGAAAAGCTAAAGCTACCCAGCTACAAACTCGACGCGGTCGTGGGA

GACGTCCTCGGCGAGCATAAGATAGACCTTCCCTATAAAGAAATACCCTCCTATTACGCG

GGAGGGCCTGACCGGAGGGGCGTAATAGGAGAGTATTGTATCCAGGACTCTAGGCTGGTG

GGCAAGCTGTTTTTTAAGTACCTCCCCCATCTGGAACTATCGGCGGTGGCCAAACTCGCC

CGTATCACCCTAACGCGGGTAATTTTTGACGGTCAGCAAATTCGCGTGTACACGTGCTTG

CTGAAACTCGCCCGCGAGAGAAATTTCATTTTGCCAGACAACAGACGCCGGTTTGACAGT

CAGGCAGACGCCGCGTCAGAGACTTCGGAGTTGGCTATGGATAGCCAAAGCCACGCCTTC

GACAGTACAGACGAACCCGACGGTGTGGACGGTACCCCGGACGCCGCAGGATCTGGCGCT

ACTTCTGAAAACGGAGGCGGGAAGCCCGGCGTCGGGAGGGCCGTGGGCTACCAGGGAGCA

AAGGTTCTAGACCCCGTATCCGGCTTTCATGTGGACCCCGTGGTTGTGTTTGACTTCGCT

AGCTTATACCCAAGCATTATCCAGGCCCATAACCTCTGTTTCACCACCCTGGCGCTCGAT

GAAGTGGATCTGGCCGGGCTTCAACCATCCGTCAACTACTCGACGTTCGAGGTGGGTGAC

CAAAAGTTATTTTTTGTCCACGCCCATATTCGCGAAAGCCTGCTTGGCATCTTGCTGCGC

GACTGGCTGGCCATGCGAAAGGCGGTGAGGGCGCGAATCCCCACCAGCACCCCCGAGGAG

GCAGTTTTACTAGATAAGCAGCAGTCTGCGATTAAGGTGATATGCAACTCGGTTTACGGA

TTCACGGGGGTGGCAAACGGCCTGTTGCCGTGTCTGAGGATAGCGGCTACCGTTACCACG

ATAGGACGCGACATGCTCCTCAAGACCCGAGATTACGTTCACTCTCGTTGGGCGACGCGC

GAGCTGCTGGAGGACAATTTTCCAGGGGCTATAGGTTTCCGAAACCACAAGCCTTACTCC

GTCAGGGTTATCTACGGAGACACCGACTCCGTGTTTATCAAGTTTGTGGGCCTGACGTAC

GAGGGGGTATCGGAGCTGGGGGATGCTATGTCGCGTCAGATTTCAGCGGACCTCTTTAGA

GCGCCCATCAAACTGGAGTGTGAGAAGACCTTTCAGCGACTGCTGCTGATCACCAAGAAG

AAGTACATAGGTGTCATAAACGGGGGGAAGATGCTCATGAAGGGGGTCGACCTGGTCCGC

AAAAATAACTGCTCTTTCATAAACTTGTACGCGCGACATCTGGTAGATCTTTTGTTGTAC

GACGAGGATGTGGCCACGGCGGCAGCAGAGGTGACAGACGTGCCTCCCGCAGAATGGGTG

GGGCGCCCGCTACCGAGCGGCTTTGACAAGTTTGGGCGAGTGCTGGTAGAGGCGTACAAC

CGTATCACTGCCCCCAACTTGGACGTGCGCGAGTTCGTTATGACTGCTGAGCTGAGCCGC

TCACCCGAATCGTATACCAACAAGCGCCTGCCGCACCTCACCGTCTACTTTAAGCTCGCC

ATGAGGAATGAAGAACTGCCCAGCGTAAAAGAGAGAATTCCGTATGTGATAGTTGCGCAG

ACCGAGGCCGCGGAACGCGAAGCGGGTGTAGTAAACTCAATGCGCGGTACCGCCCAAAAC

CCCGTGGTAACCAAGACCGCACGCCCCCAACCTAAACGCAAACTGCTGGTTTCCGACCTC

GCCGAAGACCCGACCTATGTTTCCGAGAATGACGTACCGCTAAACACAGACTACTATTTC

TCCCACCTGTTGGGTACCATAAGCGTGACCTTTAAGGCTCTATTCGGAAATGATGTGAGA

ACAACAGAAAATCTTTTAAAGCGGTTTATTCCGGAAACCCCCCACAAGACCCCCACGAAA

ACCCAGGCACTGCTTGAGCGCGCCGGCTTTGAAAAGCTGACGCCCTTTACACCGGAGGAA

GAAAGTCGTCGAATACTGCATACAGTTTTTTGTACTCTAGAAGCAGCTCCCCATCAAAGC

TGA

>KU206436.1

ATGGCGGCGCGCGAACAGGCCAACTCCGTTCGCAGGAGCGGGTTTTTTAATCCGTTTATT

GGCAAGAGGCCATTTTTCAGGCCGGGAAGCGGGCAAACCGCGGAGACAGAGCGCCCAAGG

CCCCCCCAACACTCGTACTGCACAGAGGTGGGTAGCTTTAAGTTTATAGCTCCAAGATGC

CTCGATGAAGAAGCCCCCGCCGACCAGCGACGCGGTGTACACGTGGGCACCTTGGAGCGC

CCACCCAAAGTGTACTGCGATGGCTCAGAGTACGACGTGCTGAACTTTGCCTCCGGAGGT

TGCTGGCCTCGCAGGATTCGGGTTTGGAACGGCCAGGATTTTCGGGGCGATGGATTCAAC

CCCAGATTTGAGAGATTTCACGTGTACGACATAGTAGAGACTTCGGAGAGCGCCTCACAC

GATGACCCATCCAGGTTTGCTGAGCTATCCCGCCCAAGCGGGTCTGTGGTAACACTGCTG

GGAATGAGCGAGTGTGGAAAGCGAGTCGCGGTTCACGTCTATGGTGTGCGCCATTATTTT

TACATGGCAAAGGCGGAGGTGGATAGCGCTTGTGGAATAACCACCGAGGCAGAACTCGTG

CGCGCAATGGTGGACTGCGCGCACAGCTCGGCTTTGAGCGCGGCCCTGGGAAATGGCAAC

GGCGGCAAACAGAGCGGCGGCAGCGGCGGGGGATGGTGGGGCGGAAAGCACGTGTCTGCG

GACTGCTTCAAAGTGGAGACCGTGTGCCACACGACGCTGTACTACTTTGGATCTAAGCCA

GCTCTCTACTATAGAGTATCTGCCTCCAGCAGCCGCCTGGGAGGGTTCATCTGCGACAAC

TTTCACCCGGAGATTACAAAATTCGAGGGGAGCGTGGACGTGACCACGCGGCTGTTGTTG

GACAATGAAAATTTTACCAGTTTCGGGTGGTACCGCCTGCGACCCGGCACCCACGGAGAG

CGTGTTCAACTTCGCCCCGTTGAGCGACACGTCACCTCAAGCGACGTGGAGATTAACTGT

ACTCCCGATAACCTGGAGCCGATACCAGACGAGGCTGCCTGGCCCGACTATAAGCTCATG

TGCTTTGATATAGAGTGTAAAGCTGGAACGGGTAACGAAATGGCGTTCCCAGTGGCAACT

AACCAAGAGGACCTGGTCATCCAGATCTCCTGTCTGCTGTACTCGCTTGCTACTCAGAAC

CACGAACACACCCTGCTGTTTTCCCTCGGGTCATGCGATATCTCTGAGGAATACTCGTTT

GCATGCGTCCAGCGCGGCGAGCCCAGACCGACGGTTTTGGAGTTTGACAGCGAGTACGAG

CTGCTGGTTGCCTTCCTGACCTTTCTCAAGCAGTACTCTCCCGAGTTCGCCACCGGCTAC

AACATCGTTAATTTTGACTGGGCGTACATAGTTAACAAGGTAACGTCGGTGTATAACATC

AAGCTGGACGGGTACGGCAAGTTCAACAAAGGGGGGCTGTTTAAGGTGTGGGACATCGCC

ACGAACCATTTTCAGAAGAAGAGCAAGGTGAAAATCAATGGCCTGATATCTCTAGACATG

TATTCTGTGGCGACGGAAAAGCTAAAGCTACCCAGCTACAAACTCGACGCGGTCGTGGGA

GACGTCCTCGGCGAGCATAAGATAGACCTTCCCTATAAAGAAATACCCTCCTATTACGCG

GGAGGGCCTGACCGGAGGGGCGTAATAGGAGAGTATTGTATCCAGGACTCTAGGCTGGTG

GGCAAGCTGTTTTTTAAGTACCTCCCCCATCTGGAACTATCGGCGGTGGCCAAACTCGCC

CGTATCACCCTAACGCGGGTAATTTTTGACGGTCAGCAAATTCGCGTGTACACGTGCTTG

CTGAAACTCGCCCGCGAGAGAAATTTCATTTTGCCAGACAACAGACGCCGGTTTGACAGT

CAGGCAGACGCCGCGTCAGAGACTTCGGAGTTGGCTATGGATAGCCAAAGCCACGCCTTC

GACAGTACAGACGAACCCGACGGTGTGGACGGTACCCCGGACGCCGCAGGATCTGGCGCT

ACTTCTGAAAACGGAGGCGGGAAGCCCGGCGTCGGGAGGGCCGTGGGCTACCAGGGAGCA

AAGGTTCTAGACCCCGTATCCGGCTTTCATGTGGACCCCGTGGTTGTGTTTGACTTCGCT

AGCTTATACCCAAGCATTATCCAGGCCCATAACCTCTGTTTCACCACCCTGGCGCTCGAT

GAAGTGGATCTGGCCGGGCTTCAACCATCCGTCAACTACTCGACGTTCGAGGTGGGTGAC

CAAAAGTTATTTTTTGTCCACGCCCATATTCGCGAAAGCCTGCTTGGCATCTTGCTGCGC

GACTGGCTGGCCATGCGAAAGGCGGTGAGGGCGCGAATCCCCACCAGCACCCCCGAGGAG

GCAGTTTTACTAGATAAGCAGCAGTCTGCGATTAAGGTGATATGCAACTCGGTTTACGGA

TTCACGGGGGTGGCAAACGGCCTGTTGCCGTGTCTGAGGATAGCGGCTACCGTTACCACG

ATAGGACGCGACATGCTCCTCAAGACCCGAGATTACGTTCACTCTCGTTGGGCGACGCGC

GAGCTGCTGGAGGACAATTTTCCAGGGGCTATAGGTTTCCGAAACCACAAGCCTTACTCC

GTCAGGGTTATCTACGGAGACACCGACTCCGTGTTTATCAAGTTTGTGGGCCTGACGTAC

GAGGGGGTATCGGAGCTGGGGGATGCTATGTCGCGTCAGATTTCAGCGGACCTCTTTAGA

GCGCCCATCAAACTGGAGTGTGAGAAGACCTTTCAGCGACTGCTGCTGATCACCAAGAAG

AAGTACATAGGTGTCATAAACGGGGGGAAGATGCTCATGAAGGGGGTCGACCTGGTCCGC

AAAAATAACTGCTCTTTCATAAACTTGTACGCGCGACATCTGGTAGATCTTTTGTTGTAC

GACGAGGATGTGGCCACGGCGGCAGCAGAGGTGACAGACGTGCCTCCCGCAGAATGGGTG

GGGCGCCCGCTACCGAGCGGCTTTGACAAGTTTGGGCGAGTGCTGGTAGAGGCGTACAAC

CGTATCACTGCCCCCAACTTGGACGTGCGCGAGTTCGTTATGACTGCTGAGCTGAGCCGC

TCACCCGAATCGTATACCAACAAGCGCCTGCCGCACCTCACCGTCTACTTTAAGCTCGCC

ATGAGGAATGAAGAACTGCCCAGCGTAAAAGAGAGAATTCCGTATGTGATAGTTGCGCAG

ACCGAGGCCGCGGAACGCGAAGCGGGTGTAGTAAACTCAATGCGCGGTACCGCCCAAAAC

CCCGTGGTAACCAAGACCGCACGCCCCCAACCTAAACGCAAACTGCTGGTTTCCGACCTC

GCCGAAGACCCGACCTATGTTTCCGAGAATGACGTACCGCTAAACACAGACTACTATTTC

TCCCACCTGTTGGGTACCATAAGCGTGACCTTTAAGGCTCTATTCGGAAATGATGTGAGA

ACAACAGAAAATCTTTTAAAGCGGTTTATTCCGGAAACCCCCCACAAGACCCCCACGAAA

ACCCAGGCACTGCTTGAGCGCGCCGGCTTTGAAAAGCTGACGCCCTTTACACCGGAGGAA

GAAAGTCGTCGAATACTGCATACAGTTTTTTGTACTCTAGAAGCAGCTCCCCATCAAAGC

TGA

>KU206437.1

ATGGCGGCGCGCGAACAGGCCAACTCCGTTCGCAGGAGCGGGTTTTTTAATCCGTTTATT

GGCAAGAGGCCATTTTTCAGGCCGGGAAGCGGGCAAACCGCGGAGACAGAGCGCCCAAGG

CCCCCCCAACACTCGTACTGCACAGAGGTGGGTAGCTTTAAGTTTATAGCTCCAAGATGC

CTCGATGAAGAAGCCCCCGCCGACCAGCGACGCGGTGTACACGTGGGCACCTTGGAGCGC

CCACCCAAAGTGTACTGCGATGGCTCAGAGTACGACGTGCTGAACTTTGCCTCCGGAGGT

TGCTGGCCTCGCAGGATTCGGGTTTGGAACGGCCAGGATTTTCGGGGCGATGGATTCAAC

CCCAGATTTGAGAGATTTCACGTGTACGACATAGTAGAGACTTCGGAGAGCGCCTCACAC

GATGACCCATCCAGGTTTGCTGAGCTATCCCGCCCAAGCGGGTCTGTGGTAACACTGCTG

GGAATGAGCGAGTGTGGAAAGCGAGTCGCGGTTCACGTCTATGGTGTGCGCCATTATTTT

TACATGGCAAAGGCGGAGGTGGATAGCGCTTGTGGAATAACCACCGAGGCAGAACTCGTG

CGCGCAATGGTGGACTGCGCGCACAGCTCGGCTTTGAGCGCGGCCCTGGGAAATGGCAAC

GGCGGCAAACAGAGCGGCGGCAGCGGCGGGGGATGGTGGGGCGGAAAGCACGTGTCTGCG

GACTGCTTCAAAGTGGAGACCGTGTGCCACACGACGCTGTACTACTTTGGATCTAAGCCA

GCTCTCTACTATAGAGTATCTGCCTCCAGCAGCCGCCTGGGAGGGTTCATCTGCGACAAC

TTTCACCCGGAGATTACAAAATTCGAGGGGAGCGTGGACGTGACCACGCGGCTGTTGTTG

GACAATGAAAATTTTACCAGTTTCGGGTGGTACCGCCTGCGACCCGGCACCCACGGAGAG

CGTGTTCAACTTCGCCCCGTTGAGCGACACGTCACCTCAAGCGACGTGGAGATTAACTGT

ACTCCCGATAACCTGGAGCCGATACCAGACGAGGCTGCCTGGCCCGACTATAAGCTCATG

TGCTTTGATATAGAGTGTAAAGCTGGAACGGGTAACGAAATGGCGTTCCCAGTGGCAACT

AACCAAGAGGACCTGGTCATCCAGATCTCCTGTCTGCTGTACTCGCTTGCTACTCAGAAC

CACGAACACACCCTGCTGTTTTCCCTCGGGTCATGCGATATCTCTGAGGAATACTCGTTT

GCATGCGTCCAGCGCGGCGAGCCCAGACCGACGGTTTTGGAGTTTGACAGCGAGTACGAG

CTGCTGGTTGCCTTCCTGACCTTTCTCAAGCAGTACTCTCCCGAGTTCGCCACCGGCTAC

AACATCGTTAATTTTGACTGGGCGTACATAGTTAACAAGGTAACGTCGGTGTATAACATC

AAGCTGGACGGGTACGGCAAGTTCAACAAAGGGGGGCTGTTTAAGGTGTGGGACATCGCC

ACGAACCATTTTCAGAAGAAGAGCAAGGTGAAAATCAATGGCCTGATATCTCTAGACATG

TATTCTGTGGCGACGGAAAAGCTAAAGCTACCCAGCTACAAACTCGACGCGGTCGTGGGA

GACGTCCTCGGCGAGCATAAGATAGACCTTCCCTATAAAGAAATACCCTCCTATTACGCG

GGAGGGCCTGACCGGAGGGGCGTAATAGGAGAGTATTGTATCCAGGACTCTAGGCTGGTG

GGCAAGCTGTTTTTTAAGTACCTCCCCCATCTGGAACTATCGGCGGTGGCCAAACTCGCC

CGTATCACCCTAACGCGGGTAATTTTTGACGGTCAGCAAATTCGCGTGTACACGTGCTTG

CTGAAACTCGCCCGCGAGAGAAATTTCATTTTGCCAGACAACAGACGCCGGTTTGACAGT

CAGGCAGACGCCGCGTCAGAGACTTCGGAGTTGGCTATGGATAGCCAAAGCCACGCCTTC

GACAGTACAGACGAACCCGACGGTGTGGACGGTACCCCGGACGCCGCAGGATCTGGCGCT

ACTTCTGAAAACGGAGGCGGGAAGCCCGGCGTCGGGAGGGCCGTGGGCTACCAGGGAGCA

AAGGTTCTAGACCCCGTATCCGGCTTTCATGTGGACCCCGTGGTTGTGTTTGACTTCGCT

AGCTTATACCCAAGCATTATCCAGGCCCATAACCTCTGTTTCACCACCCTGGCGCTCGAT

GAAGTGGATCTGGCCGGGCTTCAACCATCCGTCAACTACTCGACGTTCGAGGTGGGTGAC

CAAAAGTTATTTTTTGTCCACGCCCATATTCGCGAAAGCCTGCTTGGCATCTTGCTGCGC

GACTGGCTGGCCATGCGAAAGGCGGTGAGGGCGCGAATCCCCACCAGCACCCCCGAGGAG

GCAGTTTTACTAGATAAGCAGCAGTCTGCGATTAAGGTGATATGCAACTCGGTTTACGGA

TTCACGGGGGTGGCAAACGGCCTGTTGCCGTGTCTGAGGATAGCGGCTACCGTTACCACG

ATAGGACGCGACATGCTCCTCAAGACCCGAGATTACGTTCACTCTCGTTGGGCGACGCGC

GAGCTGCTGGAGGACAATTTTCCAGGGGCTATAGGTTTCCGAAACCACAAGCCTTACTCC

GTCAGGGTTATCTACGGAGACACCGACTCCGTGTTTATCAAGTTTGTGGGCCTGACGTAC

GAGGGGGTATCGGAGCTGGGGGATGCTATGTCGCGTCAGATTTCAGCGGACCTCTTTAGA

GCGCCCATCAAACTGGAGTGTGAGAAGACCTTTCAGCGACTGCTGCTGATCACCAAGAAG

AAGTACATAGGTGTCATAAACGGGGGGAAGATGCTCATGAAGGGGGTCGACCTGGTCCGC

AAAAATAACTGCTCTTTCATAAACTTGTACGCGCGACATCTGGTAGATCTTTTGTTGTAC

GACGAGGATGTGGCCACGGCGGCAGCAGAGGTGACAGACGTGCCTCCCGCAGAATGGGTG

GGGCGCCCGCTACCGAGCGGCTTTGACAAGTTTGGGCGAGTGCTGGTAGAGGCGTACAAC

CGTATCACTGCCCCCAACTTGGACGTGCGCGAGTTCGTTATGACTGCTGAGCTGAGCCGC

TCACCCGAATCGTATACCAACAAGCGCCTGCCGCACCTCACCGTCTACTTTAAGCTCGCC

ATGAGGAATGAAGAACTGCCCAGCGTAAAAGAGAGAATTCCGTATGTGATAGTTGCGCAG

ACCGAGGCCGCGGAACGCGAAGCGGGTGTAGTAAACTCAATGCGCGGTACCGCCCAAAAC

CCCGTGGTAACCAAGACCGCACGCCCCCAACCTAAACGCAAACTGCTGGTTTCCGACCTC

GCCGAAGACCCGACCTATGTTTCCGAGAATGACGTACCGCTAAACACAGACTACTATTTC

TCCCACCTGTTGGGTACCATAAGCGTGACCTTTAAGGCTCTATTCGGAAATGATGTGAGA

ACAACAGAAAATCTTTTAAAGCGGTTTATTCCGGAAACCCCCCACAAGACCCCCACGAAA

ACCCAGGCACTGCTTGAGCGCGCCGGCTTTGAAAAGCTGACGCCCTTTACACCGGAGGAA

GAAAGTCGTCGAATACTGCATACAGTTTTTTGTACTCTAGAAGCAGCTCCCCATCAAAGC

TGA

>KU206438.1

ATGGCGGCGCGCGAACAGGCCAACTCCGTTCGCAGGAGCGGGTTTTTTAATCCGTTTATT

GGCAAGAGGCCATTTTTCAGGCCGGGAAGCGGGCAAACCGCGGAGACAGAGCGCCCAAGG

CCCCCCCAACACTCGTACTGCACAGAGGTGGGTAGCTTTAAGTTTATAGCTCCAAGATGC

CTCGATGAAGAAGCCCCCGCCGACCAGCGACGCGGTGTACACGTGGGCACCTTGGAGCGC

CCACCCAAAGTGTACTGCGATGGCTCAGAGTACGACGTGCTGAACTTTGCCTCCGGAGGT

TGCTGGCCTCGCAGGATTCGGGTTTGGAACGGCCAGGATTTTCGGGGCGATGGATTCAAC

CCCAGATTTGAGAGATTTCACGTGTACGACATAGTAGAGACTTCGGAGAGCGCCTCACAC

GATGACCCATCCAGGTTTGCTGAGCTATCCCGCCCAAGCGGGTCTGTGGTAACACTGCTG

GGAATGAGCGAGTGTGGAAAGCGAGTCGCGGTTCACGTCTATGGTGTGCGCCATTATTTT

TACATGGCAAAGGCGGAGGTGGATAGCGCTTGTGGAATAACCACCGAGGCAGAACTCGTG

CGCGCAATGGTGGACTGCGCGCACAGCTCGGCTTTGAGCGCGGCCCTGGGAAATGGCAAC

GGCGGCAAACAGAGCGGCGGCAGCGGCGGGGGATGGTGGGGCGGAAAGCACGTGTCTGCG

GACTGCTTCAAAGTGGAGACCGTGTGCCACACGACGCTGTACTACTTTGGATCTAAGCCA

GCTCTCTACTATAGAGTATCTGCCTCCAGCAGCCGCCTGGGAGGGTTCATCTGCGACAAC

TTTCACCCGGAGATTACAAAATTCGAGGGGAGCGTGGACGTGACCACGCGGCTGTTGTTG

GACAATGAAAATTTTACCAGTTTCGGGTGGTACCGCCTGCGACCCGGCACCCACGGAGAG

CGTGTTCAACTTCGCCCCGTTGAGCGACACGTCACCTCAAGCGACGTGGAGATTAACTGT

ACTCCCGATAACCTGGAGCCGATACCAGACGAGGCTGCCTGGCCCGACTATAAGCTCATG

TGCTTTGATATAGAGTGTAAAGCTGGAACGGGTAACGAAATGGCGTTCCCAGTGGCAACT

AACCAAGAGGACCTGGTCATCCAGATCTCCTGTCTGCTGTACTCGCTTGCTACTCAGAAC

CACGAACACACCCTGCTGTTTTCCCTCGGGTCATGCGATATCTCTGAGGAATACTCGTTT

GCATGCGTCCAGCGCGGCGAGCCCAGACCGACGGTTTTGGAGTTTGACAGCGAGTACGAG

CTGCTGGTTGCCTTCCTGACCTTTCTCAAGCAGTACTCTCCCGAGTTCGCCACCGGCTAC

AACATCGTTAATTTTGACTGGGCGTACATAGTTAACAAGGTAACGTCGGTGTATAACATC

AAGCTGGACGGGTACGGCAAGTTCAACAAAGGGGGGCTGTTTAAGGTGTGGGACATCGCC

ACGAACCATTTTCAGAAGAAGAGCAAGGTGAAAATCAATGGCCTGATATCTCTAGACATG

TATTCTGTGGCGACGGAAAAGCTAAAGCTACCCAGCTACAAACTCGACGCGGTCGTGGGA

GACGTCCTCGGCGAGCATAAGATAGACCTTCCCTATAAAGAAATACCCTCCTATTACGCG

GGAGGGCCTGACCGGAGGGGCGTAATAGGAGAGTATTGTATCCAGGACTCTAGGCTGGTG

GGCAAGCTGTTTTTTAAGTACCTCCCCCATCTGGAACTATCGGCGGTGGCCAAACTCGCC

CGTATCACCCTAACGCGGGTAATTTTTGACGGTCAGCAAATTCGCGTGTACACGTGCTTG

CTGAAACTCGCCCGCGAGAGAAATTTCATTTTGCCAGACAACAGACGCCGGTTTGACAGT

CAGGCAGACGCCGCGTCAGAGACTTCGGAGTTGGCTATGGATAGCCAAAGCCACGCCTTC

GACAGTACAGACGAACCCGACGGTGTGGACGGTACCCCGGACGCCGCAGGATCTGGCGCT

ACTTCTGAAAACGGAGGCGGGAAGCCCGGCGTCGGGAGGGCCGTGGGCTACCAGGGAGCA

AAGGTTCTAGACCCCGTATCCGGCTTTCATGTGGACCCCGTGGTTGTGTTTGACTTCGCT

AGCTTATACCCAAGCATTATCCAGGCCCATAACCTCTGTTTCACCACCCTGGCGCTCGAT

GAAGTGGATCTGGCCGGGCTTCAACCATCCGTCAACTACTCGACGTTCGAGGTGGGTGAC

CAAAAGTTATTTTTTGTCCACGCCCATATTCGCGAAAGCCTGCTTGGCATCTTGCTGCGC

GACTGGCTGGCCATGCGAAAGGCGGTGAGGGCGCGAATCCCCACCAGCACCCCCGAGGAG

GCAGTTTTACTAGATAAGCAGCAGTCTGCGATTAAGGTGATATGCAACTCGGTTTACGGA

TTCACGGGGGTGGCAAACGGCCTGTTGCCGTGTCTGAGGATAGCGGCTACCGTTACCACG

ATAGGACGCGACATGCTCCTCAAGACCCGAGATTACGTTCACTCTCGTTGGGCGACGCGC

GAGCTGCTGGAGGACAATTTTCCAGGGGCTATAGGTTTCCGAAACCACAAGCCTTACTCC

GTCAGGGTTATCTACGGAGACACCGACTCCGTGTTTATCAAGTTTGTGGGCCTGACGTAC

GAGGGGGTATCGGAGCTGGGGGATGCTATGTCGCGTCAGATTTCAGCGGACCTCTTTAGA

GCGCCCATCAAACTGGAGTGTGAGAAGACCTTTCAGCGACTGCTGCTGATCACCAAGAAG

AAGTACATAGGTGTCATAAACGGGGGGAAGATGCTCATGAAGGGGGTCGACCTGGTCCGC

AAAAATAACTGCTCTTTCATAAACTTGTACGCGCGACATCTGGTAGATCTTTTGTTGTAC

GACGAGGATGTGGCCACGGCGGCAGCAGAGGTGACAGACGTGCCTCCCGCAGAATGGGTG

GGGCGCCCGCTACCGAGCGGCTTTGACAAGTTTGGGCGAGTGCTGGTAGAGGCGTACAAC

CGTATCACTGCCCCCAACTTGGACGTGCGCGAGTTCGTTATGACTGCTGAGCTGAGCCGC

TCACCCGAATCGTATACCAACAAGCGCCTGCCGCACCTCACCGTCTACTTTAAGCTCGCC

ATGAGGAATGAAGAACTGCCCAGCGTAAAAGAGAGAATTCCGTATGTGATAGTTGCGCAG

ACCGAGGCCGCGGAACGCGAAGCGGGTGTAGTAAACTCAATGCGCGGTACCGCCCAAAAC

CCCGTGGTAACCAAGACCGCACGCCCACAACCTAAACGCAAACTGCTGGTTTCCGACCTC

GCCGAAGACCCGACCTATGTTTCCGAGAATGACGTACCGCTAAACACAGACTACTATTTC

TCCCACCTGTTGGGTACCATAAGCGTGACCTTTAAGGCTCTATTCGGAAATGATGTGAGA

ACAACAGAAAATCTTTTAAAGCGGTTTATTCCGGAAACCCCCCACAAGACCCCCACGAAA

ACCCAGGCACTGCTTGAGCGCGCCGGCTTTGAAAAGCTGACGCCCTTTACACCGGAGGAA

GAAAGTCGTCGAATACTGCATACAGTTTTTTGTACTCTAGAAGCAGCTCCCCATCAAAGC

TGA

>KU206439.1

ATGGCGGCGCGCGAACAGGCCAACTCCGTTCGCAGGAGCGGGTTTTTTAATCCGTTTATT

GGCAAGAGGCCATTTTTCAGGCCGGGAAGCGGGCAAACCGCGGAGACAGAGCGCCCAAGG

CCCCCCCAACACTCGTACTGCACAGAGGTGGGTAGCTTTAAGTTTATAGCTCCAAGATGC

CTCGATGAAGAAGCCCCCGCCGACCAGCGACGCGGTGTACACGTGGGCACCTTGGAGCGC

CCACCCAAAGTGTACTGCGATGGCTCAGAGTACGACGTGCTGAACTTTGCCTCCGGAGGT

TGCTGGCCTCGCAGGATTCGGGTTTGGAACGGCCAGGATTTTCGGGGCGATGGATTCAAC

CCCAGATTTGAGAGATTTCACGTGTACGACATAGTAGAGACTTCGGAGAGCGCCTCACAC

GATGACCCATCCAGGTTTGCTGAGCTATCCCGCCCAAGCGGGTCTGTGGTAACACTGCTG

GGAATGAGCGAGTGTGGAAAGCGAGTCGCGGTTCACGTCTATGGTGTGCGCCATTATTTT

TACATGGCAAAGGCGGAGGTGGATAGCGCTTGTGGAATAACCACCGAGGCAGAACTCGTG

CGCGCAATGGTGGACTGCGCGCACAGCTCGGCTTTGAGCGCGGCCCTGGGAAATGGCAAC

GGCGGCAAACAGAGCGGCGGCAGCGGCGGGGGATGGTGGGGCGGAAAGCACGTGTCTGCG

GACTGCTTCAAAGTGGAGACCGTGTGCCACACGACGCTGTACTACTTTGGATCTAAGCCA

GCTCTCTACTATAGAGTATCTGCCTCCAGCAGCCGCCTGGGAGGGTTCATCTGCGACAAC

TTTCACCCGGAGATTACAAAATTCGAGGGGAGCGTGGACGTGACCACGCGGCTGTTGTTG

GACAATGAAAATTTTACCAGTTTCGGGTGGTACCGCCTGCGACCCGGCACCCACGGAGAG

CGTGTTCAACTTCGCCCCGTTGAGCGACACGTCACCTCAAGCGACGTGGAGATTAACTGT

ACTCCCGATAACCTGGAGCCGATACCAGACGAGGCTGCCTGGCCCGACTATAAGCTCATG

TGCTTTGATATAGAGTGTAAAGCTGGAACGGGTAACGAAATGGCGTTCCCAGTGGCAACT

AACCAAGAGGACCTGGTCATCCAGATCTCCTGTCTGCTGTACTCGCTTGCTACTCAGAAC

CACGAACACACCCTGCTGTTTTCCCTCGGGTCATGCGATATCTCTGAGGAATACTCGTTT

GCATGCGTCCAGCGCGGCGAGCCCAGACCGACGGTTTTGGAGTTTGACAGCGAGTACGAG

CTGCTGGTTGCCTTCCTGACCTTTCTCAAGCAGTACTCTCCCGAGTTCGCCACCGGCTAC

AACATCGTTAATTTTGACTGGGCGTACATAGTTAACAAGGTAACGTCGGTGTATAACATC

AAGCTGGACGGGTACGGCAAGTTCAACAAAGGGGGGCTGTTTAAGGTGTGGGACATCGCC

ACGAACCATTTTCAGAAGAAGAGCAAGGTGAAAATCAATGGCCTGATATCTCTAGACATG

TATTCTGTGGCGACGGAAAAGCTAAAGCTACCCAGCTACAAACTCGACGCGGTCGTGGGA

GACGTCCTCGGCGAGCATAAGATAGACCTTCCCTATAAAGAAATACCCTCCTATTACGCG

GGAGGGCCTGACCGGAGGGGCGTAATAGGAGAGTATTGTATCCAGGACTCTAGGCTGGTG

GGCAAGCTGTTTTTTAAGTACCTCCCCCATCTGGAACTATCGGCGGTGGCCAAACTCGCC

CGTATCACCCTAACGCGGGTAATTTTTGACGGTCAGCAAATTCGCGTGTACACGTGCTTG

CTGAAACTCGCCCGCGAGAGAAATTTCATTTTGCCAGACAACAGACGCCGGTTTGACAGT

CAGGCAGACGCCGCGTCAGAGACTTCGGAGTTGGCTATGGATAGCCAAAGCCACGCCTTC

GACAGTACAGACGAACCCGACGGTGTGGACGGTACCCCGGACGCCGCAGGATCTGGCGCT

ACTTCTGAAAACGGAGGCGGGAAGCCCGGCGTCGGGAGGGCCGTGGGCTACCAGGGAGCA

AAGGTTCTAGACCCCGTATCCGGCTTTCATGTGGACCCCGTGGTTGTGTTTGACTTCGCT

AGCTTATACCCAAGCATTATCCAGGCCCATAACCTCTGTTTCACCACCCTGGCGCTCGAT

GAAGTGGATCTGGCCGGGCTTCAACCATCCGTCAACTACTCGACGTTCGAGGTGGGTGAC

CAAAAGTTATTTTTTGTCCACGCCCATATTCGCGAAAGCCTGCTTGGCATCTTGCTGCGC

GACTGGCTGGCCATGCGAAAGGCGGTGAGGGCGCGAATCCCCACCAGCACCCCCGAGGAG

GCAGTTTTACTAGATAAGCAGCAGTCTGCGATTAAGGTGATATGCAACTCGGTTTACGGA

TTCACGGGGGTGGCAAACGGCCTGTTGCCGTGTCTGAGGATAGCGGCTACCGTTACCACG

ATAGGACGCGACATGCTCCTCAAGACCCGAGATTACGTTCACTCTCGTTGGGCGACGCGC

GAGCTGCTGGAGGACAATTTTCCAGGGGCTATAGGTTTCCGAAACCACAAGCCTTACTCC

GTCAGGGTTATCTACGGAGACACCGACTCCGTGTTTATCAAGTTTGTGGGCCTGACGTAC

GAGGGGGTATCGGAGCTGGGGGATGCTATGTCGCGTCAGATTTCAGCGGACCTCTTTAGA

GCGCCCATCAAACTGGAGTGTGAGAAGACCTTTCAGCGACTGCTGCTGATCACCAAGAAG

AAGTACATAGGTGTCATAAACGGGGGGAAGATGCTCATGAAGGGGGTCGACCTGGTCCGC

AAAAATAACTGCTCTTTCATAAACTTGTACGCGCGACATCTGGTAGATCTTTTGTTGTAC

GACGAGGATGTGGCCACGGCGGCAGCAAAGGTGACAGACGTGCCTCCCGCAGAATGGGTG

GGGCGCCCGCTACCGAGCGGCTTTGACAAGTTTGGGCGAGTGCTGGTAGAGGCGTACAAC

CGTATCACTGCCCCCAACTTGGACGTGCGCGAGTTCGTTATGACTGCTGAGCTGAGCCGC

TCACCCGAATCGTATACCAACAAGCGCCTGCCGCACCTCACCGTCTACTTTAAGCTCGCC

ATGAGGAATGAAGAACTGCCCAGCGTAAAAGAGAGAATTCCGTATGTGATAGTTGCGCAG

ACCGAGGCCGCGGAACGCGAAGCGGGTGTAGTAAACTCAATGCGCGGTACCGCCCAAAAC

CCCGTGGTAACCAAGACCGCACGCCCCCAACCTAAACGCAAACTGCTGGTTTCCGACCTC

GCCGAAGACCCGACCTATGTTTCCGAGAATGACGTACCGCTAAACACAGACTACTATTTC

TCCCACCTGTTGGGTACCATAAGCGTGACCTTTAAGGCTCTATTCGGAAATGATGTGAGA

ACAACAGAAAATCTTTTAAAGCGGTTTATTCCGGAAACCCCCCACAAGACCCCCACGAAA

ACCCAGGCACTGCTTGAGCGCGCCGGCTTTGAAAAGCTGACGCCCTTTACACCGGAGGAA

GAAAGTCGTCGAATACTGCATACAGTTTTTTGTACTCTAGAAGCAGCTCCCCATCAAAGC

TGA

>KU206440.1

ATGGCGGCGCGCGAACAGGCCAACTCCGTTCGCAGGAGCGGGTTTTTTAATCCGTTTATT

GGCAAGAGGCCATTTTTCAGGCCGGGAAGCGGGCAAACCGCGGAGACAGAGCGCCCAAGG

CCCCCCCAACACTCGTACTGCACAGAGGTGGGTAGCTTTAAGTTTATAGCTCCAAGATGC

CTCGATGAAGAAGCCCCCGCCGACCAGCGACGCGGTGTACACGTGGGCACCTTGGAGCGC

CCACCCAAAGTGTACTGCGATGGCTCAGAGTACGACGTGCTGAACTTTGCCTCCGGAGGT

TGCTGGCCTCGCAGGATTCGGGTTTGGAACGGCCAGGATTTTCGGGGCGATGGATTCAAC

CCCAGATTTGAGAGATTTCACGTGTACGACATAGTAGAGACTTCGGAGAGCGCCTCACAC

GATGACCCATCCAGGTTTGCTGAGCTATCCCGCCCAAGCGGGTCTGTGGTAACACTGCTG

GGAATGAGCGAGTGTGGAAAGCGAGTCGCGGTTCACGTCTATGGTGTGCGCCATTATTTT

TACATGGCAAAGGCGGAGGTGGATAGCGCTTGTGGAATAACCACCGAGGCAGAACTCGTG

CGCGCAATGGTGGACTGCGCGCACAGCTCGGCTTTGAGCGCGGCCCTGGGAAATGGCAAC

GGCGGCAAACAGAGCGGCGGCAGCGGCGGGGGATGGTGGGGCGGAAAGCACGTGTCTGCG

GACTGCTTCAAAGTGGAGACCGTGTACCACACGACGCTGTACTACTTTGGATCTAAGCCA

GCTCTCTACTATAGAGTATCTGCCTCCAGCAGCCGCCTGGGAGGGTTCATCTGCGACAAC

TTTCACCCGGAGATTACAAAATTCGAGGGGAGCGTGGACGTGACCACGCGGCTGTTGTTG

GACAATGAAAATTTTACCAGTTTCGGGTGGTACCGCCTGCGACCCGGCACCCACGGAGAG

CGTGTTCAACTTCGCCCCGTTGAGCGACACGTCACCTCAAGCGACGTGGAGATTAACTGT

ACTCCCGATAACCTGGAGCCGATACCAGACGAGGCTGCCTGGCCCGACTATAAGCTCATG

TGCTTTGATATAGAGTGTAAAGCTGGAACGGGTAACGAAATGGCGTTCCCAGTGGCAACT

AACCAAGAGGACCTGGTCATCCAGATCTCCTGTCTGCTGTACTCGCTTGCTACTCAGAAC

CACGAACACACCCTGCTGTTTTCCCTCGGGTCATGCGATATCTCTGAGGAATACTCGTTT

GCATGCGTCCAGCGCGGCGAGCCCAGACCGACGGTTTTGGAGTTTGACAGCGAGTACGAG

CTGCTGGTTGCCTTCCTGACCTTTCTCAAGCAGTACTCTCCCGAGTTCGCCACCGGCTAC

AACATCGTTAATTTTGACTGGGCGTACATAGTTAACAAGGTAACGTCGGTGTATAACATC

AAGCTGGACGGGTACGGCAAGTTCAACAAAGGGGGGCTGTTTAAGGTGTGGGACATCGCC

ACGAACCATTTTCAGAAGAAGAGCAAGGTGAAAATCAATGGCCTGATATCTCTAGACATG

TATTCTGTGGCGACGGAAAAGCTAAAGCTACCCAGCTACAAACTCGACGCGGTCGTGGGA

GACGTCCTCGGCGAGCATAAGATAGACCTTCCCTATAAAGAAATACCCTCCTATTACGCG

GGAGGGCCTGACCGGAGGGGCGTAATAGGAGAGTATTGTATCCAGGACTCTAGGCTGGTG

GGCAAGCTGTTTTTTAAGTACCTCCCCCATCTGGAACTATCGGCGGTGGCCAAACTCGCC

CGTATCACCCTAACGCGGGTAATTTTTGACGGTCAGCAAATTCGCGTGTACACGTGCTTG

CTGAAACTCGCCCGCGAGAGAAATTTCATTTTGCCAGACAACAGACGCCGGTTTGACAGT

CAGGCAGACGCCGCGTCAGAGACTTCGGAGTTGGCTATGGATAGCCAAAGCCACGCCTTC

GACAGTACAGACGAACCCGACGGTGTGGACGGTACCCCGGACGCCGCAGGATCTGGCGCT

ACTTCTGAAAACGGAGGCGGGAAGCCCGGCGTCGGGAGGGCCGTGGGCTACCAGGGAGCA

AAGGTTCTAGACCCCGTATCCGGCTTTCATGTGGACCCCGTGGTTGTGTTTGACTTCGCT

AGCTTATACCCAAGCATTATCCAGGCCCATAACCTCTGTTTCACCACCCTGGCGCTCGAT

GAAGTGGATCTGGCCGGGCTTCAACCATCCGTCGACTACTCGACGTTCGAGGTGGGTGAC

CAAAAGTTATTTTTTGTCCACGCCCATATTCGCGAAAGCCTGCTTGGCATCTTGCTGCGC

GACTGGCTGGCCATGCGAAAGGCGGTGAGGGCGCGAATCCCCACCAGCACCCCCGAGGAG

GCAGTTTTACTAGATAAGCAGCAGTCTGCGATTAAGGTGATATGCAACTCGGTTTACGGA

TTCACGGGGGTGGCAAACGGCCTGTTGCCGTGTCTGAGGATAGCGGCTACCGTTACCACG

ATAGGACGCGACATGCTCCTCAAGACCCGAGATTACGTTCACTCTCGTTGGGCGACGCGC

GAGCTGCTGGAGGACAATTTTCCAGGGGCTATAGGTTTCCGAAACCACAAGCCTTACTCC

GTCAGGGTTATCTACGGAGACACCGACTCCGTGTTTATCAAGTTTGTGGGCCTGACGTAC

GAGGGGGTATCGGAGCTGGGGGATGCTATGTCGCGTCAGATTTCAGCGGACCTCTTTAGA

GCGCCCATCAAACTGGAGTGTGAGAAGACCTTTCAGCGACTGCTTCTGATCACCAAGAAG

AAGTACATAGGTGTCATAAACGGGGGGAAGATGCTCATGAAGGGGGTCGACCTGGTCCGC

AAAAATAACTGCTCTTTCATAAACTTGTACGCGCGACATCTGGTAGATCTTTTGTTGTAC

GACGAGGATGTGGCCACGGCGGCAGCAGAGGTGACAGACGTGCCTCCCGCAGAATGGGTG

GGGCGCCCGCTACCGAGCGGCTTTGACAAGTTTGGGCGAGTGCTGGTAGAGGCGTACAAC

CGTATCACTGCCCCCAACTTGGACGTGCGCGAGTTCGTTATGACTGCTGAGCTGAGCCGC

TCACCCGAATCGTATACCAACAAGCGCCTGCCGCACCTCACCGTCTACTTTAAGCTCGCC

ATGAGGAATGAAGAACTGCCCAGCGTAAAAGAGAGAATTCCGTATGTGATAGTTGCGCAG

ACCGAGGCCGCGGAACGCGAAGCGGGTGTAGTAAACTCAATGCGCGGTACCGCCCAAAAC

CCCGTGGTAACCAAGACCGCACGCCCCCAACCTAAACGCAAACTGCTGGTTTCCGACCTC

GCCGAAGACCCGACCTATGTTTCCGAGAATGACGTACCGCTAAACACAGACTACTATTTC

TCCCACCTGTTGGGTACCATAAGCGTGACCTTTAAGGCTCTATTCGGAAATGATGTGAGA

ACAACAGAAAATCTTTTAAAGCGGTTTATTCCGGAAACCCCCCACAAGACCCCCACGAAA

ACCCAGGCACTGCTTGAGCGCGCCGGCTTTGAAAAGCTGACGCCCTTTACACCGGAGGAA

GAAAGTCGTCGAATACTGCATACAGTTTTTTGTACTCTAGAAGCAGCTCCCCATCAAAGC

TGA

>KU206441.1

ATGGCGGCGCGCGAACAGGCCAACTCCGTTCGCAGGAGCGGGTTTTTTAATCCGTTTATT

GGCAAGAGGCCATTTTTCAGGCCGGGAAGCGGGCAAACCGCGGAGACAGAGCGCCCAAGG

CCCCCCCAACACTCGTACTGCACAGAGGTGGGTAGCTTTAAGTTTATAGCTCCAAGATGC

CTCGATGAAGAAGCCCCCGCCGACCAGCGACGCGGTGTACACGTGGGCACCTTGGAGCGC

CCACCCAAAGTGTACTGCGATGGCTCAGAGTACGACGTGCTGAACTTTGCCTCCGGAGGT

TGCTGGCCTCGCAGGATTCGGGTTTGGAACGGCCAGGATTTTCGGGGCGATGGATTCAAC

CCCAGATTTGAGAGATTTCACGTGTACGACATAGTAGAGACTTCGGAGAGCGCCTCACAC

GATGACCCATCCAGGTTTGCTGAGCTATCCCGCCCAAGCGGGTCTGTGGTAACACTGCTG

GGAATGAGCGAGTGTGGAAAGCGAGTCGCGGTTCACGTCTATGGTGTGCGCCATTATTTT

TACATGGCAAAGGCGGAGGTGGATAGCGCTTGTGGAATAACCACCGAGGCAGAACTCGTG

CGCGCAATGGTGGACTGCGCGCACAGCTCGGCTTTGAGCGCGGCCCTGGGAAATGGCAAC

GGCGGCAAACAGAGCGGCGGCAGCGGCGGGGGATGGTGGGGCGGAAAGCACGTGTCTGCG

GACTGCTTCAAAGTGGAGACCGTGTGCCACACGACGCTGTACTACTTTGGATCTAAGCCA

GCTCTCTACTATAGAGTATCTGCCTCCAGCAGCCGCCTGGGAGGGTTCATCTGCGACAAC

TTTCACCCGGAGATTACAAAATTCGAGGGGAGCGTGGACGTGACCACGCGGCTGTTGTTG

GACAATGAAAATTTTACCAGTTTCGGGTGGTACCGCCTGCGACCCGGCACCCACGGAGAG

CGTGTTCAACTTCGCCCCGTTGAGCGACACGTCACCTCAAGCGACGTGGAGATTAACTGT

ACTCCCGATAACCTGGAGCCGATACCAGACGAGGCTGCCTGGCCCGACTATAAGCTCATG

TGCTTTGATATAGAGTGTAAAGCTGGAACGGGTAACGAAATGGCGTTCCCAGTGGCAACT

AACCAAGAGGACCTGGTCATCCAGATCTCCTGTCTGCTGTACTCGCTTGCTACTCAGAAC

CACGAACACACCCTGCTGTTTTCCCTCGGGTCATGCGATATCTCTGAGGAATACTCGTTT

GCATGCGTCCAGCGCGGCGAGCCCAGACCGACGGTTTTGGAGTTTGACAGCGAGTACGAG

CTGCTGGTTGCCTTCCTGACCTTTCTCAAGCAGTACTCTCCCGAGTTCGCCACCGGCTAC

AACATCGTTAATTTTGACTGGGCGTACATAGTTAACAAGGTAACGTCGGTGTATAACATC

AAGCTGGACGGGTACGGCAAGTTCAACAAAGGGGGGCTGTTTAAGGTGTGGGACATCGCC

ACGAACCATTTTCAGAAGAAGAGCAAGGTGAAAATCAATGGCCTGATATCTCTAGACATG

TATTCTGTGGCGACGGAAAAGCTAAAGCTACCCAGCTACAAACTCGACGCGGTCGTGGGA

GACGTCCTCGGCGAGCATAAGATAGACCTTCCCTATAAAGAAATACCCTCCTATTACGCG

GGAGGGCCTGACCGGAGGGGCGTAATAGGAGAGTATTGTATCCAGGACTCTAGGCTGGTG

GGCAAGCTGTTTTTTAAGTACCTCCCCCATCTGGAACTATCGGCGGTGGCCAAACTCGCC

CGTATCACCCTAACGCGGGTAATTTTTGACGGTCAGCAAATTCGCGTGTACACGTGCTTG

CTGAAACTCGCCCGCGAGAGAAATTTCATTTTGCCAGACAACAGACGCCGGTTTGACAGT

CAGGCAGACGCCGCGTCAGAGACTTCGGAGTTGGCTATGGATAGCCAAAGCCACGCCTTC

GACAGTACAGACGAACCCGACGGTGTGGACGGTACCCCGGACGCCGCAGGATCTGGCGCT

ACTTCTGAAAACGGAGGCGGGAAGCCCGGCGTCGGGAGGGCCGTGGGCTACCAGGGAGCA

AAGGTTCTAGACCCCGTATCCGGCTTTCATGTGGACCCCGTGGTTGTGTTTGACTTCGCT

AGCTTATACCCAAGCATTATCCAGGCCCATAACCTCTGTTTCACCACCCTGGCGCTCGAT

GAAGTGGATCTGGCCGGGCTTCAACCATCCGTCAACTACTCGACGTTCGAGGTGGGTGAC

CAAAAGTTATTTTTTGTCCACGCCCATATTCGCGAAAGCCTGCTTGGCATCTTGCTGCGC

GACTGGCTGGCCATGCGAAAGGCGGTGAGGGCGCGAATCCCCACCAGCACCCCCGAGGAG

GCAGTTTTACTAGATAAGCAGCAGTCTGCGATTAAGGTGATATGCAACTCGGTTTACGGA

TTCACGGGGGTGGCAAACGGCCTGTTGCCGTGTCTGAGGATAGCGGCTACCGTTACCACG

ATAGGACGCGACATGCTCCTCAAGACCCGAGATTACGTTCACTCTCGTTGGGCGACGCGC

GAGCTGCTGGAGGACAATTTTCCAGGGGCTATAGGTTTCCGAAACCACAAGCCTTACTCC

GTCAGGGTTATCTACGGAGACACCGACTCCGTGTTTATCAAGTTTGTGGGCCTGACGTAC

GAGGGGGTATCGGAGCTGGGGGATGCTATGTCGCGTCAGATTTCAGCGGACCTCTTTAGA

GCGCCCATCAAACTGGAGTGTGAGAAGACCTTTCAGCGACTGCTGCTGATCACCAAGAAG

AAGTACATAGGTGTCATAAACGGGGGGAAGATGCTCATGAAGGGGGTCGACCTGGTCCGC

AAAAATAACTGCTCTTTCATAAACTTGTACGCGCGACATCTGGTAGATCTTTTGTTGTAC

GACGAGGATGTGGCCACGGCGGCAGCAAAGGTGACAGACGTGCCTCCCGCAGAATGGGTG

GGGCGCCCGCTACCGAGCGGCTTTGACAAGTTTGGGCGAGTGCTGGTAGAGGCGTACAAC

CGTATCACTGCCCCCAACTTGGACGTGCGCGAGTTCGTTATGACTGCTGAGCTGAGCCGC

TCACCCGAATCGTATACCAACAAGCGCCTGCCGCACCTCACCGTCTACTTTAAGCTCGCC

ATGAGGAATGAAGAACTGCCCAGCGTAAAAGAGAGAATTCCGTATGTGATAGTTGCGCAG

ACCGAGGCCGCGGAACGCGAAGCGGGTGTAGTAAACTCAATGCGCGGTACCGCCCAAAAC

CCCGTGGTAACCAAGACCGCACGCCCCCAACCTAAACGCAAACTGCTGGTTTCCGACCTC

GCCGAAGACCCGACCTATGTTTCCGAGAATGACGTACCGCTAAACACAGACTACTATTTC

TCCCACCTGTTGGGTACCATAAGCGTGACCTTTAAGGCTCTATTCGGAAATGATGTGAGA

ACAACAGAAAATCTTTTAAAGCGGTTTATTCCGGAAACCCCCCACAAGACCCCCACGAAA

ACCCAGGCACTGCTTGAGCGCGCCGGCTTTGAAAAGCTGACGCCCTTTACACCGGAGGAA

GAAAGTCGTCGAATACTGCATACAGTTTTTTGTACTCTAGAAGCAGCTCCCCATCAAAGC

TGA

>KU206442.1

ATGGCGGCGCGCGAACAGGCCAACTCCGTTCGCAGGAGCGGGTTTTTTAATCCGTTTATT

GGCAAGAGGCCATTTTTCAGGCCGGGAAGCGGGCAAACCGCGGAGACAGAGCGCCCAAGG

CCCCCCCAACACTCGTACTGCACAGAGGTGGGTAGCTTTAAGTTTATAGCTCCAAGATGC

CTCGATGAAGAAGCCCCCGCCGACCAGCGACGCGGTGTACACGTGGGCACCTTGGAGCGC

CCACCCAAAGTGTACTGCGATGGCTCAGAGTACGACGTGCTGAACTTTGCCTCCGGAGGT

TGCTGGCCTCGCAGGATTCGGGTTTGGAACGGCCAGGATTTTCGGGGCGATGGATTCAAC

CCCAGATTTGAGAGATTTCACGTGTACGACATAGTAGAGACTTCGGAGAGCGCCTCACAC

GATGACCCATCCAGGTTTGCTGAGCTATCCCGCCCAAGCGGGTCTGTGGTAACACTGCTG

GGAATGAGCGAGTGTGGAAAGCGAGTCGCGGTTCACGTCTATGGTGTGCGCCATTATTTT

TACATGGCAAAGGCGGAGGTGGATAGCGCTTGTGGAATAACCACCGAGGCAGAACTCGTG

CGCGCAATGGTGGACTGCGCGCACAGCTCGGCTTTGAGCGCGGCCCTGGGAAATGGCAAC

GGCGGCAAACAGAGCGGCGGCAGCGGCGGGGGATGGTGGGGCGGAAAGCACGTGTCTGCG

GACTGCTTCAAAGTGGAGACCGTGTGCCACACGACGCTGTACTACTTTGGATCTAAGCCA

GCTCTCTACTATAGAGTATCTGCCTCCAGCAGCCGCCTGGGAGGGTTCATCTGCGACAAC

TTTCACCCGGAGATTACAAAATTCGAGGGGAGCGTGGACGTGACCACGCGGCTGTTGTTG

GACAATGAAAATTTTACCAGTTTCGGGTGGTACCGCCTGCGACCCGGCACCCACGGAGAG

CGTGTTCAACTTCGCCCCGTTGAGCGACACGTCACCTCAAGCGACGTGGAGATTAACTGT

ACTCCCGATAACCTGGAGCCGATACCAGACGAGGCTGCCTGGCCCGACTATAAGCTCATG

TGCTTTGATATAGAGTGTAAAGCTGGAACGGGTAACGAAATGGCGTTCCCAGTGGCAACT

AACCAAGAGGACCTGGTCATCCAGATCTCCTGTCTGCTGTACTCGCTTGCTACTCAGAAC

CACGAACACACCCTGCTGTTTTCCCTCGGGTCATGCGATATCTCTGAGGAATACTCGTTT

GCATGCGTCCAGCGCGGCGAGCCCAGACCGACGGTTTTGGAGTTTGACAGCGAGTACGAG

CTGCTGGTTGCCTTCCTGACCTTTCTCAAGCAGTACTCTCCCGAGTTCGCCACCGGCTAC

AACATCGTTAATTTTGACTGGGCGTACATAGTTAACAAGGTAACGTCGGTGTATAACATC

AAGCTGGACGGGTACGGCAAGTTCAACAAAGGGGGGCTGTTTAAGGTGTGGGACATCGCC

ACGAACCATTTTCAGAAGAAGAGCAAGGTGAAAATCAATGGCCTGATATCTCTAGACATG

TATTCTGTGGCGACGGAAAAGCTAAAGCTACCCAGCTACAAACTCGACGCGGTCGTGGGA

GACGTCCTCGGCGAGCATAAGATAGACCTTCCCTATAAAGAAATACCCTCCTATTACGCG

GGAGGGCCTGACCGGAGGGGCGTAATAGGAGAGTATTGTATCCAGGACTCTAGGCTGGTG

GGCAAGCTGTTTTTTAAGTACCTCCCCCATCTGGAACTATCGGCGGTGGCCAAACTCGCC

CGTATCACCCTAACGCGGGTAATTTTTGACGGTCAGCAAATTCGCGTGTACACGTGCTTG

CTGAAACTCGCCCGCGAGAGAAATTTCATTTTGCCAGACAACAGACGCCGGTTTGACAGT

CAGGCAGACGCCGCGTCAGAGACTTCGGAGTTGGCTATGGATAGCCAAAGCCACGCCTTC

GACAGTACAGACGAACCCGACGGTGTGGACGGTACCCCGGACGCCGCAGGATCTGGCGCT

ACTTCTGAAAACGGAGGCGGGAAGCCCGGCGTCGGGAGGGCCGTGGGCTACCAGGGAGCA

AAGGTTCTAGACCCCGTATCCGGCTTTCATGTGGACCCCGTGGTTGTGTTTGACTTCGCT

AGCTTATACCCAAGCATTATCCAGGCCCATAACCTCTGTTTCACCACCCTGGCGCTCGAT

GAAGTGGATCTGGCCGGGCTTCAACCATCCGTCAACTACTCGACGTTCGAGGTGGGTGAC

CAAAAGTTATTTTTTGTCCACGCCCATATTCGCGAAAGCCTGCTTGGCATCTTGCTGCGC

GACTGGCTGGCCATGCGAAAGGCGGTGAGGGCGCGAATCCCCACCAGCACCCCCGAGGAG

GCAGTTTTACTAGATAAGCAGCAGTCTGCGATTAAGGTGATATGCAACTCGGTTTACGGA

TTCACGGGGGTGGCAAACGGCCTGTTGCCGTGTCTGAGGATAGCGGCTACCGTTACCACG

ATAGGACGCGACATGCTCCTCAAGACCCGAGATTACGTTCACTCTCGTTGGGCGACGCGC

GAGCTGCTGGAGGACAATTTTCCAGGGGCTATAGGTTTCCGAAACCACAAGCCTTACTCC

GTCAGGGTTATCTACGGAGACACCGACTCCGTGTTTATCAAGTTTGTGGGCCTGACGTAC

GAGGGGGTATCGGAGCTGGGGGATGCTATGTCGCGTCAGATTTCAGCGGACCTCTTTAGA

GCGCCCATCAAACTGGAGTGTGAGAAGACCTTTCAGCGACTGCTGCTGATCACCAAGAAG

AAGTACATAGGTGTCATAAACGGGGGGAAGATGCTCATGAAGGGGGTCGACCTGGTCCGC

AAAAATAACTGCTCTTTCATAAACTTGTACGCGCGACATCTGGTAGATCTTTTGTTGTAC

GACGAGGATGTGGCCACGGCGGCAGCAAAGGTGACAGACGTGCCTCCCGCAGAATGGGTG

GGGCGCCCGCTACCGAGCGGCTTTGACAAGTTTGGGCGAGTGCTGGTAGAGGCGTACAAC

CGTATCACTGCCCCCAACTTGGACGTGCGCGAGTTCGTTATGACTGCTGAGCTGAGCCGC

TCACCCGAATCGTATACCAACAAGCGCCTGCCGCACCTCACCGTCTACTTTAAGCTCGCC

ATGAGGAATGAAGAACTGCCCAGCGTAAAAGAGAGAATTCCGTATGTGATAGTTGCGCAG

ACCGAGGCCGCGGAACGCGAAGCGGGTGTAGTAAACTCAATGCGCGGTACCGCCCAAAAC

CCCGTGGTAACCAAGACCGCACGCCCCCAACCTAAACGCAAACTGCTGGTTTCCGACCTC

GCCGAAGACCCGACCTATGTTTCCGAGAATGACGTACCGCTAAACACAGACTACTATTTC

TCCCACCTGTTGGGTACCATAAGCGTGACCTTTAAGGCTCTATTCGGAAATGATGTGAGA

ACAACAGAAAATCTTTTAAAGCGGTTTATTCCGGAAACCCCCCACAAGACCCCCACGAAA

ACCCAGGCACTGCTTGAGCGCGCCGGCTTTGAAAAGCTGACGCCCTTTACACCGGAGGAA

GAAAGTCGTCGAATACTGCATACAGTTTTTTGTACTCTAGAAGCAGCTCCCCATCAAAGC

TGA

>KU206443.1

ATGGCGGCGCGCGAACAGGCCAACTCCGTTCGCAGGAGCGGGTTTTTTAATCCGTTTATT

GGCAAGAGGCCATTTTTCAGGCCGGGAAGCGGGCAAACCGCGGAGACAGAGCGCCCAAGG

CCCCCCCAACACTCGTACTGCACAGAGGTGGGTAGCTTTAAGTTTATAGCTCCAAGGTGC

CTCGATGAAGAAGCCCCCGCCGACCAGCGACGCGGTGTACACGTGGGCACCTTGGAGCGC

CCACCCAAAGTGTACTGCGATGGCTCAGAGTACGACGTGCTGAACTTTGCCTCCGGAGGT

TGCTGGCCTCGCAGGATTCGGGTTTGGAACGGCCAGGATTTTCGGGGCGATGGATTCAAC

CCCAGATTTGAGAGATTTCACGTGTACGACATAGTAGAGACTTCGGAGAGCGCCTCACAC

GATGACCCATCCAGGTTTGCTGAGCTATCCCGCCCAAGCGGGTCTGTGGTAACACTGCTG

GGAATGAGCGAGTGTGGAAAGCGAGTCGCGGTTCACGTCTATGGTGTGCGCCATTATTTT

TACATGGCAAAGGCGGAGGTGGATAGCGCTTGTGGAATAACCACCGAGGCAGAACTCGTG

CGCGCAATGGTGGACTGCGCGCACAGCTCGGCTTTGAGCGCGGCCCTGGGAAATGGCAAC

GGCGGCAAACAGAGCGGCGGCAGCGGCGGGGGATGGTGGGGCGGAAAGCACGTGTCTGCG

GACTGCTTCAAAGTGGAGACCGTGTGCCACACGACGCTGTACTACTTTGGATCTAAGCCA

GCTCTCTACTATAGAGTATCTGCCTCCAGCAGCCGCCTGGGAGGGTTCATCTGCGACAAC

TTTCACCCGGAGATTACAAAATTCGAGGGGAGCGTGGACGTGACCACGCGGCTGTTGTTG

GACAATGAAAATTTTACCAGTTTCGGGTGGTACCGCCTGCGACCCGGCACCCACGGAGAG

CGTGTTCAACTTCGCCCCGTTGAGCGACACGTCACCTCAAGCGACGTGGAGATTAACTGT

ACTCCCGATAACCTGGAGCCGATACCAGACGAGGCTGCCTGGCCCGACTATAAGCTCATG

TGCTTTGATATAGAGTGTAAAGCTGGAACGGGTAACGAAATGGCGTTCCCAGTGGCAACT

AACCAAGAGGACCTGGTCATCCAGATCTCCTGTCTGCTGTACTCGCTTGCTACTCAGAAC

CACGAACACACCCTGCTGTTTTCCCTCGGGTCATGCGATATCTCTGAGGAATACTCGTTT

GCATGCGTCCAGCGCGGCGAGCCCAGACCGACGGTTTTGGAGTTTGACAGCGAGTACGAG

CTGCTGGTTGCCTTCCTGACCTTTCTCAAGCAGTACTCTCCCGAGTTCGCCACCGGCTAC

AACATCGTTAATTTTGACTGGGCGTACATAGTTAACAAGGTAACGTCGGTGTATAACATC

AAGCTGGACGGGTACGGCAAGTTCAACAAAGGGGGGCTGTTTAAGGTGTGGGACATCGCC

ACGAACCATTTTCAGAAGAAGAGCAAGGTGAAAATCAATGGCCTGATATCTCTAGACATG

TATTCTGTGGCGACGGAAAAGCTAAAGCTACCCAGCTACAAACTCGACGCGGTCGTGGGA

GACGTCCTCGGCGAGCATAAGATAGACCTTCCCTATAAAGAAATACCCTCCTATTACGCG

GGAGGGCCTGACCGGAGGGGCGTAATAGGAGAGTATTGTATCCAGGACTCTAGGCTGGTG

GGCAAGCTGTTTTTTAAGTACCTCCCCCATCTGGAACTATCGGCGGTGGCCAAACTCGCC

CGTATCACCCTAACGCGGGTAATTTTTGACGGTCAGCAAATTCGCGTGTACACGTGCTTG

CTGAAACTCGCCCGCGAGAGAAATTTCATTTTGCCAGACAACAGACGCCGGTTTGACAGT

CAGGCAGACGCCGCGTCAGAGACTTCGGAGTTGGCTATGGATAGCCAAAGCCACGCCTTC

GACAGTACAGACGAACCCGACGGTGTGGACGGTACCCCGGACGCCGCAGGATCTGGCGCT

ACTTCTGAAAACGGAGGCGGGAAGCCCGGCGTCGGGAGGGCCGTGGGCTACCAGGGAGCA

AAGGTTCTAGACCCCGTATCCGGCTTTCATGTGGACCCCGTGGTTGTGTTTGACTTCGCT

AGCTTATACCCAAGCATTATCCAGGCCCATAACCTCTGTTTCACCACCCTGGCGCTCGAT

GAAGTGGATCTGGCCGGGCTTCAACCATCCGTCAACTACTCGACGTTCGAGGTGGGTGAC

CAAAAGTTATTTTTTGTCCACGCCCATATTCGCGAAAGCCTGCTTGGCATCTTGCTGCGC

GACTGGCTGGCCATGCGAAAGGCGGTGAGGGCGCGAATCCCCACCAGCACCCCCGAGGAG

GCAGTTTTACTAGATAAGCAGCAGTCTGCGATTAAGGTGATATGCAACTCGGTTTACGGA

TTCACGGGGGTGGCAAACGGCCTGTTGCCGTGTCTGAGGATAGCGGCTACCGTTACCACG

ATAGGACGCGACATGCTCCTCAAGACCCGAGATTACGTTCACTCTCGTTGGGCGACGCGC

GAGCTGCTGGAGGACAATTTTCCAGGGGCTATAGGTTTCCGAAACCACAAGCCTTACTCC

GTCAGGGTTATCTACGGAGACACCGACTCCGTGTTTATCAAGTTTGTGGGCCTGACGTAC

GAGGGGGTATCGGAGCTGGGGGATGCTATGTCGCGTCAGATTTCAGCGGACCTCTTTAGA

GCGCCCATCAAACTGGAGTGTGAGAAGACCTTTCAGCGACTGCTGCTGATCACCAAGAAG

AAGTACATAGGTGTCATAAACGGGGGGAAGATGCTCATGAAGGGGGTCGACCTGGTCCGC

AAAAATAACTGCTCTTTCATAAACTTGTACGCGCGACATCTGGTAGATCTTTTGTTGTAC

GACGAGGATGTGGCCACGGCGGCAGCAAAGGTGACAGACGTGCCTCCCGCAGAATGGGTG

GGGCGCCCGCTACCGAGCGGCTTTGACAAGTTTGGGCGAGTGCTGGTAGAGGCGTACAAC

CGTATCACTGCCCCCAACTTGGACGTGCGCGAGTTCGTTATGACTGCTGAGCTGAGCCGC

TCACCCGAATCGTATACCAACAAGCGCCTGCCGCACCTCACCGTCTACTTTAAGCTCGCC

ATGAGGAATGAAGAACTGCCCAGCGTAAAAGAGAGAATTCCGTATGTGATAGTTGCGCAG

ACCGAGGCCGCGGAACGCGAAGCGGGTGTAGTAAACTCAATGCGCGGTACCGCCCAAAAC

CCCGTGGTAACCAAGACCGCACGCCCCCAACCTAAACGCAAACTGCTGGTTTCCGACCTC

GCCGAAGACCCGACCTATGTTTCCGAGAATGACGTACCGCTAAACACAGACTACTATTTC

TCCCACCTGTTGGGTACCATAAGCGTGACCTTTAAGGCTCTATTCGGAAATGATGTGAGA

ACAACAGAAAATCTTTTAAAGCGGTTTATTCCGGAAACCCCCCACAAGACCCCCACGAAA

ACCCAGGCACTGCTTGAGCGCGCCGGCTTTGAAAAGCTGACGCCCTTTACACCGGAGGAA

GAAAGTCGTCGAATACTGCATACAGTTTTTTGTACTCTAGAAGCAGCTCCCCATCAAAGC

TGA

>KU206444.1

ATGGCGGCGCGCGAACAGGCCAACTCCGTTCGCAGGAGCGGGTTTTTTAATCCGTTTATT

GGCAAGAGGCCATTTTTCAGGCCGGGAAGCGGGCAGACCGCGGAGACAGAGCGCCCAAGG

CCCCCCCAACACTCGTACTGCACAGAGGTGGGTAGCTTTAAGTTTATAGCTCCAAGATGC

CTCGATGAAGAAGCCCCCGCCGACCAGCGACGCGGTGTACACGTGGGCACCTTGGAGCGC

CCACCCAAAGTGTACTGCGATGGCTCAGAGTACGACGTGCTGAACTTTGCCTCCGGAGGT

TGCTGGCCTCGCAGGATTCGGGTTTGGAACGGCCAGGATTTTCGGGGCGATGGATTCAAC

CCCAGATTTGAGAGATTTCACGTGTACGACATAGTAGAGACTTCGGAGAGCGCCTCACAC

GATGACCCATCCAGGTTTGCTGAGCTATCCCGCCCAAGCGGGTCTGTGGTAACACTGCTG

GGAATGAGCGAGTGTGGAAAGCGAGTCGCGGTTCACGTATATGGTGTGCGCCATTATTTT

TACATGGCAAAGGCGGAGGTGGATAGCGCTTGTGGAATAACCACCGAGGCAGAACTCGTG

CGCGCAATGGTGGACTGCGCGCACAGCTCGGCTTTGAGCGCGGCCCTGGGAAATGGCAAC

GGCGGCAAACAGAGCGGCGGCAGCGGCGGGGGATGGTGGGGCGGAAAGCACGTGTCTGCG

GACTGCTTCAAAGTGGAGACCGTGTGCCACACGACGCTGTACTACTTTGGATCTAAGCCA

GCTCTCTACTATAGAGTATCTGCCTCCAGCAGCCGCCTGGGAGGGTTCATCTGCGACAAC

TTTCACCCGGAGATTACAAAATTCGAGGGGAGCGTGGACGTGACCACGCGGCTGTTGTTG

GACAATGAAAATTTTACCAGTTTCGGGTGGTACCGCCTGCGACCCGGCACCCACGGAGAG

CGTGTTCAACTTCGCCCCGTTGAGCGACACGTCACCTCAAGCGACGTGGAGATTAACTGT

ACTCCCGATAACCTGGAGCCGATACCAGACGAGGCTGCCTGGCCCGACTATAAGCTCATG

TGCTTTGATATAGAGTGTAAAGCTGGAACGGGTAACGAAATGGCGTTCCCAGTGGCAACT

AACCAAGAGGACCTGGTCATCCAGATCTCCTGTCTGCTGTACTCGCTTGCTACTCAGAAC

CACGAACACACCCTGCTGTTTTCCCTCGGGTCATGCGATATCTCTGAGGAATACTCGTTT

GCATGCGTCCAGCGCGGCGAGCCCAGACCGACGGTTTTGGAGTTTGACAGCGAGTACGAG

CTGCTGGTTGCCTTCCTGACCTTTCTCAAGCAGTACTCTCCCGAGTTCGCCACCGGCTAC

AACATCGTTAATTTTGACTGGGCGTACATAGTTAACAAGGTAACGTCGGTGTATAACATC

AAGCTGGACGGGTACGGCAAGTTCAACAAAGGGGGGCTGTTTAAGGTGTGGGACATCGCC

ACGAACCATTTTCAGAAGAAGAGCAAGGTGAAAATCAATGGCCTGATATCTCTAGACATG

TATTCTGTGGCGACGGAAAAGCTAAAGCTACCCAGCTACAAACTCGACGCGGTCGTGGGA

GACGTCCTCGGCGAGCATAAGATAGACCTTCCCTATAAAGAAATACCCTCCTATTACGCG

GGAGGGCCTGACCGGAGGGGCGTAATAGGAGAGTATTGTATCCAGGACTCTAGGCTGGTG

GGCAAGCTGTTTTTTAAGTACCTCCCCCATCTGGAACTATCGGCGGTGGCCAAACTCGCC

CGTATCACCCTAACGCGGGTAATTTTTGACGGTCAGCAAATTCGCGTGTACACGTGCTTG

CTGAAACTCGCCCGCGAGAGAAATTTCATTTTGCCAGACAACAGACGCCGGTTTGACAGT

CAGGCAGACGCCGCGTCAGAGACTTCGGAGTTGGCTATGGATAGCCAAAGCCACGCCTTC

GACAGTACAGACGAACCCGACGGTGTGGACGGTACCCCGGACGCCGCAGGATCTGGCGCT

ACTTCTGAAAACGGAGGCGGGAAGCCCGGCGTCGGGAGGGCCGTGGGCTACCAGGGAGCA

AAGGTTCTAGACCCCGTATCCGGCTTTCATGTGGACCCCGTGGTTGTGTTTGACTTCGCT

AGCTTATACCCAAGCATTATCCAGGCCCATAACCTCTGTTTCACCACCCTGGCGCTCGAT

GAAGTGGATCTGGCCGGGCTTCAACCATCCGTCGACTACTCGACGTTCGAGGTGGGTGAC

CAAAAGTTATTTTTTGTCCACGCCCATATTCGCGAAAGCCTGCTTGGCATCTTGCTGCGC

GACTGGCTGGCCATGCGAAAGGCGGTGAGGGCGCGAATCCCCACCAGCACCCCCGAGGAG

GCAGTTTTACTAGATAAGCAGCAGTCTGCGATTAAGGTGATATGCAACTCGGTTTACGGA

TTCACGGGGGTGGCAAACGGCCTGTTGCCGTGTCTGAGGATAGCGGCTACCGTTACCACG

ATAGGACGCGACATGCTCCTCAAGACCCGAGATTACGTTCACTCTCGTTGGGCGACGCGC

GAGCTGCTGGAGGACAATTTTCCAGGGGCTATAGGTTTCCGAAACCACAAGCCTTACTCC

GTCAGGGTTATCTACGGAGACACCGACTCCGTGTTTATCAAGTTTGTGGGCCTGACGTAC

GAGGGGGTATCGGAGCTGGGGGATGCTATGTCGCGTCAGATTTCAGCGGACCTCTTTAGA

GCGCCCATCAAACTGGAGTGTGAGAAGACCTTTCAGCGACTGCTGCTGATCACCAAGAAG

AAGTACATAGGTGTCATAAACGGGGGGAAGATGCTCATGAAGGGGGTCGACCTGGTCCGC

AAAAATAACTGCTCTTTCATAAACTTGTACGCGCGACATCTGGTAGATCTTTTGTTGTAC

GACGAGGATGTGGCCACGGCGGCAGCAGAGGTGACAGACGTGCCTCCCGCAGAATGGGTG

GGGCGCCCGCTACCGAGCGGCTTTGACAAGTTTGGGCGAGTGCTGGTAGAGGCGTACAAC

CGTATCACTGCCCCCAACTTGGACGTGCGCGAGTTCGTTATGACTGCTGAGCTGAGCCGC

TCACCCGAATCGTATACCAACAAGCGCCTGCCGCACCTCACCGTCTACTTTAAGCTCGCC

ATGAGGAATGAAGAACTGCCCAGCGTAAAAGAGAGAATTCCGTATGTGATAGTTGCGCAG

ACCGAGGCCGCGGAACGCGAAGCGGGTGTAGTAAACTCAATGCGCGGTACCGCCCAAAAC

CCCGTGGTAACCAAGACCGCACGCCCCCAACCTAAACGCAAACTGCTGGTTTCCGACCTC

GCCGAAGACCCGACCTATGTTTCCGAGAATGACGTACCGCTAAACACAGACTACTATTTC

TCCCACCTGTTGGGTACCATAAGCGTGACCTTTAAGGCTCTATTCGGAAATGATGTGAGA

ACAACAGAAAATCTTTTAAAGCGGTTTATTCCGGAAACCCCCCACAAGACCCCCACGAAA

ACCCAGGCACTGCTTGAGCGCGCCGGCTTTGAAAAGCTGACGCCCTTTACACCGGAGGAA

GAAAGTCGTCGAATACTGCATACAGTTTTTTGTACTCTAGAAGCAGCTCCCCATCAAAGC

TGA

>KU206445.1

ATGGCGGCGCGCGAACAGGCCAACTCCGTTCGCAGGAGCGGGTTTTTTAATCCGTTTATT

GGCAAGAGGCCATTTTTCAGGCCGGGAAGCGGGCAGACCGCGGAGACAGAGCGCCCAAGG

CCCCCCCAACACTCGTACTGCACAGAGGTGGGTAGCTTTAAGTTTATAGCTCCAAGATGC

CTCGATGAAGAAGCCCCCGCCGACCAGCGACGCGGTGTACACGTGGGCACCTTGGAGCGC

CCACCCAAAGTGTACTGCGATGGCTCAGAGTACGACGTGCTGAACTTTGCCTCCGGAGGT

TGCTGGCCTCGCAGGATTCGGGTTTGGAACGGCCAGGATTTTCGGGGCGATGGATTCAAC

CCCAGATTTGAGAGATTTCACGTGTACGACATAGTAGAGACTTCGGAGAGCGCCTCACAC

GATGACCCATCCAGGTTTGCTGAGCTATCCCGCCCAAGCGGGTCTGTGGTAACACTGCTG

GGAATGAGCGAGTGTGGAAAGCGAGTCGCGGTTCACGTCTATGGTGTGCGCCATTATTTT

TACATGGCAAAGGCGGAGGTGGATAGCGCTTGTGGAATAACCACCGAGGCAGAACTCGTG

CGCGCAATGGTGGACTGCGCGCACAGCTCGGCTTTGAGCGCGGCCCTGGGAAATGGCAAC

GGCGGCAAACAGAGCGGCGGCAGCGGCGGGGGATGGTGGGGCGGAAAGCACGTGTCTGCG

GACTGCTTCAAAGTGGAGACCGTGTGCCACACGACGCTGTACTACTTTGGATCTAAGCCA

GCTCTCTACTATAGAGTATCTGCCTCCAGCAGCCGCCTGGGAGGGTTCATCTGCGACAAC

TTTCACCCGGAGATTACAAAATTCGAGGGGAGCGTGGACGTGACCACGCGGCTGTTGTTG

GACAATGAAAATTTTACCAGTTTCGGGTGGTACCGCCTGCGACCCGGCACCCACGGAGAG

CGTGTTCAACTTCGCCCCGTTGAGCGACACGTCACCTCAAGCGACGTGGAGATTAACTGT

ACTCCCGATAACCTGGAGCCGATACCAGACGAGGCTGCCTGGCCCGACTATAAGCTCATG

TGCTTTGATATAGAGTGTAAAGCTGGAACGGGTAACGAAATGGCGTTCCCAGTGGCAACT

AACCAAGAGGACCTGGTCATCCAGATCTCCTGTCTGCTGTACTCGCTTGCTACTCAGAAC

CACGAACACACCCTGCTGTTTTCCCTCGGGTCATGCGATATCTCTGAGGAATACTCGTTT

GCATGCGTCCAGCGCGGCGAGCCCAGACCGACGGTTTTGGAGTTTGACAGCGAGTACGAG

CTGCTGGTTGCCTTCCTGACCTTTCTCAAGCAGTACTCTCCCGAGTTCGCCACCGGCTAC

AACATCGTTAATTTTGACTGGGCGTACATAGTTAACAAGGTAACGTCGGTGTATAACATC

AAGCTGGACGGGTACGGCAAGTTCAACAAAGGGGGGCTGTTTAAGGTGTGGGACATCGCC

ACGAACCATTTTCAGAAGAAGAGCAAGGTGAAAATCAATGGCCTGATATCTCTAGACATG

TATTCTGTGGCGACGGAAAAGCTAAAGCTACCCAGCTACAAACTCGACGCGGTCGTGGGA

GACGTCCTCGGCGAGCATAAGATAGACCTTCCCTATAAAGAAATACCCTCCTATTACGCG

GGAGGGCCTGACCGGAGGGGCGTAATAGGAGAGTATTGTATCCAGGACTCTAGGCTGGTG

GGCAAGCTGTTTTTTAAGTACCTCCCCCATCTGGAACTATCGGCGGTGGCCAAACTCGCC

CGTATCACCCTAACGCGGGTAATTTTTGACGGTCAGCAAATTCGCGTGTACACGTGCTTG

CTGAAACTCGCCCGCGAGAGAAATTTCATTTTGCCAGACAACAGACGCCGGTTTGACAGT

CAGGCAGACGCCGCGTCAGAGACTTCGGAGTTGGCTATGGATAGCCAAAGCCACGCCTTC

GACAGTACAGACGAACCCGACGGTGTGGACGGTACCCCGGACGCCGCAGGATCTGGCGCT

ACTTCTGAAAACGGAGGCGGGAAGCCCGGCGTCGGGAGGGCCGTGGGCTACCAGGGAGCA

AAGGTTCTAGACCCCGTATCCGGCTTTCATGTGGACCCCGTGGTTGTGTTTGACTTCGCT

AGCTTATACCCAAGCATTATCCAGGCCCATAACCTCTGTTTCACCACCCTGGCGCTCGAT

GAAGTGGATCTGGCCGGGCTTCAACCATCCGTCGACTACTCGACGTTCGAGGTGGGTGAC

CAAAAGTTATTTTTTGTCCACGCCCATATTCGCGAAAGCCTGCTTGGCATCTTGCTGCGC

GACTGGCTGGCCATGCGAAAGGCGGTGAGGGCGCGAATCCCCACCAGCACCCCCGAGGAG

GCAGTTTTACTAGATAAGCAGCAGTCTGCGATTAAGGTGATATGCAACTCGGTTTACGGA

TTCACGGGGGTGGCAAACGGCCTGTTGCCGTGTCTGAGGATAGCGGCTACCGTTACCACG

ATAGGACGCGACATGCTCCTCAAGACCCGAGATTACGTTCACTCTCGTTGGGCGACGCGC

GAGCTGCTGGAGGACAATTTTCCAGGGGCTATAGGTTTCCGAAACCACAAGCCTTACTCC

GTCAGGGTTATCTACGGAGACACCGACTCCGTGTTTATCAAGTTTGTGGGCCTGACGTAC

GAGGGGGTATCGGAGCTGGGGGATGCTATGTCGCGTCAGATTTCAGCGGACCTCTTTAGA

GCGCCCATCAAACTGGAGTGTGAGAAGACCTTTCAGCGACTGCTGCTGATCACCAAGAAG

AAGTACATAGGTGTCATAAACGGGGGGAAGATGCTCATGAAGGGGGTCGACCTGGTCCGC

AAAAATAACTGCTCTTTCATAAACTTGTACGCGCGACATCTGGTAGATCTTTTGTTGTAC

GACGAGGATGTGGCCACGGCGGCAGCAGAGGTGACAGACGTGCCTCCCGCAGAATGGGTG

GGGCGCCCGCTACCGAGCGGCTTTGACAAGTTTGGGCGAGTGCTGGTAGAGGCGTACAAC

CGTATCACTGCCCCCAACTTGGACGTGCGCGAGTTCGTTATGACTGCTGAGCTGAGCCGC

TCACCCGAATCGTATACCAACAAGCGCCTGCCGCACCTCACCGTCTACTTTAAGCTCGCC

ATGAGGAATGAAGAACTGCCCAGCGTAAAAGAGAGAATTCCGTATGTGATAGTTGCGCAG

ACCGAGGCCGCGGAACGCGAAGCGGGTGTAGTAAACTCAATGCGCGGTACCGCCCAAAAC

CCCGTGGTAACCAAGACCGCACGCCCCCAACCTAAACGCAAACTGCTGGTTTCCGACCTC

GCCGAAGACCCGACCTATGTTTCCGAGAATGACGTACCGCTAAACACAGACTACTATTTC

TCCCACCTGTTGGGTACCATAAGCGTGACCTTTAAGGCTCTATTCGGAAATGATGTGAGA

ACAACAGAAAATCTTTTAAAGCGGTTTATTCCGGAAACCCCCCACAAGACCCCCACGAAA

ACCCAGGCACTGCTTGAGCGCGCCGGCTTTGAAAAGCTGACGCCCTTTACACCGGAGGAA

GAAAGTCGTCGAATACTGCATACAGTTTTTTGTACTCTAGAAGCAGCTCCCCATCAAAGC

TGA

>KU206446.1

ATGGCGGCGCGCGAACAGGCCAACTCCGTTCGCAGGAGCGGGTTTTTTAATCCGTTTATT

GGCAAGAGGCCATTTTTCAGGCCGGGAAGCGGGCAGACCGCGGAGACAGAGCGCCCAAGG

CCCCCCCAACACTCGTACTGCACAGAGGTGGGTAGCTTTAAGTTTATAGCTCCAAGATGC

CTCGATGAAGAAGCCCCCGCCGACCAGCGACGCGGTGTACACGTGGGCACCTTGGAGCGC

CCACCCAAAGTGTACTGCGATGGCTCAGAGTACGACGTGCTGAACTTTGCCTCCGGAGGT

TGCTGGCCTCGCAGGATTCGGGTTTGGAACGGCCAGGATTTTCGGGGCGATGGATTCAAC

CCCAGATTTGAGAGATTTCACGTGTACGACATAGTAGAGACTTCGGAGAGCGCCTCACAC

GATGACCCATCCAGGTTTGCTGAGCTATCCCGCCCAAGCGGGTCTGTGGTAACACTGCTG

GGAATGAGCGAGTGTGGAAAGCGAGTCGCGGTTCACGTCTATGGTGTGCGCCATTATTTT

TACATGGCAAAGGCGGAGGTGGATAGCGCTTGTGGAATAACCACCGAGGCAGAACTCGTG

CGCGCAATGGTGGACTGCGCGCACAGCTCGGCTTTGAGCGCGGCCCTGGGAAATGGCAAC

GGCGGCAAACAGAGCGGCGGCAGCGGCGGGGGATGGTGGGGCGGAAAGCACGTGTCTGCG

GACTGCTTCAAAGTGGAGACCGTGTGCCACACGACGCTGTACTACTTTGGATCTAAGCCA

GCTCTCTACTATAGAGTATCTGCCTCCAGCAGCCGCCTGGGAGGGTTCATCTGCGACAAC

TTTCACCCGGAGATTACAAAATTCGAGGGGAGCGTGGACGTGACCACGCGGCTGTTGTTG

GACAATGAAAATTTTACCAGTTTCGGGTGGTACCGCCTGCGACCCGGCACCCACGGAGAG

CGTGTTCAACTTCGCCCCGTTGAGCGACACGTCACCTCAAGCGACGTGGAGATTAACTGT

ACTCCCGATAACCTGGAGCCGATACCAGACGAGGCTGCCTGGCCCGACTATAAGCTCATG

TGCTTTGATATAGAGTGTAAAGCTGGAACGGGTAACGAAATGGCGTTCCCAGTGGCAACT

AACCAAGAGGACCTGGTCATCCAGATCTCCTGTCTGCTGTACTCGCTTGCTACTCAGAAC

CACGAACACACCCTGCTGTTTTCCCTCGGGTCATGCGATATCTCTGAGGAATACTCGTTT

GCATGCGTCCAGCGCGGCGAGCCCAGACCGACGGTTTTGGAGTTTGACAGCGAGTACGAG

CTGCTGGTTGCCTTCCTGACCTTTCTCAAGCAGTACTCTCCCGAGTTCGCCACCGGCTAC

AACATCGTTAATTTTGACTGGGCGTACATAGTTAACAAGGTAACGTCGGTGTATAACATC

AAGCTGGACGGGTACGGCAAGTTCAACAAAGGGGGGCTGTTTAAGGTGTGGGACATCGCC

ACGAACCATTTTCAGAAGAAGAGCAAGGTGAAAATCAATGGCCTGATATCTCTAGACATG

TATTCTGTGGCGACGGAAAAGCTAAAGCTACCCAGCTACAAACTCGACGCGGTCGTGGGA

GACGTCCTCGGCGAGCATAAGATAGACCTTCCCTATAAAGAAATACCCTCCTATTACGCG

GGAGGGCCTGACCGGAGGGGCGTAATAGGAGAGTATTGTATCCAGGACTCTAGGCTGGTG

GGCAAGCTGTTTTTTAAGTACCTCCCCCATCTGGAACTATCGGCGGTGGCCAAACTCGCC

CGTATCACCCTAACGCGGGTAATTTTTGACGGTCAGCAAATTCGCGTGTACACGTGCTTG

CTGAAACTCGCCCGCGAGAGAAATTTCATTTTGCCAGACAACAGACGCCGGTTTGACAGT

CAGGCAGACGCCGCGTCAGAGACTTCGGAGTTGGCTATGGATAGCCAAAGCCACGCCTTC

GACAGTACAGACGAACCCGACGGTGTGGACGGTACCCCGGACGCCGCAGGATCTGGCGCT

ACTTCTGAAAACGGAGGCGGGAAGCCCGGCGTCGGGAGGGCCGTGGGCTACCAGGGAGCA

AAGGTTCTAGACCCCGTATCCGGCTTTCATGTGGACCCCGTGGTTGTGTTTGACTTCGCT

AGCTTATACCCAAGCATTATCCAGGCCCATAACCTCTGTTTCACCACCCTGGCGCTCGAT

GAAGTGGATCTGGCCGGGCTTCAACCATCCGTCGACTACTCGACGTTCGAGGTGGGTGAC

CAAAAGTTATTTTTTGTCCACGCCCATATTCGCGAAAGCCTGCTTGGCATCTTGCTGCGC

GACTGGCTGGCCATGCGAAAGGCGGTGAGGGCGCGAATCCCCACCAGCACCCCCGAGGAG

GCAGTTTTACTAGATAAGCAGCAGTCTGCGATTAAGGTGATATGCAACTCGGTTTACGGA

TTCACGGGGGTGGCAAACGGCCTGTTGCCGTGTCTGAGGATAGCGGCTACCGTTACCACG

ATAGGACGCGACATGCTCCTCAAGACCCGAGATTACGTTCACTCTCGTTGGGCGACGCGC

GAGCTGCTGGAGGACAATTTTCCAGGGGCTATAGGTTTCCGAAACCACAAGCCTTACTCC

GTCAGGGTTATCTACGGAGACACCGACTCCGTGTTTATCAAGTTTGTGGGCCTGACGTAC

GAGGGGGTATCGGAGCTGGGGGATGCTATGTCGCGTCAGATTTCAGCGGACCTCTTTAGA

GCGCCCATCAAACTGGAGTGTGAGAAGACCTTTCAGCGACTGCTGCTGATCACCAAGAAG

AAGTACATAGGTGTCATAAACGGGGGGAAGATGCTCATGAAGGGGGTCGACCTGGTCCGC

AAAAATAACTGCTCTTTCATAAACTTGTACGCGCGACATCTGGTAGATCTTTTGTTGTAC

GACGAGGATGTGGCCACGGCGGCAGCAGAGGTGACAGACGTGCCTCCCGCAGAATGGGTG

GGGCGCCCGCTACCGAGCGGCTTTGACAAGTTTGGGCGAGTGCTGGTAGAGGCGTACAAC

CGTATCACTGCCCCCAACTTGGACGTGCGCGAGTTCGTTATGACTGCTGAGCTGAGCCGC

TCACCCGAATCGTATACCAACAAGCGCCTGCCGCACCTCACCGTCTACTTTAAGCTCGCC

ATGAGGAATGAAGAACTGCCCAGCGTAAAAGAGAGAATTCCGTATGTGATAGTTGCGCAG

ACCGAGGCCGCGGAACGCGAAGCGGGTGTAGTAAACTCAATGCGCGGTACCGCCCAAAAC

CCCGTGGTAACCAAGACCGCACGCCCCCAACCTAAACGCAAACTGCTGGTTTCCGACCTC

GCCGAAGACCCGACCTATGTTTCCGAGAATGACGTACCGCTAAACACAGACTACTATTTC

TCCCACCTGTTGGGTACCATAAGCGTGACCTTTAAGGCTCTATTCGGAAATGATGTGAGA

ACAACAGAAAATCTTTTAAAGCGGTTTATTCCGGAAACCCCCCACAAGACCCCCACGAAA

ACCCAGGCACTGCTTGAGCGCGCCGGCTTTGAAAAGCTGACGCCCTTTACACCGGAGGAA

GAAAGTCGTCGAATACTGCATACAGTTTTTTGTACTCTAGAAGCAGCTCCCCATCAAAGC

TGA

>KU206447.1

ATGGCGGCGCGCGAACAGGCCAACTCCGTTCGCAGGAGCGGGTTTTTTAATCCGTTTATT

GGCAAGAGGCCATTTTTCAGGCCGGGAAGCGGGCAGACCGCGGAGACAGAGCGCCCAAGG

CCCCCCCAACACTCGTACTGCACAGAGGTGGGTAGCTTTAAGTTTATAGCTCCAAGATGC

CTCGATGAAGAAGCCCCCGCCGACCAGCGACGCGGTGTACACGTGGGCACCTTGGAGCGC

CCACCCAAAGTGTACTGCGATGGCTCAGAGTACGACGTGCTGAACTTTGCCTCCGGAGGT

TGCTGGCCTCGCAGGATTCGGGTTTGGAACGGCCAGGATTTTCGGGGCGATGGATTCAAC

CCCAGATTTGAGAGATTTCACGTGTACGACATAGTAGAGACTTCGGAGAGCGCCTCACAC

GATGACCCATCCAGGTTTGCTGAGCTATCCCGCCCAAGCGGGTCTGTGGTAACACTGCTG

GGAATGAGCGAGTGTGGAAAGCGAGTCGCGGTTCACGTCTATGGTGTGCGCCATTATTTT

TACATGGCAAAGGCGGAGGTGGATAGCGCTTGTGGAATAACCACCGAGGCAGAACTCGTG

CGCGCAATGGTGGACTGCGCGCACAGCTCGGCTTTGAGCGCGGCCCTGGGAAATGGCAAC

GGCGGCAAACAGAGCGGCGGCAGCGGCGGGGGATGGTGGGGCGGAAAGCACGTGTCTGCG

GACTGCTTCAAAGTGGAGACCGTGTGCCACACGACGCTGTACTACTTTGGATCTAAGCCA

GCTCTCTACTATAGAGTATCTGCCTCCAGCAGCCGCCTGGGAGGGTTCATCTGCGACAAC

TTTCACCCGGAGATTACAAAATTCGAGGGGAGCGTGGACGTGACCACGCGGCTGTTGTTG

GACAATGAAAATTTTACCAGTTTCGGGTGGTACCGCCTGCGACCCGGCACCCACGGAGAG

CGTGTTCAACTTCGCCCCGTTGAGCGACACGTCACCTCAAGCGACGTGGAGATTAACTGT

ACTCCCGATAACCTGGAGCCGATACCAGACGAGGCTGCCTGGCCCGACTATAAGCTCATG

TGCTTTGATATAGAGTGTAAAGCTGGAACGGGTAACGAAATGGCGTTCCCAGTGGCAACT

AACCAAGAGGACCTGGTCATCCAGATCTCCTGTCTGCTGTACTCGCTTGCTACTCAGAAC

CACGAACACACCCTGCTGTTTTCCCTCGGGTCATGCGATATCTCTGAGGAATACTCGTTT

GCATGCGTCCAGCGCGGCGAGCCCAGACCGACGGTTTTGGAGTTTGACAGCGAGTACGAG

CTGCTGGTTGCCTTCCTGACCTTTCTCAAGCAGTACTCTCCCGAGTTCGCCACCGGCTAC

AACATCGTTAATTTTGACTGGGCGTACATAGTTAACAAGGTAACGTCGGTGTATAACATC

AAGCTGGACGGGTACGGCAAGTTCAACAAAGGGGGGCTGTTTAAGGTGTGGGACATCGCC

ACGAACCATTTTCAGAAGAAGAGCAAGGTGAAAATCAATGGCCTGATATCTCTAGACATG

TATTCTGTGGCGACGGAAAAGCTAAAGCTACCCAGCTACAAACTCGACGCGGTCGTGGGA

GACGTCCTCGGCGAGCATAAGATAGACCTTCCCTATAAAGAAATACCCTCCTATTACGCG

GGAGGGCCTGACCGGAGGGGCGTAATAGGAGAGTATTGTATCCAGGACTCTAGGCTGGTG

GGCAAGCTGTTTTTTAAGTACCTCCCCCATCTGGAACTATCGGCGGTGGCCAAACTCGCC

CGTATCACCCTAACGCGGGTAATTTTTGACGGTCAGCAAATTCGCGTGTACACGTGCTTG

CTGAAACTCGCCCGCGAGAGAAATTTCATTTTGCCAGACAACAGACGCCGGTTTGACAGT

CAGGCAGACGCCGCGTCAGAGACTTCGGAGTTGGCTATGGATAGCCAAAGCCACGCCTTC

GACAGTACAGACGAACCCGACGGTGTGGACGGTACCCCGGACGCCGCAGGATCTGGCGCT

ACTTCTGAAAACGGAGGCGGGAAGCCCGGCGTCGGGAGGGCCGTGGGCTACCAGGGAGCA

AAGGTTCTAGACCCCGTATCCGGCTTTCATGTGGACCCCGTGGTTGTGTTTGACTTCGCT

AGCTTATACCCAAGCATTATCCAGGCCCATAACCTCTGTTTCACCACCCTGGCGCTCGAT

GAAGTGGATCTGGCCGGGCTTCAACCATCCGTCGACTACTCGACGTTCGAGGTGGGTGAC

CAAAAGTTATTTTTTGTCCACGCCCATATTCGCGAAAGCCTGCTTGGCATCTTGCTGCGC

GACTGGCTGGCCATGCGAAAGGCGGTGAGGGCGCGAATCCCCACCAGCACCCCCGAGGAG

GCAGTTTTACTAGATAAGCAGCAGTCTGCGATTAAGGTGATATGCAACTCGGTTTACGGA

TTCACGGGGGTGGCAAACGGCCTGTTGCCGTGTCTGAGGATAGCGGCTACCGTTACCACG

ATAGGACGCGACATGCTCCTCAAGACCCGAGATTACGTTCACTCTCGTTGGGCGACGCGC

GAGCTGCTGGAGGACAATTTTCCAGGGGCTATAGGTTTCCGAAACCACAAGCCTTACTCC

GTCAGGGTTATCTACGGAGACACCGACTCCGTGTTTATCAAGTTTGTGGGCCTGACGTAC

GAGGGGGTATCGGAGCTGGGGGATGCTATGTCGCGTCAGATTTCAGCGGACCTCTTTAGA

GCGCCCATCAAACTGGAGTGTGAGAAGACCTTTCAGCGACTGCTGCTGATCACCAAGAAG

AAGTACATAGGTGTCATAAACGGGGGGAAGATGCTCATGAAGGGGGTCGACCTGGTCCGC

AAAAATAACTGCTCTTTCATAAACTTGTACGCGCGACATCTGGTAGATCTTTTGTTGTAC

GACGAGGATGTGGCCACGGCGGCAGCAGAGGTGACAGACGTGCCTCCCGCAGAATGGGTG

GGGCGCCCGCTACCGAGCGGCTTTGACAAGTTTGGGCGAGTGCTGGTAGAGGCGTACAAC

CGTATCACTGCCCCCAACTTGGACGTGCGCGAGTTCGTTATGACTGCTGAGCTGAGCCGC

TCACCCGAATCGTATACCAACAAGCGCCTGCCGCACCTCACCGTCTACTTTAAGCTCGCC

ATGAGGAATGAAGAACTGCCCAGCGTAAAAGAGAGAATTCCGTATGTGATAGTTGCGCAG

ACCGAGGCCGCGGAACGCGAAGCGGGTGTAGTAAACTCAATGCGCGGTACCGCCCAAAAC

CCCGTGGTAACCAAGACCGCACGCCCCCAACCTAAACGCAAACTGCTGGTTTCCGACCTC

GCCGAAGACCCGACCTATGTTTCCGAGAATGACGTACCGCTAAACACAGACTACTATTTC

TCCCACCTGTTGGGTACCATAAGCGTGACCTTTAAGGCTCTATTCGGAAATGATGTGAGA

ACAACAGAAAATCTTTTAAAGCGGTTTATTCCGGAAACCCCCCACAAGACCCCCACGAAA

ACCCAGGCACTGCTTGAGCGCGCCGGCTTTGAAAAGCTGACGCCCTTTACACCGGAGGAA

GAAAGTCGTCGAATACTGCATACAGTTTTTTGTACTCTAGAAGCAGCTCCCCATCAAAGC

TGA

>KU206448.1

ATGGCGGCGCGCGAACAGGCCAACTCCGTTCGCAGGAGCGGGTTTTTTAATCCGTTTATT

GGCAAGAGGCCATTTTTCAGGCCGGGAAGCGGGCAGACCGCGGAGACAGAGCGCCCAAGG

CCCCCCCAACACTCGTACTGCACAGAGGTGGGTAGCTTTAAGTTTATAGCTCCAAGATGC

CTCGATGAAGAAGCCCCCGCCGACCAGCGACGCGGTGTACACGTGGGCACCTTGGAGCGC

CCACCCAAAGTGTACTGCGATGGCTCAGAGTACGACGTGCTGAACTTTGCCTCCGGAGGT

TGCTGGCCTCGCAGGATTCGGGTTTGGAACGGCCAGGATTTTCGGGGCGATGGATTCAAC

CCCAGATTTGAGAGATTTCACGTGTACGACATAGTAGAGACTTCGGAGAGCGCCTCACAC

GATGACCCATCCAGGTTTGCTGAGCTATCCCGCCCAAGCGGGTCTGTGGTAACACTGCTG

GGAATGAGCGAGTGTGGAAAGCGAGTCGCGGTTCACGTCTATGGTGTGCGCCATTATTTT

TACATGGCAAAGGCGGAGGTGGATAGCGCTTGTGGAATAACCACCGAGGCAGAACTCGTG

CGCGCAATGGTGGACTGCGCGCACAGCTCGGCTTTGAGCGCGGCCCTGGGAAATGGCAAC

GGCGGCAAACAGAGCGGCGGCAGCGGCGGGGGATGGTGGGGCGGAAAGCACGTGTCTGCG

GACTGCTTCAAAGTGGAGACCGTGTGCCACACGACGCTGTACTACTTTGGATCTAAGCCA

GCTCTCTACTATAGAGTATCTGCCTCCAGCAGCCGCCTGGGAGGGTTCATCTGCGACAAC

TTTCACCCGGAGATTACAAAATTCGAGGGGAGCGTGGACGTGACCACGCGGCTGTTGTTG

GACAATGAAAATTTTACCAGTTTCGGGTGGTACCGCCTGCGACCCGGCACCCACGGAGAG

CGTGTTCAACTTCGCCCCGTTGAGCGACACGTCACCTCAAGCGACGTGGAGATTAACTGT

ACTCCCGATAACCTGGAGCCGATACCAGACGAGGCTGCCTGGCCCGACTATAAGCTCATG

TGCTTTGATATAGAGTGTAAAGCTGGAACGGGTAACGAAATGGCGTTCCCAGTGGCAACT

AACCAAGAGGACCTGGTCATCCAGATCTCCTGTCTGCTGTACTCGCTTGCTACTCAGAAC

CACGAACACACCCTGCTGTTTTCCCTCGGGTCATGCGATATCTCTGAGGAATACTCGTTT

GCATGCGTCCAGCGCGGCGAGCCCAGACCGACGGTTTTGGAGTTTGACAGCGAGTACGAG

CTGCTGGTTGCCTTCCTGACCTTTCTCAAGCAGTACTCTCCCGAGTTCGCCACCGGCTAC

AACATCGTTAATTTTGACTGGGCGTACATAGTTAACAAGGTAACGTCGGTGTATAACATC

AAGCTGGACGGGTACGGCAAGTTCAACAAAGGGGGGCTGTTTAAGGTGTGGGACATCGCC

ACGAACCATTTTCAGAAGAAGAGCAAGGTGAAAATCAATGGCCTGATATCTCTAGACATG

TATTCTGTGGCGACGGAAAAGCTAAAGCTACCCAGCTACAAACTCGACGCGGTCGTGGGA

GACGTCCTCGGCGAGCATAAGATAGACCTTCCCTATAAAGAAATACCCTCCTATTACGCG

GGAGGGCCTGACCGGAGGGGCGTAATAGGAGAGTATTGTATCCAGGACTCTAGGCTGGTG

GGCAAGCTGTTTTTTAAGTACCTCCCCCATCTGGAACTATCGGCGGTGGCCAAACTCGCC

CGTATCACCCTAACGCGGGTAATTTTTGACGGTCAGCAAATTCGCGTGTACACGTGCTTG

CTGAAACTCGCCCGCGAGAGAAATTTCATTTTGCCAGACAACAGACGCCGGTTTGACAGT

CAGGCAGACGCCGCGTCAGAGACTTCGGAGTTGGCTATGGATAGCCAAAGCCACGCCTTC

GACAGTACAGACGAACCCGACGGTGTGGACGGTACCCCGGACGCCGCAGGATCTGGCGCT

ACTTCTGAAAACGGAGGCGGGAAGCCCGGCGTCGGGAGGGCCGTGGGCTACCAGGGAGCA

AAGGTTCTAGACCCCGTATCCGGCTTTCATGTGGACCCCGTGGTTGTGTTTGACTTCGCT

AGCTTATACCCAAGCATTATCCAGGCCCATAACCTCTGTTTCACCACCCTGGCGCTCGAT

GAAGTGGATCTGGCCGGGCTTCAACCATCCGTCGACTACTCGACGTTCGAGGTGGGTGAC

CAAAAGTTATTTTTTGTCCACGCCCATATTCGCGAAAGCCTGCTTGGCATCTTGCTGCGC

GACTGGCTGGCCATGCGAAAGGCGGTGAGGGCGCGAATCCCCACCAGCACCCCCGAGGAG

GCAGTTTTACTAGATAAGCAGCAGTCTGCGATTAAGGTGATATGCAACTCGGTTTACGGA

TTCACGGGGGTGGCAAACGGCCTGTTGCCGTGTCTGAGGATAGCGGCTACCGTTACCACG

ATAGGACGCGACATGCTCCTCAAGACCCGAGATTACGTTCACTCTCGTTGGGCGACGCGC

GAGCTGCTGGAGGACAATTTTCCAGGGGCTATAGGTTTCCGAAACCACAAGCCTTACTCC

GTCAGGGTTATCTACGGAGACACCGACTCCGTGTTTATCAAGTTTGTGGGCCTGACGTAC

GAGGGGGTATCGGAGCTGGGGGATGCTATGTCGCGTCAGATTTCAGCGGACCTCTTTAGA

GCGCCCATCAAACTGGAGTGTGAGAAGACCTTTCAGCGACTGCTGCTGATCACCAAGAAG

AAGTACATAGGTGTCATAAACGGGGGGAAGATGCTCATGAAGGGGGTCGACCTGGTCCGC

AAAAATAACTGCTCTTTCATAAACTTGTACGCGCGACATCTGGTAGATCTTTTGTTGTAC

GACGAGGATGTGGCCACGGCGGCAGCAGAGGTGACAGACGTGCCTCCCGCAGAATGGGTG

GGGCGCCCGCTACCGAGCGGCTTTGACAAGTTTGGGCGAGTGCTGGTAGAGGCGTACAAC

CGTATCACTGCCCCCAACTTGGACGTGCGCGAGTTCGTTATGACTGCTGAGCTGAGCCGC

TCACCCGAATCGTATACCAACAAGCGCCTGCCGCACCTCACCGTCTACTTTAAGCTCGCC

ATGAGGAATGAAGAACTGCCCAGCGTAAAAGAGAGAATTCCGTATGTGATAGTTGCGCAG

ACCGAGGCCGCGGAACGCGAAGCGGGTGTAGTAAACTCAATGCGCGGTACCGCCCAAAAC

CCCGTGGTAACCAAGACCGCACGCCCCCAACCTAAACGCAAACTGCTGGTTTCCGACCTC

GCCGAAGACCCGACCTATGTTTCCGAGAATGACGTACCGCTAAACACAGACTACTATTTC

TCCCACCTGTTGGGTACCATAAGCGTGACCTTTAAGGCTCTATTCGGAAATGATGTGAGA

ACAACAGAAAATCTTTTAAAGCGGTTTATTCCGGAAACCCCCCACAAGACCCCCACGAAA

ACCCAGGCACTGCTTGAGCGCGCCGGCTTTGAAAAGCTGACGCCCTTTACACCGGAGGAA

GAAAGTCGTCGAATACTGCATACAGTTTTTTGTACTCTAGAAGCAGCTCCCCATCAAAGC

TGA

>KU206449.1

ATGGCGGCGCGCGAACAGGCCAACTCCGTTCGCAGGAGCGGGTTTTTTAATCCGTTTATT

GGCAAGAGGCCATTTTTCAGGCCGGGAAGCGGGCAGACCGCGGAGACAGAGCGCCCAAGG

CCCCCCCAACACTCGTACTGCACAGAGGTGGGTAGCTTTAAGTTTATAGCTCCAAGATGC

CTCGATGAAGAAGCCCCCGCCGACCAGCGACGCGGTGTACACGTGGGCACCTTGGAGCGC

CCACCCAAAGTGTACTGCGATGGCTCAGAGTACGACGTGCTGAACTTTGCCTCCGGAGGT

TGCTGGCCTCGCAGGATTCGGGTTTGGAACGGCCAGGATTTTCGGGGCGATGGATTCAAC

CCCAGATTTGAGAGATTTCACGTGTACGACATAGTAGAGACTTCGGAGAGCGCCTCACAC

GATGACCCATCCAGGTTTGCTGAGCTATCCCGCCCAAGCGGGTCTGTGGTAACACTGCTG

GGAATGAGCGAGTGTGGAAAGCGAGTCGCGGTTCACGTCTATGGTGTGCGCCATTATTTT

TACATGGCAAAGGCGGAGGTGGATAGCGCTTGTGGAATAACCACCGAGGCAGAACTCGTG

CGCGCAATGGTGGACTGCGCGCACAGCTCGGCTTTGAGCGCGGCCCTGGGAAATGGCAAC

GGCGGCAAACAGAGCGGCGGCAGCGGCGGGGGATGGTGGGGCGGAAAGCACGTGTCTGCG

GACTGCTTCAAAGTGGAGACCGTGTGCCACACGACGCTGTACTACTTTGGATCTAAGCCA

GCTCTCTACTATAGAGTATCTGCCTCCAGCAGCCGCCTGGGAGGGTTCATCTGCGACAAC

TTTCACCCGGAGATTACAAAATTCGAGGGGAGCGTGGACGTGACCACGCGGCTGTTGTTG

GACAATGAAAATTTTACCAGTTTCGGGTGGTACCGCCTGCGACCCGGCACCCACGGAGAG

CGTGTTCAACTTCGCCCCGTTGAGCGACACGTCACCTCAAGCGACGTGGAGATTAACTGT

ACTCCCGATAACCTGGAGCCGATACCAGACGAGGCTGCCTGGCCCGACTATAAGCTCATG

TGCTTTGATATAGAGTGTAAAGCTGGAACGGGTAACGAAATGGCGTTCCCAGTGGCAACT

AACCAAGAGGACCTGGTCATCCAGATCTCCTGTCTGCTGTACTCGCTTGCTACTCAGAAC

CACGAACACACCCTGCTGTTTTCCCTCGGGTCATGCGATATCTCTGAGGAATACTCGTTT

GCATGCGTCCAGCGCGGCGAGCCCAGACCGACGGTTTTGGAGTTTGACAGCGAGTACGAG

CTGCTGGTTGCCTTCCTGACCTTTCTCAAGCAGTACTCTCCCGAGTTCGCCACCGGCTAC

AACATCGTTAATTTTGACTGGGCGTACATAGTTAACAAGGTAACGTCGGTGTATAACATC

AAGCTGGACGGGTACGGCAAGTTCAACAAAGGGGGGCTGTTTAAGGTGTGGGACATCGCC

ACGAACCATTTTCAGAAGAAGAGCAAGGTGAAAATCAATGGCCTGATATCTCTAGACATG

TATTCTGTGGCGACGGAAAAGCTAAAGCTACCCAGCTACAAACTCGACGCGGTCGTGGGA

GACGTCCTCGGCGAGCATAAGATAGACCTTCCCTATAAAGAAATACCCTCCTATTACGCG

GGAGGGCCTGACCGGAGGGGCGTAATAGGAGAGTATTGTATCCAGGACTCTAGGCTGGTG

GGCAAGCTGTTTTTTAAGTACCTCCCCCATCTGGAACTATCGGCGGTGGCCAAACTCGCC

CGTATCACCCTAACGCGGGTAATTTTTGACGGTCAGCAAATTCGCGTGTACACGTGCTTG

CTGAAACTCGCCCGCGAGAGAAATTTCATTTTGCCAGACAACAGACGCCGGTTTGACAGT

CAGGCAGACGCCGCGTCAGAGACTTCGGAGTTGGCTATGGATAGCCAAAGCCACGCCTTC

GACAGTACAGACGAACCCGACGGTGTGGACGGTACCCCGGACGCCGCAGGATCTGGCGCT

ACTTCTGAAAACGGAGGCGGGAAGCCCGGCGTCGGGAGGGCCGTGGGCTACCAGGGAGCA

AAGGTTCTAGACCCCGTATCCGGCTTTCATGTGGACCCCGTGGTTGTGTTTGACTTCGCT

AGCTTATACCCAAGCATTATCCAGGCCCATAACCTCTGTTTCACCACCCTGGCGCTCGAT

GAAGTGGATCTGGCCGGGCTTCAACCATCCGTCGACTACTCGACGTTCGAGGTGGGTGAC

CAAAAGTTATTTTTTGTCCACGCCCATATTCGCGAAAGCCTGCTTGGCATCTTGCTGCGC

GACTGGCTGGCCATGCGAAAGGCGGTGAGGGCGCGAATCCCCACCAGCACCCCCGAGGAG

GCAGTTTTACTAGATAAGCAGCAGTCTGCGATTAAGGTGATATGCAACTCGGTTTACGGA

TTCACGGGGGTGGCAAACGGCCTGTTGCCGTGTCTGAGGATAGCGGCTACCGTTACCACG

ATAGGACGCGACATGCTCCTCAAGACCCGAGATTACGTTCACTCTCGTTGGGCGACGCGC

GAGCTGCTGGAGGACAATTTTCCAGGGGCTATAGGTTTCCGAAACCACAAGCCTTACTCC

GTCAGGGTTATCTACGGAGACACCGACTCCGTGTTTATCAAGTTTGTGGGCCTGACGTAC

GAGGGGGTATCGGAGCTGGGGGATGCTATGTCGCGTCAGATTTCAGCGGACCTCTTTAGA

GCGCCCATCAAACTGGAGTGTGAGAAGACCTTTCAGCGACTGCTGCTGATCACCAAGAAG

AAGTACATAGGTGTCATAAACGGGGGGAAGATGCTCATGAAGGGGGTCGACCTGGTCCGC

AAAAATAACTGCTCTTTCATAAACTTGTACGCGCGACATCTGGTAGATCTTTTGTTGTAC

GACGAGGATGTGGCCACGGCGGCAGCAGAGGTGACAGACGTGCCTCCCGCAGAATGGGTG

GGGCGCCCGCTACCGAGCGGCTTTGACAAGTTTGGGCGAGTGCTGGTAGAGGCGTACAAC

CGTATCACTGCCCCCAACTTGGACGTGCGCGAGTTCGTTATGACTGCTGAGCTGAGCCGC

TCACCCGAATCGTATACCAACAAGCGCCTGCCGCACCTCACCGTCTACTTTAAGCTCGCC

ATGAGGAATGAAGAACTGCCCAGCGTAAAAGAGAGAATTCCGTATGTGATAGTTGCGCAG

ACCGAGGCCGCGGAACGCGAAGCGGGTGTAGTAAACTCAATGCGCGGTACCGCCCAAAAC

CCCGTGGTAACCAAGACCGCACGCCCCCAACCTAAACGCAAACTGCTGGTTTCCGACCTC

GCCGAAGACCCGACCTATGTTTCCGAGAATGACGTACCGCTAAACACAGACTACTATTTC

TCCCACCTGTTGGGTACCATAAGCGTGACCTTTAAGGCTCTATTCGGAAATGATGTGAGA

ACAACAGAAAATCTTTTAAAGCGGTTTATTCCGGAAACCCCCCACAAGACCCCCACGAAA

ACCCAGGCACTGCTTGAGCGCGCCGGCTTTGAAAAGCTGACGCCCTTTACACCGGAGGAA

GAAAGTCGTCGAATACTGCATACAGTTTTTTGTACTCTAGAAGCAGCTCCCCATCAAAGC

TGA

>KU206450.1

ATGGCGGCGCGCGAACAGGCCAACTCCGTTCGCAGGAGCGGGTTTTTTAATCCGTTTATT

GGCAAGAGGCCATTTTTCAGGCCGGGAAGCGGGCAGACCGCGGAGACAGAGCGCCCAAGG

CCCCCCCAACACTCGTACTGCACAGAGGTGGGTAGCTTTAAGTTTATAGCTCCAAGATGC

CTCGATGAAGAAGCCCCCGCCGACCAGCGACGCGGTGTACACGTGGGCACCTTGGAGCGC

CCACCCAAAGTGTACTGCGATGGCTCAGAGTACGACGTGCTGAACTTTGCCTCCGGAGGT

TGCTGGCCTCGCAGGATTCGGGTTTGGAACGGCCAGGATTTTCGGGGCGATGGATTCAAC

CCCAGATTTGAGAGATTTCACGTGTACGACATAGTAGAGACTTCGGAGAGCGCCTCACAC

GATGACCCATCCAGGTTTGCTGAGCTATCCCGCCCAAGCGGGTCTGTGGTAACACTGCTG

GGAATGAGCGAGTGTGGAAAGCGAGTCGCGGTTCACGTCTATGGTGTGCGCCATTATTTT

TACATGGCAAAGGCGGAGGTGGATAGCGCTTGTGGAATAACCACCGAGGCAGAACTCGTG

CGCGCAATGGTGGACTGCGCGCACAGCTCGGCTTTGAGCGCGGCCCTGGGAAATGGCAAC

GGCGGCAAACAGAGCGGCGGCAGCGGCGGGGGATGGTGGGGCGGAAAGCACGTGTCTGCG

GACTGCTTCAAAGTGGAGACCGTGTGCCACACGACGCTGTACTACTTTGGATCTAAGCCA

GCTCTCTACTATAGAGTATCTGCCTCCAGCAGCCGCCTGGGAGGGTTCATCTGCGACAAC

TTTCACCCGGAGATTACAAAATTCGAGGGGAGCGTGGACGTGACCACGCGGCTGTTGTTG

GACAATGAAAATTTTACCAGTTTCGGGTGGTACCGCCTGCGACCCGGCACCCACGGAGAG

CGTGTTCAACTTCGCCCCGTTGAGCGACACGTCACCTCAAGCGACGTGGAGATTAACTGT

ACTCCCGATAACCTGGAGCCGATACCAGACGAGGCTGCCTGGCCCGACTATAAGCTCATG

TGCTTTGATATAGAGTGTAAAGCTGGAACGGGTAACGAAATGGCGTTCCCAGTGGCAACT

AACCAAGAGGACCTGGTCATCCAGATCTCCTGTCTGCTGTACTCGCTTGCTACTCAGAAC

CACGAACACACCCTGCTGTTTTCCCTCGGGTCATGCGATATCTCTGAGGAATACTCGTTT

GCATGCGTCCAGCGCGGCGAGCCCAGACCGACGGTTTTGGAGTTTGACAGCGAGTACGAG

CTGCTGGTTGCCTTCCTGACCTTTCTCAAGCAGTACTCTCCCGAGTTCGCCACCGGCTAC

AACATCGTTAATTTTGACTGGGCGTACATAGTTAACAAGGTAACGTCGGTGTATAACATC

AAGCTGGACGGGTACGGCAAGTTCAACAAAGGGGGGCTGTTTAAGGTGTGGGACATCGCC

ACGAACCATTTTCAGAAGAAGAGCAAGGTGAAAATCAATGGCCTGATATCTCTAGACATG

TATTCTGTGGCGACGGAAAAGCTAAAGCTACCCAGCTACAAACTCGACGCGGTCGTGGGA

GACGTCCTCGGCGAGCATAAGATAGACCTTCCCTATAAAGAAATACCCTCCTATTACGCG

GGAGGGCCTGACCGGAGGGGCGTAATAGGAGAGTATTGTATCCAGGACTCTAGGCTGGTG

GGCAAGCTGTTTTTTAAGTACCTCCCCCATCTGGAACTATCGGCGGTGGCCAAACTCGCC

CGTATCACCCTAACGCGGGTAATTTTTGACGGTCAGCAAATTCGCGTGTACACGTGCTTG

CTGAAACTCGCCCGCGAGAGAAATTTCATTTTGCCAGACAACAGACGCCGGTTTGACAGT

CAGGCAGACGCCGCGTCAGAGACTTCGGAGTTGGCTATGGATAGCCAAAGCCACGCCTTC

GACAGTACAGACGAACCCGACGGTGTGGACGGTACCCCGGACGCCGCAGGATCTGGCGCT

ACTTCTGAAAACGGAGGCGGGAAGCCCGGCGTCGGGAGGGCCGTGGGCTACCAGGGAGCA

AAGGTTCTAGACCCCGTATCCGGCTTTCATGTGGACCCCGTGGTTGTGTTTGACTTCGCT

AGCTTATACCCAAGCATTATCCAGGCCCATAACCTCTGTTTCACCACCCTGGCGCTCGAT

GAAGTGGATCTGGCCGGGCTTCAACCATCCGTCGACTACTCGACGTTCGAGGTGGGTGAC

CAAAAGTTATTTTTTGTCCACGCCCATATTCGCGAAAGCCTGCTTGGCATCTTGCTGCGC

GACTGGCTGGCCATGCGAAAGGCGGTGAGGGCGCGAATCCCCACCAGCACCCCCGAGGAG

GCAGTTTTACTAGATAAGCAGCAGTCTGCGATTAAGGTGATATGCAACTCGGTTTACGGA

TTCACGGGGGTGGCAAACGGCCTGTTGCCGTGTCTGAGGATAGCGGCTACCGTTACCACG

ATAGGACGCGACATGCTCCTCAAGACCCGAGATTACGTTCACTCTCGTTGGGCGACGCGC

GAGCTGCTGGAGGACAATTTTCCAGGGGCTATAGGTTTCCGAAACCACAAGCCTTACTCC

GTCAGGGTTATCTACGGAGACACCGACTCCGTGTTTATCAAGTTTGTGGGCCTGACGTAC

GAGGGGGTATCGGAGCTGGGGGATGCTATGTCGCGTCAGATTTCAGCGGACCTCTTTAGA

GCGCCCATCAAACTGGAGTGTGAGAAGACCTTTCAGCGACTGCTGCTGATCACCAAGAAG

AAGTACATAGGTGTCATAAACGGGGGGAAGATGCTCATGAAGGGGGTCGACCTGGTCCGC

AAAAATAACTGCTCTTTCATAAACTTGTACGCGCGACATCTGGTAGATCTTTTGTTGTAC

GACGAGGATGTGGCCACGGCGGCAGCAGAGGTGACAGACGTGCCTCCCGCAGAATGGGTG

GGGCGCCCGCTACCGAGCGGCTTTGACAAGTTTGGGCGAGTGCTGGTAGAGGCGTACAAC

CGTATCACTGCCCCCAACTTGGACGTGCGCGAGTTCGTTATGACTGCTGAGCTGAGCCGC

TCACCCGAATCGTATACCAACAAGCGCCTGCCGCACCTCACCGTCTACTTTAAGCTCGCC

ATGAGGAATGAAGAACTGCCCAGCGTAAAAGAGAGAATTCCGTATGTGATAGTTGCGCAG

ACCGAGGCCGCGGAACGCGAAGCGGGTGTAGTAAACTCAATGCGCGGTACCGCCCAAAAC

CCCGTGGTAACCAAGACCGCACGCCCCCAACCTAAACGCAAACTGCTGGTTTCCGACCTC

GCCGAAGACCCGACCTATGTTTCCGAGAATGACGTACCGCTAAACACAGACTACTATTTC

TCCCACCTGTTGGGTACCATAAGCGTGACCTTTAAGGCTCTATTCGGAAATGATGTGAGA

ACAACAGAAAATCTTTTAAAGCGGTTTATTCCGGAAACCCCCCACAAGACCCCCACGAAA

ACCCAGGCACTGCTTGAGCGCGCCGGCTTTGAAAAGCTGACGCCCTTTACACCGGAGGAA

GAAAGTCGTCGAATACTGCATACAGTTTTTTGTACTCTAGAAGCAGCTCCCCATCAAAGC

TGA

>KU206451.1

ATGGCGGCGCGCGAACAGGCCAACTCCGTTCGCAGGAGCGGGTTTTTTAATCCGTTTATT

GGCAAGAGGCCATTTTTCAGGCCGGGAAGCGGGCAGACCGCGGAGACAGAGCGCCCAAGG

CCCCCCCAACACTCGTACTGCACAGAGGTGGGTAGCTTTAAGTTTATAGCTCCAAGATGC

CTCGATGAAGAAGCCCCCGCCGACCAGCGACGCGGTGTACACGTGGGCACCTTGGAGCGC

CCACCCAAAGTGTACTGCGATGGCTCAGAGTACGACGTGCTGAACTTTGCCTCCGGAGGT

TGCTGGCCTCGCAGGATTCGGGTTTGGAACGGCCAGGATTTTCGGGGCGATGGATTCAAC

CCCAGATTTGAGAGATTTCACGTGTACGACATAGTAGAGACTTCGGAGAGCGCCTCACAC

GATGACCCATCCAGGTTTGCTGAGCTATCCCGCCCAAGCGGGTCTGTGGTAACACTGCTG

GGAATGAGCGAGTGTGGAAAGCGAGTCGCGGTTCACGTCTATGGTGTGCGCCATTATTTT

TACATGGCAAAGGCGGAGGTGGATAGCGCTTGTGGAATAACCACCGAGGCAGAACTCGTG

CGCGCAATGGTGGACTGCGCGCACAGCTCGGCTTTGAGCGCGGCCCTGGGAAATGGCAAC

GGCGGCAAACAGAGCGGCGGCAGCGGCGGGGGATGGTGGGGCGGAAAGCACGTGTCTGCG

GACTGCTTCAAAGTGGAGACCGTGTGCCACACGACGCTGTACTACTTTGGATCTAAGCCA

GCTCTCTACTATAGAGTATCTGCCTCCAGCAGCCGCCTGGGAGGGTTCATCTGCGACAAC

TTTCACCCGGAGATTACAAAATTCGAGGGGAGCGTGGACGTGACCACGCGGCTGTTGTTG

GACAATGAAAATTTTACCAGTTTTGGGTGGTACCGCCTGCGACCCGGCACCCACGGAGAG

CGTGTTCAACTTCGCCCCGTTGAGCGACACGTCACCTCAAGCGACGTGGAGATTAACTGT

ACTCCCGATAACCTGGAGCCGATACCAGACGAGGCTGCCTGGCCCGACTATAAGCTCATG

TGCTTTGATATAGAGTGTAAAGCTGGAACGGGTAACGAAATGGCGTTCCCAGTGGCAACT

AACCAAGAGGACCTGGTCATCCAGATCTCCTGTCTGCTGTACTCGCTTGCTACTCAGAAC

CACGAACACACCCTGCTGTTTTCCCTCGGGTCATGCGATATCTCTGAGGAATACTCGTTT

GCATGCGTCCAGCGCGGCGAGCCCAGACCGACGGTTTTGGAGTTTGACAGCGAGTACGAG

CTGCTGGTTGCCTTCCTGACCTTTCTCAAGCAGTACTCTCCCGAGTTCGCCACCGGCTAC

AACATCGTTAATTTTGACTGGGCGTACATAGTTAACAAGGTAACGTCGGTGTATAACATC

AAGCTGGACGGGTACGGCAAGTTCAACAAAGGGGGGCTGTTTAAGGTGTGGGACATCGCC

ACGAACCATTTTCAGAAGAAGAGCAAGGTGAAAATCAATGGCCTGATATCTCTAGACATG

TATTCTGTGGCGACGGAAAAGCTAAAGCTACCCAGCTACAAACTCGACGCGGTCGTGGGA

GACGTCCTCGGCGAGCATAAGATAGACCTTCCCTATAAAGAAATACCCTCCTATTACGCG

GGAGGGCCTGACCGGAGGGGCGTAATAGGAGAGTATTGTATCCAGGACTCTAGGCTGGTG

GGCAAGCTGTTTTTTAAGTACCTCCCCCATCTGGAACTATCGGCGGTGGCCAAACTCGCC

CGTATCACCCTAACGCGGGTAATTTTTGACGGTCAGCAAATTCGCGTGTACACGTGCTTG

CTGAAACTCGCCCGCGAGAGAAATTTCATTTTGCCAGACAACAGACGCCGGTTTGACAGT

CAGGCAGACGCCGCGTCAGAGACTTCGGAGTTGGCTATGGATAGCCAAAGCCACGCCTTC

GACAGTACAGACGAACCCGACGGTGTGGACGGTACCCCGGACGCCGCAGGATCTGGCGCT

ACTTCTGAAAACGGAGGCGGGAAGCCCGGCGTCGGGAGGGCCGTGGGCTACCAGGGAGCA

AAGGTTCTAGACCCCGTATCCGGCTTTCATGTGGACCCCGTGGTTGTGTTTGACTTCGCT

AGCTTATACCCAAGCATTATCCAGGCCCATAACCTCTGTTTCACCACCCTGGCGCTCGAT

GAAGTGGATCTGGCCGGGCTTCAACCATCCGTCAACTACTCGACGTTCGAGGTGGGTGAC

CAAAAGTTATTTTTTGTCCACGCCCATATTCGCGAAAGCCTGCTTGGCATCTTGCTGCGC

GACTGGCTGGCCATGCGAAAGGCGGTGAGGGCGCGAATCCCCACCAGCACCCCCGAGGAG

GCAGTTTTACTAGATAAGCAGCAGTCTGCGATTAAGGTGATATGCAACTCGGTTTACGGA

TTCACGGGGGTGGCAAACGGCCTGTTGCCGTGTCTGAGGATAGCGGCTACCGTTACCACG

ATAGGACGCGACATGCTCCTCAAGACCCGAGATTACGTTCACTCTCGTTGGGCGACGCGC

GAGCTGCTGGAGGACAATTTTCCAGGGGCTATAGGTTTCCGAAACCACAAGCCTTACTCC

GTCAGGGTTATCTACGGAGACACCGACTCCGTGTTTATCAAGTTTGTGGGCCTGACGTAC

GAGGGGGTATCGGAGCTGGGGGATGCTATGTCGCGTCAGATTTCAGCGGACCTCTTTAGA

GCGCCCATCAAACTGGAGTGTGAGAAGACCTTTCAGCGACTGCTGCTGATCACCAAGAAG

AAGTACATAGGTGTCATAAACGGGGGGAAGATGCTCATGAAGGGGGTCGACCTGGTCCGC

AAAAATAACTGCTCTTTCATAAACTTGTACGCGCGACATCTGGTAGATCTTTTGTTGTAC

GACGAGGATGTGGCCACGGCGGCAGCAAAGGTGACAGACGTGCCTCCCGCAGAATGGGTG

GGGCGCCCGCTACCGAGCGGCTTTGACAAGTTTGGGCGAGTGCTGGTAGAGGCGTACAAC

CGTATCACTGCCCCCAACTTGGACGTGCGCGAGTTCGTTATGACTGCTGAGCTGAGCCGC

TCACCCGAATCGTATACCAACAAGCGCCTGCCGCACCTCACCGTCTACTTTAAGCTCGCC

ATGAGGAATGAAGAACTGCCCAGCGTAAAAGAGAGAATTCCGTATGTGATAGTTGCGCAG

ACCGAGGCCGCGGAACGCGAAGCGGGTGTAGTAAACTCAATGCGCGGTACCGCCCAAAAC

CCCGTGGTAACCAAGACCGCACGCCCCCAACCTAAACGCAAACTGCTGGTTTCCGACCTC

GCCGAAGACCCGACCTATGTTTCCGAGAATGACGTACCGCTAAACACAGACTACTATTTC

TCCCACCTGTTGGGTACCATAAGCGTGACCTTTAAGGCTCTATTCGGAAATGATGTGAGA

ACAACAGAAAATCTTTTAAAGCGGTTTATTCCGGAAACCCCCCACAAGACCCCCACGAAA

ACCCAGGCACTGCTTGAGCGCGCCGGCTTTGAAAAGCTGACGCCCTTTACACCGGAGGAA

GAAAGTCGTCGAATACTGCATACAGTTTTTTGTACTCTAGAAGCAGCTCCCCATCAAAGC

TGA

>KU206452.1

ATGGCGGCGCGCGAACAGGCCAACTCCGTTCGCAGGAGCGGGTTTTTTAATCCGTTTATT

GGCAAGAGGCCATTTTTCAGGCCGGGAAGCGGGCAGACCGCGGAGACAGAGCGCCCAAGG

CCCCCCCAACACTCGTACTGCACAGAGGTGGGTAGCTTTAAGTTTATAGCTCCAAGATGC

CTCGATGAAGAAGCCCCCGCCGACCAGCGACGCGGTGTACACGTGGGCACCTTGGAGCGC

CCACCCAAAGTGTACTGCGATGGCTCAGAGTACGACGTGCTGAACTTTGCCTCCGGAGGT

TGCTGGCCTCGCAGGATTCGGGTTTGGAACGGCCAGGATTTTCGGGGCGATGGATTCAAC

CCCAGATTTGAGAGATTTCACGTGTACGACATAGTAGAGACTTCGGAGAGCGCCTCACAC

GATGACCCATCCAGGTTTGCTGAGCTATCCCGCCCAAGCGGGTCTGTGGTAACACTGCTG

GGAATGAGCGAGTGTGGAAAGCGAGTCGCGGTTCACGTCTATGGTGTGCGCCATTATTTT

TACATGGCAAAGGCGGAGGTGGATAGCGCTTGTGGAATAACCACCGAGGCAGAACTCGTG

CGCGCAATGGTGGACTGCGCGCACAGCTCGGCTTTGAGCGCGGCCCTGGGAAATGGCAAC

GGCGGCAAACAGAGCGGCGGCAGCGGCGGGGGATGGTGGGGCGGAAAGCACGTGTCTGCG

GACTGCTTCAAAGTGGAGACCGTGTGCCACACGACGCTGTACTACTTTGGATCTAAGCCA

GCTCTCTACTATAGAGTATCTGCCTCCAGCAGCCGCCTGGGAGGGTTCATCTGCGACAAC

TTTCACCCGGAGATTACAAAATTCGAGGGGAGCGTGGACGTGACCACGCGGCTGTTGTTG

GACAATGAAAATTTTACCAGTTTTGGGTGGTACCGCCTGCGACCCGGCACCCACGGAGAG

CGTGTTCAACTTCGCCCCGTTGAGCGACACGTCACCTCAAGCGACGTGGAGATTAACTGT

ACTCCCGATAACCTGGAGCCGATACCAGACGAGGCTGCCTGGCCCGACTATAAGCTCATG

TGCTTTGATATAGAGTGTAAAGCTGGAACGGGTAACGAAATGGCGTTCCCAGTGGCAACT

AACCAAGAGGACCTGGTCATCCAGATCTCCTGTCTGCTGTACTCGCTTGCTACTCAGAAC

CACGAACACACCCTGCTGTTTTCCCTCGGGTCATGCGATATCTCTGAGGAATACTCGTTT

GCATGCGTCCAGCGCGGCGAGCCCAGACCGACGGTTTTGGAGTTTGACAGCGAGTACGAG

CTGCTGGTTGCCTTCCTGACCTTTCTCAAGCAGTACTCTCCCGAGTTCGCCACCGGCTAC

AACATCGTTAATTTTGACTGGGCGTACATAGTTAACAAGGTAACGTCGGTGTATAACATC

AAGCTGGACGGGTACGGCAAGTTCAACAAAGGGGGGCTGTTTAAGGTGTGGGACATCGCC

ACGAACCATTTTCAGAAGAAGAGCAAGGTGAAAATCAATGGCCTGATATCTCTAGACATG

TATTCTGTGGCGACGGAAAAGCTAAAGCTACCCAGCTACAAACTCGACGCGGTCGTGGGA

GACGTCCTCGGCGAGCATAAGATAGACCTTCCCTATAAAGAAATACCCTCCTATTACGCG

GGAGGGCCTGACCGGAGGGGCGTAATAGGAGAGTATTGTATCCAGGACTCTAGGCTGGTG

GGCAAGCTGTTTTTTAAGTACCTCCCCCATCTGGAACTATCGGCGGTGGCCAAACTCGCC

CGTATCACCCTAACGCGGGTAATTTTTGACGGTCAGCAAATTCGCGTGTACACGTGCTTG

CTGAAACTCGCCCGCGAGAGAAATTTCATTTTGCCAGACAACAGACGCCGGTTTGACAGT

CAGGCAGACGCCGCGTCAGAGACTTCGGAGTTGGCTATGGATAGCCAAAGCCACGCCTTC

GACAGTACAGACGAACCCGACGGTGTGGACGGTACCCCGGACGCCGCAGGATCTGGCGCT

ACTTCTGAAAACGGAGGCGGGAAGCCCGGCGTCGGGAGGGCCGTGGGCTACCAGGGAGCA

AAGGTTCTAGACCCCGTATCCGGCTTTCATGTGGACCCCGTGGTTGTGTTTGACTTCGCT

AGCTTATACCCAAGCATTATCCAGGCCCATAACCTCTGTTTCACCACCCTGGCGCTCGAT

GAAGTGGATCTGGCCGGGCTTCAACCATCCGTCAACTACTCGACGTTCGAGGTGGGTGAC

CAAAAGTTATTTTTTGTCCACGCCAATATTCGCGAAAGCCTGCTTGGCATCTTGCTGCGC

GACTGGCTGGCCATGCGAAAGGCGGTGAGGGCGCGAATCCCCACCAGCACCCCCGAGGAG

GCAGTTTTACTAGATAAGCAGCAGTCTGCGATTAAGGTGATATGCAACTCGGTTTACGGA

TTCACGGGGGTGGCAAACGGCCTGTTGCCGTGTCTGAGGATAGCGGCTACCGTTACCACG

ATAGGACGCGACATGCTCCTCAAGACCCGAGATTACGTTCACTCTCGTTGGGCGACGCGC

GAGCTGCTGGAGGACAATTTTCCAGGGGCTATAGGTTTCCGAAACCACAAGCCTTACTCC

GTCAGGGTTATCTACGGAGACACCGACTCCGTGTTTATCAAGTTTGTGGGCCTGACGTAC

GAGGGGGTATCGGAGCTGGGGGATGCTATGTCGCGTCAGATTTCAGCGGACCTCTTTAGA

GCGCCCATCAAACTGGAGTGTGAGAAGACCTTTCAGCGACTGCTGCTGATCACCAAGAAG

AAGTACATAGGTGTCATAAACGGGGGGAAGATGCTCATGAAGGGGGTCGACCTGGTCCGC

AAAAATAACTGCTCTTTCATAAACTTGTACGCGCGACATCTGGTAGATCTTTTGTTGTAC

GACGAGGATGTGGCCACGGCGGCAGCAAAGGTGACAGACGTGCCTCCCGCAGAATGGGTG

GGGCGCCCGCTACCGAGCGGCTTTGACAAGTTTGGGCGAGTGCTGGTAGAGGCGTACAAC

CGTATCACTGCCCCCAACTTGGACGTGCGCGAGTTCGTTATGACTGCTGAGCTGAGCCGC

TCACCCGAATCGTATACCAACAAGCGCCTGCCGCACCTCACCGTCTACTTTAAGCTCGCC

ATGAGGAATGAAGAACTGCCCAGCGTAAAAGAGAGAATTCCGTATGTGATAGTTGCGCAG

ACCGAGGCCGCGGAACGCGAAGCGGGTGTAGTAAACTCAATGCGCGGTACCGCCCAAAAC

CCCGTGGTAACCAAGACCGCACGCCCCCAACCTAAACGCAAACTGCTGGTTTCCGACCTC

GCCGAAGACCCGACCTATGTTTCCGAGAATGACGTACCGCTAAACACAGACTACTATTTC

TCCCACCTGTTGGGTACCATAAGCGTGACCTTTAAGGCTCTATTCGGAAATGATGTGAGA

ACAACAGAAAATCTTTTAAAGCGGTTTATTCCGGAAACCCCCCACAAGACCCCCACGAAA

ACCCAGGCACTGCTTGAGCGCGCCGGCTTTGAAAAGCTGACGCCCTTTACACCGGAGGAA

GAAAGTCGTCGAATACTGCATACAGTTTTTTGTACTCTAGAAGCAGCTCCCCATCAAAGC

TGA

>KU206453.1

ATGGCGGCGCGCGAACAGGCCAACTCCGTTCGCAGGAGCGGGTTTTTTAATCCGTTTATT

GGCAAGAGGCCATTTTTCAGGCCGGGAAGCGGGCAGACCGCGGAGACAGAGCGCCCAAGG

CCCCCCCAACACTCGTACTGCACAGAGGTGGGTAGCTTTAAGTTTATAGCTCCAAGATGC

CTCGATGAAGAAGCCCCCGCCGACCAGCGACGCGGTGTACACGTGGGCACCTTGGAGCGC

CCACCCAAAGTGTACTGCGATGGCTCAGAGTACGACGTGCTGAACTTTGCCTCCGGAGGT

TGCTGGCCTCGCAGGATTCGGGTTTGGAACGGCCAGGATTTTCGGGGCGATGGATTCAAC

CCCAGATTTGAGAGATTTCACGTGTACGACATAGTAGAGACTTCGGAGAGCGCCTCACAC

GATGACCCATCCAGGTTTGCTGAGCTATCCCGCCCAAGCGGGTCTGTGGTAACACTGCTG

GGAATGAGCGAGTGTGGAAAGCGAGTCGCGGTTCACGTCTATGGTGTGCGCCATTATTTT

TACATGGCAAAGGCGGAGGTGGATAGCGCTTGTGGAATAACCACCGAGGCAGAACTCGTG

CGCGCAATGGTGGACTGCGCGCACAGCTCGGCTTTGAGCGCGGCCCTGGGAAATGGCAAC

GGCGGCAAACAGAGCGGCGGCAGCGGCGGGGGATGGTGGGGCGGAAAGCACGTGTCTGCG

GACTGCTTCAAAGTGGAGACCGTGTGCCACACGACGCTGTACTACTTTGGATCTAAGCCA

GCTCTCTACTATAGAGTATCTGCCTCCAGCAGCCGCCTGGGAGGGTTCATCTGCGACAAC

TTTCACCCGGAGATTACAAAATTCGAGGGGAGCGTGGACGTGACCACGCGGCTGTTGTTG

GACAATGAAAATTTTACCAGTTTTGGGTGGTACCGCCTGCGACCCGGCACCCACGGAGAG

CGTGTTCAACTTCGCCCCGTTGAGCGACACGTCACCTCAAGCGACGTGGAGATTAACTGT

ACTCCCGATAACCTGGAGCCGATACCAGACGAGGCTGCCTGGCCCGACTATAAGCTCATG

TGCTTTGATATAGAGTGTAAAGCTGGAACGGGTAACGAAATGGCGTTCCCAGTGGCAACT

AACCAAGAGGACCTGGTCATCCAGATCTCCTGTCTGCTGTACTCGCTTGCTACTCAGAAC

CACGAACACACCCTGCTGTTTTCCCTCGGGTCATGCGATATCTCTGAGGAATACTCGTTT

GCATGCGTCCAGCGCGGCGAGCCCAGACCGACGGTTTTGGAGTTTGACAGCGAGTACGAG

CTGCTGGTTGCCTTCCTGACCTTTCTCAAGCAGTACTCTCCCGAGTTCGCCACCGGCTAC

AACATCGTTAATTTTGACTGGGCGTACATAGTTAACAAGGTAACGTCGGTGTATAACATC

AAGCTGGACGGGTACGGCAAGTTCAACAAAGGGGGGCTGTTTAAGGTGTGGGACATCGCC

ACGAACCATTTTCAGAAGAAGAGCAAGGTGAAAATCAATGGCCTGATATCTCTAGACATG

TATTCTGTGGCGACGGAAAAGCTAAAGCTACCCAGCTACAAACTCGACGCGGTCGTGGGA

GACGTCCTCGGCGAGCATAAGATAGACCTTCCCTATAAAGAAATACCCTCCTATTACGCG

GGAGGGCCTGACCGGAGGGGCGTAATAGGAGAGTATTGTATCCAGGACTCTAGGCTGGTG

GGCAAGCTGTTTTTTAAGTACCTCCCCCATCTGGAACTATCGGCGGTGGCCAAACTCGCC

CGTATCACCCTAACGCGGGTAATTTTTGACGGTCAGCAAATTCGCGTGTACACGTGCTTG

CTGAAACTCGCCCGCGAGAGAAATTTCATTTTGCCAGACAACAGACGCCGGTTTGACAGT

CAGGCAGACGCCGCGTCAGAGACTTCGGAGTTGGCTATGGATAGCCAAAGCCACGCCTTC

GACAGTACAGACGAACCCGACGGTGTGGACGGTACCCCGGACGCCGCAGGATCTGGCGCT

ACTTCTGAAAACGGAGGCGGGAAGCCCGGCGTCGGGAGGGCCGTGGGCTACCAGGGAGCA

AAGGTTCTAGACCCCGTATCCGGCTTTCATGTGGACCCCGTGGTTGTGTTTGACTTCGCT

AGCTTATACCCAAGCATTATCCAGGCCCATAACCTCTGTTTCACCACCCTGGCGCTCGAT

GAAGTGGATCTGGCCGGGCTTCAACCATCCGTCGACTACTCGACGTTCGAGGTGGGTGAC

CAAAAGTTATTTTTTGTCCACGCCCATATTCGCGAAAGCCTGCTTGGCATCTTGCTGCGC

GACTGGCTGGCCATGCGAAAGGCGGTGAGGGCGCGAATCCCCACCAGCACCCCCGAGGAG

GCAGTTTTACTAGATAAGCAGCAGTCTGCGATTAAGGTGATATGCAACTCGGTTTACGGA

TTCACGGGGGTGGCAAACGGCCTGTTGCCGTGTCTGAGGATAGCGGCTACCGTTACCACG

ATAGGACGCGACATGCTCCTCAAGACCCGAGATTACGTTCACTCTCGTTGGGCGACGCGC

GAGCTGCTGGAGGACAATTTTCCAGGGGCTATAGGTTTCCGAAACCACAAGCCTTACTCC

GTCAGGGTTATCTACGGAGACACCGACTCCGTGTTTATCAAGTTTGTGGGCCTGACGTAC

GAGGGGGTATCGGAGCTGGGGGATGCTATGTCGCGTCAGATTTCAGCGGACCTCTTTAGA

GCGCCCATCAAACTGGAGTGTGAGAAGACCTTTCAGCGACTGCTGCTGATCACCAAGAAG

AAGTACATAGGTGTCATAAACGGGGGGAAGATGCTCATGAAGGGGGTCGACCTGGTCCGC

AAAAATAACTGCTCTTTCATAAACTTGTACGCGCGACATCTGGTAGATCTTTTGTTGTAC

GACGAGGATGTGGCCACGGCGGCAGCAAAGGTGACAGACGTGCCTCCCGCAGAATGGGTG

GGGCGCCCGCTACCGAGCGGCTTTGACAAGTTTGGGCGAGTGCTGGTAGAGGCGTACAAC

CGTATCACTGCCCCCAACTTGGACGTGCGCGAGTTCGTTATGACTGCTGAGCTGAGCCGC

TCACCCGAATCGTATACCAACAAGCGCCTGCCGCACCTCACCGTCTACTTTAAGCTCGCC

ATGAGGAATGAAGAACTGCCCAGCGTAAAAGAGAGAATTCCGTATGTGATAGTTGCGCAG

ACCGAGGCCGCGGAACGCGAAGCGGGTGTAGTAAACTCAATGCGCGGTACCGCCCAAAAC

CCCGTGGTAACCAAGACCGCACGCCCCCAACCTAAACGCAAACTGCTGGTTTCCGACCTC

GCCGAAGACCCGACCTATGTTTCCGAGAATGACGTACCGCTAAACACAGACTACTATTTC

TCCCACCTGTTGGGTACCATAAGCGTGACCTTTAAGGCTCTATTCGGAAATGATGTGAGA

ACAACAGAAAATCTTTTAAAGCGGTTTATTCCGGAAACCCCCCACAAGACCCCCACGAAA

ACCCAGGCACTGCTTGAGCGCGCCGGCTTTGAAAAGCTGACGCCCTTTACACCGGAGGAA

GAAAGTCGTCGAATACTGCATACAGTTTTTTGTACTCTAGAAGCAGCTCCCCATCAAAGC

TGA

>KU206454.1

ATGGCGGCGCGCGAACAGGCCAACTCCGTTCGCAGGAGCGGGTTTTTTAATCCGTTTATT

GGCAAGAGGCCATTTTTCAGGCCGGGAAGCGGGCAGACCGCGGAGACAGAGCGCCCAAGG

CCCCCCCAACACTCGTACTGCACAGAGGTGGGTAGCTTTAAGTTTATAGCTCCAAGATGC

CTCGATGAAGAAGCCCCCGCCGACCAGCGACGCGGTGTACACGTGGGCACCTTGGAGCGC

CCACCCAAAGTGTACTGCGATGGCTCAGAGTACGACGTGCTGAACTTTGCCTCCGGAGGT

TGCTGGCCTCGCAGGATTCGGGTTTGGAACGGCCAGGATTTTCGGGGCGATGGATTCAAC

CCCAGATTTGAGAGATTTCACGTGTACGACATAGTAGAGACTTCGGAGAGCGCCTCACAC

GATGACCCATCCAGGTTTGCTGAGCTATCCCGCCCAAGCGGGTCTGTGGTAACACTGCTG

GGAATGAGCGAGTGTGGAAAGCGAGTCGCGGTTCACGTCTATGGTGTGCGCCATTATTTT

TACATGGCAAAGGCGGAGGTGGATAGCGCTTGTGGAATAACCACCGAGGCAGAACTCGTG

CGCGCAATGGTGGACTGCGCGCACAGCTCGGCTTTGAGCGCGGCCCTGGGAAATGGCAAC

GGCGGCAAACAGAGCGGCGGCAGCGGCGGGGGATGGTGGGGCGGAAAGCACGTGTCTGCG

GACTGCTTCAAAGTGGAGACCGTGTGCCACACGACGCTGTACTACTTTGGATCTAAGCCA

GCTCTCTACTATAGAGTATCTGCCTCCAGCAGCCGCCTGGGAGGGTTCATCTGCGACAAC

TTTCACCCGGAGATTACAAAATTCGAGGGGAGCGTGGACGTGACCACGCGGCTGTTGTTG

GACAATGAAAATTTTACCAGTTTCGGGTGGTACCGCCTGCGACCCGGCACCCACGGAGAG

CGTGTTCAACTTCGCCCCGTTGAGCGACACGTCACCTCAAGCGACGTGGAGATTAACTGT

ACTCCCGATAACCTGGAGCCGATACCAGACGAGGCTGCCTGGCCCGACTATAAGCTCATG

TGCTTTGATATAGAGTGTAAAGCTGGAACGGGTAACGAAATGGCGTTCCCAGTGGCAACT

AACCAAGAGGACCTGGTCATCCAGATCTCCTGTCTGCTGTACTCGCTTGCTACTCAGAAC

CACGAACACACCCTGCTGTTTTCCCTCGGGTCATGCGATATCTCTGAGGAATACTCGTTT

GCATGCGTCCAGCGCGGCGAGCCCAGACCGACGGTTTTGGAGTTTGACAGCGAGTACGAG

CTGCTGGTTGCCTTCCTGACCTTTCTCAAGCAGTACTCTCCCGAGTTCGCCACCGGCTAC

AACATCGTTAATTTTGACTGGGCGTACATAGTTAACAAGGTAACGTCGGTGTATAACATC

AAGCTGGACGGGTACGGCAAGTTCAACAAAGGGGGGCTGTTTAAGGTGTGGGACATCGCC

ACGAACCATTTTCAGAAGAAGAGCAAGGTGAAAATCAATGGCCTGATATCTCTAGACATG

TATTCTGTGGCGACGGAAAAGCTAAAGCTACCCAGCTACAAACTCGACGCGGTCGTGGGA

GACGTCCTCGGCGAGCATAAGATAGACCTTCCCTATAAAGAAATACCCTCCTATTACGCG

GGAGGGCCTGACCGGAGGGGCGTAATAGGAGAGTATTGTATCCAGGACTCTAGGCTGGTG

GGCAAGCTGTTTTTTAAGTACCTCCCCCATCTGGAACTATCGGCGGTGGCCAAACTCGCC

CGTATCACCCTAACGCGGGTAATTTTTGACGGTCAGCAAATTCGCGTGTACACGTGCTTG

CTGAAACTCGCCCGCGAGAGAAATTTCATTTTGCCAGACAACAGACGCCGGTTTGACAGT

CAGGCAGACGCCGCGTCAGAGACTTCGGAGTTGGCTATGGATAGCCAAAGCCACGCCTTC

GACAGTACAGACGAACCCGACGGTGTGGACGGTACCCCGGACGCCGCAGGATCTGGCGCT

ACTTCTGAAAACGGAGGCGGGAAGCCCGGCGTCGGGAGGGTCGTGGGCTACCAGGGAGCA

AAGGTTCTAGACCCCGTATCCGGCTTTCATGTGGACCCCGTGGTTGTGTTTGACTTCGCT

AGCTTATACCCAAGCATTATCCAGGCCCATAACCTCTGTTTCACCACCCTGGCGCTCGAT

GAAGTGGATCTGGCCGGGCTTCAACCATCCGTCGACTACTCGACGTTCGAGGTGGGTGAC

CAAAAGTTATTTTTTGTCCACGCCCATATTCGCGAAAGCCTGCTTGGCATCTTGCTGCGC

GACTGGCTGGCCATGCGAAAGGCGGTGAGGGCGCGAATCCCCACCAGCACCCCCGAGGAG

GCAGTTTTACTAGATAAGCAGCAGTCTGCGATTAAGGTGATATGCAACTCGGTTTACGGA

TTCACGGGGGTGGCAAACGGCCTGTTGCCGTGTCTGAGGATAGCGGCTACCGTTACCACG

ATAGGACGCGACATGCTCCTCAAGACCCGAGATTACGTTCACTCTCGTTGGGCGACGCGC

GAGCTGCTGGAGGACAATTTTCCAGGGGCTATAGGTTTCCGAAACCACAAGCCTTACTCC

GTCAGGGTTATCTACGGAGACACCGACTCCGTGTTTATCAAGTTTGTGGGCCTGACGTAC

GAGGGGGTATCGGAGCTGGGGGATGCTATGTCGCGTCAGATTTCAGCGGACCTCTTTAGA

GCGCCCATCAAACTGGAGTGTGAGAAGACCTTTCAGCGACTGCTGCTGATCACCAAGAAG

AAGTACATAGGTGTCATAAACGGGGGGAAGATGCTCATGAAGGGGGTCGACCTGGTCCGC

AAAAATAACTGCTCTTTCATAAACTTGTACGCGCGACATCTGGTAGATCTTTTGTTGTAC

GACGAGGATGTGGCCACGGCGGCAGCAGAGGTGACAGACGTGCCTCCCGCAGAATGGGTG

GGGCGCCCGCTACCGAGCGGCTTTGACAAGTTTGGGCGAGTGCTGGTAGAGGCGTACAAC

CGTATCACTGCCCCCAACTTGGACGTGCGCGAGTTCGTTATGACTGCTGAGCTGAGCCGC

TCACCCGAATCGTATACCAACAAGCGCCTGCCGCACCTCACCGTCTACTTTAAGCTCGCC

ATGAGGAATGAAGAACTGCCCAGCGTAAAAGAGAGAATTCCGTATGTGATAGTTGCGCAG

ACCGAGGCCGCGGAACGCGAAGCGGGTGTAGTAAACTCAATGCGCGGTACCGCCCAAAAC

CCCGTGGTAACCAAGACCGCACGCCCCCAACCTAAACGCAAACTGCTGGTTTCCGACCTC

GCCGAAGACCCGACCTATGTTTCCGAGAATGACGTACCGCTAAACACAGACTACTATTTC

TCCCACCTGTTGGGTACCATAAGCGTGACCTTTAAGGCTCTATTCGGAAATGATGTGAGA

ACAACAGAAAATCTTTTAAAGCGGTTTATTCCGGAAACCCCCCACAAGACCCCCACGAAA

ACCCAGGCACTGCTTGAGCGCGCCGGCTTTGAAAAGCTGACGCCCTTTACACCGGAGGAA

GAAAGTCGTCGAATACTGCATACAGTTTTTTGTACTCTAGAAGCAGCTCCCCATCAAAGC

TGA

>KU206455.1

ATGGCGGCGCGCGAACAGGCCAACTCCGTTCGCAGGAGCGGGTTTTTTAATCCGTTTATT

GGCAAGAGGCCATTTTTCAGGCCGGGAAGCGGGCAGACCGCGGAGACAGAGCGCCCAAGG

CCCCCCCAACACTCGTACTGCACAGAGGTGGGTAGCTTTAAGTTTATAGCTCCAAGATGC

CTCGATGAAGAAGCCCCCGCCGACCAGCGACGCGGTGTACACGTGGGCACCTTGGAGCGC

CCACCCAAAGTGTACTGCGATGGCTCAGAGTACGACGTGCTGAACTTTGCCTCCGGAGGT

TGCTGGCCTCGCAGGATTCGGGTTTGGAACGGCCAGGATTTTCGGGGCGATGGATTCAAC

CCCAGATTTGAGAGATTTCACGTGTACGACATAGTAGAGACTTCGGAGAGCGCCTCACAC

GATGACCCATCCAGGTTTGCTGAGCTATCCCGCCCAAGCGGGTCTGTGGTAACACTGCTG

GGAATGAGCGAGTGTGGAAAGCGAGTCGCGGTTCACGTCTATGGTGTGCGCCATTATTTT

TACATGGCAAAGGCGGAGGTGGATAGCGCTTGTGGAATAACCACCGAGGCAGAACTCGTG

CGCGCAATGGTGGACTGCGCGCACAGCTCGGCTTTGAGCGCGGCCCTGGGAAATGGCAAC

GGCGGCAAACAGAGCGGCGGCAGCGGCGGGGGATGGTGGGGCGGAAAGCACGTGTCTGCG

GACTGCTTCAAAGTGGAGACCGTGTGCCACACGACGCTGTACTACTTTGGATCTAAGCCA

GCTCTCTACTATAGAGTATCTGCCTCCAGCAGCCGCCTGGGAGGGTTCATCTGCGACAAC

TTTCACCCGGAGATTACAAAATTCGAGGGGAGCGTGGACGTGACCACGCGGCTGTTGTTG

GACAATGAAAATTTTACCAGTTTCGGGTGGTACCGCCTGCGACCCGGCACCCACGGAGAG

CGTGTTCAACTTCGCCCCGTTGAGCGACACGTCACCTCAAGCGACGTGGAGATTAACTGT

ACTCCCGATAACCTGGAGCCGATACCAGACGAGGCTGCCTGGCCCGACTATAAGCTCATG

TGCTTTGATATAGAGTGTAAAGCTGGAACGGGTAACGAAATGGCGTTCCCAGTGGCAACT

AACCAAGAGGACCTGGTCATCCAGATCTCCTGTCTGCTGTACTCGCTTGCTACTCAGAAC

CACGAACACACCCTGCTGTTTTCCCTCGGGTCATGCGATATCTCTGAGGAATACTCGTTT

GCATGCGTCCAGCGCGGCGAGCCCAGACCGACGGTTTTGGAGTTTGACAGCGAGTACGAG

CTGCTGGTTGCCTTCCTGACCTTTCTCAAGCAGTACTCTCCCGAGTTCGCCACCGGCTAC

AACATCGTTAATTTTGACTGGGCGTACATAGTTAACAAGGTAACGTCGGTGTATAACATC

AAGCTGGACGGGTACGGCAAGTTCAACAAAGGGGGGCTGTTTAAGGTGTGGGACATCGCC

ACGAACCATTTTCAGAAGAAGAGCAAGGTGAAAATCAATGGCCTGATATCTCTAGACATG

TATTCTGTGGCGACGGAAAAGCTAAAGCTACCCAGCTACAAACTCGACGCGGTCGTGGGA

GACGTCCTCGGCGAGCATAAGATAGACCTTCCCTATAAAGAAATACCCTCCTATTACGCG

GGAGGGCCTGACCGGAGGGGCGTAATAGGAGAGTATTGTATCCAGGACTCTAGGCTGGTG

GGCAAGCTGTTTTTTAAGTACCTCCCCCATCTGGAACTATCGGCGGTGGCCAAACTCGCC

CGTATCACCCTAACGCGGGTAATTTTTGACGGTCAGCAAATTCGCGTGTACACGTGCTTG

CTGAAACTCGCCCGCGAGAGAAATTTCATTTTGCCAGACAACAGACGCCGGTTTGACAGT

CAGGCAGACGCCGCGTCAGAGACTTCGGAGTTGGCTATGGATAGCCAAAGCCACGCCTTC

GACAGTACAGACGAACCCGACGGTGTGGACGGTACCCCGGACGCCGCAGGATCTGGCGCT

ACTTCTGAAAACGGAGGCGGGAAGCCCGGCGTCGGGAGGGTCGTGGGCTACCAGGGAGCA

AAGGTTCTAGACCCCGTATCCGGCTTTCATGTGGACCCCGTGGTTGTGTTTGACTTCGCT

AGCTTATACCCAAGCATTATCCAGGCCCATAACCTCTGTTTCACCACCCTGGCGCTCGAT

GAAGTGGATCTGGCCGGGCTTCAACCATCCGTCGACTACTCGACGTTCGAGGTGGGTGAC

CAAAAGTTATTTTTTGTCCACGCCCATATTCGCGAAAGCCTGCTTGGCATCTTGCTGCGC

GACTGGCTGGCCATGCGAAAGGCGGTGAGGGCGCGAATCCCCACCAGCACCCCCGAGGAG

GCAGTTTTACTAGATAAGCAGCAGTCTGCGATTAAGGTGATATGCAACTCGGTTTACGGA

TTCACGGGGGTGGCAAACGGCCTGTTGCCGTGTCTGAGGATAGCGGCTACCGTTACCACG

ATAGGACGCGACATGCTCCTCAAGACCCGAGATTACGTTCACTCTCGTTGGGCGACGCGC

GAGCTGCTGGAGGACAATTTTCCAGGGGCTATAGGTTTCCGAAACCACAAGCCTTACTCC

GTCAGGGTTATCTACGGAGACACCGACTCCGTGTTTATCAAGTTTGTGGGCCTGACGTAC

GAGGGGGTATCGGAGCTGGGGGATGCTATGTCGCGTCAGATTTCAGCGGACCTCTTTAGA

GCGCCCATCAAACTGGAGTGTGAGAAGACCTTTCAGCGACTGCTGCTGATCACCAAGAAG

AAGTACATAGGTGTCATAAACGGGGGGAAGATGCTCATGAAGGGGGTCGACCTGGTCCGC

AAAAATAACTGCTCTTTCATAAACTTGTACGCGCGACATCTGGTAGATCTTTTGTTGTAC

GACGAGGATGTGGCCACGGCGGCAGCAGAGGTGACAGACGTGCCTCCCGCAGAATGGGTG

GGGCGCCCGCTACCGAGCGGCTTTGACAAGTTTGGGCGAGTGCTGGTAGAGGCGTACAAC

CGTATCACTGCCCCCAACTTGGACGTGCGCGAGTTCGTTATGACTGCTGAGCTGAGCCGC

TCACCCGAATCGTATACCAACAAGCGCCTGCCGCACCTCACCGTCTACTTTAAGCTCGCC

ATGAGGAATGAAGAACTGCCCAGCGTAAAAGAGAGAATTCCGTATGTGATAGTTGCGCAG

ACCGAGGCCGCGGAACGCGAAGCGGGTGTAGTAAACTCAATGCGCGGTACCGCCCAAAAC

CCCGTGGTAACCAAGACCGCACGCCCCCAACCTAAACGCAAACTGCTGGTTTCCGACCTC

GCCGAAGACCCGACCTATGTTTCCGAGAATGACGTACCGCTAAACACAGACTACTATTTC

TCCCACCTGTTGGGTACCATAAGCGTGACCTTTAAGGCTCTATTCGGAAATGATGTGAGA

ACAACAGAAAATCTTTTAAAGCGGTTTATTCCGGAAACCCCCCACAAGACCCCCACGAAA

ACCCAGGCACTGCTTGAGCGCGCCGGCTTTGAAAAGCTGACGCCCTTTACACCGGAGGAA

GAAAGTCGTCGAATACTGCATACAGTTTTTTGTACTCTAGAAGCAGCTCCCCATCAAAGC

TGA

>KU206456.1

ATGGCGGCGCGCGAACAGGCCAACTCCGTTCGCAGGAGCGGGTTTTTTAATCCGTTTATT

GGCAAGAGGCCATTTTTCAGGCCGGGAAGCGGGCAGACCGCGGAGACAGAGCGCCCAAGG

CCCCCCCAACACTCGTACTGCACAGAGGTGGGTAGCTTTAAGTTTATAGCTCCAAGATGC

CTCGATGAAGAAGCCCCCGCCGACCAGCGACGCGGTGTACACGTGGGCACCTTGGAGCGC

CCACCCAAAGTGTACTGCGATGGCTCAGAGTACGACGTGCTGAACTTTGCCTCCGGAGGT

TGCTGGCCTCGCAGGATTCGGGTTTGGAACGGCCAGGATTTTCGGGGCGATGGATTCAAC

CCCAGATTTGAGAGATTTCACGTGTACGACATAGTAGAGACTTCGGAGAGCGCCTCACAC

GATGACCCATCCAGGTTTGCTGAGCTATCCCGCCCAAGCGGGTCTGTGGTAACACTGCTG

GGAATGAGCGAGTGTGGAAAGCGAGTCGCGGTTCACGTCTATGGTGTGCGCCATTATTTT

TACATGGCAAAGGCGGAGGTGGATAGCGCTTGTGGAATAACCACCGAGGCAGAACTCGTG

CGCGCAATGGTGGACTGCGCGCACAGCTCGGCTTTGAGCGCGGCCCTGGGAAATGGCAAC

GGCGGCAAACAGAGCGGCGGCAGCGGCGGGGGATGGTGGGGCGGAAAGCACGTGTCTGCG

GACTGCTTCAAAGTGGAGACCGTGTGCCACACGACGCTGTACTACTTTGGATCTAAGCCA

GCTCTCTACTATAGAGTATCTGCCTCCAGCAGCCGCCTGGGAGGGTTCATCTGCGACAAC

TTTCACCCGGAGATTACAAAATTCGAGGGGAGCGTGGACGTGACCACGCGGCTGTTGTTG

GACAATGAAAATTTTACCAGTTTCGGGTGGTACCGCCTGCGACCCGGCACCCACGGAGAG

CGTGTTCAACTTCGCCCCGTTGAGCGACACGTCACCTCAAGCGACGTGGAGATTAACTGT

ACTCCCGATAACCTGGAGCCGATACCAGACGAGGCTGCCTGGCCCGACTATAAGCTCATG

TGCTTTGATATAGAGTGTAAAGCTGGAACGGGTAACGAAATGGCGTTCCCAGTGGCAACT

AACCAAGAGGACCTGGTCATCCAGATCTCCTGTCTGCTGTACTCGCTTGCTACTCAGAAC

CACGAACACACCCTGCTGTTTTCCCTCGGGTCATGCGATATCTCTGAGGAATACTCGTTT

GCATGCGTCCAGCGCGGCGAGCCCAGACCGACGGTTTTGGAGTTTGACAGCGAGTACGAG

CTGCTGGTTGCCTTCCTGACCTTTCTCAAGCAGTACTCTCCCGAGTTCGCCACCGGCTAC

AACATCGTTAATTTTGACTGGGCGTACATAGTTAACAAGGTAACGTCGGTGTATAACATC

AAGCTGGACGGGTACGGCAAGTTCAACAAAGGGGGGCTGTTTAAGGTGTGGGACATCGCC

ACGAACCATTTTCAGAAGAAGAGCAAGGTGAAAATCAATGGCCTGATATCTCTAGACATG

TATTCTGTGGCGACGGAAAAGCTAAAGCTACCCAGCTACAAACTCGACGCGGTCGTGGGA

GACGTCCTCGGCGAGCATAAGATAGACCTTCCCTATAAAGAAATACCCTCCTATTACGCG

GGAGGGCCTGACCGGAGGGGCGTAATAGGAGAGTATTGTATCCAGGACTCTAGGCTGGTG

GGCAAGCTGTTTTTTAAGTACCTCCCCCATCTGGAACTATCGGCGGTGGCCAAACTCGCC

CGTATCACCCTAACGCGGGTAATTTTTGACGGTCAGCAAATTCGCGTGTACACGTGCTTG

CTGAAACTCGCCCGCGAGAGAAATTTCATTTTGCCAGACAACAGACGCCGGTTTGACAGT

CAGGCAGACGCCGCGTCAGAGACTTCGGAGTTGGCTATGGATAGCCAAAGCCACGCCTTC

GACAGTACAGACGAACCCGACGGTGTGGACGGTACCCCGGACGCCGCAGGATCTGGCGCT

ACTTCTGAAAACGGAGGCGGGAAGCCCGGCGTCGGGAGGGTCGTGGGCTACCAGGGAGCA

AAGGTTCTAGACCCCGTATCCGGCTTTCATGTGGACCCCGTGGTTGTGTTTGACTTCGCT

AGCTTATACCCAAGCATTATCCAGGCCCATAACCTCTGTTTCACCACCCTGGCGCTCGAT

GAAGTGGATCTGGCCGGGCTTCAACCATCCGTCGACTACTCGACGTTCGAGGTGGGTGAC

CAAAAGTTATTTTTTGTCCACGCCCATATTCGCGAAAGCCTGCTTGGCATCTTGCTGCGC

GACTGGCTGGCCATGCGAAAGGCGGTGAGGGCGCGAATCCCCACCAGCACCCCCGAGGAG

GCAGTTTTACTAGATAAGCAGCAGTCTGCGATTAAGGTGATATGCAACTCGGTTTACGGA

TTCACGGGGGTGGCAAACGGCCTGTTGCCGTGTCTGAGGATAGCGGCTACCGTTACCACG

ATAGGACGCGACATGCTCCTCAAGACCCGAGATTACGTTCACTCTCGTTGGGCGACGCGC

GAGCTGCTGGAGGACAATTTTCCAGGGGCTATAGGTTTCCGAAACCACAAGCCTTACTCC

GTCAGGGTTATCTACGGAGACACCGACTCCGTGTTTATCAAGTTTGTGGGCCTGACGTAC

GAGGGGGTATCGGAGCTGGGGGATGCTATGTCGCGTCAGATTTCAGCGGACCTCTTTAGA

GCGCCCATCAAACTGGAGTGTGAGAAGACCTTTCAGCGACTGCTGCTGATCACCAAGAAG

AAGTACATAGGTGTCATAAACGGGGGGAAGATGCTCATGAAGGGGGTCGACCTGGTCCGC

AAAAATAACTGCTCTTTCATAAACTTGTACGCGCGACATCTGGTAGATCTTTTGTTGTAC

GACGAGGATGTGGCCACGGCGGCAGCAGAGGTGACAGACGTGCCTCCCGCAGAATGGGTG

GGGCGCCCGCTACCGAGCGGCTTTGACAAGTTTGGGCGAGTGCTGGTAGAGGCGTACAAC

CGTATCACTGCCCCCAACTTGGACGTGCGCGAGTTCGTTATGACTGCTGAGCTGAGCCGC

TCACCCGAATCGTATACCAACAAGCGCCTGCCGCACCTCACCGTCTACTTTAAGCTCGCC

ATGAGGAATGAAGAACTGCCCAGCGTAAAAGAGAGAATTCCGTATGTGATAGTTGCGCAG

ACCGAGGCCGCGGAACGCGAAGCGGGTGTAGTAAACTCAATGCGCGGTACCGCCCAAAAC

CCCGTGGTAACCAAGACCGCACGCCCCCAACCTAAACGCAAACTGCTGGTTTCCGACCTC

GCCGAAGACCCGACCTATGTTTCCGAGAATGACGTACCGCTAAACACAGACTACTATTTC

TCCCACCTGTTGGGTACCATAAGCGTGACCTTTAAGGCTCTATTCGGAAATGATGTGAGA

ACAACAGAAAATCTTTTAAAGCGGTTTATTCCGGAAACCCCCCACAAGACCCCCACGAAA

ACCCAGGCACTGCTTGAGCGCGCCGGCTTTGAAAAGCTGACGCCCTTTACACCGGAGGAA

GAAAGTCGTCGAATACTGCATACAGTTTTTTGTACTCTAGAAGCAGCTCCCCATCAAAGC

TGA

>KU206457.1

ATGGCGGCGCGCGAACAGGCCAACTCCGTTCGCAGGAGCGGGTTTTTTAATCCGTTTATT

GGCAAGAGGCCATTTTTCAGGCCGGGAAGCGGGCAGACCGCGGAGACAGAGCGCCCAAGG

CCCCCCCAACACTCGTACTGCACAGAGGTGGGTAGCTTTAAGTTTATAGCTCCAAGATGC

CTCGATGAAGAAGCCCCCGCCGACCAGCGACGCGGTGTACACGTGGGCACCTTGGAGCGC

CCACCCAAAGTGTACTGCGATGGCTCAGAGTACGACGTGCTGAACTTTGCCTCCGGAGGT

TGCTGGCCTCGCAGGATTCGGGTTTGGAACGGCCAGGATTTTCGGGGCGATGGATTCAAC

CCCAGATTTGAGAGATTTCACGTGTACGACATAGTAGAGACTTCGGAGAGCGCCTCACAC

GATGACCCATCCAGGTTTGCTGAGCTATCCCGCCCAAGCGGGTCTGTGGTAACACTGCTG

GGAATGAGCGAGTGTGGAAAGCGAGTCGCGGTTCACGTCTATGGTGTGCGCCATTATTTT

TACATGGCAAAGGCGGAGGTGGATAGCGCTTGTGGAATAACCACCGAGGCAGAACTCGTG

CGCGCAATGGTGGACTGCGCGCACAGCTCGGCTTTGAGCGCGGCCCTGGGAAATGGCAAC

GGCGGCAAACAGAGCGGCGGCAGCGGCGGGGGATGGTGGGGCGGAAAGCACGTGTCTGCG

GACTGCTTCAAAGTGGAGACCGTGTGCCACACGACGCTGTACTACTTTGGATCTAAGCCA

GCTCTCTACTATAGAGTATCTGCCTCCAGCAGCCGCCTGGGAGGGTTCATCTGCGACAAC

TTTCACCCGGAGATTACAAAATTCGAGGGGAGCGTGGACGTGACCACGCGGCTGTTGTTG

GACAATGAAAATTTTACCAGTTTCGGGTGGTACCGCCTGCGACCCGGCACCCACGGAGAG

CGTGTTCAACTTCGCCCCGTTGAGCGACACGTCACCTCAAGCGACGTGGAGATTAACTGT

ACTCCCGATAACCTGGAGCCGATACCAGACGAGGCTGCCTGGCCCGACTATAAGCTCATG

TGCTTTGATATAGAGTGTAAAGCTGGAACGGGTAACGAAATGGCGTTCCCAGTGGCAACT

AACCAAGAGGACCTGGTCATCCAGATCTCCTGTCTGCTGTACTCGCTTGCTACTCAGAAC

CACGAACACACCCTGCTGTTTTCCCTCGGGTCATGCGATATCTCTGAGGAATACTCGTTT

GCATGCGTCCAGCGCGGCGAGCCCAGACCGACGGTTTTGGAGTTTGACAGCGAGTACGAG

CTGCTGGTTGCCTTCCTGACCTTTCTCAAGCAGTACTCTCCCGAGTTCGCCACCGGCTAC

AACATCGTTAATTTTGACTGGGCGTACATAGTTAACAAGGTAACGTCGGTGTATAACATC

AAGCTGGACGGGTACGGCAAGTTCAACAAAGGGGGGCTGTTTAAGGTGTGGGACATCGCC

ACGAACCATTTTCAGAAGAAGAGCAAGGTGAAAATCAATGGCCTGATATCTCTAGACATG

TATTCTGTGGCGACGGAAAAGCTAAAGCTACCCAGCTACAAACTCGACGCGGTCGTGGGA

GACGTCCTCGGCGAGCATAAGATAGACCTTCCCTATAAAGAAATACCCTCCTATTACGCG

GGAGGGCCTGACCGGAGGGGCGTAATAGGAGAGTATTGTATCCAGGACTCTAGGCTGGTG

GGCAAGCTGTTTTTTAAGTACCTCCCCCATCTGGAACTATCGGCGGTGGCCAAACTCGCC

CGTATCACCCTAACGCGGGTAATTTTTGACGGTCAGCAAATTCGCGTGTACACGTGCTTG

CTGAAACTCGCCCGCGAGAGAAATTTCATTTTGCCAGACAACAGACGCCGGTTTGACAGT

CAGGCAGACGCCGCGTCAGAGACTTCGGAGTTGGCTATGGATAGCCAAAGCCACGCCTTC

GACAGTACAGACGAACCCGACGGTGTGGACGGTACCCCGGACGCCGCAGGATCTGGCGCT

ACTTCTGAAAACGGAGGCGGGAAGCCCGGCGTCGGGAGGGTCGTGGGCTACCAGGGAGCA

AAGGTTCTAGACCCCGTATCCGGCTTTCATGTGGACCCCGTGGTTGTGTTTGACTTCGCT

AGCTTATACCCAAGCATTATCCAGGCCCATAACCTCTGTTTCACCACCCTGGCGCTCGAT

GAAGTGGATCTGGCCGGGCTTCAACCATCCGTCGACTACTCGACGTTCGAGGTGGGTGAC

CAAAAGTTATTTTTTGTCCACGCCCATATTCGCGAAAGCCTGCTTGGCATCTTGCTGCGC

GACTGGCTGGCCATGCGAAAGGCGGTGAGGGCGCGAATCCCCACCAGCACCCCCGAGGAG

GCAGTTTTACTAGATAAGCAGCAGTCTGCGATTAAGGTGATATGCAACTCGGTTTACGGA

TTCACGGGGGTGGCAAACGGCCTGTTGCCGTGTCTGAGGATAGCGGCTACCGTTACCACG

ATAGGACGCGACATGCTCCTCAAGACCCGAGATTACGTTCACTCTCGTTGGGCGACGCGC

GAGCTGCTGGAGGACAATTTTCCAGGGGCTATAGGTTTCCGAAACCACAAGCCTTACTCC

GTCAGGGTTATCTACGGAGACACCGACTCCGTGTTTATCAAGTTTGTGGGCCTGACGTAC

GAGGGGGTATCGGAGCTGGGGGATGCTATGTCGCGTCAGATTTCAGCGGACCTCTTTAGA

GCGCCCATCAAACTGGAGTGTGAGAAGACCTTTCAGCGACTGCTGCTGATCACCAAGAAG

AAGTACATAGGTGTCATAAACGGGGGGAAGATGCTCATGAAGGGGGTCGACCTGGTCCGC

AAAAATAACTGCTCTTTCATAAACTTGTACGCGCGACATCTGGTAGATCTTTTGTTGTAC

GACGAGGATGTGGCCACGGCGGCAGCAGAGGTGACAGACGTGCCTCCCGCAGAATGGGTG

GGGCGCCCGCTACCGAGCGGCTTTGACAAGTTTGGGCGAGTGCTGGTAGAGGCGTACAAC

CGTATCACTGCCCCCAACTTGGACGTGCGCGAGTTCGTTATGACTGCTGAGCTGAGCCGC

TCACCCGAATCGTATACCAACAAGCGCCTGCCGCACCTCACCGTCTACTTTAAGCTCGCC

ATGAGGAATGAAGAACTGCCCAGCGTAAAAGAGAGAATTCCGTATGTGATAGTTGCGCAG

ACCGAGGCCGCGGAACGCGAAGCGGGTGTAGTAAACTCAATGCGCGGTACCGCCCAAAAC

CCCGTGGTAACCAAGACCGCACGCCCCCAACCTAAACGCAAACTGCTGGTTTCCGACCTC

GCCGAAGACCCGACCTATGTTTCCGAGAATGACGTACCGCTAAACACAGACTACTATTTC

TCCCACCTGTTGGGTACCATAAGCGTGACCTTTAAGGCTCTATTCGGAAATGATGTGAGA

ACAACAGAAAATCTTTTAAAGCGGTTTATTCCGGAAACCCCCCACAAGACCCCCACGAAA

ACCCAGGCACTGCTTGAGCGCGCCGGCTTTGAAAAGCTGACGCCCTTTACACCGGAGGAA

GAAAGTCGTCGAATACTGCATACAGTTTTTTGTACTCTAGAAGCAGCTCCCCATCAAAGC

TGA

>KU206458.1

ATGGCGGCGCGCGAACAGGCCAACTCCGTTCGCAGGAGCGGGTTTTTTAATCCGTTTATT

GGCAAGAGGCCATTTTTCAGGCCGGGAAGCGGGCAGACCGCGGAGACAGAGCGCCCAAGG

CCCCCCCAACACTCGTACTGCACAGAGGTGGGTAGCTTTAAGTTTATAGCTCCAAGATGC

CTCGATGAAGAAGCCCCCGCCGACCAGCGACGCGGTGTACACGTGGGCACCTTGGAGCGC

CCACCCAAAGTGTACTGCGATGGCTCAGAGTACGACGTGCTGAACTTTGCCTCCGGAGGT

TGCTGGCCTCGCAGGATTCGGGTTTGGAACGGCCAGGATTTTCGGGGCGATGGATTCAAC

CCCAGATTTGAGAGATTTCACGTGTACGACATAGTAGAGACTTCGGAGAGCGCCTCACAC

GATGACCCATCCAGGTTTGCTGAGCTATCCCGCCCAAGCGGGTCTGTGGTAACACTGCTG

GGAATGAGCGAGTGTGGAAAGCGAGTCGCGGTTCACGTCTATGGTGTGCGCCATTATTTT

TACATGGCAAAGGCGGAGGTGGATAGCGCTTGTGGAATAACCACCGAGGCAGAACTCGTG

CGCGCAATGGTGGACTGCGCGCACAGCTCGGCTTTGAGCGCGGCCCTGGGAAATGGCAAC

GGCGGCAAACAGAGCGGCGGCAGCGGCGGGGGATGGTGGGGCGGAAAGCACGTGTCTGCG

GACTGCTTCAAAGTGGAGACCGTGTGCCACACGACGCTGTACTACTTTGGATCTAAGCCA

GCTCTCTACTATAGAGTATCTGCCTCCAGCAGCCGCCTGGGAGGGTTCATCTGCGACAAC

TTTCACCCGGAGATTACAAAATTCGAGGGGAGCGTGGACGTGACCACGCGGCTGTTGTTG

GACAATGAAAATTTTACCAGTTTCGGGTGGTACCGCCTGCGACCCGGCACCCACGGAGAG

CGTGTTCAACTTCGCCCCGTTGAGCGACACGTCACCTCAAGCGACGTGGAGATTAACTGT

ACTCCCGATAACCTGGAGCCGATACCAGACGAGGCTGCCTGGCCCGACTATAAGCTCATG

TGCTTTGATATAGAGTGTAAAGCTGGAACGGGTAACGAAATGGCGTTCCCAGTGGCAACT

AACCAAGAGGACCTGGTCATCCAGATCTCCTGTCTGCTGTACTCGCTTGCTACTCAGAAC

CACGAACACACCCTGCTGTTTTCCCTCGGGTCATGCGATATCTCTGAGGAATACTCGTTT

GCATGCGTCCAGCGCGGCGAGCCCAGACCGACGGTTTTGGAGTTTGACAGCGAGTACGAG

CTGCTGGTTGCCTTCCTGACCTTTCTCAAGCAGTACTCTCCCGAGTTCGCCACCGGCTAC

AACATCGTTAATTTTGACTGGGCGTACATAGTTAACAAGGTAACGTCGGTGTATAACATC

AAGCTGGACGGGTACGGCAAGTTCAACAAAGGGGGGCTGTTTAAGGTGTGGGACATCGCC

ACGAACCATTTTCAGAAGAAGAGCAAGGTGAAAATCAATGGCCTGATATCTCTAGACATG

TATTCTGTGGCGACGGAAAAGCTAAAGCTACCCAGCTACAAACTCGACGCGGTCGTGGGA

GACGTCCTCGGCGAGCATAAGATAGACCTTCCCTATAAAGAAATACCCTCCTATTACGCG

GGAGGGCCTGACCGGAGGGGCGTAATAGGAGAGTATTGTATCCAGGACTCTAGGCTGGTG

GGCAAGCTGTTTTTTAAGTACCTCCCCCATCTGGAACTATCGGCGGTGGCCAAACTCGCC

CGTATCACCCTAACGCGGGTAATTTTTGACGGTCAGCAAATTCGCGTGTACACGTGCTTG

CTGAAACTCGCCCGCGAGAGAAATTTCATTTTGCCAGACAACAGACGCCGGTTTGACAGT

CAGGCAGACGCCGCGTCAGAGACTTCGGAGTTGGCTATGGATAGCCAAAGCCACGCCTTC

GACAGTACAGACGAACCCGACGGTGTGGACGGTACCCCGGACGCCGCAGGATCTGGCGCT

ACTTCTGAAAACGGAGGCGGGAAGCCCGGCGTCGGGAGGGCCGTGGGCTACCAGGGAGCA

AAGGTTCTAGACCCCGTATCCGGCTTTCATGTGGACCCCGTGGTTGTGTTTGACTTCGCT

AGCTTATACCCAAGCATTATCCAGGCCCATAACCTCTGTTTCACCACCCTGGCGCTCGAT

GAAGTGGATCTGGCCGGGCTTCAACCATCCGTCGACTACTCGACGTTCGAGGTGGGTGAC

CAAAAGTTATTTTTTGTCCACGCCCATATTCGCGAAAGCCTGCTTGGCATCTTGCTGCGC

GACTGGCTGGCCATGCGAAAGGCGGTGAGGGCGCGAATCCCCACCAGCACCCCCGAGGAG

GCAGTTTTACTAGATAAGCAGCAGTCTGCGATTAAGGTGATATGCAACTCGGTTTACGGA

TTCACGGGGGTGGCAAACGGCCTGTTGCCGTGTCTGAGGATAGCGGCTACCGTTACCACG

ATAGGACGCGACATGCTCCTCAAGACCCGAGATTACGTTCACTCTCGTTGGGCGACGCGC

GAGCTGCTGGAGGACAATTTTCCAGGGGCTATAGGTTTCCGAAACCACAAGCCTTACTCC

GTCAGGGTTATCTACGGAGACACCGACTCCGTGTTTATCAAGTTTGTGGGCCTGACGTAC

GAGGGGGTATCGGAGCTGGGGGATGCTATGTCGCGTCAGATTTCAGCGGACCTCTTTAGA

GCGCCCATCAAACTGGAGTGTGAGAAGACCTTTCAGCGACTGCTGCTGATCACCAAGAAG

AAGTACATAGGTGTCATAAACGGGGGGAAGATGCTCATGAAGGGGGTCGACCTGGTCCGC

AAAAATAACTGCTCTTTCATAAACTTGTACGCGCGACATCTGGTAGATCTTTTGTTGTAC

GACGAGGATGTGGCCACGGCGGCAGCAGAGGTGACAGACGTGCCTCCCGCAGAATGGGTG

GGGCGCCCGCTACCGAGCGGCTTTGACAAGTTTGGGCGAGTGCTGGTAGAGGCGTACAAC

CGTATCACTGCCCCCAACTTGGACGTGCGCGAGTTCGTTATGACTGCTGAGCTGAGCCGC

TCACCCGAATCGTATACCAACAAGCGCCTGCCGCACCTCACCGTCTACTTTAAGCTCGCC

ATGAGGAATGAAGAACTGCCCAGCGTAAAAGAGAGAATTCCGTATGTGATAGTTGCGCAG

ACCGAGGCCGCGGAACGCGAAGCGGGTGTAGTAAACTCAATGCGCGGTACCGCCCAAAAC

CCCGTGGTAACCAAGACCGCACGCCCCCAACCTAAACGCAAACTGCTGGTTTCCGACCTC

GCCGAAGACCCGACCTATGTTTCCGAGAATGACGTACCGCTAAACACAGACTACTATTTC

TCCCACCTGTTGGGTACCATAAGCGTGACCTTTAAGGCTCTATTCGGAAATGATGTGAGA

ACAACAGAAAATCTTTTAAAGCGGTTTATTCCGGAAACCCCCCACAAGACCCCCACGAAA

ACCCAGGCACTGCTTGAGCGCGCCGGCTTTGAAAAGCTGACGCCCTTTACACCGGAGGAA

GAAAGTCGTCGAATACTGCATACAGTTTTTTGTACTCTAGAAGCAGCTCCCCATCAAAGC

TGA

>KU206459.1

ATGGCGGCGCGCGAACAGGCCAACTCCGTTCGCAGGAGCGGGTTTTTTAATCCGTTTATT

GGCAAGAGGCCATTTTTCAGGCCGGGAAGCGGGCAGACCGCGGAGACAGAGCGCCCAAGG

CCCCCCCAACACTCGTACTGCACAGAGGTGGGTAGCTTTAAGTTTATAGCTCCAAGATGC

CTCGATGAAGAAGCCCCCGCCGACCAGCGACGCGGTGTACACGTGGGCACCTTGGAGCGC

CCACCCAAAGTGTACTGCGATGGCTCAGAGTACGACGTGCTGAACTTTGCCTCCGGAGGT

TGCTGGCCTCGCAGGATTCGGGTTTGGAACGGCCAGGATTTTCGGGGCGATGGATTCAAC

CCCAGATTTGAGAGATTTCACGTGTACGACATAGTAGAGACTTCGGAGAGCGCCTCACAC

GATGACCCATCCAGGTTTGCTGAGCTATCCCGCCCAAGCGGGTCTGTGGTAACACTGCTG

GGAATGAGCGAGTGTGGAAAGCGAGTCGCGGTTCACGTCTATGGTGTGCGCCATTATTTT

TACATGGCAAAGGCGGAGGTGGATAGCGCTTGTGGAATAACCACCGAGGCAGAACTCGTG

CGCGCAATGGTGGACTGCGCGCACAGCTCGGCTTTGAGCGCGGCCCTGGGAAATGGCAAC

GGCGGCAAACAGAGCGGCGGCAGCGGCGGGGGATGGTGGGGCGGAAAGCACGTGTCTGCG

GACTGCTTCAAAGTGGAGACCGTGTGCCACACGACGCTGTACTACTTTGGATCTAAGCCA

GCTCTCTACTATAGAGTATCTGCCTCCAGCAGCCGCCTGGGAGGGTTCATCTGCGACAAC

TTTCACCCGGAGATTACAAAATTCGAGGGGAGCGTGGACGTGACCACGCGGCTGTTGTTG

GACAATGAAAATTTTACCAGTTTCGGGTGGTACCGCCTGCGACCCGGCACCCACGGAGAG

CGTGTTCAACTTCGCCCCGTTGAGCGACACGTCACCTCAAGCGACGTGGAGATTAACTGT

ACTCCCGATAACCTGGAGCCGATACCAGACGAGGCTGCCTGGCCCGACTATAAGCTCATG

TGCTTTGATATAGAGTGTAAAGCTGGAACGGGTAACGAAATGGCGTTCCCAGTGGCAACT

AACCAAGAGGACCTGGTCATCCAGATCTCCTGTCTGCTGTACTCGCTTGCTACTCAGAAC

CACGAACACACCCTGCTGTTTTCCCTCGGGTCATGCGATATCTCTGAGGAATACTCGTTT

GCATGCGTCCAGCGCGGCGAGCCCAGACCGACGGTTTTGGAGTTTGACAGCGAGTACGAG

CTGCTGGTTGCCTTCCTGACCTTTCTCAAGCAGTACTCTCCCGAGTTCGCCACCGGCTAC

AACATCGTTAATTTTGACTGGGCGTACATAGTTAACAAGGTAACGTCGGTGTATAACATC

AAGCTGGACGGGTACGGCAAGTTCAACAAAGGGGGGCTGTTTAAGGTGTGGGACATCGCC

ACGAACCATTTTCAGAAGAAGAGCAAGGTGAAAATCAATGGCCTGATATCTCTAGACATG

TATTCTGTGGCGACGGAAAAGCTAAAGCTACCCAGCTACAAACTCGACGCGGTCGTGGGA

GACGTCCTCGGCGAGCATAAGATAGACCTTCCCTATAAAGAAATACCCTCCTATTACGCG

GGAGGGCCTGACCGGAGGGGCGTAATAGGAGAGTATTGTATCCAGGACTCTAGGCTGGTG

GGCAAGCTGTTTTTTAAGTACCTCCCCCATCTGGAACTATCGGCGGTGGCCAAACTCGCC

CGTATCACCCTAACGCGGGTAATTTTTGACGGTCAGCAAATTCGCGTGTACACGTGCTTG

CTGAAACTCGCCCGCGAGAGAAATTTCATTTTGCCAGACAACAGACGCCGGTTTGACAGT

CAGGCAGACGCCGCGTCAGAGACTTCGGAGTTGGCTATGGATAGCCAAAGCCACGCCTTC

GACAGTACAGACGAACCCGACGGTGTGGACGGTACCCCGGACGCCGCAGGATCTGGCGCT

ACTTCTGAAAACGGAGGCGGGAAGCCCGGCGTCGGGAGGGCCGTGGGCTACCAGGGAGCA

AAGGTTCTAGACCCCGTATCCGGCTTTCATGTGGACCCCGTGGTTGTGTTTGACTTCGCT

AGCTTATACCCAAGCATTATCCAGGCCCATAACCTCTGTTTCACCACCCTGGCGCTCGAT

GAAGTGGATCTGGCCGGGCTTCAACCATCCGTCGACTACTCGACGTTCGAGGTGGGTGAC

CAAAAGTTATTTTTTGTCCACGCCCATATTCGCGAAAGCCTGCTTGGCATCTTGCTGCGC

GACTGGCTGGCCATGCGAAAGGCGGTGAGGGCGCGAATCCCCACCAGCACCCCCGAGGAG

GCAGTTTTACTAGATAAGCAGCAGTCTGCGATTAAGGTGATATGCAACTCGGTTTACGGA

TTCACGGGGGTGGCAAACGGCCTGTTGCCGTGTCTGAGGATAGCGGCTACCGTTACCACG

ATAGGACGCGACATGCTCCTCAAGACCCGAGATTACGTTCACTCTCGTTGGGCGACGCGC

GAGCTGCTGGAGGACAATTTTCCAGGGGCTATAGGTTTCCGAAACCACAAGCCTTACTCC

GTCAGGGTTATCTACGGAGACACCGACTCCGTGTTTATCAAGTTTGTGGGCCTGACGTAC

GAGGGGGTATCGGAGCTGGGGGATGCTATGTCGCGTCAGATTTCAGCGGACCTCTTTAGA

GCGCCCATCAAACTGGAGTGTGAGAAGACCTTTCAGCGACTGCTGCTGATCACCAAGAAG

AAGTACATAGGTGTCATAAACGGGGGGAAGATGCTCATGAAGGGGGTCGACCTGGTCCGC

AAAAATAACTGCTCTTTCATAAACTTGTACGCGCGACATCTGGTAGATCTTTTGTTGTAC

GACGAGGATGTGGCCACGGCGGCAGCAGAGGTGACAGACGTGCCTCCCGCAGAATGGGTG

GGGCGCCCGCTACCGAGCGGCTTTGACAAGTTTGGGCGAGTGCTGGTAGAGGCGTACAAC

CGTATCACTGCCCCCAACTTGGACGTGCGCGAGTTCGTTATGACTGCTGAGCTGAGCCGC

TCACCCGAATCGTATACCAACAAGCGCCTGCCGCACCTCACCGTCTACTTTAAGCTCGCC

ATGAGGAATGAAGAACTGCCCAGCGTAAAAGAGAGAATTCCGTATGTGATAGTTGCGCAG

ACCGAGGCCGCGGAACGCGAAGCGGGTGTAGTAAACTCAATGCGCGGTACCGCCCAAAAC

CCCGTGGTAACCAAGACCGCACGCCCCCAACCTAAACGCAAACTGCTGGTTTCCGACCTC

GCCGAAGACCCGACCTATGTTTCCGAGAATGACGTACCGCTAAACACAGACTACTATTTC

TCCCACCTGTTGGGTACCATAAGCGTGACCTTTAAGGCTCTATTCGGAAATGATGTGAGA

ACAACAGAAAATCTTTTAAAGCGGTTTATTCCGGAAACCCCCCACAAGACCCCCACGAAA

ACCCAGGCACTGCTTGAGCGCGCCGGCTTTGAAAAGCTGACGCCCTTTACACCGGAGGAA

GAAAGTCGTCGAATACTGCATACAGTTTTTTGTACTCTAGAAGCAGCTCCCCATCAAAGC

TGA

>KU206460.1

ATGGCGGCGCGCGAACAGGCCAACTCCGTTCGCAGGAGCGGGTTTTTTAATCCGTTTATT

GGCAAGAGGCCATTTTTCAGGCCGGGAAGCGGGCAGACCGCGGAGACAGAGCGCCCAAGG

CCCCCCCAACACTCGTACTGCACAGAGGTGGGTAGCTTTAAGTTTATAGCTCCAAGATGC

CTCGATGAAGAAGCCCCCGCCGACCAGCGACGCGGTGTACACGTGGGCACCTTGGAGCGC

CCACCCAAAGTGTACTGCGATGGCTCAGAGTACGACGTGCTGAACTTTGCCTCCGGAGGT

TGCTGGCCTCGCAGGATTCGGGTTTGGAACGGCCAGGATTTTCGGGGCGATGGATTCAAC

CCCAGATTTGAGAGATTTCACGTGTACGACATAGTAGAGACTTCGGAGAGCGCCTCACAC

GATGACCCATCCAGGTTTGCTGAGCTATCCAGCCCAAGCGGGTCTGTGGTAACACTGCTG

GGAATGAGCGAGTGTGGAAAGCGAGTCGCGGTTCACGTCTATGGTGTGCGCCATTATTTT

TACATGGCAAAGGCGGAGGTGGATAGCGCTTGTGGAATAACCACCGAGGCAGAACTCGTG

CGCGCAATGGTGGACTGCGCGCACAGCTCGGCTTTGAGCGCGGCCCTGGGAAATGGCAAC

GGCGGCAAACAGAGCGGCGGCAGCGGCGGGGGATGGTGGGGCGGAAAGCACGTGTCTGCG

GACTGCTTCAAAGTGGAGACCGTGTGCCACACGACGCTGTACTACTTTGGATCTAAGCCA

GCTCTCTACTATAGAGTATCTGCCTCCAGCAGCCGCCTGGGAGGGTTCATCTGCGACAAC

TTTCACCCGGAGATTACAAAATTCGAGGGGAGCGTGGACGTGACCACGCGGCTGTTGTTG

GACAATGAAAATTTTACCAGTTTCGGGTGGTACCGCCTGCGACCCGGCACCCACGGAGAG

CGTGTTCAACTTCGCCCCGTTGAGCGACACGTCACCTCAAGCGACGTGGAGATTAACTGT

ACTCCCGATAACCTGGAGCCGATACCAGACGAGGCTGCCTGGCCCGACTATAAGCTCATG

TGCTTTGATATAGAGTGTAAAGCTGGAACGGGTAACGAAATGGCGTTCCCAGTGGCAACT

AACCAAGAGGACCTGGTCATCCAGATCTCCTGTCTGCTGTACTCGCTTGCTACTCAGAAC

CACGAACACACCCTGCTGTTTTCCCTCGGGTCATGCGATATCTCTGAGGAATACTCGTTT

GCATGCGTCCAGCGCGGCGAGCCCAGACCGACGGTTTTGGAGTTTGACAGCGAGTACGAG

CTGCTGGTTGCCTTCCTGACCTTTCTCAAGCAGTACTCTCCCGAGTTCGCCACCGGCTAC

AACATCGTTAATTTTGACTGGGCGTACATAGTTAACAAGGTAACGTCGGTGTATAACATC

AAGCTGGACGGGTACGGCAAGTTCAACAAAGGGGGGCTGTTTAAGGTGTGGGACATCGCC

ACGAACCATTTTCAGAAGAAGAGCAAGGTGAAAATCAATGGCCTGATATCTCTAGACATG

TATTCTGTGGCGACGGAAAAGCTAAAGCTACCCAGCTACAAACTCGACGCGGTCGTGGGA

GACGTCCTCGGCGAGCATAAGATAGACCTTCCCTATAAAGAAATACCCTCCTATTACGCG

GGAGGGCCTGACCGGAGGGGCGTAATAGGAGAGTATTGTATCCAGGACTCTAGGCTGGTG

GGCAAGCTGTTTTTTAAGTACCTCCCCCATCTGGAACTATCGGCGGTGGCCAAACTCGCC

CGTATCACCCTAACGCGGGTAATTTTTGACGGTCAGCAAATTCGCGTGTACACGTGCTTG

CTGAAACTCGCCCGCGAGAGAAATTTCATTTTGCCAGACAACAGACGCCGGTTTGACAGT

CAGGCAGACGCCGCGTCAGAGACTTCGGAGTTGGCTATGGATAGCCAAAGCCACGCCTTC

GACAGTACAGACGAACCCGACGGTGTGGACGGTACCCCGGACGCCGCAGGATCTGGCGCT

ACTTCTGAAAACGGAGGCGGGAAGCCCGGCGTCGGGAGGGCCGTGGGCTACCAGGGAGCA

AAGGTTCTAGACCCCGTATCCGGCTTTCATGTGGACCCCGTGGTTGTGTTTGACTTCGCT

AGCTTATACCCAAGCATTATCCAGGCCCATAACCTCTGTTTCACCACCCTGGCGCTCGAT

GAAGTGGATCTGGCCGGGCTTCAACCATCCGTCGACTACTCGACGTTCGAGGTGGGTGAC

CAAAAGTTATTTTTTGTCCACGCCCATATTCGCGAAAGCCTGCTTGGCATCTTGCTGCGC

GACTGGCTGGCCATGCGAAAGGCGGTGAGGGCGCGAATCCCCACCAGCACCCCCGAGGAG

GCAGTTTTACTAGATAAGCAGCAGTCTGCGATTAAGGTGATATGCAACTCGGTTTACGGA

TTCACGGGGGTGGCAAACGGCCTGTTGCCGTGTCTGAGGATAGCGGCTACCGTTACCACG

ATAGGACGCGACATGCTCCTCAAGACCCGAGATTACGTTCACTCTCGTTGGGCGACGCGC

GAGCTGCTGGAGGACAATTTTCCAGGGGCTATAGGTTTCCGAAACCACAAGCCTTACTCC

GTCAGGGTTATCTACGGAGACACCGACTCCGTGTTTATCAAGTTTGTGGGCCTGACGTAC

GAGGGGGTATCGGAGCTGGGGGATGCTATGTCGCGTCAGATTTCAGCGGACCTCTTTAGA

GCGCCCATCAAACTGGAGTGTGAGAAGACCTTTCAGCGACTGCTGCTGATCACCAAGAAG

AAGTACATAGGTGTCATAAACGGGGGGAAGATGCTCATGAAGGGGGTCGACCTGGTCCGC

AAAAATAACTGCTCTTTCATAAACTTGTACGCGCGACATCTGGTAGATCTTTTGTTGTAC

GACGAGGATGTGGCCACGGCGGCAGCAGAGGTGACAGACGTGCCTCCCGCAGAATGGGTG

GGGCGCCCGCTACCGAGCGGCTTTGACAAGTTTGGGCGAGTGCTGGTAGAGGCGTACAAC

CGTATCACTGCCCCCAACTTGGACGTGCGCGAGTTCGTTATGACTGCTGAGCTGAGCCGC

TCACCCGAATCGTATACCAACAAGCGCCTGCCGCACCTCACCGTCTACTTTAAGCTCGCC

ATGAGGAATGAAGAACTGCCCAGCGTAAAAGAGAGAATTCCGTATGTGATAGTTGCGCAG

ACCGAGGCCGCGGAACGCGAAGCGGGTGTAGTAAACTCAATGCGCGGTACCGCCCAAAAC

CCCGTGGTAACCAAGACCGCACGCCCCCAACCTAAACGCAAACTGCTGGTTTCCGACCTC

GCCGAAGACCCGACCTATGTTTCCGAGAATGACGTACCGCTAAACACAGACTACTATTTC

TCCCACCTGTTGGGTACCATAAGCGTGACCTTTAAGGCTCTATTCGGAAATGATGTGAGA

ACAACAGAAAATCTTTTAAAGCGGTTTATTCCGGAAACCCCCCACAAGACCCCCACGAAA

ACCCAGGCACTGCTTGAGCGCGCCGGCTTTGAAAAGCTGACGCCCTTTACACCGGAGGAA

GAAAGTCGTCGAATACTGCATACAGTTTTTTGTACTCTAGAAGCAGCTCCCCATCAAAGC

TGA

>KU206461.1

ATGGCGGCGCGCGAACAGGCCAACTCCGTTCGCAGGAGCGGGTTTTTTAATCCGTTTATT

GGCAAGAGGCCATTTTTCAGGCCGGGAAGCGGGCAGACCGCGGAGACAGAGCGCCCAAGG

CCCCCCCAACACTCGTACTGCACAGAGGTGGGTAGCTTTAAGTTTATAGCTCCAAGATGC

CTCGATGAAGAAGCCCCCGCCGACCAGCGACGCGGTGTACACGTGGGCACCTTGGAGCGC

CCACCCAAAGTGTACTGCGATGGCTCAGAGTACGACGTGCTGAACTTTGCCTCCGGAGGT

TGCTGGCCTCGCAGGATTCGGGTTTGGAACGGCCAGGATTTTCGGGGCGATGGATTCAAC

CCCAGATTTGAGAGATTTCACGTGTACGACATAGTAGAGACTTCGGAGAGCGCCTCACAC

GATGACCCATCCAGGTTTGCTGAGCTATCCCGCCCAAGCGGGTCTGTGGTAACACTGCTG

GGAATGAGCGAGTGTGGAAAGCGAGTCGCGGTTCACGTCTATGGTGTGCGCCATTATTTT

TACATGGCAAAGGCGGAGGTGGATAGCGCTTGTGGAATAACCACCGAGGCAGAACTCGTG

CGCGCAATGGTGGACTGCGCGCACAGCTCGGCTTTGAGCGCGGCCCTGGGAAATGGCAAC

GGCGGCAAACAGAGCGGCGGCAGCGGCGGGGGATGGTGGGGCGGAAAGCACGTGTCTGCG

GACTGCTTCAAAGTGGAGACCGTGTGCCACACGACGCTGTACTACTTTGGATCTAAGCCA

GCTCTCTACTATAGAGTATCTGCCTCCAGCAGCCGCCTGGGAGGGTTCATCTGCGACAAC

TTTCACCCGGAGATTACAAAATTCGAGGGGAGCGTGGACGTGACCACGCGGCTGTTGTTG

GACAATGAAAATTTTACCAGTTTCGGGTGGTACCGCCTGCGACCCGGCACCCACGGAGAG

CGTGTTCAACTTCGCCCCGTTGAGCGACACGTCACCTCAAGCGACGTGGAGATTAACTGT

ACTCCCGATAACCTGGAGCCGATACCAGACGAGGCTGCCTGGCCCGACTATAAGCTCATG

TGCTTTGATATAGAGTGTAAAGCTGGAACGGGTAACGAAATGGCGTTCCCAGTGGCAACT

AACCAAGAGGACCTGGTCATCCAGATCTCCTGTCTGCTGTACTCGCTTGCTACTCAGAAC

CACGAACACACCCTGCTGTTTTCCCTCGGGTCATGCGATATCTCTGAGGAATACTCGTTT

GCATGCGTCCAGCGCGGCGAGCCCAGACCGACGGTTTTGGAGTTTGACAGCGAGTACGAG

CTGCTGGTTGCCTTCCTGACCTTTCTCAAGCAGTACTCTCCCGAGTTCGCCACCGGCTAC

AACATCGTTAATTTTGACTGGGCGTACATAGTTAACAAGGTAACGTCGGTGTATAACATC

AAGCTGGACGGGTACGGCAAGTTCAACAAAGGGGGGCTGTTTAAGGTGTGGGACATCGCC

ACGAACCATTTTCAGAAGAAGAGCAAGGTGAAAATCAATGGCCTGATATCTCTAGACATG

TATTCTGTGGCGACGGAAAAGCTAAAGCTACCCAGCTACAAACTCGACGCGGTCGTGGGA

GACGTCCTCGGCGAGCATAAGATAGACCTTCCCTATAAAGAAATACCCTCCTATTACGCG

GGAGGGCCTGACCGGAGGGGCGTAATAGGAGAGTATTGTATCCAGGACTCTAGGCTGGTG

GGCAAGCTGTTTTTTAAGTACCTCCCCCATCTGGAACTATCGGCGGTGGCCAAACTCGCC

CGTATCACCCTAACGCGGGTAATTTTTGACGGTCAGCAAATTCGCGTGTACACGTGCTTG

CTGAAACTCGCCCGCGAGAGAAATTTCATTTTGCCAGACAACAGACGCCGGTTTGACAGT

CAGGCAGACGCCGCGTCAGAGACTTCGGAGTTGGCTATGGATAGCCAAAGCCACGCCTTC

GACAGTACAGACGAACCCGACGGTGTGGACGGTACCCCGGACGCCGCAGGATCTGGCGCT

ACTTCTGAAAACGGAGGCGGGAAGCCCGGCGTCGGGAGGGCCGTGGGCTACCAGGGAGCA

AAGGTTCTAGACCCCGTATCCGGCTTTCATGTGGACCCCGTGGTTGTGTTTGACTTCGCT

AGCTTATACCCAAGCATTATCCAGGCCCATAACCTCTGTTTCACCACCCTGGCGCTCGAT

GAAGTGGATCTGGCCGGGCTTCAACCATCCGTCAACTACTCGACGTTCGAGGTGGGTGAC

CAAAAGTTATTTTTTGTCCACGCCCATATTCGCGAAAGCCTGCTTGGCATCTTGCTGCGC

GACTGGCTGGCCATGCGAAAGGCGGTGAGGGCGCGAATCCCCACCAGCACCCCCGAGGAG

GCAGTTTTACTAGATAAGCAGCAGTCTGCGATTAAGGTGATATGCAACTCGGTTTACGGA

TTCACGGGGGTGGCAAACGGCCTGTTGCCGTGTCTGAGGATAGCGGCTACCGTTACCACG

ATAGGACGCGACATGCTCCTCAAGACCCGAGATTACGTTCACTCTCGTTGGGCGACGCGC

GAGCTGCTGGAGGACAATTTTCCAGGGGCTATAGGTTTCCGAAACCACAAGCCTTACTCC

GTCAGGGTTATCTACGGAGACACCGACTCCGTGTTTATCAAGTTTGTGGGCCTGACGTAC

GAGGGGGTATCGGAGCTGGGGGATGCTATGTCGCGTCAGATTTCAGCGGACCTCTTTAGA

GCGCCCATCAAACTGGAGTGTGAGAAGACCTTTCAGCGACTGCTGCTGATCACCAAGAAG

AAGTACATAGGTGTCATAAACGGGGGGAAGATGCTCATGAAGGGGGTCGACCTGGTCCGC

AAAAATAACTGCTCTTTCATAAACTTGTACGCGCGACATCTGGTAGATCTTTTGTTGTAC

GACGAGGATGTGGCCACGGCGGCAGCAGAGGTGACAGACGTGCCTCCCGCAGAATGGGTG

GGGCGCCCGCTACCGAGCGGCTTTGACAAGTTTGGGCGAGTGCTGGTAGAGGCGTACAAC

CGTATCACTGCCCCCAACTTGGACGTGCGCGAGTTCGTTATGACTGCTGAGCTGAGCCGC

TCACCCGAATCGTATACCAACAAGCGCCTGCCGCACCTCACCGTCTACTTTAAGCTCGCC

ATGAGGAATGAAGAACTGCCCAGCGTAAAAGAGAGAATTCCGTATGTGATAGTTGCGCAG

ACCGAGGCCGCGGAACGCGAAGCGGGTGTAGTAAACTCAATGCGCGGTACCGCCCAAAAC

CCCGTGGTAACCAAGACCGCACGCCCCCAACCTAAACGCAAACTGCTGGTTTCCGACCTC

GCCGAAGACCCGACCTATGTTTCCGAGAATGACGTACCGCTAAACACAGACTACTATTTC

TCCCACCTGTTGGGTACCATAAGCGTGACCTTTAAGGCTCTATTCGGAAATGATGTGAGA

ACAACAGAAAATCTTTTAAAGCGGTTTATTCCGGAAACCCCCCACAAGACCCCCACGAAA

ACCCAGGCACTGCTTGAGCGCGCCGGCTTTGAAAAGCTGACGCCCTTTACACCGGAGGAA

GAAAGTCGTCGAATACTGCATACAGTTTTTTGTACTCTAGAAGCAGCTCCCCATCAAAGC

TGA

>KU206462.1

ATGGCGGCGCGCGAACAGGCCAACTCCGTTCGCAGGAGCGGGTTTTTTAATCCGTTTATT

GGCAAGAGGCCATTTTTCAGGCCGGGAAGCGGGCAGACCGCGGAGACAGAGCGCCCAAGG

CCCCCCCAACACTCGTACTGCACAGAGGTGGGTAGCTTTAAGTTTATAGCTCCAAGATGC

CTCGATGAAGAAGCCCCCGCCGACCAGCGACGCGGTGTACACGTGGGCACCTTGGAGCGC

CCACCCAAAGTGTACTGCGATGGCTCAGAGTACGACGTGCTGAACTTTGCCTCCGGAGGT

TGCTGGCCTCGCAGGATTCGGGTTTGGAACGGCCAGGATTTTCGGGGCGATGGATTCAAC

CCCAGATTTGAGAGATTTCACGTGTACGACATAGTAGAGACTTCGGAGAGCGCCTCACAC

GATGACCCATCCAGGTTTGCTGAGCTATCCCGCCCAAGTGGGTCTGTGGTAACACTGCTG

GGAATGAGCGAGTGTGGAAAGCGAGTCGCGGTTCACGTCTATGGTGTGCGCCATTATTTT

TACATGGCAAAGGCGGAGGTGGATAGCGCTTGTGGAATAACCACCGAGGCAGAACTCGTG

CGCGCAATGGTGGACTGCGCGCACAGCTCGGCTTTGAGCGCGGCCCTGGGAAATGGCAAC

GGCGGCAAACAGAGCGGCGGCAGCGGCGGGGGATGGTGGGGCGGAAAGCACGTGTCTGCG

GACTGCTTCAAAGTGGAGACCGTGTGCCACACGACGCTGTACTACTTTGGATCTAAGCCA

GCTCTCTACTATAGAGTATCTGCCTCCAGCAGCCGCCTGGGAGGGTTCATCTGCGACAAC

TTTCACCCGGAGATTACAAAATTCGAGGGGAGCGTGGACGTGACCACGCGGCTGTTGTTG

GACAATGAAAATTTTACCAGTTTCGGGTGGTACCGCCTGCGACCCGGCACCCACGGAGAG

CGTGTTCAACTTCGCCCCGTTGAGCGACACGTCACCTCAAGCGACGTGGAGATTAACTGT

ACTCCCGATAACCTGGAGCCGATACCAGACGAGGCTGCCTGGCCCGACTATAAGCTCATG

TGCTTTGATATAGAGTGTAAAGCTGGAACGGGTAACGAAATGGCGTTCCCAGTGGCAACT

AACCAAGAGGACCTGGTCATCCAGATCTCCTGTCTGCTGTACTCGCTTGCTACTCAGAAC

CACGAACACACCCTGCTGTTTTCCCTCGGGTCATGCGATATCTCTGAGGAATACTCGTTT

GCATGCGTCCAGCGCGGCGAGCCCAGACCGACGGTTTTGGAGTTTGACAGCGAGTACGAG

CTGCTGGTTGCCTTCCTGACCTTTCTCAAGCAGTACTCTCCCGAGTTCGCCACCGGCTAC

AACATCGTTAATTTTGACTGGGCGTACATAGTTAACAAGGTAACGTCGGTGTATAACATC

AAGCTGGACGGGTACGGCAAGTTCAACAAAGGGGGGCTGTTTAAGGTGTGGGACATCGCC

ACGAACCATTTTCAGAAGAAGAGCAAGGTGAAAATCAATGGCCTGATATCTCTAGACATG

TATTCTGTGGCGACGGAAAAGCTAAAGCTACCCAGCTACAAACTCGACGCGGTCGTGGGA

GACGTCCTCGGCGAGCATAAGATAGACCTTCCCTATAAAGAAATACCCTCCTATTACGCG

GGAGGGCCTGACCGGAGGGGCGTAATAGGAGAGTATTGTATCCAGGACTCTAGGCTGGTG

GGCAAGCTGTTTTTTAAGTACCTCCCCCATCTGGAACTATCGGCGGTGGCCAAACTCGCC

CGTATCACCCTAACGCGGGTAATTTTTGACGGTCAGCAAATTCGCGTGTACACGTGCTTG

CTGAAACTCGCCCGCGAGAGAAATTTCATTTTGCCAGACAACAGACGCCGGTTTGACAGT

CAGGCAGACGCCGCGTCAGAGACTTCGGAGTTGGCTATGGATAGCCAAAGCCACGCCTTC

GACAGTACAGACGAACCCGACGGTGTGGACGGTACCCCGGACGCCGCAGGATCTGGCGCT

ACTTCTGAAAACGGAGGCGGGAAGCCCGGCGTCGGGAGGGCCGTGGGCTACCAGGGAGCA

AAGGTTCTAGACCCCGTATCCGGCTTTCATGTGGACCCCGTGGTTGTGTTTGACTTCGCT

AGCTTATACCCAAGCATTATCCAGGCCCATAACCTCTGTTTCACCACCCTGGCGCTCGAT

GAAGTGGATCTGGCCGGGCTTCAACCATCCGTCAACTACTCGACGTTCGAGGTGGGTGAC

CAAAAGTTATTTTTTGTCCACGCCCATATTCGCGAAAGCCTGCTTGGCATCTTGCTGCGC

GACTGGCTGGCCATGCGAAAGGCGGTGAGGGCGCGAATCCCCACCAGCACCCCCGAGGAG

GCAGTTTTACTAGATAAGCAGCAGTCTGCGATTAAGGTGATATGCAACTCGGTTTACGGA

TTCACGGGGGTGGCAAACGGCCTGTTGCCGTGTCTGAGGATAGCGGCTACCGTTACCACG

ATAGGACGCGACATGCTCCTCAAGACCCGAGATTACGTTCACTCTCGTTGGGCGACGCGC

GAGCTGCTGGAGGACAATTTTCCAGGGGCTATAGGTTTCCGAAACCACAAGCCTTACTCC

GTCAGGGTTATCTACGGAGACACCGACTCCGTGTTTATCAAGTTTGTGGGCCTGACGTAC

GAGGGGGTATCGGAGCTGGGGGATGCTATGTCGCGTCAGATTTCAGCGGACCTCTTTAGA

GCGCCCATCAAACTGGAGTGTGAGAAGACCTTTCAGCGACTGCTGCTGATCACCAAGAAG

AAGTACATAGGTGTCATAAACGGGGGGAAGATGCTCATGAAGGGGGTCGACCTGGTCCGC

AAAAATAACTGCTCTTTCATAAACTTGTACGCGCGACATCTGGTAGATCTTTTGTTGTAC

GACGAGGATGTGGCCACGGCGGCAGCAGAGGTGACAGACGTGCCTCCCGCAGAATGGGTG

GGGCGCCCGCTACCGAGCGGCTTTGACAAGTTTGGGCGAGTGCTGGTAGAGGCGTACAAC

CGTATCACTGCCCCCAACTTGGACGTGCGCGAGTTCGTTATGACTGCTGAGCTGAGCCGC

TCACCCGAATCGTATACCAACAAGCGCCTGCCGCACCTCACCGTCTACTTTAAGCTCGCC

ATGAGGAATGAAGAACTGCCCAGCGTAAAAGAGAGAATTCCGTATGTGATAGTTGCGCAG

ACCGAGGCCGCGGAACGCGAAGCGGGTGTAGTAAACTCAATGCGCGGTACCGCCCAAAAC

CCCGTGGTAACCAAGACCGCACGCCCCCAACCTAAACGCAAACTGCTGGTTTCCGACCTC

GCCGAAGACCCGACCTATGTTTCCGAGAATGACGTACCGCTAAACACAGACTACTATTTC

TCCCACCTGTTGGGTACCATAAGCGTGACCTTTAAGGCTCTATTCGGAAATGATGTGAGA

ACAACAGAAAATCTTTTAAAGCGGTTTATTCCGGAAACCCCCCACAAGACCCCCACGAAA

ACCCAGGCACTGCTTGAGCGCGCCGGCTTTGAAAAGCTGACGCCCTTTACACCGGAGGAA

GAAAGTCGTCGAATACTGCATACAGTTTTTTGTACTCTAGAAGCAGCTCCCCATCAAAGC

TGA

>KU206463.1

ATGGCGGCGCGCGAACAGGCCAACTCCGTTCGCAGGAGCGGGTTTTTTAATCCGTTTATT

GGCAAGAGGCCATTTTTCAGGCCGGGAAGCGGGCAGACCGCGGAGACAGAGCGCCCAAGG

CCCCCCCAACACTCGTACTGCACAGAGGTGGGTAGCTTTAAGTTTATAGCTCCAAGATGC

CTCGATGAAGAAGCCCCCGCCGACCAGCGACGCGGTGTACACGTGGGCACCTTGGAGCGC

CCACCCAAAGTGTACTGCGATGGCTCAGAGTACGACGTGCTGAACTTTGCCTCCGGAGGT

TGCTGGCCTCGCAGGATTCGGGTTTGGAACGGCCAGGATTTTCGGGGCGATGGATTCAAC

CCCAGATTTGAGAGATTTCACGTGTACGACATAGTAGAGACTTCGGAGAGCGCCTCACAC

GATGACCCATCCAGGTTTGCTGAGCTATCCCGCCCAAGCGGGTCTGTGGTAACACTGCTG

GGAATGAGCGAGTGTGGAAAGCGAGTCGCGGTTCACGTCTATGGTGTGCGCCATTATTTT

TACATGGCAAAGGCGGAGGTGGATAGCGCTTGTGGAATAACCACCGAGGCAGAACTCGTG

CGCGCAATGGTGGACTGCGCGCACAGCTCGGCTTTGAGCGCGGCCCTGGGAAATGGCAAC

GGCGGCAAACAGAGCGGCGGCAGCGGCGGGGGATGGTGGGGCGGAAAGCACGTGTCTGCG

GACTGCTTCAAAGTGGAGACCGTGTGCCACACGACGCTGTACTACTTTGGATCTAAGCCA

GCTCTCTACTATAGAGTATCTGCCTCCAGCAGCCGCCTGGGAGGGTTCATCTGCGACAAC

TTTCACCCGGAGATTACAAAATTCGAGGGGAGCGTGGACGTGACCACGCGGCTGTTGTTG

GACAATGAAAATTTTACCAGTTTCGGGTGGTACCGCCTGCGACCCGGCACCCACGGAGAG

CGTGTTCAACTTCGCCCCGTTGAGCGACACGTCACCTCAAGCGACGTGGAGATTAACTGT

ACTCCCGATAACCTGGAGCCGATACCAGACGAGGCTGCCTGGCCCGACTATAAGCTCATG

TGCTTTGATATAGAGTGTAAAGCTGGAACGGGTAACGAAATGGCGTTCCCAGTGGCAACT

AACCAAGAGGACCTGGTCATCCAGATCTCCTGTCTGCTGTACTCGCTTGCTACTCAGAAC

CACGAACACACCCTGCTGTTTTCCCTCGGGTCATGCGATATCTCTGAGGAATACTCGTTT

GCATGCGTCCAGCGCGGCGAGCCCAGACCGACGGTTTTGGAGTTTGACAGCGAGTACGAG

CTGCTGGTTGCCTTCCTGACCTTTCTCAAGCAGTACTCTCCCGAGTTCGCCACCGGCTAC

AACATCGTTAATTTTGACTGGGCGTACATAGTTAACAAGGTAACGTCGGTGTATAACATC

AAGCTGGACGGGTACGGCAAGTTCAACAAAGGGGGGCTGTTTAAGGTGTGGGACATCGCC

ACGAACCATTTTCAGAAGAAGAGCAAGGTGAAAATCAATGGCCTGATATCTCTAGACATG

TATTCTGTGGCGACGGAAAAGCTAAAGCTACCCAGCTACAAACTCGACGCGGTCGTGGGA

GACGTCCTCGGCGAGCATAAGATAGACCTTCCCTATAAAGAAATACCCTCCTATTACGCG

GGAGGGCCTGACCGGAGGGGCGTAATAGGAGAGTATTGTATCCAGGACTCTAGGCTGGTG

GGCAAGCTGTTTTTTAAGTACCTCCCCCATCTGGAACTATCGGCGGTGGCCAAACTCGCC

CGTATCACCCTAACGCGGGTAATTTTTGACGGTCAGCAAATTCGCGTGTACACGTGCTTG

CTGAAACTCGCCCGCGAGAGAAATTTCATTTTGCCAGACAACAGACGCCGGTTTGACAGT

CAGGCAGACGCCGCGTCAGAGACTTCGGAGTTGGCTATGGATAGCCAAAGCCACGCCTTC

GACAGTACAGACGAACCCGACGGTGTGGACGGTACCCCGGACGCCGCAGGATCTGGCGCT

ACTTCTGAAAACGGAGGCGGGAAGCCCGGCGTCGGGAGGGCCGTGGGCTACCAGGGAGCA

AAGGTTCTAGACCCCGTATCCGGCTTTCATGTGGACCCCGTGGTTGTGTTTGACTTCGCT

AGCTTATACCCAAGCATTATCCAGGCCCATAACCTCTGTTTCACCACCCTGGCGCTCGAT

GAAGTGGATCTGGCCGGGCTTCAACCATCCGTCAACTACTCGACGTTCGAGGTGGGTGAC

CAAAAGTTATTTTTTGTCCACGCCCATATTCGCGAAAGCCTGCTTGGCATCTTGCTGCGC

GACTGGCTGGCCATGCGAAAGGCGGTGAGGGCGCGAATCCCCACCAGCACCCCCGAGGAG

GCAGTTTTACTAGATAAGCAGCAGTCTGCGATTAAGGTGATATGCAACTCGGTTTACGGA

TTCACGGGGGTGGCAAACGGCCTGTTGCCGTGTCTGAGGATAGCGGCTACCGTTACCACG

ATAGGACGCGACATGCTCCTCAAGACCCGAGATTACGTTCACTCTCGTTGGGCGACGCGC

GAGCTGCTGGAGGACAATTTTCCAGGGGCTATAGGTTTCCGAAACCACAAGCCTTACTCC

GTCAGGGTTATCTACGGAGACACCGACTCCGTGTTTATCAAGTTTGTGGGCCTGACGTAC

GAGGGGGTATCGGAGCTGGGGGATGCTATGTCGCGTCAGATTTCAGCGGACCTCTTTAGA

GCGCCCATCAAACTGGAGTGTGAGAAGACCTTTCAGCGACTGCTGCTGATCACCAAGAAG

AAGTACATAGGTGTCATAAACGGGGGGAAGATGCTCATGAAGGGGGTCGACCTGGTCCGC

AAAAATAACTGCTCTTTCATAAACTTGTACGCGCGACATCTGGTAGATCTTTTGTTGTAC

GACGAGGATGTGGCCACGGCGGCAGCAGAGGTGACAGACGTGCCTCCCGCAGAATGGGTG

GGGCGCCCGCTACCGAGCGGCTTTGACAAGTTTGGGCGAGTGCTGGTAGAGGCGTACAAC

CGTATCACTGCCCCCAACTTGGACGTGCGCGAGTTCGTTATGACTGCTGAGCTGAGCCGC

TCACCCGAATCGTATACCAACAAGCGCCTGCCGCACCTCACCGTCTACTTTAAGCTCGCC

ATGAGGAATGAAGAACTGCCCAGCGTAAAAGAGAGAATTCCGTATGTGATAGTTGCGCAG

ACCGAGGCCGCGGAACGCGAAGCGGGTGTAGTAAACTCAATGCGCGGTACCGCCCAAAAC

CCCGTGGTAACCAAGACCGCACGCCCCCAACCTAAACGCAAACTGCTGGTTTCCGACCTC

GCCGAAGACCCGACCTATGTTTCCGAGAATGACGTACCGCTAAACACAGACTACTATTTC

TCCCACCTGTTGGGTACCATAAGCGTGACCTTTAAGGCTCTATTCGGAAATGATGTGAGA

ACAACAGAAAATCTTTTAAAGCGGTTTATTCCGGAAACCCCCCACAAGACCCCCACGAAA

ACCCAGGCACTGCTTGAGCGCGCCGGCTTTGAAAAGCTGACGCCCTTTACACCGGAGGAA

GAAAGTCGTCGAATACTGCATACAGTTTTTTGTACTCTAGAAGCAGCTCCCCATCAAAGC

TGA

>KU206464.1

ATGGCGGCGCGCGAACAGGCCAACTCCGTTCGCAGGAGCGGGTTTTTTAATCCGTTTATT

GGCAAGAGGCCATTTTTCAGGCCGGGAAGCGGGCAGACCGCGGAGACAGAGCGCCCAAGG

CCCCCCCAACACTCGTACTGCACAGAGGTGGGTAGCTTTAAGTTTATAGCTCCAAGATGC

CTCGATGAAGAAGCCCCCGCCGACCAGCGACGCGGTGTACACGTGGGCACCTTGGAGCGC

CCACCCAAAGTGTACTGCGATGGCTCAGAGTACGACGTGCTGAACTTTGCCTCCGGAGGT

TGCTGGCCTCGCAGGATTCGGGTTTGGAACGGCCAGGATTTTCGGGGCGATGGATTCAAC

CCCAGATTTGAGAGATTTCACGTGTACGACATAGTAGAGACTTCGGAGAGCGCCTCACAC

GATGACCCATCCAGGTTTGCTGAGCTATCCCGCCCAAGCGGGTCTGTGGTAACACTGCTG

GGAATGAGCGAGTGTGGAAAGCGAGTCGCGGTTCACGTCTATGGTGTGCGCCATTATTTT

TACATGGCAAAGGCGGAGGTGGATAGCGCTTGTGGAATAACCACCGAGGCAGAACTCGTG

CGCGCAATGGTGGACTGCGCGCACAGCTCGGCTTTGAGCGCGGCCCTGGGAAATGGCAAC

GGCGGCAAACAGAGCGGCGGCAGCGGCGGGGGATGGTGGGGCGGAAAGCACGTGTCTGCG

GACTGCTTCAAAGTGGAGACCGTGTGCCACACGACGCTGTACTACTTTGGATCTAAGCCA

GCTCTCTACTATAGAGTATCTGCCTCCAGCAGCCGCCTGGGAGGGTTCATCTGCGACAAC

TTTCACCCGGAGATTACAAAATTCGAGGGGAGCGTGGACGTGACCACGCGGCTGTTGTTG

GACAATGAAAATTTTACCAGTTTCGGGTGGTACCGCCTGCGACCCGGCACCCACGGAGAG

CGTGTTCAACTTCGCCCCGTTGAGCGACACGTCACCTCAAGCGACGTGGAGATTAACTGT

ACTCCCGATAACCTGGAGCCGATACCAGACGAGGCTGCCTGGCCCGACTATAAGCTCATG

TGCTTTGATATAGAGTGTAAAGCTGGAACGGGTAACGAAATGGCGTTCCCAGTGGCAACT

AACCAAGAGGACCTGGTCATCCAGATCTCCTGTCTGCTGTACTCGCTTGCTACTCAGAAC

CACGAACACACCCTGCTGTTTTCCCTCGGGTCATGCGATATCTCTGAGGAATACTCGTTT

GCATGCGTCCAGCGCGGCGAGCCCAGACCGACGGTTTTGGAGTTTGACAGCGAGTACGAG

CTGCTGGTTGCCTTCCTGACCTTTCTCAAGCAGTACTCTCCCGAGTTCGCCACCGGCTAC

AACATCGTTAATTTTGACTGGGCGTACATAGTTAACAAGGTAACGTCGGTGTATAACATC

AAGCTGGACGGGTACGGCAAGTTCAACAAAGGGGGGCTGTTTAAGGTGTGGGACATCGCC

ACGAACCATTTTCAGAAGAAGAGCAAGGTGAAAATCAATGGCCTGATATCTCTAGACATG

TATTCTGTGGCGACGGAAAAGCTAAAGCTACCCAGCTACAAACTCGACGCGGTCGTGGGA

GACGTCCTCGGCGAGCATAAGATAGACCTTCCCTATAAAGAAATACCCTCCTATTACGCG

GGAGGGCCTGACCGGAGGGGCGTAATAGGAGAGTATTGTATCCAGGACTCTAGGCTGGTG

GGCAAGCTGTTTTTTAAGTACCTCCCCCATCTGGAACTATCGGCGGTGGCCAAACTCGCC

CGTATCACCCTAACGCGGGTAATTTTTGACGGTCAGCAAATTCGCGTGTACACGTGCTTG

CTGAAACTCGCCCGCGAGAGAAATTTCATTTTGCCAGACAACAGACGCCGGTTTGACAGT

CAGGCAGACGCCGCGTCAGAGACTTCGGAGTTGGCTATGGATAGCCAAAGCCACGCCTTC

GACAGTACAGACGAACCCGACGGTGTGGACGGTACCCCGGACGCCGCAGGATCTGGCGCT

ACTTCTGAAAACGGAGGCGGGAAGCCCGGCGTCGGGAGGGCCGTGGGCTACCAGGGAGCA

AAGGTTCTAGACCCCGTATCCGGCTTTCATGTGGACCCCGTGGTTGTGTTTGACTTCGCT

AGCTTATACCCAAGCATTATCCAGGCCCATAACCTCTGTTTCACCACCCTGGCGCTCGAT

GAAGTGGATCTGGCCGGGCTTCAACCATCCGTCGACTACTCGACGTTCGAGGTGGGTGAC

CAAAAGTTATTTTTTGTCCACGCCCATATTCGCGAAAGCCTGCTTGGCATCTTGCTGCGC

GACTGGCTGGCCATGCGAAAGGCGGTGAGGGCGCGAATCCCCACCAGCACCCCCGAGGAG

GCAGTTTTACTAGATAAGCAGCAGTCTGCGATTAAGGTGATATGCAACTCGGTTTACGGA

TTCACGGGGGTGGCAAACGGCCTGTTGCCGTGTCTGAGGATAGCGGCTACCGTTACCACG

ATAGGACGCGACATGCTCCTCAAGACCCGAGATTACGTTCACTCTCGTTGGGCGACGCGC

GAGCTGCTGGAGGACAATTTTCCAGGGGCTATAGGTTTCCGAAACCACAAGCCTTACTCC

GTCAGGGTTATCTACGGAGACACCGACTCCGTGTTTATCAAGTTTGTGGGCCTGACGTAC

GAGGGGGTATCGGAGCTGGGGGATGCTATGTCGCGTCAGATTTCAGCGGACCTCTTTAGA

GCGCCCATCAAACTGGAGTGTGAGAAGACCTTTCAGCGACTGCTGCTGATCACCAAGAAG

AAGTACATAGGTGTCATAAACGGGGGGAAGATGCTCATGAAGGGGGTCGACCTGGTCCGC

AAAAATAACTGCTCTTTCATAAACTTGTACGCGCGACATCTGGTAGATCTTTTGTTGTAC

GACGAGGATGTGGCCACGGCGGCAGCAGAGGTGACAGACGTGCCTCCCGCAGAATGGGTG

GGGCGCCCGCTACCGAGCGGCTTTGACAAGTTTGGGCGAGTGCTGGTAGAGGCGTACAAC

CGTATCACTGCCCCCAACTTGGACGTGCGCGAGTTCGTTATGACTGCTGAGCTGAGCCGC

TCACCCGAATCGTATACCAACAAGCGCCTGCCGCACCTCACCGTCTACTTTAAGCTCGCC

ATGAGGAATGAAGAACTGCCCAGCGTAAAAGAGAGAATTCCGTATGTGATAGTTGCGCAG

ACCGAGGCCGCGGAACGCGAAGCGGGTGTAGTAAACTCAATGCGCGGTACCGCCCAAAAC

CCCGTGGTAACCAAGACCGCACGCCCCCAACCTAAACGCAAACTGCTGGTTTCCGACCTC

GCCGAAGACCCGACCTATGTTTCCGAGAATGACGTACCGCTAAACACAGACTACTATTTC

TCCCACCTGTTGGGTACCATAAGCGTGACCTTTAAGGCTCTATTCGGAAATGATGTGAGA

ACAACAGAAAATCTTTTAAAGCGGTTTATTCCGGAAACCCCCCACAAGACCCCCACGAAA

ACCCAGGCACTGCTTGAGCGCGCCGGCTTTGAAAAGCTGACGCCCTTTACACCGGAGGAA

GAAAGTCGTCGAATACTGCATACAGTTTTTTGTACTCTAGAAGCAGCTCCCCATCAAAGC

TGA

>KU206465.1

ATGGCGGCGCGCGAACAGGCCAACTCCGTTCGCAGGAGCGGGTTTTTTAATCCGTTTATT

GGCAAGAGGCCATTTTTCAGGCCGGGAAGCGGGCAGACCGCGGAGACAGAGCGCCCAAGG

CCCCCCCAACACTCGTACTGCACAGAGGTGGGTAGCTTTAAGTTTATAGCTCCAAGATGC

CTCGATGAAGAAGCCCCCGCCGACCAGCGACGCGGTGTACACGTGGGCACCTTGGAGCGC

CCACCCAAAGTGTACTGCGATGGCTCAGAGTACGACGTGCTGAACTTTGCCTCCGGAGGT

TGCTGGCCTCGCAGGATTCGGGTTTGGAACGGCCAGGATTTTCGGGGCGATGGATTCAAC

CCCAGATTTGAGAGATTTCACGTGTACGACATAGTAGAGACTTCGGAGAGCGCCTCACAC

GATGACCCATCCAGGTTTGCTGAGCTATCCCGCCCAAGCGGGTCTGTGGTAACACTGCTG

GGAATGAGCGAGTGTGGAAAGCGAGTCGCGGTTCACGTCTATGGTGTGCGCCATTATTTT

TACATGGCAAAGGCGGAGGTGGATAGCGCTTGTGGAATAACCACCGAGGCAGAACTCGTG

CGCGCAATGGTGGACTGCGCGCACAGCTCGGCTTTGAGCGCGGCCCTGGGAAATGGCAAC

GGCGGCAAACAGAGCGGCGGCAGCGGCGGGGGATGGTGGGGCGGAAAGCACGTGTCTGCG

GACTGCTTCAAAGTGGAGACCGTGTGCCACACGACGCTGTACTACTTTGGATCTAAGCCA

GCTCTCTACTATAGAGTATCTGCCTCCAGCAGCCGCCTGGGAGGGTTCATCTGCGACAAC

TTTCACCCGGAGATTACAAAATTCGAGGGGAGCGTGGACGTGACCACGCGGCTGTTGTTG

GACAATGAAAATTTTACCAGTTTCGGGTGGTACCGCCTGCGACCCGGCACCCACGGAGAG

CGTGTTCAACTTCGCCCCGTTGAGCGACACGTCACCTCAAGCGACGTGGAGATTAACTGT

ACTCCCGATAACCTGGAGCCGATACCAGACGAGGCTGCCTGGCCCGACTATAAGCTCATG

TGCTTTGATATAGAGTGTAAAGCTGGAACGGGTAACGAAATGGCGTTCCCAGTGGCAACT

AACCAAGAGGACCTGGTCATCCAGATCTCCTGTCTGCTGTACTCGCTTGCTACTCAGAAC

CACGAACACACCCTGCTGTTTTCCCTCGGGTCATGCGATATCTCTGAGGAATACTCGTTT

GCATGCGTCCAGCGCGGCGAGCCCAGACCGACGGTTTTGGAGTTTGACAGCGAGTACGAG

CTGCTGGTTGCCTTCCTGACCTTTCTCAAGCAGTACTCTCCCGAGTTCGCCACCGGCTAC

AACATCGTTAATTTTGACTGGGCGTACATAGTTAACAAGGTAACGTCGGTGTATAACATC

AAGCTGGACGGGTACGGCAAGTTCAACAAAGGGGGGCTGTTTAAGGTGTGGGACATCGCC

ACGAACCATTTTCAGAAGAAGAGCAAGGTGAAAATCAATGGCCTGATATCTCTAGACATG

TATTCTGTGGCGACGGAAAAGCTAAAGCTACCCAGCTACAAACTCGACGCGGTCGTGGGA

GACGTCCTCGGCGAGCATAAGATAGACCTTCCCTATAAAGAAATACCCTCCTATTACGCG

GGAGGGCCTGACCGGAGGGGCGTAATAGGAGAGTATTGTATCCAGGACTCTAGGCTGGTG

GGCAAGCTGTTTTTTAAGTACCTCCCCCATCTGGAACTATCGGCGGTGGCCAAACTCGCC

CGTATCACCCTAACGCGGGTAATTTTTGACGGTCAGCAAATTCGCGTGTACACGTGCTTG

CTGAAACTCGCCCGCGAGAGAAATTTCATTTTGCCAGACAACAGACGCCGGTTTGACAGT

CAGGCAGACGCCGCGTCAGAGACTTCGGAGTTGGCTATGGATAGCCAAAGCCACGCCTTC

GACAGTACAGACGAACCCGACGGTGTGGACGGTACCCCGGACGCCGCAGGATCTGGCGCT

ACTTCTGAAAACGGAGGCGGGAAGCCCGGCGTCGGGAGGGCCGTGGGCTACCAGGGAGCA

AAGGTTCTAGACCCCGTATCCGGCTTTCATGTGGACCCCGTGGTTGTGTTTGACTTCGCT

AGCTTATACCCAAGCATTATCCAGGCCCATAACCTCTGTTTCACCACCCTGGCGCTCGAT

GAAGTGGATCTGGCCGGGCTTCAACCATCCGTCGACTACTCGACGTTCGAGGTGGGTGAC

CAAAAGTTATTTTTTGTCCACGCCCATATTCGCGAAAGCCTGCTTGGCATCTTGCTGCGC

GACTGGCTGGCCATGCGAAAGGCGGTGAGGGCGCGAATCCCCACCAGCACCCCCGAGGAG

GCAGTTTTACTAGATAAGCAGCAGTCTGCGATTAAGGTGATATGCAACTCGGTTTACGGA

TTCACGGGGGTGGCAAACGGCCTGTTGCCGTGTCTGAGGATAGCGGCTACCGTTACCACG

ATAGGACGCGACATGCTCCTCAAGACCCGAGATTACGTTCACTCTCGTTGGGCGACGCGC

GAGCTGCTGGAGGACAATTTTCCAGGGGCTATAGGTTTCCGAAACCACAAGCCTTACTCC

GTCAGGGTTATCTACGGAGACACCGACTCCGTGTTTATCAAGTTTGTGGGCCTGACGTAC

GAGGGGGTATCGGAGCTGGGGGATGCTATGTCGCGTCAGATTTCAGCGGACCTCTTTAGA

GCGCCCATCAAACTGGAGTGTGAGAAGACCTTTCAGCGACTGCTGCTGATCACCAAGAAG

AAGTACATAGGTGTCATAAACGGGGGGAAGATGCTCATGAAGGGGGTCGACCTGGTCCGC

AAAAATAACTGCTCTTTCATAAACTTGTACGCGCGACATCTGGTAGATCTTTTGTTGTAC

GACGAGGATGTGGCCACGGCGGCAGCAGAGGTGACAGACGTGCCTCCCGCAGAATGGGTG

GGGCGCCCGCTACCGAGCGGCTTTGACAAGTTTGGGCGAGTGCTGGTAGAGGCGTACAAC

CGTATCACTGCCCCCAACTTGGACGTGCGCGAGTTCGTTATGACTGCTGAGCTGAGCCGC

TCACCCGAATCGTATACCAACAAGCGCCTGCCGCACCTCACCGTCTACTTTAAGCTCGCC

ATGAGGAATGAAGAACTGCCCAGCGTAAAAGAGAGAATTCCGTATGTGATAGTTGCGCAG

ACCGAGGCCGCGGAACGCGAAGCGGGTGTAGTAAACTCAATGCGCGGTACCGCCCAAAAC

CCCGTGGTAACCAAGACCGCACGCCCCCAACCTAAACGCAAACTGCTGGTTTCCGACCTC

GCCGAAGACCCGACCTATGTTTCCGAGAATGACGTACCGCTAAACACAGACTACTATTTC

TCCCACCTGTTGGGTACCATAAGCGTGACCTTTAAGGCTCTATTCGGAAATGATGTGAGA

ACAACAGAAAATCTTTTAAAGCGGTTTATTCCGGAAACCCCCCACAAGACCCCCACGAAA

ACCCAGGCACTGCTTGAGCGCGCCGGCTTTGAAAAGCTGACGCCCTTTACACCGGAGGAA

GAAAGTCGTCGAATACTGCATACAGTTTTTTGTACTCTAGAAGCAGCTCCCCATCAAAGC

TGA

>KU206466.1

ATGGCGGCGCGCGAACAGGCCAACTCCGTTCGCAGGAGCGGGTTTTTTAATCCGTTTATT

GGCAAGAGGCCATTTTTCAGGCCGGGAAGCGGGCAGACCGCGGAGACAGAGCGCCCAAGG

CCCCCCCAACACTCGTACTGCACAGAGGTGGGTAGCTTTAAGTTTATAGCTCCAAGATGC

CTCGATGAAGAAGCCCCCGCCGACCAGCGACGCGGTGTACACGTGGGCACCTTGGAGCGC

CCACCCAAAGTGTACTGCGATGGCTCAGAGTACGACGTGCTGAACTTTGCCTCCGGAGGT

TGCTGGCCTCGCAGGATTCGGGTTTGGAACGGCCAGGATTTTCGGGGCGATGGATTCAAC

CCCAGATTTGAGAGATTTCACGTGTACGACATAGTAGAGACTTCGGAGAGCGCCTCACAC

GATGACCCATCCAGGTTTGCTGAGCTATCCCGCCCAAGCGGGTCTGTGGTAACACTGCTG

GGAATGAGCGAGTGTGGAAAGCGAGTCGCGGTTCACGTCTATGGTGTGCGCCATTATTTT

TACATGGCAAAGGCGGAGGTGGATAGCGCTTGTGGAATAACCACCGAGGCAGAACTCGTG

CGCGCAATGGTGGACTGCGCGCACAGCTCGGCTTTGAGCGCGGCCCTGGGAAATGGCAAC

GGCGGCAAACAGAGCGGCGGCAGCGGCGGGGGATGGTGGGGCGGAAAGCACGTGTCTGCG

GACTGCTTCAAAGTGGAGACCGTGTGCCACACGACGCTGTACTACTTTGGATCTAAGCCA

GCTCTCTACTATAGAGTATCTGCCTCCAGCAGCCGCCTGGGAGGGTTCATCTGCGACAAC

TTTCACCCGGAGATTACAAAATTCGAGGGGAGCGTGGACGTGACCACGCGGCTGTTGTTG

GACAATGAAAATTTTACCAGTTTCGGGTGGTACCGCCTGCGACCCGGCACCCACGGAGAG

CGTGTTCAACTTCGCCCCGTTGAGCGACACGTCACCTCAAGCGACGTGGAGATTAACTGT

ACTCCCGATAACCTGGAGCCGATACCAGACGAGGCTGCCTGGCCCGACTATAAGCTCATG

TGCTTTGATATAGAGTGTAAAGCTGGAACGGGTAACGAAATGGCGTTCCCAGTGGCAACT

AACCAAGAGGACCTGGTCATCCAGATCTCCTGTCTGCTGTACTCGCTTGCTACTCAGAAC

CACGAACACACCCTGCTGTTTTCCCTCGGGTCATGCGATATCTCTGAGGAATACTCGTTT

GCATGCGTCCAGCGCGGCGAGCCCAGACCGACGGTTTTGGAGTTTGACAGCGAGTACGAG

CTGCTGGTTGCCTTCCTGACCTTTCTCAAGCAGTACTCTCCCGAGTTCGCCACCGGCTAC

AACATCGTTAATTTTGACTGGGCGTACATAGTTAACAAGGTAACGTCGGTGTATAACATC

AAGCTGGACGGGTACGGCAAGTTCAACAAAGGGGGGCTGTTTAAGGTGTGGGACATCGCC

ACGAACCATTTTCAGAAGAAGAGCAAGGTGAAAATCAATGGCCTGATATCTCTAGACATG

TATTCTGTGGCGACGGAAAAGCTAAAGCTACCCAGCTACAAACTCGACGCGGTCGTGGGA

GACGTCCTCGGCGAGCATAAGATAGACCTTCCCTATAAAGAAATACCCTCCTATTACGCG

GGAGGGCCTGACCGGAGGGGCGTAATAGGAGAGTATTGTATCCAGGACTCTAGGCTGGTG

GGCAAGCTGTTTTTTAAGTACCTCCCCCATCTGGAACTATCGGCGGTGGCCAAACTCGCC

CGTATCACCCTAACGCGGGTAATTTTTGACGGTCAGCAAATTCGCGTGTACACGTGCTTG

CTGAAACTCGCCCGCGAGAGAAATTTCATTTTGCCAGACAACAGACGCCGGTTTGACAGT

CAGGCAGACGCCGCGTCAGAGACTTCGGAGTTGGCTATGGATAGCCAAAGCCACGCCTTC

GACAGTACAGACGAACCCGACGGTGTGGACGGTACCCCGGACGCCGCAGGATCTGGCGCT

ACTTCTGAAAACGGAGGCGGGAAGCCCGGCGTCGGGAGGGCCGTGGGCTACCAGGGAGCA

AAGGTTCTAGACCCCGTATCCGGCTTTCATGTGGACCCCGTGGTTGTGTTTGACTTCGCT

AGCTTATACCCAAGCATTATCCAGGCCCATAACCTCTGTTTCACCACCCTGGCGCTCGAT

GAAGTGGATCTGGCCGGGCTTCAACCATCCGTCGACTACTCGACGTTCGAGGTGGGTGAC

CAAAAGTTATTTTTTGTCCACGCCCATATTCGCGAAAGCCTGCTTGGCATCTTGCTGCGC

GACTGGCTGGCCATGCGAAAGGCGGTGAGGGCGCGAATCCCCACCAGCACCCCCGAGGAG

GCAGTTTTACTAGATAAGCAGCAGTCTGCGATTAAGGTGATATGCAACTCGGTTTACGGA

TTCACGGGGGTGGCAAACGGCCTGTTGCCGTGTCTGAGGATAGCGGCTACCGTTACCACG

ATAGGACGCGACATGCTCCTCAAGACCCGAGATTACGTTCACTCTCGTTGGGCGACGCGC

GAGCTGCTGGAGGACAATTTTCCAGGGGCTATAGGTTTCCGAAACCACAAGCCTTACTCC

GTCAGGGTTATCTACGGAGACACCGACTCCGTGTTTATCAAGTTTGTGGGCCTGACGTAC

GAGGGGGTATCGGAGCTGGGGGATGCTATGTCGCGTCAGATTTCAGCGGACCTCTTTAGA

GCGCCCATCAAACTGGAGTGTGAGAAGACCTTTCAGCGACTGCTGCTGATCACCAAGAAG

AAGTACATAGGTGTCATAAACGGGGGGAAGATGCTCATGAAGGGGGTCGACCTGGTCCGC

AAAAATAACTGCTCTTTCATAAACTTGTACGCGCGACATCTGGTAGATCTTTTGTTGTAC

GACGAGGATGTGGCCACGGCGGCAGCAGAGGTGACAGACGTGCCTCCCGCAGAATGGGTG

GGGCGCCCGCTACCGAGCGGCTTTGACAAGTTTGGGCGAGTGCTGGTAGAGGCGTACAAC

CGTATCACTGCCCCCAACTTGGACGTGCGCGAGTTCGTTATGACTGCTGAGCTGAGCCGC

TCACCCGAATCGTATACCAACAAGCGCCTGCCGCACCTCACCGTCTACTTTAAGCTCGCC

ATGAGGAATGAAGAACTGCCCAGCGTAAAAGAGAGAATTCCGTATGTGATAGTTGCGCAG

ACCGAGGCCGCGGAACGCGAAGCGGGTGTAGTAAACTCAATGCGCGGTACCGCCCAAAAC

CCCGTGGTAACCAAGACCGCACGCCCCCAACCTAAACGCAAACTGCTGGTTTCCGACCTC

GCCGAAGACCCGACCTATGTTTCCGAGAATGACGTACCGCTAAACACAGACTACTATTTC

TCCCACCTGTTGGGTACCATAAGCGTGACCTTTAAGGCTCTATTCGGAAATGATGTGAGA

ACAACAGAAAATCTTTTAAAGCGGTTTATTCCGGAAACCCCCCACAAGACCCCCACGAAA

ACCCAGGCACTGCTTGAGCGCGCCGGCTTTGAAAAGCTGACGCCCTTTACACCGGAGGAA

GAAAGTCGTCGAATACTGCATACAGTTTTTTGTACTCTAGAAGCAGCTCCCCATCAAAGC

TGA

>KU206467.1

ATGGCGGCGCGCGAACAGGCCAACTCCGTTCGCAGGAGCGGGTTTTTTAATCCGTTTATT

GGCAAGAGGCCATTTTTCAGGCCGGGAAGCGGGCAGACCGCGGAGACAGAGCGCCCAAGG

CCCCCCCAACACTCGTACTGCACAGAGGTGGGTAGCTTTAAGTTTATAGCTCCAAGATGC

CTCGATGAAGAAGCCCCCGCCGACCAGCGACGCGGTGTACACGTGGGCACCTTGGAGCGC

CCACCCAAAGTGTACTGCGATGGCTCAGAGTACGACGTGCTGAACTTTGCCTCCGGAGGT

TGCTGGCCTCGCAGGATTCGGGTTTGGAACGGCCAGGATTTTCGGGGCGATGGATTCAAC

CCCAGATTTGAGAGATTTCACGTGTACGACATAGTAGAGACTTCGGAGAGCGCCTCACAC

GATGACCCATCCAGGTTTGCTGAGCTATCCCGCCCAAGCGGGTCTGTGGTAACACTGCTG

GGAATGAGCGAGTGTGGAAAGCGAGTCGCGGTTCACGTCTATGGTGTGCGCCATTATTTT

TACATGGCAAAGGCGGAGGTGGATAGCGCTTGTGGAATAACCACCGAGGCAGAACTCGTG

CGCGCAATGGTGGACTGCGCGCACAGCTCGGCTTTGAGCGCGGCCCTGGGAAATGGCAAC

GGCGGCAAACAGAGCGGCGGCAGCGGCGGGGGATGGTGGGGCGGAAAGCACGTGTCTGCG

GACTGCTTCAAAGTGGAGACCGTGTGCCACACGACGCTGTACTACTTTGGATCTAAGCCA

GCTCTCTACTATAGAGTATCTGCCTCCAGCAGCCGCCTGGGAGGGTTCATCTGCGACAAC

TTTCACCCGGAGATTACAAAATTCGAGGGGAGCGTGGACGTGACCACGCGGCTGTTGTTG

GACAATGAAAATTTTACCAGTTTCGGGTGGTACCGCCTGCGACCCGGCACCCACGGAGAG

CGTGTTCAACTTCGCCCCGTTGAGCGACACGTCACCTCAAGCGACGTGGAGATTAACTGT

ACTCCCGATAACCTGGAGCCGATACCAGACGAGGCTGCCTGGCCCGACTATAAGCTCATG

TGCTTTGATATAGAGTGTAAAGCTGGAACGGGTAACGAAATGGCGTTCCCAGTGGCAACT

AACCAAGAGGACCTGGTCATCCAGATCTCCTGTCTGCTGTACTCGCTTGCTACTCAGAAC

CACGAACACACCCTGCTGTTTTCCCTCGGGTCATGCGATATCTCTGAGGAATACTCGTTT

GCATGCGTCCAGCGCGGCGAGCCCAGACCGACGGTTTTGGAGTTTGACAGCGAGTACGAG

CTGCTGGTTGCCTTCCTGACCTTTCTCAAGCAGTACTCTCCCGAGTTCGCCACCGGCTAC

AACATCGTTAATTTTGACTGGGCGTACATAGTTAACAAGGTAACGTCGGTGTATAACATC

AAGCTGGACGGGTACGGCAAGTTCAACAAAGGGGGGCTGTTTAAGGTGTGGGACATCGCC

ACGAACCATTTTCAGAAGAAGAGCAAGGTGAAAATCAATGGCCTGATATCTCTAGACATG

TATTCTGTGGCGACGGAAAAGCTAAAGCTACCCAGCTACAAACTCGACGCGGTCGTGGGA

GACGTCCTCGGCGAGCATAAGATAGACCTTCCCTATAAAGAAATACCCTCCTATTACGCG

GGAGGGCCTGACCGGAGGGGCGTAATAGGAGAGTATTGTATCCAGGACTCTAGGCTGGTG

GGCAAGCTGTTTTTTAAGTACCTCCCCCATCTGGAACTATCGGCGGTGGCCAAACTCGCC

CGTATCACCCTAACGCGGGTAATTTTTGACGGTCAGCAAATTCGCGTGTACACGTGCTTG

CTGAAACTCGCCCGCGAGAGAAATTTCATTTTGCCAGACAACAGACGCCGGTTTGACAGT

CAGGCAGACGCCGCGTCAGAGACTTCGGAGTTGGCTATGGATAGCCAAAGCCACGCCTTC

GACAGTACAGACGAACCCGACGGTGTGGACGGTACCCCGGACGCCGCAGGATCTGGCGCT

ACTTCTGAAAACGGAGGCGGGAAGCCCGGCGTCGGGAGGGCCGTGGGCTACCAGGGAGCA

AAGGTTCTAGACCCCGTATCCGGCTTTCATGTGGACCCCGTGGTTGTGTTTGACTTCGCT

AGCTTATACCCAAGCATTATCCAGGCCCATAACCTCTGTTTCACCACCCTGGCGCTCGAT

GAAGTGGATCTGGCCGGGCTTCAACCATCCGTCAACTACTCGACGTTCGAGGTGGGTGAC

CAAAAGTTATTTTTTGTCCACGCCCATATTCGCGAAAGCCTGCTTGGCATCTTGCTGCGC

GACTGGCTGGCCATGCGAAAGGCGGTGAGGGCGCGAATCCCCACCAGCACCCCCGAGGAG

GCAGTTTTACTAGATAAGCAGCAGTCTGCGATTAAGGTGATATGCAACTCGGTTTACGGA

TTCACGGGGGTGGCAAACGGCCTGTTGCCGTGTCTGAGGATAGCGGCTACCGTTACCACG

ATAGGACGCGACATGCTCCTCAAGACCCGAGATTACGTTCACTCTCGTTGGGCGACGCGC

GAGCTGCTGGAGGACAATTTTCCAGGGGCTATAGGTTTCCGAAACCACAAGCCTTACTCC

GTCAGGGTTATCTACGGAGACACCGACTCCGTGTTTATCAAGTTTGTGGGCCTGACGTAC

GAGGGGGTATCGGAGCTGGGGGATGCTATGTCGCGTCAGATTTCAGCGGACCTCTTTAGA

GCGCCCATCAAACTGGAGTGTGAGAAGACCTTTCAGCGACTGCTGCTGATCACCAAGAAG

AAGTACATAGGTGTCATAAACGGGGGGAAGATGCTCATGAAGGGGGTCGACCTGGTCCGC

AAAAATAACTGCTCTTTCATAAACTTGTACGCGCGACATCTGGTAGATCTTTTGTTGTAC

GACGAGGATGTGGCCACGGCGGCAGCAGAGGTGACAGACGTGCCTCCCGCAGAATGGGTG

GGGCGCCCGCTACCGAGCGGCTTTGACAAGTTTGGGCGAGTGCTGGTAGAGGCGTACAAC

CGTATCACTGCCCCCAACTTGGACGTGCGCGAGTTCGTTATGACTGCTGAGCTGAGCCGC

TCACCCGAATCGTATACCAACAAGCGCCTGCCGCACCTCACCGTCTACTTTAAGCTCGCC

ATGAGGAATGAAGAACTGCCCAGCGTAAAAGAGAGAATTCCGTATGTGATAGTTGCGCAG

ACCGAGGCCGCGGAACGCGAAGCGGGTGTAGTAAACTCAATGCGCGGTACCGCCCAAAAC

CCCGTGGTAACCAAGACCGCACGCCCCCAACCTAAACGCAAACTGCTGGTTTCCGACCTC

GCCGAAGACCCGACCTATGTTTCCGAGAATGACGTACCGCTAAACACAGACTACTATTTC

TCCCACCTGTTGGGTACCATAAGCGTGACCTTTAAGGCTCTATTCGGAAATGATGTGAGA

ACAACAGAAAATCTTTTAAAGCGGTTTATTCCGGAAACCCCCCACAAGACCCCCACGAAA

ACCCAGGCACTGCTTGAGCGCGCCGGCTTTGAAAAGCTGACGCCCTTTACACCGGAGGAA

GAAAGTCGTCGAATACTGCATACAGTTTTTTGTACTCTAGAAGCAGCTCCCCATCAAAGC

TGA

>KU206468.1

ATGGCGGCGCGCGAACAGGCCAACTCCGTTCGCAGGAGCGGGTTTTTTAATCCGTTTATT

GGCAAGAGGCCATTTTTCAGGCCGGGAAGCGGGCAGACCGCGGAGACAGAGCGCCCAAGG

CCCCCCCAACACTCGTACTGCACAGAGGTGGGTAGCTTTAAGTTTATAGCTCCAAGATGC

CTCGATGAAGAAGCCCCCGCCGACCAGCGACGCGGTGTACACGTGGGCACCTTGGAGCGC

CCACCCAAAGTGTACTGCGATGGCTCAGAGTACGACGTGCTGAACTTTGCCTCCGGAGGT

TGCTGGCCTCGCAGGATTCGGGTTTGGAACGGCCAGGATTTTCGGGGCGATGGATTCAAC

CCCAGATTTGAGAGATTTCACGTGTACGACATAGTAGAGACTTCGGAGAGCGCCTCACAC

GATGACCCATCCAGGTTTGCTGAGCTATCCCGCCCAAGCGGGTCTGTGGTAACACTGCTG

GGAATGAGCGAGTGTGGAAAGCGAGTCGCGGTTCACGTCTATGGTGTGCGCCATTATTTT

TACATGGCAAAGGCGGAGGTGGATAGCGCTTGTGGAATAACCACCGAGGCAGAACTCGTG

CGCGCAATGGTGGACTGCGCGCACAGCTCGGCTTTGAGCGCGGCCCTGGGAAATGGCAAC

GGCGGCAAACAGAGCGGCGGCAGCGGCGGGGGATGGTGGGGCGGAAAGCACGTGTCTGCG

GACTGCTTCAAAGTGGAGACCGTGTGCCACACGACGCTGTACTACTTTGGATCTAAGCCA

GCTCTCTACTATAGAGTATCTGCCTCCAGCAGCCGCCTGGGAGGGTTCATCTGCGACAAC

TTTCACCCGGAGATTACAAAATTCGAGGGGAGCGTGGACGTGACCACGCGGCTGTTGTTG

GACAATGAAAATTTTACCAGTTTCGGGTGGTACCGCCTGCGACCCGGCACCCACGGAGAG

CGTGTTCAACTTCGCCCCGTTGAGCGACACGTCACCTCAAGCGACGTGGAGATTAACTGT

ACTCCCGATAACCTGGAGCCGATACCAGACGAGGCTGCCTGGCCCGACTATAAGCTCATG

TGCTTTGATATAGAGTGTAAAGCTGGAACGGGTAACGAAATGGCGTTCCCAGTGGCAACT

AACCAAGAGGACCTGGTCATCCAGATCTCCTGTCTGCTGTACTCGCTTGCTACTCAGAAC

CACGAACACACCCTGCTGTTTTCCCTCGGGTCATGCGATATCTCTGAGGAATACTCGTTT

GCATGCGTCCAGCGCGGCGAGCCCAGACCGACGGTTTTGGAGTTTGACAGCGAGTACGAG

CTGCTGGTTGCCTTCCTGACCTTTCTCAAGCAGTACTCTCCCGAGTTCGCCACCGGCTAC

AACATCGTTAATTTTGACTGGGCGTACATAGTTAACAAGGTAACGTCGGTGTATAACATC

AAGCTGGACGGGTACGGCAAGTTCAACAAAGGGGGGCTGTTTAAGGTGTGGGACATCGCC

ACGAACCATTTTCAGAAGAAGAGCAAGGTGAAAATCAATGGCCTGATATCTCTAGACATG

TATTCTGTGGCGACGGAAAAGCTAAAGCTACCCAGCTACAAACTCGACGCGGTCGTGGGA

GACGTCCTCGGCGAGCATAAGATAGACCTTCCCTATAAAGAAATACCCTCCTATTACGCG

GGAGGGCCTGACCGGAGGGGCGTAATAGGAGAGTATTGTATCCAGGACTCTAGGCTGGTG

GGCAAGCTGTTTTTTAAGTACCTCCCCCATCTGGAACTATCGGCGGTGGCCAAACTCGCC

CGTATCACCCTAACGCGGGTAATTTTTGACGGTCAGCAAATTCGCGTGTACACGTGCTTG

CTGAAACTCGCCCGCGAGAGAAATTTCATTTTGCCAGACAACAGACGCCGGTTTGACAGT

CAGGCAGACGCCGCGTCAGAGACTTCGGAGTTGGCTATGGATAGCCAAAGCCACGCCTTC

GACAGTACAGACGAACCCGACGGTGTGGACGGTACCCCGGACGCCGCAGGATCTGGCGCT

ACTTCTGAAAACGGAGGCGGGAAGCCCGGCGTCGGGAGGGCCGTGGGCTACCAGGGAGCA

AAGGTTCTAGACCCCGTATCCGGCTTTCATGTGGACCCCGTGGTTGTGTTTGACTTCGCT

AGCTTATACCCAAGCATTATCCAGGCCCATAACCTCTGTTTCACCACCCTGGCGCTCGAT

GAAGTGGATCTGGCCGGGCTTCAACCATCCGTCGACTACTCGACGTTCGAGGTGGGTGAC

CAAAAGTTATTTTTTGTCCACGCCCATATTCGCGAAAGCCTGCTTGGCATCTTGCTGCGC

GACTGGCTGGCCATGCGAAAGGCGGTGAGGGCGCGAATCCCCACCAGCACCCCCGAGGAG

GCAGTTTTACTAGATAAGCAGCAGTCTGCGATTAAGGTGATATGCAACTCGGTTTACGGA

TTCACGGGGGTGGCAAACGGCCTGTTGCCGTGTCTGAGGATAGCGGCTACCGTTACCACG

ATAGGACGCGACATGCTCCTCAAGACCCGAGATTACGTTCACTCTCGTTGGGCGACGCGC

GAGCTGCTGGAGGACAATTTTCCAGGGGCTATAGGTTTCCGAAACCACAAGCCTTACTCC

GTCAGGGTTATCTACGGAGACACCGACTCCGTGTTTATCAAGTTTGTGGGCCTGACGTAC

GAGGGGGTATCGGAGCTGGGGGATGCTATGTCGCGTCAGATTTCAGCGGACCTCTTTAGA

GCGCCCATCAAACTGGAGTGTGAGAAGACCTTTCAGCGACTGCTGCTGATCACCAAGAAG

AAGTACATAGGTGTCATAAACGGGGGGAAGATGCTCATGAAGGGGGTCGACCTGGTCCGC

AAAAATAACTGCTCTTTCATAAACTTGTACGCGCGACATCTGGTAGATCTTTTGTTGTAC

GACGAGGATGTGGCCACGGCGGCAGCAGAGGTGACAGACGTGCCTCCCGCAGAATGGGTG

GGGCGCCCGCTACCGAGCGGCTTTGACAAGTTTGGGCGAGTGCTGGTAGAGGCGTACAAC

CGTATCACTGCCCCCAACTTGGACGTGCGCGAGTTCGTTATGACTGCTGAGCTGAGCCGC

TCACCCGAATCGTATACCAACAAGCGCCTGCCGCACCTCACCGTCTACTTTAAGCTCGCC

ATGAGGAATGAAGAACTGCCCAGCGTAAAAGAGAGAATTCCGTATGTGATAGTTGCGCAG

ACCGAGGCCGCGGAACGCGAAGCGGGTGTAGTAAACTCAATGCGCGGTACCGCCCAAAAC

CCCGTGGTAACCAAGACCGCACGCCCCCAACCTAAACGCAAACTGCTGGTTTCCGACCTC

GCCGAAGACCCGACCTATGTTTCCGAGAATGACGTACCGCTAAACACAGACTACTATTTC

TCCCACCTGTTGGGTACCATAAGCGTGACCTTTAAGGCTCTATTCGGAAATGATGTGAGA

ACAACAGAAAATCTTTTAAAGCGGTTTATTCCGGAAACCCCCCACAAGACCCCCACGAAA

ACCCAGGCACTGCTTGAGCGCGCCGGCTTTGAAAAGCTGACGCCCTTTACACCGGAGGAA

GAAAGTCGTCGAATACTGCATACAGTTTTTTGTACTCTAGAAGCAGCTCCCCATCAAAGC

TGA

>KU206469.1

ATGGCGGCGCGCGAACAGGCCAACTCCGTTCGCAGGAGCGGGTTTTTTAATCCGTTTATT

GGCAAGAGGCCATTTTTCAGGCCGGGAAGCGGGCAGACCGCGGAGACAGAGCGCCCAAGG

CCCCCCCAACACTCGTACTGCACAGAGGTGGGTAGCTTTAAGTTTATAGCTCCAAGATGC

CTCGATGAAGAAGCCCCCGCCGACCAGCGACGCGGTGTACACGTGGGCACCTTGGAGCGC

CCACCCAAAGTGTACTGCGATGGCTCAGAGTACGACGTGCTGAACTTTGCCTCCGGAGGT

TGCTGGCCTCGCAGGATTCGGGTTTGGAACGGCCAGGATTTTCGGGGCGATGGATTCAAC

CCCAGATTTGAGAGATTTCACGTGTACGACATAGTAGAGACTTCGGAGAGCGCCTCACAC

GATGACCCATCCAGGTTTGCTGAGCTATCCCGCCCAAGCGGGTCTGTGGTAACACTGCTG

GGAATGAGCGAGTGTGGAAAGCGAGTCGCGGTTCACGTCTATGGTGTGCGCCATTATTTT

TACATGGCAAAGGCGGAGGTGGATAGCGCTTGTGGAATAACCACCGAGGCAGAACTCGTG

CGCGCAATGGTGGACTGCGCGCACAGCTCGGCTTTGAGCGCGGCCCTGGGAAATGGCAAC

GGCGGCAAACAGAGCGGCGGCAGCGGCGGGGGATGGTGGGGCGGAAAGCACGTGTCTGCG

GACTGCTTCAAAGTGGAGACCGTGTGCCGCACGACGCTGTACTACTTTGGATCTAAGCCA

GCTCTCTACTATAGAGTATCTGCCTCCAGCAGCCGCCTGGGAGGGTTCATCTGCGACAAC

TTTCACCCGGAGATTACAAAATTCGAGGGGAGCGTGGACGTGACCACGCGGCTGTTGTTG

GACAATGAAAATTTTACCAGTTTCGGGTGGTACCGCCTGCGACCCGGCACCCACGGAGAG

CGTGTTCAACTTCGCCCCGTTGAGCGACACGTCACCTCAAGCGACGTGGAGATTAACTGT

ACTCCCGATAACCTGGAGCCGATACCAGACGAGGCTGCCTGGCCCGACTATAAGCTCATG

TGCTTTGATATAGAGTGTAAAGCTGGAACGGGTAACGAAATGGCGTTCCCAGTGGCAACT

AACCAAGAGGACCTGGTCATCCAGATCTCCTGTCTGCTGTACTCGCTTGCTACTCAGAAC

CACGAACACACCCTGCTGTTTTCCCTCGGGTCATGCGATATCTCTGAGGAATACTCGTTT

GCATGCGTCCAGCGCGGCGAGCCCAGACCGACGGTTTTGGAGTTTGACAGCGAGTACGAG

CTGCTGGTTGCCTTCCTGACCTTTCTCAAGCAGTACTCTCCCGAGTTCGCCACCGGCTAC

AACATCGTTAATTTTGACTGGGCGTACATAGTTAACAAGGTAACGTCGGTGTATAACATC

AAGCTGGACGGGTACGGCAAGTTCAACAAAGGGGGGCTGTTTAAGGTGTGGGACATCGCC

ACGAACCATTTTCAGAAGAAGAGCAAGGTGAAAATCAATGGCCTGATATCTCTAGACATG

TATTCTGTGGCGACGGAAAAGCTAAAGCTACCCAGCTACAAACTCGACGCGGTCGTGGGA

GACGTCCTCGGCGAGCATAAGATAGACCTTCCCTATAAAGAAATACCCTCCTATTACGCG

GGAGGGCCTGACCGGAGGGGCGTAATAGGAGAGTATTGTATCCAGGACTCTAGGCTGGTG

GGCAAGCTGTTTTTTAAGTACCTCCCCCATCTGGAACTATCGGCGGTGGCCAAACTCGCC

CGTATCACCCTAACGCGGGTAATTTTTGACGGTCAGCAAATTCGCGTGTACACGTGCTTG

CTGAAACTCGCCCGCGAGAGAAATTTCATTTTGCCAGACAACAGACGCCGGTTTGACAGT

CAGGCAGACGCCGCGTCAGAGACTTCGGAGTTGGCTATGGATAGCCAAAGCCACGCCTTC

GACAGTACAGACGAACCCGACGGTGTGGACGGTACCCCGGACGCCGCAGGATCTGGCGCT

ACTTCTGAAAACGGAGGCGGGAAGCCCGGCGTCGGGAGGGCCGTGGGCTACCAGGGAGCA

AAGGTTCTAGACCCCGTATCCGGCTTTCATGTGGACCCCGTGGTTGTGTTTGACTTCGCT

AGCTTATACCCAAGCATTATCCAGGCCCATAACCTCTGTTTCACCACCCTGGCGCTCGAT

GAAGTGGATCTGGCCGGGCTTCAACCATCCGTCAACTACTCGACGTTCGAGGTGGGTGAC

CAAAAGTTATTTTTTGTCCACGCCCATATTCGCGAAAGCCTGCTTGGCATCTTGCTGCGC

GACTGGCTGGCCATGCGAAAGGCGGTGAGGGCGCGAATCCCCACCAGCACCCCCGAGGAG

GCAGTTTTACTAGATAAGCAGCAGTCTGCGATTAAGGTGATATGCAACTCGGTTTACGGA

TTCACGGGGGTGGCAAACGGCCTGTTGCCGTGTCTGAGGATAGCGGCTACCGTTACCACG

ATAGGACGCGACATGCTCCTCAAGACCCGAGATTACGTTCACTCTCGTTGGGCGACGCGC

GAGCTGCTGGAGGACAATTTTCCAGGGGCTATAGGTTTCCGAAACCACAAGCCTTACTCC

GTCAGGGTTATCTACGGAGACACCGACTCCGTGTTTATCAAGTTTGTGGGCCTGACGTAC

GAGGGGGTATCGGAGCTGGGGGATGCTATGTCGCGTCAGATTTCAGCGGACCTCTTTAGA

GCGCCCATCAAACTGGAGTGTGAGAAGACCTTTCAGCGACTGCTGCTGATCACCAAGAAG

AAGTACATAGGTGTCATAAACGGGGGGAAGATGCTCATGAAGGGGGTCGACCTAGTCCGC

AAAAATAACTGCTCTTTCATAAACTTGTACGCGCGACATCTGGTAGATCTTTTGTTGTAC

GACGAGGATGTGGCCACGGCGGCAGCAGAGGTGACAGACGTGCCTCCCGCAGAATGGGTG

GGGCGCCCGCTACCGAGCGGCTTTGACAAGTTTGGGCGAGTGCTGGTAGAGGCGTACAAC

CGTATCACTGCCCCCAACTTGGACGTGCGCGAGTTCGTTATGACTGCTGAGCTGAGCCGC

TCACCCGAATCGTATACCAACAAGCGCCTGCCGCACCTCACCGTCTACTTTAAGCTCGCC

ATGAGGAATGAAGAACTGCCCAGCGTAAAAGAGAGAATTCCGTATGTGATAGTTGCGCAG

ACCGAGGCCGCGGAACGCGAAGCGGGTGTAGTAAACTCAATGCGCGGTACCGCCCAAAAC

CCCGTGGTAACCAAGACCGCACGCCCCCAACCTAAACGCAAACTGCTGGTTTCCGACCTC

GCCGAAGACCCGACCTATGTTTCCGAGAATGACGTACCGCTAAACACAGACTACTATTTC

TCCCACCTGTTGGGTACCATAAGCGTGACCTTTAAGGCTCTATTCGGAAATGATGTGAGA

ACAACAGAAAATCTTTTAAAGCGGTTTATTCCGGAAACCCCCCACAAGACCCCCACGAAA

ACCCAGGCACTGCTTGAGCGCGCCGGCTTTGAAAAGCTGACGCCCTTTACACCGGAGGAA

GAAAGTCGTCGAATACTGCATACAGTTTTTTGTACTCTAGAAGCAGCTCCCCATCAAAGC

TGA

>KU206470.1

ATGGCGGCGCGCGAACAGGCCAACTCCGTTCGCAGGAGCGGGTTTTTTAATCCGTTTATT

GGCAAGAGGCCATTTTTCAGGCCGGGAAGCGGGCAGACCGCGGAGACAGAGCGCCCAAGG

CCCCCCCAACACTCGTACTGCACAGAGGTGGGTAGCTTTAAGTTTATAGCTCCAAGATGC

CTCGATGAAGAAGCCCCCGCCGACCAGCGACGCGGTGTACACGTGGGCACCTTGGAGCGC

CCACCCAAAGTGTACTGCGATGGCTCAGAGTACGACGTGCTGAACTTTGCCTCCGGAGGT

TGCTGGCCTCGCAGGATTCGGGTTTGGAACGGCCAGGATTTTCGGGGCGATGGATTCAAC

CCCAGATTTGAGAGATTTCACGTGTACGACATAGTAGAGACTTCGGAGAGCGCCTCACAC

GATGACCCATCCAGGTTTGCTGAGCTATCCCGCCCAAGCGGGTCTGTGGTAACACTGCTG

GGAATGAGCGAGTGTGGAAAGCGAGTCGCGGTTCACGTCTATGGTGTGCGCCATTATTTT

TACATGGCAAAGGCGGAGGTGGATAGCGCTTGTGGAATAACCACCGAGGCAGAACTCGTG

CGCGCAATGGTGGACTGCGCGCACAGCTCGGCTTTGAGCGCGGCCCTGGGAAATGGCAAC

GGCGGCAAACAGAGCGGCGGCAGCGGCGGGGGATGGTGGGGCGGAAAGCACGTGTCTGCG

GACTGCTTCAAAGTGGAGACCGTGTGCCGCACGACGCTGTACTACTTTGGATCTAAGCCA

GCTCTCTACTATAGAGTATCTGCCTCCAGCAGCCGCCTGGGAGGGTTCATCTGCGACAAC

TTTCACCCGGAGATTACAAAATTCGAGGGGAGCGTGGACGTGACCACGCGGCTGTTGTTG

GACAATGAAAATTTTACCAGTTTCGGGTGGTACCGCCTGCGACCCGGCACCCACGGAGAG

CGTGTTCAACTTCGCCCCGTTGAGCGACACGTCACCTCAAGCGACGTGGAGATTAACTGT

ACTCCCGATAACCTGGAGCCGATACCAGACGAGGCTGCCTGGCCCGACTATAAGCTCATG

TGCTTTGATATAGAGTGTAAAGCTGGAACGGGTAACGAAATGGCGTTCCCAGTGGCAACT

AACCAAGAGGACCTGGTCATCCAGATCTCCTGTCTGCTGTACTCGCTTGCTACTCAGAAC

CACGAACACACCCTGCTGTTTTCCCTCGGGTCATGCGATATCTCTGAGGAATACTCGTTT

GCATGCGTCCAGCGCGGCGAGCCCAGACCGACGGTTTTGGAGTTTGACAGCGAGTACGAG

CTGCTGGTTGCCTTCCTGACCTTTCTCAAGCAGTACTCTCCCGAGTTCGCCACCGGCTAC

AACATCGTTAATTTTGACTGGGCGTACATAGTTAACAAGGTAACGTCGGTGTATAACATC

AAGCTGGACGGGTACGGCAAGTTCAACAAAGGGGGGCTGTTTAAGGTGTGGGACATCGCC

ACGAACCATTTTCAGAAGAAGAGCAAGGTGAAAATCAATGGCCTGATATCTCTAGACATG

TATTCTGTGGCGACGGAAAAGCTAAAGCTACCCAGCTACAAACTCGACGCGGTCGTGGGA

GACGTCCTCGGCGAGCATAAGATAGACCTTCCCTATAAAGAAATACCCTCCTATTACGCG

GGAGGGCCTGACCGGAGGGGCGTAATAGGAGAGTATTGTATCCAGGACTCTAGGCTGGTG

GGCAAGCTGTTTTTTAAGTACCTCCCCCATCTGGAACTATCGGCGGTGGCCAAACTCGCC

CGTATCACCCTAACGCGGGTAATTTTTGACGGTCAGCAAATTCGCGTGTACACGTGCTTG

CTGAAACTCGCCCGCGAGAGAAATTTCATTTTGCCAGACAACAGACGCCGGTTTGACAGT

CAGGCAGACGCCGCGTCAGAGACTTCGGAGTTGGCTATGGATAGCCAAAGCCACGCCTTC

GACAGTACAGACGAACCCGACGGTGTGGACGGTACCCCGGACGCCGCAGGATCTGGCGCT

ACTTCTGAAAACGGAGGCGGGAAGCCCGGCGTCGGGAGGGCCGTGGGCTACCAGGGAGCA

AAGGTTCTAGACCCCGTATCCGGCTTTCATGTGGACCCCGTGGTTGTGTTTGACTTCGCT

AGCTTATACCCAAGCATTATCCAGGCCCATAACCTCTGTTTCACCACCCTGGCGCTCGAT

GAAGTGGATCTGGCCGGGCTTCAACCATCCGTCAACTACTCGACGTTCGAGGTGGGTGAC

CAAAAGTTATTTTTTGTCCACGCCCATATTCGCGAAAGCCTGCTTGGCATCTTGCTGCGC

GACTGGCTGGCCATGCGAAAGGCGGTGAGGGCGCGAATCCCCACCAGCACCCCCGAGGAG

GCAGTTTTACTAGATAAGCAGCAGTCTGCGATTAAGGTGATATGCAACTCGGTTTACGGA

TTCACGGGGGTGGCAAACGGCCTGTTGCCGTGTCTGAGGATAGCGGCTACCGTTACCACG

ATAGGACGCGACATGCTCCTCAAGACCCGAGATTACGTTCACTCTCGTTGGGCGACGCGC

GAGCTGCTGGAGGACAATTTTCCAGGGGCTATAGGTTTCCGAAACCACAAGCCTTACTCC

GTCAGGGTTATCTACGGAGACACCGACTCCGTGTTTATCAAGTTTGTGGGCCTGACGTAC

GAGGGGGTATCGGAGCTGGGGGATGCTATGTCGCGTCAGATTTCAGCGGACCTCTTTAGA

GCGCCCATCAAACTGGAGTGTGAGAAGACCTTTCAGCGACTGCTGCTGATCACCAAGAAG

AAGTACATAGGTGTCATAAACGGGGGGAAGATGCTCATGAAGGGGGTCGACCTAGTCCGC

AAAAATAACTGCTCTTTCATAAACTTGTACGCGCGACATCTGGTAGATCTTTTGTTGTAC

GACGAGGATGTGGCCACGGCGGCAGCAGAGGTGACAGACGTGCCTCCCGCAGAATGGGTG

GGGCGCCCGCTACCGAGCGGCTTTGACAAGTTTGGGCGAGTGCTGGTAGAGGCGTACAAC

CGTATCACTGCCCCCAACTTGGACGTGCGCGAGTTCGTTATGACTGCTGAGCTGAGCCGC

TCACCCGAATCGTATACCAACAAGCGCCTGCCGCACCTCACCGTCTACTTTAAGCTCGCC

ATGAGGAATGAAGAACTGCCCAGCGTAAAAGAGAGAATTCCGTATGTGATAGTTGCGCAG

ACCGAGGCCGCGGAACGCGAAGCGGGTGTAGTAAACTCAATGCGCGGTACCGCCCAAAAC

CCCGTGGTAACCAAGACCGCACGCCCCCAACCTAAACGCAAACTGCTGGTTTCCGACCTC

GCCGAAGACCCGACCTATGTTTCCGAGAATGACGTACCGCTAAACACAGACTACTATTTC

TCCCACCTGTTGGGTACCATAAGCGTGACCTTTAAGGCTCTATTCGGAAATGATGTGAGA

ACAACAGAAAATCTTTTAAAGCGGTTTATTCCGGAAACCCCCCACAAGACCCCCACGAAA

ACCCAGGCACTGCTTGAGCGCGCCGGCTTTGAAAAGCTGACGCCCTTTACACCGGAGGAA

GAAAGTCGTCGAATACTGCATACAGTTTTTTGTACTCTAGAAGCAGCTCCCCATCAAAGC

TGA

>KU206471.1

ATGGCGGCGCGCGAACAGGCCAACTCCGTTCGCAGGAGCGGGTTTTTTAATCCGTTTATT

GGCAAGAGGCCATTTTTCAGGCCGGGAAGCGGGCAGACCGCGGAGACAGAGCGCCCAAGG

CCCCCCCAACACTCGTACTGCACAGAGGTGGGTAGCTTTAAGTTTATAGCTCCAAGATGC

CTCGATGAAGAAGCCCCCGCCGACCAGCGACGCGGTGTACACGTGGGCACCTTGGAGCGC

CCACCCAAAGTGTACTGCGATGGCTCAGAGTACGACGTGCTGAACTTTGCCTCCGGAGGT

TGCTGGCCTCGCAGGATTCGGGTTTGGAACGGCCAGGATTTTCGGGGCGATGGATTCAAC

CCCAGATTTGAGAGATTTCACGTGTACGACATAGTAGAGACTTCGGAGAGCGCCTCACAC

GATGACCCATCCAGGTTTGCTGAGCTATCCCGCCCAAGCGGGTCTGTGGTAACACTGCTG

GGAATGAGCGAGTGTGGAAAGCGAGTCGCGGTTCACGTCTATGGTGTGCGCCATTATTTT

TACATGGCAAAGGCGGAGGTGGATAGCGCTTGTGGAATAACCACCGAGGCAGAACTCGTG

CGCGCAATGGTGGACTGCGCGCACAGCTCGGCTTTGAGCGCGGCCCTGGGAAATGGCAAC

GGCGGCAAACAGAGCGGCGGCAGCGGCGGGGGATGGTGGGGCGGAAAGCACGTGTCTGCG

GACTGCTTCAAAGTGGAGACCGTGTGCCGCACGACGCTGTACTACTTTGGATCTAAGCCA

GCTCTCTACTATAGAGTATCTGCCTCCAGCAGCCGCCTGGGAGGGTTCATCTGCGACAAC

TTTCACCCGGAGATTACAAAATTCGAGGGGAGCGTGGACGTGACCACGCGGCTGTTGTTG

GACAATGAAAATTTTACCAGTTTCGGGTGGTACCGCCTGCGACCCGGCACCCACGGAGAG

CGTGTTCAACTTCGCCCCGTTGAGCGACACGTCACCTCAAGCGACGTGGAGATTAACTGT

ACTCCCGATAACCTGGAGCCGATACCAGACGAGGCTGCCTGGCCCGACTATAAGCTCATG

TGCTTTGATATAGAGTGTAAAGCTGGAACGGGTAACGAAATGGCGTTCCCAGTGGCAACT

AACCAAGAGGACCTGGTCATCCAGATCTCCTGTCTGCTGTACTCGCTTGCTACTCAGAAC

CACGAACACACCCTGCTGTTTTCCCTCGGGTCATGCGATATCTCTGAGGAATACTCGTTT

GCATGCGTCCAGCGCGGCGAGCCCAGACCGACGGTTTTGGAGTTTGACAGCGAGTACGAG

CTGCTGGTTGCCTTCCTGACCTTTCTCAAGCAGTACTCTCCCGAGTTCGCCACCGGCTAC

AACATCGTTAATTTTGACTGGGCGTACATAGTTAACAAGGTAACGTCGGTGTATAACATC

AAGCTGGACGGGTACGGCAAGTTCAACAAAGGGGGGCTGTTTAAGGTGTGGGACATCGCC

ACGAACCATTTTCAGAAGAAGAGCAAGGTGAAAATCAATGGCCTGATATCTCTAGACATG

TATTCTGTGGCGACGGAAAAGCTAAAGCTACCCAGCTACAAACTCGACGCGGTCGTGGGA

GACGTCCTCGGCGAGCATAAGATAGACCTTCCCTATAAAGAAATACCCTCCTATTACGCG

GGAGGGCCTGACCGGAGGGGCGTAATAGGAGAGTATTGTATCCAGGACTCTAGGCTGGTG

GGCAAGCTGTTTTTTAAGTACCTCCCCCATCTGGAACTATCGGCGGTGGCCAAACTCGCC

CGTATCACCCTAACGCGGGTAATTTTTGACGGTCAGCAAATTCGCGTGTACACGTGCTTG

CTGAAACTCGCCCGCGAGAGAAATTTCATTTTGCCAGACAACAGACGCCGGTTTGACAGT

CAGGCAGACGCCGCGTCAGAGACTTCGGAGTTGGCTATGGATAGCCAAAGCCACGCCTTC

GACAGTACAGACGAACCCGACGGTGTGGACGGTACCCCGGACGCCGCAGGATCTGGCGCT

ACTTCTGAAAACGGAGGCGGGAAGCCCGGCGTCGGGAGGGCCGTGGGCTACCAGGGAGCA

AAGGTTCTAGACCCCGTATCCGGCTTTCATGTGGACCCCGTGGTTGTGTTTGACTTCGCT

AGCTTATACCCAAGCATTATCCAGGCCCATAACCTCTGTTTCACCACCCTGGCGCTCGAT

GAAGTGGATCTGGCCGGGCTTCAACCATCCGTCAACTACTCGACGTTCGAGGTGGGTGAC

CAAAAGTTATTTTTTGTCCACGCCCATATTCGCGAAAGCCTGCTTGGCATCTTGCTGCGC

GACTGGCTGGCCATGCGAAAGGCGGTGAGGGCGCGAATCCCCACCAGCACCCCCGAGGAG

GCAGTTTTACTAGATAAGCAGCAGTCTGCGATTAAGGTGATATGCAACTCGGTTTACGGA

TTCACGGGGGTGGCAAACGGCCTGTTGCCGTGTCTGAGGATAGCGGCTACCGTTACCACG

ATAGGACGCGACATGCTCCTCAAGACCCGAGATTACGTTCACTCTCGTTGGGCGACGCGC

GAGCTGCTGGAGGACAATTTTCCAGGGGCTATAGGTTTCCGAAACCACAAGCCTTACTCC

GTCAGGGTTATCTACGGAGACACCGACTCCGTGTTTATCAAGTTTGTGGGCCTGACGTAC

GAGGGGGTATCGGAGCTGGGGGATGCTATGTCGCGTCAGATTTCAGCGGACCTCTTTAGA

GCGCCCATCAAACTGGAGTGTGAGAAGACCTTTCAGCGACTGCTGCTGATCACCAAGAAG

AAGTACATAGGTGTCATAAACGGGGGGAAGATGCTCATGAAGGGGGTCGACCTAGTCCGC

AAAAATAACTGCTCTTTCATAAACTTGTACGCGCGACATCTGGTAGATCTTTTGTTGTAC

GACGAGGATGTGGCCACGGCGGCAGCAGAGGTGACAGACGTGCCTCCCGCAGAATGGGTG

GGGCGCCCGCTACCGAGCGGCTTTGACAAGTTTGGGCGAGTGCTGGTAGAGGCGTACAAC

CGTATCACTGCCCCCAACTTGGACGTGCGCGAGTTCGTTATGACTGCTGAGCTGAGCCGC

TCACCCGAATCGTATACCAACAAGCGCCTGCCGCACCTCACCGTCTACTTTAAGCTCGCC

ATGAGGAATGAAGAACTGCCCAGCGTAAAAGAGAGAATTCCGTATGTGATAGTTGCGCAG

ACCGAGGCCGCGGAACGCGAAGCGGGTGTAGTAAACTCAATGCGCGGTACCGCCCAAAAC

CCCGTGGTAACCAAGACCGCACGCCCCCAACCTAAACGCAAACTGCTGGTTTCCGACCTC

GCCGAAGACCCGACCTATGTTTCCGAGAATGACGTACCGCTAAACACAGACTACTATTTC

TCCCACCTGTTGGGTACCATAAGCGTGACCTTTAAGGCTCTATTCGGAAATGATGTGAGA

ACAACAGAAAATCTTTTAAAGCGGTTTATTCCGGAAACCCCCCACAAGACCCCCACGAAA

ACCCAGGCACTGCTTGAGCGCGCCGGCTTTGAAAAGCTGACGCCCTTTACACCGGAGGAA

GAAAGTCGTCGAATACTGCATACAGTTTTTTGTACTCTAGAAGCAGCTCCCCATCAAAGC

TGA

>KU206472.1

ATGGCGGCGCGCGAACAGGCCAACTCCGTTCGCAGGAGCGGGTTTTTTAATCCGTTTATT

GGCAAGAGGCCATTTTTCAGGCCGGGAAGCGGGCAGACCGCGGAGACAGAGCGCCCAAGG

CCCCCCCAACACTCGTACTGCACAGAGGTGGGTAGCTTTAAGTTTATAGCTCCAAGATGC

CTCGATGAAGAAGCCCCCGCCGACCAGCGACGCGGTGTACACGTGGGCACCTTGGAGCGC

CCACCCAAAGTGTACTGCGATGGCTCAGAGTACGACGTGCTGAACTTTGCCTCCGGAGGT

TGCTGGCCTCGCAGGATTCGGGTTTGGAACGGCCAGGATTTTCGGGGCGATGGATTCAAC

CCCAGATTTGAGAGATTTCACGTGTACGACATAGTAGAGACTTCGGAGAGCGCCTCACAC

GATGACCCATCCAGGTTTGCTGAGCTATCCCGCCCAAGCGGGTCTGTGGTAACACTGCTG

GGAATGAGCGAGTGTGGAAAGCGAGTCGCGGTTCACGTCTATGGTGTGCGCCATTATTTT

TACATGGCAAAGGCGGAGGTGGATAGCGCTTGTGGAATAACCACCGAGGCAGAACTCGTG

CGCGCAATGGTGGACTGCGCGCACAGCTCGGCTTTGAGCGCGGCCCTGGGAAATGGCAAC

GGCGGCAAACAGAGCGGCGGCAGCGGCGGGGGATGGTGGGGCGGAAAGCACGTGTCTGCG

GACTGCTTCAAAGTGGAGACCGTGTGCCACACGACGCTGTACTACTTTGGATCTAAGCCA

GCTCTCTACTATAGAGTATCTGCCTCCAGCAGCCGCCTGGGAGGGTTCATCTGCGACAAC

TTTCACCCGGAGATTACAAAATTCGAGGGGAGCGTGGACGTGACCACGCGGCTGTTGTTG

GACAATGAAAATTTTACCAGTTTCGGGTGGTACCGCCTGCGACCCGGCACCCACGGAGAG

CGTGTTCAACTTCGCCCCGTTGAGCGACACGTCACCTCAAGCGACGTGGAGATTAACTGT

ACTCCCGATAACCTGGAGCCGATACCAGACGAGGCTGCCTGGCCCGACTATAAGCTCATG

TGCTTTGATATAGAGTGTAAAGCTGGAACGGGTAACGAAATGGCGTTCCCAGTGGCAACT

AACCAAGAGGACCTGGTCATCCAGATCTCCTGTCTGCTGTACTCGCTTGCTACTCAGAAC

CACGAACACACCCTGCTGTTTTCCCTCGGGTCATGCGATATCTCTGAGGAATACTCGTTT

GCATGCGTCCAGCGCGGCGAGCCCAGACCGACGGTTTTGGAGTTTGACAGCGAGTACGAG

CTGCTGGTTGCCTTCCTGACCTTTCTCAAGCAGTACTCTCCCGAGTTCGCCACCGGCTAC

AACATCGTTAATTTTGACTGGGCGTACATAGTTAACAAGGTAACGTCGGTGTATAACATC

AAGCTGGACGGGTACGGCAAGTTCAACAAAGGGGGGCTGTTTAAGGTGTGGGACATCGCC

ACGAACCATTTTCAGAAGAAGAGCAAGGTGAAAATCAATGGCCTGATATCTCTAGACATG

TATTCTGTGGCGACGGAAAAGCTAAAGCTACCCAGCTACAAACTCGACGCGGTCGTGGGA

GACGTCCTCGGCGAGCATAAGATAGACCTTCCCTATAAAGAAATACCCTCCTATTACGCG

GGAGGGCCTGACCGGAGGGGCGTAATAGGAGAGTATTGTATCCAGGACTCTAGGCTGGTG

GGCAAGCTGTTTTTTAAGTACCTCCCCCATCTGGAACTATCGGCGGTGGCCAAACTCGCC

CGTATCACCCTAACGCGGGTAATTTTTGACGGTCAGCAAATTCGCGTGTACACGTGCTTG

CTGAAACTCGCCCGCGAGAGAAATTTCATTTTGCCAGACAACAGACGCCGGTTTGACAGT

CAGGCAGACGCCGCGTCAGAGACTTCGGAGTTGGCTATGGATAGCCAAAGCCACGCCTTC

GACAGTACAGACGAACCCGACGGTGTGGACGGTACCCCGGACGCCGCAGGATCTGGCGCT

ACTTCTGAAAACGGAGGCGGGAAGCCCGGCGTCGGGAGGGCCGTGGGCTACCAGGGAGCA

AAGGTTCTAGACCCCGTATCCGGCTTTCATGTGGACCCCGTGGTTGTGTTTGACTTCGCT

AGCTTATACCCAAGCATTATCCAGGCCCATAACCTCTGTTTCACCACCCTGGCGCTCGAT

GAAGTGGATCTGGCCGGGCTTCAACCATCCGTCAACTACTCGACGTTCGAGGTGGGTGAC

CAAAAGTTATTTTTTGTCCACGCCCATATTCGCGAAAGCCTGCTTGGCATCTTGCTGCGC

GACTGGCTGGCCATGCGAAAGGCGGTGAGGGCGCGAATCCCCACCAGCACCCCCGAGGAG

GCAGTTTTACTAGATAAGCAGCAGTCTGCGATTAAGGTGATATGCAACTCGGTTTACGGA

TTCACGGGGGTGGCAAACGGCCTGTTGCCGTGTCTGAGGATAGCGGCTACCGTTACCACG

ATAGGACGCGACATGCTCCTCAAGACCCGAGATTACGTTCACTCTCGTTGGGCGACGCGC

GAGCTGCTGGAGGACAATTTTCCAGGGGCTATAGGTTTCCGAAACCACAAGCCTTACTCC

GTCAGGGTTATCTACGGAGACACCGACTCCGTGTTTATCAAGTTTGTGGGCCTGACGTAC

GAGGGGGTATCGGAGCTGGGGGATGCTATGTCGCGTCAGATTTCAGCGGACCTCTTTAGA

GCGCCCATCAAACTGGAGTGTGAGAAGACCTTTCAGCGACTGCTGCTGATCACCAAGAAG

AAGTACATAGGTGTCATAAACGGGGGGAAGATGCTCATGAAGGGGGTCGACCTAGTCCGC

AAAAATAACTGCTCTTTCATAAACTTGTACGCGCGACATCTGGTAGATCTTTTGTTGTAC

GACGAGGATGTGGCCACGGCGGCAGCAGAGGTGACAGACGTGCCTCCCGCAGAATGGGTG

GGGCGCCCGCTACCGAGCGGCTTTGACAAGTTTGGGCGAGTGCTGGTAGAGGCGTACAAC

CGTATCACTGCCCCCAACTTGGACGTGCGCGAGTTCGTTATGACTGCTGAGCTGAGCCGC

TCACCCGAATCGTATACCAACAAGCGCCTGCCGCACCTCACCGTCTACTTTAAGCTCGCC

ATGAGGAATGAAGAACTGCCCAGCGTAAAAGAGAGAATTCCGTATGTGATAGTTGCGCAG

ACCGAGGCCGCGGAACGCGAAGCGGGTGTAGTAAACTCAATGCGCGGTACCGCCCAAAAC

CCCGTGGTAACCAAGACCGCACGCCCCCAACCTAAACGCAAACTGCTGGTTTCCGACCTC

GCCGAAGACCCGACCTATGTTTCCGAGAATGACGTACCGCTAAACACAGACTACTATTTC

TCCCACCTGTTGGGTACCATAAGCGTGACCTTTAAGGCTCTATTCGGAAATGATGTGAGA

ACAACAGAAAATCTTTTAAAGCGGTTTATTCCGGAAACCCCCCACAAGACCCCCACGAAA

ACCCAGGCACTGCTTGAGCGCGCCGGCTTTGAAAAGCTGACGCCCTTTACACCGGAGGAA

GAAAGTCGTCGAATACTGCATACAGTTTTTTGTACTCTAGAAGCAGCTCCCCATCAAAGC

TGA

>KU206473.1

ATGGCGGCGCGCGAACAGGCCAACTCCGTTCGCAGGAGCGGGTTTTTTAATCCGTTTATT

GGCAAGAGGCCATTTTTCAGGCCGGGAAGCGGGCAGACCGCGGAGACAGAGCGCCCAAGG

CCCCCCCAACACTCGTACTGCACAGAGGTGGGTAGCTTTAAGTTTATAGCTCCAAGATGC

CTCGATGAAGAAGCCCCCGCCGACCAGCGACGCGGTGTACACGTGGGCACCTTGGAGCGC

CCACCCAAAGTGTACTGCGATGGCTCAGAGTACGACGTGCTGAACTTTGCCTCCGGAGGT

TGCTGGCCTCGCAGGATTCGGGTTTGGAACGGCCAGGATTTTCGGGGCGATGGATTCAAC

CCCAGATTTGAGAGATTTCACGTGTACGACATAGTAGAGACTTCGGAGAGCGCCTCACAC

GATGACCCATCCAGGTTTGCTGAGCTATCCCGCCCAAGCGGGTCTGTGGTAACACTGCTG

GGAATGAGCGAGTGTGGAAAGCGAGTCGCGGTTCACGTCTATGGTGTGCGCCATTATTTT

TACATGGCAAAGGCGGAGGTGGATAGCGCTTGTGGAATAACCACCGAGGCAGAACTCGTG

CGCGCAATGGTGGACTGCGCGCACAGCTCGGCTTTGAGCGCGGCCCTGGGAAATGGCAAC

GGCGGCAAACAGAGCGGCGGCAGCGGCGGGGGATGGTGGGGCGGAAAGCACGTGTCTGCG

GACTGCTTCAAAGTGGAGACCGTGTGCCACACGACGCTGTACTACTTTGGATCTAAGCCA

GCTCTCTACTATAGAGTATCTGCCTCCAGCAGCCGCCTGGGAGGGTTCATCTGCGACAAC

TTTCACCCAGAGATTACAAAATTCGAGGGGAGCGTGGACGTGACCACGCGGCTGTTGTTG

GACAATGAAAATTTTACCAGTTTCGGGTGGTACCGCCTGCGACCCGGCACCCACGGAGAG

CGTGTTCAACTTCGCCCCGTTGAGCGACACGTCACCTCAAGCGACGTGGAGATTAACTGT

ACTCCCGATAACCTGGAGCCGATACCAGACGAGGCTGCCTGGCCCGACTATAAGCTCATG

TGCTTTGATATAGAGTGTAAAGCTGGAACGGGTAACGAAATGGCGTTCCCAGTGGCAACT

AACCAAGAGGACCTGGTCATCCAGATCTCCTGTCTGCTGTACTCGCTTGCTACTCAGAAC

CACGAACACACCCTGCTGTTTTCCCTCGGGTCATGCGATATCTCTGAGGAATACTCGTTT

GCATGCGTCCAGCGCGGCGAGCCCAGACCGACGGTTTTGGAGTTTGACAGCGAGTACGAG

CTGCTGGTTGCCTTCCTGACCTTTCTCAAGCAGTACTCTCCCGAGTTCGCCACCGGCTAC

AACATCGTTAATTTTGACTGGGCGTACATAGTTAACAAGGTAACGTCGGTGTATAACATC

AAGCTGGACGGGTACGGCAAGTTCAACAAAGGGGGGCTGTTTAAGGTGTGGGACATCGCC

ACGAACCATTTTCAGAAGAAGAGCAAGGTGAAAATCAATGGCCTGATATCTCTAGACATG

TATTCTGTGGCGACGGAAAAGCTAAAGCTACCCAGCTACAAACTCGACGCGGTCGTGGGA

GACGTCCTCGGCGAGCATAAGATAGACCTTCCCTATAAAGAAATACCCTCCTATTACGCG

GGAGGGCCTGACCGGAGGGGCGTAATAGGAGAGTATTGTATCCAGGACTCTAGGCTGGTG

GGCAAGCTGTTTTTTAAGTACCTCCCCCATCTGGAACTATCGGCGGTGGCCAAACTCGCC

CGTATCACCCTAACGCGGGTAATTTTTGACGGTCAGCAAATTCGCGTGTACACGTGCTTG

CTGAAACTCGCCCGCGAGAGAAATTTCATTTTGCCAGACAACAGACGCCGGTTTGACAGT

CAGGCAGACGCCGCGTCAGAGACTTCGGAGTTGGCTATGGATAGCCAAAGCCACGCCTTC

GACAGTACAGACGAACCCGACGGTGTGGACGGTACCCCGGACGCCGCAGGATCTGGCGCT

ACTTCTGAAAACGGAGGCGGGAAGCCCGGCGTCGGGAGGGCCGTGGGCTACCAGGGAGCA

AAGGTTCTAGACCCCGTATCCGGCTTTCATGTGGACCCCGTGGTTGTGTTTGACTTCGCT

AGCTTATACCCAAGCATTATCCAGGCCCATAACCTCTGTTTCACCACCCTGGCGCTCGAT

GAAGTGGATCTGGCCGGGCTTCAACCATCCGTCGACTACTCGACGTTCGAGGTGGGTGAC

CAAAAGTTATTTTTTGTCCACGCCCATATTCGCGAAAGCCTGCTTGGCATCTTGCTGCGC

GACTGGCTGGCCATGCGAAAGGCGGTGAGGGCGCGAATCCCCACCAGCACCCCCGAGGAG

GCAGTTTTACTAGATAAGCAGCAGTCTGCGATTAAGGTGATATGCAACTCGGTTTACGGA

TTCACGGGGGTGGCAAACGGCCTGTTGCCGTGTCTGAGGATAGCGGCTACCGTTACCACG

ATAGGACGCGACATGCTCCTCAAGACCCGAGATTACGTTCACTCTCGTTGGGCGACGCGC

GAGCTGCTGGAGGACAATTTTCCAGGGGCTATAGGTTTCCGAAACCACAAGCCTTACTCC

GTCAGGGTTATCTACGGAGACACCGACTCCGTGTTTATCAAGTTTGTGGGCCTGACGTAC

GAGGGGGTATCGGAGCTGGGGGATGCTATGTCGCGTCAGATTTCAGCGGACCTCTTTAGA

GCGCCCATCAAACTGGAGTGTGAGAAGACCTTTCAGCGACTGCTGCTGATCACCAAGAAG

AAGTACATAGGTGTCATAAACGGGGGGAAGATGCTCATGAAGGGGGTCGACCTAGTCCGC

AAAAATAACTGCTCTTTCATAAACTTGTACGCGCGACATCTGGTAGATCTTTTGTTGTAC

GACGAGGATGTGGCCACGGCGGCAGCAGAGGTGACAGACGTGCCTCCCGCAGAATGGGTG

GGGCGCCCGCTACCGAGCGGCTTTGACAAGTTTGGGCGAGTGCTGGTAGAGGCGTACAAC

CGTATCACTGCCCCCAACTTGGACGTGCGCGAGTTCGTTATGACTGCTGAGCTGAGCCGC

TCACCCGAATCGTATACCAACAAGCGCCTGCCGCACCTCACCGTCTACTTTAAGCTCGCC

ATGAGGAATGAAGAACTGCCCAGCGTAAAAGAGAGAATTCCGTATGTGATAGTTGCGCAG

ACCGAGGCCGCGGAACGCGAAGCGGGTGTAGTAAACTCAATGCGCGGTACCGCCCAAAAC

CCCGTGGTAACCAAGACCGCACGCCCCCAACCTAAACGCAAACTGCTGGTTTCCGACCTC

GCCGAAGACCCGACCTATGTTTCCGAGAATGACGTACCGCTAAACACAGACTACTATTTC

TCCCACCTGTTGGGTACCATAAGCGTGACCTTTAAGGCTCTATTCGGAAATGATGTGAGA

ACAACAGAAAATCTTTTAAAGCGGTTTATTCCGGAAACCCCCCACAAGACCCCCACGAAA

ACCCAGGCACTGCTTGAGCGCGCCGGCTTTGAAAAGCTGACGCCCTTTACACCGGAGGAA

GAAAGTCGTCGAATACTGCATACAGTTTTTTGTACTCTAGAAGCAGCTCCCCATCAAAGC

TGA

>KU206474.1

ATGGCGGCGCGCGAACAGGCCAACTCCGTTCGCAGGAGCGGGTTTTTTAATCCGTTTATT

GGCAAGAGGCCATTTTTCAGGCCGGGAAGCGGGCAGACCGCGGAGACAGAGCGCCCAAGG

CCCCCCCAACACTCGTACTGCACAGAGGTGGGTAGCTTTAAGTTTATAGCTCCAAGATGC

CTCGATGAAGAAGCCCCCGCCGACCAGCGACGCGGTGTACACGTGGGCACCTTGGAGCGC

CCACCCAAAGTGTACTGCGATGGCTCAGAGTACGACGTGCTGAACTTTGCCTCCGGAGGT

TGCTGGCCTCGCAGGATTCGGGTTTGGAACGGCCAGGATTTTCGGGGCGATGGATTCAAC

CCCAGATTTGAGAGATTTCACGTGTACGACATAGTAGAGACTTCGGAGAGCGCCTCACAC

GATGACCCATCCAGGTTTGCTGAGCTATCCCGCCCAAGCGGGTCTGTGGTAACACTGCTG

GGAATGAGCGAGTGTGGAAAGCGAGTCGCGGTTCACGTCTATGGTGTGCGCCATTATTTT

TACATGGCAAAGGCGGAGGTGGATAGCGCTTGTGGAATAACCACCGAGGCAGAACTCGTG

CGCGCAATGGTGGACTGCGCGCACAGCTCGGCTTTGAGCGCGGCCCTGGGAAATGGCAAC

GGCGGCAAACAGAGCGGCGGCAGCGGCGGGGGATGGTGGGGCGGAAAGCACGTGTCTGCG

GACTGCTTCAAAGTGGAGACCGTGTGCCACACGACGCTGTACTACTTTGGATCTAAGCCA

GCTCTCTACTATAGAGTATCTGCCTCCAGCAGCCGCCTGGGAGGGTTCATCTGCGACAAC

TTTCACCCGGAGATTACAAAATTCGAGGGGAGCGTGGACGTGACCACGCGGCTGTTGTTG

GACAATGAAAATTTTACCAGTTTCGGGTGGTACCGCCTGCGACCCGGCACCCACGGAGAG

CGTGTTCAACTTCGCCCCGTTGAGCGACACGTCACCTCAAGCGACGTGGAGATTAACTGT

ACTCCCGATAACCTGGAGCCGATACCAGACGAGGCTGCCTGGCCCGACTATAAGCTCATG

TGCTTTGATATAGAGTGTAAAGCTGGAACGGGTAACGAAATGGCGTTCCCAGTGGCAACT

AACCAAGAGGACCTGGTCATCCAGATCTCCTGTCTGCTGTACTCGCTTGCTACTCAGAAC

CACGAACACACCCTGCTGTTTTCCCTCGGGTCATGCGATATCTCTGAGGAATACTCGTTT

GCATGCGTCCAGCGCGGCGAGCCCAGACCGACGGTTTTGGAGTTTGACAGCGAGTACGAG

CTGCTGGTTGCCTTCCTGACCTTTCTCAAGCAGTACTCTCCCGAGTTCGCCACCGGCTAC

AACATCGTTAATTTTGACTGGGCGTACATAGTTAACAAGGTAACGTCGGTGTATAACATC

AAGCTGGACGGGTACGGCAAGTTCAACAAAGGGGGGCTGTTTAAGGTGTGGGACATCGCC

ACGAACCATTTTCAGAAGAAGAGCAAGGTGAAAATCAATGGCCTGATATCTCTAGACATG

TATTCTGTGGCGACGGAAAAGCTAAAGCTACCCAGCTACAAACTCGACGCGGTCGTGGGA

GACGTCCTCGGCGAGCATAAGATAGACCTTCCCTATAAAGAAATACCCTCCTATTACGCG

GGAGGGCCTGACCGGAGGGGCGTAATAGGAGAGTATTGTATCCAGGACTCTAGGCTGGTG

GGCAAGCTGTTTTTTAAGTACCTCCCCCATCTGGAACTATCGGCGGTGGCCAAACTCGCC

CGTATCACCCTAACGCGGGTAATTTTTGACGGTCAGCAAATTCGCGTGTACACGTGCTTG

CTGAAACTCGCCCGCGAGAGAAATTTCATTTTGCCAGACAACAGACGCCGGTTTGACAGT

CAGGCAGACGCCGCGTCAGAGACTTCGGAGTTGGCTATGGATAGCCAAAGCCACGCCTTC

GACAGTACAGACGAACCCGACGGTGTGGACGGTACCCCGGACGCCGCAGGATCTGGCGCT

ACTTCTGAAAACGGAGGCGGGAAGCCCGGCGTCGGGAGGGCCGTGGGCTACCAGGGAGCA

AAGGTTCTAGACCCCGTATCCGGCTTTCATGTGGACCCCGTGGTTGTGTTTGACTTCGCT

AGCTTATACCCAAGCATTATCCAGGCCCATAACCTCTGTTTCACCACCCTGGCGCTCGAT

GAAGTGGATCTGGCCGGGCTTCAACCATCCGTCAACTACTCGACGTTCGAGGTGGGTGAC

CAAAAGTTATTTTTTGTCCACGCCCATATTCGCGAAAGCCTGCTTGGCATCTTGCTGCGC

GACTGGCTGGCCATGCGAAAGGCGGTGAGGGCGCGAATCCCCACCAGCACCCCCGAGGAG

GCAGTTTTACTAGATAAGCAGCAGTCTGCGATTAAGGTGATATGCAACTCGGTTTACGGA

TTCACGGGGGTGGCAAACGGCCTGTTGCCGTGTCTGAGGATAGCGGCTACCGTTACCACG

ATAGGACGCGACATGCTCCTCAAGACCCGAGATTACGTTCACTCTCGTTGGGCGACGCGC

GAGCTGCTGGAGGACAATTTTCCAGGGGCTATAGGTTTCCGAAACCACAAGCCTTACTCC

GTCAGGGTTATCTACGGAGACACCGACTCCGTGTTTATCAAGTTTGTGGGCCTGACGTAC

GAGGGGGTATCGGAGCTGGGGGATGCTATGTCGCGTCAGATTTCAGCGGACCTCTTTAGA

GCGCCCATCAAACTGGAGTGTGAGAAGACCTTTCAGCGACTGCTGCTGATCACCAAGAAG

AAGTACATAGGTGTCATAAACGGGGGGAAGATGCTCATGAAGGGGGTCGACCTGGTCCGC

AAAAATAACTGCTCTTTCATAAACTTGTACGCGCGACATCTGGTAGATCTTTTGTTGTAC

GACGAGGATGTGGCCACGGCGGCAGCAGAGGTGACAGACGTGCCTCCCGCAGAATGGGTG

GGGCGCCCGCTACCGAGCGGCTTTGACAAGTTTGGGCGAGTGCTGGTAGAGGCGTACAAC

CGTATCACTGCCCCCAACTTGGACGTGCGCGAGTTCGTTATGACTGCTGAGCTGAGCCGC

TCACCCGAATCGTATACCAACAAGCGCCTGCCGCACCTCACCGTCTACTTTAAGCTCGCC

ATGAGGAATGAAGAACTGCCCAGCGTAAAAGAGAGAATTCCGTATGTGATAGTTGCGCAG

ACCGAGGCCGCGGAACGCGAAGCGGGTGTAGTAAACTCAATGCGCGGTACCGCCCAAAAC

CCCGTGGTAACCAAGACCGCACGCCCCCAACCTAAACGCAAACTGCTGGTTTCCGACCTC

GCCGAAGACCCGACCTATGTTTCCGAGAATGACGTACCGCTAAACACAGACTACTATTTC

TCCCACCTGTTGGGTACCATAAGCGTGACCTTTAAGGCTCTATTCGGAAATGATGTGAGA

ACAACAGAAAATCTTTTAAAGCGGTTTATTCCGGAAACCCCCCACAAGACCCCCACGAAA

ACCCAGGCACTGCTTGAGCGCGCCGGCTTTGAAAAGCTGACGCCCTTTACACCGGAGGAA

GAAAGTCGTCGAATACTGCATACAGTTTTTTGTACTCTAGAAGCAGCTCCCCATCAAAGC

TGA

>KU206475.1

ATGGCGGCGCGCGAACAGGCCAACTCCGTTCGCAGGAGCGGGTTTTTTAATCCGTTTATT

GGCAAGAGGCCATTTTTCAGGCCGGGAAGCGGGCAGACCGCGGAGACAGAGCGCCCAAGG

CCCCCCCAACACTCGTACTGCACAGAGGTGGGTAGCTTTAAGTTTATAGCTCCAAGATGC

CTCGATGAAGAAGCCCCCGCCGACCAGCGACGCGGTGTACACGTGGGCACCTTGGAGCGC

CCACCCAAAGTGTACTGCGATGGCTCAGAGTACGACGTGCTGAACTTTGCCTCCGGAGGT

TGCTGGCCTCGCAGGATTCGGGTTTGGAACGGCCAGGATTTTCGGGGCGATGGATTCAAC

CCCAGATTTGAGAGATTTCACGTGTACGACATAGTAGAGACTTCGGAGAGCGCCTCACAC

GATGACCCATCCAGGTTTGCTGAGCTATCCCGCCCAAGCGGGTCTGTGGTAACACTGCTG

GGAATGAGCGAGTGTGGAAAGCGAGTCGCGGTTCACGTCTATGGTGTGCGCCATTATTTT

TACATGGCAAAGGCGGAGGTGGATAGCGCTTGTGGAATAACCACCGAGGCAGAACTCGTG

CGCGCAATGGTGGACTGCGCGCACAGCTCGGCTTTGAGCGCGGCCCTGGGAAATGGCAAC

GGCGGCAAACAGAGCGGCGGCAGCGGCGGGGGATGGTGGGGCGGAAAGCACGTGTCTGCG

GACTGCTTCAAAGTGGAGACCGTGTGCCACACGACGCTGTACTACTTTGGATCTAAGCCA

GCTCTCTACTATAGAGTATCTGCCTCCAGCAGCCGCCTGGGAGGGTTCATCTGCGACAAC

TTTCACCCGGAGATTACAAAATTCGAGGGGAGCGTGGACGTGACCACGCGGCTGTTGTTG

GACAATGAAAATTTTACCAGTTTCGGGTGGTACCGCCTGCGACCCGGCACCCACGGAGAG

CGTGTTCAACTTCGCCCCGTTGAGCGACACGTCACCTCAAGCGACGTGGAGATTAACTGT

ACTCCCGATAACCTGGAGCCGATACCAGACGAGGCTGCCTGGCCCGACTATAAGCTCATG

TGCTTTGATATAGAGTGTAAAGCTGGAACGGGTAACGAAATGGCGTTCCCAGTGGCAACT

AACCAAGAGGACCTGGTCATCCAGATCTCCTGTCTGCTGTACTCGCTTGCTACTCAGAAC

CACGAACACACCCTGCTGTTTTCCCTCGGGTCATGCGATATCTCTGAGGAATACTCGTTT

GCATGCGTCCAGCGCGGCGAGCCCAGACCGACGGTTTTGGAGTTTGACAGCGAGTACGAG

CTGCTGGTTGCCTTCCTGACCTTTCTCAAGCAGTACTCTCCCGAGTTCGCCACCGGCTAC

AACATCGTTAATTTTGACTGGGCGTACATAGTTAACAAGGTAACGTCGGTGTATAACATC

AAGCTGGACGGGTACGGCAAGTTCAACAAAGGGGGGCTGTTTAAGGTGTGGGACATCGCC

ACGAACCATTTTCAGAAGAAGAGCAAGGTGAAAATCAATGGCCTGATATCTCTAGACATG

TATTCTGTGGCGACGGAAAAGCTAAAGCTACCCAGCTACAAACTCGACGCGGTCGTGGGA

GACGTCCTCGGCGAGCATAAGATAGACCTTCCCTATAAAGAAATACCCTCCTATTACGCG

GGAGGGCCTGACCGGAGGGGCGTAATAGGAGAGTATTGTATCCAGGACTCTAGGCTGGTG

GGCAAGCTGTTTTTTAAGTACCTCCCCCATCTGGAACTATCGGCGGTGGCCAAACTCGCC

CGTATCACCCTAACGCGGGTAATTTTTGACGGTCAGCAAATTCGCGTGTACACGTGCTTG

CTGAAACTCGCCCGCGAGAGAAATTTCATTTTGCCAGACAACAGACGCCGGTTTGACAGT

CAGGCAGACGCCGCGTCAGAGACTTCGGAGTTGGCTATGGATAGCCAAAGCCACGCCTTC

GACAGTACAGACGAACCCGACGGTGTGGACGGTACCCCGGACGCCGCAGGATCTGGCGCT

ACTTCTGAAAACGGAGGCGGGAAGCCCGGCGTCGGGAGGGCCGTGGGCTACCAGGGAGCA

AAGGTTCTAGACCCCGTATCCGGCTTTCATGTGGACCCCGTGGTTGTGTTTGACTTCGCT

AGCTTATACCCAAGCATTATCCAGGCCCATAACCTCTGTTTCACCACCCTGGCGCTCGAT

GAAGTGGATCTGGCCGGGCTTCAACCATCCGTCAACTACTCGACGTTCGAGGTGGGTGAC

CAAAAGTTATTTTTTGTCCACGCCCATATTCGCGAAAGCCTGCTTGGCATCTTGCTGCGC

GACTGGCTGGCCATGCGAAAGGCGGTGAGGGCGCGAATCCCCACCAGCACCCCCGAGGAG

GCAGTTTTACTAGATAAGCAGCAGTCTGCGATTAAGGTGATATGCAACTCGGTTTACGGA

TTCACGGGGGTGGCAAACGGCCTGTTGCCGTGTCTGAGGATAGCGGCTACCGTTACCACG

ATAGGACGCGACATGCTCCTCAAGACCCGAGATTACGTTCACTCTCGTTGGGCGACGCGC

GAGCTGCTGGAGGACAATTTTCCAGGGGCTATAGGTTTCCGAAACCACAAGCCTTACTCC

GTCAGGGTTATCTACGGAGACACCGACTCCGTGTTTATCAAGTTTGTGGGCCTGACGTAC

GAGGGGGTATCGGAGCTGGGGGATGCTATGTCGCGTCAGATTTCAGCGGACCTCTTTAGA

GCGCCCATCAAACTGGAGTGTGAGAAGACCTTTCAGCGACTGCTGCTGATCACCAAGAAG

AAGTACATAGGTGTCATAAACGGGGGGAAGATGCTCATGAAGGGGGTCGACCTGGTCCGC

AAAAATAACTGCTCTTTCATAAACTTGTACGCGCGACATCTGGTAGATCTTTTGTTGTAC

GACGAGGATGTGGCCACGGCGGCAGCAGAGGTGACAGACGTGCCTCCCGCAGAATGGGTG

GGGCGCCCGCTACCGAGCGGCTTTGACAAGTTTGGGCGAGTGCTGGTAGAGGCGTACAAC

CGTATCACTGCCCCCAACTTGGACGTGCGCGAGTTCGTTATGACTGCTGAGCTGAGCCGC

TCACCCGAATCGTATACCAACAAGCGCCTGCCGCACCTCACCGTCTACTTTAAGCTCGCC

ATGAGGAATGAAGAACTGCCCAGCGTAAAAGAGAGAATTCCGTATGTGATAGTTGCGCAG

ACCGAGGCCGCGGAACGCGAAGCGGGTGTAGTAAACTCAATGCGCGGTACCGCCCAAAAC

CCCGTGGTAACCAAGACCGCACGCCCCCAACCTAAACGCAAACTGCTGGTTTCCGACCTC

GCCGAAGACCCGACCTATGTTTCCGAGAATGACGTACCGCTAAACACAGACTACTATTTC

TCCCACCTGTTGGGTACCATAAGCGTGACCTTTAAGGCTCTATTCGGAAATGATGTGAGA

ACAACAGAAAATCTTTTAAAGCGGTTTATTCCGGAAACCCCCCACAAGACCCCCACGAAA

ACCCAGGCACTGCTTGAGCGCGCCGGCTTTGAAAAGCTGACGCCCTTTACACCGGAGGAA

GAAAGTCGTCGAATACTGCATACAGTTTTTTGTACTCTAGAAGCAGCTCCCCATCAAAGC

TGA

>KU206476.1

ATGGCGGCGCGCGAACAGGCCAACTCCGTTCGCAGGAGCGGGTTTTTTAATCCGTTTATT

GGCAAGAGGCCATTTTTCAGGCCGGGAAGCGGGCAGACCGCGGAGACAGAGCGCCCAAGG

CCCCCCCAACACTCGTACTGCACAGAGGTGGGTAGCTTTAAGTTTATAGCTCCAAGATGC

CTCGATGAAGAAGCCCCCGCCGACCAGCGACGCGGTGTACACGTGGGCACCTTGGAGCGC

CCACCCAAAGTGTACTGCGATGGCTCAGAGTACGACGTGCTGAACTTTGCCTCCGGAGGT

TGCTGGCCTCGCAGGATTCGGGTTTGGAACGGCCAGGATTTTCGGGGCGATGGATTCAAC

CCCAGATTTGAGAGATTTCACGTGTACGACATAGTAGAGACTTCGGAGAGCGCCTCACAC

GATGACCCATCCAGGTTTGCTGAGCTATCCCGCCCAAGCGGGTCTGTGGTAACACTGCTG

GGAATGAGCGAGTGTGGAAAGCGAGTCGCGGTTCACGTCTATGGTGTGCGCCATTATTTT

TACATGGCAAAGGCGGAGGTGGATAGCGCTTGTGGAATAACCACCGAGGCAGAACTCGTG

CGCGCAATGGTGGACTGCGCGCACAGCTCGGCTTTGAGCGCGGCCCTGGGAAATGGCAAC

GGCGGCAAACAGAGCGGCGGCAGCGGCGGGGGATGGTGGGGCGGAAAGCACGTGTCTGCG

GACTGCTTCAAAGTGGAGACCGTGTGCCACACGACGCTGTACTACTTTGGATCTAAGCCA

GCTCTCTACTATAGAGTATCTGCCTCCAGCAGCCGCCTGGGAGGGTTCATCTGCGACAAC

TTTCACCCGGAGATTACAAAATTCGAGGGGAGCGTGGACGTGACCACGCGGCTGTTGTTG

GACAATGAAAATTTTACCAGTTTCGGGTGGTACCGCCTGCGACCCGGCACCCACGGAGAG

CGTGTTCAACTTCGCCCCGTTGAGCGACACGTCACCTCAAGCGACGTGGAGATTAACTGT

ACTCCCGATAACCTGGAGCCGATACCAGACGAGGCTGCCTGGCCCGACTATAAGCTCATG

TGCTTTGATATAGAGTGTAAAGCTGGAACGGGTAACGAAATGGCGTTCCCAGTGGCAACT

AACCAAGAGGACCTGGTCATCCAGATCTCCTGTCTGCTGTACTCGCTTGCTACTCAGAAC

CACGAACACACCCTGCTGTTTTCCCTCGGGTCATGCGATATCTCTGAGGAATACTCGTTT

GCATGCGTCCAGCGCGGCGAGCCCAGACCGACGGTTTTGGAGTTTGACAGCGAGTACGAG

CTGCTGGTTGCCTTCCTGACCTTTCTCAAGCAGTACTCTCCCGAGTTCGCCACCGGCTAC

AACATCGTTAATTTTGACTGGGCGTACATAGTTAACAAGGTAACGTCGGTGTATAACATC

AAGCTGGACGGGTACGGCAAGTTCAACAAAGGGGGGCTGTTTAAGGTGTGGGACATCGCC

ACGAACCATTTTCAGAAGAAGAGCAAGGTGAAAATCAATGGCCTGATATCTCTAGACATG

TATTCTGTGGCGACGGAAAAGCTAAAGCTACCCAGCTACAAACTCGACGCGGTCGTGGGA

GACGTCCTCGGCGAGCATAAGATAGACCTTCCCTATAAAGAAATACCCTCCTATTACGCG

GGAGGGCCTGACCGGAGGGGCGTAATAGGAGAGTATTGTATCCAGGACTCTAGGCTGGTG

GGCAAGCTGTTTTTTAAGTACCTCCCCCATCTGGAACTATCGGCGGTGGCCAAACTCGCC

CGTATCACCCTAACGCGGGTAATTTTTGACGGTCAGCAAATTCGCGTGTACACGTGCTTG

CTGAAACTCGCCCGCGAGAGAAATTTCATTTTGCCAGACAACAGACGCCGGTTTGACAGT

CAGGCAGACGCCGCGTCAGAGACTTCGGAGTTGGCTATGGATAGCCAAAGCCACGCCTTC

GACAGTACAGACGAACCCGACGGTGTGGACGGTACCCCGGACGCCGCAGGATCTGGCGCT

ACTTCTGAAAACGGAGGCGGGAAGCCCGGCGTCGGGAGGGCCGTGGGCTACCAGGGAGCA

AAGGTTCTAGACCCCGTATCCGGCTTTCATGTGGACCCCGTGGTTGTGTTTGACTTCGCT

AGCTTATACCCAAGCATTATCCAGGCCCATAACCTCTGTTTCACCACCCTGGCGCTCGAT

GAAGTGGATCTGGCCGGGCTTCAACCATCCGTCAACTACTCGACGTTCGAGGTGGGTGAC

CAAAAGTTATTTTTTGTCCACGCCCATATTCGCGAAAGCCTGCTTGGCATCTTGCTGCGC

GACTGGCTGGCCATGCGAAAGGCGGTGAGGGCGCGAATCCCCACCAGCACCCCCGAGGAG

GCAGTTTTACTAGATAAGCAGCAGTCTGCGATTAAGGTGATATGCAACTCGGTTTACGGA

TTCACGGGGGTGGCAAACGGCCTGTTGCCGTGTCTGAGGATAGCGGCTACCGTTACCACG

ATAGGACGCGACATGCTCCTCAAGACCCGAGATTACGTTCACTCTCGTTGGGCGACGCGC

GAGCTGCTGGAGGACAATTTTCCAGGGGCTATAGGTTTCCGAAACCACAAGCCTTACTCC

GTCAGGGTTATCTACGGAGACACCGACTCCGTGTTTATCAAGTTTGTGGGCCTGACGTAC

GAGGGGGTATCGGAGCTGGGGGATGCTATGTCGCGTCAGATTTCAGCGGACCTCTTTAGA

GCGCCCATCAAACTGGAGTGTGAGAAGACCTTTCAGCGACTGCTGCTGATCACCAAGAAG

AAGTACATAGGTGTCATAAACGGGGGGAAGATGCTCATGAAGGGGGTCGACCTGGTCCGC

AAAAATAACTGCTCTTTCATAAACTTGTACGCGCGACATCTGGTAGATCTTTTGTTGTAC

GACGAGGATGTGGCCACGGCGGCAGCAGAGGTGACAGACGTGCCTCCCGCAGAATGGGTG

GGGCGCCCGCTACCGAGCGGCTTTGACAAGTTTGGGCGAGTGCTGGTAGAGGCGTACAAC

CGTATCACTGCCCCCAACTTGGACGTGCGCGAGTTCGTTATGACTGCTGAGCTGAGCCGC

TCACCCGAATCGTATACCAACAAGCGCCTGCCGCACCTCACCGTCTACTTTAAGCTCGCC

ATGAGGAATGAAGAACTGCCCAGCGTAAAAGAGAGAATTCCGTATGTGATAGTTGCGCAG

ACCGAGGCCGCGGAACGCGAAGCGGGTGTAGTAAACTCAATGCGCGGTACCGCCCAAAAC

CCCGTGGTAACCAAGACCGCACGCCCCCAACCTAAACGCAAACTGCTGGTTTCCGACCTC

GCCGAAGACCCGACCTATGTTTCCGAGAATGACGTACCGCTAAACACAGACTACTATTTC

TCCCACCTGTTGGGTACCATAAGCGTGACCTTTAAGGCTCTATTCGGAAATGATGTGAGA

ACAACAGAAAATCTTTTAAAGCGGTTTATTCCGGAAACCCCCCACAAGACCCCCACGAAA

ACCCAGGCACTGCTTGAGCGCGCCGGCTTTGAAAAGCTGACGCCCTTTACACCGGAGGAA

GAAAGTCGTCGAATACTGCATACAGTTTTTTGTACTCTAGAAGCAGCTCCCCATCAAAGC

TGA

>KU206477.1

ATGGCGGCGCGCGAACAGGCCAACTCCGTTCGCAGGAGCGGGTTTTTTAATCCGTTTATT

GGCAAGAGGCCATTTTTCAGGCCGGGAAGCGGGCAGACCGCGGAGACAGAGCGCCCAAGG

CCCCCCCAACACTCGTACTGCACAGAGGTGGGTAGCTTTAAGTTTATAGCTCCAAGATGC

CTCGATGAAGAAGCCCCCGCCGACCAGCGACGCGGTGTACACGTGGGCACCTTGGAGCGC

CCACCCAAAGTGTACTGCGATGGCTCAGAGTACGACGTGCTGAACTTTGCCTCCGGAGGT

TGCTGGCCTCGCAGGATTCGGGTTTGGAACGGCCAGGATTTTCGGGGCGATGGATTCAAC

CCCAGATTTGAGAGATTTCACGTGTACGACATAGTAGAGACTTCGGAGAGCGCCTCACAC

GATGACCCATCCAGGTTTGCTGAGCTATCCCGCCCAAGCGGGTCTGTGGTAACACTGCTG

GGAATGAGCGAGTGTGGAAAGCGAGTCGCGGTTCACGTCTATGGTGTGCGCCATTATTTT

TACATGGCAAAGGCGGAGGTGGATAGCGCTTGTGGAATAACCACCGAGGCAGAACTCGTG

CGCGCAATGGTGGACTGCGCGCACAGCTCGGCTTTGAGCGCGGCCCTGGGAAATGGCAAC

GGCGGCAAACAGAGCGGCGGCAGCGGCGGGGGATGGTGGGGCGGAAAGCACGTGTCTGCG

GACTGCTTCAAAGTGGAGACCGTGTGCCACACGACGCTGTACTACTTTGGATCTAAGCCA

GCTCTCTACTATAGAGTATCTGCCTCCAGCAGCCGCCTGGGAGGGTTCATCTGCGACAAC

TTTCACCCGGAGATTACAAAATTCGAGGGGAGCGTGGACGTGACCACGCGGCTGTTGTTG

GACAATGAAAATTTTACCAGTTTCGGGTGGTACCGCCTGCGACCCGGCACCCACGGAGAG

CGTGTTCAACTTCGCCCCGTTGAGCGACACGTCACCTCAAGCGACGTGGAGATTAACTGT

ACTCCCGATAACCTGGAGCCGATACCAGACGAGGCTGCCTGGCCCGACTATAAGCTCATG

TGCTTTGATATAGAGTGTAAAGCTGGAACGGGTAACGAAATGGCGTTCCCAGTGGCAACT

AACCAAGAGGACCTGGTCATCCAGATCTCCTGTCTGCTGTACTCGCTTGCTACTCAGAAC

CACGAACACACCCTGCTGTTTTCCCTCGGGTCATGCGATATCTCTGAGGAATACTCGTTT

GCATGCGTCCAGCGCGGCGAGCCCAGACCGACGGTTTTGGAGTTTGACAGCGAGTACGAG

CTGCTGGTTGCCTTCCTGACCTTTCTCAAGCAGTACTCTCCCGAGTTCGCCACCGGCTAC

AACATCGTTAATTTTGACTGGGCGTACATAGTTAACAAGGTAACGTCGGTGTATAACATC

AAGCTGGACGGGTACGGCAAGTTCAACAAAGGGGGGCTGTTTAAGGTGTGGGACATCGCC

ACGAACCATTTTCAGAAGAAGAGCAAGGTGAAAATCAATGGCCTGATATCTCTAGACATG

TATTCTGTGGCGACGGAAAAGCTAAAGCTACCCAGCTACAAACTCGACGCGGTCGTGGGA

GACGTCCTCGGCGAGCATAAGATAGACCTTCCCTATAAAGAAATACCCTCCTATTACGCG

GGAGGGCCTGACCGGAGGGGCGTAATAGGAGAGTATTGTATCCAGGACTCTAGGCTGGTG

GGCAAGCTGTTTTTTAAGTACCTCCCCCATCTGGAACTATCGGCGGTGGCCAAACTCGCC

CGTATCACCCTAACGCGGGTAATTTTTGACGGTCAGCAAATTCGCGTGTACACGTGCTTG

CTGAAACTCGCCCGCGAGAGAAATTTCATTTTGCCAGACAACAGACGCCGGTTTGACAGT

CAGGCAGACGCCGCGTCAGAGACTTCGGAGTTGGCTATGGATAGCCAAAGCCACGCCTTC

GACAGTACAGACGAACCCGACGGTGTGGACGGTACCCCGGACGCCGCAGGATCTGGCGCT

ACTTCTGAAAACGGAGGCGGGAAGCCCGGCGTCGGGAGGGCCGTGGGCTACCAGGGAGCA

AAGGTTCTAGACCCCGTATCCGGCTTTCATGTGGACCCCGTGGTTGTGTTTGACTTCGCT

AGCTTATACCCAAGCATTATCCAGGCCCATAACCTCTGTTTCACCACCCTGGCGCTCGAT

GAAGTGGATCTGGCCGGGCTTCAACCATCCGTCGACTACTCGACGTTCGAGGTGGGTGAC

CAAAAGTTATTTTTTGTCCACGCCCATATTCGCGAAAGCCTGCTTGGCATCTTGCTGCGC

GACTGGCTGGCCATGCGAAAGGCGGTGAGGGCGCGAATCCCCACCAGCACCCCCGAGGAG

GCAGTTTTACTAGATAAGCAGCAGTCTGCGATTAAGGTGATATGCAACTCGGTTTACGGA

TTCACGGGGGTGGCAAACGGCCTGTTGCCGTGTCTGAGGATAGCGGCTACCGTTACCACG

ATAGGACGCGACATGCTCCTCAAGACCCGAGATTACGTTCACTCTCGTTGGGCGACGCGC

GAGCTGCTGGAGGACAATTTTCCAGGGGCTATAGGTTTCCGAAACCACAAGCCTTACTCC

GTCAGGGTTATCTACGGAGACACCGACTCCGTGTTTATCAAGTTTGTGGGCCTGACGTAC

GAGGGGGTATCGGAGCTGGGGGATGCTATGTCGCGTCAGATTTCAGCGGACCTCTTTAGA

GCGCCCATCAAACTGGAGTGTGAGAAGACCTTTCAGCGACTGCTGCTGATCACCAAGAAG

AAGTACATAGGTGTCATAAACGGGGGGAAGATGCTCATGAAGGGGGTCGACCTGGTCCGC

AAAAATAACTGCTCTTTCATAAACTTGTACGCGCGACATCTGGTAGATCTTTTGTTGTAC

GACGAGGATGTGGCCACGGCGGCAGCAGAGGTGACAGACGTGCCTCCCGCAGAATGGGTG

GGGCGCCCGCTACCGAGCGGCTTTGACAAGTTTGGGCGAGTGCTGGTAGAGGCGTACAAC

CGTATCACTGCCCCCAACTTGGACGTGCGCGAGTTCGTTATGACTGCTGAGCTGAGCCGC

TCACCCGAATCGTATACCAACAAGCGCCTGCCGCACCTCACCGTCTACTTTAAGCTCGCC

ATGAGGAATGAAGAACTGCCCAGCGTAAAAGAGAGAATTCCGTATGTGATAGTTGCGCAG

ACCGAGGCCGCGGAACGCGAAGCGGGTGTAGTAAACTCAATGCGCGGTACCGCCCAAAAC

CCCGTGGTAACCAAGACCGCACGCCCCCAACCTAAACGCAAACTGCTGGTTTCCGACCTC

GCCGAAGACCCGACCTATGTTTCCGAGAATGACGTACCGCTAAACACAGACTACTATTTC

TCCCACCTGTTGGGTACCATAAGCGTGACCTTTAAGGCTCTATTCGGAAATGATGTGAGA

ACAACAGAAAATCTTTTAAAGCGGTTTATTCCGGAAACCCCCCACAAGACCCCCACGAAA

ACCCAGGCACTGCTTGAGCGCGCCGGCTTTGAAAAGCTGACGCCCTTTACACCGGAGGAA

GAAAGTCGTCGAATACTGCATACAGTTTTTTGTACTCTAGAAGCAGCTCCCCATCAAAGC

TGA

>KU206479.1

ATGGCGGCGCGCGAACAGGCCAACTCCGTTCGCAGGAGCGGGTTTTTTAATCCGTTTATT

GGCAAGAGGCCATTTTTCAGGCCGGGAAGCGGGCAGACCGCGGAGACAGAGCGCCCAAGG

CCCCCCCAACACTCGTACTGCACAGAGGTGGGTAGCTTTAAGTTTATAGCTCCAAGATGC

CTCGATGAAGAAGCCCCCGCCGACCAGCGACGCGGTGTACACGTGGGCACCTTGGAGCGC

CCACCCAAAGTGTACTGCGATGGCTCAGAGTACGACGTGCTGAACTTTGCCTCCGGAGGT

TGCTGGCCTCGCAGGATTCGGGTTTGGAACGGCCAGGATTTTCGGGGCGATGGATTCAAC

CCCAGATTTGAGAGATTTCACGTGTACGACATAGTAGAGACTTCGGAGAGCGCCTCACAC

GATGACCCATCCAGGTTTGCTGAGCTATCCCGCCCAAGCGGGTCTGTGGTAACACTGCTG

GGAATGAGCGAGTGTGGAAAGCGAGTCGCGGTTCACGTCTATGGTGTGCGCCATTATTTT

TACATGGCAAAGGCGGAGGTGGATAGCGCTTGTGGAATAACCACCGAGGCAGAACTCGTG

CGCGCAATGGTGGACTGCGCGCACAGCTCGGCTTTGAGCGCGGCCCTGGGAAATGGCAAC

GGCGGCAAACAGAGCGGCGGCAGCGGCGGGGGATGGTGGGGCGGAAAGCACGTGTCTGCG

GACTGCTTCAAAGTGGAGACCGTGTGCCACACGACGCTGTACTACTTTGGATCTAAGCCA

GCTCTCTACTATAGAGTATCTGCCTCCAGCAGCCGCCTGGGAGGGTTCATCTGCGACAAC

TTTCACCCGGAGATTACAAAATTCGAGGGGAGCGTGGACGTGACCACGCGGCTGTTGTTG

GACAATGAAAATTTTACCAGTTTCGGGTGGTACCGCCTGCGACCCGGCACCCACGGAGAG

CGTGTTCAACTTCGCCCCGTTGAGCGACACGTCACCTCAAGCGACGTGGAGATTAACTGT

ACTCCCGATAACCTGGAGCCGATACCAGACGAGGCTGCCTGGCCCGACTATAAGCTCATG

TGCTTTGATATAGAGTGTAAAGCTGGAACGGGTAACGAAATGGCGTTCCCAGTGGCAACT

AACCAAGAGGACCTGGTCATCCAGATCTCCTGTCTGCTGTACTCGCTTGCTACTCAGAAC

CACGAACACACCCTGCTGTTTTCCCTCGGGTCATGCGATATCTCTGAGGAATACTCGTTT

GCATGCGTCCAGCGCGGCGAGCCCAGACCGACGGTTTTGGAGTTTGACAGCGAGTACGAG

CTGCTGGTTGCCTTCCTGACCTTTCTCAAGCAGTACTCTCCCGAGTTCGCCACCGGCTAC

AACATCGTTAATTTTGACTGGGCGTACATAGTTAACAAGGTAACGTCGGTGTATAACATC

AAGCTGGACGGGTACGGCAAGTTCAACAAAGGGGGGCTGTTTAAGGTGTGGGACATCGCC

ACGAACCATTTTCAGAAGAAGAGCAAGGTGAAAATCAATGGCCTGATATCTCTAGACATG

TATTCTGTGGCGACGGAAAAGCTAAAGCTACCCAGCTACAAACTCGACGCGGTCGTGGGA

GACGTCCTCGGCGAGCATAAGATAGACCTTCCCTATAAAGAAATACCATCCTATTACGCG

GGAGGGCCTGACCGGAGGGGCGTAATAGGAGAGTATTGTATCCAGGACTCTAGGCTGGTG

GGCAAGCTGTTTTTTAAGTACCTCCCCCATCTGGAACTATCGGCGGTGGCCAAACTCGCC

CGTATCACCCTAACGCGGGTAATTTTTGACGGTCAGCAAATTCGCGTGTACACGTGCTTG

CTGAAACTCGCCCGCGAGAGAAATTTCATTTTGCCAGACAACAGACGCCGGTTTGACAGT

CAGGCAGACGCCGCGTCAGAGACTTCGGAGTTGGCTATGGATAGCCAAAGCCACGCCTTC

GACAGTACAGACGAACCCGACGGTGTGGACGGTACCCCGGACGCCGCAGGATCTGGCGCT

ACTTCTGAAAACGGAGGCGGGAAGCCCGGCGTCGGGAGGGCCGTGGGCTACCAGGGAGCA

AAGGTTCTAGACCCCGTATCCGGCTTTCATGTGGACCCCGTGGTTGTGTTTGACTTCGCT

AGCTTATACCCAAGCATTATCCAGGCCCATAACCTCTGTTTCACCACCCTGGCGCTCGAT

GAAGTGGATCTGGCCGGGCTTCAACCATCCGTCGACTACTCGACGTTCGAGGTGGGTGAC

CAAAAGTTATTTTTTGTCCACGCCCATATTCGCGAAAGCCTGCTTGGCATCTTGCTGCGC

GACTGGCTGGCCATGCGAAAGGCGGTGAGGGCGCGAATCCCCACCAGCACCCCCGAGGAG

GCAGTTTTACTAGATAAGCAGCAGTCTGCGATTAAGGTGATATGCAACTCGGTTTACGGA

TTCACGGGGGTGGCAAACGGCCTGTTGCCGTGTCTGAGGATAGCGGCTACCGTTACCACG

ATAGGACGCGACATGCTCCTCAAGACCCGAGATTACGTTCACTCTCGTTGGGCGACGCGC

GAGCTGCTGGAGGACAATTTTCCAGGGGCTATAGGTTTCCGAAACCACAAGCCTTACTCC

GTCAGGGTTATCTACGGAGACACCGACTCCGTGTTTATCAAGTTTGTGGGCCTGACGTAC

GAGGGGGTATCGGAGCTGGGGGATGCTATGTCGCGTCAGATTTCAGCGGACCTCTTTAGA

GCGCCCATCAAACTGGAGTGTGAGAAGACCTTTCAGCGACTGCTGCTGATCACCAAGAAG

AAGTACATAGGTGTCATAAACGGGGGGAAGATGCTCATGAAGGGGGTCGACCTGGTCCGC

AAAAATAACTGCTCTTTCATAAACTTGTACGCGCGACATCTGGTAGATCTTTTGTTGTAC

GACGAGGATGTGGCCACGGCGGCAGCAGAGGTGACAGACGTGCCTCCCGCAGAATGGGTG

GGGCGCCCGCTACCGAGCGGCTTTGACAAGTTTGGGCGAGTGCTGGTAGAGGCGTACAAC

CGTATCACTGCCCCCAACTTGGACGTGCGCGAGTTCGTTATGACTGCTGAGCTGAGCCGC

TCACCCGAATCGTATACCAACAAGCGCCTGCCGCACCTCACCGTCTACTTTAAGCTCGCC

ATGAGGAATGAAGAACTGCCCAGCGTAAAAGAGAGAATTCCGTATGTGATAGTTGCGCAG

ACCGAGGCCGCGGAACGCGAAGCGGGTGTAGTAAACTCAATGCGCGGTACCGCCCAAAAC

CCCGTGGTAACCAAGACCGCACGCCCCCAACCTAAACGCAAACTGCTGGTTTCCGACCTC

GCCGAAGACCCGACCTATGTTTCCGAGAATGACGTACCGCTAAACACAGACTACTATTTC

TCCCACCTGTTGGGTACCATAAGCGTGACCTTTAAGGCTCTATTCGGAAATGATGTGAGA

ACAACAGAAAATCTTTTAAAGCGGTTTATTCCGGAAACCCCCCACAAGACCCCCACGAAA

ACCCAGGCACTGCTTGAGCGCGCCGGCTTTGAAAAGCTGACGCCCTTTACACCGGAGGAA

GAAAGTCGTCGAATACTGCATACAGTTTTTTGTACTCTAGAAGCAGCTCCCCATCAAAGC

TGA

>KU206480.1

ATGGCGGCGCGCGAACAGGCCAACTCCGTTCGCAGGAGCGGGTTTTTTAATCCGTTTATT

GGCAAGAGGCCATTTTTCAGGCCGGGAAGCGGGCAGACCGCGGAGACAGAGCGCCCAAGG

CCCCCCCAACACTCGTACTGCACAGAGGTGGGTAGCTTTAAGTTTATAGCTCCAAGATGC

CTCGATGAAGAAGCCCCCGCCGACCAGCGACGCGGTGTACACGTGGGCACCTTGGAGCGC

CCACCCAAAGTGTACTGCGATGGCTCAGAGTACGACGTGCTGAACTTTGCCTCCGGAGGT

TGCTGGCCTCGCAGGATTCGGGTTTGGAACGGCCAGGATTTTCGGGGCGATGGATTCAAC

CCCAGATTTGAGAGATTTCACGTGTACGACATAGTAGAGACTTCGGAGAGCGCCTCACAC

GATGACCCATCCAGGTTTGCTGAGCTATCCCGCCCAAGCGGGTCTGTGGTAACACTGCTG

GGAATGAGCGAGTGTGGAAAGCGAGTCGCGGTTCACGTCTATGGTGTGCGCCATTATTTT

TACATGGCAAAGGCGGAGGTGGATAGCGCTTGTGGAATAACCACCGAGGCAGAACTCGTG

CGCGCAATGGTGGACTGCGCGCACAGCTCGGCTTTGAGCGCGGCCCTGGGAAATGGCAAC

GGCGGCAAACAGAGCGGCGGCAGCGGCGGGGGATGGTGGGGCGGAAAGCACGTGTCTGCG

GACTGCTTCAAAGTGGAGACCGTGTGCCACACGACGCTGTACTACTTTGGATCTAAGCCA

GCTCTCTACTATAGAGTATCTGCCTCCAGCAGCCGCCTGGGAGGGTTCATCTGCGACAAC

TTTCACCCGGAGATTACAAAATTCGAGGGGAGCGTGGACGTGACCACGCGGCTGTTGTTG

GACAATGAAAATTTTACCAGTTTCGGGTGGTACCGCCTGCGACCCGGCACCCACGGAGAG

CGTGTTCAACTTCGCCCCGTTGAGCGACACGTCACCTCAAGCGACGTGGAGATTAACTGT

ACTCCCGATAACCTGGAGCCGATACCAGACGAGGCTGCCTGGCCCGACTATAAGCTCATG

TGCTTTGATATAGAGTGTAAAGCTGGAACGGGTAACGAAATGGCGTTCCCAGTGGCAACT

AACCAAGAGGACCTGGTCATCCAGATCTCCTGTCTGCTGTACTCGCTTGCTACTCAGAAC

CACGAACACACCCTGCTGTTTTCCCTCGGGTCATGCGATATCTCTGAGGAATACTCGTTT

GCATGCGTCCAGCGCGGCGAGCCCAGACCGACGGTTTTGGAGTTTGACAGCGAGTACGAG

CTGCTGGTTGCCTTCCTGACCTTTCTCAAGCAGTACTCTCCCGAGTTCGCCACCGGCTAC

AACATCGTTAATTTTGACTGGGCGTACATAGTTAACAAGGTAACGTCGGTGTATAACATC

AAGCTGGACGGGTACGGCAAGTTCAACAAAGGGGGGCTGTTTAAGGTGTGGGACATCGCC

ACGAACCATTTTCAGAAGAAGAGCAAGGTGAAAATCAATGGCCTGATATCTCTAGACATG

TATTCTGTGGCGACGGAAAAGCTAAAGCTACCCAGCTACAAACTCGACGCGGTCGTGGGA

GACGTCCTCGGCGAGCATAAGATAGACCTTCCCTATAAAGAAATACCCTCCTATTACGCG

GGAGGGCCTGACCGGAGGGGCGTAATAGGAGAGTATTGTATCCAGGACTCTAGGCTGGTG

GGCAAGCTGTTTTTTAAGTACCTCCCCCATCTGGAACTATCGGCGGTGGCCAAACTCGCC

CGTATCACCCTAACGCGGGTAATTTTTGACGGTCAGCAAATTCGCGTGTACACGTGCTTG

CTGAAACTCGCCCGCGAGAGAAATTTCATTTTGCCAGACAACAGACGCCGGTTTGACAGT

CAGGCAGACGCCGCGTCAGAGACTTCGGAGTTGGCTATGGATAGCCAAAGCCACGCCTTC

GACAGTACAGACGAACCCGACGGTGTGGACGGTACCCCGGACGCCGCAGGATCTGGCGCT

ACTTCTGAAAACGGAGGCGGGAAGCCCGGCGTCGGGAGGGCCGTGGGCTACCAGGGAGCA

AAGGTTCTAGACCCCGTATCCGGCTTTCATGTGGACCCCGTGGTTGTGTTTGACTTCGCT

AGCTTATACCCAAGCATTATCCAGGCCCATAACCTCTGTTTCACCACCCTGGCGCTCGAT

GAAGTGGATCTGGCCGGGCTTCAACCATCCGTCAACTACTCGACGTTCGAGGTGGGTGAC

CAAAAGTTATTTTTTGTCCACGCCCATATTCGCGAAAGCCTGCTTGGCATCTTGCTGCGC

GACTGGCTGGCCATGCGAAAGGCGGTGAGGGCGCGAATCCCCACCAGCACCCCCGAGGAG

GCAGTTTTACTAGATAAGCAGCAGTCTGCGATTAAGGTGATATGCAACTCGGTTTACGGA

TTCACGGGGGTGGCAAACGGCCTGTTGCCGTGTCTGAGGATAGCGGCTACCGTTACCACG

ATAGGACGCGACATGCTCCTCAAGACCCGAGATTACGTTCACTCTCGTTGGGCGACGCGC

GAGCTGCTGGAGGACAATTTTCCAGGGGCTATAGGTTTCCGAAACCACAAGCCTTACTCC

GTCAGGGTTATCTACGGAGACACCGACTCCGTGTTTATCAAGTTTGTGGGCCTGACGTAC

GAGGGGGTATCGGAGCTGGGGGATGCTATGTCGCGTCAGATTTCAGCGGACCTCTTTAGA

GCGCCCATCAAACTGGAGTGTGAGAAGACCTTTCAGCGACTGCTGCTGATCACCAAGAAG

AAGTACATAGGTGTCATAAACGGGGGGAAGATGCTCATGAAGGGGGTCGACCTGGTCCGC

AAAAATAACTGCTCTTTCATAAACTTGTACGCGCGACATCTGGTAGATCTTTTGTTGTAC

GACGAGGATGTGGCCACGGCGGCAGCAGAGGTGACAGACGTGCCTCCCGCAGAATGGGTG

GGGCGCCCGCTACCGAGCGGCTTTGACAAGTTTGGGCGAGTGCTGGTAGAGGCGTACAAC

CGTATCACTGCCCCCAACTTGGACGTGCGCGAGTTCGTTATGACTGCTGAGCTGAGCCGC

TCACCCGAATCGTATACCAACAAGCGCCTGCCGCACCTCACCGTCTACTTTAAGCTCGCC

ATGAGGAATGAAGAACTGCCCAGCGTAAAAGAGAGAATTCCGTATGTGATAGTTGCGCAG

ACCGAGGCCGCGGAACGCGAAGCGGGTGTAGTAAACTCAATGCGCGGTACCGCCCAAAAC

CCCGTGGTAACCAAGACCGCACGCCCCCAACCTAAACGCAAACTGCTGGTTTCCGACCTC

GCCGAAGACCCGACCTATGTTTCCGAGAATGACGTACCGCTAAACACAGACTACTATTTC

TCCCACCTGTTGGGTACCATAAGCGTGACCTTTAAGGCTCTATTCGGAAATGATGTGAGA

ACAACAGAAAATCTTTTAAAGCGGTTTATTCCGGAAACCCCCCACAAGACCCCCACGAAA

ACCCAGGCACTGCTTGAGCGCGCCGGCTTTGAAAAGCTGACGCCCTTTACACCGGAGGAA

GAAAGTCGTCGAATACTGCATACAGTTTTTTGTACTCTAGAAGCAGCTCCCCATCAAAGC

TGA

>KY852346.1

ATGGCGGCGCGCGAACAGGCCAACTCCGTTCGCAGGAGCGGGTTTTTTAATCCGTTTATT

GGCAAGAGGCCATTTTTCAGGCCGGGAAGCGGGCAGACCGCGGAGACAGAGCGCCCAAGG

CCCCCCCAACACTCGTACTGCACAGAGGTGGGTAGCTTTAAGTTTATAGCTCCAAGATGC

CTCGATGAAGAAGCCCCCGCCGACCAGCGACGCGGTGTACACGTGGGCACCTTGGAGCGC

CCACCCAAAGTGTACTGCGATGGCTCAGAGTACGACGTGCTGAACTTTGCCTCCGGAGGT

TGCTGGCCTCGCAGGATTCGGGTTTGGAACGGCCAGGATTTTCGGGGCGATGGATTCAAC

CCCAGATTTGAGAGATTTCACGTGTACGACATAGTAGAGACTTCGGAGAGCGCCTCACAC

GATGACCCATCCAGGTTTGCTGAGCTATCCCGCCCAAGCGGGTCTGTGGTAACACTGCTG

GGAATGAGCGAGTGTGGAAAGCGAGTCGCGGTTCACGTCTATGGTGTGCGCCATTATTTT

TACATGGCAAAGGCGGAGGTGGATAGCGCTTGTGGAATAACCACCGAGGCAGAACTCGTG

CGCGCAATGGTGGACTGCGCGCACAGCTCGGCTTTGAGCGCGGCCCTGGGAAATGGCAAC

GGCGGCAAACAGAGCGGCGGCAGCGGCGGGGGATGGTGGGGCGGAAAGCACGTGTCTGCG

GACTGCTTCAAAGTGGAGACCGTGTGCCACACGACGCTGTACTACTTTGGATCTAAGCCA

GCTCTCTACTATAGAGTATCTGCCTCCAGCAGCCGCCTGGGAGGGTTCATCTGCGACAAC

TTTCACCCGGAGATTACAAAATTCGAGGGGAGCGTGGACGTGACCACGCGGCTGTTGTTG

GACAATGAAAATTTTACCAGTTTCGGGTGGTACCGCCTGCGACCCGGCACCCACGGAGAG

CGTGTTCAACTTCGCCCCGTTGAGCGACACGTCACCTCAAGCGACGTGGAGATTAACTGT

ACTCCCGATAACCTGGAGCCGATACCAGACGAGGCTGCCTGGCCCGACTATAAGCTCATG

TGCTTTGATATAGAGTGTAAAGCTGGAACGGGTAACGAAATGGCGTTCCCAGTGGCAACT

AACCAAGAGGACCTGGTCATCCAGATCTCCTGTCTGCTGTACTCGCTTGCTACTCAGAAC

CACGAACACACCCTGCTGTTTTCCCTCGGGTCATGCGATATCTCTGAGGAATACTCGTTT

GCATGCGTCCAGCGCGGCGAGCCCAGACCGACGGTTTTGGAGTTTGACAGCGAGTACGAG

CTGCTGGTTGCCTTCCTGACCTTTCTCAAGCAGTACTCTCCCGAGTTCGCCACCGGCTAC

AACATCGTTAATTTTGACTGGGCGTACATAGTTAACAAGGTAACGTCGGTGTATAACATC

AAGCTGGACGGGTACGGCAAGTTCAACAAAGGGGGGCTGTTTAAGGTGTGGGACATCGCC

ACGAACCATTTTCAGAAGAAGAGCAAGGTGAAAATCAATGGCCTGATATCTCTAGACATG

TATTCTGTGGCGACGGAAAAGCTAAAGCTACCCAGCTACAAACTCGACGCGGTCGTGGGA

GACGTCCTCGGCGAGCATAAGATAGACCTTCCCTATAAAGAAATACCCTCCTATTACGCG

GGAGGGCCTGACCGGAGGGGCGTAATAGGAGAGTATTGTATCCAGGACTCTAGGCTGGTG

GGCAAGCTGTTTTTTAAGTACCTCCCCCATCTGGAACTATCGGCGGTGGCCAAACTCGCC

CGTATCACCCTAACGCGGGTAATTTTTGACGGTCAGCAAATTCGCGTGTACACGTGCTTG

CTGAAACTCGCCCGCGAGAGAAATTTCATTTTGCCAGACAACAGACGCCGGTTTGACAGT

CAGGCAGACGCCGCGTCAGAGACTTCGGAGTTGGCTATGGATAGCCAAAGCCACGCCTTC

GACAGTACAGACGAACCCGACGGTGTGGACGGTACCCCGGACGCCGCAGGATCTGGCGCT

ACTTCTGAAAACGGAGGCGGGAAGCCCGGCGTCGGGAGGGCCGTGGGCTACCAGGGAGCA

AAGGTTCTAGACCCCGTATCCGGCTTTCATGTGGACCCCGTGGTTGTGTTTGACTTCGCT

AGCTTATACCCAAGCATTATCCAGGCCCATAACCTCTGTTTCACCACCCTGGCGCTCGAT

GAAGTGGATCTGGCCGGGCTTCAACCATCCGTCGACTACTCGACGTTCGAGGTGGGTGAC

CAAAAGTTATTTTTTGTCCACGCCCATATTCGCGAAAGCCTGCTTGGCATCTTGCTGCGC

GACTGGCTGGCCATGCGAAAGGCGGTGAGGGCGCGAATCCCCACCAGCACCCCCGAGGAG

GCAGTTTTACTAGATAAGCAGCAGTCTGCGATTAAGGTGATATGCAACTCGGTTTACGGA

TTCACGGGGGTGGCAAACGGCCTGTTGCCGTGTCTGAGGATAGCGGCTACCGTTACCACG

ATAGGACGCGACATGCTCCTCAAGACCCGAGATTACGTTCACTCTCGTTGGGCGACGCGC

GAGCTGCTGGAGGACAATTTTCCAGGGGCTATAGGTTTCCGAAACCACAAGCCTTACTCC

GTCAGGGTTATCTACGGAGACACCGACTCCGTGTTTATCAAGTTTGTGGGCCTGACGTAC

GAGGGGGTATCGGAGCTGGGGGATGCTATGTCGCGTCAGATTTCAGCGGACCTCTTTAGA

GCGCCCATCAAACTGGAGTGTGAGAAGACCTTTCAGCGACTGCTGCTGATCACCAAGAAG

AAGTACATAGGTGTCATAAACGGGGGGAAGATGCTCATGAAGGGGGTCGACCTGGTCCGC

AAAAATAACTGCTCTTTCATAAACTTGTACGCGCGACATCTGGTAGATCTTTTGTTGTAC

GACGAGGATGTGGCCACGGCGGCAGCAGAGGTGACAGACGTGCCTCCCGCAGAATGGGTG

GGGCGCCCGCTACCGAGCGGCTTTGACAAGTTTGGGCGAGTGCTGGTAGAGGCGTACAAC

CGTATCACTGCCCCCAACTTGGACGTGCGCGAGTTCGTTATGACTGCTGAGCTGAGCCGC

TCACCCGAATCGTATACCAACAAGCGCCTGCCGCACCTCACCGTCTACTTTAAGCTCGCC

ATGAGGAATGAAGAACTGCCCAGCGTAAAAGAGAGAATTCCGTATGTGATAGTTGCGCAG

ACCGAGGCCGCGGAACGCGAAGCGGGTGTAGTAAACTCAACGCGCGGTACCGCCCAAAAC

CCCGTGGTAACCAAGACCGCACGCCCCCAACCTAAACGCAAACTGCTGGTTTCCGACCTC

GCCGAAGACCCGACCTATGTTTCCGAGAATGACGTACCGCTAAACACAGACTACTATTTC

TCCCACCTGTTGGGTACCATAAGCGTGACCTTTAAGGCTCTATTCGGAAATGATGTGAGA

ACAACAGAAAATCTTTTAAAGCGGTTTATTCCGGAAACCCCCCACAAGACCCCCACGAAA

ACCCAGGCACTGCTTGAGCGCGCCGGCTTTGAAAAGCTGACGCCCTTTACACCGGAGGAA

GAAAGTCGTCGAATACTGCATACAGTTTTTTGTACTCTAGAAGCAGCTCCCCATCAAAGC

TGA

>MN688617.1

ATGGCGGCGCGCGAACAGGCCAACTCCGTTCGCAGGAGCGGGTTTTTTAATCCGTTTATT

GGCAAGAGGCCATTTTTCAGGCCGGGAAGCGGGCAAACCGCGGAGACAGAGCGCCCAAGG

CCCCCCCAACACTCGTACTGCACAGAGGTGGGTAGCTTTAAGTTTATAGCTCCAGGATGC

CTCGATGAAGAAGCCCCCGCCGACCAGCGACGCGGTGTACACGTGGGCACCTTGGAGCGC

CCACCCAAAGTGTACTGCGATGGCTCAGAGTACGACGTGCTGAACTTTGCCTCCGGAGGT

TGCTGGCCTCGCAGGATTCGGGTTTGGAACGGCCAGGATTTTCGGGGCGATGGATTCAAC

CCCAGATTTGAGAGATTTCACGTGTACGACATAGTAGAGACTTCGGAGAGCGCCTCACAC

GATGACCCATCCAGGTTTGCTGAGCTATCCCGCCCAAGCGGGTCTGTGGTAACACTGCTG

GGAATGAGCGAGTGTGGAAAGCGAGTCGCGGTTCACGTCTATGGTGTGCGCCATTATTTT

TACATGGCAAAGGCGGAGGTGGATAGCGCTTGTGGAATAACCACCGAGGCAGAACTCGTG

CGCGCAATGGTGGACTGCGCGCACAGCTCGGCTTTGAGCGCGGCCCTGGGAAATGGCAAC

GGCGGCAAACAGAGCGGCGGCAGCGGCGGGGGATGGTGGGGCGGAAAGCACGTGTCTGCG

GACTGCTTCAAAGTGGAGACCGTGTGCCACACGACGCTGTACTACTTTGGATCTAAGCCA

GCTCTCTACTATAGAGTATCTGCCTCCAGCAGCCGCCTGGGAGGGTTCATCTGCGACAAC

TTTCACCCGGAGATTACAAAATTCGAGGGGAGCGTGGACGTGACCACGCGGCTGTTGTTG

GACAATGAAAATTTTACCAGTTTCGGGTGGTACCGCCTGCGACCCGGCACCCACGGAGAG

CGTGTTCAACTTCGCCCCGTTGAGCGACACGTCACCTCAAGCGACGTGGAGATTAACTGT

ACTCCCGATAACCTGGAGCCGATACCAGACGAGGCTGCCTGGCCCGACTATAAGCTCATG

TGCTTTGATATAGAGTGTAAAGCTGGAACGGGTAACGAAATGGCGTTCCCAGTGGCAACT

AACCAAGAGGACCTGGTCATCCAGATCTCCTGTCTGCTGTACTCGCTTGCTACTCAGAAC

CACGAACACACCCTGCTGTTTTCCCTCGGGTCATGCGATATCTCTGAGGAATACTCGTTT

GCATGCGTCCAGCGCGGCGAGCCCAGACCGACGGTTTTGGAGTTTGACAGCGAGTACGAG

CTGCTGGTTGCCTTCCTGACCTTTCTCAAGCAGTACTCTCCCGAGTTCGCCACCGGCTAC

AACATCGTTAATTTTGACTGGGCGTACATAGTTAACAAGGTAACGTCGGTGTATAACATC

AAGCTGGACGGGTACGGCAAGTTCAACAAAGGGGGGCTGTTTAAGGTGTGGGACATCGCC

ACGAACCATTTTCAGAAGAAGAGCAAGGTGAAAATCAATGGCCTGATATCTCTAGACATG

TATTCTGTGGCGACGGAAAAGCTAAAGCTACCCAGCTACAAACTCGACGCGGTCGTGGGA

GACGTCCTCGGCGAGCATAAGATAGACCTTCCCTATAAAGAAATACCCTCCTATTACGCG

GGAGGGCCTGACCGGAGGGGCGTAATAGGAGAGTATTGTATCCAGGACTCTAGGCTGGTG

GGCAAGCTGTTTTTTAAGTACCTCCCCCATCTGGAACTATCGGCGGTGGCCAAACTCGCC

CGTATCACCCTAACGCGGGTAATTTTTGACGGTCAGCAAATTCGCGTGTACACGTGCTTG

CTGAAACTCGCCCGCGAGAGAAATTTCATTTTGCCAGACAACAGACGCCGGTTTGACAGT

CAGGCAGACGCCGCGTCAGAGACTTCGGAGTTGGCTATGGATAGCCAAAGCCACGCCTTC

GACAGTACAGACGAACCCGACGGTGTGGACGGTACCCCGGACGCCGCAGGATCTGGCGCT

ACTTCTGAAAACGGAGGCGGGAAGCCCGGCGTCGGGAGGGCCGTGGGCTACCAGGGAGCA

AAGGTTCTAGACCCCGTATCCGGCTTTCATGTGGACCCCGTGGTTGTGTTTGACTTCGCT

AGCTTATACCCAAGCATTATCCAGGCCCATAACCTCTGTTTCACCACCCTGGCGCTCGAT

GAAGTGGATCTGGCCGGGCTTCAACCATCCGTCAACTACTCGACGTTCGAGGTGGGTGAC

CAAAAGTTATTTTTTGTCCACGCCCATATTCGCGAAAGCCTGCTTGGCATCTTGCTGCGC

GACTGGCTGGCCATGCGAAAGGCGGTGAGGGCGCGAATCCCCACCAGCACCCCCGAGGAG

GCAGTTTTACTAGATAAGCAGCAGTCTGCGATTAAGGTGATATGCAACTCGGTTTACGGA

TTCACGGGGGTGGCAAACGGCCTGTTGCCGTGTCTGAGGATAGCGGCTACCGTTACCACG

ATAGGACGCGACATGCTCCTCAAGACCCGAGATTACGTTCACTCTCGTTGGGCGACGCGC

GAGCTGCTGGAGGACAATTTTCCAGGGGCTATAGGTTTCCGAAACCACAAGCCTTACTCC

GTCAGGGTTATCTACGGAGACACCGACTCCGTGTTTATCAAGTTTGTGGGCCTGACGTAC

GAGGGGGTATCGGAGCTGGGGGATGCTATGTCGCGTCAGATTTCAGCGGACCTCTTTAGA

GCGCCCATCAAACTGGAGTGTGAGAAGACCTTTCAGCGACTGCTGCTGATCACCAAGAAG

AAGTACATAGGTGTCATAAACGGGGGGAAGATGCTCATGAAGGGGGTCGACCTGGTCCGC

AAAAATAACTGCTCTTTCATAAACTTGTACGCGCGACATCTGGTAGATCTTTTGTTGTAC

GACGAGGATGTGGCCACGGCGGCAGCAAAGGTGACAGACGTGCCTCCCGCAGAATGGGTG

GGGCGCCCGCTACCGAGCGGCTTTGACAAGTTTGGGCGAGTGCTGGTAGAGGCGTACAAC

CGTATCACTGCCCCCAACTTGGACGTGCGCGAGTTCGTTATGACTGCTGAGCTGAGCCGC

TCACCCGAATCGTATACCAACAAGCGCCTGCCGCACCTCACCGTCTACTTTAAGCTCGCC

ATGAGGAATGAAGAACTGCCCAGCGTAAAAGAGAGAATTCCGTATGTGATAGTTGCGCAG

ACCGAGGCCGCGGAACGCGAAGCGGGTGTAGTAAACTCAATGCGCGGTACCGCCCAAAAC

CCCGTGGTAACCAAGACCGCACGCCCCCAACCTAAACGCAAACTGCTGGTTTCCGACCTC

GCCGAAGACCCGACCTATGTTTCCGAGAATGACGTACCGCTAAACACAGACTACTATTTC

TCCCACCTGTTGGGTACCATAAGCGTGACCTTTAAGGCTCTATTCGGAAATGATGTGAGA

ACAACAGAAAATCTTTTAAAGCGGTTTATTCCGGAAACCCCCCACAAGACCCCCACGAAA

ACCCAGGCACTGCTTGAGCGCGCCGGCTTTGAAAAGCTGACGCCCTTTACACCGGAGGAA

GAAAGTCGTCGAATACTGCATACAGTTTTTTGTACTCTAGAAGCAGCTCCCCATCAAAGC

TGA

>MN688618.1

ATGGCGGCGCGCGAACAGGCCAACTCCGTTCGCAGGAGCGGGTTTTTTAATCCGTTTATT

GGCAAGAGGCCATTTTTCAGGCCGGGAAGCGGGCAGACCGCGGAGACAGAGCGCCCAAGG

CCCCCCCAACACTCGTACTGCACAGAGGTGGGTAGCTTTAAGTTTATAGCTCCAAGATGC

CTCGATGAAGAAGCCCCCGCCGACCAGCGACGCGGTGTACACGTGGGCACCTTGGAGCGC

CCACCCAAAGTGTACTGCGATGGCTCAGAGTACGACGTGCTGAACTTTGCCTCCGGAGGT

TGCTGGCCTCGCAGGATTCGGGTTTGGAACGGCCAGGATTTTCGGGGCGATGGATTCAAC

CCCAGATTTGAGAGATTTCACGTGTACGACATAGTAGAGACTTCGGAGAGCGCCTCACAC

GATGACCCATCCAGGTTTGCTGAGCTATCCCGCCCAAGCGGGTCTGTGGTAACACTGCTG

GGAATGAGCGAGTGTGGAAAGCGAGTCGCGGTTCACGTCTATGGTGTGCGCCATTATTTT

TACATGGCAAAGGCGGAGGTGGATAGCGCTTGTGGAATAACCACCGAGGCAGAACTCGTG

CGCGCAATGGTGGACTGCGCGCACAGCTCGGCTTTGAGCGCGGCCCTGGGAAATGGCAAC

GGCGGCAAACAGAGCGGCGGCAGCGGCGGGGGATGGTGGGGCGGAAAGCACGTGTCTGCG

GACTGCTTCAAAGTGGAGACCGTGTGCCACACGACGCTGTACTACTTTGGATCTAAGCCA

GCTCTCTACTATAGAGTATCTGCCTCCAGCAGCCGCCTGGGAGGGTTCATCTGCGACAAC

TTTCACCCGGAGATTACAAAATTCGAGGGGAGCGTGGACGTGACCACGCGGCTGTTGTTG

GACAATGAAAATTTTACCAGTTTCGGGTGGTACCGCCTGCGACCCGGCACCCACGGAGAG

CGTGTTCAACTTCGCCCCGTTGAGCGACACGTCACCTCAAGCGACGTGGAGATTAACTGT

ACTCCCGATAACCTGGAGCCGATACCAGACGAGGCTGCCTGGCCCGACTATAAGCTCATG

TGCTTTGATATAGAGTGTAAAGCTGGAACGGGTAACGAAATGGCGTTCCCAGTGGCAACT

AACCAAGAGGACCTGGTCATCCAGATCTCCTGTCTGCTGTACTCGCTTGCTACTCAGAAC

CACGAACACACCCTGCTGTTTTCCCTCGGGTCATGCGATATCTCTGAGGAATACTCGTTT

GCATGCGTCCAGCGCGGCGAGCCCAGACCGACGGTTTTGGAGTTTGACAGCGAGTACGAG

CTGCTGGTTGCCTTCCTGACCTTTCTCAAGCAGTACTCTCCCGAGTTCGCCACCGGCTAC

AACATCGTTAATTTTGACTGGGCGTACATAGTTAACAAGGTAACGTCGGTGTATAACATC

AAGCTGGACGGGTACGGCAAGTTCAACAAAGGGGGGCTGTTTAAGGTGTGGGACATCGCC

ACGAACCATTTTCAGAAGAAGAGCAAGGTGAAAATCAATGGCCTGATATCTCTAGACATG

TATTCTGTGGCGACGGAAAAGCTAAAGCTACCCAGCTACAAACTCGACGCGGTCGTGGGA

GACGTCCTCGGCGAGCATAAGATAGACCTTCCCTATAAAGAAATACCCTCCTATTACGCG

GGAGGGCCTGACCGGAGGGGCGTAATAGGAGAGTATTGTATCCAGGACTCTAGGCTGGTG

GGCAAGCTGTTTTTTAAGTACCTCCCCCATCTGGAACTATCGGCGGTGGCCAAACTCGCC

CGTATCACCCTAACGCGGGTAATTTTTGACGGTCAGCAAATTCGCGTGTACACGTGCTTG

CTGAAACTCGCCCGCGAGAGAAATTTCATTTTGCCAGACAACAGACGCCGGTTTGACAGT

CAGGCAGACGCCGCGTCAGAGACTTCGGAGTTGGCTATGGATAGCCAAAGCCACGCCTTC

GACAGTACAGACGAACCCGACGGTGTGGACGGTACCCCGGACGCCGCAGGATCTGGCGCT

ACTTCTGAAAACGGAGGCGGGAAGCCCGGCGTCGGGAGGGCCGTGGGCTACCAGGGAGCA

AAGGTTCTAGACCCCGTATCCGGCTTTCATGTGGACCCCGTGGTTGTGTTTGACTTCGCT

AGCTTATACCCAAGCATTATCCAGGCCCATAACCTCTGTTTCACCACCCTGGCGCTCGAT

GAAGTGGATCTGGCCGGGCTTCAACCATCCGTCAACTACTCGACGTTCGAGGTGGGTGAC

CAAAAGTTATTTTTTGTCCACGCCCATATTCGCGAAAGCCTGCTTGGCATCTTGCTGCGC

GACTGGCTGGCCATGCGAAAGGCGGTGAGGGCGCGAATCCCCACCAGCACCCCCGAGGAG

GCAGTTTTACTAGATAAGCAGCAGTCTGCGATTAAGGTGATATGCAACTCGGTTTACGGA

TTCACGGGGGTGGCAAACGGCCTGTTGCCGTGTCTGAGGATAGCGGCTACCGTTACCACG

ATAGGACGCGACATGCTCCTCAAGACCCGAGATTACGTTCACTCTCGTTGGGCGACGCGC

GAGCTGCTGGAGGACAATTTTCCAGGGGCTATAGGTTTCCGAAACCACAAGCCTTACTCC

GTCAGGGTTATCTACGGAGACACCGACTCCGTGTTTATCAAGTTTGTGGGCCTGACGTAC

GAGGGGGTATCGGAGCTGGGGGATGCTATGTCGCGTCAGATTTCAGCGGACCTCTTTAGA

GCGCCCATCAAACTGGAGTGTGAGAAGACCTTTCAGCGACTGCTGCTGATCACCAAGAAG

AAGTACATAGGTGTCATAAACGGGGGGAAGATGCTCATGAAGGGGGTCGACCTGGTCCGC

AAAAATAACTGCTCTTTCATAAACTTGTACGCGCGACATCTGGTAGATCTTTTGTTGTAC

GACGAGGATGTGGCCACGGCGGCAGCAGAGGTGACAGACGTGCCTCCCGCAGAATGGGTG

GGGCGCCCGCTACCGAGCGGCTTTGACAAGTTTGGGCGAGTGCTGGTAGAGGCGTACAAC

CGTATCACTGCCCCCAACTTGGACGTGCGCGAGTTCGTTATGACTGCTGAGCTGAGCCGC

TCACCCGAATCGTATACCAACAAGCGCCTGCCGCACCTCACCGTCTACTTTAAGCTCGCC

ATGAGGAATGAAGAACTGCCCAGCGTAAAAGAGAGAATTCCGTATGTGATAGTTGCGCAG

ACCGAGGCCGCGGAACGCGAAGCGGGTGTAGTAAACTCAATGCGCGGTACCGCCCAAAAC

CCCGTGGTAACCAAGACCGCACGCCCCCAACCTAAACGCAAACTGCTGGTTTCCGACCTC

GCCGAAGACCCGACCTATGTTTCCGAGAATGACGTACCGCTAAACACAGACTACTATTTC

TCCCACCTGTTGGGTACCATAAGCGTGACCTTTAAGGCTCTATTCGGAAATGATGTGAGA

ACAACAGAAAATCTTTTAAAGCGGTTTATTCCGGAAACCCCCCACAAGACCCCCACGAAA

ACCCAGGCACTGCTTGAGCGCGCCGGCTTTGAAAAGCTGACGCCCTTTACACCGGAGGAA

GAAAGTCGTCGAATACTGCATACAGTTTTTTGTACTCTAGAAGCAGCTCCCCATCAAAGC

TGA

>MN688619.1

ATGGCGGCGCGCGAACAGGCCAACTCCGTTCGCAGGAGCGGGTTTTTTAATCCGTTTATT

GGCAAGAGGCCATTTTTCAGGCCGGGAAGCGGGCAGACCGCGGAGACAGAGCGCCCAAGG

CCCCCCCAACACTCGTACTGCACAGAGGTGGGTAGCTTTAAGTTTATAGCTCCAAGATGC

CTCGATGAAGAAGCCCCCGCCGACCAGCGACGCGGTGTACACGTGGGCACCTTGGAGCGC

CCACCCAAAGTGTACTGCGATGGCTCAGAGTACGACGTGCTGAACTTTGCCTCCGGAGGT

TGCTGGCCTCGCAGGATTCGGGTTTGGAACGGCCAGGATTTTCGGGGCGATGGATTCAAC

CCCAGATTTGAGAGATTTCACGTGTACGACATAGTAGAGACTTCGGAGAGCGCCTCACAC

GATGACCCATCCAGGTTTGCTGAGCTATCCCGCCCAAGCGGGTCTGTGGTAACACTGCTG

GGAATGAGCGAGTGTGGAAAGCGAGTCGCGGTTCACGTCTATGGTGTGCGCCATTATTTT

TACATGGCAAAGGCGGAGGTGGATAGCGCTTGTGGAATAACCACCGAGGCAGAACTCGTG

CGCGCAATGGTGGACTGCGCGCACAGCTCGGCTTTGAGCGCGGCCCTGGGAAATGGCAAC

GGCGGCAAACAGAGCGGCGGCAGCGGCGGGGGATGGTGGGGCGGAAAGCACGTGTCTGCG

GACTGCTTCAAAGTGGAGACCGTGTGCCACACGACGCTGTACTACTTTGGATCTAAGCCA

GCTCTCTACTATAGAGTATCTGCCTCCAGCAGCCGCCTGGGAGGGTTCATCTGCGACAAC

TTTCACCCGGAGATTACAAAATTCGAGGGGAGCGTGGACGTGACCACGCGGCTGTTGTTG

GACAATGAAAATTTTACCAGTTTCGGGTGGTACCGCCTGCGACCCGGCACCCACGGAGAG

CGTGTTCAACTTCGCCCCGTTGAGCGACACGTCACCTCAAGCGACGTGGAGATTAACTGT

ACTCCCGATAACCTGGAGCCGATACCAGACGAGGCTGCCTGGCCCGACTATAAGCTCATG

TGCTTTGATATAGAGTGTAAAGCTGGAACGGGTAACGAAATGGCGTTCCCAGTGGCAACT

AACCAAGAGGACCTGGTCATCCAGATCTCCTGTCTGCTGTACTCGCTTGCTACTCAGAAC

CACGAACACACCCTGCTGTTTTCCCTCGGGTCATGCGATATCTCTGAGGAATACTCGTTT

GCATGCGTCCAGCGCGGCGAGCCCAGACCGACGGTTTTGGAGTTTGACAGCGAGTACGAG

CTGCTGGTTGCCTTCCTGACCTTTCTCAAGCAGTACTCTCCCGAGTTCGCCACCGGCTAC

AACATCGTTAATTTTGACTGGGCGTACATAGTTAACAAGGTAACGTCGGTGTATAACATC

AAGCTGGACGGGTACGGCAAGTTCAACAAAGGGGGGCTGTTTAAGGTGTGGGACATCGCC

ACGAACCATTTTCAGAAGAAGAGCAAGGTGAAAATCAATGGCCTGATATCTCTAGACATG

TATTCTGTGGCGACGGAAAAGCTAAAGCTACCCAGCTACAAACTCGACGCGGTCGTGGGA

GACGTCCTCGGCGAGCATAAGATAGACCTTCCCTATAAAGAAATACCCTCCTATTACGCG

GGAGGGCCTGACCGGAGGGGCGTAATAGGAGAGTATTGTATCCAGGACTCTAGGCTGGTG

GGCAAGCTGTTTTTTAAGTACCTCCCCCATCTGGAACTATCGGCGGTGGCCAAACTCGCC

CGTATCACCCTAACGCGGGTAATTTTTGACGGTCAGCAAATTCGCGTGTACACGTGCTTG

CTGAAACTCGCCCGCGAGAGAAATTTCATTTTGCCAGACAACAGACGCCGGTTTGACAGT

CAGGCAGACGCCGCGTCAGAGACTTCGGAGTTGGCTATGGATAGCCAAAGCCACGCCTTC

GACAGTACAGACGAACCCGACGGTGTGGACGGTACCCCGGACGCCGCAGGATCTGGCGCT

ACTTCTGAAAACGGAGGCGGGAAGCCCGGCGTCGGGAGGGCCGTGGGCTACCAGGGAGCA

AAGGTTCTAGACCCCGTATCCGGCTTTCATGTGGACCCCGTGGTTGTGTTTGACTTCGCT

AGCTTATACCCAAGCATTATCCAGGCCCATAACCTCTGTTTCACCACCCTGGCGCTCGAT

GAAGTGGATCTGGCCGGGCTTCAACCATCCGTCGACTACTCGACGTTCGAGGTGGGTGAC

CAAAAGTTATTTTTTGTCCACGCCCATATTCGCGAAAGCCTGCTTGGCATCTTGCTGCGC

GACTGGCTGGCCATGCGAAAGGCGGTGAGGGCGCGAATCCCCACCAGCACCCCCGAGGAG

GCAGTTTTACTAGATAAGCAGCAGTCTGCGATTAAGGTGATATGCAACTCGGTTTACGGA

TTCACGGGGGTGGCAAACGGCCTGTTGCCGTGTCTGAGGATAGCGGCTACCGTTACCACG

ATAGGACGCGACATGCTCCTCAAGACCCGAGATTACGTTCACTCTCGTTGGGCGACGCGC

GAGCTGCTGGAGGACAATTTTCCAGGGGCTATAGGTTTCCGAAACCACAAGCCTTACTCC

GTCAGGGTTATCTACGGAGACACCGACTCCGTGTTTATCAAGTTTGTGGGCCTGACGTAC

GAGGGGGTATCGGAGCTGGGGGATGCTATGTCGCGTCAGATTTCAGCGGACCTCTTTAGA

GCGCCCATCAAACTGGAGTGTGAGAAGACCTTTCAGCGACTGCTGCTGATCACCAAGAAG

AAGTACATAGGTGTCATAAACGGGGGGAAGATGCTCATGAAGGGGGTCGACCTGGTCCGC

AAAAATAACTGCTCTTTCATAAACTTGTACGCGCGACATCTGGTAGATCTTTTGTTGTAC

GACGAGGATGTGGCCACGGCGGCAGCAGAGGTGACAGACGTGCCTCCCGCAGAATGGGTG

GGGCGCCCGCTACCGAGCGGCTTTGACAAGTTTGGGCGAGTGCTGGTAGAGGCGTACAAC

CGTATCACTGCCCCCAACTTGGACGTGCGCGAGTTCGTTATGACTGCTGAGCTGAGCCGC

TCACCCGAATCGTATACCAACAAGCGCCTGCCGCACCTCACCGTCTACTTTAAGCTCGCC

ATGAGGAATGAAGAACTGCCCAGCGTAAAAGAGAGAATTCCGTATGTGATAGTTGCGCAG

ACCGAGGCCGCGGAACGCGAAGCGGGTGTAGTAAACTCAATGCGCGGTACCGCCCAAAAC

CCCGTGGTAACCAAGACCGCACGCCCCCAACCTAAACGCAAACTGCTGGTTTCCGACCTC

GCCGAAGACCCGACCTATGTTTCCGAGAATGACGTACCGCTAAACACAGACTACTATTTC

TCCCACCTGTTGGGTACCATAAGCGTGACCTTTAAGGCTCTATTCGGAAATGATGTGAGA

ACAACAGAAAATCTTTTAAAGCGGTTTATTCCGGAAACCCCCCACAAGACCCCCACGAAA

ACCCAGGCACTGCTTGAGCGCGCCGGCTTTGAAAAGCTGACGCCCTTTACACCGGAGGAA

GAAAGTCGTCGAATACTGCATACAGTTTTTTGTACTCTAGAAGCAGCTCCCCATCAAAGC

TGA

>MN688620.1

ATGGCGGCGCGCGAACAGGCCAACTCCGTTCGCAGGAGCGGGTTTTTTAATCCGTTTATT

GGCAAGAGGCCATTTTTCAGGCCGGGAAGCGGGCAGACCGCGGAGACAGAGCGCCCAAGG

CCCCCCCAACACTCGTACTGCACAGAGGTGGGTAGCTTTAAGTTTATAGCTCCAAGATGC

CTCGATGAAGAAGCCCCCGCCGACCAGCGACGCGGTGTACACGTGGGCACCTTGGAGCGC

CCACCCAAAGTGTACTGCGATGGCTCAGAGTACGACGTGCTGAACTTTGCCTCCGGAGGT

TGCTGGCCTCGCAGGATTCGGGTTTGGAACGGCCAGGATTTTCGGGGCGATGGATTCAAC

CCCAGATTTGAGAGATTTCACGTGTACGACATAGTAGAGACTTCGGAGAGCGCCTCACAC

GATGACCCATCCAGGTTTGCTGAGCTATCCCGCCCAAGCGGGTCTGTGGTAACACTGCTG

GGAATGAGCGAGTGTGGAAAGCGAGTCGCGGTTCACGTCTATGGTGTGCGCCATTATTTT

TACATGGCAAAGGCGGAGGTGGATAGCGCTTGTGGAATAACCACCGAGGCAGAACTCGTG

CGCGCAATGGTGGACTGCGCGCACAGCTCGGCTTTGAGCGCGGCCCTGGGAAATGGCAAC

GGCGGCAAACAGAGCGGCGGCAGCGGCGGGGGATGGTGGGGCGGAAAGCACGTGTCTGCG

GACTGCTTCAAAGTGGAGACCGTGTGCCACACGACGCTGTACTACTTTGGATCTAAGCCA

GCTCTCTACTATAGAGTATCTGCCTCCAGCAGCCGCCTGGGAGGGTTCATCTGCGACAAC

TTTCACCCGGAGATTACAAAATTCGAGGGGAGCGTGGACGTGACCACGCGGCTGTTGTTG

GACAATGAAAATTTTACCAGTTTCGGGTGGTACCGCCTGCGACCCGGCACCCACGGAGAG

CGTGTTCAACTTCGCCCCGTTGAGCGACACGTCACCTCAAGCGACGTGGAGATTAACTGT

ACTCCCGATAACCTGGAGCCGATACCAGACGAGGCTGCCTGGCCCGACTATAAGCTCATG

TGCTTTGATATAGAGTGTAAAGCTGGAACGGGTAACGAAATGGCGTTCCCAGTGGCAACT

AACCAAGAGGACCTGGTCATCCAGATCTCCTGTCTGCTGTACTCGCTTGCTACTCAGAAC

CACGAACACACCCTGCTGTTTTCCCTCGGGTCATGCGATATCTCTGAGGAATACTCGTTT

GCATGCGTCCAGCGCGGCGAGCCCAGACCGACGGTTTTGGAGTTTGACAGCGAGTACGAG

CTGCTGGTTGCCTTCCTGACCTTTCTCAAGCAGTACTCTCCCGAGTTCGCCACCGGCTAC

AACATCGTTAATTTTGACTGGGCGTACATAGTTAACAAGGTAACGTCGGTGTATAACATC

AAGCTGGACGGGTACGGCAAGTTCAACAAAGGGGGGCTGTTTAAGGTGTGGGACATCGCC

ACGAACCATTTTCAGAAGAAGAGCAAGGTGAAAATCAATGGCCTGATATCTCTAGACATG

TATTCTGTGGCGACGGAAAAGCTAAAGCTACCCAGCTACAAACTCGACGCGGTCGTGGGA

GACGTCCTCGGCGAGCATAAGATAGACCTTCCCTATAAAGAAATACCCTCCTATTACGCG

GGAGGGCCTGACCGGAGGGGCGTAATAGGAGAGTATTGTATCCAGGACTCTAGGCTGGTG

GGCAAGCTGTTTTTTAAGTACCTCCCCCATCTGGAACTATCGGCGGTGGCCAAACTCGCC

CGTATCACCCTAACGCGGGTAATTTTTGACGGTCAGCAAATTCGCGTGTACACGTGCTTG

CTGAAACTCGCCCGCGAGAGAAATTTCATTTTGCCAGACAACAGACGCCGGTTTGACAGT

CAGGCAGACGCCGCGTCAGAGACTTCGGAGTTGGCTATGGATAGCCAAAGCCACGCCTTC

GACAGTACAGACGAACCCGACGGTGTGGACGGTACCCCGGACGCCGCAGGATCTGGCGCT

ACTTCTGAAAACGGAGGCGGGAAGCCCGGCGTCGGGAGGGCCGTGGGCTACCAGGGAGCA

AAGGTTCTAGACCCCGTATCCGGCTTTCATGTGGACCCCGTGGTTGTGTTTGACTTCGCT

AGCTTATACCCAAGCATTATCCAGGCCCATAACCTCTGTTTCACCACCCTGGCGCTCGAT

GAAGTGGATCTGGCCGGGCTTCAACCATCCGTCAACTACTCGACGTTCGAGGTGGGTGAC

CAAAAGTTATTTTTTGTCCACGCCCATATTCGCGAAAGCCTGCTTGGCATCTTGCTGCGC

GACTGGCTGGCCATGCGAAAGGCGGTGAGGGCGCGAATCCCCACCAGCACCCCCGAGGAG

GCAGTTTTACTAGATAAGCAGCAGTCTGCGATTAAGGTGATATGCAACTCGGTTTACGGA

TTCACGGGGGTGGCAAACGGCCTGTTGCCGTGTCTGAGGATAGCGGCTACCGTTACCACG

ATAGGACGCGACATGCTCCTCAAGACCCGAGATTACGTTCACTCTCGTTGGGCGACGCGC

GAGCTGCTGGAGGACAATTTTCCAGGGGCTATAGGTTTCCGAAACCACAAGCCTTACTCC

GTCAGGGTTATCTACGGAGACACCGACTCCGTGTTTATCAAGTTTGTGGGCCTGACGTAC

GAGGGGGTATCGGAGCTGGGGGATGCTATGTCGCGTCAGATTTCAGCGGACCTCTTTAGA

GCGCCCATCAAACTGGAGTGTGAGAAGACCTTTCAGCGACTGCTGCTGATCACCAAGAAG

AAGTACATAGGTGTCATAAACGGGGGGAAGATGCTCATGAAGGGGGTCGACCTGGKCCGC

AAAAATAACTGCTCTTTCATAAACTTGTACGCGCGACATCTGGTAGATCTTTTGTTGTAC

GACGAGGATGTGGCCACGGCGGCAGCAGAGGTGACAGACGTGCCTCCCGCAGAATGGGTG

GGGCGCCCGCTACCGAGCGGCTTTGACAAGTTTGGGCGAGTGCTGGTAGAGGCGTACAAC

CGTATCACTGCCCCCAACTTGGACGTGCGCGAGTTCGTTATGACTGCTGAGCTGAGCCGC

TCACCCGAATCGTATACCAACAAGCGCCTGCCGCACCTCACCGTCTACTTTAAGCTCGCC

ATGAGGAATGAAGAACTGCCCAGCGTAAAAGAGAGAATTCCGTATGTGATAGTTGCGCAG

ACCGAGGCCGCGGAACGCGAAGCGGGTGTAGTAAACTCAATGCGCGGTACCGCCCAAAAC

CCCGTGGTAACCAAGACCGCACGCCCCCAACCTAAACGCAAACTGCTGGTTTCCGACCTC

GCCGAAGACCCGACCTATGTTTCCGAGAATGACGTACCGCTAAACACAGACTACTATTTC

TCCCACCTGTTGGGTACCATAAGCGTGACCTTTAAGGCTCTATTCGGAAATGATGTGAGA

ACAACAGAAAATCTTTTAAAGCGGTTTATTCCGGAAACCCCCCACAAGACCCCCACGAAA

ACCCAGGCACTGCTTGAGCGCGCCGGCTTTGAAAAGCTGACGCCCTTTACACCGGAGGAA

GAAAGTCGTCGAATACTGCATACAGTTTTTTGTACTCTAGAAGCAGCTCCCCATCAAAGC

TGA

>MN688621.1

ATGGCGGCGCGCGAACAGGCCAACTCCGTTCGCAGGAGCGGGTTTTTTAATCCGTTTATT

GGCAAGAGGCCATTTTTCAGGCCGGGAAGCGGGCAGACCGCGGAGACAGAGCGCCCAAGG

CCCCCCCAACACTCGTACTGCACAGAGGTGGGTAGCTTTAAGTTTATAGCTCCAAGATGC

CTCGATGAAGAAGCCCCCGCCGACCAGCGACGCGGTGTACACGTGGGCACCTTGGAGCGC

CCACCCAAAGTGTACTGCGATGGCTCAGAGTACGACGTGCTGAACTTTGCCTCCGGAGGT

TGCTGGCCTCGCAGGATTCGGGTTTGGAACGGCCAGGATTTTCGGGGCGATGGATTCAAC

CCCAGATTTGAGAGATTTCACGTGTACGACATAGTAGAGACTTCGGAGAGCGCCTCACAC

GATGACCCATCCAGGTTTGCTGAGCTATCCCGCCCAAGCGGGTCTGTGGTAACACTGCTG

GGAATGAGCGAGTGTGGAAAGCGAGTCGCGGTTCACGTCTATGGTGTGCGCCATTATTTT

TACATGGCAAAGGCGGAGGTGGATAGCGCTTGTGGAATAACCACCGAGGCAGAACTCGTG

CGCGCAATGGTGGACTGCGCGCACAGCTCGGCTTTGAGCGCGGCCCTGGGAAATGGCAAC

GGCGGCAAACAGAGCGGCGGCAGCGGCGGGGGATGGTGGGGCGGAAAGCACGTGTCTGCG

GACTGCTTCAAAGTGGAGACCGTGTGCCACACGACGCTGTACTACTTTGGATCTAAGCCA

GCTCTCTACTATAGAGTATCTGCCTCCAGCAGCCGCCTGGGAGGGTTCATCTGCGACAAC

TTTCACCCGGAGATTACAAAATTCGAGGGGAGCGTGGACGTGACCACGCGGCTGTTGTTG

GACAATGAAAATTTTACCAGTTTCGGGTGGTACCGCCTGCGACCCGGCACCCACGGAGAG

CGTGTTCAACTTCGCCCCGTTGAGCGACACGTCACCTCAAGCGACGTGGAGATTAACTGT

ACTCCCGATAACCTGGAGCCGATACCAGACGAGGCTGCCTGGCCCGACTATAAGCTCATG

TGCTTTGATATAGAGTGTAAAGCTGGAACGGGTAACGAAATGGCGTTCCCAGTGGCAACT

AACCAAGAGGACCTGGTCATCCAGATCTCCTGTCTGCTGTACTCGCTTGCTACTCAGAAC

CACGAACACACCCTGCTGTTTTCCCTCGGGTCATGCGATATCTCTGAGGAATACTCGTTT

GCATGCGTCCAGCGCGGCGAGCCCAAACCGACGGTTTTGGAGTTTGACAGCGAGTACGAG

CTGCTGGTTGCCTTCCTGACCTTTCTCAAGCAGTACTCTCCCGAGTTCGCCACCGGCTAC

AACATCGTTAATTTTGACTGGGCGTACATAGTTAACAAGGTAACGTCGGTGTATAACATC

AAGCTGGACGGGTACGGCAAGTTCAACAAAGGGGGGCTGTTTAAGGTGTGGGACATCGCC

ACGAACCATTTTCAGAAGAAGAGCAAGGTGAAAATCAATGGCCTGATATCTCTAGACATG

TATTCTGTGGCGACGGAAAAGCTAAAGCTACCCAGCTACAAACTCGACGCGGTCGTGGGA

GACGTCCTCGGCGAGCATAAGATAGACCTTCCCTATAAAGAAATACCCTCCTATTACGCG

GGAGGGCCTGACCGGAGGGGCGTAATAGGAGAGTATTGTATCCAGGACTCTAGGCTGGTG

GGCAAGCTGTTTTTTAAGTACCTCCCCCATCTGGAACTATCGGCGGTGGCCAAACTCGCC

CGTATCACCCTAACGCGGGTAATTTTTGACGGTCAGCAAATTCGCGTGTACACGTGCTTG

CTGAAACTCGCCCGCGAGAGAAATTTCATTTTGCCAGACAACAGACGCCGGTTTGACAGT

CAGGCAGACGCCGCGTCAGAGACTTCGGAGTTGGCTATGGATAGCCAAAGCCACGCCTTC

GACAGTACAGACGAACCCGACGGTGTGGACGGTACCCCGGACGCCGCAGGATCTGGCGCT

ACTTCTGAAAACGGAGGCGGGAAGCCCGGCGTCGGGAGGGCCGTGGGCTACCAGGGAGCA

AAGGTTCTAGACCCCGTATCCGGCTTTCATGTGGACCCCGTGGTTGTGTTTGACTTCGCT

AGCTTATACCCAAGCATTATCCAGGCCCATAACCTCTGTTTCACCACCCTGGCGCTCGAT

GAAGTGGATCTGGCCGGGCTTCAACCATCCGTCAACTACTCGACGTTCGAGGTGGGTGAC

CAAAAGTTATTTTTTGTCCACGCCCATATTCGCGAAAGCCTGCTTGGCATCTTGCTGCGC

GACTGGCTGGCCATGCGAAAGGCGGTGAGGGCGCGAATCCCCACCAGCACCCCCGAGGAG

GCAGTTTTACTAGATAAGCAGCAGTCTGCGATTAAGGTGATATGCAACTCGGTTTACGGA

TTCACGGGGGTGGCAAACGGCCTGTTGCCGTGTCTGAGGATAGCGGCTACCGTTACCACG

ATAGGACGCGACATGCTCCTCAAGACCCGAGATTACGTTCACTCTCGTTGGGCGACGCGC

GAGCTGCTGGAGGACAATTTTCCAGGGGCTATAGGTTTCCGAAACCACAAGCCTTACTCC

GTCAGGGTTATCTACGGAGACACCGACTCCGTGTTTATCAAGTTTGTGGGCCTGACGTAC

GAGGGGGTATCGGAGCTGGGGGATGCTATGTCGCGTCAGATTTCAGCGGACCTCTTTAGA

GCGCCCATCAAACTGGAGTGTGAGAAGACCTTTCAGCGACTGCTGCTGATCACCAAGAAG

AAGTACATAGGTGTCATAAACGGGGGGAAGATGCTCATGAAGGGGGTCGACCTGGTCCGC

AAAAATAACTGCTCTTTCATAAACTTGTACGCGCGACATCTGGTAGATCTTTTGTTGTAC

GACGAGGATGTGGCCACGGCGGCAGCAGAGGTGACAGACGTGCCTCCCGCAGAATGGGTG

GGGCGCCCGCTACCGAGCGGCTTTGACAAGTTTGGGCGAGTGCTGGTAGAGGCGTACAAC

CGTATCACTGCCCCCAACTTGGACGTGCGCGAGTTCGTTATGACTGCTGAGCTGAGCCGC

TCACCCGAATCGTATACCAACAAGCGCCTGCCGCACCTCACCGTCTACTTTAAGCTCGCC

ATGAGGAATGAAGAACTGCCCAGCGTAAAAGAGAGAATTCCGTATGTGATAGTTGCGCAG

ACCGAGGCCGCGGAACGCGAAGCGGGTGTAGTAAACTCAATGCGCGGTACCGCCCAAAAC

CCCGTGGTAACCAAGACCGCACGCCCCCAACCTAAACGCAAACTGCTGGTTTCCGACCTC

GCCGAAGACCCGACCTATGTTTCCGAGAATGACGTACCGCTAAACACAGACTACTATTTC

TCCCACCTGTTGGGTACCATAAGCGTGACCTTTAAGGCTCTATTCGGAAATGATGTGAGA

ACAACAGAAAATCTTTTAAAGCGGTTTATTCCGGAAACCCCCCACAAGACCCCCACGAAA

ACCCAGGCACTGCTTGAGCGCGCCGGCTTTGAAAAGCTGACGCCCTTTACACCGGAGGAA

GAAAGTCGTCGAATACTGCATACAGTTTTTTGTACTCTAGAAGCAGCTCCCCATCAAAGC

TGA

>MN688622.1

ATGGCGGCGCGCGAACAGGCCAACTCCGTTCGCAGGAGCGGGTTTTTTAATCCGTTTATT

GGCAAGAGGCCATTTTTCAGGCCGGGAAGCGGGCAGACCGCGGAGACAGAGCGCCCAAGG

CCCCCCCAACACTCGTACTGCACAGAGGTGGGTAGCTTTAAGTTTATAGCTCCAAGATGC

CTCGATGAAGAAGCCCCCGCCGACCAGCGACGCGGTGTACACGTGGGCACCTTGGAGCGC

CCACCCAAAGTGTACTGCGATGGCTCAGAGTACGACGTGCTGAACTTTGCCTCCGGAGGT

TGCTGGCCTCGCAGGATTCGGGTTTGGAACGGCCAGGATTTTCGGGGCGATGGATTCAAC

CCCAGATTTGAGAGATTTCACGTGTACGACATAGTAGAGACTTCGGAGAGCGCCTCACAC

GATGACCCATCCAGGTTTGCTGAGCTATCCCGCCCAAGCGGGTCTGTGGTAACACTGCTG

GGAATGAGCGAGTGTGGAAAGCGAGTCGCGGTTCACGTCTATGGTGTGCGCCATTATTTT

TACATGGCAAAGGCGGAGGTGGATAGCGCTTGTGGAATAACCACCGAGGCAGAACTCGTG

CGCGCAATGGTGGACTGCGCGCACAGCTCGGCTTTGAGCGCGGCCCTGGGAAATGGCAAC

GGCGGCAAACAGAGCGGCGGCAGCGGCGGGGGATGGTGGGGCGGAAAGCACGTGTCTGCG

GACTGCTTCAAAGTGGAGACCGTGTGCCACACGACGCTGTACTACTTTGGATCTAAGCCA

GCTCTCTACTATAGAGTATCTGCCTCCAGCAGCCGCCTGGGAGGGTTCATCTGCGACAAC

TTTCACCCGGAGATTACAAAATTCGAGGGGAGCGTGGACGTGACCACGCGGCTGTTGTTG

GACAATGAAAATTTTACCAGTTTCGGGTGGTACCGCCTGCGACCCGGCACCCACGGAGAG

CGTGTTCAACTTCGCCCCGTTGAGCGACACGTCACCTCAAGCGACGTGGAGATTAACTGT

ACTCCCGATAACCTGGAGCCGATACCAGACGAGGCTGCCTGGCCCGACTATAAGCTCATG

TGCTTTGATATAGAGTGTAAAGCTGGAACGGGTAACGAAATGGCGTTCCCAGTGGCAACT

AACCAAGAGGACCTGGTCATCCAGATCTCCTGTCTGCTGTACTCGCTTGCTACTCAGAAC

CACGAACACACCCTGCTGTTTTCCCTCGGGTCATGCGATATCTCTGAGGAATACTCGTTT

GCATGCGTCCAGCGCGGCGAGCCCAGACCGACGGTTTTGGAGTTTGACAGCGAGTACGAG

CTGCTGGTTGCCTTCCTGACCTTTCTCAAGCAGTACTCTCCCGAGTTCGCCACCGGCTAC

AACATCGTTAATTTTGACTGGGCGTACATAGTTAACAAGGTAACGTCGGTGTATAACATC

AAGCTGGACGGGTACGGCAAGTTCAACAAAGGGGGGCTGTTTAAGGTGTGGGACATCGCC

ACGAACCATTTTCAGAAGAAGAGCAAGGTGAAAATCAATGGCCTGATATCTCTAGACATG

TATTCTGTGGCGACGGAAAAGCTAAAGCTACCCAGCTACAAACTCGACGCGGTCGTGGGA

GACGTCCTCGGCGAGCATAAGATAGACCTTCCCTATAAAGAAATACCCTCCTATTACGCG

GGAGGGCCTGACCGGAGGGGCGTAATAGGAGAGTATTGTATCCAGGACTCTAGGCTGGTG

GGCAAGCTGTTTTTTAAGTACCTCCCCCATCTGGAACTATCGGCGGTGGCCAAACTCGCC

CGTATCACCCTAACGCGGGTAATTTTTGACGGTCAGCAAATTCGCGTGTACACGTGCTTG

CTGAAACTCGCCCGCGAGAGAAATTTCATTTTGCCAGACAACAGACGCCGGTTTGACAGT

CAGGCAGACGCCGCGTCAGAGACTTCGGAGTTGGCTATGGATAGCCAAAGCCACGCCTTC

GACAGTACAGACGAACCCGACGGTGTGGACGGTACCCCGGACGCCGCAGGATCTGGCGCT

ACTTCTGAAAACGGAGGCGGGAAGCCCGGCGTCGGGAGGGCCGTGGGCTACCAGGGAGCA

AAGGTTCTAGACCCCGTATCCGGCTTTCATGTGGACCCCGTGGTTGTGTTTGACTTCGCT

AGCTTATACCCAAGCATTATCCAGGCCCATAACCTCTGTTTCACCACCCTGGCGCTCGAT

GAAGTGGATCTGGCCGGGCTTCAACCATCCGTCAACTACTCGACGTTCGAGGTGGGTGAC

CAAAAGTTATTTTTTGTCCACGCCCATATTCGCGAAAGCCTGCTTGGCATCTTGCTGCGC

GACTGGCTGGCCATGCGAAAGGCGGTGAGGGCGCGAATCCCCACCAGCACCCCCGAGGAG

GCAGTTTTACTAGATAAGCAGCAGTCTGCGATTAAGGTGATATGCAACTCGGTTTACGGA

TTCACGGGGGTGGCAAACGGCCTGTTGCCGTGTCTGAGGATAGCGGCTACCGTTACCACG

ATAGGACGCGACATGCTCCTCAAGACCCGAGATTACGTTCACTCTCGTTGGGCGACGCGC

GAGCTGCTGGAGGACAATTTTCCAGGGGCTATAGGTTTCCGAAACCACAAGCCTTACTCC

GTCAGGGTTATCTACGGAGACACCGACTCCGTGTTTATCAAGTTTGTGGGCCTGACGTAC

GAGGGGGTATCGGAGCTGGGGGATGCTATGTCGCGTCAGATTTCAGCGGACCTCTTTAGA

GCGCCCATCAAACTGGAGTGTGAGAAGACCTTTCAGCGACTGCTGCTGATCACCAAGAAG

AAGTACATAGGTGTCATAAACGGGGGGAAGATGCTCATGAAGGGGGTCGACCTGGTCCGC

AAAAATAACTGCTCTTTCATAAACTTGTACGCGCGACATCTGGTAGATCTTTTGTTGTAC

GACGAGGATGTGGCCACGGCGGCAGCAGAGGTGACAGACGTGCCTCCCGCAGAATGGGTG

GGGCGCCCGCTACCGAGCGGCTTTGACAAGTTTGGGCGAGTGCTGGTAGAGGCGTACAAC

CGTATCACTGCCCCCAACTTGGACGTGCGCGAGTTCGTTATGACTGCTGAGCTGAGCCGC

TCACCCGAATCGTATACCAACAAGCGCCTGCCGCACCTCACCGTCTACTTTAAGCTCGCC

ATGAGGAATGAAGAACTGCCCAGCGTAAAAGAGAGAATTCCGTATGTGATAGTTGCGCAG

ACCGAGGCCGCGGAACGCGAAGCGGGTGTAGTAAACTCAATGCGCGGTACCGCCCAAAAC

CCCGTGGTAACCAAGACCGCACGCCCCCAACCTAAACGCAAACTGCTGGTTTCCGACCTC

GCCGAAGACCCGACCTATGTTTCCGAGAATGACGTACCGCTAAACACAGACTACTATTTC

TCCCACCTGTTGGGTACCATAAGCGTGACCTTTAAGGCTCTATTCGGAAATGATGTGAGA

ACAACAGAAAATCTTTTAAAGCGGTTTATTCCGGAAACCCCCCACAAGACCCCCACGAAA

ACCCAGGCACTGCTTGAGCGCGCCGGCTTTGAAAAGCTGACGCCCTTTACACCGGAGGAA

GAAAGTCGTCGAATACTGCATACAGTTTTTTGTACTCTAGAAGCAGCTCCCCATCAAAGC

TGA

>MN688624.1

ATGGCGGCGCGCGAACAGGCCAACTCCGTTCGCAGGAGCGGGTTTTTTAATCCGTTTATT

GGCAAGAGGCCATTTTTCAGGCCGGGAAGCGGGCAAACCGCGGAGACAGAGCGCCCAAGG

CCCCCCCAACACTCGTACTGCACAGAGGTGGGTAGCTTTAAGTTTATAGCTCCAAGATGC

CTCGATGAAGAAGCCCCCGCCGACCAGCGACGCGGTGTACACGTGGGCACCTTGGAGCGC

CCACCCAAAGTGTACTGCGATGGCTCAGAGTACGACGTGCTGAACTTTGCCTCCGGAGGT

TGCTGGCCTCGCAGGATTCGGGTTTGGAACGGCCAGGATTTTCGGGGCGATGGATTCAAC

CCCAGATTTGAGAGATTTCACGTGTACGACATAGTAGAGACTTCGGAGAGCGCCTCACAC

GATGACCCATCCAGGTTTGCTGAGCTATCCCGCCCAAGCGGGTCTGTGGTAACACTGCTG

GGAATGAGCGAGTGTGGAAAGCGAGTCGCGGTTCACGTCTATGGTGTGCGCCATTATTTT

TACATGGCAAAGGCGGAGGTGGATAGCGCTTGTGGAATAACCACCGAGGCAGAACTCGTG

CGCGCAATGGTGGACTGCGCGCACAGCTCGGCTTTGAGCGCGGCCCTGGGAAATGGCAAC

GGCGGCAAACAGAGCGGCGGCAGCGGCGGGGGATGGTGGGGCGGAAAGCACGTGTCTGCG

GACTGCTTCAAAGTGGAGACCGTGTGCCACACGACGCTGTACTACTTTGGATCTAAGCCA

GCTCTCTACTATAGAGTATCTGCCTCCAGCAGCCGCCTGGGAGGGTTCATCTGCGACAAC

TTTCACCCGGAGATTACAAAATTCGAGGGGAGCGTGGACGTGACCACGCGGCTGTTGTTG

GACAATGAAAATTTTACCAGTTTCGGGTGGTACCGCCTGCGACCCGGCACCCACGGAGAG

CGTGTTCAACTTCGCCCCGTTGAGCGACACGTCACCTCAAGCGACGTGGAGATTAACTGT

ACTCCCGATAACCTGGAGCCGATACCAGACGAGGCTGCCTGGCCCGACTATAAGCTCATG

TGCTTTGATATAGAGTGTAAAGCTGGAACGGGTAACGAAATGGCGTTCCCAGTGGCAACT

AACCAAGAGGACCTGGTCATCCAGATCTCCTGTCTGCTGTACTCGCTTGCTACTCAGAAC

CACGAACACACCCTGCTGTTTTCCCTCGGGTCATGCGATATCTCTGAGGAATACTCGTTT

GCATGCGTCCAGCGCGGCGAGCCCAGACCGACGGTTTTGGAGTTTGACAGCGAGTACGAG

CTGCTGGTTGCCTTCCTGACCTTTCTCAAGCAGTACTCTCCCGAGTTCGCCACCGGCTAC

AACATCGTTAATTTTGACTGGGCGTACATAGTTAACAAGGTAACGTCGGTGTATAACATC

AAGCTGGACGGGTACGGCAAGTTCAACAAAGGGGGGCTGTTTAAGGTGTGGGACATCGCC

ACGAACCATTTTCAGAAGAAGAGCAAGGTGAAAATCAATGGCCTGATATCTCTAGACATG

TATTCTGTGGCGACGGAAAAGCTAAAGCTACCCAGCTACAAACTCGACGCGGTCGTGGGA

GACGTCCTCGGCGAGCATAAGATAGACCTTCCCTATAAAGAAATACCCTCCTATTACGCG

GGAGGGCCTGACCGGAGGGGCGTAATAGGAGAGTATTGTATCCAGGACTCTAGGCTGGTG

GGCAAGCTGTTTTTTAAGTACCTCCCCCATCTGGAACTATCGGCGGTGGCCAAACTCGCC

CGTATCACCCTAACGCGGGTAATTTTTGACGGTCAGCAAATTCGCGTGTACACGTGCTTG

CTGAAACTCGCCCGCGAGAGAAATTTCATTTTGCCAGACAACAGACGCCGGTTTGACAGT

CAGGCAGACGCCGCGTCAGAGACTTCGGAGTTGGCTATGGATAGCCAAAGCCACGCCTTC

GACAGTACAGACGAACCCGACGGTGTGGACGGTACCCCGGACGCCGCAGGATCTGGCGCT

ACTTCTGAAAACGGAGGCGGGAAGCCCGGCGTCGGGAGGGCCGTGGGCTACCAGGGAGCA

AAGGTTCTAGACCCCGTATCCGGCTTTCATGTGGACCCCGTGGTTGTGTTTGACTTCGCT

AGCTTATACCCAAGCATTATCCAGGCCCATAACCTCTGTTTCACCACCCTGGCGCTCGAT

GAAGTGGATCTGGCCGGGCTTCAACCATCCGTCAACTACTCGACGTTCGAGGTGGGTGAC

CAAAAGTTATTTTTTGTCCACGCCCATATTCGCGAAAGCCTGCTTGGCATCTTGCTGCGC

GACTGGCTGGCCATGCGAAAGGCGGTGAGGGCGCGAATCCCCACCAGCACCCCCGAGGAG

GCAGTTTTACTAGATAAGCAGCAGTCTGCGATTAAGGTGATATGCAACTCGGTTTACGGA

TTCACGGGGGTGGCAAACGGCCTGTTGCCGTGTCTGAGGATAGCGGCTACCGTTACCACG

ATAGGACGCGACATGCTCCTCAAGACCCGAGATTACGTTCACTCTCGTTGGGCGACGCGC

GAGCTGCTGGAGGACAATTTTCCAGGGGCTATAGGTTTCCGAAACCACAAGCCTTACTCC

GTCAGGGTTATCTACGGAGACACCGACTCCGTGTTTATCAAGTTTGTGGGCCTGACGTAC

GAGGGGGTATCGGAGCTGGGGGATGCTATGTCGCGTCAGATTTCAGCGGACCTCTTTAGA

GCGCCCATCAAACTGGAGTGTGAGAAGACCTTTCAGCGACTGCTGCTGATCACCAAGAAG

AAGTACATAGGTGTCATAAACGGGGGGAAGATGCTCATGAAGGGGGTCGACCTGGTCCGC

AAAAATAACTGCTCTTTCATAAACTTGTACGCGCGACATCTGGTAGATCTTTTGTTGTAC

GACGAGGATGTGGCCACGGCGGCAGCAGAGGTGACAGACGTGCCTCCCGCAGAATGGGTG

GGGCGCCCGCTACCGAGCGGCTTTGACAAGTTTGGGCGAGTGCTGGTAGAGGCGTACAAC

CGTATCACTGCCCCCAACTTGGACGTGCGCGAGTTCGTTATGACTGCTGAGCTGAGCCGC

TCACCCGAATCGTATACCAACAAGCGCCTGCCGCACCTCACCGTCTACTTTAAGCTCGCC

ATGAGGAATGAAGAACTGCCCAGCGTAAAAGAGAGAATTCCGTATGTGATAGTTGCGCAG

ACCGAGGCCGCGGAACGCGAAGCGGGTGTAGTAAACTCAATGCGCGGTACCGCCCAAAAC

CCCGTGGTAACCAAGACCGCACGCCCCCAACCTAAACGCAAACTGCTGGTTTCCGACCTC

GCCGAAGACCCGACCTATGTTTCCGAGAATGACGTACCGCTAAACACAGACTACTATTTC

TCCCACCTGTTGGGTACCATAAGCGTGACCTTTAAGGCTCTATTCGGAAATGATGTGAGA

ACAACAGAAAATCTTTTAAAGCGGTTTATTCCGGAAACCCCCCACAAGACCCCCACGAAA

ACCCAGGCACTGCTTGAGCGCGCCGGCTTTGAAAAGCTGACGCCCTTTACACCGGAGGAA

GAAAGTCGTCGAATACTGCATACAGTTTTTTGTACTCTAGAAGCAGCTCCCCATCAAAGC

TGA

>MN688625.1

ATGGCGGCGCGCGAACAGGCCAACTCCGTTCGCAGGAGCGGGTTTTTTAATCCGTTTATT

GGCAAGAGGCCATTTTTCAGGCCGGGAAGCGGGCAAACCGCGGAGACAGAGCGCCCAAGG

CCCCCCCAACACTCGTACTGCACAGAGGTGGGTAGCTTTAAGTTTATAGCTCCAAGATGC

CTCGATGAAGAAGCCCCCGCCGACCAGCGACGCGGTGTACACGTGGGCACCTTGGAGCGC

CCACCCAAAGTGTACTGCGATGGCTCAGAGTACGACGTGCTGAACTTTGCCTCCGGAGGT

TGCTGGCCTCGCAGGATTCGGGTTTGGAACGGCCAGGATTTTCGGGGCGATGGATTCAAC

CCCAGATTTGAGAGATTTCACGTGTACGACATAGTAGAGACTTCGGAGAGCGCCTCACAC

GATGACCCATCCAGGTTTGCTGAGCTATCCCGCCCAAGCGGGTCTGTGGTAACACTGCTG

GGAATGAGCGAGTGTGGAAAGCGAGTCGCGGTTCACGTCTATGGTGTGCGCCATTATTTT

TACATGGCAAAGGCGGAGGTGGATAGCGCTTGTGGAATAACCACCGAGGCAGAACTCGTG

CGCGCAATGGTGGACTGCGCGCACAGCTCGGCTTTGAGCGCGGCCCTGGGAAATGGCAAC

GGCGGCAAACAGAGCGGCGGCAGCGGCGGGGGATGGTGGGGCGGAAAGCACGTGTCTGCG

GACTGCTTCAAAGTGGAGACCGTGTGCCACACGACGCTGTACTACTTTGGATCTAAGCCA

GCTCTCTACTATAGAGTATCTGCCTCCAGCAGCCGCCTGGGAGGGTTCATCTGCGACAAC

TTTCACCCGGAGATTACAAAATTCGAGGGGAGCGTGGACGTGACCACGCGGCTGTTGTTG

GACAATGAAAATTTTACCAGTTTCGGGTGGTACCGCCTGCGACCCGGCACCCACGGAGAG

CGTGTTCAACTTCGCCCCGTTGAGCGACACGTCACCTCAAGCGACGTGGAGATTAACTGT

ACTCCCGATAACCTGGAGCCGATACCAGACGAGGCTGCCTGGCCCGACTATAAGCTCATG

TGCTTTGATATAGAGTGTAAAGCTGGAACGGGTAACGAAATGGCGTTCCCAGTGGCAACT

AACCAAGAGGACCTGGTCATCCAGATCTCCTGTCTGCTGTACTCGCTTGCTACTCAGAAC

CACGAACACACCCTGCTGTTTTCCCTCGGGTCATGCGATATCTCTGAGGAATACTCGTTT

GCATGCGTCCAGCGCGGCGAGCCCAGACCGACGGTTTTGGAGTTTGACAGCGAGTACGAG

CTGCTGGTTGCCTTCCTGACCTTTCTCAAGCAGTACTCTCCCGAGTTCGCCACCGGCTAC

AACATCGTTAATTTTGACTGGGCGTACATAGTTAACAAGGTAACGTCGGTGTATAACATC

AAGCTGGACGGGTACGGCAAGTTCAACAAAGGGGGGCTGTTTAAGGTGTGGGACATCGCC

ACGAACCATTTTCAGAAGAAGAGCAAGGTGAAAATCAATGGCCTGATATCTCTAGACATG

TATTCTGTGGCGACGGAAAAGCTAAAGCTACCCAGCTACAAACTCGACGCGGTCGTGGGA

GACGTCCTCGGCGAGCATAAGATAGACCTTCCCTATAAAGAAATACCCTCCTATTACGCG

GGAGGGCCTGACCGGAGGGGCGTAATAGGAGAGTATTGTATCCAGGACTCTAGGCTGGTG

GGCAAGCTGTTTTTTAAGTACCTCCCCCATCTGGAACTATCGGCGGTGGCCAAACTCGCC

CGTATCACCCTAACGCGGGTAATTTTTGACGGTCAGCAAATTCGCGTGTACACGTGCTTG

CTGAAACTCGCCCGCGAGAGAAATTTCATTTTGCCAGACAACAGACGCCGGTTTGACAGT

CAGGCAGACGCCGCGTCAGAGACTTCGGAGTTGGCTATGGATAGCCAAAGCCACGCCTTC

GACAGTACAGACGAACCCGACGGTGTGGACGGTACCCCGGACGCCGCAGGATCTGGCGCT

ACTTCTGAAAACGGAGGCGGGAAGCCCGGCGTCGGGAGGGCCGTGGGCTACCAGGGAGCA

AAGGTTCTAGACCCCGTATCCGGCTTTCATGTGGACCCCGTGGTTGTGTTTGACTTCGCT

AGCTTATACCCAAGCATTATCCAGGCCCATAACCTCTGTTTCACCACCCTGGCGCTCGAT

GAAGTGGATCTGGCCGGGCTTCAACCATCCGTCAACTACTCGACGTTCGAGGTGGGTGAC

CAAAAGTTATTTTTTGTCCACGCCCATATTCGCGAAAGCCTGCTTGGCATCTTGCTGCGC

GACTGGCTGGCCATGCGAAAGGCGGTGAGGGCGCGAATCCCCACCAGCACCCCCGAGGAG

GCAGTTTTACTAGATAAGCAGCAGTCTGCGATTAAGGTGATATGCAACTCGGTTTACGGA

TTCACGGGGGTGGCAAACGGCCTGTTGCCGTGTCTGAGGATAGCGGCTACCGTTACCACG

ATAGGACGCGACATGCTCCTCAAGACCCGAGATTACGTTCACTCTCGTTGGGCGACGCGC

GAGCTGCTGGAGGACAATTTTCCAGGGGCTATAGGTTTCCGAAACCACAAGCCTTACTCC

GTCAGGGTTATCTACGGAGACACCGACTCCGTGTTTATCAAGTTTGTGGGCCTGACGTAC

GAGGGGGTATCGGAGCTGGGGGATGCTATGTCGCGTCAGATTTCAGCGGACCTCTTTAGA

GCGCCCATCAAACTGGAGTGTGAGAAGACCTTTCAGCGACTGCTGCTGATCACCAAGAAG

AAGTACATAGGTGTCATAAACGGGGGGAAGATGCTCATGAAGGGGGTCGACCTGGTCCGC

AAAAATAACTGCTCTTTCATAAACTTGTACGCGCGACATCTGGTAGATCTTTTGTTGTAC

GACGAGGATGTGGCCACGGCGGCAGCAAAGGTGACAGACGTGCCTCCCGCAGAATGGGTG

GGGCGCCCGCTACCGAGCGGCTTTGACAAGTTTGGGCGAGTGCTGGTAGAGGCGTACAAC

CGTATCACTGCCCCCAACTTGGACGTGCGCGAGTTCGTTATGACTGCTGAGCTGAGCCGC

TCACCCGAATCGTATACCAACAAGCGCCTGCCGCACCTCACCGTCTACTTTAAGCTCGCC

ATGAGGAATGAAGAACTGCCCAGCGTAAAAGAGAGAATTCCGTATGTGATAGTTGCGCAG

ACCGAGGCCGCGGAACGCGAAGCGGGTGTAGTAAACTCAATGCGCGGTACCGCCCAAAAC

CCCGTGGTAACCAAGACCGCACGCCCCCAACCTAAACGCAAACTGCTGGTTTCCGACCTC

GCCGAAGACCCGACCTATGTTTCCGAGAATGACGTACCGCTAAACACAGACTACTATTTC

TCCCACCTGTTGGGTACCATAAGCGTGACCTTTAAGGCTCTATTCGGAAATGATGTGAGA

ACAACAGAAAATCTTTTAAAGCGGTTTATTCCGGAAACCCCCCACAAGACCCCCACGAAA

ACCCAGGCACTGCTTGAGCGCGCCGGCTTTGAAAAGCTGACGCCCTTTACACCGGAGGAA

GAAAGTCGTCGAATACTGCATACAGTTTTTTGTACTCTAGAAGCAGCTCCCCATCAAAGC

TGA

>MN688626.1

ATGGCGGCGCGCGAACAGGCCAACTCCGTTCGCAGGAGCGGGTTTTTTAATCCGTTTATT

GGCAAGAGGCCATTTTTCAGGCCGGGAAGCGGGCAGACCGCGGAGACAGAGCGCCCAAGG

CCCCCCCAACACTCGTACTGCACAGAGGTGGGTAGCTTTAAGTTTATAGCTCCAAGATGC

CTCGATGAAGAAGCCCCCGCCGACCAGCGACGCGGTGTACACGTGGGCACCTTGGAGCGC

CCACCCAAAGTGTACTGCGATGGCTCAGAGTACGACGTGCTGAACTTTGCCTCCGGAGGT

TGCTGGCCTCGCAGGATTCGGGTTTGGAACGGCCAGGATTTTCGGGGCGATGGATTCAAC

CCCAGATTTGAGAGATTTCACGTGTACGACATAGTAGAGACTTCGGAGAGCGCCTCACAC

GATGACCCATCCAGGTTTGCTGAGCTATCCCGCCCAAGCGGGTCTGTGGTAACACTGCTG

GGAATGAGCGAGTGTGGAAAGCGAGTCGCGGTTCACGTCTATGGTGTGCGCCATTATTTT

TACATGGCAAAGGCGGAGGTGGATAGCGCTTGTGGAATAACCACCGAGGCAGAACTCGTG

CGCGCAATGGTGGACTGCGCGCACAGCTCGGCTTTGAGCGCGGCCCTGGGAAATGGCAAC

GGCGGCAAACAGAGCGGCGGCAGCGGCGGGGGATGGTGGGGCGGAAAGCACGTGTCTGCG

GACTGCTTCAAAGTGGAGACCGTGTGCCACACGACGCTGTACTACTTTGGATCTAAGCCA

GCTCTCTACTATAGAGTATCTGCCTCCAGCAGCCGCCTGGGAGGGTTCATCTGCGACAAC

TTTCACCCGGAGATTACAAAATTCGAGGGGAGCGTGGACGTGACCACGCGGCTGTTGTTG

GACAATGAAAATTTTACCAGTTTCGGGTGGTACCGCCTGCGACCCGGCACCCACGGAGAG

CGTGTTCAACTTCGCCCCGTTGAGCGACACGTCACCTCAAGCGACGTGGAGATTAACTGT

ACTCCCGATAACCTGGAGCCGATACCAGACGAGGCTGCCTGGCCCGACTATAAGCTCATG

TGCTTTGATATAGAGTGTAAAGCTGGAACGGGTAACGAAATGGCGTTCCCAGTGGCAACT

AACCAAGAGGACCTGGTCATCCAGATCTCCTGTCTGCTGTACTCGCTTGCTACTCAGAAC

CACGAACACACCCTGCTGTTTTCCCTCGGGTCATGCGATATCTCTGAGGAATACTCGTTT

GCATGCGTCCAGCGCGGCGAGCCCAGACCGACGGTTTTGGAGTTTGACAGCGAGTACGAG

CTGCTGGTTGCCTTCCTGACCTTTCTCAAGCAGTACTCTCCCGAGTTCGCCACCGGCTAC

AACATCGTTAATTTTGACTGGGCGTACATAGTTAACAAGGTAACGTCGGTGTATAACATC

AAGCTGGACGGGTACGGCAAGTTCAACAAAGGGGGGCTGTTTAAGGTGTGGGACATCGCC

ACGAACCATTTTCAGAAGAAGAGCAAGGTGAAAATCAATGGCCTGATATCTCTAGACATG

TATTCTGTGGCGACGGAAAAGCTAAAGCTACCCAGCTACAAACTCGACGCGGTCGTGGGA

GACGTCCTCGGCGAGCATAAGATAGACCTTCCCTATAAAGAAATACCCTCCTATTACGCG

GGAGGGCCTGACCGGAGGGGCGTAATAGGAGAGTATTGTATCCAGGACTCTAGGCTGGTG

GGCAAGCTGTTTTTTAAGTACCTCCCCCATCTGGAACTATCGGCGGTGGCCAAACTCGCC

CGTATCACCCTAACGCGGGTAATTTTTGACGGTCAGCAAATTCGCGTGTACACGTGCTTG

CTGAAACTCGCCCGCGAGAGAAATTTCATTTTGCCAGACAACAGACGCCGGTTTGACAGT

CAGGCAGACGCCGCGTCAGAGACTTCGGAGTTGGCTATGGATAGCCAAAGCCACGCCTTC

GACAGTACAGACGAACCCGACGGTGTGGACGGTACCCCGGACGCCGCAGGATCTGGCGCT

ACTTCTGAAAACGGAGGCGGGAAGCCCGGCGTCGGGAGGGTCGTGGGCTACCAGGGAGCA

AAGGTTCTAGACCCCGTATCCGGCTTTCATGTGGACCCCGTGGTTGTGTTTGACTTCGCT

AGCTTATACCCAAGCATTATCCAGGCCCATAACCTCTGTTTCACCACCCTGGCGCTCGAT

GAAGTGGATCTGGCCGGGCTTCAACCATCCGTCGACTACTCGACGTTCGAGGTGGGTGAC

CAAAAGTTATTTTTTGTCCACGCCCATATTCGCGAAAGCCTGCTTGGCATCTTGCTGCGC

GACTGGCTGGCCATGCGAAAGGCGGTGAGGGCGCGAATCCCCACCAGCACCCCCGAGGAG

GCAGTTTTACTAGATAAGCAGCAGTCTGCGATTAAGGTGATATGCAACTCGGTTTACGGA

TTCACGGGGGTGGCAAACGGCCTGTTGCCGTGTCTGAGGATAGCGGCTACCGTTACCACG

ATAGGACGCGACATGCTCCTCAAGACCCGAGATTACGTTCACTCTCGTTGGGCGACGCGC

GAGCTGCTGGAGGACAATTTTCCAGGGGCTATAGGTTTCCGAAACCACAAGCCTTACTCC

GTCAGGGTTATCTACGGAGACACCGACTCCGTGTTTATCAAGTTTGTGGGCCTGACGTAC

GAGGGGGTATCGGAGCTGGGGGATGCTATGTCGCGTCAGATTTCAGCGGACCTCTTTAGA

GCGCCCATCAAACTGGAGTGTGAGAAGACCTTTCAGCGACTGCTGCTGATCACCAAGAAG

AAGTACATAGGTGTCATAAACGGGGGGAAGATGCTCATGAAGGGGGTCGACCTGGTCCGC

AAAAATAACTGCTCTTTCATAAACTTGTACGCGCGACATCTGGTAGATCTTTTGTTGTAC

GACGAGGATGTGGCCACGGCGGCAGCAGAGGTGACAGACGTGCCTCCCGCAGAATGGGTG

GGGCGCCCGCTACCGAGCGGCTTTGACAAGTTTGGGCGAGTGCTGGTAGAGGCGTACAAC

CGTATCACTGCCCCCAACTTGGACGTGCGCGAGTTCGTTATGACTGCTGAGCTGAGCCGC

TCACCCGAATCGTATACCAACAAGCGCCTGCCGCACCTCACCGTCTACTTTAAGCTCGCC

ATGAGGAATGAAGAACTGCCCAGCGTAAAAGAGAGAATTCCGTATGTGATAGTTGCGCAG

ACCGAGGCCGCGGAACGCGAAGCGGGTGTAGTAAACTCAATGCGCGGTACCGCCCAAAAC

CCCGTGGTAACCAAGACCGCACGCCCCCAACCTAAACGCAAACTGCTGGTTTCCGACCTC

GCCGAAGACCCGACCTATGTTTCCGAGAATGACGTACCGCTAAACACAGACTACTATTTC

TCCCACCTGTTGGGTACCATAAGCGTGACCTTTAAGGCTCTATTCGGAAATGATGTGAGA

ACAACAGAAAATCTTTTAAAGCGGTTTATTCCGGAAACCCCCCACAAGACCCCCACGAAA

ACCCAGGCACTGCTTGAGCGCGCCGGCTTTGAAAAGCTGACGCCCTTTACACCGGAGGAA

GAAAGTCGTCGAATACTGCATACAGTTTTTTGTACTCTAGAAGCAGCTCCCCATCAAAGC

TGA

>MN688627.1

ATGGCGGCGCGCGAACAGGCCAACTCCGTTCGCAGGAGCGGGTTTTTTAATCCGTTTATT

GGCAAGAGGCCATTTTTCAGGCCGGGAAGCGGGCAGACCGCGGAGACAGAGCGCCCAAGG

CCCCCCCAACACTCGTACTGCACAGAGGTGGGTAGCTTTAAGTTTATAGCTCCAAGATGC

CTCGATGAAGAAGCCCCCGCCGACCAGCGACGCGGTGTACACGTGGGCACCTTGGAGCGC

CCACCCAAAGTGTACTGCGATGGCTCAGAGTACGACGTGCTGAACTTTGCCTCCGGAGGT

TGCTGGCCTCGCAGGATTCGGGTTTGGAACGGCCAGGATTTTCGGGGCGATGGATTCAAC

CCCAGATTTGAGAGATTTCACGTGTACGACATAGTAGAGACTTCGGAGAGCGCCTCACAC

GATGACCCATCCAGGTTTGCTGAGCTATCCCGCCCAAGCGGGTCTGTGGTAACACTGCTG

GGAATGAGCGAGTGTGGAAAGCGAGTCGCGGTTCACGTCTATGGTGTGCGCCATTATTTT

TACATGGCAAAGGCGGAGGTGGATAGCGCTTGTGGAATAACCACCGAGGCAGAACTCGTG

CGCGCAATGGTGGACTGCGCGCACAGCTCGGCTTTGAGCGCGGCCCTGGGAAATGGCAAC

GGCGGCAAACAGAGCGGCGGCAGCGGCGGGGGATGGTGGGGCGGAAAGCACGTGTCTGCG

GACTGCTTCAAAGTGGAGACCGTGTGCCACACGACGCTGTACTACTTTGGATCTAAGCCA

GCTCTCTACTATAGAGTATCTGCCTCCAGCAGCCGCCTGGGAGGGTTCATCTGCGACAAC

TTTCACCCGGAGATTACAAAATTCGAGGGGAGCGTGGACGTGACCACGCGGCTGTTGTTG

GACAATGAAAATTTTACCAGTTTCGGGTGGTACCGCCTGCGACCCGGCACCCACGGAGAG

CGTGTTCAACTTCGCCCCGTTGAGCGACACGTCACCTCAAGCGACGTGGAGATTAACTGT

ACTCCCGATAACCTGGAGCCGATACCAGACGAGGCTGCCTGGCCCGACTATAAGCTCATG

TGCTTTGATATAGAGTGTAAAGCTGGAACGGGTAACGAAATGGCGTTCCCAGTGGCAACT

AACCAAGAGGACCTGGTCATCCAGATCTCCTGTCTGCTGTACTCGCTTGCTACTCAGAAC

CACGAACACACCCTGCTGTTTTCCCTCGGGTCATGCGATATCTCTGAGGAATACTTGTTT

GCATGCGTCCAGCGCGGCGAGCCCAGACCGACGGTTTTGGAGTTTGACAGCGAGTACGAG

CTGCTGGTTGCCTTCCTGACCTTTCTCAAGCAGTACTCTCCCGAGTTCGCCACCGGCTAC

AACATCGTTAATTTTGACTGGGCGTACATAGTTAACAAGGTAACGTCGGTGTATAACATC

AAGCTGGACGGGTACGGCAAGTTCAACAAAGGGGGGCTGTTTAAGGTGTGGGACATCGCC

ACGAACCATTTTCAGAAGAAGAGCAAGGTGAAAATCAATGGCCTGATATCTCTAGACATG

TATTCTGTGGCGACGGAAAAGCTAAAGCTACCCAGCTACAAACTCGACGCGGTCGTGGGA

GACGTCCTCGGCGAGCATAAGATAGACCTTCCCTATAAAGAAATACCCTCCTATTACGCG

GGAGGGCCTGACCGGAGGGGCGTAATAGGAGAGTATTGTATCCAGGACTCTAGGCTGGTG

GGCAAGCTGTTTTTTAAGTACCTCCCCCATCTGGAACTATCGGCGGTGGCCAAACTCGCC

CGTATCACCCTAACGCGGGTAATTTTTGACGGTCAGCAAATTCGCGTGTACACGTGCTTG

CTGAAACTCGCCCGCGAGAGAAATTTCATTTTGCCAGACAACAGACGCCGGTTTGACAGT

CAGGCAGACGCCGCGTCAGAGACTTCGGAGTTGGCTATGGATAGCCAAAGCCACGCCTTC

GACAGTACAGACGAACCCGACGGTGTGGACGGTACCCCGGACGCCGCAGGATCTGGCGCT

ACTTCTGAAAACGGAGGCGGGAAGCCCGGCGTCGGGAGGGCCGTGGGCTACCAGGGAGCA

AAGGTTCTAGACCCCGTATCCGGCTTTCATGTGGACCCCGTGGTTGTGTTTGACTTCGCT

AGCTTATACCCAAGCATTATCCAGGCCCATAACCTCTGTTTCACCACCCTGGCGCTCGAT

GAAGTGGATCTGGCCGGGCTTCAACCATCCGTCAACTACTCGACGTTCGAGGTGGGTGAC

CAAAAGTTATTTTTTGTCCACGCCCATATTCGCGAAAGCCTGCTTGGCATCTTGCTGCGC

GACTGGCTGGCCATGCGAAAGGCGGTGAGGGCGCGAATCCCCACCAGCACCCCCGAGGAG

GCAGTTTTACTAGATAAGCAGCAGTCTGCGATTAAGGTGATATGCAACTCGGTTTACGGA

TTCACGGGGGTGGCAAACGGCCTGTTGCCGTGTCTGAGGATAGCGGCTACCGTTACCACG

ATAGGACGCGACATGCTCCTCAAGACCCGAGATTACGTTCACTCTCGTTGGGCGACGCGC

GAGCTGCTGGAGGACAATTTTCCAGGGGCTATAGGTTTCCGAAACCACAAGCCTTACTCC

GTCAGGGTTATCTACGGAGACACCGACTCCGTGTTTATCAAGTTTGTGGGCCTGACGTAC

GAGGGGGTATCGGAGCTGGGGGATGCTATGTCGCGTCAGATTTCAGCGGACCTCTTTAGA

GCGCCCATCAAACTGGAGTGTGAGAAGACCTTTCAGCGACTGCTGCTGATCACCAAGAAG

AAGTACATAGGTGTCATAAACGGGGGGAAGATGCTCATGAAGGGGGTCGACCTGGTCCGC

AAAAATAACTGCTCTTTCATAAACTTGTACGCGCGACATCTGGTAGATCTTTTGTTGTAC

GACGAGGATGTGGCCACGGCGGCAGCAGAGGTGACAGACGTGCCTCCCGCAGAATGGGTG

GGGCGCCCGCTACCGAGCGGCTTTGACAAGTTTGGGCGAGTGCTGGTAGAGGCGTACAAC

CGTATCACTGCCCCCAACTTGGACGTGCGCGAGTTCGTTATGACTGCTGAGCTGAGCCGC

TCACCCGAATCGTATACCAACAAGCGCCTGCCGCACCTCACCGTCTACTTTAAGCTCGCC

ATGAGGAATGAAGAACTGCCCAGCGTAAAAGAGAGAATTCCGTATGTGATAGTTGCGCAG

ACCGAGGCCGCGGAACGCGAAGCGGGTGTAGTAAACTCAATGCGCGGTACCGCCCAAAAC

CCCGTGGTAACCAAGACCGCACGCCCCCAACCTAAACGCAAACTGCTGGTTTCCGACCTC

GCCGAAGACCCGACCTATGTTTCCGAGAATGACGTACCGCTAAACACAGACTACTATTTC

TCCCACCTGTTGGGTACCATAAGCGTGACCTTTAAGGCTCTATTCGGAAATGATGTGAGA

ACAACAGAAAATCTTTTAAAGCGGTTTATTCCGGAAACCCCCCACAAGACCCCCACGAAA

ACCCAGGCACTGCTTGAGCGCGCCGGCTTTGAAAAGCTGACGCCCTTTACACCGGAGGAA

GAAAGTCGTCGAATACTGCATACAGTTTTTTGTACTCTAGAAGCAGCTCCCCATCAAAGC

TGA

>MN688628.1

ATGGCGGCGCGCGAACAGGCCAACTCCGTTCGCAGGAGCGGGTTTTTTAATCCGTTTATT

GGCAAGAGGCCATTTTTCAGGCCGGGAAGCGGGCAAACCGCGGAGACAGAGCGCCCAAGG

CCCCCCCAACACTCGTACTGCACAGAGGTGGGTAGCTTTAAGTTTATAGCTCCAAGATGC

CTCGATGAAGAAGCCCCCGCCGACCAGCGACGCGGTGTACACGTGGGCACCTTGGAGCGC

CCACCCAAAGTGTACTGCGATGGCTCAGAGTACGACGTGCTGAACTTTGCCTCCGGAGGT

TGCTGGCCTCGCAGGATTCGGGTTTGGAACGGCCAGGATTTTCGGGGCGATGGATTCAAC

CCCAGATTTGAGAGATTTCACGTGTACGACATAGTAGAGACTTCGGAGAGCGCCTCACAC

GATGACCCATCCAGGTTTGCTGAGCTATCCCGCCCAAGCGGGTCTGTGGTAACACTGCTG

GGAATGAGCGAGTGTGGAAAGCGAGTCGCGGTTCACGTCTATGGTGTGCGCCATTATTTT

TACATGGCAAAGGCGGAGGTGGATAGCGCTTGTGGAATAACCACCGAGGCAGAACTCGTG

CGCGCAATGGTGGACTGCGCGCACAGCTCGGCTTTGAGCGCGGCCCTGGGAAATGGCAAC

GGCGGCAAACAGAGCGGCGGCAGCGGCGGGGGATGGTGGGGCGGAAAGCACGTGTCTGCG

GACTGCTTCAAAGTGGAGACCGTGTGCCACACGACGCTGTACTACTTTGGATCTAAGCCA

GCTCTCTACTATAGAGTATCTGCCTCCAGCAGCCGCCTGGGAGGGTTCATCTGCGACAAC

TTTCACCCGGAGATTACAAAATTCGAGGGGAGCGTGGACGTGACCACGCGGCTGTTGTTG

GACAATGAAAATTTTACCAGTTTCGGGTGGTACCGCCTGCGACCCGGCACCCACGGAGAG

CGTGTTCAACTTCGCCCCGTTGAGCGACACGTCACCTCAAGCGACGTGGAGATTAACTGT

ACTCCCGATAACCTGGAGCCGATACCAGACGAGGCTGCCTGGCCCGACTATAAGCTCATG

TGCTTTGATATAGAGTGTAAAGCTGGAACGGGTAACGAAATGGCGTTCCCAGTGGCAACT

AACCAAGAGGACCTGGTCATCCAGATCTCCTGTCTGCTGTACTCGCTTGCTACTCAGAAC

CACGAACACACCCTGCTGTTTTCCCTCGGGTCATGCGATATCTCTGAGGAATACTCGTTT

GCATGCGTCCAGCGCGGCGAGCCCAGACCGACGGTTTTGGAGTTTGACAGCGAGTACGAG

CTGCTGGTTGCCTTCCTGACCTTTCTCAAGCAGTACTCTCCCGAGTTCGCCACCGGCTAC

AACATCGTTAATTTTGACTGGGCGTACATAGTTAACAAGGTAACGTCGGTGTATAACATC

AAGCTGGACGGGTACGGCAAGTTCAACAAAGGGGGGCTGTTTAAGGTGTGGGACATCGCC

ACGAACCATTTTCAGAAGAAGAGCAAGGTGAAAATCAATGGCCTGATATCTCTAGACATG

TATTCTGTGGCGACGGAAAAGCTAAAGCTACCCAGCTACAAACTCGACGCGGTCGTGGGA

GACGTCCTCGGCGAGCATAAGATAGACCTTCCCTATAAAGAAATACCCTCCTATTACGCG

GGAGGGCCTGACCGGAGGGGCGTAATAGGAGAGTATTGTATCCAGGACTCTAGGCTGGTG

GGCAAGCTGTTTTTTAAGTACCTCCCCCATCTGGAACTATCGGCGGTGGCCAAACTCGCC

CGTATCACCCTAACGCGGGTAATTTTTGACGGTCAGCAAATTCGCGTGTACACGTGCTTG

CTGAAACTCGCCCGCGAGAGAAATTTCATTTTGCCAGACAACAGACGCCGGTTTGACAGT

CAGGCAGACGCCGCGTCAGAGACTTCGGAGTTGGCTATGGATAGCCAAAGCCACGCCTTC

GACAGTACAGACGAACCCGACGGTGTGGACGGTACCCCGGACGCCGCAGGATCTGGCGCT

ACTTCTGAAAACGGAGGCGGGAAGCCCGGCGTCGGGAGGGCCGTGGGCTACCAGGGAGCA

AAGGTTCTAGACCCCGTATCCGGCTTTCATGTGGACCCCGTGGTTGTGTTTGACTTCGCT

AGCTTATACCCAAGCATTATCCAGGCCCATAACCTCTGTTTCACCACCCTGGCGCTCGAT

GAAGTGGATCTGGCCGGGCTTCAACCATCCGTCAACTACTCGACGTTCGAGGTGGGTGAC

CAAAAGTTATTTTTTGTCCACGCCCATATTCGCGAAAGCCTGCTTGGCATCTTGCTGCGC

GACTGGCTGGCCATGCGAAAGGCGGTGAGGGCGCGAATCCCCACCAGCACCCCCGAGGAG

GCAGTTTTACTAGATAAGCAGCAGTCTGCGATTAAGGTGATATGCAACTCGGTTTACGGA

TTCACGGGGGTGGCAAACGGCCTGTTGCCGTGTCTGAGGATAGCGGCTACCGTTACCACG

ATAGGACGCGACATGCTCCTCAAGACCCGAGATTACGTTCACTCTCGTTGGGCGACGCGC

GAGCTGCTGGAGGACAATTTTCCAGGGGCTATAGGTTTCCGAAACCACAAGCCTTACTCC

GTCAGGGTTATCTACGGAGACACCGACTCCGTGTTTATCAAGTTTGTGGGCCTGACGTAC

GAGGGGGTATCGGAGCTGGGGGATGCTATGTCGCGTCAGATTTCAGCGGACCTCTTTAGA

GCGCCCATCAAACTGGAGTGTGAGAAGACCTTTCAGCGACTGCTGCTGATCACCAAGAAG

AAGTACATAGGTGTCATAAACGGGGGGAAGATGCTCATGAAGGGGGTCGACCTGGTCCGC

AAAAATAACTGCTCTTTCATAAACTTGTACGCGCGACATCTGGTAGATCTTTTGTTGTAC

GACGAGGATGTGGCCACGGCGGCAGCAAAGGTGACAGACGTGCCTCCCGCAGAATGGGTG

GGGCGCCCGCTACCGAGCGGCTTTGACAAGTTTGGGCGAGTGCTGGTAGAGGCGTACAAC

CGTATCACTGCCCCCAACTTGGACGTGCGCGAGTTCGTTATGACTGCTGAGCTGAGCCGC

TCACCCGAATCGTATACCAACAAGCGCCTGCCGCACCTCACCGTCTACTTTAAGCTCGCC

ATGAGGAATGAAGAACTGCCCAGCGTAAAAGAGAGAATTCCGTATGTGATAGTTGCGCAG

ACCGAGGCCGCGGAACGCGAAGCGGGTGTAGTAAACTCAATGCGCGGTACCGCCCAAAAC

CCCGTGGTAACCAAGACCGCACGCCCCCAACCTAAACGCAAACTGCTGGTTTCCGACCTC

GCCGAAGACCCGACCTATGTTTCCGAGAATGACGTACCGCTAAACACAGACTACTATTTC

TCCCACCTGTTGGGTACCATAAGCGTGACCTTTAAGGCTCTATTCGGAAATGATGTGAGA

ACAACAGAAAATCTTTTAAAGCGGTTTATTCCGGAAACCCCCCACAAGACCCCCACGAAA

ACCCAGGCACTGCTTGAGCGCGCCGGCTTTGAAAAGCTGACGCCCTTTACACCGGAGGAA

GAAAGTCGTCGAATACTGCATACAGTTTTTTGTACTCTAGAAGCAGCTCCCCATCAAAGC

TGA

>MN688629.1

ATGGCGGCGCGCGAACAGGCCAACTCCGTTCGCAGGAGCGGGTTTTTTAATCCGTTTATT

GGCAAGAGGCCATTTTTCAGGCCGGGAAGCGGGCAAACCGCGGAGACAGAGCGCCCAAGG

CCCCCCCAACACTCGTACTGCACAGAGGTGGGTAGCTTTAAGTTTATAGCTCCAAGATGC

CTCGATGAAGAAGCCCCCGCCGACCAGCGACGCGGTGTACACGTGGGCACCTTGGAGCGC

CCACCCAAAGTGTACTGCGATGGCTCAGAGTACGACGTGCTGAACTTTGCCTCCGGAGGT

TGCTGGCCTCGCAGGATTCGGGTTTGGAACGGCCAGGATTTTCGGGGCGATGGATTCAAC

CCCAGATTTGAGAGATTTCACGTGTACGACATAGTAGAGACTTCGGAGAGCGCCTCACAC

GATGACCCATCCAGGTTTGCTGAGCTATCCCGCCCAAGCGGGTCTGTGGTAACACTGCTG

GGAATGAGCGAGTGTGGAAAGCGAGTCGCGGTTCACGTCTATGGTGTGCGCCATTATTTT

TACATGGCAAAGGCGGAGGTGGATAGCGCTTGTGGAATAACCACCGAGGCAGAACTCGTG

CGCGCAATGGTGGACTGCGCGCACAGCTCGGCTTTGAGCGCGGCCCTGGGAAATGGCAAC

GGCGGCAAACAGAGCGGCGGCAGCGGCGGGGGATGGTGGGGCGGAAAGCACGTGTCTGCG

GACTGCTTCAAAGTGGAGACCGTGTGCCACACGACGCTGTACTACTTTGGATCTAAGCCA

GCTCTCTACTATAGAGTATCTGCCTCCAGCAGCCGCCTGGGAGGGTTCATCTGCGACAAC

TTTCACCCGGAGATTACAAAATTCGAGGGGAGCGTGGACGTGACCACGCGGCTGTTGTTG

GACAATGAAAATTTTACCAGTTTCGGGTGGTACCGCCTGCGACCCGGCACCCACGGAGAG

CGTGTTCAACTTCGCCCCGTTGAGCGACACGTCACCTCAAGCGACGTGGAGATTAACTGT

ACTCCCGATAACCTGGAGCCGATACCAGACGAGGCTGCCTGGCCCGACTATAAGCTCATG

TGCTTTGATATAGAGTGTAAAGCTGGAACGGGTAACGAAATGGCGTTCCCAGTGGCAACT

AACCAAGAGGACCTGGTCATCCAGATCTCCTGTCTGCTGTACTCGCTTGCTACTCAGAAC

CACGAACACACCCTGCTGTTTTCCCTCGGGTCATGCGATATCTCTGAGGAATACTCGTTT

GCATGCGTCCAGCGCGGCGAGCCCAGACCGACGGTTTTGGAGTTTGACAGCGAGTACGAG

CTGCTGGTTGCCTTCCTGACCTTTCTCAAGCAGTACTCTCCCGAGTTCGCCACCGGCTAC

AACATCGTTAATTTTGACTGGGCGTACATAGTTAACAAGGTAACGTCGGTGTATAACATC

AAGCTGGACGGGTACGGCAAGTTCAACAAAGGGGGGCTGTTTAAGGTGTGGGACATCGCC

ACGAACCATTTTCAGAAGAAGAGCAAGGTGAAAATCAATGGCCTGATATCTCTAGACATG

TATTCTGTGGCGACGGAAAAGCTAAAGCTACCCAGCTACAAACTCGACGCGGTCGTGGGA

GACGTCCTCGGCGAGCATAAGATAGACCTTCCCTATAAAGAAATACCCTCCTATTACGCG

GGAGGGCCTGACCGGAGGGGCGTAATAGGAGAGTATTGTATCCAGGACTCTAGGCTGGTG

GGCAAGCTGTTTTTTAAGTACCTCCCCCATCTGGAACTATCGGCGGTGGCCAAACTCGCC

CGTATCACCCTAACGCGGGTAATTTTTGACGGTCAGCAAATTCGCGTGTACACGTGCTTG

CTGAAACTCGCCCGCGAGAGAAATTTCATTTTGCCAGACAACAGACGCCGGTTTGACAGT

CAGGCAGACGCCGCGTCAGAGACTTCGGAGTTGGCTATGGATAGCCAAAGCCACGCCTTC

GACAGTACAGACGAACCCGACGGTGTGGACGGTACCCCGGACGCCGCAGGATCTGGCGCT

ACTTCTGAAAACGGAGGCGGGAAGCCCGGCGTCGGGAGGGCCGTGGGCTACCAGGGAGCA

AAGGTTCTAGACCCCGTATCCGGCTTTCATGTGGACCCCGTGGTTGTGTTTGACTTCGCT

AGCTTATACCCAAGCATTATCCAGGCCCATAACCTCTGTTTCACCACCCTGGCGCTCGAT

GAAGTGGATCTGGCCGGGCTTCAACCATCCGTCAACTACTCGACGTTCGAGGTGGGTGAC

CAAAAGTTATTTTTTGTCCACGCCCATATTCGCGAAAGCCTGCTTGGCATCTTGCTGCGC

GACTGGCTGGCCATGCGAAAGGCGGTGAGGGCGCGAATCCCCACCAGCACCCCCGAGGAG

GCAGTTTTACTAGATAAGCAGCAGTCTGCGATTAAGGTGATATGCAACTCGGTTTACGGA

TTCACGGGGGTGGCAAACGGCCTGTTGCCGTGTCTGAGGATAGCGGCTACCGTTACCACG

ATAGGACGCGACATGCTCCTCAAGACCCGAGATTACGTTCACTCTCGTTGGGCGACGCGC

GAGCTGCTGGAGGACAATTTTCCAGGGGCTATAGGTTTCCGAAACCACAAGCCTTACTCC

GTCAGGGTTATCTACGGAGACACCGACTCCGTGTTTATCAAGTTTGTGGGCCTGACGTAC

GAGGGGGTATCGGAGCTGGGGGATGCTATGTCGCGTCAGATTTCAGCGGACCTCTTTAGA

GCGCCCATCAAACTGGAGTGTGAGAAGACCTTTCAGCGACTGCTGCTGATCACCAAGAAG

AAGTACATAGGTGTCATAAACGGGGGGAAGATGCTCATGAAGGGGGTCGACCTGGTCCGC

AAAAATAACTGCTCTTTCATAAACTTGTACGCGCGACATCTGGTAGATCTTTTGTTGTAC

GACGAGGATGTGGCCACGGCGGCAGCAAAGGTGACAGACGTGCCTCCCGCAGAATGGGTG

GGGCGCCCGCTACCGAGCGGCTTTGACAAGTTTGGGCGAGTGCTGGTAGAGGCGTACAAC

CGTATCACTGCCCCCAACTTGGACGTGCGCGAGTTCGTTATGACTGCTGAGCTGAGCCGC

TCACCCGAATCGTATACCAACAAGCGCCTGCCGCACCTCACCGTCTACTTTAAGCTCGCC

ATGAGGAATGAAGAACTGCCCAGCGTAAAAGAGAGAATTCCGTATGTGATAGTTGCGCAG

ACCGAGGCCGCGGAACGCGAAGCGGGTGTAGTAAACTCAATGCGCGGTACCGCCCAAAAC

CCCGTGGTAACCAAGACCGCACGCCCCCAACCTAAACGCAAACTGCTGGTTTCCGACCTC

GCCGAAGACCCGACCTATGTTTCCGAGAATGACGTACCGCTAAACACAGACTACTATTTC

TCCCACCTGTTGGGTACCATAAGCGTGACCTTTAAGGCTCTATTCGGAAATGATGTGAGA

ACAACAGAAAATCTTTTAAAGCGGTTTATTCCGGAAACCCCCCACAAGACCCCCACGAAA

ACCCAGGCACTGCTTGAGCGCGCCGGCTTTGAAAAGCTGACGCCCTTTACACCGGAGGAA

GAAAGTCGTCGAATACTGCATACAGTTTTTTGTACTCTAGAAGCAGCTCCCCATCAAAGC

TGA

>MT968035.1

ATGGCGGCGCGCGAACAGGCCAACTCCGTTCGCAGGAGCGGGTTTTTTAATCCGTTTATT

GGCAAGAGGCCATTTTTCAGGCCGGGAAGCGGGCAAACCGCGGAGACAGAGCGCCCAAGG

CCCCCCCAACACTCGTACTGCACAGAGGTGGGTAGCTTTAAGTTTATAGCTCCAAGATGC

CTCGATGAAGAAGCCCCCGCCGACCAGCGACGCGGTGTACACGTGGGCACCTTGGAGCGC

CCACCCAAAGTGTACTGCGATGGCTCAGAGTACGACGTGCTGAACTTTGCCTCCGGAGGT

TGCTGGCCTCGCAGGATTCGGGTTTGGAACGGCCAGGATTTTCGGGGCGATGGATTCAAC

CCCAGATTTGAGAGATTTCACGTGTACGACATAGTAGAGACTTCGGAGAGCGCCTCACAC

GATGACCCATCCAGGTTTGCTGAGCTATCCCGCCCAAGCGGGTCTGTGGTAACACTGCTG

GGAATGAGCGAGTGTGGAAAGCGAGTCGCGGTTCACGTCTATGGTGTGCGCCATTATTTT

TACATGGCAAAGGCGGAGGTGGATAGCGCTTGTGGAATAACCACCGAGGCAGAACTCGTG

CGCGCAATGGTGGACTGCGCGCACAGCTCGGCTTTGAGCGCGGCCCTGGGAAATGGCAAC

GGCGGCAAACAGAGCGGCGGCAGCGGCGGGGGATGGTGGGGCGGAAAGCACGTGTCTGCG

GACTGCTTCAAAGTGGAGACCGTGTGCCACACGACGCTGTACTACTTTGGATCTAAGCCA

GCTCTCTACTATAGAGTATCTGCCTCCAGCAGCCGCCTGGGAGGGTTCATCTGCGACAAC

TTTCACCCGGAGATTACAAAATTCGAGGGGAGCGTGGACGTGACCACGCGGCTGTTGTTG

GACAATGAAAATTTTACCAGTTTCGGGTGGTACCGCCTGCGACCCGGCACCCACGGAGAG

CGTGTTCAACTTCGCCCCGTTGAGCGACACGTCACCTCAAGCGACGTGGAGATTAACTGT

ACTCCCGATAACCTGGAGCCGATACCAGACGAGGCTGCCTGGCCCGACTATAAGCTCATG

TGCTTTGATATAGAGTGTAAAGCTGGAACGGGTAACGAAATGGCGTTCCCAGTGGCAACT

AACCAAGAGGACCTGGTCATCCAGATCTCCTGTCTGCTGTACTCGCTTGCTACTCAGAAC

CACGAACACACCCTGCTGTTTTCCCTCGGGTCATGCGATATCTCTGAGGAATACTCGTTT

GCATGCGTCCAGCGCGGCGAGCCCAGACCGACGGTTTTGGAGTTTGACAGCGAGTACGAG

CTGCTGGTTGCCTTCCTGACCTTTCTCAAGCAGTACTCTCCCGAGTTCGCCACCGGCTAC

AACATCGTTAATTTTGACTGGGCGTACATAGTTAACAAGGTAACGTCGGTGTATAACATC

AAGCTGGACGGGTACGGCAAGTTCAACAAAGGGGGGCTGTTTAAGGTGTGGGACATCGCC

ACGAACCATTTTCAGAAGAAGAGCAAGGTGAAAATCAATGGCCTGATATCTCTAGACATG

TATTCTGTGGCGACGGAAAAGCTAAAGCTACCCAGCTACAAACTCGACGCGGTCGTGGGA

GACGTCCTCGGCGAGCATAAGATAGACCTTCCCTATAAAGAAATACCCTCCTATTACGCG

GGAGGGCCTGACCGGAGGGGCGTAATAGGAGAGTATTGTATCCAGGACTCTAGGCTGGTG

GGCAAGCTGTTTTTTAAGTACCTCCCCCATCTGGAACTATCGGCGGTGGCCAAACTCGCC

CGTATCACCCTAACGCGGGTAATTTTTGACGGTCAGCAAATTCGCGTGTACACGTGCTTG

CTGAAACTCGCCCGCGAGAGAAATTTCATTTTGCCAGACAACAGACGCCGGTTTGACAGT

CAGGCAGACGCCGCGTCAGAGACTTCGGAGTTGGCTATGGATAGCCAAAGCCACGCCTTC

GACAGTACAGACGAACCCGACGGTGTGGACGGTACCCCGGACGCCGCAGGATCTGGCGCT

ACTTCTGAAAACGGAGGCGGGAAGCCCGGCGTCGGGAGGGCCGTGGGCTACCAGGGAGCA

AAGGTTCTAGACCCCGTATCCGGCTTTCATGTGGACCCCGTGGTTGTGTTTGACTTCGCT

AGCTTATACCCAAGCATTATCCAGGCCCATAACCTCTGTTTCACCACCCTGGCGCTCGAT

GAAGTGGATCTGGCCGGGCTTCAACCATCCGTCCACTACTCGACGTTCGAGGTGGGTGAC

CAAAAGTTATTTTTTGTCCACGCCCATATTCGCGAAAGCCTGCTTGGCATCTTGCTGCGC

GACTGGCTGGCCATGCGAAAGGCGGTGAGGGCGCGAATCCCCACCAGCACCCCCGAGGAG

GCAGTTTTACTAGATAAGCAGCAGTCTGCGATTAAGGTGATATGCAACTCGGTTTACGGA

TTCACGGGGGTGGCAAACGGCCTGTTGCCGTGTCTGAGGATAGCGGCTACCGTTACCACG

ATAGGACGCGACATGCTCCTCAAGACCCGAGATTACGTTCACTCTCGTTGGGCGACGCGC

GAGCTGCTGGAGGACAATTTTCCAGGGGCTATAGGTTTCCGAAACCACAAGCCTTACTCC

GTCAGGGTTATCTACGGAGACACCGACTCCGTGTTTATCAAGTTTGTGGGCCTGACGTAC

GAGGGGGTATCGGAGCTGGGGGATGCTATGTCGCGTCAGATTTCAGCGGACCTCTTTAGA

GCGCCCATCAAACTGGAGTGTGAGAAGACCTTTCAGCGACTGCTGCTGATCACCAAGAAG

AAGTACATAGGTGTCATAAACGGGGGGAAGATGCTCATGAAGGGGGTCGACCTGGTCCGC

AAAAATAACTGCTCTTTCATAAACTTGTACGCGCGACATCTGGTAGATCTTTTGTTGTAC

GACGAGGATGTGGCCACGGCGGCAGCAAAGGTGACAGACGTGCCTCCCGCAGAATGGGTG

GGGCGCCCGCTACCGAGCGGCTTTGACAAGTTTGGGCGAGTGCTGGTAGAGGCGTACAAC

CGTATCACTGCCCCCAACTTGGACGTGCGCGAGTTCGTTATGACTGCTGAGCTGAGCCGC

TCACCCGAATCGTATACCAACAAGCGCCTGCCGCACCTCACCGTCTACTTTAAGCTCGCC

ATGAGGAATGAAGAACTGCCCAGCGTAAAAGAGAGAATTCCGTATGTGATAGTTGCGCAG

ACCGAGGCCGCGGAACGCGAAGCGGGTGTAGTAAACTCAATGCGCGGTACCGCCCAAAAC

CCCGTGGTAACCAAGACCGCACGCCCCCAACCTAAACGCAAACTGCTGGTTTCCGACCTC

GCCGAAGACCCGACCTATGTTTCCGAGAATGACGTACCGCTAAACACAGACTACTATTTC

TCCCACCTGTTGGGTACCATAAGCGTGACCTTTAAGGCTCTATTCGGAAATGATGTGAGA

ACAACAGAAAATCTTTTAAAGCGGTTTATTCCGGAAACCCCCCACAAGACCCCCACGAAA

ACCCAGGCACTGCTTGAGCGCGCCGGCTTTGAAAAGCTGACGCCCTTTACACCGGAGGAA

GAAAGTCGTCGAATACTGCATACAGTTTTTTGTACTCTAGAAGCAGCTCCCCATCAAAGC

TGA

>MW396756.1

ATGGCGGCGCGCGAACAGGCCAACTCCGTTCGCAGGAGCGGGTTTTTTAATCCGTTTATT

GGCAAGAGGCCATTTTTCAGGCCGGGAAGCGGGCAGACCGCGGAGACAGAGCGCCCAAGG

CCCCCCCAACACTCGTACTGCACAGAGGTGGGTAGCTTTAAGTTTATAGCTCCAAGATGC

CTCGATGAAGAAGCCCCCGCCGACCAGCGACGCGGTGTACACGTGGGCACCTTGGAGCGC

CCACCCAAAGTGTACTGCGATGGCTCAGAGTACGACGTGCTGAACTTTGCCTCCGGAGGT

TGCTGGCCTCGCAGGATTCGGGTTTGGAACGGCCAGGATTTTCGGGGCGATGGATTCAAC

CCCAGATTTGAGAGATTTCACGTGTACGACATAGTAGAGACTTCGGAGAGCGCCTCACAC

GATGACCCATCCAGGTTTGCTGAGCTATCCCGCCCAAGCGGGTCTGTGGTAACACTGCTG

GGAATGAGCGAGTGTGGAAAGCGAGTCGCGGTTCACGTCTATGGTGTGCGCCATTATTTT

TACATGGCAAAGGCGGAGGTGGATAGCGCTTGTGGAATAACCACCGAGGCAGAACTCGTG

CGCGCAATGGTGGACTGCGCGCACAGCTCGGCTTTGAGCGCGGCCCTGGGAAATGGCAAC

GGCGGCAAACAGAGCGGCGGCAGCGGCGGGGGATGGTGGGGCGGAAAGCACGTGTCTGCG

GACTGCTTCAAAGTGGAGACCGTGTGCCACACGACGCTGTACTACTTTGGATCTAAGCCA

GCTCTCTACTATAGAGTATCTGCCTCCAGCAGCCGCCTGGGAGGGTTCATCTGCGACAAC

TTTCACCCGGAGATTACAAAATTCGAGGGGAGCGTGGACGTGACCACGCGGCTGTTGTTG

GACAATGAAAATTTTACCAGTTTCGGGTGGTACCGCCTGCGACCCGGCACCCACGGAGAG

CGTGTTCAACTTCGCCCCGTTGAGCGACACGTCACCTCAAGCGACGTGGAGATTAACTGT

ACTCCCGATAACCTGGAGCCGATACCAGACGAGGCTGCCTGGCCCGACTATAAGCTCATG

TGCTTTGATATAGAGTGTAAAGCTGGAACGGGTAACGAAATGGCGTTCCCAGTGGCAACT

AACCAAGAGGACCTGGTCATCCAGATCTCCTGTCTGCTGTACTCGCTTGCTACTCAGAAC

CACGAACACACCCTGCTGTTTTCCCTCGGGTCATGCGATATCTCTGAGGAATACTCGTTT

GCATGCGTCCAGCGCGGCGAGCCCAGACCGACGGTTTTGGAGTTTGACAGCGAGTACGAG

CTGCTGGTTGCCTTCCTGACCTTTCTCAAGCAGTACTCTCCCGAGTTCGCCACCGGCTAC

AACATCGTTAATTTTGACTGGGCGTACATAGTTAACAAGGTAACGTCGGTGTATAACATC

AAGCTGGACGGGTACGGCAAGTTCAACAAAGGGGGGCTGTTTAAGGTGTGGGACATCGCC

ACGAACCATTTTCAGAAGAAGAGCAAGGTGAAAATCAATGGCCTGATATCTCTAGACATG

TATTCTGTGGCGACGGAAAAGCTAAAGCTACCCAGCTACAAACTCGACGCGGTCGTGGGA

GACGTCCTCGGCGAGCATAAGATAGACCTTCCCTATAAAGAAATACCCTCCTATTACGCG

GGAGGGCCTGACCGGAGGGGCGTAATAGGAGAGTATTGTATCCAGGACTCTAGGCTGGTG

GGCAAGCTGTTTTTTAAGTACCTCCCCCATCTGGAACTATCGGCGGTGGCCAAACTCGCC

CGTATCACCCTAACGCGGGTAATTTTTGACGGTCAGCAAATTCGCGTGTACACGTGCTTG

CTGAAACTCGCCCGCGAGAGAAATTTCATTTTGCCAGACAACAGACGCCGGTTTGACAGT

CAGGCAGACGCCGCGTCAGAGACTTCGGAGTTGGCTATGGATAGCCAAAGCCACGCCTTC

GACAGTACAGACGAACCCGACGGTGTGGACGGTACCCCGGACGCCGCAGGATCTGGCGCT

ACTTCTGAAAACGGAGGCGGGAAGCCCGGCGTCGGGAGGGCCGTGGGCTACCAGGGAGCA

AAGGTTCTAGACCCCGTATCCGGCTTTCATGTGGACCCCGTGGTTGTGTTTGACTTCGCT

AGCTTATACCCAAGCATTATCCAGGCCCATAACCTCTGTTTCACCACCCTGGCGCTCGAT

GAAGTGGATCTGGCCGGGCTTCAACCATCCGTCAACTACTCGACGTTCGAGGTGGGTGAC

CAAAAGTTATTTTTTGTCCACGCCCATATTCGCGAAAGCCTGCTTGGCATCTTGCTGCGC

GACTGGCTGGCCATGCGAAAGGCGGTGAGGGCGCGAATCCCCACCAGCACCCCCGAGGAG

GCAGTTTTACTAGATAAGCAGCAGTCTGCGATTAAGGTGATATGCAACTCGGTTTACGGA

TTCACGGGGGTGGCAAACGGCCTGTTGCCGTGTCTGAGGATAGCGGCTACCGTTACCACG

ATAGGACGCGACATGCTCCTCAAGACCCGAGATTACGTTCACTCTCGTTGGGCGACGCGC

GAGCTGCTGGAGGACAATTTTCCAGGGGCTATAGGTTTCCGAAACCACAAGCCTTACTCC

GTCAGGGTTATCTACGGAGACACCGACTCCGTGTTTATCAAGTTTGTGGGCCTGACGTAC

GAGGGGGTATCGGAGCTGGGGGATGCTATGTCGCGTCAGATTTCAGCGGACCTCTTTAGA

GCGCCCATCAAACTGGAGTGTGAGAAGACCTTTCAGCGACTGCTGCTGATCACCAAGAAG

AAGTACATAGGTGTCATAAACGGGGGGAAGATGCTCATGAAGGGGGTCGACCTGGTCCGC

AAAAATAACTGCTCTTTCATAAACTTGTACGCGCGACATCTGGTAGATCTTTTGTTGTAC

GACGAGGATGTGGCCACGGCGGCAGCAGAGGTGACAGACGTGCCTCCCGCAGAATGGGTG

GGGCGCCCGCTACCGAGCGGCTTTGACAAGTTTGGGCGAGTGCTGGTAGAGGCGTACAAC

CGTATCACTGCCCCCAACTTGGACGTGCGCGAGTTCGTTATGACTGCTGAGCTGAGCCGC

TCACCCGAATCGTATACCAACAAGCGCCTGCCGCACCTCACCGTCTACTTTAAGCTCGCC

ATGAGGAATGAAGAACTGCCCAGCGTAAAAGAGAGAATTCCGTATGTGATAGTTGCGCAG

ACCGAGGCCGCGGAACGCGAAGCGGGTGTAGTAAACTCAATGCGCGGTACCGCCCAAAAC

CCCGTGGTAACCAAGACCGCACGCCCCCAACCTAAACGCAAACTGCTGGTTTCCGACCTC

GCCGAAGACCCGACCTATGTTTCCGAGAATGACGTACCGCTAAACACAGACTACTATTTC

TCCCACCTGTTGGGTACCATAAGCGTGACCTTTAAGGCTCTATTCGGAAATGATGTGAGA

ACAACAGAAAATCTTTTAAAGCGGTTTATTCCGGAAACCCCCCACAAGACCCCCACGAAA

ACCCAGGCACTGCTTGAGCGCGCCGGCTTTGAAAAGCTGACGCCCTTTACACCGGAGGAA

GAAAGTCGTCGAATACTGCATACAGTTTTTTGTACTCTAGAAGCAGCTCCCCATCAAAGC

TGA

>MW396757.1

ATGGCGGCGCGCGAACAGGCCAACTCCGTTCGCAGGAGCGGGTTTTTTAATCCGTTTATT

GGCAAGAGGCCATTTTTCAGGCCGGGAAGCGGGCAGACCGCGGAGACAGAGCGCCCAAGG

CCCCCCCAACACTCGTACTGCACAGAGGTGGGTAGCTTTAAGTTTATAGCTCCAAGATGC

CTCGATGAAGAAGCCCCCGCCGACCAGCGACGCGGTGTACACGTGGGCACCTTGGAGCGC

CCACCCAAAGTGTACTGCGATGGCTCAGAGTACGACGTGCTGAACTTTGCCTCCGGAGGT

TGCTGGCCTCGCAGGATTCGGGTTTGGAACGGCCAGGATTTTCGGGGCGATGGATTCAAC

CCCAGATTTGAGAGATTTCACGTGTACGACATAGTAGAGACTTCGGAGAGCGCCTCACAC

GATGACCCATCCAGGTTTGCTGAGCTATCCCGCCCAAGCGGGTCTGTGGTAACACTGCTG

GGAATGAGCGAGTGTGGAAAGCGAGTCGCGGTTCACGTCTATGGTGTGCGCCATTATTTT

TACATGGCAAAGGCGGAGGTGGATAGCGCTTGTGGAATAACCACCGAGGCAGAACTCGTG

CGCGCAATGGTGGACTGCGCGCACAGCTCGGCTTTGAGCGCGGCCCTGGGAAATGGCAAC

GGCGGCAAACAGAGCGGCGGCAGCGGCGGGGGATGGTGGGGCGGAAAGCACGTGTCTGCG

GACTGCTTCAAAGTGGAGACCGTGTGCCACACGACGCTGTACTACTTTGGATCTAAGCCA

GCTCTCTACTATAGAGTATCTGCCTCCAGCAGCCGCCTGGGAGGGTTCATCTGCGACAAC

TTTCACCCGGAGATTACAAAATTCGAGGGGAGCGTGGACGTGACCACGCGGCTGTTGTTG

GACAATGAAAATTTTACCAGTTTCGGGTGGTACCGCCTGCGACCCGGCACCCACGGAGAG

CGTGTTCAACTTCGCCCCGTTGAGCGACACGTCACCTCAAGCGACGTGGAGATTAACTGT

ACTCCCGATAACCTGGAGCCGATACCAGACGAGGCTGCCTGGCCCGACTATAAGCTCATG

TGCTTTGATATAGAGTGTAAAGCTGGAACGGGTAACGAAATGGCGTTCCCAGTGGCAACT

AACCAAGAGGACCTGGTCATCCAGATCTCCTGTCTGCTGTACTCGCTTGCTACTCAGAAC

CACGAACACACCCTGCTGTTTTCCCTCGGGTCATGCGATATCTCTGAGGAATACTCGTTT

GCATGCGTCCAGCGCGGCGAGCCCAGACCGACGGTTTTGGAGTTTGACAGCGAGTACGAG

CTGCTGGTTGCCTTCCTGACCTTTCTCAAGCAGTACTCTCCCGAGTTCGCCACCGGCTAC

AACATCGTTAATTTTGACTGGGCGTACATAGTTAACAAGGTAACGTCGGTGTATAACATC

AAGCTGGACGGGTACGGCAAGTTCAACAAAGGGGGGCTGTTTAAGGTGTGGGACATCGCC

ACGAACCATTTTCAGAAGAAGAGCAAGGTGAAAATCAATGGCCTGATATCTCTAGACATG

TATTCTGTGGCGACGGAAAAGCTAAAGCTACCCAGCTACAAACTCGACGCGGTCGTGGGA

GACGTCCTCGGCGAGCATAAGATAGACCTTCCCTATAAAGAAATACCCTCCTATTACGCG

GGAGGGCCTGACCGGAGGGGCGTAATAGGAGAGTATTGTATCCAGGACTCTAGGCTGGTG

GGCAAGCTGTTTTTTAAGTACCTCCCCCATCTGGAACTATCGGCGGTGGCCAAACTCGCC

CGTATCACCCTAACGCGGGTAATTTTTGACGGTCAGCAAATTCGCGTGTACACGTGCTTG

CTGAAACTCGCCCGCGAGAGAAATTTCATTTTGCCAGACAACAGACGCCGGTTTGACAGT

CAGGCAGACGCCGCGTCAGAGACTTCGGAGTTGGCTATGGATAGCCAAAGCCACGCCTTC

GACAGTACAGACGAACCCGACGGTGTGGACGGTACCCCGGACGCCGCAGGATCTGGCGCT

ACTTCTGAAAACGGAGGCGGGAAGCCCGGCGTCGGGAGGGCCGTGGGCTACCAGGGAGCA

AAGGTTCTAGACCCCGTATCCGGCTTTCATGTGGACCCCGTGGTTGTGTTTGACTTCGCT

AGCTTATACCCAAGCATTATCCAGGCCCATAACCTCTGTTTCACCACCCTGGCGCTCGAT

GAAGTGGATCTGGCCGGGCTTCAACCATCCGTCAACTACTCGACGTTCGAGGTGGGTGAC

CAAAAGTTATTTTTTGTCCACGCCCATATTCGCGAAAGCCTGCTTGGCATCTTGCTGCGC

GACTGGCTGGCCATGCGAAAGGCGGTGAGGGCGCGAATCCCCACCAGCACCCCCGAGGAG

GCAGTTTTACTAGATAAGCAGCAGTCTGCGATTAAGGTGATATGCAACTCGGTTTACGGA

TTCACGGGGGTGGCAAACGGCCTGTTGCCGTGTCTGAGGATAGCGGCTACCGTTACCACG

ATAGGACGCGACATGCTCCTCAAGACCCGAGATTACGTTCACTCTCGTTGGGCGACGCGC

GAGCTGCTGGAGGACAATTTTCCAGGGGCTATAGGTTTCCGAAACCACAAGCCTTACTCC

GTCAGGGTTATCTACGGAGACACCGACTCCGTGTTTATCAAGTTTGTGGGCCTGACGTAC

GAGGGGGTATCGGAGCTGGGGGATGCTATGTCGCGTCAGATTTCAGCGGACCTCTTTAGA

GCGCCCATCAAACTGGAGTGTGAGAAGACCTTTCAGCGACTGCTGCTGATCACCAAGAAG

AAGTACATAGGTGTCATAAACGGGGGGAAGATGCTCATGAAGGGGGTCGACCTGGTCCGC

AAAAATAACTGCTCTTTCATAAACTTGTACGCGCGACATCTGGTAGATCTTTTGTTGTAC

GACGAGGATGTGGCCACGGCGGCAGCAGAGGTGACAGACGTGCCTCCCGCAGAATGGGTG

GGGCGCCCGCTACCGAGCGGCTTTGACAAGTTTGGGCGAGTGCTGGTAGAGGCGTACAAC

CGTATCACTGCCCCCAACTTGGACGTGCGCGAGTTCGTTATGACTGCTGAGCTGAGCCGC

TCACCCGAATCGTATACCAACAAGCGCCTGCCGCACCTCACCGTCTACTTTAAGCTCGCC

ATGAGGAATGAAGAACTGCCCAGCGTAAAAGAGAGAATTCCGTATGTGATAGTTGCGCAG

ACCGAGGCCGCGGAACGCGAAGCGGGTGTAGTAAACTCAATGCGCGGTACCGCCCAAAAC

CCCGTGGTAACCAAGACCGCACGCCCCCAACCTAAACGCAAACTGCTGGTTTCCGACCTC

GCCGAAGACCCGACCTATGTTTCCGAGAATGACGTACCGCTAAACACAGACTACTATTTC

TCCCACCTGTTGGGTACCATAAGCGTGACCTTTAAGGCTCTATTCGGAAATGATGTGAGA

ACAACAGAAAATCTTTTAAAGCGGTTTATTCCGGAAACCCCCCACAAGACCCCCACGAAA

ACCCAGGCACTGCTTGAGCGCGCCGGCTTTGAAAAGCTGACGCCCTTTACACCGGAGGAA

GAAAGTCGTCGAATACTGCATACAGTTTTTTGTACTCTAGAAGCAGCTCCCCATCAAAGC

TGA

>MW396758.1

ATGGCGGCGCGCGAACAGGCCAACTCCGTTCGCAGGAGCGGGTTTTTTAATCCGTTTATT

GGCAAGAGGCCATTTTTCAGGCCGGGAAGCGGGCAGACCGCGGAGACAGAGCGCCCAAGG

CCCCCCCAACACTCGTACTGCACAGAGGTGGGTAGCTTTAAGTTTATAGCTCCAAGATGC

CTCGATGAAGAAGCCCCCGCCGACCAGCGACGCGGTGTACACGTGGGCACCTTGGAGCGC

CCACCCAAAGTGTACTGCGATGGCTCAGAGTACGACGTGCTGAACTTTGCCTCCGGAGGT

TGCTGGCCTCGCAGGATTCGGGTTTGGAACGGCCAGGATTTTCGGGGCGATGGATTCAAC

CCCAGATTTGAGAGATTTCACGTGTACGACATAGTAGAGACTTCGGAGAGCGCCTCACAC

GATGACCCATCCAGGTTTGCTGAGCTATCCCGCCCAAGCGGGTCTGTGGTAACACTGCTG

GGAATGAGCGAGTGTGGAAAGCGAGTCGCGGTTCACGTCTATGGTGTGCGCCATTATTTT

TACATGGCAAAGGCGGAGGTGGATAGCGCTTGTGGAATAACCACCGAGGCAGAACTCGTG

CGCGCAATGGTGGACTGCGCGCACAGCTCGGCTTTGAGCGCGGCCCTGGGAAATGGCAAC

GGCGGCAAACAGAGCGGCGGCAGCGGCGGGGGATGGTGGGGCGGAAAGCACGTGTCTGCG

GACTGCTTCAAAGTGGAGACCGTGTGCCACACGACGCTGTACTACTTTGGATCTAAGCCA

GCTCTCTACTATAGAGTATCTGCCTCCAGCAGCCGCCTGGGAGGGTTCATCTGCGACAAC

TTTCACCCGGAGATTACAAAATTCGAGGGGAGCGTGGACGTGACCACGCGGCTGTTGTTG

GACAATGAAAATTTTACCAGTTTCGGGTGGTACCGCCTGCGACCCGGCACCCACGGAGAG

CGTGTTCAACTTCGCCCCGTTGAGCGACACGTCACCTCAAGCGACGTGGAGATTAACTGT

ACTCCCGATAACCTGGAGCCGATACCAGACGAGGCTGCCTGGCCCGACTATAAGCTCATG

TGCTTTGATATAGAGTGTAAAGCTGGAACGGGTAACGAAATGGCGTTCCCAGTGGCAACT

AACCAAGAGGACCTGGTCATCCAGATCTCCTGTCTGCTGTACTCGCTTGCTACTCAGAAC

CACGAACACACCCTGCTGTTTTCCCTCGGGTCATGCGATATCTCTGAGGAATACTCGTTT

GCATGCGTCCAGCGCGGCGAGCCCAGACCGACGGTTTTGGAGTTTGACAGCGAGTACGAG

CTGCTGGTTGCCTTCCTGACCTTTCTCAAGCAGTACTCTCCCGAGTTCGCCACCGGCTAC

AACATCGTTAATTTTGACTGGGCGTACATAGTTAACAAGGTAACGTCGGTGTATAACATC

AAGCTGGACGGGTACGGCAAGTTCAACAAAGGGGGGCTGTTTAAGGTGTGGGACATCGCC

ACGAACCATTTTCAGAAGAAGAGCAAGGTGAAAATCAATGGCCTGATATCTCTAGACATG

TATTCTGTGGCGACGGAAAAGCTAAAGCTACCCAGCTACAAACTCGACGCGGTCGTGGGA

GACGTCCTCGGCGAGCATAAGATAGACCTTCCCTATAAAGAAATACCCTCCTATTACGCG

GGAGGGCCTGACCGGAGGGGCGTAATAGGAGAGTATTGTATCCAGGACTCTAGGCTGGTG

GGCAAGCTGTTTTTTAAGTACCTCCCCCATCTGGAACTATCGGCGGTGGCCAAACTCGCC

CGTATCACCCTAACGCGGGTAATTTTTGACGGTCAGCAAATTCGCGTGTACACGTGCTTG

CTGAAACTCGCCCGCGAGAGAAATTTCATTTTGCCAGACAACAGACGCCGGTTTGACAGT

CAGGCAGACGCCGCGTCAGAGACTTCGGAGTTGGCTATGGATAGCCAAAGCCACGCCTTC

GACAGTACAGACGAACCCGACGGTGTGGACGGTACCCCGGACGCCGCAGGATCTGGCGCT

ACTTCTGAAAACGGAGGCGGGAAGCCCGGCGTCGGGAGGGCCGTGGGCTACCAGGGAGCA

AAGGTTCTAGACCCCGTATCCGGCTTTCATGTGGACCCCGTGGTTGTGTTTGACTTCGCT

AGCTTATACCCAAGCATTATCCAGGCCCATAACCTCTGTTTCACCACCCTGGCGCTCGAT

GAAGTGGATCTGGCCGGGCTTCAACCATCCGTCAACTACTCGACGTTCGAGGTGGGTGAC

CAAAAGTTATTTTTTGTCCACGCCCATATTCGCGAAAGCCTGCTTGGCATCTTGCTGCGC

GACTGGCTGGCCATGCGAAAGGCGGTGAGGGCGCGAATCCCCACCAGCACCCCCGAGGAG

GCAGTTTTACTAGATAAGCAGCAGTCTGCGATTAAGGTGATATGCAACTCGGTTTACGGA

TTCACGGGGGTGGCAAACGGCCTGTTGCCGTGTCTGAGGATAGCGGCTACCGTTACCACG

ATAGGACGCGACATGCTCCTCAAGACCCGAGATTACGTTCACTCTCGTTGGGCGACGCGC

GAGCTGCTGGAGGACAATTTTCCAGGGGCTATAGGTTTCCGAAACCACAAGCCTTACTCC

GTCAGGGTTATCTACGGAGACACCGACTCCGTGTTTATCAAGTTTGTGGGCCTGACGTAC

GAGGGGGTATCGGAGCTGGGGGATGCTATGTCGCGTCAGATTTCAGCGGACCTCTTTAGA

GCGCCCATCAAACTGGAGTGTGAGAAGACCTTTCAGCGACTGCTGCTGATCACCAAGAAG

AAGTACATAGGTGTCATAAACGGGGGGAAGATGCTCATGAAGGGGGTCGACCTAGTCCGC

AAAAATAACTGCTCTTTCATAAACTTGTACGCGCGACATCTGGTAGATCTTTTGTTGTAC

GACGAGGATGTGGCCACGGCGGCAGCAGAGGTGACAGACGTGCCTCCCGCAGAATGGGTG

GGGCGCCCGCTACCGAGCGGCTTTGACAAGTTTGGGCGAGTGCTGGTAGAGGCGTACAAC

CGTATCACTGCCCCCAACTTGGACGTGCGCGAGTTCGTTATGACTGCTGAGCTGAGCCGC

TCACCCGAATCGTATACCAACAAGCGCCTGCCGCACCTCACCGTCTACTTTAAGCTCGCC

ATGAGGAATGAAGAACTGCCCAGCGTAAAAGAGAGAATTCCGTATGTGATAGTTGCGCAG

ACCGAGGCCGCGGAACGCGAAGCGGGTGTAGTAAACTCAATGCGCGGTACCGCCCAAAAC

CCCGTGGTAACCAAGACCGCACGCCCCCAACCTAAACGCAAACTGCTGGTTTCCGACCTC

GCCGAAGACCCGACCTATGTTTCCGAGAATGACGTACCGCTAAACACAGACTACTATTTC

TCCCACCTGTTGGGTACCATAAGCGTGACCTTTAAGGCTCTATTCGGAAATGATGTGAGA

ACAACAGAAAATCTTTTAAAGCGGTTTATTCCGGAAACCCCCCACAAGACCCCCACGAAA

ACCCAGGCACTGCTTGAGCGCGCCGGCTTTGAAAAGCTGACGCCCTTTACACCGGAGGAA

GAAAGTCGTCGAATACTGCATACAGTTTTTTGTACTCTAGAAGCAGCTCCCCATCAAAGC

TGA

>MW396759.1

ATGGCGGCGCGCGAACAGGCCAACTCCGTTCGCAGGAGCGGGTTTTTTAATCCGTTTATT

GGCAAGAGGCCATTTTTCAGGCCGGGAAGCGGGCAGACCGCGGAGACAGAGCGCCCAAGG

CCCCCCCAACACTCGTACTGCACAGAGGTGGGTAGCTTTAAGTTTATAGCTCCAAGATGC

CTCGATGAAGAAGCCCCCGCCGACCAGCGACGCGGTGTACACGTGGGCACCTTGGAGCGC

CCACCCAAAGTGTACTGCGATGGCTCAGAGTACGACGTGCTGAACTTTGCCTCCGGAGGT

TGCTGGCCTCGCAGGATTCGGGTTTGGAACGGCCAGGATTTTCGGGGCGATGGATTCAAC

CCCAGATTTGAGAGATTTCACGTGTACGACATAGTAGAGACTTCGGAGAGCGCCTCACAC

GATGACCCATCCAGGTTTGCTGAGCTATCCCGCCCAAGCGGGTCTGTGGTAACACTGCTG

GGAATGAGCGAGTGTGGAAAGCGAGTCGCGGTTCACGTCTATGGTGTGCGCCATTATTTT

TACATGGCAAAGGCGGAGGTGGATAGCGCTTGTGGAATAACCACCGAGGCAGAACTCGTG

CGCGCAATGGTGGACTGCGCGCACAGCTCGGCTTTGAGCGCGGCCCTGGGAAATGGCAAC

GGCGGCAAACAGAGCGGCGGCAGCGGCGGGGGATGGTGGGGCGGAAAGCACGTGTCTGCG

GACTGCTTCAAAGTGGAGACCGTGTGCCACACGACGCTGTACTACTTTGGATCTAAGCCA

GCTCTCTACTATAGAGTATCTGCCTCCAGCAGCCGCCTGGGAGGGTTCATCTGCGACAAC

TTTCACCCGGAGATTACAAAATTCGAGGGGAGCGTGGACGTGACCACGCGGCTGTTGTTG

GACAATGAAAATTTTACCAGTTTCGGGTGGTACCGCCTGCGACCCGGCACCCACGGAGAG

CGTGTTCAACTTCGCCCCGTTGAGCGACACGTCACCTCAAGCGACGTGGAGATTAACTGT

ACTCCCGATAACCTGGAGCCGATACCAGACGAGGCTGCCTGGCCCGACTATAAGCTCATG

TGCTTTGATATAGAGTGTAAAGCTGGAACGGGTAACGAAATGGCGTTCCCAGTGGCAACT

AACCAAGAGGACCTGGTCATCCAGATCTCCTGTCTGCTGTACTCGCTTGCTACTCAGAAC

CACGAACACACCCTGCTGTTTTCCCTCGGGTCATGCGATATCTCTGAGGAATACTCGTTT

GCATGCGTCCAGCGCGGCGAGCCCAGACCGACGGTTTTGGAGTTTGACAGCGAGTACGAG

CTGCTGGTTGCCTTCCTGACCTTTCTCAAGCAGTACTCTCCCGAGTTCGCCACCGGCTAC

AACATCGTTAATTTTGACTGGGCGTACATAGTTAACAAGGTAACGTCGGTGTATAACATC

AAGCTGGACGGGTACGGCAAGTTCAACAAAGGGGGGCTGTTTAAGGTGTGGGACATCGCC

ACGAACCATTTTCAGAAGAAGAGCAAGGTGAAAATCAATGGCCTGATATCTCTAGACATG

TATTCTGTGGCGACGGAAAAGCTAAAGCTACCCAGCTACAAACTCGACGCGGTCGTGGGA

GACGTCCTCGGCGAGCATAAGATAGACCTTCCCTATAAAGAAATACCCTCCTATTACGCG

GGAGGGCCTGACCGGAGGGGCGTAATAGGAGAGTATTGTATCCAGGACTCTAGGCTGGTG

GGCAAGCTGTTTTTTAAGTACCTCCCCCATCTGGAACTATCGGCGGTGGCCAAACTCGCC

CGTATCACCCTAACGCGGGTAATTTTTGACGGTCAGCAAATTCGCGTGTACACGTGCTTG

CTGAAACTCGCCCGCGAGAGAAATTTCATTTTGCCAGACAACAAACGCCGGTTTGACAGT

CAGGCAGACGCCGCGTCAGAGACTTCGGAGTTGGCTATGGATAGCCAAAGCCACGCCTTC

GACAGTACAGACGAACCCGACGGTGTGGACGGTACCCCGGACGCCGCAGGATCTGGCGCT

ACTTCTGAAAACGGAGGCGGGAAGCCCGGCGTCGGGAGGGCCGTGGGCTACCAGGGAGCA

AAGGTTCTAGACCCCGTATCCGGCTTTCATGTGGACCCCGTGGTTGTGTTTGACTTCGCT

AGCTTATACCCAAGCATTATCCAGGCCCATAACCTCTGTTTCACCACCCTGGCGCTCGAT

GAAGTGGATCTGGCCGGGCTTCAACCATCCGTCAACTACTCGACGTTCGAGGTGGGTGAC

CAAAAGTTATTTTTTGTCCACGCCCATATTCGCGAAAGCCTGCTTGGCATCTTGCTGCGC

GACTGGCTGGCCATGCGAAAGGCGGTGAGGGCGCGAATCCCCACCAGCACCCCCGAGGAG

GCAGTTTTACTAGATAAGCAGCAGTCTGCGATTAAGGTGATATGCAACTCGGTTTACGGA

TTCACGGGGGTGGCAAACGGCCTGTTGCCGTGTCTGAGGATAGCGGCTACCGTTACCACG

ATAGGACGCGACATGCTCCTCAAGACCCGAGATTACGTTCACTCTCGTTGGGCGACGCGC

GAGCTGCTGGAGGACAATTTTCCAGGGGCTATAGGTTTCCGAAACCACAAGCCTTACTCC

GTCAGGGTTATCTACGGAGACACCGACTCCGTGTTTATCAAGTTTGTGGGCCTGACGTAC

GAGGGGGTATCGGAGCTGGGGGATGCTATGTCGCGTCAGATTTCAGCGGACCTCTTTAGA

GCGCCCATCAAACTGGAGTGTGAGAAGACCTTTCAGCGACTGCTGCTGATCACCAAGAAG

AAGTACATAGGTGTCATAAACGGGGGGAAGATGCTCATGAAGGGGGTCGACCTAGTCCGC

AAAAATAACTGCTCTTTCATAAACTTGTACGCGCGACATCTGGTAGATCTTTTGTTGTAC

GACGAGGATGTGGCCACGGCGGCAGCAGAGGTGACAGACGTGCCTCCCGCAGAATGGGTG

GGGCGCCCGCTACCGAGCGGCTTTGACAAGTTTGGGCGAGTGCTGGTAGAGGCGTACAAC

CGTATCACTGCCCCCAACTTGGACGTGCGCGAGTTCGTTATGACTGCTGAGCTGAGCCGC

TCACCCGAATCGTATACCAACAAGCGCCTGCCGCACCTCACCGTCTACTTTAAGCTCGCC

ATGAGGAATGAAGAACTGCCCAGCGTAAAAGAGAGAATTCCGTATGTGATAGTTGCGCAG

ACCGAGGCCGCGGAACGCGAAGCGGGTGTAGTAAACTCAATGCGCGGTACCGCCCAAAAC

CCCGTGGTAACCAAGACCGCACGCCCCCAACCTAAACGCAAACTGCTGGTTTCCGACCTC

GCCGAAGACCCGACCTATGTTTCCGAGAATGACGTACCGCTAAACACAGACTACTATTTC

TCCCACCTGTTGGGTACCATAAGCGTGACCTTTAAGGCTCTATTCGGAAATGATGTGAGA

ACAACAGAAAATCTTTTAAAGCGGTTTATTCCGGAAACCCCCCACAAGACCCCCACGAAA

ACCCAGGCACTGCTTGAGCGCGCCGGCTTTGAAAAGCTGACGCCCTTTACACCGGAGGAA

GAAAGTCGTCGAATACTGCATACAGTTTTTTGTACTCTAGAAGCAGCTCCCCATCAAAGC

TGA

>MW396760.1

ATGGCGGCGCGCGAACAGGCCAACTCCGTTCGCAGGAGCGGGTTTTTTAATCCGTTTATT

GGCAAGAGGCCATTTTTCAGGCCGGGAAGCGGGCAGACCGCGGAGACAGAGCGCCCAAGG

CCCCCCCAACACTCGTACTGCACAGAGGTGGGTAGCTTTAAGTTTATAGCTCCAAGATGC

CTCGATGAAGAAGCCCCCGCCGACCAGCGACGCGGTGTACACGTGGGCACCTTGGAGCGC

CCACCCAAAGTGTACTGCGATGGCTCAGAGTACGACGTGCTGAACTTTGCCTCCGGAGGT

TGCTGGCCTCGCAGGATTCGGGTTTGGAACGGCCAGGATTTTCGGGGCGATGGATTCAAC

CCCAGATTTGAGAGATTTCACGTGTACGACATAGTAGAGACTTCGGAGAGCGCCTCACAC

GATGACCCATCCAGGTTTGCTGAGCTATCCCGCCCAAGCGGGTCTGTGGTAACACTGCTG

GGAATGAGCGAGTGTGGAAAGCGAGTCGCGGTTCACGTCTATGGTGTGCGCCATTATTTT

TACATGGCAAAGGCGGAGGTGGATAGCGCTTGTGGAATAACCACCGAGGCAGAACTCGTG

CGCGCAATGGTGGACTGCGCGCACAGCTCGGCTTTGAGCGCGGCCCTGGGAAATGGCAAC

GGCGGCAAACAGAGCGGCGGCAGCGGCGGGGGATGGTGGGGCGGAAAGCACGTGTCTGCG

GACTGCTTCAAAGTGGAGACCGTGTGCCGCACGACGCTGTACTACTTTGGATCTAAGCCA

GCTCTCTACTATAGAGTATCTGCCTCCAGCAGCCGCCTGGGAGGGTTCATCTGCGACAAC

TTTCACCCGGAGATTACAAAATTCGAGGGGAGCGTGGACGTGACCACGCGGCTGTTGTTG

GACAATGAAAATTTTACCAGTTTCGGGTGGTACCGCCTGCGACCCGGCACCCACGGAGAG

CGTGTTCAACTTCGCCCCGTTGAGCGACACGTCACCTCAAGCGACGTGGAGATTAACTGT

ACTCCCGATAACCTGGAGCCGATACCAGACGAGGCTGCCTGGCCCGACTATAAGCTCATG

TGCTTTGATATAGAGTGTAAAGCTGGAACGGGTAACGAAATGGCGTTCCCAGTGGCAACT

AACCAAGAGGACCTGGTCATCCAGATCTCCTGTCTGCTGTACTCGCTTGCTACTCAGAAC

CACGAACACACCCTGCTGTTTTCCCTCGGGTCATGCGATATCTCTGAGGAATACTCGTTT

GCATGCGTCCAGCGCGGCGAGCCCAGACCGACGGTTTTGGAGTTTGACAGCGAGTACGAG

CTGCTGGTTGCCTTCCTGACCTTTCTCAAGCAGTACTCTCCCGAGTTCGCCACCGGCTAC

AACATCGTTAATTTTGACTGGGCGTACATAGTTAACAAGGTAACGTCGGTGTATAACATC

AAGCTGGACGGGTACGGCAAGTTCAACAAAGGGGGGCTGTTTAAGGTGTGGGACATCGCC

ACGAACCATTTTCAGAAGAAGAGCAAGGTGAAAATCAATGGCCTGATATCTCTAGACATG

TATTCTGTGGCGACGGAAAAGCTAAAGCTACCCAGCTACAAACTCGACGCGGTCGTGGGA

GACGTCCTCGGCGAGCATAAGATAGACCTTCCCTATAAAGAAATACCCTCCTATTACGCG

GGAGGGCCTGACCGGAGGGGCGTAATAGGAGAGTATTGTATCCAGGACTCTAGGCTGGTG

GGCAAGCTGTTTTTTAAGTACCTCCCCCATCTGGAACTATCGGCGGTGGCCAAACTCGCC

CGTATCACCCTAACGCGGGTAATTTTTGACGGTCAGCAAATTCGCGTGTACACGTGCTTG

CTGAAACTCGCCCGCGAGAGAAATTTCATTTTGCCAGACAACAAACGCCGGTTTGACAGT

CAGGCAGACGCCGCGTCAGAGACTTCGGAGTTGGCTATGGATAGCCAAAGCCACGCCTTC

GACAGTACAGACGAACCCGACGGTGTGGACGGTACCCCGGACGCCGCAGGATCTGGCGCT

ACTTCTGAAAACGGAGGCGGGAAGCCCGGCGTCGGGAGGGCCGTGGGCTACCAGGGAGCA

AAGGTTCTAGACCCCGTATCCGGCTTTCATGTGGACCCCGTGGTTGTGTTTGACTTCGCT

AGCTTATACCCAAGCATTATCCAGGCCCATAACCTCTGTTTCACCACCCTGGCGCTCGAT

GAAGTGGATCTGGCCGGGCTTCAACCATCCGTCAACTACTCGACGTTCGAGGTGGGTGAC

CAAAAGTTATTTTTTGTCCACGCCCATATTCGCGAAAGCCTGCTTGGCATCTTGCTGCGC

GACTGGCTGGCCATGCGAAAGGCGGTGAGGGCGCGAATCCCCACCAGCACCCCCGAGGAG

GCAGTTTTACTAGATAAGCAGCAGTCTGCGATTAAGGTGATATGCAACTCGGTTTACGGA

TTCACGGGGGTGGCAAACGGCCTGTTGCCGTGTCTGAGGATAGCGGCTACCGTTACCACG

ATAGGACGCGACATGCTCCTCAAGACCCGAGATTACGTTCACTCTCGTTGGGCGACGCGC

GAGCTGCTGGAGGACAATTTTCCAGGGGCTATAGGTTTCCGAAACCACAAGCCTTACTCC

GTCAGGGTTATCTACGGAGACACCGACTCCGTGTTTATCAAGTTTGTGGGCCTGACGTAC

GAGGGGGTATCGGAGCTGGGGGATGCTATGTCGCGTCAGATTTCAGCGGACCTCTTTAGA

GCGCCCATCAAACTGGAGTGTGAGAAGACCTTTCAGCGACTGCTGCTGATCACCAAGAAG

AAGTACATAGGTGTCATAAACGGGGGGAAGATGCTCATGAAGGGGGTCGACCTAGTCCGC

AAAAATAACTGCTCTTTCATAAACTTGTACGCGCGACATCTGGTAGATCTTTTGTTGTAC

GACGAGGATGTGGCCACGGCGGCAGCAGAGGTGACAGACGTGCCTCCCGCAGAATGGGTG

GGGCGCCCGCTACCGAGCGGCTTTGACAAGTTTGGGCGAGTGCTGGTAGAGGCGTACAAC

CGTATCACTGCCCCCAACTTGGACGTGCGCGAGTTCGTTATGACTGCTGAGCTGAGCCGC

TCACCCGAATCGTATACCAACAAGCGCCTGCCGCACCTCACCGTCTACTTTAAGCTCGCC

ATGAGGAATGAAGAACTGCCCAGCGTAAAAGAGAGAATTCCGTATGTGATAGTTGCGCAG

ACCGAGGCCGCGGAACGCGAAGCGGGTGTAGTAAACTCAATGCGCGGTACCGCCCAAAAC

CCCGTGGTAACCAAGACCGCACGCCCCCAACCTAAACGCAAACTGCTGGTTTCCGACCTC

GCCGAAGACCCGACCTATGTTTCCGAGAATGACGTACCGCTAAACACAGACTACTATTTC

TCCCACCTGTTGGGTACCATAAGCGTGACCTTTAAGGCTCTATTCGGAAATGATGTGAGA

ACAACAGAAAATCTTTTAAAGCGGTTTATTCCGGAAACCCCCCACAAGACCCCCACGAAA

ACCCAGGCACTGCTTGAGCGCGCCGGCTTTGAAAAGCTGACGCCCTTTACACCGGAGGAA

GAAAGTCGTCGAATACTGCATACAGTTTTTTGTACTCTAGAAGCAGCTCCCCATCAAAGC

TGA

>PP856397.1

ATGGCGGCGCGCGAACAGGCCAACTCCGTTCGCAGGAGCGGGTTTTTTAATCCGTTTATT

GGCAAGAGGCCATTTTTCAGGCCGGGAAGCGGGCAGACCGCGGAGACAGAGCGCCCAAGG

CCCCCCCAACACTCGTACTGCACAGAGGTGGGTAGCTTTAAGTTTATAGCTCCAAGATGC

CTCGATGAAGAAGCCCCCGCCGACCAGCGACGCGGTGTACACGTGGGCACCTTGGAGCGC

CCACCCAAAGTGTACTGCGATGGCTCAGAGTACGACGTGCTGAACTTTGCCTCCGGAGGT

TGCTGGCCTCGCAGGATTCGGGTTTGGAACGGCCAGGATTTTCGGGGCGATGGATTCAAC

CCCAGATTTGAGAGATTTCACGTGTACGACATAGTAGAGACTTCGGAGAGCGCCTCACAC

GATGACCCATCCAGGTTTGCTGAGCTATCCCGCCCAAGCGGGTCTGTGGTAACACTGCTG

GGAATGAGCGAGTGTGGAAAGCGAGTCGCGGTTCACGTATATGGTGTGCGCCATTATTTT

TACATGGCAAAGGCGGAGGTGGATAGCGCTTGTGGAATAACCACCGAGGCAGAACTCGTG

CGCGCAATGGTGGACTGCGCGCACAGCTCGGCTTTGAGCGCGGCCCTGGGAAATGGCAAC

GGCGGCAAACAGAGCGGCGGCAGCGGCGGGGGATGGTGGGGCGGAAAGCACGTGTCTGCG

GACTGCTTCAAAGTGGAGACCGTGTGCCACACGACGCTGTACTACTTTGGATCTAAGCCA

GCTCTCTACTATAGAGTATCTGCCTCCAGCAGCCGCCTGGGAGGGTTCATCTGCGACAAC

TTTCACCCGGAGATTACAAAATTCGAGGGGAGCGTGGACGTGACCACGCGGCTGTTGTTG

GACAATGAAAATTTTACCAGTTTCGGGTGGTACCGCCTGCGACCCGGCACCCACGGAGAG

CGTGTTCAACTTCGCCCCGTTGAGCGACACGTCACCTCAAGCGACGTGGAGATTAACTGT

ACTCCCGATAACCTGGAGCCGATACCAGACGAGGCTGCCTGGCCCGACTATAAGCTCATG

TGCTTTGATATAGAGTGTAAAGCTGGAACGGGTAACGAAATGGCGTTCCCAGTGGCAACT

AACCAAGAGGACCTGGTCATCCAGATCTCCTGTCTGCTGTACTCGCTTGCTACTCAGAAC

CACGAACACACCCTGCTGTTTTCCCTCGGGTCATGCGATATCTCTGAGGAATACTCGTTT

GCATGCGTCCAGCGCGGCGAGCCCAGACCGACGGTTTTGGAGTTTGACAGCGAGTACGAG

CTGCTGGTTGCCTTCCTGACCTTTCTCAAGCAGTACTCTCCCGAGTTCGCCACCGGCTAC

AACATCGTTAATTTTGACTGGGCGTACATAGTTAACAAGGTAACGTCGGTGTATAACATC

AAGCTGGACGGGTACGGCAAGTTCAACAAAGGGGGGCTGTTTAAGGTGTGGGACATCGCC

ACGAACCATTTTCAGAAGAAGAGCAAGGTGAAAATCAATGGCCTGATATCTCTAGACATG

TATTCTGTGGCGACGGAAAAGCTAAAGCTACCCAGCTACAAACTCGACGCGGTCGTGGGA

GACGTCCTCGGCGAGCATAAGATAGACCTTCCCTATAAAGAAATACCCTCCTATTACGCG

GGAGGGCCTGACCGGAGGGGCGTAATAGGAGAGTATTGTATCCAGGACTCTAGGCTGGTG

GGCAAGCTGTTTTTTAAGTACCTCCCCCATCTGGAACTATCGGCGGTGGCCAAACTCGCC

CGTATCACCCTAACGCGGGTAATTTTTGACGGTCAGCAAATTCGCGTGTACACGTGCTTG

CTGAAACTCGCCCGCGAGAGAAATTTCATTTTGCCAGACAACAGACGCCGGTTTGACAGT

CAGGCAGACGCCGCGTCAGAGACTTCGGAGTTGGCTATGGATAGCCAAAGCCACGCCTTC

GACAGTACAGACGAACCCGACGGTGTGGACGGTACCCCGGACGCCGCAGGATCTGGCGCT

ACTTCTGAAAACGGAGGCGGGAAGCCCGGCGTCGGGAGGGCCGTGGGCTACCAGGGAGCA

AAGGTTCTAGACCCCGTATCCGGCTTTCATGTGGACCCCGTGGTTGTGTTTGACTTCGCT

AGCTTATACCCAAGCATTATCCAGGCCCATAACCTCTGTTTCACCACCCTGGCGCTCGAT

GAAGTGGATCTGGCCGGGCTTCAACCATCCGTCGACTACTCGACGTTCGAGGTGGGTGAC

CAAAAGTTATTTTTTGTCCACGCCCATATTCGCGAAAGCCTGCTTGGCATCTTGCTGCGC

GACTGGCTGGCCATGCGAAAGGCGGTGAGGGCGCGAATCCCCACCAGCACCCCCGAGGAG

GCAGTTTTACTAGATAAGCAGCAGTCTGCGATTAAGGTGATATGCAACTCGGTTTACGGA

TTCACGGGGGTGGCAAACGGCCTGTTGCCGTGTCTGAGGATAGCGGCTACCGTTACCACG

ATAGGACGCGACATGCTCCTCAAGACCCGAGATTACGTTCACTCTCGTTGGGCGACGCGC

GAGCTGCTGGAGGACAATTTTCCAGGGGCTATAGGTTTCCGAAACCACAAGCCTTACTCC

GTCAGGGTTATCTACGGAGACACCGACTCCGTGTTTATCAAGTTTGTGGGCCTGACGTAC

GAGGGGGTATCGGAGCTGGGGGATGCTATGTCGCGTCAGATTTCAGCGGACCTCTTTAGA

GCGCCCATCAAACTGGAGTGTGAGAAGACCTTTCAGCGACTGCTGCTGATCACCAAGAAG

AAGTACATAGGTGTCATAAACGGGGGGAAGATGCTCATGAAGGGGGTCGACCTGGTCCGC

AAAAATAACTGCTCTTTCATAAACTTGTACGCGCGACATCTGGTAGATCTTTTGTTGTAC

GACGAGGATGTGGCCACGGCGGCAGCAGAGGTGACAGACGTGCCTCCCGCAGAATGGGTG

GGGCGCCCGCTACCGAGCGGCTTTGACAAGTTTGGGCGAGTGCTGGTAGAGGCGTACAAC

CGTATCACTGCCCCCAACTTGGACGTGCGCGAGTTCGTTATGACTGCTGAGCTGAGCCGC

TCACCCGAATCGTATACCAACAAGCGCCTGCCGCACCTCACCGTCTACTTTAAGCTCGCC

ATGAGGAATGAAGAACTGCCCAGCGTAAAAGAGAGAATTCCGTATGTGATAGTTGCGCAG

ACCGAGGCCGCGGAACGCGAAGCGGGTGTAGTAAACTCAATGCGCGGTACCGCCCAAAAC

CCCGTGGTAACCAAGACCGCACGCCCCCAACCTAAACGCAAACTGCTGGTTTCCGACCTC

GCCGAAGACCCGACCTATGTTTCCGAGAATGACGTACCGCTAAACACAGACTACTATTTC

TCCCACCTGTTGGGTACCATAAGCGTGACCTTTAAGGCTCTATTCGGAAATGATGTGAGA

ACAACAGAAAATCTTTTAAAGCGGTTTATTCCGGAAACCCCCCACAAGACCCCCACGAAA

ACCCAGGCACTGCTTGAGCGCGCCGGCTTTGAAAAGCTGACGCCCTTTACACCGGAGGAA

GAAAGTCGTCGAATACTGCATACAGTTTTTTGTACTCTAGAAGCAGCTCCCCATCAAAGC

TGA

>LC834900.1

ATGGCGGCGCGCGAACAGGCCAACTCCGTTCGCAGGAGCGGGTTTTTTAATCCGTTTATT

GGCAAGAGGCCATTTTTCAGGCCGGGAAGCGGGCAGACCGCGGAGACAGAGCGCCCAAGG

CCCCCCCAACACTCGTACTGCACAGAGGTGGGTAGCTTTAAGTTTATAGCTCCAAGATGC

CTCGATGAAGAAGCCCCCGCCGACCAGCGACGCGGTGTACACGTGGGCACCTTGGAGCGC

CCACCCAAAGTGTACTGCGATGGCTCAGAGTACGACGTGCTGAACTTTGCCTCCGGAGGT

TGCTGGCCTCGCAGGATTCGGGTTTGGAACGGCCAGGATTTTCGGGGCGATGGATTCAAC

CCCAGATTTGAGAGATTTCACGTGTACGACATAGTAGAGACTTCGGAGAGCGCCTCACAC

GATGACCCATCCAGGTTTGCTGAGCTATCCCGCCCAAGCGGGTCTGTGGTAACACTGCTG

GGAATGAGCGAGTGTGGAAAGCGAGTCGCGGTTCACGTCTATGGTGTGCGCCATTATTTT

TACATGGCAAAGGCGGAGGTGGATAGCGCTTGTGGAATAACCACCGAGGCAGAACTCGTG

CGCGCAATGGTGGACTGCGCGCACAGCTCGGCTTTGAGCGCGGCCCTGGGAAATGGCAAC

GGCGGCAAACAGAGCGGCGGCAGCGGCGGGGGATGGTGGGGCGGAAAGCACGTGTCTGCG

GACTGCTTCAAAGTGGAGACCGTGTGCCACACGACGCTGTACTACTTTGGATCTAAGCCA

GCTCTCTACTATAGAGTATCTGCCTCCAGCAGCCGCCTGGGAGGGTTCATCTGCGACAAC

TTTCACCCGGAGATTACAAAATTCGAGGGGAGCGTGGACGTGACCACGCGGCTGTTGTTG

GACAATGAAAATTTTACCAGTTTCGGGTGGTACCGCCTGCGACCCGGCACCCACGGAGAG

CGTGTTCAACTTCGCCCCGTTGAGCGACACGTCACCTCAAGCGACGTGGAGATTAACTGT

ACTCCCGATAACCTGGAGCCGATACCAGACGAGGCTGCCTGGCCCGACTATAAGCTCATG

TGCTTTGATATAGAGTGTAAAGCTGGAACGGGTAACGAAATGGCGTTCCCAGTGGCAACT

AACCAAGAGGACCTGGTCATCCAGATCTCCTGTCTGCTGTACTCGCTTGCTACTCAGAAC

CACGAACACACCCTGCTGTTTTCCCTCGGGTCATGCGATATCTCTGAGGAATACTCGTTT

GCATGCGTCCAGCGCGGCGAGCCCAGACCGACGGTTTTGGAGTTTGACAGCGAGTACGAG

CTGCTGGTTGCCTTCCTGACCTTTCTCAAGCAGTACTCTCCCGAGTTCGCCACCGGCTAC

AACATCGTTAATTTTGACTGGGCGTACATAGTTAACAAGGTAACGTCGGTGTATAACATC

AAGCTGGACGGGTACGGCAAGTTCAACAAAGGGGGGCTGTTTAAGGTGTGGGACATCGCC

ACGAACCATTTTCAGAAGAAGAGCAAGGTGAAAATCAATGGCCTGATATCTCTAGACATG

TATTCTGTGGCGACGGAAAAGCTAAAGCTACCCAGCTACAAACTCGACGCGGTCGTGGGA

GACGTCCTCGGCGAGCATAAGATAGACCTTCCCTATAAAGAAATACCCTCCTATTACGCG

GGAGGGCCTGACCGGAGGGGCGTAATAGGAGAGTATTGTATCCAGGACTCTAGGCTGGTG

GGCAAGCTGTTTTTTAAGTACCTCCCCCATCTGGAACTATCGGCGGTGGCCAAACTCGCC

CGTATCACCCTAACGCGGGTAATTTTTGACGGTCAGCAAATTCGCGTGTACACGTGCTTG

CTGAAACTCGCCCGCGAGAGAAATTTCATTTTGCCAGACAACAGACGCCGGTTTGACAGT

CAGGCAGACGCCGCGTCAGAGACTTCGGAGTTGGCTATGGATAGCCAAAGCCACGCCTTC

GACAGTACAGACGAACCCGACGGTGTGGACGGTACCCCGGACGCCGCAGGATCTGGCGCT

ACTTCTGAAAACGGAGGCGGGAAGCCCGGCGTCGGGAGGGCCGTGGGCTACCAGGGAGCA

AAGGTTCTAGACCCCGTATCCGGCTTTCATGTGGACCCCGTGGTTGTGTTTGACTTCGCT

AGCTTATACCCAAGCATTATCCAGGCCCATAACCTCTGTTTCACCACCCTGGCGCTCGAT

GAAGTGGATCTGGCCGGGCTTCAACCATCCGTCAACTACTCGACGTTCGAGGTGGGTGAC

CAAAAGTTATTTTTTGTCCACGCCCATATTCGCGAAAGCCTGCTTGGCATCTTGCTGCGC

GACTGGCTGGCCATGCGAAAGGCGGTGAGGGCGCGAATCCCCACCAGCACCCCCGAGGAG

GCAGTTTTACTAGATAAGCAGCAGTCTGCGATTAAGGTGATATGCAACTCGGTTTACGGA

TTCACGGGGGTGGCAAACGGCCTGTTGCCGTGTCTGAGGATAGCGGCTACCGTTACCACG

ATAGGACGCGACATGCTCCTCAAGACCCGAGATTACGTTCACTCTCGTTGGGCGACGCGC

GAGCTGCTGGAGGACAATTTTCCAGGGGCTATAGGTTTCCGAAACCACAAGCCTTACTCC

GTCAGGGTTATCTACGGAGACACCGACTCCGTGTTTATCAAGTTTGTGGGCCTGACGTAC

GAGGGGGTATCGGAGCTGGGGGATGCTATGTCGCGTCAGATTTCAGCGGACCTCTTTAGA

GCCCCCATCAAACTGGAGTGTGAGAAGACCTTTCAGCGACTGCTGCTGATCACCAAGAAG

AAGTACATAGGTGTCATAAACGGGGGGAAGATGCTCATGAAGGGGGTCGACCTGGTCCGC

AAAAATAACTGCTCTTTCATAAACTTGTACGCGCGACATCTGGTAGATCTTTTGTTGTAC

GACGAGGATGTGGCCACGGCGGCAGCAGAGGTGACAGACGTGCCTCCCGCAGAATGGGTG

GGGCGCCCGCTACCGAGCGGCTTTGACAAGTTTGGGCGAGTGCTGGTAGAGGCGTACAAC

CGTATCACTGCCCCCAACTTGGACGTGCGCGAGTTCGTTATGACTGCTGAGCTGAGCCGC

TCACCCGAATCGTATACCAACAAGCGCCTGCCGCACCTCACCGTCTACTTTAAGCTCGCC

ATGAGGAATGAAGAACTGCCCAGCGTAAAAGAGAGAATTCCGTATGTGATAGTTGCGCAG

ACCGAGGCCGCGGAACGCGAAGCGGGTGTAGTAAACTCAATGCGCGGTACCGCCCAAAAC

CCCGTGGTAACCAAGACCGCACGCCCCCAACCTAAACGCAAACTGCTGGTTTCCGACCTC

GCCGAAGACCCGACCTATGTTTCCGAGAATGACGTACCGCTAAACACAGACTACTATTTC

TCCCACCTGTTGGGTACCATAAGCGTGACCTTTAAGGCTCTATTCGGAAATGATGTGAGA

ACAACAGAAAATCTTTTAAAGCGGTTTATTCCGGAAACCCCCCACAAGACCCCCACGAAA

ACCCAGGCACTGCTTGAGCGCGCCGGCTTTGAAAAGCTGACGCCCTTTACACCGGAGGAA

GAAAGTCGTCGAATACTGCATACAGTTTTTTGTACTCTAGAAGCAGCTCCCCATCAAAGC

TGA

>LC834901.1

ATGGCGGCGCGCGAACAGGCCAACTCCGTTCGCAGGAGCGGGTTTTTTAATCCGTTTATT

GGCAAGAGGCCATTTTTCAGGCCGGGAAGCGGGCAGACCGCGGAGACAGAGCGCCCAAGG

CCCCCCCAACACTCGTACTGCACAGAGGTGGGTAGCTTTAAGTTTATAGCTCCAAGATGC

CTCGATGAAGAAGCCCCCGCCGACCAGCGACGCGGTGTACACGTGGGCACCTTGGAGCGC

CCACCCAAAGTGTACTGCGATGGCTCAGAGTACGACGTGCTGAACTTTGCCTCCGGAGGT

TGCTGGCCTCGCAGGATTCGGGTTTGGAACGGCCAGGATTTTCGGGGCGATGGATTCAAC

CCCAGATTTGAGAGATTTCACGTGTACGACATAGTAGAGACTTCGGAGAGCGCCTCACAC

GATGACCCATCCAGGTTTGCTGAGCTATCCCGCCCAAGCGGGTCTGTGGTAACACTGCTG

GGAATGAGCGAGTGTGGAAAGCGAGTCGCGGTTCACGTCTATGGTGTGCGCCATTATTTT

TACATGGCAAAGGCGGAGGTGGATAGCGCTTGTGGAATAACCACCGAGGCAGAACTCGTG

CGCGCAATGGTGGACTGCGCGCACAGCTCGGCTTTGAGCGCGGCCCTGGGAAATGGCAAC

GGCGGCAAACAGAGCGGCGGCAGCGGCGGGGGATGGTGGGGCGGAAAGCACGTGTCTGCG

GACTGCTTCAAAGTGGAGACCGTGTGCCACACGACGCTGTACTACTTTGGATCTAAGCCA

GCTCTCTACTATAGAGTATCTGCCTCCAGCAGCCGCCTGGGAGGGTTCATCTGCGACAAC

TTTCACCCGGAGATTACAAAATTCGAGGGGAGCGTGGACGTGACCACGCGGCTGTTGTTG

GACAATGAAAATTTTACCAGTTTCGGGTGGTACCGCCTGCGACCCGGCACCCACGGAGAG

CGTGTTCAACTTCGCCCCGTTGAGCGACACGTCACCTCAAGCGACGTGGAGATTAACTGT

ACTCCCGATAACCTGGAGCCGATACCAGACGAGGCTGCCTGGCCCGACTATAAGCTCATG

TGCTTTGATATAGAGTGTAAAGCTGGAACGGGTAACGAAATGGCGTTCCCAGTGGCAACT

AACCAAGAGGACCTGGTCATCCAGATCTCCTGTCTGCTGTACTCGCTTGCTACTCAGAAC

CACGAACACACCCTGCTGTTTTCCCTCGGGTCATGCGATATCTCTGAGGAATACTCGTTT

GCATGCGTCCAGCGCGGCGAGCCCAGACCGACGGTTTTGGAGTTTGACAGCGAGTACGAG

CTGCTGGTTGCCTTCCTGACCTTTCTCAAGCAGTACTCTCCCGAGTTCGCCACCGGCTAC

AACATCGTTAATTTTGACTGGGCGTACATAGTTAACAAGGTAACGTCGGTGTATAACATC

AAGCTGGACGGGTACGGCAAGTTCAACAAAGGGGGGCTGTTTAAGGTGTGGGACATCGCC

ACGAACCATTTTCAGAAGAAGAGCAAGGTGAAAATCAATGGCCTGATATCTCTAGACATG

TATTCTGTGGCGACGGAAAAGCTAAAGCTACCCAGCTACAAACTCGACGCGGTCGTGGGA

GACGTCCTCGGCGAGCATAAGATAGACCTTCCCTATAAAGAAATACCCTCCTATTACGCG

GGAGGGCCTGACCGGAGGGGCGTAATAGGAGAGTATTGTATCCAGGACTCTAGGCTGGTG

GGCAAGCTGTTTTTTAAGTACCTCCCCCATCTGGAACTATCGGCGGTGGCCAAACTCGCC

CGTATCACCCTAACGCGGGTAATTTTTGACGGTCAGCAAATTCGCGTGTACACGTGCTTG

CTGAAACTCGCCCGCGAGAGAAATTTCATTTTGCCAGACAACAGACGCCGGTTTGACAGT

CAGGCAGACGCCGCGTCAGAGACTTCGGAGTTGGCTATGGATAGCCAAAGCCACGCCTTC

GACAGTACAGACGAACCCGACGGTGTGGACGGTACCCCGGACGCCGCAGGATCTGGCGCT

ACTTCTGAAAACGGAGGCGGGAAGCCCGGCGTCGGGAGGGCCGTGGGCTACCAGGGAGCA

AAGGTTCTAGACCCCGTATCCGGCTTTCATGTGGACCCCGTGGTTGTGTTTGACTTCGCT

AGCTTATACCCAAGCATTATCCAGGCCCATAACCTCTGTTTCACCACCCTGGCGCTCGAT

GAAGTGGATCTGGCCGGGCTTCAACCATCCGTCAACTACTCGACGTTCGAGGTGGGTGAC

CAAAAGTTATTTTTTGTCCACGCCCATATTCGCGAAAGCCTGCTTGGCATCTTGCTGCGC

GACTGGCTGGCCATGCGAAAGGCGGTGAGGGCGCGAATCCCCACCAGCACCCCCGAGGAG

GCAGTTTTACTAGATAAGCAGCAGTCTGCGATTAAGGTGATATGCAACTCGGTTTACGGA

TTCACGGGGGTGGCAAACGGCCTGTTGCCGTGTCTGAGGATAGCGGCTACCGTTACCACG

ATAGGACGCGACATGCTCCTCAAGACCCGAGATTACGTTCACTCTCGTTGGGCGACGCGC

GAGCTGCTGGAGGACAATTTTCCAGGGGCTATAGGTTTCCGAAACCACAAGCCTTACTCC

GTCAGGGTTATCTACGGAGACACCGACTCCGTGTTTATCAAGTTTGTGGGCCTGACGTAC

GAGGGGGTATCGGAGCTGGGGGATGCTATGTCGCGTCAGATTTCAGCGGACCTCTTTAGA

GCCCCCATCAAACTGGAGTGTGAGAAGACCTTTCAGCGACTGCTGCTGATCACCAAGAAG

AAGTACATAGGTGTCATAAACGGGGGGAAGATGCTCATGAAGGGGGTCGACCTGGTCCGC

AAAAATAACTGCTCTTTCATAAACTTGTACGCGCGACATCTGGTAGATCTTTTGTTGTAC

GACGAGGATGTGGCCACGGCGGCAGCAGAGGTGACAGACGTGCCTCCCGCAGAATGGGTG

GGGCGCCCGCTACCGAGCGGCTTTGACAAGTTTGGGCGAGTGCTGGTAGAGGCGTACAAC

CGTATCACTGCCCCCAACTTGGACGTGCGCGAGTTCGTTATGACTGCTGAGCTGAGCCGC

TCACCCGAATCGTATACCAACAAGCGCCTGCCGCACCTCACCGTCTACTTTAAGCTCGCC

ATGAGGAATGAAGAACTGCCCAGCGTAAAAGAGAGAATTCCGTATGTGATAGTTGCGCAG

ACCGAGGCCGCGGAACGCGAAGCGGGTGTAGTAAACTCAATGCGCGGTACCGCCCAAAAC

CCCGTGGTAACCAAGACCGCACGCCCCCAACCTAAACGCAAACTGCTGGTTTCCGACCTC

GCCGAAGACCCGACCTATGTTTCCGAGAATGACGTACCGCTAAACACAGACTACTATTTC

TCCCACCTGTTGGGTACCATAAGCGTGACCTTTAAGGCTCTATTCGGAAATGATGTGAGA

ACAACAGAAAATCTTTTAAAGCGGTTTATTCCGGAAACCCCCCACAAGACCCCCACGAAA

ACCCAGGCACTGCTTGAGCGCGCCGGCTTTGAAAAGCTGACGCCCTTTACACCGGAGGAA

GAAAGTCGTCGAATACTGCATACAGTTTTTTGTACTCTAGAAGCAGCTCCCCATCAAAGC

TGA

>LC834902.1

ATGGCGGCGCGCGAACAGGCCAACTCCGTTCGCAGGAGCGGGTTTTTTAATCCGTTTATT

GGCAAGAGGCCATTTTTCAGGCCGGGAAGCGGGCAAACCGCGGAGACAGAGCGCCCAAGG

CCCCCCCAACACTCGTACTGCACAGAGGTGGGTAGCTTTAAGTTTATAGCTCCAAGATGC

CTCGATGAAGAAGCCCCCGCCGACCAGCGACGCGGTGTACACGTGGGCACCTTGGAGCGC

CCACCCAAAGTGTACTGCGATGGCTCAGAGTACGACGTGCTGAACTTTGCCTCCGGAGGT

TGCTGGCCTCGCAGGATTCGGGTTTGGAACGGCCAGGATTTTCGGGGCGATGGATTCAAC

CCCAGATTTGAGAGATTTCACGTGTACGACATAGTAGAGACTTCGGAGAGCGCCTCACAC

GATGACCCATCCAGGTTTGCTGAGCTATCCCGCCCAAGCGGGTCTGTGGTAACACTGCTG

GGAATGAGCGAGTGTGGAAAGCGAGTCGCGGTTCACGTCTATGGTGTGCGCCATTATTTT

TACATGGCAAAGGCGGAGGTGGATAGCGCTTGTGGAATAACCACCGAGGCAGAACTCGTG

CGCGCAATGGTGGACTGCGCGCACAGCTCGGCTTTGAGCGCGGCCCTGGGAAATGGCAAC

GGCGGCAAACAGAGCGGCGGCAGCGGCGGGGGATGGTGGGGCGGAAAGCACGTGTCTGCG

GACTGCTTCAAAGTGGAGACCGTGTGCCACACGACGCTGTACTACTTTGGATCTAAGCCA

GCTCTCTACTATAGAGTATCTGCCTCCAGCAGCCGCCTGGGAGGGTTCATCTGCGACAAC

TTTCACCCGGAGATTACAAAATTCGAGGGGAGCGTGGACGTGACCACGCGGCTGTTGTTG

GACAATGAAAATTTTACCAGTTTCGGGTGGTACCGCCTGCGACCCGGCACCCACGGAGAG

CGTGTTCAACTTCGCCCCGTTGAGCGACACGTCACCTCAAGCGACGTGGAGATTAACTGT

ACTCCCGATAACCTGGAGCCGATACCAGACGAGGCTGCCTGGCCCGACTATAAGCTCATG

TGCTTTGATATAGAGTGTAAAGCTGGAACGGGTAACGAAATGGCGTTCCCAGTGGCAACT

AACCAAGAGGACCTGGTCATCCAGATCTCCTGTCTGCTGTACTCGCTTGCTACTCAGAAC

CACGAACACACCCTGCTGTTTTCCCTCGGGTCATGCGATATCTCTGAGGAATACTCGTTT

GCATGCGTCCAGCGCGGCGAGCCCAGACCGACGGTTTTGGAGTTTGACAGCGAGTACGAG

CTGCTGGTTGCCTTCCTGACCTTTCTCAAGCAGTACTCTCCCGAGTTCGCCACCGGCTAC

AACATCGTTAATTTTGACTGGGCGTACATAGTTAACAAGGTAACGTCGGTGTATAACATC

AAGCTGGACGGGTACGGCAAGTTCAACAAAGGGGGGCTGTTTAAGGTGTGGGACATCGCC

ACGAACCATTTTCAGAAGAAGAGCAAGGTGAAAATCAATGGCCTGATATCTCTAGACATG

TATTCTGTGGCGACGGAAAAGCTAAAGCTACCCAGCTACAAACTCGACGCGGTCGTGGGA

GACGTCCTCGGCGAGCATAAGATAGACCTTCCCTATAAAGAAATACCCTCCTATTACGCG

GGAGGGCCTGACCGGAGGGGCGTAATAGGAGAGTATTGTATCCAGGACTCTAGGCTGGTG

GGCAAGCTGTTTTTTAAGTACCTCCCCCATCTGGAACTATCGGCGGTGGCCAAACTCGCC

CGTATCACCCTAACGCGGGTAATTTTTGACGGTCAGCAAATTCGCGTGTACACGTGCTTG

CTGAAACTCGCCCGCGAGAGAAATTTCATTTTGCCAGACAACAGACGCCGGTTTGACAGT

CAGGCAGACGCCGCGTCAGAGACTTCGGAGTTGGCTATGGATAGCCAAAGCCACGCCTTC

GACAGTACAGACGAACCCGACGGTGTGGACGGTACCCCGGACGCCGCAGGATCTGGCGCT

ACTTCTGAAAACGGAGGCGGGAAGCCCGGCGTCGGGAGGGCCGTGGGCTACCAGGGAGCA

AAGGTTCTAGACCCCGTATCCGGCTTTCATGTGGACCCCGTGGTTGTGTTTGACTTCGCT

AGCTTATACCCAAGCATTATCCAGGCCCATAACCTCTGTTTCACCACCCTGGCGCTCGAT

GAAGTGGATCTGGCCGGGCTTCAACCATCCGTCAACTACTCGACGTTCGAGGTGGGTGAC

CAAAAGTTATTTTTTGTCCACGCCCATATTCGCGAAAGCCTGCTTGGCATCTTGCTGCGC

GACTGGCTGGCCATGCGAAAGGCGGTGAGGGCGCGAATCCCCACCAGCACCCCCGAGGAG

GCAGTTTTACTAGATAAGCAGCAGTCTGCGATTAAGGTGATATGCAACTCGGTTTACGGA

TTCACGGGGGTGGCAAACGGCCTGTTGCCGTGTCTGAGGATAGCGGCTACCGTTACCACG

ATAGGACGCGACATGCTCCTCAAGACCCGAGATTACGTTCACTCTCGTTGGGCGACGCGC

GAGCTGCTGGAGGACAATTTTCCAGGGGCTATAGGTTTCCGAAACCACAAGCCTTACTCC

GTCAGGGTTATCTACGGAGACACCGACTCCGTGTTTATCAAGTTTGTGGGCCTGACGTAC

GAGGGGGTATCGGAGCTGGGGGATGCTATGTCGCGTCAGATTTCAGCGGACCTCTTTAGA

GCGCCCATCAAACTGGAGTGTGAGAAGACCTTTCAGCGACTGCTGCTGATCACCAAGAAG

AAGTACATAGGTGTCATAAACGGGGGGAAGATGCTCATGAAGGGGGTCGACCTGGTCCGC

AAAAATAACTGCTCTTTCATAAACTTGTACGCGCGACATCTGGTAGATCTTTTGTTGTAC

GACGAGGATGTGGCCACGGCGGCAGCAGAGGTGACAGACGTGCCTCCCGCAGAATGGGTG

GGGCGCCCGCTACCGAGCGGCTTTGACAAGTTTGGGCGAGTGCTGGTAGAGGCGTACAAC

CGTATCACTGCCCCCAACTTGGACGTGCGCGAGTTCGTTATGACTGCTGAGCTGAGCCGC

TCACCCGAATCGTATACCAACAAGCGCCTGCCGCACCTCACCGTCTACTTTAAGCTCGCC

ATGAGGAATGAAGAACTGCCCAGCGTAAAAGAGAGAATTCCGTATGTGATAGTTGCGCAG

ACCGAGGCCGCGGAACGCGAAGCGGGTGTAGTAAACTCAATGCGCGGTACCGCCCAAAAC

CCCGTGGTAACCAAGACCGCACGCCCCCAACCTAAACGCAAACTGCTGGTTTCCGACCTC

GCCGAAGACCCGACCTATGTTTCCGAGAATGACGTACCGCTAAACACAGACTACTATTTC

TCCCACCTGTTGGGTACCATAAGCGTGACCTTTAAGGCTCTATTCGGAAATGATGTGAGA

ACAACAGAAAATCTTTTAAAGCGGTTTATTCCGGAAACCCCCCACAAGACCCCCACGAAA

ACCCAGGCACTGCTTGAGCGCGCCGGCTTTGAAAAGCTGACGCCCTTTACACCGGAGGAA

GAAAGTCGTCGAATACTGCATACAGTTTTTTGTACTCTAGAAGCAGCTCCCCATCAAAGC

TGA

>LC834903.1

ATGGCGGCGCGCGAACAGGCCAACTCCGTTCGCAGGAGCGGGTTTTTTAATCCGTTTATT

GGCAAGAGGCCATTTTTCAGGCCGGGAAGCGGGCAAACCGCGGAGACAGAGCGCCCAAGG

CCCCCCCAACACTCGTACTGCACAGAGGTGGGTAGCTTTAAGTTTATAGCTCCAAGATGC

CTCGATGAAGAAGCCCCCGCCGACCAGCGACGCGGTGTACACGTGGGCACCTTGGAGCGC

CCACCCAAAGTGTACTGCGATGGCTCAGAGTACGACGTGCTGAACTTTGCCTCCGGAGGT

TGCTGGCCTCGCAGGATTCGGGTTTGGAACGGCCAGGATTTTCGGGGCGATGGATTCAAC

CCCAGATTTGAGAGATTTCACGTGTACGACATAGTAGAGACTTCGGAGAGCGCCTCACAC

GATGACCCATCCAGGTTTGCTGAGCTATCCCGCCCAAGCGGGTCTGTGGTAACACTGCTG

GGAATGAGCGAGTGTGGAAAGCGAGTCGCGGTTCACGTCTATGGTGTGCGCCATTATTTT

TACATGGCAAAGGCGGAGGTGGATAGCGCTTGTGGAATAACCACCGAGGCAGAACTCGTG

CGCGCAATGGTGGACTGCGCGCACAGCTCGGCTTTGAGCGCGGCCCTGGGAAATGGCAAC

GGCGGCAAACAGAGCGGCGGCAGCGGCGGGGGATGGTGGGGCGGAAAGCACGTGTCTGCG

GACTGCTTCAAAGTGGAGACCGTGTGCCACACGACGCTGTACTACTTTGGATCTAAGCCA

GCTCTCTACTATAGAGTATCTGCCTCCAGCAGCCGCCTGGGAGGGTTCATCTGCGACAAC

TTTCACCCGGAGATTACAAAATTCGAGGGGAGCGTGGACGTGACCACGCGGCTGTTGTTG

GACAATGAAAATTTTACCAGTTTCGGGTGGTACCGCCTGCGACCCGGCACCCACGGAGAG

CGTGTTCAACTTCGCCCCGTTGAGCGACACGTCACCTCAAGCGACGTGGAGATTAACTGT

ACTCCCGATAACCTGGAGCCGATACCAGACGAGGCTGCCTGGCCCGACTATAAGCTCATG

TGCTTTGATATAGAGTGTAAAGCTGGAACGGGTAACGAAATGGCGTTCCCAGTGGCAACT

AACCAAGAGGACCTGGTCATCCAGATCTCCTGTCTGCTGTACTCGCTTGCTACTCAGAAC

CACGAACACACCCTGCTGTTTTCCCTCGGGTCATGCGATATCTCTGAGGAATACTCGTTT

GCATGCGTCCAGCGCGGCGAGCCCAGACCGACGGTTTTGGAGTTTGACAGCGAGTACGAG

CTGCTGGTTGCCTTCCTGACCTTTCTCAAGCAGTACTCTCCCGAGTTCGCCACCGGCTAC

AACATCGTTAATTTTGACTGGGCGTACATAGTTAACAAGGTAACGTCGGTGTATAACATC

AAGCTGGACGGGTACGGCAAGTTCAACAAAGGGGGGCTGTTTAAGGTGTGGGACATCGCC

ACGAACCATTTTCAGAAGAAGAGCAAGGTGAAAATCAATGGCCTGATATCTCTAGACATG

TATTCTGTGGCGACGGAAAAGCTAAAGCTACCCAGCTACAAACTCGACGCGGTCGTGGGA

GACGTCCTCGGCGAGCATAAGATAGACCTTCCCTATAAAGAAATACCCTCCTATTACGCG

GGAGGGCCTGACCGGAGGGGCGTAATAGGAGAGTATTGTATCCAGGACTCTAGGCTGGTG

GGCAAGCTGTTTTTTAAGTACCTCCCCCATCTGGAACTATCGGCGGTGGCCAAACTCGCC

CGTATCACCCTAACGCGGGTAATTTTTGACGGTCAGCAAATTCGCGTGTACACGTGCTTG

CTGAAACTCGCCCGCGAGAGAAATTTCATTTTGCCAGACAACAGACGCCGGTTTGACAGT

CAGGCAGACGCCGCGTCAGAGACTTCGGAGTTGGCTATGGATAGCCAAAGCCACGCCTTC

GACAGTACAGACGAACCCGACGGTGTGGACGGTACCCCGGACGCCGCAGGATCTGGCGCT

ACTTCTGAAAACGGAGGCGGGAAGCCCGGCGTCGGGAGGGCCGTGGGCTACCAGGGAGCA

AAGGTTCTAGACCCCGTATCCGGCTTTCATGTGGACCCCGTGGTTGTGTTTGACTTCGCT

AGCTTATACCCAAGCATTATCCAGGCCCATAACCTCTGTTTCACCACCCTGGCGCTCGAT

GAAGTGGATCTGGCCGGGCTTCAACCATCCGTCAACTACTCGACGTTCGAGGTGGGTGAC

CAAAAGTTATTTTTTGTCCACGCCCATATTCGCGAAAGCCTGCTTGGCATCTTGCTGCGC

GACTGGCTGGCCATGCGAAAGGCGGTGAGGGCGCGAATCCCCACCAGCACCCCCGAGGAG

GCAGTTTTACTAGATAAGCAGCAGTCTGCGATTAAGGTGATATGCAACTCGGTTTACGGA

TTCACGGGGGTGGCAAACGGCCTGTTGCCGTGTCTGAGGATAGCGGCTACCGTTACCACG

ATAGGACGCGACATGCTCCTCAAGACCCGAGATTACGTTCACTCTCGTTGGGCGACGCGC

GAGCTGCTGGAGGACAATTTTCCAGGGGCTATAGGTTTCCGAAACCACAAGCCTTACTCC

GTCAGGGTTATCTACGGAGACACCGACTCCGTGTTTATCAAGTTTGTGGGCCTGACGTAC

GAGGGGGTATCGGAGCTGGGGGATGCTATGTCGCGTCAGATTTCAGCGGACCTCTTTAGA

GCGCCCATCAAACTGGAGTGTGAGAAGACCTTTCAGCGACTGCTGCTGATCACCAAGAAG

AAGTACATAGGTGTCATAAACGGGGGGAAGATGCTCATGAAGGGGGTCGACCTGGTCCGC

AAAAATAACTGCTCTTTCATAAACTTGTACGCGCGACATCTGGTAGATCTTTTGTTGTAC

GACGAGGATGTGGCCACGGCGGCAGCAGAGGTGACAGACGTGCCTCCCGCAGAATGGGTG

GGGCGCCCGCTACCGAGCGGCTTTGACAAGTTTGGGCGAGTGCTGGTAGAGGCGTACAAC

CGTATCACTGCCCCCAACTTGGACGTGCGCGAGTTCGTTATGACTGCTGAGCTGAGCCGC

TCACCCGAATCGTATACCAACAAGCGCCTGCCGCACCTCACCGTCTACTTTAAGCTCGCC

ATGAGGAATGAAGAACTGCCCAGCGTAAAAGAGAGAATTCCGTATGTGATAGTTGCGCAG

ACCGAGGCCGCGGAACGCGAAGCGGGTGTAGTAAACTCAATGCGCGGTACCGCCCAAAAC

CCCGTGGTAACCAAGACCGCACGCCCCCAACCTAAACGCAAACTGCTGGTTTCCGACCTC

GCCGAAGACCCGACCTATGTTTCCGAGAATGACGTACCGCTAAACACAGACTACTATTTC

TCCCACCTGTTGGGTACCATAAGCGTGACCTTTAAGGCTCTATTCGGAAATGATGTGAGA

ACAACAGAAAATCTTTTAAAGCGGTTTATTCCGGAAACCCCCCACAAGACCCCCACGAAA

ACCCAGGCACTGCTTGAGCGCGCCGGCTTTGAAAAGCTGACGCCCTTTACACCGGAGGAA

GAAAGTCGTCGAATACTGCATACAGTTTTTTGTACTCTAGAAGCAGCTCCCCATCAAAGC

TGA

>LC834904.1

ATGGCGGCGCGCGAACAGGCCAACTCCGTTCGCAGGAGCGGGTTTTTTAATCCGTTTATT

GGCAAGAGGCCATTTTTCAGGCCGGGAAGCGGGCAAACCGCGGAGACAGAGCGCCCAAGG

CCCCCCCAACACTCGTACTGCACAGAGGTGGGTAGCTTTAAGTTTATAGCTCCAAGATGC

CTCGATGAAGAAGCCCCCGCCGACCAGCGACGCGGTGTACACGTGGGCACCTTGGAGCGC

CCACCCAAAGTGTACTGCGATGGCTCAGAGTACGACGTGCTGAACTTTGCCTCCGGAGGT

TGCTGGCCTCGCAGGATTCGGGTTTGGAACGGCCAGGATTTTCGGGGCGATGGATTCAAC

CCCAGATTTGAGAGATTTCACGTGTACGACATAGTAGAGACTTCGGAGAGCGCCTCACAC

GATGACCCATCCAGGTTTGCTGAGCTATCCCGCCCAAGCGGGTCTGTGGTAACACTGCTG

GGAATGAGCGAGTGTGGAAAGCGAGTCGCGGTTCACGTCTATGGTGTGCGCCATTATTTT

TACATGGCAAAGGCGGAGGTGGATAGCGCTTGTGGAATAACCACCGAGGCAGAACTCGTG

CGCGCAATGGTGGACTGCGCGCACAGCTCGGCTTTGAGCGCGGCCCTGGGAAATGGCAAC

GGCGGCAAACAGAGCGGCGGCAGCGGCGGGGGATGGTGGGGCGGAAAGCACGTGTCTGCG

GACTGCTTCAAAGTGGAGACCGTGTGCCACACGACGCTGTACTACTTTGGATCTAAGCCA

GCTCTCTACTATAGAGTATCTGCCTCCAGCAGCCGCCTGGGAGGGTTCATCTGCGACAAC

TTTCACCCGGAGATTACAAAATTCGAGGGGAGCGTGGACGTGACCACGCGGCTGTTGTTG

GACAATGAAAATTTTACCAGTTTCGGGTGGTACCGCCTGCGACCCGGCACCCACGGAGAG

CGTGTTCAACTTCGCCCCGTTGAGCGACACGTCACCTCAAGCGACGTGGAGATTAACTGT

ACTCCCGATAACCTGGAGCCGATACCAGACGAGGCTGCCTGGCCCGACTATAAGCTCATG

TGCTTTGATATAGAGTGTAAAGCTGGAACGGGTAACGAAATGGCGTTCCCAGTGGCAACT

AACCAAGAGGACCTGGTCATCCAGATCTCCTGTCTGCTGTACTCGCTTGCTACTCAGAAC

CACGAACACACCCTGCTGTTTTCCCTCGGGTCATGCGATATCTCTGAGGAATACTCGTTT

GCATGCGTCCAGCGCGGCGAGCCCAGACCGACGGTTTTGGAGTTTGACAGCGAGTACGAG

CTGCTGGTTGCCTTCCTGACCTTTCTCAAGCAGTACTCTCCCGAGTTCGCCACCGGCTAC

AACATCGTTAATTTTGACTGGGCGTACATAGTTAACAAGGTAACGTCGGTGTATAACATC

AAGCTGGACGGGTACGGCAAGTTCAACAAAGGGGGGCTGTTTAAGGTGTGGGACATCGCC

ACGAACCATTTTCAGAAGAAGAGCAAGGTGAAAATCAATGGCCTGATATCTCTAGACATG

TATTCTGTGGCGACGGAAAAGCTAAAGCTACCCAGCTACAAACTCGACGCGGTCGTGGGA

GACGTCCTCGGCGAGCATAAGATAGACCTTCCCTATAAAGAAATACCCTCCTATTACGCG

GGAGGGCCTGACCGGAGGGGCGTAATAGGAGAGTATTGTATCCAGGACTCTAGGCTGGTG

GGCAAGCTGTTTTTTAAGTACCTCCCCCATCTGGAACTATCGGCGGTGGCCAAACTCGCC

CGTATCACCCTAACGCGGGTAATTTTTGACGGTCAGCAAATTCGCGTGTACACGTGCTTG

CTGAAACTCGCCCGCGAGAGAAATTTCATTTTGCCAGACAACAGACGCCGGTTTGACAGT

CAGGCAGACGCCGCGTCAGAGACTTCGGAGTTGGCTATGGATAGCCAAAGCCACGCCTTC

GACAGTACAGACGAACCCGACGGTGTGGACGGTACCCCGGACGCCGCAGGATCTGGCGCT

ACTTCTGAAAACGGAGGCGGGAAGCCCGGCGTCGGGAGGGCCGTGGGCTACCAGGGAGCA

AAGGTTCTAGACCCCGTATCCGGCTTTCATGTGGACCCCGTGGTTGTGTTTGACTTCGCT

AGCTTATACCCAAGCATTATCCAGGCCCATAACCTCTGTTTCACCACCCTGGCGCTCGAT

GAAGTGGATCTGGCCGGGCTTCAACCATCCGTCAACTACTCGACGTTCGAGGTGGGTGAC

CAAAAGTTATTTTTTGTCCACGCCCATATTCGCGAAAGCCTGCTTGGCATCTTGCTGCGC

GACTGGCTGGCCATGCGAAAGGCGGTGAGGGCGCGAATCCCCACCAGCACCCCCGAGGAG

GCAGTTTTACTAGATAAGCAGCAGTCTGCGATTAAGGTGATATGCAACTCGGTTTACGGA

TTCACGGGGGTGGCAAACGGCCTGTTGCCGTGTCTGAGGATAGCGGCTACCGTTACCACG

ATAGGACGCGACATGCTCCTCAAGACCCGAGATTACGTTCACTCTCGTTGGGCGACGCGC

GAGCTGCTGGAGGACAATTTTCCAGGGGCTATAGGTTTCCGAAACCACAAGCCTTACTCC

GTCAGGGTTATCTACGGAGACACCGACTCCGTGTTTATCAAGTTTGTGGGCCTGACGTAC

GAGGGGGTATCGGAGCTGGGGGATGCTATGTCGCGTCAGATTTCAGCGGACCTCTTTAGA

GCGCCCATCAAACTGGAGTGTGAGAAGACCTTTCAGCGACTGCTGCTGATCACCAAGAAG

AAGTACATAGGTGTCATAAACGGGGGGAAGATGCTCATGAAGGGGGTCGACCTGGTCCGC

AAAAATAACTGCTCTTTCATAAACTTGTACGCGCGACATCTGGTAGATCTTTTGTTGTAC

GACGAGGATGTGGCCACGGCGGCAGCAGAGGTGACAGACGTGCCTCCCGCAGAATGGGTG

GGGCGCCCGCTACCGAGCGGCTTTGACAAGTTTGGGCGAGTGCTGGTAGAGGCGTACAAC

CGTATCACTGCCCCCAACTTGGACGTGCGCGAGTTCGTTATGACTGCTGAGCTGAGCCGC

TCACCCGAATCGTATACCAACAAGCGCCTGCCGCACCTCACCGTCTACTTTAAGCTCGCC

ATGAGGAATGAAGAACTGCCCAGCGTAAAAGAGAGAATTCCGTATGTGATAGTTGCGCAG

ACCGAGGCCGCGGAACGCGAAGCGGGTGTAGTAAACTCAATGCGCGGTACCGCCCAAAAC

CCCGTGGTAACCAAGACCGCACGCCCCCAACCTAAACGCAAACTGCTGGTTTCCGACCTC

GCCGAAGACCCGACCTATGTTTCCGAGAATGACGTACCGCTAAACACAGACTACTATTTC

TCCCACCTGTTGGGTACCATAAGCGTGACCTTTAAGGCTCTATTCGGAAATGATGTGAGA

ACAACAGAAAATCTTTTAAAGCGGTTTATTCCGGAAACCCCCCACAAGACCCCCACGAAA

ACCCAGGCACTGCTTGAGCGCGCCGGCTTTGAAAAGCTGACGCCCTTTACACCGGAGGAA

GAAAGTCGTCGAATACTGCATACAGTTTTTTGTACTCTAGAAGCAGCTCCCCATCAAAGC

TGA

>LC834905.1

ATGGCGGCGCGCGAACAGGCCAACTCCGTTCGCAGGAGCGGGTTTTTTAATCCGTTTATT

GGCAAGAGGCCATTTTTCAGGCCGGGAAGCGGGCAGACCGCGGAGACAGAGCGCCCAAGG

CCCCCCCAACACTCGTACTGCACAGAGGTGGGTAGCTTTAAGTTTATAGCTCCAAGATGC

CTCGATGAAGAAGCCCCCGCCGACCAGCGACGCGGTGTACACGTGGGCACCTTGGAGCGC

CCACCCAAAGTGTACTGCGATGGCTCAGAGTACGACGTGCTGAACTTTGCCTCCGGAGGT

TGCTGGCCTCGCAGGATTCGGGTTTGGAACGGCCAGGATTTTCGGGGCGATGGATTCAAC

CCCAGATTTGAGAGATTTCACGTGTACGACATAGTAGAGACTTCGGAGAGCGCCTCACAC

GATGACCCATCCAGGTTTGCTGAGCTATCCCGCCCAAGCGGGTCTGTGGTAACACTGCTG

GGAATGAGCGAGTGTGGAAAGCGAGTCGCGGTTCACGTCTATGGTGTGCGCCATTATTTT

TACATGGCAAAGGCGGAGGTGGATAGCGCTTGTGGAATAACCACCGAGGCAGAACTCGTG

CGCGCAATGGTGGACTGCGCGCACAGCTCGGCTTTGAGCGCGGCCCTGGGAAATGGCAAC

GGCGGCAAACAGAGCGGCGGCAGCGGCGGGGGATGGTGGGGCGGAAAGCACGTGTCTGCG

GACTGCTTCAAAGTGGAGACCGTGTGCCGCACGACGCTGTACTACTTTGGATCTAAGCCA

GCTCTCTACTATAGAGTATCTGCCTCCAGCAGCCGCCTGGGAGGGTTCATCTGCGACAAC

TTTCACCCGGAGATTACAAAATTCGAGGGGAGCGTGGACGTGACCACGCGGCTGTTGTTG

GACAATGAAAATTTTACCAGTTTCGGGTGGTACCGCCTGCGACCCGGCACCCACGGAGAG

CGTGTTCAACTTCGCCCCGTTGAGCGACACGTCACCTCAAGCGACGTGGAGATTAACTGT

ACTCCCGATAACCTGGAGCCGATACCAGACGAGGCTGCCTGGCCCGACTATAAGCTCATG

TGCTTTGATATAGAGTGTAAAGCTGGAACGGGTAACGAAATGGCGTTCCCAGTGGCAACT

AACCAAGAGGACCTGGTCATCCAGATCTCCTGTCTGCTGTACTCGCTTGCTACTCAGAAC

CACGAACACACCCTGCTGTTTTCCCTCGGGTCATGCGATATCTCTGAGGAATACTCGTTT

GCATGCGTCCAGCGCGGCGAGCCCAGACCGACGGTTTTGGAGTTTGACAGCGAGTACGAG

CTGCTGGTTGCCTTCCTGACCTTTCTCAAGCAGTACTCTCCCGAGTTCGCCACCGGCTAC

AACATCGTTAATTTTGACTGGGCGTACATAGTTAACAAGGTAACGTCGGTGTATAACATC

AAGCTGGACGGGTACGGCAAGTTCAACAAAGGGGGGCTGTTTAAGGTGTGGGACATCGCC

ACGAACCATTTTCAGAAGAAGAGCAAGGTGAAAATCAATGGCCTGATATCTCTAGACATG

TATTCTGTGGCGACGGAAAAGCTAAAGCTACCCAGCTACAAACTCGACGCGGTCGTGGGA

GACGTCCTCGGCGAGCATAAGATAGACCTTCCCTATAAAGAAATACCCTCCTATTACGCG

GGAGGGCCTGACCGGAGGGGCGTAATAGGAGAGTATTGTATCCAGGACTCTAGGCTGGTG

GGCAAGCTGTTTTTTAAGTACCTCCCCCATCTGGAACTATCGGCGGTGGCCAAACTCGCC

CGTATCACCCTAACGCGGGTAATTTTTGACGGTCAGCAAATTCGCGTGTACACGTGCTTG

CTGAAACTCGCCCGCGAGAGAAATTTCATTTTGCCAGACAACAGACGCCGGTTTGACAGT

CAGGCAGACGCCGCGTCAGAGACTTCGGAGTTGGCTATGGATAGCCAAAGCCACGCCTTC

GACAGTACAGACGAACCCGACGGTGTGGACGGTACCCCGGACGCCGCAGGATCTGGCGCT

ACTTCTGAAAACGGAGGCGGGAAGCCCGGCGTCGGGAGGGCCGTGGGCTACCAGGGAGCA

AAGGTTCTAGACCCCGTATCCGGCTTTCATGTGGACCCCGTGGTTGTGTTTGACTTCGCT

AGCTTATACCCAAGCATTATCCAGGCCCATAACCTCTGTTTCACCACCCTGGCGCTCGAT

GAAGTGGATCTGGCCGGGCTTCAACCATCCGTCAACTACTCGACGTTCGAGGTGGGTGAC

CAAAAGTTATTTTTTGTCCACGCCCATATTCGCGAAAGCCTGCTTGGCATCTTGCTGCGC

GACTGGCTGGCCATGCGAAAGGCGGTGAGGGCGCGAATCCCCACCAGCACCCCCGAGGAG

GCAGTTTTACTAGATAAGCAGCAGTCTGCGATTAAGGTGATATGCAACTCGGTTTACGGA

TTCACGGGGGTGGCAAACGGCCTGTTGCCGTGTCTGAGGATAGCGGCTACCGTTACCACG

ATAGGACGCGACATGCTCCTCAAGACCCGAGATTACGTTCACTCTCGTTGGGCGACGCGC

GAGCTGCTGGAGGACAATTTTCCAGGGGCTATAGGTTTCCGAAACCACAAGCCTTACTCC

GTCAGGGTTATCTACGGAGACACCGACTCCGTGTTTATCAAGTTTGTGGGCCTGACGTAC

GAGGGGGTATCGGAGCTGGGGGATGCTATGTCGCGTCAGATTTCAGCGGACCTCTTTAGA

GCGCCCATCAAACTGGAGTGTGAGAAGACCTTTCAGCGACTGCTGCTGATCACCAAGAAG

AAGTACATAGGTGTCATAAACGGGGGGAAGATGCTCATGAAGGGGGTCGACCTGGTCCGC

AAAAATAACTGCTCTTTCATAAACTTGTACGCGCGACATCTGGTAGATCTTTTGTTGTAC

GACGAGGATGTGGCCACGGCGGCAGCAGAGGTGACAGACGTGCCTCCCGCAGAATGGGTG

GGGCGCCCGCTACCGAGCGGCTTTGACAAGTTTGGGCGAGTGCTGGTAGAGGCGTACAAC

CGTATCACTGCCCCCAACTTGGACGTGCGCGAGTTCGTTATGACTGCTGAGCTGAGCCGC

TCACCCGAATCGTATACCAACAAGCGCCTGCCGCACCTCACCGTCTACTTTAAGCTCGCC

ATGAGGAATGAAGAACTGCCCAGCGTAAAAGAGAGAATTCCGTATGTGATAGTTGCGCAG

ACCGAGGCCGCGGAACGCGAAGCGGGTGTAGTAAACTCAATGCGCGGTACCGCCCAAAAC

CCCGTGGTAACCAAGACCGCACGCCCCCAACCTAAACGCAAACTGCTGGTTTCCGACCTC

GCCGAAGACCCGACCTATGTTTCCGAGAATGACGTACCGCTAAACACAGACTACTATTTC

TCCCACCTGTTGGGTACCATAAGCGTGACCTTTAAGGCTCTATTCGGAAATGATGTGAGA

ACAACAGAAAATCTTTTAAAGCGGTTTATTCCGGAAACCCCCCACAAGACCCCCACGAAA

ACCCAGGCACTGCTTGAGCGCGCCGGCTTTGAAAAGCTGACGCCCTTTACACCGGAGGAA

GAAAGTCGTCGAATACTGCATACAGTTTTTTGTACTCTAGAAGCAGCTCCCCATCAAAGC

TGA

>LC834906.1

ATGGCGGCGCGCGAACAGGCCAACTCCGTTCGCAGGAGCGGGTTTTTTAATCCGTTTATT

GGCAAGAGGCCATTTTTCAGGCCGGGAAGCGGGCAGACCGCGGAGACAGAGCGCCCAAGG

CCCCCCCAACACTCGTACTGCACAGAGGTGGGTAGCTTTAAGTTTATAGCTCCAAGATGC

CTCGATGAAGAAGCCCCCGCCGACCAGCGACGCGGTGTACACGTGGGCACCTTGGAGCGC

CCACCCAAAGTGTACTGCGATGGCTCAGAGTACGACGTGCTGAACTTTGCCTCCGGAGGT

TGCTGGCCTCGCAGGATTCGGGTTTGGAACGGCCAGGATTTTCGGGGCGATGGATTCAAC

CCCAGATTTGAGAGATTTCACGTGTACGACATAGTAGAGACTTCGGAGAGCGCCTCACAC

GATGACCCATCCAGGTTTGCTGAGCTATCCCGCCCAAGCGGGTCTGTGGTAACACTGCTG

GGAATGAGCGAGTGTGGAAAGCGAGTCGCGGTTCACGTCTATGGTGTGCGCCATTATTTT

TACATGGCAAAGGCGGAGGTGGATAGCGCTTGTGGAATAACCACCGAGGCAGAACTCGTG

CGCGCAATGGTGGACTGCGCGCACAGCTCGGCTTTGAGCGCGGCCCTGGGAAATGGCAAC

GGCGGCAAACAGAGCGGCGGCAGCGGCGGGGGATGGTGGGGCGGAAAGCACGTGTCTGCG

GACTGCTTCAAAGTGGAGACCGTGTGCCACACGACGCTGTACTACTTTGGATCTAAGCCA

GCTCTCTACTATAGAGTATCTGCCTCCAGCAGCCGCCTGGGAGGGTTCATCTGCGACAAC

TTTCACCCGGAGATTACAAAATTCGAGGGGAGCGTGGACGTGACCACGCGGCTGTTGTTG

GACAATGAAAATTTTACCAGTTTCGGGTGGTACCGCCTGCGACCCGGCACCCACGGAGAG

CGTGTTCAACTTCGCCCCGTTGAGCGACACGTCACCTCAAGCGACGTGGAGATTAACTGT

ACTCCCGATAACCTGGAGCCGATACCAGACGAGGCTGCCTGGCCCGACTATAAGCTCATG

TGCTTTGATATAGAGTGTAAAGCTGGAACGGGTAACGAAATGGCGTTCCCAGTGGCAACT

AACCAAGAGGACCTGGTCATCCAGATCTCCTGTCTGCTGTACTCGCTTGCTACTCAGAAC

CACGAACACACCCTGCTGTTTTCCCTCGGGTCATGCGATATCTCTGAGGAATACTCGTTT

GCATGCGTCCAGCGCGGCGAGCCCAGACCGACGGTTTTGGAGTTTGACAGCGAGTACGAG

CTGCTGGTTGCCTTCCTGACCTTTCTCAAGCAGTACTCTCCCGAGTTCGCCACCGGCTAC

AACATCGTTAATTTTGACTGGGCGTACATAGTTAACAAGGTAACGTCGGTGTATAACATC

AAGCTGGACGGGTACGGCAAGTTCAACAAAGGGGGGCTGTTTAAGGTGTGGGACATCGCC

ACGAACCATTTTCAGAAGAAGAGCAAGGTGAAAATCAATGGCCTGATATCTCTAGACATG

TATTCTGTGGCGACGGAAAAGCTAAAGCTACCCAGCTACAAACTCGACGCGGTCGTGGGA

GACGTCCTCGGCGAGCATAAGATAGACCTTCCCTATAAAGAAATACCCTCCTATTACGCG

GGAGGGCCTGACCGGAGGGGCGTAATAGGAGAGTATTGTATCCAGGACTCTAGGCTGGTG

GGCAAGCTGTTTTTTAAGTACCTCCCCCATCTGGAACTATCGGCGGTGGCCAAACTCGCC

CGTATCACCCTAACGCGGGTAATTTTTGACGGTCAGCAAATTCGCGTGTACACGTGCTTG

CTGAAACTCGCCCGCGAGAGAAATTTCATTTTGCCAGACAACAGACGCCGGTTTGACAGT

CAGGCAGACGCCGCGTCAGAGACTTCGGAGTTGGCTATGGATAGCCAAAGCCACGCCTTC

GACAGTACAGACGAACCCGACGGTGTGGACGGTACCCCGGACGCCGCAGGATCTGGCGCT

ACTTCTGAAAACGGAGGCGGGAAGCCCGGCGTCGGGAGGGCCGTGGGCTACCAGGGAGCA

AAGGTTCTAGACCCCGTATCCGGCTTTCATGTGGACCCCGTGGTTGTGTTTGACTTCGCT

AGCTTATACCCAAGCATTATCCAGGCCCATAACCTCTGTTTCACCACCCTGGCGCTCGAT

GAAGCGGATCTGGCCGGGCTTCAACCATCCGTCAACTACTCGACGTTCGAGGTGGGTGAC

CAAAAGTTATTTTTTGTCCACGCCCATATTCGCGAAAGCCTGCTTGGCATCTTGCTGCGC

GACTGGCTGGCCATGCGAAAGGCGGTGAGGGCGCGAATCCCCACCAGCACCCCCGAGGAG

GCAGTTTTACTAGATAAGCAGCAGTCTGCGATTAAGGTGATATGCAACTCGGTTTACGGA

TTCACGGGGGTGGCAAACGGCCTGTTGCCGTGTCTGAGGATAGCGGCTACCGTTACCACG

ATAGGACGCGACATGCTCCTCAAGACCCGAGATTACGTTCACTCTCGTTGGGCGACGCGC

GAGCTGCTGGAGGACAATTTTCCAGGGGCTATAGGTTTCCGAAACCACAAGCCTTACTCC

GTCAGGGTTATCTACGGAGACACCGACTCCGTGTTTATCAAGTTTGTGGGCCTGACGTAC

GAGGGGGTATCGGAGCTGGGGGATGCTATGTCGCGTCAGATTTCAGCGGACCTCTTTAGA

GCGCCCATCAAACTGGAGTGTGAGAAGACCTTTCAGCGACTGCTGCTGATCACCAAGAAG

AAGTACATAGGTGTCATAAACGGGGGGAAGATGCTCATGAAGGGGGTCGACCTGGTCCGC

AAAAATAACTGCTCTTTCATAAACTTGTACGCGCGACATCTGGTAGATCTTTTGTTGTAC

GACGAGGATGTGGCCACGGCGGCAGCAGAGGTGACAGACGTGCCTCCCGCAGAATGGGTG

GGGCGCCCGCTACCGAGCGGCTTTGACAAGTTTGGGCGAGTGCTGGTAGAGGCGTACAAC

CGTATCACTGCCCCCAACTTGGACGTGCGCGAGTTCGTTATGACTGCTGAGCTGAGCCGC

TCACCCGAATCGTATACCAACAAGCGCCTGCCGCACCTCACCGTCTACTTTAAGCTCGCC

ATGAGGAATGAAGAACTGCCCAGCGTAAAAGAGAGAATTCCGTATGTGATAGTTGCGCAG

ACCGAGGCCGCGGAACGCGAAGCGGGTGTAGTAAACTCAATGCGCGGTACCGCCCAAAAC

CCCGTGGTAACCAAGACCGCACGCCCCCAACCTAAACGCAAACTGCTGGTTTCCGACCTC

GCCGAAGACCCGACCTATGTTTCCGAGAATGACGTACCGCTAAACACAGACTACTATTTC

TCCCACCTGTTGGGTACCATAAGCGTGACCTTTAAGGCTCTATTCGGAAATGATGTGAGA

ACAACAGAAAATCTTTTAAAGCGGTTTATTCCGGAAACCCCCCACAAGACCCCCACGAAA

ACCCAGGCACTGCTTGAGCGCGCCGGCTTTGAAAAGCTGACGCCCTTTACACCGGAGGAA

GAAAGTCGTCGAATACTGCATACAGTTTTTTGTACTCTAGAAGCAGCTCCCCATCAAAGC

TGA

>LC834907.1

ATGGCGGCGCGCGAACAGGCCAACTCCGTTCGCAGGAGCGGGTTTTTTAATCCGTTTATT

GGCAAGAGGCCATTTTTCAGGCCGGGAAGCGGGCAGACCGCGGAGACAGAGCGCCCAAGG

CCCCCCCAACACTCGTACTGCACAGAGGTGGGTAGCTTTAAGTTTATAGCTCCAAGATGC

CTCGATGAAGAAGCCCCCGCCGACCAGCGACGCGGTGTACACGTGGGCACCTTGGAGCGC

CCACCCAAAGTGTACTGCGATGGCTCAGAGTACGACGTGCTGAACTTTGCCTCCGGAGGT

TGCTGGCCTCGCAGGATTCGGGTTTGGAACGGCCAGGATTTTCGGGGCGATGGATTCAAC

CCCAGATTTGAGAGATTTCACGTGTACGACATAGTAGAGACTTCGGAGAGCGCCTCACAC

GATGACCCATCCAGGTTTGCTGAGCTATCCCGCCCAAGCGGGTCTGTGGTAACACTGCTG

GGAATGAGCGAGTGTGGAAAGCGAGTCGCGGTTCACGTCTATGGTGTGCGCCATTATTTT

TACATGGCAAAGGCGGAGGTGGATAGCGCTTGTGGAATAACCACCGAGGCAGAACTCGTG

CGCGCAATGGTGGACTGCGCGCACAGCTCGGCTTTGAGCGCGGCCCTGGGAAATGGCAAC

GGCGGCAAACAGAGCGGCGGCAGCGGCGGGGGATGGTGGGGCGGAAAGCACGTGTCTGCG

GACTGCTTCAAAGTGGAGACCGTGTGCCACACGACGCTGTACTACTTTGGATCTAAGCCA

GCTCTCTACTATAGAGTATCTGCCTCCAGCAGCCGCCTGGGAGGGTTCATCTGCGACAAC

TTTCACCCGGAGATTACAAAATTCGAGGGGAGCGTGGACGTGACCACGCGGCTGTTGTTG

GACAATGAAAATTTTACCAGTTTCGGGTGGTACCGCCTGCGACCCGGCACCCACGGAGAG

CGTGTTCAACTTCGCCCCGTTGAGCGACACGTCACCTCAAGCGACGTGGAGATTAACTGT

ACTCCCGATAACCTGGAGCCGATACCAGACGAGGCTGCCTGGCCCGACTATAAGCTCATG

TGCTTTGATATAGAGTGTAAAGCTGGAACGGGTAACGAAATGGCGTTCCCAGTGGCAACT

AACCAAGAGGACCTGGTCATCCAGATCTCCTGTCTGCTGTACTCGCTTGCTACTCAGAAC

CACGAACACACCCTGCTGTTTTCCCTCGGGTCATGCGATATCTCTGAGGAATACTCGTTT

GCATGCGTCCAGCGCGGCGAGCCCAGACCGACGGTTTTGGAGTTTGACAGCGAGTACGAG

CTGCTGGTTGCCTTCCTGACCTTTCTCAAGCAGTACTCTCCCGAGTTCGCCACCGGCTAC

AACATCGTTAATTTTGACTGGGCGTACATAGTTAACAAGGTAACGTCGGTGTATAACATC

AAGCTGGACGGGTACGGCAAGTTCAACAAAGGGGGGCTGTTTAAGGTGTGGGACATCGCC

ACGAACCATTTTCAGAAGAAGAGCAAGGTGAAAATCAATGGCCTGATATCTCTAGACATG

TATTCTGTGGCGACGGAAAAGCTAAAGCTACCCAGCTACAAACTCGACGCGGTCGTGGGA

GACGTCCTCGGCGAGCATAAGATAGACCTTCCCTATAAAGAAATACCCTCCTATTACGCG

GGAGGGCCTGACCGGAGGGGCGTAATAGGAGAGTATTGTATCCAGGACTCTAGGCTGGTG

GGCAAGCTGTTTTTTAAGTACCTCCCCCATCTGGAACTATCGGCGGTGGCCAAACTCGCC

CGTATCACCCTAACGCGGGTAATTTTTGACGGTCAGCAAATTCGCGTGTACACGTGCTTG

CTGAAACTCGCCCGCGAGAGAAATTTCATTTTGCCAGACAACAGACGCCGGTTTGACAGT

CAGGCAGACGCCGCGTCAGAGACTTCGGAGTTGGCTATGGATAGCCAAAGCCACGCCTTC

GACAGTACAGACGAACCCGACGGTGTGGACGGTACCCCGGACGCCGCAGGATCTGGCGCT

ACTTCTGAAAACGGAGGCGGGAAGCCCGGCGTCGGGAGGGCCGTGGGCTACCAGGGAGCA

AAGGTTCTAGACCCCGTATCCGGCTTTCATGTGGACCCCGTGGTTGTGTTTGACTTCGCT

AGCTTATACCCAAGCATTATCCAGGCCCATAACCTCTGTTTCACCACCCTGGCGCTCGAT

GAAGCGGATCTGGCCGGGCTTCAACCATCCGTCAACTACTCGACGTTCGAGGTGGGTGAC

CAAAAGTTATTTTTTGTCCACGCCCATATTCGCGAAAGCCTGCTTGGCATCTTGCTGCGC

GACTGGCTGGCCATGCGAAAGGCGGTGAGGGCGCGAATCCCCACCAGCACCCCCGAGGAG

GCAGTTTTACTAGATAAGCAGCAGTCTGCGATTAAGGTGATATGCAACTCGGTTTACGGA

TTCACGGGGGTGGCAAACGGCCTGTTGCCGTGTCTGAGGATAGCGGCTACCGTTACCACG

ATAGGACGCGACATGCTCCTCAAGACCCGAGATTACGTTCACTCTCGTTGGGCGACGCGC

GAGCTGCTGGAGGACAATTTTCCAGGGGCTATAGGTTTCCGAAACCACAAGCCTTACTCC

GTCAGGGTTATCTACGGAGACACCGACTCCGTGTTTATCAAGTTTGTGGGCCTGACGTAC

GAGGGGGTATCGGAGCTGGGGGATGCTATGTCGCGTCAGATTTCAGCGGACCTCTTTAGA

GCGCCCATCAAACTGGAGTGTGAGAAGACCTTTCAGCGACTGCTGCTGATCACCAAGAAG

AAGTACATAGGTGTCATAAACGGGGGGAAGATGCTCATGAAGGGGGTCGACCTGGTCCGC

AAAAATAACTGCTCTTTCATAAACTTGTACGCGCGACATCTGGTAGATCTTTTGTTGTAC

GACGAGGATGTGGCCACGGCGGCAGCAGAGGTGACAGACGTGCCTCCCGCAGAATGGGTG

GGGCGCCCGCTACCGAGCGGCTTTGACAAGTTTGGGCGAGTGCTGGTAGAGGCGTACAAC

CGTATCACTGCCCCCAACTTGGACGTGCGCGAGTTCGTTATGACTGCTGAGCTGAGCCGC

TCACCCGAATCGTATACCAACAAGCGCCTGCCGCACCTCACCGTCTACTTTAAGCTCGCC

ATGAGGAATGAAGAACTGCCCAGCGTAAAAGAGAGAATTCCGTATGTGATAGTTGCGCAG

ACCGAGGCCGCGGAACGCGAAGCGGGTGTAGTAAACTCAATGCGCGGTACCGCCCAAAAC

CCCGTGGTAACCAAGACCGCACGCCCCCAACCTAAACGCAAACTGCTGGTTTCCGACCTC

GCCGAAGACCCGACCTATGTTTCCGAGAATGACGTACCGCTAAACACAGACTACTATTTC

TCCCACCTGTTGGGTACCATAAGCGTGACCTTTAAGGCTCTATTCGGAAATGATGTGAGA

ACAACAGAAAATCTTTTAAAGCGGTTTATTCCGGAAACCCCCCACAAGACCCCCACGAAA

ACCCAGGCACTGCTTGAGCGCGCCGGCTTTGAAAAGCTGACGCCCTTTACACCGGAGGAA

GAAAGTCGTCGAATACTGCATACAGTTTTTTGTACTCTAGAAGCAGCTCCCCATCAAAGC

TGA

>LC834908.1

ATGGCGGCGCGCGAACAGGCCAACTCCGTTCGCAGGAGCGGGTTTTTTAATCCGTTTATT

GGCAAGAGGCCATTTTTCAGGCCGGGAAGCGGGCAGACCGCGGAGACAGAGCGCCCAAGG

CCCCCCCAACACTCGTACTGCACAGAGGTGGGTAGCTTTAAGTTTATAGCTCCAAGATGC

CTCGATGAAGAAGCCCCCGCCGACCAGCGACGCGGTGTACACGTGGGCACCTTGGAGCGC

CCACCCAAAGTGTACTGCGATGGCTCAGAGTACGACGTGCTGAACTTTGCCTCCGGAGGT

TGCTGGCCTCGCAGGATTCGGGTTTGGAACGGCCAGGATTTTCGGGGCGATGGATTCAAC

CCCAGATTTGAGAGATTTCACGTGTACGACATAGTAGAGACTTCGGAGAGCGCCTCACAC

GATGACCCATCCAGGTTTGCTGAGCTATCCCGCCCAAGCGGGTCTGTGGTAACACTGCTG

GGAATGAGCGAGTGTGGAAAGCGAGTCGCGGTTCACGTCTATGGTGTGCGCCATTATTTT

TACATGGCAAAGGCGGAGGTGGATAGCGCTTGTGGAATAACCACCGAGGCAGAACTCGTG

CGCGCAATGGTGGACTGCGCGCACAGCTCGGCTTTGAGCGCGGCCCTGGGAAATGGCAAC

GGCGGCAAACAGAGCGGCGGCAGCGGCGGGGGATGGTGGGGCGGAAAGCACGTGTCTGCG

GACTGCTTCAAAGTGGAGACCGTGTGCCACACGACGCTGTACTACTTTGGATCTAAGCCA

GCTCTCTACTATAGAGTATCTGCCTCCAGCAGCCGCCTGGGAGGGTTCATCTGCGACAAC

TTTCACCCGGAGATTACAAAATTCGAGGGGAGCGTGGACGTGACCACGCGGCTGTTGTTG

GACAATGAAAATTTTACCAGTTTCGGGTGGTACCGCCTGCGACCCGGCACCCACGGAGAG

CGTGTTCAACTTCGCCCCGTTGAGCGACACGTCACCTCAAGCGACGTGGAGATTAACTGT

ACTCCCGATAACCTGGAGCCGATACCAGACGAGGCTGCCTGGCCCGACTATAAGCTCATG

TGCTTTGATATAGAGTGTAAAGCTGGAACGGGTAACGAAATGGCGTTCCCAGTGGCAACT

AACCAAGAGGACCTGGTCATCCAGATCTCCTGTCTGCTGTACTCGCTTGCTACTCAGAAC

CACGAACACACCCTGCTGTTTTCCCTCGGGTCATGCGATATCTCTGAGGAATACTCGTTT

GCATGCGTCCAGCGCGGCGAGCCCAGACCGACGGTTTTGGAGTTTGACAGCGAGTACGAG

CTGCTGGTTGCCTTCCTGACCTTTCTCAAGCAGTACTCTCCCGAGTTCGCCACCGGCTAC

AACATCGTTAATTTTGACTGGGCGTACATAGTTAACAAGGTAACGTCGGTGTATAACATC

AAGCTGGACGGGTACGGCAAGTTCAACAAAGGGGGGCTGTTTAAGGTGTGGGACATCGCC

ACGAACCATTTTCAGAAGAAGAGCAAGGTGAAAATCAATGGCCTGATATCTCTAGACATG

TATTCTGTGGCGACGGAAAAGCTAAAGCTACCCAGCTACAAACTCGACGCGGTCGTGGGA

GACGTCCTCGGCGAGCATAAGATAGACCTTCCCTATAAAGAAATACCCTCCTATTACGCG

GGAGGGCCTGACCGGAGGGGCGTAATAGGAGAGTATTGTATCCAGGACTCTAGGCTGGTG

GGCAAGCTGTTTTTTAAGTACCTCCCCCATCTGGAACTATCGGCGGTGGCCAAACTCGCC

CGTATCACCCTAACGCGGGTAATTTTTGACGGTCAGCAAATTCGCGTGTACACGTGCTTG

CTGAAACTCGCCCGCGAGAGAAATTTCATTTTGCCAGACAACAGACGCCGGTTTGACAGT

CAGGCAGACGCCGCGTCAGAGACTTCGGAGTTGGCTATGGATAGCCAAAGCCACGCCTTC

GACAGTACAGACGAACCCGACGGTGTGGACGGTACCCCGGACGCCGCAGGATCTGGCGCT

ACTTCTGAAAACGGAGGCGGGAAGCCCGGCGTCGGGAGGGCCGTGGGCTACCAGGGAGCA

AAGGTTCTAGACCCCGTATCCGGCTTTCATGTGGACCCCGTGGTTGTGTTTGACTTCGCT

AGCTTATACCCAAGCATTATCCAGGCCCATAACCTCTGTTTCACCACCCTGGCGCTCGAT

GAAGTGGATCTGGCCGGGCTTCAACCATCCGTCAACTACTCGACGTTCGAGGTGGGTGGC

CAAAAGTTATTTTTTGTCCACGCCCATATTCGCGAAAGCCTGCTTGGCATCTTGCTGCGC

GACTGGCTGGCCATGCGAAAGGCGGTGAGGGCGCGAATCCCCACCAGCACCCCCGAGGAG

GCAGTTTTACTAGATAAGCAGCAGTCTGCGATTAAGGTGATATGCAACTCGGTTTACGGA

TTCACGGGGGTGGCAAACGGCCTGTTGCCGTGTCTGAGGATAGCGGCTACCGTTACCACG

ATAGGACGCGACATGCTCCTCAAGACCCGAGATTACGTTCACTCTCGTTGGGCGACGCGC

GAGCTGCTGGAGGACAATTTTCCAGGGGCTATAGGTTTCCGAAACCACAAGCCTTACTCC

GTCAGGGTTATCTACGGAGACACCGACTCCGTGTTTATCAAGTTTGTGGGCCTGACGTAC

GAGGGGGTATCGGAGCTGGGGGATGCTATGTCGCGTCAGATTTCAGCGGACCTCTTTAGA

GCGCCCATCAAACTGGAGTGTGAGAAGACCTTTCAGCGACTGCTGCTGATCACCAAGAAG

AAGTACATAGGTGTCATAAACGGGGGGAAGATGCTCATGAAGGGGGTCGACCTGGTCCGC

AAAAATAACTGCTCTTTCATAAACTTGTACGCGCGACATCTGGTAGATCTTTTGTTGTAC

GACGAGGATGTGGCCACGGCGGCAGCAGAGGTGACAGACGTGCCTCCCGCAGAATGGGTG

GGGCGCCCGCTACCGAGCGGCTTTGACAAGTTTGGGCGAGTGCTGGTAGAGGCGTACAAC

CGTATCACTGCCCCCAACTTGGACGTGCGCGAGTTCGTTATGACTGCTGAGCTGAGCCGC

TCACCCGAATCGTATACCAACAAGCGCCTGCCGCACCTCACCGTCTACTTTAAGCTCGCC

ATGAGGAATGAAGAACTGCCCAGCGTAAAAGAGAGAATTCCGTATGTGATAGTTGCGCAG

ACCGAGGCCGCGGAACGCGAAGCGGGTGTAGTAAACTCAATGCGCGGTACCGCCCAAAAC

CCCGTGGTAACCAAGACCGCACGCCCCCAACCTAAACGCAAACTGCTGGTTTCCGACCTC

GCCGAAGACCCGACCTATGTTTCCGAGAATGACGTACCGCTAAACACAGACTACTATTTC

TCCCACCTGTTGGGTACCATAAGCGTGACCTTTAAGGCTCTATTCGGAAATGATGTGAGA

ACAACAGAAAATCTTTTAAAGCGGTTTATTCCGGAAACCCCCCACAAGACCCCCACGAAA

ACCCAGGCACTGCTTGAGCGCGCCGGCTTTGAAAAGCTGACGCCCTTTACACCGGAGGAA

GAAAGTCGTCGAATACTGCATACAGTTTTTTGTACTCTAGAAGCAGCTCCCCATCAAAGC

TGA

>LC834909.1

ATGGCGGCGCGCGAACAGGCCAACTCCGTTCGCAGGAGCGGGTTTTTTAATCCGTTTATT

GGCAAGAGGCCATTTTTCAGGCCGGGAAGCGGGCAAACCGCGGAGACAGAGCGCCCAAGG

CCCCCCCAACACTCGTACTGCACAGAGGTGGGTAGCTTTAAGTTTATAGCTCCAAGATGC

CTCGATGAAGAAGCCCCCGCCGACCAGCGACGCGGTGTACACGTGGGCACCTTGGAGCGC

CCACCCAAAGTGTACTGCGATGGCTCAGAGTACGACGTGCTGAACTTTGCCTCCGGAGGT

TGCTGGCCTCGCAGGATTCGGGTTTGGAACGGCCAGGATTTTCGGGGCGATGGATTCAAC

CCCAGATTTGAGAGATTTCACGTGTACGACATAGTAGAGACTTCGGAGAGCGCCTCACAC

GATGACCCATCCAGGTTTGCTGAGCTATCCCGCCCAAGCGGGTCTGTGGTAACACTGCTG

GGAATGAGCGAGTGTGGAAAGCGAGTCGCGGTTCACGTCTATGGTGTGCGCCATTATTTT

TACATGGCAAAGGCGGAGGTGGATAGCGCTTGTGGAATAACCACCGAGGCAGAACTCGTG

CGCGCAATGGTGGACTGCGCGCACAGCTCGGCTTTGAGCGCGGCCCTGGGAAATGGCAAC

GGCGGCAAACAGAGCGGCGGCAGCGGCGGGGGATGGTGGGGCGGAAAGCACGTGTCTGCG

GACTGCTTCAAAGTGGAGACCGTGTGCCACACGACGCTGTACTACTTTGGATCTAAGCCA

GCTCTCTACTATAGAGTATCTGCCTCCAGCAGCCGCCTGGGAGGGTTCATCTGCGACAAC

TTTCACCCGGAGATTACAAAATTCGAGGGGAGCGTGGACGTGACCACGCGGCTGTTGTTG

GACAATGAAAATTTTACCAGTTTCGGGTGGTACCGCCTGCGACCCGGCACCCACGGAGAG

CGTGTTCAACTTCGCCCCGTTGAGCGACACGTCACCTCAAGCGACGTGGAGATTAACTGT

ACTCCCGATAACCTGGAGCCGATACCAGACGAGGCTGCCTGGCCCGACTATAAGCTCATG

TGCTTTGATATAGAGTGTAAAGCTGGAACGGGTAACGAAATGGCGTTCCCAGTGGCAACT

AACCAAGAGGACCTGGTCATCCAGATCTCCTGTCTGCTGTACTCGCTTGCTACTCAGAAC

CACGAACACACCCTGCTGTTTTCCCTCGGGTCATGCGATATCTCTGAGGAATACTCGTTT

GCATGCGTCCAGCGCGGCGAGCCCAGACCGACGGTTTTGGAGTTTGACAGCGAGTACGAG

CTGCTGGTTGCCTTCCTGACCTTTCTCAAGCAGTACTCTCCCGAGTTCGCCACCGGCTAC

AACATCGTTAATTTTGACTGGGCGTACATAGTTAACAAGGTAACGTCGGTGTATAACATC

AAGCTGGACGGGTACGGCAAGTTCAACAAAGGGGGGCTGTTTAAGGTGTGGGACATCGCC

ACGAACCATTTTCAGAAGAAGAGCAAGGTGAAAATCAATGGCCTGATATCTCTAGACATG

TATTCTGTGGCGACGGAAAAGCTAAAGCTACCCAGCTACAAACTCGACGCGGTCGTGGGA

GACGTCCTCGGCGAGCATAAGATAGACCTTCCCTATAAAGAAATACCCTCCTATTACGCG

GGAGGGCCTGACCGGAGGGGCGTAATAGGAGAGTATTGTATCCAGGACTCTAGGCTGGTG

GGCAAGCTGTTTTTTAAGTACCTCCCCCATCTGGAACTATCGGCGGTGGCCAAACTCGCC

CGTATCACCCTAACGCGGGTAATTTTTGACGGTCAGCAAATTCGCGTGTACACGTGCTTG

CTGAAACTCGCCCGCGAGAGAAATTTCATTTTGCCAGACAACAGACGCCGGTTTGACAGT

CAGGCAGACGCCGCGTCAGAGACTTCGGAGTTGGCTATGGATAGCCAAAGCCACGCCTTC

GACAGTACAGACGAACCCGACGGTGTGGACGGTACCCCGGACGCCGCAGGATCTGGCGCT

ACTTCTGAAAACGGAGGCGGGAAGCCCGGCGTCGGGAGGGCCGTGGGCTACCAGGGAGCA

AAGGTTCTAGACCCCGTATCCGGCTTTCATGTGGACCCCGTGGTTGTGTTTGACTTCGCT

AGCTTATACCCAAGCATTATCCAGGCCCATAACCTCTGTTTCACCACCCTGGCGCTCGAT

GAAGTGGATCTGGCCGGGCTTCAACCATCCGTCAACTACTCGACGTTCGAGGTGGGTGAC

CAAAAGTTATTTTTTGTCCACGCCCATATTCGCGAAAGCCTGCTTGGCATCTTGCTGCGC

GACTGGCTGGCCATGCGAAAGGCGGTGAGGGCGCGAATCCCCACCAGCACCCCCGAGGAG

GCAGTTTTACTAGATAAGCAGCAGTCTGCGATTAAGGTGATATGCAACTCGGTTTACGGA

TTCACGGGGGTGGCAAACGGCCTGTTGCCGTGTCTGAGGATAGCGGCTACCGTTACCACG

ATAGGACGCGACATGCTCCTCAAGACCCGAGATTACGTTCACTCTCGTTGGGCGACGCGC

GAGCTGCTGGAGGACAATTTTCCAGGGGCTATAGGTTTCCGAAACCACAAGCCTTACTCC

GTCAGGGTTATCTACGGAGACACCGACTCCGTGTTTATCAAGTTTGTGGGCCTGACGTAC

GAGGGGGTATCGGAGCTGGGGGATGCTATGTCGCGTCAGATTTCAGCGGACCTCTTTAGA

GCGCCCATCAAACTGGAGTGTGAGAAGACCTTTCAGCGACTGCTGCTGATCACCAAGAAG

AAGTACATAGGTGTCATAAACGGGGGGAAGATGCTCATGAAGGGGGTCGACCTGGTCCGC

AAAAATAACTGCTCTTTCATAAACTTGTACGCGCGACATCTGGTAGATCTTTTGTTGTAC

GACGAGGATGTGGCCACGGCGGCAGCAGAGGTGACAGACGTGCCTCCCGCAGAATGGGTG

GGGCGCCCGCTACCGAGCGGCTTTGACAAGTTTGGGCGAGTGCTGGTAGAGGCGTACAAC

CGTATCACTGCCCCCAACTTGGACGTGCGCGAGTTCGTTATGACTGCTGAGCTGAGCCGC

TCACCCGAATCGTATACCAACAAGCGCCTGCCGCACCTCACCGTCTACTTTAAGCTCGCC

ATGAGGAATGAAGAACTGCCCAGCGTAAAAGAGAGAATTCCGTATGTGATAGTTGCGCAG

ACCGAGGCCGCGGAACGCGAAGCGGGTGTAGTAAACTCAATGCGCGGTACCGCCCAAAAC

CCCGTGGTAACCAAGACCGCACGCCCCCAACCTAAACGCAAACTGCTGGTTTCCGACCTC

GCCGAAGACCCGACCTATGTTTCCGAGAATGACGTACCGCTAAACACAGACTACTATTTC

TCCCACCTGTTGGGTACCATAAGCGTGACCTTTAAGGCTCTATTCGGAAATGATGTGAGA

ACAACAGAAAATCTTTTAAAGCGGTTTATTCCGGAAACCCCCCACAAGACCCCCACGAAA

ACCCAGGCACTGCTTGAGCGCGCCGGCTTTGAAAAGCTGACGCCCTTTACACCGGAGGAA

GAAAGTCGTCGAATACTGCATACAGTTTTTTGTACTCTAGAAGCAGCTCCCCATCAAAGC

TGA

>LC834910.1

ATGGCGGCGCGCGAACAGGCCAACTCCGTTCGCAGGAGCGGGTTTTTTAATCCGTTTATT

GGCAAGAGGCCATTTTTCAGGCCGGGAAGCGGGCAGACCGCGGAGACAGAGCGCCCAAGG

CCCCCCCAACACTCGTACTGCACAGAGGTGGGTAGCTTTAAGTTTATAGCTCCAAGATGC

CTCGATGAAGAAGCCCCCGCCGACCAGCGACGCGGTGTACACGTGGGCACCTTGGAGCGC

CCACCCAAAGTGTACTGCGATGGCTCAGAGTACGACGTGCTGAACTTTGCCTCCGGAGGT

TGCTGGCCTCGCAGGATTCGGGTTTGGAACGGCCAGGATTTTCGGGGCGATGGATTCAAC

CCCAGATTTGAGAGATTTCACGTGTACGACATAGTAGAGACTTCGGAGAGCGCCTCACAC

GATGACCCATCCAGGTTTGCTGAGCTATCCCGCCCAAGCGGGTCTGTGGTAACACTGCTG

GGAATGAGCGAGTGTGGAAAGCGAGTCGCGGTTCACGTCTATGGTGTGCGCCATTATTTT

TACATGGCAAAGGCGGAGGTGGATAGCGCTTGTGGAATAACCACCGAGGCAGAACTCGTG

CGCGCAATGGTGGACTGCGCGCACAGCTCGGCTTTGAGCGCGGCCCTGGGAAATGGCAAC

GGCGGCAAACAGAGCGGCGGCAGCGGCGGGGGATGGTGGGGCGGAAAGCACGTGTCTGCG

GACTGCTTCAAAGTGGAGACCGTGTGCCACACGACGCTGTACTACTTTGGATCTAAGCCA

GCTCTCTACTATAGAGTATCTGCCTCCAGCAGCCGCCTGGGAGGGTTCATCTGCGACAAC

TTTCACCCGGAGATTACAAAATTCGAGGGGATCGTGGACGTGACCACGCGGCTGTTGTTG

GACAATGAAAATTTTACCAGTTTCGGGTGGTACCGCCTGCGACCCGGCACCCACGGAGAG

CGTGTTCAACTTCGCCCCGTTGAGCGACACGTCACCTCAAGCGACGTGGAGATTAACTGT

ACTCCCGATAACCTGGAGCCGATACCAGACGAGGCTGCCTGGCCCGACTATAAGCTCATG

TGCTTTGATATAGAGTGTAAAGCTGGAACGGGTAACGAAATGGCGTTCCCAGTGGCAACT

AACCAAGAGGACCTGGTCATCCAGATCTCCTGTCTGCTGTACTCGCTTGCTACTCAGAAC

CACGAACACACCCTGCTGTTTTCCCTCGGGTCATGCGATATCTCTGAGGAATACTCGTTT

GCATGCGTCCAGCGCGGCGAGCCCAGACCGACGGTTTTGGAGTTTGACAGCGAGTACGAG

CTGCTGGTTGCCTTCCTGACCTTTCTCAAGCAGTACTCTCCCGAGTTCGCCACCGGCTAC

AACATCGTTAATTTTGACTGGGCGTACATAGTTAACAAGGTAACGTCGGTGTATAACATC

AAGCTGGACGGGTACGGCAAGTTCAACAAAGGGGGGCTGTTTAAGGTGTGGGACATCGCC

ACGAACCATTTTCAGAAGAAGAGCAAGGTGAAAATCAATGGCCTGATATCTCTAGACATG

TATTCTGTGGCGACGGAAAAGCTAAAGCTACCCAGCTACAAACTCGACGCGGTCGTGGGA

GACGTCCTCGGCGAGCATAAGATAGACCTTCCCTATAAAGAAATACCCTCCTATTACGCG

GGAGGGCCTGACCGGAGGGGCGTAATAGGAGAGTATTGTATCCAGGACTCTAGGCTGGTG

GGCAAGCTGTTTTTTAAGTACCTCCCCCATCTGGAACTATCGGCGGTGGCCAAACTCGCC

CGTATCACCCTAACGCGGGTAATTTTTGACGGTCAGCAAATTCGCGTGTACACGTGCTTG

CTGAAACTCGCCCGCGAGAGAAATTTCATTTTGCCAGACAACAGACGCCGGTTTGACAGT

CAGGCAGACGCCGCGTCAGAGACTTCGGAGTTGGCTATGGATAGCCAAAGCCACGCCTTC

GACAGTACAGACGAACCCGACGGTGTGGACGGTACCCCGGACGCCGCAGGATCTGGCGCT

ACTTCTGAAAACGGAGGCGGGAAGCCCGGCGTCGGGAGGGCCGTGGGCTACCAGGGAGCA

AAGGTTCTAGACCCCGTATCCGGCTTTCATGTGGACCCCGTGGTTGTGTTTGACTTCGCT

AGCTTATACCCAAGCATTATCCAGGCCCATAACCTCTGTTTCACCACCCTGGCGCTCGAT

GAAGTGGATCTGGCCGGGCTTCAACCATCCGTCAACTACTCGACGTTCGAGGTGGGTGAC

CAAAAGTTATTTTTTGTCCACGCCCATATTCGCGAAAGCCTGCTTGGCATCTTGCTGCGC

GACTGGCTGGCCATGCGAAAGGCGGTGAGGGCGCGAATCCCCACCAGCACCCCCGAGGAG

GCAGTTTTACTAGATAAGCAGCAGTCTGCGATTAAGGTGATATGCAACTCGGTTTACGGA

TTCACGGGGGTGGCAAACGGCCTGTTGCCGTGTCTGAGGATAGCGGCTACCGTTACCACG

ATAGGACGCGACATGCTCCTCAAGACCCGAGATTACGTTCACTCTCGTTGGGCGACGCGC

GAGCTGCTGGAGGACAATTTTCCAGGGGCTATAGGTTTCCGAAACCACAAGCCTTACTCC

GTCAGGGTTATCTACGGAGACACCGACTCCGTGTTTATCAAGTTTGTGGGCCTGACGTAC

GAGGGGGTATCGGAGCTGGGGGATGCTATGTCGCGTCAGATTTCAGCGGACCTCTTTAGA

GCCCCCATCAAACTGGAGTGTGAGAAGACCTTTCAGCGACTGCTGCTGATCACCAAGAAG

AAGTACATAGGTGTCATAAACGGGGGGAAGATGCTCATGAAGGGGGTCGACCTGGTCCGC

AAAAATAACTGCTCTTTCATAAACTTGTACGCGCGACATCTGGTAGATCTTTTGTTGTAC

GACGAGGATGTGGCCACGGCGGCAGCAGAGGTGACAGACGTGCCTCCCGCAGAATGGGTG

GGGCGCCCGCTACCGAGCGGCTTTGACAAGTTTGGGCGAGTGCTGGTAGAGGCGTACAAC

CGTATCACTGCCCCCAACTTGGACGTGCGCGAGTTCGTTATGACTGCTGAGCTGAGCCGC

TCACCCGAATCGTATACCAACAAGCGCCTGCCGCACCTCACCGTCTACTTTAAGCTCGCC

ATGAGGAATGAAGAACTGCCCAGCGTAAAAGAGAGAATTCCGTATGTGATAGTTGCGCAG

ACCGAGGCCGCGGAACGCGAAGCGGGTGTAGTAAACTCAATGCGCGGTACCGCCCAAAAC

CCCGTGGTAACCAAGACCGCACGCCCCCAACCTAAACGCAAACTGCTGGTTTCCGACCTC

GCCGAAGACCCGACCTATGTTTCCGAGAATGACGTACCGCTAAACACAGACTACTATTTC

TCCCACCTGTTGGGTACCATAAGCGTGACCTTTAAGGCTCTATTCGGAAATGATGTGAGA

ACAACAGAAAATCTTTTAAAGCGGTTTATTCCGGAAACCCCCCACAAGACCCCCACGAAA

ACCCAGGCACTGCTTGAGCGCGCCGGCTTTGAAAAGCTGACGCCCTTTACACCGGAGGAA

GAAAGTCGTCGAATACTGCATACAGTTTTTTGTACTCTAGAAGCAGCTCCCCATCAAAGC

TGA

>LC834911.1

ATGGCGGCGCGCGAACAGGCCAACTCCGTTCGCAGGAGCGGGTTTTTTAATCCGTTTATT

GGCAAGAGGCCATTTTTCAGGCCGGGAAGCGGGCAAACCGCGGAGACAGAGCGCCCAAGG

CCCCCCCAACACTCGTACTGCACAGAGGTGGGTAGCTTTAAGTTTATAGCTCCAAGATGC

CTCGATGAAGAAGCCCCCGCCGACCAGCGACGCGGTGTACACGTGGGCACCTTGGAGCGC

CCACCCAAAGTGTACTGCGATGGCTCAGAGTACGACGTGCTGAACTTTGCCTCCGGAGGT

TGCTGGCCTCGCAGGATTCGGGTTTGGAACGGCCAGGATTTTCGGGGCGATGGATTCAAC

CCCAGATTTGAGAGATTTCACGTGTACGACATAGTAGAGACTTCGGAGAGCGCCTCACAC

GATGACCCATCCAGGTTTGCTGAGCTATCCCGCCCAAGCGGGTCTGTGGTAACACTGCTG

GGAATGAGCGAGTGTGGAAAGCGAGTCGCGGTTCACGTCTATGGTGTGCGCCATTATTTT

TACATGGCAAAGGCGGAGGTGGATAGCGCTTGTGGAATAACCACCGAGGCAGAACTCGTG

CGCGCAATGGTGGACTGCGCGCACAGCTCGGCTTTGAGCGCGGCCCTGGGAAATGGCAAC

GGCGGCAAACAGAGCGGCGGCAGCGGCGGGGGATGGTGGGGCGGAAAGCACGTGTCTGCG

GACTGCTTCAAAGTGGAGACCGTGTGCCACACGACGCTGTACTACTTTGGATCTAAGCCA

GCTCTCTACTATAGAGTATCTGCCTCCAGCAGCCGCCTGGGAGGGTTCATCTGCGACAAC

TTTCACCCGGAGATTACAAAATTCGAGGGGAGCGTGGACGTGACCACGCGGCTGTTGTTG

GACAATGAAAATTTTACCAGTTTCGGGTGGTACCGCCTGCGACCCGGCACCCACGGAGAG

CGTGTTCAACTTCGCCCCGTTGAGCGACACGTCACCTCAAGCGACGTGGAGATTAACTGT

ACTCCCGATAACCTGGAGCCGATACCAGACGAGGCTGCCTGGCCCGACTATAAGCTCATG

TGCTTTGATATAGAGTGTAAAGCTGGAACGGGTAACGAAATGGCGTTCCCAGTGGCAACT

AACCAAGAGGACCTGGTCATCCAGATCTCCTGTCTGCTGTACTCGCTTGCTACTCAGAAC

CACGAACACACCCTGCTGTTTTCCCTCGGGTCATGCGATATCTCTGAGGAATACTCGTTT

GCATGCGTCCAGCGCGGCGAGCCCAGACCGACGGTTTTGGAGTTTGACAGCGAGTACGAG

CTGCTGGTTGCCTTCCTGACCTTTCTCAAGCAGTACTCTCCCGAGTTCGCCACCGGCTAC

AACATCGTTAATTTTGACTGGGCGTACATAGTTAACAAGGTAACGTCGGTGTATAACATC

AAGCTGGACGGGTACGGCAAGTTCAACAAAGGGGGGCTGTTTAAGGTGTGGGACATCGCC

ACGAACCATTTTCAGAAGAAGAGCAAGGTGAAAATCAATGGCCTGATATCTCTAGACATG

TATTCTGTGGCGACGGAAAAGCTAAAGCTACCCAGCTACAAACTCGACGCGGTCGTGGGA

GACGTCCTCGGCGAGCATAAGATAGACCTTCCCTATAAAGAAATACCCTCCTATTACGCG

GGAGGGCCTGACCGGAGGGGCGTAATAGGAGAGTATTGTATCCAGGACTCTAGGCTGGTG

GGCAAGCTGTTTTTTAAGTACCTCCCCCATCTGGAACTATCGGCGGTGGCCAAACTCGCC

CGTATCACCCTAACGCGGGTAATTTTTGACGGTCAGCAAATTCGCGTGTACACGTGCTTG

CTGAAACTCGCCCGCGAGAGAAATTTCATTTTGCCAGACAACAGACGCCGGTTTGACAGT

CAGGCAGACGCCGCGTCAGAGACTTCGGAGTTGGCTATGGATAGCCAAAGCCACGCCTTC

GACAGTACAGACGAACCCGACGGTGTGGACGGTACCCCGGACGCCGCAGGATCTGGCGCT

ACTTCTGAAAACGGAGGCGGGAAGCCCGGCGTCGGGAGGGCCGTGGGCTACCAGGGAGCA

AAGGTTCTAGACCCCGTATCCGGCTTTCATGTGGACCCCGTGGTTGTGTTTGACTTCGCT

AGCTTATACCCAAGCATTATCCAGGCCCATAACCTCTGTTTCACCACCCTGGCGCTCGAT

GAAGTGGATCTGGCCGGGCTTCAACCATCCGTCAACTACTCGACGTTCGAGGTGGGTGAC

CAAAAGTTATTTTTTGTCCACGCCCATATTCGCGAAAGCCTGCTTGGCATCTTGCTGCGC

GACTGGCTGGCCATGCGAAAGGCGGTGAGGGCGCGAATCCCCACCAGCACCCCCGAGGAG

GCAGTTTTACTAGATAAGCAGCAGTCTGCGATTAAGGTGATATGCAACTCGGTTTACGGA

TTCACGGGGGTGGCAAACGGCCTGTTGCCGTGTCTGAGGATAGCGGCTACCGTTACCACG

ATAGGACGCGACATGCTCCTCAAGACCCGAGATTACGTTCACTCTCGTTGGGCGACGCGC

GAGCTGCTGGAGGACAATTTTCCAGGGGCTATAGGTTTCCGAAACCACAAGCCTTACTCC

GTCAGGGTTATCTACGGAGACACCGACTCCGTGTTTATCAAGTTTGTGGGCCTGACGTAC

GAGGGGGTATCGGAGCTGGGGGATGCTATGTCGCGTCAGATTTCAGCGGACCTCTTTAGA

GCGCCCATCAAACTGGAGTGTGAGAAGACCTTTCAGCGACTGCTGCTGATCACCAAGAAG

AAGTACATAGGTGTCATAAACGGGGGGAAGATGCTCATGAAGGGGGTCGACCTGGTCCGC

AAAAATAACTGCTCTTTCATAAACTTGTACGCGCGACATCTGGTAGATCTTTTGTTGTAC

GACGAGGATGTGGCCACGGCGGCAGCAGAGGTGACAGACGTGCCTCCCGCAGAATGGGTG

GGGCGCCCGCTACCGAGCGGCTTTGACAAGTTTGGGCGAGTGCTGGTAGAGGCGTACAAC

CGTATCACTGCCCCCAACTTGGACGTGCGCGAGTTCGTTATGACTGCTGAGCTGAGCCGC

TCACCCGAATCGTATACCAACAAGCGCCTGCCGCACCTCACCGTCTACTTTAAGCTCGCC

ATGAGGAATGAAGAACTGCCCAGCGTAAAAGAGAGAATTCCGTATGTGATAGTTGCGCAG

ACCGAGGCCGCGGAACGCGAAGCGGGTGTAGTAAACTCAATGCGCGGTACCGCCCAAAAC

CCCGTGGTAACCAAGACCGCACGCCCCCAACCTAAACGCAAACTGCTGGTTTCCGACCTC

GCCGAAGACCCGACCTATGTTTCCGAGAATGACGTACCGCTAAACACAGACTACTATTTC

TCCCACCTGTTGGGTACCATAAGCGTGACCTTTAAGGCTCTATTCGGAAATGATGTGAGA

ACAACAGAAAATCTTTTAAAGCGGTTTATTCCGGAAACCCCCCACAAGACCCCCACGAAA

ACCCAGGCACTGCTTGAGCGCGCCGGCTTTGAAAAGCTGACGCCCTTTACACCGGAGGAA

GAAAGTCGTCGAATACTGCATACAGTTTTTTGTACTCTAGAAGCAGCTCCCCATCAAAGC

TGA

>LC834912.1

ATGGCGGCGCGCGAACAGGCCAACTCCGTTCGCAGGAGCGGGTTTTTTAATCCGTTTATT

GGCAAGAGGCCATTTTTCAGGCCGGGAAGCGGGCAGACCGCGGAGACAGAGCGCCCAAGG

CCCCCCCAACACTCGTACTGCACAGAGGTGGGTAGCTTTAAGTTTATAGCTCCAAGATGC

CTCGATGAAGAAGCCCCCGCCGACCAGCGACGCGGTGTACACGTGGGCACCTTGGAGCGC

CCACCCAAAGTGTACTGCGATGGCTCAGAGTACGACGTGCTGAACTTTGCCTCCGGAGGT

TGCTGGCCTCGCAGGATTCGGGTTTGGAACGGCCAGGATTTTCGGGGCGATGGATTCAAC

CCCAGATTTGAGAGATTTCACGTGTACGACATAGTAGAGACTTCGGAGAGCGCCTCACAC

GATGACCCATCCAGGTTTGCTGAGCTATCCCGCCCAAGCGGGTCTGTGGTAACACTGCTG

GGAATGAGCGAGTGTGGAAAGCGAGTCGCGGTTCACGTCTATGGTGTGCGCCATTATTTT

TACATGGCAAAGGCGGAGGTGGATAGCGCTTGTGGAATAACCACCGAGGCAGAACTCGTG

CGCGCAATGGTGGACTGCGCGCACAGCTCGGCTTTGAGCGCGGCCCTGGGAAATGGCAAC

GGCGGCAAACAGAGCGGCGGCAGCGGCGGGGGATGGTGGGGCGGAAAGCACGTGTCTGCG

GACTGCTTCAAAGTGGAGACCGTGTGCCACACGACGCTGTACTACTTTGGATCTAAGCCA

GCTCTCTACTATAGAGTATCTGCCTCCAGCAGCCGCCTGGGAGGGTTCATCTGCGACAAC

TTTCACCCGGAGATTACAAAATTCGAGGGGAGCGTGGACGTGACCACGCGGCTGTTGTTG

GACAATGAAAATTTTACCAGTTTCGGGTGGTACCGCCTGCGACCCGGCACCCACGGAGAG

CGTGTTCAACTTCGCCCCGTTGAGCGACACGTCACCTCAAGCGACGTGGAGATTAACTGT

ACTCCCGATAACCTGGAGCCGATACCAGACGAGGCTGCCTGGCCCGACTATAAGCTCATG

TGCTTTGATATAGAGTGTAAAGCTGGAACGGGTAACGAAATGGCGTTCCCAGTGGCAACT

AACCAAGAGGACCTGGTCATCCAGATCTCCTGTCTGCTGTACTCGCTTGCTACTCAGAAC

CACGAACACACCCTGCTGTTTTCCCTCGGGTCATGCGATATCTCTGAGGAATACTCGTTT

GCATGCGTCCAGCGCGGCGAGCCCAGACCGACGGTTTTGGAGTTTGACAGCGAGTACGAG

CTGCTGGTTGCCTTCCTGACCTTTCTCAAGCAGTACTCTCCCGAGTTCGCCACCGGCTAC

AACATCGTTAATTTTGACTGGGCGTACATAGTTAACAAGGTAACGTCGGTGTATAACATC

AAGCTGGACGGGTACGGCAAGTTCAACAAAGGGGGGCTGTTTAAGGTGTGGGACATCGCC

ACGAACCATTTTCAGAAGAAGAGCAAGGTGAAAATCAATGGCCTGATATCTCTAGACATG

TATTCTGTGGCGACGGAAAAGCTAAAGCTACCCAGCTACAAACTCGACGCGGTCGTGGGA

GACGTCCTCGGCGAGCATAAGATAGACCTTCCCTATAAAGAAATACCCTCCTATTACGCG

GGAGGGCCTGACCGGAGGGGCGTAATAGGAGAGTATTGTATCCAGGACTCTAGGCTGGTG

GGCAAGCTGTTTTTTAAGTACCTCCCCCATCTGGAACTATCGGCGGTGGCCAAACTCGCC

CGTATCACCCTAACGCGGGTAATTTTTGACGGTCAGCAAATTCGCGTGTACACGTGCTTG

CTGAAACTCGCCCGCGAGAGAAATTTCATTTTGCCAGACAACAGACGCCGGTTTGACAGT

CAGGCAGACGCCGCGTCAGAGACTTCGGAGTTGGCTATGGATAGCCAAAGCCACGCCTTC

GACAGTACAGACGAACCCGACGGTGTGGACGGTACCCCGGACGCCGCAGGATCTGGCGCT

ACTTCTGAAAACGGAGGCGGGAAGCCCGGCGTCGGGAGGGCCGTGGGCTACCAGGGAGCA

AAGGTTCTAGACCCCGTATCCGGCTTTCATGTGGACCCCGTGGTTGTGTTTGACTTCGCT

AGCTTATACCCAAGCATTATCCAGGCCCATAACCTCTGTTTCACCACCCTGGCGCTCGAT

GAAGTGGATCTGGCCGGGCTTCAACCATCCGTCAACTACTCGACGTTCGAGGTGGGTGAC

CAAAAGTTATTTTTTGTCCACGCCCATATTCGCGAAAGCCTGCTTGGCATCTTGCTGCGC

GACTGGCTGGCCATGCGAAAGGCGGTGAGGGCGCGAATCCCCACCAGCACCCCCGAGGAG

GCAGTTTTACTAGATAAGCAGCAGTCTGCGATTAAGGTGATATGCAACTCGGTTTACGGA

TTCACGGGGGTGGCAAACGGCCTGTTGCCGTGTCTGAGGATAGCGGCTACCGTTACCACG

ATAGGACGCGACATGCTCCTCAAGACCCGAGATTACGTTCACTCTCGTTGGGCGACGCGC

GAGCTGCTGGAGGACAATTTTCCAGGGGCTATAGGTTTCCGAAACCACAAGCCTTACTCC

GTCAGGGTTATCTACGGAGACACCGACTCCGTGTTTATCAAGTTTGTGGGCCTGACGTAC

GAGGGGGTATCGGAGCTGGGGGATGCTATGTCGCGTCAGATTTCAGCGGACCTCTTTAGA

GCGCCCATCAAACTGGAGTGTGAGAAGACCTTTCAGCGACTGCTGCTGATCACCAAGAAG

AAGTACATAGGTGTCATAAACGGGGGGAAGATGCTCATGAAGGGGGTCGACCTGGTCCGC

AAAAATAACTGCTCTTTCATAAACTTGTACGCGCGACATCTGGTAGATCTTTTGTTGTAC

GACGAGGATGTGGCCACGGCGGCAGCAGAGGTGACAGACGTGCCTCCCGCAGAATGGGTG

GGGCGCCCGCTACCGAGCGGCTTTGACAAGTTTGGGCGAGTGCTGGTAGAGGCGTACAAC

CGTATCACTGCCCCCAACTTGGACGTGCGCGAGTTCGTTATGACTGCTGAGCTGAGCCGC

TCACCCGAATCGTATACCAACAAGCGCCTGCCGCACCTCACCGTCTACTTTAAGCTCGCC

ATGAGGAATGAAGAACTGCCCAGCGTAAAAGAGAGAATTCCGTATGTGATAGTTGCGCAG

ACCGAGGCCGCGGAACGCGAAGCGGGTGTAGTAAACTCAATGCGCGGTACCGCCCAAAAC

CCCGTGGTAACCAAGACCGCACGCCCCCAACCTAAACGCAAACTGCTGGTTTCCGACCTC

GCCGAAGACCCGACCTATGTTTCCGAGAATGACGTACCGCTAAACACAGACTACTATTTC

TCCCACCTGTTGGGTACCATAAGCGTGACCTTTAAGGCTCTATTCGGAAATGATGTGAGA

ACAACAGAAAATCTTTTAAAGCGGTTTATTCCGGAAACCCCCCACAAGACCCCCACGAAA

ACCCAGGCACTGCTTGAGCGCGCCGGCTTTGAAAAGCTGACGCCCTTTACACCGGAGGAA

GAAAGTCGTCGAATACTGCATACAGTTTTTTGTACTCTAGAAGCAGCTCCCCATCAAAGC

TGA

>LC834913.1

ATGGCGGCGCGCGAACAGGCCAACTCCGTTCGCAGGAGCGGGTTTTTTAATCCGTTTATT

GGCAAGAGGCCATTTTTCAGGCCGGGAAGCGGGCAGACCGCGGAGACAGAGCGCCCAAGG

CCCCCCCAACACTCGTACTGCACAGAGGTGGGTAGCTTTAAGTTTATAGCTCCAAGATGC

CTCGATGAAGAAGCCCCCGCCGACCAGCGACGCGGTGTACACGTGGGCACCTTGGAGCGC

CCACCCAAAGTGTACTGCGATGGCTCAGAGTACGACGTGCTGAACTTTGCCTCCGGAGGT

TGCTGGCCTCGCAGGATTCGGGTTTGGAACGGCCAGGATTTTCGGGGCGATGGATTCAAC

CCCAGATTTGAGAGATTTCACGTGTACGACATAGTAGAGACTTCGGAGAGCGCCTCACAC

GATGACCCATCCAGGTTTGCTGAGCTATCCAGCCCAAGCGGGTCTGTGGTAACACTGCTG

GGAATGAGCGAGTGTGGAAAGCGAGTCGCGGTTCACGTCTATGGTGTGCGCCATTATTTT

TACATGGCAAAGGCGGAGGTGGATAGCGCTTGTGGAATAACCACCGAGGCAGAACTCGTG

CGCGCAATGGTGGACTGCGCGCACAGCTCGGCTTTGAGCGCGGCCCTGGGAAATGGCAAC

GGCGGCAAACAGAGCGGCGGCAGCGGCGGGGGATGGTGGGGCGGAAAGCACGTGTCTGCG

GACTGCTTCAAAGTGGAGACCGTGTGCCACACGACGCTGTACTACTTTGGATCTAAGCCA

GCTCTCTACTATAGAGTATCTGCCTCCAGCAGCCGCCTGGGAGGGTTCATCTGCGACAAC

TTTCACCCGGAGATTACAAAATTCGAGGGGAGCGTGGACGTGACCACGCGGCTGTTGTTG

GACAATGAAAATTTTACCAGTTTCGGGTGGTACCGCCTGCGACCCGGCACCCACGGAGAG

CGTGTTCAACTTCGCCCCGTTGAGCGACACGTCACCTCAAGCGACGTGGAGATTAACTGT

ACTCCCGATAACCTGGAGCCGATACCAGACGAGGCTGCCTGGCCCGACTATAAGCTCATG

TGCTTTGATATAGAGTGTAAAGCTGGAACGGGTAACGAAATGGCGTTCCCAGTGGCAACT

AACCAAGAGGACCTGGTCATCCAGATCTCCTGTCTGCTGTACTCGCTTGCTACTCAGAAC

CACGAACACACCCTGCTGTTTTCCCTCGGGTCATGCGATATCTCTGAGGAATACTCGTTT

GCATGCGTCCAGCGCGGCGAGCCCAGACCGACGGTTTTGGAGTTTGACAGCGAGTACGAG

CTGCTGGTTGCCTTCCTGACCTTTCTCAAGCAGTACTCTCCCGAGTTCGCCACCGGCTAC

AACATCGTTAATTTTGACTGGGCGTACATAGTTAACAAGGTAACGTCGGTGTATAACATC

AAGCTGGACGGGTACGGCAAGTTCAACAAAGGGGGGCTGTTTAAGGTGTGGGACATCGCC

ACGAACCATTTTCAGAAGAAGAGCAAGGTGAAAATCAATGGCCTGATATCTCTAGACATG

TATTCTGTGGCGACGGAAAAGCTAAAGCTACCCAGCTACAAACTCGACGCGGTCGTGGGA

GACGTCCTCGGCGAGCATAAGATAGACCTTCCCTATAAAGAAATACCCTCCTATTACGCG

GGAGGGCCTGACCGGAGGGGCGTAATAGGAGAGTATTGTATCCAGGACTCTAGGCTGGTG

GGCAAGCTGTTTTTTAAGTACCTCCCCCATCTGGAACTATCGGCGGTGGCCAAACTCGCC

CGTATCACCCTAACGCGGGTAATTTTTGACGGTCAGCAAATTCGCGTGTACACGTGCTTG

CTGAAACTCGCCCACGAGAGAAATTTCATTTTGCCAGACAACAGACGCCGGTTTGACAGT

CAGGCAGACGCCGCGTCAGAGACTTCGGAGTTGGCTATGGATAGCCAAAGCCACGCCTTC

GACAGTACAGACGAACCCGACGGTGTGGACGGTACCCCGGACGCCGCAGGATCTGGCGCT

ACTTCTAAAAACGGAGGCGGGAAGCCCGGCGTCGGGAGGGCCGTGGGCTACCAGGGAGCA

AAGGTTCTAGACCCCGTATCCGGCTTTCATGTGGACCCCGTGGTTGTGTTTGACTTCGCT

AGCTTATACCCAAGCATTATCCAGGCCCATAACCTCTGTTTCACCACCCTGGCGCTCGAT

GAAGTGGATCTGGCCGGGCTTCAACCATCCGTCAACTACTCGACGTTCGAGGTGGGTGAC

CAAAAGTTATTTTTTGTCCACGCCCATATTCGCGAAAGCCTGCTTGGCATCTTGCTGCGC

GACTGGCTGGCCATGCGAAAGGCGGTGAGGGCGCGAATCCCCACCAGCACCCCCGAGGAG

GCAGTTTTACTAGATAAGCAGCAGTCTGCGATTAAGGTGATATGCAACTCGGTTTACGGA

TTCACGGGGGTGGCAAACGGCCTGTTGCCGTGTCTGAGGATAGCGGCTACCGTTACCACG

ATAGGACGCGACATGCTCCTCAAGACCCGAGATTACGTTCACTCTCGTTGGGCGACGCGC

GAGCTGCTGGAGGACAATTTTCCAGGGGCTATAGGTTTCCGAAACCACAAGCCTTACTCC

GTCAGGGTTATCTACGGAGACACCGACTCCGTGTTTATCAAGTTTGTGGGCCTGACGTAC

GAGGGGGTATCGGAGCTGGGGGATGCTATGTCGCGTCAGATTTCAGCGGACCTCTTTAGA

GCCCCCATCAAACTGGAGTGTGAGAAGACCTTTCAGCGACTGCTGCTGATCACCAAGAAG

AAGTACATAGGTGTCATAAACGGGGGGAAGATGCTCATGAAGGGGGTCGACCTGGTCCGC

AAAAATAACTGCTCTTTCATAAACTTGTACGCGCGACATCTGGTAGATCTTTTGTTGTAC

GACGAGGATGTGGCCACGGCGGCAGCAGAGGTGACAGACGTGCCTCCCGCAGAATGGGTG

GGGCGCCCGCTACCGAGCGGCTTTGACAAGTTTGGGCGAGTGCTGGTAGAGGCGTACAAC

CGTATCACTGCCCCCAACTTGGACGTGCGCGAGTTCGTTATGACTGCTGAGCTGAGCCGC

TCACCCGAATCGTATACCAACAAGCGCCTGCCGCACCTCACCGTCTACTTTAAGCTCGCC

ATGAGGAATGAAGAACTGCCCAGCGTAAAAGAGAGAATTCCGTATGTGATAGTTGCGCAG

ACCGAGGCCGCGGAACGCGAAGCGGGTGTAGTAAACTCAATGCGCGGTACCGCCCAAAAC

CCCGTGGTAACCAAGACCACACGCCCCCAACCTAAACGCAAACTGCTGGTTTCCGACCTC

GCCGAAGACCCGACCTATGTTTCCGAGAATGACGTACCGCTAAACACAGACTACTATTTC

TCCCACCTGTTGGGTACCATAAGCGTGACCTTTAAGGCTCTATTCGGAAATGATGTGAGA

ACAACAGAAAATCTTTTAAAGCGGTTTATTCCGGAAACCCCCCACAAGACCCCCACGAAA

ACCCAGGCACTGCTTGAGCGCGCCGGCTTTGAAAAGCTGACGCCCTTTACACCGGAGGAA

GAAAGTCGTCGAATACTGCATACAGTTTTTTGTACTCTAGAAGCAGCTCCCCATCAAAGC

TGA

ORF30_nt_matched_by_commonsites.aln.fasta

>AY464052.1

atggcggcgcgcgaacaggccaactccgttcgcaggagcgggttttttaatccgtttatt

ggcaagaggccatttttcaggccgggaagcgggcagaccgcggagacagagcgcccaagg

cccccccaacactcgtactgcacagaggtgggtagctttaagtttatagctccaagatgc

ctcgatgaagaagcccccgccgaccagcgacgcggtgtacacgtgggcaccttggagcgc

ccacccaaagtgtactgcgatggctcagagtacgacgtgctgaactttgcctccggaggt

tgctggcctcgcaggattcgggtttggaacggccaggattttcggggcgatggattcaac

cccagatttgagagatttcacgtgtacgacatagtagagacttcggagagcgcctcacac

gatgacccatccaggtttgctgagctatcccgcccaagcgggtctgtggtaacactgctg

ggaatgagcgagtgtggaaagcgagtcgcggttcacgtctatggtgtgcgccattatttt

tacatggcaaaggcggaggtggatagcgcttgtggaataaccaccgaggcagaactcgtg

cgcgcaatggtggactgcgcgcacagctcggctttgagcgcggccctgggaaatggcaac

ggcggcaaacagagcggcggcagcggcgggggatggtggggcggaaagcacgtgtctgcg

gactgcttcaaagtggagaccgtgtgccacacgacgctgtactactttggatctaagcca

gctctctactatagagtatctgcctccagcagccgcctgggagggttcatctgcgacaac

tttcacccggagattacaaaattcgaggggagcgtggacgtgaccacgcggctgttgttg

gacaatgaaaattttaccagttttgggtggtaccgcctgcgacccggcacccacggagag

cgtgttcaacttcgccccgttgagcgacacgtcacctcaagcgacgtggagattaactgt

actcccgataacctggagccgataccagacgaggctgcctggcccgactataagctcatg

tgctttgatatagagtgtaaagctggaacgggtaacgaaatggcgttcccagtggcaact

aaccaagaggacctggtcatccagatctcctgtctgctgtactcgcttgctactcagaac

cacgaacacaccctgctgttttccctcgggtcatgcgatatctctgaggaatactcgttt

gcatgcgtccagcgcggcgagcccagaccgacggttttggagtttgacagcgagtacgag

ctgctggttgccttcctgacctttctcaagcagtactctcccgagttcgccaccggctac

aacatcgttaattttgactgggcgtacatagttaacaaggtaacgtcggtgtataacatc

aagctggacgggtacggcaagttcaacaaaggggggctgtttaaggtgtgggacatcgcc

acgaaccattttcagaagaagagcaaggtgaaaatcaatggcctgatatctctagacatg

tattctgtggcgacggaaaagctaaagctacccagctacaaactcgacgcggtcgtggga

gacgtcctcggcgagcataagatagaccttccctataaagaaataccctcctattacgcg

ggagggcctgaccggaggggcgtaataggagagtattgtatccaggactctaggctggtg

ggcaagctgttttttaagtacctcccccatctggaactatcggcggtggccaaactcgcc

cgtatcaccctaacgcgggtaatttttgacggtcagcaaattcgcgtgtacacgtgcttg

ctgaaactcgcccgcgagagaaatttcattttgccagacaacagacgccggtttgacagt

caggcagacgccgcgtcagagacttcggagttggctatggatagccaaagccacgccttc

gacagtacagacgaacccgacggtgtggacggtaccccggacgccgcaggatctggcgct

acttctgaaaacggaggcgggaagcccggcgtcgggagggccgtgggctaccagggagca

aaggttctagaccccgtatccggctttcatgtggaccccgtggttgtgtttgacttcgct

agcttatacccaagcattatccaggcccataacctctgtttcaccaccctggcgctcgat

gaagtggatctggccgggcttcaaccatccgtcaactactcgacgttcgaggtgggtgac

caaaagttattttttgtccacgcccatattcgcgaaagcctgcttggcatcttgctgcgc

gactggctggccatgcgaaaggcggtgagggcgcgaatccccaccagcacccccgaggag

gcagttttactagataagcagcagtctgcgattaaggtgatatgcaactcggtttacgga

ttcacgggggtggcaaacggcctgttgccgtgtctgaggatagcggctaccgttaccacg

ataggacgcgacatgctcctcaagacccgagattacgttcactctcgttgggcgacgcgc

gagctgctggaggacaattttccaggggctataggtttccgaaaccacaagccttactcc

gtcagggttatctacggagacaccgactccgtgtttatcaagtttgtgggcctgacgtac

gagggggtatcggagctgggggatgctatgtcgcgtcagatttcagcggacctctttaga

gcgcccatcaaactggagtgtgagaagacctttcagcgactgctgctgatcaccaagaag

aagtacataggtgtcataaacggggggaagatgctcatgaagggggtcgacctggtccgc

aaaaataactgctctttcataaacttgtacgcgcgacatctggtagatcttttgttgtac

gacgaggatgtggccacggcggcagcaaaggtgacagacgtgcctcccgcagaatgggtg

gggcgcccgctaccgagcggctttgacaagtttgggcgagtgctggtagaggcgtacaac

cgtatcactgcccccaacttggacgtgcgcgagttcgttatgactgctgagctgagccgc

tcacccgaatcgtataccaacaagcgcctgccgcacctcaccgtctactttaagctcgcc

atgaggaatgaagaactgcccagcgtaaaagagagaattccgtatgtgatagttgcgcag

accgaggccgcggaacgcgaagcgggtgtagtaaactcaatgcgcggtaccgcccaaaac

cccgtggtaaccaagaccgcacgcccccaacctaaacgcaaactgctggtttccgacctc

gccgaagacccgacctatgtttccgagaatgacgtaccgctaaacacagactactatttc

tcccacctgttgggtaccataagcgtgacctttaaggctctattcggaaatgatgtgaga

acaacagaaaatcttttaaagcggtttattccggaaaccccccacaagacccccacgaaa

acccaggcactgcttgagcgcgccggctttgaaaagctgacgccctttacaccggaggaa

gaaagtcgtcgaatactgcatacagttttttgtactctagaagcagctccccatcaaagc

tga

>AY665713.1

atggcggcgcgcgaacaggccaactccgttcgcaggagcgggttttttaatccgtttatt

ggcaagaggccatttttcaggccgggaagcgggcagaccgcggagacagagcgcccaagg

cccccccaacactcgtactgcacagaggtgggtagctttaagtttatagctccaagatgc

ctcgatgaagaagcccccgccgaccagcgacgcggtgtacacgtgggcaccttggagcgc

ccacccaaagtgtactgcgatggctcagagtacgacgtgctgaactttgcctccggaggt

tgctggcctcgcaggattcgggtttggaacggccaggattttcggggcgatggattcaac

cccagatttgagagatttcacgtgtacgacatagtagagacttcggagagcgcctcacac

gatgacccatccaggtttgctgagctatcccgcccaagcgggtctgtggtaacactgctg

ggaatgagcgagtgtggaaagcgagtcgcggttcacgtctatggtgtgcgccattatttt

tacatggcaaaggcggaggtggatagcgcttgtggaataaccaccgaggcagaactcgtg

cgcgcaatggtggactgcgcgcacagctcggctttgagcgcggccctgggaaatggcaac

ggcggcaaacagagcggcggcagcggcgggggatggtggggcggaaagcacgtgtctgcg

gactgcttcaaagtggagaccgtgtgccacacgacgctgtactactttggatctaagcca

gctctctactatagagtatctgcctccagcagccgcctgggagggttcatctgcgacaac

tttcacccggagattacaaaattcgaggggagcgtggacgtgaccacgcggctgttgttg

gacaatgaaaattttaccagtttcgggtggtaccgcctgcgacccggcacccacggagag

cgtgttcaacttcgccccgttgagcgacacgtcacctcaagcgacgtggagattaactgt

actcccgataacctggagccgataccagacgaggctgcctggcccgactataagctcatg

tgctttgatatagagtgtaaagctggaacgggtaacgaaatggcgttcccagtggcaact

aaccaagaggacctggtcatccagatctcctgtctgctgtactcgcttgctactcagaac

cacgaacacaccctgctgttttccctcgggtcatgcgatatctctgaggaatactcgttt

gcatgcgtccagcgcggcgagcccagaccgacggttttggagtttgacagcgagtacgag

ctgctggttgccttcctgacctttctcaagcagtactctcccgagttcgccaccggctac

aacatcgttaattttgactgggcgtacatagttaacaaggtaacgtcggtgtataacatc

aagctggacgggtacggcaagttcaacaaaggggggctgtttaaggtgtgggacatcgcc

acgaaccattttcagaagaagagcaaggtgaaaatcaatggcctgatatctctagacatg

tattctgtggcgacggaaaagctaaagctacccagctacaaactcgacgcggtcgtggga

gacgtcctcggcgagcataagatagaccttccctataaagaaataccctcctattacgcg

ggagggcctgaccggaggggcgtaataggagagtattgtatccaggactctaggctggtg

ggcaagctgttttttaagtacctcccccatctggaactatcggcggtggccaaactcgcc

cgtatcaccctaacgcgggtaatttttgacggtcagcaaattcgcgtgtacacgtgcttg

ctgaaactcgcccgcgagagaaatttcattttgccagacaacagacgccggtttgacagt

caggcagacgccgcgtcagagacttcggagttggctatggatagccaaagccacgccttc

gacagtacagacgaacccgacggtgtggacggtaccccggacgccgcaggatctggcgct

acttctgaaaacggaggcgggaagcccggcgtcgggagggccgtgggctaccagggagca

aaggttctagaccccgtatccggctttcatgtggaccccgtggttgtgtttgacttcgct

agcttatacccaagcattatccaggcccataacctctgtttcaccaccctggcgctcgat

gaagtggatctggccgggcttcaaccatccgtcgactactcgacgttcgaggtgggtgac

caaaagttattttttgtccacgcccatattcgcgaaagcctgcttggcatcttgctgcgc

gactggctggccatgcgaaaggcggtgagggcgcgaatccccaccagcacccccgaggag

gcagttttactagataagcagcagtctgcgattaaggtgatatgcaactcggtttacgga

ttcacgggggtggcaaacggcctgttgccgtgtctgaggatagcggctaccgttaccacg

ataggacgcgacatgctcctcaagacccgagattacgttcactctcgttgggcgacgcgc

gagctgctggaggacaattttccaggggctataggtttccgaaaccacaagccttactcc

gtcagggttatctacggagacaccgactccgtgtttatcaagtttgtgggcctgacgtac

gagggggtatcggagctgggggatgctatgtcgcgtcagatttcagcggacctctttaga

gcgcccatcaaactggagtgtgagaagacctttcagcgactgctgctgatcaccaagaag

aagtacataggtgtcataaacggggggaagatgctcatgaagggggtcgacctggtccgc

aaaaataactgctctttcataaacttgtacgcgcgacatctggtagatcttttgttgtac

gacgaggatgtggccacggcggcagcagaggtgacagacgtgcctcccgcagaatgggtg

gggcgcccgctaccgagcggctttgacaagtttgggcgagtgctggtagaggcgtacaac

cgtatcactgcccccaacttggacgtgcgcgagttcgttatgactgctgagctgagccgc

tcacccgaatcgtataccaacaagcgcctgccgcacctcaccgtctactttaagctcgcc

atgaggaatgaagaactgcccagcgtaaaagagagaattccgtatgtgatagttgcgcag

accgaggccgcggaacgcgaagcgggtgtagtaaactcaatgcgcggtaccgcccaaaac

cccgtggtaaccaagaccgcacgcccccaacctaaacgcaaactgctggtttccgacctc

gccgaagacccgacctatgtttccgagaatgacgtaccgctaaacacagactactatttc

tcccacctgttgggtaccataagcgtgacctttaaggctctattcggaaatgatgtgaga

acaacagaaaatcttttaaagcggtttattccggaaaccccccacaagacccccacgaaa

acccaggcactgcttgagcgcgccggctttgaaaagctgacgccctttacaccggaggaa

gaaagtcgtcgaatactgcatacagttttttgtactctagaagcagctccccatcaaagc

tga

>KF644566.1

atggcggcgcgcgaacaggccaactccgttcgcaggagcgggttttttaatccgtttatt

ggcaagaggccatttttcaggccgggaagcgggcagaccgcggagacagagcgcccaagg

cccccccaacactcgtactgcacagaggtgggtagctttaagtttatagctccaagatgc

ctcgatgaagaagcccccgccgaccagcgacgcggtgtacacgtgggcaccttggagcgc

ccacccaaagtgtactgcgatggctcagagtacgacgtgctgaactttgcctccggaggt

tgctggcctcgcaggattcgggtttggaacggccaggattttcggggcgatggattcaac

cccagatttgagagatttcacgtgtacgacatagtagagacttcggagagcgcctcacac

gatgacccatccaggtttgctgagctatcccgcccaagcgggtctgtggtaacactgctg

ggaatgagcgagtgtggaaagcgagtcgcggttcacgtctatggtgtgcgccattatttt

tacatggcaaaggcggaggtggatagcgcttgtggaataaccaccgaggcagaactcgtg

cgcgcaatggtggactgcgcgcacagctcggctttgagcgcggccctgggaaatggcaac

ggcggcaaacagagcggcggcagcggcgggggatggtggggcggaaagcacgtgtccgcg

gactgcttcaaagtggagaccgtgtgccacacgacgctgtactactttggatctaagcca

gctctctactatagagtatctgcctccagcagccgcctgggagggttcatctgcgacaac

tttcacccggagattacaaaattcgaggggagcgtggacgtgaccacgcggctgttgttg

gacaatgaaaattttaccagtttcgggtggtaccgcctgcgacccggcacccacggagag

cgtgttcaacttcgccccgttgagcgacacgtcacctcaagcgacgtggagattaactgt

actcccgataacctggagccgataccagacgaggctgcctggcccgactataagctcatg

tgctttgatatagagtgtaaagctggaacgggtaacgaaatggcgttcccagtggcaact

aaccaagaggacctggtcatccagatctcctgtctgctgtactcgcttgctactcagaac

cacgaacacaccctgctgttttccctcgggtcatgcgatatctctgaggaatactcgttt

gcatgcgtccagcgcggcgagcccagaccgacggttttggagtttgacagcgagtacgag

ctgctggttgccttcctgacctttctcaagcagtactctcccgagttcgccaccggctac

aacatcgttaattttgactgggcgtacatagttaacaaggtaacgtcggtgtataacatc

aagctggacgggtacggcaagttcaacaaaggggggctgtttaaggtgtgggacatcgcc

acgaaccattttcagaagaagagcaaggtgaaaatcaatggcctgatatctctagacatg

tattctgtggcgacggaaaagctaaagctacccagctacaaactcgacgcggtcgtggga

gacgtcctcggcgagcataagatagaccttccctataaagaaataccctcctattacgcg

ggagggcctgaccggaggggcgtaataggagagtattgtatccaggactctaggctggtg

ggcaagctgttttttaagtacctcccccatctggaactatcggcggtggccaaactcgcc

cgtatcaccctaacgcgggtaatttttgacggtcagcaaattcgcgtgtacacgtgcttg

ctgaaactcgcccgcgagagaaatttcattttgccagacaacagacgccggtttgacagt

caggcagacgccgcgtcagagacttcggagttggctatggatagccaaagccacgccttc

gacagtacagacgaacccgacggtgtggacggtaccccggacgccgcaggatctggcgct

acttctgaaaacggaggcgggaagcccggcgtcgggagggccgtgggctaccagggagca

aaggttctagaccccgtatccggctttcatgtggaccccgtggttgtgtttgacttcgct

agcttatacccaagcattatccaggcccataacctctgtttcaccaccctggcgctcgat

gaagtggatctggccgggcttcaaccatccgtcaactactcgacgttcgaggtgggtgac

caaaagttattttttgtccacgcccatattcgcgaaagcctgcttggcatcttgctgcgc

gactggctggccatgcgaaaggcggtgagggcgcgaatccccaccagcacccccgaggag

gcagttttactagataagcagcagtctgcgattaaggtgatatgcaactcggtttacgga

ttcacgggggtggcaaacggcctgttgccgtgtctgaggatagcggctaccgttaccacg

ataggacgcgacatgctcctcaagacccgagattacgttcactctcgttgggcgacgcgc

gagctgctggaggacaattttccaggggctataggtttccgaaaccacaagccttactcc

gtcagggttatctacggagacaccgactccgtgtttatcaagtttgtgggcctgacgtac

gagggggtatcggagctgggggatgctatgtcgcgtcagatttcagcggacctctttaga

gcccccatcaaactggagtgtgagaagacctttcagcgactgctgctgatcaccaagaag

aagtacataggtgtcataaacggggggaagatgctcatgaagggggtcgacctggtccgc

aaaaataactgctctttcataaacttgtacgcgcgacatctggtagatcttttgttgtac

gacgaggatgtggccacggcggcagcagaggtgacagacgtgcctcccgcagaatgggtg

gggcgcccgctaccgagcggctttgacaagtttgggcgagtgctggtagaggcgtacaac

cgtatcactgcccccaacttggacgtgcgcgagttcgttatgactgctgagctgagccgc

tcacccgaatcgtataccaacaagcgcctgccgcacctcaccgtctactttaagctcgcc

atgaggaatgaagaactgcccagcgtaaaagagagaattccgtatgtgatagttgcgcag

accgaggccgcggaacgcgaagcgggtgtagtaaactcaatgcgcggtaccgcccaaaac

cccgtggtaaccaagaccgcacgcccccaacctaaacgcaaactgctggtttccgacctc

gccgaagacccgacctatgtttccgagaatgacgtaccgctaaacacagactactatttc

tcccacctgttgggtaccataagcgtgacctttaaggctctattcggaaatgatgtgaga

acaacagaaaatcttttaaagcggtttattccggaaaccccccacaagacccccacgaaa

acccaggcactgcttgagcgcgccggctttgaaaagctgacgccctttacaccggaggaa

gaaagtcgtcgaatactgcatacagttttttgtactctagaagcagctccccatcaaagc

tga

>KF644567.1

atggcggcgcgcgaacaggccaactccgttcgcaggagcgggttttttaatccgtttatt

ggcaagaggccatttttcaggccgggaagcgggcaaaccgcggagacagagcgcccaagg

cccccccaacactcgtactgcacagaggtgggtagctttaagtttatagctccaagatgc

ctcgatgaagaagcccccgccgaccagcgacgcggtgtacacgtgggcaccttggagcgc

ccacccaaagtgtactgcgatggctcagagtacgacgtgctgaactttgcctccggaggt

tgctggcctcgcaggattcgggtttggaacggccaggattttcggggcgatggattcaac

cccagatttgagagatttcacgtgtacgacatagtagagacttcggagagcgcctcacac

gatgacccatccaggtttgctgagctatcccgcccaagcgggtctgtggtaacactgctg

ggaatgagcgagtgtggaaagcgagtcgcggttcacgtctatggtgtgcgccattatttt

tacatggcaaaggcggaggtggatagcgcttgtggaataaccaccgaggcagaactcgtg

cgcgcaatggtggactgcgcgcacagctcggctttgagcgcggccctgggaaatggcaac

ggcggcaaacagagcggcggcagcggcgggggatggtggggcggaaagcacgtgtctgcg

gactgcttcaaagtggagaccgtgtgccacacgacgctgtactactttggatctaagcca

gctctctactatagagtatctgcctccagcagccgcctgggagggttcatctgcgacaac

tttcacccggagattacaaaattcgaggggagcgtggacgtgaccacgcggctgttgttg

gacaatgaaaattttaccagtttcgggtggtaccgcctgcgacccggcacccacggagag

cgtgttcaacttcgccccgttgagcgacacgtcacctcaagcgacgtggagattaactgt

actcccgataacctggagccgataccagacgaggctgcctggcccgactataagctcatg

tgctttgatatagagtgtaaagctggaacgggtaacgaaatggcgttcccagtggcaact

aaccaagaggacctggtcatccagatctcctgtctgctgtactcgcttgctactcagaac

cacgaacacaccctgctgttttccctcgggtcatgcgatatctctgaggaatactcgttt

gcatgcgtccagcgcggcgagcccagaccgacggttttggagtttgacagcgagtacgag

ctgctggttgccttcctgacctttctcaagcagtactctcccgagttcgccaccggctac

aacatcgttaattttgactgggcgtacatagttaacaaggtaacgtcggtgtataacatc

aagctggacgggtacggcaagttcaacaaaggggggctgtttaaggtgtgggacatcgcc

acgaaccattttcagaagaagagcaaggtgaaaatcaatggcctgatatctctagacatg

tattctgtggcgacggaaaagctaaagctacccagctacaaactcgacgcggtcgtggga

gacgtcctcggcgagcataagatagaccttccctataaagaaataccctcctattacgcg

ggagggcctgaccggaggggcgtaataggagagtattgtatccaggactctaggctggtg

ggcaagctgttttttaagtacctcccccatctggaactatcggcggtggccaaactcgcc

cgtatcaccctaacgcgggtaatttttgacggtcagcaaattcgcgtgtacacgtgcttg

ctgaaactcgcccgcgagagaaatttcattttgccagacaacagacgccggtttgacagt

caggcagacgccgcgtcagagacttcggagttggctatggatagccaaagccacgccttc

gacagtacagacgaacccgacggtgtggacggtaccccggacgccgcaggatctggcgct

acttctgaaaacggaggcgggaagcccggcgtcgggagggccgtgggctaccagggagca

aaggttctagaccccgtatccggctttcatgtggaccccgtggttgtgtttgacttcgct

agcttatacccaagcattatccaggcccataacctctgtttcaccaccctggcgctcgat

gaagtggatctggccgggcttcaaccatccgtcgactactcgacgttcgaggtgggtgac

caaaagttattttttgtccacgcccatattcgcgaaagcctgcttggcatcttgctgcgc

gactggctggccatgcgaaaggcggtgagggcgcgaatccccaccagcacccccgaggag

gcagttttactagataagcagcagtctgcgattaaggtgatatgcaactcggtttacgga

ttcacgggggtggcaaacggcctgttgccgtgtctgaggatagcggctaccgttaccacg

ataggacgcgacatgctcctcaagacccgagattacgttcactctcgttgggcgacgcgc

gagctgctggaggacaattttccaggggctataggtttccgaaaccacaagccttactcc

gtcagggttatctacggagacaccgactccgtgtttatcaagtttgtgggcctgacgtac

gagggggtatcggagctgggggatgctatgtcgcgtcagatttcagcggacctctttaga

gcgcccatcaaactggagtgtgagaagacctttcagcgactgctgctgatcaccaagaag

aagtacataggtgtcataaacggggggaagatgctcatgaagggggtcgacctggtccgc

aaaaataactgctctttcataaacttgtacgcgcgacatctggtagatcttttgttgtac

gacgaggatgtggccacggcggcagcagaggtgacagacgtgcctcccgcagaatgggtg

gggcgcccgctaccgagcggctttgacaagtttgggcgagtgctggtagaggcgtacaac

cgtatcactgcccccaacttggacgtgcgcgagttcgttatgactgctgagctgagccgc

tcacccgaatcgtataccaacaagcgcctgccgcacctcaccgtctactttaagctcgcc

atgaggaatgaagaactgcccagcgtaaaagagagaattccgtatgtgatagttgcgcag

accgaggccgcggaacgcgaagcgggtgtagtaaactcaatgcgcggtaccgcccaaaac

cccgtggtaaccaagaccgcacgcccccaacctaaacgcaaactgctggtttccgacctc

gccgaagacccgacctatgtttccgagaatgacgtaccgctaaacacagactactatttc

tcccacctgttgggtaccataagcgtgacctttaaggctctattcggaaatgatgtgaga

acaacagaaaatcttttaaagcggtttattccggaaaccccccacaagacccccacgaaa

acccaggcactgcttgagcgcgccggctttgaaaagctgacgccctttacaccggaggaa

gaaagtcgtcgaatactgcatacagttttttgtactctagaagcagctccccatcaaagc

tga

>KF644568.1

atggcggcgcgcgaacaggccaactccgttcgcaggagcgggttttttaatccgtttatt

ggcaagaggccatttttcaggccgggaagcgggcagaccgcggagacagagcgcccaagg

cccccccaacactcgtactgcacagaggtgggtagctttaagtttatagctccaagatgc

ctcgatgaagaagcccccgccgaccagcgacgcggtgtacacgtgggcaccttggagcgc

ccacccaaagtgtactgcgatggctcagagtacgacgtgctgaactttgcctccggaggt

tgctggcctcgcaggattcgggtttggaacggccaggattttcggggcgatggattcaac

cccagatttgagagatttcacgtgtacgacatagtagagacttcggagagcgcctcacac

gatgacccatccaggtttgctgagctatcccgcccaagcgggtctgtggtaacactgctg

ggaatgagcgagtgtggaaagcgagtcgcggttcacgtctatggtgtgcgccattatttt

tacatggcaaaggcggaggtggatagcgcttgtggaataaccaccgaggcagaactcgtg

cgcgcaatggtggactgcgcgcacagctcggctttgagcgcggccctgggaaatggcaac

ggcggcaaacagagcggcggcagcggcgggggatggtggggcggaaagcacgtgtctgcg

gactgcttcaaagtggagaccgtgtgccacacgacgctgtactactttggatctaagcca

gctctctactatagagtatctgcctccagcagccgcctgggagggttcatctgcgacaac

tttcacccggagattacaaaattcgaggggagcgtggacgtgaccacgcggctgttgttg

gacaatgaaaattttaccagtttcgggtggtaccgcctgcgacccggcacccacggagag

cgtgttcaacttcgccccgttgagcgacacgtcacctcaagcgacgtggagattaactgt

actcccgataacctggagccgataccagacgaggctgcctggcccgactataagctcatg

tgctttgatatagagtgtaaagctggaacgggtaacgaaatggcgttcccagtggcaact

aaccaagaggacctggtcatccagatctcctgtctgctgtactcgcttgctactcagaac

cacgaacacaccctgctgttttccctcgggtcatgcgatatctctgaggaatactcgttt

gcatgcgtccagcgcggcgagcccagaccgacggttttggagtttgacagcgagtacgag

ctgctggttgccttcctgacctttctcaagcagtactctcccgagttcgccaccggctac

aacatcgttaattttgactgggcgtacatagttaacaaggtaacgtcggtgtataacatc

aagctggacgggtacggcaagttcaacaaaggggggctgtttaaggtgtgggacatcgcc

acgaaccattttcagaagaagagcaaggtgaaaatcaatggcctgatatctctagacatg

tattctgtggcgacggaaaagctaaagctacccagctacaaactcgacgcggtcgtggga

gacgtcctcggcgagcataagatagaccttccctataaagaaataccctcctattacgcg

ggagggcctgaccggaggggcgtaataggagagtattgtatccaggactctaggctggtg

ggcaagctgttttttaagtacctcccccatctggaactatcggcggtggccaaactcgcc

cgtatcaccctaacgcgggtaatttttgacggtcagcaaattcgcgtgtacacgtgcttg

ctgaaactcgcccgcgagagaaatttcattttgccagacaacagacgccggtttgacagt

caggcagacgccgcgtcagagacttcggagttggctatggatagccaaagccacgccttc

gacagtacagacgaacccgacggtgtggacggtaccccggacgccgcaggatctggcgct

acttctgaaaacggaggcgggaagcccggcgtcgggagggtcgtgggctaccagggagca

aaggttctagaccccgtatccggctttcatgtggaccccgtggttgtgtttgacttcgct

agcttatacccaagcattatccaggcccataacctctgtttcaccaccctggcgctcgat

gaagtggatctggccgggcttcaaccatccgtcgactactcgacgttcgaggtgggtgac

caaaagttattttttgtccacgcccatattcgcgaaagcctgcttggcatcttgctgcgc

gactggctggccatgcgaaaggcggtgagggcgcgaatccccaccagcacccccgaggag

gcagttttactagataagcagcagtctgcgattaaggtgatatgcaactcggtttacgga

ttcacgggggtggcaaacggcctgttgccgtgtctgaggatagcggctaccgttaccacg

ataggacgcgacatgctcctcaagacccgagattacgttcactctcgttgggcgacgcgc

gagctgctggaggacaattttccaggggctataggtttccgaaaccacaagccttactcc

gtcagggttatctacggagacaccgactccgtgtttatcaagtttgtgggcctgacgtac

gagggggtatcggagctgggggatgctatgtcgcgtcagatttcagcggacctctttaga

gcgcccatcaaactggagtgtgagaagacctttcagcgactgctgctgatcaccaagaag

aagtacataggtgtcataaacggggggaagatgctcatgaagggggtcgacctggtccgc

aaaaataactgctctttcataaacttgtacgcgcgacatctggtagatcttttgttgtac

gacgaggatgtggccacggcggcagcagaggtgacagacgtgcctcccgcagaatgggtg

gggcgcccgctaccgagcggctttgacaagtttgggcgagtgctggtagaggcgtacaac

cgtatcactgcccccaacttggacgtgcgcgagttcgttatgactgctgagctgagccgc

tcacccgaatcgtataccaacaagcgcctgccgcacctcaccgtctactttaagctcgcc

atgaggaatgaagaactgcccagcgtaaaagagagaattccgtatgtgatagttgcgcag

accgaggccgcggaacgcgaagcgggtgtagtaaactcaatgcgcggtaccgcccaaaac

cccgtggtaaccaagaccgcacgcccccaacctaaacgcaaactgctggtttccgacctc

gccgaagacccgacctatgtttccgagaatgacgtaccgctaaacacagactactatttc

tcccacctgttgggtaccataagcgtgacctttaaggctctattcggaaatgatgtgaga

acaacagaaaatcttttaaagcggtttattccggaaaccccccacaagacccccacgaaa

acccaggcactgcttgagcgcgccggctttgaaaagctgacgccctttacaccggaggaa

gaaagtcgtcgaatactgcatacagttttttgtactctagaagcagctccccatcaaagc

tga

>KF644569.1

atggcggcgcgcgaacaggccaactccgttcgcaggagcgggttttttaatccgtttatt

ggcaagaggccatttttcaggccgggaagcgggcagaccgcggagacagagcgcccaagg

cccccccaacactcgtactgcacagaggtgggtagctttaagtttatagctccaagatgc

ctcgatgaagaagcccccgccgaccagcgacgcggtgtacacgtgggcaccttggagcgc

ccacccaaagtgtactgcgatggctcagagtacgacgtgctgaactttgcctccggaggt

tgctggcctcgcaggattcgggtttggaacggccaggattttcggggcgatggattcaac

cccagatttgagagatttcacgtgtacgacatagtagagacttcggagagcgcctcacac

gatgacccatccaggtttgctgagctatcccgcccaagcgggtctgtggtaacactgctg

ggaatgagcgagtgtggaaagcgagtcgcggttcacgtctatggtgtgcgccattatttt

tacatggcaaaggcggaggtggatagcgcttgtggaataaccaccgaggcagaactcgtg

cgcgcaatggtggactgcgcgcacagctcggctttgagcgcggccctgggaaatggcaac

ggcggcaaacagagcggcggcagcggcgggggatggtggggcggaaagcacgtgtctgcg

gactgcttcaaagtggagaccgtgtgccacacgacgctgtactactttggatctaagcca

gctctctactatagagtatctgcctccagcagccgcctgggagggttcatctgcgacaac

tttcacccggagattacaaaattcgaggggagcgtggacgtgaccacgcggctgttgttg

gacaatgaaaattttaccagtttcgggtggtaccgcctgcgacccggcacccacggagag

cgtgttcaacttcgccccgttgagcgacacgtcacctcaagcgacgtggagattaactgt

actcccgataacctggagccgataccagacgaggctgcctggcccgactataagctcatg

tgctttgatatagagtgtaaagctggaacgggtaacgaaatggcgttcccagtggcaact

aaccaagaggacctggtcatccagatctcctgtctgctgtactcgcttgctactcagaac

cacgaacacaccctgctgttttccctcgggtcatgcgatatctctgaggaatactcgttt

gcatgcgtccagcgcggcgagcccagaccgacggttttggagtttgacagcgagtacgag

ctgctggttgccttcctgacctttctcaagcagtactctcccgagttcgccaccggctac

aacatcgttaattttgactgggcgtacatagttaacaaggtaacgtcggtgtataacatc

aagctggacgggtacggcaagttcaacaaaggggggctgtttaaggtgtgggacatcgcc

acgaaccattttcagaagaagagcaaggtgaaaatcaatggcctgatatctctagacatg

tattctgtggcgacggaaaagctaaagctacccagctacaaactcgacgcggtcgtggga

gacgtcctcggcgagcataagatagaccttccctataaagaaataccctcctattacgcg

ggagggcctgaccggaggggcgtaataggagagtattgtatccaggactctaggctggtg

ggcaagctgttttttaagtacctcccccatctggaactatcggcggtggccaaactcgcc

cgtatcaccctaacgcgggtaatttttgacggtcagcaaattcgcgtgtacacgtgcttg

ctgaaactcgcccgcgagagaaatttcattttgccagacaacagacgccggtttgacagt

caggcagacgccgcgtcagagacttcggagttggctatggatagccaaagccacgccttc

gacagtacagacgaacccgacggtgtggacggtaccccggacgccgcaggatctggcgct

acttctgaaaacggaggcgggaagcccggcgtcgggagggccgtgggctaccagggagca

aaggttctagaccccgtatccggctttcatgtggaccccgtggttgtgtttgacttcgct

agcttatacccaagcattatccaggcccataacctctgtttcaccaccctggcgctcgat

gaagtggatctggccgggcttcaaccatccgtcaactactcgacgttcgaggtgggtgac

caaaagttattttttgtccacgcccatattcgcgaaagcctgcttggcatcttgctgcgc

gactggctggccatgcgaaaggcggtgagggcgcgaatccccaccagcacccccgaggag

gcagttttactagataagcagcagtctgcgattaaggtgatatgcaactcggtttacgga

ttcacgggggtggcaaacggcctgttgccgtgtctgaggatagcggctaccgttaccacg

ataggacgcgacatgctcctcaagacccgagattacgttcactctcgttgggcgacgcgc

gagctgctggaggacaattttccaggggctataggtttccgaaaccacaagccttactcc

gtcagggttatctacggagacaccgactccgtgtttatcaagtttgtgggcctgacgtac

gagggggtatcggagctgggggatgctatgtcgcgtcagatttcagcggacctctttaga

gcgcccatcaaactggagtgtgagaagacctttcagcgactgctgctgatcaccaagaag

aagtacataggtgtcataaacggggggaagatgctcatgaagggggtcgacctggtccgc

aaaaataactgctctttcataaacttgtacgcgcgacatctggtagatcttttgttgtac

gacgaggatgtggccacggcggcagcagaggtgacagacgtgcctcccgcagaatgggtg

gggcgcccgctaccgagcggctttgacaagtttgggcgagtgctggtagaggcgtacaac

cgtatcactgcccccaacttggacgtgcgcgagttcgttatgactgctgagctgagccgc

tcacccgaattgtataccaacaagcgcctgccgcacctcaccgtctactttaagctcgcc

atgaggaatgaagaactgcccagcgtaaaagagagaattccgtatgtgatagttgcgcag

accgaggccgcggaacgcgaagcgggtgtagtaaactcaatgcgcggtaccgcccaaaac

cccgtggtaaccaagaccgcacgcccccaacctaaacgcaaactgctggtttccgacctc

gccgaagacccgacctatgtttccgagaatgacgtaccgctaaacacagactactatttc

tcccacctgttgggtaccataagcgtgacctttaaggctctattcggaaatgatgtgaga

acaacagaaaatcttttaaagcggtttattccggaaaccccccacaagacccccacgaaa

acccaggcactgcttgagcgcgccggctttgaaaagctgacgccctttacaccggaggaa

gaaagtcgtcgaatactgcatacagttttttgtactctagaagcagctccccatcaaagc

tga

>KF644570.1

atggcggcgcgcgaacaggccaactccgttcgcaggagcgggttttttaatccgtttatt

ggcaagaggccatttttcaggccgggaagcgggcagaccgcggagacagagcgcccaagg

cccccccaacactcgtactgcacagaggtgggtagctttaagtttatagctccaagatgc

ctcgatgaagaagcccccgccgaccagcgacgcggtgtacacgtgggcaccttggagcgc

ccacccaaagtgtactgcgatggctcagagtacgacgtgctgaactttgcctccggaggt

tgctggcctcgcaggattcgggtttggaacggccaggattttcggggcgatggattcaac

cccagatttgagagatttcacgtgtacgacatagtagagacttcggagagcgcctcacac

gatgacccatccaggtttgctgagctatcccgcccaagcgggtctgtggtaacactgctg

ggaatgagcgagtgtggaaagcgagtcgcggttcacgtctatggtgtgcgccattatttt

tacatggcaaaggcggaggtggatagcgcttgtggaataaccaccgaggcagaactcgtg

cgcgcaatggtggactgcgcgcacagctcggctttgagcgcggccctgggaaatggcaac

ggcggcaaacagagcggcggcagcggcgggggatggtggggcggaaagcacgtgtctgcg

gactgcttcaaagtggagaccgtgtgccacacgacgctgtactactttggatctaagcca

gctctctactatagagtatctgcctccagcagccgcctgggagggttcatctgcgacaac

tttcacccggagattacaaaattcgaggggagcgtggacgtgaccacgcggctgttgttg

gacaatgaaaattttaccagtttcgggtggtaccgcctgcgacccggcacccacggagag

cgtgttcaacttcgccccgttgagcgacacgtcacctcaagcgacgtggagattaactgt

actcccgataacctggagccgataccagacgaggctgcctggcccgactataagctcatg

tgctttgatatagagtgtaaagctggaacgggtaacgaaatggcgttcccagtggcaact

aaccaagaggacctggtcatccagatctcctgtctgctgtactcgcttgctactcagaac

cacgaacacaccctgctgttttccctcgggtcatgcgatatctctgaggaatactcgttt

gcatgcgtccagcgcggcgagcccagaccgacggttttggagtttgacagcgagtacgag

ctgctggttgccttcctgacctttctcaagcagtactctcccgagttcgccaccggctac

aacatcgttaattttgactgggcgtacatagttaacaaggtaacgtcggtgtataacatc

aagctggacgggtacggcaagttcaacaaaggggggctgtttaaggtgtgggacatcgcc

acgaaccattttcagaagaagagcaaggtgaaaatcaatggcctgatatctctagacatg

tattctgtggcgacggaaaagctaaagctacccagctacaaactcgacgcggtcgtggga

gacgtcctcggcgagcataagatagaccttccctataaagaaataccctcctattacgcg

ggagggcctgaccggaggggcgtaataggagagtattgtatccaggactctaggctggtg

ggcaagctgttttttaagtacctcccccatctggaactatcggcggtggccaaactcgcc

cgtatcaccctaacgcgggtaatttttgacggtcagcaaattcgcgtgtacacgtgcttg

ctgaaactcgcccgcgagagaaatttcattttgccagacaacagacgccggtttgacagt

caggcagacgccgcgtcagagacttcggagttggctatggatagccaaagccacgccttc

gacagtacagacgaacccgacggtgtggacggtaccccggacgccgcaggatctggcgct

acttctgaaaacggaggcgggaagcccggcgtcgggagggccgtgggctaccagggagca

aaggttctagaccccgtatccggctttcatgtggaccccgtggttgtgtttgacttcgct

agcttatacccaagcattatccaggcccataacctctgtttcaccaccctggcgctcgat

gaagtggatctggccgggcttcaaccatccgtcgactactcgacgttcgaggtgggtgac

caaaagttattttttgtccacgcccatattcgcgaaagcctgcttggcatcttgctgcgc

gactggctggccatgcgaaaggcggtgagggcgcgaatccccaccagcacccccgaggag

gcagttttactagataagcagcagtctgcgattaaggtgatatgcaactcggtttacgga

ttcacgggggtggcaaacggcctgttgccgtgtctgaggatagcggctaccgttaccacg

ataggacgcgacatgctcctcaagacccgagattacgttcactctcgttgggcgacgcgc

gagctgctggaggacaattttccaggggctataggtttccgaaaccacaagccttactcc

gtcagggttatctacggagacaccgactccgtgtttatcaagtttgtgggcctgacgtac

gagggggtatcggagctgggggatgctatgtcgcgtcagatttcagcggacctctttaga

gcgcccatcaaactggagtgtgagaagacctttcagcgactgctgctgatcaccaagaag

aagtacataggtgtcataaacggggggaagatgctcatgaagggggtcgacctggtccgc

aaaaataactgctctttcataaacttgtacgcgcgacatctggtagatcttttgttgtac

gacgaggatgtggccacggcggcagcagaggtgacagacgtgcctcccgcagaatgggtg

gggcgcccgctaccgagcggctttgacaagtttgggcgagtgctggtagaggcgtacaac

cgtatcactgcccccaacttggacgtgcgcgagttcgttatgactgctgagctgagccgc

tcacccgaatcgtataccaacaagcgcctgccgcacctcaccgtctactttaagctcgcc

atgaggaatgaagaactgcccagcgtaaaagagagaattccgtatgtgatagttgcgcag

accgaggccgcggaacgcgaagcgggtgtagtaaactcaatgcgcggtaccgcccaaaac

cccgtggtaaccaagaccgcacgcccccaacctaaacgcaaactgctggtttccgacctc

gccgaagacccgacctatgtttccgagaatgacgtaccgctaaacacagactactatttc

tcccacctgttgggtaccataagcgtgacctttaaggctctattcggaaatgatgtgaga

acaacagaaaatcttttaaagcggtttattccggaaaccccccacaagacccccacgaaa

acccaggcactgcttgagcgcgccggctttgaaaagctgacgccctttacaccggaggaa

gaaagtcgtcgaatactgcatacagttttttgtactctagaagcagctccccatcaaagc

tga

>KF644572.1

atggcggcgcgcgaacaggccaactccgttcgcaggagcgggttttttaatccgtttatt

ggcaagaggccatttttcaggccgggaagcgggcagaccgcggagacagagcgcccaagg

cccccccaacactcgtactgcacagaggtgggtagctttaagtttatagctccaagatgc

ctcgatgaagaagcccccgccgaccagcgacgcggtgtacacgtgggcaccttggagcgc

ccacccaaagtgtactgcgatggctcagagtacgacgtgctgaactttgcctccggaggt

tgctggcctcgcaggattcgggtttggaacggccaggattttcggggcgatggattcaac

cccagatttgagagatttcacgtgtacgacatagtagagacttcggagagcgcctcacac

gatgacccatccaggtttgctgagctatcccgcccaagcgggtctgtggtaacactgctg

ggaatgagcgagtgtggaaagcgagtcgcggttcacgtctatggtgtgcgccattatttt

tacatggcaaaggcggaggtggatagcgcttgtggaataaccaccgaggcagaactcgtg

cgcgcaatggtggactgcgcgcacagctcggctttgagcgcggccctgggaaatggcaac

ggcggcaaacagagcggcggcagcggcgggggatggtggggcggaaagcacgtgtctgcg

gactgcttcaaagtggagaccgtgtgccacacgacgctgtactactttggatctaagcca

gctctctactatagagtatctgcctccagcagccgcctgggagggttcatctgcgacaac

tttcacccggagattacaaaattcgaggggagcgtggacgtgaccacgcggctgttgttg

gacaatgaaaattttaccagtttcgggtggtaccgcctgcgacccggcacccacggagag

cgtgttcaacttcgccccgttgagcgacacgtcacctcaagcgacgtggagattaactgt

actcccgataacctggagccgataccagacgaggctgcctggcccgactataagctcatg

tgctttgatatagagtgtaaagctggaacgggtaacgaaatggcgttcccagtggcaact

aaccaagaggacctggtcatccagatctcctgtctgctgtactcgcttgctactcagaac

cacgaacacaccctgctgttttccctcgggtcatgcgatatctctgaggaatactcgttt

gcatgcgtccagcgcggcgagcccagaccgacggttttggagtttgacagcgagtacgag

ctgctggttgccttcctgacctttctcaagcagtactctcccgagttcgccaccggctac

aacatcgttaattttgactgggcgtacatagttaacaaggtaacgtcggtgtataacatc

aagctggacgggtacggcaagttcaacaaaggggggctgtttaaggtgtgggacatcgcc

acgaaccattttcagaagaagagcaaggtgaaaatcaatggcctgatatctctagacatg

tattctgtggcgacggaaaagctaaagctacccagctacaaactcgacgcggtcgtggga

gacgtcctcggcgagcataagatagaccttccctataaagaaataccctcctattacgcg

ggagggcctgaccggaggggcgtaataggagagtattgtatccaggactctaggctggtg

ggcaagctgttttttaagtacctcccccatctggaactatcggcggtggccaaactcgcc

cgtatcaccctaacgcgggtaatttttgacggtcagcaaattcgcgtgtacacgtgcttg

ctgaaactcgcccgcgagagaaatttcattttgccagacaacagacgccggtttgacagt

caggcagacgccgcgtcagagacttcggagttggctatggatagccaaagccacgccttc

gacagtacagacgaacccgacggtgtggacggtaccccggacgccgcaggatctggcgct

acttctgaaaacggaggcgggaagcccggcgtcgggagggccgtgggctaccagggagca

aaggttctagaccccgtatccggctttcatgtggaccccgtggttgtgtttgacttcgct

agcttatacccaagcattatccaggcccataacctctgtttcaccaccctggcgctcgat

gaagtggatctggccgggcttcaaccatccgtcaactactcgacgttcgaggtgggtggc

caaaagttattttttgtccacgcccatattcgcgaaagcctgcttggcatcttgctgcgc

gactggctggccatgcgaaaggcggtgagggcgcgaatccccaccagcacccccgaggag

gcagttttactagataagcagcagtctgcgattaaggtgatatgcaactcggtttacgga

ttcacgggggtggcaaacggcctgttgccgtgtctgaggatagcggctaccgttaccacg

ataggacgcgacatgctcctcaagacccgagattacgttcactctcgttgggcgacgcgc

gagctgctggaggacaattttccaggggctataggtttccgaaaccacaagccttactcc

gtcagggttatctacggagacaccgactccgtgtttatcaagtttgtgggcctgacgtac

gagggggtatcggagctgggggatgctatgtcgcgtcagatttcagcggacctctttaga

gcgcccatcaaactggagtgtgagaagacctttcagcgactgctgctgatcaccaagaag

aagtacataggtgtcataaacggggggaagatgctcatgaagggggtcgacctggtccgc

aaaaataactgctctttcataaacttgtacgcgcgacatctggtagatcttttgttgtac

gacgaggatgtggccacggcggcagcagaggtgacagacgtgcctcccgcagaatgggtg

gggcgcccgctaccgagcggctttgacaagtttgggcgagtgctggtagaggcgtacaac

cgtatcactgcccccaacttggacgtgcgcgagttcgttatgactgctgagctgagccgc

tcacccgaatcgtataccaacaagcgcctgccgcacctcaccgtctactttaagctcgcc

atgaggaatgaagaactgcccagcgtaaaagagagaattccgtatgtgatagttgcgcag

accgaggccgcggaacgcgaagcgggtgtagtaaactcaatgcgcggtaccgcccaaaac

cccgtggtaaccaagaccgcacgcccccaacctaaacgcaaactgctggtttccgacctc

gccgaagacccgacctatgtttccgagaatgacgtaccgctaaacacagactactatttc

tcccacctgttgggtaccataagcgtgacctttaaggctctattcggaaatgatgtgaga

acaacagaaaatcttttaaagcggtttattccggaaaccccccacaagacccccacgaaa

acccaggcactgcttgagcgcgccggctttgaaaagctgacgccctttacaccggaggaa

gaaagtcgtcgaatactgcatacagttttttgtactctagaagcagctccccatcaaagc

tga

>KF644576.1

atggcggcgcgcgaacaggccaactccgttcgcaggagcgggttttttaatccgtttatt

ggcaagaggccatttttcaggccgggaagcgggcagaccgcggagacagagcgcccaagg

cccccccaacactcgtactgcacagaggtgggtagctttaagtttatagctccaagatgc

ctcgatgaagaagcccccgccgaccagcgacgcggtgtacacgtgggcaccttggagcgc

ccacccaaagtgtactgcgatggctcagagtacgacgtgctgaactttgcctccggaggt

tgctggcctcgcaggattcgggtttggaacggccaggattttcggggcgatggattcaac

cccagatttgagagatttcacgtgtacgacatagtagagacttcggagagcgcctcacac

gatgacccatccaggtttgctgagctatcccgcccaagcgggtctgtggtaacactgctg

ggaatgagcgagtgtggaaagcgagtcgcggttcacgtctatggtgtgcgccattatttt

tacatggcaaaggcggaggtggatagcgcttgtggaataaccaccgaggcagaactcgtg

cgcgcaatggtggactgcgcgcacagctcggctttgagcgcggccctgggaaatggcaac

ggcggcaaacagagcggcggcagcggcgggggatggtggggcggaaagcacgtgtctgcg

gactgcttcaaagtggagaccgtgtgccacacgacgctgtactactttggatctaagcca

gctctctactatagagtatctgcctccagcagccgcctgggagggttcatctgcgacaac

tttcacccggagattacaaaattcgaggggagcgtggacgtgaccacgcggctgttgttg

gacaatgaaaattttaccagtttcgggtggtaccgcctgcgacccggcacccacggagag

cgtgttcaacttcgccccgttgagcgacacgtcacctcaagcgacgtggagattaactgt

actcccgataacctggagccgataccagacgaggctgcctggcccgactataagctcatg

tgctttgatatagagtgtaaagctggaacgggtaacgaaatggcgttcccagtggcaact

aaccaagaggacctggtcatccagatctcctgtctgctgtactcgcttgctactcagaac

cacgaacacaccctgctgttttccctcgggtcatgcgatatctctgaggaatactcgttt

gcatgcgtccagcgcggcgagcccagaccgacggttttggagtttgacagcgagtacgag

ctgctggttgccttcctgacctttctcaagcagtactctcccgagttcgccaccggctac

aacatcgttaattttgactgggcgtacatagttaacaaggtaacgtcggtgtataacatc

aagctggacgggtacggcaagttcaacaaaggggggctgtttaaggtgtgggacatcgcc

acgaaccattttcagaagaagagcaaggtgaaaatcaatggcctgatatctctagacatg

tattctgtggcgacggaaaagctaaagctacccagctacaaactcgacgcggtcgtggga

gacgtcctcggcgagcataagatagaccttccctataaagaaataccctcctattacgcg

ggagggcctgaccggaggggcgtaataggagagtattgtatccaggactctaggctggtg

ggcaagctgttttttaagtacctcccccatctggaactatcggcggtggccaaactcgcc

cgtatcaccctaacgcgggtaatttttgacggtcagcaaattcgcgtgtacacgtgcttg

ctgaaactcgcccgcgagagaaatttcattttgccagacaacagacgccggtttgacagt

caggcagacgccgcgtcagagacttcggagttggctatggatagccaaagccacgccttc

gacagtacagacgaacccgacggtgtggacggtaccccggacgccgcaggatctggcgct

acttctgaaaacggaggcgggaagcccggcgtcgggagggccgtgggctaccagggagca

aaggttctagaccccgtatccggctttcatgtggaccccgtggttgtgtttgacttcgct

agcttatacccaagcattatccaggcccataacctctgtttcaccaccctggcgctcgat

gaagtggatctggccgggcttcaaccatccgtcaactactcgacgttcgaggtgggtgac

caaaagttattttttgtccacgcccatattcgcgaaagcctgcttggcatcttgctgcgc

gactggctggccatgcgaaaggcggtgagggcgcgaatccccaccagcacccccgaggag

gcagttttactagataagcagcagtctgcgattaaggtgatatgcaactcggtttacgga

ttcacgggggtggcaaacggcctgttgccgtgtctgaggatagcggctaccgttaccacg

ataggacgcgacatgctcctcaaaacccgagattacgttcactctcgttgggcgacgcgc

gagctgctggaggacaattttccaggggctataggtttccgaaaccacaagccttactcc

gtcagggttatctacggagacaccgactccgtgtttatcaagtttgtgggcctgacgtac

gagggggtatcggagctgggggatgctatgtcgcgtcagatttcagcggacctctttaga

gcccccatcaaactggagtgtgagaagacctttcagcgactgctgctgatcaccaagaag

aagtacataggtgtcataaacggggggaagatgctcatgaagggggtcgacctggtccgc

aaaaataactgctctttcataaacttgtacgcgcgacatctggtagatcttttgttgtac

gacgaggatgtggccacggcggcagcagaggtgacagacgtgcctcccgcagaatgggtg

gggcgcccgctaccgagcggctttgacaagtttgggcgagtgctggtagaggcgtacaac

cgtatcactgcccccaacttggacgtgcgcgagttcgttatgactgctgagctgagccgc

tcacccgaatcgtataccaacaagcgcctgccgcacctcaccgtctactttaagctcgcc

atgaggaatgaagaactgcccagcgtaaaagagagaattccgtatgtgatagttgcgcag

accgaggccgcggaacgcgaagcgggtgtagtaaactcaatgcgcggtaccgcccaaaac

cccgtggtaaccaagaccgcacgcccccaacctaaacgcaaactgctggtttccgacctc

gccgaagacccgacctatgtttccgagaatgacgtaccgctaaacacagactactatttc

tcccacctgttgggtaccataagcgtgacctttaaggctctattcggaaatgatgtgaga

acaacagaaaatcttttaaagcggtttattccggaaaccccccacaagacccccacgaaa

acccaggcactgcttgagcgcgccggctttgaaaagctgacgccctttacaccggaggaa

gaaagtcgtcgaatactgcatacagttttttgtactctagaagcagctccccatcaaagc

tga

>KF644577.1

atggcggcgcgcgaacaggccaactccgttcgcaggagcgggttttttaatccgtttatt

ggcaagaggccatttttcaggccgggaagcgggcagaccgcggagacagagcgcccaagg

cccccccaacactcgtactgcacagaggtgggtagctttaagtttatagctccaagatgc

ctcgatgaagaagcccccgccgaccagcgacgcggtgtacacgtgggcaccttggagcgc

ccacccaaagtgtactgcgatggctcagagtacgacgtgctgaactttgcctccggaggt

tgctggcctcgcaggattcgggtttggaacggccaggattttcggggcgatggattcaac

cccagatttgagagatttcacgtgtacgacatagtagagacttcggagagcgcctcacac

gatgacccatccaggtttgctgagctatcccgcccaagcgggtctgtggtaacactgctg

ggaatgagcgagtgtggaaagcgagtcgcggttcacgtctatggtgtgcgccattatttt

tacatggcaaaggcggaggtggatagcgcttgtggaataaccaccgaggcagaactcgtg

cgcgcaatggtggactgcgcgcacagctcggctttgagcgcggccctgggaaatggcaac

ggcggcaaacagagcggcggcagcggcgggggatggtggggcggaaagcacgtgtctgcg

gactgcttcaaagtggagaccgtgtgccacacgacgctgtactactttggatctaagcca

gctctctactatagagtatctgcctccagcagccgcctgggagggttcatctgcgacaac

tttcacccggagattacaaaattcgaggggagcgtggacgtgaccacgcggctgttgttg

gacaatgaaaattttaccagtttcgggtggtaccgcctgcgacccggcacccacggagag

cgtgttcaacttcgccccgttgagcgacacgtcacctcaagcgacgtggagattaactgt

actcccgataacctggagccgataccagacgaggctgcctggcccgactataagctcatg

tgctttgatatagagtgtaaagctggaacgggtaacgaaatggcgttcccagtggcaact

aaccaagaggacctggtcatccagatctcctgtctgctgtactcgcttgctactcagaac

cacgaacacaccctgctgttttccctcgggtcatgcgatatctctgaggaatactcgttt

gcatgcgtccagcgcggcgagcccagaccgacggttttggagtttgacagcgagtacgag

ctgctggttgccttcctgacctttctcaagcagtactctcccgagttcgccaccggctac

aacatcgttaattttgactgggcgtacatagttaacaaggtaacgtcggtgtataacatc

aagctggacgggtacggcaagttcaacaaaggggggctgtttaaggtgtgggacatcgcc

acgaaccattttcagaagaagagcaaggtgaaaatcaatggcctgatatctctagacatg

tattctgtggcgacggaaaagctaaagctacccagctacaaactcgacgcggtcgtggga

gacgtcctcggcgagcataagatagaccttccctataaagaaataccctcctattacgcg

ggagggcctgaccggaggggcgtaataggagagtattgtatccaggactctaggctggtg

ggcaagctgttttttaagtacctcccccatctggaactatcggcggtggccaaactcgcc

cgtatcaccctaacgcgggtaatttttgacggtcagcaaattcgcgtgtacacgtgcttg

ctgaaactcgcccgcgagagaaatttcattttgccagacaacagacgccggtttgacagt

caggcagacgccgcgtcagagacttcggagttggctatggatagccaaagccacgccttc

gacagtacagacgaacccgacggtgtggacggtaccccggacgccgcaggatctggcgct

acttctgaaaacggaggcgggaagcccggcgtcgggagggccgtgggctaccagggagca

aaggttctagaccccgtatccggctttcatgtggaccccgtggttgtgtttgacttcgct

agcttatacccaagcattatccaggcccataacctctgtttcaccaccctggcgctcgat

gaagcggatctggccgggcttcaaccatccgtcaactactcgacgttcgaggtgggtgac

caaaagttattttttgtccacgcccatattcgcgaaagcctgcttggcatcttgctgcgc

gactggctggccatgcgaaaggcggtgagggcgcgaatccccaccagcacccccgaggag

gcagttttactagataagcagcagtctgcgattaaggtgatatgcaactcggtttacgga

ttcacgggggtggcaaacggcctgttgccgtgtctgaggatagcggctaccgttaccacg

ataggacgcgacatgctcctcaagacccgagattacgttcactctcgttgggcgacgcgc

gagctgctggaggacaattttccaggggctataggtttccgaaaccacaagccttactcc

gtcagggttatctacggagacaccgactccgtgtttatcaagtttgtgggcctgacgtac

gagggggtatcggagctgggggatgctatgtcgcgtcagatttcagcggacctctttaga

gcccccatcaaactggagtgtgagaagacctttcagcgactgctgctgatcaccaagaag

aagtacataggtgtcataaacggggggaagatgctcatgaagggggtcgacctggtccgc

aaaaataactgctctttcataaacttgtacgcgcgacatctggtagatcttttgttgtac

gacgaggatgtggccacggcggcagcagaggtgacagacgtgcctcccgcagaatgggtg

gggcgcccgctaccgagcggctttgacaagtttgggcgagtgctggtagaggcgtacaac

cgtatcactgcccccaacttggacgtgcgcgagttcgttatgactgctgagctgagccgc

tcacccgaatcgtataccaacaagcgcctgccgcacctcaccgtctactttaagctcgcc

atgaggaatgaagaactgcccagcgtaaaagagagaattccgtatgtgatagttgcgcag

accgaggccgcggaacgcgaagcgggtgtagtaaactcaatgcgcggtaccgcccaaaac

cccgtggtaaccaagaccgcacgcccccaacctaaacgcaaactgctggtttccgacctc

gccgaagacccgacctatgtttccgagaatgacgtaccgctaaacacagactactatttc

tcccacctgttgggtaccataagcgtgacctttaaggctctattcggaaatgatgtgaga

acaacagaaaatcttttaaagcggtttattccggaaaccccccacaagacccccacgaaa

acccaggcactgcttgagcgcgccggctttgaaaagctgacgccctttacaccggaggaa

gaaagtcgtcgaatactgcatacagttttttgtactctagaagcagctccccatcaaagc

tga

>KF644578.1

atggcggcgcgcgaacaggccaactccgttcgcaggagcgggttttttaatccgtttatt

ggcaagaggccatttttcaggccgggaagcgggcagaccgcggagacagagcgcccaagg

cccccccaacactcgtactgcacagaggtgggtagctttaagtttatagctccaagatgc

ctcgatgaagaagcccccgccgaccagcgacgcggtgtacacgtgggcaccttggagcgc

ccacccaaagtgtactgcgatggctcagagtacgacgtgctgaactttgcctccggaggt

tgctggcctcgcaggattcgggtttggaacggccaggattttcggggcgatggattcaac

cccagatttgagagatttcacgtgtacgacatagtagagacttcggagagcgcctcacac

gatgacccatccaggtttgctgagctatcccgcccaagcgggtctgtggtaacactgctg

ggaatgagcgagtgtggaaagcgagtcgcggttcacgtctatggtgtgcgccattatttt

tacatggcaaaggcggaggtggatagcgcttgtggaataaccaccgaggcagaactcgtg

cgcgcaatggtggactgcgcgcacagctcggctttgagcgcggccctgggaaatggcaac

ggcggcaaacagagcggcggcagcggcgggggatggtggggcggaaagcacgtgtccgcg

gactgcttcaaagtggagaccgtgtgccacacgacgctgtactactttggatctaagcca

gctctctactatagagtatctgcctccagcagccgcctgggagggttcatctgcgacaac

tttcacccggagattacaaaattcgaggggagcgtggacgtgaccacgcggctgttgttg

gacaatgaaaattttaccagtttcgggtggtaccgcctgcgacccggcacccacggagag

cgtgttcaacttcgccccgttgagcgacacgtcacctcaagcgacgtggagattaactgt

actcccgataacctggagccgataccagacgaggctgcctggcccgactataagctcatg

tgctttgatatagagtgtaaagctggaacgggtaacgaaatggcgttcccagtggcaact

aaccaagaggacctggtcatccagatctcctgtctgctgtactcgcttgctactcagaac

cacgaacacaccctgctgttttccctcgggtcatgcgatatctctgaggaatactcgttt

gcatgcgtccagcgcggcgagcccagaccgacggttttggagtttgacagcgagtacgag

ctgctggttgccttcctgacctttctcaagcagtactctcccgagttcgccaccggctac

aacatcgttaattttgactgggcgtacatagttaacaaggtaacgtcggtgtataacatc

aagctggacgggtacggcaagttcaacaaaggggggctgtttaaggtgtgggacatcgcc

acgaaccattttcagaagaagagcaaggtgaaaatcaatggcctgatatctctagacatg

tattctgtggcgacggaaaagctaaagctacccagctacaaactcgacgcggtcgtggga

gacgtcctcggcgagcataagatagaccttccctataaagaaataccctcctattacgcg

ggagggcctgaccggaggggcgtaataggagagtattgtatccaggactctaggctggtg

ggcaagctgttttttaagtacctcccccatctggaactatcggcggtggccaaactcgcc

cgtatcaccctaacgcgggtaatttttgacggtcagcaaattcgcgtgtacacgtgcttg

ctgaaactcgcccgcgagagaaatttcattttgccagacaacagacgccggtttgacagt

caggcagacgccgcgtcagagacttcggagttggctatggatagccaaagccacgccttc

gacagtacagacgaacccgacggtgtggacggtaccccggacgccgcaggatctggcgct

acttctgaaaacggaggcgggaagcccggcgtcgggagggccgtgggctaccagggagca

aaggttctagaccccgtatccggctttcatgtggaccccgtggttgtgtttgacttcgct

agcttatacccaagcattatccaggcccataacctctgtttcaccaccctggcgctcgat

gaagtggatctggccgggcttcaaccatccgtcgactactcgacgttcgaggtgggtgac

caaaagttattttttgtccacgcccatattcgcgaaagcctgcttggcatcttgctgcgc

gactggctggccatgcgaaaggcggtgagggcgcgaatccccaccagcacccccgaggag

gcagttttactagataagcagcagtctgcgattaaggtgatatgcaactcggtttacgga

ttcacgggggtggcaaacggcctgttgccgtgtctgaggatagcggctaccgttaccacg

ataggacgcgacatgctcctcaagacccgagattacgttcactctcgttgggcgacgcgc

gagctgctggaggacaattttccaggggctataggtttccgaaaccacaagccttactcc

gtcagggttatctacggagacaccgactccgtgtttatcaagtttgtgggcctgacgtac

gagggggtatcggagctgggggatgctatgtcgcgtcagatttcagcggacctctttaga

gcccccatcaaactggagtgtgagaagacctttcagcgactgctgctgatcaccaagaag

aagtacataggtgtcataaacggggggaagatgctcatgaagggggtcgacctggtccgc

aaaaataactgctctttcataaacttgtacgcgcgacatctggtagatcttttgttgtac

gacgaggatgtggccacggcggcagcagaggtgacagacgtgcctcccgcagaatgggtg

gggcgcccgctaccgagcggctttgacaagtttgggcgagtgctggtagaggcgtacaac

cgtatcactgcccccaacttggacgtgcgcgagttcgttatgactgctgagctgagccgc

tcacccgaatcgtataccaacaagcgcctgccgcacctcaccgtctactttaagctcgcc

atgaggaatgaagaactgcccagcgtaaaagagagaattccgtatgtgatagttgcgcag

accgaggccgcggaacgcgaagcgggtgtagtaaactcaatgcgcggtaccgcccaaaac

cccgtggtaaccaagaccgcacgcccccaacctaaacgcaaactgctggtttccgacctc

gccgaagacccgacctatgtttccgagaatgacgtaccgctaaacacagactactatttc

tcccacctgttgggtaccataagcgtgacctttaaggctctattcggaaatgatgtgaga

acaacagaaaatcttttaaagcggtttattccggaaaccccccacaagacccccacgaaa

acccaggcactgcttgagcgcgccggctttgaaaagctgacgccctttacaccggaggaa

gaaagtcgtcgaatactgcatacagttttttgtactctagaagcagctccccatcaaagc

tga

>KF644579.1

atggcggcgcgcgaacaggccaactccgttcgcaggagcgggttttttaatccgtttatt

ggcaagaggccatttttcaggccgggaagcgggcagaccgcggagacagagcgcccaagg

cccccccaacactcgtactgcacagaggtgggtagctttaagtttatagctccaagatgc

ctcgatgaagaagcccccgccgaccagcgacgcggtgtacacgtgggcaccttggagcgc

ccacccaaagtgtactgcgatggctcagagtacgacgtgctgaactttgcctccggaggt

tgctggcctcgcaggattcgggtttggaacggccaggattttcggggcgatggattcaac

cccagatttgagagatttcacgtgtacgacatagtagagacttcggagagcgcctcacac

gatgacccatccaggtttgctgagctatcccgcccaagcgggtctgtggtaacactgctg

ggaatgagcgagtgtggaaagcgagtcgcggttcacgtctatggtgtgcgccattatttt

tacatggcaaaggcggaggtggatagcgcttgtggaataaccaccgaggcagaactcgtg

cgcgcaatggtggactgcgcgcacagctcggctttgagcgcggccctgggaaatggcaac

ggcggcaaacagagcggcggcagcggcgggggatggtggggcggaaagcacgtgtctgcg

gactgcttcaaagtggagaccgtgtgccacacgacgctgtactactttggatctaagcca

gctctctactatagagtatctgcctccagcagccgcctgggagggttcatctgcgacaac

tttcacccggagattacaaaattcgaggggagcgtggacgtgaccacgcggctgttgttg

gacaatgaaaattttaccagtttcgggtggtaccgcctgcgacccggcacccacggagag

cgtgttcaacttcgccccgttgagcgacacgtcacctcaagcgacgtggagattaactgt

actcccgataacctggagccgataccagacgaggctgcctggcccgactataagctcatg

tgctttgatatagagtgtaaagctggaacgggtaacgaaatggcgttcccagtggcaact

aaccaagaggacctggtcatccagatctcctgtctgctgtactcgcttgctactcagaac

cacgaacacaccctgctgttttccctcgggtcatgcgatatctctgaggaatactcgttt

gcatgcgtccagcgcggcgagcccagaccgacggttttggagtttgacagcgagtacgag

ctgctggttgccttcctgacctttctcaagcagtactctcccgagttcgccaccggctac

aacatcgttaattttgactgggcgtacatagttaacaaggtaacgtcggtgtataacatc

aagctggacgggtacggcaagttcaacaaaggggggctgtttaaggtgtgggacatcgcc

acgaaccattttcagaagaagagcaaggtgaaaatcaatggcctgatatctctagacatg

tattctgtggcgacggaaaagctaaagctacccagctacaaactcgacgcggtcgtggga

gacgtcctcggcgagcataagatagaccttccctataaagaaataccctcctattacgcg

ggagggcctgaccggaggggcgtaataggagagtattgtatccaggactctaggctggtg

ggcaagctgttttttaagtacctcccccatctggaactatcggcggtggccaaactcgcc

cgtatcaccctaacgcgggtaatttttgacggtcagcaaattcgcgtgtacacgtgcttg

ctgaaactcgcccgcgagagaaatttcattttgccagacaacagacgccggtttgacagt

caggcagacgccgcgtcagagacttcggagttggctatggatagccaaagccacgccttc

gacagtacagacgaacccgacggtgtggacggtaccccggacgccgcaggatctggcgct

acttctgaaaacggaggcgggaagcccggcgtcgggagggccgtgggctaccagggagca

aaggttctagaccccgtatccggctttcatgtggaccccgtggttgtgtttgacttcgct

agcttatacccaagcattatccaggcccataacctctgtttcaccaccctggcgctcgat

gaagcggatctggccgggcttcaaccatccgtcaactactcgacgttcgaggtgggtgac

caaaagttattttttgtccacgcccatattcgcgaaagcctgcttggcatcttgctgcgc

gactggctggccatgcgaaaggcggtgagggcgcgaatccccaccagcacccccgaggag

gcagttttactagataagcagcagtctgcgattaaggtgatatgcaactcggtttacgga

ttcacgggggtggcaaacggcctgttgccgtgtctgaggatagcggctaccgttaccacg

ataggacgcgacatgctcctcaagacccgagattacgttcactctcgttgggcgacgcgc

gagctgctggaggacaattttccaggggctataggtttccgaaaccacaagccttactcc

gtcagggttatctacggagacaccgactccgtgtttatcaagtttgtgggcctgacgtac

gagggggtatcggagctgggggatgctatgtcgcgtcagatttcagcggacctctttaga

gcccccatcaaactggagtgtgagaagacctttcagcgactgctgctgatcaccaagaag

aagtacataggtgtcataaacggggggaagatgctcatgaagggggtcgacctggtccgc

aaaaataactgctctttcataaacttgtacgcgcgacatctggtagatcttttgttgtac

gacgaggatgtggccacggcggcagcagaggtgacagacgtgcctcccgcagaatgggtg

gggcgcccgctaccgagcggctttgacaagtttgggcgagtgctggtagaggcgtacaac

cgtatcactgcccccaacttggacgtgcgcgagttcgttatgactgctgagctgagccgc

tcacccgaatcgtataccaacaagcgcctgccgcacctcaccgtctactttaagctcgcc

atgaggaatgaagaactgcccagcgtaaaagagagaattccgtatgtgatagttgcgcag

accgaggccgcggaacgcgaagcgggtgtagtaaactcaatgcgcggtaccgcccaaaac

cccgtggtaaccaagaccgcacgcccccaacctaaacgcaaactgctggtttccgacctc

gccgaagacccgacctatgtttccgagaatgacgtaccgctaaacacagactactatttc

tcccacctgttgggtaccataagcgtgacctttaaggctctattcggaaatgatgtgaga

acaacagaaaatcttttaaagcggtttattccggaaaccccccacaagacccccacgaaa

acccaggcactgcttgagcgcgccggctttgaaaagctgacgccctttacaccggaggaa

gaaagtcgtcgaatactgcatacagttttttgtactctagaagcagctccccatcaaagc

tga

>AB992258.1

atggcggcgcgcgaacaggccaactccgttcgcaggagcgggttttttaatccgtttatt

ggcaagaggccatttttcaggccgggaagcgggcagaccgcggagacagagcgcccaagg

cccccccaacactcgtactgcacagaggtgggtagctttaagtttatagctccaagatgc

ctcgatgaagaagcccccgccgaccagcgacgcggtgtacacgtgggcaccttggagcgc

ccacccaaagtgtactgcgatggctcagagtacgacgtgctgaactttgcctccggaggt

tgctggcctcgcaggattcgggtttggaacggccaggattttcggggcgatggattcaac

cccagatttgagagatttcacgtgtacgacatagtagagacttcggagagcgcctcacac

gatgacccatccaggtttgctgagctatcccgcccaagcgggtctgtggtaacactgctg

ggaatgagcgagtgtggaaagcgagtcgcggttcacgtctatggtgtgcgccattatttt

tacatggcaaaggcggaggtggatagcgcttgtggaataaccaccgaggcagaactcgtg

cgcgcaatggtggactgcgcgcacagctcggctttgagcgcggccctgggaaatggcaac

ggcggcaaacagagcggcggcagcggcgggggatggtggggcggaaagcacgtgtctgcg

gactgcttcaaagtggagaccgtgtgccacacgacgctgtactactttggatctaagcca

gctctctactatagagtatctgcctccagcagccgcctgggagggttcatctgcgacaac

tttcacccggagattacaaaattcgaggggagcgtggacgtgaccacgcggctgttgttg

gacaatgaaaattttaccagtttcgggtggtaccgcctgcgacccggcacccacggagag

cgtgttcaacttcgccccgttgagcgacacgtcacctcaagcgacgtggagattaactgt

actcccgataacctggagccgataccagacgaggctgcctggcccgactataagctcatg

tgctttgatatagagtgtaaagctggaacgggtaacgaaatggcgttcccagtggcaact

aaccaagaggacctggtcatccagatctcctgtctgctgtactcgcttgctactcagaac

cacgaacacaccctgctgttttccctcgggtcatgcgatatctctgaggaatactcgttt

gcatgcgtccagcgcggcgagcccagaccgacggttttggagtttgacagcgagtacgag

ctgctggttgccttcctgacctttctcaagcagtactctcccgagttcgccaccggctac

aacatcgttaattttgactgggcgtacatagttaacaaggtaacgtcggtgtataacatc

aagctggacgggtacggcaagttcaacaaaggggggctgtttaaggtgtgggacatcgcc

acgaaccattttcagaagaagagcaaggtgaaaatcaatggcctgatatctctagacatg

tattctgtggcgacggaaaagctaaagctacccagctacaaactcgacgcggtcgtggga

gacgtcctcggcgagcataagatagaccttccctataaagaaataccctcctattacgcg

ggagggcctgaccggaggggcgtaataggagagtattgtatccaggactctaggctggtg

ggcaagctgttttttaagtacctcccccatctggaactatcggcggtggccaaactcgcc

cgtatcaccctaacgcgggtaatttttgacggtcagcaaattcgcgtgtacacgtgcttg

ctgaaactcgcccgcgagagaaatttcattttgccagacaacagacgccggtttgacagt

caggcagacgccgcgtcagagacttcggagttggctatggatagccaaagccacgccttc

gacagtacagacgaacccgacggtgtggacggtaccccggacgccgcaggatctggcgct

acttctgaaaacggaggcgggaagcccggcgtcgggagggccgtgggctaccagggagca

aaggttctagaccccgtatccggctttcatgtggaccccgtggttgtgtttgacttcgct

agcttatacccaagcattatccaggcccataacctctgtttcaccaccctggcgctcgat

gaagtggatctggccgggcttcaaccatccgtcaactactcgacgttcgaggtgggtgac

caaaagttattttttgtccacgcccatattcgcgaaagcctgcttggcatcttgctgcgc

gactggctggccatgcgaaaggcggtgagggcgcgaatccccaccagcacccccgaggag

gcagttttactagataagcagcagtctgcgattaaggtgatatgcaactcggtttacgga

ttcacgggggtggcaaacggcctgttgccgtgtctgaggatagcggctaccgttaccacg

ataggacgcgacatgctcctcaagacccgagattacgttcactctcgttgggcgacgcgc

gagctgctggaggacaattttccaggggctataggtttccgaaaccacaagccttactcc

gtcagggttatctacggagacaccgactccgtgtttatcaagtttgtgggcctgacgtac

gagggggtatcggagctgggggatgctatgtcgcgtcagatttcagcggacctctttaga

gcgcccatcaaactggagtgtgagaagacctttcagcgactgctgctgatcaccaagaag

aagtacataggtgtcataaacggggggaagatgctcatgaagggggtcgacctggtccgc

aaaaataactgctctttcataaacttgtacgcgcgacatctggtagatcttttgttgtac

gacgaggatgtggccacggcggcagcagaggtgacagacgtgcctcccgcagaatgggtg

gggcgcccgctaccgagcggctttgacaagtttgggcgagtgctggtagaggcgtacaac

cgtatcactgcccccaacttggacgtgcgcgagttcgttatgactgctgagctgagccgc

tcacccgaatcgtataccaacaagcgcctgccgcacctcaccgtctactttaagctcgcc

atgaggaatgaagaactgcccagcgtaaaagagagaattccgtatgtgatagttgcgcag

accgaggccgcggaacgcgaagcgggtgtagtaaactcaatgcgcggtaccgcccaaaac

cccgtggtaaccaagaccgcacgcccccaacctaaacgcaaactgctggtttccgacctc

gccgaagacccgacctatgtttccgagaatgacgtaccgctaaacacagactactatttc

tcccacctgttgggtaccataagcgtgacctttaaggctctattcggaaatgatgtgaga

acaacagaaaatcttttaaagcggtttattccggaaaccccccacaagacccccacgaaa

acccaggcactgcttgagcgcgccggctttgaaaagctgacgccctttacaccggaggaa

gaaagtcgtcgaatactgcatacagttttttgtactctagaagcagctccccatcaaagc

tga

>KM593996.1

atggcggcgcgcgaacaggccaactccgttcgcaggagcgggttttttaatccgtttatt

ggcaagaggccatttttcaggccgggaagcgggcagaccgcggagacagagcgcccaagg

cccccccaacactcgtactgcacagaggtgggtagctttaagtttatagctccaagatgc

ctcgatgaagaagcccccgccgaccagcgacgcggtgtacacgtgggcaccttggagcgc

ccacccaaagtgtactgcgatggctcagagtacgacgtgctgaactttgcctccggaggt

tgctggcctcgcaggattcgggtttggaacggccaggattttcggggcgatggattcaac

cccagatttgagagatttcacgtgtacgacatagtagagacttcggagagcgcctcacac

gatgacccatccaggtttgctgagctatcccgcccaagcgggtctgtggtaacactgctg

ggaatgagcgagtgtggaaagcgagtcgcggttcacgtctatggtgtgcgccattatttt

tacatggcaaaggcggaggtggatagcgcttgtggaataaccaccgaggcagaactcgtg

cgcgcaatggtggactgcgcgcacagctcggctttgagcgcggccctgggaaatggcaac

ggcggcaaacagagcggcggcagcggcgggggatggtggggcggaaagcacgtgtctgcg

gactgcttcaaagtggagaccgtgtgccacacgacgctgtactactttggatctaagcca

gctctctactatagagtatctgcctccagcagccgcctgggagggttcatctgcgacaac

tttcacccggagattacaaaattcgaggggagcgtggacgtgaccacgcggctgttgttg

gacaatgaaaattttaccagtttcgggtggtaccgcctgcgacccggcacccacggagag

cgtgttcaacttcgccccgttgagcgacacgtcacctcaagcgacgtggagattaactgt

actcccgataacctggagccgataccagacgaggctgcctggcccgactataagctcatg

tgctttgatatagagtgtaaagctggaacgggtaacgaaatggcgttcccagtggcaact

aaccaagaggacctggtcatccagatctcctgtctgctgtactcgcttgctactcagaac

cacgaacacaccctgctgttttccctcgggtcatgcgatatctctgaggaatactcgttt

gcatgcgtccagcgcggcgagcccagaccgacggttttggagtttgacagcgagtacgag

ctgctggttgccttcctgacctttctcaagcagtactctcccgagttcgccaccggctac

aacatcgttaattttgactgggcgtacatagttaacaaggtaacgtcggtgtataacatc

aagctggacgggtacggcaagttcaacaaaggggggctgtttaaggtgtgggacatcgcc

acgaaccattttcagaagaagagcaaggtgaaaatcaatggcctgatatctctagacatg

tattctgtggcgacggaaaagctaaagctacccagctacaaactcgacgcggtcgtggga

gacgtcctcggcgagcataagatagaccttccctataaagaaataccctcctattacgcg

ggagggcctgaccggaggggcgtaataggagagtattgtatccaggactctaggctggtg

ggcaagctgttttttaagtacctcccccatctggaactatcggcggtggccaaactcgcc

cgtatcaccctaacgcgggtaatttttgacggtcagcaaattcgcgtgtacacgtgcttg

ctgaaactcgcccgcgagagaaatttcattttgccagacaacagacgccggtttgacagt

caggcagacgccgcgtcagagacttcggagttggctatggatagccaaagccacgccttc

gacagtacagacgaacccgacggtgtggacggtaccccggacgccgcaggatctggcgct

acttctgaaaacggaggcgggaagcccggcgtcgggagggccgtgggctaccagggagca

aaggttctagaccccgtatccggctttcatgtggaccccgtggttgtgtttgacttcgct

agcttatacccaagcattatccaggcccataacctctgtttcaccaccctggcgctcgat

gaagtggatctggccgggcttcaaccatccgtcgactactcgacgttcgaggtgggtgac

caaaagttattttttgtccacgcccatattcgcgaaagcctgcttggcatcttgctgcgc

gactggctggccatgcgaaaggcggtgagggcgcgaatccccaccagcacccccgaggag

gcagttttactagataagcagcagtctgcgattaaggtgatatgcaactcggtttacgga

ttcacgggggtggcaaacggcctgttgccgtgtctgaggatagcggctaccgttaccacg

ataggacgcgacatgctcctcaagacccgagattacgttcactctcgttgggcgacgcgc

gagctgctggaggacaattttccaggggctataggtttccgaaaccacaagccttactcc

gtcagggttatctacggagacaccgactccgtgtttatcaagtttgtgggcctgacgtac

gagggggtatcggagctgggggatgctatgtcgcgtcagatttcagcggacctctttaga

gcgcccatcaaactggagtgtgagaagacctttcagcgactgctgctgatcaccaagaag

aagtacataggtgtcataaacggggggaagatgctcatgaagggggtcgacctagtccgc

aaaaataactgctctttcataaacttgtacgcgcgacatctggtagatcttttgttgtac

gacgaggatgtggccacggcggcagcagaggtgacagacgtgcctcccgcagaatgggtg

gggcgcccgctaccgagcggctttgacaagtttgggcgagtgctggtagaggcgtacaac

cgtatcactgcccccaacttggacgtgcgcgagttcgttatgactgctgagctgagccgc

tcacccgaatcgtataccaacaagcgcctgccgcacctcaccgtctactttaagctcgcc

atgaggaatgaagaactgcccagcgtaaaagagagaattccgtatgtgatagttgcgcag

accgaggccgcggaacgcgaagcgggtgtagtaaactcaatgcgcggtaccgcccaaaac

cccgtggtaaccaagaccgcacgcccccaacctaaacgcaaactgctggtttccgacctc

gccgaagacccgacctatgtttccgagaatgacgtaccgctaaacacagactactatttc

tcccacctgttgggtaccataagcgtgacctttaaggctctattcggaaatgatgtgaga

acaacagaaaatcttttaaagcggtttattccggaaaccccccacaagacccccacgaaa

acccaggcactgcttgagcgcgccggctttgaaaagctgacgccctttacaccggaggaa

gaaagtcgtcgaatactgcatacagttttttgtactctagaagcagctccccatcaaagc

tga

>KT324724.1

atggcggcgcgcgaacaggccaactccgttcgcaggagcgggttttttaatccgtttatt

ggcaagaggccatttttcaggccgggaagcgggcagaccgcggagacagagcgcccaagg

cccccccaacactcgtactgcacagaggtgggtagctttaagtttatagctccaagatgc

ctcgatgaagaagcccccgccgaccagcgacgcggtgtacacgtgggcaccttggagcgc

ccacccaaagtgtactgcgatggctcagagtacgacgtgctgaactttgcctccggaggt

tgctggcctcgcaggattcgggtttggaacggccaggattttcggggcgatggattcaac

cccagatttgagagatttcacgtgtacgacatagtagagacttcggagagcgcctcacac

gatgacccatccaggtttgctgagctatcccgcccaagcgggtctgtggtaacactgctg

ggaatgagcgagtgtggaaagcgagtcgcggttcacgtctatggtgtgcgccattatttt

tacatggcaaaggcggaggtggatagcgcttgtggaataaccaccgaggcagaactcgtg

cgcgcaatggtggactgcgcgcacagctcggctttgagcgcggccctgggaaatggcaac

ggcggcaaacagagcggcggcagcggcgggggatggtggggcggaaagcacgtgtctgcg

gactgcttcaaagtggagaccgtgtgccacacgacgctgtactactttggatctaagcca

gctctctactatagagtatctgcctccagcagccgcctgggagggttcatctgcgacaac

tttcacccggagattacaaaattcgaggggagcgtggacgtgaccacgcggctgttgttg

gacaatgaaaattttaccagtttcgggtggtaccgcctgcgacccggcacccacggagag

cgtgttcaacttcgccccgttgagcgacacgtcacctcaagcgacgtggagattaactgt

actcccgataacctggagccgataccagacgaggctgcctggcccgactataagctcatg

tgctttgatatagagtgtaaagctggaacgggtaacgaaatggcgttcccagtggcaact

aaccaagaggacctggtcatccagatctcctgtctgctgtactcgcttgctactcagaac

cacgaacacaccctgctgttttccctcgggtcatgcgatatctctgaggaatactcgttt

gcatgcgtccagcgcggcgagcccagaccgacggttttggagtttgacagcgagtacgag

ctgctggttgccttcctgacctttctcaagcagtactctcccgagttcgccaccggctac

aacatcgttaattttgactgggcgtacatagttaacaaggtaacgtcggtgtataacatc

aagctggacgggtacggcaagttcaacaaaggggggctgtttaaggtgtgggacatcgcc

acgaaccattttcagaagaagagcaaggtgaaaatcaatggcctgatatctctagacatg

tattctgtggcgacggaaaagctaaagctacccagctacaaactcgacgcggtcgtggga

gacgtcctcggcgagcataagatagaccttccctataaagaaataccctcctattacgcg

ggagggcctgaccggaggggcgtaataggagagtattgtatccaggactctaggctggtg

ggcaagctgttttttaagtacctcccccatctggaactatcggcggtggccaaactcgcc

cgtatcaccctaacgcgggtaatttttgacggtcagcaaattcgcgtgtacacgtgcttg

ctgaaactcgcccgcgagagaaatttcattttgccagacaacagacgccggtttgacagt

caggcagacgccgcgtcagagacttcggagttggctatggatagccaaagccacgccttc

gacagtacagacgaacccgacggtgtggacggtaccccggacgccgcaggatctggcgct

acttctgaaaacggaggcgggaagcccggcgtcgggagggccgtgggctaccagggagca

aaggttctagaccccgtatccggctttcatgtggaccccgtggttgtgtttgacttcgct

agcttatacccaagcattatccaggcccataacctctgtttcaccaccctggcgctcgat

gaagtggatctggccgggcttcaaccatccgtcaactactcgacgttcgaggtgggtgac

caaaagttattttttgtccacgcccatattcgcgaaagcctgcttggcatcttgctgcgc

gactggctggccatgcgaaaggcggtgagggcgcgaatccccaccagcacccccgaggag

gcagttttactagataagcagcagtctgcgattaaggtgatatgcaactcggtttacgga

ttcacgggggtggcaaacggcctgttgccgtgtctgaggatagcggctaccgttaccacg

ataggacgcgacatgctcctcaagacccgagattacgttcactctcgttgggcgacgcgc

gagctgctggaggacaattttccaggggctataggtttccgaaaccacaagccttactcc

gtcagggttatctacggagacaccgactccgtgtttatcaagtttgtgggcctgacgtac

gagggggtatcggagctgggggatgctatgtcgcgtcagatttcagcggacctctttaga

gcgcccatcaaactggagtgtgagaagacctttcagcgactgctgctgatcaccaagaag

aagtacataggtgtcataaacggggggaagatgctcatgaagggggtcgacctggtccgc

aaaaataactgctctttcataaacttgtacgcgcgacatctggtagatcttttgttgtac

gacgaggatgtggccacggcggcagcagaggtgacagacgtgcctcccgcagaatgggtg

gggcgcccgctaccgagcggctttgacaagtttgggcgagtgctggtagaggcgtacaac

cgtatcactgcccccaacttggacgtgcgcgagttcgttatgactgctgagctgagccgc

tcacccgaatcgtataccaacaagcgcctgccgcacctcaccgtctactttaagctcgcc

atgaggaatgaagaactgcccagcgtaaaagagagaattccgtatgtgatagttgcgcag

accgaggccgcggaacgcgaagcgggtgtagtaaactcaatgcgcggtaccgcccaaaac

cccgtggtaaccaagaccgcacgcccccaacctaaacgcaaactgctggtttccgacctc

gccgaagacccgacctatgtttccgagaatgacgtaccgctaaacacagactactatttc

tcccacctgttgggtaccataagcgtgacctttaaggctctattcggaaatgatgtgaga

acaacagaaaatcttttaaagcggtttattccggaaaccccccacaagacccccacgaaa

acccaggcactgcttgagcgcgccggctttgaaaagctgacgccctttacaccggaggaa

gaaagtcgtcgaatactgcatacagttttttgtactctagaagcagctccccatcaaagc

tga

>KT324725.1

atggcggcgcgcgaacaggccaactccgttcgcaggagcgggttttttaatccgtttatt

ggcaagaggccatttttcaggccgggaagcgggcagaccgcggagacagagcgcccaagg

cccccccaacactcgtactgcacagaggtgggtagctttaagtttatagctccaagatgc

ctcgatgaagaagcccccgccgaccagcgacgcggtgtacacgtgggcaccttggagcgc

ccacccaaagtgtactgcgatggctcagagtacgacgtgctgaactttgcctccggaggt

tgctggcctcgcaggattcgggtttggaacggccaggattttcggggcgatggattcaac

cccagatttgagagatttcacgtgtacgacatagtagagacttcggagagcgcctcacac

gatgacccatccaggtttgctgagctatcccgcccaagcgggtctgtggtaacactgctg

ggaatgagcgagtgtggaaagcgagtcgcggttcacgtctatggtgtgcgccattatttt

tacatggcaaaggcggaggtggatagcgcttgtggaataaccaccgaggcagaactcgtg

cgcgcaatggtggactgcgcgcacagctcggctttgagcgcggccctgggaaatggcaac

ggcggcaaacagagcggcggcagcggcgggggatggtggggcggaaagcacgtgtctgcg

gactgcttcaaagtggagaccgtgtgccacacgacgctgtactactttggatctaagcca

gctctctactatagagtatctgcctccagcagccgcctgggagggttcatctgcgacaac

tttcacccggagattacaaaattcgaggggagcgtggacgtgaccacgcggctgttgttg

gacaatgaaaattttaccagtttcgggtggtaccgcctgcgacccggcacccacggagag

cgtgttcaacttcgccccgttgagcgacacgtcacctcaagcgacgtggagattaactgt

actcccgataacctggagccgataccagacgaggctgcctggcccgactataagctcatg

tgctttgatatagagtgtaaagctggaacgggtaacgaaatggcgttcccagtggcaact

aaccaagaggacctggtcatccagatctcctgtctgctgtactcgcttgctactcagaac

cacgaacacaccctgctgttttccctcgggtcatgcgatatctctgaggaatactcgttt

gcatgcgtccagcgcggcgagcccagaccgacggttttggagtttgacagcgagtacgag

ctgctggttgccttcctgacctttctcaagcagtactctcccgagttcgccaccggctac

aacatcgttaattttgactgggcgtacatagttaacaaggtaacgtcggtgtataacatc

aagctggacgggtacggcaagttcaacaaaggggggctgtttaaggtgtgggacatcgcc

acgaaccattttcagaagaagagcaaggtgaaaatcaatggcctgatatctctagacatg

tattctgtggcgacggaaaagctaaagctacccagctacaaactcgacgcggtcgtggga

gacgtcctcggcgagcataagatagaccttccctataaagaaataccctcctattacgcg

ggagggcctgaccggaggggcgtaataggagagtattgtatccaggactctaggctggtg

ggcaagctgttttttaagtacctcccccatctggaactatcggcggtggccaaactcgcc

cgtatcaccctaacgcgggtaatttttgacggtcagcaaattcgcgtgtacacgtgcttg

ctgaaactcgcccgcgagagaaatttcattttgccagacaacagacgccggtttgacagt

caggcagacgccgcgtcagagacttcggagttggctatggatagccaaagccacgccttc

gacagtacagacgaacccgacggtgtggacggtaccccggacgccgcaggatctggcgct

acttctgaaaacggaggcgggaagcccggcgtcgggagggccgtgggctaccagggagca

aaggttctagaccccgtatccggctttcatgtggaccccgtggttgtgtttgacttcgct

agcttatacccaagcattatccaggcccataacctctgtttcaccaccctggcgctcgat

gaagtggatctggccgggcttcaaccatccgtcaactactcgacgttcgaggtgggtgac

caaaagttattttttgtccacgcccatattcgcgaaagcctgcttggcatcttgctgcgc

gactggctggccatgcgaaaggcggtgagggcgcgaatccccaccagcacccccgaggag

gcagttttactagataagcagcagtctgcgattaaggtgatatgcaactcggtttacgga

ttcacgggggtggcaaacggcctgttgccgtgtctgaggatagcggctaccgttaccacg

ataggacgcgacatgctcctcaagacccgagattacgttcactctcgttgggcgacgcgc

gagctgctggaggacaattttccaggggctataggtttccgaaaccacaagccttactcc

gtcagggttatctacggagacaccgactccgtgtttatcaagtttgtgggcctgacgtac

gagggggtatcggagctgggggatgctatgtcgcgtcagatttcagcggacctctttaga

gcgcccatcaaactggagtgtgagaagacctttcagcgactgctgctgatcaccaagaag

aagtacataggtgtcataaacggggggaagatgctcatgaagggggtcgacctggtccgc

aaaaataactgctctttcataaacttgtacgcgcgacatctggtagatcttttgttgtac

gacgaggatgtggccacggcggcagcagaggtgacagacgtgcctcccgcagaatgggtg

gggcgcccgctaccgagcggctttgacaagtttgggcgagtgctggtagaggcgtacaac

cgtatcactgcccccaacttggacgtgcgcaagttcgttatgactgctgagctgagccgc

tcacccgaatcgtataccaacaagcgcctgccgcacctcaccgtctactttaagctcgcc

atgaggaatgaagaactgcccagcgtaaaagagagaattccgtatgtgatagttgcgcag

accgaggccgcggaacgcgaagcgggtgtagtaaactcaatgcgcggtaccgcccaaaac

cccgtggtaaccaagaccgcacgcccccaacctaaacgcaaactgctggtttccgacctc

gccgaagacccgacctatgtttccgagaatgacgtaccgctaaacacagactactatttc

tcccacctgttgggtaccataagcgtgacctttaaggctctattcggaaatgatgtgaga

acaacagaaaatcttttaaagcggtttattccggaaaccccccacaagacccccacgaaa

acccaggcactgcttgagcgcgccggctttgaaaagctgacgccctttacaccggaggaa

gaaagtcgtcgaatactgcatacagttttttgtactctagaagcagctccccatcaaagc

tga

>KT324726.1

atggcggcgcgcgaacaggccaactccgttcgcaggagcgggttttttaatccgtttatt

ggcaagaggccatttttcaggccgggaagcgggcaaaccgcggagacagagcgcccaagg

cccccccaacactcgtactgcacagaggtgggtagctttaagtttatagctccaagatgc

ctcgatgaagaagcccccgccgaccagcgacgcggtgtacacgtgggcaccttggagcgc

ccacccaaagtgtactgcgatggctcagagtacgacgtgctgaactttgcctccggaggt

tgctggcctcgcaggattcgggtttggaacggccaggattttcggggcgatggattcaac

cccagatttgagagatttcacgtgtacgacatagtagagacttcggagagcgcctcacac

gatgacccatccaggtttgctgagctatcccgcccaagcgggtctgtggtaacactgctg

ggaatgagcgagtgtggaaagcgagtcgcggttcacgtctatggtgtgcgccattatttt

tacatggcaaaggcggaggtggatagcgcttgtggaataaccaccgaggcagaactcgtg

cgcgcaatggtggactgcgcgcacagctcggctttgagcgcggccctgggaaatggcaac

ggcggcaaacagagcggcggcagcggcgggggatggtggggcggaaagcacgtgtctgcg

gactgcttcaaagtggagaccgtgtgccacacgacgctgtactactttggatctaagcca

gctctctactatagagtatctgcctccagcagccgcctgggagggttcatctgcgacaac

tttcacccggagattacaaaattcgaggggagcgtggacgtgaccacgcggctgttgttg

gacaatgaaaattttaccagtttcgggtggtaccgcctgcgacccggcacccacggagag

cgtgttcaacttcgccccgttgagcgacacgtcacctcaagcgacgtggagattaactgt

actcccgataacctggagccgataccagacgaggctgcctggcccgactataagctcatg

tgctttgatatagagtgtaaagctggaacgggtaacgaaatggcgttcccagtggcaact

aaccaagaggacctggtcatccagatctcctgtctgctgtactcgcttgctactcagaac

cacgaacacaccctgctgttttccctcgggtcatgcgatatctctgaggaatactcgttt

gcatgcgtccagcgcggcgagcccagaccgacggttttggagtttgacagcgagtacgag

ctgctggttgccttcctgacctttctcaagcagtactctcccgagttcgccaccggctac

aacatcgttaattttgactgggcgtacatagttaacaaggtaacgtcggtgtataacatc

aagctggacgggtacggcaagttcaacaaaggggggctgtttaaggtgtgggacatcgcc

acgaaccattttcagaagaagagcaaggtgaaaatcaatggcctgatatctctagacatg

tattctgtggcgacggaaaagctaaagctacccagctacaaactcgacgcggtcgtggga

gacgtcctcggcgagcataagatagaccttccctataaagaaataccctcctattacgcg

ggagggcctgaccggaggggcgtaataggagagtattgtatccaggactctaggctggtg

ggcaagctgttttttaagtacctcccccatctggaactatcggcggtggccaaactcgcc

cgtatcaccctaacgcgggtaatttttgacggtcagcaaattcgcgtgtacacgtgcttg

ctgaaactcgcccgcgagagaaatttcattttgccagacaacagacgccggtttgacagt

caggcagacgccgcgtcagagacttcggagttggctatggatagccaaagccacgccttc

gacagtacagacgaacccgacggtgtggacggtaccccggacgccgcaggatctggcgct

acttctgaaaacggaggcgggaagcccggcgtcgggagggccgtgggctaccagggagca

aaggttctagaccccgtatccggctttcatgtggaccccgtggttgtgtttgacttcgct

agcttatacccaagcattatccaggcccataacctctgtttcaccaccctggcgctcgat

gaagtggatctggccgggcttcaaccatccgtcaactactcgacgttcgaggtgggtgac

caaaagttattttttgtccacgcccatattcgcgaaagcctgcttggcatcttgctgcgc

gactggctggccatgcgaaaggcggtgagggcgcgaatccccaccagcacccccgaggag

gcagttttactagataagcagcagtctgcgattaaggtgatatgcaactcggtttacgga

ttcacgggggtggcaaacggcctgttgccgtgtctgaggatagcggctaccgttaccacg

ataggacgcgacatgctcctcaagacccgagattacgttcactctcgttgggcgacgcgc

gagctgctggaggacaattttccaggggctataggtttccgaaaccacaagccttactcc

gtcagggttatctacggagacaccgactccgtgtttatcaagtttgtgggcctgacgtac

gagggggtatcggagctgggggatgctatgtcgcgtcagatttcagcggacctctttaga

gcgcccatcaaactggagtgtgagaagacctttcagcgactgctgctgatcaccaagaag

aagtacataggtgtcataaacggggggaagatgctcatgaagggggtcgacctggtccgc

aaaaataactgctctttcataaacttgtacgcgcgacatctggtagatcttttgttgtac

gacgaggatgtggccacggcggcagcagaggtgacagacgtgcctcccgcagaatgggtg

gggcgcccgctaccgagcggctttgacaagtttgggcgagtgctggtagaggcgtacaac

cgtatcactgcccccaacttggacgtgcgcgagttcgttatgactgctgagctgagccgc

tcacccgaatcgtataccaacaagcgcctgccgcacctcaccgtctactttaagctcgcc

atgaggaatgaagaactgcccagcgtaaaagagagaattccgtatgtgatagttgcgcag

accgaggccgcggaacgcgaagcgggtgtagtaaactcaatgcgcggtaccgcccaaaac

cccgtggtaaccaagaccgcacgcccccaacctaaacgcaaactgctggtttccgacctc

gccgaagacccgacctatgtttccgagaatgacgtaccgctaaacacagactactatttc

tcccacctgttgggtaccataagcgtgacctttaaggctctattcggaaatgatgtgaga

acaacagaaaatcttttaaagcggtttattccggaaaccccccacaagacccccacgaaa

acccaggcactgcttgagcgcgccggctttgaaaagctgacgccctttacaccggaggaa

gaaagtcgtcgaatactgcatacagttttttgtactctagaagcagctccccatcaaagc

tga

>KT324728.1

atggcggcgcgcgaacaggccaactccgttcgcaggagcgggttttttaatccgtttatt

ggcaagaggccatttttcaggccgggaagcgggcaaaccgcggagacagagcgcccaagg

cccccccaacactcgtactgcacagaggtgggtagctttaagtttatagctccaagatgc

ctcgatgaagaagcccccgccgaccagcgacgcggtgtacacgtgggcaccttggagcgc

ccacccaaagtgtactgcgatggctcagagtacgacgtgctgaactttgcctccggaggt

tgctggcctcgcaggattcgggtttggaacggccaggattttcggggcgatggattcaac

cccagatttgagagatttcacgtgtacgacatagtagagacttcggagagcgcctcacac

gatgacccatccaggtttgctgagctatcccgcccaagcgggtctgtggtaacactgctg

ggaatgagcgagtgtggaaagcgagtcgcggttcacgtctatggtgtgcgccattatttt

tacatggcaaaggcggaggtggatagcgcttgtggaataaccaccgaggcagaactcgtg

cgcgcaatggtggactgcgcgcacagctcggctttgagcgcggccctgggaaatggcaac

ggcggcaaacagagcggcggcagcggcgggggatggtggggcggaaagcacgtgtctgcg

gactgcttcaaagtggagaccgtgtgccacacgacgctgtactactttggatctaagcca

gctctctactatagagtatctgcctccagcagccgcctgggagggttcatctgcgacaac

tttcacccggagattacaaaattcgaggggagcgtggacgtgaccacgcggctgttgttg

gacaatgaaaattttaccagtttcgggtggtaccgcctgcgacccggcacccacggagag

cgtgttcaacttcgccccgttgagcgacacgtcacctcaagcgacgtggagattaactgt

actcccgataacctggagccgataccagacgaggctgcctggcccgactataagctcatg

tgctttgatatagagtgtaaagctggaacgggtaacgaaatggcgttcccagtggcaact

aaccaagaggacctggtcatccagatctcctgtctgctgtactcgcttgctactcagaac

cacgaacacaccctgctgttttccctcgggtcatgcgatatctctgaggaatactcgttt

gcatgcgtccagcgcggcgagcccagaccgacggttttggagtttgacagcgagtacgag

ctgctggttgccttcctgacctttctcaagcagtactctcccgagttcgccaccggctac

aacatcgttaattttgactgggcgtacatagttaacaaggtaacgtcggtgtataacatc

aagctggacgggtacggcaagttcaacaaaggggggctgtttaaggtgtgggacatcgcc

acgaaccattttcagaagaagagcaaggtgaaaatcaatggcctgatatctctagacatg

tattctgtggcgacggaaaagctaaagctacccagctacaaactcgacgcggtcgtggga

gacgtcctcggcgagcataagatagaccttccctataaagaaataccctcctattacgcg

ggagggcctgaccggaggggcgtaataggagagtattgtatccaggactctaggctggtg

ggcaagctgttttttaagtacctcccccatctggaactatcggcggtggccaaactcgcc

cgtatcaccctaacgcgggtaatttttgacggtcagcaaattcgcgtgtacacgtgcttg

ctgaaactcgcccgcgagagaaatttcattttgccagacaacagacgccggtttgacagt

caggcagacgccgcgtcagagacttcggagttggctatggatagccaaagccacgccttc

gacagtacagacgaacccgacggtgtggacggtaccccggacgccgcaggatctggcgct

acttctgaaaacggaggcgggaagcccggcgtcgggagggccgtgggctaccagggagca

aaggttctagaccccgtatccggctttcatgtggaccccgtggttgtgtttgacttcgct

agcttatacccaagcattatccaggcccataacctctgtttcaccaccctggcgctcgat

gaagtggatctggccgggcttcaaccatccgtcaactactcgacgttcgaggtgggtgac

caaaagttattttttgtccacgcccatattcgcgaaagcctgcttggcatcttgctgcgc

gactggctggccatgcgaaaggcggtgagggcgcgaatccccaccagcacccccgaggag

gcagttttactagataagcagcagtctgcgattaaggtgatatgcaactcggtttacgga

ttcacgggggtggcaaacggcctgttgccgtgtctgaggatagcggctaccgttaccacg

ataggacgcgacatgctcctcaagacccgagattacgttcactctcgttgggcgacgcgc

gagctgctggaggacaattttccaggggctataggtttccgaaaccacaagccttactcc

gtcagggttatctacggagacaccgactccgtgtttatcaagtttgtgggcctgacgtac

gagggggtatcggagctgggggatgctatgtcgcgtcagatttcagcggacctctttaga

gcgcccatcaaactggagtgtgagaagacctttcagcgactgctgctgatcaccaagaag

aagtacataggtgtcataaacggggggaagatgctcatgaagggggtcgacctggtccgc

aaaaataactgctctttcataaacttgtacgcgcgacatctggtagatcttttgttgtac

gacgaggatgtggccacggcggcagcagaggtgacagacgtgcctcccgcagaatgggtg

gggcgcccgctaccgagcggctttgacaagtttgggcgagtgctggtagaggcgtacaac

cgtatcactgcccccaacttggacgtgcgcgagttcgttatgactgctgagctgagccgc

tcacccgaatcgtataccaacaagcgcctgccgcacctcaccgtctactttaagctcgcc

atgaggaatgaagaactgcccagcgtaaaagagagaattccgtatgtgatagttgcgcag

accgaggccgcggaacgcgaagcgggtgtagtaaactcaatgcgcggtaccgcccaaaac

cccgtggtaaccaagaccgcacgcccccaacctaaacgcaaactgctggtttccgacctc

gccgaagacccgacctatgtttccgagaatgacgtaccgctaaacacagactactatttc

tcccacctgttgggtaccataagcgtgacctttaaggctctattcggaaatgatgtgaga

acaacagaaaatcttttaaagcggtttattccggaaaccccccacaagacccccacgaaa

acccaggcactgcttgagcgcgccggctttgaaaagctgacgccctttacaccggaggaa

gaaagtcgtcgaatactgcatacagttttttgtactctagaagcagctccccatcaaagc

tga

>KT324729.1

atggcggcgcgcgaacaggccaactccgttcgcaggagcgggttttttaatccgtttatt

ggcaagaggccatttttcaggccgggaagcgggcaaaccgcggagacagagcgcccaagg

cccccccaacactcgtactgcacagaggtgggtagctttaagtttatagctccaagatgc

ctcgatgaagaagcccccgccgaccagcgacgcggtgtacacgtgggcaccttggagcgc

ccacccaaagtgtactgcgatggctcagagtacgacgtgctgaactttgcctccggaggt

tgctggcctcgcaggattcgggtttggaacggccaggattttcggggcgatggattcaac

cccagatttgagagatttcacgtgtacgacatagtagagacttcggagagcgcctcacac

gatgacccatccaggtttgctgagctatcccgcccaagcgggtctgtggtaacactgctg

ggaatgagcgagtgtggaaagcgagtcgcggttcacgtctatggtgtgcgccattatttt

tacatggcaaaggcggaggtggatagcgcttgtggaataaccaccgaggcagaactcgtg

cgcgcaatggtggactgcgcgcacagctcggctttgagcgcggccctgggaaatggcaac

ggcggcaaacagagcggcggcagcggcgggggatggtggggcggaaagcacgtgtctgcg

gactgcttcaaagtggagaccgtgtgccacacgacgctgtactactttggatctaagcca

gctctctactatagagtatctgcctccagcagccgcctgggagggttcatctgcgacaac

tttcacccggagattacaaaattcgaggggagcgtggacgtgaccacgcggctgttgttg

gacaatgaaaattttaccagtttcgggtggtaccgcctgcgacccggcacccacggagag

cgtgttcaacttcgccccgttgagcgacacgtcacctcaagcgacgtggagattaactgt

actcccgataacctggagccgataccagacgaggctgcctggcccgactataagctcatg

tgctttgatatagagtgtaaagctggaacgggtaacgaaatggcgttcccagtggcaact

aaccaagaggacctggtcatccagatctcctgtctgctgtactcgcttgctactcagaac

cacgaacacaccctgctgttttccctcgggtcatgcgatatctctgaggaatactcgttt

gcatgcgtccagcgcggcgagcccagaccgacggttttggagtttgacagcgagtacgag

ctgctggttgccttcctgacctttctcaagcagtactctcccgagttcgccaccggctac

aacatcgttaattttgactgggcgtacatagttaacaaggtaacgtcggtgtataacatc

aagctggacgggtacggcaagttcaacaaaggggggctgtttaaggtgtgggacatcgcc

acgaaccattttcagaagaagagcaaggtgaaaatcaatggcctgatatctctagacatg

tattctgtggcgacggaaaagctaaagctacccagctacaaactcgacgcggtcgtggga

gacgtcctcggcgagcataagatagaccttccctataaagaaataccctcctattacgcg

ggagggcctgaccggaggggcgtaataggagagtattgtatccaggactctaggctggtg

ggcaagctgttttttaagtacctcccccatctggaactatcggcggtggccaaactcgcc

cgtatcaccctaacgcgggtaatttttgacggtcagcaaattcgcgtgtacacgtgcttg

ctgaaactcgcccgcgagagaaatttcattttgccagacaacagacgccggtttgacagt

caggcagacgccgcgtcagagacttcggagttggctatggatagccaaagccacgccttc

gacagtacagacgaacccgacggtgtggacggtaccccggacgccgcaggatctggcgct

acttctgaaaacggaggcgggaagcccggcgtcgggagggccgtgggctaccagggagca

aaggttctagaccccgtatccggctttcatgtggaccccgtggttgtgtttgacttcgct

agcttatacccaagcattatccaggcccataacctctgtttcaccaccctggcgctcgat

gaagtggatctggccgggcttcaaccatccgtcaactactcgacgttcgaggtgggtgac

caaaagttattttttgtccacgcccatattcgcgaaagcctgcttggcatcttgctgcgc

gactggctggccatgcgaaaggcggtgagggcgcgaatccccaccagcacccccgaggag

gcagttttactagataagcagcagtctgcgattaaggtgatatgcaactcggtttacgga

ttcacgggggtggcaaacggcctgttgccgtgtctgaggatagcggctaccgttaccacg

ataggacgcgacatgctcctcaagacccgagattacgttcactctcgttgggcgacgcgc

gagctgctggaggacaattttccaggggctataggtttccgaaaccacaagccttactcc

gtcagggttatctacggagacaccgactccgtgtttatcaagtttgtgggcctgacgtac

gagggggtatcggagctgggggatgctatgtcgcgtcagatttcagcggacctctttaga

gcgcccatcaaactggagtgtgagaagacctttcagcgactgctgctgatcaccaagaag

aagtacataggtgtcataaacggggggaagatgctcatgaagggggtcgacctggtccgc

aaaaataactgctctttcataaacttgtacgcgcgacatctggtagatcttttgttgtac

gacgaggatgtggccacggcggcagcagaggtgacagacgtgcctcccgcagaatgggtg

gggcgcccgctaccgagcggctttgacaagtttgggcgagtgctggtagaggcgtacaac

cgtatcactgcccccaacttggacgtgcgcgagttcgttatgactgctgagctgagccgc

tcacccgaatcgtataccaacaagcgcctgccgcacctcaccgtctactttaagctcgcc

atgaggaatgaagaactgcccagcgtaaaagagagaattccgtatgtgatagttgcgcag

accgaggccgcggaacgcgaagcgggtgtagtaaactcaatgcgcggtaccgcccaaaac

cccgtggtaaccaagaccgcacgcccccaacctaaacgcaaactgctggtttccgacctc

gccgaagacccgacctatgtttccgagaatgacgtaccgctaaacacagactactatttc

tcccacctgttgggtaccataagcgtgacctttaaggctctattcggaaatgatgtgaga

acaacagaaaatcttttaaagcggtttattccggaaaccccccacaagacccccacgaaa

acccaggcactgcttgagcgcgccggctttgaaaagctgacgccctttacaccggaggaa

gaaagtcgtcgaatactgcatacagttttttgtactctagaagcagctccccatcaaagc

tga

>KT324730.1

atggcggcgcgcgaacaggccaactccgttcgcaggagcgggttttttaatccgtttatt

ggcaagaggccatttttcaggccgggaagcgggcagaccgcggagacagagcgcccaagg

cccccccaacactcgtactgcacagaggtgggtagctttaagtttatagctccaagatgc

ctcgatgaagaagcccccgccgaccagcgacgcggtgtacacgtgggcaccttggagcgc

ccacccaaagtgtactgcgatggctcagagtacgacgtgctgaactttgcctccggaggt

tgctggcctcgcaggattcgggtttggaacggccaggattttcggggcgatggattcaac

cccagatttgagagatttcacgtgtacgacatagtagagacttcggagagcgcctcacac

gatgacccatccaggtttgctgagctatcccgcccaagtgggtctgtggtaacactgctg

ggaatgagcgagtgtggaaagcgagtcgcggttcacgtctatggtgtgcgccattatttt

tacatggcaaaggcggaggtggatagcgcttgtggaataaccaccgaggcagaactcgtg

cgcgcaatggtggactgcgcgcacagctcggctttgagcgcggccctgggaaatggcaac

ggcggcaaacagagcggcggcagcggcgggggatggtggggcggaaagcacgtgtctgcg

gactgcttcaaagtggagaccgtgtgccacacgacgctgtactactttggatctaagcca

gctctctactatagagtatctgcctccagcagccgcctgggagggttcatctgcgacaac

tttcacccggagattacaaaattcgaggggagcgtggacgtgaccacgcggctgttgttg

gacaatgaaaattttaccagtttcgggtggtaccgcctgcgacccggcacccacggagag

cgtgttcaacttcgccccgttgagcgacacgtcacctcaagcgacgtggagattaactgt

actcccgataacctggagccgataccagacgaggctgcctggcccgactataagctcatg

tgctttgatatagagtgtaaagctggaacgggtaacgaaatggcgttcccagtggcaact

aaccaagaggacctggtcatccagatctcctgtctgctgtactcgcttgctactcagaac

cacgaacacaccctgctgttttccctcgggtcatgcgatatctctgaggaatactcgttt

gcatgcgtccagcgcggcgagcccagaccgacggttttggagtttgacagcgagtacgag

ctgctggttgccttcctgacctttctcaagcagtactctcccgagttcgccaccggctac

aacatcgttaattttgactgggcgtacatagttaacaaggtaacgtcggtgtataacatc

aagctggacgggtacggcaagttcaacaaaggggggctgtttaaggtgtgggacatcgcc

acgaaccattttcagaagaagagcaaggtgaaaatcaatggcctgatatctctagacatg

tattctgtggcgacggaaaagctaaagctacccagctacaaactcgacgcggtcgtggga

gacgtcctcggcgagcataagatagaccttccctataaagaaataccctcctattacgcg

ggagggcctgaccggaggggcgtaataggagagtattgtatccaggactctaggctggtg

ggcaagctgttttttaagtacctcccccatctggaactatcggcggtggccaaactcgcc

cgtatcaccctaacgcgggtaatttttgacggtcagcaaattcgcgtgtacacgtgcttg

ctgaaactcgcccgcgagagaaatttcattttgccagacaacagacgccggtttgacagt

caggcagacgccgcgtcagagacttcggagttggctatggatagccaaagccacgccttc

gacagtacagacgaacccgacggtgtggacggtaccccggacgccgcaggatctggcgct

acttctgaaaacggaggcgggaagcccggcgtcgggagggccgtgggctaccagggagca

aaggttctagaccccgtatccggctttcatgtggaccccgtggttgtgtttgacttcgct

agcttatacccaagcattatccaggcccataacctctgtttcaccaccctggcgctcgat

gaagtggatctggccgggcttcaaccatccgtcaactactcgacgttcgaggtgggtgac

caaaagttattttttgtccacgcccatattcgcgaaagcctgcttggcatcttgctgcgc

gactggctggccatgcgaaaggcggtgagggcgcgaatccccaccagcacccccgaggag

gcagttttactagataagcagcagtctgcgattaaggtgatatgcaactcggtttacgga

ttcacgggggtggcaaacggcctgttgccgtgtctgaggatagcggctaccgttaccacg

ataggacgcgacatgctcctcaagacccgagattacgttcactctcgttgggcgacgcgc

gagctgctggaggacaattttccaggggctataggtttccgaaaccacaagccttactcc

gtcagggttatctacggagacaccgactccgtgtttatcaagtttgtgggcctgacgtac

gagggggtatcggagctgggggatgctatgtcgcgtcagatttcagcggacctctttaga

gcgcccatcaaactggagtgtgagaagacctttcagcgactgctgctgatcaccaagaag

aagtacataggtgtcataaacggggggaagatgctcatgaagggggtcgacctggtccgc

aaaaataactgctctttcataaacttgtacgcgcgacatctggtagatcttttgttgtac

gacgaggatgtggccacggcggcagcagaggtgacagacgtgcctcccgcagaatgggtg

gggcgcccgctaccgagcggctttgacaagtttgggcgagtgctggtagaggcgtacaac

cgtatcactgcccccaacttggacgtgcgcgagttcgttatgactgctgagctgagccgc

tcacccgaatcgtataccaacaagcgcctgccgcacctcaccgtctactttaagctcgcc

atgaggaatgaagaactgcccagcgtaaaagagagaattccgtatgtgatagttgcgcag

accgaggccgcggaacgcgaagcgggtgtagtaaactcaatgcgcggtaccgcccaaaac

cccgtggtaaccaagaccgcacgcccccaacctaaacgcaaactgctggtttccgacctc

gccgaagacccgacctatgtttccgagaatgacgtaccgctaaacacagactactatttc

tcccacctgttgggtaccataagcgtgacctttaaggctctattcggaaatgatgtgaga

acaacagaaaatcttttaaagcggtttattccggaaaccccccacaagacccccacgaaa

acccaggcactgcttgagcgcgccggctttgaaaagctgacgccctttacaccggaggaa

gaaagtcgtcgaatactgcatacagttttttgtactctagaagcagctccccatcaaagc

tga

>KT324733.1

atggcggcgcgcgaacaggccaactccgttcgcaggagcgggttttttaatccgtttatt

ggcaagaggccatttttcaggccgggaagcgggcagaccgcggagacagagcgcccaagg

cccccccaacactcgtactgcacagaggtgggtagctttaagtttatagctccaagatgc

ctcgatgaagaagcccccgccgaccagcgacgcggtgtacacgtgggcaccttggagcgc

ccacccaaagtgtactgcgatggctcagagtacgacgtgctgaactttgcctccggaggt

tgctggcctcgcaggattcgggtttggaacggccaggattttcggggcgatggattcaac

cccagatttgagagatttcacgtgtacgacatagtagagacttcggagagcgcctcacac

gatgacccatccaggtttgctgagctatcccgcccaagcgggtctgtggtaacactgctg

ggaatgagcgagtgtggaaagcgagtcgcggttcacgtctatggtgtgcgccattatttt

tacatggcaaaggcggaggtggatagcgcttgtggaataaccaccgaggcagaactcgtg

cgcgcaatggtggactgcgcgcacagctcggctttgagcgcggccctgggaaatggcaac

ggcggcaaacagagcggcggcagcggcgggggatggtggggcggaaagcacgtgtctgcg

gactgcttcaaagtggagaccgtgtgccacacgacgctgtactactttggatctaagcca

gctctctactatagagtatctgcctccagcagccgcctgggagggttcatctgcgacaac

tttcacccggagattacaaaattcgaggggagcgtggacgtgaccacgcggctgttgttg

gacaatgaaaattttaccagtttcgggtggtaccgcctgcgacccggcacccacggagag

cgtgttcaacttcgccccgttgagcgacacgtcacctcaagcgacgtggagattaactgt

actcccgataacctggagccgataccagacgaggctgcctggcccgactataagctcatg

tgctttgatatagagtgtaaagctggaacgggtaacgaaatggcgttcccagtggcaact

aaccaagaggacctggtcatccagatctcctgtctgctgtactcgcttgctactcagaac

cacgaacacaccctgctgttttccctcgggtcatgcgatatctctgaggaatactcgttt

gcatgcgtccagcgcggcgagcccagaccgacggttttggagtttgacagcgagtacgag

ctgctggttgccttcctgacctttctcaagcagtactctcccgagttcgccaccggctac

aacatcgttaattttgactgggcgtacatagttaacaaggtaacgtcggtgtataacatc

aagctggacgggtacggcaagttcaacaaaggggggctgtttaaggtgtgggacatcgcc

acgaaccattttcagaagaagagcaaggtgaaaatcaatggcctgatatctctagacatg

tattctgtggcgacggaaaagctaaagctacccagctacaaactcgacgcggtcgtggga

gacgtcctcggcgagcataagatagaccttccctataaagaaataccctcctattacgcg

ggagggcctgaccggaggggcgtaataggagagtattgtatccaggactctaggctggtg

ggcaagctgttttttaagtacctcccccatctggaactatcggcggtggccaaactcgcc

cgtatcaccctaacgcgggtaatttttgacggtcagcaaattcgcgtgtacacgtgcttg

ctgaaactcgcccgcgagagaaatttcattttgccagacaacagacgccggtttgacagt

caggcagacgccgcgtcagagacttcggagttggctatggatagccaaagccacgccttc

gacagtacagacgaacccgacggtgtggacggtaccccggacgccgcaggatctggcgct

acttctgaaaacggaggcgggaagcccggcgtcgggagggccgtgggctaccagggagca

aaggttctagaccccgtatccggctttcatgtggaccccgtggttgtgtttgacttcgct

agcttatacccaagcattatccaggcccataacctctgtttcaccaccctggcgctcgat

gaagtggatctggccgggcttcaaccatccgtcaactactcgacgttcgaggtgggtgac

caaaagttattttttgtccacgcccatattcgcgaaagcctgcttggcatcttgctgcgc

gactggctggccatgcgaaaggcggtgagggcgcgaatccccaccagcacccccgaggag

gcagttttactagataagcagcagtctgcgattaaggtgatatgcaactcggtttacgga

ttcacgggggtggcaaacggcctgttgccgtgtctgaggatagcggctaccgttaccacg

ataggacgcgacatgctcctcaagacccgagattacgttcactctcgttgggcgacgcgc

gagctgctggaggacaattttccaggggctataggtttccgaaaccacaagccttactcc

gtcagggttatctacggagacaccgactccgtgtttatcaagtttgtgggcctgacgtac

gagggggtatcggagctgggggatgctatgtcgcgtcagatttcagcggacctctttaga

gcgcccatcaaactggagtgtgagaagacctttcagcgactgctgctgatcaccaagaag

aagtacataggtgtcataaacggggggaagatgctcatgaagggggtcgacctggtccgc

aaaaataactgctctttcataaacttgtacgcgcgacatctggtagatcttttgttgtac

gacgaggatgtggccacggcggcagcagaggtgacagacgtgcctcccgcagaatgggtg

gggcgcccgctaccgagcggctttgacaagtttgggcgagtgctggtagaggcgtacaac

cgtatcactgcccccaacttggacgtgcgcaagttcgttatgactgctgagctgagccgc

tcacccgaatcgtataccaacaagcgcctgccgcacctcaccgtctactttaagctcgcc

atgaggaatgaagaactgcccagcgtaaaagagagaattccgtatgtgatagttgcgcag

accgaggccgcggaacgcgaagcgggtgtagtaaactcaatgcgcggtaccgcccaaaac

cccgtggtaaccaagaccgcacgcccccaacctaaacgcaaactgctggtttccgacctc

gccgaagacccgacctatgtttccgagaatgacgtaccgctaaacacagactactatttc

tcccacctgttgggtaccataagcgtgacctttaaggctctattcggaaatgatgtgaga

acaacagaaaatcttttaaagcggtttattccggaaaccccccacaagacccccacgaaa

acccaggcactgcttgagcgcgccggctttgaaaagctgacgccctttacaccggaggaa

gaaagtcgtcgaatactgcatacagttttttgtactctagaagcagctccccatcaaagc

tga

>MF975656.1

atggcggcgcgcgaacaggccaactccgttcgcaggagcgggttttttaatccgtttatt

ggcaagaggccatttttcaggccgggaagcgggcagaccgcggagacagagcgcccaagg

cccccccaacactcgtactgcacagaggtgggtagctttaagtttatagctccaagatgc

ctcgatgaagaagcccccgccgaccagcgacgcggtgtacacgtgggcaccttggagcgc

ccacccaaagtgtactgcgatggctcagagtacgacgtgctgaactttgcctccggaggt

tgctggcctcgcaggattcgggtttggaacggccaggattttcggggcgatggattcaac

cccagatttgagagatttcacgtgtacgacatagtagagacttcggagagcgcctcacac

gatgacccatccaggtttgctgagctatcccgcccaagcgggtctgtggtaacactgctg

ggaatgagcgagtgtggaaagcgagtcgcggttcacgtctatggtgtgcgccattatttt

tacatggcaaaggcggaggtggatagcgcttgtggaataaccaccgaggcagaactcgtg

cgcgcaatggtggactgcgcgcacagctcggctttgagcgcggccctgggaaatggcaac

ggcggcaaacagagcggcggcagcggcgggggatggtggggcggaaagcacgtgtctgcg

gactgcttcaaagtggagaccgtgtgccgcacgacgctgtactactttggatctaagcca

gctctctactatagagtatctgcctccagcagccgcctgggagggttcatctgcgacaac

tttcacccggagattacaaaattcgaggggagcgtggacgtgaccacgcggctgttgttg

gacaatgaaaattttaccagtttcgggtggtaccgcctgcgacccggcacccacggagag

cgtgttcaacttcgccccgttgagcgacacgtcacctcaagcgacgtggagattaactgt

actcccgataacctggagccgataccagacgaggctgcctggcccgactataagctcatg

tgctttgatatagagtgtaaagctggaacgggtaacgaaatggcgttcccagtggcaact

aaccaagaggacctggtcatccagatctcctgtctgctgtactcgcttgctactcagaac

cacgaacacaccctgctgttttccctcgggtcatgcgatatctctgaggaatactcgttt

gcatgcgtccagcgcggcgagcccagaccgacggttttggagtttgacagcgagtacgag

ctgctggttgccttcctgacctttctcaagcagtactctcccgagttcgccaccggctac

aacatcgttaattttgactgggcgtacatagttaacaaggtaacgtcggtgtataacatc

aagctggacgggtacggcaagttcaacaaaggggggctgtttaaggtgtgggacatcgcc

acgaaccattttcagaagaagagcaaggtgaaaatcaatggcctgatatctctagacatg

tattctgtggcgacggaaaagctaaagctacccagctacaaactcgacgcggtcgtggga

gacgtcctcggcgagcataagatagaccttccctataaagaaataccctcctattacgcg

ggagggcctgaccggaggggcgtaataggagagtattgtatccaggactctaggctggtg

ggcaagctgttttttaagtacctcccccatctggaactatcggcggtggccaaactcgcc

cgtatcaccctaacgcgggtaatttttgacggtcagcaaattcgcgtgtacacgtgcttg

ctgaaactcgcccgcgagagaaatttcattttgccagacaacagacgccggtttgacagt

caggcagacgccgcgtcagagacttcggagttggctatggatagccaaagccacgccttc

gacagtacagacgaacccgacggtgtggacggtaccccggacgccgcaggatctggcgct

acttctgaaaacggaggcgggaagcccggcgtcgggagggccgtgggctaccagggagca

aaggttctagaccccgtatccggctttcatgtggaccccgtggttgtgtttgacttcgct

agcttatacccaagcattatccaggcccataacctctgtttcaccaccctggcgctcgat

gaagtggatctggccgggcttcaaccatccgtcgactactcgacgttcgaggtgggtgac

caaaagttattttttgtccacgcccatattcgcgaaagcctgcttggcatcttgctgcgc

gactggctggccatgcgaaaggcggtgagggcgcgaatccccaccagcacccccgaggag

gcagttttactagataagcagcagtctgcgattaaggtgatatgcaactcggtttacgga

ttcacgggggtggcaaacggcctgttgccgtgtctgaggatagcggctaccgttaccacg

ataggacgcgacatgctcctcaagacccgagattacgttcactctcgttgggcgacgcgc

gagctgctggaggacaattttccaggggctataggtttccgaaaccacaagccttactcc

gtcagggttatctacggagacaccgactccgtgtttatcaagtttgtgggcctgacgtac

gagggggtatcggagctgggggatgctatgtcgcgtcagatttcagcggacctctttaga

gcgcccatcaaactggagtgtgagaagacctttcagcgactgctgctgatcaccaagaag

aagtacataggtgtcataaacggggggaagatgctcatgaagggggtcgacctggtccgc

aaaaataactgctctttcataaacttgtacgcgcgacatctggtagatcttttgttgtac

gacgaggatgtggccacggcggcagcagaggtgacagacgtgcctcccgcagaatgggtg

gggcgcccgctaccgagcggctttgacaagtttgggcgagtgctggtagaggcgtacaac

cgtatcactgcccccaacttggacgtgcgcgagttcgttatgactgctgagctgagccgc

tcacccgaatcgtataccaacaagcgcctgccgcacctcaccgtctactttaagctcgcc

atgaggaatgaagaactgcccagcgtaaaagagagaattccgtatgtgatagttgcgcag

accgaggccgcggaacgcgaagcgggtgtagtaaactcaatgcgcggtaccgcccaaaac

cccgtggtaaccaagaccgcacgcccccaacctaaacgcaaactgctggtttccgacctc

gccgaagacccgacctatgtttccgagaatgacgtaccgctaaacacagactactatttc

tcccacctgttgggtaccataagcgtgacctttaaggctctattcggaaatgatgtgaga

acaacagaaaatcttttaaagcggtttattccggaaaccccccacaagacccccacgaaa

acccaggcactgcttgagcgcgccggctttgaaaagctgacgccctttacaccggaggaa

gaaagtcgtcgaatactgcatacagttttttgtactctagaagcagctccccatcaaagc

tga

>MN912433.1

atggcggcgcgcgaacaggccaactccgttcgcaggagcgggttttttaatccgtttatt

ggcaagaggccatttttcaggccgggaagcgggcagaccgcggagacagagcgcccaagg

cccccccaacactcgtactgcacagaggtgggtagctttaagtttatagctccaagatgc

ctcgatgaagaagcccccgccgaccagcgacgcggtgtacacgtgggcaccttggagcgc

ccacccaaagtgtactgcgatggctcagagtacgacgtgctgaactttgcctccggaggt

tgctggcctcgcaggattcgggtttggaacggccaggattttcggggcgatggattcaac

cccagatttgagagatttcacgtgtacgacatagtagagacttcggagagcgcctcacac

gatgacccatccaggtttgctgagctatcccgcccaagcgggtctgtggtaacactgctg

ggaatgagcgagtgtggaaagcgagtcgcggttcacgtctatggtgtgcgccattatttt

tacatggcaaaggcggaggtggatagcgcttgtggaataaccaccgaggcagaactcgtg

cgcgcaatggtggactgcgcgcacagctcggctttgagcgcggccctgggaaatggcaac

ggcggcaaacagagcggcggcagcggcgggggatggtggggcggaaagcacgtgtctgcg

gactgcttcaaagtggagaccgtgtgccacacgacgctgtactactttggatctaagcca

gctctctactatagagtatctgcctccagcagccgcctgggagggttcatctgcgacaac

tttcacccggagattacaaaattcgaggggagcgtggacgtgaccacgcggctgttgttg

gacaatgaaaattttaccagtttcgggtggtaccgcctgcgacccggcacccacggagag

cgtgttcaacttcgccccgttgagcgacacgtcacctcaagcgacgtggagattaactgt

actcccgataacctggagccgataccagacgaggctgcctggcccgactataagctcatg

tgctttgatatagagtgtaaagctggaacgggtaacgaaatggcgttcccagtggcaact

aaccaagaggacctggtcatccagatctcctgtctgctgtactcgcttgctactcagaac

cacgaacacaccctgctgttttccctcgggtcatgcgatatctctgaggaatactcgttt

gcatgcgtccagcgcggcgagcccagaccgacggttttggagtttgacagcgagtacgag

ctgctggttgccttcctgacctttctcaagcagtactctcccgagttcgccaccggctac

aacatcgttaattttgactgggcgtacatagttaacaaggtaacgtcggtgtataacatc

aagctggacgggtacggcaagttcaacaaaggggggctgtttaaggtgtgggacatcgcc

acgaaccattttcagaagaagagcaaggtgaaaatcaatggcctgatatctctagacatg

tattctgtggcgacggaaaagctaaagctacccagctacaaactcgacgcggtcgtggga

gacgtcctcggcgagcataagatagaccttccctataaagaaataccctcctattacgcg

ggagggcctgaccggaggggcgtaataggagagtattgtatccaggactctaggctggtg

ggcaagctgttttttaagtacctcccccatctggaactatcggcggtggccaaactcgcc

cgtatcaccctaacgcgggtaatttttgacggtcagcaaattcgcgtgtacacgtgcttg

ctgaaactcgcccgcgagagaaatttcattttgccagacaacaaacgccggtttgacagt

caggcagacgccgcgtcagagacttcggagttggctatggatagccaaagccacgccttc

gacagtacagacgaacccgacggtgtggacggtaccccggacgccgcaggatctggcgct

acttctgaaaacggaggcgggaagcccggcgtcgggagggccgtgggctaccagggagca

aaggttctagaccccgtatccggctttcatgtggaccccgtggttgtgtttgacttcgct

agcttatacccaagcattatccaggcccataacctctgtttcaccaccctggcgctcgat

gaagtggatctggccgggcttcaaccatccgtcaactactcgacgttcgaggtgggtgac

caaaagttattttttgtccacgcccatattcgcgaaagcctgcttggcatcttgctgcgc

gactggctggccatgcgaaaggcggtgagggcgcgaatccccaccagcacccccgaggag

gcagttttactagataagcagcagtctgcgattaaggtgatatgcaactcggtttacgga

ttcacgggggtggcaaacggcctgttgccgtgtctgaggatagcggctaccgttaccacg

ataggacgcgacatgctcctcaagacccgagattacgttcactctcgttgggcgacgcgc

gagctgctggaggacaattttccaggggctataggtttccgaaaccacaagccttactcc

gtcagggttatctacggagacaccgactccgtgtttatcaagtttgtgggcctgacgtac

gagggggtatcggagctgggggatgctatgtcgcgtcagatttcagcggacctctttaga

gcgcccatcaaactggagtgtgagaagacctttcagcgactgctgctgatcaccaagaag

aagtacataggtgtcataaacggggggaagatgctcatgaagggggtcgacctagtccgc

aaaaataactgctctttcataaacttgtacgcgcgacatctggtagatcttttgttgtac

gacgaggatgtggccacggcggcagcagaggtgacagacgtgcctcccgcagaatgggtg

gggcgcccgctaccgagcggctttgacaagtttgggcgagtgctggtagaggcgtacaac

cgtatcactgcccccaacttggacgtgcgcgagttcgttatgactgctgagctgagccgc

tcacccgaatcgtataccaacaagcgcctgccgcacctcaccgtctactttaagctcgcc

atgaggaatgaagaactgcccagcgtaaaagagagaattccgtatgtgatagttgcgcag

accgaggccgcggaacgcgaagcgggtgtagtaaactcaatgcgcggtaccgcccaaaac

cccgtggtaaccaagaccgcacgcccccaacctaaacgcaaactgctggtttccgacctc

gccgaagacccgacctatgtttccgagaatgacgtaccgctaaacacagactactatttc

tcccacctgttgggtaccataagcgtgacctttaaggctctattcggaaatgatgtgaga

acaacagaaaatcttttaaagcggtttattccggaaaccccccacaagacccccacgaaa

acccaggcactgcttgagcgcgccggctttgaaaagctgacgccctttacaccggaggaa

gaaagtcgtcgaatactgcatacagttttttgtactctagaagcagctccccatcaaagc

tga

>MT077857.1

atggcggcgcgcgaacaggccaactccgttcgcaggagcgggttttttaatccgtttatt

ggcaagaggccatttttcaggccgggaagcgggcagaccgcggagacagagcgcccaagg

cccccccaacactcgtactgcacagaggtgggtagctttaagtttatagctccaagatgc

ctcgatgaagaagcccccgccgaccagcgacgcggtgtacacgtgggcaccttggagcgc

ccacccaaagtgtactgcgatggctcagagtacgacgtgctgaactttgcctccggaggt

tgctggcctcgcaggattcgggtttggaacggccaggattttcggggcgatggattcaac

cccagatttgagagatttcacgtgtacgacatagtagagacttcggagagcgcctcacac

gatgacccatccaggtttgctgagctatcccgcccaagcgggtctgtggtaacactgctg

ggaatgagcgagtgtggaaagcgagtcgcggttcacgtctatggtgtgcgccattatttt

tacatggcaaaggcggaggtggatagcgcttgtggaataaccaccgaggcagaactcgtg

cgcgcaatggtggactgcgcgcacagctcggctttgagcgcggccctgggaaatggcaac

ggcggcaaacagagcggcggcagcggcgggggatggtggggcggaaagcacgtgtctgcg

gactgcttcaaagtggagaccgtgtgccacacgacgctgtactactttggatctaagcca

gctctctactatagagtatctgcctccagcagccgcctgggagggttcatctgcgacaac

tttcacccggagattacaaaattcgaggggagcgtggacgtgaccacgcggctgttgttg

gacaatgaaaattttaccagtttcgggtggtaccgcctgcgacccggcacccacggagag

cgtgttcaacttcgccccgttgagcgacacgtcacctcaagcgacgtggagattaactgt

actcccgataacctggagccgataccagacgaggctgcctggcccgactataagctcatg

tgctttgatatagagtgtaaagctggaacgggtaacgaaatggcgttcccagtggcaact

aaccaagaggacctggtcatccagatctcctgtctgctgtactcgcttgctactcagaac

cacgaacacaccctgctgttttccctcgggtcatgcgatatctctgaggaatactcgttt

gcatgcgtccagcgcggcgagcccagaccgacggttttggagtttgacagcgagtacgag

ctgctggttgccttcctgacctttctcaagcagtactctcccgagttcgccaccggctac

aacatcgttaattttgactgggcgtacatagttaacaaggtaacgtcggtgtataacatc

aagctggacgggtacggcaagttcaacaaaggggggctgtttaaggtgtgggacatcgcc

acgaaccattttcagaagaagagcaaggtgaaaatcaatggcctgatatctctagacatg

tattctgtggcgacggaaaagctaaagctacccagctacaaactcgacgcggtcgtggga

gacgtcctcggcgagcataagatagaccttccctataaagaaataccctcctattacgcg

ggagggcctgaccggaggggcgtaataggagagtattgtatccaggactctaggctggtg

ggcaagctgttttttaagtacctcccccatctggaactatcggcggtggccaaactcgcc

cgtatcaccctaacgcgggtaatttttgacggtcagcaaattcgcgtgtacacgtgcttg

ctgaaactcgcccgcgagagaaatttcattttgccagacaacagacgccggtttgacagt

caggcagacgccgcgtcagagacttcggagttggctatggatagccaaagccacgccttc

gacagtacagacgaacccgacggtgtggacggtaccccggacgccgcaggatctggcgct

acttctgaaaacggaggcgggaagcccggcgtcgggagggccgtgggctaccagggagca

aaggttctagaccccgtatccggctttcatgtggaccccgtggttgtgtttgacttcgct

agcttatacccaagcattatccaggcccataacctctgtttcaccaccctggcgctcgat

gaagtggatctggccgggcttcaaccatccgtcaactactcgacgttcgaggtgggtgac

caaaagttattttttgtccacgcccatattcgcgaaagcctgcttggcatcttgctgcgc

gactggctggccatgcgaaaggcggtgagggcgcgaatccccaccagcacccccgaggag

gcagttttactagataagcagcagtctgcgattaaggtgatatgcaactcggtttacgga

ttcacgggggtggcaaacggcctgttgccgtgtctgaggatagcggctaccgttaccacg

ataggacgcgacatgctcctcaagacccgagattacgttcactctcgttgggcgacgcgc

gagctgctggaggacaattttccaggggctataggtttccgaaaccacaagccttactcc

gtcagggttatctacggagacaccgactccgtgtttatcaagtttgtgggcctgacgtac

gagggggtatcggagctgggggatgctatgtcgcgtcagatttcagcggacctctttaga

gcgcccatcaaactggagtgtgagaagacctttcagcgactgctgctgatcaccaagaag

aagtacataggtgtcataaacggggggaagatgctcatgaagggggtcgacctagtccgc

aaaaataactgctctttcataaacttgtacgcgcgacatctggtagatcttttgttgtac

gacgaggatgtggccacggcggcagcagaggtgacagacgtgcctcccgcagaatgggtg

gggcgcccgctaccgagcggctttgacaagtttgggcgagtgctggtagaggcgtacaac

cgtatcactgcccccaacttggacgtgcgcgagttcgttatgactgctgagctgagccgc

tcacccgaatcgtataccaacaagcgcctgccgcacctcaccgtctactttaagctcgcc

atgaggaatgaagaactgcccagcgtaaaagagagaattccgtatgtgatagttgcgcag

accgaggccgcggaacgcgaagcgggtgtagtaaactcaatgcgcggtaccgcccaaaac

cccgtggtaaccaagaccgcacgcccccaacctaaacgcaaactgctggtttccgacctc

gccgaagacccgacctatgtttccgagaatgacgtaccgctaaacacagactactatttc

tcccacctgttgggtaccataagcgtgacctttaaggctctattcggaaatgatgtgaga

acaacagaaaatcttttaaagcggtttattccggaaaccccccacaagacccccacgaaa

acccaggcactgcttgagcgcgccggctttgaaaagctgacgccctttacaccggaggaa

gaaagtcgtcgaatactgcatacagttttttgtactctagaagcagctccccatcaaagc

tga

>MT063054.1

atggcggcgcgcgaacaggccaactccgttcgcaggaacgggttttttaatccgtttatt

ggcaagaggccatttttcaggccgggaagcgggcagaccgcggagacagagcgcccaagg

cccccccaacactcgtactgcacagaggtgggtagctttaagtttatagctccaagatgc

ctcgatgaagaagcccccgccgaccagcgacgcggtgtacacgtgggcaccttggagcgc

ccacccaaagtgtactgcgatggctcagagtacgacgtgctgaactttgcctccggaggt

tgctggcctcgcaggattcgggtttggaacggccaggattttcggggcgatggattcaac

cccagatttgagagatttcacgtgtacgacatagtagagacttcggagagcgcctcacac

gatgacccatccaggtttgctgagctatcccgcccaagcgggtctgtggtaacactgctg

ggaatgagcgagtgtggaaagcgagtcgcggttcacgtctatggtgtgcgccattatttt

tacatggcaaaggcggaggtggatagcgcttgtggaataaccaccgaggcagaactcgtg

cgcgcaatggtggactgcgcgcacagctcggctttgagcgcggccctgggaaatggcaac

ggcggcaaacagagcggcggcagcggcgggggatggtggggcggaaagcacgtgtctgcg

gactgcttcaaggtggagaccgtgtgccacacgacgctgtactactttggatctaagcca

gctctctactatagagtatctgcctccagcagccgcctgggaggattcatctgcgacaac

tttcacccggagattacaaaattcgaggggagcgtggacgtgaccacgcggctgttgttg

gacaatgaaaattttaccagtttcgggtggtaccgcctgcgacccggcacccacggagag

cgtgttcaacttcgccccgttgagcgacacgtcacctcaagcgacgtggagattaactgt

actcccgataacctggagccgataccagacgaggctgcctggcccgactataagctcatg

tgctttgatatagagtgtaaagctggaacgggtaacgaaatggcgttcccagtggcaact

aaccaagaggacctggtcatccagatctcctgtctgctgtactcgcttgctactcagaac

cacgaacacaccctgctgttttccctcgggtcatgcgatatctctgaggaatactcgttt

gcatgcgtccagcgcggcgagcccagacagacggttttggagtttgacagcgagtacgag

ctgctggttgccttcctgacctttctcaagcagtactctcccgagttcgccaccggctac

aacatcgttaattttgactgggcgtacatagttaacaaggtaacgtcggtgtataacatc

aagctggacgggtacggcaagttcaacaaaggggggctgtttaaggtgtgggacatcgcc

acgaaccattttcagaagaagagcaaggtgaaaatcaatggcctgatatctctagacatg

tattctgtggcgacggaaaagctaaagctacccagctacaaactcgacgcggtcgtggga

gacgtcctcggcgagcataagatagaccttccctataaagaaataccctcctattacgcg

ggagggcctgaccggaggggcgtaataggagagtattgtatccaggactctaggctggtg

ggcaagctgttttttaagtacctcccccatctggaactatcggcggtggccaaactcgcc

cgtatcaccctaacgcgggtaatttttgacggtcagcaaattcgcgtgtccacgtgcttg

ctgaaactcgcccgcgagagaaatttcattttgccagacaacagacgccggtttgacagt

caggcagacgccgcgtcagagacttcggagttggctatggatagccaaagccacgccttc

gacagtacagacgaacccgacggtgtggacggtaccccggacgccgcaggatctggcgct

acttctgaaaacggaggcgggaagcccggcgtcgggagggccgtgggctaccagggagca

aaggttctagaccccgtatccggctttcatgtggaccccgtggttgtgtttgacttcgct

agcttatacccaagcattatccaggcccataacctctgtttcaccaccctggcgctcgat

gaagtggatctggccgggcttcaaccatccgtcaactactcgacgttcgaggtgggtgac

caaaagttattttttgtccacgcccatattcgcgaaagcctgcttggcatcttgctgcgc

gactggctggccatgcgaaaggcggtgagggcgcgaatccccaccagcacccccgaggag

gcagttttactagataagcagcagtctgcgattaaggtgatatgcaactcggtttacgga

ttcacgggggtggcaaacggcctgttgccgtgtctgaggatagcggctaccgttaccacg

ataggacgcgacatgctcctcaagacccgagattacgttcactctcgttgggcgacgcgc

gagctgctggaggacaattttccaggggctataggtttccgaaaccacaagccttactcc

gtcagggttatctacggagacaccgactccgtgtttatcaagtttgtgggcctgacgtac

gagggggtatcggagctgggggatgctatgtcgcgtcagatttcagcggacctctttaga

gcgcccatcaaactggagtgtgagaagacctttcagcgactgctgctgatcaccaagaag

aagtacataggtgtcataaacggggggaagatgctcatgaagggggtcgacctggtccgc

aaaaataactgctctttcataaacttgtacgcgcgacatctggtagatcttttgttgtac

gacgaggatgtggccacggcggcagcagaggtgacagacgtgcctcccgcagaatgggtg

gggcgcccgctaccgagcggctttgacaagtttgggcgagtgctggtagaggcgtacaac

cgtatcactgcccccaacttggacgtgcgcgagttcgttatgactgctgagctgagccgc

tcacccgaatcgtataccaacaagcgcctgccgcacctcaccgtctactttaagctcgcc

atgaggaatgaagaactgcccagcgtaaaagagagaattccgtatgtgatagttgcgcag

accgaggccgcggaacgcgaagcgggtgtagtaaactcaatgcgcggtaccgcccaaaac

cccgtggtaaccaagaccgcacgcccccaacctaaacgcaaactgctggtttccgacctc

gccgaagacccgacctatgtttccgagaatgacgtaccgctaaacacagactactatttc

tcccacctgttgggtaccataagcgtgacctttaaggctctattcggaaatgatgtgaga

acaacagaaaatcttttaaagcggtttattccggaaaccccccacaagacccccacgaaa

acccaggcactgcttgagcgcgccggctttgaaaagctgacgccctttacaccggaggaa

gaaagtcgtcgaatactgcatacagttttttgtactctagaagcagctccccatcaaagc

tga

>MW855958.1

atggcggcgcgcgaacaggccaactccgttcgcaggagcgggttttttaatccgtttatt

ggcaagaggccatttttcaggccgggaagcgggcaaaccgcggagacagagcgcccaagg

cccccccaacactcgtactgcacagaggtgggtagctttaagtttatagctccaagatgc

ctcgatgaagaagcccccgccgaccagcgacgcggtgtacacgtgggcaccttggagcgc

ccacccaaagtgtactgcgatggctcagagtacgacgtgctgaactttgcctccggaggt

tgctggcctcgcaggattcgggtttggaacggccaggattttcggggcgatggattcaac

cccagatttgagagatttcacgtgtacgacatagtagagacttcggagagcgcctcacac

gatgacccatccaggtttgctgagctatcccgcccaagcgggtctgtggtaacactgctg

ggaatgagcgagtgtggaaagcgagtcgcggttcacgtctatggtgtgcgccattatttt

tacatggcaaaggcggaggtggatagcgcttgtggaataaccaccgaggcagaactcgtg

cgcgcaatggtggactgcgcgcacagctcggctttgagcgcggccctgggaaatggcaac

ggcggcaaacagagcggcggcagcggcgggggatggtggggcggaaagcacgtgtctgcg

gactgcttcaaagtggagaccgtgtgccacacgacgctgtactactttggatctaagcca

gctctctactatagagtatctgcctccagcagccgcctgggagggttcatctgcgacaac

tttcacccggagattacaaaattcgaggggatcgtggacgtgaccacgcggctgttgttg

gacaatgaaaattttaccagtttcgggtggtaccgcctgcgacccggcacccacggagag

cgtgttcaacttcgccccgttgagcgacacgtcacctcaagcgacgtggagattaactgt

actcccgataacctggagccgataccagacgaggctgcctggcccgactataagctcatg

tgctttgatatagagtgtaaagctggaacgggtaacgaaatggcgttcccagtggcaact

aaccaagaggacctggtcatccagatctcctgtctgctgtactcgcttgctactcagaac

cacgaacacaccctgctgttttccctcgggtcatgcgatatctctgaggaatactcgttt

gcatgcgtccagcgcggcgagcccagaccgacggttttggagtttgacagcgagtacgag

ctgctggttgccttcctgacctttctcaagcagtactctcccgagttcgccaccggctac

aacatcgttaattttgactgggcgtacatagttaacaaggtaacgtcggtgtataacatc

aagctggacgggtacggcaagttcaacaaagggggnctgtttaaggtgtgggacatcgcc

acgaaccattttcagaagaagagcaaggtgaaaatcaatggcctgatatctctagacatg

tattctgtggcgacggaaaagctaaagctacccagctacaaactcgacgcggtcgtggga

gacgtcctcggcgagcataagatagaccttccctataaagaaataccctcctattacgcg

ggagggcctgaccggaggggcgtaataggagagtattgtatccaggactctaggctggtg

ggcaagctgttttttaagtacctcccccatctggaactatcggcggtggccaaactcgcc

cgtatcaccctaacgcgggtaatttttgacggtcagcaaattcgcgtgtacacgtgcttg

ctgaaactcgcccgcgagagaaatttcattttgccagacaacagacgccggtttgacagt

caggcagacgccgcgtcagagacttcggagttggctatggatagccaaagccacgccttc

gacagtacagacgaacccgacggtgtggacggtaccccggacgccgcaggatctggcgct

acttctgaaaacggaggcgggaagcccggcgtcgggagggccgtgggctaccagggagca

aaggttctagaccccgtatccggctttcatgtggaccccgtggttgtgtttgacttcgct

agcttatacccaagcattatccaggcccataacctctgtttcaccaccctggcgctcgat

gaagtggatctggccgggcttcaaccatccgtcaactactcgacgttcgaggtgggtgac

caaaagttattttttgtccacgcccatattcgcgaaagcctgcttggcatcttgctgcgc

gactggctggccatgcgaaaggcggtgagggcgcgaatccccaccagcacccccgaggag

gcagttttactagataagcagcagtctgcgattaaggtgatatgcaactcggtttacgga

ttcacgggggtggcaaacggcctgttgccgtgtctgaggatagcggctaccgttaccacg

ataggacgcgacatgctcctcaagacccgagattacgttcactctcgttgggcgacgcgc

gagctgctggaggacaattttccaggggctataggtttccgaaaccacaagccttactcc

gtcagggttatctacggagacaccgactccgtgtttatcaagtttgtgggcctgacgtac

gagggggtatcggagctgggggatgctatgtcgcgtcagatttcagcggacctctttaga

gcgcccatcaaactggagtgtgagaagacctttcagcgactgcttctgatcaccaagaag

aagtacataggtgtcataaacggggggaagatgctcatgaagggggtcgacctggtccgc

aaaaataactgctctttcataaacttgtacgcgcgacatctggtagatcttttgttgtac

gacgaggatgtggccacggcggcagcagaggtgacagacgtgcctcccgcagaatgggtg

gggcgcccgctaccgagcggctttgacaagtttgggcgagtgctggtagaggcgtacaac

cgtatcactgcccccaacttggacgtgcgcgagttcgttatgactgctgagctgagccgc

tcacccgaatcgtataccaacaagcgcctgccgcacctcaccgtctactttaagctcgcc

atgaggaatgaagaactgcccagcgtaaaagagagaattccgtatgtgatagttgcgcag

accgaggccgcggaacgcgaagcgggtgtagtaaactcaatgcgcggtaccgcccaaaac

cccgtggtaaccaagaccgcacgcccccaacctaaacgcaaactgctggtttccgacctc

gccgaagacccgacctatgtttccgagaatgacgtaccgctaaacacagactactatttc

tcccacctgttgggtaccataagcgtgacctttaaggctctattcggaaatgatgtgaga

acaacagaaaatcttttaaagcggtttattccggaaaccccccacaagacccccacgaaa

acccaggcactgcttgagcgcgccggctttgaaaagctgacgccctttacaccggaggaa

gaaagtcgtcgaatactgcatacagttttttgtactctagaagcagctccccatcaaagc

tga

>MW855959.1

atggcggcgcgcgaacaggccaactccgttcgcaggagcgggttttttaatccgtttatt

ggcaagaggccatttttcaggccgggaagcgggcaaaccgcggagacagagcgcccaagg

cccccccaacactcgtactgcacagaggtgggtagctttaagtttatagctccaagatgc

ctcgatgaagaagcccccgccgaccagcgacgcggtgtacacgtgggcaccttggagcgc

ccacccaaagtgtactgcgatggctcagagtacgacgtgctgaactttgcctccggaggt

tgctggcctcgcaggattcgggtttggaacggccaggattttcggggcgatggattcaac

cccagatttgagagatttcacgtgtacgacatagtagagacttcggagagcgcctcacac

gatgacccatccaggtttgctgagctatcccgcccaagcgggtctgtggtaacactgctg

ggaatgagcgagtgtggaaagcgagtcgcggttcacgtctatggtgtgcgccattatttt

tacatggcaaaggcggaggtggatagcgcttgtggaataaccaccgaggcagaactcgtg

cgcgcaatggtggactgcgcgcacagctcggctttgagcgcggccctgggaaatggcaac

ggcggcaaacagagcggcggcagcggcgggggatggtggggcggaaagcacgtgtctgcg

gactgcttcaaagtggagaccgtgtgccacacgacgctgtactactttggatctaagcca

gctctctactatagagtatctgcctccagcagccgcctgggagggttcatctgcgacaac

tttcacccggagattacaaaattcgaggggatcgtggacgtgaccacgcggctgttgttg

gacaatgaaaattttaccagtttcgggtggtaccgcctgcgacccggcacccacggagag

cgtgttcaacttcgccccgttgagcgacacgtcacctcaagcgacgtggagattaactgt

actcccgataacctggagccgataccagacgaggctgcctggcccgactataagctcatg

tgctttgatatagagtgtaaagctggaacgggtaacgaaatggcgttcccagtggcaact

aaccaagaggacctggtcatccagatctcctgtctgctgtactcgcttgctactcagaac

cacgaacacaccctgctgttttccctcgggtcatgcgatatctctgaggaatactcgttt

gcatgcgtccagcgcggcgagcccagaccgacggttttggagtttgacagcgagtacgag

ctgctggttgccttcctgacctttctcaagcagtactctcccgagttcgccaccggctac

aacatcgttaattttgactgggcgtacatagttaacaaggtaacgtcggtgtataacatc

aagctggacgggtacggcaagttcaacaaaggggggctgtttaaggtgtgggacatcgcc

acgaaccattttcagaagaagagcaaggtgaaaatcaatggcctgatatctctagacatg

tattctgtggcgacggaaaagctaaagctacccagctacaaactcgacgcggtcgtggga

gacgtcctcggcgagcataagatagaccttccctataaagaaataccctcctattacgcg

ggagggcctgaccggaggggcgtaataggagagtattgtatccaggactctaggctggtg

ggcaagctgttttttaagtacctcccccatctggaactatcggcggtggccaaactcgcc

cgtatcaccctaacgcgggtaatttttgacggtcagcaaattcgcgtgtacacgtgcttg

ctgaaactcgcccgcgagagaaatttcattttgccagacaacagacgccggtttgacagt

caggcagacgccgcgtcagagacttcggagttggctatggatagccaaagccacgccttc

gacagtacagacgaacccgacggtgtggacggtaccccggacgccgcaggatctggcgct

acttctgaaaacggaggcgggaagcccggcgtcgggagggccgtgggctaccagggagca

aaggttctagaccccgtatccggctttcatgtggaccccgtggttgtgtttgacttcgct

agcttatacccaagcattatccaggcccataacctctgtttcaccaccctggcgctcgat

gaagtggatctggccgggcttcaaccatccgtcaactactcgacgttcgaggtgggtgac

caaaagttattttttgtccacgcccatattcgcgaaagcctgcttggcatcttgctgcgc

gactggctggccatgcgaaaggcggtgagggcgcgaatccccaccagcacccccgaggag

gcagttttactagataagcagcagtctgcgattaaggtgatatgcaactcggtttacgga

ttcacgggggtggcaaacggcctgttgccgtgtctgaggatagcggctaccgttaccacg

ataggacgcgacatgctcctcaagacccgagattacgttcactctcgttgggcgacgcgc

gagctgctggaggacaattttccaggggctataggtttccgaaaccacaagccttactcc

gtcagggttatctacggagacaccgactccgtgtttatcaagtttgtgggcctgacgtac

gagggggtatcggagctgggggatgctatgtcgcgtcagatttcagcggacctctttaga

gcgcccatcaaactggagtgtgagaagacctttcagcgactgcttctgatcaccaagaag

aagtacataggtgtcataaacggggggaagatgctcatgaagggggtcgacctggtccgc

aaaaataactgctctttcataaacttgtacgcgcgacatctggtagatcttttgttgtac

gacgaggatgtggccacggcggcagcagaggtgacagacgtgcctcccgcagaatgggtg

gggcgcccgctaccgagcggctttgacaagtttgggcgagtgctggtagaggcgtacaac

cgtatcactgcccccaacttggacgtgcgcgagttcgttatgactgctgagctgagccgc

tcacccgaatcgtataccaacaagcgcctgccgcacctcaccgtctactttaagctcgcc

atgaggaatgaagaactgcccagcgtaaaagagagaattccgtatgtgatagttgcgcag

accgaggccgcggaacgcgaagcgggtgtagtaaactcaatgcgcggtaccgcccaaaac

cccgtggtaaccaagaccgcacgcccccaacctaaacgcaaactgctggtttccgacctc

gccgaagacccgacctatgtttccgagaatgacgtaccgctaaacacagactactatttc

tcccacctgttgggtaccataagcgtgacctttaaggctctattcggaaatgatgtgaga

acaacagaaaatcttttaaagcggtttattccggaaaccccccacaagacccccacgaaa

acccaggcactgcttgagcgcgccggctttgaaaagctgacgccctttacaccggaggaa

gaaagtcgtcgaatactgcatacagttttttgtactctagaagcagctccccatcaaagc

tga

>MW855960.1

atggcggcgcgcgaacaggccaactccgttcgcaggagcgggttttttaatccgtttatt

ggcaagaggccatttttcaggccgggaagcgggcaaaccgcggagacagagcgcccaagg

ccccccnaacactcgtactgcacagaggtgggtagctttaagtttatagctccaagatgc

ctcgatgaagaagcccccgccgaccagcgacgcggtgtacacgtgggcaccttggagcgc

ccacccaaagtgtactgcgatggctcagagtacgacgtgctgaactttgcctccggaggt

tgctggcctcgcaggattcgggtttggaacggccaggattttcggggcgatggattcaac

cccagatttgagagatttcacgtgtacgacatagtagagacttcggagagcgcctcacac

gatgacccatccaggtttgctgagctatcccgcccaagcgggtctgtggtaacactgctg

ggaatgagcgagtgtggaaagcgagtcgcggttcacgtctatggtgtgcgccattatttt

tacatggcaaaggcggaggtggatagcgcttgtggaataaccaccgaggcagaactcgtg

cgcgcaatggtggactgcgcgcacagctcggctttgagcgcggccctgggaaatggcaac

ggcggcaaacagagcggcggcagcggcggggnatggtggggcggaaagcacgtgtctgcg

gactgcttcaaagtggagaccgtgtgccacacgacgctgtactactttggatctaagcca

gctctctactatagagtatctgcctccagcagccgcctgggagggttcatctgcgacaac

tttcacccggagattacaaaattcgaggggatcgtggacgtgaccacgcggctgttgttg

gacaatgaaaattttaccagtttcgggtggtaccgcctgcgacccggcacccacggagag

cgtgttcaacttcgccccgttgagcgacacgtcacctcaagcgacgtggagattaactgt

actcccgataacctggagccgataccagacgaggctgcctggcccgactataagctcatg

tgctttgatatagagtgtaaagctggaacgggtaacgaaatggcgttcccagtggcaact

aaccaagaggacctggtcatccagatctcctgtctgctgtactcgcttgctactcagaac

cacgaacacaccctgctgttttccctcgggtcatgcgatatctctgaggaatactcgttt

gcatgcgtccagcgcggcgagcccagaccgacggttttggagtttgacagcgagtacgag

ctgctggttgccttcctgacctttctcaagcagtactctcccgagttcgccaccggctac

aacatcgttaattttgactgggcgtacatagttaacaaggtaacgtcggtgtataacatc

aagctggacgggtacggcaagttcaacaaaggggggctgtttaaggtgtgggacatcgcc

acgaaccattttcagaagaagagcaaggtgaaaatcaatggcctgatatctctagacatg

tattctgtggcgacggaaaagctaaagctacccagctacaaactcgacgcggtcgtggga

gacgtcctcggcgagcataagatagaccttccctataaagaaataccctcctattacgcg

ggagggcctgaccggaggggcgtaataggagagtattgtatccaggactctaggctggtg

ggcaagctgttttttaagtacctcccccatctggaactatcggcggtggccaaactcgcc

cgtatcaccctaacgcgggtaatttttgacggtcagcaaattcgcgtgtacacgtgcttg

ctgaaactcgcccgcgagagaaatttcattttgccagacaacagacgccggtttgacagt

caggcagacgccgcgtcagagacttcggagttggctatggatagccaaagccacgccttc

gacagtacagacgaacccgacggtgtggacggtaccccggacgccgcaggatctggcgct

acttctgaaaacggaggcgggaagcccggcgtcgggagggccgtgggctaccagggagca

aaggttctagaccccgtatccggctttcatgtggaccccgtggttgtgtttgacttcgct

agcttatacccaagcattatccaggcccataacctctgtttcaccaccctggcgctcgat

gaagtggatctggccgggcttcaaccatccgtcaactactcgacgttcgaggtgggtgac

caaaagttattttttgtccacgcccatattcgcgaaagcctgcttggcatcttgctgcgc

gactggctggccatgcgaaaggcggtgagggcgcgaatccccaccagcacccccgaggag

gcagttttactagataagcagcagtctgcgattaaggtgatatgcaactcggtttacgga

ttcacgggggtggcaaacggcctgttgccgtgtctgaggatagcggctaccgttaccacg

ataggacgcgacatgctcctcaagacccgagattacgttcactctcgttgggcgacgcgc

gagctgctggaggacaattttccaggggctataggtttccgaaaccacaagccttactcc

gtcagggttatctacggagacaccgactccgtgtttatcaagtttgtgggcctgacgtac

gagggggtatcggagctgggggatgctatgtcgcgtcagatttcagcggacctctttaga

gcgcccatcaaactggagtgtgagaagacctttcagcgactgcttctgatcaccaagaag

aagtacataggtgtcataaacggggggaagatgctcatgaagggggtcgacctggtccgc

aaaaataactgctctttcataaacttgtacgcgcgacatctggtagatcttttgttgtac

gacgaggatgtggccacggcggcagcagaggtgacagacgtgcctcccgcagaatgggtg

gggcgcccgctaccgagcggctttgacaagtttgggcgagtgctggtagaggcgtacaac

cgtatcactgcccccaacttggacgtgcgcgagttcgttatgactgctgagctgagccgc

tcacccgaatcgtataccaacaagcgcctgccgcacctcaccgtctactttaagctcgcc

atgaggaatgaagaactgcccagcgtaaaagagagaattccgtatgtgatagttgcgcag

accgaggccgcggaacgcgaagcgggtgtagtaaactcaatgcgcggtaccgcccaaaac

cccgtggtaaccaagaccgcacgcccccaacctaaacgcaaactgctggtttccgacctc

gccgaagacccgacctatgtttccgagaatgacgtaccgctaaacacagactactatttc

tcccacctgttgggtaccataagcgtgacctttaaggctctattcggaaatgatgtgaga

acaacagaaaatcttttaaagcggtttattccggaaaccccccacaagacccccacgaaa

acccaggcactgcttgagcgcgccggctttgaaaagctgacgccctttacaccggaggaa

gaaagtcgtcgaatactgcatacagttttttgtactctagaagcagctccccatcaaagc

tga

>MW855961.1

atggcggcgcgcgaacaggccaactccgttcgcaggagcgggttttttaatccgtttatt

ggcaagaggccatttttcaggccgggaagcgggcaaaccgcggagacagagcgcccaagg

cccccccaacactcgtactgcacagaggtgggtagctttaagtttatagctccaagatgc

ctcgatgaagaagcccccgccgaccagcgacgcggtgtacacgtgggcaccttggagcgc

ccacccaaagtgtactgcgatggctcagagtacgacgtgctgaactttgcctccggaggt

tgctggcctcgcaggattcgggtttggaacggccaggattttcggggcgatggattcaac

cccagatttgagagatttcacgtgtacgacatagtagagacttcggagagcgcctcacac

gatgacccatccaggtttgctgagctatcccgcccaagcgggtctgtggtaacactgctg

ggaatgagcgagtgtggaaagcgagtcgcggttcacgtctatggtgtgcgccattatttt

tacatggcaaaggcggaggtggatagcgcttgtggaataaccaccgaggcagaactcgtg

cgcgcaatggtggactgcgcgcacagctcggctttgagcgcggccctgggaaatggcaac

ggcggcaaacagagcggcggcagcggcgggggatggtggggcggaaagcacgtgtctgcg

gactgcttcaaagtggagaccgtgtgccacacgacgctgtactactttggatctaagcca

gctctctactatagagtatctgcctccagcagccgcctgggagggttcatctgcgacaac

tttcacccggagattacaaaattcgaggggatcgtggacgtgaccacgcggctgttgttg

gacaatgaaaattttaccagtttcgggtggtaccgcctgcgacccggcacccacggagag

cgtgttcaacttcgccccgttgagcgacacgtcacctcaagcgacgtggagattaactgt

actcccgataacctggagccgataccagacgaggctgcctggcccgactataagctcatg

tgctttgatatagagtgtaaagctggaacgggtaacgaaatggcgttcccagtggcaact

aaccaagaggacctggtcatccagatctcctgtctgctgtactcgcttgctactcagaac

cacgaacacaccctgctgttttccctcgggtcatgcgatatctctgaggaatactcgttt

gcatgcgtccagcgcggcgagcccagaccgacggttttggagtttgacagcgagtacgag

ctgctggttgccttcctgacctttctcaagcagtactctcccgagttcgccaccggctac

aacatcgttaattttgactgggcgtacatagttaacaaggtaacgtcggtgtataacatc

aagctggacgggtacggcaagttcaacaaaggggggctgtttaaggtgtgggacatcgcc

acgaaccattttcagaagaagagcaaggtgaaaatcaatggcctgatatctctagacatg

tattctgtggcgacggaaaagctaaagctacccagctacaaactcgacgcggtcgtggga

gacgtcctcggcgagcataagatagaccttccctataaagaaataccctcctattacgcg

ggagggcctgaccggaggggcgtaataggagagtattgtatccaggactctaggctggtg

ggcaagctgttttttaagtacctcccccatctggaactatcggcggtggccaaactcgcc

cgtatcaccctaacgcgggtaatttttgacggtcagcaaattcgcgtgtacacgtgcttg

ctgaaactcgcccgcgagagaaatttcattttgccagacaacagacgccggtttgacagt

caggcagacgccgcgtcagagacttcggagttggctatggatagccaaagccacgccttc

gacagtacagacgaacccgacggtgtggacggtaccccggacgccgcaggatctggcgct

acttctgaaaacggaggcgggaagcccggcgtcgggagggccgtgggctaccagggagca

aaggttctagaccccgtatccggctttcatgtggaccccgtggttgtgtttgacttcgct

agcttatacccaagcattatccaggcccataacctctgtttcaccaccctggcgctcgat

gaagtggatctggccgggcttcaaccatccgtcaactactcgacgttcgaggtgggtgac

caaaagttattttttgtccacgcccatattcgcgaaagcctgcttggcatcttgctgcgc

gactggctggccatgcgaaaggcggtgagggcgcgaatccccaccagcacccccgaggag

gcagttttactagataagcagcagtctgcgattaaggtgatatgcaactcggtttacgga

ttcacgggggtggcaaacggcctgttgccgtgtctgaggatagcggctaccgttaccacg

ataggacgcgacatgctcctcaagacccgagattacgttcactctcgttgggcgacgcgc

gagctgctggaggacaattttccaggggctataggtttccgaaaccacaagccttactcc

gtcagggttatctacggagacaccgactccgtgtttatcaagtttgtgggcctgacgtac

gagggggtatcggagctgggggatgctatgtcgcgtcagatttcagcggacctctttaga

gcgcccatcaaactggagtgtgagaagacctttcagcgactgcttctgatcaccaagaag

aagtacataggtgtcataaacggggggaagatgctcatgaagggggtcgacctggtccgc

aaaaataactgctctttcataaacttgtacgcgcgacatctggtagatcttttgttgtac

gacgaggatgtggccacggcggcagcagaggtgacagacgtgcctcccgcagaatgggtg

gggcgcccgctaccgagcggctttgacaagtttgggcgagtgctggtagaggcgtacaac

cgtatcactgcccccaacttggacgtgcgcgagttcgttatgactgctgagctgagccgc

tcacccgaatcgtataccaacaagcgcctgccgcacctcaccgtctactttaagctcgcc

atgaggaatgaagaactgcccagcgtaaaagagagaattccgtatgtgatagttgcgcag

accgaggccgcggaacgcgaagcgggtgtagtaaactcaatgcgcggtaccgcccaaaac

cccgtggtaaccaagaccgcacgcccccaacctaaacgcaaactgctggtttccgacctc

gccgaagacccgacctatgtttccgagaatgacgtaccgctaaacacagactactatttc

tcccacctgttgggtaccataagcgtgacctttaaggctctattcggaaatgatgtgaga

acaacagaaaatcttttaaagcggtttattccggaaaccccccacaagacccccacgaaa

acccaggcactgcttgagcgcgccggctttgaaaagctgacgccctttacaccggaggaa

gaaagtcgtcgaatactgcatacagttttttgtactctagaagcagctccccatcaaagc

tga

>MW855962.1

atggcggcgcgcgaacaggccaactccgttcgcaggagcgggttttttaatccgtttatt

ggcaagaggccatttttcaggccgggaagcgggcaaaccgcggagacagagcgcccaagg

cccccccaacactcgtactgcacagaggtgggtagctttaagtttatagctccaagatgc

ctcgatgaagaagcccccgccgaccagcgacgcggtgtacacgtgggcaccttggagcgc

ccacccaaagtgtactgcgatggctcagagtacgacgtgctgaactttgcctccggaggt

tgctggcctcgcaggattcgggtttggaacggccaggattttcggggcgatggattcaac

cccagatttgagagatttcacgtgtacgacatagtagagacttcggagagcgcctcacac

gatgacccatccaggtttgctgagctatcccgcccaagcgggtctgtggtaacactgctg

ggaatgagcgagtgtggaaagcgagtcgcggttcacgtctatggtgtgcgccattatttt

tacatggcaaaggcggaggtggatagcgcttgtggaataaccaccgaggcagaactcgtg

cgcgcaatggtggactgcgcgcacagctcggctttgagcgcggccctgggaaatggcaac

ggcggcaaacagagcggcggcagcggcgggggatggtggggcggaaagcacgtgtctgcg

gactgcttcaaagtggagaccgtgtgccacacgacgctgtactactttggatctaagcca

gctctctactatagagtatctgcctccagcagccgcctgggagggttcatctgcgacaac

tttcacccggagattacaaaattcgaggggatcgtggacgtgaccacgcggctgttgttg

gacaatgaaaattttaccagtttcgggtggtaccgcctgcgacccggcacccacggagag

cgtgttcaacttcgccccgttgagcgacacgtcacctcaagcgacgtggagattaactgt

actcccgataacctggagccgataccagacgaggctgcctggcccgactataagctcatg

tgctttgatatagagtgtaaagctggaacgggtaacgaaatggcgttcccagtggcaact

aaccaagaggacctggtcatccagatctcctgtctgctgtactcgcttgctactcagaac

cacgaacacaccctgctgttttccctcgggtcatgcgatatctctgaggaatactcgttt

gcatgcgtccagcgcggcgagcccagaccgacggttttggagtttgacagcgagtacgag

ctgctggttgccttcctgacctttctcaagcagtactctcccgagttcgccaccggctac

aacatcgttaattttgactgggcgtacatagttaacaaggtaacgtcggtgtataacatc

aagctggacgggtacggcaagttcaacaaaggggggctgtttaaggtgtgggacatcgcc

acgaaccattttcagaagaagagcaaggtgaaaatcaatggcctgatatctctagacatg

tattctgtggcgacggaaaagctaaagctacccagctacaaactcgacgcggtcgtggga

gacgtcctcggcgagcataagatagaccttccctataaagaaataccctcctattacgcg

ggagggcctgaccggaggggcgtaataggagagtattgtatccaggactctaggctggtg

ggcaagctgttttttaagtacctcccccatctggaactatcggcggtggccaaactcgcc

cgtatcaccctaacgcgggtaatttttgacggtcagcaaattcgcgtgtacacgtgcttg

ctgaaactcgcccgcgagagaaatttcattttgccagacaacagacgccggtttgacagt

caggcagacgccgcgtcagagacttcggagttggctatggatagccaaagccacgccttc

gacagtacagacgaacccgacggtgtggacggtaccccggacgccgcaggatctggcgct

acttctgaaaacggaggcgggaagcccggcgtcgggagggccgtgggctaccagggagca

aaggttctagaccccgtatccggctttcatgtggaccccgtggttgtgtttgacttcgct

agcttatacccaagcattatccaggcccataacctctgtttcaccaccctggcgctcgat

gaagtggatctggccgggcttcaaccatccgtcaactactcgacgttcgaggtgggtgac

caaaagttattttttgtccacgcccatattcgcgaaagcctgcttggcatcttgctgcgc

gactggctggccatgcgaaaggcggtgagggcgcgaatccccaccagcacccccgaggag

gcagttttactagataagcagcagtctgcgattaaggtgatatgcaactcggtttacgga

ttcacgggggtggcaaacggcctgttgccgtgtctgaggatagcggctaccgttaccacg

ataggacgcgacatgctcctcaagacccgagattacgttcactctcgttgggcgacgcgc

gagctgctggaggacaattttccaggggctataggtttccgaaaccacaagccttactcc

gtcagggttatctacggagacaccgactccgtgtttatcaagtttgtgggcctgacgtac

gagggggtatcggagctgggggatgctatgtcgcgtcagatttcagcggacctctttaga

gcgcccatcaaactggagtgtgagaagacctttcagcgactgcttctgatcaccaagaag

aagtacataggtgtcataaacggggggaagatgctcatgaagggggtcgacctggtccgc

aaaaataactgctctttcataaacttgtacgcgcgacatctggtagatcttttgttgtac

gacgaggatgtggccacggcggcagcagaggtgacagacgtgcctcccgcagaatgggtg

gggcgcccgctaccgagcggctttgacaagtttgggcgagtgctggtagaggcgtacaac

cgtatcactgcccccaacttggacgtgcgcgagttcgttatgactgctgagctgagccgc

tcacccgaatcgtataccaacaagcgcctgccgcacctcaccgtctactttaagctcgcc

atgaggaatgaagaactgcccagcgtaaaagagagaattccgtatgtgatagttgcgcag

accgaggccgcggaacgcgaagcgggtgtagtaaactcaatgcgcggtaccgcccaaaac

cccgtggtaaccaagaccgcacgcccccaacctaaacgcaaactgctggtttccgacctc

gccgaagacccgacctatgtttccgagaatgacgtaccgctaaacacagactactatttc

tcccacctgttgggtaccataagcgtgacctttaaggctctattcggaaatgatgtgaga

acaacagaaaatcttttaaagcggtttattccggaaaccccccacaagacccccacgaaa

acccaggcactgcttgagcgcgccggctttgaaaagctgacgccctttacaccggaggaa

gaaagtcgtcgaatactgcatacagttttttgtactctagaagcagctccccatcaaagc

tga

>MZ357402.1

atggcggcgcgcgaacaggccaactccgttcgcaggagcgggttttttaatccgtttatt

ggcaagaggccatttttcaggccgggaagcgggcaaaccgcggagacagagcgcccaagg

cccccccaacactcgtactgcacagaggtgggtagctttaagtttatagctccaagatgc

ctcgatgaagaagcccccgccgaccagcgacgcggtgtacacgtgggcaccttggagcgc

ccacccaaagtgtactgcgatggctcagagtacgacgtgctgaactttgcctccggaggt

tgctggcctcgcaggattcgggtttggaacggccaggattttcggggcgatggattcaac

cccagatttgagagatttcacgtgtacgacatagtagagacttcggagagcgcctcacac

gatgacccatccaggtttgctgagctatcccgcccaagcgggtctgtggtaacactgctg

ggaatgagcgagtgtggaaagcgagtcgcggttcacgtctatggtgtgcgccattatttt

tacatggcaaaggcggaggtggatagcgcttgtggaataaccaccgaggcagaactcgtg

cgcgcaatggtggactgcgcgcacagctcggctttgagcgcggccctgggaaatggcaac

ggcggcaaacagagcggcggcagcggcgggggatggtggggcggaaagcacgtgtctgcg

gactgcttcaaagtggagaccgtgtgccacacgacgctgtactactttggatctaagcca

gctctctactatagagtatctgcctccagcagccgcctgggagggttcatctgcgacaac

tttcacccggagattacaaaattcgaggggatcgtggacgtgaccacgcggctgttgttg

gacaatgaaaattttaccagnnnnnnnnnnnnnnnnnnnnnnnnnnnnnnnnnnnnnnnn

nnnnnnnnnnnnnnnnnnnnnnnnnnnnnnnnnnnnnnnnnnnnnnnnnnnnnnnnnnnn

nnnnnnnnnnnnnnnnnnnnnnnnnnnnnnnnnnnnnnnnnnnnnnnnnnnnnnnnnntg

tgctttgatatagagtgtaaagctggaacgggtaacgaaatggcgttcccagtggcaact

aaccaagaggacctggtcatccagatctcctgtctgctgtactcgcttgctactcagaac

cacgaacacaccctgctgttttccctcggnnnnnnnnnnnnnnnnnnnnnnnnnnnnnnn

nnnnnnnnncagcgcggcgagcccagaccgacggttttggagtttgacagcgagtacgag

ctgctggttgccttcctgacctttctcaagcagtactctcccgagttcgccaccggctac

aacatcgttaattttgactgggcgtacatagttaacaaggnnnnnnnnnnnnnnnnnnnn

nnnnnngacgggtacggcaagttcaacaaaggggggctgtttaaggtgtgggacatcgcc

acgaaccattttcagaagaagagcaaggtgaaaatcaatggcctgagatctctagacatg

tattctgtggcgacggaaaagctaaagctacccagctacaaactcgacgcggtcgtggga

gacgtcctcggcgagcataagatagaccttccctataaagaaataccctcctattacgcg

ggagggcctgaccggaggggcgtaataggagagtattgtatccaggactctaggctggtg

ggcaagctgttttttaagtacctcccccatctggaactatcggcggtggccaaactcgcc

cgtatcaccctaacgcgggtaatttttgacggtcagcaaattcgcgtgtacacgtgcttg

ctgaaactcgcccgcgagagaaatttcattttgccagacaacagacgccggtttgacagt

caggcagacgccgcgtcagagacttcggagttggctatggatagccaaagccacgccttc

gacagtacagacgaacccgacggtgtggacggtaccccggacgccgcaggatctggcgct

acttctgaaaacggaggcgggaagcccggcgtcgggagggccgtgggctaccagggagca

aaggttctagaccccgtatccggctttcatgtggaccccgtggttgtgtttgacttcgct

agcttatacccaagcattatccaggcccataacctctgtttcaccaccctggcgctcgat

gaagtggatctggccgggcttcaaccatccgtcaactactcgacgttcgaggtgggtgac

caaaagttattttttgtccacgcccatattcgcgaaagcctgcttggcatcttgctgcgc

gactggctggccatgcgaaaggcggtgagggcgcgaatccccaccagcacccccgaggag

gcagttttactagataagcagcagtctgcgattaaggtgatatgcaactcggtttacgga

ttcacgggggtggcaaacggcctgttgccgtgtctgaggatagcggctaccgttaccacg

ataggacgcgacatgctcctcaagacccgagattacgttcactctcgttgggcgacgcgc

gagctgctggaggacaattttccaggggctataggtttccgaaaccacaagccttactcc

gtcagggttatctacggagacaccgactccgtgtttatcaagtttgtgggcctgacgtac

gagggggtatcggagctgggggatgctatgtcgcgtcagatttcagcggacctctttaga

gcgcccatcaaactggagtgtgagaagacctttcagcgactgcttctgatcaccaagaag

aagnnnnnnnnnnnnnnnnnnnnnnnnnnnnnnnnnnnnnnnnnngtcgacctggtccgc

aaaaataactgctctttcataaacttgtacgcgcgacatctggtagatcttttgttgtac

gacgaggatgtggccacggcggcagcagaggtgacagacgtgcctcccgcagaatgggtg

gggcgcccgctaccgagcggctttgacaagtttgggcgagtgctggtagaggcgtacaac

cgtatcactgcccccaacttggacgtgcgcgagttcgttatgactgctgagctgagccgc

tcacccgaatcgtataccaacaagcgcctgccgcacctcaccgtctactttaagctcgcc

atgaggaatgaagaactgcccagcgtaaaagagagaattccgtatgtgatagttgcgcag

accgaggccgcggaacgcgaagcgggtgtagtaaactcaatgcgcggtaccgcccaaaac

cccgtggtaaccaagaccgcacgcccccnnnnnnnnnnnnnnnnnnnnnnnnnnnnnnnn

nnnnnnnnnnnnnnctatgtttccgagaatgacgtaccgctaaacacagactactatttc

tcccacctgttgggtaccataagcgtgacctttaaggctctattcggaaatgatgtgaga

acaacagaaaatcttttaaagcggtttattccggaaaccccccacaagacccccacgaaa

acccaggcactgcttgagcgcgccggctttgaaaagctgacgccctttacaccggaggaa

gaaagtcgtcgaatactgcatacagttttttgtactctagaagcagctccccatcaaagc

tga

>MZ318700.1

atggcggcgcgcgaacaggccaactccgttcgcaggagcgggttttttaatccgtttatt

ggcaagaggccatttttcaggccgggaagcgggcaaaccgcggagacagagcgcccaagg

cccccccaacactcgtactgcacagaggtgggtagctttaagtttatagctccaagatgc

ctcgatgaagaagcccccgccgaccagcgacgcggtgtacacgtgggcaccttggagcgc

ccacccaaagtgtactgcgatggctcagagtacgacgtgctgaactttgcctccggaggt

tgctggcctcgcaggattcgggtttggaacggccaggattttcggggcgatggattcaac

cccagatttgagagatttcacgtgtacgacatagtagagacttcggagagcgcctcacac

gatgacccatccaggtttgctgagctatcccgcccaagcgggtctgtggtaacactgctg

ggaatgagcgagtgtggaaagcgagtcgcggttcacgtctatggtgtgcgccattatttt

tacatggcaaaggcggaggtggatagcgcttgtggaataaccaccgaggcagaactcgtg

cgcgcaatggtggactgcgcgcacagctcggctttgagcgcggccctgggaaatggcaac

ggcggcaaacagagcggcggcagcggcgggggatggtggggcggaaagcacgtgtctgcg

gactgcttcaaagtggagaccgtgtgccacacgacgctgtactactttggatctaagcca

gctctctactatagagtatctgcctccagcagccgcctgggagggttcatctgcgacaac

tttcacccggagattacaaaattcgaggggagcgtggacgtgaccacgcggctgttgttg

gacaatgaaaattttaccagtttcgggtggtaccgcctgcgacccggcacccacggagag

cgtgttcaacttcgccccgttgagcgacacgtcacctcaagcgacgtggagattaactgt

actcccgataacctggagccgataccagacgaggctgcctggcccgactataagctcatg

tgctttgatatagagtgtaaagctggaacgggtaacgaaatggcgttcccagtggcaact

aaccaagaggacctggtcatccagatctcctgtctgctgtactcgcttgctactcagaac

cacgaacacaccctgctgttttccctcgggtcatgcgatatctctgaggaatactcgttt

gcatgcgtccagcgcggcgagcccagaccgacggttttggagtttgacagcgagtacgag

ctgctggttgccttcctgacctttctcaagcagtactctcccgagttcgccaccggctac

aacatcgttaattttgactgggcgtacatagttaacaaggtaacgtcggtgtataacatc

aagctggacgggtacggcaagttcaacaaaggggggctgtttaaggtgtgggacatcgcc

acgaaccattttcagaagaagagcaaggtgaaaatcaatggcctgatatctctagacatg

tattctgtggcgacggaaaagctaaagctacccagctacaaactcgacgcggtcgtggga

gacgtcctcggcgagcataagatagaccttccctataaagaaataccctcctattacgcg

ggagggcctgaccggaggggcgtaataggagagtattgtatccaggactctaggctggtg

ggcaagctgttttttaagtacctcccccatctggaactatcggcggtggccaaactcgcc

cgtatcaccctaacgcgggtaatttttgacggtcagcaaattcgcgtgtacacgtgcttg

ctgaaactcgcccgcgagagaaatttcattttgccagacaacagacgccggtttgacagt

caggcagacgccgcgtcagagacttcggagttggctatggatagccaaagccacgccttc

gacagtacagacgaacccgacggtgtggacggtaccccggacgccgcaggatctggcgct

acttctgaaaacggaggcgggaagcccggcgtcgggagggccgtgggctaccagggagca

aaggttctagaccccgtatccggctttcatgtggaccccgtggttgtgtttgacttcgct

agcttatacccaagcattatccaggcccataacctctgtttcaccaccctggcgctcgat

gaagtggatctggccgggcttcaaccatccgtcaactactcgacgttcgaggtgggtgac

caaaagttattttttgtccacgcccatattcgcgaaagcctgcttggcatcttgctgcgc

gactggctggccatgcgaaaggcggtgagggcgcgaatccccaccagcacccccgaggag

gcagttttactagataagcagcagtctgcgattaaggtgatatgcaactcggtttacgga

ttcacgggggtggcaaacggcctgttgccgtgtctgaggatagcggctaccgttaccacg

ataggacgcgacatgctcctcaagacccgagattacgttcactctcgttgggcgacgcgc

gagctgctggaggacaattttccaggggctataggtttccgaaaccacaagccttactcc

gtcagggttatctacggagacaccgactccgtgtttatcaagtttgtgggcctgacgtac

gagggggtatcggagctgggggatgctatgtcgcgtcagatttcagcggacctctttaga

gcgcccatcaaactggagtgtgagaagacctttcagcgactgctgctgatcaccaagaag

aagtacataggtgtcataaacggggggaagatgctcatgaagggggtcgacctggtccgc

aaaaataactgctctttcataaacttgtacgcgcgacatctggtagatcttttgttgtac

gacgaggatgtggccacggcggcagcaaaggtgacagacgtgcctcccgcagaatgggtg

gggcgcccgctaccgagcggctttgacaagtttgggcgagtgctggtagaggcgtacaac

cgtatcactgcccccaacttggacgtgcgcgagttcgttatgactgctgagctgagccgc

tcacccgaatcgtataccaacaagcgcctgccgcacctcaccgtctactttaagctcgcc

atgaggaatgaagaactgcccagcgtaaaagagagaattccgtatgtgatagttgcgcag

accgaggccgcggaacgcgaagcgggtgtagtaaactcaatgcgcggtaccgcccaaaac

cccgtggtaaccaagaccgcacgcccccaacctaaacgcaaactgctggtttccgacctc

gccgaagacccgacctatgtttccgagaatgacgtaccgctaaacacagactactatttc

tcccacctgttgggtaccataagcgtgacctttaaggctctattcggaaatgatgtgaga

acaacagaaaatcttttaaagcggtttattccggaaaccccccacaagacccccacgaaa

acccaggcactgcttgagcgcgccggctttgaaaagctgacgccctttacaccggaggaa

gaaagtcgtcgaatactgcatacagttttttgtactctagaagcagctccccatcaaagc

tga

>MZ318701.1

atggcggcgcgcgaacaggccaactccgttcgcaggagcgggttttttaatccgtttatt

ggcaagaggccatttttcaggccgggaagcgggcaaaccgcggagacagagcgcccaagg

cccccccaacactcgtactgcacagaggtgggtagctttaagtttatagctccaagatgc

ctcgatgaagaagcccccgccgaccagcgacgcggtgtacacgtgggcaccttggagcgc

ccacccaaagtgtactgcgatggctcagagtacgacgtgctgaactttgcctccggaggt

tgctggcctcgcaggattcgggtttggaacggccaggattttcggggcgatggattcaac

cccagatttgagagatttcacgtgtacgacatagtagagacttcggagagcgcctcacac

gatgacccatccaggtttgctgagctatcccgcccaagcgggtctgtggtaacactgctg

ggaatgagcgagtgtggaaagcgagtcgcggttcacgtctatggtgtgcgccattatttt

tacatggcaaaggcggaggtggatagcgcttgtggaataaccaccgaggcagaactcgtg

cgcgcaatggtggactgcgcgcacagctcggctttgagcgcggccctgggaaatggcaac

ggcggcaaacagagcggcggcagcggcgggggatggtggggcggaaagcacgtgtctgcg

gactgcttcaaagtggagaccgtgtgccacacgacgctgtactactttggatctaagcca

gctctctactatagagtatctgcctccagcagccgcctgggagggttcatctgcgacaac

tttcacccggagattacaaaattcgaggggagcgtggacgtgaccacgcggctgttgttg

gacaatgaaaattttaccagtttcgggtggtaccgcctgcgacccggcacccacggagag

cgtgttcaacttcgccccgttgagcgacacgtcacctcaagcgacgtggagattaactgt

actcccgataacctggagccgataccagacgaggctgcctggcccgactataagctcatg

tgctttgatatagagtgtaaagctggaacgggtaacgaaatggcgttcccagtggcaact

aaccaagaggacctggtcatccagatctcctgtctgctgtactcgcttgctactcagaac

cacgaacacaccctgctgttttccctcgggtcatgcgatatctctgaggaatactcgttt

gcatgcgtccagcgcggcgagcccagaccgacggttttggagtttgacagcgagtacgag

ctgctggttgccttcctgacctttctcaagcagtactctcccgagttcgccaccggctac

aacatcgttaattttgactgggcgtacatagttaacaaggtaacgtcggtgtataacatc

aagctggacgggtacggcaagttcaacaaangggggctgtttaaggtgtgggacatcgcc

acgaaccattttcagaagaagagcaaggtgaaaatcaatggcctgatatctctagacatg

tattctgtggcgacggaaaagctaaagctacccagctacaaactcgacgcggtcgtggga

gacgtcctcggcgagcataagatagaccttccctataaagaaataccctcctattacgcg

ggagggcctgaccggaggggcgtaataggagagtattgtatccaggactctaggctggtg

ggcaagctgntttttaagtacctcccccatctggaactatcggcggtggccaaactcgcc

cgtatcaccctaacgcgggtaatttttgacggtcagcaaattcgcgtgtacacgtgcttg

ctgaaactcgcccgcgagagaaatttcattttgccagacaacagacgccggtttgacagt

caggcagacgccgcgtcagagacttcggagttggctatggatagccaaagccacgccttc

gacagtacagacgaacccgacggtgtggacggtaccccggacgccgcaggatctggcgct

acttctgaaaacggaggcgggaagcccggcgtcgggagggccgtgggctaccagggagca

aaggttctagaccccgtatccggctttcatgtggaccccgtggttgtgtttgacttcgct

agcttatacccaagcattatccaggcccataacctctgtttcaccaccctggcgctcgat

gaagtggatctggccgggcttcaaccatccgtcaactactcgacgttcgaggtgggtgac

caaaagttattttttgtccacgcccatattcgcgaaagcctgcttggcatcttgctgcgc

gactggctggccatgcgaaaggcggtgagggcgcgaatccccaccagcacccccgaggag

gcagttttactagataagcagcagtctgcgattaaggtgatatgcaactcggtttacgga

ttcacgggggtggcaaacggcctgttgccgtgtctgaggatagcggctaccgttaccacg

ataggacgcgacatgctcctcaagacccgagattacgttcactctcgttgggcgacgcgc

gagctgctggaggacaattttccaggggctataggtttccgaaaccacaagccttactcc

gtcagggttatctacggagacaccgactccgtgtttatcaagtttgtgggcctgacgtac

gagggggtatcggagctgggggatgctatgtcgcgtcagatttcagcggacctctttaga

gcgcccatcaaactggagtgtgagaagacctttcagcgactgctgctgatcaccaagaag

aagtacataggtgtcataaacggggggaagatgctcatgaagggggtcgacctggtccgc

aaaaataactgctctttcataaacttgtacgcgcgacatctggtagatcttttgttgtac

gacgaggatgtggccacggcggcagcaaaggtgacagacgtgcctcccgcagaatgggtg

gggcgcccgctaccgagcggctttgacaagtttgggcgagtgctggtagaggcgtacaac

cgtatcactgcccccaacttggacgtgcgcgagttcgttatgactgctgagctgagccgc

tcacccgaatcgtataccaacaagcgcctgccgcacctcaccgtctactttaagctcgcc

atgaggaatgaagaactgcccagcgtaaaagagagaattccgtatgtgatagttgcgcag

accgaggccgcggaacgcgaagcgggtgtagtaaactcaatgcgcggtaccgcccaaaac

cccgtggtaaccaagaccgcacgcccccaacctaaacgcaaactgctggtttccgacctc

gccgaagacccgacctatgtttccgagaatgacgtaccgctaaacacagactactatttc

tcccacctgttgggtaccataagcgtgacctttaaggctctattcggaaatgatgtgaga

acaacagaaaatcttttaaagcggtttattccggaaaccccccacaagacccccacgaaa

acccaggcactgcttgagcgcgccggctttgaaaagctgacgccctttacaccggaggaa

gaaagtcgtcgaatactgcatacagttttttgtactctagaagcagctccccatcaaagc

tga

>MZ318702.1

atggcggcgcgcgaacaggccaactccgttcgcaggagcgggttttttaatccgtttatt

ggcaagaggccatttttcaggccgggaagcgggcaaaccgcggagacagagcgcccaagg

nccccccaacactcgtactgcacagaggtgggtagctttaagtttatagctccaagatgc

ctcgatgaagaagcccccgccgaccagcgacgcggtgtacacgtgggcaccttggagcgc

ccacccaaagtgtactgcgatggctcagagtacgacgtgctgaactttgcctccggaggt

tgctggcctcgcaggattcgggtttggaacggccaggattttcggggcgatggattcaac

cccagatttgagagatttcacgtgtacgacatagtagagacttcggagagcgcctcacac

gatgacccatccaggtttgctgagctatcccgcccaagcgggtctgtggtaacactgctg

ggaatgagcgagtgtggaaagcgagtcgcggttcacgtctatggtgtgcgccattatttt

tacatggcaaaggcggaggtggatagcgcttgtggaataaccaccgaggcagaactcgtg

cgcgcaatggtggactgcgcgcacagctcggctttgagcgcggccctgggaaatggcaac

ggcggcaaacagagcggcggcagcggcnggggatggtggggcggaaagcacgtgtctgcg

gactgcttcaaagtggagaccgtgtgccacacgacgctgtactactttggatctaagcca

gctctctactatagagtatctgcctccagcagccgcctgggagggttcatctgcgacaac

tttcacccggagattacaaaattcgaggggagcgtggacgtgaccacgcggctgttgttg

gacaatgaaaattttaccagtttcgggtggtaccgcctgcgacccggcacccacggagag

cgtgttcaacttcgccccgttgagcgacacgtcacctcaagcgacgtggagattaactgt

actcccgataacctggagccgataccagacgaggctgcctggcccgactataagctcatg

tgctttgatatagagtgtaaagctggaacgggtaacgaaatggcgttcccagtggcaact

aaccaagaggacctggtcatccagatctcctgtctgctgtactcgcttgctactcagaac

cacgaacacaccctgctgttttccctcgggtcatgcgatatctctgaggaatactcgttt

gcatgcgtccagcgcggcgagcccagaccgacggttttggagtttgacagcgagtacgag

ctgctggttgccttcctgacctttctcaagcagtactctcccgagttcgccaccggctac

aacatcgttaattttgactgggcgtacatagttaacaaggtaacgtcggtgtataacatc

aagctggacgggtacggcaagttcaacaaangggggctgtttaaggtgtgggacatcgcc

acgaaccattttcagaagaagagcaaggtgaaaatcaatggcctgatatctctagacatg

tattctgtggcgacggaaaagctaaagctacccagctacaaactcgacgcggtcgtggga

gacgtcctcggcgagcataagatagaccttccctataaagaaataccctcctattacgcg

ggagggcctgaccggaggggcgtaataggagagtattgtatccaggactctaggctggtg

ggcaagctgttttttaagtacctcccccatctggaactatcggcggtggccaaactcgcc

cgtatcaccctaacgcgggtaatttttgacggtcagcaaattcgcgtgtacacgtgcttg

ctgaaactcgcccgcgagagaaatttcattttgccagacaacagacgccggtttgacagt

caggcagacgccgcgtcagagacttcggagttggctatggatagccaaagccacgccttc

gacagtacagacgaacccgacggtgtggacggtaccccggacgccgcaggatctggcgct

acttctgaaaacggaggcgggaagcccggcgtcgggagggccgtgggctaccagggagca

aaggttctagaccccgtatccggctttcatgtggaccccgtggttgtgtttgacttcgct

agcttatacccaagcattatccaggcccataacctctgtttcaccaccctggcgctcgat

gaagtggatctggccgggcttcaaccatccgtcaactactcgacgttcgaggtgggtgac

caaaagttattttttgtccacgcccatattcgcgaaagcctgcttggcatcttgctgcgc

gactggctggccatgcgaaaggcggtgagggcgcgaatccccaccagcacccccgaggag

gcagttttactagataagcagcagtctgcgattaaggtgatatgcaactcggtttacgga

ttcacgggggtggcaaacggcctgttgccgtgtctgaggatagcggctaccgttaccacg

ataggacgcgacatgctcctcaagacccgagattacgttcactctcgttgggcgacgcgc

gagctgctggaggacaattttccaggggctataggtttccgaaaccacaagccttactcc

gtcagggttatctacggagacaccgactccgtgtttatcaagtttgtgggcctgacgtac

gagggggtatcggagctgggggatgctatgtcgcgtcagatttcagcggacctctttaga

gcgcccatcaaactggagtgtgagaagacctttcagcgactgctgctgatcaccaagaag

aagtacataggtgtcataaacggggggaagatgctcatgaagggggtcgacctggtccgc

aaaaataactgctctttcataaacttgtacgcgcgacatctggtagatcttttgttgtac

gacgaggatgtggccacggcggcagcaaaggtgacagacgtgcctcccgcagaatgggtg

gggcgcccgctaccgagcggctttgacaagtttgggcgagtgctggtagaggcgtacaac

cgtatcactgcccccaacttggacgtgcgcgagttcgttatgactgctgagctgagccgc

tcacccgaatcgtataccaacaagcgcctgccgcacctcaccgtctactttaagctcgcc

atgaggaatgaagaactgcccagcgtaaaagagagaattccgtatgtgatagttgcgcag

accgaggccgcggaacgcgaagcgggtgtagtaaactcaatgcgcggtaccgcccaaaac

cccgtggtaaccaagaccgcacgcccccaacctaaacgcaaactgctggtttccgacctc

gccgaagacccgacctatgtttccgagaatgacgtaccgctaaacacagactactatttc

tcccacctgttgggtaccataagcgtgacctttaaggctctattcggaaatgatgtgaga

acaacagaaaatcttttaaagcggtttattccggaaaccccccacaagacccccacgaaa

acccaggcactgcttgagcgcgccggctttgaaaagctgacgccctttacaccggaggaa

gaaagtcgtcgaatactgcatacagttttttgtactctagaagcagctccccatcaaagc

tga

>MZ318703.1

atggcggcgcgcgaacaggccaactccgttcgcaggagcgggttttttaatccgtttatt

ggcaagaggccatttttcaggccgggaagcgggcaaaccgcggagacagagcgcccaagg

cccccccaacactcgtactgcacagaggtgggtagctttaagtttatagctccaagatgc

ctcgatgaagaagcccccgccgaccagcgacgcggtgtacacgtgggcaccttggagcgc

ccacccaaagtgtactgcgatggctcagagtacgacgtgctgaactttgcctccggaggt

tgctggcctcgcaggattcgggtttggaacggccaggattttcggggcgatggattcaac

cccagatttgagagatttcacgtgtacgacatagtagagacttcggagagcgcctcacac

gatgacccatccaggtttgctgagctatcccgcccaagcgggtctgtggtaacactgctg

ggaatgagcgagtgtggaaagcgagtcgcggttcacgtctatggtgtgcgccattatttt

tacatggcaaaggcggaggtggatagcgcttgtggaataaccaccgaggcagaactcgtg

cgcgcaatggtggactgcgcgcacagctcggctttgagcgcggccctgggaaatggcaac

ggcggcaaacagagcggcggcagcggcgggggatggtggggcggaaagcacgtgtctgcg

gactgcttcaaagtggagaccgtgtgccacacgacgctgtactactttggatctaagcca

gctctctactatagagtatctgcctccagcagccgcctgggagggttcatctgcgacaac

tttcacccggagattacaaaattcgaggggagcgtggacgtgaccacgcggctgttgttg

gacaatgaaaattttaccagtttcgggtggtaccgcctgcgacccggcacccacggagag

cgtgttcaacttcgccccgttgagcgacacgtcacctcaagcgacgtggagattaactgt

actcccgataacctggagccgataccagacgaggctgcctggcccgactataagctcatg

tgctttgatatagagtgtaaagctggaacgggtaacgaaatggcgttcccagtggcaact

aaccaagaggacctggtcatccagatctcctgtctgctgtactcgcttgctactcagaac

cacgaacacaccctgctgttttccctcgggtcatgcgatatctctgaggaatactcgttt

gcatgcgtccagcgcggcgagcccagaccgacggttttggagtttgacagcgagtacgag

ctgctggttgccttcctgacctttctcaagcagtactctcccgagttcgccaccggctac

aacatcgttaattttgactgggcgtacatagttaacaaggtaacgtcggtgtataacatc

aagctggacgggtacggcaagttcaacaaangggggctgtttaaggtgtgggacatcgcc

acgaaccattttcagaagaagagcaaggtgaaaatcaatggcctgatatctctagacatg

tattctgtggcgacggaaaagctaaagctacccagctacaaactcgacgcggtcgtggga

gacgtcctcggcgagcataagatagaccttccctataaagaaataccctcctattacgcg

ggagggcctgaccggaggggcgtaataggagagtattgtatccaggactctaggctggtg

ggcaagctgttttttaagtacctcccccatctggaactatcggcggtggccaaactcgcc

cgtatcaccctaacgcgggtaatttttgacggtcagcaaattcgcgtgtacacgtgcttg

ctgaaactcgcccgcgagagaaatttcattttgccagacaacagacgccggtttgacagt

caggcagacgccgcgtcagagacttcggagttggctatggatagccaaagccacgccttc

gacagtacagacgaacccgacggtgtggacggtaccccggacgccgcaggatctggcgct

acttctgaaaacggaggcgggaagcccggcgtcgggagggccgtgggctaccagggagca

aaggttctagaccccgtatccggctttcatgtggaccccgtggttgtgtttgacttcgct

agcttatacccaagcattatccaggcccataacctctgtttcaccaccctggcgctcgat

gaagtggatctggccgggcttcaaccatccgtcaactactcgacgttcgaggtgggtgac

caaaagttattttttgtccacgcccatattcgcgaaagcctgcttggcatcttgctgcgc

gactggctggccatgcgaaaggcggtgagggcgcgaatccccaccagcacccccgaggag

gcagttttactagataagcagcagtctgcgattaaggtgatatgcaactcggtttacgga

ttcacgggggtggcaaacggcctgttgccgtgtctgaggatagcggctaccgttaccacg

ataggacgcgacatgctcctcaagacccgagattacgttcactctcgttgggcgacgcgc

gagctgctggaggacaattttccaggggctataggtttccgaaaccacaagccttactcc

gtcagggttatctacggagacaccgactccgtgtttatcaagtttgtgggcctgacgtac

gagggggtatcggagctgggggatgctatgtcgcgtcagatttcagcggacctctttaga

gcgcccatcaaactggagtgtgagaagacctttcagcgactgctgctgatcaccaagaag

aagtacataggtgtcataaacngggggaagatgctcatgaagggggtcgacctggtccgc

aaaaataactgctctttcataaacttgtacgcgcgacatctggtagatcttttgttgtac

gacgaggatgtggccacggcggcagcaaaggtgacagacgtgcctcccgcagaatgggtg

gggcgcccgctaccgagcggctttgacaagtttgggcgagtgctggtagaggcgtacaac

cgtatcactgcccccaacttggacgtgcgcgagttcgttatgactgctgagctgagccgc

tcacccgaatcgtataccaacaagcgcctgccgcacctcaccgtctactttaagctcgcc

atgaggaatgaagaactgcccagcgtnaaagagagaattccgtatgtgatagttgcgcag

accgaggccgcggaacgcgaagcgggtgtagtaaactcaatgcgcggtaccgcccaaaac

cccgtggtaaccaagaccgcacgcccccaacctaaacgcaaactgctggtttccgacctc

gccgaagacccgacctatgtttccgagaatgacgtaccgctaaacacagactactatttc

tcccacctgttgggtaccataagcgtgacctttaaggctctattcggaaatgatgtgaga

acaacagaaaatcttttaaagcggtttattccggaaancccccacaagacccccacgaaa

acccaggcactgcttgagcgcgccggctttgaaaagctgacgccctttacaccggaggaa

gaaagtcgtcgaatactgcatacagttttttgtactctagaagcagctccccatcaaagc

tga

>MZ318704.1

atggcggcgcgcgaacaggccaactccgttcgcaggagcgggttttttaatccgtttatt

ggcaagaggccatttttcaggccgggaagcgggcaaaccgcggagacagagcgcccaagg

cccccccaacactcgtactgcacagaggtgggtagctttaagtttatagctccaagatgc

ctcgatgaagaagcccccgccgaccagcgacgcggtgtacacgtgggcaccttggagcgc

ccacccaaagtgtactgcgatggctcagagtacgacgtgctgaactttgcctccggaggt

tgctggcctcgcaggattcgggtttggaacggccaggattttcggggcgatggattcaac

cccagatttgagagatttcacgtgtacgacatagtagagacttcggagagcgcctcacac

gatgacccatccaggtttgctgagctatcccgcccaagcgggtctgtggtaacactgctg

ggaatgagcgagtgtggaaagcgagtcgcggttcacgtctatggtgtgcgccattatttt

tacatggcaaaggcggaggtggatagcgcttgtggaataaccaccgaggcagaactcgtg

cgcgcaatggtggactgcgcgcacagctcggctttgagcgcggccctgggaaatggcaac

ggcggcaaacagagcggcggcagcggcgggggatggtggggcggaaagcacgtgtctgcg

gactgcttcaaagtggagaccgtgtgccacacgacgctgtactactttggatctaagcca

gctctctactatagagtatctgcctccagcagccgcctgggagggttcatctgcgacaac

tttcacccggagattacaaaattcgaggggagcgtggacgtgaccacgcggctgttgttg

gacaatgaaaattttaccagtttcgggtggtaccgcctgcgacccggcacccacggagag

cgtgttcaacttcgccccgttgagcgacacgtcacctcaagcgacgtggagattaactgt

actcccgataacctggagccgataccagacgaggctgcctggcccgactataagctcatg

tgctttgatatagagtgtaaagctggaacgggtaacgaaatggcgttcccagtggcaact

aaccaagaggacctggtcatccagatctcctgtctgctgtactcgcttgctactcagaac

cacgaacacaccctgctgttttccctcgggtcatgcgatatctctgaggaatactcgttt

gcatgcgtccagcgcggcgagcccagaccgacggttttggagtttgacagcgagtacgag

ctgctggttgccttcctgacctttctcaagcagtactctcccgagttcgccaccggctac

aacatcgttaattttgactgggcgtacatagttaacaaggtaacgtcggtgtataacatc

aagctggacgggtacggcaagttcaacaaaggggggctgtttaaggtgtgggacatcgcc

acgaaccattttcagaagaagagcaaggtgaaaatcaatggcctgatatctctagacatg

tattctgtggcgacggaaaagctaaagctacccagctacaaactcgacgcggtcgtggga

gacgtcctcggcgagcataagatagaccttccctataaagaaataccctcctattacgcg

ggagggcctgaccggaggggcgtaataggagagtattgtatccaggactctaggctggtg

ggcaagctgttttttaagtacctcccccatctggaactatcggcggtggccaaactcgcc

cgtatcaccctaacgcgggtaatttttgacggtcagcaaattcgcgtgtacacgtgcttg

ctgaaactcgcccgcgagagaaatttcattttgccagacaacagacgccggtttgacagt

caggcagacgccgcgtcagagacttcggagttggctatggatagccaaagccacgccttc

gacagtacagacgaacccgacggtgtggacggtaccccggacgccgcaggatctggcgct

acttctgaaaacggaggcgggaagcccggcgtcgggagggccgtgggctaccagggagca

aaggttctagaccccgtatccggctttcatgtggaccccgtggttgtgtttgacttcgct

agcttatacccaagcattatccaggcccataacctctgtttcaccaccctggcgctcgat

gaagtggatctggccgggcttcaaccatccgtcaactactcgacgttcgaggtgggtgac

caaaagttattttttgtccacgcccatattcgcgaaagcctgcttggcatcttgctgcgc

gactggctggccatgcgaaaggcggtgagggcgcgaatccccaccagcacccccgaggag

gcagttttactagataagcagcagtctgcgattaaggtgatatgcaactcggtttacgga

ttcacgggggtggcaaacggcctgttgccgtgtctgaggatagcggctaccgttaccacg

ataggacgcgacatgctcctcaagacccgagattacgttcactctcgttgggcgacgcgc

gagctgctggaggacaattttccaggggctataggtttccgaaaccacaagccttactcc

gtcagggttatctacggagacaccgactccgtgtttatcaagtttgtgggcctgacgtac

gagggggtatcggagctgggggatgctatgtcgcgtcagatttcagcggacctctttaga

gcgcccatcaaactggagtgtgagaagacctttcagcgactgctgctgatcaccaagaag

aagtacataggtgtcataaacggggggaagatgctcatgaagggggtcgacctggtccgc

aaaaataactgctctttcataaacttgtacgcgcgacatctggtagatcttttgttgtac

gacgaggatgtggccacggcggcagcaaaggtgacagacgtgcctcccgcagaatgggtg

gggcgcccgctaccgagcggctttgacaagtttgggcgagtgctggtagaggcgtacaac

cgtatcactgcccccaacttggacgtgcgcgagttcgttatgactgctgagctgagccgc

tcacccgaatcgtataccaacaagcgcctgccgcacctcaccgtctactttaagctcgcc

atgaggaatgaagaactgcccagcgtaaaagagagaattccgtatgtgatagttgcgcag

accgaggccgcggaacgcgaagcgggtgtagtaaactcaatgcgcggtaccgcccaaaac

cccgtggtaaccaagaccgcacgcccccaacctaaacgcaaactgctggtttccgacctc

gccgaagacccgacctatgtttccgagaatgacgtaccgctaaacacagactactatttc

tcccacctgttgggtaccataagcgtgacctttaaggctctattcggaaatgatgtgaga

acaacagaaaatcttttaaagcggtttattccggaaancccccacaagacccccacgaaa

acccaggcactgcttgagcgcgccggctttgaaaagctgacgccctttacaccggaggaa

gaaagtcgtcgaatactgcatacagttttttgtactctagaagcagctccccatcaaagc

tga

>MZ318705.1

atggcggcgcgcgaacaggccaactccgttcgcaggagcgggttttttaatccgtttatt

ggcaagaggccatttttcaggccgggaagcgggcaaaccgcggagacagagcgcccaagg

nccccccaacactcgtactgcacagaggtgggtagctttaagtttatagctccaagatgc

ctcgatgaagaagcccccgccgaccagcgacgcggtgtacacgtgggcaccttggagcgc

ccacccaaagtgtactgcgatggctcagagtacgacgtgctgaactttgcctccggaggt

tgctggcctcgcaggattcgggtttggaacggccaggattttcggggcgatggattcaac

cccagatttgagagatttcacgtgtacgacatagtagagacttcggagagcgcctcacac

gatgacccatccaggtttgctgagctatcccgcccaagcgggtctgtggtaacactgctg

ggaatgagcgagtgtggaaagcgagtcgcggttcacgtctatggtgtgcgccattatttt

tacatggcaaaggcggaggtggatagcgcttgtggaataaccaccgaggcagaactcgtg

cgcgcaatggtggactgcgcgcacagctcggctttgagcgcggccctgggaaatggcaac

ggcggcaaacagagcggcggcagcggcgggggatggtggggcggaaagcacgtgtctgcg

gactgcttcaaagtggagaccgtgtgccacacgacgctgtactactttggatctaagcca

gctctctactatagagtatctgcctccagcagccgcctgggagggttcatctgcgacaac

tttcacccggagattacaaaattcgaggggagcgtggacgtgaccacgcggctgttgttg

gacaatgaaaattttaccagtttcgggtggtaccgcctgcgacccggcacccacggagag

cgtgttcaacttcgccccgttgagcgacacgtcacctcaagcgacgtggagattaactgt

actcccgataacctggagccgataccagacgaggctgcctggcccgactataagctcatg

tgctttgatatagagtgtaaagctggaacgggtaacgaaatggcgttcccagtggcaact

aaccaagaggacctggtcatccagatctcctgtctgctgtactcgcttgctactcagaac

cacgaacacaccctgctgttttccctcgggtcatgcgatatctctgaggaatactcgttt

gcatgcgtccagcgcggcgagcccagaccgacggttttggagtttgacagcgagtacgag

ctgctggttgccttcctgacctttctcaagcagtactctcccgagttcgccaccggctac

aacatcgttaattttgactgggcgtacatagttaacaaggtaacgtcggtgtataacatc

aagctggacgggtacggcaagttcaacaaaggggggctgtttaaggtgtgggacatcgcc

acgaaccattttcagaagaagagcaaggtgaaaatcaatggcctgatatctctagacatg

tattctgtggcgacggaaaagctaaagctacccagctacaaactcgacgcggtcgtggga

gacgtcctcggcgagcataagatagaccttccctataaagaaataccctcctattacgcg

ggagggcctgaccggaggggcgtaataggagagtattgtatccaggactctaggctggtg

ggcaagctgttttttaagtacctcccccatctggaactatcggcggtggccaaactcgcc

cgtatcaccctaacgcgggtaatttttgacggtcagcaaattcgcgtgtacacgtgcttg

ctgaaactcgcccgcgagagaaatttcattttgccagacaacagacgccggtttgacagt

caggcagacgccgcgtcagagacttcggagttggctatggatagccaaagccacgccttc

gacagtacagacgaacccgacggtgtggacggtaccccggacgccgcaggatctggcgct

acttctgaaaacggaggcgggaagcccggcgtcgggagggccgtgggctaccagggagca

aaggttctagaccccgtatccggctttcatgtggaccccgtggttgtgtttgacttcgct

agcttatacccaagcattatccaggcccataacctctgtttcaccaccctggcgctcgat

gaagtggatctggccgggcttcaaccatccgtcaactactcgacgttcgaggtgggtgac

caaaagttattttttgtccacgcccatattcgcgaaagcctgcttggcatcttgctgcgc

gactggctggccatgcgaaaggcggtgagggcgcgaatccccaccagcacccccgaggag

gcagttttactagataagcagcagtctgcgattaaggtgatatgcaactcggtttacgga

ttcacgggggtggcaaacggcctgttgccgtgtctgaggatagcggctaccgttaccacg

ataggacgcgacatgctcctcaagacccgagattacgttcactctcgttgggcgacgcgc

gagctgctggaggacaattttccaggggctataggtttccgaaaccacaagccttactcc

gtcagggttatctacggagacaccgactccgtgtttatcaagtttgtgggcctgacgtac

gagggggtatcggagctgggggatgctatgtcgcgtcagatttcagcggacctctttaga

gcgcccatcaaactggagtgtgagaagacctttcagcgactgctgctgatcaccaagaag

aagtacataggtgtcataaacggggggaagatgctcatgaagggggtcgacctggtccgc

aaaaataactgctctttcataaacttgtacgcgcgacatctggtagatcttttgttgtac

gacgaggatgtggccacggcggcagcaaaggtgacagacgtgcctcccgcagaatgggtg

gggcgcccgctaccgagcggctttgacaagtttgggcgagtgctggtagaggcgtacaac

cgtatcactgcccccaacttggacgtgcgcgagttcgttatgactgctgagctgagccgc

tcacccgaatcgtataccaacaagcgcctgccgcacctcaccgtctactttaagctcgcc

atgaggaatgaagaactgcccagcgtaaaagagagaattccgtatgtgatagttgcgcag

accgaggccgcggaacgcgaagcgggtgtagtaaactcaatgcgcggtaccgcccaaaac

cccgtggtaaccaagaccgcacgcccccaacctaaacgcaaactgctggtttccgacctc

gccgaagacccgacctatgtttccgagaatgacgtaccgctaaacacagactactatttc

tcccacctgttgggtaccataagcgtgacctttaaggctctattcggaaatgatgtgaga

acaacagaaaatcttttaaagcggtttattccggaaaccccccacaagacccccacgaaa

acccaggcactgcttgagcgcgccggctttgaaaagctgacgccctttacaccggaggaa

gaaagtcgtcgaatactgcatacagttttttgtactctagaagcagctccccatcaaagc

tga

>MZ318706.1

atggcggcgcgcgaacaggccaactccgttcgcaggagcgggttttttaatccgtttatt

ggcaagaggccatttttcaggccgggaagcgggcaaaccgcggagacagagcgcccaagg

nccccccaacactcgtactgcacagaggtgggtagctttaagtttatagctccaaggtgc

ctcgatgaagaagcccccgccgaccagcgacgcggtgtacacgtgggcaccttggagcgc

ccacccaaagtgtactgcgatggctcagagtacgacgtgctgaactttgcctccggaggt

tgctggcctcgcaggattcgggtttggaacggccaggattttcggggcgatggattcaac

cccagatttgagagatttcacgtgtacgacatagtagagacttcggagagcgcctcacac

gatgacccatccaggtttgctgagctatcccgcccaagcgggtctgtggtaacactgctg

ggaatgagcgagtgtggaaagcgagtcgcggttcacgtctatggtgtgcgccattatttt

tacatggcaaaggcggaggtggatagcgcttgtggaataaccaccgaggcagaactcgtg

cgcgcaatggtggactgcgcgcacagctcggctttgagcgcggccctgggaaatggcaac

ggcggcaaacagagcggcggcagcggcnggggatggtggggcggaaagcacgtgtctgcg

gactgcttcaaagtggagaccgtgtgccacacgacgctgtactactttggatctaagcca

gctctctactatagagtatctgcctccagcagccgcctgggagggttcatctgcgacaac

tttcacccggagattacaaaattcgaggggagcgtggacgtgaccacgcggctgttgttg

gacaatgaaaattttaccagtttcgggtggtaccgcctgcgacccggcacccacggagag

cgtgttcaacttcgccccgttgagcgacacgtcacctcaagcgacgtggagattaactgt

actcccgataacctggagccgataccagacgaggctgcctggcccgactataagctcatg

tgctttgatatagagtgtaaagctggaacgggtaacgaaatggcgttcccagtggcaact

aaccaagaggacctggtcatccagatctcctgtctgctgtactcgcttgctactcagaac

cacgaacacaccctgctgttttccctcgggtcatgcgatatctctgaggaatactcgttt

gcatgcgtccagcgcggcgagcccagaccgacggttttggagtttgacagcgagtacgag

ctgctggttgccttcctgacctttctcaagcagtactctcccgagttcgccaccggctac

aacatcgttaattttgactgggcgtacatagttaacaaggtaacgtcggtgtataacatc

aagctggacgggtacggcaagttcaacaaaggggggctgtttaaggtgtgggacatcgcc

acgaaccattttcagaagaagagcaaggtgaaaatcaatggcctgatatctctagacatg

tattctgtggcgacggaaaagctaaagctacccagctacaaactcgacgcggtcgtggga

gacgtcctcggcgagcataagatagaccttccctataaagaaataccctcctattacgcg

ggagggcctgaccggaggggcgtaataggagagtattgtatccaggactctaggctggtg

ggcaagctgttttttaagtacctcccccatctggaactatcggcggtggccaaactcgcc

cgtatcaccctaacgcgggtaatttttgacggtcagcaaattcgcgtgtacacgtgcttg

ctgaaactcgcccgcgagagaaatttcattttgccagacaacagacgccggtttgacagt

caggcagacgccgcgtcagagacttcggagttggctatggatagccaaagccacgccttc

gacagtacagacgaacccgacggtgtggacggtaccccggacgccgcaggatctggcgct

acttctgaaaacggaggcgggaagcccggcgtcgggagggccgtgggctaccagggagca

aaggttctagaccccgtatccggctttcatgtggaccccgtggttgtgtttgacttcgct

agcttatacccaagcattatccaggcccataacctctgtttcaccaccctggcgctcgat

gaagtggatctggccgggcttcaaccatccgtcaactactcgacgttcgaggtgggtgac

caaaagttatnntttgtccacgcccatattcgcgaaagcctgcttggcatcttgctgcgc

gactggctggccatgcgaaaggcggtgagggcgcgaatccccaccagcacccccgaggag

gcagttttactagataagcagcagtctgcgattaaggtgatatgcaactcggtttacgga

ttcacgggggtggcaaacggcctgttgccgtgtctgaggatagcggctaccgttaccacg

ataggacgcgacatgctcctcaagacccgagattacgttcactctcgttgggcgacgcgc

gagctgctggaggacaattttccaggggctataggtttccgaaaccacaagccttactcc

gtcagggttatctacggagacaccgactccgtgtttatcaagtttgtgggcctgacgtac

gagggggtatcggagctgggggatgctatgtcgcgtcagatttcagcggacctctttaga

gcgcccatcaaactggagtgtgagaagacctttcagcgactgctgctgatcaccaagaag

aagtacataggtgtcataaacggggggaagatgctcatgaagggggtcgacctggtccgc

aaaaataactgctctttcataaacttgtacgcgcgacatctggtagatcttttgttgtac

gacgaggatgtggccacggcggcagcaaaggtgacagacgtgcctcccgcagaatgggtg

gggcgcccgctaccgagcggctttgacaagtttgggcgagtgctggtagaggcgtacaac

cgtatcactgcccccaacttggacgtgcgcgagttcgttatgactgctgagctgagccgc

tcacccgaatcgtataccaacaagcgcctgccgcacctcaccgtctactttaagctcgcc

atgaggaatgaagaactgcccagcgtaaaagagagaattccgtatgtgatagttgcgcag

accgaggccgcggaacgcgaagcgggtgtagtaaactcaatgcgcggtaccgcccaaaac

cccgtggtaaccaagaccgcacgcccccaacctaaacgcaaactgctggtttccgacctc

gccgaagacccgacctatgtttccgagaatgacgtaccgctaaacacagactactatttc

tcccacctgttgggtaccataagcgtgacctttaaggctctattcggaaatgatgtgaga

acaacagaaaatcttttaaagcggtttattccggaaaccccccacaagacccccacgaaa

acccaggcactgcttgagcgcgccggctttgaaaagctgacgccctttacaccggaggaa

gaaagtcgtcgaatactgcatacagttttttgtactctagaagcagctccccatcaaagc

tga

>OR085496.1

atggcggcgcgcgaacaggccaactccgttcgcaggagcgggttttttaatccgtttatt

ggcaagaggccatttttcaggccgggaagcgggcagaccgcggagacagagcgcccaagg

cccccccaacactcgtactgcacagaggtgggtagctttaagtttatagctccaagatgc

ctcgatgaagaagcccccgccgaccagcgacgcggtgtacacgtgggcaccttggagcgc

ccacccaaagtgtactgcgatggctcagagtacgacgtgctgaactttgcctccggaggt

tgctggcctcgcaggattcgggtttggaacggccaggattttcggggcgatggattcaac

cccagatttgagagatttcacgtgtacgacatagtagagacttcggagagcgcctcacac

gatgacccatccaggtttgctgagctatcccgcccaagcgggtctgtggtaacactgctg

ggaatgagcgagtgtggaaagcgagtcgcggttcacgtctatggtgtgcgccattatttt

tacatggcaaaggcggaggtggatagcgcttgtggaataaccaccgaggcagaactcgtg

cgcgcaatggtggactgcgcgcacagctcggctttgagcgcggccctgggaaatggcaac

ggcggcaaacagagcggcggcagcggcgggggatggtggggcggaaagcacgtgtctgcg

gactgcttcaaagtggagaccgtgtgccacacgacgctgtactactttggatctaagcca

gctctctactatagagtatctgcctccagcagccgcctgggagggttcatctgcgacaac

tttcacccggagattacaaaattcgaggggagcgtggacgtgaccacgcggctgttgttg

gacaatgaaaattttaccagtttcgggtggtaccgcctgcgacccggcacccacggagag

cgtgttcaacttcgccccgttgagcgacacgtcacctcaagcgacgtggagattaactgt

actcccgataacctggagccgataccagacgaggctgcctggcccgactataagctcatg

tgctttgatatagagtgtaaagctggaacgggtaacgaaatggcgttcccagtggcaact

aaccaagaggacctggtcatccagatctcctgtctgctgtactcgcttgctactcagaac

cacgaacacaccctgctgttttccctcgggtcatgcgatatctctgaggaatactcgttt

gcatgcgtccagcgcggcgagcccagaccgacggttttggagtttgacagcgagtacgag

ctgctggttgccttcctgacctttctcaagcagtactctcccgagttcgccaccggctac

aacatcgttaattttgactgggcgtacatagttaacaaggtaacgtcggtgtataacatc

aagctggacgggtacggcaagttcaacaaaggggggctgtttaaggtgtgggacatcgcc

acgaaccattttcagaagaagagcaaggtgaaaatcaatggcctgatatctctagacatg

tattctgtggcgacggaaaagctaaagctacccagctacaaactcgacgcggtcgtggga

gacgtcctcggcgagcataagatagaccttccctataaagaaataccctcctattacgcg

ggagggcctgaccggaggggcgtaataggagagtattgtatccaggactctaggctggtg

ggcaagctgttttttaagtacctcccccatctggaactatcggcggtggccaaactcgcc

cgtatcaccctaacgcgggtaatttttgacggtcagcaaattcgcgtgtacacgtgcttg

ctgaaactcgcccgcgagagaaatttcattttgccagacaacagacgccggtttgacagt

caggcagacgccgcgtcagagacttcggagttggctatggatagccaaagccacgccttc

gacagtacagacgaacccgacggtgtggacggtaccccggacgccgcaggatctggcgct

acttctgaaaacggaggcgggaagcccggcgtcgggagggccgtgggctaccagggagca

aaggttctagaccccgtatccggctttcatgtggaccccgtggttgtgtttgacttcgct

agcttatacccaagcattatccaggcccataacctctgtttcaccaccctggcgctcgat

gaagtggatctggccgggcttcaaccatccgtcgactactcgacgttcgaggtgggtgac

caaaagttattttttgtccacgcccatattcgcgaaagcctgcttggcatcttgctgcgc

gactggctggccatgcgaaaggcggtgagggcgcgaatccccaccagcacccccgaggag

gcagttttactagataagcagcagtctgcgattaaggtgatatgcaactcggtttacgga

ttcacgggggtggcaaacggcctgttgccgtgtctgaggatagcggctaccgttaccacg

ataggacgcgacatgctcctcaagacccgagattacgttcactctcgttgggcgacgcgc

gagctgctggaggacaattttccaggggctataggtttccgaaaccacaagccttactcc

gtcagggttatctacggagacaccgactccgtgtttatcaagtttgtgggcctgacgtac

gagggggtatcggagctgggggatgctatgtcgcgtcagatttcagcggacctctttaga

gcgcccatcaaactggagtgtgagaagacctttcagcgactgctgctgatcaccaagaag

aagtacataggtgtcataaacggggggaagatgctcatgaagggggtcgacctggtccgc

aaaaataactgctctttcataaacttgtacgcgcgacatctggtagatcttttgttgtac

gacgaggatgtggccacggcggcagcagaggtgacagacgtgcctcccgcagaatgggtg

gggcgcccgctaccgagcggctttgacaagtttgggcgagtgctggtagaggcgtacaac

cgtatcactgcccccaacttggacgtgcgcgagttcgttatgactgctgagctgagccgc

tcacccgaatcgtataccaacaagcgcctgccgcacctcaccgtctactttaagctcgcc

atgaggaatgaagaactgcccagcgtaaaagagagaattccgtatgtgatagttgcgcag

accgaggccgcggaacgcgaagcgggtgtagtaaactcaatgcgcggtaccgcccaaaac

cccgtggtaaccaagaccgcacgcccccaacctaaacgcaaactgctggtttccgacctc

gccgaagacccgacctatgtttccgagaatgacgtaccgctaaacacagactactatttc

tcccacctgttgggtaccataagcgtgacctttaaggctctattcggaaatgatgtgaga

acaacagaaaatcttttaaagcggtttattccggaaaccccccacaagacccccacgaaa

acccaggcactgcttgagcgcgccggctttgaaaagctgacgccctttacaccggaggaa

gaaagtcgtcgaatactgcatacagttttttgtactctagaagcagctccccatcaaagc

tga

>OR085497.1

atggcggcgcgcgaacaggccaactccgttcgcaggagcgggttttttaatccgtttatt

ggcaagaggccatttttcaggccgggaagcgggcagaccgcggagacagagcgcccaagg

cccccccaacactcgtactgcacagaggtgggtagctttaagtttatagctccaagatgc

ctcgatgaagaagcccccgccgaccagcgacgcggtgtacacgtgggcaccttggagcgc

ccacccaaagtgtactgcgatggctcagagtacgacgtgctgaactttgcctccggaggt

tgctggcctcgcaggattcgggtttggaacggccaggattttcggggcgatggattcaac

cccagatttgagagatttcacgtgtacgacatagtagagacttcggagagcgcctcacac

gatgacccatccaggtttgctgagctatcccgcccaagcgggtctgtggtaacactgctg

ggaatgagcgagtgtggaaagcgagtcgcggttcacgtctatggtgtgcgccattatttt

tacatggcaaaggcggaggtggatagcgcttgtggaataaccaccgaggcagaactcgtg

cgcgcaatggtggactgcgcgcacagctcggctttgagcgcggccctgggaaatggcaac

ggcggcaaacagagcggcggcagcggcgggggatggtggggcggaaagcacgtgtctgcg

gactgcttcaaagtggagaccgtgtgccacacgacgctgtactactttggatctaagcca

gctctctactatagagtatctgcctccagcagccgcctgggagggttcatctgcgacaac

tttcacccggagattacaaaattcgaggggagcgtggacgtgaccacgcggctgttgttg

gacaatgaaaattttaccagtttcgggtggtaccgcctgcgacccggcacccacggagag

cgtgttcaacttcgccccgttgagcgacacgtcacctcaagcgacgtggagattaactgt

actcccgataacctggagccgataccagacgaggctgcctggcccgactataagctcatg

tgctttgatatagagtgtaaagctggaacgggtaacgaaatggcgttcccagtggcaact

aaccaagaggacctggtcatccagatctcctgtctgctgtactcgcttgctactcagaac

cacgaacacaccctgctgttttccctcgggtcatgcgatatctctgaggaatactcgttt

gcatgcgtccagcgcggcgagcccagaccgacggttttggagtttgacagcgagtacgag

ctgctggttgccttcctgacctttctcaagcagtactctcccgagttcgccaccggctac

aacatcgttaattttgactgggcgtacatagttaacaaggtaacgtcggtgtataacatc

aagctggacgggtacggcaagttcaacaaaggggggctgtttaaggtgtgggacatcgcc

acgaaccattttcagaagaagagcaaggtgaaaatcaatggcctgatatctctagacatg

tattctgtggcgacggaaaagctaaagctacccagctacaaactcgacgcggtcgtggga

gacgtcctcggcgagcataagatagaccttccctataaagaaataccctcctattacgcg

ggagggcctgaccggaggggcgtaataggagagtattgtatccaggactctaggctggtg

ggcaagctgttttttaagtacctcccccatctggaactatcggcggtggccaaactcgcc

cgtatcaccctaacgcgggtaatttttgacggtcagcaaattcgcgtgtacacgtgcttg

ctgaaactcgcccgcgagagaaatttcattttgccagacaacagacgccggtttgacagt

caggcagacgccgcgtcagagacttcggagttggctatggatagccaaagccacgccttc

gaccgtacagacgaacccgacggtgtggacggtaccccggacgccgcaggatctggcgct

acttctgaaaacggaggcgggaagcccggcgtcgggagggccgtgggctaccagggagca

aaggttctagaccccgtatccggctttcatgtggaccccgtggttgtgtttgacttcgct

agcttatacccaagcattatccaggcccataacctctgtttcaccaccctggcgctcgat

gaagtggatctggccgggcttcaaccatccgtcaactactcgacgttcgaggtgggtgac

caaaagttattttttgtccacgcccatattcgcgaaagcctgcttggcatcttgctgcgc

gactggctggccatgcgaaaggcggtgagggcgcgaatccccaccagcacccccgaggag

gcagttttactagataagcagcagtctgcgattaaggtgatatgcaactcggtttacgga

ttcacgggggtggcaaacggcctgttgccgtgtctgaggatagcggctaccgttaccacg

ataggacgcgacatgctcctcaagacccgagattacgttcactctcgttgggcgacgcgc

gagctgctggaggacaattttccaggggctataggtttccgaaaccacaagccttactcc

gtcagggttatctacggagacaccgactccgtgtttatcaagtttgtgggcctgacgtac

gagggggtatcggagctgggggatgctatgtcgcgtcagatttcagcggacctctttaga

gcgcccatcaaactggagtgtgagaagacctttcagcgactgctgctgatcaccaagaag

aagtacataggtgtcataaacggggggaagatgctcatgaagggggtcgacctggtccgc

aaaaataactgctctttcataaacttgtacgcgcgacatctggtagatcttttgttgtac

gacgaggatgtggccacggcggcagcagaggtgacagacgtgcctcccgcagaatgggtg

gggcgcccgctaccgagcggctttgacaagtttgggcgagtgctggtagaggcgtacaac

cgtatcactgcccccaacttggacgtgcgcgagttcgttatgactgctgagctgagccgc

tcacccgaatcgtataccaacaagcgcctgccgcacctcaccgtctactttaagctcgcc

atgaggaatgaagaactgcccagcgtaaaagagagaattccgtatgtgatagttgcgcag

accgaggccgcggaacgcgaagcgggtgtagtaaactcaatgcgcggtaccgcccaaaac

cccgtggtaaccaagaccgcacgcccccaacctaaacgcaaactgctggtttccgacctc

gccgaagacccgacctatgtttccgagaatgacgtaccgctaaacacagactactatttc

tcccacctgttgggtaccataagcgtgacctttaaggctctattcggaaatgatgtgaga

acaacagaaaatcttttaaagcggtttattccggaaaccccccacaagacccccacgaaa

acccaggcactgcttgagcgcgccggctttgaaaagctgacgccctttacaccggaggaa

gaaagtcgtcgaatactgcatacagttttttgtactctagaagcagctccccatcaaagc

tga

>OR085498.1

atggcggcgcgcgaacaggccaactccgttcgcaggagcgggttttttaatccgtttatt

ggcaagaggccatttttcaggccgggaagcgggcagaccgcggagacagagcgcccaagg

cccccccaacactcgtactgcacagaggtgggtagctttaagtttatagctccaagatgc

ctcgatgaagaagcccccgccgaccagcgacgcggtgtacacgtgggcaccttggagcgc

ccacccaaagtgtactgcgatggctcagagtacgacgtgctgaactttgcctccggaggt

tgctggcctcgcaggattcgggtttggaacggccaggattttcggggcgatggattcaac

cccagatttgagagatttcacgtgtacgacatagtagagacttcggagagcgcctcacac

gatgacccatccaggtttgctgagctatcccgcccaagcgggtctgtggtaacactgctg

ggaatgagcgagtgtggaaagcgagtcgcggttcacgtctatggtgtgcgccattatttt

tacatggcaaaggcggaggtggatagcgcttgtggaataaccaccgaggcagaactcgtg

cgcgcaatggtggactgcgcgcacagctcggctttgagcgcggccctgggaaatggcaac

ggcggcaaacagagcggcggcagcggcgggggatggtggggcggaaagcacgtgtctgcg

gactgcttcaaagtggagaccgtgtgccacacgacgctgtactactttggatctaagcca

gctctctactatagagtatctgcctccagcagccgcctgggagggttcatctgcgacaac

tttcacccggagattacaaaattcgaggggagcgtggacgtgaccacgcggctgttgttg

gacaatgaaaattttaccagtttcgggtggtaccgcctgcgacccggcacccacggagag

cgtgttcaacttcgccccgttgagcgacacgtcacctcaagcgacgtggagattaactgt

actcccgataacctggagccgataccagacgaggctgcctggcccgactataagctcatg

tgctttgatatagagtgtaaagctggaacgggtaacgaaatggcgttcccagtggcaact

aaccaagaggacctggtcatccagatctcctgtctgctgtactcgcttgctactcagaac

cacgaacacaccctgctgttttccctcgggtcatgcgatatctctgaggaatactcgttt

gcatgcgtccagcgcggcgagcccagaccgacggttttggagtttgacagcgagtacgag

ctgctggttgccttcctgacctttctcaagcagtactctcccgagttcgccaccggctac

aacatcgttaattttgactgggcgtacatagttaacaaggtaacgtcggtgtataacatc

aagctggacgggtacggcaagttcaacaaaggggggctgtttaaggtgtgggacatcgcc

acgaaccattttcagaagaagagcaaggtgaaaatcaatggcctgatatctctagacatg

tattctgtggcgacggaaaagctaaagctacccagctacaaactcgacgcggtcgtggga

gacgtcctcggcgagcataagatagaccttccctataaagaaataccctcctattacgcg

ggagggcctgaccggaggggcgtaataggagagtattgtatccaggactctaggctggtg

ggcaagctgttttttaagtacctcccccatctggaactatcggcggtggccaaactcgcc

cgtatcaccctaacgcgggtaatttttgacggtcagcaaattcgcgtgtacacgtgcttg

ctgaaactcgcccgcgagagaaatttcattttgccagacaacagacgccggtttgacagt

caggcagacgccgcgtcagagacttcggagttggctatggatagccaaagccacgccttc

gacagtacagacgaacccgacggtgtggacggtaccccggacgccgcaggatctggcgct

acttctgaaaacggaggcgggaagcccggcgtcgggagggccgtgggctaccagggagca

aaggttctagaccccgtatccggctttcatgtggaccccgtggttgtgtttgacttcgct

agcttatacccaagcattatccaggcccataacctctgtttcaccaccctggcgctcgat

gaagtggatctggccgggcttcaaccatccgtcaactactcgacgttcgaggtgggtgac

caaaagttattttttgtccacgcccatattcgcgaaagcctgcttggcatcttgctgcgc

gactggctggccatgcgaaaggcggtgagggcgcgaatccccaccagcacccccgaggag

gcagttttactagataagcagcagtctgcgattaaggtgatatgcaactcggtttacgga

ttcacgggggtggcaaacggcctgttgccgtgtctgaggatagcggctaccgttaccacg

ataggacgcgacatgctcctcaagacccgagattacgttcactctcgttgggcgacgcgc

gagctgctggaggacaattttccaggggctataggtttccgaaaccacaagccttactcc

gtcagggttatctacggagacaccgactccgtgtttatcaagtttgtgggcctgacgtac

gagggggtatcggagctgggggatgctatgtcgcgtcagatttcagcggacctctttaga

gcgcccatcaaactggagtgtgagaagacctttcagcgactgctgctgatcaccaagaag

aagtacataggtgtcataaacggggggaagatgctcatgaagggggtcgacctggtccgc

aaaaataactgctctttcataaacttgtacgcgcgacatctggtagatcttttgttgtac

gacgaggatgtggccacggcggcagcagaggtgacagacgtgcctcccgcagaatgggtg

gggcgcccgctaccgagcggctttgacaagtttgggcgagtgctggtagaggcgtacaac

cgtatcactgcccccaacttggacgtgcgcgagttcgttatgactgctgagctgagccgc

tcacccgaatcgtataccaacaagcgcctgccgcacctcaccgtctactttaagctcgcc

atgaggaatgaagaactgcccagcgtaaaagagagaattccgtatgtgatagttgcgcag

accgaggccgcggaacgcgaagcgggtgtagtaaactcaatgcgcggtaccgcccaaaac

cccgtggtaaccaagaccgcacgcccccaacctaaacgcaaactgctggtttccgacctc

gccgaagacccgacctatgtttccgagaatgacgtaccgctaaacacagactactatttc

tcccacctgttgggtaccataagcgtgacctttaaggctctattcggaaatgatgtgaga

acaacagaaaatcttttaaagcggtttattccggaaaccccccacaagacccccacgaaa

acccaggcactgcttgagcgcgccggctttgaaaagctgacgccctttacaccggaggaa

gaaagtcgtcgaatactgcatacagttttttgtactctagaagcagctccccatcaaagc

tga

>OR085499.1

atggcggcgcgcgaacaggccaactccgttcgcaggagcgggttttttaatccgtttatt

ggcaagaggccatttttcaggccgggaagcgggcaaaccgcggagacagagcgcccaagg

cccccccaacactcgtactgcacagaggtgggtagctttaagtttatagctccaagatgc

ctcgatgaagaagcccccgccgaccagcgacgcggtgtacacgtgggcaccttggagcgc

ccacccaaagtgtactgcgatggctcagagtacgacgtgctgaactttgcctccggaggt

tgctggcctcgcaggattcgggtttggaacggccaggattttcggggcgatggattcaac

cccagatttgagagatttcacgtgtacgacatagtagagacttcggagagcgcctcacac

gatgacccatccaggtttgctgagctatcccgcccaagcgggtctgtggtaacactgctg

ggaatgagcgagtgtggaaagcgagtcgcggttcacgtctatggtgtgcgccattatttt

tacatggcaaaggcggaggtggatagcgcttgtggaataaccaccgaggcagaactcgtg

cgcgcaatggtggactgcgcgcacagctcggctttgagcgcggccctgggaaatggcaac

ggcggcaaacagagcggcggcagcggcgggggatggtggggcggaaagcacgtgtctgcg

gactgcttcaaagtggagaccgtgtgccacacgacgctgtactactttggatctaagcca

gctctctactatagagtatctgcctccagcagccgcctgggagggttcatctgcgacaac

tttcacccggagattacaaaattcgaggggagcgtggacgtgaccacgcggctgttgttg

gacaatgaaaattttaccagtttcgggtggtaccgcctgcgacccggcacccacggagag

cgtgttcaacttcgccccgttgagcgacacgtcacctcaagcgacgtggagattaactgt

actcccgataacctggagccgataccagacgaggctgcctggcccgactataagctcatg

tgctttgatatagagtgtaaagctggaacgggtaacgaaatggcgttcccagtggcaact

aaccaagaggacctggtcatccagatctcctgtctgctgtactcgcttgctactcagaac

cacgaacacaccctgctgttttccctcgggtcatgcgatatctctgaggaatactcgttt

gcatgcgtccagcgcggcgagcccagaccgacggttttggagtttgacagcgagtacgag

ctgctggttgccttcctgacctttctcaagcagtactctcccgagttcgccaccggctac

aacatcgttaattttgactgggcgtacatagttaacaaggtaacgtcggtgtataacatc

aagctggacgggtacggcaagttcaacaaaggggggctgtttaaggtgtgggacatcgcc

acgaaccattttcagaagaagagcaaggtgaaaatcaatggcctgatatctctagacatg

tattctgtggcgacggaaaagctaaagctacccagctacaaactcgacgcggtcgtggga

gacgtcctcggcgagcataagatagaccttccctataaagaaataccctcctattacgcg

ggagggcctgaccggaggggcgtaataggagagtattgtatccaggactctaggctggtg

ggcaagctgttttttaagtacctcccccatctggaactatcggcggtggccaaactcgcc

cgtatcaccctaacgcgggtaatttttgacggtcagcaaattcgcgtgtacacgtgcttg

ctgaaactcgcccgcgagagaaatttcattttgccagacaacagacgccggtttgacagt

caggcagacgccgcgtcagagacttcggagttggctatggatagccaaagccacgccttc

gacagtacagacgaacccgacggtgtggacggtaccccggacgccgcaggatctggcgct

acttctgaaaacggaggcgggaagcccggcgtcgggagggccgtgggctaccagggagca

aaggttctagaccccgtatccggctttcatgtggaccccgtggttgtgtttgacttcgct

agcttatacccaagcattatccaggcccataacctctgtttcaccaccctggcgctcgat

gaagtggatctggccgggcttcaaccatccgtcaactactcgacgttcgaggtgggtgac

caaaagttattttttgtccacgcccatattcgcgaaagcctgcttggcatcttgctgcgc

gactggctggccatgcgaaaggcggtgagggcgcgaatccccaccagcacccccgaggag

gcagttttactagataagcagcagtctgcgattaaggtgatatgcaactcggtttacgga

ttcacgggggtggcaaacggcctgttgccgtgtctgaggatagcggctaccgttaccacg

ataggacgcgacatgctcctcaagacccgagattacgttcactctcgttgggcgacgcgc

gagctgctggaggacaattttccaggggctataggtttccgaaaccacaagccttactcc

gtcagggttatctacggagacaccgactccgtgtttatcaagtttgtgggcctgacgtac

gagggggtatcggagctgggggatgctatgtcgcgtcagatttcagcggacctctttaga

gcgcccatcaaactggagtgtgagaagacctttcagcgactgcttctgatcaccaagaag

aagtacataggtgtcataaacggggggaagatgctcatgaagggggtcgacctggtccgc

aaaaataactgctctttcataaacttgtacgcgcgacatctggtagatcttttgttgtac

gacgaggatgtggccacggcggcagcagaggtgacagacgtgcctcccgcagaatgggtg

gggcgcccgctaccgagcggctttgacaagtttgggcgagtgctggtagaggcgtacaac

cgtatcactgcccccaacttggacgtgcgcgagttcgttatgactgctgagctgagccgc

tcacccgaatcgtataccaacaagcgcctgccgcacctcaccgtctactttaagctcgcc

atgaggaatgaagaactgcccagcgtaaaagagagaattccgtatgtgatagttgcgcag

accgaggccgcggaacgcgaagcgggtgtagtaaactcaatgcgcggtaccgcccaaaac

cccgtggtaaccaagaccgcacgcccccaacctaaacgcaaactgctggtttccgacctc

gccgaagacccgacctatgtttccgagaatgacgtaccgctaaacacagactactatttc

tcccacctgttgggtaccataagcgtgacctttaaggctctattcggaaatgatgtgaga

acaacagaaaatcttttaaagcggtttattccggaaaccccccacaagacccccacgaaa

acccaggcactgcttgagcgcgccggctttgaaaagctgacgccctttacaccggaggaa

gaaagtcgtcgaatactgcatacagttttttgtactctagaagcagctccccatcaaagc

tga

>OR085500.1

atggcggcgcgcgaacaggccaactccgttcgcaggagcgggttttttaatccgtttatt

ggcaagaggccatttttcaggccgggaagcgggcaaaccgcggagacagagcgcccaagg

cccccccaacactcgtactgcacagaggtgggtagctttaagtttatagctccaagatgc

ctcgatgaagaagcccccgccgaccagcgacgcggtgtacacgtgggcaccttggagcgc

ccacccaaagtgtactgcgatggctcagagtacgacgtgctgaactttgcctccggaggt

tgctggcctcgcaggattcgggtttggaacggccaggattttcggggcgatggattcaac

cccagatttgagagatttcacgtgtacgacatagtagagacttcggagagcgcctcacac

gatgacccatccaggtttgctgagctatcccgcccaagcgggtctgtggtaacactgctg

ggaatgagcgagtgtggaaagcgagtcgcggttcacgtctatggtgtgcgccattatttt

tacatggcaaaggcggaggtggatagcgcttgtggaataaccaccgaggcagaactcgtg

cgcgcaatggtggactgcgcgcacagctcggctttgagcgcggccctgggaaatggcaac

ggcggcaaacagagcggcggcagcggcgggggatggtggggcggaaagcacgtgtctgcg

gactgcttcaaagtggagaccgtgtgccacacgacgctgtactactttggatctaagcca

gctctctactatagagtatctgcctccagcagccgcctgggagggttcatctgcgacaac

tttcacccggagattacaaaattcgaggggagcgtggacgtgaccacgcggctgttgttg

gacaatgaaaattttaccagtttcgggtggtaccgcctgcgacccggcacccacggagag

cgtgttcaacttcgccccgttgagcgacacgtcacctcaagcgacgtggagattaactgt

actcccgataacctggagccgataccagacgaggctgcctggcccgactataagctcatg

tgctttgatatagagtgtaaagctggaacgggtaacgaaatggcgttcccagtggcaact

aaccaagaggacctggtcatccagatctcctgtctgctgtactcgcttgctactcagaac

cacgaacacaccctgctgttttccctcgggtcatgcgatatctctgaggaatactcgttt

gcatgcgtccagcgcggcgagcccagaccgacggttttggagtttgacagcgagtacgag

ctgctggttgccttcctgacctttctcaagcagtactctcccgagttcgccaccggctac

aacatcgttaattttgactgggcgtacatagttaacaaggtaacgtcggtgtataacatc

aagctggacgggtacggcaagttcaacaaaggggggctgtttaaggtgtgggacatcgcc

acgaaccattttcagaagaagagcaaggtgaaaatcaatggcctgatatctctagacatg

tattctgtggcgacggaaaagctaaagctacccagctacaaactcgacgcggtcgtggga

gacgtcctcggcgagcataagatagaccttccctataaagaaataccctcctattacgcg

ggagggcctgaccggaggggcgtaataggagagtattgtatccaggactctaggctggtg

ggcaagctgttttttaagtacctcccccatctggaactatcggcggtggccaaactcgcc

cgtatcaccctaacgcgggtaatttttgacggtcagcaaattcgcgtgtacacgtgcttg

ctgaaactcgcccgcgagagaaatttcattttgccagacaacagacgccggtttgacagt

caggcagacgccgcgtcagagacttcggagttggctatggatagccaaagccacgccttc

gacagtacagacgaacccgacggtgtggacggtaccccggacgccgcaggatctggcgct

acttctgaaaacggaggcgggaagcccggcgtcgggagggccgtgggctaccagggagca

aaggttctagaccccgtatccggctttcatgtggaccccgtggttgtgtttgacttcgct

agcttatacccaagcattatccaggcccataacctctgtttcaccaccctggcgctcgat

gaagtggatctggccgggcttcaaccatccgtcaactactcgacgttcgaggtgggtgac

caaaagttattttttgtccacgcccatattcgcgaaagcctgcttggcatcttgctgcgc

gactggctggccatgcgaaaggcggtgagggcgcgaatccccaccagcacccccgaggag

gcagttttactagataagcagcagtctgcgattaaggtgatatgcaactcggtttacgga

ttcacgggggtggcaaacggcctgttgccgtgtctgaggatagcggctaccgttaccacg

ataggacgcgacatgctcctcaagacccgagattacgttcactctcgttgggcgacgcgc

gagctgctggaggacaattttccaggggctataggtttccgaaaccacaagccttactcc

gtcagggttatctacggagacaccgactccgtgtttatcaagtttgtgggcctgacgtac

gagggggtatcggagctgggggatgctatgtcgcgtcagatttcagcggacctctttaga

gcgcccatcaaactggagtgtgagaagacctttcagcgactgcttctgatcaccaagaag

aagtacataggtgtcataaacggggggaagatgctcatgaagggggtcgacctggtccgc

aaaaataactgctctttcataaacttgtacgcgcgacatctggtagatcttttgttgtac

gacgaggatgtggccacggcggcagcagaggtgacagacgtgcctcccgcagaatgggtg

gggcgcccgctaccgagcggctttgacaagtttgggcgagtgctggtagaggcgtacaac

cgtatcactgcccccaacttggacgtgcgcgagttcgttatgactgctgagctgagccgc

tcacccgaatcgtataccaacaagcgcctgccgcacctcaccgtctactttaagctcgcc

atgaggaatgaagaactgcccagcgtaaaagagagaattccgtatgtgatagttgcgcag

accgaggccgcggaacgcgaagcgggtgtagtaaactcaatgcgcggtaccgcccaaaac

cccgtggtaaccaagaccgcacgcccccaacctaaacgcaaactgctggtttccgacctc

gccgaagacccgacctatgtttccgagaatgacgtaccgctaaacacagactactatttc

tcccacctgttgggtaccataagcgtgacctttaaggctctattcggaaatgatgtgaga

acaacagaaaatcttttaaagcggtttattccggaaaccccccacaagacccccacgaaa

acccaggcactgcttgagcgcgccggctttgaaaagctgacgccctttacaccggaggaa

gaaagtcgtcgaatactgcatacagttttttgtactctagaagcagctccccatcaaagc

tga

>OR085501.1

atggcggcgcgcgaacaggccaactccgttcgcaggagcgggttttttaatccgtttatt

ggcaagaggccatttttcaggccgggaagcgggcaaaccgcggagacagagcgcccaagg

cccccccaacactcgtactgcacagaggtgggtagctttaagtttatagctccaagatgc

ctcgatgaagaagcccccgccgaccagcgacgcggtgtacacgtgggcaccttggagcgc

ccacccaaagtgtactgcgatggctcagagtacgacgtgctgaactttgcctccggaggt

tgctggcctcgcaggattcgggtttggaacggccaggattttcggggcgatggattcaac

cccagatttgagagatttcacgtgtacgacatagtagagacttcggagagcgcctcacac

gatgacccatccaggtttgctgagctatcccgcccaagcgggtctgtggtaacactgctg

ggaatgagcgagtgtggaaagcgagtcgcggttcacgtctatggtgtgcgccattatttt

tacatggcaaaggcggaggtggatagcgcttgtggaataaccaccgaggcagaactcgtg

cgcgcaatggtggactgcgcgcacagctcggctttgagcgcggccctgggaaatggcaac

ggcggcaaacagagcggcggcagcggcgggggatggtggggcggaaagcacgtgtctgcg

gactgcttcaaagtggagaccgtgtgccacacgacgctgtactactttggatctaagcca

gctctctactatagagtatctgcctccagcagccgcctgggagggttcatctgcgacaac

tttcacccggagattacaaaattcgaggggagcgtggacgtgaccacgcggctgttgttg

gacaatgaaaattttaccagtttcgggtggtaccgcctgcgacccggcacccacggagag

cgtgttcaacttcgccccgttgagcgacacgtcacctcaagcgacgtggagattaactgt

actcccgataacctggagccgataccagacgaggctgcctggcccgactataagctcatg

tgctttgatatagagtgtaaagctggaacgggtaacgaaatggcgttcccagtggcaact

aaccaagaggacctggtcatccagatctcctgtctgctgtactcgcttgctactcagaac

cacgaacacaccctgctgttttccctcgggtcatgcgatatctctgaggaatactcgttt

gcatgcgtccagcgcggcgagcccagaccgacggttttggagtttgacagcgagtacgag

ctgctggttgccttcctgacctttctcaagcagtactctcccgagttcgccaccggctac

aacatcgttaattttgactgggcgtacatagttaacaaggtaacgtcggtgtataacatc

aagctggacgggtacggcaagttcaacaaaggggggctgtttaaggtgtgggacatcgcc

acgaaccattttcagaagaagagcaaggtgaaaatcaatggcctgatatctctagacatg

tattctgtggcgacggaaaagctaaagctacccagctacaaactcgacgcggtcgtggga

gacgtcctcggcgagcataagatagaccttccctataaagaaataccctcctattacgcg

ggagggcctgaccggaggggcgtaataggagagtattgtatccaggactctaggctggtg

ggcaagctgttttttaagtacctcccccatctggaactatcggcggtggccaaactcgcc

cgtatcaccctaacgcgggtaatttttgacggtcagcaaattcgcgtgtacacgtgcttg

ctgaaactcgcccgcgagagaaatttcattttgccagacaacagacgccggtttgacagt

caggcagacgccgcgtcagagacttcggagttggctatggatagccaaagccacgccttc

gacagtacagacgaacccgacggtgtggacggtaccccggacgccgcaggatctggcgct

acttctgaaaacggaggcgggaagcccggcgtcgggagggccgtgggctaccagggagca

aaggttctagaccccgtatccggctttcatgtggaccccgtggttgtgtttgacttcgct

agcttatacccaagcattatccaggcccataacctctgtttcaccaccctggcgctcgat

gaagtggatctggccgggcttcaaccatccgtcaactactcgacgttcgaggtgggtgac

caaaagttattttttgtccacgcccatattcgcgaaagcctgcttggcatcttgctgcgc

gactggctggccatgcgaaaggcggtgagggcgcgaatccccaccagcacccccgaggag

gcagttttactagataagcagcagtctgcgattaaggtgatatgcaactcggtttacgga

ttcacgggggtggcaaacggcctgttgccgtgtctgaggatagcggctaccgttaccacg

ataggacgcgacatgctcctcaagacccgagattacgttcactctcgttgggcgacgcgc

gagctgctggaggacaattttccaggggctataggtttccgaaaccacaagccttactcc

gtcagggttatctacggagacaccgactccgtgtttatcaagtttgtgggcctgacgtac

gagggggtatcggagctgggggatgctatgtcgcgtcagatttcagcggacctctttaga

gcgcccatcaaactggagtgtgagaagacctttcagcgactgcttctgatcaccaagaag

aagtacataggtgtcataaacggggggaagatgctcatgaagggggtcgacctggtccgc

aaaaataactgctctttcataaacttgtacgcgcgacatctggtagatcttttgttgtac

gacgaggatgtggccacggcggcagcagaggtgacagacgtgcctcccgcagaatgggtg

gggcgcccgctaccgagcggctttgacaagtttgggcgagtgctggtagaggcgtacaac

cgtatcactgcccccaacttggacgtgcgcgagttcgttatgactgctgagctgagccgc

tcacccgaatcgtataccaacaagcgcctgccgcacctcaccgtctactttaagctcgcc

atgaggaatgaagaactgcccagcgtaaaagagagaattccgtatgtgatagttgcgcag

accgaggccgcggaacgcgaagcgggtgtagtaaactcaatgcgcggtaccgcccaaaac

cccgtggtaaccaagaccgcacgcccccaacctaaacgcaaactgctggtttccgacctc

gccgaagacccgacctatgtttccgagaatgacgtaccgctaaacacagactactatttc

tcccacctgttgggtaccataagcgtgacctttaaggctctattcggaaatgatgtgaga

acaacagaaaatcttttaaagcggtttattccggaaaccccccacaagacccccacgaaa

acccaggcactgcttgagcgcgccggctttgaaaagctgacgccctttacaccggaggaa

gaaagtcgtcgaatactgcatacagttttttgtactctagaagcagctccccatcaaagc

tga

>OR085502.1

atggcggcgcgcgaacaggccaactccgttcgcaggagcgggttttttaatccgtttatt

ggcaagaggccatttttcaggccgggaagcgggcaaaccgcggagacagagcgcccaagg

cccccccaacactcgtactgcacagaggtgggtagctttaagtttatagctccaagatgc

ctcgatgaagaagcccccgccgaccagcgacgcggtgtacacgtgggcaccttggagcgc

ccacccaaagtgtactgcgatggctcagagtacgacgtgctgaactttgcctccggaggt

tgctggcctcgcaggattcgggtttggaacggccaggattttcggggcgatggattcaac

cccagatttgagagatttcacgtgtacgacatagtagagacttcggagagcgcctcacac

gatgacccatccaggtttgctgagctatcccgcccaagcgggtctgtggtaacactgctg

ggaatgagcgagtgtggaaagcgagtcgcggttcacgtctatggtgtgcgccattatttt

tacatggcaaaggcggaggtggatagcgcttgtggaataaccaccgaggcagaactcgtg

cgcgcaatggtggactgcgcgcacagctcggctttgagcgcggccctgggaaatggcaac

ggcggcaaacagagcggcggcagcggcgggggatggtggggcggaaagcacgtgtctgcg

gactgcttcaaagtggagaccgtgtgccacacgacgctgtactactttggatctaagcca

gctctctactatagagtatctgcctccagcagccgcctgggagggttcatctgcgacaac

tttcacccggagattacaaaattcgaggggagcgtggacgtgaccacgcggctgttgttg

gacaatgaaaattttaccagtttcgggtggtaccgcctgcgacccggcacccacggagag

cgtgttcaacttcgccccgttgagcgacacgtcacctcaagcgacgtggagattaactgt

actcccgataacctggagccgataccagacgaggctgcctggcccgactataagctcatg

tgctttgatatagagtgtaaagctggaacgggtaacgaaatggcgttcccagtggcaact

aaccaagaggacctggtcatccagatctcctgtctgctgtactcgcttgctactcagaac

cacgaacacaccctgctgttttccctcgggtcatgcgatatctctgaggaatactcgttt

gcatgcgtccagcgcggcgagcccagaccgacggttttggagtttgacagcgagtacgag

ctgctggttgccttcctgacctttctcaagcagtactctcccgagttcgccaccggctac

aacatcgttaattttgactgggcgtacatagttaacaaggtaacgtcggtgtataacatc

aagctggacgggtacggcaagttcaacaaaggggggctgtttaaggtgtgggacatcgcc

acgaaccattttcagaagaagagcaaggtgaaaatcaatggcctgatatctctagacatg

tattctgtggcgacggaaaagctaaagctacccagctacaaactcgacgcggtcgtggga

gacgtcctcggcgagcataagatagaccttccctataaagaaataccctcctattacgcg

ggagggcctgaccggaggggcgtaataggagagtattgtatccaggactctaggctggtg

ggcaagctgttttttaagtacctcccccatctggaactatcggcggtggccaaactcgcc

cgtatcaccctaacgcgggtaatttttgacggtcagcaaattcgcgtgtacacgtgcttg

ctgaaactcgcccgcgagagaaatttcattttgccagacaacagacgccggtttgacagt

caggcagacgccgcgtcagagacttcggagttggctatggatagccaaagccacgccttc

gacagtacagacgaacccgacggtgtggacggtaccccggacgccgcaggatctggcgct

acttctgaaaacggaggcgggaagcccggcgtcgggagggccgtgggctaccagggagca

aaggttctagaccccgtatccggctttcatgtggaccccgtggttgtgtttgacttcgct

agcttatacccaagcattatccaggcccataacctctgtttcaccaccctggcgctcgat

gaagtggatctggccgggcttcaaccatccgtcaactactcgacgttcgaggtgggtgac

caaaagttattttttgtccacgcccatattcgcgaaagcctgcttggcatcttgctgcgc

gactggctggccatgcgaaaggcggtgagggcgcgaatccccaccagcacccccgaggag

gcagttttactagataagcagcagtctgcgattaaggtgatatgcaactcggtttacgga

ttcacgggggtggcaaacggcctgttgccgtgtctgaggatagcggctaccgttaccacg

ataggacgcgacatgctcctcaagacccgagattacgttcactctcgttgggcgacgcgc

gagctgctggaggacaattttccaggggctataggtttccgaaaccacaagccttactcc

gtcagggttatctacggagacaccgactccgtgtttatcaagtttgtgggcctgacgtac

gagggggtatcggagctgggggatgctatgtcgcgtcagatttcagcggacctctttaga

gcgcccatcaaactggagtgtgagaagacctttcagcgactgcttctgatcaccaagaag

aagtacataggtgtcataaacggggggaagatgctcatgaagggggtcgacctggtccgc

aaaaataactgctctttcataaacttgtacgcgcgacatctggtagatcttttgttgtac

gacgaggatgtggccacggcggcagcagaggtgacagacgtgcctcccgcagaatgggtg

gggcgcccgctaccgagcggctttgacaagtttgggcgagtgctggtagaggcgtacaac

cgtatcactgcccccaacttggacgtgcgcgagttcgttatgactgctgagctgagccgc

tcacccgaatcgtataccaacaagcgcctgccgcacctcaccgtctactttaagctcgcc

atgaggaatgaagaactgcccagcgtaaaagagagaattccgtatgtgatagttgcgcag

accgaggccgcggaacgcgaagcgggtgtagtaaactcaatgcgcggtaccgcccaaaac

cccgtggtaaccaagaccgcacgcccccaacctaaacgcaaactgctggtttccgacctc

gccgaagacccgacctatgtttccgagaatgacgtaccgctaaacacagactactatttc

tcccacctgttgggtaccataagcgtgacctttaaggctctattcggaaatgatgtgaga

acaacagaaaatcttttaaagcggtttattccggaaaccccccacaagacccccacgaaa

acccaggcactgcttgagcgcgccggctttgaaaagctgacgccctttacaccggaggaa

gaaagtcgtcgaatactgcatacagttttttgtactctagaagcagctccccatcaaagc

tga

>OR085503.1

atggcggcgcgcgaacaggccaactccgttcgcaggagcgggttttttaatccgtttatt

ggcaagaggccatttttcaggccgggaagcgggcagaccgcggagacagagcgcccaagg

cccccccaacactcgtactgcacagaggtgggtagctttaagtttatagctccaagatgc

ctcgatgaagaagcccccgccgaccagcgacgcggtgtacacgtgggcaccttggagcgc

ccacccaaagtgtactgcgatggctcagagtacgacgtgctgaactttgcctccggaggt

tgctggcctcgcaggattcgggtttggaacggccaggattttcggggcgatggattcaac

cccagatttgagagatttcacgtgtacgacatagtagagacttcggagagcgcctcacac

gatgacccatccaggtttgctgagctatcccgcccaagcgggtctgtggtaacactgctg

ggaatgagcgagtgtggaaagcgagtcgcggttcacgtctatggtgtgcgccattatttt

tacatggcaaaggcggaggtggatagcgcttgtggaataaccaccgaggcagaactcgtg

cgcgcaatggtggactgcgcgcacagctcggctttgagcgcggccctgggaaatggcaac

ggcggcaaacagagcggcggcagcggcgggggatggtggggcggaaagcacgtgtctgcg

gactgcttcaaagtggagaccgtgtgccacacgacgctgtactactttggatctaagcca

gctctctactatagagtatctgcctccagcagccgcctgggagggttcatctgcgacaac

tttcacccggagattacaaaattcgaggggagcgtggacgtgaccacgcggctgttgttg

gacaatgaaaattttaccagtttcgggtggtaccgcctgcgacccggcacccacggagag

cgtgttcaacttcgccccgttgagcgacacgtcacctcaagcgacgtggagattaactgt

actcccgataacctggagccgataccagacgaggctgcctggcccgactataagctcatg

tgctttgatatagagtgtaaagctggaacgggtaacgaaatggcgttcccagtggcaact

aaccaagaggacctggtcatccagatctcctgtctgctgtactcgcttgctactcagaac

cacgaacacaccctgctgttttccctcgggtcatgcgatatctctgaggaatactcgttt

gcatgcgtccagcgcggcgagcccagaccgacggttttggagtttgacagcgagtacgag

ctgctggttgccttcctgacctttctcaagcagtactctcccgagttcgccaccggctac

aacatcgttaattttgactgggcgtacatagttaacaaggtaacgtcggtgtataacatc

aagctggacgggtacggcaagttcaacaaaggggggctgtttaaggtgtgggacatcgcc

acgaaccattttcagaagaagagcaaggtgaaaatcaatggcctgatatctctagacatg

tattctgtggcgacggaaaagctaaagctacccagctacaaactcgacgcggtcgtggga

gacgtcctcggcgagcataagatagaccttccctataaagaaataccctcctattacgcg

ggagggcctgaccggaggggcgtaataggagagtattgtatccaggactctaggctggtg

ggcaagctgttttttaagtacctcccccatctggaactatcggcggtggccaaactcgcc

cgtatcaccctaacgcgggtaatttttgacggtcagcaaattcgcgtgtacacgtgcttg

ctgaaactcgcccgcgagagaaatttcattttgccagacaacagacgccggtttgacagt

caggcagacgccgcgtcagagacttcggagttggctatggatagccaaagccacgccttc

gacagtacagacgaacccgacggtgtggacggtaccccggacgccgcaggatctggcgct

acttctgaaaacggaggcgggaagcccggcgtcgggagggccgtgggctaccagggagca

aaggttctagaccccgtatccggctttcatgtggaccccgtggttgtgtttgacttcgct

agcttatacccaagcattatccaggcccataacctctgtttcaccaccctggcgctcgat

gaagtggatctggccgggcttcaaccatccgtcaactactcgacgttcgaggtgggtgac

caaaagttattttttgtccacgcccatattcgcgaaagcctgcttggcatcttgctgcgc

gactggctggccatgcgaaaggcggtgagggcgcgaatccccaccagcacccccgaggag

gcagttttactagataagcagcagtctgcgattaaggtgatatgcaactcggtttacgga

ttcacgggggtggcaaacggcctgttgccgtgtctgaggatagcggctaccgttaccacg

ataggacgcgacatgctcctcaagacccgagattacgttcactctcgttgggcgacgcgc

gagctgctggaggacaattttccaggggctataggtttccgaaaccacaagccttactcc

gtcagggttatctacggagacaccgactccgtgtttatcaagtttgtgggcctgacgtac

gagggggtatcggagctgggggatgctatgtcgcgtcagatttcagcggacctctttaga

gcgcccatcaaactggagtgtgagaagacctttcagcgactgctgctgatcaccaagaag

aagtacataggtgtcataaacggggggaagatgctcatgaagggggtcgacctggtccgc

aaaaataactgctctttcataaacttgtacgcgcgacatctggtagatcttttgttgtac

gacgaggatgtggccacggcggcagcagaggtgacagacgtgcctcccgcagaatgggtg

gggcgcccgctaccgagcggctttgacaagtttgggcgagtgctggtagaggcgtacaac

cgtatcactgcccccaacttggacgtgcgcgagttcgttatgactgctgagctgagccgc

tcacccgaatcgtataccaacaagcgcctgccgcacctcaccgtctactttaagctcgcc

atgaggaatgaagaactgcccagcgtaaaagagagaattccgtatgtgatagttgcgcag

accgaggccgcggaacgcgaagcgggtgtagtaaactcaatgcgcggtaccgcccaaaac

cccgtggtaaccaagaccgcacgcccccaacctaaacgcaaactgctggtttccgacctc

gccgaagacccgacctatgtttccgagaatgacgtaccgctaaacacagactactatttc

tcccacctgttgggtaccataagcgtgacctttaaggctctattcggaaatgatgtgaga

acaacagaaaatcttttaaagcggtttattccggaaaccccccacaagacccccacgaaa

acccaggcactgcttgagcgcgccggctttgaaaagctgacgccctttacaccggaggaa

gaaagtcgtcgaatactgcatacagttttttgtactctagaagcagctccccatcaaagc

tga

>OR085504.1

atggcggcgcgcgaacaggccaactccgttcgcaggagcgggttttttaatccgtttatt

ggcaagaggccatttttcaggccgggaagcgggcaaaccgcggagacagagcgcccaagg

cccccccaacactcgtactgcacagaggtgggtagctttaagtttatagctccaagatgc

ctcgatgaagaagcccccgccgaccagcgacgcggtgtacacgtgggcaccttggagcgc

ccacccaaagtgtactgcgatggctcagagtacgacgtgctgaactttgcctccggaggt

tgctggcctcgcaggattcgggtttggaacggccaggattttcggggcgatggattcaac

cccagatttgagagatttcacgtgtacgacatagtagagacttcggagagcgcctcacac

gatgacccatccaggtttgctgagctatcccgcccaagcgggtctgtggtaacactgctg

ggaatgagcgagtgtggaaagcgagtcgcggttcacgtctatggtgtgcgccattatttt

tacatggcaaaggcggaggtggatagcgcttgtggaataaccaccgaggcagaactcgtg

cgcgcaatggtggactgcgcgcacagctcggctttgagcgcggccctgggaaatggcaac

ggcggcaaacagagcggcggcagcggcgggggatggtggggcggaaagcacgtgtctgcg

gactgcttcaaagtggagaccgtgtgccacacgacgctgtactactttggatctaagcca

gctctctactatagagtatctgcctccagcagccgcctgggagggttcatctgcgacaac

tttcacccggagattacaaaattcgaggggagcgtggacgtgaccacgcggctgttgttg

gacaatgaaaattttaccagtttcgggtggtaccgcctgcgacccggcacccacggagag

cgtgttcaacttcgccccgttgagcgacacgtcacctcaagcgacgtggagattaactgt

actcccgataacctggagccgataccagacgaggctgcctggcccgactataagctcatg

tgctttgatatagagtgtaaagctggaacgggtaacgaaatggcgttcccagtggcaact

aaccaagaggacctggtcatccagatctcctgtctgctgtactcgcttgctactcagaac

cacgaacacaccctgctgttttccctcgggtcatgcgatatctctgaggaatactcgttt

gcatgcgtccagcgcggcgagcccagaccgacggttttggagtttgacagcgagtacgag

ctgctggttgccttcctgacctttctcaagcagtactctcccgagttcgccaccggctac

aacatcgttaattttgactgggcgtacatagttaacaaggtaacgtcggtgtataacatc

aagctggacgggtacggcaagttcaacaaaggggggctgtttaaggtgtgggacatcgcc

acgaaccattttcagaagaagagcaaggtgaaaatcaatggcctgatatctctagacatg

tattctgtggcgacggaaaagctaaagctacccagctacaaactcgacgcggtcgtggga

gacgtcctcggcgagcataagatagaccttccctataaagaaataccctcctattacgcg

ggagggcctgaccggaggggcgtaataggagagtattgtatccaggactctaggctggtg

ggcaagctgttttttaagtacctcccccatctggaactatcggcggtggccaaactcgcc

cgtatcaccctaacgcgggtaatttttgacggtcagcaaattcgcgtgtacacgtgcttg

ctgaaactcgcccgcgagagaaatttcattttgccagacaacagacgccggtttgacagt

caggcagacgccgcgtcagagacttcggagttggctatggatagccaaagccacgccttc

gacagtacagacgaacccgacggtgtggacggtaccccggacgccgcaggatctggcgct

acttctgaaaacggaggcgggaagcccggcgtcgggagggccgtgggctaccagggagca

aaggttctagaccccgtatccggctttcatgtggaccccgtggttgtgtttgacttcgct

agcttatacccaagcattatccaggcccataacctctgtttcaccaccctggcgctcgat

gaagtggatctggccgggcttcaaccatccgtcaactactcgacgttcgaggtgggtgac

caaaagttattttttgtccacgcccatattcgcgaaagcctgcttggcatcttgctgcgc

gactggctggccatgcgaaaggcggtgagggcgcgaatccccaccagcacccccgaggag

gcagttttactagataagcagcagtctgcgattaaggtgatatgcaactcggtttacgga

ttcacgggggtggcaaacggcctgttgccgtgtctgaggatagcggctaccgttaccacg

ataggacgcgacatgctcctcaagacccgagattacgttcactctcgttgggcgacgcgc

gagctgctggaggacaattttccaggggctataggtttccgaaaccacaagccttactcc

gtcagggttatctacggagacaccgactccgtgtttatcaagtttgtgggcctgacgtac

gagggggtatcggagctgggggatgctatgtcgcgtcagatttcagcggacctctttaga

gcgcccatcaaactggagtgtgagaagacctttcagcgactgcttctgatcaccaagaag

aagtacataggtgtcataaacggggggaagatgctcatgaagggggtcgacctggtccgc

aaaaataactgctctttcataaacttgtacgcgcgacatctggtagatcttttgttgtac

gacgaggatgtggccacggcggcagcagaggtgacagacgtgcctcccgcagaatgggtg

gggcgcccgctaccgagcggctttgacaagtttgggcgagtgctggtagaggcgtacaac

cgtatcactgcccccaacttggacgtgcgcgagttcgttatgactgctgagctgagccgc

tcacccgaatcgtataccaacaagcgcctgccgcacctcaccgtctactttaagctcgcc

atgaggaatgaagaactgcccagcgtaaaagagagaattccgtatgtgatagttgcgcag

accgaggccgcggaacgcgaagcgggtgtagtaaactcaatgcgcggtaccgcccaaaac

cccgtggtaaccaagaccgcacgcccccaacctaaacgcaaactgctggtttccgacctc

gccgaagacccgacctatgtttccgagaatgacgtaccgctaaacacagactactatttc

tcccacctgttgggtaccataagcgtgacctttaaggctctattcggaaatgatgtgaga

acaacagaaaatcttttaaagcggtttattccggaaaccccccacaagacccccacgaaa

acccaggcactgcttgagcgcgccggctttgaaaagctgacgccctttacaccggaggaa

gaaagtcgtcgaatactgcatacagttttttgtactctagaagcagctccccatcaaagc

tga

>OR085505.1

atggcggcgcgcgaacaggccaactccgttcgcaggagcgggttttttaatccgtttatt

ggcaagaggccatttttcaggccgggaagcgggcaaaccgcggagacagagcgcccaagg

cccccccaacactcgtactgcacagaggtgggtagctttaagtttatagctccaagatgc

ctcgatgaagaagcccccgccgaccagcgacgcggtgtacacgtgggcaccttggagcgc

ccacccaaagtgtactgcgatggctcagagtacgacgtgctgaactttgcctccggaggt

tgctggcctcgcaggattcgggtttggaacggccaggattttcggggcgatggattcaac

cccagatttgagagatttcacgtgtacgacatagtagagacttcggagagcgcctcacac

gatgacccatccaggtttgctgagctatcccgcccaagcgggtctgtggtaacactgctg

ggaatgagcgagtgtggaaagcgagtcgcggttcacgtctatggtgtgcgccattatttt

tacatggcaaaggcggaggtggatagcgcttgtggaataaccaccgaggcagaactcgtg

cgcgcaatggtggactgcgcgcacagctcggctttgagcgcggccctgggaaatggcaac

ggcggcaaacagagcggcggcagcggcgggggatggtggggcggaaagcacgtgtctgcg

gactgcttcaaagtggagaccgtgtgccacacgacgctgtactactttggatctaagcca

gctctctactatagagtatctgcctccagcagccgcctgggagggttcatctgcgacaac

tttcacccggagattacaaaattcgaggggagcgtggacgtgaccacgcggctgttgttg

gacaatgaaaattttaccagtttcgggtggtaccgcctgcgacccggcacccacggagag

cgtgttcaacttcgccccgttgagcgacacgtcacctcaagcgacgtggagattaactgt

actcccgataacctggagccgataccagacgaggctgcctggcccgactataagctcatg

tgctttgatatagagtgtaaagctggaacgggtaacgaaatggcgttcccagtggcaact

aaccaagaggacctggtcatccagatctcctgtctgctgtactcgcttgctactcagaac

cacgaacacaccctgctgttttccctcgggtcatgcgatatctctgaggaatactcgttt

gcatgcgtccagcgcggcgagcccagaccgacggttttggagtttgacagcgagtacgag

ctgctggttgccttcctgacctttctcaagcagtactctcccgagttcgccaccggctac

aacatcgttaattttgactgggcgtacatagttaacaaggtaacgtcggtgtataacatc

aagctggacgggtacggcaagttcaacaaaggggggctgtttaaggtgtgggacatcgcc

acgaaccattttcagaagaagagcaaggtgaaaatcaatggcctgatatctctagacatg

tattctgtggcgacggaaaagctaaagctacccagctacaaactcgacgcggtcgtggga

gacgtcctcggcgagcataagatagaccttccctataaagaaataccctcctattacgcg

ggagggcctgaccggaggggcgtaataggagagtattgtatccaggactctaggctggtg

ggcaagctgttttttaagtacctcccccatctggaactatcggcggtggccaaactcgcc

cgtatcaccctaacgcgggtaatttttgacggtcagcaaattcgcgtgtacacgtgcttg

ctgaaactcgcccgcgagagaaatttcattttgccagacaacagacgccggtttgacagt

caggcagacgccgcgtcagagacttcggagttggctatggatagccaaagccacgccttc

gacagtacagacgaacccgacggtgtggacggtaccccggacgccgcaggatctggcgct

acttctgaaaacggaggcgggaagcccggcgtcgggagggccgtgggctaccagggagca

aaggttctagaccccgtatccggctttcatgtggaccccgtggttgtgtttgacttcgct

agcttatacccaagcattatccaggcccataacctctgtttcaccaccctggcgctcgat

gaagtggatctggccgggcttcaaccatccgtcaactactcgacgttcgaggtgggtgac

caaaagttattttttgtccacgcccatattcgcgaaagcctgcttggcatcttgctgcgc

gactggctggccatgcgaaaggcggtgagggcgcgaatccccaccagcacccccgaggag

gcagttttactagataagcagcagtctgcgattaaggtgatatgcaactcggtttacgga

ttcacgggggtggcaaacggcctgttgccgtgtctgaggatagcggctaccgttaccacg

ataggacgcgacatgctcctcaagacccgagattacgttcactctcgttgggcgacgcgc

gagctgctggaggacaattttccaggggctataggtttccgaaaccacaagccttactcc

gtcagggttatctacggagacaccgactccgtgtttatcaagtttgtgggcctgacgtac

gagggggtatcggagctgggggatgctatgtcgcgtcagatttcagcggacctctttaga

gcgcccatcaaactggagtgtgagaagacctttcagcgactgctgctgatcaccaagaag

aagtacataggtgtcataaacggggggaagatgctcatgaagggggtcgacctagtccgc

aaaaataactgctctttcataaacttgtacgcgcgacatctggtagatcttttgttgtac

gacgaggatgtggccacggcggcagcagaggtgacagacgtgcctcccgcagaatgggtg

gggcgcccgctaccgagcggctttgacaagtttgggcgagtgctggtagaggcgtacaac

cgtatcactgcccccaacttggacgtgcgcgagttcgttatgactgctgagctgagccgc

tcacccgaatcgtataccaacaagcgcctgccgcacctcaccgtctactttaagctcgcc

atgaggaatgaagaactgcccagcgtaaaagagagaattccgtatgtgatagttgcgcag

accgaggccgcggaacgcgaagcgggtgtagtaaactcaatgcgcggtaccgcccaaaac

cccgtggtaaccaagaccgcacgcccccaacctaaacgcaaactgctggtttccgacctc

gccgaagacccgacctatgtttccgagaatgacgtaccgctaaacacagactactatttc

tcccacctgttgggtaccataagcgtgacctttaaggctctattcggaaatgatgtgaga

acaacagaaaatcttttaaagcggtttattccggaaaccccccacaagacccccacgaaa

acccaggcactgcttgagcgcgccggctttgaaaagctgacgccctttacaccggaggaa

gaaagtcgtcgaatactgcatacagttttttgtactctagaagcagctccccatcaaagc

tga

>OR085506.1

atggcggcgcgcgaacaggccaactccgttcgcaggagcgggttttttaatccgtttatt

ggcaagaggccatttttcaggccgggaagcgggcagaccgcggagacagagcgcccaagg

cccccccaacactcgtactgcacagaggtgggtagctttaagtttatagctccaagatgc

ctcgatgaagaagcccccgccgaccagcgacgcggtgtacacgtgggcaccttggagcgc

ccacccaaagtgtactgcgatggctcagagtacgacgtgctgaactttgcctccggaggt

tgctggcctcgcaggattcgggtttggaacggccaggattttcggggcgatggattcaac

cccagatttgagagatttcacgtgtacgacatagtagagacttcggagagcgcctcacac

gatgacccatccaggtttgctgagctatcccgcccaagcgggtctgtggtaacactgctg

ggaatgagcgagtgtggaaagcgagtcgcggttcacgtctatggtgtgcgccattatttt

tacatggcaaaggcggaggtggatagcgcttgtggaataaccaccgaggcagaactcgtg

cgcgcaatggtggactgcgcgcacagctcggctttgagcgcggccctgggaaatggcaac

ggcggcaaacagagcggcggcagcggcgggggatggtggggcggaaagcacgtgtctgcg

gactgcttcaaagtggagaccgtgtgccacacgacgctgtactactttggatctaagcca

gctctctactatagagtatctgcctccagcagccgcctgggagggttcatctgcgacaac

tttcacccggagattacaaaattcgaggggagcgtggacgtgaccacgcggctgttgttg

gacaatgaaaattttaccagtttcgggtggtaccgcctgcgacccggcacccacggagag

cgtgttcaacttcgccccgttgagcgacacgtcacctcaagcgacgtggagattaactgt

actcccgataacctggagccgataccagacgaggctgcctggcccgactataagctcatg

tgctttgatatagagtgtaaagctggaacgggtaacgaaatggcgttcccagtggcaact

aaccaagaggacctggtcatccagatctcctgtctgctgtactcgcttgctactcagaac

cacgaacacaccctgctgttttccctcgggtcatgcgatatctctgaggaatactcgttt

gcatgcgtccagcgcggcgagcccaaaccgacggttttggagtttgacagcgagtacgag

ctgctggttgccttcctgacctttctcaagcagtactctcccgagttcgccaccggctac

aacatcgttaattttgactgggcgtacatagttaacaaggtaacgtcggtgtataacatc

aagctggacgggtacggcaagttcaacaaaggggggctgtttaaggtgtgggacatcgcc

acgaaccattttcagaagaagagcaaggtgaaaatcaatggcctgatatctctagacatg

tattctgtggcgacggaaaagctaaagctacccagctacaaactcgacgcggtcgtggga

gacgtcctcggcgagcataagatagaccttccctataaagaaataccctcctattacgcg

ggagggcctgaccggaggggcgtaataggagagtattgtatccaggactctaggctggtg

ggcaagctgttttttaagtacctcccccatctggaactatcggcggtggccaaactcgcc

cgtatcaccctaacgcgggtaatttttgacggtcagcaaattcgcgtgtacacgtgcttg

ctgaaactcgcccgcgagagaaatttcattttgccagacaacagacgccggtttgacagt

caggcagacgccgcgtcagagacttcggagttggctatggatagccaaagccacgccttc

gacagtacagacgaacccgacggtgtggacggtaccccggacgccgcaggatctggcgct

acttctgaaaacggaggcgggaagcccggcgtcgggagggccgtgggctaccagggagca

aaggttctagaccccgtatccggctttcatgtggaccccgtggttgtgtttgacttcgct

agcttatacccaagcattatccaggcccataacctctgtttcaccaccctggcgctcgat

gaagtggatctggccgggcttcaaccatccgtcaactactcgacgttcgaggtgggtgac

caaaagttattttttgtccacgcccatattcgcgaaagcctgcttggcatcttgctgcgc

gactggctggccatgcgaaaggcggtgagggcgcgaatccccaccagcacccccgaggag

gcagttttactagataagcagcagtctgcgattaaggtgatatgcaactcggtttacgga

ttcacgggggtggcaaacggcctgttgccgtgtctgaggatagcggctaccgttaccacg

ataggacgcgacatgctcctcaagacccgagattacgttcactctcgttgggcgacgcgc

gagctgctggaggacaattttccaggggctataggtttccgaaaccacaagccttactcc

gtcagggttatctacggagacaccgactccgtgtttatcaagtttgtgggcctgacgtac

gagggggtatcggagctgggggatgctatgtcgcgtcagatttcagcggacctctttaga

gcgcccatcaaactggagtgtgagaagacctttcagcgactgctgctgatcaccaagaag

aagtacataggtgtcataaacggggggaagatgctcatgaagggggtcgacctggtccgc

aaaaataactgctctttcataaacttgtacgcgcgacatctggtagatcttttgttgtac

gacgaggatgtggccacggcggcagcagaggtgacagacgtgcctcccgcagaatgggtg

gggcgcccgctaccgagcggctttgacaagtttgggcgagtgctggtagaggcgtacaac

cgtatcactgcccccaacttggacgtgcgcgagttcgttatgactgctgagctgagccgc

tcacccgaatcgtataccaacaagcgcctgccgcacctcaccgtctactttaagctcgcc

atgaggaatgaagaactgcccagcgtaaaagagagaattccgtatgtgatagttgcgcag

accgaggccgcggaacgcgaagcgggtgtagtaaactcaatgcgcggtaccgcccaaaac

cccgtggtaaccaagaccgcacgcccccaacctaaacgcaaactgctggtttccgacctc

gccgaagacccgacctatgtttccgagaatgacgtaccgctaaacacagactactatttc

tcccacctgttgggtaccataagcgtgacctttaaggctctattcggaaatgatgtgaga

acaacagaaaatcttttaaagcggtttattccggaaaccccccacaagacccccacgaaa

acccaggcactgcttgagcgcgccggctttgaaaagctgacgccctttacaccggaggaa

gaaagtcgtcgaatactgcatacagttttttgtactctagaagcagctccccatcaaagc

tga

>OR085507.1

atggcggcgcgcgaacaggccaactccgttcgcaggagcgggttttttaatccgtttatt

ggcaagaggccatttttcaggccgggaagcgggcagaccgcggagacagagcgcccaagg

cccccccaacactcgtactgcacagaggtgggtagctttaagtttatagctccaagatgc

ctcgatgaagaagcccccgccgaccagcgacgcggtgtacacgtgggcaccttggagcgc

ccacccaaagtgtactgcgatggctcagagtacgacgtgctgaactttgcctccggaggt

tgctggcctcgcaggattcgggtttggaacggccaggattttcggggcgatggattcaac

cccagatttgagagatttcacgtgtacgacatagtagagacttcggagagcgcctcacac

gatgacccatccaggtttgctgagctatcccgcccaagcgggtctgtggtaacactgctg

ggaatgagcgagtgtggaaagcgagtcgcggttcacgtctatggtgtgcgccattatttt

tacatggcaaaggcggaggtggatagcgcttgtggaataaccaccgaggcagaactcgtg

cgcgcaatggtggactgcgcgcacagctcggctttgagcgcggccctgggaaatggcaac

ggcggcaaacagagcggcggcagcggcgggggatggtggggcggaaagcacgtgtctgcg

gactgcttcaaagtggagaccgtgtgccacacgacgctgtactactttggatctaagcca

gctctctactatagagtatctgcctccagcagccgcctgggagggttcatctgcgacaac

tttcacccggagattacaaaattcgaggggagcgtggacgtgaccacgcggctgttgttg

gacaatgaaaattttaccagtttcgggtggtaccgcctgcgacccggcacccacggagag

cgtgttcaacttcgccccgttgagcgacacgtcacctcaagcgacgtggagattaactgt

actcccgataacctggagccgataccagacgaggctgcctggcccgactataagctcatg

tgctttgatatagagtgtaaagctggaacgggtaacgaaatggcgttcccagtggcaact

aaccaagaggacctggtcatccagatctcctgtctgctgtactcgcttgctactcagaac

cacgaacacaccctgctgttttccctcgggtcatgcgatatctctgaggaatactcgttt

gcatgcgtccagcgcggcgagcccagaccgacggttttggagtttgacagcgagtacgag

ctgctggttgccttcctgacctttctcaagcagtactctcccgagttcgccaccggctac

aacatcgttaattttgactgggcgtacatagttaacaaggtaacgtcggtgtataacatc

aagctggacgggtacggcaagttcaacaaaggggggctgtttaaggtgtgggacatcgcc

acgaaccattttcagaagaagagcaaggtgaaaatcaatggcctgatatctctagacatg

tattctgtggcgacggaaaagctaaagctacccagctacaaactcgacgcggtcgtggga

gacgtcctcggcgagcataagatagaccttccctataaagaaataccctcctattacgcg

ggagggcctgaccggaggggcgtaataggagagtattgtatccaggactctaggctggtg

ggcaagctgttttttaagtacctcccccatctggaactatcggcggtggccaaactcgcc

cgtatcaccctaacgcgggtaatttttgacggtcagcaaattcgcgtgtacacgtgcttg

ctgaaactcgcccgcgagagaaatttcattttgccagacaacagacgccggtttgacagt

caggcagacgccgcgtcagagacttcggagttggctatggatagccaaagccacgccttc

gacagtacagacgaacccgacggtgtggacggtaccccggacgccgcaggatctggcgct

acttctgaaaacggaggcgggaagcccggcgtcgggagggccgtgggctaccagggagca

aaggttctagaccccgtatccggctttcatgtggaccccgtggttgtgtttgacttcgct

agcttatacccaagcattatccaggcccataacctctgtttcaccaccctggcgctcgat

gaagtggatctggccgggcttcaaccatccgtcaactactcgacgttcgaggtgggtgac

caaaagttattttttgtccacgcccatattcgcgaaagcctgcttggcatcttgctgcgc

gactggctggccatgcgaaaggcggtgagggcgcgaatccccaccagcacccccgaggag

gcagttttactagataagcagcagtctgcgattaaggtgatatgcaactcggtttacgga

ttcacgggggtggcaaacggcctgttgccgtgtctgaggatagcggctaccgttaccacg

ataggacgcgacatgctcctcaagacccgagattacgttcactctcgttgggcgacgcgc

gagctgctggaggacaattttccaggggctataggtttccgaaaccacaagccttactcc

gtcagggttatctacggagacaccgactccgtgtttatcaagtttgtgggcctgacgtac

gagggggtatcggagctgggggatgctatgtcgcgtcagatttcagcggacctctttaga

gcgcccatcaaactggagtgtgagaagacctttcagcgactgctgctgatcaccaagaag

aagtacataggtgtcataaacggggggaagatgctcatgaagggggtcgacctggtccgc

aaaaataactgctctttcataaacttgtacgcgcgacatctggtagatcttttgttgtac

gacgaggatgtggccacggcggcagcagaggtgacagacgtgcctcccgcagaatgggtg

gggcgcccgctaccgagcggctttgacaagtttgggcgagtgctggtagaggcgtacaac

cgtatcactgcccccaacttggacgtgcgcgagttcgttatgactgctgagctgagccgc

tcacccgaatcgtataccaacaagcgcctgccgcacctcaccgtctactttaagctcgcc

atgaggaatgaagaactgcccagcgtaaaagagagaattccgtatgtgatagttgcgcag

accgaggccgcggaacgcgaagcgggtgtagtaaactcaatgcgcggtaccgcccaaaac

cccgtggtaaccaagaccgcacgcccccaacctaaacgcaaactgctggtttccgacctc

gccgaagacccgacctatgtttccgagaatgacgtaccgctaaacacagactactatttc

tcccacctgttgggtaccataagcgtgacctttaaggctctattcggaaatgatgtgaga

acaacagaaaatcttttaaagcggtttattccggaaaccccccacaagacccccacgaaa

acccaggcactgcttgagcgcgccggctttgaaaagctgacgccctttacaccggaggaa

gaaagtcgtcgaatactgcatacagttttttgtactctagaagcagctccccatcaaagc

tga

>OR085508.1

atggcggcgcgcgaacaggccaactccgttcgcaggagcgggttttttaatccgtttatt

ggcaagaggccatttttcaggccgggaagcgggcagaccgcggagacagagcgcccaagg

cccccccaacactcgtactgcacagaggtgggtagctttaagtttatagctccaagatgc

ctcgatgaagaagcccccgccgaccagcgacgcggtgtacacgtgggcaccttggagcgc

ccacccaaagtgtactgcgatggctcagagtacgacgtgctgaactttgcctccggaggt

tgctggcctcgcaggattcgggtttggaacggccaggattttcggggcgatggattcaac

cccagatttgagagatttcacgtgtacgacatagtagagacttcggagagcgcctcacac

gatgacccatccaggtttgctgagctatcccgcccaagcgggtctgtggtaacactgctg

ggaatgagcgagtgtggaaagcgagtcgcggttcacgtctatggtgtgcgccattatttt

tacatggcaaaggcggaggtggatagcgcttgtggaataaccaccgaggcagaactcgtg

cgcgcaatggtggactgcgcgcacagctcggctttgagcgcggccctgggaaatggcaac

ggcggcaaacagagcggcggcagcggcgggggatggtggggcggaaagcacgtgtctgcg

gactgcttcaaagtggagaccgtgtgccacacgacgctgtactactttggatctaagcca

gctctctactatagagtatctgcctccagcagccgcctgggagggttcatctgcgacaac

tttcacccggagattacaaaattcgaggggagcgtggacgtgaccacgcggctgttgttg

gacaatgaaaattttaccagtttcgggtggtaccgcctgcgacccggcacccacggagag

cgtgttcaacttcgccccgttgagcgacacgtcacctcaagcgacgtggagattaactgt

actcccgataacctggagccgataccagacgaggctgcctggcccgactataagctcatg

tgctttgatatagagtgtaaagctggaacgggtaacgaaatggcgttcccagtggcaact

aaccaagaggacctggtcatccagatctcctgtctgctgtactcgcttgctactcagaac

cacgaacacaccctgctgttttccctcgggtcatgcgatatctctgaggaatactcgttt

gcatgcgtccagcgcggcgagcccaaaccgacggttttggagtttgacagcgagtacgag

ctgctggttgccttcctgacctttctcaagcagtactctcccgagttcgccaccggctac

aacatcgttaattttgactgggcgtacatagttaacaaggtaacgtcggtgtataacatc

aagctggacgggtacggcaagttcaacaaaggggggctgtttaaggtgtgggacatcgcc

acgaaccattttcagaagaagagcaaggtgaaaatcaatggcctgatatctctagacatg

tattctgtggcgacggaaaagctaaagctacccagctacaaactcgacgcggtcgtggga

gacgtcctcggcgagcataagatagaccttccctataaagaaataccctcctattacgcg

ggagggcctgaccggaggggcgtaataggagagtattgtatccaggactctaggctggtg

ggcaagctgttttttaagtacctcccccatctggaactatcggcggtggccaaactcgcc

cgtatcaccctaacgcgggtaatttttgacggtcagcaaattcgcgtgtacacgtgcttg

ctgaaactcgcccgcgagagaaatttcattttgccagacaacagacgccggtttgacagt

caggcagacgccgcgtcagagacttcggagttggctatggatagccaaagccacgccttc

gacagtacagacgaacccgacggtgtggacggtaccccggacgccgcaggatctggcgct

acttctgaaaacggaggcgggaagcccggcgtcgggagggccgtgggctaccagggagca

aaggttctagaccccgtatccggctttcatgtggaccccgtggttgtgtttgacttcgct

agcttatacccaagcattatccaggcccataacctctgtttcaccaccctggcgctcgat

gaagtggatctggccgggcttcaaccatccgtcaactactcgacgttcgaggtgggtgac

caaaagttattttttgtccacgcccatattcgcgaaagcctgcttggcatcttgctgcgc

gactggctggccatgcgaaaggcggtgagggcgcgaatccccaccagcacccccgaggag

gcagttttactagataagcagcagtctgcgattaaggtgatatgcaactcggtttacgga

ttcacgggggtggcaaacggcctgttgccgtgtctgaggatagcggctaccgttaccacg

ataggacgcgacatgctcctcaagacccgagattacgttcactctcgttgggcgacgcgc

gagctgctggaggacaattttccaggggctataggtttccgaaaccacaagccttactcc

gtcagggttatctacggagacaccgactccgtgtttatcaagtttgtgggcctgacgtac

gagggggtatcggagctgggggatgctatgtcgcgtcagatttcagcggacctctttaga

gcgcccatcaaactggagtgtgagaagacctttcagcgactgctgctgatcaccaagaag

aagtacataggtgtcataaacggggggaagatgctcatgaagggggtcgacctggtccgc

aaaaataactgctctttcataaacttgtacgcgcgacatctggtagatcttttgttgtac

gacgaggatgtggccacggcggcagcagaggtgacagacgtgcctcccgcagaatgggtg

gggcgcccgctaccgagcggctttgacaagtttgggcgagtgctggtagaggcgtacaac

cgtatcactgcccccaacttggacgtgcgcgagttcgttatgactgctgagctgagccgc

tcacccgaatcgtataccaacaagcgcctgccgcacctcaccgtctactttaagctcgcc

atgaggaatgaagaactgcccagcgtaaaagagagaattccgtatgtgatagttgcgcag

accgaggccgcggaacgcgaagcgggtgtagtaaactcaatgcgcggtaccgcccaaaac

cccgtggtaaccaagaccgcacgcccccaacctaaacgcaaactgctggtttccgacctc

gccgaagacccgacctatgtttccgagaatgacgtaccgctaaacacagactactatttc

tcccacctgttgggtaccataagcgtgacctttaaggctctattcggaaatgatgtgaga

acaacagaaaatcttttaaagcggtttattccggaaaccccccacaagacccccacgaaa

acccaggcactgcttgagcgcgccggctttgaaaagctgacgccctttacaccggaggaa

gaaagtcgtcgaatactgcatacagttttttgtactctagaagcagctccccatcaaagc

tga

>OR085509.1

atggcggcgcgcgaacaggccaactccgttcgcaggagcgggttttttaatccgtttatt

ggcaagaggccatttttcaggccgggaagcgggcagaccgcggagacagagcgcccaagg

cccccccaacactcgtactgcacagaggtgggtagctttaagtttatagctccaagatgc

ctcgatgaagaagcccccgccgaccagcgacgcggtgtacacgtgggcaccttggagcgc

ccacccaaagtgtactgcgatggctcagagtacgacgtgctgaactttgcctccggaggt

tgctggcctcgcaggattcgggtttggaacggccaggattttcggggcgatggattcaac

cccagatttgagagatttcacgtgtacgacatagtagagacttcggagagcgcctcacac

gatgacccatccaggtttgctgagctatcccgcccaagcgggtctgtggtaacactgctg

ggaatgagcgagtgtggaaagcgagtcgcggttcacgtctatggtgtgcgccattatttt

tacatggcaaaggcggaggtggatagcgcttgtggaataaccaccgaggcagaactcgtg

cgcgcaatggtggactgcgcgcacagctcggctttgagcgcggccctgggaaatggcaac

ggcggcaaacagagcggcggcagcggcgggggatggtggggcggaaagcacgtgtctgcg

gactgcttcaaagtggagaccgtgtgccacacgacgctgtactactttggatctaagcca

gctctctactatagagtatctgcctccagcagccgcctgggagggttcatctgcgacaac

tttcacccggagattacaaaattcgaggggagcgtggacgtgaccacgcggctgttgttg

gacaatgaaaattttaccagtttcgggtggtaccgcctgcgacccggcacccacggagag

cgtgttcaacttcgccccgttgagcgacacgtcacctcaagcgacgtggagattaactgt

actcccgataacctggagccgataccagacgaggctgcctggcccgactataagctcatg

tgctttgatatagagtgtaaagctggaacgggtaacgaaatggcgttcccagtggcaact

aaccaagaggacctggtcatccagatctcctgtctgctgtactcgcttgctactcagaac

cacgaacacaccctgctgttttccctcgggtcatgcgatatctctgaggaatactcgttt

gcatgcgtccagcgcggcgagcccagaccgacggttttggagtttgacagcgagtacgag

ctgctggttgccttcctgacctttctcaagcagtactctcccgagttcgccaccggctac

aacatcgttaattttgactgggcgtacatagttaacaaggtaacgtcggtgtataacatc

aagctggacgggtacggcaagttcaacaaaggggggctgtttaaggtgtgggacatcgcc

acgaaccattttcagaagaagagcaaggtgaaaatcaatggcctgatatctctagacatg

tattctgtggcgacggaaaagctaaagctacccagctacaaactcgacgcggtcgtggga

gacgtcctcggcgagcataagatagaccttccctataaagaaataccctcctattacgcg

ggagggcctgaccggaggggcgtaataggagagtattgtatccaggactctaggctggtg

ggcaagctgttttttaagtacctcccccatctggaactatcggcggtggccaaactcgcc

cgtatcaccctaacgcgggtaatttttgacggtcagcaaattcgcgtgtacacgtgcttg

ctgaaactcgcccgcgagagaaatttcattttgccagacaacagacgccggtttgacagt

caggcagacgccgcgtcagagacttcggagttggctatggatagccaaagccacgccttc

gacagtacagacgaacccgacggtgtggacggtaccccggacgccgcaggatctggcgct

acttctgaaaacggaggcgggaagcccggcgtcgggagggccgtgggctaccagggagca

aaggttctagaccccgtatccggctttcatgtggaccccgtggttgtgtttgacttcgct

agcttatacccaagcattatccaggcccataacctctgtttcaccaccctggcgctcgat

gaagtggatctggccgggcttcaaccatccgtcaactactcgacgttcgaggtgggtgac

caaaagttattttttgtccacgcccatattcgcgaaagcctgcttggcatcttgctgcgc

gactggctggccatgcgaaaggcggtgagggcgcgaatccccaccagcacccccgaggag

gcagttttactagataagcagcagtctgcgattaaggtgatatgcaactcggtttacgga

ttcacgggggtggcaaacggcctgttgccgtgtctgaggatagcggctaccgttaccacg

ataggacgcgacatgctcctcaagacccgagattacgttcactctcgttgggcgacgcgc

gagctgctggaggacaattttccaggggctataggtttccgaaaccacaagccttactcc

gtcagggttatctacggagacaccgactccgtgtttatcaagtttgtgggcctgacgtac

gagggggtatcggagctgggggatgctatgtcgcgtcagatttcagcggacctctttaga

gcgcccatcaaactggagtgtgagaagacctttcagcgactgctgctgatcaccaagaag

aagtacataggtgtcataaacggggggaagatgctcatgaagggggtcgacctggtccgc

aaaaataactgctctttcataaacttgtacgcgcgacatctggtagatcttttgttgtac

gacgaggatgtggccacggcggcagcagaggtgacagacgtgcctcccgcagaatgggtg

gggcgcccgctaccgagcggctttgacaagtttgggcgagtgctggtagaggcgtacaac

cgtatcactgcccccaacttggacgtgcgcgagttcgttatgactgctgagctgagccgc

tcacccgaatcgtataccaacaagcgcctgccgcacctcaccgtctactttaagctcgcc

atgaggaatgaagaactgcccagcgtaaaagagagaattccgtatgtgatagttgcgcag

accgaggccgcggaacgcgaagcgggtgtagtaaactcaatgcgcggtaccgcccaaaac

cccgtggtaaccaagaccgcacgcccccaacctaaacgcaaactgctggtttccgacctc

gccgaagacccgacctatgtttccgagaatgacgtaccgctaaacacagactactatttc

tcccacctgttgggtaccataagcgtgacctttaaggctctattcggaaatgatgtgaga

acaacagaaaatcttttaaagcggtttattccggaaaccccccacaagacccccacgaaa

acccaggcactgcttgagcgcgccggctttgaaaagctgacgccctttacaccggaggaa

gaaagtcgtcgaatactgcatacagttttttgtactctagaagcagctccccatcaaagc

tga

>OR085510.1

atggcggcgcgcgaacaggccaactccgttcgcaggagcgggttttttaatccgtttatt

ggcaagaggccatttttcaggccgggaagcgggcagaccgcggagacagagcgcccaagg

cccccccaacactcgtactgcacagaggtgggtagctttaagtttatagctccaagatgc

ctcgatgaagaagcccccgccgaccagcgacgcggtgtacacgtgggcaccttggagcgc

ccacccaaagtgtactgcgatggctcagagtacgacgtgctgaactttgcctccggaggt

tgctggcctcgcaggattcgggtttggaacggccaggattttcggggcgatggattcaac

cccagatttgagagatttcacgtgtacgacatagtagagacttcggagagcgcctcacac

gatgacccatccaggtttgctgagctatcccgcccaagcgggtctgtggtaacactgctg

ggaatgagcgagtgtggaaagcgagtcgcggttcacgtctatggtgtgcgccattatttt

tacatggcaaaggcggaggtggatagcgcttgtggaataaccaccgaggcagaactcgtg

cgcgcaatggtggactgcgcgcacagctcggctttgagcgcggccctgggaaatggcaac

ggcggcaaacagagcggcggcagcggcgggggatggtggggcggaaagcacgtgtctgcg

gactgcttcaaagtggagaccgtgtgccacacgacgctgtactactttggatctaagcca

gctctctactatagagtatctgcctccagcagccgcctgggagggttcatctgcgacaac

tttcacccggagattacaaaattcgaggggagcgtggacgtgaccacgcggctgttgttg

gacaatgaaaattttaccagtttcgggtggtaccgcctgcgacccggcacccacggagag

cgtgttcaacttcgccccgttgagcgacacgtcacctcaagcgacgtggagattaactgt

actcccgataacctggagccgataccagacgaggctgcctggcccgactataagctcatg

tgctttgatatagagtgtaaagctggaacgggtaacgaaatggcgttcccagtggcaact

aaccaagaggacctggtcatccagatctcctgtctgctgtactcgcttgctactcagaac

cacgaacacaccctgctgttttccctcgggtcatgcgatatctctgaggaatactcgttt

gcatgcgtccagcgcggcgagcccagaccgacggttttggagtttgacagcgagtacgag

ctgctggttgccttcctgacctttctcaagcagtactctcccgagttcgccaccggctac

aacatcgttaattttgactgggcgtacatagttaacaaggtaacgtcggtgtataacatc

aagctggacgggtacggcaagttcaacaaaggggggctgtttaaggtgtgggacatcgcc

acgaaccattttcagaagaagagcaaggtgaaaatcaatggcctgatatctctagacatg

tattctgtggcgacggaaaagctaaagctacccagctacaaactcgacgcggtcgtggga

gacgtcctcggcgagcataagatagaccttccctataaagaaataccctcctattacgcg

ggagggcctgaccggaggggcgtaataggagagtattgtatccaggactctaggctggtg

ggcaagctgttttttaagtacctcccccatctggaactatcggcggtggccaaactcgcc

cgtatcaccctaacgcgggtaatttttgacggtcagcaaattcgcgtgtacacgtgcttg

ctgaaactcgcccgcgagagaaatttcattttgccagacaacagacgccggtttgacagt

caggcagacgccgcgtcagagacttcggagttggctatggatagccaaagccacgccttc

gacagtacagacgaacccgacggtgtggacggtaccccggacgccgcaggatctggcgct

acttctgaaaacggaggcgggaagcccggcgtcgggagggccgtgggctaccagggagca

aaggttctagaccccgtatccggctttcatgtggaccccgtggttgtgtttgacttcgct

agcttatacccaagcattatccaggcccataacctctgtttcaccaccctggcgctcgat

gaagtggatctggccgggcttcaaccatccgtcaactactcgacgttcgaggtgggtgac

caaaagttattttttgtccacgcccatattcgcgaaagcctgcttggcatcttgctgcgc

gactggctggccatgcgaaaggcggtgagggcgcgaatccccaccagcacccccgaggag

gcagttttactagataagcagcagtctgcgattaaggtgatatgcaactcggtttacgga

ttcacgggggtggcaaacggcctgttgccgtgtctgaggatagcggctaccgttaccacg

ataggacgcgacatgctcctcaagacccgagattacgttcactctcgttgggcgacgcgc

gagctgctggaggacaattttccaggggctataggtttccgaaaccacaagccttactcc

gtcagggttatctacggagacaccgactccgtgtttatcaagtttgtgggcctgacgtac

gagggggtatcggagctgggggatgctatgtcgcgtcagatttcagcggacctctttaga

gcgcccatcaaactggagtgtgagaagacctttcagcgactgctgctgatcaccaagaag

aagtacataggtgtcataaacggggggaagatgctcatgaagggggtcgacctggtccgc

aaaaataactgctctttcataaacttgtacgcgcgacatctggtagatcttttgttgtac

gacgaggatgtggccacggcggcagcagaggtgacagacgtgcctcccgcagaatgggtg

gggcgcccgctaccgagcggctttgacaagtttgggcgagtgctggtagaggcgtacaac

cgtatcactgcccccaacttggacgtgcgcgagttcgttatgactgctgagctgagccgc

tcacccgaatcgtataccaacaagcgcctgccgcacctcaccgtctactttaagctcgcc

atgaggaatgaagaactgcccagcgtaaaagagagaattccgtatgtgatagttgcgcag

accgaggccgcggaacgcgaagcgggtgtagtaaactcaatgcgcggtaccgcccaaaac

cccgtggtaaccaagaccgcacgcccccaacctaaacgcaaactgctggtttccgacctc

gccgaagacccgacctatgtttccgagaatgacgtaccgctaaacacagactactatttc

tcccacctgttgggtaccataagcgtgacctttaaggctctattcggaaatgatgtgaga

acaacagaaaatcttttaaagcggtttattccggaaaccccccacaagacccccacgaaa

acccaggcactgcttgagcgcgccggctttgaaaagctgacgccctttacaccggaggaa

gaaagtcgtcgaatactgcatacagttttttgtactctagaagcagctccccatcaaagc

tga

>OR085511.1

atggcggcgcgcgaacaggccaactccgttcgcaggagcgggttttttaatccgtttatt

ggcaagaggccatttttcaggccgggaagcgggcagaccgcggagacagagcgcccaagg

cccccccaacactcgtactgcacagaggtgggtagctttaagtttatagctccaagatgc

ctcgatgaagaagcccccgccgaccagcgacgcggtgtacacgtgggcaccttggagcgc

ccacccaaagtgtactgcgatggctcagagtacgacgtgctgaactttgcctccggaggt

tgctggcctcgcaggattcgggtttggaacggccaggattttcggggcgatggattcaac

cccagatttgagagatttcacgtgtacgacatagtagagacttcggagagcgcctcacac

gatgacccatccaggtttgctgagctatcccgcccaagcgggtctgtggtaacactgctg

ggaatgagcgagtgtggaaagcgagtcgcggttcacgtctatggtgtgcgccattatttt

tacatggcaaaggcggaggtggatagcgcttgtggaataaccaccgaggcagaactcgtg

cgcgcaatggtggactgcgcgcacagctcggctttgagcgcggccctgggaaatggcaac

ggcggcaaacagagcggcggcagcggcgggggatggtggggcggaaagcacgtgtctgcg

gactgcttcaaagtggagaccgtgtgccacacgacgctgtactactttggatctaagcca

gctctctactatagagtatctgcctccagcagccgcctgggagggttcatctgcgacaac

tttcacccggagattacaaaattcgaggggagcgtggacgtgaccacgcggctgttgttg

gacaatgaaaattttaccagtttcgggtggtaccgcctgcgacccggcacccacggagag

cgtgttcaacttcgccccgttgagcgacacgtcacctcaagcgacgtggagattaactgt

actcccgataacctggagccgataccagacgaggctgcctggcccgactataagctcatg

tgctttgatatagagtgtaaagctggaacgggtaacgaaatggcgttcccagtggcaact

aaccaagaggacctggtcatccagatctcctgtctgctgtactcgcttgctactcagaac

cacgaacacaccctgctgttttccctcgggtcatgcgatatctctgaggaatactcgttt

gcatgcgtccagcgcggcgagcccagaccgacggttttggagtttgacagcgagtacgag

ctgctggttgccttcctgacctttctcaagcagtactctcccgagttcgccaccggctac

aacatcgttaattttgactgggcgtacatagttaacaaggtaacgtcggtgtataacatc

aagctggacgggtacggcaagttcaacaaaggggggctgtttaaggtgtgggacatcgcc

acgaaccattttcagaagaagagcaaggtgaaaatcaatggcctgatatctctagacatg

tattctgtggcgacggaaaagctaaagctacccagctacaaactcgacgcggtcgtggga

gacgtcctcggcgagcataagatagaccttccctataaagaaataccctcctattacgcg

ggagggcctgaccggaggggcgtaataggagagtattgtatccaggactctaggctggtg

ggcaagctgttttttaagtacctcccccatctggaactatcggcggtggccaaactcgcc

cgtatcaccctaacgcgggtaatttttgacggtcagcaaattcgcgtgtacacgtgcttg

ctgaaactcgcccgcgagagaaatttcattttgccagacaacagacgccggtttgacagt

caggcagacgccgcgtcagagacttcggagttggctatggatagccaaagccacgccttc

gacagtacagacgaacccgacggtgtggacggtaccccggacgccgcaggatctggcgct

acttctgaaaacggaggcgggaagcccggcgtcgggagggccgtgggctaccagggagca

aaggttctagaccccgtatccggctttcatgtggaccccgtggttgtgtttgacttcgct

agcttatacccaagcattatccaggcccataacctctgtttcaccaccctggcgctcgat

gaagtggatctggccgggcttcaaccatccgtcaactactcgacgttcgaggtgggtgac

caaaagttattttttgtccacgcccatattcgcgaaagcctgcttggcatcttgctgcgc

gactggctggccatgcgaaaggcggtgagggcgcgaatccccaccagcacccccgaggag

gcagttttactagataagcagcagtctgcgattaaggtgatatgcaactcggtttacgga

ttcacgggggtggcaaacggcctgttgccgtgtctgaggatagcggctaccgttaccacg

ataggacgcgacatgctcctcaagacccgagattacgttcactctcgttgggcgacgcgc

gagctgctggaggacaattttccaggggctataggtttccgaaaccacaagccttactcc

gtcagggttatctacggagacaccgactccgtgtttatcaagtttgtgggcctgacgtac

gagggggtatcggagctgggggatgctatgtcgcgtcagatttcagcggacctctttaga

gcgcccatcaaactggagtgtgagaagacctttcagcgactgctgctgatcaccaagaag

aagtacataggtgtcataaacggggggaagatgctcatgaagggggtcgacctggtccgc

aaaaataactgctctttcataaacttgtacgcgcgacatctggtagatcttttgttgtac

gacgaggatgtggccacggcggcagcagaggtgacagacgtgcctcccgcagaatgggtg

gggcgcccgctaccgagcggctttgacaagtttgggcgagtgctggtagaggcgtacaac

cgtatcactgcccccaacttggacgtgcgcgagttcgttatgactgctgagctgagccgc

tcacccgaatcgtataccaacaagcgcctgccgcacctcaccgtctactttaagctcgcc

atgaggaatgaagaactgcccagcgtaaaagagagaattccgtatgtgatagttgcgcag

accgaggccgcggaacgcgaagcgggtgtagtaaactcaatgcgcggtaccgcccaaaac

cccgtggtaaccaagaccgcacgcccccaacctaaacgcaaactgctggtttccgacctc

gccgaagacccgacctatgtttccgagaatgacgtaccgctaaacacagactactatttc

tcccacctgttgggtaccataagcgtgacctttaaggctctattcggaaatgatgtgaga

acaacagaaaatcttttaaagcggtttattccggaaaccccccacaagacccccacgaaa

acccaggcactgcttgagcgcgccggctttgaaaagctgacgccctttacaccggaggaa

gaaagtcgtcgaatactgcatacagttttttgtactctagaagcagctccccatcaaagc

tga

>OR085512.1

atggcggcgcgcgaacaggccaactccgttcgcaggagcgggttttttaatccgtttatt

ggcaagaggccatttttcaggccgggaagcgggcagaccgcggagacagagcgcccaagg

cccccccaacactcgtactgcacagaggtgggtagctttaagtttatagctccaagatgc

ctcgatgaagaagcccccgccgaccagcgacgcggtgtacacgtgggcaccttggagcgc

ccacccaaagtgtactgcgatggctcagagtacgacgtgctgaactttgcctccggaggt

tgctggcctcgcaggattcgggtttggaacggccaggattttcggggcgatggattcaac

cccagatttgagagatttcacgtgtacgacatagtagagacttcggagagcgcctcacac

gatgacccatccaggtttgctgagctatcccgcccaagcgggtctgtggtaacactgctg

ggaatgagcgagtgtggaaagcgagtcgcggttcacgtctatggtgtgcgccattatttt

tacatggcaaaggcggaggtggatagcgcttgtggaataaccaccgaggcagaactcgtg

cgcgcaatggtggactgcgcgcacagctcggctttgagcgcggccctgggaaatggcaac

ggcggcaaacagagcggcggcagcggcgggggatggtggggcggaaagcacgtgtctgcg

gactgcttcaaagtggagaccgtgtgccacacgacgctgtactactttggatctaagcca

gctctctactatagagtatctgcctccagcagccgcctgggagggttcatctgcgacaac

tttcacccggagattacaaaattcgaggggagcgtggacgtgaccacgcggctgttgttg

gacaatgaaaattttaccagtttcgggtggtaccgcctgcgacccggcacccacggagag

cgtgttcaacttcgccccgttgagcgacacgtcacctcaagcgacgtggagattaactgt

actcccgataacctggagccgataccagacgaggctgcctggcccgactataagctcatg

tgctttgatatagagtgtaaagctggaacgggtaacgaaatggcgttcccagtggcaact

aaccaagaggacctggtcatccagatctcctgtctgctgtactcgcttgctactcagaac

cacgaacacaccctgctgttttccctcgggtcatgcgatatctctgaggaatactcgttt

gcatgcgtccagcgcggcgagcccagaccgacggttttggagtttgacagcgagtacgag

ctgctggttgccttcctgacctttctcaagcagtactctcccgagttcgccaccggctac

aacatcgttaattttgactgggcgtacatagttaacaaggtaacgtcggtgtataacatc

aagctggacgggtacggcaagttcaacaaaggggggctgtttaaggtgtgggacatcgcc

acgaaccattttcagaagaagagcaaggtgaaaatcaatggcctgatatctctagacatg

tattctgtggcgacggaaaagctaaagctacccagctacaaactcgacgcggtcgtggga

gacgtcctcggcgagcataagatagaccttccctataaagaaataccctcctattacgcg

ggagggcctgaccggaggggcgtaataggagagtattgtatccaggactctaggctggtg

ggcaagctgttttttaagtacctcccccatctggaactatcggcggtggccaaactcgcc

cgtatcaccctaacgcgggtaatttttgacggtcagcaaattcgcgtgtacacgtgcttg

ctgaaactcgcccgcgagagaaatttcattttgccagacaacagacgccggtttgacagt

caggcagacgccgcgtcagagacttcggagttggctatggatagccaaagccacgccttc

gaccgtacagacgaacccgacggtgtggacggtaccccggacgccgcaggatctggcgct

acttctgaaaacggaggcgggaagcccggcgtcgggagggccgtgggctaccagggagca

aaggttctagaccccgtatccggctttcatgtggaccccgtggttgtgtttgacttcgct

agcttatacccaagcattatccaggcccataacctctgtttcaccaccctggcgctcgat

gaagtggatctggccgggcttcaaccatccgtcaactactcgacgttcgaggtgggtgac

caaaagttattttttgtccacgcccatattcgcgaaagcctgcttggcatcttgctgcgc

gactggctggccatgcgaaaggcggtgagggcgcgaatccccaccagcacccccgaggag

gcagttttactagataagcagcagtctgcgattaaggtgatatgcaactcggtttacgga

ttcacgggggtggcaaacggcctgttgccgtgtctgaggatagcggctaccgttaccacg

ataggacgcgacatgctcctcaagacccgagattacgttcactctcgttgggcgacgcgc

gagctgctggaggacaattttccaggggctataggtttccgaaaccacaagccttactcc

gtcagggttatctacggagacaccgactccgtgtttatcaagtttgtgggcctgacgtac

gagggggtatcggagctgggggatgctatgtcgcgtcagatttcagcggacctctttaga

gcgcccatcaaactggagtgtgagaagacctttcagcgactgctgctgatcaccaagaag

aagtacataggtgtcataaacggggggaagatgctcatgaagggggtcgacctggtccgc

aaaaataactgctctttcataaacttgtacgcgcgacatctggtagatcttttgttgtac

gacgaggatgtggccacggcggcagcagaggtgacagacgtgcctcccgcagaatgggtg

gggcgcccgctaccgagcggctttgacaagtttgggcgagtgctggtagaggcgtacaac

cgtatcactgcccccaacttggacgtgcgcgagttcgttatgactgctgagctgagccgc

tcacccgaatcgtataccaacaagcgcctgccgcacctcaccgtctactttaagctcgcc

atgaggaatgaagaactgcccagcgtaaaagagagaattccgtatgtgatagttgcgcag

accgaggccgcggaacgcgaagcgggtgtagtaaactcaatgcgcggtaccgcccaaaac

cccgtggtaaccaagaccgcacgcccccaacctaaacgcaaactgctggtttccgacctc

gccgaagacccgacctatgtttccgagaatgacgtaccgctaaacacagactactatttc

tcccacctgttgggtaccataagcgtgacctttaaggctctattcggaaatgatgtgaga

acaacagaaaatcttttaaagcggtttattccggaaaccccccacaagacccccacgaaa

acccaggcactgcttgagcgcgccggctttgaaaagctgacgccctttacaccggaggaa

gaaagtcgtcgaatactgcatacagttttttgtactctagaagcagctccccatcaaagc

tga

>OR085513.1

atggcggcgcgcgaacaggccaactccgttcgcaggagcgggttttttaatccgtttatt

ggcaagaggccatttttcaggccgggaagcgggcagaccgcggagacagagcgcccaagg

cccccccaacactcgtactgcacagaggtgggtagctttaagtttatagctccaagatgc

ctcgatgaagaagcccccgccgaccagcgacgcggtgtacacgtgggcaccttggagcgc

ccacccaaagtgtactgcgatggctcagagtacgacgtgctgaactttgcctccggaggt

tgctggcctcgcaggattcgggtttggaacggccaggattttcggggcgatggattcaac

cccagatttgagagatttcacgtgtacgacatagtagagacttcggagagcgcctcacac

gatgacccatccaggtttgctgagctatcccgcccaagcgggtctgtggtaacactgctg

ggaatgagcgagtgtggaaagcgagtcgcggttcacgtctatggtgtgcgccattatttt

tacatggcaaaggcggaggtggatagcgcttgtggaataaccaccgaggcagaactcgtg

cgcgcaatggtggactgcgcgcacagctcggctttgagcgcggccctgggaaatggcaac

ggcggcaaacagagcggcggcagcggcgggggatggtggggcggaaagcacgtgtctgcg

gactgcttcaaagtggagaccgtgtgccacacgacgctgtactactttggatctaagcca

gctctctactatagagtatctgcctccagcagccgcctgggagggttcatctgcgacaac

tttcacccggagattacaaaattcgaggggagcgtggacgtgaccacgcggctgttgttg

gacaatgaaaattttaccagtttcgggtggtaccgcctgcgacccggcacccacggagag

cgtgttcaacttcgccccgttgagcgacacgtcacctcaagcgacgtggagattaactgt

actcccgataacctggagccgataccagacgaggctgcctggcccgactataagctcatg

tgctttgatatagagtgtaaagctggaacgggtaacgaaatggcgttcccagtggcaact

aaccaagaggacctggtcatccagatctcctgtctgctgtactcgcttgctactcagaac

cacgaacacaccctgctgttttccctcgggtcatgcgatatctctgaggaatactcgttt

gcatgcgtccagcgcggcgagcccagaccgacggttttggagtttgacagcgagtacgag

ctgctggttgccttcctgacctttctcaagcagtactctcccgagttcgccaccggctac

aacatcgttaattttgactgggcgtacatagttaacaaggtaacgtcggtgtataacatc

aagctggacgggtacggcaagttcaacaaaggggggctgtttaaggtgtgggacatcgcc

acgaaccattttcagaagaagagcaaggtgaaaatcaatggcctgatatctctagacatg

tattctgtggcgacggaaaagctaaagctacccagctacaaactcgacgcggtcgtggga

gacgtcctcggcgagcataagatagaccttccctataaagaaataccctcctattacgcg

ggagggcctgaccggaggggcgtaataggagagtattgtatccaggactctaggctggtg

ggcaagctgttttttaagtacctcccccatctggaactatcggcggtggccaaactcgcc

cgtatcaccctaacgcgggtaatttttgacggtcagcaaattcgcgtgtacacgtgcttg

ctgaaactcgcccgcgagagaaatttcattttgccagacaacagacgccggtttgacagt

caggcagacgccgcgtcagagacttcggagttggctatggatagccaaagccacgccttc

gacagtacagacgaacccgacggtgtggacggtaccccggacgccgcaggatctggcgct

acttctgaaaacggaggcgggaagcccggcgtcgggagggccgtgggctaccagggagca

aaggttctagaccccgtatccggctttcatgtggaccccgtggttgtgtttgacttcgct

agcttatacccaagcattatccaggcccataacctctgtttcaccaccctggcgctcgat

gaagtggatctggccgggcttcaaccatccgtcaactactcgacgttcgaggtgggtgac

caaaagttattttttgtccacgcccatattcgcgaaagcctgcttggcatcttgctgcgc

gactggctggctatgcgaaaggcggtgagggcgcgaatccccaccagcacccccgaggag

gcagttttactagataagcagcagtctgcgattaaggtgatatgcaactcggtttacgga

ttcacgggggtggcaaacggcctgttgccgtgtctgaggatagcggctaccgttaccacg

ataggacgcgacatgctcctcaagacccgagattacgttcactctcgttgggcgacgcgc

gagctgctggaggacaattttccaggggctataggtttccgaaaccacaagccttactcc

gtcagggttatctacggagacaccgactccgtgtttatcaagtttgtgggcctgacgtac

gagggggtatcggagctgggggatgctatgtcgcgtcagatttcagcggacctctttaga

gcgcccatcaaactggagtgtgagaagacctttcagcgactgctgctgatcaccaagaag

aagtacataggtgtcataaacggggggaagatgctcatgaagggggtcgacctggtccgc

aaaaataactgctctttcataaacttgtacgcgcgacatctggtagatcttttgttgtac

gacgaggatgtggccacggcggcagcagaggtgacagacgtgcctcccgcagaatgggtg

gggcgcccgctaccgagcggctttgacaagtttgggcgagtgctggtagaggcgtacaac

cgtatcactgcccccaacttggacgtgcgcgagttcgttatgactgctgagctgagccgc

tcacccgaatcgtataccaacaagcgcctgccgcacctcaccgtctactttaagctcgcc

atgaggaatgaagaactgcccagcgtaaaagagagaattccgtatgtgatagttgcgcag

accgaggccgcggaacgcgaagcgggtgtagtaaactcaatgcgcggtaccgcccaaaac

cccgtggtaaccaagaccgcacgcccccaacctaaacgcaaactgctggtttccgacctc

gccgaagacccgacctatgtttccgagaatgacgtaccgctaaacacagactactatttc

tcccacctgttgggtaccataagcgtgacctttaaggctctattcggaaatgatgtgaga

acaacagaaaatcttttaaagcggtttattccggaaaccccccacaagacccccacgaaa

acccaggcactgcttgagcgcgccggctttgaaaagctgacgccctttacaccggaggaa

gaaagtcgtcgaatactgcatacagttttttgtactctagaagcagctccccatcaaagc

tga

>OR085514.1

atggcggcgcgcgaacaggccaactccgttcgcaggagcgggttttttaatccgtttatt

ggcaagaggccatttttcaggccgggaagcgggcagaccgcggagacagagcgcccaagg

cccccccaacactcgtactgcacagaggtgggtagctttaagtttatagctccaagatgc

ctcgatgaagaagcccccgccgaccagcgacgcggtgtacacgtgggcaccttggagcgc

ccacccaaagtgtactgcgatggctcagagtacgacgtgctgaactttgcctccggaggt

tgctggcctcgcaggattcgggtttggaacggccaggattttcggggcgatggattcaac

cccagatttgagagatttcacgtgtacgacatagtagagacttcggagagcgcctcacac

gatgacccatccaggtttgctgagctatcccgcccaagcgggtctgtggtaacactgctg

ggaatgagcgagtgtggaaagcgagtcgcggttcacgtctatggtgtgcgccattatttt

tacatggcaaaggcggaggtggatagcgcttgtggaataaccaccgaggcagaactcgtg

cgcgcaatggtggactgcgcgcacagctcggctttgagcgcggccctgggaaatggcaac

ggcggcaaacagagcggcggcagcggcgggggatggtggggcggaaagcacgtgtctgcg

gactgcttcaaagtggagaccgtgtgccacacgacgctgtactactttggatctaagcca

gctctctactatagagtatctgcctccagcagccgcctgggagggttcatctgcgacaac

tttcacccggagattacaaaattcgaggggagcgtggacgtgaccacgcggctgttgttg

gacaatgaaaattttaccagtttcgggtggtaccgcctgcgacccggcacccacggagag

cgtgttcaacttcgccccgttgagcgacacgtcacctcaagcgacgtggagattaactgt

actcccgataacctggagccgataccagacgaggctgcctggcccgactataagctcatg

tgctttgatatagagtgtaaagctggaacgggtaacgaaatggcgttcccagtggcaact

aaccaagaggacctggtcatccagatctcctgtctgctgtactcgcttgctactcagaac

cacgaacacaccctgctgttttccctcgggtcatgcgatatctctgaggaatactcgttt

gcatgcgtccagcgcggcgagcccagaccgacggttttggagtttgacagcgagtacgag

ctgctggttgccttcctgacctttctcaagcagtactctcccgagttcgccaccggctac

aacatcgttaattttgactgggcgtacatagttaacaaggtaacgtcggtgtataacatc

aagctggacgggtacggcaagttcaacaaaggggggctgtttaaggtgtgggacatcgcc

acgaaccattttcagaagaagagcaaggtgaaaatcaatggcctgatatctctagacatg

tattctgtggcgacggaaaagctaaagctacccagctacaaactcgacgcggtcgtggga

gacgtcctcggcgagcataagatagaccttccctataaagaaataccctcctattacgcg

ggagggcctgaccggaggggcgtaataggagagtattgtatccaggactctaggctggtg

ggcaagctgttttttaagtacctcccccatctggaactatcggcggtggccaaactcgcc

cgtatcaccctaacgcgggtaatttttgacggtcagcaaattcgcgtgtacacgtgcttg

ctgaaactcgcccgcgagagaaatttcattttgccagacaacagacgccggtttgacagt

caggcagacgccgcgtcagagacttcggagttggctatggatagccaaagccacgccttc

gacagtacagacgaacccgacggtgtggacggtaccccggacgccgcaggatctggcgct

acttctgaaaacggaggcgggaagcccggcgtcgggagggccgtgggctaccagggagca

aaggttctagaccccgtatccggctttcatgtggaccccgtggttgtgtttgacttcgct

agcttatacccaagcattatccaggcccataacctctgtttcaccaccctggcgctcgat

gaagtggatctggccgggcttcaaccatccgtcaactactcgacgttcgaggtgggtgac

caaaagttattttttgtccacgcccatattcgcgaaagcctgcttggcatcttgctgcgc

gactggctggccatgcgaaaggcggtgagggcgcgaatccccaccagcacccccgaggag

gcagttttactagataagcagcagtctgcgattaaggtgatatgcaactcggtttacgga

ttcacgggggtggcaaacggcctgttgccgtgtctgaggatagcggctaccgttaccacg

ataggacgcgacatgctcctcaagacccgagattacgttcactctcgttgggcgacgcgc

gagctgctggaggacaattttccaggggctataggtttccgaaaccacaagccttactcc

gtcagggttatctacggagacaccgactccgtgtttatcaagtttgtgggcctgacgtac

gagggggtatcggagctgggggatgctatgtcgcgtcagatttcagcggacctctttaga

gcgcccatcaaactggagtgtgagaagacctttcagcgactgctgctgatcaccaagaag

aagtacataggtgtcataaacggggggaagatgctcatgaagggggtcgacctggtccgc

aaaaataactgctctttcataaacttgtacgcgcgacatctggtagatcttttgttgtac

gacgaggatgtggccacggcggcagcagaggtgacagacgtgcctcccgcagaatgggtg

gggcgcccgctaccgagcggctttgacaagtttgggcgagtgctggtagaggcgtacaac

cgtatcactgcccccaacttggacgtgcgcgagttcgttatgactgctgagctgagccgc

tcacccgaatcgtataccaacaagcgcctgccgcacctcaccgtctactttaagctcgcc

atgaggaatgaagaactgcccagcgtaaaagagagaattccgtatgtgatagttgcgcag

accgaggccgcggaacgcgaagcgggtgtagtaaactcaatgcgcggtaccgcccaaaac

cccgtggtaaccaagaccgcacgcccccaacctaaacgcaaactgctggtttccgacctc

gccgaagacccgacctatgtttccgagaatgacgtaccgctaaacacagactactatttc

tcccacctgttgggtaccataagcgtgacctttaaggctctattcggaaatgatgtgaga

acaacagaaaatcttttaaagcggtttattccggaaaccccccacaagacccccacgaaa

acccaggcactgcttgagcgcgccggctttgaaaagctgacgccctttacaccggaggaa

gaaagtcgtcgaatactgcatacagttttttgtactctagaagcagctccccatcaaagc

tga

>OR085515.1

atggcggcgcgcgaacaggccaactccgttcgcaggagcgggttttttaatccgtttatt

ggcaagaggccatttttcaggccgggaagcgggcagaccgcggagacagagcgcccaagg

cccccccaacactcgtactgcacagaggtgggtagctttaagtttatagctccaagatgc

ctcgatgaagaagcccccgccgaccagcgacgcggtgtacacgtgggcaccttggagcgc

ccacccaaagtgtactgcgatggctcagagtacgacgtgctgaactttgcctccggaggt

tgctggcctcgcaggattcgggtttggaacggccaggattttcggggcgatggattcaac

cccagatttgagagatttcacgtgtacgacatagtagagacttcggagagcgcctcacac

gatgacccatccaggtttgctgagctatcccgcccaagcgggtctgtggtaacactgctg

ggaatgagcgagtgtggaaagcgagtcgcggttcacgtctatggtgtgcgccattatttt

tacatggcaaaggcggaggtggatagcgcttgtggaataaccaccgaggcagaactcgtg

cgcgcaatggtggactgcgcgcacagctcggctttgagcgcggccctgggaaatggcaac

ggcggcaaacagagcggcggcagcggcgggggatggtggggcggaaagcacgtgtctgcg

gactgcttcaaagtggagaccgtgtgccacacgacgctgtactactttggatctaagcca

gctctctactatagagtatctgcctccagcagccgcctgggagggttcatctgcgacaac

tttcacccggagattacaaaattcgaggggagcgtggacgtgaccacgcggctgttgttg

gacaatgaaaattttaccagtttcgggtggtaccgcctgcgacccggcacccacggagag

cgtgttcaacttcgccccgttgagcgacacgtcacctcaagcgacgtggagattaactgt

actcccgataacctggagccgataccagacgaggctgcctggcccgactataagctcatg

tgctttgatatagagtgtaaagctggaacgggtaacgaaatggcgttcccagtggcaact

aaccaagaggacctggtcatccagatctcctgtctgctgtactcgcttgctactcagaac

cacgaacacaccctgctgttttccctcgggtcatgcgatatctctgaggaatactcgttt

gcatgcgtccagcgcggcgagcccagaccgacggttttggagtttgacagcgagtacgag

ctgctggttgccttcctgacctttctcaagcagtactctcccgagttcgccaccggctac

aacatcgttaattttgactgggcgtacatagttaacaaggtaacgtcggtgtataacatc

aagctggacgggtacggcaagttcaacaaaggggggctgtttaaggtgtgggacatcgcc

acgaaccattttcagaagaagagcaaggtgaaaatcaatggcctgatatctctagacatg

tattctgtggcgacggaaaagctaaagctacccagctacaaactcgacgcggtcgtggga

gacgtcctcggcgagcataagatagaccttccctataaagaaataccctcctattacgcg

ggagggcctgaccggaggggcgtaataggagagtattgtatccaggactctaggctggtg

ggcaagctgttttttaagtacctcccccatctggaactatcggcggtggccaaactcgcc

cgtatcaccctaacgcgggtaatttttgacggtcagcaaattcgcgtgtacacgtgcttg

ctgaaactcgcccgcgagagaaatttcattttgccagacaacagacgccggtttgacagt

caggcagacgccgcgtcagagacttcggagttggctatggatagccaaagccacgccttc

gacagtacagacgaacccgacggtgtggacggtaccccggacgccgcaggatctggcgct

acttctgaaaacggaggcgggaagcccggcgtcgggagggccgtgggctaccagggagca

aaggttctagaccccgtatccggctttcatgtggaccccgtggttgtgtttgacttcgct

agcttatacccaagcattatccaggcccataacctctgtttcaccaccctggcgctcgat

gaagtggatctggccgggcttcaaccatccgtcaactactcgacgttcgaggtgggtggc

caaaagttattttttgtccacgcccatattcgcgaaagcctgcttggcatcttgctgcgc

gactggctggccatgcgaaaggcggtgagggcgcgaatccccaccagcacccccgaggag

gcagttttactagataagcagcagtctgcgattaaggtgatatgcaactcggtttacgga

ttcacgggggtggcaaacggcctgttgccgtgtctgaggatagcggctaccgttaccacg

ataggacgcgacatgctcctcaagacccgagattacgttcactctcgttgggcgacgcgc

gagctgctggaggacaattttccaggggctataggtttccgaaaccacaagccttactcc

gtcagggttatctacggagacaccgactccgtgtttatcaagtttgtgggcctgacgtac

gagggggtatcggagctgggggatgctatgtcgcgtcagatttcagcggacctctttaga

gcgcccatcaaactggagtgtgagaagacctttcagcgactgctgctgatcaccaagaag

aagtacataggtgtcataaacggggggaagatgctcatgaagggggtcgacctggtccgc

aaaaataactgctctttcataaacttgtacgcgcgacatctggtagatcttttgttgtac

gacgaggatgtggccacggcggcagcagaggtgacagacgtgcctcccgcagaatgggtg

gggcgcccgctaccgagcggctttgacaagtttgggcgagtgctggtagaggcgtacaac

cgtatcactgcccccaacttggacgtgcgcgagttcgttatgactgctgagctgagccgc

tcacccgaatcgtataccaacaagcgcctgccgcacctcaccgtctactttaagctcgcc

atgaggaatgaagaactgcccagcgtaaaagagagaattccgtatgtgatagttgcgcag

accgaggccgcggaacgcgaagcgggtgtagtaaactcaatgcgcggtaccgcccaaaac

cccgtggtaaccaagaccgcacgcccccaacctaaacgcaaactgctggtttccgacctc

gccgaagacccgacctatgtttccgagaatgacgtaccgctaaacacagactactatttc

tcccacctgttgggtaccataagcgtgacctttaaggctctattcggaaatgatgtgaga

acaacagaaaatcttttaaagcggtttattccggaaaccccccacaagacccccacgaaa

acccaggcactgcttgagcgcgccggctttgaaaagctgacgccctttacaccggaggaa

gaaagtcgtcgaatactgcatacagttttttgtactctagaagcagctccccatcaaagc

tga

>OR085516.1

atggcggcgcgcgaacaggccaactccgttcgcaggagcgggttttttaatccgtttatt

ggcaagaggccatttttcaggccgggaagcgggcaaaccgcggagacagagcgcccaagg

cccccccaacactcgtactgcacagaggtgggtagctttaagtttatagctccaagatgc

ctcgatgaagaagcccccgccgaccagcgacgcggtgtacacgtgggcaccttggagcgc

ccacccaaagtgtactgcgatggctcagagtacgacgtgctgaactttgcctccggaggt

tgctggcctcgcaggattcgggtttggaacggccaggattttcggggcgatggattcaac

cccagatttgagagatttcacgtgtacgacatagtagagacttcggagagcgcctcacac

gatgacccatccaggtttgctgagctatcccgcccaagcgggtctgtggtaacactgctg

ggaatgagcgagtgtggaaagcgagtcgcggttcacgtctatggtgtgcgccattatttt

tacatggcaaaggcggaggtggatagcgcttgtggaataaccaccgaggcagaactcgtg

cgcgcaatggtggactgcgcgcacagctcggctttgagcgcggccctgggaaatggcaac

ggcggcaaacagagcggcggcagcggcgggggatggtggggcggaaagcacgtgtctgcg

gactgcttcaaagtggagaccgtgtgccacacgacgctgtactactttggatctaagcca

gctctctactatagagtatctgcctccagcagccgcctgggagggttcatctgcgacaac

tttcacccggagattacaaaattcgaggggagcgtggacgtgaccacgcggctgttgttg

gacaatgaaaattttaccagtttcgggtggtaccgcctgcgacccggcacccacggagag

cgtgttcaacttcgccccgttgagcgacacgtcacctcaagcgacgtggagattaactgt

actcccgataacctggagccgataccagacgaggctgcctggcccgactataagctcatg

tgctttgatatagagtgtaaagctggaacgggtaacgaaatggcgttcccagtggcaact

aaccaagaggacctggtcatccagatctcctgtctgctgtactcgcttgctactcagaac

cacgaacacaccctgctgttttccctcgggtcatgcgatatctctgaggaatactcgttt

gcatgcgtccagcgcggcgagcccagaccgacggttttggagtttgacagcgagtacgag

ctgctggttgccttcctgacctttctcaagcagtactctcccgagttcgccaccggctac

aacatcgttaattttgactgggcgtacatagttaacaaggtaacgtcggtgtataacatc

aagctggacgggtacggcaagttcaacaaaggggggctgtttaaggtgtgggacatcgcc

acgaaccattttcagaagaagagcaaggtgaaaatcaatggcctgatatctctagacatg

tattctgtggcgacggaaaagctaaagctacccagctacaaactcgacgcggtcgtggga

gacgtcctcggcgagcataagatagaccttccctataaagaaataccctcctattacgcg

ggagggcctgaccggaggggcgtaataggagagtattgtatccaggactctaggctggtg

ggcaagctgttttttaagtacctcccccatctggaactatcggcggtggccaaactcgcc

cgtatcaccctaacgcgggtaatttttgacggtcagcaaattcgcgtgtacacgtgcttg

ctgaaactcgcccgcgagagaaatttcattttgccagacaacagacgccggtttgacagt

caggcagacgccgcgtcagagacttcggagttggctatggatagccaaagccacgccttc

gacagtacagacgaacccgacggtgtggacggtaccccggacgccgcaggatctggcgct

acttctgaaaacggaggcgggaagcccggcgtcgggagggccgtgggctaccagggagca

aaggttctagaccccgtatccggctttcatgtggaccccgtggttgtgtttgacttcgct

agcttatacccaagcattatccaggcccataacctctgtttcaccaccctggcgctcgat

gaagtggatctggccgggcttcaaccatccgtcaactactcgacgttcgaggtgggtgac

caaaagttattttttgtccacgcccatattcgcgaaagcctgcttggcatcttgctgcgc

gactggctggccatgcgaaaggcggtgagggcgcgaatccccaccagcacccccgaggag

gcagttttactagataagcagcagtctgcgattaaggtgatatgcaactcggtttacgga

ttcacgggggtggcaaacggcctgttgccgtgtctgaggatagcggctaccgttaccacg

ataggacgcgacatgctcctcaagacccgagattacgttcactctcgttgggcgacgcgc

gagctgctggaggacaattttccaggggctataggtttccgaaaccacaagccttactcc

gtcagggttatctacggagacaccgactccgtgtttatcaagtttgtgggcctgacgtac

gagggggtatcggagctgggggatgctatgtcgcgtcagatttcagcggacctctttaga

gcgcccatcaaactggagtgtgagaagacctttcagcgactgctgctgatcaccaagaag

aagtacataggtgtcataaacggggggaagatgctcatgaagggggtcgacctggtccgc

aaaaataactgctctttcataaacttgtacgcgcgacatctggtagatcttttgttgtac

gacgaggatgtggccacggcggcagcaaaggtgacagacgtgcctcccgcagaatgggtg

gggcgcccgctaccgagcggctttgacaagtttgggcgagtgctggtagaggcgtacaac

cgtatcactgcccccaacttggacgtgcgcgagttcgttatgactgctgagctgagccgc

tcacccgaatcgtataccaacaagcgcctgccgcacctcaccgtctactttaagctcgcc

atgaggaatgaagaactgcccagcgtaaaagagagaattccgtatgtgatagttgcgcag

accgaggccgcggaacgcgaagcgggtgtagtaaactcaatgcgcggtaccgcccaaaac

cccgtggtaaccaagaccgcacgcccccaacctaaacgcaaactgctggtttccgacctc

gccgaagacccgacctatgtttccgagaatgacgtaccgctaaacacagactactatttc

tcccacctgttgggtaccataagcgtgacctttaaggctctattcggaaatgatgtgaga

acaacagaaaatcttttaaagcggtttattccggaaaccccccacaagacccccacgaaa

acccaggcactgcttgagcgcgccggctttgaaaagctgacgccctttacaccggaggaa

gaaagtcgtcgaatactgcatacagttttttgtactctagaagcagctccccatcaaagc

tga

>OR085517.1

atggcggcgcgcgaacaggccaactccgttcgcaggagcgggttttttaatccgtttatt

ggcaagaggccatttttcaggccgggaagcgggcagaccgcggagacagagcgcccaagg

cccccccaacactcgtactgcacagaggtgggtagctttaagtttatagctccaagatgc

ctcgatgaagaagcccccgccgaccagcgacgcggtgtacacgtgggcaccttggagcgc

ccacccaaagtgtactgcgatggctcagagtacgacgtgctgaactttgcctccggaggt

tgctggcctcgcaggattcgggtttggaacggccaggattttcggggcgatggattcaac

cccagatttgagagatttcacgtgtacgacatagtagagacttcggagagcgcctcacac

gatgacccatccaggtttgctgagctatcccgcccaagcgggtctgtggtaacactgctg

ggaatgagcgagtgtggaaagcgagtcgcggttcacgtctatggtgtgcgccattatttt

tacatggcaaaggcggaggtggatagcgcttgtggaataaccaccgaggcagaactcgtg

cgcgcaatggtggactgcgcgcacagctcggctttgagcgcggccctgggaaatggcaac

ggcggcaaacagagcggcggcagcggcgggggatggtggggcggaaagcacgtgtctgcg

gactgcttcaaagtggagaccgtgtgccacacgacgctgtactactttggatctaagcca

gctctctactatagagtatctgcctccagcagccgcctgggagggttcatctgcgacaac

tttcacccggagattacaaaattcgaggggagcgtggacgtgaccacgcggctgttgttg

gacaatgaaaattttaccagtttcgggtggtaccgcctgcgacccggcacccacggagag

cgtgttcaacttcgccccgttgagcgacacgtcacctcaagcgacgtggagattaactgt

actcccgataacctggagccgataccagacgaggctgcctggcccgactataagctcatg

tgctttgatatagagtgtaaagctggaacgggtaacgaaatggcgttcccagtggcaact

aaccaagaggacctggtcatccagatctcctgtctgctgtactcgcttgctactcagaac

cacgaacacaccctgctgttttccctcgggtcatgcgatatctctgaggaatactcgttt

gcatgcgtccagcgcggcgagcccagaccgacggttttggagtttgacagcgagtacgag

ctgctggttgccttcctgacctttctcaagcagtactctcccgagttcgccaccggctac

aacatcgttaattttgactgggcgtacatagttaacaaggtaacgtcggtgtataacatc

aagctggacgggtacggcaagttcaacaaaggggggctgtttaaggtgtgggacatcgcc

acgaaccattttcagaagaagagcaaggtgaaaatcaatggcctgatatctctagacatg

tattctgtggcgacggaaaagctaaagctacccagctacaaactcgacgcggtcgtggga

gacgtcctcggcgagcataagatagaccttccctataaagaaataccctcctattacgcg

ggagggcctgaccggaggggcgtaataggagagtattgtatccaggactctaggctggtg

ggcaagctgttttttaagtacctcccccatctggaactatcggcggtggccaaactcgcc

cgtatcaccctaacgcgggtaatttttgacggtcagcaaattcgcgtgtacacgtgcttg

ctgaaactcgcccgcgagagaaatttcattttgccagacaacagacgccggtttgacagt

caggcagacgccgcgtcagagacttcggagttggctatggatagccaaagccacgccttc

gacagtacagacgaacccgacggtgtggacggtaccccggacgccgcaggatctggcgct

acttctgaaaacggaggcgggaagcccggcgtcgggagggccgtgggctaccagggagca

aaggttctagaccccgtatccggctttcatgtggaccccgtggttgtgcttgacttcgct

agcttatacccaagcattatccaggcccataacctctgtttcaccaccctggcgctcgat

gaagtggatctggccgggcttcaaccatccgtcaactactcgacgttcgaggtgggtgac

caaaagttattttttgtccacgcccatattcgcgaaagcctgcttggcatcttgctgcgc

gactggctggccatgcgaaaggcggtgagggcgcgaatccccaccagcacccccgaggag

gcagttttactagataagcagcagtctgcgattaaggtgatatgcaactcggtttacgga

ttcacgggggtggcaaacggcctgttgccgtgtctgaggatagcggctaccgttaccacg

ataggacgcgacatgctcctcaagacccgagattacgttcactctcgttgggcgacgcgc

gagctgctggaggacaattttccaggggctataggtttccgaaaccacaagccttactcc

gtcagggttatctacggagacaccgactccgtgtttatcaagtttgtgggcctgacgtac

gagggggtatcggagctgggggatgctatgtcgcgtcagatttcagcggacctctttaga

gcgcccatcaaactggagtgtgagaagacctttcagcgactgctgctgatcaccaagaag

aagtacataggtgtcataaacggggggaagatgctcatgaagggggtcgacctggtccgc

aaaaataactgctctttcataaacttgtacgcgcgacatctggtagatcttttgttgtac

gacgaggatgtggccacggcggcagcagaggtgacagacgtgcctcccgcagaatgggtg

gggcgcccgctaccgagcggctttgacaagtttgggcgagtgctggtagaggcgtacaac

cgtatcactgcccccaacttggacgtgcgcgagttcgttatgactgctgagctgagccgc

tcacccgaattgtataccaacaagcgcctgccgcacctcaccgtctactttaagctcgcc

atgaggaatgaagaactgcccagcgtaaaagagagaattccgtatgtgatagttgcgcag

accgaggccgcggaacgcgaagcgggtgtagtaaactcaatgcgcggtaccgcccaaaac

cccgtggtaaccaagaccgcacgcccccaacctaaacgcaaactgctggtttccgacctc

gccgaagacccgacctatgtttccgagaatgacgtaccgctaaacacagactactatttc

tcccacctgttgggtaccataagcgtgacctttaaggctctattcggaaatgatgtgaga

acaacagaaaatcttttaaagcggtttattccggaaaccccccacaagacccccacgaaa

acccaggcactgcttgagcgcgccggctttgaaaagctgacgccctttacaccggaggaa

gaaagtcgtcgaatactgcatacagttttttgtactctagaagcagctccccatcaaagc

tga

>OR085518.1

atggcggcgcgcgaacaggccaactccgttcgcaggagcgggttttttaatccgtttatt

ggcaagaggccatttttcaggccgggaagcgggcagaccgcggagacagagcgcccaagg

cccccccaacactcgtactgcacagaggtgggtagctttaagtttatagctccaagatgc

ctcgatgaagaagcccccgccgaccagcgacgcggtgtacacgtgggcaccttggagcgc

ccacccaaagtgtactgcgatggctcagagtacgacgtgctgaactttgcctccggaggt

tgctggcctcgcaggattcgggtttggaacggccaggattttcggggcgatggattcaac

cccagatttgagagatttcacgtgtacgacatagtagagacttcggagagcgcctcacac

gatgacccatccaggtttgctgagctatcccgcccaagcgggtctgtggtaacactgctg

ggaatgagcgagtgtggaaagcgagtcgcggttcacgtctatggtgtgcgccattatttt

tacatggcaaaggcggaggtggatagcgcttgtggaataaccaccgaggcagaactcgtg

cgcgcaatggtggactgcgcgcacagctcggctttgagcgcggccctgggaaatggcaac

ggcggcaaacagagcggcggcagcggcgggggatggtggggcggaaagcacgtgtctgcg

gactgcttcaaagtggagaccgtgtgccacacgacgctgtactactttggatctaagcca

gctctctactatagagtatctgcctccagcagccgcctgggagggttcatctgcgacaac

tttcacccggagattacaaaattcgaggggagcgtggacgtgaccacgcggctgttgttg

gacaatgaaaattttaccagtttcgggtggtaccgcctgcgacccggcacccacggagag

cgtgttcaacttcgccccgttgagcgacacgtcacctcaagcgacgtggagattaactgt

actcccgataacctggagccgataccagacgaggctgcctggcccgactataagctcatg

tgctttgatatagagtgtaaagctggaacgggtaacgaaatggcgttcccagtggcaact

aaccaagaggacctggtcatccagatctcctgtctgctgtactcgcttgctactcagaac

cacgaacacaccctgctgttttccctcgggtcatgcgatatctctgaggaatactcgttt

gcatgcgtccagcgcggcgagcccagaccgacggttttggagtttgacagcgagtacgag

ctgctggttgccttcctgacctttctcaagcagtactctcccgagttcgccaccggctac

aacatcgttaattttgactgggcgtacatagttaacaaggtaacgtcggtgtataacatc

aagctggacgggtacggcaagttcaacaaaggggggctgtttaaggtgtgggacatcgcc

acgaaccattttcagaagaagagcaaggtgaaaatcaatggcctgatatctctagacatg

tattctgtggcgacggaaaagctaaagctacccagctacaaactcgacgcggtcgtggga

gacgtcctcggcgagcataagatagaccttccctataaagaaataccctcctattacgcg

ggagggcctgaccggaggggcgtaataggagagtattgtatccaggactctaggctggtg

ggcaagctgttttttaagtacctcccccatctggaactatcggcggtggccaaactcgcc

cgtatcaccctaacgcgggtaatttttgacggtcagcaaattcgcgtgtacacgtgcttg

ctgaaactcgcccgcgagagaaatttcattttgccagacaacagacgccggtttgacagt

caggcagacgccgcgtcagagacttcggagttggctatggatagccaaagccacgccttc

gacagtacagacgaacccgacggtgtggacggtaccccggacgccgcaggatctggcgct

acttctgaaaacggaggcgggaagcccggcgtcgggagggccgtgggctaccagggagca

aaggttctagaccccgtatccggctttcatgtggaccccgtggttgtgtttgacttcgct

agcttatacccaagcattatccaggcccataacctctgtttcaccaccctggcgctcgat

gaagtggatctggccgggcttcaaccatccgtcaactactcgacgttcgaggtgggtgac

caaaagttattttttgtccacgcccatattcgcgaaagcctgcttggcatcttgctgcgc

gactggctggccatgcgaaaggcggtgagggcgcgaatccccaccagcacccccgaggag

gcagttttactagataagcagcagtctgcgattaaggtgatatgcaactcggtttacgga

ttcacgggggtggcaaacggcctgttgccgtgtctgaggatagcggctaccgttaccacg

ataggacgcgacatgctcctcaagacccgagattacgttcactctcgttgggcgacgcgc

gagctgctggaggacaattttccaggggctataggtttccgaaaccacaagccttactcc

gtcagggttatctacggagacaccgactccgtgtttatcaagtttgtgggcctgacgtac

gagggggtatcggagctgggggatgctatgtcgcgtcagatttcagcggacctctttaga

gcgcccatcaaactggagtgtgagaagacctttcagcgactgctgctgatcaccaagaag

aagtacataggtgtcataaacggggggaagatgctcatgaagggggtcgacctggtccgc

aaaaataactgctctttcataaacttgtacgcgcgacatctggtagatcttttgttgtac

gacgaggatgtggccacggcggcagcagaggtgacagacgtgcctcccgcagaatgggtg

gggcgcccgctaccgagcggctttgacaagtttgggcgagtgctggtagaggcgtacaac

cgtatcactgcccccaacttggacgtgcgcgagttcgttatgactgctgagctgagccgc

tcacccgaatcgtataccaacaagcgcctgccgcacctcaccgtctactttaagctcgcc

atgaggaatgaagaactgcccagcgtaaaagagagaattccgtatgtgatagttgcgcag

accgaggccgcggaacgcgaagcgggtgtagtaaactcaatgcgcggtaccgcccaaaac

cccgtggtaaccaagaccgcacgcccccaacctaaacgcaaactgctggtttccgacctc

gccgaagacccgacctatgtttccgagaatgacgtaccgctaaacacagactactatttc

tcccacctgttgggtaccataagcgtgacctttaaggctctattcggaaatgatgtgaga

acaacagaaaatcttttaaagcggtttattccggaaaccccccacaagacccccacgaaa

acccaggcactgcttgagcgcgccggctttgaaaagctgacgccctttacaccggaggaa

gaaagtcgtcgaatactgcatacagttttttgtactctagaagcagctccccatcaaagc

tga

>PP481413.1

atggcggcgcgcgaacaggccaactccgttcgcaggagcgggttttttaatccgtttatt

ggcaagaggccatttttcaggccgggaagcgggcagaccgcggagacagagcgcccaagg

cccccccaacactcgtactgcacagaggtgggtagctttaagtttatagctccaagatgc

ctcgatgaagaagcccccgccgaccagcgacgcggtgtacacgtgggcaccttggagcgc

ccacccaaagtgtactgcgatggctcagagtacgacgtgctgaactttgcctccggaggt

tgctggcctcgcaggattcgggtttggaacggccaggattttcggggcgatggattcaac

cccagatttgagagatttcacgtgtacgacatagtagagacttcggagagcgcctcacac

gatgacccatccaggtttgctgagctatcccgcccaagcgggtctgtggtaacactgctg

ggaatgagcgagtgtggaaagcgagtcgcggttcacgtctatggtgtgcgccattatttt

tacatggcaaaggcggaggtggatagcgcttgtggaataaccaccgaggcagaactcgtg

cgcgcaatggtggactgcgcgcacagctcggctttgagcgcggccctgggaaatggcaac

ggcggcaaacagagcggcggcagcggcgggggatggtggggcggaaagcacgtgtctgcg

gactgcttcaaagtggagaccgtgtgccacacgacgctgtactactttggatctaagcca

gctctctactatagagtatctgcctccagcagccgcctgggagggttcatctgcgacaac

tttcacccggagattacaaaattcgaggggagcgtggacgtgaccacgcggctgttgttg

gacaatgaaaattttaccagtttcgggtggtaccgcctgcgacccggcacccacggagag

cgtgttcaacttcgccccgttgagcgacacgtcacctcaagcgacgtggagattaactgt

actcccgataacctggagccgataccagacgaggctgcctggcccgactataagctcatg

tgctttgatatagagtgtaaagctggaacgggtaacgaaatggcgttcccagtggcaact

aaccaagaggacctggtcatccagatctcctgtctgctgtactcgcttgctactcagaac

cacgaacacaccctgctgttttccctcgggtcatgcgatatctctgaggaatactcgttt

gcatgcgtccagcgcggcgagcccagaccgacggttttggagtttgacagcgagtacgag

ctgctggttgccttcctgacctttctcaagcagtactctcccgagttcgccaccggctac

aacatcgttaattttgactgggcgtacatagttaacaaggtaacgtcggtgtataacatc

aagctggacgggtacggcaagttcaacaaaggggggctgtttaaggtgtgggacatcgcc

acgaaccattttcagaagaagagcaaggtgaaaatcaatggcctgatatctctagacatg

tattctgtggcgacggaaaagctaaagctacccagctacaaactcgacgcggtcgtggga

gacgtcctcggcgagcataagatagaccttccctataaagaaataccctcctattacgcg

ggagggcctgaccggaggggcgtaataggagagtattgtatccaggactctaggctggtg

ggcaagctgttttttaagtacctcccccatctggaactatcggcggtggccaaactcgcc

cgtatcaccctaacgcgggtaatttttgacggtcagcaaattcgcgtgtacacgtgcttg

ctgaaactcgcccgcgagagaaatttcattttgccagacaacagacgccggtttgacagt

caggcagacgccgcgtcagagacttcggagttggctatggatagccaaagccacgccttc

gacagtacagacgaacccgacggtgtggacggtaccccggacgccgcaggatctggcgct

acttctgaaaacggaggcgggaagcccggcgtcgggagggtcgtgggctaccagggagca

aaggttctagaccccgtatccggctttcatgtggaccccgtggttgtgtttgacttcgct

agcttatacccaagcattatccaggcccataacctctgtttcaccaccctggcgctcgat

gaagtggatctggccgggcttcaaccatccgtcgactactcgacgttcgaggtgggtgac

caaaagttattttttgtccacgcccatattcgcgaaagcctgcttggcatcttgctgcgc

gactggctggccatgcgaaaggcggtgagggcgcgaatccccaccagcacccccgaggag

gcagttttactagataagcagcagtctgcgattaaggtgatatgcaactcggtttacgga

ttcacgggggtggcaaacggcctgttgccgtgtctgaggatagcggctaccgttaccacg

ataggacgcgacatgctcctcaagacccgagattacgttcactctcgttgggcgacgcgc

gagctgctggaggacaattttccaggggctataggtttccgaaaccacaagccttactcc

gtcagggttatctacggagacaccgactccgtgtttatcaagtttgtgggcctgacgtac

gagggggtatcggagctgggggatgctatgtcgcgtcagatttcagcggacctctttaga

gcgcccatcaaactggagtgtgagaagacctttcagcgactgctgctgatcaccaagaag

aagtacataggtgtcataaacggggggaagatgctcatgaagggggtcgacctggtccgc

aaaaataactgctctttcataaacttgtacgcgcgacatctggtagatcttttgttgtac

gacgaggatgtggccacggcggcagcagaggtgacagacgtgcctcccgcagaatgggtg

gggcgcccgctaccgagcggctttgacaagtttgggcgagtgctggtagaggcgtacaac

cgtatcactgcccccaacttggacgtgcgcgagttcgttatgactgctgagctgagccgc

tcacccgaatcgtataccaacaagcgcctgccgcacctcaccgtctactttaagctcgcc

atgaggaatgaagaactgcccagcgtaaaagagagaattccgtatgtgatagttgcgcag

accgaggccgcggaacgcgaagcgggtgtagtaaactcaatgcgcggtaccgcccaaaac

cccgtggtaaccaagaccgcacgcccccaacctaaacgcaaactgctggtttccgacctc

gccgaagacccgacctatgtttccgagaatgacgtaccgctaaacacagactactatttc

tcccacctgttgggtaccataagcgtgacctttaaggctctattcggaaatgatgtgaga

acaacagaaaatcttttaaagcggtttattccggaaaccccccacaagacccccacgaaa

acccaggcactgcttgagcgcgccggctttgaaaagctgacgccctttacaccggaggaa

gaaagtcgtcgaatactgcatacagttttttgtactctagaagcagctccccatcaaagc

tga

>OR801338.1

atggcggcgcgcgaacaggccaactccgttcgcaggagcgggttttttaatccgtttatt

ggcaagaggccatttttcaggccgggaagcgggcagaccgcggagacagagcgcccaagg

cccccccaacactcgtactgcacagaggtgggtagctttaagtttatagctccaagatgc

ctcgatgaagaagcccccgccgaccagcgacgcggtgtacacgtgggcaccttggagcgc

ccacccaaagtgtactgcgatggctcagagtacgacgtgctgaactttgcctccggaggt

tgctggcctcgcaggattcgggtttggaacggccaggattttcggggcgatggattcaac

cccagatttgagagatttcacgtgtacgacatagtagagacttcggagagcgcctcacac

gatgacccatccaggtttgctgagctatcccgcccaagcgggtctgtggtaacactgctg

ggaatgagcgagtgtggaaagcgagtcgcggttcacgtctatggtgtgcgccattatttt

tacatggcaaaggcggaggtggatagcgcttgtggaataaccaccgaggcagaactcgtg

cgcgcaatggtggactgcgcgcacagctcggctttgagcgcggccctgggaaatggcaac

ggcggcaaacagagcggcggcagcggcgggggatggtggggcggaaagcacgtgtctgcg

gactgcttcaaagtggagaccgtgtgccacacgacgctgtactactttggatctaagcca

gctctctactatagagtatctgcctccagcagccgcctgggagggttcatctgcgacaac

tttcacccggagattacaaaattcgaggggagcgtggacgtgaccacgcggctgttgttg

gacaatgaaaattttaccagtttcgggtggtaccgcctgcgacccggcacccacggagag

cgtgttcaacttcgccccgttgagcgacacgtcacctcaagcgacgtggagattaactgt

actcccgataacctggagccgataccagacgaggctgcctggcccgactataagctcatg

tgctttgatatagagtgtaaagctggaacgggtaacgaaatggcgttcccagtggcaact

aaccaagaggacctggtcatccagatctcctgtctgctgtactcgcttgctactcagaac

cacgaacacaccctgctgttttccctcgggtcatgcgatatctctgaggaatactcgttt

gcatgcgtccagcgcggcgagcccagaccgacggttttggagtttgacagcgagtacgag

ctgctggttgccttcctgacctttctcaagcagtactctcccgagttcgccaccggctac

aacatcgttaattttgactgggcgtacatagttaacaaggtaacgtcggtgtataacatc

aagctggacgggtacggcaagttcaacaaaggggggctgtttaaggtgtgggacatcgcc

acgaaccattttcagaagaagagcaaggtgaaaatcaatggcctgatatctctagacatg

tattctgtggcgacggaaaagctaaagctacccagctacaaactcgacgcggtcgtggga

gacgtcctcggcgagcataagatagaccttccctataaagaaataccctcctattacgcg

ggagggcctgaccggaggggcgtaataggagagtattgtatccaggactctaggctggtg

ggcaagctgttttttaagtacctcccccatctggaactatcggcggtggccaaactcgcc

cgtatcaccctaacgcgggtaatttttgacggtcagcaaattcgcgtgtacacgtgcttg

ctgaaactcgcccgcgagagaaatttcattttgccagacaacagacgccggtttgacagt

caggcagacgccgcgtcagagacttcggagttggctatggatagccaaagccacgccttc

gacagtacagacgaacccgacggtgtggacggtaccccggacgccgcaggatctggcgct

acttctgaaaacggaggcgggaagcccggcgtcgggagggccgtgggctaccagggagca

aaggttctagaccccgtatccggctttcatgtggaccccgtggttgtgtttgacttcgct

agcttatacccaagcattatccaggcccataacctctgtttcaccaccctggcgctcgat

gaagtggatctggccgggcttcaaccatccgtcgactactcgacgttcgaggtgggtgac

caaaagttattttttgtccacgcccatattcgcgaaagcctgcttggcatcttgctgcgc

gactggctggccatgcgaaaggcggtgagggcgcgaatccccaccagcacccccgaggag

gcagttttactagataagcagcagtctgcgattaaggtgatatgcaactcggtttacgga

ttcacgggggtggcaaacggcctgttgccgtgtctgaggatagcggctaccgttaccacg

ataggacgcgacatgctcctcaagacccgagattacgttcactctcgttgggcgacgcgc

gagctgctggaggacaattttccaggggctataggtttccgaaaccacaagccttactcc

gtcagggttatctacggagacaccgactccgtgtttatcaagtttgtgggcctgacgtac

gagggggtatcggagctgggggatgctatgtcgcgtcagatttcagcggacctctttaga

gcgcccatcaaactggagtgtgagaagacctttcagcgactgctgctgatcaccaagaag

aagtacataggtgtcataaacggggggaagatgctcatgaagggggtcgacctggtccgc

aaaaataactgctctttcataaacttgtacgcgcgacatctggtagatcttttgttgtac

gacgaggatgtggccacggcggcagcagaggtgacagacgtgcctcccgcagaatgggtg

gggcgcccgctaccgagcggctttgacaagtttgggcgagtgctggtagaggcgtacaac

cgtatcactgcccccaacttggacgtgcgcgagttcgttatgactgctgagctgagccgc

tcacccgaatcgtataccaacaagcgcctgccgcacctcaccgtctactttaagctcgcc

atgaggaatgaagaactgcccagcgtaaaagagagaattccgtatgtgatagttgcgcag

accgaggccgcggaacgcgaagcgggtgtagtaaactcaatgcgcggtaccgcccaaaac

cccgtggtaaccaagaccgcacgcccccaacctaaacgcaaactgctggtttccgacctc

gccgaagacccgacctatgtttccgagaatgacgtaccgctaaacacagactactatttc

tcccacctgttgggtaccataagcgtgacctttaaggctctattcggaaatgatgtgaga

acaacagaaaatcttttaaagcggtttattccggaaaccccccacaagacccccacgaaa

acccaggcactgcttgagcgcgccggctttgaaaagctgacgccctttacaccggaggaa

gaaagtcgtcgaatactgcatacagttttttgtactctagaagcagctccccatcaaagc

tga

>PP084325.1

atggcggcgcgcgaacaggccaactccgttcgcaggagcgggttttttaatccgtttatt

ggcaagaggccatttttcaggccgggaagcgggcagaccgcggagacagagcgcccaagg

cccccccaacactcgtactgcacagaggtgggtagctttaagtttatagctccaagatgc

ctcgatgaagaagcccccgccgaccagcgacgcggtgtacacgtgggcaccttggagcgc

ccacccaaagtgtactgcgatggctcagagtacgacgtgctgaactttgcctccggaggt

tgctggcctcgcaggattcgggtttggaacggccaggattttcggggcgatggattcaac

cccagatttgagagatttcacgtgtacgacatagtagagacttcggagagcgcctcacac

gatgacccatccaggtttgctgagctatcccgcccaagcgggtctgtggtaacactgctg

ggaatgagcgagtgtggaaagcgagtcgcggttcacgtctatggtgtgcgccattatttt

tacatggcaaaggcggaggtggatagcgcttgtggaataaccaccgaggcagaactcgtg

cgcgcaatggtggactgcgcgcacagctcggctttgagcgcggccctgggaaatggcaac

ggcggcaaacagagcggcggcagcggcgggggatggtggggcggaaagcacgtgtctgcg

gactgcttcaaagtggagaccgtgtgccacacgacgctgtactactttggatctaagcca

gctctctactatagagtatctgcctccagcagccgcctgggagggttcatctgcgacaac

tttcacccggagattacaaaattcgaggggagcgtggacgtgaccacgcggctgttgttg

gacaatgaaaattttaccagtttcgggtggtaccgcctgcgacccggcacccacggagag

cgtgttcaacttcgccccgttgagcgacacgtcacctcaagcgacgtggagattaactgt

actcccgataacctggagccgataccagacgaggctgcctggcccgactataagctcatg

tgctttgatatagagtgtaaagctggaacgggtaacgaaatggcgttcccagtggcaact

aaccaagaggacctggtcatccagatctcctgtctgctgtactcgcttgctactcagaac

cacgaacacaccctgctgttttccctcgggtcatgcgatatctctgaggaatactcgttt

gcatgcgtccagcgcggcgagcccagaccgacggttttggagtttgacagcgagtacgag

ctgctggttgccttcctgacctttctcaagcagtactctcccgagttcgccaccggctac

aacatcgttaattttgactgggcgtacatagttaacaaggtaacgtcggtgtataacatc

aagctggacgggtacggcaagttcaacaaaggggggctgtttaaggtgtgggacatcgcc

acgaaccattttcagaagaagagcaaggtgaaaatcaatggcctgatatctctagacatg

tattctgtggcgacggaaaagctaaagctacccagctacaaactcgacgcggtcgtggga

gacgtcctcggcgagcataagatagaccttccctataaagaaataccctcctattacgcg

ggagggcctgaccggaggggcgtaataggagagtattgtatccaggactctaggctggtg

ggcaagctgttttttaagtacctcccccatctggaactatcggcggtggccaaactcgcc

cgtatcaccctaacgcgggtaatttttgacggtcagcaaattcgcgtgtacacgtgcttg

ctgaaactcgcccgcgagagaaatttcattttgccagacaacagacgccggtttgacagt

caggcagacgccgcgtcagagacttcggagttggctatggatagccaaagccacgccttc

gacagtacagacgaacccgacggtgtggacggtaccccggacgccgcaggatctggcgct

acttctgaaaacggaggcgggaagcccggcgtcgggagggccgtgggctaccagggagca

aaggttctagaccccgtatccggctttcatgtggaccccgtggttgtgtttgacttcgct

agcttatacccaagcattatccaggcccataacctctgtttcaccaccctggcgctcgat

gaagtggatctggccgggcttcaaccatccgtcaactactcgacgttcgaggtgggtgac

caaaagttattttttgtccacgcccatattcgcgaaagcctgcttggcatcttgctgcgc

gactggctggccatgcgaaaggcggtgagggcgcgaatccccaccagcacccccgaggag

gcagttttactagataagcagcagtctgcgattaaggtgatatgcaactcggtttacgga

ttcacgggggtggcaaacggcctgttgccgtgtctgaggatagcggctaccgttaccacg

ataggacgcgacatgctcctcaagacccgagattacgttcactctcgttgggcgacgcgc

gagctgctggaggacaattttccaggggctataggtttccgaaaccacaagccttactcc

gtcagggttatctacggagacaccgactccgtgtttatcaagtttgtgggcctgacgtac

gagggggtatcggagctgggggatgctatgtcgcgtcagatttcagcggacctctttaga

gcgcccatcaaactggagtgtgagaagacctttcagcgactgctgctgatcaccaagaag

aagtacataggtgtcataaacggggggaagatgctcatgaagggggtcgacctggtccgc

aaaaataactgctctttcataaacttgtacgcgcgacatctggtagatcttttgttgtac

gacgaggatgtggccacggcggcagcagaggtgacagacgtgcctcccgcagaatgggtg

gggcgcccgctaccgagcggctttgacaagtttgggcgagtgctggtagaggcgtacaac

cgtatcactgcccccaacttggacgtgcgcgagttcgttatgactgctgagctgagccgc

tcacccgaatcgtataccaacaagcgcctgccgcacctcaccgtctactttaagctcgcc

atgaggaatgaagaactgcccagcgtaaaagagagaattccgtatgtgatagttgcgcag

accgaggccgcggaacgcgaagcgggtgtagtaaactcaatgcgcggtaccgcccaaaac

cccgtggtaaccaagaccgcacgcccccaacctaaacgcaaactgctggtttccgacctc

gccgaagacccgacctatgtttccgagaatgacgtaccgctaaacacagactactatttc

tcccacctgttgggtaccataagcgtgacctttaaggctctattcggaaatgatgtgaga

acaacagaaaatcttttaaagcggtttattccggaaaccccccacaagacccccacgaaa

acccaggcactgcttgagcgcgccggctttgaaaagctgacgccctttacaccggaggaa

gaaagtcgtcgaatactgcatacagttttttgtactctagaagcagctccccatcaaagc

tga

>PP084326.1

atggcggcgcgcgaacaggccaactccgttcgcaggagcgggttttttaatccgtttatt

ggcaagaggccatttttcaggccgggaagcgggcagaccgcggagacagagcgcccaagg

cccccccaacactcgtactgcacagaggtgggtagctttaagtttatagctccaagatgc

ctcgatgaagaagcccccgccgaccagcgacgcggtgtacacgtgggcaccttggagcgc

ccacccaaagtgtactgcgatggctcagagtacgacgtgctgaactttgcctccggaggt

tgctggcctcgcaggattcgggtttggaacggccaggattttcggggcgatggattcaac

cccagatttgagagatttcacgtgtacgacatagtagagacttcggagagcgcctcacac

gatgacccatccaggtttgctgagctatcccgcccaagcgggtctgtggtaacactgctg

ggaatgagcgagtgtggaaagcgagtcgcggttcacgtctatggtgtgcgccattatttt

tacatggcaaaggcggaggtggatagcgcttgtggaataaccaccgaggcagaactcgtg

cgcgcaatggtggactgcgcgcacagctcggctttgagcgcggccctgggaaatggcaac

ggcggcaaacagagcggcggcagcggcgggggatggtggggcggaaagcacgtgtctgcg

gactgcttcaaagtggagaccgtgtgccacacgacgctgtactactttggatctaagcca

gctctctactatagagtatctgcctccagcagccgcctgggagggttcatctgcgacaac

tttcacccggagattacaaaattcgaggggagcgtggacgtgaccacgcggctgttgttg

gacaatgaaaattttaccagtttcgggtggtaccgcctgcgacccggcacccacggagag

cgtgttcaacttcgccccgttgagcgacacgtcacctcaagcgacgtggagattaactgt

actcccgataacctggagccgataccagacgaggctgcctggcccgactataagctcatg

tgctttgatatagagtgtaaagctggaacgggtaacgaaatggcgttcccagtggcaact

aaccaagaggacctggtcatccagatctcctgtctgctgtactcgcttgctactcagaac

cacgaacacaccctgctgttttccctcgggtcatgcgatatctctgaggaatactcgttt

gcatgcgtccagcgcggcgagcccagaccgacggttttggagtttgacagcgagtacgag

ctgctggttgccttcctgacctttctcaagcagtactctcccgagttcgccaccggctac

aacatcgttaattttgactgggcgtacatagttaacaaggtaacgtcggtgtataacatc

aagctggacgggtacggcaagttcaacaaaggggggctgtttaaggtgtgggacatcgcc

acgaaccattttcagaagaagagcaaggtgaaaatcaatggcctgatatctctagacatg

tattctgtggcgacggaaaagctaaagctacccagctacaaactcgacgcggtcgtggga

gacgtcctcggcgagcataagatagaccttccctataaagaaataccctcctattacgcg

ggagggcctgaccggaggggcgtaataggagagtattgtatccaggactctaggctggtg

ggcaagctgttttttaagtacctcccccatctggaactatcggcggtggccaaactcgcc

cgtatcaccctaacgcgggtaatttttgacggtcagcaaattcgcgtgtacacgtgcttg

ctgaaactcgcccgcgagagaaatttcattttgccagacaacagacgccggtttgacagt

caggcagacgccgcgtcagagacttcggagttggctatggatagccaaagccacgccttc

gacagtacagacgaacccgacggtgtggacggtaccccggacgccgcaggatctggcgct

acttctgaaaacggaggcgggaagcccggcgtcgggagggccgtgggctaccagggagca

aaggttctagaccccgtatccggctttcatgtggaccccgtggttgtgtttgacttcgct

agcttatacccaagcattatccaggcccataacctctgtttcaccaccctggcgctcgat

gaagtggatctggccgggcttcaaccatccgtcaactactcgacgttcgaggtgggtgac

caaaagttattttttgtccacgcccatattcgcgaaagcctgcttggcatcttgctgcgc

gactggctggccatgcgaaaggcggtgagggcgcgaatccccaccagcacccccgaggag

gcagttttactagataagcagcagtctgcgattaaggtgatatgcaactcggtttacgga

ttcacgggggtggcaaacggcctgttgccgtgtctgaggatagcggctaccgttaccacg

ataggacgcgacatgctcctcaagacccgagattacgttcactctcgttgggcgacgcgc

gagctgctggaggacaattttccaggggctataggtttccgaaaccacaagccttactcc

gtcagggttatctacggagacaccgactccgtgtttatcaagtttgtgggcctgacgtac

gagggggtatcggagctgggggatgctatgtcgcgtcagatttcagcggacctctttaga

gcgcccatcaaactggagtgtgagaagacctttcagcgactgctgctgatcaccaagaag

aagtacataggtgtcataaacggggggaagatgctcatgaagggggtcgacctggtccgc

aaaaataactgctctttcataaacttgtacgcgcgacatctggtagatcttttgttgtac

gacgaggatgtggccacggcggcagcagaggtgacagacgtgcctcccgcagaatgggtg

gggcgcccgctaccgagcggctttgacaagtttgggcgagtgctggtagaggcgtacaac

cgtatcactgcccccaacttggacgtgcgcgagttcgttatgactgctgagctgagccgc

tcacccgaatcgtataccaacaagcgcctgccgcacctcaccgtctactttaagctcgcc

atgaggaatgaagaactgcccagcgtaaaagagagaattccgtatgtgatagttgcgcag

accgaggccgcggaacgcgaagcgggtgtagtaaactcaatgcgcggtaccgcccaaaac

cccgtggtaaccaagaccgcacgcccccaacctaaacgcaaactgctggtttccgacctc

gccgaagacccgacctatgtttccgagaatgacgtaccgctaaacacagactactatttc

tcccacctgttgggtaccataagcgtgacctttaaggctctattcggaaatgatgtgaga

acaacagaaaatcttttaaagcggtttattccggaaaccccccacaagacccccacgaaa

acccaggcactgcttgagcgcgccggctttgaaaagctgacgccctttacaccggaggaa

gaaagtcgtcgaatactgcatacagttttttgtactctagaagcagctccccatcaaagc

tga

>LC109623.1

atggcggcgcgcgaacaggccaactccgttcgcaggagcgggttttttaatccgtttatt

ggcaagaggccatttttcaggccgggaagcgggcagaccgcggagacagagcgcccaagg

cccccccaacactcgtactgcacagaggtgggtagctttaagtttatagctccaagatgc

ctcgatgaagaagcccccgccgaccagcgacgcggtgtacacgtgggcaccttggagcgc

ccacccaaagtgtactgcgatggctcagagtacgacgtgctgaactttgcctccggaggt

tgctggcctcgcaggattcgggtttggaacggccaggattttcggggcgatggattcaac

cccagatttgagagatttcacgtgtacgacatagtagagacttcggagagcgcctcacac

gatgacccatccaggtttgctgagctatcccgcccaagcgggtctgtggtaacactgctg

ggaatgagcgagtgtggaaagcgagtcgcggttcacgtctatggtgtgcgccattatttt

tacatggcaaaggcggaggtggatagcgcttgtggaataaccaccgaggcagaactcgtg

cgcgcaatggtggactgcgcgcacagctcggctttgagcgcggccctgggaaatggcaac

ggcggcaaacagagcggcggcagcggcgggggatggtggggcggaaagcacgtgtctgcg

gactgcttcaaagtggagaccgtgtgccacacgacgctgtactactttggatctaagcca

gctctctactatagagtatctgcctccagcagccgcctgggagggttcatctgcgacaac

tttcacccggagattacaaaattcgaggggagcgtggacgtgaccacgcggctgttgttg

gacaatgaaaattttaccagtttcgggtggtaccgcctgcgacccggcacccacggagag

cgtgttcaacttcgccccgttgagcgacacgtcacctcaagcgacgtggagattaactgt

actcccgataacctggagccgataccagacgaggctgcctggcccgactataagctcatg

tgctttgatatagagtgtaaagctggaacgggtaacgaaatggcgttcccagtggcaact

aaccaagaggacctggtcatccagatctcctgtctgctgtactcgcttgctactcagaac

cacgaacacaccctgctgttttccctcgggtcatgcgatatctctgaggaatactcgttt

gcatgcgtccagcgcggcgagcccagaccgacggttttggagtttgacagcgagtacgag

ctgctggttgccttcctgacctttctcaagcagtactctcccgagttcgccaccggctac

aacatcgttaattttgactgggcgtacatagttaacaaggtaacgtcggtgtataacatc

aagctggacgggtacggcaagttcaacaaaggggggctgtttaaggtgtgggacatcgcc

acgaaccattttcagaagaagagcaaggtgaaaatcaatggcctgatatctctagacatg

tattctgtggcgacggaaaagctaaagctacccagctacaaactcgacgcggtcgtggga

gacgtcctcggcgagcataagatagaccttccctataaagaaataccctcctattacgcg

ggagggcctgaccggaggggcgtaataggagagtattgtatccaggactctaggctggtg

ggcaagctgttttttaagtacctcccccatctggaactatcggcggtggccaaactcgcc

cgtatcaccctaacgcgggtaatttttgacggtcagcaaattcgcgtgtacacgtgcttg

ctgaaactcgcccgcgagagaaatttcattttgccagacaacagacgccggtttgacagt

caggcagacgccgcgtcagagacttcggagttggctatggatagccaaagccacgccttc

gacagtacagacgaacccgacggtgtggacggtaccccggacgccgcaggatctggcgct

acttctgaaaacggaggcgggaagcccggcgtcgggagggccgtgggctaccagggagca

aaggttctagaccccgtatccggctttcatgtggaccccgtggttgtgtttgacttcgct

agcttatacccaagcattatccaggcccataacctctgtttcaccaccctggcgctcgat

gaagtggatctggccgggcttcaaccatccgtcaactactcgacgttcgaggtgggtgac

caaaagttattttttgtccacgcccatattcgcgaaagcctgcttggcatcttgctgcgc

gactggctggccatgcgaaaggcggtgagggcgcgaatccccaccagcacccccgaggag

gcagttttactagataagcagcagtctgcgattaaggtgatatgcaactcggtttacgga

ttcacgggggtggcaaacggcctgttgccgtgtctgaggatagcggctaccgttaccacg

ataggacgcgacatgctcctcaagacccgagattacgttcactctcgttgggcgacgcgc

gagctgctggaggacaattttccaggggctataggtttccgaaaccacaagccttactcc

gtcagggttatctacggagacaccgactccgtgtttatcaagtttgtgggcctgacgtac

gagggggtatcggagctgggggatgctatgtcgcgtcagatttcagcggacctctttaga

gcccccatcaaactggagtgtgagaagacctttcagcgactgctgctgatcaccaagaag

aagtacataggtgtcataaacggggggaagatgctcatgaagggggtcgacctggtccgc

aaaaataactgctctttcataaacttgtacgcgcgacatctggtagatcttttgttgtac

gacgaggatgtggccacggcggcagcagaggtgacagacgtgcctcccgcagaatgggtg

gggcgcccgctaccgagcggctttgacaagtttgggcgagtgctggtagaggcgtacaac

cgtatcactgcccccaacttggacgtgcgcgagttcgttatgactgctgagctgagccgc

tcacccgaatcgtataccaacaagcgcctgccgcacctcaccgtctactttaagctcgcc

atgaggaatgaagaactgcccagcgtaaaagagagaattccgtatgtgatagttgcgcag

accgaggccgcggaacgcgaagcgggtgtagtaaactcaatgcgcggtaccgcccaaaac

cccgtggtaaccaagaccgcacgcccccaacctaaacgcaaactgctggtttccgacctc

gccgaagacccgacctatgtttccgagaatgacgtaccgctaaacacagactactatttc

tcccacctgttgggtaccataagcgtgacctttaaggctctattcggaaatgatgtgaga

acaacagaaaatcttttaaagcggtttattccggaaaccccccacaagacccccacgaaa

acccaggcactgcttgagcgcgccggctttgaaaagctgacgccctttacaccggaggaa

gaaagtcgtcgaatactgcatacagttttttgtactctagaagcagctccccatcaaagc

tga

>LC109624.1

atggcggcgcgcgaacaggccaactccgttcgcaggagcgggttttttaatccgtttatt

ggcaagaggccatttttcaggccgggaagcgggcagaccgcggagacagagcgcccaagg

cccccccaacactcgtactgcacagaggtgggtagctttaagtttatagctccaagatgc

ctcgatgaagaagcccccgccgaccagcgacgcggtgtacacgtgggcaccttggagcgc

ccacccaaagtgtactgcgatggctcagagtacgacgtgctgaactttgcctccggaggt

tgctggcctcgcaggattcgggtttggaacggccaggattttcggggcgatggattcaac

cccagatttgagagattccacgtgtacgacatagtagagacttcggagagcgcctcacac

gatgacccatccaggtttgctgagctatcccgcccaagcgggtctgtggtaacactgctg

ggaatgagcgagtgtggaaagcgagtcgcggttcacgtctatggtgtgcgccattatttt

tacatggcaaaggcggaggtggatagcgcttgtggaataaccaccgaggcagaactcgtg

cgcgcaatggtggactgcgcgcacagctcggctttgagcgcggccctgggaaatggcaac

ggcggcaaacagagcggcggcagcggcgggggatggtggggcggaaagcacgtgtctgcg

gactgcttcaaagtggagaccgtgtgccacacgacgctgtactactttggatctaagcca

gctctctactatagagtatctgcctccagcagccgcctgggagggttcatctgcgacaac

tttcacccggagattacaaaattcgaggggagcgtggacgtgaccacgcggctgttgttg

gacaatgaaaattttaccagtttcgggtggtaccgcctgcgacccggcacccacggagag

cgtgttcaacttcgccccgttgagcgacacgtcacctcaagcgacgtggagattaactgt

actcccgataacctggagccgataccagacgaggctgcctggcccgactataagctcatg

tgctttgatatagagtgtaaagctggaacgggtaacgaaatggcgttcccagtggcaact

aaccaagaggacctggtcatccagatctcctgtctgctgtactcgcttgctactcagaac

cacgaacacaccctgctgttttccctcgggtcatgcgatatctctgaggaatactcgttt

gcatgcgtccagcgcggcgagcccagaccgacggttttggagtttgacagcgagtacgag

ctgctggttgccttcctgacctttctcaagcagtactctcccgagttcgccaccggctac

aacatcgttaattttgactgggcgtacatagttaacaaggtaacgtcggtgtataacatc

aagctggacgggtacggcaagttcaacaaaggggggctgtttaaggtgtgggacatcgcc

acgaaccattttcagaagaagagcaaggtgaaaatcaatggcctgatatctctagacatg

tattctgtggcgacggaaaagctaaagctacccagctacaaactcgacgcggtcgtggga

gacgtcctcggcgagcataagatagaccttccctataaagaaataccctcctattacgcg

ggagggcctgaccggaggggcgtaataggagagtattgtatccaggactctaggctggtg

ggcaagctgttttttaagtacctcccccatctggaactatcggcggtggccaaactcgcc

cgtatcaccctaacgcgggtaatttttgacggtcagcaaattcgcgtgtacacgtgcttg

ctgaaactcgcccgcgagagaaatttcattttgccagacaacagacgccggtttgacagt

caggcagacgccgcgtcagagacttcggagttggctatggatagccaaagccacgccttc

gacagtacagacgaacccgacggtgtggacggtaccccggacgccgcaggatctggcgct

acttctgaaaacggaggcgggaagcccggcgtcgggagggccgtgggctaccagggagca

aaggttctagaccccgtatccggctttcatgtggaccccgtggttgtgtttgacttcgct

agcttatacccaagcattatccaggcccataacctctgtttcaccaccctggcgctcgat

gaagtggatctggccgggcttcaaccatccgtcaactactcgacgttcgaggtgggtgac

caaaagttattttttgtccacgcccatattcgcgaaagcctgcttggcatcttgctgcgc

gactggctggccatgcgaaaggcggtgagggcgcgaatccccaccagcacccccgaggag

gcagttttactagataagcagcagtctgcgattaaggtgatatgcaactcggtttacgga

ttcacgggggtggcaaacggcctgttgccgtgtctgaggatagcggctaccgttaccacg

ataggacgcgacatgctcctcaagacccgagattacgttcactctcgttgggcgacgcgc

gagctgctggaggacaattttccaggggctataggtttccgaaaccacaagccttactcc

gtcagggttatctacggagacaccgactccgtgtttatcaagtttgtgggcctgacgtac

gagggggtatcggagctgggggatgctatgtcgcgtcagatttcagcggacctctttaga

gcccccatcaaactggagtgtgagaagacctttcagcgactgctgctgatcaccaagaag

aagtacataggtgtcataaacggggggaagatgctcatgaagggggtcgacctggtccgc

aaaaataactgctctttcataaacttgtacgcgcgacatctggtagatcttttgttgtac

gacgaggatgtggccacggcggcagcagaggtgacagacgtgcctcccgcagaatgggtg

gggcgcccgctaccgagcggctttgacaagtttgggcgagtgctggtagaggcgtacaac

cgtatcactgcccccaacttggacgtgcgcgagttcgttatgactgctgagctgagccgc

tcacccgaatcgtataccaacaagcgcctgccgcacctcaccgtctactttaagctcgcc

atgaggaatgaagaactgcccagcgtaaaagagagaattccgtatgtgatagttgcgcag

accgaggccgcggaacgcgaagcgggtgtagtaaactcaatgcgcggtaccgcccaaaac

cccgtggtaaccaagaccgcacgcccccaacctaaacgcaaactgctggtttccgacctc

gccgaagacccgacctatgtttccgagaatgacgtaccgctaaacacagactactatttc

tcccacctgttgggtaccataagcgtgacctttaaggctctattcggaaatgatgtgaga

acaacagaaaatcttttaaagcggtttattccggaaaccccccacaagacccccacgaaa

acccaggcactgcttgagcgcgccggctttgaaaagctgacgccctttacaccggaggaa

gaaagtcgtcgaatactgcatacagttttttgtactctagaagcagctccccatcaaagc

tga

>LC109625.1

atggcggcgcgcgaacaggccaactccgttcgcaggagcgggttttttaatccgtttatt

ggcaagaggccatttttcaggccgggaagcgggcagaccgcggagacagagcgcccaagg

cccccccaacactcgtactgcacagaggtgggtagctttaagtttatagctccaagatgc

ctcgatgaagaagcccccgccgaccagcgacgcggtgtacacgtgggcaccttggagcgc

ccacccaaagtgtactgcgatggctcagagtacgacgtgctgaactttgcctccggaggt

tgctggcctcgcaggattcgggtttggaacggccaggattttcggggcgatggattcaac

cccagatttgagagatttcacgtgtacgacatagtagagacttcggagagcgcctcacac

gatgacccatccaggtttgctgagctatcccgcccaagcgggtctgtggtaacactgctg

ggaatgagcgagtgtggaaagcgagtcgcggttcacgtctatggtgtgcgccattatttt

tacatggcaaaggcggaggtggatagcgcttgtggaataaccaccgaggcagaactcgtg

cgcgcaatggtggactgcgcgcacagctcggctttgagcgcggccctgggaaatggcaac

ggcggcaaacagagcggcggcagcggcgggggatggtggggcggaaagcacgtgtctgcg

gactgcttcaaagtggagaccgtgtgccacacgacgctgtactactttggatctaagcca

gctctctactatagagtatctgcctccagcagccgcctgggagggttcatctgcgacaac

tttcacccggagattacaaaattcgaggggagcgtggacgtgaccacgcggctgttgttg

gacaatgaaaattttaccagtttcgggtggtaccgcctgcgacccggcacccacggagag

cgtgttcaacttcgccccgttgagcgacacgtcacctcaagcgacgtggagattaactgt

actcccgataacctggagccgataccagacgaggctgcctggcccgactataagctcatg

tgctttgatatagagtgtaaagctggaacgggtaacgaaatggcgttcccagtggcaact

aaccaagaggacctggtcatccagatctcctgtctgctgtactcgcttgctactcagaac

cacgaacacaccctgctgttttccctcgggtcatgcgatatctctgaggaatactcgttt

gcatgcgtccagcgcggcgagcccagaccgacggttttggagtttgacagcgagtacgag

ctgctggttgccttcctgacctttctcaagcagtactctcccgagttcgccaccggctac

aacatcgttaattttgactgggcgtacatagttaacaaggtaacgtcggtgtataacatc

aagctggacgggtacggcaagttcaacaaaggggggctgtttaaggtgtgggacatcgcc

acgaaccattttcagaagaagagcaaggtgaaaatcaatggcctgatatctctagacatg

tattctgtggcgacggaaaagctaaagctacccagctacaaactcgacgcggtcgtggga

gacgtcctcggcgagcataagatagaccttccctataaagaaataccctcctattacgcg

ggagggcctgaccggaggggcgtaataggagagtattgtatccaggactctaggctggtg

ggcaagctgttttttaagtacctcccccatctggaactatcggcggtggccaaactcgcc

cgtatcaccctaacgcgggtaatttttgacggtcagcaaattcgcgtgtacacgtgcttg

ctgaaactcgcccgcgagagaaatttcattttgccagacaacagacgccggtttgacagt

caggcagacgccgcgtcagagacttcggagttggctatggatagccaaagccacgccttc

gacagtacagacgaacccgacggtgtggacggtaccccggacgccgcaggatctggcgct

acttctgaaaacggaggcgggaagcccggcgtcgggagggccgtgggctaccagggagca

aaggttctagaccccgtatccggctttcatgtggaccccgtggttgtgtttgacttcgct

agcttatacccaagcattatccaggcccataacctctgtttcaccaccctggcgctcgat

gaagtggatctggccgggcttcaaccatccgtcaactactcgacgttcgaggtgggtgac

caaaagttattttttgtccacgcccatattcgcgaaagcctgcttggcatcttgctgcgc

gactggctggccatgcgaaaggcggtgagggcgcgaatccccaccagcacccccgaggag

gcagttttactagataagcagcagtctgcgattaaggtgatatgcaactcggtttacgga

ttcacgggggtggcaaacggcctgttgccgtgtctgaggatagcggctaccgttaccacg

ataggacgcgacatgctcctcaagacccgagattacgttcactctcgttgggcgacgcgc

gagctgctggaggacaattttccaggggctataggtttccgaaaccacaagccttactcc

gtcagggttatctacggagacaccgactccgtgtttatcaagtttgtgggcctgacgtac

gagggggtatcggagctgggggatgctatgtcgcgtcagatttcagcggacctctttaga

gcccccatcaaactggagtgtgagaagacctttcagcgactgctgctgatcaccaagaag

aagtacataggtgtcataaacggggggaagatgctcatgaagggggtcgacctggtccgc

aaaaataactgctctttcataaacttgtacgcgcgacatctggtagatcttttgttgtac

gacgaggatgtggccacggcggcagcagaggtgacagacgtgcctcccgcagaatgggtg

gggcgcccgctaccgagcggctttgacaagtttgggcgagtgctggtagaggcgtacaac

cgtatcactgcccccaacttggacgtgcgcgagttcgttatgactgctgagctgagccgc

tcacccgaatcgtataccaacaagcgcctgccgcacctcaccgtctactttaagctcgcc

atgaggaatgaagaactgcccagcgtaaaagagagaattccgtatgtgatagttgcgcag

accgaggccgcggaacgcgaagcgggtgtagtaaactcaatgcgcggtaccgcccaaaac

cccgtggtaaccaagaccgcacgcccccaacctaaacgcaaactgctggtttccgacctc

gccgaagacccgacctatgtttccgagaatgacgtaccgctaaacacagactactatttc

tcccacctgttgggtaccataagcgtgacctttaaggctctattcggaaatgatgtgaga

acaacagaaaatcttttaaagcggtttattccggaaaccccccacaagacccccacgaaa

acccaggcactgcttgagcgcgccggctttgaaaagctgacgccctttacaccggaggaa

gaaagtcgtcgaatactgcatacagttttttgtactctagaagcagctccccatcaaagc

tga

>LC109626.1

atggcggcgcgcgaacaggccaactccgttcgcaggagcgggttttttaatccgtttatt

ggcaagaggccatttttcaggccgggaagcgggcagaccgcggagacagagcgcccaagg

cccccccaacactcgtactgcacagaggtgggtagctttaagtttatagctccaagatgc

ctcgatgaagaagcccccgccgaccagcgacgcggtgtacacgtgggcaccttggagcgc

ccacccaaagtgtactgcgatggctcagagtacgacgtgctgaactttgcctccggaggt

tgctggcctcgcaggattcgggtttggaacggccaggattttcggggcgatggattcaac

cccagatttgagagatttcacgtgtacgacatagtagagacttcggagagcgcctcacac

gatgacccatccaggtttgctgagctatcccgcccaagcgggtctgtggtaacactgctg

ggaatgagcgagtgtggaaagcgagtcgcggttcacgtctatggtgtgcgccattatttt

tacatggcaaaggcggaggtggatagcgcttgtggaataaccaccgaggcagaactcgtg

cgcgcaatggtggactgcgcgcacagctcggctttgagcgcggccctgggaaatggcaac

ggcggcaaacagagcggcggcagcggcgggggatggtggggcggaaagcacgtgtctgcg

gactgcttcaaagtggagaccgtgtgccacacgacgctgtactactttggatctaagcca

gctctctactatagagtatctgcctccagcagccgcctgggagggttcatctgcgacaac

tttcacccggagattacaaaattcgaggggagcgtggacgtgaccacgcggctgttgttg

gacaatgaaaattttaccagtttcgggtggtaccgcctgcgacccggcacccacggagag

cgtgttcaacttcgccccgttgagcgacacgtcacctcaagcgacgtggagattaactgt

actcccgataacctggagccgataccagacgaggctgcctggcccgactataagctcatg

tgctttgatatagagtgtaaagctggaacgggtaacgaaatggcgttcccagtggcaact

aaccaagaggacctggtcatccagatctcctgtctgctgtactcgcttgctactcagaac

cacgaacacaccctgctgttttccctcgggtcatgcgatatctctgaggaatactcgttt

gcatgcgtccagcgcggcgagcccagaccgacggttttggagtttgacagcgagtacgag

ctgctggttgccttcctgacctttctcaagcagtactctcccgagttcgccaccggctac

aacatcgttaattttgactgggcgtacatagttaacaaggtaacgtcggtgtataacatc

aagctggacgggtacggcaagttcaacaaaggggggctgtttaaggtgtgggacatcgcc

acgaaccattttcagaagaagagcaaggtgaaaatcaatggcctgatatctctagacatg

tattctgtggcgacggaaaagctaaagctacccagctacaaactcgacgcggtcgtggga

gacgtcctcggcgagcataagatagaccttccctataaagaaataccctcctattacgcg

ggagggcctgaccggaggggcgtaataggagagtattgtatccaggactctaggctggtg

ggcaagctgttttttaagtacctcccccatctggaactatcggcggtggccaaactcgcc

cgtatcaccctaacgcgggtaatttttgacggtcagcaaattcgcgtgtacacgtgcttg

ctgaaactcgcccgcgagagaaatttcattttgccagacaacagacgccggtttgacagt

caggcagacgccgcgtcagagacttcggagttggctatggatagccaaagccacgccttc

gacagtacagacgaacccgacggtgtggacggtaccccggacgccgcaggatctggcgct

acttctgaaaacggaggcgggaagcccggcgtcgggagggccgtgggctaccagggagca

aaggttctagaccccgtatccggctttcatgtggaccccgtggttgtgtttgacttcgct

agcttatacccaagcattatccaggcccataacctctgtttcaccaccctggcgctcgat

gaagtggatctggccgggcttcaaccatccgtcaactactcgacgttcgaggtgggtgac

caaaagttattttttgtccacgcccatattcgcgaaagcctgcttggcatcttgctgcgc

gactggctggccatgcgaaaggcggtgagggcgcgaatccccaccagcacccccgaggag

gcagttttactagataagcagcagtctgcgattaaggtgatatgcaactcggtttacgga

ttcacgggggtggcaaacggcctgttgccgtgtctgaggatagcggctaccgttaccacg

ataggacgcgacatgctcctcaagacccgagattacgttcactctcgttgggcgacgcgc

gagctgctggaggacaattttccaggggctataggtttccgaaaccacaagccttactcc

gtcagggttatctacggagacaccgactccgtgtttatcaagtttgtgggcctgacgtac

gagggggtatcggagctgggggatgctatgtcgcgtcagatttcagcggacctctttaga

gcccccatcaaactggagtgtgagaagacctttcagcgactgctgctgatcaccaagaag

aagtacataggtgtcataaacggggggaagatgctcatgaagggggtcgacctggtccgc

aaaaataactgctctttcataaacttgtacgcgcgacatctggtagatcttttgttgtac

gacgaggatgtggccacggcggcagcagaggtgacagacgtgcctcccgcagaatgggtg

gggcgcccgctaccgagcggctttgacaagtttgggcgagtgctggtagaggcgtacaac

cgtatcactgcccccaacttggacgtgcgcgagttcgttatgactgctgagctgagccgc

tcacccgaatcgtataccaacaagcgcctgccgcacctcaccgtctactttaagctcgcc

atgaggaatgaagaactgcccagcgtaaaagagagaattccgtatgtgatagttgcgcag

accgaggccgcggaacgcgaagcgggtgtagtaaactcaatgcgcggtaccgcccaaaac

cccgtggtaaccaagaccgcacgcccccaacctaaacgcaaactgctggtttccgacctc

gccgaagacccgacctatgtttccgagaatgacgtaccgctaaacacagactactatttc

tcccacctgttgggtaccataagcgtgacctttaaggctctattcggaaatgatgtgaga

acaacagaaaatcttttaaagcggtttattccggaaaccccccacaagacccccacgaaa

acccaggcactgcttgagcgcgccggctttgaaaagctgacgccctttacaccggaggaa

gaaagtcgtcgaatactgcatacagttttttgtactctagaagcagctccccatcaaagc

tga

>LC109627.1

atggcggcgcgcgaacaggccaactccgttcgcaggagcgggttttttaatccgtttatt

ggcaagaggccatttttcaggccgggaagcgggcagaccgcggagacagagcgcccaagg

cccccccaacactcgtactgcacagaggtgggtagctttaagtttatagctccaagatgc

ctcgatgaagaagcccccgccgaccagcgacgcggtgtacacgtgggcaccttggagcgc

ccacccaaagtgtactgcgatggctcagagtacgacgtgctgaactttgcctccggaggt

tgctggcctcgcaggattcgggtttggaacggccaggattttcggggcgatggattcaac

cccagatttgagagatttcacgtgtacgacatagtagagacttcggagagcgcctcacac

gatgacccatccaggtttgctgagctatcccgcccaagcgggtctgtggtaacactgctg

ggaatgagcgagtgtggaaagcgagtcgcggttcacgtctatggtgtgcgccattatttt

tacatggcaaaggcggaggtggatagcgcttgtggaataaccaccgaggcagaactcgtg

cgcgcaatggtggactgcgcgcacagctcggctttgagcgcggccctgggaaatggcaac

ggcggcaaacagagcggcggcagcggcgggggatggtggggcggaaagcacgtgtctgcg

gactgcttcaaagtggagaccgtgtgccacacgacgctgtactactttggatctaagcca

gctctctactatagagtatctgcctccagcagccgcctgggagggttcatctgcgacaac

tttcacccggagattacaaaattcgaggggagcgtggacgtgaccacgcggctgttgttg

gacaatgaaaattttaccagtttcgggtggtaccgcctgcgacccggcacccacggagag

cgtgttcaacttcgccccgttgagcgacacgtcacctcaagcgacgtggagattaactgt

actcccgataacctggagccgataccagacgaggctgcctggcccgactataagctcatg

tgctttgatatagagtgtaaagctggaacgggtaacgaaatggcgttcccagtggcaact

aaccaagaggacctggtcatccagatctcctgtctgctgtactcgcttgctactcagaac

cacgaacacaccctgctgttttccctcgggtcatgcgatatctctgaggaatactcgttt

gcatgcgtccagcgcggcgagcccagaccgacggttttggagtttgacagcgagtacgag

ctgctggttgccttcctgacctttctcaagcagtactctcccgagttcgccaccggctac

aacatcgttaattttgactgggcgtacatagttaacaaggtaacgtcggtgtataacatc

aagctggacgggtacggcaagttcaacaaaggggggctgtttaaggtgtgggacatcgcc

acgaaccattttcagaagaagagcaaggtgaaaatcaatggcctgatatctctagacatg

tattctgtggcgacggaaaagctaaagctacccagctacaaactcgacgcggtcgtggga

gacgtcctcggcgagcataagatagaccttccctataaagaaataccctcctattacgcg

ggagggcctgaccggaggggcgtaataggagagtattgtatccaggactctaggctggtg

ggcaagctgttttttaagtacctcccccatctggaactatcggcggtggccaaactcgcc

cgtatcaccctaacgcgggtaatttttgacggtcagcaaattcgcgtgtacacgtgcttg

ctgaaactcgcccgcgagagaaatttcattttgccagacaacagacgccggtttgacagt

caggcagacgccgcgtcagagacttcggagttggctatggatagccaaagccacgccttc

gacagtacagacgaacccgacggtgtggacggtaccccggacgccgcaggatctggcgct

acttctgaaaacggaggcgggaagcccggcgtcgggagggccgtgggctaccagggagca

aaggttctagaccccgtatccggctttcatgtggaccccgtggttgtgtttgacttcgct

agcttatacccaagcattatccaggcccataacctctgtttcaccaccctggcgctcgat

gaagtggatctggccgggcttcaaccatccgtcaactactcgacgttcgaggtgggtgac

caaaagttattttttgtccacgcccatattcgcgaaagcctgcttggcatcttgctgcgc

gactggctggccatgcgaaaggcggtgagggcgcgaatccccaccagcacccccgaggag

gcagttttactagataagcagcagtctgcgattaaggtgatatgcaactcggtttacgga

ttcacgggggtggcaaacggcctgttgccgtgtctgaggatagcggctaccgttaccacg

ataggacgcgacatgctcctcaagacccgagattacgttcactctcgttgggcgacgcgc

gagctgctggaggacaattttccaggggctataggtttccgaaaccacaagccttactcc

gtcagggttatctacggagacaccgactccgtgtttatcaagtttgtgggcctgacgtac

gagggggtatcggagctgggggatgctatgtcgcgtcagatttcagcggacctctttaga

gcccccatcaaactggagtgtgagaagacctttcagcgactgctgctgatcaccaagaag

aagtacataggtgtcataaacggggggaagatgctcatgaagggggtcgacctggtccgc

aaaaataactgctctttcataaacttgtacgcgcgacatctggtagatcttttgttgtac

gacgaggatgtggccacggcggcagcagaggtgacagacgtgcctcccgcagaatgggtg

gggcgcccgctaccgagcggctttgacaagtttgggcgagtgctggtagaggcgtacaac

cgtatcactgcccccaacttggacgtgcgcgagttcgttatgactgctgagctgagccgc

tcacccgaatcgtataccaacaagcgcctgccgcacctcaccgtctactttaagctcgcc

atgaggaatgaagaactgcccagcgtaaaagagagaattccgtatgtgatagttgcgcag

accgaggccgcggaacgcgaagcgggtgtagtaaactcaatgcgcggtaccgcccaaaac

cccgtggtaaccaagaccgcacgcccccaacctaaacgcaaactgctggtttccgacctc

gccgaagacccgacctatgtttccgagaatgacgtaccgctaaacacagactactatttc

tcccacctgttgggtaccataagcgtgacctttaaggctctattcggaaatgatgtgaga

acaacagaaaatcttttaaagcggtttattccggaaaccccccacaagacccccacgaaa

acccaggcactgcttgagcgcgccggctttgaaaagctgacgccctttacaccggaggaa

gaaagtcgtcgaatactgcatacagttttttgtactctagaagcagctccccatcaaagc

tga

>LC109628.1

atggcggcgcgcgaacaggccaactccgttcgcaggagcgggttttttaatccgtttatt

ggcaagaggccatttttcaggccgggaagcgggcagaccgcggagacagagcgcccaagg

cccccccaacactcgtactgcacagaggtgggtagctttaagtttatagctccaagatgc

ctcgatgaagaagcccccgccgaccagcgacgcggtgtacacgtgggcaccttggagcgc

ccacccaaagtgtactgcgatggctcagagtacgacgtgctgaactttgcctccggaggt

tgctggcctcgcaggattcgggtttggaacggccaggattttcggggcgatggattcaac

cccagatttgagagatttcacgtgtacgacatagtagagacttcggagagcgcctcacac

gatgacccatccaggtttgctgagctatcccgcccaagcgggtctgtggtaacactgctg

ggaatgagcgagtgtggaaagcgagtcgcggttcacgtctatggtgtgcgccattatttt

tacatggcaaaggcggaggtggatagcgcttgtggaataaccaccgaggcagaactcgtg

cgcgcaatggtggactgcgcgcacagctcggctttgagcgcggccctgggaaatggcaac

ggcggcaaacagagcggcggcagcggcgggggatggtggggcggaaagcacgtgtctgcg

gactgcttcaaagtggagaccgtgtgccacacgacgctgtactactttggatctaagcca

gctctctactatagagtatctgcctccagcagccgcctgggagggttcatctgcgacaac

tttcacccggagattacaaaattcgaggggagcgtggacgtgaccacgcggctgttgttg

gacaatgaaaattttaccagtttcgggtggtaccgcctgcgacccggcacccacggagag

cgtgttcaacttcgccccgttgagcgacacgtcacctcaagcgacgtggagattaactgt

actcccgataacctggagccgataccagacgaggctgcctggcccgactataagctcatg

tgctttgatatagagtgtaaagctggaacgggtaacgaaatggcgttcccagtggcaact

aaccaagaggacctggtcatccagatctcctgtctgctgtactcgcttgctactcagaac

cacgaacacaccctgctgttttccctcgggtcatgcgatatctctgaggaatactcgttt

gcatgcgtccagcgcggcgagcccagaccgacggttttggagtttgacagcgagtacgag

ctgctggttgccttcctgacctttctcaagcagtactctcccgagttcgccaccggctac

aacatcgttaattttgactgggcgtacatagttaacaaggtaacgtcggtgtataacatc

aagctggacgggtacggcaagttcaacaaaggggggctgtttaaggtgtgggacatcgcc

acgaaccattttcagaagaagagcaaggtgaaaatcaatggcctgatatctctagacatg

tattctgtggcgacggaaaagctaaagctacccagctacaaactcgacgcggtcgtggga

gacgtcctcggcgagcataagatagaccttccctataaagaaataccctcctattacgcg

ggagggcctgaccggaggggcgtaataggagagtattgtatccaggactctaggctggtg

ggcaagctgttttttaagtacctcccccatctggaactatcggcggtggccaaactcgcc

cgtatcaccctaacgcgggtaatttttgacggtcagcaaattcgcgtgtacacgtgcttg

ctgaaactcgcccgcgagagaaatttcattttgccagacaacagacgccggtttgacagt

caggcagacgccgcgtcagagacttcggagttggctatggatagccaaagccacgccttc

gacagtacagacgaacccgacggtgtggacggtaccccggacgccgcaggatctggcgct

acttctgaaaacggaggcgggaagcccggcgtcgggagggccgtgggctaccagggagca

aaggttctagaccccgtatccggctttcatgtggaccccgtggttgtgtttgacttcgct

agcttatacccaagcattatccaggcccataacctctgtttcaccaccctggcgctcgat

gaagtggatctggccgggcttcaaccatccgtcaactactcgacgttcgaggtgggtgac

caaaagttattttttgtccacgcccatattcgcgaaagcctgcttggcatcttgctgcgc

gactggctggccatgcgaaaggcggtgagggcgcgaatccccaccagcacccccgaggag

gcagttttactagataagcagcagtctgcgattaaggtgatatgcaactcggtttacgga

ttcacgggggtggcaaacggcctgttgccgtgtctgaggatagcggctaccgttaccacg

ataggacgcgacatgctcctcaagacccgagattacgttcactctcgttgggcgacgcgc

gagctgctggaggacaattttccaggggctataggtttccgaaaccacaagccttactcc

gtcagggttatctacggagacaccgactccgtgtttatcaagtttgtgggcctgacgtac

gagggggtatcggagctgggggatgctatgtcgcgtcagatttcagcggacctctttaga

gcccccatcaaactggagtgtgagaagacctttcagcgactgctgctgatcaccaagaag

aagtacataggtgtcataaacggggggaagatgctcatgaagggggtcgacctggtccgc

aaaaataactgctctttcataaacttgtacgcgcgacatctggtagatcttttgttgtac

gacgaggatgtggccacggcggcagcagaggtgacagacgtgcctcccgcagaatgggtg

gggcgcccgctaccgagcggctttgacaagtttgggcgagtgctggtagaggcgtacaac

cgtatcactgcccccaacttggacgtgcgcgagttcgttatgactgctgagctgagccgc

tcacccgaatcgtataccaacaagcgcctgccgcacctcaccgtctactttaagctcgcc

atgaggaatgaagaactgcccagcgtaaaagagagaattccgtatgtgatagttgcgcag

accgaggccgcggaacgcgaagcgggtgtagtaaactcaatgcgcggtaccgcccaaaac

cccgtggtaaccaagaccgcacgcccccaacctaaacgcaaactgctggtttccgacctc

gccgaagacccgacctatgtttccgagaatgacgtaccgctaaacacagactactatttc

tcccacctgttgggtaccataagcgtgacctttaaggctctattcggaaatgatgtgaga

acaacagaaaatcttttaaagcggtttattccggaaaccccccacaagacccccacgaaa

acccaggcactgcttgagcgcgccggctttgaaaagctgacgccctttacaccggaggaa

gaaagtcgtcgaatactgcatacagttttttgtactctagaagcagctccccatcaaagc

tga

>LC109629.1

atggcggcgcgcgaacaggccaactccgttcgcaggagcgggttttttaatccgtttatt

ggcaagaggccatttttcaggccgggaagcgggcagaccgcggagacagagcgcccaagg

cccccccaacactcgtactgcacagaggtgggtagctttaagtttatagctccaagatgc

ctcgatgaagaagcccccgccgaccagcgacgcggtgtacacgtgggcaccttggagcgc

ccacccaaagtgtactgcgatggctcagagtacgacgtgctgaactttgcctccggaggt

tgctggcctcgcaggattcgggtttggaacggccaggattttcggggcgatggattcaac

cccagatttgagagatttcacgtgtacgacatagtagagacttcggagagcgcctcacac

gatgacccatccaggtttgctgagctatcccgcccaagcgggtctgtggtaacactgctg

ggaatgagcgagtgtggaaagcgagtcgcggttcacgtctatggtgtgcgccattatttt

tacatggcaaaggcggaggtggatagcgcttgtggaataaccaccgaggcagaactcgtg

cgcgcaatggtggactgcgcgcacagctcggctttgagcgcggccctgggaaatggcaac

ggcggcaaacagagcggcggcagcggcgggggatggtggggcggaaagcacgtgtccgcg

gactgcttcaaagtggagaccgtgtgccacacgacgctgtactactttggatctaagcca

gctctctactatagagtatctgcctccagcagccgcctgggagggttcatctgcgacaac

tttcacccggagattacaaaattcgaggggagcgtggacgtgaccacgcggctgttgttg

gacaatgaaaattttaccagtttcgggtggtaccgcctgcgacccggcacccacggagag

cgtgttcaacttcgccccgttgagcgacacgtcacctcaagcgacgtggagattaactgt

actcccgataacctggagccgataccagacgaggctgcctggcccgactataagctcatg

tgctttgatatagagtgtaaagctggaacgggtaacgaaatggcgttcccagtggcaact

aaccaagaggacctggtcatccagatctcctgtctgctgtactcgcttgctactcagaac

cacgaacacaccctgctgttttccctcgggtcatgcgatatctctgaggaatactcgttt

gcatgcgtccagcgcggcgagcccagaccgacggttttggagtttgacagcgagtacgag

ctgctggttgccttcctgacctttctcaagcagtactctcccgagttcgccaccggctac

aacatcgttaattttgactgggcgtacatagttaacaaggtaacgtcggtgtataacatc

aagctggacgggtacggcaagttcaacaaaggggggctgtttaaggtgtgggacatcgcc

acgaaccattttcagaagaagagcaaggtgaaaatcaatggcctgatatctctagacatg

tattctgtggcgacggaaaagctaaagctacccagctacaaactcgacgcggtcgtggga

gacgtcctcggcgagcataagatagaccttccctataaagaaataccctcctattacgcg

ggagggcctgaccggaggggcgtaataggagagtattgtatccaggactctaggctggtg

ggcaagctgttttttaagtacctcccccatctggaactatcggcggtggccaaactcgcc

cgtatcaccctaacgcgggtaatttttgacggtcagcaaattcgcgtgtacacgtgcttg

ctgaaactcgcccgcgagagaaatttcattttgccagacaacagacgccggtttgacagt

caggcagacgccgcgtcagagacttcggagttggctatggatagccaaagccacgccttc

gacagtacagacgaacccgacggtgtggacggtaccccggacgccgcaggatctggcgct

acttctgaaaacggaggcgggaagcccggcgtcgggagggccgtgggctaccagggagca

aaggttctagaccccgtatccggctttcatgtggaccccgtggttgtgtttgacttcgct

agcttatacccaagcattatccaggcccataacctctgtttcaccaccctggcgctcgat

gaagtggatctggccgggcttcaaccatccgtcaactactcgacgttcgaggtgggtgac

caaaagttattttttgtccacgcccatattcgcgaaagcctgcttggcatcttgctgcgc

gactggctggccatgcgaaaggcggtgagggcgcgaatccccaccagcacccccgaggag

gcagttttactagataagcagcagtctgcgattaaggtgatatgcaactcggtttacgga

ttcacgggggtggcaaacggcctgttgccgtgtctgaggatagcggctaccgttaccacg

ataggacgcgacatgctcctcaagacccgagattacgttcactctcgttgggcgacgcgc

gagctgctggaggacaattttccaggggctataggtttccgaaaccacaagccttactcc

gtcagggttatctacggagacaccgactccgtgtttatcaagtttgtgggcctgacgtac

gagggggtatcggagctgggggatgctatgtcgcgtcagatttcagcggacctctttaga

gcccccatcaaactggagtgtgagaagacctttcagcgactgctgctgatcaccaagaag

aagtacataggtgtcataaacggggggaagatgctcatgaagggggtcgacctggtccgc

aaaaataactgctctttcataaacttgtacgcgcgacatctggtagatcttttgttgtac

gacgaggatgtggccacggcggcagcagaggtgacagacgtgcctcccgcagaatgggtg

gggcgcccgctaccgagcggctttgacaagtttgggcgagtgctggtagaggcgtacaac

cgtatcactgcccccaacttggacgtgcgcgagttcgttatgactgctgagctgagccgc

tcacccgaatcgtataccaacaagcgcctgccgcacctcaccgtctactttaagctcgcc

atgaggaatgaagaactgcccagcgtaaaagagagaattccgtatgtgatagttgcgcag

accgaggccgcggaacgcgaagcgggtgtagtaaactcaatgcgcggtaccgcccaaaac

cccgtggtaaccaagaccgcacgcccccaacctaaacgcaaactgctggtttccgacctc

gccgaagacccgacctatgtttccgagaatgacgtaccgctaaacacagactactatttc

tcccacctgttgggtaccataagcgtgacctttaaggctctattcggaaatgatgtgaga

acaacagaaaatcttttaaagcggtttattccggaaaccccccacaagacccccacgaaa

acccaggcactgcttgagcgcgccggctttgaaaagctgacgccctttacaccggaggaa

gaaagtcgtcgaatactgcatacagttttttgtactctagaagcagctccccatcaaagc

tga

>LC109630.1

atggcggcgcgcgaacaggccaactccgttcgcaggagcgggttttttaatccgtttatt

ggcaagaggccatttttcaggccgggaagcgggcagaccgcggagacagagcgcccaagg

cccccccaacactcgtactgcacagaggtgggtagctttaagtttatagctccaagatgc

ctcgatgaagaagcccccgccgaccagcgacgcggtgtacacgtgggcaccttggagcgc

ccacccaaagtgtactgcgatggctcagagtacgacgtgctgaactttgcctccggaggt

tgctggcctcgcaggattcgggtttggaacggccaggattttcggggcgatggattcaac

cccagatttgagagatttcacgtgtacgacatagtagagacttcggagagcgcctcacac

gatgacccatccaggtttgctgagctatcccgcccaagcgggtctgtggtaacactgctg

ggaatgagcgagtgtggaaagcgagtcgcggttcacgtctatggtgtgcgccattatttt

tacatggcaaaggcggaggtggatagcgcttgtggaataaccaccgaggcagaactcgtg

cgcgcaatggtggactgcgcgcacagctcggctttgagcgcggccctgggaaatggcaac

ggcggcaaacagagcggcggcagcggcgggggatggtggggcggaaagcacgtgtctgcg

gactgcttcaaagtggagaccgtgtgccacacgacgctgtactactttggatctaagcca

gctctctactatagagtatctgcctccagcagccgcctgggagggttcatctgcgacaac

tttcacccggagattacaaaattcgaggggagcgtggacgtgaccacgcggctgttgttg

gacaatgaaaattttaccagtttcgggtggtaccgcctgcgacccggcacccacggagag

cgtgttcaacttcgccccgttgagcgacacgtcacctcaagcgacgtggagattaactgt

actcccgataacctggagccgataccagacgaggctgcctggcccgactataagctcatg

tgctttgatatagagtgtaaagctggaacgggtaacgaaatggcgttcccagtggcaact

aaccaagaggacctggtcatccagatctcctgtctgctgtactcgcttgctactcagaac

cacgaacacaccctgctgttttccctcgggtcatgcgatatctctgaggaatactcgttt

gcatgcgtccagcgcggcgagcccagaccgacggttttggagtttgacagcgagtacgag

ctgctggttgccttcctgacctttctcaagcagtactctcccgagttcgccaccggctac

aacatcgttaattttgactgggcgtacatagttaacaaggtaacgtcggtgtataacatc

aagctggacgggtacggcaagttcaacaaaggggggctgtttaaggtgtgggacatcgcc

acgaaccattttcagaagaagagcaaggtgaaaatcaatggcctgatatctctagacatg

tattctgtggcgacggaaaagctaaagctacccagctacaaactcgacgcggtcgtggga

gacgtcctcggcgagcataagatagaccttccctataaagaaataccctcctattacgcg

ggagggcctgaccggaggggcgtaataggagagtattgtatccaggactctaggctggtg

ggcaagctgttttttaagtacctcccccatctggaactatcggcggtggccaaactcgcc

cgtatcaccctaacgcgggtaatttttgacggtcagcaaattcgcgtgtacacgtgcttg

ctgaaactcgcccgcgagagaaatttcattttgccagacaacagacgccggtttgacagt

caggcagacgccgcgtcagagacttcggagttggctatggatagccaaagccacgccttc

gacagtacagacgaacccgacggtgtggacggtaccccggacgccgcaggatctggcgct

acttctgaaaacggaggcgggaagcccggcgtcgggagggccgtgggctaccagggagca

aaggttctagaccccgtatccggctttcatgtggaccccgtggttgtgtttgacttcgct

agcttatacccaagcattatccaggcccataacctctgtttcaccaccctggcgctcgat

gaagtggatctggccgggcttcaaccatccgtcaactactcgacgttcgaggtgggtgac

caaaagttattttttgtccacgcccatattcgcgaaagcctgcttggcatcttgctgcgc

gactggctggccatgcgaaaggcggtgagggcgcgaatccccaccagcacccccgaggag

gcagttttactagataagcagcagtctgcgattaaggtgatatgcaactcggtttacgga

ttcacgggggtggcaaacggcctgttgccgtgtctgaggatagcggctaccgttaccacg

ataggacgcgacatgctcctcaagacccgagattacgttcactctcgttgggcgacgcgc

gagctgctggaggacaattttccaggggctataggtttccgaaaccacaagccttactcc

gtcagggttatctacggagacaccgactccgtgtttatcaagtttgtgggcctgacgtac

gagggggtatcggagctgggggatgctatgtcgcgtcagatttcagcggacctctttaga

gcgcccatcaaactggagtgtgagaagacctttcagcgactgctgctgatcaccaagaag

aagtacataggtgtcataaacggggggaagatgctcatgaagggggtcgacctggtccgc

aaaaataactgctctttcataaacttgtacgcgcgacatctggtagatcttttgttgtac

gacgaggatgtggccacggcggcagcagaggtgacagacgtgcctcccgcagaatgggtg

gggcgcccgctaccgagcggctttgacaagtttgggcgagtgctggtagaggcgtacaac

cgtatcactgcccccaacttggacgtgcgcgagttcgttatgactgctgagctgagccgc

tcacccgaatcgtataccaacaagcgcctgccgcacctcaccgtctactttaagctcgcc

atgaggaatgaagaactgcccagcgtaaaagagagaattccgtatgtgatagttgcgcag

accgaggccgcggaacgcgaagcgggtgtagtaaactcaatgcgcggtaccgcccaaaac

cccgtggtaaccaagaccgcacgcccccaacctaaacgcaaactgctggtttccgacctc

gccgaagacccgacctatgtttccgagaatgacgtaccgctaaacacagactactatttc

tcccacctgttgggtaccataagcgtgacctttaaggctctattcggaaatgatgtgaga

acaacagaaaatcttttaaagcggtttattccggaaaccccccacaagacccccacgaaa

acccaggcactgcttgagcgcgccggctttgaaaagctgacgccctttacaccggaggaa

gaaagtcgtcgaatactgcatacagttttttgtactctagaagcagctccccatcaaagc

tga

>LC109631.1

atggcggcgcgcgaacaggccaactccgttcgcaggagcgggttttttaatccgtttatt

ggcaagaggccatttttcaggccgggaagcgggcaaaccgcggagacagagcgcccaagg

cccccccaacactcgtactgcacagaggtgggtagctttaagtttatagctccaagatgc

ctcgatgaagaagcccccgccgaccagcgacgcggtgtacacgtgggcaccttggagcgc

ccacccaaagtgtactgcgatggctcagagtacgacgtgctgaactttgcctccggaggt

tgctggcctcgcaggattcgggtttggaacggccaggattttcggggcgatggattcaac

cccagatttgagagatttcacgtgtacgacatagtagagacttcggagagcgcctcacac

gatgacccatccaggtttgctgagctatcccgcccaagcgggtctgtggtaacactgctg

ggaatgagcgagtgtggaaagcgagtcgcggttcacgtctatggtgtgcgccattatttt

tacatggcaaaggcggaggtggatagcgcttgtggaataaccaccgaggcagaactcgtg

cgcgcaatggtggactgcgcgcacagctcggctttgagcgcggccctgggaaatggcaac

ggcggcaaacagagcggcggcagcggcgggggatggtggggcggaaagcacgtgtctgcg

gactgcttcaaagtggagaccgtgtgccacacgacgctgtactactttggatctaagcca

gctctctactatagagtatctgcctccagcagccgcctgggagggttcatctgcgacaac

tttcacccggagattacaaaattcgaggggagcgtggacgtgaccacgcggctgttgttg

gacaatgaaaattttaccagtttcgggtggtaccgcctgcgacccggcacccacggagag

cgtgttcaacttcgccccgttgagcgacacgtcacctcaagcgacgtggagattaactgt

actcccgataacctggagccgataccagacgaggctgcctggcccgactataagctcatg

tgctttgatatagagtgtaaagctggaacgggtaacgaaatggcgttcccagtggcaact

aaccaagaggacctggtcatccagatctcctgtctgctgtactcgcttgctactcagaac

cacgaacacaccctgctgttttccctcgggtcatgcgatatctctgaggaatactcgttt

gcatgcgtccagcgcggcgagcccagaccgacggttttggagtttgacagcgagtacgag

ctgctggttgccttcctgacctttctcaagcagtactctcccgagttcgccaccggctac

aacatcgttaattttgactgggcgtacatagttaacaaggtaacgtcggtgtataacatc

aagctggacgggtacggcaagttcaacaaaggggggctgtttaaggtgtgggacatcgcc

acgaaccattttcagaagaagagcaaggtgaaaatcaatggcctgatatctctagacatg

tattctgtggcgacggaaaagctaaagctacccagctacaaactcgacgcggtcgtggga

gacgtcctcggcgagcataagatagaccttccctataaagaaataccctcctattacgcg

ggagggcctgaccggaggggcgtaataggagagtattgtatccaggactctaggctggtg

ggcaagctgttttttaagtacctcccccatctggaactatcggcggtggccaaactcgcc

cgtatcaccctaacgcgggtaatttttgacggtcagcaaattcgcgtgtacacgtgcttg

ctgaaactcgcccgcgagagaaatttcattttgccagacaacagacgccggtttgacagt

caggcagacgccgcgtcagagacttcggagttggctatggatagccaaagccacgccttc

gacagtacagacgaacccgacggtgtggacggtaccccggacgccgcaggatctggcgct

acttctgaaaacggaggcgggaagcccggcgtcgggagggccgtgggctaccagggagca

aaggttctagaccccgtatccggctttcatgtggaccccgtggttgtgtttgacttcgct

agcttatacccaagcattatccaggcccataacctctgtttcaccaccctggcgctcgat

gaagtggatctggccgggcttcaaccatccgtcaactactcgacgttcgaggtgggtgac

caaaagttattttttgtccacgcccatattcgcgaaagcctgcttggcatcttgctgcgc

gactggctggccatgcgaaaggcggtgagggcgcgaatccccaccagcacccccgaggag

gcagttttactagataagcagcagtctgcgattaaggtgatatgcaactcggtttacgga

ttcacgggggtggcaaacggcctgttgccgtgtctgaggatagcggctaccgttaccacg

ataggacgcgacatgctcctcaagacccgagattacgttcactctcgttgggcgacgcgc

gagctgctggaggacaattttccaggggctataggtttccgaaaccacaagccttactcc

gtcagggttatctacggagacaccgactccgtgtttatcaagtttgtgggcctgacgtac

gagggggtatcggagctgggggatgctatgtcgcgtcagatttcagcggacctctttaga

gcgcccatcaaactggagtgtgagaagacctttcagcgactgctgctgatcaccaagaag

aagtacataggtgtcataaacggggggaagatgctcatgaagggggtcgacctggtccgc

aaaaataactgctctttcataaacttgtacgcgcgacatctggtagatcttttgttgtac

gacgaggatgtggccacggcggcagcagaggtgacagacgtgcctcccgcagaatgggtg

gggcgcccgctaccgagcggctttgacaagtttgggcgagtgctggtagaggcgtacaac

cgtatcactgcccccaacttggacgtgcgcgagttcgttatgactgctgagctgagccgc

tcacccgaatcgtataccaacaagcgcctgccgcacctcaccgtctactttaagctcgcc

atgaggaatgaagaactgcccagcgtaaaagagagaattccgtatgtgatagttgcgcag

accgaggccgcggaacgcgaagcgggtgtagtaaactcaatgcgcggtaccgcccaaaac

cccgtggtaaccaagaccgcacgcccccaacctaaacgcaaactgctggtttccgacctc

gccgaagacccgacctatgtttccgagaatgacgtaccgctaaacacagactactatttc

tcccacctgttgggtaccataagcgtgacctttaaggctctattcggaaatgatgtgaga

acaacagaaaatcttttaaagcggtttattccggaaaccccccacaagacccccacgaaa

acccaggcactgcttgagcgcgccggctttgaaaagctgacgccctttacaccggaggaa

gaaagtcgtcgaatactgcatacagttttttgtactctagaagcagctccccatcaaagc

tga

>LC109632.1

atggcggcgcgcgaacaggccaactccgttcgcaggagcgggttttttaatccgtttatt

ggcaagaggccatttttcaggccgggaagcgggcaaaccgcggagacagagcgcccaagg

cccccccaacactcgtactgcacagaggtgggtagctttaagtttatagctccaagatgc

ctcgatgaagaagcccccgccgaccagcgacgcggtgtacacgtgggcaccttggagcgc

ccacccaaagtgtactgcgatggctcagagtacgacgtgctgaactttgcctccggaggt

tgctggcctcgcaggattcgggtttggaacggccaggattttcggggcgatggattcaac

cccagatttgagagatttcacgtgtacgacatagtagagacttcggagagcgcctcacac

gatgacccatccaggtttgctgagctatcccgcccaagcgggtctgtggtaacactgctg

ggaatgagcgagtgtggaaagcgagtcgcggttcacgtctatggtgtgcgccattatttt

tacatggcaaaggcggaggtggatagcgcttgtggaataaccaccgaggcagaactcgtg

cgcgcaatggtggactgcgcgcacagctcggctttgagcgcggccctgggaaatggcaac

ggcggcaaacagagcggcggcagcggcgggggatggtggggcggaaagcacgtgtctgcg

gactgcttcaaagtggagaccgtgtgccacacgacgctgtactactttggatctaagcca

gctctctactatagagtatctgcctccagcagccgcctgggagggttcatctgcgacaac

tttcacccggagattacaaaattcgaggggagcgtggacgtgaccacgcggctgttgttg

gacaatgaaaattttaccagtttcgggtggtaccgcctgcgacccggcacccacggagag

cgtgttcaacttcgccccgttgagcgacacgtcacctcaagcgacgtggagattaactgt

actcccgataacctggagccgataccagacgaggctgcctggcccgactataagctcatg

tgctttgatatagagtgtaaagctggaacgggtaacgaaatggcgttcccagtggcaact

aaccaagaggacctggtcatccagatctcctgtctgctgtactcgcttgctactcagaac

cacgaacacaccctgctgttttccctcgggtcatgcgatatctctgaggaatactcgttt

gcatgcgtccagcgcggcgagcccagaccgacggttttggagtttgacagcgagtacgag

ctgctggttgccttcctgacctttctcaagcagtactctcccgagttcgccaccggctac

aacatcgttaattttgactgggcgtacatagttaacaaggtaacgtcggtgtataacatc

aagctggacgggtacggcaagttcaacaaaggggggctgtttaaggtgtgggacatcgcc

acgaaccattttcagaagaagagcaaggtgaaaatcaatggcctgatatctctagacatg

tattctgtggcgacggaaaagctaaagctacccagctacaaactcgacgcggtcgtggga

gacgtcctcggcgagcataagatagaccttccctataaagaaataccctcctattacgcg

ggagggcctgaccggaggggcgtaataggagagtattgtatccaggactctaggctggtg

ggcaagctgttttttaagtacctcccccatctggaactatcggcggtggccaaactcgcc

cgtatcaccctaacgcgggtaatttttgacggtcagcaaattcgcgtgtacacgtgcttg

ctgaaactcgcccgcgagagaaatttcattttgccagacaacagacgccggtttgacagc

caggcagacgccgcgtcagagacttcggagttggctatggatagccaaagccacgccttc

gacagtacagacgaacccgacggtgtggacggtaccccggacgccgcaggatctggcgct

acttctgaaaacggaggcgggaagcccggcgtcgggagggccgtgggctaccagggagca

aaggttctagaccccgtatccggctttcatgtggaccccgtggttgtgtttgacttcgct

agcttatacccaagcattatccaggcccataacctctgtttcaccaccctggcgctcgat

gaagtggatctggccgggcttcaaccatccgtcaactactcgacgttcgaggtgggtgac

caaaagttattttttgtccacgcccatattcgcgaaagcctgcttggcatcttgctgcgc

gactggctggccatgcgaaaggcggtgagggcgcgaatccccaccagcacccccgaggag

gcagttttactagataagcagcagtctgcgattaaggtgatatgcaactcggtttacgga

ttcacgggggtggcaaacggcctgttgccgtgtctgaggatagcggctaccgttaccacg

ataggacgcgacatgctcctcaagacccgagattacgttcactctcgttgggcgacgcgc

gagctgctggaggacaattttccaggggctataggtttccgaaaccacaagccttactcc

gtcagggttatctacggagacaccgactccgtgtttatcaagtttgtgggcctgacgtac

gagggggtatcggagctgggggatgctatgtcgcgtcagatttcagcggacctctttaga

gcgcccatcaaactggagtgtgagaagacctttcagcgactgctgctgatcaccaagaag

aagtacataggtgtcataaacggggggaagatgctcatgaagggggtcgacctggtccgc

aaaaataactgctctttcataaacttgtacgcgcgacatctggtagatcttttgttgtac

gacgaggatgtggccacggcggcagcagaggtgacagacgtgcctcccgcagaatgggtg

gggcgcccgctaccgagcggctttgacaagtttgggcgagtgctggtagaggcgtacaac

cgtatcactgcccccaacttggacgtgcgcgagttcgttatgactgctgagctgagccgc

tcacccgaatcgtataccaacaagcgcctgccgcacctcaccgtctactttaagctcgcc

atgaggaatgaagaactgcccagcgtaaaagagagaattccgtatgtgatagttgcgcag

accgaggccgcggaacgcgaagcgggtgtagtaaactcaatgcgcggtaccgcccaaaac

cccgtggtaaccaagaccgcacgcccccaacctaaacgcaaactgctggtttccgacctc

gccgaagacccgacctatgtttccgagaatgacgtaccgctaaacacagactactatttc

tcccacctgttgggtaccataagcgtgacctttaaggctctattcggaaatgatgtgaga

acaacagaaaatcttttaaagcggtttattccggaaaccccccacaagacccccacgaaa

acccaggcactgcttgagcgcgccggctttgaaaagctgacgccctttacaccggaggaa

gaaagtcgtcgaatactgcatacagttttttgtactctagaagcagctccccatcaaagc

tga

>LC109633.1

atggcggcgcgcgaacaggccaactccgttcgcaggagcgggttttttaatccgtttatt

ggcaagaggccatttttcaggccgggaagcgggcagaccgcggagacagagcgcccaagg

cccccccaacactcgtactgcacagaggtgggtagctttaaatttatagctccaagatgc

ctcgatgaagaagcccccgccgaccagcgacgcggtgtacacgtgggcaccttggagcgc

ccacccaaagtgtactgcgatggctcagagtacgacgtgctgaactttgcctccggaggt

tgctggcctcgcaggattcgggtttggaacggccaggattttcggggcgatggattcaac

cccagatttgagagatttcacgtgtacgacatagtagagacttcggagagcgcctcacac

gatgacccatccaggtttgctgagctatcccgcccaagcgggtctgtggtaacactgctg

ggaatgagcgagtgtggaaagcgagtcgcggttcacgtctatggtgtgcgccattatttt

tacatggcaaaggcggaggtggatagcgcttgtggaataaccaccgaggcagaactcgtg

cgcgcaatggtggactgcgcgcacagctcggctttgagcgcggccctgggaaatggcaac

ggcggcaaacagagcggcggcagcggcgggggatggtggggcggaaagcacgtgtccgcg

gactgcttcaaagtggagaccgtgtgccacacgacgctgtactactttggatctaagcca

gctctctactatagagtatctgcctccagcagccgcctgggagggttcatctgcgacaac

tttcacccggagattacaaaattcgaggggagcgtggacgtgaccacgcggctgttgttg

gacaatgaaaattttaccagtttcgggtggtaccgcctgcgacccggcacccacggagag

cgtgttcaacttcgccccgttgagcgacacgtcacctcaagcgacgtggagattaactgt

actcccgataacctggagccgataccagacgaggctgcctggcccgactataagctcatg

tgctttgatatagagtgtaaagctggaacgggtaacgaaatggcgttcccagtggcaact

aaccaagaggacctggtcatccagatctcctgtctgctgtactcgcttgctactcagaac

cacgaacacaccctgctgttttccctcgggtcatgcgatatctctgaggaatactcgttt

gcatgcgtccagcgcggcgagcccagaccgacggttttggagtttgacagcgagtacgag

ctgctggttgccttcctgacctttctcaagcagtactctcccgagttcgccaccggctac

aacatcgttaattttgactgggcgtacatagttaacaaggtaacgtcggtgtataacatc

aagctggacgggtacggcaagttcaacaaaggggggctgtttaaggtgtgggacatcgcc

acgaaccattttcagaagaagagcaaggtgaaaatcaatggcctgatatctctagacatg

tattctgtggcgacggaaaagctaaagctacccagctacaaactcgacgcggtcgtggga

gacgtcctcggcgagcataagatagaccttccctataaagaaataccctcctattacgcg

ggagggcctgaccggaggggcgtaataggagagtattgtatccaggactctaggctggtg

ggcaagctgttttttaagtacctcccccatctggaactatcggcggtggccaaactcgcc

cgtatcaccctaacgcgggtaatttttgacggtcagcaaattcgcgtgtacacgtgcttg

ctgaaactcgcccgcgagagaaatttcattttgccagacaacagacgccggtttgacagt

caggcagacgccgcgtcagagacttcggagttggctatggatagccaaagccacgccttc

gacagtacagacgaacccgacggtgtggacggtaccccggacgccgcaggatctggcgct

acttctgaaaacggaggcgggaagcccggcgtcgggagggccgtgggctaccagggagca

aaggttctagaccccgtatccggctttcatgtggaccccgtggttgtgtttgacttcgct

agcttatacccaagcattatccaggcccataacctctgtttcaccaccctggcgctcgat

gaagtggatctggccgggcttcaaccatccgtcaactactcgacgttcgaggtgggtgac

caaaagttattttttgtccacgcccatattcgcgaaagcctgcttggcatcttgctgcgc

gactggctggccatgcgaaaggcggtgagggcgcgaatccccaccagcacccccgaggag

gcagttttactagataagcagcagtctgcgattaaggtgatatgcaactcggtttacgga

ttcacgggggtggcaaacggcctgttgccgtgtctgaggatagcggctaccgttaccacg

ataggacgcgacatgctcctcaagacccgagattacgttcactctcgttgggcgacgcgc

gagctgctggaggacaattttccaggggctataggtttccgaaaccacaagccttactcc

gtcagggttatctacggagacaccgactccgtgtttatcaagtttgtgggcctgacgtac

gagggggtatcggagctgggggatgctatgtcgcgtcagatttcagcggacctctttaga

gcccccatcaaactggagtgtgagaagacctttcagcgactgctgctgatcaccaagaag

aagtacataggtgtcataaacggggggaagatgctcatgaagggggtcgacctggtccgc

aaaaataactgctctttcataaacttgtacgcgcgacatctggtagatcttttgttgtac

gacgaggatgtggccacggcggcagcagaggtgacagacgtgcctcccgcagaatgggtg

gggcgcccgctaccgagcggctttgacaagtttgggcgagtgctggtagaggcgtacaac

cgtatcactgcccccaacttggacgtgcgcgagttcgttatgactgctgagctgagccgc

tcacccgaatcgtataccaacaagcgcctgccgcacctcaccgtctactttaagctcgcc

atgaggaatgaagaactgcccagcgtaaaagagagaattccgtatgtgatagttgcgcag

accgaggccgcggaacgcgaagcgggtgtagtaaactcaatgcgcggtaccgcccaaaac

cccgtggtaaccaagaccgcacgcccccaacctaaacgcaaactgctggtttccgacctc

gccgaagacccgacctatgtttccgagaatgacgtaccgctaaacacagactactatttc

tcccacctgttgggtaccataagcgtgacctttaaggctctattcggaaatgatgtgaga

acaacagaaaatcttttaaagcggtttattccggaaaccccccacaagacccccacgaaa

acccaggcactgcttgagcgcgccggctttgaaaagctgacgccctttacaccggaggaa

gaaagtcgtcgaatactgcatacagttttttgtactctagaagcagctccccatcaaagc

tga

>LC109634.1

atggcggcgcgcgaacaggccaactccgttcgcaggagcgggttttttaatccgtttatt

ggcaagaggccatttttcaggccgggaagcgggcagaccgcggagacagagcgcccaagg

cccccccaacactcgtactgcacagaggtgggtagctttaagtttatagctccaagatgc

ctcgatgaagaagcccccgccgaccagcgacgcggtgtacacgtgggcaccttggagcgc

ccacccaaagtgtactgcgatggctcagagtacgacgtgctgaactttgcctccggaggt

tgctggcctcgcaggattcgggtttggaacggccaggattttcggggcgatggattcaac

cccagatttgagagatttcacgtgtacgacatagtagagacttcggagagcgcctcacac

gatgacccatccaggtttgctgagctatcccgcccaagcgggtctgtggtaacactgctg

ggaatgagcgagtgtggaaagcgagtcgcggttcacgtctatggtgtgcgccattatttt

tacatggcaaaggcggaggtggatagcgcttgtggaataaccaccgaggcagaactcgtg

cgcgcaatggtggactgcgcgcacagctcggctttgagcgcggccctgggaaatggcaac

ggcggcaaacagagcggcggcagcggcgggggatggtggggcggaaagcacgtgtctgcg

gactgcttcaaagtggagaccgtgtgccacacgacgctgtactactttggatctaagcca

gctctctactatagagtatctgcctccagcagccgcctgggagggttcatctgcgacaac

tttcacccggagattacaaaattcgaggggagcgtggacgtgaccacgcggctgttgttg

gacaatgaaaattttaccagtttcgggtggtaccgcctgcgacccggcacccacggagag

cgtgttcaacttcgccccgttgagcgacacgtcacctcaagcgacgtggagattaactgt

actcccgataacctggagccgataccagacgaggctgcctggcccgactataagctcatg

tgctttgatatagagtgtaaagctggaacgggtaacgaaatggcgttcccagtggcaact

aaccaagaggacctggtcatccagatctcctgtctgctgtactcgcttgctactcagaac

cacgaacacaccctgctgttttccctcgggtcatgcgatatctctgaggaatactcgttt

gcatgcgtccagcgcggcgagcccagaccgacggttttggagtttgacagcgagtacgag

ctgctggttgccttcctgacctttctcaagcagtactctcccgagttcgccaccggctac

aacatcgttaattttgactgggcgtacatagttaacaaggtaacgtcggtgtataacatc

aagctggacgggtacggcaagttcaacaaaggggggctgtttaaggtgtgggacatcgcc

acgaaccattttcagaagaagagcaaggtgaaaatcaatggcctgatatctctagacatg

tattctgtggcgacggaaaagctaaagctacccagctacaaactcgacgcggtcgtggga

gacgtcctcggcgagcataagatagaccttccctataaagaaataccctcctattacgcg

ggagggcctgaccggaggggcgtaataggagagtattgtatccaggactctaggctggtg

ggcaagctgttttttaagtacctcccccatctggaactatcggcggtggccaaactcgcc

cgtatcaccctaacgcgggtaatttttgacggtcagcaaattcgcgtgtacacgtgcttg

ctgaaactcgcccgcgagagaaatttcattttgccagacaacagacgccggtttgacagt

caggcagacgccgcgtcagagacttcggagttggctatggatagccaaagccacgccttc

gacagtacagacgaacccgacggtgtggacggtaccccggacgccgcaggatctggcgct

acttctgaaaacggaggcgggaagcccggcgtcgggagggccgtgggctaccagggagca

aaggttctagaccccgtatccggctttcatgtggaccccgtggttgtgtttgacttcgct

agcttatacccaagcattatccaggcccataacctctgtttcaccaccctggcgctcgat

gaagtggatctggccgggcttcaaccatccgtcaactactcgacgttcgaggtgggtgac

caaaagttattttttgtccacgcccatattcgcgaaagcctgcttggcatcttgctgcgc

gactggctggccatgcgaaaggcggtgagggcgcgaatccccaccagcacccccgaggag

gcagttttactagataagcagcagtctgcgattaaggtgatatgcaactcggtttacgga

ttcacgggggtggcaaacggcctgttgccgtgtctgaggatagcggctaccgttaccacg

ataggacgcgacatgctcctcaagacccgagattacgttcactctcgttgggcgacgcgc

gagctgctggaggacaattttccaggggctataggtttccgaaaccacaagccttactcc

gtcagggttatctacggagacaccgactccgtgtttatcaagtttgtgggcctgacgtac

gagggggtatcggagctgggggatgctatgtcgcgtcagatttcagcggacctctttaga

gcccccatcaaactggagtgtgagaagacctttcagcgactgctgctgatcaccaagaag

aagtacataggtgtcataaacggggggaagatgctcatgaagggggtcgacctggtccgc

aaaaataactgctctttcataaacttgtacgcgcgacatctggtagatcttttgttgtac

gacgaggatgtggccacggcggcagcagaggtgacagacgtgcctcccgcagaatgggtg

gggcgcccgctaccgagcggctttgacaagtttgggcgagtgctggtagaggcgtacaac

cgtatcactgcccccaacttggacgtgcgcgagttcgttatgactgctgagctgagccgc

tcacccgaatcgtataccaacaagcgcctgccgcacctcaccgtctactttaagctcgcc

atgaggaatgaagaactgcccagcgtaaaagagagaattccgtatgtgatagttgcgcag

accgaggccgcggaacgcgaagcgggtgtagtaaactcaatgcgcggtaccgcccaaaac

cccgtggtaaccaagaccgcacgcccccaacctaaacgcaaactgctggtttccgacctc

gccgaagacccgacctatgtttccgagaatgacgtaccgctaaacacagactactatttc

tcccacctgttgggtaccataagcgtgacctttaaggctctattcggaaatgatgtgaga

acaacagaaaatcttttaaagcggtttattccggaaaccccccacaagacccccacgaaa

acccaggcactgcttgagcgcgccggctttgaaaagctgacgccctttacaccggaggaa

gaaagtcgtcgaatactgcatacagttttttgtactctagaagcagctccccatcaaagc

tga

>LC109635.1

atggcggcgcgcgaacaggccaactccgttcgcaggagcgggttttttaatccgtttatt

ggcaagaggccatttttcaggccgggaagcgggcagaccgcggagacagagcgcccaagg

cccccccaacactcgtactgcacagaggtgggtagctttaagtttatagctccaagatgc

ctcgatgaagaagcccccgccgaccagcgacgcggtgtacacgtgggcaccttggagcgc

ccacccaaagtgtactgcgatggctcagagtacgacgtgctgaactttgcctccggaggt

tgctggcctcgcaggattcgggtttggaacggccaggattttcggggcgatggattcaac

cccagatttgagagatttcacgtgtacgacatagtagagacttcggagagcgcctcacac

gatgacccatccaggtttgctgagctatcccgcccaagcgggtctgtggtaacactgctg

ggaatgagcgagtgtggaaagcgagtcgcggttcacgtctatggtgtgcgccattatttt

tacatggcaaaggcggaggtggatagcgcttgtggaataaccaccgaggcagaactcgtg

cgcgcaatggtggactgcgcgcacagctcggctttgagcgcggccctgggaaatggcaac

ggcggcaaacagagcggcggcagcggcgggggatggtggggcggaaagcacgtgtctgcg

gactgcttcaaagtggagaccgtgtgccacacgacgctgtactactttggatctaagcca

gctctctactatagagtatctgcctccagcagccgcctgggagggttcatctgcgacaac

tttcacccggagattacaaaattcgaggggagcgtggacgtgaccacgcggctgttgttg

gacaatgaaaattttaccagtttcgggtggtaccgcctgcgacccggcacccacggagag

cgtgttcaacttcgccccgttgagcgacacgtcacctcaagcgacgtggagattaactgt

actcccgataacctggagccgataccagacgaggctgcctggcccgactataagctcatg

tgctttgatatagagtgtaaagctggaacgggtaacgaaatggcgttcccagtggcaact

aaccaagaggacctggtcatccagatctcctgtctgctgtactcgcttgctactcagaac

cacgaacacaccctgctgttttccctcgggtcatgcgatatctctgaggaatactcgttt

gcatgcgtccagcgcggcgagcccagaccgacggttttggagtttgacagcgagtacgag

ctgctggttgccttcctgacctttctcaagcagtactctcccgagttcgccaccggctac

aacatcgttaattttgactgggcgtacatagttaacaaggtaacgtcggtgtataacatc

aagctggacgggtacggcaagttcaacaaaggggggctgtttaaggtgtgggacatcgcc

acgaaccattttcagaagaagagcaaggtgaaaatcaatggcctgatatctctagacatg

tattctgtggcgacggaaaagctaaagctacccagctacaaactcgacgcggtcgtggga

gacgtcctcggcgagcataagatagaccttccctataaagaaataccctcctattacgcg

ggagggcctgaccggaggggcgtaataggagagtattgtatccaggactctaggctggtg

ggcaagctgttttttaagtacctcccccatctggaactatcggcggtggccaaactcgcc

cgtatcaccctaacgcgggtaatttttgacggtcagcaaattcgcgtgtacacgtgcttg

ctgaaactcgcccgcgagagaaatttcattttgccagacaacagacgccggtttgacagt

caggcagacgccgcgtcagagacttcggagttggctatggatagccaaagccacgccttc

gacagtacagacgaacccgacggtgtggacggtaccccggacgccgcaggatctggcgct

acttctgaaaacggaggcgggaagcccggcgtcgggagggccgtgggctaccagggagca

aaggttctagaccccgtatccggctttcatgtggaccccgtggttgtgtttgacttcgct

agcttatacccaagcattatccaggcccataacctctgtttcaccaccctggcgctcgat

gaagtggatctggccgggcttcaaccatccgtcaactactcgacgttcgaggtgggtgac

caaaagttattttttgtccacgcccatattcgcgaaagcctgcttggcatcttgctgcgc

gactggctggccatgcgaaaggcggtgagggcgcgaatccccaccagcacccccgaggag

gcagttttactagataagcagcagtctgcgattaaggtgatatgcaactcggtttacgga

ttcacgggggtggcaaacggcctgttgccgtgtctgaggatagcggctaccgttaccacg

ataggacgcgacatgctcctcaagacccgagattacgttcactctcgttgggcgacgcgc

gagctgctggaggacaattttccaggggctataggtttccgaaaccacaagccttactcc

gtcagggttatctacggagacaccgactccgtgtttatcaagtttgtgggcctgacgtac

gagggggtatcggagctgggggatgctatgtcgcgtcagatttcagcggacctctttaga

gcgcccatcaaactggagtgtgagaagacctttcagcgactgctgctgatcaccaagaag

aagtacataggtgtcataaacggggggaagatgctcatgaagggggtcgacctggtccgc

aaaaataactgctctttcataaacttgtacgcgcgacatctggtagatcttttgttgtac

gacgaggatgtggccacggcggcagcagaggtgacagacgtgcctcccgcagaatgggtg

gggcgcccgctaccgagcggctttgacaagtttgggcgagtgctggtagaggcgtacaac

cgtatcactgcccccaacttggacgtgcgcgagttcgttatgactgctgagctgagccgc

tcacccgaatcgtataccaacaagcgcctgccgcacctcaccgtctactttaagctcgcc

atgaggaatgaagaactgcccagcgtaaaagagagaattccgtatgtgatagttgcgcag

accgaggccgcggaacgcgaagcgggtgtagtaaactcaatgcgcggtaccgcccaaaac

cccgtggtaaccaagaccgcacgcccccaacctaaacgcaaactgctggtttccgacctc

gccgaagacccgacctatgtttccgagaatgacgtaccgctaaacacagactactatttc

tcccacctgttgggtaccataagcgtgacctttaaggctctattcggaaatgatgtgaga

acaacagaaaatcttttaaagcggtttattccggaaaccccccacaagacccccacgaaa

acccaggcactgcttgagcgcgccggctttgaaaagctgacgccctttacaccggaggaa

gaaagtcgtcgaatactgcatacagttttttgtactctagaagcagctccccatcaaagc

tga

>LC109636.1

atggcggcgcgcgaacaggccaactccgttcgcaggagcgggttttttaatccgtttatt

ggcaagaggccatttttcaggccgggaagcgggcagaccgcggagacagagcgcccaagg

cccccccaacactcgtactgcacagaggtgggtagctttaagtttatagctccaagatgc

ctcgatgaagaagcccccgccgaccagcgacgcggtgtacacgtgggcaccttggagcgc

ccacccaaagtgtactgcgatggctcagagtacgacgtgctgaactttgcctccggaggt

tgctggcctcgcaggattcgggtttggaacggccaggattttcggggcgatggattcaac

cccagatttgagagatttcacgtgtacgacatagtagagacttcggagagcgcctcacac

gatgacccatccaggtttgctgagctatcccgcccaagcgggtctgtggtaacactgctg

ggaatgagcgagtgtggaaagcgagtcgcggttcacgtctatggtgtgcgccattatttt

tacatggcaaaggcggaggtggatagcgcttgtggaataaccaccgaggcagaactcgtg

cgcgcaatggtggactgcgcgcacagctcggctttgagcgcggccctgggaaatggcaac

ggcggcaaacagagcggcggcagcggcgggggatggtggggcggaaagcacgtgtctgcg

gactgcttcaaagtggagaccgtgtgccacacgacgctgtactactttggatctaagcca

gctctctactatagagtatctgcctccagcagccgcctgggagggttcatctgcgacaac

tttcacccggagattacaaaattcgaggggagcgtggacgtgaccacgcggctgttgttg

gacaatgaaaattttaccagtttcgggtggtaccgcctgcgacccggcacccacggagag

cgtgttcaacttcgccccgttgagcgacacgtcacctcaagcgacgtggagattaactgt

actcccgataacctggagccgataccagacgaggctgcctggcccgactataagctcatg

tgctttgatatagagtgtaaagctggaacgggtaacgaaatggcgttcccagtggcaact

aaccaagaggacctggtcatccagatctcctgtctgctgtactcgcttgctactcagaac

cacgaacacaccctgctgttttccctcgggtcatgcgatatctctgaggaatactcgttt

gcatgcgtccagcgcggcgagcccagaccgacggttttggagtttgacagcgagtacgag

ctgctggttgccttcctgacctttctcaagcagtactctcccgagttcgccaccggctac

aacatcgttaattttgactgggcgtacatagttaacaaggtaacgtcggtgtataacatc

aagctggacgggtacggcaagttcaacaaaggggggctgtttaaggtgtgggacatcgcc

acgaaccattttcagaagaagagcaaggtgaaaatcaatggcctgatatctctagacatg

tattctgtggcgacggaaaagctaaagctacccagctacaaactcgacgcggtcgtggga

gacgtcctcggcgagcataagatagaccttccctataaagaaataccctcctattacgcg

ggagggcctgaccggaggggcgtaataggagagtattgtatccaggactctaggctggtg

ggcaagctgttttttaagtacctcccccatctggaactatcggcggtggccaaactcgcc

cgtatcaccctaacgcgggtaatttttgacggtcagcaaattcgcgtgtacacgtgcttg

ctgaaactcgcccgcgagagaaatttcattttgccagacaacagacgccggtttgacagt

caggcagacgccgcgtcagagacttcggagttggctatggatagccaaagccacgccttc

gacagtacagacgaacccgacggtgtggacggtaccccggacgccgcaggatctggcgct

acttctgaaaacggaggcgggaagcccggcgtcgggagggccgtgggctaccagggagca

aaggttctagaccccgtatccggctttcatgtggaccccgtggttgtgtttgacttcgct

agcttatacccaagcattatccaggcccataacctctgtttcaccaccctggcgctcgat

gaagtggatctggccgggcttcaaccatccgtcaactactcgacgttcgaggtgggtgac

caaaagttattttttgtccacgcccatattcgcgaaagcctgcttggcatcttgctgcgc

gactggctggccatgcgaaaggcggtgagggcgcgaatccccaccagcacccccgaggag

gcagttttactagataagcagcagtctgcgattaaggtgatatgcaactcggtttacgga

ttcacgggggtggcaaacggcctgttgccgtgtctgaggatagcggctaccgttaccacg

ataggacgcgacatgctcctcaagacccgagattacgttcactctcgttgggcgacgcgc

gagctgctggaggacaattttccaggggctataggtttccgaaaccacaagccttactcc

gtcagggttatctacggagacaccgactccgtgtttatcaagtttgtgggcctgacgtac

gagggggtatcggagctgggggatgctatgtcgcgtcagatttcagcggacctctttaga

gcccccatcaaactggagtgtgagaagacctttcagcgactgctgctgatcaccaagaag

aagtacataggtgtcataaacggggggaagatgctcatgaagggggtcgacctggtccgc

aaaaataactgctctttcataaacttgtacgcgcgacatctggtagatcttttgttgtac

gacgaggatgtggccacggcggcagcagaggtgacagacgtgcctcccgcagaatgggtg

gggcgcccgctaccgagcggctttgacaagtttgggcgagtgctggtagaggcgtacaac

cgtatcactgcccccaacttggacgtgcgcgagttcgttatgactgctgagctgagccgc

tcacccgaatcgtataccaacaagcgcctgccgcacctcaccgtctactttaagctcgcc

atgaggaatgaagaactgcccagcgtaaaagagagaattccgtatgtgatagttgcgcag

accgaggccgcggaacgcgaagcgggtgtagtaaactcaatgcgcggtaccgcccaaaac

cccgtggtaaccaagaccgcacgcccccaacctaaacgcaaactgctggtttccgacctc

gccgaagacccgacctatgtttccgagaatgacgtaccgctaaacacagactactatttc

tcccacctgttgggtaccataagcgtgacctttaaggctctattcggaaatgatgtgaga

acaacagaaaatcttttaaagcggtttattccggaaaccccccacaagacccccacgaaa

acccaggcactgcttgagcgcgccggctttgaaaagctgacgccctttacaccggaggaa

gaaagtcgtcgaatactgcatacagttttttgtactctagaagcagctccccatcaaagc

tga

>LC109637.1

atggcggcgcgcgaacaggccaactccgttcgcaggagcgggttttttaatccgtttatt

ggcaagaggccatttttcaggccgggaagcgggcagaccgcggagacagagcgcccaagg

cccccccaacactcgtactgcacagaggtgggtagctttaagtttatagctccaagatgc

ctcgatgaagaagcccccgccgaccagcgacgcggtgtacacgtgggcaccttggagcgc

ccacccaaagtgtactgcgatggctcagagtacgacgtgctgaactttgcctccggaggt

tgctggcctcgcaggattcgggtttggaacggccaggattttcggggcgatggattcaac

cccagatttgagagatttcacgtgtacgacatagtagagacttcggagagcgcctcacac

gatgacccatccaggtttgctgagctatcccgcccaagcgggtctgtggtaacactgctg

ggaatgagcgagtgtggaaagcgagtcgcggttcacgtctatggtgtgcgccattatttt

tacatggcaaaggcggaggtggatagcgcttgtggaataaccaccgaggcagaactcgtg

cgcgcaatggtggactgcgcgcacagctcggctttgagcgcggccctgggaaatggcaac

ggcggcaaacagagcggcggcagcggcgggggatggtggggcggaaagcacgtgtctgcg

gactgcttcaaagtggagaccgtgtgccacacgacgctgtactactttggatctaagcca

gctctctactatagagtatctgcctccagcagccgcctgggagggttcatctgcgacaac

tttcacccggagattacaaaattcgaggggagcgtggacgtgaccacgcggctgttgttg

gacaatgaaaattttaccagtttcgggtggtaccgcctgcgacccggcacccacggagag

cgtgttcaacttcgccccgttgagcgacacgtcacctcaagcgacgtggagattaactgt

actcccgataacctggagccgataccagacgaggctgcctggcccgactataagctcatg

tgctttgatatagagtgtaaagctggaacgggtaacgaaatggcgttcccagtggcaact

aaccaagaggacctggtcatccagatctcctgtctgctgtactcgcttgctactcagaac

cacgaacacaccctgctgttttccctcgggtcatgcgatatctctgaggaatactcgttt

gcatgcgtccagcgcggcgagcccagaccgacggttttggagtttgacagcgagtacgag

ctgctggttgccttcctgacctttctcaagcagtactctcccgagttcgccaccggctac

aacatcgttaattttgactgggcgtacatagttaacaaggtaacgtcggtgtataacatc

aagctggacgggtacggcaagttcaacaaaggggggctgtttaaggtgtgggacatcgcc

acgaaccattttcagaagaagagcaaggtgaaaatcaatggcctgatatctctagacatg

tattctgtggcgacggaaaagctaaagctacccagctacaaactcgacgcggtcgtggga

gacgtcctcggcgagcataagatagaccttccctataaagaaataccctcctattacgcg

ggagggcctgaccggaggggcgtaataggagagtattgtatccaggactctaggctggtg

ggcaagctgttttttaagtacctcccccatctggaactatcggcggtggccaaactcgcc

cgtatcaccctaacgcgggtaatttttgacggtcagcaaattcgcgtgtacacgtgcttg

ctgaaactcgcccgcgagagaaatttcattttgccagacaacagacgccggtttgacagt

caggcagacgccgcgtcagagacttcggagttggctatggatagccaaagccacgccttc

gacagtacagacgaacccgacggtgtggacggtaccccggacgccgcaggatctggcgct

acttctgaaaacggaggcgggaagcccggcgtcgggagggccgtgggctaccagggagca

aaggttctagaccccgtatccggctttcatgtggaccccgtggttgtgtttgacttcgct

agcttatacccaagcattatccaggcccataacctctgtttcaccaccctggcgctcgat

gaagtggatctggccgggcttcaaccatccgtcaactactcgacgttcgaggtgggtgac

caaaagttattttttgtccacgcccatattcgcgaaagcctgcttggcatcttgctgcgc

gactggctggccatgcgaaaggcggtgagggcgcgaatccccaccagcacccccgaggag

gcagttttactagataagcagcagtctgcgattaaggtgatatgcaactcggtttacgga

ttcacgggggtggcaaacggcctgttgccgtgtctgaggatagcggctaccgttaccacg

ataggacgcgacatgctcctcaagacccgagattacgttcactctcgttgggcgacgcgc

gagctgctggaggacaattttccaggggctataggtttccgaaaccacaagccttactcc

gtcagggttatctacggagacaccgactccgtgtttatcaagtttgtgggcctgacgtac

gagggggtatcggagctgggggatgctatgtcgcgtcagatttcagcggacctctttaga

gcgcccatcaaactggagtgtgagaagacctttcagcgactgctgctgatcaccaagaag

aagtacataggtgtcataaacggggggaagatgctcatgaagggggtcgacctggtccgc

aaaaataactgctctttcataaacttgtacgcgcgacatctggtagatcttttgttgtac

gacgaggatgtggccacggcggcagcagaggtgacagacgtgcctcccgcagaatgggtg

gggcgcccgctaccgagcggctttgacaagtttgggcgagtgctggtagaggcgtacaac

cgtatcactgcccccaacttggacgtgcgcgagttcgttatgactgctgagctgagccgc

tcacccgaatcgtataccaacaagcgcctgccgcacctcaccgtctactttaagctcgcc

atgaggaatgaagaactgcccagcgtaaaagagagaattccgtatgtgatagttgcgcag

accgaggccgcggaacgcgaagcgggtgtagtaaactcaatgcgcggtaccgcccaaaac

cccgtggtaaccaagaccgcacgcccccaacctaaacgcaaactgctggtttccgacctc

gccgaagacccgacctatgtttccgagaatgacgtaccgctaaacacagactactatttc

tcccacctgttgggtaccataagcgtgacctttaaggctctattcggaaatgatgtgaga

acaacagaaaatcttttaaagcggtttattccggaaaccccccacaagacccccacgaaa

acccaggcactgcttgagcgcgccggctttgaaaagctgacgccctttacaccggaggaa

gaaagtcgtcgaatactgcatacagttttttgtactctagaagcagctccccatcaaagc

tga

>LC109638.1

atggcggcgcgcgaacaggccaactccgttcgcaggagcgggttttttaatccgtttatt

ggcaagaggccatttttcaggccgggaagcgggcaaaccgcggagacagagcgcccaagg

cccccccaacactcgtactgcacagaggtgggtagctttaagtttatagctccaagatgc

ctcgatgaagaagcccccgccgaccagcgacgcggtgtacacgtgggcaccttggagcgc

ccacccaaagtgtactgcgatggctcagagtacgacgtgctgaactttgcctccggaggt

tgctggcctcgcaggattcgggtttggaacggccaggattttcggggcgatggattcaac

cccagatttgagagatttcacgtgtacgacatagtagagacttcggagagcgcctcacac

gatgacccatccaggtttgctgagctatcccgcccaagcgggtctgtggtaacactgctg

ggaatgagcgagtgtggaaagcgagtcgcggttcacgtctatggtgtgcgccattatttt

tacatggcaaaggcggaggtggatagcgcttgtggaataaccaccgaggcagaactcgtg

cgcgcaatggtggactgcgcgcacagctcggctttgagcgcggccctgggaaatggcaac

ggcggcaaacagagcggcggcagcggcgggggatggtggggcggaaagcacgtgtctgcg

gactgcttcaaagtggagaccgtgtgccacacgacgctgtactactttggatctaagcca

gctctctactatagagtatctgcctccagcagccgcctgggagggttcatctgcgacaac

tttcacccggagattacaaaattcgaggggagcgtggacgtgaccacgcggctgttgttg

gacaatgaaaattttaccagtttcgggtggtaccgcctgcgacccggcacccacggagag

cgtgttcaacttcgccccgttgagcgacacgtcacctcaagcgacgtggagattaactgt

actcccgataacctggagccgataccagacgaggctgcctggcccgactataagctcatg

tgctttgatatagagtgtaaagctggaacgggtaacgaaatggcgttcccagtggcaact

aaccaagaggacctggtcatccagatctcctgtctgctgtactcgcttgctactcagaac

cacgaacacaccctgctgttttccctcgggtcatgcgatatctctgaggaatactcgttt

gcatgcgtccagcgcggcgagcccagaccgacggttttggagtttgacagcgagtacgag

ctgctggttgccttcctgacctttctcaagcagtactctcccgagttcgccaccggctac

aacatcgttaattttgactgggcgtacatagttaacaaggtaacgtcggtgtataacatc

aagctggacgggtacggcaagttcaacaaaggggggctgtttaaggtgtgggacatcgcc

acgaaccattttcagaagaagagcaaggtgaaaatcaatggcctgatatctctagacatg

tattctgtggcgacggaaaagctaaagctacccagctacaaactcgacgcggtcgtggga

gacgtcctcggcgagcataagatagaccttccctataaagaaataccctcctattacgcg

ggagggcctgaccggaggggcgtaataggagagtattgtatccaggactctaggctggtg

ggcaagctgttttttaagtacctcccccatctggaactatcggcggtggccaaactcgcc

cgtatcaccctaacgcgggtaatttttgacggtcagcaaattcgcgtgtacacgtgcttg

ctgaaactcgcccgcgagagaaatttcattttgccagacaacagacgccggtttgacagt

caggcagacgccgcgtcagagacttcggagttggctatggatagccaaagccacgccttc

gacagtacagacgaacccgacggtgtggacggtaccccggacgccgcaggatctggcgct

acttctgaaaacggaggcgggaagcccggcgtcgggagggccgtgggctaccagggagca

aaggttctagaccccgtatccggctttcatgtggaccccgtggttgtgtttgacttcgct

agcttatacccaagcattatccaggcccataacctctgtttcaccaccctggcgctcgat

gaagtggatctggccgggcttcaaccatccgtcaactactcgacgttcgaggtgggtgac

caaaagttattttttgtccacgcccatattcgcgaaagcctgcttggcatcttgctgcgc

gactggctggccatgcgaaaggcggtgagggcgcgaatccccaccagcacccccgaggag

gcagttttactagataagcagcagtctgcgattaaggtgatatgcaactcggtttacgga

ttcacgggggtggcaaacggcctgttgccgtgtctgaggatagcggctaccgttaccacg

ataggacgcgacatgctcctcaagacccgagattacgttcactctcgttgggcgacgcgc

gagctgctggaggacaattttccaggggctataggtttccgaaaccacaagccttactcc

gtcagggttatctacggagacaccgactccgtgtttatcaagtttgtgggcctgacgtac

gagggggtatcggagctgggggatgctatgtcgcgtcagatttcagcggacctctttaga

gcgcccatcaaactggagtgtgagaagacctttcagcgactgctgctgatcaccaagaag

aagtacataggtgtcataaacggggggaagatgctcatgaagggggtcgacctggtccgc

aaaaataactgctctttcataaacttgtacgcgcgacatctggtagatcttttgttgtac

gacgaggatgtggccacggcggcagcagaggtgacagacgtgcctcccgcagaatgggtg

gggcgcccgctaccgagcggctttgacaagtttgggcgagtgctggtagaggcgtacaac

cgtatcactgcccccaacttggacgtgcgcgagttcgttatgactgctgagctgagccgc

tcacccgaatcgtataccaacaagcgcctgccgcacctcaccgtctactttaagctcgcc

atgaggaatgaagaactgcccagcgtaaaagagagaattccgtatgtgatagttgcgcag

accgaggccgcggaacgcgaagcgggtgtagtaaactcaatgcgcggtaccgcccaaaac

cccgtggtaaccaagaccgcacgcccccaacctaaacgcaaactgctggtttccgacctc

gccgaagacccgacctatgtttccgagaatgacgtaccgctaaacacagactactatttc

tcccacctgttgggtaccataagcgtgacctttaaggctctattcggaaatgatgtgaga

acaacagaaaatcttttaaagcggtttattccggaaaccccccacaagacccccacgaaa

acccaggcactgcttgagcgcgccggctttgaaaagctgacgccctttacaccggaggaa

gaaagtcgtcgaatactgcatacagttttttgtactctagaagcagctccccatcaaagc

tga

>LC109639.1

atggcggcgcgcgaacaggccaactccgttcgcaggagcgggttttttaatccgtttatt

ggcaagaggccatttttcaggccgggaagcgggcagaccgcggagacagagcgcccaagg

cccccccaacactcgtactgcacagaggtgggtagctttaagtttatagctccaagatgc

ctcgatgaagaagcccccgccgaccagcgacgcggtgtacacgtgggcaccttggagcgc

ccacccaaagtgtactgcgatggctcagagtacgacgtgctgaactttgcctccggaggt

tgctggcctcgcaggattcgggtttggaacggccaggattttcggggcgatggattcaac

cccagatttgagagatttcacgtgtacgacatagtagagacttcggagagcgcctcacac

gatgacccatccaggtttgctgagctatcccgcccaagcgggtctgtggtaacactgctg

ggaatgagcgagtgtggaaagcgagtcgcggttcacgtctatggtgtgcgccattatttt

tacatggcaaaggcggaggtggatagcgcttgtggaataaccaccgaggcagaactcgtg

cgcgcaatggtggactgcgcgcacagctcggctttgagcgcggccctgggaaatggcaac

ggcggcaaacagagcggcggcagcggcgggggatggtggggcggaaagcacgtgtccgcg

gactgcttcaaagtggagaccgtgtgccacacgacgctgtactactttggatctaagcca

gctctctactatagagtatctgcctccagcagccgcctgggagggttcatctgcgacaac

tttcacccggagattacaaaattcgaggggagcgtggacgtgaccacgcggctgttgttg

gacaatgaaaattttaccagtttcgggtggtaccgcctgcgacccggcacccacggagag

cgtgttcaacttcgccccgttgagcgacacgtcacctcaagcgacgtggagattaactgt

actcccgataacctggagccgataccagacgaggctgcctggcccgactataagctcatg

tgctttgatatagagtgtaaagctggaacgggtaacgaaatggcgttcccagtggcaact

aaccaagaggacctggtcatccagatctcctgtctgctgtactcgcttgctactcagaac

cacgaacacaccctgctgttttccctcgggtcatgcgatatctctgaggaatactcgttt

gcatgcgtccagcgcggcgagcccagaccgacggttttggagtttgacagcgagtacgag

ctgctggttgccttcctgacctttctcaagcagtactctcccgagttcgccaccggctac

aacatcgttaattttgactgggcgtacatagttaacaaggtaacgtcggtgtataacatc

aagctggacgggtacggcaagttcaacaaaggggggctgtttaaggtgtgggacatcgcc

acgaaccattttcagaagaagagcaaggtgaaaatcaatggcctgatatctctagacatg

tattctgtggcgacggaaaagctaaagctacccagctacaaactcgacgcggtcgtggga

gacgtcctcggcgagcataagatagaccttccctataaagaaataccctcctattacgcg

ggagggcctgaccggaggggcgtaataggagagtattgtatccaggactctaggctggtg

ggcaagctgttttttaagtacctcccccatctggaactatcggcggtggccaaactcgcc

cgtatcaccctaacgcgggtaatttttgacggtcagcaaattcgcgtgtacacgtgcttg

ctgaaactcgcccgcgagagaaatttcattttgccagacaacagacgccggtttgacagt

caggcagacgccgcgtcagagacttcggagttggctatggatagccaaagccacgccttc

gacagtacagacgaacccgacggtgtggacggtaccccggacgccgcaggatctggcgct

acttctgaaaacggaggcgggaagcccggcgtcgggagggccgtgggctaccagggagca

aaggttctagaccccgtatccggctttcatgtggaccccgtggttgtgtttgacttcgct

agcttatacccaagcattatccaggcccataacctctgtttcaccaccctggcgctcgat

gaagtggatctggccgggcttcaaccatccgtcaactactcgacgttcgaggtgggtgac

caaaagttattttttgtccacgcccatattcgcgaaagcctgcttggcatcttgctgcgc

gactggctggccatgcgaaaggcggtgagggcgcgaatccccaccagcacccccgaggag

gcagttttactagataagcagcagtctgcgattaaggtgatatgcaactcggtttacgga

ttcacgggggtggcaaacggcctgttgccgtgtctgaggatagcggctaccgttaccacg

ataggacgcgacatgctcctcaagacccgagattacgttcactctcgttgggcgacgcgc

gagctgctggaggacaattttccaggggctataggtttccgaaaccacaagccttactcc

gtcagggttatctacggagacaccgactccgtgtttatcaagtttgtgggcctgacgtac

gagggggtatcggagctgggggatgctatgtcgcgtcagatttcagcggacctctttaga

gcccccatcaaactggagtgtgagaagacctttcagcgactgctgctgatcaccaagaag

aagtacataggtgtcataaacggggggaagatgctcatgaagggggtcgacctggtccgc

aaaaataactgctctttcataaacttgtacgcgcgacatctggtagatcttttgttgtac

gacgaggatgtggccacggcggcagcagaggtgacagacgtgcctcccgcagaatgggtg

gggcgcccgctaccgagcggctttgacaagtttgggcgagtgctggtagaggcgtacaac

cgtatcactgcccccaacttggacgtgcgcgagttcgttatgactgctgagctgagccgc

tcacccgaatcgtataccaacaagcgcctgccgcacctcaccgtctactttaagctcgcc

atgaggaatgaagaactgcccagcgtaaaagagagaattccgtatgtgatagttgcgcag

accgaggccgcggaacgcgaagcgggtgtagtaaactcaatgcgcggtaccgcccaaaac

cccgtggtaaccaagaccgcacgcccccaacctaaacgcaaactgctggtttccgacctc

gccgaagacccgacctatgtttccgagaatgacgtaccgctaaacacagactactatttc

tcccacctgttgggtaccataagcgtgacctttaaggctctattcggaaatgatgtgaga

acaacagaaaatcttttaaagcggtttattccggaaaccccccacaagacccccacgaaa

acccaggcactgcttgagcgcgccggctttgaaaagctgacgccctttacaccggaggaa

gaaagtcgtcgaatactgcatacagttttttgtactctagaagcagctccccatcaaagc

tga

>LC109640.1

atggcggcgcgcgaacaggccaactccgttcgcaggagcgggttttttaatccgtttatt

ggcaagaggccatttttcaggccgggaagcgggcagaccgcggagacagagcgcccaagg

cccccccaacactcgtactgcacagaggtgggtagctttaagtttatagctccaagatgc

ctcgatgaagaagcccccgccgaccagcgacgcggtgtacacgtgggcaccttggagcgc

ccacccaaagtgtactgcgatggctcagagtacgacgtgctgaactttgcctccggaggt

tgctggcctcgcaggattcgggtttggaacggccaggattttcggggcgatggattcaac

cccagatttgagagatttcacgtgtacgacatagtagagacttcggagagcgcctcacac

gatgacccatccaggtttgctgagctatcccgcccaagcgggtctgtggtaacactgctg

ggaatgagcgagtgtggaaagcgagtcgcggttcacgtctatggtgtgcgccattatttt

tacatggcaaaggcggaggtggatagcgcttgtggaataaccaccgaggcagaactcgtg

cgcgcaatggtggactgcgcgcacagctcggctttgagcgcggccctgggaaatggcaac

ggcggcaaacagagcggcggcagcggcgggggatggtggggcggaaagcacgtgtccgcg

gactgcttcaaagtggagaccgtgtgccacacgacgctgtactactttggatctaagcca

gctctctactatagagtatctgcctccagcagccgcctgggagggttcatctgcgacaac

tttcacccggagattacaaaattcgaggggagcgtggacgtgaccacgcggctgttgttg

gacaatgaaaattttaccagtttcgggtggtaccgcctgcgacccggcacccacggagag

cgtgttcaacttcgccccgttgagcgacacgtcacctcaagcgacgtggagattaactgt

actcccgataacctggagccgataccagacgaggctgcctggcccgactataagctcatg

tgctttgatatagagtgtaaagctggaacgggtaacgaaatggcgttcccagtggcaact

aaccaagaggacctggtcatccagatctcctgtctgctgtactcgcttgctactcagaac

cacgaacacaccctgctgttttccctcgggtcatgcgatatctctgaggaatactcgttt

gcatgcgtccagcgcggcgagcccagaccgacggttttggagtttgacagcgagtacgag

ctgctggttgccttcctgacctttctcaagcagtactctcccgagttcgccaccggctac

aacatcgttaattttgactgggcgtacatagttaacaaggtaacgtcggtgtataacatc

aagctggacgggtacggcaagttcaacaaaggggggctgtttaaggtgtgggacatcgcc

acgaaccattttcagaagaagagcaaggtgaaaatcaatggcctgatatctctagacatg

tattctgtggcgacggaaaagctaaagctacccagctacaaactcgacgcggtcgtggga

gacgtcctcggcgagcataagatagaccttccctataaagaaataccctcctattacgcg

ggagggcctgaccggaggggcgtaataggagagtattgtatccaggactctaggctggtg

ggcaagctgttttttaagtacctcccccatctggaactatcggcggtggccaaactcgcc

cgtatcaccctaacgcgggtaatttttgacggtcagcaaattcgcgtgtacacgtgcttg

ctgaaactcgcccgcgagagaaatttcattttgccagacaacagacgccggtttgacagt

caggcagacgccgcgtcagagacttcggagttggctatggatagccaaagccacgccttc

gacagtacagacgaacccgacggtgtggacggtaccccggacgccgcaggatctggcgct

acttctgaaaacggaggcgggaagcccggcgtcgggagggccgtgggctaccagggagca

aaggttctagaccccgtatccggctttcatgtggaccccgtggttgtgtttgacttcgct

agcttatacccaagcattatccaggcccataacctctgtttcaccaccctggcgctcgat

gaagtggatctggccgggcttcaaccatccgtcaactactcgacgttcgaggtgggtgac

caaaagttattttttgtccacgcccatattcgcgaaagcctgcttggcatcttgctgcgc

gactggctggccatgcgaaaggcggtgagggcgcgaatccccaccagcacccccgaggag

gcagttttactagataagcagcagtctgcgattaaggtgatatgcaactcggtttacgga

ttcacgggggtggcaaacggcctgttgccgtgtctgaggatagcggctaccgttaccacg

ataggacgcgacatgctcctcaagacccgagattacgttcactctcgttgggcgacgcgc

gagctgctggaggacaattttccaggggctataggtttccgaaaccacaagccttactcc

gtcagggttatctacggagacaccgactccgtgtttatcaagtttgtgggcctgacgtac

gagggggtatcggagctgggggatgctatgtcgcgtcagatttcagcggacctctttaga

gcccccatcaaactggagtgtgagaagacctttcagcgactgctgctgatcaccaagaag

aagtacataggtgtcataaacggggggaagatgctcatgaagggggtcgacctggtccgc

aaaaataactgctctttcataaacttgtacgcgcgacatctggtagatcttttgttgtac

gacgaggatgtggccacggcggcagcagaggtgacagacgtgcctcccgcagaatgggtg

gggcgcccgctaccgagcggctttgacaagtttgggcgagtgctggtagaggcgtacaac

cgtatcactgcccccaacttggacgtgcgcgagttcgttatgactgctgagctgagccgc

tcacccgaatcgtataccaacaagcgcctgccgcacctcaccgtctactttaagctcgcc

atgaggaatgaagaactgcccagcgtaaaagagagaattccgtatgtgatagttgcgcag

accgaggccgcggaacgcgaagcgggtgtagtaaactcaatgcgcggtaccgcccaaaac

cccgtggtaaccaagaccgcacgcccccaacctaaacgcaaactgctggtttccgacctc

gccgaagacccgacctatgtttccgagaatgacgtaccgctaaacacagactactatttc

tcccacctgttgggtaccataagcgtgacctttaaggctctattcggaaatgatgtgaga

acaacagaaaatcttttaaagcggtttattccggaaaccccccacaagacccccacgaaa

acccaggcactgcttgagcgcgccggctttgaaaagctgacgccctttacaccggaggaa

gaaagtcgtcgaatactgcatacagttttttgtactctagaagcagctccccatcaaagc

tga

>LC109641.1

atggcggcgcgcgaacaggccaactccgttcgcaggagcgggttttttaatccgtttatt

ggcaagaggccatttttcaggccgggaagcgggcagaccgcggagacagagcgcccaagg

cccccccaacactcgtactgcacagaggtgggtagctttaagtttatagctccaagatgc

ctcgatgaagaagcccccgccgaccagcgacgcggtgtacacgtgggcaccttggagcgc

ccacccaaagtgtactgcgatggctcagagtacgacgtgctgaactttgcctccggaggt

tgctggcctcgcaggattcgggtttggaacggccaggattttcggggcgatggattcaac

cccagatttgagagatttcacgtgtacgacatagtagagacttcggagagcgcctcacac

gatgacccatccaggtttgctgagctatcccgcccaagcgggtctgtggtaacactgctg

ggaatgagcgagtgtggaaagcgagtcgcggttcacgtctatggtgtgcgccattatttt

tacatggcaaaggcggaggtggatagcgcttgtggaataaccaccgaggcagaactcgtg

cgcgcaatggtggactgcgcgcacagctcggctttgagcgcggccctgggaaatggcaac

ggcggcaaacagagcggcggcagcggcgggggatggtggggcggaaagcacgtgtctgcg

gactgcttcaaagtggagaccgtgtgccacacgacgctgtactactttggatctaagcca

gctctctactatagagtatctgcctccagcagccgcctgggagggttcatctgcgacaac

tttcacccggagattacaaaattcgaggggagcgtggacgtgaccacgcggctgttgttg

gacaatgaaaattttaccagtttcgggtggtaccgcctgcgacccggcacccacggagag

cgtgttcaacttcgccccgttgagcgacacgtcacctcaagcgacgtggagattaactgt

actcccgataacctggagccgataccagacgaggctgcctggcccgactataagctcatg

tgctttgatatagagtgtaaagctggaacgggtaacgaaatggcgttcccagtggcaact

aaccaagaggacctggtcatccagatctcctgtctgctgtactcgcttgctactcagaac

cacgaacacaccctgctgttttccctcgggtcatgcgatatctctgaggaatactcgttt

gcatgcgtccagcgcggcgagcccagaccgacggttttggagtttgacagcgagtacgag

ctgctggttgccttcctgacctttctcaagcagtactctcccgagttcgccaccggctac

aacatcgttaattttgactgggcgtacatagttaacaaggtaacgtcggtgtataacatc

aagctggacgggtacggcaagttcaacaaaggggggctgtttaaggtgtgggacatcgcc

acgaaccattttcagaagaagagcaaggtgaaaatcaatggcctgatatctctagacatg

tattctgtggcgacggaaaagctaaagctacccagctacaaactcgacgcggtcgtggga

gacgtcctcggcgagcataagatagaccttccctataaagaaataccctcctattacgcg

ggagggcctgaccggaggggcgtaataggagagtattgtatccaggactctaggctggtg

ggcaagctgttttttaagtacctcccccatctggaactatcggcggtggccaaactcgcc

cgtatcaccctaacgcgggtaatttttgacggtcagcaaattcgcgtgtacacgtgcttg

ctgaaactcgcccgcgagagaaatttcattttgccagacaacagacgccggtttgacagt

caggcagacgccgcgtcagagacttcggagttggctatggatagccaaagccacgccttc

gacagtacagacgaacccgacggtgtggacggtaccccggacgccgcaggatctggcgct

acttctaaaaacggaggcgggaagcccggcgtcgggagggccgtgggctaccagggagca

aaggttctagaccccgtatccggctttcatgtggaccccgtggttgtgtttgacttcgct

agcttatacccaagcattatccaggcccataacctctgtttcaccaccctggcgctcgat

gaagtggatctggccgggcttcaaccatccgtcaactactcgacgttcgaggtgggtgac

caaaagttattttttgtccacgccaatattcgcgaaagcctgcttggcatcttgctgcgc

gactggctggccatgcgaaaggcggtgagggcgcgaatccccaccagcacccccgaggag

gcagttttactagataagcagcagtctgcgattaaggtgatatgcaactcggtttacgga

ttcacgggggtggcaaacggcctgttgccgtgtctgaggatagcggctaccgttaccacg

ataggacgcgacatgctcctcaagacccgagattacgttcactctcgttgggcgacgcgc

gagctgctggaggacaattttccaggggctataggtttccgaaaccacaagccttactcc

gtcagggttatctacggagacaccgactccgtgtttatcaagtttgtgggcctgacgtac

gagggggtatcggagctgggggatgctatgtcgcgtcagatttcagcggacctctttaga

gcccccatcaaactggagtgtgagaagacctttcagcgactgctgctgatcaccaagaag

aagtacataggtgtcataaacggggggaagatgctcatgaagggggtcgacctggtccgc

aaaaataactgctctttcataaacttgtacgcgcgacatctggtagatcttttgttgtac

gacgaggatgtggccacggcggcagcagaggtgacagacgtgcctcccgcagaatgggtg

gggcgcccgctaccgagcggctttgacaagtttgggcgagtgctggtagaggcgtacaac

cgtatcactgcccccaacttggacgtgcgcgagttcgttatgactgctgagctgagccgc

tcacccgaatcgtataccaacaagcgcctgccgcacctcaccgtctactttaagctcgcc

atgaggaatgaagaactgcccagcgtaaaagagagaattccgtatgtgatagttgcgcag

accgaggccgcggaacgcgaagcgggtgtagtaaactcaatgcgcggtaccgcccaaaac

cccgtggtaaccaagaccacacgcccccaacctaaacgcaaactgctggtttccgacctc

gccgaagacccgacctatgtttccgagaatgacgtaccgctaaacacagactactatttc

tcccacctgttgggtaccataagcgtgacctttaaggctctattcggaaatgatgtgaga

acaacagaaaatcttttaaagcggtttattccggaaaccccccacaagacccccacgaaa

acccaggcactgcttgagcgcgccggctttgaaaagctgacgccctttacaccggaggaa

gaaagtcgtcgaatactgcatacagttttttgtactctagaagcagctccccatcaaagc

tga

>LC109642.1

atggcggcgcgcgaacaggccaactccgttcgcaggagcgggttttttaatccgtttatt

ggcaagaggccatttttcaggccgggaagcgggcagaccgcggagacagagcgcccaagg

cccccccaacactcgtactgcacagaggtgggtagctttaagtttatagctccaagatgc

ctcgatgaagaagcccccgccgaccagcgacgcggtgtacacgtgggcaccttggagcgc

ccacccaaagtgtactgcgatggctcagagtacgacgtgctgaactttgcctccggaggt

tgctggcctcgcaggattcgggtttggaacggccaggattttcggggcgatggattcaac

cccagatttgagagatttcacgtgtacgacatagtagagacttcggagagcgcctcacac

gatgacccatccaggtttgctgagctatcccgcccaagcgggtctgtggtaacactgctg

ggaatgagcgagtgtggaaagcgagtcgcggttcacgtctatggtgtgcgccattatttt

tacatggcaaaggcggaggtggatagcgcttgtggaataaccaccgaggcagaactcgtg

cgcgcaatggtggactgcgcgcacagctcggctttgagcgcggccctgggaaatggcaac

ggcggcaaacagagcggcggcagcggcgggggatggtggggcggaaagcacgtgtctgcg

gactgcttcaaagtggagaccgtgtgccacacgacgctgtactactttggatctaagcca

gctctctactatagagtatctgcctccagcagccgcctgggagggttcatctgcgacaac

tttcacccggagattacaaaattcgaggggagcgtggacgtgaccacgcggctgttgttg

gacaatgaaaattttaccagtttcgggtggtaccgcctgcgacccggcacccacggagag

cgtgttcaacttcgccccgttgagcgacacgtcacctcaagcgacgtggagattaactgt

actcccgataacctggagccgataccagacgaggctgcctggcccgactataagctcatg

tgctttgatatagagtgtaaagctggaacgggtaacgaaatggcgttcccagtggcaact

aaccaagaggacctggtcatccagatctcctgtctgctgtactcgcttgctactcagaac

cacgaacacaccctgctgttttccctcgggtcatgcgatatctctgaggaatactcgttt

gcatgcgtccagcgcggcgagcccagaccgacggttttggagtttgacagcgagtacgag

ctgctggttgccttcctgacctttctcaagcagtactctcccgagttcgccaccggctac

aacatcgttaattttgactgggcgtacatagttaacaaggtaacgtcggtgtataacatc

aagctggacgggtacggcaagttcaacaaaggggggctgtttaaggtgtgggacatcgcc

acgaaccattttcagaagaagagcaaggtgaaaatcaatggcctgatatctctagacatg

tattctgtggcgacggaaaagctaaagctacccagctacaaactcgacgcggtcgtggga

gacgtcctcggcgagcataagatagaccttccctataaagaaataccctcctattacgcg

ggagggcctgaccggaggggcgtaataggagagtattgtatccaggactctaggctggtg

ggcaagctgttttttaagtacctcccccatctggaactatcggcggtggccaaactcgcc

cgtatcaccctaacgcgggtaatttttgacggtcagcaaattcgcgtgtacacgtgcttg

ctgaaactcgcccgcgagagaaatttcattttgccagacaacagacgccggtttgacagt

caggcagacgccgcgtcagagacttcggagttggctatggatagccaaagccacgccttc

gacagtacagacgaacccgacggtgtggacggtaccccggacgccgcaggatctggcgct

acttctgaaaacggaggcgggaagcccggcgtcgggagggccgtgggctaccagggagca

aaggttctagaccccgtatccggctttcatgtggaccccgtggttgtgtttgacttcgct

agcttatacccaagcattatccaggcccataacctctgtttcaccaccctggcgctcgat

gaagtggatctggccgggcttcaaccatccgtcaactactcgacgttcgaggtgggtgac

caaaagttattttttgtccacgcccatattcgcgaaagcctgcttggcatcttgctgcgc

gactggctggccatgcgaaaggcggtgagggcgcgaatccccaccagcacccccgaggag

gcagttttactagataagcagcagtctgcgattaaggtgatatgcaactcggtttacgga

ttcacgggggtggcaaacggcctgttgccgtgtctgaggatagcggctaccgttaccacg

ataggacgcgacatgctcctcaagacccgagattacgttcactctcgttgggcgacgcgc

gagctgctggaggacaattttccaggggctataggtttccgaaaccacaagccttactcc

gtcagggttatctacggagacaccgactccgtgtttatcaagtttgtgggcctgacgtac

gagggggtatcggagctgggggatgctatgtcgcgtcagatttcagcggacctctttaga

gcgcccatcaaactggagtgtgagaagacctttcagcgactgctgctgatcaccaagaag

aagtacataggtgtcataaacggggggaagatgctcatgaagggggtcgacctggtccgc

aaaaataactgctctttcataaacttgtacgcgcgacatctggtagatcttttgttgtac

gacgaggatgtggccacggcggcagcagaggtgacagacgtgcctcccgcagaatgggtg

gggcgcccgctaccgagcggctttgacaagtttgggcgagtgctggtagaggcgtacaac

cgtatcactgcccccaacttggacgtgcgcgagttcgttatgactgctgagctgagccgc

tcacccgaatcgtataccaacaagcgcctgccgcacctcaccgtctactttaagctcgcc

atgaggaatgaagaactgcccagcgtaaaagagagaattccgtatgtgatagttgcgcag

accgaggccgcggaacgcgaagcgggtgtagtaaactcaatgcgcggtaccgcccaaaac

cccgtggtaaccaagaccgcacgcccccaacctaaacgcaaactgctggtttccgacctc

gccgaagacccgacctatgtttccgagaatgacgtaccgctaaacacagactactatttc

tcccacctgttgggtaccataagcgtgacctttaaggctctattcggaaatgatgtgaga

acaacagaaaatcttttaaagcggtttattccggaaaccccccacaagacccccacgaaa

acccaggcactgcttgagcgcgccggctttgaaaagctgacgccctttacaccggaggaa

gaaagtcgtcgaatactgcatacagttttttgtactctagaagcagctccccatcaaagc

tga

>LC109643.1

atggcggcgcgcgaacaggccaactccgttcgcaggagcgggttttttaatccgtttatt

ggcaagaggccatttttcaggccgggaagcgggcagaccgcggagacagagcgcccaagg

cccccccaacactcgtactgcacagaggtgggtagctttaagtttatagctccaagatgc

ctcgatgaagaagcccccgccgaccagcgacgcggtgtacacgtgggcaccttggagcgc

ccacccaaagtgtactgcgatggctcagagtacgacgtgctgaactttgcctccggaggt

tgctggcctcgcaggattcgggtttggaacggccaggattttcggggcgatggattcaac

cccagatttgagagatttcacgtgtacgacatagtagagacttcggagagcgcctcacac

gatgacccatccaggtttgctgagctatcccgcccaagcgggtctgtggtaacactgctg

ggaatgagcgagtgtggaaagcgagtcgcggttcacgtctatggtgtgcgccattatttt

tacatggcaaaggcggaggtggatagcgcttgtggaataaccaccgaggcagaactcgtg

cgcgcaatggtggactgcgcgcacagctcggctttgagcgcggccctgggaaatggcaac

ggcggcaaacagagcggcggcagcggcgggggatggtggggcggaaagcacgtgtctgcg

gactgcttcaaagtggagaccgtgtgccacacgacgctgtactactttggatctaagcca

gctctctactatagagtatctgcctccagcagccgcctgggagggttcatctgcgacaac

tttcacccggagattacaaaattcgaggggagcgtggacgtgaccacgcggctgttgttg

gacaatgaaaattttaccagtttcgggtggtaccgcctgcgacccggcacccacggagag

cgtgttcaacttcgccccgttgagcgacacgtcacctcaagcgacgtggagattaactgt

actcccgataacctggagccgataccagacgaggctgcctggcccgactataagctcatg

tgctttgatatagagtgtaaagctggaacgggtaacgaaatggcgttcccagtggcaact

aaccaagaggacctggtcatccagatctcctgtctgctgtactcgcttgctactcagaac

cacgaacacaccctgctgttttccctcgggtcatgcgatatctctgaggaatactcgttt

gcatgcgtccagcgcggcgagcccagaccgacggttttggagtttgacagcgagtacgag

ctgctggttgccttcctgacctttctcaagcagtactctcccgagttcgccaccggctac

aacatcgttaattttgactgggcgtacatagttaacaaggtaacgtcggtgtataacatc

aagctggacgggtacggcaagttcaacaaaggggggctgtttaaggtgtgggacatcgcc

acgaaccattttcagaagaagagcaaggtgaaaatcaatggcctgatatctctagacatg

tattctgtggcgacggaaaagctaaagctacccagctacaaactcgacgcggtcgtggga

gacgtcctcggcgagcataagatagaccttccctataaagaaataccctcctattacgcg

ggagggcctgaccggaggggcgtaataggagagtattgtatccaggactctaggctggtg

ggcaagctgttttttaagtacctcccccatctggaactatcggcggtggccaaactcgcc

cgtatcaccctaacgcgggtaatttttgacggtcagcaaattcgcgtgtacacgtgcttg

ctgaaactcgcccgcgagagaaatttcattttgccagacaacagacgccggtttgacagt

caggcagacgccgcgtcagagacttcggagttggctatggatagccaaagccacgccttc

gacagtacagacgaacccgacggtgtggacggtaccccggacgccgcaggatctggcgct

acttctgaaaacggaggcgggaagcccggcgtcgggagggccgtgggctaccagggagca

aaggttctagaccccgtatccggctttcatgtggaccccgtggttgtgtttgacttcgct

agcttatacccaagcattatccaggcccataacctctgtttcaccaccctggcgctcgat

gaagtggatctggccgggcttcaaccatccgtcgactactcgacgttcgaggtgggtgac

caaaagttattttttgtccacgcccatattcgcgaaagcctgcttggcatcttgctgcgc

gactggctggccatgcgaaaggcggtgagggcgcgaatccccaccagcacccccgaggag

gcagttttactagataagcagcagtctgcgattaaggtgatatgcaactcggtttacgga

ttcacgggggtggcaaacggcctgttgccgtgtctgaggatagcggctaccgttaccacg

ataggacgcgacatgctcctcaagacccgagattacgttcactctcgttgggcgacgcgc

gagctgctggaggacaattttccaggggctataggtttccgaaaccacaagccttactcc

gtcagggttatctacggagacaccgactccgtgtttatcaagtttgtgggcctgacgtac

gagggggtatcggagctgggggatgctatgtcgcgtcagatttcagcggacctctttaga

gcccccatcaaactggagtgtgagaagacctttcagcgactgctgctgatcaccaagaag

aagtacataggtgtcataaacggggggaagatgctcatgaagggggtcgacctggtccgc

aaaaataactgctctttcataaacttgtacgcgcgacatctggtagatcttttgttgtac

gacgaggatgtggccacggcggcagcagaggtgacagacgtgcctcccgcagaatgggtg

gggcgcccgctaccgagcggctttgacaagtttgggcgagtgctggtagaggcgtacaac

cgtatcactgcccccaacttggacgtgcgcgagttcgttatgactgctgagctgagccgc

tcacccgaatcgtataccaacaagcgcctgccgcacctcaccgtctactttaagctcgcc

atgaggaatgaagaactgcccagcgtaaaagagagaattccgtatgtgatagttgcgcag

accgaggccgcggaacgcgaagcgggtgtagtaaactcaatgcgcggtaccgcccaaaac

cccgtggtaaccaagaccgcacgcccccaacctaaacgcaaactgctggtttccgacctc

gccgaagacccgacctatgtttccgagaatgacgtaccgctaaacacagactactatttc

tcccacctgttgggtaccataagcgtgacctttaaggctctattcggaaatgatgtgaga

acaacagaaaatcttttaaagcggtttattccggaaaccccccacaagacccccacgaaa

acccaggcactgcttgagcgcgccggctttgaaaagctgacgccctttacaccggaggaa

gaaagtcgtcgaatactgcatacagttttttgtactctagaagcagctccccatcaaagc

tga

>LC109644.1

atggcggcgcgcgaacaggccaactccgttcgcaggagcgggttttttaatccgtttatt

ggcaagaggccatttttcaggccgggaagcgggcagaccgcggagacagagcgcccaagg

cccccccaacactcgtactgcacagaggtgggtagctttaagtttatagctccaagatgc

ctcgatgaagaagcccccgccgaccagcgacgcggtgtacacgtgggcaccttggagcgc

ccacccaaagtgtactgcgatggctcagagtacgacgtgctgaactttgcctccggaggt

tgctggcctcgcaggattcgggtttggaacggccaggattttcggggcgatggattcaac

cccagatttgagagatttcacgtgtacgacatagtagagacttcggagagcgcctcacac

gatgacccatccaggtttgctgagctatcccgcccaagcgggtctgtggtaacactgctg

ggaatgagcgagtgtggaaagcgagtcgcggttcacgtctatggtgtgcgccattatttt

tacatggcaaaggcggaggtggatagcgcttgtggaataaccaccgaggcagaactcgtg

cgcgcaatggtggactgcgcgcacagctcggctttgagcgcggccctgggaaatggcaac

ggcggcaaacagagcggcggcagcggcgggggatggtggggcggaaagcacgtgtctgcg

gactgcttcaaagtggagaccgtgtgccacacgacgctgtactactttggatctaagcca

gctctctactatagagtatctgcctccagcagccgcctgggagggttcatctgcgacaac

tttcacccggagattacaaaattcgaggggagcgtggacgtgaccacgcggctgttgttg

gacaatgaaaattttaccagtttcgggtggtaccgcctgcgacccggcacccacggagag

cgtgttcaacttcgccccgttgagcgacacgtcacctcaagcgacgtggagattaactgt

actcccgataacctggagccgataccagacgaggctgcctggcccgactataagctcatg

tgctttgatatagagtgtaaagctggaacgggtaacgaaatggcgttcccagtggcaact

aaccaagaggacctggtcatccagatctcctgtctgctgtactcgcttgctactcagaac

cacgaacacaccctgctgttttccctcgggtcatgcgatatctctgaggaatactcgttt

gcatgcgtccagcgcggcgagcccagaccgacggttttggagtttgacagcgagtacgag

ctgctggttgccttcctgacctttctcaagcagtactctcccgagttcgccaccggctac

aacatcgttaattttgactgggcgtacatagttaacaaggtaacgtcggtgtataacatc

aagctggacgggtacggcaagttcaacaaaggggggctgtttaaggtgtgggacatcgcc

acgaaccattttcagaagaagagcaaggtgaaaatcaatggcctgatatctctagacatg

tattctgtggcgacggaaaagctaaagctacccagctacaaactcgacgcggtcgtggga

gacgtcctcggcgagcataagatagaccttccctataaagaaataccctcctattacgcg

ggagggcctgaccggaggggcgtaataggagagtattgtatccaggactctaggctggtg

ggcaagctgttttttaagtacctcccccatctggaactatcggcggtggccaaactcgcc

cgtatcaccctaacgcgggtaatttttgacggtcagcaaattcgcgtgtacacgtgcttg

ctgaaactcgcccgcgagagaaatttcattttgccagacaacagacgccggtttgacagt

caggcagacgccgcgtcagagacttcggagttggctatggatagccaaagccacgccttc

gacagtacagacgaacccgacggtgtggacggtaccccggacgccgcaggatctggcgct

acttctgaaaacggaggcgggaagcccggcgtcgggagggccgtgggctaccagggagca

aaggttctagaccccgtatccggctttcatgtggaccccgtggttgtgtttgacttcgct

agcttatacccaagcattatccaggcccataacctctgtttcaccaccctggcgctcgat

gaagtggatctggccgggcttcaaccatccgtcaactactcgacgttcgaggtgggtgac

caaaagttattttttgtccacgcccatattcgcgaaagcctgcttggcatcttgctgcgc

gactggctggccatgcgaaaggcggtgagggcgcgaatccccaccagcacccccgaggag

gcagttttactagataagcagcagtctgcgattaaggtgatatgcaactcggtttacgga

ttcacgggggtggcaaacggcctgttgccgtgtctgaggatagcggctaccgttaccacg

ataggacgcgacatgctcctcaagacccgagattacgttcactctcgttgggcgacgcgc

gagctgctggaggacaattttccaggggctataggtttccgaaaccacaagccttactcc

gtcagggttatctacggagacaccgactccgtgtttatcaagtttgtgggcctgacgtac

gagggggtatcggagctgggggatgctatgtcgcgtcagatttcagcggacctctttaga

gcccccatcaaactggagtgtgagaagacctttcagcgactgctgctgatcaccaagaag

aagtacataggtgtcataaacggggggaagatgctcatgaagggggtcgacctggtccgc

aaaaataactgctctttcataaacttgtacgcgcgacatctggtagatcttttgttgtac

gacgaggatgtggccacggcggcagcagaggtgacagacgtgcctcccgcagaatgggtg

gggcgcccgctaccgagcggctttgacaagtttgggcgagtgctggtagaggcgtacaac

cgtatcactgcccccaacttggacgtgcgcgagttcgttatgactgctgagctgagccgc

tcacccgaatcgtataccaacaagcgcctgccgcacctcaccgtctactttaagctcgcc

atgaggaatgaagaactgcccagcgtaaaagagagaattccgtatgtgatagttgcgcag

accgaggccgcggaacgcgaagcgggtgtagtaaactcaatgcgcggtaccgcccaaaac

cccgtggtaaccaagaccgcacgcccccaacctaaacgcaaactgctggtttccgacctc

gccgaagacccgacctatgtttccgagaatgacgtaccgctaaacacagactactatttc

tcccacctgttgggtaccataagcgtgacctttaaggctctattcggaaatgatgtgaga

acaacagaaaatcttttaaagcggtttattccggaaaccccccacaagacccccacgaaa

acccaggcactgcttgagcgcgccggctttgaaaagctgacgccctttacaccggaggaa

gaaagtcgtcgaatactgcatacagttttttgtactctagaagcagctccccatcaaagc

tga

>LC109645.1

atggcggcgcgcgaacaggccaactccgttcgcaggagcgggttttttaatccgtttatt

ggcaagaggccatttttcaggccgggaagcgggcagaccgcggagacagagcgcccaagg

cccccccaacactcgtactgcacagaggtgggtagctttaagtttatagctccaagatgc

ctcgatgaagaagcccccgccgaccagcgacgcggtgtacacgtgggcaccttggagcgc

ccacccaaagtgtactgcgatggctcagagtacgacgtgctgaactttgcctccggaggt

tgctggcctcgcaggattcgggtttggaacggccaggattttcggggcgatggattcaac

cccagatttgagagatttcacgtgtacgacatagtagagacttcggagagcgcctcacac

gatgacccatccaggtttgctgagctatcccgcccaagcgggtctgtggtaacactgctg

ggaatgagcgagtgtggaaagcgagtcgcggttcacgtctatggtgtgcgccattatttt

tacatggcaaaggcggaggtggatagcgcttgtggaataaccaccgaggcagaactcgtg

cgcgcaatggtggactgcgcgcacagctcggctttgagcgcggccctgggaaatggcaac

ggcggcaaacagagcggcggcagcggcgggggatggtggggcggaaagcacgtgtctgcg

gactgcttcaaagtggagaccgtgtgccacacgacgctgtactactttggatctaagcca

gctctctactatagagtatctgcctccagcagccgcctgggagggttcatctgcgacaac

tttcacccggagattacaaaattcgaggggagcgtggacgtgaccacgcggctgttgttg

gacaatgaaaattttaccagtttcgggtggtaccgcctgcgacccggcacccacggagag

cgtgttcaacttcgccccgttgagcgacacgtcacctcaagcgacgtggagattaactgt

actcccgataacctggagccgataccagacgaggctgcctggcccgactataagctcatg

tgctttgatatagagtgtaaagctggaacgggtaacgaaatggcgttcccagtggcaact

aaccaagaggacctggtcatccagatctcctgtctgctgtactcgcttgctactcagaac

cacgaacacaccctgctgttttccctcgggtcatgcgatatctctgaggaatactcgttt

gcatgcgtccagcgcggcgagcccagaccgacggttttggagtttgacagcgagtacgag

ctgctggttgccttcctgacctttctcaagcagtactctcccgagttcgccaccggctac

aacatcgttaattttgactgggcgtacatagttaacaaggtaacgtcggtgtataacatc

aagctggacgggtacggcaagttcaacaaaggggggctgtttaaggtgtgggacatcgcc

acgaaccattttcagaagaagagcaaggtgaaaatcaatggcctgatatctctagacatg

tattctgtggcgacggaaaagctaaagctacccagctacaaactcgacgcggtcgtggga

gacgtcctcggcgagcataagatagaccttccctataaagaaataccctcctattacgcg

ggagggcctgaccggaggggcgtaataggagagtattgtatccaggactctaggctggtg

ggcaagctgttttttaagtacctcccccatctggaactatcggcggtggccaaactcgcc

cgtatcaccctaacgcgggtaatttttgacggtcagcaaattcgcgtgtacacgtgcttg

ctgaaactcgcccgcgagagaaatttcattttgccagacaacagacgccggtttgacagt

caggcagacgccgcgtcagagacttcggagttggctatggatagccaaagccacgccttc

gacagtacagacgaacccgacggtgtggacggtaccccggacgccgcaggatctggcgct

acttctgaaaacggaggcgggaagcccggcgtcgggagggccgtgggctaccagggagca

aaggttctagaccccgtatccggctttcatgtggaccccgtggttgtgtttgacttcgct

agcttatacccaagcattatccaggcccataacctctgtttcaccaccctggcgctcgat

gaagtggatctggccgggcttcaaccatccgtcaactactcgacgttcgaggtgggtgac

caaaagttattttttgtccacgcccatattcgcgaaagcctgcttggcatcttgctgcgc

gactggctggccatgcgaaaggcggtgagggcgcgaatccccaccagcacccccgaggag

gcagttttactagataagcagcagtctgcgattaaggtgatatgcaactcggtttacgga

ttcacgggggtggcaaacggcctgttgccgtgtctgaggatagcggctaccgttaccacg

ataggacgcgacatgctcctcaagacccgagattacgttcactctcgttgggcgacgcgc

gagctgctggaggacaattttccaggggctataggtttccgaaaccacaagccttactcc

gtcagggttatctacggagacaccgactccgtgtttatcaagtttgtgggcctgacgtac

gagggggtatcggagctgggggatgctatgtcgcgtcagatttcagcggacctctttaga

gcgcccatcaaactggagtgtgagaagacctttcagcgactgctgctgatcaccaagaag

aagtacataggtgtcataaacggggggaagatgctcatgaagggggtcgacctggtccgc

aaaaataactgctctttcataaacttgtacgcgcgacatctggtagatcttttgttgtac

gacgaggatgtggccacggcggcagcagaggtgacagacgtgcctcccgcagaatgggtg

gggcgcccgctaccgagcggctttgacaagtttgggcgagtgctggtagaggcgtacaac

cgtatcactgcccccaacttggacgtgcgcgagttcgttatgactgctgagctgagccgc

tcacccgaatcgtataccaacaagcgcctgccgcacctcaccgtctactttaagctcgcc

atgaggaatgaagaactgcccagcgtaaaagagagaattccgtatgtgatagttgcgcag

accgaggccgcggaacgcgaagcgggtgtagtaaactcaatgcgcggtaccgcccaaaac

cccgtggtaaccaagaccgcacgcccccaacctaaacgcaaactgctggtttccgacctc

gccgaagacccgacctatgtttccgagaatgacgtaccgctaaacacagactactatttc

tcccacctgttgggtaccataagcgtgacctttaaggctctattcggaaatgatgtgaga

acaacagaaaatcttttaaagcggtttattccggaaaccccccacaagacccccacgaaa

acccaggcactgcttgagcgcgccggctttgaaaagctgacgccctttacaccggaggaa

gaaagtcgtcgaatactgcatacagttttttgtactctagaagcagctccccatcaaagc

tga

>LC109646.1

atggcggcgcgcgaacaggccaactccgttcgcaggagcgggttttttaatccgtttatt

ggcaagaggccatttttcaggccgggaagcgggcaaaccgcggagacagagcgcccaagg

cccccccaacactcgtactgcacagaggtgggtagctttaagtttatagctccaagatgc

ctcgatgaagaagcccccgccgaccagcgacgcggtgtacacgtgggcaccttggagcgc

ccacccaaagtgtactgcgatggctcagagtacgacgtgctgaactttgcctccggaggt

tgctggcctcgcaggattcgggtttggaacggccaggattttcggggcgatggattcaac

cccagatttgagagatttcacgtgtacgacatagtagagacttcggagagcgcctcacac

gatgacccatccaggtttgctgagctatcccgcccaagcgggtctgtggtaacactgctg

ggaatgagcgagtgtggaaagcgagtcgcggttcacgtctatggtgtgcgccattatttt

tacatggcaaaggcggaggtggatagcgcttgtggaataaccaccgaggcagaactcgtg

cgcgcaatggtggactgcgcgcacagctcggctttgagcgcggccctgggaaatggcaac

ggcggcaaacagagcggcggcagcggcgggggatggtggggcggaaagcacgtgtctgcg

gactgcttcaaagtggagaccgtgtgccacacgacgctgtactactttggatctaagcca

gctctctactatagagtatctgcctccagcagccgcctgggagggttcatctgcgacaac

tttcacccggagattacaaaattcgaggggagcgtggacgtgaccacgcggctgttgttg

gacaatgaaaattttaccagtttcgggtggtaccgcctgcgacccggcacccacggagag

cgtgttcaacttcgccccgttgagcgacacgtcacctcaagcgacgtggagattaactgt

actcccgataacctggagccgataccagacgaggctgcctggcccgactataagctcatg

tgctttgatatagagtgtaaagctggaacgggtaacgaaatggcgttcccagtggcaact

aaccaagaggacctggtcatccagatctcctgtctgctgtactcgcttgctactcagaac

cacgaacacaccctgctgttttccctcgggtcatgcgatatctctgaggaatactcgttt

gcatgcgtccagcgcggcgagcccagaccgacggttttggagtttgacagcgagtacgag

ctgctggttgccttcctgacctttctcaagcagtactctcccgagttcgccaccggctac

aacatcgttaattttgactgggcgtacatagttaacaaggtaacgtcggtgtataacatc

aagctggacgggtacggcaagttcaacaaaggggggctgtttaaggtgtgggacatcgcc

acgaaccattttcagaagaagagcaaggtgaaaatcaatggcctgatatctctagacatg

tattctgtggcgacggaaaagctaaagctacccagctacaaactcgacgcggtcgtggga

gacgtcctcggcgagcataagatagaccttccctataaagaaataccctcctattacgcg

ggagggcctgaccggaggggcgtaataggagagtattgtatccaggactctaggctggtg

ggcaagctgttttttaagtacctcccccatctggaactatcggcggtggccaaactcgcc

cgtatcaccctaacgcgggtaatttttgacggtcagcaaattcgcgtgtacacgtgcttg

ctgaaactcgcccgcgagagaaatttcattttgccagacaacagacgccggtttgacagt

caggcagacgccgcgtcagagacttcggagttggctatggatagccaaagccacgccttc

gacagtacagacgaacccgacggtgtggacggtaccccggacgccgcaggatctggcgct

acttctgaaaacggaggcgggaagcccggcgtcgggagggccgtgggctaccagggagca

aaggttctagaccccgtatccggctttcatgtggaccccgtggttgtgtttgacttcgct

agcttatacccaagcattatccaggcccataacctctgtttcaccaccctggcgctcgat

gaagtggatctggccgggcttcaaccatccgtcaactactcgacgttcgaggtgggtgac

caaaagttattttttgtccacgcccatattcgcgaaagcctgcttggcatcttgctgcgc

gactggctggccatgcgaaaggcggtgagggcgcgaatccccaccagcacccccgaggag

gcagttttactagataagcagcagtctgcgattaaggtgatatgcaactcggtttacgga

ttcacgggggtggcaaacggcctgttgccgtgtctgaggatagcggctaccgttaccacg

ataggacgcgacatgctcctcaagacccgagattacgttcactctcgttgggcgacgcgc

gagctgctggaggacaattttccaggggctataggtttccgaaaccacaagccttactcc

gtcagggttatctacggagacaccgactccgtgtttatcaagtttgtgggcctgacgtac

gagggggtatcggagctgggggatgctatgtcgcgtcagatttcagcggacctctttaga

gcgcccatcaaactggagtgtgagaagacctttcagcgactgctgctgatcaccaagaag

aagtacataggtgtcataaacggggggaagatgctcatgaagggggtcgacctggtccgc

aaaaataactgctctttcataaacttgtacgcgcgacatctggtagatcttttgttgtac

gacgaggatgtggccacggcggcagcagaggtgacagacgtgcctcccgcagaatgggtg

gggcgcccgctaccgagcggctttgacaagtttgggcgagtgctggtagaggcgtacaac

cgtatcactgcccccaacttggacgtgcgcgagttcgttatgactgctgagctgagccgc

tcacccgaatcgtataccaacaagcgcctgccgcacctcaccgtctactttaagctcgcc

atgaggaatgaagaactgcccagcgtaaaagagagaattccgtatgtgatagttgcgcag

accgaggccgcggaacgcgaagcgggtgtagtaaactcaatgcgcggtaccgcccaaaac

cccgtggtaaccaagaccgcacgcccccaacctaaacgcaaactgctggtttccgacctc

gccgaagacccgacctatgtttccgagaatgacgtaccgctaaacacagactactatttc

tcccacctgttgggtaccataagcgtgacctttaaggctctattcggaaatgatgtgaga

acaacagaaaatcttttaaagcggtttattccggaaaccccccacaagacccccacgaaa

acccaggcactgcttgagcgcgccggctttgaaaagctgacgccctttacaccggaggaa

gaaagtcgtcgaatactgcatacagttttttgtactctagaagcagctccccatcaaagc

tga

>LC109647.1

atggcggcgcgcgaacaggccaactccgttcgcaggagcgggttttttaatccgtttatt

ggcaagaggccatttttcaggccgggaagcgggcagaccgcggagacagagcgcccaagg

cccccccaacactcgtactgcacagaggtgggtagctttaagtttatagctccaagatgc

ctcgatgaagaagcccccgccgaccagcgacgcggtgtacacgtgggcaccttggagcgc

ccacccaaagtgtactgcgatggctcagagtacgacgtgctgaactttgcctccggaggt

tgctggcctcgcaggattcgggtttggaacggccaggattttcggggcgatggattcaac

cccagatttgagagatttcacgtgtacgacatagtagagacttcggagagcgcctcacac

gatgacccatccaggtttgctgagctatcccgcccaagcgggtctgtggtaacactgctg

ggaatgagcgagtgtggaaagcgagtcgcggttcacgtctatggtgtgcgccattatttt

tacatggcaaaggcggaggtggatagcgcttgtggaataaccaccgaggcagaactcgtg

cgcgcaatggtggactgcgcgcacagctcggctttgagcgcggccctgggaaatggcaac

ggcggcaaacagagcggcggcagcggcgggggatggtggggcggaaagcacgtgtctgcg

gactgcttcaaagtggagaccgtgtgccacacgacgctgtactactttggatctaagcca

gctctctactatagagtatctgcctccagcagccgcctgggagggttcatctgcgacaac

tttcacccggagattacaaaattcgaggggagcgtggacgtgaccacgcggctgttgttg

gacaatgaaaattttaccagtttcgggtggtaccgcctgcgacccggcacccacggagag

cgtgttcaacttcgccccgttgagcgacacgtcacctcaagcgacgtggagattaactgt

actcccgataacctggagccgataccagacgaggctgcctggcccgactataagctcatg

tgctttgatatagagtgtaaagctggaacgggtaacgaaatggcgttcccagtggcaact

aaccaagaggacctggtcatccagatctcctgtctgctgtactcgcttgctactcagaac

cacgaacacaccctgctgttttccctcgggtcatgcgatatctctgaggaatactcgttt

gcatgcgtccagcgcggcgagcccagaccgacggttttggagtttgacagcgagtacgag

ctgctggttgccttcctgacctttctcaagcagtactctcccgagttcgccaccggctac

aacatcgttaattttgactgggcgtacatagttaacaaggtaacgtcggtgtataacatc

aagctggacgggtacggcaagttcaacaaaggggggctgtttaaggtgtgggacatcgcc

acgaaccattttcagaagaagagcaaggtgaaaatcaatggcctgatatctctagacatg

tattctgtggcgacggaaaagctaaagctacccagctacaaactcgacgcggtcgtggga

gacgtcctcggcgagcataagatagaccttccctataaagaaataccctcctattacgcg

ggagggcctgaccggaggggcgtaataggagagtattgtatccaggactctaggctggtg

ggcaagctgttttttaagtacctcccccatctggaactatcggcggtggccaaactcgcc

cgtatcaccctaacgcgggtaatttttgacggtcagcaaattcgcgtgtacacgtgcttg

ctgaaactcgcccgcgagagaaatttcattttgccagacaacagacgccggtttgacagt

caggcagacgccgcgtcagagacttcggagttggctatggatagccaaagccacgccttc

gacagtacagacgaacccgacggtgtggacggtaccccggacgccgcaggatctggcgct

acttctgaaaacggaggcgggaagcccggcgtcgggagggccgtgggctaccagggagca

aaggttctagaccccgtatccggctttcatgtggaccccgtggttgtgtttgacttcgct

agcttatacccaagcattatccaggcccataacctctgtttcaccaccctggcgctcgat

gaagtggatctggccgggcttcaaccatccgtcaactactcgacgttcgaggtgggtgac

caaaagttattttttgtccacgcccatattcgcgaaagcctgcttggcatcttgctgcgc

gactggctggccatgcgaaaggcggtgagggcgcgaatccccaccagcacccccgaggag

gcagttttactagataagcagcagtctgcgattaaggtgatatgcaactcggtttacgga

ttcacgggggtggcaaacggcctgttgccgtgtctgaggatagcggctaccgttaccacg

ataggacgcgacatgctcctcaagacccgagattacgttcactctcgttgggcgacgcgc

gagctgctggaggacaattttccaggggctataggtttccgaaaccacaagccttactcc

gtcagggttatctacggagacaccgactccgtgtttatcaagtttgtgggcctgacgtac

gagggggtatcggagctgggggatgctatgtcgcgtcagatttcagcggacctctttaga

gcccccatcaaactggagtgtgagaagacctttcagcgactgctgctgatcaccaagaag

aagtacataggtgtcataaacggggggaagatgctcatgaagggggtcgacctggtccgc

aaaaataactgctctttcataaacttgtacgcgcgacatctggtagatcttttgttgtac

gacgaggatgtggccacggcggcagcagaggtgacagacgtgcctcccgcagaatgggtg

gggcgcccgctaccgagcggctttgacaagtttgggcgagtgctggtagaggcgtacaac

cgtatcactgcccccaacttggacgtgcgcgagttcgttatgactgctgagctgagccgc

tcacccgaatcgtataccaacaagcgcctgccgcacctcaccgtctactttaagctcgcc

atgaggaatgaagaactgcccagcgtaaaagagagaattccgtatgtgatagttgcgcag

accgaggccgcggaacgcgaagcgggtgtagtaaactcaatgcgcggtaccgcccaaaac

cccgtggtaaccaagaccgcacgcccccaacctaaacgcaaactgctggtttccgacctc

gccgaagacccgacctatgtttccgagaatgacgtaccgctaaacacagactactatttc

tcccacctgttgggtaccataagcgtgacctttaaggctctattcggaaatgatgtgaga

acaacagaaaatcttttaaagcggtttattccggaaaccccccacaagacccccacgaaa

acccaggcactgcttgagcgcgccggctttgaaaagctgacgccctttacaccggaggaa

gaaagtcgtcgaatactgcatacagttttttgtactctagaagcagctccccatcaaagc

tga

>LC109648.1

atggcggcgcgcgaacaggccaactccgttcgcaggagcgggttttttaatccgtttatt

ggcaagaggccatttttcaggccgggaagcgggcagaccgcggagacagagcgcccaagg

cccccccaacactcgtactgcacagaggtgggtagctttaagtttatagctccaagatgc

ctcgatgaagaagcccccgccgaccagcgacgcggtgtacacgtgggcaccttggagcgc

ccacccaaagtgtactgcgatggctcagagtacgacgtgctgaactttgcctccggaggt

tgctggcctcgcaggattcgggtttggaacggccaggattttcggggcgatggattcaac

cccagatttgagagatttcacgtgtacgacatagtagagacttcggagagcgcctcacac

gatgacccatccaggtttgctgagctatcccgcccaagcgggtctgtggtaacactgctg

ggaatgagcgagtgtggaaagcgagtcgcggttcacgtctatggtgtgcgccattatttt

tacatggcaaaggcggaggtggatagcgcttgtggaataaccaccgaggcagaactcgtg

cgcgcaatggtggactgcgcgcacagctcggctttgagcgcggccctgggaaatggcaac

ggcggcaaacagagcggcggcagcggcgggggatggtggggcggaaagcacgtgtctgcg

gactgcttcaaagtggagaccgtgtgccacacgacgctgtactactttggatctaagcca

gctctctactatagagtatctgcctccagcagccgcctgggagggttcatctgcgacaac

tttcacccggagattacaaaattcgaggggagcgtggacgtgaccacgcggctgttgttg

gacaatgaaaattttaccagtttcgggtggtaccgcctgcgacccggcacccacggagag

cgtgttcaacttcgccccgttgagcgacacgtcacctcaagcgacgtggagattaactgt

actcccgataacctggagccgataccagacgaggctgcctggcccgactataagctcatg

tgctttgatatagagtgtaaagctggaacgggtaacgaaatggcgttcccagtggcaact

aaccaagaggacctggtcatccagatctcctgtctgctgtactcgcttgctactcagaac

cacgaacacaccctgctgttttccctcgggtcatgcgatatctctgaggaatactcgttt

gcatgcgtccagcgcggcgagcccagaccgacggttttggagtttgacagcgagtacgag

ctgctggttgccttcctgacctttctcaagcagtactctcccgagttcgccaccggctac

aacatcgttaattttgactgggcgtacatagttaacaaggtaacgtcggtgtataacatc

aagctggacgggtacggcaagttcaacaaaggggggctgtttaaggtgtgggacatcgcc

acgaaccattttcagaagaagagcaaggtgaaaatcaatggcctgatatctctagacatg

tattctgtggcgacggaaaagctaaagctacccagctacaaactcgacgcggtcgtggga

gacgtcctcggcgagcataagatagaccttccctataaagaaataccctcctattacgcg

ggagggcctgaccggaggggcgtaataggagagtattgtatccaggactctaggctggtg

ggcaagctgttttttaagtacctcccccatctggaactatcggcggtggccaaactcgcc

cgtatcaccctaacgcgggtaatttttgacggtcagcaaattcgcgtgtacacgtgcttg

ctgaaactcgcccgcgagagaaatttcattttgccagacaacagacgccggtttgacagt

caggcagacgccgcgtcagagacttcggagttggctatggatagccaaagccacgccttc

gacagtacagacgaacccgacggtgtggacggtaccccggacgccgcaggatctggcgct

acttctgaaaacggaggcgggaagcccggcgtcgggagggccgtgggctaccagggagca

aaggttctagaccccgtatccggctttcatgtggaccccgtggttgtgtttgacttcgct

agcttatacccaagcattatccaggcccataacctctgtttcaccaccctggcgctcgat

gaagtggatctggccgggcttcaaccatccgtcaactactcgacgttcgaggtgggtgac

caaaagttattttttgtccacgcccatattcgcgaaagcctgcttggcatcttgctgcgc

gactggctggccatgcgaaaggcggtgagggcgcgaatccccaccagcacccccgaggag

gcagttttactagataagcagcagtctgcgattaaggtgatatgcaactcggtttacgga

ttcacgggggtggcaaacggcctgttgccgtgtctgaggatagcggctaccgttaccacg

ataggacgcgacatgctcctcaagacccgagattacgttcactctcgttgggcgacgcgc

gagctgctggaggacaattttccaggggctataggtttccgaaaccacaagccttactcc

gtcagggttatctacggagacaccgactccgtgtttatcaagtttgtgggcctgacgtac

gagggggtatcggagctgggggatgctatgtcgcgtcagatttcagcggacctctttaga

gcgcccatcaaactggagtgtgagaagacctttcagcgactgctgctgatcaccaagaag

aagtacataggtgtcgtaaacggggggaagatgctcatgaagggggtcgacctggtccgc

aaaaataactgctctttcataaacttgtacgcgcgacatctggtagatcttttgttgtac

gacgaggatgtggccacggcggcagcagaggtgacagacgtgcctcccgcagaatgggtg

gggcgcccgctaccgagcggctttgacaagtttgggcgagtgctggtagaggcgtacaac

cgtatcactgcccccaacttggacgtgcgcgagttcgttatgactgctgagctgagccgc

tcacccgaatcgtataccaacaagcgcctgccgcacctcaccgtctactttaagctcgcc

atgaggaatgaagaactgcccagcgtaaaagagagaattccgtatgtgatagttgcgcag

accgaggccgcggaacgcgaagcgggtgtagtaaactcaatgcgcggtaccgcccaaaac

cccgtggtaaccaagaccgcacgcccccaacctaaacgcaaactgctggtttccgacctc

gccgaagacccgacctatgtttccgagaatgacgtaccgctaaacacagactactatttc

tcccacctgttgggtaccataagcgtgacctttaaggctctattcggaaatgatgtgaga

acaacagaaaatcttttaaagcggtttattccggaaaccccccacaagacccccacgaaa

acccaggcactgcttgagcgcgccggctttgaaaagctgacgccctttacaccggaggaa

gaaagtcgtcgaatactgcatacagttttttgtactctagaagcagctccccatcaaagc

tga

>LC109649.1

atggcggcgcgcgaacaggccaactccgttcgcaggagcgggttttttaatccgtttatt

ggcaagaggccatttttcaggccgggaagcgggcagaccgcggagacagagcgcccaagg

cccccccaacactcgtactgcacagaggtgggtagctttaagtttatagctccaagatgc

ctcgatgaagaagcccccgccgaccagcgacgcggtgtacacgtgggcaccttggagcgc

ccacccaaagtgtactgcgatggctcagagtacgacgtgctgaactttgcctccggaggt

tgctggcctcgcaggattcgggtttggaacggccaggattttcggggcgatggattcaac

cccagatttgagagatttcacgtgtacgacatagtagagacttcggagagcgcctcacac

gatgacccatccaggtttgctgagctatcccgcccaagcgggtctgtggtaacactgctg

ggaatgagcgagtgtggaaagcgagtcgcggttcacgtctatggtgtgcgccattatttt

tacatggcaaaggcggaggtggatagcgcttgtggaataaccaccgaggcagaactcgtg

cgcgcaatggtggactgcgcgcacagctcggctttgagcgcggccctgggaaatggcaac

ggcggcaaacagagcggcggcagcggcgggggatggtggggcggaaagcacgtgtctgcg

gactgcttcaaagtggagaccgtgtgccacacgacgctgtactactttggatctaagcca

gctctctactatagagtatctgcctccagcagccgcctgggagggttcatctgcgacaac

tttcacccggagattacaaaattcgaggggagcgtggacgtgaccacgcggctgttgttg

gacaatgaaaattttaccagtttcgggtggtaccgcctgcgacccggcacccacggagag

cgtgttcaacttcgccccgttgagcgacacgtcacctcaagcgacgtggagattaactgt

actcccgataacctggagccgataccagacgaggctgcctggcccgactataagctcatg

tgctttgatatagagtgtaaagctggaacgggtaacgaaatggcgttcccagtggcaact

aaccaagaggacctggtcatccagatctcctgtctgctgtactcgcttgctactcagaac

cacgaacacaccctgctgttttccctcgggtcatgcgatatctctgaggaatactcgttt

gcatgcgtccagcgcggcgagcccagaccgacggttttggagtttgacagcgagtacgag

ctgctggttgccttcctgacctttctcaagcagtactctcccgagttcgccaccggctac

aacatcgttaattttgactgggcgtacatagttaacaaggtaacgtcggtgtataacatc

aagctggacgggtacggcaagttcaacaaaggggggctgtttaaggtgtgggacatcgcc

acgaaccattttcagaagaagagcaaggtgaaaatcaatggcctgatatctctagacatg

tattctgtggcgacggaaaagctaaagctacccagctacaaactcgacgcggtcgtggga

gacgtcctcggcgagcataagatagaccttccctataaagaaataccctcctattacgcg

ggagggcctgaccggaggggcgtaataggagagtattgtatccaggactctaggctggtg

ggcaagctgttttttaagtacctcccccatctggaactatcggcggtggccaaactcgcc

cgtatcaccctaacgcgggtaatttttgacggtcagcaaattcgcgtgtacacgtgcttg

ctgaaactcgcccgcgagagaaatttcattttgccagacaacagacgccggtttgacagt

caggcagacgccgcgtcagagacttcggagttggctatggatagccaaagccacgccttc

gacagtacagacgaacccgacggtgtggacggtaccccggacgccgcaggatctggcgct

acttctgaaaacggaggcgggaagcccggcgtcgggagggccgtgggctaccagggagca

aaggttctagaccccgtatccggctttcatgtggaccccgtggttgtgtttgacttcgct

agcttatacccaagcattatccaggcccataacctctgtttcaccaccctggcgctcgat

gaagtggatctggccgggcttcaaccatccgtcaactactcgacgttcgaggtgggtgac

caaaagttattttttgtccacgcccatattcgcgaaagcctgcttggcatcttgctgcgc

gactggctggccatgcgaaaggcggtgagggcgcgaatccccaccagcacccccgaggag

gcagttttactagataagcagcagtctgcgattaaggtgatatgcaactcggtttacgga

ttcacgggggtggcaaacggcctgttgccgtgtctgaggatagcggctaccgttaccacg

ataggacgcgacatgctcctcaagacccgagattacgttcactctcgttgggcgacgcgc

gagctgctggaggacaattttccaggggctataggtttccgaaaccacaagccttactcc

gtcagggttatctacggagacaccgactccgtgtttatcaagtttgtgggcctgacgtac

gagggggtatcggagctgggggatgctatgtcgcgtcagatttcagcggacctctttaga

gcgcccatcaaactggagtgtgagaagacctttcagcgactgctgctgatcaccaagaag

aagtacataggtgtcataaacggggggaagatgctcatgaagggggtcgacctggtccgc

aaaaataactgctctttcataaacttgtacgcgcgacatctggtagatcttttgttgtac

gacgaggatgtggccacggcggcagcagaggtgacagacgtgcctcccgcagaatgggtg

gggcgcccgctaccgagcggctttgacaagtttgggcgagtgctggtagaggcgtacaac

cgtatcactgcccccaacttggacgtgcgcgagttcgttatgactgctgagctgagccgc

tcacccgaatcgtataccaacaagcgcctgccgcacctcaccgtctactttaagctcgcc

atgaggaatgaagaactgcccagcgtaaaagagagaattccgtatgtgatagttgcgcag

accgaggccgcggaacgcgaagcgggtgtagtaaactcaatgcgcggtaccgcccaaaac

cccgtggtaaccaagaccgcacgcccccaacctaaacgcaaactgctggtttccgacctc

gccgaagacccgacctatgtttccgagaatgacgtaccgctaaacacagactactatttc

tcccacctgttgggtaccataagcgtgacctttaaggctctattcggaaatgatgtgaga

acaacagaaaatcttttaaagcggtttattccggaaaccccccacaagacccccacgaaa

acccaggcactgcttgagcgcgccggctttgaaaagctgacgccctttacaccggaggaa

gaaagtcgtcgaatactgcatacagttttttgtactctagaagcagctccccatcaaagc

tga

>LC109650.1

atggcggcgcgcgaacaggccaactccgttcgcaggagcgggttttttaatccgtttatt

ggcaagaggccatttttcaggccgggaagcgggcagaccgcggagacagagcgcccaagg

cccccccaacactcgtactgcacagaggtgggtagctttaagtttatagctccaagatgc

ctcgatgaagaagcccccgccgaccagcgacgcggtgtacacgtgggcaccttggagcgc

ccacccaaagtgtactgcgatggctcagagtacgacgtgctgaactttgcctccggaggt

tgctggcctcgcaggattcgggtttggaacggccaggattttcggggcgatggattcaac

cccagatttgagagatttcacgtgtacgacatagtagagacttcggagagcgcctcacac

gatgacccatccaggtttgctgagctatcccgcccaagcgggtctgtggtaacactgctg

ggaatgagcgagtgtggaaagcgagtcgcggttcacgtctatggtgtgcgccattatttt

tacatggcaaaggcggaggtggatagcgcttgtggaataaccaccgaggcagaactcgtg

cgcgcaatggtggactgcgcgcacagctcggctttgagcgcggccctgggaaatggcaac

ggcggcaaacagagcggcggcagcggcgggggatggtggggcggaaagcacgtgtctgcg

gactgcttcaaagtggagaccgtgtgccacacgacgctgtactactttggatctaagcca

gctctctactatagagtatctgcctccagcagccgcctgggagggttcatctgcgacaac

tttcacccggagattacaaaattcgaggggagcgtggacgtgaccacgcggctgttgttg

gacaatgaaaattttaccagtttcgggtggtaccgcctgcgacccggcacccacggagag

cgtgttcaacttcgccccgttgagcgacacgtcacctcaagcgacgtggagattaactgt

actcccgataacctggagccgataccagacgaggctgcctggcccgactataagctcatg

tgctttgatatagagtgtaaagctggaacgggtaacgaaatggcgttcccagtggcaact

aaccaagaggacctggtcatccagatctcctgtctgctgtactcgcttgctactcagaac

cacgaacacaccctgctgttttccctcgggtcatgcgatatctctgaggaatactcgttt

gcatgcgtccagcgcggcgagcccagaccgacggttttggagtttgacagcgagtacgag

ctgctggttgccttcctgacctttctcaagcagtactctcccgagttcgccaccggctac

aacatcgttaattttgactgggcgtacatagttaacaaggtaacgtcggtgtataacatc

aagctggacgggtacggcaagttcaacaaaggggggctgtttaaggtgtgggacatcgcc

acgaaccattttcagaagaagagcaaggtgaaaatcaatggcctgatatctctagacatg

tattctgtggcgacggaaaagctaaagctacccagctacaaactcgacgcggtcgtggga

gacgtcctcggcgagcataagatagaccttccctataaagaaataccctcctattacgcg

ggagggcctgaccggaggggcgtaataggagagtattgtatccaggactctaggctggtg

ggcaagctgttttttaagtacctcccccatctggaactatcggcggtggccaaactcgcc

cgtatcaccctaacgcgggtaatttttgacggtcagcaaattcgcgtgtacacgtgcttg

ctgaaactcgcccgcgagagaaatttcattttgccagacaacagacgccggtttgacagt

caggcagacgccgcgtcagagacttcggagttggctatggatagccaaagccacgccttc

gacagtacagacgaacccgacggtgtggacggtaccccggacgccgcaggatctggcgct

acttctgaaaacggaggcgggaagcccggcgtcgggagggccgtgggctaccagggagca

aaggttctagaccccgtatccggctttcatgtggaccccgtggttgtgtttgacttcgct

agcttatacccaagcattatccaggcccataacctctgtttcaccaccctggcgctcgat

gaagtggatctggccgggcttcaaccatccgtcaactactcgacgttcgaggtgggtgac

caaaagttattttttgtccacgcccatattcgcgaaagcctgcttggcatcttgctgcgc

gactggctggccatgcgaaaggcggtgagggcgcgaatccccaccagcacccccgaggag

gcagttttactagataagcagcagtctgcgattaaggtgatatgcaactcggtttacgga

ttcacgggggtggcaaacggcctgttgccgtgtctgaggatagcggctaccgttaccacg

ataggacgcgacatgctcctcaagacccgagattacgttcactctcgttgggcgacgcgc

gagctgctggaggacaattttccaggggctataggtttccgaaaccacaagccttactcc

gtcagggttatctacggagacaccgactccgtgtttatcaagtttgtgggcctgacgtac

gagggggtatcggagctgggggatgctatgtcgcgtcagatttcagcggacctctttaga

gcccccatcaaactggagtgtgagaagacctttcagcgactgctgctgatcaccaagaag

aagtacataggtgtcataaacggggggaagatgctcatgaagggggtcgacctggtccgc

aaaaataactgctctttcataaacttgtacgcgcgacatctggtagatcttttgttgtac

gacgaggatgtggccacggcggcagcagaggtgacagacgtgcctcccgcagaatgggtg

gggcgcccgctaccgagcggctttgacaagtttgggcgagtgctggtagaggcgtacaac

cgtatcactgcccccaacttggacgtgcgcgagttcgttatgactgctgagctgagccgc

tcacccgaatcgtataccaacaagcgcctgccgcacctcaccgtctactttaagctcgcc

atgaggaatgaagaactgcccagcgtaaaagagagaattccgtatgtgatagttgcgcag

accgaggccgcggaacgcgaagcgggtgtagtaaactcaatgcgcggtaccgcccaaaac

cccgtggtaaccaagaccgcacgcccccaacctaaacgcaaactgctggtttccgacctc

gccgaagacccgacctatgtttccgagaatgacgtaccgctaaacacagactactatttc

tcccacctgttgggtaccataagcgtgacctttaaggctctattcggaaatgatgtgaga

acaacagaaaatcttttaaagcggtttattccggaaaccccccacaagacccccacgaaa

acccaggcactgcttgagcgcgccggctttgaaaagctgacgccctttacaccggaggaa

gaaagtcgtcgaatactgcatacagttttttgtactctagaagcagctccccatcaaagc

tga

>LC109651.1

atggcggcgcgcgaacaggccaactccgttcgcaggagcgggttttttaatccgtttatt

ggcaagaggccatttttcaggccgggaagcgggcagaccgcggagacagagcgcccaagg

cccccccaacactcgtactgcacagaggtgggtagctttaagtttatagctccaagatgc

ctcgatgaagaagcccccgccgaccagcgacgcggtgtacacgtgggcaccttggagcgc

ccacccaaagtgtactgcgatggctcagagtacgacgtgctgaactttgcctccggaggt

tgctggcctcgcaggattcgggtttggaacggccaggattttcggggcgatggattcaac

cccagatttgagagatttcacgtgtacgacatagtagagacttcggagagcgcctcacac

gatgacccatccaggtttgctgagctatcccgcccaagcgggtctgtggtaacactgctg

ggaatgagcgagtgtggaaagcgagtcgcggttcacgtctatggtgtgcgccattatttt

tacatggcaaaggcggaggtggatagcgcttgtggaataaccaccgaggcagaactcgtg

cgcgcaatggtggactgcgcgcacagctcggctttgagcgcggccctgggaaatggcaac

ggcggcaaacagagcggcggcagcggcgggggatggtggggcggaaagcacgtgtctgcg

gactgcttcaaagtggagaccgtgtgccacacgacgctgtactactttggatctaagcca

gctctctactatagagtatctgcctccagcagccgcctgggagggttcatctgcgacaac

tttcacccggagattacaaaattcgaggggagcgtggacgtgaccacgcggctgttgttg

gacaatgaaaattttaccagtttcgggtggtaccgcctgcgacccggcacccacggagag

cgtgttcaacttcgccccgttgagcgacacgtcacctcaagcgacgtggagattaactgt

actcccgataacctggagccgataccagacgaggctgcctggcccgactataagctcatg

tgctttgatatagagtgtaaagctggaacgggtaacgaaatggcgttcccagtggcaact

aaccaagaggacctggtcatccagatctcctgtctgctgtactcgcttgctactcagaac

cacgaacacaccctgctgttttccctcgggtcatgcgatatctctgaggaatactcgttt

gcatgcgtccagcgcggcgagcccagaccgacggttttggagtttgacagcgagtacgag

ctgctggttgccttcctgacctttctcaagcagtactctcccgagttcgccaccggctac

aacatcgttaattttgactgggcgtacatagttaacaaggtaacgtcggtgtataacatc

aagctggacgggtacggcaagttcaacaaaggggggctgtttaaggtgtgggacatcgcc

acgaaccattttcagaagaagagcaaggtgaaaatcaatggcctgatatctctagacatg

tattctgtggcgacggaaaagctaaagctacccagctacaaactcgacgcggtcgtggga

gacgtcctcggcgagcataagatagaccttccctataaagaaataccctcctattacgcg

ggagggcctgaccggaggggcgtaataggagagtattgtatccaggactctaggctggtg

ggcaagctgttttttaagtacctcccccatctggaactatcggcggtggccaaactcgcc

cgtatcaccctaacgcgggtaatttttgacggtcagcaaattcgcgtgtacacgtgcttg

ctgaaactcgcccgcgagagaaatttcattttgccagacaacagacgccggtttgacagt

caggcagacgccgcgtcagagacttcggagttggctatggatagccaaagccacgccttc

gacagtacagacgaacccgacggtgtggacggtaccccggacgccgcaggatctggcgct

acttctgaaaacggaggcgggaagcccggcgtcgggagggccgtgggctaccagggagca

aaggttctagaccccgtatccggctttcatgtggaccccgtggttgtgtttgacttcgct

agcttatacccaagcattatccaggcccataacctctgtttcaccaccctggcgctcgat

gaagtggatctggccgggcttcaaccatccgtcaactactcgacgttcgaggtgggtgac

caaaagttattttttgtccacgcccatattcgcgaaagcctgcttggcatcttgctgcgc

gactggctggccatgcgaaaggcggtgagggcgcgaatccccaccagcacccccgaggag

gcagttttactagataagcagcagtctgcgattaaggtgatatgcaactcggtttacgga

ttcacgggggtggcaaacggcctgttgccgtgtctgaggatagcggctaccgttaccacg

ataggacgcgacatgctcctcaagacccgagattacgttcactctcgttgggcgacgcgc

gagctgctggaggacaattttccaggggctataggtttccgaaaccacaagccttactcc

gtcagggttatctacggagacaccgactccgtgtttatcaagtttgtgggcctgacgtac

gagggggtatcggagctgggggatgctatgtcgcgtcagatttcagcggacctctttaga

gcgcccatcaaactggagtgtgagaagacctttcagcgactgctgctgatcaccaagaag

aagtacataggtgtcataaacggggggaagatgctcatgaagggggtcgacctggtccgc

aaaaataactgctctttcataaacttgtacgcgcgacatctggtagatcttttgttgtac

gacgaggatgtggccacggcggcagcagaggtgacagacgtgcctcccgcagaatgggtg

gggcgcccgctaccgagcggctttgacaagtttgggcgagtgctggtagaggcgtacaac

cgtatcactgcccccaacttggacgtgcgcgagttcgttatgactgctgagctgagccgc

tcacccgaatcgtataccaacaagcgcctgccgcacctcaccgtctactttaagctcgcc

atgaggaatgaagaactgcccagcgtaaaagagagaattccgtatgtgatagttgcgcag

accgaggccgcggaacgcgaagcgggtgtagtaaactcaatgcgcggtaccgcccaaaac

cccgtggtaaccaagaccgcacgcccccaacctaaacgcaaactgctggtttccgacctc

gccgaagacccgacctatgtttccgagaatgacgtaccgctaaacacagactactatttc

tcccacctgttgggtaccataagcgtgacctttaaggctctattcggaaatgatgtgaga

acaacagaaaatcttttaaagcggtttattccggaaaccccccacaagacccccacgaaa

acccaggcactgcttgagcgcgccggctttgaaaagctgacgccctttacaccggaggaa

gaaagtcgtcgaatactgcatacagttttttgtactctagaagcagctccccatcaaagc

tga

>LC109652.1

atggcggcgcgcgaacaggccaactccgttcgcaggagcgggttttttaatccgtttatt

ggcaagaggccatttttcaggccgggaagcgggcagaccgcggagacagagcgcccaagg

cccccccaacactcgtactgcacagaggtgggtagctttaagtttatagctccaagatgc

ctcgatgaagaagcccccgccgaccagcgacgcggtgtacacgtgggcaccttggagcgc

ccacccaaagtgtactgcgatggctcagagtacgacgtgctgaactttgcctccggaggt

tgctggcctcgcaggattcgggtttggaacggccaggattttcggggcgatggattcaac

cccagatttgagagatttcacgtgtacgacatagtagagacttcggagagcgcctcacac

gatgacccatccaggtttgctgagctatcccgcccaagcgggtctgtggtaacactgctg

ggaatgagcgagtgtggaaagcgagtcgcggttcacgtctatggtgtgcgccattatttt

tacatggcaaaggcggaggtggatagcgcttgtggaataaccaccgaggcagaactcgtg

cgcgcaatggtggactgcgcgcacagctcggctttgagcgcggccctgggaaatggcaac

ggcggcaaacagagcggcggcagcggcgggggatggtggggcggaaagcacgtgtctgcg

gactgcttcaaagtggagaccgtgtgccacacgacgctgtactactttggatctaagcca

gctctctactatagagtatctgcctccagcagccgcctgggagggttcatctgcgacaac

tttcacccggagattacaaaattcgaggggagcgtggacgtgaccacgcggctgttgttg

gacaatgaaaattttaccagtttcgggtggtaccgcctgcgacccggcacccacggagag

cgtgttcaacttcgccccgttgagcgacacgtcacctcaagcgacgtggagattaactgt

actcccgataacctggagccgataccagacgaggctgcctggcccgactataagctcatg

tgctttgatatagagtgtaaagctggaacgggtaacgaaatggcgttcccagtggcaact

aaccaagaggacctggtcatccagatctcctgtctgctgtactcgcttgctactcagaac

cacgaacacaccctgctgttttccctcgggtcatgcgatatctctgaggaatactcgttt

gcatgcgtccagcgcggcgagcccagaccgacggttttggagtttgacagcgagtacgag

ctgctggttgccttcctgacctttctcaagcagtactctcccgagttcgccaccggctac

aacatcgttaattttgactgggcgtacatagttaacaaggtaacgtcggtgtataacatc

aagctggacgggtacggcaagttcaacaaaggggggctgtttaaggtgtgggacatcgcc

acgaaccattttcagaagaagagcaaggtgaaaatcaatggcctgatatctctagacatg

tattctgtggcgacggaaaagctaaagctacccagctacaaactcgacgcggtcgtggga

gacgtcctcggcgagcataagatagaccttccctataaagaaataccctcctattacgcg

ggagggcctgaccggaggggcgtaataggagagtattgtatccaggactctaggctggtg

ggcaagctgttttttaagtacctcccccatctggaactatcggcggtggccaaactcgcc

cgtatcaccctaacgcgggtaatttttgacggtcagcaaattcgcgtgtacacgtgcttg

ctgaaactcgcccgcgagagaaatttcattttgccagacaacagacgccggtttgacagt

caggcagacgccgcgtcagagacttcggagttggctatggatagccaaagccacgccttc

gacagtacagacgaacccgacggtgtggacggtaccccggacgccgcaggatctggcgct

acttctgaaaacggaggcgggaagcccggcgtcgggagggccgtgggctaccagggagca

aaggttctagaccccgtatccggctttcatgtggaccccgtggttgtgtttgacttcgct

agcttatacccaagcattatccaggcccataacctctgtttcaccaccctggcgctcgat

gaagtggatctggccgggcttcaaccatccgtcaactactcgacgttcgaggtgggtgac

caaaagttattttttgtccacgcccatattcgcgaaagcctgcttggcatcttgctgcgc

gactggctggccatgcgaaaggcggtgagggcgcgaatccccaccagcacccccgaggag

gcagttttactagataagcagcagtctgcgattaaggtgatatgcaactcggtttacgga

ttcacgggggtggcaaacggcctgttgccgtgtctgaggatagcggctaccgttaccacg

ataggacgcgacatgctcctcaagacccgagattacgttcactctcgttgggcgacgcgc

gagctgctggaggacaattttccaggggctataggtttccgaaaccacaagccttactcc

gtcagggttatctacggagacaccgactccgtgtttatcaagtttgtgggcctgacgtac

gagggggtatcggagctgggggatgctatgtcgcgtcagatttcagcggacctctttaga

gcgcccatcaaactggagtgtgagaagacctttcagcgactgctgctgatcaccaagaag

aagtacataggtgtcataaacggggggaagatgctcatgaagggggtcgacctggtccgc

aaaaataactgctctttcataaacttgtacgcgcgacatctggtagatcttttgttgtac

gacgaggatgtggccacggcggcagcagaggtgacagacgtgcctcccgcagaatgggtg

gggcgcccgctaccgagcggctttgacaagtttgggcgagtgctggtagaggcgtacaac

cgtatcactgcccccaacttggacgtgcgcgagttcgttatgactgctgagctgagccgc

tcacccgaatcgtataccaacaagcgcctgccgcacctcaccgtctactttaagctcgcc

atgaggaatgaagaactgcccagcgtaaaagagagaattccgtatgtgatagttgcgcag

accgaggccgcggaacgcgaagcgggtgtagtaaactcaatgcgcggtaccgcccaaaac

cccgtggtaaccaagaccgcacgcccccaacctaaacgcaaactgctggtttccgacctc

gccgaagacccgacctatgtttccgagaatgacgtaccgctaaacacagactactatttc

tcccacctgttgggtaccataagcgtgacctttaaggctctattcggaaatgatgtgaga

acaacagaaaatcttttaaagcggtttattccggaaaccccccacaagacccccacgaaa

acccaggcactgcttgagcgcgccggctttgaaaagctgacgccctttacaccggaggaa

gaaagtcgtcgaatactgcatacagttttttgtactctagaagcagctccccatcaaagc

tga

>LC109653.1

atggcggcgcgcgaacaggccaactccgttcgcaggagcgggttttttaatccgtttatt

ggcaagaggccatttttcaggccgggaagcgggcagaccgcggagacagagcgcccaagg

cccccccaacactcgtactgcacagaggtgggtagctttaagtttatagctccaagatgc

ctcgatgaagaagcccccgccgaccagcgacgcggtgtacacgtgggcaccttggagcgc

ccacccaaagtgtactgcgatggctcagagtacgacgtgctgaactttgcctccggaggt

tgctggcctcgcaggattcgggtttggaacggccaggattttcggggcgatggattcaac

cccagatttgagagatttcacgtgtacgacatagtagagacttcggagagcgcctcacac

gatgacccatccaggtttgctgagctatcccgcccaagcgggtctgtggtaacactgctg

ggaatgagcgagtgtggaaagcgagtcgcggttcacgtctatggtgtgcgccattatttt

tacatggcaaaggcggaggtggatagcgcttgtggaataaccaccgaggcagaactcgtg

cgcgcaatggtggactgcgcgcacagctcggctttgagcgcggccctgggaaatggcaac

ggcggcaaacagagcggcggcagcggcgggggatggtggggcggaaagcacgtgtctgcg

gactgcttcaaagtggagaccgtgtgccacacgacgctgtactactttggatctaagcca

gctctctactatagagtatctgcctccagcagccgcctgggagggttcatctgcgacaac

tttcacccggagattacaaaattcgaggggagcgtggacgtgaccacgcggctgttgttg

gacaatgaaaattttaccagtttcgggtggtaccgcctgcgacccggcacccacggagag

cgtgttcaacttcgccccgttgagcgacacgtcacctcaagcgacgtggagattaactgt

actcccgataacctggagccgataccagacgaggctgcctggcccgactataagctcatg

tgctttgatatagagtgtaaagctggaacgggtaacgaaatggcgttcccagtggcaact

aaccaagaggacctggtcatccagatctcctgtctgctgtactcgcttgctactcagaac

cacgaacacaccctgctgttttccctcgggtcatgcgatatctctgaggaatactcgttt

gcatgcgtccagcgcggcgagcccagaccgacggttttggagtttgacagcgagtacgag

ctgctggttgccttcctgacctttctcaagcagtactctcccgagttcgccaccggctac

aacatcgttaattttgactgggcgtacatagttaacaaggtaacgtcggtgtataacatc

aagctggacgggtacggcaagttcaacaaaggggggctgtttaaggtgtgggacatcgcc

acgaaccattttcagaagaagagcaaggtgaaaatcaatggcctgatatctctagacatg

tattctgtggcgacggaaaagctaaagctacccagctacaaactcgacgcggtcgtggga

gacgtcctcggcgagcataagatagaccttccctataaagaaataccctcctattacgcg

ggagggcctgaccggaggggcgtaataggagagtattgtatccaggactctaggctggtg

ggcaagctgttttttaagtacctcccccatctggaactatcggcggtggccaaactcgcc

cgtatcaccctaacgcgggtaatttttgacggtcagcaaattcgcgtgtacacgtgcttg

ctgaaactcgcccgcgagagaaatttcattttgccagacaacagacgccggtttgacagt

caggcagacgccgcgtcagagacttcggagttggctatggatagccaaagccacgccttc

gacagtacagacgaacccgacggtgtggacggtaccccggacgccgcaggatctggcgct

acttctgaaaacggaggcgggaagcccggcgtcgggagggccgtgggctaccagggagca

aaggttctagaccccgtatccggctttcatgtggaccccgtggttgtgtttgacttcgct

agcttatacccaagcattatccaggcccataacctctgtttcaccaccctggcgctcgat

gaagtggatctggccgggcttcaaccatccgtcaactactcgacgttcgaggtgggtgac

caaaagttattttttgtccacgcccatattcgcgaaagcctgcttggcatcttgctgcgc

gactggctggccatgcgaaaggcggtgagggcgcgaatccccaccagcacccccgaggag

gcagttttactagataagcagcagtctgcgattaaggtgatatgcaactcggtttacgga

ttcacgggggtggcaaacggcctgttgccgtgtctgaggatagcggctaccgttaccacg

ataggacgcgacatgctcctcaagacccgagattacgttcactctcgttgggcgacgcgc

gagctgctggaggacaattttccaggggctataggtttccgaaaccacaagccttactcc

gtcagggttatctacggagacaccgactccgtgtttatcaagtttgtgggcctgacgtac

gagggggtatcggagctgggggatgctatgtcgcgtcagatttcagcggacctctttaga

gcccccatcaaactggagtgtgagaagacctttcagcgactgctgctgatcaccaagaag

aagtacataggtgtcataaacggggggaagatgctcatgaagggggtcgacctggtccgc

aaaaataactgctctttcataaacttgtacgcgcgacatctggtagatcttttgttgtac

gacgaggatgtggccacggcggcagcagaggtgacagacgtgcctcccgcagaatgggtg

gggcgcccgctaccgagcggctttgacaagtttgggcgagtgctggtagaggcgtacaac

cgtatcactgcccccaacttggacgtgcgcgagttcgttatgactgctgagctgagccgc

tcacccgaatcgtataccaacaagcgcctgccgcacctcaccgtctactttaagctcgcc

atgaggaatgaagaactgcccagcgtaaaagagagaattccgtatgtgatagttgcgcag

accgaggccgcggaacgcgaagcgggtgtagtaaactcaatgcgcggtaccgcccaaaac

cccgtggtaaccaagaccgcacgcccccaacctaaacgcaaactgctggtttccgacctc

gccgaagacccgacctatgtttccgagaatgacgtaccgctaaacacagactactatttc

tcccacctgttgggtaccataagcgtgacctttaaggctctattcggaaatgatgtgaga

acaacagaaaatcttttaaagcggtttattccggaaaccccccacaagacccccacgaaa

acccaggcactgcttgagcgcgccggctttgaaaagctgacgccctttacaccggaggaa

gaaagtcgtcgaatactgcatacagttttttgtactctagaagcagctccccatcaaagc

tga

>LC109654.1

atggcggcgcgcgaacaggccaactccgttcgcaggagcgggttttttaatccgtttatt

ggcaagaggccatttttcaggccgggaagcgggcagaccgcggagacagagcgcccaagg

cccccccaacactcgtactgcacagaggtgggtagctttaagtttatagctccaagatgc

ctcgatgaagaagcccccgccgaccagcgacgcggtgtacacgtgggcaccttggagcgc

ccacccaaagtgtactgcgatggctcagagtacgacgtgctgaactttgcctccggaggt

tgctggcctcgcaggattcgggtttggaacggccaggattttcggggcgatggattcaac

cccagatttgagagatttcacgtgtacgacatagtagagacttcggagagcgcctcacac

gatgacccatccaggtttgctgagctatcccgcccaagcgggtctgtggtaacactgctg

ggaatgagcgagtgtggaaagcgagtcgcggttcacgtctatggtgtgcgccattatttt

tacatggcaaaggcggaggtggatagcgcttgtggaataaccaccgaggcagaactcgtg

cgcgcaatggtggactgcgcgcacagctcggctttgagcgcggccctgggaaatggcaac

ggcggcaaacagagcggcggcagcggcgggggatggtggggcggaaagcacgtgtctgcg

gactgcttcaaagtggagaccgtgtgccacacgacgctgtactactttggatctaagcca

gctctctactatagagtatctgcctccagcagccgcctgggagggttcatctgcgacaac

tttcacccggagattacaaaattcgaggggagcgtggacgtgaccacgcggctgttgttg

gacaatgaaaattttaccagtttcgggtggtaccgcctgcgacccggcacccacggagag

cgtgttcaacttcgccccgttgagcgacacgtcacctcaagcgacgtggagattaactgt

actcccgataacctggagccgataccagacgaggctgcctggcccgactataagctcatg

tgctttgatatagagtgtaaagctggaacgggtaacgaaatggcgttcccagtggcaact

aaccaagaggacctggtcatccagatctcctgtctgctgtactcgcttgctactcagaac

cacgaacacaccctgctgttttccctcgggtcatgcgatatctctgaggaatactcgttt

gcatgcgtccagcgcggcgagcccagaccgacggttttggagtttgacagcgagtacgag

ctgctggttgccttcctgacctttctcaagcagtactctcccgagttcgccaccggctac

aacatcgttaattttgactgggcgtacatagttaacaaggtaacgtcggtgtataacatc

aagctggacgggtacggcaagttcaacaaaggggggctgtttaaggtgtgggacatcgcc

acgaaccattttcagaagaagagcaaggtgaaaatcaatggcctgatatctctagacatg

tattctgtggcgacggaaaagctaaagctacccagctacaaactcgacgcggtcgtggga

gacgtcctcggcgagcataagatagaccttccctataaagaaataccctcctattacgcg

ggagggcctgaccggaggggcgtaataggagagtattgtatccaggactctaggctggtg

ggcaagctgttttttaagtacctcccccatctggaactatcggcggtggccaaactcgcc

cgtatcaccctaacgcgggtaatttttgacggtcagcaaattcgcgtgtacacgtgcttg

ctgaaactcgcccgcgagagaaatttcattttgccagacaacagacgccggtttgacagt

caggcagacgccgcgtcagagacttcggagttggctatggatagccaaagccacgccttc

gacagtacagacgaacccgacggtgtggacggtaccccggacgccgcaggatctggcgct

acttctgaaaacggaggcgggaagcccggcgtcgggagggccgtgggctaccagggagca

aaggttctagaccccgtatccggctttcatgtggaccccgtggttgtgtttgacttcgct

agcttatacccaagcattatccaggcccataacctctgtttcaccaccctggcgctcgat

gaagtggatctggccgggcttcaaccatccgtcaactactcgacgttcgaggtgggtgac

caaaagttattttttgtccacgcccatattcgcgaaagcctgcttggcatcttgctgcgc

gactggctggccatgcgaaaggcggtgagggcgcgaatccccaccagcacccccgaggag

gcagttttactagataagcagcagtctgcgattaaggtgatatgcaactcggtttacgga

ttcacgggggtggcaaacggcctgttgccgtgtctgaggatagcggctaccgttaccacg

ataggacgcgacatgctcctcaagacccgagattacgttcactctcgttgggcgacgcgc

gagctgctggaggacaattttccaggggctataggtttccgaaaccacaagccttactcc

gtcagggttatctacggagacaccgactccgtgtttatcaagtttgtgggcctgacgtac

gagggggtatcggagctgggggatgctatgtcgcgtcagatttcagcggacctctttaga

gcgcccatcaaactggagtgtgagaagacctttcagcgactgctgctgatcaccaagaag

aagtacataggtgtcataaacggggggaagatgctcatgaagggggtcgacctggtccgc

aaaaataactgctctttcataaacttgtacgcgcgacatctggtagatcttttgttgtac

gacgaggatgtggccacggcggcagcagaggtgacagacgtgcctcccgcagaatgggtg

gggcgcccgctaccgagcggctttgacaagtttgggcgagtgctggtagaggcgtacaac

cgtatcactgcccccaacttggacgtgcgcgagttcgttatgactgctgagctgagccgc

tcacccgaatcgtataccaacaagcgcctgccgcacctcaccgtctactttaagctcgcc

atgaggaatgaagaactgcccagcgtaaaagagagaattccgtatgtgatagttgcgcag

accgaggccgcggaacgcgaagcgggtgtagtaaactcaatgcgcggtaccgcccaaaac

cccgtggtaaccaagaccgcacgcccccaacctaaacgcaaactgctggtttccgacctc

gccgaagacccgacctatgtttccgagaatgacgtaccgctaaacacagactactatttc

tcccacctgttgggtaccataagcgtgacctttaaggctctattcggaaatgatgtgaga

acaacagaaaatcttttaaagcggtttattccggaaaccccccacaagacccccacgaaa

acccaggcactgcttgagcgcgccggctttgaaaagctgacgccctttacaccggaggaa

gaaagtcgtcgaatactgcatacagttttttgtactctagaagcagctccccatcaaagc

tga

>LC109655.1

atggcggcgcgcgaacaggccaactccgttcgcaggagcgggttttttaatccgtttatt

ggcaagaggccatttttcaggccgggaagcgggcagaccgcggagacagagcgcccaagg

cccccccaacactcgtactgcacagaggtgggtagctttaagtttatagctccaagatgc

ctcgatgaagaagcccccgccgaccagcgacgcggtgtacacgtgggcaccttggagcgc

ccacccaaagtgtactgcgatggctcagagtacgacgtgctgaactttgcctccggaggt

tgctggcctcgcaggattcgggtttggaacggccaggattttcggggcgatggattcaac

cccagatttgagagatttcacgtgtacgacatagtagagacttcggagagcgcctcacac

gatgacccatccaggtttgctgagctatcccgcccaagcgggtctgtggtaacactgctg

ggaatgagcgagtgtggaaagcgagtcgcggttcacgtctatggtgtgcgccattatttt

tacatggcaaaggcggaggtggatagcgcttgtggaataaccaccgaggcagaactcgtg

cgcgcaatggtggactgcgcgcacagctcggctttgagcgcggccctgggaaatggcaac

ggcggcaaacagagcggcggcagcggcgggggatggtggggcggaaagcacgtgtctgcg

gactgcttcaaagtggagaccgtgtgccacacgacgctgtactactttggatctaagcca

gctctctactatagagtatctgcctccagcagccgcctgggagggttcatctgcgacaac

tttcacccggagattacaaaattcgaggggagcgtggacgtgaccacgcggctgttgttg

gacaatgaaaattttaccagtttcgggtggtaccgcctgcgacccggcacccacggagag

cgtgttcaacttcgccccgttgagcgacacgtcacctcaagcgacgtggagattaactgt

actcccgataacctggagccgataccagacgaggctgcctggcccgactataagctcatg

tgctttgatatagagtgtaaagctggaacgggtaacgaaatggcgttcccagtggcaact

aaccaagaggacctggtcatccagatctcctgtctgctgtactcgcttgctactcagaac

cacgaacacaccctgctgttttccctcgggtcatgcgatatctctgaggaatactcgttt

gcatgcgtccagcgcggcgagcccagaccgacggttttggagtttgacagcgagtacgag

ctgctggttgccttcctgacctttctcaagcagtactctcccgagttcgccaccggctac

aacatcgttaattttgactgggcgtacatagttaacaaggtaacgtcggtgtataacatc

aagctggacgggtacggcaagttcaacaaaggggggctgtttaaggtgtgggacatcgcc

acgaaccattttcagaagaagagcaaggtgaaaatcaatggcctgatatctctagacatg

tattctgtggcgacggaaaagctaaagctacccagctacaaactcgacgcggtcgtggga

gacgtcctcggcgagcataagatagaccttccctataaagaaataccctcctattacgcg

ggagggcctgaccggaggggcgtaataggagagtattgtatccaggactctaggctggtg

ggcaagctgttttttaagtacctcccccatctggaactatcggcggtggccaaactcgcc

cgtatcaccctaacgcgggtaatttttgacggtcagcaaattcgcgtgtacacgtgcttg

ctgaaactcgcccgcgagagaaatttcattttgccagacaacagacgccggtttgacagt

caggcagacgccgcgtcagagacttcggagttggctatggatagccaaagccacgccttc

gacagtacagacgaacccgacggtgtggacggtaccccggacgccgcaggatctggcgct

acttctgaaaacggaggcgggaagcccggcgtcgggagggccgtgggctaccagggagca

aaggttctagaccccgtatccggctttcatgtggaccccgtggttgtgtttgacttcgct

agcttatacccaagcattatccaggcccataacctctgtttcaccaccctggcgctcgat

gaagtggatctggccgggcttcaaccatccgtcaactactcgacgttcgaggtgggtgac

caaaagttattttttgtccacgcccatattcgcgaaagcctgcttggcatcttgctgcgc

gactggctggccatgcgaaaggcggtgagggcgcgaatccccaccagcacccccgaggag

gcagttttactagataagcagcagtctgcgattaaggtgatatgcaactcggtttacgga

ttcacgggggtggcaaacggcctgttgccgtgtctgaggatagcggctaccgttaccacg

ataggacgcgacatgctcctcaagacccgagattacgttcactctcgttgggcgacgcgc

gagctgctggaggacaattttccaggggctataggtttccgaaaccacaagccttactcc

gtcagggttatctacggagacaccgactccgtgtttatcaagtttgtgggcctgacgtac

gagggggtatcggagctgggggatgctatgtcgcgtcagatttcagcggacctctttaga

gcccccatcaaactggagtgtgagaagacctttcagcgactgctgctgatcaccaagaag

aagtacataggtgtcataaacggggggaagatgctcatgaagggggtcgacctggtccgc

aaaaataactgctctttcataaacttgtacgcgcgacatctggtagatcttttgttgtac

gacgaggatgtggccacggcggcagcagaggtgacagacgtgcctcccgcagaatgggtg

gggcgcccgctaccgagcggctttgacaagtttgggcgagtgctggtagaggcgtacaac

cgtatcactgcccccaacttggacgtgcgcgagttcgttatgactgctgagctgagccgc

tcacccgaatcgtataccaacaagcgcctgccgcacctcaccgtctactttaagctcgcc

atgaggaatgaagaactgcccagcgtaaaagagagaattccgtatgtgatagttgcgcag

accgaggccgcggaacgcgaagcgggtgtagtaaactcaatgcgcggtaccgcccaaaac

cccgtggtaaccaagaccgcacgcccccaacctaaacgcaaactgctggtttccgacctc

gccgaagacccgacctatgtttccgagaatgacgtaccgctaaacacagactactatttc

tcccacctgttgggtaccataagcgtgacctttaaggctctattcggaaatgatgtgaga

acaacagaaaatcttttaaagcggtttattccggaaaccccccacaagacccccacgaaa

acccaggcactgcttgagcgcgccggctttgaaaagctgacgccctttacaccggaggaa

gaaagtcgtcgaatactgcatacagttttttgtactctagaagcagctccccatcaaagc

tga

>LC109656.1

atggcggcgcgcgaacaggccaactccgttcgcaggagcgggttttttaatccgtttatt

ggcaagaggccatttttcaggccgggaagcgggcagaccgcggagacagagcgcccaagg

cccccccaacactcgtactgcacagaggtgggtagctttaagtttatagctccaagatgc

ctcgatgaagaagcccccgccgaccagcgacgcggtgtacacgtgggcaccttggagcgc

ccacccaaagtgtactgcgatggctcagagtacgacgtgctgaactttgcctccggaggt

tgctggcctcgcaggattcgggtttggaacggccaggattttcggggcgatggattcaac

cccagatttgagagatttcacgtgtacgacatagtagagacttcggagagcgcctcacac

gatgacccatccaggtttgctgagctatcccgcccaagcgggtctgtggtaacactgctg

ggaatgagcgagtgtggaaagcgagtcgcggttcacgtctatggtgtgcgccattatttt

tacatggcaaaggcggaggtggatagcgcttgtggaataaccaccgaggcagaactcgtg

cgcgcaatggtggactgcgcgcacagctcggctttgagcgcggccctgggaaatggcaac

ggcggcaaacagagcggcggcagcggcgggggatggtggggcggaaagcacgtgtctgcg

gactgcttcaaagtggagaccgtgtgccacacgacgctgtactactttggatctaagcca

gctctctactatagagtatctgcctccagcagccgcctgggagggttcatctgcgacaac

tttcacccggagattacaaaattcgaggggagcgtggacgtgaccacgcggctgttgttg

gacaatgaaaattttaccagtttcgggtggtaccgcctgcgacccggcacccacggagag

cgtgttcaacttcgccccgttgagcgacacgtcacctcaagcgacgtggagattaactgt

actcccgataacctggagccgataccagacgaggctgcctggcccgactataagctcatg

tgctttgatatagagtgtaaagctggaacgggtaacgaaatggcgttcccagtggcaact

aaccaagaggacctggtcatccagatctcctgtctgctgtactcgcttgctactcagaac

cacgaacacaccctgctgttttccctcgggtcatgcgatatctctgaggaatactcgttt

gcatgcgtccagcgcggcgagcccagaccgacggttttggagtttgacagcgagtacgag

ctgctggttgccttcctgacctttctcaagcagtactctcccgagttcgccaccggctac

aacatcgttaattttgactgggcgtacatagttaacaaggtaacgtcggtgtataacatc

aagctggacgggtacggcaagttcaacaaaggggggctgtttaaggtgtgggacatcgcc

acgaaccattttcagaagaagagcaaggtgaaaatcaatggcctgatatctctagacatg

tattctgtggcgacggaaaagctaaagctacccagctacaaactcgacgcggtcgtggga

gacgtcctcggcgagcataagatagaccttccctataaagaaataccctcctattacgcg

ggagggcctgaccggaggggcgtaataggagagtattgtatccaggactctaggctggtg

ggcaagctgttttttaagtacctcccccatctggaactatcggcggtggccaaactcgcc

cgtatcaccctaacgcgggtaatttttgacggtcagcaaattcgcgtgtacacgtgcttg

ctgaaactcgcccgcgagagaaatttcattttgccagacaacagacgccggtttgacagt

caggcagacgccgcgtcagagacttcggagttggctatggatagccaaagccacgccttc

gacagtacagacgaacccgacggtgtggacggtaccccggacgccgcaggatctggcgct

acttctgaaaacggaggcgggaagcccggcgtcgggagggccgtgggctaccagggagca

aaggttctagaccccgtatccggctttcatgtggaccccgtggttgtgtttgacttcgct

agcttatacccaagcattatccaggcccataacctctgtttcaccaccctggcgctcgat

gaagtggatctggccgggcttcaaccatccgtcaactactcgacgttcgaggtgggtgac

caaaagttattttttgtccacgcccatattcgcgaaagcctgcttggcatcttgctgcgc

gactggctggccatgcgaaaggcggtgagggcgcgaatccccaccagcacccccgaggag

gcagttttactagataagcagcagtctgcgattaaggtgatatgcaactcggtttacgga

ttcacgggggtggcaaacggcctgttgccgtgtctgaggatagcggctaccgttaccacg

ataggacgcgacatgctcctcaagacccgagattacgttcactctcgttgggcgacgcgc

gagctgctggaggacaattttccaggggctataggtttccgaaaccacaagccttactcc

gtcagggttatctacggagacaccgactccgtgtttatcaagtttgtgggcctgacgtac

gagggggtatcggagctgggggatgctatgtcgcgtcagatttcagcggacctctttaga

gcgcccatcaaactggagtgtgagaagacctttcagcgactgctgctgatcaccaagaag

aagtacataggtgtcataaacggggggaagatgctcatgaagggggtcgacctggtccgc

aaaaataactgctctttcataaacttgtacgcgcgacatctggtagatcttttgttgtac

gacgaggatgtggccacggcggcagcagaggtgacagacgtgcctcccgcagaatgggtg

gggcgcccgctaccgagcggctttgacaagtttgggcgagtgctggtagaggcgtacaac

cgtatcactgcccccaacttggacgtgcgcgagttcgttatgactgctgagctgagccgc

tcacccgaatcgtataccaacaagcgcctgccgcacctcaccgtctactttaagctcgcc

atgaggaatgaagaactgcccagcgtaaaagagagaattccgtatgtgatagttgcgcag

accgaggccgcggaacgcgaagcgggtgtagtaaactcaatgcgcggtaccgcccaaaac

cccgtggtaaccaagaccgcacgcccccaacctaaacgcaaactgctggtttccgacctc

gccgaagacccgacctatgtttccgagaatgacgtaccgctaaacacagactactatttc

tcccacctgttgggtaccataagcgtgacctttaaggctctattcggaaatgatgtgaga

acaacagaaaatcttttaaagcggtttattccggaaaccccccacaagacccccacgaaa

acccaggcactgcttgagcgcgccggctttgaaaagctgacgccctttacaccggaggaa

gaaagtcgtcgaatactgcatacagttttttgtactctagaagcagctccccatcaaagc

tga

>LC109657.1

atggcggcgcgcgaacaggccaactccgttcgcaggagcgggttttttaatccgtttatt

ggcaagaggccatttttcaggccgggaagcgggcagaccgcggagacagagcgcccaagg

cccccccaacactcgtactgcacagaggtgggtagctttaagtttatagctccaagatgc

ctcgatgaagaagcccccgccgaccagcgacgcggtgtacacgtgggcaccttggagcgc

ccacccaaagtgtactgcgatggctcagagtacgacgtgctgaactttgcctccggaggt

tgctggcctcgcaggattcgggtttggaacggccaggattttcggggcgatggattcaac

cccagatttgagagatttcacgtgtacgacatagtagagacttcggagagcgcctcacac

gatgacccatccaggtttgctgagctatcccgcccaagcgggtctgtggtaacactgctg

ggaatgagcgagtgtggaaagcgagtcgcggttcacgtctatggtgtgcgccattatttt

tacatggcaaaggcggaggtggatagcgcttgtggaataaccaccgaggcagaactcgtg

cgcgcaatggtggactgcgcgcacagctcggctttgagcgcggccctgggaaatggcaac

ggcggcaaacagagcggcggcagcggcgggggatggtggggcggaaagcacgtgtctgcg

gactgcttcaaagtggagaccgtgtgccacacgacgctgtactactttggatctaagcca

gctctctactatagagtatctgcctccagcagccgcctgggagggttcatctgcgacaac

tttcacccggagattacaaaattcgaggggagcgtggacgtgaccacgcggctgttgttg

gacaatgaaaattttaccagtttcgggtggtaccgcctgcgacccggcacccacggagag

cgtgttcaacttcgccccgttgagcgacacgtcacctcaagcgacgtggagattaactgt

actcccgataacctggagccgataccagacgaggctgcctggcccgactataagctcatg

tgctttgatatagagtgtaaagctggaacgggtaacgaaatggcgttcccagtggcaact

aaccaagaggacctggtcatccagatctcctgtctgctgtactcgcttgctactcagaac

cacgaacacaccctgctgttttccctcgggtcatgcgatatctctgaggaatactcgttt

gcatgcgtccagcgcggcgagcccagaccgacggttttggagtttgacagcgagtacgag

ctgctggttgccttcctgacctttctcaagcagtactctcccgagttcgccaccggctac

aacatcgttaattttgactgggcgtacatagttaacaaggtaacgtcggtgtataacatc

aagctggacgggtacggcaagttcaacaaaggggggctgtttaaggtgtgggacatcgcc

acgaaccattttcagaagaagagcaaggtgaaaatcaatggcctgatatctctagacatg

tattctgtggcgacggaaaagctaaagctacccagctacaaactcgacgcggtcgtggga

gacgtcctcggcgagcataagatagaccttccctataaagaaataccctcctattacgcg

ggagggcctgaccggaggggcgtaataggagagtattgtatccaggactctaggctggtg

ggcaagctgttttttaagtacctcccccatctggaactatcggcggtggccaaactcgcc

cgtatcaccctaacgcgggtaatttttgacggtcagcaaattcgcgtgtacacgtgcttg

ctgaaactcgcccgcgagagaaatttcattttgccagacaacagacgccggtttgacagt

caggcagacgccgcgtcagagacttcggagttggctatggatagccaaagccacgccttc

gacagtacagacgaacccgacggtgtggacggtaccccggacgccgcaggatctggcgct

acttctgaaaacggaggcgggaagcccggcgtcgggagggccgtgggctaccagggagca

aaggttctagaccccgtatccggctttcatgtggaccccgtggttgtgtttgacttcgct

agcttatacccaagcattatccaggcccataacctctgtttcaccaccctggcgctcgat

gaagtggatctggccgggcttcaaccatccgtcaactactcgacgttcgaggtgggtgac

caaaagttattttttgtccacgcccatattcgcgaaagcctgcttggcatcttgctgcgc

gactggctggccatgcgaaaggcggtgagggcgcgaatccccaccagcacccccgaggag

gcagttttactagataagcagcagtctgcgattaaggtgatatgcaactcggtttacgga

ttcacgggggtggcaaacggcctgttgccgtgtctgaggatagcggctaccgttaccacg

ataggacgcgacatgctcctcaagacccgagattacgttcactctcgttgggcgacgcgc

gagctgctggaggacaattttccaggggctataggtttccgaaaccacaagccttactcc

gtcagggttatctacggagacaccgactccgtgtttatcaagtttgtgggcctgacgtac

gagggggtatcggagctgggggatgctatgtcgcgtcagatttcagcggacctctttaga

gcccccatcaaactggagtgtgagaagacctttcagcgactgctgctgatcaccaagaag

aagtacataggtgtcataaacggggggaagatgctcatgaagggggtcgacctggtccgc

aaaaataactgctctttcataaacttgtacgcgcgacatctggtagatcttttgttgtac

gacgaggatgtggccacggcggcagcagaggtgacagacgtgcctcccgcagaatgggtg

gggcgcccgctaccgagcggctttgacaagtttgggcgagtgctggtagaggcgtacaac

cgtatcactgcccccaacttggacgtgcgcgagttcgttatgactgctgagctgagccgc

tcacccgaatcgtataccaacaagcgcctgccgcacctcaccgtctactttaagctcgcc

atgaggaatgaagaactgcccagcgtaaaagagagaattccgtatgtgatagttgcgcag

accgaggccgcggaacgcgaagcgggtgtagtaaactcaatgcgcggtaccgcccaaaac

cccgtggtaaccaagaccgcacgcccccaacctaaacgcaaactgctggtttccgacctc

gccgaagacccgacctatgtttccgagaatgacgtaccgctaaacacagactactatttc

tcccacctgttgggtaccataagcgtgacctttaaggctctattcggaaatgatgtgaga

acaacagaaaatcttttaaagcggtttattccggaaaccccccacaagacccccacgaaa

acccaggcactgcttgagcgcgccggctttgaaaagctgacgccctttacaccggaggaa

gaaagtcgtcgaatactgcatacagttttttgtactctagaagcagctccccatcaaagc

tga

>LC109658.1

atggcggcgcgcgaacaggccaactccgttcgcaggagcgggttttttaatccgtttatt

ggcaagaggccatttttcaggccgggaagcgggcagaccgcggagacagagcgcccaagg

cccccccaacactcgtactgcacagaggtgggtagctttaagtttatagctccaagatgc

ctcgatgaagaagcccccgccgaccagcgacgcggtgtacacgtgggcaccttggagcgc

ccacccaaagtgtactgcgatggctcagagtacgacgtgctgaactttgcctccggaggt

tgctggcctcgcaggattcgggtttggaacggccaggattttcggggcgatggattcaac

cccagatttgagagatttcacgtgtacgacatagtagagacttcggagagcgcctcacac

gatgacccatccaggtttgctgagctatcccgcccaagcgggtctgtggtaacactgctg

ggaatgagcgagtgtggaaagcgagtcgcggttcacgtctatggtgtgcgccattatttt

tacatggcaaaggcggaggtggatagcgcttgtggaataaccaccgaggcagaactcgtg

cgcgcaatggtggactgcgcgcacagctcggctttgagcgcggccctgggaaatggcaac

ggcggcaaacagagcggcggcagcggcgggggatggtggggcggaaagcacgtgtctgcg

gactgcttcaaagtggagaccgtgtgccacacgacgctgtactactttggatctaagcca

gctctctactatagagtatctgcctccagcagccgcctgggagggttcatctgcgacaac

tttcacccggagattacaaaattcgaggggagcgtggacgtgaccacgcggctgttgttg

gacaatgaaaattttaccagtttcgggtggtaccgcctgcgacccggcacccacggagag

cgtgttcaacttcgccccgttgagcgacacgtcacctcaagcgacgtggagattaactgt

actcccgataacctggagccgataccagacgaggctgcctggcccgactataagctcatg

tgctttgatatagagtgtaaagctggaacgggtaacgaaatggcgttcccagtggcaact

aaccaagaggacctggtcatccagatctcctgtctgctgtactcgcttgctactcagaac

cacgaacacaccctgctgttttccctcgggtcatgcgatatctctgaggaatactcgttt

gcatgcgtccagcgcggcgagcccagaccgacggttttggagtttgacagcgagtacgag

ctgctggttgccttcctgacctttctcaagcagtactctcccgagttcgccaccggctac

aacatcgttaattttgactgggcgtacatagttaacaaggtaacgtcggtgtataacatc

aagctggacgggtacggcaagttcaacaaaggggggctgtttaaggtgtgggacatcgcc

acgaaccattttcagaagaagagcaaggtgaaaatcaatggcctgatatctctagacatg

tattctgtggcgacggaaaagctaaagctacccagctacaaactcgacgcggtcgtggga

gacgtcctcggcgagcataagatagaccttccctataaagaaataccctcctattacgcg

ggagggcctgaccggaggggcgtaataggagagtattgtatccaggactctaggctggtg

ggcaagctgttttttaagtacctcccccatctggaactatcggcggtggccaaactcgcc

cgtatcaccctaacgcgggtaatttttgacggtcagcaaattcgcgtgtacacgtgcttg

ctgaaactcgcccgcgagagaaatttcattttgccagacaacagacgccggtttgacagt

caggcagacgccgcgtcagagacttcggagttggctatggatagccaaagccacgccttc

gacagtacagacgaacccgacggtgtggacggtaccccggacgccgcaggatctggcgct

acttctgaaaacggaggcgggaagcccggcgtcgggagggccgtgggctaccagggagca

aaggttctagaccccgtatccggctttcatgtggaccccgtggttgtgtttgacttcgct

agcttatacccaagcattatccaggcccataacctctgtttcaccaccctggcgctcgat

gaagtggatctggccgggcttcaaccatccgtcaactactcgacgttcgaggtgggtgac

caaaagttattttttgtccacgcccatattcgcgaaagcctgcttggcatcttgctgcgc

gactggctggccatgcgaaaggcggtgagggcgcgaatccccaccagcacccccgaggag

gcagttttactagataagcagcagtctgcgattaaggtgatatgcaactcggtttacgga

ttcacgggggtggcaaacggcctgttgccgtgtctgaggatagcggctaccgttaccacg

ataggacgcgacatgctcctcaagacccgagattacgttcactctcgttgggcgacgcgc

gagctgctggaggacaattttccaggggctataggtttccgaaaccacaagccttactcc

gtcagggttatctacggagacaccgactccgtgtttatcaagtttgtgggcctgacgtac

gagggggtatcggagctgggggatgctatgtcgcgtcagatttcagcggacctctttaga

gcgcccatcaaactggagtgtgagaagacctttcagcgactgctgctgatcaccaagaag

aagtacataggtgtcataaacggggggaagatgctcatgaagggggtcgacctggtccgc

aaaaataactgctctttcataaacttgtacgcgcgacatctggtagatcttttgttgtac

gacgaggatgtggccacggcggcagcagaggtgacagacgtgcctcccgcagaatgggtg

gggcgcccgctaccgagcggctttgacaagtttgggcgagtgctggtagaggcgtacaac

cgtatcactgcccccaacttggacgtgcgcgagttcgttatgactgctgagctgagccgc

tcacccgaatcgtataccaacaagcgcctgccgcacctcaccgtctactttaagctcgcc

atgaggaatgaagaactgcccagcgtaaaagagagaattccgtatgtgatagttgcgcag

accgaggccgcggaacgcgaagcgggtgtagtaaactcaatgcgcggtaccgcccaaaac

cccgtggtaaccaagaccgcacgcccccaacctaaacgcaaactgctggtttccgacctc

gccgaagacccgacctatgtttccgagaatgacgtaccgctaaacacagactactatttc

tcccacctgttgggtaccataagcgtgacctttaaggctctattcggaaatgatgtgaga

acaacagaaaatcttttaaagcggtttattccggaaaccccccacaagacccccacgaaa

acccaggcactgcttgagcgcgccggctttgaaaagctgacgccctttacaccggaggaa

gaaagtcgtcgaatactgcatacagttttttgtactctagaagcagctccccatcaaagc

tga

>LC109659.1

atggcggcgcgcgaacaggccaactccgttcgcaggagcgggttttttaatccgtttatt

ggcaagaggccatttttcaggccgggaagcgggcaaaccgcggagacagagcgcccaagg

cccccccaacactcgtactgcacagaggtgggtagctttaagtttatagctccaagatgc

ctcgatgaagaagcccccgccgaccagcgacgcggtgtacacgtgggcaccttggagcgc

ccacccaaagtgtactgcgatggctcagagtacgacgtgctgaactttgcctccggaggt

tgctggcctcgcaggattcgggtttggaacggccaggattttcggggcgatggattcaac

cccagatttgagagatttcacgtgtacgacatagtagagacttcggagagcgcctcacac

gatgacccatccaggtttgctgagctatcccgcccaagcgggtctgtggtaacactgctg

ggaatgagcgagtgtggaaagcgagtcgcggttcacgtctatggtgtgcgccattatttt

tacatggcaaaggcggaggtggatagcgcttgtggaataaccaccgaggcagaactcgtg

cgcgcaatggtggactgcgcgcacagctcggctttgagcgcggccctgggaaatggcaac

ggcggcaaacagagcggcggcagcggcgggggatggtggggcggaaagcacgtgtctgcg

gactgcttcaaagtggagaccgtgtgccacacgacgctgtactactttggatctaagcca

gctctctactatagagtatctgcctccagcagccgcctgggagggttcatctgcgacaac

tttcacccggagattacaaaattcgaggggagcgtggacgtgaccacgcggctgttgttg

gacaatgaaaattttaccagtttcgggtggtaccgcctgcgacccggcacccacggagag

cgtgttcaacttcgccccgttgagcgacacgtcacctcaagcgacgtggagattaactgt

actcccgataacctggagccgataccagacgaggctgcctggcccgactataagctcatg

tgctttgatatagagtgtaaagctggaacgggtaacgaaatggcgttcccagtggcaact

aaccaagaggacctggtcatccagatctcctgtctgctgtactcgcttgctactcagaac

cacgaacacaccctgctgttttccctcgggtcatgcgatatctctgaggaatactcgttt

gcatgcgtccagcgcggcgagcccagaccgacggttttggagtttgacagcgagtacgag

ctgctggttgccttcctgacctttctcaagcagtactctcccgagttcgccaccggctac

aacatcgttaattttgactgggcgtacatagttaacaaggtaacgtcggtgtataacatc

aagctggacgggtacggcaagttcaacaaaggggggctgtttaaggtgtgggacatcgcc

acgaaccattttcagaagaagagcaaggtgaaaatcaatggcctgatatctctagacatg

tattctgtggcgacggaaaagctaaagctacccagctacaaactcgacgcggtcgtggga

gacgtcctcggcgagcataagatagaccttccctataaagaaataccctcctattacgcg

ggagggcctgaccggaggggcgtaataggagagtattgtatccaggactctaggctggtg

ggcaagctgttttttaagtacctcccccatctggaactatcggcggtggccaaactcgcc

cgtatcaccctaacgcgggtaatttttgacggtcagcaaattcgcgtgtacacgtgcttg

ctgaaactcgcccgcgagagaaatttcattttgccagacaacagacgccggtttgacagt

caggcagacgccgcgtcagagacttcggagttggctatggatagccaaagccacgccttc

gacagtacagacgaacccgacggtgtggacggtaccccggacgccgcaggatctggcgct

acttctgaaaacggaggcgggaagcccggcgtcgggagggccgtgggctaccagggagca

aaggttctagaccccgtatccggctttcatgtggaccccgtggttgtgtttgacttcgct

agcttatacccaagcattatccaggcccataacctctgtttcaccaccctggcgctcgat

gaagtggatctggccgggcttcaaccatccgtcaactactcgacgttcgaggtgggtgac

caaaagttattttttgtccacgcccatattcgcgaaagcctgcttggcatcttgctgcgc

gactggctggccatgcgaaaggcggtgagggcgcgaatccccaccagcacccccgaggag

gcagttttactagataagcagcagtctgcgattaaggtgatatgcaactcggtttacgga

ttcacgggggtggcaaacggcctgttgccgtgtctgaggatagcggctaccgttaccacg

ataggacgcgacatgctcctcaagacccgagattacgttcactctcgttgggcgacgcgc

gagctgctggaggacaattttccaggggctataggtttccgaaaccacaagccttactcc

gtcagggttatctacggagacaccgactccgtgtttatcaagtttgtgggcctgacgtac

gagggggtatcggagctgggggatgctatgtcgcgtcagatttcagcggacctctttaga

gcgcccatcaaactggagtgtgagaagacctttcagcgactgctgctgatcaccaagaag

aagtacataggtgtcataaacggggggaagatgctcatgaagggggtcgacctggtccgc

aaaaataactgctctttcataaacttgtacgcgcgacatctggtagatcttttgttgtac

gacgaggatgtggccacggcggcagcagaggtgacagacgtgcctcccgcagaatgggtg

gggcgcccgctaccgagcggctttgacaagtttgggcgagtgctggtagaggcgtacaac

cgtatcactgcccccaacttggacgtgcgcgagttcgttatgactgctgagctgagccgc

tcacccgaatcgtataccaacaagcgcctgccgcacctcaccgtctactttaagctcgcc

atgaggaatgaagaactgcccagcgtaaaagagagaattccgtatgtgatagttgcgcag

accgaggccgcggaacgcgaagcgggtgtagtaaactcaatgcgcggtaccgcccaaaac

cccgtggtaaccaagaccgcacgcccccaacctaaacgcaaactgctggtttccgacctc

gccgaagacccgacctatgtttccgagaatgacgtaccgctaaacacagactactatttc

tcccacctgttgggtaccataagcgtgacctttaaggctctattcggaaatgatgtgaga

acaacagaaaatcttttaaagcggtttattccggaaaccccccacaagacccccacgaaa

acccaggcactgcttgagcgcgccggctttgaaaagctgacgccctttacaccggaggaa

gaaagtcgtcgaatactgcatacagttttttgtactctagaagcagctccccatcaaagc

tga

>LC109660.1

atggcggcgcgcgaacaggccaactccgttcgcaggagcgggttttttaatccgtttatt

ggcaagaggccatttttcaggccgggaagcgggcagaccgcggagacagagcgcccaagg

cccccccaacactcgtactgcacagaggtgggtagctttaagtttatagctccaagatgc

ctcgatgaagaagcccccgccgaccagcgacgcggtgtacacgtgggcaccttggagcgc

ccacccaaagtgtactgcgatggctcagagtacgacgtgctgaactttgcctccggaggt

tgctggcctcgcaggattcgggtttggaacggccaggattttcggggcgatggattcaac

cccagatttgagagatttcacgtgtacgacatagtagagacttcggagagcgcctcacac

gatgacccatccaggtttgctgagctatcccgcccaagcgggtctgtggtaacactgctg

ggaatgagcgagtgtggaaagcgagtcgcggttcacgtctatggtgtgcgccattatttt

tacatggcaaaggcggaggtggatagcgcttgtggaataaccaccgaggcagaactcgtg

cgcgcaatggtggactgcgcgcacagctcggctttgagcgcggccctgggaaatggcaac

ggcggcaaacagagcggcggcagcggcgggggatggtggggcggaaagcacgtgtccgcg

gactgcttcaaagtggagaccgtgtgccacacgacgctgtactactttggatctaagcca

gctctctactatagagtatctgcctccagcagccgcctgggagggttcatctgcgacaac

tttcacccggagattacaaaattcgaggggagcgtggacgtgaccacgcggctgttgttg

gacaatgaaaattttaccagtttcgggtggtaccgcctgcgacccggcacccacggagag

cgtgttcaacttcgccccgttgagcgacacgtcacctcaagcgacgtggagattaactgt

actcccgataacctggagccgataccagacgaggctgcctggcccgactataagctcatg

tgctttgatatagagtgtaaagctggaacgggtaacgaaatggcgttcccagtggcaact

aaccaagaggacctggtcatccagatctcctgtctgctgtactcgcttgctactcagaac

cacgaacacaccctgctgttttccctcgggtcatgcgatatctctgaggaatactcgttt

gcatgcgtccagcgcggcgagcccagaccgacggttttggagtttgacagcgagtacgag

ctgctggttgccttcctgacctttctcaagcagtactctcccgagttcgccaccggctac

aacatcgttaattttgactgggcgtacatagttaacaaggtaacgtcggtgtataacatc

aagctggacgggtacggcaagttcaacaaaggggggctgtttaaggtgtgggacatcgcc

acgaaccattttcagaagaagagcaaggtgaaaatcaatggcctgatatctctagacatg

tattctgtggcgacggaaaagctaaagctacccagctacaaactcgacgcggtcgtggga

gacgtcctcggcgagcataagatagaccttccctataaagaaataccctcctattacgcg

ggagggcctgaccggaggggcgtaataggagagtattgtatccaggactctaggctggtg

ggcaagctgttttttaagtacctcccccatctggaactatcggcggtggccaaactcgcc

cgtatcaccctaacgcgggtaatttttgacggtcagcaaattcgcgtgtacacgtgcttg

ctgaaactcgcccgcgagagaaatttcattttgccagacaacagacgccggtttgacagt

caggcagacgccgcgtcagagacttcggagttggctatggatagccaaagccacgccttc

gacagtacagacgaacccgacggtgtggacggtaccccggacgccgcaggatctggcgct

acttctgaaaacggaggcgggaagcccggcgtcgggagggccgtgggctaccagggagca

aaggttctagaccccgtatccggctttcatgtggaccccgtggttgtgtttgacttcgct

agcttatacccaagcattatccaggcccataacctctgtttcaccaccctggcgctcgat

gaagtggatctggccgggcttcaaccatccgtcaactactcgacgttcgaggtgggtgac

caaaagttattttttgtccacgcccatattcgcgaaagcctgcttggcatcttgctgcgc

gactggctggccatgcgaaaggcggtgagggcgcgaatccccaccagcacccccgaggag

gcagttttactagataagcagcagtctgcgattaaggtgatatgcaactcggtttacgga

ttcacgggggtggcaaacggcctgttgccgtgtctgaggatagcggctaccgttaccacg

ataggacgcgacatgctcctcaagacccgagattacgttcactctcgttgggcgacgcgc

gagctgctggaggacaattttccaggggctataggtttccgaaaccacaagccttactcc

gtcagggttatctacggagacaccgactccgtgtttatcaagtttgtgggcctgacgtac

gagggggtatcggagctgggggatgctatgtcgcgtcagatttcagcggacctctttaga

gcccccatcaaactggagtgtgagaagacctttcagcgactgctgctgatcaccaagaag

aagtacataggtgtcataaacggggggaagatgctcatgaagggggtcgacctggtccgc

aaaaataactgctctttcataaacttgtacgcgcgacatctggtagatcttttgttgtac

gacgaggatgtggccacggcggcagcagaggtgacagacgtgcctcccgcagaatgggtg

gggcgcccgctaccgagcggctttgacaagtttgggcgagtgctggtagaggcgtacaac

cgtatcactgcccccaacttggacgtgcgcgagttcgttatgactgctgagctgagccgc

tcacccgaatcgtataccaacaagcgcctgccgcacctcaccgtctactttaagctcgcc

atgaggaatgaagaactgcccagcgtaaaagagagaattccgtatgtgatagttgcgcag

accgaggccgcggaacgcgaagcgggtgtagtaaactcaatgcgcggtaccgcccaaaac

cccgtggtaaccaagaccgcacgcccccaacctaaacgcaaactgctggtttccgacctc

gccgaagacccgacctatgtttccgagaatgacgtaccgctaaacacagactactatttc

tcccacctgttgggtaccataagcgtgacctttaaggctctattcggaaatgatgtgaga

acaacagaaaatcttttaaagcggtttattccggaaaccccccacaagacccccacgaaa

acccaggcactgcttgagcgcgccggctttgaaaagctgacgccctttacaccggaggaa

gaaagtcgtcgaatactgcatacagttttttgtactctagaagcagctccccatcaaagc

tga

>LC109662.1

atggcggcgcgcgaacaggccaactccgttcgcaggagcgggttttttaatccgtttatt

ggcaagaggccatttttcaggccgggaagcgggcagaccgcggagacagagcgcccaagg

cccccccaacactcgtactgcacagaggtgggtagctttaagtttatagctccaagatgc

ctcgatgaagaagcccccgccgaccagcgacgcggtgtacacgtgggcaccttggagcgc

ccacccaaagtgtactgcgatggctcagagtacgacgtgctgaactttgcctccggaggt

tgctggcctcgcaggattcgggtttggaacggccaggattttcggggcgatggattcaac

cccagatttgagagatttcacgtgtacgacatagtagagacttcggagagcgcctcacac

gatgacccatccaggtttgctgagctatcccgcccaagcgggtctgtggtaacactgctg

ggaatgagcgagtgtggaaagcgagtcgcggttcacgtctatggtgtgcgccattatttt

tacatggcaaaggcggaggtggatagcgcttgtggaataaccaccgaggcagaactcgtg

cgcgcaatggtggactgcgcgcacagctcggctttgagcgcggccctgggaaatggcaac

ggcggcaaacagagcggcggcagcggcgggggatggtggggcggaaagcacgtgtctgcg

gactgcttcaaagtggagaccgtgtgccgcacgacgctgtactactttggatctaagcca

gctctctactatagagtatctgcctccagcagccgcctgggagggttcatctgcgacaac

tttcacccggagattacaaaattcgaggggagcgtggacgtgaccacgcggctgttgttg

gacaatgaaaattttaccagtttcgggtggtaccgcctgcgacccggcacccacggagag

cgtgttcaacttcgccccgttgagcgacacgtcacctcaagcgacgtggagattaactgt

actcccgataacctggagccgataccagacgaggctgcctggcccgactataagctcatg

tgctttgatatagagtgtaaagctggaacgggtaacgaaatggcgttcccagtggcaact

aaccaagaggacctggtcatccagatctcctgtctgctgtactcgcttgctactcagaac

cacgaacacaccctgctgttttccctcgggtcatgcgatatctctgaggaatactcgttt

gcatgcgtccagcgcggcgagcccagaccgacggttttggagtttgacagcgagtacgag

ctgctggttgccttcctgacctttctcaagcagtactctcccgagttcgccaccggctac

aacatcgttaattttgactgggcgtacatagttaacaaggtaacgtcggtgtataacatc

aagctggacgggtacggcaagttcaacaaaggggggctgtttaaggtgtgggacatcgcc

acgaaccattttcagaagaagagcaaggtgaaaatcaatggcctgatatctctagacatg

tattctgtggcgacggaaaagctaaagctacccagctacaaactcgacgcggtcgtggga

gacgtcctcggcgagcataagatagaccttccctataaagaaataccctcctattacgcg

ggagggcctgaccggaggggcgtaataggagagtattgtatccaggactctaggctggtg

ggcaagctgttttttaagtacctcccccatctggaactatcggcggtggccaaactcgcc

cgtatcaccctaacgcgggtaatttttgacggtcagcaaattcgcgtgtacacgtgcttg

ctgaaactcgcccgcgagagaaatttcattttgccagacaacagacgccggtttgacagt

caggcagacgccgcgtcagagacttcggagttggctatggatagccaaagccacgccttc

gacagtacagacgaacccgacggtgtggacggtaccccggacgccgcaggatctggcgct

acttctgaaaacggaggcgggaagcccggcgtcgggagggccgtgggctaccagggagca

aaggttctagaccccgtatccggctttcatgtggaccccgtggttgtgtttgacttcgct

agcttatacccaagcattatccaggcccataacctctgtttcaccaccctggcgctcgat

gaagtggatctggccgggcttcaaccatccgtcgactactcgacgttcgaggtgggtgac

caaaagttattttttgtccacgcccatattcgcgaaagcctgcttggcatcttgctgcgc

gactggctggccatgcgaaaggcggtgagggcgcgaatccccaccagcacccccgaggag

gcagttttactagataagcagcagtctgcgattaaggtgatatgcaactcggtttacgga

ttcacgggggtggcaaacggcctgttgccgtgtctgaggatagcggctaccgttaccacg

ataggacgcgacatgctcctcaagacccgagattacgttcactctcgttgggcgacgcgc

gagctgctggaggacaattttccaggggctataggtttccgaaaccacaagccttactcc

gtcagggttatctacggagacaccgactccgtgtttatcaagtttgtgggcctgacgtac

gagggggtatcggagctgggggatgctatgtcgcgtcagatttcagcggacctctttaga

gcgcccatcaaactggagtgtgagaagacctttcagcgactgctgctgatcaccaagaag

aagtacataggtgtcataaacggggggaagatgctcatgaagggggtcgacctggtccgc

aaaaataactgctctttcataaacttgtacgcgcgacatctggtagatcttttgttgtac

gacgaggatgtggccacggcggcagcagaggtgacagacgtgcctcccgcagaatgggtg

gggcgcccgctaccgagcggctttgacaagtttgggcgagtgctggtagaggcgtacaac

cgtatcactgcccccaacttggacgtgcgcgagttcgttatgactgctgagctgagccgc

tcacccgaatcgtataccaacaagcgcctgccgcacctcaccgtctactttaagctcgcc

atgaggaatgaagaactgcccagcgtaaaagagagaattccgtatgtgatagttgcgcag

accgaggccgcggaacgcgaagcgggtgtagtaaactcaatgcgcggtaccgcccaaaac

cccgtggtaaccaagaccgcacgcccccaacctaaacgcaaactgctggtttccgacctc

gccgaagacccgacctatgtttccgagaatgacgtaccgctaaacacagactactatttc

tcccacctgttgggtaccataagcgtgacctttaaggctctattcggaaatgatgtgaga

acaacagaaaatcttttaaagcggtttattccggaaaccccccacaagacccccacgaaa

acccaggcactgcttgagcgcgccggctttgaaaagctgacgccctttacaccggaggaa

gaaagtcgtcgaatactgcatacagttttttgtactctagaagcagctccccatcaaagc

tga

>LC109663.1

atggcggcgcgcgaacaggccaactccgttcgcaggagcgggttttttaatccgtttatt

ggcaagaggccatttttcaggccgggaagcgggcagaccgcggagacagagcgcccaagg

cccccccaacactcgtactgcacagaggtgggtagctttaagtttatagctccaagatgc

ctcgatgaagaagcccccgccgaccagcgacgcggtgtacacgtgggcaccttggagcgc

ccacccaaagtgtactgcgatggctcagagtacgacgtgctgaactttgcctccggaggt

tgctggcctcgcaggattcgggtttggaacggccaggattttcggggcgatggattcaac

cccagatttgagagatttcacgtgtacgacatagtagagacttcggagagcgcctcacac

gatgacccatccaggtttgctgagctatcccgcccaagcgggtctgtggtaacactgctg

ggaatgagcgagtgtggaaagcgagtcgcggttcacgtctatggtgtgcgccattatttt

tacatggcaaaggcggaggtggatagcgcttgtggaataaccaccgaggcagaactcgtg

cgcgcaatggtggactgcgcgcacagctcggctttgagcgcggccctgggaaatggcaac

ggcggcaaacagagcggcggcagcggcgggggatggtggggcggaaagcacgtgtctgcg

gactgcttcaaagtggagaccgtgtgccgcacgacgctgtactactttggatctaagcca

gctctctactatagagtatctgcctccagcagccgcctgggagggttcatctgcgacaac

tttcacccggagattacaaaattcgaggggagcgtggacgtgaccacgcggctgttgttg

gacaatgaaaattttaccagtttcgggtggtaccgcctgcgacccggcacccacggagag

cgtgttcaacttcgccccgttgagcgacacgtcacctcaagcgacgtggagattaactgt

actcccgataacctggagccgataccagacgaggctgcctggcccgactataagctcatg

tgctttgatatagagtgtaaagctggaacgggtaacgaaatggcgttcccagtggcaact

aaccaagaggacctggtcatccagatctcctgtctgctgtactcgcttgctactcagaac

cacgaacacaccctgctgttttccctcgggtcatgcgatatctctgaggaatactcgttt

gcatgcgtccagcgcggcgagcccagaccgacggttttggagtttgacagcgagtacgag

ctgctggttgccttcctgacctttctcaagcagtactctcccgagttcgccaccggctac

aacatcgttaattttgactgggcgtacatagttaacaaggtaacgtcggtgtataacatc

aagctggacgggtacggcaagttcaacaaaggggggctgtttaaggtgtgggacatcgcc

acgaaccattttcagaagaagagcaaggtgaaaatcaatggcctgatatctctagacatg

tattctgtggcgacggaaaagctaaagctacccagctacaaactcgacgcggtcgtggga

gacgtcctcggcgagcataagatagaccttccctataaagaaataccctcctattacgcg

ggagggcctgaccggaggggcgtaataggagagtattgtatccaggactctaggctggtg

ggcaagctgttttttaagtacctcccccatctggaactatcggcggtggccaaactcgcc

cgtatcaccctaacgcgggtaatttttgacggtcagcaaattcgcgtgtacacgtgcttg

ctgaaactcgcccgcgagagaaatttcattttgccagacaacagacgccggtttgacagt

caggcagacgccgcgtcagagacttcggagttggctatggatagccaaagccacgccttc

gacagtacagacgaacccgacggtgtggacggtaccccggacgccgcaggatctggcgct

acttctgaaaacggaggcgggaagcccggcgtcgggagggccgtgggctaccagggagca

aaggttctagaccccgtatccggctttcatgtggaccccgtggttgtgtttgacttcgct

agcttatacccaagcattatccaggcccataacctctgtttcaccaccctggcgctcgat

gaagtggatctggccgggcttcaaccatccgtcgactactcgacgttcgaggtgggtgac

caaaagttattttttgtccacgcccatattcgcgaaagcctgcttggcatcttgctgcgc

gactggctggccatgcgaaaggcggtgagggcgcgaatccccaccagcacccccgaggag

gcagttttactagataagcagcagtctgcgattaaggtgatatgcaactcggtttacgga

ttcacgggggtggcaaacggcctgttgccgtgtctgaggatagcggctaccgttaccacg

ataggacgcgacatgctcctcaagacccgagattacgttcactctcgttgggcgacgcgc

gagctgctggaggacaattttccaggggctataggtttccgaaaccacaagccttactcc

gtcagggttatctacggagacaccgactccgtgtttatcaagtttgtgggcctgacgtac

gagggggtatcggagctgggggatgctatgtcgcgtcagatttcagcggacctctttaga

gcgcccatcaaactggagtgtgagaagacctttcagcgactgctgctgatcaccaagaag

aagtacataggtgtcataaacggggggaagatgctcatgaagggggtcgacctggtccgc

aaaaataactgctctttcataaacttgtacgcgcgacatctggtagatcttttgttgtac

gacgaggatgtggccacggcggcagcagaggtgacagacgtgcctcccgcagaatgggtg

gggcgcccgctaccgagcggctttgacaagtttgggcgagtgctggtagaggcgtacaac

cgtatcactgcccccaacttggacgtgcgcgagttcgttatgactgctgagctgagccgc

tcacccgaatcgtataccaacaagcgcctgccgcacctcaccgtctactttaagctcgcc

atgaggaatgaagaactgcccagcgtaaaagagagaattccgtatgtgatagttgcgcag

accgaggccgcggaacgcgaagcgggtgtagtaaactcaatgcgcggtaccgcccaaaac

cccgtggtaaccaagaccgcacgcccccaacctaaacgcaaactgctggtttccgacctc

gccgaagacccgacctatgtttccgagaatgacgtaccgctaaacacagactactatttc

tcccacctgttgggtaccataagcgtgacctttaaggctctattcggaaatgatgtgaga

acaacagaaaatcttttaaagcggtttattccggaaaccccccacaagacccccacgaaa

acccaggcactgcttgagcgcgccggctttgaaaagctgacgccctttacaccggaggaa

gaaagtcgtcgaatactgcatacagttttttgtactctagaagcagctccccatcaaagc

tga

>PP856396.1

atggcggcgcgcgaacaggccaactccgttcgcaggagcgggttttttaatccgtttatt

ggcaagaggccatttttcaggccgggaagcgggcaaaccgcggagacagagcgcccaagg

cccccccaacactcgtactgcacagaggtgggtagctttaagtttatagctccaagatgc

ctcgatgaagaagcccccgccgaccagcgacgcggtgtacacgtgggcaccttggagcgc

ccacccaaagtgtactgcgatggctcagagtacgacgtgctgaactttgcctccggaggt

tgctggcctcgcaggattcgggtttggaacggccaggattttcggggcgatggattcaac

cccagatttgagagatttcacgtgtacgacatagtagagacttcggagagcgcctcacac

gatgacccatccaggtttgctgagctatcccgcccaagcgggtctgtggtaacactgctg

ggaatgagcgagtgtggaaagcgagtcgcggttcacgtctatggtgtgcgccattatttt

tacatggcaaaggcggaggtggatagcgcttgtggaataaccaccgaggcagaactcgtg

cgcgcaatggtggactgcgcgcacagctcggctttgagcgcggccctgggaaatggcaac

ggcggcaaacagagcggcggcagcggcgggggatggtggggcggaaagcacgtgtctgcg

gactgcttcaaagtggagaccgtgtgccacacgacgctgtactactttggatctaagcca

gctctctactatagagtatctgcctccagcagccgcctgggagggttcatctgcgacaac

tttcacccggagattacaaaattcgaggggagcgtggacgtgaccacgcggctgttgttg

gacaatgaaaattttaccagtttcgggtggtaccgcctgcgacccggcacccacggagag

cgtgttcaacttcgccccgttgagcgacacgtcacctcaagcgacgtggagattaactgt

actcccgataacctggagccgataccagacgaggctgcctggcccgactataagctcatg

tgctttgatatagagtgtaaagctggaacgggtaacgaaatggcgttcccagtggcaact

aaccaagaggacctggtcatccagatctcctgtctgctgtactcgcttgctactcagaac

cacgaacacaccctgctgttttccctcgggtcatgcgatatctctgaggaatactcgttt

gcatgcgtccagcgcggcgagcccagaccgacggttttggagtttgacagcgagtacgag

ctgctggttgccttcctgacctttctcaagcagtactctcccgagttcgccaccggctac

aacatcgttaattttgactgggcgtacatagttaacaaggtaacgtcggtgtataacatc

aagctggacgggtacggcaagttcaacaaaggggggctgtttaaggtgtgggacatcgcc

acgaaccattttcagaagaagagcaaggtgaaaatcaatggcctgatatctctagacatg

tattctgtggcgacggaaaagctaaagctacccagctacaaactcgacgcggtcgtggga

gacgtcctcggcgagcataagatagaccttccctataaagaaataccctcctattacgcg

ggagggcctgaccggaggggcgtaataggagagtattgtatccaggactctaggctggtg

ggcaagctgttttttaagtacctcccccatctggaactatcggcggtggccaaactcgcc

cgtatcaccctaacgcgggtaatttttgacggtcagcaaattcgcgtgtacacgtgcttg

ctgaaactcgcccgcgagagaaatttcattttgccagacaacagacgccggtttgacagt

caggcagacgccgcgtcagagacttcggagttggctatggatagccaaagccacgccttc

gacagtacagacgaacccgacggtgtggacggtaccccggacgccgcaggatctggcgct

acttctgaaaacggaggcgggaagcccggcgtcgggagggccgtgggctaccagggagca

aaggttctagaccccgtatccggctttcatgtggaccccgtggttgtgtttgacttcgct

agcttatacccaagcattatccaggcccataacctctgtttcaccaccctggcgctcgat

gaagtggatctggccgggcttcaaccatccgtcaactactcgacgttcgaggtgggtgac

caaaagttattttttgtccacgcccatattcgcgaaagcctgcttggcatcttgctgcgc

gactggctggccatgcgaaaggcggtgagggcgcgaatccccaccagcacccccgaggag

gcagttttactagataagcagcagtctgcgattaaggtgatatgcaactcggtttacgga

ttcacgggggtggcaaacggcctgttgccgtgtctgaggatagcggctaccgttaccacg

ataggacgcgacatgctcctcaagacccgagattacgttcactctcgttgggcgacgcgc

gagctgctggaggacaattttccaggggctataggtttccgaaaccacaagccttactcc

gtcagggttatctacggagacaccgactccgtgtttatcaagtttgtgggcctgacgtac

gagggggtatcggagctgggggatgctatgtcgcgtcagatttcagcggacctctttaga

gcgcccatcaaactggagtgtgagaagacctttcagcgactgcttctgatcaccaagaag

aagtacataggtgtcataaacggggggaagatgctcatgaagggggtcgacctggtccgc

aaaaataactgctctttcataaacttgtacgcgcgacatctggtagatcttttgttgtac

gacgaggatgtggccacggcggcagcagaggtgacagacgtgcctcccgcagaatgggtg

gggcgcccgctaccgagcggctttgacaagtttgggcgagtgctggtagaggcgtacaac

cgtatcactgcccccaacttggacgtgcgcgagttcgttatgactgctgagctgagccgc

tcacccgaatcgtataccaacaagcgcctgccgcacctcaccgtctactttaagctcgcc

atgaggaatgaagaactgcccagcgtaaaagagagaattccgtatgtgatagttgcgcag

accgaggccgcggaacgcgaagcgggtgtagtaaactcaatgcgcggtaccgcccaaaac

cccgtggtaaccaagaccgcacgcccccaacctaaacgcaaactgctggtttccgacctc

gccgaagacccgacctatgtttccgagaatgacgtaccgctaaacacagactactatttc

tcccacctgttgggtaccataagcgtgacctttaaggctctattcggaaatgatgtgaga

acaacagaaaatcttttaaagcggtttattccggaaaccccccacaagacccccacgaaa

acccaggcactgcttgagcgcgccggctttgaaaagctgacgccctttacaccggaggaa

gaaagtcgtcgaatactgcatacagttttttgtactctagaagcagctccccatcaaagc

tga

>PQ630625.1

atggcggcgcgcgaacaggccaactccgttcgcaggagcgggttttttaatccgtttatt

ggcaagaggccatttttcaggccgggaagcgggcagaccgcggagacagagcgcccaagg

cccccccaacactcgtactgcacagaggtgggtagctttaagtttatagctccaagatgc

ctcgatgaagaagcccccgccgaccagcgacgcggtgtacacgtgggcaccttggagcgc

ccacccaaagtgtactgcgatggctcagagtacgacgtgctgaactttgcctccggaggt

tgctggcctcgcaggattcgggtttggaacggccaggattttcggggcgatggattcaac

cccagatttgagagatttcacgtgtacgacatagtagagacttcggagagcgcctcacac

gatgacccatccaggtttgctgagctatcccgcccaagcgggtctgtggtaacactgctg

ggaatgagcgagtgtggaaagcgagtcgcggttcacgtctatggtgtgcgccattatttt

tacatggcaaaggcggaggtggatagcgcttgtggaataaccaccgaggcagaactcgtg

cgcgcaatggtggactgcgcgcacagctcggctttgagcgcggccctgggaaatggcaac

ggcggcaaacagagcggcggcagcggcgggggatggtggggcggaaagcacgtgtctgcg

gactgcttcaaagtggagaccgtgtgccacacgacgctgtactactttggatctaagcca

gctctctactatagagtatctgcctccagcagccgcctgggagggttcatctgcgacaac

tttcacccggagattacaaaattcgaggggagcgtggacgtgaccacgcggctgttgttg

gacaatgaaaattttaccagtttcgggtggtaccgcctgcgacccggcacccacggagag

cgtgttcaacttcgccccgttgagcgacacgtcacctcaagcgacgtggagattaactgt

actcccgataacctggagccgataccagacgaggctgcctggcccgactataagctcatg

tgctttgatatagagtgtaaagctggaacgggtaacgaaatggcgttcccagtggcaact

aaccaagaggacctggtcatccagatctcctgtctgctgtactcgcttgctactcagaac

cacgaacacaccctgctgttttccctcgggtcatgcgatatctctgaggaatactcgttt

gcatgcgtccagcgcggcgagcccagaccgacggttttggagtttgacagcgagtacgag

ctgctggttgccttcctgacctttctcaagcagtactctcccgagttcgccaccggctac

aacatcgttaattttgactgggcgtacatagttaacaaggtaacgtcggtgtataacatc

aagctggacgggtacggcaagttcaacaaaggggggctgtttaaggtgtgggacatcgcc

acgaaccattttcagaagaagagcaaggtgaaaatcaatggcctgatatctctagacatg

tattctgtggcgacggaaaagctaaagctacccagctacaaactcgacgcggtcgtggga

gacgtcctcggcgagcataagatagaccttccctataaagaaataccctcctattacgcg

ggagggcctgaccggaggggcgtaataggagagtattgtatccaggactctaggctggtg

ggcaagctgttttttaagtacctcccccatctggaactatcggcggtggccaaactcgcc

cgtatcaccctaacgcgggtaatttttgacggtcagcaaattcgcgtgtacacgtgcttg

ctgaaactcgcccgcgagagaaatttcattttgccagacaacagacgccggtttgacagt

caggcagacgccgcgtcagagacttcggagttggctatggatagccaaagccacgccttc

gacagtacagacgaacccgacggtgtggacggtaccccggacgccgcaggatctggcgct

acttctgaaaacggaggcgggaagcccggcgtcgggagggccgtgggctaccagggagca

aaggttctagaccccgtatccggctttcatgtggaccccgtggttgtgcttgacttcgct

agcttatacccaagcattatccaggcccataacctctgtttcaccaccctggcgctcgat

gaagtggatctggccgggcttcaaccatccgtcaactactcgacgttcgaggtgggtgac

caaaagttattttttgtccacgcccatattcgcgaaagcctgcttggcatcttgctgcgc

gactggctggccatgcgaaaggcggtgagggcgcgaatccccaccagcacccccgaggag

gcagttttactagataagcagcagtctgcgattaaggtgatatgcaactcggtttacgga

ttcacgggggtggcaaacggcctgttgccgtgtctgaggatagcggctaccgttaccacg

ataggacgcgacatgctcctcaagacccgagattacgttcactctcgttgggcgacgcgc

gagctgctggaggacaattttccaggggctataggtttccgaaaccacaagccttactcc

gtcagggttatctacggagacaccgactccgtgtttatcaagtttgtgggcctgacgtac

gagggggtatcggagctgggggatgctatgtcgcgtcagatttcagcggacctctttaga

gcgcccatcaaactggagtgtgagaagacctttcagcgactgctgctgatcaccaagaag

aagtacataggtgtcataaacggggggaagatgctcatgaagggggtcgacctggtccgc

aaaaataactgctctttcataaacttgtacgcgcgacatctggtagatcttttgttgtac

gacgaggatgtggccacggcggcagcagaggtgacagacgtgcctcccgcagaatgggtg

gggcgcccgctaccgagcggctttgacaagtttgggcgagtgctggtagaggcgtacaac

cgtatcactgcccccaacttggacgtgcgcgagttcgttatgactgctgagctgagccgc

tcacccgaattgtataccaacaagcgcctgccgcacctcaccgtctactttaagctcgcc

atgaggaatgaagaactgcccagcgtaaaagagagaattccgtatgtgatagttgcgcag

accgaggccgcggaacgcgaagcgggtgtagtaaactcaatgcgcggtaccgcccaaaac

cccgtggtaaccaagaccgcacgcccccaacctaaacgcaaactgctggtttccgacctc

gccgaagacccgacctatgtttccgagaatgacgtaccgctaaacacagactactatttc

tcccacctgttgggtaccataagcgtgacctttaaggctctattcggaaatgatgtgaga

acaacagaaaatcttttaaagcggtttattccggaaaccccccacaagacccccacgaaa

acccaggcactgcttgagcgcgccggctttgaaaagctgacgccctttacaccggaggaa

gaaagtcgtcgaatactgcatacagttttttgtactctagaagcagctccccatcaaagc

tga

>PQ630626.1

atggcggcgcgcgaacaggccaactccgttcgcaggagcgggttttttaatccgtttatt

ggcaagaggccatttttcaggccgggaagcgggcagaccgcggagacagagcgcccaagg

cccccccaacactcgtactgcacagaggtgggtagctttaagtttatagctccaagatgc

ctcgatgaagaagcccccgccgaccagcgacgcggtgtacacgtgggcaccttggagcgc

ccacccaaagtgtactgcgatggctcagagtacgacgtgctgaactttgcctccggaggt

tgctggcctcgcaggattcgggtttggaacggccaggattttcggggcgatggattcaac

cccagatttgagagatttcacgtgtacgacatagtagagacttcggagagcgcctcacac

gatgacccatccaggtttgctgagctatcccgcccaagcgggtctgtggtaacactgctg

ggaatgagcgagtgtggaaagcgagtcgcggttcacgtctatggtgtgcgccattatttt

tacatggcaaaggcggaggtggatagcgcttgtggaataaccaccgaggcagaactcgtg

cgcgcaatggtggactgcgcgcacagctcggctttgagcgcggccctgggaaatggcaac

ggcggcaaacagagcggcggcagcggcgggggatggtggggcggaaagcacgtgtctgcg

gactgcttcaaagtggagaccgtgtgccacacgacgctgtactactttggatctaagcca

gctctctactatagagtatctgcctccagcagccgcctgggagggttcatctgcgacaac

tttcacccggagattacaaaattcgaggggagcgtggacgtgaccacgcggctgttgttg

gacaatgaaaattttaccagtttcgggtggtaccgcctgcgacccggcacccacggagag

cgtgttcaacttcgccccgttgagcgacacgtcacctcaagcgacgtggagattaactgt

actcccgataacctggagccgataccagacgaggctgcctggcccgactataagctcatg

tgctttgatatagagtgtaaagctggaacgggtaacgaaatggcgttcccagtggcaact

aaccaagaggacctggtcatccagatctcctgtctgctgtactcgcttgctactcagaac

cacgaacacaccctgctgttttccctcgggtcatgcgatatctctgaggaatactcgttt

gcatgcgtccagcgcggcgagcccagaccgacggttttggagtttgacagcgagtacgag

ctgctggttgccttcctgacctttctcaagcagtactctcccgagttcgccaccggctac

aacatcgttaattttgactgggcgtacatagttaacaaggtaacgtcggtgtataacatc

aagctggacgggtacggcaagttcaacaaaggggggctgtttaaggtgtgggacatcgcc

acgaaccattttcagaagaagagcaaggtgaaaatcaatggcctgatatctctagacatg

tattctgtggcgacggaaaagctaaagctacccagctacaaactcgacgcggtcgtggga

gacgtcctcggcgagcataagatagaccttccctataaagaaataccctcctattacgcg

ggagggcctgaccggaggggcgtaataggagagtattgtatccaggactctaggctggtg

ggcaagctgttttttaagtacctcccccatctggaactatcggcggtggccaaactcgcc

cgtatcaccctaacgcgggtaatttttgacggtcagcaaattcgcgtgtacacgtgcttg

ctgaaactcgcccgcgagagaaatttcattttgccagacaacagacgccggtttgacagt

caggcagacgccgcgtcagagacttcggagttggctatggatagccaaagccacgccttc

gacagtacagacgaacccgacggtgtggacggtaccccggacgccgcaggatctggcgct

acttctgaaaacggaggcgggaagcccggcgtcgggagggccgtgggctaccagggagca

aaggttctagaccccgtatccggctttcatgtggaccccgtggttgtgtttgacttcgct

agcttatacccaagcattatccaggcccataacctctgtttcaccaccctggcgctcgat

gaagtggatctggccgggcttcaaccatccgtcaactactcgacgttcgaggtgggtgac

caaaagttattttttgtccacgcccatattcgcgaaagcctgcttggcatcttgctgcgc

gactggctggccatgcgaaaggcggtgagggcgcgaatccccaccagcacccccgaggag

gcagttttactagataagcagcagtctgcgattaaggtgatatgcaactcggtttacgga

ttcacgggggtggcaaacggcctgttgccgtgtctgaggatagcggctaccgttaccacg

ataggacgcgacatgctcctcaagacccgagattacgttcactctcgttgggcgacgcgc

gagctgctggaggacaattttccaggggctataggtttccgaaaccacaagccttactcc

gtcagggttatctacggagacaccgactccgtgtttatcaagtttgtgggcctgacgtac

gagggggtatcggagctgggggatgctatgtcgcgtcagatttcagcggacctctttaga

gcgcccatcaaactggagtgtgagaagacctttcagcgactgctgctgatcaccaagaag

aagtacataggtgtcataaacggggggaagatgctcatgaagggggtcgacctggtccgc

aaaaataactgctctttcataaacttgtacgcgcgacatctggtagatcttttgttgtac

gacgaggatgtggccacggcggcagcagaggtgacagacgtgcctcccgcagaatgggtg

gggcgcccgctaccgagcggctttgacaagtttgggcgagtgctggtagaggcgtacaac

cgtatcactgcccccaacttggacgtgcgcgagttcgttatgactgctgagctgagccgc

tcacccgaatcgtataccaacaagcgcctgccgcacctcaccgtctactttaagctcgcc

atgaggaatgaagaactgcccagcgtaaaagagagaattccgtatgtgatagttgcgcag

accgaggccgcggaacgcgaagcgggtgtagtaaactcaatgcgcggtaccgcccaaaac

cccgtggtaaccaagaccgcacgcccccaacctaaacgcaaactgctggtttccgacctc

gccgaagacccgacctatgtttccgagaatgacgtaccgctaaacacagactactatttc

tcccacctgttgggtaccataagcgtgacctttaaggctctattcggaaatgatgtgaga

acaacagaaaatcttttaaagcggtttattccggaaaccccccacaagacccccacgaaa

acccaggcactgcttgagcgcgccggctttgaaaagctgacgccctttacaccggaggaa

gaaagtcgtcgaatactgcatacagttttttgtactctagaagcagctccccatcaaagc

tga

>PQ630627.1

atggcggcgcgcgaacaggccaactccgttcgcaggagcgggttttttaatccgtttatt

ggcaagaggccatttttcaggccgggaagcgggcagaccgcggagacagagcgcccaagg

cccccccaacactcgtactgcacagaggtgggtagctttaagtttatagctccaagatgc

ctcgatgaagaagcccccgccgaccagcgacgcggtgtacacgtgggcaccttggagcgc

ccacccaaagtgtactgcgatggctcagagtacgacgtgctgaactttgcctccggaggt

tgctggcctcgcaggattcgggtttggaacggccaggattttcggggcgatggattcaac

cccagatttgagagatttcacgtgtacgacatagtagagacttcggagagcgcctcacac

gatgacccatccaggtttgctgagctatcccgcccaagcgggtctgtggtaacactgctg

ggaatgagcgagtgtggaaagcgagtcgcggttcacgtctatggtgtgcgccattatttt

tacatggcaaaggcggaggtggatagcgcttgtggaataaccaccgaggcagaactcgtg

cgcgcaatggtggactgcgcgcacagctcggctttgagcgcggccctgggaaatggcaac

ggcggcaaacagagcggcggcagcggcgggggatggtggggcggaaagcacgtgtctgcg

gactgcttcaaagtggagaccgtgtgccacacgacgctgtactactttggatctaagcca

gctctctactatagagtatctgcctccagcagccgcctgggagggttcatctgcgacaac

tttcacccggagattacaaaattcgaggggagcgtggacgtgaccacgcggctgttgttg

gacaatgaaaattttaccagtttcgggtggtaccgcctgcgacccggcacccacggagag

cgtgttcaacttcgccccgttgagcgacacgtcacctcaagcgacgtggagattaactgt

actcccgataacctggagccgataccagacgaggctgcctggcccgactataagctcatg

tgctttgatatagagtgtaaagctggaacgggtaacgaaatggcgttcccagtggcaact

aaccaagaggacctggtcatccagatctcctgtctgctgtactcgcttgctactcagaac

cacgaacacaccctgctgttttccctcgggtcatgcgatatctctgaggaatactcgttt

gcatgcgtccagcgcggcgagcccagaccgacggttttggagtttgacagcgagtacgag

ctgctggttgccttcctgacctttctcaagcagtactctcccgagttcgccaccggctac

aacatcgttaattttgactgggcgtacatagttaacaaggtaacgtcggtgtataacatc

aagctggacgggtacggcaagttcaacaaaggggggctgtttaaggtgtgggacatcgcc

acgaaccattttcagaagaagagcaaggtgaaaatcaatggcctgatatctctagacatg

tattctgtggcgacggaaaagctaaagctacccagctacaaactcgacgcggtcgtggga

gacgtcctcggcgagcataagatagaccttccctataaagaaataccctcctattacgcg

ggagggcctgaccggaggggcgtaataggagagtattgtatccaggactctaggctggtg

ggcaagctgttttttaagtacctcccccatctggaactatcggcggtggccaaactcgcc

cgtatcaccctaacgcgggtaatttttgacggtcagcaaattcgcgtgtacacgtgcttg

ctgaaactcgcccgcgagagaaatttcattttgccagacaacagacgccggtttgacagt

caggcagacgccgcgtcagagacttcggagttggctatggatagccaaagccacgccttc

gaccgtacagacgaacccgacggtgtggacggtaccccggacgccgcaggatctggcgct

acttctgaaaacggaggcgggaagcccggcgtcgggagggccgtgggctaccagggagca

aaggttctagaccccgtatccggctttcatgtggaccccgtggttgtgtttgacttcgct

agcttatacccaagcattatccaggcccataacctctgtttcaccaccctggcgctcgat

gaagtggatctggccgggcttcaaccatccgtcgactactcgacgttcgaggtgggtgac

caaaagttattttttgtccacgcccatattcgcgaaagcctgcttggcatcttgctgcgc

gactggctggccatgcgaaaggcggtgagggcgcgaatccccaccagcacccccgaggag

gcagttttactagataagcagcagtctgcgattaaggtgatatgcaactcggtttacgga

ttcacgggggtggcaaacggcctgttgccgtgtctgaggatagcggctaccgttaccacg

ataggacgcgacatgctcctcaagacccgagattacgttcactctcgttgggcgacgcgc

gagctgctggaggacaattttccaggggctataggtttccgaaaccacaagccttactcc

gtcagggttatctacggagacaccgactccgtgtttatcaagtttgtgggcctgacgtac

gagggggtatcggagctgggggatgctatgtcgcgtcagatttcagcggacctctttaga

gcgcccatcaaactggagtgtgagaagacctttcagcgactgctgctgatcaccaagaag

aagtacataggtgtcataaacggggggaagatgctcatgaagggggtcgacctggtccgc

aaaaataactgctctttcataaacttgtacgcgcgacatctggtagatcttttgttgtac

gacgaggatgtggccacggcggcagcagaggtgacagacgtgcctcccgcagaatgggtg

gggcgcccgctaccgagcggctttgacaagtttgggcgagtgctggtagaggcgtacaac

cgtatcactgcccccaacttggacgtgcgcgagttcgttatgactgctgagctgagccgc

tcacccgaatcgtataccaacaagcgcctgccgcacctcaccgtctactttaagctcgcc

atgaggaatgaagaactgcccagcgtaaaagagagaattccgtatgtgatagttgcgcag

accgaggccgcggaacgcgaagcgggtgtagtaaactcaatgcgcggtaccgcccaaaac

cccgtggtaaccaagaccgcacgcccccaacctaaacgcaaactgctggtttccgacctc

gccgaagacccgacctatgtttccgagaatgacgtaccgctaaacacagactactatttc

tcccacctgttgggtaccataagcgtgacctttaaggctctattcggaaatgatgtgaga

acaacagaaaatcttttaaagcggtttattccggaaaccccccacaagacccccacgaaa

acccaggcactgcttgagcgcgccggctttgaaaagctgacgccctttacaccggaggaa

gaaagtcgtcgaatactgcatacagttttttgtactctagaagcagctccccatcaaagc

tga

>PQ630628.1

atggcggcgcgcgaacaggccaactccgttcgcaggagcgggttttttaatccgtttatt

ggcaagaggccatttttcaggccgggaagcgggcagaccgcggagacagagcgcccaagg

cccccccaacactcgtactgcacagaggtgggtagctttaagtttatagctccaagatgc

ctcgatgaagaagcccccgccgaccagcgacgcggtgtacacgtgggcaccttggagcgc

ccacccaaagtgtactgcgatggctcagagtacgacgtgctgaactttgcctccggaggt

tgctggcctcgcaggattcgggtttggaacggccaggattttcggggcgatggattcaac

cccagatttgagagatttcacgtgtacgacatagtagagacttcggagagcgcctcacac

gatgacccatccaggtttgctgagctatcccgcccaagcgggtctgtggtaacactgctg

ggaatgagcgagtgtggaaagcgagtcgcggttcacgtctatggtgtgcgccattatttt

tacatggcaaaggcggaggtggatagcgcttgtggaataaccaccgaggcagaactcgtg

cgcgcaatggtggactgcgcgcacagctcggctttgagcgcggccctgggaaatggcaac

ggcggcaaacagagcggcggcagcggcgggggatggtggggcggaaagcacgtgtctgcg

gactgcttcaaagtggagaccgtgtgccacacgacgctgtactactttggatctaagcca

gctctctactatagagtatctgcctccagcagccgcctgggagggttcatctgcgacaac

tttcacccggagattacaaaattcgaggggagcgtggacgtgaccacgcggctgttgttg

gacaatgaaaattttaccagtttcgggtggtaccgcctgcgacccggcacccacggagag

cgtgttcaacttcgccccgttgagcgacacgtcacctcaagcgacgtggagattaactgt

actcccgataacctggagccgataccagacgaggctgcctggcccgactataagctcatg

tgctttgatatagagtgtaaagctggaacgggtaacgaaatggcgttcccagtggcaact

aaccaagaggacctggtcatccagatctcctgtctgctgtactcgcttgctactcagaac

cacgaacacaccctgctgttttccctcgggtcatgcgatatctctgaggaatactcgttt

gcatgcgtccagcgcggcgagcccagaccgacggttttggagtttgacagcgagtacgag

ctgctggttgccttcctgacctttctcaagcagtactctcccgagttcgccaccggctac

aacatcgttaattttgactgggcgtacatagttaacaaggtaacgtcggtgtataacatc

aagctggacgggtacggcaagttcaacaaaggggggctgtttaaggtgtgggacatcgcc

acgaaccattttcagaagaagagcaaggtgaaaatcaatggcctgatatctctagacatg

tattctgtggcgacggaaaagctaaagctacccagctacaaactcgacgcggtcgtggga

gacgtcctcggcgagcataagatagaccttccctataaagaaataccctcctattacgcg

ggagggcctgaccggaggggcgtaataggagagtattgtatccaggactctaggctggtg

ggcaagctgttttttaagtacctcccccatctggaactatcggcggtggccaaactcgcc

cgtatcaccctaacgcgggtaatttttgacggtcagcaaattcgcgtgtacacgtgcttg

ctgaaactcgcccgcgagagaaatttcattttgccagacaacagacgccggtttgacagt

caggcagacgccgcgtcagagacttcggagttggctatggatagccaaagccacgccttc

gacagtacagacgaacccgacggtgtggacggtaccccggacgccgcaggatctggcgct

acttctgaaaacggaggcgggaagcccggcgtcgggagggccgtgggctaccagggagca

aaggttctagaccccgtatccggctttcatgtggaccccgtggttgtgtttgacttcgct

agcttatacccaagcattatccaggcccataacctctgtttcaccaccctggcgctcgat

gaagtggatctggccgggcttcaaccatccgtcaactactcgacgttcgaggtgggtgac

caaaagttattttttgtccacgcccatattcgcgaaagcctgcttggcatcttgctgcgc

gactggctggccatgcgaaaggcggtgagggcgcgaatccccaccagcacccccgaggag

gcagttttactagataagcagcagtctgcgattaaggtgatatgcaactcggtttacgga

ttcacgggggtggcaaacggcctgttgccgtgtctgaggatagcggctaccgttaccacg

ataggacgcgacatgctcctcaagacccgagattacgttcactctcgttgggcgacgcgc

gagctgctggaggacaattttccaggggctataggtttccgaaaccacaagccttactcc

gtcagggttatctacggagacaccgactccgtgtttatcaagtttgtgggcctgacgtac

gagggggtatcggagctgggggatgctatgtcgcgtcagatttcagcggacctctttaga

gcgcccatcaaactggagtgtgagaagacctttcagcgactgctgctgatcaccaagaag

aagtacataggtgtcataaacggggggaagatgctcatgaagggggtcgacctggtccgc

aaaaataactgctctttcataaacttgtacgcgcgacatctggtagatcttttgttgtac

gacgaggatgtggccacggcggcagcagaggtgacagacgtgcctcccgcagaatgggtg

gggcgcccgctaccgagcggctttgacaagtttgggcgagtgctggtagaggcgtacaac

cgtatcactgcccccaacttggacgtgcgcgagttcgttatgactgctgagctgagccgc

tcacccgaatcgtataccaacaagcgcctgccgcacctcaccgtctactttaagctcgcc

atgaggaatgaagaactgcccagcgtaaaagagagaattccgtatgtgatagttgcgcag

accgaggccgcggaacgcgaagcgggtgtagtaaactcaatgcgcggtaccgcccaaaac

cccgtggtaaccaagaccgcacgcccccaacctaaacgcaaactgctggtttccgacctc

gccgaagacccgacctatgtttccgagaatgacgtaccgctaaacacagactactatttc

tcccacctgttgggtaccataagcgtgacctttaaggctctattcggaaatgatgtgaga

acaacagaaaatcttttaaagcggtttattccggaaaccccccacaagacccccacgaaa

acccaggcactgcttgagcgcgccggctttgaaaagctgacgccctttacaccggaggaa

gaaagtcgtcgaatactgcatacagttttttgtactctagaagcagctccccatcaaagc

tga

>PQ630629.1

atggcggcgcgcgaacaggccaactccgttcgcaggagcgggttttttaatccgtttatt

ggcaagaggccatttttcaggccgggaagcgggcagaccgcggagacagagcgcccaagg

cccccccaacactcgtactgcacagaggtgggtagctttaagtttatagctccaagatgc

ctcgatgaagaagcccccgccgaccagcgacgcggtgtacacgtgggcaccttggagcgc

ccacccaaagtgtactgcgatggctcagagtacgacgtgctgaactttgcctccggaggt

tgctggcctcgcaggattcgggtttggaacggccaggattttcggggcgatggattcaac

cccagatttgagagatttcacgtgtacgacatagtagagacttcggagagcgcctcacac

gatgacccatccaggtttgctgagctatcccgcccaagcgggtctgtggtaacactgctg

ggaatgagcgagtgtggaaagcgagtcgcggttcacgtctatggtgtgcgccattatttt

tacatggcaaaggcggaggtggatagcgcttgtggaataaccaccgaggcagaactcgtg

cgcgcaatggtggactgcgcgcacagctcggctttgagcgcggccctgggaaatggcaac

ggcggcaaacagagcggcggcagcggcgggggatggtggggcggaaagcacgtgtctgcg

gactgcttcaaagtggagaccgtgtgccacacgacgctgtactactttggatctaagcca

gctctctactatagagtatctgcctccagcagccgcctgggagggttcatctgcgacaac

tttcacccggagattacaaaattcgaggggagcgtggacgtgaccacgcggctgttgttg

gacaatgaaaattttaccagtttcgggtggtaccgcctgcgacccggcacccacggagag

cgtgttcaacttcgccccgttgagcgacacgtcacctcaagcgacgtggagattaactgt

actcccgataacctggagccgataccagacgaggctgcctggcccgactataagctcatg

tgctttgatatagagtgtaaagctggaacgggtaacgaaatggcgttcccagtggcaact

aaccaagaggacctggtcatccagatctcctgtctgctgtactcgcttgctactcagaac

cacgaacacaccctgctgttttccctcgggtcatgcgatatctctgaggaatactcgttt

gcatgcgtccagcgcggcgagcccagaccgacggttttggagtttgacagcgagtacgag

ctgctggttgccttcctgacctttctcaagcagtactctcccgagttcgccaccggctac

aacatcgttaattttgactgggcgtacatagttaacaaggtaacgtcggtgtataacatc

aagctggacgggtacggcaagttcaacaaaggggggctgtttaaggtgtgggacatcgcc

acgaaccattttcagaagaagagcaaggtgaaaatcaatggcctgatatctctagacatg

tattctgtggcgacggaaaagctaaagctacccagctacaaactcgacgcggtcgtggga

gacgtcctcggcgagcataagatagaccttccctataaagaaataccctcctattacgcg

ggagggcctgaccggaggggcgtaataggagagtattgtatccaggactctaggctggtg

ggcaagctgttttttaagtacctcccccatctggaactatcggcggtggccaaactcgcc

cgtatcaccctaacgcgggtaatttttgacggtcagcaaattcgcgtgtacacgtgcttg

ctgaaactcgcccgcgagagaaatttcattttgccagacaacagacgccggtttgacagt

caggcagacgccgcgtcagagacttcggagttggctatggatagccaaagccacgccttc

gacagtacagacgaacccgacggtgtggacggtaccccggacgccgcaggatctggcgct

acttctgaaaacggaggcgggaagcccggcgtcgggagggccgtgggctaccagggagca

aaggttctagaccccgtatccggctttcatgtggaccccgtggttgtgtttgacttcgct

agcttatacccaagcattatccaggcccataacctctgtttcaccaccctggcgctcgat

gaagtggatctggccgggcttcaaccatccgtcaactactcgacgttcgaggtgggtgac

caaaagttattttttgtccacgcccatattcgcgaaagcctgcttggcatcttgctgcgc

gactggctggccatgcgaaaggcggtgagggcgcgaatccccaccagcacccccgaggag

gcagttttactagataagcagcagtctgcgattaaggtgatatgcaactcggtttacgga

ttcacgggggtggcaaacggcctgttgccgtgtctgaggatagcggctaccgttaccacg

ataggacgcgacatgctcctcaagacccgagattacgttcactctcgttgggcgacgcgc

gagctgctggaggacaattttccaggggctataggtttccgaaaccacaagccttactcc

gtcagggttatctacggagacaccgactccgtgtttatcaagtttgtgggcctgacgtac

gagggggtatcggagctgggggatgctatgtcgcgtcagatttcagcggacctctttaga

gcgcccatcaaactggagtgtgagaagacctttcagcgactgctgctgatcaccaagaag

aagtacataggtgtcataaacggggggaagatgctcatgaagggggtcgacctggtccgc

aaaaataactgctctttcataaacttgtacgcgcgacatctggtagatcttttgttgtac

gacgaggatgtggccacggcggcagcagaggtgacagacgtgcctcccgcagaatgggtg

gggcgcccgctaccgagcggctttgacaagtttgggcgagtgctggtagaggcgtacaac

cgtatcactgcccccaacttggacgtgcgcgagttcgttatgactgctgagctgagccgc

tcacccgaatcgtataccaacaagcgcctgccgcacctcaccgtctactttaagctcgcc

atgaggaatgaagaactgcccagcgtaaaagagagaattccgtatgtgatagttgcgcag

accgaggccgcggaacgcgaagcgggtgtagtaaactcaatgcgcggtaccgcccaaaac

cccgtggtaaccaggaccgcacgcccccaacctaaacgcaaactgctggtttccgacctc

gccgaagacccgacctatgtttccgagaatgacgtaccgctaaacacagactactatttc

tcccacctgttgggtaccataagcgtgacctttaaggctctattcggaaatgatgtgaga

acaacagaaaatcttttaaagcggtttattccggaaaccccccacaagacccccacgaaa

acccaggcactgcttgagcgcgccggctttgaaaagctgacgccctttacaccggaggaa

gaaagtcgtcgaatactgcatacagttttttgtactctagaagcagctccccatcaaagc

tga

>PQ630630.1

atggcggcgcgcgaacaggccaactccgttcgcaggagcgggttttttaatccgtttatt

ggcaagaggccatttttcaggccgggaagcgggcagaccgcggagacagagcgcccaagg

cccccccaacactcgtactgcacagaggtgggtagctttaagtttatagctccaagatgc

ctcgatgaagaagcccccgccgaccagcgacgcggtgtacacgtgggcaccttggagcgc

ccacccaaagtgtactgcgatggctcagagtacgacgtgctgaactttgcctccggaggt

tgctggcctcgcaggattcgggtttggaacggccaggattttcggggcgatggattcaac

cccagatttgagagatttcacgtgtacgacatagtagagacttcggagagcgcctcacac

gatgacccatccaggtttgctgagctatcccgcccaagcgggtctgtggtaacactgctg

ggaatgagcgagtgtggaaagcgagtcgcggttcacgtctatggtgtgcgccattatttt

tacatggcaaaggcggaggtggatagcgcttgtggaataaccaccgaggcagaactcgtg

cgcgcaatggtggactgcgcgcacagctcggctttgagcgcggccctgggaaatggcaac

ggcggcaaacagagcggcggcagcggcgggggatggtggggcggaaagcacgtgtctgcg

gactgcttcaaagtggagaccgtgtgccacacgacgctgtactactttggatctaagcca

gctctctactatagagtatctgcctccagcagccgcctgggagggttcatctgcgacaac

tttcacccggagattacaaaattcgaggggagcgtggacgtgaccacgcggctgttgttg

gacaatgaaaattttaccagtttcgggtggtaccgcctgcgacccggcacccacggagag

cgtgttcaacttcgccccgttgagcgacacgtcacctcaagcgacgtggagattaactgt

actcccgataacctggagccgataccagacgaggctgcctggcccgactataagctcatg

tgctttgatatagagtgtaaagctggaacgggtaacgaaatggcgttcccagtggcaact

aaccaagaggacctggtcatccagatctcctgtctgctgtactcgcttgctactcagaac

cacgaacacaccctgctgttttccctcgggtcatgcgatatctctgaggaatactcgttt

gcatgcgtccagcgcggcgagcccagaccgacggttttggagtttgacagcgagtacgag

ctgctggttgccttcctgacctttctcaagcagtactctcccgagttcgccaccggctac

aacatcgttaattttgactgggcgtacatagttaacaaggtaacgtcggtgtataacatc

aagctggacgggtacggcaagttcaacaaaggggggctgtttaaggtgtgggacatcgcc

acgaaccattttcagaagaagagcaaggtgaaaatcaatggcctgatatctctagacatg

tattctgtggcgacggaaaagctaaagctacccagctacaaactcgacgcggtcgtggga

gacgtcctcggcgagcataagatagaccttccctataaagaaataccctcctattacgcg

ggagggcctgaccggaggggcgtaataggagagtattgtatccaggactctaggctggtg

ggcaagctgttttttaagtacctcccccatctggaactatcggcggtggccaaactcgcc

cgtatcaccctaacgcgggtaatttttgacggtcagcaaattcgcgtgtacacgtgcttg

ctgaaactcgcccgcgagagaaatttcattttgccagacaacagacgccggtttgacagt

caggcagacgccgcgtcagagacttcggagttggctatggatagccaaagccacgccttc

gacagtacagacgaacccgacggtgtggacggtaccccggacgccgcaggatctggcgct

acttctgaaaacggaggcgggaagcccggcgtcgggagggccgtgggctaccagggagca

aaggttctagaccccgtatccggctttcatgtggaccccgtggttgtgtttgacttcgct

agcttatacccaagcattatccaggcccataacctctgtttcaccaccctggcgctcgat

gaagtggatctggccgggcttcaaccatccgtcaactactcgacgttcgaggtgggtgac

caaaagttattttttgtccacgcccatattcgcgaaagcctgcttggcatcttgctgcgc

gactggctggccatgcgaaaggcggtgagggcgcgaatccccaccagcacccccgaggag

gcagttttactagataagcagcagtctgcgattaaggtgatatgcaactcggtttacgga

ttcacgggggtggcaaacggcctgttgccgtgtctgaggatagcggctaccgttaccacg

ataggacgcgacatgctcctcaagacccgagattacgttcactctcgttgggcgacgcgc

gagctgctggaggacaattttccaggggctataggtttccgaaaccacaagccttactcc

gtcagggttatctacggagacaccgactccgtgtttatcaagtttgtgggcctgacgtac

gagggggtatcggagctgggggatgctatgtcgcgtcagatttcagcggacctctttaga

gcgcccatcaaactggagtgtgagaagacctttcagcgactgctgctgatcaccaagaag

aagtacataggtgtcataaacggggggaagatgctcatgaagggggtcgacctggtccgc

aaaaataactgctctttcataaacttgtacgcgcgacatctggtagatcttttgttgtac

gacgaggatgtggccacggcggcagcagaggtgacagacgtgcctcccgcagaatgggtg

gggcgcccgctaccgagcggctttgacaagtttgggcgagtgctggtagaggcgtacaac

cgtatcactgcccccaacttggacgtgcgcgagttcgttatgactgctgagctgagccgc

tcacccgaatcgtataccaacaagcgcctgccgcacctcaccgtctactttaagctcgcc

atgaggaatgaagaactgcccagcgtaaaagagagaattccgtatgtgatagttgcgcag

accgaggccgcggaacgcgaagcgggtgtagtaaactcaatgcgcggtaccgcccaaaac

cccgtggtaaccaagaccgcacgcccccaacctaaacgcaaactgctggtttccgacctc

gccgaagacccgacctatgtttccgagaatgacgtaccgctaaacacagactactatttc

tcccacctgttgggtaccataagcgtgacctttaaggctctattcggaaatgatgtgaga

acaacagaaaatcttttaaagcggtttattccggaaaccccccacaagacccccacgaaa

acccaggcactgcttgagcgcgccggctttgaaaagctgacgccctttacaccggaggaa

gaaagtcgtcgaatactgcatacagttttttgtactctagaagcagctccccatcaaagc

tga

>PQ630631.1

atggcggcgcgcgaacaggccaactccgttcgcaggagcgggttttttaatccgtttatt

ggcaagaggccatttttcaggccgggaagcgggcagaccgcggagacagagcgcccaagg

cccccccaacactcgtactgcacagaggtgggtagctttaagtttatagctccaagatgc

ctcgatgaagaagcccccgccgaccagcgacgcggtgtacacgtgggcaccttggagcgc

ccacccaaagtgtactgcgatggctcagagtacgacgtgctgaactttgcctccggaggt

tgctggcctcgcaggattcgggtttggaacggccaggattttcggggcgatggattcaac

cccagatttgagagatttcacgtgtacgacatagtagagacttcggagagcgcctcacac

gatgacccatccaggtttgctgagctatcccgcccaagcgggtctgtggtaacactgctg

ggaatgagcgagtgtggaaagcgagtcgcggttcacgtctatggtgtgcgccattatttt

tacatggcaaaggcggaggtggatagcgcttgtggaataaccaccgaggcagaactcgtg

cgcgcaatggtggactgcgcgcacagctcggctttgagcgcggccctgggaaatggcaac

ggcggcaaacagagcggcggcagcggcgggggatggtggggcggaaagcacgtgtctgcg

gactgcttcaaagtggagaccgtgtgccacacgacgctgtactactttggatctaagcca

gctctctactatagagtatctgcctccagcagccgcctgggagggttcatctgcgacaac

tttcacccggagattacaaaattcgaggggagcgtggacgtgaccacgcggctgttgttg

gacaatgaaaattttaccagtttcgggtggtaccgcctgcgacccggcacccacggagag

cgtgttcaacttcgccccgttgagcgacacgtcacctcaagcgacgtggagattaactgt

actcccgataacctggagccgataccagacgaggctgcctggcccgactataagctcatg

tgctttgatatagagtgtaaagctggaacgggtaacgaaatggcgttcccagtggcaact

aaccaagaggacctggtcatccagatctcctgtctgctgtactcgcttgctactcagaac

cacgaacacaccctgctgttttccctcgggtcatgcgatatctctgaggaatactcgttt

gcatgcgtccagcgcggcgagcccagaccgacggttttggagtttgacagcgagtacgag

ctgctggttgccttcctgacctttctcaagcagtactctcccgagttcgccaccggctac

aacatcgttaattttgactgggcgtacatagttaacaaggtaacgtcggtgtataacatc

aagctggacgggtacggcaagttcaacaaaggggggctgtttaaggtgtgggacatcgcc

acgaaccattttcagaagaagagcaaggtgaaaatcaatggcctgatatctctagacatg

tattctgtggcgacggaaaagctaaagctacccagctacaaactcgacgcggtcgtggga

gacgtcctcggcgagcataagatagaccttccctataaagaaataccctcctattacgcg

ggagggcctgaccggaggggcgtaataggagagtattgtatccaggactctaggctggtg

ggcaagctgttttttaagtacctcccccatctggaactatcggcggtggccaaactcgcc

cgtatcaccctaacgcgggtaatttttgacggtcagcaaattcgcgtgtacacgtgcttg

ctgaaactcgcccgcgagagaaatttcattttgccagacaacagacgccggtttgacagt

caggcagacgccgcgtcagagacttcggagttggctatggatagccaaagccacgccttc

gacagtacagacgaacccgacggtgtggacggtaccccggacgccgcaggatctggcgct

acttctgaaaacggaggcgggaagcccggcgtcgggagggccgtgggctaccagggagca

aaggttctagaccccgtatccggctttcatgtggaccccgtggttgtgtttgacttcgct

agcttatacccaagcattatccaggcccataacctctgtttcaccaccctggcgctcgat

gaagtggatctggccgggcttcaaccatccgtcaactactcgacgttcgaggtgggtgac

caaaagttattttttgtccacgcccatattcgcgaaagcctgcttggcatcttgctgcgc

gactggctggccatgcgaaaggcggtgagggcgcgaatccccaccagcacccccgaggag

gcagttttactagataagcagcagtctgcgattaaggtgatatgcaactcggtttacgga

ttcacgggggtggcaaacggcctgttgccgtgtctgaggatagcggctaccgttaccacg

ataggacgcgacatgctcctcaagacccgagattacgttcactctcgttgggcgacgcgc

gagctgctggaggacaattttccaggggctataggtttccgaaaccacaagccttactcc

gtcagggttatctacggagacaccgactccgtgtttatcaagtttgtgggcctgacgtac

gagggggtatcggagctgggggatgctatgtcgcgtcagatttcagcggacctctttaga

gcgcccatcaaactggagtgtgagaagacctttcagcgactgctgctgatcaccaagaag

aagtacataggtgtcataaacggggggaagatgctcatgaagggggtcgacctggtccgc

aaaaataactgctctttcataaacttgtacgcgcgacatctggtagatcttttgttgtac

gacgaggatgtggccacggcggcagcagaggtgacagacgtgcctcccgcagaatgggtg

gggcgcccgctaccgagcggctttgacaagtttgggcgagtgctggtagaggcgtacaac

cgtatcactgcccccaacttggacgtgcgcgagttcgttatgactgctgagctgagccgc

tcacccgaatcgtataccaacaagcgcctgccgcacctcaccgtctactttaagctcgcc

atgaggaatgaagaactgcccagcgtaaaagagagaattccgtatgtgatagttgcgcag

accgaggccgcggaacgcgaagcgggtgtagtaaactcaatgcgcggtaccgcccaaaac

cccgtggtaaccaagaccgcacgcccccaacctaaacgcaaactgctggtttccgacctc

gccgaagacccgacctatgtttccgagaatgacgtaccgctaaacacagactactatttc

tcccacctgttgggtaccataagcgtgacctttaaggctctattcggaaatgatgtgaga

acaacagaaaatcttttaaagcggtttattccggaaaccccccacaagacccccacgaaa

acccaggcactgcttgagcgcgccggctttgaaaagctgacgccctttacaccggaggaa

gaaagtcgtcgaatactgcatacagttttttgtactctagaagcagctccccatcaaagc

tga

>KF434369.1

atggcggcgcgcgaacaggccaactccgttcgcaggagcgggttttttaatccgtttatt

ggcaagaggccatttttcaggccgggaagcgggcagaccgcggagacagagcgcccaagg

cccccccaacactcgtactgcacagaggtgggtagctttaagtttatagctccaagatgc

ctcgatgaagaagcccccgccgaccagcgacgcggtgtacacgtgggcaccttggagcgc

ccacccaaagtgtactgcgatggctcagagtacgacgtgctgaactttgcctccggaggt

tgctggcctcgcaggattcgggtttggaacggccaggattttcggggcgatggattcaac

cccagatttgagagatttcacgtgtacgacatagtagagacttcggagagcgcctcacac

gatgacccatccaggtttgctgagctatcccgcccaagcgggtctgtggtaacactgctg

ggaatgagcgagtgtggaaagcgagtcgcggttcacgtctatggtgtgcgccattatttt

tacatggcaaaggcggaggtggatagcgcttgtggaataaccaccgaggcagaactcgtg

cgcgcaatggtggactgcgcgcacagctcggctttgagcgcggccctgggaaatggcaac

ggcggcaaacagagcggcggcagcggcgggggatggtggggcggaaagcacgtgtctgcg

gactgcttcaaagtggagaccgtgtgccacacgacgctgtactactttggatctaagcca

gctctctactatagagtatctgcctccagcagccgcctgggagggttcatctgcgacaac

tttcacccggagattacaaaattcgaggggagcgtggacgtgaccacgcggctgttgttg

gacaatgaaaattttaccagtttcgggtggtaccgcctgcgacccggcacccacggagag

cgtgttcaacttcgccccgttgagcgacacgtcacctcaagcgacgtggagattaactgt

actcccgataacctggagccgataccagacgaggctgcctggcccgactataagctcatg

tgctttgatatagagtgtaaagctggaacgggtaacgaaatggcgttcccagtggcaact

aaccaagaggacctggtcatccagatctcctgtctgctgtactcgcttgctactcagaac

cacgaacacaccctgctgttttccctcgggtcatgcgatatctctgaggaatactcgttt

gcatgcgtccagcgcggcgagcccagaccgacggttttggagtttgacagcgagtacgag

ctgctggttgccttcctgacctttctcaagcagtactctcccgagttcgccaccggctac

aacatcgttaattttgactgggcgtacatagttaacaaggtaacgtcggtgtataacatc

aagctggacgggtacggcaagttcaacaaaggggggctgtttaaggtgtgggacatcgcc

acgaaccattttcagaagaagagcaaggtgaaaatcaatggcctgatatctctagacatg

tattctgtggcgacggaaaagctaaagctacccagctacaaactcgacgcggtcgtggga

gacgtcctcggcgagcataagatagaccttccctataaagaaataccctcctattacgcg

ggagggcctgaccggaggggcgtaataggagagtattgtatccaggactctaggctggtg

ggcaagctgttttttaagtacctcccccatctggaactatcggcggtggccaaactcgcc

cgtatcaccctaacgcgggtaatttttgacggtcagcaaattcgcgtgtacacgtgcttg

ctgaaactcgcccgcgagagaaatttcattttgccagacaacagacgccggtttgacagt

caggcagacgccgcgtcagagacttcggagttggctatggatagccaaagccacgccttc

gacagtacagacgaacccgacggtgtggacggtaccccggacgccgcaggatctggcgct

acttctgaaaacggaggcgggaagcccggcgtcgggagggccgtgggctaccagggagca

aaggttctagaccccgtatccggctttcatgtggaccccgtggttgtgtttgacttcgct

agcttatacccaagcattatccaggcccataacctctgtttcaccaccctggcgctcgat

gaagtggatctggccgggcttcaaccatccgtcaactactcgacgttcgaggtgggtgac

caaaagttattttttgtccacgcccatattcgcgaaagcctgcttggcatcttgctgcgc

gactggctggccatgcgaaaggcggtgagggcgcgaatccccaccagcacccccgaggag

gcagttttactagataagcagcagtctgcgattaaggtgatatgcaactcggtttacgga

ttcacgggggtggcaaacggcctgttgccgtgtctgaggatagcggctaccgttaccacg

ataggacgcgacatgctcctcaagacccgagattacgttcactctcgttgggcgacgcgc

gagctgctggaggacaattttccaggggctataggtttccgaaaccacaagccttactcc

gtcagggttatctacggagacaccgactccgtgtttatcaagtttgtgggcctgacgtac

gagggggtatcggagctgggggatgctatgtcgcgtcagatttcagcggacctctttaga

gcgcccatcaaactggagtgtgagaagacctttcagcgactgctgctgatcaccaagaag

aagtacataggtgtcataaacggggggaagatgctcatgaagggggtcgacctggtccgc

aaaaataactgctctttcataaacttgtacgcgcgacatctggtagatcttttgttgtac

gacgaggatgtggccacggcggcagcagaggtgacagacgtgcctcccgcagaatgggtg

gggcgcccgctaccgagcggctttgacaagtttgggcgagtgctggtagaggcgtacaac

cgtatcactgcccccaacttggacgtgcgcaagttcgttatgactgctgagctgagccgc

tcacccgaatcgtataccaacaagcgcctgccgcacctcaccgtctactttaagctcgcc

atgaggaatgaagaactgcccagcgtaaaagagagaattccgtatgtgatagttgcgcag

accgaggccgcggaacgcgaagcgggtgtagtaaactcaatgcgcggtaccgcccaaaac

cccgtggtaaccaagaccgcacgcccccaacctaaacgcaaactgctggtttccgacctc

gccgaagacccgacctatgtttccgagaatgacgtaccgctaaacacagactactatttc

tcccacctgttgggtaccataagcgtgacctttaaggctctattcggaaatgatgtgaga

acaacagaaaatcttttaaagcggtttattccggaaaccccccacaagacccccacgaaa

acccaggcactgcttgagcgcgccggctttgaaaagctgacgccctttacaccggaggaa

gaaagtcgtcgaatactgcatacagttttttgtactctagaagcagctccccatcaaagc

tga

>KF434370.1

atggcggcgcgcgaacaggccaactccgttcgcaggagcgggttttttaatccgtttatt

ggcaagaggccatttttcaggccgggaagcgggcagaccgcggagacagagcgcccaagg

cccccccaacactcgtactgcacagaggtgggtagctttaagtttatagctccaagatgc

ctcgatgaagaagcccccgccgaccagcgacgcggtgtacacgtgggcaccttggagcgc

ccacccaaagtgtactgcgatggctcagagtacgacgtgctgaactttgcctccggaggt

tgctggcctcgcaggattcgggtttggaacggccaggattttcggggcgatggattcaac

cccagatttgagagatttcacgtgtacgacatagtagagacttcggagagcgcctcacac

gatgacccatccaggtttgctgagctatcccgcccaagcgggtctgtggtaacactgctg

ggaatgagcgagtgtggaaagcgagtcgcggttcacgtctatggtgtgcgccattatttt

tacatggcaaaggcggaggtggatagcgcttgtggaataaccaccgaggcagaactcgtg

cgcgcaatggtggactgcgcgcacagctcggctttgagcgcggccctgggaaatggcaac

ggcggcaaacagagcggcggcagcggcgggggatggtggggcggaaagcacgtgtctgcg

gactgcttcaaagtggagaccgtgtgccacacgacgctgtactactttggatctaagcca

gctctctactatagagtatctgcctccagcagccgcctgggagggttcatctgcgacaac

tttcacccggagattacaaaattcgaggggagcgtggacgtgaccacgcggctgttgttg

gacaatgaaaattttaccagttttgggtggtaccgcctgcgacccggcacccacggagag

cgtgttcaacttcgccccgttgagcgacacgtcacctcaagcgacgtggagattaactgt

actcccgataacctggagccgataccagacgaggctgcctggcccgactataagctcatg

tgctttgatatagagtgtaaagctggaacgggtaacgaaatggcgttcccagtggcaact

aaccaagaggacctggtcatccagatctcctgtctgctgtactcgcttgctactcagaac

cacgaacacaccctgctgttttccctcgggtcatgcgatatctctgaggaatactcgttt

gcatgcgtccagcgcggcgagcccagaccgacggttttggagtttgacagcgagtacgag

ctgctggttgccttcctgacctttctcaagcagtactctcccgagttcgccaccggctac

aacatcgttaattttgactgggcgtacatagttaacaaggtaacgtcggtgtataacatc

aagctggacgggtacggcaagttcaacaaaggggggctgtttaaggtgtgggacatcgcc

acgaaccattttcagaagaagagcaaggtgaaaatcaatggcctgatatctctagacatg

tattctgtggcgacggaaaagctaaagctacccagctacaaactcgacgcggtcgtggga

gacgtcctcggcgagcataagatagaccttccctataaagaaataccctcctattacgcg

ggagggcctgaccggaggggcgtaataggagagtattgtatccaggactctaggctggtg

ggcaagctgttttttaagtacctcccccatctggaactatcggaggtggccaaactcgcc

cgtatcaccctaacgcgggtaatttttgacggtcagcaaattcgcgtgtacacgtgcttg

ctgaaactcgcccgcgagagaaatttcattttgccagacaacagacgccggtttgacagt

caggcagacgccgcgtcagagacttcggagttggctatggatagccaaagccacgccttc

gacagtacagacgaacccgacggtgtggacggtaccccggacgccgcaggatctggcgct

acttctgaaaacggaggcgggaagcccggcgtcgggagggccgtgggctaccagggagca

aaggttctagaccccgtatccggctttcatgtggaccccgtggttgtgtttgacttcgct

agcttatacccaagcattatccaggcccataacctctgtttcaccaccctggcgctcgat

gaagtggatctggccgggcttcaaccatccgtcaactactcgacgttcgaggtgggtgac

caaaagttattttttgtccacgcccatattcgcgaaagcctgcttggcatcttgctgcgc

gactggctggccatgcgaaaggcggtgagggcgcgaatccccaccagcacccccgaggag

gcagttttactagataagcagcagtctgcgattaaggtgatatgcaactcggtttacgga

ttcacgggggtggcaaacggcctgttgccgtgtctgaggatagcggctaccgttaccacg

ataggacgcgacatgctcctcaagacccgagattacgttcactctcgttgggcgacgcgc

gagctgctggaggacaattttccaggggctataggtttccgaaaccacaagccttactcc

gtcagggttatctacggagacaccgactccgtgtttatcaagtttgtgggcctgacgtac

gagggggtatcggagctgggggatgctatgtcgcgtcagatttcagcggacctctttaga

gcgcccatcaaactggagtgtgagaagacctttcagcgactgctgctgatcaccaagaag

aagtacataggtgtcataaacggggggaagatgctcatgaagggggtcgacctggtccgc

aaaaataactgctctttcataaacttgtacgcgcgacatctggtagatcttttgttgtac

gacgaggatgtggccacggcggcagcaaaggtgacagacgtgcctcccgcagaatgggtg

gggcgcccgctaccgagcggctttgacaagtttgggcgagtgctggtagaggcgtacaac

cgtatcactgcccccaacttggacgtgcgcgagttcgttatgactgctgagctgagccgc

tcacccgaatcgtataccaacaagcgcctgccgcacctcaccgtctactttaagctcgcc

atgaggaatgaagaactgcccagcgtaaaagagagaattccgtatgtgatagttgcgcag

accgaggccgcggaacgcgaagcgggtgtagtaaactcaatgcgcggtaccgcccaaaac

cccgtggtaaccaagaccgcacgcccccaacctaaacgcaaactgctggtttccgacctc

gccgaagacccgacctatgtttccgagaatgacgtaccgctaaacacagactactatttc

tcccacctgttgggtaccataagcgtgacctttaaggctctattcggaaatgatgtgaga

acaacagaaaatcttttaaagcggtttattccggaaaccccccacaagacccccacgaaa

acccaggcactgcttgagcgcgccggctttgaaaagctgacgccctttacaccggaggaa

gaaagtcgtcgaatactgcatacagttttttgtactctagaagcagctccccatcaaagc

tga

>KF434371.1

atggcggcgcgcgaacaggccaactccgttcgcaggagcgggttttttaatccgtttatt

ggcaagaggccatttttcaggccgggaagcgggcaaaccgcggagacagagcgcccaagg

cccccccaacactcgtactgcacagaggtgggtagctttaagtttatagctccaagatgc

ctcgatgaagaagcccccgccgaccagcgacgcggtgtacacgtgggcaccttggagcgc

ccacccaaagtgtactgcgatggctcagagtacgacgtgctgaactttgcctccggaggt

tgctggcctcgcaggattcgggtttggaacggccaggattttcggggcgatggattcaac

cccagatttgagagatttcacgtgtacgacatagtagagacttcggagagcgcctcacac

gatgacccatccaggtttgctgagctatcccgcccaagcgggtctgtggtaacactgctg

ggaatgagcgagtgtggaaagcgagtcgcggttcacgtctatggtgtgcgccattatttt

tacatggcaaaggcggaggtggatagcgcttgtggaataaccaccgaggcagaactcgtg

cgcgcaatggtggactgcgcgcacagctcggctttgagcgcggccctgggaaatggcaac

ggcggcaaacagagcggcggcagcggcgggggatggtggggcggaaagcacgtgtctgcg

gactgcttcaaagtggagaccgtgtgccacacgacgctgtactactttggatctaagcca

gctctctactatagagtatctgcctccagcagccgcctgggagggttcatctgcgacaac

tttcacccggagattacaaaattcgaggggagcgtggacgtgaccacgcggctgttgttg

gacaatgaaaattttaccagtttcgggtggtaccgcctgcgacccggcacccacggagag

cgtgttcaacttcgccccgttgagcgacacgtcacctcaagcgacgtggagattaactgt

actcccgataacctggagccgataccagacgaggctgcctggcccgactataagctcatg

tgctttgatatagagtgtaaagctggaacgggtaacgaaatggcgttcccagtggcaact

aaccaagaggacctggtcatccagatctcctgtctgctgtactcgcttgctactcagaac

cacgaacacaccctgctgttttccctcgggtcatgcgatatctctgaggaatactcgttt

gcatgcgtccagcgcggcgagcccagaccgacggttttggagtttgacagcgagtacgag

ctgctggttgccttcctgacctttctcaagcagtactctcccgagttcgccaccggctac

aacatcgttaattttgactgggcgtacatagttaacaaggtaacgtcggtgtataacatc

aagctggacgggtacggcaagttcaacaaaggggggctgtttaaggtgtgggacatcgcc

acgaaccattttcagaagaagagcaaggtgaaaatcaatggcctgatatctctagacatg

tattctgtggcgacggaaaagctaaagctacccagctacaaactcgacgcggtcgtggga

gacgtcctcggcgagcataagatagaccttccctataaagaaataccctcctattacgcg

ggagggcctgaccggaggggcgtaataggagagtattgtatccaggactctaggctggtg

ggcaagctgttttttaagtacctcccccatctggaactatcggcggtggccaaactcgcc

cgtatcaccctaacgcgggtaatttttgacggtcagcaaattcgcgtgtacacgtgcttg

ctgaaactcgcccgcgagagaaatttcattttgccagacaacagacgccggtttgacagt

caggcagacgccgcgtcagagacttcggagttggctatggatagccaaagccacgccttc

gacagtacagacgaacccgacggtgtggacggtaccccggacgccgcaggatctggcgct

acttctgaaaacggaggcgggaagcccggcgtcgggagggccgtgggctaccagggagca

aaggttctagaccccgtatccggctttcatgtggaccccgtggttgtgtttgacttcgct

agcttatacccaagcattatccaggcccataacctctgtttcaccaccctggcgctcgat

gaagtggatctggccgggcttcaaccatccgtcaactactcgacgttcgaggtgggtgac

caaaagttattttttgtccacgcccatattcgcgaaagcctgcttggcatcttgctgcgc

gactggctggccatgcgaaaggcggtgagggcgcgaatccccaccagcacccccgaggag

gcagttttactagataagcagcagtctgcgattaaggtgatatgcaactcggtttacgga

ttcacgggggtggcaaacggcctgttgccgtgtctgaggatagcggctaccgttaccacg

ataggacgcgacatgctcctcaagacccgagattacgttcactctcgttgggcgacgcgc

gagctgctggaggacaattttccaggggctataggtttccgaaaccacaagccttactcc

gtcagggttatctacggagacaccgactccgtgtttatcaagtttgtgggcctgacgtac

gagggggtatcggagctgggggatgctatgtcgcgtcagatttcagcggacctctttaga

gcgcccatcaaactggagtgtgagaagacctttcagcgactgctgctgatcaccaagaag

aagtacataggtgtcataaacggggggaagatgctcatgaagggggtcgacctggtccgc

aaaaataactgctctttcataaacttgtacgcgcgacatctggtagatcttttgttgtac

gacgaggatgtggccacggcggcagcagaggtgacagacgtgcctcccgcagaatgggtg

gggcgcccgctaccgagcggctttgacaagtttgggcgagtgctggtagaggcgtacaac

cgtatcactgcccccaacttggacgtgcgcgagttcgttatgactgctgagctgagccgc

tcacccgaatcgtataccaacaagcgcctgccgcacctcaccgtctactttaagctcgcc

atgaggaatgaagaactgcccagcgtaaaagagagaattccgtatgtgatagttgcgcag

accgaggccgcggaacgcgaagcgggtgtagtaaactcaatgcgcggtaccgcccaaaac

cccgtggtaaccaagaccgcacgcccccaacctaaacgcaaactgctggtttccgacctc

gccgaagacccgacctatgtttccgagaatgacgtaccgctaaacacagactactatttc

tcccacctgttgggtaccataagcgtgacctttaaggctctattcggaaatgatgtgaga

acaacagaaaatcttttaaagcggtttattccggaaaccccccacaagacccccacgaaa

acccaggcactgcttgagcgcgccggctttgaaaagctgacgccctttacaccggaggaa

gaaagtcgtcgaatactgcatacagttttttgtactctagaagcagctccccatcaaagc

tga

>KF434372.1

atggcggcgcgcgaacaggccaactccgttcgcaggagcgggttttttaatccgtttatt

ggcaagaggccatttttcaggccgggaagcgggcagaccgcggagacagagcgcccaagg

cccccccaacactcgtactgcacagaggtgggtagctttaagtttatagctccaagatgc

ctcgatgaagaagcccccgccgaccagcgacgcggtgtacacgtgggcaccttggagcgc

ccacccaaagtgtactgcgatggctcagagtacgacgtgctgaactttgcctccggaggt

tgctggcctcgcaggattcgggtttggaacggccaggattttcggggcgatggattcaac

cccagatttgagagatttcacgtgtacgacatagtagagacttcggagagcgcctcacac

gatgacccatccaggtttgctgagctatcccgcccaagcgggtctgtggtaacactgctg

ggaatgagcgagtgtggaaagcgagtcgcggttcacgtctatggtgtgcgccattatttt

tacatggcaaaggcggaggtggatagcgcttgtggaataaccaccgaggcagaactcgtg

cgcgcaatggtggactgcgcgcacagctcggctttgagcgcggccctgggaaatggcaac

ggcggcaaacagagcggcggcagcggcgggggatggtggggcggaaagcacgtgtctgcg

gactgcttcaaagtggagaccgtgtgccacacgacgctgtactactttggatctaagcca

gctctctactatagagtatctgcctccagcagccgcctgggagggttcatctgcgacaac

tttcacccggagattacaaaattcgaggggagcgtggacgtgaccacgcggctgttgttg

gacaatgaaaattttaccagtttcgggtggtaccgcctgcgacccggcacccacggagag

cgtgttcaacttcgccccgttgagcgacacgtcacctcaagcgacgtggagattaactgt

actcccgataacctggagccgataccagacgaggctgcctggcccgactataagctcatg

tgctttgatatagagtgtaaagctggaacgggtaacgaaatggcgttcccagtggcaact

aaccaagaggacctggtcatccagatctcctgtctgctgtactcgcttgctactcagaac

cacgaacacaccctgctgttttccctcgggtcatgcgatatctctgaggaatactcgttt

gcatgcgtccagcgcggcgagcccagaccgacggttttggagtttgacagcgagtacgag

ctgctggttgccttcctgacctttctcaagcagtactctcccgagttcgccaccggctac

aacatcgttaattttgactgggcgtacatagttaacaaggtaacgtcggtgtataacatc

aagctggacgggtacggcaagttcaacaaaggggggctgtttaaggtgtgggacatcgcc

acgaaccattttcagaagaagagcaaggtgaaaatcaatggcctgatatctctagacatg

tattctgtggcgacggaaaagctaaagctacccagctacaaactcgacgcggtcgtggga

gacgtcctcggcgagcataagatagaccttccctataaagaaataccctcctattacgcg

ggagggcctgaccggaggggcgtaataggagagtattgtatccaggactctaggctggtg

ggcaagctgttttttaagtacctcccccatctggaactatcggcggtggccaaactcgcc

catatcaccctaacgcgggtaatttttgacggtcagcaaattcgcgtgtacacgtgcttg

ctgaaactcgcccgcgagagaaatttcattttgccagacaacagacgccggtttgacagt

caggcagacgccgcgtcagagacttcggagttggctatggatagccaaagccacgccttc

gacagtacagacgaacccgacggtgtggacggtaccccggacgccgcaggatctggcgct

acttctgaaaacggaggcgggaagcccggcgtcgggagggccgtgggctaccagggagca

aaggttctagaccccgtatccggctttcatgtggaccccgtggttgtgtttgacttcgct

agcttatacccaagcattatccaggcccataacctctgtttcaccaccctggcgctcgat

gaagtggatctggccgggcttcaaccatccgtcaactactcgacgttcgaggtgggtgac

caaaagttattttttgtccacgcccatattcgcgaaagcctgcttggcatcttgctgcgc

gactggctggccatgcgaaaggcggtgagggcgcgaatccccaccagcacccccgaggag

gcagttttactagataagcagcagtctgcgattaaggtgatatgcaactcggtttacgga

ttcacgggggtggcaaacggcctgttgccgtgtctgaggatagcggctaccgttaccacg

ataggacgcgacatgctcctcaagacccgagattacgttcactctcgttgggcgacgcgc

gagctgctggaggacaattttccaggggctataggtttccgaaaccacaagccttactcc

gtcagggttatctacggagacaccgactccgtgtttatcaagtttgtgggcctgacgtac

gagggggtatcggagctgggggatgctatgtcgcgtcagatttcagcggacctctttaga

gcgcccatcaaactggagtgtgagaagacctttcagcgactgctgctgatcaccaagaag

aagtacataggtgtcataaacggggggaagatgctcatgaagggggtcgacctggtccgc

aaaaataactgctctttcataaacttgtacgcgcgacatctggtagatcttttgttgtac

gacgaggatgtggccacggcggcagcagaggtgacagacgtgcctcccgcagaatgggtg

gggcgcccgctaccgagcggctttgacaagtttgggcgagtgctggtagaggcgtacaac

cgtatcactgcccccaacttggacgtgcgcgagttcgttatgactgctgagctgagccgc

tcacccgaatcgtataccaacaagcgcctgccgcacctcaccgtctactttaagctcgcc

atgaggaatgaagaactgcccagcgtaaaagagagaattccgtatgtgatagttgcgcag

accgaggccgcggaacgcgaagcgggtgtagtaaactcaatgcgcggtaccgcccaaaac

cccgtggtaaccaagaccgcacgcccccaacctaaacgcaaactgctggtttccgacctc

gccgaagacccgacctatgtttccgagaatgacgtaccgctaaacacagactactatttc

tcccacctgttgggtaccataagcgtgacctttaaggctctattcggaaatgatgtgaga

acaacagaaaatcttttaaagcggtttattccggaaaccccccacaagacccccacgaaa

acccaggcactgcttgagcgcgccggctttgaaaagctgacgccctttacaccggaggaa

gaaagtcgtcgaatactgcatacagttttttgtactctagaagcagctccccatcaaagc

tga

>KF434373.1

atggcggcgcgcgaacaggccaactccgttcgcaggagcgggttttttaatccgtttatt

ggcaagaggccatttttcaggccgggaagcgggcagaccgcggagacagagcgcccaagg

cccccccaacactcgtactgcacagaggtgggtagctttaagtttatagctccaagatgc

ctcgatgaagaagcccccgccgaccagcgacgcggtgtacacgtgggcaccttggagcgc

ccacccaaagtgtactgcgatggctcagagtacgacgtgctgaactttgcctccggaggt

tgctggcctcgcaggattcgggtttggaacggccaggattttcggggcgatggattcaac

cccagatttgagagatttcacgtgtacgacatagtagagacttcggagagcgcctcacac

gatgacccatccaggtttgctgagctatcccgcccaagcgggtctgtggtaacactgctg

ggaatgagcgagtgtggaaagcgagtcgcggttcacgtctatggtgtgcgccattatttt

tacatggcaaaggcggaggtggatagcgcttgtggaataaccaccgaggcagaactcgtg

cgcgcaatggtggactgcgcgcacagctcggctttgagcgcggccctgggaaatggcaac

ggcggcaaacagagcggcggcagcggcgggggatggtggggcggaaagcacgtgtctgcg

gactgcttcaaagtggagaccgtgtgccacacgacgctgtactactttggatctaagcca

gctctctactatagagtatctgcctccagcagccgcctgggagggttcatctgcgacaac

tttcacccggagattacaaaattcgaggggagcgtggacgtgaccacgcggctgttgttg

gacaatgaaaattttaccagtttcgggtggtaccgcctgcgacccggcacccacggagag

cgtgttcaacttcgccccgttgagcgacacgtcacctcaagcgacgtggagattaactgt

actcccgataacctggagccgataccagacgaggctgcctggcccgactataagctcatg

tgctttgatatagagtgtaaagctggaacgggtaacgaaatggcgttcccagtggcaact

aaccaagaggacctggtcatccagatctcctgtctgctgtactcgcttgctactcagaac

cacgaacacaccctgctgttttccctcgggtcatgcgatatctctgaggaatactcgttt

gcatgcgtccagcgcggcgagcccagaccgacggttttggagtttgacagcgagtacgag

ctgctggttgccttcctgacctttctcaagcagtactctcccgagttcgccaccggctac

aacatcgttaattttgactgggcgtacatagttaacaaggtaacgtcggtgtataacatc

aagctggacgggtacggcaagttcaacaaaggggggctgtttaaggtgtgggacatcgcc

acgaaccattttcagaagaagagcaaggtgaaaatcaatggcctgatatctctagacatg

tattctgtggcgacggaaaagctaaagctacccagctacaaactcgacgcggtcgtggga

gacgtcctcggcgagcataagatagaccttccctataaagaaataccctcctattacgcg

ggagggcctgaccggaggggcgtaataggagagtattgtatccaggactctaggctggtg

ggcaagctgttttttaagtacctcccccatctggaactatcggcggtggccaaactcgcc

cgtatcaccctaacgcgggtaatttttgacggtcagcaaattcgcgtgtacacgtgcttg

ctgaaactcgcccgcgagagaaatttcattttgccagacaacagacgccggtttgacagt

caggcagacgccgcgtcagagacttcggagttggctatggatagccaaagccacgccttc

gacagtacagacgaacccgacggtgtggacggtaccccggacgccgcaggatctggcgct

acttctgaaaacggaggcgggaagcccggcgtcgggagggccgtgggctaccagggagca

aaggttctagaccccgtatccggctttcatgtggaccccgtggttgtgtttgacttcgct

agcttatacccaagcattatccaggcccataacctctgtttcaccaccctggcgctcgat

gaagtggatctggccgggcttcaaccatccgtcaactactcgacgttcgaggtgggtgac

caaaagttattttttgtccacgcccatattcgcgaaagcctgcttggcatcttgctgcgc

gactggctggccatgcgaaaggcggtgagggcgcgaatccccaccagcacccccgaggag

gcagttttactagataagcagcagtctgcgattaaggtgatatgcaactcggtttacgga

ttcacgggggtggcaaacggcctgttgccgtgtctgaggatagcggctaccgttaccacg

ataggacgcgacatgctcctcaagacccgagattacgttcactctcgttgggcgacgcgc

gagctgctggaggacaattttccaggggctataggtttccgaaaccacaagccttactcc

gtcagggttatctacggagacaccgactccgtgtttatcaagtttgtgggcctgacgtac

gagggggtatcggagctgggggatgctatgtcgcgtcagatttcagcggacctctttaga

gcgcccatcaaactggagtgtgagaagacctttcagcgactgctgctgatcaccaagaag

aagtacataggtgtcataaacggggggaagatgctcatgaagggggtcgacctggtccgc

aaaaataactgctctttcataaacttgtacgcgcgacatctggtagatcttttgttgtac

gacgaggatgtggccacggcggcagcagaggtgacagacgtgcctcccgcagaatgggtg

gggcgcccgctaccgagcggctttgacaagtttgggcgagtgctggtagaggcgtacaac

cgtatcactgcccccaacttggacgtgcgcgagttcgttatgactgctgagctgagccgc

tcacccgaatcgtataccaacaagcgcctgccgcacctcaccgtctactttaagctcgcc

atgaggaatgaagaactgcccagcgtaaaagagagaattccgtatgtgatagttgcgcag

accgaggccgcggaacgcgaagcgggtgtagtaaactcaatgcgcggtaccgcccaaaac

cccgtggtaaccaagaccgcacgcccccaacctaaacgcaaactgctggtttccgacctc

gccgaagacccgacctatgtttccgagaatgacgtaccgctaaacacagactactatttc

tcccacctgttgggtaccataagcgtgacctttaaggctctattcggaaatgatgtgaga

acaacagaaaatcttttaaagcggtttattccggaaaccccccacaagacccccacgaaa

acccaggcactgcttgagcgcgccggctttgaaaagctgacgccctttacaccggaggaa

gaaagtcgtcgaatactgcatacagttttttgtactctagaagcagctccccatcaaagc

tga

>KF434374.1

atggcggcgcgcgaacaggccaactccgttcgcaggagcgggttttttaatccgtttatt

ggcaagaggccatttttcaggccgggaagcgggcagaccgcggagacagagcgcccaagg

cccccccaacactcgtactgcacagaggtgggtagctttaagtttatagctccaagatgc

ctcgatgaagaagcccccgccgaccagcgacgcggtgtacacgtgggcaccttggagcgc

ccacccaaagtgtactgcgatggctcagagtacgacgtgctgaactttgcctccggaggt

tgctggcctcgcaggattcgggtttggaacggccaggattttcggggcgatggattcaac

cccagatttgagagatttcacgtgtacgacatagtagagacttcggagagcgcctcacac

gatgacccatccaggtttgctgagctatcccgcccaagtgggtctgtggtaacactgctg

ggaatgagcgagtgtggaaagcgagtcgcggttcacgtctatggtgtgcgccattatttt

tacatggcaaaggcggaggtggatagcgcttgtggaataaccaccgaggcagaactcgtg

cgcgcaatggtggactgcgcgcacagctcggctttgagcgcggccctgggaaatggcaac

ggcggcaaacagagcggcggcagcggcgggggatggtggggcggaaagcacgtgtctgcg

gactgcttcaaagtggagaccgtgtgccacacgacgctgtactactttggatctaagcca

gctctctactatagagtatctgcctccagcagccgcctgggagggttcatctgcgacaac

tttcacccggagattacaaaattcgaggggagcgtggacgtgaccacgcggctgttgttg

gacaatgaaaattttaccagtttcgggtggtaccgcctgcgacccggcacccacggagag

cgtgttcaacttcgccccgttgagcgacacgtcacctcaagcgacgtggagattaactgt

actcccgataacctggagccgataccagacgaggctgcctggcccgactataagctcatg

tgctttgatatagagtgtaaagctggaacgggtaacgaaatggcgttcccagtggcaact

aaccaagaggacctggtcatccagatctcctgtctgctgtactcgcttgctactcagaac

cacgaacacaccctgctgttttccctcgggtcatgcgatatctctgaggaatactcgttt

gcatgcgtccagcgcggcgagcccagaccgacggttttggagtttgacagcgagtacgag

ctgctggttgccttcctgacctttctcaagcagtactctcccgagttcgccaccggctac

aacatcgttaattttgactgggcgtacatagttaacaaggtaacgtcggtgtataacatc

aagctggacgggtacggcaagttcaacaaaggggggctgtttaaggtgtgggacatcgcc

acgaaccattttcagaagaagagcaaggtgaaaatcaatggcctgatatctctagacatg

tattctgtggcgacggaaaagctaaagctacccagctacaaactcgacgcggtcgtggga

gacgtcctcggcgagcataagatagaccttccctataaagaaataccctcctattacgcg

ggagggcctgaccggaggggcgtaataggagagtattgtatccaggactctaggctggtg

ggcaagctgttttttaagtacctcccccatctggaactatcggcggtggccaaactcgcc

cgtatcaccctaacgcgggtaatttttgacggtcagcaaattcgcgtgtacacgtgcttg

ctgaaactcgcccgcgagagaaatttcattttgccagacaacagacgccggtttgacagt

caggcagacgccgcgtcagagacttcggagttggctatggatagccaaagccacgccttc

gacagtacagacgaacccgacggtgtggacggtaccccggacgccgcaggatctggcgct

acttctgaaaacggaggcgggaagcccggcgtcgggagggccgtgggctaccagggagca

aaggttctagaccccgtatccggctttcatgtggaccccgtggttgtgtttgacttcgct

agcttatacccaagcattatccaggcccataacctctgtttcaccaccctggcgctcgat

gaagtggatctggccgggcttcaaccatccgtcaactactcgacgttcgaggtgggtgac

caaaagttattttttgtccacgcccatattcgcgaaagcctgcttggcatcttgctgcgc

gactggctggccatgcgaaaggcggtgagggcgcgaatccccaccagcacccccgaggag

gcagttttactagataagcagcagtctgcgattaaggtgatatgcaactcggtttacgga

ttcacgggggtggcaaacggcctgttgccgtgtctgaggatagcggctaccgttaccacg

ataggacgcgacatgctcctcaagacccgagattacgttcactctcgttgggcgacgcgc

gagctgctggaggacaattttccaggggctataggtttccgaaaccacaagccttactcc

gtcagggttatctacggagacaccgactccgtgtttatcaagtttgtgggcctgacgtac

gagggggtatcggagctgggggatgctatgtcgcgtcagatttcagcggacctctttaga

gcgcccatcaaactggagtgtgagaagacctttcagcgactgctgctgatcaccaagaag

aagtacataggtgtcataaacggggggaagatgctcatgaagggggtcgacctggtccgc

aaaaataactgctctttcataaacttgtacgcgcgacatctggtagatcttttgttgtac

gacgaggatgtggccacggcggcagcagaggtgacagacgtgcctcccgcagaatgggtg

gggcgcccgctaccgagcggctttgacaagtttgggcgagtgctggtagaggcgtacaac

cgtatcactgcccccaacttggacgtgcgcgagttcgttatgactgctgagctgagccgc

tcacccgaatcgtataccaacaagcgcctgccgcacctcaccgtctactttaagctcgcc

atgaggaatgaagaactgcccagcgtaaaagagagaattccgtatgtgatagttgcgcag

accgaggccgcggaacgcgaagcgggtgtagtaaactcaatgcgcggtaccgcccaaaac

cccgtggtaaccaagaccgcacgcccccaacctaaacgcaaactgctggtttccgacctc

gccgaagacccgacctatgtttccgagaatgacgtaccgctaaacacagactactatttc

tcccacctgttgggtaccataagcgtgacctttaaggctctattcggaaatgatgtgaga

acaacagaaaatcttttaaagcggtttattccggaaaccccccacaagacccccacgaaa

acccaggcactgcttgagcgcgccggctttgaaaagctgacgccctttacaccggaggaa

gaaagtcgtcgaatactgcatacagttttttgtactctagaagcagctccccatcaaagc

tga

>KF434375.1

atggcggcgcgcgaacaggccaactccgttcgcaggagcgggttttttaatccgtttatt

ggcaagaggccatttttcaggccgggaagcgggcagaccgcggagacagagcgcccaagg

cccccccaacactcgtactgcacagaggtgggtagctttaagtttatagctccaagatgc

ctcgatgaagaagcccccgccgaccagcgacgcggtgtacacgtgggcaccttggagcgc

ccacccaaagtgtactgcgatggctcagagtacgacgtgctgaactttgcctccggaggt

tgctggcctcgcaggattcgggtttggaacggccaggattttcggggcgatggattcaac

cccagatttgagagatttcacgtgtacgacatagtagagacttcggagagcgcctcacac

gatgacccatccaggtttgctgagctatcccgcccaagcgggtctgtggtaacactgctg

ggaatgagcgagtgtggaaagcgagtcgcggttcacgtctatggtgtgcgccattatttt

tacatggcaaaggcggaggtggatagcgcttgtggaataaccaccgaggcagaactcgtg

cgcgcaatggtggactgcgcgcacagctcggctttgagcgcggccctgggaaatggcaac

ggcggcaaacagagcggcggcagcggcgggggatggtggggcggaaagcacgtgtctgcg

gactgcttcaaagtggagaccgtgtgccacacgacgctgtactactttggatctaagcca

gctctctactatagagtatctgcctccagcagccgcctgggagggttcatctgcgacaac

tttcacccggagattacaaaattcgaggggagcgtggacgtgaccacgcggctgttgttg

gacaatgaaaattttaccagtttcgggtggtaccgcctgcgacccggcacccacggagag

cgtgttcaacttcgccccgttgagcgacacgtcacctcaagcgacgtggagattaactgt

actcccgataacctggagccgataccagacgaggctgcctggcccgactataagctcatg

tgctttgatatagagtgtaaagctggaacgggtaacgaaatggcgttcccagtggcaact

aaccaagaggacctggtcatccagatctcctgtctgctgtactcgcttgctactcagaac

cacgaacacaccctgctgttttccctcgggtcatgcgatatctctgaggaatactcgttt

gcatgcgtccagcgcggcgagcccagaccgacggttttggagtttgacagcgagtacgag

ctgctggttgccttcctgacctttctcaagcagtactctcccgagttcgccaccggctac

aacatcgttaattttgactgggcgtacatagttaacaaggtaacgtcggtgtataacatc

aagctggacgggtacggcaagttcaacaaaggggggctgtttaaggtgtgggacatcgcc

acgaaccattttcagaagaagagcaaggtgaaaatcaatggcctgatatctatagacatg

tattctgtggcgacggaaaagctaaagctacccagctacaaactcgacgcggtcgtggga

gacgtcctcggcgagcataagatagaccttccctataaagaaataccctcctattacgcg

ggagggcctgaccggaggggcgtaataggagagtattgtatccaggactctaggctggtg

ggcaagctgttttttaagtacctcccccatctggaactatcggcggtggccaaactcgcc

cgtatcaccctaacgcgggtaatttttgacggtcagcaaattcgcgtgtacacgtgcttg

ctgaaactcgcccgcgagagaaatttcattttgccagacaacagacgccggtttgacagt

caggcagacgccgcgtcagagacttcggagttggctatggatagccaaagccacgccttc

gacagtacagacgaacccgacggtgtggacggtaccccggacgccgcaggatctggcgct

acttctgaaaacggaggcgggaagcccggcgtcgggagggccgtgggctaccagggagca

aaggttctagaccccgtatccggctttcatgtggaccccgtggttgtgtttgacttcgct

agcttatacccaagcattatccaggcccataacctctgtttcaccaccctggcgctcgat

gaagtggatctggccgggcttcaaccatccgtcaactactcgacgttcgaggtgggtgac

caaaagttattttttgtccacgcccatattcgcgaaagcctgcttggcatcttgctgcgc

gactggctggccatgcgaaaggcggtgagggcgcgaatccccaccagcacccccgaggag

gcagttttactagataagcagcagtctgcgattaaggtgatatgcaactcggtttacgga

ttcacgggggtggcaaacggcctgttgccgtgtctgaggatagcggctaccgttaccacg

ataggacgcgacatgctcctcaagacccgagattacgttcactctcgttgggcgacgcgc

gagctgctggaggacaattttccaggggctataggtttccgaaaccacaagccttactcc

gtcagggttatctacggagacaccgactccgtgtttatcaagtttgtgggcctgacgtac

gagggggtatcggagctgggggatgctatgtcgcgtcagatttcagcggacctctttaga

gcgcccatcaaactggagtgtgagaagacctttcagcgactgctgctgatcaccaagaag

aagtacataggtgtcataaacggggggaagatgctcatgaagggggtcgacctggtccgc

aaaaataactgctctttcataaacttgtacgcgcgacatctggtagatcttttgttgtac

gacgaggatgtggccacggcggcagcagaggtgacagacgtgcctcccgcagaatgggtg

gggcgcccgctaccgagcggctttgacaagtttgggcgagtgctggtagaggcgtacaac

cgtatcactgcccccaacttggacgtgcgcgagttcgttatgactgctgagctgagccgc

tcacccgaatcgtataccaacaagcgcctgccgcacctcaccgtctactttaagctcgcc

atgaggaatgaagaactgcccagcgtaaaagagagaattccgtatgtgatagttgcgcag

accgaggccgcggaacgcgaagcgggtgtagtaaactcaatgcgcggtaccgcccaaaac

cccgtggtaaccaagaccgcacgcccccaacctaaacgcaaactgctggtttccgacctc

gccgaagacccgacctatgtttccgagaatgacgtaccgctaaacacagactactatttc

tcccacctgttgggtaccataagcgtgacctttaaggctctattcggaaatgatgtgaga

acaacagaaaatcttttaaagcggtttattccggaaaccccccacaagacccccacgaaa

acccaggcactgcttgagcgcgccggctttgaaaagctgacgccctttacaccggaggaa

gaaagtcgtcgaatactgcatacagttttttgtactctagaagcagctccccatcaaagc

tga

>KF434376.1

atggcggcgcgcgaacaggccaactccgttcgcaggagcgggttttttaatccgtttatt

ggcaagaggccatttttcaggccgggaagcgggcagaccgcggagacagagcgcccaagg

cccccccaacactcgtactgcacagaggtgggtagctttaagtttatagctccaagatgc

ctcgatgaagaagcccccgccgaccagcgacgcggtgtacacgtgggcaccttggagcgc

ccacccaaagtgtactgcgatggctcagagtacgacgtgctgaactttgcctccggaggt

tgctggcctcgcaggattcgggtttggaacggccaggattttcggggcgatggattcaac

cccagatttgagagatttcacgtgtacgacatagtagagacttcggagagcgcctcacac

gatgacccatccaggtttgctgagctatcccgcccaagcgggtctgtggtaacactgctg

ggaatgagcgagtgtggaaagcgagtcgcggttcacgtctatggtgtgcgccattatttt

tacatggcaaaggcggaggtggatagcgcttgtggaataaccaccgaggcagaactcgtg

cgcgcaatggtggactgcgcgcacagctcggctttgagcgcggccctgggaaatggcaac

ggcggcaaacagagcggcggcagcggcgggggatggtggggcggaaagcacgtgtctgcg

gactgcttcaaagtggagaccgtgtgccacacgacgctgtactactttggatctaagcca

gctctctactatagagtatctgcctccagcagccgcctgggagggttcatctgcgacaac

tttcacccggagattacaaaattcgaggggagcgtggacgtgaccacgcggctgttgttg

gacaatgaaaattttaccagtttcgggtggtaccgcctgcgacccggcacccacggagag

cgtgttcaacttcgccccgttgagcgacacgtcacctcaagcgacgtggagattaactgt

actcccgataacctggagccgataccagacgaggctgcctggcccgactataagctcatg

tgctttgatatagagtgtaaagctggaacgggtaacgaaatggcgttcccagtggcaact

aaccaagaggacctggtcatccagatctcctgtctgctgtactcgcttgctactcagaac

cacgaacacaccctgctgttttccctcgggtcatgcgatatctctgaggaatactcgttt

gcatgcgtccagcgcggcgagcccagaccgacggttttggagtttgacagcgagtacgag

ctgctggttgccttcctgacctttctcaagcagtactctcccgagttcgccaccggctac

aacatcgttaattttgactgggcgtacatagttaacaaggtaacgtcggtgtataacatc

aagctggacgggtacggcaagttcaacaaaggggggctgtttaaggtgtgggacatcgcc

acgaaccattttcagaagaagagcaaggtgaaaatcaatggcctgatatctctagacatg

tattctgtggcgacggaaaagctaaagctacccagctacaaactcgacgcggtcgtggga

gacgtcctcggcgagcataagatagaccttccctataaagaaataccctcctattacgcg

ggagggcctgaccggaggggcgtaataggagagtattgtatccaggactctaggctggtg

ggcaagctgttttttaagtacctcccccatctggaactatcggcggtggccaaactcgcc

cgtatcaccctaacgcgggtaatttttgacggtcagcaaattcgcgtgtacacgtgcttg

ctgaaactcgcccgcgagagaaatttcattttgccagacaacagacgccggtttgacagt

caggcagacgccgcgtcagagacttcggagttggctatggatagccaaagccacgccttc

gacagtacagacgaacccgacggtgtggacggtaccccggacgccgcaggatctggcgct

acttctgaaaacggaggcgggaagcccggcgtcgggagggccgtgggctaccagggagca

aaggttctagaccccgtatccggctttcatgtggaccccgtggttgtgtttgacttcgct

agcttatacccaagcattatccaggcccataacctctgtttcaccaccctggcgctcgat

gaagtggatctggccgggcttcaaccatccgtcgactactcgacgttcgaggtgggtgac

caaaagttattttttgtccacgcccatattcgcgaaagcctgcttggcatcttgctgcgc

gactggctggccatgcgaaaggcggtgagggcgcgaatccccaccagcacccccgaggag

gcagttttactagataagcagcagtctgcgattaaggtgatatgcaactcggtttacgga

ttcacgggggtggcaaacggcctgttgccgtgtctgaggatagcggctaccgttaccacg

ataggacgcgacatgctcctcaagacccgagattacgttcactctcgttgggcgacgcgc

gagctgctggaggacaattttccaggggctataggtttccgaaaccacaagccttactcc

gtcagggttatctacggagacaccgactccgtgtttatcaagtttgtgggcctgacgtac

gagggggtatcggagctgggggatgctatgtcgcgtcagatttcagcggacctctttaga

gcgcccatcaaactggagtgtgagaagacctttcagcgactgctgctgatcaccaagaag

aagtacataggtgtcataaacggggggaagatgctcatgaagggggtcgacctggtccgc

aaaaataactgctctttcataaacttgtacgcgcgacatctggtagatcttttgttgtac

gacgaggatgtggccacggcggcagcagaggtgacagacgtgcctcccgcagaatgggtg

gggcgcccgctaccgagcggctttgacaagtttgggcgagtgctggtagaggcgtacaac

cgtatcactgcccccaacttggacgtgcgcgagttcgttatgactgctgagctgagccgc

tcacccgaattgtataccaacaagcgcctgccgcacctcaccgtctactttaagctcgcc

atgaggaatgaagaactgcccagcgtaaaagagagaattccgtatgtgatagttgcgcag

accgaggccgcggaacgcgaagcgggtgtagtaaactcaatgcgcggtaccgcccaaaac

cccgtggtaaccaagaccgcacgcccccaacctaaacgcaaactgctggtttccgacctc

gccgaagacccgacctatgtttccgagaatgacgtaccgctaaacacagactactatttc

tcccacctgttgggtaccataagcgtgacctttaaggctctattcggaaatgatgtgaga

acaacagaaaatcttttaaagcggtttattccggaaaccccccacaagacccccacgaaa

acccaggcactgcttgagcgcgccggctttgaaaagctgacgccctttacaccggaggaa

gaaagtcgtcgaatactgcatacagttttttgtactctagaagcagctccccatcaaagc

tga

>KF434377.1

atggcggcgcgcgaacaggccaactccgttcgcaggagcgggttttttaatccgtttatt

ggcaagaggccatttttcaggccgggaagcgggcaaaccgcggagacagagcgcccaagg

cccccccaacactcgtactgcacagaggtgggtagctttaagtttatagctccaagatgc

ctcgatgaagaagcccccgccgaccagcgacgcggtgtacacgtgggcaccttggagcgc

ccacccaaagtgtactgcgatggctcagagtacgacgtgctgaactttgcctccggaggt

tgctggcctcgcaggattcgggtttggaacggccaggattttcggggcgatggattcaac

cccagatttgagagatttcacgtgtacgacatagtagagacttcggagagcgcctcacac

gatgacccatccaggtttgctgagctatcccgcccaagcgggtctgtggtaacactgctg

ggaatgagcgagtgtggaaagcgagtcgcggttcacgtctatggtgtgcgccattatttt

tacatggcaaaggcggaggtggatagcgcttgtggaataaccaccgaggcagaactcgtg

cgcgcaatggtggactgcgcgcacagctcggctttgagcgcggccctgggaaatggcaac

ggcggcaaacagagcggcggcagcggcgggggatggtggggcggaaagcacgtgtctgcg

gactgcttcaaagtggagaccgtgtgccacacgacgctgtactactttggatctaagcca

gctctctactatagagtatctgcctccagcagccgcctgggagggttcatctgcgacaac

tttcacccggagattacaaaattcgaggggagcgtggacgtgaccacgcggctgttgttg

gacaatgaaaattttaccagtttcgggtggtaccgcctgcgacccggcacccacggagag

cgtgttcaacttcgccccgttgagcgacacgtcacctcaagcgacgtggagattaactgt

actcccgataacctggagccgataccagacgaggctgcctggcccgactataagctcatg

tgctttgatatagagtgtaaagctggaacgggtaacgaaatggcgttcccagtggcaact

aaccaagaggacctggtcatccagatctcctgtctgctgtactcgcttgctactcagaac

cacgaacacaccctgctgttttccctcgggtcatgcgatatctctgaggaatactcgttt

gcatgcgtccagcgcggcgagcccagaccgacggttttggagtttgacagcgagtacgag

ctgctggttgccttcctgacctttctcaagcagtactctcccgagttcgccaccggctac

aacatcgttaattttgactgggcgtacatagttaacaaggtaacgtcggtgtataacatc

aagctggacgggtacggcaagttcaacaaaggggggctgtttaaggtgtgggacatcgcc

acgaaccattttcagaagaagagcaaggtgaaaatcaatggcctgatatctctagacatg

tattctgtggcgacggaaaagctaaagctacccagctacaaactcgacgcggtcgtggga

gacgtcctcggcgagcataagatagaccttccctataaagaaataccctcctattacgcg

ggagggcctgaccggaggggcgtaataggagagtattgtatccaggactctaggctggtg

ggcaagctgttttttaagtacctcccccatctggaactatcggcggtggccaaactcgcc

cgtatcaccctaacgcgggtaatttttgacggtcagcaaattcgcgtgtacacgtgcttg

ctgaaactcgcccgcgagagaaatttcattttgccagacaacagacgccggtttgacagt

caggcagacgccgcgtcagagacttcggagttggctatggatagccaaagccacgccttc

gacagtacagacgaacccgacggtgtggacggtaccccggacgccgcaggatctggcgct

acttctgaaaacggaggcgggaagcccggcgtcgggagggccgtgggctaccagggagca

aaggttctagaccccgtatccggctttcatgtggaccccgtggttgtgtttgacttcgct

agcttatacccaagcattatccaggcccataacctctgtttcaccaccctggcgctcgat

gaagtggatctggccgggcttcaaccatccgtcgactactcgacgttcgaggtgggtgac

caaaagttattttttgtccacgcccatattcgcgaaagcctgcttggcatcttgctgcgc

gactggctggccatgcgaaaggcggtgagggcgcgaatccccaccagcacccccgaggag

gcagttttactagataagcagcagtctgcgattaaggtgatatgcaactcggtttacgga

ttcacgggggtggcaaacggcctgttgccgtgtctgaggatagcggctaccgttaccacg

ataggacgcgacatgctcctcaagacccgagattacgttcactctcgttgggcgacgcgc

gagctgctggaggacaattttccaggggctataggtttccgaaaccacaagccttactcc

gtcagggttatctacggagacaccgactccgtgtttatcaagtttgtgggcctgacgtac

gagggggtatcggagctgggggatgctatgtcgcgtcagatttcagcggacctctttaga

gcgcccatcaaactggagtgtgagaagacctttcagcgactgctgctgatcaccaagaag

aagtacataggtgtcataaacggggggaagatgctcatgaagggggtcgacctggtccgc

aaaaataactgctctttcataaacttgtacgcgcgacatctggtagatcttttgttgtac

gacgaggatgtggccacggcggcagcagaggtgacagacgtgcctcccgcagaatgggtg

gggcgcccgctaccgagcggctttgacaagtttgggcgagtgctggtagaggcgtacaac

cgtatcactgcccccaacttggacgtgcgcgagttcgttatgactgctgagctgagccgc

tcacccgaatcgtataccaacaagcgcctgccgcacctcaccgtctactttaagctcgcc

atgaggaatgaagaactgcccagcgtaaaagagagaattccgtatgtgatagttgcgcag

accgaggccgcggaacgcgaagcgggtgtagtaaactcaatgcgcggtaccgcccaaaac

cccgtggtaaccaagaccgcacgcccccaacctaaacgcaaactgctggtttccgacctc

gccgaagacccgacctatgtttccgagaatgacgtaccgctaaacacagactactatttc

tcccacctgttgggtaccataagcgtgacctttaaggctctattcggaaatgatgtgaga

acaacagaaaatcttttaaagcggtttattccggaaaccccccacaagacccccacgaaa

acccaggcactgcttgagcgcgccggctttgaaaagctgacgccctttacaccggaggaa

gaaagtcgtcgaatactgcatacagttttttgtactctagaagcagctccccatcaaagc

tga

>KF434378.1

atggcggcgcgcgaacaggccaactccgttcgcaggagcgggttttttaatccgtttatt

ggcaagaggccatttttcaggccgggaagcgggcaaaccgcggagacagagcgcccaagg

cccccccaacactcgtactgcacagaggtgggtagctttaagtttatagctccaagatgc

ctcgatgaagaagcccccgccgaccagcgacgcggtgtacacgtgggcaccttggagcgc

ccacccaaagtgtactgcgatggctcagagtacgacgtgctgaactttgcctccggaggt

tgctggcctcgcaggattcgggtttggaacggccaggattttcggggcgatggattcaac

cccagatttgagagatttcacgtgtacgacatagtagagacttcggagagcgcctcacac

gatgacccatccaggtttgctgagctatcccgcccaagcgggtctgtggtaacactgctg

ggaatgagcgagtgtggaaagcgagtcgcggttcacgtctatggtgtgcgccattatttt

tacatggcaaaggcggaggtggatagcgcttgtggaataaccaccgaggcagaactcgtg

cgcgcaatggtggactgcgcgcacagctcggctttgagcgcggccctgggaaatggcaac

ggcggcaaacagagcggcggcagcggcgggggatggtggggcggaaagcacgtgtctgcg

gactgcttcaaagtggagaccgtgtgccacacgacgctgtactactttggatctaagcca

gctctctactatagagtatctgcctccagcagccgcctgggagggttcatctgcgacaac

tttcacccggagattacaaaattcgaggggagcgtggacgtgaccacgcggctgttgttg

gacaatgaaaattttaccagtttcgggtggtaccgcctgcgacccggcacccacggagag

cgtgttcaacttcgccccgttgagcgacacgtcacctcaagcgacgtggagattaactgt

actcccgataacctggagccgataccagacgaggctgcctggcccgactataagctcatg

tgctttgatatagagtgtaaagctggaacgggtaacgaaatggcgttcccagtggcaact

aaccaagaggacctggtcatccagatctcctgtctgctgtactcgcttgctactcagaac

cacgaacacaccctgctgttttccctcgggtcatgcgatatctctgaggaatactcgttt

gcatgcgtccagcgcggcgagcccagaccgacggttttggagtttgacagcgagtacgag

ctgctggttgccttcctgacctttctcaagcagtactctcccgagttcgccaccggctac

aacatcgttaattttgactgggcgtacatagttaacaaggtaacgtcggtgtataacatc

aagctggacgggtacggcaagttcaacaaaggggggctgtttaaggtgtgggacatcgcc

acgaaccattttcagaagaagagcaaggtgaaaatcaatggcctgatatctctagacatg

tattctgtggcgacggaaaagctaaagctacccagctacaaactcgacgcggtcgtggga

gacgtcctcggcgagcataagatagaccttccctataaagaaataccctcctattacgcg

ggagggcctgaccggaggggcgtaataggagagtattgtatccaggactctaggctggtg

ggcaagctgttttttaagtacctcccccatctggaactatcggcggtggccaaactcgcc

cgtatcaccctaacgcgggtaatttttgacggtcagcaaattcgcgtgtacacgtgcttg

ctgaaactcgcccgcgagagaaatttcattttgccagacaacagacgccggtttgacagt

caggcagacgccgcgtcagagacttcggagttggctatggatagccaaagccacgccttc

gacagtacagacgaacccgacggtgtggacggtaccccggacgccgcaggatctggcgct

acttctgaaaacggaggcgggaagcccggcgtcgggagggccgtgggctaccagggagca

aaggttctagaccccgtatccggctttcatgtggaccccgtggttgtgtttgacttcgct

agcttatacccaagcattatccaggcccataacctctgtttcaccaccctggcgctcgat

gaagtggatctggccgggcttcaaccatccgtcaactactcgacgttcgaggtgggtgac

caaaagttattttttgtccacgcccatattcgcgaaagcctgcttggcatcttgctgcgc

gactggctggccatgcgaaaggcggtgagggcgcgaatccccaccagcacccccgaggag

gcagttttactagataagcagcagtctgcgattaaggtgatatgcaactcggtttacgga

ttcacgggggtggcaaacggcctgttgccgtgtctggggatagcggctaccgttaccacg

ataggacgcgacatgctcctcaagacccgagattacgttcactctcgttgggcgacgcgc

gagctgctggaggacaattttccaggggctataggtttccgaaaccacaagccttactcc

gtcagggttatctacggagacaccgactccgtgtttatcaagtttgtgggcctgacgtac

gagggggtatcggagctgggggatgctatgtcgcgtcagatttcagcggacctctttaga

gcgcccatcaaactggagtgtgagaagacctttcagcgactgctgctgatcaccaagaag

aagtacataggtgtcataaacggggggaagatgctcatgaagggggtcgacctggtccgc

aaaaataactgctctttcataaacttgtacgcgcgacatctggtagatcttttgttgtac

gacgaggatgtggccacggcggcagcagaggtgacagacgtgcctcccgcagaatgggtg

gggcgcccgctaccgagcggctttgacaagtttgggcgagtgctggtagaggcgtacaac

cgtatcactgcccccaacttggacgtgcgcgagttcgttatgactgctgagctgagccgc

tcacccgaatcgtataccaacaagcgcctgccgcacctcaccgtctactttaagctcgcc

atgaggaatgaagaactgcccagcgtaaaagagagaattccgtatgtgatagttgcgcag

accgaggccgcggaacgcgaagcgggtgtagtaaactcaatgcgcggtaccgcccaaaac

cccgtggtaaccaagaccgcacgcccccaacctaaacgcaaactgctggtttccgacctc

gccgaagacccgacctatgtttccgagaatgacgtaccgctaaacacagactactatttc

tcccacctgttgggtaccataagcgtgacctttaaggctctattcggaaatgatgtgaga

acaacagaaaatcttttaaagcggtttattccggaaaccccccacaagacccccacgaaa

acccaggcactgcttgagcgcgccggctttgaaaagctgacgccctttacaccggaggaa

gaaagtcgtcgaatactgcatacagttttttgtactctagaagcagctccccatcaaagc

tga

>KF434379.1

atggcggcgcgcgaacaggccaactccgttcgcaggagcgggttttttaatccgtttatt

ggcaagaggccatttttcaggccgggaagcgggcagaccgcggagacagagcgcccaagg

cccccccaacactcgtactgcacagaggtgggtagctttaagtttatagctccaagatgc

ctcgatgaagaagcccccgccgaccagcgacgcggtgtacacgtgggcaccttggagcgc

ccacccaaagtgtactgcgatggctcagagtacgacgtgctgaactttgcctccggaggt

tgctggcctcgcaggattcgggtttggaacggccaggattttcggggcgatggattcaac

cccagatttgagagatttcacgtgtacgacatagtagagacttcggagagcgcctcacac

gatgacccatccaggtttgctgagctatcccgcccaagcgggtctgtggtaacactgctg

ggaatgagcgagtgtggaaagcgagtcgcggttcacgtctatggtgtgcgccattatttt

tacatggcaaaggcggaggtggatagcgcttgtggaataaccaccgaggcagaactcgtg

cgcgcaatggtggactgcgcgcacagctcggctttgagcgcggccctgggaaatggcaac

ggcggcaaacagagcggcggcagcggcgggggatggtggggcggaaagcacgtgtctgcg

gactgcttcaaagtggagaccgtgtgccgcacgacgctgtactactttggatctaagcca

gctctctactatagagtatctgcctccagcagccgcctgggagggttcatctgcgacaac

tttcacccggagattacaaaattcgaggggagcgtggacgtgaccacgcggctgttgttg

gacaatgaaaattttaccagtttcgggtggtaccgcctgcgacccggcacccacggagag

cgtgttcaacttcgccccgttgagcgacacgtcacctcaagcgacgtggagattaactgt

actcccgataacctggagccgataccagacgaggctgcctggcccgactataagctcatg

tgctttgatatagagtgtaaagctggaacgggtaacgaaatggcgttcccagtggcaact

aaccaagaggacctggtcatccagatctcctgtctgctgtactcgcttgctactcagaac

cacgaacacaccctgctgttttccctcgggtcatgcgatatctctgaggaatactcgttt

gcatgcgtccagcgcggcgagcccagaccgacggttttggagtttgacagcgagtacgag

ctgctggttgccttcctgacctttctcaagcagtactctcccgagttcgccaccggctac

aacatcgttaattttgactgggcgtacatagttaacaaggtaacgtcggtgtataacatc

aagctggacgggtacggcaagttcaacaaaggggggctgtttaaggtgtgggacatcgcc

acgaaccattttcagaagaagagcaaggtgaaaatcaatggcctgatatctctagacatg

tattctgtggcgacggaaaagctaaagctacccagctacaaactcgacgcggtcgtggga

gacgtcctcggcgagcataagatagaccttccctataaagaaataccctcctattacgcg

ggagggcctgaccggaggggcgtaataggagagtattgtatccaggactctaggctggtg

ggcaagctgttttttaagtacctcccccatctggaactatcggcggtggccaaactcgcc

cgtatcaccctaacgcgggtaatttttgacggtcagcaaattcgcgtgtacacgtgcttg

ctgaaactcgcccgcgagagaaatttcattttgccagacaacagacgccggtttgacagt

caggcagacgccgcgtcagagacttcggagttggctatggatagccaaagccacgccttc

gacagtacagacgaacccgacggtgtggacggtaccccggacgccgcaggatctggcgct

acttctgaaaacggaggcgggaagcccggcgtcgggagggccgtgggctaccagggagca

aaggttctagaccccgtatccggctttcatgtggaccccgtggttgtgtttgacttcgct

agcttatacccaagcattatccaggcccataacctctgtttcaccaccctggcgctcgat

gaagtggatctggccgggcttcaaccatccgtcaactactcgacgttcgaggtgggtgac

caaaagttattttttgtccacgcccatattcgcgaaagcctgcttggcatcttgctgcgc

gactggctggccatgcgaaaggcggtgagggcgcgaatccccaccagcacccccgaggag

gcagttttactagataagcagcagtctgcgattaaggtgatatgcaactcggtttacgga

ttcacgggggtggcaaacggcctgttgccgtgtctgaggatagcggctaccgttaccacg

ataggacgcgacatgctcctcaagacccgagattacgttcactctcgttgggcgacgcgc

gagctgctggaggacaattttccaggggctataggtttccgaaaccacaagccttactcc

gtcagggttatctacggagacaccgactccgtgtttatcaagtttgtgggcctgacgtac

gagggggtatcggagctgggggatgctatgtcgcgtcagatttcagcggacctctttaga

gcgcccatcaaactggagtgtgagaagacctttcagcgactgctgctgatcaccaagaag

aagtacataggtgtcataaacggggggaagatgctcatgaagggggtcgacctggtccgc

aaaaataactgctctttcataaacttgtacgcgcgacatctggtagatcttttgttgtac

gacgaggatgtggccacggcggcagcagaggtgacagacgtgcctcccgcagaatgggtg

gggcgcccgctaccgagcggctttgacaagtttgggcgagtgctggtagaggcgtacaac

cgtatcactgcccccaacttggacgtgcgcgagttcgttatgactgctgagctgagccgc

tcacccgaatcgtataccaacaagcgcctgccgcacctcaccgtctactttaagctcgcc

atgaggaatgaagaactgcccagcgtaaaagagagaattccgtatgtgatagttgcgcag

accgaggccgcggaacgcgaagcgggtgtagtaaactcaatgcgcggtaccgcccaaaac

cccgtggtaaccaagaccgcacgcccccaacctaaacgcaaactgctggtttccgacctc

gccgaagacccgacctatgtttccgagaatgacgtaccgctaaacacagactactatttc

tcccacctgttgggtaccataagcgtgacctttaaggctctattcggaaatgatgtgaga

acaacagaaaatcttttaaagcggtttattccggaaaccccccacaagacccccacgaaa

acccaggcactgcttgagcgcgccggctttgaaaagctgacgccctttacaccggaggaa

gaaagtcgtcgaatactgcatacagttttttgtactctagaagcagctccccatcaaagc

tga

>KF434380.1

atggcggcgcgcgaacaggccaactccgttcgcaggagcgggttttttaatccgtttatt

ggcaagaggccatttttcaggccgggaagcgggcagaccgcggagacagagcgcccaagg

cccccccaacactcgtactgcacagaggtgggcagctttaagtttatagctccaagatgc

ctcgatgaagaagcccccgccgaccagcgacgcggtgtacacgtgggcaccttggagcgc

ccacccaaagtgtactgcgatggctcagagtacgacgtgctgaactttgcctccggaggt

tgctggcctcgcaggattcgggtttggaacggccaggattttcggggcgatggattcaac

cccagatttgagagatttcacgtgtacgacatagtagagacttcggagagcgcctcacac

gatgacccatccaggtttgctgagctatcccgcccaagcgggtctgtggtaacactgctg

ggaatgagcgagtgtggaaagcgagtcgcggttcacgtctatggtgtgcgccattatttt

tacatggcaaaggcggaggtggatagcgcttgtggaataaccaccgaggcagaactcgtg

cgcgcaatggtggactgcgcgcacagctcggctttgagcgcggccctgggaaatggcaac

ggcggcaaacagagcggcggcagcggcgggggatggtggggcggaaagcacgtgtctgcg

gactgcttcaaagtggagaccgtgtgccacacgacgctgtactactttggatctaagcca

gctctctactatagagtatctgcctccagcagccgcctgggagggttcatctgcgacaac

tttcacccggagattacaaaattcgaggggagcgtggacgtgaccacgcggctgttgttg

gacaatgaaaattttaccagtttcgggtggtaccgcctgcgacccggcacccacggagag

cgtgttcaacttcgccccgttgagcgacacgtcacctcaagcgacgtggagattaactgt

actcccgataacctggagccgataccagacgaggctgcctggcccgactataagctcatg

tgctttgatatagagtgtaaagctggaacgggtaacgaaatggcgttcccagtggcaact

aaccaagaggacctggtcatccagatctcctgtctgctgtactcgcttgctactcagaac

cacgaacacaccctgctgttttccctcgggtcatgcgatatctctgaggaatactcgttt

gcatgcgtccagcgcggcgagcccagaccgacggttttggagtttgacagcgagtacgag

ctgctggttgccttcctgacctttctcaagcagtactctcccgagttcgccaccggctac

aacatcgttaattttgactgggcgtacatagttaacaaggtaacgtcggtgtataacatc

aagctggacgggtacggcaagttcaacaaaggggggctgtttaaggtgtgggacatcgcc

acgaaccattttcagaagaagagcaaggtgaaaatcaatggcctgatatctctagacatg

tattctgtggcgacggaaaagctaaagctacccagctacaaactcgacgcggtcgtggga

gacgtcctcggcgagcataagatagaccttccctataaagaaataccctcctattacgcg

ggagggcctgaccggaggggcgtaataggagagtattgtatccaggactctaggctggtg

ggcaagctgttttttaagtacctcccccatctggaactatcggcggtggccaaactcgcc

cgtatcaccctaacgcgggtaatttttgacggtcagcaaattcgcgtgtacacgtgcttg

ctgaaactcgcccgcgagagaaatttcattttgccagacaacagacgccggtttgacagt

caggcagacgccgcgtcagagacttcggagttggctatggatagccaaagccacgccttc

gacagtacagacgaacccgacggtgtggacggtaccccggacgccgcaggatctggcgct

acttctgaaaacggaggcgggaagcccggcgtcgggagggccgtgggctaccagggagca

aaggttctagaccccgtatccggctttcatgtggaccccgtggttgtgtttgacttcgct

agcttatacccaagcattatccaggcccataacctctgtttcaccaccctggcgctcgat

gaagtggatctggccgggcttcaaccatccgtcaactactcgacgttcgaggtgggtgac

caaaagttattttttgtccacgcccatattcgcgaaagcctgcttggcatcttgctgcgc

gactggctggccatgcgaaaggcggtgagggcgcgaatccccaccagcacccccgaggag

gcagttttactagataagcagcagtctgcgattaaggtgatatgcaactcggtttacgga

ttcacgggggtggcaaacggcctgttgccgtgtctgaggatagcggctaccgttaccacg

ataggacgcgacatgctcctcaagacccgagattacgttcactctcgttgggcgacgcgc

gagctgctggaggacaattttccaggggctataggtttccgaaaccacaagccttactcc

gtcagggttatctacggagacaccgactccgtgtttatcaagtttgtgggcctgacgtac

gagggggtatcggagctgggggatgctatgtcgcgtcagatttcagcggacctctttaga

gcgcccatcaaactggagtgtgagaagacctttcagcgactgctgctgatcaccaagaag

aagtacataggtgtcataaacggggggaagatgctcatgaagggggtcgacctggtccgc

aaaaataactgctctttcataaacttgtacgcgcgacatctggtagatcttttgttgtac

gacgaggatgtggccacggcggcagcagaggtgacagacgtgcctcccgcagaatgggtg

gggcgcccgctaccgagcggctttgacaagtttgggcgagtgctggtagaggcgtacaac

cgtatcactgcccccaacttggacgtgcgcgagttcgttatgactgctgagctgagccgc

tcacccgaatcgtataccaacaagcgcctgccgcacctcaccgtctactttaagctcgcc

atgaggaatgaagaactgcccagcgtaaaagagagaattccgtatgtgatagttgcgcag

accgaggccgcggaacgcgaagcgggtgtagtaaactcaatgcgcggtaccgcccaaaac

cccgtggtaaccaagaccgcacgcccccaacctaaacgcaaactgctggtttccgacctc

gccgaagacccgacctatgtttccgagaatgacgtaccgctaaacacagactactatttc

tcccacctgttgggtaccataagcgtgacctttaaggctctattcggaaatgatgtgaga

acaacagaaaatcttttaaagcggtttattccggaaaccccccacaagacccccacgaaa

acccaggcactgcttgagcgcgccggctttgaaaagctgacgccctttacaccggaggaa

gaaagtcgtcgaatactgcatacagttttttgtactctagaagcagctccccatcaaagc

tga

>KF434381.1

atggcggcgcgcgaacaggccaactccgttcgcaggagcgggttttttaatccgtttatt

ggcaagaggccatttttcaggccgggaagcgggcagaccgcggagacagagcgcccaagg

cccccccaacactcgtactgcacagaggtgggtagctttaagtttatagctccaagatgc

ctcgatgaagaagcccccgccgaccagcgacgcggtgtacacgtgggcaccttggagcgc

ccacccaaagtgtactgcgatggctcagagtacgacgtgctgaactttgcctccggaggt

tgctggcctcgcaggattcgggtttggaacggccaggattttcggggcgatggattcaac

cccagatttgagagatttcacgtgtacgacatagtagagacttcggagagcgcctcacac

gatgacccatccaggtttgctgagctatcccgcccaagcgggtctgtggtaacactgctg

ggaatgagcgagtgtggaaagcgagtcgcggttcacgtctatggtgtgcgccattatttt

tacatggcaaaggcggaggtggatagcgcttgtggaataaccaccgaggcagaactcgtg

cgcgcaatggtggactgcgcgcacagctcggctttgagcgcggccctgggaaatggcaac

ggcggcaaacagagcggcggcagcggcgggggatggtggggcggaaagcacgtgtctgcg

gactgcttcaaagtggagaccgtgtgccacacgacgctgtactactttggatctaagcca

gctctctactatagagtatctgcctccagcagccgcctgggagggttcatctgcgacaac

tttcacccggagattacaaaattcgaggggagcgtggacgtgaccacgcggctgttgttg

gacaatgaaaattttaccagtttcgggtggtaccgcctgcgacccggcacccacggagag

cgtgttcaacttcgccccgttgagcgacacgtcacctcaagcgacgtggagattaactgt

actcccgataacctggagccgataccagacgaggctgcctggcccgactataagctcatg

tgctttgatatagagtgtaaagctggaacgggtaacgaaatggcgttcccagtggcaact

aaccaagaggacctggtcatccagatctcctgtctgctgtactcgcttgctactcagaac

cacgaacacaccctgctgttttccctcgggtcatgcgatatctctgaggaatactcgttt

gcatgcgtccagcgcggcgagcccagaccgacggttttggagtttgacagcgagtacgag

atgctggttgccttcctgacctttctcaagcagtactctcccgagttcgccaccggctac

aacatcgttaattttgactgggcgtacatagttaacaaggtaacgtcggtgtataacatc

aagctggacgggtacggcaagttcaacaaaggggggctgtttaaggtgtgggacatcgcc

acgaaccattttcagaagaagagcaaggtgaaaatcaatggcctgatatctctagacatg

tattctgtggcgacggaaaagctaaagctacccagctacaaactcgacgcggtcgtggga

gacgtcctcggcgagcataagatagaccttccctataaagaaataccctcctattacgcg

ggagggcctgaccggaggggcgtaataggagagtattgtatccaggactctaggctggtg

ggcaagctgttttttaagtacctcccccatctggaactatcggcggtggccaaactcgcc

cgtatcaccctaacgcgggtaatttttgacggtcagcaaattcgcgtgtacacgtgcttg

ctgaaactcgcccgcgagagaaatttcattttgccagacaacagacgccggtttgacagt

caggcagacgccgcgtcagagacttcggagttggctatggatagccaaagccacgccttc

gacagtacagacgaacccgacggtgtggacggtaccccggacgccgcaggatctggcgct

acttctgaaaacggaggcgggaagcccggcgtcgggagggccgtgggctaccagggagca

aaggttctagaccccgtatccggctttcatgtggaccccgtggttgtgtttgacttcgct

agcttatacccaagcattatccaggcccataacctctgtttcaccaccctggcgctcgat

gaagtggatctggccgggcttcaaccatccgtcaactactcgacgttcgaggtgggtgac

caaaagttattttttgtccacgcccatattcgcgaaagcctgcttggcatcttgctgcgc

gactggctggccatgcgaaaggcggtgagggcgcgaatccccaccagcacccccgaggag

gcagttttactagataagcagcagtctgcgattaaggtgatatgcaactcggtttacgga

ttcacgggggtggcaaacggcctgttgccgtgtctgaggatagcggctaccgttaccacg

ataggacgcgacatgctcctcaagacccgagattacgttcactctcgttgggcgacgcgc

gagctgctggaggacaattttccaggggctataggtttccgaaaccacaagccttactcc

gtcagggttatctacggagacaccgactccgtgtttatcaagtttgtgggcctgacgtac

gagggggtatcggagctgggggatgctatgtcgcgtcagatttcagcggacctctttaga

gcgcccatcaaactggagtgtgagaagacctttcagcgactgctgctgatcaccaagaag

aagtacataggtgtcataaacggggggaagatgctcatgaagggggtcgacctggtccgc

aaaaataactgctctttcataaacttgtacgcgcgacatctggtagatcttttgttgtac

gacgaggatgtggccacggcggcagcagaggtgacagacgtgcctcccgcagaatgggtg

gggcgcccgctaccgagcggctttgacaagtttgggcgagtgctggtagaggcgtacaac

cgtatcactgcccccaacttggacgtgcgcgagttcgttatgactgctgagctgagccgc

tcacccgaatcgtataccaacaagcgcctgccgcacctcaccgtctactttaagctcgcc

atgaggaatgaagaactgcccagcgtaaaagagagaattccgtatgtgatagttgcgcag

accgaggccgcggaacgcgaagcgggtgtagtaaactcaatgcgcggtaccgcccaaaac

cccgtggtaaccaagaccgcacgcccccaacctaaacgcaaactgctggtttccgacctc

gccgaagacccgacctatgtttccgagaatgacgtaccgctaaacacagactactatttc

tcccacctgttgggtaccataagcgtgacctttaaggctctattcggaaatgatgtgaga

acaacagaaaatcttttaaagcggtttattccggaaaccccccacaagacccccacgaaa

acccaggcactgcttgagcgcgccggctttgaaaagctgacgccctttacaccggaggaa

gaaagtcgtcgaatactgcatacagttttttgtactctagaagcagctccccatcaaagc

tga

>KF434382.1

atggcggcgcgcgaacaggccaactccgttcgcaggagcgggttttttaatccgtttatt

ggcaagaggccatttttcaggccgggaagcgggcaaaccgcggagacagagcgcccaagg

cccccccaacactcgtactgcacagaggtgggtagctttaagtttatagctccaagatgc

ctcgatgaagaagcccccgccgaccagcgacgcggtgtacacgtgggcaccttggagcgc

ccacccaaagtgtactgcgatggctcagagtacgacgtgctgaactttgcctccggaggt

tgctggcctcgcaggattcgggtttggaacggccaggattttcggggcgatggattcaac

cccagatttgagagatttcacgtgtacgacatagtagagacttcggagagcgcctcacac

gatgacccatccaggtttgctgagctatcccgcccaagcgggtctgtggtaacactgctg

ggaatgagcgagtgtggaaagcgagtcgcggttcacgtctatggtgtgcgccattatttt

tacatggcaaaggcggaggtggatagcgcttgtggaataaccaccgaggcagaactcgtg

cgcgcaatggtggactgcgcgcacagctcggctttgagcgcggccctgggaaatggcaac

ggcggcaaacagagcggcggcagcggcgggggatggtggggcggaaagcacgtgtctgcg

gactgcttcaaagtggagaccgtgtgccacacgacgctgtactactttggatctaagcca

gctctctactatagagtatctgcctccagcagccgcctgggagggttcatctgcgacaac

tttcacccggagattacaaaattcgaggggagcgtggacgtgaccacgcggctgttgttg

gacaatgaaaattttaccagtttcgggtggtaccgcctgcgacccggcacccacggagag

cgtgttcaacttcgccccgttgagcgacacgtcacctcaagcgacgtggagattaactgt

actcccgataacctggagccgataccagacgaggctgcctggcccgactataagctcatg

tgctttgatatagagtgtaaagctggaacgggtaacgaaatggcgttcccagtggcaact

aaccaagaggacctggtcatccagatctcctgtctgctgtactcgcttgctactcagaac

cacgaacacaccctgctgttttccctcgggtcatgcgatatctctgaggaatactcgttt

gcatgcgtccagcgcggcgagcccagaccgacggttttggagtttgacagcgagtacgag

ctgctggttgccttcctgacctttctcaagcagtactctcccgagttcgccaccggctac

aacatcgttaattttgactgggcgtacatagttaacaaggtaacgtcggtgtataacatc

aagctggacgggtacggcaagttcaacaaaggggggctgtttaaggtgtgggacatcgcc

acgaaccattttcagaagaagagcaaggtgaaaatcaatggcctgatatctctagacatg

tattctgtggcgacggaaaagctaaagctacccagctacaaactcgacgcggtcgtggga

gacgtcctcggcgagcataagatagaccttccctataaagaaataccctcctattacgcg

ggagggcctgaccggaggggcgtaataggagagtattgtatccaggactctaggctggtg

ggcaagctgttttttaagtacctcccccatctggaactatcggcggtggccaaactcgcc

cgtatcaccctaacgcgggtaatttttgacggtcagcaaattcgcgtgtacacgtgcttg

ctgaaactcgcccgcgagagaaatttcattttgccagacaacagacgccggtttgacagt

caggcagacgccgcgtcagagacttcggagttggctatggatagccaaagccacgccttc

gacagtacagacgaacccgacggtgtggacggtaccccggacgccgcaggatctggcgct

acttctgaaaacggaggcgggaagcccggcgtcgggagggccgtgggctaccagggagca

aaggttctagaccccgtatccggctttcatgtggaccccgtggttgtgtttgacttcgct

agcttatacccaagcattatccaggcccataacctctgtttcaccaccctggcgctcgat

gaagcggatctggccgggcttcaaccatccgtcaactactcgacgttcgaggtgggtgac

caaaagttattttttgtccacgcccatattcgcgaaagcctgcttggcatcttgctgcgc

gactggctggccatgcgaaaggcggtgagggcgcgaatccccaccagcacccccgaggag

gcagttttactagataagcagcagtctgcgattaaggtgatatgcaactcggtttacgga

ttcacgggggtggcaaacggcctgttgccgtgtctgaggatagcggctaccgttaccacg

ataggacgcgacatgctcctcaagacccgagattacgttcactctcgttgggcgacgcgc

gagctgctggaggacaattttccaggggctataggtttccgaaaccacaagccttactcc

gtcagggttatctacggagacaccgactccgtgtttatcaagtttgtgggcctgacgtac

gagggggtatcggagctgggggatgctatgtcgcgtcagatttcagcggacctctttaga

gcgcccatcaaactggagtgtgagaagacctttcagcgactgctgctgatcaccaagaag

aagtacataggtgtcataaacggggggaagatgctcatgaagggggtcgacctggtccgc

aaaaataactgctctttcataaacttgtacgcgcgacatctggtagatcttttgttgtac

gacgaggatgtggccacggcggcagcagaggtgacagacgtgcctcccgcagaatgggtg

gggcgcccgctaccgagcggctttgacaagtttgggcgagtgctggtagaggcgtacaac

cgtatcactgcccccaacttggacgtgcgcgagttcgttatgactgctgagctgagccgc

tcacccgaatcgtataccaacaagcgcctgccgcacctcaccgtctactttaagctcgcc

atgaggaatgaagaactgcccagcgtaaaagagagaattccgtatgtgatagttgcgcag

accgaggccgcggaacgcgaagcgggtgtagtaaactcaatgcgcggtaccgcccaaaac

cccgtggtaaccaagaccgcacgcccccaacctaaacgcaaactgctggtttccgacctc

gccgaagacccgacctatgtttccgagaatgacgtaccgctaaacacagactactatttc

tcccacctgttgggtaccataagcgtgacctttaaggctctattcggaaatgatgtgaga

acaacagaaaatcttttaaagcggtttattccggaaaccccccacaagacccccacgaaa

acccaggcactgcttgagcgcgccggctttgaaaagctgacgccctttacaccggaggaa

gaaagtcgtcgaatactgcatacagttttttgtactctagaagcagctccc---------

---

>KF515985.1

atggcggcgcgcgaacaggccaactccgttcgcaggagcgggttttttaatccgtttatt

ggcaagaggccatttttcaggccgggaagcgggcagaccgcggagacagagcgcccaagg

cccccccaacactcgtactgcacagaggtgggtagctttaagtttatagctccaagatgc

ctcgatgaagaagcccccgccgaccagcgacgcggtgtacacgtgggcaccttggagcgc

ccacccaaagtgtactgcgatggctcagagtacgacgtgctgaactttgcctccggaggt

tgctggcctcgcaggattcgggtttggaacggccaggattttcggggcgatggattcaac

cccagatttgagagatttcacgtgtacgacatagtagagacttcggagagcgcctcacac

gatgacccatccaggtttgctgagctatcccgcccaagtgggtctgtggtaacactgctg

ggaatgagcgagtgtggaaagcgagtcgcggttcacgtctatggtgtgcgccattatttt

tacatggcaaaggcggaggtggatagcgcttgtggaataaccaccgaggcagaactcgtg

cgcgcaatggtggactgcgcgcacagctcggctttgagcgcggccctgggaaatggcaac

ggcggcaaacagagcggcggcagcggcgggggatggtggggcggaaagcacgtgtctgcg

gactgcttcaaagtggagaccgtgtgccacacgacgctgtactactttggatctaagcca

gctctctactatagagtatctgcctccagcagccgcctgggagggttcatctgcgacaac

tttcacccggagattacaaaattcgaggggagcgtggacgtgaccacgcggctgttgttg

gacaatgaaaattttaccagtttcgggtggtaccgcctgcgacccggcacccacggagag

cgtgttcaacttcgccccgttgagcgacacgtcacctcaagcgacgtggagattaactgt

actcccgataacctggagccgataccagacgaggctgcctggcccgactataagctcatg

tgctttgatatagagtgtaaagctggaacgggtaacgaaatggcgttcccagtggcaact

aaccaagaggacctggtcatccagatctcctgtctgctgtactcgcttgctactcagaac

cacgaacacaccctgctgttttccctcgggtcatgcgatatctctgaggaatactcgttt

gcatgcgtccagcgcggcgagcccagaccgacggttttggagtttgacagcgagtacgag

ctgctggttgccttcctgacctttctcaagcagtactctcccgagttcgccaccggctac

aacatcgttaattttgactgggcgtacatagttaacaaggtaacgtcggtgtataacatc

aagctggacgggtacggcaagttcaacaaaggggggctgtttaaggtgtgggacatcgcc

acgaaccattttcagaagaagagcaaggtgaaaatcaatggcctgatatctctagacatg

tattctgtggcgacggaaaagctaaagctacccagctacaaactcgacgcggtcgtggga

gacgtcctcggcgagcataagatagaccttccctataaagaaataccctcctattacgcg

ggagggcctgaccggaggggcgtaataggagagtattgtatccaggactctaggctggtg

ggcaagctgttttttaagtacctcccccatctggaactatcggcggtggccaaactcgcc

cgtatcaccctaacgcgggtaatttttgacggtcagcaaattcgcgtgtacacgtgcttg

ctgaaactcgcccgcgagagaaatttcattttgccagacaacagacgccggtttgacagt

caggcagacgccgcgtcagagacttcggagttggctatggatagccaaagccacgccttc

gacagtacagacgaacccgacggtgtggacggtaccccggacgccgcaggatctggcgct

acttctgaaaacggaggcgggaagcccggcgtcgggagggccgtgggctaccagggagca

aaggttctagaccccgtatccggctttcatgtggaccccgtggttgtgtttgacttcgct

agcttatacccaagcattatccaggcccataacctctgtttcaccaccctggcgctcgat

gaagtggatctggccgggcttcaaccatccgtcaactactcgacgttcgaggtgggtgac

caaaagttattttttgtccacgcccatattcgcgaaagcctgcttggcatcttgctgcgc

gactggctggccatgcgaaaggcggtgagggcgcgaatccccaccagcacccccgaggag

gcagttttactagataagcagcagtctgcgattaaggtgatatgcaactcggtttacgga

ttcacgggggtggcaaacggcctgttgccgtgtctgaggatagcggctaccgttaccacg

ataggacgcgacatgctcctcaagacccgagattacgttcactctcgttgggcgacgcgc

gagctgctggaggacaattttccaggggctataggtttccgaaaccacaagccttactcc

gtcagggttatctacggagacaccgactccgtgtttatcaagtttgtgggcctgacgtac

gagggggtatcggagctgggggatgctatgtcgcgtcagatttcagcggacctctttaga

gcgcccatcaaactggagtgtgagaagacctttcagcgactgctgctgatcaccaagaag

aagtacataggtgtcataaacggggggaagatgctcatgaagggggtcgacctggtccgc

aaaaataactgctctttcataaacttgtacgcgcgacatctggtagatcttttgttgtac

gacgaggatgtggccacggcggcagcagaggtgacagacgtgcctcccgcagaatgggtg

gggcgcccgctaccgagcggctttgacaagtttgggcgagtgctggtagaggcgtacaac

cgtatcactgcccccaacttggacgtgcgcgagttcgttatgactgctgagctgagccgc

tcacccgaatcgtataccaacaagcgcctgccgcacctcaccgtctactttaagctcgcc

atgaggaatgaagaactgcccagcgtaaaagagagaattccgtatgtgatagttgcgcag

accgaggccgcggaacgcgaagcgggtgtagtaaactcaatgcgcggtaccgcccaaaac

cccgtggtaaccaagaccgcacgcccccaacctaaacgcaaactgctggtttccgacctc

gccgaagacccgacctatgtttccgagaatgacgtaccgctaaacacagactactatttc

tcccacctgttgggtaccataagcgtgacctttaaggctctattcggaaatgatgtgaga

acaacagaaaatcttttaaagcggtttattccggaaaccccccacaagacccccacgaaa

acccaggcactgcttgagcgcgccggctttgaaaagctgacgccttttacaccggaggaa

gaaagtcgtcgaatactgcatacagttttttgtactctagaagcagctccccatcaaagc

tga

>KF515986.1

atggcggcgcgcgaacaggccaactccgttcgcaggagcgggttttttaatccgtttatt

ggcaagaggccatttttcaggccgggaagcgggcaaaccgcggagacagagcgcccaagg

cccccccaacactcgtactgcacagaggtgggtagctttaagtttatagctccaagatgc

ctcgatgaagaagcccccgccgaccagcgacgcggtgtacacgtgggcaccttggagcgc

ccacccaaagtgtactgcgatggctcagagtacgacgtgctgaactttgcctccggaggt

tgctggcctcgcaggattcgggtttggaacggccaggattttcggggcgatggattcaac

cccagatttgagagatttcacgtgtacgacatagtagagacttcggagagcgcctcacac

gatgacccatccaggtttgctgagctatcccgcccaagcgggtctgtggtaacactgctg

ggaatgagcgagtgtggaaagcgagtcgcggttcacgtctatggtgtgcgccattatttt

tacatggcaaaggcggaggtggatagcgcttgtggaataaccaccgaggcagaactcgtg

cgcgcaatggtggactgcgcgcacagctcggctttgagcgcggccctgggaaatggcaac

ggcggcaaacagagcggcggcagcggcgggggatggtggggcggaaagcacgtgtctgcg

gactgcttcaaagtggagaccgtgtgccacacgacgctgtactactttggatctaagcca

gctctctactatagagtatctgcctccagcagccgcctgggagggttcatctgcgacaac

tttcacccggagattacaaaattcgaggggagcgtggacgtgaccacgcggctgttgttg

gacaatgaaaattttaccagtttcgggtggtaccgcctgcgacccggcacccacggagag

cgtgttcaacttcgccccgttgagcgacacgtcacctcaagcgacgtggagattaactgt

actcccgataacctggagccgataccagacgaggctgcctggcccgactataagctcatg

tgctttgatatagagtgtaaagctggaacgggtaacgaaatggcgttcccagtggcaact

aaccaagaggacctggtcatccagatctcctgtctgctgtactcgcttgctactcagaac

cacgaacacaccctgctgttttccctcgggtcatgcgatatctctgaggaatactcgttt

gcatgcgtccagcgcggcgagcccagaccgacggttttggagtttgacagcgagtacgag

ctgctggttgccttcctgacctttctcaagcagtactctcccgagttcgccaccggctac

aacatcgttaattttgactgggcgtacatagttaacaaggtaacgtcggtgtataacatc

aagctggacgggtacggcaagttcaacaaaggggggctgtttaaggtgtgggacatcgcc

acgaaccattttcagaagaagagcaaggtgaaaatcaatggcctgatatctctagacatg

tattctgtggcgacggaaaagctaaagctacccagctacaaactcgacgcggtcgtggga

gacgtcctcggcgagcataagatagaccttccctataaagaaataccctcctattacgcg

ggagggcctgaccggaggggcgtaataggagagtattgtatccaggactctaggctggtg

ggcaagctgttttttaagtacctcccccatctggaactatcggcggtggccaaactcgcc

cgtatcaccctaacgcgggtaatttttgacggtcagcaaattcgcgtgtacacgtgcttg

ctgaaactcgcccgcgagagaaatttcattttgccagacaacagacgccggtttgacagt

caggcagacgccgcgtcagagacttcggagttggctatggatagccaaagccacgccttc

gacagtacagacgaacccgacggtgtggacggtaccccggacgccgcaggatctggcgct

acttctgaaaacggaggcgggaagcccggcgtcgggagggccgtgggctaccagggagca

aaggttctagaccccgtatccggctttcatgtggaccccgtggttgtgtttgacttcgct

agcttatacccaagcattatccaggcccataacctctgtttcaccaccctggcgctcgat

gaagtggatctggccgggcttcaaccatccgtcgactactcgacgttcgaggtgggtgac

caaaagttattttttgtccacgcccatattcgcgaaagcctgcttggcatcttgctgcgc

gactggctggccatgcgaaaggcggtgagggcgcgaatccccaccagcacccccgaggag

gcagttttactagataagcagcagtctgcgattaaggtgatatgcaactcggtttacgga

ttcacgggggtggcaaacggcctgttgccgtgtctgaggatagcggctaccgttaccacg

ataggacgcgacatgctcctcaagacccgagattacgttcactctcgttgggcgacgcgc

gagctgctggaggacaattttccaggggctataggtttccgaaaccacaagccttactcc

gtcagggttatctacggagacaccgactccgtgtttatcaagtttgtgggcctgacgtac

gagggggtatcggagctgggggatgctatgtcgcgtcagatttcagcggacctctttaga

gcgcccatcaaactggagtgtgagaagacctttcagcgactgctgctgatcaccaagaag

aagtacataggtgtcataaacggggggaagatgctcatgaagggggtcgacctggtccgc

aaaaataactgctctttcataaacttgtacgcgcgacatctggtagatcttttgctgtac

gacgaggatgtggccacggcggcagcagaggtgacagacgtgcctcccgcagaatgggtg

gggcgcccgctaccgagcggctttgacaagtttgggcgagtgctggtagaggcgtacaac

cgtatcactgcccccaacttggacgtgcgcgagttcgttatgactgctgagctgagccgc

tcacccgaatcgtataccaacaagcgcctgccgcacctcaccgtctactttaagctcgcc

atgaggaatgaagaactgcccagcgtaaaagagagaattccgtatgtgatagttgcgcag

accgaggccgcggaacgcgaagcgggtgtagtaaactcaatgcgcggtaccgcccaaaac

cccgtggtaaccaagaccgcacgcccccaacctaaacgcaaactgctggtttccgacctc

gccgaagacccgacctatgtttccgagaatgacgtaccgctaaacacagactactatttc

tcccacctgttgggtaccataagcgtgacctttaaggctctattcggaaatgatgtgaga

acaacagaaaatcttttaaagcggtttattccggaaaccccccacaagacccccacgaaa

acccaggcactgcttgagcgcgccggctttgaaaagctgacgccctttacaccggaggaa

gaaagtcgtcgaatactgcatacagttttttgtactctagaagcagctccccatcaaagc

tga

>KC924782.1

atggcggcgcgcgaacaggccaactccgttcgcaggagcgggttttttaatccgtttatt

ggcaagaggccatttttcaggccgggaagcgggcagaccgcggagacagagcgcccaagg

cccccccaacactcgtactgcacagaggtgggtagctttaagtttatagctccaagatgc

ctcgatgaagaagcccccgccgaccagcgacgcggtgtacacgtgggcaccttggagcgc

ccacccaaagtgtactgcgatggctcagagtacgacgtgctgaactttgcctccggaggt

tgctggcctcgcaggattcgggtttggaacggccaggattttcggggcgatggattcaac

cccagatttgagagatttcacgtgtacgacatagtagagacttcggagagcgcctcacac

gatgacccatccaggtttgctgagctatcccgcccaagcgggtctgtggtaacactgctg

ggaatgagcgagtgtggaaagcgagtcgcggttcacgtctatggtgtgcgccattatttt

tacatggcaaaggcggaggtggatagcgcttgtggaataaccaccgaggcagaactcgtg

cgcgcaatggtggactgcgcgcacagctcggctttgagcgcggccctgggaaatggcaac

ggcggcaaacagagcggcggcagcggcgggggatggtggggcggaaagcacgtgtctgcg

gactgcttcaaagtggagaccgtgtgccacacgacgctgtactactttggatctaagcca

gctctctactatagagtatctgcctccagcagccgcctgggagggttcatctgcgacaac

tttcacccggagattacaaaattcgaggggagcgtggacgtgaccacgcggctgttgttg

gacaatgaaaattttaccagtttcgggtggtaccgcctgcgacccggcacccacggagag

cgtgttcaacttcgccccgttgagcgacacgtcacctcaagcgacgtggagattaactgt

actcccgataacctggagccgataccagacgaggctgcctggcccgactataagctcatg

tgctttgatatagagtgtaaagctggaacgggtaacgaaatggcgttcccagtggcaact

aaccaagaggacctggtcatccagatctcctgtctgctgtactcgcttgctactcagaac

cacgaacacaccctgctgttttccctcgggtcatgcgatatctctgaggaatactcgttt

gcatgcgtccagcgcggcgagcccagaccgacggttttggagtttgacagcgagtacgag

ctgctggttgccttcctgacctttctcaagcagtactctcccgagttcgccaccggctac

aacatcgttaattttgactgggcgtacatagttaacaaggtaacgtcggtgtataacatc

aagctggacgggtacggcaagttcaacaaaggggggctgtttaaggtgtgggacatcgcc

acgaaccattttcagaagaagagcaaggtgaaaatcaatggcctgatatctctagacatg

tattctgtggcgacggaaaagctaaagctacccagctacaaactcgacgcggtcgtggga

gacgtcctcggcgagcataagatagaccttccctataaagaaataccctcctattacgcg

ggagggcctgaccggaggggcgtaataggagagtattgtatccaggactctaggctggtg

ggcaagctgttttttaagtacctcccccatctggaactatcggcggtggccaaactcgcc

cgtatcaccctaacgcgggtaatttttgacggtcagcaaattcgcgtgtacacgtgcttg

ctgaaactcgcccgcgagagaaatttcattttgccagacaacagacgccggtttgacagt

caggcagacgccgcgtcagagacttcggagttggctatggatagccaaagccacgccttc

gacagtacagacgaacccgacggtgtggacggtaccccggacgccgcaggatctggcgct

acttctgaaaacggaggcgggaagcccggcgtcgggagggccgtgggctaccagggagca

aaggttctagaccccgtatccggctttcatgtggaccccgtggttgtgtttgacttcgct

agcttatacccaagcattatccaggcccataacctctgtttcaccaccctggcgctcgat

gaagtggatctggccgggcttcaaccatccgtcgactactcgacgttcgaggtgggtgac

caaaagttattttttgtccacgcccatattcgcgaaagcctgcttggcatcttgctgcgc

gactggctggccatgcgaaaggcggtgagggcgcgaatccccaccagcacccccgaggag

gcagttttactagataagcagcagtctgcgattaaggtgatatgcaactcggtttacgga

ttcacgggggtggcaaacggcctgttgccgtgtctgaggatagcggctaccgttaccacg

ataggacgcgacatgctcctcaagacccgagattacgttcactctcgttgggcgacgcgc

gagctgctggaggacaattttccaggggctataggtttccgaaaccacaagccttactcc

gtcagggttatctacggagacaccgactccgtgtttatcaagtttgtgggcctgacgtac

gagggggtatcggagctgggggatgctatgtcgcgtcagatttcagcggacctctttaga

gcgcccatcaaactggagtgtgagaagacctttcagcgactgctgctgatcaccaagaag

aagtacataggtgtcataaacggggggaagatgctcatgaagggggtcgacctggtccgc

aaaaataactgctctttcataaacttgtacgcgcgacatctggtagatcttttgttgtac

gacgaggatgtggccacggcggcagcagaggtgacagacgtgcctcccgcagaatgggtg

gggcgcccgctaccgagcggctttgacaagtttgggcgagtgctggtagaggcgtacaac

cgtatcactgcccccaacttggacgtgcgcgagttcgttatgactgctgagctgagccgc

tcacccgaatcgtataccaacaagcgcctgccgcacctcaccgtctactttaagctcgcc

atgaggaatgaagaactgcccagcgtaaaagagagaattccgtatgtgatagttgcgcag

accgaggccgcggaacgcgaagcgggtgtagtaaactcaatgcgcggtaccgcccaaaac

cccgtggtaaccaagaccgcacgcccccaacctaaacgcaaactgctggtttccgacctc

gccgaagacccgacctatgtttccgagaatgacgtaccgctaaacacagactactatttc

tcccacctgttgggtaccataagcgtgacctttaaggctctattcggaaatgatgtgaga

acaacagaaaatcttttaaagcggtttattccggaaaccccccacaagacccccacgaaa

acccaggcactgcttgagcgcgccggctttgaaaagctgacgccctttacaccggaggaa

gaaagtcgtcgaatactgcatacagttttttgtactctagaagcagctccccatcaaagc

tga

>KC924783.1

atggcggcgcgcgaacaggccaactccgttcgcaggagcgggttttttaatccgtttatt

ggcaagaggccatttttcaggccgggaagcgggcagaccgcggagacagagcgcccaagg

cccccccaacactcgtactgcacagaggtgggtagctttaagtttatagctccaagatgc

ctcgatgaagaagcccccgccgaccagcgacgcggtgtacacgtgggcaccttggagcgc

ccacccaaagtgtactgcgatggctcagagtacgacgtgctgaactttgcctccggaggt

tgctggcctcgcaggattcgggtttggaacggccaggattttcggggcgatggattcaac

cccagatttgagagatttcacgtgtacgacatagtagagacttcggagagcgcctcacac

gatgacccatccaggtttgctgagctatcccgcccaagcgggtctgtggtaacactgctg

ggaatgagcgagtgtggaaagcgagtcgcggttcacgtctatggtgtgcgccattatttt

tacatggcaaaggcggaggtggatagcgcttgtggaataaccaccgaggcagaactcgtg

cgcgcaatggtggactgcgcgcacagctcggctttgagcgcggccctgggaaatggcaac

ggcggcaaacagagcggcggcagcggcgggggatggtggggcggaaagcacgtgtctgcg

gactgcttcaaagtggagaccgtgtgccacacgacgctgtactactttggatctaagcca

gctctctactatagagtatctgcctccagcagccgcctgggagggttcatctgcgacaac

tttcacccggagattacaaaattcgaggggagcgtggacgtgaccacgcggctgttgttg

gacaatgaaaattttaccagtttcgggtggtaccgcctgcgacccggcacccacggagag

cgtgttcaacttcgccccgttgagcgacacgtcacctcaagcgacgtggagattaactgt

actcccgataacctggagccgataccagacgaggctgcctggcccgactataagctcatg

tgctttgatatagagtgtaaagctggaacgggtaacgaaatggcgttcccagtggcaact

aaccaagaggacctggtcatccagatctcctgtctgctgtactcgcttgctactcagaac

cacgaacacaccctgctgttttccctcgggtcatgcgatatctctgaggaatactcgttt

gcatgcgtccagcgcggcgagcccagaccgacggttttggagtttgacagcgagtacgag

ctgctggttgccttcctgacctttctcaagcagtactctcccgagttcgccaccggctac

aacatcgttaattttgactgggcgtacatagttaacaaggtaacgtcggtgtataacatc

aagctggacgggtacggcaagttcaacaaaggggggctgtttaaggtgtgggacatcgcc

acgaaccattttcagaagaagagcaaggtgaaaatcaatggcctgatatctctagacatg

tattctgtggcgacggaaaagctaaagctacccagctacaaactcgacgcggtcgtggga

gacgtcctcggcgagcataagatagaccttccctataaagaaataccctcctattacgcg

ggagggcctgaccggaggggcgtaataggagagtattgtatccaggactctaggctggtg

ggcaagctgttttttaagtacctcccccatctggaactatcggcggtggccaaactcgcc

cgtatcaccctaacgcgggtaatttttgacggtcagcaaattcgcgtgtacacgtgcttg

ctgaaactcgcccgcgagagaaatttcattttgccagacaacagacgccggtttgacagt

caggcagacgccgcgtcagagacttcggagttggctatggatagccaaagccacgccttc

gacagtacagacgaacccgacggtgtggacggtaccccggacgccgcaggatctggcgct

acttctgaaaacggaggcgggaagcccggcgtcgggagggccgtgggctaccagggagca

aaggttctagaccccgtatccggctttcatgtggaccccgtggttgtgtttgacttcgct

agcttatacccaagcattatccaggcccataacctctgtttcaccaccctggcgctcgat

gaagtggatctggccgggcttcaaccatccgtcgactactcgacgttcgaggtgggtgac

caaaagttattttttgtccacgcccatattcgcgaaagcctgcttggcatcttgctgcgc

gactggctggccatgcgaaaggcggtgagggcgcgaatccccaccagcacccccgaggag

gcagttttactagataagcagcagtctgcgattaaggtgatatgcaactcggtttacgga

ttcacgggggtggcaaacggcctgttgccgtgtctgaggatagcggctaccgttaccacg

ataggacgcgacatgctcctcaagacccgagattacgttcactctcgttgggcgacgcgc

gagctgctggaggacaattttccaggggctataggtttccgaaaccacaagccttactcc

gtcagggttatctacggagacaccgactccgtgtttatcaagtttgtgggcctgacgtac

gagggggtatcggagctgggggatgctatgtcgcgtcagatttcagcggacctctttaga

gcgcccatcaaactggagtgtgagaagacctttcagcgactgctgctgatcaccaagaag

aagtacataggtgtcataaacggggggaagatgctcatgaagggggtcgacctggtccgc

aaaaataactgctctttcataaacttgtacgcgcgacatctggtagatcttttgttgtac

gacgaggatgtggccacggcggcagcagaggtgacagacgtgcctcccgcagaatgggtg

gggcgcccgctaccgagcggctttgacaagtttgggcgagtgctggtagaggcgtacaac

cgtatcactgcccccaacttggacgtgcgcgagttcgttatgactgctgagctgagccgc

tcacccgaatcgtataccaacaagcgcctgccgcacctcaccgtctactttaagctcgcc

atgaggaatgaagaactgcccagcgtaaaagagagaattccgtatgtgatagttgcgcag

accgaggccgcggaacgcgaagcgggtgtagtaaactcaatgcgcggtaccgcccaaaac

cccgtggtaaccaagaccgcacgcccccaacctaaacgcaaactgctggtttccgacctc

gccgaagacccgacctatgtttccgagaatgacgtaccgctaaacacagactactatttc

tcccacctgttgggtaccataagcgtgacctttaaggctctattcggaaatgatgtgaga

acaacagaaaatcttttaaagcggtttattccggaaaccccccacaagacccccacgaaa

acccaggcactgcttgagcgcgccggctttgaaaagctgacgccctttacaccggaggaa

gaaagtcgtcgaatactgcatacagttttttgtactctagaagcagctccccatcaaagc

tga

>KC924784.1

atggcggcgcgcgaacaggccaactccgttcgcaggagcgggttttttaatccgtttatt

ggcaagaggccatttttcaggccgggaagcgggcagaccgcggagacagagcgcccaagg

cccccccaacactcgtactgcacagaggtgggtagctttaagtttatagctccaagatgc

ctcgatgaagaagcccccgccgaccagcgacgcggtgtacacgtgggcaccttggagcgc

ccacccaaagtgtactgcgatggctcagagtacgacgtgctgaactttgcctccggaggt

tgctggcctcgcaggattcgggtttggaacggccaggattttcggggcgatggattcaac

cccagatttgagagatttcacgtgtacgacatagtagagacttcggagagcgcctcacac

gatgacccatccaggtttgctgagctatcccgcccaagcgggtctgtggtaacactgctg

ggaatgagcgagtgtggaaagcgagtcgcggttcacgtctatggtgtgcgccattatttt

tacatggcaaaggcggaggtggatagcgcttgtggaataaccaccgaggcagaactcgtg

cgcgcaatggtggactgcgcgcacagctcggctttgagcgcggccctgggaaatggcaac

ggcggcaaacagagcggcggcagcggcgggggatggtggggcggaaagcacgtgtctgcg

gactgcttcaaagtggagaccgtgtgccacacgacgctgtactactttggatctaagcca

gctctctactatagagtatctgcctccagcagccgcctgggagggttcatctgcgacaac

tttcacccggagattacaaaattcgaggggagcgtggacgtgaccacgcggctgttgttg

gacaatgaaaattttaccagtttcgggtggtaccgcctgcgacccggcacccacggagag

cgtgttcaacttcgccccgttgagcgacacgtcacctcaagcgacgtggagattaactgt

actcccgataacctggagccgataccagacgaggctgcctggcccgactataagctcatg

tgctttgatatagagtgtaaagctggaacgggtaacgaaatggcgttcccagtggcaact

aaccaagaggacctggtcatccagatctcctgtctgctgtactcgcttgctactcagaac

cacgaacacaccctgctgttttccctcgggtcatgcgatatctctgaggaatactcgttt

gcatgcgtccagcgcggcgagcccagaccgacggttttggagtttgacagcgagtacgag

ctgctggttgccttcctgacctttctcaagcagtactctcccgagttcgccaccggctac

aacatcgttaattttgactgggcgtacatagttaacaaggtaacgtcggtgtataacatc

aagctggacgggtacggcaagttcaacaaaggggggctgtttaaggtgtgggacatcgcc

acgaaccattttcagaagaagagcaaggtgaaaatcaatggcctgatatctctagacatg

tattctgtggcgacggaaaagctaaagctacccagctacaaactcgacgcggtcgtggga

gacgtcctcggcgagcataagatagaccttccctataaagaaataccctcctattacgcg

ggagggcctgaccggaggggcgtaataggagagtattgtatccaggactctaggctggtg

ggcaagctgttttttaagtacctcccccatctggaactatcggcggtggccaaactcgcc

cgtatcaccctaacgcgggtaatttttgacggtcagcaaattcgcgtgtacacgtgcttg

ctgaaactcgcccgcgagagaaatttcattttgccagacaacagacgccggtttgacagt

caggcagacgccgcgtcagagacttcggagttggctatggatagccaaagccacgccttc

gacagtacagacgaacccgacggtgtggacggtaccccggacgccgcaggatctggcgct

acttctgaaaacggaggcgggaagcccggcgtcgggagggccgtgggctaccagggagca

aaggttctagaccccgtatccggctttcatgtggaccccgtggttgtgtttgacttcgct

agcttatacccaagcattatccaggcccataacctctgtttcaccaccctggcgctcgat

gaagtggatctggccgggcttcaaccatccgtcaactactcgacgttcgaggtgggtgac

caaaagttattttttgtccacgcccatattcgcgaaagcctgcttggcatcttgctgcgc

gactggctggccatgcgaaaggcggtgagggcgcgaatccccaccagcacccccgaggag

gcagttttactagataagcagcagtctgcgattaaggtgatatgcaactcggtttacgga

ttcacgggggtggcaaacggcctgttgccgtgtctgaggatagcggctaccgttaccacg

ataggacgcgacatgctcctcaagacccgagattacgttcactctcgttgggcgacgcgc

gagctgctggaggacaattttccaggggctataggtttccgaaaccacaagccttactcc

gtcagggttatctacggagacaccgactccgtgtttatcaagtttgtgggcctgacgtac

gagggggtatcggagctgggggatgctatgtcgcgtcagatttcagcggacctctttaga

gcgcccatcaaactggagtgtgagaagacctttcagcgactgctgctgatcaccaagaag

aagtacataggtgtcataaacggggggaagatgctcatgaagggggtcgacctggtccgc

aaaaataactgctctttcataaacttgtacgcgcgacatctggtagatcttttgttgtac

gacgaggatgtggccacggcggcagcagaggtgacagacgtgcctcccgcagaatgggtg

gggcgcccgctaccgagcggctttgacaagtttgggcgagtgctggtagaggcgtacaac

cgtatcactgcccccaacttggacgtgcgcgagttcgttatgactgctgagctgagccgc

tcacccgaatcgtataccaacaagcgcctgccgcacctcaccgtctactttaagctcgcc

atgaggaatgaagaactgcccagcgtaaaagagagaattccgtatgtgatagttgcgcag

accgaggccgcggaacgcgaagcgggtgtagtaaactcaatgcgcggtaccgcccaaaac

cccgtggtaaccaagaccgcacgcccccaacctaaacgcaaactgctggtttccgacctc

gccgaagacccgacctatgtttccgagaatgacgtaccgctaaacacagactactatttc

tcccacctgttgggtaccataagcgtgacctttaaggctctattcggaaatgatgtgaga

acaacagaaaatcttttaaagcggtttattccggaaaccccccacaagacccccacgaaa

acccaggcactgcttgagcgcgccggctttgaaaagctgacgccctttacaccggaggaa

gaaagtcgtcgaatactgcatacagttttttgtactctagaagcagctccccatcaaagc

tga

>KC924787.1

atggcggcgcgcgaacaggccaactccgttcgcaggagcgggttttttaatccgtttatt

ggcaagaggccatgtttcaggccaggaggcgggcagaccgcggagaccgatcgccccagg

cccccccaacactcgtactgcaccgaggtgggtagctttaagtttatagcccccagatgc

ctcgatgaagaagcccccgcagaccagcgatgtggtgtacacgtgggcaccttggagcgc

ccacccaaagtgtactgcgatggcacagagtacgacgtgcttgactttgccgccggatgt

tgctggcctcgcaggattcgggtttggaacggccaggatttttggggcgatggattcaac

cccagatttgagagatttcacgtgtacgacatagtagagacttcggagagcgccgcccac

gatgacccatccaggtttgctgagctatcccgcccaagcgggtctgtggtaacgctgctg

ggaatgagcgagtgcggaaagcgagtcgcggttcacgtctatggcgtgcgccattatttt

tacatggcaaaggcggaggttgacagcgcttgcggaataaccaccgaggccgaactcgtg

cgcgccatggtggactgcgcgcacagctcggctttgagcgcggccctgggaaatggcaac

ggcggcaaacagagcggcggcagcggcgggggatggtggggcggaaagcacgtgtctgcg

gactgcttcagagttgatactgtgtgccacacgacgctgtactactttggatctaagcca

gctctctactatagagtttctgcatccagcagccgcctgggcgggttcatttgcgacaac

tttcacccggagattacaaaattcgaggggagcgtggacgtgaccacgcggctgttgttg

gacaatgaaaattttaccagtttcgggtggtaccgcctgcggcccggcgcccacggagag

cgtgttcaacttcgccccgttgagcgacacgtcacctcaagcgacgtggagattaactgt

actcccgataacctggagccgatacccgacgaggctgcctggcccgactataagctcatg

tgctttgatatagagtgtaaagctggaacggggaacgaaatggcgttccccgtggcaact

aaccaagaggacctggtcatccagatatcctgtctgctgtactcgcttgctactcagaaa

cacgaacacaccctgctgttttccctagggtcgtgcgacatctctgaggaatgctcgtcc

gcatgcgtccaacgcggcgagcccagaccgacggttttggagtttgacagcgagtacgag

ctgctggttgccttcctgacctttctcaagcagtactctcccgagttcgccaccggctac

aacatcgtgaattttgactgggcgtacatagttaacaaggtaacgtcggtgtataacatc

aagctggacgggtacggcaagttcaacaaaggggggctgttcaaggtgtgggacatcgcc

acgaaccactttcagaagaagagcaaggtgaagatcaacggcctgatatctctagacatg

tattctgtggcgacggaaaagctaaagctacccagctacaaactcgacgcggtcgtggga

gacgtcctcggcgagcataagatagaccttccctataaagaaataccctcctattacgcg

ggagggcctgaccggaggggcgtaataggagagtattgtatccaggactctaggctggtg

ggcaagctgttttttaagtacctcccccatctggaactatccgcggtggccaaactcgcc

cgtataaccctaacgcgggtgatttttgacggtcagcaaattcgcgtgtacacgtgcttg

ctgaaactcgcccgcgagagaaatttcattttgcccgacaaccgacgccggtttgacggt

caggcagacgccgcgtccgagacttcggagttggctctggatagccagagccacgccttc

gacagtaccgacgaaccagacggtgtggacggtaccccggacgccgcgggatctggcgcg

acttctgaaaatggaggcggaaagcccggcgtcgggagggccgtgggctaccagggagca

aaggtgctagaccccgtatccggctttcatgtggaccccgtggttgtgtttgacttcgct

agcttataccccagcattatccaggcccataacctctgtttcaccaccctggcgcttgat

gaagtggatctggccgggcttcaaccatccgtcgactactcgacgttcgaggtgggtgac

caaaagttattttttgtccacgcccatattcgcgaaagcctgcttggcatcttgctgcgc

gactggctggccatgcgaaagtccgtaagggcgcggatccccaccagcacccccgaggag

gcagttttactagataaacagcagtctgcgattaaggtgatatgcaactccgtttacgga

ttcacgggggtggcaaacggcctgttgccgtgtctgaggatagcggctaccgttaccacg

ataggacgcgacatgctcctcaagacccgagattacgttcactctcgttgggcgacgcgc

gagctgctggaggacaatttcccaggggctacagctttccgaaaccacaagccttactcc

gtcagggttatctacggagacaccgactccgtgtttatcaagtttgtgggcctgacgtac

gagggggtatcggagctgggggatgctatgtcgcgtcagatttcagcggacctctttaga

gcgcccatcaaactggagtgtgagaagacctttcagcgcctgctgctgatcaccaaaaag

aagtacataggtgtcataaacggggggaagatgctcatgaagggggtcgacctggtccgc

aaaaataactgctctttcataaactcgtacgcgcggcatctggtagatcttttgttgtac

gacgaggatgtggccacggcggccgcagaggtgacagacgtgcctccctcagagtgggtg

gggcgtccgctaccgagcgggtttgacaagtttgggcgagtgctggtagaggcgtacaac

cgtatcactgcgcccaacttggacgtgcgcgagttcgttatgacggctgagctgagccgc

tcgcccgaatcgtatactaacaagcgcctgccgcacctcaccgtctactttaagctagcc

atgaggaatgaagaactgcccagcgtaaaagagagaattccgtacgtgatagttgctcag

accgaggccgcagaacgcgaagcgggtgtggtaaactcaatgcgcggtaccgcccaaaac

cccttggtcaccaataccgcacgcccccaacctaaacgcaaactgcttgtttccgacctc

gccgaagacccgacctatgtttccgagaatgacataccgctaaacacagactactatttc

tcccacctgttgggtaccataagcgtaacctttaaggctctattcggaaatgatgtgaga

acaacagaaaatcttttgaagcggtttattccggaaaccccccacaagacctccacaaaa

acccaggcactgctggagcgcgcaggctttgaaaagctgacgccctctacaccggaggaa

gaaagtcgtcgaatactgcatacagttttttgtactctagaagcagctccccatcaaagc

tga

>KC924794.1

atggcggcgcgcgaacaggccaactccgttcgcaggagcgggttttttaatccgtttatt

ggcaagaggccatttttcaggccgggaagcgggcagaccgcggagacagagcgcccaagg

cccccccaacactcgtactgcacagaggtgggtagctttaagtttatagctccaagatgc

ctcgatgaagaagcccccgccgaccagcgacgcggtgtacacgtgggcaccttggagcgc

ccacccaaagtgtactgcgatggctcagagtacgacgtgctgaactttgcctccggaggt

tgctggcctcgcaggattcgggtttggaacggccaggattttcggggcgatggattcaac

cccagatttgagagatttcacgtgtacgacatagtagagacttcggagagcgcctcacac

gatgacccatccaggtttgctgagctatcccgcccaagcgggtctgtggtaacactgctg

ggaatgagcgagtgtggaaagcgagtcgcggttcacgtctatggtgtgcgccattatttt

tacatggcaaaggcggaggtggatagcgcttgtggaataaccaccgaggcagaactcgtg

cgcgcaatggtggactgcgcgcacagctcggctttgagcgcggccctgggaaatggcaac

ggcggcaaacagagcggcggcagcggcgggggatggtggggcggaaagcacgtgtctgcg

gactgcttcaaagtggagaccgtgtgccgcacgacgctgtactactttggatctaagcca

gctctctactatagagtatctgcctccagcggccgcctgggagggttcatctgcgacaac

tttcacccggagattacaaaattcgaggggagcgtggacgtgaccacgcggctgttgttg

gacaatgaaaattttaccagtttcgggtggtaccgcctgcgacccggcacccacggagag

cgtgttcaacttcgccccgttgagcgacacgtcacctcaagcgacgtggagattaactgt

actcccgataacctggagccgataccagacgaggctgcctggcccgactataagctcatg

tgctttgatatagagtgtaaagctggaacgggtaacgaaatggcgttcccagtggcaact

aaccaagaggacctggtcatccagatctcctgtctgctgtactcgcttgctactcagaac

cacgaacacaccctgctgttttccctcgggtcatgcgatatctctgaggaatactcgttt

gcatgcgtccagcgcggcgagcccagaccgacggttttggagtttgacagcgagtacgag

ctgctggttgccttcctgacctttctcaagcagtactctcccgagttcgccaccggctac

aacatcgttaattttgactgggcgtacatagttaacaaggtaacgtcggtgtataacatc

aagctggacgggtacggcaagttcaacaaaggggggctgtttaaggtgtgggacatcgcc

acgaaccattttcagaagaagagcaaggtgaaaatcaatggcctgatatctctagacatg

tattctgtggcgacggaaaagctaaagctacccagctacaaactcgacgcggtcgtggga

gacgtcctcggcgagcataagatagaccttccctataaagaaataccctcctattacgcg

ggagggcctgaccggaggggcgtaataggagagtattgtatccaggactctaggctggtg

ggcaagctgttttttaagtacctcccccatctggaactatcggcggtggccaaactcgcc

cgtatcaccctaacgcgggtaatttttgacggtcagcaaattcgcgtgtacacgtgcttg

ctgaaactcgcccgcgagagaaatttcattttgccagacaacagacgccggtttgacagt

caggcagacgccgcgtcagagacttcggagttggctatggatagccaaagccacgccttc

gacagtacagacgaacccgacggtgtggacggtaccccggacgccgcaggatctggcgct

acttctgaaaacggaggcgggaagcccggcgtcgggagggccgtgggctaccagggagca

aaggttctagaccccgtatccggctttcatgtggaccccgtggttgtgtttgacttcgct

agcttatacccaagcattatccaggcccataacctctgtttcaccaccctggcgctcgat

gaagtggatctggccgggcttcaaccatccgtcgactcctcgacgttcgaggtgggtgac

caaaagttattttttgtccacgcccatattcgcgaaagcctgcttggcatcttgctgcgc

gactggctggccatgcgaaaggcggtgagggcgcgaatccccaccagcacccccgaggag

gcagttttactagataagcagcagtctgcgattaaggtgatatgcaactcggtttacgga

ttcacgggggtggcaaacggcctgttgccgtgtctgaggatagcggctaccgttaccacg

ataggacgcgacatgctcctcaagacccgagattacgttcactctcgttgggcgacgcgc

gagctgctggaggacaattttccaggggctataggtttccgaaaccacaagccttactcc

gtcagggttatctacggagacaccgactccgtgtttatcaagtttgtgggcctgacgtac

gagggggtatcggagctgggggatgctatgtcgcgtcagatttcagcggacctctttaga

gcgcccatcaaactggagtgtgagaagacctttcagcgactgctgctgatcaccaagaag

aagtacataggtgtcataaacggggggaagatgctcatgaagggggtcgacctggtccgc

aaaaataactgctctttcataaacttgtacgcgcgacatctggtagatcttttgttgtac

gacgaggatgtggccacggcggcagcagaggtgacagacgtgcctcccgcagaatgggtg

gggcgcccgctaccgagcggctttgacaagtttgggcgagtgctggtagaggcgtacaac

cgtatcactgcccccaacttggacgtgcgcgagttcgttatgactgctgagctgagccgc

tcacccgaatcgtataccaacaagcgcctgccgcacctcaccgtctactttaagctcgcc

atgaggaatgaagaactgcccagcgtaaaagagagaattccgtatgtgatagttgcgcag

accgaggccgcggaacgcgaagcgggtgtagtaaactcaatgcgcggtaccgcccaaaac

cccgtggtaaccaagaccgcacgcccccaacctaaacgcaaactgctggtttccgacctc

gccgaagacccgacctatgtttccgagaatgacgtaccgctaaacacagactactatttc

tcccacctgttgggtaccataagcgtgacctttaaggctctattcggaaatgatgtgaga

acaacagaaaatcttttaaagcggtttattccggaaaccccccacaagacccccacgaaa

acccaggcactgcttgagcgcgccggctttgaaaagctgacgccctttacaccggaggaa

gaaagtcgtcgaatactgcatacagttttttgtactctagaagcagctccccatcaaagc

tga

>KC924795.1

atggcggcgcgcgaacaggccaactccgttcgcaggagcgggttttttaatccgtttatt

ggcaagaggccatttttcaggccgggaagcgggcagaccgcggagacagagcgcccaagg

cccccccaacactcgtactgcacagaggtgggtagctttaagtttatagctccaagatgc

ctcgatgaagaagcccccgccgaccagcgacgcggtgtacacgtgggcaccttggagcgc

ccacccaaagtgtactgcgatggctcagagtacgacgtgctgaactttgcctccggaggt

tgctggcctcgcaggattcgggtttggaacggccaggattttcggggcgatggattcaac

cccagatttgagagatttcacgtgtacgacatagtagagacttcggagagcgcctcacac

gatgacccatccaggtttgctgagctatcccgcccaagcgggtctgtggtaacactgctg

ggaatgagcgagtgtggaaagcgagtcgcggttcacgtctatggtgtgcgccattatttt

tacatggcaaaggcggaggtggatagcgcttgtggaataaccaccgaggcagaactcgtg

cgcgcaatggtggactgcgcgcacagctcggctttgagcgcggccctgggaaatggcaac

ggcggcaaacagagcggcggcagcggcgggggatggtggggcggaaagcacgtgtctgcg

gactgcttcaaagtggagaccgtgtgccgcacgacgctgtactactttggatctaagcca

gctctctactatagagtatctgcctccagcggccgcctgggagggttcatctgcgacaac

tttcacccggagattacaaaattcgaggggagcgtggacgtgaccacgcggctgttgttg

gacaatgaaaattttaccagtttcgggtggtaccgcctgcgacccggcacccacggagag

cgtgttcaacttcgccccgttgagcgacacgtcacctcaagcgacgtggagattaactgt

actcccgataacctggagccgataccagacgaggctgcctggcccgactataagctcatg

tgctttgatatagagtgtaaagctggaacgggtaacgaaatggcgttcccagtggcaact

aaccaagaggacctggtcatccagatctcctgtctgctgtactcgcttgctactcagaac

cacgaacacaccctgctgttttccctcgggtcatgcgatatctctgaggaatactcgttt

gcatgcgtccagcgcggcgagcccagaccgacggttttggagtttgacagcgagtacgag

ctgctggttgccttcctgacctttctcaagcagtactctcccgagttcgccaccggctac

aacatcgttaattttgactgggcgtacatagttaacaaggtaacgtcggtgtataacatc

aagctggacgggtacggcaagttcaacaaaggggggctgtttaaggtgtgggacatcgcc

acgaaccattttcagaagaagagcaaggtgaaaatcaatggcctgatatctctagacatg

tattctgtggcgacggaaaagctaaagctacccagctacaaactcgacgcggtcgtggga

gacgtcctcggcgagcataagatagaccttccctataaagaaataccctcctattacgcg

ggagggcctgaccggaggggcgtaataggagagtattgtatccaggactctaggctggtg

ggcaagctgttttttaagtacctcccccatctggaactatcggcggtggccaaactcgcc

cgtatcaccctaacgcgggtaatttttgacggtcagcaaattcgcgtgtacacgtgcttg

ctgaaactcgcccgcgagagaaatttcattttgccagacaacagacgccggtttgacagt

caggcagacgccgcgtcagagacttcggagttggctatggatagccaaagccacgccttc

gacagtacagacgaacccgacggtgtggacggtaccccggacgccgcaggatctggcgct

acttctgaaaacggaggcgggaagcccggcgtcgggagggccgtgggctaccagggagca

aaggttctagaccccgtatccggctttcatgtggaccccgtggttgtgtttgacttcgct

agcttatacccaagcattatccaggcccataacctctgtttcaccaccctggcgctcgat

gaagtggatctggccgggcttcaaccatccgtcgactcctcgacgttcgaggtgggtgac

caaaagttattttttgtccacgcccatattcgcgaaagcctgcttggcatcttgctgcgc

gactggctggccatgcgaaaggcggtgagggcgcgaatccccaccagcacccccgaggag

gcagttttactagataagcagcagtctgcgattaaggtgatatgcaactcggtttacgga

ttcacgggggtggcaaacggcctgttgccgtgtctgaggatagcggctaccgttaccacg

ataggacgcgacatgctcctcaagacccgagattacgttcactctcgttgggcgacgcgc

gagctgctggaggacaattttccaggggctataggtttccgaaaccacaagccttactcc

gtcagggttatctacggagacaccgactccgtgtttatcaagtttgtgggcctgacgtac

gagggggtatcggagctgggggatgctatgtcgcgtcagatttcagcggacctctttaga

gcgcccatcaaactggagtgtgagaagacctttcagcgactgctgctgatcaccaagaag

aagtacataggtgtcataaacggggggaagatgctcatgaagggggtcgacctggtccgc

aaaaataactgctctttcataaacttgtacgcgcgacatctggtagatcttttgttgtac

gacgaggatgtggccacggcggcagcagaggtgacagacgtgcctcccgcagaatgggtg

gggcgcccgctaccgagcggctttgacaagtttgggcgagtgctggtagaggcgtacaac

cgtatcactgcccccaacttggacgtgcgcgagttcgttatgactgctgagctgagccgc

tcacccgaatcgtataccaacaagcgcctgccgcacctcaccgtctactttaagctcgcc

atgaggaatgaagaactgcccagcgtaaaagagagaattccgtatgtgatagttgcgcag

accgaggccgcggaacgcgaagcgggtgtagtaaactcaatgcgcggtaccgcccaaaac

cccgtggtaaccaagaccgcacgcccccaacctaaacgcaaactgctggtttccgacctc

gccgaagacccgacctatgtttccgagaatgacgtaccgctaaacacagactactatttc

tcccacctgttgggtaccataagcgtgacctttaaggctctattcggaaatgatgtgaga

acaacagaaaatcttttaaagcggtttattccggaaaccccccacaagacccccacgaaa

acccaggcactgcttgagcgcgccggctttgaaaagctgacgccctttacaccggaggaa

gaaagtcgtcgaatactgcatacagttttttgtactctagaagcagctccccatcaaagc

tga

>KC924796.1

atggcggcgcgcgaacaggccaactccgttcgcaggagcgggttttttaatccgtttatt

ggcaagaggccatttttcaggccgggaagcgggcagaccgcggagacagagcgcccaagg

cccccccaacactcgtactgcacagaggtgggtagctttaagtttatagctccaagatgc

ctcgatgaagaagcccccgccgaccagcgacgcggtgtacacgtgggcaccttggagcgc

ccacccaaagtgtactgcgatggctcagagtacgacgtgctgaactttgcctccggaggt

tgctggcctcgcaggattcgggtttggaacggccaggattttcggggcgatggattcaac

cccagatttgagagatttcacgtgtacgacatagtagagacttcggagagcgcctcacac

gatgacccatccaggtttgctgagctatcccgcccaagcgggtctgtggtaacactgctg

ggaatgagcgagtgtggaaagcgagtcgcggttcacgtctatggtgtgcgccattatttt

tacatggcaaaggcggaggtggatagcgcttgtggaataaccaccgaggcagaactcgtg

cgcgcaatggtggactgcgcgcacagctcggctttgagcgcggccctgggaaatggcaac

ggcggcaaacagagcggcggcagcggcgggggatggtggggcggaaagcacgtgtctgcg

gactgcttcaaagtggagaccgtgtgccacacgacgctgtactactttggatctaagcca

gctctctactatagagtatctgcctccagcagccgcctgggagggttcatctgcgacaac

tttcacccggagattacaaaattcgaggggagcgtggacgtgaccacgcggctgttgttg

gacaatgaaaattttaccagtttcgggtggtaccgcctgcgacccggcacccacggagag

cgtgttcaacttcgccccgttgagcgacacgtcacctcaagcgacgtggagattaactgt

actcccgataacctggagccgataccagacgaggctgcctggcccgactataagctcatg

tgctttgatatagagtgtaaagctggaacgggtaacgaaatggcgttcccagtggcaact

aaccaagaggacctggtcatccagatctcctgtctgctgtactcgcttgctactcagaac

cacgaacacaccctgctgttttccctcgggtcatgcgatatctctgaggaatactcgttt

gcatgcgtccagcgcggcgagcccagaccgacggttttggagtttgacagcgagtacgag

ctgctggttgccttcctgacctttctcaagcagtactctcccgagttcgccaccggctac

aacatcgttaattttgactgggcgtacatagttaacaaggtaacgtcggtgtataacatc

aagctggacgggtacggcaagttcaacaaaggggggctgtttaaggtgtgggacatcgcc

acgaaccattttcagaagaagagcaaggtgaaaatcaatggcctgatatctctagacatg

tattctgtggcgacggaaaagctaaagctacccagctacaaactcgacgcggtcgtggga

gacgtcctcggcgagcataagatagaccttccctataaagaaataccctcctattacgcg

ggagggcctgaccggaggggcgtaataggagagtattgtatccaggactctaggctggtg

ggcaagctgttttttaagtacctcccccatctggaactatcggcggtggccaaactcgcc

cgtatcaccctaacgcgggtaatttttgacggtcagcaaattcgcgtgtacacgtgcttg

ctgaaactcgcccgcgagagaaatttcattttgccagacaacagacgccggtttgacagt

caggcagacgccgcgtcagagacttcggagttggctatggatagccaaagccacgccttc

gacagtacagacgaacccgacggtgtggacggtaccccggacgccgcaggatctggcgct

acttctgaaaacggaggcgggaagcccggcgtcgggagggccgtgggctaccagggagca

aaggttctagaccccgtatccggctttcatgtggaccccgtggttgtgtttgacttcgct

agcttatacccaagcattatccaggcccataacctctgtttcaccaccctggcgctcgat

gaagtggatctggccgggcttcaaccatccgtcaactactcgacgttcgaggtgggtgac

caaaagttattttttgtccacgcccatattcgcgaaagcctgcttggcatcttgctgcgc

gactggctggccatgcgaaaggcggtgagggcgcgaatccccaccagcacccccgaggag

gcagttttactagataagcagcagtctgcgattaaggtgatatgcaactcggtttacgga

ttcacgggggtggcaaacggcctgttgccgtgtctgaggatagcggctaccgttaccacg

ataggacgcgacatgctcctcaagacccgagattacgttcactctcgttgggcgacgcgc

gagctgctggaggacaattttccaggggctataggtttccgaaaccacaagccttactcc

gtcagggttatctacggagacaccgactccgtgtttatcaagtttgtgggcctgacgtac

gagggggtatcggagctgggggatgctatgtcgcgtcagatttcagcggacctctttaga

gcgcccatcaaactggagtgtgagaagacctttcagcgactgctgctgatcaccaagaag

aagtacataggtgtcataaacggggggaagatgctcatgaagggggtcgacctggtccgc

aaaaataactgctctttcataaacttgtacgcgcgacatctggtagatcttttgttgtac

gacgaggatgtggccacggcggcagcagaggtgacagacgtgcctcccgcagaatgggtg

gggcgcccgctaccgagcggctttgacaagtttgggcgagtgctggtagaggcgtacaac

cgtatcactgcccccaacttggacgtgcgcgagttcgttatgactgctgagctgagccgc

tcacccgaatcgtataccaacaagcgcctgccgcacctcaccgtctactttaagctcgcc

atgaggaatgaagaactgcccagcgtaaaagagagaattccgtatgtgatagttgcgcag

accgaggccgcggaacgcgaagcgggtgtagtaaactcaatgcgcggtaccgcccaaaac

cccgtggtaaccaagaccgcacgcccccaacctaaacgcaaactgctggtttccgacctc

gccgaagacccgacctatgtttccgagaatgacgtaccgctaaacacagactactatttc

tcccacctgttgggtaccataagcgtgacctttaaggctctattcggaaatgatgtgaga

acaacagaaaatcttttaaagcggtttattccggaaaccccccacaagacccccacgaaa

acccaggcactgcttgagcgcgccggctttgaaaagctgacgccctttacaccggaggaa

gaaagtcgtcgaatactgcatacagttttttgtactctagaagcagctccccatcaaagc

tga

>KC924797.1

atggcggcgcgcgaacaggccaactccgttcgcaggagcgggttttttaatccgtttatt

ggcaagaggccatttttcaggccgggaagcgggcagaccgcggagacagagcgcccaagg

cccccccaacactcgtactgcacagaggtgggtagctttaagtttatagctccaagatgc

ctcgatgaagaagcccccgccgaccagcgacgcggtgtacacgtgggcaccttggagcgc

ccacccaaagtgtactgcgatggctcagagtacgacgtgctgaactttgcctccggaggt

tgctggcctcgcaggattcgggtttggaacggccaggattttcggggcgatggattcaac

cccagatttgagagatttcacgtgtacgacatagtagagacttcggagagcgcctcacac

gatgacccatccaggtttgctgagctatcccgcccaagcgggtctgtggtaacactgctg

ggaatgagcgagtgtggaaagcgagtcgcggttcacgtctatggtgtgcgccattatttt

tacatggcaaaggcggaggtggatagcgcttgtggaataaccaccgaggcagaactcgtg

cgcgcaatggtggactgcgcgcacagctcggctttgagcgcggccctgggaaatggcaac

ggcggcaaacagagcggcggcagcggcgggggatggtggggcggaaagcacgtgtctgcg

gactgcttcaaagtggagaccgtgtgccacacgacgctgtactactttggatctaagcca

gctctctactatagagtatctgcctccagcagccgcctgggagggttcatctgcgacaac

tttcacccggagattacaaaattcgaggggagcgtggacgtgaccacgcggctgttgttg

gacaatgaaaattttaccagtttcgggtggtaccgcctgcgacccggcacccacggagag

cgtgttcaacttcgccccgttgagcgacacgtcacctcaagcgacgtggagattaactgt

actcccgataacctggagccgataccagacgaggctgcctggcccgactataagctcatg

tgctttgatatagagtgtaaagctggaacgggtaacgaaatggcgttcccagtggcaact

aaccaagaggacctggtcatccagatctcctgtctgctgtactcgcttgctactcagaac

cacgaacacaccctgctgttttccctcgggtcatgcgatatctctgaggaatactcgttt

gcatgcgtccagcgcggcgagcccagaccgacggttttggagtttgacagcgagtacgag

ctgctggttgccttcctgacctttctcaagcagtactctcccgagttcgccaccggctac

aacatcgttaattttgactgggcgtacatagttaacaaggtaacgtcggtgtataacatc

aagctggacgggtacggcaagttcaacaaaggggggctgtttaaggtgtgggacatcgcc

acgaaccattttcagaagaagagcaaggtgaaaatcaatggcctgatatctctagacatg

tattctgtggcgacggaaaagctaaagctacccagctacaaactcgacgcggtcgtggga

gacgtcctcggcgagcataagatagaccttccctataaagaaataccctcctattacgcg

ggagggcctgaccggaggggcgtaataggagagtattgtatccaggactctaggctggtg

ggcaagctgttttttaagtacctcccccatctggaactatcggcggtggccaaactcgcc

cgtatcaccctaacgcgggtaatttttgacggtcagcaaattcgcgtgtacacgtgcttg

ctgaaactcgcccgcgagagaaatttcattttgccagacaacagacgccggtttgacagt

caggcagacgccgcgtcagagacttcggagttggctatggatagccaaagccacgccttc

gacagtacagacgaacccgacggtgtggacggtaccccggacgccgcaggatctggcgct

acttctgaaaacggaggcgggaagcccggcgtcgggagggccgtgggctaccagggagca

aaggttctagaccccgtatccggctttcatgtggaccccgtggttgtgtttgacttcgct

agcttatacccaagcattatccaggcccataacctctgtttcaccaccctggcgctcgat

gaagtggatctggccgggcttcaaccatccgtcaactactcgacgttcgaggtgggtgac

caaaagttattttttgtccacgcccatattcgcgaaagcctgcttggcatcttgctgcgc

gactggctggccatgcgaaaggcggtgagggcgcgaatccccaccagcacccccgaggag

gcagttttactagataagcagcagtctgcgattaaggtgatatgcaactcggtttacgga

ttcacgggggtggcaaacggcctgttgccgtgtctgaggatagcggctaccgttaccacg

ataggacgcgacatgctcctcaagacccgagattacgttcactctcgttgggcgacgcgc

gagctgctggaggacaattttccaggggctataggtttccgaaaccacaagccttactcc

gtcagggttatctacggagacaccgactccgtgtttatcaagtttgtgggcctgacgtac

gagggggtatcggagctgggggatgctatgtcgcgtcagatttcagcggacctctttaga

gcgcccatcaaactggagtgtgagaagacctttcagcgactgctgctgatcaccaagaag

aagtacataggtgtcataaacggggggaagatgctcatgaagggggtcgacctggtccgc

aaaaataactgctctttcataaacttgtacgcgcgacatctggtagatcttttgttgtac

gacgaggatgtggccacggcggcagcagaggtgacagacgtgcctcccgcagaatgggtg

gggcgcccgctaccgagcggctttgacaagtttgggcgagtgctggtagaggcgtacaac

cgtatcactgcccccaacttggacgtgcgcgagttcgttatgactgctgagctgagccgc

tcacccgaatcgtataccaacaagcgcctgccgcacctcaccgtctactttaagctcgcc

atgaggaatgaagaactgcccagcgtaaaagagagaattccgtatgtgatagttgcgcag

accgaggccgcggaacgcgaagcgggtgtagtaaactcaatgcgcggtaccgcccaaaac

cccgtggtaaccaagaccgcacgcccccaacctaaacgcaaactgctggtttccgacctc

gccgaagacccgacctatgtttccgagaatgacgtaccgctaaacacagactactatttc

tcccacctgttgggtaccataagcgtgacctttaaggctctattcggaaatgatgtgaga

acaacagaaaatcttttaaagcggtttattccggaaaccccccacaagacccccacgaaa

acccaggcactgcttgagcgcgccggctttgaaaagctgacgccctttacaccggaggaa

gaaagtcgtcgaatactgcatacagttttttgtactctagaagcagctccccatcaaagc

tga

>KC924798.1

atggcggcgcgcgaacaggccaactccgttcgcaggagcgggttttttaatccgtttatt

ggcaagaggccatttttcaggccgggaagcgggcagaccgcggagacagagcgcccaagg

cccccccaacactcgtactgcacagaggtgggtagctttaagtttatagctccaagatgc

ctcgatgaagaagcccccgccgaccagcgacgcggtgtacacgtgggcaccttggagcgc

ccacccaaagtgtactgcgatggctcagagtacgacgtgctgaactttgcctccggaggt

tgctggcctcgcaggattcgggtttggaacggccaggattttcggggcgatggattcaac

cccagatttgagagatttcacgtgtacgacatagtagagacttcggagagcgcctcacac

gatgacccatccaggtttgctgagctatcccgcccaagcgggtctgtggtaacactgctg

ggaatgagcgagtgtggaaagcgagtcgcggttcacgtctatggtgtgcgccattatttt

tacatggcaaaggcggaggtggatagcgcttgtggaataaccaccgaggcagaactcgtg

cgcgcaatggtggactgcgcgcacagctcggctttgagcgcggccctgggaaatggcaac

ggcggcaaacagagcggcggcagcggcgggggatggtggggcggaaagcacgtgtctgcg

gactgcttcaaagtggagaccgtgtgccacacgacgctgtactactttggatctaagcca

gctctctactatagagtatctgcctccagcagccgcctgggagggttcatctgcgacaac

tttcacccggagattacaaaattcgaggggagcgtggacgtgaccacgcggctgttgttg

gacaatgaaaattttaccagtttcgggtggtaccgcctgcgacccggcacccacggagag

cgtgttcaacttcgccccgttgagcgacacgtcacctcaagcgacgtggagattaactgt

actcccgataacctggagccgataccagacgaggctgcctggcccgactataagctcatg

tgctttgatatagagtgtaaagctggaacgggtaacgaaatggcgttcccagtggcaact

aaccaagaggacctggtcatccagatctcctgtctgctgtactcgcttgctactcagaac

cacgaacacaccctgctgttttccctcgggtcatgcgatatctctgaggaatactcgttt

gcatgcgtccagcgcggcgagcccagaccgacggttttggagtttgacagcgagtacgag

ctgctggttgccttcctgacctttctcaagcagtactctcccgagttcgccaccggctac

aacatcgttaattttgactgggcgtacatagttaacaaggtaacgtcggtgtataacatc

aagctggacgggtacggcaagttcaacaaaggggggctgtttaaggtgtgggacatcgcc

acgaaccattttcagaagaagagcaaggtgaaaatcaatggcctgatatctctagacatg

tattctgtggcgacggaaaagctaaagctacccagctacaaactcgacgcggtcgtggga

gacgtcctcggcgagcataagatagaccttccctataaagaaataccctcctattacgcg

ggagggcctgaccggaggggcgtaataggagagtattgtatccaggactctaggctggtg

ggcaagctgttttttaagtacctcccccatctggaactatcggcggtggccaaactcgcc

cgtatcaccctaacgcgggtaatttttgacggtcagcaaattcgcgtgtacacgtgcttg

ctgaaactcgcccgcgagagaaatttcattttgccagacaacagacgccggtttgacagt

caggcagacgccgcgtcagagacttcggagttggctatggatagccaaagccacgccttc

gacagtacagacgaacccgacggtgtggacggtaccccggacgccgcaggatctggcgct

acttctgaaaacggaggcgggaagcccggcgtcgggagggccgtgggctaccagggagca

aaggttctagaccccgtatccggctttcatgtggaccccgtggttgtgtttgacttcgct

agcttatacccaagcattatccaggcccataacctctgtttcaccaccctggcgctcgat

gaagtggatctggccgggcttcaaccatccgtcgactactcgacgttcgaggtgggtgac

caaaagttattttttgtccacgcccatattcgcgaaagcctgcttggcatcttgctgcgc

gactggctggccatgcgaaaggcggtgagggcgcgaatccccaccagcacccccgaggag

gcagttttactagataagcagcagtctgcgattaaggtgatatgcaactcggtttacgga

ttcacgggggtggcaaacggcctgttgccgtgtctgaggatagcggctaccgttaccacg

ataggacgcgacatgctcctcaagacccgagattacgttcactctcgttgggcgacgcgc

gagctgctggaggacaattttccaggggctataggtttccgaaaccacaagccttactcc

gtcagggttatctacggagacaccgactccgtgtttatcaagtttgtgggcctgacgtac

gagggggtatcggagctgggggatgctatgtcgcgtcagatttcagcggacctctttaga

gcgcccatcaaactggagtgtgagaagacctttcagcgactgctgctgatcaccaagaag

aagtacataggtgtcataaacggggggaagatgctcatgaagggggtcgacctggtccgc

aaaaataactgctctttcataaacttgtacgcgcgacatctggtagatcttttgttgtac

gacgaggatgtggccacggcggcagcagaggtgacagacgtgcctcccgcagaatgggtg

gggcgcccgctaccgagcggctttgacaagtttgggcgagtgctggtagaggcgtacaac

cgtatcactgcccccaacttggacgtgcgcgagttcgttatgactgctgagctgagccgc

tcacccgaatcgtataccaacaagcgcctgccgcacctcaccgtctactttaagctcgcc

atgaggaatgaagaactgcccagcgtaaaagagagaattccgtatgtgatagttgcgcag

accgaggccgcggaacgcgaagcgggtgtagtaaactcaatgcgcggtaccgcccaaaac

cccgtggtaaccaagaccgcacgcccccaacctaaacgcaaactgctggtttccgacctc

gccgaagacccgacctatgtttccgagaatgacgtaccgctaaacacagactactatttc

tcccacctgttgggtaccataagcgtgacctttaaggctctattcggaaatgatgtgaga

acaacagaaaatcttttaaagcggtttattccggaaaccccccacaagacccccacgaaa

acccaggcactgcttgagcgcgccggctttgaaaagctgacgccctttacaccggaggaa

gaaagtcgtcgaatactgcatacagttttttgtactctagaagcagctccccatcaaagc

tga

>KC924799.1

atggcggcgcgcgaacaggccaactccgttcgcaggagcgggttttttaatccgtttatt

ggcaagaggccatttttcaggccgggaagcgggcagaccgcggagacagagcgcccaagg

cccccccaacactcgtactgcacagaggtgggtagctttaagtttatagctccaagatgc

ctcgatgaagaagcccccgccgaccagcgacgcggtgtacacgtgggcaccttggagcgc

ccacccaaagtgtactgcgatggctcagagtacgacgtgctgaactttgcctccggaggt

tgctggcctcgcaggattcgggtttggaacggccaggattttcggggcgatggattcaac

cccagatttgagagatttcacgtgtacgacatagtagagacttcggagagcgcctcacac

gatgacccatccaggtttgctgagctatcccgcccaagcgggtctgtggtaacactgctg

ggaatgagcgagtgtggaaagcgagtcgcggttcacgtctatggtgtgcgccattatttt

tacatggcaaaggcggaggtggatagcgcttgtggaataaccaccgaggcagaactcgtg

cgcgcaatggtggactgcgcgcacagctcggctttgagcgcggccctgggaaatggcaac

ggcggcaaacagagcggcggcagcggcgggggatggtggggcggaaagcacgtgtctgcg

gactgcttcaaagtggagaccgtgtgccacacgacgctgtactactttggatctaagcca

gctctctactatagagtatctgcctccagcagccgcctgggagggttcatctgcgacaac

tttcacccggagattacaaaattcgaggggagcgtggacgtgaccacgcggctgttgttg

gacaatgaaaattttaccagtttcgggtggtaccgcctgcgacccggcacccacggagag

cgtgttcaacttcgccccgttgagcgacacgtcacctcaagcgacgtggagattaactgt

actcccgataacctggagccgataccagacgaggctgcctggcccgactataagctcatg

tgctttgatatagagtgtaaagctggaacgggtaacgaaatggcgttcccagtggcaact

aaccaagaggacctggtcatccagatctcctgtctgctgtactcgcttgctactcagaac

cacgaacacaccctgctgttttccctcgggtcatgcgatatctctgaggaatactcgttt

gcatgcgtccagcgcggcgagcccagaccgacggttttggagtttgacagcgagtacgag

ctgctggttgccttcctgacctttctcaagcagtactctcccgagttcgccaccggctac

aacatcgttaattttgactgggcgtacatagttaacaaggtaacgtcggtgtataacatc

aagctggacgggtacggcaagttcaacaaaggggggctgtttaaggtgtgggacatcgcc

acgaaccattttcagaagaagagcaaggtgaaaatcaatggcctgatatctctagacatg

tattctgtggcgacggaaaagctaaagctacccagctacaaactcgacgcggtcgtggga

gacgtcctcggcgagcataagatagaccttccctataaagaaataccctcctattacgcg

ggagggcctgaccggaggggcgtaataggagagtattgtatccaggactctaggctggtg

ggcaagctgttttttaagtacctcccccatctggaactatcggcggtggccaaactcgcc

cgtatcaccctaacgcgggtaatttttgacggtcagcaaattcgcgtgtacacgtgcttg

ctgaaactcgcccgcgagagaaatttcattttgccagacaacagacgccggtttgacagt

caggcagacgccgcgtcagagacttcggagttggctatggatagccaaagccacgccttc

gacagtacagacgaacccgacggtgtggacggtaccccggacgccgcaggatctggcgct

acttctgaaaacggaggcgggaagcccggcgtcgggagggccgtgggctaccagggagca

aaggttctagaccccgtatccggctttcatgtggaccccgtggttgtgtttgacttcgct

agcttatacccaagcattatccaggcccataacctctgtttcaccaccctggcgctcgat

gaagtggatctggccgggcttcaaccatccgtcaactactcgacgttcgaggtgggtgac

caaaagttattttttgtccacgcccatattcgcgaaagcctgcttggcatcttgctgcgc

gactggctggccatgcgaaaggcggtgagggcgcgaatccccaccagcacccccgaggag

gcagttttactagataagcagcagtctgcgattaaggtgatatgcaactcggtttacgga

ttcacgggggtggcaaacggcctgttgccgtgtctgaggatagcggctaccgttaccacg

ataggacgcgacatgctcctcaagacccgagattacgttcactctcgttgggcgacgcgc

gagctgctggaggacaattttccaggggctataggtttccgaaaccacaagccttactcc

gtcagggttatctacggagacaccgactccgtgtttatcaagtttgtgggcctgacgtac

gagggggtatcggagctgggggatgctatgtcgcgtcagatttcagcggacctctttaga

gcgcccatcaaactggagtgtgagaagacctttcagcgactgctgctgatcaccaagaag

aagtacataggtgtcataaacggggggaagatgctcatgaagggggtcgacctggtccgc

aaaaataactgctctttcataaacttgtacgcgcgacatctggtagatcttttgttgtac

gacgaggatgtggccacggcggcagcagaggtgacagacgtgcctcccgcagaatgggtg

gggcgcccgctaccgagcggctttgacaagtttgggcgagtgctggtagaggcgtacaac

cgtatcactgcccccaacttggacgtgcgcgagttcgttatgactgctgagctgagccgc

tcacccgaatcgtataccaacaagcgcctgccgcacctcaccgtctactttaagctcgcc

atgaggaatgaagaactgcccagcgtaaaagagagaattccgtatgtgatagttgcgcag

accgaggccgcggaacgcgaagcgggtgtagtaaactcaatgcgcggtaccgcccaaaac

cccgtggtaaccaagaccgcacgcccccaacctaaacgcaaactgctggtttccgacctc

gccgaagacccgacctatgtttccgagaatgacgtaccgctaaacacagactactatttc

tcccacctgttgggtaccataagcgtgacctttaaggctctattcggaaatgatgtgaga

acaacagaaaatcttttaaagcggtttattccggaaaccccccacaagacccccacgaaa

acccaggcactgcttgagcgcgccggctttgaaaagctgacgccctttacaccggaggaa

gaaagtcgtcgaatactgcatacagttttttgtactctagaagcagctccccatcaaagc

tga

>KC924800.1

atggcggcgcgcgaacaggccaactccgttcgcaggagcgggttttttaatccgtttatt

ggcaagaggccatttttcaggccgggaagcgggcagaccgcggagacagagcgcccaagg

cccccccaacactcgtactgcacagaggtgggtagctttaagtttatagctccaagatgc

ctcgatgaagaagcccccgccgaccagcgacgcggtgtacacgtgggcaccttggagcgc

ccacccaaagtgtactgcgatggctcagagtacgacgtgctgaactttgcctccggaggt

tgctggcctcgcaggattcgggtttggaacggccaggattttcggggcgatggattcaac

cccagatttgagagatttcacgtgtacgacatagtagagacttcggagagcgcctcacac

gatgacccatccaggtttgctgagctatcccgcccaagcgggtctgtggtaacactgctg

ggaatgagcgagtgtggaaagcgagtcgcggttcacgtctatggtgtgcgccattatttt

tacatggcaaaggcggaggtggatagcgcttgtggaataaccaccgaggcagaactcgtg

cgcgcaatggtggactgcgcgcacagctcggctttgagcgcggccctgggaaatggcaac

ggcggcaaacagagcggcggcagcggcgggggatggtggggcggaaagcacgtgtctgcg

gactgcttcaaagtggagaccgtgtgccacacgacgctgtactactttggatctaagcca

gctctctactatagagtatctgcctccagcagccgcctgggagggttcatctgcgacaac

tttcacccggagattacaaaattcgaggggagcgtggacgtgaccacgcggctgttgttg

gacaatgaaaattttaccagtttcgggtggtaccgcctgcgacccggcacccacggagag

cgtgttcaacttcgccccgttgagcgacacgtcacctcaagcgacgtggagattaactgt

actcccgataacctggagccgataccagacgaggctgcctggcccgactataagctcatg

tgctttgatatagagtgtaaagctggaacgggtaacgaaatggcgttcccagtggcaact

aaccaagaggacctggtcatccagatctcctgtctgctgtactcgcttgctactcagaac

cacgaacacaccctgctgttttccctcgggtcatgcgatatctctgaggaatactcgttt

gcatgcgtccagcgcggcgagcccagaccgacggttttggagtttgacagcgagtacgag

ctgctggttgccttcctgacctttctcaagcagtactctcccgagttcgccaccggctac

aacatcgttaattttgactgggcgtacatagttaacaaggtaacgtcggtgtataacatc

aagctggacgggtacggcaagttcaacaaaggggggctgtttaaggtgtgggacatcgcc

acgaaccattttcagaagaagagcaaggtgaaaatcaatggcctgatatctctagacatg

tattctgtggcgacggaaaagctaaagctacccagctacaaactcgacgcggtcgtggga

gacgtcctcggcgagcataagatagaccttccctataaagaaataccctcctattacgcg

ggagggcctgaccggaggggcgtaataggagagtattgtatccaggactctaggctggtg

ggcaagctgttttttaagtacctcccccatctggaactatcggcggtggccaaactcgcc

cgtatcaccctaacgcgggtaatttttgacggtcagcaaattcgcgtgtacacgtgcttg

ctgaaactcgcccgcgagagaaatttcattttgccagacaacagacgccggtttgacagt

caggcagacgccgcgtcagagacttcggagttggctatggatagccaaagccacgccttc

gacagtacagacgaacccgacggtgtggacggtaccccggacgccgcaggatctggcgct

acttctgaaaacggaggcgggaagcccggcgtcgggagggccgtgggctaccagggagca

aaggttctagaccccgtatccggctttcatgtggaccccgtggttgtgtttgacttcgct

agcttatacccaagcattatccaggcccataacctctgtttcaccaccctggcgctcgat

gaagtggatctggccgggcttcaaccatccgtcaactactcgacgttcgaggtgggtgac

caaaagttattttttgtccacgcccatattcgcgaaagcctgcttggcatcttgctgcgc

gactggctggccatgcgaaaggcggtgagggcgcgaatccccaccagcacccccgaggag

gcagttttactagataagcagcagtctgcgattaaggtgatatgcaactcggtttacgga

ttcacgggggtggcaaacggcctgttgccgtgtctgaggatagcggctaccgttaccacg

ataggacgcgacatgctcctcaagacccgagattacgttcactctcgttgggcgacgcgc

gagctgctggaggacaattttccaggggctataggtttccgaaaccacaagccttactcc

gtcagggttatctacggagacaccgactccgtgtttatcaagtttgtgggcctgacgtac

gagggggtatcggagctgggggatgctatgtcgcgtcagatttcagcggacctctttaga

gcgcccatcaaactggagtgtgagaagacctttcagcgactgctgctgatcaccaagaag

aagtacataggtgtcataaacggggggaagatgctcatgaagggggtcgacctggtccgc

aaaaataactgctctttcataaacttgtacgcgcgacatctggtagatcttttgttgtac

gacgaggatgtggccacggcggcagcagaggtgacagacgtgcctcccgcagaatgggtg

gggcgcccgctaccgagcggctttgacaagtttgggcgagtgctggtagaggcgtacaac

cgtatcactgcccccaacttggacgtgcgcgagttcgttatgactgctgagctgagccgc

tcacccgaatcgtataccaacaagcgcctgccgcacctcaccgtctactttaagctcgcc

atgaggaatgaagaactgcccagcgtaaaagagagaattccgtatgtgatagttgcgcag

accgaggccgcggaacgcgaagcgggtgtagtaaactcaatgcgcggtaccgcccaaaac

cccgtggtaaccaagaccgcacgcccccaacctaaacgcaaactgctggtttccgacctc

gccgaagacccgacctatgtttccgagaatgacgtaccgctaaacacagactactatttc

tcccacctgttgggtaccataagcgtgacctttaaggctctattcggaaatgatgtgaga

acaacagaaaatcttttaaagcggtttattccggaaaccccccacaagacccccacgaaa

acccaggcactgcttgagcgcgccggctttgaaaagctgacgccctttacaccggaggaa

gaaagtcgtcgaatactgcatacagttttttgtactctagaagcagctccccatcaaagc

tga

>KC924801.1

atggcggcgcgcgaacaggccaactccgttcgcaggagcgggttttttaatccgtttatt

ggcaagaggccatttttcaggccgggaagcgggcagaccgcggagacagagcgcccaagg

cccccccaacactcgtactgcacagaggtgggtagctttaagtttatagctccaagatgc

ctcgatgaagaagcccccgccgaccagcgacgcggtgtacacgtgggcaccttggagcgc

ccacccaaagtgtactgcgatggctcagagtacgacgtgctgaactttgcctccggaggt

tgctggcctcgcaggattcgggtttggaacggccaggattttcggggcgatggattcaac

cccagatttgagagatttcacgtgtacgacatagtagagacttcggagagcgcctcacac

gatgacccatccaggtttgctgagctatcccgcccaagcgggtctgtggtaacactgctg

ggaatgagcgagtgtggaaagcgagtcgcggttcacgtctatggtgtgcgccattatttt

tacatggcaaaggcggaggtggatagcgcttgtggaataaccaccgaggcagaactcgtg

cgcgcaatggtggactgcgcgcacagctcggctttgagcgcggccctgggaaatggcaac

ggcggcaaacagagcggcggcagcggcgggggatggtggggcggaaagcacgtgtctgcg

gactgcttcaaagtggagaccgtgtgccacacgacgctgtactactttggatctaagcca

gctctctactatagagtatctgcctccagcagccgcctgggagggttcatctgcgacaac

tttcacccggagattacaaaattcgaggggagcgtggacgtgaccacgcggctgttgttg

gacaatgaaaattttaccagtttcgggtggtaccgcctgcgacccggcacccacggagag

cgtgttcaacttcgccccgttgagcgacacgtcacctcaagcgacgtggagattaactgt

actcccgataacctggagccgataccagacgaggctgcctggcccgactataagctcatg

tgctttgatatagagtgtaaagctggaacgggtaacgaaatggcgttcccagtggcaact

aaccaagaggacctggtcatccagatctcctgtctgctgtactcgcttgctactcagaac

cacgaacacaccctgctgttttccctcgggtcatgcgatatctctgaggaatactcgttt

gcatgcgtccagcgcggcgagcccagaccgacggttttggagtttgacagcgagtacgag

ctgctggttgccttcctgacctttctcaagcagtactctcccgagttcgccaccggctac

aacatcgttaattttgactgggcgtacatagttaacaaggtaacgtcggtgtataacatc

aagctggacgggtacggcaagttcaacaaaggggggctgtttaaggtgtgggacatcgcc

acgaaccattttcagaagaagagcaaggtgaaaatcaatggcctgatatctctagacatg

tattctgtggcgacggaaaagctaaagctacccagctacaaactcgacgcggtcgtggga

gacgtcctcggcgagcataagatagaccttccctataaagaaataccctcctattacgcg

ggagggcctgaccggaggggcgtaataggagagtattgtatccaggactctaggctggtg

ggcaagctgttttttaagtacctcccccatctggaactatcggcggtggccaaactcgcc

cgtatcaccctaacgcgggtaatttttgacggtcagcaaattcgcgtgtacacgtgcttg

ctgaaactcgcccgcgagagaaatttcattttgccagacaacagacgccggtttgacagt

caggcagacgccgcgtcagagacttcggagttggctatggatagccaaagccacgccttc

gacagtacagacgaacccgacggtgtggacggtaccccggacgccgcaggatctggcgct

acttctgaaaacggaggcgggaagcccggcgtcgggagggccgtgggctaccagggagca

aaggttctagaccccgtatccggctttcatgtggaccccgtggttgtgtttgacttcgct

agcttatacccaagcattatccaggcccataacctctgtttcaccaccctggcgctcgat

gaagtggatctggccgggcttcaaccatccgtcgactactcgacgttcgaggtgggtgac

caaaagttattttttgtccacgcccatattcgcgaaagcctgcttggcatcttgctgcgc

gactggctggccatgcgaaaggcggtgagggcgcgaatccccaccagcacccccgaggag

gcagttttactagataagcagcagtctgcgattaaggtgatatgcaactcggtttacgga

ttcacgggggtggcaaacggcctgttgccgtgtctgaggatagcggctaccgttaccacg

ataggacgcgacatgctcctcaagacccgagattacgttcactctcgttgggcgacgcgc

gagctgctggaggacaattttccaggggctataggtttccgaaaccacaagccttactcc

gtcagggttatctacggagacaccgactccgtgtttatcaagtttgtgggcctgacgtac

gagggggtatcggagctgggggatgctatgtcgcgtcagatttcagcggacctctttaga

gcgcccatcaaactggagtgtgagaagacctttcagcgactgctgctgatcaccaagaag

aagtacataggtgtcataaacggggggaagatgctcatgaagggggtcgacctggtccgc

aaaaataactgctctttcataaacttgtacgcgcgacatctggtagatcttttgttgtac

gacgaggatgtggccacggcggcagcagaggtgacagacgtgcctcccgcagaatgggtg

gggcgcccgctaccgagcggctttgacaagtttgggcgagtgctggtagaggcgtacaac

cgtatcactgcccccaacttggacgtgcgcgagttcgttatgactgctgagctgagccgc

tcacccgaatcgtataccaacaagcgcctgccgcacctcaccgtctactttaagctcgcc

atgaggaatgaagaactgcccagcgtaaaagagagaattccgtatgtgatagttgcgcag

accgaggccgcggaacgcgaagcgggtgtagtaaactcaatgcgcggtaccgcccaaaac

cccgtggtaaccaagaccgcacgcccccaacctaaacgcaaactgctggtttccgacctc

gccgaagacccgacctatgtttccgagaatgacgtaccgctaaacacagactactatttc

tcccacctgttgggtaccataagcgtgacctttaaggctctattcggaaatgatgtgaga

acaacagaaaatcttttaaagcggtttattccggaaaccccccacaagacccccacgaaa

acccaggcactgcttgagcgcgccggctttgaaaagctgacgccctttacaccggaggaa

gaaagtcgtcgaatactgcatacagttttttgtactctagaagcagctccccatcaaagc

tga

>KC924802.1

atggcggcgcgcgaacaggccaactccgttcgcaggagcgggttttttaatccgtttatt

ggcaagaggccatttttcaggccgggaagcgggcagaccgcggagacagagcgcccaagg

cccccccaacactcgtactgcacagaggtgggtagctttaagtttatagctccaagatgc

ctcgatgaagaagcccccgccgaccagcgacgcggtgtacacgtgggcaccttggagcgc

ccacccaaagtgtactgcgatggctcagagtacgacgtgctgaactttgcctccggaggt

tgctggcctcgcaggattcgggtttggaacggccaggattttcggggcgatggattcaac

cccagatttgagagatttcacgtgtacgacatagtagagacttcggagagcgcctcacac

gatgacccatccaggtttgctgagctatcccgcccaagcgggtctgtggtaacactgctg

ggaatgagcgagtgtggaaagcgagtcgcggttcacgtctatggtgtgcgccattatttt

tacatggcaaaggcggaggtggatagcgcttgtggaataaccaccgaggcagaactcgtg

cgcgcaatggtggactgcgcgcacagctcggctttgagcgcggccctgggaaatggcaac

ggcggcaaacagagcggcggcagcggcgggggatggtggggcggaaagcacgtgtctgcg

gactgcttcaaagtggagaccgtgtgccacacgacgctgtactactttggatctaagcca

gctctctactatagagtatctgcctccagcagccgcctgggagggttcatctgcgacaac

tttcacccggagattacaaaattcgaggggagcgtggacgtgaccacgcggctgttgttg

gacaatgaaaattttaccagtttcgggtggtaccgcctgcgacccggcacccacggagag

cgtgttcaacttcgccccgttgagcgacacgtcacctcaagcgacgtggagattaactgt

actcccgataacctggagccgataccagacgaggctgcctggcccgactataagctcatg

tgctttgatatagagtgtaaagctggaacgggtaacgaaatggcgttcccagtggcaact

aaccaagaggacctggtcatccagatctcctgtctgctgtactcgcttgctactcagaac

cacgaacacaccctgctgttttccctcgggtcatgcgatatctctgaggaatactcgttt

gcatgcgtccagcgcggcgagcccagaccgacggttttggagtttgacagcgagtacgag

ctgctggttgccttcctgacctttctcaagcagtactctcccgagttcgccaccggctac

aacatcgttaattttgactgggcgtacatagttaacaaggtaacgtcggtgtataacatc

aagctggacgggtacggcaagttcaacaaaggggggctgtttaaggtgtgggacatcgcc

acgaaccattttcagaagaagagcaaggtgaaaatcaatggcctgatatctctagacatg

tattctgtggcgacggaaaagctaaagctacccagctacaaactcgacgcggtcgtggga

gacgtcctcggcgagcataagatagaccttccctataaagaaataccctcctattacgcg

ggagggcctgaccggaggggcgtaataggagagtattgtatccaggactctaggctggtg

ggcaagctgttttttaagtacctcccccatctggaactatcggcggtggccaaactcgcc

cgtatcaccctaacgcgggtaatttttgacggtcagcaaattcgcgtgtacacgtgcttg

ctgaaactcgcccgcgagagaaatttcattttgccagacaacagacgccggtttgacagt

caggcagacgccgcgtcagagacttcggagttggctatggatagccaaagccacgccttc

gacagtacagacgaacccgacggtgtggacggtaccccggacgccgcaggatctggcgct

acttctgaaaacggaggcgggaagcccggcgtcgggagggccgtgggctaccagggagca

aaggttctagaccccgtatccggctttcatgtggaccccgtggttgtgtttgacttcgct

agcttatacccaagcattatccaggcccataacctctgtttcaccaccctggcgctcgat

gaagtggatctggccgggcttcaaccatccgtcaactactcgacgttcgaggtgggtgac

caaaagttattttttgtccacgcccatattcgcgaaagcctgcttggcatcttgctgcgc

gactggctggccatgcgaaaggcggtgagggcgcgaatccccaccagcacccccgaggag

gcagttttactagataagcagcagtctgcgattaaggtgatatgcaactcggtttacgga

ttcacgggggtggcaaacggcctgttgccgtgtctgaggatagcggctaccgttaccacg

ataggacgcgacatgctcctcaagacccgagattacgttcactctcgttgggcgacgcgc

gagctgctggaggacaattttccaggggctataggtttccgaaaccacaagccttactcc

gtcagggttatctacggagacaccgactccgtgtttatcaagtttgtgggcctgacgtac

gagggggtatcggagctgggggatgctatgtcgcgtcagatttcagcggacctctttaga

gcgcccatcaaactggagtgtgagaagacctttcagcgactgctgctgatcaccaagaag

aagtacataggtgtcataaacggggggaagatgctcatgaagggggtcgacctggtccgc

aaaaataactgctctttcataaacttgtacgcgcgacatctggtagatcttttgttgtac

gacgaggatgtggccacggcggcagcagaggtgacagacgtgcctcccgcagaatgggtg

gggcgcccgctaccgagcggctttgacaagtttgggcgagtgctggtagaggcgtacaac

cgtatcactgcccccaacttggacgtgcgcgagttcgttatgactgctgagctgagccgc

tcacccgaatcgtataccaacaagcgcctgccgcacctcaccgtctactttaagctcgcc

atgaggaatgaagaactgcccagcgtaaaagagagaattccgtatgtgatagttgcgcag

accgaggccgcggaacgcgaagcgggtgtagtaaactcaatgcgcggtaccgcccaaaac

cccgtggtaaccaagaccgcacgcccccaacctaaacgcaaactgctggtttccgacctc

gccgaagacccgacctatgtttccgagaatgacgtaccgctaaacacagactactatttc

tcccacctgttgggtaccataagcgtgacctttaaggctctattcggaaatgatgtgaga

acaacagaaaatcttttaaagcggtttattccggaaaccccccacaagacccccacgaaa

acccaggcactgcttgagcgcgccggctttgaaaagctgacgccctttacaccggaggaa

gaaagtcgtcgaatactgcatacagttttttgtactctagaagcagctccccatcaaagc

tga

>KC924803.1

atggcggcgcgcgaacaggccaactccgttcgcaggagcgggttttttaatccgtttatt

ggcaagaggccatttttcaggccgggaagcgggcagaccgcggagacagagcgcccaagg

cccccccaacactcgtactgcacagaggtgggtagctttaagtttatagctccaagatgc

ctcgatgaagaagcccccgccgaccagcgacgcggtgtacacgtgggcaccttggagcgc

ccacccaaagtgtactgcgatggctcagagtacgacgtgctgaactttgcctccggaggt

tgctggcctcgcaggattcgggtttggaacggccaggattttcggggcgatggattcaac

cccagatttgagagatttcacgtgtacgacatagtagagacttcggagagcgcctcacac

gatgacccatccaggtttgctgagctatcccgcccaagcgggtctgtggtaacactgctg

ggaatgagcgagtgtggaaagcgagtcgcggttcacgtctatggtgtgcgccattatttt

tacatggcaaaggcggaggtggatagcgcttgtggaataaccaccgaggcagaactcgtg

cgcgcaatggtggactgcgcgcacagctcggctttgagcgcggccctgggaaatggcaac

ggcggcaaacagagcggcggcagcggcgggggatggtggggcggaaagcacgtgtctgcg

gactgcttcaaagtggagaccgtgtgccacacgacgctgtactactttggatctaagcca

gctctctactatagagtatctgcctccagcagccgcctgggagggttcatctgcgacaac

tttcacccggagattacaaaattcgaggggagcgtggacgtgaccacgcggctgttgttg

gacaatgaaaattttaccagtttcgggtggtaccgcctgcgacccggcacccacggagag

cgtgttcaacttcgccccgttgagcgacacgtcacctcaagcgacgtggagattaactgt

actcccgataacctggagccgataccagacgaggctgcctggcccgactataagctcatg

tgctttgatatagagtgtaaagctggaacgggtaacgaaatggcgttcccagtggcaact

aaccaagaggacctggtcatccagatctcctgtctgctgtactcgcttgctactcagaac

cacgaacacaccctgctgttttccctcgggtcatgcgatatctctgaggaatactcgttt

gcatgcgtccagcgcggcgagcccagaccgacggttttggagtttgacagcgagtacgag

ctgctggttgccttcctgacctttctcaagcagtactctcccgagttcgccaccggctac

aacatcgttaattttgactgggcgtacatagttaacaaggtaacgtcggtgtataacatc

aagctggacgggtacggcaagttcaacaaaggggggctgtttaaggtgtgggacatcgcc

acgaaccattttcagaagaagagcaaggtgaaaatcaatggcctgatatctctagacatg

tattctgtggcgacggaaaagctaaagctacccagctacaaactcgacgcggtcgtggga

gacgtcctcggcgagcataagatagaccttccctataaagaaataccctcctattacgcg

ggagggcctgaccggaggggcgtaataggagagtattgtatccaggactctaggctggtg

ggcaagctgttttttaagtacctcccccatctggaactatcggcggtggccaaactcgcc

cgtatcaccctaacgcgggtaatttttgacggtcagcaaattcgcgtgtacacgtgcttg

ctgaaactcgcccgcgagagaaatttcattttgccagacaacagacgccggtttgacagt

caggcagacgccgcgtcagagacttcggagttggctatggatagccaaagccacgccttc

gacagtacagacgaacccgacggtgtggacggtaccccggacgccgcaggatctggcgct

acttctgaaaacggaggcgggaagcccggcgtcgggagggccgtgggctaccagggagca

aaggttctagaccccgtatccggctttcatgtggaccccgtggttgtgtttgacttcgct

agcttatacccaagcattatccaggcccataacctctgtttcaccaccctggcgctcgat

gaagtggatctggccgggcttcaaccatccgtcaactactcgacgttcgaggtgggtgac

caaaagttattttttgtccacgcccatattcgcgaaagcctgcttggcatcttgctgcgc

gactggctggccatgcgaaaggcggtgagggcgcgaatccccaccagcacccccgaggag

gcagttttactagataagcagcagtctgcgattaaggtgatatgcaactcggtttacgga

ttcacgggggtggcaaacggcctgttgccgtgtctgaggatagcggctaccgttaccacg

ataggacgcgacatgctcctcaagacccgagattacgttcactctcgttgggcgacgcgc

gagctgctggagaacaattttccaggggctataggtttccgaaaccacaagccttactcc

gtcagggttatctacggagacaccgactccgtgtttatcaagtttgtgggcctgacgtac

gagggggtatcggagctgggggatgctatgtcgcgtcagatttcagcggacctctttaga

gcgcccatcaaactggagtgtgagaagacctttcagcgactgctgctgatcaccaagaag

aagtacataggtgtcataaacggggggaagatgctcatgaagggggtcgacctggtccgc

aaaaataactgctctttcataaacttgtacgcgcgacatctggtagatcttttgttgtac

gacgaggatgtggccacggcggcagcagaggtgacagacgtgcctcccgcagaatgggtg

gggcgcccgctaccgagcggctttgacaagtttgggcgagtgctggtagaggcgtacaac

cgtatcactgcccccaacttggacgtgcgcgagttcgttatgactgctgagctgagccgc

tcacccgaatcgtataccaacaagcgcctgccgcacctcaccgtctactttaagctcgcc

atgaggaatgaagaactgcccagcgtaaaagagagaattccgtatgtgatagttgcgcag

accgaggccgcggaacgcgaagcgggtgtagtaaactcaatgcgcggtaccgcccaaaac

cccgtggtaaccaagaccgcacgcccccaacctaaacgcaaactgctggtttccgacctc

gccgaagacccgacctatgtttccgagaatgacgtaccgctaaacacagactactatttc

tcccacctgttgggtaccataagcgtgacctttaaggctctattcggaaatgatgtgaga

acaacagaaaatcttttaaagcggtttattccggaaaccccccacaagacccccacgaaa

acccaggcactgcttgagcgcgccggctttgaaaagctgacgccctttacaccggaggaa

gaaagtcgtcgaatactgcatacagttttttgtactctagaagcagctccccatcaaagc

tga

>KC924804.1

atggcggcgcgcgaacaggccaactccgttcgcaggagcgggttttttaatccgtttatt

ggcaagaggccatttttcaggccgggaagcgggcagaccgcggagacagagcgcccaagg

cccccccaacactcgtactgcacagaggtgggtagctttaagtttatagctccaagatgc

ctcgatgaagaagcccccgccgaccagcgacgcggtgtacacgtgggcaccttggagcgc

ccacccaaagtgtactgcgatggctcagagtacgacgtgctgaactttgcctccggaggt

tgctggcctcgcaggattcgggtttggaacggccaggattttcggggcgatggattcaac

cccagatttgagagatttcacgtgtacgacatagtagagacttcggagagcgcctcacac

gatgacccatccaggtttgctgagctatcccgcccaagcgggtctgtggtaacactgctg

ggaatgagcgagtgtggaaagcgagtcgcggttcacgtctatggtgtgcgccattatttt

tacatggcaaaggcggaggtggatagcgcttgtggaataaccaccgaggcagaactcgtg

cgcgcaatggtggactgcgcgcacagctcggctttgagcgcggccctgggaaatggcaac

ggcggcaaacagagcggcggcagcggcgggggatggtggggcggaaagcacgtgtctgcg

gactgcttcaaagtggagaccgtgtgccacacgacgctgtactactttggatctaagcca

gctctctactatagagtatctgcctccagcagccgcctgggagggttcatctgcgacaac

tttcacccggagattacaaaattcgaggggagcgtggacgtgaccacgcggctgttgttg

gacaatgaaaattttaccagtttcgggtggtaccgcctgcgacccggcacccacggagag

cgtgttcaacttcgccccgttgagcgacacgtcacctcaagcgacgtggagattaactgt

actcccgataacctggagccgataccagacgaggctgcctggcccgactataagctcatg

tgctttgatatagagtgtaaagctggaacgggtaacgaaatggcgttcccagtggcaact

aaccaagaggacctggtcatccagatctcctgtctgctgtactcgcttgctactcagaac

cacgaacacaccctgctgttttccctcgggtcatgcgatatctctgaggaatactcgttt

gcatgcgtccagcgcggcgagcccagaccgacggttttggagtttgacagcgagtacgag

ctgctggttgccttcctgacctttctcaagcagtactctcccgagttcgccaccggctac

aacatcgttaattttgactgggcgtacatagttaacaaggtaacgtcggtgtataacatc

aagctggacgggtacggcaagttcaacaaaggggggctgtttaaggtgtgggacatcgcc

acgaaccattttcagaagaagagcaaggtgaaaatcaatggcctgatatctctagacatg

tattctgtggcgacggaaaagctaaagctacccagctacaaactcgacgcggtcgtggga

gacgtcctcggcgagcataagatagaccttccctataaagaaataccctcctattacgcg

ggagggcctgaccggaggggcgtaataggagagtattgtatccaggactctaggctggtg

ggcaagctgttttttaagtacctcccccatctggaactatcggcggtggccaaactcgcc

cgtatcaccctaacgcgggtaatttttgacggtcagcaaattcgcgtgtacacgtgcttg

ctgaaactcgcccgcgagagaaatttcattttgccagacaacagacgccggtttgacagt

caggcagacgccgcgtcagagacttcggagttggctatggatagccaaagccacgccttc

gacagtacagacgaacccgacggtgtggacggtaccccggacgccgcaggatctggcgct

acttctgaaaacggaggcgggaagcccggcgtcgggagggccgtgggctaccagggagca

aaggttctagaccccgtatccggctttcatgtggaccccgtggttgtgtttgacttcgct

agcttatacccaagcattatccaggcccataacctctgtttcaccaccctggcgctcgat

gaagtggatctggccgggcttcaaccatccgtcaactactcgacgttcgaggtgggtgac

caaaagttattttttgtccacgcccatattcgcgaaagcctgcttggcatcttgctgcgc

gactggctggccatgcgaaaggcggtgagggcgcgaatccccaccagcacccccgaggag

gcagttttactagataagcagcagtctgcgattaaggtgatatgcaactcggtttacgga

ttcacgggggtggcaaacggcctgttgccgtgtctgaggatagcggctaccgttaccacg

ataggacgcgacatgctcctcaagacccgagattacgttcactctcgttgggcgacgcgc

gagctgctggaggacaattttccaggggctataggtttccgaaaccacaagccttactcc

gtcagggttatctacggagacaccgactccgtgtttatcaagtttgtgggcctgacgtac

gagggggtatcggagctgggggatgctatgtcgcgtcagatttcagcggacctctttaga

gcgcccatcaaactggagtgtgagaagacctttcagcgactgctgctgatcaccaagaag

aagtacataggtgtcataaacggggggaagatgctcatgaagggggtcgacctggtccgc

aaaaataactgctctttcataaacttgtacgcgcgacatctggtagatcttttgttgtac

gacgaggatgtggccacggcggcagcagaggtgacagacgtgcctcccgcagaatgggtg

gggcgcccgctaccgagcggctttgacaagtttgggcgagtgctggtagaggcgtacaac

cgtatcactgcccccaacttggacgtgcgcgagttcgttatgactgctgagctgagccgc

tcacccgaatcgtataccaacaagcgcctgccgcacctcaccgtctactttaagctcgcc

atgaggaatgaagaactgcccagcgtaaaagagagaattccgtatgtgatagttgcgcag

accgaggccgcggaacgcgaagcgggtgtagtaaactcaatgcgcggtaccgcccaaaac

cccgtggtaaccaagaccgcacgcccccaacctaaacgcaaactgctggtttccgacctc

gccgaagacccgacctatgtttccgagaatgacgtaccgctaaacacagactactatttc

tcccacctgttgggtaccataagcgtgacctttaaggctctattcggaaatgatgtgaga

acaacagaaaatcttttaaagcggtttattccggaaaccccccacaagacccccacgaaa

acccaggcactgcttgagcgcgccggctttgaaaagctgacgccctttacaccggaggaa

gaaagtcgtcgaatactgcatacagttttttgtactctagaagcagctccccatcaaagc

tga

>KC924805.1

atggcggcgcgcgaacaggccaactccgttcgcaggagcgggttttttaatccgtttatt

ggcaagaggccatttttcaggccgggaagcgggcagaccgcggagacagagcgcccaagg

cccccccaacactcgtactgcacagaggtgggtagctttaagtttatagctccaagatgc

ctcgatgaagaagcccccgccgaccagcgacgcggtgtacacgtgggcaccttggagcgc

ccacccaaagtgtactgcgatggctcagagtacgacgtgctgaactttgcctccggaggt

tgctggcctcgcaggattcgggtttggaacggccaggattttcggggcgatggattcaac

cccagatttgagagatttcacgtgtacgacatagtagagacttcggagagcgcctcacac

gatgacccatccaggtttgctgagctatcccgcccaagcgggtctgtggtaacactgctg

ggaatgagcgagtgtggaaagcgagtcgcggttcacgtctatggtgtgcgccattatttt

tacatggcaaaggcggaggtggatagcgcttgtggaataaccaccgaggcagaactcgtg

cgcgcaatggtggactgcgcgcacagctcggctttgagcgcggccctgggaaatggcaac

ggcggcaaacagagcggcggcagcggcgggggatggtggggcggaaagcacgtgtctgcg

gactgcttcaaagtggagaccgtgtgccacacgacgctgtactactttggatctaagcca

gctctctactatagagtatctgcctccagcagccgcctgggagggttcatctgcgacaac

tttcacccggagattacaaaattcgaggggagcgtggacgtgaccacgcggctgttgttg

gacaatgaaaattttaccagtttcgggtggtaccgcctgcgacccggcacccacggagag

cgtgttcaacttcgccccgttgagcgacacgtcacctcaagcgacgtggagattaactgt

actcccgataacctggagccgataccagacgaggctgcctggcccgactataagctcatg

tgctttgatatagagtgtaaagctggaacgggtaacgaaatggcgttcccagtggcaact

aaccaagaggacctggtcatccagatctcctgtctgctgtactcgcttgctactcagaac

cacgaacacaccctgctgttttccctcgggtcatgcgatatctctgaggaatactcgttt

gcatgcgtccagcgcggcgagcccagaccgacggttttggagtttgacagcgagtacgag

ctgctggttgccttcctgacctttctcaagcagtactctcccgagttcgccaccggctac

aacatcgttaattttgactgggcgtacatagttaacaaggtaacgtcggtgtataacatc

aagctggacgggtacggcaagttcaacaaaggggggctgtttaaggtgtgggacatcgcc

acgaaccattttcagaagaagagcaaggtgaaaatcaatggcctgatatctctagacatg

tattctgtggcgacggaaaagctaaagctacccagctacaaactcgacgcggtcgtggga

gacgtcctcggcgagcataagatagaccttccctataaagaaataccctcctattacgcg

ggagggcctgaccggaggggcgtaataggagagtattgtatccaggactctaggctggtg

ggcaagctgttttttaagtacctcccccatctggaactatcggcggtggccaaactcgcc

cgtatcaccctaacgcgggtaatttttgacggtcagcaaattcgcgtgtacacgtgcttg

ctgaaactcgcccgcgagagaaatttcattttgccagacaacagacgccggtttgacagt

caggcagacgccgcgtcagagacttcggagttggctatggatagccaaagccacgccttc

gacagtacagacgaacccgacggtgtggacggtaccccggacgccgcaggatctggcgct

acttctgaaaacggaggcgggaagcccggcgtcgggagggccgtgggctaccagggagca

aaggttctagaccccgtatccggctttcatgtggaccccgtggttgtgtttgacttcgct

agcttatacccaagcattatccaggcccataacctctgtttcaccaccctggcgctcgat

gaagtggatctggccgggcttcaaccatccgtcaactactcgacgttcgaggtgggtgac

caaaagttattttttgtccacgcccatattcgcgaaagcctgcttggcatcttgctgcgc

gactggctggccatgcgaaaggcggtgagggcgcgaatccccaccagcacccccgaggag

gcagttttactagataagcagcagtctgcgattaaggtgatatgcaactcggtttacgga

ttcacgggggtggcaaacggcctgttgccgtgtctgaggatagcggctaccgttaccacg

ataggacgcgacatgctcctcaagacccgagattacgttcactctcgttgggcgacgcgc

gagctgctggaggacaattttccaggggctataggtttccgaaaccacaagccttactcc

gtcagggttatctacggagacaccgactccgtgtttatcaagtttgtgggcctgacgtac

gagggggtatcggagctgggggatgctatgtcgcgtcagatttcagcggacctctttaga

gcgcccatcaaactggagtgtgagaagacctttcagcgactgctgctgatcaccaagaag

aagtacataggtgtcataaacggggggaagatgctcatgaagggggtcgacctggtccgc

aaaaataactgctctttcataaacttgtacgcgcgacatctggtagatcttttgttgtac

gacgaggatgtggccacggcggcagcagaggtgacagacgtgcctcccgcagaatgggtg

gggcgcccgctaccgagcggctttgacaagtttgggcgagtgctggtagaggcgtacaac

cgtatcactgcccccaacttggacgtgcgcgagttcgttatgactgctgagctgagccgc

tcacccgaattgtataccaacaagcgcctgccgcacctcaccgtctactttaagctcgcc

atgaggaatgaagaactgcccagcgtaaaagagagaattccgtatgtgatagttgcgcag

accgaggccgcggaacgcgaagcgggtgtagtaaactcaatgcgcggtaccgcccaaaac

cccgtggtaaccaagaccgcacgcccccaacctaaacgcaaactgctggtttccgacctc

gccgaagacccgacctatgtttccgagaatgacgtaccgctaaacacagactactatttc

tcccacctgttgggtaccataagcgtgacctttaaggctctattcggaaatgatgtgaga

acaacagaaaatcttttaaagcggtttattccggaaaccccccacaagacccccacgaaa

acccaggcactgcttgagcgcgccggctttgaaaagctgacgccctttacaccggaggaa

gaaagtcgtcgaatactgcatacagttttttgtactctagaagcagctccccatcaaagc

tga

>KC924806.1

atggcggcgcgcgaacaggccaactccgttcgcaggagcgggttttttaatccgtttatt

ggcaagaggccatttttcaggccgggaagcgggcagaccgcggagacagagcgcccaagg

cccccccaacactcgtactgcacagaggtgggtagctttaagtttatagctccaagatgc

ctcgatgaagaagcccccgccgaccagcgacgcggtgtacacgtgggcaccttggagcgc

ccacccaaagtgtactgcgatggctcagagtacgacgtgctgaactttgcctccggaggt

tgctggcctcgcaggattcgggtttggaacggccaggattttcggggcgatggattcaac

cccagatttgagagatttcacgtgtacgacatagtagagacttcggagagcgcctcacac

gatgacccatccaggtttgctgagctatcccgcccaagcgggtctgtggtaacactgctg

ggaatgagcgagtgtggaaagcgagtcgcggttcacgtctatggtgtgcgccattatttt

tacatggcaaaggcggaggtggatagcgcttgtggaataaccaccgaggcagaactcgtg

cgcgcaatggtggactgcgcgcacagctcggctttgagcgcggccctgggaaatggcaac

ggcggcaaacagagcggcggcagcggcgggggatggtggggcggaaagcacgtgtctgcg

gactgcttcaaagtggagaccgtgtgccacacgacgctgtactactttggatctaagcca

gctctctactatagagtatctgcctccagcagccgcctgggagggttcatctgcgacaac

tttcacccggagattacaaaattcgaggggagcgtggacgtgaccacgcggctgttgttg

gacaatgaaaattttaccagtttcgggtggtaccgcctgcgacccggcacccacggagag

cgtgttcaacttcgccccgttgagcgacacgtcacctcaagcgacgtggagattaactgt

actcccgataacctggagccgataccagacgaggctgcctggcccgactataagctcatg

tgctttgatatagagtgtaaagctggaacgggtaacgaaatggcgttcccagtggcaact

aaccaagaggacctggtcatccagatctcctgtctgctgtactcgcttgctactcagaac

cacgaacacaccctgctgttttccctcgggtcatgcgatatctctgaggaatactcgttt

gcatgcgtccagcgcggcgagcccagaccgacggttttggagtttgacagcgagtacgag

ctgctggttgccttcctgacctttctcaagcagtactctcccgagttcgccaccggctac

aacatcgttaattttgactgggcgtacatagttaacaaggtaacgtcggtgtataacatc

aagctggacgggtacggcaagttcaacaaaggggggctgtttaaggtgtgggacatcgcc

acgaaccattttcagaagaagagcaaggtgaaaatcaatggcctgatatctctagacatg

tattctgtggcgacggaaaagctaaagctacccagctacaaactcgacgcggtcgtggga

gacgtcctcggcgagcataagatagaccttccctataaagaaataccctcctattacgcg

ggagggcctgaccggaggggcgtaataggagagtattgtatccaggactctaggctggtg

ggcaagctgttttttaagtacctcccccatctggaactatcggcggtggccaaactcgcc

cgtatcaccctaacgcgggtaatttttgacggtcagcaaattcgcgtgtacacgtgcttg

ctgaaactcgcccgcgagagaaatttcattttgccagacaacagacgccggtttgacagt

caggcagacgccgcgtcagagacttcggagttggctatggatagccaaagccacgccttc

gacagtacagacgaacccgacggtgtggacggtaccccggacgccgcaggatctggcgct

acttctgaaaacggaggcgggaagcccggcgtcgggagggccgtgggctaccagggagca

aaggttctagaccccgtatccggctttcatgtggaccccgtggttgtgtttgacttcgct

agcttatacccaagcattatccaggcccataacctctgtttcaccaccctggcgctcgat

gaagtggatctggccgggcttcaaccatccgtcgactactcgacgttcgaggtgggtgac

caaaagttattttttgtccacgcccatattcgcgaaagcctgcttggcatcttgctgcgc

gactggctggccatgcgaaaggcggtgagggcgcgaatccccaccagcacccccgaggag

gcagttttactagataagcagcagtctgcgattaaggtgatatgcaactcggtttacgga

ttcacgggggtggcaaacggcctgttgccgtgtctgaggatagcggctaccgttaccacg

ataggacgcgacatgctcctcaagacccgagattacgttcactctcgttgggcgacgcgc

gagctgctggaggacaattttccaggggctataggtttccgaaaccacaagccttactcc

gtcagggttatctacggagacaccgactccgtgtttatcaagtttgtgggcctgacgtac

gagggggtatcggagctgggggatgctatgtcgcgtcagatttcagcggacctctttaga

gcgcccatcaaactggagtgtgagaagacctttcagcgactgctgctgatcaccaagaag

aagtacataggtgtcataaacggggggaagatgctcatgaagggggtcgacctggtccgc

aaaaataactgctctttcataaacttgtacgcgcgacatctggtagatcttttgttgtac

gacgaggatgtggccacggcggcagcagaggtgacagacgtgcctcccgcagaatgggtg

gggcgcccgctaccgagcggctttgacaagtttgggcgagtgctggtagaggcgtacaac

cgtatcactgcccccaacttggacgtgcgcgagttcgttatgactgctgagctgagccgc

tcacccgaatcgtataccaacaagcgcctgccgcacctcaccgtctactttaagctcgcc

atgaggaatgaagaactgcccagcgtaaaagagagaattccgtatgtgatagttgcgcag

accgaggccgcggaacgcgaagcgggtgtagtaaactcaatgcgcggtaccgcccaaaac

cccgtggtaaccaagaccgcacgcccccaacctaaacgcaaactgctggtttccgacctc

gccgaagacccgacctatgtttccgagaatgacgtaccgctaaacacagactactatttc

tcccacctgttgggtaccataagcgtgacctttaaggctctattcggaaatgatgtgaga

acaacagaaaatcttttaaagcggtttattccggaaaccccccacaagacccccacgaaa

acccaggcactgcttgagcgcgccggctttgaaaagctgacgccctttacaccggaggaa

gaaagtcgtcgaatactgcatacagttttttgtactctagaagcagctccccatcaaagc

tga

>KC924807.1

atggcggcgcgcgaacaggccaactccgttcgcaggagcgggttttttaatccgtttatt

ggcaagaggccatttttcaggccgggaagcgggcagaccgcggagacagagcgcccaagg

cccccccaacactcgtactgcacagaggtgggtagctttaagtttatagctccaagatgc

ctcgatgaagaagcccccgccgaccagcgacgcggtgtacacgtgggcaccttggagcgc

ccacccaaagtgtactgcgatggctcagagtacgacgtgttgaactttgcctccggaggt

tgctggcctcgcaggattcgggtttggaacggccaggattttcggggcgatggattcaac

cccagatttgagagatttcacgtgtacgacatagtagagacttcggagagcgcctcacac

gatgacccatccaggtttgctgagctatcccgcccaagcgggtctgtggtaacactgctg

ggaatgagcgagtgtggaaagcgagtcgcggttcacgtctatggtgtgcgccattatttt

tacatggcaaaggcggaggtggatagcgcttgtggaataaccaccgaggcagaactcgtg

cgcgcaatggtggactgcgcgcacagctcggctttgagcgcggccctgggaaatggcaac

ggcggcaaacagagcggcggcagcggcgggggatggtggggcggaaagcacgtgtctgcg

gactgcttcaaagtggagaccgtgtgccacacgacgctgtactactttggatctaagcca

gctctctactatagagtatctgcctccagcagccgcctgggagggttcatctgcgacaac

tttcacccggagattacaaaattcgaggggagcgtggacgtgaccacgcggctgttgttg

gacaatgaaaattttaccagtttcgggtggtaccgcctgcgacccggcacccacggagag

cgtgttcaacttcgccccgttgagcgacacgtcacctcaagcgacgtggagattaactgt

actcccgataacctggagccgataccagacgaggctgcctggcccgactataagctcatg

tgctttgatatagagtgtaaagctggaacgggtaacgaaatggcgttcccagtggcaact

aaccaagaggacctggtcatccagatctcctgtctgctgtactcgcttgctactcagaac

cacgaacacaccctgctgttttccctcgggtcatgcgatatctctgaggaatactcgttt

gcatgcgtccagcgcggcgagcccagaccgacggttttggagtttgacagcgagtacgag

ctgctggttgccttcctgacctttctcaagcagtactctcccgagttcgccaccggctac

aacatcgttaattttgactgggcgtacatagttaacaaggtaacgtcggtgtataacatc

aagctggacgggtacggcaagttcaacaaaggggggctgtttaaggtgtgggacatcgcc

acgaaccattttcagaagaagagcaaggtgaaaatcaatggcctgatatctctagacatg

tattctgtggcgacggaaaagctaaagctacccagctacaaactcgacgcggtcgtggga

gacgtcctcggcgagcataagatagaccttccctataaagaaataccctcctattacgcg

ggagggcctgaccggaggggcgtaataggagagtattgtatccaggactctaggctggtg

ggcaagctgttttttaagtacctcccccatctggaactatcggcggtggccaaactcgcc

cgtatcaccctaacgcgggtaatttttgacggtcagcaaattcgcgtgtacacgtgcttg

ctgaaactcgcccgcgagagaaatttcattttgccagacaacagacgccggtttgacagt

caggcagacgccgcgtcagagacttcggagttggctatggatagccaaagccacgccttc

gacagtacagacgaacccgacggtgtggacggtaccccggacgccgcaggatctggcgct

acttctgaaaacggaggcgggaagcccggcgtcgggagggtcgtgggctaccagggagca

aaggttctagaccccgtatccggctttcatgtggaccccgtggttgtgtttgacttcgct

agcttatacccaagcattatccaggcccataacctctgtttcaccaccctggcgctcgat

gaagtggatctggccgggcttcaaccatccgtcaactactcgacgttcgaggtgggtgac

caaaagttattttttgtccacgcccatattcgcgaaagcctgcttggcatcttgctgcgc

gactggctggccatgcgaaaggcggtgagggcgcgaatccccaccagcacccccgaggag

gcagttttactagataagcagcagtctgcgattaaggtgatatgcaactcggtttacgga

ttcacgggggtggcaaacggcctgttgccgtgtctgaggatagcggctaccgttaccacg

ataggacgcgacatgctcctcaagacccgagattacgttcactctcgttgggcgacgcgc

gagctgctggaggacaattttccaggggctataggtttccgaaaccacaagccttactcc

gtcagggttatctacggagacaccgactccgtgtttatcaagtttgtgggcctgacgtac

gagggggtatcggagctgggggatgctatgtcgcgtcagatttcagcggacctctttaga

gcgcccatcaaactggagtgtgagaagacctttcagcgactgctgctgatcaccaagaag

aagtacataggtgtcataaacggggggaagatgctcatgaagggggtcgacctggtccgc

aaaaataactgctctttcataaacttgtacgcgcgacatctggtagatcttttgttgtac

gacgaggatgtggccacggcggcagcagaggtgacagacgtgcctcccgcagaatgggtg

gggcgcccgctaccgagcggctttgacaagtttgggcgagtgctggtagaggcgtacaac

cgtatcactgcccccaacttggacgtgcgcgagttcgttatgactgctgagctgagccgc

tcacccgaatcgtataccaacaagcgcctgccgcacctcaccgtctactttaagctcgcc

atgaggaatgaagaactgcccagcgtaaaagagagaattccgtatgtgatagttgcgcag

accgaggccgcggaacgcgaagcgggtgtagtaaactcaatgcgcggtaccgcccaaaac

cccgtggtaaccaagaccgcacgcccccaacctaaacgcaaactgctggtttccgacctc

gccgaagacccgacctatgtttccgagaatgacgtaccgctaaacacagactactatttc

tcccacctgttgggtaccataagcgtgacctttaaggctctattcggaaatgatgtgaga

acaacagaaaatcttttaaagcggtttattccggaaaccccccacaagacccccacgaaa

acccaggcactgcttgagcgcgccggctttgaaaagctgacgccctttacaccggaggaa

gaaagtcgtcgaatactgcatacagttttttgtactctagaagcagctccccatcaaagc

tga

>KC924808.1

atggcggcgtgcgaacaggccaactccgttcgcaggagcgggttttttaatccgtttatt

ggcaagaggccatttttcaggccgggaagcgggcagaccgcggagacagagcgcccaagg

cccccccaacactcgtactgcacagaggtgggtagctttaagtttatagctccaagatgc

ctcgatgaagaagcccccgccgaccagcgacgcggtgtacacgtgggcaccttggagcgc

ccacccaaagtgtactgcgatggctcagagtacgacgtgctgaactttgcctccggaggt

tgctggcctcgcaggattcgggtttggaacggccaggattttcggggcgatggattcaac

cccagatttgagagatttcacgtgtacgacatagtagagacttcggagagcgcctcacac

gatgacccatccaggtttgctgagctatcccgcccaagcgggtctgtggtaacactgctg

ggaatgagcgagtgtggaaagcgagtcgcggttcacgtctatggtgtgcgccattatttt

tacatggcaaaggcggaggtggatagcgcttgtggaataaccaccgaggcagaactcgtg

cgcgcaatggtggactgcgcgcacagctcggctttgagcgcggccctgggaaatggcaac

ggcggcaaacagagcggcggcagcggcgggggatggtggggcggaaagcacgtgtctgcg

gactgcttcaaagtggagaccgtgtgccacacgacgctgtactactttggatctaagcca

gctctctactatagagtatctgcctccagcagccgcctgggagggttcatctgcgacaac

tttcacccggagattacaaaattcgaggggagcgtggacgtgaccacgcggctgttgttg

gacaatgaaaattttaccagtttcgggtggtaccgcctgcgacccggcacccacggagag

cgtgttcaacttcgccccgttgagcgacacgtcacctcaagcgacgtggagattaactgt

actcccgataacctggagccgataccagacgaggctgcctggcccgactataagctcatg

tgctttgatatagagtgtaaagctggaacgggtaacgaaatggcgttcccagtggcaact

aaccaagaggacctggtcatccagatctcctgtctgctgtactcgcttgctactcagaac

cacgaacacaccctgctgttttccctcgggtcatgcgatatctctgaggaatactcgttt

gcatgcgtccagcgcggcgagcccagaccgacggttttggagtttgacagcgagtacgag

ctgctggttgccttcctgacctttctcaagcagtactctcccgagttcgccaccggctac

aacatcgttaattttgactgggcgtacatagttaacaaggtaacgtcggtgtataacatc

aagctggacgggtacggcaagttcaacaaaggggggctgtttaaggtgtgggacatcgcc

acgaaccattttcagaagaagagcaaggtgaaaatcaatggcctgatatctctagacatg

tattctgtggcgacggaaaagctaaagctacccagctacaaactcgacgcggtcgtggga

gacgtcctcggcgagcataagatagaccttccctataaagaaataccctcctattacgcg

ggagggcctgaccggaggggcgtaataggagagtattgtatccaggactctaggctggtg

ggcaagctgttttttaagtacctcccccatctggaactatcggcggtggccaaactcgcc

cgtatcaccctaacgcgggtaatttttgacggtcagcaaattcgcgtgtacacgtgcttg

ctgaaactcgcccgcgagagaaatttcattttgccagacaacagacgccggtttgacagt

caggcagacgccgcgtcagagacttcggagttggctatggatagccaaagccacgccttc

gacagtacagacgaacccgacggtgtggacggtaccccggacgccgcaggatctggcgct

acttctgaaaacggaggcgggaagcccggcgtcgggagggccgtgggctaccagggagca

aaggttctagaccccgtatccggctttcatgtggaccccgtggttgtgtttgacttcgct

agcttatacccaagcattatccaggcccataacctctgtttcaccaccctggcgctcgat

gaagtggatctggccgggcttcaaccatccgtcaactactcgacgttcgaggtgggtgac

caaaagttattttttgtccacgcccatattcgcgaaagcctgcttggcatcttgctgcgc

gactggctggccatgcgaaaggcggtgagggcgcgaatccccaccagcacccccgaggag

gcagttttactagataagcagcagtctgcgattaaggtgatatgcaactcggtttacgga

ttcacgggggtggcaaacggcctgttgccgtgtctgaggatagcggctaccgttaccacg

ataggacgcgacatgctcctcaagacccgagattacgttcactctcgttgggcgacgcgc

gagctgctggaggacaattttccaggggctataggtttccgaaaccacaagccttactcc

gtcagggttatctacggagacaccgactccgtgtttatcaagtttgtgggcctgacgtac

gagggggtatcggagctgggggatgctatgtcgcgtcagatttcagcggacctctttaga

gcgcccatcaaactggagtgtgagaagacctttcagcgactgctgctgatcaccaagaag

aagtacataggtgtcataaacggggggaagatgctcatgaagggggtcgacctggtccgc

aaaaataactgctctttcataaacttgtacgcgcgacatctggtagatcttttgttgtac

gacgaggatgtggccacggcggcagcagaggtgacagacgtgcctcccgcagaatgggtg

gggcgcccgctaccgagcggctttgacaagtttgggcgagtgctggtagaggcgtacaac

cgtatcactgcccccaacttggacgtgcgcgagttcgttatgactgctgagctgagccgc

tcacccgaatcgtataccaacaagcgcctgccgcacctcaccgtctactttaagctcgcc

atgaggaatgaagaactgcccagcgtaaaagagagaattccgtatgtgatagttgcgcag

accgaggccgcggaacgcgaagcgggtgtagtaaactcaatgcgcggtaccgcccaaaac

cccgtggtaaccaagaccgcacgcccccaacctaaacgcaaactgctggtttccgacctc

gccgaagacccgacctatgtttccgagaatgacgtaccgctaaacacagactactatttc

tcccacctgttgggtaccataagcgtgacctttaaggctctattcggaaatgatgtgaga

acaacagaaaatcttttaaagcggtttattccggaaaccccccacaagacccccacgaaa

acccaggcactgcttgagcgcgccggctttgaaaagctgacgccctttacaccggaggaa

gaaagtcgtcgaatactgcatacagttttttgtactctagaagcagctccccatcaaagc

tga

>KC924809.1

atggcggcgcgcgaacaggccaactccgttcgcaggagcgggttttttaatccgtttatt

ggcaagaggccatttttcaggccgggaagcgggcagaccgcggagacagagcgcccaagg

cccccccaacactcgtactgcacagaggtgggtagctttaagtttatagctccaagatgc

ctcgatgaagaagcccccgccgaccagcgacgcggtgtacacgtgggcaccttggagcgc

ccacccaaagtgtactgcgatggctcagagtacgacgtgctgaactttgcctccggaggt

tgctggcctcgcaggattcgggtttggaacggccaggattttcggggcgatggattcaac

cccagatttgagagatttcacgtgtacgacatagtagagacttcggagagcgcctcacac

gatgacccatccaggtttgctgagctatcccgcccaagcgggtctgtggtaacactgctg

ggaatgagcgagtgtggaaagcgagtcgcggttcacgtctatggtgtgcgccattatttt

tacatggcaaaggcggaggtggatagcgcttgtggaataaccaccgaggcagaactcgtg

cgcgcaatggtggactgcgcgcacagctcggctttgagcgcggccctgggaaatggcaac

ggcggcaaacagagcggcggcagcggcgggggatggtggggcggaaagcacgtgtctgcg

gactgcttcaaagtggagaccgtgtgccacacgacgctgtactactttggatctaagcca

gctctctactatagagtatctgcctccagcagccgcctgggagggttcatctgcgacaac

tttcacccggagattacaaaattcgaggggagcgtggacgtgaccacgcggctgttgttg

gacaatgaaaattttaccagtttcgggtggtaccgcctgcgacccggcacccacggagag

cgtgttcaacttcgccccgttgagcgacacgtcacctcaagcgacgtggagattaactgt

actcccgataacctggagccgataccagacgaggctgcctggcccgactataagctcatg

tgctttgatatagagtgtaaagctggaacgggtaacgaaatggcgttcccagtggcaact

aaccaagaggacctggtcatccagatctcctgtctgctgtactcgcttgctactcagaac

cacgaacacaccctgctgttttccctcgggtcatgcgatatctctgaggaatactcgttt

gcatgcgtccagcgcggcgagcccagaccgacggttttggagtttgacagcgagtacgag

ctgctggttgccttcctgacctttctcaagcagtactctcccgagttcgccaccggctac

aacatcgttaattttgactgggcgtacatagttaacaaggtaacgtcggtgtataacatc

aagctggacgggtacggcaagttcaacaaaggggggctgtttaaggtgtgggacatcgcc

acgaaccattttcagaagaagagcaaggtgaaaatcaatggcctgatatctctagacatg

tattctgtggcgacggaaaagctaaagctacccagctacaaactcgacgcggtcgtggga

gacgtcctcggcgagcataagatagaccttccctataaagaaataccctcctattacgcg

ggagggcctgaccggaggggcgtaataggagagtattgtatccaggactctaggctggtg

ggcaagctgttttttaagtacctcccccatctggaactatcggcggtggccaaactcgcc

cgtatcaccctaacgcgggtaatttttgacggtcagcaaattcgcgtgtacacgtgcttg

ctgaaactcgcccgcgagagaaatttcattttgccagacaacagacgccggtttgacagt

caggcagacgccgcgtcagagacttcggagttggctatggatagccaaagccacgccttc

gacagtacagacgaacccgacggtgtggacggtaccccggacgccgcaggatctggcgct

acttctgaaaacggaggcgggaagcccggcgtcgggagggccgtgggctaccagggagca

aaggttctagaccccgtatccggctttcatgtggaccccgtggttgtgtttgacttcgct

agcttatacccaagcattatccaggcccataacctctgtttcaccaccctggcgctcgat

gaagtggatctggccgggcttcaaccatccgtcgactactcgacgttcgaggtgggtgac

caaaagttattttttgtccacgcccatattcgcgaaagcctgcttggcatcttgctgcgc

gactggctggccatgcgaaaggcggtgagggcgcgaatccccaccagcacccccgaggag

gcagttttactagataagcagcagtctgcgattaaggtgatatgcaactcggtttacgga

ttcacgggggtggcaaacggcctgttgccgtgtctgaggatagcggctaccgttaccacg

ataggacgcgacatgctcctcaagacccgagattacgttcactctcgttgggcgacgcgc

gagctgctggaggacaattttccaggggctataggtttccgaaaccacaagccttactcc

gtcagggttatctacggagacaccgactccgtgtttatcaagtttgtgggcctgacgtac

gagggggtatcggagctgggggatgctatgtcgcgtcagatttcagcggacctctttaga

gcgcccatcaaactggagtgtgagaagacctttcagcgactgctgctgatcaccaagaag

aagtacataggtgtcataaacggggggaagatgctcatgaagggggtcgacctggtccgc

aaaaataactgctctttcataaacttgtacgcgcgacatctggtagatcttttgttgtac

gacgaggatgtggccacggcggcagcagaggtgacagacgtgcctcccgcagaatgggtg

gggcgcccgctaccgagcggctttgacaagtttgggcgagtgctggtagaggcgtacaac

cgtatcactgcccccaacttggacgtgcgcgagttcgttatgactgctgagctgagccgc

tcacccgaatcgtataccaacaagcgcctgccgcacctcaccgtctactttaagctcgcc

atgaggaatgaagaactgcccagcgtaaaagagagaattccgtatgtgatagttgcgcag

accgaggccgcggaacgcgaagcgggtgtagtaaactcaatgcgcggtaccgcccaaaac

cccgtggtaaccaagaccgcacgcccccaacctaaacgcaaactgctggtttccgacctc

gccgaagacccgacctatgtttccgagaatgacgtaccgctaaacacagactactatttc

tcccacctgttgggtaccataagcgtgacctttaaggctctattcggaaatgatgtgaga

acaacagaaaatcttttaaagcggtttattccggaaaccccccacaagacccccacgaaa

acccaggcactgcttgagcgcgccggctttgaaaagctgacgccctttacaccggaggaa

gaaagtcgtcgaatactgcatacagttttttgtactctagaagcagctccccatcaaagc

tga

>KC924810.1

atggcggcgcgcgaacaggccaactccgttcgcaggagcgggttttttaatccgtttatt

ggcaagaggccatttttcaggccgggaagcgggcagaccgcggagacagagcgcccaagg

cccccccaacactcgtactgcacagaggtgggtagctttaagtttatagctccaagatgc

ctcgatgaagaagcccccgccgaccagcgacgcggtgtacacgtgggcaccttggagcgc

ccacccaaagtgtactgcgatggctcagagtacgacgtgctgaactttgcctccggaggt

tgctggcctcgcaggattcgggtttggaacggccaggattttcggggcgatggattcaac

cccagatttgagagatttcacgtgtacgacatagtagagacttcggagagcgcctcacac

gatgacccatccaggtttgctgagctatcccgcccaagcgggtctgtggtaacactgctg

ggaatgagcgagtgtggaaagcgagtcgcggttcacgtctatggtgtgcgccattatttt

tacatggcaaaggcggaggtggatagcgcttgtggaataaccaccgaggcagaactcgtg

cgcgcaatggtggactgcgcgcacagctcggctttgagcgcggccctgggaaatggcaac

ggcggcaaacagagcggcggcagcggcgggggatggtggggcggaaagcacgtgtctgcg

gactgcttcaaagtggagaccgtgtgccacacgacgctgtactactttggatctaagcca

gctctctactatagagtatctgcctccagcagccgcctgggagggttcatctgcgacaac

tttcacccggagattacaaaattcgaggggagcgtggacgtgaccacgcggctgttgttg

gacaatgaaaattttaccagtttcgggtggtaccgcctgcgacccggcacccacggagag

cgtgttcaacttcgccccgttgagcgacacgtcacctcaagcgacgtggagattaactgt

actcccgataacctggagccgataccagacgaggctgcctggcccgactataagctcatg

tgctttgatatagagtgtaaagctggaacgggtaacgaaatggcgttcccagtggcaact

aaccaagaggacctggtcatccagatctcctgtctgctgtactcgcttgctactcagaac

cacgaacacaccctgctgttttccctcgggtcatgcgatatctctgaggaatactcgttt

gcatgcgtccagcgcggcgagcccagaccgacggttttggagtttgacagcgagtacgag

ctgctggttgccttcctgacctttctcaagcagtactctcccgagttcgccaccggctac

aacatcgttaattttgactgggcgtacatagttaacaaggtaacgtcggtgtataacatc

aagctggacgggtacggcaagttcaacaaaggggggctgtttaaggtgtgggacatcgcc

acgaaccattttcagaagaagagcaaggtgaaaatcaatggcctgatatctctagacatg

tattctgtggcgacggaaaagctaaagctacccagctacaaactcgacgcggtcgtggga

gacgtcctcggcgagcataagatagaccttccctataaagaaataccctcctattacgcg

ggagggcctgaccggaggggcgtaataggagagtattgtatccaggactctaggctggtg

ggcaagctgttttttaagtacctcccccatctggaactatcggcggtggccaaactcgcc

cgtatcaccctaacgcgggtaatttttgacggtcagcaaattcgcgtgtacacgtgcttg

ctgaaactcgcccgcgagagaaatttcattttgccagacaacagacgccggtttgacagt

caggcagacgccgcgtcagagacttcggagttggctatggatagccaaagccacgccttc

gacagtacagacgaacccgacggtgtggacggtaccccggacgccgcaggatctggcgct

acttctgaaaacggaggcgggaagcccggcgtcgggagggccgtgggctaccagggagca

aaggttctagaccccgtatccggctttcatgtggaccccgtggttgtgtttgacttcgct

agcttatacccaagcattatccaggcccataacctctgtttcaccaccctggcgctcgat

gaagtggatctggccgggcttcaaccatccgtcgactactcgacgttcgaggtgggtgac

caaaagttattttttgtccacgcccatattcgcgaaagcctgcttggcatcttgctgcgc

gactggctggccatgcgaaaggcggtgagggcgcgaatccccaccagcacccccgaggag

gcagttttactagataagcagcagtctgcgattaaggtgatatgcaactcggtttacgga

ttcacgggggtggcaaacggcctgttgccgtgtctgaggatagcggctaccgttaccacg

ataggacgcgacatgctcctcaagacccgagattacgttcactctcgttgggcgacgcgc

gagctgctggaggacaattttccaggggctataggtttccgaaaccacaagccttactcc

gtcagggttatctacggagacaccgactccgtgtttatcaagtttgtgggcctgacgtac

gagggggtatcggagctgggggatgctatgtcgcgtcagatttcagcggacctctttaga

gcgcccatcaaactggagtgtgagaagacctttcagcgactgctgctgatcaccaagaag

aagtacataggtgtcataaacggggggaagatgctcatgaagggggtcgacctggtccgc

aaaaataactgctctttcataaacttgtacgcgcgacatctggtagatcttttgttgtac

gacgaggatgtggccacggcggcagcagaggtgacagacgtgcctcccgcagaatgggtg

gggcgcccgctaccgagcggctttgacaagtttgggcgagtgctggtagaggcgtacaac

cgtatcactgcccccaacttggacgtgcgcgagttcgttatgactgctgagctgagccgc

tcacccgaatcgtataccaacaagcgcctgccgcacctcaccgtctactttaagctcgcc

atgaggaatgaagaactgcccagcgtaaaagagagaattccgtatgtgatagttgcgcag

accgaggccgcggaacgcgaagcgggtgtagtaaactcaatgcgcggtaccgcccaaaac

cccgtggtaaccaagaccgcacgcccccaacctaaacgcaaactgctggtttccgacctc

gccgaagacccgacctatgtttccgagaatgacgtaccgctaaacacagactactatttc

tcccacctgttgggtaccataagcgtgacctttaaggctctattcggaaatgatgtgaga

acaacagaaaatcttttaaagcggtttattccggaaaccccccacaagacccccacgaaa

acccaggcactgcttgagcgcgccggctttgaaaagctgacgccctttacaccggaggaa

gaaagtcgtcgaatactgcatacagttttttgtactctagaagcagctccccatcaaagc

tga

>KC924812.1

atggcggcgcgcgaacaggccaactccgttcgcaggagcgggttttttaatccgtttatt

ggcaagaggccatttttcaggccgggaagcgggcagaccgcggagacagagcgcccaagg

cccccccaacactcgtactgcacagaggtgggtagctttaagtttatagctccaagatgc

ctcgatgaagaagcccccgccgaccagcgacgcggtgtacacgtgggcaccttggagcgc

ccacccaaagtgtactgcgatggctcagagtacgacgtgctgaactttgcctccggaggt

tgctggcctcgcaggattcgggtttggaacggccaggattttcggggcgatggattcaac

cccagatttgagagatttcacgtgtacgacatagtagagacttcggagagcgcctcacac

gatgacccatccaggtttgctgagctatcccgcccaagcgggtctgtggtaacactgctg

ggaatgagcgagtgtggaaagcgagtcgcggttcacgtctatggtgtgcgccattatttt

tacatggcaaaggcggaggtggatagcgcttgtggaataaccaccgaggcagaactcgtg

cgcgcaatggtggactgcgcgcacagctcggctttgagcgcggccctgggaaatggcaac

ggcggcaaacagagcggcggcagcggcgggggatggtggggcggaaagcacgtgtctgcg

gactgcttcaaagtggagaccgtgtgccacacgacgctgtactactttggatctaagcca

gctctctactatagagtatctgcctccagcagccgcctgggagggttcatctgcgacaac

tttcacccggagattacaaaattcgaggggagcgtggacgtgaccacgcggctgttgttg

gacaatgaaaattttaccagtttcgggtggtaccgcctgcgacccggcacccacggagag

cgtgttcaacttcgccccgttgagcgacacgtcacctcaagcgacgtggagattaactgt

actcccgataacctggagccgataccagacgaggctgcctggcccgactataagctcatg

tgctttgatatagagtgtaaagctggaacgggtaacgaaatggcgttcccagtggcaact

aaccaagaggacctggtcatccagatctcctgtctgctgtactcgcttgctactcagaac

cacgaacacaccctgctgttttccctcgggtcatgcgatatctctgaggaatactcgttt

gcatgcgtccagcgcggcgagcccagaccgacggttttggagtttgacagcgagtacgag

ctgctggttgccttcctgacctttctcaagcagtactctcccgagttcgccaccggctac

aacatcgttaattttgactgggcgtacatagttaacaaggtaacgtcggtgtataacatc

aagctggacgggtacggcaagttcaacaaaggggggctgtttaaggtgtgggacatcgcc

acgaaccattttcagaagaagagcaaggtgaaaatcaatggcctgatatctctagacatg

tattctgtggcgacggaaaagctaaagctacccagctacaaactcgacgcggtcgtggga

gacgtcctcggcgagcataagatagaccttccctataaagaaataccctcctattacgcg

ggagggcctgaccggaggggcgtaataggagagtattgtatccaggactctaggctggtg

ggcaagctgttttttaagtacctcccccatctggaactatcggcggtggccaaactcgcc

cgtatcaccctaacgcgggtaatttttgacggtcagcaaattcgcgtgtacacgtgcttg

ctgaaactcgcccgcgagagaaatttcattttgccagacaacagacgccggtttgacagt

caggcagacgccgcgtcagagacttcggagttggctatggatagccaaagccacgccttc

gacagtacagacgaacccgacggtgtggacggtaccccggacgccgcaggatctggcgct

acttctgaaaacggaggcgggaagcccggcgtcgggagggccgtgggctaccagggagca

aaggttctagaccccgtatccggctttcatgtggaccccgtggttgtgtttgacttcgct

agcttatacccaagcattatccaggcccataacctctgtttcaccaccctggcgctcgat

gaagtggatctggccgggcttcaaccatccgtcgactactcgacgttcgaggtgggtgac

caaaagttattttttgtccacgcccatattcgcgaaagcctgcttggcatcttgctgcgc

gactggctggccatgcgaaaggcggtgagggcgcgaatccccaccagcacccccgaggag

gcagttttactagataagcagcagtctgcgattaaggtgatatgcaactcggtttacgga

ttcacgggggtggcaaacggcctgttgccgtgtctgaggatagcggctaccgttaccacg

ataggacgcgacatgctcctcaagacccgagattacgttcactctcgttgggcgacgcgc

gagctgctggaggacaattttccaggggctataggtttccgaaaccacaagccttactcc

gtcagggttatctacggagacaccgactccgtgtttatcaagtttgtgggcctgacgtac

gagggggtatcggagctgggggatgctatgtcgcgtcagatttcagcggacctctttaga

gcgcccatcaaactggagtgtgagaagacctttcagcgactgctgctgatcaccaagaag

aagtacataggtgtcataaacggggggaagatgctcatgaagggggtcgacctagtccgc

aaaaataactgctctttcataaacttgtacgcgcgacatctggtagatcttttgttgtac

gacgaggatgtggccacggcggcagcagaggtgacagacgtgcctcccgcagaatgggtg

gggcgcccgctaccgagcggctttgacaagtttgggcgagtgctggtagaggcgtacaac

cgtatcactgcccccaacttggacgtgcgcgagttcgttatgactgctgagctgagccgc

tcacccgaatcgtataccaacaagcgcctgccgcacctcaccgtctactttaagctcgcc

atgaggaatgaagaactgcccagcgtaaaagagagaattccgtatgtgatagttgcgcag

accgaggccgcggaacgcgaagcgggtgtagtaaactcaatgcgcggtaccgcccaaaac

cccgtggtaaccaagaccgcacgcccccaacctaaacgcaaactgctggtttccgacctc

gccgaagacccgacctatgtttccgagaatgacgtaccgctaaacacagactactatttc

tcccacctgttgggtaccataagcgtgacctttaaggctctattcggaaatgatgtgaga

acaacagaaaatcttttaaagcggtttattccggaaaccccccacaagacccccacgaaa

acccaggcactgcttgagcgcgccggctttgaaaagctgacgccctttacaccggaggaa

gaaagtcgtcgaatactgcatacagttttttgtactctagaagcagctccccatcaaagc

tga

>KC924813.1

atggcggcgcgcgaacaggccaactccgttcgcaggagcgggttttttaatccgtttatt

ggcaagaggccatttttcaggccgggaagcgggcagaccgcggagacagagcgcccaagg

cccccccaacactcgtactgcacagaggtgggtagctttaagtttatagctccaagatgc

ctcgatgaagaagcccccgccgaccagcgacgcggtgtacacgtgggcaccttggagcgc

ccacccaaagtgtactgcgatggctcagagtacgacgtgctgaactttgcctccggaggt

tgctggcctcgcaggattcgggtttggaacggccaggattttcggggcgatggattcaac

cccagatttgagagatttcacgtgtacgacatagtagagacttcggagagcgcctcacac

gatgacccatccaggtttgctgagctatcccgcccaagcgggtctgtggtaacactgctg

ggaatgagcgagtgtggaaagcgagtcgcggttcacgtctatggtgtgcgccattatttt

tacatggcaaaggcggaggtggatagcgcttgtggaataaccaccgaggcagaactcgtg

cgcgcaatggtggactgcgcgcacagctcggctttgagcgcggccctgggaaatggcaac

ggcggcaaacagagcggcggcagcggcgggggatggtggggcggaaagcacgtgtctgcg

gactgcttcaaagtggagaccgtgtgccacacgacgctgtactactttggatctaagcca

gctctctactatagagtatctgcctccagcagccgcctgggagggttcatctgcgacaac

tttcacccggagattacaaaattcgaggggagcgtggacgtgaccacgcggctgttgttg

gacaatgaaaattttaccagtttcgggtggtaccgcctgcgacccggcacccacggagag

cgtgttcaacttcgccccgttgagcgacacgtcacctcaagcgacgtggagattaactgt

actcccgataacctggagccgataccagacgaggctgcctggcccgactataagctcatg

tgctttgatatagagtgtaaagctggaacgggtaacgaaatggcgttcccagtggcaact

aaccaagaggacctggtcatccagatctcctgtctgctgtactcgcttgctactcagaac

cacgaacacaccctgctgttttccctcgggtcatgcgatatctctgaggaatactcgttt

gcatgcgtccagcgcggcgagcccagaccgacggttttggagtttgacagcgagtacgag

ctgctggttgccttcctgacctttctcaagcagtactctcccgagttcgccaccggctac

aacatcgttaattttgactgggcgtacatagttaacaaggtaacgtcggtgtataacatc

aagctggacgggtacggcaagttcaacaaaggggggctgtttaaggtgtgggacatcgcc

acgaaccattttcagaagaagagcaaggtgaaaatcaatggcctgatatctctagacatg

tattctgtggcgacggaaaagctaaagctacccagctacaaactcgacgcggtcgtggga

gacgtcctcggcgagcataagatagaccttccctataaagaaataccctcctattacgcg

ggagggcctgaccggaggggcgtaataggagagtattgtatccaggactctaggctggtg

ggcaagctgttttttaagtacctcccccatctggaactatcggcggtggccaaactcgcc

cgtatcaccctaacgcgggtaatttttgacggtcagcaaattcgcgtgtacacgtgcttg

ctgaaactcgcccgcgagagaaatttcattttgccagacaacagacgccggtttgacagt

caggcagacgccgcgtcagagacttcggagttggctatggatagccaaagccacgccttc

gacagtacagacgaacccgacggtgtggacggtaccccggacgccgcaggatctggcgct

acttctgaaaacggaggcgggaagcccggcgtcgggagggccgtgggctaccagggagca

aaggttctagaccccgtatccggctttcatgtggaccccgtggttgtgtttgacttcgct

agcttatacccaagcattatccaggcccataacctctgtttcaccaccctggcgctcgat

gaagtggatctggccgggcttcaaccatccgtcgactactcgacgttcgaggtgggtgac

caaaagttattttttgtccacgcccatattcgcgaaagcctgcttggcatcttgctgcgc

gactggctggccatgcgaaaggcggtgagggcgcgaatccccaccagcacccccgaggag

gcagttttactagataagcagcagtctgcgattaaggtgatatgcaactcggtttacgga

ttcacgggggtggcaaacggcctgttgccgtgtctgaggatagcggctaccgttaccacg

ataggacgcgacatgctcctcaagacccgagattacgttcactctcgttgggcgacgcgc

gagctgctggaggacaattttccaggggctataggtttccgaaaccacaagccttactcc

gtcagggttatctacggagacaccgactccgtgtttatcaagtttgtgggcctgacgtac

gagggggtatcggagctgggggatgctatgtcgcgtcagatttcagcggacctctttaga

gcgcccatcaaactggagtgtgagaagacctttcagcgactgctgctgatcaccaagaag

aagtacataggtgtcataaacggggggaagatgctcatgaagggggtcgacctagtccgc

aaaaataactgctctttcataaacttgtacgcgcgacatctggtagatcttttgttgtac

gacgaggatgtggccacggcggcagcagaggtgacagacgtgcctcccgcagaatgggtg

gggcgcccgctaccgagcggctttgacaagtttgggcgagtgctggtagaggcgtacaac

cgtatcactgcccccaacttggacgtgcgcgagttcgttatgactgctgagctgagccgc

tcacccgaatcgtataccaacaagcgcctgccgcacctcaccgtctactttaagctcgcc

atgaggaatgaagaactgcccagcgtaaaagagagaattccgtatgtgatagttgcgcag

accgaggccgcggaacgcgaagcgggtgtagtaaactcaatgcgcggtaccgcccaaaac

cccgtggtaaccaagaccgcacgcccccaacctaaacgcaaactgctggtttccgacctc

gccgaagacccgacctatgtttccgagaatgacgtaccgctaaacacagactactatttc

tcccacctgttgggtaccataagcgtgacctttaaggctctattcggaaatgatgtgaga

acaacagaaaatcttttaaagcggtttattccggaaaccccccacaagacccccacgaaa

acccaggcactgcttgagcgcgccggctttgaaaagctgacgccctttacaccggaggaa

gaaagtcgtcgaatactgcatacagttttttgtactctagaagcagctccccatcaaagc

tga

>KC924814.1

atggcggcgcgcgaacaggccaactccgttcgcaggagcgggttttttaatccgtttatt

ggcaagaggccatttttcaggccgggaagcgggcagaccgcggagacagagcgcccaagg

cccccccaacactcgtactgcacagaggtgggtagctttaagtttatagctccaagatgc

ctcgatgaagaagcccccgccgaccagcgacgcggtgtacacgtgggcaccttggagcgc

ccacccaaagtgtactgcgatggctcagagtacgacgtgctgaactttgcctccggaggt

tgctggcctcgcaggattcgggtttggaacggccaggattttcggggcgatggattcaac

cccagatttgagagatttcacgtgtacgacatagtagagacttcggagagcgcctcacac

gatgacccatccaggtttgctgagctatcccgcccaagcgggtctgtggtaacactgctg

ggaatgagcgagtgtggaaagcgagtcgcggttcacgtctatggtgtgcgccattatttt

tacatggcaaaggcggaggtggatagcgcttgtggaataaccaccgaggcagaactcgtg

cgcgcaatggtggactgcgcgcacagctcggctttgagcgcggccctgggaaatggcaac

ggcggcaaacagagcggcggcagcggcgggggatggtggggcggaaagcacgtgtctgcg

gactgcttcaaagtggagaccgtgtgccacacgacgctgtactactttggatctaagcca

gctctctactatagagtatctgcctccagcagccgcctgggagggttcatctgcgacaac

tttcacccggagattacaaaattcgaggggagcgtggacgtgaccacgcggctgttgttg

gacaatgaaaattttaccagtttcgggtggtaccgcctgcgacccggcacccacggagag

cgtgttcaacttcgccccgttgagcgacacgtcacctcaagcgacgtggagattaactgt

actcccgataacctggagccgataccagacgaggctgcctggcccgactataagctcatg

tgctttgatatagagtgtaaagctggaacgggtaacgaaatggcgttcccagtggcaact

aaccaagaggacctggtcatccagatctcctgtctgctgtactcgcttgctactcagaac

cacgaacacaccctgctgttttccctcgggtcatgcgatatctctgaggaatactcgttt

gcatgcgtccagcgcggcgagcccagaccgacggttttggagtttgacagcgagtacgag

ctgctggttgccttcctgacctttctcaagcagtactctcccgagttcgccaccggctac

aacatcgttaattttgactgggcgtacatagttaacaaggtaacgtcggtgtataacatc

aagctggacgggtacggcaagttcaacaaaggggggctgtttaaggtgtgggacatcgcc

acgaaccattttcagaagaagagcaaggtgaaaatcaatggcctgatatctctagacatg

tattctgtggcgacggaaaagctaaagctacccagctacaaactcgacgcggtcgtggga

gacgtcctcggcgagcataagatagaccttccctataaagaaataccctcctattacgcg

ggagggcctgaccggaggggcgtaataggagagtattgtatccaggactctaggctggtg

ggcaagctgttttttaagtacctcccccatctggaactatcggcggtggccaaactcgcc

cgtatcaccctaacgcgggtaatttttgacggtcagcaaattcgcgtgtacacgtgcttg

ctgaaactcgcccgcgagagaaatttcattttgccagacaacagacgccggtttgacagt

caggcagacgccgcgtcagagacttcggagttggctatggatagccaaagccacgccttc

gacagtacagacgaacccgacggtgtggacggtaccccggacgccgcaggatctggcgct

acttctgaaaacggaggcgggaagcccggcgtcgggagggccgtgggctaccagggagca

aaggttctagaccccgtatccggctttcatgtggaccccgtggttgtgtttgacttcgct

agcttatacccaagcattatccaggcccataacctctgtttcaccaccctggcgctcgat

gaagtggatctggccgggcttcaaccatccgtcgactactcgacgttcgaggtgggtgac

caaaagttattttttgtccacgcccatattcgcgaaagcctgcttggcatcttgctgcgc

gactggctggccatgcgaaaggcggtgagggcgcgaatccccaccagcacccccgaggag

gcagttttactagataagcagcagtctgcgattaaggtgatatgcaactcggtttacgga

ttcacgggggtggcaaacggcctgttgccgtgtctgaggatagcggctaccgttaccacg

ataggacgcgacatgctcctcaagacccgagattacgttcactctcgttgggcgacgcgc

gagctgctggaggacaattttccaggggctataggtttccgaaaccacaagccttactcc

gtcagggttatctacggagacaccgactccgtgtttatcaagtttgtgggcctgacgtac

gagggggtatcggagctgggggatgctatgtcgcgtcagatttcagcggacctctttaga

gcgcccatcaaactggagtgtgagaagacctttcagcgactgctgctgatcaccaagaag

aagtacataggtgtcataaacggggggaagatgctcatgaagggggtcgacctagtccgc

aaaaataactgctctttcataaacttgtacgcgcgacatctggtagatcttttgttgtac

gacgaggatgtggccacggcggcagcagaggtgacagacgtgcctcccgcagaatgggtg

gggcgcccgctaccgagcggctttgacaagtttgggcgagtgctggtagaggcgtacaac

cgtatcactgcccccaacttggacgtgcgcgagttcgttatgactgctgagctgagccgc

tcacccgaatcgtataccaacaagcgcctgccgcacctcaccgtctactttaagctcgcc

atgaggaatgaagaactgcccagcgtaaaagagagaattccgtatgtgatagttgcgcag

accgaggccgcggaacgcgaagcgggtgtagtaaactcaatgcgcggtaccgcccaaaac

cccgtggtaaccaagaccgcacgcccccaacctaaacgcaaactgctggtttccgacctc

gccgaagacccgacctatgtttccgagaatgacgtaccgctaaacacagactactatttc

tcccacctgttgggtaccataagcgtgacctttaaggctctattcggaaatgatgtgaga

acaacagaaaatcttttaaagcggtttattccggaaaccccccacaagacccccacgaaa

acccaggcactgcttgagcgcgccggctttgaaaagctgacgccctttacaccggaggaa

gaaagtcgtcgaatactgcatacagttttttgtactctagaagcagctccccatcaaagc

tga

>KC924815.1

atggcggcgcgcgaacaggccaactccgttcgcaggagcgggttttttaatccgtttatt

ggcaagaggccatttttcaggccgggaagcgggcagaccgcggagacagagcgcccaagg

cccccccaacactcgtactgcacagaggtgggtagctttaagtttatagctccaagatgc

ctcgatgaagaagcccccgccgaccagcgacgcggtgtacacgtgggcaccttggagcgc

ccacccaaagtgtactgcgatggctcagagtacgacgtgctgaactttgcctccggaggt

tgctggcctcgcaggattcgggtttggaacggccaggattttcggggcgatggattcaac

cccagatttgagagatttcacgtgtacgacatagtagagacttcggagagcgcctcacac

gatgacccatccaggtttgctgagctatcccgcccaagcgggtctgtggtaacactgctg

ggaatgagcgagtgtggaaagcgagtcgcggttcacgtctatggtgtgcgccattatttt

tacatggcaaaggcggaggtggatagcgcttgtggaataaccaccgaggcagaactcgtg

cgcgcaatggtggactgcgcgcacagctcggctttgagcgcggccctgggaaatggcaac

ggcggcaaacagagcggcggcagcggcgggggatggtggggcggaaagcacgtgtctgcg

gactgcttcaaagtggagaccgtgtgccacacgacgctgtactactttggatctaagcca

gctctctactatagagtatctgcctccagcagccgcctgggagggttcatctgcgacaac

tttcacccggagattacaaaattcgaggggagcgtggacgtgaccacgcggctgttgttg

gacaatgaaaattttaccagtttcgggtggtaccgcctgcgacccggcacccacggagag

cgtgttcaacttcgccccgttgagcgacacgtcacctcaagcgacgtggagattaactgt

actcccgataacctggagccgataccagacgaggctgcctggcccgactataagctcatg

tgctttgatatagagtgtaaagctggaacgggtaacgaaatggcgttcccagtggcaact

aaccaagaggacctggtcatccagatctcctgtctgctgtactcgcttgctactcagaac

cacgaacacaccctgctgttttccctcgggtcatgcgatatctctgaggaatactcgttt

gcatgcgtccagcgcggcgagcccagaccgacggttttggagtttgacagcgagtacgag

ctgctggttgccttcctgacctttctcaagcagtactctcccgagttcgccaccggctac

aacatcgttaattttgactgggcgtacatagttaacaaggtaacgtcggtgtataacatc

aagctggacgggtacggcaagttcaacaaaggggggctgtttaaggtgtgggacatcgcc

acgaaccattttcagaagaagagcaaggtgaaaatcaatggcctgatatctctagacatg

tattctgtggcgacggaaaagctaaagctacccagctacaaactcgacgcggtcgtggga

gacgtcctcggcgagcataagatagaccttccctataaagaaataccctcctattacgcg

ggagggcctgaccggaggggcgtaataggagagtattgtatccaggactctaggctggtg

ggcaagctgttttttaagtacctcccccatctggaactatcggcggtggccaaactcgcc

cgtatcaccctaacgcgggtaatttttgacggtcagcaaattcgcgtgtacacgtgcttg

ctgaaactcgcccgcgagagaaatttcattttgccagacaacagacgccggtttgacagt

caggcagacgccgcgtcagagacttcggagttggctatggatagccaaagccacgccttc

gacagtacagacgaacccgacggtgtggacggtaccccggacgccgcaggatctggcgct

acttctgaaaacggaggcgggaagcccggcgtcgggagggccgtgggctaccagggagca

aaggttctagaccccgtatccggctttcatgtggaccccgtggttgtgtttgacttcgct

agcttatacccaagcattatccaggcccataacctctgtttcaccaccctggcgctcgat

gaagtggatctggccgggcttcaaccatccgtcgactactcgacgttcgaggtgggtgac

caaaagttattttttgtccacgcccatattcgcgaaagcctgcttggcatcttgctgcgc

gactggctggccatgcgaaaggcggtgagggcgcgaatccccaccagcacccccgaggag

gcagttttactagataagcagcagtctgcgattaaggtgatatgcaactcggtttacgga

ttcacgggggtggcaaacggcctgttgccgtgtctgaggatagcggctaccgttaccacg

ataggacgcgacatgctcctcaagacccgagattacgttcactctcgttgggcgacgcgc

gagctgctggaggacaattttccaggggctataggtttccgaaaccacaagccttactcc

gtcagggttatctacggagacaccgactccgtgtttatcaagtttgtgggcctgacgtac

gagggggtatcggagctgggggatgctatgtcgcgtcagatttcagcggacctctttaga

gcgcccatcaaactggagtgtgagaagacctttcagcgactgctgctgatcaccaagaag

aagtacataggtgtcataaacggggggaagatgctcatgaagggggtcgacctggtccgc

aaaaataactgctctttcataaacttgtacgcgcgacatctggtagatcttttgttgtac

gacgaggatgtggccacggcggcagcagaggtgacagacgtgcctcccgcagaatgggtg

gggcgcccgctaccgagcggctttgacaagtttgggcgagtgctggtagaggcgtacaac

cgtatcactgcccccaacttggacgtgcgcgagttcgttatgactgctgagctgagccgc

tcacccgaatcgtataccaacaagcgcctgccgcacctcaccgtctactttaagctcgcc

atgaggaatgaagaactgcccagcgtaaaagagagaattccgtatgtgatagttgcgcag

accgaggccgcggaacgcgaagcgggtgtagtaaactcaatgcgcggtaccgcccaaaac

cccgtggtaaccaagaccgcacgcccccaacctaaacgcaaactgctggtttccgacctc

gccgaagacccgacctatgtttccgagaatgacgtaccgctaaacacagactactatttc

tcccacctgttgggtaccataagcgtgacctttaaggctctattcggaaatgatgtgaga

acaacagaaaatcttttaaagcggtttattccggaaaccccccacaagacccccacgaaa

acccaggcactgcttgagcgcgccggctttgaaaagctgacgccctttacaccggaggaa

gaaagtcgtcgaatactgcatacagttttttgtactctagaagcagctccccatcaaagc

tga

>KC924816.1

atggcggcgcgcgaacaggccaactccgttcgcaggagcgggttttttaatccgtttatt

ggcaagaggccatttttcaggccgggaagcgggcagaccgcggagacagagcgcccaagg

cccccccaacactcgtactgcacagaggtgggtagctttaagtttatagctccaagatgc

ctcgatgaagaagcccccgccgaccagcgacgcggtgtacacgtgggcaccttggagcgc

ccacccaaagtgtactgcgatggctcagagtacgacgtgctgaactttgcctccggaggt

tgctggcctcgcaggattcgggtttggaacggccaggattttcggggcgatggattcaac

cccagatttgagagatttcacgtgtacgacatagtagagacttcggagagcgcctcacac

gatgacccatccaggtttgctgagctatcccgcccaagcgggtctgtggtaacactgctg

ggaatgagcgagtgtggaaagcgagtcgcggttcacgtctatggtgtgcgccattatttt

tacatggcaaaggcggaggtggatagcgcttgtggaataaccaccgaggcagaactcgtg

cgcgcaatggtggactgcgcgcacagctcggctttgagcgcggccctgggaaatggcaac

ggcggcaaacagagcggcggcagcggcgggggatggtggggcggaaagcacgtgtctgcg

gactgcttcaaagtggagaccgtgtgccacacgacgctgtactactttggatctaagcca

gctctctactatagagtatctgcctccagcagccgcctgggagggttcatctgcgacaac

tttcacccggagattacaaaattcgaggggagcgtggacgtgaccacgcggctgttgttg

gacaatgaaaattttaccagtttcgggtggtaccgcctgcgacccggcacccacggagag

cgtgttcaacttcgccccgttgagcgacacgtcacctcaagcgacgtggagattaactgt

actcccgataacctggagccgataccagacgaggctgcctggcccgactataagctcatg

tgctttgatatagagtgtaaagctggaacgggtaacgaaatggcgttcccagtggcaact

aaccaagaggacctggtcatccagatctcctgtctgctgtactcgcttgctactcagaac

cacgaacacaccctgctgttttccctcgggtcatgcgatatctctgaggaatactcgttt

gcatgcgtccagcgcggcgagcccagaccgacggttttggagtttgacagcgagtacgag

ctgctggttgccttcctgacctttctcaagcagtactctcccgagttcgccaccggctac

aacatcgttaattttgactgggcgtacatagttaacaaggtaacgtcggtgtataacatc

aagctggacgggtacggcaagttcaacaaaggggggctgtttaaggtgtgggacatcgcc

acgaaccattttcagaagaagagcaaggtgaaaatcaatggcctgatatctctagacatg

tattctgtggcgacggaaaagctaaagctacccagctacaaactcgacgcggtcgtggga

gacgtcctcggcgagcataagatagaccttccctataaagaaataccctcctattacgcg

ggagggcctgaccggaggggcgtaataggagagtattgtatccaggactctaggctggtg

ggcaagctgttttttaagtacctcccccatctggaactatcggcggtggccaaactcgcc

cgtatcaccctaacgcgggtaatttttgacggtcagcaaattcgcgtgtacacgtgcttg

ctgaaactcgcccgcgagagaaatttcattttgccagacaacagacgccggtttgacagt

caggcagacgccgcgtcagagacttcggagttggctatggatagccaaagccacgccttc

gacagtacagacgaacccgacggtgtggacggtaccccggacgccgcaggatctggcgct

acttctgaaaacggaggcgggaagcccggcgtcgggagggccgtgggctaccagggagca

aaggttctagaccccgtatccggctttcatgtggaccccgtggttgtgtttgacttcgct

agcttatacccaagcattatccaggcccataacctctgtttcaccaccctggcgctcgat

gaagtggatctggccgggcttcaaccatccgtcgactactcgacgttcgaggtgggtgac

caaaagttattttttgtccacgcccatattcgcgaaagcctgcttggcatcttgctgcgc

gactggctggccatgcgaaaggcggtgagggcgcgaatccccaccagcacccccgaggag

gcagttttactagataagcagcagtctgcgattaaggtgatatgcaactcggtttacgga

ttcacgggggtggcaaacggcctgttgccgtgtctgaggatagcggctaccgttaccacg

ataggacgcgacatgctcctcaagacccgagattacgttcactctcgttgggcgacgcgc

gagctgctggaggacaattttccaggggctataggtttccgaaaccacaagccttactcc

gtcagggttatctacggagacaccgactccgtgtttatcaagtttgtgggcctgacgtac

gagggggtatcggagctgggggatgctatgtcgcgtcagatttcagcggacctctttaga

gcgcccatcaaactggagtgtgagaagacctttcagcgactgctgctgatcaccaagaag

aagtacataggtgtcataaacggggggaagatgctcatgaagggggtcgacctggtccgc

aaaaataactgctctttcataaacttgtacgcgcgacatctggtagatcttttgttgtac

gacgaggatgtggccacggcggcagcagaggtgacagacgtgcctcccgcagaatgggtg

gggcgcccgctaccgagcggctttgacaagtttgggcgagtgctggtagaggcgtacaac

cgtatcactgcccccaacttggacgtgcgcgagttcgttatgactgctgagctgagccgc

tcacccgaatcgtataccaacaagcgcctgccgcacctcaccgtctactttaagctcgcc

atgaggaatgaagaactgcccagcgtaaaagagagaattccgtatgtgatagttgcgcag

accgaggccgcggaacgcgaagcgggtgtagtaaactcaatgcgcggtaccgcccaaaac

cccgtggtaaccaagaccgcacgcccccaacctaaacgcaaactgctggtttccgacctc

gccgaagacccgacctatgtttccgagaatgacgtaccgctaaacacagactactatttc

tcccacctgttgggtaccataagcgtgacctttaaggctctattcggaaatgatgtgaga

acaacagaaaatcttttaaagcggtttattccggaaaccccccacaagacccccacgaaa

acccaggcactgcttgagcgcgccggctttgaaaagctgacgccctttacaccggaggaa

gaaagtcgtcgaatactgcatacagttttttgtactctagaagcagctccccatcaaagc

tga

>KC924817.1

atggcggcgcgcgaacaggccaactccgttcgcaggagcgggttttttaatccgtttatt

ggcaagaggccatttttcaggccgggaagcgggcagaccgcggagacagagcgcccaagg

cccccccaacactcgtactgcacagaggtgggtagctttaagtttatagctccaagatgc

ctcgatgaagaagcccccgccgaccagcgacgcggtgtacacgtgggcaccttggagcgc

ccacccaaagtgtactgcgatggctcagagtacgacgtgctgaactttgcctccggaggt

tgctggcctcgcaggattcgggtttggaacggccaggattttcggggcgatggattcaac

cccagatttgagagatttcacgtgtacgacatagtagagacttcggagagcgcctcacac

gatgacccatccaggtttgctgagctatcccgcccaagcgggtctgtggtaacactgctg

ggaatgagcgagtgtggaaagcgagtcgcggttcacgtctatggtgtgcgccattatttt

tacatggcaaaggcggaggtggatagcgcttgtggaataaccaccgaggcagaactcgtg

cgcgcaatggtggactgcgcgcacagctcggctttgagcgcggccctgggaaatggcaac

ggcggcaaacagagcggcggcagcggcgggggatggtggggcggaaagcacgtgtctgcg

gactgcttcaaagtggagaccgtgtgccacacgacgctgtactactttggatctaagcca

gctctctactatagagtatctgcctccagcagccgcctgggagggttcatctgcgacaac

tttcacccggagattacaaaattcgaggggagcgtggacgtgaccacgcggctgttgttg

gacaatgaaaattttaccagtttcgggtggtaccgcctgcgacccggcacccacggagag

cgtgttcaacttcgccccgttgagcgacacgtcacctcaagcgacgtggagattaactgt

actcccgataacctggagccgataccagacgaggctgcctggcccgactataagctcatg

tgctttgatatagagtgtaaagctggaacgggtaacgaaatggcgttcccagtggcaact

aaccaagaggacctggtcatccagatctcctgtctgctgtactcgcttgctactcagaac

cacgaacacaccctgctgttttccctcgggtcatgcgatatctctgaggaatactcgttt

gcatgcgtccagcgcggcgagcccagaccgacggttttggagtttgacagcgagtacgag

ctgctggttgccttcctgacctttctcaagcagtactctcccgagttcgccaccggctac

aacatcgttaattttgactgggcgtacatagttaacaaggtaacgtcggtgtataacatc

aagctggacgggtacggcaagttcaacaaaggggggctgtttaaggtgtgggacatcgcc

acgaaccattttcagaagaagagcaaggtgaaaatcaatggcctgatatctctagacatg

tattctgtggcgacggaaaagctaaagctacccagctacaaactcgacgcggtcgtggga

gacgtcctcggcgagcataagatagaccttccctataaagaaataccctcctattacgcg

ggagggcctgaccggaggggcgtaataggagagtattgtatccaggactctaggctggtg

ggcaagctgttttttaagtacctcccccatctggaactatcggcggtggccaaactcgcc

cgtatcaccctaacgcgggtaatttttgacggtcagcaaattcgcgtgtacacgtgcttg

ctgaaactcgcccgcgagagaaatttcattttgccagacaacagacgccggtttgacagt

caggcagacgccgcgtcagagacttcggagttggctatggatagccaaagccacgccttc

gacagtacagacgaacccgacggtgtggacggtaccccggacgccgcaggatctggcgct

acttctgaaaacggaggcgggaagcccggcgtcgggagggccgtgggctaccagggagca

aaggttctagaccccgtatccggctttcatgtggaccccgtggttgtgtttgacttcgct

agcttatacccaagcattatccaggcccataacctctgtttcaccaccctggcgctcgat

gaagtggatctggccgggcttcaaccatccgtcgactactcgacgttcgaggtgggtgac

caaaagttattttttgtccacgcccatattcgcgaaagcctgcttggcatcttgctgcgc

gactggctggccatgcgaaaggcggtgagggcgcgaatccccaccagcacccccgaggag

gcagttttactagataagcagcagtctgcgattaaggtgatatgcaactcggtttacgga

ttcacgggggtggcaaacggcctgttgccgtgtctgaggatagcggctaccgttaccacg

ataggacgcgacatgctcctcaagacccgagattacgttcactctcgttgggcgacgcgc

gagctgctggaggacaattttccaggggctataggtttccgaaaccacaagccttactcc

gtcagggttatctacggagacaccgactccgtgtttatcaagtttgtgggcctgacgtac

gagggggtatcggagctgggggatgctatgtcgcgtcagatttcagcggacctctttaga

gcgcccatcaaactggagtgtgagaagacctttcagcgactgctgctgatcaccaagaag

aagtacataggtgtcataaacggggggaagatgctcatgaagggggtcgacctggtccgc

aaaaataactgctctttcataaacttgtacgcgcgacatctggtagatcttttgttgtac

gacgaggatgtggccacggcggcagcagaggtgacagacgtgcctcccgcagaatgggtg

gggcgcccgctaccgagcggctttgacaagtttgggcgagtgctggtagaggcgtacaac

cgtatcactgcccccaacttggacgtgcgcgagttcgttatgactgctgagctgagccgc

tcacccgaatcgtataccaacaagcgcctgccgcacctcaccgtctactttaagctcgcc

atgaggaatgaagaactgcccagcgtaaaagagagaattccgtatgtgatagttgcgcag

accgaggccgcggaacgcgaagcgggtgtagtaaactcaatgcgcggtaccgcccaaaac

cccgtggtaaccaagaccgcacgcccccaacctaaacgcaaactgctggtttccgacctc

gccgaagacccgacctatgttttcgagaatgacgtaccgctaaacacagactactatttc

tcccacctgttgggtaccataagcgtgacctttaaggctctattcggaaatgatgtgaga

acaacagaaaatcttttaaagcggtttattccggaaaccccccacaagacccccacgaaa

acccaggcactgcttgagcgcgccggctttgaaaagctgacgccctttacaccggaggaa

gaaagtcgtcgaatactgcatacagttttttgtactctagaagcagctccccatcaaagc

tga

>KU206404.1

atggcggcgcgcgaacaggccaactccgttcgcaggagcgggttttttaatccgtttatt

ggcaagaggccatttttcaggccgggaagcgggcaaaccgcggagacagagcgcccaagg

cccccccaacactcgtactgcacagaggtgggtagctttaagtttatagctccaagatgc

ctcgatgaagaagcccccgccgaccagcgacgcggtgtacacgtgggcaccttggagcgc

ccacccaaagtgtactgcgatggctcagagtacgacgtgctgaactttgcctccggaggt

tgctggcctcgcaggattcgggtttggaacggccaggattttcggggcgatggattcaac

cccagatttgagagatttcacgtgtacgacatagtagagacttcggagagcgcctcacac

gatgacccatccaggtttgctgagctatcccgcccaagcgggtctgtggtaacactgctg

ggaatgagcgagtgtggaaagcgagtcgcggttcacgtctatggtgtgcgccattatttt

tacatggcaaaggcggaggtggatagcgcttgtggaataaccaccgaggcagaactcgtg

cgcgcaatggtggactgcgcgcacagctcggctttgagcgcggccctgggaaatggcaac

ggcggcaaacagagcggcggcagcggcgggggatggtggggcggaaagcacgtgtctgcg

gactgcttcaaagtggagaccgtgtgccacacgacgctgtactactttggatctaagcca

gctctctactatagagtatctgcctccagcagccgcctgggagggttcatctgcgacaac

tttcacccggagattacaaaattcgaggggagcgtggacgtgaccacgcggctgttgttg

gacaatgaaaattttaccagtttcgggtggtaccgcctgcgacccggcacccacggagag

cgtgttcaacttcgccccgttgagcgacacgtcacctcaagcgacgtggagattaactgt

actcccgataacctggagccgataccagacgaggctgcctggcccgactataagctcatg

tgctttgatatagagtgtaaagctggaacgggtaacgaaatggcgttcccagtggcaact

aaccaagaggacctggtcatccagatctcctgtctgctgtactcgcttgctactcagaac

cacgaacacaccctgctgttttccctcgggtcatgcgatatctctgaggaatactcgttt

gcatgcgtccagcgcggcgagcccagaccgacggttttggagtttgacagcgagtacgag

ctgctggttgccttcctgacctttctcaagcagtactctcccgagttcgccaccggctac

aacatcgttaattttgactgggcgtacatagttaacaaggtaacgtcggtgtataacatc

aagctggacgggtacggcaagttcaacaaaggggggctgtttaaggtgtgggacatcgcc

acgaaccattttcagaagaagagcaaggtgaaaatcaatggcctgatatctctagacatg

tattctgtggcgacggaaaagctaaagctacccagctacaaactcgacgcggtcgtggga

gacgtcctcggcgagcataagatagaccttccctataaagaaataccctcctattacgcg

ggagggcctgaccggaggggcgtaataggagagtattgtatccaggactctaggctggtg

ggcaagctgttttttaagtacctcccccatctggaactatcggcggtggccaaactcgcc

cgtatcaccctaacgcgggtaatttttgacggtcagcaaattcgcgtgtacacgtgcttg

ctgaaactcgcccgcgagagaaatttcattttgccagacaacagacgccggtttgacagt

caggcagacgccgcgtcagagacttcggagttggctatggatagccaaagccacgccttc

gacagtacagacgaacccgacggtgtggacggtaccccggacgccgcaggatctggcgct

acttctgaaaacggaggcgggaagcccggcgtcgggagggccgtgggctaccagggagca

aaggttctagaccccgtatccggctttcatgtggaccccgtggttgtgtttgacttcgct

agcttatacccaagcattatccaggcccataacctctgtttcaccaccctggcgctcgat

gaagtggatctggccgggcttcaaccatccgtcaactactcgacgttcgaggtgggtgac

caaaagttattttttgtccacgcccatattcgcgaaagcctgcttggcatcttgctgcgc

gactggctggccatgcgaaaggcggtgagggcgcgaatccccaccagcacccccgaggag

gcagttttactagataagcagcagtctgcgattaaggtgatatgcaactcggtttacgga

ttcacgggggtggcaaacggcctgttgccgtgtctgaggatagcggctaccgttaccacg

ataggacgcgacatgctcctcaagacccgagattacgttcactctcgttgggcgacgcgc

gagctgctggaggacaattttccaggggctataggtttccgaaaccacaagccttactcc

gtcagggttatctacggagacaccgactccgtgtttatcaagtttgtgggcctgacgtac

gagggggtatcggagctgggggatgctatgtcgcgtcagatttcagcggacctctttaga

gcgcccatcaaactggagtgtgagaagacctttcagcgactgctgctgatcaccaagaag

aagtacataggtgtcataaacggggggaagatgctcatgaagggggtcgacctggtccgc

aaaaataactgctctttcataaacttgtacgcgcgacatctggtagatcttttgttgtac

gacgaggatgtggccacggcggcagcagaggtgacagacgtgcctcccgcagaatgggtg

gggcgcccgctaccgagcggctttgacaagtttgggcgagtgctggtagaggcgtacaac

cgtatcactgcccccaacttggacgtgcgcgagttcgttatgactgctgagctgagccgc

tcacccgaatcgtataccaacaagcgcctgccgcacctcaccgtctactttaagctcgcc

atgaggaatgaagaactgcccagcgtaaaagagagaattccgtatgtgatagttgcgcag

accgaggccgcggaacgcgaagcgggtgtagtaaactcaatgcgcggtaccgcccaaaac

cccgtggtaaccaagaccgcacgcccccaacctaaacgcaaactgctggtttccgacctc

gccgaagacccgacctatgtttccgagaatgacgtaccgctaaacacagactactatttc

tcccacctgttgggtaccataagcgtgacctttaaggctctattcggaaatgatgtgaga

acaacagaaaatcttttaaagcggtttattccggaaaccccccacaagacccccacgaaa

acccaggcactgcttgagcgcgccggctttgaaaagctgacgccctttacaccggaggaa

gaaagtcgtcgaatactgcatacagttttttgtactctagaagcagctccccatcaaagc

tga

>KU206405.1

atggcggcgcgcgaacaggccaactccgttcgcaggagcgggttttttaatccgtttatt

ggcaagaggccatttttcaggccgggaagcgggcaaaccgcggagacagagcgcccaagg

cccccccaacactcgtactgcacagaggtgggtagctttaagtttatagctccaagatgc

ctcgatgaagaagcccccgccgaccagcgacgcggtgtacacgtgggcaccttggagcgc

ccacccaaagtgtactgcgatggctcagagtacgacgtgctgaactttgcctccggaggt

tgctggcctcgcaggattcgggtttggaacggccaggattttcggggcgatggattcaac

cccagatttgagagatttcacgtgtacgacatagtagagacttcggagagcgcctcacac

gatgacccatccaggtttgctgagctatcccgcccaagcgggtctgtggtaacactgctg

ggaatgagcgagtgtggaaagcgagtcgcggttcacgtctatggtgtgcgccattatttt

tacatggcaaaggcggaggtggatagcgcttgtggaataaccaccgaggcagaactcgtg

cgcgcaatggtggactgcgcgcacagctcggctttgagcgcggccctgggaaatggcaac

ggcggcaaacagagcggcggcagcggcgggggatggtggggcggaaagcacgtgtctgcg

gactgcttcaaagtggagaccgtgtgccacacgacgctgtactactttggatctaagcca

gctctctactatagagtatctgcctccagcagccgcctgggagggttcatctgcgacaac

tttcacccggagattacaaaattcgaggggagcgtggacgtgaccacgcggctgttgttg

gacaatgaaaattttaccagtttcgggtggtaccgcctgcgacccggcacccacggagag

cgtgttcaacttcgccccgttgagcgacacgtcacctcaagcgacgtggagattaactgt

actcccgataacctggagccgataccagacgaggctgcctggcccgactataagctcatg

tgctttgatatagagtgtaaagctggaacgggtaacgaaatggcgttcccagtggcaact

aaccaagaggacctggtcatccagatctcctgtctgctgtactcgcttgctactcagaac

cacgaacacaccctgctgttttccctcgggtcatgcgatatctctgaggaatactcgttt

gcatgcgtccagcgcggcgagcccagaccgacggttttggagtttgacagcgagtacgag

ctgctggttgccttcctgacctttctcaagcagtactctcccgagttcgccaccggctac

aacatcgttaattttgactgggcgtacatagttaacaaggtaacgtcggtgtataacatc

aagctggacgggtacggcaagttcaacaaaggggggctgtttaaggtgtgggacatcgcc

acgaaccattttcagaagaagagcaaggtgaaaatcaatggcctgatatctctagacatg

tattctgtggcgacggaaaagctaaagctacccagctacaaactcgacgcggtcgtggga

gacgtcctcggcgagcataagatagaccttccctataaagaaataccctcctattacgcg

ggagggcctgaccggaggggcgtaataggagagtattgtatccaggactctaggctggtg

ggcaagctgttttttaagtacctcccccatctggaactatcggcggtggccaaactcgcc

cgtatcaccctaacgcgggtaatttttgacggtcagcaaattcgcgtgtacacgtgcttg

ctgaaactcgcccgcgagagaaatttcattttgccagacaacagacgccggtttgacagt

caggcaaacgccgcgtcagagacttcggagttggctatggatagccaaagccacgccttc

gacagtacagacgaacccgacggtgtggacggtaccccggacgccgcaggatctggcgct

acttctgaaaacggaggcgggaagcccggcgtcgggagggccgtgggctaccagggagca

aaggttctagaccccgtatccggctttcatgtggaccccgtggttgtgtttgacttcgct

agcttatacccaagcattatccaggcccataacctctgtttcaccaccctggcgctcgat

gaagtggatctggccgggcttcaaccatccgtcaactactcgacgttcgaggtgggtgac

caaaagttattttttgtccacgcccatattcgcgaaagcctgcttggcatcttgctgcgc

gactggctggccatgcgaaaggcggtgagggcgcgaatccccaccagcacccccgaggag

gcagttttactagataagcagcagtctgcgattaaggtgatatgcaactcggtttacgga

ttcacgggggtggcaaacggcctgttgccgtgtctgaggatagcggctaccgttaccacg

ataggacgcgacatgctcctcaagacccgagattacgttcactctcgttgggcgacgcgc

gagctgctggaggacaattttccaggggctataggtttccgaaaccacaagccttactcc

gtcagggttatctacggagacaccgactccgtgtttatcaagtttgtgggcctgacgtac

gagggggtatcggagctgggggatgctatgtcgcgtcagatttcagcggacctctttaga

gcgcccatcaaactggagtgtgagaagacctttcagcgactgctgctgatcaccaagaag

aagtacataggtgtcataaacggggggaagatgctcatgaagggggtcgacctggtccgc

aaaaataactgctctttcataaacttgtacgcgcgacatctggtagatcttttgttgtac

gacgaggatgtggccacggcggcagcagaggtgacagacgtgcctcccgcagaatgggtg

gggcgcccgctaccgagcggctttgacaagtttgggcgagtgctggtagaggcgtacaac

cgtatcactgcccccaacttggacgtgcgcgagttcgttatgactgctgagctgagccgc

tcacccgaatcgtataccaacaagcgcctgccgcacctcaccgtctactttaagctcgcc

atgaggaatgaagaactgcccagcgtaaaagagagaattccgtatgtgatagttgcgcag

accgaggccgcggaacgcgaagcgggtgtagtaaactcaatgcgcggtaccgcccaaaac

cccgtggtaaccaagaccgcacgcccccaacctaaacgcaaactgctggtttccgacctc

gccgaagacccgacctatgtttccgagaatgacgtaccgctaaacacagactactatttc

tcccacctgttgggtaccataagcgtgacctttaaggctctattcggaaatgatgtgaga

acaacagaaaatcttttaaagcggtttattccggaaaccccccacaagacccccacgaaa

acccaggcactgcttgagcgcgccggctttgaaaagctgacgccctttacaccggaggaa

gaaagtcgtcgaatactgcatacagttttttgtactctagaagcagctccccatcaaagc

tga

>KU206406.1

atggcggcgcgcgaacaggccaactccgttcgcaggagcgggttttttaatccgtttatt

ggcaagaggccatttttcaggccgggaagcgggcaaaccgcggagacagagcgcccaagg

cccccccaacactcgtactgcacagaggtgggtagctttaagtttatagctccaagatgc

ctcgatgaagaagcccccgccgaccagcgacgcggtgtacacgtgggcaccttggagcgc

ccacccaaagtgtactgcgatggctcagagtacgacgtgctgaactttgcctccggaggt

tgctggcctcgcaggattcgggtttggaacggccaggattttcggggcgatggattcaac

cccagatttgagagatttcacgtgtacgacatagtagagacttcggagagcgcctcacac

gatgacccatccaggtttgctgagctatcccgcccaagcgggtctgtggtaacactgctg

ggaatgagcgagtgtggaaagcgagtcgcggttcacgtctatggtgtgcgccattatttt

tacatggcaaaggcggaggtggatagcgcttgtggaataaccaccgaggcagaactcgtg

cgcgcaatggtggactgcgcgcacagctcggctttgagcgcggccctgggaaatggcaac

ggcggcaaacagagcggcggcagcggcgggggatggtggggcggaaagcacgtgtctgcg

gactgcttcaaagtggagaccgtgtgccacacgacgctgtactactttggatctaagcca

gctctctactatagagtatctgcctccagcagccgcctgggagggttcatctgcgacaac

tttcacccggagattacaaaattcgaggggagcgtggacgtgaccacgcggctgttgttg

gacaatgaaaattttaccagtttcgggtggtaccgcctgcgacccggcacccacggagag

cgtgttcaacttcgccccgttgagcgacacgtcacctcaagcgacgtggagattaactgt

actcccgataacctggagccgataccagacgaggctgcctggcccgactataagctcatg

tgctttgatatagagtgtaaagctggaacgggtaacgaaatggcgttcccagtggcaact

aaccaagaggacctggtcatccagatctcctgtctgctgtactcgcttgctactcagaac

cacgaacacaccctgctgttttccctcgggtcatgcgatatctctgaggaatactcgttt

gcatgcgtccagcgcggcgagcccagaccgacggttttggagtttgacagcgagtacgag

ctgctggttgccttcctgacctttctcaagcagtactctcccgagttcgccaccggctac

aacatcgttaattttgactgggcgtacatagttaacaaggtaacgtcggtgtataacatc

aagctggacgggtacggcaagttcaacaaaggggggctgtttaaggtgtgggacatcgcc

acgaaccattttcagaagaagagcaaggtgaaaatcaatggcctgatatctctagacatg

tattctgtggcgacggaaaagctaaagctacccagctacaaactcgacgcggtcgtggga

gacgtcctcggcgagcataagatagaccttccctataaagaaataccctcctattacgcg

ggagggcctgaccggaggggcgtaataggagagtattgtatccaggactctaggctggtg

ggcaagctgttttttaagtacctcccccatctggaactatcggcggtggccaaactcgcc

cgtatcaccctaacgcgggtaatttttgacggtcagcaaattcgcgtgtacacgtgcttg

ctgaaactcgcccgcgagagaaatttcattttgccagacaacagacgccggtttgacagt

caggcaaacgccgcgtcagagacttcggagttggctatggatagccaaagccacgccttc

gacagtacagacgaacccgacggtgtggacggtaccccggacgccgcaggatctggcgct

acttctgaaaacggaggcgggaagcccggcgtcgggagggccgtgggctaccagggagca

aaggttctagaccccgtatccggctttcatgtggaccccgtggttgtgtttgacttcgct

agcttatacccaagcattatccaggcccataacctctgtttcaccaccctggcgctcgat

gaagtggatctggccgggcttcaaccatccgtcaactactcgacgttcgaggtgggtgac

caaaagttattttttgtccacgcccatattcgcgaaagcctgcttggcatcttgctgcgc

gactggctggccatgcgaaaggcggtgagggcgcgaatccccaccagcacccccgaggag

gcagttttactagataagcagcagtctgcgattaaggtgatatgcaactcggtttacgga

ttcacgggggtggcaaacggcctgttgccgtgtctgaggatagcggctaccgttaccacg

ataggacgcgacatgctcctcaagacccgagattacgttcactctcgttgggcgacgcgc

gagctgctggaggacaattttccaggggctataggtttccgaaaccacaagccttactcc

gtcagggttatctacggagacaccgactccgtgtttatcaagtttgtgggcctgacgtac

gagggggtatcggagctgggggatgctatgtcgcgtcagatttcagcggacctctttaga

gcgcccatcaaactggagtgtgagaagacctttcagcgactgctgctgatcaccaagaag

aagtacataggtgtcataaacggggggaagatgctcatgaagggggtcgacctggtccgc

aaaaataactgctctttcataaacttgtacgcgcgacatctggtagatcttttgttgtac

gacgaggatgtggccacggcggcagcagaggtgacagacgtgcctcccgcagaatgggtg

gggcgcccgctaccgagcggctttgacaagtttgggcgagtgctggtagaggcgtacaac

cgtatcactgcccccaacttggacgtgcgcgagttcgttatgactgctgagctgagccgc

tcacccgaatcgtataccaacaagcgcctgccgcacctcaccgtctactttaagctcgcc

atgaggaatgaagaactgcccagcgtaaaagagagaattccgtatgtgatagttgcgcag

accgaggccgcggaacgcgaagcgggtgtagtaaactcaatgcgcggtaccgcccaaaac

cccgtggtaaccaagaccgcacgcccccaacctaaacgcaaactgctggtttccgacctc

gccgaagacccgacctatgtttccgagaatgacgtaccgctaaacacagactactatttc

tcccacctgttgggtaccataagcgtgacctttaaggctctattcggaaatgatgtgaga

acaacagaaaatcttttaaagcggtttattccggaaaccccccacaagacccccacgaaa

acccaggcactgcttgagcgcgccggctttgaaaagctgacgccctttacaccggaggaa

gaaagtcgtcgaatactgcatacagttttttgtactctagaagcagctccccatcaaagc

tga

>KU206407.1

atggcggcgcgcgaacaggccaactccgttcgcaggagcgggttttttaatccgtttatt

ggcaagaggccatttttcaggccgggaagcgggcaaaccgcggagacagagcgcccaagg

cccccccaacactcgtactgcacagaggtgggtagctttaagtttatagctccaagatgc

ctcgatgaagaagcccccgccgaccagcgacgcggtgtacacgtgggcaccttggagcgc

ccacccaaagtgtactgcgatggctcagagtacgacgtgctgaactttgcctccggaggt

tgctggcctcgcaggattcgggtttggaacggccaggattttcggggcgatggattcaac

cccagatttgagagatttcacgtgtacgacatagtagagacttcggagagcgcctcacac

gatgacccatccaggtttgctgagctatcccgcccaagcgggtctgtggtaacactgctg

ggaatgagcgagtgtggaaagcgagtcgcggttcacgtctatggtgtgcgccattatttt

tacatggcaaaggcggaggtggatagcgcttgtggaataaccaccgaggcagaactcgtg

cgcgcaatggtggactgcgcgcacagctcggctttgagcgcggccctgggaaatggcaac

ggcggcaaacagagcggcggcagcggcgggggatggtggggcggaaagcacgtgtctgcg

gactgcttcaaagtggagaccgtgtgccacacgacgctgtactactttggatctaagcca

gctctctactatagagtatctgcctccagcagccgcctgggagggttcatctgcgacaac

tttcacccggagattacaaaattcgaggggagcgtggacgtgaccacgcggctgttgttg

gacaatgaaaattttaccagtttcgggtggtaccgcctgcgacccggcacccacggagag

cgtgttcaacttcgccccgttgagcgacacgtcacctcaagcgacgtggagattaactgt

actcccgataacctggagccgataccagacgaggctgcctggcccgactataagctcatg

tgctttgatatagagtgtaaagctggaacgggtaacgaaatggcgttcccagtggcaact

aaccaagaggacctggtcatccagatctcctgtctgctgtactcgcttgctactcagaac

cacgaacacaccctgctgttttccctcgggtcatgcgatatctctgaggaatactcgttt

gcatgcgtccagcgcggcgagcccagaccgacggttttggagtttgacagcgagtacgag

ctgctggttgccttcctgacctttctcaagcagtactctcccgagttcgccaccggctac

aacatcgttaattttgactgggcgtacatagttaacaaggtaacgtcggtgtataacatc

aagctggacgggtacggcaagttcaacaaaggggggctgtttaaggtgtgggacatcgcc

acgaaccattttcagaagaagagcaaggtgaaaatcaatggcctgatatctctagacatg

tattctgtggcgacggaaaagctaaagctacccagctacaaactcgacgcggtcgtggga

gacgtcctcggcgagcataagatagaccttccctataaagaaataccctcctattacgcg

ggagggcctgaccggaggggcgtaataggagagtattgtatccaggactctaggctggtg

ggcaagctgttttttaagtacctcccccatctggaactatcggcggtggccaaactcgcc

cgtatcaccctaacgcgggtaatttttgacggtcagcaaattcgcgtgtacacgtgcttg

ctgaaactcgcccgcgagagaaatttcattttgccagacaacagacgccggtttgacagt

caggcagacgccgcgtcagagacttcggagttggctatggatagccaaagccacgccttc

gacagtacagacgaacccgacggtgtggacggtaccccggacgccgcaggatctggcgct

acttctgaaaacggaggcgggaagcccggcgtcgggagggccgtgggctaccagggagca

aaggttctagaccccgtatccggctttcatgtggaccccgtggttgtgtttgacttcgct

agcttatacccaagcattatccaggcccataacctctgtttcaccaccctggcgctcgat

gaagtggatctggccgggcttcaaccatccgtcaactactcgacgttcgaggtgggtgac

caaaagttattttttgtccacgcccatattcgcgaaagcctgcttggcatcttgctgcgc

gactggctggccatgcgaaaggcggtgagggcgcgaatccccaccagcacccccgaggag

gcagttttactagataagcagcagtctgcgattaaggtgatatgcaactcggtttacgga

ttcacgggggtggcaaacggcctgttgccgtgtctgaggatagcggctaccgttaccacg

ataggacgcgacatgctcctcaagacccgagattacgttcactctcgttgggcgacgcgc

gagctgctggaggacaattttccaggggctataggtttccgaaaccacaagccttactcc

gtcagggttatctacggagacaccgactccgtgtttatcaagtttgtgggcctgacgtac

gagggggtatcggagctgggggatgctatgtcgcgtcagatttcagcggacctctttaga

gcgcccatcaaactggagtgtgagaagacctttcagcgactgctgctgatcaccaagaag

aagtacataggtgtcataaacggggggaagatgctcatgaagggggtcgacctggtccgc

aaaaataactgctctttcataaacttgtacgcgcgacatctggtagatcttttgttgtac

gacgaggatgtggccacggcggcagcagaggtgacagacgtgcctcccgcagaatgggtg

gggcgcccgctaccgagcggctttgacaagtttgggcgagtgctggtagaggcgtacaac

cgtatcactgcccccaacttggacgtgcgcgagttcgttatgactgctgagctgagccgc

tcacccgaatcgtataccaacaagcgcctgccgcacctcaccgtctactttaagctcgcc

atgaggaatgaagaactgcccagcgtaaaagagagaattccgtatgtgatagttgcgcag

accgaggccgcggaacgcgaagcgggtgtagtaaactcaatgcgcggtaccgcccaaaac

cccgtggtaaccaagaccgcacgcccccaacctaaacgcaaactgctggtttccgacctc

gccgaagacccgacctatgtttccgagaatgacgtaccgctaaacacagactactatttc

tcccacctgttgggtaccataagcgtgacctttaaggctctattcggaaatgatgtgaga

acaacagaaaatcttttaaagcggtttattccggaaaccccccacaagacccccacgaaa

acccaggcactgcttgagcgcgccggctttgaaaagctgacgccctttacaccggaggaa

gaaagtcgtcgaatactgcatacagttttttgtactctagaagcagctccccatcaaagc

tga

>KU206408.1

atggcggcgcgcgaacaggccaactccgttcgcaggagcgggttttttaatccgtttatt

ggcaagaggccatttttcaggccgggaagcgggcaaaccgcggagacagagcgcccaagg

cccccccaacactcgtactgcacagaggtgggtagctttaagtttatagctccaagatgc

ctcgatgaagaagcccccgccgaccagcgacgcggtgtacacgtgggcaccttggagcgc

ccacccaaagtgtactgcgatggctcagagtacgacgtgctgaactttgcctccggaggt

tgctggcctcgcaggattcgggtttggaacggccaggattttcggggcgatggattcaac

cccagatttgagagatttcacgtgtacgacatagtagagacttcggagagcgcctcacac

gatgacccatccaggtttgctgagctatcccgcccaagcgggtctgtggtaacactgctg

ggaatgagcgagtgtggaaagcgagtcgcggttcacgtctatggtgtgcgccattatttt

tacatggcaaaggcggaggtggatagcgcttgtggaataaccaccgaggcagaactcgtg

cgcgcaatggtggactgcgcgcacagctcggctttgagcgcggccctgggaaatggcaac

ggcggcaaacagagcggcggcagcggcgggggatggtggggcggaaagcacgtgtctgcg

gactgcttcaaagtggagaccgtgtgccacacgacgctgtactactttggatctaagcca

gctctctactatagagtatctgcctccagcagccgcctgggagggttcatctgcgacaac

tttcacccggagattacaaaattcgaggggagcgtggacgtgaccacgcggctgttgttg

gacaatgaaaattttaccagtttcgggtggtaccgcctgcgacccggcacccacggagag

cgtgttcaacttcgccccgttgagcgacacgtcacctcaagcgacgtggagattaactgt

actcccgataacctggagccgataccagacgaggctgcctggcccgactataagctcatg

tgctttgatatagagtgtaaagctggaacgggtaacgaaatggcgttcccagtggcaact

aaccaagaggacctggtcatccagatctcctgtctgctgtactcgcttgctactcagaac

cacgaacacaccctgctgttttccctcgggtcatgcgatatctctgaggaatactcgttt

gcatgcgtccagcgcggcgagcccagaccgacggttttggagtttgacagcgagtacgag

ctgctggttgccttcctgacctttctcaagcagtactctcccgagttcgccaccggctac

aacatcgttaattttgactgggcgtacatagttaacaaggtaacgtcggtgtataacatc

aagctggacgggtacggcaagttcaacaaaggggggctgtttaaggtgtgggacatcgcc

acgaaccattttcagaagaagagcaaggtgaaaatcaatggcctgatatctctagacatg

tattctgtggcgacggaaaagctaaagctacccagctacaaactcgacgcggtcgtggga

gacgtcctcggcgagcataagatagaccttccctataaagaaataccctcctattacgcg

ggagggcctgaccggaggggcgtaataggagagtattgtatccaggactctaggctggtg

ggcaagctgttttttaagtacctcccccatctggaactatcggcggtggccaaactcgcc

cgtatcaccctaacgcgggtaatttttgacggtcagcaaattcgcgtgtacacgtgcttg

ctgaaactcgcccgcgagagaaatttcattttgccagacaacagacgccggtttgacagt

caggcagacgccgcgtcagagacttcggagttggctatggatagccaaagccacgccttc

gacagtacagacgaacccgacggtgtggacggtaccccggacgccgcaggatctggcgct

acttctgaaaacggaggcgggaagcccggcgtcgggagggccgtgggctaccagggagca

aaggttctagaccccgtatccggctttcatgtggaccccgtggttgtgtttgacttcgct

agcttatacccaagcattatccaggcccataacctctgtttcaccaccctggcgctcgat

gaagtggatctggccgggcttcaaccatccgtcaactactcgacgttcgaggtgggtgac

caaaagttattttttgtccacgcccatattcgcgaaagcctgcttggcatcttgctgcgc

gactggctggccatgcgaaaggcggtgagggcgcgaatccccaccagcacccccgaggag

gcagttttactagataagcagcagtctgcgattaaggtgatatgcaactcggtttacgga

ttcacgggggtggcaaacggcctgttgccgtgtctgaggatagcggctaccgttaccacg

ataggacgcgacatgctcctcaagacccgagattacgttcactctcgttgggcgacgcgc

gagctgctggaggacaattttccaggggctataggtttccgaaaccacaagccttactcc

gtcagggttatctacggagacaccgactccgtgtttatcaagtttgtgggcctgacgtac

gagggggtatcggagctgggggatgctatgtcgcgtcagatttcagcggacctctttaga

gcgcccatcaaactggagtgtgagaagacctttcagcgactgctgctgatcaccaagaag

aagtacataggtgtcataaacggggggaagatgctcatgaagggggtcgacctggtccgc

aaaaataactgctctttcataaacttgtacgcgcgacatctggtagatcttttgttgtac

gacgaggatgtggccacggcggcagcagaggtgacagacgtgcctcccgcagaatgggtg

gggcgcccgctaccgagcggctttgacaagtttgggcgagtgctggtagaggcgtacaac

cgtatcactgcccccaacttggacgtgcgcgagttcgttatgactgctgagctgagccgc

tcacccgaatcgtataccaacaagcgcctgccgcacctcaccgtctactttaagctcgcc

atgaggaatgaagaactgcccagcgtaaaagagagaattccgtatgtgatagttgcgcag

accgaggccgcggaacgcgaagcgggtgtagtaaactcaatgcgcggtaccgcccaaaac

cccgtggtaaccaagaccgcacgcccccaacctaaacgcaaactgctggtttccgacctc

gccgaagacccgacctatgtttccgagaatgacgtaccgctaaacacagactactatttc

tcccacctgttgggtaccataagcgtgacctttaaggctctattcggaaatgatgtgaga

acaacagaaaatcttttaaagcggtttattccggaaaccccccacaagacccccacgaaa

acccaggcactgcttgagcgcgccggctttgaaaagctgacgccctttacaccggaggaa

gaaagtcgtcgaatactgcatacagttttttgtactctagaagcagctccccatcaaagc

tga

>KU206409.1

atggcggcgcgcgaacaggccaactccgttcgcaggagcgggttttttaatccgtttatt

ggcaagaggccatttttcaggccgggaagcgggcaaaccgcggagacagagcgcccaagg

cccccccaacactcgtactgcacagaggtgggtagctttaagtttatagctccaagatgc

ctcgatgaagaagcccccgccgaccagcgacgcggtgtacacgtgggcaccttggagcgc

ccacccaaagtgtactgcgatggctcagagtacgacgtgctgaactttgcctccggaggt

tgctggcctcgcaggattcgggtttggaacggccaggattttcggggcgatggattcaac

cccagatttgagagatttcacgtgtacgacatagtagagacttcggagagcgcctcacac

gatgacccatccaggtttgctgagctatcccgcccaagcgggtctgtggtaacactgctg

ggaatgagcgagtgtggaaagcgagtcgcggttcacgtctatggtgtgcgccattatttt

tacatggcaaaggcggaggtggatagcgcttgtggaataaccaccgaggcagaactcgtg

cgcgcaatggtggactgcgcgcacagctcggctttgagcgcggccctgggaaatggcaac

ggcggcaaacagagcggcggcagcggcgggggatggtggggcggaaagcacgtgtctgcg

gactgcttcaaagtggagaccgtgtgccacacgacgctgtactactttggatctaagcca

gctctctactatagagtatctgcctccagcagccgcctgggagggttcatctgcgacaac

tttcacccggagattacaaaattcgaggggagcgtggacgtgaccacgcggctgttgttg

gacaatgaaaattttaccagtttcgggtggtaccgcctgcgacccggcacccacggagag

cgtgttcaacttcgccccgttgagcgacacgtcacctcaagcgacgtggagattaactgt

actcccgataacctggagccgataccagacgaggctgcctggcccgactataagctcatg

tgctttgatatagagtgtaaagctggaacgggtaacgaaatggcgttcccagtggcaact

aaccaagaggacctggtcatccagatctcctgtctgctgtactcgcttgctactcagaac

cacgaacacaccctgctgttttccctcgggtcatgcgatatctctgaggaatactcgttt

gcatgcgtccagcgcggcgagcccagaccgacggttttggagtttgacagcgagtacgag

ctgctggttgccttcctgacctttctcaagcagtactctcccgagttcgccaccggctac

aacatcgttaattttgactgggcgtacatagttaacaaggtaacgtcggtgtataacatc

aagctggacgggtacggcaagttcaacaaaggggggctgtttaaggtgtgggacatcgcc

acgaaccattttcagaagaagagcaaggtgaaaatcaatggcctgatatctctagacatg

tattctgtggcgacggaaaagctaaagctacccagctacaaactcgacgcggtcgtggga

gacgtcctcggcgagcataagatagaccttccctataaagaaataccctcctattacgcg

ggagggcctgaccggaggggcgtaataggagagtattgtatccaggactctaggctggtg

ggcaagctgttttttaagtacctcccccatctggaactatcggcggtggccaaactcgcc

cgtatcaccctaacgcgggtaatttttgacggtcagcaaattcgcgtgtacacgtgcttg

ctgaaactcgcccgcgagagaaatttcattttgccagacaacagacgccggtttgacagt

caggcagacgccgcgtcagagacttcggagttggctatggatagccaaagccacgccttc

gacagtacagacgaacccgacggtgtggacggtaccccggacgccgcaggatctggcgct

acttctgaaaacggaggcgggaagcccggcgtcgggagggccgtgggctaccagggagca

aaggttctagaccccgtatccggctttcatgtggaccccgtggttgtgtttgacttcgct

agcttatacccaagcattatccaggcccataacctctgtttcaccaccctggcgctcgat

gaagtggatctggccgggcttcaaccatccgtcaactactcgacgttcgaggtgggtgac

caaaagttattttttgtccacgcccatattcgcgaaagcctgcttggcatcttgctgcgc

gactggctggccatgcgaaaggcggtgagggcgcgaatccccaccagcacccccgaggag

gcagttttactagataagcagcagtctgcgattaaggtgatatgcaactcggtttacgga

ttcacgggggtggcaaacggcctgttgccgtgtctgaggatagcggctaccgttaccacg

ataggacgcgacatgctcctcaagacccgagattacgttcactctcgttgggcgacgcgc

gagctgctggaggacaattttccaggggctataggtttccgaaaccacaagccttactcc

gtcagggttatctacggagacaccgactccgtgtttatcaagtttgtgggcctgacgtac

gagggggtatcggagctgggggatgctatgtcgcgtcagatttcagcggacctctttaga

gcgcccatcaaactggagtgtgagaagacctttcagcgactgctgctgatcaccaagaag

aagtacataggtgtcataaacggggggaagatgctcatgaagggggtcgacctggtccgc

aaaaataactgctctttcataaacttgtacgcgcgacatctggtagatcttttgttgtac

gacgaggatgtggccacggcggcagcagaggtgacagacgtgcctcccgcagaatgggtg

gggcgcccgctaccgagcggctttgacaagtttgggcgagtgctggtagaggcgtacaac

cgtatcactgcccccaacttggacgtgcgcgagttcgttatgactgctgagctgagccgc

tcacccgaatcgtataccaacaagcgcctgccgcacctcaccgtctactttaagctcgcc

atgaggaatgaagaactgcccagcgtaaaagagagaattccgtatgtgatagttgcgcag

accgaggccgcggaacgcgaagcgggtgtagtaaactcaatgcgcggtaccgcccaaaac

cccgtggtaaccaagaccgcacgcccccaacctaaacgcaaactgctggtttccgacctc

gccgaagacccgacctatgtttccgagaatgacgtaccgctaaacacagactactatttc

tcccacctgttgggtaccataagcgtgacctttaaggctctattcggaaatgatgtgaga

acaacagaaaatcttttaaagcggtttattccggaaaccccccacaagacccccacgaaa

acccaggcactgcttgagcgcgccggctttgaaaagctgacgccctttacaccggaggaa

gaaagtcgtcgaatactgcatacagttttttgtactctagaagcagctccccatcaaagc

tga

>KU206410.1

atggcggcgcgcgaacaggccaactccgttcgcaggagcgggttttttaatccgtttatt

ggcaagaggccatttttcaggccgggaagcgggcaaaccgcggagacagagcgcccaagg

cccccccaacactcgtactgcacagaggtgggtagctttaagtttatagctccaagatgc

ctcgatgaagaagcccccgccgaccagcgacgcggtgtacacgtgggcaccttggagcgc

ccacccaaagtgtactgcgatggctcagagtacgacgtgctgaactttgcctccggaggt

tgctggcctcgcaggattcgggtttggaacggccaggattttcggggcgatggattcaac

cccagatttgagagatttcacgtgtacgacatagtagagacttcggagagcgcctcacac

gatgacccatccaggtttgctgagctatcccgcccaagcgggtctgtggtaacactgctg

ggaatgagcgagtgtggaaagcgagtcgcggttcacgtctatggtgtgcgccattatttt

tacatggcaaaggcggaggtggatagcgcttgtggaataaccaccgaggcagaactcgtg

cgcgcaatggtggactgcgcgcacagctcggctttgagcgcggccctgggaaatggcaac

ggcggcaaacagagcggcggcagcggcgggggatggtggggcggaaagcacgtgtctgcg

gactgcttcaaagtggagaccgtgtgccacacgacgctgtactactttggatctaagcca

gctctctactatagagtatctgcctccagcagccgcctgggagggttcatctgcgacaac

tttcacccggagattacaaaattcgaggggagcgtggacgtgaccacgcggctgttgttg

gacaatgaaaattttaccagtttcgggtggtaccgcctgcgacccggcacccacggagag

cgtgttcaacttcgccccgttgagcgacacgtcacctcaagcgacgtggagattaactgt

actcccgataacctggagccgataccagacgaggctgcctggcccgactataagctcatg

tgctttgatatagagtgtaaagctggaacgggtaacgaaatggcgttcccagtggcaact

aaccaagaggacctggtcatccagatctcctgtctgctgtactcgcttgctactcagaac

cacgaacacaccctgctgttttccctcgggtcatgcgatatctctgaggaatactcgttt

gcatgcgtccagcgcggcgagcccagaccgacggttttggagtttgacagcgagtacgag

ctgctggttgccttcctgacctttctcaagcagtactctcccgagttcgccaccggctac

aacatcgttaattttgactgggcgtacatagttaacaaggtaacgtcggtgtataacatc

aagctggacgggtacggcaagttcaacaaaggggggctgtttaaggtgtgggacatcgcc

acgaaccattttcagaagaagagcaaggtgaaaatcaatggcctgatatctctagacatg

tattctgtggcgacggaaaagctaaagctacccagctacaaactcgacgcggtcgtggga

gacgtcctcggcgagcataagatagaccttccctataaagaaataccctcctattacgcg

ggagggcctgaccggaggggcgtaataggagagtattgtatccaggactctaggctggtg

ggcaagctgttttttaagtacctcccccatctggaactatcggcggtggccaaactcgcc

cgtatcaccctaacgcgggtaatttttgacggtcagcaaattcgcgtgtacacgtgcttg

ctgaaactcgcccgcgagagaaatttcattttgccagacaacagacgccggtttgacagt

caggcagacgccgcgtcagagacttcggagttggctatggatagccaaagccacgccttc

gacagtacagacgaacccgacggtgtggacggtaccccggacgccgcaggatctggcgct

acttctgaaaacggaggcgggaagcccggcgtcgggagggccgtgggctaccagggagca

aaggttctagaccccgtatccggctttcatgtggaccccgtggttgtgtttgacttcgct

agcttatacccaagcattatccaggcccataacctctgtttcaccaccctggcgctcgat

gaagtggatctggccgggcttcaaccatccgtcaactactcgacgttcgaggtgggtgac

caaaagttattttttgtccacgcccatattcgcgaaagcctgcttggcatcttgctgcgc

gactggctggccatgcgaaaggcggtgagggcgcgaatccccaccagcacccccgaggag

gcagttttactagataagcagcagtctgcgattaaggtgatatgcaactcggtttacgga

ttcacgggggtggcaaacggcctgttgccgtgtctgaggatagcggctaccgttaccacg

ataggacgcgacatgctcctcaagacccgagattacgttcactctcgttgggcgacgcgc

gagctgctggaggacaattttccaggggctataggtttccgaaaccacaagccttactcc

gtcagggttatctacggagacaccgactccgtgtttatcaagtttgtgggcctgacgtac

gagggggtatcggagctgggggatgctatgtcgcgtcagatttcagcggacctctttaga

gcgcccatcaaactggagtgtgagaagacctttcagcgactgctgctgatcaccaagaag

aagtacataggtgtcataaacggggggaagatgctcatgaagggggtcgacctggtccgc

aaaaataactgctctttcataaacttgtacgcgcgacatctggtagatcttttgttgtac

gacgaggatgtggccacggcggcagcagaggtgacagacgtgcctcccgcagaatgggtg

gggcgcccgctaccgagcggctttgacaagtttgggcgagtgctggtagaggcgtacaac

cgtatcactgcccccaacttggacgtgcgcgagttcgttatgactgctgagctgagccgc

tcacccgaatcgtataccaacaagcgcctgccgcacctcaccgtctactttaagctcgcc

atgaggaatgaagaactgcccagcgtaaaagagagaattccgtatgtgatagttgcgcag

accgaggccgcggaacgcgaagcgggtgtagtaaactcaatgcgcggtaccgcccaaaac

cccgtggtaaccaagaccgcacgcccccaacctaaacgcaaactgctggtttccgacctc

gccgaagacccgacctatgtttccgagaatgacgtaccgctaaacacagactactatttc

tcccacctgttgggtaccataagcgtgacctttaaggctctattcggaaatgatgtgaga

acaacagaaaatcttttaaagcggtttattccggaaaccccccacaagacccccacgaaa

acccaggcactgcttgagcgcgccggctttgaaaagctgacgccctttacaccggaggaa

gaaagtcgtcgaatactgcatacagttttttgtactctagaagcagctccccatcaaagc

tga

>KU206411.1

atggcggcgcgcgaacaggccaactccgttcgcaggagcgggttttttaatccgtttatt

ggcaagaggccatttttcaggccgggaagcgggcaaaccgcggagacagagcgcccaagg

cccccccaacactcgtactgcacagaggtgggtagctttaagtttatagctccaagatgc

ctcgatgaagaagcccccgccgaccagcgacgcggtgtacacgtgggcaccttggagcgc

ccacccaaagtgtactgcgatggctcagagtacgacgtgctgaactttgcctccggaggt

tgctggcctcgcaggattcgggtttggaacggccaggattttcggggcgatggattcaac

cccagatttgagagatttcacgtgtacgacatagtagagacttcggagagcgcctcacac

gatgacccatccaggtttgctgagctatcccgcccaagcgggtctgtggtaacactgctg

ggaatgagcgagtgtggaaagcgagtcgcggttcacgtctatggtgtgcgccattatttt

tacatggcaaaggcggaggtggatagcgcttgtggaataaccaccgaggcagaactcgtg

cgcgcaatggtggactgcgcgcacagctcggctttgagcgcggccctgggaaatggcaac

ggcggcaaacagagcggcggcagcggcgggggatggtggggcggaaagcacgtgtctgcg

gactgcttcaaagtggagaccgtgtgccacacgacgctgtactactttggatctaagcca

gctctctactatagagtatctgcctccagcagccgcctgggagggttcatctgcgacaac

tttcacccggagattacaaaattcgaggggagcgtggacgtgaccacgcggctgttgttg

gacaatgaaaattttaccagtttcgggtggtaccgcctgcgacccggcacccacggagag

cgtgttcaacttcgccccgttgagcgacacgtcacctcaagcgacgtggagattaactgt

actcccgataacctggagccgataccagacgaggctgcctggcccgactataagctcatg

tgctttgatatagagtgtaaagctggaacgggtaacgaaatggcgttcccagtggcaact

aaccaagaggacctggtcatccagatctcctgtctgctgtactcgcttgctactcagaac

cacgaacacaccctgctgttttccctcgggtcatgcgatatctctgaggaatactcgttt

gcatgcgtccagcgcggcgagcccagaccgacggttttggagtttgacagcgagtacgag

ctgctggttgccttcctgacctttctcaagcagtactctcccgagttcgccaccggctac

aacatcgttaattttgactgggcgtacatagttaacaaggtaacgtcggtgtataacatc

aagctggacgggtacggcaagttcaacaaaggggggctgtttaaggtgtgggacatcgcc

acgaaccattttcagaagaagagcaaggtgaaaatcaatggcctgatatctctagacatg

tattctgtggcgacggaaaagctaaagctacccagctacaaactcgacgcggtcgtggga

gacgtcctcggcgagcataagatagaccttccctataaagaaataccctcctattacgcg

ggagggcctgaccggaggggcgtaataggagagtattgtatccaggactctaggctggtg

ggcaagctgttttttaagtacctcccccatctggaactatcggcggtggccaaactcgcc

cgtatcaccctaacgcgggtaatttttgacggtcagcaaattcgcgtgtacacgtgcttg

ctgaaactcgcccgcgagagaaatttcattttgccagacaacagacgccggtttgacagt

caggcagacgccgcgtcagagacttcggagttggctatggatagccaaagccacgccttc

gacagtacagacgaacccgacggtgtggacggtaccccggacgccgcaggatctggcgct

acttctgaaaacggaggcgggaagcccggcgtcgggagggccgtgggctaccagggagca

aaggttctagaccccgtatccggctttcatgtggaccccgtggttgtgtttgacttcgct

agcttatacccaagcattatccaggcccataacctctgtttcaccaccctggcgctcgat

gaagtggatctggccgggcttcaaccatccgtcaactactcgacgttcgaggtgggtgac

caaaagttattttttgtccacgcccatattcgcgaaagcctgcttggcatcttgctgcgc

gactggctggccatgcgaaaggcggtgagggcgcgaatccccaccagcacccccgaggag

gcagttttactagataagcagcagtctgcgattaaggtgatatgcaactcggtttacgga

ttcacgggggtggcaaacggcctgttgccgtgtctgaggatagcggctaccgttaccacg

ataggacgcgacatgctcctcaagacccgagattacgttcactctcgttgggcgacgcgc

gagctgctggaggacaattttccaggggctataggtttccgaaaccacaagccttactcc

gtcagggttatctacggagacaccgactccgtgtttatcaagtttgtgggcctgacgtac

gagggggtatcggagctgggggatgctatgtcgcgtcagatttcagcggacctctttaga

gcgcccatcaaactggagtgtgagaagacctttcagcgactgctgctgatcaccaagaag

aagtacataggtgtcataaacggggggaagatgctcatgaagggggtcgacctggtccgc

aaaaataactgctctttcataaacttgtacgcgcgacatctggtagatcttttgttgtac

gacgaggatgtggccacggcggcagcagaggtgacagacgtgcctcccgcagaatgggtg

gggcgcccgctaccgagcggctttgacaagtttgggcgagtgctggtagaggcgtacaac

cgtatcactgcccccaacttggacgtgcgcgagttcgttatgactgctgagctgagccgc

tcacccgaatcgtataccaacaagcgcctgccgcacctcaccgtctactttaagctcgcc

atgaggaatgaagaactgcccagcgtaaaagagagaattccgtatgtgatagttgcgcag

accgaggccgcggaacgcgaagcgggtgtagtaaactcaatgcgcggtaccgcccaaaac

cccgtggtaaccaagaccgcacgcccccaacctaaacgcaaactgctggtttccgacctc

gccgaagacccgacctatgtttccgagaatgacgtaccgctaaacacagactactatttc

tcccacctgttgggtaccataagcgtgacctttaaggctctattcggaaatgatgtgaga

acaacagaaaatcttttaaagcggtttattccggaaaccccccacaagacccccacgaaa

acccaggcactgcttgagcgcgccggctttgaaaagctgacgccctttacaccggaggaa

gaaagtcgtcgaatactgcatacagttttttgtactctagaagcagctccccatcaaagc

tga

>KU206412.1

atggcggcgcgcgaacaggccaactccgttcgcaggagcgggttttttaatccgtttatt

ggcaagaggccatttttcaggccgggaagcgggcaaaccgcggagacagagcgcccaagg

cccccccaacactcgtactgcacagaggtgggtagctttaagtttatagctccaagatgc

ctcgatgaagaagcccccgccgaccagcgacgcggtgtacacgtgggcaccttggagcgc

ccacccaaagtgtactgcgatggctcagagtacgacgtgctgaactttgcctccggaggt

tgctggcctcgcaggattcgggtttggaacggccaggattttcggggcgatggattcaac

cccagatttgagagatttcacgtgtacgacatagtagagacttcggagagcgcctcacac

gatgacccatccaggtttgctgagctatcccgcccaagcgggtctgtggtaacactgctg

ggaatgagcgagtgtggaaagcgagtcgcggttcacgtctatggtgtgcgccattatttt

tacatggcaaaggcggaggtggatagcgcttgtggaataaccaccgaggcagaactcgtg

cgcgcaatggtggactgcgcgcacagctcggctttgagcgcggccctgggaaatggcaac

ggcggcaaacagagcggcggcagcggcgggggatggtggggcggaaagcacgtgtctgcg

gactgcttcaaagtggagaccgtgtgccacacgacgctgtactactttggatctaagcca

gctctctactatagagtatctgcctccagcagccgcctgggagggttcatctgcgacaac

tttcacccggagattacaaaattcgaggggagcgtggacgtgaccacgcggctgttgttg

gacaatgaaaattttaccagtttcgggtggtaccgcctgcgacccggcacccacggagag

cgtgttcaacttcgccccgttgagcgacacgtcacctcaagcgacgtggagattaactgt

actcccgataacctggagccgataccagacgaggctgcctggcccgactataagctcatg

tgctttgatatagagtgtaaagctggaacgggtaacgaaatggcgttcccagtggcaact

aaccaagaggacctggtcatccagatctcctgtctgctgtactcgcttgctactcagaac

cacgaacacaccctgctgttttccctcgggtcatgcgatatctctgaggaatactcgttt

gcatgcgtccagcgcggcgagcccagaccgacggttttggagtttgacagcgagtacgag

ctgctggttgccttcctgacctttctcaagcagtactctcccgagttcgccaccggctac

aacatcgttaattttgactgggcgtacatagttaacaaggtaacgtcggtgtataacatc

aagctggacgggtacggcaagttcaacaaaggggggctgtttaaggtgtgggacatcgcc

acgaaccattttcagaagaagagcaaggtgaaaatcaatggcctgatatctctagacatg

tattctgtggcgacggaaaagctaaagctacccagctacaaactcgacgcggtcgtggga

gacgtcctcggcgagcataagatagaccttccctataaagaaataccctcctattacgcg

ggagggcctgaccggaggggcgtaataggagagtattgtatccaggactctaggctggtg

ggcaagctgttttttaagtacctcccccatctggaactatcggcggtggccaaactcgcc

cgtatcaccctaacgcgggtaatttttgacggtcagcaaattcgcgtgtacacgtgcttg

ctgaaactcgcccgcgagagaaatttcattttgccagacaacagacgccggtttgacagt

caggcagacgccgcgtcagagacttcggagttggctatggatagccaaagccacgccttc

gacagtacagacgaacccgacggtgtggacggtaccccggacgccgcaggatctggcgct

acttctgaaaacggaggcgggaagcccggcgtcgggagggccgtgggctaccagggagca

aaggttctagaccccgtatccggctttcatgtggaccccgtggttgtgtttgacttcgct

agcttatacccaagcattatccaggcccataacctctgtttcaccaccctggcgctcgat

gaagtggatctggccgggcttcaaccatccgtcaactactcgacgttcgaggtgggtgac

caaaagttattttttgtccacgcccatattcgcgaaagcctgcttggcatcttgctgcgc

gactggctggccatgcgaaaggcggtgagggcgcgaatccccaccagcacccccgaggag

gcagttttactagataagcagcagtctgcgattaaggtgatatgcaactcggtttacgga

ttcacgggggtggcaaacggcctgttgccgtgtctgaggatagcggctaccgttaccacg

ataggacgcgacatgctcctcaagacccgagattacgttcactctcgttgggcgacgcgc

gagctgctggaggacaattttccaggggctataggtttccgaaaccacaagccttactcc

gtcagggttatctacggagacaccgactccgtgtttatcaagtttgtgggcctgacgtac

gagggggtatcggagctgggggatgctatgtcgcgtcagatttcagcggacctctttaga

gcgcccatcaaactggagtgtgagaagacctttcagcgactgctgctgatcaccaagaag

aagtacataggtgtcataaacggggggaagatgctcatgaagggggtcgacctggtccgc

aaaaataactgctctttcataaacttgtacgcgcgacatctggtagatcttttgttgtac

gacgaggatgtggccacggcggcagcagaggtgacagacgtgcctcccgcagaatgggtg

gggcgcccgctaccgagcggctttgacaagtttgggcgagtgctggtagaggcgtacaac

cgtatcactgcccccaacttggacgtgcgcgagttcgttatgactgctgagctgagccgc

tcacccgaatcgtataccaacaagcgcctgccgcacctcaccgtctactttaagctcgcc

atgaggaatgaagaactgcccagcgtaaaagagagaattccgtatgtgatagttgcgcag

accgaggccgcggaacgcgaagcgggtgtagtaaactcaatgcgcggtaccgcccaaaac

cccgtggtaaccaagaccgcacgcccccaacctaaacgcaaactgctggtttccgacctc

gccgaagacccgacctatgtttccgagaatgacgtaccgctaaacacagactactatttc

tcccacctgttgggtaccataagcgtgacctttaaggctctattcggaaatgatgtgaga

acaacagaaaatcttttaaagcggtttattccggaaaccccccacaagacccccacgaaa

acccaggcactgcttgagcgcgccggctttgaaaagctgacgccctttacaccggaggaa

gaaagtcgtcgaatactgcatacagttttttgtactctagaagcagctccccatcaaagc

tga

>KU206413.1

atggcggcgcgcgaacaggccaactccgttcgcaggagcgggttttttaatccgtttatt

ggcaagaggccatttttcaggccgggaagcgggcaaaccgcggagacagagcgcccaagg

cccccccaacactcgtactgcacagaggtgggtagctttaagtttatagctccaagatgc

ctcgatgaagaagcccccgccgaccagcgacgcggtgtacacgtgggcaccttggagcgc

ccacccaaagtgtactgcgatggctcagagtacgacgtgctgaactttgcctccggaggt

tgctggcctcgcaggattcgggtttggaacggccaggattttcggggcgatggattcaac

cccagatttgagagatttcacgtgtacgacatagtagagacttcggagagcgcctcacac

gatgacccatccaggtttgctgagctatcccgcccaagcgggtctgtggtaacactgctg

ggaatgagcgagtgtggaaagcgagtcgcggttcacgtctatggtgtgcgccattatttt

tacatggcaaaggcggaggtggatagcgcttgtggaataaccaccgaggcagaactcgtg

cgcgcaatggtggactgcgcgcacagctcggctttgagcgcggccctgggaaatggcaac

ggcggcaaacagagcggcggcagcggcgggggatggtggggcggaaagcacgtgtctgcg

gactgcttcaaagtggagaccgtgtgccacacgacgctgtactactttggatctaagcca

gctctctactatagagtatctgcctccagcagccgcctgggagggttcatctgcgacaac

tttcacccggagattacaaaattcgaggggagcgtggacgtgaccacgcggctgttgttg

gacaatgaaaattttaccagtttcgggtggtaccgcctgcgacccggcacccacggagag

cgtgttcaacttcgccccgttgagcgacacgtcacctcaagcgacgtggagattaactgt

actcccgataacctggagccgataccagacgaggctgcctggcccgactataagctcatg

tgctttgatatagagtgtaaagctggaacgggtaacgaaatggcgttcccagtggcaact

aaccaagaggacctggtcatccagatctcctgtctgctgtactcgcttgctactcagaac

cacgaacacaccctgctgttttccctcgggtcatgcgatatctctgaggaatactcgttt

gcatgcgtccagcgcggcgagcccagaccgacggttttggagtttgacagcgagtacgag

ctgctggttgccttcctgacctttctcaagcagtactctcccgagttcgccaccggctac

aacatcgttaattttgactgggcgtacatagttaacaaggtaacgtcggtgtataacatc

aagctggacgggtacggcaagttcaacaaaggggggctgtttaaggtgtgggacatcgcc

acgaaccattttcagaagaagagcaaggtgaaaatcaatggcctgatatctctagacatg

tattctgtggcgacggaaaagctaaagctacccagctacaaactcgacgcggtcgtggga

gacgtcctcggcgagcataagatagaccttccctataaagaaataccctcctattacgcg

ggagggcctgaccggaggggcgtaataggagagtattgtatccaggactctaggctggtg

ggcaagctgttttttaagtacctcccccatctggaactatcggcggtggccaaactcgcc

cgtatcaccctaacgcgggtaatttttgacggtcagcaaattcgcgtgtacacgtgcttg

ctgaaactcgcccgcgagagaaatttcattttgccagacaacagacgccggtttgacagt

caggcagacgccgcgtcagagacttcggagttggctatggatagccaaagccacgccttc

gacagtacagacgaacccgacggtgtggacggtaccccggacgccgcaggatctggcgct

acttctgaaaacggaggcgggaagcccggcgtcgggagggccgtgggctaccagggagca

aaggttctagaccccgtatccggctttcatgtggaccccgtggttgtgtttgacttcgct

agcttatacccaagcattatccaggcccataacctctgtttcaccaccctggcgctcgat

gaagtggatctggccgggcttcaaccatccgtcaactactcgacgttcgaggtgggtgac

caaaagttattttttgtccacgcccatattcgcgaaagcctgcttggcatcttgctgcgc

gactggctggccatgcgaaaggcggtgagggcgcgaatccccaccagcacccccgaggag

gcagttttactagataagcagcagtctgcgattaaggtgatatgcaactcggtttacgga

ttcacgggggtggcaaacggcctgttgccgtgtctgaggatagcggctaccgttaccacg

ataggacgcgacatgctcctcaagacccgagattacgttcactctcgttgggcgacgcgc

gagctgctggaggacaattttccaggggctataggtttccgaaaccacaagccttactcc

gtcagggttatctacggagacaccgactccgtgtttatcaagtttgtgggcctgacgtac

gagggggtatcggagctgggggatgctatgtcgcgtcagatttcagcggacctctttaga

gcgcccatcaaactggagtgtgagaagacctttcagcgactgctgctgatcaccaagaag

aagtacataggtgtcataaacggggggaagatgctcatgaagggggtcgacctggtccgc

aaaaataactgctctttcataaacttgtacgcgcgacatctggtagatcttttgttgtac

gacgaggatgtggccacggcggcagcagaggtgacagacgtgcctcccgcagaatgggtg

gggcgcccgctaccgagcggctttgacaagtttgggcgagtgctggtagaggcgtacaac

cgtatcactgcccccaacttggacgtgcgcgagttcgttatgactgctgagctgagccgc

tcacccgaatcgtataccaacaagcgcctgccgcacctcaccgtctactttaagctcgcc

atgaggaatgaagaactgcccagcgtaaaagagagaattccgtatgtgatagttgcgcag

accgaggccgcggaacgcgaagcgggtgtagtaaactcaatgcgcggtaccgcccaaaac

cccgtggtaaccaagaccgcacgcccccaacctaaacgcaaactgctggtttccgacctc

gccgaagacccgacctatgtttccgagaatgacgtaccgctaaacacagactactatttc

tcccacctgttgggtaccataagcgtgacctttaaggctctattcggaaatgatgtgaga

acaacagaaaatcttttaaagcggtttattccggaaaccccccacaagacccccacgaaa

acccaggcactgcttgagcgcgccggctttgaaaagctgacgccctttacaccggaggaa

gaaagtcgtcgaatactgcatacagttttttgtactctagaagcagctccccatcaaagc

tga

>KU206414.1

atggcggcgcgcgaacaggccaactccgttcgcaggagcgggttttttaatccgtttatt

ggcaagaggccatttttcaggccgggaagcgggcaaaccgcggagacagagcgcccaagg

cccccccaacactcgtactgcacagaggtgggtagctttaagtttatagctccaagatgc

ctcgatgaagaagcccccgccgaccagcgacgcggtgtacacgtgggcaccttggagcgc

ccacccaaagtgtactgcgatggctcagagtacgacgtgctgaactttgcctccggaggt

tgctggcctcgcaggattcgggtttggaacggccaggattttcggggcgatggattcaac

cccagatttgagagatttcacgtgtacgacatagtagagacttcggagagcgcctcacac

gatgacccatccaggtttgctgagctatcccgcccaagcgggtctgtggtaacactgctg

ggaatgagcgagtgtggaaagcgagtcgcggttcacgtctatggtgtgcgccattatttt

tacatggcaaaggcggaggtggatagcgcttgtggaataaccaccgaggcagaactcgtg

cgcgcaatggtggactgcgcgcacagctcggctttgagcgcggccctgggaaatggcaac

ggcggcaaacagagcggcggcagcggcgggggatggtggggcggaaagcacgtgtctgcg

gactgcttcaaagtggagaccgtgtgccacacgacgctgtactactttggatctaagcca

gctctctactatagagtatctgcctccagcagccgcctgggagggttcatctgcgacaac

tttcacccggagattacaaaattcgaggggagcgtggacgtgaccacgcggctgttgttg

gacaatgaaaattttaccagtttcgggtggtaccgcctgcgacccggcacccacggagag

cgtgttcaacttcgccccgttgagcgacacgtcacctcaagcgacgtggagattaactgt

actcccgataacctggagccgataccagacgaggctgcctggcccgactataagctcatg

tgctttgatatagagtgtaaagctggaacgggtaacgaaatggcgttcccagtggcaact

aaccaagaggacctggtcatccagatctcctgtctgctgtactcgcttgctactcagaac

cacgaacacaccctgctgttttccctcgggtcatgcgatatctctgaggaatactcgttt

gcatgcgtccagcgcggcgagcccagaccgacggttttggagtttgacagcgagtacgag

ctgctggttgccttcctgacctttctcaagcagtactctcccgagttcgccaccggctac

aacatcgttaattttgactgggcgtacatagttaacaaggtaacgtcggtgtataacatc

aagctggacgggtacggcaagttcaacaaaggggggctgtttaaggtgtgggacatcgcc

acgaaccattttcagaagaagagcaaggtgaaaatcaatggcctgatatctctagacatg

tattctgtggcgacggaaaagctaaagctacccagctacaaactcgacgcggtcgtggga

gacgtcctcggcgagcataagatagaccttccctataaagaaataccctcctattacgcg

ggagggcctgaccggaggggcgtaataggagagtattgtatccaggactctaggctggtg

ggcaagctgttttttaagtacctcccccatctggaactatcggcggtggccaaactcgcc

cgtatcaccctaacgcgggtaatttttgacggtcagcaaattcgcgtgtacacgtgcttg

ctgaaactcgcccgcgagagaaatttcattttgccagacaacagacgccggtttgacagt

caggcagacgccgcgtcagagacttcggagttggctatggatagccaaagccacgccttc

gacagtacagacgaacccgacggtgtggacggtaccccggacgccgcaggatctggcgct

acttctgaaaacggaggcgggaagcccggcgtcgggagggccgtgggctaccagggagca

aaggttctagaccccgtatccggctttcatgtggaccccgtggttgtgtttgacttcgct

agcttatacccaagcattatccaggcccataacctctgtttcaccaccctggcgctcgat

gaagtggatctggccgggcttcaaccatccgtcaactactcgacgttcgaggtgggtgac

caaaagttattttttgtccacgcccatattcgcgaaagcctgcttggcatcttgctgcgc

gactggctggccatgcgaaaggcggtgagggcgcgaatccccaccagcacccccgaggag

gcagttttactagataagcagcagtctgcgattaaggtgatatgcaactcggtttacgga

ttcacgggggtggcaaacggcctgttgccgtgtctgaggatagcggctaccgttaccacg

ataggacgcgacatgctcctcaagacccgagattacgttcactctcgttgggcgacgcgc

gagctgctggaggacaattttccaggggctataggtttccgaaaccacaagccttactcc

gtcagggttatctacggagacaccgactccgtgtttatcaagtttgtgggcctgacgtac

gagggggtatcggagctgggggatgctatgtcgcgtcagatttcagcggacctctttaga

gcgcccatcaaactggagtgtgagaagacctttcagcgactgctgctgatcaccaagaag

aagtacataggtgtcataaacggggggaagatgctcatgaagggggtcgacctggtccgc

aaaaataactgctctttcataaacttgtacgcgcgacatctggtagatcttttgttgtac

gacgaggatgtggccacggcggcagcagaggtgacagacgtgcctcccgcagaatgggtg

gggcgcccgctaccgagcggctttgacaagtttgggcgagtgctggtagaggcgtacaac

cgtatcactgcccccaacttggacgtgcgcgagttcgttatgactgctgagctgagccgc

tcacccgaatcgtataccaacaagcgcctgccgcacctcaccgtctactttaagctcgcc

atgaggaatgaagaactgcccagcgtaaaagagagaattccgtatgtgatagttgcgcag

accgaggccgcggaacgcgaagcgggtgtagtaaactcaatgcgcggtaccgcccaaaac

cccgtggtaaccaagaccgcacgcccccaacctaaacgcaaactgctggtttccgacctc

gccgaagacccgacctatgtttccgagaatgacgtaccgctaaacacagactactatttc

tcccacctgttgggtaccataagcgtgacctttaaggctctattcggaaatgatgtgaga

acaacagaaaatcttttaaagcggtttattccggaaaccccccacaagacccccacgaaa

acccaggcactgcttgagcgcgccggctttgaaaagctgacgccctttacaccggaggaa

gaaagtcgtcgaatactgcatacagttttttgtactctagaagcagctccccatcaaagc

tga

>KU206415.1

atggcggcgcgcgaacaggccaactccgttcgcaggagcgggttttttaatccgtttatt

ggcaagaggccatttttcaggccgggaagcgggcaaaccgcggagacagagcgcccaagg

cccccccaacactcgtactgcacagaggtgggtagctttaagtttatagctccaagatgc

ctcgatgaagaagcccccgccgaccagcgacgcggtgtacacgtgggcaccttggagcgc

ccacccaaagtgtactgcgatggctcagagtacgacgtgctgaactttgcctccggaggt

tgctggcctcgcaggattcgggtttggaacggccaggattttcggggcgatggattcaac

cccagatttgagagatttcacgtgtacgacatagtagagacttcggagagcgcctcacac

gatgacccatccaggtttgctgagctatcccgcccaagcgggtctgtggtaacactgctg

ggaatgagcgagtgtggaaagcgagtcgcggttcacgtctatggtgtgcgccattatttt

tacatggcaaaggcggaggtggatagcgcttgtggaataaccaccgaggcagaactcgtg

cgcgcaatggtggactgcgcgcacagctcggctttgagcgcggccctgggaaatggcaac

ggcggcaaacagagcggcggcagcggcgggggatggtggggcggaaagcacgtgtctgcg

gactgcttcaaagtggagaccgtgtgccacacgacgctgtactactttggatctaagcca

gctctctactatagagtatctgcctccagcagccgcctgggagggttcatctgcgacaac

tttcacccggagattacaaaattcgaggggagcgtggacgtgaccacgcggctgttgttg

gacaatgaaaattttaccagtttcgggtggtaccgcctgcgacccggcacccacggagag

cgtgttcaacttcgccccgttgagcgacacgtcacctcaagcgacgtggagattaactgt

actcccgataacctggagccgataccagacgaggctgcctggcccgactataagctcatg

tgctttgatatagagtgtaaagctggaacgggtaacgaaatggcgttcccagtggcaact

aaccaagaggacctggtcatccagatctcctgtctgctgtactcgcttgctactcagaac

cacgaacacaccctgctgttttccctcgggtcatgcgatatctctgaggaatactcgttt

gcatgcgtccagcgcggcgagcccagaccgacggttttggagtttgacagcgagtacgag

ctgctggttgccttcctgacctttctcaagcagtactctcccgagttcgccaccggctac

aacatcgttaattttgactgggcgtacatagttaacaaggtaacgtcggtgtataacatc

aagctggacgggtacggcaagttcaacaaaggggggctgtttaaggtgtgggacatcgcc

acgaaccattttcagaagaagagcaaggtgaaaatcaatggcctgatatctctagacatg

tattctgtggcgacggaaaagctaaagctacccagctacaaactcgacgcggtcgtggga

gacgtcctcggcgagcataagatagaccttccctataaagaaataccctcctattacgcg

ggagggcctgaccggaggggcgtaataggagagtattgtatccaggactctaggctggtg

ggcaagctgttttttaagtacctcccccatctggaactatcggcggtggccaaactcgcc

cgtatcaccctaacgcgggtaatttttgacggtcagcaaattcgcgtgtacacgtgcttg

ctgaaactcgcccgcgagagaaatttcattttgccagacaacagacgccggtttgacagt

caggcagacgccgcgtcagagacttcggagttggctatggatagccaaagccacgccttc

gacagtacagacgaacccgacggtgtggacggtaccccggacgccgcaggatctggcgct

acttctgaaaacggaggcgggaagcccggcgtcgggagggccgtgggctaccagggagca

aaggttctagaccccgtatccggctttcatgtggaccccgtggttgtgtttgacttcgct

agcttatacccaagcattatccaggcccataacctctgtttcaccaccctggcgctcgat

gaagtggatctggccgggcttcaaccatccgtcaactactcgacgttcgaggtgggtgac

caaaagttattttttgtccacgcccatattcgcgaaagcctgcttggcatcttgctgcgc

gactggctggccatgcgaaaggcggtgagggcgcgaatccccaccagcacccccgaggag

gcagttttactagataagcagcagtctgcgattaaggtgatatgcaactcggtttacgga

ttcacgggggtggcaaacggcctgttgccgtgtctgaggatagcggctaccgttaccacg

ataggacgcgacatgctcctcaagacccgagattacgttcactctcgttgggcgacgcgc

gagctgctggaggacaattttccaggggctataggtttccgaaaccacaagccttactcc

gtcagggttatctacggagacaccgactccgtgtttatcaagtttgtgggcctgacgtac

gagggggtatcggagctgggggatgctatgtcgcgtcagatttcagcggacctctttaga

gcgcccatcaaactggagtgtgagaagacctttcagcgactgctgctgatcaccaagaag

aagtacataggtgtcataaacggggggaagatgctcatgaagggggtcgacctggtccgc

aaaaataactgctctttcataaacttgtacgcgcgacatctggtagatcttttgttgtac

gacgaggatgtggccacggcggcagcagaggtgacagacgtgcctcccgcagaatgggtg

gggcgcccgctaccgagcggctttgacaagtttgggcgagtgctggtagaggcgtacaac

cgtatcactgcccccaacttggacgtgcgcgagttcgttatgactgctgagctgagccgc

tcacccgaatcgtataccaacaagcgcctgccgcacctcaccgtctactttaagctcgcc

atgaggaatgaagaactgcccagcgtaaaagagagaattccgtatgtgatagttgcgcag

accgaggccgcggaacgcgaagcgggtgtagtaaactcaatgcgcggtaccgcccaaaac

cccgtggtaaccaagaccgcacgcccccaacctaaacgcaaactgctggtttccgacctc

gccgaagacccgacctatgtttccgagaatgacgtaccgctaaacacagactactatttc

tcccacctgttgggtaccataagcgtgacctttaaggctctattcggaaatgatgtgaga

acaacagaaaatcttttaaagcggtttattccggaaaccccccacaagacccccacgaaa

acccaggcactgcttgagcgcgccggctttgaaaagctgacgccctttacaccggaggaa

gaaagtcgtcgaatactgcatacagttttttgtactctagaagcagctccccatcaaagc

tga

>KU206416.1

atggcggcgcgcgaacaggccaactccgttcgcaggagcgggttttttaatccgtttatt

ggcaagaggccatttttcaggccgggaagcgggcaaaccgcggagacagagcgcccaagg

cccccccaacactcgtactgcacagaggtgggtagctttaagtttatagctccaagatgc

ctcgatgaagaagcccccgccgaccagcgacgcggtgtacacgtgggcaccttggagcgc

ccacccaaagtgtactgcgatggctcagagtacgacgtgctgaactttgcctccggaggt

tgctggcctcgcaggattcgggtttggaacggccaggattttcggggcgatggattcaac

cccagatttgagagatttcacgtgtacgacatagtagagacttcggagagcgcctcacac

gatgacccatccaggtttgctgagctatcccgcccaagcgggtctgtggtaacactgctg

ggaatgagcgagtgtggaaagcgagtcgcggttcacgtctatggtgtgcgccattatttt

tacatggcaaaggcggaggtggatagcgcttgtggaataaccaccgaggcagaactcgtg

cgcgcaatggtggactgcgcgcacagctcggctttgagcgcggccctgggaaatggcaac

ggcggcaaacagagcggcggcagcggcgggggatggtggggcggaaagcacgtgtctgcg

gactgcttcaaagtggagaccgtgtgccacacgacgctgtactactttggatctaagcca

gctctctactatagagtatctgcctccagcagccgcctgggagggttcatctgcgacaac

tttcacccggagattacaaaattcgaggggagcgtggacgtgaccacgcggctgttgttg

gacaatgaaaattttaccagtttcgggtggtaccgcctgcgacccggcacccacggagag

cgtgttcaacttcgccccgttgagcgacacgtcacctcaagcgacgtggagattaactgt

actcccgataacctggagccgataccagacgaggctgcctggcccgactataagctcatg

tgctttgatatagagtgtaaagctggaacgggtaacgaaatggcgttcccagtggcaact

aaccaagaggacctggtcatccagatctcctgtctgctgtactcgcttgctactcagaac

cacgaacacaccctgctgttttccctcgggtcatgcgatatctctgaggaatactcgttt

gcatgcgtccagcgcggcgagcccagaccgacggttttggagtttgacagcgagtacgag

ctgctggttgccttcctgacctttctcaagcagtactctcccgagttcgccaccggctac

aacatcgttaattttgactgggcgtacatagttaacaaggtaacgtcggtgtataacatc

aagctggacgggtacggcaagttcaacaaaggggggctgtttaaggtgtgggacatcgcc

acgaaccattttcagaagaagagcaaggtgaaaatcaatggcctgatatctctagacatg

tattctgtggcgacggaaaagctaaagctacccagctacaaactcgacgcggtcgtggga

gacgtcctcggcgagcataagatagaccttccctataaagaaataccctcctattacgcg

ggagggcctgaccggaggggcgtaataggagagtattgtatccaggactctaggctggtg

ggcaagctgttttttaagtacctcccccatctggaactatcggcggtggccaaactcgcc

cgtatcaccctaacgcgggtaatttttgacggtcagcaaattcgcgtgtacacgtgcttg

ctgaaactcgcccgcgagagaaatttcattttgccagacaacagacgccggtttgacagc

caggcagacgccgcgtcagagacttcggagttggctatggatagccaaagccacgccttc

gacagtacagacgaacccgacggtgtggacggtaccccggacgccgcaggatctggcgct

acttctgaaaacggaggcgggaagcccggcgtcgggagggccgtgggctaccagggagca

aaggttctagaccccgtatccggctttcatgtggaccccgtggttgtgtttgacttcgct

agcttatacccaagcattatccaggcccataacctctgtttcaccaccctggcgctcgat

gaagtggatctggccgggcttcaaccatccgtcaactactcgacgttcgaggtgggtgac

caaaagttattttttgtccacgcccatattcgcgaaagcctgcttggcatcttgctgcgc

gactggctggccatgcgaaaggcggtgagggcgcgaatccccaccagcacccccgaggag

gcagttttactagataagcagcagtctgcgattaaggtgatatgcaactcggtttacgga

ttcacgggggtggcaaacggcctgttgccgtgtctgaggatagcggctaccgttaccacg

ataggacgcgacatgctcctcaagacccgagattacgttcactctcgttgggcgacgcgc

gagctgctggaggacaattttccaggggctataggtttccgaaaccacaagccttactcc

gtcagggttatctacggagacaccgactccgtgtttatcaagtttgtgggcctgacgtac

gagggggtatcggagctgggggatgctatgtcgcgtcagatttcagcggacctctttaga

gcgcccatcaaactggagtgtgagaagacctttcagcgactgctgctgatcaccaagaag

aagtacataggtgtcataaacggggggaagatgctcatgaagggggtcgacctggtccgc

aaaaataactgctctttcataaacttgtacgcgcgacatctggtagatcttttgttgtac

gacgaggatgtggccacggcggcagcagaggtgacagacgtgcctcccgcagaatgggtg

gggcgcccgctaccgagcggctttgacaagtttgggcgagtgctggtagaggcgtacaac

cgtatcactgcccccaacttggacgtgcgcgagttcgttatgactgctgagctgagccgc

tcacccgaatcgtataccaacaagcgcctgccgcacctcaccgtctactttaagctcgcc

atgaggaatgaagaactgcccagcgtaaaagagagaattccgtatgtgatagttgcgcag

accgaggccgcggaacgcgaagcgggtgtagtaaactcaatgcgcggtaccgcccaaaac

cccgtggtaaccaagaccgcacgcccccaacctaaacgcaaactgctggtttccgacctc

gccgaagacccgacctatgtttccgagaatgacgtaccgctaaacacagactactatttc

tcccacctgttgggtaccataagcgtgacctttaaggctctattcggaaatgatgtgaga

acaacagaaaatcttttaaagcggtttattccggaaaccccccacaagacccccacgaaa

acccaggcactgcttgagcgcgccggctttgaaaagctgacgccctttacaccggaggaa

gaaagtcgtcgaatactgcatacagttttttgtactctagaagcagctccccatcaaagc

tga

>KU206417.1

atggcggcgcgcgaacaggccaactccgttcgcaggagcgggttttttaatccgtttatt

ggcaagaggccatttttcaggccgggaagcgggcaaaccgcggagacagagcgcccaagg

cccccccaacactcgtactgcacagaggtgggtagctttaagtttatagctccaagatgc

ctcgatgaagaagcccccgccgaccagcgacgcggtgtacacgtgggcaccttggagcgc

ccacccaaagtgtactgcgatggctcagagtacgacgtgctgaactttgcctccggaggt

tgctggcctcgcaggattcgggtttggaacggccaggattttcggggcgatggattcaac

cccagatttgagagatttcacgtgtacgacatagtagagacttcggagagcgcctcacac

gatgacccatccaggtttgctgagctatcccgcccaagcgggtctgtggtaacactgctg

ggaatgagcgagtgtggaaagcgagtcgcggttcacgtctatggtgtgcgccattatttt

tacatggcaaaggcggaggtggatagcgcttgtggaataaccaccgaggcagaactcgtg

cgcgcaatggtggactgcgcgcacagctcggctttgagcgcggccctgggaaatggcaac

ggcggcaaacagagcggcggcagcggcgggggatggtggggcggaaagcacgtgtctgcg

gactgcttcaaagtggagaccgtgtgccacacgacgctgtactactttggatctaagcca

gctctctactatagagtatctgcctccagcagccgcctgggagggttcatctgcgacaac

tttcacccggagattacaaaattcgaggggagcgtggacgtgaccacgcggctgttgttg

gacaatgaaaattttaccagtttcgggtggtaccgcctgcgacccggcacccacggagag

cgtgttcaacttcgccccgttgagcgacacgtcacctcaagcgacgtggagattaactgt

actcccgataacctggagccgataccagacgaggctgcctggcccgactataagctcatg

tgctttgatatagagtgtaaagctggaacgggtaacgaaatggcgttcccagtggcaact

aaccaagaggacctggtcatccagatctcctgtctgctgtactcgcttgctactcagaac

cacgaacacaccctgctgttttccctcgggtcatgcgatatctctgaggaatactcgttt

gcatgcgtccagcgcggcgagcccagaccgacggttttggagtttgacagcgagtacgag

ctgctggttgccttcctgacctttctcaagcagtactctcccgagttcgccaccggctac

aacatcgttaattttgactgggcgtacatagttaacaaggtaacgtcggtgtataacatc

aagctggacgggtacggcaagttcaacaaaggggggctgtttaaggtgtgggacatcgcc

acgaaccattttcagaagaagagcaaggtgaaaatcaatggcctgatatctctagacatg

tattctgtggcgacggaaaagctaaagctacccagctacaaactcgacgcggtcgtggga

gacgtcctcggcgagcataagatagaccttccctataaagaaataccctcctattacgcg

ggagggcctgaccggaggggcgtaataggagagtattgtatccaggactctaggctggtg

ggcaagctgttttttaagtacctcccccatctggaactatcggcggtggccaaactcgcc

cgtatcaccctaacgcgggtaatttttgacggtcagcaaattcgcgtgtacacgtgcttg

ctgaaactcgcccgcgagagaaatttcattttgccagacaacagacgccggtttgacagt

caggcagacgccgcgtcagagacttcggagttggctatggatagccaaagccacgccttc

gacagtacagacgaacccgacggtgtggacggtaccccggacgccgcaggatctggcgct

acttctgaaaacggaggcgggaagcccggcgtcgggagggccgtgggctaccagggagca

aaggttctagaccccgtatccggctttcatgtggaccccgtggttgtgtttgacttcgct

agcttatacccaagcattatccaggcccataacctctgtttcaccaccctggcgctcgat

gaagtggatctggccgggcttcaaccatccgtcaactactcgacgttcgaggtgggtgac

caaaagttattttttgtccacgcccatattcgcgaaagcctgcttggcatcttgctgcgc

gactggctggccatgcgaaaggcggtgagggcgcgaatccccaccagcacccccgaggag

gcagttttactagataagcagcagtctgcgattaaggtgatatgcaactcggtttacgga

ttcacgggggtggcaaacggcctgttgccgtgtctgaggatagcggctaccgttaccacg

ataggacgcgacatgctcctcaagacccgagattacgttcactctcgttgggcgacgcgc

gagctgctggaggacaattttccaggggctataggtttccgaaaccacaagccttactcc

gtcagggttatctacggagacaccgactccgtgtttatcaagtttgtgggcctgacgtac

gagggggtatcggagctgggggatgctatgtcgcgtcagatttcagcggacctctttaga

gcgcccatcaaactggagtgtgagaagacctttcagcgactgctgctgatcaccaagaag

aagtacataggtgtcataaacggggggaagatgctcatgaagggggtcgacctggtccgc

aaaaataactgctctttcataaacttgtacgcgcgacatctggtagatcttttgttgtac

gacgaggatgtggccacggcggcagcagaggtgacagacgtgcctcccgcagaatgggtg

gggcgcccgctaccgagcggctttgacaagtttgggcgagtgctggtagaggcgtacaac

cgtatcactgcccccaacttggacgtgcgcgagttcgttatgactgctgagctgagccgc

tcacccgaatcgtataccaacaagcgcctgccgcacctcaccgtctactttaagctcgcc

atgaggaatgaagaactgcccagcgtaaaagagagaattccgtatgtgatagttgcgcag

accgaggccgcggaacgcgaagcgggtgtagtaaactcaatgcgcggtaccgcccaaaac

cccgtggtaaccaagaccgcacgcccccaacctaaacgcaaactgctggtttccgacctc

gccgaagacccgacctatgtttccgagaatgacgtaccgctaaacacagactactatttc

tcccacctgttgggtaccataagcgtgacctttaaggctctattcggaaatgatgtgaga

acaacagaaaatcttttaaagcggtttattccggaaaccccccacaagacccccacgaaa

acccaggcactgcttgagcgcgccggctttgaaaagctgacgccctttacaccggaggaa

gaaagtcgtcgaatactgcatacagttttttgtactctagaagcagctccccatcaaagc

tga

>KU206418.1

atggcggcgcgcgaacaggccaactccgttcgcaggagcgggttttttaatccgtttatt

ggcaagaggccatttttcaggccgggaagcgggcaaaccgcggagacagagcgcccaagg

cccccccaacactcgtactgcacagaggtgggtagctttaagtttatagctccaagatgc

ctcgatgaagaagcccccgccgaccagcgacgcggtgtacacgtgggcaccttggagcgc

ccacccaaagtgtactgcgatggctcagagtacgacgtgctgaactttgcctccggaggt

tgctggcctcgcaggattcgggtttggaacggccaggattttcggggcgatggattcaac

cccagatttgagagatttcacgtgtacgacatagtagagacttcggagagcgcctcacac

gatgacccatccaggtttgctgagctatcccgcccaagcgggtctgtggtaacactgctg

ggaatgagcgagtgtggaaagcgagtcgcggttcacgtctatggtgtgcgccattatttt

tacatggcaaaggcggaggtggatagcgcttgtggaataaccaccgaggcagaactcgtg

cgcgcaatggtggactgcgcgcacagctcggctttgagcgcggccctgggaaatggcaac

ggcggcaaacagagcggcggcagcggcgggggatggtggggcggaaagcacgtgtctgcg

gactgcttcaaagtggagaccgtgtgccacacgacgctgtactactttggatctaagcca

gctctctactatagagtatctgcctccagcagccgcctgggagggttcatctgcgacaac

tttcacccggagattacaaaattcgaggggagcgtggacgtgaccacgcggctgttgttg

gacaatgaaaattttaccagtttcgggtggtaccgcctgcgacccggcacccacggagag

cgtgttcaacttcgccccgttgagcgacacgtcacctcaagcgacgtggagattaactgt

actcccgataacctggagccgataccagacgaggctgcctggcccgactataagctcatg

tgctttgatatagagtgtaaagctggaacgggtaacgaaatggcgttcccagtggcaact

aaccaagaggacctggtcatccagatctcctgtctgctgtactcgcttgctactcagaac

cacgaacacaccctgctgttttccctcgggtcatgcgatatctctgaggaatactcgttt

gcatgcgtccagcgcggcgagcccagaccgacggttttggagtttgacagcgagtacgag

ctgctggttgccttcctgacctttctcaagcagtactctcccgagttcgccaccggctac

aacatcgttaattttgactgggcgtacatagttaacaaggtaacgtcggtgtataacatc

aagctggacgggtacggcaagttcaacaaaggggggctgtttaaggtgtgggacatcgcc

acgaaccattttcagaagaagagcaaggtgaaaatcaatggcctgatatctctagacatg

tattctgtggcgacggaaaagctaaagctacccagctacaaactcgacgcggtcgtggga

gacgtcctcggcgagcataagatagaccttccctataaagaaataccctcctattacgcg

ggagggcctgaccggaggggcgtaataggagagtattgtatccaggactctaggctggtg

ggcaagctgttttttaagtacctcccccatctggaactatcggcggtggccaaactcgcc

cgtatcaccctaacgcgggtaatttttgacggtcagcaaattcgcgtgtacacgtgcttg

ctgaaactcgcccgcgagagaaatttcattttgccagacaacagacgccggtttgacagt

caggcagacgccgcgtcagagacttcggagttggctatggatagccaaagccacgccttc

gacagtacagacgaacccgacggtgtggacggtaccccggacgccgcaggatctggcgct

acttctgaaaacggaggcgggaagcccggcgtcgggagggccgtgggctaccagggagca

aaggttctagaccccgtatccggctttcatgtggaccccgtggttgtgtttgacttcgct

agcttatacccaagcattatccaggcccataacctctgtttcaccaccctggcgctcgat

gaagtggatctggccgggcttcaaccatccgtcaactactcgacgttcgaggtgggtgac

caaaagttattttttgtccacgcccatattcgcgaaagcctgcttggcatcttgctgcgc

gactggctggccatgcgaaaggcggtgagggcgcgaatccccaccagcacccccgaggag

gcagttttactagataagcagcagtctgcgattaaggtgatatgcaactcggtttacgga

ttcacgggggtggcaaacggcctgttgccgtgtctgaggatagcggctaccgttaccacg

ataggacgcgacatgctcctcaagacccgagattacgttcactctcgttgggcgacgcgc

gagctgctggaggacaattttccaggggctataggtttccgaaaccacaagccttactcc

gtcagggttatctacggagacaccgactccgtgtttatcaagtttgtgggcctgacgtac

gagggggtatcggagctgggggatgctatgtcgcgtcagatttcagcggacctctttaga

gcgcccatcaaactggagtgtgagaagacctttcagcgactgctgctgatcaccaagaag

aagtacataggtgtcataaacggggggaagatgctcatgaagggggtcgacctggtccgc

aaaaataactgctctttcataaacttgtacgcgcgacatctggtagatcttttgttgtac

gacgaggatgtggccacggcggcagcagaggtgacagacgtgcctcccgcagaatgggtg

gggcgcccgctaccgagcggctttgacaagtttgggcgagtgctggtagaggcgtacaac

cgtatcactgcccccaacttggacgtgcgcgagttcgttatgactgctgagctgagccgc

tcacccgaatcgtataccaacaagcgcctgccgcacctcaccgtctactttaagctcgcc

atgaggaatgaagaactgcccagcgtaaaagagagaattccgtatgtgatagttgcgcag

accgaggccgcggaacgcgaagcgggtgtagtaaactcaatgcgcggtaccgcccaaaac

cccgtggtaaccaagaccgcacgcccccaacctaaacgcaaactgctggtttccgacctc

gccgaagacccgacctatgtttccgagaatgacgtaccgctaaacacagactactatttc

tcccacctgttgggtaccataagcgtgacctttaaggctctattcggaaatgatgtgaga

acaacagaaaatcttttaaagcggtttattccggaaaccccccacaagacccccacgaaa

acccaggcactgcttgagcgcgccggctttgaaaagctgacgccctttacaccggaggaa

gaaagtcgtcgaatactgcatacagttttttgtactctagaagcagctccccatcaaagc

tga

>KU206419.1

atggcggcgcgcgaacaggccaactccgttcgcaggagcgggttttttaatccgtttatt

ggcaagaggccatttttcaggccgggaagcgggcaaaccgcggagacagagcgcccaagg

cccccccaacactcgtactgcacagaggtgggtagctttaagtttatagctccaagatgc

ctcgatgaagaagcccccgccgaccagcgacgcggtgtacacgtgggcaccttggagcgc

ccacccaaagtgtactgcgatggctcagagtacgacgtgctgaactttgcctccggaggt

tgctggcctcgcaggattcgggtttggaacggccaggattttcggggcgatggattcaac

cccagatttgagagatttcacgtgtacgacatagtagagacttcggagagcgcctcacac

gatgacccatccaggtttgctgagctatcccgcccaagcgggtctgtggtaacactgctg

ggaatgagcgagtgtggaaagcgagtcgcggttcacgtctatggtgtgcgccattatttt

tacatggcaaaggcggaggtggatagcgcttgtggaataaccaccgaggcagaactcgtg

cgcgcaatggtggactgcgcgcacagctcggctttgagcgcggccctgggaaatggcaac

ggcggcaaacagagcggcggcagcggcgggggatggtggggcggaaagcacgtgtctgcg

gactgcttcaaagtggagaccgtgtgccacacgacgctgtactactttggatctaagcca

gctctctactatagagtatctgcctccagcagccgcctgggagggttcatctgcgacaac

tttcacccggagattacaaaattcgaggggagcgtggacgtgaccacgcggctgttgttg

gacaatgaaaattttaccagtttcgggtggtaccgcctgcgacccggcacccacggagag

cgtgttcaacttcgccccgttgagcgacacgtcacctcaagcgacgtggagattaactgt

actcccgataacctggagccgataccagacgaggctgcctggcccgactataagctcatg

tgctttgatatagagtgtaaagctggaacgggtaacgaaatggcgttcccagtggcaact

aaccaagaggacctggtcatccagatctcctgtctgctgtactcgcttgctactcagaac

cacgaacacaccctgctgttttccctcgggtcatgcgatatctctgaggaatactcgttt

gcatgcgtccagcgcggcgagcccagaccgacggttttggagtttgacagcgagtacgag

ctgctggttgccttcctgacctttctcaagcagtactctcccgagttcgccaccggctac

aacatcgttaattttgactgggcgtacatagttaacaaggtaacgtcggtgtataacatc

aagctggacgggtacggcaagttcaacaaaggggggctgtttaaggtgtgggacatcgcc

acgaaccattttcagaagaagagcaaggtgaaaatcaatggcctgatatctctagacatg

tattctgtggcgacggaaaagctaaagctacccagctacaaactcgacgcggtcgtggga

gacgtcctcggcgagcataagatagaccttccctataaagaaataccctcctattacgcg

ggagggcctgaccggaggggcgtaataggagagtattgtatccaggactctaggctggtg

ggcaagctgttttttaagtacctcccccatctggaactatcggcggtggccaaactcgcc

cgtatcaccctaacgcgggtaatttttgacggtcagcaaattcgcgtgtacacgtgcttg

ctgaaactcgcccgcgagagaaatttcattttgccagacaacagacgccggtttgacagt

caggcagacgccgcgtcagagacttcggagttggctatggatagccaaagccacgccttc

gacagtacagacgaacccgacggtgtggacggtaccccggacgccgcaggatctggcgct

acttctgaaaacggaggcgggaagcccggcgtcgggagggccgtgggctaccagggagca

aaggttctagaccccgtatccggctttcatgtggaccccgtggttgtgtttgacttcgct

agcttatacccaagcattatccaggcccataacctctgtttcaccaccctggcgctcgat

gaagtggatctggccgggcttcaaccatccgtcaactactcgacgttcgaggtgggtgac

caaaagttattttttgtccacgcccatattcgcgaaagcctgcttggcatcttgctgcgc

gactggctggccatgcgaaaggcggtgagggcgcgaatccccaccagcacccccgaggag

gcagttttactagataagcagcagtctgcgattaaggtgatatgcaactcggtttacgga

ttcacgggggtggcaaacggcctgttgccgtgtctgaggatagcggctaccgttaccacg

ataggacgcgacatgctcctcaagacccgagattacgttcactctcgttgggcgacgcgc

gagctgctggaggacaattttccaggggctataggtttccgaaaccacaagccttactcc

gtcagggttatctacggagacaccgactccgtgtttatcaagtttgtgggcctgacgtac

gagggggtatcggagctgggggatgctatgtcgcgtcagatttcagcggacctctttaga

gcgcccatcaaactggagtgtgagaagacctttcagcgactgctgctgatcaccaagaag

aagtacataggtgtcataaacggggggaagatgctcatgaagggggtcgacctggtccgc

aaaaataactgctctttcataaacttgtacgcgcgacatctggtagatcttttgttgtac

gacgaggatgtggccacggcggcagcagaggtgacagacgtgcctcccgcagaatgggtg

gggcgcccgctaccgagcggctttgacaagtttgggcgagtgctggtagaggcgtacaac

cgtatcactgcccccaacttggacgtgcgcgagttcgttatgactgctgagctgagccgc

tcacccgaatcgtataccaacaagcgcctgccgcacctcaccgtctactttaagctcgcc

atgaggaatgaagaactgcccagcgtaaaagagagaattccgtatgtgatagttgcgcag

accgaggccgcggaacgcgaagcgggtgtagtaaactcaatgcgcggtaccgcccaaaac

cccgtggtaaccaagaccgcacgcccccaacctaaacgcaaactgctggtttccgacctc

gccgaagacccgacctatgtttccgagaatgacgtaccgctaaacacagactactatttc

tcccacctgttgggtaccataagcgtgacctttaaggctctattcggaaatgatgtgaga

acaacagaaaatcttttaaagcggtttattccggaaaccccccacaagacccccacgaaa

acccaggcactgcttgagcgcgccggctttgaaaagctgacgccctttacaccggaggaa

gaaagtcgtcgaatactgcatacagttttttgtactctagaagcagctccccatcaaagc

tga

>KU206420.1

atggcggcgcgcgaacaggccaactccgttcgcaggagcgggttttttaatccgtttatt

ggcaagaggccatttttcaggccgggaagcgggcaaaccgcggagacagagcgcccaagg

cccccccaacactcgtactgcacagaggtgggtagctttaagtttatagctccaagatgc

ctcgatgaagaagcccccgccgaccagcgacgcggtgtacacgtgggcaccttggagcgc

ccacccaaagtgtactgcgatggctcagagtacgacgtgctgaactttgcctccggaggt

tgctggcctcgcaggattcgggtttggaacggccaggattttcggggcgatggattcaac

cccagatttgagagatttcacgtgtacgacatagtagagacttcggagagcgcctcacac

gatgacccatccaggtttgctgagctatcccgcccaagcgggtctgtggtaacactgctg

ggaatgagcgagtgtggaaagcgagtcgcggttcacgtctatggtgtgcgccattatttt

tacatggcaaaggcggaggtggatagcgcttgtggaataaccaccgaggcagaactcgtg

cgcgcaatggtggactgcgcgcacagctcggctttgagcgcggccctgggaaatggcaac

ggcggcaaacagagcggcggcagcggcgggggatggtggggcggaaagcacgtgtctgcg

gactgcttcaaagtggagaccgtgtgccacacgacgctgtactactttggatctaagcca

gctctctactatagagtatctgcctccagcagccgcctgggagggttcatctgcgacaac

tttcacccggagattacaaaattcgaggggagcgtggacgtgaccacgcggctgttgttg

gacaatgaaaattttaccagtttcgggtggtaccgcctgcgacccggcacccacggagag

cgtgttcaacttcgccccgttgagcgacacgtcacctcaagcgacgtggagattaactgt

actcccgataacctggagccgataccagacgaggctgcctggcccgactataagctcatg

tgctttgatatagagtgtaaagctggaacgggtaacgaaatggcgttcccagtggcaact

aaccaagaggacctggtcatccagatctcctgtctgctgtactcgcttgctactcagaac

cacgaacacaccctgctgttttccctcgggtcatgcgatatctctgaggaatactcgttt

gcatgcgtccagcgcggcgagcccagaccgacggttttggagtttgacagcgagtacgag

ctgctggttgccttcctgacctttctcaagcagtactctcccgagttcgccaccggctac

aacatcgttaattttgactgggcgtacatagttaacaaggtaacgtcggtgtataacatc

aagctggacgggtacggcaagttcaacaaaggggggctgtttaaggtgtgggacatcgcc

acgaaccattttcagaagaagagcaaggtgaaaatcaatggcctgatatctctagacatg

tattctgtggcgacggaaaagctaaagctacccagctacaaactcgacgcggtcgtggga

gacgtcctcggcgagcataagatagaccttccctataaagaaataccctcctattacgcg

ggagggcctgaccggaggggcgtaataggagagtattgtatccaggactctaggctggtg

ggcaagctgttttttaagtacctcccccatctggaactatcggcggtggccaaactcgcc

cgtatcaccctaacgcgggtaatttttgacggtcagcaaattcgcgtgtacacgtgcttg

ctgaaactcgcccgcgagagaaatttcattttgccagacaacagacgccggtttgacagt

caggcagacgccgcgtcagagacttcggagttggctatggatagccaaagccacgccttc

gacagtacagacgaacccgacggtgtggacggtaccccggacgccgcaggatctggcgct

acttctgaaaacggaggcgggaagcccggcgtcgggagggccgtgggctaccagggagca

aaggttctagaccccgtatccggctttcatgtggaccccgtggttgtgtttgacttcgct

agcttatacccaagcattatccaggcccataacctctgtttcaccaccctggcgctcgat

gaagtggatctggccgggcttcaaccatccgtcaactactcgacgttcgaggtgggtgac

caaaagttattttttgtccacgcccatattcgcgaaagcctgcttggcatcttgctgcgc

gactggctggccatgcgaaaggcggtgagggcgcgaatccccaccagcacccccgaggag

gcagttttactagataagcagcagtctgcgattaaggtgatatgcaactcggtttacgga

ttcacgggggtggcaaacggcctgttgccgtgtctgaggatagcggctaccgttaccacg

ataggacgcgacatgctcctcaagacccgagattacgttcactctcgttgggcgacgcgc

gagctgctggaggacaattttccaggggctataggtttccgaaaccacaagccttactcc

gtcagggttatctacggagacaccgactccgtgtttatcaagtttgtgggcctgacgtac

gagggggtatcggagctgggggatgctatgtcgcgtcagatttcagcggacctctttaga

gcgcccatcaaactggagtgtgagaagacctttcagcgactgctgctgatcaccaagaag

aagtacataggtgtcataaacggggggaagatgctcatgaagggggtcgacctggtccgc

aaaaataactgctctttcataaacttgtacgcgcgacatctggtagatcttttgttgtac

gacgaggatgtggccacggcggcagcagaggtgacagacgtgcctcccgcagaatgggtg

gggcgcccgctaccgagcggctttgacaagtttgggcgagtgctggtagaggcgtacaac

cgtatcactgcccccaacttggacgtgcgcgagttcgttatgactgctgagctgagccgc

tcacccgaatcgtataccaacaagcgcctgccgcacctcaccgtctactttaagctcgcc

atgaggaatgaagaactgcccagcgtaaaagagagaattccgtatgtgatagttgcgcag

accgaggccgcggaacgcgaagcgggtgtagtaaactcaatgcgcggtaccgcccaaaac

cccgtggtaaccaagaccgcacgcccccaacctaaacgcaaactgctggtttccgacctc

gccgaagacccgacctatgtttccgagaatgacgtaccgctaaacacagactactatttc

tcccacctgttgggtaccataagcgtgacctttaaggctctattcggaaatgatgtgaga

acaacagaaaatcttttaaagcggtttattccggaaaccccccacaagacccccacgaaa

acccaggcactgcttgagcgcgccggctttgaaaagctgacgccctttacaccggaggaa

gaaagtcgtcgaatactgcatacagttttttgtactctagaagcagctccccatcaaagc

tga

>KU206421.1

atggcggcgcgcgaacaggccaactccgttcgcaggagcgggttttttaatccgtttatt

ggcaagaggccatttttcaggccgggaagcgggcaaaccgcggagacagagcgcccaagg

cccccccaacactcgtactgcacagaggtgggtagctttaagtttatagctccaagatgc

ctcgatgaagaagcccccgccgaccagcgacgcggtgtacacgtgggcaccttggagcgc

ccacccaaagtgtactgcgatggctcagagtacgacgtgctgaactttgcctccggaggt

tgctggcctcgcaggattcgggtttggaacggccaggattttcggggcgatggattcaac

cccagatttgagagatttcacgtgtacgacatagtagagacttcggagagcgcctcacac

gatgacccatccaggtttgctgagctatcccgcccaagcgggtctgtggtaacactgctg

ggaatgagcgagtgtggaaagcgagtcgcggttcacgtctatggtgtgcgccattatttt

tacatggcaaaggcggaggtggatagcgcttgtggaataaccaccgaggcagaactcgtg

cgcgcaatggtggactgcgcgcacagctcggctttgagcgcggccctgggaaatggcaac

ggcggcaaacagagcggcggcagcggcgggggatggtggggcggaaagcacgtgtctgcg

gactgcttcaaagtggagaccgtgtgccacacgacgctgtactactttggatctaagcca

gctctctactatagagtatctgcctccagcagccgcctgggagggttcatctgcgacaac

tttcacccggagattacaaaattcgaggggagcgtggacgtgaccacgcggctgttgttg

gacaatgaaaattttaccagtttcgggtggtaccgcctgcgacccggcacccacggagag

cgtgttcaacttcgccccgttgagcgacacgtcacctcaagcgacgtggagattaactgt

actcccgataacctggagccgataccagacgaggctgcctggcccgactataagctcatg

tgctttgatatagagtgtaaagctggaacgggtaacgaaatggcgttcccagtggcaact

aaccaagaggacctggtcatccagatctcctgtctgctgtactcgcttgctactcagaac

cacgaacacaccctgctgttttccctcgggtcatgcgatatctctgaggaatactcgttt

gcatgcgtccagcgcggcgagcccagaccgacggttttggagtttgacagcgagtacgag

ctgctggttgccttcctgacctttctcaagcagtactctcccgagttcgccaccggctac

aacatcgttaattttgactgggcgtacatagttaacaaggtaacgtcggtgtataacatc

aagctggacgggtacggcaagttcaacaaaggggggctgtttaaggtgtgggacatcgcc

acgaaccattttcagaagaagagcaaggtgaaaatcaatggcctgatatctctagacatg

tattctgtggcgacggaaaagctaaagctacccagctacaaactcgacgcggtcgtggga

gacgtcctcggcgagcataagatagaccttccctataaagaaataccctcctattacgcg

ggagggcctgaccggaggggcgtaataggagagtattgtatccaggactctaggctggtg

ggcaagctgttttttaagtacctcccccatctggaactatcggcggtggccaaactcgcc

cgtatcaccctaacgcgggtaatttttgacggtcagcaaattcgcgtgtacacgtgcttg

ctgaaactcgcccgcgagagaaatttcattttgccagacaacagacgccggtttgacagt

caggcagacgccgcgtcagagacttcggagttggctatggatagccaaagccacgccttc

gacagtacagacgaacccgacggtgtggacggtaccccggacgccgcaggatctggcgct

acttctgaaaacggaggcgggaagcccggcgtcgggagggccgtgggctaccagggagca

aaggttctagaccccgtatccggctttcatgtggaccccgtggttgtgtttgacttcgct

agcttatacccaagcattatccaggcccataacctctgtttcaccaccctggcgctcgat

gaagtggatctggccgggcttcaaccatccgtcaactactcgacgttcgaggtgggtgac

caaaagttattttttgtccacgcccatattcgcgaaagcctgcttggcatcttgctgcgc

gactggctggccatgcgaaaggcggtgagggcgcgaatccccaccagcacccccgaggag

gcagttttactagataagcagcagtctgcgattaaggtgatatgcaactcggtttacgga

ttcacgggggtggcaaacggcctgttgccgtgtctgaggatagcggctaccgttaccacg

ataggacgcgacatgctcctcaagacccgagattacgttcactctcgttgggcgacgcgc

gagctgctggaggacaattttccaggggctataggtttccgaaaccacaagccttactcc

gtcagggttatctacggagacaccgactccgtgtttatcaagtttgtgggcctgacgtac

gagggggtatcggagctgggggatgctatgtcgcgtcagatttcagcggacctctttaga

gcgcccatcaaactggagtgtgagaagacctttcagcgactgctgctgatcaccaagaag

aagtacataggtgtcataaacggggggaagatgctcatgaagggggtcgacctggtccgc

aaaaataactgctctttcataaacttgtacgcgcgacatctggtagatcttttgttgtac

gacgaggatgtggccacggcggcagcagaggtgacagacgtgcctcccgcagaatgggtg

gggcgcccgctaccgagcggctttgacaagtttgggcgagtgctggtagaggcgtacaac

cgtatcactgcccccaacttggacgtgcgcgagttcgttatgactgctgagctgagccgc

tcacccgaatcgtataccaacaagcgcctgccgcacctcaccgtctactttaagctcgcc

atgaggaatgaagaactgcccagcgtaaaagagagaattccgtatgtgatagttgcgcag

accgaggccgcggaacgcgaagcgggtgtagtaaactcaatgcgcggtaccgcccaaaac

cccgtggtaaccaagaccgcacgcccccaacctaaacgcaaactgctggtttccgacctc

gccgaagacccgacctatgtttccgagaatgacgtaccgctaaacacagactactatttc

tcccacctgttgggtaccataagcgtgacctttaaggctctattcggaaatgatgtgaga

acaacagaaaatcttttaaagcggtttattccggaaaccccccacaagacccccacgaaa

acccaggcactgcttgagcgcgccggctttgaaaagctgacgccctttacaccggaggaa

gaaagtcgtcgaatactgcatacagttttttgtactctagaagcagctccccatcaaagc

tga

>KU206422.1

atggcggcgcgcgaacaggccaactccgttcgcaggagcgggttttttaatccgtttatt

ggcaagaggccatttttcaggccgggaagcgggcaaaccgcggagacagagcgcccaagg

cccccccaacactcgtactgcacagaggtgggtagctttaagtttatagctccaagatgc

ctcgatgaagaagcccccgccgaccagcgacgcggtgtacacgtgggcaccttggagcgc

ccacccaaagtgtactgcgatggctcagagtacgacgtgctgaactttgcctccggaggt

tgctggcctcgcaggattcgggtttggaacggccaggattttcggggcgatggattcaac

cccagatttgagagatttcacgtgtacgacatagtagagacttcggagagcgcctcacac

gatgacccatccaggtttgctgagctatcccgcccaagcgggtctgtggtaacactgctg

ggaatgagcgagtgtggaaagcgagtcgcggttcacgtctatggtgtgcgccattatttt

tacatggcaaaggcggaggtggatagcgcttgtggaataaccaccgaggcagaactcgtg

cgcgcaatggtggactgcgcgcacagctcggctttgagcgcggccctgggaaatggcaac

ggcggcaaacagagcggcggcagcggcgggggatggtggggcggaaagcacgtgtctgcg

gactgcttcaaagtggagaccgtgtgccacacgacgctgtactactttggatctaagcca

gctctctactatagagtatctgcctccagcagccgcctgggagggttcatctgcgacaac

tttcacccggagattacaaaattcgaggggagcgtggacgtgaccacgcggctgttgttg

gacaatgaaaattttaccagtttcgggtggtaccgcctgcgacccggcacccacggagag

cgtgttcaacttcgccccgttgagcgacacgtcacctcaagcgacgtggagattaactgt

actcccgataacctggagccgataccagacgaggctgcctggcccgactataagctcatg

tgctttgatatagagtgtaaagctggaacgggtaacgaaatggcgttcccagtggcaact

aaccaagaggacctggtcatccagatctcctgtctgctgtactcgcttgctactcagaac

cacgaacacaccctgctgttttccctcgggtcatgcgatatctctgaggaatactcgttt

gcatgcgtccagcgcggcgagcccagaccgacggttttggagtttgacagcgagtacgag

ctgctggttgccttcctgacctttctcaagcagtactctcccgagttcgccaccggctac

aacatcgttaattttgactgggcgtacatagttaacaaggtaacgtcggtgtataacatc

aagctggacgggtacggcaagttcaacaaaggggggctgtttaaggtgtgggacatcgcc

acgaaccattttcagaagaagagcaaggtgaaaatcaatggcctgatatctctagacatg

tattctgtggcgacggaaaagctaaagctacccagctacaaactcgacgcggtcgtggga

gacgtcctcggcgagcataagatagaccttccctataaagaaataccctcctattacgcg

ggagggcctgaccggaggggcgtaataggagagtattgtatccaggactctaggctggtg

ggcaagctgttttttaagtacctcccccatctggaactatcggcggtggccaaactcgcc

cgtatcaccctaacgcgggtaatttttgacggtcagcaaattcgcgtgtacacgtgcttg

ctgaaactcgcccgcgagagaaatttcattttgccagacaacagacgccggtttgacagt

caggcagacgccgcgtcagagacttcggagttggctatggatagccaaagccacgccttc

gacagtacagacgaacccgacggtgtggacggtaccccggacgccgcaggatctggcgct

acttctgaaaacggaggcgggaagcccggcgtcgggagggccgtgggctaccagggagca

aaggttctagaccccgtatccggctttcatgtggaccccgtggttgtgtttgacttcgct

agcttatacccaagcattatccaggcccataacctctgtttcaccaccctggcgctcgat

gaagtggatctggccgggcttcaaccatccgtcaactactcgacgttcgaggtgggtgac

caaaagttattttttgtccacgcccatattcgcgaaagcctgcttggcatcttgctgcgc

gactggctggccatgcgaaaggcggtgagggcgcgaatccccaccagcacccccgaggag

gcagttttactagataagcagcagtctgcgattaaggtgatatgcaactcggtttacgga

ttcacgggggtggcaaacggcctgttgccgtgtctgaggatagcggctaccgttaccacg

ataggacgcgacatgctcctcaagacccgagattacgttcactctcgttgggcgacgcgc

gagctgctggaggacaattttccaggggctataggtttccgaaaccacaagccttactcc

gtcagggttatctacggagacaccgactccgtgtttatcaagtttgtgggcctgacgtac

gagggggtatcggagctgggggatgctatgtcgcgtcagatttcagcggacctctttaga

gcgcccatcaaactggagtgtgagaagacctttcagcgactgctgctgatcaccaagaag

aagtacataggtgtcataaacggggggaagatgctcatgaagggggtcgacctggtccgc

aaaaataactgctctttcataaacttgtacgcgcgacatctggtagatcttttgttgtac

gacgaggatgtggccacggcggcagcagaggtgacagacgtgcctcccgcagaatgggtg

gggcgcccgctaccgagcggctttgacaagtttgggcgagtgctggtagaggcgtacaac

cgtatcactgcccccaacttggacgtgcgcgagttcgttatgactgctgagctgagccgc

tcacccgaatcgtataccaacaagcgcctgccgcacctcaccgtctactttaagctcgcc

atgaggaatgaagaactgcccagcgtaaaagagagaattccgtatgtgatagttgcgcag

accgaggccgcggaacgcgaagcgggtgtagtaaactcaatgcgcggtaccgcccaaaac

cccgtggtaaccaagaccgcacgcccccaacctaaacgcaaactgctggtttccgacctc

gccgaagacccgacctatgtttccgagaatgacgtaccgctaaacacagactactatttc

tcccacctgttgggtaccataagcgtgacctttaaggctctattcggaaatgatgtgaga

acaacagaaaatcttttaaagcggtttattccggaaaccccccacaagacccccacgaaa

acccaggcactgcttgagcgcgccggctttgaaaagctgacgccctttacaccggaggaa

gaaagtcgtcgaatactgcatacagttttttgtactctagaagcagctccccatcaaagc

tga

>KU206423.1

atggcggcgcgcgaacaggccaactccgttcgcaggagcgggttttttaatccgtttatt

ggcaagaggccatttttcaggccgggaagcgggcaaaccgcggagacagagcgcccaagg

cccccccaacactcgtactgcacagaggtgggtagctttaagtttatagctccaagatgc

ctcgatgaagaagcccccgccgaccagcgacgcggtgtacacgtgggcaccttggagcgc

ccacccaaagtgtactgcgatggctcagagtacgacgtgctgaactttgcctccggaggt

tgctggcctcgcaggattcgggtttggaacggccaggattttcggggcgatggattcaac

cccagatttgagagatttcacgtgtacgacatagtagagacttcggagagcgcctcacac

gatgacccatccaggtttgctgagctatcccgcccaagcgggtctgtggtaacactgctg

ggaatgagcgagtgtggaaagcgagtcgcggttcacgtctatggtgtgcgccattatttt

tacatggcaaaggcggaggtggatagcgcttgtggaataaccaccgaggcagaactcgtg

cgcgcaatggtggactgcgcgcacagctcggctttgagcgcggccctgggaaatggcaac

ggcggcaaacagagcggcggcagcggcgggggatggtggggcggaaagcacgtgtctgcg

gactgcttcaaagtggagaccgtgtgccacacgacgctgtactactttggatctaagcca

gctctctactatagagtatctgcctccagcagccgcctgggagggttcatctgcgacaac

tttcacccggagattacaaaattcgaggggagcgtggacgtgaccacgcggctgttgttg

gacaatgaaaattttaccagtttcgggtggtaccgcctgcgacccggcacccacggagag

cgtgttcaacttcgccccgttgagcgacacgtcacctcaagcgacgtggagattaactgt

actcccgataacctggagccgataccagacgaggctgcctggcccgactataagctcatg

tgctttgatatagagtgtaaagctggaacgggtaacgaaatggcgttcccagtggcaact

aaccaagaggacctggtcatccagatctcctgtctgctgtactcgcttgctactcagaac

cacgaacacaccctgctgttttccctcgggtcatgcgatatctctgaggaatactcgttt

gcatgcgtccagcgcggcgagcccagaccgacggttttggagtttgacagcgagtacgag

ctgctggttgccttcctgacctttctcaagcagtactctcccgagttcgccaccggctac

aacatcgttaattttgactgggcgtacatagttaacaaggtaacgtcggtgtataacatc

aagctggacgggtacggcaagttcaacaaaggggggctgtttaaggtgtgggacatcgcc

acgaaccattttcagaagaagagcaaggtgaaaatcaatggcctgatatctctagacatg

tattctgtggcgacggaaaagctaaagctacccagctacaaactcgacgcggtcgtggga

gacgtcctcggcgagcataagatagaccttccctataaagaaataccctcctattacgcg

ggagggcctgaccggaggggcgtaataggagagtattgtatccaggactctaggctggtg

ggcaagctgttttttaagtacctcccccatctggaactatcggcggtggccaaactcgcc

cgtatcaccctaacgcgggtaatttttgacggtcagcaaattcgcgtgtacacgtgcttg

ctgaaactcgcccgcgagagaaatttcattttgccagacaacagacgccggtttgacagt

caggcagacgccgcgtcagagacttcggagttggctatggatagccaaagccacgccttc

gacagtacagacgaacccgacggtgtggacggtaccccggacgccgcaggatctggcgct

acttctgaaaacggaggcgggaagcccggcgtcgggagggccgtgggctaccagggagca

aaggttctagaccccgtatccggctttcatgtggaccccgtggttgtgtttgacttcgct

agcttatacccaagcattatccaggcccataacctctgtttcaccaccctggcgctcgat

gaagtggatctggccgggcttcaaccatccgtcaactactcgacgttcgaggtgggtgac

caaaagttattttttgtccacgcccatattcgcgaaagcctgcttggcatcttgctgcgc

gactggctggccatgcgaaaggcggtgagggcgcgaatccccaccagcacccccgaggag

gcagttttactagataagcagcagtctgcgattaaggtgatatgcaactcggtttacgga

ttcacgggggtggcaaacggcctgttgccgtgtctgaggatagcggctaccgttaccacg

ataggacgcgacatgctcctcaagacccgagattacgttcactctcgttgggcgacgcgc

gagctgctggaggacaattttccaggggctataggtttccgaaaccacaagccttactcc

gtcagggttatctacggagacaccgactccgtgtttatcaagtttgtgggcctgacgtac

gagggggtatcggagctgggggatgctatgtcgcgtcagatttcagcggacctctttaga

gcgcccatcaaactggagtgtgagaagacctttcagcgactgctgctgatcaccaagaag

aagtacataggtgtcataaacggggggaagatgctcatgaagggggtcgacctggtccgc

aaaaataactgctctttcataaacttgtacgcgcgacatctggtagatcttttgttgtac

gacgaggatgtggccacggcggcagcagaggtgacagacgtgcctcccgcagaatgggtg

gggcgcccgctaccgagcggctttgacaagtttgggcgagtgctggtagaggcgtacaac

cgtatcactgcccccaacttggacgtgcgcgagttcgttatgactgctgagctgagccgc

tcacccgaatcgtataccaacaagcgcctgccgcacctcaccgtctactttaagctcgcc

atgaggaatgaagaactgcccagcgtaaaagagagaattccgtatgtgatagttgcgcag

accgaggccgcggaacgcgaagcgggtgtagtaaactcaatgcgcggtaccgcccaaaac

cccgtggtaaccaagaccgcacgcccccaacctaaacgcaaactgctggtttccgacctc

gccgaagacccgacctatgtttccgagaatgacgtaccgctaaacacagactactatttc

tcccacctgttgggtaccataagcgtgacctttaaggctctattcggaaatgatgtgaga

acaacagaaaatcttttaaagcggtttattccggaaaccccccacaagacccccacgaaa

acccaggcactgcttgagcgcgccggctttgaaaagctgacgccctttacaccggaggaa

gaaagtcgtcgaatactgcatacagttttttgtactctagaagcagctccccatcaaagc

tga

>KU206424.1

atggcggcgcgcgaacaggccaactccgttcgcaggagcgggttttttaatccgtttatt

ggcaagaggccatttttcaggccgggaagcgggcaaaccgcggagacagagcgcccaagg

cccccccaacactcgtactgcacagaggtgggtagctttaagtttatagctccaagatgc

ctcgatgaagaagcccccgccgaccagcgacgcggtgtacacgtgggcaccttggagcgc

ccacccaaagtgtactgcgatggctcagagtacgacgtgctgaactttgcctccggaggt

tgctggcctcgcaggattcgggtttggaacggccaggattttcggggcgatggattcaac

cccagatttgagagatttcacgtgtacgacatagtagagacttcggagagcgcctcacac

gatgacccatccaggtttgctgagctatcccgcccaagcgggtctgtggtaacactgctg

ggaatgagcgagtgtggaaagcgagtcgcggttcacgtctatggtgtgcgccattatttt

tacatggcaaaggcggaggtggatagcgcttgtggaataaccaccgaggcagaactcgtg

cgcgcaatggtggactgcgcgcacagctcggctttgagcgcggccctgggaaatggcaac

ggcggcaaacagagcggcggcagcggcgggggatggtggggcggaaagcacgtgtctgcg

gactgcttcaaagtggagaccgtgtgccacacgacgctgtactactttggatctaagcca

gctctctactatagagtatctgcctccagcagccgcctgggagggttcatctgcgacaac

tttcacccggagattacaaaattcgaggggagcgtggacgtgaccacgcggctgttgttg

gacaatgaaaattttaccagtttcgggtggtaccgcctgcgacccggcacccacggagag

cgtgttcaacttcgccccgttgagcgacacgtcacctcaagcgacgtggagattaactgt

actcccgataacctggagccgataccagacgaggctgcctggcccgactataagctcatg

tgctttgatatagagtgtaaagctggaacgggtaacgaaatggcgttcccagtggcaact

aaccaagaggacctggtcatccagatctcctgtctgctgtactcgcttgctactcagaac

cacgaacacaccctgctgttttccctcgggtcatgcgatatctctgaggaatactcgttt

gcatgcgtccagcgcggcgagcccagaccgacggttttggagtttgacagcgagtacgag

ctgctggttgccttcctgacctttctcaagcagtactctcccgagttcgccaccggctac

aacatcgttaattttgactgggcgtacatagttaacaaggtaacgtcggtgtataacatc

aagctggacgggtacggcaagttcaacaaaggggggctgtttaaggtgtgggacatcgcc

acgaaccattttcagaagaagagcaaggtgaaaatcaatggcctgatatctctagacatg

tattctgtggcgacggaaaagctaaagctacccagctacaaactcgacgcggtcgtggga

gacgtcctcggcgagcataagatagaccttccctataaagaaataccctcctattacgcg

ggagggcctgaccggaggggcgtaataggagagtattgtatccaggactctaggctggtg

ggcaagctgttttttaagtacctcccccatctggaactatcggcggtggccaaactcgcc

cgtatcaccctaacgcgggtaatttttgacggtcagcaaattcgcgtgtacacgtgcttg

ctgaaactcgcccgcgagagaaatttcattttgccagacaacagacgccggtttgacagt

caggcagacgccgcgtcagagacttcggagttggctatggatagccaaagccacgccttc

gacagtacagacgaacccgacggtgtggacggtaccccggacgccgcaggatctggcgct

acttctgaaaacggaggcgggaagcccggcgtcgggagggccgtgggctaccagggagca

aaggttctagaccccgtatccggctttcatgtggaccccgtggttgtgtttgacttcgct

agcttatacccaagcattatccaggcccataacctctgtttcaccaccctggcgctcgat

gaagtggatctggccgggcttcaaccatccgtcaactactcgacgttcgaggtgggtgac

caaaagttattttttgtccacgcccatattcgcgaaagcctgcttggcatcttgctgcgc

gactggctggccatgcgaaaggcggtgagggcgcgaatccccaccagcacccccgaggag

gcagttttactagataagcagcagtctgcgattaaggtgatatgcaactcggtttacgga

ttcacgggggtggcaaacggcctgttgccgtgtctgaggatagcggctaccgttaccacg

ataggacgcgacatgctcctcaagacccgagattacgttcactctcgttgggcgacgcgc

gagctgctggaggacaattttccaggggctataggtttccgaaaccacaagccttactcc

gtcagggttatctacggagacaccgactccgtgtttatcaagtttgtgggcctgacgtac

gagggggtatcggagctgggggatgctatgtcgcgtcagatttcagcggacctctttaga

gcgcccatcaaactggagtgtgagaagacctttcagcgactgctgctgatcaccaagaag

aagtacataggtgtcataaacggggggaagatgctcatgaagggggtcgacctggtccgc

aaaaataactgctctttcataaacttgtacgcgcgacatctggtagatcttttgttgtac

gacgaggatgtggccacggcggcagcagaggtgacagacgtgcctcccgcagaatgggtg

gggcgcccgctaccgagcggctttgacaagtttgggcgagtgctggtagaggcgtacaac

cgtatcactgcccccaacttggacgtgcgcgagttcgttatgactgctgagctgagccgc

tcacccgaatcgtataccaacaagcgcctgccgcacctcaccgtctactttaagctcgcc

atgaggaatgaagaactgcccagcgtaaaagagagaattccgtatgtgatagttgcgcag

accgaggccgcggaacgcgaagcgggtgtagtaaactcaatgcgcggtaccgcccaaaac

cccgtggtaaccaagaccgcacgcccccaacctaaacgcaaactgctggtttccgacctc

gccgaagacccgacctatgtttccgagaatgacgtaccgctaaacacagactactatttc

tcccacctgttgggtaccataagcgtgacctttaaggctctattcggaaatgatgtgaga

acaacagaaaatcttttaaagcggtttattccggaaaccccccacaagacccccacgaaa

acccaggcactgcttgagcgcgccggctttgaaaagctgacgccctttacaccggaggaa

gaaagtcgtcgaatactgcatacagttttttgtactctagaagcagctccccatcaaagc

tga

>KU206425.1

atggcggcgcgcgaacaggccaactccgttcgcaggagcgggttttttaatccgtttatt

ggcaagaggccatttttcaggccgggaagcgggcaaaccgcggagacagagcgcccaagg

cccccccaacactcgtactgcacagaggtgggtagctttaagtttatagctccaagatgc

ctcgatgaagaagcccccgccgaccagcgacgcggtgtacacgtgggcaccttggagcgc

ccacccaaagtgtactgcgatggctcagagtacgacgtgctgaactttgcctccggaggt

tgctggcctcgcaggattcgggtttggaacggccaggattttcggggcgatggattcaac

cccagatttgagagatttcacgtgtacgacatagtagagacttcggagagcgcctcacac

gatgacccatccaggtttgctgagctatcccgcccaagcgggtctgtggtaacactgctg

ggaatgagcgagtgtggaaagcgagtcgcggttcacgtctatggtgtgcgccattatttt

tacatggcaaaggcggaggtggatagcgcttgtggaataaccaccgaggcagaactcgtg

cgcgcaatggtggactgcgcgcacagctcggctttgagcgcggccctgggaaatggcaac

ggcggcaaacagagcggcggcagcggcgggggatggtggggcggaaagcacgtgtctgcg

gactgcttcaaagtggagaccgtgtgccacacgacgctgtactactttggatctaagcca

gctctctactatagagtatctgcctccagcagccgcctgggagggttcatctgcgacaac

tttcacccggagattacaaaattcgaggggagcgtggacgtgaccacgcggctgttgttg

gacaatgaaaattttaccagtttcgggtggtaccgcctgcgacccggcacccacggagag

cgtgttcaacttcgccccgttgagcgacacgtcacctcaagcgacgtggagattaactgt

actcccgataacctggagccgataccagacgaggctgcctggcccgactataagctcatg

tgctttgatatagagtgtaaagctggaacgggtaacgaaatggcgttcccagtggcaact

aaccaagaggacctggtcatccagatctcctgtctgctgtactcgcttgctactcagaac

cacgaacacaccctgctgttttccctcgggtcatgcgatatctctgaggaatactcgttt

gcatgcgtccagcgcggcgagcccagaccgacggttttggagtttgacagcgagtacgag

ctgctggttgccttcctgacctttctcaagcagtactctcccgagttcgccaccggctac

aacatcgttaattttgactgggcgtacatagttaacaaggtaacgtcggtgtataacatc

aagctggacgggtacggcaagttcaacaaaggggggctgtttaaggtgtgggacatcgcc

acgaaccattttcagaagaagagcaaggtgaaaatcaatggcctgatatctctagacatg

tattctgtggcgacggaaaagctaaagctacccagctacaaactcgacgcggtcgtggga

gacgtcctcggcgagcataagatagaccttccctataaagaaataccctcctattacgcg

ggagggcctgaccggaggggcgtaataggagagtattgtatccaggactctaggctggtg

ggcaagctgttttttaagtacctcccccatctggaactatcggcggtggccaaactcgcc

cgtatcaccctaacgcgggtaatttttgacggtcagcaaattcgcgtgtacacgtgcttg

ctgaaactcgcccgcgagagaaatttcattttgccagacaacagacgccggtttgacagt

caggcagacgccgcgtcagagacttcggagttggctatggatagccaaagccacgccttc

gacagtacagacgaacccgacggtgtggacggtaccccggacgccgcaggatctggcgct

acttctgaaaacggaggcgggaagcccggcgtcgggagggccgtgggctaccagggagca

aaggttctagaccccgtatccggctttcatgtggaccccgtggttgtgtttgacttcgct

agcttatacccaagcattatccaggcccataacctctgtttcaccaccctggcgctcgat

gaagtggatctggccgggcttcaaccatccgtcaactactcgacgttcgaggtgggtgac

caaaagttattttttgtccacgcccatattcgcgaaagcctgcttggcatcttgctgcgc

gactggctggccatgcgaaaggcggtgagggcgcgaatccccaccagcacccccgaggag

gcagttttactagataagcagcagtctgcgattaaggtgatatgcaactcggtttacgga

ttcacgggggtggcaaacggcctgttgccgtgtctgaggatagcggctaccgttaccacg

ataggacgcgacatgctcctcaagacccgagattacgttcactctcgttgggcgacgcgc

gagctgctggaggacaattttccaggggctataggtttccgaaaccacaagccttactcc

gtcagggttatctacggagacaccgactccgtgtttatcaagtttgtgggcctgacgtac

gagggggtatcggagctgggggatgctatgtcgcgtcagatttcagcggacctctttaga

gcgcccatcaaactggagtgtgagaagacctttcagcgactgctgctgatcaccaagaag

aagtacataggtgtcataaacggggggaagatgctcatgaagggggtcgacctggtccgc

aaaaataactgctctttcataaacttgtacgcgcgacatctggtagatcttttgttgtac

gacgaggatgtggccacggcggcagcagaggtgacagacgtgcctcccgcagaatgggtg

gggcgcccgctaccgagcggctttgacaagtttgggcgagtgctggtagaggcgtacaac

cgtatcactgcccccaacttggacgtgcgcgagttcgttatgactgctgagctgagccgc

tcacccgaatcgtataccaacaagcgcctgccgcacctcaccgtctactttaagctcgcc

atgaggaatgaagaactgcccagcgtaaaagagagaattccgtatgtgatagttgcgcag

accgaggccgcggaacgcgaagcgggtgtagtaaactcaatgcgcggtaccgcccaaaac

cccgtggtaaccaagaccgcacgcccccaacctaaacgcaaactgctggtttccgacctc

gccgaagacccgacctatgtttccgagaatgacgtaccgctaaacacagactactatttc

tcccacctgttgggtaccataagcgtgacctttaaggctctattcggaaatgatgtgaga

acaacagaaaatcttttaaagcggtttattccggaaaccccccacaagacccccacgaaa

acccaggcactgcttgagcgcgccggctttgaaaagctgacgccctttacaccggaggaa

gaaagtcgtcgaatactgcatacagttttttgtactctagaagcagctccccatcaaagc

tga

>KU206426.1

atggcggcgcgcgaacaggccaactccgttcgcaggagcgggttttttaatccgtttatt

ggcaagaggccatttttcaggccgggaagcgggcaaaccgcggagacagagcgcccaagg

cccccccaacactcgtactgcacagaggtgggtagctttaagtttatagctccaagatgc

ctcgatgaagaagcccccgccgaccagcgacgcggtgtacacgtgggcaccttggagcgc

ccacccaaagtgtactgcgatggctcagagtacgacgtgctgaactttgcctccggaggt

tgctggcctcgcaggattcgggtttggaacggccaggattttcggggcgatggattcaac

cccagatttgagagatttcacgtgtacgacatagtagagacttcggagagcgcctcacac

gatgacccatccaggtttgctgagctatcccgcccaagcgggtctgtggtaacactgctg

ggaatgagcgagtgtggaaagcgagtcgcggttcacgtctatggtgtgcgccattatttt

tacatggcaaaggcggaggtggatagcgcttgtggaataaccaccgaggcagaactcgtg

cgcgcaatggtggactgcgcgcacagctcggctttgagcgcggccctgggaaatggcaac

ggcggcaaacagagcggcggcagcggcgggggatggtggggcggaaagcacgtgtctgcg

gactgcttcaaagtggagaccgtgtgccacacgacgctgtactactttggatctaagcca

gctctctactatagagtatctgcctccagcagccgcctgggagggttcatctgcgacaac

tttcacccggagattacaaaattcgaggggagcgtggacgtgaccacgcggctgttgttg

gacaatgaaaattttaccagtttcgggtggtaccgcctgcgacccggcacccacggagag

cgtgttcaacttcgccccgttgagcgacacgtcacctcaagcgacgtggagattaactgt

actcccgataacctggagccgataccagacgaggctgcctggcccgactataagctcatg

tgctttgatatagagtgtaaagctggaacgggtaacgaaatggcgttcccagtggcaact

aaccaagaggacctggtcatccagatctcctgtctgctgtactcgcttgctactcagaac

cacgaacacaccctgctgttttccctcgggtcatgcgatatctctgaggaatactcgttt

gcatgcgtccagcgcggcgagcccagaccgacggttttggagtttgacagcgagtacgag

ctgctggttgccttcctgacctttctcaagcagtactctcccgagttcgccaccggctac

aacatcgttaattttgactgggcgtacatagttaacaaggtaacgtcggtgtataacatc

aagctggacgggtacggcaagttcaacaaaggggggctgtttaaggtgtgggacatcgcc

acgaaccattttcagaagaagagcaaggtgaaaatcaatggcctgatatctctagacatg

tattctgtggcgacggaaaagctaaagctacccagctacaaactcgacgcggtcgtggga

gacgtcctcggcgagcataagatagaccttccctataaagaaataccctcctattacgcg

ggagggcctgaccggaggggcgtaataggagagtattgtatccaggactctaggctggtg

ggcaagctgttttttaagtacctcccccatctggaactatcggcggtggccaaactcgcc

cgtatcaccctaacgcgggtaatttttgacggtcagcaaattcgcgtgtacacgtgcttg

ctgaaactcgcccgcgagagaaatttcattttgccagacaacagacgccggtttgacagt

caggcagacgccgcgtcagagacttcggagttggctatggatagccaaagccacgccttc

gacagtacagacgaacccgacggtgtggacggtaccccggacgccgcaggatctggcgct

acttctgaaaacggaggcgggaagcccggcgtcgggagggccgtgggctaccagggagca

aaggttctagaccccgtatccggctttcatgtggaccccgtggttgtgtttgacttcgct

agcttatacccaagcattatccaggcccataacctctgtttcaccaccctggcgctcgat

gaagtggatctggccgggcttcaaccatccgtcaactactcgacgttcgaggtgggtgac

caaaagttattttttgtccacgcccatattcgcgaaagcctgcttggcatcttgctgcgc

gactggctggccatgcgaaaggcggtgagggcgcgaatccccaccagcacccccgaggag

gcagttttactagataagcagcagtctgcgattaaggtgatatgcaactcggtttacgga

ttcacgggggtggcaaacggcctgttgccgtgtctgaggatagcggctaccgttaccacg

ataggacgcgacatgctcctcaagacccgagattacgttcactctcgttgggcgacgcgc

gagctgctggaggacaattttccaggggctataggtttccgaaaccacaagccttactcc

gtcagggttatctacggagacaccgactccgtgtttatcaagtttgtgggcctgacgtac

gagggggtatcggagctgggggatgctatgtcgcgtcagatttcagcggacctctttaga

gcgcccatcaaactggagtgtgagaagacctttcagcgactgctgctgatcaccaagaag

aagtacataggtgtcataaacggggggaagatgctcatgaagggggtcgacctggtccgc

aaaaataactgctctttcataaacttgtacgcgcgacatctggtagatcttttgttgtac

gacgaggatgtggccacggcggcagcagaggtgacagacgtgcctcccgcagaatgggtg

gggcgcccgctaccgagcggctttgacaagtttgggcgagtgctggtagaggcgtacaac

cgtatcactgcccccaacttggacgtgcgcgagttcgttatgactgctgagctgagccgc

tcacccgaatcgtataccaacaagcgcctgccgcacctcaccgtctactttaagctcgcc

atgaggaatgaagaactgcccagcgtaaaagagagaattccgtatgtgatagttgcgcag

accgaggccgcggaacgcgaagcgggtgtagtaaactcaatgcgcggtaccgcccaaaac

cccgtggtaaccaagaccgcacgcccccaacctaaacgcaaactgctggtttccgacctc

gccgaagacccgacctatgtttccgagaatgacgtaccgctaaacacagactactatttc

tcccacctgttgggtaccataagcgtgacctttaaggctctattcggaaatgatgtgaga

acaacagaaaatcttttaaagcggtttattccggaaaccccccacaagacccccacgaaa

acccaggcactgcttgagcgcgccggctttgaaaagctgacgccctttacaccggaggaa

gaaagtcgtcgaatactgcatacagttttttgtactctagaagcagctccccatcaaagc

tga

>KU206427.1

atggcggcgcgcgaacaggccaactccgttcgcaggagcgggttttttaatccgtttatt

ggcaagaggccatttttcaggccgggaagcgggcaaaccgcggagacagagcgcccaagg

cccccccaacactcgtactgcacagaggtgggtagctttaagtttatagctccaagatgc

ctcgatgaagaagcccccgccgaccagcgacgcggtgtacacgtgggcaccttggagcgc

ccacccaaagtgtactgcgatggctcagagtacgacgtgctgaactttgcctccggaggt

tgctggcctcgcaggattcgggtttggaacggccaggattttcggggcgatggattcaac

cccagatttgagagatttcacgtgtacgacatagtagagacttcggagagcgcctcacac

gatgacccatccaggtttgctgagctatcccgcccaagcgggtctgtggtaacactgctg

ggaatgagcgagtgtggaaagcgagtcgcggttcacgtctatggtgtgcgccattatttt

tacatggcaaaggcggaggtggatagcgcttgtggaataaccaccgaggcagaactcgtg

cgcgcaatggtggactgcgcgcacagctcggctttgagcgcggccctgggaaatggcaac

ggcggcaaacagagcggcggcagcggcgggggatggtggggcggaaagcacgtgtctgcg

gactgcttcaaagtggagaccgtgtgccacacgacgctgtactactttggatctaagcca

gctctctactatagagtatctgcctccagcagccgcctgggagggttcatctgcgacaac

tttcacccggagattacaaaattcgaggggagcgtggacgtgaccacgcggctgttgttg

gacaatgaaaattttaccagtttcgggtggtaccgcctgcgacccggcacccacggagag

cgtgttcaacttcgccccgttgagcgacacgtcacctcaagcgacgtggagattaactgt

actcccgataacctggagccgataccagacgaggctgcctggcccgactataagctcatg

tgctttgatatagagtgtaaagctggaacgggtaacgaaatggcgttcccagtggcaact

aaccaagaggacctggtcatccagatctcctgtctgctgtactcgcttgctactcagaac

cacgaacacaccctgctgttttccctcgggtcatgcgatatctctgaggaatactcgttt

gcatgcgtccagcgcggcgagcccagaccgacggttttggagtttgacagcgagtacgag

ctgctggttgccttcctgacctttctcaagcagtactctcccgagttcgccaccggctac

aacatcgttaattttgactgggcgtacatagttaacaaggtaacgtcggtgtataacatc

aagctggacgggtacggcaagttcaacaaaggggggctgtttaaggtgtgggacatcgcc

acgaaccattttcagaagaagagcaaggtgaaaatcaatggcctgatatctctagacatg

tattctgtggcgacggaaaagctaaagctacccagctacaaactcgacgcggtcgtggga

gacgtcctcggcgagcataagatagaccttccctataaagaaataccctcctattacgcg

ggagggcctgaccggaggggcgtaataggagagtattgtatccaggactctaggctggtg

ggcaagctgttttttaagtacctcccccatctggaactatcggcggtggccaaactcgcc

cgtatcaccctaacgcgggtaatttttgacggtcagcaaattcgcgtgtacacgtgcttg

ctgaaactcgcccgcgagagaaatttcattttgccagacaacagacgccggtttgacagt

caggcagacgccgcgtcagagacttcggagttggctatggatagccaaagccacgccttc

gacagtacagacgaacccgacggtgtggacggtaccccggacgccgcaggatctggcgct

acttctgaaaacggaggcgggaagcccggcgtcgggagggccgtgggctaccagggagca

aaggttctagaccccgtatccggctttcatgtggaccccgtggttgtgtttgacttcgct

agcttatacccaagcattatccaggcccataacctctgtttcaccaccctggcgctcgat

gaagtggatctggccgggcttcaaccatccgtcaactactcgacgttcgaggtgggtgac

caaaagttattttttgtccacgcccatattcgcgaaagcctgcttggcatcttgctgcgc

gactggctggccatgcgaaaggcggtgagggcgcgaatccccaccagcacccccgaggag

gcagttttactagataagcagcagtctgcgattaaggtgatatgcaactcggtttacgga

ttcacgggggtggcaaacggcctgttgccgtgtctgaggatagcggctaccgttaccacg

ataggacgcgacatgctcctcaagacccgagattacgttcactctcgttgggcgacgcgc

gagctgctggaggacaattttccaggggctataggtttccgaaaccacaagccttactcc

gtcagggttatctacggagacaccgactccgtgtttatcaagtttgtgggcctgacgtac

gagggggtatcggagctgggggatgctatgtcgcgtcagatttcagcggacctctttaga

gcgcccatcaaactggagtgtgagaagacctttcagcgactgctgctgatcaccaagaag

aagtacataggtgtcataaacggggggaagatgctcatgaagggggtcgacctggtccgc

aaaaataactgctctttcataaacttgtacgcgcgacatctggtagatcttttgttgtac

gacgaggatgtggccacggcggcagcagaggtgacagacgtgcctcccgcagaatgggtg

gggcgcccgctaccgagcggctttgacaagtttgggcgagtgctggtagaggcgtacaac

cgtatcactgcccccaacttggacgtgcgcgagttcgttatgactgctgagctgagccgc

tcacccgaatcgtataccaacaagcgcctgccgcacctcaccgtctactttaagctcgcc

atgaggaatgaagaactgcccagcgtaaaagagagaattccgtatgtgatagttgcgcag

accgaggccgcggaacgcgaagcgggtgtagtaaactcaatgcgcggtaccgcccaaaac

cccgtggtaaccaagaccgcacgcccccaacctaaacgcaaactgctggtttccgacctc

gccgaagacccgacctatgtttccgagaatgacgtaccgctaaacacagactactatttc

tcccacctgttgggtaccataagcgtgacctttaaggctctattcggaaatgatgtgaga

acaacagaaaatcttttaaagcggtttattccggaaaccccccacaagacccccacgaaa

acccaggcactgcttgagcgcgccggctttgaaaagctgacgccctttacaccggaggaa

gaaagtcgtcgaatactgcatacagttttttgtactctagaagcagctccccatcaaagc

tga

>KU206428.1

atggcggcgcgcgaacaggccaactccgttcgcaggagcgggttttttaatccgtttatt

ggcaagaggccatttttcaggccgggaagcgggcaaaccgcggagacagagcgcccaagg

cccccccaacactcgtactgcacagaggtgggtagctttaagtttatagctccaagatgc

ctcgatgaagaagcccccgccgaccagcgacgcggtgtacacgtgggcaccttggagcgc

ccacccaaagtgtactgcgatggctcagagtacgacgtgctgaactttgcctccggaggt

tgctggcctcgcaggattcgggtttggaacggccaggattttcggggcgatggattcaac

cccagatttgagagatttcacgtgtacgacatagtagagacttcggagagcgcctcacac

gatgacccatccaggtttgctgagctatcccgcccaagcgggtctgtggtaacactgctg

ggaatgagcgagtgtggaaagcgagtcgcggttcacgtctatggtgtgcgccattatttt

tacatggcaaaggcggaggtggatagcgcttgtggaataaccaccgaggcagaactcgtg

cgcgcaatggtggactgcgcgcacagctcggctttgagcgcggccctgggaaatggcaac

ggcggcaaacagagcggcggcagcggcgggggatggtggggcggaaagcacgtgtctgcg

gactgcttcaaagtggagaccgtgtgccacacgacgctgtactactttggatctaagcca

gctctctactatagagtatctgcctccagcagccgcctgggagggttcatctgcgacaac

tttcacccggagattacaaaattcgaggggagcgtggacgtgaccacgcggctgttgttg

gacaatgaaaattttaccagtttcgggtggtaccgcctgcgacccggcacccacggagag

cgtgttcaacttcgccccgttgagcgacacgtcacctcaagcgacgtggagattaactgt

actcccgataacctggagccgataccagacgaggctgcctggcccgactataagctcatg

tgctttgatatagagtgtaaagctggaacgggtaacgaaatggcgttcccagtggcaact

aaccaagaggacctggtcatccagatctcctgtctgctgtactcgcttgctactcagaac

cacgaacacaccctgctgttttccctcgggtcatgcgatatctctgaggaatactcgttt

gcatgcgtccagcgcggcgagcccagaccgacggttttggagtttgacagcgagtacgag

ctgctggttgccttcctgacctttctcaagcagtactctcccgagttcgccaccggctac

aacatcgttaattttgactgggcgtacatagttaacaaggtaacgtcggtgtataacatc

aagctggacgggtacggcaagttcaacaaaggggggctgtttaaggtgtgggacatcgcc

acgaaccattttcagaagaagagcaaggtgaaaatcaatggcctgatatctctagacatg

tattctgtggcgacggaaaagctaaagctacccagctacaaactcgacgcggtcgtggga

gacgtcctcggcgagcataagatagaccttccctataaagaaataccctcctattacgcg

ggagggcctgaccggaggggcgtaataggagagtattgtatccaggactctaggctggtg

ggcaagctgttttttaagtacctcccccatctggaactatcggcggtggccaaactcgcc

cgtatcaccctaacgcgggtaatttttgacggtcagcaaattcgcgtgtacacgtgcttg

ctgaaactcgcccgcgagagaaatttcattttgccagacaacagacgccggtttgacagt

caggcagacgccgcgtcagagacttcggagttggctatggatagccaaagccacgccttc

gacagtacagacgaacccgacggtgtggacggtaccccggacgccgcaggatctggcgct

acttctgaaaacggaggcgggaagcccggcgtcgggagggccgtgggctaccagggagca

aaggttctagaccccgtatccggctttcatgtggaccccgtggttgtgtttgacttcgct

agcttatacccaagcattatccaggcccataacctctgtttcaccaccctggcgctcgat

gaagtggatctggccgggcttcaaccatccgtcaactactcgacgttcgaggtgggtgac

caaaagttattttttgtccacgcccatattcgcgaaagcctgcttggcatcttgctgcgc

gactggctggccatgcgaaaggcggtgagggcgcgaatccccaccagcacccccgaggag

gcagttttactagataagcagcagtctgcgattaaggtgatatgcaactcggtttacgga

ttcacgggggtggcaaacggcctgttgccgtgtctgaggatagcggctaccgttaccacg

ataggacgcgacatgctcctcaagacccgagattacgttcactctcgttgggcgacgcgc

gagctgctggaggacaattttccaggggctataggtttccgaaaccacaagccttactcc

gtcagggttatctacggagacaccgactccgtgtttatcaagtttgtgggcctgacgtac

gagggggtatcggagctgggggatgctatgtcgcgtcagatttcagcggacctctttaga

gcgcccatcaaactggagtgtgagaagacctttcagcgactgctgctgatcaccaagaag

aagtacataggtgtcataaacggggggaagatgctcatgaagggggtcgacctggtccgc

aaaaataactgctctttcataaacttgtacgcgcgacatctggtagatcttttgttgtac

gacgaggatgtggccacggcggcagcagaggtgacagacgtgcctcccgcagaatgggtg

gggcgcccgctaccgagcggctttgacaagtttgggcgagtgctggtagaggcgtacaac

cgtatcactgcccccaacttggacgtgcgcgagttcgttatgactgctgagctgagccgc

tcacccgaatcgtataccaacaagcgcctgccgcacctcaccgtctactttaagctcgcc

atgaggaatgaagaactgcccagcgtaaaagagagaattccgtatgtgatagttgcgcag

accgaggccgcggaacgcgaagcgggtgtagtaaactcaatgcgcggtaccgcccaaaac

cccgtggtaaccaagaccgcacgcccccaacctaaacgcaaactgctggtttccgacctc

gccgaagacccgacctatgtttccgagaatgacgtaccgctaaacacagactactatttc

tcccacctgttgggtaccataagcgtgacctttaaggctctattcggaaatgatgtgaga

acaacagaaaatcttttaaagcggtttattccggaaaccccccacaagacccccacgaaa

acccaggcactgcttgagcgcgccggctttgaaaagctgacgccctttacaccggaggaa

gaaagtcgtcgaatactgcatacagttttttgtactctagaagcagctccccatcaaagc

tga

>KU206429.1

atggcggcgcgcgaacaggccaactccgttcgcaggagcgggttttttaatccgtttatt

ggcaagaggccatttttcaggccgggaagcgggcaaaccgcggagacagagcgcccaagg

cccccccaacactcgtactgcacagaggtgggtagctttaagtttatagctccaagatgc

ctcgatgaagaagcccccgccgaccagcgacgcggtgtacacgtgggcaccttggagcgc

ccacccaaagtgtactgcgatggctcagagtacgacgtgctgaactttgcctccggaggt

tgctggcctcgcaggattcgggtttggaacggccaggattttcggggcgatggattcaac

cccagatttgagagatttcacgtgtacgacatagtagagacttcggagagcgcctcacac

gatgacccatccaggtttgctgagctatcccgcccaagcgggtctgtggtaacactgctg

ggaatgagcgagtgtggaaagcgagtcgcggttcacgtctatggtgtgcgccattatttt

tacatggcaaaggcggaggtggatagcgcttgtggaataaccaccgaggcagaactcgtg

cgcgcaatggtggactgcgcgcacagctcggctttgagcgcggccctgggaaatggcaac

ggcggcaaacagagcggcggcagcggcgggggatggtggggcggaaagcacgtgtctgcg

gactgcttcaaagtggagaccgtgtgccacacgacgctgtactactttggatctaagcca

gctctctactatagagtatctgcctccagcagccgcctgggagggttcatctgcgacaac

tttcacccggagattacaaaattcgaggggagcgtggacgtgaccacgcggctgttgttg

gacaatgaaaattttaccagtttcgggtggtaccgcctgcgacccggcacccacggagag

cgtgttcaacttcgccccgttgagcgacacgtcacctcaagcgacgtggagattaactgt

actcccgataacctggagccgataccagacgaggctgcctggcccgactataagctcatg

tgctttgatatagagtgtaaagctggaacgggtaacgaaatggcgttcccagtggcaact

aaccaagaggacctggtcatccagatctcctgtctgctgtactcgcttgctactcagaac

cacgaacacaccctgctgttttccctcgggtcatgcgatatctctgaggaatactcgttt

gcatgcgtccagcgcggcgagcccagaccgacggttttggagtttgacagcgagtacgag

ctgctggttgccttcctgacctttctcaagcagtactctcccgagttcgccaccggctac

aacatcgttaattttgactgggcgtacatagttaacaaggtaacgtcggtgtataacatc

aagctggacgggtacggcaagttcaacaaaggggggctgtttaaggtgtgggacatcgcc

acgaaccattttcagaagaagagcaaggtgaaaatcaatggcctgatatctctagacatg

tattctgtggcgacggaaaagctaaagctacccagctacaaactcgacgcggtcgtggga

gacgtcctcggcgagcataagatagaccttccctataaagaaataccctcctattacgcg

ggagggcctgaccggaggggcgtaataggagagtattgtatccaggactctaggctggtg

ggcaagctgttttttaagtacctcccccatctggaactatcggcggtggccaaactcgcc

cgtatcaccctaacgcgggtaatttttgacggtcagcaaattcgcgtgtacacgtgcttg

ctgaaactcgcccgcgagagaaatttcattttgccagacaacagacgccggtttgacagt

caggcagacgccgcgtcagagacttcggagttggctatggatagccaaagccacgccttc

gacagtacagacgaacccgacggtgtggacggtaccccggacgccgcaggatctggcgct

acttctgaaaacggaggcgggaagcccggcgtcgggagggccgtgggctaccagggagca

aaggttctagaccccgtatccggctttcatgtggaccccgtggttgtgtttgacttcgct

agcttatacccaagcattatccaggcccataacctctgtttcaccaccctggcgctcgat

gaagtggatctggccgggcttcaaccatccgtcaactactcgacgttcgaggtgggtgac

caaaagttattttttgtccacgcccatattcgcgaaagcctgcttggcatcttgctgcgc

gactggctggccatgcgaaaggcggtgagggcgcgaatccccaccagcacccccgaggag

gcagttttactagataagcagcagtctgcgattaaggtgatatgcaactcggtttacgga

ttcacgggggtggcaaacggcctgttgccgtgtctgaggatagcggctaccgttaccacg

ataggacgcgacatgctcctcaagacccgagattacgttcactctcgttgggcgacgcgc

gagctgctggaggacaattttccaggggctataggtttccgaaaccacaagccttactcc

gtcagggttatctacggagacaccgactccgtgtttatcaagtttgtgggcctgacgtac

gagggggtatcggagctgggggatgctatgtcgcgtcagatttcagcggacctctttaga

gcgcccatcaaactggagtgtgagaagacctttcagcgactgctgctgatcaccaagaag

aagtacataggtgtcataaacggggggaagatgctcatgaagggggtcgacctggtccgc

aaaaataactgctctttcataaacttgtacgcgcgacatctggtagatcttttgttgtac

gacgaggatgtggccacggcggcagcagaggtgacagacgtgcctcccgcagaatgggtg

gggcgcccgctaccgagcggctttgacaagtttgggcgagtgctggtagaggcgtacaac

cgtatcactgcccccaacttggacgtgcgcgagttcgttatgactgctgagctgagccgc

tcacccgaatcgtataccaacaagcgcctgccgcacctcaccgtctactttaagctcgcc

atgaggaatgaagaactgcccagcgtaaaagagagaattccgtatgtgatagttgcgcag

accgaggccgcggaacgcgaagcgggtgtagtaaactcaatgcgcggtaccgcccaaaac

cccgtggtaaccaagaccgcacgcccccaacctaaacgcaaactgctggtttccgacctc

gccgaagacccgacctatgtttccgagaatgacgtaccgctaaacacagactactatttc

tcccacctgttgggtaccataagcgtgacctttaaggctctattcggaaatgatgtgaga

acaacagaaaatcttttaaagcggtttattccggaaaccccccacaagacccccacgaaa

acccaggcactgcttgagcgcgccggctttgaaaagctgacgccctttacaccggaggaa

gaaagtcgtcgaatactgcatacagttttttgtactctagaagcagctccccatcaaagc

tga

>KU206430.1

atggcggcgcgcgaacaggccaactccgttcgcaggagcgggttttttaatccgtttatt

ggcaagaggccatttttcaggccgggaagcgggcaaaccgcggagacagagcgcccaagg

cccccccaacactcgtactgcacagaggtgggtagctttaagtttatagctccaagatgc

ctcgatgaagaagcccccgccgaccagcgacgcggtgtacacgtgggcaccttggagcgc

acacccaaagtgtactgcgatggctcagagtacgacgtgctgaactttgcctccggaggt

tgctggcctcgcaggattcgggtttggaacggccaggattttcggggcgatggattcaac

cccagatttgagagatttcacgtgtacgacatagtagagacttcggagagcgcctcacac

gatgacccatccaggtttgctgagctatcccgcccaagcgggtctgtggtaacactgctg

ggaatgagcgagtgtggaaagcgagtcgcggttcacgtctatggtgtgcgccattatttt

tacatggcaaaggcggaggtggatagcgcttgtggaataaccaccgaggcagaactcgtg

cgcgcaatggtggactgcgcgcacagctcggctttgagcgcggccctgggaaatggcaac

ggcggcaaacagagcggcggcagcggcgggggatggtggggcggaaagcacgtgtctgcg

gactgcttcaaagtggagaccgtgtgccacacgacgctgtactactttggatctaagcca

gctctctactatagagtatctgcctccagcagccgcctgggagggttcatctgcgacaac

tttcacccagagattacaaaattcgaggggagcgtggacgtgaccacgcggctgttgttg

gacaatgaaaattttaccagtttcgggtggtaccgcctgcgacccggcacccacggagag

cgtgttcaacttcgccccgttgagcgacacgtcacctcaagcgacgtggagattaactgt

actcccgataacctggagccgataccagacgaggctgcctggcccgactataagctcatg

tgctttgatatagagtgtaaagctggaacgggtaacgaaatggcgttcccagtggcaact

aaccaagaggacctggtcatccagatctcctgtctgctgtactcgcttgctactcagaac

cacgaacacaccctgctgttttccctcgggtcatgcgatatctctgaggaatactcgttt

gcatgcgtccagcgcggcgagcccagaccgacggttttggagtttgacagcgagtacgag

ctgctggttgccttcctgacctttctcaagcagtactctcccgagttcgccaccggctac

aacatcgttaattttgactgggcgtacatagttaacaaggtaacgtcggtgtataacatc

aagctggacgggtacggcaagttcaacaaaggggggctgtttaaggtgtgggacatcgcc

acgaaccattttcagaagaagagcaaggtgaaaatcaatggcctgatatctctagacatg

tattctgtggcgacggaaaagctaaagctacccagctacaaactcgacgcggtcgtggga

gacgtcctcggcgagcataagatagaccttccctataaagaaataccctcctattacgcg

ggagggcctgaccggaggggcgtaataggagagtattgtatccaggactctaggctggtg

ggcaagctgttttttaagtacctcccccatctggaactatcggcggtggccaaactcgcc

cgtatcaccctaacgcgggtaatttttgacggtcagcaaattcgcgtgtacacgtgcttg

ctgaaactcgcccgcgagagaaatttcattttgccagacaacagacgccggtttgacagt

caggcagacgccgcgtcagagacttcggagttggctatggatagccaaagccacgccttc

gacagtacagacgaacccgacggtgtggacggtaccccggacgccgcaggatctggcgct

acttctgaaaacggaggcgggaagcccggcgtcgggagggccgtgggctaccagggagca

aaggttctagaccccgtatccggctttcatgtggaccccgtggttgtgtttgacttcgct

agcttatacccaagcattatccaggcccataacctctgtttcaccaccctggcgctcgat

gaagtggatctggccgggcttcaaccatccgtcaactactcgacgttcgaggtgggtgac

caaaagttattttttgtccacgcccatattcgcgaaagcctgcttggcatcttgctgcgc

gactggctggccatgcgaaaggcggtgagggcgcgaatccccaccagcacccccgaggag

gcagttttactagataagcagcagtctgcgattaaggtgatatgcaactcggtttacgga

ttcacgggggtggcaaacggcctgttgccgtgtctgaggatagcggctaccgttaccacg

ataggacgcgacatgctcctcaagacccgagattacgttcactctcgttgggcgacgcgc

gagctgctggaggacaattttccaggggctataggtttccgaaaccacaagccttactcc

gtcagggttatctacggagacaccgactccgtgtttatcaagtttgtgggcctgacgtac

gagggggtatcggagctgggggatgctatgtcgcgtcagatttcagcggacctctttaga

gcgcccatcaaactggagtgtgagaagacctttcagcgactgctgctgatcaccaagaag

aagtacataggtgtcataaacggggggaagatgctcatgaagggggtcgacctggtccgc

aaaaataactgctctttcataaacttgtacgcgcgacatctggtagatcttttgttgtac

gacgaggatgtggccacggcggcagcagaggtgacagacgtgcctcccgcagaatgggtg

gggcgcccgctaccgagcggctttgacaagtttgggcgagtgctggtagaggcgtacaac

cgtatcactgcccccaacttggacgtgcgcgagttcgttatgactgctgagctgagccgc

tcacccgaatcgtataccaacaagcgcctgccgcacctcaccgtctactttaagctcgcc

atgaggaatgaagaactgcccagcgtaaaagagagaattccgtatgtgatagttgcgcag

accgaggccgcggaacgcgaagcgggtgtagtaaactcaatgcgcggtaccgcccaaaac

cccgtggtaaccaagaccgcacgcccccaacctaaacgcaaactgctggtttccgacctc

gccgaagacccgacctatgtttccgagaatgacgtaccgctaaacacagactactatttc

tcccacctgttgggtaccataagcgtgacctttaaggctctattcggaaatgatgtgaga

acaacagaaaatcttttaaagcggtttattccggaaaccccccacaagacccccacgaaa

acccaggcactgcttgagcgcgccggctttgaaaagctgacgccctttacaccggaggaa

gaaagtcgtcgaatactgcatacagttttttgtactctagaagcagctccccatcaaagc

tga

>KU206431.1

atggcggcgcgcgaacaggccaactccgttcgcaggagcgggttttttaatccgtttatt

ggcaagaggccatttttcaggccgggaagcgggcaaaccgcggagacagagcgcccaagg

cccccccaacactcgtactgcacagaggtgggtagctttaagtttatagctccaagatgc

ctcgatgaagaagcccccgccgaccagcgacgcggtgtacacgtgggcaccttggagcgc

ccacccaaagtgtactgcgatggctcagagtacgacgtgctgaactttgcctccggaggt

tgctggcctcgcaggattcgggtttggaacggccaggattttcggggcgatggattcaac

cccagatttgagagatttcacgtgtacgacatagtagagacttcggagagcgcctcacac

gatgacccatccaggtttgctgagctatcccgcccaagcgggtctgtggtaacactgctg

ggaatgagcgagtgtggaaagcgagtcgcggttcacgtctatggtgtgcgccattatttt

tacatggcaaaggcggaggtggatagcgcttgtggaataaccaccgaggcagaactcgtg

cgcgcaatggtggactgcgcgcacagctcggctttgagcgcggccctgggaaatggcaac

ggcggcaaacagagcggcggcagcggcgggggatggtggggcggaaagcacgtgtctgcg

gactgcttcaaagtggagaccgtgtgccacacgacgctgtactactttggatctaagcca

gctctctactatagagtatctgcctccagcagccgcctgggagggttcatctgcgacaac

tttcacccggagattacaaaattcgaggggagcgtggacgtgaccacgcggctgttgttg

gacaatgaaaattttaccagtttcgggtggtaccgcctgcgacccggcacccacggagag

cgtgttcaacttcgccccgttgagcgacacgtcacctcaagcgacgtggagattaactgt

actcccgataacctggagccgataccagacgaggctgcctggcccgactataagctcatg

tgctttgatatagagtgtaaagctggaacgggtaacgaaatggcgttcccagtggcaact

aaccaagaggacctggtcatccagatctcctgtctgctgtactcgcttgctactcagaac

cacgaacacaccctgctgttttccctcgggtcatgcgatatctctgaggaatactcgttt

gcatgcgtccagcgcggcgagcccagaccgacggttttggagtttgacagcgagtacgag

ctgctggttgccttcctgacctttctcaagcagtactctcccgagttcgccaccggctac

aacatcgttaattttgactgggcgtacatagttaacaaggtaacgtcggtgtataacatc

aagctggacgggtacggcaagttcaacaaaggggggctgtttaaggtgtgggacatcgcc

acgaaccattttcagaagaagagcaaggtgaaaatcaatggcctgatatctctagacatg

tattctgtggcgacggaaaagctaaagctacccagctacaaactcgacgcggtcgtggga

gacgtcctcggcgagcataagatagaccttccctataaagaaataccctcctattacgcg

ggagggcctgaccggaggggcgtaataggagagtattgtatccaggactctaggctggtg

ggcaagctgttttttaagtacctcccccatctggaactatcggcggtggccaaactcgcc

cgtatcaccctaacgcgggtaatttttgacggtcagcaaattcgcgtgtacacgtgcttg

ctgaaactcgcccgcgagagaaatttcattttgccagacaacagacgccggtttgacagt

caggcagacgccgcgtcagagacttcggagttggctatggatagccaaagccacgccttc

gacagtacagacgaacccgacggtgtggacggtaccccggacgccgcaggatctggcgct

acttctgaaaacggaggcgggaagcccggcgtcgggagggccgtgggctaccagggagca

aaggttctagaccccgtatccggctttcatgtggaccccgtggttgtgtttgacttcgct

agcttatacccaagcattatccaggcccataacctctgtttcaccaccctggcgctcgat

gaagtggatctggccgggcttcaaccatccgtcaactactcgacgttcgaggtgggtgac

caaaagttattttttgtccacgcccatattcgcgaaagcctgcttggcatcttgctgcgc

gactggctggccatgcgaaaggcggtgagggcgcgaatccccaccagcacccccgaggag

gcagttttactagataagcagcagtctgcgattaaggtgatatgcaactcggtttacgga

ttcacgggggtggcaaacggcctgttgccgtgtctgaggatagcggctaccgttaccacg

ataggacgcgacatgctcctcaagacccgagattacgttcactctcgttgggcgacgcgc

gagctgctggaggacaattttccaggggctataggtttccgaaaccacaagccttactcc

gtcagggttatctacggagacaccgactccgtgtttatcaagtttgtgggcctgacgtac

gagggggtatcggagctgggggatgctatgtcgcgtcagatttcagcggacctctttaga

gcgcccatcaaactggagtgtgagaagacctttcagcgactgctgctgatcaccaagaag

aagtacataggtgtcataaacggggggaagatgctcatgaagggggtcgacctggtccgc

aaaaataactgctctttcataaacttgtacgcgcgacatctggtagatcttttgttgtac

gacgaggatgtggccacggcggcagcagaggtgacagacgtgcctcccgcagaatgggtg

gggcgcccgctaccgagcggctttgacaagtttgggcgagtgctggtagaggcgtacaac

cgtatcactgcccccaacttggacgtgcgcgagttcgttatgactgctgagctgagccgc

tcacccgaatcgtataccaacaagcgcctgccgcacctcaccgtctactttaagctcgcc

atgaggaatgaagaactgcccagcgtaaaagagagaattccgtatgtgatagttgcgcag

accgaggccgcggaacgcgaagcgggtgtagtaaactcaatgcgcggtaccgcccaaaac

cccgtggtaaccaagaccgcacgcccccaacctaaacgcaaactgctggtttccgacctc

gccgaagacccgacctatgtttccgagaatgacgtaccgctaaacacagactactatttc

tcccacctgttgggtaccataagcgtgacctttaaggctctattcggaaatgatgtgaga

acaacagaaaatcttttaaagcggtttattccggaaaccccccacaagacccccacgaaa

acccaggcactgcttgagcgcgccggctttgaaaagctgacgccctttacaccggaggaa

gaaagtcgtcgaatactgcatacagttttttgtactctagaagcagctccccatcaaagc

tga

>KU206432.1

atggcggcgcgcgaacaggccaactccgttcgcaggagcgggttttttaatccgtttatt

ggcaagaggccatttttcaggccgggaagcgggcaaaccgcggagacagagcgcccaagg

cccccccaacactcgtactgcacagaggtgggtagctttaagtttatagctccaagatgc

ctcgatgaagaagcccccgccgaccagcgacgcggtgtacacgtgggcaccttggagcgc

ccacccaaagtgtactgcgatggctcagagtacgacgtgctgaactttgcctccggaggt

tgctggcctcgcaggattcgggtttggaacggccaggattttcggggcgatggattcaac

cccagatttgagagatttcacgtgtacgacatagtagagacttcggagagcgcctcacac

gatgacccatccaggtttgctgagctatcccgcccaagcgggtctgtggtaacactgctg

ggaatgagcgagtgtggaaagcgagtcgcggttcacgtctatggtgtgcgccattatttt

tacatggcaaaggcggaggtggatagcgcttgtggaataaccaccgaggcagaactcgtg

cgcgcaatggtggactgcgcgcacagctcggctttgagcgcggccctgggaaatggcaac

ggcggcaaacagagcggcggcagcggcgggggatggtggggcggaaagcacgtgtctgcg

gactgcttcaaagtggagaccgtgtgccacacgacgctgtactactttggatctaagcca

gctctctactatagagtatctgcctccagcagccgcctgggagggttcatctgcgacaac

tttcacccggagattacaaaattcgaggggagcgtggacgtgaccacgcggctgttgttg

gacaatgaaaattttaccagtttcgggtggtaccgcctgcgacccggcacccacggagag

cgtgttcaacttcgccccgttgagcgacacgtcacctcaagcgacgtggagattaactgt

actcccgataacctggagccgataccagacgaggctgcctggcccgactataagctcatg

tgctttgatatagagtgtaaagctggaacgggtaacgaaatggcgttcccagtggcaact

aaccaagaggacctggtcatccagatctcctgtctgctgtactcgcttgctactcagaac

cacgaacacaccctgctgttttccctcgggtcatgcgatatctctgaggaatactcgttt

gcatgcgtccagcgcggcgagcccagaccgacggttttggagtttgacagcgagtacgag

ctgctggttgccttcctgacctttctcaagcagtactctcccgagttcgccaccggctac

aacatcgttaattttgactgggcgtacatagttaacaaggtaacgtcggtgtataacatc

aagctggacgggtacggcaagttcaacaaaggggggctgtttaaggtgtgggacatcgcc

acgaaccattttcagaagaagagcaaggtgaaaatcaatggcctgatatctctagacatg

tattctgtggcgacggaaaagctaaagctacccagctacaaactcgacgcggtcgtggga

gacgtcctcggcgagcataagatagaccttccctataaagaaataccctcctattacgcg

ggagggcctgaccggaggggcgtaataggagagtattgtatccaggactctaggctggtg

ggcaagctgttttttaagtacctcccccatctggaactatcggcggtggccaaactcgcc

cgtatcaccctaacgcgggtaatttttgacggtcagcaaattcgcgtgtacacgtgcttg

ctgaaactcgcccgcgagagaaatttcattttgccagacaacagacgccggtttgacagt

caggcagacgccgcgtcagagacttcggagttggctatggatagccaaagccacgccttc

gacagtacagacgaacccgacggtgtggacggtaccccggacgccgcaggatctggcgct

acttctgaaaacggaggcgggaagcccggcgtcgggagggccgtgggctaccagggagca

aaggttctagaccccgtatccggctttcatgtggaccccgtggttgtgtttgacttcgct

agcttatacccaagcattatccaggcccataacctctgtttcaccaccctggcgctcgat

gaagtggatctggccgggcttcaaccatccgtcaactactcgacgttcgaggtgggtgac

caaaagttattttttgtccacgcccatattcgcgaaagcctgcttggcatcttgctgcgc

gactggctggccatgcgaaaggcggtgagggcgcgaatccccaccagcacccccgaggag

gcagttttactagataagcagcagtctgcgattaaggtgatatgcaactcggtttacgga

ttcacgggggtggcaaacggcctgttgccgtgtctgaggatagcggctaccgttaccacg

ataggacgcgacatgctcctcaagacccgagattacgttcactctcgttgggcgacgcgc

gagctgctggaggacaattttccaggggctataggtttccgaaaccacaagccttactcc

gtcagggttatctacggagacaccgactccgtgtttatcaagtttgtgggcctgacgtac

gagggggtatcggagctgggggatgctatgtcgcgtcagatttcagcggacctctttaga

gcgcccatcaaactggagtgtgagaagacctttcagcgactgctgctgatcaccaagaag

aagtacataggtgtcataaacggggggaagatgctcatgaagggggtcgacctggtccgc

aaaaataactgctctttcataaacttgtacgcgcgacatctggtagatcttttgttgtac

gacgaggatgtggccacggcggcagcagaggtgacagacgtgcctcccgcagaatgggtg

gggcgcccgctaccgagcggctttgacaagtttgggcgagtgctggtagaggcgtacaac

cgtatcactgcccccaacttggacgtgcgcgagttcgttatgactgctgagctgagccgc

tcacccgaatcgtataccaacaagcgcctgccgcacctcaccgtctactttaagctcgcc

atgaggaatgaagaactgcccagcgtaaaagagagaattccgtatgtgatagttgcgcag

accgaggccgcggaacgcgaagcgggtgtagtaaactcaatgcgcggtaccgcccaaaac

cccgtggtaaccaagaccgcacgcccccaacctaaacgcaaactgctggtttccgacctc

gccgaagacccgacctatgtttccgagaatgacgtaccgctaaacacagactactatttc

tcccacctgttgggtaccataagcgtgacctttaaggctctattcggaaatgatgtgaga

acaacagaaaatcttttaaagcggtttattccggaaaccccccacaagacccccacgaaa

acccaggcactgcttgagcgcgccggctttgaaaagctgacgccctttacaccggaggaa

gaaagtcgtcgaatactgcatacagttttttgtactctagaagcagctccccatcaaagc

tga

>KU206433.1

atggcggcgcgcgaacaggccaactccgttcgcaggagcgggttttttaatccgtttatt

ggcaagaggccatttttcaggccgggaagcgggcaaaccgcggagacagagcgcccaagg

cccccccaacactcgtactgcacagaggtgggtagctttaagtttatagctccaagatgc

ctcgatgaagaagcccccgccgaccagcgacgcggtgtacacgtgggcaccttggagcgc

ccacccaaagtgtactgcgatggctcagagtacgacgtgctgaactttgcctccggaggt

tgctggcctcgcaggattcgggtttggaacggccaggattttcggggcgatggattcaac

cccagatttgagagatttcacgtgtacgacatagtagagacttcggagagcgcctcacac

gatgacccatccaggtttgctgagctatcccgcccaagcgggtctgtggtaacactgctg

ggaatgagcgagtgtggaaagcgagtcgcggttcacgtctatggtgtgcgccattatttt

tacatggcaaaggcggaggtggatagcgcttgtggaataaccaccgaggcagaactcgtg

cgcgcaatggtggactgcgcgcacagctcggctttgagcgcggccctgggaaatggcaac

ggcggcaaacagagcggcggcagcggcgggggatggtggggcggaaagcacgtgtctgcg

gactgcttcaaagtggagaccgtgtgccacacgacgctgtactactttggatctaagcca

gctctctactatagagtatctgcctccagcagccgcctgggagggttcatctgcgacaac

tttcacccggagattacaaaattcgaggggagcgtggacgtgaccacgcggctgttgttg

gacaatgaaaattttaccagtttcgggtggtaccgcctgcgacccggcacccacggagag

cgtgttcaacttcgccccgttgagcgacacgtcacctcaagcgacgtggagattaactgt

actcccgataacctggagccgataccagacgaggctgcctggcccgactataagctcatg

tgctttgatatagagtgtaaagctggaacgggtaacgaaatggcgttcccagtggcaact

aaccaagaggacctggtcatccagatctcctgtctgctgtactcgcttgctactcagaac

cacgaacacaccctgctgttttccctcgggtcatgcgatatctctgaggaatactcgttt

gcatgcgtccagcgcggcgagcccagaccgacggttttggagtttgacagcgagtacgag

ctgctggttgccttcctgacctttctcaagcagtactctcccgagttcgccaccggctac

aacatcgttaattttgactgggcgtacatagttaacaaggtaacgtcggtgtataacatc

aagctggacgggtacggcaagttcaacaaaggggggctgtttaaggtgtgggacatcgcc

acgaaccattttcagaagaagagcaaggtgaaaatcaatggcctgatatctctagacatg

tattctgtggcgacggaaaagctaaagctacccagctacaaactcgacgcggtcgtggga

gacgtcctcggcgagcataagatagaccttccctataaagaaataccctcctattacgcg

ggagggcctgaccggaggggcgtaataggagagtattgtatccaggactctaggctggtg

ggcaagctgttttttaagtacctcccccatctggaactatcggcggtggccaaactcgcc

cgtatcaccctaacgcgggtaatttttgacggtcagcaaattcgcgtgtacacgtgcttg

ctgaaactcgcccgcgagagaaatttcattttgccagacaacagacgccggtttgacagt

caggcagacgccgcgtcagagacttcggagttggctatggatagccaaagccacgccttc

gacagtacagacgaacccgacggtgtggacggtaccccggacgccgcaggatctggcgct

acttctgaaaacggaggcgggaagcccggcgtcgggagggccgtgggctaccagggagca

aaggttctagaccccgtatccggctttcatgtggaccccgtggttgtgtttgacttcgct

agcttatacccaagcattatccaggcccataacctctgtttcaccaccctggcgctcgat

gaagtggatctggccgggcttcaaccatccgtcaactactcgacgttcgaggtgggtgac

caaaagttattttttgtccacgcccatattcgcgaaagcctgcttggcatcttgctgcgc

gactggctggccatgcgaaaggcggtgagggcgcgaatccccaccagcacccccgaggag

gcagttttactagataagcagcagtctgcgattaaggtgatatgcaactcggtttacgga

ttcacgggggtggcaaacggcctgttgccgtgtctgaggatagcggctaccgttaccacg

ataggacgcgacatgctcctcaagacccgagattacgttcactctcgttgggcgacgcgc

gagctgctggaggacaattttccaggggctataggtttccgaaaccacaagccttactcc

gtcagggttatctacggagacaccgactccgtgtttatcaagtttgtgggcctgacgtac

gagggggtatcggagctgggggatgctatgtcgcgtcagatttcagcggacctctttaga

gcgcccatcaaactggagtgtgagaagacctttcagcgactgctgctgatcaccaagaag

aagtacataggtgtcataaacggggggaagatgctcatgaagggggtcgacctggtccgc

aaaaataactgctctttcataaacttgtacgcgcgacatctggtagatcttttgttgtac

gacgaggatgtggccacggcggcagcagaggtgacagacgtgcctcccgcagaatgggtg

gggcgcccgctaccgagcggctttgacaagtttgggcgagtgctggtagaggcgtacaac

cgtatcactgcccccaacttggacgtgcgcgagttcgttatgactgctgagctgagccgc

tcacccgaatcgtataccaacaagcgcctgccgcacctcaccgtctactttaagctcgcc

atgaggaatgaagaactgcccagcgtaaaagagagaattccgtatgtgatagttgcgcag

accgaggccgcggaacgcgaagcgggtgtagtaaactcaatgcgcggtaccgcccaaaac

cccgtggtaaccaagaccgcacgcccccaacctaaacgcaaactgctggtttccgacctc

gccgaagacccgacctatgtttccgagaatgacgtaccgctaaacacagactactatttc

tcccacctgttgggtaccataagcgtgacctttaaggctctattcggaaatgatgtgaga

acaacagaaaatcttttaaagcggtttattccggaaaccccccacaagacccccacgaaa

acccaggcactgcttgagcgcgccggctttgaaaagctgacgccctttacaccggaggaa

gaaagtcgtcgaatactgcatacagttttttgtactctagaagcagctccccatcaaagc

tga

>KU206434.1

atggcggcgcgcgaacaggccaactccgttcgcaggagcgggttttttaatccgtttatt

ggcaagaggccatttttcaggccgggaagcgggcaaaccgcggagacagagcgcccaagg

cccccccaacactcgtactgcacagaggtgggtagctttaagtttatagctccaagatgc

ctcgatgaagaagcccccgccgaccagcgacgcggtgtacacgtgggcaccttggagcgc

ccacccaaagtgtactgcgatggctcagagtacgacgtgctgaactttgcctccggaggt

tgctggcctcgcaggattcgggtttggaacggccaggattttcggggcgatggattcaac

cccagatttgagagatttcacgtgtacgacatagtagagacttcggagagcgcctcacac

gatgacccatccaggtttgctgagctatcccgcccaagcgggtctgtggtaacactgctg

ggaatgagcgagtgtggaaagcgagtcgcggttcacgtctatggtgtgcgccattatttt

tacatggcaaaggcggaggtggatagcgcttgtggaataaccaccgaggcagaactcgtg

cgcgcaatggtggactgcgcgcacagctcggctttgagcgcggccctgggaaatggcaac

ggcggcaaacagagcggcggcagcggcgggggatggtggggcggaaagcacgtgtctgcg

gactgcttcaaagtggagaccgtgtgccacacgacgctgtactactttggatctaagcca

gctctctactatagagtatctgcctccagcagccgcctgggagggttcatctgcgacaac

tttcacccggagattacaaaattcgaggggagcgtggacgtgaccacgcggctgttgttg

gacaatgaaaattttaccagtttcgggtggtaccgcctgcgacccggcacccacggagag

cgtgttcaacttcgccccgttgagcgacacgtcacctcaagcgacgtggagattaactgt

actcccgataacctggagccgataccagacgaggctgcctggcccgactataagctcatg

tgctttgatatagagtgtaaagctggaacgggtaacgaaatggcgttcccagtggcaact

aaccaagaggacctggtcatccagatctcctgtctgctgtactcgcttgctactcagaac

cacgaacacaccctgctgttttccctcgggtcatgcgatatctctgaggaatactcgttt

gcatgcgtccagcgcggcgagcccagaccgacggttttggagtttgacagcgagtacgag

ctgctggttgccttcctgacctttctcaagcagtactctcccgagttcgccaccggctac

aacatcgttaattttgactgggcgtacatagttaacaaggtaacgtcggtgtataacatc

aagctggacgggtacggcaagttcaacaaaggggggctgtttaaggtgtgggacatcgcc

acgaaccattttcagaagaagagcaaggtgaaaatcaatggcctgatatctctagacatg

tattctgtggcgacggaaaagctaaagctacccagctacaaactcgacgcggtcgtggga

gacgtcctcggcgagcataagatagaccttccctataaagaaataccctcctattacgcg

ggagggcctgaccggaggggcgtaataggagagtattgtatccaggactctaggctggtg

ggcaagctgttttttaagtacctcccccatctggaactatcggcggtggccaaactcgcc

cgtatcaccctaacgcgggtaatttttgacggtcagcaaattcgcgtgtacacgtgcttg

ctgaaactcgcccgcgagagaaatttcattttgccagacaacagacgccggtttgacagt

caggcagacgccgcgtcagagacttcggagttggctatggatagccaaagccacgccttc

gacagtacagacgaacccgacggtgtggacggtaccccggacgccgcaggatctggcgct

acttctgaaaacggaggcgggaagcccggcgtcgggagggccgtgggctaccagggagca

aaggttctagaccccgtatccggctttcatgtggaccccgtggttgtgtttgacttcgct

agcttatacccaagcattatccaggcccataacctctgtttcaccaccctggcgctcgat

gaagtggatctggccgggcttcaaccatccgtcaactactcgacgttcgaggtgggtgac

caaaagttattttttgtccacgcccatattcgcgaaagcctgcttggcatcttgctgcgc

gactggctggccatgcgaaaggcggtgagggcgcgaatccccaccagcacccccgaggag

gcagttttactagataagcagcagtctgcgattaaggtgatatgcaactcggtttacgga

ttcacgggggtggcaaacggcctgttgccgtgtctgaggatagcggctaccgttaccacg

ataggacgcgacatgctcctcaagacccgagattacgttcactctcgttgggcgacgcgc

gagctgctggaggacaattttccaggggctataggtttccgaaaccacaagccttactcc

gtcagggttatctacggagacaccgactccgtgtttatcaagtttgtgggcctgacgtac

gagggggtatcggagctgggggatgctatgtcgcgtcagatttcagcggacctctttaga

gcgcccatcaaactggagtgtgagaagacctttcagcgactgctgctgatcaccaagaag

aagtacataggtgtcataaacggggggaagatgctcatgaagggggtcgacctggtccgc

aaaaataactgctctttcataaacttgtacgcgcgacatctggtagatcttttgttgtac

gacgaggatgtggccacggcggcagcagaggtgacagacgtgcctcccgcagaatgggtg

gggcgcccgctaccgagcggctttgacaagtttgggcgagtgctggtagaggcgtacaac

cgtatcactgcccccaacttggacgtgcgcgagttcgttatgactgctgagctgagccgc

tcacccgaatcgtataccaacaagcgcctgccgcacctcaccgtctactttaagctcgcc

atgaggaatgaagaactgcccagcgtaaaagagagaattccgtatgtgatagttgcgcag

accgaggccgcggaacgcgaagcgggtgtagtaaactcaatgcgcggtaccgcccaaaac

cccgtggtaaccaagaccgcacgcccccaacctaaacgcaaactgctggtttccgacctc

gccgaagacccgacctatgtttccgagaatgacgtaccgctaaacacagactactatttc

tcccacctgttgggtaccataagcgtgacctttaaggctctattcggaaatgatgtgaga

acaacagaaaatcttttaaagcggtttattccggaaaccccccacaagacccccacgaaa

acccaggcactgcttgagcgcgccggctttgaaaagctgacgccctttacaccggaggaa

gaaagtcgtcgaatactgcatacagttttttgtactctagaagcagctccccatcaaagc

tga

>KU206435.1

atggcggcgcgcgaacaggccaactccgttcgcaggagcgggttttttaatccgtttatt

ggcaagaggccatttttcaggccgggaagcgggcaaaccgcggagacagagcgcccaagg

cccccccaacactcgtactgcacagaggtgggtagctttaagtttatagctccaagatgc

ctcgatgaagaagcccccgccgaccagcgacgcggtgtacacgtgggcaccttggagcgc

ccacccaaagtgtactgcgatggctcagagtacgacgtgctgaactttgcctccggaggt

tgctggcctcgcaggattcgggtttggaacggccaggattttcggggcgatggattcaac

cccagatttgagagatttcacgtgtacgacatagtagagacttcggagagcgcctcacac

gatgacccatccaggtttgctgagctatcccgcccaagcgggtctgtggtaacactgctg

ggaatgagcgagtgtggaaagcgagtcgcggttcacgtctatggtgtgcgccattatttt

tacatggcaaaggcggaggtggatagcgcttgtggaataaccaccgaggcagaactcgtg

cgcgcaatggtggactgcgcgcacagctcggctttgagcgcggccctgggaaatggcaac

ggcggcaaacagagcggcggcagcggcgggggatggtggggcggaaagcacgtgtctgcg

gactgcttcaaagtggagaccgtgtgccacacgacgctgtactactttggatctaagcca

gctctctactatagagtatctgcctccagcagccgcctgggagggttcatctgcgacaac

tttcacccggagattacaaaattcgaggggagcgtggacgtgaccacgcggctgttgttg

gacaatgaaaattttaccagtttcgggtggtaccgcctgcgacccggcacccacggagag

cgtgttcaacttcgccccgttgagcgacacgtcacctcaagcgacgtggagattaactgt

actcccgataacctggagccgataccagacgaggctgcctggcccgactataagctcatg

tgctttgatatagagtgtaaagctggaacgggtaacgaaatggcgttcccagtggcaact

aaccaagaggacctggtcatccagatctcctgtctgctgtactcgcttgctactcagaac

cacgaacacaccctgctgttttccctcgggtcatgcgatatctctgaggaatactcgttt

gcatgcgtccagcgcggcgagcccagaccgacggttttggagtttgacagcgagtacgag

ctgctggttgccttcctgacctttctcaagcagtactctcccgagttcgccaccggctac

aacatcgttaattttgactgggcgtacatagttaacaaggtaacgtcggtgtataacatc

aagctggacgggtacggcaagttcaacaaaggggggctgtttaaggtgtgggacatcgcc

acgaaccattttcagaagaagagcaaggtgaaaatcaatggcctgatatctctagacatg

tattctgtggcgacggaaaagctaaagctacccagctacaaactcgacgcggtcgtggga

gacgtcctcggcgagcataagatagaccttccctataaagaaataccctcctattacgcg

ggagggcctgaccggaggggcgtaataggagagtattgtatccaggactctaggctggtg

ggcaagctgttttttaagtacctcccccatctggaactatcggcggtggccaaactcgcc

cgtatcaccctaacgcgggtaatttttgacggtcagcaaattcgcgtgtacacgtgcttg

ctgaaactcgcccgcgagagaaatttcattttgccagacaacagacgccggtttgacagt

caggcagacgccgcgtcagagacttcggagttggctatggatagccaaagccacgccttc

gacagtacagacgaacccgacggtgtggacggtaccccggacgccgcaggatctggcgct

acttctgaaaacggaggcgggaagcccggcgtcgggagggccgtgggctaccagggagca

aaggttctagaccccgtatccggctttcatgtggaccccgtggttgtgtttgacttcgct

agcttatacccaagcattatccaggcccataacctctgtttcaccaccctggcgctcgat

gaagtggatctggccgggcttcaaccatccgtcaactactcgacgttcgaggtgggtgac

caaaagttattttttgtccacgcccatattcgcgaaagcctgcttggcatcttgctgcgc

gactggctggccatgcgaaaggcggtgagggcgcgaatccccaccagcacccccgaggag

gcagttttactagataagcagcagtctgcgattaaggtgatatgcaactcggtttacgga

ttcacgggggtggcaaacggcctgttgccgtgtctgaggatagcggctaccgttaccacg

ataggacgcgacatgctcctcaagacccgagattacgttcactctcgttgggcgacgcgc

gagctgctggaggacaattttccaggggctataggtttccgaaaccacaagccttactcc

gtcagggttatctacggagacaccgactccgtgtttatcaagtttgtgggcctgacgtac

gagggggtatcggagctgggggatgctatgtcgcgtcagatttcagcggacctctttaga

gcgcccatcaaactggagtgtgagaagacctttcagcgactgctgctgatcaccaagaag

aagtacataggtgtcataaacggggggaagatgctcatgaagggggtcgacctggtccgc

aaaaataactgctctttcataaacttgtacgcgcgacatctggtagatcttttgttgtac

gacgaggatgtggccacggcggcagcagaggtgacagacgtgcctcccgcagaatgggtg

gggcgcccgctaccgagcggctttgacaagtttgggcgagtgctggtagaggcgtacaac

cgtatcactgcccccaacttggacgtgcgcgagttcgttatgactgctgagctgagccgc

tcacccgaatcgtataccaacaagcgcctgccgcacctcaccgtctactttaagctcgcc

atgaggaatgaagaactgcccagcgtaaaagagagaattccgtatgtgatagttgcgcag

accgaggccgcggaacgcgaagcgggtgtagtaaactcaatgcgcggtaccgcccaaaac

cccgtggtaaccaagaccgcacgcccccaacctaaacgcaaactgctggtttccgacctc

gccgaagacccgacctatgtttccgagaatgacgtaccgctaaacacagactactatttc

tcccacctgttgggtaccataagcgtgacctttaaggctctattcggaaatgatgtgaga

acaacagaaaatcttttaaagcggtttattccggaaaccccccacaagacccccacgaaa

acccaggcactgcttgagcgcgccggctttgaaaagctgacgccctttacaccggaggaa

gaaagtcgtcgaatactgcatacagttttttgtactctagaagcagctccccatcaaagc

tga

>KU206436.1

atggcggcgcgcgaacaggccaactccgttcgcaggagcgggttttttaatccgtttatt

ggcaagaggccatttttcaggccgggaagcgggcaaaccgcggagacagagcgcccaagg

cccccccaacactcgtactgcacagaggtgggtagctttaagtttatagctccaagatgc

ctcgatgaagaagcccccgccgaccagcgacgcggtgtacacgtgggcaccttggagcgc

ccacccaaagtgtactgcgatggctcagagtacgacgtgctgaactttgcctccggaggt

tgctggcctcgcaggattcgggtttggaacggccaggattttcggggcgatggattcaac

cccagatttgagagatttcacgtgtacgacatagtagagacttcggagagcgcctcacac

gatgacccatccaggtttgctgagctatcccgcccaagcgggtctgtggtaacactgctg

ggaatgagcgagtgtggaaagcgagtcgcggttcacgtctatggtgtgcgccattatttt

tacatggcaaaggcggaggtggatagcgcttgtggaataaccaccgaggcagaactcgtg

cgcgcaatggtggactgcgcgcacagctcggctttgagcgcggccctgggaaatggcaac

ggcggcaaacagagcggcggcagcggcgggggatggtggggcggaaagcacgtgtctgcg

gactgcttcaaagtggagaccgtgtgccacacgacgctgtactactttggatctaagcca

gctctctactatagagtatctgcctccagcagccgcctgggagggttcatctgcgacaac

tttcacccggagattacaaaattcgaggggagcgtggacgtgaccacgcggctgttgttg

gacaatgaaaattttaccagtttcgggtggtaccgcctgcgacccggcacccacggagag

cgtgttcaacttcgccccgttgagcgacacgtcacctcaagcgacgtggagattaactgt

actcccgataacctggagccgataccagacgaggctgcctggcccgactataagctcatg

tgctttgatatagagtgtaaagctggaacgggtaacgaaatggcgttcccagtggcaact

aaccaagaggacctggtcatccagatctcctgtctgctgtactcgcttgctactcagaac

cacgaacacaccctgctgttttccctcgggtcatgcgatatctctgaggaatactcgttt

gcatgcgtccagcgcggcgagcccagaccgacggttttggagtttgacagcgagtacgag

ctgctggttgccttcctgacctttctcaagcagtactctcccgagttcgccaccggctac

aacatcgttaattttgactgggcgtacatagttaacaaggtaacgtcggtgtataacatc

aagctggacgggtacggcaagttcaacaaaggggggctgtttaaggtgtgggacatcgcc

acgaaccattttcagaagaagagcaaggtgaaaatcaatggcctgatatctctagacatg

tattctgtggcgacggaaaagctaaagctacccagctacaaactcgacgcggtcgtggga

gacgtcctcggcgagcataagatagaccttccctataaagaaataccctcctattacgcg

ggagggcctgaccggaggggcgtaataggagagtattgtatccaggactctaggctggtg

ggcaagctgttttttaagtacctcccccatctggaactatcggcggtggccaaactcgcc

cgtatcaccctaacgcgggtaatttttgacggtcagcaaattcgcgtgtacacgtgcttg

ctgaaactcgcccgcgagagaaatttcattttgccagacaacagacgccggtttgacagt

caggcagacgccgcgtcagagacttcggagttggctatggatagccaaagccacgccttc

gacagtacagacgaacccgacggtgtggacggtaccccggacgccgcaggatctggcgct

acttctgaaaacggaggcgggaagcccggcgtcgggagggccgtgggctaccagggagca

aaggttctagaccccgtatccggctttcatgtggaccccgtggttgtgtttgacttcgct

agcttatacccaagcattatccaggcccataacctctgtttcaccaccctggcgctcgat

gaagtggatctggccgggcttcaaccatccgtcaactactcgacgttcgaggtgggtgac

caaaagttattttttgtccacgcccatattcgcgaaagcctgcttggcatcttgctgcgc

gactggctggccatgcgaaaggcggtgagggcgcgaatccccaccagcacccccgaggag

gcagttttactagataagcagcagtctgcgattaaggtgatatgcaactcggtttacgga

ttcacgggggtggcaaacggcctgttgccgtgtctgaggatagcggctaccgttaccacg

ataggacgcgacatgctcctcaagacccgagattacgttcactctcgttgggcgacgcgc

gagctgctggaggacaattttccaggggctataggtttccgaaaccacaagccttactcc

gtcagggttatctacggagacaccgactccgtgtttatcaagtttgtgggcctgacgtac

gagggggtatcggagctgggggatgctatgtcgcgtcagatttcagcggacctctttaga

gcgcccatcaaactggagtgtgagaagacctttcagcgactgctgctgatcaccaagaag

aagtacataggtgtcataaacggggggaagatgctcatgaagggggtcgacctggtccgc

aaaaataactgctctttcataaacttgtacgcgcgacatctggtagatcttttgttgtac

gacgaggatgtggccacggcggcagcagaggtgacagacgtgcctcccgcagaatgggtg

gggcgcccgctaccgagcggctttgacaagtttgggcgagtgctggtagaggcgtacaac

cgtatcactgcccccaacttggacgtgcgcgagttcgttatgactgctgagctgagccgc

tcacccgaatcgtataccaacaagcgcctgccgcacctcaccgtctactttaagctcgcc

atgaggaatgaagaactgcccagcgtaaaagagagaattccgtatgtgatagttgcgcag

accgaggccgcggaacgcgaagcgggtgtagtaaactcaatgcgcggtaccgcccaaaac

cccgtggtaaccaagaccgcacgcccccaacctaaacgcaaactgctggtttccgacctc

gccgaagacccgacctatgtttccgagaatgacgtaccgctaaacacagactactatttc

tcccacctgttgggtaccataagcgtgacctttaaggctctattcggaaatgatgtgaga

acaacagaaaatcttttaaagcggtttattccggaaaccccccacaagacccccacgaaa

acccaggcactgcttgagcgcgccggctttgaaaagctgacgccctttacaccggaggaa

gaaagtcgtcgaatactgcatacagttttttgtactctagaagcagctccccatcaaagc

tga

>KU206437.1

atggcggcgcgcgaacaggccaactccgttcgcaggagcgggttttttaatccgtttatt

ggcaagaggccatttttcaggccgggaagcgggcaaaccgcggagacagagcgcccaagg

cccccccaacactcgtactgcacagaggtgggtagctttaagtttatagctccaagatgc

ctcgatgaagaagcccccgccgaccagcgacgcggtgtacacgtgggcaccttggagcgc

ccacccaaagtgtactgcgatggctcagagtacgacgtgctgaactttgcctccggaggt

tgctggcctcgcaggattcgggtttggaacggccaggattttcggggcgatggattcaac

cccagatttgagagatttcacgtgtacgacatagtagagacttcggagagcgcctcacac

gatgacccatccaggtttgctgagctatcccgcccaagcgggtctgtggtaacactgctg

ggaatgagcgagtgtggaaagcgagtcgcggttcacgtctatggtgtgcgccattatttt

tacatggcaaaggcggaggtggatagcgcttgtggaataaccaccgaggcagaactcgtg

cgcgcaatggtggactgcgcgcacagctcggctttgagcgcggccctgggaaatggcaac

ggcggcaaacagagcggcggcagcggcgggggatggtggggcggaaagcacgtgtctgcg

gactgcttcaaagtggagaccgtgtgccacacgacgctgtactactttggatctaagcca

gctctctactatagagtatctgcctccagcagccgcctgggagggttcatctgcgacaac

tttcacccggagattacaaaattcgaggggagcgtggacgtgaccacgcggctgttgttg

gacaatgaaaattttaccagtttcgggtggtaccgcctgcgacccggcacccacggagag

cgtgttcaacttcgccccgttgagcgacacgtcacctcaagcgacgtggagattaactgt

actcccgataacctggagccgataccagacgaggctgcctggcccgactataagctcatg

tgctttgatatagagtgtaaagctggaacgggtaacgaaatggcgttcccagtggcaact

aaccaagaggacctggtcatccagatctcctgtctgctgtactcgcttgctactcagaac

cacgaacacaccctgctgttttccctcgggtcatgcgatatctctgaggaatactcgttt

gcatgcgtccagcgcggcgagcccagaccgacggttttggagtttgacagcgagtacgag

ctgctggttgccttcctgacctttctcaagcagtactctcccgagttcgccaccggctac

aacatcgttaattttgactgggcgtacatagttaacaaggtaacgtcggtgtataacatc

aagctggacgggtacggcaagttcaacaaaggggggctgtttaaggtgtgggacatcgcc

acgaaccattttcagaagaagagcaaggtgaaaatcaatggcctgatatctctagacatg

tattctgtggcgacggaaaagctaaagctacccagctacaaactcgacgcggtcgtggga

gacgtcctcggcgagcataagatagaccttccctataaagaaataccctcctattacgcg

ggagggcctgaccggaggggcgtaataggagagtattgtatccaggactctaggctggtg

ggcaagctgttttttaagtacctcccccatctggaactatcggcggtggccaaactcgcc

cgtatcaccctaacgcgggtaatttttgacggtcagcaaattcgcgtgtacacgtgcttg

ctgaaactcgcccgcgagagaaatttcattttgccagacaacagacgccggtttgacagt

caggcagacgccgcgtcagagacttcggagttggctatggatagccaaagccacgccttc

gacagtacagacgaacccgacggtgtggacggtaccccggacgccgcaggatctggcgct

acttctgaaaacggaggcgggaagcccggcgtcgggagggccgtgggctaccagggagca

aaggttctagaccccgtatccggctttcatgtggaccccgtggttgtgtttgacttcgct

agcttatacccaagcattatccaggcccataacctctgtttcaccaccctggcgctcgat

gaagtggatctggccgggcttcaaccatccgtcaactactcgacgttcgaggtgggtgac

caaaagttattttttgtccacgcccatattcgcgaaagcctgcttggcatcttgctgcgc

gactggctggccatgcgaaaggcggtgagggcgcgaatccccaccagcacccccgaggag

gcagttttactagataagcagcagtctgcgattaaggtgatatgcaactcggtttacgga

ttcacgggggtggcaaacggcctgttgccgtgtctgaggatagcggctaccgttaccacg

ataggacgcgacatgctcctcaagacccgagattacgttcactctcgttgggcgacgcgc

gagctgctggaggacaattttccaggggctataggtttccgaaaccacaagccttactcc

gtcagggttatctacggagacaccgactccgtgtttatcaagtttgtgggcctgacgtac

gagggggtatcggagctgggggatgctatgtcgcgtcagatttcagcggacctctttaga

gcgcccatcaaactggagtgtgagaagacctttcagcgactgctgctgatcaccaagaag

aagtacataggtgtcataaacggggggaagatgctcatgaagggggtcgacctggtccgc

aaaaataactgctctttcataaacttgtacgcgcgacatctggtagatcttttgttgtac

gacgaggatgtggccacggcggcagcagaggtgacagacgtgcctcccgcagaatgggtg

gggcgcccgctaccgagcggctttgacaagtttgggcgagtgctggtagaggcgtacaac

cgtatcactgcccccaacttggacgtgcgcgagttcgttatgactgctgagctgagccgc

tcacccgaatcgtataccaacaagcgcctgccgcacctcaccgtctactttaagctcgcc

atgaggaatgaagaactgcccagcgtaaaagagagaattccgtatgtgatagttgcgcag

accgaggccgcggaacgcgaagcgggtgtagtaaactcaatgcgcggtaccgcccaaaac

cccgtggtaaccaagaccgcacgcccccaacctaaacgcaaactgctggtttccgacctc

gccgaagacccgacctatgtttccgagaatgacgtaccgctaaacacagactactatttc

tcccacctgttgggtaccataagcgtgacctttaaggctctattcggaaatgatgtgaga

acaacagaaaatcttttaaagcggtttattccggaaaccccccacaagacccccacgaaa

acccaggcactgcttgagcgcgccggctttgaaaagctgacgccctttacaccggaggaa

gaaagtcgtcgaatactgcatacagttttttgtactctagaagcagctccccatcaaagc

tga

>KU206438.1

atggcggcgcgcgaacaggccaactccgttcgcaggagcgggttttttaatccgtttatt

ggcaagaggccatttttcaggccgggaagcgggcaaaccgcggagacagagcgcccaagg

cccccccaacactcgtactgcacagaggtgggtagctttaagtttatagctccaagatgc

ctcgatgaagaagcccccgccgaccagcgacgcggtgtacacgtgggcaccttggagcgc

ccacccaaagtgtactgcgatggctcagagtacgacgtgctgaactttgcctccggaggt

tgctggcctcgcaggattcgggtttggaacggccaggattttcggggcgatggattcaac

cccagatttgagagatttcacgtgtacgacatagtagagacttcggagagcgcctcacac

gatgacccatccaggtttgctgagctatcccgcccaagcgggtctgtggtaacactgctg

ggaatgagcgagtgtggaaagcgagtcgcggttcacgtctatggtgtgcgccattatttt

tacatggcaaaggcggaggtggatagcgcttgtggaataaccaccgaggcagaactcgtg

cgcgcaatggtggactgcgcgcacagctcggctttgagcgcggccctgggaaatggcaac

ggcggcaaacagagcggcggcagcggcgggggatggtggggcggaaagcacgtgtctgcg

gactgcttcaaagtggagaccgtgtgccacacgacgctgtactactttggatctaagcca

gctctctactatagagtatctgcctccagcagccgcctgggagggttcatctgcgacaac

tttcacccggagattacaaaattcgaggggagcgtggacgtgaccacgcggctgttgttg

gacaatgaaaattttaccagtttcgggtggtaccgcctgcgacccggcacccacggagag

cgtgttcaacttcgccccgttgagcgacacgtcacctcaagcgacgtggagattaactgt

actcccgataacctggagccgataccagacgaggctgcctggcccgactataagctcatg

tgctttgatatagagtgtaaagctggaacgggtaacgaaatggcgttcccagtggcaact

aaccaagaggacctggtcatccagatctcctgtctgctgtactcgcttgctactcagaac

cacgaacacaccctgctgttttccctcgggtcatgcgatatctctgaggaatactcgttt

gcatgcgtccagcgcggcgagcccagaccgacggttttggagtttgacagcgagtacgag

ctgctggttgccttcctgacctttctcaagcagtactctcccgagttcgccaccggctac

aacatcgttaattttgactgggcgtacatagttaacaaggtaacgtcggtgtataacatc

aagctggacgggtacggcaagttcaacaaaggggggctgtttaaggtgtgggacatcgcc

acgaaccattttcagaagaagagcaaggtgaaaatcaatggcctgatatctctagacatg

tattctgtggcgacggaaaagctaaagctacccagctacaaactcgacgcggtcgtggga

gacgtcctcggcgagcataagatagaccttccctataaagaaataccctcctattacgcg

ggagggcctgaccggaggggcgtaataggagagtattgtatccaggactctaggctggtg

ggcaagctgttttttaagtacctcccccatctggaactatcggcggtggccaaactcgcc

cgtatcaccctaacgcgggtaatttttgacggtcagcaaattcgcgtgtacacgtgcttg

ctgaaactcgcccgcgagagaaatttcattttgccagacaacagacgccggtttgacagt

caggcagacgccgcgtcagagacttcggagttggctatggatagccaaagccacgccttc

gacagtacagacgaacccgacggtgtggacggtaccccggacgccgcaggatctggcgct

acttctgaaaacggaggcgggaagcccggcgtcgggagggccgtgggctaccagggagca

aaggttctagaccccgtatccggctttcatgtggaccccgtggttgtgtttgacttcgct

agcttatacccaagcattatccaggcccataacctctgtttcaccaccctggcgctcgat

gaagtggatctggccgggcttcaaccatccgtcaactactcgacgttcgaggtgggtgac

caaaagttattttttgtccacgcccatattcgcgaaagcctgcttggcatcttgctgcgc

gactggctggccatgcgaaaggcggtgagggcgcgaatccccaccagcacccccgaggag

gcagttttactagataagcagcagtctgcgattaaggtgatatgcaactcggtttacgga

ttcacgggggtggcaaacggcctgttgccgtgtctgaggatagcggctaccgttaccacg

ataggacgcgacatgctcctcaagacccgagattacgttcactctcgttgggcgacgcgc

gagctgctggaggacaattttccaggggctataggtttccgaaaccacaagccttactcc

gtcagggttatctacggagacaccgactccgtgtttatcaagtttgtgggcctgacgtac

gagggggtatcggagctgggggatgctatgtcgcgtcagatttcagcggacctctttaga

gcgcccatcaaactggagtgtgagaagacctttcagcgactgctgctgatcaccaagaag

aagtacataggtgtcataaacggggggaagatgctcatgaagggggtcgacctggtccgc

aaaaataactgctctttcataaacttgtacgcgcgacatctggtagatcttttgttgtac

gacgaggatgtggccacggcggcagcagaggtgacagacgtgcctcccgcagaatgggtg

gggcgcccgctaccgagcggctttgacaagtttgggcgagtgctggtagaggcgtacaac

cgtatcactgcccccaacttggacgtgcgcgagttcgttatgactgctgagctgagccgc

tcacccgaatcgtataccaacaagcgcctgccgcacctcaccgtctactttaagctcgcc

atgaggaatgaagaactgcccagcgtaaaagagagaattccgtatgtgatagttgcgcag

accgaggccgcggaacgcgaagcgggtgtagtaaactcaatgcgcggtaccgcccaaaac

cccgtggtaaccaagaccgcacgcccacaacctaaacgcaaactgctggtttccgacctc

gccgaagacccgacctatgtttccgagaatgacgtaccgctaaacacagactactatttc

tcccacctgttgggtaccataagcgtgacctttaaggctctattcggaaatgatgtgaga

acaacagaaaatcttttaaagcggtttattccggaaaccccccacaagacccccacgaaa

acccaggcactgcttgagcgcgccggctttgaaaagctgacgccctttacaccggaggaa

gaaagtcgtcgaatactgcatacagttttttgtactctagaagcagctccccatcaaagc

tga

>KU206439.1

atggcggcgcgcgaacaggccaactccgttcgcaggagcgggttttttaatccgtttatt

ggcaagaggccatttttcaggccgggaagcgggcaaaccgcggagacagagcgcccaagg

cccccccaacactcgtactgcacagaggtgggtagctttaagtttatagctccaagatgc

ctcgatgaagaagcccccgccgaccagcgacgcggtgtacacgtgggcaccttggagcgc

ccacccaaagtgtactgcgatggctcagagtacgacgtgctgaactttgcctccggaggt

tgctggcctcgcaggattcgggtttggaacggccaggattttcggggcgatggattcaac

cccagatttgagagatttcacgtgtacgacatagtagagacttcggagagcgcctcacac

gatgacccatccaggtttgctgagctatcccgcccaagcgggtctgtggtaacactgctg

ggaatgagcgagtgtggaaagcgagtcgcggttcacgtctatggtgtgcgccattatttt

tacatggcaaaggcggaggtggatagcgcttgtggaataaccaccgaggcagaactcgtg

cgcgcaatggtggactgcgcgcacagctcggctttgagcgcggccctgggaaatggcaac

ggcggcaaacagagcggcggcagcggcgggggatggtggggcggaaagcacgtgtctgcg

gactgcttcaaagtggagaccgtgtgccacacgacgctgtactactttggatctaagcca

gctctctactatagagtatctgcctccagcagccgcctgggagggttcatctgcgacaac

tttcacccggagattacaaaattcgaggggagcgtggacgtgaccacgcggctgttgttg

gacaatgaaaattttaccagtttcgggtggtaccgcctgcgacccggcacccacggagag

cgtgttcaacttcgccccgttgagcgacacgtcacctcaagcgacgtggagattaactgt

actcccgataacctggagccgataccagacgaggctgcctggcccgactataagctcatg

tgctttgatatagagtgtaaagctggaacgggtaacgaaatggcgttcccagtggcaact

aaccaagaggacctggtcatccagatctcctgtctgctgtactcgcttgctactcagaac

cacgaacacaccctgctgttttccctcgggtcatgcgatatctctgaggaatactcgttt

gcatgcgtccagcgcggcgagcccagaccgacggttttggagtttgacagcgagtacgag

ctgctggttgccttcctgacctttctcaagcagtactctcccgagttcgccaccggctac

aacatcgttaattttgactgggcgtacatagttaacaaggtaacgtcggtgtataacatc

aagctggacgggtacggcaagttcaacaaaggggggctgtttaaggtgtgggacatcgcc

acgaaccattttcagaagaagagcaaggtgaaaatcaatggcctgatatctctagacatg

tattctgtggcgacggaaaagctaaagctacccagctacaaactcgacgcggtcgtggga

gacgtcctcggcgagcataagatagaccttccctataaagaaataccctcctattacgcg

ggagggcctgaccggaggggcgtaataggagagtattgtatccaggactctaggctggtg

ggcaagctgttttttaagtacctcccccatctggaactatcggcggtggccaaactcgcc

cgtatcaccctaacgcgggtaatttttgacggtcagcaaattcgcgtgtacacgtgcttg

ctgaaactcgcccgcgagagaaatttcattttgccagacaacagacgccggtttgacagt

caggcagacgccgcgtcagagacttcggagttggctatggatagccaaagccacgccttc

gacagtacagacgaacccgacggtgtggacggtaccccggacgccgcaggatctggcgct

acttctgaaaacggaggcgggaagcccggcgtcgggagggccgtgggctaccagggagca

aaggttctagaccccgtatccggctttcatgtggaccccgtggttgtgtttgacttcgct

agcttatacccaagcattatccaggcccataacctctgtttcaccaccctggcgctcgat

gaagtggatctggccgggcttcaaccatccgtcaactactcgacgttcgaggtgggtgac

caaaagttattttttgtccacgcccatattcgcgaaagcctgcttggcatcttgctgcgc

gactggctggccatgcgaaaggcggtgagggcgcgaatccccaccagcacccccgaggag

gcagttttactagataagcagcagtctgcgattaaggtgatatgcaactcggtttacgga

ttcacgggggtggcaaacggcctgttgccgtgtctgaggatagcggctaccgttaccacg

ataggacgcgacatgctcctcaagacccgagattacgttcactctcgttgggcgacgcgc

gagctgctggaggacaattttccaggggctataggtttccgaaaccacaagccttactcc

gtcagggttatctacggagacaccgactccgtgtttatcaagtttgtgggcctgacgtac

gagggggtatcggagctgggggatgctatgtcgcgtcagatttcagcggacctctttaga

gcgcccatcaaactggagtgtgagaagacctttcagcgactgctgctgatcaccaagaag

aagtacataggtgtcataaacggggggaagatgctcatgaagggggtcgacctggtccgc

aaaaataactgctctttcataaacttgtacgcgcgacatctggtagatcttttgttgtac

gacgaggatgtggccacggcggcagcaaaggtgacagacgtgcctcccgcagaatgggtg

gggcgcccgctaccgagcggctttgacaagtttgggcgagtgctggtagaggcgtacaac

cgtatcactgcccccaacttggacgtgcgcgagttcgttatgactgctgagctgagccgc

tcacccgaatcgtataccaacaagcgcctgccgcacctcaccgtctactttaagctcgcc

atgaggaatgaagaactgcccagcgtaaaagagagaattccgtatgtgatagttgcgcag

accgaggccgcggaacgcgaagcgggtgtagtaaactcaatgcgcggtaccgcccaaaac

cccgtggtaaccaagaccgcacgcccccaacctaaacgcaaactgctggtttccgacctc

gccgaagacccgacctatgtttccgagaatgacgtaccgctaaacacagactactatttc

tcccacctgttgggtaccataagcgtgacctttaaggctctattcggaaatgatgtgaga

acaacagaaaatcttttaaagcggtttattccggaaaccccccacaagacccccacgaaa

acccaggcactgcttgagcgcgccggctttgaaaagctgacgccctttacaccggaggaa

gaaagtcgtcgaatactgcatacagttttttgtactctagaagcagctccccatcaaagc

tga

>KU206440.1

atggcggcgcgcgaacaggccaactccgttcgcaggagcgggttttttaatccgtttatt

ggcaagaggccatttttcaggccgggaagcgggcaaaccgcggagacagagcgcccaagg

cccccccaacactcgtactgcacagaggtgggtagctttaagtttatagctccaagatgc

ctcgatgaagaagcccccgccgaccagcgacgcggtgtacacgtgggcaccttggagcgc

ccacccaaagtgtactgcgatggctcagagtacgacgtgctgaactttgcctccggaggt

tgctggcctcgcaggattcgggtttggaacggccaggattttcggggcgatggattcaac

cccagatttgagagatttcacgtgtacgacatagtagagacttcggagagcgcctcacac

gatgacccatccaggtttgctgagctatcccgcccaagcgggtctgtggtaacactgctg

ggaatgagcgagtgtggaaagcgagtcgcggttcacgtctatggtgtgcgccattatttt

tacatggcaaaggcggaggtggatagcgcttgtggaataaccaccgaggcagaactcgtg

cgcgcaatggtggactgcgcgcacagctcggctttgagcgcggccctgggaaatggcaac

ggcggcaaacagagcggcggcagcggcgggggatggtggggcggaaagcacgtgtctgcg

gactgcttcaaagtggagaccgtgtaccacacgacgctgtactactttggatctaagcca

gctctctactatagagtatctgcctccagcagccgcctgggagggttcatctgcgacaac

tttcacccggagattacaaaattcgaggggagcgtggacgtgaccacgcggctgttgttg

gacaatgaaaattttaccagtttcgggtggtaccgcctgcgacccggcacccacggagag

cgtgttcaacttcgccccgttgagcgacacgtcacctcaagcgacgtggagattaactgt

actcccgataacctggagccgataccagacgaggctgcctggcccgactataagctcatg

tgctttgatatagagtgtaaagctggaacgggtaacgaaatggcgttcccagtggcaact

aaccaagaggacctggtcatccagatctcctgtctgctgtactcgcttgctactcagaac

cacgaacacaccctgctgttttccctcgggtcatgcgatatctctgaggaatactcgttt

gcatgcgtccagcgcggcgagcccagaccgacggttttggagtttgacagcgagtacgag

ctgctggttgccttcctgacctttctcaagcagtactctcccgagttcgccaccggctac

aacatcgttaattttgactgggcgtacatagttaacaaggtaacgtcggtgtataacatc

aagctggacgggtacggcaagttcaacaaaggggggctgtttaaggtgtgggacatcgcc

acgaaccattttcagaagaagagcaaggtgaaaatcaatggcctgatatctctagacatg

tattctgtggcgacggaaaagctaaagctacccagctacaaactcgacgcggtcgtggga

gacgtcctcggcgagcataagatagaccttccctataaagaaataccctcctattacgcg

ggagggcctgaccggaggggcgtaataggagagtattgtatccaggactctaggctggtg

ggcaagctgttttttaagtacctcccccatctggaactatcggcggtggccaaactcgcc

cgtatcaccctaacgcgggtaatttttgacggtcagcaaattcgcgtgtacacgtgcttg

ctgaaactcgcccgcgagagaaatttcattttgccagacaacagacgccggtttgacagt

caggcagacgccgcgtcagagacttcggagttggctatggatagccaaagccacgccttc

gacagtacagacgaacccgacggtgtggacggtaccccggacgccgcaggatctggcgct

acttctgaaaacggaggcgggaagcccggcgtcgggagggccgtgggctaccagggagca

aaggttctagaccccgtatccggctttcatgtggaccccgtggttgtgtttgacttcgct

agcttatacccaagcattatccaggcccataacctctgtttcaccaccctggcgctcgat

gaagtggatctggccgggcttcaaccatccgtcgactactcgacgttcgaggtgggtgac

caaaagttattttttgtccacgcccatattcgcgaaagcctgcttggcatcttgctgcgc

gactggctggccatgcgaaaggcggtgagggcgcgaatccccaccagcacccccgaggag

gcagttttactagataagcagcagtctgcgattaaggtgatatgcaactcggtttacgga

ttcacgggggtggcaaacggcctgttgccgtgtctgaggatagcggctaccgttaccacg

ataggacgcgacatgctcctcaagacccgagattacgttcactctcgttgggcgacgcgc

gagctgctggaggacaattttccaggggctataggtttccgaaaccacaagccttactcc

gtcagggttatctacggagacaccgactccgtgtttatcaagtttgtgggcctgacgtac

gagggggtatcggagctgggggatgctatgtcgcgtcagatttcagcggacctctttaga

gcgcccatcaaactggagtgtgagaagacctttcagcgactgcttctgatcaccaagaag

aagtacataggtgtcataaacggggggaagatgctcatgaagggggtcgacctggtccgc

aaaaataactgctctttcataaacttgtacgcgcgacatctggtagatcttttgttgtac

gacgaggatgtggccacggcggcagcagaggtgacagacgtgcctcccgcagaatgggtg

gggcgcccgctaccgagcggctttgacaagtttgggcgagtgctggtagaggcgtacaac

cgtatcactgcccccaacttggacgtgcgcgagttcgttatgactgctgagctgagccgc

tcacccgaatcgtataccaacaagcgcctgccgcacctcaccgtctactttaagctcgcc

atgaggaatgaagaactgcccagcgtaaaagagagaattccgtatgtgatagttgcgcag

accgaggccgcggaacgcgaagcgggtgtagtaaactcaatgcgcggtaccgcccaaaac

cccgtggtaaccaagaccgcacgcccccaacctaaacgcaaactgctggtttccgacctc

gccgaagacccgacctatgtttccgagaatgacgtaccgctaaacacagactactatttc

tcccacctgttgggtaccataagcgtgacctttaaggctctattcggaaatgatgtgaga

acaacagaaaatcttttaaagcggtttattccggaaaccccccacaagacccccacgaaa

acccaggcactgcttgagcgcgccggctttgaaaagctgacgccctttacaccggaggaa

gaaagtcgtcgaatactgcatacagttttttgtactctagaagcagctccccatcaaagc

tga

>KU206441.1

atggcggcgcgcgaacaggccaactccgttcgcaggagcgggttttttaatccgtttatt

ggcaagaggccatttttcaggccgggaagcgggcaaaccgcggagacagagcgcccaagg

cccccccaacactcgtactgcacagaggtgggtagctttaagtttatagctccaagatgc

ctcgatgaagaagcccccgccgaccagcgacgcggtgtacacgtgggcaccttggagcgc

ccacccaaagtgtactgcgatggctcagagtacgacgtgctgaactttgcctccggaggt

tgctggcctcgcaggattcgggtttggaacggccaggattttcggggcgatggattcaac

cccagatttgagagatttcacgtgtacgacatagtagagacttcggagagcgcctcacac

gatgacccatccaggtttgctgagctatcccgcccaagcgggtctgtggtaacactgctg

ggaatgagcgagtgtggaaagcgagtcgcggttcacgtctatggtgtgcgccattatttt

tacatggcaaaggcggaggtggatagcgcttgtggaataaccaccgaggcagaactcgtg

cgcgcaatggtggactgcgcgcacagctcggctttgagcgcggccctgggaaatggcaac

ggcggcaaacagagcggcggcagcggcgggggatggtggggcggaaagcacgtgtctgcg

gactgcttcaaagtggagaccgtgtgccacacgacgctgtactactttggatctaagcca

gctctctactatagagtatctgcctccagcagccgcctgggagggttcatctgcgacaac

tttcacccggagattacaaaattcgaggggagcgtggacgtgaccacgcggctgttgttg

gacaatgaaaattttaccagtttcgggtggtaccgcctgcgacccggcacccacggagag

cgtgttcaacttcgccccgttgagcgacacgtcacctcaagcgacgtggagattaactgt

actcccgataacctggagccgataccagacgaggctgcctggcccgactataagctcatg

tgctttgatatagagtgtaaagctggaacgggtaacgaaatggcgttcccagtggcaact

aaccaagaggacctggtcatccagatctcctgtctgctgtactcgcttgctactcagaac

cacgaacacaccctgctgttttccctcgggtcatgcgatatctctgaggaatactcgttt

gcatgcgtccagcgcggcgagcccagaccgacggttttggagtttgacagcgagtacgag

ctgctggttgccttcctgacctttctcaagcagtactctcccgagttcgccaccggctac

aacatcgttaattttgactgggcgtacatagttaacaaggtaacgtcggtgtataacatc

aagctggacgggtacggcaagttcaacaaaggggggctgtttaaggtgtgggacatcgcc

acgaaccattttcagaagaagagcaaggtgaaaatcaatggcctgatatctctagacatg

tattctgtggcgacggaaaagctaaagctacccagctacaaactcgacgcggtcgtggga

gacgtcctcggcgagcataagatagaccttccctataaagaaataccctcctattacgcg

ggagggcctgaccggaggggcgtaataggagagtattgtatccaggactctaggctggtg

ggcaagctgttttttaagtacctcccccatctggaactatcggcggtggccaaactcgcc

cgtatcaccctaacgcgggtaatttttgacggtcagcaaattcgcgtgtacacgtgcttg

ctgaaactcgcccgcgagagaaatttcattttgccagacaacagacgccggtttgacagt

caggcagacgccgcgtcagagacttcggagttggctatggatagccaaagccacgccttc

gacagtacagacgaacccgacggtgtggacggtaccccggacgccgcaggatctggcgct

acttctgaaaacggaggcgggaagcccggcgtcgggagggccgtgggctaccagggagca

aaggttctagaccccgtatccggctttcatgtggaccccgtggttgtgtttgacttcgct

agcttatacccaagcattatccaggcccataacctctgtttcaccaccctggcgctcgat

gaagtggatctggccgggcttcaaccatccgtcaactactcgacgttcgaggtgggtgac

caaaagttattttttgtccacgcccatattcgcgaaagcctgcttggcatcttgctgcgc

gactggctggccatgcgaaaggcggtgagggcgcgaatccccaccagcacccccgaggag

gcagttttactagataagcagcagtctgcgattaaggtgatatgcaactcggtttacgga

ttcacgggggtggcaaacggcctgttgccgtgtctgaggatagcggctaccgttaccacg

ataggacgcgacatgctcctcaagacccgagattacgttcactctcgttgggcgacgcgc

gagctgctggaggacaattttccaggggctataggtttccgaaaccacaagccttactcc

gtcagggttatctacggagacaccgactccgtgtttatcaagtttgtgggcctgacgtac

gagggggtatcggagctgggggatgctatgtcgcgtcagatttcagcggacctctttaga

gcgcccatcaaactggagtgtgagaagacctttcagcgactgctgctgatcaccaagaag

aagtacataggtgtcataaacggggggaagatgctcatgaagggggtcgacctggtccgc

aaaaataactgctctttcataaacttgtacgcgcgacatctggtagatcttttgttgtac

gacgaggatgtggccacggcggcagcaaaggtgacagacgtgcctcccgcagaatgggtg

gggcgcccgctaccgagcggctttgacaagtttgggcgagtgctggtagaggcgtacaac

cgtatcactgcccccaacttggacgtgcgcgagttcgttatgactgctgagctgagccgc

tcacccgaatcgtataccaacaagcgcctgccgcacctcaccgtctactttaagctcgcc

atgaggaatgaagaactgcccagcgtaaaagagagaattccgtatgtgatagttgcgcag

accgaggccgcggaacgcgaagcgggtgtagtaaactcaatgcgcggtaccgcccaaaac

cccgtggtaaccaagaccgcacgcccccaacctaaacgcaaactgctggtttccgacctc

gccgaagacccgacctatgtttccgagaatgacgtaccgctaaacacagactactatttc

tcccacctgttgggtaccataagcgtgacctttaaggctctattcggaaatgatgtgaga

acaacagaaaatcttttaaagcggtttattccggaaaccccccacaagacccccacgaaa

acccaggcactgcttgagcgcgccggctttgaaaagctgacgccctttacaccggaggaa

gaaagtcgtcgaatactgcatacagttttttgtactctagaagcagctccccatcaaagc

tga

>KU206442.1

atggcggcgcgcgaacaggccaactccgttcgcaggagcgggttttttaatccgtttatt

ggcaagaggccatttttcaggccgggaagcgggcaaaccgcggagacagagcgcccaagg

cccccccaacactcgtactgcacagaggtgggtagctttaagtttatagctccaagatgc

ctcgatgaagaagcccccgccgaccagcgacgcggtgtacacgtgggcaccttggagcgc

ccacccaaagtgtactgcgatggctcagagtacgacgtgctgaactttgcctccggaggt

tgctggcctcgcaggattcgggtttggaacggccaggattttcggggcgatggattcaac

cccagatttgagagatttcacgtgtacgacatagtagagacttcggagagcgcctcacac

gatgacccatccaggtttgctgagctatcccgcccaagcgggtctgtggtaacactgctg

ggaatgagcgagtgtggaaagcgagtcgcggttcacgtctatggtgtgcgccattatttt

tacatggcaaaggcggaggtggatagcgcttgtggaataaccaccgaggcagaactcgtg

cgcgcaatggtggactgcgcgcacagctcggctttgagcgcggccctgggaaatggcaac

ggcggcaaacagagcggcggcagcggcgggggatggtggggcggaaagcacgtgtctgcg

gactgcttcaaagtggagaccgtgtgccacacgacgctgtactactttggatctaagcca

gctctctactatagagtatctgcctccagcagccgcctgggagggttcatctgcgacaac

tttcacccggagattacaaaattcgaggggagcgtggacgtgaccacgcggctgttgttg

gacaatgaaaattttaccagtttcgggtggtaccgcctgcgacccggcacccacggagag

cgtgttcaacttcgccccgttgagcgacacgtcacctcaagcgacgtggagattaactgt

actcccgataacctggagccgataccagacgaggctgcctggcccgactataagctcatg

tgctttgatatagagtgtaaagctggaacgggtaacgaaatggcgttcccagtggcaact

aaccaagaggacctggtcatccagatctcctgtctgctgtactcgcttgctactcagaac

cacgaacacaccctgctgttttccctcgggtcatgcgatatctctgaggaatactcgttt

gcatgcgtccagcgcggcgagcccagaccgacggttttggagtttgacagcgagtacgag

ctgctggttgccttcctgacctttctcaagcagtactctcccgagttcgccaccggctac

aacatcgttaattttgactgggcgtacatagttaacaaggtaacgtcggtgtataacatc

aagctggacgggtacggcaagttcaacaaaggggggctgtttaaggtgtgggacatcgcc

acgaaccattttcagaagaagagcaaggtgaaaatcaatggcctgatatctctagacatg

tattctgtggcgacggaaaagctaaagctacccagctacaaactcgacgcggtcgtggga

gacgtcctcggcgagcataagatagaccttccctataaagaaataccctcctattacgcg

ggagggcctgaccggaggggcgtaataggagagtattgtatccaggactctaggctggtg

ggcaagctgttttttaagtacctcccccatctggaactatcggcggtggccaaactcgcc

cgtatcaccctaacgcgggtaatttttgacggtcagcaaattcgcgtgtacacgtgcttg

ctgaaactcgcccgcgagagaaatttcattttgccagacaacagacgccggtttgacagt

caggcagacgccgcgtcagagacttcggagttggctatggatagccaaagccacgccttc

gacagtacagacgaacccgacggtgtggacggtaccccggacgccgcaggatctggcgct

acttctgaaaacggaggcgggaagcccggcgtcgggagggccgtgggctaccagggagca

aaggttctagaccccgtatccggctttcatgtggaccccgtggttgtgtttgacttcgct

agcttatacccaagcattatccaggcccataacctctgtttcaccaccctggcgctcgat

gaagtggatctggccgggcttcaaccatccgtcaactactcgacgttcgaggtgggtgac

caaaagttattttttgtccacgcccatattcgcgaaagcctgcttggcatcttgctgcgc

gactggctggccatgcgaaaggcggtgagggcgcgaatccccaccagcacccccgaggag

gcagttttactagataagcagcagtctgcgattaaggtgatatgcaactcggtttacgga

ttcacgggggtggcaaacggcctgttgccgtgtctgaggatagcggctaccgttaccacg

ataggacgcgacatgctcctcaagacccgagattacgttcactctcgttgggcgacgcgc

gagctgctggaggacaattttccaggggctataggtttccgaaaccacaagccttactcc

gtcagggttatctacggagacaccgactccgtgtttatcaagtttgtgggcctgacgtac

gagggggtatcggagctgggggatgctatgtcgcgtcagatttcagcggacctctttaga

gcgcccatcaaactggagtgtgagaagacctttcagcgactgctgctgatcaccaagaag

aagtacataggtgtcataaacggggggaagatgctcatgaagggggtcgacctggtccgc

aaaaataactgctctttcataaacttgtacgcgcgacatctggtagatcttttgttgtac

gacgaggatgtggccacggcggcagcaaaggtgacagacgtgcctcccgcagaatgggtg

gggcgcccgctaccgagcggctttgacaagtttgggcgagtgctggtagaggcgtacaac

cgtatcactgcccccaacttggacgtgcgcgagttcgttatgactgctgagctgagccgc

tcacccgaatcgtataccaacaagcgcctgccgcacctcaccgtctactttaagctcgcc

atgaggaatgaagaactgcccagcgtaaaagagagaattccgtatgtgatagttgcgcag

accgaggccgcggaacgcgaagcgggtgtagtaaactcaatgcgcggtaccgcccaaaac

cccgtggtaaccaagaccgcacgcccccaacctaaacgcaaactgctggtttccgacctc

gccgaagacccgacctatgtttccgagaatgacgtaccgctaaacacagactactatttc

tcccacctgttgggtaccataagcgtgacctttaaggctctattcggaaatgatgtgaga

acaacagaaaatcttttaaagcggtttattccggaaaccccccacaagacccccacgaaa

acccaggcactgcttgagcgcgccggctttgaaaagctgacgccctttacaccggaggaa

gaaagtcgtcgaatactgcatacagttttttgtactctagaagcagctccccatcaaagc

tga

>KU206443.1

atggcggcgcgcgaacaggccaactccgttcgcaggagcgggttttttaatccgtttatt

ggcaagaggccatttttcaggccgggaagcgggcaaaccgcggagacagagcgcccaagg

cccccccaacactcgtactgcacagaggtgggtagctttaagtttatagctccaaggtgc

ctcgatgaagaagcccccgccgaccagcgacgcggtgtacacgtgggcaccttggagcgc

ccacccaaagtgtactgcgatggctcagagtacgacgtgctgaactttgcctccggaggt

tgctggcctcgcaggattcgggtttggaacggccaggattttcggggcgatggattcaac

cccagatttgagagatttcacgtgtacgacatagtagagacttcggagagcgcctcacac

gatgacccatccaggtttgctgagctatcccgcccaagcgggtctgtggtaacactgctg

ggaatgagcgagtgtggaaagcgagtcgcggttcacgtctatggtgtgcgccattatttt

tacatggcaaaggcggaggtggatagcgcttgtggaataaccaccgaggcagaactcgtg

cgcgcaatggtggactgcgcgcacagctcggctttgagcgcggccctgggaaatggcaac

ggcggcaaacagagcggcggcagcggcgggggatggtggggcggaaagcacgtgtctgcg

gactgcttcaaagtggagaccgtgtgccacacgacgctgtactactttggatctaagcca

gctctctactatagagtatctgcctccagcagccgcctgggagggttcatctgcgacaac

tttcacccggagattacaaaattcgaggggagcgtggacgtgaccacgcggctgttgttg

gacaatgaaaattttaccagtttcgggtggtaccgcctgcgacccggcacccacggagag

cgtgttcaacttcgccccgttgagcgacacgtcacctcaagcgacgtggagattaactgt

actcccgataacctggagccgataccagacgaggctgcctggcccgactataagctcatg

tgctttgatatagagtgtaaagctggaacgggtaacgaaatggcgttcccagtggcaact

aaccaagaggacctggtcatccagatctcctgtctgctgtactcgcttgctactcagaac

cacgaacacaccctgctgttttccctcgggtcatgcgatatctctgaggaatactcgttt

gcatgcgtccagcgcggcgagcccagaccgacggttttggagtttgacagcgagtacgag

ctgctggttgccttcctgacctttctcaagcagtactctcccgagttcgccaccggctac

aacatcgttaattttgactgggcgtacatagttaacaaggtaacgtcggtgtataacatc

aagctggacgggtacggcaagttcaacaaaggggggctgtttaaggtgtgggacatcgcc

acgaaccattttcagaagaagagcaaggtgaaaatcaatggcctgatatctctagacatg

tattctgtggcgacggaaaagctaaagctacccagctacaaactcgacgcggtcgtggga

gacgtcctcggcgagcataagatagaccttccctataaagaaataccctcctattacgcg

ggagggcctgaccggaggggcgtaataggagagtattgtatccaggactctaggctggtg

ggcaagctgttttttaagtacctcccccatctggaactatcggcggtggccaaactcgcc

cgtatcaccctaacgcgggtaatttttgacggtcagcaaattcgcgtgtacacgtgcttg

ctgaaactcgcccgcgagagaaatttcattttgccagacaacagacgccggtttgacagt

caggcagacgccgcgtcagagacttcggagttggctatggatagccaaagccacgccttc

gacagtacagacgaacccgacggtgtggacggtaccccggacgccgcaggatctggcgct

acttctgaaaacggaggcgggaagcccggcgtcgggagggccgtgggctaccagggagca

aaggttctagaccccgtatccggctttcatgtggaccccgtggttgtgtttgacttcgct

agcttatacccaagcattatccaggcccataacctctgtttcaccaccctggcgctcgat

gaagtggatctggccgggcttcaaccatccgtcaactactcgacgttcgaggtgggtgac

caaaagttattttttgtccacgcccatattcgcgaaagcctgcttggcatcttgctgcgc

gactggctggccatgcgaaaggcggtgagggcgcgaatccccaccagcacccccgaggag

gcagttttactagataagcagcagtctgcgattaaggtgatatgcaactcggtttacgga

ttcacgggggtggcaaacggcctgttgccgtgtctgaggatagcggctaccgttaccacg

ataggacgcgacatgctcctcaagacccgagattacgttcactctcgttgggcgacgcgc

gagctgctggaggacaattttccaggggctataggtttccgaaaccacaagccttactcc

gtcagggttatctacggagacaccgactccgtgtttatcaagtttgtgggcctgacgtac

gagggggtatcggagctgggggatgctatgtcgcgtcagatttcagcggacctctttaga

gcgcccatcaaactggagtgtgagaagacctttcagcgactgctgctgatcaccaagaag

aagtacataggtgtcataaacggggggaagatgctcatgaagggggtcgacctggtccgc

aaaaataactgctctttcataaacttgtacgcgcgacatctggtagatcttttgttgtac

gacgaggatgtggccacggcggcagcaaaggtgacagacgtgcctcccgcagaatgggtg

gggcgcccgctaccgagcggctttgacaagtttgggcgagtgctggtagaggcgtacaac

cgtatcactgcccccaacttggacgtgcgcgagttcgttatgactgctgagctgagccgc

tcacccgaatcgtataccaacaagcgcctgccgcacctcaccgtctactttaagctcgcc

atgaggaatgaagaactgcccagcgtaaaagagagaattccgtatgtgatagttgcgcag

accgaggccgcggaacgcgaagcgggtgtagtaaactcaatgcgcggtaccgcccaaaac

cccgtggtaaccaagaccgcacgcccccaacctaaacgcaaactgctggtttccgacctc

gccgaagacccgacctatgtttccgagaatgacgtaccgctaaacacagactactatttc

tcccacctgttgggtaccataagcgtgacctttaaggctctattcggaaatgatgtgaga

acaacagaaaatcttttaaagcggtttattccggaaaccccccacaagacccccacgaaa

acccaggcactgcttgagcgcgccggctttgaaaagctgacgccctttacaccggaggaa

gaaagtcgtcgaatactgcatacagttttttgtactctagaagcagctccccatcaaagc

tga

>KU206444.1

atggcggcgcgcgaacaggccaactccgttcgcaggagcgggttttttaatccgtttatt

ggcaagaggccatttttcaggccgggaagcgggcagaccgcggagacagagcgcccaagg

cccccccaacactcgtactgcacagaggtgggtagctttaagtttatagctccaagatgc

ctcgatgaagaagcccccgccgaccagcgacgcggtgtacacgtgggcaccttggagcgc

ccacccaaagtgtactgcgatggctcagagtacgacgtgctgaactttgcctccggaggt

tgctggcctcgcaggattcgggtttggaacggccaggattttcggggcgatggattcaac

cccagatttgagagatttcacgtgtacgacatagtagagacttcggagagcgcctcacac

gatgacccatccaggtttgctgagctatcccgcccaagcgggtctgtggtaacactgctg

ggaatgagcgagtgtggaaagcgagtcgcggttcacgtatatggtgtgcgccattatttt

tacatggcaaaggcggaggtggatagcgcttgtggaataaccaccgaggcagaactcgtg

cgcgcaatggtggactgcgcgcacagctcggctttgagcgcggccctgggaaatggcaac

ggcggcaaacagagcggcggcagcggcgggggatggtggggcggaaagcacgtgtctgcg

gactgcttcaaagtggagaccgtgtgccacacgacgctgtactactttggatctaagcca

gctctctactatagagtatctgcctccagcagccgcctgggagggttcatctgcgacaac

tttcacccggagattacaaaattcgaggggagcgtggacgtgaccacgcggctgttgttg

gacaatgaaaattttaccagtttcgggtggtaccgcctgcgacccggcacccacggagag

cgtgttcaacttcgccccgttgagcgacacgtcacctcaagcgacgtggagattaactgt

actcccgataacctggagccgataccagacgaggctgcctggcccgactataagctcatg

tgctttgatatagagtgtaaagctggaacgggtaacgaaatggcgttcccagtggcaact

aaccaagaggacctggtcatccagatctcctgtctgctgtactcgcttgctactcagaac

cacgaacacaccctgctgttttccctcgggtcatgcgatatctctgaggaatactcgttt

gcatgcgtccagcgcggcgagcccagaccgacggttttggagtttgacagcgagtacgag

ctgctggttgccttcctgacctttctcaagcagtactctcccgagttcgccaccggctac

aacatcgttaattttgactgggcgtacatagttaacaaggtaacgtcggtgtataacatc

aagctggacgggtacggcaagttcaacaaaggggggctgtttaaggtgtgggacatcgcc

acgaaccattttcagaagaagagcaaggtgaaaatcaatggcctgatatctctagacatg

tattctgtggcgacggaaaagctaaagctacccagctacaaactcgacgcggtcgtggga

gacgtcctcggcgagcataagatagaccttccctataaagaaataccctcctattacgcg

ggagggcctgaccggaggggcgtaataggagagtattgtatccaggactctaggctggtg

ggcaagctgttttttaagtacctcccccatctggaactatcggcggtggccaaactcgcc

cgtatcaccctaacgcgggtaatttttgacggtcagcaaattcgcgtgtacacgtgcttg

ctgaaactcgcccgcgagagaaatttcattttgccagacaacagacgccggtttgacagt

caggcagacgccgcgtcagagacttcggagttggctatggatagccaaagccacgccttc

gacagtacagacgaacccgacggtgtggacggtaccccggacgccgcaggatctggcgct

acttctgaaaacggaggcgggaagcccggcgtcgggagggccgtgggctaccagggagca

aaggttctagaccccgtatccggctttcatgtggaccccgtggttgtgtttgacttcgct

agcttatacccaagcattatccaggcccataacctctgtttcaccaccctggcgctcgat

gaagtggatctggccgggcttcaaccatccgtcgactactcgacgttcgaggtgggtgac

caaaagttattttttgtccacgcccatattcgcgaaagcctgcttggcatcttgctgcgc

gactggctggccatgcgaaaggcggtgagggcgcgaatccccaccagcacccccgaggag

gcagttttactagataagcagcagtctgcgattaaggtgatatgcaactcggtttacgga

ttcacgggggtggcaaacggcctgttgccgtgtctgaggatagcggctaccgttaccacg

ataggacgcgacatgctcctcaagacccgagattacgttcactctcgttgggcgacgcgc

gagctgctggaggacaattttccaggggctataggtttccgaaaccacaagccttactcc

gtcagggttatctacggagacaccgactccgtgtttatcaagtttgtgggcctgacgtac

gagggggtatcggagctgggggatgctatgtcgcgtcagatttcagcggacctctttaga

gcgcccatcaaactggagtgtgagaagacctttcagcgactgctgctgatcaccaagaag

aagtacataggtgtcataaacggggggaagatgctcatgaagggggtcgacctggtccgc

aaaaataactgctctttcataaacttgtacgcgcgacatctggtagatcttttgttgtac

gacgaggatgtggccacggcggcagcagaggtgacagacgtgcctcccgcagaatgggtg

gggcgcccgctaccgagcggctttgacaagtttgggcgagtgctggtagaggcgtacaac

cgtatcactgcccccaacttggacgtgcgcgagttcgttatgactgctgagctgagccgc

tcacccgaatcgtataccaacaagcgcctgccgcacctcaccgtctactttaagctcgcc

atgaggaatgaagaactgcccagcgtaaaagagagaattccgtatgtgatagttgcgcag

accgaggccgcggaacgcgaagcgggtgtagtaaactcaatgcgcggtaccgcccaaaac

cccgtggtaaccaagaccgcacgcccccaacctaaacgcaaactgctggtttccgacctc

gccgaagacccgacctatgtttccgagaatgacgtaccgctaaacacagactactatttc

tcccacctgttgggtaccataagcgtgacctttaaggctctattcggaaatgatgtgaga

acaacagaaaatcttttaaagcggtttattccggaaaccccccacaagacccccacgaaa

acccaggcactgcttgagcgcgccggctttgaaaagctgacgccctttacaccggaggaa

gaaagtcgtcgaatactgcatacagttttttgtactctagaagcagctccccatcaaagc

tga

>KU206445.1

atggcggcgcgcgaacaggccaactccgttcgcaggagcgggttttttaatccgtttatt

ggcaagaggccatttttcaggccgggaagcgggcagaccgcggagacagagcgcccaagg

cccccccaacactcgtactgcacagaggtgggtagctttaagtttatagctccaagatgc

ctcgatgaagaagcccccgccgaccagcgacgcggtgtacacgtgggcaccttggagcgc

ccacccaaagtgtactgcgatggctcagagtacgacgtgctgaactttgcctccggaggt

tgctggcctcgcaggattcgggtttggaacggccaggattttcggggcgatggattcaac

cccagatttgagagatttcacgtgtacgacatagtagagacttcggagagcgcctcacac

gatgacccatccaggtttgctgagctatcccgcccaagcgggtctgtggtaacactgctg

ggaatgagcgagtgtggaaagcgagtcgcggttcacgtctatggtgtgcgccattatttt

tacatggcaaaggcggaggtggatagcgcttgtggaataaccaccgaggcagaactcgtg

cgcgcaatggtggactgcgcgcacagctcggctttgagcgcggccctgggaaatggcaac

ggcggcaaacagagcggcggcagcggcgggggatggtggggcggaaagcacgtgtctgcg

gactgcttcaaagtggagaccgtgtgccacacgacgctgtactactttggatctaagcca

gctctctactatagagtatctgcctccagcagccgcctgggagggttcatctgcgacaac

tttcacccggagattacaaaattcgaggggagcgtggacgtgaccacgcggctgttgttg

gacaatgaaaattttaccagtttcgggtggtaccgcctgcgacccggcacccacggagag

cgtgttcaacttcgccccgttgagcgacacgtcacctcaagcgacgtggagattaactgt

actcccgataacctggagccgataccagacgaggctgcctggcccgactataagctcatg

tgctttgatatagagtgtaaagctggaacgggtaacgaaatggcgttcccagtggcaact

aaccaagaggacctggtcatccagatctcctgtctgctgtactcgcttgctactcagaac

cacgaacacaccctgctgttttccctcgggtcatgcgatatctctgaggaatactcgttt

gcatgcgtccagcgcggcgagcccagaccgacggttttggagtttgacagcgagtacgag

ctgctggttgccttcctgacctttctcaagcagtactctcccgagttcgccaccggctac

aacatcgttaattttgactgggcgtacatagttaacaaggtaacgtcggtgtataacatc

aagctggacgggtacggcaagttcaacaaaggggggctgtttaaggtgtgggacatcgcc

acgaaccattttcagaagaagagcaaggtgaaaatcaatggcctgatatctctagacatg

tattctgtggcgacggaaaagctaaagctacccagctacaaactcgacgcggtcgtggga

gacgtcctcggcgagcataagatagaccttccctataaagaaataccctcctattacgcg

ggagggcctgaccggaggggcgtaataggagagtattgtatccaggactctaggctggtg

ggcaagctgttttttaagtacctcccccatctggaactatcggcggtggccaaactcgcc

cgtatcaccctaacgcgggtaatttttgacggtcagcaaattcgcgtgtacacgtgcttg

ctgaaactcgcccgcgagagaaatttcattttgccagacaacagacgccggtttgacagt

caggcagacgccgcgtcagagacttcggagttggctatggatagccaaagccacgccttc

gacagtacagacgaacccgacggtgtggacggtaccccggacgccgcaggatctggcgct

acttctgaaaacggaggcgggaagcccggcgtcgggagggccgtgggctaccagggagca

aaggttctagaccccgtatccggctttcatgtggaccccgtggttgtgtttgacttcgct

agcttatacccaagcattatccaggcccataacctctgtttcaccaccctggcgctcgat

gaagtggatctggccgggcttcaaccatccgtcgactactcgacgttcgaggtgggtgac

caaaagttattttttgtccacgcccatattcgcgaaagcctgcttggcatcttgctgcgc

gactggctggccatgcgaaaggcggtgagggcgcgaatccccaccagcacccccgaggag

gcagttttactagataagcagcagtctgcgattaaggtgatatgcaactcggtttacgga

ttcacgggggtggcaaacggcctgttgccgtgtctgaggatagcggctaccgttaccacg

ataggacgcgacatgctcctcaagacccgagattacgttcactctcgttgggcgacgcgc

gagctgctggaggacaattttccaggggctataggtttccgaaaccacaagccttactcc

gtcagggttatctacggagacaccgactccgtgtttatcaagtttgtgggcctgacgtac

gagggggtatcggagctgggggatgctatgtcgcgtcagatttcagcggacctctttaga

gcgcccatcaaactggagtgtgagaagacctttcagcgactgctgctgatcaccaagaag

aagtacataggtgtcataaacggggggaagatgctcatgaagggggtcgacctggtccgc

aaaaataactgctctttcataaacttgtacgcgcgacatctggtagatcttttgttgtac

gacgaggatgtggccacggcggcagcagaggtgacagacgtgcctcccgcagaatgggtg

gggcgcccgctaccgagcggctttgacaagtttgggcgagtgctggtagaggcgtacaac

cgtatcactgcccccaacttggacgtgcgcgagttcgttatgactgctgagctgagccgc

tcacccgaatcgtataccaacaagcgcctgccgcacctcaccgtctactttaagctcgcc

atgaggaatgaagaactgcccagcgtaaaagagagaattccgtatgtgatagttgcgcag

accgaggccgcggaacgcgaagcgggtgtagtaaactcaatgcgcggtaccgcccaaaac

cccgtggtaaccaagaccgcacgcccccaacctaaacgcaaactgctggtttccgacctc

gccgaagacccgacctatgtttccgagaatgacgtaccgctaaacacagactactatttc

tcccacctgttgggtaccataagcgtgacctttaaggctctattcggaaatgatgtgaga

acaacagaaaatcttttaaagcggtttattccggaaaccccccacaagacccccacgaaa

acccaggcactgcttgagcgcgccggctttgaaaagctgacgccctttacaccggaggaa

gaaagtcgtcgaatactgcatacagttttttgtactctagaagcagctccccatcaaagc

tga

>KU206446.1

atggcggcgcgcgaacaggccaactccgttcgcaggagcgggttttttaatccgtttatt

ggcaagaggccatttttcaggccgggaagcgggcagaccgcggagacagagcgcccaagg

cccccccaacactcgtactgcacagaggtgggtagctttaagtttatagctccaagatgc

ctcgatgaagaagcccccgccgaccagcgacgcggtgtacacgtgggcaccttggagcgc

ccacccaaagtgtactgcgatggctcagagtacgacgtgctgaactttgcctccggaggt

tgctggcctcgcaggattcgggtttggaacggccaggattttcggggcgatggattcaac

cccagatttgagagatttcacgtgtacgacatagtagagacttcggagagcgcctcacac

gatgacccatccaggtttgctgagctatcccgcccaagcgggtctgtggtaacactgctg

ggaatgagcgagtgtggaaagcgagtcgcggttcacgtctatggtgtgcgccattatttt

tacatggcaaaggcggaggtggatagcgcttgtggaataaccaccgaggcagaactcgtg

cgcgcaatggtggactgcgcgcacagctcggctttgagcgcggccctgggaaatggcaac

ggcggcaaacagagcggcggcagcggcgggggatggtggggcggaaagcacgtgtctgcg

gactgcttcaaagtggagaccgtgtgccacacgacgctgtactactttggatctaagcca

gctctctactatagagtatctgcctccagcagccgcctgggagggttcatctgcgacaac

tttcacccggagattacaaaattcgaggggagcgtggacgtgaccacgcggctgttgttg

gacaatgaaaattttaccagtttcgggtggtaccgcctgcgacccggcacccacggagag

cgtgttcaacttcgccccgttgagcgacacgtcacctcaagcgacgtggagattaactgt

actcccgataacctggagccgataccagacgaggctgcctggcccgactataagctcatg

tgctttgatatagagtgtaaagctggaacgggtaacgaaatggcgttcccagtggcaact

aaccaagaggacctggtcatccagatctcctgtctgctgtactcgcttgctactcagaac

cacgaacacaccctgctgttttccctcgggtcatgcgatatctctgaggaatactcgttt

gcatgcgtccagcgcggcgagcccagaccgacggttttggagtttgacagcgagtacgag

ctgctggttgccttcctgacctttctcaagcagtactctcccgagttcgccaccggctac

aacatcgttaattttgactgggcgtacatagttaacaaggtaacgtcggtgtataacatc

aagctggacgggtacggcaagttcaacaaaggggggctgtttaaggtgtgggacatcgcc

acgaaccattttcagaagaagagcaaggtgaaaatcaatggcctgatatctctagacatg

tattctgtggcgacggaaaagctaaagctacccagctacaaactcgacgcggtcgtggga

gacgtcctcggcgagcataagatagaccttccctataaagaaataccctcctattacgcg

ggagggcctgaccggaggggcgtaataggagagtattgtatccaggactctaggctggtg

ggcaagctgttttttaagtacctcccccatctggaactatcggcggtggccaaactcgcc

cgtatcaccctaacgcgggtaatttttgacggtcagcaaattcgcgtgtacacgtgcttg

ctgaaactcgcccgcgagagaaatttcattttgccagacaacagacgccggtttgacagt

caggcagacgccgcgtcagagacttcggagttggctatggatagccaaagccacgccttc

gacagtacagacgaacccgacggtgtggacggtaccccggacgccgcaggatctggcgct

acttctgaaaacggaggcgggaagcccggcgtcgggagggccgtgggctaccagggagca

aaggttctagaccccgtatccggctttcatgtggaccccgtggttgtgtttgacttcgct

agcttatacccaagcattatccaggcccataacctctgtttcaccaccctggcgctcgat

gaagtggatctggccgggcttcaaccatccgtcgactactcgacgttcgaggtgggtgac

caaaagttattttttgtccacgcccatattcgcgaaagcctgcttggcatcttgctgcgc

gactggctggccatgcgaaaggcggtgagggcgcgaatccccaccagcacccccgaggag

gcagttttactagataagcagcagtctgcgattaaggtgatatgcaactcggtttacgga

ttcacgggggtggcaaacggcctgttgccgtgtctgaggatagcggctaccgttaccacg

ataggacgcgacatgctcctcaagacccgagattacgttcactctcgttgggcgacgcgc

gagctgctggaggacaattttccaggggctataggtttccgaaaccacaagccttactcc

gtcagggttatctacggagacaccgactccgtgtttatcaagtttgtgggcctgacgtac

gagggggtatcggagctgggggatgctatgtcgcgtcagatttcagcggacctctttaga

gcgcccatcaaactggagtgtgagaagacctttcagcgactgctgctgatcaccaagaag

aagtacataggtgtcataaacggggggaagatgctcatgaagggggtcgacctggtccgc

aaaaataactgctctttcataaacttgtacgcgcgacatctggtagatcttttgttgtac

gacgaggatgtggccacggcggcagcagaggtgacagacgtgcctcccgcagaatgggtg

gggcgcccgctaccgagcggctttgacaagtttgggcgagtgctggtagaggcgtacaac

cgtatcactgcccccaacttggacgtgcgcgagttcgttatgactgctgagctgagccgc

tcacccgaatcgtataccaacaagcgcctgccgcacctcaccgtctactttaagctcgcc

atgaggaatgaagaactgcccagcgtaaaagagagaattccgtatgtgatagttgcgcag

accgaggccgcggaacgcgaagcgggtgtagtaaactcaatgcgcggtaccgcccaaaac

cccgtggtaaccaagaccgcacgcccccaacctaaacgcaaactgctggtttccgacctc

gccgaagacccgacctatgtttccgagaatgacgtaccgctaaacacagactactatttc

tcccacctgttgggtaccataagcgtgacctttaaggctctattcggaaatgatgtgaga

acaacagaaaatcttttaaagcggtttattccggaaaccccccacaagacccccacgaaa

acccaggcactgcttgagcgcgccggctttgaaaagctgacgccctttacaccggaggaa

gaaagtcgtcgaatactgcatacagttttttgtactctagaagcagctccccatcaaagc

tga

>KU206447.1

atggcggcgcgcgaacaggccaactccgttcgcaggagcgggttttttaatccgtttatt

ggcaagaggccatttttcaggccgggaagcgggcagaccgcggagacagagcgcccaagg

cccccccaacactcgtactgcacagaggtgggtagctttaagtttatagctccaagatgc

ctcgatgaagaagcccccgccgaccagcgacgcggtgtacacgtgggcaccttggagcgc

ccacccaaagtgtactgcgatggctcagagtacgacgtgctgaactttgcctccggaggt

tgctggcctcgcaggattcgggtttggaacggccaggattttcggggcgatggattcaac

cccagatttgagagatttcacgtgtacgacatagtagagacttcggagagcgcctcacac

gatgacccatccaggtttgctgagctatcccgcccaagcgggtctgtggtaacactgctg

ggaatgagcgagtgtggaaagcgagtcgcggttcacgtctatggtgtgcgccattatttt

tacatggcaaaggcggaggtggatagcgcttgtggaataaccaccgaggcagaactcgtg

cgcgcaatggtggactgcgcgcacagctcggctttgagcgcggccctgggaaatggcaac

ggcggcaaacagagcggcggcagcggcgggggatggtggggcggaaagcacgtgtctgcg

gactgcttcaaagtggagaccgtgtgccacacgacgctgtactactttggatctaagcca

gctctctactatagagtatctgcctccagcagccgcctgggagggttcatctgcgacaac

tttcacccggagattacaaaattcgaggggagcgtggacgtgaccacgcggctgttgttg

gacaatgaaaattttaccagtttcgggtggtaccgcctgcgacccggcacccacggagag

cgtgttcaacttcgccccgttgagcgacacgtcacctcaagcgacgtggagattaactgt

actcccgataacctggagccgataccagacgaggctgcctggcccgactataagctcatg

tgctttgatatagagtgtaaagctggaacgggtaacgaaatggcgttcccagtggcaact

aaccaagaggacctggtcatccagatctcctgtctgctgtactcgcttgctactcagaac

cacgaacacaccctgctgttttccctcgggtcatgcgatatctctgaggaatactcgttt

gcatgcgtccagcgcggcgagcccagaccgacggttttggagtttgacagcgagtacgag

ctgctggttgccttcctgacctttctcaagcagtactctcccgagttcgccaccggctac

aacatcgttaattttgactgggcgtacatagttaacaaggtaacgtcggtgtataacatc

aagctggacgggtacggcaagttcaacaaaggggggctgtttaaggtgtgggacatcgcc

acgaaccattttcagaagaagagcaaggtgaaaatcaatggcctgatatctctagacatg

tattctgtggcgacggaaaagctaaagctacccagctacaaactcgacgcggtcgtggga

gacgtcctcggcgagcataagatagaccttccctataaagaaataccctcctattacgcg

ggagggcctgaccggaggggcgtaataggagagtattgtatccaggactctaggctggtg

ggcaagctgttttttaagtacctcccccatctggaactatcggcggtggccaaactcgcc

cgtatcaccctaacgcgggtaatttttgacggtcagcaaattcgcgtgtacacgtgcttg

ctgaaactcgcccgcgagagaaatttcattttgccagacaacagacgccggtttgacagt

caggcagacgccgcgtcagagacttcggagttggctatggatagccaaagccacgccttc

gacagtacagacgaacccgacggtgtggacggtaccccggacgccgcaggatctggcgct

acttctgaaaacggaggcgggaagcccggcgtcgggagggccgtgggctaccagggagca

aaggttctagaccccgtatccggctttcatgtggaccccgtggttgtgtttgacttcgct

agcttatacccaagcattatccaggcccataacctctgtttcaccaccctggcgctcgat

gaagtggatctggccgggcttcaaccatccgtcgactactcgacgttcgaggtgggtgac

caaaagttattttttgtccacgcccatattcgcgaaagcctgcttggcatcttgctgcgc

gactggctggccatgcgaaaggcggtgagggcgcgaatccccaccagcacccccgaggag

gcagttttactagataagcagcagtctgcgattaaggtgatatgcaactcggtttacgga

ttcacgggggtggcaaacggcctgttgccgtgtctgaggatagcggctaccgttaccacg

ataggacgcgacatgctcctcaagacccgagattacgttcactctcgttgggcgacgcgc

gagctgctggaggacaattttccaggggctataggtttccgaaaccacaagccttactcc

gtcagggttatctacggagacaccgactccgtgtttatcaagtttgtgggcctgacgtac

gagggggtatcggagctgggggatgctatgtcgcgtcagatttcagcggacctctttaga

gcgcccatcaaactggagtgtgagaagacctttcagcgactgctgctgatcaccaagaag

aagtacataggtgtcataaacggggggaagatgctcatgaagggggtcgacctggtccgc

aaaaataactgctctttcataaacttgtacgcgcgacatctggtagatcttttgttgtac

gacgaggatgtggccacggcggcagcagaggtgacagacgtgcctcccgcagaatgggtg

gggcgcccgctaccgagcggctttgacaagtttgggcgagtgctggtagaggcgtacaac

cgtatcactgcccccaacttggacgtgcgcgagttcgttatgactgctgagctgagccgc

tcacccgaatcgtataccaacaagcgcctgccgcacctcaccgtctactttaagctcgcc

atgaggaatgaagaactgcccagcgtaaaagagagaattccgtatgtgatagttgcgcag

accgaggccgcggaacgcgaagcgggtgtagtaaactcaatgcgcggtaccgcccaaaac

cccgtggtaaccaagaccgcacgcccccaacctaaacgcaaactgctggtttccgacctc

gccgaagacccgacctatgtttccgagaatgacgtaccgctaaacacagactactatttc

tcccacctgttgggtaccataagcgtgacctttaaggctctattcggaaatgatgtgaga

acaacagaaaatcttttaaagcggtttattccggaaaccccccacaagacccccacgaaa

acccaggcactgcttgagcgcgccggctttgaaaagctgacgccctttacaccggaggaa

gaaagtcgtcgaatactgcatacagttttttgtactctagaagcagctccccatcaaagc

tga

>KU206448.1

atggcggcgcgcgaacaggccaactccgttcgcaggagcgggttttttaatccgtttatt

ggcaagaggccatttttcaggccgggaagcgggcagaccgcggagacagagcgcccaagg

cccccccaacactcgtactgcacagaggtgggtagctttaagtttatagctccaagatgc

ctcgatgaagaagcccccgccgaccagcgacgcggtgtacacgtgggcaccttggagcgc

ccacccaaagtgtactgcgatggctcagagtacgacgtgctgaactttgcctccggaggt

tgctggcctcgcaggattcgggtttggaacggccaggattttcggggcgatggattcaac

cccagatttgagagatttcacgtgtacgacatagtagagacttcggagagcgcctcacac

gatgacccatccaggtttgctgagctatcccgcccaagcgggtctgtggtaacactgctg

ggaatgagcgagtgtggaaagcgagtcgcggttcacgtctatggtgtgcgccattatttt

tacatggcaaaggcggaggtggatagcgcttgtggaataaccaccgaggcagaactcgtg

cgcgcaatggtggactgcgcgcacagctcggctttgagcgcggccctgggaaatggcaac

ggcggcaaacagagcggcggcagcggcgggggatggtggggcggaaagcacgtgtctgcg

gactgcttcaaagtggagaccgtgtgccacacgacgctgtactactttggatctaagcca

gctctctactatagagtatctgcctccagcagccgcctgggagggttcatctgcgacaac

tttcacccggagattacaaaattcgaggggagcgtggacgtgaccacgcggctgttgttg

gacaatgaaaattttaccagtttcgggtggtaccgcctgcgacccggcacccacggagag

cgtgttcaacttcgccccgttgagcgacacgtcacctcaagcgacgtggagattaactgt

actcccgataacctggagccgataccagacgaggctgcctggcccgactataagctcatg

tgctttgatatagagtgtaaagctggaacgggtaacgaaatggcgttcccagtggcaact

aaccaagaggacctggtcatccagatctcctgtctgctgtactcgcttgctactcagaac

cacgaacacaccctgctgttttccctcgggtcatgcgatatctctgaggaatactcgttt

gcatgcgtccagcgcggcgagcccagaccgacggttttggagtttgacagcgagtacgag

ctgctggttgccttcctgacctttctcaagcagtactctcccgagttcgccaccggctac

aacatcgttaattttgactgggcgtacatagttaacaaggtaacgtcggtgtataacatc

aagctggacgggtacggcaagttcaacaaaggggggctgtttaaggtgtgggacatcgcc

acgaaccattttcagaagaagagcaaggtgaaaatcaatggcctgatatctctagacatg

tattctgtggcgacggaaaagctaaagctacccagctacaaactcgacgcggtcgtggga

gacgtcctcggcgagcataagatagaccttccctataaagaaataccctcctattacgcg

ggagggcctgaccggaggggcgtaataggagagtattgtatccaggactctaggctggtg

ggcaagctgttttttaagtacctcccccatctggaactatcggcggtggccaaactcgcc

cgtatcaccctaacgcgggtaatttttgacggtcagcaaattcgcgtgtacacgtgcttg

ctgaaactcgcccgcgagagaaatttcattttgccagacaacagacgccggtttgacagt

caggcagacgccgcgtcagagacttcggagttggctatggatagccaaagccacgccttc

gacagtacagacgaacccgacggtgtggacggtaccccggacgccgcaggatctggcgct

acttctgaaaacggaggcgggaagcccggcgtcgggagggccgtgggctaccagggagca

aaggttctagaccccgtatccggctttcatgtggaccccgtggttgtgtttgacttcgct

agcttatacccaagcattatccaggcccataacctctgtttcaccaccctggcgctcgat

gaagtggatctggccgggcttcaaccatccgtcgactactcgacgttcgaggtgggtgac

caaaagttattttttgtccacgcccatattcgcgaaagcctgcttggcatcttgctgcgc

gactggctggccatgcgaaaggcggtgagggcgcgaatccccaccagcacccccgaggag

gcagttttactagataagcagcagtctgcgattaaggtgatatgcaactcggtttacgga

ttcacgggggtggcaaacggcctgttgccgtgtctgaggatagcggctaccgttaccacg

ataggacgcgacatgctcctcaagacccgagattacgttcactctcgttgggcgacgcgc

gagctgctggaggacaattttccaggggctataggtttccgaaaccacaagccttactcc

gtcagggttatctacggagacaccgactccgtgtttatcaagtttgtgggcctgacgtac

gagggggtatcggagctgggggatgctatgtcgcgtcagatttcagcggacctctttaga

gcgcccatcaaactggagtgtgagaagacctttcagcgactgctgctgatcaccaagaag

aagtacataggtgtcataaacggggggaagatgctcatgaagggggtcgacctggtccgc

aaaaataactgctctttcataaacttgtacgcgcgacatctggtagatcttttgttgtac

gacgaggatgtggccacggcggcagcagaggtgacagacgtgcctcccgcagaatgggtg

gggcgcccgctaccgagcggctttgacaagtttgggcgagtgctggtagaggcgtacaac

cgtatcactgcccccaacttggacgtgcgcgagttcgttatgactgctgagctgagccgc

tcacccgaatcgtataccaacaagcgcctgccgcacctcaccgtctactttaagctcgcc

atgaggaatgaagaactgcccagcgtaaaagagagaattccgtatgtgatagttgcgcag

accgaggccgcggaacgcgaagcgggtgtagtaaactcaatgcgcggtaccgcccaaaac

cccgtggtaaccaagaccgcacgcccccaacctaaacgcaaactgctggtttccgacctc

gccgaagacccgacctatgtttccgagaatgacgtaccgctaaacacagactactatttc

tcccacctgttgggtaccataagcgtgacctttaaggctctattcggaaatgatgtgaga

acaacagaaaatcttttaaagcggtttattccggaaaccccccacaagacccccacgaaa

acccaggcactgcttgagcgcgccggctttgaaaagctgacgccctttacaccggaggaa

gaaagtcgtcgaatactgcatacagttttttgtactctagaagcagctccccatcaaagc

tga

>KU206449.1

atggcggcgcgcgaacaggccaactccgttcgcaggagcgggttttttaatccgtttatt

ggcaagaggccatttttcaggccgggaagcgggcagaccgcggagacagagcgcccaagg

cccccccaacactcgtactgcacagaggtgggtagctttaagtttatagctccaagatgc

ctcgatgaagaagcccccgccgaccagcgacgcggtgtacacgtgggcaccttggagcgc

ccacccaaagtgtactgcgatggctcagagtacgacgtgctgaactttgcctccggaggt

tgctggcctcgcaggattcgggtttggaacggccaggattttcggggcgatggattcaac

cccagatttgagagatttcacgtgtacgacatagtagagacttcggagagcgcctcacac

gatgacccatccaggtttgctgagctatcccgcccaagcgggtctgtggtaacactgctg

ggaatgagcgagtgtggaaagcgagtcgcggttcacgtctatggtgtgcgccattatttt

tacatggcaaaggcggaggtggatagcgcttgtggaataaccaccgaggcagaactcgtg

cgcgcaatggtggactgcgcgcacagctcggctttgagcgcggccctgggaaatggcaac

ggcggcaaacagagcggcggcagcggcgggggatggtggggcggaaagcacgtgtctgcg

gactgcttcaaagtggagaccgtgtgccacacgacgctgtactactttggatctaagcca

gctctctactatagagtatctgcctccagcagccgcctgggagggttcatctgcgacaac

tttcacccggagattacaaaattcgaggggagcgtggacgtgaccacgcggctgttgttg

gacaatgaaaattttaccagtttcgggtggtaccgcctgcgacccggcacccacggagag

cgtgttcaacttcgccccgttgagcgacacgtcacctcaagcgacgtggagattaactgt

actcccgataacctggagccgataccagacgaggctgcctggcccgactataagctcatg

tgctttgatatagagtgtaaagctggaacgggtaacgaaatggcgttcccagtggcaact

aaccaagaggacctggtcatccagatctcctgtctgctgtactcgcttgctactcagaac

cacgaacacaccctgctgttttccctcgggtcatgcgatatctctgaggaatactcgttt

gcatgcgtccagcgcggcgagcccagaccgacggttttggagtttgacagcgagtacgag

ctgctggttgccttcctgacctttctcaagcagtactctcccgagttcgccaccggctac

aacatcgttaattttgactgggcgtacatagttaacaaggtaacgtcggtgtataacatc

aagctggacgggtacggcaagttcaacaaaggggggctgtttaaggtgtgggacatcgcc

acgaaccattttcagaagaagagcaaggtgaaaatcaatggcctgatatctctagacatg

tattctgtggcgacggaaaagctaaagctacccagctacaaactcgacgcggtcgtggga

gacgtcctcggcgagcataagatagaccttccctataaagaaataccctcctattacgcg

ggagggcctgaccggaggggcgtaataggagagtattgtatccaggactctaggctggtg

ggcaagctgttttttaagtacctcccccatctggaactatcggcggtggccaaactcgcc

cgtatcaccctaacgcgggtaatttttgacggtcagcaaattcgcgtgtacacgtgcttg

ctgaaactcgcccgcgagagaaatttcattttgccagacaacagacgccggtttgacagt

caggcagacgccgcgtcagagacttcggagttggctatggatagccaaagccacgccttc

gacagtacagacgaacccgacggtgtggacggtaccccggacgccgcaggatctggcgct

acttctgaaaacggaggcgggaagcccggcgtcgggagggccgtgggctaccagggagca

aaggttctagaccccgtatccggctttcatgtggaccccgtggttgtgtttgacttcgct

agcttatacccaagcattatccaggcccataacctctgtttcaccaccctggcgctcgat

gaagtggatctggccgggcttcaaccatccgtcgactactcgacgttcgaggtgggtgac

caaaagttattttttgtccacgcccatattcgcgaaagcctgcttggcatcttgctgcgc

gactggctggccatgcgaaaggcggtgagggcgcgaatccccaccagcacccccgaggag

gcagttttactagataagcagcagtctgcgattaaggtgatatgcaactcggtttacgga

ttcacgggggtggcaaacggcctgttgccgtgtctgaggatagcggctaccgttaccacg

ataggacgcgacatgctcctcaagacccgagattacgttcactctcgttgggcgacgcgc

gagctgctggaggacaattttccaggggctataggtttccgaaaccacaagccttactcc

gtcagggttatctacggagacaccgactccgtgtttatcaagtttgtgggcctgacgtac

gagggggtatcggagctgggggatgctatgtcgcgtcagatttcagcggacctctttaga

gcgcccatcaaactggagtgtgagaagacctttcagcgactgctgctgatcaccaagaag

aagtacataggtgtcataaacggggggaagatgctcatgaagggggtcgacctggtccgc

aaaaataactgctctttcataaacttgtacgcgcgacatctggtagatcttttgttgtac

gacgaggatgtggccacggcggcagcagaggtgacagacgtgcctcccgcagaatgggtg

gggcgcccgctaccgagcggctttgacaagtttgggcgagtgctggtagaggcgtacaac

cgtatcactgcccccaacttggacgtgcgcgagttcgttatgactgctgagctgagccgc

tcacccgaatcgtataccaacaagcgcctgccgcacctcaccgtctactttaagctcgcc

atgaggaatgaagaactgcccagcgtaaaagagagaattccgtatgtgatagttgcgcag

accgaggccgcggaacgcgaagcgggtgtagtaaactcaatgcgcggtaccgcccaaaac

cccgtggtaaccaagaccgcacgcccccaacctaaacgcaaactgctggtttccgacctc

gccgaagacccgacctatgtttccgagaatgacgtaccgctaaacacagactactatttc

tcccacctgttgggtaccataagcgtgacctttaaggctctattcggaaatgatgtgaga

acaacagaaaatcttttaaagcggtttattccggaaaccccccacaagacccccacgaaa

acccaggcactgcttgagcgcgccggctttgaaaagctgacgccctttacaccggaggaa

gaaagtcgtcgaatactgcatacagttttttgtactctagaagcagctccccatcaaagc

tga

>KU206450.1

atggcggcgcgcgaacaggccaactccgttcgcaggagcgggttttttaatccgtttatt

ggcaagaggccatttttcaggccgggaagcgggcagaccgcggagacagagcgcccaagg

cccccccaacactcgtactgcacagaggtgggtagctttaagtttatagctccaagatgc

ctcgatgaagaagcccccgccgaccagcgacgcggtgtacacgtgggcaccttggagcgc

ccacccaaagtgtactgcgatggctcagagtacgacgtgctgaactttgcctccggaggt

tgctggcctcgcaggattcgggtttggaacggccaggattttcggggcgatggattcaac

cccagatttgagagatttcacgtgtacgacatagtagagacttcggagagcgcctcacac

gatgacccatccaggtttgctgagctatcccgcccaagcgggtctgtggtaacactgctg

ggaatgagcgagtgtggaaagcgagtcgcggttcacgtctatggtgtgcgccattatttt

tacatggcaaaggcggaggtggatagcgcttgtggaataaccaccgaggcagaactcgtg

cgcgcaatggtggactgcgcgcacagctcggctttgagcgcggccctgggaaatggcaac

ggcggcaaacagagcggcggcagcggcgggggatggtggggcggaaagcacgtgtctgcg

gactgcttcaaagtggagaccgtgtgccacacgacgctgtactactttggatctaagcca

gctctctactatagagtatctgcctccagcagccgcctgggagggttcatctgcgacaac

tttcacccggagattacaaaattcgaggggagcgtggacgtgaccacgcggctgttgttg

gacaatgaaaattttaccagtttcgggtggtaccgcctgcgacccggcacccacggagag

cgtgttcaacttcgccccgttgagcgacacgtcacctcaagcgacgtggagattaactgt

actcccgataacctggagccgataccagacgaggctgcctggcccgactataagctcatg

tgctttgatatagagtgtaaagctggaacgggtaacgaaatggcgttcccagtggcaact

aaccaagaggacctggtcatccagatctcctgtctgctgtactcgcttgctactcagaac

cacgaacacaccctgctgttttccctcgggtcatgcgatatctctgaggaatactcgttt

gcatgcgtccagcgcggcgagcccagaccgacggttttggagtttgacagcgagtacgag

ctgctggttgccttcctgacctttctcaagcagtactctcccgagttcgccaccggctac

aacatcgttaattttgactgggcgtacatagttaacaaggtaacgtcggtgtataacatc

aagctggacgggtacggcaagttcaacaaaggggggctgtttaaggtgtgggacatcgcc

acgaaccattttcagaagaagagcaaggtgaaaatcaatggcctgatatctctagacatg

tattctgtggcgacggaaaagctaaagctacccagctacaaactcgacgcggtcgtggga

gacgtcctcggcgagcataagatagaccttccctataaagaaataccctcctattacgcg

ggagggcctgaccggaggggcgtaataggagagtattgtatccaggactctaggctggtg

ggcaagctgttttttaagtacctcccccatctggaactatcggcggtggccaaactcgcc

cgtatcaccctaacgcgggtaatttttgacggtcagcaaattcgcgtgtacacgtgcttg

ctgaaactcgcccgcgagagaaatttcattttgccagacaacagacgccggtttgacagt

caggcagacgccgcgtcagagacttcggagttggctatggatagccaaagccacgccttc

gacagtacagacgaacccgacggtgtggacggtaccccggacgccgcaggatctggcgct

acttctgaaaacggaggcgggaagcccggcgtcgggagggccgtgggctaccagggagca

aaggttctagaccccgtatccggctttcatgtggaccccgtggttgtgtttgacttcgct

agcttatacccaagcattatccaggcccataacctctgtttcaccaccctggcgctcgat

gaagtggatctggccgggcttcaaccatccgtcgactactcgacgttcgaggtgggtgac

caaaagttattttttgtccacgcccatattcgcgaaagcctgcttggcatcttgctgcgc

gactggctggccatgcgaaaggcggtgagggcgcgaatccccaccagcacccccgaggag

gcagttttactagataagcagcagtctgcgattaaggtgatatgcaactcggtttacgga

ttcacgggggtggcaaacggcctgttgccgtgtctgaggatagcggctaccgttaccacg

ataggacgcgacatgctcctcaagacccgagattacgttcactctcgttgggcgacgcgc

gagctgctggaggacaattttccaggggctataggtttccgaaaccacaagccttactcc

gtcagggttatctacggagacaccgactccgtgtttatcaagtttgtgggcctgacgtac

gagggggtatcggagctgggggatgctatgtcgcgtcagatttcagcggacctctttaga

gcgcccatcaaactggagtgtgagaagacctttcagcgactgctgctgatcaccaagaag

aagtacataggtgtcataaacggggggaagatgctcatgaagggggtcgacctggtccgc

aaaaataactgctctttcataaacttgtacgcgcgacatctggtagatcttttgttgtac

gacgaggatgtggccacggcggcagcagaggtgacagacgtgcctcccgcagaatgggtg

gggcgcccgctaccgagcggctttgacaagtttgggcgagtgctggtagaggcgtacaac

cgtatcactgcccccaacttggacgtgcgcgagttcgttatgactgctgagctgagccgc

tcacccgaatcgtataccaacaagcgcctgccgcacctcaccgtctactttaagctcgcc

atgaggaatgaagaactgcccagcgtaaaagagagaattccgtatgtgatagttgcgcag

accgaggccgcggaacgcgaagcgggtgtagtaaactcaatgcgcggtaccgcccaaaac

cccgtggtaaccaagaccgcacgcccccaacctaaacgcaaactgctggtttccgacctc

gccgaagacccgacctatgtttccgagaatgacgtaccgctaaacacagactactatttc

tcccacctgttgggtaccataagcgtgacctttaaggctctattcggaaatgatgtgaga

acaacagaaaatcttttaaagcggtttattccggaaaccccccacaagacccccacgaaa

acccaggcactgcttgagcgcgccggctttgaaaagctgacgccctttacaccggaggaa

gaaagtcgtcgaatactgcatacagttttttgtactctagaagcagctccccatcaaagc

tga

>KU206451.1

atggcggcgcgcgaacaggccaactccgttcgcaggagcgggttttttaatccgtttatt

ggcaagaggccatttttcaggccgggaagcgggcagaccgcggagacagagcgcccaagg

cccccccaacactcgtactgcacagaggtgggtagctttaagtttatagctccaagatgc

ctcgatgaagaagcccccgccgaccagcgacgcggtgtacacgtgggcaccttggagcgc

ccacccaaagtgtactgcgatggctcagagtacgacgtgctgaactttgcctccggaggt

tgctggcctcgcaggattcgggtttggaacggccaggattttcggggcgatggattcaac

cccagatttgagagatttcacgtgtacgacatagtagagacttcggagagcgcctcacac

gatgacccatccaggtttgctgagctatcccgcccaagcgggtctgtggtaacactgctg

ggaatgagcgagtgtggaaagcgagtcgcggttcacgtctatggtgtgcgccattatttt

tacatggcaaaggcggaggtggatagcgcttgtggaataaccaccgaggcagaactcgtg

cgcgcaatggtggactgcgcgcacagctcggctttgagcgcggccctgggaaatggcaac

ggcggcaaacagagcggcggcagcggcgggggatggtggggcggaaagcacgtgtctgcg

gactgcttcaaagtggagaccgtgtgccacacgacgctgtactactttggatctaagcca

gctctctactatagagtatctgcctccagcagccgcctgggagggttcatctgcgacaac

tttcacccggagattacaaaattcgaggggagcgtggacgtgaccacgcggctgttgttg

gacaatgaaaattttaccagttttgggtggtaccgcctgcgacccggcacccacggagag

cgtgttcaacttcgccccgttgagcgacacgtcacctcaagcgacgtggagattaactgt

actcccgataacctggagccgataccagacgaggctgcctggcccgactataagctcatg

tgctttgatatagagtgtaaagctggaacgggtaacgaaatggcgttcccagtggcaact

aaccaagaggacctggtcatccagatctcctgtctgctgtactcgcttgctactcagaac

cacgaacacaccctgctgttttccctcgggtcatgcgatatctctgaggaatactcgttt

gcatgcgtccagcgcggcgagcccagaccgacggttttggagtttgacagcgagtacgag

ctgctggttgccttcctgacctttctcaagcagtactctcccgagttcgccaccggctac

aacatcgttaattttgactgggcgtacatagttaacaaggtaacgtcggtgtataacatc

aagctggacgggtacggcaagttcaacaaaggggggctgtttaaggtgtgggacatcgcc

acgaaccattttcagaagaagagcaaggtgaaaatcaatggcctgatatctctagacatg

tattctgtggcgacggaaaagctaaagctacccagctacaaactcgacgcggtcgtggga

gacgtcctcggcgagcataagatagaccttccctataaagaaataccctcctattacgcg

ggagggcctgaccggaggggcgtaataggagagtattgtatccaggactctaggctggtg

ggcaagctgttttttaagtacctcccccatctggaactatcggcggtggccaaactcgcc

cgtatcaccctaacgcgggtaatttttgacggtcagcaaattcgcgtgtacacgtgcttg

ctgaaactcgcccgcgagagaaatttcattttgccagacaacagacgccggtttgacagt

caggcagacgccgcgtcagagacttcggagttggctatggatagccaaagccacgccttc

gacagtacagacgaacccgacggtgtggacggtaccccggacgccgcaggatctggcgct

acttctgaaaacggaggcgggaagcccggcgtcgggagggccgtgggctaccagggagca

aaggttctagaccccgtatccggctttcatgtggaccccgtggttgtgtttgacttcgct

agcttatacccaagcattatccaggcccataacctctgtttcaccaccctggcgctcgat

gaagtggatctggccgggcttcaaccatccgtcaactactcgacgttcgaggtgggtgac

caaaagttattttttgtccacgcccatattcgcgaaagcctgcttggcatcttgctgcgc

gactggctggccatgcgaaaggcggtgagggcgcgaatccccaccagcacccccgaggag

gcagttttactagataagcagcagtctgcgattaaggtgatatgcaactcggtttacgga

ttcacgggggtggcaaacggcctgttgccgtgtctgaggatagcggctaccgttaccacg

ataggacgcgacatgctcctcaagacccgagattacgttcactctcgttgggcgacgcgc

gagctgctggaggacaattttccaggggctataggtttccgaaaccacaagccttactcc

gtcagggttatctacggagacaccgactccgtgtttatcaagtttgtgggcctgacgtac

gagggggtatcggagctgggggatgctatgtcgcgtcagatttcagcggacctctttaga

gcgcccatcaaactggagtgtgagaagacctttcagcgactgctgctgatcaccaagaag

aagtacataggtgtcataaacggggggaagatgctcatgaagggggtcgacctggtccgc

aaaaataactgctctttcataaacttgtacgcgcgacatctggtagatcttttgttgtac

gacgaggatgtggccacggcggcagcaaaggtgacagacgtgcctcccgcagaatgggtg

gggcgcccgctaccgagcggctttgacaagtttgggcgagtgctggtagaggcgtacaac

cgtatcactgcccccaacttggacgtgcgcgagttcgttatgactgctgagctgagccgc

tcacccgaatcgtataccaacaagcgcctgccgcacctcaccgtctactttaagctcgcc

atgaggaatgaagaactgcccagcgtaaaagagagaattccgtatgtgatagttgcgcag

accgaggccgcggaacgcgaagcgggtgtagtaaactcaatgcgcggtaccgcccaaaac

cccgtggtaaccaagaccgcacgcccccaacctaaacgcaaactgctggtttccgacctc

gccgaagacccgacctatgtttccgagaatgacgtaccgctaaacacagactactatttc

tcccacctgttgggtaccataagcgtgacctttaaggctctattcggaaatgatgtgaga

acaacagaaaatcttttaaagcggtttattccggaaaccccccacaagacccccacgaaa

acccaggcactgcttgagcgcgccggctttgaaaagctgacgccctttacaccggaggaa

gaaagtcgtcgaatactgcatacagttttttgtactctagaagcagctccccatcaaagc

tga

>KU206452.1

atggcggcgcgcgaacaggccaactccgttcgcaggagcgggttttttaatccgtttatt

ggcaagaggccatttttcaggccgggaagcgggcagaccgcggagacagagcgcccaagg

cccccccaacactcgtactgcacagaggtgggtagctttaagtttatagctccaagatgc

ctcgatgaagaagcccccgccgaccagcgacgcggtgtacacgtgggcaccttggagcgc

ccacccaaagtgtactgcgatggctcagagtacgacgtgctgaactttgcctccggaggt

tgctggcctcgcaggattcgggtttggaacggccaggattttcggggcgatggattcaac

cccagatttgagagatttcacgtgtacgacatagtagagacttcggagagcgcctcacac

gatgacccatccaggtttgctgagctatcccgcccaagcgggtctgtggtaacactgctg

ggaatgagcgagtgtggaaagcgagtcgcggttcacgtctatggtgtgcgccattatttt

tacatggcaaaggcggaggtggatagcgcttgtggaataaccaccgaggcagaactcgtg

cgcgcaatggtggactgcgcgcacagctcggctttgagcgcggccctgggaaatggcaac

ggcggcaaacagagcggcggcagcggcgggggatggtggggcggaaagcacgtgtctgcg

gactgcttcaaagtggagaccgtgtgccacacgacgctgtactactttggatctaagcca

gctctctactatagagtatctgcctccagcagccgcctgggagggttcatctgcgacaac

tttcacccggagattacaaaattcgaggggagcgtggacgtgaccacgcggctgttgttg

gacaatgaaaattttaccagttttgggtggtaccgcctgcgacccggcacccacggagag

cgtgttcaacttcgccccgttgagcgacacgtcacctcaagcgacgtggagattaactgt

actcccgataacctggagccgataccagacgaggctgcctggcccgactataagctcatg

tgctttgatatagagtgtaaagctggaacgggtaacgaaatggcgttcccagtggcaact

aaccaagaggacctggtcatccagatctcctgtctgctgtactcgcttgctactcagaac

cacgaacacaccctgctgttttccctcgggtcatgcgatatctctgaggaatactcgttt

gcatgcgtccagcgcggcgagcccagaccgacggttttggagtttgacagcgagtacgag

ctgctggttgccttcctgacctttctcaagcagtactctcccgagttcgccaccggctac

aacatcgttaattttgactgggcgtacatagttaacaaggtaacgtcggtgtataacatc

aagctggacgggtacggcaagttcaacaaaggggggctgtttaaggtgtgggacatcgcc

acgaaccattttcagaagaagagcaaggtgaaaatcaatggcctgatatctctagacatg

tattctgtggcgacggaaaagctaaagctacccagctacaaactcgacgcggtcgtggga

gacgtcctcggcgagcataagatagaccttccctataaagaaataccctcctattacgcg

ggagggcctgaccggaggggcgtaataggagagtattgtatccaggactctaggctggtg

ggcaagctgttttttaagtacctcccccatctggaactatcggcggtggccaaactcgcc

cgtatcaccctaacgcgggtaatttttgacggtcagcaaattcgcgtgtacacgtgcttg

ctgaaactcgcccgcgagagaaatttcattttgccagacaacagacgccggtttgacagt

caggcagacgccgcgtcagagacttcggagttggctatggatagccaaagccacgccttc

gacagtacagacgaacccgacggtgtggacggtaccccggacgccgcaggatctggcgct

acttctgaaaacggaggcgggaagcccggcgtcgggagggccgtgggctaccagggagca

aaggttctagaccccgtatccggctttcatgtggaccccgtggttgtgtttgacttcgct

agcttatacccaagcattatccaggcccataacctctgtttcaccaccctggcgctcgat

gaagtggatctggccgggcttcaaccatccgtcaactactcgacgttcgaggtgggtgac

caaaagttattttttgtccacgccaatattcgcgaaagcctgcttggcatcttgctgcgc

gactggctggccatgcgaaaggcggtgagggcgcgaatccccaccagcacccccgaggag

gcagttttactagataagcagcagtctgcgattaaggtgatatgcaactcggtttacgga

ttcacgggggtggcaaacggcctgttgccgtgtctgaggatagcggctaccgttaccacg

ataggacgcgacatgctcctcaagacccgagattacgttcactctcgttgggcgacgcgc

gagctgctggaggacaattttccaggggctataggtttccgaaaccacaagccttactcc

gtcagggttatctacggagacaccgactccgtgtttatcaagtttgtgggcctgacgtac

gagggggtatcggagctgggggatgctatgtcgcgtcagatttcagcggacctctttaga

gcgcccatcaaactggagtgtgagaagacctttcagcgactgctgctgatcaccaagaag

aagtacataggtgtcataaacggggggaagatgctcatgaagggggtcgacctggtccgc

aaaaataactgctctttcataaacttgtacgcgcgacatctggtagatcttttgttgtac

gacgaggatgtggccacggcggcagcaaaggtgacagacgtgcctcccgcagaatgggtg

gggcgcccgctaccgagcggctttgacaagtttgggcgagtgctggtagaggcgtacaac

cgtatcactgcccccaacttggacgtgcgcgagttcgttatgactgctgagctgagccgc

tcacccgaatcgtataccaacaagcgcctgccgcacctcaccgtctactttaagctcgcc

atgaggaatgaagaactgcccagcgtaaaagagagaattccgtatgtgatagttgcgcag

accgaggccgcggaacgcgaagcgggtgtagtaaactcaatgcgcggtaccgcccaaaac

cccgtggtaaccaagaccgcacgcccccaacctaaacgcaaactgctggtttccgacctc

gccgaagacccgacctatgtttccgagaatgacgtaccgctaaacacagactactatttc

tcccacctgttgggtaccataagcgtgacctttaaggctctattcggaaatgatgtgaga

acaacagaaaatcttttaaagcggtttattccggaaaccccccacaagacccccacgaaa

acccaggcactgcttgagcgcgccggctttgaaaagctgacgccctttacaccggaggaa

gaaagtcgtcgaatactgcatacagttttttgtactctagaagcagctccccatcaaagc

tga

>KU206453.1

atggcggcgcgcgaacaggccaactccgttcgcaggagcgggttttttaatccgtttatt

ggcaagaggccatttttcaggccgggaagcgggcagaccgcggagacagagcgcccaagg

cccccccaacactcgtactgcacagaggtgggtagctttaagtttatagctccaagatgc

ctcgatgaagaagcccccgccgaccagcgacgcggtgtacacgtgggcaccttggagcgc

ccacccaaagtgtactgcgatggctcagagtacgacgtgctgaactttgcctccggaggt

tgctggcctcgcaggattcgggtttggaacggccaggattttcggggcgatggattcaac

cccagatttgagagatttcacgtgtacgacatagtagagacttcggagagcgcctcacac

gatgacccatccaggtttgctgagctatcccgcccaagcgggtctgtggtaacactgctg

ggaatgagcgagtgtggaaagcgagtcgcggttcacgtctatggtgtgcgccattatttt

tacatggcaaaggcggaggtggatagcgcttgtggaataaccaccgaggcagaactcgtg

cgcgcaatggtggactgcgcgcacagctcggctttgagcgcggccctgggaaatggcaac

ggcggcaaacagagcggcggcagcggcgggggatggtggggcggaaagcacgtgtctgcg

gactgcttcaaagtggagaccgtgtgccacacgacgctgtactactttggatctaagcca

gctctctactatagagtatctgcctccagcagccgcctgggagggttcatctgcgacaac

tttcacccggagattacaaaattcgaggggagcgtggacgtgaccacgcggctgttgttg

gacaatgaaaattttaccagttttgggtggtaccgcctgcgacccggcacccacggagag

cgtgttcaacttcgccccgttgagcgacacgtcacctcaagcgacgtggagattaactgt

actcccgataacctggagccgataccagacgaggctgcctggcccgactataagctcatg

tgctttgatatagagtgtaaagctggaacgggtaacgaaatggcgttcccagtggcaact

aaccaagaggacctggtcatccagatctcctgtctgctgtactcgcttgctactcagaac

cacgaacacaccctgctgttttccctcgggtcatgcgatatctctgaggaatactcgttt

gcatgcgtccagcgcggcgagcccagaccgacggttttggagtttgacagcgagtacgag

ctgctggttgccttcctgacctttctcaagcagtactctcccgagttcgccaccggctac

aacatcgttaattttgactgggcgtacatagttaacaaggtaacgtcggtgtataacatc

aagctggacgggtacggcaagttcaacaaaggggggctgtttaaggtgtgggacatcgcc

acgaaccattttcagaagaagagcaaggtgaaaatcaatggcctgatatctctagacatg

tattctgtggcgacggaaaagctaaagctacccagctacaaactcgacgcggtcgtggga

gacgtcctcggcgagcataagatagaccttccctataaagaaataccctcctattacgcg

ggagggcctgaccggaggggcgtaataggagagtattgtatccaggactctaggctggtg

ggcaagctgttttttaagtacctcccccatctggaactatcggcggtggccaaactcgcc

cgtatcaccctaacgcgggtaatttttgacggtcagcaaattcgcgtgtacacgtgcttg

ctgaaactcgcccgcgagagaaatttcattttgccagacaacagacgccggtttgacagt

caggcagacgccgcgtcagagacttcggagttggctatggatagccaaagccacgccttc

gacagtacagacgaacccgacggtgtggacggtaccccggacgccgcaggatctggcgct

acttctgaaaacggaggcgggaagcccggcgtcgggagggccgtgggctaccagggagca

aaggttctagaccccgtatccggctttcatgtggaccccgtggttgtgtttgacttcgct

agcttatacccaagcattatccaggcccataacctctgtttcaccaccctggcgctcgat

gaagtggatctggccgggcttcaaccatccgtcgactactcgacgttcgaggtgggtgac

caaaagttattttttgtccacgcccatattcgcgaaagcctgcttggcatcttgctgcgc

gactggctggccatgcgaaaggcggtgagggcgcgaatccccaccagcacccccgaggag

gcagttttactagataagcagcagtctgcgattaaggtgatatgcaactcggtttacgga

ttcacgggggtggcaaacggcctgttgccgtgtctgaggatagcggctaccgttaccacg

ataggacgcgacatgctcctcaagacccgagattacgttcactctcgttgggcgacgcgc

gagctgctggaggacaattttccaggggctataggtttccgaaaccacaagccttactcc

gtcagggttatctacggagacaccgactccgtgtttatcaagtttgtgggcctgacgtac

gagggggtatcggagctgggggatgctatgtcgcgtcagatttcagcggacctctttaga

gcgcccatcaaactggagtgtgagaagacctttcagcgactgctgctgatcaccaagaag

aagtacataggtgtcataaacggggggaagatgctcatgaagggggtcgacctggtccgc

aaaaataactgctctttcataaacttgtacgcgcgacatctggtagatcttttgttgtac

gacgaggatgtggccacggcggcagcaaaggtgacagacgtgcctcccgcagaatgggtg

gggcgcccgctaccgagcggctttgacaagtttgggcgagtgctggtagaggcgtacaac

cgtatcactgcccccaacttggacgtgcgcgagttcgttatgactgctgagctgagccgc

tcacccgaatcgtataccaacaagcgcctgccgcacctcaccgtctactttaagctcgcc

atgaggaatgaagaactgcccagcgtaaaagagagaattccgtatgtgatagttgcgcag

accgaggccgcggaacgcgaagcgggtgtagtaaactcaatgcgcggtaccgcccaaaac

cccgtggtaaccaagaccgcacgcccccaacctaaacgcaaactgctggtttccgacctc

gccgaagacccgacctatgtttccgagaatgacgtaccgctaaacacagactactatttc

tcccacctgttgggtaccataagcgtgacctttaaggctctattcggaaatgatgtgaga

acaacagaaaatcttttaaagcggtttattccggaaaccccccacaagacccccacgaaa

acccaggcactgcttgagcgcgccggctttgaaaagctgacgccctttacaccggaggaa

gaaagtcgtcgaatactgcatacagttttttgtactctagaagcagctccccatcaaagc

tga

>KU206454.1

atggcggcgcgcgaacaggccaactccgttcgcaggagcgggttttttaatccgtttatt

ggcaagaggccatttttcaggccgggaagcgggcagaccgcggagacagagcgcccaagg

cccccccaacactcgtactgcacagaggtgggtagctttaagtttatagctccaagatgc

ctcgatgaagaagcccccgccgaccagcgacgcggtgtacacgtgggcaccttggagcgc

ccacccaaagtgtactgcgatggctcagagtacgacgtgctgaactttgcctccggaggt

tgctggcctcgcaggattcgggtttggaacggccaggattttcggggcgatggattcaac

cccagatttgagagatttcacgtgtacgacatagtagagacttcggagagcgcctcacac

gatgacccatccaggtttgctgagctatcccgcccaagcgggtctgtggtaacactgctg

ggaatgagcgagtgtggaaagcgagtcgcggttcacgtctatggtgtgcgccattatttt

tacatggcaaaggcggaggtggatagcgcttgtggaataaccaccgaggcagaactcgtg

cgcgcaatggtggactgcgcgcacagctcggctttgagcgcggccctgggaaatggcaac

ggcggcaaacagagcggcggcagcggcgggggatggtggggcggaaagcacgtgtctgcg

gactgcttcaaagtggagaccgtgtgccacacgacgctgtactactttggatctaagcca

gctctctactatagagtatctgcctccagcagccgcctgggagggttcatctgcgacaac

tttcacccggagattacaaaattcgaggggagcgtggacgtgaccacgcggctgttgttg

gacaatgaaaattttaccagtttcgggtggtaccgcctgcgacccggcacccacggagag

cgtgttcaacttcgccccgttgagcgacacgtcacctcaagcgacgtggagattaactgt

actcccgataacctggagccgataccagacgaggctgcctggcccgactataagctcatg

tgctttgatatagagtgtaaagctggaacgggtaacgaaatggcgttcccagtggcaact

aaccaagaggacctggtcatccagatctcctgtctgctgtactcgcttgctactcagaac

cacgaacacaccctgctgttttccctcgggtcatgcgatatctctgaggaatactcgttt

gcatgcgtccagcgcggcgagcccagaccgacggttttggagtttgacagcgagtacgag

ctgctggttgccttcctgacctttctcaagcagtactctcccgagttcgccaccggctac

aacatcgttaattttgactgggcgtacatagttaacaaggtaacgtcggtgtataacatc

aagctggacgggtacggcaagttcaacaaaggggggctgtttaaggtgtgggacatcgcc

acgaaccattttcagaagaagagcaaggtgaaaatcaatggcctgatatctctagacatg

tattctgtggcgacggaaaagctaaagctacccagctacaaactcgacgcggtcgtggga

gacgtcctcggcgagcataagatagaccttccctataaagaaataccctcctattacgcg

ggagggcctgaccggaggggcgtaataggagagtattgtatccaggactctaggctggtg

ggcaagctgttttttaagtacctcccccatctggaactatcggcggtggccaaactcgcc

cgtatcaccctaacgcgggtaatttttgacggtcagcaaattcgcgtgtacacgtgcttg

ctgaaactcgcccgcgagagaaatttcattttgccagacaacagacgccggtttgacagt

caggcagacgccgcgtcagagacttcggagttggctatggatagccaaagccacgccttc

gacagtacagacgaacccgacggtgtggacggtaccccggacgccgcaggatctggcgct

acttctgaaaacggaggcgggaagcccggcgtcgggagggtcgtgggctaccagggagca

aaggttctagaccccgtatccggctttcatgtggaccccgtggttgtgtttgacttcgct

agcttatacccaagcattatccaggcccataacctctgtttcaccaccctggcgctcgat

gaagtggatctggccgggcttcaaccatccgtcgactactcgacgttcgaggtgggtgac

caaaagttattttttgtccacgcccatattcgcgaaagcctgcttggcatcttgctgcgc

gactggctggccatgcgaaaggcggtgagggcgcgaatccccaccagcacccccgaggag

gcagttttactagataagcagcagtctgcgattaaggtgatatgcaactcggtttacgga

ttcacgggggtggcaaacggcctgttgccgtgtctgaggatagcggctaccgttaccacg

ataggacgcgacatgctcctcaagacccgagattacgttcactctcgttgggcgacgcgc

gagctgctggaggacaattttccaggggctataggtttccgaaaccacaagccttactcc

gtcagggttatctacggagacaccgactccgtgtttatcaagtttgtgggcctgacgtac

gagggggtatcggagctgggggatgctatgtcgcgtcagatttcagcggacctctttaga

gcgcccatcaaactggagtgtgagaagacctttcagcgactgctgctgatcaccaagaag

aagtacataggtgtcataaacggggggaagatgctcatgaagggggtcgacctggtccgc

aaaaataactgctctttcataaacttgtacgcgcgacatctggtagatcttttgttgtac

gacgaggatgtggccacggcggcagcagaggtgacagacgtgcctcccgcagaatgggtg

gggcgcccgctaccgagcggctttgacaagtttgggcgagtgctggtagaggcgtacaac

cgtatcactgcccccaacttggacgtgcgcgagttcgttatgactgctgagctgagccgc

tcacccgaatcgtataccaacaagcgcctgccgcacctcaccgtctactttaagctcgcc

atgaggaatgaagaactgcccagcgtaaaagagagaattccgtatgtgatagttgcgcag

accgaggccgcggaacgcgaagcgggtgtagtaaactcaatgcgcggtaccgcccaaaac

cccgtggtaaccaagaccgcacgcccccaacctaaacgcaaactgctggtttccgacctc

gccgaagacccgacctatgtttccgagaatgacgtaccgctaaacacagactactatttc

tcccacctgttgggtaccataagcgtgacctttaaggctctattcggaaatgatgtgaga

acaacagaaaatcttttaaagcggtttattccggaaaccccccacaagacccccacgaaa

acccaggcactgcttgagcgcgccggctttgaaaagctgacgccctttacaccggaggaa

gaaagtcgtcgaatactgcatacagttttttgtactctagaagcagctccccatcaaagc

tga

>KU206455.1

atggcggcgcgcgaacaggccaactccgttcgcaggagcgggttttttaatccgtttatt

ggcaagaggccatttttcaggccgggaagcgggcagaccgcggagacagagcgcccaagg

cccccccaacactcgtactgcacagaggtgggtagctttaagtttatagctccaagatgc

ctcgatgaagaagcccccgccgaccagcgacgcggtgtacacgtgggcaccttggagcgc

ccacccaaagtgtactgcgatggctcagagtacgacgtgctgaactttgcctccggaggt

tgctggcctcgcaggattcgggtttggaacggccaggattttcggggcgatggattcaac

cccagatttgagagatttcacgtgtacgacatagtagagacttcggagagcgcctcacac

gatgacccatccaggtttgctgagctatcccgcccaagcgggtctgtggtaacactgctg

ggaatgagcgagtgtggaaagcgagtcgcggttcacgtctatggtgtgcgccattatttt

tacatggcaaaggcggaggtggatagcgcttgtggaataaccaccgaggcagaactcgtg

cgcgcaatggtggactgcgcgcacagctcggctttgagcgcggccctgggaaatggcaac

ggcggcaaacagagcggcggcagcggcgggggatggtggggcggaaagcacgtgtctgcg

gactgcttcaaagtggagaccgtgtgccacacgacgctgtactactttggatctaagcca

gctctctactatagagtatctgcctccagcagccgcctgggagggttcatctgcgacaac

tttcacccggagattacaaaattcgaggggagcgtggacgtgaccacgcggctgttgttg

gacaatgaaaattttaccagtttcgggtggtaccgcctgcgacccggcacccacggagag

cgtgttcaacttcgccccgttgagcgacacgtcacctcaagcgacgtggagattaactgt

actcccgataacctggagccgataccagacgaggctgcctggcccgactataagctcatg

tgctttgatatagagtgtaaagctggaacgggtaacgaaatggcgttcccagtggcaact

aaccaagaggacctggtcatccagatctcctgtctgctgtactcgcttgctactcagaac

cacgaacacaccctgctgttttccctcgggtcatgcgatatctctgaggaatactcgttt

gcatgcgtccagcgcggcgagcccagaccgacggttttggagtttgacagcgagtacgag

ctgctggttgccttcctgacctttctcaagcagtactctcccgagttcgccaccggctac

aacatcgttaattttgactgggcgtacatagttaacaaggtaacgtcggtgtataacatc

aagctggacgggtacggcaagttcaacaaaggggggctgtttaaggtgtgggacatcgcc

acgaaccattttcagaagaagagcaaggtgaaaatcaatggcctgatatctctagacatg

tattctgtggcgacggaaaagctaaagctacccagctacaaactcgacgcggtcgtggga

gacgtcctcggcgagcataagatagaccttccctataaagaaataccctcctattacgcg

ggagggcctgaccggaggggcgtaataggagagtattgtatccaggactctaggctggtg

ggcaagctgttttttaagtacctcccccatctggaactatcggcggtggccaaactcgcc

cgtatcaccctaacgcgggtaatttttgacggtcagcaaattcgcgtgtacacgtgcttg

ctgaaactcgcccgcgagagaaatttcattttgccagacaacagacgccggtttgacagt

caggcagacgccgcgtcagagacttcggagttggctatggatagccaaagccacgccttc

gacagtacagacgaacccgacggtgtggacggtaccccggacgccgcaggatctggcgct

acttctgaaaacggaggcgggaagcccggcgtcgggagggtcgtgggctaccagggagca

aaggttctagaccccgtatccggctttcatgtggaccccgtggttgtgtttgacttcgct

agcttatacccaagcattatccaggcccataacctctgtttcaccaccctggcgctcgat

gaagtggatctggccgggcttcaaccatccgtcgactactcgacgttcgaggtgggtgac

caaaagttattttttgtccacgcccatattcgcgaaagcctgcttggcatcttgctgcgc

gactggctggccatgcgaaaggcggtgagggcgcgaatccccaccagcacccccgaggag

gcagttttactagataagcagcagtctgcgattaaggtgatatgcaactcggtttacgga

ttcacgggggtggcaaacggcctgttgccgtgtctgaggatagcggctaccgttaccacg

ataggacgcgacatgctcctcaagacccgagattacgttcactctcgttgggcgacgcgc

gagctgctggaggacaattttccaggggctataggtttccgaaaccacaagccttactcc

gtcagggttatctacggagacaccgactccgtgtttatcaagtttgtgggcctgacgtac

gagggggtatcggagctgggggatgctatgtcgcgtcagatttcagcggacctctttaga

gcgcccatcaaactggagtgtgagaagacctttcagcgactgctgctgatcaccaagaag

aagtacataggtgtcataaacggggggaagatgctcatgaagggggtcgacctggtccgc

aaaaataactgctctttcataaacttgtacgcgcgacatctggtagatcttttgttgtac

gacgaggatgtggccacggcggcagcagaggtgacagacgtgcctcccgcagaatgggtg

gggcgcccgctaccgagcggctttgacaagtttgggcgagtgctggtagaggcgtacaac

cgtatcactgcccccaacttggacgtgcgcgagttcgttatgactgctgagctgagccgc

tcacccgaatcgtataccaacaagcgcctgccgcacctcaccgtctactttaagctcgcc

atgaggaatgaagaactgcccagcgtaaaagagagaattccgtatgtgatagttgcgcag

accgaggccgcggaacgcgaagcgggtgtagtaaactcaatgcgcggtaccgcccaaaac

cccgtggtaaccaagaccgcacgcccccaacctaaacgcaaactgctggtttccgacctc

gccgaagacccgacctatgtttccgagaatgacgtaccgctaaacacagactactatttc

tcccacctgttgggtaccataagcgtgacctttaaggctctattcggaaatgatgtgaga

acaacagaaaatcttttaaagcggtttattccggaaaccccccacaagacccccacgaaa

acccaggcactgcttgagcgcgccggctttgaaaagctgacgccctttacaccggaggaa

gaaagtcgtcgaatactgcatacagttttttgtactctagaagcagctccccatcaaagc

tga

>KU206456.1

atggcggcgcgcgaacaggccaactccgttcgcaggagcgggttttttaatccgtttatt

ggcaagaggccatttttcaggccgggaagcgggcagaccgcggagacagagcgcccaagg

cccccccaacactcgtactgcacagaggtgggtagctttaagtttatagctccaagatgc

ctcgatgaagaagcccccgccgaccagcgacgcggtgtacacgtgggcaccttggagcgc

ccacccaaagtgtactgcgatggctcagagtacgacgtgctgaactttgcctccggaggt

tgctggcctcgcaggattcgggtttggaacggccaggattttcggggcgatggattcaac

cccagatttgagagatttcacgtgtacgacatagtagagacttcggagagcgcctcacac

gatgacccatccaggtttgctgagctatcccgcccaagcgggtctgtggtaacactgctg

ggaatgagcgagtgtggaaagcgagtcgcggttcacgtctatggtgtgcgccattatttt

tacatggcaaaggcggaggtggatagcgcttgtggaataaccaccgaggcagaactcgtg

cgcgcaatggtggactgcgcgcacagctcggctttgagcgcggccctgggaaatggcaac

ggcggcaaacagagcggcggcagcggcgggggatggtggggcggaaagcacgtgtctgcg

gactgcttcaaagtggagaccgtgtgccacacgacgctgtactactttggatctaagcca

gctctctactatagagtatctgcctccagcagccgcctgggagggttcatctgcgacaac

tttcacccggagattacaaaattcgaggggagcgtggacgtgaccacgcggctgttgttg

gacaatgaaaattttaccagtttcgggtggtaccgcctgcgacccggcacccacggagag

cgtgttcaacttcgccccgttgagcgacacgtcacctcaagcgacgtggagattaactgt

actcccgataacctggagccgataccagacgaggctgcctggcccgactataagctcatg

tgctttgatatagagtgtaaagctggaacgggtaacgaaatggcgttcccagtggcaact

aaccaagaggacctggtcatccagatctcctgtctgctgtactcgcttgctactcagaac

cacgaacacaccctgctgttttccctcgggtcatgcgatatctctgaggaatactcgttt

gcatgcgtccagcgcggcgagcccagaccgacggttttggagtttgacagcgagtacgag

ctgctggttgccttcctgacctttctcaagcagtactctcccgagttcgccaccggctac

aacatcgttaattttgactgggcgtacatagttaacaaggtaacgtcggtgtataacatc

aagctggacgggtacggcaagttcaacaaaggggggctgtttaaggtgtgggacatcgcc

acgaaccattttcagaagaagagcaaggtgaaaatcaatggcctgatatctctagacatg

tattctgtggcgacggaaaagctaaagctacccagctacaaactcgacgcggtcgtggga

gacgtcctcggcgagcataagatagaccttccctataaagaaataccctcctattacgcg

ggagggcctgaccggaggggcgtaataggagagtattgtatccaggactctaggctggtg

ggcaagctgttttttaagtacctcccccatctggaactatcggcggtggccaaactcgcc

cgtatcaccctaacgcgggtaatttttgacggtcagcaaattcgcgtgtacacgtgcttg

ctgaaactcgcccgcgagagaaatttcattttgccagacaacagacgccggtttgacagt

caggcagacgccgcgtcagagacttcggagttggctatggatagccaaagccacgccttc

gacagtacagacgaacccgacggtgtggacggtaccccggacgccgcaggatctggcgct

acttctgaaaacggaggcgggaagcccggcgtcgggagggtcgtgggctaccagggagca

aaggttctagaccccgtatccggctttcatgtggaccccgtggttgtgtttgacttcgct

agcttatacccaagcattatccaggcccataacctctgtttcaccaccctggcgctcgat

gaagtggatctggccgggcttcaaccatccgtcgactactcgacgttcgaggtgggtgac

caaaagttattttttgtccacgcccatattcgcgaaagcctgcttggcatcttgctgcgc

gactggctggccatgcgaaaggcggtgagggcgcgaatccccaccagcacccccgaggag

gcagttttactagataagcagcagtctgcgattaaggtgatatgcaactcggtttacgga

ttcacgggggtggcaaacggcctgttgccgtgtctgaggatagcggctaccgttaccacg

ataggacgcgacatgctcctcaagacccgagattacgttcactctcgttgggcgacgcgc

gagctgctggaggacaattttccaggggctataggtttccgaaaccacaagccttactcc

gtcagggttatctacggagacaccgactccgtgtttatcaagtttgtgggcctgacgtac

gagggggtatcggagctgggggatgctatgtcgcgtcagatttcagcggacctctttaga

gcgcccatcaaactggagtgtgagaagacctttcagcgactgctgctgatcaccaagaag

aagtacataggtgtcataaacggggggaagatgctcatgaagggggtcgacctggtccgc

aaaaataactgctctttcataaacttgtacgcgcgacatctggtagatcttttgttgtac

gacgaggatgtggccacggcggcagcagaggtgacagacgtgcctcccgcagaatgggtg

gggcgcccgctaccgagcggctttgacaagtttgggcgagtgctggtagaggcgtacaac

cgtatcactgcccccaacttggacgtgcgcgagttcgttatgactgctgagctgagccgc

tcacccgaatcgtataccaacaagcgcctgccgcacctcaccgtctactttaagctcgcc

atgaggaatgaagaactgcccagcgtaaaagagagaattccgtatgtgatagttgcgcag

accgaggccgcggaacgcgaagcgggtgtagtaaactcaatgcgcggtaccgcccaaaac

cccgtggtaaccaagaccgcacgcccccaacctaaacgcaaactgctggtttccgacctc

gccgaagacccgacctatgtttccgagaatgacgtaccgctaaacacagactactatttc

tcccacctgttgggtaccataagcgtgacctttaaggctctattcggaaatgatgtgaga

acaacagaaaatcttttaaagcggtttattccggaaaccccccacaagacccccacgaaa

acccaggcactgcttgagcgcgccggctttgaaaagctgacgccctttacaccggaggaa

gaaagtcgtcgaatactgcatacagttttttgtactctagaagcagctccccatcaaagc

tga

>KU206457.1

atggcggcgcgcgaacaggccaactccgttcgcaggagcgggttttttaatccgtttatt

ggcaagaggccatttttcaggccgggaagcgggcagaccgcggagacagagcgcccaagg

cccccccaacactcgtactgcacagaggtgggtagctttaagtttatagctccaagatgc

ctcgatgaagaagcccccgccgaccagcgacgcggtgtacacgtgggcaccttggagcgc

ccacccaaagtgtactgcgatggctcagagtacgacgtgctgaactttgcctccggaggt

tgctggcctcgcaggattcgggtttggaacggccaggattttcggggcgatggattcaac

cccagatttgagagatttcacgtgtacgacatagtagagacttcggagagcgcctcacac

gatgacccatccaggtttgctgagctatcccgcccaagcgggtctgtggtaacactgctg

ggaatgagcgagtgtggaaagcgagtcgcggttcacgtctatggtgtgcgccattatttt

tacatggcaaaggcggaggtggatagcgcttgtggaataaccaccgaggcagaactcgtg

cgcgcaatggtggactgcgcgcacagctcggctttgagcgcggccctgggaaatggcaac

ggcggcaaacagagcggcggcagcggcgggggatggtggggcggaaagcacgtgtctgcg

gactgcttcaaagtggagaccgtgtgccacacgacgctgtactactttggatctaagcca

gctctctactatagagtatctgcctccagcagccgcctgggagggttcatctgcgacaac

tttcacccggagattacaaaattcgaggggagcgtggacgtgaccacgcggctgttgttg

gacaatgaaaattttaccagtttcgggtggtaccgcctgcgacccggcacccacggagag

cgtgttcaacttcgccccgttgagcgacacgtcacctcaagcgacgtggagattaactgt

actcccgataacctggagccgataccagacgaggctgcctggcccgactataagctcatg

tgctttgatatagagtgtaaagctggaacgggtaacgaaatggcgttcccagtggcaact

aaccaagaggacctggtcatccagatctcctgtctgctgtactcgcttgctactcagaac

cacgaacacaccctgctgttttccctcgggtcatgcgatatctctgaggaatactcgttt

gcatgcgtccagcgcggcgagcccagaccgacggttttggagtttgacagcgagtacgag

ctgctggttgccttcctgacctttctcaagcagtactctcccgagttcgccaccggctac

aacatcgttaattttgactgggcgtacatagttaacaaggtaacgtcggtgtataacatc

aagctggacgggtacggcaagttcaacaaaggggggctgtttaaggtgtgggacatcgcc

acgaaccattttcagaagaagagcaaggtgaaaatcaatggcctgatatctctagacatg

tattctgtggcgacggaaaagctaaagctacccagctacaaactcgacgcggtcgtggga

gacgtcctcggcgagcataagatagaccttccctataaagaaataccctcctattacgcg

ggagggcctgaccggaggggcgtaataggagagtattgtatccaggactctaggctggtg

ggcaagctgttttttaagtacctcccccatctggaactatcggcggtggccaaactcgcc

cgtatcaccctaacgcgggtaatttttgacggtcagcaaattcgcgtgtacacgtgcttg

ctgaaactcgcccgcgagagaaatttcattttgccagacaacagacgccggtttgacagt

caggcagacgccgcgtcagagacttcggagttggctatggatagccaaagccacgccttc

gacagtacagacgaacccgacggtgtggacggtaccccggacgccgcaggatctggcgct

acttctgaaaacggaggcgggaagcccggcgtcgggagggtcgtgggctaccagggagca

aaggttctagaccccgtatccggctttcatgtggaccccgtggttgtgtttgacttcgct

agcttatacccaagcattatccaggcccataacctctgtttcaccaccctggcgctcgat

gaagtggatctggccgggcttcaaccatccgtcgactactcgacgttcgaggtgggtgac

caaaagttattttttgtccacgcccatattcgcgaaagcctgcttggcatcttgctgcgc

gactggctggccatgcgaaaggcggtgagggcgcgaatccccaccagcacccccgaggag

gcagttttactagataagcagcagtctgcgattaaggtgatatgcaactcggtttacgga

ttcacgggggtggcaaacggcctgttgccgtgtctgaggatagcggctaccgttaccacg

ataggacgcgacatgctcctcaagacccgagattacgttcactctcgttgggcgacgcgc

gagctgctggaggacaattttccaggggctataggtttccgaaaccacaagccttactcc

gtcagggttatctacggagacaccgactccgtgtttatcaagtttgtgggcctgacgtac

gagggggtatcggagctgggggatgctatgtcgcgtcagatttcagcggacctctttaga

gcgcccatcaaactggagtgtgagaagacctttcagcgactgctgctgatcaccaagaag

aagtacataggtgtcataaacggggggaagatgctcatgaagggggtcgacctggtccgc

aaaaataactgctctttcataaacttgtacgcgcgacatctggtagatcttttgttgtac

gacgaggatgtggccacggcggcagcagaggtgacagacgtgcctcccgcagaatgggtg

gggcgcccgctaccgagcggctttgacaagtttgggcgagtgctggtagaggcgtacaac

cgtatcactgcccccaacttggacgtgcgcgagttcgttatgactgctgagctgagccgc

tcacccgaatcgtataccaacaagcgcctgccgcacctcaccgtctactttaagctcgcc

atgaggaatgaagaactgcccagcgtaaaagagagaattccgtatgtgatagttgcgcag

accgaggccgcggaacgcgaagcgggtgtagtaaactcaatgcgcggtaccgcccaaaac

cccgtggtaaccaagaccgcacgcccccaacctaaacgcaaactgctggtttccgacctc

gccgaagacccgacctatgtttccgagaatgacgtaccgctaaacacagactactatttc

tcccacctgttgggtaccataagcgtgacctttaaggctctattcggaaatgatgtgaga

acaacagaaaatcttttaaagcggtttattccggaaaccccccacaagacccccacgaaa

acccaggcactgcttgagcgcgccggctttgaaaagctgacgccctttacaccggaggaa

gaaagtcgtcgaatactgcatacagttttttgtactctagaagcagctccccatcaaagc

tga

>KU206458.1

atggcggcgcgcgaacaggccaactccgttcgcaggagcgggttttttaatccgtttatt

ggcaagaggccatttttcaggccgggaagcgggcagaccgcggagacagagcgcccaagg

cccccccaacactcgtactgcacagaggtgggtagctttaagtttatagctccaagatgc

ctcgatgaagaagcccccgccgaccagcgacgcggtgtacacgtgggcaccttggagcgc

ccacccaaagtgtactgcgatggctcagagtacgacgtgctgaactttgcctccggaggt

tgctggcctcgcaggattcgggtttggaacggccaggattttcggggcgatggattcaac

cccagatttgagagatttcacgtgtacgacatagtagagacttcggagagcgcctcacac

gatgacccatccaggtttgctgagctatcccgcccaagcgggtctgtggtaacactgctg

ggaatgagcgagtgtggaaagcgagtcgcggttcacgtctatggtgtgcgccattatttt

tacatggcaaaggcggaggtggatagcgcttgtggaataaccaccgaggcagaactcgtg

cgcgcaatggtggactgcgcgcacagctcggctttgagcgcggccctgggaaatggcaac

ggcggcaaacagagcggcggcagcggcgggggatggtggggcggaaagcacgtgtctgcg

gactgcttcaaagtggagaccgtgtgccacacgacgctgtactactttggatctaagcca

gctctctactatagagtatctgcctccagcagccgcctgggagggttcatctgcgacaac

tttcacccggagattacaaaattcgaggggagcgtggacgtgaccacgcggctgttgttg

gacaatgaaaattttaccagtttcgggtggtaccgcctgcgacccggcacccacggagag

cgtgttcaacttcgccccgttgagcgacacgtcacctcaagcgacgtggagattaactgt

actcccgataacctggagccgataccagacgaggctgcctggcccgactataagctcatg

tgctttgatatagagtgtaaagctggaacgggtaacgaaatggcgttcccagtggcaact

aaccaagaggacctggtcatccagatctcctgtctgctgtactcgcttgctactcagaac

cacgaacacaccctgctgttttccctcgggtcatgcgatatctctgaggaatactcgttt

gcatgcgtccagcgcggcgagcccagaccgacggttttggagtttgacagcgagtacgag

ctgctggttgccttcctgacctttctcaagcagtactctcccgagttcgccaccggctac

aacatcgttaattttgactgggcgtacatagttaacaaggtaacgtcggtgtataacatc

aagctggacgggtacggcaagttcaacaaaggggggctgtttaaggtgtgggacatcgcc

acgaaccattttcagaagaagagcaaggtgaaaatcaatggcctgatatctctagacatg

tattctgtggcgacggaaaagctaaagctacccagctacaaactcgacgcggtcgtggga

gacgtcctcggcgagcataagatagaccttccctataaagaaataccctcctattacgcg

ggagggcctgaccggaggggcgtaataggagagtattgtatccaggactctaggctggtg

ggcaagctgttttttaagtacctcccccatctggaactatcggcggtggccaaactcgcc

cgtatcaccctaacgcgggtaatttttgacggtcagcaaattcgcgtgtacacgtgcttg

ctgaaactcgcccgcgagagaaatttcattttgccagacaacagacgccggtttgacagt

caggcagacgccgcgtcagagacttcggagttggctatggatagccaaagccacgccttc

gacagtacagacgaacccgacggtgtggacggtaccccggacgccgcaggatctggcgct

acttctgaaaacggaggcgggaagcccggcgtcgggagggccgtgggctaccagggagca

aaggttctagaccccgtatccggctttcatgtggaccccgtggttgtgtttgacttcgct

agcttatacccaagcattatccaggcccataacctctgtttcaccaccctggcgctcgat

gaagtggatctggccgggcttcaaccatccgtcgactactcgacgttcgaggtgggtgac

caaaagttattttttgtccacgcccatattcgcgaaagcctgcttggcatcttgctgcgc

gactggctggccatgcgaaaggcggtgagggcgcgaatccccaccagcacccccgaggag

gcagttttactagataagcagcagtctgcgattaaggtgatatgcaactcggtttacgga

ttcacgggggtggcaaacggcctgttgccgtgtctgaggatagcggctaccgttaccacg

ataggacgcgacatgctcctcaagacccgagattacgttcactctcgttgggcgacgcgc

gagctgctggaggacaattttccaggggctataggtttccgaaaccacaagccttactcc

gtcagggttatctacggagacaccgactccgtgtttatcaagtttgtgggcctgacgtac

gagggggtatcggagctgggggatgctatgtcgcgtcagatttcagcggacctctttaga

gcgcccatcaaactggagtgtgagaagacctttcagcgactgctgctgatcaccaagaag

aagtacataggtgtcataaacggggggaagatgctcatgaagggggtcgacctggtccgc

aaaaataactgctctttcataaacttgtacgcgcgacatctggtagatcttttgttgtac

gacgaggatgtggccacggcggcagcagaggtgacagacgtgcctcccgcagaatgggtg

gggcgcccgctaccgagcggctttgacaagtttgggcgagtgctggtagaggcgtacaac

cgtatcactgcccccaacttggacgtgcgcgagttcgttatgactgctgagctgagccgc

tcacccgaatcgtataccaacaagcgcctgccgcacctcaccgtctactttaagctcgcc

atgaggaatgaagaactgcccagcgtaaaagagagaattccgtatgtgatagttgcgcag

accgaggccgcggaacgcgaagcgggtgtagtaaactcaatgcgcggtaccgcccaaaac

cccgtggtaaccaagaccgcacgcccccaacctaaacgcaaactgctggtttccgacctc

gccgaagacccgacctatgtttccgagaatgacgtaccgctaaacacagactactatttc

tcccacctgttgggtaccataagcgtgacctttaaggctctattcggaaatgatgtgaga

acaacagaaaatcttttaaagcggtttattccggaaaccccccacaagacccccacgaaa

acccaggcactgcttgagcgcgccggctttgaaaagctgacgccctttacaccggaggaa

gaaagtcgtcgaatactgcatacagttttttgtactctagaagcagctccccatcaaagc

tga

>KU206459.1

atggcggcgcgcgaacaggccaactccgttcgcaggagcgggttttttaatccgtttatt

ggcaagaggccatttttcaggccgggaagcgggcagaccgcggagacagagcgcccaagg

cccccccaacactcgtactgcacagaggtgggtagctttaagtttatagctccaagatgc

ctcgatgaagaagcccccgccgaccagcgacgcggtgtacacgtgggcaccttggagcgc

ccacccaaagtgtactgcgatggctcagagtacgacgtgctgaactttgcctccggaggt

tgctggcctcgcaggattcgggtttggaacggccaggattttcggggcgatggattcaac

cccagatttgagagatttcacgtgtacgacatagtagagacttcggagagcgcctcacac

gatgacccatccaggtttgctgagctatcccgcccaagcgggtctgtggtaacactgctg

ggaatgagcgagtgtggaaagcgagtcgcggttcacgtctatggtgtgcgccattatttt

tacatggcaaaggcggaggtggatagcgcttgtggaataaccaccgaggcagaactcgtg

cgcgcaatggtggactgcgcgcacagctcggctttgagcgcggccctgggaaatggcaac

ggcggcaaacagagcggcggcagcggcgggggatggtggggcggaaagcacgtgtctgcg

gactgcttcaaagtggagaccgtgtgccacacgacgctgtactactttggatctaagcca

gctctctactatagagtatctgcctccagcagccgcctgggagggttcatctgcgacaac

tttcacccggagattacaaaattcgaggggagcgtggacgtgaccacgcggctgttgttg

gacaatgaaaattttaccagtttcgggtggtaccgcctgcgacccggcacccacggagag

cgtgttcaacttcgccccgttgagcgacacgtcacctcaagcgacgtggagattaactgt

actcccgataacctggagccgataccagacgaggctgcctggcccgactataagctcatg

tgctttgatatagagtgtaaagctggaacgggtaacgaaatggcgttcccagtggcaact

aaccaagaggacctggtcatccagatctcctgtctgctgtactcgcttgctactcagaac

cacgaacacaccctgctgttttccctcgggtcatgcgatatctctgaggaatactcgttt

gcatgcgtccagcgcggcgagcccagaccgacggttttggagtttgacagcgagtacgag

ctgctggttgccttcctgacctttctcaagcagtactctcccgagttcgccaccggctac

aacatcgttaattttgactgggcgtacatagttaacaaggtaacgtcggtgtataacatc

aagctggacgggtacggcaagttcaacaaaggggggctgtttaaggtgtgggacatcgcc

acgaaccattttcagaagaagagcaaggtgaaaatcaatggcctgatatctctagacatg

tattctgtggcgacggaaaagctaaagctacccagctacaaactcgacgcggtcgtggga

gacgtcctcggcgagcataagatagaccttccctataaagaaataccctcctattacgcg

ggagggcctgaccggaggggcgtaataggagagtattgtatccaggactctaggctggtg

ggcaagctgttttttaagtacctcccccatctggaactatcggcggtggccaaactcgcc

cgtatcaccctaacgcgggtaatttttgacggtcagcaaattcgcgtgtacacgtgcttg

ctgaaactcgcccgcgagagaaatttcattttgccagacaacagacgccggtttgacagt

caggcagacgccgcgtcagagacttcggagttggctatggatagccaaagccacgccttc

gacagtacagacgaacccgacggtgtggacggtaccccggacgccgcaggatctggcgct

acttctgaaaacggaggcgggaagcccggcgtcgggagggccgtgggctaccagggagca

aaggttctagaccccgtatccggctttcatgtggaccccgtggttgtgtttgacttcgct

agcttatacccaagcattatccaggcccataacctctgtttcaccaccctggcgctcgat

gaagtggatctggccgggcttcaaccatccgtcgactactcgacgttcgaggtgggtgac

caaaagttattttttgtccacgcccatattcgcgaaagcctgcttggcatcttgctgcgc

gactggctggccatgcgaaaggcggtgagggcgcgaatccccaccagcacccccgaggag

gcagttttactagataagcagcagtctgcgattaaggtgatatgcaactcggtttacgga

ttcacgggggtggcaaacggcctgttgccgtgtctgaggatagcggctaccgttaccacg

ataggacgcgacatgctcctcaagacccgagattacgttcactctcgttgggcgacgcgc

gagctgctggaggacaattttccaggggctataggtttccgaaaccacaagccttactcc

gtcagggttatctacggagacaccgactccgtgtttatcaagtttgtgggcctgacgtac

gagggggtatcggagctgggggatgctatgtcgcgtcagatttcagcggacctctttaga

gcgcccatcaaactggagtgtgagaagacctttcagcgactgctgctgatcaccaagaag

aagtacataggtgtcataaacggggggaagatgctcatgaagggggtcgacctggtccgc

aaaaataactgctctttcataaacttgtacgcgcgacatctggtagatcttttgttgtac

gacgaggatgtggccacggcggcagcagaggtgacagacgtgcctcccgcagaatgggtg

gggcgcccgctaccgagcggctttgacaagtttgggcgagtgctggtagaggcgtacaac

cgtatcactgcccccaacttggacgtgcgcgagttcgttatgactgctgagctgagccgc

tcacccgaatcgtataccaacaagcgcctgccgcacctcaccgtctactttaagctcgcc

atgaggaatgaagaactgcccagcgtaaaagagagaattccgtatgtgatagttgcgcag

accgaggccgcggaacgcgaagcgggtgtagtaaactcaatgcgcggtaccgcccaaaac

cccgtggtaaccaagaccgcacgcccccaacctaaacgcaaactgctggtttccgacctc

gccgaagacccgacctatgtttccgagaatgacgtaccgctaaacacagactactatttc

tcccacctgttgggtaccataagcgtgacctttaaggctctattcggaaatgatgtgaga

acaacagaaaatcttttaaagcggtttattccggaaaccccccacaagacccccacgaaa

acccaggcactgcttgagcgcgccggctttgaaaagctgacgccctttacaccggaggaa

gaaagtcgtcgaatactgcatacagttttttgtactctagaagcagctccccatcaaagc

tga

>KU206460.1

atggcggcgcgcgaacaggccaactccgttcgcaggagcgggttttttaatccgtttatt

ggcaagaggccatttttcaggccgggaagcgggcagaccgcggagacagagcgcccaagg

cccccccaacactcgtactgcacagaggtgggtagctttaagtttatagctccaagatgc

ctcgatgaagaagcccccgccgaccagcgacgcggtgtacacgtgggcaccttggagcgc

ccacccaaagtgtactgcgatggctcagagtacgacgtgctgaactttgcctccggaggt

tgctggcctcgcaggattcgggtttggaacggccaggattttcggggcgatggattcaac

cccagatttgagagatttcacgtgtacgacatagtagagacttcggagagcgcctcacac

gatgacccatccaggtttgctgagctatccagcccaagcgggtctgtggtaacactgctg

ggaatgagcgagtgtggaaagcgagtcgcggttcacgtctatggtgtgcgccattatttt

tacatggcaaaggcggaggtggatagcgcttgtggaataaccaccgaggcagaactcgtg

cgcgcaatggtggactgcgcgcacagctcggctttgagcgcggccctgggaaatggcaac

ggcggcaaacagagcggcggcagcggcgggggatggtggggcggaaagcacgtgtctgcg

gactgcttcaaagtggagaccgtgtgccacacgacgctgtactactttggatctaagcca

gctctctactatagagtatctgcctccagcagccgcctgggagggttcatctgcgacaac

tttcacccggagattacaaaattcgaggggagcgtggacgtgaccacgcggctgttgttg

gacaatgaaaattttaccagtttcgggtggtaccgcctgcgacccggcacccacggagag

cgtgttcaacttcgccccgttgagcgacacgtcacctcaagcgacgtggagattaactgt

actcccgataacctggagccgataccagacgaggctgcctggcccgactataagctcatg

tgctttgatatagagtgtaaagctggaacgggtaacgaaatggcgttcccagtggcaact

aaccaagaggacctggtcatccagatctcctgtctgctgtactcgcttgctactcagaac

cacgaacacaccctgctgttttccctcgggtcatgcgatatctctgaggaatactcgttt

gcatgcgtccagcgcggcgagcccagaccgacggttttggagtttgacagcgagtacgag

ctgctggttgccttcctgacctttctcaagcagtactctcccgagttcgccaccggctac

aacatcgttaattttgactgggcgtacatagttaacaaggtaacgtcggtgtataacatc

aagctggacgggtacggcaagttcaacaaaggggggctgtttaaggtgtgggacatcgcc

acgaaccattttcagaagaagagcaaggtgaaaatcaatggcctgatatctctagacatg

tattctgtggcgacggaaaagctaaagctacccagctacaaactcgacgcggtcgtggga

gacgtcctcggcgagcataagatagaccttccctataaagaaataccctcctattacgcg

ggagggcctgaccggaggggcgtaataggagagtattgtatccaggactctaggctggtg

ggcaagctgttttttaagtacctcccccatctggaactatcggcggtggccaaactcgcc

cgtatcaccctaacgcgggtaatttttgacggtcagcaaattcgcgtgtacacgtgcttg

ctgaaactcgcccgcgagagaaatttcattttgccagacaacagacgccggtttgacagt

caggcagacgccgcgtcagagacttcggagttggctatggatagccaaagccacgccttc

gacagtacagacgaacccgacggtgtggacggtaccccggacgccgcaggatctggcgct

acttctgaaaacggaggcgggaagcccggcgtcgggagggccgtgggctaccagggagca

aaggttctagaccccgtatccggctttcatgtggaccccgtggttgtgtttgacttcgct

agcttatacccaagcattatccaggcccataacctctgtttcaccaccctggcgctcgat

gaagtggatctggccgggcttcaaccatccgtcgactactcgacgttcgaggtgggtgac

caaaagttattttttgtccacgcccatattcgcgaaagcctgcttggcatcttgctgcgc

gactggctggccatgcgaaaggcggtgagggcgcgaatccccaccagcacccccgaggag

gcagttttactagataagcagcagtctgcgattaaggtgatatgcaactcggtttacgga

ttcacgggggtggcaaacggcctgttgccgtgtctgaggatagcggctaccgttaccacg

ataggacgcgacatgctcctcaagacccgagattacgttcactctcgttgggcgacgcgc

gagctgctggaggacaattttccaggggctataggtttccgaaaccacaagccttactcc

gtcagggttatctacggagacaccgactccgtgtttatcaagtttgtgggcctgacgtac

gagggggtatcggagctgggggatgctatgtcgcgtcagatttcagcggacctctttaga

gcgcccatcaaactggagtgtgagaagacctttcagcgactgctgctgatcaccaagaag

aagtacataggtgtcataaacggggggaagatgctcatgaagggggtcgacctggtccgc

aaaaataactgctctttcataaacttgtacgcgcgacatctggtagatcttttgttgtac

gacgaggatgtggccacggcggcagcagaggtgacagacgtgcctcccgcagaatgggtg

gggcgcccgctaccgagcggctttgacaagtttgggcgagtgctggtagaggcgtacaac

cgtatcactgcccccaacttggacgtgcgcgagttcgttatgactgctgagctgagccgc

tcacccgaatcgtataccaacaagcgcctgccgcacctcaccgtctactttaagctcgcc

atgaggaatgaagaactgcccagcgtaaaagagagaattccgtatgtgatagttgcgcag

accgaggccgcggaacgcgaagcgggtgtagtaaactcaatgcgcggtaccgcccaaaac

cccgtggtaaccaagaccgcacgcccccaacctaaacgcaaactgctggtttccgacctc

gccgaagacccgacctatgtttccgagaatgacgtaccgctaaacacagactactatttc

tcccacctgttgggtaccataagcgtgacctttaaggctctattcggaaatgatgtgaga

acaacagaaaatcttttaaagcggtttattccggaaaccccccacaagacccccacgaaa

acccaggcactgcttgagcgcgccggctttgaaaagctgacgccctttacaccggaggaa

gaaagtcgtcgaatactgcatacagttttttgtactctagaagcagctccccatcaaagc

tga

>KU206461.1

atggcggcgcgcgaacaggccaactccgttcgcaggagcgggttttttaatccgtttatt

ggcaagaggccatttttcaggccgggaagcgggcagaccgcggagacagagcgcccaagg

cccccccaacactcgtactgcacagaggtgggtagctttaagtttatagctccaagatgc

ctcgatgaagaagcccccgccgaccagcgacgcggtgtacacgtgggcaccttggagcgc

ccacccaaagtgtactgcgatggctcagagtacgacgtgctgaactttgcctccggaggt

tgctggcctcgcaggattcgggtttggaacggccaggattttcggggcgatggattcaac

cccagatttgagagatttcacgtgtacgacatagtagagacttcggagagcgcctcacac

gatgacccatccaggtttgctgagctatcccgcccaagcgggtctgtggtaacactgctg

ggaatgagcgagtgtggaaagcgagtcgcggttcacgtctatggtgtgcgccattatttt

tacatggcaaaggcggaggtggatagcgcttgtggaataaccaccgaggcagaactcgtg

cgcgcaatggtggactgcgcgcacagctcggctttgagcgcggccctgggaaatggcaac

ggcggcaaacagagcggcggcagcggcgggggatggtggggcggaaagcacgtgtctgcg

gactgcttcaaagtggagaccgtgtgccacacgacgctgtactactttggatctaagcca

gctctctactatagagtatctgcctccagcagccgcctgggagggttcatctgcgacaac

tttcacccggagattacaaaattcgaggggagcgtggacgtgaccacgcggctgttgttg

gacaatgaaaattttaccagtttcgggtggtaccgcctgcgacccggcacccacggagag

cgtgttcaacttcgccccgttgagcgacacgtcacctcaagcgacgtggagattaactgt

actcccgataacctggagccgataccagacgaggctgcctggcccgactataagctcatg

tgctttgatatagagtgtaaagctggaacgggtaacgaaatggcgttcccagtggcaact

aaccaagaggacctggtcatccagatctcctgtctgctgtactcgcttgctactcagaac

cacgaacacaccctgctgttttccctcgggtcatgcgatatctctgaggaatactcgttt

gcatgcgtccagcgcggcgagcccagaccgacggttttggagtttgacagcgagtacgag

ctgctggttgccttcctgacctttctcaagcagtactctcccgagttcgccaccggctac

aacatcgttaattttgactgggcgtacatagttaacaaggtaacgtcggtgtataacatc

aagctggacgggtacggcaagttcaacaaaggggggctgtttaaggtgtgggacatcgcc

acgaaccattttcagaagaagagcaaggtgaaaatcaatggcctgatatctctagacatg

tattctgtggcgacggaaaagctaaagctacccagctacaaactcgacgcggtcgtggga

gacgtcctcggcgagcataagatagaccttccctataaagaaataccctcctattacgcg

ggagggcctgaccggaggggcgtaataggagagtattgtatccaggactctaggctggtg

ggcaagctgttttttaagtacctcccccatctggaactatcggcggtggccaaactcgcc

cgtatcaccctaacgcgggtaatttttgacggtcagcaaattcgcgtgtacacgtgcttg

ctgaaactcgcccgcgagagaaatttcattttgccagacaacagacgccggtttgacagt

caggcagacgccgcgtcagagacttcggagttggctatggatagccaaagccacgccttc

gacagtacagacgaacccgacggtgtggacggtaccccggacgccgcaggatctggcgct

acttctgaaaacggaggcgggaagcccggcgtcgggagggccgtgggctaccagggagca

aaggttctagaccccgtatccggctttcatgtggaccccgtggttgtgtttgacttcgct

agcttatacccaagcattatccaggcccataacctctgtttcaccaccctggcgctcgat

gaagtggatctggccgggcttcaaccatccgtcaactactcgacgttcgaggtgggtgac

caaaagttattttttgtccacgcccatattcgcgaaagcctgcttggcatcttgctgcgc

gactggctggccatgcgaaaggcggtgagggcgcgaatccccaccagcacccccgaggag

gcagttttactagataagcagcagtctgcgattaaggtgatatgcaactcggtttacgga

ttcacgggggtggcaaacggcctgttgccgtgtctgaggatagcggctaccgttaccacg

ataggacgcgacatgctcctcaagacccgagattacgttcactctcgttgggcgacgcgc

gagctgctggaggacaattttccaggggctataggtttccgaaaccacaagccttactcc

gtcagggttatctacggagacaccgactccgtgtttatcaagtttgtgggcctgacgtac

gagggggtatcggagctgggggatgctatgtcgcgtcagatttcagcggacctctttaga

gcgcccatcaaactggagtgtgagaagacctttcagcgactgctgctgatcaccaagaag

aagtacataggtgtcataaacggggggaagatgctcatgaagggggtcgacctggtccgc

aaaaataactgctctttcataaacttgtacgcgcgacatctggtagatcttttgttgtac

gacgaggatgtggccacggcggcagcagaggtgacagacgtgcctcccgcagaatgggtg

gggcgcccgctaccgagcggctttgacaagtttgggcgagtgctggtagaggcgtacaac

cgtatcactgcccccaacttggacgtgcgcgagttcgttatgactgctgagctgagccgc

tcacccgaatcgtataccaacaagcgcctgccgcacctcaccgtctactttaagctcgcc

atgaggaatgaagaactgcccagcgtaaaagagagaattccgtatgtgatagttgcgcag

accgaggccgcggaacgcgaagcgggtgtagtaaactcaatgcgcggtaccgcccaaaac

cccgtggtaaccaagaccgcacgcccccaacctaaacgcaaactgctggtttccgacctc

gccgaagacccgacctatgtttccgagaatgacgtaccgctaaacacagactactatttc

tcccacctgttgggtaccataagcgtgacctttaaggctctattcggaaatgatgtgaga

acaacagaaaatcttttaaagcggtttattccggaaaccccccacaagacccccacgaaa

acccaggcactgcttgagcgcgccggctttgaaaagctgacgccctttacaccggaggaa

gaaagtcgtcgaatactgcatacagttttttgtactctagaagcagctccccatcaaagc

tga

>KU206462.1

atggcggcgcgcgaacaggccaactccgttcgcaggagcgggttttttaatccgtttatt

ggcaagaggccatttttcaggccgggaagcgggcagaccgcggagacagagcgcccaagg

cccccccaacactcgtactgcacagaggtgggtagctttaagtttatagctccaagatgc

ctcgatgaagaagcccccgccgaccagcgacgcggtgtacacgtgggcaccttggagcgc

ccacccaaagtgtactgcgatggctcagagtacgacgtgctgaactttgcctccggaggt

tgctggcctcgcaggattcgggtttggaacggccaggattttcggggcgatggattcaac

cccagatttgagagatttcacgtgtacgacatagtagagacttcggagagcgcctcacac

gatgacccatccaggtttgctgagctatcccgcccaagtgggtctgtggtaacactgctg

ggaatgagcgagtgtggaaagcgagtcgcggttcacgtctatggtgtgcgccattatttt

tacatggcaaaggcggaggtggatagcgcttgtggaataaccaccgaggcagaactcgtg

cgcgcaatggtggactgcgcgcacagctcggctttgagcgcggccctgggaaatggcaac

ggcggcaaacagagcggcggcagcggcgggggatggtggggcggaaagcacgtgtctgcg

gactgcttcaaagtggagaccgtgtgccacacgacgctgtactactttggatctaagcca

gctctctactatagagtatctgcctccagcagccgcctgggagggttcatctgcgacaac

tttcacccggagattacaaaattcgaggggagcgtggacgtgaccacgcggctgttgttg

gacaatgaaaattttaccagtttcgggtggtaccgcctgcgacccggcacccacggagag

cgtgttcaacttcgccccgttgagcgacacgtcacctcaagcgacgtggagattaactgt

actcccgataacctggagccgataccagacgaggctgcctggcccgactataagctcatg

tgctttgatatagagtgtaaagctggaacgggtaacgaaatggcgttcccagtggcaact

aaccaagaggacctggtcatccagatctcctgtctgctgtactcgcttgctactcagaac

cacgaacacaccctgctgttttccctcgggtcatgcgatatctctgaggaatactcgttt

gcatgcgtccagcgcggcgagcccagaccgacggttttggagtttgacagcgagtacgag

ctgctggttgccttcctgacctttctcaagcagtactctcccgagttcgccaccggctac

aacatcgttaattttgactgggcgtacatagttaacaaggtaacgtcggtgtataacatc

aagctggacgggtacggcaagttcaacaaaggggggctgtttaaggtgtgggacatcgcc

acgaaccattttcagaagaagagcaaggtgaaaatcaatggcctgatatctctagacatg

tattctgtggcgacggaaaagctaaagctacccagctacaaactcgacgcggtcgtggga

gacgtcctcggcgagcataagatagaccttccctataaagaaataccctcctattacgcg

ggagggcctgaccggaggggcgtaataggagagtattgtatccaggactctaggctggtg

ggcaagctgttttttaagtacctcccccatctggaactatcggcggtggccaaactcgcc

cgtatcaccctaacgcgggtaatttttgacggtcagcaaattcgcgtgtacacgtgcttg

ctgaaactcgcccgcgagagaaatttcattttgccagacaacagacgccggtttgacagt

caggcagacgccgcgtcagagacttcggagttggctatggatagccaaagccacgccttc

gacagtacagacgaacccgacggtgtggacggtaccccggacgccgcaggatctggcgct

acttctgaaaacggaggcgggaagcccggcgtcgggagggccgtgggctaccagggagca

aaggttctagaccccgtatccggctttcatgtggaccccgtggttgtgtttgacttcgct

agcttatacccaagcattatccaggcccataacctctgtttcaccaccctggcgctcgat

gaagtggatctggccgggcttcaaccatccgtcaactactcgacgttcgaggtgggtgac

caaaagttattttttgtccacgcccatattcgcgaaagcctgcttggcatcttgctgcgc

gactggctggccatgcgaaaggcggtgagggcgcgaatccccaccagcacccccgaggag

gcagttttactagataagcagcagtctgcgattaaggtgatatgcaactcggtttacgga

ttcacgggggtggcaaacggcctgttgccgtgtctgaggatagcggctaccgttaccacg

ataggacgcgacatgctcctcaagacccgagattacgttcactctcgttgggcgacgcgc

gagctgctggaggacaattttccaggggctataggtttccgaaaccacaagccttactcc

gtcagggttatctacggagacaccgactccgtgtttatcaagtttgtgggcctgacgtac

gagggggtatcggagctgggggatgctatgtcgcgtcagatttcagcggacctctttaga

gcgcccatcaaactggagtgtgagaagacctttcagcgactgctgctgatcaccaagaag

aagtacataggtgtcataaacggggggaagatgctcatgaagggggtcgacctggtccgc

aaaaataactgctctttcataaacttgtacgcgcgacatctggtagatcttttgttgtac

gacgaggatgtggccacggcggcagcagaggtgacagacgtgcctcccgcagaatgggtg

gggcgcccgctaccgagcggctttgacaagtttgggcgagtgctggtagaggcgtacaac

cgtatcactgcccccaacttggacgtgcgcgagttcgttatgactgctgagctgagccgc

tcacccgaatcgtataccaacaagcgcctgccgcacctcaccgtctactttaagctcgcc

atgaggaatgaagaactgcccagcgtaaaagagagaattccgtatgtgatagttgcgcag

accgaggccgcggaacgcgaagcgggtgtagtaaactcaatgcgcggtaccgcccaaaac

cccgtggtaaccaagaccgcacgcccccaacctaaacgcaaactgctggtttccgacctc

gccgaagacccgacctatgtttccgagaatgacgtaccgctaaacacagactactatttc

tcccacctgttgggtaccataagcgtgacctttaaggctctattcggaaatgatgtgaga

acaacagaaaatcttttaaagcggtttattccggaaaccccccacaagacccccacgaaa

acccaggcactgcttgagcgcgccggctttgaaaagctgacgccctttacaccggaggaa

gaaagtcgtcgaatactgcatacagttttttgtactctagaagcagctccccatcaaagc

tga

>KU206463.1

atggcggcgcgcgaacaggccaactccgttcgcaggagcgggttttttaatccgtttatt

ggcaagaggccatttttcaggccgggaagcgggcagaccgcggagacagagcgcccaagg

cccccccaacactcgtactgcacagaggtgggtagctttaagtttatagctccaagatgc

ctcgatgaagaagcccccgccgaccagcgacgcggtgtacacgtgggcaccttggagcgc

ccacccaaagtgtactgcgatggctcagagtacgacgtgctgaactttgcctccggaggt

tgctggcctcgcaggattcgggtttggaacggccaggattttcggggcgatggattcaac

cccagatttgagagatttcacgtgtacgacatagtagagacttcggagagcgcctcacac

gatgacccatccaggtttgctgagctatcccgcccaagcgggtctgtggtaacactgctg

ggaatgagcgagtgtggaaagcgagtcgcggttcacgtctatggtgtgcgccattatttt

tacatggcaaaggcggaggtggatagcgcttgtggaataaccaccgaggcagaactcgtg

cgcgcaatggtggactgcgcgcacagctcggctttgagcgcggccctgggaaatggcaac

ggcggcaaacagagcggcggcagcggcgggggatggtggggcggaaagcacgtgtctgcg

gactgcttcaaagtggagaccgtgtgccacacgacgctgtactactttggatctaagcca

gctctctactatagagtatctgcctccagcagccgcctgggagggttcatctgcgacaac

tttcacccggagattacaaaattcgaggggagcgtggacgtgaccacgcggctgttgttg

gacaatgaaaattttaccagtttcgggtggtaccgcctgcgacccggcacccacggagag

cgtgttcaacttcgccccgttgagcgacacgtcacctcaagcgacgtggagattaactgt

actcccgataacctggagccgataccagacgaggctgcctggcccgactataagctcatg

tgctttgatatagagtgtaaagctggaacgggtaacgaaatggcgttcccagtggcaact

aaccaagaggacctggtcatccagatctcctgtctgctgtactcgcttgctactcagaac

cacgaacacaccctgctgttttccctcgggtcatgcgatatctctgaggaatactcgttt

gcatgcgtccagcgcggcgagcccagaccgacggttttggagtttgacagcgagtacgag

ctgctggttgccttcctgacctttctcaagcagtactctcccgagttcgccaccggctac

aacatcgttaattttgactgggcgtacatagttaacaaggtaacgtcggtgtataacatc

aagctggacgggtacggcaagttcaacaaaggggggctgtttaaggtgtgggacatcgcc

acgaaccattttcagaagaagagcaaggtgaaaatcaatggcctgatatctctagacatg

tattctgtggcgacggaaaagctaaagctacccagctacaaactcgacgcggtcgtggga

gacgtcctcggcgagcataagatagaccttccctataaagaaataccctcctattacgcg

ggagggcctgaccggaggggcgtaataggagagtattgtatccaggactctaggctggtg

ggcaagctgttttttaagtacctcccccatctggaactatcggcggtggccaaactcgcc

cgtatcaccctaacgcgggtaatttttgacggtcagcaaattcgcgtgtacacgtgcttg

ctgaaactcgcccgcgagagaaatttcattttgccagacaacagacgccggtttgacagt

caggcagacgccgcgtcagagacttcggagttggctatggatagccaaagccacgccttc

gacagtacagacgaacccgacggtgtggacggtaccccggacgccgcaggatctggcgct

acttctgaaaacggaggcgggaagcccggcgtcgggagggccgtgggctaccagggagca

aaggttctagaccccgtatccggctttcatgtggaccccgtggttgtgtttgacttcgct

agcttatacccaagcattatccaggcccataacctctgtttcaccaccctggcgctcgat

gaagtggatctggccgggcttcaaccatccgtcaactactcgacgttcgaggtgggtgac

caaaagttattttttgtccacgcccatattcgcgaaagcctgcttggcatcttgctgcgc

gactggctggccatgcgaaaggcggtgagggcgcgaatccccaccagcacccccgaggag

gcagttttactagataagcagcagtctgcgattaaggtgatatgcaactcggtttacgga

ttcacgggggtggcaaacggcctgttgccgtgtctgaggatagcggctaccgttaccacg

ataggacgcgacatgctcctcaagacccgagattacgttcactctcgttgggcgacgcgc

gagctgctggaggacaattttccaggggctataggtttccgaaaccacaagccttactcc

gtcagggttatctacggagacaccgactccgtgtttatcaagtttgtgggcctgacgtac

gagggggtatcggagctgggggatgctatgtcgcgtcagatttcagcggacctctttaga

gcgcccatcaaactggagtgtgagaagacctttcagcgactgctgctgatcaccaagaag

aagtacataggtgtcataaacggggggaagatgctcatgaagggggtcgacctggtccgc

aaaaataactgctctttcataaacttgtacgcgcgacatctggtagatcttttgttgtac

gacgaggatgtggccacggcggcagcagaggtgacagacgtgcctcccgcagaatgggtg

gggcgcccgctaccgagcggctttgacaagtttgggcgagtgctggtagaggcgtacaac

cgtatcactgcccccaacttggacgtgcgcgagttcgttatgactgctgagctgagccgc

tcacccgaatcgtataccaacaagcgcctgccgcacctcaccgtctactttaagctcgcc

atgaggaatgaagaactgcccagcgtaaaagagagaattccgtatgtgatagttgcgcag

accgaggccgcggaacgcgaagcgggtgtagtaaactcaatgcgcggtaccgcccaaaac

cccgtggtaaccaagaccgcacgcccccaacctaaacgcaaactgctggtttccgacctc

gccgaagacccgacctatgtttccgagaatgacgtaccgctaaacacagactactatttc

tcccacctgttgggtaccataagcgtgacctttaaggctctattcggaaatgatgtgaga

acaacagaaaatcttttaaagcggtttattccggaaaccccccacaagacccccacgaaa

acccaggcactgcttgagcgcgccggctttgaaaagctgacgccctttacaccggaggaa

gaaagtcgtcgaatactgcatacagttttttgtactctagaagcagctccccatcaaagc

tga

>KU206464.1

atggcggcgcgcgaacaggccaactccgttcgcaggagcgggttttttaatccgtttatt

ggcaagaggccatttttcaggccgggaagcgggcagaccgcggagacagagcgcccaagg

cccccccaacactcgtactgcacagaggtgggtagctttaagtttatagctccaagatgc

ctcgatgaagaagcccccgccgaccagcgacgcggtgtacacgtgggcaccttggagcgc

ccacccaaagtgtactgcgatggctcagagtacgacgtgctgaactttgcctccggaggt

tgctggcctcgcaggattcgggtttggaacggccaggattttcggggcgatggattcaac

cccagatttgagagatttcacgtgtacgacatagtagagacttcggagagcgcctcacac

gatgacccatccaggtttgctgagctatcccgcccaagcgggtctgtggtaacactgctg

ggaatgagcgagtgtggaaagcgagtcgcggttcacgtctatggtgtgcgccattatttt

tacatggcaaaggcggaggtggatagcgcttgtggaataaccaccgaggcagaactcgtg

cgcgcaatggtggactgcgcgcacagctcggctttgagcgcggccctgggaaatggcaac

ggcggcaaacagagcggcggcagcggcgggggatggtggggcggaaagcacgtgtctgcg

gactgcttcaaagtggagaccgtgtgccacacgacgctgtactactttggatctaagcca

gctctctactatagagtatctgcctccagcagccgcctgggagggttcatctgcgacaac

tttcacccggagattacaaaattcgaggggagcgtggacgtgaccacgcggctgttgttg

gacaatgaaaattttaccagtttcgggtggtaccgcctgcgacccggcacccacggagag

cgtgttcaacttcgccccgttgagcgacacgtcacctcaagcgacgtggagattaactgt

actcccgataacctggagccgataccagacgaggctgcctggcccgactataagctcatg

tgctttgatatagagtgtaaagctggaacgggtaacgaaatggcgttcccagtggcaact

aaccaagaggacctggtcatccagatctcctgtctgctgtactcgcttgctactcagaac

cacgaacacaccctgctgttttccctcgggtcatgcgatatctctgaggaatactcgttt

gcatgcgtccagcgcggcgagcccagaccgacggttttggagtttgacagcgagtacgag

ctgctggttgccttcctgacctttctcaagcagtactctcccgagttcgccaccggctac

aacatcgttaattttgactgggcgtacatagttaacaaggtaacgtcggtgtataacatc

aagctggacgggtacggcaagttcaacaaaggggggctgtttaaggtgtgggacatcgcc

acgaaccattttcagaagaagagcaaggtgaaaatcaatggcctgatatctctagacatg

tattctgtggcgacggaaaagctaaagctacccagctacaaactcgacgcggtcgtggga

gacgtcctcggcgagcataagatagaccttccctataaagaaataccctcctattacgcg

ggagggcctgaccggaggggcgtaataggagagtattgtatccaggactctaggctggtg

ggcaagctgttttttaagtacctcccccatctggaactatcggcggtggccaaactcgcc

cgtatcaccctaacgcgggtaatttttgacggtcagcaaattcgcgtgtacacgtgcttg

ctgaaactcgcccgcgagagaaatttcattttgccagacaacagacgccggtttgacagt

caggcagacgccgcgtcagagacttcggagttggctatggatagccaaagccacgccttc

gacagtacagacgaacccgacggtgtggacggtaccccggacgccgcaggatctggcgct

acttctgaaaacggaggcgggaagcccggcgtcgggagggccgtgggctaccagggagca

aaggttctagaccccgtatccggctttcatgtggaccccgtggttgtgtttgacttcgct

agcttatacccaagcattatccaggcccataacctctgtttcaccaccctggcgctcgat

gaagtggatctggccgggcttcaaccatccgtcgactactcgacgttcgaggtgggtgac

caaaagttattttttgtccacgcccatattcgcgaaagcctgcttggcatcttgctgcgc

gactggctggccatgcgaaaggcggtgagggcgcgaatccccaccagcacccccgaggag

gcagttttactagataagcagcagtctgcgattaaggtgatatgcaactcggtttacgga

ttcacgggggtggcaaacggcctgttgccgtgtctgaggatagcggctaccgttaccacg

ataggacgcgacatgctcctcaagacccgagattacgttcactctcgttgggcgacgcgc

gagctgctggaggacaattttccaggggctataggtttccgaaaccacaagccttactcc

gtcagggttatctacggagacaccgactccgtgtttatcaagtttgtgggcctgacgtac

gagggggtatcggagctgggggatgctatgtcgcgtcagatttcagcggacctctttaga

gcgcccatcaaactggagtgtgagaagacctttcagcgactgctgctgatcaccaagaag

aagtacataggtgtcataaacggggggaagatgctcatgaagggggtcgacctggtccgc

aaaaataactgctctttcataaacttgtacgcgcgacatctggtagatcttttgttgtac

gacgaggatgtggccacggcggcagcagaggtgacagacgtgcctcccgcagaatgggtg

gggcgcccgctaccgagcggctttgacaagtttgggcgagtgctggtagaggcgtacaac

cgtatcactgcccccaacttggacgtgcgcgagttcgttatgactgctgagctgagccgc

tcacccgaatcgtataccaacaagcgcctgccgcacctcaccgtctactttaagctcgcc

atgaggaatgaagaactgcccagcgtaaaagagagaattccgtatgtgatagttgcgcag

accgaggccgcggaacgcgaagcgggtgtagtaaactcaatgcgcggtaccgcccaaaac

cccgtggtaaccaagaccgcacgcccccaacctaaacgcaaactgctggtttccgacctc

gccgaagacccgacctatgtttccgagaatgacgtaccgctaaacacagactactatttc

tcccacctgttgggtaccataagcgtgacctttaaggctctattcggaaatgatgtgaga

acaacagaaaatcttttaaagcggtttattccggaaaccccccacaagacccccacgaaa

acccaggcactgcttgagcgcgccggctttgaaaagctgacgccctttacaccggaggaa

gaaagtcgtcgaatactgcatacagttttttgtactctagaagcagctccccatcaaagc

tga

>KU206465.1

atggcggcgcgcgaacaggccaactccgttcgcaggagcgggttttttaatccgtttatt

ggcaagaggccatttttcaggccgggaagcgggcagaccgcggagacagagcgcccaagg

cccccccaacactcgtactgcacagaggtgggtagctttaagtttatagctccaagatgc

ctcgatgaagaagcccccgccgaccagcgacgcggtgtacacgtgggcaccttggagcgc

ccacccaaagtgtactgcgatggctcagagtacgacgtgctgaactttgcctccggaggt

tgctggcctcgcaggattcgggtttggaacggccaggattttcggggcgatggattcaac

cccagatttgagagatttcacgtgtacgacatagtagagacttcggagagcgcctcacac

gatgacccatccaggtttgctgagctatcccgcccaagcgggtctgtggtaacactgctg

ggaatgagcgagtgtggaaagcgagtcgcggttcacgtctatggtgtgcgccattatttt

tacatggcaaaggcggaggtggatagcgcttgtggaataaccaccgaggcagaactcgtg

cgcgcaatggtggactgcgcgcacagctcggctttgagcgcggccctgggaaatggcaac

ggcggcaaacagagcggcggcagcggcgggggatggtggggcggaaagcacgtgtctgcg

gactgcttcaaagtggagaccgtgtgccacacgacgctgtactactttggatctaagcca

gctctctactatagagtatctgcctccagcagccgcctgggagggttcatctgcgacaac

tttcacccggagattacaaaattcgaggggagcgtggacgtgaccacgcggctgttgttg

gacaatgaaaattttaccagtttcgggtggtaccgcctgcgacccggcacccacggagag

cgtgttcaacttcgccccgttgagcgacacgtcacctcaagcgacgtggagattaactgt

actcccgataacctggagccgataccagacgaggctgcctggcccgactataagctcatg

tgctttgatatagagtgtaaagctggaacgggtaacgaaatggcgttcccagtggcaact

aaccaagaggacctggtcatccagatctcctgtctgctgtactcgcttgctactcagaac

cacgaacacaccctgctgttttccctcgggtcatgcgatatctctgaggaatactcgttt

gcatgcgtccagcgcggcgagcccagaccgacggttttggagtttgacagcgagtacgag

ctgctggttgccttcctgacctttctcaagcagtactctcccgagttcgccaccggctac

aacatcgttaattttgactgggcgtacatagttaacaaggtaacgtcggtgtataacatc

aagctggacgggtacggcaagttcaacaaaggggggctgtttaaggtgtgggacatcgcc

acgaaccattttcagaagaagagcaaggtgaaaatcaatggcctgatatctctagacatg

tattctgtggcgacggaaaagctaaagctacccagctacaaactcgacgcggtcgtggga

gacgtcctcggcgagcataagatagaccttccctataaagaaataccctcctattacgcg

ggagggcctgaccggaggggcgtaataggagagtattgtatccaggactctaggctggtg

ggcaagctgttttttaagtacctcccccatctggaactatcggcggtggccaaactcgcc

cgtatcaccctaacgcgggtaatttttgacggtcagcaaattcgcgtgtacacgtgcttg

ctgaaactcgcccgcgagagaaatttcattttgccagacaacagacgccggtttgacagt

caggcagacgccgcgtcagagacttcggagttggctatggatagccaaagccacgccttc

gacagtacagacgaacccgacggtgtggacggtaccccggacgccgcaggatctggcgct

acttctgaaaacggaggcgggaagcccggcgtcgggagggccgtgggctaccagggagca

aaggttctagaccccgtatccggctttcatgtggaccccgtggttgtgtttgacttcgct

agcttatacccaagcattatccaggcccataacctctgtttcaccaccctggcgctcgat

gaagtggatctggccgggcttcaaccatccgtcgactactcgacgttcgaggtgggtgac

caaaagttattttttgtccacgcccatattcgcgaaagcctgcttggcatcttgctgcgc

gactggctggccatgcgaaaggcggtgagggcgcgaatccccaccagcacccccgaggag

gcagttttactagataagcagcagtctgcgattaaggtgatatgcaactcggtttacgga

ttcacgggggtggcaaacggcctgttgccgtgtctgaggatagcggctaccgttaccacg

ataggacgcgacatgctcctcaagacccgagattacgttcactctcgttgggcgacgcgc

gagctgctggaggacaattttccaggggctataggtttccgaaaccacaagccttactcc

gtcagggttatctacggagacaccgactccgtgtttatcaagtttgtgggcctgacgtac

gagggggtatcggagctgggggatgctatgtcgcgtcagatttcagcggacctctttaga

gcgcccatcaaactggagtgtgagaagacctttcagcgactgctgctgatcaccaagaag

aagtacataggtgtcataaacggggggaagatgctcatgaagggggtcgacctggtccgc

aaaaataactgctctttcataaacttgtacgcgcgacatctggtagatcttttgttgtac

gacgaggatgtggccacggcggcagcagaggtgacagacgtgcctcccgcagaatgggtg

gggcgcccgctaccgagcggctttgacaagtttgggcgagtgctggtagaggcgtacaac

cgtatcactgcccccaacttggacgtgcgcgagttcgttatgactgctgagctgagccgc

tcacccgaatcgtataccaacaagcgcctgccgcacctcaccgtctactttaagctcgcc

atgaggaatgaagaactgcccagcgtaaaagagagaattccgtatgtgatagttgcgcag

accgaggccgcggaacgcgaagcgggtgtagtaaactcaatgcgcggtaccgcccaaaac

cccgtggtaaccaagaccgcacgcccccaacctaaacgcaaactgctggtttccgacctc

gccgaagacccgacctatgtttccgagaatgacgtaccgctaaacacagactactatttc

tcccacctgttgggtaccataagcgtgacctttaaggctctattcggaaatgatgtgaga

acaacagaaaatcttttaaagcggtttattccggaaaccccccacaagacccccacgaaa

acccaggcactgcttgagcgcgccggctttgaaaagctgacgccctttacaccggaggaa

gaaagtcgtcgaatactgcatacagttttttgtactctagaagcagctccccatcaaagc

tga

>KU206466.1

atggcggcgcgcgaacaggccaactccgttcgcaggagcgggttttttaatccgtttatt

ggcaagaggccatttttcaggccgggaagcgggcagaccgcggagacagagcgcccaagg

cccccccaacactcgtactgcacagaggtgggtagctttaagtttatagctccaagatgc

ctcgatgaagaagcccccgccgaccagcgacgcggtgtacacgtgggcaccttggagcgc

ccacccaaagtgtactgcgatggctcagagtacgacgtgctgaactttgcctccggaggt

tgctggcctcgcaggattcgggtttggaacggccaggattttcggggcgatggattcaac

cccagatttgagagatttcacgtgtacgacatagtagagacttcggagagcgcctcacac

gatgacccatccaggtttgctgagctatcccgcccaagcgggtctgtggtaacactgctg

ggaatgagcgagtgtggaaagcgagtcgcggttcacgtctatggtgtgcgccattatttt

tacatggcaaaggcggaggtggatagcgcttgtggaataaccaccgaggcagaactcgtg

cgcgcaatggtggactgcgcgcacagctcggctttgagcgcggccctgggaaatggcaac

ggcggcaaacagagcggcggcagcggcgggggatggtggggcggaaagcacgtgtctgcg

gactgcttcaaagtggagaccgtgtgccacacgacgctgtactactttggatctaagcca

gctctctactatagagtatctgcctccagcagccgcctgggagggttcatctgcgacaac

tttcacccggagattacaaaattcgaggggagcgtggacgtgaccacgcggctgttgttg

gacaatgaaaattttaccagtttcgggtggtaccgcctgcgacccggcacccacggagag

cgtgttcaacttcgccccgttgagcgacacgtcacctcaagcgacgtggagattaactgt

actcccgataacctggagccgataccagacgaggctgcctggcccgactataagctcatg

tgctttgatatagagtgtaaagctggaacgggtaacgaaatggcgttcccagtggcaact

aaccaagaggacctggtcatccagatctcctgtctgctgtactcgcttgctactcagaac

cacgaacacaccctgctgttttccctcgggtcatgcgatatctctgaggaatactcgttt

gcatgcgtccagcgcggcgagcccagaccgacggttttggagtttgacagcgagtacgag

ctgctggttgccttcctgacctttctcaagcagtactctcccgagttcgccaccggctac

aacatcgttaattttgactgggcgtacatagttaacaaggtaacgtcggtgtataacatc

aagctggacgggtacggcaagttcaacaaaggggggctgtttaaggtgtgggacatcgcc

acgaaccattttcagaagaagagcaaggtgaaaatcaatggcctgatatctctagacatg

tattctgtggcgacggaaaagctaaagctacccagctacaaactcgacgcggtcgtggga

gacgtcctcggcgagcataagatagaccttccctataaagaaataccctcctattacgcg

ggagggcctgaccggaggggcgtaataggagagtattgtatccaggactctaggctggtg

ggcaagctgttttttaagtacctcccccatctggaactatcggcggtggccaaactcgcc

cgtatcaccctaacgcgggtaatttttgacggtcagcaaattcgcgtgtacacgtgcttg

ctgaaactcgcccgcgagagaaatttcattttgccagacaacagacgccggtttgacagt

caggcagacgccgcgtcagagacttcggagttggctatggatagccaaagccacgccttc

gacagtacagacgaacccgacggtgtggacggtaccccggacgccgcaggatctggcgct

acttctgaaaacggaggcgggaagcccggcgtcgggagggccgtgggctaccagggagca

aaggttctagaccccgtatccggctttcatgtggaccccgtggttgtgtttgacttcgct

agcttatacccaagcattatccaggcccataacctctgtttcaccaccctggcgctcgat

gaagtggatctggccgggcttcaaccatccgtcgactactcgacgttcgaggtgggtgac

caaaagttattttttgtccacgcccatattcgcgaaagcctgcttggcatcttgctgcgc

gactggctggccatgcgaaaggcggtgagggcgcgaatccccaccagcacccccgaggag

gcagttttactagataagcagcagtctgcgattaaggtgatatgcaactcggtttacgga

ttcacgggggtggcaaacggcctgttgccgtgtctgaggatagcggctaccgttaccacg

ataggacgcgacatgctcctcaagacccgagattacgttcactctcgttgggcgacgcgc

gagctgctggaggacaattttccaggggctataggtttccgaaaccacaagccttactcc

gtcagggttatctacggagacaccgactccgtgtttatcaagtttgtgggcctgacgtac

gagggggtatcggagctgggggatgctatgtcgcgtcagatttcagcggacctctttaga

gcgcccatcaaactggagtgtgagaagacctttcagcgactgctgctgatcaccaagaag

aagtacataggtgtcataaacggggggaagatgctcatgaagggggtcgacctggtccgc

aaaaataactgctctttcataaacttgtacgcgcgacatctggtagatcttttgttgtac

gacgaggatgtggccacggcggcagcagaggtgacagacgtgcctcccgcagaatgggtg

gggcgcccgctaccgagcggctttgacaagtttgggcgagtgctggtagaggcgtacaac

cgtatcactgcccccaacttggacgtgcgcgagttcgttatgactgctgagctgagccgc

tcacccgaatcgtataccaacaagcgcctgccgcacctcaccgtctactttaagctcgcc

atgaggaatgaagaactgcccagcgtaaaagagagaattccgtatgtgatagttgcgcag

accgaggccgcggaacgcgaagcgggtgtagtaaactcaatgcgcggtaccgcccaaaac

cccgtggtaaccaagaccgcacgcccccaacctaaacgcaaactgctggtttccgacctc

gccgaagacccgacctatgtttccgagaatgacgtaccgctaaacacagactactatttc

tcccacctgttgggtaccataagcgtgacctttaaggctctattcggaaatgatgtgaga

acaacagaaaatcttttaaagcggtttattccggaaaccccccacaagacccccacgaaa

acccaggcactgcttgagcgcgccggctttgaaaagctgacgccctttacaccggaggaa

gaaagtcgtcgaatactgcatacagttttttgtactctagaagcagctccccatcaaagc

tga

>KU206467.1

atggcggcgcgcgaacaggccaactccgttcgcaggagcgggttttttaatccgtttatt

ggcaagaggccatttttcaggccgggaagcgggcagaccgcggagacagagcgcccaagg

cccccccaacactcgtactgcacagaggtgggtagctttaagtttatagctccaagatgc

ctcgatgaagaagcccccgccgaccagcgacgcggtgtacacgtgggcaccttggagcgc

ccacccaaagtgtactgcgatggctcagagtacgacgtgctgaactttgcctccggaggt

tgctggcctcgcaggattcgggtttggaacggccaggattttcggggcgatggattcaac

cccagatttgagagatttcacgtgtacgacatagtagagacttcggagagcgcctcacac

gatgacccatccaggtttgctgagctatcccgcccaagcgggtctgtggtaacactgctg

ggaatgagcgagtgtggaaagcgagtcgcggttcacgtctatggtgtgcgccattatttt

tacatggcaaaggcggaggtggatagcgcttgtggaataaccaccgaggcagaactcgtg

cgcgcaatggtggactgcgcgcacagctcggctttgagcgcggccctgggaaatggcaac

ggcggcaaacagagcggcggcagcggcgggggatggtggggcggaaagcacgtgtctgcg

gactgcttcaaagtggagaccgtgtgccacacgacgctgtactactttggatctaagcca

gctctctactatagagtatctgcctccagcagccgcctgggagggttcatctgcgacaac

tttcacccggagattacaaaattcgaggggagcgtggacgtgaccacgcggctgttgttg

gacaatgaaaattttaccagtttcgggtggtaccgcctgcgacccggcacccacggagag

cgtgttcaacttcgccccgttgagcgacacgtcacctcaagcgacgtggagattaactgt

actcccgataacctggagccgataccagacgaggctgcctggcccgactataagctcatg

tgctttgatatagagtgtaaagctggaacgggtaacgaaatggcgttcccagtggcaact

aaccaagaggacctggtcatccagatctcctgtctgctgtactcgcttgctactcagaac

cacgaacacaccctgctgttttccctcgggtcatgcgatatctctgaggaatactcgttt

gcatgcgtccagcgcggcgagcccagaccgacggttttggagtttgacagcgagtacgag

ctgctggttgccttcctgacctttctcaagcagtactctcccgagttcgccaccggctac

aacatcgttaattttgactgggcgtacatagttaacaaggtaacgtcggtgtataacatc

aagctggacgggtacggcaagttcaacaaaggggggctgtttaaggtgtgggacatcgcc

acgaaccattttcagaagaagagcaaggtgaaaatcaatggcctgatatctctagacatg

tattctgtggcgacggaaaagctaaagctacccagctacaaactcgacgcggtcgtggga

gacgtcctcggcgagcataagatagaccttccctataaagaaataccctcctattacgcg

ggagggcctgaccggaggggcgtaataggagagtattgtatccaggactctaggctggtg

ggcaagctgttttttaagtacctcccccatctggaactatcggcggtggccaaactcgcc

cgtatcaccctaacgcgggtaatttttgacggtcagcaaattcgcgtgtacacgtgcttg

ctgaaactcgcccgcgagagaaatttcattttgccagacaacagacgccggtttgacagt

caggcagacgccgcgtcagagacttcggagttggctatggatagccaaagccacgccttc

gacagtacagacgaacccgacggtgtggacggtaccccggacgccgcaggatctggcgct

acttctgaaaacggaggcgggaagcccggcgtcgggagggccgtgggctaccagggagca

aaggttctagaccccgtatccggctttcatgtggaccccgtggttgtgtttgacttcgct

agcttatacccaagcattatccaggcccataacctctgtttcaccaccctggcgctcgat

gaagtggatctggccgggcttcaaccatccgtcaactactcgacgttcgaggtgggtgac

caaaagttattttttgtccacgcccatattcgcgaaagcctgcttggcatcttgctgcgc

gactggctggccatgcgaaaggcggtgagggcgcgaatccccaccagcacccccgaggag

gcagttttactagataagcagcagtctgcgattaaggtgatatgcaactcggtttacgga

ttcacgggggtggcaaacggcctgttgccgtgtctgaggatagcggctaccgttaccacg

ataggacgcgacatgctcctcaagacccgagattacgttcactctcgttgggcgacgcgc

gagctgctggaggacaattttccaggggctataggtttccgaaaccacaagccttactcc

gtcagggttatctacggagacaccgactccgtgtttatcaagtttgtgggcctgacgtac

gagggggtatcggagctgggggatgctatgtcgcgtcagatttcagcggacctctttaga

gcgcccatcaaactggagtgtgagaagacctttcagcgactgctgctgatcaccaagaag

aagtacataggtgtcataaacggggggaagatgctcatgaagggggtcgacctggtccgc

aaaaataactgctctttcataaacttgtacgcgcgacatctggtagatcttttgttgtac

gacgaggatgtggccacggcggcagcagaggtgacagacgtgcctcccgcagaatgggtg

gggcgcccgctaccgagcggctttgacaagtttgggcgagtgctggtagaggcgtacaac

cgtatcactgcccccaacttggacgtgcgcgagttcgttatgactgctgagctgagccgc

tcacccgaatcgtataccaacaagcgcctgccgcacctcaccgtctactttaagctcgcc

atgaggaatgaagaactgcccagcgtaaaagagagaattccgtatgtgatagttgcgcag

accgaggccgcggaacgcgaagcgggtgtagtaaactcaatgcgcggtaccgcccaaaac

cccgtggtaaccaagaccgcacgcccccaacctaaacgcaaactgctggtttccgacctc

gccgaagacccgacctatgtttccgagaatgacgtaccgctaaacacagactactatttc

tcccacctgttgggtaccataagcgtgacctttaaggctctattcggaaatgatgtgaga

acaacagaaaatcttttaaagcggtttattccggaaaccccccacaagacccccacgaaa

acccaggcactgcttgagcgcgccggctttgaaaagctgacgccctttacaccggaggaa

gaaagtcgtcgaatactgcatacagttttttgtactctagaagcagctccccatcaaagc

tga

>KU206468.1

atggcggcgcgcgaacaggccaactccgttcgcaggagcgggttttttaatccgtttatt

ggcaagaggccatttttcaggccgggaagcgggcagaccgcggagacagagcgcccaagg

cccccccaacactcgtactgcacagaggtgggtagctttaagtttatagctccaagatgc

ctcgatgaagaagcccccgccgaccagcgacgcggtgtacacgtgggcaccttggagcgc

ccacccaaagtgtactgcgatggctcagagtacgacgtgctgaactttgcctccggaggt

tgctggcctcgcaggattcgggtttggaacggccaggattttcggggcgatggattcaac

cccagatttgagagatttcacgtgtacgacatagtagagacttcggagagcgcctcacac

gatgacccatccaggtttgctgagctatcccgcccaagcgggtctgtggtaacactgctg

ggaatgagcgagtgtggaaagcgagtcgcggttcacgtctatggtgtgcgccattatttt

tacatggcaaaggcggaggtggatagcgcttgtggaataaccaccgaggcagaactcgtg

cgcgcaatggtggactgcgcgcacagctcggctttgagcgcggccctgggaaatggcaac

ggcggcaaacagagcggcggcagcggcgggggatggtggggcggaaagcacgtgtctgcg

gactgcttcaaagtggagaccgtgtgccacacgacgctgtactactttggatctaagcca

gctctctactatagagtatctgcctccagcagccgcctgggagggttcatctgcgacaac

tttcacccggagattacaaaattcgaggggagcgtggacgtgaccacgcggctgttgttg

gacaatgaaaattttaccagtttcgggtggtaccgcctgcgacccggcacccacggagag

cgtgttcaacttcgccccgttgagcgacacgtcacctcaagcgacgtggagattaactgt

actcccgataacctggagccgataccagacgaggctgcctggcccgactataagctcatg

tgctttgatatagagtgtaaagctggaacgggtaacgaaatggcgttcccagtggcaact

aaccaagaggacctggtcatccagatctcctgtctgctgtactcgcttgctactcagaac

cacgaacacaccctgctgttttccctcgggtcatgcgatatctctgaggaatactcgttt

gcatgcgtccagcgcggcgagcccagaccgacggttttggagtttgacagcgagtacgag

ctgctggttgccttcctgacctttctcaagcagtactctcccgagttcgccaccggctac

aacatcgttaattttgactgggcgtacatagttaacaaggtaacgtcggtgtataacatc

aagctggacgggtacggcaagttcaacaaaggggggctgtttaaggtgtgggacatcgcc

acgaaccattttcagaagaagagcaaggtgaaaatcaatggcctgatatctctagacatg

tattctgtggcgacggaaaagctaaagctacccagctacaaactcgacgcggtcgtggga

gacgtcctcggcgagcataagatagaccttccctataaagaaataccctcctattacgcg

ggagggcctgaccggaggggcgtaataggagagtattgtatccaggactctaggctggtg

ggcaagctgttttttaagtacctcccccatctggaactatcggcggtggccaaactcgcc

cgtatcaccctaacgcgggtaatttttgacggtcagcaaattcgcgtgtacacgtgcttg

ctgaaactcgcccgcgagagaaatttcattttgccagacaacagacgccggtttgacagt

caggcagacgccgcgtcagagacttcggagttggctatggatagccaaagccacgccttc

gacagtacagacgaacccgacggtgtggacggtaccccggacgccgcaggatctggcgct

acttctgaaaacggaggcgggaagcccggcgtcgggagggccgtgggctaccagggagca

aaggttctagaccccgtatccggctttcatgtggaccccgtggttgtgtttgacttcgct

agcttatacccaagcattatccaggcccataacctctgtttcaccaccctggcgctcgat

gaagtggatctggccgggcttcaaccatccgtcgactactcgacgttcgaggtgggtgac

caaaagttattttttgtccacgcccatattcgcgaaagcctgcttggcatcttgctgcgc

gactggctggccatgcgaaaggcggtgagggcgcgaatccccaccagcacccccgaggag

gcagttttactagataagcagcagtctgcgattaaggtgatatgcaactcggtttacgga

ttcacgggggtggcaaacggcctgttgccgtgtctgaggatagcggctaccgttaccacg

ataggacgcgacatgctcctcaagacccgagattacgttcactctcgttgggcgacgcgc

gagctgctggaggacaattttccaggggctataggtttccgaaaccacaagccttactcc

gtcagggttatctacggagacaccgactccgtgtttatcaagtttgtgggcctgacgtac

gagggggtatcggagctgggggatgctatgtcgcgtcagatttcagcggacctctttaga

gcgcccatcaaactggagtgtgagaagacctttcagcgactgctgctgatcaccaagaag

aagtacataggtgtcataaacggggggaagatgctcatgaagggggtcgacctggtccgc

aaaaataactgctctttcataaacttgtacgcgcgacatctggtagatcttttgttgtac

gacgaggatgtggccacggcggcagcagaggtgacagacgtgcctcccgcagaatgggtg

gggcgcccgctaccgagcggctttgacaagtttgggcgagtgctggtagaggcgtacaac

cgtatcactgcccccaacttggacgtgcgcgagttcgttatgactgctgagctgagccgc

tcacccgaatcgtataccaacaagcgcctgccgcacctcaccgtctactttaagctcgcc

atgaggaatgaagaactgcccagcgtaaaagagagaattccgtatgtgatagttgcgcag

accgaggccgcggaacgcgaagcgggtgtagtaaactcaatgcgcggtaccgcccaaaac

cccgtggtaaccaagaccgcacgcccccaacctaaacgcaaactgctggtttccgacctc

gccgaagacccgacctatgtttccgagaatgacgtaccgctaaacacagactactatttc

tcccacctgttgggtaccataagcgtgacctttaaggctctattcggaaatgatgtgaga

acaacagaaaatcttttaaagcggtttattccggaaaccccccacaagacccccacgaaa

acccaggcactgcttgagcgcgccggctttgaaaagctgacgccctttacaccggaggaa

gaaagtcgtcgaatactgcatacagttttttgtactctagaagcagctccccatcaaagc

tga

>KU206469.1

atggcggcgcgcgaacaggccaactccgttcgcaggagcgggttttttaatccgtttatt

ggcaagaggccatttttcaggccgggaagcgggcagaccgcggagacagagcgcccaagg

cccccccaacactcgtactgcacagaggtgggtagctttaagtttatagctccaagatgc

ctcgatgaagaagcccccgccgaccagcgacgcggtgtacacgtgggcaccttggagcgc

ccacccaaagtgtactgcgatggctcagagtacgacgtgctgaactttgcctccggaggt

tgctggcctcgcaggattcgggtttggaacggccaggattttcggggcgatggattcaac

cccagatttgagagatttcacgtgtacgacatagtagagacttcggagagcgcctcacac

gatgacccatccaggtttgctgagctatcccgcccaagcgggtctgtggtaacactgctg

ggaatgagcgagtgtggaaagcgagtcgcggttcacgtctatggtgtgcgccattatttt

tacatggcaaaggcggaggtggatagcgcttgtggaataaccaccgaggcagaactcgtg

cgcgcaatggtggactgcgcgcacagctcggctttgagcgcggccctgggaaatggcaac

ggcggcaaacagagcggcggcagcggcgggggatggtggggcggaaagcacgtgtctgcg

gactgcttcaaagtggagaccgtgtgccgcacgacgctgtactactttggatctaagcca

gctctctactatagagtatctgcctccagcagccgcctgggagggttcatctgcgacaac

tttcacccggagattacaaaattcgaggggagcgtggacgtgaccacgcggctgttgttg

gacaatgaaaattttaccagtttcgggtggtaccgcctgcgacccggcacccacggagag

cgtgttcaacttcgccccgttgagcgacacgtcacctcaagcgacgtggagattaactgt

actcccgataacctggagccgataccagacgaggctgcctggcccgactataagctcatg

tgctttgatatagagtgtaaagctggaacgggtaacgaaatggcgttcccagtggcaact

aaccaagaggacctggtcatccagatctcctgtctgctgtactcgcttgctactcagaac

cacgaacacaccctgctgttttccctcgggtcatgcgatatctctgaggaatactcgttt

gcatgcgtccagcgcggcgagcccagaccgacggttttggagtttgacagcgagtacgag

ctgctggttgccttcctgacctttctcaagcagtactctcccgagttcgccaccggctac

aacatcgttaattttgactgggcgtacatagttaacaaggtaacgtcggtgtataacatc

aagctggacgggtacggcaagttcaacaaaggggggctgtttaaggtgtgggacatcgcc

acgaaccattttcagaagaagagcaaggtgaaaatcaatggcctgatatctctagacatg

tattctgtggcgacggaaaagctaaagctacccagctacaaactcgacgcggtcgtggga

gacgtcctcggcgagcataagatagaccttccctataaagaaataccctcctattacgcg

ggagggcctgaccggaggggcgtaataggagagtattgtatccaggactctaggctggtg

ggcaagctgttttttaagtacctcccccatctggaactatcggcggtggccaaactcgcc

cgtatcaccctaacgcgggtaatttttgacggtcagcaaattcgcgtgtacacgtgcttg

ctgaaactcgcccgcgagagaaatttcattttgccagacaacagacgccggtttgacagt

caggcagacgccgcgtcagagacttcggagttggctatggatagccaaagccacgccttc

gacagtacagacgaacccgacggtgtggacggtaccccggacgccgcaggatctggcgct

acttctgaaaacggaggcgggaagcccggcgtcgggagggccgtgggctaccagggagca

aaggttctagaccccgtatccggctttcatgtggaccccgtggttgtgtttgacttcgct

agcttatacccaagcattatccaggcccataacctctgtttcaccaccctggcgctcgat

gaagtggatctggccgggcttcaaccatccgtcaactactcgacgttcgaggtgggtgac

caaaagttattttttgtccacgcccatattcgcgaaagcctgcttggcatcttgctgcgc

gactggctggccatgcgaaaggcggtgagggcgcgaatccccaccagcacccccgaggag

gcagttttactagataagcagcagtctgcgattaaggtgatatgcaactcggtttacgga

ttcacgggggtggcaaacggcctgttgccgtgtctgaggatagcggctaccgttaccacg

ataggacgcgacatgctcctcaagacccgagattacgttcactctcgttgggcgacgcgc

gagctgctggaggacaattttccaggggctataggtttccgaaaccacaagccttactcc

gtcagggttatctacggagacaccgactccgtgtttatcaagtttgtgggcctgacgtac

gagggggtatcggagctgggggatgctatgtcgcgtcagatttcagcggacctctttaga

gcgcccatcaaactggagtgtgagaagacctttcagcgactgctgctgatcaccaagaag

aagtacataggtgtcataaacggggggaagatgctcatgaagggggtcgacctagtccgc

aaaaataactgctctttcataaacttgtacgcgcgacatctggtagatcttttgttgtac

gacgaggatgtggccacggcggcagcagaggtgacagacgtgcctcccgcagaatgggtg

gggcgcccgctaccgagcggctttgacaagtttgggcgagtgctggtagaggcgtacaac

cgtatcactgcccccaacttggacgtgcgcgagttcgttatgactgctgagctgagccgc

tcacccgaatcgtataccaacaagcgcctgccgcacctcaccgtctactttaagctcgcc

atgaggaatgaagaactgcccagcgtaaaagagagaattccgtatgtgatagttgcgcag

accgaggccgcggaacgcgaagcgggtgtagtaaactcaatgcgcggtaccgcccaaaac

cccgtggtaaccaagaccgcacgcccccaacctaaacgcaaactgctggtttccgacctc

gccgaagacccgacctatgtttccgagaatgacgtaccgctaaacacagactactatttc

tcccacctgttgggtaccataagcgtgacctttaaggctctattcggaaatgatgtgaga

acaacagaaaatcttttaaagcggtttattccggaaaccccccacaagacccccacgaaa

acccaggcactgcttgagcgcgccggctttgaaaagctgacgccctttacaccggaggaa

gaaagtcgtcgaatactgcatacagttttttgtactctagaagcagctccccatcaaagc

tga

>KU206470.1

atggcggcgcgcgaacaggccaactccgttcgcaggagcgggttttttaatccgtttatt

ggcaagaggccatttttcaggccgggaagcgggcagaccgcggagacagagcgcccaagg

cccccccaacactcgtactgcacagaggtgggtagctttaagtttatagctccaagatgc

ctcgatgaagaagcccccgccgaccagcgacgcggtgtacacgtgggcaccttggagcgc

ccacccaaagtgtactgcgatggctcagagtacgacgtgctgaactttgcctccggaggt

tgctggcctcgcaggattcgggtttggaacggccaggattttcggggcgatggattcaac

cccagatttgagagatttcacgtgtacgacatagtagagacttcggagagcgcctcacac

gatgacccatccaggtttgctgagctatcccgcccaagcgggtctgtggtaacactgctg

ggaatgagcgagtgtggaaagcgagtcgcggttcacgtctatggtgtgcgccattatttt

tacatggcaaaggcggaggtggatagcgcttgtggaataaccaccgaggcagaactcgtg

cgcgcaatggtggactgcgcgcacagctcggctttgagcgcggccctgggaaatggcaac

ggcggcaaacagagcggcggcagcggcgggggatggtggggcggaaagcacgtgtctgcg

gactgcttcaaagtggagaccgtgtgccgcacgacgctgtactactttggatctaagcca

gctctctactatagagtatctgcctccagcagccgcctgggagggttcatctgcgacaac

tttcacccggagattacaaaattcgaggggagcgtggacgtgaccacgcggctgttgttg

gacaatgaaaattttaccagtttcgggtggtaccgcctgcgacccggcacccacggagag

cgtgttcaacttcgccccgttgagcgacacgtcacctcaagcgacgtggagattaactgt

actcccgataacctggagccgataccagacgaggctgcctggcccgactataagctcatg

tgctttgatatagagtgtaaagctggaacgggtaacgaaatggcgttcccagtggcaact

aaccaagaggacctggtcatccagatctcctgtctgctgtactcgcttgctactcagaac

cacgaacacaccctgctgttttccctcgggtcatgcgatatctctgaggaatactcgttt

gcatgcgtccagcgcggcgagcccagaccgacggttttggagtttgacagcgagtacgag

ctgctggttgccttcctgacctttctcaagcagtactctcccgagttcgccaccggctac

aacatcgttaattttgactgggcgtacatagttaacaaggtaacgtcggtgtataacatc

aagctggacgggtacggcaagttcaacaaaggggggctgtttaaggtgtgggacatcgcc

acgaaccattttcagaagaagagcaaggtgaaaatcaatggcctgatatctctagacatg

tattctgtggcgacggaaaagctaaagctacccagctacaaactcgacgcggtcgtggga

gacgtcctcggcgagcataagatagaccttccctataaagaaataccctcctattacgcg

ggagggcctgaccggaggggcgtaataggagagtattgtatccaggactctaggctggtg

ggcaagctgttttttaagtacctcccccatctggaactatcggcggtggccaaactcgcc

cgtatcaccctaacgcgggtaatttttgacggtcagcaaattcgcgtgtacacgtgcttg

ctgaaactcgcccgcgagagaaatttcattttgccagacaacagacgccggtttgacagt

caggcagacgccgcgtcagagacttcggagttggctatggatagccaaagccacgccttc

gacagtacagacgaacccgacggtgtggacggtaccccggacgccgcaggatctggcgct

acttctgaaaacggaggcgggaagcccggcgtcgggagggccgtgggctaccagggagca

aaggttctagaccccgtatccggctttcatgtggaccccgtggttgtgtttgacttcgct

agcttatacccaagcattatccaggcccataacctctgtttcaccaccctggcgctcgat

gaagtggatctggccgggcttcaaccatccgtcaactactcgacgttcgaggtgggtgac

caaaagttattttttgtccacgcccatattcgcgaaagcctgcttggcatcttgctgcgc

gactggctggccatgcgaaaggcggtgagggcgcgaatccccaccagcacccccgaggag

gcagttttactagataagcagcagtctgcgattaaggtgatatgcaactcggtttacgga

ttcacgggggtggcaaacggcctgttgccgtgtctgaggatagcggctaccgttaccacg

ataggacgcgacatgctcctcaagacccgagattacgttcactctcgttgggcgacgcgc

gagctgctggaggacaattttccaggggctataggtttccgaaaccacaagccttactcc

gtcagggttatctacggagacaccgactccgtgtttatcaagtttgtgggcctgacgtac

gagggggtatcggagctgggggatgctatgtcgcgtcagatttcagcggacctctttaga

gcgcccatcaaactggagtgtgagaagacctttcagcgactgctgctgatcaccaagaag

aagtacataggtgtcataaacggggggaagatgctcatgaagggggtcgacctagtccgc

aaaaataactgctctttcataaacttgtacgcgcgacatctggtagatcttttgttgtac

gacgaggatgtggccacggcggcagcagaggtgacagacgtgcctcccgcagaatgggtg

gggcgcccgctaccgagcggctttgacaagtttgggcgagtgctggtagaggcgtacaac

cgtatcactgcccccaacttggacgtgcgcgagttcgttatgactgctgagctgagccgc

tcacccgaatcgtataccaacaagcgcctgccgcacctcaccgtctactttaagctcgcc

atgaggaatgaagaactgcccagcgtaaaagagagaattccgtatgtgatagttgcgcag

accgaggccgcggaacgcgaagcgggtgtagtaaactcaatgcgcggtaccgcccaaaac

cccgtggtaaccaagaccgcacgcccccaacctaaacgcaaactgctggtttccgacctc

gccgaagacccgacctatgtttccgagaatgacgtaccgctaaacacagactactatttc

tcccacctgttgggtaccataagcgtgacctttaaggctctattcggaaatgatgtgaga

acaacagaaaatcttttaaagcggtttattccggaaaccccccacaagacccccacgaaa

acccaggcactgcttgagcgcgccggctttgaaaagctgacgccctttacaccggaggaa

gaaagtcgtcgaatactgcatacagttttttgtactctagaagcagctccccatcaaagc

tga

>KU206471.1

atggcggcgcgcgaacaggccaactccgttcgcaggagcgggttttttaatccgtttatt

ggcaagaggccatttttcaggccgggaagcgggcagaccgcggagacagagcgcccaagg

cccccccaacactcgtactgcacagaggtgggtagctttaagtttatagctccaagatgc

ctcgatgaagaagcccccgccgaccagcgacgcggtgtacacgtgggcaccttggagcgc

ccacccaaagtgtactgcgatggctcagagtacgacgtgctgaactttgcctccggaggt

tgctggcctcgcaggattcgggtttggaacggccaggattttcggggcgatggattcaac

cccagatttgagagatttcacgtgtacgacatagtagagacttcggagagcgcctcacac

gatgacccatccaggtttgctgagctatcccgcccaagcgggtctgtggtaacactgctg

ggaatgagcgagtgtggaaagcgagtcgcggttcacgtctatggtgtgcgccattatttt

tacatggcaaaggcggaggtggatagcgcttgtggaataaccaccgaggcagaactcgtg

cgcgcaatggtggactgcgcgcacagctcggctttgagcgcggccctgggaaatggcaac

ggcggcaaacagagcggcggcagcggcgggggatggtggggcggaaagcacgtgtctgcg

gactgcttcaaagtggagaccgtgtgccgcacgacgctgtactactttggatctaagcca

gctctctactatagagtatctgcctccagcagccgcctgggagggttcatctgcgacaac

tttcacccggagattacaaaattcgaggggagcgtggacgtgaccacgcggctgttgttg

gacaatgaaaattttaccagtttcgggtggtaccgcctgcgacccggcacccacggagag

cgtgttcaacttcgccccgttgagcgacacgtcacctcaagcgacgtggagattaactgt

actcccgataacctggagccgataccagacgaggctgcctggcccgactataagctcatg

tgctttgatatagagtgtaaagctggaacgggtaacgaaatggcgttcccagtggcaact

aaccaagaggacctggtcatccagatctcctgtctgctgtactcgcttgctactcagaac

cacgaacacaccctgctgttttccctcgggtcatgcgatatctctgaggaatactcgttt

gcatgcgtccagcgcggcgagcccagaccgacggttttggagtttgacagcgagtacgag

ctgctggttgccttcctgacctttctcaagcagtactctcccgagttcgccaccggctac

aacatcgttaattttgactgggcgtacatagttaacaaggtaacgtcggtgtataacatc

aagctggacgggtacggcaagttcaacaaaggggggctgtttaaggtgtgggacatcgcc

acgaaccattttcagaagaagagcaaggtgaaaatcaatggcctgatatctctagacatg

tattctgtggcgacggaaaagctaaagctacccagctacaaactcgacgcggtcgtggga

gacgtcctcggcgagcataagatagaccttccctataaagaaataccctcctattacgcg

ggagggcctgaccggaggggcgtaataggagagtattgtatccaggactctaggctggtg

ggcaagctgttttttaagtacctcccccatctggaactatcggcggtggccaaactcgcc

cgtatcaccctaacgcgggtaatttttgacggtcagcaaattcgcgtgtacacgtgcttg

ctgaaactcgcccgcgagagaaatttcattttgccagacaacagacgccggtttgacagt

caggcagacgccgcgtcagagacttcggagttggctatggatagccaaagccacgccttc

gacagtacagacgaacccgacggtgtggacggtaccccggacgccgcaggatctggcgct

acttctgaaaacggaggcgggaagcccggcgtcgggagggccgtgggctaccagggagca

aaggttctagaccccgtatccggctttcatgtggaccccgtggttgtgtttgacttcgct

agcttatacccaagcattatccaggcccataacctctgtttcaccaccctggcgctcgat

gaagtggatctggccgggcttcaaccatccgtcaactactcgacgttcgaggtgggtgac

caaaagttattttttgtccacgcccatattcgcgaaagcctgcttggcatcttgctgcgc

gactggctggccatgcgaaaggcggtgagggcgcgaatccccaccagcacccccgaggag

gcagttttactagataagcagcagtctgcgattaaggtgatatgcaactcggtttacgga

ttcacgggggtggcaaacggcctgttgccgtgtctgaggatagcggctaccgttaccacg

ataggacgcgacatgctcctcaagacccgagattacgttcactctcgttgggcgacgcgc

gagctgctggaggacaattttccaggggctataggtttccgaaaccacaagccttactcc

gtcagggttatctacggagacaccgactccgtgtttatcaagtttgtgggcctgacgtac

gagggggtatcggagctgggggatgctatgtcgcgtcagatttcagcggacctctttaga

gcgcccatcaaactggagtgtgagaagacctttcagcgactgctgctgatcaccaagaag

aagtacataggtgtcataaacggggggaagatgctcatgaagggggtcgacctagtccgc

aaaaataactgctctttcataaacttgtacgcgcgacatctggtagatcttttgttgtac

gacgaggatgtggccacggcggcagcagaggtgacagacgtgcctcccgcagaatgggtg

gggcgcccgctaccgagcggctttgacaagtttgggcgagtgctggtagaggcgtacaac

cgtatcactgcccccaacttggacgtgcgcgagttcgttatgactgctgagctgagccgc

tcacccgaatcgtataccaacaagcgcctgccgcacctcaccgtctactttaagctcgcc

atgaggaatgaagaactgcccagcgtaaaagagagaattccgtatgtgatagttgcgcag

accgaggccgcggaacgcgaagcgggtgtagtaaactcaatgcgcggtaccgcccaaaac

cccgtggtaaccaagaccgcacgcccccaacctaaacgcaaactgctggtttccgacctc

gccgaagacccgacctatgtttccgagaatgacgtaccgctaaacacagactactatttc

tcccacctgttgggtaccataagcgtgacctttaaggctctattcggaaatgatgtgaga

acaacagaaaatcttttaaagcggtttattccggaaaccccccacaagacccccacgaaa

acccaggcactgcttgagcgcgccggctttgaaaagctgacgccctttacaccggaggaa

gaaagtcgtcgaatactgcatacagttttttgtactctagaagcagctccccatcaaagc

tga

>KU206472.1

atggcggcgcgcgaacaggccaactccgttcgcaggagcgggttttttaatccgtttatt

ggcaagaggccatttttcaggccgggaagcgggcagaccgcggagacagagcgcccaagg

cccccccaacactcgtactgcacagaggtgggtagctttaagtttatagctccaagatgc

ctcgatgaagaagcccccgccgaccagcgacgcggtgtacacgtgggcaccttggagcgc

ccacccaaagtgtactgcgatggctcagagtacgacgtgctgaactttgcctccggaggt

tgctggcctcgcaggattcgggtttggaacggccaggattttcggggcgatggattcaac

cccagatttgagagatttcacgtgtacgacatagtagagacttcggagagcgcctcacac

gatgacccatccaggtttgctgagctatcccgcccaagcgggtctgtggtaacactgctg

ggaatgagcgagtgtggaaagcgagtcgcggttcacgtctatggtgtgcgccattatttt

tacatggcaaaggcggaggtggatagcgcttgtggaataaccaccgaggcagaactcgtg

cgcgcaatggtggactgcgcgcacagctcggctttgagcgcggccctgggaaatggcaac

ggcggcaaacagagcggcggcagcggcgggggatggtggggcggaaagcacgtgtctgcg

gactgcttcaaagtggagaccgtgtgccacacgacgctgtactactttggatctaagcca

gctctctactatagagtatctgcctccagcagccgcctgggagggttcatctgcgacaac

tttcacccggagattacaaaattcgaggggagcgtggacgtgaccacgcggctgttgttg

gacaatgaaaattttaccagtttcgggtggtaccgcctgcgacccggcacccacggagag

cgtgttcaacttcgccccgttgagcgacacgtcacctcaagcgacgtggagattaactgt

actcccgataacctggagccgataccagacgaggctgcctggcccgactataagctcatg

tgctttgatatagagtgtaaagctggaacgggtaacgaaatggcgttcccagtggcaact

aaccaagaggacctggtcatccagatctcctgtctgctgtactcgcttgctactcagaac

cacgaacacaccctgctgttttccctcgggtcatgcgatatctctgaggaatactcgttt

gcatgcgtccagcgcggcgagcccagaccgacggttttggagtttgacagcgagtacgag

ctgctggttgccttcctgacctttctcaagcagtactctcccgagttcgccaccggctac

aacatcgttaattttgactgggcgtacatagttaacaaggtaacgtcggtgtataacatc

aagctggacgggtacggcaagttcaacaaaggggggctgtttaaggtgtgggacatcgcc

acgaaccattttcagaagaagagcaaggtgaaaatcaatggcctgatatctctagacatg

tattctgtggcgacggaaaagctaaagctacccagctacaaactcgacgcggtcgtggga

gacgtcctcggcgagcataagatagaccttccctataaagaaataccctcctattacgcg

ggagggcctgaccggaggggcgtaataggagagtattgtatccaggactctaggctggtg

ggcaagctgttttttaagtacctcccccatctggaactatcggcggtggccaaactcgcc

cgtatcaccctaacgcgggtaatttttgacggtcagcaaattcgcgtgtacacgtgcttg

ctgaaactcgcccgcgagagaaatttcattttgccagacaacagacgccggtttgacagt

caggcagacgccgcgtcagagacttcggagttggctatggatagccaaagccacgccttc

gacagtacagacgaacccgacggtgtggacggtaccccggacgccgcaggatctggcgct

acttctgaaaacggaggcgggaagcccggcgtcgggagggccgtgggctaccagggagca

aaggttctagaccccgtatccggctttcatgtggaccccgtggttgtgtttgacttcgct

agcttatacccaagcattatccaggcccataacctctgtttcaccaccctggcgctcgat

gaagtggatctggccgggcttcaaccatccgtcaactactcgacgttcgaggtgggtgac

caaaagttattttttgtccacgcccatattcgcgaaagcctgcttggcatcttgctgcgc

gactggctggccatgcgaaaggcggtgagggcgcgaatccccaccagcacccccgaggag

gcagttttactagataagcagcagtctgcgattaaggtgatatgcaactcggtttacgga

ttcacgggggtggcaaacggcctgttgccgtgtctgaggatagcggctaccgttaccacg

ataggacgcgacatgctcctcaagacccgagattacgttcactctcgttgggcgacgcgc

gagctgctggaggacaattttccaggggctataggtttccgaaaccacaagccttactcc

gtcagggttatctacggagacaccgactccgtgtttatcaagtttgtgggcctgacgtac

gagggggtatcggagctgggggatgctatgtcgcgtcagatttcagcggacctctttaga

gcgcccatcaaactggagtgtgagaagacctttcagcgactgctgctgatcaccaagaag

aagtacataggtgtcataaacggggggaagatgctcatgaagggggtcgacctagtccgc

aaaaataactgctctttcataaacttgtacgcgcgacatctggtagatcttttgttgtac

gacgaggatgtggccacggcggcagcagaggtgacagacgtgcctcccgcagaatgggtg

gggcgcccgctaccgagcggctttgacaagtttgggcgagtgctggtagaggcgtacaac

cgtatcactgcccccaacttggacgtgcgcgagttcgttatgactgctgagctgagccgc

tcacccgaatcgtataccaacaagcgcctgccgcacctcaccgtctactttaagctcgcc

atgaggaatgaagaactgcccagcgtaaaagagagaattccgtatgtgatagttgcgcag

accgaggccgcggaacgcgaagcgggtgtagtaaactcaatgcgcggtaccgcccaaaac

cccgtggtaaccaagaccgcacgcccccaacctaaacgcaaactgctggtttccgacctc

gccgaagacccgacctatgtttccgagaatgacgtaccgctaaacacagactactatttc

tcccacctgttgggtaccataagcgtgacctttaaggctctattcggaaatgatgtgaga

acaacagaaaatcttttaaagcggtttattccggaaaccccccacaagacccccacgaaa

acccaggcactgcttgagcgcgccggctttgaaaagctgacgccctttacaccggaggaa

gaaagtcgtcgaatactgcatacagttttttgtactctagaagcagctccccatcaaagc

tga

>KU206473.1

atggcggcgcgcgaacaggccaactccgttcgcaggagcgggttttttaatccgtttatt

ggcaagaggccatttttcaggccgggaagcgggcagaccgcggagacagagcgcccaagg

cccccccaacactcgtactgcacagaggtgggtagctttaagtttatagctccaagatgc

ctcgatgaagaagcccccgccgaccagcgacgcggtgtacacgtgggcaccttggagcgc

ccacccaaagtgtactgcgatggctcagagtacgacgtgctgaactttgcctccggaggt

tgctggcctcgcaggattcgggtttggaacggccaggattttcggggcgatggattcaac

cccagatttgagagatttcacgtgtacgacatagtagagacttcggagagcgcctcacac

gatgacccatccaggtttgctgagctatcccgcccaagcgggtctgtggtaacactgctg

ggaatgagcgagtgtggaaagcgagtcgcggttcacgtctatggtgtgcgccattatttt

tacatggcaaaggcggaggtggatagcgcttgtggaataaccaccgaggcagaactcgtg

cgcgcaatggtggactgcgcgcacagctcggctttgagcgcggccctgggaaatggcaac

ggcggcaaacagagcggcggcagcggcgggggatggtggggcggaaagcacgtgtctgcg

gactgcttcaaagtggagaccgtgtgccacacgacgctgtactactttggatctaagcca

gctctctactatagagtatctgcctccagcagccgcctgggagggttcatctgcgacaac

tttcacccagagattacaaaattcgaggggagcgtggacgtgaccacgcggctgttgttg

gacaatgaaaattttaccagtttcgggtggtaccgcctgcgacccggcacccacggagag

cgtgttcaacttcgccccgttgagcgacacgtcacctcaagcgacgtggagattaactgt

actcccgataacctggagccgataccagacgaggctgcctggcccgactataagctcatg

tgctttgatatagagtgtaaagctggaacgggtaacgaaatggcgttcccagtggcaact

aaccaagaggacctggtcatccagatctcctgtctgctgtactcgcttgctactcagaac

cacgaacacaccctgctgttttccctcgggtcatgcgatatctctgaggaatactcgttt

gcatgcgtccagcgcggcgagcccagaccgacggttttggagtttgacagcgagtacgag

ctgctggttgccttcctgacctttctcaagcagtactctcccgagttcgccaccggctac

aacatcgttaattttgactgggcgtacatagttaacaaggtaacgtcggtgtataacatc

aagctggacgggtacggcaagttcaacaaaggggggctgtttaaggtgtgggacatcgcc

acgaaccattttcagaagaagagcaaggtgaaaatcaatggcctgatatctctagacatg

tattctgtggcgacggaaaagctaaagctacccagctacaaactcgacgcggtcgtggga

gacgtcctcggcgagcataagatagaccttccctataaagaaataccctcctattacgcg

ggagggcctgaccggaggggcgtaataggagagtattgtatccaggactctaggctggtg

ggcaagctgttttttaagtacctcccccatctggaactatcggcggtggccaaactcgcc

cgtatcaccctaacgcgggtaatttttgacggtcagcaaattcgcgtgtacacgtgcttg

ctgaaactcgcccgcgagagaaatttcattttgccagacaacagacgccggtttgacagt

caggcagacgccgcgtcagagacttcggagttggctatggatagccaaagccacgccttc

gacagtacagacgaacccgacggtgtggacggtaccccggacgccgcaggatctggcgct

acttctgaaaacggaggcgggaagcccggcgtcgggagggccgtgggctaccagggagca

aaggttctagaccccgtatccggctttcatgtggaccccgtggttgtgtttgacttcgct

agcttatacccaagcattatccaggcccataacctctgtttcaccaccctggcgctcgat

gaagtggatctggccgggcttcaaccatccgtcgactactcgacgttcgaggtgggtgac

caaaagttattttttgtccacgcccatattcgcgaaagcctgcttggcatcttgctgcgc

gactggctggccatgcgaaaggcggtgagggcgcgaatccccaccagcacccccgaggag

gcagttttactagataagcagcagtctgcgattaaggtgatatgcaactcggtttacgga

ttcacgggggtggcaaacggcctgttgccgtgtctgaggatagcggctaccgttaccacg

ataggacgcgacatgctcctcaagacccgagattacgttcactctcgttgggcgacgcgc

gagctgctggaggacaattttccaggggctataggtttccgaaaccacaagccttactcc

gtcagggttatctacggagacaccgactccgtgtttatcaagtttgtgggcctgacgtac

gagggggtatcggagctgggggatgctatgtcgcgtcagatttcagcggacctctttaga

gcgcccatcaaactggagtgtgagaagacctttcagcgactgctgctgatcaccaagaag

aagtacataggtgtcataaacggggggaagatgctcatgaagggggtcgacctagtccgc

aaaaataactgctctttcataaacttgtacgcgcgacatctggtagatcttttgttgtac

gacgaggatgtggccacggcggcagcagaggtgacagacgtgcctcccgcagaatgggtg

gggcgcccgctaccgagcggctttgacaagtttgggcgagtgctggtagaggcgtacaac

cgtatcactgcccccaacttggacgtgcgcgagttcgttatgactgctgagctgagccgc

tcacccgaatcgtataccaacaagcgcctgccgcacctcaccgtctactttaagctcgcc

atgaggaatgaagaactgcccagcgtaaaagagagaattccgtatgtgatagttgcgcag

accgaggccgcggaacgcgaagcgggtgtagtaaactcaatgcgcggtaccgcccaaaac

cccgtggtaaccaagaccgcacgcccccaacctaaacgcaaactgctggtttccgacctc

gccgaagacccgacctatgtttccgagaatgacgtaccgctaaacacagactactatttc

tcccacctgttgggtaccataagcgtgacctttaaggctctattcggaaatgatgtgaga

acaacagaaaatcttttaaagcggtttattccggaaaccccccacaagacccccacgaaa

acccaggcactgcttgagcgcgccggctttgaaaagctgacgccctttacaccggaggaa

gaaagtcgtcgaatactgcatacagttttttgtactctagaagcagctccccatcaaagc

tga

>KU206474.1

atggcggcgcgcgaacaggccaactccgttcgcaggagcgggttttttaatccgtttatt

ggcaagaggccatttttcaggccgggaagcgggcagaccgcggagacagagcgcccaagg

cccccccaacactcgtactgcacagaggtgggtagctttaagtttatagctccaagatgc

ctcgatgaagaagcccccgccgaccagcgacgcggtgtacacgtgggcaccttggagcgc

ccacccaaagtgtactgcgatggctcagagtacgacgtgctgaactttgcctccggaggt

tgctggcctcgcaggattcgggtttggaacggccaggattttcggggcgatggattcaac

cccagatttgagagatttcacgtgtacgacatagtagagacttcggagagcgcctcacac

gatgacccatccaggtttgctgagctatcccgcccaagcgggtctgtggtaacactgctg

ggaatgagcgagtgtggaaagcgagtcgcggttcacgtctatggtgtgcgccattatttt

tacatggcaaaggcggaggtggatagcgcttgtggaataaccaccgaggcagaactcgtg

cgcgcaatggtggactgcgcgcacagctcggctttgagcgcggccctgggaaatggcaac

ggcggcaaacagagcggcggcagcggcgggggatggtggggcggaaagcacgtgtctgcg

gactgcttcaaagtggagaccgtgtgccacacgacgctgtactactttggatctaagcca

gctctctactatagagtatctgcctccagcagccgcctgggagggttcatctgcgacaac

tttcacccggagattacaaaattcgaggggagcgtggacgtgaccacgcggctgttgttg

gacaatgaaaattttaccagtttcgggtggtaccgcctgcgacccggcacccacggagag

cgtgttcaacttcgccccgttgagcgacacgtcacctcaagcgacgtggagattaactgt

actcccgataacctggagccgataccagacgaggctgcctggcccgactataagctcatg

tgctttgatatagagtgtaaagctggaacgggtaacgaaatggcgttcccagtggcaact

aaccaagaggacctggtcatccagatctcctgtctgctgtactcgcttgctactcagaac

cacgaacacaccctgctgttttccctcgggtcatgcgatatctctgaggaatactcgttt

gcatgcgtccagcgcggcgagcccagaccgacggttttggagtttgacagcgagtacgag

ctgctggttgccttcctgacctttctcaagcagtactctcccgagttcgccaccggctac

aacatcgttaattttgactgggcgtacatagttaacaaggtaacgtcggtgtataacatc

aagctggacgggtacggcaagttcaacaaaggggggctgtttaaggtgtgggacatcgcc

acgaaccattttcagaagaagagcaaggtgaaaatcaatggcctgatatctctagacatg

tattctgtggcgacggaaaagctaaagctacccagctacaaactcgacgcggtcgtggga

gacgtcctcggcgagcataagatagaccttccctataaagaaataccctcctattacgcg

ggagggcctgaccggaggggcgtaataggagagtattgtatccaggactctaggctggtg

ggcaagctgttttttaagtacctcccccatctggaactatcggcggtggccaaactcgcc

cgtatcaccctaacgcgggtaatttttgacggtcagcaaattcgcgtgtacacgtgcttg

ctgaaactcgcccgcgagagaaatttcattttgccagacaacagacgccggtttgacagt

caggcagacgccgcgtcagagacttcggagttggctatggatagccaaagccacgccttc

gacagtacagacgaacccgacggtgtggacggtaccccggacgccgcaggatctggcgct

acttctgaaaacggaggcgggaagcccggcgtcgggagggccgtgggctaccagggagca

aaggttctagaccccgtatccggctttcatgtggaccccgtggttgtgtttgacttcgct

agcttatacccaagcattatccaggcccataacctctgtttcaccaccctggcgctcgat

gaagtggatctggccgggcttcaaccatccgtcaactactcgacgttcgaggtgggtgac

caaaagttattttttgtccacgcccatattcgcgaaagcctgcttggcatcttgctgcgc

gactggctggccatgcgaaaggcggtgagggcgcgaatccccaccagcacccccgaggag

gcagttttactagataagcagcagtctgcgattaaggtgatatgcaactcggtttacgga

ttcacgggggtggcaaacggcctgttgccgtgtctgaggatagcggctaccgttaccacg

ataggacgcgacatgctcctcaagacccgagattacgttcactctcgttgggcgacgcgc

gagctgctggaggacaattttccaggggctataggtttccgaaaccacaagccttactcc

gtcagggttatctacggagacaccgactccgtgtttatcaagtttgtgggcctgacgtac

gagggggtatcggagctgggggatgctatgtcgcgtcagatttcagcggacctctttaga

gcgcccatcaaactggagtgtgagaagacctttcagcgactgctgctgatcaccaagaag

aagtacataggtgtcataaacggggggaagatgctcatgaagggggtcgacctggtccgc

aaaaataactgctctttcataaacttgtacgcgcgacatctggtagatcttttgttgtac

gacgaggatgtggccacggcggcagcagaggtgacagacgtgcctcccgcagaatgggtg

gggcgcccgctaccgagcggctttgacaagtttgggcgagtgctggtagaggcgtacaac

cgtatcactgcccccaacttggacgtgcgcgagttcgttatgactgctgagctgagccgc

tcacccgaatcgtataccaacaagcgcctgccgcacctcaccgtctactttaagctcgcc

atgaggaatgaagaactgcccagcgtaaaagagagaattccgtatgtgatagttgcgcag

accgaggccgcggaacgcgaagcgggtgtagtaaactcaatgcgcggtaccgcccaaaac

cccgtggtaaccaagaccgcacgcccccaacctaaacgcaaactgctggtttccgacctc

gccgaagacccgacctatgtttccgagaatgacgtaccgctaaacacagactactatttc

tcccacctgttgggtaccataagcgtgacctttaaggctctattcggaaatgatgtgaga

acaacagaaaatcttttaaagcggtttattccggaaaccccccacaagacccccacgaaa

acccaggcactgcttgagcgcgccggctttgaaaagctgacgccctttacaccggaggaa

gaaagtcgtcgaatactgcatacagttttttgtactctagaagcagctccccatcaaagc

tga

>KU206475.1

atggcggcgcgcgaacaggccaactccgttcgcaggagcgggttttttaatccgtttatt

ggcaagaggccatttttcaggccgggaagcgggcagaccgcggagacagagcgcccaagg

cccccccaacactcgtactgcacagaggtgggtagctttaagtttatagctccaagatgc

ctcgatgaagaagcccccgccgaccagcgacgcggtgtacacgtgggcaccttggagcgc

ccacccaaagtgtactgcgatggctcagagtacgacgtgctgaactttgcctccggaggt

tgctggcctcgcaggattcgggtttggaacggccaggattttcggggcgatggattcaac

cccagatttgagagatttcacgtgtacgacatagtagagacttcggagagcgcctcacac

gatgacccatccaggtttgctgagctatcccgcccaagcgggtctgtggtaacactgctg

ggaatgagcgagtgtggaaagcgagtcgcggttcacgtctatggtgtgcgccattatttt

tacatggcaaaggcggaggtggatagcgcttgtggaataaccaccgaggcagaactcgtg

cgcgcaatggtggactgcgcgcacagctcggctttgagcgcggccctgggaaatggcaac

ggcggcaaacagagcggcggcagcggcgggggatggtggggcggaaagcacgtgtctgcg

gactgcttcaaagtggagaccgtgtgccacacgacgctgtactactttggatctaagcca

gctctctactatagagtatctgcctccagcagccgcctgggagggttcatctgcgacaac

tttcacccggagattacaaaattcgaggggagcgtggacgtgaccacgcggctgttgttg

gacaatgaaaattttaccagtttcgggtggtaccgcctgcgacccggcacccacggagag

cgtgttcaacttcgccccgttgagcgacacgtcacctcaagcgacgtggagattaactgt

actcccgataacctggagccgataccagacgaggctgcctggcccgactataagctcatg

tgctttgatatagagtgtaaagctggaacgggtaacgaaatggcgttcccagtggcaact

aaccaagaggacctggtcatccagatctcctgtctgctgtactcgcttgctactcagaac

cacgaacacaccctgctgttttccctcgggtcatgcgatatctctgaggaatactcgttt

gcatgcgtccagcgcggcgagcccagaccgacggttttggagtttgacagcgagtacgag

ctgctggttgccttcctgacctttctcaagcagtactctcccgagttcgccaccggctac

aacatcgttaattttgactgggcgtacatagttaacaaggtaacgtcggtgtataacatc

aagctggacgggtacggcaagttcaacaaaggggggctgtttaaggtgtgggacatcgcc

acgaaccattttcagaagaagagcaaggtgaaaatcaatggcctgatatctctagacatg

tattctgtggcgacggaaaagctaaagctacccagctacaaactcgacgcggtcgtggga

gacgtcctcggcgagcataagatagaccttccctataaagaaataccctcctattacgcg

ggagggcctgaccggaggggcgtaataggagagtattgtatccaggactctaggctggtg

ggcaagctgttttttaagtacctcccccatctggaactatcggcggtggccaaactcgcc

cgtatcaccctaacgcgggtaatttttgacggtcagcaaattcgcgtgtacacgtgcttg

ctgaaactcgcccgcgagagaaatttcattttgccagacaacagacgccggtttgacagt

caggcagacgccgcgtcagagacttcggagttggctatggatagccaaagccacgccttc

gacagtacagacgaacccgacggtgtggacggtaccccggacgccgcaggatctggcgct

acttctgaaaacggaggcgggaagcccggcgtcgggagggccgtgggctaccagggagca

aaggttctagaccccgtatccggctttcatgtggaccccgtggttgtgtttgacttcgct

agcttatacccaagcattatccaggcccataacctctgtttcaccaccctggcgctcgat

gaagtggatctggccgggcttcaaccatccgtcaactactcgacgttcgaggtgggtgac

caaaagttattttttgtccacgcccatattcgcgaaagcctgcttggcatcttgctgcgc

gactggctggccatgcgaaaggcggtgagggcgcgaatccccaccagcacccccgaggag

gcagttttactagataagcagcagtctgcgattaaggtgatatgcaactcggtttacgga

ttcacgggggtggcaaacggcctgttgccgtgtctgaggatagcggctaccgttaccacg

ataggacgcgacatgctcctcaagacccgagattacgttcactctcgttgggcgacgcgc

gagctgctggaggacaattttccaggggctataggtttccgaaaccacaagccttactcc

gtcagggttatctacggagacaccgactccgtgtttatcaagtttgtgggcctgacgtac

gagggggtatcggagctgggggatgctatgtcgcgtcagatttcagcggacctctttaga

gcgcccatcaaactggagtgtgagaagacctttcagcgactgctgctgatcaccaagaag

aagtacataggtgtcataaacggggggaagatgctcatgaagggggtcgacctggtccgc

aaaaataactgctctttcataaacttgtacgcgcgacatctggtagatcttttgttgtac

gacgaggatgtggccacggcggcagcagaggtgacagacgtgcctcccgcagaatgggtg

gggcgcccgctaccgagcggctttgacaagtttgggcgagtgctggtagaggcgtacaac

cgtatcactgcccccaacttggacgtgcgcgagttcgttatgactgctgagctgagccgc

tcacccgaatcgtataccaacaagcgcctgccgcacctcaccgtctactttaagctcgcc

atgaggaatgaagaactgcccagcgtaaaagagagaattccgtatgtgatagttgcgcag

accgaggccgcggaacgcgaagcgggtgtagtaaactcaatgcgcggtaccgcccaaaac

cccgtggtaaccaagaccgcacgcccccaacctaaacgcaaactgctggtttccgacctc

gccgaagacccgacctatgtttccgagaatgacgtaccgctaaacacagactactatttc

tcccacctgttgggtaccataagcgtgacctttaaggctctattcggaaatgatgtgaga

acaacagaaaatcttttaaagcggtttattccggaaaccccccacaagacccccacgaaa

acccaggcactgcttgagcgcgccggctttgaaaagctgacgccctttacaccggaggaa

gaaagtcgtcgaatactgcatacagttttttgtactctagaagcagctccccatcaaagc

tga

>KU206476.1

atggcggcgcgcgaacaggccaactccgttcgcaggagcgggttttttaatccgtttatt

ggcaagaggccatttttcaggccgggaagcgggcagaccgcggagacagagcgcccaagg

cccccccaacactcgtactgcacagaggtgggtagctttaagtttatagctccaagatgc

ctcgatgaagaagcccccgccgaccagcgacgcggtgtacacgtgggcaccttggagcgc

ccacccaaagtgtactgcgatggctcagagtacgacgtgctgaactttgcctccggaggt

tgctggcctcgcaggattcgggtttggaacggccaggattttcggggcgatggattcaac

cccagatttgagagatttcacgtgtacgacatagtagagacttcggagagcgcctcacac

gatgacccatccaggtttgctgagctatcccgcccaagcgggtctgtggtaacactgctg

ggaatgagcgagtgtggaaagcgagtcgcggttcacgtctatggtgtgcgccattatttt

tacatggcaaaggcggaggtggatagcgcttgtggaataaccaccgaggcagaactcgtg

cgcgcaatggtggactgcgcgcacagctcggctttgagcgcggccctgggaaatggcaac

ggcggcaaacagagcggcggcagcggcgggggatggtggggcggaaagcacgtgtctgcg

gactgcttcaaagtggagaccgtgtgccacacgacgctgtactactttggatctaagcca

gctctctactatagagtatctgcctccagcagccgcctgggagggttcatctgcgacaac

tttcacccggagattacaaaattcgaggggagcgtggacgtgaccacgcggctgttgttg

gacaatgaaaattttaccagtttcgggtggtaccgcctgcgacccggcacccacggagag

cgtgttcaacttcgccccgttgagcgacacgtcacctcaagcgacgtggagattaactgt

actcccgataacctggagccgataccagacgaggctgcctggcccgactataagctcatg

tgctttgatatagagtgtaaagctggaacgggtaacgaaatggcgttcccagtggcaact

aaccaagaggacctggtcatccagatctcctgtctgctgtactcgcttgctactcagaac

cacgaacacaccctgctgttttccctcgggtcatgcgatatctctgaggaatactcgttt

gcatgcgtccagcgcggcgagcccagaccgacggttttggagtttgacagcgagtacgag

ctgctggttgccttcctgacctttctcaagcagtactctcccgagttcgccaccggctac

aacatcgttaattttgactgggcgtacatagttaacaaggtaacgtcggtgtataacatc

aagctggacgggtacggcaagttcaacaaaggggggctgtttaaggtgtgggacatcgcc

acgaaccattttcagaagaagagcaaggtgaaaatcaatggcctgatatctctagacatg

tattctgtggcgacggaaaagctaaagctacccagctacaaactcgacgcggtcgtggga

gacgtcctcggcgagcataagatagaccttccctataaagaaataccctcctattacgcg

ggagggcctgaccggaggggcgtaataggagagtattgtatccaggactctaggctggtg

ggcaagctgttttttaagtacctcccccatctggaactatcggcggtggccaaactcgcc

cgtatcaccctaacgcgggtaatttttgacggtcagcaaattcgcgtgtacacgtgcttg

ctgaaactcgcccgcgagagaaatttcattttgccagacaacagacgccggtttgacagt

caggcagacgccgcgtcagagacttcggagttggctatggatagccaaagccacgccttc

gacagtacagacgaacccgacggtgtggacggtaccccggacgccgcaggatctggcgct

acttctgaaaacggaggcgggaagcccggcgtcgggagggccgtgggctaccagggagca

aaggttctagaccccgtatccggctttcatgtggaccccgtggttgtgtttgacttcgct

agcttatacccaagcattatccaggcccataacctctgtttcaccaccctggcgctcgat

gaagtggatctggccgggcttcaaccatccgtcaactactcgacgttcgaggtgggtgac

caaaagttattttttgtccacgcccatattcgcgaaagcctgcttggcatcttgctgcgc

gactggctggccatgcgaaaggcggtgagggcgcgaatccccaccagcacccccgaggag

gcagttttactagataagcagcagtctgcgattaaggtgatatgcaactcggtttacgga

ttcacgggggtggcaaacggcctgttgccgtgtctgaggatagcggctaccgttaccacg

ataggacgcgacatgctcctcaagacccgagattacgttcactctcgttgggcgacgcgc

gagctgctggaggacaattttccaggggctataggtttccgaaaccacaagccttactcc

gtcagggttatctacggagacaccgactccgtgtttatcaagtttgtgggcctgacgtac

gagggggtatcggagctgggggatgctatgtcgcgtcagatttcagcggacctctttaga

gcgcccatcaaactggagtgtgagaagacctttcagcgactgctgctgatcaccaagaag

aagtacataggtgtcataaacggggggaagatgctcatgaagggggtcgacctggtccgc

aaaaataactgctctttcataaacttgtacgcgcgacatctggtagatcttttgttgtac

gacgaggatgtggccacggcggcagcagaggtgacagacgtgcctcccgcagaatgggtg

gggcgcccgctaccgagcggctttgacaagtttgggcgagtgctggtagaggcgtacaac

cgtatcactgcccccaacttggacgtgcgcgagttcgttatgactgctgagctgagccgc

tcacccgaatcgtataccaacaagcgcctgccgcacctcaccgtctactttaagctcgcc

atgaggaatgaagaactgcccagcgtaaaagagagaattccgtatgtgatagttgcgcag

accgaggccgcggaacgcgaagcgggtgtagtaaactcaatgcgcggtaccgcccaaaac

cccgtggtaaccaagaccgcacgcccccaacctaaacgcaaactgctggtttccgacctc

gccgaagacccgacctatgtttccgagaatgacgtaccgctaaacacagactactatttc

tcccacctgttgggtaccataagcgtgacctttaaggctctattcggaaatgatgtgaga

acaacagaaaatcttttaaagcggtttattccggaaaccccccacaagacccccacgaaa

acccaggcactgcttgagcgcgccggctttgaaaagctgacgccctttacaccggaggaa

gaaagtcgtcgaatactgcatacagttttttgtactctagaagcagctccccatcaaagc

tga

>KU206477.1

atggcggcgcgcgaacaggccaactccgttcgcaggagcgggttttttaatccgtttatt

ggcaagaggccatttttcaggccgggaagcgggcagaccgcggagacagagcgcccaagg

cccccccaacactcgtactgcacagaggtgggtagctttaagtttatagctccaagatgc

ctcgatgaagaagcccccgccgaccagcgacgcggtgtacacgtgggcaccttggagcgc

ccacccaaagtgtactgcgatggctcagagtacgacgtgctgaactttgcctccggaggt

tgctggcctcgcaggattcgggtttggaacggccaggattttcggggcgatggattcaac

cccagatttgagagatttcacgtgtacgacatagtagagacttcggagagcgcctcacac

gatgacccatccaggtttgctgagctatcccgcccaagcgggtctgtggtaacactgctg

ggaatgagcgagtgtggaaagcgagtcgcggttcacgtctatggtgtgcgccattatttt

tacatggcaaaggcggaggtggatagcgcttgtggaataaccaccgaggcagaactcgtg

cgcgcaatggtggactgcgcgcacagctcggctttgagcgcggccctgggaaatggcaac

ggcggcaaacagagcggcggcagcggcgggggatggtggggcggaaagcacgtgtctgcg

gactgcttcaaagtggagaccgtgtgccacacgacgctgtactactttggatctaagcca

gctctctactatagagtatctgcctccagcagccgcctgggagggttcatctgcgacaac

tttcacccggagattacaaaattcgaggggagcgtggacgtgaccacgcggctgttgttg

gacaatgaaaattttaccagtttcgggtggtaccgcctgcgacccggcacccacggagag

cgtgttcaacttcgccccgttgagcgacacgtcacctcaagcgacgtggagattaactgt

actcccgataacctggagccgataccagacgaggctgcctggcccgactataagctcatg

tgctttgatatagagtgtaaagctggaacgggtaacgaaatggcgttcccagtggcaact

aaccaagaggacctggtcatccagatctcctgtctgctgtactcgcttgctactcagaac

cacgaacacaccctgctgttttccctcgggtcatgcgatatctctgaggaatactcgttt

gcatgcgtccagcgcggcgagcccagaccgacggttttggagtttgacagcgagtacgag

ctgctggttgccttcctgacctttctcaagcagtactctcccgagttcgccaccggctac

aacatcgttaattttgactgggcgtacatagttaacaaggtaacgtcggtgtataacatc

aagctggacgggtacggcaagttcaacaaaggggggctgtttaaggtgtgggacatcgcc

acgaaccattttcagaagaagagcaaggtgaaaatcaatggcctgatatctctagacatg

tattctgtggcgacggaaaagctaaagctacccagctacaaactcgacgcggtcgtggga

gacgtcctcggcgagcataagatagaccttccctataaagaaataccctcctattacgcg

ggagggcctgaccggaggggcgtaataggagagtattgtatccaggactctaggctggtg

ggcaagctgttttttaagtacctcccccatctggaactatcggcggtggccaaactcgcc

cgtatcaccctaacgcgggtaatttttgacggtcagcaaattcgcgtgtacacgtgcttg

ctgaaactcgcccgcgagagaaatttcattttgccagacaacagacgccggtttgacagt

caggcagacgccgcgtcagagacttcggagttggctatggatagccaaagccacgccttc

gacagtacagacgaacccgacggtgtggacggtaccccggacgccgcaggatctggcgct

acttctgaaaacggaggcgggaagcccggcgtcgggagggccgtgggctaccagggagca

aaggttctagaccccgtatccggctttcatgtggaccccgtggttgtgtttgacttcgct

agcttatacccaagcattatccaggcccataacctctgtttcaccaccctggcgctcgat

gaagtggatctggccgggcttcaaccatccgtcgactactcgacgttcgaggtgggtgac

caaaagttattttttgtccacgcccatattcgcgaaagcctgcttggcatcttgctgcgc

gactggctggccatgcgaaaggcggtgagggcgcgaatccccaccagcacccccgaggag

gcagttttactagataagcagcagtctgcgattaaggtgatatgcaactcggtttacgga

ttcacgggggtggcaaacggcctgttgccgtgtctgaggatagcggctaccgttaccacg

ataggacgcgacatgctcctcaagacccgagattacgttcactctcgttgggcgacgcgc

gagctgctggaggacaattttccaggggctataggtttccgaaaccacaagccttactcc

gtcagggttatctacggagacaccgactccgtgtttatcaagtttgtgggcctgacgtac

gagggggtatcggagctgggggatgctatgtcgcgtcagatttcagcggacctctttaga

gcgcccatcaaactggagtgtgagaagacctttcagcgactgctgctgatcaccaagaag

aagtacataggtgtcataaacggggggaagatgctcatgaagggggtcgacctggtccgc

aaaaataactgctctttcataaacttgtacgcgcgacatctggtagatcttttgttgtac

gacgaggatgtggccacggcggcagcagaggtgacagacgtgcctcccgcagaatgggtg

gggcgcccgctaccgagcggctttgacaagtttgggcgagtgctggtagaggcgtacaac

cgtatcactgcccccaacttggacgtgcgcgagttcgttatgactgctgagctgagccgc

tcacccgaatcgtataccaacaagcgcctgccgcacctcaccgtctactttaagctcgcc

atgaggaatgaagaactgcccagcgtaaaagagagaattccgtatgtgatagttgcgcag

accgaggccgcggaacgcgaagcgggtgtagtaaactcaatgcgcggtaccgcccaaaac

cccgtggtaaccaagaccgcacgcccccaacctaaacgcaaactgctggtttccgacctc

gccgaagacccgacctatgtttccgagaatgacgtaccgctaaacacagactactatttc

tcccacctgttgggtaccataagcgtgacctttaaggctctattcggaaatgatgtgaga

acaacagaaaatcttttaaagcggtttattccggaaaccccccacaagacccccacgaaa

acccaggcactgcttgagcgcgccggctttgaaaagctgacgccctttacaccggaggaa

gaaagtcgtcgaatactgcatacagttttttgtactctagaagcagctccccatcaaagc

tga

>KU206479.1

atggcggcgcgcgaacaggccaactccgttcgcaggagcgggttttttaatccgtttatt

ggcaagaggccatttttcaggccgggaagcgggcagaccgcggagacagagcgcccaagg

cccccccaacactcgtactgcacagaggtgggtagctttaagtttatagctccaagatgc

ctcgatgaagaagcccccgccgaccagcgacgcggtgtacacgtgggcaccttggagcgc

ccacccaaagtgtactgcgatggctcagagtacgacgtgctgaactttgcctccggaggt

tgctggcctcgcaggattcgggtttggaacggccaggattttcggggcgatggattcaac

cccagatttgagagatttcacgtgtacgacatagtagagacttcggagagcgcctcacac

gatgacccatccaggtttgctgagctatcccgcccaagcgggtctgtggtaacactgctg

ggaatgagcgagtgtggaaagcgagtcgcggttcacgtctatggtgtgcgccattatttt

tacatggcaaaggcggaggtggatagcgcttgtggaataaccaccgaggcagaactcgtg

cgcgcaatggtggactgcgcgcacagctcggctttgagcgcggccctgggaaatggcaac

ggcggcaaacagagcggcggcagcggcgggggatggtggggcggaaagcacgtgtctgcg

gactgcttcaaagtggagaccgtgtgccacacgacgctgtactactttggatctaagcca

gctctctactatagagtatctgcctccagcagccgcctgggagggttcatctgcgacaac

tttcacccggagattacaaaattcgaggggagcgtggacgtgaccacgcggctgttgttg

gacaatgaaaattttaccagtttcgggtggtaccgcctgcgacccggcacccacggagag

cgtgttcaacttcgccccgttgagcgacacgtcacctcaagcgacgtggagattaactgt

actcccgataacctggagccgataccagacgaggctgcctggcccgactataagctcatg

tgctttgatatagagtgtaaagctggaacgggtaacgaaatggcgttcccagtggcaact

aaccaagaggacctggtcatccagatctcctgtctgctgtactcgcttgctactcagaac

cacgaacacaccctgctgttttccctcgggtcatgcgatatctctgaggaatactcgttt

gcatgcgtccagcgcggcgagcccagaccgacggttttggagtttgacagcgagtacgag

ctgctggttgccttcctgacctttctcaagcagtactctcccgagttcgccaccggctac

aacatcgttaattttgactgggcgtacatagttaacaaggtaacgtcggtgtataacatc

aagctggacgggtacggcaagttcaacaaaggggggctgtttaaggtgtgggacatcgcc

acgaaccattttcagaagaagagcaaggtgaaaatcaatggcctgatatctctagacatg

tattctgtggcgacggaaaagctaaagctacccagctacaaactcgacgcggtcgtggga

gacgtcctcggcgagcataagatagaccttccctataaagaaataccatcctattacgcg

ggagggcctgaccggaggggcgtaataggagagtattgtatccaggactctaggctggtg

ggcaagctgttttttaagtacctcccccatctggaactatcggcggtggccaaactcgcc

cgtatcaccctaacgcgggtaatttttgacggtcagcaaattcgcgtgtacacgtgcttg

ctgaaactcgcccgcgagagaaatttcattttgccagacaacagacgccggtttgacagt

caggcagacgccgcgtcagagacttcggagttggctatggatagccaaagccacgccttc

gacagtacagacgaacccgacggtgtggacggtaccccggacgccgcaggatctggcgct

acttctgaaaacggaggcgggaagcccggcgtcgggagggccgtgggctaccagggagca

aaggttctagaccccgtatccggctttcatgtggaccccgtggttgtgtttgacttcgct

agcttatacccaagcattatccaggcccataacctctgtttcaccaccctggcgctcgat

gaagtggatctggccgggcttcaaccatccgtcgactactcgacgttcgaggtgggtgac

caaaagttattttttgtccacgcccatattcgcgaaagcctgcttggcatcttgctgcgc

gactggctggccatgcgaaaggcggtgagggcgcgaatccccaccagcacccccgaggag

gcagttttactagataagcagcagtctgcgattaaggtgatatgcaactcggtttacgga

ttcacgggggtggcaaacggcctgttgccgtgtctgaggatagcggctaccgttaccacg

ataggacgcgacatgctcctcaagacccgagattacgttcactctcgttgggcgacgcgc

gagctgctggaggacaattttccaggggctataggtttccgaaaccacaagccttactcc

gtcagggttatctacggagacaccgactccgtgtttatcaagtttgtgggcctgacgtac

gagggggtatcggagctgggggatgctatgtcgcgtcagatttcagcggacctctttaga

gcgcccatcaaactggagtgtgagaagacctttcagcgactgctgctgatcaccaagaag

aagtacataggtgtcataaacggggggaagatgctcatgaagggggtcgacctggtccgc

aaaaataactgctctttcataaacttgtacgcgcgacatctggtagatcttttgttgtac

gacgaggatgtggccacggcggcagcagaggtgacagacgtgcctcccgcagaatgggtg

gggcgcccgctaccgagcggctttgacaagtttgggcgagtgctggtagaggcgtacaac

cgtatcactgcccccaacttggacgtgcgcgagttcgttatgactgctgagctgagccgc

tcacccgaatcgtataccaacaagcgcctgccgcacctcaccgtctactttaagctcgcc

atgaggaatgaagaactgcccagcgtaaaagagagaattccgtatgtgatagttgcgcag

accgaggccgcggaacgcgaagcgggtgtagtaaactcaatgcgcggtaccgcccaaaac

cccgtggtaaccaagaccgcacgcccccaacctaaacgcaaactgctggtttccgacctc

gccgaagacccgacctatgtttccgagaatgacgtaccgctaaacacagactactatttc

tcccacctgttgggtaccataagcgtgacctttaaggctctattcggaaatgatgtgaga

acaacagaaaatcttttaaagcggtttattccggaaaccccccacaagacccccacgaaa

acccaggcactgcttgagcgcgccggctttgaaaagctgacgccctttacaccggaggaa

gaaagtcgtcgaatactgcatacagttttttgtactctagaagcagctccccatcaaagc

tga

>KU206480.1

atggcggcgcgcgaacaggccaactccgttcgcaggagcgggttttttaatccgtttatt

ggcaagaggccatttttcaggccgggaagcgggcagaccgcggagacagagcgcccaagg

cccccccaacactcgtactgcacagaggtgggtagctttaagtttatagctccaagatgc

ctcgatgaagaagcccccgccgaccagcgacgcggtgtacacgtgggcaccttggagcgc

ccacccaaagtgtactgcgatggctcagagtacgacgtgctgaactttgcctccggaggt

tgctggcctcgcaggattcgggtttggaacggccaggattttcggggcgatggattcaac

cccagatttgagagatttcacgtgtacgacatagtagagacttcggagagcgcctcacac

gatgacccatccaggtttgctgagctatcccgcccaagcgggtctgtggtaacactgctg

ggaatgagcgagtgtggaaagcgagtcgcggttcacgtctatggtgtgcgccattatttt

tacatggcaaaggcggaggtggatagcgcttgtggaataaccaccgaggcagaactcgtg

cgcgcaatggtggactgcgcgcacagctcggctttgagcgcggccctgggaaatggcaac

ggcggcaaacagagcggcggcagcggcgggggatggtggggcggaaagcacgtgtctgcg

gactgcttcaaagtggagaccgtgtgccacacgacgctgtactactttggatctaagcca

gctctctactatagagtatctgcctccagcagccgcctgggagggttcatctgcgacaac

tttcacccggagattacaaaattcgaggggagcgtggacgtgaccacgcggctgttgttg

gacaatgaaaattttaccagtttcgggtggtaccgcctgcgacccggcacccacggagag

cgtgttcaacttcgccccgttgagcgacacgtcacctcaagcgacgtggagattaactgt

actcccgataacctggagccgataccagacgaggctgcctggcccgactataagctcatg

tgctttgatatagagtgtaaagctggaacgggtaacgaaatggcgttcccagtggcaact

aaccaagaggacctggtcatccagatctcctgtctgctgtactcgcttgctactcagaac

cacgaacacaccctgctgttttccctcgggtcatgcgatatctctgaggaatactcgttt

gcatgcgtccagcgcggcgagcccagaccgacggttttggagtttgacagcgagtacgag

ctgctggttgccttcctgacctttctcaagcagtactctcccgagttcgccaccggctac

aacatcgttaattttgactgggcgtacatagttaacaaggtaacgtcggtgtataacatc

aagctggacgggtacggcaagttcaacaaaggggggctgtttaaggtgtgggacatcgcc

acgaaccattttcagaagaagagcaaggtgaaaatcaatggcctgatatctctagacatg

tattctgtggcgacggaaaagctaaagctacccagctacaaactcgacgcggtcgtggga

gacgtcctcggcgagcataagatagaccttccctataaagaaataccctcctattacgcg

ggagggcctgaccggaggggcgtaataggagagtattgtatccaggactctaggctggtg

ggcaagctgttttttaagtacctcccccatctggaactatcggcggtggccaaactcgcc

cgtatcaccctaacgcgggtaatttttgacggtcagcaaattcgcgtgtacacgtgcttg

ctgaaactcgcccgcgagagaaatttcattttgccagacaacagacgccggtttgacagt

caggcagacgccgcgtcagagacttcggagttggctatggatagccaaagccacgccttc

gacagtacagacgaacccgacggtgtggacggtaccccggacgccgcaggatctggcgct

acttctgaaaacggaggcgggaagcccggcgtcgggagggccgtgggctaccagggagca

aaggttctagaccccgtatccggctttcatgtggaccccgtggttgtgtttgacttcgct

agcttatacccaagcattatccaggcccataacctctgtttcaccaccctggcgctcgat

gaagtggatctggccgggcttcaaccatccgtcaactactcgacgttcgaggtgggtgac

caaaagttattttttgtccacgcccatattcgcgaaagcctgcttggcatcttgctgcgc

gactggctggccatgcgaaaggcggtgagggcgcgaatccccaccagcacccccgaggag

gcagttttactagataagcagcagtctgcgattaaggtgatatgcaactcggtttacgga

ttcacgggggtggcaaacggcctgttgccgtgtctgaggatagcggctaccgttaccacg

ataggacgcgacatgctcctcaagacccgagattacgttcactctcgttgggcgacgcgc

gagctgctggaggacaattttccaggggctataggtttccgaaaccacaagccttactcc

gtcagggttatctacggagacaccgactccgtgtttatcaagtttgtgggcctgacgtac

gagggggtatcggagctgggggatgctatgtcgcgtcagatttcagcggacctctttaga

gcgcccatcaaactggagtgtgagaagacctttcagcgactgctgctgatcaccaagaag

aagtacataggtgtcataaacggggggaagatgctcatgaagggggtcgacctggtccgc

aaaaataactgctctttcataaacttgtacgcgcgacatctggtagatcttttgttgtac

gacgaggatgtggccacggcggcagcagaggtgacagacgtgcctcccgcagaatgggtg

gggcgcccgctaccgagcggctttgacaagtttgggcgagtgctggtagaggcgtacaac

cgtatcactgcccccaacttggacgtgcgcgagttcgttatgactgctgagctgagccgc

tcacccgaatcgtataccaacaagcgcctgccgcacctcaccgtctactttaagctcgcc

atgaggaatgaagaactgcccagcgtaaaagagagaattccgtatgtgatagttgcgcag

accgaggccgcggaacgcgaagcgggtgtagtaaactcaatgcgcggtaccgcccaaaac

cccgtggtaaccaagaccgcacgcccccaacctaaacgcaaactgctggtttccgacctc

gccgaagacccgacctatgtttccgagaatgacgtaccgctaaacacagactactatttc

tcccacctgttgggtaccataagcgtgacctttaaggctctattcggaaatgatgtgaga

acaacagaaaatcttttaaagcggtttattccggaaaccccccacaagacccccacgaaa

acccaggcactgcttgagcgcgccggctttgaaaagctgacgccctttacaccggaggaa

gaaagtcgtcgaatactgcatacagttttttgtactctagaagcagctccccatcaaagc

tga

>KY852346.1

atggcggcgcgcgaacaggccaactccgttcgcaggagcgggttttttaatccgtttatt

ggcaagaggccatttttcaggccgggaagcgggcagaccgcggagacagagcgcccaagg

cccccccaacactcgtactgcacagaggtgggtagctttaagtttatagctccaagatgc

ctcgatgaagaagcccccgccgaccagcgacgcggtgtacacgtgggcaccttggagcgc

ccacccaaagtgtactgcgatggctcagagtacgacgtgctgaactttgcctccggaggt

tgctggcctcgcaggattcgggtttggaacggccaggattttcggggcgatggattcaac

cccagatttgagagatttcacgtgtacgacatagtagagacttcggagagcgcctcacac

gatgacccatccaggtttgctgagctatcccgcccaagcgggtctgtggtaacactgctg

ggaatgagcgagtgtggaaagcgagtcgcggttcacgtctatggtgtgcgccattatttt

tacatggcaaaggcggaggtggatagcgcttgtggaataaccaccgaggcagaactcgtg

cgcgcaatggtggactgcgcgcacagctcggctttgagcgcggccctgggaaatggcaac

ggcggcaaacagagcggcggcagcggcgggggatggtggggcggaaagcacgtgtctgcg

gactgcttcaaagtggagaccgtgtgccacacgacgctgtactactttggatctaagcca

gctctctactatagagtatctgcctccagcagccgcctgggagggttcatctgcgacaac

tttcacccggagattacaaaattcgaggggagcgtggacgtgaccacgcggctgttgttg

gacaatgaaaattttaccagtttcgggtggtaccgcctgcgacccggcacccacggagag

cgtgttcaacttcgccccgttgagcgacacgtcacctcaagcgacgtggagattaactgt

actcccgataacctggagccgataccagacgaggctgcctggcccgactataagctcatg

tgctttgatatagagtgtaaagctggaacgggtaacgaaatggcgttcccagtggcaact

aaccaagaggacctggtcatccagatctcctgtctgctgtactcgcttgctactcagaac

cacgaacacaccctgctgttttccctcgggtcatgcgatatctctgaggaatactcgttt

gcatgcgtccagcgcggcgagcccagaccgacggttttggagtttgacagcgagtacgag

ctgctggttgccttcctgacctttctcaagcagtactctcccgagttcgccaccggctac

aacatcgttaattttgactgggcgtacatagttaacaaggtaacgtcggtgtataacatc

aagctggacgggtacggcaagttcaacaaaggggggctgtttaaggtgtgggacatcgcc

acgaaccattttcagaagaagagcaaggtgaaaatcaatggcctgatatctctagacatg

tattctgtggcgacggaaaagctaaagctacccagctacaaactcgacgcggtcgtggga

gacgtcctcggcgagcataagatagaccttccctataaagaaataccctcctattacgcg

ggagggcctgaccggaggggcgtaataggagagtattgtatccaggactctaggctggtg

ggcaagctgttttttaagtacctcccccatctggaactatcggcggtggccaaactcgcc

cgtatcaccctaacgcgggtaatttttgacggtcagcaaattcgcgtgtacacgtgcttg

ctgaaactcgcccgcgagagaaatttcattttgccagacaacagacgccggtttgacagt

caggcagacgccgcgtcagagacttcggagttggctatggatagccaaagccacgccttc

gacagtacagacgaacccgacggtgtggacggtaccccggacgccgcaggatctggcgct

acttctgaaaacggaggcgggaagcccggcgtcgggagggccgtgggctaccagggagca

aaggttctagaccccgtatccggctttcatgtggaccccgtggttgtgtttgacttcgct

agcttatacccaagcattatccaggcccataacctctgtttcaccaccctggcgctcgat

gaagtggatctggccgggcttcaaccatccgtcgactactcgacgttcgaggtgggtgac

caaaagttattttttgtccacgcccatattcgcgaaagcctgcttggcatcttgctgcgc

gactggctggccatgcgaaaggcggtgagggcgcgaatccccaccagcacccccgaggag

gcagttttactagataagcagcagtctgcgattaaggtgatatgcaactcggtttacgga

ttcacgggggtggcaaacggcctgttgccgtgtctgaggatagcggctaccgttaccacg

ataggacgcgacatgctcctcaagacccgagattacgttcactctcgttgggcgacgcgc

gagctgctggaggacaattttccaggggctataggtttccgaaaccacaagccttactcc

gtcagggttatctacggagacaccgactccgtgtttatcaagtttgtgggcctgacgtac

gagggggtatcggagctgggggatgctatgtcgcgtcagatttcagcggacctctttaga

gcgcccatcaaactggagtgtgagaagacctttcagcgactgctgctgatcaccaagaag

aagtacataggtgtcataaacggggggaagatgctcatgaagggggtcgacctggtccgc

aaaaataactgctctttcataaacttgtacgcgcgacatctggtagatcttttgttgtac

gacgaggatgtggccacggcggcagcagaggtgacagacgtgcctcccgcagaatgggtg

gggcgcccgctaccgagcggctttgacaagtttgggcgagtgctggtagaggcgtacaac

cgtatcactgcccccaacttggacgtgcgcgagttcgttatgactgctgagctgagccgc

tcacccgaatcgtataccaacaagcgcctgccgcacctcaccgtctactttaagctcgcc

atgaggaatgaagaactgcccagcgtaaaagagagaattccgtatgtgatagttgcgcag

accgaggccgcggaacgcgaagcgggtgtagtaaactcaacgcgcggtaccgcccaaaac

cccgtggtaaccaagaccgcacgcccccaacctaaacgcaaactgctggtttccgacctc

gccgaagacccgacctatgtttccgagaatgacgtaccgctaaacacagactactatttc

tcccacctgttgggtaccataagcgtgacctttaaggctctattcggaaatgatgtgaga

acaacagaaaatcttttaaagcggtttattccggaaaccccccacaagacccccacgaaa

acccaggcactgcttgagcgcgccggctttgaaaagctgacgccctttacaccggaggaa

gaaagtcgtcgaatactgcatacagttttttgtactctagaagcagctccccatcaaagc

tga

>MN688617.1

atggcggcgcgcgaacaggccaactccgttcgcaggagcgggttttttaatccgtttatt

ggcaagaggccatttttcaggccgggaagcgggcaaaccgcggagacagagcgcccaagg

cccccccaacactcgtactgcacagaggtgggtagctttaagtttatagctccaggatgc

ctcgatgaagaagcccccgccgaccagcgacgcggtgtacacgtgggcaccttggagcgc

ccacccaaagtgtactgcgatggctcagagtacgacgtgctgaactttgcctccggaggt

tgctggcctcgcaggattcgggtttggaacggccaggattttcggggcgatggattcaac

cccagatttgagagatttcacgtgtacgacatagtagagacttcggagagcgcctcacac

gatgacccatccaggtttgctgagctatcccgcccaagcgggtctgtggtaacactgctg

ggaatgagcgagtgtggaaagcgagtcgcggttcacgtctatggtgtgcgccattatttt

tacatggcaaaggcggaggtggatagcgcttgtggaataaccaccgaggcagaactcgtg

cgcgcaatggtggactgcgcgcacagctcggctttgagcgcggccctgggaaatggcaac

ggcggcaaacagagcggcggcagcggcgggggatggtggggcggaaagcacgtgtctgcg

gactgcttcaaagtggagaccgtgtgccacacgacgctgtactactttggatctaagcca

gctctctactatagagtatctgcctccagcagccgcctgggagggttcatctgcgacaac

tttcacccggagattacaaaattcgaggggagcgtggacgtgaccacgcggctgttgttg

gacaatgaaaattttaccagtttcgggtggtaccgcctgcgacccggcacccacggagag

cgtgttcaacttcgccccgttgagcgacacgtcacctcaagcgacgtggagattaactgt

actcccgataacctggagccgataccagacgaggctgcctggcccgactataagctcatg

tgctttgatatagagtgtaaagctggaacgggtaacgaaatggcgttcccagtggcaact

aaccaagaggacctggtcatccagatctcctgtctgctgtactcgcttgctactcagaac

cacgaacacaccctgctgttttccctcgggtcatgcgatatctctgaggaatactcgttt

gcatgcgtccagcgcggcgagcccagaccgacggttttggagtttgacagcgagtacgag

ctgctggttgccttcctgacctttctcaagcagtactctcccgagttcgccaccggctac

aacatcgttaattttgactgggcgtacatagttaacaaggtaacgtcggtgtataacatc

aagctggacgggtacggcaagttcaacaaaggggggctgtttaaggtgtgggacatcgcc

acgaaccattttcagaagaagagcaaggtgaaaatcaatggcctgatatctctagacatg

tattctgtggcgacggaaaagctaaagctacccagctacaaactcgacgcggtcgtggga

gacgtcctcggcgagcataagatagaccttccctataaagaaataccctcctattacgcg

ggagggcctgaccggaggggcgtaataggagagtattgtatccaggactctaggctggtg

ggcaagctgttttttaagtacctcccccatctggaactatcggcggtggccaaactcgcc

cgtatcaccctaacgcgggtaatttttgacggtcagcaaattcgcgtgtacacgtgcttg

ctgaaactcgcccgcgagagaaatttcattttgccagacaacagacgccggtttgacagt

caggcagacgccgcgtcagagacttcggagttggctatggatagccaaagccacgccttc

gacagtacagacgaacccgacggtgtggacggtaccccggacgccgcaggatctggcgct

acttctgaaaacggaggcgggaagcccggcgtcgggagggccgtgggctaccagggagca

aaggttctagaccccgtatccggctttcatgtggaccccgtggttgtgtttgacttcgct

agcttatacccaagcattatccaggcccataacctctgtttcaccaccctggcgctcgat

gaagtggatctggccgggcttcaaccatccgtcaactactcgacgttcgaggtgggtgac

caaaagttattttttgtccacgcccatattcgcgaaagcctgcttggcatcttgctgcgc

gactggctggccatgcgaaaggcggtgagggcgcgaatccccaccagcacccccgaggag

gcagttttactagataagcagcagtctgcgattaaggtgatatgcaactcggtttacgga

ttcacgggggtggcaaacggcctgttgccgtgtctgaggatagcggctaccgttaccacg

ataggacgcgacatgctcctcaagacccgagattacgttcactctcgttgggcgacgcgc

gagctgctggaggacaattttccaggggctataggtttccgaaaccacaagccttactcc

gtcagggttatctacggagacaccgactccgtgtttatcaagtttgtgggcctgacgtac

gagggggtatcggagctgggggatgctatgtcgcgtcagatttcagcggacctctttaga

gcgcccatcaaactggagtgtgagaagacctttcagcgactgctgctgatcaccaagaag

aagtacataggtgtcataaacggggggaagatgctcatgaagggggtcgacctggtccgc

aaaaataactgctctttcataaacttgtacgcgcgacatctggtagatcttttgttgtac

gacgaggatgtggccacggcggcagcaaaggtgacagacgtgcctcccgcagaatgggtg

gggcgcccgctaccgagcggctttgacaagtttgggcgagtgctggtagaggcgtacaac

cgtatcactgcccccaacttggacgtgcgcgagttcgttatgactgctgagctgagccgc

tcacccgaatcgtataccaacaagcgcctgccgcacctcaccgtctactttaagctcgcc

atgaggaatgaagaactgcccagcgtaaaagagagaattccgtatgtgatagttgcgcag

accgaggccgcggaacgcgaagcgggtgtagtaaactcaatgcgcggtaccgcccaaaac

cccgtggtaaccaagaccgcacgcccccaacctaaacgcaaactgctggtttccgacctc

gccgaagacccgacctatgtttccgagaatgacgtaccgctaaacacagactactatttc

tcccacctgttgggtaccataagcgtgacctttaaggctctattcggaaatgatgtgaga

acaacagaaaatcttttaaagcggtttattccggaaaccccccacaagacccccacgaaa

acccaggcactgcttgagcgcgccggctttgaaaagctgacgccctttacaccggaggaa

gaaagtcgtcgaatactgcatacagttttttgtactctagaagcagctccccatcaaagc

tga

>MN688618.1

atggcggcgcgcgaacaggccaactccgttcgcaggagcgggttttttaatccgtttatt

ggcaagaggccatttttcaggccgggaagcgggcagaccgcggagacagagcgcccaagg

cccccccaacactcgtactgcacagaggtgggtagctttaagtttatagctccaagatgc

ctcgatgaagaagcccccgccgaccagcgacgcggtgtacacgtgggcaccttggagcgc

ccacccaaagtgtactgcgatggctcagagtacgacgtgctgaactttgcctccggaggt

tgctggcctcgcaggattcgggtttggaacggccaggattttcggggcgatggattcaac

cccagatttgagagatttcacgtgtacgacatagtagagacttcggagagcgcctcacac

gatgacccatccaggtttgctgagctatcccgcccaagcgggtctgtggtaacactgctg

ggaatgagcgagtgtggaaagcgagtcgcggttcacgtctatggtgtgcgccattatttt

tacatggcaaaggcggaggtggatagcgcttgtggaataaccaccgaggcagaactcgtg

cgcgcaatggtggactgcgcgcacagctcggctttgagcgcggccctgggaaatggcaac

ggcggcaaacagagcggcggcagcggcgggggatggtggggcggaaagcacgtgtctgcg

gactgcttcaaagtggagaccgtgtgccacacgacgctgtactactttggatctaagcca

gctctctactatagagtatctgcctccagcagccgcctgggagggttcatctgcgacaac

tttcacccggagattacaaaattcgaggggagcgtggacgtgaccacgcggctgttgttg

gacaatgaaaattttaccagtttcgggtggtaccgcctgcgacccggcacccacggagag

cgtgttcaacttcgccccgttgagcgacacgtcacctcaagcgacgtggagattaactgt

actcccgataacctggagccgataccagacgaggctgcctggcccgactataagctcatg

tgctttgatatagagtgtaaagctggaacgggtaacgaaatggcgttcccagtggcaact

aaccaagaggacctggtcatccagatctcctgtctgctgtactcgcttgctactcagaac

cacgaacacaccctgctgttttccctcgggtcatgcgatatctctgaggaatactcgttt

gcatgcgtccagcgcggcgagcccagaccgacggttttggagtttgacagcgagtacgag

ctgctggttgccttcctgacctttctcaagcagtactctcccgagttcgccaccggctac

aacatcgttaattttgactgggcgtacatagttaacaaggtaacgtcggtgtataacatc

aagctggacgggtacggcaagttcaacaaaggggggctgtttaaggtgtgggacatcgcc

acgaaccattttcagaagaagagcaaggtgaaaatcaatggcctgatatctctagacatg

tattctgtggcgacggaaaagctaaagctacccagctacaaactcgacgcggtcgtggga

gacgtcctcggcgagcataagatagaccttccctataaagaaataccctcctattacgcg

ggagggcctgaccggaggggcgtaataggagagtattgtatccaggactctaggctggtg

ggcaagctgttttttaagtacctcccccatctggaactatcggcggtggccaaactcgcc

cgtatcaccctaacgcgggtaatttttgacggtcagcaaattcgcgtgtacacgtgcttg

ctgaaactcgcccgcgagagaaatttcattttgccagacaacagacgccggtttgacagt

caggcagacgccgcgtcagagacttcggagttggctatggatagccaaagccacgccttc

gacagtacagacgaacccgacggtgtggacggtaccccggacgccgcaggatctggcgct

acttctgaaaacggaggcgggaagcccggcgtcgggagggccgtgggctaccagggagca

aaggttctagaccccgtatccggctttcatgtggaccccgtggttgtgtttgacttcgct

agcttatacccaagcattatccaggcccataacctctgtttcaccaccctggcgctcgat

gaagtggatctggccgggcttcaaccatccgtcaactactcgacgttcgaggtgggtgac

caaaagttattttttgtccacgcccatattcgcgaaagcctgcttggcatcttgctgcgc

gactggctggccatgcgaaaggcggtgagggcgcgaatccccaccagcacccccgaggag

gcagttttactagataagcagcagtctgcgattaaggtgatatgcaactcggtttacgga

ttcacgggggtggcaaacggcctgttgccgtgtctgaggatagcggctaccgttaccacg

ataggacgcgacatgctcctcaagacccgagattacgttcactctcgttgggcgacgcgc

gagctgctggaggacaattttccaggggctataggtttccgaaaccacaagccttactcc

gtcagggttatctacggagacaccgactccgtgtttatcaagtttgtgggcctgacgtac

gagggggtatcggagctgggggatgctatgtcgcgtcagatttcagcggacctctttaga

gcgcccatcaaactggagtgtgagaagacctttcagcgactgctgctgatcaccaagaag

aagtacataggtgtcataaacggggggaagatgctcatgaagggggtcgacctggtccgc

aaaaataactgctctttcataaacttgtacgcgcgacatctggtagatcttttgttgtac

gacgaggatgtggccacggcggcagcagaggtgacagacgtgcctcccgcagaatgggtg

gggcgcccgctaccgagcggctttgacaagtttgggcgagtgctggtagaggcgtacaac

cgtatcactgcccccaacttggacgtgcgcgagttcgttatgactgctgagctgagccgc

tcacccgaatcgtataccaacaagcgcctgccgcacctcaccgtctactttaagctcgcc

atgaggaatgaagaactgcccagcgtaaaagagagaattccgtatgtgatagttgcgcag

accgaggccgcggaacgcgaagcgggtgtagtaaactcaatgcgcggtaccgcccaaaac

cccgtggtaaccaagaccgcacgcccccaacctaaacgcaaactgctggtttccgacctc

gccgaagacccgacctatgtttccgagaatgacgtaccgctaaacacagactactatttc

tcccacctgttgggtaccataagcgtgacctttaaggctctattcggaaatgatgtgaga

acaacagaaaatcttttaaagcggtttattccggaaaccccccacaagacccccacgaaa

acccaggcactgcttgagcgcgccggctttgaaaagctgacgccctttacaccggaggaa

gaaagtcgtcgaatactgcatacagttttttgtactctagaagcagctccccatcaaagc

tga

>MN688619.1

atggcggcgcgcgaacaggccaactccgttcgcaggagcgggttttttaatccgtttatt

ggcaagaggccatttttcaggccgggaagcgggcagaccgcggagacagagcgcccaagg

cccccccaacactcgtactgcacagaggtgggtagctttaagtttatagctccaagatgc

ctcgatgaagaagcccccgccgaccagcgacgcggtgtacacgtgggcaccttggagcgc

ccacccaaagtgtactgcgatggctcagagtacgacgtgctgaactttgcctccggaggt

tgctggcctcgcaggattcgggtttggaacggccaggattttcggggcgatggattcaac

cccagatttgagagatttcacgtgtacgacatagtagagacttcggagagcgcctcacac

gatgacccatccaggtttgctgagctatcccgcccaagcgggtctgtggtaacactgctg

ggaatgagcgagtgtggaaagcgagtcgcggttcacgtctatggtgtgcgccattatttt

tacatggcaaaggcggaggtggatagcgcttgtggaataaccaccgaggcagaactcgtg

cgcgcaatggtggactgcgcgcacagctcggctttgagcgcggccctgggaaatggcaac

ggcggcaaacagagcggcggcagcggcgggggatggtggggcggaaagcacgtgtctgcg

gactgcttcaaagtggagaccgtgtgccacacgacgctgtactactttggatctaagcca

gctctctactatagagtatctgcctccagcagccgcctgggagggttcatctgcgacaac

tttcacccggagattacaaaattcgaggggagcgtggacgtgaccacgcggctgttgttg

gacaatgaaaattttaccagtttcgggtggtaccgcctgcgacccggcacccacggagag

cgtgttcaacttcgccccgttgagcgacacgtcacctcaagcgacgtggagattaactgt

actcccgataacctggagccgataccagacgaggctgcctggcccgactataagctcatg

tgctttgatatagagtgtaaagctggaacgggtaacgaaatggcgttcccagtggcaact

aaccaagaggacctggtcatccagatctcctgtctgctgtactcgcttgctactcagaac

cacgaacacaccctgctgttttccctcgggtcatgcgatatctctgaggaatactcgttt

gcatgcgtccagcgcggcgagcccagaccgacggttttggagtttgacagcgagtacgag

ctgctggttgccttcctgacctttctcaagcagtactctcccgagttcgccaccggctac

aacatcgttaattttgactgggcgtacatagttaacaaggtaacgtcggtgtataacatc

aagctggacgggtacggcaagttcaacaaaggggggctgtttaaggtgtgggacatcgcc

acgaaccattttcagaagaagagcaaggtgaaaatcaatggcctgatatctctagacatg

tattctgtggcgacggaaaagctaaagctacccagctacaaactcgacgcggtcgtggga

gacgtcctcggcgagcataagatagaccttccctataaagaaataccctcctattacgcg

ggagggcctgaccggaggggcgtaataggagagtattgtatccaggactctaggctggtg

ggcaagctgttttttaagtacctcccccatctggaactatcggcggtggccaaactcgcc

cgtatcaccctaacgcgggtaatttttgacggtcagcaaattcgcgtgtacacgtgcttg

ctgaaactcgcccgcgagagaaatttcattttgccagacaacagacgccggtttgacagt

caggcagacgccgcgtcagagacttcggagttggctatggatagccaaagccacgccttc

gacagtacagacgaacccgacggtgtggacggtaccccggacgccgcaggatctggcgct

acttctgaaaacggaggcgggaagcccggcgtcgggagggccgtgggctaccagggagca

aaggttctagaccccgtatccggctttcatgtggaccccgtggttgtgtttgacttcgct

agcttatacccaagcattatccaggcccataacctctgtttcaccaccctggcgctcgat

gaagtggatctggccgggcttcaaccatccgtcgactactcgacgttcgaggtgggtgac

caaaagttattttttgtccacgcccatattcgcgaaagcctgcttggcatcttgctgcgc

gactggctggccatgcgaaaggcggtgagggcgcgaatccccaccagcacccccgaggag

gcagttttactagataagcagcagtctgcgattaaggtgatatgcaactcggtttacgga

ttcacgggggtggcaaacggcctgttgccgtgtctgaggatagcggctaccgttaccacg

ataggacgcgacatgctcctcaagacccgagattacgttcactctcgttgggcgacgcgc

gagctgctggaggacaattttccaggggctataggtttccgaaaccacaagccttactcc

gtcagggttatctacggagacaccgactccgtgtttatcaagtttgtgggcctgacgtac

gagggggtatcggagctgggggatgctatgtcgcgtcagatttcagcggacctctttaga

gcgcccatcaaactggagtgtgagaagacctttcagcgactgctgctgatcaccaagaag

aagtacataggtgtcataaacggggggaagatgctcatgaagggggtcgacctggtccgc

aaaaataactgctctttcataaacttgtacgcgcgacatctggtagatcttttgttgtac

gacgaggatgtggccacggcggcagcagaggtgacagacgtgcctcccgcagaatgggtg

gggcgcccgctaccgagcggctttgacaagtttgggcgagtgctggtagaggcgtacaac

cgtatcactgcccccaacttggacgtgcgcgagttcgttatgactgctgagctgagccgc

tcacccgaatcgtataccaacaagcgcctgccgcacctcaccgtctactttaagctcgcc

atgaggaatgaagaactgcccagcgtaaaagagagaattccgtatgtgatagttgcgcag

accgaggccgcggaacgcgaagcgggtgtagtaaactcaatgcgcggtaccgcccaaaac

cccgtggtaaccaagaccgcacgcccccaacctaaacgcaaactgctggtttccgacctc

gccgaagacccgacctatgtttccgagaatgacgtaccgctaaacacagactactatttc

tcccacctgttgggtaccataagcgtgacctttaaggctctattcggaaatgatgtgaga

acaacagaaaatcttttaaagcggtttattccggaaaccccccacaagacccccacgaaa

acccaggcactgcttgagcgcgccggctttgaaaagctgacgccctttacaccggaggaa

gaaagtcgtcgaatactgcatacagttttttgtactctagaagcagctccccatcaaagc

tga

>MN688620.1

atggcggcgcgcgaacaggccaactccgttcgcaggagcgggttttttaatccgtttatt

ggcaagaggccatttttcaggccgggaagcgggcagaccgcggagacagagcgcccaagg

cccccccaacactcgtactgcacagaggtgggtagctttaagtttatagctccaagatgc

ctcgatgaagaagcccccgccgaccagcgacgcggtgtacacgtgggcaccttggagcgc

ccacccaaagtgtactgcgatggctcagagtacgacgtgctgaactttgcctccggaggt

tgctggcctcgcaggattcgggtttggaacggccaggattttcggggcgatggattcaac

cccagatttgagagatttcacgtgtacgacatagtagagacttcggagagcgcctcacac

gatgacccatccaggtttgctgagctatcccgcccaagcgggtctgtggtaacactgctg

ggaatgagcgagtgtggaaagcgagtcgcggttcacgtctatggtgtgcgccattatttt

tacatggcaaaggcggaggtggatagcgcttgtggaataaccaccgaggcagaactcgtg

cgcgcaatggtggactgcgcgcacagctcggctttgagcgcggccctgggaaatggcaac

ggcggcaaacagagcggcggcagcggcgggggatggtggggcggaaagcacgtgtctgcg

gactgcttcaaagtggagaccgtgtgccacacgacgctgtactactttggatctaagcca

gctctctactatagagtatctgcctccagcagccgcctgggagggttcatctgcgacaac

tttcacccggagattacaaaattcgaggggagcgtggacgtgaccacgcggctgttgttg

gacaatgaaaattttaccagtttcgggtggtaccgcctgcgacccggcacccacggagag

cgtgttcaacttcgccccgttgagcgacacgtcacctcaagcgacgtggagattaactgt

actcccgataacctggagccgataccagacgaggctgcctggcccgactataagctcatg

tgctttgatatagagtgtaaagctggaacgggtaacgaaatggcgttcccagtggcaact

aaccaagaggacctggtcatccagatctcctgtctgctgtactcgcttgctactcagaac

cacgaacacaccctgctgttttccctcgggtcatgcgatatctctgaggaatactcgttt

gcatgcgtccagcgcggcgagcccagaccgacggttttggagtttgacagcgagtacgag

ctgctggttgccttcctgacctttctcaagcagtactctcccgagttcgccaccggctac

aacatcgttaattttgactgggcgtacatagttaacaaggtaacgtcggtgtataacatc

aagctggacgggtacggcaagttcaacaaaggggggctgtttaaggtgtgggacatcgcc

acgaaccattttcagaagaagagcaaggtgaaaatcaatggcctgatatctctagacatg

tattctgtggcgacggaaaagctaaagctacccagctacaaactcgacgcggtcgtggga

gacgtcctcggcgagcataagatagaccttccctataaagaaataccctcctattacgcg

ggagggcctgaccggaggggcgtaataggagagtattgtatccaggactctaggctggtg

ggcaagctgttttttaagtacctcccccatctggaactatcggcggtggccaaactcgcc

cgtatcaccctaacgcgggtaatttttgacggtcagcaaattcgcgtgtacacgtgcttg

ctgaaactcgcccgcgagagaaatttcattttgccagacaacagacgccggtttgacagt

caggcagacgccgcgtcagagacttcggagttggctatggatagccaaagccacgccttc

gacagtacagacgaacccgacggtgtggacggtaccccggacgccgcaggatctggcgct

acttctgaaaacggaggcgggaagcccggcgtcgggagggccgtgggctaccagggagca

aaggttctagaccccgtatccggctttcatgtggaccccgtggttgtgtttgacttcgct

agcttatacccaagcattatccaggcccataacctctgtttcaccaccctggcgctcgat

gaagtggatctggccgggcttcaaccatccgtcaactactcgacgttcgaggtgggtgac

caaaagttattttttgtccacgcccatattcgcgaaagcctgcttggcatcttgctgcgc

gactggctggccatgcgaaaggcggtgagggcgcgaatccccaccagcacccccgaggag

gcagttttactagataagcagcagtctgcgattaaggtgatatgcaactcggtttacgga

ttcacgggggtggcaaacggcctgttgccgtgtctgaggatagcggctaccgttaccacg

ataggacgcgacatgctcctcaagacccgagattacgttcactctcgttgggcgacgcgc

gagctgctggaggacaattttccaggggctataggtttccgaaaccacaagccttactcc

gtcagggttatctacggagacaccgactccgtgtttatcaagtttgtgggcctgacgtac

gagggggtatcggagctgggggatgctatgtcgcgtcagatttcagcggacctctttaga

gcgcccatcaaactggagtgtgagaagacctttcagcgactgctgctgatcaccaagaag

aagtacataggtgtcataaacggggggaagatgctcatgaagggggtcgacctggkccgc

aaaaataactgctctttcataaacttgtacgcgcgacatctggtagatcttttgttgtac

gacgaggatgtggccacggcggcagcagaggtgacagacgtgcctcccgcagaatgggtg

gggcgcccgctaccgagcggctttgacaagtttgggcgagtgctggtagaggcgtacaac

cgtatcactgcccccaacttggacgtgcgcgagttcgttatgactgctgagctgagccgc

tcacccgaatcgtataccaacaagcgcctgccgcacctcaccgtctactttaagctcgcc

atgaggaatgaagaactgcccagcgtaaaagagagaattccgtatgtgatagttgcgcag

accgaggccgcggaacgcgaagcgggtgtagtaaactcaatgcgcggtaccgcccaaaac

cccgtggtaaccaagaccgcacgcccccaacctaaacgcaaactgctggtttccgacctc

gccgaagacccgacctatgtttccgagaatgacgtaccgctaaacacagactactatttc

tcccacctgttgggtaccataagcgtgacctttaaggctctattcggaaatgatgtgaga

acaacagaaaatcttttaaagcggtttattccggaaaccccccacaagacccccacgaaa

acccaggcactgcttgagcgcgccggctttgaaaagctgacgccctttacaccggaggaa

gaaagtcgtcgaatactgcatacagttttttgtactctagaagcagctccccatcaaagc

tga

>MN688621.1

atggcggcgcgcgaacaggccaactccgttcgcaggagcgggttttttaatccgtttatt

ggcaagaggccatttttcaggccgggaagcgggcagaccgcggagacagagcgcccaagg

cccccccaacactcgtactgcacagaggtgggtagctttaagtttatagctccaagatgc

ctcgatgaagaagcccccgccgaccagcgacgcggtgtacacgtgggcaccttggagcgc

ccacccaaagtgtactgcgatggctcagagtacgacgtgctgaactttgcctccggaggt

tgctggcctcgcaggattcgggtttggaacggccaggattttcggggcgatggattcaac

cccagatttgagagatttcacgtgtacgacatagtagagacttcggagagcgcctcacac

gatgacccatccaggtttgctgagctatcccgcccaagcgggtctgtggtaacactgctg

ggaatgagcgagtgtggaaagcgagtcgcggttcacgtctatggtgtgcgccattatttt

tacatggcaaaggcggaggtggatagcgcttgtggaataaccaccgaggcagaactcgtg

cgcgcaatggtggactgcgcgcacagctcggctttgagcgcggccctgggaaatggcaac

ggcggcaaacagagcggcggcagcggcgggggatggtggggcggaaagcacgtgtctgcg

gactgcttcaaagtggagaccgtgtgccacacgacgctgtactactttggatctaagcca

gctctctactatagagtatctgcctccagcagccgcctgggagggttcatctgcgacaac

tttcacccggagattacaaaattcgaggggagcgtggacgtgaccacgcggctgttgttg

gacaatgaaaattttaccagtttcgggtggtaccgcctgcgacccggcacccacggagag

cgtgttcaacttcgccccgttgagcgacacgtcacctcaagcgacgtggagattaactgt

actcccgataacctggagccgataccagacgaggctgcctggcccgactataagctcatg

tgctttgatatagagtgtaaagctggaacgggtaacgaaatggcgttcccagtggcaact

aaccaagaggacctggtcatccagatctcctgtctgctgtactcgcttgctactcagaac

cacgaacacaccctgctgttttccctcgggtcatgcgatatctctgaggaatactcgttt

gcatgcgtccagcgcggcgagcccaaaccgacggttttggagtttgacagcgagtacgag

ctgctggttgccttcctgacctttctcaagcagtactctcccgagttcgccaccggctac

aacatcgttaattttgactgggcgtacatagttaacaaggtaacgtcggtgtataacatc

aagctggacgggtacggcaagttcaacaaaggggggctgtttaaggtgtgggacatcgcc

acgaaccattttcagaagaagagcaaggtgaaaatcaatggcctgatatctctagacatg

tattctgtggcgacggaaaagctaaagctacccagctacaaactcgacgcggtcgtggga

gacgtcctcggcgagcataagatagaccttccctataaagaaataccctcctattacgcg

ggagggcctgaccggaggggcgtaataggagagtattgtatccaggactctaggctggtg

ggcaagctgttttttaagtacctcccccatctggaactatcggcggtggccaaactcgcc

cgtatcaccctaacgcgggtaatttttgacggtcagcaaattcgcgtgtacacgtgcttg

ctgaaactcgcccgcgagagaaatttcattttgccagacaacagacgccggtttgacagt

caggcagacgccgcgtcagagacttcggagttggctatggatagccaaagccacgccttc

gacagtacagacgaacccgacggtgtggacggtaccccggacgccgcaggatctggcgct

acttctgaaaacggaggcgggaagcccggcgtcgggagggccgtgggctaccagggagca

aaggttctagaccccgtatccggctttcatgtggaccccgtggttgtgtttgacttcgct

agcttatacccaagcattatccaggcccataacctctgtttcaccaccctggcgctcgat

gaagtggatctggccgggcttcaaccatccgtcaactactcgacgttcgaggtgggtgac

caaaagttattttttgtccacgcccatattcgcgaaagcctgcttggcatcttgctgcgc

gactggctggccatgcgaaaggcggtgagggcgcgaatccccaccagcacccccgaggag

gcagttttactagataagcagcagtctgcgattaaggtgatatgcaactcggtttacgga

ttcacgggggtggcaaacggcctgttgccgtgtctgaggatagcggctaccgttaccacg

ataggacgcgacatgctcctcaagacccgagattacgttcactctcgttgggcgacgcgc

gagctgctggaggacaattttccaggggctataggtttccgaaaccacaagccttactcc

gtcagggttatctacggagacaccgactccgtgtttatcaagtttgtgggcctgacgtac

gagggggtatcggagctgggggatgctatgtcgcgtcagatttcagcggacctctttaga

gcgcccatcaaactggagtgtgagaagacctttcagcgactgctgctgatcaccaagaag

aagtacataggtgtcataaacggggggaagatgctcatgaagggggtcgacctggtccgc

aaaaataactgctctttcataaacttgtacgcgcgacatctggtagatcttttgttgtac

gacgaggatgtggccacggcggcagcagaggtgacagacgtgcctcccgcagaatgggtg

gggcgcccgctaccgagcggctttgacaagtttgggcgagtgctggtagaggcgtacaac

cgtatcactgcccccaacttggacgtgcgcgagttcgttatgactgctgagctgagccgc

tcacccgaatcgtataccaacaagcgcctgccgcacctcaccgtctactttaagctcgcc

atgaggaatgaagaactgcccagcgtaaaagagagaattccgtatgtgatagttgcgcag

accgaggccgcggaacgcgaagcgggtgtagtaaactcaatgcgcggtaccgcccaaaac

cccgtggtaaccaagaccgcacgcccccaacctaaacgcaaactgctggtttccgacctc

gccgaagacccgacctatgtttccgagaatgacgtaccgctaaacacagactactatttc

tcccacctgttgggtaccataagcgtgacctttaaggctctattcggaaatgatgtgaga

acaacagaaaatcttttaaagcggtttattccggaaaccccccacaagacccccacgaaa

acccaggcactgcttgagcgcgccggctttgaaaagctgacgccctttacaccggaggaa

gaaagtcgtcgaatactgcatacagttttttgtactctagaagcagctccccatcaaagc

tga

>MN688622.1

atggcggcgcgcgaacaggccaactccgttcgcaggagcgggttttttaatccgtttatt

ggcaagaggccatttttcaggccgggaagcgggcagaccgcggagacagagcgcccaagg

cccccccaacactcgtactgcacagaggtgggtagctttaagtttatagctccaagatgc

ctcgatgaagaagcccccgccgaccagcgacgcggtgtacacgtgggcaccttggagcgc

ccacccaaagtgtactgcgatggctcagagtacgacgtgctgaactttgcctccggaggt

tgctggcctcgcaggattcgggtttggaacggccaggattttcggggcgatggattcaac

cccagatttgagagatttcacgtgtacgacatagtagagacttcggagagcgcctcacac

gatgacccatccaggtttgctgagctatcccgcccaagcgggtctgtggtaacactgctg

ggaatgagcgagtgtggaaagcgagtcgcggttcacgtctatggtgtgcgccattatttt

tacatggcaaaggcggaggtggatagcgcttgtggaataaccaccgaggcagaactcgtg

cgcgcaatggtggactgcgcgcacagctcggctttgagcgcggccctgggaaatggcaac

ggcggcaaacagagcggcggcagcggcgggggatggtggggcggaaagcacgtgtctgcg

gactgcttcaaagtggagaccgtgtgccacacgacgctgtactactttggatctaagcca

gctctctactatagagtatctgcctccagcagccgcctgggagggttcatctgcgacaac

tttcacccggagattacaaaattcgaggggagcgtggacgtgaccacgcggctgttgttg

gacaatgaaaattttaccagtttcgggtggtaccgcctgcgacccggcacccacggagag

cgtgttcaacttcgccccgttgagcgacacgtcacctcaagcgacgtggagattaactgt

actcccgataacctggagccgataccagacgaggctgcctggcccgactataagctcatg

tgctttgatatagagtgtaaagctggaacgggtaacgaaatggcgttcccagtggcaact

aaccaagaggacctggtcatccagatctcctgtctgctgtactcgcttgctactcagaac

cacgaacacaccctgctgttttccctcgggtcatgcgatatctctgaggaatactcgttt

gcatgcgtccagcgcggcgagcccagaccgacggttttggagtttgacagcgagtacgag

ctgctggttgccttcctgacctttctcaagcagtactctcccgagttcgccaccggctac

aacatcgttaattttgactgggcgtacatagttaacaaggtaacgtcggtgtataacatc

aagctggacgggtacggcaagttcaacaaaggggggctgtttaaggtgtgggacatcgcc

acgaaccattttcagaagaagagcaaggtgaaaatcaatggcctgatatctctagacatg

tattctgtggcgacggaaaagctaaagctacccagctacaaactcgacgcggtcgtggga

gacgtcctcggcgagcataagatagaccttccctataaagaaataccctcctattacgcg

ggagggcctgaccggaggggcgtaataggagagtattgtatccaggactctaggctggtg

ggcaagctgttttttaagtacctcccccatctggaactatcggcggtggccaaactcgcc

cgtatcaccctaacgcgggtaatttttgacggtcagcaaattcgcgtgtacacgtgcttg

ctgaaactcgcccgcgagagaaatttcattttgccagacaacagacgccggtttgacagt

caggcagacgccgcgtcagagacttcggagttggctatggatagccaaagccacgccttc

gacagtacagacgaacccgacggtgtggacggtaccccggacgccgcaggatctggcgct

acttctgaaaacggaggcgggaagcccggcgtcgggagggccgtgggctaccagggagca

aaggttctagaccccgtatccggctttcatgtggaccccgtggttgtgtttgacttcgct

agcttatacccaagcattatccaggcccataacctctgtttcaccaccctggcgctcgat

gaagtggatctggccgggcttcaaccatccgtcaactactcgacgttcgaggtgggtgac

caaaagttattttttgtccacgcccatattcgcgaaagcctgcttggcatcttgctgcgc

gactggctggccatgcgaaaggcggtgagggcgcgaatccccaccagcacccccgaggag

gcagttttactagataagcagcagtctgcgattaaggtgatatgcaactcggtttacgga

ttcacgggggtggcaaacggcctgttgccgtgtctgaggatagcggctaccgttaccacg

ataggacgcgacatgctcctcaagacccgagattacgttcactctcgttgggcgacgcgc

gagctgctggaggacaattttccaggggctataggtttccgaaaccacaagccttactcc

gtcagggttatctacggagacaccgactccgtgtttatcaagtttgtgggcctgacgtac

gagggggtatcggagctgggggatgctatgtcgcgtcagatttcagcggacctctttaga

gcgcccatcaaactggagtgtgagaagacctttcagcgactgctgctgatcaccaagaag

aagtacataggtgtcataaacggggggaagatgctcatgaagggggtcgacctggtccgc

aaaaataactgctctttcataaacttgtacgcgcgacatctggtagatcttttgttgtac

gacgaggatgtggccacggcggcagcagaggtgacagacgtgcctcccgcagaatgggtg

gggcgcccgctaccgagcggctttgacaagtttgggcgagtgctggtagaggcgtacaac

cgtatcactgcccccaacttggacgtgcgcgagttcgttatgactgctgagctgagccgc

tcacccgaatcgtataccaacaagcgcctgccgcacctcaccgtctactttaagctcgcc

atgaggaatgaagaactgcccagcgtaaaagagagaattccgtatgtgatagttgcgcag

accgaggccgcggaacgcgaagcgggtgtagtaaactcaatgcgcggtaccgcccaaaac

cccgtggtaaccaagaccgcacgcccccaacctaaacgcaaactgctggtttccgacctc

gccgaagacccgacctatgtttccgagaatgacgtaccgctaaacacagactactatttc

tcccacctgttgggtaccataagcgtgacctttaaggctctattcggaaatgatgtgaga

acaacagaaaatcttttaaagcggtttattccggaaaccccccacaagacccccacgaaa

acccaggcactgcttgagcgcgccggctttgaaaagctgacgccctttacaccggaggaa

gaaagtcgtcgaatactgcatacagttttttgtactctagaagcagctccccatcaaagc

tga

>MN688624.1

atggcggcgcgcgaacaggccaactccgttcgcaggagcgggttttttaatccgtttatt

ggcaagaggccatttttcaggccgggaagcgggcaaaccgcggagacagagcgcccaagg

cccccccaacactcgtactgcacagaggtgggtagctttaagtttatagctccaagatgc

ctcgatgaagaagcccccgccgaccagcgacgcggtgtacacgtgggcaccttggagcgc

ccacccaaagtgtactgcgatggctcagagtacgacgtgctgaactttgcctccggaggt

tgctggcctcgcaggattcgggtttggaacggccaggattttcggggcgatggattcaac

cccagatttgagagatttcacgtgtacgacatagtagagacttcggagagcgcctcacac

gatgacccatccaggtttgctgagctatcccgcccaagcgggtctgtggtaacactgctg

ggaatgagcgagtgtggaaagcgagtcgcggttcacgtctatggtgtgcgccattatttt

tacatggcaaaggcggaggtggatagcgcttgtggaataaccaccgaggcagaactcgtg

cgcgcaatggtggactgcgcgcacagctcggctttgagcgcggccctgggaaatggcaac

ggcggcaaacagagcggcggcagcggcgggggatggtggggcggaaagcacgtgtctgcg

gactgcttcaaagtggagaccgtgtgccacacgacgctgtactactttggatctaagcca

gctctctactatagagtatctgcctccagcagccgcctgggagggttcatctgcgacaac

tttcacccggagattacaaaattcgaggggagcgtggacgtgaccacgcggctgttgttg

gacaatgaaaattttaccagtttcgggtggtaccgcctgcgacccggcacccacggagag

cgtgttcaacttcgccccgttgagcgacacgtcacctcaagcgacgtggagattaactgt

actcccgataacctggagccgataccagacgaggctgcctggcccgactataagctcatg

tgctttgatatagagtgtaaagctggaacgggtaacgaaatggcgttcccagtggcaact

aaccaagaggacctggtcatccagatctcctgtctgctgtactcgcttgctactcagaac

cacgaacacaccctgctgttttccctcgggtcatgcgatatctctgaggaatactcgttt

gcatgcgtccagcgcggcgagcccagaccgacggttttggagtttgacagcgagtacgag

ctgctggttgccttcctgacctttctcaagcagtactctcccgagttcgccaccggctac

aacatcgttaattttgactgggcgtacatagttaacaaggtaacgtcggtgtataacatc

aagctggacgggtacggcaagttcaacaaaggggggctgtttaaggtgtgggacatcgcc

acgaaccattttcagaagaagagcaaggtgaaaatcaatggcctgatatctctagacatg

tattctgtggcgacggaaaagctaaagctacccagctacaaactcgacgcggtcgtggga

gacgtcctcggcgagcataagatagaccttccctataaagaaataccctcctattacgcg

ggagggcctgaccggaggggcgtaataggagagtattgtatccaggactctaggctggtg

ggcaagctgttttttaagtacctcccccatctggaactatcggcggtggccaaactcgcc

cgtatcaccctaacgcgggtaatttttgacggtcagcaaattcgcgtgtacacgtgcttg

ctgaaactcgcccgcgagagaaatttcattttgccagacaacagacgccggtttgacagt

caggcagacgccgcgtcagagacttcggagttggctatggatagccaaagccacgccttc

gacagtacagacgaacccgacggtgtggacggtaccccggacgccgcaggatctggcgct

acttctgaaaacggaggcgggaagcccggcgtcgggagggccgtgggctaccagggagca

aaggttctagaccccgtatccggctttcatgtggaccccgtggttgtgtttgacttcgct

agcttatacccaagcattatccaggcccataacctctgtttcaccaccctggcgctcgat

gaagtggatctggccgggcttcaaccatccgtcaactactcgacgttcgaggtgggtgac

caaaagttattttttgtccacgcccatattcgcgaaagcctgcttggcatcttgctgcgc

gactggctggccatgcgaaaggcggtgagggcgcgaatccccaccagcacccccgaggag

gcagttttactagataagcagcagtctgcgattaaggtgatatgcaactcggtttacgga

ttcacgggggtggcaaacggcctgttgccgtgtctgaggatagcggctaccgttaccacg

ataggacgcgacatgctcctcaagacccgagattacgttcactctcgttgggcgacgcgc

gagctgctggaggacaattttccaggggctataggtttccgaaaccacaagccttactcc

gtcagggttatctacggagacaccgactccgtgtttatcaagtttgtgggcctgacgtac

gagggggtatcggagctgggggatgctatgtcgcgtcagatttcagcggacctctttaga

gcgcccatcaaactggagtgtgagaagacctttcagcgactgctgctgatcaccaagaag

aagtacataggtgtcataaacggggggaagatgctcatgaagggggtcgacctggtccgc

aaaaataactgctctttcataaacttgtacgcgcgacatctggtagatcttttgttgtac

gacgaggatgtggccacggcggcagcagaggtgacagacgtgcctcccgcagaatgggtg

gggcgcccgctaccgagcggctttgacaagtttgggcgagtgctggtagaggcgtacaac

cgtatcactgcccccaacttggacgtgcgcgagttcgttatgactgctgagctgagccgc

tcacccgaatcgtataccaacaagcgcctgccgcacctcaccgtctactttaagctcgcc

atgaggaatgaagaactgcccagcgtaaaagagagaattccgtatgtgatagttgcgcag

accgaggccgcggaacgcgaagcgggtgtagtaaactcaatgcgcggtaccgcccaaaac

cccgtggtaaccaagaccgcacgcccccaacctaaacgcaaactgctggtttccgacctc

gccgaagacccgacctatgtttccgagaatgacgtaccgctaaacacagactactatttc

tcccacctgttgggtaccataagcgtgacctttaaggctctattcggaaatgatgtgaga

acaacagaaaatcttttaaagcggtttattccggaaaccccccacaagacccccacgaaa

acccaggcactgcttgagcgcgccggctttgaaaagctgacgccctttacaccggaggaa

gaaagtcgtcgaatactgcatacagttttttgtactctagaagcagctccccatcaaagc

tga

>MN688625.1

atggcggcgcgcgaacaggccaactccgttcgcaggagcgggttttttaatccgtttatt

ggcaagaggccatttttcaggccgggaagcgggcaaaccgcggagacagagcgcccaagg

cccccccaacactcgtactgcacagaggtgggtagctttaagtttatagctccaagatgc

ctcgatgaagaagcccccgccgaccagcgacgcggtgtacacgtgggcaccttggagcgc

ccacccaaagtgtactgcgatggctcagagtacgacgtgctgaactttgcctccggaggt

tgctggcctcgcaggattcgggtttggaacggccaggattttcggggcgatggattcaac

cccagatttgagagatttcacgtgtacgacatagtagagacttcggagagcgcctcacac

gatgacccatccaggtttgctgagctatcccgcccaagcgggtctgtggtaacactgctg

ggaatgagcgagtgtggaaagcgagtcgcggttcacgtctatggtgtgcgccattatttt

tacatggcaaaggcggaggtggatagcgcttgtggaataaccaccgaggcagaactcgtg

cgcgcaatggtggactgcgcgcacagctcggctttgagcgcggccctgggaaatggcaac

ggcggcaaacagagcggcggcagcggcgggggatggtggggcggaaagcacgtgtctgcg

gactgcttcaaagtggagaccgtgtgccacacgacgctgtactactttggatctaagcca

gctctctactatagagtatctgcctccagcagccgcctgggagggttcatctgcgacaac

tttcacccggagattacaaaattcgaggggagcgtggacgtgaccacgcggctgttgttg

gacaatgaaaattttaccagtttcgggtggtaccgcctgcgacccggcacccacggagag

cgtgttcaacttcgccccgttgagcgacacgtcacctcaagcgacgtggagattaactgt

actcccgataacctggagccgataccagacgaggctgcctggcccgactataagctcatg

tgctttgatatagagtgtaaagctggaacgggtaacgaaatggcgttcccagtggcaact

aaccaagaggacctggtcatccagatctcctgtctgctgtactcgcttgctactcagaac

cacgaacacaccctgctgttttccctcgggtcatgcgatatctctgaggaatactcgttt

gcatgcgtccagcgcggcgagcccagaccgacggttttggagtttgacagcgagtacgag

ctgctggttgccttcctgacctttctcaagcagtactctcccgagttcgccaccggctac

aacatcgttaattttgactgggcgtacatagttaacaaggtaacgtcggtgtataacatc

aagctggacgggtacggcaagttcaacaaaggggggctgtttaaggtgtgggacatcgcc

acgaaccattttcagaagaagagcaaggtgaaaatcaatggcctgatatctctagacatg

tattctgtggcgacggaaaagctaaagctacccagctacaaactcgacgcggtcgtggga

gacgtcctcggcgagcataagatagaccttccctataaagaaataccctcctattacgcg

ggagggcctgaccggaggggcgtaataggagagtattgtatccaggactctaggctggtg

ggcaagctgttttttaagtacctcccccatctggaactatcggcggtggccaaactcgcc

cgtatcaccctaacgcgggtaatttttgacggtcagcaaattcgcgtgtacacgtgcttg

ctgaaactcgcccgcgagagaaatttcattttgccagacaacagacgccggtttgacagt

caggcagacgccgcgtcagagacttcggagttggctatggatagccaaagccacgccttc

gacagtacagacgaacccgacggtgtggacggtaccccggacgccgcaggatctggcgct

acttctgaaaacggaggcgggaagcccggcgtcgggagggccgtgggctaccagggagca

aaggttctagaccccgtatccggctttcatgtggaccccgtggttgtgtttgacttcgct

agcttatacccaagcattatccaggcccataacctctgtttcaccaccctggcgctcgat

gaagtggatctggccgggcttcaaccatccgtcaactactcgacgttcgaggtgggtgac

caaaagttattttttgtccacgcccatattcgcgaaagcctgcttggcatcttgctgcgc

gactggctggccatgcgaaaggcggtgagggcgcgaatccccaccagcacccccgaggag

gcagttttactagataagcagcagtctgcgattaaggtgatatgcaactcggtttacgga

ttcacgggggtggcaaacggcctgttgccgtgtctgaggatagcggctaccgttaccacg

ataggacgcgacatgctcctcaagacccgagattacgttcactctcgttgggcgacgcgc

gagctgctggaggacaattttccaggggctataggtttccgaaaccacaagccttactcc

gtcagggttatctacggagacaccgactccgtgtttatcaagtttgtgggcctgacgtac

gagggggtatcggagctgggggatgctatgtcgcgtcagatttcagcggacctctttaga

gcgcccatcaaactggagtgtgagaagacctttcagcgactgctgctgatcaccaagaag

aagtacataggtgtcataaacggggggaagatgctcatgaagggggtcgacctggtccgc

aaaaataactgctctttcataaacttgtacgcgcgacatctggtagatcttttgttgtac

gacgaggatgtggccacggcggcagcaaaggtgacagacgtgcctcccgcagaatgggtg

gggcgcccgctaccgagcggctttgacaagtttgggcgagtgctggtagaggcgtacaac

cgtatcactgcccccaacttggacgtgcgcgagttcgttatgactgctgagctgagccgc

tcacccgaatcgtataccaacaagcgcctgccgcacctcaccgtctactttaagctcgcc

atgaggaatgaagaactgcccagcgtaaaagagagaattccgtatgtgatagttgcgcag

accgaggccgcggaacgcgaagcgggtgtagtaaactcaatgcgcggtaccgcccaaaac

cccgtggtaaccaagaccgcacgcccccaacctaaacgcaaactgctggtttccgacctc

gccgaagacccgacctatgtttccgagaatgacgtaccgctaaacacagactactatttc

tcccacctgttgggtaccataagcgtgacctttaaggctctattcggaaatgatgtgaga

acaacagaaaatcttttaaagcggtttattccggaaaccccccacaagacccccacgaaa

acccaggcactgcttgagcgcgccggctttgaaaagctgacgccctttacaccggaggaa

gaaagtcgtcgaatactgcatacagttttttgtactctagaagcagctccccatcaaagc

tga

>MN688626.1

atggcggcgcgcgaacaggccaactccgttcgcaggagcgggttttttaatccgtttatt

ggcaagaggccatttttcaggccgggaagcgggcagaccgcggagacagagcgcccaagg

cccccccaacactcgtactgcacagaggtgggtagctttaagtttatagctccaagatgc

ctcgatgaagaagcccccgccgaccagcgacgcggtgtacacgtgggcaccttggagcgc

ccacccaaagtgtactgcgatggctcagagtacgacgtgctgaactttgcctccggaggt

tgctggcctcgcaggattcgggtttggaacggccaggattttcggggcgatggattcaac

cccagatttgagagatttcacgtgtacgacatagtagagacttcggagagcgcctcacac

gatgacccatccaggtttgctgagctatcccgcccaagcgggtctgtggtaacactgctg

ggaatgagcgagtgtggaaagcgagtcgcggttcacgtctatggtgtgcgccattatttt

tacatggcaaaggcggaggtggatagcgcttgtggaataaccaccgaggcagaactcgtg

cgcgcaatggtggactgcgcgcacagctcggctttgagcgcggccctgggaaatggcaac

ggcggcaaacagagcggcggcagcggcgggggatggtggggcggaaagcacgtgtctgcg

gactgcttcaaagtggagaccgtgtgccacacgacgctgtactactttggatctaagcca

gctctctactatagagtatctgcctccagcagccgcctgggagggttcatctgcgacaac

tttcacccggagattacaaaattcgaggggagcgtggacgtgaccacgcggctgttgttg

gacaatgaaaattttaccagtttcgggtggtaccgcctgcgacccggcacccacggagag

cgtgttcaacttcgccccgttgagcgacacgtcacctcaagcgacgtggagattaactgt

actcccgataacctggagccgataccagacgaggctgcctggcccgactataagctcatg

tgctttgatatagagtgtaaagctggaacgggtaacgaaatggcgttcccagtggcaact

aaccaagaggacctggtcatccagatctcctgtctgctgtactcgcttgctactcagaac

cacgaacacaccctgctgttttccctcgggtcatgcgatatctctgaggaatactcgttt

gcatgcgtccagcgcggcgagcccagaccgacggttttggagtttgacagcgagtacgag

ctgctggttgccttcctgacctttctcaagcagtactctcccgagttcgccaccggctac

aacatcgttaattttgactgggcgtacatagttaacaaggtaacgtcggtgtataacatc

aagctggacgggtacggcaagttcaacaaaggggggctgtttaaggtgtgggacatcgcc

acgaaccattttcagaagaagagcaaggtgaaaatcaatggcctgatatctctagacatg

tattctgtggcgacggaaaagctaaagctacccagctacaaactcgacgcggtcgtggga

gacgtcctcggcgagcataagatagaccttccctataaagaaataccctcctattacgcg

ggagggcctgaccggaggggcgtaataggagagtattgtatccaggactctaggctggtg

ggcaagctgttttttaagtacctcccccatctggaactatcggcggtggccaaactcgcc

cgtatcaccctaacgcgggtaatttttgacggtcagcaaattcgcgtgtacacgtgcttg

ctgaaactcgcccgcgagagaaatttcattttgccagacaacagacgccggtttgacagt

caggcagacgccgcgtcagagacttcggagttggctatggatagccaaagccacgccttc

gacagtacagacgaacccgacggtgtggacggtaccccggacgccgcaggatctggcgct

acttctgaaaacggaggcgggaagcccggcgtcgggagggtcgtgggctaccagggagca

aaggttctagaccccgtatccggctttcatgtggaccccgtggttgtgtttgacttcgct

agcttatacccaagcattatccaggcccataacctctgtttcaccaccctggcgctcgat

gaagtggatctggccgggcttcaaccatccgtcgactactcgacgttcgaggtgggtgac

caaaagttattttttgtccacgcccatattcgcgaaagcctgcttggcatcttgctgcgc

gactggctggccatgcgaaaggcggtgagggcgcgaatccccaccagcacccccgaggag

gcagttttactagataagcagcagtctgcgattaaggtgatatgcaactcggtttacgga

ttcacgggggtggcaaacggcctgttgccgtgtctgaggatagcggctaccgttaccacg

ataggacgcgacatgctcctcaagacccgagattacgttcactctcgttgggcgacgcgc

gagctgctggaggacaattttccaggggctataggtttccgaaaccacaagccttactcc

gtcagggttatctacggagacaccgactccgtgtttatcaagtttgtgggcctgacgtac

gagggggtatcggagctgggggatgctatgtcgcgtcagatttcagcggacctctttaga

gcgcccatcaaactggagtgtgagaagacctttcagcgactgctgctgatcaccaagaag

aagtacataggtgtcataaacggggggaagatgctcatgaagggggtcgacctggtccgc

aaaaataactgctctttcataaacttgtacgcgcgacatctggtagatcttttgttgtac

gacgaggatgtggccacggcggcagcagaggtgacagacgtgcctcccgcagaatgggtg

gggcgcccgctaccgagcggctttgacaagtttgggcgagtgctggtagaggcgtacaac

cgtatcactgcccccaacttggacgtgcgcgagttcgttatgactgctgagctgagccgc

tcacccgaatcgtataccaacaagcgcctgccgcacctcaccgtctactttaagctcgcc

atgaggaatgaagaactgcccagcgtaaaagagagaattccgtatgtgatagttgcgcag

accgaggccgcggaacgcgaagcgggtgtagtaaactcaatgcgcggtaccgcccaaaac

cccgtggtaaccaagaccgcacgcccccaacctaaacgcaaactgctggtttccgacctc

gccgaagacccgacctatgtttccgagaatgacgtaccgctaaacacagactactatttc

tcccacctgttgggtaccataagcgtgacctttaaggctctattcggaaatgatgtgaga

acaacagaaaatcttttaaagcggtttattccggaaaccccccacaagacccccacgaaa

acccaggcactgcttgagcgcgccggctttgaaaagctgacgccctttacaccggaggaa

gaaagtcgtcgaatactgcatacagttttttgtactctagaagcagctccccatcaaagc

tga

>MN688627.1

atggcggcgcgcgaacaggccaactccgttcgcaggagcgggttttttaatccgtttatt

ggcaagaggccatttttcaggccgggaagcgggcagaccgcggagacagagcgcccaagg

cccccccaacactcgtactgcacagaggtgggtagctttaagtttatagctccaagatgc

ctcgatgaagaagcccccgccgaccagcgacgcggtgtacacgtgggcaccttggagcgc

ccacccaaagtgtactgcgatggctcagagtacgacgtgctgaactttgcctccggaggt

tgctggcctcgcaggattcgggtttggaacggccaggattttcggggcgatggattcaac

cccagatttgagagatttcacgtgtacgacatagtagagacttcggagagcgcctcacac

gatgacccatccaggtttgctgagctatcccgcccaagcgggtctgtggtaacactgctg

ggaatgagcgagtgtggaaagcgagtcgcggttcacgtctatggtgtgcgccattatttt

tacatggcaaaggcggaggtggatagcgcttgtggaataaccaccgaggcagaactcgtg

cgcgcaatggtggactgcgcgcacagctcggctttgagcgcggccctgggaaatggcaac

ggcggcaaacagagcggcggcagcggcgggggatggtggggcggaaagcacgtgtctgcg

gactgcttcaaagtggagaccgtgtgccacacgacgctgtactactttggatctaagcca

gctctctactatagagtatctgcctccagcagccgcctgggagggttcatctgcgacaac

tttcacccggagattacaaaattcgaggggagcgtggacgtgaccacgcggctgttgttg

gacaatgaaaattttaccagtttcgggtggtaccgcctgcgacccggcacccacggagag

cgtgttcaacttcgccccgttgagcgacacgtcacctcaagcgacgtggagattaactgt

actcccgataacctggagccgataccagacgaggctgcctggcccgactataagctcatg

tgctttgatatagagtgtaaagctggaacgggtaacgaaatggcgttcccagtggcaact

aaccaagaggacctggtcatccagatctcctgtctgctgtactcgcttgctactcagaac

cacgaacacaccctgctgttttccctcgggtcatgcgatatctctgaggaatacttgttt

gcatgcgtccagcgcggcgagcccagaccgacggttttggagtttgacagcgagtacgag

ctgctggttgccttcctgacctttctcaagcagtactctcccgagttcgccaccggctac

aacatcgttaattttgactgggcgtacatagttaacaaggtaacgtcggtgtataacatc

aagctggacgggtacggcaagttcaacaaaggggggctgtttaaggtgtgggacatcgcc

acgaaccattttcagaagaagagcaaggtgaaaatcaatggcctgatatctctagacatg

tattctgtggcgacggaaaagctaaagctacccagctacaaactcgacgcggtcgtggga

gacgtcctcggcgagcataagatagaccttccctataaagaaataccctcctattacgcg

ggagggcctgaccggaggggcgtaataggagagtattgtatccaggactctaggctggtg

ggcaagctgttttttaagtacctcccccatctggaactatcggcggtggccaaactcgcc

cgtatcaccctaacgcgggtaatttttgacggtcagcaaattcgcgtgtacacgtgcttg

ctgaaactcgcccgcgagagaaatttcattttgccagacaacagacgccggtttgacagt

caggcagacgccgcgtcagagacttcggagttggctatggatagccaaagccacgccttc

gacagtacagacgaacccgacggtgtggacggtaccccggacgccgcaggatctggcgct

acttctgaaaacggaggcgggaagcccggcgtcgggagggccgtgggctaccagggagca

aaggttctagaccccgtatccggctttcatgtggaccccgtggttgtgtttgacttcgct

agcttatacccaagcattatccaggcccataacctctgtttcaccaccctggcgctcgat

gaagtggatctggccgggcttcaaccatccgtcaactactcgacgttcgaggtgggtgac

caaaagttattttttgtccacgcccatattcgcgaaagcctgcttggcatcttgctgcgc

gactggctggccatgcgaaaggcggtgagggcgcgaatccccaccagcacccccgaggag

gcagttttactagataagcagcagtctgcgattaaggtgatatgcaactcggtttacgga

ttcacgggggtggcaaacggcctgttgccgtgtctgaggatagcggctaccgttaccacg

ataggacgcgacatgctcctcaagacccgagattacgttcactctcgttgggcgacgcgc

gagctgctggaggacaattttccaggggctataggtttccgaaaccacaagccttactcc

gtcagggttatctacggagacaccgactccgtgtttatcaagtttgtgggcctgacgtac

gagggggtatcggagctgggggatgctatgtcgcgtcagatttcagcggacctctttaga

gcgcccatcaaactggagtgtgagaagacctttcagcgactgctgctgatcaccaagaag

aagtacataggtgtcataaacggggggaagatgctcatgaagggggtcgacctggtccgc

aaaaataactgctctttcataaacttgtacgcgcgacatctggtagatcttttgttgtac

gacgaggatgtggccacggcggcagcagaggtgacagacgtgcctcccgcagaatgggtg

gggcgcccgctaccgagcggctttgacaagtttgggcgagtgctggtagaggcgtacaac

cgtatcactgcccccaacttggacgtgcgcgagttcgttatgactgctgagctgagccgc

tcacccgaatcgtataccaacaagcgcctgccgcacctcaccgtctactttaagctcgcc

atgaggaatgaagaactgcccagcgtaaaagagagaattccgtatgtgatagttgcgcag

accgaggccgcggaacgcgaagcgggtgtagtaaactcaatgcgcggtaccgcccaaaac

cccgtggtaaccaagaccgcacgcccccaacctaaacgcaaactgctggtttccgacctc

gccgaagacccgacctatgtttccgagaatgacgtaccgctaaacacagactactatttc

tcccacctgttgggtaccataagcgtgacctttaaggctctattcggaaatgatgtgaga

acaacagaaaatcttttaaagcggtttattccggaaaccccccacaagacccccacgaaa

acccaggcactgcttgagcgcgccggctttgaaaagctgacgccctttacaccggaggaa

gaaagtcgtcgaatactgcatacagttttttgtactctagaagcagctccccatcaaagc

tga

>MN688628.1

atggcggcgcgcgaacaggccaactccgttcgcaggagcgggttttttaatccgtttatt

ggcaagaggccatttttcaggccgggaagcgggcaaaccgcggagacagagcgcccaagg

cccccccaacactcgtactgcacagaggtgggtagctttaagtttatagctccaagatgc

ctcgatgaagaagcccccgccgaccagcgacgcggtgtacacgtgggcaccttggagcgc

ccacccaaagtgtactgcgatggctcagagtacgacgtgctgaactttgcctccggaggt

tgctggcctcgcaggattcgggtttggaacggccaggattttcggggcgatggattcaac

cccagatttgagagatttcacgtgtacgacatagtagagacttcggagagcgcctcacac

gatgacccatccaggtttgctgagctatcccgcccaagcgggtctgtggtaacactgctg

ggaatgagcgagtgtggaaagcgagtcgcggttcacgtctatggtgtgcgccattatttt

tacatggcaaaggcggaggtggatagcgcttgtggaataaccaccgaggcagaactcgtg

cgcgcaatggtggactgcgcgcacagctcggctttgagcgcggccctgggaaatggcaac

ggcggcaaacagagcggcggcagcggcgggggatggtggggcggaaagcacgtgtctgcg

gactgcttcaaagtggagaccgtgtgccacacgacgctgtactactttggatctaagcca

gctctctactatagagtatctgcctccagcagccgcctgggagggttcatctgcgacaac

tttcacccggagattacaaaattcgaggggagcgtggacgtgaccacgcggctgttgttg

gacaatgaaaattttaccagtttcgggtggtaccgcctgcgacccggcacccacggagag

cgtgttcaacttcgccccgttgagcgacacgtcacctcaagcgacgtggagattaactgt

actcccgataacctggagccgataccagacgaggctgcctggcccgactataagctcatg

tgctttgatatagagtgtaaagctggaacgggtaacgaaatggcgttcccagtggcaact

aaccaagaggacctggtcatccagatctcctgtctgctgtactcgcttgctactcagaac

cacgaacacaccctgctgttttccctcgggtcatgcgatatctctgaggaatactcgttt

gcatgcgtccagcgcggcgagcccagaccgacggttttggagtttgacagcgagtacgag

ctgctggttgccttcctgacctttctcaagcagtactctcccgagttcgccaccggctac

aacatcgttaattttgactgggcgtacatagttaacaaggtaacgtcggtgtataacatc

aagctggacgggtacggcaagttcaacaaaggggggctgtttaaggtgtgggacatcgcc

acgaaccattttcagaagaagagcaaggtgaaaatcaatggcctgatatctctagacatg

tattctgtggcgacggaaaagctaaagctacccagctacaaactcgacgcggtcgtggga

gacgtcctcggcgagcataagatagaccttccctataaagaaataccctcctattacgcg

ggagggcctgaccggaggggcgtaataggagagtattgtatccaggactctaggctggtg

ggcaagctgttttttaagtacctcccccatctggaactatcggcggtggccaaactcgcc

cgtatcaccctaacgcgggtaatttttgacggtcagcaaattcgcgtgtacacgtgcttg

ctgaaactcgcccgcgagagaaatttcattttgccagacaacagacgccggtttgacagt

caggcagacgccgcgtcagagacttcggagttggctatggatagccaaagccacgccttc

gacagtacagacgaacccgacggtgtggacggtaccccggacgccgcaggatctggcgct

acttctgaaaacggaggcgggaagcccggcgtcgggagggccgtgggctaccagggagca

aaggttctagaccccgtatccggctttcatgtggaccccgtggttgtgtttgacttcgct

agcttatacccaagcattatccaggcccataacctctgtttcaccaccctggcgctcgat

gaagtggatctggccgggcttcaaccatccgtcaactactcgacgttcgaggtgggtgac

caaaagttattttttgtccacgcccatattcgcgaaagcctgcttggcatcttgctgcgc

gactggctggccatgcgaaaggcggtgagggcgcgaatccccaccagcacccccgaggag

gcagttttactagataagcagcagtctgcgattaaggtgatatgcaactcggtttacgga

ttcacgggggtggcaaacggcctgttgccgtgtctgaggatagcggctaccgttaccacg

ataggacgcgacatgctcctcaagacccgagattacgttcactctcgttgggcgacgcgc

gagctgctggaggacaattttccaggggctataggtttccgaaaccacaagccttactcc

gtcagggttatctacggagacaccgactccgtgtttatcaagtttgtgggcctgacgtac

gagggggtatcggagctgggggatgctatgtcgcgtcagatttcagcggacctctttaga

gcgcccatcaaactggagtgtgagaagacctttcagcgactgctgctgatcaccaagaag

aagtacataggtgtcataaacggggggaagatgctcatgaagggggtcgacctggtccgc

aaaaataactgctctttcataaacttgtacgcgcgacatctggtagatcttttgttgtac

gacgaggatgtggccacggcggcagcaaaggtgacagacgtgcctcccgcagaatgggtg

gggcgcccgctaccgagcggctttgacaagtttgggcgagtgctggtagaggcgtacaac

cgtatcactgcccccaacttggacgtgcgcgagttcgttatgactgctgagctgagccgc

tcacccgaatcgtataccaacaagcgcctgccgcacctcaccgtctactttaagctcgcc

atgaggaatgaagaactgcccagcgtaaaagagagaattccgtatgtgatagttgcgcag

accgaggccgcggaacgcgaagcgggtgtagtaaactcaatgcgcggtaccgcccaaaac

cccgtggtaaccaagaccgcacgcccccaacctaaacgcaaactgctggtttccgacctc

gccgaagacccgacctatgtttccgagaatgacgtaccgctaaacacagactactatttc

tcccacctgttgggtaccataagcgtgacctttaaggctctattcggaaatgatgtgaga

acaacagaaaatcttttaaagcggtttattccggaaaccccccacaagacccccacgaaa

acccaggcactgcttgagcgcgccggctttgaaaagctgacgccctttacaccggaggaa

gaaagtcgtcgaatactgcatacagttttttgtactctagaagcagctccccatcaaagc

tga

>MN688629.1

atggcggcgcgcgaacaggccaactccgttcgcaggagcgggttttttaatccgtttatt

ggcaagaggccatttttcaggccgggaagcgggcaaaccgcggagacagagcgcccaagg

cccccccaacactcgtactgcacagaggtgggtagctttaagtttatagctccaagatgc

ctcgatgaagaagcccccgccgaccagcgacgcggtgtacacgtgggcaccttggagcgc

ccacccaaagtgtactgcgatggctcagagtacgacgtgctgaactttgcctccggaggt

tgctggcctcgcaggattcgggtttggaacggccaggattttcggggcgatggattcaac

cccagatttgagagatttcacgtgtacgacatagtagagacttcggagagcgcctcacac

gatgacccatccaggtttgctgagctatcccgcccaagcgggtctgtggtaacactgctg

ggaatgagcgagtgtggaaagcgagtcgcggttcacgtctatggtgtgcgccattatttt

tacatggcaaaggcggaggtggatagcgcttgtggaataaccaccgaggcagaactcgtg

cgcgcaatggtggactgcgcgcacagctcggctttgagcgcggccctgggaaatggcaac

ggcggcaaacagagcggcggcagcggcgggggatggtggggcggaaagcacgtgtctgcg

gactgcttcaaagtggagaccgtgtgccacacgacgctgtactactttggatctaagcca

gctctctactatagagtatctgcctccagcagccgcctgggagggttcatctgcgacaac

tttcacccggagattacaaaattcgaggggagcgtggacgtgaccacgcggctgttgttg

gacaatgaaaattttaccagtttcgggtggtaccgcctgcgacccggcacccacggagag

cgtgttcaacttcgccccgttgagcgacacgtcacctcaagcgacgtggagattaactgt

actcccgataacctggagccgataccagacgaggctgcctggcccgactataagctcatg

tgctttgatatagagtgtaaagctggaacgggtaacgaaatggcgttcccagtggcaact

aaccaagaggacctggtcatccagatctcctgtctgctgtactcgcttgctactcagaac

cacgaacacaccctgctgttttccctcgggtcatgcgatatctctgaggaatactcgttt

gcatgcgtccagcgcggcgagcccagaccgacggttttggagtttgacagcgagtacgag

ctgctggttgccttcctgacctttctcaagcagtactctcccgagttcgccaccggctac

aacatcgttaattttgactgggcgtacatagttaacaaggtaacgtcggtgtataacatc

aagctggacgggtacggcaagttcaacaaaggggggctgtttaaggtgtgggacatcgcc

acgaaccattttcagaagaagagcaaggtgaaaatcaatggcctgatatctctagacatg

tattctgtggcgacggaaaagctaaagctacccagctacaaactcgacgcggtcgtggga

gacgtcctcggcgagcataagatagaccttccctataaagaaataccctcctattacgcg

ggagggcctgaccggaggggcgtaataggagagtattgtatccaggactctaggctggtg

ggcaagctgttttttaagtacctcccccatctggaactatcggcggtggccaaactcgcc

cgtatcaccctaacgcgggtaatttttgacggtcagcaaattcgcgtgtacacgtgcttg

ctgaaactcgcccgcgagagaaatttcattttgccagacaacagacgccggtttgacagt

caggcagacgccgcgtcagagacttcggagttggctatggatagccaaagccacgccttc

gacagtacagacgaacccgacggtgtggacggtaccccggacgccgcaggatctggcgct

acttctgaaaacggaggcgggaagcccggcgtcgggagggccgtgggctaccagggagca

aaggttctagaccccgtatccggctttcatgtggaccccgtggttgtgtttgacttcgct

agcttatacccaagcattatccaggcccataacctctgtttcaccaccctggcgctcgat

gaagtggatctggccgggcttcaaccatccgtcaactactcgacgttcgaggtgggtgac

caaaagttattttttgtccacgcccatattcgcgaaagcctgcttggcatcttgctgcgc

gactggctggccatgcgaaaggcggtgagggcgcgaatccccaccagcacccccgaggag

gcagttttactagataagcagcagtctgcgattaaggtgatatgcaactcggtttacgga

ttcacgggggtggcaaacggcctgttgccgtgtctgaggatagcggctaccgttaccacg

ataggacgcgacatgctcctcaagacccgagattacgttcactctcgttgggcgacgcgc

gagctgctggaggacaattttccaggggctataggtttccgaaaccacaagccttactcc

gtcagggttatctacggagacaccgactccgtgtttatcaagtttgtgggcctgacgtac

gagggggtatcggagctgggggatgctatgtcgcgtcagatttcagcggacctctttaga

gcgcccatcaaactggagtgtgagaagacctttcagcgactgctgctgatcaccaagaag

aagtacataggtgtcataaacggggggaagatgctcatgaagggggtcgacctggtccgc

aaaaataactgctctttcataaacttgtacgcgcgacatctggtagatcttttgttgtac

gacgaggatgtggccacggcggcagcaaaggtgacagacgtgcctcccgcagaatgggtg

gggcgcccgctaccgagcggctttgacaagtttgggcgagtgctggtagaggcgtacaac

cgtatcactgcccccaacttggacgtgcgcgagttcgttatgactgctgagctgagccgc

tcacccgaatcgtataccaacaagcgcctgccgcacctcaccgtctactttaagctcgcc

atgaggaatgaagaactgcccagcgtaaaagagagaattccgtatgtgatagttgcgcag

accgaggccgcggaacgcgaagcgggtgtagtaaactcaatgcgcggtaccgcccaaaac

cccgtggtaaccaagaccgcacgcccccaacctaaacgcaaactgctggtttccgacctc

gccgaagacccgacctatgtttccgagaatgacgtaccgctaaacacagactactatttc

tcccacctgttgggtaccataagcgtgacctttaaggctctattcggaaatgatgtgaga

acaacagaaaatcttttaaagcggtttattccggaaaccccccacaagacccccacgaaa

acccaggcactgcttgagcgcgccggctttgaaaagctgacgccctttacaccggaggaa

gaaagtcgtcgaatactgcatacagttttttgtactctagaagcagctccccatcaaagc

tga

>MT968035.1

atggcggcgcgcgaacaggccaactccgttcgcaggagcgggttttttaatccgtttatt

ggcaagaggccatttttcaggccgggaagcgggcaaaccgcggagacagagcgcccaagg

cccccccaacactcgtactgcacagaggtgggtagctttaagtttatagctccaagatgc

ctcgatgaagaagcccccgccgaccagcgacgcggtgtacacgtgggcaccttggagcgc

ccacccaaagtgtactgcgatggctcagagtacgacgtgctgaactttgcctccggaggt

tgctggcctcgcaggattcgggtttggaacggccaggattttcggggcgatggattcaac

cccagatttgagagatttcacgtgtacgacatagtagagacttcggagagcgcctcacac

gatgacccatccaggtttgctgagctatcccgcccaagcgggtctgtggtaacactgctg

ggaatgagcgagtgtggaaagcgagtcgcggttcacgtctatggtgtgcgccattatttt

tacatggcaaaggcggaggtggatagcgcttgtggaataaccaccgaggcagaactcgtg

cgcgcaatggtggactgcgcgcacagctcggctttgagcgcggccctgggaaatggcaac

ggcggcaaacagagcggcggcagcggcgggggatggtggggcggaaagcacgtgtctgcg

gactgcttcaaagtggagaccgtgtgccacacgacgctgtactactttggatctaagcca

gctctctactatagagtatctgcctccagcagccgcctgggagggttcatctgcgacaac

tttcacccggagattacaaaattcgaggggagcgtggacgtgaccacgcggctgttgttg

gacaatgaaaattttaccagtttcgggtggtaccgcctgcgacccggcacccacggagag

cgtgttcaacttcgccccgttgagcgacacgtcacctcaagcgacgtggagattaactgt

actcccgataacctggagccgataccagacgaggctgcctggcccgactataagctcatg

tgctttgatatagagtgtaaagctggaacgggtaacgaaatggcgttcccagtggcaact

aaccaagaggacctggtcatccagatctcctgtctgctgtactcgcttgctactcagaac

cacgaacacaccctgctgttttccctcgggtcatgcgatatctctgaggaatactcgttt

gcatgcgtccagcgcggcgagcccagaccgacggttttggagtttgacagcgagtacgag

ctgctggttgccttcctgacctttctcaagcagtactctcccgagttcgccaccggctac

aacatcgttaattttgactgggcgtacatagttaacaaggtaacgtcggtgtataacatc

aagctggacgggtacggcaagttcaacaaaggggggctgtttaaggtgtgggacatcgcc

acgaaccattttcagaagaagagcaaggtgaaaatcaatggcctgatatctctagacatg

tattctgtggcgacggaaaagctaaagctacccagctacaaactcgacgcggtcgtggga

gacgtcctcggcgagcataagatagaccttccctataaagaaataccctcctattacgcg

ggagggcctgaccggaggggcgtaataggagagtattgtatccaggactctaggctggtg

ggcaagctgttttttaagtacctcccccatctggaactatcggcggtggccaaactcgcc

cgtatcaccctaacgcgggtaatttttgacggtcagcaaattcgcgtgtacacgtgcttg

ctgaaactcgcccgcgagagaaatttcattttgccagacaacagacgccggtttgacagt

caggcagacgccgcgtcagagacttcggagttggctatggatagccaaagccacgccttc

gacagtacagacgaacccgacggtgtggacggtaccccggacgccgcaggatctggcgct

acttctgaaaacggaggcgggaagcccggcgtcgggagggccgtgggctaccagggagca

aaggttctagaccccgtatccggctttcatgtggaccccgtggttgtgtttgacttcgct

agcttatacccaagcattatccaggcccataacctctgtttcaccaccctggcgctcgat

gaagtggatctggccgggcttcaaccatccgtccactactcgacgttcgaggtgggtgac

caaaagttattttttgtccacgcccatattcgcgaaagcctgcttggcatcttgctgcgc

gactggctggccatgcgaaaggcggtgagggcgcgaatccccaccagcacccccgaggag

gcagttttactagataagcagcagtctgcgattaaggtgatatgcaactcggtttacgga

ttcacgggggtggcaaacggcctgttgccgtgtctgaggatagcggctaccgttaccacg

ataggacgcgacatgctcctcaagacccgagattacgttcactctcgttgggcgacgcgc

gagctgctggaggacaattttccaggggctataggtttccgaaaccacaagccttactcc

gtcagggttatctacggagacaccgactccgtgtttatcaagtttgtgggcctgacgtac

gagggggtatcggagctgggggatgctatgtcgcgtcagatttcagcggacctctttaga

gcgcccatcaaactggagtgtgagaagacctttcagcgactgctgctgatcaccaagaag

aagtacataggtgtcataaacggggggaagatgctcatgaagggggtcgacctggtccgc

aaaaataactgctctttcataaacttgtacgcgcgacatctggtagatcttttgttgtac

gacgaggatgtggccacggcggcagcaaaggtgacagacgtgcctcccgcagaatgggtg

gggcgcccgctaccgagcggctttgacaagtttgggcgagtgctggtagaggcgtacaac

cgtatcactgcccccaacttggacgtgcgcgagttcgttatgactgctgagctgagccgc

tcacccgaatcgtataccaacaagcgcctgccgcacctcaccgtctactttaagctcgcc

atgaggaatgaagaactgcccagcgtaaaagagagaattccgtatgtgatagttgcgcag

accgaggccgcggaacgcgaagcgggtgtagtaaactcaatgcgcggtaccgcccaaaac

cccgtggtaaccaagaccgcacgcccccaacctaaacgcaaactgctggtttccgacctc

gccgaagacccgacctatgtttccgagaatgacgtaccgctaaacacagactactatttc

tcccacctgttgggtaccataagcgtgacctttaaggctctattcggaaatgatgtgaga

acaacagaaaatcttttaaagcggtttattccggaaaccccccacaagacccccacgaaa

acccaggcactgcttgagcgcgccggctttgaaaagctgacgccctttacaccggaggaa

gaaagtcgtcgaatactgcatacagttttttgtactctagaagcagctccccatcaaagc

tga

>MW396756.1

atggcggcgcgcgaacaggccaactccgttcgcaggagcgggttttttaatccgtttatt

ggcaagaggccatttttcaggccgggaagcgggcagaccgcggagacagagcgcccaagg

cccccccaacactcgtactgcacagaggtgggtagctttaagtttatagctccaagatgc

ctcgatgaagaagcccccgccgaccagcgacgcggtgtacacgtgggcaccttggagcgc

ccacccaaagtgtactgcgatggctcagagtacgacgtgctgaactttgcctccggaggt

tgctggcctcgcaggattcgggtttggaacggccaggattttcggggcgatggattcaac

cccagatttgagagatttcacgtgtacgacatagtagagacttcggagagcgcctcacac

gatgacccatccaggtttgctgagctatcccgcccaagcgggtctgtggtaacactgctg

ggaatgagcgagtgtggaaagcgagtcgcggttcacgtctatggtgtgcgccattatttt

tacatggcaaaggcggaggtggatagcgcttgtggaataaccaccgaggcagaactcgtg

cgcgcaatggtggactgcgcgcacagctcggctttgagcgcggccctgggaaatggcaac

ggcggcaaacagagcggcggcagcggcgggggatggtggggcggaaagcacgtgtctgcg

gactgcttcaaagtggagaccgtgtgccacacgacgctgtactactttggatctaagcca

gctctctactatagagtatctgcctccagcagccgcctgggagggttcatctgcgacaac

tttcacccggagattacaaaattcgaggggagcgtggacgtgaccacgcggctgttgttg

gacaatgaaaattttaccagtttcgggtggtaccgcctgcgacccggcacccacggagag

cgtgttcaacttcgccccgttgagcgacacgtcacctcaagcgacgtggagattaactgt

actcccgataacctggagccgataccagacgaggctgcctggcccgactataagctcatg

tgctttgatatagagtgtaaagctggaacgggtaacgaaatggcgttcccagtggcaact

aaccaagaggacctggtcatccagatctcctgtctgctgtactcgcttgctactcagaac

cacgaacacaccctgctgttttccctcgggtcatgcgatatctctgaggaatactcgttt

gcatgcgtccagcgcggcgagcccagaccgacggttttggagtttgacagcgagtacgag

ctgctggttgccttcctgacctttctcaagcagtactctcccgagttcgccaccggctac

aacatcgttaattttgactgggcgtacatagttaacaaggtaacgtcggtgtataacatc

aagctggacgggtacggcaagttcaacaaaggggggctgtttaaggtgtgggacatcgcc

acgaaccattttcagaagaagagcaaggtgaaaatcaatggcctgatatctctagacatg

tattctgtggcgacggaaaagctaaagctacccagctacaaactcgacgcggtcgtggga

gacgtcctcggcgagcataagatagaccttccctataaagaaataccctcctattacgcg

ggagggcctgaccggaggggcgtaataggagagtattgtatccaggactctaggctggtg

ggcaagctgttttttaagtacctcccccatctggaactatcggcggtggccaaactcgcc

cgtatcaccctaacgcgggtaatttttgacggtcagcaaattcgcgtgtacacgtgcttg

ctgaaactcgcccgcgagagaaatttcattttgccagacaacagacgccggtttgacagt

caggcagacgccgcgtcagagacttcggagttggctatggatagccaaagccacgccttc

gacagtacagacgaacccgacggtgtggacggtaccccggacgccgcaggatctggcgct

acttctgaaaacggaggcgggaagcccggcgtcgggagggccgtgggctaccagggagca

aaggttctagaccccgtatccggctttcatgtggaccccgtggttgtgtttgacttcgct

agcttatacccaagcattatccaggcccataacctctgtttcaccaccctggcgctcgat

gaagtggatctggccgggcttcaaccatccgtcaactactcgacgttcgaggtgggtgac

caaaagttattttttgtccacgcccatattcgcgaaagcctgcttggcatcttgctgcgc

gactggctggccatgcgaaaggcggtgagggcgcgaatccccaccagcacccccgaggag

gcagttttactagataagcagcagtctgcgattaaggtgatatgcaactcggtttacgga

ttcacgggggtggcaaacggcctgttgccgtgtctgaggatagcggctaccgttaccacg

ataggacgcgacatgctcctcaagacccgagattacgttcactctcgttgggcgacgcgc

gagctgctggaggacaattttccaggggctataggtttccgaaaccacaagccttactcc

gtcagggttatctacggagacaccgactccgtgtttatcaagtttgtgggcctgacgtac

gagggggtatcggagctgggggatgctatgtcgcgtcagatttcagcggacctctttaga

gcgcccatcaaactggagtgtgagaagacctttcagcgactgctgctgatcaccaagaag

aagtacataggtgtcataaacggggggaagatgctcatgaagggggtcgacctggtccgc

aaaaataactgctctttcataaacttgtacgcgcgacatctggtagatcttttgttgtac

gacgaggatgtggccacggcggcagcagaggtgacagacgtgcctcccgcagaatgggtg

gggcgcccgctaccgagcggctttgacaagtttgggcgagtgctggtagaggcgtacaac

cgtatcactgcccccaacttggacgtgcgcgagttcgttatgactgctgagctgagccgc

tcacccgaatcgtataccaacaagcgcctgccgcacctcaccgtctactttaagctcgcc

atgaggaatgaagaactgcccagcgtaaaagagagaattccgtatgtgatagttgcgcag

accgaggccgcggaacgcgaagcgggtgtagtaaactcaatgcgcggtaccgcccaaaac

cccgtggtaaccaagaccgcacgcccccaacctaaacgcaaactgctggtttccgacctc

gccgaagacccgacctatgtttccgagaatgacgtaccgctaaacacagactactatttc

tcccacctgttgggtaccataagcgtgacctttaaggctctattcggaaatgatgtgaga

acaacagaaaatcttttaaagcggtttattccggaaaccccccacaagacccccacgaaa

acccaggcactgcttgagcgcgccggctttgaaaagctgacgccctttacaccggaggaa

gaaagtcgtcgaatactgcatacagttttttgtactctagaagcagctccccatcaaagc

tga

>MW396757.1

atggcggcgcgcgaacaggccaactccgttcgcaggagcgggttttttaatccgtttatt

ggcaagaggccatttttcaggccgggaagcgggcagaccgcggagacagagcgcccaagg

cccccccaacactcgtactgcacagaggtgggtagctttaagtttatagctccaagatgc

ctcgatgaagaagcccccgccgaccagcgacgcggtgtacacgtgggcaccttggagcgc

ccacccaaagtgtactgcgatggctcagagtacgacgtgctgaactttgcctccggaggt

tgctggcctcgcaggattcgggtttggaacggccaggattttcggggcgatggattcaac

cccagatttgagagatttcacgtgtacgacatagtagagacttcggagagcgcctcacac

gatgacccatccaggtttgctgagctatcccgcccaagcgggtctgtggtaacactgctg

ggaatgagcgagtgtggaaagcgagtcgcggttcacgtctatggtgtgcgccattatttt

tacatggcaaaggcggaggtggatagcgcttgtggaataaccaccgaggcagaactcgtg

cgcgcaatggtggactgcgcgcacagctcggctttgagcgcggccctgggaaatggcaac

ggcggcaaacagagcggcggcagcggcgggggatggtggggcggaaagcacgtgtctgcg

gactgcttcaaagtggagaccgtgtgccacacgacgctgtactactttggatctaagcca

gctctctactatagagtatctgcctccagcagccgcctgggagggttcatctgcgacaac

tttcacccggagattacaaaattcgaggggagcgtggacgtgaccacgcggctgttgttg

gacaatgaaaattttaccagtttcgggtggtaccgcctgcgacccggcacccacggagag

cgtgttcaacttcgccccgttgagcgacacgtcacctcaagcgacgtggagattaactgt

actcccgataacctggagccgataccagacgaggctgcctggcccgactataagctcatg

tgctttgatatagagtgtaaagctggaacgggtaacgaaatggcgttcccagtggcaact

aaccaagaggacctggtcatccagatctcctgtctgctgtactcgcttgctactcagaac

cacgaacacaccctgctgttttccctcgggtcatgcgatatctctgaggaatactcgttt

gcatgcgtccagcgcggcgagcccagaccgacggttttggagtttgacagcgagtacgag

ctgctggttgccttcctgacctttctcaagcagtactctcccgagttcgccaccggctac

aacatcgttaattttgactgggcgtacatagttaacaaggtaacgtcggtgtataacatc

aagctggacgggtacggcaagttcaacaaaggggggctgtttaaggtgtgggacatcgcc

acgaaccattttcagaagaagagcaaggtgaaaatcaatggcctgatatctctagacatg

tattctgtggcgacggaaaagctaaagctacccagctacaaactcgacgcggtcgtggga

gacgtcctcggcgagcataagatagaccttccctataaagaaataccctcctattacgcg

ggagggcctgaccggaggggcgtaataggagagtattgtatccaggactctaggctggtg

ggcaagctgttttttaagtacctcccccatctggaactatcggcggtggccaaactcgcc

cgtatcaccctaacgcgggtaatttttgacggtcagcaaattcgcgtgtacacgtgcttg

ctgaaactcgcccgcgagagaaatttcattttgccagacaacagacgccggtttgacagt

caggcagacgccgcgtcagagacttcggagttggctatggatagccaaagccacgccttc

gacagtacagacgaacccgacggtgtggacggtaccccggacgccgcaggatctggcgct

acttctgaaaacggaggcgggaagcccggcgtcgggagggccgtgggctaccagggagca

aaggttctagaccccgtatccggctttcatgtggaccccgtggttgtgtttgacttcgct

agcttatacccaagcattatccaggcccataacctctgtttcaccaccctggcgctcgat

gaagtggatctggccgggcttcaaccatccgtcaactactcgacgttcgaggtgggtgac

caaaagttattttttgtccacgcccatattcgcgaaagcctgcttggcatcttgctgcgc

gactggctggccatgcgaaaggcggtgagggcgcgaatccccaccagcacccccgaggag

gcagttttactagataagcagcagtctgcgattaaggtgatatgcaactcggtttacgga

ttcacgggggtggcaaacggcctgttgccgtgtctgaggatagcggctaccgttaccacg

ataggacgcgacatgctcctcaagacccgagattacgttcactctcgttgggcgacgcgc

gagctgctggaggacaattttccaggggctataggtttccgaaaccacaagccttactcc

gtcagggttatctacggagacaccgactccgtgtttatcaagtttgtgggcctgacgtac

gagggggtatcggagctgggggatgctatgtcgcgtcagatttcagcggacctctttaga

gcgcccatcaaactggagtgtgagaagacctttcagcgactgctgctgatcaccaagaag

aagtacataggtgtcataaacggggggaagatgctcatgaagggggtcgacctggtccgc

aaaaataactgctctttcataaacttgtacgcgcgacatctggtagatcttttgttgtac

gacgaggatgtggccacggcggcagcagaggtgacagacgtgcctcccgcagaatgggtg

gggcgcccgctaccgagcggctttgacaagtttgggcgagtgctggtagaggcgtacaac

cgtatcactgcccccaacttggacgtgcgcgagttcgttatgactgctgagctgagccgc

tcacccgaatcgtataccaacaagcgcctgccgcacctcaccgtctactttaagctcgcc

atgaggaatgaagaactgcccagcgtaaaagagagaattccgtatgtgatagttgcgcag

accgaggccgcggaacgcgaagcgggtgtagtaaactcaatgcgcggtaccgcccaaaac

cccgtggtaaccaagaccgcacgcccccaacctaaacgcaaactgctggtttccgacctc

gccgaagacccgacctatgtttccgagaatgacgtaccgctaaacacagactactatttc

tcccacctgttgggtaccataagcgtgacctttaaggctctattcggaaatgatgtgaga

acaacagaaaatcttttaaagcggtttattccggaaaccccccacaagacccccacgaaa

acccaggcactgcttgagcgcgccggctttgaaaagctgacgccctttacaccggaggaa

gaaagtcgtcgaatactgcatacagttttttgtactctagaagcagctccccatcaaagc

tga

>MW396758.1

atggcggcgcgcgaacaggccaactccgttcgcaggagcgggttttttaatccgtttatt

ggcaagaggccatttttcaggccgggaagcgggcagaccgcggagacagagcgcccaagg

cccccccaacactcgtactgcacagaggtgggtagctttaagtttatagctccaagatgc

ctcgatgaagaagcccccgccgaccagcgacgcggtgtacacgtgggcaccttggagcgc

ccacccaaagtgtactgcgatggctcagagtacgacgtgctgaactttgcctccggaggt

tgctggcctcgcaggattcgggtttggaacggccaggattttcggggcgatggattcaac

cccagatttgagagatttcacgtgtacgacatagtagagacttcggagagcgcctcacac

gatgacccatccaggtttgctgagctatcccgcccaagcgggtctgtggtaacactgctg

ggaatgagcgagtgtggaaagcgagtcgcggttcacgtctatggtgtgcgccattatttt

tacatggcaaaggcggaggtggatagcgcttgtggaataaccaccgaggcagaactcgtg

cgcgcaatggtggactgcgcgcacagctcggctttgagcgcggccctgggaaatggcaac

ggcggcaaacagagcggcggcagcggcgggggatggtggggcggaaagcacgtgtctgcg

gactgcttcaaagtggagaccgtgtgccacacgacgctgtactactttggatctaagcca

gctctctactatagagtatctgcctccagcagccgcctgggagggttcatctgcgacaac

tttcacccggagattacaaaattcgaggggagcgtggacgtgaccacgcggctgttgttg

gacaatgaaaattttaccagtttcgggtggtaccgcctgcgacccggcacccacggagag

cgtgttcaacttcgccccgttgagcgacacgtcacctcaagcgacgtggagattaactgt

actcccgataacctggagccgataccagacgaggctgcctggcccgactataagctcatg

tgctttgatatagagtgtaaagctggaacgggtaacgaaatggcgttcccagtggcaact

aaccaagaggacctggtcatccagatctcctgtctgctgtactcgcttgctactcagaac

cacgaacacaccctgctgttttccctcgggtcatgcgatatctctgaggaatactcgttt

gcatgcgtccagcgcggcgagcccagaccgacggttttggagtttgacagcgagtacgag

ctgctggttgccttcctgacctttctcaagcagtactctcccgagttcgccaccggctac

aacatcgttaattttgactgggcgtacatagttaacaaggtaacgtcggtgtataacatc

aagctggacgggtacggcaagttcaacaaaggggggctgtttaaggtgtgggacatcgcc

acgaaccattttcagaagaagagcaaggtgaaaatcaatggcctgatatctctagacatg

tattctgtggcgacggaaaagctaaagctacccagctacaaactcgacgcggtcgtggga

gacgtcctcggcgagcataagatagaccttccctataaagaaataccctcctattacgcg

ggagggcctgaccggaggggcgtaataggagagtattgtatccaggactctaggctggtg

ggcaagctgttttttaagtacctcccccatctggaactatcggcggtggccaaactcgcc

cgtatcaccctaacgcgggtaatttttgacggtcagcaaattcgcgtgtacacgtgcttg

ctgaaactcgcccgcgagagaaatttcattttgccagacaacagacgccggtttgacagt

caggcagacgccgcgtcagagacttcggagttggctatggatagccaaagccacgccttc

gacagtacagacgaacccgacggtgtggacggtaccccggacgccgcaggatctggcgct

acttctgaaaacggaggcgggaagcccggcgtcgggagggccgtgggctaccagggagca

aaggttctagaccccgtatccggctttcatgtggaccccgtggttgtgtttgacttcgct

agcttatacccaagcattatccaggcccataacctctgtttcaccaccctggcgctcgat

gaagtggatctggccgggcttcaaccatccgtcaactactcgacgttcgaggtgggtgac

caaaagttattttttgtccacgcccatattcgcgaaagcctgcttggcatcttgctgcgc

gactggctggccatgcgaaaggcggtgagggcgcgaatccccaccagcacccccgaggag

gcagttttactagataagcagcagtctgcgattaaggtgatatgcaactcggtttacgga

ttcacgggggtggcaaacggcctgttgccgtgtctgaggatagcggctaccgttaccacg

ataggacgcgacatgctcctcaagacccgagattacgttcactctcgttgggcgacgcgc

gagctgctggaggacaattttccaggggctataggtttccgaaaccacaagccttactcc

gtcagggttatctacggagacaccgactccgtgtttatcaagtttgtgggcctgacgtac

gagggggtatcggagctgggggatgctatgtcgcgtcagatttcagcggacctctttaga

gcgcccatcaaactggagtgtgagaagacctttcagcgactgctgctgatcaccaagaag

aagtacataggtgtcataaacggggggaagatgctcatgaagggggtcgacctagtccgc

aaaaataactgctctttcataaacttgtacgcgcgacatctggtagatcttttgttgtac

gacgaggatgtggccacggcggcagcagaggtgacagacgtgcctcccgcagaatgggtg

gggcgcccgctaccgagcggctttgacaagtttgggcgagtgctggtagaggcgtacaac

cgtatcactgcccccaacttggacgtgcgcgagttcgttatgactgctgagctgagccgc

tcacccgaatcgtataccaacaagcgcctgccgcacctcaccgtctactttaagctcgcc

atgaggaatgaagaactgcccagcgtaaaagagagaattccgtatgtgatagttgcgcag

accgaggccgcggaacgcgaagcgggtgtagtaaactcaatgcgcggtaccgcccaaaac

cccgtggtaaccaagaccgcacgcccccaacctaaacgcaaactgctggtttccgacctc

gccgaagacccgacctatgtttccgagaatgacgtaccgctaaacacagactactatttc

tcccacctgttgggtaccataagcgtgacctttaaggctctattcggaaatgatgtgaga

acaacagaaaatcttttaaagcggtttattccggaaaccccccacaagacccccacgaaa

acccaggcactgcttgagcgcgccggctttgaaaagctgacgccctttacaccggaggaa

gaaagtcgtcgaatactgcatacagttttttgtactctagaagcagctccccatcaaagc

tga

>MW396759.1

atggcggcgcgcgaacaggccaactccgttcgcaggagcgggttttttaatccgtttatt

ggcaagaggccatttttcaggccgggaagcgggcagaccgcggagacagagcgcccaagg

cccccccaacactcgtactgcacagaggtgggtagctttaagtttatagctccaagatgc

ctcgatgaagaagcccccgccgaccagcgacgcggtgtacacgtgggcaccttggagcgc

ccacccaaagtgtactgcgatggctcagagtacgacgtgctgaactttgcctccggaggt

tgctggcctcgcaggattcgggtttggaacggccaggattttcggggcgatggattcaac

cccagatttgagagatttcacgtgtacgacatagtagagacttcggagagcgcctcacac

gatgacccatccaggtttgctgagctatcccgcccaagcgggtctgtggtaacactgctg

ggaatgagcgagtgtggaaagcgagtcgcggttcacgtctatggtgtgcgccattatttt

tacatggcaaaggcggaggtggatagcgcttgtggaataaccaccgaggcagaactcgtg

cgcgcaatggtggactgcgcgcacagctcggctttgagcgcggccctgggaaatggcaac

ggcggcaaacagagcggcggcagcggcgggggatggtggggcggaaagcacgtgtctgcg

gactgcttcaaagtggagaccgtgtgccacacgacgctgtactactttggatctaagcca

gctctctactatagagtatctgcctccagcagccgcctgggagggttcatctgcgacaac

tttcacccggagattacaaaattcgaggggagcgtggacgtgaccacgcggctgttgttg

gacaatgaaaattttaccagtttcgggtggtaccgcctgcgacccggcacccacggagag

cgtgttcaacttcgccccgttgagcgacacgtcacctcaagcgacgtggagattaactgt

actcccgataacctggagccgataccagacgaggctgcctggcccgactataagctcatg

tgctttgatatagagtgtaaagctggaacgggtaacgaaatggcgttcccagtggcaact

aaccaagaggacctggtcatccagatctcctgtctgctgtactcgcttgctactcagaac

cacgaacacaccctgctgttttccctcgggtcatgcgatatctctgaggaatactcgttt

gcatgcgtccagcgcggcgagcccagaccgacggttttggagtttgacagcgagtacgag

ctgctggttgccttcctgacctttctcaagcagtactctcccgagttcgccaccggctac

aacatcgttaattttgactgggcgtacatagttaacaaggtaacgtcggtgtataacatc

aagctggacgggtacggcaagttcaacaaaggggggctgtttaaggtgtgggacatcgcc

acgaaccattttcagaagaagagcaaggtgaaaatcaatggcctgatatctctagacatg

tattctgtggcgacggaaaagctaaagctacccagctacaaactcgacgcggtcgtggga

gacgtcctcggcgagcataagatagaccttccctataaagaaataccctcctattacgcg

ggagggcctgaccggaggggcgtaataggagagtattgtatccaggactctaggctggtg

ggcaagctgttttttaagtacctcccccatctggaactatcggcggtggccaaactcgcc

cgtatcaccctaacgcgggtaatttttgacggtcagcaaattcgcgtgtacacgtgcttg

ctgaaactcgcccgcgagagaaatttcattttgccagacaacaaacgccggtttgacagt

caggcagacgccgcgtcagagacttcggagttggctatggatagccaaagccacgccttc

gacagtacagacgaacccgacggtgtggacggtaccccggacgccgcaggatctggcgct

acttctgaaaacggaggcgggaagcccggcgtcgggagggccgtgggctaccagggagca

aaggttctagaccccgtatccggctttcatgtggaccccgtggttgtgtttgacttcgct

agcttatacccaagcattatccaggcccataacctctgtttcaccaccctggcgctcgat

gaagtggatctggccgggcttcaaccatccgtcaactactcgacgttcgaggtgggtgac

caaaagttattttttgtccacgcccatattcgcgaaagcctgcttggcatcttgctgcgc

gactggctggccatgcgaaaggcggtgagggcgcgaatccccaccagcacccccgaggag

gcagttttactagataagcagcagtctgcgattaaggtgatatgcaactcggtttacgga

ttcacgggggtggcaaacggcctgttgccgtgtctgaggatagcggctaccgttaccacg

ataggacgcgacatgctcctcaagacccgagattacgttcactctcgttgggcgacgcgc

gagctgctggaggacaattttccaggggctataggtttccgaaaccacaagccttactcc

gtcagggttatctacggagacaccgactccgtgtttatcaagtttgtgggcctgacgtac

gagggggtatcggagctgggggatgctatgtcgcgtcagatttcagcggacctctttaga

gcgcccatcaaactggagtgtgagaagacctttcagcgactgctgctgatcaccaagaag

aagtacataggtgtcataaacggggggaagatgctcatgaagggggtcgacctagtccgc

aaaaataactgctctttcataaacttgtacgcgcgacatctggtagatcttttgttgtac

gacgaggatgtggccacggcggcagcagaggtgacagacgtgcctcccgcagaatgggtg

gggcgcccgctaccgagcggctttgacaagtttgggcgagtgctggtagaggcgtacaac

cgtatcactgcccccaacttggacgtgcgcgagttcgttatgactgctgagctgagccgc

tcacccgaatcgtataccaacaagcgcctgccgcacctcaccgtctactttaagctcgcc

atgaggaatgaagaactgcccagcgtaaaagagagaattccgtatgtgatagttgcgcag

accgaggccgcggaacgcgaagcgggtgtagtaaactcaatgcgcggtaccgcccaaaac

cccgtggtaaccaagaccgcacgcccccaacctaaacgcaaactgctggtttccgacctc

gccgaagacccgacctatgtttccgagaatgacgtaccgctaaacacagactactatttc

tcccacctgttgggtaccataagcgtgacctttaaggctctattcggaaatgatgtgaga

acaacagaaaatcttttaaagcggtttattccggaaaccccccacaagacccccacgaaa

acccaggcactgcttgagcgcgccggctttgaaaagctgacgccctttacaccggaggaa

gaaagtcgtcgaatactgcatacagttttttgtactctagaagcagctccccatcaaagc

tga

>MW396760.1

atggcggcgcgcgaacaggccaactccgttcgcaggagcgggttttttaatccgtttatt

ggcaagaggccatttttcaggccgggaagcgggcagaccgcggagacagagcgcccaagg

cccccccaacactcgtactgcacagaggtgggtagctttaagtttatagctccaagatgc

ctcgatgaagaagcccccgccgaccagcgacgcggtgtacacgtgggcaccttggagcgc

ccacccaaagtgtactgcgatggctcagagtacgacgtgctgaactttgcctccggaggt

tgctggcctcgcaggattcgggtttggaacggccaggattttcggggcgatggattcaac

cccagatttgagagatttcacgtgtacgacatagtagagacttcggagagcgcctcacac

gatgacccatccaggtttgctgagctatcccgcccaagcgggtctgtggtaacactgctg

ggaatgagcgagtgtggaaagcgagtcgcggttcacgtctatggtgtgcgccattatttt

tacatggcaaaggcggaggtggatagcgcttgtggaataaccaccgaggcagaactcgtg

cgcgcaatggtggactgcgcgcacagctcggctttgagcgcggccctgggaaatggcaac

ggcggcaaacagagcggcggcagcggcgggggatggtggggcggaaagcacgtgtctgcg

gactgcttcaaagtggagaccgtgtgccgcacgacgctgtactactttggatctaagcca

gctctctactatagagtatctgcctccagcagccgcctgggagggttcatctgcgacaac

tttcacccggagattacaaaattcgaggggagcgtggacgtgaccacgcggctgttgttg

gacaatgaaaattttaccagtttcgggtggtaccgcctgcgacccggcacccacggagag

cgtgttcaacttcgccccgttgagcgacacgtcacctcaagcgacgtggagattaactgt

actcccgataacctggagccgataccagacgaggctgcctggcccgactataagctcatg

tgctttgatatagagtgtaaagctggaacgggtaacgaaatggcgttcccagtggcaact

aaccaagaggacctggtcatccagatctcctgtctgctgtactcgcttgctactcagaac

cacgaacacaccctgctgttttccctcgggtcatgcgatatctctgaggaatactcgttt

gcatgcgtccagcgcggcgagcccagaccgacggttttggagtttgacagcgagtacgag

ctgctggttgccttcctgacctttctcaagcagtactctcccgagttcgccaccggctac

aacatcgttaattttgactgggcgtacatagttaacaaggtaacgtcggtgtataacatc

aagctggacgggtacggcaagttcaacaaaggggggctgtttaaggtgtgggacatcgcc

acgaaccattttcagaagaagagcaaggtgaaaatcaatggcctgatatctctagacatg

tattctgtggcgacggaaaagctaaagctacccagctacaaactcgacgcggtcgtggga

gacgtcctcggcgagcataagatagaccttccctataaagaaataccctcctattacgcg

ggagggcctgaccggaggggcgtaataggagagtattgtatccaggactctaggctggtg

ggcaagctgttttttaagtacctcccccatctggaactatcggcggtggccaaactcgcc

cgtatcaccctaacgcgggtaatttttgacggtcagcaaattcgcgtgtacacgtgcttg

ctgaaactcgcccgcgagagaaatttcattttgccagacaacaaacgccggtttgacagt

caggcagacgccgcgtcagagacttcggagttggctatggatagccaaagccacgccttc

gacagtacagacgaacccgacggtgtggacggtaccccggacgccgcaggatctggcgct

acttctgaaaacggaggcgggaagcccggcgtcgggagggccgtgggctaccagggagca

aaggttctagaccccgtatccggctttcatgtggaccccgtggttgtgtttgacttcgct

agcttatacccaagcattatccaggcccataacctctgtttcaccaccctggcgctcgat

gaagtggatctggccgggcttcaaccatccgtcaactactcgacgttcgaggtgggtgac

caaaagttattttttgtccacgcccatattcgcgaaagcctgcttggcatcttgctgcgc

gactggctggccatgcgaaaggcggtgagggcgcgaatccccaccagcacccccgaggag

gcagttttactagataagcagcagtctgcgattaaggtgatatgcaactcggtttacgga

ttcacgggggtggcaaacggcctgttgccgtgtctgaggatagcggctaccgttaccacg

ataggacgcgacatgctcctcaagacccgagattacgttcactctcgttgggcgacgcgc

gagctgctggaggacaattttccaggggctataggtttccgaaaccacaagccttactcc

gtcagggttatctacggagacaccgactccgtgtttatcaagtttgtgggcctgacgtac

gagggggtatcggagctgggggatgctatgtcgcgtcagatttcagcggacctctttaga

gcgcccatcaaactggagtgtgagaagacctttcagcgactgctgctgatcaccaagaag

aagtacataggtgtcataaacggggggaagatgctcatgaagggggtcgacctagtccgc

aaaaataactgctctttcataaacttgtacgcgcgacatctggtagatcttttgttgtac

gacgaggatgtggccacggcggcagcagaggtgacagacgtgcctcccgcagaatgggtg

gggcgcccgctaccgagcggctttgacaagtttgggcgagtgctggtagaggcgtacaac

cgtatcactgcccccaacttggacgtgcgcgagttcgttatgactgctgagctgagccgc

tcacccgaatcgtataccaacaagcgcctgccgcacctcaccgtctactttaagctcgcc

atgaggaatgaagaactgcccagcgtaaaagagagaattccgtatgtgatagttgcgcag

accgaggccgcggaacgcgaagcgggtgtagtaaactcaatgcgcggtaccgcccaaaac

cccgtggtaaccaagaccgcacgcccccaacctaaacgcaaactgctggtttccgacctc

gccgaagacccgacctatgtttccgagaatgacgtaccgctaaacacagactactatttc

tcccacctgttgggtaccataagcgtgacctttaaggctctattcggaaatgatgtgaga

acaacagaaaatcttttaaagcggtttattccggaaaccccccacaagacccccacgaaa

acccaggcactgcttgagcgcgccggctttgaaaagctgacgccctttacaccggaggaa

gaaagtcgtcgaatactgcatacagttttttgtactctagaagcagctccccatcaaagc

tga

>PP856397.1

atggcggcgcgcgaacaggccaactccgttcgcaggagcgggttttttaatccgtttatt

ggcaagaggccatttttcaggccgggaagcgggcagaccgcggagacagagcgcccaagg

cccccccaacactcgtactgcacagaggtgggtagctttaagtttatagctccaagatgc

ctcgatgaagaagcccccgccgaccagcgacgcggtgtacacgtgggcaccttggagcgc

ccacccaaagtgtactgcgatggctcagagtacgacgtgctgaactttgcctccggaggt

tgctggcctcgcaggattcgggtttggaacggccaggattttcggggcgatggattcaac

cccagatttgagagatttcacgtgtacgacatagtagagacttcggagagcgcctcacac

gatgacccatccaggtttgctgagctatcccgcccaagcgggtctgtggtaacactgctg

ggaatgagcgagtgtggaaagcgagtcgcggttcacgtatatggtgtgcgccattatttt

tacatggcaaaggcggaggtggatagcgcttgtggaataaccaccgaggcagaactcgtg

cgcgcaatggtggactgcgcgcacagctcggctttgagcgcggccctgggaaatggcaac

ggcggcaaacagagcggcggcagcggcgggggatggtggggcggaaagcacgtgtctgcg

gactgcttcaaagtggagaccgtgtgccacacgacgctgtactactttggatctaagcca

gctctctactatagagtatctgcctccagcagccgcctgggagggttcatctgcgacaac

tttcacccggagattacaaaattcgaggggagcgtggacgtgaccacgcggctgttgttg

gacaatgaaaattttaccagtttcgggtggtaccgcctgcgacccggcacccacggagag

cgtgttcaacttcgccccgttgagcgacacgtcacctcaagcgacgtggagattaactgt

actcccgataacctggagccgataccagacgaggctgcctggcccgactataagctcatg

tgctttgatatagagtgtaaagctggaacgggtaacgaaatggcgttcccagtggcaact

aaccaagaggacctggtcatccagatctcctgtctgctgtactcgcttgctactcagaac

cacgaacacaccctgctgttttccctcgggtcatgcgatatctctgaggaatactcgttt

gcatgcgtccagcgcggcgagcccagaccgacggttttggagtttgacagcgagtacgag

ctgctggttgccttcctgacctttctcaagcagtactctcccgagttcgccaccggctac

aacatcgttaattttgactgggcgtacatagttaacaaggtaacgtcggtgtataacatc

aagctggacgggtacggcaagttcaacaaaggggggctgtttaaggtgtgggacatcgcc

acgaaccattttcagaagaagagcaaggtgaaaatcaatggcctgatatctctagacatg

tattctgtggcgacggaaaagctaaagctacccagctacaaactcgacgcggtcgtggga

gacgtcctcggcgagcataagatagaccttccctataaagaaataccctcctattacgcg

ggagggcctgaccggaggggcgtaataggagagtattgtatccaggactctaggctggtg

ggcaagctgttttttaagtacctcccccatctggaactatcggcggtggccaaactcgcc

cgtatcaccctaacgcgggtaatttttgacggtcagcaaattcgcgtgtacacgtgcttg

ctgaaactcgcccgcgagagaaatttcattttgccagacaacagacgccggtttgacagt

caggcagacgccgcgtcagagacttcggagttggctatggatagccaaagccacgccttc

gacagtacagacgaacccgacggtgtggacggtaccccggacgccgcaggatctggcgct

acttctgaaaacggaggcgggaagcccggcgtcgggagggccgtgggctaccagggagca

aaggttctagaccccgtatccggctttcatgtggaccccgtggttgtgtttgacttcgct

agcttatacccaagcattatccaggcccataacctctgtttcaccaccctggcgctcgat

gaagtggatctggccgggcttcaaccatccgtcgactactcgacgttcgaggtgggtgac

caaaagttattttttgtccacgcccatattcgcgaaagcctgcttggcatcttgctgcgc

gactggctggccatgcgaaaggcggtgagggcgcgaatccccaccagcacccccgaggag

gcagttttactagataagcagcagtctgcgattaaggtgatatgcaactcggtttacgga

ttcacgggggtggcaaacggcctgttgccgtgtctgaggatagcggctaccgttaccacg

ataggacgcgacatgctcctcaagacccgagattacgttcactctcgttgggcgacgcgc

gagctgctggaggacaattttccaggggctataggtttccgaaaccacaagccttactcc

gtcagggttatctacggagacaccgactccgtgtttatcaagtttgtgggcctgacgtac

gagggggtatcggagctgggggatgctatgtcgcgtcagatttcagcggacctctttaga

gcgcccatcaaactggagtgtgagaagacctttcagcgactgctgctgatcaccaagaag

aagtacataggtgtcataaacggggggaagatgctcatgaagggggtcgacctggtccgc

aaaaataactgctctttcataaacttgtacgcgcgacatctggtagatcttttgttgtac

gacgaggatgtggccacggcggcagcagaggtgacagacgtgcctcccgcagaatgggtg

gggcgcccgctaccgagcggctttgacaagtttgggcgagtgctggtagaggcgtacaac

cgtatcactgcccccaacttggacgtgcgcgagttcgttatgactgctgagctgagccgc

tcacccgaatcgtataccaacaagcgcctgccgcacctcaccgtctactttaagctcgcc

atgaggaatgaagaactgcccagcgtaaaagagagaattccgtatgtgatagttgcgcag

accgaggccgcggaacgcgaagcgggtgtagtaaactcaatgcgcggtaccgcccaaaac

cccgtggtaaccaagaccgcacgcccccaacctaaacgcaaactgctggtttccgacctc

gccgaagacccgacctatgtttccgagaatgacgtaccgctaaacacagactactatttc

tcccacctgttgggtaccataagcgtgacctttaaggctctattcggaaatgatgtgaga

acaacagaaaatcttttaaagcggtttattccggaaaccccccacaagacccccacgaaa

acccaggcactgcttgagcgcgccggctttgaaaagctgacgccctttacaccggaggaa

gaaagtcgtcgaatactgcatacagttttttgtactctagaagcagctccccatcaaagc

tga

>LC834900.1

atggcggcgcgcgaacaggccaactccgttcgcaggagcgggttttttaatccgtttatt

ggcaagaggccatttttcaggccgggaagcgggcagaccgcggagacagagcgcccaagg

cccccccaacactcgtactgcacagaggtgggtagctttaagtttatagctccaagatgc

ctcgatgaagaagcccccgccgaccagcgacgcggtgtacacgtgggcaccttggagcgc

ccacccaaagtgtactgcgatggctcagagtacgacgtgctgaactttgcctccggaggt

tgctggcctcgcaggattcgggtttggaacggccaggattttcggggcgatggattcaac

cccagatttgagagatttcacgtgtacgacatagtagagacttcggagagcgcctcacac

gatgacccatccaggtttgctgagctatcccgcccaagcgggtctgtggtaacactgctg

ggaatgagcgagtgtggaaagcgagtcgcggttcacgtctatggtgtgcgccattatttt

tacatggcaaaggcggaggtggatagcgcttgtggaataaccaccgaggcagaactcgtg

cgcgcaatggtggactgcgcgcacagctcggctttgagcgcggccctgggaaatggcaac

ggcggcaaacagagcggcggcagcggcgggggatggtggggcggaaagcacgtgtctgcg

gactgcttcaaagtggagaccgtgtgccacacgacgctgtactactttggatctaagcca

gctctctactatagagtatctgcctccagcagccgcctgggagggttcatctgcgacaac

tttcacccggagattacaaaattcgaggggagcgtggacgtgaccacgcggctgttgttg

gacaatgaaaattttaccagtttcgggtggtaccgcctgcgacccggcacccacggagag

cgtgttcaacttcgccccgttgagcgacacgtcacctcaagcgacgtggagattaactgt

actcccgataacctggagccgataccagacgaggctgcctggcccgactataagctcatg

tgctttgatatagagtgtaaagctggaacgggtaacgaaatggcgttcccagtggcaact

aaccaagaggacctggtcatccagatctcctgtctgctgtactcgcttgctactcagaac

cacgaacacaccctgctgttttccctcgggtcatgcgatatctctgaggaatactcgttt

gcatgcgtccagcgcggcgagcccagaccgacggttttggagtttgacagcgagtacgag

ctgctggttgccttcctgacctttctcaagcagtactctcccgagttcgccaccggctac

aacatcgttaattttgactgggcgtacatagttaacaaggtaacgtcggtgtataacatc

aagctggacgggtacggcaagttcaacaaaggggggctgtttaaggtgtgggacatcgcc

acgaaccattttcagaagaagagcaaggtgaaaatcaatggcctgatatctctagacatg

tattctgtggcgacggaaaagctaaagctacccagctacaaactcgacgcggtcgtggga

gacgtcctcggcgagcataagatagaccttccctataaagaaataccctcctattacgcg

ggagggcctgaccggaggggcgtaataggagagtattgtatccaggactctaggctggtg

ggcaagctgttttttaagtacctcccccatctggaactatcggcggtggccaaactcgcc

cgtatcaccctaacgcgggtaatttttgacggtcagcaaattcgcgtgtacacgtgcttg

ctgaaactcgcccgcgagagaaatttcattttgccagacaacagacgccggtttgacagt

caggcagacgccgcgtcagagacttcggagttggctatggatagccaaagccacgccttc

gacagtacagacgaacccgacggtgtggacggtaccccggacgccgcaggatctggcgct

acttctgaaaacggaggcgggaagcccggcgtcgggagggccgtgggctaccagggagca

aaggttctagaccccgtatccggctttcatgtggaccccgtggttgtgtttgacttcgct

agcttatacccaagcattatccaggcccataacctctgtttcaccaccctggcgctcgat

gaagtggatctggccgggcttcaaccatccgtcaactactcgacgttcgaggtgggtgac

caaaagttattttttgtccacgcccatattcgcgaaagcctgcttggcatcttgctgcgc

gactggctggccatgcgaaaggcggtgagggcgcgaatccccaccagcacccccgaggag

gcagttttactagataagcagcagtctgcgattaaggtgatatgcaactcggtttacgga

ttcacgggggtggcaaacggcctgttgccgtgtctgaggatagcggctaccgttaccacg

ataggacgcgacatgctcctcaagacccgagattacgttcactctcgttgggcgacgcgc

gagctgctggaggacaattttccaggggctataggtttccgaaaccacaagccttactcc

gtcagggttatctacggagacaccgactccgtgtttatcaagtttgtgggcctgacgtac

gagggggtatcggagctgggggatgctatgtcgcgtcagatttcagcggacctctttaga

gcccccatcaaactggagtgtgagaagacctttcagcgactgctgctgatcaccaagaag

aagtacataggtgtcataaacggggggaagatgctcatgaagggggtcgacctggtccgc

aaaaataactgctctttcataaacttgtacgcgcgacatctggtagatcttttgttgtac

gacgaggatgtggccacggcggcagcagaggtgacagacgtgcctcccgcagaatgggtg

gggcgcccgctaccgagcggctttgacaagtttgggcgagtgctggtagaggcgtacaac

cgtatcactgcccccaacttggacgtgcgcgagttcgttatgactgctgagctgagccgc

tcacccgaatcgtataccaacaagcgcctgccgcacctcaccgtctactttaagctcgcc

atgaggaatgaagaactgcccagcgtaaaagagagaattccgtatgtgatagttgcgcag

accgaggccgcggaacgcgaagcgggtgtagtaaactcaatgcgcggtaccgcccaaaac

cccgtggtaaccaagaccgcacgcccccaacctaaacgcaaactgctggtttccgacctc

gccgaagacccgacctatgtttccgagaatgacgtaccgctaaacacagactactatttc

tcccacctgttgggtaccataagcgtgacctttaaggctctattcggaaatgatgtgaga

acaacagaaaatcttttaaagcggtttattccggaaaccccccacaagacccccacgaaa

acccaggcactgcttgagcgcgccggctttgaaaagctgacgccctttacaccggaggaa

gaaagtcgtcgaatactgcatacagttttttgtactctagaagcagctccccatcaaagc

tga

>LC834901.1

atggcggcgcgcgaacaggccaactccgttcgcaggagcgggttttttaatccgtttatt

ggcaagaggccatttttcaggccgggaagcgggcagaccgcggagacagagcgcccaagg

cccccccaacactcgtactgcacagaggtgggtagctttaagtttatagctccaagatgc

ctcgatgaagaagcccccgccgaccagcgacgcggtgtacacgtgggcaccttggagcgc

ccacccaaagtgtactgcgatggctcagagtacgacgtgctgaactttgcctccggaggt

tgctggcctcgcaggattcgggtttggaacggccaggattttcggggcgatggattcaac

cccagatttgagagatttcacgtgtacgacatagtagagacttcggagagcgcctcacac

gatgacccatccaggtttgctgagctatcccgcccaagcgggtctgtggtaacactgctg

ggaatgagcgagtgtggaaagcgagtcgcggttcacgtctatggtgtgcgccattatttt

tacatggcaaaggcggaggtggatagcgcttgtggaataaccaccgaggcagaactcgtg

cgcgcaatggtggactgcgcgcacagctcggctttgagcgcggccctgggaaatggcaac

ggcggcaaacagagcggcggcagcggcgggggatggtggggcggaaagcacgtgtctgcg

gactgcttcaaagtggagaccgtgtgccacacgacgctgtactactttggatctaagcca

gctctctactatagagtatctgcctccagcagccgcctgggagggttcatctgcgacaac

tttcacccggagattacaaaattcgaggggagcgtggacgtgaccacgcggctgttgttg

gacaatgaaaattttaccagtttcgggtggtaccgcctgcgacccggcacccacggagag

cgtgttcaacttcgccccgttgagcgacacgtcacctcaagcgacgtggagattaactgt

actcccgataacctggagccgataccagacgaggctgcctggcccgactataagctcatg

tgctttgatatagagtgtaaagctggaacgggtaacgaaatggcgttcccagtggcaact

aaccaagaggacctggtcatccagatctcctgtctgctgtactcgcttgctactcagaac

cacgaacacaccctgctgttttccctcgggtcatgcgatatctctgaggaatactcgttt

gcatgcgtccagcgcggcgagcccagaccgacggttttggagtttgacagcgagtacgag

ctgctggttgccttcctgacctttctcaagcagtactctcccgagttcgccaccggctac

aacatcgttaattttgactgggcgtacatagttaacaaggtaacgtcggtgtataacatc

aagctggacgggtacggcaagttcaacaaaggggggctgtttaaggtgtgggacatcgcc

acgaaccattttcagaagaagagcaaggtgaaaatcaatggcctgatatctctagacatg

tattctgtggcgacggaaaagctaaagctacccagctacaaactcgacgcggtcgtggga

gacgtcctcggcgagcataagatagaccttccctataaagaaataccctcctattacgcg

ggagggcctgaccggaggggcgtaataggagagtattgtatccaggactctaggctggtg

ggcaagctgttttttaagtacctcccccatctggaactatcggcggtggccaaactcgcc

cgtatcaccctaacgcgggtaatttttgacggtcagcaaattcgcgtgtacacgtgcttg

ctgaaactcgcccgcgagagaaatttcattttgccagacaacagacgccggtttgacagt

caggcagacgccgcgtcagagacttcggagttggctatggatagccaaagccacgccttc

gacagtacagacgaacccgacggtgtggacggtaccccggacgccgcaggatctggcgct

acttctgaaaacggaggcgggaagcccggcgtcgggagggccgtgggctaccagggagca

aaggttctagaccccgtatccggctttcatgtggaccccgtggttgtgtttgacttcgct

agcttatacccaagcattatccaggcccataacctctgtttcaccaccctggcgctcgat

gaagtggatctggccgggcttcaaccatccgtcaactactcgacgttcgaggtgggtgac

caaaagttattttttgtccacgcccatattcgcgaaagcctgcttggcatcttgctgcgc

gactggctggccatgcgaaaggcggtgagggcgcgaatccccaccagcacccccgaggag

gcagttttactagataagcagcagtctgcgattaaggtgatatgcaactcggtttacgga

ttcacgggggtggcaaacggcctgttgccgtgtctgaggatagcggctaccgttaccacg

ataggacgcgacatgctcctcaagacccgagattacgttcactctcgttgggcgacgcgc

gagctgctggaggacaattttccaggggctataggtttccgaaaccacaagccttactcc

gtcagggttatctacggagacaccgactccgtgtttatcaagtttgtgggcctgacgtac

gagggggtatcggagctgggggatgctatgtcgcgtcagatttcagcggacctctttaga

gcccccatcaaactggagtgtgagaagacctttcagcgactgctgctgatcaccaagaag

aagtacataggtgtcataaacggggggaagatgctcatgaagggggtcgacctggtccgc

aaaaataactgctctttcataaacttgtacgcgcgacatctggtagatcttttgttgtac

gacgaggatgtggccacggcggcagcagaggtgacagacgtgcctcccgcagaatgggtg

gggcgcccgctaccgagcggctttgacaagtttgggcgagtgctggtagaggcgtacaac

cgtatcactgcccccaacttggacgtgcgcgagttcgttatgactgctgagctgagccgc

tcacccgaatcgtataccaacaagcgcctgccgcacctcaccgtctactttaagctcgcc

atgaggaatgaagaactgcccagcgtaaaagagagaattccgtatgtgatagttgcgcag

accgaggccgcggaacgcgaagcgggtgtagtaaactcaatgcgcggtaccgcccaaaac

cccgtggtaaccaagaccgcacgcccccaacctaaacgcaaactgctggtttccgacctc

gccgaagacccgacctatgtttccgagaatgacgtaccgctaaacacagactactatttc

tcccacctgttgggtaccataagcgtgacctttaaggctctattcggaaatgatgtgaga

acaacagaaaatcttttaaagcggtttattccggaaaccccccacaagacccccacgaaa

acccaggcactgcttgagcgcgccggctttgaaaagctgacgccctttacaccggaggaa

gaaagtcgtcgaatactgcatacagttttttgtactctagaagcagctccccatcaaagc

tga

>LC834902.1

atggcggcgcgcgaacaggccaactccgttcgcaggagcgggttttttaatccgtttatt

ggcaagaggccatttttcaggccgggaagcgggcaaaccgcggagacagagcgcccaagg

cccccccaacactcgtactgcacagaggtgggtagctttaagtttatagctccaagatgc

ctcgatgaagaagcccccgccgaccagcgacgcggtgtacacgtgggcaccttggagcgc

ccacccaaagtgtactgcgatggctcagagtacgacgtgctgaactttgcctccggaggt

tgctggcctcgcaggattcgggtttggaacggccaggattttcggggcgatggattcaac

cccagatttgagagatttcacgtgtacgacatagtagagacttcggagagcgcctcacac

gatgacccatccaggtttgctgagctatcccgcccaagcgggtctgtggtaacactgctg

ggaatgagcgagtgtggaaagcgagtcgcggttcacgtctatggtgtgcgccattatttt

tacatggcaaaggcggaggtggatagcgcttgtggaataaccaccgaggcagaactcgtg

cgcgcaatggtggactgcgcgcacagctcggctttgagcgcggccctgggaaatggcaac

ggcggcaaacagagcggcggcagcggcgggggatggtggggcggaaagcacgtgtctgcg

gactgcttcaaagtggagaccgtgtgccacacgacgctgtactactttggatctaagcca

gctctctactatagagtatctgcctccagcagccgcctgggagggttcatctgcgacaac

tttcacccggagattacaaaattcgaggggagcgtggacgtgaccacgcggctgttgttg

gacaatgaaaattttaccagtttcgggtggtaccgcctgcgacccggcacccacggagag

cgtgttcaacttcgccccgttgagcgacacgtcacctcaagcgacgtggagattaactgt

actcccgataacctggagccgataccagacgaggctgcctggcccgactataagctcatg

tgctttgatatagagtgtaaagctggaacgggtaacgaaatggcgttcccagtggcaact

aaccaagaggacctggtcatccagatctcctgtctgctgtactcgcttgctactcagaac

cacgaacacaccctgctgttttccctcgggtcatgcgatatctctgaggaatactcgttt

gcatgcgtccagcgcggcgagcccagaccgacggttttggagtttgacagcgagtacgag

ctgctggttgccttcctgacctttctcaagcagtactctcccgagttcgccaccggctac

aacatcgttaattttgactgggcgtacatagttaacaaggtaacgtcggtgtataacatc

aagctggacgggtacggcaagttcaacaaaggggggctgtttaaggtgtgggacatcgcc

acgaaccattttcagaagaagagcaaggtgaaaatcaatggcctgatatctctagacatg

tattctgtggcgacggaaaagctaaagctacccagctacaaactcgacgcggtcgtggga

gacgtcctcggcgagcataagatagaccttccctataaagaaataccctcctattacgcg

ggagggcctgaccggaggggcgtaataggagagtattgtatccaggactctaggctggtg

ggcaagctgttttttaagtacctcccccatctggaactatcggcggtggccaaactcgcc

cgtatcaccctaacgcgggtaatttttgacggtcagcaaattcgcgtgtacacgtgcttg

ctgaaactcgcccgcgagagaaatttcattttgccagacaacagacgccggtttgacagt

caggcagacgccgcgtcagagacttcggagttggctatggatagccaaagccacgccttc

gacagtacagacgaacccgacggtgtggacggtaccccggacgccgcaggatctggcgct

acttctgaaaacggaggcgggaagcccggcgtcgggagggccgtgggctaccagggagca

aaggttctagaccccgtatccggctttcatgtggaccccgtggttgtgtttgacttcgct

agcttatacccaagcattatccaggcccataacctctgtttcaccaccctggcgctcgat

gaagtggatctggccgggcttcaaccatccgtcaactactcgacgttcgaggtgggtgac

caaaagttattttttgtccacgcccatattcgcgaaagcctgcttggcatcttgctgcgc

gactggctggccatgcgaaaggcggtgagggcgcgaatccccaccagcacccccgaggag

gcagttttactagataagcagcagtctgcgattaaggtgatatgcaactcggtttacgga

ttcacgggggtggcaaacggcctgttgccgtgtctgaggatagcggctaccgttaccacg

ataggacgcgacatgctcctcaagacccgagattacgttcactctcgttgggcgacgcgc

gagctgctggaggacaattttccaggggctataggtttccgaaaccacaagccttactcc

gtcagggttatctacggagacaccgactccgtgtttatcaagtttgtgggcctgacgtac

gagggggtatcggagctgggggatgctatgtcgcgtcagatttcagcggacctctttaga

gcgcccatcaaactggagtgtgagaagacctttcagcgactgctgctgatcaccaagaag

aagtacataggtgtcataaacggggggaagatgctcatgaagggggtcgacctggtccgc

aaaaataactgctctttcataaacttgtacgcgcgacatctggtagatcttttgttgtac

gacgaggatgtggccacggcggcagcagaggtgacagacgtgcctcccgcagaatgggtg

gggcgcccgctaccgagcggctttgacaagtttgggcgagtgctggtagaggcgtacaac

cgtatcactgcccccaacttggacgtgcgcgagttcgttatgactgctgagctgagccgc

tcacccgaatcgtataccaacaagcgcctgccgcacctcaccgtctactttaagctcgcc

atgaggaatgaagaactgcccagcgtaaaagagagaattccgtatgtgatagttgcgcag

accgaggccgcggaacgcgaagcgggtgtagtaaactcaatgcgcggtaccgcccaaaac

cccgtggtaaccaagaccgcacgcccccaacctaaacgcaaactgctggtttccgacctc

gccgaagacccgacctatgtttccgagaatgacgtaccgctaaacacagactactatttc

tcccacctgttgggtaccataagcgtgacctttaaggctctattcggaaatgatgtgaga

acaacagaaaatcttttaaagcggtttattccggaaaccccccacaagacccccacgaaa

acccaggcactgcttgagcgcgccggctttgaaaagctgacgccctttacaccggaggaa

gaaagtcgtcgaatactgcatacagttttttgtactctagaagcagctccccatcaaagc

tga

>LC834903.1

atggcggcgcgcgaacaggccaactccgttcgcaggagcgggttttttaatccgtttatt

ggcaagaggccatttttcaggccgggaagcgggcaaaccgcggagacagagcgcccaagg

cccccccaacactcgtactgcacagaggtgggtagctttaagtttatagctccaagatgc

ctcgatgaagaagcccccgccgaccagcgacgcggtgtacacgtgggcaccttggagcgc

ccacccaaagtgtactgcgatggctcagagtacgacgtgctgaactttgcctccggaggt

tgctggcctcgcaggattcgggtttggaacggccaggattttcggggcgatggattcaac

cccagatttgagagatttcacgtgtacgacatagtagagacttcggagagcgcctcacac

gatgacccatccaggtttgctgagctatcccgcccaagcgggtctgtggtaacactgctg

ggaatgagcgagtgtggaaagcgagtcgcggttcacgtctatggtgtgcgccattatttt

tacatggcaaaggcggaggtggatagcgcttgtggaataaccaccgaggcagaactcgtg

cgcgcaatggtggactgcgcgcacagctcggctttgagcgcggccctgggaaatggcaac

ggcggcaaacagagcggcggcagcggcgggggatggtggggcggaaagcacgtgtctgcg

gactgcttcaaagtggagaccgtgtgccacacgacgctgtactactttggatctaagcca

gctctctactatagagtatctgcctccagcagccgcctgggagggttcatctgcgacaac

tttcacccggagattacaaaattcgaggggagcgtggacgtgaccacgcggctgttgttg

gacaatgaaaattttaccagtttcgggtggtaccgcctgcgacccggcacccacggagag

cgtgttcaacttcgccccgttgagcgacacgtcacctcaagcgacgtggagattaactgt

actcccgataacctggagccgataccagacgaggctgcctggcccgactataagctcatg

tgctttgatatagagtgtaaagctggaacgggtaacgaaatggcgttcccagtggcaact

aaccaagaggacctggtcatccagatctcctgtctgctgtactcgcttgctactcagaac

cacgaacacaccctgctgttttccctcgggtcatgcgatatctctgaggaatactcgttt

gcatgcgtccagcgcggcgagcccagaccgacggttttggagtttgacagcgagtacgag

ctgctggttgccttcctgacctttctcaagcagtactctcccgagttcgccaccggctac

aacatcgttaattttgactgggcgtacatagttaacaaggtaacgtcggtgtataacatc

aagctggacgggtacggcaagttcaacaaaggggggctgtttaaggtgtgggacatcgcc

acgaaccattttcagaagaagagcaaggtgaaaatcaatggcctgatatctctagacatg

tattctgtggcgacggaaaagctaaagctacccagctacaaactcgacgcggtcgtggga

gacgtcctcggcgagcataagatagaccttccctataaagaaataccctcctattacgcg

ggagggcctgaccggaggggcgtaataggagagtattgtatccaggactctaggctggtg

ggcaagctgttttttaagtacctcccccatctggaactatcggcggtggccaaactcgcc

cgtatcaccctaacgcgggtaatttttgacggtcagcaaattcgcgtgtacacgtgcttg

ctgaaactcgcccgcgagagaaatttcattttgccagacaacagacgccggtttgacagt

caggcagacgccgcgtcagagacttcggagttggctatggatagccaaagccacgccttc

gacagtacagacgaacccgacggtgtggacggtaccccggacgccgcaggatctggcgct

acttctgaaaacggaggcgggaagcccggcgtcgggagggccgtgggctaccagggagca

aaggttctagaccccgtatccggctttcatgtggaccccgtggttgtgtttgacttcgct

agcttatacccaagcattatccaggcccataacctctgtttcaccaccctggcgctcgat

gaagtggatctggccgggcttcaaccatccgtcaactactcgacgttcgaggtgggtgac

caaaagttattttttgtccacgcccatattcgcgaaagcctgcttggcatcttgctgcgc

gactggctggccatgcgaaaggcggtgagggcgcgaatccccaccagcacccccgaggag

gcagttttactagataagcagcagtctgcgattaaggtgatatgcaactcggtttacgga

ttcacgggggtggcaaacggcctgttgccgtgtctgaggatagcggctaccgttaccacg

ataggacgcgacatgctcctcaagacccgagattacgttcactctcgttgggcgacgcgc

gagctgctggaggacaattttccaggggctataggtttccgaaaccacaagccttactcc

gtcagggttatctacggagacaccgactccgtgtttatcaagtttgtgggcctgacgtac

gagggggtatcggagctgggggatgctatgtcgcgtcagatttcagcggacctctttaga

gcgcccatcaaactggagtgtgagaagacctttcagcgactgctgctgatcaccaagaag

aagtacataggtgtcataaacggggggaagatgctcatgaagggggtcgacctggtccgc

aaaaataactgctctttcataaacttgtacgcgcgacatctggtagatcttttgttgtac

gacgaggatgtggccacggcggcagcagaggtgacagacgtgcctcccgcagaatgggtg

gggcgcccgctaccgagcggctttgacaagtttgggcgagtgctggtagaggcgtacaac

cgtatcactgcccccaacttggacgtgcgcgagttcgttatgactgctgagctgagccgc

tcacccgaatcgtataccaacaagcgcctgccgcacctcaccgtctactttaagctcgcc

atgaggaatgaagaactgcccagcgtaaaagagagaattccgtatgtgatagttgcgcag

accgaggccgcggaacgcgaagcgggtgtagtaaactcaatgcgcggtaccgcccaaaac

cccgtggtaaccaagaccgcacgcccccaacctaaacgcaaactgctggtttccgacctc

gccgaagacccgacctatgtttccgagaatgacgtaccgctaaacacagactactatttc

tcccacctgttgggtaccataagcgtgacctttaaggctctattcggaaatgatgtgaga

acaacagaaaatcttttaaagcggtttattccggaaaccccccacaagacccccacgaaa

acccaggcactgcttgagcgcgccggctttgaaaagctgacgccctttacaccggaggaa

gaaagtcgtcgaatactgcatacagttttttgtactctagaagcagctccccatcaaagc

tga

>LC834904.1

atggcggcgcgcgaacaggccaactccgttcgcaggagcgggttttttaatccgtttatt

ggcaagaggccatttttcaggccgggaagcgggcaaaccgcggagacagagcgcccaagg

cccccccaacactcgtactgcacagaggtgggtagctttaagtttatagctccaagatgc

ctcgatgaagaagcccccgccgaccagcgacgcggtgtacacgtgggcaccttggagcgc

ccacccaaagtgtactgcgatggctcagagtacgacgtgctgaactttgcctccggaggt

tgctggcctcgcaggattcgggtttggaacggccaggattttcggggcgatggattcaac

cccagatttgagagatttcacgtgtacgacatagtagagacttcggagagcgcctcacac

gatgacccatccaggtttgctgagctatcccgcccaagcgggtctgtggtaacactgctg

ggaatgagcgagtgtggaaagcgagtcgcggttcacgtctatggtgtgcgccattatttt

tacatggcaaaggcggaggtggatagcgcttgtggaataaccaccgaggcagaactcgtg

cgcgcaatggtggactgcgcgcacagctcggctttgagcgcggccctgggaaatggcaac

ggcggcaaacagagcggcggcagcggcgggggatggtggggcggaaagcacgtgtctgcg

gactgcttcaaagtggagaccgtgtgccacacgacgctgtactactttggatctaagcca

gctctctactatagagtatctgcctccagcagccgcctgggagggttcatctgcgacaac

tttcacccggagattacaaaattcgaggggagcgtggacgtgaccacgcggctgttgttg

gacaatgaaaattttaccagtttcgggtggtaccgcctgcgacccggcacccacggagag

cgtgttcaacttcgccccgttgagcgacacgtcacctcaagcgacgtggagattaactgt

actcccgataacctggagccgataccagacgaggctgcctggcccgactataagctcatg

tgctttgatatagagtgtaaagctggaacgggtaacgaaatggcgttcccagtggcaact

aaccaagaggacctggtcatccagatctcctgtctgctgtactcgcttgctactcagaac

cacgaacacaccctgctgttttccctcgggtcatgcgatatctctgaggaatactcgttt

gcatgcgtccagcgcggcgagcccagaccgacggttttggagtttgacagcgagtacgag

ctgctggttgccttcctgacctttctcaagcagtactctcccgagttcgccaccggctac

aacatcgttaattttgactgggcgtacatagttaacaaggtaacgtcggtgtataacatc

aagctggacgggtacggcaagttcaacaaaggggggctgtttaaggtgtgggacatcgcc

acgaaccattttcagaagaagagcaaggtgaaaatcaatggcctgatatctctagacatg

tattctgtggcgacggaaaagctaaagctacccagctacaaactcgacgcggtcgtggga

gacgtcctcggcgagcataagatagaccttccctataaagaaataccctcctattacgcg

ggagggcctgaccggaggggcgtaataggagagtattgtatccaggactctaggctggtg

ggcaagctgttttttaagtacctcccccatctggaactatcggcggtggccaaactcgcc

cgtatcaccctaacgcgggtaatttttgacggtcagcaaattcgcgtgtacacgtgcttg

ctgaaactcgcccgcgagagaaatttcattttgccagacaacagacgccggtttgacagt

caggcagacgccgcgtcagagacttcggagttggctatggatagccaaagccacgccttc

gacagtacagacgaacccgacggtgtggacggtaccccggacgccgcaggatctggcgct

acttctgaaaacggaggcgggaagcccggcgtcgggagggccgtgggctaccagggagca

aaggttctagaccccgtatccggctttcatgtggaccccgtggttgtgtttgacttcgct

agcttatacccaagcattatccaggcccataacctctgtttcaccaccctggcgctcgat

gaagtggatctggccgggcttcaaccatccgtcaactactcgacgttcgaggtgggtgac

caaaagttattttttgtccacgcccatattcgcgaaagcctgcttggcatcttgctgcgc

gactggctggccatgcgaaaggcggtgagggcgcgaatccccaccagcacccccgaggag

gcagttttactagataagcagcagtctgcgattaaggtgatatgcaactcggtttacgga

ttcacgggggtggcaaacggcctgttgccgtgtctgaggatagcggctaccgttaccacg

ataggacgcgacatgctcctcaagacccgagattacgttcactctcgttgggcgacgcgc

gagctgctggaggacaattttccaggggctataggtttccgaaaccacaagccttactcc

gtcagggttatctacggagacaccgactccgtgtttatcaagtttgtgggcctgacgtac

gagggggtatcggagctgggggatgctatgtcgcgtcagatttcagcggacctctttaga

gcgcccatcaaactggagtgtgagaagacctttcagcgactgctgctgatcaccaagaag

aagtacataggtgtcataaacggggggaagatgctcatgaagggggtcgacctggtccgc

aaaaataactgctctttcataaacttgtacgcgcgacatctggtagatcttttgttgtac

gacgaggatgtggccacggcggcagcagaggtgacagacgtgcctcccgcagaatgggtg

gggcgcccgctaccgagcggctttgacaagtttgggcgagtgctggtagaggcgtacaac

cgtatcactgcccccaacttggacgtgcgcgagttcgttatgactgctgagctgagccgc

tcacccgaatcgtataccaacaagcgcctgccgcacctcaccgtctactttaagctcgcc

atgaggaatgaagaactgcccagcgtaaaagagagaattccgtatgtgatagttgcgcag

accgaggccgcggaacgcgaagcgggtgtagtaaactcaatgcgcggtaccgcccaaaac

cccgtggtaaccaagaccgcacgcccccaacctaaacgcaaactgctggtttccgacctc

gccgaagacccgacctatgtttccgagaatgacgtaccgctaaacacagactactatttc

tcccacctgttgggtaccataagcgtgacctttaaggctctattcggaaatgatgtgaga

acaacagaaaatcttttaaagcggtttattccggaaaccccccacaagacccccacgaaa

acccaggcactgcttgagcgcgccggctttgaaaagctgacgccctttacaccggaggaa

gaaagtcgtcgaatactgcatacagttttttgtactctagaagcagctccccatcaaagc

tga

>LC834905.1

atggcggcgcgcgaacaggccaactccgttcgcaggagcgggttttttaatccgtttatt

ggcaagaggccatttttcaggccgggaagcgggcagaccgcggagacagagcgcccaagg

cccccccaacactcgtactgcacagaggtgggtagctttaagtttatagctccaagatgc

ctcgatgaagaagcccccgccgaccagcgacgcggtgtacacgtgggcaccttggagcgc

ccacccaaagtgtactgcgatggctcagagtacgacgtgctgaactttgcctccggaggt

tgctggcctcgcaggattcgggtttggaacggccaggattttcggggcgatggattcaac

cccagatttgagagatttcacgtgtacgacatagtagagacttcggagagcgcctcacac

gatgacccatccaggtttgctgagctatcccgcccaagcgggtctgtggtaacactgctg

ggaatgagcgagtgtggaaagcgagtcgcggttcacgtctatggtgtgcgccattatttt

tacatggcaaaggcggaggtggatagcgcttgtggaataaccaccgaggcagaactcgtg

cgcgcaatggtggactgcgcgcacagctcggctttgagcgcggccctgggaaatggcaac

ggcggcaaacagagcggcggcagcggcgggggatggtggggcggaaagcacgtgtctgcg

gactgcttcaaagtggagaccgtgtgccgcacgacgctgtactactttggatctaagcca

gctctctactatagagtatctgcctccagcagccgcctgggagggttcatctgcgacaac

tttcacccggagattacaaaattcgaggggagcgtggacgtgaccacgcggctgttgttg

gacaatgaaaattttaccagtttcgggtggtaccgcctgcgacccggcacccacggagag

cgtgttcaacttcgccccgttgagcgacacgtcacctcaagcgacgtggagattaactgt

actcccgataacctggagccgataccagacgaggctgcctggcccgactataagctcatg

tgctttgatatagagtgtaaagctggaacgggtaacgaaatggcgttcccagtggcaact

aaccaagaggacctggtcatccagatctcctgtctgctgtactcgcttgctactcagaac

cacgaacacaccctgctgttttccctcgggtcatgcgatatctctgaggaatactcgttt

gcatgcgtccagcgcggcgagcccagaccgacggttttggagtttgacagcgagtacgag

ctgctggttgccttcctgacctttctcaagcagtactctcccgagttcgccaccggctac

aacatcgttaattttgactgggcgtacatagttaacaaggtaacgtcggtgtataacatc

aagctggacgggtacggcaagttcaacaaaggggggctgtttaaggtgtgggacatcgcc

acgaaccattttcagaagaagagcaaggtgaaaatcaatggcctgatatctctagacatg

tattctgtggcgacggaaaagctaaagctacccagctacaaactcgacgcggtcgtggga

gacgtcctcggcgagcataagatagaccttccctataaagaaataccctcctattacgcg

ggagggcctgaccggaggggcgtaataggagagtattgtatccaggactctaggctggtg

ggcaagctgttttttaagtacctcccccatctggaactatcggcggtggccaaactcgcc

cgtatcaccctaacgcgggtaatttttgacggtcagcaaattcgcgtgtacacgtgcttg

ctgaaactcgcccgcgagagaaatttcattttgccagacaacagacgccggtttgacagt

caggcagacgccgcgtcagagacttcggagttggctatggatagccaaagccacgccttc

gacagtacagacgaacccgacggtgtggacggtaccccggacgccgcaggatctggcgct

acttctgaaaacggaggcgggaagcccggcgtcgggagggccgtgggctaccagggagca

aaggttctagaccccgtatccggctttcatgtggaccccgtggttgtgtttgacttcgct

agcttatacccaagcattatccaggcccataacctctgtttcaccaccctggcgctcgat

gaagtggatctggccgggcttcaaccatccgtcaactactcgacgttcgaggtgggtgac

caaaagttattttttgtccacgcccatattcgcgaaagcctgcttggcatcttgctgcgc

gactggctggccatgcgaaaggcggtgagggcgcgaatccccaccagcacccccgaggag

gcagttttactagataagcagcagtctgcgattaaggtgatatgcaactcggtttacgga

ttcacgggggtggcaaacggcctgttgccgtgtctgaggatagcggctaccgttaccacg

ataggacgcgacatgctcctcaagacccgagattacgttcactctcgttgggcgacgcgc

gagctgctggaggacaattttccaggggctataggtttccgaaaccacaagccttactcc

gtcagggttatctacggagacaccgactccgtgtttatcaagtttgtgggcctgacgtac

gagggggtatcggagctgggggatgctatgtcgcgtcagatttcagcggacctctttaga

gcgcccatcaaactggagtgtgagaagacctttcagcgactgctgctgatcaccaagaag

aagtacataggtgtcataaacggggggaagatgctcatgaagggggtcgacctggtccgc

aaaaataactgctctttcataaacttgtacgcgcgacatctggtagatcttttgttgtac

gacgaggatgtggccacggcggcagcagaggtgacagacgtgcctcccgcagaatgggtg

gggcgcccgctaccgagcggctttgacaagtttgggcgagtgctggtagaggcgtacaac

cgtatcactgcccccaacttggacgtgcgcgagttcgttatgactgctgagctgagccgc

tcacccgaatcgtataccaacaagcgcctgccgcacctcaccgtctactttaagctcgcc

atgaggaatgaagaactgcccagcgtaaaagagagaattccgtatgtgatagttgcgcag

accgaggccgcggaacgcgaagcgggtgtagtaaactcaatgcgcggtaccgcccaaaac

cccgtggtaaccaagaccgcacgcccccaacctaaacgcaaactgctggtttccgacctc

gccgaagacccgacctatgtttccgagaatgacgtaccgctaaacacagactactatttc

tcccacctgttgggtaccataagcgtgacctttaaggctctattcggaaatgatgtgaga

acaacagaaaatcttttaaagcggtttattccggaaaccccccacaagacccccacgaaa

acccaggcactgcttgagcgcgccggctttgaaaagctgacgccctttacaccggaggaa

gaaagtcgtcgaatactgcatacagttttttgtactctagaagcagctccccatcaaagc

tga

>LC834906.1

atggcggcgcgcgaacaggccaactccgttcgcaggagcgggttttttaatccgtttatt

ggcaagaggccatttttcaggccgggaagcgggcagaccgcggagacagagcgcccaagg

cccccccaacactcgtactgcacagaggtgggtagctttaagtttatagctccaagatgc

ctcgatgaagaagcccccgccgaccagcgacgcggtgtacacgtgggcaccttggagcgc

ccacccaaagtgtactgcgatggctcagagtacgacgtgctgaactttgcctccggaggt

tgctggcctcgcaggattcgggtttggaacggccaggattttcggggcgatggattcaac

cccagatttgagagatttcacgtgtacgacatagtagagacttcggagagcgcctcacac

gatgacccatccaggtttgctgagctatcccgcccaagcgggtctgtggtaacactgctg

ggaatgagcgagtgtggaaagcgagtcgcggttcacgtctatggtgtgcgccattatttt

tacatggcaaaggcggaggtggatagcgcttgtggaataaccaccgaggcagaactcgtg

cgcgcaatggtggactgcgcgcacagctcggctttgagcgcggccctgggaaatggcaac

ggcggcaaacagagcggcggcagcggcgggggatggtggggcggaaagcacgtgtctgcg

gactgcttcaaagtggagaccgtgtgccacacgacgctgtactactttggatctaagcca

gctctctactatagagtatctgcctccagcagccgcctgggagggttcatctgcgacaac

tttcacccggagattacaaaattcgaggggagcgtggacgtgaccacgcggctgttgttg

gacaatgaaaattttaccagtttcgggtggtaccgcctgcgacccggcacccacggagag

cgtgttcaacttcgccccgttgagcgacacgtcacctcaagcgacgtggagattaactgt

actcccgataacctggagccgataccagacgaggctgcctggcccgactataagctcatg

tgctttgatatagagtgtaaagctggaacgggtaacgaaatggcgttcccagtggcaact

aaccaagaggacctggtcatccagatctcctgtctgctgtactcgcttgctactcagaac

cacgaacacaccctgctgttttccctcgggtcatgcgatatctctgaggaatactcgttt

gcatgcgtccagcgcggcgagcccagaccgacggttttggagtttgacagcgagtacgag

ctgctggttgccttcctgacctttctcaagcagtactctcccgagttcgccaccggctac

aacatcgttaattttgactgggcgtacatagttaacaaggtaacgtcggtgtataacatc

aagctggacgggtacggcaagttcaacaaaggggggctgtttaaggtgtgggacatcgcc

acgaaccattttcagaagaagagcaaggtgaaaatcaatggcctgatatctctagacatg

tattctgtggcgacggaaaagctaaagctacccagctacaaactcgacgcggtcgtggga

gacgtcctcggcgagcataagatagaccttccctataaagaaataccctcctattacgcg

ggagggcctgaccggaggggcgtaataggagagtattgtatccaggactctaggctggtg

ggcaagctgttttttaagtacctcccccatctggaactatcggcggtggccaaactcgcc

cgtatcaccctaacgcgggtaatttttgacggtcagcaaattcgcgtgtacacgtgcttg

ctgaaactcgcccgcgagagaaatttcattttgccagacaacagacgccggtttgacagt

caggcagacgccgcgtcagagacttcggagttggctatggatagccaaagccacgccttc

gacagtacagacgaacccgacggtgtggacggtaccccggacgccgcaggatctggcgct

acttctgaaaacggaggcgggaagcccggcgtcgggagggccgtgggctaccagggagca

aaggttctagaccccgtatccggctttcatgtggaccccgtggttgtgtttgacttcgct

agcttatacccaagcattatccaggcccataacctctgtttcaccaccctggcgctcgat

gaagcggatctggccgggcttcaaccatccgtcaactactcgacgttcgaggtgggtgac

caaaagttattttttgtccacgcccatattcgcgaaagcctgcttggcatcttgctgcgc

gactggctggccatgcgaaaggcggtgagggcgcgaatccccaccagcacccccgaggag

gcagttttactagataagcagcagtctgcgattaaggtgatatgcaactcggtttacgga

ttcacgggggtggcaaacggcctgttgccgtgtctgaggatagcggctaccgttaccacg

ataggacgcgacatgctcctcaagacccgagattacgttcactctcgttgggcgacgcgc

gagctgctggaggacaattttccaggggctataggtttccgaaaccacaagccttactcc

gtcagggttatctacggagacaccgactccgtgtttatcaagtttgtgggcctgacgtac

gagggggtatcggagctgggggatgctatgtcgcgtcagatttcagcggacctctttaga

gcgcccatcaaactggagtgtgagaagacctttcagcgactgctgctgatcaccaagaag

aagtacataggtgtcataaacggggggaagatgctcatgaagggggtcgacctggtccgc

aaaaataactgctctttcataaacttgtacgcgcgacatctggtagatcttttgttgtac

gacgaggatgtggccacggcggcagcagaggtgacagacgtgcctcccgcagaatgggtg

gggcgcccgctaccgagcggctttgacaagtttgggcgagtgctggtagaggcgtacaac

cgtatcactgcccccaacttggacgtgcgcgagttcgttatgactgctgagctgagccgc

tcacccgaatcgtataccaacaagcgcctgccgcacctcaccgtctactttaagctcgcc

atgaggaatgaagaactgcccagcgtaaaagagagaattccgtatgtgatagttgcgcag

accgaggccgcggaacgcgaagcgggtgtagtaaactcaatgcgcggtaccgcccaaaac

cccgtggtaaccaagaccgcacgcccccaacctaaacgcaaactgctggtttccgacctc

gccgaagacccgacctatgtttccgagaatgacgtaccgctaaacacagactactatttc

tcccacctgttgggtaccataagcgtgacctttaaggctctattcggaaatgatgtgaga

acaacagaaaatcttttaaagcggtttattccggaaaccccccacaagacccccacgaaa

acccaggcactgcttgagcgcgccggctttgaaaagctgacgccctttacaccggaggaa

gaaagtcgtcgaatactgcatacagttttttgtactctagaagcagctccccatcaaagc

tga

>LC834907.1

atggcggcgcgcgaacaggccaactccgttcgcaggagcgggttttttaatccgtttatt

ggcaagaggccatttttcaggccgggaagcgggcagaccgcggagacagagcgcccaagg

cccccccaacactcgtactgcacagaggtgggtagctttaagtttatagctccaagatgc

ctcgatgaagaagcccccgccgaccagcgacgcggtgtacacgtgggcaccttggagcgc

ccacccaaagtgtactgcgatggctcagagtacgacgtgctgaactttgcctccggaggt

tgctggcctcgcaggattcgggtttggaacggccaggattttcggggcgatggattcaac

cccagatttgagagatttcacgtgtacgacatagtagagacttcggagagcgcctcacac

gatgacccatccaggtttgctgagctatcccgcccaagcgggtctgtggtaacactgctg

ggaatgagcgagtgtggaaagcgagtcgcggttcacgtctatggtgtgcgccattatttt

tacatggcaaaggcggaggtggatagcgcttgtggaataaccaccgaggcagaactcgtg

cgcgcaatggtggactgcgcgcacagctcggctttgagcgcggccctgggaaatggcaac

ggcggcaaacagagcggcggcagcggcgggggatggtggggcggaaagcacgtgtctgcg

gactgcttcaaagtggagaccgtgtgccacacgacgctgtactactttggatctaagcca

gctctctactatagagtatctgcctccagcagccgcctgggagggttcatctgcgacaac

tttcacccggagattacaaaattcgaggggagcgtggacgtgaccacgcggctgttgttg

gacaatgaaaattttaccagtttcgggtggtaccgcctgcgacccggcacccacggagag

cgtgttcaacttcgccccgttgagcgacacgtcacctcaagcgacgtggagattaactgt

actcccgataacctggagccgataccagacgaggctgcctggcccgactataagctcatg

tgctttgatatagagtgtaaagctggaacgggtaacgaaatggcgttcccagtggcaact

aaccaagaggacctggtcatccagatctcctgtctgctgtactcgcttgctactcagaac

cacgaacacaccctgctgttttccctcgggtcatgcgatatctctgaggaatactcgttt

gcatgcgtccagcgcggcgagcccagaccgacggttttggagtttgacagcgagtacgag

ctgctggttgccttcctgacctttctcaagcagtactctcccgagttcgccaccggctac

aacatcgttaattttgactgggcgtacatagttaacaaggtaacgtcggtgtataacatc

aagctggacgggtacggcaagttcaacaaaggggggctgtttaaggtgtgggacatcgcc

acgaaccattttcagaagaagagcaaggtgaaaatcaatggcctgatatctctagacatg

tattctgtggcgacggaaaagctaaagctacccagctacaaactcgacgcggtcgtggga

gacgtcctcggcgagcataagatagaccttccctataaagaaataccctcctattacgcg

ggagggcctgaccggaggggcgtaataggagagtattgtatccaggactctaggctggtg

ggcaagctgttttttaagtacctcccccatctggaactatcggcggtggccaaactcgcc

cgtatcaccctaacgcgggtaatttttgacggtcagcaaattcgcgtgtacacgtgcttg

ctgaaactcgcccgcgagagaaatttcattttgccagacaacagacgccggtttgacagt

caggcagacgccgcgtcagagacttcggagttggctatggatagccaaagccacgccttc

gacagtacagacgaacccgacggtgtggacggtaccccggacgccgcaggatctggcgct

acttctgaaaacggaggcgggaagcccggcgtcgggagggccgtgggctaccagggagca

aaggttctagaccccgtatccggctttcatgtggaccccgtggttgtgtttgacttcgct

agcttatacccaagcattatccaggcccataacctctgtttcaccaccctggcgctcgat

gaagcggatctggccgggcttcaaccatccgtcaactactcgacgttcgaggtgggtgac

caaaagttattttttgtccacgcccatattcgcgaaagcctgcttggcatcttgctgcgc

gactggctggccatgcgaaaggcggtgagggcgcgaatccccaccagcacccccgaggag

gcagttttactagataagcagcagtctgcgattaaggtgatatgcaactcggtttacgga

ttcacgggggtggcaaacggcctgttgccgtgtctgaggatagcggctaccgttaccacg

ataggacgcgacatgctcctcaagacccgagattacgttcactctcgttgggcgacgcgc

gagctgctggaggacaattttccaggggctataggtttccgaaaccacaagccttactcc

gtcagggttatctacggagacaccgactccgtgtttatcaagtttgtgggcctgacgtac

gagggggtatcggagctgggggatgctatgtcgcgtcagatttcagcggacctctttaga

gcgcccatcaaactggagtgtgagaagacctttcagcgactgctgctgatcaccaagaag

aagtacataggtgtcataaacggggggaagatgctcatgaagggggtcgacctggtccgc

aaaaataactgctctttcataaacttgtacgcgcgacatctggtagatcttttgttgtac

gacgaggatgtggccacggcggcagcagaggtgacagacgtgcctcccgcagaatgggtg

gggcgcccgctaccgagcggctttgacaagtttgggcgagtgctggtagaggcgtacaac

cgtatcactgcccccaacttggacgtgcgcgagttcgttatgactgctgagctgagccgc

tcacccgaatcgtataccaacaagcgcctgccgcacctcaccgtctactttaagctcgcc

atgaggaatgaagaactgcccagcgtaaaagagagaattccgtatgtgatagttgcgcag

accgaggccgcggaacgcgaagcgggtgtagtaaactcaatgcgcggtaccgcccaaaac

cccgtggtaaccaagaccgcacgcccccaacctaaacgcaaactgctggtttccgacctc

gccgaagacccgacctatgtttccgagaatgacgtaccgctaaacacagactactatttc

tcccacctgttgggtaccataagcgtgacctttaaggctctattcggaaatgatgtgaga
[truncated: 299,200 more chars]
